# Multiomic screening of invasive GBM cells reveals targetable transsulfuration pathway alterations

**DOI:** 10.1172/JCI170397

**Published:** 2024-02-01

**Authors:** Joseph H. Garcia, Erin A. Akins, Saket Jain, Kayla J. Wolf, Jason Zhang, Nikita Choudhary, Meeki Lad, Poojan Shukla, Jennifer Rios, Kyounghee Seo, Sabraj A. Gill, William H. Carson, Luis R. Carette, Allison C. Zheng, David R. Raleigh, Sanjay Kumar, Manish K. Aghi

**Affiliations:** 1 Department of Neurosurgery, UCSF, San Francisco, California, USA.; 2 Department of Bioengineering, UC Berkeley, Berkeley, California, USA.; 3 Graduate Program in Bioengineering, UC Berkeley–UCSF, San Francisco, California, USA.; 4 Department of Chemical and Biomolecular Engineering, UC Berkeley, Berkeley, California, USA.; 5 Department of Bioengineering and Therapeutic Sciences, UCSF, San Francisco, California, USA.; 6 California Institute for Quantitative Biosciences at UC Berkeley (QB3-Berkeley), Berkeley, California, USA.

**Keywords:** Metabolism, Oncology, Amino acid metabolism, Bioenergetics, Brain cancer

## Abstract

While the poor prognosis of glioblastoma arises from the invasion of a subset of tumor cells, little is known of the metabolic alterations within these cells that fuel invasion. We integrated spatially addressable hydrogel biomaterial platforms, patient site–directed biopsies, and multiomics analyses to define metabolic drivers of invasive glioblastoma cells. Metabolomics and lipidomics revealed elevations in the redox buffers cystathionine, hexosylceramides, and glucosyl ceramides in the invasive front of both hydrogel-cultured tumors and patient site–directed biopsies, with immunofluorescence indicating elevated reactive oxygen species (ROS) markers in invasive cells. Transcriptomics confirmed upregulation of ROS-producing and response genes at the invasive front in both hydrogel models and patient tumors. Among oncologic ROS, H_2_O_2_ specifically promoted glioblastoma invasion in 3D hydrogel spheroid cultures. A CRISPR metabolic gene screen revealed cystathionine γ-lyase (CTH), which converts cystathionine to the nonessential amino acid cysteine in the transsulfuration pathway, to be essential for glioblastoma invasion. Correspondingly, supplementing CTH knockdown cells with exogenous cysteine rescued invasion. Pharmacologic CTH inhibition suppressed glioblastoma invasion, while CTH knockdown slowed glioblastoma invasion in vivo. Our studies highlight the importance of ROS metabolism in invasive glioblastoma cells and support further exploration of the transsulfuration pathway as a mechanistic and therapeutic target.

## Abstract 2

<p>Multi-omic analysis reveals oxidative stress during glioblastoma invasion in tumor models and CRISPR screening identifies cystathionine gamma lyase as a targetable glioblastoma-invasion mediator.</p>

## Graphical abstract

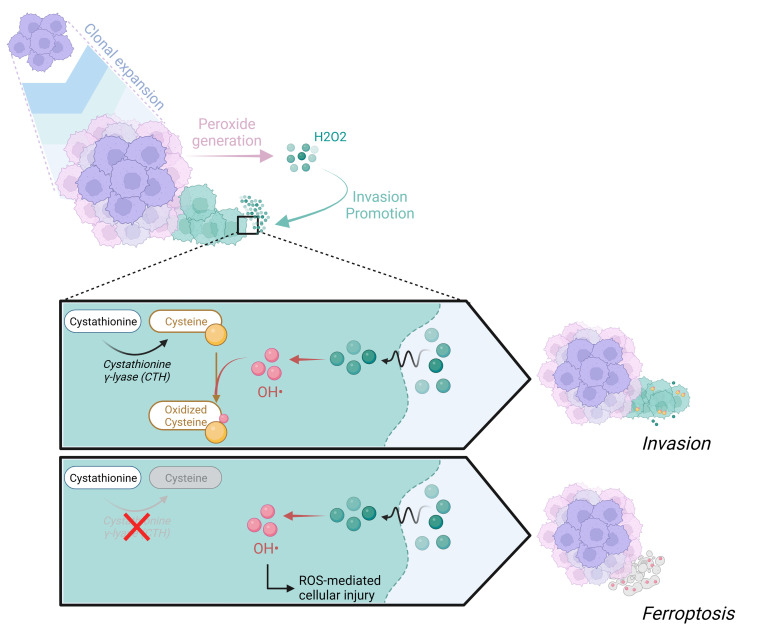

## Introduction

Glioblastoma (GBM) is the most common and lethal adult brain tumor (1) and is characterized by an unparalleled invasive capacity (2). Current therapeutic strategies are insufficient to control the disease, with a dismal median survival of less than 15 months from the time of diagnosis (1). The current standard of care consists of maximal surgical resection followed by radiation and temozolomide chemotherapy (3). Unfortunately, tumor invasiveness impedes these treatment modalities, as it renders complete resection impossible; spreads tumor cells outside of the field of radiation; and enables tumor cells to escape the area of MRI enhancement where the blood-brain barrier (BBB) is disrupted into regions outside the enhancement where the BBB is intact, making these invasive cells less accessible to systemic chemotherapy (2).

While numerous hypotheses have been proposed regarding pathways that regulate GBM invasion (2, 4), when investigating biological processes that might drive invasion in tumors, it is logical to examine the tumor’s cellular metabolism (5), as the process of invasion is likely to create a need for GBM cells to shift their metabolic profile in response to the bioenergetic demands of the invasive process and the limited nutrient availability of the surrounding brain (4, 6, 7). Unfortunately, the mechanisms enabling GBM cells to fuel their invasive capacity remain understudied (5, 8).

Historically, studies investigating metabolic reprogramming in GBM, as with other cancer types, have focused on glucose metabolism (6, 9). GBM cells, like all cancer cells, often metabolize glucose into lactate, even when oxygen is present, a process known as the Warburg effect (9). This metabolic shift is hypothesized to allow tumor cells to use glucose-derived carbons for the synthesis of essential cellular ingredients while still generating sufficient ATP to fuel cellular reactions (9). While glucose metabolism is undoubtedly important for numerous cellular processes in GBM, recent studies have painted a more complex picture of metabolic reprogramming in this tumor (9). Besides a shift toward glycolysis, GBM cells also increase intracellular lipid, amino acid, and nucleotide stores through a variety of molecular mechanisms, including increased extracellular uptake, de novo synthesis, and fluxing carbons through numerous biochemical pathways, such as the use of glycolysis to provide carbon substrates for the synthesis of nucleic acids (8). Importantly, these metabolic adaptations respond not only to the tumor’s genotype, but also to features of the surrounding microenvironment, such as hypoxia, which alters the transcription of metabolic genes (9). More recently, fluctuations in oxygen have been shown to drive ferroptosis, iron-dependent cell death mediated by lipid peroxidation (10). GBM cells guard against ferroptosis via glutathione (11), an antioxidant generated from the amino acid cysteine, and indirectly from methionine via the transsulfuration pathway. Findings like these have had translational implications by revealing that alterations in dietary lipids and amino acids affect the survival of mice carrying GBM tumors (11, 12).

Despite these advances in our understanding of the metabolic adjustments that enable GBM cells to meet their nutritional demands, the metabolic alterations needed for GBM invasion, a defining hallmark of this cancer, remain unknown. To address this knowledge gap, we employed a multiomics approach in microdissectable biomimetic 3D invasion devices and site-directed biopsies of patient GBMs to define metabolic changes in invasive GBM cells. After validating that our 3D hydrogel platforms adequately and reproducibly reflect the metabolic changes associated with GBM invasion, we then performed a CRISPR screen of metabolic genes in these platforms and discovered targetable metabolic factors that mediate invasion in this devastating disease.

## Results

### Metabolomics reveals increased cystathionine and other oxidative stress metabolites in invasive GBM cells in 3D hydrogels and patient specimens.

**Figure 1:**
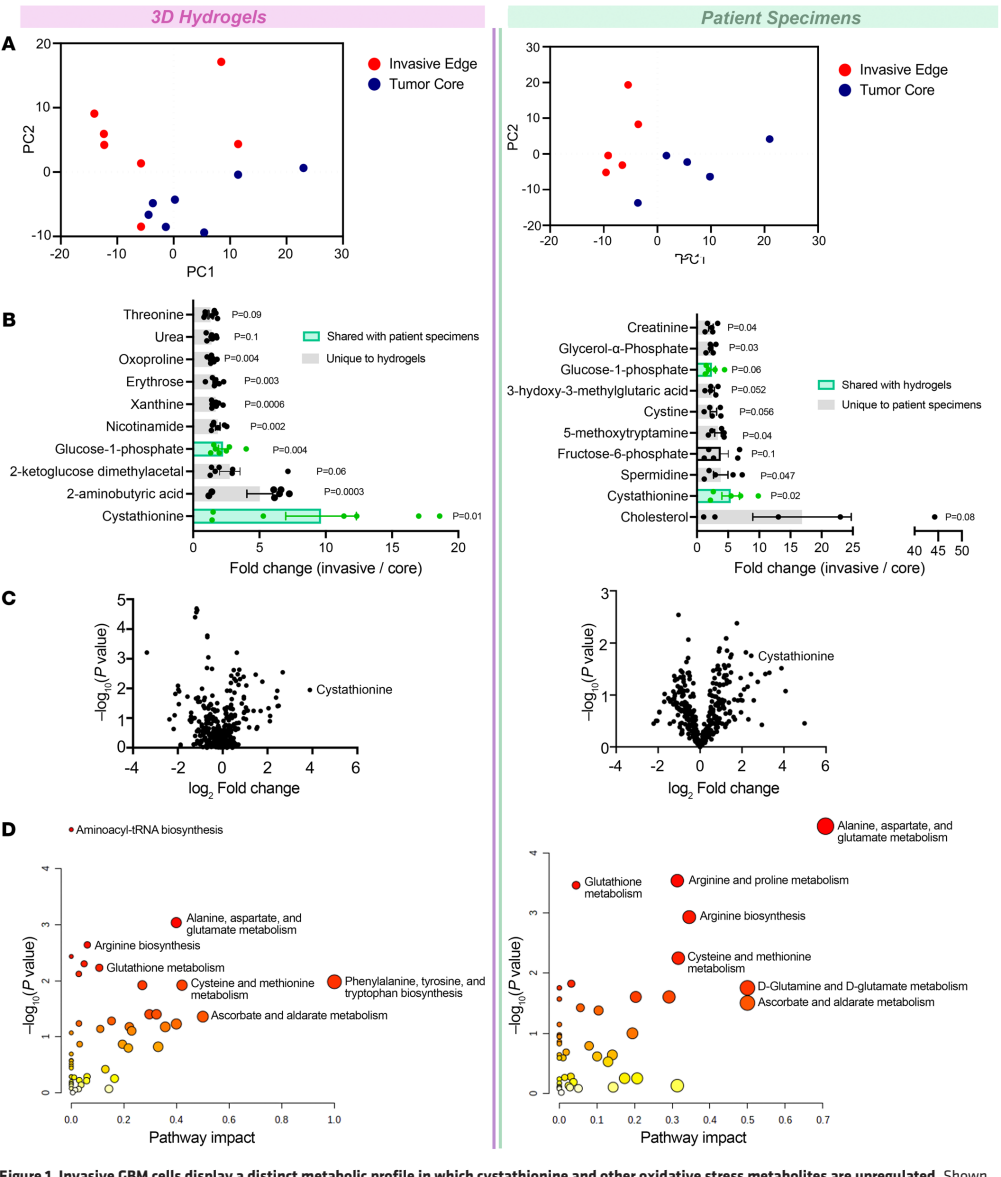
Invasive GBM cells display a distinct metabolic profile in which cystathionine and other oxidative stress metabolites are upregulated. Shown are results from metabolomic analysis of cells from the invasive front and tumor core of GBM43 cells in 3D hydrogels (left) and site-directed biopsies (right) of patient GBMs. (**A**) PCAs from hydrogels (left, *n* = 7/group) and site-directed patient biopsies (right, *n* = 5/group). (**B**) Bar graphs displaying 10 most enriched metabolites by *t* test at the invasive tumor front versus core of hydrogels (left) and patient tumors (right). (**C**) Volcano plots displaying fold change for metabolites in the invasive front of hydrogels (left) and patient tumors (right) compared with the tumor core. (**D**) MetaboAnalyst identified pathways upregulated at the invasive tumor front of hydrogels (left) and patient GBMs (right). Pathways are plotted according to significance (*y* axis) and pathway impact value (*x* axis). Node color is based on *P* value (darker colors = more significance), and node radius is based on pathway impact values (larger circles = greater pathway enrichment). Most contributing pathways are in the top right corner.

To comprehensively analyze the metabolic perturbations in invasive GBM cells, we performed metabolomic analysis of invasive and core GBM cells isolated from 3D hydrogel invasion devices and site-directed patient biopsies. The 3D hydrogel invasion devices are a modified version of our previously published invasion devices and contain hyaluronic acid (HA) hydrogels decorated with integrin-binding peptides (RGD) and crosslinked with protease-cleavable crosslinkers (13) (Supplemental Figure 1, A–D; supplemental material available online with this article; https://doi.org/10.1172/JCI170397DS1). The benefit of our devices over traditional 3D invasion platforms (spheroid invasion assays) is the ability to isolate large quantities of highly invasive and noninvasive cells from the same device. After long-term culture (28 days) of GBM43 cells isolated from a patient-derived xenograft (PDX) in the devices, the devices were disassembled, and the hydrogel and cells were microdissected to isolate invasive and noninvasive core cell fractions (*n* = 7; Supplemental Figure 1, E and F). Following separation, tumor cells in each of the fractions underwent metabolomic analysis (Supplemental Table 1). In parallel, site-directed biopsies from the invasive edge and central core of patient IDH WT GBMs distributed across 4 previously described molecular GBM subtypes (14) (*n* = 5; Supplemental Figure 1G and Supplemental Table 2) underwent metabolomic analysis (Supplemental Table 1). Principal-component analysis (PCA) confirmed distinct metabolic profiles for invasive and core tumor fractions in both 3D hydrogels and patient tumors (Figure 1A and Supplemental Figure 1H). Heatmaps were generated to explore the heterogeneity of relative metabolite levels within tumor groups and to determine whether differences seen between tumor fractions were driven by only a subset of devices or tumors (Supplemental Figure 2A). In 3D hydrogels, the 10 most upregulated metabolites in invasive GBM cells included cystathionine (9.6-fold change; *P* = 0.01), a metabolic precursor to cysteine in the transsulfuration pathway that has been implicated in redox homeostasis (15); 2-aminobutyric acid (5.0-fold change; *P* = 0.003), which is generated by an amino group transfer to 2-oxobutyric acid, a byproduct of cysteine biosynthesis from cystathionine, and modulates glutathione homeostasis (16); nicotinamide (1.9-fold change; *P* = 0.002), a precursor of nicotinamide-adenine dinucleotide (NAD^+^), which suppresses reactive oxygen species (ROS) production and enhances mitochondrial quality, thereby protecting against oxidative stress (17); and glucose-1-phosphate (2.25-fold change; *P* = 0.004), which is produced from glycogen and has been implicated in GBM invasion (18). In patient biopsies, the top upregulated metabolites in invasive samples included cystathionine (5.4-fold change; *P* = 0.02); spermidine (3.8-fold; *P* = 0.047), a polyamine that supports immunosuppressive myeloid cells and GBM invasion in vivo (19); cystine (2.6-fold change; *P* = 0.06), the oxidized form of cysteine which protects against ferroptosis (20); and glucose-1-phosphate (2.4-fold change; *P* = 0.06) (Figure 1B). Notably, only cystathionine and glucose-1-phosphate appeared among the 10 most enriched metabolites in the invasive fractions of hydrogels and patient tumors (Figure 1B), with only cystathionine achieving significance in hydrogels and patient tumors, as seen in volcano plots (Figure 1C).

MetaboAnalyst pathway analysis (https://www.metaboanalyst.ca/) revealed several transsulfuration-related changes shared between invasive GBM cells relative to core GBM cells in hydrogels and patient specimens, including increased metabolism of glutathione (*P* = 0.01 and impact = 0.1 hydrogels; *P* = 0.0003 and impact = 0.04 patient specimens) and several amino acids, including cysteine and methionine (*P* = 0.0002 and impact = 0.4 hydrogels; *P* = 0.005 and impact = 0.3 patient specimens) (Figure 1D). Thus, the metabolomic signature of invasive GBM cells differed from that of core GBM cells, and our analysis suggested that the 3D hydrogel models produced invasive and core GBM cells with metabolic profiles similar to those from site-directed patient GBM biopsies. These metabolic profiles implicated several metabolites related to the cellular response to oxidative stress, particularly metabolites from the transsulfuration pathway, in GBM invasion in hydrogels and patients.

### Lipidomics indicates increased oxidative stress, lipid peroxidation, and apoptotic signaling at the invasive tumor front.

**Figure 2:**
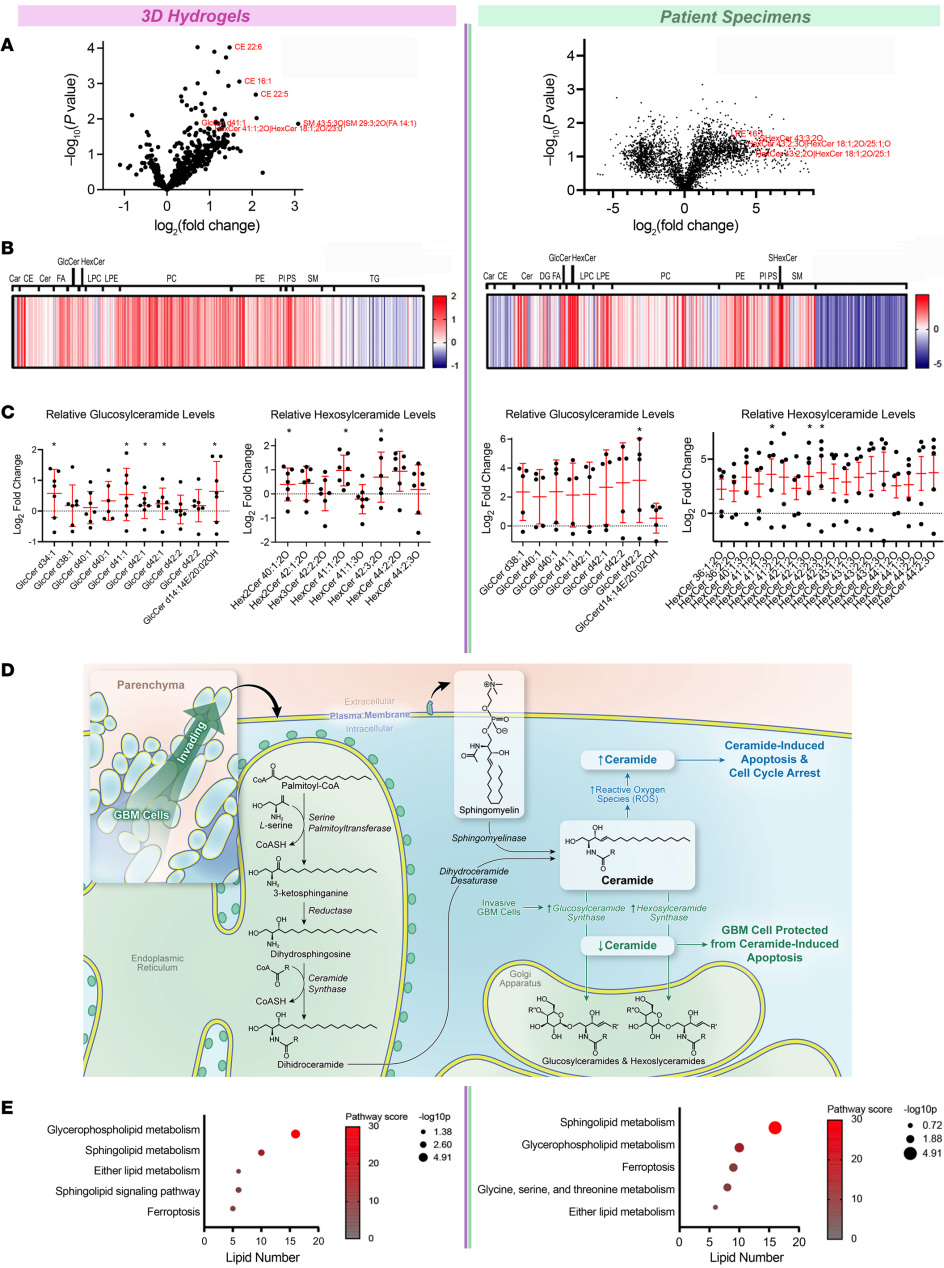
Lipidomic profiling indicates increased oxidative stress, lipid peroxidation, and apoptotic signaling at the invasive GBM front. Shown are results from unbiased lipidomic analysis of cells from the invasive front and tumor core of GBM43 cells in 3D hydrogels and site-directed biopsies of patient GBMs. (**A**) Volcano plots displaying relative fold change for individual lipid abundance at the invasive front of hydrogels (left) and patient specimens (right) versus tumor core. (**B**) Heatmaps displaying relative abundance of lipids in hydrogels (left) and patient specimens (right) organized by lipid classification. (**C**) Relative fold change of hexosylceramide and glucosylceramide species at the invasive tumor front in hydrogels (left) and patient tumors (right). Data are represented as mean ± SD. *P < 0.05, *t* test. (**D**) Illustration of pathways enabling hexosylceramide and glucosylceramide species to protect against apoptosis in invasive GBM cells exposed to oxidative stress. (**E**) KEGG pathway enrichment analysis of untargeted lipidomics displaying lipid pathways upregulated at the invasive tumor front of hydrogels (left) and patient tumors (right) using bubble plots.

To further define metabolic changes associated with GBM invasion, metabolomic analysis was supplemented with high-throughput lipidomic analysis of invasive and core GBM cells in 3D hydrogels and patient specimens (Supplemental Table 3). Volcano plots (Figure 2A) and heatmaps (Figure 2B) for hydrogels and patient specimens profiling 691 lipids revealed strong overlap in lipid perturbations between invasive and core GBM cells in hydrogels and patient specimens. Of note, patient specimens amplified the magnitude of change seen with hydrogels. Previous studies have shown that lipid production is higher in 3D than 2D cultures due to higher nutrient and oxygen gradients driving lipid biosynthesis in 3D culture (21). It is possible that, while 3D culture better replicates these gradients than 2D culture, the gradients in vivo still exceed those in 3D culture, leading to greater lipid perturbations in GBM cells invading in vivo compared with in hydrogels.

Changes observed in the invasive tumor front in hydrogels and patient specimens included elevated phosphatidylserines (e.g., phosphatidylserine 36:1 2.3-fold, *P* = 0.046 in devices; phosphatidylserine 35:1 18.6-fold, *P* = 0.04 in patients), glucosylceramides (e.g., glucosylceramide d42:1 1.2-fold change, *P* = 0.048 in devices; glucosylceramide d42:2 11.6-fold, *P* = 0.04 in patients), and hexosylceramides (HexCer) (e.g., hexosylceramide 41:1;O2 2.0-fold change, *P* = 0.02 and hexosylceramide 42:3;O2 1.7-fold change, *P* = 0.02 in devices; hexosylceramide 41:1;O3 17.0-fold, *P* = 0.04 and HexCer 42:1;O3 19.2-fold, *P* = 0.03 in patients) (Figure 2C and Supplemental Table 4). Interestingly, each of these 3 lipids has functions that could be relevant during invasion. Phosphatidylserines trigger phagocytic removal of invasive GBM cells (22). Increased hexosylceramides and glucosylceramides could reflect adaptation to oxidative stress in invasive GBM cells, as they represent ceramide modifications cancer cells utilize to prevent ceramide-induced apoptosis during oxidative stress (Figure 2D) (23).

To examine the physiologic role of these lipid differences between the invasive fraction and tumor core, KEGG metabolic pathway analysis (https://www.genome.jp/kegg/pathway.html) was performed on individual lipids upregulated in invasive GBM cells from both hydrogels and patient specimens (Figure 2E). Pathway analysis revealed upregulated cellular pathways involved in ceramide production from hydrolysis of membrane sphingomyelin before ceramide undergoes modifications during oxidative stress (Figure 2D); cancer cell synthesis of glycerophospholipids for their membranes; ether lipid synthesis for tumor cell membranes to increase membrane fluidity; and ferroptosis in hydrogels and patient specimens (Figure 2E). Together, these lipidomic changes corroborated the finding of changes associated with oxidative stress from our metabolomic analysis of invasive GBM cells in devices and patient specimens.

### Transcriptomic profiling of invasive GBM cells reveals upregulated genes producing and responding to oxidative stress.

**Figure 3:**
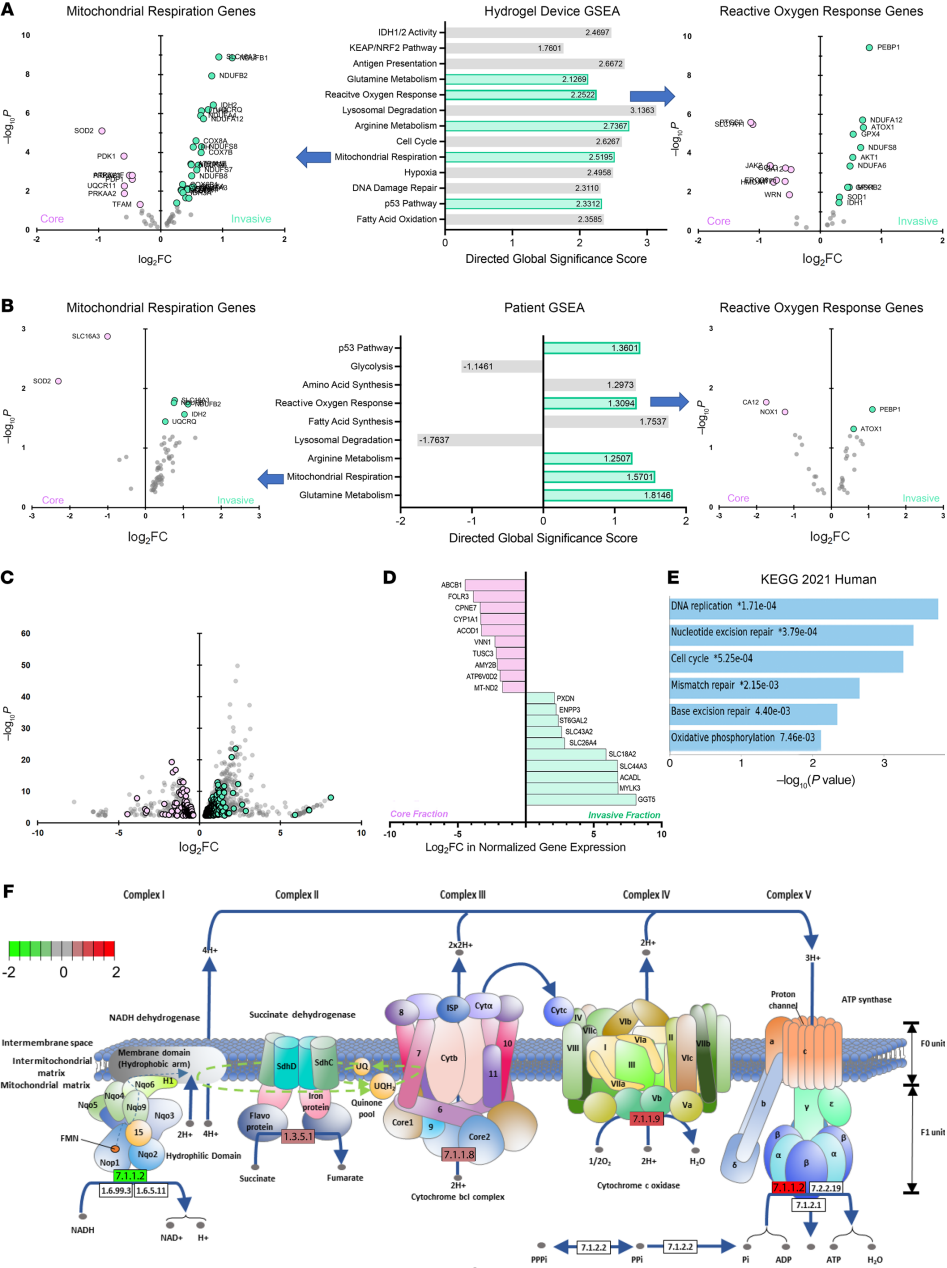
Gene-expression profiling demonstrates upregulated pathways involved in adapting to oxidative stress in invasive GBM cells. (**A** and **B**) RNA from invasive and core (**A**) GBM43 cells from hydrogel invasion devices or (**B**) site-directed biopsies of patient GBMs were assessed using the NanoString nCounter panel, which analyzes expression of 770 genes from 34 metabolic pathways, with GSEA revealing enriched metabolic pathways, including 5 shared between GBM43 cells in hydrogels and patient specimens (green). Volcano plots (P and FC = probability of significance and fold change invasive versus core) are shown for genes in 2 of these pathways — mitochondrial respiration (left) and ROS response genes (right) — highlighting genes in invasive (log_2_FC > 0) and core (log_2_FC < 0) samples. (**C**–**F**) Bulk RNA-Seq on invasive and core GBM43 cells isolated from hydrogels revealed the following: (**C**) Of 2,172 up- or downregulated (P_adjusted_ < 0.05) genes in invasive versus core GBM43 cells (gray dots on volcano plot), 344 (16%) were involved in cellular metabolism (green dots = upregulated genes, pink dots = downregulated genes). (**D**) Among 2,172 up- or downregulated (P_adjusted_ < 0.05) genes in invasive versus core GBM43 cells (gray dots on volcano plot), shown are the 10 most up- and downregulated metabolic genes (green dots = upregulated genes, pink dots = downregulated genes and listed accordingly in the graph to the right). (**E**) KEGG pathway analysis of genes enriched in invasive GBM cells implicated pathways involved in the production of and response to ROS. (**F**) Gene-expression changes overlaid on an oxidative phosphorylation schematic revealed upregulated genes encoding mitochondrial complexes II–V in invasive GBM43 cells versus those in the core.

To identify gene-expression changes associated with the altered hydrophilic metabolites and lipidomes identified by metabolomic and lipidomic analyses, we extracted RNA from invasive and core GBM43 cells from hydrogels. Samples were transcriptomically assessed using the NanoString nCounter panel consisting of a multiplex to analyze expression of 770 genes across 34 annotated metabolic pathways (Supplemental Table 5 and Supplemental Figure 2B). PCA revealed that cells in the invasive front clustered together, but apart from cells in the tumor core (Supplemental Figure 2C), indicating a consistent gene-expression pattern differentiating cells in the invasive fraction relative to cells in the core fraction. A heatmap (Supplemental Figure 2D) and volcano plot (Supplemental Figure 2E) revealed the most differentially expressed genes (DEGs) in the invasive fraction relative to the core, including thrombospondin (*THBS1*) log_2_FC = 1.99; P_adjusted_ = 6.2 × 10^-20^), which encodes a glycoprotein involved in GBM invasion (24); NAD(P)H dehydrogenase (quinone 1/*NQO1*) (log_2_FC = 1.69; P_adjusted_ = 3.1 × 10^-19^), which encodes a cytoplasmic 2-electron reductase protecting against oxidative stress (25); and acyl-CoA-acyltransferase 2 (*ACAT2*) (log_2_FC = 1.18; P_adjusted_ = 1.1 × 10^-9^), whose product esterifies cholesterol to provide cholesteryl ester for cytoplasmic lipid droplets that suppress ferroptosis (26). Gene set enrichment analysis (GSEA) revealed upregulated pathways related to the cellular response to oxidative stress, such as the ROS response (Supplemental Figure 2F and Figure 3A), as well as pathways generating ROS, such as mitochondrial respiration (Figure 3A), in invasive GBM43 cells relative to core cells.

To determine whether these findings were reflective of patient GBMs, the same multiplex platform was used to analyze metabolic gene expression in RNA from matched specimens taken from the invasive edge and tumor core of patient GBMs (*n* = 3) (Supplemental Table 6). This analysis yielded a volcano plot delineating upregulated genes with functions similar to those of the upregulated genes from invasive GBM cells in hydrogels (Supplemental Figure 2G), including acetyl-CoA carboxylase 2 (*ACACB*) (log_2_FC = 0.8, *P* = 0.04), whose fatty acid oxidation and ferroptosis roles are phosphorylation dependent (27), and mitochondrial electron transport chain genes *NDUFA4* (log_2_FC = 0.7, *P* = 0.02), *NDUFB1* (log_2_FC = 0.7, *P* = 0.006), and *NDUFB8*, (log_2_FC = 1.0, *P* = 0.02).

GSEA of patient GBM specimens from the invasive front revealed 5 upregulated metabolic pathways also upregulated in invasive GBM43 cells from 3D hydrogels, including genes in ROS response, mitochondrial respiration, and glutamine metabolism (Figure 3B and Supplemental Figure 2H). GSEA also revealed that invasive cells in patient GBMs upregulated genes in the amino acid synthesis pathway (Figure 3B), which assesses production of 15 amino acids, including sulfur-containing amino acids (cystathionine and methionine) produced via the transsulfuration pathway whose components were identified in our metabolomic analysis. Thus, metabolic transcript analysis revealed increased production of and adaptation to oxidative stress in invasive GBM cells in hydrogels and patient GBMs.

To determine how these metabolic gene-expression changes fit in with broader transcriptomic changes in invasive GBM cells, we performed bulk RNA-Seq on invasive and core GBM43 cells isolated from our hydrogel invasion devices (Supplemental Table 7). This analysis revealed 2,172 genes up- or downregulated (*P* < 0.05) in invasive versus core GBM43 cells, of which 344 (16%) were involved in metabolism based on the human metabolic atlas (28) (Figure 3C), underscoring the important role of metabolism in the broader transcriptomic changes occurring during GBM invasion. Two of the three most upregulated metabolic genes based on fold change in invasive GBM43 cells had roles concordant with our metabolomic and lipidomic findings: LCAD-acyl-CoA dehydrogenase (*ACADL*) (log_2_FC = 6.8, *P* = 0.002) and γ-glutamyltransferase 5 (*GGT5*) (log_2_FC = 8.1, *P* = 6.7 × 10^-7^) (Figure 3D). *ACADL* mediates fatty acid oxidation, which protects against ferroptosis (29), a finding consistent with our lipidomic findings (Figure 2E), and *GGT5* hydrolyzes glutathione before it is recycled to cysteine, a potential response to the increased flux through the transsulfuration pathway suggested by the increased cystathionine identified in our metabolomic analysis of invasive GBM cells (Figure 1C).

We then analyzed the upregulated pathways from bulk RNA-Seq of invasive GBM43 cells in hydrogel devices and found pathways that could generate ROS, such as oxidative phosphorylation, or that could help cells cope with oxidative stress, such as mismatch repair, nucleotide excision repair, and base excision repair (Figure 3E). A more detailed interrogation of oxidative phosphorylation genes revealed upregulated genes in mitochondrial complexes II–V in invasive GBM43 cells in hydrogel devices (Figure 3F), which was of interest in light of our data suggesting increased production of and adaptation to oxidative stress in invasive GBM cells because of studies supporting complex III being a major source of the ROS superoxide and hydrogen peroxide (H_2_O_2_) in mitochondria (30).

### Invasive GBM cells exhibit increased ROS.

**Figure 4:**
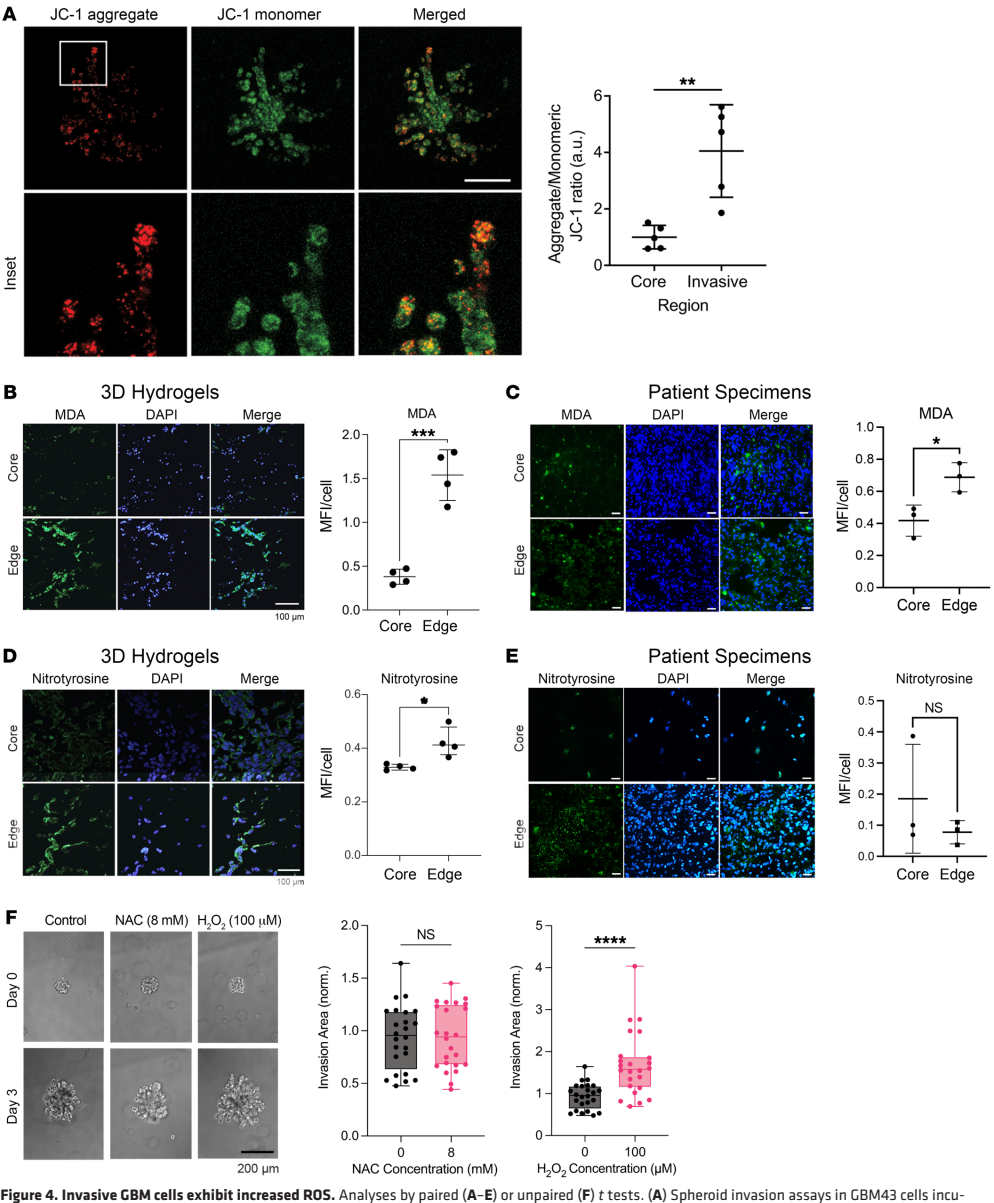
Invasive GBM cells exhibit increased ROS. Analyses by paired (**A**–**E**) or unpaired (**F**) *t* tests. (**A**) Spheroid invasion assays in GBM43 cells incubated with JC-1 dye revealed increased mitochondrial membrane potential in invasive GBM43 cells (*P* < 0.01; *n* = 5 pairs). (**B** and **C**) MDA staining of (**B**) hydrogels and (**C**) patient specimens revealed increasing MDA in the edge versus the core of hydrogels (*P* < 0.001; *n* = 4 pairs) and patient specimens (*P* = 0.025; *n* = 3 pairs). (**D** and **E**) Nitrotyrosine staining of (**D**) hydrogels and (**E**) patient specimens revealed increased staining in the edge versus the core in the hydrogels (*P* < 0.05; *n* = 4 pairs), but not in the patient specimens (*P* = 0.5; *n* = 3 pairs). (**F**) While H_2_O_2_ increased invasion of GBM43 cells in HA hydrogels (*P* < 0.0001), ROS scavenger NAC did not affect invasion (*P* = NS) of GBM spheroids in HA hydrogel invasion assays (*n* = 24 spheres, collected across 3 independent experiments). *P < 0.05; **P < 0.01; ***P < 0.001; ****P < 0.0001. Original magnification, ×20 (**B**, **C**, **D**, **E**); ×10 (**F**). Scale bars: 200 μm (**A**); 100 μm (**B**, **D**); 50 μm (**C**, **E**); 200 μm (**F**).

Because multiomic analysis of invasive GBM cells in hydrogels and patient biopsies reflected heightened ability to produce and adapt to oxidative stress, we then corroborated these findings by interrogating functional markers of that stress. We first functionally corroborated our findings of increased transcription of mitochondrial complexes II–V by using JC-1 dye to measure mitochondrial membrane potential, which is generated by proton pumps in complexes I, III, and IV. Spheroid invasion assays of GBM43 cells incubated with JC-1 revealed increased mitochondrial membrane potential in invasive GBM43 cells relative to core cells (*P* < 0.01; Figure 4A). Because mitochondrial oxidative phosphorylation can generate ROS, we then asked whether invasive GBM cells exhibit higher levels of ROS than core GBM cells. While direct ROS assessment in tissues is challenging, ROS presence can be inferred from biomarkers of oxidative damage arising from the effects of ROS on protein, carbohydrates, nucleic acids, and lipids (31). We therefore assessed ROS markers in invasive GBM cells in hydrogels and in patient GBM biopsies: fatty acid peroxidation product malondialdehyde (MDA) (32) and tyrosine oxidation product nitrotyrosine (33). Immunostaining revealed increased MDA in the invasive edge compared with the tumor core of 3D hydrogels (*P* < 0.001; Figure 4B) and patient GBMs (*P* = 0.025; Figure 4C). Immunostaining also revealed elevated nitrotyrosine in invasive GBM cells compared with those in the core of hydrogels (*P* < 0.05; Figure 4D), although there was unchanged nitrotyrosine between the tumor core and invasive edge of patient specimens (*P* = 0.5; Figure 4E).

To determine whether these elevated ROS could promote invasion rather than merely being a byproduct of the invasive process, we assessed the impact of ROS manipulation on GBM43 spheroid invasion in hydrogels (Supplemental Figure 3A), focusing on the 3 most common ROS in cancer: superoxide (O_2_^–^), H_2_O_2_, and hydroxyl free radicals (OH·).^3^ Short-term (30 minutes every 2 days) H_2_O_2_ exposure increased GBM43 spheroid invasion in 3D hydrogels (*P* < 0.001; Figure 4F) at multiple nontoxic concentrations up to 100 μM (Supplemental Figure 3B), which is the H_2_O_2_ concentration in malignant cells (34). Similarly, MnTBAP, a metalloporphyrin superoxide dismutase (SOD) mimetic that converts O_2_^–^ (*P* < 0.001; Supplemental Figure 3C) to H_2_O_2_, also increased GBM43 spheroid invasion in HA hydrogels (*P* < 0.001; Supplemental Figure 3, D and E). In contrast, N-acetylcysteine (NAC) did not affect invasion (*P* > 0.05; Figure 4F) at concentrations just below the maximum tolerable dose (Supplemental Figure 3B), but also did not affect superoxide levels in GBM43 cells (Supplemental Figure 3F). This suggests that, among the most common oncologic ROS, H_2_O_2_ promoted GBM invasion in hydrogels.

### CRISPR metabolic gene screen links transsulfuration pathway to invasion.

**Figure 5:**
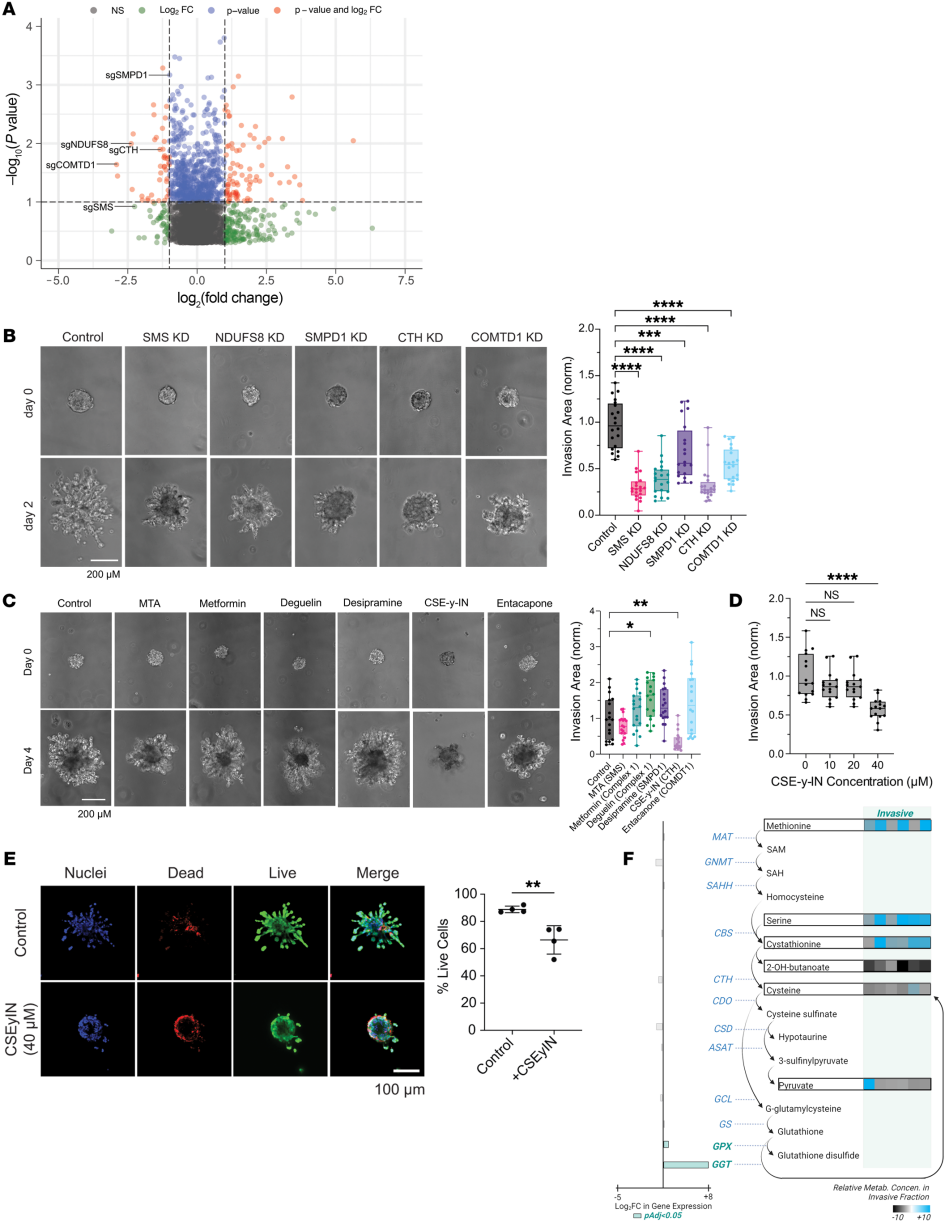
Metabolic CRISPR screen to identify metabolic genes essential to GBM invasion reveals that ROS response genes including CTH are necessary for GBM cell invasion. Analyses used ANOVA with post hoc Tukey’s (**B**–**D**) or *t* test (**E**). (**A**) Volcano plot displaying enrichment of sgRNAs for metabolic genes in the core (log_2_fold change < 0) and invasive front (log_2_fold change > 0) of GBM 3D invasion devices, with labeling of the 5 genes (*COMTD1*, *SMS*, *CTH*, *SMPD1*, and *NDUFS8*) selected for further evaluation. (**B**) Quantification and representative images of spheroid invasion assays of 5 knockdown GBM43 cell lines selected from CRISPR screen hits compared with control cells expressing dCas9 (*n* = 20 spheres from 3 independent experiments). Original magnification, ×10. Scale bar: 200 μm. (**C**) Spheroid invasion assays of GBM43 cells treated with inhibitors of the 5 metabolic enzymes encoded by genes chosen from the CRISPR screen (*n* = 18 spheres from 3 independent experiments). Original magnification, ×10. Scale bar: 200 μm. (**D**) CTH inhibitor CSE-γ-IN slowed GBM43 tumorsphere invasion at 40 μM (*P* < 0.0001; *n* = 15 spheres across 3 independent experiments). (**E**) GBM43 cells treated with 40 μM CSE-γ-IN in neurosphere invasion assays exhibited cell death specifically at the spheroid edge (*P* < 0.01; *n* = 4 spheres/group). Original magnification, ×10. Scale bar: 100 μm. (**F**) Integrated depiction of multiomic findings from invasive GBM43 cells related to the transsulfuration pathway. Metabolites: fold changes in metabolites in each invasive versus paired core fraction are indicated in the heatmap to the right (blue, upregulated; gray, downregulated), with unboxed metabolites undetected. Enzymes: log_2_FC for DEGs (green or pink bars for genes with P_adjusted_ < 0.05; gray bars for genes with P_adjusted_ > 0.05) are indicated with green or pink representing up- or downregulation in invasive cells relative to core cells, respectively. *P < 0.05; **P < 0.01; ***P < 0.001; ****P < 0.0001.

To determine which upregulated metabolic pathways identified by our multiomics analysis have functional importance in GBM cell invasion, we performed a CRISPR/Cas9 knockout screen with 29,790 sgRNAs targeting 2,981 metabolic genes (35) to identify metabolic genes crucial to GBM invasion. GBM43 cells expressing Cas9 and the sgRNA library were seeded in 3D hydrogels (*n* = 6) and cultured for 28 days. Afterwards, devices were disassembled and microdissected to isolate invasive and core cells for DNA sequencing (Supplemental Figure 4A). sgRNAs enriched in the core relative to the invasive fraction (indicating genes whose knockout disrupted invasion) and in the invasive fraction compared with the core (indicating genes whose knockout enabled invasion) were scored based on their abundance compared with nontargeting sgRNAs in the library (Supplemental Tables 8–10 and Supplemental Figure 4B). We chose 5 genes based on enrichment of sgRNAs targeting them in the core (Figure 5A and Supplemental Table 10) and their overlap with our multiomic data sets: (a) NADH:ubiquinone oxidoreductase core subunit S8 (*NDUFS8*) (log_2_FC = –2.4, *P* = 0.01), a subunit of electron transport chain complex I whose other subunits were transcriptomically upregulated in patient GBMs (Supplemental Figure 2G); (b) sphingomyelin phosphodiesterase 1 (*SMPD1*) (log_2_FC = –1.0, *P* = 0.0007), which converts sphingomyelin, whose metabolism was an upregulated lipidomic pathway in devices and patient GBMs (Figure 2E), to ceramide; (c) cystathionine γ-lyase (CTH/*CSE*) (log_2_FC = –1.2, *P* = 0.01), which converts cystathionine, the only metabolite enhanced in invasive GBM cells in devices and patient specimens (Figure 1, B and C), into cysteine in the last step of the transsulfuration pathway; (d) catechol-*O*-methyltransferase domain containing 1 (*COMTD1*) (log_2_FC = –2.9, *P* = 0.02), which clears oxidized dopamine, an inhibitor of cancer cell invasion (36); and (5) spermine synthase (*SMS*) (log_2_FC = –1.8, *P* = 0.09), which converts spermidine, an enhanced metabolite in the invasive front of patient GBMs (Figure 1B), into spermine, an antioxidant. We performed single-gene knockdowns of these 5 genes using CRISPRi (Supplemental Table 17) to test the effect of gene silencing on tumor spheroid invasion (Supplemental Figure 4C). Compared with control GBM43 cells expressing dCas9, all 5 knockdown cell lines exhibited decreased spheroid invasion in HA-RGD hydrogels (*P* < 0.001; Figure 5B and Supplemental Table 16).

We then assessed the effect of pharmacologically inhibiting the proteins encoded by these 5 genes on GBM43 spheroid invasion assays (Supplemental Table 18). Only one inhibitor, cystathionine-γ-lyase-IN-1 (CSE-γ-IN), a small molecule inhibitor of CTH, slowed invasion (*P* < 0.01; Figure 5C). While this finding could have reflected different abilities of the drugs to inhibit their targets, the efficacy of CSE-γ-IN at slowing invasion combined with our multiomic data implicating the transsulfuration pathway in GBM invasion led us to choose CTH for further mechanistic studies in GBM invasion.

We first confirmed that the antiinvasive effects of targeting CTH did not reflect effects on cell proliferation or viability. GBM43/CTHkd cells expanded over 5 days in culture at varying seeding densities to the same degree as control GBM43 cells (*P* = 0.1–0.9; Supplemental Figure 4D). Similarly, to confirm that the antiinvasive effects of CSE-γ-IN did not reflect effects on cell survival, we assessed the concentration window for which CSE- γ-IN inhibited GBM spheroid invasion without cytotoxicity and found that the invasion inhibitory effect of CSE-γ-IN on spheroids derived from GBM43 cells, which are of the proneural GBM subtype (37), began at 40 μM (*P* < 0.0001; Figure 5D) with 40 μM CSE-γ-IN also inhibiting spheroid invasion in classical subtype U251 cells (38) (*P* < 0.001; Supplemental Figure 4E). Concentrations of CSE-γ-IN above 100 μM began to affect the viability of GBM43 or U251 cells (Supplemental Figure 4F). Interestingly, while 40 μM CSE-γ-IN was nontoxic to GBM43 cells in 2D culture, GBM43 cells treated with 40 μM CSE-γ-IN in 3D spheroid invasion assays exhibited cell death specifically at the spheroid edge (*P* = 0.006; Figure 5E), confirming that CTH and the transsulfuration pathway are particularly important for 3D invasion such that when CTH is inhibited by 40 μM CSE-γ-IN, cells are unable to survive the high oxidative and metabolic stresses associated with invasion.

We then investigated whether CTH targeting by CSE-γ-IN could slow GBM invasion in cell-culture models distinct from the HA-based 3D model that our CRISPR screen was performed in. We cocultured GPMP017 GBM cells derived from PDXs with human-induced pluripotent stem cell–derived (hiPSC-derived) cerebral organoids in CSE-γ-IN or DMSO vehicle and found that 40 μM CSE-γ-IN slowed the invasion of GPMP017 GBM cells into organoids after 7 days (*P* < 0.001; Supplemental Figure 4G).

The ability of CTH to play a functional role in invading GBM cells was further supported by our finding from Ivy GAP analysis that pyridoxal kinase (PDXK), the enzyme that converts pyridoxine and other vitamin B6 precursors into pyridoxal-5′-phosphate (PLP), the bioactive form of CTH cofactor vitamin B6 (39), was enriched at the leading edge of the tumor relative to the core (*P* < 0.001; Supplemental Figure 4H).

We then integrated our multiomic data to interrogate the roles of the transsulfuration pathway and glutathione turnover in GBM invasion (Figure 5F). This analysis revealed cystathionine accumulation in invasive GBM cells without changes in gene expression of transsulfuration enzymes, suggesting that CTH was a rate-limiting step during transsulfuration in invasive GBM cells. The concomitant upregulation of glutathione turnover enzymes glutathione peroxidase 8 (*GPX8*) (log_2_FC = 0.8, *P* = 0.0002) and *GGT5* (log_2_FC = 8.1, *P* = 6.7 × 10^-7^) in invasive GBM cells was suggestive of increased flux through the transsulfuration pathway. The CRISPR screen also highlighted that invasive GBM cells were dependent on this increased transsulfuration such that the rate-limiting CTH step represented a therapeutic vulnerability as the only step whose targeting slowed invasion.

### Transsulfuration pathway inhibition slows GBM invasion.

**Figure 6:**
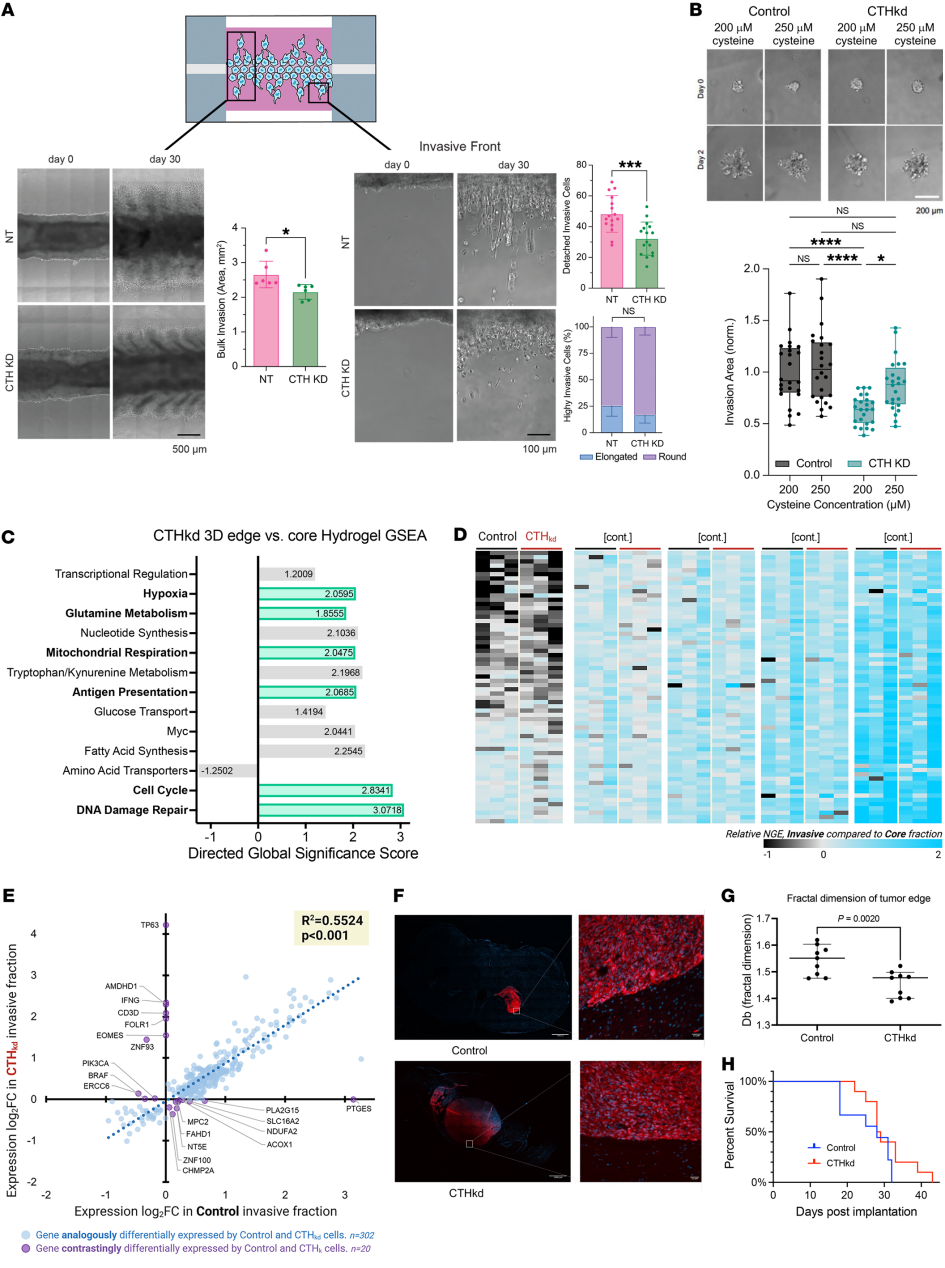
Targeting CTH inhibits GBM invasion. Analyses used *t* test (**A** and **G**), ANOVA with post hoc Tukey’s test (**B**), Pearson’s correlation (**E**), or Kaplan-Meier test (**H**). (**A**) GBM43 cells with knockdown were less invasive in 3D hydrogels based on bulk invasive area (left; *P* < 0.05; *n* = 6 regions of interest across 3 devices) and number of detached invasive cells (right; *P* < 0.001), with invasive cell morphology unaffected by CTHkd (right; *P* > 0.05; *n* = 16 regions of interest across 3 devices). (**B**) Spheroid invasion assays revealed that increasing cysteine from 200 to 250 μM reversed the slowed invasion caused by CTHkd (*n* = 24 spheres across 3 independent experiments). (**C**–**E**) GBM43 cells with CTH knockdown of CTH were seeded into invasion devices, after which cells from core and invasive fractions were assessed using the NanoString 770 metabolic gene platform. (**C**) GSEA: 6/13 upregulated pathways were shared with control cells invading hydrogels (green). (**D**) Heatmap depicting normalized gene expression (NGE) of cells in the invasive versus core hydrogel fractions for CTHkd (red bars) and control GBM43 cells (black bars) (*n* = 3/group), with uniform gene-expression changes across control GBM43 versus CTHkd cells suggesting similar transcriptional profiles among invasive GBM cells regardless of CTH expression. (**E**) Scatter plot depicting gene expression fold change for individual genes in invasive versus core fractions for GBM43 control (*x* axis) and GBM43 CTHkd (*y* axis). The high correlation between fold change in invasive GBM43 control versus CTHkd cells (*P* < 0.001) means that metabolic transcriptional patterns change during invasion similarly regardless of CTH expression. Purple dots indicate genes with discordant expression changes in control GBM43 versus CTHkd cells, which are scant (20/322 total genes = 6.2%). (**F**–**H**) Intracranial GBM43 PDXs expressing mCherry along with dCas9 or dCas9 with sgRNA targeting CTH (**F** and **G**) were less invasive with CTHkd (median ± 95% CI shown; *P* = 0.002; *n* = 9/group) based on fractal analysis of images of tumors and their surrounding brain, which yields fractal dimension, a measure of invasive tumor growth as a continuous number between 1 and 2, with higher numbers representing greater invasiveness and (**H**) exhibited unchanged survival with CTHkd (*P* = 0.1; *n* = 9–10/group). Original magnification, ×10 (left); ×20 (right). Scale bars: 25 mm (left); 1,000 mm (right). *P < 0.05; **P < 0.01; ***P < 0.001; ****P < 0.0001.

Because CTH was the only metabolic gene emerging from our CRISPR screen whose pharmacologic targeting inhibited invasion in spheroid invasion assays (Figure 5C) and because cystathionine, a precursor to cysteine in glutathione synthesis in the transsulfuration pathway, was enriched in the invasive fraction of both patient-derived tumor biopsies and 3D hydrogels (Figure 1C), we focused further investigation on the specific role of CTH in GBM invasion. We first expanded upon the effects of CTH knockdown (CTHkd) on invasion (see complete unedited blots in the supplemental material) by demonstrating that CTHkd slowed long-term GBM43 invasion in 3D hydrogel devices (28-day culture period), resulting in decreased bulk invasion area (*P* < 0.05) and fewer detached invasive cells (*P* < 0.001) with unchanged invasive cell morphology (Figure 6A and Supplemental Figure 5A).

To investigate whether CTH enabled GBM cells to cope with the oxidative stress we identified in GBM cells invading in hydrogels and patient specimens (Figure 4, B–E), we assessed ROS levels using the CellROX reagent, which measures hydroxyl radical and superoxide anion (40), in cultured GBM43 cells with or without CTHkd at varying oxygen levels. CTHkd led to higher ROS levels in normoxia (18.6% oxygen) and, at 2% oxygen, a level of hypoxia comparable to that in patient GBMs (41) in cultured GBM43 cells (*P* = 0.01–0.02; Supplemental Figure 5B). CTHkd did not alter superoxide levels in cultured GBM43 cells in normal (200 μM) and low (100 μM) cysteine concentrations, as measured by the MitoSOX probe (*P* = 0.2–0.8; Supplemental Figure 5C), suggesting that hydroxyl radical accumulates in cells deprived of cysteine due to CTHkd. These results are consistent with cell-free chemistry studies implicating cysteine disulfides in the antioxidant response to hydroxyl radical attack (42).

We next asked whether CTHkd altered the ability of GBM cells to cope with long-term (2 days) exposure to H_2_O_2_, since short-term (30 minutes) exposure to H_2_O_2_ increased invasion of control GBM43 cells in a spheroid invasion assay. While control and CTHkd cells exhibited similar sensitivity to high (>50 μM) and low (<1 μM) H_2_O_2_ (Supplemental Figure 5D), at moderate H_2_O_2_ (10 μM), CTHkd cells were less viable than controls (*P* < 0.001; Supplemental Figure 5D). These results suggest that inhibiting the transsulfuration pathway through CTH targeting may sensitize GBM cells to otherwise manageable H_2_O_2_ levels, the ROS formed from superoxide that gives rise to hydroxyl radical. In summary, CTHkd not only led to ROS accumulation, but also increased ROS sensitivity.

We then determined whether CTHkd caused morphologic changes in GBM cells that could affect invasion. While CTHkd did not alter the morphology of GBM43 cells in 2D culture (Supplemental Figure 5E) and CSE-γ-IN did not alter the morphology of GBM43 cells in the core of 3D neurosphere invasion assays (Supplemental Figure 5F), CSE-γ-IN raised the form factor of invasive GBM43 cells, conferring a less mesenchymal morphology that is less conducive to invasion (*P* < 0.001; Supplemental Figure 5F). These results are consistent with a study in which oxidative stress, which we found to occur with CTH targeting, destabilizes the actin cytoskeleton of lung cancer cells in a manner that reduces invasiveness (43). Together, these findings suggest that, while H_2_O_2_ drives GBM invasion (Figure 4E), CTH is needed in invasive GBM cells to quench the hydroxyl radical generated from H_2_O_2_. Thus, while H_2_O_2_ drives GBM invasion, CTH is needed to prevent hydroxyl generated from this H_2_O_2_ from reaching toxic levels. Of note, hydroxyl is produced from H_2_O_2_ when enzymes responsible for converting H_2_O_2_ to water cannot keep up with H_2_O_2_ levels, suggesting that the upregulation of these enzymes seen in invasive GBM43 cells (Supplemental Table 7), such as peroxiredoxin 3 (*PRDX3*) (log_2_FC = 0.6, *P* = 0.001) and 4 (*PRDX4*) (log_2_FC = 0.5, *P* = 0.003) and *GPX8* (log_2_FC = 0.8, *P* = 0.0002), is insufficient to address the H_2_O_2_ in invading GBM cells.

### The transsulfuration pathway is necessary for GBM invasion because of its role in de novo cysteine synthesis.

Because the transsulfuration pathway is the primary route for biosynthesis of the antioxidants cysteine and glutathione and because CTH and its downstream partner CBS also produce the protumoral gaseous transmitter hydrogen sulfide (H_2_S), another ROS scavenger, as a byproduct of their enzymatic activity, we next investigated whether GBM cells lacking CTH are less invasive due to a limited supply of these 3 factors produced downstream of CTH.

We performed a spheroid invasion assay in the presence of 50 μM additional cysteine to see if exogenous cysteine reverses the decreased invasion of CTHkd cells. In standard culture media containing 200 μM cysteine, the cysteine concentration in normal cells (44), GBM43 spheroids with CTHkd were less invasive than control spheroids; however, increasing cysteine concentration to 250 μM “rescued” the decreased invasive capacity of GBM43 spheroids with CTHkd without altering the invasive capacity of control GBM43 spheroids (*P* = 0.01; Figure 6B).

We then examined the effects of limiting cysteine concentration on GBM invasion. In a spheroid invasion assay, control GBM43 cells in low cysteine (100 μM) were slightly less invasive than control GBM43 cells in normal cysteine (200 μM) (*P* < 0.01; Supplemental Figure 5G). CTHkd cells were not sensitive to microenvironmental cysteine deprivation and remained less invasive than control cells at both cysteine concentrations (Supplemental Figure 5G). The ability of control GBM43 cells in low microenvironmental cysteine to remain more invasive than CTHkd GBM43 cells suggests that an active transsulfuration pathway can overcome microenvironmental cysteine deficiency. We then asked whether the reduced invasiveness of control GBM cells in media containing low cysteine correlated with altered ROS levels in these cells. Growth in 100 μM cysteine increased ROS in CTHkd and control cells while preserving the elevated ROS in CTHkd (*P* < 0.001; Supplemental Figure 5H), confirming that low cysteine increased ROS and slowed invasion in control cells in a manner that approached but was not as severe as CTHkd.

Because of our findings that invasive GBM cells exhibit metabolomic, lipidomic, and transcriptomic changes protecting against ferroptosis (Figure 1D, Figure 2E, and Figure 3D), we then investigated whether cysteine supplementation or CTH targeting affected the sensitivity of GBM cells in 2D culture to the ferroptosis inducer erastin. We found that cysteine supplementation protects GBM43 cells from erastin-induced cell death (*P* < 0.001) and that inhibiting the transsulfuration pathway with CSE-γ-IN (*P* < 0.05) makes GBM43 cells more sensitive to erastin (*P* < 0.05; Supplemental Figure 6A).

To determine whether cysteine promotes invasion by serving as a precursor to glutathione, we performed invasion assays with control and CTHkd cells with glutathione supplementation. Surprisingly, glutathione supplementation did not rescue the invasive ability of CTHkd cells (*P* > 0.05; Supplemental Figure 6, B and C). In fact, GBM43 CTHkd cells had more glutathione than control GBM43 cells (*P* = 0.01–0.03; Supplemental Figure 6D), suggesting that GBM cells do not fully rely on de novo cysteine production to synthesize glutathione (45) and that cysteine drives invasion through glutathione-independent pathways.

To test the possibility that CTH promotes invasion through H_2_S synthesis, we performed a spheroid invasion assay of GBM43 cells with or without CTHkd with H_2_S supplementation using the potent and fast-acting chemical donor sodium hydrosulfide (NaHS). However, rather than rescuing invasion in CTHkd cells, H_2_S supplementation decreased invasion in control and CTHkd cells (*P* < 0.001; Supplemental Figure 6E). To further investigate the invasion-suppressing effects of H_2_S, we performed a NaHS dose-response viability curve on control and CTHkd cells. We observed that GBM cell viability was suppressed in NaHS concentrations above 1 μM (Supplemental Figure 6F), corroborating a study that explored the tumor-suppressive functions of H_2_S (12). Together, these findings suggest that CTHkd slowed GBM invasion because of its role in cysteine production rather than its roles in glutathione or H_2_S production.

### Upregulation of other transsulfuration enzymes in GBM cells invading despite CTHkd reveals the importance of cysteine for GBM invasion.

While CTHkd considerably slowed GBM43 invasion through 3D hydrogels in spheroid invasion assays and long-term invasion devices (Figure 5B and Figure 6A), a population of GBM43 CTHkd cells remained moderately invasive in our assays. We therefore investigated whether invasive CTHkd GBM43 cells relied on metabolic genes and pathways similar to those of invasive control GBM43 cells or whether they utilized compensatory pathways to invade. First, we performed Ki-67 staining of the core and invasive fractions of GBM43 control and CTHkd cells cultured in the invasion devices. There were no differences in the percentages of Ki-67^+^ cells between control and CTHkd cells in the core or invasive fractions of the devices (Supplemental Figure 7A), confirming that invasive GBM cells with or without CTHkd did not exhibit proliferative differences. Then, cells isolated from core and invasive fractions from devices containing control and CTHkd GBM cells were transcriptomically assessed using the NanoString nCounter platform and the 770 metabolic gene multiplex described previously (Supplemental Tables 11 and 12 and Supplemental Figure 7B). PCA revealed that cells in the invasive front clustered together, but apart from cells in the core (Supplemental Figure 7C). A volcano plot (Supplemental Figure 7D) and heatmap (Supplemental Figure 7E) revealed enriched metabolic genes (Supplemental Figure 7F) in the invasive fractions relative to the core fractions of the hydrogels, with GSEA revealing that genes in hypoxia response and mitochondrial respiration were enriched in invasive CTHkd and invasive control cells, while genes involved in fatty acid synthesis, glucose transport, amino acid transporters, tryptophan metabolism, and glutamine metabolism were enriched in invasive CTHkd cells, but not in invasive control cells (Figure 6C).

We then compared these upregulated metabolic genes in invasive CTHkd GBM43 cells to those upregulated in invasive control GBM43 cells. A heatmap revealing DEGs across invasive samples from CTHkd versus control GBM43 cells revealed an unchanged general pattern of metabolic gene expression between CTHkd and control GBM43 cells (Figure 6D). Similarly, a scatter plot comparing the fold changes in gene expression for individual genes in invasive compared with core fractions of both cell lines (CTHkd and control) revealed a high correlation between fold change values in CTHkd and control samples (*P* < 0.001), with only 6.2% (20/322) of the genes having discordant expression changes in CTHkd versus control cells in the invasive fractions relative to core fractions (Figure 6E). This finding was corroborated in a volcano plot demonstrating negligible differences in gene expression within the 770 metabolic gene multiplex in the invasive fractions of CTHkd versus control GBM43 cells (Supplemental Figure 7G). Together, these findings revealed that transcriptional patterns related to metabolism change similarly regardless of CTH expression.

Having demonstrated no differences in expression of the 770 metabolic genes in the multiplex between invasive CTHkd versus control GBM43 cells, we then expanded this transcriptomic comparison using bulk RNA-Seq and found that invasive CTHkd cells upregulated another enzyme in the transsulfuration pathway, cystathionine β-synthase (CBS) (Supplemental Figure 7H and Supplemental Tables 13–15). Because CBS catalyzes the first step of transsulfuration by condensing serine with homocysteine to generate cystathionine, which is then converted by CTH into cysteine, our finding of CBS upregulation in invasive CTHkd cells underscored the essential nature of cysteine for GBM invasion.

### CTHkd slows GBM invasiveness in vivo.

Finally, we analyzed the invasiveness of intracranially implanted GBM43 cells with or without CTHkd (Supplemental Figure 8A). Invasiveness in vivo was assessed by fractal analysis of images of tumors and their surrounding brain, yielding the fractal dimension, a numeric description of invasive tumor growth pattern as a number between 1 and 2, with higher numbers representing greater invasiveness. This method revealed that CTHkd reduced PDX invasiveness (*P* = 0.03; Figure 6, F and G), with examples of tumor metastasizing to the brain stem in mice with GBM43 cells lacking CTHkd (Supplemental Figure 8B). Despite this reduced invasiveness in vivo, CTHkd did not alter survival (*P* = 0.2; Figure 6H). The unchanged survival could reflect our finding that CTHkd created larger tumors (*P* = 0.02; Supplemental Figure 8C), suggesting that a compensatory shift from an invasive to proliferative phenotype with CTHkd prevented CTHkd from affecting survival.

## Discussion

While the hallmark of GBM and a defining contributor to its poor prognosis is invasion into the surrounding white matter, studies to date have emphasized mechanisms driving this invasion more than the metabolic requirements needed to sustain it. To close this knowledge gap, we developed a bioengineered 3D hydrogel invasion platform for high-throughput screening of invasion mediators. The spatially dissectable nature of our hydrogel-based invasion devices allowed us to perform a multiomic analysis of tumor cells in the invasive front versus noninvasive core from individual invasion assays. We then benchmarked these findings from our 3D hydrogel models against site-directed biopsies from the core versus invasive edge of patient GBMs. Finally, we performed a CRISPR screen using GBM cells invading 3D hydrogels in a long-term assay, narrowed down our list of key hits using insights from our multiomics analysis, and used shorter term 3D spheroid invasion assays to validate genes emerging from our screen. This study represents one of the closest integrations to date of 3D biomaterial models and patient data and illustrates the value of reductionist paradigms for identifying biomarkers and mechanisms of cancer invasion.

By emphasizing the previously understudied role of metabolic reprogramming in GBM invasion, we produced several insights linking oxidative stress to GBM invasion. First, we found that the invasive front of GBM produces elevated ROS and that invasive tumor cells exhibit metabolomic, lipidomic, and transcriptomic profiles reflecting their exposure to this oxidative stress. Second, we found that the ROS H_2_O_2_ promotes GBM cell invasion, building upon prior reports linking ROS to invasiveness in other cancers (46). Third, our unbiased metabolic CRISPR knockout screen identified 5 candidate genes whose necessity for GBM invasion was validated in clonal KD cell lines, with each of these genes having demonstrated roles in the cellular response to oxidative stress.

Several of our findings implicated the transsulfuration pathway, which synthesizes the nonessential amino acid cysteine via the intermediate cystathionine, as being critical for GBM invasion. First, cystathionine, the central metabolite in the transsulfuration pathway, was among the 2 most enriched metabolites in the invasive edge of 3D hydrogels and GBM patient specimens, with 2-aminobutyric acid, which is generated by an amino group transfer to 2-oxobutyric acid, a byproduct of cysteine biosynthesis from cystathionine (16), being the second most enriched metabolite in the invasive edge of 3D hydrogels. Second, CTH, which converts cystathionine into the nonessential amino acid cysteine in the last step of the transsulfuration pathway, was identified by our metabolic CRISPR screen as crucial for invasion of cultured GBM cells. Besides slowing GBM invasion, CTHkd led to ROS accumulation in GBM cells, with both the ROS accumulation and lost invasion “rescued” through cysteine supplementation. These results suggest that the importance of the transsulfuration pathway and CTH in particular in GBM invasion arises from its production of the antioxidant cysteine. Indeed, neither glutathione nor H_2_S, other ROS scavengers produced downstream of CTH in the transsulfuration pathway, rescued lost invasion in CTHkd cells.

Prior studies have suggested that cancer cells have an increased cysteine demand that exceeds the amino acid’s availability in the microenvironment (47, 48). This activates the transsulfuration pathway to meet the metabolic requirements of the cancer cells (48). A similar phenomenon could occur in GBM cells invading adjacent tissue, where increased demand for cysteine likely arises from the ROS-induced oxidative stress we identified at the invasive GBM front, with the transsulfuration pathway providing this cysteine due to insufficient cysteine available in the invaded white matter for uptake through membrane transporters (48, 49).

Our finding that cysteine deficiency drives the diminished invasiveness caused by CTHkd builds upon studies in which cysteine depletion induces ferroptosis in cancer cells (11, 50) by suggesting that cysteine depletion slows GBM invasion by impairing the ability of invasive GBM cells to cope with oxidative stress and evade ferroptosis. Ferroptosis-relevant findings from our multiomic analysis included a lipidomic profile reflecting ferroptosis pathway enhancement in invasive GBM cells in hydrogels and patients. In terms of individual metabolites and genes relevant to ferroptosis, there were greater changes in hydrogels than in patient specimens, which could reflect interpatient variability not encountered with hydrogels. The ferroptosis-relevant changes we found in invasive GBM cells in hydrogels included increased cystine and cholesterol esters, along with expression of *ACAT2* (which generates cholesterol esters that we detected) and *ACADL*, all of which protect against ferroptosis. In contrast, in patient specimens, the ferroptosis-relevant change in invasive GBM cells was upregulation of *ACACB*, which exerts phosphorylation-dependent effects on ferroptosis (27).

Interestingly, in healthy brain, the transsulfuration pathway has been confirmed to be intact but inefficient at later steps, with cystathionine present at higher levels in the brain compared with other organs (51). This inefficiency is exacerbated in neurodegenerative conditions such as Parkinson’s and Alzheimer’s diseases (51). While gene expression changes might not correlate with enzymatic activity and the relationship between enzyme activities and metabolite levels can be nonintuitive (52), our studies suggest that this inefficiency is also exacerbated in invasive GBM cells. Specifically, integration of our multiomic findings (Figure 5E) suggested increased flux through the transsulfuration pathway in invasive GBM cells based on upregulated glutathione turnover enzymes, with CTH a rate-limiting targetable transsulfuration step in invasive GBM cells based on the accumulation of cystathionine in these cells without changes in transsulfuration enzyme gene expression. Thus, invasive GBM cells upregulate and rely on the transsulfuration pathway beyond the level seen in healthy brain to generate cysteine, enabling them to cope with the oxidative stress associated with invasion identified by us in patient GBMs and by others in prostate cancer (46).

ROS at the invasive GBM edge likely derive from intrinsic and extrinsic sources (53). Intrinsically, GBM cell invasion could generate ROS, a possibility supported by our finding of upregulation of the components of mitochondrial oxidative phosphorylation most associated with ROS production at the invasive front. Extrinsically, ROS at the invasive tumor edge could arise from greater oxygen at the tumor edge than the relatively hypoxic core (54). Regardless, for GBM cells to continue brain invasion, ROS generated during invasion must be detoxified (55).

Having shown that invasive GBM cells produce and adapt to oxidative stress during invasion, we then investigated whether these ROS directly promote invasion. Prior reports have demonstrated that ROS promote metastases, tumor proliferation, apoptosis suppression, and angiogenesis (53, 55, 56). The role of ROS in tumor cell invasion has been less investigated (57). Our work addresses this knowledge gap by demonstrating that exposure to H_2_O_2_, 1 of 3 predominant cancer ROS (58), increased invasion in 3D hydrogels. This finding has translational implications that should be accounted for when considering therapeutic strategies such as SOD mimetics that are in clinical trials for GBM, since we found that the ability of these agents to convert superoxide to H_2_O_2_ promoted GBM invasion.

Further work will be needed to determine how H_2_O_2_ drives GBM cell invasion (55). Transcription-independent mechanisms promoting degradation of proteins suppressing invasion via the ubiquitin/proteasome pathway mediate ROS-promoted lung cancer invasion (59). Furthermore, ROS-regulated oncologic processes often depend on ROS levels, where moderate ROS levels promote tumor growth and survival, and high ROS levels induce tumor cell apoptosis (54, 55). Indeed, we found a similar dose-response relationship between H_2_O_2_ concentration and GBM invasion (Supplemental Figure 3B).

Although our patient specimens spanned the 4 GBM subtypes and our cell lines represented 2 of them, further studies are needed to prove that our findings of the dependence of GBM invasion on the transsulfuration pathway are independent of GBM molecular subtype. Further studies are also needed to investigate the efficacy of targeting GBM invasion via its metabolic dependence on the transsulfuration pathway. Notably, we found upregulation of CBS, the enzyme just upstream of CTH, in GBM cells invading despite CTHkd. This finding, along with another study implicating 3-mercaptopyruvate sulfurtransferase (MPST), an enzyme downstream of CTH that generates H_2_S from cysteine, in GBM cell motility (60), suggests that targeting the transsulfuration pathway at multiple steps may be needed to prevent invasive escape from targeting a single step.

As mentioned above, we also found that, while CTHkd slowed invasion of intracranial GBM PDXs in vivo, this was not enough to improve the survival of tumor-bearing mice, as the resulting tumors were larger. Interestingly, a prior study demonstrated that a high-fat diet inhibits H_2_S production, which increases tumor proliferation and chemotherapy resistance (12). While our finding that CTHkd slowed GBM invasion was linked to cysteine and not H_2_S based on rescue studies, it is possible that CTHkd lowering H_2_S could have caused some of the tumor growth we noticed in the setting of reduced invasion in vivo with CTHkd, a potential mechanism by which CTH could contribute to the “go or grow” hypothesis in which invasive GBM cells suppress proliferative programs and vice versa (61). Our finding of expansile tumor growth when invasion is inhibited with CTHkd in vivo contrasts with our finding of unchanged Ki-67 labeling when GBM cells with CTHkd invade hydrogels in culture, suggesting that proliferation occurring with CTHkd involves an in vivo mechanism. This finding also suggests that targeting invasion via the transsulfuration pathway may be more effective if this approach is combined with traditional cytotoxic chemotherapy targeting proliferating cells.

## Methods

### Cell culture

U-251 MG (UC Berkeley Tissue Culture Facility from ATCC) cells were cultured in DMEM (Thermo Fisher) supplemented with 10% (vol/vol) fetal bovine serum (Corning, MT 35-010-CV), 1% (vol/vol) penicillin-streptomycin (Thermo Fisher), 1% (vol/vol) MEM nonessential amino acids (Thermo Fisher), and 1% (vol/vol) sodium pyruvate (Thermo Fisher). GBM43 cells (Mayo Clinic) were cultured in DMEM (Thermo Fisher) supplemented with 10% (vol/vol) fetal bovine serum (Corning, MT 35-010-CV), 1% (vol/vol) penicillin-streptomycin (Thermo Fisher), and 1% (vol/vol) Glutamax (Thermo Fisher, 35-050-061). GPMP017 cells were obtained from a GBM PDX established by the Raleigh lab (UCSF) and were grown in DMEM/F12 with 0.5% N2 supplement, 0.5% B27 without vitamin A, 1% antimycotic/antibiotic, 20 ng/ml rhEGF, and 20 ng/mL rhFGFb. Cells were harvested using 0.25% Trypsin-EDTA (Thermo Fisher) and passaged under 30 times. Cells were screened bimonthly for mycoplasma and validated every 6 months by short tandem repeat (STR) analysis at the University of California Cell Culture Facility. To generate media with 100 μM cysteine, normal media were mixed 1:1 with cysteine-free media. Cysteine-free media were made by adding l-methionine (MilliporeSigma, M5308, 0.201 mM) and l-glutamine (Gibco, Thermo Fisher Scientific, 25030081, 4 mM) to high-glucose DMEM lacking glucose, glutamine, methionine, or cystine (Gibco, Thermo Fisher Scientific, 21013024). Additional cell culture reagents were used at concentrations described in Supplemental Table 19.

### 3D hydrogels

#### Me-HA synthesis.

HA hydrogels were synthesized as described (62). Methacrylic anhydride (Sigma-Aldrich, 94%) was used to functionalize sodium hyaluronate (Lifecore Biomedical, Research Grade, 66–99 kDa) with methacrylate groups (Me-HA). The extent of methacrylation per disaccharide was quantified by ^1^H nuclear magnetic resonance spectroscopy (NMR) and was approximately 85% for materials used in this study. To add integrin-adhesive functionality, Me-HA was conjugated via Michael addition with cysteine-containing RGD peptide Ac-GCGYGRGDSPG-NH2 (Anaspec) at 0.5 mmol/l.

#### HA hydrogel rheological characterization.

Hydrogel stiffness was characterized by shear rheology via a Physica MCR 301 rheometer (Anton Paar) with 8 mm parallel plate geometry for γ = 0.5% and *f* = 1 Hz. Frequency was controlled to 50-1 Hz for the frequency sweep at a constant strain (γ = 0.5%), and the modulus saturation curve with time was obtained under oscillation with constant strain (γ = 0.5%) and frequency (*f* = 1 Hz). Gel solution temperature was controlled (*T* = 37°C) with a Peltier element (Anton Paar), and the sample remained humidified throughout the experiment.

#### Tumorsphere invasion assays.

Tumorspheres were fabricated using AggreWell Microwell Plates (STEMCELL Technologies). Briefly, 1.2 × 10^5^ cells were seeded into a single cell of the AggreWell plate to form spheroids with 100 cells. After 48 hours, spheroids were resuspended in phenol red–free serum-free DMEM (Thermo Fisher, 21-063-029) at 1.5 spheroids/μL and used as solvent for HA hydrogel crosslinking. To form hydrogels, 6 wt.% Me-HA was crosslinked in phenol red–free serum-free DMEM (Thermo Fisher, 21-063-029) with a protease-cleavable peptide (KKCG-GPQGIWGQ-GCKK, Genscript). HA-RGD gels were crosslinked with peptide crosslinkers at varying ratios to yield hydrogels with a shear modulus of approximately 300 Pa and a final 1.5 wt.% Me-HA (Supplemental Figure 1). Unless otherwise mentioned, 3.405 mM peptide crosslinker was selected to yield a 300 Pa shear modulus. After 1 hour crosslinking in a humidified 37˚C chamber, cell-culture medium was added to hydrogels and, unless noted, replenished every 2 days.

#### Invasion devices.

To fabricate invasion devices, the device base, lid, and spacers were laser cut out of 1.5 mm thick CLAREX acrylic glass (Astra Products). Pieces were assembled and fastened with epoxy, UV-treated for 10 minutes, and stored in a cold room. On the day of the experiment, devices were brought to room temperature and a 22 gauge × 1.5 inch bevel needle (BD Precision Glide) was inserted into the device as a channel mold. HA hydrogel solution was casted around the wire and incubated for 1 hour in a humidified 37˚C chamber. After crosslinking, devices with hydrogels and needles were submerged in culture medium for at least 10 minutes, before removing the needle, which left an open channel. Afterwards, 4 million cells were seeded into the open channel and channel ends were plugged with vacuum grease. Unless stated, devices were cultured for 28 days and media were replenished every 3 days, with 28 days chosen based on experiments revealing it to be when control GBM43 cells fully invade through hydrogels.

### Invasion quantification

For invasion analysis of spheroids in HA hydrogels, spheroids were imaged every 2 days using the Eclipse TE2000 Nikon Microscope with a Plan Fluor Ph1 ×10 objective. Images were acquired using NIS-Elements software (NIS-ELEMENTS AR 5.42.02). For each spheroid, invasion was calculated as (*A_f_* – *A_i_*)/*A_i_*) where *A_f_* = final spheroid area and *A_i_* = initial spheroid area. Spheroid area was measured using ImageJ (NIH), and invasion was normalized to control spheroids.

To analyze cells invading in devices, cells were imaged every 7 days using the Eclipse TE2000 Nikon Microscope with a Plan Fluor Ph1 ×10 objective. Images were acquired and stitched using NIS-Elements software (NIS-ELEMENTS AR 5.42.02). For each device, total cell reservoir area was outlined in ImageJ at each time point, and invasion was calculated using the same equation as above. Detached cells were defined as single cells without neighboring cells within 10 μm. Highly invasive cells were defined as cells invading over 200 μm from the channel’s edge. Cells with aspect ratio of 2 or more were labeled elongated and those with aspect ratio of less than 2 were labeled round.

### CRISPR knockout screen

Metabolism-focused sgRNA libraries were designed and screens performed as described (63). Oligonucleotides for sgRNAs were synthesized by Genewiz and amplified by PCR, and 2.4 × 10^7^ GBM43 cells expressing sgRNAs were seeded into 6 3D hydrogel devices. After culture, invasive and noninvasive “core” cells were isolated by carefully disassembling the devices and isolating fractions by microdissection. Invasive cells were defined as those invading a distance greater than 200 μm from the channel wall. Genomic DNA (gDNA) was extracted using the Monarch Genomic DNA Purification Kit (New England BioLabs, T3010S) per the manufacturer’s protocol. gDNAs from 6 invasion devices were pooled and amplified by PCR. PCR amplicons were sequenced together with the initial and in vitro samples. We performed PCA on normalized counts from each in vitro sample. Sequencing counts from samples were summed, normalized (count/million), and analyzed as single conditions. Fitness scores for each guide were calculated as the log_2_ ratio of normalized counts. The median of the guides was used as the fitness score for each gene, and *t* test assessed whether guides significantly deviated from 0.

### NanoString multiplex transcriptomic analysis

A bioanalyzer was used to assess quantity and quality of RNA from paired biopsies from the core and edge of 3 hydrogel devices and 3 patient GBMs. RNA (100 ng) was used for the metabolic pathways panel. RNA from each sample was hybridized with the code set for 18 hours, and 30 μL of the reaction was loaded into the nCounter cartridge and run on the nCounter SPRINT Profiler. Enrichr software (Enrichr, version 3.2) was used to analyze the expression of pathways from the KEGG 2019 Human Database and their significance.

### ROS measurements

Live-cell ROS measurements were performed using Molecular Probes CellROX Deep Red (Thermo Fisher, C10422) or MitoSOX Green (Thermo Fisher, M36006). Cells were cultured in normoxia or hypoxia in 6-well plates for 48 hours or until 80% confluence, followed by staining in stock solutions for 30 minutes. Cells were harvested and assessed by flow cytometry following the manufacturer’s recommendations. For cells in hydrogels, 10 μM probe and 30-minute incubation were used to enhance probe diffusion through hydrogel. Fluorescence imaging was performed on a Zeiss LSM 710 Laser Scanning Confocal Microscope.

### Statistics

Invasion, proliferation, viability, morphology, and quantitative PCR (qPCR) assays were done with 3 technical and biological replicates. To compare multiple groups, 1-way ANOVA (parametric) or Kruskal-Wallis (nonparametric) tests were used for continuous outcome variables, with χ^2^ and Fisher’s exact tests used for categorical outcome variables. ANOVA or Kruskal-Wallis tests were followed by Tukey’s or pairwise Wilcoxon’s post hoc tests for comparisons between groups, respectively. Nonparametric 2-tailed *t* tests were used to compare 2 groups. NanoString data were analyzed using the DESeq2 package in R, through which a geometric mean is calculated for each gene across replicates and counts in each replicate are divided by the mean, with count outliers removed using Cook’s distance analysis and the Wald test used to assess significance. Kaplan-Meier analysis was carried out for in vivo survival studies.

### Study approval

Animal experiments were approved by the UCSF IACUC (approval AN105170-02). Patient biopsies were performed with informed consent under UCSF IRB approval (11-06160).

### Data availability

RNA-Seq data are available in the NCBI’s Gene Expression Omnibus database (GEO GSE246697). Values for all data points in graphs are reported in the Supporting Data Values file.

## Author contributions

JHG, EAA, and SJ designed the project, conducted experiments, processed data, interpreted results, and edited the manuscript. KJW, JZ, NC, ML, PS, JR, KS, SG, WC, LC, AZ, and DRR conducted experiments, processed data, and interpreted results. SK and MKA procured funding, designed experiments, interpreted results, and wrote and edited the manuscript.

## Supplementary Material

Supplemental data

Unedited blot and gel images

Supplemental table 1

Supplemental table 10

Supplemental table 11

Supplemental table 12

Supplemental table 13

Supplemental table 14

Supplemental table 15

Supplemental table 16

Supplemental table 17

Supplemental table 18

Supplemental table 19

Supplemental table 2

Supplemental table 3

Supplemental table 4

Supplemental table 5

Supplemental table 6

Supplemental table 7

Supplemental table 8

Supplemental table 9

Supporting data values

## Version 1 11/16/2023

In-Press Preview

## Version 2 02/01/2024

Electronic publication
