# Supplementary material for: Multiomic screening of invasive GBM cells reveals targetable transsulfuration pathway alterations
Source: J Clin Invest. 2024 Feb 1;134(3):e170397. doi: 10.1172/JCI170397 (PMC10849762; doi:10.1172/JCI170397)
Supplement: Supplemental data [file jci-134-170397-s069.pdf]

SUPPLEMENTAL FIGURES

Supplemental Figure 1. Setup for 3D hydrogel invasion devices and patient site-directed biopsies. (**a**) Shown is an illustration of the invasion device which consists of a cell reservoir within a hyaluronic acid (HA) hydrogel on an acrylic slide. (**b**) HA-methacrylate (Me-HA) was functionalized with an integrin binding (RGD) peptide using the Michael-type addition reaction between the methacrylate groups on the polymer and the cysteine thiol groups on the peptide. The same addition reaction with the methacrylate groups was used to induce crosslinking with protease-sensitive peptide crosslinkers to form HA hydrogels. (**c**) Hydrogel storage modulus (G') (in Pascals) versus time after the gel is casted. Time=0 represents the intersection of storage modulus (G') and loss modulus (G'') (n=3 hydrogels). (**d**) Phase image (10x magnification) of single cells of invasive fraction in HA hydrogel invasion device; scale bar=100 μm. (**e**) Illustration of invasion device (**f**) Phase image of invasion device with dotted lines indicating microdissection boundaries to isolate core and invasive cells. (**g**) Shown are representative locations of site-directed biopsies taken from the central core vs. enhancing edge of a newly diagnosed glioblastoma. (**h**) PCA in which metabolomics results from the core and edge of 3D hydrogels and the core and edge of patient GBMs are on the same plot, revealing that patient site-directed biopsies clustered together and 3D hydrogels clustered together given the different sources of these samples (patient vs. cell line in 3D culture), with core vs. edge from each source clustering separately only when patient GBMs and 3D hydrogels were placed on separate PCA plots **(Fig. 1a**).

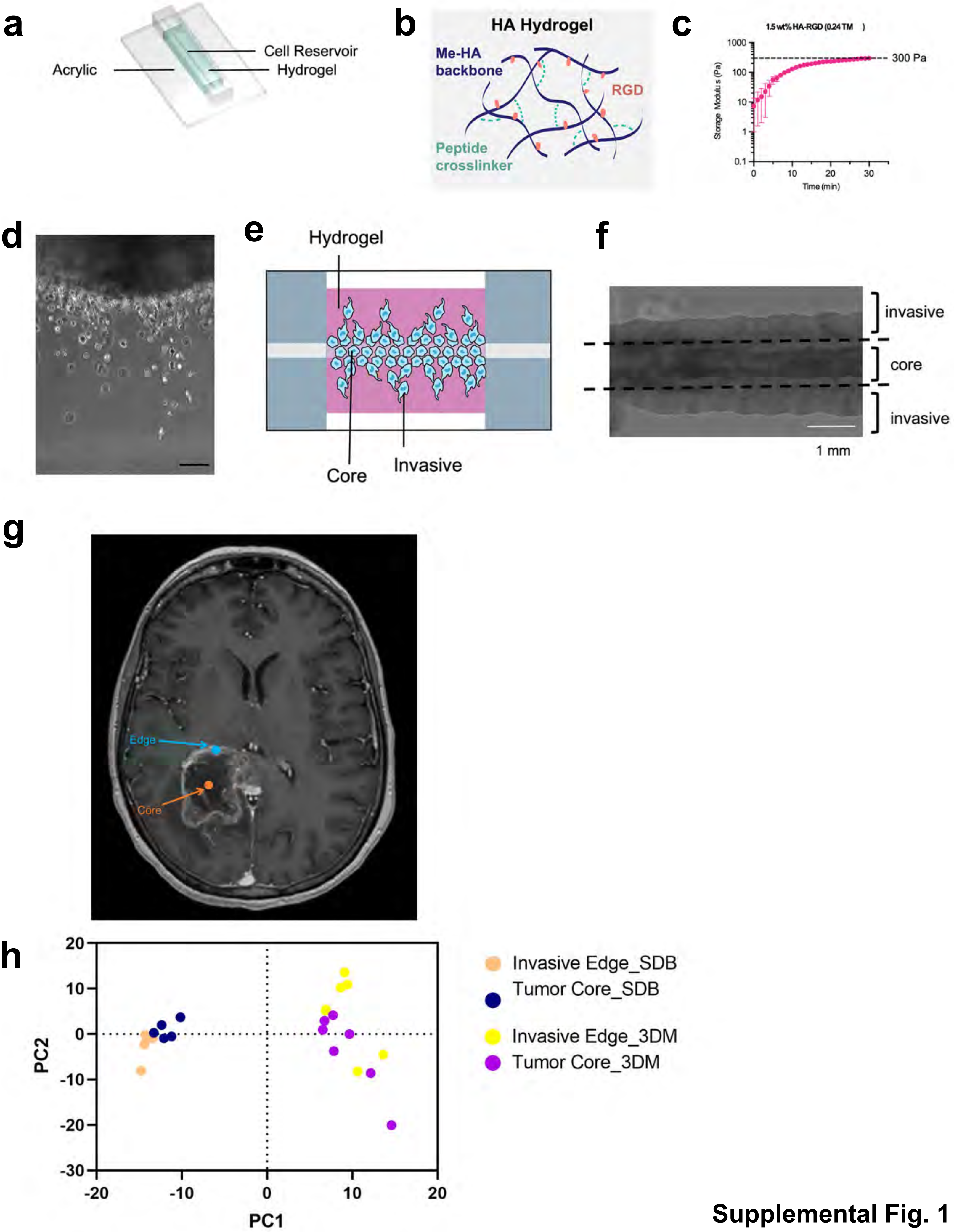

Supplemental Fig. 1

Supplemental Figure 2. Metabolomic and transcriptomic analysis of invasive versus core GBM cells in 3D hydrogels and patient specimens. (**a**) Shown are levels of individual metabolites in the invasive fraction vs. core of 3D hydrogels after long-term culture (n=7 paired samples; left) and site-directed biopsies from patient tumor cores vs. invasive front (n=5 paired samples; right). (**b-e**) After GBM43 cells invaded 3D hydrogels during long-term culture, tumor cells in the invasive vs. core fractions were profiled (n=3 devices) using a 770 gene multiplex to analyze expression of metabolism genes, revealing: (**b**) an MA plot, in which the y-axis represents log_2(fold change), the x-axis represents normalized RNA read counts of a particular gene, and each dot represents a gene, with < 0 indicating an increase in log_2(fold change) in the Invasive fraction as compared to the Core fraction; (**c**) principal component analysis (PCA) of the data, showing that cells in the invasive front clustered together, but separate from cells in the tumor core; (**d**) a heatmap of the top 85 differentially expressed genes; (**e**) a Volcano plot illustrating differentially expressed genes in the invasive (right) and core (left) fractions; and (**f**) upregulated genes in the invasive fraction in the ROS response pathway. (**g-h**) Paired specimens from the invasive front vs. core of patient GBMs (n=3 pairs) were profiled using a 770 gene multiplex to analyze expression of metabolism genes, revealing (**g**) a Volcano plot illustrating differentially expressed genes in the invasive (right) and core (left) fractions; and (**h**) upregulated genes in the invasive fraction in the glutamine metabolism and mitochondrial respiration pathways.

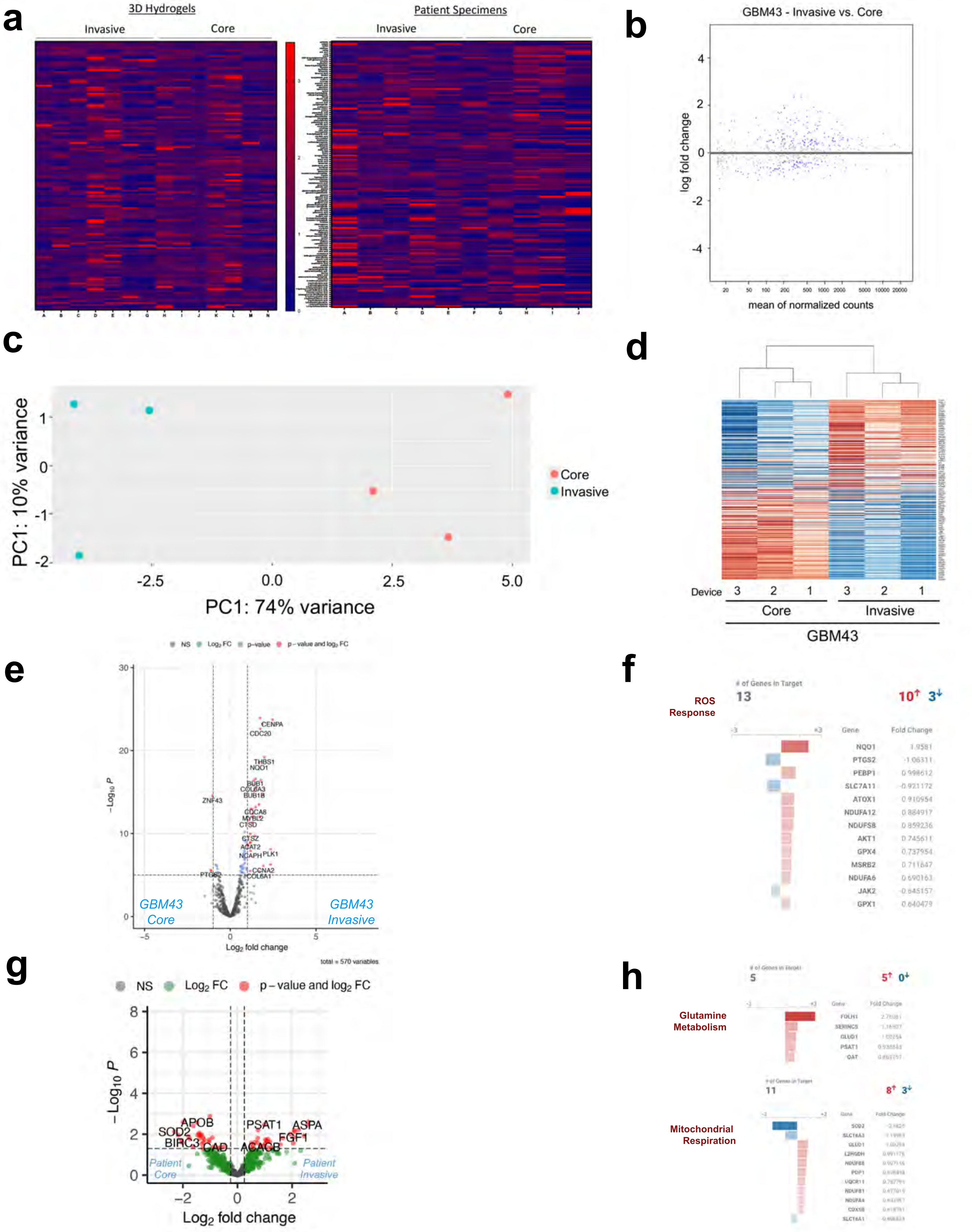

Supplemental Fig. 2

Supplemental Figure 3. Hydrogen peroxide increases GBM invasion in 3D hydrogels. (**a**) Tumorsphere invasion assay schematic. **(b)** GBM43 spheroid invasion assay with various NAC and H_2O_2 concentrations (n=24 spheres, collected from 3 independent experiments). **(c)** Treating cultured GBM43 cells with 30 μM SOD mimetic MnTBAP lowered superoxide levels as assessed with the MitoSOX™ dye by 80% within 3 hours (individual points shown with line connecting the means, n=3-6/group, t-test). (**d**) 30 μM SOD mimetic MnTBAP increased invasion of GBM43 cells in tumorsphere invasion assay. **(e)** Quantification of data from (**d**) (n=30 spheres, collected from 3 independent experiments). (**f**) NAC did not alter superoxide levels in GBM43 cells assessed by MitoSOX™. Individual data points shown (n=3/group, t-test) with horizontal line at mean, vertical lines represent standard deviations. *P< 0.05; **P< 0.01; ***P<0.001; ****P<0.0001; (NAC=N-acetylcysteine).

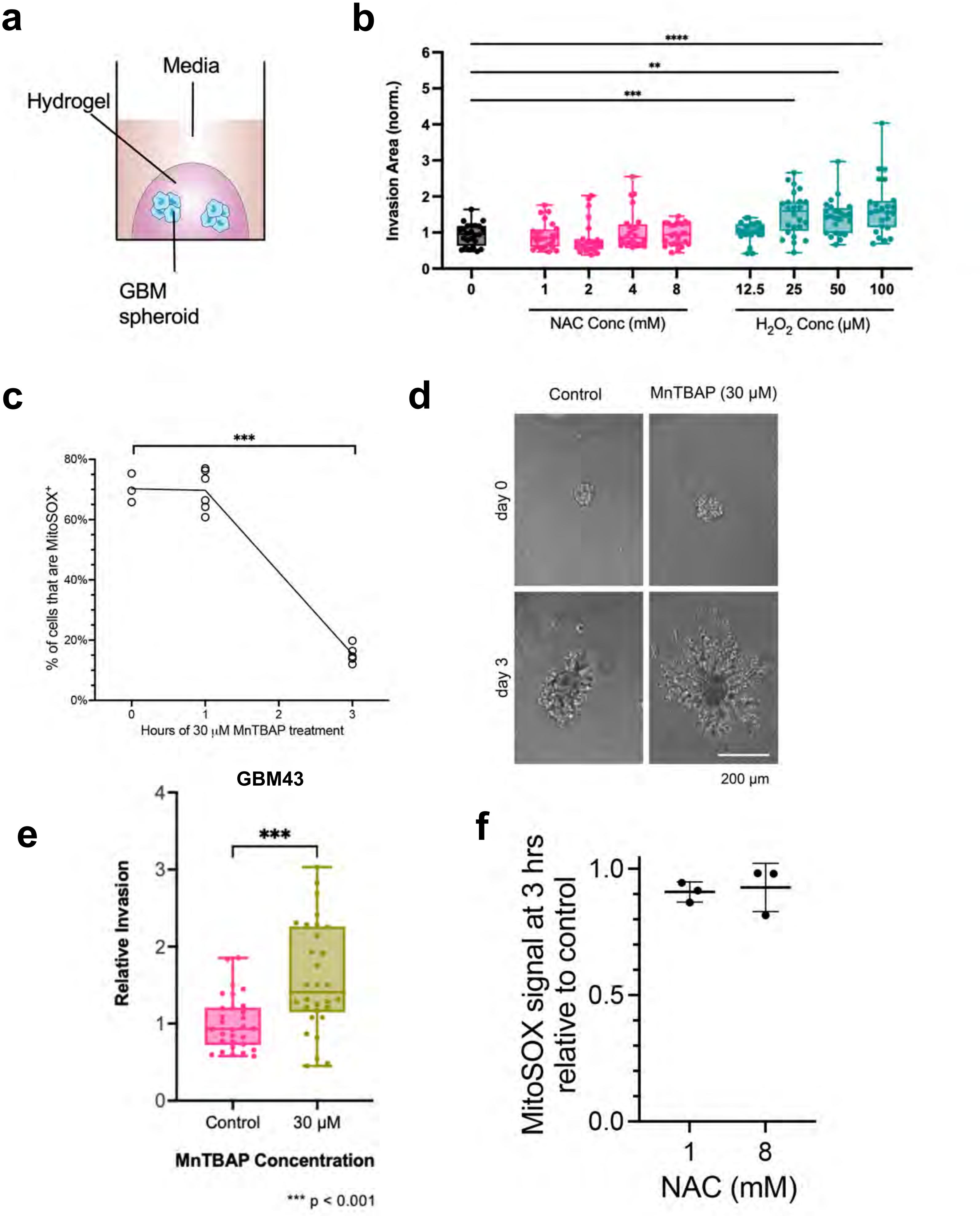

Supplemental Fig. 3

Supplemental Figure 4. CRISPR knockout screen of metabolic genes reveals a role for CTH in GBM invasion. (**a**) CRISPR Screening schematic. **(b)** Volcano plot displaying the enrichment of sgRNAs for metabolic genes in the core (left) and invasive front (right) of GBM 3D tumor models. (**c**) Results of qPCRs validating CRISPRi knockdown of five metabolic genes chosen for further evaluation from CRISPR screen of 3,000 metabolic genes. (**d**) GBM43/CTHkd cells expanded to the same amount over five days in culture at varying seeding densities as control GBM43 cells (P=0.1-0.9). (**e**) Results from tumorsphere invasion assays in U251 GBM cells treated with vehicle or 40 μM CSE-γ-IN, a CTH small molecule inhibitor. (n=30 spheres, collected from 3 independent experiments). (**f**) Dose response curve for GBM43 (left) and U251 (right) GBM cells grown in varying concentrations of CSE-γ-IN for 48 hours after which cell viability was assessed (n=3 biological replicates). Based on these results, 40 μM of CSE-γ-IN was used for subsequent experiments. (**g**) *Left*: microscopy images of GFP^+ GBM organoids being invaded by RFP^+ GPMP017 GBM cells in DMSO vs. 40 μM of CSE-γ-IN, 10x magnification, scale bar=300 μm. Right: quantification of RFP intensity that is within the GFP organoid (n=8/group, box from 25th to 75th percentile with whiskers showing min to max; t-test). (**h**) Expression of pyridoxal kinase (PDXK), the enzyme that converts pyridoxine and other vitamin B6 precursors into pyridoxal-5′-phosphate (PLP), the bioactive form of CTH cofactor vitamin B6, in the invasive front versus core of patient GBMs was assessed using the Ivy Glioblastoma Atlas Project which sampled GBMs from 10 patients. *P< 0.05; **P< 0.01; ***P < 0.001; ****P<0.0001; CSE-γ-IN=Cystathionine-γ-lyase inhibitor.

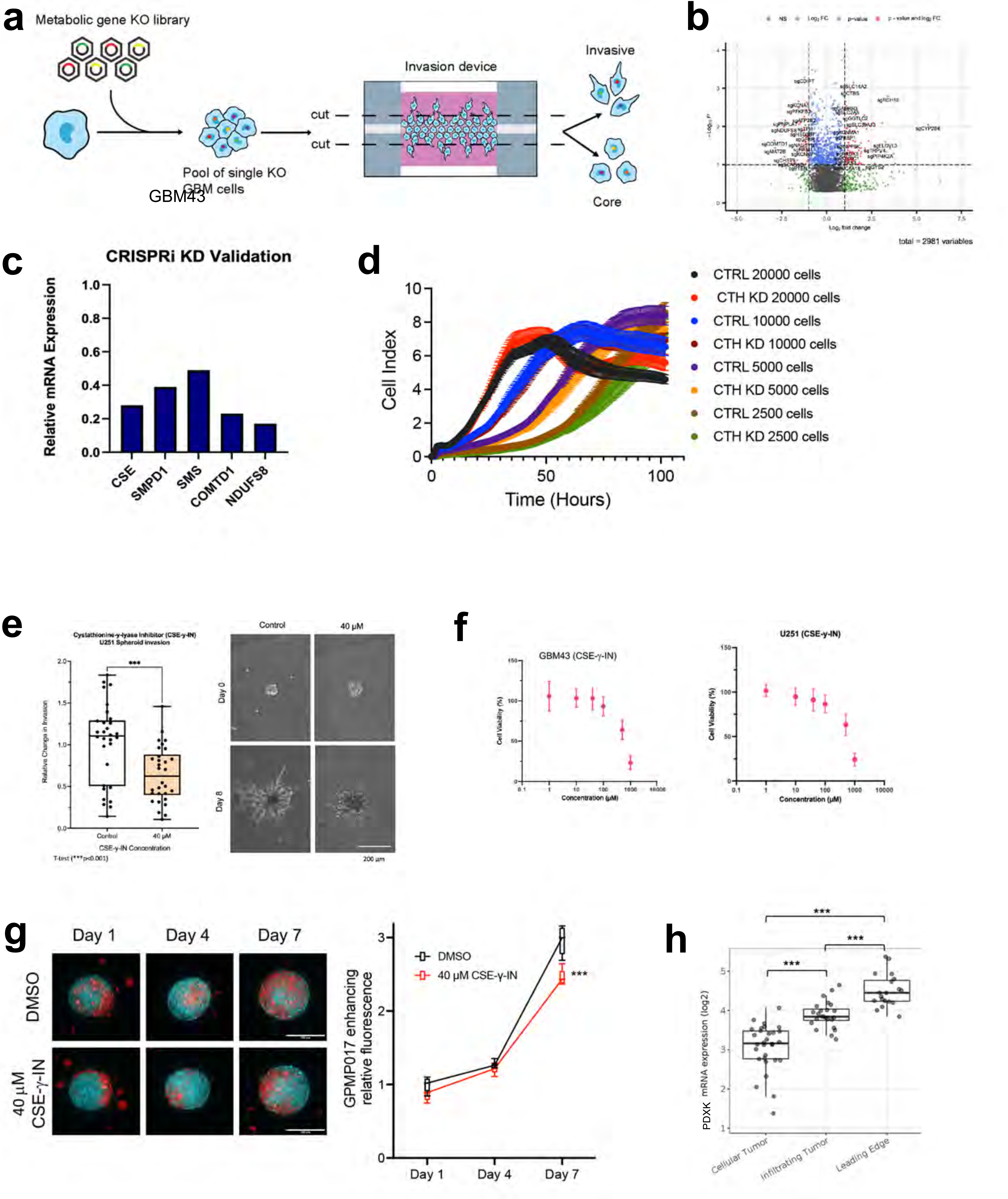

Supplemental Fig. 4

Supplemental Figure 5. Effect of CTH knockdown on GBM ROS levels during invasion. (**a**) Western blot validating CRISPRi knockdown of CTH in GBM43 cells (three replicates of GBM43/dCas9 in the left most lanes, three replicates of GBM43/CTHkd in the right most lanes). (**b**) CTH knockdown increases ROS levels in normoxic (left=18.6% O_2; P=0.01, n=3/group) and hypoxic (right=2% O_2; P=0.02; n=5/group) GBM43 cells as determined using the CellROX reagent and measuring percent of cells that are CellROX$^+$. Shown are dot plots with horizontal bars=means and vertical lines=standard deviation. (**c**) CTH knockdown did not affect superoxide levels in GBM43 cells in 200 or 100 μM cysteine, as determined using the MitoSOX reagent and measuring percent of cells that are MitoSOX positive (n=3/group; shown are dot plots with horizontal bars=means and vertical lines=standard deviation; t-test). (**d**) CTH knockdown led to decreased survival of GBM43 cells exposed to 10 μM peroxide for 48 hours (P<0.001; n=4/group). (**e**) CTH knockdown did not alter the morphology of GBM43 cells in 2D culture (P=ns; t-test; n=64-68/group). (**f**) While CSE-γ-IN did not alter the morphology of GBM43 cells in the core of 3D neurosphere invasion assays (n=38 cells/group collected across 3 independent experiments; P=ns), CSE-γ-IN raised the form factor of invasive GBM43 cells, conferring a less mesenchymal morphology that is less conducive to invasion (n=38 cells/group collected across 3 independent experiments; ANOVA with post-hoc Tukey). (**g**) In spheroid invasion assays, control GBM43 cells cultured in low cysteine concentration (100 μM) were slightly less invasive than control GBM43 cells cultured in normal cysteine concentration (200 μM) (n=52 spheres collected across 3 independent experiments; ANOVA with post-hoc Tukey), while CTH knockdown cells were not sensitive to cysteine deprivation and remained less invasive than control cells at both concentrations of

cysteine (n=52 spheres; ANOVA with post-hoc Tukey). (**h**) Cysteine deprivation increased ROS accumulation in GBM43 cells expressing dCas9-KRAB with or without sgRNAs targeting CTH (n=3/group; ANOVA with post-hoc Tukey). (**f**). *P< 0.05; **P< 0.01; ***P < 0.001; ****P<0.0001**.**

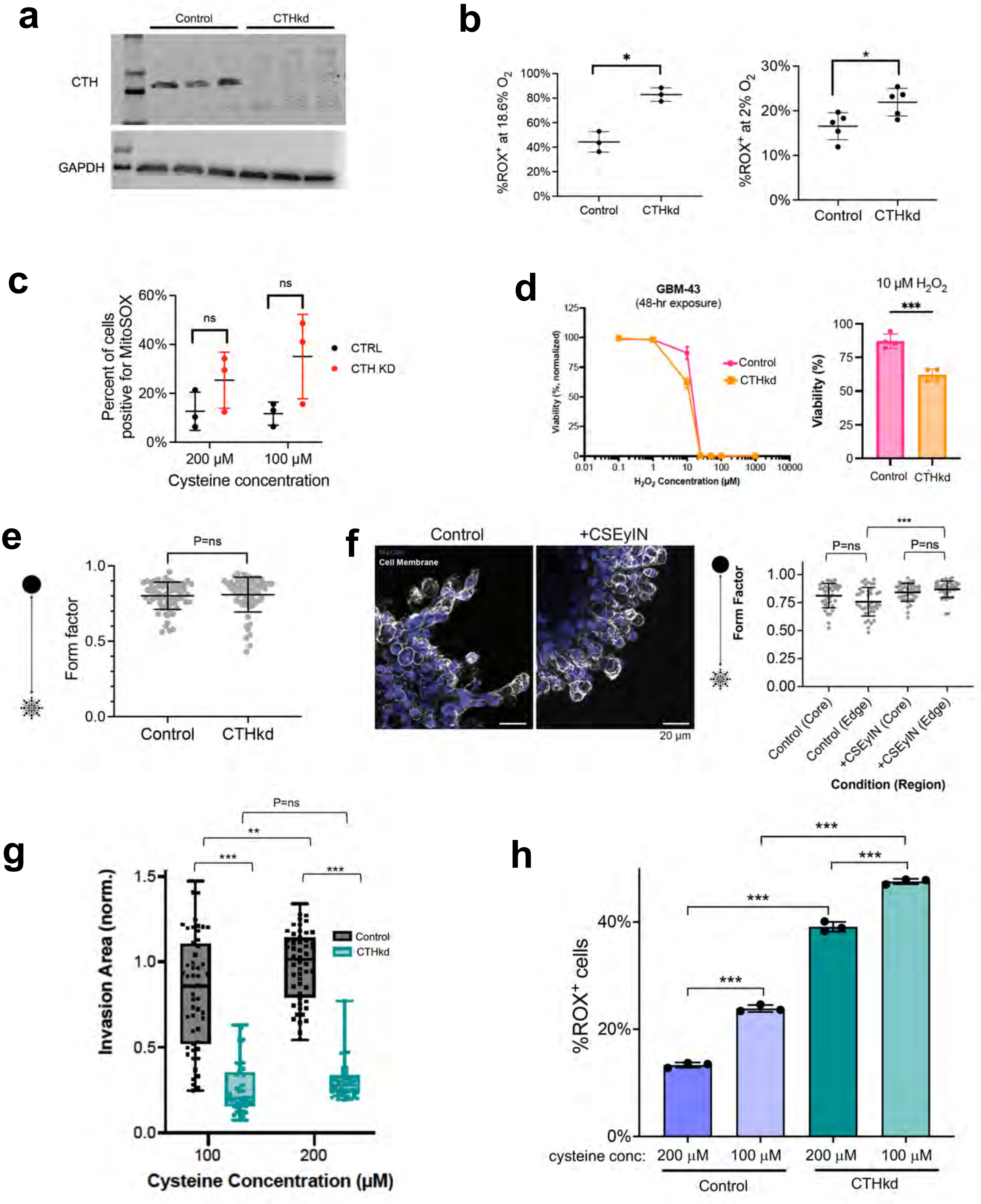

Supplemental Fig. 5

Supplemental Figure 6. CTH drives GBM invasion due to cysteine not glutathione or H_2S biosynthesis. (**a**) *Left*: Baseline cell viability was not affected by CTH knockdown, treatment with 40 μM CSE-y-IN, or 50 μM additional cysteine (n=4 biological replicates; ANOVA with post-hoc Tukey). *Middle*: The effect of CTH knockdown, treatment with 40 μM CSE-y-IN, or 50 μM additional cysteine (total cysteine=250 μM) was assessed on the response of GBM43 cells to ferroptosis inducer erastin (n=4 biological replicates). *Right*: Cell viability in 10 nM erastin was reduced by 40 μM CSE-y-IN (ANOVA with post-hoc Tukey) and increased by 50 μM additional cysteine (n=4 biological replicates; ANOVA with post-hoc Tukey). (**b-c**) The impact of glutathione supplementation on invasiveness of GBM43 cells with CTH knockdown was assessed through **(b)** images of GBM43 control and CTH KD spheroid invasion assay in 3D Hydrogels with glutathione supplementation (10x, scale bar=200 μm), from which (**c**) invasion was quantified and normalized relative to control cells without glutathione (n=26 spheres, collected from 3 independent experiments; ANOVA with post-hoc Tukey). (**d**) When GBM43 cells expressing Krab-dCAS9 and sgRNA targeting CTH were compared to GBM43 cells expressing just Krab-dCAS9, oxidized (left; P=0.03; t-test) and total (right; P=0.01; t-test) glutathione levels were slightly elevated. (**e**) NaHS, a generator of H_2S in biological tissues, reduces invasion in GBM43 cells with or without CTH knockdown (ANOVA with post-hoc Tukey; n=38 spheres/group collected from 3 independent experiments). (**f**) Dose-response curve showing cell viability declines in GBM43 cells with or without CTH knockdown as they are exposed to increasing concentrations of NaHS (n=4 biological replicates).*P< 0.05; **P< 0.01; ***P < 0.001; ****P<0.0001.

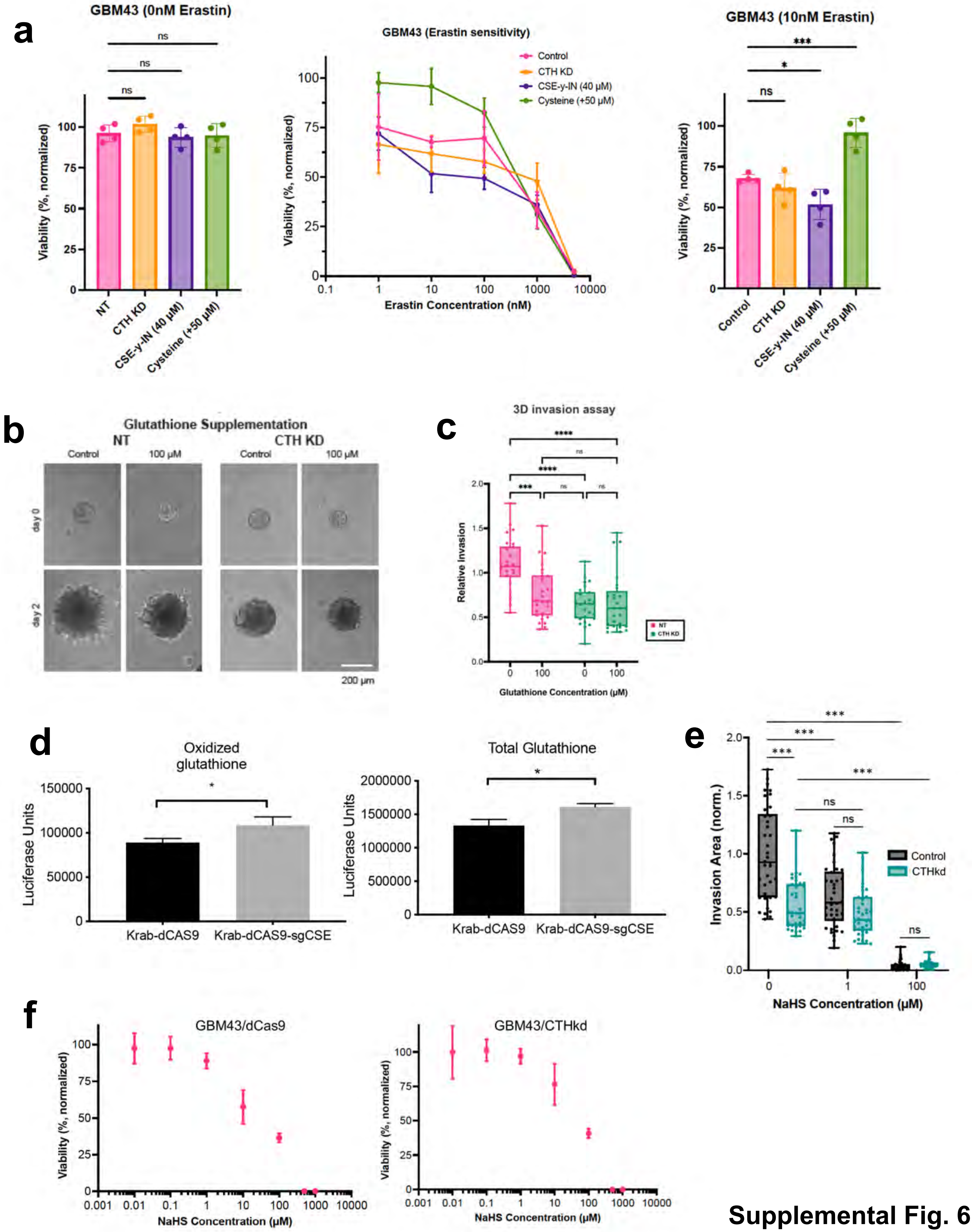

Supplemental Fig. 6

Supplemental Figure 7. CTH knockdown slows invasion without altering GBM proliferation and creates a subset of invasive GBM cells upregulating CBS. (**a**) Ki-67 staining of control and CTHkd GBM43 cells in hydrogel invasion devices revealed no differences in proliferation between core vs. edge fractions or between control and CTH knockdown cells (n=4/group; ANOVA with post-hoc Tukey). (**b-f**) GBM43 cells with CRISPRi targeting of CTH were seeded in devices, after which cells isolated from core vs. invasive fractions were transcriptomically assessed using the NanoString nCounter platform and the 770 metabolic gene multiplex, revealing (**b**) an MA plot, in which the y-axis represents $\log_2$(fold change) and the x-axis represents normalized RNA read counts of a gene, with each dot representing a gene, and < 0 indicating an increase in $\log_2$(fold change) in the Invasive fraction vs. the Core fraction; (**c**) PCA plots in which cells in the invasive front clustered together and apart from cells in the core; (**d**) Volcano plot from CTHkd core ($\log_2$fold change<0) vs. CTHkd invasive ($\log_2$fold change>0) comparison; (**e**) heatmap identified enriched metabolic genes in invasive fractions vs. core fractions of GBM43 CTHkd cells from hydrogels; and (**f**) GSEA identified upregulated pathways in CTHkd cells in invasive vs. core fractions, including DNA repair genes listed here. (**g**) Upregulated genes from the 770 metabolic gene multiplex in GBM43/CTHkd cells in the invasive fraction were similar to genes upregulated in control GBM43 cells in the invasive fraction, creating a Volcano plot with log2fold change>0 representing genes upregulated in invasive CTHkd cells vs. invasive control GBM43 cells and log2 fold change<0 representing genes upregulated in invasive CTHkd cells vs. invasive control cells, revealing few differentially expressed genes between invasive fractions of the two cell

types. (**h**) Bulk RNA-seq was used to make the same comparison made in the 770 metabolic gene multiplex in (**g**).

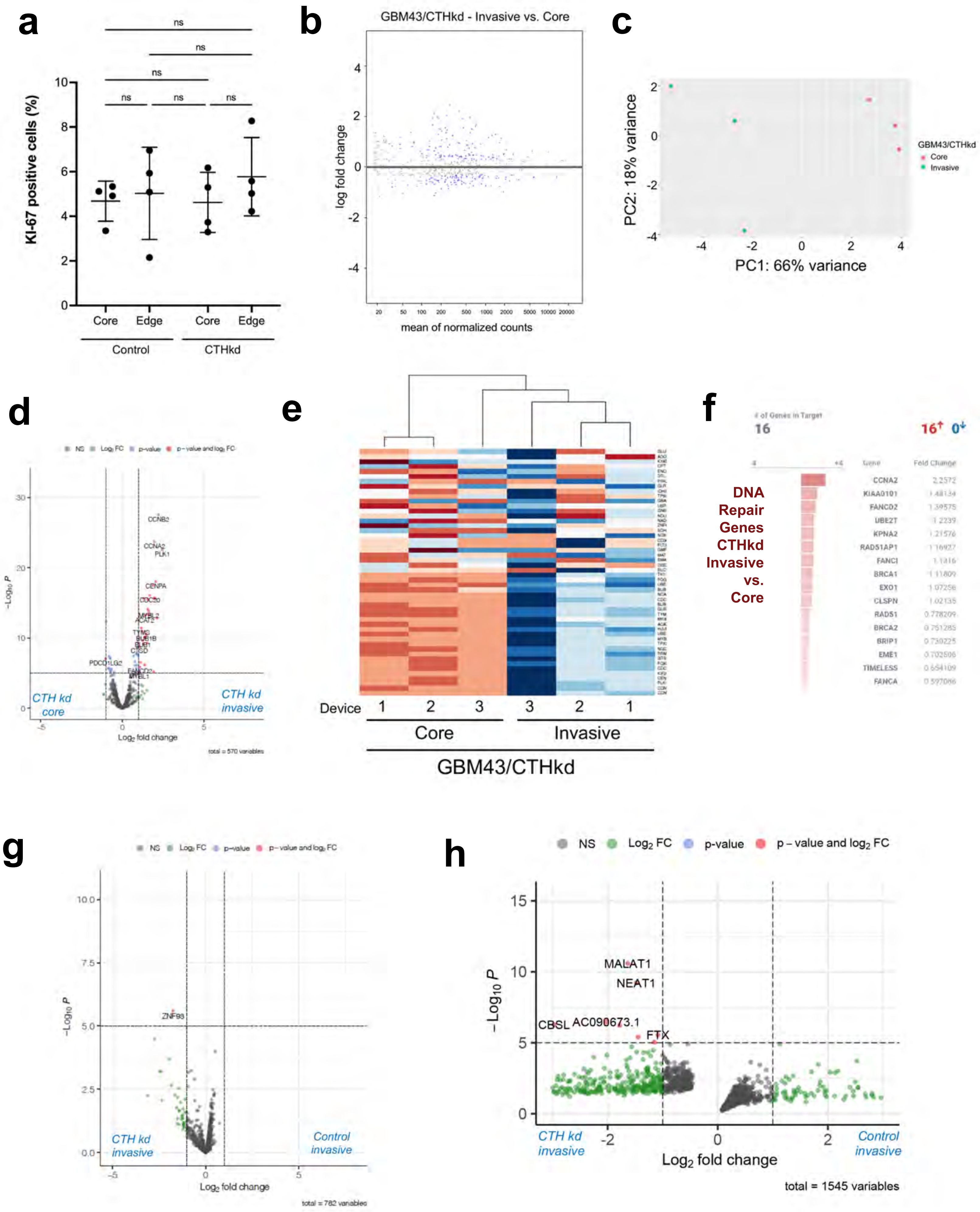

Supplemental Fig. 7

Supplemental Figure 8. CTH knockdown slows GBM43 invasion *in vivo*. (**a**) After mice carrying intracranial GBM43 xenografts with CRISPRi targeting of CTH reached endpoint, the tumors maintained their knockdown of CTH, as assessed by qPCR of explanted tumors. Horizontal lines at mean, vertical bars represent standard deviation; unpaired t-test; ***P<0.001. (**b-c**) Compared to intracranial control GBM43 xenografts, GBM43 xenografts with CRISPRi targeting of CTH (**b**) were less invasive, as measured in **Fig. 6f** and exemplified here by brainstem invasion seen in 25% of intracranial GBM43/dCas9 xenografts vs. no GBM43/CTHkd xenografts, but (**c**) had a larger maximal cross-sectional area when assessed just before endpoint (n=8-9/group; P=0.017; unpaired t-test). 10x magnification, 1000 μm scale bars.

a

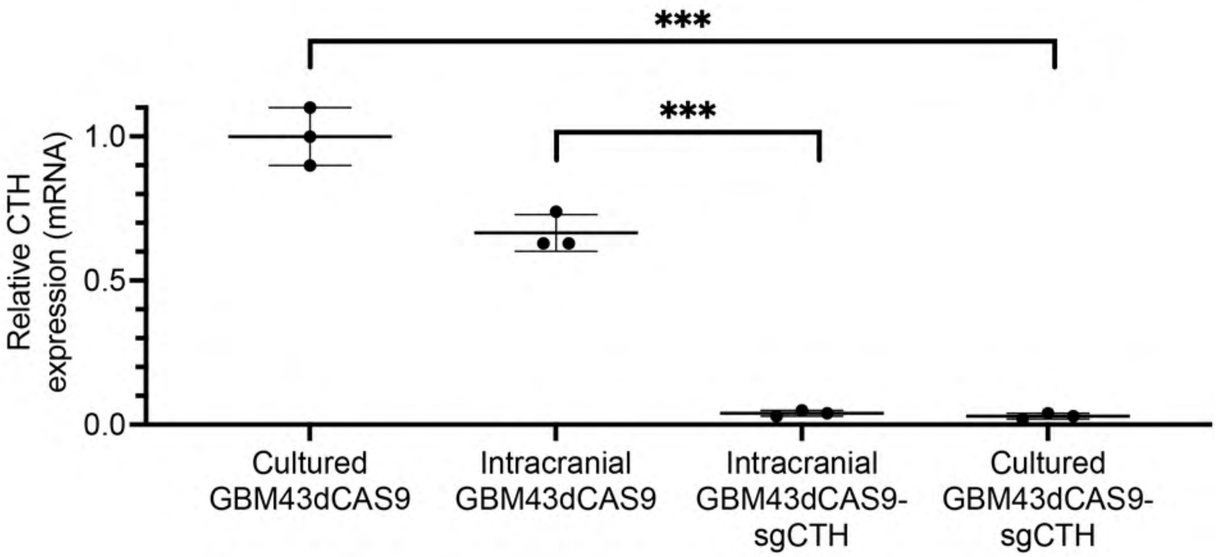

b

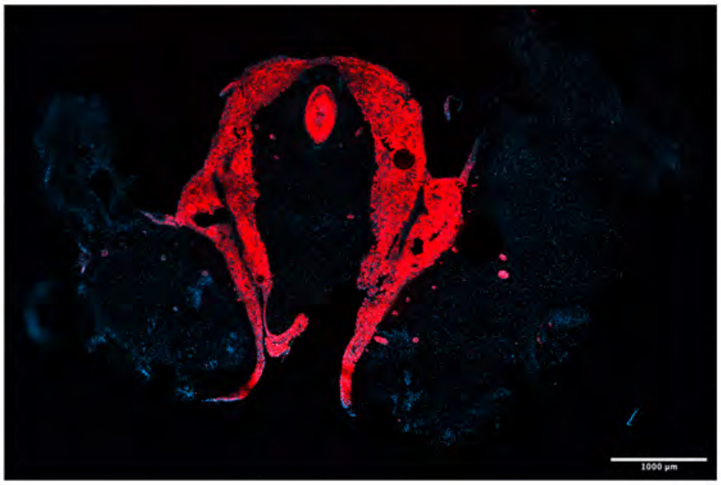

GBM43/dCas9 with brainstem invasion

c

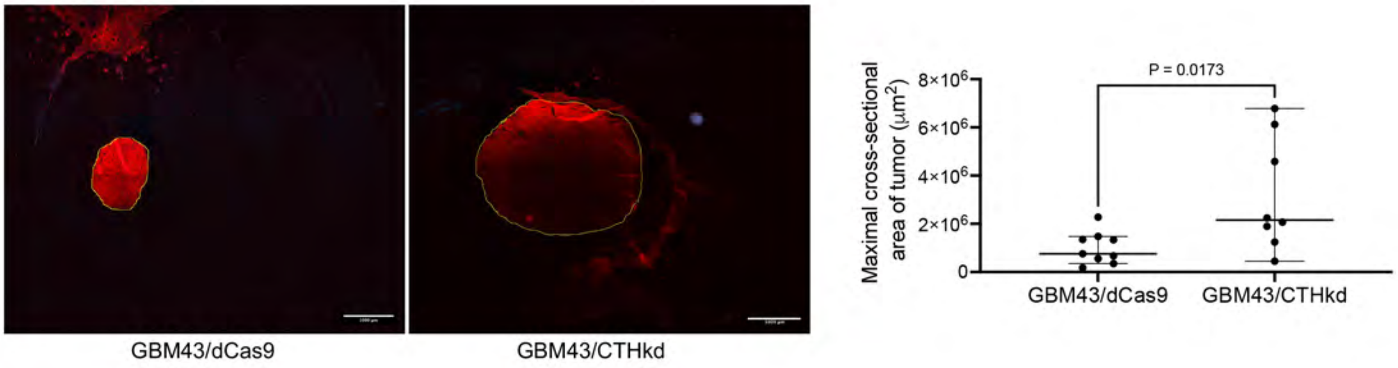

Supplemental Fig. 8

SUPPLEMENTAL METHODS

Site directed biopsies

Biopsies were performed with three-dimensional intraoperative navigation for sampling the enhancing edge of the tumor and the central tumor core inside the enhancement before tumor resection commenced. Samples were flash frozen in liquid nitrogen and stored at -80°C.

Metabolomics

Brain tumor tissue (4 mg) was homogenized in extraction solution by mixing acetonitrile, isopropanol and water in proportions 3:3:2 (JT Baker, Center Valley PA), then vortexed for 45 seconds and then 5 minutes at 4C. Following centrifugation for 2 minutes at 14,000 rcf, two aliquots of the supernatant (500 µL each aliquot) were made for analysis and one for backup. One aliquot was dried via evaporation overnight in the Labconco Centrivap cold trap concentrator (Labconco, Kansas City MO). The dried aliquot was then resuspended with 500µL 50% acetonitrile (degassed as given), then centrifuged for 2 minutes at 14,000 rcf using the centrifuge Eppendorf 5415. The supernatant was moved to a new Eppendorf tube and again evaporated to dryness. Internal standards (C08-C30, fatty acid methyl esters) were then added and the sample was derivatized by methoxyamine hydrochloride in pyridine and subsequently by N-methyl-N-trimethylsilyltrifluoroacetamide for trimethylsilylation of acidic protons. Data were acquired as previously described (1). Briefly, metabolites were measured using a Restek corporation rtx5Sil-MS column (Restek Corporation; Bellefonte PA; 30 m length x 0.25

mm internal diameter with 0.25µm film made of 95% dimethyl/5%diphenylpolysiloxane) protected by a 10m long empty guard column which is cut by 20cm intervals whenever the reference mixture QC samples indicate problems caused by column contaminations. This sequence of column cuts has been validated by UC Davis Metabolomics Core with no detrimental effects detected with respect to peak shapes, absolute or relative metabolite retention times or reproducibility of quantifications. This chromatography method yields excellent retention and separation of primary metabolite classes (amino acids, hydroxyl acids, carbohydrates, sugar acids, sterols, aromatics, nucleosides, amines and miscellaneous compounds) with arrow peak widths of 2–3 seconds and very good within-series retention time reproducibility of better than 0.2s absolute deviation of retention times. The mobile phase consisted of helium, with a flow rate of 1 mL/min, and injection volume of 0.5 µL. The following mass spectrometry parameters were used: a Leco Pegasus IV mass spectrometer with unit mass resolution at 17 spectra s-1 from 80-500 Da at -70 eV for elution of metabolites. As a quality control, for each sequence of sample extractions, one blank negative control was performed by applying the total procedure (i.e. all materials and plastic ware) without biological sample. Result files were transformed by calculating the sum intensities of all structurally identified compounds for each sample (i.e. those signals that had been positively identified in the data pre-processing schema outlined above), and subsequently dividing all data associated with a sample by the corresponding metabolite sum. The resulting data were multiplied by a constant factor to obtain values without decimal places. Intensities of identified metabolites with more than one peak (e.g. for the syn- and anti-forms of methoximated reducing sugars) were summed to only one value in the transformed data set. The original

nontransformed data set was retained. The general concept of this data transformation is to normalize data to the 'total metabolite content', but disregarding unknowns that might potentially comprise artifact peaks or chemical contaminants.

Metabolic pathways analysis

Pathway enrichment analyses were performed for metabolites enriched in the invasive tumor front via MetaboAnalyst (version 5.0, www.metaboanalyst.ca)) to depict the most relevant metabolic pathways involving the identified features of the untargeted metabolomics dataset. The summary plot of the metabolite set enrichment analysis was implemented using hypergeometric testing to evaluate whether a particular metabolite set was represented more than expected by chance within the provided compound list. One-tailored p-values were provided after adjusting for multiple testing (Holm-Bonferroni method). The pathway analysis algorithm offers two parameters to determine relevant pathways within the comparison groups: statistical p-values derived from the quantitative enrichment analysis, and the pathway impact value calculated by the topological analysis with the relative-betweenness centrality.

Lipidomics

Extraction of tumor tissue was carried out using a biphasic solvent system of cold methanol, methyl tertbutyl ether (MTBE), and water with some modifications. In more detail, 300 µL of cold methanol containing a mixture of odd chain and deuterated lipid internal standards [LPE(17:1), LPC(17:0), PC(12:0/13:0), PE(17:0/17:0), PG(17:0/17:0), d7-cholesterol, SM(d18:1/17:0), Cer(d18:1/17:0), sphingosine (d17:1),

DG(12:0/12:0/0:0), DG(18:1/2:0/0:0), and d5-TG- (17:0/17:1/17:0)] was added to tissue aliquots in a 2 mL Eppendorf tube and then vortexed (10 s). Then, 1000 μL of cold MTBE containing CE 22:1 (internal standard) was added, followed by vortexing (10 s) and shaking (6 min) at 4 °C. Phase separation was induced by adding 250 μL of LC−MSgrade water followed by centrifugation at 14000 rpm for 2 min. The concentration of internal standards has been previously reported. . Ten aliquots (each 100 μL) of the upper organic phase were collected and evaporated. The volumes of tissue samples and extraction solvents were used to ensure an aliquot for each platform and one backup. For a single platform, the method can be scaled as reported previously (2, 3). Dried lipid extracts were resuspended using a methanol/toluene (9:1, v/v) mixture containing an internal standard CUDA (150 ng/mL), vortexed for (10 s), and centrifuged at 14000 rpm for 2 min prior to LC− MS analysis.

RNA isolation from flash-frozen tumor samples

Brain tumor samples were homogenized in liquid nitrogen with mortar and pestle. Following homogenization, RNA was isolated from tissue using RNAeasy Mini Kit (Qiagen) following manufacturer's instructions. Following extraction, RNA quantity and quality were assessed using NanoDrop™ 2000/2000c Spectrophotometers.

Oxidative Phosphorylation Gene Expression Figure Production

Pathway level expression changes for genes in the oxidative phosphorylation pathway (Kyoto Encyclopedia of Genes and Genomes entry HSA 00190) were visualized in R studio version 4.2.0 using the *pathview* function from the pathview package version

1.36.1. Briefly, the log2 fold change was computed for tumor invasive cells relative to tumor core cells averaged across bulk RNA-sequencing of three samples of invasive and core cells each. Differentially expressed genes were sorted in descending order by log2 fold change, and gene names were converted to ENTREZ IDs using utilities from the clusterProfiler package version 4.4.4. Upper and lower limits of the color scale were manually set to 2 and -2, respectively.

Online data set analysis

RNA-seq data for specific tumor anatomic structures in GBM, identified by H&E staining, was based on the Ivy Glioblastoma Atlas Project (Ivy GAP) (https://glioblastoma.alleninstitute.org/ and http://gliovis.bioinfo.cnio.es accessed on 27 Apr 2022).

Cryosectioning

Prior to immunostaining, invasion devices were carefully disassembled. Hydrogels were fixed for 20 minutes in 4% PFA and rinsed twice with 1X PBS. Samples were cryoprotected by incubating hydrogels in 10% sucrose for 1 hour at room temperature followed by an overnight incubation in 30% sucrose at 4˚C. Samples were equilibrated for 1 hour at room temperature in Tissue-Tek O.C.T. (Sakura Finetek #4583) and then frozen at -80˚C. Cryostar NX70 (Thermo Scientific) was used to make 10 μm-thick sections, which were immediately adhered to superfrost plus microscope slides (Thermo Fisher Sci, 22-037-246) and stored at -80˚C until use. Samples were thawed for 10 min at room temperature, rinsed twice with 1X PBS, permeabilized with 0.05% Triton X-100

(Millipore Sigma, 9002-93-1) and blocked for 1 hour at room temperature with 5% Goat Serum in PBS prior to primary and secondary antibody incubation.

Mitochondrial Membrane Potential Quantification

Three-day sphere invasion assays were performed and afterwards samples were rinsed twice with 1 X PBS and incubated with JC-1 (MedChemExpress, CBIC2, 10 μM) for 30 minutes at room temperature. Fluorescence imaging was performed on a Zeiss LSM 710 Laser Scanning Confocal Microscope and JC-1 ratio was calculated by dividing the intensity of aggregate JC-1 (Ex=585nm, Em=590nm) by the intensity of monomeric JC-1 (Ex=514nm, Em=529nm).

Generating CRISPRi knockdown cells

A lentiviral plasmid containing a dCAS9/KRAB/MeCP2 cassette was obtained from Addgene. Lenti-X 293T cells were transfected using this plasmid and virus containing the plasmid was generated and appropriate titers were determined. GBM43 cells were transduced with the virus and selected using 5 μg/ml blasticidin to obtain pure dCAS9/KRAB/MeCP2^{+} cells. GBM43 cells expressing dCAS9/KRAB/MeCP2 were transfected with the plasmid containing sgRNAs (**Supplementary Table 16**) and a puromycin resistance gene, followed by selection using 5 μg/ml Blasticidin and 5 μg/ml Puromycin. Gene knockdown was confirmed using qPCR and Western blot.

qPCR

cDNA was created using qScript XLT cDNA Supermix (Quanta Bio), following the manufacturer's protocol. cDNA was diluted to a constant concentration for all samples to ensure similar nucleic acid loading. Quantitative PCR was carried out using Power Syber Green Master Mix (Applied Biosystems), primer sequences in **Supplementary Table 17**, and an Applied Biosystems StepOne Real-Time PCR cycler following Applied Biosystems Syber guidelines: 95°C for 10 minutes, followed by 40 cycles of 95°C/15 sec and 60°C/1 min. Ct values were calculated using StepOne software (Applied Biosystems).

Western Blots

Cell preparations were harvested in 1× radio immunoprecipitation buffer (10× RIPA; 9806, Cell Signaling) and one tablet each of PhoStop and Complete Mini (04906845001 and 04693124001, Roche). Insoluble materials were removed by centrifugation at 300×g for 20 min at 4°C. Protein concentration was determined using the bicinchronic acid (BCA) assay (23225, Thermo Scientific). Samples were prepared with 30 μg of protein in RIPA buffer with 4× LDS loading buffer (LP0001, Life Technologies). Samples were electrophoresed on SDS/PAGE gels, transferred to PVDF membranes, and probed with anti-CTH (Cell Signaling #30068, 1:1000) and anti-GAPDH (Cell Signaling #5174, 1:50000) overnight at 4°C. Membranes were detected using anti-rabbit HRP-conjugated secondary antibodies (Cell Signaling #5127, 1:8000).

Viability Assays

Small molecule inhibitors were reconstituted and handled per manufacturer recommendations. For 2D cell viability assays, cells were seeded at 20k cells/ml in 48

well plates and incubated in a humidified 37˚C chamber for 4-6 hours before replacing maintenance media with media containing pharmacologic inhibitors. After a 48-hour incubation, media and non-adherent cells were removed and rinsed with 1X PBS. Adherent cells were fixed with 4% paraformaldehyde (PFA, Sigma-Aldrich) in 1X PBS for 15 min at room temperature and rinsed twice with 1X PBS. Cell nuclei were visualized using 4′,6-diamidino-2-phenylindole dihydrochloride (DAPI, Sigma-Aldrich, 1 μg/ml). Fluorescence imaging was performed on a Nikon Eclipse Ti-E epifluorescence microscope. Nuclei were counted using ImageJ and counts were normalized to control wells. Viability was confirmed for working concentrations using LIVE/DEAD Cell Imaging Kit (ThermoFisher) with the highest dose that didn't affect cell viability (**Supplementary Table 18)** after a 48-hour incubation used for invasion assays. For 3D cell viability assays, spheroids were encapsulated in HA-RGD hydrogels and viability was assessed at the end of the invasion assay (~day 3). Samples were incubated with components from the LIVE/DEAD Cell Imaging Kit (ThermoFisher, R37601) for 45 minutes at room temperature with gentle agitation. Hoechst (Thermo, H1399, 5 μg/ml) was added the last 10 minutes to visualize nuclei. Fluorescence imaging was performed on a Zeiss LSM 710 Laser Scanning Confocal Microscope and viability was quantified from a z-stack using the equation Viability (%)=100*[($Area_{total}$–$Area_{dead}$)/$Area_{total}$] where $Area_{total=}$$Area_{live}$+$Area_{dead}$. All inhibitors and additional cell culture reagents (**Supplementary Table 19**) were replenished during media changes every 2 days unless otherwise noted.

Immunofluorescence Staining

Fluorescence imaging was performed on a Zeiss LSM 710 Laser Scanning Confocal Microscope. Immunostaining was performed to measure indirect ROS markers in frozen sections of hydrogel devices and patient tumors. Samples were probed with mouse anti-Nitrotyrosine (R&D, MAB3248, 1:20) or mouse anti-Malondialdehyde (Thermo Fisher, MA527559, 1:100) and later probed with goat anti-mouse Alexa Fluor 488 (Thermo Fisher, A-11001, 1:200). Nuclei were visualized using 4′,6-diamidino-2-phenylindole dihydrochloride (DAPI, Sigma-Aldrich, 1 μg/ml) and Mean Fluorescence Intensity was quantified using ImageJ and normalized to cell number.

Organoid Invasion Quantification

Organoid cultures were established as described (4). To quantify invasion of RFP^+ GBM cells into GFP^+ organoids, images were obtained with a Zeiss Cell Observer Spinning Disk confocal microscope (Carl Zeiss AG). In ImageJ, projections were generated from Z stack images. The GFP channel was duplicated and thressholded with the Li method, with the particle/composite ROI analyzed. The RFP channel was duplicated with the ROI from the GFP channel analyzed and measured for red fluorescence.

Form Factor Quantification

For 2D experiments, 15,000 cells/well were seeded in 2-chamber slides (Millipore Sigma), incubated in regular media overnight at 37°C, stained with CytoTracker Green (Thermo Fisher) supplemented media for 30 minutes, fixed using 4% paraformaldehyde in PBS, and mounted in DAPI. Cells were imaged at 20x on a Zeiss Spinning Disc

Confocal Microscope using ZEN Blue software. Individual cell form factor was quantified using the shape descriptor plugin on ImageJ.

For 3D experiments, 3-day neurosphere invasion assays were performed and afterwards samples were rinsed twice with 1X PBS and fixed 4% PFA (20 minutes). Samples were incubated with Wheat Germ Agglutinin (WGA, Invitrogen, W11262, 20 μg/ml) and DAPI (Sigma-Aldrich, 1 μg/ml) for 30 minutes at room temperature. Fluorescence imaging was performed on a Zeiss LSM 710 Laser Scanning Confocal Microscope and form factor was quantified with ImageJ. "Edge" cells were defined as cells at the spheroid edge directly in contact with one or fewer cells and "core" cells were defined as cells directly in contact with two or more cells.

RNA-sequencing

Invasion devices were disassembled, and invasive cells were separated from non-invasive "core" cells using microdissection. Samples were treated with 10k U/ml Hyaluronidase from bovine testes, Type IV-S (Sigma-Aldrich, H3884) for 15 min with agitation followed by 800 U/ml Proteinase K solution (Thermo Fisher, AM2548) until the hydrogel was fully degraded. Afterwards, RNA was extracted using RNeasy Mini Kit (Qiagen, 74104). Invasive and Core RNA libraries were prepared and Illumina HiSeq NGS was performed (UC Davis Core) per standard protocols. RNA-Seq raw files were processed via FASTQC for quality control followed by adaptor trimming using "cutadapt". Files were aligned by STAR alignment. Read counts were generated using the FeatureCounts. Differential gene expression analysis was carried out using the DESeq2 package in R. To quantify what percent of the top 250 genes enriched in the

invasive fraction had roles in metabolism or managing oxidative stress, we highlighted genes with the keywords “metabolism” or “oxidative stress” in their DAVID descriptions (https://david.ncifcrf.gov).

Murine Studies

75,000 luciferase- and mCherry-expressing GBM43/dCas9 or GBM43/CTHkd cells were implanted intracranially into the right frontal lobes of athymic mice (6-8 weeks, female) stereotactically. Mice for invasion fractal analysis were euthanized when BLI suggested endpoint was imminent (2-4 weeks), while separate tumors were taken to endpoint for Kaplan-Meier analysis. Intracranial PDX fractal analysis was performed on 20X images of the tumor edge using the open-source imaging processing software Fiji (https://fiji.sc/). Images were converted to grayscale using a built-in ImageJ function.

Fractal analysis was performed using the Frac-Lac plug-in which utilizes a box-counting algorithm allowing for quantification of the morphological complexity of the tumor border. Four representative images were taken from each quadrant of the tumor edge to obtain an average value of the fractal dimension for each coronal section, with 3 sections imaged per tumor.

REFERENCES

1. Fiehn O, Wohlgemuth G, Scholz M, Kind T, Lee DY, Lu Y, et al. Quality control for plant metabolomics: reporting MSI-compliant studies. *Plant J.* 2008;53(4):691-704.
2. Cajka T, Smilowitz JT, and Fiehn O. Validating Quantitative Untargeted Lipidomics Across Nine Liquid Chromatography-High-Resolution Mass Spectrometry Platforms. *Anal Chem.* 2017;89(22):12360-8.
3. Contrepois K, Mahmoudi S, Ubhi BK, Papsdorf K, Hornburg D, Brunet A, et al. Cross-Platform Comparison of Untargeted and Targeted Lipidomics Approaches on Aging Mouse Plasma. *Scientific Reports.* 2018;8(1):17747.
4. Magill ST, Vasudevan HN, Seo K, Villanueva-Meyer JE, Choudhury A, John Liu S, et al. Multiplatform genomic profiling and magnetic resonance imaging identify mechanisms underlying intratumor heterogeneity in meningioma. *Nat Commun.* 2020;11(1):4803.
