# Supplementary material for: Multiomic screening of invasive GBM cells reveals targetable transsulfuration pathway alterations
Source: J Clin Invest. 2024 Feb 1;134(3):e170397. doi: 10.1172/JCI170397 (PMC10849762; doi:10.1172/JCI170397)
Supplement: Unedited blot and gel images [file jci-134-170397-s070.pdf]

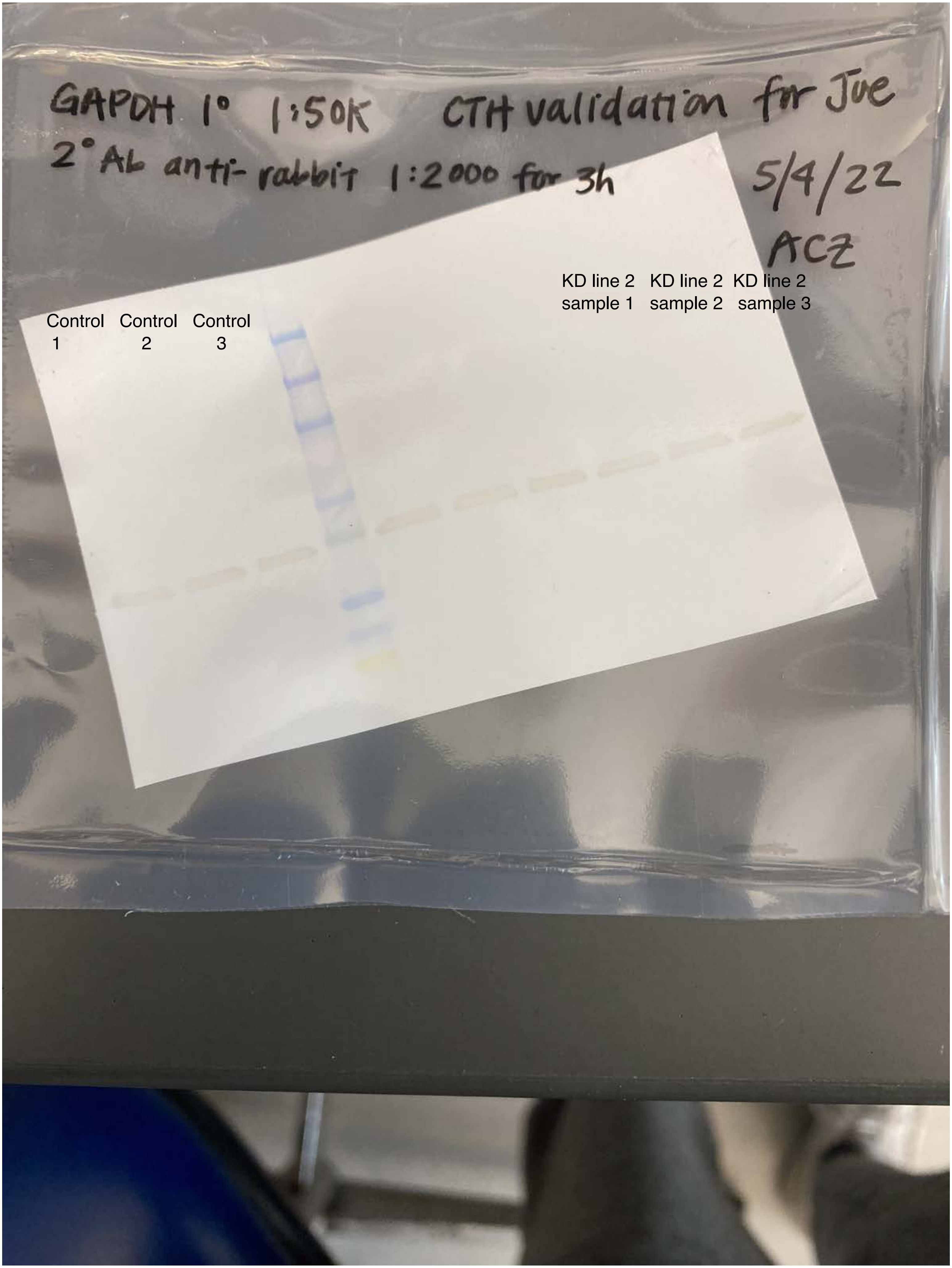
GAPDH 1° 1:50K
CTH validation for Joe
2° Ab anti-rabbit 1:2000 for 3h
5/4/22
ACZ
KD line 2 sample 1
KD line 2 sample 2
KD line 2 sample 3
Control 1
Control 2
Control 3

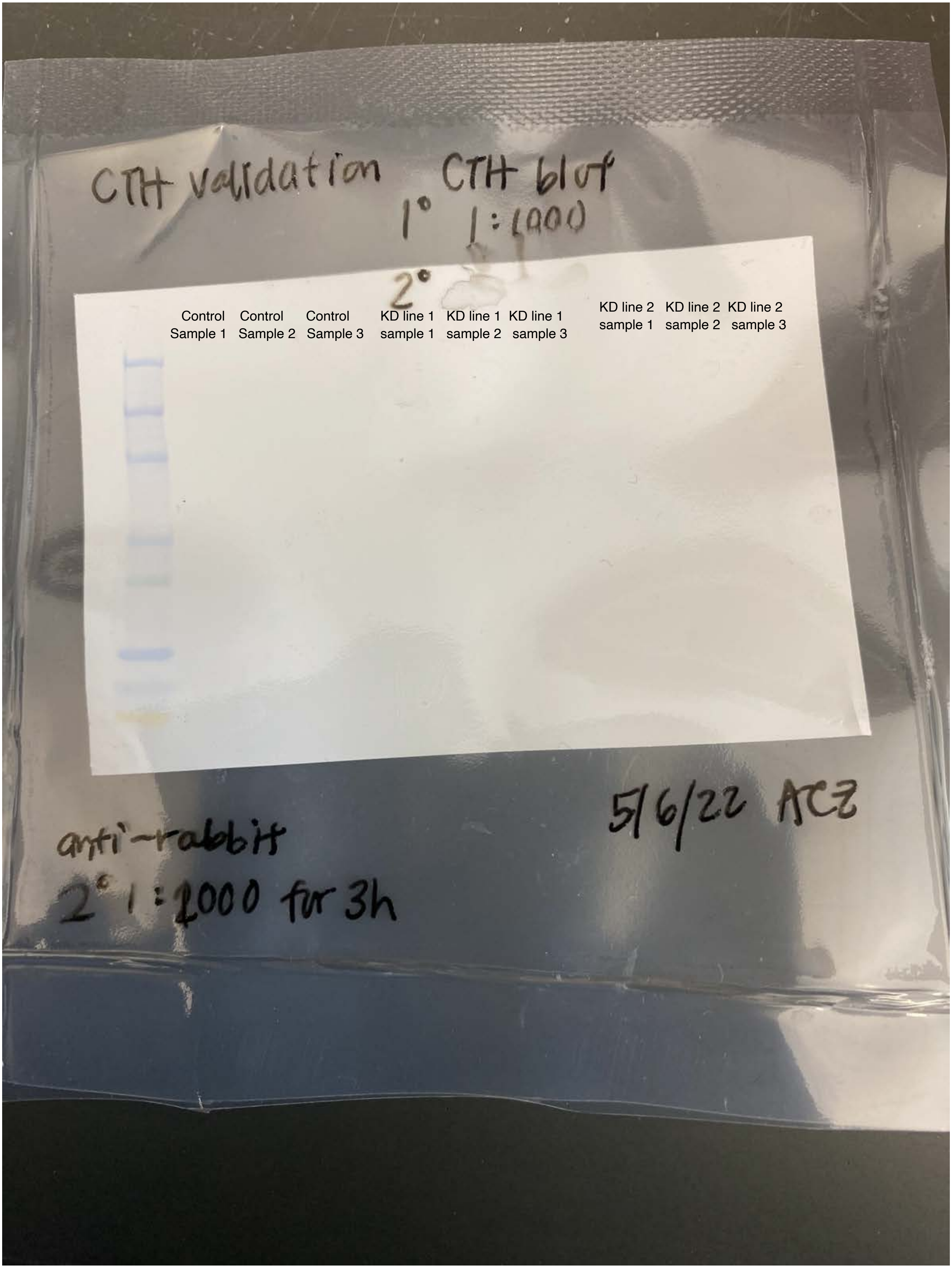
CTH validation CTH blot
1° 1:1000
2°
Control Sample 1
Control Sample 2
Control Sample 3
KD line 1 sample 1
KD line 1 sample 2
KD line 1 sample 3
KD line 2 sample 1
KD line 2 sample 2
KD line 2 sample 3
5/6/22 ACZ
anti-rabbit
2° 1:2000 for 3h

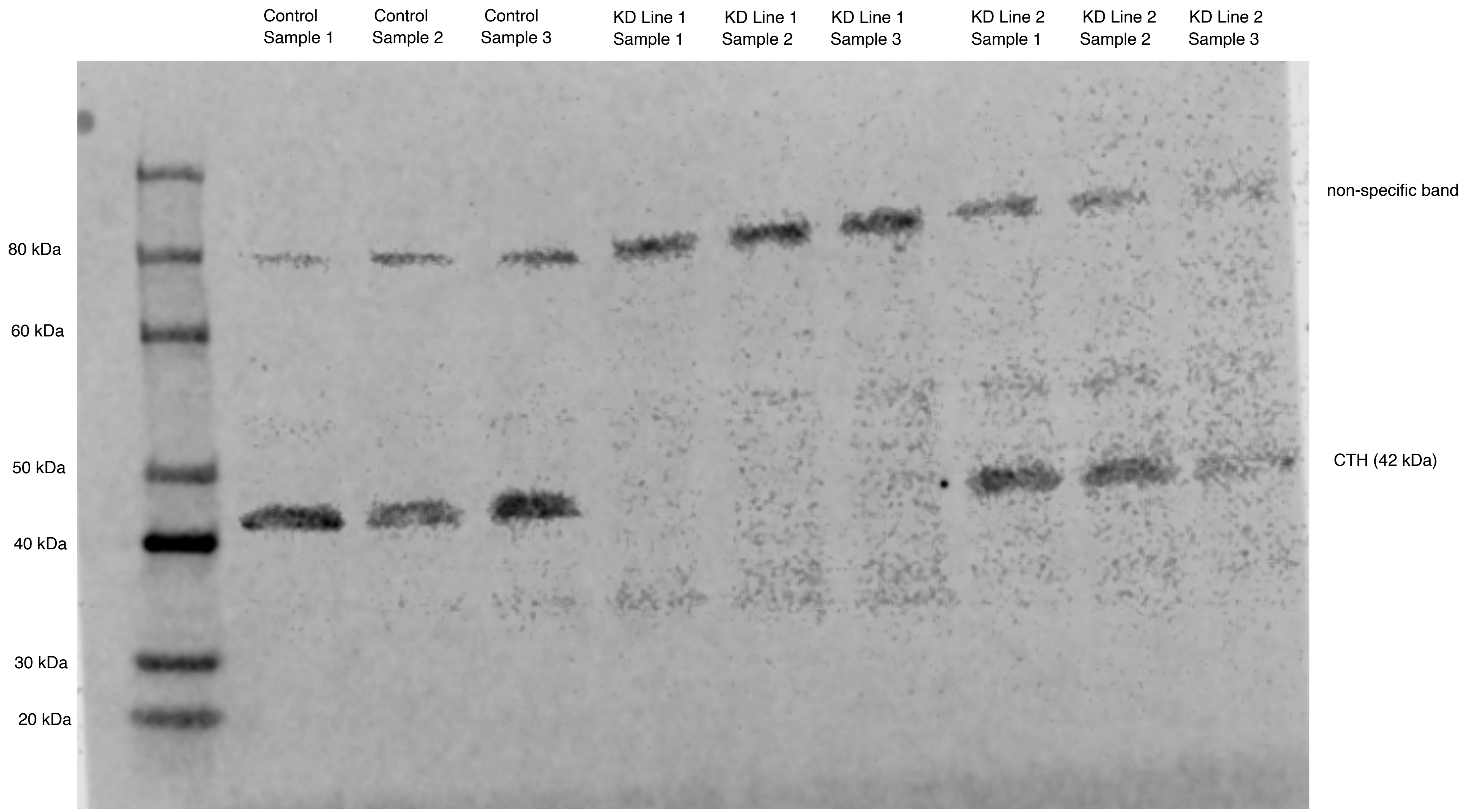
Control Sample 1
Control Sample 2
Control Sample 3
KD Line 1 Sample 1
KD Line 1 Sample 2
KD Line 1 Sample 3
KD Line 2 Sample 1
KD Line 2 Sample 2
KD Line 2 Sample 3
80 kDa
60 kDa
50 kDa
40 kDa
30 kDa
20 kDa
non-specific band
CTH (42 kDa)

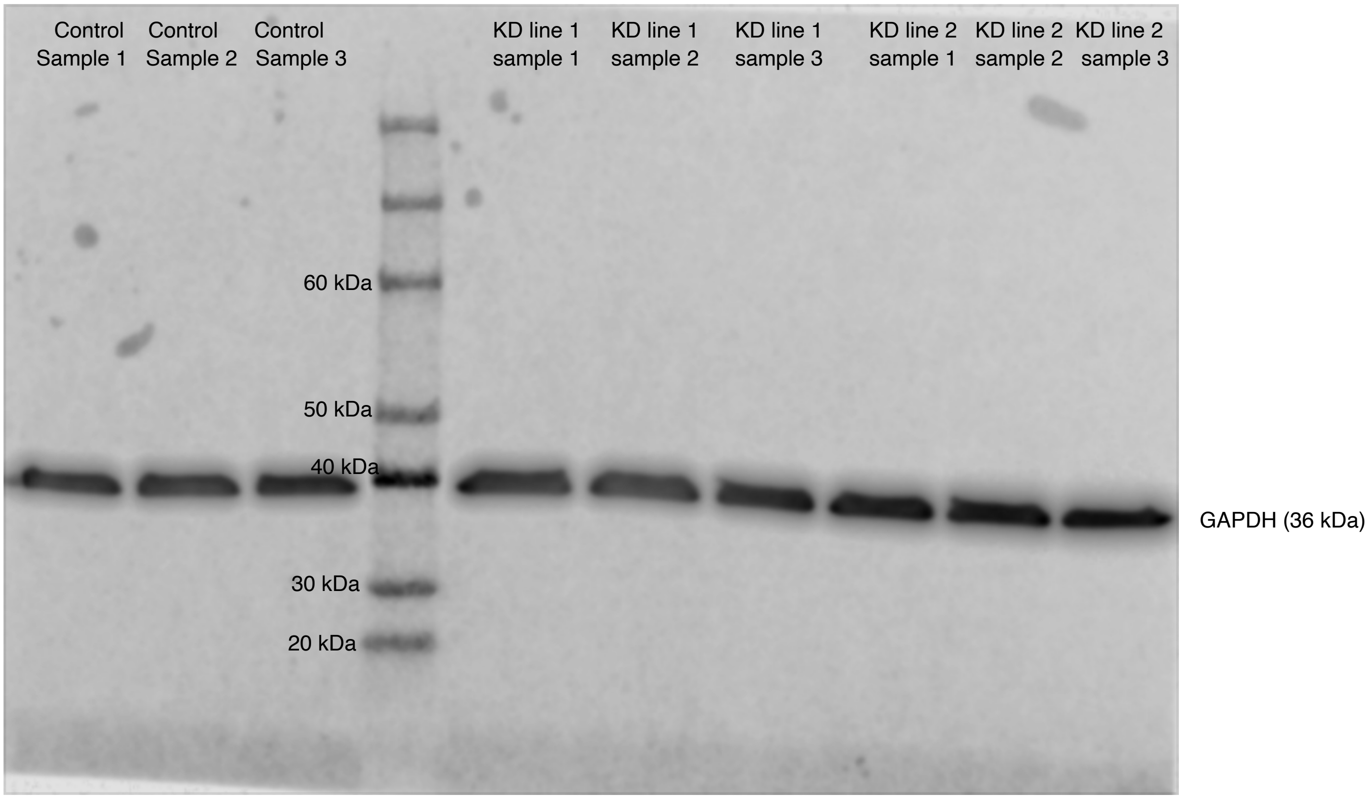
Control Sample 1
Control Sample 2
Control Sample 3
KD line 1 sample 1
KD line 1 sample 2
KD line 1 sample 3
KD line 2 sample 1
KD line 2 sample 2
KD line 2 sample 3
60 kDa
50 kDa
40 kDa
30 kDa
20 kDa
GAPDH (36 kDa)
