# Supplementary material for: Multiomic screening of invasive GBM cells reveals targetable transsulfuration pathway alterations
Source: J Clin Invest. 2024 Feb 1;134(3):e170397. doi: 10.1172/JCI170397 (PMC10849762; doi:10.1172/JCI170397)
Supplement: Supplemental table 13 [file jci-134-170397-s075.pdf]

Supplemental Table 11. Bulk RNA-seq results of edge vs. core fractions in GBM43 CTH knockdown cells invading 3D hydrogels.

gene_id	CTHKD_INV1	CTHKD_INV2	CTHKD_INV3	CTHKD_CORE1	CTHKD_CORE2	CTHKD_CORE3	CTH_KD_Inv	CTH_KD_Core	log2FoldChange	pvalue	padj	gene_name	gene_chr	gene_start	gene_end	gene_strand	gene_length	gene_biotype	gene_description	tf_family	CTHKD_INV1_count	CTHKD_INV2_count	CTHKD_INV3_count	CTHKD_CORE1_count	CTHKD_CORE2_count	CTHKD_CORE3_count	CTHKD_INV1_fpkm	CTHKD_INV2_fpkm	CTHKD_INV3_fpkm	CTHKD_CORE1_fpkm	CTHKD_CORE2_fpkm	CTHKD_CORE3_fpkm
ENSG00000131747	10741.2322	9550.377604	14883.06969	2203.040041	2268.200216	2206.753703	11724.89316	2225.997987	2.397045276	1.03E-50	1.58E-46	TOP2A	17	40388516	40417950	-	6437	protein_coding	DNA topoisomerase II alpha [Source:HGNC Symbol;Acc:HGNC:11989]	-	9284	10303	15221	2193	2482	2249	78.87626463	70.56903012	109.5373254	16.47237949	16.80261459	16.30992568
ENSG00000011426	9040.498534	7242.269263	9847.407849	1905.684887	2172.244929	2040.92828	8710.058549	2039.619365	2.094231832	3.77E-48	2.88E-44	ANLN	7	36389806	36453791	+	6012	protein_coding	anillin actin binding protein [Source:HGNC Symbol;Acc:HGNC:14082]	-	7814	7813	10071	1897	2377	2080	71.08028291	57.29712229	77.598992	15.25631248	17.22934659	16.15066462
ENSG00000072571	1162.746484	1087.313688	1631.945557	178.8149235	168.1502174	215.8674143	1294.00191	187.6108517	2.786596573	4.47E-40	2.28E-36	HMMR	5	163460203	163491945	+	3936	protein_coding	hyaluronan mediated motility receptor [Source:HGNC Symbol;Acc:HGNC:5012]	-	1005	1173	1669	178	184	220	13.96386639	13.13944073	19.64281393	2.186583814	2.037142132	2.609237592
ENSG00000170312	2010.799392	1859.463989	2359.427578	514.3440497	462.413098	548.4994754	2076.563653	508.4188743	2.030422938	2.70E-36	1.03E-32	CDK1	10	60778331	60794852	+	3931	protein_coding	cyclin dependent kinase 1 [Source:HGNC Symbol;Acc:HGNC:1722]	-	1738	2006	2413	512	506	559	24.17917291	22.49892888	28.43522925	6.297499383	5.609266457	6.638268277
ENSG00000143228	1648.670388	1440.482073	1948.752243	303.3826231	381.0795689	258.0596816	1679.301568																									

314.1739579	2.417354048	6.11E-36	1.87E-32	NUF2
	1	163266576	163355764	+	3362
protein_coding	"NUF2, NDC80 kinetochore complex component
[Source:HGNC Symbol;Acc:HGNC:14621]"	-	1425	1554	1993
	302	417	263	23.17991654	20.37920619
27.46072793	4.343206217	5.405015499	3.651775539
ENSG00000066279 3180.487647	2451.785768	4983.838527
582.6553688	498.9674931	358.1436646	3538.70398
479.9221755	2.882415714	1.52E-35	3.87E-32	ASPM
	1	197084128	197146694	-	10887
protein_coding	abnormal spindle microtubule assembly [Source:HGNC
Symbol;Acc:HGNC:19048]	-	2749	2645	5097	580	546
	365	13.80896821	10.71152761	21.68746876
2.57585248	2.185460782	1.565058867
ENSG00000112984 1314.308464	1322.759278	2191.246251
274.2498546	246.7421669	285.5337162	1609.437997
268.8419126	2.582256429	4.53E-33	9.19E-30	KIF20A
	5	138178719	138187715	+	3958
protein_coding	kinesin family member 20A [Source:HGNC
Symbol;Acc:HGNC:9787]	-	1136	1427	2241	273	270
	291	15.69629868	15.8957909	26.22820216
3.334940375	2.972669122	3.432126092
ENSG00000126787 1228.6933	1117.903076	1757.103754
222.0117871	248.5698867	266.8906213	1367.900043
245.8240983	2.47618958	4.81E-33	9.19E-30	DLGAP5
	14	55148112	55191678	-	3667
protein_coding	DLG associated protein 5 [Source:HGNC
Symbol;Acc:HGNC:16864]	-	1062	1206	1797	221	272
	272	15.83829105	14.50007858	22.70071953
2.913953252	3.232336689	3.462613596
ENSG00000164104 3198.999033	4128.640381	5450.248372
1027.683521	964.1221707	1029.29508	4259.295929
1007.033591	2.080784239	3.45E-31	5.86E-28	HMGB2
	4	173331695	173335125	-	2775
protein_coding	high mobility group box 2 [Source:HGNC
Symbol;Acc:HGNC:5000]	HMG	2765	4454	5574	1023	1055
	1049	54.49126135	70.76543542	93.04786931
17.82435662	16.56716013	17.64649822
ENSG00000118193 1168.531292	1080.82503	1486.253593
301.3734666	242.1728675	276.7027765	1245.203305
273.4163702	2.187908608	5.98E-30	9.15E-27	KIF14
	1	200551497	200620734	-	7277
protein_coding	kinesin family member 14 [Source:HGNC
Symbol;Acc:HGNC:19181]	-	1010	1166	1520	300	265
	282	7.590383372	7.064478919	9.675951188
1.993288202	1.586909269	1.809017505
ENSG00000166851 1811.801984	2178.335181	3182.733845
323.4741875	447.7913399	521.0254408	2390.957003
430.7636561	2.472585103	3.01E-29	4.19E-26	PLK1
	16	23677656	23690367	+	4760

protein_coding polo like kinase 1 [Source:HGNC Symbol;Acc:HGNC:9077]
- 1566 2350 3255 322 490 531
17.99200312 21.76681407 31.6771788 3.270770977
4.485880757 5.207551867
ENSG00000137807 1969.148772 1642.557422 2560.854052
530.4173012 491.656614 464.1149407 2057.520082
495.3962853 2.054308954 5.36E-29 6.82E-26 KIF23
15 69414246 69448427 + 6576
protein_coding kinesin family member 23 [Source:HGNC
Symbol;Acc:HGNC:6392] - 1702 1772 2619 528 538
473 14.15443257 11.88053152 18.44914099
3.882159142 3.565160358 3.357726916
ENSG00000113810 5745.471682 4838.684955 6340.04493
2022.215961 1828.633615 1885.896228 5641.400522
1912.248601 1.56079422 8.30E-29 9.76E-26 SMC4
3 160399274 160434962 + 8457
protein_coding structural maintenance of chromosomes 4 [Source:HGNC
Symbol;Acc:HGNC:14013] - 4966 5220 6484 2013 2001
1922 32.1133119 27.21373368 35.51641577
11.50876337 10.31072861 10.60921104
ENSG00000145386 1986.503197 2264.541637 3090.820793
591.6965728 568.4208438 390.5237767 2447.288543
516.8803978 2.24338173 1.73E-28 1.83E-25 CCNA2
4 121816444 121823933 - 2798
protein_coding cyclin A2 [Source:HGNC Symbol;Acc:HGNC:1578] -
1717 2443 3161 589 622 398 33.55964033
38.49547783 52.33343501 10.17814893 9.687267053
6.64020363
ENSG00000109805 2250.290459 1750.083754 2524.675511
580.6462123 623.2524364 534.7624581 2175.016575
579.5537023 1.907649958 1.80E-28 1.83E-25 NCAPG
4 17810902 17844862 + 4921
protein_coding non-SMC condensin I complex subunit G [Source:HGNC
Symbol;Acc:HGNC:24304] - 1945 1888 2582 578 682
545 21.61528436 16.91541211 24.30554304
5.679049968 6.039341771 5.169983691
ENSG00000138180 1502.893217 1354.275617 1736.569987
434.9823701 409.4092251 330.6696301 1531.246274
391.6870751 1.966870859 4.51E-28 4.31E-25 CEP55
10 93496632 93529092 + 3271
protein_coding centrosomal protein 55 [Source:HGNC
Symbol;Acc:HGNC:1161] - 1299 1461 1776 433 448
337 21.71817457 19.69262586 25.15155689
6.400421401 5.96837452 4.809449762
ENSG00000198830 13740.07686 13586.32287 17412.6342
5173.577843 5575.459112 5599.79697 14913.01131
5449.611308 1.452351544 2.04E-27 1.83E-24 HMGN2
1 26472450 26475972 + 2509
protein_coding high mobility group nucleosomal binding domain 2
[Source:HGNC Symbol;Acc:HGNC:4986] - 11876 14657 17808

5150 6101 5707 258.8595789 257.5599811
328.7887442 99.24480533 105.9641507 106.1825735
ENSG00000134057 3061.320594 2934.727312 4276.890272
879.0059443 958.6390114 1042.050882 3424.312726
959.8986125 1.834896303 2.65E-27 2.25E-24 CCNB1
5 69167010 69178245 + 2558
protein_coding cyclin B1 [Source:HGNC Symbol;Acc:HGNC:1579] -
2646 3166 4374 875 1049 1062 56.56971783
54.56879056 79.21012422 16.53898028 17.87037005
19.3807247
ENSG00000140416 12895.49484 10360.5329 14291.50165
4403.066347 3835.469906 4503.779234 12515.84313
4247.438496 1.559109297 3.63E-27 2.92E-24 TPM1
15 63042632 63071915 + 12876
protein_coding tropomyosin 1 [Source:HGNC Symbol;Acc:HGNC:12010]
- 11146 11177 14616 4383 4197 4590
47.34049378 38.27174293 52.58356446 16.45855561
14.20419412 16.64093612
ENSG00000171241 1322.407195 1110.487467 1627.056564
354.6161124 293.3490207 328.707199 1353.317076
325.557444 2.055914112 7.61E-27 5.82E-24 SHCBP1
16 46580554 46621626 - 4459
protein_coding SHC binding and spindle associated 1 [Source:HGNC
Symbol;Acc:HGNC:29547] - 1143 1198 1664 353 321
335 14.01856213 11.84549745 17.2869472
3.827704912 3.137084073 3.507142163
ENSG00000089685 2543.001763 2593.609292 4092.086372
752.4290883 608.6306784 798.7094328 3076.232476
719.9230665 2.095656073 8.50E-27 6.19E-24 BIRC5
17 78214186 78225636 + 3815
protein_coding baculoviral IAP repeat containing 5 [Source:HGNC
Symbol;Acc:HGNC:593] - 2198 2798 4185 749 666
814 31.50850943 32.33606177 50.81633926
9.49267237 7.607435601 9.96037979
ENSG00000117650 963.7490759 897.2887043 1253.53757
251.1445555 239.4312879 231.5668626 1038.191783
240.7142353 2.108874732 9.33E-27 6.48E-24 NEK2
1 211658657 211675630 - 3095
protein_coding NIMA related kinase 2 [Source:HGNC
Symbol;Acc:HGNC:7745] - 833 968 1282 250 262
236 14.7190256 13.78950453 19.18800521
3.905535339 3.688919989 3.559568751
ENSG00000138778 1384.883126 1151.273317 1784.482109
321.465031 237.6035681 157.9756986 1440.212851
239.0147659 2.591466288 1.35E-26 8.99E-24 CENPE
4 103105806 103198409 - 9444
protein_coding centromere protein E [Source:HGNC
Symbol;Acc:HGNC:1856] - 1197 1242 1825 320 260
161 6.931590291 5.79828523 8.951778883
1.638306734 1.199709139 0.795821987

ENSG00000164176 3323.950894 2544.480882 3698.03361
934.2577465 958.6390114 1016.539278 3188.821795
969.812012 1.717098861 2.71E-26 1.73E-23 EDIL3
5 83940554 84384793 - 5738
protein_coding EGF like repeats and discoidin domains 3 [Source:HGNC
Symbol;Acc:HGNC:3173] - 2873 2745 3782 930 1049
1036 27.38229184 21.09190288 30.53257527
7.836526763 7.966609724 8.428402113
ENSG00000157456 1225.222415 1082.678933 1676.924284
272.2406982 260.4500651 337.5381387 1328.27521
290.0763006 2.195386016 2.88E-26 1.76E-23 CCNB2
15 59105078 59125045 + 2801
protein_coding cyclin B2 [Source:HGNC Symbol;Acc:HGNC:1580] -
1059 1168 1715 271 285 344 20.67652563
18.38500244 28.36308532 4.677969636 4.433945482
5.733124455
ENSG00000101447 1143.078136 1065.993812 1820.660651
276.2590111 249.4837465 282.5900696 1343.244199
269.4442757 2.318114995 3.14E-26 1.85E-23 FAM83D
20 38926312 38953106 + 2475
protein_coding family with sequence similarity 83 member D
[Source:HGNC Symbol;Acc:HGNC:16122] - 988 1150 1862
275 273 288 21.83114198 20.48597296
34.85033276 5.372280832 4.806689199 5.432044516
ENSG00000169679 1744.698207 1446.04378 1998.619963
417.9045404 536.435748 438.6033372 1729.787316
464.3145419 1.896750212 7.39E-26 4.19E-23 BUB1
2 110637698 110678114 - 6032
protein_coding BUB1 mitotic checkpoint serine/threonine kinase
[Source:HGNC Symbol;Acc:HGNC:1148] - 1508 1560 2044
416 587 447 13.67208411 11.40242498
15.69719359 3.334519137 4.240678673 3.459331855
ENSG00000080986 1088.700937 1050.235643 1612.389588
271.2361199 272.3302435 182.5060866 1250.442056
242.02415 2.369084925 9.20E-26 5.02E-23 NDC80
18 2571511 2616635 + 3600
protein_coding "NDC80, kinetochore complex component [Source:HGNC
Symbol;Acc:HGNC:16909]" - 941 1133 1649 270 298
186 14.2949235 13.87590657 21.21878981
3.626289562 3.607217764 2.411884349
ENSG00000112742 1330.505927 973.2986979 1482.342399
321.465031 279.6411225 304.176811 1262.049008
301.7609882 2.064338886 1.79E-25 9.41E-23 TTK
6 80003887 80042527 + 4579
protein_coding TTK protein kinase [Source:HGNC Symbol;Acc:HGNC:12401]
- 1150 1050 1516 320 306 310
13.73478639 10.11003394 15.3366679 3.378940554
2.912120762 3.160363855
ENSG00000088325 5677.210943 5294.744917 8317.153328
1791.16297 1888.034507 2004.623306 6429.703062

1894.606928	1.762884343	2.20E-25	1.12E-22	TPX2
	20	31739271	31801805	+	3605
protein_coding	"TPX2, microtubule nucleation factor [Source:HGNC
Symbol;Acc:HGNC:1249]"	-	4907	5712	8506	1783	2066
	2043	74.43985211	69.85811901	109.3006048
23.9137283	24.97374344	26.4550833
ENSG00000138160 2199.384146	1887.272523	2951.973418
576.6278994	701.8443859	555.367984	2346.210029
611.2800898	1.94009167	3.53E-25	1.74E-22	KIF11
	10	92593286	92655395	+	4860
protein_coding	kinesin family member 11 [Source:HGNC
Symbol;Acc:HGNC:6388]	-	1901	2036	3019	574	768
	566	21.39146658	18.47036434	28.77592544
5.710535552	6.8862621	5.436585191
ENSG00000137801 1092.171822	1835.363259	2315.426649
353.6115342	288.7797213	338.5193542	1747.65391
326.9702032	2.419046661	3.73E-25	1.78E-22	THBS1
	15	39581079	39599466	+	9158
protein_coding	thrombospondin 1 [Source:HGNC Symbol;Acc:HGNC:11785]
	-	944	1980	2368	352	316	345
5.637234068	9.532317714	11.9779781	1.858417305
1.503644054	1.758589564
ENSG00000137812 1283.070498	974.225649	1812.838264
287.3093715	286.0381416	248.2475264	1356.71147
273.8650132	2.308440825	4.30E-25	1.99E-22	KNL1
	15	40594020	40664342	+	10250
protein_coding	kinetochore scaffold 1 [Source:HGNC
Symbol;Acc:HGNC:24054]	-	1109	1051	1854	286	313
	253	5.91701123	4.520774125	8.378925331
1.349097645	1.330696669	1.152239321
ENSG00000087586 1765.523517	1962.355565	3077.131616
516.3532061	514.503111	582.8420186	2268.336899
537.8994452	2.076526476	1.06E-24	4.76E-22	AURKA
	20	56369389	56392337	-	2928
protein_coding	aurora kinase A [Source:HGNC Symbol;Acc:HGNC:11393]
	-	1526	2117	3147	514	563	594
28.50218627	31.87746251	49.78839499	8.487763364
8.37907196	9.470249227
ENSG00000117724 3585.424233	2869.840732	5004.372294
1102.02231	919.3430367	1021.445356	3819.879086
1014.270234	1.913229481	2.30E-24	1.00E-21	CENPF
	1	214603195	214664588	+	11690
protein_coding	centromere protein F [Source:HGNC
Symbol;Acc:HGNC:1857]	-	3099	3096	5118	1097	1006
	1041	14.4977891	11.67670743	20.28094682
4.537256515	3.750093252	4.157021342
ENSG00000138182 1789.819712	1391.353662	1851.9502
501.2845328	508.1060918	388.5613457	1677.707858
465.9839901	1.847699076	2.64E-24	1.12E-21	KIF20B
	10	89701610	89774939	+	6851

protein_coding kinesin family member 20B [Source:HGNC
Symbol;Acc:HGNC:7212] - 1547 1501 1894 499 556
396 12.34897919 9.65963425 12.80644129
3.521663001 3.5365469 2.698281491
ENSG00000166803 1004.242735 723.948841 1004.198973
229.0438346 168.1502174 207.0364746 910.7968496
201.4101756 2.177481188 7.17E-24 2.96E-21 PCLAF
15 64364311 64387687 - 3734
protein_coding PCNA clamp associated factor [Source:HGNC
Symbol;Acc:HGNC:28961] - 868 781 1027 228 184
211 12.71276808 9.221693387 12.74085537
2.952308588 2.147346393 2.637874791
ENSG00000183856 1484.38183 1616.60279 2313.471053
496.2616417 378.3379893 479.814389 1804.818557
451.47134 1.999883093 8.81E-24 3.54E-21 IQGAP3
1 156525405 156572604 - 6742
protein_coding IQ motif containing GTPase activating protein 3
[Source:HGNC Symbol;Acc:HGNC:20669] - 1283 1744 2366
494 414 489 10.40716933 11.40490548
16.25655239 3.542741113 2.675901917 3.385837781
ENSG00000163453 335.518886 842.598587 1059.933483
64.29300621 65.79791117 87.32818123 746.0169854
72.47303287 3.364849826 2.39E-23 9.35E-21 IGFBP7
4 57030773 57110385 - 1930
protein_coding insulin like growth factor binding protein 7
[Source:HGNC Symbol;Acc:HGNC:5476] - 290 909 1084
64 72 89 8.217418428 20.76541104
26.01803113 1.603333554 1.625675217 2.15267632
ENSG00000139734 1690.321008 1370.960737 1994.708769
430.9640572 539.1773277 421.9226734 1685.330172
464.0213528 1.860146899 2.45E-23 9.38E-21 DIAPH3
13 59665583 60163987 - 7442
protein_coding diaphanous related formin 3 [Source:HGNC
Symbol;Acc:HGNC:15480] - 1461 1479 2040 429 590
430 10.73631544 8.762185966 12.69822323
2.787204554 3.454784351 2.697272507
ENSG00000090889 2676.052356 2155.161402 3614.920745
756.4474012 852.6312656 703.5315275 2815.378168
770.8700648 1.868564691 2.61E-23 9.72E-21 KIF4A
X 70290090 70420832 + 4533
protein_coding kinesin family member 4A [Source:HGNC
Symbol;Acc:HGNC:13339] - 2313 2325 3697 753 933
717 27.90516703 22.6136776 37.7803683
8.031755394 8.969216815 7.38379231
ENSG00000156970 1204.397104 1179.081851 1750.259165
391.7855066 348.1806133 334.5944921 1377.912707
358.1868707 1.944083158 6.65E-23 2.42E-20 BUB1B
15 40161023 40221136 + 4476
protein_coding BUB1 mitotic checkpoint serine/threonine kinase B
[Source:HGNC Symbol;Acc:HGNC:1149] - 1041 1272 1790

390 381 341 12.7190702 12.52942069
18.52530679 4.21284757 3.709313119 3.556397833
ENSG00000068489 1477.44006 1130.880392 2098.355401
407.8587581 315.2816577 307.1204576 1568.891951
343.4202911 2.192038103 1.20E-22 4.27E-20 PRR11
17 59155499 59204705 + 4080
protein_coding proline rich 11 [Source:HGNC Symbol;Acc:HGNC:25619]
- 1277 1220 2146 406 345 313
17.11691314 13.18358809 24.36531387 4.81135151
3.684830622 3.581213478
ENSG00000156802 4622.061894 4049.849534 4893.881073
1416.455293 1673.277436 1882.952582 4521.930834
1657.56177 1.447740973 2.16E-22 7.51E-20 ATAD2
8 123319850 123416350 - 7118
protein_coding "ATPase family, AAA domain containing 2 [Source:HGNC
Symbol;Acc:HGNC:30123]" - 3995 4369 5005 1410 1831
1919 30.69400169 27.06188343 32.57230959
9.577724609 11.20957013 12.58528419
ENSG00000186185 1140.764212 1261.580503 2168.756887
292.3322626 364.6300911 307.1204576 1523.700534
321.3609371 2.245197124 4.37E-22 1.49E-19 KIF18B
17 44924709 44947711 - 4655
protein_coding kinesin family member 18B [Source:HGNC
Symbol;Acc:HGNC:27102] - 986 1361 2218 291 399
313 11.58382379 12.8905782 22.0721334
3.022557143 3.735182345 3.138850911
ENSG00000177283 1729.657705 2422.123331 1652.479323
6065.643305 5112.132154 6742.91305 1934.753453
5973.562836 -1.626021683 6.41E-22 2.13E-19 FZD8
10 35638249 35642278 - 4030
protein_coding frizzled class receptor 8 [Source:HGNC
Symbol;Acc:HGNC:4046] - 1495 2613 1690 6038 5594
6872 20.28760868 28.58698288 19.42603265
72.44180769 60.48894551 79.60202951
ENSG00000163535 1507.521063 1054.870398 1379.673565
353.6115342 423.1171232 349.3127249 1314.021676
375.3471274 1.806689745 2.11E-21 6.85E-19 SGO2
2 200510008 200583782 + 6022
protein_coding shugoshin 2 [Source:HGNC Symbol;Acc:HGNC:30812] -
1303 1138 1411 352 463 356 11.83309571
8.331735395 10.85397254 2.826201541 3.350416799
2.759658119
ENSG00000146918 2103.356326 1925.27752 2681.123257
629.8705452 814.2491508 710.4000361 2236.585701
718.173244 1.638571192 4.05E-21 1.29E-18 NCAPG2
7 158631311 158704829 - 6988
protein_coding non-SMC condensin II complex subunit G2 [Source:HGNC
Symbol;Acc:HGNC:21904] - 1818 2077 2742 627 891
724 14.22773263 13.10441259 18.17678247
4.338262842 5.556270817 4.83650572

ENSG00000135476 866.5642951 897.2887043 1369.895581
255.1628684 262.2777848 251.191173 1044.58286
256.2106087 2.027767719 4.24E-21 1.32E-18 ESPL1
12 53268299 53293643 + 8890
protein_coding "extra spindle pole bodies like 1, separase
[Source:HGNC Symbol;Acc:HGNC:16856]" - 749 968 1401
254 287 256 4.607599999 4.800733018
7.300268443 1.381443643 1.406820647 1.344262985
ENSG00000123485 990.3591944 1022.427108 1393.362743
339.547439 276.8995429 320.8574749 1135.383015
312.4348189 1.862338071 5.01E-21 1.53E-18 HJURP
2 233833416 233854566 - 3821
protein_coding Holliday junction recognition protein [Source:HGNC
Symbol;Acc:HGNC:25444] - 856 1103 1425 338 303
327 12.25156137 12.72718722 17.27588413
4.277016041 3.455605755 3.994999698
ENSG00000162511 3820.287453 3923.784179 6080.92835
1469.697939 1184.362401 1495.372452 4608.333328
1383.144264 1.736548288 5.23E-21 1.57E-18 LAPTM5
1 30732469 30757820 - 3274
protein_coding lysosomal protein transmembrane 5 [Source:HGNC
Symbol;Acc:HGNC:29612] - 3302 4233 6219 1463 1296
1524 55.15604365 57.00376636 87.9922327
21.60562667 17.24983416 21.72962981
ENSG00000175063 1796.761482 1997.579709 3129.93273
539.4585052 618.683137 677.0387084 2308.091307
611.7267836 1.915881709 6.85E-21 2.01E-18 UBE2C
20 45812576 45816957 + 1286
protein_coding ubiquitin conjugating enzyme E2 C [Source:HGNC
Symbol;Acc:HGNC:15937] - 1553 2155 3201 537 677
690 66.04275776 73.88227568 115.3047401
20.189917 22.94068057 25.04691016
ENSG00000111206 1595.450151 1789.942653 3021.397106
577.6324777 489.8288943 497.4762684 2135.596637
521.6458801 2.033918969 1.07E-20 3.08E-18 FOXM1
12 2857681 2877155 - 4899 protein_coding
forkhead box M1 [Source:HGNC Symbol;Acc:HGNC:3818] Fork 1379
1931 3090 575 536 507 15.39400203
17.37836011 29.21820257 5.674944541 4.767777151
4.83110588
ENSG00000114346 3137.680065 2788.269032 3436.961433
1071.884963 1301.336465 1245.162494 3120.970176
1206.127974 1.371349167 1.42E-20 4.02E-18 ECT2
3 172750682 172821474 + 5499
protein_coding epithelial cell transforming 2 [Source:HGNC
Symbol;Acc:HGNC:3155] - 2712 3008 3515 1067 1424
1269 26.97122539 24.11726582 29.61038577
9.381708099 11.28456617 10.77268444
ENSG00000168078 1103.741439 1137.36905 1415.852107
250.1399773 384.7350084 208.9989056 1218.987532

281.2912971	2.114480034	1.45E-20	4.04E-18	PBK
	8	27809620	27838095	-	2165
protein_coding	PDZ binding kinase [Source:HGNC Symbol;Acc:HGNC:18282]
	-	954	1227	1448	249	421	213
24.09823231	24.98737416	30.98226163	5.560868982
8.47388943	4.592696413
ENSG00000197594 3635.173585	2245.075663	4343.380565
735.3512585	771.2977365	1048.91939	3407.876604
851.8561285	2.000118872	1.55E-20	4.23E-18	ENPP1
	6	131808016	131895155	+	9959
protein_coding	ectonucleotide pyrophosphatase/phosphodiesterase 1
[Source:HGNC Symbol;Acc:HGNC:3356]	-	3142	2422	4442
	732	844	1069	17.25381595	10.72240888
20.66166319	3.553829313	3.693051058	5.010810845
ENSG00000076382 1847.667796	1792.723506	2793.570075
550.5088657	609.5445382	702.5503119	2144.653792
620.8679053	1.788485321	2.82E-20	7.57E-18	SPAG5
	17	28577565	28599279	-	5472
protein_coding	sperm associated antigen 5 [Source:HGNC
Symbol;Acc:HGNC:13452]	-	1597	1934	2857	548	667
	716	15.96075901	15.58275846	24.18614251
4.84212154	5.311758762	6.108196073
ENSG00000024526 1925.184228	1359.837324	2212.757816
211.9660048	448.7051998	440.5657682	1832.593123
367.078991	2.318897634	3.40E-20	8.95E-18	DEPDC1
	1	68474152	68497221	-	5904
protein_coding	DEP domain containing 1 [Source:HGNC
Symbol;Acc:HGNC:22949]	-	1664	1467	2263	211	491
	449	15.41351488	10.95513473	17.75581703
1.727974475	3.62404633	3.550144481
ENSG00000092969 275.3568788	394.881186	433.1646984
38.17397244	24.67421669	59.85414669	367.8009211
40.9007786	3.172627192	7.26E-20	1.88E-17	TGFB2
	1	218346235	218444619	+	5151
protein_coding	transforming growth factor beta 2 [Source:HGNC
Symbol;Acc:HGNC:11768]	-	238	426	443	38	27
	61	2.526853829	3.646296723	3.983956907
0.356691913	0.228418256	0.552820731
ENSG00000198763 38766.31487	49660.48042	42059.02107
106074.419	93575.59601	93991.61583	43495.27212
97880.54362	-1.170137182	1.50E-19	3.79E-17	MT-ND2
	MT	4470	5511	+	1042	protein_coding
mitochondrially encoded NADH:ubiquinone oxidoreductase core subunit 2
[Source:HGNC Symbol;Acc:HGNC:7456]	-	33507	53574	43014
	105591	102396	95791	1758.581659	2266.837103
1912.249551	4899.597455	4282.268265	4291.439793
ENSG00000075218 857.3086017	1085.459786	1860.750386
216.988896	284.2104219	269.8342679	1267.839591
257.0111952	2.302474542	1.51E-19	3.79E-17	GTSE1
	22	46296741	46330810	+	4242

protein_coding	G2 and S-phase expressed 1 [Source:HGNC Symbol;Acc:HGNC:13698]	-	741	1171	1903	216	311	275	9.553054525	12.17082979	20.78119798	2.461978769	3.194834184	3.026272738
ENSG00000165304	1914.771573	1735.252536	2291.959487	703.2047554	776.7808958	640.7337342	1980.661199	706.9064618	1.486154228	1.75E-19	4.31E-17	MELK	9	36572862	36677683	+	3087	protein_coding	maternal embryonic leucine zipper kinase [Source:HGNC Symbol;Acc:HGNC:16870]	-	1655	1872	2344	700	850	653	29.3194677	26.73641495	35.17413561	10.96383843	11.99888508	9.874669925
ENSG00000115884	1066.718665	1085.459786	1627.056564	285.3002151	328.075696	394.4486388	1259.745005	335.9415166	1.907012386	3.85E-19	9.34E-17	SDC1	2	20200797	20225433	-	3595	protein_coding	syndecan 1 [Source:HGNC Symbol;Acc:HGNC:10658]	-	922	1171	1664	284	359	402	14.02577085	14.3612406	21.44158485	3.819624425	4.351651939	5.220032347
ENSG00000112972	1693.791893	1508.149506	1884.217548	621.8339194	683.5671883	592.6541738	1695.386316	632.6850938	1.421732786	5.67E-19	1.35E-16	HMGCS1	5	43289395	43313512	-	4702	protein_coding	3-hydroxy-3-methylglutaryl-CoA synthase 1 [Source:HGNC Symbol;Acc:HGNC:5007]	-	1464	1627	1927	619	748	604	17.02758922	15.25593698	18.98460413	6.365158766	6.932303543	5.99653468
ENSG00000010292	4131.510144	4339.05829	6325.377954	1775.089718	1833.202914	1523.827702	4931.982129	1710.706778	1.527628869	7.02E-19	1.65E-16	NCAPD2	12	6493356	6531955	+	6487	protein_coding	non-SMC condensin I complex subunit D2 [Source:HGNC Symbol;Acc:HGNC:24305]	-	3571	4681	6469	1767	2006	1553	30.10514096	31.81476365	46.19507845	13.17024543	13.47552296	11.17567063
ENSG00000114251	4903.203582	2763.241351	4437.249213	1387.322525	1195.32872	1029.29508	4034.564715	1203.982108	1.7444207	8.04E-19	1.86E-16	WNT5A	3	55465715	55490539	-	7397	protein_coding	Wnt family member 5A [Source:HGNC Symbol;Acc:HGNC:12784]	-	4238	2981	4538	1381	1308	1049	31.33286059	17.76807241	28.41916599	9.026913406	7.705675622	6.620120664
ENSG00000142945	1914.771573	2246.002614	3531.807879	632.8842799	609.5445382	826.1834674	2564.194022	689.5374285	1.895152339	8.36E-19	1.90E-16	KIF2C	1	44739818	44767767	+	3646	protein_coding	kinesin family member 2C [Source:HGNC Symbol;Acc:HGNC:6393]	-	1655	2423	3612	630	667

842	24.82424487	29.3002083	45.89163525
8.354589227	7.972008762	10.78056352
ENSG00000184661 881.6047969	748.9765218	1317.094467
158.7233591	263.1916447	209.9801212	982.5585951
210.6317083	2.220796626	8.46E-19	1.90E-16	CDCA2
8	25458997	25507920	+	3908
protein_coding	cell division cycle associated 2 [Source:HGNC
Symbol;Acc:HGNC:14623]	-	762	808	1347	158	288
214	10.66338597	9.115715526	15.96671217
1.954806383	3.211415731	2.556261353
ENSG00000144554 1317.779349	1036.331375	1456.91964
418.9091186	435.9111615	385.6176992	1270.343455
413.4793264	1.618914586	9.78E-19	2.17E-16	FANCD2
3	10026414	10101930	+	8378
protein_coding	FA complementation group D2 [Source:HGNC
Symbol;Acc:HGNC:3585]	-	1139	1118	1490	417	477
393	7.434950489	5.883495227	8.238504214
2.406561228	2.481056308	2.189768631
ENSG00000178999 1023.911083	1588.794255	2199.068638
363.6573164	379.2518491	431.7348286	1603.924659
391.547998	2.034859302	3.16E-18	6.90E-16	AURKB
17	8204733 8210600 -	2241	protein_coding	aurora
kinase B [Source:HGNC Symbol;Acc:HGNC:11390]	-	885	1714
2249	362	415	440	21.5971342	33.72119208
46.48898397	7.810303861	8.069838399	9.165514647
ENSG00000101255 2930.583925	3885.779182	2618.544158
8478.640194	8116.903431	7363.041258	3144.969088
7986.194961	-1.344251409	6.55E-18	1.41E-15	TRIB3
20	362835	397559	+	3434	protein_coding
tribbles pseudokinase 3 [Source:HGNC Symbol;Acc:HGNC:16228]	-
2533	4192	2678	8440	8882	7504	40.33941648
53.82139389	36.12540608	118.8347269	112.7117196
102.0090188
ENSG00000121621 743.9263575	623.8381178	892.729954
185.8469711	228.4649694	212.9237677	753.4981431
209.0785694	1.848847348	9.40E-18	2.00E-15	KIF18A
11	28020620	28108308	-	4668
protein_coding	kinesin family member 18A [Source:HGNC
Symbol;Acc:HGNC:29441]	-	643	673	913	185	250
217	7.533119179	6.356501808	9.060295993
1.916205567	2.333822159	2.170075892
ENSG00000163808 1064.404742	950.1249194	1543.943699
349.5932213	248.5698867	348.3315094	1186.157787
315.4982058	1.911382501	1.06E-17	2.22E-15	KIF15
3	44761717	44873376	+	5285
protein_coding	kinesin family member 15 [Source:HGNC
Symbol;Acc:HGNC:17273]	-	920	1025	1579	348	272
355	9.520013154	8.550919772	13.84011167
3.183724421	2.242758494	3.135663114
ENSG00000182481 7996.919103	7446.198514	9730.072039

3890.731454   3789.776912   3469.578077   8391.063219
3716.695481   1.174842108   1.29E-17   2.66E-15   KPNA2
17   68035519   68046842   +   3140
protein_coding karyopherin subunit alpha 2 [Source:HGNC
Symbol;Acc:HGNC:6395]   -   6912   8033   9951   3873   4147
3536   120.3840068   112.7929815   146.804556
59.63744999   57.55233985   52.56887029
ENSG00000157168 883.9187202   1264.361356   1424.652292
201.9202226   288.7797213   373.8431129   1190.977456
288.1810189   2.047312956   1.97E-17   4.02E-15   NRG1
8   31639386   32767959   +   11328
protein_coding neuregulin 1 [Source:HGNC Symbol;Acc:HGNC:7997] -
764   1364   1457   201   316   381   3.688372973
5.308784397   5.958113433   0.85791455   1.215604894
1.570065502
ENSG00000228716 2009.642431   1856.683135   2437.651451
822.7495638   880.9609218   806.559157   2101.325672
836.7565476   1.328318397   2.57E-17   5.16E-15   DHFR
5   80626228   80654983   -   4409
protein_coding dihydrofolate reductase [Source:HGNC
Symbol;Acc:HGNC:2861]   -   1737   2003   2493   819   964
822   21.5453936   20.02971678   26.19296336
8.98142028   9.527863636   8.703175768
ENSG00000210082 103074.8727   107162.9675   85848.74534
232863.241   199427.0717   198837.4377   98695.52849
210375.9168   -1.09191336   3.93E-17   7.79E-15   MT-
RNR2   MT   1671   3229   +   1559   Mt_rRNA
mitochondrially encoded 16S RNA [Source:HGNC Symbol;Acc:HGNC:7471]
-       89091   115608 87798   231802 218225 202644
3125.233214   3269.45776   2608.801068   7189.062844
6099.81951   6067.833706
ENSG00000115163 315.8505375   367.9996029   605.2572196
35.16023777   78.59194946   61.81657773   429.7024533
58.52292165   2.873936416   4.99E-17   9.78E-15   CENPA
2   26764289   26801067   +   1983
protein_coding centromere protein A [Source:HGNC
Symbol;Acc:HGNC:1851]   -   273   397   619   35   86
63   7.528954032   8.826768786   14.46006984
0.853388029   1.88988046   1.483077585
ENSG00000163430 6440.805649   5737.827562   6673.47419
2477.289896   3204.90659   2777.821136   6284.0358
2820.005874   1.155798114   1.03E-16   2.00E-14   FSTL1
3   120392293   120451253   -   8727
protein_coding follistatin like 1 [Source:HGNC Symbol;Acc:HGNC:3972]
-       5567   6190   6825   2466   3507   2831
34.88598246   31.27228613   36.22764768   13.66247288
17.51174351   15.14331406
ENSG00000171848 3370.229361   2606.586608   3233.579362
785.5801696   1312.302784   1047.938175   3070.131777
1048.607043   1.549255851   1.06E-16   2.02E-14   RRM2

2	10120698	10131419	+	5323
protein_coding	ribonucleotide reductase regulatory subunit M2
[Source:HGNC Symbol;Acc:HGNC:10452]	-	2913	2812	3307
782	1436	1068	29.92807139	23.2912506
28.77929723	7.103158463	11.75591863	9.366143823
ENSG00000120437 1272.657843	1322.759278	1469.631019
539.4585052	442.3081807	558.3116306	1355.016047
513.3594388	1.400907074	1.09E-16	2.06E-14	ACAT2
6	159760328	159779055	+	4299
protein_coding	acetyl-CoA acetyltransferase 2 [Source:HGNC
Symbol;Acc:HGNC:94]	-	1100	1427	1503	537	484
569	13.99329381	14.63492449	16.19548623
6.03959834	4.906101335	6.178611055
ENSG00000189403 10129.19947	10526.45716	12961.6958
5543.262629	4943.981937	5105.264348	11205.78414
5197.502971	1.108434067	1.49E-16	2.77E-14	HMGB1
13	30456704	30617597	-	7723
protein_coding	high mobility group box 1 [Source:HGNC
Symbol;Acc:HGNC:4983]	HMG	8755	11356	13256	5518	5410
5203	61.99616541	64.82959477	79.51131139
34.54592913	30.52599495	31.44950592
ENSG00000117399 2684.151088	3401.910687	5128.552693
1121.109296	1069.216057	1326.603382	3738.204822
1172.309578	1.673259778	1.63E-16	3.01E-14	CDC20
1	43358955	43363203	+	2038
protein_coding	cell division cycle 20 [Source:HGNC
Symbol;Acc:HGNC:1723]	-	2320	3670	5245	1116	1170
1352	62.2556136	79.39549168	119.2185317
26.47654008	25.01729097	30.96838431
ENSG00000134222 753.1820509	840.7446847	1314.161071
210.9614266	293.3490207	168.7690693	969.3626023
224.3598389	2.110613067	2.02E-16	3.68E-14	PSRC1
1	109279556	109283186	-	2855
protein_coding	proline and serine rich coiled-coil 1 [Source:HGNC
Symbol;Acc:HGNC:24472]	-	651	907	1344	210	321
172	12.47009002	14.0066776	21.80698435
3.556431094	4.899564932	2.812343537
ENSG00000181019 1055.149048	995.5455253	1277.98253
289.3185279	419.4616837	370.8994664	1109.559034
359.893226	1.623677115	2.14E-16	3.84E-14	NQO1
16	69706996	69726951	-	3254
protein_coding	NAD(P)H quinone dehydrogenase 1 [Source:HGNC
Symbol;Acc:HGNC:2874]	-	912	1074	1307	288	459
378	15.32752392	14.55193314	18.60632038
4.279333718	6.146865844	5.422758791
ENSG00000163507 2046.665204	1480.340972	2166.80129
702.2001772	632.3910352	741.7989327	1897.935822
692.1300484	1.455214082	2.44E-16	4.33E-14	CIP2A
3	108549869	108589644	-	4579
protein_coding	cell proliferation regulating inhibitor of protein

phosphatase 2A [Source:HGNC Symbol;Acc:HGNC:29302]	-	1769
	1597	2216	699	692	756	21.12768446
15.37688019	22.41824279	7.380873273	6.585580286
7.707209917
ENSG00000182628 1406.865398	1219.867701	1380.651364
526.3989883	575.7317228	501.4011304	1335.794821
534.5106139	1.320873178	2.71E-16	4.77E-14	SKA2
	17	59109951	59155269	-	3148
protein_coding	spindle and kinetochore associated complex subunit 2
[Source:HGNC Symbol;Acc:HGNC:28006]	-	1216	1316	1412
	524	630	511	21.1248466	18.43126416
20.77793718	8.048181832	8.72096259	7.577609351
ENSG00000113721 3783.26468	2847.593905	6232.487105
1364.217226	1164.257484	1219.650891	4287.781896
1249.3752	1.779112033	4.14E-16	7.19E-14	PDGFRB
	5	150113837	150155872	-	7137
protein_coding	platelet derived growth factor receptor beta
[Source:HGNC Symbol;Acc:HGNC:8804]	-	3270	3072	6374
	1358	1274	1243	25.05686705	18.97752065
41.37126669	9.199946243	7.778795047	8.130204465
ENSG00000119403 1404.551474	1499.806946	2227.424792
586.6736817	581.214882	579.898372	1710.594404
582.5956452	1.554219869	4.60E-16	7.90E-14	PHF19
	9	120855652	120894896	-	6733
protein_coding	PHD finger protein 19 [Source:HGNC
Symbol;Acc:HGNC:24566]	-	1214	1618	2278	584	636
	591	9.860632768	10.59507074	15.67283499
4.193778116	4.116300757	4.097556043
ENSG00000237649 1559.584339	1629.580106	2505.119542
565.577539	718.2938636	502.382346	1898.094662
595.4179162	1.672397118	9.07E-16	1.54E-13	KIFC1
	6	33391536	33409924	+	3345
protein_coding	kinesin family member C1 [Source:HGNC
Symbol;Acc:HGNC:6389]	-	1348	1758	2562	563	786
	512	22.03882736	23.17163648	35.48015097
8.137921369	10.23964786	7.145290245
ENSG00000134690 1362.900854	1436.774268	2295.870681
466.124295	530.0387289	593.6353893	1698.515268
529.9328044	1.680563934	1.06E-15	1.78E-13	CDCA8
	1	37692418	37709719	+	2469
protein_coding	cell division cycle associated 8 [Source:HGNC
Symbol;Acc:HGNC:14629]	-	1178	1550	2348	464	580
	605	26.09269353	27.67862847	44.05340263
9.086530886	10.23683024	11.43879615
ENSG00000112029 998.4579261	894.5078509	1080.46725
312.423827	379.2518491	386.5989147	991.1443423
359.4248636	1.462913444	1.55E-15	2.57E-13	FBXO5
	6	152970519	152983579	-	3833
protein_coding	F-box protein 5 [Source:HGNC Symbol;Acc:HGNC:13584]
	-	863	965	1105	311	415	394

12.31307966 11.09998654 13.35444718 3.923040451
4.718107971 4.798477231
ENSG00000171320 1175.473063 818.4978574 1159.668922
216.988896 370.1132504 311.0453197 1051.21328
299.3824887 1.810664075 2.49E-15 4.10E-13 ESCO2
8 27771949 27812640 + 7973
protein_coding establishment of sister chromatid cohesion N-
acetyltransferase 2 [Source:HGNC Symbol;Acc:HGNC:27230] - 1016
883 1186 216 405 317 6.968938896
4.88284454 6.89073234 1.309885105 2.213562847
1.85602377
ENSG00000051341 931.354149 888.9461441 927.9306969
360.6435817 266.8470842 346.3690784 916.0769967
324.6199148 1.497432324 2.57E-15 4.17E-13 POLQ
3 121431427 121546641 - 9400
protein_coding DNA polymerase theta [Source:HGNC
Symbol;Acc:HGNC:9186] - 805 959 949 359 292
353 4.683416045 4.498054483 4.67671403
1.846578656 1.353672467 1.753044286
ENSG00000265415 207.09614 155.7277917 265.9611692
17.07782977 15.53561792 31.39889662 209.5950336
21.3374481 3.29806785 2.63E-15 4.23E-13
AC099850.3 17 59202677 59203829 - 321
antisense "novel transcript, antisense to PRR11" -
179 168 272 17 17 32 30.49598823
23.07481391 39.25241008 2.560619836 2.307822944
4.653620691
ENSG00000117525 1138.450289 652.5736032 835.0398474
252.1491337 241.2590076 265.9094058 875.3545798
253.105849 1.789249723 2.75E-15 4.38E-13 F3
1 94529225 94541800 - 2875
protein_coding "coagulation factor III, tissue factor [Source:HGNC
Symbol;Acc:HGNC:3541]" - 984 704 854 251 264
271 18.71767758 10.79614647 13.76012812
4.22121127 4.001517086 4.400251277
ENSG00000177000 345.931541 359.6570426 294.3173233
1178.370254 899.2381194 860.5260106 333.301969
979.3781281 -1.554981226 3.47E-15 5.47E-13 MTHFR
1 11785723 11806920 - 11978
protein_coding methylenetetrahydrofolate reductase [Source:HGNC
Symbol;Acc:HGNC:7436] - 299 388 301 1173 984
877 1.365153831 1.42817483 1.164084865
4.734944794 3.57988757 3.417915491
ENSG00000035499 554.1846427 537.6316617 607.2128164
138.6317946 137.9928415 199.1867505 566.3430403
158.6037955 1.836706714 3.89E-15 6.07E-13
DEPDC1B 5 60596912 60700190 - 3806
protein_coding DEP domain containing 1B [Source:HGNC
Symbol;Acc:HGNC:24902] - 479 580 621 138 151
203 6.882741238 6.718822495 7.55831995

1.753119494	1.728887607	2.489850607
ENSG00000154734 3185.115493	3651.260544	2403.428507
7286.205844	6697.679042	8454.152916	3079.934848
7479.345934	-1.279963574	4.18E-15	6.46E-13
ADAMTS1 21	26835747	26845409	-	7063
protein_coding	ADAM metallopeptidase with thrombospondin type 1 motif
1 [Source:HGNC Symbol;Acc:HGNC:217]	-	2753	3939	2458
7253	7329	8616	21.3162948	24.58842621
16.12111693	49.65119296	45.21828775	56.9459077
ENSG00000188641 2123.024675	1847.413624	2131.600547
941.289794	884.6163613	958.6475625	2034.012949
928.1845726	1.131763179	4.42E-15	6.75E-13	DPYD
1	97077743	97995000	-	7131
protein_coding	dihydropyrimidine dehydrogenase [Source:HGNC
Symbol;Acc:HGNC:3012]	-	1835	1993	2180	937	968
977	14.07279447	12.32227283	14.16147575
6.353168456	5.915391849	6.395730614
ENSG00000164611 1360.586931	1218.94075	1717.991817
515.3486279	506.2783721	592.6541738	1432.506499
538.0937246	1.412744402	4.51E-15	6.83E-13	PTTG1
5	160421822	160428744	+	2013
protein_coding	pituitary tumor-transforming 1 [Source:HGNC
Symbol;Acc:HGNC:9690]	-	1176	1315	1757	513	554
604	31.94907285	28.80155499	40.43248577
12.32181848	11.99291036	14.00680877
ENSG00000130766 373.6986213	559.8784891	412.6309316
1287.869281	1249.246452	1202.970227	448.736014
1246.69532	-1.472431389	5.12E-15	7.67E-13	SESN2
1	28259527	28282491	+	3453
protein_coding	sestrin 2 [Source:HGNC Symbol;Acc:HGNC:20746]	-
323	604	422	1282	1367	1226	5.115648036
7.712129584	5.661328145	17.95116601	17.25164584
16.5744806
ENSG00000116962 655.9972701	746.1956684	1541.010304
236.0758822	128.8542427	210.9613367	981.0677475
191.9638205	2.354887864	8.29E-15	1.23E-12	NID1
1	235975830	236065162	-	5864
protein_coding	nidogen 1 [Source:HGNC Symbol;Acc:HGNC:7821]	-
567	805	1576	235	141	215	5.287907019
6.052515032	12.44986687	1.937649038	1.047812919
1.711553717
ENSG00000185697 1138.450289	945.4901637	1371.851178
456.0785128	396.6151868	387.5801302	1151.930543
413.4246099	1.478463712	9.55E-15	1.40E-12	MYBL1
8	66562175	66614247	-	7239
protein_coding	MYB proto-oncogene like 1 [Source:HGNC
Symbol;Acc:HGNC:7547]	MYB	984	1020	1403	454	434
395	7.433806195	6.212344814	8.978040294
3.032344175	2.612580919	2.547208849
ENSG00000167900 2543.001763	3001.467794	3875.992922

974.4408754	1320.527523	1287.354762	3140.15416
1194.10772	1.394917394	1.36E-14	1.98E-12	TK1
	17	78174075	78187233	-	1861
protein_coding	thymidine kinase 1 [Source:HGNC Symbol;Acc:HGNC:11830]
	-	2198	3238	3964	970	1445	1312
64.59159778	76.71219848	98.67104517	25.20151084
33.83608223	32.91042553
ENSG00000171604 1420.748938	1209.671239	1658.346114
581.6507906	572.9901431	441.5469838	1429.588763
532.0626392	1.425729851	1.39E-14	2.01E-12	CXXC5
	5	139647299	139683882	+	3852
protein_coding	CXXC finger protein 5 [Source:HGNC
Symbol;Acc:HGNC:26943]	-	1228	1305	1696	579	627
	450	17.43439178	14.9368215	20.39586014
7.267641593	7.093161695	5.453461747
ENSG00000142731 1011.184505	685.9438442	920.1083096
318.4512964	264.1055046	283.5712851	872.4122195
288.709362	1.595091524	1.64E-14	2.34E-12	PLK4
	4	127880861	127899195	+	4482
protein_coding	polo like kinase 4 [Source:HGNC Symbol;Acc:HGNC:11397]
	-	874	740	941	317	289	289
10.66434762	7.279370519	9.72568562	3.419704867
2.809859394	3.010038333
ENSG00000120802 4906.674467	4225.970251	5442.425984
2157.834021	2388.82972	2409.865316	4858.356901
2318.843019	1.066949112	1.73E-14	2.45E-12	TMPO
	12	98515512	98550379	+	8593
protein_coding	thymopoietin [Source:HGNC Symbol;Acc:HGNC:11875]
	-	4241	4559	5566	2148	2614	2456
26.99095017	23.39153588	30.00549848	12.08622519
13.25620981	13.34226575
ENSG00000136108 4082.917754	3158.122537	4467.560963
1591.251904	1859.704851	1640.592349	3902.867085
1697.183034	1.201140709	1.91E-14	2.69E-12	CKAP2
	13	52455429	52476628	+	4547
protein_coding	cytoskeleton associated protein 2 [Source:HGNC
Symbol;Acc:HGNC:1990]	-	3529	3407	4569	1584	2035
	1672	42.44449951	33.03551924	46.54774641
16.84346504	19.50284895	17.16553544
ENSG00000170017 4349.018939	3147.926074	3964.972578
1461.661313	1473.142122	1880.008935	3820.639197
1604.937457	1.251127703	1.95E-14	2.71E-12	ALCAM
	3	105366909	105576900	+	7179
protein_coding	activated leukocyte cell adhesion molecule
[Source:HGNC Symbol;Acc:HGNC:400]	-	3759	3396	4055
	1455	1612	1916	28.63538886	20.85632022
26.16551945	9.799417398	9.784974132	12.45883939
ENSG00000140525 2569.611882	2235.806152	2365.294369
1231.6129	1028.092362	1006.727123	2390.237467
1088.810795	1.134372596	2.02E-14	2.78E-12	FANCI

15 89243949 89317261 + 7766
protein_coding FA complementation group I [Source:HGNC
Symbol;Acc:HGNC:25568] - 2221 2412 2419 1226 1125
1026 15.64032903 13.69348137 14.4291563
7.632983094 6.312679407 6.167313289
ENSG00000161888 529.8884475 469.0372773 625.7909863
89.40746176 175.4610965 119.7082934 541.572237
128.1922839 2.076675214 2.86E-14 3.90E-12 SPC24
19 11131520 11155808 - 2839
protein_coding "SPC24, NDC80 kinetochore complex component
[Source:HGNC Symbol;Acc:HGNC:26913]" - 458 506 640
89 192 122 8.822563609 7.858127703
10.44280185 1.515743905 2.947097024 2.00604409
ENSG00000152234 8449.291118 7119.911713 8072.703724
4044.431922 4290.572124 3943.505173 7880.635518
4092.836406 0.945075576 2.90E-14 3.92E-12
ATP5F1A 18 46080248 46104334 - 10908
protein_coding ATP synthase F1 subunit alpha [Source:HGNC
Symbol;Acc:HGNC:823] - 7303 7681 8256 4026 4695
4019 36.61431252 31.04606724 35.06121895
17.84554673 18.75638182 17.19962241
ENSG00000143401 3917.472234 3397.275931 4388.359292
1854.451398 1938.2968 1617.043176 3901.035819
1803.263791 1.11311265 3.00E-14 4.02E-12 ANP32E
1 150218417 150236156 - 3605
protein_coding acidic nuclear phosphoprotein 32 family member E
[Source:HGNC Symbol;Acc:HGNC:16673] - 3386 3665 4488
1846 2121 1648 51.36607688 44.82318035
57.6700111 24.75868897 25.63858172 21.34017488
ENSG00000095752 654.8403085 1256.018796 1041.355313
233.0621475 289.6935811 311.0453197 984.0714725
277.9336828 1.824645404 3.52E-14 4.68E-12 IL11
19 55364389 55370463 - 2671
protein_coding interleukin 11 [Source:HGNC Symbol;Acc:HGNC:5966]
- 566 1355 1065 232 317 317
11.58876766 22.3665688 18.47047974 4.199671426
5.171826796 5.540276121
ENSG00000136824 2554.57138 2047.63507 2581.387819
1166.315316 958.6390114 1042.050882 2394.531423
1055.668403 1.18158336 3.69E-14 4.87E-12 SMC2
9 104094260 104141417 + 6468
protein_coding structural maintenance of chromosomes 2 [Source:HGNC
Symbol;Acc:HGNC:14011] - 2208 2209 2640 1161 1049
1062 18.6691167 15.05773545 18.9075985
8.678874833 7.067471644 7.664794956
ENSG00000198826 1721.558973 1516.492066 2323.249037
687.1315039 784.0917748 620.1282083 1853.766692
697.1171623 1.410786819 4.39E-14 5.73E-12
ARHGAP11A 15 32615144 32639949 + 6428
protein_coding Rho GTPase activating protein 11A [Source:HGNC

Symbol;Acc:HGNC:15783] -   1488   1636   2376   684   858
        632   12.65965224   11.2212539   17.12273061
5.144953454   5.816610963   4.589731141
ENSG00000168268 9789.052738   8499.21501   12451.28503
4812.934262   4307.935462   4871.735054   10246.51759
4664.201593   1.135466314   5.60E-14   7.26E-12   NT5DC2
        3   52524385   52535054   -   4719
protein_coding 5'-nucleotidase domain containing 2 [Source:HGNC
Symbol;Acc:HGNC:25717] -   8461   9169   12734   4791   4714
        4965   98.05425186   85.66549986   125.0021043
49.08823421   43.53095536   49.11513152
ENSG00000105894 695.3339671   447.717401   665.8807214
139.6363729   187.3412749   92.23425883   602.9773632
139.7373022   2.107472441   7.85E-14   1.01E-11   PTN
        7   137227341   137343865   -   1713
protein_coding pleiotrophin [Source:HGNC Symbol;Acc:HGNC:9630] -
        601   483   681   139   205   94   19.18720969
        12.43150548   18.41586848   3.923364461   5.215009403
        2.561630741
ENSG00000132205 186.2708298   275.3044888   293.3395248
41.1877071   22.84649694   47.09834493   251.6382812
37.04418299   2.768521659   8.58E-14   1.09E-11
EMILIN2 18   2847030 2915993 +   6435   protein_coding
elastin microfibril interfacer 2 [Source:HGNC Symbol;Acc:HGNC:19881]
        -       161   297   300   41   25   48
1.368270733   2.034894304   2.159609152   0.308060859
0.169297309   0.348207982
ENSG00000135451 404.9365865   531.1430037   807.6614917
131.5997471   99.61072664   160.9193452   581.2470273
130.7099396   2.154449526   9.03E-14   1.14E-11   TROAP
        12   49323236   49331731   +   3954
protein_coding trophinin associated protein [Source:HGNC
Symbol;Acc:HGNC:12327] -   350   573   826   131   109
        164   4.840899785   6.389279914   9.677113575
1.601901644   1.201291573   1.936213389
ENSG00000129195 598.1491863   899.1426066   1401.18513
270.2315417   223.89567   247.2663109   966.1589744
247.1311742   1.967956095   1.09E-13   1.36E-11   PIMREG
        17   6444415 6451469 +   2823   protein_coding PICALM
interacting mitotic regulator [Source:HGNC Symbol;Acc:HGNC:25483]
        -       517   970   1433   269   245   252
10.01554018   15.14937842   23.51460934   4.607258907
3.781932767   4.16711704
ENSG00000104341 4235.636695   4007.209782   4509.606295
2221.122449   2316.634789   2166.523867   4250.817591
2234.760368   0.927547308   1.11E-13   1.38E-11
LAPTM4B 8   97775057   97853013   +   3197
protein_coding lysosomal protein transmembrane 4 beta [Source:HGNC
Symbol;Acc:HGNC:13646] -   3661   4323   4612   2211   2535
        2208   62.62558639   59.61788394   66.82656191

33.43854122	34.55364962	32.24055198
ENSG00000182010 533.3593325	428.2514271	491.8326033
139.6363729	116.0602044	163.8629918	484.481121
139.8531897	1.792816888	1.21E-13	1.49E-11	RTKN2
10	62183035	62268707	-	9548
protein_coding	rhotekin 2 [Source:HGNC Symbol;Acc:HGNC:19364]	-
461	462	503	139	127	167	2.6404821
2.133356931	2.440383222	0.703888073	0.579628736
0.816488546
ENSG00000162063 900.1161837	723.948841	1258.426562
273.2452764	332.6449954	309.0828886	960.8305288
304.9910535	1.655134509	1.51E-13	1.85E-11	CCNF
16	2429394 2458854 +	6309	protein_coding	cyclin
F [Source:HGNC Symbol;Acc:HGNC:1591]	-	778	781	1287
272	364	315	6.743941313	5.457886053
9.449753399	2.084536928	2.514197869	2.330751982
ENSG00000169607 807.5592496	634.9615315	1127.401574
206.9431137	306.1430589	197.2243194	856.640785
236.770164	1.854189988	1.97E-13	2.39E-11	CKAP2L
2	112736607	112764677	-	5219
protein_coding	cytoskeleton associated protein 2 like [Source:HGNC
Symbol;Acc:HGNC:26877]	-	698	685	1153	206	335
201	7.314132751	5.786783475	10.2339777
1.908451555	2.797152264	1.797855555
ENSG00000168615 5340.535095	4316.811463	5725.009727
2471.262426	2214.282483	2629.657592	5127.452095
2438.400834	1.072294534	2.06E-13	2.48E-11	ADAM9
8	38996869	39105144	+	6913
protein_coding	ADAM metallopeptidase domain 9 [Source:HGNC
Symbol;Acc:HGNC:216]	-	4616	4657	5855	2460	2423
2680	36.51690452	29.70117557	39.23401902
17.20559781	15.27374216	18.0973203
ENSG00000184445 1727.343782	1543.37365	1738.525584
830.7861896	776.7808958	732.967993	1669.747672
780.1783595	1.097639046	2.17E-13	2.60E-11	KNTC1
12	122527246	122626396	+	11325
protein_coding	kinetochore associated 1 [Source:HGNC
Symbol;Acc:HGNC:17255]	-	1493	1665	1778	827	850
747	7.209685324	6.482014305	7.272705501
3.530762582	3.27068947	3.079132849
ENSG00000233922 727.728894	738.7800593	694.2368755
1658.558645	1452.123345	1517.940409	720.2486096
1542.874133	-1.09896188	2.30E-13	2.72E-11
LINC01694	21	45593654	45603056	+	6810
lincRNA long intergenic non-protein coding RNA 1694
[Source:HGNC Symbol;Acc:HGNC:52481]	-	629	797	710
1651	1589	1547	5.051242822	5.159946128
4.829628132	11.72198545	10.16799638	10.60447659
ENSG00000105011 1775.936172	1694.466685	2063.154658
765.4886052	689.0503476	924.3050193	1844.519172

792.9479907 1.21821851 2.85E-13 3.35E-11 ASF1B
19 14119509 14136956 - 2304
protein_coding anti-silencing function 1B histone chaperone
[Source:HGNC Symbol;Acc:HGNC:20996] - 1535 1828 2110
762 754 942 36.4351547 34.98063385
42.42310348 15.99092967 14.26091755 19.08597998
ENSG00000167657 1398.766666 1594.355962 1439.319269
2789.713723 3124.486921 2869.074179 1477.480632
2927.758274 -0.986398185 2.92E-13 3.41E-11 DAPK3
19 3958453 3971123 - 3549 protein_coding death
associated protein kinase 3 [Source:HGNC Symbol;Acc:HGNC:2676] -
1209 1720 1472 2777 3419 2924 18.63009263
21.36763249 19.21340186 37.83302757 41.98089558
38.46071952
ENSG00000198888 19795.61427 26047.32706 25163.64224
50338.41013 43889.03447 45882.61891 23668.86119
46703.3545 -0.980477935 3.37E-13 3.91E-11 MT-ND1
MT 3307 4262 + 956 protein_coding
mitochondrially encoded NADH:ubiquinone oxidoreductase core subunit 1
[Source:HGNC Symbol;Acc:HGNC:7455] - 17110 28100 25735
50109 48026 46761 978.7839292 1295.932512
1247.006554 2534.306048 2189.158074 2283.347092
ENSG00000117519 4435.791065 3967.350883 4764.811682
1767.053093 2379.691121 2067.4211 4389.317876
2071.388438 1.083157327 3.45E-13 3.97E-11 CNN3
1 94896949 94927278 - 2838
protein_coding calponin 3 [Source:HGNC Symbol;Acc:HGNC:2157] -
3834 4280 4873 1759 2604 2107 73.88128326
66.49137835 79.54016289 29.96778642 39.98408725
34.65757568
ENSG00000197299 885.0756819 680.3821374 839.9288395
264.2040724 285.1242818 328.707199 801.7955529
292.6785177 1.45332128 3.56E-13 4.06E-11 BLM
15 90717327 90816165 + 6674
protein_coding Bloom syndrome RecQ like helicase [Source:HGNC
Symbol;Acc:HGNC:1058] - 765 734 859 263 312
335 6.268591155 4.848906564 5.962239498
1.905332444 2.037168675 2.343174544
ENSG00000140945 2485.153679 3134.021807 3819.280614
1092.981106 1283.973128 1475.748141 3146.152033
1284.234125 1.292875736 4.11E-13 4.65E-11 CDH13
16 82626803 83800640 + 11187
protein_coding cadherin 13 [Source:HGNC Symbol;Acc:HGNC:1753] -
2148 3381 3906 1088 1405 1504 10.5006299
13.32494701 16.17413474 4.702366489 5.472947522
6.275960969
ENSG00000154839 879.2908735 633.1076292 944.55327
298.3597319 169.9779372 253.153604 818.9839243
240.4970911 1.768613124 4.33E-13 4.87E-11 SKA1
18 50374995 50394173 + 2950

protein_coding spindle and kinetochore associated complex subunit 1
[Source:HGNC Symbol;Acc:HGNC:28109]   -   760   683   966
        297   186   258   14.08919854   10.20781159
15.16902148   4.867832768   2.747574809   4.082664813
ENSG00000162745 573.8529912   557.0976356   1082.422847
220.0026306   111.490905   163.8629918   737.7911578
165.1188425   2.161294373   4.60E-13   5.14E-11
OLFML2B 1   161983192   162023854   -   3415
protein_coding olfactomedin like 2B [Source:HGNC
Symbol;Acc:HGNC:24558] -   496   601   1107   219   122
        167   7.94302046   7.759213858   15.01617665
3.100663403   1.556781709   2.282820684
ENSG00000101057 2656.384007   3131.240954   4804.901417
1278.828077   1222.744516   1473.78571   3530.842126
1325.119434   1.414138033   5.37E-13   5.95E-11   MYBL2
        20   43667019   43716496   +   2777
protein_coding MYB proto-oncogene like 2 [Source:HGNC
Symbol;Acc:HGNC:7548]   MYB   2296   3378   4914   1273   1338
        1502   45.2158518   53.63122581   81.97128216
22.16428574   20.99610961   25.24876201
ENSG00000164109 2325.492968   1800.139116   2459.163017
874.9876314   1078.354655   849.7326399   2194.9317
934.3583089   1.231631599   5.47E-13   6.02E-11   MAD2L1
        4   120055608   120067074   -   5748
protein_coding mitotic arrest deficient 2 like 1 [Source:HGNC
Symbol;Acc:HGNC:6763]   -   2010   1942   2515   871   1180
        866   19.12379197   14.89588889   20.26859691
7.3266022   8.945896012   7.033106088
ENSG00000185950 836.4832915   1140.149903   906.4191318
2675.191805   2037.907527   2105.688505   961.0174422
2272.929279   -1.241058183   6.50E-13   7.09E-11   IRS2
        13   109752698   109786568   -   8138
protein_coding insulin receptor substrate 2 [Source:HGNC
Symbol;Acc:HGNC:6126]   -   723   1230   927   2663   2230
        2146   4.858646749   6.66379125   5.276725525
15.82175654   11.94113959   12.31000043
ENSG00000118705 4353.646786   3880.217476   5108.996724
2275.369673   1775.629742   2177.317238   4447.620329
2076.105551   1.09929936   7.22E-13   7.82E-11   RPN2
        20   37178410   37241623   +   2819
protein_coding ribophorin II [Source:HGNC Symbol;Acc:HGNC:10382]
        -       3763   4186   5225   2265   1943   2219
73.00184817   65.4693616   85.8605517   38.84850825
30.03560073   36.74584698
ENSG00000035862 12381.80385   7443.417661   12964.6292
4408.089238   4833.404892   4074.988052   10929.95024
4438.827394   1.299933346   7.27E-13   7.82E-11   TIMP2
        17   78852977   78925387   -   5633
protein_coding TIMP metallopeptidase inhibitor 2 [Source:HGNC
Symbol;Acc:HGNC:11821] -   10702   8030   13259   4388   5289

4153	103.9010432	62.85064689	109.0368949
37.66414249	40.91592874	34.41661992
ENSG00000161800 2360.201818	1986.456295	2770.102913
1011.61027	1032.661661	1156.853097	2372.253675
1067.041676	1.152583452	8.47E-13	9.01E-11
RACGAP1 12	49976923	50033136	-	4487
protein_coding	Rac GTPase activating protein 1 [Source:HGNC
Symbol;Acc:HGNC:9804]	-	2040	2143	2833	1007	1130
1179	24.86387482	21.05717272	29.2477836
10.85112128	10.9744047	12.26602291
ENSG00000121152 1425.376784	1357.05647	1795.237892
721.2871634	487.0873147	440.5657682	1525.890382
549.6467488	1.47355933	8.49E-13	9.01E-11	NCAPH
2	96335787	96373845	+	3278
protein_coding	non-SMC condensin I complex subunit H [Source:HGNC
Symbol;Acc:HGNC:1112]	-	1232	1464	1836	718	533
449	20.55400564	19.69092352	25.94574727
10.59050602	7.085603685	6.394158944
ENSG00000091986 1035.4807	1082.678933	1736.569987
374.7076768	283.296562	500.4199149	1284.909873
386.1413846	1.735203286	8.72E-13	9.19E-11	CCDC80
3	112596794	112649530	-	13071
protein_coding	coiled-coil domain containing 80 [Source:HGNC
Symbol;Acc:HGNC:30649]	-	895	1168	1776	373	310
510	3.744630182	3.939743849	6.294142956
1.37975264	1.03350237	1.821408661
ENSG00000138587 358.6581195	351.3144824	431.2091015
108.494448	112.4047649	108.9149227	380.3939011
109.9380452	1.790825051	9.42E-13	9.85E-11	MNS1
15	56421544	56465137	-	2504
protein_coding	meiosis specific nuclear structural 1 [Source:HGNC
Symbol;Acc:HGNC:29636]	-	310	379	441	108	123
111	6.770520884	6.673272254	8.158432426
2.085406138	2.140569754	2.06935352
ENSG00000129116 2943.310503	2769.730009	3911.193665
1536.000101	1444.812466	1414.912779	3208.078059
1465.241782	1.130672739	9.47E-13	9.85E-11	PALLD
4	168497066	168928457	+	10114
protein_coding	"palladin, cytoskeletal associated protein
[Source:HGNC Symbol;Acc:HGNC:17068]"	-	2544	2988	4000
1529	1581	1442	13.75589558	13.02541598
18.32059178	7.309467722	6.811888308	6.655617376
ENSG00000126890 242.9619519	199.2944953	234.6716199
44.20144177	49.34843338	38.26740526	225.642689
43.93909347	2.358838433	1.03E-12	1.06E-10	CTAG2
X	154651972	154653579	-	993
protein_coding	cancer/testis antigen 2 [Source:HGNC
Symbol;Acc:HGNC:2492]	-	210	215	240	44	54
39	11.56550922	9.546038232	11.1960402
2.142420151	2.369753149	1.833417593

ENSG00000204054 431.546705 424.5436225 494.7659986
1086.953636 986.0548077 1045.975744 450.285442
1039.661396 -1.206875408 1.05E-12 1.08E-10
LINC00963 9 129483451 129513686 + 13171
processed_transcript long intergenic non-protein coding RNA
963 [Source:HGNC Symbol;Acc:HGNC:48716] - 373 458 506
1082 1079 1066 1.548762394 1.533136016
1.779648459 3.97200446 3.569943088 3.77819606
ENSG00000111665 470.883402 524.6543457 753.8825789
180.82408 93.2137075 162.8817762 583.1401089
145.6398546 2.003508781 1.67E-12 1.71E-10 CDCA3
12 6844793 6852066 - 5653 protein_coding cell
division cycle associated 3 [Source:HGNC Symbol;Acc:HGNC:14624] -
407 566 771 180 102 166 3.937405436
4.41439716 6.317974204 1.539553325 0.786284626
1.370803291
ENSG00000168003 7819.903966 10119.5256 7741.230061
18496.29422 15754.94429 16489.3268 8560.219877
16913.52177 -0.982345487 2.27E-12 2.29E-10 SLC3A2
11 62856102 62888875 + 5178
protein_coding solute carrier family 3 member 2 [Source:HGNC
Symbol;Acc:HGNC:11026] - 6759 10917 7917 18412 17240
16805 71.3863395 92.95552802 70.82736005
171.9254369 145.0887747 151.5034444
ENSG00000167325 4786.350452 4032.237463 4984.816326
1832.350677 2503.062204 2229.32166 4601.134747
2188.244847 1.071956656 2.47E-12 2.49E-10 RRM1
11 4094707 4138876 + 4177 protein_coding
ribonucleotide reductase catalytic subunit M1 [Source:HGNC
Symbol;Acc:HGNC:10451] - 4137 4350 5098 1824 2739
2272 54.1646272 45.91543886 56.53765764
21.11356527 28.57499445 25.39158946
ENSG00000151503 3815.659607 3710.585417 4361.958734
2240.209435 1872.498889 1979.111703 3962.734586
2030.606676 0.964747222 2.55E-12 2.54E-10 NCAPD3
11 134150119 134225454 - 9114
protein_coding non-SMC condensin II complex subunit D3 [Source:HGNC
Symbol;Acc:HGNC:28952] - 3298 4003 4461 2230 2049
2017 19.78956918 19.36468888 22.67387013
11.83033534 9.796964445 10.33101304
ENSG00000092853 1682.222276 1547.081454 1764.926141
798.6396865 834.3540681 827.1646829 1664.743291
820.0528125 1.021338843 2.66E-12 2.64E-10 CLSPN
1 35720218 35769967 - 5411
protein_coding claspin [Source:HGNC Symbol;Acc:HGNC:19715] -
1454 1669 1805 795 913 843 14.69540587
13.59918121 15.45262012 7.103801396 7.352784564
7.272706754
ENSG00000119630 1211.338874 999.2533298 1258.426562
417.9045404 487.0873147 540.6497512 1156.339589

481.8805354	1.262381649	3.12E-12	3.08E-10	PGF
	14	74941834	74955784	-	5962
protein_coding	placental growth factor [Source:HGNC
Symbol;Acc:HGNC:8893]	-	1047	1078	1287	416	533
	551	9.603939658	7.971879898	9.999747433
3.373669815	3.89577472	4.314253529
ENSG00000090530 474.354287	467.183375	585.7012513
155.7096244	119.7156439	187.4121642	509.0796378
154.2791442	1.723563918	3.33E-12	3.26E-10	P3H2
	3	189956728	190122437	-	5605
protein_coding	prolyl 3-hydroxylase 2 [Source:HGNC
Symbol;Acc:HGNC:19317]	-	410	504	599	155	131
	191	4.000395707	3.964504156	4.950552395
1.337079708	1.018484154	1.590756792
ENSG00000119326 1913.614612	1787.1618	1732.658793
538.453927	933.0509348	649.5646739	1811.145068
707.0231786	1.356306148	3.88E-12	3.78E-10
CTNNAL1 9	108942569	109013529	-	3131
protein_coding	catenin alpha like 1 [Source:HGNC
Symbol;Acc:HGNC:2512]	-	1654	1928	1772	536	1021
	662	28.88997396	27.1492552	26.21700674
8.27719027	14.2102354	9.870086474
ENSG00000109674 348.2454644	298.4782674	419.4755205
81.37083598	90.47212786	109.8961382	355.3997508
93.91303401	1.919871268	4.18E-12	4.04E-10	NEIL3
	4	177309836	177362943	+	2959
protein_coding	nei like DNA glycosylase 3 [Source:HGNC
Symbol;Acc:HGNC:24573]	-	301	322	429	81	99
	112	5.563092013	4.797830097	6.716063332
1.323552797	1.457970804	1.766928978
ENSG00000162413 4552.644194	6126.22009	4145.865285
12052.92951	10603.51616	9404.950754	4941.576523
10687.13214	-1.112686095	4.43E-12	4.26E-10	KLHL21
	1	6590724 6614607 -	6901	protein_coding	kelch
like family member 21 [Source:HGNC Symbol;Acc:HGNC:29041]	-
	3935	6609	4240	11998	11603	9585	31.18368408
	42.22383565	28.46140171	84.0616764	73.26842612
	64.83747979
ENSG00000259781 1306.209732	1299.585499	1672.035292
679.0948781	627.8217358	551.4431219	1425.943508
619.4532453	1.203060707	4.72E-12	4.51E-10
HMGB1P6 15	71164770	71165415	-	646
processed_pseudogene	high mobility group box 1 pseudogene 6
[Source:HGNC Symbol;Acc:HGNC:4998]	-	1129	1402	1710
	676	687	562	95.57760345	95.68623214
122.6213373	50.59590803	46.34285835	40.61157507
ENSG00000136153 2458.543561	2316.450901	3510.296314
1167.319894	867.2530237	1270.674098	2761.763592
1101.749005	1.3261256	5.06E-12	4.80E-10	LMO7
	13	75620434	75859870	+	14367

protein_coding LIM domain 7 [Source:HGNC Symbol;Acc:HGNC:6646] -
2125 2499 3590 1162 949 1295 8.088864406
7.668918499 11.57526154 3.910580702 2.878450189
4.207747906
ENSG00000175216 7531.820509 7101.37269 9203.038693
4395.029721 4031.949779 4035.739432 7945.410631
4154.239644 0.935583989 5.72E-12 5.40E-10 CKAP5
11 46743048 46846308 - 8538
protein_coding cytoskeleton associated protein 5 [Source:HGNC
Symbol;Acc:HGNC:28959] - 6510 7661 9412 4375 4412
4113 41.69841534 39.56063645 51.06557469
24.77553968 22.51842187 22.48788413
ENSG00000225630 2037.409511 1916.934959 1805.993675
4535.670672 3545.776324 3507.845482 1920.112715
3863.097493 -1.008665596 5.88E-12 5.52E-10
MTND2P28 1 629640 630683 + 1044
unprocessed_pseudogene MT-ND2 pseudogene 28 [Source:HGNC
Symbol;Acc:HGNC:42129] - 1761 2068 1847 4515 3880
3575 92.24728013 87.33412909 81.95375415
209.1021376 161.9533086 159.8532916
ENSG00000116830 2151.948717 2115.302503 2642.01132
1197.457241 1001.590426 1121.529339 2303.087514
1106.859002 1.057399598 6.98E-12 6.50E-10 TTF2
1 117060303 117107453 + 10181
protein_coding transcription termination factor 2 [Source:HGNC
Symbol;Acc:HGNC:12398] - 1860 2282 2702 1192 1096
1143 9.991190037 9.882325641 12.2941176
5.660920221 4.691143461 5.240851194
ENSG00000128606 756.6529359 480.160691 737.2600058
127.5814342 236.6897083 188.3933797 658.0245442
184.2215074 1.834816724 7.17E-12 6.64E-10 LRRC17
7 102912991 102944949 + 2674
protein_coding leucine rich repeat containing 17 [Source:HGNC
Symbol;Acc:HGNC:16895] - 654 518 754 127 259
192 13.3755318 8.540873997 13.06208179
2.29637883 4.220821236 3.351859929
ENSG00000129810 622.4453815 441.228743 569.0786782
208.9522702 157.1838989 141.2950348 544.2509343
169.1437346 1.68591323 7.41E-12 6.82E-10 SGO1
3 20160593 20186292 - 3828
protein_coding shugoshin 1 [Source:HGNC Symbol;Acc:HGNC:25088] -
538 476 582 208 172 144 7.686082811
5.48237807 7.042932261 2.627196896 1.958010947
1.756048874
ENSG00000113140 10905.52076 8698.509506 12687.91225
5499.061187 5417.361353 5199.461038 10763.98084
5371.961193 1.002655536 7.48E-12 6.85E-10 SPARC
5 151661096 151687165 - 4831
protein_coding secreted protein acidic and cysteine rich [Source:HGNC
Symbol;Acc:HGNC:11219] - 9426 9384 12976 5474 5928

5299	106.7050837	85.64162902	124.4246006
54.78592165	53.47240964	51.20388551
ENSG00000136205 3984.576011	4217.627691	6354.711907
2063.403668	2154.881591	2190.073039	4852.305203
2136.119433	1.183780634	7.54E-12	6.86E-10	TNS3
7	47275154	47582558	-	11797
protein_coding	tensin 3 [Source:HGNC Symbol;Acc:HGNC:21616]	-
3444	4550	6499	2054	2358	2232	15.96563793
17.00488797	25.51980863	8.418410059	8.710253988
8.832194953
ENSG00000111247 726.5719323	759.1729844	761.7049662
172.7874542	319.8509571	253.153604	749.149961
248.5973384	1.590279225	8.60E-12	7.78E-10
RAD51AP1	12	4538798 4560048 +	2558	protein_coding
RAD51 associated protein 1 [Source:HGNC Symbol;Acc:HGNC:16956]
-	628	819	779	172	350	258
13.42622177	14.11618429	14.1071529	3.251090981
5.962468559	4.708311649
ENSG00000102038 4809.489686	4078.58502	4924.192824
2192.994259	2204.230024	2663.01892	4604.089176
2353.414401	0.968097882	9.09E-12	8.18E-10
SMARCA1 X	129446501	129523500	-	4363
protein_coding	"SWI/SNF related, matrix associated, actin dependent regulator of chromatin, subfamily a, member 1 [Source:HGNC Symbol;Acc:HGNC:11097]" -	4157	4400	5036	2183	2412
2714	52.10621466	44.46327228	53.46911111
24.19188666	24.09077038	29.03826513
ENSG00000183741 1405.708436	1981.821539	1625.100968
3737.030986	3262.479762	3416.592439	1670.876981
3472.034396	-1.0545042	9.65E-12	8.63E-10	CBX6
22	38861450	38872314	-	8030
protein_coding	chromobox 6 [Source:HGNC Symbol;Acc:HGNC:1556]	-
1215	2138	1662	3720	3570	3482	8.274760743
11.73886515	9.587777125	22.39899904	19.37364192
20.24227205
ENSG00000106333 1661.396966	1579.524744	3215.978991
604.7560897	606.8029586	845.8077778	2152.300234
685.788942	1.650270057	9.78E-12	8.69E-10	PCOLCE
7	100602177	100608175	+	3532
protein_coding	procollagen C-endopeptidase enhancer [Source:HGNC Symbol;Acc:HGNC:8738]	-	1436	1704	3289	602	664
862	22.23455581	21.27075246	43.1365725
8.240944946	8.192302538	11.39285596
ENSG00000137831 2585.809345	1850.194477	2575.521028
1139.191704	969.6053299	1070.506132	2337.17495
1059.767722	1.140878355	9.93E-12	8.78E-10	UACA
15	70654554	70763593	-	11031
protein_coding	uveal autoantigen with coiled-coil domains and ankyrin repeats [Source:HGNC Symbol;Acc:HGNC:15947]	-	2235	1996
2634	1134	1061	1091	11.08044891	7.977735079

11.06122794 4.970492084 4.191399884 4.616957766
ENSG00000138119 3651.371049 3833.869919 3902.393479
1699.746352 1675.105155 2248.945971 3795.878149
1874.599159 1.0179883 1.01E-11 8.85E-10 MYOF
10 93306429 93482317 - 8473
protein_coding myoferlin [Source:HGNC Symbol;Acc:HGNC:3656] -
3156 4136 3991 1692 1833 2292 20.37016284
21.5217351 21.81960967 9.655268797 9.427224648
12.62767636
ENSG00000087074 2043.194319 3959.008323 2560.854052
6639.257469 6999.252801 7769.264483 2854.352231
7135.924918 -1.321504681 1.13E-11 9.89E-10
PPP1R15A 19 48872392 48876057 + 3093
protein_coding protein phosphatase 1 regulatory subunit 15A
[Source:HGNC Symbol;Acc:HGNC:14375] - 1766 4271 2619
6609 7659 7918 31.22521893 60.88125683
39.22455581 103.3134938 107.9072804 119.5037719
ENSG00000166949 1876.591838 1383.011102 2848.326786
832.7953461 653.4098124 643.6773808 2035.976575
709.9608464 1.520065798 1.61E-11 1.40E-09 SMAD3
15 67063763 67195195 + 9113
protein_coding SMAD family member 3 [Source:HGNC
Symbol;Acc:HGNC:6769] MH1 1622 1492 2913 829 715
656 9.733839756 7.218407753 14.80749416
4.398396499 3.419032817 3.36038088
ENSG00000122966 1077.13132 1075.263323 1667.1463
506.3074239 467.8962572 522.9878719 1273.180314
499.063851 1.35154468 2.03E-11 1.75E-09 CIT
12 119685790 119877291 - 13230
protein_coding citron rho-interacting serine/threonine kinase
[Source:HGNC Symbol;Acc:HGNC:1985] - 931 1160 1705
504 512 533 3.84843849 3.86573521
5.969899154 1.841924857 1.686431535 1.880673481
ENSG00000142039 858.4655634 982.5682093 934.7752859
2234.181966 1684.243754 1900.614461 925.2696862
1939.68006 -1.067253454 2.08E-11 1.79E-09 CCDC97
19 41310189 41324883 + 3821
protein_coding coiled-coil domain containing 97 [Source:HGNC
Symbol;Acc:HGNC:28289] - 742 1060 956 2224 1843
1937 10.61992819 12.23102308 11.58999665
28.14225939 21.01875051 23.66457008
ENSG00000158402 233.7062585 228.0299806 330.4958647
34.15565955 70.36721056 51.02320701 264.0773679
51.84869237 2.345802352 2.52E-11 2.15E-09 CDC25C
5 138285265 138338355 - 3338
protein_coding cell division cycle 25C [Source:HGNC
Symbol;Acc:HGNC:1727] - 202 246 338 34 77
52 3.309479916 3.249247046 4.690647787
0.4924859 1.005224328 0.727215366
ENSG00000050165 1137.293327 1201.328679 1849.016805

455.0739346 409.4092251 627.9779325 1395.879604
497.4870307 1.488960231 2.65E-11 2.25E-09 DKK3
11 11963106 12009769 - 5059
protein_coding dickkopf WNT signaling pathway inhibitor 3
[Source:HGNC Symbol;Acc:HGNC:2893] - 983 1296 1891
453 448 640 10.62633618 11.29468908
17.31527149 4.329470044 3.858974709 5.90556332
ENSG00000126777 5580.026162 4803.460812 5762.166066
3068.986468 2941.714945 2940.702912 5381.884347
2983.801442 0.850889594 2.73E-11 2.30E-09 KTN1
14 55559072 55701526 + 17575
protein_coding kinectin 1 [Source:HGNC Symbol;Acc:HGNC:6467] -
4823 5182 5893 3055 3219 2997 15.00778644
12.99978091 15.53257872 8.404600938 7.981494905
7.960446815
ENSG00000123219 822.5997514 547.8281242 786.1499266
277.2635893 271.4163836 225.6795695 718.8592674
258.1198474 1.476840775 2.97E-11 2.50E-09 CENPK
5 65517766 65563171 - 4312
protein_coding centromere protein K [Source:HGNC
Symbol;Acc:HGNC:29479] - 711 591 804 276 297
230 9.017487756 6.042862142 8.637334767
3.094792576 3.001485813 2.489975762
ENSG00000146670 1771.308326 1761.207168 2376.050151
956.3584674 943.1033935 849.7326399 1969.521881
916.3981669 1.103930728 3.00E-11 2.51E-09 CDCA5
11 65066300 65084164 - 3925
protein_coding cell division cycle associated 5 [Source:HGNC
Symbol;Acc:HGNC:14626] - 1531 1900 2430 952 1032
866 21.33193454 21.34262815 28.67933443
11.72731266 11.45773132 10.29969269
ENSG00000145934 372.5416596 550.6089777 890.7743571
149.6821551 83.16124884 172.6939314 604.6416648
135.1791118 2.163169769 3.51E-11 2.92E-09 TENM2
5 167284799 168264157 + 11042
protein_coding teneurin transmembrane protein 2 [Source:HGNC
Symbol;Acc:HGNC:29943] - 322 594 911 149 91
176 1.594787568 2.371770484 3.821845179
0.652438743 0.359130465 0.744065145
ENSG00000164442 1664.867851 2327.574315 1365.984387
3674.747136 4666.168534 4126.992475 1786.142184
4155.969382 -1.218088463 3.65E-11 3.01E-09 CITED2
6 139371807 139374620 - 2535
protein_coding Cbp/p300 interacting transactivator with Glu/Asp rich
carboxy-terminal domain 2 [Source:HGNC Symbol;Acc:HGNC:1987] -
1439 2511 1397 3658 5106 4206 31.04399058
43.67196236 25.52824142 69.76971581 87.77310149
77.45283887
ENSG00000165480 845.7389849 696.1403068 777.3497408
360.6435817 270.5025237 229.6044316 773.0763442

286.9168457	1.430036562	4.06E-11	3.34E-09	SKA3
	13	21153595	21176602	-	3048
protein_coding	spindle and kinetochore associated complex subunit 3
[Source:HGNC Symbol;Acc:HGNC:20262]	-	731	751	795
	359	296	234	13.11587071	10.86322894
12.08243929	5.69482919	4.231899507	3.583826121
ENSG00000204262 3056.692747	1801.066067	3962.039182
977.45461	947.6726929	1227.500615	2939.932665
1050.875973	1.484131277	4.35E-11	3.55E-09	COL5A2
	2	189031896	189225312	-	7453
protein_coding	collagen type V alpha 2 chain [Source:HGNC
Symbol;Acc:HGNC:2210]	-	2642	1943	4052	973	1037
	1251	19.38636587	11.49411763	25.18493134
6.312231752	6.063260575	7.8355994
ENSG00000113368 3946.396276	3848.701137	5338.779352
2247.241483	1869.757309	2234.227738	4377.958922
2117.07551	1.048369784	4.71E-11	3.83E-09	LMNB1
	5	126776623	126837020	+	3633
protein_coding	lamin B1 [Source:HGNC Symbol;Acc:HGNC:6637]	-
	3411	4152	5460	2237	2046	2277	51.34652231
	50.38785895	69.61930775	29.77157446	24.54137147
	29.25793485
ENSG00000139618 2345.161317	1786.234848	1977.108397
1084.94448	913.8598774	953.7414849	2036.168188
984.1819474	1.048613393	4.92E-11	3.98E-09	BRCA2
	13	32315474	32400266	+	12273
protein_coding	"BRCA2, DNA repair associated [Source:HGNC
Symbol;Acc:HGNC:1101]"	-	2027	1927	2022	1080	1000
	972	9.032286967	6.922531465	7.631903543
4.254751869	3.550649992	3.697103141
ENSG00000140961 231.3923351	382.8308212	336.3626552
874.9876314	738.398781	1020.46414	316.8619372
877.9501842	-1.467743454	6.21E-11	5.00E-09	OSGIN1
	16	83948282	83966332	+	3460
protein_coding	oxidative stress induced growth inhibitor 1
[Source:HGNC Symbol;Acc:HGNC:30093]	-	200	413	344
	871	808	1040	3.161175516	5.262691482
4.605584974	12.17147672	10.17639275	14.03147337
ENSG00000013810 1869.650068	2103.252139	2872.771747
1139.191704	692.7057871	883.0939675	2281.891318
904.9971528	1.334824561	7.16E-11	5.73E-09	TACC3
	4	1721490 1745176 +	6528	protein_coding
transforming acidic coiled-coil containing protein 3 [Source:HGNC
Symbol;Acc:HGNC:11524]	-	1616	2269	2938	1134	758
	900	13.53804407	15.32457038	20.8484658
8.399126559	5.059966687	6.435886841
ENSG00000128487 746.2402808	781.4198117	1215.403431
377.7214115	320.764817	293.3834403	914.354508
330.6232229	1.468144494	7.81E-11	6.22E-09	SPECC1
	17	20009344	20319026	+	13676

protein_coding sperm antigen with calponin homology and coiled-coil
domains 1 [Source:HGNC Symbol;Acc:HGNC:30615]   -   645   843
        1243   376   351   299   2.57926126   2.717705795
        4.210314059   1.329321317   1.118424371   1.020606035
ENSG00000166033 6846.899197   4003.501977   8315.197731
1717.82876   1124.047649   1270.674098   6388.532968
1370.850169   2.220459214   8.01E-11   6.35E-09   HTRA1
        10   122458551   122514908   +   2279
protein_coding HtrA serine peptidase 1 [Source:HGNC
Symbol;Acc:HGNC:9476]   -   5918   4319   8504   1710   1230
        1295   142.0120996   83.55507567   172.8547754
36.27880738   23.5190288   26.52598252
ENSG00000182158 1624.374193   1404.330978   2351.605191
807.6808905   633.304895   750.6298724   1793.436787
730.5385526   1.296007392   8.88E-11   6.99E-09
CREB3L2 7   137874979   138002067   -   8788
protein_coding cAMP responsive element binding protein 3 like 2
[Source:HGNC Symbol;Acc:HGNC:23720]   TF_bZIP 1404   1515   2405
        804   693   765   8.737189844   7.600751652
12.67732103   4.423512074   3.436384758   4.063660549
ENSG00000167004 5304.669283   4046.14173   6033.016228
2143.769926   2301.099171   2813.144894   5127.942414
2419.337997   1.083694919   9.82E-11   7.70E-09   PDIA3
        15   43746392   43773279   +   4240
protein_coding protein disulfide isomerase family A member 3
[Source:HGNC Symbol;Acc:HGNC:4606]   -   4585   4365   6170
        2134   2518   2867   59.13821286   45.38918131
67.40960204   24.33491171   25.87905818   31.56515107
ENSG00000106003 559.969451   832.4021245   1284.827119
261.1903377   187.3412749   313.9889662   892.3995648
254.1735263   1.813089145   1.04E-10   8.12E-09   LFNG
        7   2512529 2529177 +   3418   protein_coding LFNG
O-fucosylpeptide 3-beta-N-acetylglucosaminyltransferase [Source:HGNC
Symbol;Acc:HGNC:6560]   -   484   898   1314   260   205
        320   7.74404764   11.58345821   17.80843529
3.677921927   2.613607697   4.370427858
ENSG00000211459 19399.93338   32750.11076   20191.53729
56149.89514   48453.76456   54994.18622   24113.86048
53199.28197   -1.141494258   1.05E-10   8.17E-09   MT-
RNR1   MT   648   1601   +   954   Mt_rRNA
mitochondrially encoded 12S RNA [Source:HGNC Symbol;Acc:HGNC:7470]
        -       16768   35331   20650   55894   53021   56047
961.2306345   1632.832038   1002.707208   2832.813819
2421.910765   2742.521446
ENSG00000012048 2381.027129   2055.050679   2715.346202
1317.002049   1128.616949   1097.980166   2383.808003
1181.199721   1.013065195   1.21E-10   9.37E-09   BRCA1
        17   43044295   43170245   -   9187
protein_coding "BRCA1, DNA repair associated [Source:HGNC
Symbol;Acc:HGNC:1100]" -   2058   2217   2777   1311   1235

1119 12.25085407 10.63961553 14.00246898
6.899699743 5.858033339 5.685941747
ENSG00000258791 72.88858557 78.79084697 44.97872714
350.5977995 458.7576585 187.4121642 65.5527199
332.2558741 -2.342630926 1.28E-10 9.82E-09
LINC00520 14 55781132 55796731 - 3482
lincRNA long intergenic non-protein coding RNA 520
[Source:HGNC Symbol;Acc:HGNC:19843] - 63 85 46
349 502 191 0.989478804 1.076277142
0.611971956 4.846161429 6.282515201 2.560652446
ENSG00000102317 4408.023984 4142.544648 4479.294544
2310.529911 2606.32837 2541.348195 4343.287726
2486.068825 0.804788262 1.32E-10 1.01E-08 RBM3
X 48574449 48579066 + 3731
protein_coding RNA binding motif protein 3 [Source:HGNC
Symbol;Acc:HGNC:9900] - 3810 4469 4581 2300 2852
2590 55.84630442 52.81035217 56.87710703
29.8060073 33.31063179 32.40563611
ENSG00000169919 970.6908459 928.8050431 1240.82619
472.1517644 456.0160788 478.8331735 1046.774026
469.0003389 1.158518583 1.55E-10 1.18E-08 GUSB
7 65960684 65982314 - 3992
protein_coding glucuronidase beta [Source:HGNC Symbol;Acc:HGNC:4696]
- 839 1002 1269 470 499 488
11.4938663 11.06652188 14.72561851 5.692572125
5.447140919 5.706572316
ENSG00000075702 1049.36424 1050.235643 1829.460837
533.4310359 413.0646646 498.4574839 1309.686906
481.6510615 1.443741938 1.73E-10 1.31E-08 WDR62
19 36054881 36105106 + 9812
protein_coding WD repeat domain 62 [Source:HGNC
Symbol;Acc:HGNC:24502] - 907 1133 1871 531 452
508 5.055271213 5.091037878 8.833213017
2.616605187 2.007425761 2.416863977
ENSG00000197019 579.6377995 653.5005543 548.5449115
1229.603744 1197.156439 1190.214425 593.8944218
1205.658203 -1.021118797 1.80E-10 1.36E-08
SERTAD1 19 40421592 40426025 - 2114
protein_coding SERTA domain containing 1 [Source:HGNC
Symbol;Acc:HGNC:17932] - 501 705 561 1224 1310
1213 12.96067008 14.70341083 12.29306942
27.99481819 27.00380172 26.78562928
ENSG00000173545 1249.51861 1203.182581 924.9973017
2627.976629 2188.694406 2156.711712 1125.899497
2324.460916 -1.046092236 1.85E-10 1.38E-08 ZNF622
5 16451519 16465792 - 1699
protein_coding zinc finger protein 622 [Source:HGNC
Symbol;Acc:HGNC:30958] - 1080 1298 946 2616 2395
2198 34.76362763 33.6833495 25.79290231
74.44672155 61.42861684 60.3921297

ENSG00000142627 3033.553514 3398.202882 5671.230814
1564.128292 1347.943319 1884.915013 4034.32907
1598.995541 1.335395386 1.88E-10 1.41E-08 EPHA2
1 16124337 16156087 - 4159
protein_coding EPH receptor A2 [Source:HGNC Symbol;Acc:HGNC:3386]
- 2622 3666 5800 1557 1475 1921
34.47771535 38.8631052 64.60134037 18.10093083
15.4547398 21.56177354
ENSG00000158109 1367.528701 1512.784262 1045.266507
2481.308208 2760.77069 2767.027765 1308.52649
2669.702221 -1.028775149 2.11E-10 1.57E-08 TPRG1L
1 3625002 3630127 + 2414 protein_coding tumor
protein p63 regulated 1 like [Source:HGNC Symbol;Acc:HGNC:27007]
- 1182 1632 1069 2470 3021 2820
26.77780185 29.80690248 20.51364782 49.47216359
54.53459061 54.53281019
ENSG00000103187 4667.1834 5583.953672 6696.941352
2245.232326 2446.402892 3255.673094 5649.359475
2649.102771 1.09271859 2.34E-10 1.72E-08 COTL1
16 84565594 84618077 - 6762
protein_coding coactosin like F-actin binding protein 1 [Source:HGNC
Symbol;Acc:HGNC:18304] - 4034 6024 6849 2235 2677
3318 32.62536959 39.27749253 46.9196167
15.98098624 17.25169623 22.90589424
ENSG00000196262 29628.63155 26976.1321 31622.97858
15273.60729 18203.1749 17648.14233 29409.24741
17041.64151 0.787179585 2.53E-10 1.86E-08 PPIA
7 44796680 44824564 + 4432
protein_coding peptidylprolyl isomerase A [Source:HGNC
Symbol;Acc:HGNC:9253] - 25609 29102 32341 15204 19919
17986 316.0003627 289.5056492 338.0307029
165.8667464 195.8512635 189.4440079
ENSG00000205336 327.4201542 118.649746 267.916766
44.20144177 31.07123583 43.17348286 237.9955554
39.48205349 2.591576054 2.74E-10 2.00E-08 ADGRG1
16 57610652 57665580 + 8659
protein_coding adhesion G protein-coupled receptor G1 [Source:HGNC
Symbol;Acc:HGNC:4512] - 283 128 274 44 34
44 1.787365655 0.651742719 1.465835647
0.245689249 0.171107787 0.237208896
ENSG00000117632 8978.022604 11194.78893 12492.35256
4616.03693 6213.333307 5698.899737 10888.38803
5509.423325 0.982838363 2.92E-10 2.13E-08 STMN1
1 25884181 25906991 - 6261
protein_coding stathmin 1 [Source:HGNC Symbol;Acc:HGNC:6510] -
7760 12077 12776 4595 6799 5808 67.78174265
85.04510477 94.52651685 35.48485447 47.32165611
43.3040923
ENSG00000040275 1512.14891 1176.300998 1406.074122
714.2551159 643.3573537 611.2972686 1364.841343

656.3032461	1.055952183	2.96E-10	2.15E-08	SPDL1
	5	169583634	169604778	+	4777
protein_coding	spindle apparatus coiled-coil protein 1 [Source:HGNC
Symbol;Acc:HGNC:26010]	-	1307	1269	1438	711	704
	623	14.96287539	11.71225013	13.94460127
7.196404657	6.4220845	6.088058841
ENSG00000140451 179.3290597	283.6470491	484.010216
40.18312888	44.77913399	82.42210364	315.6621083
55.79478884	2.501694993	3.08E-10	2.22E-08	PIF1
	15	64815632	64825668	-	4656
protein_coding	PIF1 5'-to-3' DNA helicase [Source:HGNC
Symbol;Acc:HGNC:26220]	-	155	306	495	40	49
	84	1.820595392	2.897626554	4.924869002
0.415382539	0.458608084	0.842194402
ENSG00000164032 6050.909564	7099.518788	7492.869263
3600.408348	3732.203739	4180.959328	6881.099205
3837.857139	0.842467997	3.09E-10	2.22E-08	H2AFZ
	4	99948086	99950388	-	2303
protein_coding	H2A histone family member Z [Source:HGNC
Symbol;Acc:HGNC:4741]	-	5230	7659	7663	3584	4084
	4261	124.1945287	146.6263722	154.1371567
75.2445899	77.2770248	86.37014169
ENSG00000224051 923.2554172	1649.04608	1297.538498
3116.201645	2861.295276	2783.708429	1289.946665
2920.401783	-1.177887732	3.22E-10	2.30E-08	CPTP
	1	1324756 1328897 +	2556	protein_coding
ceramide-1-phosphate transfer protein [Source:HGNC
Symbol;Acc:HGNC:28116]	-	798	1779	1327	3102	3131
	2837	17.07405809	30.68662008	24.0498587
58.67892656	53.38027611	51.81368997
ENSG00000160049 2201.698069	2239.513956	1833.37203
4369.915266	4043.829958	3455.84106	2091.528018
3956.528761	-0.919756118	3.32E-10	2.36E-08	DFFA
	1	10456522	10472526	-	6622
protein_coding	DNA fragmentation factor subunit alpha [Source:HGNC
Symbol;Acc:HGNC:2772]	-	1903	2416	1875	4350	4425
	3522	15.71608339	16.0857647	13.11639695
31.76152138	29.11941838	24.82825579
ENSG00000148082 999.6148878	875.9688281	634.5911721
1785.135501	1815.839576	1841.74153	836.7249627
1814.238869	-1.117342679	3.76E-10	2.66E-08	SHC3
	9	89005771	89178767	-	9929
protein_coding	SHC adaptor protein 3 [Source:HGNC
Symbol;Acc:HGNC:18181]	-	864	945	649	1777	1987
	1877	4.758860175	4.196239386	3.027900794
8.65332736	8.720692119	8.8247985
ENSG00000128708 2082.531016	1801.066067	1864.66158
1093.985684	947.6726929	996.9149678	1916.086221
1012.857781	0.919613719	3.82E-10	2.69E-08	HAT1
	2	171922448	171983682	+	6790

protein_coding histone acetyltransferase 1 [Source:HGNC Symbol;Acc:HGNC:4821] - 1800 1943 1907 1089 1037 1016 14.49764441 12.61644458 13.01018208 7.754598592 6.655299126 6.985057242
ENSG00000152253 354.0302728 427.3244759 617.968599 72.32963199 166.3224977 113.8210003 466.4411159 117.4910433 1.987725574 4.33E-10 3.03E-08 SPC25 2 168834132 168913371 - 1841
protein_coding "SPC25, NDC80 kinetochore complex component [Source:HGNC Symbol;Acc:HGNC:24031]" - 306 461 632 72 182 116 9.089967924 11.0403056 15.9025125 1.890949473 4.308004986 2.941373914
ENSG00000124207 9283.460486 7664.958984 8040.436376 4187.082029 5182.499365 4517.516252 8329.618615 4629.032549 0.847352301 4.34E-10 3.03E-08 CSE1L 20 49046246 49096960 + 3800
protein_coding chromosome segregation 1 like [Source:HGNC Symbol;Acc:HGNC:2431] - 8024 8269 8223 4168 5671 4604 115.4787399 95.94080393 100.2418676 53.03289437 65.03312874 56.55848323
ENSG00000174371 1747.01213 1525.761578 1745.370173 517.3577843 838.9233675 820.2961743 1672.714627 725.5257754 1.20453963 4.65E-10 3.23E-08 EXO1 1 241847967 241895148 + 4140
protein_coding exonuclease 1 [Source:HGNC Symbol;Acc:HGNC:3511] - 1510 1646 1785 515 918 836 19.94671208 17.52925455 19.97286356 6.014618758 9.662754326 9.426532564
ENSG00000114268 1056.30601 613.6416552 1012.999159 328.4970786 319.8509571 365.0121732 894.3156081 337.7867363 1.404055829 4.84E-10 3.35E-08 PFKFB4 3 48517684 48562015 - 6107
protein_coding "6-phosphofructo-2-kinase/fructose-2,6-biphosphatase 4 [Source:HGNC Symbol;Acc:HGNC:8875]" - 913 662 1036 327 350 372 8.175937639 4.779297085 7.858402898 2.588934418 2.49746104 2.843551222
ENSG00000136869 513.690984 293.8435116 549.5227099 111.5081826 148.95916 134.4265262 452.3524018 131.6312896 1.779409261 4.86E-10 3.35E-08 TLR4 9 117704175 117724730 + 13272
protein_coding toll like receptor 4 [Source:HGNC Symbol;Acc:HGNC:11850] - 444 317 562 111 163 137 1.829537476 1.053069046 1.96156362 0.404378281 0.535192266 0.481870378
ENSG00000168461 6136.524728 4347.400851 4822.501789 2668.159758 2832.96562 2410.846531 5102.142456 2637.32397 0.951731433 5.00E-10 3.43E-08 RAB31 18 9708165 9862551 + 5586
protein_coding "RAB31, member RAS oncogene family [Source:HGNC Symbol;Acc:HGNC:9771]" - 5304 4690 4932 2656 3100

2457 51.92748593 37.01739638 40.90012096
22.98943806 24.18351142 20.53289853
ENSG00000168159 2008.485469 2790.122934 2256.758745
4556.766815 4195.530697 4581.29526 2351.789049
4444.530924 -0.917785785 5.85E-10 3.99E-08 RNF187
1 228487061 228495766 + 3285
protein_coding ring finger protein 187 [Source:HGNC
Symbol;Acc:HGNC:27146] - 1736 3010 2308 4536 4591
4669 28.90074644 40.39848508 32.54639466
66.7634681 60.90185439 66.34902356
ENSG00000133216 1403.394513 1065.066861 1486.253593
680.0994563 359.1469318 441.5469838 1318.238322
493.5977906 1.41768338 6.20E-10 4.21E-08 EPHB2
1 22710839 22921500 + 12516
protein_coding EPH receptor B2 [Source:HGNC Symbol;Acc:HGNC:3393]
- 1213 1149 1520 677 393 450
5.300171947 4.047514676 5.625750783 2.615316963
1.368313443 1.678390432
ENSG00000138448 1993.444967 1430.28561 2117.911369
960.3767803 520.9001301 684.8884326 1847.213982
722.0551143 1.355478559 6.28E-10 4.25E-08 ITGAV
2 186590065 186680901 + 8211
protein_coding integrin subunit alpha V [Source:HGNC
Symbol;Acc:HGNC:6150] - 1723 1543 2166 956 570
698 11.47582556 8.285215937 12.21982133
5.629412286 3.02508374 3.968308309
ENSG00000124496 892.017452 800.8857857 1228.114811
441.0098395 376.5102695 401.3171475 973.6726828
406.2790855 1.261332689 6.40E-10 4.31E-08 TRERF1
6 42224931 42452051 - 7732
protein_coding transcriptional regulating factor 1 [Source:HGNC
Symbol;Acc:HGNC:18273] zf-C2H2 771 864 1256 439 412
409 5.453273071 4.926697021 7.524891631
2.745199375 2.322009373 2.469320708
ENSG00000186871 821.4427898 761.0268866 1035.488523
333.5199697 421.2894035 364.0309577 872.652733
372.946777 1.226019071 6.53E-10 4.37E-08 ERCC6L
X 72204657 72239047 - 4364
protein_coding "ERCC excision repair 6 like, spindle assembly
checkpoint helicase [Source:HGNC Symbol;Acc:HGNC:20794]" -
710 821 1059 332 461 371 8.897506615
8.294541286 11.24122587 3.67836277 4.60335832
3.968580586
ENSG00000124762 1035.4807 1584.1595 789.0833218
2638.022411 3370.315228 2555.085213 1136.241174
2854.474284 -1.328574692 6.54E-10 4.37E-08 CDKN1A
6 36676460 36687339 + 3050
protein_coding cyclin dependent kinase inhibitor 1A [Source:HGNC
Symbol;Acc:HGNC:1784] - 895 1709 807 2626 3688
2604 16.04788889 24.70450642 12.25677323

41.62901154	52.69260514	39.85540038
ENSG00000121957 1583.880534	1411.746588	1750.259165
699.1864425	857.200565	814.4088812	1581.962095
790.2652962	1.000947328	6.78E-10	4.51E-08	GPSM2
1	108875350	108934545	+	9130
protein_coding	G protein signaling modulator 2 [Source:HGNC
Symbol;Acc:HGNC:29501]	-	1369	1523	1790	696	938
830	8.200255485	7.354668185	9.082067163
3.685867156	4.477036742	4.243784778
ENSG00000100234 6404.939837	6280.093979	7077.304936
2893.185279	3592.383178	4073.025621	6587.446251
3519.53136	0.904299373	7.35E-10	4.87E-08	TIMP3
22	32801701	32863043	+	4603
protein_coding	TIMP metallopeptidase inhibitor 3 [Source:HGNC
Symbol;Acc:HGNC:11822]	-	5536	6775	7238	2880	3931
4151	65.77332837	64.89366203	72.84169778
30.2519051	37.21522651	42.09764431
ENSG00000167703 89.08604903	64.88657986	111.4690194
1.004578222	9.138598774	1.962431039	88.48054944
4.035202678	4.429481469	7.62E-10	5.02E-08
SLC43A2 17	1569267 1628886 -	10606	protein_coding	solute
carrier family 43 member 2 [Source:HGNC Symbol;Acc:HGNC:23087]	-
77	70	114	1	10	2	0.397039591
0.290991548	0.497915544	0.00455879	0.041087241
0.008802872
ENSG00000082781 4344.391092	3718.927977	5010.239084
2371.809182	2065.323323	2481.494049	4357.852718
2306.208851	0.91814583	7.65E-10	5.02E-08	ITGB5
3	124761948	124901418	-	6447
protein_coding	integrin subunit beta 5 [Source:HGNC
Symbol;Acc:HGNC:6160]	-	3755	4012	5124	2361	2260
2529	31.85275373	27.4370372	36.81746704
17.70677763	15.27599004	18.31205968
ENSG00000138758 5622.833744	4721.889111	5816.922778
2827.887695	3317.311355	2801.370308	5387.215211
2982.189786	0.852974977	8.15E-10	5.32E-08	11-Sep
4	76949703	77040384	+	9787
protein_coding	septin 11 [Source:HGNC Symbol;Acc:HGNC:25589]	-
4860	5094	5949	2815	3630	2855	27.15697507
22.94791911	28.15767788	13.90689025	16.1627641
13.6176623
ENSG00000103995 704.5896605	640.5232383	754.8603773
180.82408	321.6786769	273.7591299	699.991092
258.7539622	1.434583693	8.62E-10	5.61E-08	CEP152
15	48712928	48811146	-	8168
protein_coding	centrosomal protein 152 [Source:HGNC
Symbol;Acc:HGNC:29298]	-	609	691	772	180	352
279	4.077521656	3.729892166	4.378284989
1.065511135	1.877956517	1.594536665
ENSG00000100065 258.0024537	260.4732706	359.8298171

82.37541421 95.04142725 73.59116396 292.7685138
83.66933514 1.806599592 9.43E-10 6.11E-08 CARD10
22 37490362 37519542 - 5652
protein_coding caspase recruitment domain family member 10
[Source:HGNC Symbol;Acc:HGNC:16422] - 223 281 368
82 104 75 2.157731604 2.19198776
3.016116561 0.70147616 0.801843815 0.619448415
ENSG00000081923 424.604935 312.3825345 440.9870857
74.33878843 97.78300688 152.0884055 392.658185
108.0700669 1.860774036 9.59E-10 6.19E-08 ATP8B1
18 57646426 57803315 - 7028
protein_coding ATPase phospholipid transporting 8B1 [Source:HGNC
Symbol;Acc:HGNC:3706] - 367 337 451 74 107
155 2.85580812 2.11413203 2.972673728
0.509097757 0.663453703 1.029546535
ENSG00000197461 1163.903446 1403.404027 1898.884524
514.3440497 700.930526 666.2453377 1488.730666
627.1733045 1.247234671 1.03E-09 6.59E-08 PDGFA
7 497258 520296 - 4151 protein_coding
platelet derived growth factor subunit A [Source:HGNC
Symbol;Acc:HGNC:8799] - 1006 1514 1942 512 767
679 13.25378618 16.0807796 21.6719978
5.963736467 8.051952946 7.635950013
ENSG00000102384 815.6579814 583.9792187 996.3765861
352.6069559 284.2104219 264.9281903 798.6712621
300.581856 1.409874648 1.14E-09 7.28E-08 CENPI
X 101098218 101163681 + 4321
protein_coding centromere protein I [Source:HGNC
Symbol;Acc:HGNC:3968] - 705 630 1019 351 311
270 8.922767226 6.428212739 10.92426869
3.927571199 3.136423654 2.91692682
ENSG00000167106 391.0530464 532.996906 412.6309316
900.1020869 986.0548077 1089.149227 445.5602947
991.7687071 -1.15312279 1.14E-09 7.28E-08
FAM102A 9 127940579 127980513 - 6100
protein_coding family with sequence similarity 102 member A
[Source:HGNC Symbol;Acc:HGNC:31419] - 338 575 422
896 1079 1110 3.030271756 4.155965825
3.204682965 7.10197912 7.708150888 8.49452658
ENSG00000164932 851.5237933 786.0545674 933.7974874
233.0621475 323.5063966 428.791182 857.1252827
328.453242 1.383550232 1.17E-09 7.43E-08 CTHRC1
8 103371515 103382997 + 1776
protein_coding collagen triple helix repeat containing 1 [Source:HGNC
Symbol;Acc:HGNC:18831] - 736 848 955 232 354
437 22.66363492 21.05168431 24.90937702
6.316059898 8.685981466 11.48641522
ENSG00000083720 1055.149048 967.736991 1224.203617
538.453927 357.3192121 506.307208 1082.363219
467.3601157 1.212246783 1.27E-09 8.04E-08 OXCT1

5 41730065 41870519 - 4194
protein_coding 3-oxoacid CoA-transferase 1 [Source:HGNC
Symbol;Acc:HGNC:8527] - 912 1044 1252 536 391
516 11.89217044 10.97503795 13.82860458
6.179275807 4.062626799 5.743376824
ENSG00000124795 7212.499086 5302.160526 5881.457473
3327.163071 3490.944732 3332.207904 6132.039028
3383.438569 0.857641527 1.30E-09 8.21E-08 DEK
6 18223868 18264823 - 4361
protein_coding DEK proto-oncogene [Source:HGNC Symbol;Acc:HGNC:2768]
- 6234 5720 6015 3312 3820 3396
78.17635618 57.82876268 63.89281177 36.72023551
38.17120534 36.35194317
ENSG00000166833 1499.422332 1191.132216 1958.530228
710.236803 425.8587029 678.019924 1549.694925
604.7051433 1.358149842 1.34E-09 8.37E-08 NAV2
11 19350724 20121598 + 14673
protein_coding neuron navigator 2 [Source:HGNC Symbol;Acc:HGNC:15997]
- 1296 1285 2003 707 466 691
4.83037443 3.861163287 6.323601409 2.329709189
1.383966561 2.198392155
ENSG00000132780 6654.843559 6141.051308 6178.708192
3886.713141 3636.248452 3991.584733 6324.867686
3838.182109 0.720578202 1.51E-09 9.43E-08 NASP
1 45583846 45618904 + 8304
protein_coding nuclear autoantigenic sperm protein [Source:HGNC
Symbol;Acc:HGNC:7644] - 5752 6625 6319 3869 3979
4068 37.88141994 35.17486992 35.25035302
22.52747963 20.88070686 22.86860323
ENSG00000178202 3957.965893 3134.948758 3770.390693
2014.179335 1571.838989 2073.308393 3621.101781
1886.442239 0.940766842 1.57E-09 9.77E-08 KDELC2
11 108472105 108498432 - 4993
protein_coding KDEL motif containing 2 [Source:HGNC
Symbol;Acc:HGNC:28496] - 3421 3382 3856 2005 1720
2113 37.47021809 29.86386373 35.7748577
19.41574357 15.01154798 19.75531536
ENSG00000134508 385.268238 535.7777594 782.2387329
211.9660048 125.1988032 171.7127159 567.7615768
169.6258413 1.744788096 1.68E-09 1.04E-07
CABLES1 18 23134564 23260467 + 6654
protein_coding Cdk5 and Abl enzyme substrate 1 [Source:HGNC
Symbol;Acc:HGNC:25097] - 333 578 800 211 137
175 2.736882481 3.829825618 5.569415848
1.533207289 0.897214675 1.227725533
ENSG00000225614 695.3339671 494.9919092 1284.827119
241.0987733 245.828307 261.9845437 825.0509984
249.637208 1.724735117 1.69E-09 1.04E-07 ZNF469
16 88427471 88440757 + 13287
protein_coding zinc finger protein 469 [Source:HGNC

Symbol;Acc:HGNC:23216] zf-C2H2 601   534   1314   240   269
        267   2.473672778   1.771937013   4.581111751
0.873344367   0.882234309   0.938059449
ENSG00000178773 2.313923351   8.342560268   1.955596832
40.18312888   119.7156439   113.8210003   4.204026817
91.23992436   -4.411598298   1.75E-09   1.07E-07   CPNE7
        16   89575768   89597246   +   3048
protein_coding copine 7 [Source:HGNC Symbol;Acc:HGNC:2320]   -
        2   9   2   40   131   116   0.035884735
        0.130185167   0.030396074   0.634521358   1.872901471
        1.776597564
ENSG00000129534 957.9642675   764.7346912   1029.621732
386.7626155   462.413098   317.9138283   917.4402303
389.0298473   1.236933985   1.80E-09   1.10E-07
MIS18BP1   14   45203190   45253540   -   6250
        protein_coding MIS18 binding protein 1 [Source:HGNC
Symbol;Acc:HGNC:20190] -   828   825   1053   385   506
        324   7.24511081   5.819797708   7.804602876
2.978392494   3.528004231   2.419975832
ENSG00000228253 753.1820509   1038.185278   1002.243377
1852.442241   1930.985921   1754.413349   931.2035684
1845.94717   -0.986188016   1.88E-09   1.15E-07   MT-
ATP8   MT   8366   8572   +   207   protein_coding
mitochondrially encoded ATP synthase membrane subunit 8 [Source:HGNC
Symbol;Acc:HGNC:7415]   -   651   1120   1025   1844   2113
        1788   171.9908552   238.5512163   229.3802257
430.7167763   444.823527   403.221058
ENSG00000079616 1389.510973   1426.577806   1849.016805
720.2825852   665.2899908   852.6762864   1555.035194
746.0829541   1.059929681   1.94E-09   1.18E-07   KIF22
        16   29790719   29805385   +   4312
protein_coding kinesin family member 22 [Source:HGNC
Symbol;Acc:HGNC:6391]   -   1201   1539   1891   717   728
        869   15.23207144   15.73598111   20.31492543
8.039732888   7.357177345   9.407777989
ENSG00000082497 566.9112211   364.2917984   697.1702707
154.7050462   203.7907527   174.6563625   542.7910967
177.7173871   1.609723335   2.01E-09   1.21E-07
SERTAD4 1   210232799   210246631   +   5467
protein_coding SERTA domain containing 4 [Source:HGNC
Symbol;Acc:HGNC:25236] -   490   393   713   154   223
        178   4.90164347   3.169402779   6.041474013
1.36198669   1.777519554   1.519906822
ENSG00000130706 3610.87739   4318.665365   3936.616423
7850.778805   6461.903193   6518.214696   3955.386393
6943.632231   -0.811646579   2.18E-09   1.31E-07   ADRM1
        20   62302093   62308862   +   2142
protein_coding adhesion regulating molecule 1 [Source:HGNC
Symbol;Acc:HGNC:15759] -   3121   4659   4026   7815   7071
        6643   79.68361251   95.89748155   87.06763738

176.4049357	143.8533462	144.7740827
ENSG00000185567 1067.875627	1636.068764	798.861306
2717.384091	2499.406765	3499.014542	1167.601899
2905.268466	-1.314666982	2.30E-09	1.38E-07	AHNAK2
	14	104937244	104978357	-	18771
protein_coding	AHNAK nucleoprotein 2 [Source:HGNC
Symbol;Acc:HGNC:20125]	-	923	1765	817	2705	2735
	3566	2.689112702	4.145636869	2.016216213
6.967565759	6.34933905	8.868291604
ENSG00000000971 584.2656462	374.4882609	689.3478834
185.8469711	184.5996952	195.2618884	549.3672635
188.5695182	1.54220899	2.34E-09	1.40E-07	CFH
	1	196651878	196747504	+	8145
protein_coding	complement factor H [Source:HGNC Symbol;Acc:HGNC:4883]
	-	505	404	705	185	202	199
3.390744002	2.186876383	4.009594782	1.098201054
1.080734159	1.140533441
ENSG00000069020 539.1441409	522.8004434	464.4542477
1216.544227	923.9123361	1054.806683	508.7996107
1065.087749	-1.065925215	2.43E-09	1.44E-07	MAST4
	5	66596361	67169595	+	14675
protein_coding	microtubule associated serine/threonine kinase family
member 4 [Source:HGNC Symbol;Acc:HGNC:19037]	-	466	564
	475	1211	1011	1075	1.736610888	1.694474166
	1.49940155	3.989948129	3.002144856	3.419608518
ENSG00000182551 1596.607112	1624.94535	1801.104683
843.8457065	866.3391638	988.0840281	1674.219048
899.4229661	0.896570824	2.67E-09	1.58E-07	ADI1
	2	3497361 3519736 -	5560	protein_coding
acireductone dioxygenase 1 [Source:HGNC Symbol;Acc:HGNC:30576]	-
	1380	1753	1842	840	948	1007	13.57372379
	13.90084121	15.3467808	7.304755952	7.430056966
	8.454748918
ENSG00000085117 1797.918444	2004.995318	2765.213921
651.9712661	1172.482223	881.1315365	2189.375894
901.8616751	1.279302839	2.80E-09	1.65E-07	CD82
	11	44564427	44620363	+	4054
protein_coding	CD82 molecule [Source:HGNC Symbol;Acc:HGNC:6210]
	-	1554	2163	2828	649	1283	898
20.96341263	23.52375963	32.3145503	7.740377983
13.79118263	10.34043069
ENSG00000118515 666.4099252	659.9892123	350.051833
1251.704465	1559.044951	1413.931564	558.8169902
1408.226993	-1.334108432	3.02E-09	1.78E-07	SGK1
	6	134169246	134318112	-	9992
protein_coding	serum/glucocorticoid regulated kinase 1 [Source:HGNC
Symbol;Acc:HGNC:10810]	-	576	712	358	1246	1706
	1441	3.152570235	3.141676954	1.659713235
6.029299165	7.440210095	6.732209018
ENSG00000148841 2072.118361	2768.803058	2209.824421

4454.299836	5096.596536	3869.914009	2350.248613
4473.603461	-0.928335803	3.36E-09	1.97E-07	ITPRIP
	10	104309698	104338448	-	7279
protein_coding	"inositol 1,4,5-trisphosphate receptor interacting
protein [Source:HGNC Symbol;Acc:HGNC:29370]"	-	1791	2987
	2260	4434	5577	3944	13.45608058	18.09245334
	14.38265873	29.45270489	33.38777844	25.29363358
ENSG00000141753 2102.199365	2584.339781	4878.236298
612.7927154	541.9189073	697.6442343	3188.258481
617.4519524	2.368489883	3.39E-09	1.98E-07	IGFBP4
	17	40443461	40457731	+	2200
protein_coding	insulin like growth factor binding protein 4
[Source:HGNC Symbol;Acc:HGNC:5473]	-	1817	2788	4989
	610	593	711	45.16759423	55.8732645
105.0493281	13.40628262	11.7460166	15.08665488
ENSG00000152952 8029.314029	4962.896408	6472.047717
3051.908639	3177.490794	3578.493	6488.086051
3269.297477	0.988613844	3.52E-09	2.04E-07	PLOD2
	3	146069440	146163653	-	7646
protein_coding	"procollagen-lysine,2-oxoglutarate 5-dioxygenase 2
[Source:HGNC Symbol;Acc:HGNC:9082]"	-	6940	5354	6619
	3038	3477	3647	49.63864175	30.872943
40.10149312	19.2112088	19.81659323	22.26627177
ENSG00000119865 922.0984555	940.855408	1057.977886
391.7855066	492.5704739	506.307208	973.6439166
463.5543962	1.070484533	3.53E-09	2.05E-07	CNRIP1
	2	68284171	68320051	-	3548
protein_coding	cannabinoid receptor interacting protein 1
[Source:HGNC Symbol;Acc:HGNC:24546]	-	797	1015	1082
	390	539	516	12.28483769	12.61294172
14.1268751	5.314742312	6.620087836	6.789098759
ENSG00000132510 470.883402	538.5586128	554.411702
1226.590009	950.4142725	1053.825468	521.2845723
1076.94325	-1.045821131	3.56E-09	2.05E-07	KDM6B
	17	7839904 7854796 +	7258	protein_coding	lysine
demethylase 6B [Source:HGNC Symbol;Acc:HGNC:29012]	-	407
	581	567	1221	1040	1074	3.066706107
3.529336979	3.618833074	8.133920373	6.244173663
6.907698176
ENSG00000122952 1476.283098	1561.912672	2123.77816
821.7449856	738.398781	915.4740797	1720.657977
825.2059487	1.060558716	3.58E-09	2.06E-07	ZWINT
	10	56357228	56361275	-	2378
protein_coding	ZW10 interacting kinetochore protein [Source:HGNC
Symbol;Acc:HGNC:13195]	-	1276	1685	2172	818	808
	933	29.34496101	31.24079038	42.31072104
16.63193082	14.80669424	18.3153756
ENSG00000100526 443.1163218	495.9188604	583.7456544
122.5585431	233.9481286	132.4640951	507.5936122
162.9902556	1.637467853	3.83E-09	2.19E-07	CDKN3

14	54396849	54420218	+	1836
protein_coding	cyclin dependent kinase inhibitor 3 [Source:HGNC Symbol;Acc:HGNC:1791]	-	383	535	597	122	256
135	11.40829676	12.84739459	15.06274452
3.212834616	6.076113618	3.432472982
ENSG00000118523 974.161731	1308.855011	1476.475608
543.4768181	424.944843	641.7149497	1253.164117
536.7122036	1.224286358	3.96E-09	2.25E-07	CTGF
6	131948176	131951373	-	2339
protein_coding	connective tissue growth factor [Source:HGNC Symbol;Acc:HGNC:2500]	-	842	1412	1510	541	465
654	19.68686587	26.6157331	29.90537009
11.18325582	8.663259606	13.05249581
ENSG00000166598 11162.36625	8740.222307	12946.05103
5045.996409	5125.840052	6662.453377	10949.54653
5611.429946	0.964414658	3.96E-09	2.25E-07
HSP90B1 12	103930107	103953645	+	4695
protein_coding	heat shock protein 90 beta family member 1 [Source:HGNC Symbol;Acc:HGNC:12028]	-	9648	9429	13240
5023	5609	6790	112.3819105	88.54499079
130.6335847	51.72837052	52.06051274	67.5118818
ENSG00000004700 1698.41974	1429.358659	1461.808632
824.7587203	730.1740421	860.5260106	1529.862344
805.1529243	0.925881565	4.17E-09	2.36E-07	RECQL
12	21468911	21501669	-	3778
protein_coding	RecQ like helicase [Source:HGNC Symbol;Acc:HGNC:9948]
-	1468	1542	1495	821	799	877
21.24999415	17.99518758	18.33081164	10.50708922
9.216020316	10.83636627
ENSG00000105373 4192.829113	5867.600722	4755.033698
8504.759228	8526.312656	10614.78949	4938.487844
9215.287125	-0.899740449	4.50E-09	2.54E-07	NOP53
19	47745522	47757058	+	5341
protein_coding	NOP53 ribosome biogenesis factor [Source:HGNC Symbol;Acc:HGNC:4333]	-	3624	6330	4863	8466	9330
10818	37.10738274	52.25346447	42.17782178
76.64024822	76.12330991	94.55193802
ENSG00000215030 3118.011716	2812.369761	2712.412806
4576.85838	5142.28953	4771.651071	2880.931428
4830.266327	-0.745841556	4.62E-09	2.60E-07
RPL13P12	17	17383377	17384012	-	636
processed_pseudogene	ribosomal protein L13 pseudogene 12 [Source:HGNC Symbol;Acc:HGNC:35701]	-	2695	3034	2774
4556	5627	4863	231.7375262	210.3257367
202.0467164	346.360068	385.5479491	356.9383319
ENSG00000185480 592.364378	498.6997138	503.5661843
248.1308208	212.9293514	204.092828	531.5434254
221.7176668	1.261142525	4.74E-09	2.65E-07	PARPBP
12	102120185	102197520	+	4745
protein_coding	PARP1 binding protein [Source:HGNC

Symbol;Acc:HGNC:26074] -   512   538   515   247   233
        208   5.90103862   4.998964088   5.027747608
2.516876774   2.139825221   2.046318139
ENSG00000168036 12982.26696   12171.79543   13389.97151
7395.704871   8406.597012   8142.126381   12848.0113
7981.476088   0.686768347   5.19E-09   2.90E-07   CTNNB1
        3   41194741   41260096   +   11927
protein_coding catenin beta 1 [Source:HGNC Symbol;Acc:HGNC:2514]
        -       11221   13131   13694   7362   9199   8298
51.4511464   48.54008584   53.18651813   29.84460329
33.60996013   32.47792294
ENSG00000069275 16178.95207   13531.63275   18993.73423
9338.559152   9635.738547   8504.194907   16234.77302
9159.497536   0.825719606   5.82E-09   3.24E-07   NUCKS1
        1   205712819   205750276   -   6546
protein_coding nuclear casein kinase and cyclin dependent kinase
substrate 1 [Source:HGNC Symbol;Acc:HGNC:29923] -   13984   14598
        19425   9296   10544   8667   116.8288568   98.3221386
        137.4635269   68.6627717   70.19206093   61.80716611
ENSG00000104142 1045.893355   940.855408   1099.04542
1711.80129   2143.915272   1880.990151   1028.598061
1912.235571   -0.894938968   5.96E-09   3.30E-07   VPS18
        15   40894430   40903975   +   4049
protein_coding "VPS18, CORVET/HOPS core subunit [Source:HGNC
Symbol;Acc:HGNC:15972]" -   904   1015   1124   1704   2346
        1917   12.20999164   11.05228877   12.85940843
20.34806096   25.24868876   22.10142989
ENSG00000196950 1000.771849   605.299095   739.2156026
323.4741875   333.5588553   313.0077507   781.7621824
323.3469312   1.272430665   6.02E-09   3.32E-07
SLC39A10   2   195575977   195737702   +   6917
        protein_coding solute carrier family 39 member 10
[Source:HGNC Symbol;Acc:HGNC:20861]   -   865   653   756
        322   365   319   6.839006941   4.162261516
5.062983075   2.25081247   2.299501443   2.152875638
ENSG00000176890 1251.832533   1376.522444   1477.453407
618.8201848   799.6273927   673.1138464   1368.602795
697.1871413   0.972938776   6.13E-09   3.37E-07   TYMS
        18   657604 673578 +   2470   protein_coding
thymidylate synthetase [Source:HGNC Symbol;Acc:HGNC:12441]   -
        1082   1485   1511   616   875   686   23.9565911
        26.5071758   28.3380503   12.0582692   15.43724147
        12.96502022
ENSG00000106049 969.5338842   1161.469779   1153.802131
510.3257368   554.7129456   593.6353893   1094.935265
552.8913572   0.986202337   6.27E-09   3.44E-07   HIBADH
        7   27525442   27662995   -   2323
protein_coding 3-hydroxyisobutyrate dehydrogenase [Source:HGNC
Symbol;Acc:HGNC:4907]   -   838   1253   1180   508   607
        605   19.72829356   23.78131246   23.53072202

10.57342573   11.38670525   12.15772178
ENSG00000142173 42749.73392   28062.51884   46788.63201
15571.96702   18141.94629   22730.83872   39200.29492
18814.91734   1.058969884   6.67E-09   3.64E-07   COL6A2
21   46098097   46132849   +   5386
protein_coding collagen type VI alpha 2 chain [Source:HGNC
Symbol;Acc:HGNC:2212]   -   36950   30274   47851   15501   19852
23166   375.1827017   247.8206065   411.5542822
139.153644   160.6188511   200.7847567
ENSG00000166963 930.1971873   890.8000464   794.9501123
2173.907272   1450.295625   1708.296219   871.9824487
1777.499706   -1.027524529   6.74E-09   3.66E-07   MAP1A
15   43510958   43531620   +   10629
protein_coding microtubule associated protein 1A [Source:HGNC
Symbol;Acc:HGNC:6835]   -   804   961   813   2164   1587
1741   4.136741226   3.98625373   3.543240198
9.843874447   6.506435329   7.646318777
ENSG00000143870 3307.753431   3497.386654   4241.689529
1827.327786   1776.543602   2299.969178   3682.276538
1967.946855   0.904091468   6.79E-09   3.68E-07   PDIA6
2   10783391   10837977   -   4101
protein_coding protein disulfide isomerase family A member 6
[Source:HGNC Symbol;Acc:HGNC:30168]   -   2859   3773   4338
1819   1944   2344   38.12581172   40.56308652
49.00069436   21.44589356   20.65689724   26.68172317
ENSG00000163041 4226.381001   4003.501977   4171.288043
2327.60774   2608.15609   2587.465325   4133.723674
2507.743052   0.720918873   7.06E-09   3.81E-07   H3F3A
1   226061851   226072001   +   3146
protein_coding H3 histone family member 3A [Source:HGNC
Symbol;Acc:HGNC:4764]   -   3653   4319   4266   2317   2854
2637   63.50174602   60.52829544   62.81517711
35.6097178   39.53246073   39.12888272
ENSG00000203760 355.1872344   409.7124043   505.5217812
53.24264577   147.1314403   132.4640951   423.4738066
110.9460604   1.931042558   7.21E-09   3.88E-07   CENPW
6   126340174   126348875   +   795
protein_coding centromere protein W [Source:HGNC
Symbol;Acc:HGNC:21488] -   307   442   517   53   161
135   21.11864061   24.51258421   30.12491778
3.223368499   8.825053464   7.927069679
ENSG00000142230 4639.41632   4984.216284   5872.657287
3092.091767   2879.572474   3069.242145   5165.429964
3013.635462   0.777545344   7.33E-09   3.92E-07   SAE1
19   47113274   47210636   +   3396
protein_coding SUMO1 activating enzyme subunit 1 [Source:HGNC
Symbol;Acc:HGNC:30660] -   4010   5377   6006   3078   3151
3128   64.57603919   69.80817958   81.92568892
43.8230046   40.43331222   42.99768747
ENSG00000122545 8390.286072   6969.745628   7873.232847

4025.344936	5074.663899	4292.817898	7744.421516
4464.275578	0.794548722	7.33E-09	3.92E-07	7-Sep
	7	35800932	35907105	+	10906
protein_coding	septin 7 [Source:HGNC Symbol;Acc:HGNC:1717]	-
	7252	7519	8052	4007	5553	4375	36.36528661
	30.39684784	34.20115153	17.76458497	22.18813389
	18.72658559
ENSG00000137573 319.3214225	178.9015702	356.8964219
85.38914887	46.60685375	73.59116396	285.0398049
68.52905553	2.057573461	7.40E-09	3.94E-07	SULF1
	8	69466624	69660915	+	9404
protein_coding	sulfatase 1 [Source:HGNC Symbol;Acc:HGNC:20391] -
	276	193	365	85	51	75	1.60505964
	0.904854283	1.797971071	0.437026248	0.23632853
	0.372301408
ENSG00000091136 4167.375956	2717.820745	5668.297418
1889.611636	1269.35137	1956.543746	4184.49804
1705.168917	1.295238156	7.57E-09	4.02E-07	LAMB1
	7	107923799	108003255	-	8140
protein_coding	laminin subunit beta 1 [Source:HGNC
Symbol;Acc:HGNC:6486]	-	3602	2932	5797	1881	1389
	1994	24.19992479	15.88084178	32.98992675
11.17289216	7.435949618	11.43527952
ENSG00000137135 264.9442237	246.5690035	356.8964219
75.34336665	99.61072664	90.27182779	289.469883
88.40864036	1.710387871	7.77E-09	4.11E-07
ARHGEF39	9	35658875	35675866	-	6496
	protein_coding	Rho guanine nucleotide exchange factor 39
[Source:HGNC Symbol;Acc:HGNC:25909]	-	229	266	365
	75	109	92	1.92789856	1.805383955
2.602850978	0.558234231	0.731204877	0.661131496
ENSG00000087303 263.7872621	210.417909	363.7410108
91.4166182	26.50193645	47.09834493	279.3153939
55.00563319	2.347698859	8.25E-09	4.35E-07	NID2
	14	52004803	52069228	-	6392
protein_coding	nidogen 2 [Source:HGNC Symbol;Acc:HGNC:13389]	-
	228	227	372	91	29	48	1.950710373
	1.565752266	2.695930111	0.688344493	0.197705991
	0.350550432
ENSG00000153904 1033.166776	823.1326131	1087.311839
471.1471861	437.7388813	508.2696391	981.2037428
472.3852355	1.054424323	8.60E-09	4.52E-07	DDAH1
	1	85318481	85578363	-	4568
protein_coding	dimethylarginine dimethylaminohydrolase 1 [Source:HGNC
Symbol;Acc:HGNC:2715]	-	893	888	1112	469	479
	518	10.691043	8.570789493	11.27667718
4.964185069	4.569492995	5.293582676
ENSG00000077097 5968.765285	4990.704942	5821.81177
3636.573164	3077.880067	2867.111748	5593.760666
3193.854993	0.808473514	9.31E-09	4.87E-07	TOP2B

3	25597905	25664907	-	6044
protein_coding	DNA topoisomerase II beta [Source:HGNC
Symbol;Acc:HGNC:11990]	-	5159	5384	5954	3620	3368
2922	46.68053072	39.27485167	45.63382057
28.95911805	24.28321723	22.56845307
ENSG00000019549 1430.004631	1011.303695	1180.202688
579.6416341	559.282245	632.8840101	1207.170338
590.6026297	1.030759482	9.84E-09	5.13E-07	SNAI2
8	48917604	48921740	-	3392
protein_coding	snail family transcriptional repressor 2 [Source:HGNC
Symbol;Acc:HGNC:11094]	zf-C2H2 1236	1091	1207	577	612
645	19.9277075	14.18086965	16.48366889
8.224721216	7.862382648	8.876666568
ENSG00000064393 2080.217093	2022.607389	1955.596832
3494.927634	3137.280959	3381.26868	2019.473772
3337.825758	-0.724972292	1.12E-08	5.82E-07	HIPK2
7	139561570	139777778	-	15402
protein_coding	homeodomain interacting protein kinase 2 [Source:HGNC
Symbol;Acc:HGNC:14402]	-	1798	2182	2000	3479	3433
3446	6.38421172	6.246138144	6.015272862
10.92140535	9.713042346	10.44441669
ENSG00000184226 520.6327541	542.2664174	350.051833
1239.649526	1118.56449	890.9436917	470.9836682
1083.052569	-1.201682119	1.20E-08	6.24E-07	PCDH9
13	66302834	67230445	-	30944
protein_coding	protocadherin 9 [Source:HGNC Symbol;Acc:HGNC:8661]
-	450	585	358	1234	1224	908
0.795299618	0.833514908	0.535931187	1.928146036
1.723707468	1.369794544
ENSG00000123473 1509.834987	1339.444399	1675.946485
572.6095866	824.3016094	802.6342949	1508.408624
733.1818303	1.040342138	1.23E-08	6.38E-07	STIL
1	47250139	47314147	-	7291
protein_coding	"STIL, centriolar assembly protein [Source:HGNC
Symbol;Acc:HGNC:10879]" -	1305	1445	1714	570	902
818	9.78854465	8.738053649	10.88995726
3.779975404	5.391108061	5.23735776
ENSG00000213949 497.4935206	232.6647364	489.8770065
114.5219173	132.5096822	110.8773537	406.6784211
119.3029844	1.767696377	1.30E-08	6.69E-07	ITGA1
5	52787896	52959210	+	18566
protein_coding	integrin subunit alpha 1 [Source:HGNC
Symbol;Acc:HGNC:6134]	-	430	251	501	114	145
113	1.26661557	0.596059115	1.250034028
0.296884635	0.340336285	0.284122831
ENSG00000166801 1899.731072	1631.434008	1759.059351
1034.715569	988.7963874	800.6718639	1763.408143
941.3946066	0.90519849	1.33E-08	6.81E-07
FAM111A 11	59142748	59155039	+	7221
protein_coding	family with sequence similarity 111 member A

[Source:HGNC Symbol;Acc:HGNC:24725]	-	1642	1760	1799
	1030	1082	816	12.43570813	10.74606049
11.54080955	6.896696208	6.529629109	5.275199026
ENSG00000268858 363.2859662	389.3194792	303.117509
956.3584674	630.5633154	860.5260106	351.9076514
815.8159311	-1.212701688	1.33E-08	6.81E-07
AL118506.1	20	63861212	63864293	-	3082
	antisense	"novel transcript, antisense to ABHD16B"
	-	314	420	310	952	690	877
5.571751343	6.008286231	4.659416307	14.93501044
9.756073288	13.28351452
ENSG00000116584 3652.52801	5574.684161	3886.748704
8636.358975	8567.436351	7632.875526	4371.320292
8278.890284	-0.92112111	1.34E-08	6.82E-07
ARHGEF2 1	155946851	156007070	-	9135
protein_coding	Rho/Rac guanine nucleotide exchange factor 2
[Source:HGNC Symbol;Acc:HGNC:682]	-	3157	6014	3975
	8597	9375	7779	18.89995382	29.02610955
20.15723863	45.50295401	44.72201083	39.75220795
ENSG00000093009 1096.799669	1274.557819	1369.895581
632.8842799	680.8256087	657.414398	1247.084356
657.0414289	0.924842735	1.35E-08	6.86E-07	CDC45
	22	19479459	19520612	+	2846
protein_coding	cell division cycle 45 [Source:HGNC
Symbol;Acc:HGNC:1739]	-	948	1375	1401	630	745
	670	18.2166349	21.30108672	22.80371977
10.70303314	11.40722413	10.98970267
ENSG00000174442 1937.910807	1560.985721	1725.814205
1054.807133	838.9233675	858.5635795	1741.570244
917.43136	0.924595326	1.38E-08	6.96E-07	ZWILCH
	15	66504959	66550128	+	4849
protein_coding	zwilch kinetochore protein [Source:HGNC
Symbol;Acc:HGNC:25468]	-	1675	1684	1765	1050	918
	875	18.89110405	15.31171585	16.86145242
10.46979869	8.249907798	8.423680859
ENSG00000076706 1885.847531	2641.810751	3327.44801
1147.22833	1052.766579	1410.987917	2618.368764
1203.660942	1.121674534	1.39E-08	6.99E-07	MCAM
	11	119308529	119321521	-	7422
protein_coding	melanoma cell adhesion molecule [Source:HGNC
Symbol;Acc:HGNC:6934]	-	1630	2850	3403	1142	1152
	1438	12.01050773	16.93003547	21.23945922
7.43954492	6.763790179	9.044487687
ENSG00000160570 542.6150259	979.7873559	708.9038517
1673.627318	1746.386226	1512.053116	743.7687445
1644.02222	-1.142940579	1.45E-08	7.30E-07	DEDD2
	19	42198598	42220140	-	2631
protein_coding	death effector domain containing 2 [Source:HGNC
Symbol;Acc:HGNC:24450]	-	469	1057	725	1666	1911
	1541	9.748700033	17.71283582	12.76496459

30.61648757	31.65180174	27.3418456
ENSG00000183087 2495.566335	2417.488575	3485.851354
1414.446137	1060.991318	1567.001185	2799.635421
1347.479546	1.055281112	1.49E-08	7.45E-07	GAS6
13	113820549	113864067	-	6006
protein_coding	growth arrest specific 6 [Source:HGNC
Symbol;Acc:HGNC:4168]	-	2157	2608	3565	1408	1161
1597	19.64081613	19.14503732	27.49645224
11.3349222	8.423750393	12.41268185
ENSG00000148773 5632.089437	5614.54306	11594.73362
1505.862755	1201.725739	1813.28628	7613.788705
1506.958258	2.337026035	1.52E-08	7.59E-07	MKI67
10	128096659	128126385	-	12807
protein_coding	marker of proliferation Ki-67 [Source:HGNC
Symbol;Acc:HGNC:7107]	-	4868	6057	11858	1499	1315
1848	20.7872899	20.85182743	42.89103164
5.659205178	4.474421993	6.735976963
ENSG00000204388 2570.768843	2867.98683	2254.803148
6745.742761	3849.177804	5228.897503	2564.519607
5274.606023	-1.040239056	1.53E-08	7.60E-07	HSPA1B
6	31827735	31830255	+	2521
protein_coding	heat shock protein family A (Hsp70) member 1B
[Source:HGNC Symbol;Acc:HGNC:5233]	-	2222	3094	2306
6715	4212	5329	48.20209582	54.11048439
42.37297073	128.7877002	72.80716399	98.67767548
ENSG00000154898 38.1797353	36.15109449	59.64570339	0
0	0	44.65884439	0	7.974179537
1.63E-08	8.09E-07	CCDC144CP	17	20321164
20403557	+	8145
transcribed_processed_pseudogene	"coiled-coil domain containing
144C, pseudogene [Source:HGNC Symbol;Acc:HGNC:29073]"	-	33
39	61	0	0	0	0.22157337
0.211109354	0.346929478	0	0	0
ENSG00000151725 1586.194457	1268.069161	1371.851178
477.1746555	814.2491508	629.9403635	1408.704932
640.4547232	1.136262199	1.77E-08	8.75E-07	CENPU
4	184694618	184734133	-	2953
protein_coding	centromere protein U [Source:HGNC
Symbol;Acc:HGNC:21348]	-	1371	1368	1403	475	891
642	25.39035193	20.42474337	22.00881601
7.777345262	13.1483984	10.1488683
ENSG00000139289 8466.645543	7781.754827	6301.910792
13026.3658	14594.34224	11805.00391	7516.770387
13141.90399	-0.806098384	1.85E-08	9.14E-07	PHLDA1
12	76025447	76033932	-	8069
protein_coding	pleckstrin homology like domain family A member 1
[Source:HGNC Symbol;Acc:HGNC:8933]	-	7318	8395	6445
12967	15970	12031	49.59836981	45.87065515
37.0003355	77.69999876	86.24696045	69.60301416
ENSG00000119574 694.1770054	831.4751733	727.4820216

1380.290477	1350.684899	1382.532667	751.0447335
1371.169348	-0.867718662	1.91E-08	9.41E-07	ZBTB45
	19	58513530	58538911	-	3418
protein_coding	zinc finger and BTB domain containing 45 [Source:HGNC
Symbol;Acc:HGNC:23715]	ZBTB	600	897	744	1374	1478
	1409	9.600059058	11.57055904	10.08331496
19.4364028	18.84347403	19.24354016
ENSG00000143815 2185.500605	2156.088354	2900.150102
1320.015784	1292.197867	1324.640951	2413.91302
1312.284867	0.879447039	1.96E-08	9.59E-07	LBR
	1	225401502	225428925	-	5257
protein_coding	lamin B receptor [Source:HGNC Symbol;Acc:HGNC:6518]
	-	1889	2326	2966	1314	1414	1350
19.65118271	19.50768307	26.13578961	12.08533253
11.72114477	11.98786455
ENSG00000128595 7914.774824	6950.279654	8345.509482
4876.22269	4890.064204	4485.136139	7736.854653
4750.474344	0.703612399	2.11E-08	1.03E-06	CALU
	7	128739292	128771807	+	4537
protein_coding	calumenin [Source:HGNC Symbol;Acc:HGNC:1458]	-
	6841	7498	8535	4854	5351	4571	82.46041647
	72.86359832	87.14394208	51.72877682	51.39546142
	47.03146185
ENSG00000231607 215.1948717	236.3725409	214.1378531
33.15108133	73.10879019	59.85414669	221.9017552
55.3713394	2.000200363	2.16E-08	1.05E-06	DLEU2
	13	49982552	50125720	-	5103
antisense	deleted in lymphocytic leukemia 2 [Source:HGNC
Symbol;Acc:HGNC:13748]	-	186	255	219	33	80
	61	1.993343244	2.203172845	1.988021159
0.312672429	0.683160923	0.558020691
ENSG00000104881 1126.880672	1881.710816	1375.762372
2910.263109	2715.991556	3241.936076	1461.451287
2956.06358	-1.015476771	2.22E-08	1.08E-06
PPP1R13L	19	45379634	45406349	-	5032
	protein_coding	protein phosphatase 1 regulatory subunit 13
like [Source:HGNC Symbol;Acc:HGNC:18838]	-	974	2030
	1407	2897	2972	3304	10.58554048	17.78645359
	12.95256919	27.83614431	25.73752434	30.65105603
ENSG00000122566 23173.94236	25406.80382	29312.44092
16385.67538	13498.62425	16272.47817	25964.3957
15385.5926	0.755003385	2.23E-08	1.08E-06
HNRNPA2B1	7	26173057	26201529	-	9267
	protein_coding	heterogeneous nuclear ribonucleoprotein A2/B1
[Source:HGNC Symbol;Acc:HGNC:5033]	-	20030	27409	29978
	16311	14771	16584	118.2051774	130.403121
149.8531747	85.10256328	69.45912897	83.54032527
ENSG00000101868 1055.149048	1095.656248	1088.289637
564.5729608	503.5367925	628.959148	1079.698311
565.6896337	0.932961081	2.26E-08	1.09E-06	POLA1

X 24693919 24996986 + 6336
protein_coding "DNA polymerase alpha 1, catalytic subunit
[Source:HGNC Symbol;Acc:HGNC:9173]" - 912 1182 1113
562 551 641 7.871806002 8.225006805
8.137339797 4.288667369 3.789614453 4.722684102
ENSG00000203805 500.9644056 225.2491272 374.4967934
79.36167954 30.15737595 116.7646468 366.9034421
75.42790077 2.283392488 2.29E-08 1.10E-06 PLPP4
10 120456954 120589855 + 1785
protein_coding phospholipid phosphatase 4 [Source:HGNC
Symbol;Acc:HGNC:23531] - 433 243 383 79 33
119 13.26613427 6.002083198 9.939465013
2.13988329 0.805627565 3.112108837
ENSG00000115461 142.3062861 99.18377207 80.17947013
282.2864804 319.8509571 444.4906303 107.2231761
348.8760226 -1.706006096 2.39E-08 1.15E-06 IGFBP5
2 216672105 216695525 - 6442
protein_coding insulin like growth factor binding protein 5
[Source:HGNC Symbol;Acc:HGNC:5474] - 123 107 82
281 350 453 1.044188976 0.732313458
0.589651744 2.109049709 2.367586863 3.282641966
ENSG00000117906 2577.710613 2174.627376 2350.627392
1258.736512 1502.385638 1136.247572 2367.655127
1299.123241 0.865445367 2.43E-08 1.16E-06 RCN2
15 76931619 76954392 + 10019
protein_coding reticulocalbin 2 [Source:HGNC Symbol;Acc:HGNC:9935]
- 2228 2346 2404 1253 1644 1158
12.16145459 10.32375262 11.11507871 6.046832114
7.150493799 5.395481635
ENSG00000179300 149.2480562 177.974619 301.1619122
32.1465031 24.67421669 62.79779325 209.4615291
39.87283768 2.39582265 2.51E-08 1.19E-06 RTL3
X 78656069 78659328 - 2648
protein_coding retrotransposon Gag like 3 [Source:HGNC
Symbol;Acc:HGNC:22997] - 129 192 308 32 27
64 2.664197658 3.196812803 5.388094345
0.584296405 0.444328715 1.12825698
ENSG00000075426 1759.738709 1713.005708 2623.433151
996.5415962 764.9007174 1086.20558 2032.059189
949.2159646 1.098525186 2.57E-08 1.22E-06 FOSL2
2 28392448 28417312 + 7157
protein_coding "FOS like 2, AP-1 transcription factor subunit
[Source:HGNC Symbol;Acc:HGNC:3798]" TF_bZIP 1521 1848 2683
992 837 1107 11.62232216 11.38426267
17.36569269 6.701651987 5.096277155 7.220422976
ENSG00000137563 1989.974082 1776.965337 1782.526513
673.0674088 1139.583267 864.4508727 1849.821977
892.3671828 1.051013204 2.74E-08 1.29E-06 GGH
8 63015079 63039171 - 3130
protein_coding gamma-glutamyl hydrolase [Source:HGNC

Symbol;Acc:HGNC:4248]   -   1720   1917   1823   670   1247
        881   30.05237657   27.0029824   26.98017653
10.34979342   17.36123892   13.13946271
ENSG00000186815 1221.75153   1477.560119   1977.108397
780.5572785   662.5484111   787.9160621   1558.806682
743.6739173   1.06832556   2.76E-08   1.30E-06   TPCN1
        12   113221050   113298585   +   8728
protein_coding two pore segment channel 1 [Source:HGNC
Symbol;Acc:HGNC:18182] -   1056   1594   2022   777   725
        803   6.616737313   8.052069921   10.73170855
4.304349205   3.619777421   4.294838559
ENSG00000102054 3006.943395   2849.447807   3055.620051
1450.610953   1831.375194   1839.779099   2970.670418
1707.255082   0.798906005   2.90E-08   1.37E-06   RBBP7
        X   16839283   16870414   -   7392
protein_coding "RB binding protein 7, chromatin remodeling factor
[Source:HGNC Symbol;Acc:HGNC:9890]"   -   2599   3074   3125
        1444   2004   1875   19.22821786   18.33478674
19.58350933   9.445097632   11.81392901   11.84091735
ENSG00000210135 258.0024537   557.0976356   362.7632124
1120.104718   858.1144249   1073.449778   392.6211006
1017.222974   -1.371037488   2.94E-08   1.38E-06   MT-TN
        MT   5657   5729   -   73   Mt_tRNA
mitochondrially encoded tRNA asparagine [Source:HGNC
Symbol;Acc:HGNC:7493]   -   223   601   371   1115   939
        1094   167.0616305   362.9824017   235.4255021
738.5046322   560.5331861   699.5850139
ENSG00000071539 1566.526109   1540.592796   1648.56813
873.9830532   965.9498904   723.1558378   1585.229012
854.3629271   0.891534394   2.97E-08   1.39E-06   TRIP13
        5   892643 919357 +   3560   protein_coding
thyroid hormone receptor interactor 13 [Source:HGNC
Symbol;Acc:HGNC:12307] -   1354   1662   1686   870   1057
        737   20.80000211   20.58329884   21.93865649
11.8159997   12.93848978   9.664146964
ENSG00000147862 317.0074991   350.3875312   508.4551764
149.6821551   141.648281   127.5580175   391.9500689
139.6294845   1.489813873   2.98E-08   1.39E-06   NFIB
        9   14081843   14398983   -   12765
protein_coding nuclear factor I B [Source:HGNC Symbol;Acc:HGNC:7785]
        CTF/NFI 274   378   520   149   155   130
1.173882035   1.305584359   1.887056834   0.564373568
0.52913864   0.475410281
ENSG00000173039 2493.252411   3481.628485   2914.817079
5263.989883   5046.334243   5185.72402   2963.232658
5165.349382   -0.801282712   3.03E-08   1.41E-06   RELA
        11   65653596   65663094   -   5464
protein_coding "RELA proto-oncogene, NF-kB subunit [Source:HGNC
Symbol;Acc:HGNC:9955]" RHD   2155   3756   2981   5240   5522
        5285   21.56906387   30.30741186   25.27282215

46.36836824	44.03969569	45.15234774
ENSG00000135636 67.10377719	106.5993812	69.42368755	0
	10.05245865	2.943646558	81.04228198	4.33203507
	4.202819519	3.14E-08	1.45E-06	DYSF	2
	71453722	71686768	+	8386	protein_coding
	dysferlin [Source:HGNC Symbol;Acc:HGNC:3097]	-	58
	115	71	0	11	3	0.378240343
0.604612247	0.392198516	0	0.057160553	0.016699845
ENSG00000225190 826.0706365	1016.865402	931.8418906
1543.032149	1896.259246	1662.17909	924.9259762
1700.490162	-0.878016839	3.15E-08	1.45E-06
PLEKHM1 17	45435900	45490749	-	9233
protein_coding	pleckstrin homology and RUN domain containing M1
[Source:HGNC Symbol;Acc:HGNC:29017]	-	714	1097	953
	1536	2075	1694	4.229120785	5.238389051
4.781371856	8.043583909	9.793408346	8.564787777
ENSG00000082898 6211.727237	5458.815268	6209.997741
3872.649046	3674.630567	3633.441069	5960.180082
3726.906894	0.677310497	3.26E-08	1.50E-06	XPO1
	2	61477849	61538626	-	11708
protein_coding	exportin 1 [Source:HGNC Symbol;Acc:HGNC:12825]	-
	5369	5889	6351	3855	4021	3703	25.0787221
	22.17648947	25.12822747	15.91999346	14.96614529
	14.76444186
ENSG00000146834 1528.346374	1956.793858	2165.823492
3779.223271	3036.756373	3657.971457	1883.654575
3491.317033	-0.889674661	3.38E-08	1.55E-06	MEPCE
	7	100428790	100434126	+	3420
protein_coding	methylphosphate capping enzyme [Source:HGNC
Symbol;Acc:HGNC:20247]	-	1321	2111	2215	3762	3323
	3728	21.12376972	27.21423215	30.00199127
53.18558024	42.34116789	50.88570941
ENSG00000154096 422.2910116	454.206059	693.2590771
136.6226382	193.738294	224.698354	523.2520492
185.0197621	1.499880718	3.47E-08	1.59E-06	THY1
	11	119417378	119424985	-	5925
protein_coding	Thy-1 cell surface antigen [Source:HGNC
Symbol;Acc:HGNC:11801]	-	365	490	709	136	212
	229	3.368986126	3.646210046	5.543197294
1.109818015	1.559215358	1.80423525
ENSG00000154133 726.5719323	977.0065025	732.3710137
1301.933376	2011.40559	1818.192358	811.9831495
1710.510441	-1.07446448	3.60E-08	1.64E-06	ROBO4
	11	124883691	124898500	-	7033
protein_coding	roundabout guidance receptor 4 [Source:HGNC
Symbol;Acc:HGNC:17985]	-	628	1054	749	1296	2201
	1853	4.883303751	6.607451004	4.933369632
8.909751689	13.63760235	12.29931254
ENSG00000112186 399.1517781	355.9492381	398.9417538
169.7737195	122.4572236	156.0132676	384.6809233

149.4147369 1.365100733 3.70E-08 1.68E-06 CAP2
6 17393216 17557792 + 3637
protein_coding cyclase associated actin cytoskeleton regulatory
protein 2 [Source:HGNC Symbol;Acc:HGNC:20039] - 345 384
408 169 134 159 5.187648081 4.655023538
5.196600345 2.246697593 1.605536174 2.040797244
ENSG00000229689 570.3821061 533.9238571 352.0074298
1190.425193 996.1072664 1008.689554 485.4377977
1065.074004 -1.134315132 3.75E-08 1.70E-06
AC009237.3 2 95525345 95532405 + 4978
transcribed_unprocessed_pseudogene Ankyrin repeat domain-
containing protein pseudogene - 493 576 360 1185
1090 1028 5.416100816 5.101542971 3.350040553
11.50971777 9.541797673 9.640160359
ENSG00000137845 1836.098179 1522.053773 1996.664366
1043.756773 822.4738897 972.3845798 1784.938773
946.2050807 0.915759743 3.77E-08 1.71E-06 ADAM10
15 58588807 58749978 - 16168
protein_coding ADAM metallopeptidase domain 10 [Source:HGNC
Symbol;Acc:HGNC:188] - 1587 1642 2042 1039 900
991 5.368035002 4.477656874 5.850620022
3.107137436 2.425743111 2.861299967
ENSG00000188643 8328.967103 9085.048131 7252.330853
12311.10611 14712.23017 13819.43938 8222.115363
13614.25855 -0.727544555 3.81E-08 1.72E-06
S100A16 1 153606886 153613145 - 1701
protein_coding S100 calcium binding protein A16 [Source:HGNC
Symbol;Acc:HGNC:20441] - 7199 9801 7417 12255 16099
14084 231.4528712 254.0387889 201.9883964
348.3455111 412.4327885 386.5162333
ENSG00000203706 77.51643227 104.7454789 94.84644637
8.036625776 11.88017841 15.69944831 92.36945252
11.87208416 2.959667438 3.91E-08 1.76E-06
SERTAD4-AS1 1 210231456 210234047 - 1004
antisense SERTAD4 antisense RNA 1 [Source:HGNC
Symbol;Acc:HGNC:32019] - 67 113 97 8 13
16 3.649520459 4.962250551 4.475488827
0.385263167 0.564245673 0.743930399
ENSG00000188610 285.7695339 369.8535052 551.4783067
157.7187809 98.69686676 124.614371 402.3671153
127.0100062 1.665600416 3.99E-08 1.79E-06 FAM72B
1 121167646 121185539 - 3033
protein_coding family with sequence similarity 72 member B
[Source:HGNC Symbol;Acc:HGNC:24805] - 247 399 564
157 108 127 4.453682525 5.800086137
8.614084933 2.502813326 1.551707799 1.954687548
ENSG00000179750 189.7417148 420.8358179 439.0314889
61.27927154 109.6631853 102.046414 349.8696739
90.99629029 1.943195616 4.14E-08 1.85E-06
APOBEC3B 22 38982347 38992804 + 1896

protein_coding apolipoprotein B mRNA editing enzyme catalytic subunit 3B [Source:HGNC Symbol;Acc:HGNC:17352] - 164 454
449 61 120 104 4.730425725 10.55726633
10.9700969 1.555581317 2.758046035 2.560595879
ENSG00000134243 1551.485607 1820.532041 2267.514527
1015.628582 980.5716485 654.4707515 1879.844058
883.5569941 1.089411728 4.37E-08 1.95E-06 SORT1
1 109309568 109397951 - 9160
protein_coding sortilin 1 [Source:HGNC Symbol;Acc:HGNC:11186] -
1341 1964 2319 1011 1073 667 8.006229165
9.453224411 11.72756182 5.336504726 5.104613281
3.39919748
ENSG00000038427 440.8023984 324.4328993 622.8575911
113.5173391 120.6295038 201.1491815 462.6976296
145.0986748 1.673227989 4.54E-08 2.02E-06 VCAN
5 83471465 83582303 + 14831
protein_coding versican [Source:HGNC Symbol;Acc:HGNC:2464] -
381 350 637 113 132 205 1.404912425
1.040474803 1.989626026 0.368391181 0.38784848
0.645252153
ENSG00000107438 1126.880672 1065.066861 1369.895581
572.6095866 640.6157741 648.5834584 1187.281038
620.6029397 0.935849757 4.61E-08 2.04E-06 PDLIM1
10 95237572 95291024 - 2239
protein_coding PDZ and LIM domain 1 [Source:HGNC Symbol;Acc:HGNC:2067] - 974 1149 1401 570 701
661 23.79028123 22.62558896 28.98588051
12.30897752 13.64339718 13.78140202
ENSG00000129173 340.1467327 354.0953358 525.0777495
166.7599849 133.4235421 79.47845708 406.4399393
126.5539947 1.683890038 4.84E-08 2.14E-06 E2F8
11 19224063 19241620 - 4140
protein_coding E2F transcription factor 8 [Source:HGNC Symbol;Acc:HGNC:24727] E2F 294 382 537 166 146
81 3.883664471 4.068150206 6.008642986
1.938692648 1.536777921 0.913336289
ENSG00000197363 274.1999171 327.2137527 310.9398963
644.9392185 583.9564617 701.5690964 304.1178554
643.4882589 -1.079954169 4.87E-08 2.14E-06 ZNF517
8 144798876 144811169 + 5308
protein_coding zinc finger protein 517 [Source:HGNC Symbol;Acc:HGNC:27984] zf-C2H2 237 353 318 642 639
715 2.441811555 2.932093054 2.775227955
5.847972617 5.246003086 6.288124958
ENSG00000144057 505.5922523 239.1533943 638.5023658
176.8057671 85.90282848 93.21547435 461.0826708
118.6413566 1.959202241 5.10E-08 2.24E-06
ST6GAL2 2 106801600 106887108 - 7708
protein_coding "ST6 beta-galactoside alpha-2,6-sialyltransferase 2 [Source:HGNC Symbol;Acc:HGNC:10861]" - 437 258 653

176	94	95	3.100519333	1.475747166
3.924406001	1.104007893	0.531428382	0.575344459
ENSG00000173207	1876.591838	2415.634673	2275.336914
1047.775086	1285.800848	1273.617744	2189.187808
1202.397892	0.864689421	5.18E-08	2.27E-06	CKS1B
1	154974653	154979249	+	2338
protein_coding	CDC28 protein kinase regulatory subunit 1B
[Source:HGNC Symbol;Acc:HGNC:19083]	-	1622	2606	2327
1043	1407	1298	37.94032579	49.14324866
46.10566944	21.5695467	26.22455868	25.91649233
ENSG00000131473	10987.66503	11129.90235	13073.16482
7526.300039	6501.199168	7598.532983	11730.24407
7208.677397	0.702498462	5.47E-08	2.39E-06	ACLY
17	41866908	41930542	-	5217
protein_coding	ATP citrate lyase [Source:HGNC Symbol;Acc:HGNC:115]
-	9497	12007	13370	7492	7114	7744
99.5543667	101.4723298	118.7170309	69.43495342
59.42259613	69.29318814
ENSG00000106462	1118.78194	1196.693923	1364.028791
652.9758443	713.7245643	610.3160531	1226.501551
659.0054872	0.896266577	5.50E-08	2.39E-06	EZH2
7	148807383	148884321	-	4522
protein_coding	enhancer of zeste 2 polycomb repressive complex 2
subunit [Source:HGNC Symbol;Acc:HGNC:3527]	-	967	1291
1395	650	781	622	11.69474156	12.58721476
14.2904566	6.949987366	7.526257511	6.421047202
ENSG00000121060	2346.318278	2723.382452	2366.272167
4932.47907	3694.735484	4274.174803	2478.657632
4300.463119	-0.79465757	5.89E-08	2.56E-06	TRIM25
17	56836387	56914080	-	9501
protein_coding	tripartite motif containing 25 [Source:HGNC
Symbol;Acc:HGNC:12932]	-	2028	2938	2420	4910	4043
4356	11.67329189	13.6337847	11.79908972
24.98695821	18.54355604	21.4025041
ENSG00000150093	12239.49757	10246.51791	12085.58842
6955.699609	7149.125821	7601.476629	11523.86797
7235.43402	0.671409455	5.91E-08	2.56E-06	ITGB1
10	32900319	33005792	-	6011
protein_coding	integrin subunit beta 1 [Source:HGNC
Symbol;Acc:HGNC:6153]	-	10579	11054	12360	6924	7823
7747	96.24819682	81.07868386	95.2520209
55.69440232	56.71333676	60.16346821
ENSG00000198836	3306.596469	2934.727312	3400.782891
2024.225117	1998.611552	1724.976883	3214.035558
1915.937851	0.746200525	6.14E-08	2.65E-06	OPA1
3	193593144	193697823	+	9052
protein_coding	"OPA1, mitochondrial dynamin like GTPase [Source:HGNC
Symbol;Acc:HGNC:8140]"	-	2858	3166	3478	2015	2187
1758	17.2668212	15.42056631	17.79866743
10.76295989	10.52841113	9.066096999

ENSG00000179218 13833.79076 15382.75418 20156.33655
7886.943621 8824.230976 10454.85136 16457.62716
9055.341986 0.86195169 6.36E-08 2.74E-06 CALR
19 12938578 12944489 + 2714
protein_coding calreticulin [Source:HGNC Symbol;Acc:HGNC:1455] -
11957 16595 20614 7851 9656 10655 240.9389973
269.5885056 351.8478359 139.8673513 155.0408039
183.2692686
ENSG00000166451 1084.07309 843.5255382 908.3747286
446.0327306 514.503111 481.7768201 945.3244523
480.7708872 0.974488711 6.51E-08 2.80E-06 CENPN
16 81006498 81033114 + 3568
protein_coding centromere protein N [Source:HGNC
Symbol;Acc:HGNC:30873] - 937 910 929 444 563
491 14.36181929 11.24476813 12.0612779
6.016713623 6.876099411 6.423957843
ENSG00000104415 2200.541107 1392.280614 2617.56636
513.3394715 183.6858354 294.3646558 2070.12936
330.4633209 2.647476809 6.66E-08 2.85E-06 WISP1
8 133191039 133231690 + 5190
protein_coding WNT1 inducible signaling pathway protein 1
[Source:HGNC Symbol;Acc:HGNC:12769] - 1902 1502 2677
511 201 300 20.04185277 12.75958451
23.89370344 4.760523998 1.687669094 2.698360263
ENSG00000146576 1641.728618 1894.688132 1589.900225
3011.72551 2848.501238 2714.042127 1708.772325
2858.089625 -0.741815864 6.96E-08 2.97E-06
C7orf26 7 6590017 6608726 + 2728 protein_coding
chromosome 7 open reading frame 26 [Source:HGNC
Symbol;Acc:HGNC:21702] - 1419 2044 1626 2998 3117
2766 28.44675564 33.03470884 27.61077717
53.1359536 49.79102125 47.3318899
ENSG00000113645 439.6454368 582.1253165 794.9501123
266.2132288 230.2926891 229.6044316 605.5736219
242.0367832 1.324201647 7.31E-08 3.11E-06 WWC1
5 168291651 168472303 + 10076
protein_coding WW and C2 domain containing 1 [Source:HGNC
Symbol;Acc:HGNC:29435] - 380 628 813 265 252
234 2.062481922 2.747928592 3.73770346
1.271624631 1.089860668 1.084110959
ENSG00000138166 1014.65539 1121.61088 792.0167171
1752.988997 1938.2968 1749.507271 976.094329
1813.59769 -0.893820805 7.49E-08 3.18E-06 DUSP5
10 110497838 110511544 + 2615
protein_coding dual specificity phosphatase 5 [Source:HGNC
Symbol;Acc:HGNC:3071] - 877 1210 810 1745 2121
1783 18.34098319 20.40082052 14.34880658
32.26450114 35.34496639 31.82919726
ENSG00000131408 2024.682933 2417.488575 2157.023306
3599.40377 3403.214183 3922.899647 2199.731605

3641.8392	-0.726981703	7.91E-08	3.35E-06	NR1H2
	19	50329653	50382982	+	3753
protein_coding	nuclear receptor subfamily 1 group H member 2
[Source:HGNC Symbol;Acc:HGNC:7965]	THR-like	1750	2608
	2206	3583	3724	3998	25.50082301	30.63818122
	27.22885627	46.16038902	43.24040028	49.72906127
ENSG00000158792 376.0125446	701.7020136	531.9223384
1144.214595	1346.115599	1114.66083	536.5456322
1201.663675	-1.161568746	7.94E-08	3.35E-06
SPATA2L 16	89696343	89701705	-	2482
protein_coding	spermatogenesis associated 2 like [Source:HGNC
Symbol;Acc:HGNC:28393]	-	325	757	544	1139	1473
	1136	7.161043247	13.44708222	10.15312138
22.18825577	25.86184875	21.36596881
ENSG00000104738 9320.48326	8920.97778	9745.716814
6017.42355	5580.028411	6400.468833	9329.059284
5999.306932	0.63696265	8.79E-08	3.70E-06	MCM4
	8	47960185	47978160	+	6973
protein_coding	minichromosome maintenance complex component 4
[Source:HGNC Symbol;Acc:HGNC:6947]	-	8056	9624	9967
	5990	6106	6523	63.18216525	60.8513065
66.21360731	41.53444137	38.15888995	43.6690505
ENSG00000177606 3695.335592	3758.786876	3419.361061
5607.555635	5599.219469	5619.42128	3624.49451
5608.732128	-0.629915133	9.00E-08	3.78E-06	JUN
	1	58780788	58784327	-	3540
protein_coding	"Jun proto-oncogene, AP-1 transcription factor subunit
[Source:HGNC Symbol;Acc:HGNC:6204]"	TF_bZIP 3194	4055	3497
	5582	6127	5727	49.34309225	50.50350905
45.76092832	76.24086002	75.42289811	75.5213869
ENSG00000160298 393.3669697	456.0599613	645.3469547
207.947692	172.7195168	209.9801212	498.2579619
196.8824433	1.340787175	9.29E-08	3.89E-06
C21orf58	21	46300181	46323875	-	5315
	protein_coding	chromosome 21 open reading frame 58
[Source:HGNC Symbol;Acc:HGNC:1300]	-	340	492	660
	207	189	214	3.498407222	4.08127437
5.752321123	1.883077929	1.54959117	1.879561499
ENSG00000179776 214.03791	87.13340724	320.7178805
27.12361199	22.84649694	53.96685357	207.2963992
34.64565417	2.581812176	1.04E-07	4.34E-06	CDH5
	16	66366622	66404786	+	6082
protein_coding	cadherin 5 [Source:HGNC Symbol;Acc:HGNC:1764]	-
	185	94	328	27	25	55	1.663489352
	0.681420815	2.498215414	0.214643907	0.179123345
	0.422145641
ENSG00000169744 337.8328093	231.7377852	441.9648841
128.5860124	106.0077458	93.21547435	337.1784929
109.2697442	1.625639732	1.07E-07	4.46E-06	LDB2
	4	16501541	16898809	-	5586

protein_coding LIM domain binding 2 [Source:HGNC
Symbol;Acc:HGNC:6533]   -   292   250   452   128   116
        95   2.858752996   1.973208762   3.748348474
1.107924726   0.904931395   0.793905316
ENSG00000005893 4718.089714   4189.819157   5213.621155
2712.361199   3001.115837   2927.94711   4707.176675
2880.474716   0.70845329   1.15E-07   4.78E-06   LAMP2
        X   120427827   120469365   -   5526
protein_coding lysosomal associated membrane protein 2 [Source:HGNC
Symbol;Acc:HGNC:6501]   -   4078   4520   5332   2700   3284
        2984   40.35813174   36.06297179   44.69734386
23.62403623   25.89708401   25.20774367
ENSG00000146648 3011.571242   1920.642764   3207.178805
1423.487341   970.5191898   1438.461952   2713.130937
1277.489494   1.086676468   1.15E-07   4.78E-06   EGFR
        7   55019021   55211628   +   12521
protein_coding epidermal growth factor receptor [Source:HGNC
Symbol;Acc:HGNC:3236]   -   2603   2072   3280   1417   1062
        1466   11.36919892   7.295997785   12.13493024
5.471823134   3.696103286   5.465639592
ENSG00000087086 42860.80224   57238.306   33562.93063
74266.4588   102422.6735   80546.01956   44554.01296
85745.05061   -0.944492205   1.24E-07   5.13E-06   FTL
        19   48965301   48966878   +   878
protein_coding ferritin light chain [Source:HGNC
Symbol;Acc:HGNC:3999]   -   37046   61749   34325   73928   112077
        82088   2307.498988   3100.768695   1811.000148
4071.136442   5562.635196   4364.466803
ENSG00000214357 118.0100909   119.5766972   304.0953074
26.11903377   31.07123583   43.17348286   180.5606985
33.45458415   2.433026554   1.25E-07   5.15E-06
NEURL1B 5   172641266   172691540   +   6424
protein_coding neuralized E3 ubiquitin protein ligase 1B [Source:HGNC
Symbol;Acc:HGNC:35422] -   102   129   311   26   34
        44   0.86833909   0.88535641   2.242628374
0.19569018   0.230638594   0.319737209
ENSG00000178295 1145.392059   848.1602939   968.020432
535.4401923   469.723977   515.1381477   987.1909283
506.767439   0.961504835   1.28E-07   5.25E-06   GEN1
        2   17753858   17788941   +   11827
protein_coding "GEN1, Holliday junction 5' flap endonuclease
[Source:HGNC Symbol;Acc:HGNC:26881]"   -   990   915   990
        533   514   525   4.577784144   3.410990071
3.877600419   2.178982933   1.893856723   2.072195577
ENSG00000029993 1839.569064   2016.118731   2833.65981
773.525231   1261.126631   1120.548123   2229.782535
1051.733328   1.084030506   1.36E-07   5.56E-06   HMGB3
        X   150980509   150990775   +   3967
protein_coding high mobility group box 3 [Source:HGNC
Symbol;Acc:HGNC:5004]   HMG   1590   2175   2898   770   1380

1142 21.91944919 24.17302598 33.84064534
9.384901984 15.15917211 13.4384735
ENSG00000136492 1080.602205 894.5078509 1096.112025
494.2524852 594.9227802 526.912734 1023.740694
538.6959998 0.925603444 1.44E-07 5.88E-06 BRIP1
17 61681266 61863521 - 7991
protein_coding BRCA1 interacting protein C-terminal helicase 1
[Source:HGNC Symbol;Acc:HGNC:20473] - 934 965 1121
492 651 537 6.39205434 5.324270855
6.498407444 2.976906461 3.550082581 3.137033748
ENSG00000100109 1831.470333 1925.27752 1734.61439
3390.451499 2789.100346 2915.191308 1830.454081
3031.581051 -0.72772634 1.46E-07 5.97E-06 TFIP11
22 26491225 26512505 - 5436
protein_coding tuftelin interacting protein 11 [Source:HGNC
Symbol;Acc:HGNC:17165] - 1583 2077 1774 3375 3052
2971 15.92561379 16.84577541 15.11738325
30.01895333 24.46603986 25.51345297
ENSG00000162390 288.0834573 358.7300915 237.6050151
681.1040345 631.4771753 617.1845617 294.806188
643.2552572 -1.124835581 1.52E-07 6.19E-06 ACOT11
1 54542257 54639192 + 9768
protein_coding acyl-CoA thioesterase 11 [Source:HGNC
Symbol;Acc:HGNC:18156] - 249 387 243 678 691
629 1.394082286 1.746784269 1.152399546
3.356025557 3.082698096 3.006014218
ENSG00000167778 477.8251721 660.9161634 549.5227099
1148.232908 1131.358528 987.1028126 562.7546818
1088.898083 -0.951050686 1.54E-07 6.26E-06 SPRYD3
12 53064316 53079420 - 3479
protein_coding SPRY domain containing 3 [Source:HGNC
Symbol;Acc:HGNC:25920] - 413 713 562 1143 1238
1006 6.492176759 9.035850963 7.483148137
15.88521211 15.50689384 13.49862672
ENSG00000122565 9613.194564 7860.545674 8064.881337
4616.03693 5686.036157 5242.634521 8512.873858
5181.569203 0.716080379 1.58E-07 6.38E-06 CBX3
7 26201162 26213356 + 4641
protein_coding chromobox 3 [Source:HGNC Symbol;Acc:HGNC:1553] -
8309 8480 8248 4595 6222 5343 97.91109403
80.55977449 82.32647864 47.87129365 58.42208283
53.74271983
ENSG00000029534 247.5897986 232.6647364 399.9195522
66.30216265 46.60685375 116.7646468 293.3913624
76.55788774 1.939927162 1.59E-07 6.41E-06 ANK1
8 41653220 41896762 - 10797
protein_coding ankyrin 1 [Source:HGNC Symbol;Acc:HGNC:492] -
214 251 409 66 51 119 1.083940353
1.02495448 1.754779946 0.295557545 0.205838056
0.514505351

ENSG00000079459 4918.244083 4989.777991 6598.183712
3362.323309 3108.037443 3265.485249 5502.068596
3245.282 0.761747839 1.63E-07 6.57E-06 FDFT1
8 11795573 11839309 + 5202
protein_coding farnesyl-diphosphate farnesyltransferase 1
[Source:HGNC Symbol;Acc:HGNC:3629] - 4251 5383 6748
3347 3401 3328 44.69052637 45.62343601
60.09068875 31.10903797 28.49015958 29.86475839
ENSG00000130522 2414.579017 3771.764192 3180.778248
4964.625573 6229.782784 6015.83235 3122.373819
5736.746902 -0.877239848 1.69E-07 6.78E-06 JUND
19 18279760 18281622 - 1863
protein_coding "JunD proto-oncogene, AP-1 transcription factor
subunit [Source:HGNC Symbol;Acc:HGNC:6206]" TF_bZIP 2087 4069
3253 4942 6817 6131 61.263853 96.29612093
80.88605683 128.2599607 159.4553286 153.6259201
ENSG00000141458 4012.343091 4521.667665 3231.623765
6657.339877 6473.783372 6593.768291 3921.878174
6574.963847 -0.745401212 1.79E-07 7.16E-06 NPC1
18 23506184 23586898 - 10102
protein_coding NPC intracellular cholesterol transporter 1
[Source:HGNC Symbol;Acc:HGNC:7897] - 3468 4878 3305
6627 7084 6720 18.77441603 21.28964353
15.15537041 31.71836722 30.55834193 31.05331328
ENSG00000164754 10855.7714 10065.76244 10950.36446
6821.086128 7441.560982 6509.383756 10623.9661
6924.010288 0.617576117 1.84E-07 7.34E-06 RAD21
8 116845935 116874866 - 6192
protein_coding RAD21 cohesin complex component [Source:HGNC
Symbol;Acc:HGNC:9811] - 9383 10859 11199 6790 8143
6634 82.87155374 77.32017769 83.78200567
53.02003903 57.30758205 50.01388087
ENSG00000174276 432.7036667 606.2260461 442.9426825
1015.628582 1032.661661 902.7182779 493.9574651
983.6695073 -0.992611356 1.96E-07 7.82E-06 ZNHIT2
11 65116403 65117708 - 1306
protein_coding zinc finger HIT-type containing 2 [Source:HGNC
Symbol;Acc:HGNC:1177] - 374 654 453 1011 1130
920 15.66113157 22.07844738 16.06783935
37.42908369 37.70455889 32.88445785
ENSG00000111110 163.1315963 228.0299806 309.9620979
21.09614266 48.4345735 78.49724156 233.7078916
49.34265257 2.243827763 2.05E-07 8.13E-06 PPM1H
12 62643982 62935037 - 7063
protein_coding "protein phosphatase, Mg2+/Mn2+ dependent 1H
[Source:HGNC Symbol;Acc:HGNC:18583]" - 141 246 317
21 53 80 1.091753566 1.535606207
2.079086277 0.143757763 0.326998124 0.528745661
ENSG00000174738 1442.73121 1114.195271 1378.695767
717.2688505 741.1403606 719.2309758 1311.874083

725.8800623 0.853281792 2.09E-07 8.29E-06 NR1D2
3 23945260 23980618 + 5820
protein_coding nuclear receptor subfamily 1 group D member 2
[Source:HGNC Symbol;Acc:HGNC:7963] THR-like 1247 1202
1410 714 811 733 11.71758686 9.105744098
11.22273179 5.931662651 6.07234541 5.879319014
ENSG00000170558 2148.477832 1712.078757 2232.313784
1250.699886 1145.980286 1067.562485 2030.956791
1154.747553 0.814393133 2.36E-07 9.32E-06 CDH2
18 27950966 28177446 - 4950
protein_coding cadherin 2 [Source:HGNC Symbol;Acc:HGNC:1759] -
1857 1847 2283 1245 1254 1088 20.51641227
16.45112698 21.36501334 12.16089025 11.03953893
10.26052853
ENSG00000075711 2906.287729 2485.156009 2662.545087
1551.068775 1768.318863 1589.569142 2684.662942
1636.318926 0.713902275 2.37E-07 9.33E-06 DLG1
3 197042560 197299300 - 11001
protein_coding discs large MAGUK scaffold protein 1 [Source:HGNC
Symbol;Acc:HGNC:2900] - 2512 2681 2723 1544 1935
1620 12.48769213 10.74480671 11.46615828
6.786038946 7.664916047 6.874306403
ENSG00000166670 26.61011854 50.05536161 47.91212239
152.6958897 137.0789816 254.1348195 41.52586751
181.3032303 -2.119526718 2.43E-07 9.55E-06 MMP10
11 102770503 102780628 - 1758
protein_coding matrix metallopeptidase 10 [Source:HGNC
Symbol;Acc:HGNC:7156] - 23 54 49 152 150
259 0.715490181 1.354281192 1.291158816
4.180477917 3.718184928 6.877441884
ENSG00000133119 2857.695339 2454.566621 2401.47291
1590.247325 1629.412161 1377.626589 2571.244957
1532.428692 0.746299476 2.52E-07 9.87E-06 RFC3
13 33818049 33966558 + 2874
protein_coding replication factor C subunit 3 [Source:HGNC
Symbol;Acc:HGNC:9971] - 2470 2648 2456 1583 1783
1404 47.00076235 40.62236228 39.58622187
26.631484 27.034801 22.80480588
ENSG00000178913 2324.336007 2274.7381 1981.019591
3464.790288 3861.057982 3343.001275 2193.364566
3556.283182 -0.6974348 2.61E-07 1.02E-05 TAF7
5 141260225 141320821 - 2723
protein_coding TATA-box binding protein associated factor 7
[Source:HGNC Symbol;Acc:HGNC:11541] - 2009 2454 2026
3449 4225 3407 40.34846415 39.73387078
34.46626759 61.24163398 67.61416198 58.40775693
ENSG00000087245 21981.11488 15031.4397 23492.58475
11298.49126 11270.63387 11942.37409 20168.37977
11503.83307 0.809943729 2.67E-07 1.04E-05 MMP2
16 55389700 55506691 + 4933

protein_coding matrix metallopeptidase 2 [Source:HGNC
Symbol;Acc:HGNC:7166] - 18999 16216 24026 11247 12333
12171 210.6271445 144.9327652 225.6175158
110.2368503 108.9472353 115.1757855
ENSG00000122483 650.2124618 525.5812969 616.9908006
287.3093715 281.4688422 309.0828886 597.5948531
292.6203675 1.029631749 2.84E-07 1.10E-05 CCDC18
1 93179919 93278730 + 5633
protein_coding coiled-coil domain containing 18 [Source:HGNC
Symbol;Acc:HGNC:30370] - 562 567 631 286 308
315 5.456212511 4.437897483 5.189100283
2.454864346 2.382701087 2.610458771
ENSG00000242802 1423.062861 1992.944953 1592.83362
3089.078033 2722.388575 2913.228877 1669.613811
2908.231828 -0.799981319 2.84E-07 1.10E-05 AP5Z1
7 4775615 4794395 + 8396 protein_coding
adaptor related protein complex 5 subunit zeta 1 [Source:HGNC
Symbol;Acc:HGNC:22197] - 1230 2150 1629 3075 2979
2969 8.011750097 11.29015718 8.987752617
17.70817914 15.46167966 16.50759493
ENSG00000077152 1100.270554 996.4724764 937.7086811
475.165499 586.6980413 570.0862168 1011.483904
543.9832524 0.894147839 2.84E-07 1.10E-05 UBE2T
1 202331657 202341980 - 1652
protein_coding ubiquitin conjugating enzyme E2 T [Source:HGNC
Symbol;Acc:HGNC:25009] - 951 1075 959 473 642
581 31.4822082 28.69012096 26.89125184
13.8437043 16.9349369 16.41769281
ENSG00000152402 540.3011026 365.2187495 603.3016228
246.1216644 84.9889686 118.7270779 502.9404916
149.9459036 1.747279943 2.87E-07 1.11E-05
GUCY1A2 11 106674012 107018524 - 16361
protein_coding guanylate cyclase 1 soluble subunit alpha 2
[Source:HGNC Symbol;Acc:HGNC:4684] - 467 394 617
245 93 121 1.560995851 1.061745271
1.74693914 0.724031492 0.247703248 0.345240361
ENSG00000182220 1626.688116 1604.552425 1621.189774
934.2577465 922.9984762 1047.938175 1617.476772
968.3981325 0.740120754 2.92E-07 1.12E-05
ATP6AP2 X 40579372 40606848 + 9560
protein_coding ATPase H+ transporting accessory protein 2
[Source:HGNC Symbol;Acc:HGNC:18305] - 1406 1731 1658
930 1010 1068 8.043075421 7.983128751
8.033949356 4.703555499 4.603859689 5.215061043
ENSG00000101096 414.1922799 115.8688926 256.183185
48.21975466 61.22861179 69.66630188 262.0814525
59.70488944 2.131667392 3.06E-07 1.18E-05 NFATC2
20 51386957 51562831 - 8286
protein_coding nuclear factor of activated T cells 2 [Source:HGNC
Symbol;Acc:HGNC:7776] RHD 358 125 262 48 67

71	2.362831818	0.665118522	1.464734187
0.280089949	0.352361517	0.399999507
ENSG00000145833 3723.102672	3413.034101	3974.750562
2556.651575	2027.855068	2123.350384	3703.629112
2235.952342	0.728146946	3.10E-07	1.19E-05	DDX46
5	134758771	134855133	+	9222
protein_coding	DEAD-box helicase 46 [Source:HGNC
Symbol;Acc:HGNC:18681]	-	3218	3682	4065	2545	2219
2164	19.08339478	17.60324057	20.41916074
13.3433195	10.48553954	10.95413716
ENSG00000176531 107.5974358	157.5816939	145.691964
314.4329835	372.85483	357.1624491	136.9570313
348.1500875	-1.343117653	3.16E-07	1.21E-05	PHLDB3
19	43474954	43504935	-	4553
protein_coding	pleckstrin homology like domain family B member 3
[Source:HGNC Symbol;Acc:HGNC:30499]	-	93	170	149
313	408	364	1.117069029	1.646209975
1.51597163	3.32389965	3.90500065	3.732069899
ENSG00000161681 130.7366694	343.8988733	213.1600547
635.8980145	560.1961049	739.8365017	229.2651991
645.310207	-1.489459404	3.17E-07	1.21E-05	SHANK1
19	50661827	50719450	-	7567
protein_coding	SH3 and multiple ankyrin repeat domains 1 [Source:HGNC
Symbol;Acc:HGNC:15474]	-	113	371	218	633	613
754	0.816675303	2.16164381	1.334551124
4.044652293	3.530167711	4.651506667
ENSG00000070081 937.1389573	767.5155446	814.5060807
437.9961048	477.034856	410.1480871	839.7201942
441.7263493	0.925882402	3.20E-07	1.22E-05	NUCB2
11	17208153	17349980	+	9025
protein_coding	nucleobindin 2 [Source:HGNC Symbol;Acc:HGNC:8044]
-	810	828	833	436	522	418
4.908316068	4.044986571	4.27563129	2.335826037
2.520472075	2.162096666
ENSG00000178896 1151.176867	1258.799649	1055.044491
1763.03478	1986.731374	2224.415583	1155.007003
1991.393912	-0.785787937	3.27E-07	1.24E-05	EXOSC4
8	144078626	144080647	+	1407
protein_coding	exosome component 4 [Source:HGNC
Symbol;Acc:HGNC:18189]	-	995	1358	1079	1755	2174
2267	38.67440991	42.55392565	35.52464961
60.30929335	67.33239152	75.21482658
ENSG00000099998 47.4354287	89.91426066	176.9815133	0
6.397019142	15.69944831	104.7770676	7.365489151
3.829633887	3.30E-07	1.25E-05	GGT5	22
24219654	24245142	-	6054	protein_coding
gamma-glutamyltransferase 5 [Source:HGNC Symbol;Acc:HGNC:4260]
-	41	97	181	0	7	16
0.3703703	0.70642047	1.384964412	0	0.050386503
0.123373988

ENSG00000204291 68.26073887 172.4129122 145.691964
24.10987733 27.41579632 15.69944831 128.7885384
22.40837399 2.523783453 3.32E-07 1.25E-05
COL15A1 9 98943179 99070792 + 6653
protein_coding collagen type XV alpha 1 chain [Source:HGNC
Symbol;Acc:HGNC:2192] - 59 186 149 24 30
16 0.484985999 1.232620479 1.037459617
0.174419459 0.196499898 0.112266064
ENSG00000127022 12226.77099 10593.19764 14635.68669
7249.03645 7214.923732 8215.717545 12485.21844
7559.892576 0.723781882 3.40E-07 1.28E-05 CANX
5 179678628 179730925 + 6235
protein_coding calnexin [Source:HGNC Symbol;Acc:HGNC:1473] -
10568 11428 14968 7216 7895 8373 92.69387962
80.81048846 111.2063976 55.95788394 55.17905701
62.68890287
ENSG00000099901 4913.616237 5353.142838 5847.234529
3243.783079 3191.198692 3692.314 5371.331201
3375.765257 0.670205361 3.42E-07 1.29E-05 RANBP1
22 20115938 20127357 + 4622
protein_coding RAN binding protein 1 [Source:HGNC
Symbol;Acc:HGNC:9847] - 4247 5775 5980 3229 3492
3763 50.25126889 55.08787316 59.93406005
33.77841914 32.92326454 38.00583801
ENSG00000198899 36632.87754 40050.77794 47179.75138
64055.92575 62096.77867 67991.36699 41287.80229
64714.69047 -0.648357509 3.46E-07 1.30E-05 MT-
ATP6 MT 8527 9207 + 681 protein_coding
mitochondrially encoded ATP synthase membrane subunit 6 [Source:HGNC
Symbol;Acc:HGNC:7414] - 31663 43207 48251 63764 67950
69293 2542.726573 2797.312326 3282.174465
4527.199758 4348.114249 4749.941798
ENSG00000171425 342.460656 526.508248 425.342311
953.3447327 741.1403606 1081.299502 431.4370717
925.2615319 -1.098924272 3.48E-07 1.30E-05 ZNF581
19 55635459 55645622 + 1736
protein_coding zinc finger protein 581 [Source:HGNC
Symbol;Acc:HGNC:25017] zf-C2H2 296 568 435 949 811
1102 9.324739392 14.42555639 11.60758819
26.43125034 20.35774786 29.63315615
ENSG00000108387 37.02277362 44.49365476 85.06846221
2.009156444 4.569299387 2.943646558 55.52829686
3.17403413 4.12071414 3.61E-07 1.35E-05 4-Sep
17 58520250 58540818 - 8012
protein_coding septin 4 [Source:HGNC Symbol;Acc:HGNC:9165] -
32 48 87 2 5 3 0.218425707
0.264140049 0.503014805 0.012069528 0.027194912
0.017479393
ENSG00000115524 7952.954559 7261.735237 7593.5825
5028.918579 4972.311593 5199.461038 7602.757432

5066.89707	0.585360972	3.70E-07	1.37E-05	SF3B1
	2	197389784	197435091	-	9164
protein_coding	splicing factor 3b subunit 1 [Source:HGNC
Symbol;Acc:HGNC:10768]	-	6874	7834	7766	5006	5441
	5299	41.02222006	37.69054737	39.2567879
26.41234621	25.87332496	26.99323122
ENSG00000073008 2033.938626	2283.08066	2326.182432
3609.449552	3261.565902	3710.957095	2214.400573
3527.324183	-0.671320158	3.74E-07	1.39E-05	PVR
	19	44643798	44663583	+	3519
protein_coding	poliovirus receptor [Source:HGNC Symbol;Acc:HGNC:9705]
	-	1758	2463	2379	3593	3569	3782
27.3208569	30.85880492	31.31681819	49.36727629
44.19629654	50.17048432
ENSG00000073282 56.69112211	85.27950496	190.6706912
15.06867333	14.62175804	16.68066383	110.8804394
15.45703173	2.844686001	3.79E-07	1.40E-05	TP63
	3	189631416	189897279	+	7454
protein_coding	tumor protein p63 [Source:HGNC Symbol;Acc:HGNC:15979]
	P53	49	92	195	15	16	17
0.359502077	0.544167245	1.211846684	0.097297815
0.09353824	0.106464684
ENSG00000148848 1913.614612	1751.937656	1618.256379
1038.733882	901.0658391	1126.435416	1761.269549
1022.078379	0.785084393	4.28E-07	1.58E-05	ADAM12
	10	126012381	126388455	-	9860
protein_coding	ADAM metallopeptidase domain 12 [Source:HGNC
Symbol;Acc:HGNC:190]	-	1654	1890	1655	1034	986
	1148	9.173885239	8.451209101	7.7754143
5.070430571	4.357712735	5.435143425
ENSG00000170540 3607.406505	3059.865716	4222.133561
2070.435716	2344.050586	1973.22441	3629.801927
2129.236904	0.769400197	4.78E-07	1.76E-05
ARL6IP1 16	18791667	18801678	-	3666
protein_coding	ADP ribosylation factor like GTPase 6 interacting
protein 1 [Source:HGNC Symbol;Acc:HGNC:697]	-	3118	3301
	4318	2061	2565	2011	46.51342962	39.69968142
	54.56229548	27.1823342	30.48972495	25.60740946
ENSG00000159111 1588.508381	1687.051076	1478.431205
2590.807235	2697.714358	2369.63548	1584.663554
2552.719024	-0.68784334	4.82E-07	1.77E-05	MRPL10
	17	47823272	47831534	-	2764
protein_coding	mitochondrial ribosomal protein L10 [Source:HGNC
Symbol;Acc:HGNC:14055]	-	1373	1820	1512	2579	2952
	2415	27.16609476	29.03135506	25.34056001
45.11433083	46.54112878	40.78731643
ENSG00000060339 2125.338598	2108.813845	2379.961345
1392.345416	1379.014555	1348.190124	2204.704596
1373.183365	0.68313161	4.92E-07	1.80E-05	CCAR1
	10	68721012	68792377	+	6059

protein_coding cell division cycle and apoptosis regulator 1
[Source:HGNC Symbol;Acc:HGNC:24236]   -   1837   2275   2434
        1386   1509   1374   16.5807021   16.55443666
18.60895892   11.06021309   10.85292708   10.58599821
ENSG00000198853 608.5618414   814.7900528   618.9463974
1388.327103   1106.684312   1350.152555   680.7660972
1281.721323   -0.911822699   4.97E-07   1.82E-05   RUSC2
        9   35490127   35561898   +   5682
protein_coding RUN and SH3 domain containing 2 [Source:HGNC
Symbol;Acc:HGNC:23625] -   526   879   633   1382   1211
        1376   5.062665428   6.820584656   5.160656305
11.76001918   9.287557414   11.30480929
ENSG00000162591 126.1088227   280.8661957   364.7188092
72.32963199   24.67421669   67.70387084   257.2312759
54.90257317   2.232071626   5.01E-07   1.83E-05   MEGF6
        1   3489920 3611495 -   7039   protein_coding
multiple EGF like domains 6 [Source:HGNC Symbol;Acc:HGNC:3232] -
        109   303   373   72   27   69   0.846857319
        1.897866331   2.454710738   0.494564282   0.16715193
        0.457598046
ENSG00000115216 2909.758614   2797.538543   2779.880897
4527.634047   4215.635615   4183.902975   2829.059352
4309.057545   -0.607112655   5.07E-07   1.84E-05   NRBP1
        2   27427790   27442259   +   5329
protein_coding nuclear receptor binding protein 1 [Source:HGNC
Symbol;Acc:HGNC:7993]   -   2515   3018   2843   4507   4613
        4264   25.80993922   24.96936358   24.71346241
40.89244275   37.72214083   37.35231398
ENSG00000005022 11659.85977   10042.58866   12743.64676
7788.494955   7158.26442   6949.949524   11482.03173
7298.902967   0.653610237   5.07E-07   1.84E-05
SLC25A5 X   119468400   119471319   +   1500
protein_coding solute carrier family 25 member 5 [Source:HGNC
Symbol;Acc:HGNC:10991] -   10078   10834   13033   7753   7833
        7083   367.432703   318.4428706   402.4904609
249.9077598   227.559759   220.4306689
ENSG00000076003 3758.968484   3949.738811   3852.52576
1978.014519   2693.145059   2324.499566   3853.744352
2331.886381   0.724619506   5.18E-07   1.87E-05   MCM6
        2   135839626   135876426   -   4118
protein_coding minichromosome maintenance complex component 6
[Source:HGNC Symbol;Acc:HGNC:6949]   -   3249   4261   3940
        1969   2947   2369   43.14774285   45.62040641
44.32128418   23.11854993   31.18547701   26.85497511
ENSG00000196611 1124.566749   1126.245636   660.9917293
1822.304895   1978.506635   1947.712806   970.6013714
1916.174779   -0.981666114   5.44E-07   1.97E-05   MMP1
        11   102789920   102798160   -   1970
protein_coding matrix metallopeptidase 1 [Source:HGNC
Symbol;Acc:HGNC:7155]   -   972   1215   676   1814   2165

1985	26.98328072	27.19217896	15.89581961
44.52175476	47.89059935	47.03707646
ENSG00000163527 6097.188031	4797.899105	5790.522221
3393.465234	3554.001063	3551.018965	5561.869786
3499.495087	0.668240395	5.46E-07	1.97E-05	STT3B
3	31532638	31637622	+	5012
protein_coding	"STT3B, catalytic subunit of the
oligosaccharyltransferase complex [Source:HGNC
Symbol;Acc:HGNC:30611]" -	5270	5176	5922	3378	3889
3619	57.5034982	45.53204572	54.73432877
32.5874066	33.81313812	33.7072682
ENSG00000168385 8081.377305	5888.920598	6872.945067
4218.223954	4516.295514	3946.448819	6947.747657
4226.989429	0.716705032	5.50E-07	1.98E-05	2-Sep
2	241315100	241354027	+	7378
protein_coding	septin 2 [Source:HGNC Symbol;Acc:HGNC:7729]	-
6985	6353	7029	4199	4942	4022	51.77528192
37.96419211	44.13237992	27.51746611	29.18923331
25.44775361
ENSG00000185085 935.9819957	1153.127219	1229.092609
1822.304895	1887.120647	1960.468608	1106.067275
1889.964717	-0.772190571	5.58E-07	2.00E-05	INTS5
11	62646848	62653302	-	3285
protein_coding	integrator complex subunit 5 [Source:HGNC
Symbol;Acc:HGNC:29352]	-	809	1244	1257	1814	2065
1998	13.46814739	16.69625098	17.72565775
26.69949981	27.39323226	28.39266418
ENSG00000108582 2593.908077	2264.541637	3309.847639
1628.421298	1557.217231	1474.766926	2722.765784
1553.468485	0.809594326	5.66E-07	2.03E-05	CPD
17	30378905	30469989	+	12532
protein_coding	carboxypeptidase D [Source:HGNC Symbol;Acc:HGNC:2301]
-	2242	2443	3385	1621	1704	1503
9.783853358	8.594825007	12.51240354	6.254085945
5.925265322	5.598666911
ENSG00000138346 719.6301623	512.6039809	557.3450972
317.4467182	161.7531983	241.3790178	596.5264135
240.1929781	1.312824715	5.67E-07	2.03E-05	DNA2
10	68414064	68472121	-	4867
protein_coding	DNA replication helicase/nuclease 2 [Source:HGNC
Symbol;Acc:HGNC:2939]	-	622	553	570	316	177
246	6.989140181	5.009538781	5.42520265
3.139257589	1.584785605	2.359498995
ENSG00000149177 768.2225527	701.7020136	869.262792
405.8496017	285.1242818	433.6972596	779.7291194
374.890381	1.05716297	5.70E-07	2.03E-05	PTPRJ
11	47980558	48170841	+	9620
protein_coding	"protein tyrosine phosphatase, receptor type J
[Source:HGNC Symbol;Acc:HGNC:9673]"	-	664	757	889
404	312	442	3.774745883	3.469403126

4.280841466   2.030521113   1.413312239   2.144831766
ENSG00000075223 2062.862668   926.9511408   1315.13887
659.0033136   600.4059395   640.7337342   1434.984226
633.3809958   1.179159478   5.84E-07   2.08E-05   SEMA3C
7   80742538   80922359   -   6268
protein_coding semaphorin 3C [Source:HGNC Symbol;Acc:HGNC:10725]
-       1783   1000   1345   656   657   653
15.55668536   7.034042211   9.940214413   5.060297708
4.56767273   4.863290692
ENSG00000277443 3526.419188   3861.678453   6002.704477
2242.218592   2495.751325   2378.466419   4463.600706
2372.145445   0.91211312   5.97E-07   2.12E-05   MARCKS
6   113857362   113863471   +   4275
protein_coding myristoylated alanine rich protein kinase C substrate
[Source:HGNC Symbol;Acc:HGNC:6759]   -   3048   4166   6139
2232   2731   2424   38.99182443   42.96522639
66.52179662   25.24406488   27.83839411   26.46930463
ENSG00000164465 1037.794623   965.8830888   1101.001017
534.4356141   652.4959525   498.4574839   1034.892909
561.7963502   0.880828392   6.02E-07   2.13E-05   DCBLD1
6   117453817   117569858   +   6819
protein_coding "discoidin, CUB and LCCL domain containing 1
[Source:HGNC Symbol;Acc:HGNC:21479]"   -   897   1042   1126
532   714   508   7.193934269   6.737223991
7.649272909   3.77217783   4.562849235   3.477675515
ENSG00000121210 340.1467327   213.1987624   408.719738
124.5676995   106.0077458   104.9900606   320.688411
111.8551686   1.519266883   6.12E-07   2.16E-05
TMEM131L   4   153466346   153636711   +   6393
protein_coding transmembrane 131 like [Source:HGNC
Symbol;Acc:HGNC:29146] -   294   230   418   124   116
107   2.514996232   1.586196874   3.028823967
0.937817208   0.790700262   0.781313105
ENSG00000125845 27.76708022   43.56670362   21.51156516
147.6729986   102.3523063   178.5812245   30.94844966
142.8688432   -2.202285531   6.65E-07   2.34E-05   BMP2
20   6767664 6780280 +   3601   protein_coding bone
morphogenetic protein 2 [Source:HGNC Symbol;Acc:HGNC:1069]   -
24   47   22   147   112   182   0.364487663
0.575451458   0.283010152   1.973764938   1.355356363
2.35936049
ENSG00000149485 8223.683591   7778.973974   10076.21268
5465.910106   4770.34856   5791.133996   8692.956748
5342.464221   0.702406513   6.66E-07   2.34E-05   FADS1
11   61799625   61829318   -   10230
protein_coding fatty acid desaturase 1 [Source:HGNC
Symbol;Acc:HGNC:3574]   -   7108   8392   10305   5441   5220
5902   37.99850394   36.16794216   46.66323227
25.71605279   22.23583625   26.93206211
ENSG00000100335 1906.672842   1915.081057   1820.660651

2949.44166	2871.347735	2858.280808	1880.80485
2893.023401	-0.621247704	7.22E-07	2.53E-05	MIEF1
	22	39499432	39518132	+	7555
protein_coding	mitochondrial elongation factor 1 [Source:HGNC
Symbol;Acc:HGNC:25979]	-	1648	2066	1862	2936	3142
	2913	11.92936842	12.05673753	11.41688598
18.78982776	18.12300915	17.9991523
ENSG00000085662 4927.499777	6279.167028	6876.856261
3469.813179	3851.005523	3634.422284	6027.841022
3651.746995	0.723194329	7.25E-07	2.54E-05	AKR1B1
	7	134442350	134459284	-	3660
protein_coding	aldo-keto reductase family 1 member B [Source:HGNC
Symbol;Acc:HGNC:381]	-	4259	6774	7033	3454	4214
	3704	63.63869532	81.6014855	89.01475233
45.62915901	50.17322805	47.24283251
ENSG00000178177 588.8934929	561.7323914	611.1240101
246.1216644	345.4390337	234.5105092	587.2499648
275.3570691	1.09164879	7.29E-07	2.54E-05	LCORL
	4	17841199	18021876	-	14858
protein_coding	ligand dependent nuclear receptor corepressor like
[Source:HGNC Symbol;Acc:HGNC:30776]	HTH	509	606	625
	245	378	239	1.873493286	1.798234097
1.948597402	0.797272798	1.108638723	0.750902556
ENSG00000168243 915.1566855	586.7600722	965.0870368
315.4375617	417.633964	400.3359319	822.3345981
377.8024859	1.121162097	7.46E-07	2.60E-05	GNG4
	1	235547687	235650754	-	5401
protein_coding	G protein subunit gamma 4 [Source:HGNC
Symbol;Acc:HGNC:4407]	-	791	633	987	314	457
	408	8.009345328	5.167297792	8.465359989
2.810973085	3.687233327	3.526403644
ENSG00000189320 333.2049626	373.5613098	238.5828135
858.9143798	559.282245	702.5503119	315.116362
706.9156456	-1.165346226	7.57E-07	2.63E-05
FAM180A 7	135728348	135748846	-	2526
protein_coding	family with sequence similarity 180 member A
[Source:HGNC Symbol;Acc:HGNC:33773]	-	288	403	244
	855	612	716	6.235249759	7.03405335
4.474648607	16.36567736	10.55787884	13.23200669
ENSG00000164761 583.1086846	832.4021245	815.4838791
289.3185279	336.3004349	420.9414579	743.664896
348.8534736	1.092804458	8.08E-07	2.80E-05
TNFRSF11B	8	118923557	118952200	-	2827
	protein_coding	TNF receptor superfamily member 11b
[Source:HGNC Symbol;Acc:HGNC:11909]	-	504	898	834
	288	368	429	9.749883821	14.00504428
13.66604033	4.925699299	5.672579719	7.08398315
ENSG00000128050 8464.33162	8082.086997	8623.204232
5129.376402	5887.08533	5732.261065	8389.874283
5582.907599	0.587566378	8.39E-07	2.90E-05	PAICS

4 56435741 56464579 + 7517
protein_coding phosphoribosylaminoimidazole carboxylase and
phosphoribosylaminoimidazolesuccinocarboxamide synthase [Source:HGNC
Symbol;Acc:HGNC:8587] - 7316 8719 8819 5106 6442
5842 53.22600364 51.13945382 54.34720929
32.8425959 37.34519814 36.27964579
ENSG00000115159 1719.24505 1725.983024 2070.977045
1227.594587 981.4855083 961.5912091 1838.73504
1056.890435 0.799208807 8.63E-07 2.98E-05 GPD2
2 156435290 156613735 + 7155
protein_coding glycerol-3-phosphate dehydrogenase 2 [Source:HGNC
Symbol;Acc:HGNC:4456] - 1486 1862 2118 1222 1074
980 11.35805282 11.47371337 13.71256734
8.257770034 6.541136936 6.393850441
ENSG00000031691 813.344058 750.8304241 623.8353895
374.7076768 405.7537856 349.3127249 729.3366239
376.5913958 0.952863267 8.75E-07 3.01E-05 CENPQ
6 49463378 49493107 + 1733
protein_coding centromere protein Q [Source:HGNC
Symbol;Acc:HGNC:21347] - 703 810 638 373 444
356 22.18459349 20.60726776 17.05393376
10.40666287 11.16459581 9.589533289
ENSG00000131043 1764.366555 2100.471285 1595.767015
3284.970786 2800.980524 2991.726119 1820.201619
3025.892476 -0.73297454 8.98E-07 3.09E-05 AAR2
20 36236459 36270918 + 3101
protein_coding AAR2 splicing factor homolog [Source:HGNC
Symbol;Acc:HGNC:15886] - 1525 2266 1632 3270 3065
3049 26.89445761 32.21751929 24.37927824
50.98556108 43.07123358 45.89883833
ENSG00000145217 195.5265232 261.4002217 251.294193
630.8751234 446.8774801 492.5701908 236.073646
523.4409314 -1.14663378 9.29E-07 3.18E-05
SLC26A1 4 979073 993440 - 4293 protein_coding solute
carrier family 26 member 1 [Source:HGNC Symbol;Acc:HGNC:10993] -
169 282 257 628 489 502 2.152883498
2.896157511 2.773158489 7.072939964 4.963711921
5.458695444
ENSG00000144567 1722.715935 1751.010705 1643.679138
2699.301683 2640.141186 2567.841014 1705.801926
2635.761294 -0.627772375 9.30E-07 3.18E-05
RETREG2 2 219176225 219185479 + 5689
protein_coding reticulophagy regulator family member 2 [Source:HGNC
Symbol;Acc:HGNC:28450] - 1489 1889 1681 2687 2889
2617 14.31375162 14.63962601 13.68781843
22.8366791 22.12942888 21.47404331
ENSG00000143799 7336.293986 7994.95359 9792.651138
5230.838802 4480.654979 5580.172659 8374.632904
5097.222147 0.716442599 9.40E-07 3.21E-05 PARP1
1 226360691 226408079 - 7169

protein_coding	poly(ADP-ribose) polymerase 1 [Source:HGNC Symbol;Acc:HGNC:270]	Others	6341	8625	10015	5207	4903	5687	48.37198233	53.04378198	64.71349106	35.11803552	29.80313229	37.03144711
ENSG00000076242 1292.326192	1442.335975	1490.164786	624.8476541	830.6986286	879.1691054	1408.275651	778.2384627	0.855642007	9.48E-07	3.23E-05	MLH1	3	36993332	37050918	+	3532	protein_coding	mutL homolog 1 [Source:HGNC Symbol;Acc:HGNC:7127]	-	1117	1556	1524	622	909	896	17.29526381	19.42329274	19.98787975	8.514730493	11.21506477	11.84222615
ENSG00000145715 2534.903031	2481.448204	2793.570075	1509.881068	1548.992492	1793.66197	2603.307104	1617.511843	0.686626692	9.60E-07	3.26E-05	RASA1	5	87267888	87391931	+	5232	protein_coding	RAS p21 protein activator 1 [Source:HGNC Symbol;Acc:HGNC:9871]	-	2191	2677	2857	1503	1695	1828	22.90178615	22.55872727	25.29559859	13.88968708	14.11759	16.31001994
ENSG00000058804 3260.318002	2713.185989	3436.961433	2045.32126	1753.697105	1955.56253	3136.821808	1918.193632	0.709533846	9.67E-07	3.28E-05	NDC1	1	53765460	53838860	-	5135	protein_coding	NDC1 transmembrane nucleoporin [Source:HGNC Symbol;Acc:HGNC:25525]	-	2818	2927	3515	2036	1919	1993	30.01202182	25.13137395	31.70934982	19.17072521	16.28520105	18.11810977
ENSG00000164904 1420.748938	1585.086451	1856.839192	879.0059443	886.4440811	1032.238726	1620.891527	932.5629173	0.797895111	9.90E-07	3.35E-05	ALDH7A1 5	126531200	126595418	-	17970	protein_coding	aldehyde dehydrogenase 7 family member A1 [Source:HGNC Symbol;Acc:HGNC:877]	-	1228	1710	1899	875	970	1052	3.737188488	4.195483247	4.895300355	2.354296692	2.35224338	2.732836808
ENSG00000173846 537.9871792	828.6943199	493.7882002	1122.113874	1530.715295	1205.913873	620.1565664	1286.247681	-1.051706155	9.92E-07	3.35E-05	PLK3	1	44800225	44805990	+	3479	protein_coding	polo like kinase 3 [Source:HGNC Symbol;Acc:HGNC:2154]	-	465	894	505	1117	1675	1229	7.309593688	11.32966446	6.724181155	15.5238687	20.980652	16.49086703
ENSG00000161381 281.1416872	46.34755704	260.0943787	44.20144177	26.50193645	20.60552591	195.8612076	30.43630137	2.6858776	9.99E-07	3.36E-05	PLXDC1	17	39063303	39154394	-	9277	protein_coding	plexin domain containing 1 [Source:HGNC Symbol;Acc:HGNC:20945]	-	243	50	266	44	29

21	1.432496039	0.23762734	1.328239942
0.229322325	0.13622256	0.105671476
ENSG00000185215 699.9618138	914.900776	1268.204546
452.0601999	474.2932764	449.3967079	961.0223785
458.5833947	1.067985868	1.03E-06	3.45E-05
TNFAIP2 14	103123442	103137439	+	5029
protein_coding	TNF alpha induced protein 2 [Source:HGNC
Symbol;Acc:HGNC:11895]	-	605	987	1297	450	519
458	6.579129756	8.653055216	11.94705316
4.326454041	4.497221932	4.251379541
ENSG00000140937 194.3695615	55.61706845	220.9824421
12.05493866	5.483159264	39.24862078	156.9896907
18.92890624	3.054445038	1.03E-06	3.46E-05	CDH11
16	64943753	65126112	-	13381
protein_coding	cadherin 11 [Source:HGNC Symbol;Acc:HGNC:1750]	-
168	60	226	12	6	40	0.686618378
0.197695433	0.782388258	0.043360461	0.019539852
0.139546021
ENSG00000254087 388.739123	406.9315508	641.435761
193.8835969	197.3937335	215.8674143	479.0354783
202.3815816	1.243659651	1.07E-06	3.58E-05	LYN
8	55879813	56014168	+	6185
protein_coding	"LYN proto-oncogene, Src family tyrosine kinase
[Source:HGNC Symbol;Acc:HGNC:6735]"	-	336	439	656
193	216	220	2.970942771	3.129383398
4.913224301	1.508755345	1.52185279	1.660462274
ENSG00000258807 18.51138681	17.61207168	16.62257307
86.39372709	155.3561792	66.72265532	17.58201052
102.8241872	-2.550166745	1.12E-06	3.72E-05
AL359237.1	14	87710419	87872291	-	1845
lincRNA novel transcript	-	16	19	17
86	170	68	0.474261996	0.45403694
0.426830069	2.253737325	4.015236667	1.720515455
ENSG00000147853 1263.40215	1172.593193	1229.092609
643.9346403	784.0917748	712.3624671	1221.695984
713.4629608	0.775471447	1.13E-06	3.75E-05	AK3
9	4709559 4742043 -	4483	protein_coding
adenylate kinase 3 [Source:HGNC Symbol;Acc:HGNC:17376]	-	1092
1265	1257	641	858	726	13.32136145
12.44101302	12.98879895	6.913381245	8.340213087
7.559862868
ENSG00000065328 1404.551474	1471.071461	1442.252664
952.3401545	773.1254563	845.8077778	1439.291866
857.0911295	0.748245602	1.15E-06	3.81E-05	MCM10
10	13161554	13211104	+	5454
protein_coding	minichromosome maintenance 10 replication initiation
factor [Source:HGNC Symbol;Acc:HGNC:18043]	-	1214	1587
1475	948	846	862	12.17301805	12.82908702
12.5279307	8.404162094	6.759488401	7.377991797
ENSG00000165672 3139.993988	3095.089859	2886.460925

1857.465133	2092.739119	1935.93822	3040.514924
1962.047491	0.631776136	1.15E-06	3.81E-05	PRDX3
	10	119167703	119178833	-	3417
protein_coding	peroxiredoxin 3 [Source:HGNC Symbol;Acc:HGNC:9354]
	-	2714	3339	2952	1849	2290	1973
43.43697544	43.08294656	40.01970013	26.16333782
29.20445468	26.95430525
ENSG00000145040 273.0429555	155.7277917	342.2294457
104.4761351	51.17615314	52.98563805	257.0000643
69.54597543	1.886965719	1.16E-06	3.84E-05	UCN2
	3	48561727	48563773	-	1481
protein_coding	urocortin 2 [Source:HGNC Symbol;Acc:HGNC:18414] -
	236	168	350	104	56	54	8.714684266
	5.001360746	10.9475123	3.395310506	1.647750933
	1.702098689
ENSG00000166401 1691.47797	1672.219858	1181.180487
2730.443607	2629.174867	2496.212282	1514.959438
2618.610252	-0.789770686	1.25E-06	4.11E-05
SERPINB8	18	63969925	64019779	+	5868
	protein_coding	serpin family B member 8 [Source:HGNC
Symbol;Acc:HGNC:8952]	-	1462	1804	1208	2718	2877
	2544	13.62548532	13.55440275	9.536286385
22.39548973	21.36526847	20.23825379
ENSG00000204389 1876.591838	1779.74619	1211.492238
4207.173594	2239.87056	3573.586922	1622.610089
3340.210358	-1.041704109	1.28E-06	4.22E-05	HSPA1A
	6	31815464	31817946	+	2483
protein_coding	heat shock protein family A (Hsp70) member 1A
[Source:HGNC Symbol;Acc:HGNC:5232]	-	1622	1920	1239
	4188	2451	3642	35.72472078	34.09247001
23.11516738	81.55135285	43.0155212	68.47140788
ENSG00000196878 1096.799669	944.5632125	1680.835477
574.618743	508.1060918	689.7945102	1240.732786
590.8397817	1.070649782	1.28E-06	4.22E-05	LAMB3
	1	209614870	209652466	-	4688
protein_coding	laminin subunit beta 3 [Source:HGNC
Symbol;Acc:HGNC:6490]	-	948	1019	1719	572	556
	703	11.05898953	9.583420378	16.98598474
5.89942443	5.16827705	7.000253346
ENSG00000267317 320.4783842	335.556313	357.8742203
632.8842799	620.5108568	644.6585963	337.9696392
632.6845776	-0.904069832	1.34E-06	4.39E-05
AC027307.2	19	1457670 1458580 -	911	antisense
	"novel transcript, antisense to APC2"	-	277	362
	366	630	679	657	16.62861602	17.5195986
	18.61080524	33.43669849	32.47954937	33.666117
ENSG00000134352 3800.619105	3630.867619	4559.474015
2482.312787	2459.19693	2639.469747	3996.986913
2526.993155	0.661547378	1.36E-06	4.44E-05	IL6ST
	5	55935095	55994993	-	9292

protein_coding interleukin 6 signal transducer [Source:HGNC
Symbol;Acc:HGNC:6021]   -   3285   3917   4663   2471   2691
        2690   19.33396311   18.58567457   23.24655864
12.85774359   12.62010866   13.51416181
ENSG00000173706 1874.277915   1713.005708   3195.445224
977.45461   969.6053299   1308.941503   2260.909616
1085.333814   1.058938831   1.37E-06   4.47E-05   HEG1
        3   124965710   125055958   -   9925
protein_coding heart development protein with EGF like domains 1
[Source:HGNC Symbol;Acc:HGNC:29227]   -   1620   1848   3268
        973   1061   1334   8.926458944   8.20928644
15.25295497   4.740056751   4.65847175   6.274387691
ENSG00000001036 1336.290735   1543.37365   2206.891025
758.4565576   605.8890987   1047.938175   1695.51847
804.0946104   1.076806794   1.42E-06   4.63E-05   FUCA2
        6   143494811   143511690   -   2793
protein_coding alpha-L-fucosidase 2 [Source:HGNC
Symbol;Acc:HGNC:4008]   -   1155   1665   2257   755   663
        1068   22.61547747   26.28314071   37.4337279
13.0700495   10.34430198   17.85033425
ENSG00000140534 775.1643227   575.6366585   1053.088894
429.959479   263.1916447   345.3878629   801.2966251
346.1796622   1.211356236   1.42E-06   4.63E-05   TICRR
        15   89575482   89631056   +   8402
protein_coding TOPBP1 interacting checkpoint and replication
regulator [Source:HGNC Symbol;Acc:HGNC:28704]   -   670   621
        1077   428   288   352   4.361007547   3.258688747
        5.937935583   2.46298807   1.493717291   1.955717051
ENSG00000134910 4462.401183   3772.691143   5145.175266
2848.983838   2367.810942   2851.4123   4460.089197
2689.40236   0.729836593   1.43E-06   4.64E-05   STT3A
        11   125591712   125625215   +   7393
protein_coding "STT3A, catalytic subunit of the
oligosaccharyltransferase complex [Source:HGNC Symbol;Acc:HGNC:6172]"
        -       3857   4070   5262   2836   2591   2906
28.53143698   24.27211723   32.97103599   18.54755796
15.27233017   18.34936077
ENSG00000177565 4233.322771   3217.44741   4016.795894
2488.340256   2405.279197   2020.322755   3822.522025
2304.647403   0.729756169   1.44E-06   4.65E-05
TBL1XR1 3   177019340   177228000   -   11603
protein_coding transducin beta like 1 X-linked receptor 1
[Source:HGNC Symbol;Acc:HGNC:29529]   -   3659   3471   4108
        2477   2632   2059   17.24593838   13.1891947
16.40070808   10.32183544   9.88494348   8.283847404
ENSG00000169220 223.2936034   268.8158308   228.8048294
442.0144177   489.8288943   578.9171565   240.3047545
503.5868228   -1.066379332   1.44E-06   4.65E-05   RGS14
        5   177357837   177372601   +   3797
protein_coding regulator of G protein signaling 14 [Source:HGNC

Symbol;Acc:HGNC:9996]	-	193	290	234	440	536
	590	2.779786392	3.367374034	2.854813331
5.602905477	6.151524957	7.253664263
ENSG00000122591 2441.189136	2261.760784	2473.829993
1436.546857	1542.595473	1605.26859	2392.259971
1528.136973	0.646450872	1.48E-06	4.76E-05
FAM126A 7	22889371	23014130	-	14499
protein_coding	family with sequence similarity 126 member A
[Source:HGNC Symbol;Acc:HGNC:24587]	-	2110	2440	2530
	1430	1688	1636	7.958644725	7.419689555
8.083229828	4.768691242	5.073328573	5.267339186
ENSG00000106799 2265.330961	2665.911481	2577.476625
1611.343468	1638.55076	1301.091779	2502.906356
1516.995336	0.722552463	1.49E-06	4.81E-05	TGFBR1
	9	99104038	99154192	+	6844
protein_coding	transforming growth factor beta receptor 1
[Source:HGNC Symbol;Acc:HGNC:11772]	-	1958	2876	2636
	1604	1793	1326	15.64578649	18.52733008
17.84176689	11.33171334	11.41639236	9.044395787
ENSG00000168487 1117.624979	821.2787108	1714.080624
624.8476541	350.9221929	556.3491995	1217.661438
510.7063489	1.25403166	1.52E-06	4.89E-05	BMP1
	8	22164736	22212326	+	7471
protein_coding	bone morphogenetic protein 1 [Source:HGNC
Symbol;Acc:HGNC:1067]	-	966	886	1753	622	384
	567	7.071199705	5.228642437	10.86940161
4.02543543	2.239809517	3.542830365
ENSG00000221955 573.8529912	499.6266649	393.0749633
940.2852158	1122.219929	816.3713122	488.8515398
959.6254858	-0.974254017	1.54E-06	4.93E-05
SLC12A8 3	125082636	125212864	-	5586
protein_coding	solute carrier family 12 member 8 [Source:HGNC
Symbol;Acc:HGNC:15595]	-	496	539	402	936	1228
	832	4.855963994	4.254238091	3.333708156
8.101699558	9.579790976	6.952939185
ENSG00000075420 2818.358642	2609.367461	2951.973418
1752.988997	1868.843449	1802.492909	2793.233174
1808.108452	0.627320553	1.55E-06	4.93E-05	FNDC3B
	3	172039628	172401665	+	13270
protein_coding	fibronectin type III domain containing 3B [Source:HGNC
Symbol;Acc:HGNC:24670]	-	2436	2815	3019	1745	2045
	1837	10.03924548	9.352795408	10.53888453
6.358076147	6.715540726	6.462257649
ENSG00000108798 108.7543975	168.7051076	275.7391534
16.07325155	60.31475191	32.38011214	184.3995528
36.25603853	2.343456922	1.55E-06	4.93E-05	ABI3
	17	49210227	49223225	+	2456
protein_coding	ABI family member 3 [Source:HGNC
Symbol;Acc:HGNC:29859]	-	94	182	282	16	66
	33	2.093120368	3.267209502	5.318916857

0.31498715	1.171046582	0.62723692
ENSG00000115306 3137.680065	2915.261338	3838.836582
2088.518124	1746.386226	2115.50066	3297.259328
1983.468336	0.733399343	1.56E-06	4.95E-05	SPTBN1
	2	54456285	54671445	+	18504
protein_coding	"spectrin beta, non-erythrocytic 1 [Source:HGNC
Symbol;Acc:HGNC:11275]" -	2712	3145	3926	2079	1911
	2156	8.015281474	7.493573786	9.828497495
5.432379305	4.500426414	5.439126664
ENSG00000226950 811.0301347	805.5205414	957.2646494
400.8267106	519.0724104	464.1149407	857.9384418
461.3380206	0.894729994	1.56E-06	4.95E-05	DANCR
	4	52712404	52720351	+	2285
processed_transcript	differentiation antagonizing non-protein
coding RNA [Source:HGNC Symbol;Acc:HGNC:28964]	-	701	869
	979	399	568	473	16.77747214	16.76746969
	19.84718616	8.442827339	10.83230124	9.663200087
ENSG00000284946 93.71389573	54.69011731	123.2026004
14.06409511	15.53561792	14.71823279	90.53553783
14.77264861	2.614172564	1.57E-06	4.97E-05
AC068831.7	15	90966190	91022566	-	7148
	protein_coding	novel transcript	-	81	59
	126	14	17	15	0.619719537	0.363916231
	0.816560668	0.094698851	0.103638943	0.097960897
ENSG00000226674 155.0328645	127.9192574	202.4042721
60.27469332	31.07123583	26.49281903	161.7854647
39.27958273	2.044475532	1.60E-06	5.08E-05	TEX41
	2	144667967	145262988	+	15056
lincRNA testis expressed 41 [Source:HGNC Symbol;Acc:HGNC:48667] -
	134	138	207	60	34	27	0.486732006
	0.404113574	0.636888189	0.192682761	0.098407434
	0.083714405
ENSG00000173334 441.9593601	456.0599613	362.7632124
755.442823	731.0879019	883.0939675	420.2608446
789.8748975	-0.910449328	1.62E-06	5.11E-05	TRIB1
	8	125430321	125438405	+	4138
protein_coding	tribbles pseudokinase 1 [Source:HGNC
Symbol;Acc:HGNC:16891]	-	382	492	371	752	800
	900	5.048560782	5.242139506	4.153229012
8.786756084	8.424770876	10.15308586
ENSG00000006327 2704.976398	5157.556148	3771.368491
6599.07434	7244.167248	8034.192673	3877.967012
7292.478087	-0.910776026	1.63E-06	5.15E-05
TNFRSF12A	16	3018445 3022383 +	1848	protein_coding
	TNF receptor superfamily member 12A [Source:HGNC
Symbol;Acc:HGNC:18152]	-	2338	5564	3857	6569	7927
	8188	69.1890317	132.7452875	96.68300223
171.8693805	186.9241821	206.8339867
ENSG00000104313 321.6353458	324.4328993	466.4098445
167.7645631	115.1463446	162.8817762	370.8260299

148.5975613	1.320797831	1.64E-06	5.17E-05	EYA1
	8	71197433	71592025	-	9418
protein_coding	EYA transcriptional coactivator and phosphatase 1
[Source:HGNC Symbol;Acc:HGNC:3519]	-	278	350	477
	167	126	166	1.614287272	1.638488193
2.346184432	0.857351677	0.583002553	0.822802188
ENSG00000128567 1684.5362	1790.869604	2624.410949
1092.981106	1178.879242	1066.58127	2033.272251
1112.813872	0.869757847	1.65E-06	5.19E-05	PODXL
	7	131500262	131558217	-	6874
protein_coding	podocalyxin like [Source:HGNC Symbol;Acc:HGNC:9171]
	-	1456	1932	2684	1088	1290	1087
11.58368022	12.39171888	18.0873707	7.652803886
8.177843219	7.381864211
ENSG00000130724 1833.784256	2166.284816	1810.882667
2881.130341	3115.348322	3168.344912	1936.983913
3054.941192	-0.657081197	1.67E-06	5.22E-05	CHMP2A
	19	58551566	58555124	-	2129
protein_coding	charged multivesicular body protein 2A [Source:HGNC
Symbol;Acc:HGNC:30216]	-	1585	2337	1852	2868	3409
	3229	40.71442614	48.39684033	40.29654176
65.13354291	69.77662149	70.80084148
ENSG00000162490 255.6885303	53.76316617	234.6716199
48.21975466	13.70789816	22.56795695	181.3744388
28.16520326	2.689256132	1.70E-06	5.33E-05	DRAXIN
	1	11691729	11725857	+	7346
protein_coding	dorsal inhibitory axon guidance protein [Source:HGNC
Symbol;Acc:HGNC:25054]	-	221	58	240	48	15
	23	1.645265771	0.348105614	1.513431516
0.315930482	0.088981338	0.14615812
ENSG00000175305 485.9239038	486.6493489	494.7659986
190.8698622	272.3302435	236.4729402	489.1130838
233.2243486	1.067606637	1.72E-06	5.36E-05	CCNE2
	8	94879770	94896678	-	4397
protein_coding	cyclin E2 [Source:HGNC Symbol;Acc:HGNC:1590]	-
	420	525	506	190	298	241	5.223811986
	5.264253515	5.330850547	2.089288202	2.953373653
	2.558624846
ENSG00000176393 1170.845216	1459.021096	1779.593117
721.2871634	783.1779149	888.0000451	1469.81981
797.4883745	0.882563396	1.73E-06	5.40E-05	RNPEP
	1	201982372	202006147	+	3479
protein_coding	arginyl aminopeptidase [Source:HGNC
Symbol;Acc:HGNC:10078]	-	1012	1574	1820	718	857
	905	15.90819099	19.94730633	24.23368258
9.978637177	10.73457837	12.1433968
ENSG00000152818 888.5465669	817.5709062	1203.66985
378.7259897	337.2142948	603.4475445	969.9291078
439.795943	1.141458698	1.74E-06	5.40E-05	UTRN
	6	144285701	144853034	+	14604

protein_coding utrophin [Source:HGNC Symbol;Acc:HGNC:12635] -
768 882 1231 377 369 615 2.875968391
2.662751996 3.904709099 1.248161385 1.101065461
1.965845249
ENSG00000137804 166.6024813 209.4909578 363.7410108
36.16481599 69.45335068 90.27182779 246.6114833
65.29666482 1.917152968 1.77E-06 5.49E-05 NUSAP1
15 41332694 41381050 + 2875
protein_coding nucleolar and spindle associated protein 1
[Source:HGNC Symbol;Acc:HGNC:18538] - 144 226 372
36 76 92 2.739172329 3.465808385
5.993873137 0.605432692 1.151951888 1.493812242
ENSG00000108055 3827.229223 3539.099456 3581.675598
2542.58748 2235.30126 2409.865316 3649.334759
2395.918019 0.607040186 1.81E-06 5.61E-05 SMC3
10 110567691 110604636 + 4275
protein_coding structural maintenance of chromosomes 3 [Source:HGNC
Symbol;Acc:HGNC:2468] - 3308 3818 3663 2531 2446
2456 42.31789869 39.37619644 39.69202492
28.62577429 24.93325228 26.8187344
ENSG00000041357 3689.550784 3210.031801 3308.86984
2223.131605 2303.840751 2119.425522 3402.817475
2215.465959 0.61886243 1.82E-06 5.62E-05 PSMA4
15 78540405 78552419 + 6996
protein_coding proteasome subunit alpha 4 [Source:HGNC
Symbol;Acc:HGNC:9533] - 3189 3463 3384 2213 2521
2160 24.92868852 21.82411536 22.40696364
15.29441357 15.70296427 14.41285396
ENSG00000065534 3576.16854 3507.583117 5065.973594
2265.323891 2245.353719 2578.634385 4049.908417
2363.103998 0.777319205 1.85E-06 5.70E-05 MYLK
3 123610049 123884331 - 22167
protein_coding myosin light chain kinase [Source:HGNC
Symbol;Acc:HGNC:7590] - 3091 3784 5181 2255 2457
2628 7.625824329 7.526241755 10.82702468
4.91859248 4.830107904 5.534322658
ENSG00000132646 4669.497323 4205.577326 4498.850513
2982.592741 3026.703914 2961.308438 4457.975054
2990.201698 0.576015006 1.85E-06 5.70E-05 PCNA
20 5114953 5126626 - 1471 protein_coding
proliferating cell nuclear antigen [Source:HGNC Symbol;Acc:HGNC:8729]
- 4036 4537 4601 2969 3312 3018
150.0490318 135.9847053 144.8912023 97.58852218
98.11519089 95.77509657
ENSG00000172167 636.3289216 464.4025216 632.6355753
273.2452764 302.4876194 275.721561 577.7890062
283.8181523 1.024590175 1.86E-06 5.70E-05 MTBP
8 120445400 120542133 + 8326
protein_coding MDM2 binding protein [Source:HGNC
Symbol;Acc:HGNC:7417] - 550 501 647 272 331

281	3.612609301	2.652987949	3.599733336
1.579551222	1.732408018	1.575491082
ENSG00000008300 333.2049626	341.1180198	463.4764493
777.5435438	715.552284	715.3061137	379.2664772
736.1339805	-0.955982675	1.88E-06	5.74E-05	CELSR3
3	48636469	48662915	-	13873
protein_coding	cadherin EGF LAG seven-pass G-type receptor 3
[Source:HGNC Symbol;Acc:HGNC:3230]	-	288	368	474
774	783	729	1.135316146	1.169530064
1.582743036	2.69756421	2.45951782	2.453031798
ENSG00000033100 1269.186958	1675.927663	1769.815133
2885.148654	2627.347148	2467.757031	1571.643251
2660.084278	-0.758542768	1.88E-06	5.74E-05	CHPF2
7	151232489	151238827	+	4205
protein_coding	chondroitin polymerizing factor 2 [Source:HGNC
Symbol;Acc:HGNC:29270]	-	1097	1808	1810	2872	2875
2515	14.26708801	18.95685918	19.9395352
33.0232378	29.79411204	27.92016787
ENSG00000112992 1389.510973	1314.416718	1316.116668
802.6579994	869.9946033	783.0099845	1340.014786
818.5541957	0.710757853	1.91E-06	5.83E-05	NNT
5	43602692	43707405	+	8245
protein_coding	nicotinamide nucleotide transhydrogenase [Source:HGNC
Symbol;Acc:HGNC:7863]	-	1201	1418	1346	799	952
798	7.96612396	7.582624134	7.562351432
4.685515035	5.031585839	4.518125261
ENSG00000007376 1166.217369	1487.756581	1516.565343
2305.50702	2210.627043	2333.330505	1390.179765
2283.154856	-0.715045038	1.92E-06	5.84E-05	RPUSD1
16	784974	788397	-	2769	protein_coding	RNA
pseudouridylate synthase domain containing 1 [Source:HGNC
Symbol;Acc:HGNC:14173]	-	1008	1605	1551	2295	2419
2378	19.90821348	25.55559748	25.94724771
40.07383915	38.06900364	40.08989607
ENSG00000105968 4398.768291	3346.293618	4434.315817
2551.628684	2563.376956	2430.470842	4059.792576
2515.158827	0.690557037	1.95E-06	5.91E-05	H2AFV
7	44826791	44848083	-	4749
protein_coding	H2A histone family member V [Source:HGNC
Symbol;Acc:HGNC:20664]	-	3802	3610	4535	2540	2805
2477	43.78291327	33.51498199	44.23617603
25.86025265	25.73885918	24.34836889
ENSG00000187951 183.9569064	178.9015702	244.449604
56.25638043	52.09001301	84.38453467	202.4360269
64.24364271	1.656943392	1.95E-06	5.91E-05
AC091057.1	15	30658717	30772993	+	5637
processed_transcript	OTU deubiquitinase 7A pseudogene
[Source:NCBI gene;Acc:100288637]	-	159	193	250
56	57	86	1.542566169	1.509535157
2.054444577	0.480331655	0.440641522	0.712190953

ENSG00000197063 1565.369147 1642.557422 1754.170359
2584.779765 2511.286943 2538.404549 1654.032309
2544.823752 -0.621343402 1.97E-06 5.95E-05 MAFG
17 81918270 81927714 - 5220
protein_coding MAF bZIP transcription factor G [Source:HGNC
Symbol;Acc:HGNC:6781] TF_bZIP 1353 1772 1794 2573 2748
2587 14.17496536 14.96673856 15.92041526
23.83254928 22.94060268 23.13513092
ENSG00000109685 4668.340362 4718.181307 6063.327979
3447.712458 2833.87948 3215.443257 5149.949882
3165.678398 0.702205857 2.00E-06 6.03E-05 NSD2
4 1871424 1982207 + 19776 protein_coding
nuclear receptor binding SET domain protein 2 [Source:HGNC
Symbol;Acc:HGNC:12766] HMG 4035 5090 6201 3432 3101
3277 11.15834534 11.34784217 14.52532083
8.390928922 6.833165045 7.735422223
ENSG00000182718 21647.90991 18583.51647 19340.85267
10901.68287 13876.96224 13636.93329 19857.42635
12805.1928 0.632886523 2.22E-06 6.70E-05 ANXA2
15 60347134 60402883 - 8824
protein_coding annexin A2 [Source:HGNC Symbol;Acc:HGNC:537] -
18711 20048 19780 10852 15185 13898 115.9648077
100.170424 103.8396567 59.46282008 74.9907841
73.52462933
ENSG00000076716 401.4657015 400.4428928 497.6993938
205.9385355 186.427415 220.7734919 433.2026627
204.3798141 1.08443573 2.23E-06 6.70E-05 GPC4
X 133300103 133415490 - 4960
protein_coding glypican 4 [Source:HGNC Symbol;Acc:HGNC:4452] -
347 432 509 205 204 225 3.825978375
3.840042476 4.753774335 1.998358495 1.792285077
2.11761438
ENSG00000232112 577.3238762 622.9111666 847.7512268
300.3688884 344.5251738 366.9746043 682.6620899
337.2895555 1.017513126 2.26E-06 6.78E-05 TMA7
3 48440257 48444208 + 1139
protein_coding translation machinery associated 7 homolog
[Source:HGNC Symbol;Acc:HGNC:26932] - 499 672 867
299 377 374 23.95915705 26.01234509
35.26126018 12.69254409 14.42368482 15.32829726
ENSG00000132002 4638.259358 4568.015222 4087.19738
7627.76244 6067.115726 6860.658912 4431.15732
6851.845693 -0.62880953 2.26E-06 6.79E-05 DNAJB1
19 14514770 14529770 - 3672
protein_coding DnaJ heat shock protein family (Hsp40) member B1
[Source:HGNC Symbol;Acc:HGNC:5270] - 4009 4928 4180
7593 6639 6992 59.70739127 59.17005659
52.73222118 99.97972637 78.78773107 88.88833737
ENSG00000094916 6893.177664 5290.110161 7051.882177
4276.489491 3876.5936 3914.068707 6411.723334

4022.383933 0.672578023 2.27E-06 6.80E-05 CBX5
12 54230940 54280133 - 15040
protein_coding chromobox 5 [Source:HGNC Symbol;Acc:HGNC:1555] -
5958 5707 7212 4257 4242 3989 21.6644354
16.72992501 22.21315963 13.68538534 12.29083605
12.38118566
ENSG00000165704 2633.244774 2203.362862 2216.669009
1384.30879 1495.988619 1537.564719 2351.092215
1472.620709 0.674544364 2.28E-06 6.82E-05 HPRT1
X 134460153 134520513 + 1624
protein_coding hypoxanthine phosphoribosyltransferase 1 [Source:HGNC
Symbol;Acc:HGNC:5157] - 2276 2377 2267 1378 1637
1567 76.64449121 64.53229565 64.66480184
41.02649439 43.92595904 45.04317624
ENSG00000154229 3149.249681 2017.045682 3168.066868
1447.597218 1368.962096 1739.695116 2778.120744
1518.751477 0.871026141 2.38E-06 7.08E-05 PRKCA
17 66302636 66810743 + 11152
protein_coding protein kinase C alpha [Source:HGNC
Symbol;Acc:HGNC:9393] - 2722 2176 3240 1441 1498
1773 13.34842645 8.602805186 13.45843946
6.247588784 5.853527329 7.421676338
ENSG00000133316 1055.149048 1563.766575 1239.848392
2468.248692 1878.895908 2719.92942 1286.254672
2355.69134 -0.872202126 2.38E-06 7.09E-05 WDR74
11 62832342 62841809 - 4247
protein_coding WD repeat domain 74 [Source:HGNC
Symbol;Acc:HGNC:25529] - 912 1687 1268 2457 2056
2772 11.74376332 17.5132513 13.83054991
27.97203816 21.09596747 30.46891581
ENSG00000138385 2519.86253 2741.921475 2588.232408
1679.654787 1521.576696 1818.192358 2616.672137
1673.14128 0.64542893 2.42E-06 7.19E-05 SSB
2 169791933 169812064 + 5113
protein_coding Sjogren syndrome antigen B [Source:HGNC
Symbol;Acc:HGNC:11316] - 2178 2958 2647 1672 1665
1853 23.29575528 25.50682103 23.98173526
15.81108585 14.19047859 16.91786918
ENSG00000067064 1666.024813 1537.811943 1795.237892
950.330998 1112.167471 995.9337523 1666.358216
1019.477407 0.708577538 2.44E-06 7.23E-05 IDI1
10 1039152 1049170 - 4345 protein_coding
isopentenyl-diphosphate delta isomerase 1 [Source:HGNC
Symbol;Acc:HGNC:5387] - 1440 1659 1836 946 1217
1015 18.12455799 16.8341256 19.57425997
10.52695029 12.20560736 10.90491531
ENSG00000249992 16051.68629 22990.2422 13573.79761
26276.75255 30771.48979 41500.5104 17538.57537
32849.58425 -0.905312071 2.45E-06 7.25E-05
TMEM158 3 45224466 45226278 - 1813

protein_coding transmembrane protein 158 (gene/pseudogene) [Source:HGNC Symbol;Acc:HGNC:30293] - 13874 24802 13882
26157 33672 42295 418.5030224 603.1465626
354.6963274 697.5757019 809.3375798 1089.023524
ENSG00000059122 578.4808379 702.6289648 671.7475119
1100.013153 1082.923955 1140.172434 650.9524382
1107.70318 -0.766007724 2.46E-06 7.27E-05
FLYWCH1 16 2911937 2951208 + 8419 protein_coding
FLYWCH-type zinc finger 1 [Source:HGNC Symbol;Acc:HGNC:25404] -
500 758 687 1095 1185 1162 3.247911654
3.96956259 3.780059913 6.288612377 6.133613958
6.443052266
ENSG00000103257 12232.5558 16518.26933 16180.60819
26914.65972 22630.826 22460.02324 14977.14444
24001.83632 -0.680292249 2.48E-06 7.31E-05 SLC7A5
16 87830023 87869488 - 4745
protein_coding solute carrier family 7 member 5 [Source:HGNC Symbol;Acc:HGNC:11063] - 10573 17820 16548 26792 24764
22890 121.8587526 165.5790707 161.5517814
273.0047065 227.4276042 225.193376
ENSG00000162840 205.9391783 243.78815 224.8936357
426.9457444 493.4843338 452.3403545 224.8736547
457.5901442 -1.023996031 2.52E-06 7.41E-05 MT2P1
4 68376323 68376505 + 183
processed_pseudogene metallothionein 2 pseudogene 1 [Source:HGNC Symbol;Acc:HGNC:7407] - 178 263 230 425 540
461 53.19411959 63.36342098 58.22093854
112.2894764 128.5882447 117.5968995
ENSG00000187123 440.8023984 331.8485084 448.809473
134.6134818 178.2026761 208.9989056 407.15346
173.9383545 1.226014367 2.54E-06 7.45E-05 LYPD6
2 149329985 149474148 + 5033
protein_coding LY6/PLAUR domain containing 6 [Source:HGNC Symbol;Acc:HGNC:28751] - 381 358 459 134 195
213 4.139927713 3.136101096 4.22462545
1.28729797 1.688364759 1.975598596
ENSG00000139354 945.2376891 804.5935903 986.5986019
445.0281524 571.1624234 485.7016821 912.1432937
500.6307526 0.864770467 2.62E-06 7.67E-05 GAS2L3
12 100573683 100628286 + 6799
protein_coding growth arrest specific 2 like 3 [Source:HGNC Symbol;Acc:HGNC:27475] - 817 868 1009 443 625
495 6.571609187 5.628706997 6.874618159
3.150357947 4.00583977 3.398648053
ENSG00000185049 748.5542042 959.3944308 1007.132369
1596.274795 1559.958811 1468.879633 905.0270012
1541.704413 -0.767569899 2.62E-06 7.67E-05 NELFA
4 1982714 2041903 - 5935 protein_coding
negative elongation factor complex member A [Source:HGNC Symbol;Acc:HGNC:12768] - 647 1035 1030 1589 1707

1497	5.961811908	7.688711838	8.039313362
12.94506962	12.53347201	11.77462577
ENSG00000129636 747.3972425	630.3267758	692.2812786
395.8038195	386.5627281	315.9513973	690.0017656
366.1059816	0.913746367	2.65E-06	7.72E-05	ITFG1
16	47154387	47464149	-	7770
protein_coding	integrin alpha FG-GAP repeat containing 1 [Source:HGNC
Symbol;Acc:HGNC:30697]	-	646	680	708	394	423
322	4.546803775	3.858529739	4.22099361
2.45175133	2.372345543	1.934554142
ENSG00000120738 205.9391783	564.5132448	338.318252
859.9189581	936.7063744	929.2110969	369.590225
908.6121431	-1.295301631	2.66E-06	7.75E-05	EGR1
5	138465490	138469315	+	3138
protein_coding	early growth response 1 [Source:HGNC
Symbol;Acc:HGNC:3238]	zf-C2H2 178	609	346	856	1025
947	3.102142729	8.556542491	5.107702755
13.18930897	14.23408398	14.0877967
ENSG00000186854 163.1315963	175.1937656	230.7604262
63.28842799	70.36721056	53.96685357	189.6952627
62.54083071	1.600778331	2.68E-06	7.76E-05
TRABD2A 2	84821650	84907008	-	8771
protein_coding	TraB domain containing 2A [Source:HGNC
Symbol;Acc:HGNC:27013]	-	141	189	236	63	77
55	0.87915351	0.950050413	1.246422694
0.347290301	0.382560575	0.292724865
ENSG00000277142 38.1797353	69.52133556	56.71230814
225.0255217	219.3263706	129.5204486	54.80445967
191.2907803	-1.798315184	2.68E-06	7.76E-05
LINC00235	16	525155	527407	-	2253	lincRNA long
intergenic non-protein coding RNA 235 [Source:HGNC
Symbol;Acc:HGNC:14138]	-	33	75	58	224	240
132	0.801027564	1.467688967	1.192529847
4.807154087	4.642037534	2.735009098
ENSG00000084234 6314.696826	5722.069392	7834.12091
4036.395296	3712.098822	4513.59139	6623.629043
4087.361836	0.696501879	2.68E-06	7.76E-05	APLP2
11	130068147	130144811	+	7463
protein_coding	amyloid beta precursor like protein 2 [Source:HGNC
Symbol;Acc:HGNC:598]	-	5458	6173	8012	4018	4062
4600	39.99583817	36.46840702	49.73131635
26.03141089	23.71838286	28.77334983
ENSG00000188070 401.4657015	635.8884826	527.0333463
893.0700394	1043.62798	1063.637623	521.4625101
1000.111881	-0.937978598	2.74E-06	7.92E-05
C11orf95	11	63759892	63768775	-	5948
protein_coding	chromosome 11 open reading frame 95
[Source:HGNC Symbol;Acc:HGNC:28449]	-	347	686	539
889	1142	1084	3.190459439	5.084954999
4.197785675	7.226566735	8.36669123	8.507547022

ENSG00000204356 2120.710752 2296.984927 1863.683781
3064.968155 3619.798974 3244.879723 2093.793153
3309.882284 -0.660700728 2.84E-06 8.19E-05 NELFE
6 31952087 31959110 - 2719
protein_coding negative elongation factor complex member E
[Source:HGNC Symbol;Acc:HGNC:13974] - 1833 2478 1906
3051 3961 3307 36.86786343 40.18149142
32.47253133 54.2543065 63.48253087 56.77681459
ENSG00000134775 668.7238486 813.8631017 871.2183888
410.8724928 396.6151868 464.1149407 784.6017797
423.8675401 0.889179908 2.88E-06 8.29E-05 FHOD3
18 36297714 36780055 + 7965
protein_coding formin homology 2 domain containing 3 [Source:HGNC
Symbol;Acc:HGNC:26178] - 578 878 891 409 434
473 3.96859491 4.860071894 5.181963859
2.482782893 2.374447366 2.772179812
ENSG00000213694 531.0454092 377.2691143 636.5467689
220.0026306 266.8470842 158.9569142 514.9537641
215.2688763 1.257292309 2.90E-06 8.33E-05 S1PR3
9 88990863 89005010 + 13578
protein_coding sphingosine-1-phosphate receptor 3 [Source:HGNC
Symbol;Acc:HGNC:3167] - 459 407 651 219 292
162 1.848721934 1.321577277 2.220995303
0.779847218 0.937142524 0.55696159
ENSG00000074696 3754.340638 3052.450107 3017.485912
1956.918377 2211.540903 2020.322755 3274.758886
2062.927345 0.666300319 2.92E-06 8.38E-05 HACD3
15 65530418 65578352 + 7097
protein_coding 3-hydroxyacyl-CoA dehydratase 3 [Source:HGNC
Symbol;Acc:HGNC:24175] - 3245 3293 3086 1948 2420
2059 25.00544621 20.45742103 20.14297308
13.27135798 14.85932763 13.543396
ENSG00000135956 2091.78671 2017.972634 2176.579274
3567.257266 2967.303022 3165.401266 2095.446206
3233.320518 -0.625694617 2.94E-06 8.40E-05
TMEM127 2 96248516 96265994 - 6367
protein_coding transmembrane protein 127 [Source:HGNC
Symbol;Acc:HGNC:26038] - 1808 2177 2226 3551 3247
3226 15.52952918 15.07500751 16.19544054
26.96603159 22.22317143 23.65241819
ENSG00000087460 19376.79414 15874.03829 18239.85166
11018.21394 12835.16198 11663.70888 17830.22803
11839.02827 0.590699712 3.22E-06 9.21E-05 GNAS
20 58839718 58911192 + 13320
protein_coding GNAS complex locus [Source:HGNC Symbol;Acc:HGNC:4392]
- 16748 17125 18654 10968 14045 11887
68.76278217 56.68397702 64.87392933 39.81295687
45.94900553 41.65950197
ENSG00000120708 45986.91269 17588.8979 40045.73413
11360.77511 6893.245055 8811.315365 34540.51491

9021.778511 1.936804137 3.26E-06 9.31E-05 TGFBI
5 136028895 136063818 + 7810
protein_coding transforming growth factor beta induced [Source:HGNC
Symbol;Acc:HGNC:11771] - 39748 18975 40955 11309 7543
8980 278.3293209 107.1185558 242.9172479
70.01230671 42.08735872 53.6749117
ENSG00000120837 696.4909288 718.3871342 684.4588913
302.3780448 437.7388813 356.1812336 699.7789848
365.4327199 0.936580396 3.36E-06 9.55E-05 NFYB
12 104117077 104138289 - 4956
protein_coding nuclear transcription factor Y subunit beta
[Source:HGNC Symbol;Acc:HGNC:7805] NF-YB 602 775 700
301 479 363 6.642933521 6.894525191
6.54288366 2.936543336 4.21175222 3.419175266
ENSG00000145107 53.22023708 56.54401959 33.24514615
181.8286582 217.4986508 116.7646468 47.66980094
172.0306519 -1.85284363 3.39E-06 9.64E-05
TM4SF19 3 196319342 196338503 - 1899
protein_coding transmembrane 4 L six family member 19 [Source:HGNC
Symbol;Acc:HGNC:25167] - 46 61 34 181 238
119 1.324730635 1.41624643 0.829385442
4.608449434 5.461483049 2.925283978
ENSG00000166845 676.8225803 423.6166714 691.3034802
304.3872013 247.6560268 279.646423 597.2475773
277.2298837 1.106861975 3.41E-06 9.67E-05
C18orf54 18 54357917 54385218 + 6165
protein_coding chromosome 18 open reading frame 54
[Source:HGNC Symbol;Acc:HGNC:13796] - 585 457 707
303 271 285 5.189404187 3.2682636
5.312375788 2.376351959 1.915555801 2.158031675
ENSG00000148296 1386.040088 1580.451695 1318.072265
2292.447503 2109.188597 2378.466419 1428.188016
2260.034173 -0.661844509 3.53E-06 9.98E-05 SURF6
9 133328774 133336398 - 4626
protein_coding surfeit 6 [Source:HGNC Symbol;Acc:HGNC:11478] -
1198 1705 1348 2282 2308 2424 14.16269499
16.24997559 13.49853757 23.85125459 21.74146345
24.46093327
ENSG00000117385 1398.766666 1345.933057 1968.308212
878.001366 668.0315704 963.5536401 1571.002645
836.5288589 0.909636146 3.71E-06 0.00010482 P3H1
1 42746335 42767084 - 6766
protein_coding prolyl 3-hydroxylase 1 [Source:HGNC
Symbol;Acc:HGNC:19316] - 1209 1452 2013 874 731
982 9.772125147 9.461687081 13.7820632
6.245693324 4.708081599 6.775253203
ENSG00000135862 6629.390402 5037.052499 8150.927597
4008.267106 3381.281546 4125.030044 6605.790166
3838.192899 0.783313559 3.74E-06 0.00010552 LAMC1
1 183023460 183145592 + 8406

protein_coding laminin subunit gamma 1 [Source:HGNC Symbol;Acc:HGNC:6492] - 5730 5434 8336 3990 3700 4204 37.27863047 28.50126961 45.93786172 22.95010762 19.18098634 23.34636965
ENSG00000105048 194.3695615 282.720098 174.0481181 406.8541799 465.1546776 680.9635705 217.0459259 517.657476 -1.252573292 3.76E-06 0.000105832 TNNT1 19 55132794 55149354 - 2171 protein_coding "troponin T1, slow skeletal type [Source:HGNC Symbol;Acc:HGNC:11948]" - 168 305 178 405 509 694 4.2319855 6.194039547 3.798067114 9.01978979 10.21683916 14.92264072
ENSG00000101150 7679.911603 8273.965883 6902.27902 11202.05175 11616.0729 10873.83039 7618.718835 11230.65168 -0.559813507 3.79E-06 0.000106476 TPD52L2 20 63865228 63891545 + 4694 protein_coding tumor protein D52 like 2 [Source:HGNC Symbol;Acc:HGNC:12007] - 6638 8926 7059 11151 12711 11082 77.33727679 83.83932155 69.66306083 114.8608292 118.0035931 110.2100239
ENSG00000128564 65.94681552 176.1207168 83.11286537 220.0026306 482.5180153 387.5801302 108.3934659 363.3669254 -1.741326137 3.93E-06 0.000110232 VGF 7 101162509 101165593 - 2704 protein_coding VGF nerve growth factor inducible [Source:HGNC Symbol;Acc:HGNC:12684] - 57 190 85 219 528 395 1.15282366 3.097996135 1.456178767 3.91596358 8.509143211 6.819247359
ENSG00000109943 21.98227184 25.95463194 28.35615407 0 0 0 25.43101928 0 7.162062725 3.99E-06 0.000111657 CRTAM 11 122838500 122872639 + 2675 protein_coding cytotoxic and regulatory T cell molecule [Source:HGNC Symbol;Acc:HGNC:24313] - 19 28 29 0 0 0 0.38844052 0.461496278 0.502199953 0 0 0
ENSG00000156876 838.7972149 862.9915121 968.020432 441.0098395 531.8664487 538.6873202 889.9363863 503.8545361 0.820630448 4.00E-06 0.000111657 SASS6 1 100083563 100132955 - 3972 protein_coding SAS-6 centriolar assembly protein [Source:HGNC Symbol;Acc:HGNC:25403] - 725 931 990 439 582 549 9.98213593 10.33414139 11.54591645 5.343877535 6.385168207 6.452219605
ENSG00000136104 1510.991948 1389.49976 1511.676351 875.9922096 757.5898384 1001.821045 1470.722687 878.4676978 0.743594256 4.10E-06 0.000114259 RNASEH2B 13 50909678 51024120 + 26291 protein_coding ribonuclease H2 subunit B [Source:HGNC Symbol;Acc:HGNC:25671] - 1306 1499 1546 872 829 1021 2.716631828 2.513787056 2.723985806

1.603653721	1.374061031	1.812861696
ENSG00000147799 469.7264403	607.1529973	518.2331606
1088.962793	802.3689724	1053.825468	531.7041994
981.7190777	-0.883468226	4.11E-06	0.000114259
ARHGAP39	8	144529179	144605816	-	4888
	protein_coding	Rho GTPase activating protein 39 [Source:HGNC
Symbol;Acc:HGNC:29351]	-	406	655	530	1084	878
	1074	4.542443656	5.908048621	5.022814371
10.72258016	7.827479095	10.25697082
ENSG00000100629 267.2581471	288.2818048	299.2063153
130.5951689	128.8542427	104.9900606	284.9154224
121.4798241	1.230040156	4.12E-06	0.000114259	CEP128
	14	80476983	80959517	-	9400
protein_coding	centrosomal protein 128 [Source:HGNC
Symbol;Acc:HGNC:20359]	-	231	311	306	130	141
	107	1.343936778	1.458701714	1.507981552
0.668677508	0.65365691	0.53137603
ENSG00000130821 5111.456683	6458.068598	5345.623941
7528.309196	9398.134979	12465.36196	5638.383074
9797.268712	-0.797009186	4.12E-06	0.000114259	SLC6A8
	X	153688099	153696593	+	5498
protein_coding	solute carrier family 6 member 8 [Source:HGNC
Symbol;Acc:HGNC:11055]	-	4418	6967	5467	7494	10284
	12704	43.94562938	55.86953194	46.06242459
65.90375646	81.51094538	107.8653074
ENSG00000170961 857.3086017	605.299095	419.4755205
1101.017731	1232.796975	1755.394564	627.3610724
1363.069757	-1.120670799	4.15E-06	0.000115061	HAS2
	8	121612116	121641390	-	4190
protein_coding	hyaluronan synthase 2 [Source:HGNC
Symbol;Acc:HGNC:4819]	-	741	653	429	1096	1349
	1789	9.67161272	6.871208331	4.742919188
12.64729788	14.02996296	19.93160874
ENSG00000116459 5370.616099	5214.100167	5186.242799
3023.780448	3528.412987	3815.947155	5256.986355
3456.046863	0.60503337	4.21E-06	0.000116289	ATP5PB
	1	111448864	111462773	+	3383
protein_coding	ATP synthase peripheral stalk-membrane subunit b
[Source:HGNC Symbol;Acc:HGNC:840]	-	4642	5625	5304
	3010	3861	3889	75.04086838	73.30852594
72.62798135	43.01953525	49.73434488	53.66386905
ENSG00000162627 1578.095726	1270.850014	1380.651364
450.0510435	931.2232151	679.982355	1409.865701
687.0855378	1.036141469	4.26E-06	0.000117664	SNX7
	1	98661701	98760500	+	2067
protein_coding	sorting nexin 7 [Source:HGNC Symbol;Acc:HGNC:14971]
	-	1364	1371	1412	448	1019	693
36.08848132	29.24360682	31.64438618	10.47945637
21.48287023	15.65088116
ENSG00000171606 321.6353458	393.9542349	320.7178805

596.7194639	713.7245643	639.7525187	345.4358204
650.0655156	-0.91146467	4.31E-06	0.000118691	ZNF274
19	58183029	58213562	+	8333
protein_coding	zinc finger protein 274 [Source:HGNC
Symbol;Acc:HGNC:13068]	zf-C2H2	278	425	328	594	781
652	1.824475882	2.248648151	1.823370473
3.446563462	4.084211744	3.652517032
ENSG00000216775 325.1062309	276.23144	321.6956789
95.43493109	143.4760008	142.2762503	307.6777833
127.0623941	1.274481822	4.40E-06	0.000121058
AL109918.1	6	52664366	52669155	+	3821
transcribed_unprocessed_pseudogene	uncharacterized
LOC730101 [Source:NCBI gene;Acc:730101]	-	281	298	329
95	157	145	4.021832645	3.438532903
3.988607633	1.202119893	1.790528394	1.771483046
ENSG00000261371 194.3695615	194.6597396	201.4264737
69.31589732	58.48703215	83.40331915	196.8185916
70.40208288	1.484187787	4.42E-06	0.000121258	PECAM1
17	64319415	64413776	-	7759
protein_coding	platelet and endothelial cell adhesion molecule 1
[Source:HGNC Symbol;Acc:HGNC:8823]	-	168	210	206
69	64	85	1.184126888	1.193294121
1.229883356	0.429976337	0.359445309	0.511398217
ENSG00000123416 13243.7403	12428.5609	15910.73583
8298.820692	9356.097425	9540.358496	13861.01234
9065.092204	0.612629172	4.43E-06	0.000121272	TUBA1B
12	49127782	49131397	-	3574
protein_coding	tubulin alpha 1b [Source:HGNC Symbol;Acc:HGNC:18809]
-	11447	13408	16272	8261	10238	9723
175.1587541	165.403011	210.9059554	111.7581722
124.8300587	126.9965062
ENSG00000116786 2183.186682	2926.384752	2759.34713
4526.629468	4418.512507	3749.2245	2622.972855
4231.455492	-0.689559761	4.59E-06	0.000125508
PLEKHM2 1	15684332	15734769	+	4122
protein_coding	pleckstrin homology and RUN domain containing M2
[Source:HGNC Symbol;Acc:HGNC:29131]	-	1887	3157	2822
4506	4835	3821	25.03563582	33.76762782
31.71403329	52.85479788	51.11484977	43.27280883
ENSG00000127863 739.2985108	596.0295836	1013.976958
412.8816493	310.7123583	408.1856561	783.101684
377.2598879	1.05406862	4.60E-06	0.000125508
TNFRSF19	13	23570370	23676104	+	5113
protein_coding	TNF receptor superfamily member 19
[Source:HGNC Symbol;Acc:HGNC:11915]	-	639	643	1037
411	340	416	6.834705062	5.54458618
9.395186802	3.886576726	2.897755388	3.798075326
ENSG00000173848 992.6731178	844.4524893	1091.223032
570.6004301	575.7317228	384.6364836	976.1162132
510.3228788	0.935294737	4.63E-06	0.000126131	NET1

10	5412551 5458463 +	4175	protein_coding
neuroepithelial cell transforming 1 [Source:HGNC
Symbol;Acc:HGNC:14592]	-	858	911	1116	568	630
392	11.23894435	9.620460374	12.38255229
6.577987932	6.575710235	4.383041907
ENSG00000158290 4200.927845	3799.572726	4080.352791
2312.539067	2821.085442	2751.328317	4026.95112
2628.317608	0.615342091	4.69E-06	0.000127483	CUL4B
X	120524609	120575794	-	7031
protein_coding	cullin 4B [Source:HGNC Symbol;Acc:HGNC:2555]	-
3631	4099	4173	2302	3087	2804	28.24254723
25.70364878	27.49373501	15.83031067	19.13278227
18.61688205
ENSG00000260032 7495.954697	5351.288936	7193.662948
12444.71501	10795.42673	9554.095513	6680.302194
10931.41242	-0.710616659	4.72E-06	0.000128121	NORAD
20	36045622	36050960	-	5339
lincRNA non-coding RNA activated by DNA damage [Source:HGNC
Symbol;Acc:HGNC:44311]	-	6479	5773	7357	12388	11813
9737	66.36556129	47.67334164	63.83271122
112.1869891	96.41816921	85.13561644
ENSG00000088888 2142.693023	2049.488972	2471.874396
3330.176806	3459.873496	3357.719508	2221.352131
3382.589937	-0.606671576	4.87E-06	0.00013208	MAVS
20	3846799 3876123 +	11771	protein_coding
mitochondrial antiviral signaling protein [Source:HGNC
Symbol;Acc:HGNC:29233]	-	1852	2211	2528	3315	3786
3422	8.604434549	8.281506381	9.948696121
13.61668496	14.01605676	13.57102596
ENSG00000149781 237.1771435	371.7074075	161.3367387
562.5638043	740.2265007	559.2928461	256.7404299
620.6943837	-1.272591722	4.96E-06	0.000134272	FERMT3
11	64206678	64223886	+	3066
protein_coding	fermitin family member 3 [Source:HGNC
Symbol;Acc:HGNC:23151]	-	205	401	165	560	810
570	3.656591314	5.766418789	2.492953911
8.831146574	11.51254832	8.678581395
ENSG00000111716 572.6960295	708.1906716	741.1711995
325.4833439	347.2667534	399.3547164	674.0193002
357.3682713	0.916107747	5.01E-06	0.00013528	LDHB
12	21635342	21757857	-	2983
protein_coding	lactate dehydrogenase B [Source:HGNC
Symbol;Acc:HGNC:6541]	-	495	764	758	324	380
407	9.075000514	11.29208304	11.77113683
5.251616127	5.551226414	6.369233808
ENSG00000073969 1558.427377	1287.535135	1590.878023
818.7312509	986.9686676	843.8453467	1478.946845
883.1817551	0.743238035	5.07E-06	0.000136789	NSF
17	46590669	46757464	+	5005
protein_coding	"N-ethylmaleimide sensitive factor, vesicle fusing

ATPase [Source:HGNC Symbol;Acc:HGNC:8016]"	-	1347	1389
1627	815	1080	860	14.71831952	12.23579302
15.0586461	7.873262719	9.403256252	8.02121958
ENSG00000139318 1343.232506	1346.860008	881.9741714
1749.975263	2613.639249	2282.307298	1190.688895
2215.30727	-0.896077182	5.17E-06	0.000139113	DUSP6
12	89347232	89353271	-	4632
protein_coding	dual specificity phosphatase 6 [Source:HGNC
Symbol;Acc:HGNC:3072]	-	1161	1453	902	1742	2860
2326	13.70750401	13.83028156	9.020704213
18.18363966	26.9064301	23.441597
ENSG00000143127 2685.308049	1880.783865	3202.289813
1245.676995	1149.635726	1702.408926	2589.460576
1365.907216	0.922764072	5.18E-06	0.000139223	ITGA10
1	145891208	145910189	-	6320
protein_coding	integrin subunit alpha 10 [Source:HGNC
Symbol;Acc:HGNC:6135]	-	2321	2029	3275	1240	1258
1735	20.08411849	14.15464321	24.00472206
9.4864959	8.67405478	12.81528995
ENSG00000101003 1218.280645	972.3717467	1191.936269
708.2276465	656.151392	616.2033462	1127.529554
660.1941282	0.771828983	5.28E-06	0.000141503	GINS1
20	25407727	25452628	+	4068
protein_coding	GINS complex subunit 1 [Source:HGNC
Symbol;Acc:HGNC:28980]	-	1053	1049	1219	705	718
628	14.15605169	11.36916323	13.88114265
8.379331829	7.691341554	7.206505714
ENSG00000150764 1629.002039	1119.756978	1682.791074
850.8777541	864.511444	768.2917517	1477.183364
827.8936499	0.83485889	5.29E-06	0.000141503	DIXDC1
11	111927144	112022584	+	8509
protein_coding	DIX domain containing 1 [Source:HGNC
Symbol;Acc:HGNC:23695]	-	1408	1208	1721	847	946
783	9.049380385	6.259251017	9.369249462
4.812891854	4.844748205	4.295653813
ENSG00000160606 151.5619795	176.1207168	148.6253593
403.8404453	414.8923843	275.721561	158.7693518
364.8181302	-1.199632709	5.30E-06	0.000141638	TLCD1
17	28724348	28727935	-	1311
protein_coding	TLC domain containing 1 [Source:HGNC
Symbol;Acc:HGNC:25177]	-	131	190	152	402	454
281	5.464662145	6.389764722	5.370854065
14.82601987	15.09078247	10.00575038
ENSG00000130589 491.7087122	953.8327239	498.6771922
1193.438928	1892.603806	1237.31277	648.0728761
1441.118501	-1.152076167	5.31E-06	0.000141676	HELZ2
20	63558086	63574239	-	12066
protein_coding	helicase with zinc finger 2 [Source:HGNC
Symbol;Acc:HGNC:30021]	-	425	1029	510	1188	2071
1261	1.92628402	3.759984129	1.957984777

4.760519365	7.47954838	4.878629094
ENSG00000136631 1159.275599	1152.200268	1237.892795
602.7469332	737.4849211	766.3293207	1183.122887
702.1870583	0.75249027	5.37E-06	0.000143107	VPS45
	1	150067279	150145329	+	6637
protein_coding	vacuolar protein sorting 45 homolog [Source:HGNC
Symbol;Acc:HGNC:14579]	-	1002	1243	1266	600	807
	781	8.256397936	8.257208842	8.83617572
4.370998417	5.298589992	5.493197985
ENSG00000008324 485.9239038	441.228743	470.3210382
242.1033515	189.1689946	258.0596816	465.8245617
229.7773426	1.019987181	5.46E-06	0.000145129	SS18L2
	3	42581840	42595114	+	1298
protein_coding	SS18 like 2 [Source:HGNC Symbol;Acc:HGNC:15593] -
	420	476	481	241	207	263	17.69576371
	16.16836922	17.16614749	8.977255104	6.949511065
	9.458605055
ENSG00000091651 1000.771849	808.3013948	736.2822074
433.9777919	527.2971493	395.4298543	848.4518172
452.2349318	0.906625409	5.56E-06	0.000147623	ORC6
	16	46689643	46698394	+	4309
protein_coding	origin recognition complex subunit 6 [Source:HGNC
Symbol;Acc:HGNC:17151]	-	865	872	753	432	577
	403	10.97828058	8.922240979	8.095076139
4.847395655	5.835229168	4.365908081
ENSG00000140450 229.0784118	240.0803455	172.0925212
500.2799546	423.1171232	419.9602423	213.7504262
447.7857734	-1.067049295	5.59E-06	0.000148031	ARRDC4
	15	97960698	97973838	+	4072
protein_coding	arrestin domain containing 4 [Source:HGNC
Symbol;Acc:HGNC:28087]	-	198	259	176	498	463
	428	2.659206929	2.804309561	2.002199526
5.913203018	4.954864923	4.906615603
ENSG00000167526 53342.87502	72048.20437	61826.19385
85194.2607	98232.62594	113635.5505	62405.75775
99020.81239	-0.666036858	5.74E-06	0.000151765	RPL13
	16	89560657	89566828	+	6172
protein_coding	ribosomal protein L13 [Source:HGNC
Symbol;Acc:HGNC:10303]	-	46106	77726	63230	84806	107492
	115811	408.5321516	555.2318347	474.5693875
664.3575558	758.9424131	875.9310674
ENSG00000117298 2320.865121	2257.126028	4018.75149
1640.476237	1185.276261	1588.587926	2865.58088
1471.446808	0.961870979	5.78E-06	0.000152439	ECE1
	1	21217247	21345572	-	7333
protein_coding	endothelin converting enzyme 1 [Source:HGNC
Symbol;Acc:HGNC:3146]	-	2006	2435	4110	1633	1297
	1619	14.96042587	14.64034256	25.96346148
10.76727279	7.70755955	10.30649981
ENSG00000140795 33.5518886	38.93194792	59.64570339	0

4.569299387 0 44.04317997 1.523099796
4.810934319 5.78E-06 0.000152439 MYLK3 16
46703369 46790407 - 9522 protein_coding myosin
light chain kinase 3 [Source:HGNC Symbol;Acc:HGNC:29826] -
29 42 61 0 5 0 0.16655763
0.1944711 0.296759147 0 0.02288234 0
ENSG00000109586 709.2175072 708.1906716 853.6180173
212.9705831 446.8774801 372.8618974 757.008732
344.2366535 1.136142846 5.84E-06 0.000153656 GALNT7
4 173168753 173323967 + 6865
protein_coding polypeptide N-acetylgalactosaminyltransferase 7
[Source:HGNC Symbol;Acc:HGNC:4129] - 613 764 873
212 489 380 4.883313945 4.906669149
5.890825498 1.493126268 3.104037185 2.583979661
ENSG00000105447 4115.312681 4609.728023 4735.477729
7012.960568 6170.381892 6736.044541 4486.839478
6639.795667 -0.5652459 5.93E-06 0.000155806 GRWD1
19 48445773 48457022 + 5763
protein_coding glutamate rich WD repeat containing 1 [Source:HGNC
Symbol;Acc:HGNC:21270] - 3557 4973 4843 6981 6752
6865 33.75436625 38.04554394 38.92855697
58.56932716 51.05548567 55.60808737
ENSG00000101188 128.422746 241.0072966 89.95745429
315.4375617 464.2408177 541.6309667 153.1291656
440.4364487 -1.522157204 6.28E-06 0.000164514 NTSR1
20 62708837 62762771 + 4237
protein_coding neurotensin receptor 1 [Source:HGNC
Symbol;Acc:HGNC:8039] - 111 260 92 314 508
552 1.432713086 2.705508121 1.005846755
3.583211148 5.224729926 6.081723193
ENSG00000142892 1292.326192 935.2937011 1232.026004
754.4382447 572.9901431 562.2364927 1153.215299
629.8882935 0.872304089 6.28E-06 0.000164514 PIGK
1 77088990 77219430 - 5513
protein_coding phosphatidylinositol glycan anchor biosynthesis class
K [Source:HGNC Symbol;Acc:HGNC:8965] - 1117 1009 1260
751 627 573 11.08051366 8.069323593
10.58729486 6.586476718 4.956078152 4.851909206
ENSG00000163755 1104.8984 1087.313688 1334.694838
532.4264577 620.5108568 792.8221397 1175.635642
648.5864847 0.858144586 6.37E-06 0.000166539 HPS3
3 149129584 149173732 + 6256
protein_coding "HPS3, biogenesis of lysosomal organelles complex 2
subunit 1 [Source:HGNC Symbol;Acc:HGNC:15597]" - 955 1173
1365 530 679 808 8.348363378 8.266758109
10.10737472 4.096192387 4.729678624 6.029213412
ENSG00000148840 2249.133498 2581.558927 2547.164874
4271.4666 3279.8431 4039.664294 2459.285766
3863.657998 -0.651393328 6.66E-06 0.000173619 PPRC1
10 102132994 102150331 + 6095

protein_coding "peroxisome proliferator-activated receptor gamma, coactivator-related 1 [Source:HGNC Symbol;Acc:HGNC:30025]"   -
        1944   2785   2605   4252   3589   4117   17.44284266
        20.14584311   19.79869081   33.73034338   25.66010009
        31.53212161
ENSG00000078053 443.1163218   363.3648472   400.8973506
108.494448   233.9481286   136.3889572   402.4595065
159.6105113   1.332034825   6.76E-06   0.000176111   AMPH
        7   38383704   38631567   -   5636
protein_coding amphiphysin [Source:HGNC Symbol;Acc:HGNC:471]   -
        383   392   410   108   256   139   3.716400435
        3.066542871   3.369886921   0.92651827   1.979372712
        1.151303571
ENSG00000119397 521.7897157   482.0145932   679.5698992
314.4329835   198.3075934   278.6652075   561.1247361
263.8019281   1.089871221   6.93E-06   0.000180258   CNTRL
        9   121074863   121177610   +   9417
protein_coding centriolin [Source:HGNC Symbol;Acc:HGNC:1858]   -
        451   520   695   313   217   284   2.619139825
        2.434583819   3.41880783   1.607063301   1.004166575
        1.407835154
ENSG00000177352 849.20987   909.3390692   889.7965587
1345.130239   1400.033332   1423.743719   882.7818326
1389.635763   -0.654314879   6.98E-06   0.000181122   CCDC71
        3   49162535   49166321   -   1781
protein_coding coiled-coil domain containing 71 [Source:HGNC Symbol;Acc:HGNC:25760] -   734   981   910   1339   1532
        1451   22.5385957   24.28505246   23.66900104
36.35112651   37.48464857   38.03203191
ENSG00000143479 795.9896329   578.4175119   440.0092873
1245.676995   1186.190121   1062.656408   604.8054774
1164.841175   -0.947023312   7.03E-06   0.000182104   DYRK3
        1   206635536   206684419   +   4336
protein_coding dual specificity tyrosine phosphorylation regulated kinase 3 [Source:HGNC Symbol;Acc:HGNC:3094]   -   688   624
        450   1240   1298   1083   8.677485116   6.344965633
        4.807570881   13.82718037   13.04499799   11.65964208
ENSG00000231991 1939.067768   1402.477076   1217.359028
824.7587203   853.5451255   874.2630278   1519.634624
850.8556245   0.836035414   7.08E-06   0.000183066
ANXA2P2 9   33624274   33625293   +   1020
processed_pseudogene   annexin A2 pseudogene 2 [Source:HGNC Symbol;Acc:HGNC:539]   -   1676   1513   1245   821   934
        891   89.860443   65.39924193   56.54206109
38.91743438   39.9029774   40.77777903
ENSG00000079819 2305.82462   2398.022601   2417.117685
1571.160339   1410.085791   1671.991245   2373.654969
1551.079125   0.614069883   7.17E-06   0.000185165
EPB41L2 6   130839347   131063322   -   10320
protein_coding erythrocyte membrane protein band 4.1 like 2

[Source:HGNC Symbol;Acc:HGNC:3379]	-	1993	2587	2472
	1564	1543	1704	10.56142001	11.05224973
11.09612205	7.327541182	6.515456154	7.707897468
ENSG00000165119 13191.67703	14212.94184	15546.99482
9247.142534	10057.94181	10227.20936	14317.20456
9844.097901	0.540449466	7.21E-06	0.000185909	HNRNPK
	9	83968083	83980616	-	5114
protein_coding	heterogeneous nuclear ribonucleoprotein K [Source:HGNC
Symbol;Acc:HGNC:5044]	-	11402	15333	15900	9205	11006
	10423	121.9312499	132.190538	144.0253225
87.02905858	93.78370427	95.14326479
ENSG00000164604 313.5366141	343.8988733	439.0314889
182.8332364	170.8917971	135.4077417	365.4889921
163.0442584	1.165161426	7.25E-06	0.000186509	GPR85
	7	113078331	113087778	-	7396
protein_coding	G protein-coupled receptor 85 [Source:HGNC
Symbol;Acc:HGNC:4536]	-	271	371	449	182	187
	138	2.003858731	2.211622324	2.812236848
1.18980476	1.101801354	0.871020186
ENSG00000205426 107.5974358	138.11572	135.9139798
39.17855066	36.5543951	14.71823279	127.2090452
30.15039285	2.077078965	7.29E-06	0.000187355	KRT81
	12	52285913	52291534	-	1929
protein_coding	keratin 81 [Source:HGNC Symbol;Acc:HGNC:6458]	-
	93	149	139	39	40	15	2.6366072
	3.405555786	3.337989978	0.977537881	0.903621096
	0.362998698
ENSG00000144354 1064.404742	1332.955741	1316.116668
852.8869105	597.6643598	603.4475445	1237.825717
684.6662716	0.855148011	7.33E-06	0.000187944	CDCA7
	2	173354820	173368997	+	3934
protein_coding	cell division cycle associated 7 [Source:HGNC
Symbol;Acc:HGNC:14628]	-	920	1438	1346	849	654
	615	12.78934151	16.11604563	15.84941219
10.43456986	7.244392804	7.297713274
ENSG00000168077 263.7872621	219.6874204	404.8085443
141.6455293	89.55826799	90.27182779	296.0944089
107.1585417	1.467489075	7.42E-06	0.000189935	SCARA3
	8	27633868	27676776	+	4031
protein_coding	scavenger receptor class A member 3 [Source:HGNC
Symbol;Acc:HGNC:19000]	-	228	237	414	141	98
	92	3.093262393	2.592205966	4.757622712
1.691248915	1.059429045	1.06542054
ENSG00000137710 4181.259496	3261.014114	3257.046524
2083.495232	2485.698867	2141.012263	3566.440045
2236.735454	0.672676916	7.54E-06	0.000192793	RDX
	11	109864295	110296722	-	8528
protein_coding	radixin [Source:HGNC Symbol;Acc:HGNC:9944]	-
	3614	3518	3331	2074	2720	2182	23.17584989
	18.18790183	18.09380464	11.75879386	13.89889615

11.94410439
ENSG00000059588 802.9314029 763.8077401 837.9732427
404.8450235 473.3794165 499.4386994 801.5707952
459.2210465 0.803355417 7.65E-06 0.000195314 TARBP1
1 234391313 234479103 - 7743
protein_coding TAR (HIV-1) RNA binding protein 1 [Source:HGNC
Symbol;Acc:HGNC:11568] - 694 824 857 403 518
509 4.901679644 4.691934173 5.127126331
2.516500398 2.915272113 3.068700887
ENSG00000134291 1826.842486 1878.003011 1926.26288
1100.013153 1354.340338 982.196735 1877.036126
1145.516742 0.7122242 7.67E-06 0.000195314
TMEM106C 12 47963569 47968878 + 3475
protein_coding transmembrane protein 106C [Source:HGNC
Symbol;Acc:HGNC:28775] - 1579 2026 1970 1095 1482
1001 24.84975057 25.70505811 26.26115802
15.23563384 18.58454755 13.44699689
ENSG00000060762 387.5821614 367.9996029 372.5411966
175.8011889 207.4461922 149.144759 376.0409869
177.4640467 1.082432715 7.68E-06 0.000195314 MPC1
6 166364919 166383013 - 1546
protein_coding mitochondrial pyruvate carrier 1 [Source:HGNC
Symbol;Acc:HGNC:21606] - 335 397 381 175 227
152 11.8503187 11.32178687 11.41610467
5.473054535 6.398452723 4.589655982
ENSG00000146410 260.316377 253.9846126 267.916766
108.494448 121.5433637 107.9337071 260.7392519
112.6571729 1.210150171 7.78E-06 0.000197517 MTFR2
6 136231024 136250335 - 2450
protein_coding mitochondrial fission regulator 2 [Source:HGNC
Symbol;Acc:HGNC:21115] - 225 274 274 108 133
110 5.022398244 4.930811911 5.180681988
2.131370191 2.365615485 2.095910033
ENSG00000100714 2983.804162 3485.33629 3922.927246
2151.806552 1793.906939 2419.677471 3464.022566
2121.796987 0.707489733 7.85E-06 0.00019893 MTHFD1
14 64388031 64463457 + 9377
protein_coding "methylenetetrahydrofolate dehydrogenase,
cyclohydrolase and formyltetrahydrofolate synthetase 1 [Source:HGNC
Symbol;Acc:HGNC:7432]" - 2579 3760 4012 2142 1963
2466 15.04118798 17.67900778 19.81980896
11.0447723 9.122523301 12.27651764
ENSG00000160888 709.2175072 1142.930757 866.3293967
1665.590692 1447.554046 1759.319426 906.1592202
1624.154721 -0.840687588 7.90E-06 0.000199984 IER2
19 13150415 13154908 + 3072
protein_coding immediate early response 2 [Source:HGNC
Symbol;Acc:HGNC:28871] - 613 1233 886 1658 1584
1793 10.91274422 17.69602908 13.36026174
26.09543443 22.46945629 27.24614817

ENSG00000170759 6209.413314 5744.31622 5907.85803
4167.995043 4311.590902 3759.036655 5953.862521
4079.540867 0.545311177 7.97E-06 0.000201135 KIF5B
10 32009010 32056431 - 5877
protein_coding kinesin family member 5B [Source:HGNC
Symbol;Acc:HGNC:6324] - 5367 6197 6042 4149 4718
3831 49.94253899 46.49002325 47.62417726
34.13413962 34.98330557 30.43003817
ENSG00000186193 426.9188583 662.7700657 1169.446906
293.3368408 227.5511095 362.0685267 753.0452766
294.3188257 1.356420112 7.97E-06 0.000201135 SAPCD2
9 137062124 137070588 - 3848
protein_coding suppressor APC domain containing 2 [Source:HGNC
Symbol;Acc:HGNC:28055] - 369 715 1196 292 249
369 5.244281742 8.19228281 14.39788075
3.669010922 2.819829707 4.476487114
ENSG00000161091 1585.037496 1915.081057 1985.908583
2947.432503 2692.231199 2865.149317 1828.675712
2834.937673 -0.631993484 8.04E-06 0.000202375 MFSD12
19 3538261 3574290 - 5277 protein_coding major
facilitator superfamily domain containing 12 [Source:HGNC
Symbol;Acc:HGNC:28299] - 1370 2066 2031 2934 2946
2920 14.19803315 17.26144628 17.82893021
26.88278334 24.32787894 25.83103412
ENSG00000018510 3114.540831 2764.168302 3120.154746
2052.353308 2053.443145 1897.670815 2999.621293
2001.155756 0.583783081 8.51E-06 0.000213968 AGPS
2 177392644 177559299 + 8965
protein_coding alkylglycerone phosphate synthase [Source:HGNC
Symbol;Acc:HGNC:327] - 2692 2982 3191 2043 2247
1934 16.42175144 14.66531187 16.48841714
11.01841915 10.92223147 10.07052731
ENSG00000137809 217.508795 154.8008405 380.3635839
96.43950931 78.59194946 64.76022428 250.8910731
79.93056102 1.650670892 8.58E-06 0.000214905 ITGA11
15 68296533 68432162 - 10864
protein_coding integrin subunit alpha 11 [Source:HGNC
Symbol;Acc:HGNC:6136] - 188 167 389 96 86
66 0.94637401 0.677736183 1.658678824
0.427250611 0.344958851 0.283596074
ENSG00000166147 2982.6472 1454.38634 2948.062225
1364.217226 964.1221707 1292.260839 2461.698588
1206.866745 1.028258775 8.58E-06 0.000214905 FBN1
15 48408306 48645849 - 16057
protein_coding fibrillin 1 [Source:HGNC Symbol;Acc:HGNC:3603] -
2578 1569 3015 1358 1055 1317 8.780378111
4.308166647 8.698119399 4.089183305 2.863166803
3.828841631
ENSG00000134779 2488.624564 2143.111038 2384.850337
1630.430454 1459.434224 1485.560296 2338.86198

1525.141658	0.616730581	8.72E-06	0.000218068	TPGS2
	18	36777647	36829216	-	10571
protein_coding	tubulin polyglutamylase complex subunit 2 [Source:HGNC Symbol;Acc:HGNC:24561]	-	2151	2312	2439	1623	1597
	1514	11.12804954	9.642856745	10.68804278
7.42341369	6.583357524	6.685837829
ENSG00000111581 2414.579017	2425.831136	2551.076068
1771.071405	1488.67774	1593.494004	2463.82874
1617.747716	0.607122455	8.88E-06	0.00022185	NUP107
	12	68686734	68745809	+	8320
protein_coding	nucleoporin 107 [Source:HGNC Symbol;Acc:HGNC:29914]
	-	2087	2617	2609	1763	1629	1624
13.71809593	13.86801665	14.52623978	10.24543028
8.532108228	9.111895585
ENSG00000100934 2293.098041	1930.839226	2164.845693
1332.070722	1467.658963	1319.734874	2129.59432
1373.154853	0.632669413	8.90E-06	0.000221957	SEC23A
	14	39031919	39109646	-	8945
protein_coding	"Sec23 homolog A, coat complex II component [Source:HGNC Symbol;Acc:HGNC:10701]"	-	1982	2083	2214
	1326	1606	1345	12.11763922	10.26698395
11.46567764	7.167445439	7.823909059	7.019205789
ENSG00000091483 2827.614335	2731.725012	3085.931801
1845.410194	2041.562966	1871.177996	2881.75705
1919.383719	0.586211892	9.03E-06	0.000224799	FH
	1	241497603	241519761	-	2142
protein_coding	fumarate hydratase [Source:HGNC Symbol;Acc:HGNC:3700]
	-	2444	2947	3156	1837	2234	1907
62.39883018	60.65891353	68.25272319	41.46588189
45.44878735	41.56016493
ENSG00000068308 1279.599613	1369.106835	1372.828976
2305.50702	1914.536443	2020.322755	1340.511808
2080.122072	-0.633536763	9.16E-06	0.000227122	OTUD5
	X	48922028	48958386	-	3592
protein_coding	OTU deubiquitinase 5 [Source:HGNC Symbol;Acc:HGNC:25402]	-	1106	1477	1404	2295	2095
	2059	16.83889201	18.12917851	18.10644691
30.89211041	25.41594705	26.75876432
ENSG00000100626 136.5214777	145.5313291	200.4486753
65.29758443	27.41579632	14.71823279	160.8338274
35.81053785	2.170096108	9.16E-06	0.000227122
GALNT16 14	69259277	69357033	+	10802
protein_coding	polypeptide N-acetylgalactosaminyltransferase 16 [Source:HGNC Symbol;Acc:HGNC:23233]	-	118	157	205
	65	30	15	0.597410081	0.640810232
0.879128064	0.290944666	0.121025164	0.064823596
ENSG00000164919 1336.290735	1432.139513	1399.229534
611.7881372	922.0846163	859.5447951	1389.219927
797.8058495	0.799884442	9.17E-06	0.000227122	COX6C
	8	99873200	99894062	-	3066

protein_coding cytochrome c oxidase subunit 6C [Source:HGNC
Symbol;Acc:HGNC:2285] - 1155 1545 1431 609 1009
876 20.60177057 22.21724945 21.62070937
9.603871899 14.34093982 13.3376093
ENSG00000123700 18.51138681 41.71280134 55.73450972 0
0 1.962431039 38.65289929 0.65414368
5.904443926 9.41E-06 0.000232857 KCNJ2 17
70168673 70180048 + 5577 protein_coding
potassium voltage-gated channel subfamily J member 2 [Source:HGNC
Symbol;Acc:HGNC:6263] - 16 45 57 0 0
2 0.156896787 0.355750752 0.473452776 0
0 0.016740768
ENSG00000063177 14027.00336 16830.65186 15635.97447
21268.93012 22009.40129 25804.00573 15497.87656
23027.44571 -0.571226629 9.52E-06 0.000235024 RPL18
19 48615328 48619536 - 4209
protein_coding ribosomal protein L18 [Source:HGNC
Symbol;Acc:HGNC:10310] - 12124 18157 15991 21172 24084
26298 157.5294348 190.1950132 175.9945233
243.2115391 249.3493788 291.6687035
ENSG00000102189 1499.422332 1300.512451 1290.693909
908.1387127 770.3838767 844.8265623 1363.542897
841.1163839 0.696818511 9.53E-06 0.000235024 EEA1
12 92770637 92929331 - 10150
protein_coding early endosome antigen 1 [Source:HGNC
Symbol;Acc:HGNC:3185] - 1296 1403 1320 904 843
861 6.98286542 6.094324664 6.024352072
4.306293286 3.619262893 3.95989021
ENSG00000181830 854.9946784 868.553219 990.5097956
1405.404933 1517.007397 1391.363607 904.6858976
1437.925312 -0.668340798 9.59E-06 0.000235967
SLC35C1 11 45804072 45813015 + 4505
protein_coding solute carrier family 35 member C1 [Source:HGNC
Symbol;Acc:HGNC:20197] - 739 937 1013 1399 1660
1418 8.97107228 9.170198858 10.41638698
15.01495848 16.05727667 14.69357491
ENSG00000163156 1196.298373 1160.542828 1111.756799
1839.382725 1757.352544 1707.315004 1156.199333
1768.016758 -0.612892926 9.68E-06 0.000237796 SCNM1
1 151156664 151170297 + 3265
protein_coding sodium channel modifier 1 [Source:HGNC
Symbol;Acc:HGNC:23136] - 1034 1252 1137 1831 1923
1740 17.31936903 16.9065542 16.13168507
27.11479811 25.66579354 24.87780724
ENSG00000104067 1372.156547 918.6085806 1291.671708
724.3008981 599.4920796 675.0762774 1194.145612
666.2897517 0.841397336 9.74E-06 0.000239009 TJP1
15 29699367 29968865 - 11265
protein_coding tight junction protein 1 [Source:HGNC
Symbol;Acc:HGNC:11827] - 1186 991 1321 721 656

688	5.757689038	3.878612711	5.432179063
3.094605444	2.537647185	2.851039787
ENSG00000116774	196.6834849	167.7781565	259.1165803
87.39830532	45.69299387	74.57237948	207.8594072
69.22122622	1.588390627	9.82E-06	0.000240617	OLFML3
1	113979391	114035572	+	2821
protein_coding	olfactomedin like 3 [Source:HGNC
Symbol;Acc:HGNC:24956]	-	170	181	265	87	50
76	3.295645939	2.82884692	4.351562681
1.491136438	0.772370212	1.257640579
ENSG00000135829	9141.1542	9175.889343	9425.976732
6941.635514	6161.243294	6362.201428	9247.673425
6488.360079	0.511284061	9.84E-06	0.000240728	DHX9
1	182839369	182887751	+	5645
protein_coding	DExH-box helicase 9 [Source:HGNC Symbol;Acc:HGNC:2750]
-	7901	9899	9640	6910	6742	6484
76.54429515	77.31456842	79.10711448	59.18549955
52.04552571	53.61978839
ENSG00000130592	468.5694787	309.601681	716.726239
259.1811813	110.5770452	162.8817762	498.2991329
177.5466676	1.489882442	9.89E-06	0.000241409	LSP1
11	1852970	1892267	+	5399	protein_coding
lymphocyte specific protein 1 [Source:HGNC Symbol;Acc:HGNC:6707]
-	405	334	733	258	121	166
4.102384933	2.727514684	6.289166653	2.31050863
0.976631304	1.435293759
ENSG00000011275	3475.512874	4135.129039	3628.609922
5575.409132	5451.174169	5452.614642	3746.417279
5493.065981	-0.551886508	1.00E-05	0.000244995	RNF216
7	5620047	5781739	-	7619	protein_coding	ring
finger protein 216 [Source:HGNC Symbol;Acc:HGNC:21698]	-	3004
4461	3711	5550	5965	5557	21.56237861
25.81476689	22.56292691	35.2205575	34.11701859
34.04775326
ENSG00000128245	1816.429831	1839.071063	1911.595904
1056.81629	1281.231548	1231.425477	1855.698933
1189.824438	0.641065717	1.02E-05	0.000248401	YWHAH
22	31944461	31957603	+	2453
protein_coding	tyrosine 3-monooxygenase/tryptophan 5-monooxygenase
activation protein eta [Source:HGNC Symbol;Acc:HGNC:12853]	-
1570	1984	1955	1052	1402	1255	35.00231887
35.65973222	36.91914794	20.73573376	24.9062913
23.88318339
ENSG00000228672	68.26073887	171.4859611	158.4033434
455.0739346	312.5400781	332.6320611	132.7166811
366.7486913	-1.461697878	1.02E-05	0.000248409	PROB1
5	139390592	139395713	-	5122
protein_coding	proline rich basic protein 1 [Source:HGNC
Symbol;Acc:HGNC:41906]	-	59	185	162	453	342
339	0.629951552	1.592451126	1.465135854

4.27621807	2.909679335	3.08962777
ENSG00000162521 6015.043752	5431.933685	5343.668344
3332.185962	4160.804022	3680.539414	5596.881927
3724.509799	0.58736836	1.05E-05	0.000253653	RBBP4
	1	32651142	32686211	+	9470
protein_coding	"RB binding protein 4, chromatin remodeling factor
[Source:HGNC Symbol;Acc:HGNC:9887]"	-	5199	5860	5465
	3317	4553	3751	30.02372345	27.28233862
26.73268882	16.93544875	20.95107295	18.49026438
ENSG00000152078 244.1189136	222.4682738	239.560612
40.18312888	111.490905	78.49724156	235.3825998
76.72375849	1.614473818	1.07E-05	0.000259936	TMEM56
	1	95117338	95197607	+	7402
protein_coding	transmembrane protein 56 [Source:HGNC
Symbol;Acc:HGNC:26477]	-	211	240	245	40	122
	80	1.558935286	1.429539365	1.533272899
0.261283585	0.718239602	0.504529938
ENSG00000104763 1568.840032	1449.751584	1416.829905
813.7083598	877.3054823	1041.069666	1478.473841
910.6945028	0.698853951	1.07E-05	0.000259936	ASAH1
	8	18055992	18084998	-	15378
protein_coding	N-acylsphingosine amidohydrolase 1 [Source:HGNC
Symbol;Acc:HGNC:735]	-	1356	1564	1449	810	960
	1061	4.822303564	4.484054166	4.364866695
2.546750375	2.720382511	3.220783726
ENSG00000170275 8209.800051	7955.094691	9263.662195
5208.738081	5528.852258	6341.595902	8476.185645
5693.062081	0.574221501	1.08E-05	0.000261577	CRTAP
	3	33113979	33147773	+	6695
protein_coding	cartilage associated protein [Source:HGNC
Symbol;Acc:HGNC:2379]	-	7096	8582	9474	5185	6050
	6463	57.96391864	56.51606121	65.55189559
37.44547947	39.37888282	45.06398674
ENSG00000139687 5330.12244	4559.672662	4448.982794
3201.590794	3384.936986	3016.256507	4779.592632
3200.928095	0.578164572	1.09E-05	0.000262378	RB1
	13	48303726	48599436	+	6452
protein_coding	RB transcriptional corepressor 1 [Source:HGNC
Symbol;Acc:HGNC:9884]	-	4607	4919	4550	3187	3704
	3074	39.04977773	33.6137079	32.66777034
23.88300234	25.01699934	22.241063
ENSG00000170801 293.8682656	584.9061699	935.7530843
201.9202226	204.7046125	268.8530523	604.8425066
225.1592958	1.426675125	1.12E-05	0.000269661	HTRA3
	4	8269765 8307111 +	3321	protein_coding	HtrA
serine peptidase 3 [Source:HGNC Symbol;Acc:HGNC:30406]	-	254
	631	957	201	224	274	4.182727327
8.377114309	13.34890118	2.926364356	2.939258214
3.851480675
ENSG00000143742 6050.909564	5208.53846	5140.286274

3271.911269	4084.039792	3377.343818	5466.578099
3577.76496	0.611333243	1.16E-05	0.00027771	SRP9
	1	225777813	225790466	+	1728
protein_coding	signal recognition particle 9 [Source:HGNC
Symbol;Acc:HGNC:11304]	-	5230	5619	5257	3257	4469
	3442	165.5208331	143.3670179	140.9278073
91.13290975	112.7003369	92.98505744
ENSG00000112312 1183.571794	1019.646255	1039.399716
601.742355	727.4324624	608.3536221	1080.872589
645.8428132	0.742147216	1.16E-05	0.00027771	GMNN
	6	24774931	24786099	+	2476
protein_coding	"geminin, DNA replication inhibitor [Source:HGNC
Symbol;Acc:HGNC:17493]" -	1023	1100	1063	599	796
	620	22.59538294	19.58736439	19.8877238
11.69707834	14.00944805	11.68926179
ENSG00000103264 1575.781802	1651.826933	1953.641235
2996.656836	2568.860115	2538.404549	1727.083324
2701.307167	-0.645015286	1.17E-05	0.000280186	FBXO31
	16	87326987	87392142	-	6897
protein_coding	F-box protein 31 [Source:HGNC Symbol;Acc:HGNC:16510]
	-	1362	1782	1998	2983	2811	2587
10.79969758	11.39151357	13.41954261	20.91193613
17.76066478	17.50984246
ENSG00000180340 854.9946784	1254.164894	1321.00566
608.7744025	498.0536332	715.3061137	1143.388411
607.3780498	0.913632086	1.18E-05	0.000281815	FZD2
	17	44557459	44559570	+	2112
protein_coding	frizzled class receptor 2 [Source:HGNC
Symbol;Acc:HGNC:4040]	-	739	1353	1351	606	545
	729	19.13573893	28.24475687	29.63219964
13.87330476	11.2450447	16.11312033
ENSG00000105245 894.3313753	1377.449395	992.4653924
1965.95958	1899.000825	1743.619978	1088.082054
1869.526795	-0.780015628	1.19E-05	0.000283582	NUMBL
	19	40665905	40690972	-	5823
protein_coding	"NUMB like, endocytic adaptor protein [Source:HGNC
Symbol;Acc:HGNC:8061]"	-	773	1486	1015	1957	2078
	1777	7.259846138	11.25138478	8.074612838
16.24969643	15.55096525	14.24579444
ENSG00000165490 1286.541383	998.3263787	1118.601388
502.289111	770.3838767	603.4475445	1134.489717
625.3735107	0.85823188	1.19E-05	0.000284445	DDIAS
	11	82899975	82958277	+	5339
protein_coding	DNA damage induced apoptosis suppressor [Source:HGNC
Symbol;Acc:HGNC:26351]	-	1112	1077	1144	500	843
	615	11.39041583	8.893848769	9.925869463
4.528050898	6.880599056	5.377262413
ENSG00000180998 141.1493244	88.06035838	132.0027862
23.10529911	21.93263706	43.17348286	120.4041563
29.40380634	2.033768713	1.19E-05	0.000284501

GPR137C 14 52553148 52637713 + 6193
protein_coding G protein-coupled receptor 137C [Source:HGNC
Symbol;Acc:HGNC:25445] - 122 95 135 23 24
44 1.077341683 0.676326623 1.009799484
0.179567598 0.168876321 0.331663464
ENSG00000138623 2599.692885 3121.971442 2478.718985
3593.3763 4627.786419 5027.748322 2733.461104
4416.30368 -0.69200111 1.20E-05 0.000285821 SEMA7A
15 74409289 74433958 - 3800
protein_coding semaphorin 7A (John Milton Hagen blood group)
[Source:HGNC Symbol;Acc:HGNC:10741] - 2247 3368 2535
3577 5064 5124 32.33807683 39.07711061
30.90272825 45.51311496 58.07225603 62.94649611
ENSG00000138658 578.4808379 354.0953358 599.3904291
261.1903377 168.1502174 248.2475264 510.6555343
225.8626939 1.177012354 1.20E-05 0.000285821 ZGRF1
4 112539333 112636995 - 9103
protein_coding zinc finger GRF-type containing 1 [Source:HGNC
Symbol;Acc:HGNC:25654] - 500 382 613 260 184
253 3.003863365 1.850174871 3.119452576
1.380988372 0.880829554 1.29742426
ENSG00000283041 2877.363688 3634.575423 3765.501701
4647.178855 5673.242119 5888.274332 3425.813604
5402.898435 -0.657050591 1.22E-05 0.000289255
AC008038.1 7 133034607 133035920 - 1314
processed_pseudogene eukaryotic translation elongation
factor 1 gamma (- 2487 3921 3851 4626 6208
6001 103.5082897 131.563505 135.7627446
170.2203502 205.8803703 213.1936659
ENSG00000140545 507.9061756 587.6870233 837.9732427
309.4100924 275.985683 364.0309577 644.5221472
316.4755777 1.027077338 1.22E-05 0.000289812 MFGE8
15 88898683 88913411 - 6837
protein_coding milk fat globule-EGF factor 8 protein [Source:HGNC
Symbol;Acc:HGNC:7036] - 439 634 857 308 302
371 3.511507926 4.088440069 5.806543686
2.178142821 1.924863604 2.533111844
ENSG00000140391 4558.429002 3868.167111 4011.906902
2695.28337 2985.58022 2673.812291 4146.167672
2784.89196 0.573880346 1.23E-05 0.000290092 TSPAN3
15 77041404 77083984 - 8506
protein_coding tetraspanin 3 [Source:HGNC Symbol;Acc:HGNC:17752]
- 3940 4173 4103 2683 3267 2725
25.33177117 21.63002216 22.34490921 15.25093643
16.73718258 14.95502571
ENSG00000071575 2310.452466 1924.350568 2362.360973
1417.459871 1471.314403 1372.720512 2199.054669
1420.498262 0.630203949 1.23E-05 0.000290163 TRIB2
2 12716889 12742734 + 4588
protein_coding tribbles pseudokinase 2 [Source:HGNC

Symbol;Acc:HGNC:30809] -   1997   2076   2416   1411   1610
        1399   23.80396858   19.94977022   24.39360441
14.86978951   15.29188645   14.23443852
ENSG00000165055 860.7794867   980.714307   844.8178316
1394.354572   1550.820212   1343.284046   895.4372084
1429.486277   -0.674594502   1.24E-05   0.000292805
METTL2B 7   128476729   128506602   +   6749
protein_coding methyltransferase like 2B [Source:HGNC
Symbol;Acc:HGNC:18272] -   744   1058   864   1388   1697
        1369   6.028763121   6.911625488   5.930301451
9.943774212   10.9572359   9.469129496
ENSG00000110237 1283.070498   1510.003408   2590.188004
961.3813585   621.4247166   994.9525367   1794.420637
859.2528706   1.062913703   1.25E-05   0.000294673
ARHGEF17   11   73308289   73369388   +   9908
        protein_coding Rho guanine nucleotide exchange factor 17
[Source:HGNC Symbol;Acc:HGNC:21726]   -   1109   1629   2649
        957   680   1014   6.121252029   7.248848854
12.38506859   4.670110498   2.990759649   4.777470268
ENSG00000105640 17353.26817   21737.00425   20696.08128
28182.43744   27848.05204   38161.43398   19928.78457
31397.30782   -0.655741168   1.26E-05   0.000296455   RPL18A
        19   17859876   17864153   +   3527
protein_coding ribosomal protein L18a [Source:HGNC
Symbol;Acc:HGNC:10311] -   14999   23450   21166   28054   30473
        38892   232.5688569   293.1374768   277.9942339
384.5834132   376.5029209   514.7553313
ENSG00000175505 188.5847531   215.0526647   302.1397106
84.38457065   63.05633154   107.9337071   235.2590428
85.12486978   1.468560336   1.27E-05   0.000298091   CLCF1
        11   67364168   67374177   -   1975
protein_coding cardiotrophin like cytokine factor 1 [Source:HGNC
Symbol;Acc:HGNC:17412] -   163   232   309   84   69
        110   4.513518399   5.179106515   7.247593641
2.056427498   1.522441411   2.599989661
ENSG00000104419 15251.06881   20703.45373   8122.571443
37808.30596   51001.6059   52614.73858   14692.36466
47141.55015   -1.681930057   1.27E-05   0.000298091   NDRG1
        8   133237171   133302022   -   9823
protein_coding N-myc downstream regulated 1 [Source:HGNC
Symbol;Acc:HGNC:7679]   -   13182   22335   8307   37636   55809
        53622   73.38915309   100.2480124   39.17441522
185.2509878   247.5817877   254.8266824
ENSG00000178951 1077.13132   1440.482073   1530.254521
2376.832073   2074.461922   2186.148177   1349.289305
2212.480724   -0.71267308   1.29E-05   0.000300726   ZBTB7A
        19   4044364 4066945 -   5725   protein_coding zinc
finger and BTB domain containing 7A [Source:HGNC
Symbol;Acc:HGNC:18078] ZBTB   931   1554   1565   2366   2270
        2228   8.893422047   11.96766659   12.66313704

19.98206953	17.27861644	18.16710521
ENSG00000198157 483.6099804	445.8634987	510.4107732
263.1994942	249.4837465	257.0784661	479.9614175
256.5872356	0.903448318	1.29E-05	0.000300726	HMGN5
X	81113701	81201942	-	2416
protein_coding	high mobility group nucleosome binding domain 5
[Source:HGNC Symbol;Acc:HGNC:8013]	-	418	481	522
262	273	262	9.461806551	8.777727705
10.00866213	5.243310514	4.924071096	5.062329358
ENSG00000143458 301.9669974	334.6293618	267.916766
793.6167954	601.3197993	459.2088631	301.5043751
618.0484859	-1.035169091	1.29E-05	0.000300726	GABPB2
1	151070578	151125542	+	9189
protein_coding	GA binding protein transcription factor subunit beta 2
[Source:HGNC Symbol;Acc:HGNC:28441]	-	261	361	274
790	658	468	1.553341583	1.732099787
1.38128968	4.156808871	3.120442899	2.377517035
ENSG00000164949 1660.240005	1606.406327	1001.265578
2324.594006	2991.977239	2341.180229	1422.637303
2552.583825	-0.843776505	1.29E-05	0.000300931	GEM
8	94249253	94262350	-	2648
protein_coding	GTP binding protein overexpressed in skeletal muscle
[Source:HGNC Symbol;Acc:HGNC:4234]	-	1435	1733	1024
2314	3274	2386	29.63661736	28.85456556
17.91366431	42.25193377	53.8789709	42.06283054
ENSG00000160214 1551.485607	1846.486673	1688.657865
2643.045302	2390.657439	2715.023342	1695.543381
2582.908695	-0.606776889	1.30E-05	0.000301196	RRP1
21	43789513	43805293	+	4251
protein_coding	ribosomal RNA processing 1 [Source:HGNC
Symbol;Acc:HGNC:18785]	-	1341	1992	1727	2631	2616
2767	17.2517194	20.66008895	18.81930966
29.92477954	26.81669376	30.38533928
ENSG00000112559 819.1288664	1018.719304	2228.40259
348.588643	128.8542427	163.8629918	1355.41692
213.7686258	2.665031898	1.35E-05	0.000312064	MDFI
6	41636882	41654246	+	2523
protein_coding	MyoD family inhibitor [Source:HGNC
Symbol;Acc:HGNC:6967]	-	708	1099	2279	347	141
167	15.34654863	19.20500391	41.84364708
6.649874372	2.43534481	3.08990592
ENSG00000131171 2531.432146	1788.088751	1893.017734
968.413406	1392.722453	1207.876304	2070.84621
1189.670721	0.798945821	1.35E-05	0.000312064
SH3BGRL X	81201943	81298547	+	2304
protein_coding	SH3 domain binding glutamate rich protein like
[Source:HGNC Symbol;Acc:HGNC:10823]	-	2188	1929	1936
964	1524	1231	51.9349306	36.91337128
38.92470537	20.22999501	28.82445403	24.94144517
ENSG00000154146 352.8733111	300.3321696	617.968599

152.6958897	201.9630329	164.8442073	423.7246932
173.16771	1.290634104	1.35E-05	0.000312396	NRGN
	11	124739846	124747210	+	1309
protein_coding	neurogranin [Source:HGNC Symbol;Acc:HGNC:8000]	-
	305	324	632	152	221	168	12.74250772
	10.91287854	22.36556571	5.61442336	7.357177345
	5.991225569
ENSG00000121211 320.4783842	242.8611989	317.7844853
127.5814342	131.5958223	54.94806909	293.7080228
104.7084419	1.486851473	1.38E-05	0.000317666	MND1
	4	153344649	153415118	+	1511
protein_coding	meiotic nuclear divisions 1 [Source:HGNC
Symbol;Acc:HGNC:24839]	-	277	262	325	127	144
	56	10.02559179	7.644881975	9.963716282
4.063876235	4.152949265	1.730093596
ENSG00000100911 1027.381968	1172.593193	1397.273937
606.7652461	707.3275451	776.1414759	1199.083033
696.7447557	0.783534313	1.41E-05	0.000324831	PSME2
	14	24143362	24147570	-	3582
protein_coding	proteasome activator subunit 2 [Source:HGNC
Symbol;Acc:HGNC:9569]	-	888	1265	1429	604	774
	791	13.55757754	15.57036889	18.48030366
8.1529086	9.416163197	10.30853472
ENSG00000104549 2509.449875	2173.700425	2704.590419
1551.068775	1577.322148	1685.728262	2462.58024
1604.706395	0.61774232	1.41E-05	0.000324831	SQLE
	8	124998497	125022283	+	4305
protein_coding	squalene epoxidase [Source:HGNC Symbol;Acc:HGNC:11279]
	-	2169	2345	2766	1544	1726	1718
27.55377508	24.01616448	29.763327	17.34104865
17.47134072	18.62927868
ENSG00000128039 820.2858281	913.9738249	582.767856
1188.416037	1552.647932	1414.912779	772.342503
1385.325582	-0.843098689	1.43E-05	0.000328102	SRD5A3
	4	55346109	55373096	+	4420
protein_coding	steroid 5 alpha-reductase 3 [Source:HGNC
Symbol;Acc:HGNC:25812]	-	709	986	596	1183	1699
	1442	8.772405097	9.835322468	6.246351883
12.94087648	16.75057452	15.22961858
ENSG00000125871 844.5820233	686.8707954	784.1943298
339.547439	480.6902955	445.4718458	771.8823828
421.9031935	0.870439717	1.44E-05	0.000329911	MGME1
	20	17968913	17991122	+	2336
protein_coding	mitochondrial genome maintenance exonuclease 1
[Source:HGNC Symbol;Acc:HGNC:16205]	-	730	741	802
	338	526	454	17.09010513	13.98554283
15.90391279	6.995923927	9.812315491	9.072543313
ENSG00000130764 3139.993988	3662.383957	3111.35456
5117.321463	4710.033808	4763.801347	3304.577502
4863.718873	-0.557390691	1.45E-05	0.000331755	LRRC47

1 3778558 3796504 - 4516 protein_coding
leucine rich repeat containing 47 [Source:HGNC Symbol;Acc:HGNC:29207]
- 2714 3951 3182 5094 5154 4855
32.86628545 38.57332304 32.63989086 54.5388811
49.73350628 50.18585608
ENSG00000112578 1610.490653 1956.793858 1691.59126
2611.903377 2568.860115 2811.182463 1752.95859
2663.981985 -0.603368515 1.46E-05 0.000333564 BYSL
6 41921188 41933046 + 2248
protein_coding bystin like [Source:HGNC Symbol;Acc:HGNC:1157] -
1392 2111 1730 2600 2811 2865 33.8639521
41.40243503 35.64940223 55.92142859 54.49079404
59.49416249
ENSG00000161203 11234.09787 10417.07692 11858.73919
7324.379817 7634.385416 8371.730812 11169.97133
7776.832015 0.522351828 1.49E-05 0.000339952 AP2M1
3 184174689 184184091 + 4756
protein_coding adaptor related protein complex 2 subunit mu 1
[Source:HGNC Symbol;Acc:HGNC:564] - 9710 11238 12128
7291 8354 8532 111.6534371 104.1792292
118.1271696 74.12188729 76.54401218 83.74425756
ENSG00000132967 859.6225251 1107.706613 1484.297996
696.1727079 537.3496079 598.5414669 1150.542378
610.6879276 0.914650548 1.49E-05 0.000340064
HMGB1P5 3 22381819 22382929 + 1111
transcribed_processed_pseudogene high mobility group box 1
pseudogene 5 [Source:HGNC Symbol;Acc:HGNC:4997] - 743 1195
1518 693 588 610 36.57374795 47.4228668
63.29365397 30.15923992 23.06332213 25.63077935
ENSG00000157613 1501.736255 1561.912672 2453.296226
915.1707603 994.2795466 1141.153649 1838.981718
1016.867985 0.854995531 1.52E-05 0.000345158
CREB3L1 11 46277661 46321422 + 4037
protein_coding cAMP responsive element binding protein 3 like 1
[Source:HGNC Symbol;Acc:HGNC:18856] TF_bZIP 1298 1685 2509
911 1088 1163 17.5837158 18.40242743
28.79017917 10.91090675 11.74434346 13.448288
ENSG00000176022 1637.100771 1791.796555 2010.353544
2697.292526 2720.560855 2780.764782 1813.083623
2732.872721 -0.59164662 1.52E-05 0.000346884
B3GALT6 1 1232226 1235041 + 2816
protein_coding "beta-1,3-galactosyltransferase 6 [Source:HGNC
Symbol;Acc:HGNC:17978]" - 1415 1933 2056 2685 2977
2834 27.48011223 30.26447618 33.82150396
46.10126645 46.06857533 46.98002367
ENSG00000145794 41.65062033 17.61207168 72.3570828 0
4.569299387 0 43.87325827 1.523099796
4.807046129 1.54E-05 0.000348863 MEGF10 5
127290831 127465737 + 9007 protein_coding
multiple EGF like domains 10 [Source:HGNC Symbol;Acc:HGNC:29634]

- 36 19 74 0 5 0
0.218583336 0.093005235 0.380587055 0 0.0241907
0
ENSG00000198756 148.0910945 136.2618177 124.1803989
286.3047933 309.7984984 285.5337162 136.1777703
293.8790026 -1.110977501 1.59E-05 0.000360456
COLGALT2 1 183929854 184037729 - 7806
protein_coding collagen beta(1-O)galactosyltransferase 2
[Source:HGNC Symbol;Acc:HGNC:16790] - 128 147 127
285 339 291 0.896759808 0.8302765
0.753663755 1.765295969 1.892473248 1.740245334
ENSG00000164985 2356.730933 2877.256341 3020.419308
1547.050462 1769.232723 1917.295125 2751.468861
1744.526103 0.657601562 1.59E-05 0.000360456 PSIP1
9 15464066 15511019 - 6768
protein_coding PC4 and SFRS1 interacting protein 1 [Source:HGNC
Symbol;Acc:HGNC:9527] - 2037 3104 3089 1540 1936
1954 16.45983175 20.22065971 21.1426789
11.00174532 12.46532485 13.47752808
ENSG00000166710 15352.88144 14983.23824 13739.04555
6796.97625 11536.56709 8230.435777 14691.72174
8854.659707 0.730415581 1.63E-05 0.000367684 B2M
15 44711487 44718877 + 6079
protein_coding beta-2-microglobulin [Source:HGNC Symbol;Acc:HGNC:914]
- 13270 16164 14051 6766 12624 8388
119.3805271 117.2332099 107.0724022 53.81471772
90.49476159 64.41282018
ENSG00000197256 2733.90044 2615.856119 3500.51833
1902.671153 1509.696517 2001.67966 2950.09163
1804.682443 0.709242899 1.65E-05 0.000372504 KANK2
19 11164267 11197791 - 7638
protein_coding KN motif and ankyrin repeat domains 2 [Source:HGNC
Symbol;Acc:HGNC:29300] - 2363 2822 3580 1894 1652
2040 16.91915933 16.2896335 21.71229987
11.98951284 9.425165539 12.46799298
ENSG00000161011 8075.592496 9868.321845 7825.320725
12328.18394 13233.60488 12364.29676 8589.745022
12642.02853 -0.557467994 1.66E-05 0.000373996 SQSTM1
5 179806398 179838078 + 7654
protein_coding sequestosome 1 [Source:HGNC Symbol;Acc:HGNC:11280]
- 6980 10646 8003 12272 14481 12601
49.87256184 61.32420996 48.43583765 77.52255989
82.44582979 76.85331223
ENSG00000263465 1168.531292 1102.144906 981.7096098
1685.682257 1608.393384 1739.695116 1084.128603
1677.923586 -0.630456085 1.67E-05 0.000376756 SRSF8
11 95067197 95071224 + 4028
protein_coding serine and arginine rich splicing factor 8
[Source:HGNC Symbol;Acc:HGNC:16988] - 1010 1189 1004
1678 1760 1773 13.71281524 13.01446593

11.54640287	20.14205192	19.04065148	20.54779904
ENSG00000071859 2928.270001	3139.583514	2811.170446
4386.993096	4211.980175	4186.846622	2959.674654
4261.939964	-0.52598118	1.68E-05	0.000378318	FAM50A
X	154444126	154450654	+	2567
protein_coding	family with sequence similarity 50 member A
[Source:HGNC Symbol;Acc:HGNC:18786]	-	2531	3387	2875
4367	4609	4267	53.92137885	58.17324444
51.88172844	82.25428654	78.24190883	77.596621
ENSG00000066056 672.1947336	637.7423849	1477.453407
315.4375617	356.4053522	500.4199149	929.1301751
390.7542763	1.249935282	1.69E-05	0.000379842	TIE1
1	43300993	43323108	+	6694
protein_coding	tyrosine kinase with immunoglobulin like and EGF like
domains 1 [Source:HGNC Symbol;Acc:HGNC:11809]	-	581	688
1511	314	390	510	4.746627348	4.53144474
10.45637649	2.268011	2.538852654	3.556562982
ENSG00000148606 1859.237413	1725.056073	1622.167572
2891.176123	2415.331656	2586.484109	1735.487019
2630.997296	-0.600421399	1.74E-05	0.000389502	POLR3A
10	77969251	78029545	-	8917
protein_coding	RNA polymerase III subunit A [Source:HGNC
Symbol;Acc:HGNC:30074]	-	1607	1861	1659	2878	2643
2636	9.855798659	9.201562164	8.618468034
15.60534015	12.91626641	13.79979628
ENSG00000100644 5254.919931	4434.534258	4629.875501
2621.949159	3669.147408	2700.30511	4773.109896
2997.133892	0.671057429	1.75E-05	0.000391954	HIF1A
14	61695513	61748259	+	5151
protein_coding	hypoxia inducible factor 1 subunit alpha [Source:HGNC
Symbol;Acc:HGNC:4910]	Others	4542	4784	4735	2610	4015
2752	48.2225634	40.94808339	42.58247394
24.49910246	33.96664072	24.94037134
ENSG00000198176 5971.079208	5988.10437	6465.203128
4276.489491	4278.691946	4462.568183	6141.462235
4339.249873	0.501172709	1.77E-05	0.000395552	TFDP1
13	113584721	113641470	+	3741
protein_coding	transcription factor Dp-1 [Source:HGNC
Symbol;Acc:HGNC:11749]	E2F	5161	6460	6612	4257	4682
4548	75.44680682	76.13402104	81.8742986
55.01956577	54.5383882	56.75168801
ENSG00000079246 15507.9143	12636.19795	14311.05762
10281.8581	9643.049426	9468.729763	14151.72329
9797.879097	0.530376221	1.77E-05	0.000395552	XRCC5
2	216107464	216206303	+	5463
protein_coding	X-ray repair cross complementing 5 [Source:HGNC
Symbol;Acc:HGNC:12833]	-	13404	13632	14636	10235	10552
9650	134.1831341	110.0176426	124.1062508
90.58532837	84.17094048	82.45977561
ENSG00000114473 746.2402808	794.3971277	616.9908006

1135.173391	1273.920669	1144.097296	719.209403
1184.397119	-0.719835601	1.78E-05	0.000396426	IQCG
3	197889075	197960142	-	7474
protein_coding	IQ motif containing G [Source:HGNC
Symbol;Acc:HGNC:25251]	-	645	857	631	1130	1394
1166	4.719558068	5.055471732	3.910918102
7.310154678	8.127711471	7.282684447
ENSG00000143384 13071.35301	17054.97404	10632.57998
20230.19624	25307.52159	21115.75798	13586.30234
22217.82527	-0.709542022	1.78E-05	0.000397175	MCL1
1	150574551	150579738	-	4371
protein_coding	"MCL1, BCL2 family apoptosis regulator [Source:HGNC
Symbol;Acc:HGNC:6943]"	-	11298	18399	10874	20138	27693
21520	141.3564001	185.5869228	115.2420507
222.7597627	276.0881693	229.8304124
ENSG00000136271 3984.576011	4321.446219	3703.9004
6650.30783	5692.433176	5483.032323	4003.307543
5941.924443	-0.569656045	1.80E-05	0.000399895	DDX56
7	44565417	44575051	-	4068
protein_coding	DEAD-box helicase 56 [Source:HGNC
Symbol;Acc:HGNC:18193]	-	3444	4662	3788	6620	6229
5588	46.29956506	50.5272059	43.13516681
78.68252016	66.72613724	64.12413046
ENSG00000100162 297.3391507	482.0145932	598.4126307
204.9339573	214.7570712	195.2618884	459.2554582
204.9843056	1.16495896	1.84E-05	0.000408285	CENPM
22	41938721	41947164	-	1778
protein_coding	centromere protein M [Source:HGNC
Symbol;Acc:HGNC:18352]	-	257	520	612	204	235
199	7.904894524	12.89453083	15.9449118
5.547529589	5.759631568	5.224772148
ENSG00000164877 1169.688254	1185.570509	1937.018662
846.8594412	615.0276975	807.5403725	1430.759142
756.4758371	0.919947918	1.92E-05	0.000425384
MICALL2 7	1428465 1459502 -	8843	protein_coding	MICAL
like 2 [Source:HGNC Symbol;Acc:HGNC:29672]	-	1011	1279
1981	843	673	823	6.252392642	6.37683056
10.37737011	4.609238344	3.316454451	4.344564468
ENSG00000004660 556.498566	703.5559159	420.4533189
1057.820868	970.5191898	1029.29508	560.169267
1019.211713	-0.863041322	1.93E-05	0.000426673	CAMKK1
17	3860315 3894891 -	7424	protein_coding
calcium/calmodulin dependent protein kinase kinase 1 [Source:HGNC
Symbol;Acc:HGNC:1469]	-	481	759	430	1053	1062
1049	3.543250246	4.507521124	2.683075837
6.857907523	6.233689285	6.596044255
ENSG00000104824 4364.059441	5047.248962	5164.731234
3370.359935	2942.628805	3487.239956	4858.679879
3266.742899	0.572949818	1.96E-05	0.00043241	HNRNPL
19	38836388	38852347	-	6821

protein_coding heterogeneous nuclear ribonucleoprotein L [Source:HGNC Symbol;Acc:HGNC:5045] - 3772 5445 5282 3355 3220
3554 30.24254582 35.19522877 35.87176974
23.78185306 20.57152178 24.32290311
ENSG00000269190 1044.736393 1428.431708 1134.246163
1870.524649 1855.135551 2086.064194 1202.471421
1937.241465 -0.687273846 1.96E-05 0.000432769 FBXO17
19 38941401 38975910 - 4960
protein_coding F-box protein 17 [Source:HGNC Symbol;Acc:HGNC:18754]
- 903 1541 1160 1862 2030 2126
9.956364475 13.6979293 10.83374898 18.15094399
17.83499365 20.00910299
ENSG00000172175 1382.569202 1010.376744 1215.403431
753.4336665 702.7582457 717.2685447 1202.783126
724.486819 0.730769204 1.98E-05 0.00043558 MALT1
18 58671386 58754477 + 11527
protein_coding MALT1 paracaspase [Source:HGNC Symbol;Acc:HGNC:6819]
- 1195 1090 1243 750 769 731
5.669520433 4.169117764 4.995250722 3.145909223
2.907158058 2.96037767
ENSG00000150687 4964.52255 3655.8953 4899.747863
2698.297104 2553.324497 3234.086352 4506.721904
2828.569318 0.671891293 1.99E-05 0.000438602 PRSS23
11 86791059 86952910 + 11725
protein_coding serine protease 23 [Source:HGNC Symbol;Acc:HGNC:14370]
- 4291 3944 5011 2686 2794 3296
20.01429865 14.8305758 19.79766664 11.07629142
10.38417858 13.122615
ENSG00000108861 3332.049626 3542.80726 2751.524743
4749.645834 5106.648995 4602.882002 3208.793876
4819.72561 -0.58696893 2.02E-05 0.000442935 DUSP3
17 43766121 43778988 - 5012
protein_coding dual specificity phosphatase 3 [Source:HGNC Symbol;Acc:HGNC:3069] - 2880 3822 2814 4728 5588
4691 31.42506164 33.62122851 26.00851083
45.61079289 48.58519307 43.69184723
ENSG00000114446 1387.197049 1083.605884 1101.978815
649.9621096 744.7958001 755.53595 1190.927249
716.7646199 0.731708947 2.03E-05 0.000444992 IFT57
3 108160812 108222570 - 3766
protein_coding intraflagellar transport 57 [Source:HGNC Symbol;Acc:HGNC:17367] - 1199 1169 1127 647 815
770 17.41139548 13.68573585 13.86264354
8.30663603 9.430525436 9.544571712
ENSG00000121064 608.5618414 579.344463 750.9491836
363.6573164 371.0271102 350.2939405 646.2851627
361.6594557 0.837601623 2.04E-05 0.00044677 SCPEP1
17 56978105 57006768 + 3853
protein_coding serine carboxypeptidase 1 [Source:HGNC Symbol;Acc:HGNC:29507] - 526 625 768 362 406

357	7.46588761	7.151793502	9.233464139
4.542665703	4.591828109	4.325290117
ENSG00000151348 2177.401874	1846.486673	2632.233336
1253.713621	1202.639599	1552.282952	2218.707294
1336.212057	0.731605793	2.04E-05	0.00044677	EXT2
11	44095549	44245429	+	5286
protein_coding	exostosin glycosyltransferase 2 [Source:HGNC
Symbol;Acc:HGNC:3513]	-	1882	1992	2692	1248	1316
1582	19.47095141	16.61483885	23.59121739
11.41533453	10.84894052	13.97093127
ENSG00000133067 17.35442514	15.75816939	31.28954932	0
0	0	21.46738128	0	6.917485676
2.07E-05	1	LGR6	1	202193901	202319781
+	4583	protein_coding	leucine rich repeat containing
G protein-coupled receptor 6 [Source:HGNC Symbol;Acc:HGNC:19719]
-	15	17	32	0	0	0
0.178993028	0.1635434	0.32344659	0	0	0
ENSG00000280287 144.6202095	121.4305995	188.7150943
360.6435817	319.8509571	316.9326128	151.5886344
332.4757172	-1.13308269	2.08E-05	0.000454396
AC131212.3	12	132550729	132554947	+	4219
TEC	novel transcript	-	125	131	193
359	350	323	1.620299136	1.368975665
2.119094086	4.114207009	3.615073376	3.573872319
ENSG00000128656 488.2378272	564.5132448	657.0805357
292.3322626	316.1955176	319.8762593	569.9438692
309.4680132	0.881591686	2.09E-05	0.000455993	CHN1
2	174799363	175005369	-	4971
protein_coding	chimerin 1 [Source:HGNC Symbol;Acc:HGNC:1943]	-
422	609	672	291	346	326	4.642622807
5.40141427	6.262214879	2.830417119	3.033129363
3.061398554
ENSG00000136718 1763.209594	2144.037989	1873.461765
2897.203592	2865.864576	2850.431084	1926.903116
2871.166417	-0.574955493	2.12E-05	0.000461943	IMP4
2	130342225	130347810	+	3861
protein_coding	"IMP4, U3 small nucleolar ribonucleoprotein
[Source:HGNC Symbol;Acc:HGNC:30856]"	-	1524	2313	1916
2884	3136	2905	21.58638299	26.41251697
22.98783964	36.11575273	35.39442408	35.12306205
ENSG00000122642 2685.308049	2550.042588	3403.716287
1719.837916	1755.524825	2010.510599	2879.688975
1828.624447	0.655235116	2.13E-05	0.000463748	FKBP9
7	32957404	33006931	+	5170
protein_coding	FK506 binding protein 9 [Source:HGNC
Symbol;Acc:HGNC:3725]	-	2321	2751	3481	1712	1921
2049	24.55157231	23.46032398	31.19004031
16.01085166	16.19181076	18.50109577
ENSG00000143226 38.1797353	7.415609127	22.48936357	0
0	0	22.69490267	0	6.993809159

2.16E-05	1	FCGR2A	1	161505430	161524013
	+	5314	protein_coding	Fc fragment of IgG receptor
IIa [Source:HGNC Symbol;Acc:HGNC:3616]	-	33	8	23
	0	0	0	0.339615187	0.066374673
0.200497398	0	0	0
ENSG00000142192 10254.15133	9315.858966	13328.37021
7082.276465	5357.046601	7682.917517	10966.12684
6707.413528	0.709287072	2.16E-05	0.000468681	APP
	21	25880550	26171128	-	6316
protein_coding	amyloid beta precursor protein [Source:HGNC
Symbol;Acc:HGNC:620]	-	8863	10050	13631	7050	5862
	7830	76.74204018	70.15488198	99.9742264
53.96947733	40.44476259	57.87162491
ENSG00000115414 41839.20508	14581.8684	41520.25415
10152.26751	5104.821275	8438.453467	32647.10921
7898.514085	2.047304022	2.18E-05	0.000472987	FN1
	2	215360440	215436172	-	17315
protein_coding	fibronectin 1 [Source:HGNC Symbol;Acc:HGNC:3778]
	-	36163	15731	42463	10106	5586	8600
114.2185568	40.05601981	113.603218	28.22006531
14.05843681	23.18579497
ENSG00000187676 843.4250616	694.2864045	791.0389187
478.1792337	459.6715183	433.6972596	776.2501283
457.1826705	0.763217736	2.18E-05	0.000473039	B3GLCT
	13	31199936	31332276	+	4566
protein_coding	beta 3-glucosyltransferase [Source:HGNC
Symbol;Acc:HGNC:20207]	-	729	749	809	476	503
	442	8.731449246	7.232357218	8.207578974
5.04048425	4.800546443	4.518896537
ENSG00000105321 507.9061756	796.25103	864.3737999
1363.212647	1161.515904	1427.668581	722.8436685
1317.465711	-0.864622681	2.20E-05	0.000476032	CCDC9
	19	47255980	47273701	+	3845
protein_coding	coiled-coil domain containing 9 [Source:HGNC
Symbol;Acc:HGNC:24560]	-	439	859	884	1357	1271
	1455	6.24399992	9.849876328	10.65021504
17.06415236	14.40481895	17.66496108
ENSG00000135631 1221.75153	1592.50206	1366.962186
2614.917112	1957.487857	2196.941548	1393.738592
2256.448839	-0.694434623	2.23E-05	0.000481473
RAB11FIP5	2	73073382	73156721	-	7058
	protein_coding	RAB11 family interacting protein 5
[Source:HGNC Symbol;Acc:HGNC:24845]	-	1056	1718	1398
	2603	2142	2239	8.182329735	10.73187149
9.175462681	17.8317403	13.22502221	14.80875252
ENSG00000110492 1439.260325	1658.315591	2560.854052
917.1799167	1220.916796	897.8122003	1886.143322
1011.969638	0.898325204	2.25E-05	0.000484553	MDK
	11	46380756	46383837	+	2196
protein_coding	midkine [Source:HGNC Symbol;Acc:HGNC:6972]	-

1244 1789 2619 913 1336 915 30.98009587
35.91798484 55.24660798 20.10201803 26.51140353
19.45068023
ENSG00000001617 627.0732282 1057.651252 1064.822475
300.3688884 493.4843338 521.0254408 916.5156517
438.2928877 1.064636874 2.26E-05 0.000485933 SEMA3F
3 50155045 50189075 + 4826
protein_coding semaphorin 3F [Source:HGNC Symbol;Acc:HGNC:10728]
- 542 1141 1089 299 540 531
6.141955729 10.42394917 10.45304976 2.995608728
4.876015079 5.136333793
ENSG00000136143 1122.252825 977.0065025 968.020432
604.7560897 660.7206914 629.9403635 1022.426587
631.8057148 0.693799301 2.26E-05 0.000485933 SUCLA2
13 47745736 48037968 - 8928
protein_coding succinate-CoA ligase ADP-forming beta subunit
[Source:HGNC Symbol;Acc:HGNC:11448] - 970 1054 990
602 723 642 5.94172114 5.204995846
5.136691325 3.260194618 3.528927316 3.356810943
ENSG00000163719 1057.462972 1149.419415 1084.378444
1512.894802 1797.562379 1770.112797 1097.086943
1693.523326 -0.626226631 2.26E-05 0.000485933 MTMR14
3 9649433 9702393 + 6019 protein_coding
myotubularin related protein 14 [Source:HGNC Symbol;Acc:HGNC:26190]
- 914 1240 1109 1506 1967 1804
8.304558813 9.083041528 8.535120533 12.0976731
14.24093861 13.99130506
ENSG00000105671 2288.470195 2922.676947 2702.634822
4643.160542 3587.813879 4051.43888 2637.927321
4094.137767 -0.63374274 2.27E-05 0.000486218 DDX49
19 18919675 18928633 + 2780
protein_coding DEAD-box helicase 49 [Source:HGNC
Symbol;Acc:HGNC:18684] - 1978 3153 2764 4622 3926
4129 38.91134153 50.00496559 46.05700557
80.38710002 61.54093597 69.33397871
ENSG00000174307 509.0631373 971.4447956 688.370085
1311.979158 1231.883115 1560.132676 722.9593393
1367.998316 -0.918654587 2.27E-05 0.000486218 PHLDA3
1 201464383 201469237 - 3138
protein_coding pleckstrin homology like domain family A member 3
[Source:HGNC Symbol;Acc:HGNC:8934] - 440 1048 704
1306 1348 1590 7.668217983 14.72455916
10.39255127 20.12294101 18.7195563 23.65321726
ENSG00000137509 1471.655252 1333.882692 1799.149086
836.8136589 976.916209 995.9337523 1534.895676
936.5545401 0.712581522 2.29E-05 0.000489179 PRCP
11 82823502 82970584 - 12798
protein_coding prolylcarboxypeptidase [Source:HGNC
Symbol;Acc:HGNC:9344] - 1272 1439 1840 833 1069
1015 5.43550273 4.957384974 6.660060479

3.147053399	3.639939767	3.702286064
ENSG00000125352 345.931541	469.0372773	324.6290742
631.8797017	744.7958001	723.1558378	379.8659642
699.9437799	-0.880787179	2.30E-05	0.000491603
RNF113A X	119870475	119871827	-	1353
protein_coding	ring finger protein 113A [Source:HGNC
Symbol;Acc:HGNC:12974]	-	299	506	332	629	815
737	12.08559689	16.48870994	11.36691842
22.47781359	26.24934131	25.42820635
ENSG00000143195 590.0504546	303.1130231	603.3016228
258.1766031	142.5621409	216.8486298	498.8217001
205.8624579	1.27698402	2.31E-05	0.000492077	ILDR2
1	166895711	166975482	-	13884
protein_coding	immunoglobulin like domain containing receptor 2
[Source:HGNC Symbol;Acc:HGNC:18131]	-	510	327	617
257	156	221	2.008862833	1.038405801
2.05860496	0.894993198	0.489630644	0.743059694
ENSG00000153956 742.7693958	581.1983653	661.9695277
410.8724928	316.1955176	369.9182508	661.9790963
365.6620871	0.85609725	2.32E-05	0.000493826
CACNA2D1	7	81946444	82443798	-	9010
protein_coding	calcium voltage-gated channel auxiliary
subunit alpha2delta 1 [Source:HGNC Symbol;Acc:HGNC:1399]	-
642	627	677	409	346	377	3.896771586
3.068150845	3.480697918	2.194824167	1.673439075
1.953271418
ENSG00000087995 1140.764212	1139.222952	978.7762146
1675.636474	1766.491143	1581.719417	1086.25446
1674.615678	-0.624715333	2.36E-05	0.000501741
METTL2A 17	62423867	62450822	+	3843
protein_coding	methyltransferase like 2A [Source:HGNC
Symbol;Acc:HGNC:25755]	-	986	1229	1001	1668	1933
1612	14.03140768	14.09988129	12.06607857
20.98586517	21.91896622	19.58126247
ENSG00000170962 141.1493244	61.1787753	131.0249878
28.12819022	29.24351608	22.56795695	111.1176958
26.64655441	2.057585301	2.40E-05	0.000510163	PDGFD
11	103907186	104164379	-	4266
protein_coding	platelet derived growth factor D [Source:HGNC
Symbol;Acc:HGNC:30620]	-	122	66	134	28	32
23	1.563988993	0.682114124	1.455078431
0.317349923	0.32687953	0.251682501
ENSG00000175602 2763.981443	4710.765698	3776.257483
5945.093918	6054.321688	6617.317463	3750.334875
6205.57769	-0.726183246	2.41E-05	0.0005108
CCDC85B 11	65890112	65891635	+	1524
protein_coding	coiled-coil domain containing 85B [Source:HGNC
Symbol;Acc:HGNC:24926]	-	2389	5082	3862	5918	6625
6744	85.72863237	147.0224487	117.3896366
187.7548699	189.4346908	206.5754134

ENSG00000127483 5932.899473   5305.86833   5430.692403
3724.976047   3638.076172   4180.959328   5556.486736
3848.003849   0.529984627   2.43E-05   0.000515279   HP1BP3
        1   20742661   20787323   -   7442
protein_coding heterochromatin protein 1 binding protein 3
[Source:HGNC Symbol;Acc:HGNC:24973]   -   5128   5724   5554
        3708   3981   4261   37.68365886   33.91125928
34.57153521   24.090803   23.31101102   26.72808873
ENSG00000103043 1959.893079   2319.231754   2004.486753
3762.145441   2935.317926   3021.162584   2094.537195
3239.541984   -0.628822431   2.45E-05   0.000516775   VAC14
        16   70687439   70801161   -   8276
protein_coding "Vac14, PIKFYVE complex component [Source:HGNC
Symbol;Acc:HGNC:25507]" -   1694   2502   2050   3745   3212
        3079   11.19406016   13.32909862   11.47455455
21.87925634   16.91272753   17.36741743
ENSG00000143621 7241.423128   6790.844058   7619.983057
5252.939523   5007.038268   5114.095287   7217.416748
5124.691026   0.49401326   2.45E-05   0.000516775   ILF2
        1   153661788   153671048   -   2281
protein_coding interleukin enhancer binding factor 2 [Source:HGNC
Symbol;Acc:HGNC:6037]   -   6259   7326   7793   5229   5479
        5212   150.0632607   141.6040214   158.2638939
110.8395038   104.6729859   106.6657908
ENSG00000124201 1759.738709   1643.484373   1500.920569
2756.562641   2480.215707   2243.058678   1634.71455
2493.279009   -0.609229046   2.46E-05   0.000518518   ZNFX1
        20   49237946   49278426   -   9340
protein_coding zinc finger NFX1-type containing 1 [Source:HGNC
Symbol;Acc:HGNC:29271] -   1521   1773   1535   2744   2714
        2286   8.905884338   8.369428766   7.61314251
14.20490872   12.66256142   11.4255045
ENSG00000120868 480.1390954   376.3421632   514.3219669
134.6134818   267.7609441   208.9989056   456.9344085
203.7911105   1.163316772   2.47E-05   0.000518518   APAF1
        12   98645141   98735433   +   7908
protein_coding apoptotic peptidase activating factor 1 [Source:HGNC
Symbol;Acc:HGNC:576]   -   415   406   526   134   293
        213   2.869962016   2.263566881   3.081211707
0.819293207   1.614579959   1.257358085
ENSG00000167601 6754.342263   8419.497212   12315.37105
1990.069458   2601.759071   4012.190259   9163.070176
2868.006263   1.675800112   2.47E-05   0.000518518   AXL
        19   41219203   41261766   +   5154
protein_coding AXL receptor tyrosine kinase [Source:HGNC
Symbol;Acc:HGNC:905]   -   5838   9083   12595   1981   2847
        4089   61.94615989   77.6996134   113.2025509
18.58408905   24.07141668   37.03554434
ENSG00000165072 170.0733663   106.5993812   253.2497898
13.05951689   53.00387289   56.91050013   176.6408458

40.99129664 2.104996843 2.47E-05 0.000518662 MAMDC2
9 70043581 70226970 + 3854
protein_coding MAM domain containing 2 [Source:HGNC
Symbol;Acc:HGNC:23673] - 147 115 259 13 58
58 2.085932915 1.315588559 3.113081636
0.163092075 0.655805238 0.702525866
ENSG00000164308 1210.181913 888.0191929 1512.65415
816.7220945 477.034856 510.2320701 1203.618419
601.3296735 1.001442159 2.47E-05 0.000518662 ERAP2
5 96875939 96919716 + 7422
protein_coding endoplasmic reticulum aminopeptidase 2 [Source:HGNC
Symbol;Acc:HGNC:29499] - 1046 958 1547 813 522
520 7.707356495 5.690868063 9.655434443
5.296278476 3.064842425 3.270607509
ENSG00000154319 77.51643227 71.37523785 329.5180662
48.21975466 18.27719755 9.812155195 159.4699121
25.43636913 2.651538036 2.50E-05 0.000522964
FAM167A 8 11421463 11475908 - 5076
protein_coding family with sequence similarity 167 member A
[Source:HGNC Symbol;Acc:HGNC:15549] - 67 77 337
48 20 10 0.721851564 0.66881048
3.075464676 0.45721539 0.171698689 0.091965391
ENSG00000255874 150.4050178 88.06035838 127.1137941
40.18312888 16.44947779 35.3237587 121.8597234
30.65212179 1.993200472 2.53E-05 0.000528681
LINC00346 13 110863987 110870251 - 6265
lincRNA long intergenic non-protein coding RNA 346
[Source:HGNC Symbol;Acc:HGNC:27492] - 130 95 130
40 18 36 1.134793892 0.668553994
0.961224281 0.30870249 0.125201643 0.268242422
ENSG00000261786 240.6480285 215.0526647 268.8945644
59.2701151 87.73054823 125.5955865 241.5317526
90.86541661 1.409920594 2.54E-05 0.000530275
AC006058.1 3 44117299 44122365 + 5067
lincRNA novel transcript - 208 232 275
59 96 128 2.244952433 2.01869654
2.514109826 0.56299213 0.82561757 1.179247872
ENSG00000101160 2545.315687 3164.611195 3918.038254
1701.755508 2083.600521 2065.458668 3209.321712
1950.271566 0.718764558 2.56E-05 0.000533789 CTSZ
20 58995185 59007247 - 2038
protein_coding cathepsin Z [Source:HGNC Symbol;Acc:HGNC:2547] -
2200 3414 4007 1694 2280 2105 59.03549566
73.85727755 91.07886681 40.18930008 48.75164395
48.2163084
ENSG00000112893 769.3795144 688.7246976 820.3728712
464.1151386 362.8023713 472.9458804 759.4923611
433.2877968 0.810056082 2.56E-05 0.000534045 MAN2A1
5 109689366 109869625 + 11815
protein_coding mannosidase alpha class 2A member 1 [Source:HGNC

Symbol;Acc:HGNC:6824] - 665 743 839 462 397
482 3.078099342 2.772611663 3.289506059
1.890642717 1.464250492 1.904405154
ENSG00000160469 177.0151364 269.742782 192.626288
477.1746555 371.0271102 479.814389 213.1280688
442.6720516 -1.052075587 2.58E-05 0.000535848 BRSK1
19 55282072 55312533 + 3841
protein_coding BR serine/threonine kinase 1 [Source:HGNC
Symbol;Acc:HGNC:18994] - 153 291 197 475 406
489 2.178421108 3.340278205 2.375879306
5.979302411 4.606173836 5.943066472
ENSG00000120539 732.3567407 565.4401959 680.5476976
138.6317946 390.2181677 303.1955955 659.4482114
277.3485193 1.248192937 2.62E-05 0.000544184 MASTL
10 27154824 27186924 + 3631
protein_coding microtubule associated serine/threonine kinase like
[Source:HGNC Symbol;Acc:HGNC:19042] - 633 610 696
138 427 309 9.533934718 7.406918125
8.879437332 1.837612998 5.124602969 3.972631358
ENSG00000078804 686.0782737 596.0295836 733.3488121
382.7443026 350.0083331 417.0165958 671.8188898
383.2564105 0.80977004 2.62E-05 0.000544213
TP53INP2 20 34704290 34713439 + 4228
protein_coding tumor protein p53 inducible nuclear protein 2
[Source:HGNC Symbol;Acc:HGNC:16104] - 593 643 750
381 383 425 7.670336685 6.705172455
8.217292392 4.357036654 3.947502312 4.692453604
ENSG00000185585 652.5263851 480.160691 962.1536415
308.4055142 310.7123583 385.6176992 698.2802392
334.9118572 1.060053132 2.66E-05 0.000550867
OLFML2A 9 124777158 124814885 + 6746
protein_coding olfactomedin like 2A [Source:HGNC
Symbol;Acc:HGNC:27270] - 564 518 984 307 340
393 4.572223799 3.385457615 6.756957968
2.200357536 2.196297554 2.719519952
ENSG00000139722 846.8959466 944.5632125 865.3515983
1342.116505 1334.235421 1413.931564 885.6035858
1363.42783 -0.62210202 2.67E-05 0.000553168 VPS37B
12 122865328 122896444 - 5953
protein_coding "VPS37B, ESCRT-I subunit [Source:HGNC
Symbol;Acc:HGNC:25754]" - 732 1019 885 1336 1460
1441 6.724653497 7.546963671 6.886679059
10.85105069 10.6874863 11.29988787
ENSG00000203666 415.3492416 334.6293618 484.9880144
216.988896 163.5809181 215.8674143 411.6555393
198.8124094 1.050469179 2.70E-05 0.000558621 EFCAB2
1 244969705 245127164 + 8607
protein_coding EF-hand calcium binding domain 2 [Source:HGNC
Symbol;Acc:HGNC:28166] - 359 361 496 216 179
220 2.281063411 1.8492233 2.669514778

1.213397692	0.906274636	1.19321008
ENSG00000166173 817.9719047	1075.263323	731.3932153
1405.404933	1704.348671	1363.889572	874.8761478
1491.214392	-0.768961218	2.72E-05	0.000560756	LARP6
	15	70829130	70854159	-	5554
protein_coding	La ribonucleoprotein domain family member 6
[Source:HGNC Symbol;Acc:HGNC:24012]	-	707	1160	748
	1399	1865	1390	6.961586939	9.208440193
6.238758553	12.17903996	14.63293888	11.68301571
ENSG00000099821 2391.439784	3263.794967	2735.879968
4812.934262	4026.46662	4148.579216	2797.03824
4329.326699	-0.629829707	2.72E-05	0.000561251	POLRMT
	19	617224	633604	-	5124	protein_coding	RNA
polymerase mitochondrial [Source:HGNC Symbol;Acc:HGNC:9200]	-
	2067	3521	2798	4791	4406	4228	22.06104438
	30.29638855	25.2953705	45.20830937	37.47088664
	38.51872413
ENSG00000183876 129.5797077	164.0703519	302.1397106
63.28842799	28.3296562	75.553595	198.5965901
55.72389306	1.836625228	2.76E-05	0.000568568	ARSI
	5	150296343	150339307	-	3269
protein_coding	arylsulfatase family member I [Source:HGNC
Symbol;Acc:HGNC:32521]	-	112	177	309	63	31
	77	1.873690327	2.387219227	4.378708302
0.931808881	0.413242872	1.099567362
ENSG00000160957 937.1389573	1240.260626	1602.611604
592.701151	551.971366	840.9017002	1260.003729
661.8580724	0.929499732	2.82E-05	0.000579228	RECQL4
	8	144511288	144517845	-	4236
protein_coding	RecQ like helicase 4 [Source:HGNC
Symbol;Acc:HGNC:9949]	-	810	1338	1639	590	604
	857	10.45740144	13.92624784	17.92360886
6.734374698	6.21354696	9.444324622
ENSG00000263513 99.49870411	131.627062	130.0471893
29.13276844	46.60685375	18.64309487	120.3909852
31.46090569	1.933915556	2.82E-05	0.000579228	FAM72C
	1	143955364	143971965	-	2297
protein_coding	family with sequence similarity 72 member C
[Source:HGNC Symbol;Acc:HGNC:30602]	-	86	142	133
	29	51	19	2.047538935	2.725594895
2.682212002	0.610433303	0.967537438	0.386134531
ENSG00000008083 443.1163218	473.672033	374.4967934
983.4820794	800.5412526	617.1845617	430.4283827
800.4026312	-0.894857029	2.84E-05	0.000581228	JARID2
	6	15246296	15522040	+	6015
protein_coding	jumonji and AT-rich interaction domain containing 2
[Source:HGNC Symbol;Acc:HGNC:6196]	ARID	383	511	383
	979	876	629	3.482233226	3.745581285
2.949616799	7.869520601	6.346394607	4.881587178
ENSG00000077232 2658.697931	2215.413227	2433.740258

1799.199596	1441.157027	1493.410021	2435.950472
1577.922214	0.626371804	2.88E-05	0.000589404
DNAJC10 2	182716041	182794464	+	21835
protein_coding	DnaJ heat shock protein family (Hsp40) member C10
[Source:HGNC Symbol;Acc:HGNC:24637]	-	2298	2390	2489
	1791	1577	1522	5.755612417	4.825903825
5.280489169	3.965916865	3.147292413	3.253924651
ENSG00000186472 345.931541	357.8031404	116.3580115
1273.805186	1644.947779	914.4928641	273.364231
1277.74861	-2.225015614	2.93E-05	0.000598072	PCLO
	7	82754013	83162930	-	22873
protein_coding	piccolo presynaptic cytomatrix protein [Source:HGNC
Symbol;Acc:HGNC:13406]	-	299	386	119	1268	1800
	932	0.714895842	0.744043167	0.24100513
2.680385995	3.429319689	1.902123969
ENSG00000120833 93.71389573	101.9646255	72.3570828
20.09156444	21.93263706	23.54917247	89.34520134
21.85779132	2.030943959	2.95E-05	0.000602585	SOCS2
	12	93569814	93583487	+	6776
protein_coding	suppressor of cytokine signaling 2 [Source:HGNC
Symbol;Acc:HGNC:19382]	-	81	110	74	20	24
	24	0.65374192	0.715736633	0.505895456
0.14271112	0.154346378	0.165342264
ENSG00000136997 1183.571794	1447.897682	1068.733669
2000.11524	2171.331069	1766.187935	1233.401048
1979.211415	-0.682040625	2.98E-05	0.000605894	MYC
	8	127735434	127741434	+	3001
protein_coding	"MYC proto-oncogene, bHLH transcription factor
[Source:HGNC Symbol;Acc:HGNC:7553]"	bHLH	1023	1562	1093
	1991	2376	1800	18.64250855	22.94821933
16.87161367	32.07794077	34.50158434	27.99964632
ENSG00000134369 4573.469504	3098.797664	5076.729377
2680.214696	2513.114663	2570.784661	4249.665515
2588.038007	0.715367459	2.98E-05	0.000605894	NAV1
	1	201622885	201826969	+	22453
protein_coding	neuron navigator 1 [Source:HGNC Symbol;Acc:HGNC:15989]
	-	3953	3343	5192	2668	2750	2620
9.628245398	6.564413927	10.71180759	5.745299396
5.337242249	5.447195354
ENSG00000198355 983.4174244	1827.94765	1305.360886
2451.170862	2372.380242	2400.053161	1372.241987
2407.868088	-0.810345117	2.98E-05	0.000606545	PIM3
	22	49960513	49964080	+	2461
protein_coding	"Pim-3 proto-oncogene, serine/threonine kinase
[Source:HGNC Symbol;Acc:HGNC:19310]"	-	850	1972	1335
	2440	2596	2446	18.88869808	35.32882999
25.12882071	47.9379468	45.96758334	46.39710412
ENSG00000266028 1333.976812	1221.721604	1712.125027
707.2230683	874.5639027	933.135959	1422.607814
838.3076433	0.762886826	3.02E-05	0.000612304	SRGAP2

1 206203345 206464443 + 15675
protein_coding SLIT-ROBO Rho GTPase activating protein 2 [Source:HGNC
Symbol;Acc:HGNC:19751] - 1153 1318 1751 704 957
951 4.022689117 3.707164168 5.174650856
2.171532463 2.660498302 2.832167946
ENSG00000166825 1037.794623 807.3744437 1197.80306
398.8175541 545.5743468 665.2641222 1014.324042
536.5520077 0.918348139 3.03E-05 0.000612765 ANPEP
15 89784889 89815401 - 4871
protein_coding "alanyl aminopeptidase, membrane [Source:HGNC
Symbol;Acc:HGNC:500]" - 897 871 1225 397 597
678 10.07091722 7.883770684 11.64985218
3.940701994 5.340904338 6.497669241
ENSG00000170522 1065.561703 924.1702874 873.1739856
442.0144177 646.0989333 550.4619064 954.3019921
546.1917525 0.804065846 3.03E-05 0.000612765 ELOVL6
4 110045846 110199199 - 7297
protein_coding ELOVL fatty acid elongase 6 [Source:HGNC
Symbol;Acc:HGNC:15829] - 921 997 893 440 707
561 6.902556921 6.023997321 5.669040615
2.91547651 4.222150067 3.588926389
ENSG00000116863 1368.685662 1876.149109 1675.946485
2527.518807 2337.653566 3033.918386 1640.260419
2633.030253 -0.682178675 3.03E-05 0.000612765
ADPRHL2 1 36088875 36093932 + 1668
protein_coding ADP-ribosylhydrolase like 2 [Source:HGNC
Symbol;Acc:HGNC:21304] - 1183 2024 1714 2516 2558
3092 38.7867518 53.49933945 47.60112612
72.93161101 66.82871209 86.53453709
ENSG00000179431 686.0782737 1127.172587 1106.867807
1592.256482 1741.816926 1745.582409 973.3728894
1693.218606 -0.797612505 3.03E-05 0.000612765 FJX1
11 35618419 35620868 + 2450
protein_coding four jointed box 1 [Source:HGNC Symbol;Acc:HGNC:17166]
- 593 1216 1132 1585 1906 1779
13.23680959 21.88272731 21.4034015 31.27983105
33.90122642 33.89658135
ENSG00000006576 1900.888033 1718.567415 1922.351686
1044.761351 1358.909638 1137.228787 1847.269045
1180.299925 0.645806814 3.08E-05 0.000621244 PHTF2
7 77798792 77957503 + 8646
protein_coding putative homeodomain transcription factor 2
[Source:HGNC Symbol;Acc:HGNC:13411] - 1643 1854 1966
1040 1487 1159 10.39242849 9.454279919
10.53345243 5.81593206 7.494701408 6.257692821
ENSG00000167671 2033.938626 2810.515859 2238.180575
3287.984521 3766.930415 4056.344957 2360.878353
3703.753298 -0.649294078 3.14E-05 0.000632417 UBXN6
19 4444999 4457822 - 5275 protein_coding UBX
domain protein 6 [Source:HGNC Symbol;Acc:HGNC:14928] - 1758

3032 2289 3273 4122 4134 18.22598966
25.34198859 20.10137587 30.00024199 34.05211734
36.58424057
ENSG00000110031 1189.356603 1065.066861 708.9038517
1852.442241 1835.030634 1506.165822 987.7757717
1731.212899 -0.810126392 3.15E-05 0.000633334 LPXN
11 58526871 58578220 - 2467
protein_coding leupaxin [Source:HGNC Symbol;Acc:HGNC:14061] -
1028 1149 725 1844 2008 1535 22.78865418
20.53453332 13.61354756 36.14040239 35.46934403
29.04592865
ENSG00000103316 34.70885027 87.13340724 33.24514615
246.1216644 213.8432113 111.8585692 51.69580122
190.607815 -1.876889905 3.17E-05 0.000637736 CRYM
16 21238874 21303083 - 3782
protein_coding crystallin mu [Source:HGNC Symbol;Acc:HGNC:2418]
- 30 94 34 245 234 114
0.43380489 1.095822686 0.416447106 3.132173251
2.69620513 1.407114255
ENSG00000169957 584.2656462 769.3694469 869.262792
1334.079879 1184.362401 1230.444261 740.9659617
1249.628847 -0.752861161 3.19E-05 0.000639032 ZNF768
16 30524001 30526821 - 2395
protein_coding zinc finger protein 768 [Source:HGNC
Symbol;Acc:HGNC:26273] zf-C2H2 505 830 889 1328 1296
1254 11.53136113 15.27940817 17.19486217
26.80981232 23.58077539 24.442074
ENSG00000106012 1066.718665 1116.049174 1309.272079
2129.705831 1626.670582 1809.361418 1164.013306
1855.245943 -0.672061244 3.21E-05 0.00064232 IQCE
7 2558972 2614734 + 9240 protein_coding IQ
motif containing E [Source:HGNC Symbol;Acc:HGNC:29171] - 922
1204 1339 2120 1780 1844 5.456996341
5.744979372 6.712913663 11.09341107 8.394727997
9.316118012
ENSG00000204256 4047.051942 5439.349294 4913.437041
7590.593046 6681.229564 7285.525232 4799.946092
7185.782614 -0.581852147 3.25E-05 0.000650337 BRD2
6 32968660 32981505 + 7337
protein_coding bromodomain containing 2 [Source:HGNC
Symbol;Acc:HGNC:1103] - 3498 5868 5025 7556 7311
7425 26.07329983 35.26188657 31.7263421
49.79372846 43.42270384 47.2415322
ENSG00000164916 2042.037358 2315.52395 2683.078854
4429.185381 3132.71166 3676.614551 2346.880054
3746.170531 -0.674326822 3.28E-05 0.000654801 FOXK1
7 4682309 4771443 + 13093 protein_coding
forkhead box K1 [Source:HGNC Symbol;Acc:HGNC:23480] Fork 1765
2498 2744 4409 3428 3747 7.372253403
8.411766799 9.70839404 16.28178994 11.40933266

13.35951097
ENSG00000104679 802.9314029 913.9738249 967.0426336
1374.263008 1400.947192 1380.570236 894.6492871
1385.260145 -0.630164982 3.31E-05 0.00066021 R3HCC1
8 23270120 23296279 + 2630
protein_coding R3H domain and coiled-coil containing 1 [Source:HGNC
Symbol;Acc:HGNC:27329] - 694 986 989 1368 1533
1407 14.43106672 16.52932521 17.41979336
25.14962799 25.40066016 24.97378593
ENSG00000092964 2602.006809 2482.375155 2302.71527
1275.814342 1847.824672 1524.808917 2462.365745
1549.482644 0.667873249 3.33E-05 0.000662766 DPYSL2
8 26514022 26658178 + 7305
protein_coding dihydropyrimidinase like 2 [Source:HGNC
Symbol;Acc:HGNC:3014] - 2249 2678 2355 1270 2022
1554 16.83697038 16.16308699 14.93389684
8.405909639 12.06200568 9.930630663
ENSG00000125844 2306.981581 2724.309403 4151.732075
1834.359833 1203.553459 1900.614461 3061.007686
1646.175918 0.895249025 3.34E-05 0.000665207 RRBP1
20 17613678 17682295 - 6980
protein_coding ribosome binding protein 1 [Source:HGNC
Symbol;Acc:HGNC:10448] - 1994 2939 4246 1826 1317
1937 15.62300041 18.56428048 28.17909382
12.64871966 8.222217296 12.95448743
ENSG00000058056 1604.705844 1674.07376 1859.772588
1182.388567 1156.946605 985.1403815 1712.850731
1108.158518 0.628371515 3.35E-05 0.000665207 USP13
3 179652755 179789401 + 8440
protein_coding ubiquitin specific peptidase 13 [Source:HGNC
Symbol;Acc:HGNC:12611] - 1387 1806 1902 1177 1266
1004 8.987289411 9.43429077 10.43927941
6.742721666 6.536569103 5.553123114
ENSG00000126432 1881.219685 2170.919572 2123.77816
2900.217327 2877.744754 3909.16263 2058.639139
3229.04157 -0.649134294 3.40E-05 0.000675923 PRDX5
11 64318088 64321811 + 893
protein_coding peroxiredoxin 5 [Source:HGNC Symbol;Acc:HGNC:9355]
- 1626 2342 2172 2887 3149 3984
99.57809075 115.6296976 112.6706547 156.3135195
153.6667122 208.2638568
ENSG00000108509 721.9440856 1021.500157 1004.198973
1562.119135 1445.726326 1495.372452 915.8810721
1501.072638 -0.71164354 3.41E-05 0.000675932 CAMTA2
17 4967992 4987652 - 5999 protein_coding
calmodulin binding transcription activator 2 [Source:HGNC
Symbol;Acc:HGNC:18807] CG-1 624 1102 1027 1555 1582
1524 5.688535078 8.099098681 7.930380722
12.53293386 11.4917512 11.85911119
ENSG00000062485 5060.55037 4647.73302 5254.688688

3772.191224	3299.948017	3301.790223	4987.657359
3457.976488	0.528445469	3.42E-05	0.000676233	CS
	12	56271699	56300392	-	6402
protein_coding	citrate synthase [Source:HGNC Symbol;Acc:HGNC:2422]
	-	4374	5014	5374	3755	3611	3365
37.36438356	34.53048019	38.88520994	28.35929877
24.57935128	24.53665941
ENSG00000146112 2574.239728	3091.382055	2986.196363
4135.84854	4021.897321	4489.061002	2883.939382
4215.602287	-0.547372276	3.42E-05	0.000676233
PPP1R18 6	30676389	30687895	-	4599
protein_coding	protein phosphatase 1 regulatory subunit 18
[Source:HGNC Symbol;Acc:HGNC:29413]	-	2225	3335	3054
	4117	4401	4575	26.45826235	31.9717484
30.76154038	43.28313148	41.70100837	46.43802325
ENSG00000176208 901.2731454	812.9361505	792.9945155
563.5683826	413.0646646	488.6453287	835.7346038
488.4261253	0.775086495	3.46E-05	0.000684232	ATAD5
	17	30831970	30895869	+	7075
protein_coding	"ATPase family, AAA domain containing 5 [Source:HGNC
Symbol;Acc:HGNC:25752]" -	779	877	811	561	452
	498	6.021514358	5.465213182	5.310028669
3.833872215	2.784008702	3.285859083
ENSG00000132842 1988.817121	1750.083754	2058.265666
1185.402302	1290.370147	1350.152555	1932.388847
1275.308335	0.599312332	3.50E-05	0.000690168	AP3B1
	5	78000525	78294755	-	7310
protein_coding	adaptor related protein complex 3 subunit beta 1
[Source:HGNC Symbol;Acc:HGNC:566]	-	1719	1888	2105
	1180	1412	1376	12.86036256	11.38724254
13.33942713	7.804873111	8.417360304	8.787130834
ENSG00000102265 5615.891974	6148.466917	4243.645126
7839.728445	8304.244706	8311.876665	5336.001339
8151.949939	-0.611403708	3.50E-05	0.000690168	TIMP1
	X	47582313	47586789	+	1541
protein_coding	TIMP metallopeptidase inhibitor 1 [Source:HGNC
Symbol;Acc:HGNC:11820]	-	4854	6633	4340	7804	9087
	8471	172.2629364	189.7760122	130.4636566
244.8588686	256.9664869	256.6126602
ENSG00000128335 295.0252273	213.1987624	206.3154658
495.2570635	407.5815053	499.4386994	238.1798185
467.4257561	-0.97476647	3.60E-05	0.000707928	APOL2
	22	36226203	36239954	-	3484
protein_coding	apolipoprotein L2 [Source:HGNC Symbol;Acc:HGNC:619]
	-	255	230	211	493	446	509
4.002734153	2.910607524	2.805477337	6.84179393
5.578472675	6.82002037
ENSG00000160445 848.0529083	826.8404176	885.885365
1423.487341	1305.905765	1225.538184	853.592897
1318.31043	-0.627029774	3.63E-05	0.000712646	ZER1

9	128729786	128772414	-	4370
protein_coding	zyg-11 related cell cycle regulator [Source:HGNC
Symbol;Acc:HGNC:30960]	-	733	892	906	1417	1429
1249	9.173123708	8.999479155	9.603935098
15.6779628	14.24982036	13.34218743
ENSG00000006125 9688.397072	8538.146958	10328.48467
6655.330721	6206.022428	7037.277706	9518.3429
6632.876951	0.521066181	3.64E-05	0.000714697	AP2B1
17	35578046	35726409	+	7587
protein_coding	adaptor related protein complex 2 subunit beta 1
[Source:HGNC Symbol;Acc:HGNC:563]	-	8374	9211	10563
6625	6791	7172	60.3611611	53.52672304
64.49405023	42.21988199	39.0051762	44.12820201
ENSG00000146247 2260.703114	1948.451298	2119.866966
1301.933376	1493.24704	1424.724934	2109.673793
1406.635117	0.584367825	3.65E-05	0.00071607	PHIP
6	78935867	79078236	-	13252
protein_coding	pleckstrin homology domain interacting protein
[Source:HGNC Symbol;Acc:HGNC:15673]	-	1954	2102	2168
1296	1634	1452	8.063764669	6.993349651
7.578448549	4.728515215	5.373153192	5.11483025
ENSG00000091137 98.34174244	81.57170039	101.6910353
24.10987733	0	10.79337071	93.86815937	11.63441601
3.021152347	3.71E-05	0.000726594	SLC26A4 7
107660635	107717809	+	5800	protein_coding
solute carrier family 26 member 4 [Source:HGNC
Symbol;Acc:HGNC:8818]	-	85	88	104	24	0
11	0.801466999	0.668942265	0.830630361
0.200071148	0	0.088534131
ENSG00000138795 697.6478905	642.3771406	900.5523413
420.918275	460.5853782	376.7867595	746.8591241
419.4301376	0.832256429	3.78E-05	0.000738817	LEF1
4	108047545	108168956	-	5361
protein_coding	lymphoid enhancer binding factor 1 [Source:HGNC
Symbol;Acc:HGNC:6551]	HMG	603	693	921	419	504
384	6.15129022	5.699298259	7.958226194
3.77893509	4.096786455	3.343731935
ENSG00000275183 116.8531292	113.0880392	141.7807703
204.9339573	382.9072886	298.2895179	123.9073129
295.3769213	-1.253669957	3.82E-05	0.000745656	LENG9
19	54461796	54463711	-	1916
protein_coding	leukocyte receptor cluster member 9 [Source:HGNC
Symbol;Acc:HGNC:16306]	-	101	122	145	204	419
304	2.882840282	2.807361139	3.505701652
5.14796848	9.529653633	7.40668909
ENSG00000189184 362.1290045	214.1257135	487.9214097
142.6501075	159.9254785	136.3889572	354.7253759
146.3215144	1.276760257	3.83E-05	0.000746046	PCDH18
4	137518918	137532494	-	6686
protein_coding	protocadherin 18 [Source:HGNC Symbol;Acc:HGNC:14268]

- 313 231 499 142 175 139
2.560192836 1.523279388 3.457296521 1.02688826
1.140591877 0.970497596
ENSG00000187688 85.615164 107.5263323 109.5134226
222.0117871 227.5511095 245.3038799 100.884973
231.6222588 -1.196809933 3.83E-05 0.000746071 TRPV2
17 16415542 16437003 + 3410
protein_coding transient receptor potential cation channel subfamily
V member 2 [Source:HGNC Symbol;Acc:HGNC:18082] - 74 116
112 221 249 250 1.186785013 1.49981457
1.52147948 3.133567911 3.182024842 3.422407079
ENSG00000149823 2035.095588 2937.508165 2664.500684
4007.262528 3844.608504 3939.580311 2545.701479
3930.483781 -0.626136936 3.85E-05 0.000748812 VPS51
11 65089324 65111860 + 4878
protein_coding "VPS51, GARP complex subunit [Source:HGNC
Symbol;Acc:HGNC:1172]" - 1759 3169 2725 3989 4207
4015 19.72053788 28.64272947 25.87778894
39.53879749 37.58281566 38.42286894
ENSG00000108821 38319.72766 24612.40669 64413.44846
14697.98397 8874.49327 12084.65034 42448.52761
11885.70919 1.836490685 3.87E-05 0.000749822 COL1A1
17 50183289 50201632 - 9819
protein_coding collagen type I alpha 1 chain [Source:HGNC
Symbol;Acc:HGNC:2197] - 33121 26552 65876 14631 9711
12316 184.4721857 119.2240683 310.786694
72.04568365 43.0978189 58.55290626
ENSG00000048740 76.3594706 110.3071858 144.7141656
39.17855066 17.36333767 25.51160351 110.460274
27.35116395 2.018631849 3.87E-05 0.000749822 CELF2
10 10798397 11336675 + 10978
protein_coding CUGBP Elav-like family member 2 [Source:HGNC
Symbol;Acc:HGNC:2550] - 66 119 148 39 19
26 0.328787594 0.477922738 0.624512226
0.171768134 0.075420424 0.110559523
ENSG00000179833 784.4200161 750.8304241 626.7687848
1272.800607 1101.201152 1112.698399 720.673075
1162.233386 -0.689805606 3.87E-05 0.000749822
SERTAD2 2 64631621 64751005 - 6274
protein_coding SERTA domain containing 2 [Source:HGNC
Symbol;Acc:HGNC:30784] - 678 810 641 1267 1205
1134 5.909896733 5.692125443 4.732776228
9.764124694 8.369531154 8.437515352
ENSG00000074416 2192.442375 2767.876107 2085.644022
3346.250058 3512.877369 3950.373681 2348.654168
3603.167036 -0.617167478 3.88E-05 0.000751408 MGLL
3 127689062 128052190 - 11849
protein_coding monoglyceride lipase [Source:HGNC
Symbol;Acc:HGNC:17038] - 1895 2986 2133 3331 3844
4026 8.74625686 11.11071639 8.338954646

13.5923375 14.13709828 15.86127544
ENSG00000101596 2866.951032 2506.475885 2667.434079
1866.506337 1794.820799 1872.159211 2680.286999
1844.495449 0.538975186 3.89E-05 0.000751842 SMCHD1
18 2655738 2805017 + 12435 protein_coding
structural maintenance of chromosomes flexible hinge domain containing
1 [Source:HGNC Symbol;Acc:HGNC:29090] - 2478 2704 2728
1858 1964 1908 10.89808586 9.587267734
10.16251108 7.22438923 6.882627915 7.162730592
ENSG00000176994 1504.050178 1328.320985 1464.742027
2737.475655 2101.877718 1964.39347 1432.371064
2267.915614 -0.663075127 3.99E-05 0.000770408 SMCR8
17 18315310 18328055 + 8279
protein_coding "Smith-Magenis syndrome chromosome region, candidate 8
[Source:HGNC Symbol;Acc:HGNC:17921]" - 1300 1433 1498
2725 2300 2002 8.587370137 7.631365701
8.38178249 15.91438428 12.1062197 11.28839575
ENSG00000184867 947.5516124 853.7220007 569.0786782
1284.855546 1680.588315 1278.523822 790.1174304
1414.655894 -0.841108114 4.00E-05 0.000770408 ARMCX2
X 101655281 101659891 - 3589
protein_coding armadillo repeat containing X-linked 2 [Source:HGNC
Symbol;Acc:HGNC:16869] - 819 921 582 1279 1839
1303 12.47972904 11.31410305 7.51193778
17.23051676 22.32887635 16.94794294
ENSG00000205208 1286.541383 920.4624829 1070.689266
536.4447706 767.642297 535.7436736 1092.564377
613.2769137 0.831987015 4.01E-05 0.000771552
C4orf46 4 158666679 158672255 - 3730
protein_coding chromosome 4 open reading frame 46 [Source:HGNC
Symbol;Acc:HGNC:27320] - 1112 993 1095 534 840
546 16.30386866 11.73746674 13.59902409
6.922032622 9.813615812 6.833289913
ENSG00000132128 3509.064762 3533.537749 3086.9096
5245.907475 4539.142011 4834.448864 3376.504037
4873.166117 -0.529331202 4.01E-05 0.000771636 LRRC41
1 46261196 46303608 - 8606
protein_coding leucine rich repeat containing 41 [Source:HGNC
Symbol;Acc:HGNC:16917] - 3033 3812 3157 5222 4967
4927 19.27373047 19.52924745 16.99322063
29.3384214 25.15077755 26.72558722
ENSG00000182054 1550.328645 1532.250236 2164.845693
1000.559909 1078.354655 1133.303925 1749.141525
1070.739496 0.708178176 4.04E-05 0.000775969 IDH2
15 90083045 90102504 - 2814
protein_coding "isocitrate dehydrogenase (NADP(+)) 2, mitochondrial
[Source:HGNC Symbol;Acc:HGNC:5383]" - 1340 1653 2214
996 1180 1155 26.04206497 25.89898347
36.44651262 17.11340632 18.27328013 19.16037157
ENSG00000117592 5254.919931 4478.100961 5036.639642

2780.672519	3479.064553	3588.305155	4923.220178
3282.680742	0.584540069	4.06E-05	0.000778329	PRDX6
	1	173477266	173488807	+	2367
protein_coding	peroxiredoxin 6 [Source:HGNC Symbol;Acc:HGNC:16753]
	-	4542	4831	5151	2768	3807	3657
104.9406101	89.98554214	100.8081739	56.54172374
70.08792726	72.12282647
ENSG00000144366 269.5720704	215.9796158	217.0712484
523.3852537	477.034856	370.8994664	234.2076449
457.1065253	-0.966272627	4.07E-05	0.000779262	GULP1
	2	188291669	188595931	+	11979
protein_coding	"GULP, engulfment adaptor PTB domain containing 1
[Source:HGNC Symbol;Acc:HGNC:18649]"	-	233	233	222
	521	522	378	1.063726721	0.857569475
0.858489258	2.102898808	1.898928164	1.473049262
ENSG00000170604 902.4301071	1896.542034	1940.929856
3235.746453	2671.212422	3063.354852	1579.967332
2990.104576	-0.919534178	4.10E-05	0.000784419
IRF2BP1 19	45883607	45886170	-	2564
protein_coding	interferon regulatory factor 2 binding protein 1
[Source:HGNC Symbol;Acc:HGNC:21728]	-	780	2046	1985
	3221	2923	3122	16.63685742	35.18208443
35.86286208	60.73987873	49.67860501	56.8408958
ENSG00000179134 3719.631787	3937.688446	3972.794965
6019.432706	4872.700866	6094.329591	3876.705066
5662.154388	-0.546370218	4.16E-05	0.000795402	SAMD4B
	19	39342396	39385710	+	8057
protein_coding	sterile alpha motif domain containing 4B [Source:HGNC
Symbol;Acc:HGNC:25492]	-	3215	4248	4063	5992	5332
	6211	21.82239067	23.24583241	23.36016546
35.95834191	28.83867979	35.98605186
ENSG00000156603 736.9845874	907.4851669	819.3950727
1300.928798	1182.534681	1442.386814	821.2882757
1308.616764	-0.671252733	4.17E-05	0.000795402	MED19
	11	57703714	57712323	-	1877
protein_coding	mediator complex subunit 19 [Source:HGNC
Symbol;Acc:HGNC:29600]	-	637	979	838	1295	1294
	1470	18.55965387	22.99600409	20.68150798
33.35851524	30.04198338	36.55940323
ENSG00000106070 821.4427898	739.7070104	847.7512268
497.2662199	496.2259134	484.7204666	802.967009
492.7375333	0.704276746	4.21E-05	0.000803305	GRB10
	7	50590063	50793462	-	9795
protein_coding	growth factor receptor bound protein 10 [Source:HGNC
Symbol;Acc:HGNC:4564]	-	710	798	867	495	543
	494	3.964136689	3.591967587	4.100313971
2.443441665	2.415761118	2.354336547
ENSG00000186642 129.5797077	195.5866907	128.0915925
265.2086506	338.1281546	433.6972596	151.085997
345.6780216	-1.192115161	4.23E-05	0.000805164	PDE2A

11 72576141 72674591 - 7182
protein_coding phosphodiesterase 2A [Source:HGNC
Symbol;Acc:HGNC:8777] - 112 211 131 264 370
442 0.852839554 1.29530193 0.844944826
1.777295915 2.244992637 2.872915844
ENSG00000106089 983.4174244 1107.706613 989.5319972
1715.819603 1548.992492 1454.1614 1026.885345
1572.991165 -0.614847161 4.34E-05 0.00082543 STX1A
7 73699206 73719672 - 3693
protein_coding syntaxin 1A [Source:HGNC Symbol;Acc:HGNC:11433] -
850 1195 1012 1708 1695 1482 12.58735065
14.26666802 12.69415102 22.36195531 20.00087486
18.73332777
ENSG00000090520 1548.014722 1229.137213 1717.991817
905.124978 860.8560045 1005.745907 1498.381251
923.9089633 0.697450658 4.36E-05 0.00082818
DNAJB11 3 186567403 186585800 + 5731
protein_coding DnaJ heat shock protein family (Hsp40) member B11
[Source:HGNC Symbol;Acc:HGNC:14889] - 1338 1326 1757
901 942 1025 12.76792779 10.20110161
14.20181362 7.601435224 7.162738434 8.349096732
ENSG00000184281 433.8606284 667.4048214 667.8363182
1174.351942 891.0133805 1111.717184 589.7005893
1059.027502 -0.843075859 4.37E-05 0.00082883 TSSC4
11 2400488 2403878 + 2659 protein_coding tumor
suppressing subtransferable candidate 4 [Source:HGNC
Symbol;Acc:HGNC:12386] - 375 720 683 1169 975
1133 7.712721385 11.93845473 11.89884541
21.25677572 15.97882631 19.89104539
ENSG00000144724 2846.125722 2801.246348 3890.659898
2260.301 1830.461334 1900.614461 3179.343989
1997.125598 0.671010458 4.38E-05 0.00082883 PTPRG
3 61561569 62297613 + 14629
protein_coding "protein tyrosine phosphatase, receptor type G
[Source:HGNC Symbol;Acc:HGNC:9671]" - 2460 3022 3979
2250 2003 1937 9.196343402 9.10780614
12.59974498 7.436508774 5.966572294 6.181032351
ENSG00000117640 1236.792031 1065.993812 1017.888151
1554.082509 1875.240468 1713.202297 1106.891332
1714.175092 -0.631710626 4.41E-05 0.000833741 MTFR1L
1 25818640 25832942 + 5817
protein_coding mitochondrial fission regulator 1 like [Source:HGNC
Symbol;Acc:HGNC:28836] - 1069 1150 1041 1547 2052
1746 10.05016875 8.71631134 8.289992192
12.85856387 15.37223059 14.0117123
ENSG00000142856 1056.30601 850.0141962 964.1092383
524.3898319 662.5484111 522.9878719 956.8098148
569.9753716 0.746375004 4.42E-05 0.000835931
ITGB3BP 1 63440770 63593721 - 5459
protein_coding integrin subunit beta 3 binding protein [Source:HGNC

Symbol;Acc:HGNC:6157]	-	913	917	986	522	725
	533	9.146446448	7.406110702	8.366932714
4.623369729	5.787400134	4.557851283
ENSG00000138696 369.0707746	283.6470491	158.4033434
625.8522323	767.642297	453.32157	270.3737224
615.6053664	-1.18905173	4.47E-05	0.000844498	BMPR1B
	4	94757968	95158448	+	6312
protein_coding	bone morphogenetic protein receptor type 1B
[Source:HGNC Symbol;Acc:HGNC:1077]	-	319	306	162
	623	840	462	2.763875051	2.137412743
1.188914107	4.77223996	5.799237481	3.416811508
ENSG00000111602 1542.229914	1662.023396	1831.416433
1244.672417	799.6273927	977.2906574	1678.556581
1007.196822	0.737437937	4.49E-05	0.000846744
TIMELESS	12	56416373	56449403	-	5818
	protein_coding	timeless circadian regulator [Source:HGNC
Symbol;Acc:HGNC:11813]	-	1333	1793	1873	1239	875
	996	12.53000214	13.58753046	14.91305145
10.2967177	6.553796225	7.991561709
ENSG00000106266 1843.039949	2074.516653	2109.111184
2809.805287	2813.774563	3271.372542	2008.889262
2964.98413	-0.561314075	4.53E-05	0.000852144	SNX8
	7	2251770 2354318 -	5773	protein_coding
sorting nexin 8 [Source:HGNC Symbol;Acc:HGNC:14972]	-	1593
	2238	2157	2797	3079	3334	15.09068421
17.0919842	17.30816567	23.42567563	23.24163782
26.95939077
ENSG00000065154 1460.085635	1535.95804	1630.967758
934.2577465	898.3242595	1135.266356	1542.337144
989.2827873	0.640960355	4.57E-05	0.0008587	OAT
	10	124397303	124418976	-	3388
protein_coding	ornithine aminotransferase [Source:HGNC
Symbol;Acc:HGNC:8091]	-	1262	1657	1668	930	983
	1157	20.37092107	21.56319274	22.80631405
13.27213417	12.64354079	15.94174996
ENSG00000101384 1870.80703	1625.872301	2818.015035
1431.523966	922.9984762	1104.848675	2104.898122
1153.123706	0.868502259	4.70E-05	0.000883402	JAG1
	20	10637684	10673999	-	9298
protein_coding	jagged 1 [Source:HGNC Symbol;Acc:HGNC:6188]	-
	1617	1754	2882	1425	1010	1126	9.510759304
	8.317139871	14.35842786	7.410142146	4.733587721
	5.653206954
ENSG00000011132 313.5366141	429.1783782	438.0536904
792.6122172	709.1552649	620.1282083	393.5895609
707.2985635	-0.843995336	4.76E-05	0.000892287	APBA3
	19	3750819 3761699 -	5556	protein_coding
amyloid beta precursor protein binding family A member 3 [Source:HGNC
Symbol;Acc:HGNC:580]	-	271	463	448	789	776
	632	2.667483652	3.674114715	3.7352376

6.866192619	6.086366239	5.310077713
ENSG00000166881 1822.214639	1500.733897	1766.881738
1215.539649	1032.661661	1040.088451	1696.610091
1096.096587	0.63012625	4.77E-05	0.000893872	NEMP1
	12	57055643	57088063	-	5708
protein_coding	nuclear envelope integral membrane protein 1
[Source:HGNC Symbol;Acc:HGNC:29001]	-	1575	1619	1807
	1210	1130	1060	15.09007181	12.50537854
14.66481687	10.24949864	8.626866487	8.668978716
ENSG00000187164 594.6783013	299.4052185	374.4967934
196.8973315	193.738294	187.4121642	422.8601044
192.6825966	1.132135639	4.81E-05	0.000900562	SHTN1
	10	116881482	117126586	-	8247
protein_coding	shootin 1 [Source:HGNC Symbol;Acc:HGNC:29319]	-
	514	323	383	196	212	191	3.408488532
	1.72679382	2.151321092	1.149109178	1.120207469
	1.081143667
ENSG00000167889 298.4961123	355.9492381	447.8316746
197.9019097	112.4047649	181.5248711	367.425675
163.9438486	1.166226788	4.86E-05	0.000908138	MGAT5B
	17	76868456	76950393	+	6386
protein_coding	"alpha-1,6-mannosylglycoprotein 6-beta-N-
acetylglucosaminyltransferase B [Source:HGNC Symbol;Acc:HGNC:24140]"
	-	258	384	458	197	123	185
2.209456749	2.651162012	3.322301327	1.491552445
0.839333959	1.352349205
ENSG00000089006 2227.151226	1957.720809	2028.931714
1439.560592	1313.216644	1448.274107	2071.267916
1400.350448	0.564572136	4.87E-05	0.000908138	SNX5
	20	17941597	17968980	-	7953
protein_coding	sorting nexin 5 [Source:HGNC Symbol;Acc:HGNC:14969]
	-	1925	2112	2075	1433	1437	1476
13.23714921	11.70838216	12.08619437	8.711971067
7.873800076	8.66366021
ENSG00000085999 622.4453815	591.3948279	711.837247
361.6481599	416.7201041	289.4585782	641.8924855
355.9422808	0.850252302	4.88E-05	0.000909564	RAD54L
	1	46247688	46278473	+	3212
protein_coding	RAD54 like [Source:HGNC Symbol;Acc:HGNC:9826]	-
	538	638	728	360	456	295	9.16012609
	8.757478909	10.49925052	5.41911267	6.186541119
	4.287385306
ENSG00000071794 3180.487647	2985.709625	2830.726415
2187.971368	1922.761182	2100.782427	2998.974562
2070.504992	0.534457613	5.04E-05	0.000938107	HLTF
	3	149030127	149086554	-	6024
protein_coding	helicase like transcription factor [Source:HGNC
Symbol;Acc:HGNC:11099]	-	2749	3221	2895	2178	2104
	2141	24.95654662	23.5743496	22.26209649
17.48131621	15.22016533	16.59119776

ENSG00000196235 4296.955664   5326.261255   4610.319532
6947.662984   6340.35983   7391.496508   4744.51215
6893.173107   -0.538694374   5.06E-05   0.000940868   SUPT5H
        19   39436156   39476670   +   8568
protein_coding "SPT5 homolog, DSIF elongation factor subunit
[Source:HGNC Symbol;Acc:HGNC:11469]"   -   3714   5746   4715
        6916   6938   7533   23.70593855   29.56787556
25.49204609   39.02804017   35.28689421   41.0425698
ENSG00000238227 887.3896053   1266.215258   1504.831762
2258.291843   1874.326609   2071.345962   1219.478875
2067.988138   -0.761107413   5.11E-05   0.000948495
TMEM250 9   136114581   136118863   -   3396
protein_coding transmembrane protein 250 [Source:HGNC
Symbol;Acc:HGNC:31009] -   767   1366   1539   2248   2051
        2111   12.35157657   17.73441944   20.99294626
32.0058851   26.31822385   29.01794061
ENSG00000130816 6656.00052   7840.152749   9506.156202
5799.430076   3924.114314   5202.404684   8000.769824
4975.316358   0.685519681   5.14E-05   0.000953153   DNMT1
        19   10133345   10231286   -   9796
protein_coding DNA methyltransferase 1 [Source:HGNC
Symbol;Acc:HGNC:2976]   -   5753   8458   9722   5773   4294
        5302   32.1173948   38.06736904   45.97368291
28.49403789   19.10169302   25.26602856
ENSG00000248323 758.9668593   977.9334536   477.1656271
1194.443506   2024.199628   1249.087356   738.02198
1489.243497   -1.012887084   5.18E-05   0.000960403   LUCAT1
        5   91054834   91314547   -   16538
antisense   lung cancer associated transcript 1 [Source:HGNC
Symbol;Acc:HGNC:48498] -   656   1055   488   1189   2215
        1273   2.169279762   2.812570582   1.366908016
3.476162607   5.836457679   3.593283241
ENSG00000198682 673.3516953   491.2841046   631.6577768
302.3780448   213.8432113   383.6552681   598.7645256
299.9588414   0.997342203   5.27E-05   0.000975893   PAPSS2
        10   87659613   87747705   +   4083
protein_coding 3'-phosphoadenosine 5'-phosphosulfate synthase 2
[Source:HGNC Symbol;Acc:HGNC:8604]   -   582   530   646
        301   234   391   7.795398433   5.723088314
7.329183477   3.564415571   2.497440069   4.470369417
ENSG00000116133 6716.162528   6964.183921   9492.467024
5385.543848   4132.474366   5221.047779   7724.271158
4913.021998   0.652915832   5.40E-05   0.000998423   DHCR24
        1   54849627   54887271   -   6290
protein_coding 24-dehydrocholesterol reductase [Source:HGNC
Symbol;Acc:HGNC:2859]   -   5805   7513   9708   5361   4522
        5321   50.47150922   52.6619215   71.49597252
41.20940825   31.32842129   39.49013781
ENSG00000087111 1119.938902   1406.184881   1691.59126
795.6259518   880.9609218   878.1878899   1405.905014

851.5915879 0.723713377 5.41E-05 0.000999187 PIGS
17 28553383 28571872 - 5990
protein_coding phosphatidylinositol glycan anchor biosynthesis class S [Source:HGNC Symbol;Acc:HGNC:14937] - 968 1517 1730
792 964 895 8.837781246 11.16587383
13.37894094 6.392924503 7.013080262 6.97496847
ENSG00000121005 192.0556382 117.7227949 208.2710626
55.25180221 61.22861179 68.68508636 172.6831652
61.72183345 1.483034672 5.42E-05 0.000999647
CRISPLD1 8 74984515 75034558 + 4543
protein_coding cysteine rich secretory protein LCCL domain containing 1 [Source:HGNC Symbol;Acc:HGNC:18206] - 166
127 213 55 67 70 1.998297127
1.232522744 2.171897485 0.585357476 0.642673901
0.719285556
ENSG00000137494 558.8124894 640.5232383 523.1221526
1084.94448 821.5600298 965.5160712 574.1526268
957.3401936 -0.736961469 5.43E-05 0.001000562
ANKRD42 11 83193739 83260694 + 6724
protein_coding ankyrin repeat domain 42 [Source:HGNC Symbol;Acc:HGNC:26752] - 483 691 535 1080 899
984 3.928385856 4.530898158 3.685772565
7.765997872 5.826269704 6.831458423
ENSG00000235655 1041.265508 817.5709062 932.819689
567.5866954 620.5108568 453.32157 930.5520345
547.1397074 0.765350225 5.53E-05 0.00101643
H3F3AP4 2 174719908 174720318 + 411
processed_pseudogene "H3 histone, family 3A, pseudogene 4 [Source:HGNC Symbol;Acc:HGNC:42980]" - 900 882 954
565 679 462 119.7554812 94.6151585
107.5248904 66.46727015 71.99238315 52.47424389
ENSG00000196352 101.8126275 125.138404 98.75764003
306.3963577 198.3075934 251.191173 108.5695572
251.9650414 -1.21278037 5.54E-05 0.00101866 CD55
1 207321376 207386804 + 5908
protein_coding CD55 molecule (Cromer blood group) [Source:HGNC Symbol;Acc:HGNC:2665] - 88 135 101 305 217
256 0.814585918 1.007458673 0.791923705
2.49609189 1.600581692 2.022765392
ENSG00000128590 627.0732282 505.1883718 365.6966076
786.5847478 964.1221707 979.2530884 499.3194025
909.986669 -0.867278088 5.60E-05 0.001027924 DNAJB9
7 108569568 108574850 + 2837
protein_coding DnaJ heat shock protein family (Hsp40) member B9 [Source:HGNC Symbol;Acc:HGNC:6968] - 542 545 374
783 1055 998 10.44803608 8.469760393
6.106814417 13.34454107 16.20510023 16.421667
ENSG00000069424 351.7163494 318.8711925 348.0962362
626.8568105 529.124869 599.5226824 339.5612593
585.1681207 -0.785408548 5.63E-05 0.00103218 KCNAB2

1	5991466 6101193 +	9534	protein_coding
potassium voltage-gated channel subfamily A regulatory beta subunit 2
[Source:HGNC Symbol;Acc:HGNC:6229]	-	304	344	356
624	579	611	1.743785848	1.590806119
1.729725971	3.164540503	2.646439767	2.991659061
ENSG00000159261 30.08100357	75.08304241	17.60037149
154.7050462	222.9818101	115.7834313	40.92147249
164.4900959	-2.001903842	5.68E-05	0.001040565	CLDN14
21	36460621	36576569	-	2853
protein_coding	claudin 14 [Source:HGNC Symbol;Acc:HGNC:2035]	-
26	81	18	154	244	118	0.498386522
1.251748862	0.292262563	2.609877755	3.726890667
1.930751013
ENSG00000136518 2315.080313	2236.733103	2464.052009
1506.867333	1657.741818	1663.160306	2338.621808
1609.256485	0.539176872	5.84E-05	0.001067848	ACTL6A
3	179562880	179588408	+	3675
protein_coding	actin like 6A [Source:HGNC Symbol;Acc:HGNC:24124]
-	2001	2413	2520	1500	1814	1695
29.77724114	28.94902468	31.76476547	19.73490918
21.50990722	21.53071216
ENSG00000127526 1995.758891	2220.047982	2147.245322
3093.096346	3044.067252	2929.909541	2121.017398
3022.357713	-0.51066725	5.88E-05	0.001073567
SLC35E1 19	16549831	16572382	-	7257
protein_coding	solute carrier family 35 member E1 [Source:HGNC
Symbol;Acc:HGNC:20803]	-	1725	2395	2196	3079	3331
2986	12.99950122	14.5506486	14.01772929
20.51416207	20.00212363	19.20784826
ENSG00000158480 500.9644056	411.5663065	549.5227099
836.8136589	769.4700168	838.9392691	487.3511407
815.074315	-0.742373094	5.90E-05	0.001077089	SPATA2
20	49903391	49915508	-	4270
protein_coding	spermatogenesis associated 2 [Source:HGNC
Symbol;Acc:HGNC:14681]	-	433	444	562	833	842
855	5.545679081	4.584469134	6.096925613
9.432316019	8.592960476	9.347258978
ENSG00000213923 1004.242735	1488.683532	1258.426562
1941.849703	1930.072061	2095.87635	1250.450943
1989.266038	-0.668914733	5.92E-05	0.001079128	CSNK1E
22	38290691	38318084	-	6246
protein_coding	casein kinase 1 epsilon [Source:HGNC
Symbol;Acc:HGNC:2453]	-	868	1606	1287	1933	2112
2136	7.599980151	11.33646154	9.545067915
14.96342774	14.73501328	15.96413179
ENSG00000144395 186.2708298	143.6774268	195.5596832
26.11903377	84.07510872	50.04199149	175.1693133
53.41204466	1.709733055	5.94E-05	0.001081119
CCDC150 2	196639554	196763490	+	9683
protein_coding	coiled-coil domain containing 150 [Source:HGNC

Symbol;Acc:HGNC:26834]	-	161	155	200	26	92
	51	0.909307257	0.705757861	0.956802981
0.129826884	0.414034464	0.245870418
ENSG00000071282 335.518886	341.1180198	489.8770065
218.9980524	174.5472366	178.5812245	388.8379708
190.7088378	1.028759632	6.01E-05	0.001090641	LMCD1
	3	8501707 8574673 +	9130	protein_coding	LIM
and cysteine rich domains 1 [Source:HGNC Symbol;Acc:HGNC:6633]	-
	290	368	501	218	191	182	1.737088452
	1.777096449	2.54196405	1.154481379	0.911635413
	0.930564855
ENSG00000099250 5275.745241	4314.03061	4194.755205
6502.634831	6400.674581	7116.756163	4594.843685
6673.355192	-0.538622678	6.01E-05	0.001090641	NRP1
	10	33177492	33336262	-	11522
protein_coding	neuropilin 1 [Source:HGNC Symbol;Acc:HGNC:8004] -
	4560	4654	4290	6473	7004	7253	21.64370892
	17.80871017	17.2477273	27.16307624	26.48968929
	29.38568659
ENSG00000136122 389.8960847	437.5209385	538.7669273
245.1170862	258.6223453	144.2386814	455.3946502
215.9927043	1.076197875	6.01E-05	0.001090641	BORA
	13	72727749	72756198	+	3189
protein_coding	"bora, aurora kinase A activator [Source:HGNC
Symbol;Acc:HGNC:24724]" -	337	472	551	244	283
	147	5.779231539	6.525614846	8.003860956
3.699444562	3.867145513	2.151834426
ENSG00000150995 4382.570828	5442.130148	2502.186147
6540.808804	7564.018205	7922.334104	4108.962374
7342.387038	-0.837467763	6.04E-05	0.001093417	ITPR1
	3	4493345 4847840 +	32454
protein_coding	"inositol 1,4,5-trisphosphate receptor type 1
[Source:HGNC Symbol;Acc:HGNC:6180]"	-	3788	5871	2559
	6511	8277	8074	6.383170592	7.975865221
3.652620144	9.700199806	11.11381904	11.61359158
ENSG00000136982 1294.640115	1159.615877	1140.112953
824.7587203	724.6908828	774.1790449	1198.122982
774.5428826	0.62919498	6.05E-05	0.001093417	DSCC1
	8	119833976	119856010	-	2603
protein_coding	DNA replication and sister chromatid cohesion 1
[Source:HGNC Symbol;Acc:HGNC:24453]	-	1119	1251	1166
	821	793	789	23.50989184	21.1893239
20.75041745	15.25001271	13.27570572	14.14975339
ENSG00000164692 17593.9162	7898.550671	21867.48378
5889.842116	4233.912812	5385.891986	15786.65022
5169.882305	1.610500667	6.05E-05	0.001093417	COL1A2
	7	94394561	94431232	+	11156
protein_coding	collagen type I alpha 2 chain [Source:HGNC
Symbol;Acc:HGNC:2198]	-	15207	8521	22364	5863	4633
	5489	74.5469283	33.67565237	92.86315482

25.41046457	18.09724194	22.96840096
ENSG00000111725 790.2048245	982.5682093	773.4385472
1306.956267	1304.991905	1378.607805	848.7371937
1330.185326	-0.647614838	6.09E-05	0.001098605	PRKAB1
12	119667753	119681630	+	6246
protein_coding	protein kinase AMP-activated non-catalytic subunit
beta 1 [Source:HGNC Symbol;Acc:HGNC:9378]	-	683	1060
791	1301	1428	1405	5.980168713	7.48234697
5.866471422	10.0710913	9.9628783	10.50075148
ENSG00000169136 882.7617586	944.5632125	980.7318114
1490.794081	1307.733485	1461.029908	936.0189275
1419.852492	-0.600661704	6.11E-05	0.001101977	ATF5
19	49928702	49933935	+	2954
protein_coding	activating transcription factor 5 [Source:HGNC
Symbol;Acc:HGNC:790]	TF_bZIP 763	1019	1003	1484	1431
1489	14.12566036	15.20889463	15.72870249
24.28983846	21.10997605	23.53045053
ENSG00000075239 1464.713481	1587.867304	1719.947414
957.3630456	1000.676566	1154.890666	1590.842733
1037.643426	0.616747653	6.13E-05	0.0011033	ACAT1
11	108121516	108147776	+	5584
protein_coding	acetyl-CoA acetyltransferase 1 [Source:HGNC
Symbol;Acc:HGNC:93]	-	1266	1713	1759	953	1095
1177	12.39889576	13.525269	14.59227097
8.251800269	8.545299866	9.839591962
ENSG00000123395 307.7518057	538.5586128	454.6762635
780.5572785	726.5186025	912.5304331	433.6622274
806.5354381	-0.893115362	6.15E-05	0.001106254	ATG101
12	52069246	52077494	+	3199
protein_coding	autophagy related 101 [Source:HGNC
Symbol;Acc:HGNC:25679]	-	266	581	465	777	795
930	4.547389025	8.007479773	6.733504716
11.74378239	10.82957682	13.57109043
ENSG00000119912 1515.619795	1245.822333	1390.429348
985.4912358	822.4738897	850.7138554	1383.957159
886.226327	0.642850625	6.17E-05	0.001107694	IDE
10	92451684	92574076	-	7607
protein_coding	insulin degrading enzyme [Source:HGNC
Symbol;Acc:HGNC:5381]	-	1310	1344	1422	981	900
867	9.417867849	7.789683465	8.659416642
6.235292161	5.15570062	5.320491051
ENSG00000198890 1357.116046	1590.648158	1341.539427
2248.246061	2443.661312	1893.745953	1429.767877
2195.217775	-0.61838616	6.17E-05	0.001107694	PRMT6
1	107056679	107067636	+	4335
protein_coding	protein arginine methyltransferase 6 [Source:HGNC
Symbol;Acc:HGNC:18241]	-	1173	1716	1372	2238	2674
1930	14.79802045	17.45268056	14.6611307
24.9615872	26.88010116	20.78328739
ENSG00000106608 1256.46038	1376.522444	1362.073194

2045.32126	1901.742405	1898.65203	1331.685339
1948.571898	-0.548801563	6.24E-05	0.001117779	URGCP
	7	43875894	43926411	-	7830
protein_coding	upregulator of cell proliferation [Source:HGNC
Symbol;Acc:HGNC:30890]	-	1086	1485	1393	2036	2081
	1935	7.585125589	8.361778317	8.241225737
12.57237216	11.58160945	11.53626552
ENSG00000133466 260.316377	199.2944953	339.2960504
123.5631213	97.78300688	122.6519399	266.3023076
114.6660227	1.216093095	6.36E-05	0.00113922
C1QTNF6 22	37180167	37199385	-	7143
protein_coding	C1q and TNF related 6 [Source:HGNC
Symbol;Acc:HGNC:14343]	-	225	215	347	123	107
	125	1.722648145	1.327063694	2.250356273
0.832579432	0.652772312	0.816912231
ENSG00000160352 195.5265232	182.6093747	287.4727344
92.42119643	71.28107044	104.0088451	221.8695441
89.23703731	1.315305497	6.46E-05	0.001154347	ZNF714
	19	21082159	21125270	+	8033
protein_coding	zinc finger protein 714 [Source:HGNC
Symbol;Acc:HGNC:27124]	zf-C2H2 169	197	294	92	78
	106	1.150545109	1.08124078	1.695399377
0.55374686	0.423131574	0.61599067
ENSG00000146950 189.7417148	291.9896094	372.5411966
94.43035287	128.8542427	131.4828796	284.7575069
118.2558251	1.268693236	6.66E-05	0.001190001
SHROOM2 X	9786456 9949443 +	8191	protein_coding	shroom
family member 2 [Source:HGNC Symbol;Acc:HGNC:630]	-	164
	315	381	94	141	134	1.094968523
1.695538228	2.154718327	0.554871149	0.750137341
0.763684381
ENSG00000084636 1309.680617	1199.474776	2961.751403
462.1059821	229.3788292	512.1945012	1823.635599
401.2264375	2.184545783	6.72E-05	0.001198328
COL16A1 1	31652247	31704319	-	9269
protein_coding	collagen type XVI alpha 1 chain [Source:HGNC
Symbol;Acc:HGNC:2193]	-	1132	1294	3029	460	251
	522	6.678951002	6.155103387	15.13801206
2.3995299	1.180047358	2.628958053
ENSG00000253729 15112.23341	14627.289	18782.52978
11830.91772	8243.016094	11316.35859	16174.0174
10463.4308	0.628377831	6.73E-05	0.001198858	PRKDC
	8	47773108	47960183	-	15417
protein_coding	"protein kinase, DNA-activated, catalytic subunit
[Source:HGNC Symbol;Acc:HGNC:9413]"	-	13062	15780	19209
	11777	9020	11533	46.33450415	45.12748021
57.71747718	36.93482275	25.49560153	34.92114343
ENSG00000150556 111.0683209	126.9923063	234.6716199
65.29758443	29.24351608	45.1359139	157.5774157
46.5590048	1.762310699	6.81E-05	0.001211877	LYPD6B

2 149038107 149215262 + 3771
protein_coding LY6/PLAUR domain containing 6B [Source:HGNC
Symbol;Acc:HGNC:27018] - 96 137 240 65 32
46 1.392224953 1.601762024 2.948201516
0.833408721 0.369787344 0.569439167
ENSG00000233968 70.57466222 34.29719221 46.93432398
8.036625776 5.483159264 7.849724156 50.60205947
7.123169732 2.828892811 6.86E-05 0.001218729
AL157895.1 10 19710328 19728550 - 2151
antisense uncharacterized LOC101928834 [Source:NCBI
gene;Acc:101928834] - 61 37 48 8 6
8 1.550901219 0.758394669 1.033720866
0.1798253 0.121554051 0.173618345
ENSG00000123933 529.8884475 846.3063916 605.2572196
1284.855546 1023.523063 1137.228787 660.4840196
1148.535799 -0.796817302 6.91E-05 0.001226 MXD4
4 2247432 2262294 - 7058 protein_coding MAX
dimerization protein 4 [Source:HGNC Symbol;Acc:HGNC:13906] bHLH
458 913 619 1279 1120 1159 3.548775586
5.703258829 4.062669099 8.761734863 6.915044295
7.665629375
ENSG00000152377 2401.852439 1265.288307 2040.665295
1229.603744 975.0884892 985.1403815 1902.602014
1063.277538 0.839077074 6.94E-05 0.00123119 SPOCK1
5 136975298 137598379 - 6222
protein_coding "SPARC (osteonectin), cwcv and kazal like domains
proteoglycan 1 [Source:HGNC Symbol;Acc:HGNC:11251]" - 2076
1365 2087 1224 1067 1004 18.2470245
9.672452432 15.53799216 9.511579179 7.47296607
7.532683877
ENSG00000183160 915.1566855 1043.746985 1529.276723
626.8568105 616.8554173 733.9492086 1162.726798
659.2204788 0.819202275 7.00E-05 0.001239658
TMEM119 12 108589846 108598320 - 3189
protein_coding transmembrane protein 119 [Source:HGNC
Symbol;Acc:HGNC:27884] - 791 1126 1564 624 675
748 13.56490251 15.56746254 22.71876322
9.460874617 9.223756966 10.94947041
ENSG00000231500 28671.82425 36138.11718 33144.43291
41159.57891 47581.02838 61945.11696 32651.45811
50228.57475 -0.621338652 7.03E-05 0.001244066 RPS18
6 33272010 33276510 + 1898
protein_coding ribosomal protein S18 [Source:HGNC
Symbol;Acc:HGNC:10401] - 24782 38986 33897 40972 52066
63131 714.0602494 905.6208827 827.308547
1043.739627 1195.409227 1552.717674
ENSG00000187601 127.2657843 173.3398633 61.60130022
235.071304 371.9409701 355.200018 120.7356493
320.7374307 -1.409284714 7.07E-05 0.001250001 MAGEH1
X 55452105 55453566 + 1462

protein_coding MAGE family member H1 [Source:HGNC
Symbol;Acc:HGNC:24092] -   110   187   63   234   407
        362   4.114717515   5.639338865   1.996161305
7.738730118   12.13125228   11.5586532
ENSG00000145247 859.6225251   1186.49746   906.4191318
1516.913115   1481.366861   1722.033237   984.1797057
1573.437738   -0.676114028   7.09E-05   0.001250001   OCIAD2
        4   48885019   48906937   -   2483
protein_coding OCIA domain containing 2 [Source:HGNC
Symbol;Acc:HGNC:28685] -   743   1280   927   1510   1621
        1755   16.36465323   22.72831334   17.29439884
29.40366352   28.44886164   32.99487118
ENSG00000143322 5561.514775   4892.448121   4456.805181
7634.794487   6936.19647   6808.65449   4970.256026
7126.548482   -0.520037031   7.09E-05   0.001250001   ABL2
        1   179099327   179229684   -   13257
protein_coding "ABL proto-oncogene 2, non-receptor tyrosine kinase
[Source:HGNC Symbol;Acc:HGNC:77]"   -   4807   5278   4558
        7600   7590   6939   19.83003947   17.5532722
15.9269098   27.71848902   24.94911342   24.43417427
ENSG00000148926 3239.492692   3628.086765   2405.384104
4334.755028   6150.276975   4608.769295   3090.987854
5031.267099   -0.702929601   7.11E-05   0.001250702   ADM
        11   10304680   10307397   +   2718
protein_coding adrenomedullin [Source:HGNC Symbol;Acc:HGNC:259]
        -       2800   3914   2460   4315   6730   4697
56.33824209   63.48999997   41.92645185   76.75957547
107.9006869   80.67094485
ENSG00000150991 13751.64648   12081.88117   10502.53279
17656.46683   17402.63365   16807.24063   12112.02015
17288.78037   -0.513476826   7.11E-05   0.001250702   UBC
        12   124911604   124917368   -   3898
protein_coding ubiquitin C [Source:HGNC Symbol;Acc:HGNC:12468] -
        11886   13034   10741   17576   19043   17129   166.7587396
        147.4245599   127.6454497   218.0115113   212.8884649
        205.1333207
ENSG00000183137 500.9644056   329.9946061   445.8760778
194.8881751   202.8768928   247.2663109   425.6116965
215.0104596   0.984083911   7.13E-05   0.001253426
CEP57L1 6   109095110   109163932   +   6432
protein_coding centrosomal protein 57 like 1 [Source:HGNC
Symbol;Acc:HGNC:21561] -   433   356   456   194   222
        252   3.681599763   2.440270221   3.284136977
1.458333696   1.504061299   1.828944559
ENSG00000101224 1620.903308   1675.927663   2408.317499
1178.370254   1138.669407   1207.876304   1901.716156
1174.971989   0.694949501   7.16E-05   0.001256393   CDC25B
        20   3786772 3806121 +   4314   protein_coding cell
division cycle 25B [Source:HGNC Symbol;Acc:HGNC:1726]   -   1401
        1808   2463   1173   1246   1231   17.76039855

18.4778843	26.44762795	13.14677069	12.58625421
13.32060493
ENSG00000108786 126.1088227	89.91426066	123.2026004
387.7671937	205.6184724	234.5105092	113.0752279
275.9653918	-1.288248795	7.20E-05	0.001262195
HSD17B1 17	42549214	42555213	+	5658
protein_coding	hydroxysteroid 17-beta dehydrogenase 1 [Source:HGNC
Symbol;Acc:HGNC:5210]	-	109	97	126	386	225
239	1.053557559	0.755862412	1.03159697
3.298569037	1.732918638	1.971882322
ENSG00000131351 401.4657015	535.7777594	600.3682275
316.4421399	257.7084854	221.7547074	512.5372295
265.3017776	0.951243448	7.26E-05	0.001271288	HAUS8
19	17049729	17075625	-	5533
protein_coding	HAUS augmin like complex subunit 8 [Source:HGNC
Symbol;Acc:HGNC:30532]	-	347	578	614	315	282
226	3.429758312	4.605758117	5.140556735
2.752650671	2.220992213	1.906750218
ENSG00000146263 1218.280645	930.6589454	1062.866878
599.7331985	579.3871623	756.5171655	1070.602156
645.2125088	0.7301515	7.27E-05	0.001271288	MMS22L
6	97142161	97283217	-	21793
protein_coding	"MMS22 like, DNA repair protein [Source:HGNC
Symbol;Acc:HGNC:21475]" -	1053	1004	1087	597	634
771	2.64244566	2.031190478	2.310547925
1.324520025	1.267741878	1.651518318
ENSG00000198554 1192.827488	1120.683929	1167.491309
719.278007	812.421431	745.7237948	1160.334242
759.1410776	0.611717652	7.30E-05	0.001275493	WDHD1
14	54938950	55027105	-	6385
protein_coding	WD repeat and HMG-box DNA binding protein 1
[Source:HGNC Symbol;Acc:HGNC:23170]	HMG	1031	1209	1194
716	889	760	8.830646024	8.348325181
8.662552526	5.421922895	6.067355711	5.556466835
ENSG00000167363 128.422746	139.0426711	102.6688337
218.9980524	312.5400781	283.5712851	123.3780836
271.7031385	-1.139440336	7.42E-05	0.001294712	FN3K
17	82735575	82751197	+	3872
protein_coding	fructosamine 3 kinase [Source:HGNC
Symbol;Acc:HGNC:24822]	-	111	150	105	218	342
289	1.567769975	1.708007874	1.256193108
2.722214616	3.849012798	3.484243752
ENSG00000138172 490.5517505	417.1280134	676.636504
252.1491337	256.7946256	311.0453197	528.1054226
273.329693	0.950321881	7.43E-05	0.001295489	CALHM2
10	103446786	103452402	-	3108
protein_coding	calcium homeostasis modulator family member 2
[Source:HGNC Symbol;Acc:HGNC:23493]	-	424	450	692
251	281	317	7.460699693	6.383596995
10.31400981	3.904756242	3.939888284	4.761286203

ENSG00000132383 3741.614059 3745.80956 4003.106716
2551.628684 2954.508984 2552.141566 3830.176778
2686.093078 0.511815985 7.49E-05 0.001304379 RPA1
17 1829702 1900082 + 5492 protein_coding
replication protein A1 [Source:HGNC Symbol;Acc:HGNC:10289] -
3234 4041 4094 2540 3233 2601 32.2035834
32.44085411 34.53184362 22.36167877 25.65274085
22.10832598
ENSG00000149503 1755.110862 2209.85152 3421.316658
1496.821551 1187.103981 1462.99234 2462.093013
1382.305957 0.833215556 7.59E-05 0.001319395 INCENP
11 62123973 62153163 + 4473
protein_coding inner centromere protein [Source:HGNC
Symbol;Acc:HGNC:6058] - 1517 2384 3499 1490 1299
1491 18.54732984 23.49856333 36.23660485
16.10603308 12.65519527 15.56054419
ENSG00000142949 1409.179321 1862.244842 2265.55893
1123.118452 804.1966921 1241.237632 1845.661031
1056.184259 0.805916054 7.65E-05 0.001326817 PTPRF
1 43525187 43623666 + 10712
protein_coding "protein tyrosine phosphatase, receptor type F
[Source:HGNC Symbol;Acc:HGNC:9670]" - 1218 2009 2317
1118 880 1265 6.218296655 8.268816052
10.01977399 5.046292918 3.579898438 5.512720797
ENSG00000100242 1066.718665 1343.152203 2190.268452
698.1818643 735.6572013 979.2530884 1533.379773
804.3640514 0.931214229 7.65E-05 0.001326817 SUN2
22 38734725 38794143 - 6489
protein_coding Sad1 and UNC84 domain containing 2 [Source:HGNC
Symbol;Acc:HGNC:14210] - 922 1449 2240 695 805
998 7.770480227 9.845200595 15.99089236
5.178550872 5.406008248 7.179576096
ENSG00000143933 8400.698727 7937.482619 8208.617704
5648.743342 6407.071601 5674.369349 8182.26635
5910.061431 0.4692372 7.75E-05 0.001343186 CALM2
2 47160082 47176601 - 6466
protein_coding calmodulin 2 [Source:HGNC Symbol;Acc:HGNC:1445] -
7261 8563 8395 5623 7011 5783 61.41231222
58.38808098 60.14332802 42.04686299 47.25011443
41.75067755
ENSG00000174840 2537.216955 2462.909181 2158.001104
3415.565955 3666.405828 3232.123921 2386.042414
3438.031901 -0.527165202 7.78E-05 0.001347106 PDE12
3 57556276 57566844 + 8947
protein_coding phosphodiesterase 12 [Source:HGNC
Symbol;Acc:HGNC:25386] - 2193 2657 2207 3400 4012
3294 13.40466322 13.09326853 11.42687171
18.37395702 19.54078853 17.18668801
ENSG00000126062 968.3769226 1799.212164 1505.809561
2347.699305 2311.15163 2664.981351 1424.466216

2441.277429	-0.7763446	7.81E-05	0.001350362
TMEM115 3	50354749	50359610	-	2197
protein_coding	transmembrane protein 115 [Source:HGNC
Symbol;Acc:HGNC:30055]	-	837	1941	1540	2337	2529
2716	20.83483732	38.95197084	32.4708098
51.43158068	50.1622918	57.70929177
ENSG00000178719 3408.409097	4281.58732	3836.880985
5261.980727	5474.934526	5814.683168	3842.292467
5517.199474	-0.521715134	7.91E-05	0.001366351	GRINA
8	143990058	143993415	+	2527
protein_coding	glutamate ionotropic receptor NMDA type subunit
associated protein 1 [Source:HGNC Symbol;Acc:HGNC:4589] -	2946
4619	3924	5238	5991	5926	63.75616903
80.58916914	71.93267527	100.2216316	103.3124535
109.4718459
ENSG00000188985 322.7923075	366.1457006	416.5421253
190.8698622	199.2214533	190.3558108	368.4933778
193.4823754	0.930024702	7.95E-05	0.001372334	DHFRP1
18	26170726	26171284	-	559
processed_pseudogene	dihydrofolate reductase pseudogene 1
[Source:HGNC Symbol;Acc:HGNC:2862]	-	279	395	426
190	218	194	27.29525199	31.15438953
35.30207612	16.43398967	16.99430011	16.20078124
ENSG00000136378 59.00504546	27.80853423	78.22387329
12.05493866	2.741579632	5.887293117	55.01248433
6.894603804	3.003834252	7.97E-05	0.001373739
ADAMTS7 15	78759203	78811431	-	7730
protein_coding	ADAM metallopeptidase with thrombospondin type 1 motif
7 [Source:HGNC Symbol;Acc:HGNC:223]	-	51	30	80
12	3	6	0.360815674	0.171110129
0.479416469	0.075059034	0.01691221	0.036234126
ENSG00000104524 425.7618967	532.996906	571.034275
978.4591883	749.3650995	868.3757347	509.9310259
865.4000075	-0.761717908	8.00E-05	0.001376671	PYCR3
8	143603913	143609773	-	3244
protein_coding	pyrroline-5-carboxylate reductase 3 [Source:HGNC
Symbol;Acc:HGNC:25846]	-	368	575	584	974	820
885	6.203855674	7.81485559	8.339393319
14.51708193	11.01518016	12.73527892
ENSG00000155959 3037.024399	2407.292113	2453.296226
1704.769243	1897.173106	1675.916107	2632.537579
1759.286152	0.580985572	8.03E-05	0.001380316	VBP1
X	155197007	155239817	+	2018
protein_coding	VHL binding protein 1 [Source:HGNC
Symbol;Acc:HGNC:12662]	-	2625	2597	2509	1697	2076
1708	71.13819779	56.73940088	57.59462504
40.65948719	44.82959187	39.51051953
ENSG00000179598 189.7417148	307.7477788	116.3580115
503.2936892	498.0536332	414.0729492	204.615835
471.8067572	-1.203985612	8.04E-05	0.001380316	PLD6

17 17200995 17206315 - 2560
protein_coding phospholipase D family member 6 [Source:HGNC
Symbol;Acc:HGNC:30447] - 164 332 119 501 545
422 3.503471553 5.717841025 2.153324352
9.462349326 9.277161878 7.695175367
ENSG00000125304 3130.738294 2685.377455 3475.095571
2241.214013 2020.544189 2011.491815 3097.07044
2091.083339 0.566622854 8.07E-05 0.001384886 TM9SF2
13 99446311 99564006 + 5524
protein_coding transmembrane 9 superfamily member 2 [Source:HGNC
Symbol;Acc:HGNC:11865] - 2706 2897 3554 2231 2211
2050 26.78976075 23.12218029 29.80342729
19.52752115 17.44189511 17.32392229
ENSG00000111846 892.017452 866.6993167 1095.134226
581.6507906 598.5782197 598.5414669 951.2836649
592.9234924 0.68214474 8.11E-05 0.001389753 GCNT2
6 10492223 10629368 + 9072
protein_coding glucosaminyl (N-acetyl) transferase 2 (I blood group)
[Source:HGNC Symbol;Acc:HGNC:4204] - 771 935 1120
579 655 610 4.647785206 4.544043993
5.718964977 3.085863692 3.146276281 3.138866387
ENSG00000183963 798.3035562 1366.325982 1016.910353
1836.36899 1625.756722 1905.520539 1060.513297
1789.215417 -0.753491959 8.21E-05 0.001405764 SMTN
22 31064105 31104757 + 10242
protein_coding smoothelin [Source:HGNC Symbol;Acc:HGNC:11126] -
690 1474 1040 1828 1779 1942 3.684334323
6.345219789 4.703823566 8.629639158 7.569196403
8.851369891
ENSG00000144959 1397.609704 1022.427108 1406.074122
742.3833061 657.0652519 890.9436917 1275.370312
763.4640832 0.740091282 8.26E-05 0.001412001 NCEH1
3 172630249 172711218 - 4808
protein_coding neutral cholesterol ester hydrolase 1 [Source:HGNC
Symbol;Acc:HGNC:29260] - 1208 1103 1438 739 719
908 13.74033078 10.1145138 13.85469223
7.431580661 6.516629486 8.815915632
ENSG00000104823 1585.037496 1689.83193 1541.010304
2332.630632 2462.85237 2168.486298 1605.293243
2321.3231 -0.53206243 8.29E-05 0.001416062 ECH1
19 38815422 38832005 - 2467
protein_coding enoyl-CoA hydratase 1 [Source:HGNC
Symbol;Acc:HGNC:3149] - 1370 1823 1576 2322 2695
2210 30.3700936 32.58002979 29.5930358
45.50868457 47.60452299 41.81856828
ENSG00000134824 8993.063105 10560.75435 15766.99946
6282.632201 6431.745817 8431.584959 11773.60564
7048.654326 0.740204583 8.31E-05 0.001416748 FADS2
11 61792980 61867354 + 11026
protein_coding fatty acid desaturase 2 [Source:HGNC

Symbol;Acc:HGNC:3575]	-	7773	11393	16125	6254	7038
	8593	38.5536404	45.55688984	67.74608317
27.42465073	27.81569221	36.38085149
ENSG00000198492 3780.950756	3823.673456	3532.785678
4968.643886	5825.856719	4972.800253	3712.469963
5255.766952	-0.501608892	8.31E-05	0.001416748	YTHDF2
	1	28736621	28769775	+	3859
protein_coding	YTH N6-methyladenosine RNA binding protein 2
[Source:HGNC Symbol;Acc:HGNC:31675]	-	3268	4125	3613
	4946	6375	5068	46.31290061	47.12844737
43.37062082	61.96986498	71.98864651	61.30668924
ENSG00000182287 1669.495698	1389.49976	1307.316482
885.0334136	1039.97254	851.6950709	1455.437314
925.5670083	0.652318867	8.35E-05	0.001421372	AP1S2
	X	15825806	15854931	-	5371
protein_coding	adaptor related protein complex 1 subunit sigma 2
[Source:HGNC Symbol;Acc:HGNC:560]	-	1443	1499	1337
	881	1138	868	14.69284481	12.30496658
11.53131168	7.930890844	9.233061055	7.544155103
ENSG00000183688 897.8022604	756.3921309	984.6430051
545.4859746	581.214882	440.5657682	879.6124655
522.4222083	0.751195634	8.38E-05	0.001424379	RFLNB
	17	439978	445939	-	3621	protein_coding
refilin B [Source:HGNC Symbol;Acc:HGNC:28705]	-	776	816
	1007	543	636	449	11.72000803	9.935634159
	12.88259642	7.250576202	7.65397763	5.788470869
ENSG00000140365 860.7794867	1108.633564	1245.715182
610.783559	675.3424494	667.2265532	1071.709411
651.1175205	0.719497025	8.45E-05	0.001435312	COMMD4
	15	75335891	75343224	+	6779
protein_coding	COMM domain containing 4 [Source:HGNC
Symbol;Acc:HGNC:26027]	-	744	1196	1274	608	739
	680	6.002083243	7.778565332	8.70575117
4.336498114	4.750478996	4.682624301
ENSG00000112977 3351.717975	2958.828042	3860.348147
2090.52728	2404.365337	2357.860893	3390.298054
2284.25117	0.569572316	8.52E-05	0.001445686	DAP
	5	10679230	10761272	-	3361
protein_coding	death associated protein [Source:HGNC
Symbol;Acc:HGNC:2672]	-	2897	3192	3948	2081	2631
	2403	47.1383846	41.87244571	54.41405451
29.93675921	34.11229458	33.37577002
ENSG00000002822 1986.503197	2320.158706	2155.067709
3286.979942	2938.059506	3055.505128	2153.909871
3093.514859	-0.521919046	8.59E-05	0.001456133	MAD1L1
	7	1815793 2233243 -	7230	protein_coding
mitotic arrest deficient 1 like 1 [Source:HGNC Symbol;Acc:HGNC:6762]
	-	1717	2503	2204	3272	3215	3114
12.98753439	15.26358362	14.12133477	21.88145587
19.3776576	20.10603095

ENSG00000019505 19.66834849 15.75816939 17.60037149 0
0 0 17.67562979 0 6.635961258
8.61E-05 1 SYT13 11 45240301 45286319
- 5424 protein_coding synaptotagmin 13 [Source:HGNC
Symbol;Acc:HGNC:14962] - 17 17 18 0 0
0 0.171405184 0.13818573 0.153728815 0
0 0
ENSG00000182473 2246.819574 2011.483976 1945.818848
2954.464551 2901.505111 2977.007886 2068.040799
2944.325849 -0.50997786 8.71E-05 0.00147503 EXOC7
17 76081017 76121576 - 10086
protein_coding exocyst complex component 7 [Source:HGNC
Symbol;Acc:HGNC:23214] - 1942 2170 1990 2941 3175
3034 10.52991764 9.485816694 9.139797389
14.09864182 13.71776515 14.04244231
ENSG00000134986 1217.123683 600.6643393 793.9723139
471.1471861 448.7051998 447.4342769 870.5867787
455.7622209 0.932558595 8.75E-05 0.001478917 NREP
5 111662621 111997464 - 9337
protein_coding neuronal regeneration related protein [Source:HGNC
Symbol;Acc:HGNC:16834] - 1052 648 812 469 491
456 6.161736096 3.059860343 4.028571966
2.42865989 2.291567905 2.279835541
ENSG00000114520 806.402288 845.3794405 957.2646494
322.4696093 497.1397733 597.5602514 869.6821259
472.389878 0.880385451 8.76E-05 0.00147911 SNX4
3 125446644 125520197 - 2709
protein_coding sorting nexin 4 [Source:HGNC Symbol;Acc:HGNC:11175]
- 697 912 979 321 544 609
14.07079014 14.84293519 16.74079748 5.729243014
8.750814795 10.4943205
ENSG00000255031 6.941770054 48.20145932 57.69010655 0
0 3.924862078 37.61111198 1.308287359
4.859798869 8.78E-05 0.001482242 AP002807.1 11
68050740 68053762 + 1209 antisense
"novel transcript, antisense to CHKA" - 6 52
59 0 0 4 0.271406136 1.896317272
2.260623129 0 0 0.154447089
ENSG00000105559 314.6935758 554.3167822 384.2747776
568.5912737 953.1558521 985.1403815 417.7617119
835.6291691 -0.998922333 8.84E-05 0.001489464
PLEKHA4 19 48837097 48868632 - 3851
protein_coding pleckstrin homology domain containing A4 [Source:HGNC
Symbol;Acc:HGNC:14339] - 272 598 393 566 1043
1004 3.86269216 6.846389819 4.727390603
7.106309676 11.8023744 12.17043861
ENSG00000118785 48.59239038 22.24682738 9.777984162
177.8103453 79.50580934 107.9337071 26.87240064
121.7499539 -2.190508135 8.89E-05 0.001496772 SPP1
4 87975650 87983426 + 2321

protein_coding secreted phosphoprotein 1 [Source:HGNC
Symbol;Acc:HGNC:11255] -   42   24   10   177   87
        110   0.989620909   0.45590049   0.199584732
3.687222476   1.633438208   2.212399647
ENSG00000171223 1217.123683   2021.680438   1324.916854
2210.072088   3093.415685   2502.099575   1521.240325
2601.862449   -0.773816954   8.91E-05   0.001498872   JUNB
        19   12791496   12793315   +   1820
protein_coding "JunB proto-oncogene, AP-1 transcription factor
subunit [Source:HGNC Symbol;Acc:HGNC:6205]"   TF_bZIP 1052   2181
        1355   2200   3385   2550   31.6110604   52.8345771
        34.48818687   58.44569257   81.04866818   65.40558407
ENSG00000177084 3870.036805   4240.801469   4925.170622
3134.284053   2341.309006   3081.016731   4345.336299
2852.203263   0.607647642   8.98E-05   0.001508647   POLE
        12   132623753   132687365   -   16609
protein_coding "DNA polymerase epsilon, catalytic subunit
[Source:HGNC Symbol;Acc:HGNC:9177]"   -   3345   4575   5037
        3120   2562   3140   11.01405776   12.14455403
14.04853124   9.082644697   6.721933908   8.825355303
ENSG00000213380 1047.050317   1064.13991   1166.51351
1650.522019   1621.187423   1553.264167   1092.567912
1608.324536   -0.557652947   8.99E-05   0.001508647   COG8
        16   69320140   69339667   -   7450
protein_coding component of oligomeric golgi complex 8 [Source:HGNC
Symbol;Acc:HGNC:18623] -   905   1148   1193   1643   1774
        1583   6.643348251   6.793906619   7.417996545
10.66307606   10.37662066   9.919063669
ENSG00000105287 638.642845   899.1426066   836.0176458
1160.287846   1347.029459   1325.622167   791.2676991
1277.646491   -0.690241767   9.01E-05   0.001510951   PRKD2
        19   46674275   46717127   -   5446
protein_coding protein kinase D2 [Source:HGNC Symbol;Acc:HGNC:17293]
        -       552   970   855   1155   1474   1351
5.543143906   7.852863621   7.272620629   10.25428925
11.79447039   11.58040499
ENSG00000081320 3342.462281   2355.382849   2359.427578
1393.349994   1963.884877   1573.869693   2685.757569
1643.701521   0.707792317   9.05E-05   0.001515412   STK17B
        2   196133566   196176503   -   6580
protein_coding serine/threonine kinase 17b [Source:HGNC
Symbol;Acc:HGNC:11396] -   2889   2541   2413   1387   2149
        1604   24.01133799   17.02600393   16.98767267
10.19182092   14.23210436   11.37953474
ENSG00000138376 767.065591   733.2183524   746.0601915
418.9091186   481.6041554   489.6265442   748.7813783
463.3799394   0.69197089   9.22E-05   0.001541478   BARD1
        2   214725646   214809711   -   6067
protein_coding BRCA1 associated RING domain 1 [Source:HGNC
Symbol;Acc:HGNC:952]   -   663   791   763   417   527

499	5.97632554	5.748260569	5.825765493
3.323252013	3.785255664	3.839481563
ENSG00000004399 3062.477556	3057.084863	6165.019014
1687.691413	1188.931701	1346.227693	4094.860477
1407.616935	1.540638265	9.30E-05	0.001554031	PLXND1
3	129555175	129606818	-	9738
protein_coding	plexin D1 [Source:HGNC Symbol;Acc:HGNC:9107]	-
2647	3298	6305	1680	1301	1372	14.86547818
14.93189197	29.99285283	8.341434195	5.821918534
6.577038392
ENSG00000147251 835.3263299	673.8934794	857.529211
511.330315	491.656614	386.5989147	788.9163401
463.1952812	0.767788547	9.31E-05	0.001554031	DOCK11
X	118495898	118686163	+	6721
protein_coding	dedicator of cytokinesis 11 [Source:HGNC
Symbol;Acc:HGNC:23483]	-	722	727	877	509	538
394	5.874866672	4.769078526	6.044608169
3.661719758	3.488244981	2.736581346
ENSG00000108424 15303.13208	14644.90107	16366.38989
12012.74638	10399.7254	11186.83814	15438.14102
11199.76997	0.463054183	9.34E-05	0.00155746	KPNB1
17	47649476	47685505	+	7678
protein_coding	karyopherin subunit beta 1 [Source:HGNC
Symbol;Acc:HGNC:6400]	-	13227	15799	16738	11958	11380
11401	94.2123764	90.72259189	100.9852422
75.30289239	64.58813614	69.31717801
ENSG00000132561 185.1138681	141.8235245	215.1156516
96.43950931	35.64053522	37.28618974	180.6843481
56.45541142	1.680505707	9.41E-05	0.001566734	MATN2
8	97868840	98036716	+	6933
protein_coding	matrilin 2 [Source:HGNC Symbol;Acc:HGNC:6908]	-
160	153	220	96	39	38	1.262099211
0.972980617	1.46995465	0.66950103	0.245133127
0.255863556
ENSG00000212719 939.4528807	946.4171148	910.3303254
1540.018414	1356.168058	1305.997856	932.0667736
1400.72811	-0.587596135	9.44E-05	0.001568421
C17orf51	17	21428381	21574517	-	14794
protein_coding	chromosome 17 open reading frame 51
[Source:HGNC Symbol;Acc:HGNC:27904]	-	812	1021	931
1533	1484	1331	3.001685087	3.042804751
2.915187697	5.010231083	4.371262471	4.199895426
ENSG00000126243 696.4909288	842.598587	810.594887
1156.269534	1330.579982	1161.759175	783.2281343
1216.202897	-0.634285903	9.44E-05	0.001568421	LRFN3
19	35935358	35945767	+	4044
protein_coding	leucine rich repeat and fibronectin type III domain
containing 3 [Source:HGNC Symbol;Acc:HGNC:28370]	-	602
909	829	1151	1456	1184	8.141043158
9.910297554	9.496112246	13.76148792	15.68948997

13.6674216
ENSG00000108107 15872.35723 23625.20373 22240.02498
29458.25178 31215.62569 31841.42482 20579.19531
30838.4341 -0.583474547 9.46E-05 0.00157049 RPL28
19 55385345 55403250 + 6933
protein_coding ribosomal protein L28 [Source:HGNC
Symbol;Acc:HGNC:10330] - 13719 25487 22745 29324 34158
32451 108.2171192 162.0807646 151.973266
204.5046687 214.6989061 218.500744
ENSG00000183255 9023.144109 9887.787819 11013.92136
5812.489593 7576.812244 7097.131852 9974.951096
6828.81123 0.546677996 9.47E-05 0.00157049
PTTG1IP 21 44849585 44873903 - 3537
protein_coding PTTG1 interacting protein [Source:HGNC
Symbol;Acc:HGNC:13524] - 7799 10667 11264 5786 8291
7233 120.5864676 132.9661804 147.5231027
79.09419058 102.1481377 95.46176089
ENSG00000067955 4041.267133 3889.486987 4251.467513
3006.702619 2970.044602 2753.290748 4060.740545
2910.012656 0.480694008 9.60E-05 0.001589698 CBFB
16 67029116 67101058 + 4129
protein_coding core-binding factor subunit beta [Source:HGNC
Symbol;Acc:HGNC:1539] CBF 3493 4196 4348 2993 3250
2806 46.2645578 44.80480119 48.78059669
35.04798469 34.30023345 31.72406417
ENSG00000145147 2222.523379 1279.192574 2616.588562
1153.255799 772.2115964 1289.317193 2039.434838
1071.594863 0.928431491 9.65E-05 0.001596482 SLIT2
4 20253260 20620561 + 9712
protein_coding slit guidance ligand 2 [Source:HGNC
Symbol;Acc:HGNC:11086] - 1921 1380 2676 1148 845
1314 10.81716374 6.264759028 12.76379708
5.715239452 3.791461348 6.315863383
ENSG00000112144 1755.110862 1293.096841 1473.542213
992.5232834 1024.436923 838.9392691 1507.249972
951.9664917 0.662269103 9.68E-05 0.001599173 ICK
6 53001279 53061802 - 6259
protein_coding intestinal cell kinase [Source:HGNC
Symbol;Acc:HGNC:21219] - 1517 1395 1507 988 1121
855 13.25486601 9.82659855 11.15348934
7.63226093 7.804754715 6.376864648
ENSG00000111328 469.7264403 454.206059 431.2091015
257.1720248 260.4500651 256.0972506 451.713867
257.9064468 0.808278361 9.74E-05 0.001608601
CDK2AP1 12 123260971 123272334 - 3117
protein_coding cyclin dependent kinase 2 associated protein 1
[Source:HGNC Symbol;Acc:HGNC:14002] - 406 490 441
256 285 261 7.123344431 6.930957499
6.553966889 3.971041077 3.984434166 3.908856624
ENSG00000272899 61.31896881 113.0880392 97.77984162

342.5611737   244.0005873   151.10719   90.72894987
245.8896503   -1.434516647   9.76E-05   0.001609346
ATP6V1FNB   7   128866308   128872044   +   3902
        protein_coding ATP6V1F neighbor [Source:HGNC
Symbol;Acc:HGNC:52392] -   53   122   100   341   267
        154   0.742819536   1.378499216   1.187176225
4.225404888   2.981828038   1.842381193
ENSG00000204525 1715.774165   2050.415924   2174.623678
1059.830024   1421.052109   1324.640951   1980.271255
1268.507695   0.642656085   9.77E-05   0.001609346   HLA-C
        6   31268749   31272130   -   2507
protein_coding "major histocompatibility complex, class I, C
[Source:HGNC Symbol;Acc:HGNC:4933]"   -   1483   2212   2224
        1055   1555   1350   32.35053966   38.9013566
41.09442468   20.34695114   27.0292912   25.13769603
ENSG00000112655 2558.042265   2128.279819   3487.80695
1601.297686   1421.052109   1907.48297   2724.709678
1643.277588   0.729622868   9.79E-05   0.001610621   PTK7
        6   43076268   43161719   +   9024
protein_coding protein tyrosine kinase 7 (inactive) [Source:HGNC
Symbol;Acc:HGNC:9618]   -   2211   2296   3567   1594   1555
        1944   13.39936966   11.21777578   18.31076456
8.540640605   7.509134866   10.05641552
ENSG00000160447 423.4479733   460.694717   743.1267963
247.1262426   216.5847909   329.6884145   542.4231622
264.4664827   1.037262952   9.82E-05   0.001614969   PKN3
        9   128702523   128720918   +   3375
protein_coding protein kinase N3 [Source:HGNC Symbol;Acc:HGNC:17999]
        -       366   497   760   246   237   336
5.930646262   6.492568936   10.43139212   3.524216226
3.060082721   4.647415863
ENSG00000122884 1924.027267   1142.930757   1520.476537
784.5755914   950.4142725   964.5348556   1529.144854
899.8415732   0.764178412   9.91E-05   0.001625885   P4HA1
        10   73007217   73096974   -   3093
protein_coding prolyl 4-hydroxylase subunit alpha 1 [Source:HGNC
Symbol;Acc:HGNC:8546]   -   1663   1233   1555   781   1040
        983   29.40404251   17.57588145   23.28911198
12.20878176   14.65250968   14.83609596
ENSG00000204231 1339.76162   1599.917669   1503.853964
2062.39909   2255.406177   2173.392376   1481.177751
2163.732548   -0.546360891   9.91E-05   0.001625885   RXRB
        6   33193588   33200688   -   3920
protein_coding retinoid X receptor beta [Source:HGNC
Symbol;Acc:HGNC:10478] RXR-like   1158   1726   1538   2053
        2468   2215   16.15538102   19.41282244   18.17492905
        25.32235534   27.43580365   26.37750411
ENSG00000143369 822.5997514   672.9665283   1236.914996
420.918275   517.2446906   532.8000271   910.827092
490.3209976   0.893327466   1.00E-04   0.0016378   ECM1

1	150508062	150513789	+	3360
protein_coding	extracellular matrix protein 1 [Source:HGNC
Symbol;Acc:HGNC:3153]	-	711	726	1265	419	566
543	11.57244262	9.526454582	17.44029007
6.029425899	7.340670858	7.544085261
ENSG00000100567 2381.027129	1658.315591	1693.546857
1012.614848	1336.977001	1166.665253	1910.963192
1172.0857	0.704459667	0.000100292	0.001641496	PSMA3
14	58244831	58272012	+	3119
protein_coding	proteasome subunit alpha 3 [Source:HGNC
Symbol;Acc:HGNC:9532]	-	2058	1789	1732	1008	1463
1189	36.08483372	25.28884088	25.72379078
15.62594797	20.44031334	17.7955951
ENSG00000114270 4803.704878	4885.959463	9060.280124
2326.603162	1594.685486	2690.492954	6249.981488
2203.927201	1.503836181	0.000100528	0.001643608	COL7A1
3	48564073	48595267	-	11071
protein_coding	collagen type VII alpha 1 chain [Source:HGNC
Symbol;Acc:HGNC:2214]	-	4152	5271	9266	2316	1745
2742	20.50997858	20.99133809	38.77108019
10.11469801	6.868583437	11.56183149
ENSG00000060656 1025.068045	1106.779662	1603.589403
809.690047	732.0017618	652.5083204	1245.145703
731.4000431	0.767997393	0.000100657	0.001643959	PTPRU
1	29236516	29326813	+	6682
protein_coding	"protein tyrosine phosphatase, receptor type U
[Source:HGNC Symbol;Acc:HGNC:9683]"	-	886	1194	1640
806	801	665	7.251401688	7.878287285
11.36945986	5.832164795	5.223777164	4.64580749
ENSG00000163781 4986.504822	4297.345489	4261.245498
3340.222588	3062.344449	3179.138283	4515.031936
3193.901774	0.499274658	0.000101092	0.001649301	TOPBP1
3	133598175	133662380	-	7304
protein_coding	DNA topoisomerase II binding protein 1 [Source:HGNC
Symbol;Acc:HGNC:17008]	-	4310	4636	4358	3325	3351
3240	32.27091046	27.98444001	27.63941948
22.01061116	19.99273737	20.70762452
ENSG00000185730 397.9948164	617.3494598	488.8992081
972.4317189	748.4512396	907.6243555	501.4144948
876.1691047	-0.803494597	0.000102297	0.001667187	ZNF696
8	143289676	143298061	+	4012
protein_coding	zinc finger protein 696 [Source:HGNC
Symbol;Acc:HGNC:25872]	zf-C2H2 344	666	500	968	819
925	4.689129544	7.318924427	5.773132641
11.66583016	8.895729637	10.76283901
ENSG00000156504 2300.039811	2048.562021	2772.05851
1713.810447	1552.647932	1309.922718	2373.553447
1525.460366	0.637815594	0.000103071	0.001675459
FAM122B X	134769566	134797232	-	4404
protein_coding	family with sequence similarity 122B [Source:HGNC

Symbol;Acc:HGNC:30490]	-	1988	2210	2835	1706	1699
	1335	24.68674224	22.12477798	29.82003911
18.72979108	16.81143038	14.15076736
ENSG00000127666 435.0175901	793.4701766	602.3238244
1212.525914	997.9349861	1052.844252	610.2705303
1087.768384	-0.832175146	0.000103099	0.001675459	TICAM1
	19	4815932 4831704 -	2676	protein_coding	toll
like receptor adaptor molecule 1 [Source:HGNC Symbol;Acc:HGNC:18348]
	-	376	856	616	1207	1092	1073
7.684160874	14.10332823	10.66343335	21.80832836
17.78259457	18.71800887
ENSG00000137936 1427.690708	1439.555122	1170.424704
1900.661996	1983.075934	2136.106186	1345.890178
2006.614705	-0.5763722	0.000103134	0.001675459	BCAR3
	1	93561786	93847150	-	6866
protein_coding	"BCAR3, NSP family adaptor protein [Source:HGNC
Symbol;Acc:HGNC:973]"	-	1234	1553	1197	1892	2170
	2177	9.828926181	9.972444193	8.075934857
13.32350685	13.77255554	14.80132742
ENSG00000122376 1325.87808	1188.351363	1265.271151
823.7541421	883.7025015	806.559157	1259.833531
838.0052668	0.587775272	0.000103334	0.001676936	SHLD2
	10	87094161	87191468	+	4242
protein_coding	shieldin complex subunit 2 [Source:HGNC
Symbol;Acc:HGNC:28773]	-	1146	1282	1294	820	967
	822	14.77435963	13.32451221	14.13077782
9.346400883	9.933777028	9.045804328
ENSG00000136720 521.7897157	588.6139744	704.0148596
964.3950931	1005.245865	947.8541918	604.8061833
972.4983834	-0.684473574	0.000103685	0.001680845	HS6ST1
	2	128236716	128318577	-	4683
protein_coding	heparan sulfate 6-O-sulfotransferase 1 [Source:HGNC
Symbol;Acc:HGNC:5201]	-	451	635	720	960	1100
	966	5.266803274	5.978380125	7.122144725
9.911703266	10.23592571	9.629395056
ENSG00000277957 5.784808379	28.73548537	20.53376674	0
	0	0	18.35135349	0	6.694086386
0.000105098	1	SENP3-EIF4A1	17	7563287 7578715 +
	3519	protein_coding	SENP3-EIF4A1 readthrough (NMD
candidate) [Source:HGNC Symbol;Acc:HGNC:49182]	-	5	31
	21	0	0	0	0.077704371	0.388397463
	0.276441018	0	0	0
ENSG00000011028 3488.239452	2842.959149	7165.306794
1444.583483	1053.680439	1468.879633	4498.835132
1322.381185	1.766465642	0.000105571	0.001709218	MRC2
	17	62627401	62693597	+	6860
protein_coding	mannose receptor C type 2 [Source:HGNC
Symbol;Acc:HGNC:16875]	-	3015	3067	7328	1438	1153
	1497	24.03576302	19.711679	49.48388635
10.1352855	7.324260618	10.18693935

ENSG00000133134 20.82531016 16.68512054 0.977798416
106.4852915 60.31475191 82.42210364 12.8294097
83.07404903 -2.703713516 0.000105659 0.001709218 BEX2
X 103309346 103311046 - 1176
protein_coding brain expressed X-linked 2 [Source:HGNC
Symbol;Acc:HGNC:30933] - 18 18 1 106 66
84 0.837066374 0.674837397 0.03939083
4.358125777 2.445655107 3.334402325
ENSG00000101152 3565.755885 4134.202088 4295.468442
6434.323512 5434.724691 5403.553866 3998.475472
5757.534023 -0.525769293 0.000106165 0.001715581 DNAJC5
20 63895182 63936031 + 5287
protein_coding DnaJ heat shock protein family (Hsp40) member C5
[Source:HGNC Symbol;Acc:HGNC:16235] - 3082 4460 4393
6405 5947 5507 31.87997974 37.19285408
38.49057054 58.57483045 49.01705625 48.6241253
ENSG00000121680 551.8707193 778.6389583 702.0592628
1117.090983 1044.54184 1111.717184 677.5229801
1091.116669 -0.686139648 0.000108463 0.001750866 PEX16
11 45909669 45918812 - 3013
protein_coding peroxisomal biogenesis factor 16 [Source:HGNC
Symbol;Acc:HGNC:8857] - 477 840 718 1112 1143
1133 8.657927805 12.29176114 11.03895005
17.84460225 16.5312501 17.5540291
ENSG00000169499 1123.409787 1075.263323 1370.873379
442.0144177 872.7361829 634.8464411 1189.84883
649.8656806 0.872007831 0.0001086 0.001751233
PLEKHA2 8 38901235 38973909 + 6325
protein_coding pleckstrin homology domain containing A2 [Source:HGNC
Symbol;Acc:HGNC:14336] - 971 1160 1402 440 955
647 8.39563236 8.085956811 10.26809645
3.363514956 6.579629506 4.775180437
ENSG00000023697 876.9769502 792.5432254 669.7919151
515.3486279 428.6002825 465.0961562 779.7706969
469.6816889 0.731166935 0.000110988 0.001787862 DERA
12 15911172 16037282 + 2844
protein_coding deoxyribose-phosphate aldolase [Source:HGNC
Symbol;Acc:HGNC:24269] - 758 855 685 513 469
474 14.57586463 13.25471764 11.1574111
8.721455908 7.186242169 7.780272093
ENSG00000105723 1544.543837 2071.7358 1903.773516
2602.862173 2777.220167 2840.618929 1840.017718
2740.233757 -0.574029443 0.000111389 0.001792422 GSK3A
19 42230186 42242625 - 4126
protein_coding glycogen synthase kinase 3 alpha [Source:HGNC
Symbol;Acc:HGNC:4616] - 1335 2235 1947 2591 3039
2895 17.69484468 23.88263613 21.85944764
30.3626313 32.09667718 32.7540781
ENSG00000147145 75.20250892 67.66743328 101.6910353
25.11445555 18.27719755 18.64309487 81.52032583

20.67824932	1.980664492	0.000112636	0.001810586	LPAR4
	X	78747709	78757094	+	3113
protein_coding	lysophosphatidic acid receptor 4 [Source:HGNC
Symbol;Acc:HGNC:4478]	-	65	73	104	25	20
	19	1.141902303	1.033898005	1.547592707
0.388295274	0.279968695	0.284918413
ENSG00000198053 341.3036943	290.1357071	411.6531332
178.8149235	180.9442557	175.637578	347.6975115
178.4655857	0.96196814	0.000112979	0.001814176	SIRPA
	20	1894167 1940592 +	5068	protein_coding	signal
regulatory protein alpha [Source:HGNC Symbol;Acc:HGNC:9662]	-
	295	313	421	178	198	179	3.183318715
	2.722962681	3.848114141	1.698183483	1.70250024
	1.64877905
ENSG00000171135 1234.478108	1407.111832	1010.065764
1852.442241	1865.18801	1866.271918	1217.218568
1861.300723	-0.61260902	0.000113097	0.001814176	JAGN1
	3	9890572 9894349 +	1781	protein_coding
jagunal homolog 1 [Source:HGNC Symbol;Acc:HGNC:26926]	-	1067
	1518	1033	1844	2041	1902	32.76387141
37.57870502	26.86821766	50.06084935	49.93875178
49.85315279
ENSG00000131943 994.9870411	1192.059167	1150.868736
1645.499128	1668.708136	1623.911685	1112.638315
1646.03965	-0.564408688	0.000113857	0.001824458
C19orf12	19	29698886	29715789	-	5378
	protein_coding	chromosome 19 open reading frame 12
[Source:HGNC Symbol;Acc:HGNC:25443]	-	860	1286	1177
	1638	1826	1655	8.745252758	10.54275535
10.13813618	14.7263228	14.79580412	14.36558235
ENSG00000087510 90.24301071	142.7504757	109.5134226
48.21975466	21.93263706	9.812155195	114.1689697
26.65484897	2.10219712	0.000114071	0.001825972	TFAP2C
	20	56629302	56639283	+	3005
protein_coding	transcription factor AP-2 gamma [Source:HGNC
Symbol;Acc:HGNC:11744]	AP-2	78	154	112	48	24
	10	1.419530862	2.25948885	1.726537447
0.772321238	0.348036957	0.155346531
ENSG00000138095 5171.61869	4159.229769	4393.248284
3198.577059	3251.513444	3196.800162	4574.698914
3215.630222	0.508328929	0.000114235	0.001826684	LRPPRC
	2	43886508	43996005	-	9760
protein_coding	leucine rich pentatricopeptide repeat containing
[Source:HGNC Symbol;Acc:HGNC:15714]	-	4470	4487	4493
	3184	3558	3258	25.04680982	20.26936811
21.32500083	15.7733688	15.88600606	15.58286464
ENSG00000204103 2877.363688	2031.876901	1816.749457
4012.285419	3110.779023	3703.10737	2241.996682
3608.723937	-0.687153476	0.000114489	0.001828829	MAFB
	20	40685848	40689240	-	3393

protein_coding MAF bZIP transcription factor B [Source:HGNC Symbol;Acc:HGNC:6408]   TF_bZIP 2487   2192   1858   3994   3404
        3774   40.08543846   28.48332256   25.36671945
56.91482664   43.71840304   51.92351349
ENSG00000167693 33.5518886   124.2114529   119.2914068
9.041203998   8.224738897   30.4176811   92.35158275
15.89454133   2.543001274   0.000116126   0.001851608   NXN
        17   799313 979770 -   5880   protein_coding
nucleoredoxin [Source:HGNC Symbol;Acc:HGNC:18008]   -   29
        134   122   9   9   31   0.269721387
1.004757902   0.961136257   0.074005909   0.066699685
0.246110648
ENSG00000179115 5332.436363   7303.448039   6538.538009
10211.53763   8080.349036   10543.16076   6391.474137
9611.682473   -0.588438984   0.000116157   0.001851608   FARSA
        19   12922479   12934037   -   2435
protein_coding phenylalanyl-tRNA synthetase subunit alpha [Source:HGNC Symbol;Acc:HGNC:3592]   -   4609   7879   6687
        10165   8842   10745   103.5148019   142.6612723
127.2139722   201.8411137   158.2377659   205.9934874
ENSG00000123992 1026.225006   1424.723903   1100.023218
1891.620792   1776.543602   1810.342633   1183.657376
1826.169009   -0.624806934   0.000117432   0.001869981   DNPEP
        2   219373527   219400022   -   4753
protein_coding aspartyl aminopeptidase [Source:HGNC Symbol;Acc:HGNC:2981]   -   887   1537   1125   1883   1944
        1845   10.20588143   14.2573894   10.96445789
19.15506907   17.82325596   18.12068421
ENSG00000167395 498.6504822   710.0445739   709.8816501
1178.370254   1007.073585   995.9337523   639.5255688
1060.459197   -0.728217623   0.000117864   0.001874916   ZNF646
        16   31074422   31084196   +   8635
protein_coding zinc finger protein 646 [Source:HGNC Symbol;Acc:HGNC:29004] zf-C2H2 431   766   726   1173   1102
        1015   2.729666821   3.911113197   3.894724429
6.568056601   5.561319553   5.487186687
ENSG00000112118 8708.450533   8506.63062   8298.575158
6649.303252   5703.399495   6217.962747   8504.552104
6190.221831   0.458273705   0.000118526   0.001883478   MCM3
        6   52264009   52284881   -   3650
protein_coding minichromosome maintenance complex component 3 [Source:HGNC Symbol;Acc:HGNC:6945]   -   7527   9177   8487
        6619   6241   6337   112.7778379   110.8515641
107.7119265   87.68003876   74.51091831   81.04698781
ENSG00000179151 1252.989495   1313.489767   1284.827119
1817.282004   1825.892035   1872.159211   1283.768793
1838.444417   -0.517941364   0.000119031   0.001889547   EDC3
        15   74630558   74696292   -   5103
protein_coding enhancer of mRNA decapping 3 [Source:HGNC Symbol;Acc:HGNC:26114] -   1083   1417   1314   1809   1998

1908	11.60640179	12.2427291	11.92812695
17.14013408	17.06194404	17.45415538
ENSG00000168393 1254.146456	1352.421714	1585.011233
987.5003922	903.8074188	824.2210364	1397.193135
905.1762825	0.626623937	0.000119424	0.001893819	DTYMK
2	241675742	241686991	-	2451
protein_coding	deoxythymidylate kinase [Source:HGNC
Symbol;Acc:HGNC:3061]	-	1084	1459	1621	983	989
840	24.18692643	26.24496142	30.63671238
19.39150083	17.58375314	15.99860112
ENSG00000077463 389.8960847	762.8807889	616.0130022
1222.571696	901.979699	1145.078511	589.5966253
1089.876635	-0.884601698	0.000120251	0.001904955	SIRT6
19	4174109 4182604 -	2787	protein_coding
sirtuin 6 [Source:HGNC Symbol;Acc:HGNC:14934]	-	337	823
630	1217	987	1167	6.612834366	13.0195755
10.47143103	21.11323716	15.4325887	19.54699146
ENSG00000244038 5651.757786	7014.239283	8724.895267
4100.688302	4651.546776	5105.264348	7130.297445
4619.166475	0.626440903	0.000120553	0.001906615	DDOST
1	20651767	20661544	-	2721
protein_coding	dolichyl-diphosphooligosaccharide--protein
glycosyltransferase non-catalytic subunit [Source:HGNC
Symbol;Acc:HGNC:2728]	-	4885	7567	8923	4082	5090
5203	98.18174328	122.6109197	151.9094555
72.53467594	81.5169343	89.26296736
ENSG00000104320 3806.403913	3274.918381	3180.778248
2476.285317	2417.159376	2306.837686	3420.70018
2400.094126	0.510956901	0.000120606	0.001906615	NBN
8	89933336	90003228	-	6156
protein_coding	nibrin [Source:HGNC Symbol;Acc:HGNC:7652]	-
3290	3533	3253	2465	2645	2351	29.22752223
25.30340602	24.47867509	19.36063195	18.72344085
17.82789443
ENSG00000111674 2377.556244	3156.268635	1938.974259
3344.240901	4757.554522	4126.011259	2490.933046
4075.935561	-0.710349528	0.000121177	0.001913663	ENO2
12	6913745 6923698 +	4281	protein_coding
enolase 2 [Source:HGNC Symbol;Acc:HGNC:3353]	-	2055	3405
1983	3329	5206	4205	26.25193445	35.06758403
21.45754056	37.59843635	52.99288134	45.8529
ENSG00000204227 926.7263022	1138.296001	1057.977886
1495.816973	1511.524237	1696.521633	1041.000063
1567.954281	-0.590234653	0.000121871	0.001922642	RING1
6	33208495	33212722	+	1818
protein_coding	ring finger protein 1 [Source:HGNC
Symbol;Acc:HGNC:10018]	-	801	1228	1082	1489	1654
1729	24.09535615	29.78094304	27.56994106
39.60062455	39.64607736	44.39633811
ENSG00000267209 3.470885027	2.780853423	0	53.24264577

47.52071363	9.812155195	2.083912817	36.85850486
-4.165768144	0.000122447	0.00192862	LINC01897
18	57961231	57964434	-	405
lincRNA long intergenic non-protein coding RNA 1897 [Source:HGNC Symbol;Acc:HGNC:52716]	-	3	3	0	53	52
10	0.405098788	0.326587975	0	6.32735298
5.595087957	1.152632903
ENSG00000136938 3248.748385	3753.225169	4037.32966
2429.070141	2507.631504	2731.704006	3679.767738
2556.135217	0.525865341	0.000122502	0.00192862	ANP32B
9	97983361	98015943	+	1850
protein_coding	acidic nuclear phosphoprotein 32 family member B [Source:HGNC Symbol;Acc:HGNC:16677]	-	2808	4049	4129
2418	2744	2784	83.00802632	96.49615447
103.3893037	63.1954462	64.63547971	70.24954867
ENSG00000100949 660.6251168	600.6643393	620.9019943
851.8823323	1096.631853	1032.238726	627.3971501
993.5843039	-0.663906288	0.000122755	0.001930581
RABGGTA 14	24265538	24271739	-	3545
protein_coding	Rab geranylgeranyltransferase subunit alpha [Source:HGNC Symbol;Acc:HGNC:9795]	-	571	648	635
848	1200	1052	8.808756023	8.059214675
8.297742273	11.56593718	14.75107273	13.85305429
ENSG00000069399 289.2404189	542.2664174	677.6143024
236.0758822	229.3788292	242.3602333	503.0403796
235.9383149	1.093657154	0.000122879	0.001930581	BCL3
19	44747705	44760044	+	3242
protein_coding	B cell CLL/lymphoma 3 [Source:HGNC Symbol;Acc:HGNC:998]	-	250	585	693	235	251
247	4.217175851	7.955670974	9.90199448
3.504742122	3.373799804	3.55655868
ENSG00000125868 6935.985246	5297.52577	5567.584182
3669.724245	4544.62517	3795.341629	5933.698399
4003.230348	0.567498542	0.00012357	0.00193944	DSTN
20	17569863	17609919	+	3851
protein_coding	"destrin, actin depolymerizing factor [Source:HGNC Symbol;Acc:HGNC:15750]" -	5995	5715	5694	3653	4973
3868	85.13543934	65.42996291	68.49303331
45.86457464	56.27344958	46.88770572
ENSG00000163902 7067.878877	5935.268155	7673.76197
4524.620312	4883.667185	5038.541692	6892.303001
4815.60973	0.517187425	0.000124427	0.001950891	RPN1
3	128619970	128681075	-	3992
protein_coding	ribophorin I [Source:HGNC Symbol;Acc:HGNC:10381]
-	6109	6403	7848	4504	5344	5135
83.6901421	70.71750457	91.06907335	54.55179755
58.33571357	60.04764107
ENSG00000075790 2512.92076	1785.307897	1805.015876
1391.340838	1328.752262	1130.360278	2034.414844
1283.484459	0.664008362	0.000124567	0.001951081	BCAP29

7	107579977	107629170	+	8409
protein_coding	B cell receptor associated protein 29 [Source:HGNC
Symbol;Acc:HGNC:24131]	-	2172	1926	1846	1385	1454
1152	14.12570659	10.09824465	10.16927051
7.963548647	7.534920106	6.395200464
ENSG00000149554	1741.227322	1692.612783	1643.679138
1066.862072	1207.208898	1204.932658	1692.506414
1159.667876	0.54521767	0.000125256	0.001959859	CHEK1
11	125625136	125676255	+	6558
protein_coding	checkpoint kinase 1 [Source:HGNC Symbol;Acc:HGNC:1925]
-	1505	1826	1681	1062	1321	1228
12.55046452	12.27618201	11.87404682	7.829865843
8.777887349	8.741238911
ENSG00000025039	21.98227184	56.54401959	7.822387329
127.5814342	213.8432113	76.53481052	28.78289292
139.3198187	-2.269957037	0.000125992	0.001969367	RRAGD
6	89364636	89412270	-	5072
protein_coding	Ras related GTP binding D [Source:HGNC
Symbol;Acc:HGNC:19903]	-	19	61	8	127	234
78	0.204865614	0.530254726	0.073065641
1.210669754	2.010458951	0.717895769
ENSG00000031698	5064.021255	5102.86603	4546.762635
6833.141066	6496.629869	6850.846757	4904.549973
6726.872564	-0.455832415	0.000126218	0.001969695	SARS
1	109213918	109238169	+	3836
protein_coding	seryl-tRNA synthetase [Source:HGNC
Symbol;Acc:HGNC:10537]	-	4377	5505	4650	6802	7109
6982	62.40115969	63.27216321	56.15349736
85.73521585	80.75855015	84.96641254
ENSG00000117569	918.6275705	638.669336	816.4616775
503.2936892	484.345735	397.3922854	791.2528614
461.6772365	0.776406692	0.000126271	0.001969695	PTBP2
1	96721665	96823738	+	12937
protein_coding	polypyrimidine tract binding protein 2 [Source:HGNC
Symbol;Acc:HGNC:17662]	-	794	689	835	501	530
405	3.356461245	2.348116291	2.989890981
1.872429023	1.785257594	1.461394542
ENSG00000168040	362.1290045	515.3848343	387.2081728
730.3283674	762.1591378	668.2077688	421.5740039
720.231758	-0.771360796	0.000127041	0.001979687	FADD
11	70203163	70207390	+	1841
protein_coding	Fas associated via death domain [Source:HGNC
Symbol;Acc:HGNC:3573]	-	313	556	396	727	834
681	9.297908367	13.3154228	9.964232515
19.09333704	19.74107779	17.26789341
ENSG00000221829	572.6960295	692.4325022	780.2831361
330.506235	425.8587029	424.8663199	681.8038893
393.7437526	0.792420237	0.000127715	0.001988165	FANCG
9	35073835	35080016	-	3614
protein_coding	FA complementation group G [Source:HGNC

Symbol;Acc:HGNC:3588]   -   495   747   798   329   466
        433   7.490516473   9.113105784   10.22862364
4.401583715   5.618965508   5.593012423
ENSG00000196821 2714.232091   3474.212876   3453.584006
4787.819806   4353.628456   4856.035606   3214.009658
4665.827956   -0.537386374   0.000128373   0.001996381
C6orf106   6   34587288   34696859   -   4551
        protein_coding chromosome 6 open reading frame 106
[Source:HGNC Symbol;Acc:HGNC:21215]   -   2346   3748   3532
        4766   4764   4949   28.19135075   36.31003811
35.95144206   50.63472073   45.61666331   50.7640957
ENSG00000136156 3291.555967   2706.697331   2724.146387
1948.881751   2111.930177   1967.337117   2907.466562
2009.383015   0.532616254   0.000130304   0.002024344   ITM2B
        13   48232612   48270357   +   10706
protein_coding integral membrane protein 2B [Source:HGNC
Symbol;Acc:HGNC:6174]   -   2845   2920   2786   1940   2311
        2005   14.53281498   12.02512419   12.05469784
8.761444362   9.40657027   8.742450334
ENSG00000142621 229.0784118   289.2087559   197.5152801
433.9777919   386.5627281   555.367984   238.6008159
458.636168   -0.941712955   0.000132787   0.00206082   FHAD1
        1   15247272   15400283   +   12805
protein_coding forkhead associated phosphopeptide binding domain 1
[Source:HGNC Symbol;Acc:HGNC:29408]   -   198   312   202
        432   423   566   0.84562988   1.074258922
0.730759117   1.631193118   1.439525566   2.063397425
ENSG00000179772 61.31896881   43.56670362   27.37835565   0
        1.827719755   8.830939675   44.08800936   3.552886477
        3.633648977   0.000134137   0.002079668   FOXS1   20
        31844301   31845619   -   1319   protein_coding
        forkhead box S1 [Source:HGNC Symbol;Acc:HGNC:3735]   Fork
        53   47   28   0   2   9   2.19748433
        1.571039196   0.983367139   0   0.066076008
0.318525165
ENSG00000155660 3472.041989   4085.073678   5294.778424
2528.523385   2535.0473   3154.607895   4283.964697
2739.39286   0.645290211   0.00013457   0.002084274   PDIA4
        7   149003062   149028641   -   3277
protein_coding protein disulfide isomerase family A member 4
[Source:HGNC Symbol;Acc:HGNC:30167]   -   3001   4407   5415
        2517   2774   3215   50.08230016   59.29260988
76.54634798   37.1371003   36.88829761   45.79842803
ENSG00000167522 7830.316621   9194.428366   10273.72796
15580.00365   11711.11433   12972.65038   9099.490982
13421.25612   -0.560547568   0.000140368   0.002171864
ANKRD11 16   89267619   89490561   -   19538
protein_coding ankyrin repeat domain 11 [Source:HGNC
Symbol;Acc:HGNC:21316] -   6768   9919   10507   15509   12815
        13221   18.94414275   22.38317772   24.91156908

38.37999441	28.58229538	31.58858962
ENSG00000166189 458.1568236	685.0168931	800.8169028
982.4775011	1221.830656	1178.439839	647.9968732
1127.582665	-0.798017139	0.000142382	0.002199725	HPS6
10	102065390	102068038	+	2649
protein_coding	"HPS6, biogenesis of lysosomal organelles complex 2 subunit 3 [Source:HGNC Symbol;Acc:HGNC:18817]"	-	396	739
819	978	1337	1201	8.175379852	12.29975436
14.32202407	17.85081762	21.99419376	21.16445477
ENSG00000149548 673.3516953	649.7927497	775.394144
425.9411661	405.7537856	458.2276476	699.512863
429.9741998	0.702347029	0.000142456	0.002199725	CCDC15
11	124954121	125041489	+	4823
protein_coding	coiled-coil domain containing 15 [Source:HGNC Symbol;Acc:HGNC:25798]	-	582	701	793	424	444
467	6.59933896	6.408180175	7.616551469
4.250595823	4.011661734	4.520075141
ENSG00000104147 138.8354011	149.2391337	162.3145371
37.16939421	47.52071363	75.553595	150.1296906
53.41456761	1.491335491	0.000142852	0.002203613	OIP5
15	41309268	41332621	-	1236
protein_coding	Opa interacting protein 5 [Source:HGNC Symbol;Acc:HGNC:20300]	-	120	161	166	37	52
77	5.309547226	5.743033681	6.22145656
1.447386341	1.833341928	2.908159957
ENSG00000211592 10.41265508	21.31987624	63.55689705	0
0	2.943646558	31.76314279	0.981215519
5.031797525	0.000143569	0.002211169	IGKC	2
88857161	88857683	-	523	IG_C_gene
immunoglobulin kappa constant [Source:HGNC Symbol;Acc:HGNC:5716]
-	9	23	65	0	0	3
0.94109948	1.938920959	5.757237209	0	0
0.267772271
ENSG00000243742 155.0328645	141.8235245	55.73450972
386.7626155	239.4312879	288.4773627	117.5302996
304.890422	-1.376843072	0.000143631	0.002211169
RPLP0P2 11	61615036	61639449	+	3909
transcribed_processed_pseudogene	ribosomal protein lateral stalk subunit P0 pseudogene 2 [Source:HGNC Symbol;Acc:HGNC:17960]
-	134	153	57	385	262	294
1.874708898	1.725677825	0.675478672	4.762075488
2.920748879	3.510974666
ENSG00000095002 2611.262502	2209.85152	2047.509883
1689.700569	1513.351957	1388.41996	2289.541302
1530.490829	0.580785502	0.000144239	0.002218298	MSH2
2	47402969	47663146	+	13777
protein_coding	mutS homolog 2 [Source:HGNC Symbol;Acc:HGNC:7325]
-	2257	2384	2094	1682	1656	1415
8.959249134	7.629315073	7.040839991	5.902996824
5.237985258	4.794549616

ENSG00000163359 33677.99742	17053.12014	39222.42787
12643.6215	8442.237548	12901.02165	29984.51514
11328.96023	1.404202378	0.00014462	0.002221922	COL6A3
2	237324003	237414375	-	19633
protein_coding	collagen type VI alpha 3 chain [Source:HGNC
Symbol;Acc:HGNC:2213]	-	29109	18397	40113	12586	9238
13148	81.08403123	41.3137198	94.64570983
30.99575913	20.50453331	31.26216599
ENSG00000160360 917.4706088	1267.14221	1291.671708
1734.906589	1680.588315	2077.233255	1158.761509
1830.909386	-0.659129152	0.00014572	0.002236571	GPSM1
9	136327476	136359605	+	5138
protein_coding	G protein signaling modulator 1 [Source:HGNC
Symbol;Acc:HGNC:17858]	-	793	1367	1321	1727	1839
2117	8.44060934	11.73027983	11.90998387
16.2517246	15.59718513	19.23414093
ENSG00000113328 1333.976812	1154.981121	1478.431205
575.6233212	995.1934065	745.7237948	1322.463046
772.1801742	0.77559844	0.000147242	0.002257664	CCNG1
5	163437569	163446151	+	3096
protein_coding	cyclin G1 [Source:HGNC Symbol;Acc:HGNC:1592]	-
1153	1246	1512	573	1089	760	20.36681263
17.74398037	22.62316146	8.948595689	15.32800119
11.45931549
ENSG00000105810 3783.26468	2613.075266	3695.100215
2404.960264	2040.649106	2121.387953	3363.813387
2188.999108	0.619653156	0.000149655	0.002292354	CDK6
7	92604921	92836594	-	12260
protein_coding	cyclin dependent kinase 6 [Source:HGNC
Symbol;Acc:HGNC:1777]	-	3270	2819	3779	2394	2233
2162	14.58653019	10.13767966	14.27870686
9.441367277	7.937008596	8.232111712
ENSG00000164077 698.8048521	667.4048214	746.0601915
1192.43435	959.5528713	1114.66083	704.089955
1088.882684	-0.628831229	0.000149814	0.002292496	MON1A
3	49907160	49930173	-	6446
protein_coding	"MON1 homolog A, secretory trafficking associated
[Source:HGNC Symbol;Acc:HGNC:28207]"	-	604	720	763
1187	1050	1136	5.124380268	4.924658879
5.483232896	8.903517861	7.098353044	8.226859228
ENSG00000124813 1482.067907	1062.286007	1202.692052
879.0059443	646.0989333	771.2353983	1249.015322
765.4467586	0.706128591	0.000150614	0.002302441	RUNX2
6	45328157	45664349	+	8192
protein_coding	runt related transcription factor 2 [Source:HGNC
Symbol;Acc:HGNC:10472]	Runt	1281	1146	1230	875	707
786	8.55172839	6.167776558	6.955328133
5.1643935	3.760867803	4.478974999
ENSG00000164211 1081.759167	729.5105478	935.7530843
382.7443026	585.7841814	576.9547254	915.6742663

515.1610698	0.828743729	0.000151774	0.002317844	STARD4
	5	111496033	111512590	-	5684
protein_coding	StAR related lipid transfer domain containing 4
[Source:HGNC Symbol;Acc:HGNC:18058]	-	935	787	957
	381	641	588	8.996058157	6.104563576
7.799384379	3.240948447	4.914310104	4.829134402
ENSG00000149273 28826.85711	35936.04183	32414.99529
41329.35263	44346.87827	55747.75974	32392.63141
47141.33021	-0.541300682	0.000152149	0.002321255	RPS3
	11	75399486	75422280	+	5894
protein_coding	ribosomal protein S3 [Source:HGNC
Symbol;Acc:HGNC:10420]	-	24916	38768	33151	41141	48527
	56815	231.1867307	289.9994827	260.5487283
337.4939008	358.7830436	449.98591
ENSG00000185838 291.5543423	498.6997138	428.2757063
608.7744025	757.5898384	880.150321	406.1765874
748.8381873	-0.880785295	0.000153022	0.002332256	GNB1L
	22	19783224	19854939	-	6988
protein_coding	G protein subunit beta 1 like [Source:HGNC
Symbol;Acc:HGNC:4397]	-	252	538	438	606	829
	897	1.972160959	3.39440249	2.903512299
4.192962172	5.169639178	5.992190098
ENSG00000081803 387.5821614	225.2491272	359.8298171
76.34794487	173.6333767	151.10719	324.2203686
133.6961705	1.275785873	0.000153681	0.00233996	CADPS2
	7	122318425	122886759	-	6351
protein_coding	calcium dependent secretion activator 2 [Source:HGNC
Symbol;Acc:HGNC:16018]	-	335	243	368	76	190
	154	2.884678429	1.686934106	2.684158527
0.578592362	1.303677247	1.131943224
ENSG00000157954 2880.834573	2713.185989	2805.303656
3953.015304	3855.574823	3722.731681	2799.774739
3843.773936	-0.457316962	0.000154034	0.002342996	WIPI2
	7	5190188 5233826 +	8977	protein_coding	"WD
repeat domain, phosphoinositide interacting 2 [Source:HGNC
Symbol;Acc:HGNC:32225]" -	2490	2927	2869	3935	4219
	3794	15.16920549	14.37558263	14.80477389
21.19408774	20.48032754	19.72932092
ENSG00000140538 38.1797353	32.44328993	44.00092873
5.02289111	0	4.906077597	38.20798465	3.309656236
	3.54436376	0.000155361	0.002360831	NTRK3	15
	87859751	88256768	-	26750	protein_coding
	neurotrophic receptor tyrosine kinase 3 [Source:HGNC
Symbol;Acc:HGNC:8033]	-	33	35	45	5	0
	5	0.067465985	0.057687035	0.077927579
0.009037482	0	0.008725539
ENSG00000263528 350.5593877	397.6620394	525.0777495
211.9660048	213.8432113	249.2287419	424.4330589
225.0126527	0.916389672	0.000156489	0.002375611	IKBKE
	1	206470476	206496889	+	3791

protein_coding inhibitor of nuclear factor kappa B kinase subunit epsilon [Source:HGNC Symbol;Acc:HGNC:14552] - 303 429 537 211 234 254 4.371027681 4.989275271 6.561799515 2.691100317 2.689804221 3.127706323
ENSG00000169855 2055.920898 1522.053773 2185.37946 1324.034097 1069.216057 1269.692882 1921.118044 1220.981012 0.653822941 0.000157621 0.002388345 ROBO1 3 78597240 79767815 - 10875 protein_coding roundabout guidance receptor 1 [Source:HGNC Symbol;Acc:HGNC:10249] - 1777 1642 2235 1318 1170 1294 8.936199893 6.656989089 9.520301835 5.859861631 4.688297839 5.554577704
ENSG00000074695 3564.598923 2856.863416 3522.029895 2004.133553 2195.091426 2523.686316 3314.497411 2240.970432 0.564465942 0.00015764 0.002388345 LMAN1 18 59327823 59359962 - 6767 protein_coding "lectin, mannose binding 1 [Source:HGNC Symbol;Acc:HGNC:6631]" - 3081 3082 3602 1995 2402 2572 24.89947754 20.08031013 24.65755371 14.25436713 15.46804491 17.74274552
ENSG00000189052 17.35442514 15.75816939 15.64477466 0 0 0 16.2524564 0 6.515156856 0.000160838 1 CGB5 19 49043884 49045311 + 841 protein_coding chorionic gonadotropin subunit beta 5 [Source:HGNC Symbol;Acc:HGNC:16452] - 15 17 16 0 0 0 0.975416226 0.891224021 0.881305423 0 0 0
ENSG00000152661 7100.273804 5527.409653 6914.012601 3819.4064 4797.764356 4703.9472 6513.898686 4440.372652 0.552666198 0.000165082 0.002498628 GJA1 6 121435577 121449727 + 3413 protein_coding gap junction protein alpha 1 [Source:HGNC Symbol;Acc:HGNC:4274] - 6137 5963 7071 3802 5250 4794 98.33645493 77.03045782 95.97254349 53.86132597 67.03191286 65.57039158
ENSG00000271590 30.08100357 32.44328993 27.37835565 0 0 3.924862078 29.96754972 1.308287359 4.529985881 0.000166701 0.002520635 AC108463.3 2 111210995 111212476 - 1482 lincRNA novel transcript - 26 35 28 0 0 4 0.959444499 1.041247085 0.875210025 0 0 0.125996309
ENSG00000108963 922.0984555 1120.683929 892.729954 1697.737195 1240.107854 1745.582409 978.5041129 1561.142486 -0.673363984 0.000166888 0.002520965 DPH1 17 2030110 2043430 + 5757 protein_coding diphthamide biosynthesis 1 [Source:HGNC Symbol;Acc:HGNC:3003] - 797 1209 913 1690 1357 1779 7.571062035 9.258998833 7.346441149 14.19357156 10.27169738 14.42532991

ENSG00000182752 205.9391783 119.5766972 68.44588913
317.4467182 358.2330719 295.3458714 131.3205882
323.6752205 -1.305939039 0.000169009 0.00254898 PAPPA
9 116153804 116402322 + 11573
protein_coding pappalysin 1 [Source:HGNC Symbol;Acc:HGNC:8602] -
178 129 70 316 392 301 0.841140922
0.491448162 0.280191233 1.320207957 1.476041988
1.214133881
ENSG00000132394 834.1693682 907.4851669 766.5939583
1253.713621 1247.418733 1239.275201 836.0828311
1246.802518 -0.576352375 0.000169076 0.00254898 EEFSEC
3 128153454 128408646 + 2421
protein_coding "eukaryotic elongation factor, selenocysteine-tRNA
specific [Source:HGNC Symbol;Acc:HGNC:24614]" - 721 979
784 1248 1365 1263 16.28677843 17.82878962
15.00112152 24.92418765 24.56950799 24.35311934
ENSG00000212907 12325.11273 9612.483331 13897.44889
18226.06268 16392.81848 17628.51802 11945.01498
17415.79973 -0.544020567 0.000172229 0.002593951 MT-
ND4L MT 10470 10766 + 297 protein_coding
mitochondrially encoded NADH:ubiquinone oxidoreductase core subunit 4L
[Source:HGNC Symbol;Acc:HGNC:7460] - 10653 10370 14213
18143 17938 17966 1961.598815 1539.416953
2216.826797 2953.614883 2631.941113 2823.845827
ENSG00000149639 2173.930989 1903.030692 3637.410108
1583.215278 1215.433637 1612.137098 2571.457263
1470.262004 0.806726119 0.000172923 0.002601844 SOGA1
20 36777442 36863686 - 15128
protein_coding "suppressor of glucose, autophagy associated 1
[Source:HGNC Symbol;Acc:HGNC:16111]" - 1879 2053 3720
1576 1330 1643 6.792661565 5.983308442
11.39105319 5.037045963 3.831146178 5.069931405
ENSG00000145604 1167.374331 1053.943447 1390.429348
789.5984825 679.9117488 830.1083295 1203.915709
766.5395203 0.65152318 0.000173095 0.002601879 SKP2
5 36151989 36184319 + 3837
protein_coding S-phase kinase associated protein 2 [Source:HGNC
Symbol;Acc:HGNC:10901] - 1009 1137 1422 786 744
846 14.38116535 13.06479572 17.16762637
9.904486476 8.449669729 10.29258826
ENSG00000228624 3.470885027 0.926951141 0 25.11445555
20.1049173 43.17348286 1.465945389 29.46428524
-4.395147731 0.000173499 0.002605383 HDAC2-AS2
6 113969701 114471705 + 8063
antisense HDAC2 and HS3ST5 antisense RNA 2 [Source:HGNC
Symbol;Acc:HGNC:43590] - 3 1 0 25 22
44 0.020347887 0.005468111 0 0.149914819
0.118900757 0.254742879
ENSG00000110203 11.56961676 24.10072966 2.933395248
43.19686355 102.3523063 86.34696571 12.86791389

77.29871184	-2.581023547	0.000173956	0.002608608	FOLR3
	11	72114869	72139892	+	1396
protein_coding	folate receptor 3 [Source:HGNC Symbol;Acc:HGNC:3795]
	-	10	26	3	43	112	88
0.391750261	0.821148847	0.099549319	1.489307079
3.496159215	2.942681708
ENSG00000128791 1943.695615	1395.061467	1422.696696
847.8640194	1145.980286	958.6475625	1587.151259
984.1639561	0.688599251	0.000174225	0.002608608	TWSG1
	18	9334767 9402420 +	4435	protein_coding
twisted gastrulation BMP signaling modulator 1 [Source:HGNC
Symbol;Acc:HGNC:12429]	-	1680	1505	1455	844	1254
	977	20.71621312	14.96155846	15.19748855
9.201317971	12.32146961	10.28364262
ENSG00000104907 1538.759029	1459.948047	1333.71704
2431.079297	1845.996952	2177.317238	1444.141372
2151.464496	-0.575156867	0.000174226	0.002608608	TRMT1
	19	13104902	13117567	-	3364
protein_coding	tRNA methyltransferase 1 [Source:HGNC
Symbol;Acc:HGNC:25980]	-	1330	1575	1364	2420	2020
	2219	21.62172635	20.64232108	18.78282183
34.78248411	26.16700275	30.79267023
ENSG00000072274 9126.113698	9134.176542	11652.42373
7393.695714	6100.928542	7189.366111	9970.904655
6894.663456	0.532325766	0.000175569	0.002626143	TFRC
	3	196027183	196082189	-	7887
protein_coding	transferrin receptor [Source:HGNC
Symbol;Acc:HGNC:11763]	-	7888	9854	11917	7360	6676
	7327	54.69527042	55.08516759	69.99347478
45.1198025	36.88612935	43.36710051
ENSG00000164054 2787.120677	3418.595807	3028.241695
4048.450235	4698.15363	4374.258786	3077.98606
4373.620883	-0.506631514	0.000176141	0.002632131	SHISA5
	3	48467798	48504826	-	4664
protein_coding	shisa family member 5 [Source:HGNC
Symbol;Acc:HGNC:30376]	-	2409	3688	3097	4030	5141
	4458	28.24704169	34.86312625	30.75991418
41.77800725	48.03387901	44.61979372
ENSG00000136167 3157.348413	3904.318205	3069.309228
5011.84075	4848.94051	4609.75051	3376.991949
4823.51059	-0.5141357	0.000177185	0.002640961	LCP1
	13	46125920	46211871	-	5061
protein_coding	lymphocyte cytosolic protein 1 [Source:HGNC
Symbol;Acc:HGNC:6528]	-	2729	4212	3139	4989	5306
	4698	29.48912668	36.69323338	28.73144272
47.66267174	45.68667017	43.33339454
ENSG00000137834 247.5897986	292.9165605	534.8557336
164.7508284	116.9740643	183.4873021	358.4540309
155.0707316	1.210428183	0.000177186	0.002640961	SMAD6
	15	66702228	66782848	+	8563

protein_coding SMAD family member 6 [Source:HGNC
Symbol;Acc:HGNC:6772]   MH1   214   316   547   164   128
        187   1.366729417   1.627028261   2.959128591
0.926017343   0.651392304   1.019440066
ENSG00000157557 2091.78671   1977.186783   2455.251823
1446.59264   1582.805308   1437.480736   2174.741772
1488.959561   0.546467962   0.000177563   0.002640961   ETS2
        21   38805307   38824955   +   4595
protein_coding "ETS proto-oncogene 2, transcription factor
[Source:HGNC Symbol;Acc:HGNC:3489]"   ETS   1808   2133   2511
        1440   1732   1465   21.51828341   20.4662982
25.3141677   15.15228718   16.42558968   14.88326261
ENSG00000148356 358.6581195   345.7527755   395.0305601
738.3649932   607.7168185   528.875165   366.480485
624.9856589   -0.769892107   0.000177584   0.002640961   LRSAM1
        9   127451486   127503501   +   4365
protein_coding leucine rich repeat and sterile alpha motif containing
1 [Source:HGNC Symbol;Acc:HGNC:25135]   -   310   373   404
        735   665   539   3.883936837   3.767545811
4.287454981   8.141497756   6.638897982   5.764352794
ENSG00000149948 3274.201542   3685.557736   2612.677368
4659.233794   4831.577172   4536.159347   3190.812215
4675.656771   -0.551229719   0.000177596   0.002640961   HMGA2
        12   65824131   65966295   +   15403
protein_coding high mobility group AT-hook 2 [Source:HGNC
Symbol;Acc:HGNC:5009]   HMGI/HMGY   2830   3976   2672   4638
        5287   4623   10.04791223   11.38085836   8.035882801
        14.55883571   14.95762334   14.0108542
ENSG00000067704 5576.555277   5110.281639   5307.489803
3946.987834   3970.721167   3877.763733   5331.44224
3931.824245   0.439225332   0.000178085   0.002645662   IARS2
        1   220094102   220148041   +   4165
protein_coding "isoleucyl-tRNA synthetase 2, mitochondrial
[Source:HGNC Symbol;Acc:HGNC:29685]"   -   4820   5513   5428
        3929   4345   3952   63.2887831   58.35887949
60.37084978   45.61085775   45.46041257   44.29431257
ENSG00000139117 635.17196   507.042274   477.1656271
299.3643102   322.5925367   317.9138283   539.793287
313.2902251   0.783764057   0.000178278   0.002645958   CPNE8
        12   38646822   38907430   -   5178
protein_coding copine 8 [Source:HGNC Symbol;Acc:HGNC:23498]   -
        549   547   488   298   353   324   5.798357802
        4.657568364   4.365763762   2.782629817   2.970785237
        2.920982802
ENSG00000125650 99.49870411   168.7051076   141.7807703
359.6390035   259.5362052   265.9094058   136.6615274
295.0282048   -1.106634348   0.000178893   0.002652505   PSPN
        19   6375148 6379058 -   3911   protein_coding
persephin [Source:HGNC Symbol;Acc:HGNC:9579]   -   86   182
        145   358   284   271   1.202556107   2.051717345

1.717444225 4.425847313 3.164383577 3.234651604
ENSG00000125170 137.6784394 214.1257135 162.3145371
255.1628684 409.4092251 418.9790268 171.3728967
361.1837068 -1.073762283 0.000179202 0.002654511 DOK4
16 57471922 57487327 - 4788
protein_coding docking protein 4 [Source:HGNC Symbol;Acc:HGNC:19868]
- 119 231 166 254 448 427
1.35921304 2.127119045 1.606040165 2.564961149
4.077392033 4.16312805
ENSG00000276043 4823.373226 6003.862539 5973.370524
3793.287366 3370.315228 4345.803536 5600.202097
3836.46871 0.545924702 0.000179874 0.002661885 UHRF1
19 4903080 4962154 + 5145 protein_coding
ubiquitin like with PHD and ring finger domains 1 [Source:HGNC
Symbol;Acc:HGNC:12556] - 4169 6477 6109 3776 3688
4429 44.3140281 55.50376912 55.00310438
35.48524622 31.23662695 40.18521877
ENSG00000145241 715.0023156 633.1076292 701.0814644
427.9503226 461.4992381 331.6508456 683.0638031
407.0334688 0.746251763 0.000180337 0.002666163 CENPC
4 67468748 67545606 - 8613
protein_coding centromere protein C [Source:HGNC
Symbol;Acc:HGNC:1854] - 618 683 717 426 505
338 3.923997668 3.496231766 3.856267607
2.391422816 2.555027205 1.831927529
ENSG00000111237 915.1566855 780.4928606 814.5060807
506.3074239 590.3534808 476.8707425 836.7185422
524.5105491 0.672905147 0.000181361 0.002678711 VPS29
12 110491097 110502117 - 3211
protein_coding "VPS29, retromer complex component [Source:HGNC
Symbol;Acc:HGNC:14340]" - 791 842 833 504 646
486 13.47196329 11.56127533 12.01730688
7.589120478 8.766996036 7.065485338
ENSG00000169764 1588.508381 1279.192574 1269.182344
603.7515114 973.2607694 900.7558469 1378.9611
825.9227093 0.738740388 0.000182817 0.002697615 UGP2
2 63840940 63891562 + 6501
protein_coding UDP-glucose pyrophosphorylase 2 [Source:HGNC
Symbol;Acc:HGNC:12527] - 1373 1380 1298 601 1065
918 11.55008244 9.359073939 9.249046912
4.469876484 7.138846429 6.591868741
ENSG00000237765 542.6150259 433.8131339 381.3413823
292.3322626 228.4649694 188.3933797 452.5898474
236.3968706 0.936432197 0.000183008 0.002697821
FAM200B 4 15681662 15705565 + 4817
protein_coding family with sequence similarity 200 member B
[Source:HGNC Symbol;Acc:HGNC:27740] - 469 468 390
291 250 192 5.324648077 4.283543334
3.750510766 2.920905854 2.261632103 1.86067541
ENSG00000166886 497.4935206 853.7220007 687.3922866

1078.91701	1131.358528	1227.500615	679.5359359
1145.925385	-0.752440648	0.000184428	0.002716132	NAB2
	12	57088894	57095476	+	3010
protein_coding	NGFI-A binding protein 2 [Source:HGNC
Symbol;Acc:HGNC:7627]	-	430	921	703	1074	1238
	1251	7.81261949	13.49047038	10.81910374
17.25198223	17.92308427	19.40156888
ENSG00000064666 3092.558559	3832.942967	4559.474015
2202.035463	2491.182026	2813.144894	3828.32518
2502.120794	0.613760538	0.000184812	0.002719174	CNN2
	19	1026581 1039068 +	3645	protein_coding
calponin 2 [Source:HGNC Symbol;Acc:HGNC:2156]	-	2673	4135
	4663	2192	2726	2867	40.10478005	50.01634353
	59.26118597	29.07664095	32.59019182	36.71776146
ENSG00000196141 2567.297958	2362.798458	2705.568218
1826.323208	1742.730786	1849.591254	2545.221545
1806.215083	0.494798238	0.000185119	0.002721068
SPATS2L 2	200305881	200482263	+	10437
protein_coding	spermatogenesis associated serine rich 2 like
[Source:HGNC Symbol;Acc:HGNC:24574]	-	2219	2549	2767
	1818	1907	1885	11.62723182	10.76782801
12.28106221	8.42208096	7.962209626	8.431050816
ENSG00000205809 139.9923628	104.7454789	122.224802
46.21059821	40.20983461	45.1359139	122.3208812
43.85211557	1.479354083	0.00018566	0.002726403	KLRC2
	12	10426854	10442300	-	1499
protein_coding	killer cell lectin like receptor C2 [Source:HGNC
Symbol;Acc:HGNC:6375]	-	121	113	125	46	44
	46	4.414468785	3.323615446	3.86287661
1.483738669	1.279115146	1.432525082
ENSG00000172164 369.0707746	339.2641176	411.6531332
204.9339573	214.7570712	198.2055349	373.3293418
205.9655211	0.857773378	0.000187089	0.002744745	SNTB1
	8	120535745	120813273	-	6868
protein_coding	syntrophin beta 1 [Source:HGNC Symbol;Acc:HGNC:11168]
	-	319	366	421	204	235	202
2.54012512	2.349550354	2.839581023	1.436154282
1.491063618	1.372989193
ENSG00000181649 317.0074991	442.1556942	540.7225241
155.7096244	161.7531983	286.5149317	433.2952392
201.3259181	1.106987524	0.000188215	0.002758624	PHLDA2
	11	2928273 2929455 -	955	protein_coding
pleckstrin homology like domain family A member 2 [Source:HGNC
Symbol;Acc:HGNC:12385]	-	274	477	553	155	177
	292	15.69068501	22.02160485	26.8240417
7.847467813	8.076598473	14.27333687
ENSG00000136231 1406.865398	1342.225252	1433.452478
1004.578222	965.9498904	885.0563986	1394.181043
951.8615037	0.550525124	0.000190149	0.002784304
IGF2BP3 7	23310209	23470467	-	8511

protein_coding insulin like growth factor 2 mRNA binding protein 3
[Source:HGNC Symbol;Acc:HGNC:28868] - 1216 1448 1466
1000 1057 902 7.813537434 7.501047737
7.979135415 5.68094554 5.411940267 4.947342564
ENSG00000102554 264.9442237 279.0122934 205.3376674
414.8908057 575.7317228 408.1856561 249.7647282
466.2693949 -0.901269754 0.000190433 0.002785791 KLF5
13 73054976 73077542 + 3893
protein_coding Kruppel like factor 5 [Source:HGNC
Symbol;Acc:HGNC:6349] zf-C2H2 229 301 210 413 630
416 3.216960967 3.408914038 2.498833657
5.129403507 7.052039618 4.988327548
ENSG00000186111 1420.748938 1510.003408 1989.819777
2515.463868 2336.739707 2564.897368 1640.190708
2472.366981 -0.591667047 0.000193705 0.002830939
PIP5K1C 19 3630183 3700479 - 6963 protein_coding
phosphatidylinositol-4-phosphate 5-kinase type 1 gamma [Source:HGNC
Symbol;Acc:HGNC:8996] - 1228 1629 2035 2504 2557
2614 9.644876797 10.31474859 13.53849766
17.38758019 16.00268773 17.5248869
ENSG00000175040 1211.338874 1828.874601 1740.481181
2237.1957 3312.742056 2320.574704 1593.564885
2623.504153 -0.718819365 0.000196875 0.002874522 CHST2
3 143119331 143124014 + 4582
protein_coding carbohydrate sulfotransferase 2 [Source:HGNC
Symbol;Acc:HGNC:1970] - 1047 1973 1780 2227 3625
2365 12.49644004 18.98479703 17.99564318
23.4999181 34.47557544 24.09473177
ENSG00000078269 2959.507966 2669.619286 3012.59692
2140.756191 2042.476826 2000.698444 2880.574724
2061.310487 0.4827044 0.000199754 0.002913775 SYNJ2
6 157981887 158099176 + 12493
protein_coding synaptojanin 2 [Source:HGNC Symbol;Acc:HGNC:11504]
- 2558 2880 3081 2131 2235 2039
11.19769187 10.16388414 11.42424252 8.24741648
7.795956106 7.618974528
ENSG00000114315 281.1416872 230.8108341 436.0980936
137.6272164 162.6670582 140.3138193 316.0168716
146.8693646 1.105174556 0.000202338 0.002948662 HES1
3 194136145 194138732 + 2062
protein_coding hes family bHLH transcription factor 1 [Source:HGNC
Symbol;Acc:HGNC:5192] bHLH 243 249 446 137 178
143 6.444842751 5.324080877 10.01956008
3.212425929 3.761750082 3.237378009
ENSG00000049860 3038.18136 3063.57352 3010.641323
2237.1957 2095.480699 2277.401221 3037.465401
2203.359207 0.463239788 0.00020308 0.002956644 HEXB
5 74640023 74722647 + 5067
protein_coding hexosaminidase subunit beta [Source:HGNC
Symbol;Acc:HGNC:4879] - 2626 3305 3079 2227 2293

2321	28.34252447	28.75772441	28.14888783
21.25056734	19.72021966	21.38308055
ENSG00000122862 809.873173	905.6312646	765.6161599
447.0373088	591.2673407	498.4574839	827.0401992
512.2540445	0.690803124	0.00020426	0.002971008	SRGN
10	69088106	69104811	+	1220
protein_coding	serglycin [Source:HGNC Symbol;Acc:HGNC:9361]	-
700	977	783	445	647	508	31.37855369
35.3076401	29.73064883	17.63605306	23.11016508
19.43792569
ENSG00000196154 5971.079208	6671.267361	8162.661178
2656.104819	4357.283896	5308.37596	6935.002582
4107.254892	0.755726667	0.000205461	0.002985636	S100A4
1	153543613	153550136	-	1025
protein_coding	S100 calcium binding protein A4 [Source:HGNC
Symbol;Acc:HGNC:10494]	-	5161	7197	8348	2644	4768
5410	275.3624432	309.5719446	377.2776088
124.7207753	202.7080422	246.3879338
ENSG00000143153 573.8529912	467.183375	534.8557336
319.4558746	332.6449954	262.9657592	525.2973666
305.0222097	0.783334518	0.000207305	0.003009558	ATP1B1
1	169105697	169132722	+	2962
protein_coding	ATPase Na+/K+ transporting subunit beta 1 [Source:HGNC
Symbol;Acc:HGNC:804]	-	496	504	547	318	364
268	9.157803805	7.502041119	8.554698894
5.190907408	5.355190532	4.223726376
ENSG00000177363 91.39997238	229.8838829	141.7807703
349.5932213	320.764817	377.767975	154.3548752
349.3753377	-1.174334929	0.000208663	0.003025025
LRRN4CL 11	62686402	62689899	-	2585
protein_coding	LRRN4 C-terminal like [Source:HGNC
Symbol;Acc:HGNC:33724]	-	79	248	145	348	351
385	1.671326336	4.229851215	2.598423352
6.509084552	5.917049014	6.952583572
ENSG00000138071 9038.184611	8074.671388	8452.089509
6421.263995	6271.820339	6326.87767	8521.648503
6339.987334	0.426580489	0.000208766	0.003025025	ACTR2
2	65227753	65271253	+	4347
protein_coding	ARP2 actin related protein 2 homolog [Source:HGNC
Symbol;Acc:HGNC:169]	-	7812	8711	8644	6392	6863
6448	98.28048866	88.35117538	92.1144098
71.09651984	68.79913159	69.24388468
ENSG00000230838 24.29619519	4.634755704	21.51156516	0
0	0	16.81417202	0	6.561924333
0.000208773	1	LINC01614	2	215718043
215719424	+	648	lincRNA long intergenic non-protein
coding RNA 1614 [Source:HGNC Symbol;Acc:HGNC:51847]	-	21
5	22	0	0	0	1.772307199
0.340195807	1.572715369	0	0	0
ENSG00000187498 2942.153541	1999.433611	6012.482461

1202.480132	706.4136852	927.2486659	3651.356538
945.3808276	1.94953022	0.0002099	0.003038586	COL4A1
	13	110148963	110307233	-	14738
protein_coding	collagen type IV alpha 1 chain [Source:HGNC
Symbol;Acc:HGNC:2202]	-	2543	2157	6149	1197	773
	945	9.436316973	6.45276057	19.32717578
3.926963048	2.28559638	2.993224506
ENSG00000128272 5141.537687	5577.465014	4738.411125
6847.205161	7245.994968	7025.503119	5152.471275
7039.56775	-0.450199109	0.000211175	0.003054149	ATF4
	22	39519695	39522685	+	2514
protein_coding	activating transcription factor 4 [Source:HGNC
Symbol;Acc:HGNC:786]	TF_bZIP 4444	6017	4846	6816	7929
	7160	96.67262017	105.5233806	89.2936534
131.088781	137.4395556	132.9516663
ENSG00000185269 87.92908735	49.12841046	159.3811418
11.05036044	14.62175804	37.28618974	98.81287988
20.98610274	2.235927122	0.000212247	0.003063951	NOTUM
	17	81952507	81961840	-	3019
protein_coding	"notum, palmitoleoyl-protein carboxylesterase
[Source:HGNC Symbol;Acc:HGNC:27106]"	-	76	53	163
	11	16	38	1.376718638	0.774010255
2.501076336	0.176169527	0.230948671	0.587579343
ENSG00000140199 945.2376891	932.5128477	771.4829504
1324.034097	1373.531396	1268.711667	883.077829
1322.092386	-0.582555985	0.000212254	0.003063951
SLC12A6 15	34229996	34338060	-	10056
protein_coding	solute carrier family 12 member 6 [Source:HGNC
Symbol;Acc:HGNC:10914]	-	817	1006	789	1318	1503
	1293	4.443155416	4.410691412	3.634579681
6.337111698	6.513168498	6.002322086
ENSG00000075618 9558.817365	10424.49253	8588.003489
11647.07991	13787.40397	15368.77868	9523.771128
13601.08752	-0.514113093	0.000212751	0.003068237	FSCN1
	7	5592823 5606655 +	3692	protein_coding	fascin
actin-bundling protein 1 [Source:HGNC Symbol;Acc:HGNC:11148]	-
	8262	11246	8783	11594	15087	15663	122.3821873
	134.2982473	110.2005206	151.8353239	178.0737054
	198.0429065
ENSG00000185090 350.5593877	636.8154338	402.8529475
805.6717341	825.2154693	852.6762864	463.4092563
827.8544966	-0.835354451	0.000213098	0.003070334	MANEAL
	1	37793802	37801137	+	3018
protein_coding	mannosidase endo-alpha like [Source:HGNC
Symbol;Acc:HGNC:26452]	-	303	687	412	802	903
	869	5.490578509	10.03624974	6.323833638
12.84861599	13.03848443	13.44146411
ENSG00000103047 667.5668869	803.6666391	674.6809072
1080.926167	1105.770452	1067.562485	715.3048111
1084.753035	-0.600132571	0.0002144	0.003086196	TANGO6

16	68843604	69085180	+	6839
protein_coding	transport and golgi organization 6 homolog
[Source:HGNC Symbol;Acc:HGNC:25749]	-	577	867	690
1076	1210	1088	4.614003527	5.589339011
4.673679669	7.607130806	7.709946498	7.426468229
ENSG00000111843 3183.958532	2915.261338	3323.536817
1984.041988	2291.960573	2366.691833	3140.918895
2214.231465	0.504233113	0.000218308	0.003139482
TMEM14C 6	10722915	10731129	+	1321
protein_coding	transmembrane protein 14C [Source:HGNC
Symbol;Acc:HGNC:20952]	-	2752	3145	3399	1975	2508
2412	113.9305843	104.9667595	119.1930143
72.28788175	82.7338648	85.23550168
ENSG00000149016 503.2783289	674.8204305	532.9001368
922.2028078	961.380591	848.7514243	570.3329654
910.7782744	-0.674323808	0.000218889	0.003144878	TUT1
11	62575045	62592177	-	4082
protein_coding	"terminal uridylyl transferase 1, U6 snRNA-specific
[Source:HGNC Symbol;Acc:HGNC:26184]"	-	435	728	545
918	1052	865	5.827884946	7.86307353
6.184804236	10.87353852	11.23055805	9.892114689
ENSG00000107984 122.6379376	136.2618177	229.7826278
66.30216265	53.91773277	63.77900877	162.8941277
61.33296806	1.410964417	0.000221246	0.003175295	DKK1
10	52314296	52318042	+	2206
protein_coding	dickkopf WNT signaling pathway inhibitor 1
[Source:HGNC Symbol;Acc:HGNC:2891]	-	106	147	235
66	59	65	2.62781671	2.937959364
4.934746071	1.446570632	1.165480741	1.375478747
ENSG00000168569 764.7516676	904.7043135	771.4829504
1081.930745	1483.194581	1249.087356	813.6463105
1271.404227	-0.643767438	0.000221421	0.003175295
TMEM223 11	62771629	62792021	-	2280
protein_coding	transmembrane protein 223 [Source:HGNC
Symbol;Acc:HGNC:28464]	-	661	976	789	1077	1623
1273	15.85482034	18.87334717	16.03040933
22.83926233	31.02003408	26.06391151
ENSG00000155755 1198.612296	1036.331375	966.0648352
603.7515114	721.9493032	731.9867775	1067.002836
685.895864	0.636798091	0.000222057	0.003181431
TMEM237 2	201620184	201643570	-	7647
protein_coding	transmembrane protein 237 [Source:HGNC
Symbol;Acc:HGNC:14432]	-	1036	1118	988	601	790
746	7.409064541	6.44591644	5.985057267
3.800008765	4.501887094	4.554007831
ENSG00000177602 380.6403913	386.5386257	461.5208524
195.8927533	239.4312879	246.2850954	409.5666232
227.2030455	0.850050178	0.000222326	0.003182298	HASPIN
17	3723903 3726699 +	2797	protein_coding
histone H3 associated protein kinase [Source:HGNC

Symbol;Acc:HGNC:19682]	-	329	417	472	195	262
	251	6.432771786	6.573210595	7.817213765
3.370880537	4.081947575	4.189163308
ENSG00000014641 2230.622111	2405.43821	2381.916942
1593.26106	1782.026761	1606.249805	2339.325754
1660.512542	0.49448882	0.000222684	0.003184438	MDH1
	2	63588609	63607197	+	4990
protein_coding	malate dehydrogenase 1 [Source:HGNC
Symbol;Acc:HGNC:6970]	-	1928	2595	2436	1586	1950
	1637	21.13008269	22.92824293	22.61409406
15.36752237	17.02913794	15.31419489
ENSG00000267519 485.9239038	586.7600722	552.4561051
926.2211207	717.3800038	1014.576847	541.7133604
886.0593239	-0.708842355	0.00022331	0.003190416
AC020916.1	19	13823880	13842928	-	19049
	antisense	novel transcript	-	420	633
	565	922	785	1034	1.205790399	1.465093988
	1.373974656	2.340237616	1.795792166	2.533928713
ENSG00000160285 2473.584063	3265.648869	4432.36022
2090.52728	1899.000825	2296.044316	3390.531051
2095.190807	0.694744632	0.000223602	0.003191598	LSS
	21	46188141	46228824	-	7238
protein_coding	lanosterol synthase [Source:HGNC Symbol;Acc:HGNC:6708]
	-	2138	3523	4533	2081	2078	2340
16.15413972	21.45991623	29.01146073	13.90127766
12.51081385	15.091879
ENSG00000205903 1529.503335	2556.531246	2677.212063
3858.584951	3193.940272	3989.622302	2254.415548
3680.715842	-0.706666051	0.000224171	0.003196743	ZNF316
	7	6637322 6656432 +	5811	protein_coding	zinc
finger protein 316 [Source:HGNC Symbol;Acc:HGNC:13843]	-	1322
	2758	2738	3841	3495	4066	12.44157301
20.92557229	21.82654646	31.95910792	26.20926865
32.66348614
ENSG00000152767 1003.085773	755.4651798	999.3099813
552.5180221	580.3010222	599.5226824	919.286978
577.4472422	0.670214334	0.000224593	0.003199765	FARP1
	13	98142562	98455176	+	36228
protein_coding	"FERM, ARH/RhoGEF and pleckstrin domain protein 1
[Source:HGNC Symbol;Acc:HGNC:3591]"	-	867	815	1022
	550	635	611	1.308788442	0.991852763
1.306799599	0.734039696	0.763814615	0.787304778
ENSG00000132031 82.14427898	41.71280134	63.55689705
10.04578222	17.36333767	1.962431039	62.47132579
9.790516977	2.665972544	0.00022496	0.003202016	MATN3
	2	19992111	20012694	-	3118
protein_coding	matrilin 3 [Source:HGNC Symbol;Acc:HGNC:6909]	-
	71	45	65	10	19	2	1.245308495
	0.636312362	0.965694375	0.155069043	0.265543752
	0.029943318

ENSG00000151693 610.8757648 449.5713033 589.6124449
317.4467182 281.4688422 350.2939405 550.0198377
316.403167 0.797285082 0.000227466 0.003234671 ASAP2
2 9206765 9405683 + 7257
protein_coding "ArfGAP with SH3 domain, ankyrin repeat and PH domain
2 [Source:HGNC Symbol;Acc:HGNC:2721]" - 528 485 603
316 308 357 3.978977764 2.946582285
3.849130582 2.105383311 1.849490867 2.296450713
ENSG00000225697 643.2706917 647.0118963 878.0629777
467.1288732 356.4053522 449.3967079 722.7818552
424.3103111 0.769214127 0.000231231 0.003285154
SLC26A6 3 48625723 48635493 - 6373
protein_coding solute carrier family 26 member 6 [Source:HGNC
Symbol;Acc:HGNC:14472] - 556 698 898 465 390
458 4.771177633 4.828869426 6.527319543
3.527851135 2.666731472 3.354807424
ENSG00000051825 613.1896881 461.6216681 840.9066379
370.6893639 307.0569188 363.0497422 638.5726647
346.9320083 0.880376648 0.000233093 0.00330853
MPHOSPH9 12 123152320 123244014 - 10304
protein_coding M-phase phosphoprotein 9 [Source:HGNC
Symbol;Acc:HGNC:7215] - 530 498 860 369 336
370 2.812967615 2.130872432 3.866295616
1.731496957 1.420993283 1.676262039
ENSG00000109472 894.3313753 680.3821374 796.9057092
424.9365879 408.4953652 574.0110789 790.5397406
469.1476773 0.75239073 0.000236477 0.003353449 CPE
4 165361194 165498320 + 2958
protein_coding carboxypeptidase E [Source:HGNC Symbol;Acc:HGNC:2303]
- 773 734 815 423 447 585
14.29144154 10.94036593 12.76326819 6.914223505
6.58518456 9.23216871
ENSG00000196586 1291.16923 1256.945747 1155.757728
906.1295563 827.9570489 718.2497603 1234.624235
817.4454552 0.594777635 0.000236988 0.003357586 MYO6
6 75749192 75919537 + 8785
protein_coding myosin VI [Source:HGNC Symbol;Acc:HGNC:7605] -
1116 1356 1182 902 906 732 6.947317411
6.805372185 6.232727886 4.964391098 4.494123777
3.889693231
ENSG00000273319 13.88354011 12.97731597 19.55596832 0
0 0 15.4722748 0 6.444776214
0.000238037 1 AC058791.1 7 130936464
130939661 + 3198 lincRNA novel transcript -
12 14 20 0 0 0 0.205209518
0.193011655 0.289703667 0 0 0
ENSG00000054654 988.0452711 793.4701766 1434.430277
588.6828381 541.9189073 703.5315275 1071.981908
611.3777576 0.810304956 0.000239722 0.003393177 SYNE2
14 63852983 64226433 + 31374

protein_coding spectrin repeat containing nuclear envelope protein 2
[Source:HGNC Symbol;Acc:HGNC:17084]   -   854   856   1467
        586   593   717   1.48861603   1.202923005
2.166021073   0.903085648   0.82365132   1.066830195
ENSG00000104228 1008.870581   1192.059167   935.7530843
1464.675048   1572.752849   1629.798978   1045.560944
1555.742292   -0.573015031   0.000240405   0.003399691   TRIM35
        8   27284887   27311319   -   4269
protein_coding tripartite motif containing 35 [Source:HGNC
Symbol;Acc:HGNC:16285] -   872   1286   957   1458   1721
        1661   11.17081972   13.28155031   10.38456332
16.51325113   17.56763555   18.16308074
ENSG00000148300 923.2554172   1348.71391   1264.293352
1877.556697   1729.022888   1828.004513   1178.754226
1811.528033   -0.618984043   0.00024121   0.003407934   REXO4
        9   133406059   133418096   -   2654
protein_coding "REX4 homolog, 3'-5' exonuclease [Source:HGNC
Symbol;Acc:HGNC:12820]" -   798   1455   1293   1869   1892
        1863   16.44359174   24.17107872   22.56836318
34.04941066   31.06553314   32.76860642
ENSG00000118473 37.02277362   27.80853423   42.04533189
3.013734666   6.397019142   1.962431039   35.62554658
3.791061616   3.221964246   0.000243496   0.003437055   SGIP1
        1   66533383   66748299   +   10477
protein_coding SH3 domain GRB2 like endophilin interacting protein 1
[Source:HGNC Symbol;Acc:HGNC:25412]   -   32   30   43
        3   7   2   0.167035102   0.126246187
0.190122697   0.013844763   0.029115194   0.008911259
ENSG00000129911 705.7466222   1341.298301   1297.538498
1732.897433   1971.195756   2019.341539   1114.86114
1907.811576   -0.774076093   0.000243974   0.003440615   KLF16
        19   1852399 1863568 -   2955   protein_coding
Kruppel like factor 16 [Source:HGNC Symbol;Acc:HGNC:16857]   zf-
C2H2   610   1447   1327   1725   2157   2058   11.28930126
        21.58962027   20.80251738   28.22492722   31.80909093
        32.51126897
ENSG00000173692 4125.725336   3424.157514   4293.512845
3085.05972   2537.78888   2555.085213   3947.798565
2725.977937   0.534244767   0.000244283   0.003441804   PSMD1
        2   231056864   231172827   +   5187
protein_coding "proteasome 26S subunit, non-ATPase 1 [Source:HGNC
Symbol;Acc:HGNC:9554]" -   3566   3694   4391   3071   2777
        2604   37.59757234   31.3989121   39.21476754
28.62627143   23.33018752   23.43531351
ENSG00000047410 6370.230986   6174.421549   6933.568569
4867.181486   4208.324735   5012.048873   6492.740368
4695.851698   0.467514642   0.000244803   0.003445953   TPR
        1   186311652   186375693   -   12350
protein_coding "translocated promoter region, nuclear basket protein
[Source:HGNC Symbol;Acc:HGNC:12017]"   -   5506   6661   7091

4845	4605	5108	24.38169882	23.77970343
26.59763265	18.96828386	16.24879931	19.30767442
ENSG00000142546 2238.720843	2998.686941	2909.928086
3930.914583	3590.555458	4562.652166	2715.778623
4028.040736	-0.568292036	0.000246957	0.003473071	NOSIP
19	49555711	49590262	-	4248
protein_coding	nitric oxide synthase interacting protein [Source:HGNC Symbol;Acc:HGNC:17946]	-	1935	3235	2976	3913	3929
4650	24.91100071	33.57559634	32.45270295
44.53757394	40.30473949	51.09924468
ENSG00000119669 1181.257871	1748.229852	1527.321126
2684.233009	2498.492905	1893.745953	1485.60295
2358.823956	-0.666410613	0.000249085	0.003499779
IRF2BPL 14	77024543	77028699	-	4157
protein_coding	interferon regulatory factor 2 binding protein like [Source:HGNC Symbol;Acc:HGNC:14282]	-	1021	1886	1562
2672	2734	1930	13.43199218	20.00302243
17.40617962	31.07832799	28.66005922	21.67321406
ENSG00000182580 76.3594706	56.54401959	199.4708769
30.13734666	27.41579632	27.47403455	110.7914557
28.34239251	1.967769201	0.000249406	0.00350107	EPHB3
3	184561784	184582409	+	4532
protein_coding	EPH receptor B3 [Source:HGNC Symbol;Acc:HGNC:3394]
-	66	61	204	30	30	28
0.796432084	0.593436004	2.085176021	0.320060862
0.288462891	0.288412558
ENSG00000131844 1793.290597	1666.658151	2018.175931
1311.979158	1265.69593	1210.819951	1826.04156
1262.83168	0.532053938	0.000250509	0.003512343	MCCC2
5	71587288	71658704	+	6034
protein_coding	methylcrotonoyl-CoA carboxylase 2 [Source:HGNC Symbol;Acc:HGNC:6937]	-	1550	1798	2064	1306	1385
1234	14.0482137	13.13766972	15.84553266
10.4649965	10.00237345	9.54675747
ENSG00000172340 1896.260186	2028.169096	2073.910441
1234.626635	1537.112314	1370.758081	1999.446574
1380.832343	0.533991424	0.000250668	0.003512343	SUCLG2
3	67360460	67654614	-	3090
protein_coding	succinate-CoA ligase GDP-forming beta subunit [Source:HGNC Symbol;Acc:HGNC:11450]	-	1639	2188	2121
1229	1682	1397	29.00782635	31.21927377
31.79689003	19.23067906	23.72062402	21.10493226
ENSG00000130830 1077.13132	1196.693923	1305.360886
703.2047554	864.511444	783.9912001	1193.062043
783.9024665	0.605995962	0.000250983	0.003513535	MPP1
X	154778684	154821007	-	5396
protein_coding	membrane palmitoylated protein 1 [Source:HGNC Symbol;Acc:HGNC:7219]	-	931	1291	1335	700	946
799	9.43566368	10.54844054	11.46071679
6.272307125	7.639726182	6.912272871

ENSG00000129038 2173.930989 2411.926868 3068.33143
1659.563223 1623.015142 1831.929375 2551.396429
1704.835913 0.581939858 0.000254622 0.003561217 LOXL1
15 73925989 73952137 + 3637
protein_coding lysyl oxidase like 1 [Source:HGNC
Symbol;Acc:HGNC:6665] - 1879 2602 3138 1652 1776
1867 28.25388621 31.54263345 39.9679703
21.96180132 21.27934511 23.96332361
ENSG00000100985 167.759443 282.720098 939.6642779
66.30216265 68.53949081 83.40331915 463.381273
72.7483242 2.671499408 0.00025611 0.003578745 MMP9
20 46008908 46016561 + 2336
protein_coding matrix metallopeptidase 9 [Source:HGNC
Symbol;Acc:HGNC:7176] - 145 305 961 66 75
85 3.394609924 5.756532473 19.05693291
1.366067986 1.399094414 1.698603924
ENSG00000131876 866.5642951 825.9134665 928.9084954
598.7286203 564.7654042 557.3304151 873.795419
573.6081465 0.607278179 0.000258167 0.003602266 SNRPA1
15 101281510 101295282 - 3669
protein_coding small nuclear ribonucleoprotein polypeptide
A' [Source:HGNC Symbol;Acc:HGNC:11152] - 749 891 950
596 618 568 11.16423112 10.70690502
11.99439507 7.854160367 7.340055793 7.226810382
ENSG00000180182 1874.277915 1682.416321 1805.993675
1330.061566 1148.721866 1253.012218 1787.562637
1243.931883 0.523048378 0.000258264 0.003602266 MED14
X 40648306 40735858 - 9656
protein_coding mediator complex subunit 14 [Source:HGNC
Symbol;Acc:HGNC:2370] - 1620 1815 1847 1324 1257
1277 9.175135151 8.287305146 8.860782863
6.629670505 5.672788845 6.173616899
ENSG00000184678 87.92908735 86.2064561 93.86864795
258.1766031 251.3114663 132.4640951 89.33473047
213.9840548 -1.260341702 0.00025948 0.003615929
HIST2H2BE 1 149884459 149886652 - 2194
protein_coding histone cluster 2 H2B family member e
[Source:HGNC Symbol;Acc:HGNC:4760] - 76 93 96
257 275 135 1.894399986 1.868875124
2.026922136 5.663667076 5.462037385 2.872388512
ENSG00000131174 1565.369147 1699.101441 1925.285081
1012.614848 1262.040491 1205.913873 1729.918557
1160.189737 0.576390067 0.000259724 0.003616026 COX7B
X 77899438 77907373 + 3024
protein_coding cytochrome c oxidase subunit 7B [Source:HGNC
Symbol;Acc:HGNC:2291] - 1353 1833 1969 1008 1381
1229 24.46869021 26.7248106 30.16243403
16.1168425 19.90079791 18.97213175
ENSG00000144645 238.3341052 222.4682738 278.6725486
118.5402302 101.4384464 138.3513882 246.4916425

119.4433549	1.045903589	0.000260046	0.003617213
OSBPL10 3	31657890	32077580	-	11976
protein_coding	oxysterol binding protein like 10 [Source:HGNC
Symbol;Acc:HGNC:16395]	-	206	240	285	118	111
	141	0.940697838	0.883554641	1.102390669
0.476399653	0.40389622	0.549608399
ENSG00000010165 2514.077721	2473.105644	2411.250894
3459.767397	3312.742056	3300.809007	2466.144753
3357.77282	-0.445285698	0.000260837	0.003622482
METTL13 1	171781664	171814023	+	3827
protein_coding	methyltransferase like 13 [Source:HGNC
Symbol;Acc:HGNC:24248]	-	2173	2668	2466	3444	3625
	3364	31.05245756	30.73698895	29.84950035
43.51168453	41.277002	41.03397228
ENSG00000119705 1349.017314	1062.286007	1128.379372
701.195599	805.110552	807.5403725	1179.894231
771.2821745	0.612549341	0.000260898	0.003622482	SLIRP
	14	77708071	77761104	+	2631
protein_coding	SRA stem-loop interacting RNA binding protein
[Source:HGNC Symbol;Acc:HGNC:20495]	-	1166	1146	1154
	698	881	823	24.23664017	19.20426665
20.31830225	12.82731592	14.59196093	14.6024263
ENSG00000066044 3955.651969	3935.834544	4275.912474
2825.878539	3002.029697	3084.941593	4055.799662
2970.949943	0.449066413	0.000262679	0.003643896	ELAVL1
	19	7958579 8005659 -	6956	protein_coding	ELAV
like RNA binding protein 1 [Source:HGNC Symbol;Acc:HGNC:3312]	-
	3419	4246	4373	2813	3285	3144	26.88030797
	26.91252055	29.12207794	19.55290883	20.57948007
	21.09934628
ENSG00000177839 17.35442514	16.68512054	23.46716199
89.40746176	86.81668835	51.02320701	19.16890255
75.74911904	-1.98159224	0.000264151	0.003660989	PCDHB9
	5	141187127	141191541	+	4415
protein_coding	protocadherin beta 9 [Source:HGNC
Symbol;Acc:HGNC:8694]	-	15	18	24	89	95
	52	0.185804088	0.179752838	0.251815808
0.974676545	0.937673182	0.549817643
ENSG00000074410 3551.872344	4428.972551	2451.340629
4377.951892	6705.90378	6269.967169	3477.395175
5784.607614	-0.734211379	0.000268183	0.003713506	CA12
	15	63321378	63382161	-	7313
protein_coding	carbonic anhydrase 12 [Source:HGNC
Symbol;Acc:HGNC:1371]	-	3070	4778	2507	4358	7338
	6390	22.95818308	28.80610437	15.88039192
28.81329124	43.7260988	40.78977602
ENSG00000172172 1202.083181	1233.771968	975.8428193
556.536335	700.0166661	841.8829157	1137.232656
699.4786389	0.700958861	0.000269345	0.003726228	MRPL13
	8	120380761	120445402	-	4209

protein_coding mitochondrial ribosomal protein L13 [Source:HGNC Symbol;Acc:HGNC:14278] - 1039 1331 998 554 766
858 13.49992434 13.94225712 10.9838368
6.364027615 7.930643752 9.515999224
ENSG00000100353 6392.213258 6558.179321 5618.429699
8276.719971 8685.324275 8212.773898 6189.607426
8391.606048 -0.439134689 0.000270392 0.00373733 EIF3D
22 36510850 36529436 - 4070
protein_coding eukaryotic translation initiation factor 3 subunit D [Source:HGNC Symbol;Acc:HGNC:3278] - 5525 7075 5746
8239 9504 8370 74.23908078 76.64185241
65.39938559 97.8771489 101.7584812 96.00129349
ENSG00000196550 203.6252549 101.0376744 206.3154658
62.28384977 40.20983461 78.49724156 170.3261317
60.33030864 1.497464192 0.000271152 0.003744438 FAM72A
1 206186179 206204414 - 3783
protein_coding family with sequence similarity 72 member A [Source:HGNC Symbol;Acc:HGNC:24044] - 176 109 211
62 44 80 2.544315943 1.270352114
2.583738578 0.792422074 0.506844727 0.987187577
ENSG00000261221 754.3390126 1146.638561 1360.117597
1892.62537 1538.026174 2034.059772 1087.031724
1821.570439 -0.74384921 0.000271571 0.003746848 ZNF865
19 55605405 55617269 + 4005
protein_coding zinc finger protein 865 [Source:HGNC Symbol;Acc:HGNC:38705] zf-C2H2 652 1237 1391 1884 1683
2073 8.903070001 13.61761768 16.08892641
22.74466761 18.3121861 24.16255288
ENSG00000174945 128.422746 160.3625474 222.9380389
83.37999243 52.09001301 17.66187935 170.5744441
51.0439616 1.74205015 0.000272075 0.003747685 AMZ1
7 2679522 2775500 + 7011 protein_coding
archaelysin family metallopeptidase 1 [Source:HGNC Symbol;Acc:HGNC:22231] - 111 173 228 83 57
18 0.865840157 1.087927849 1.506459067
0.572399627 0.354285588 0.119850148
ENSG00000189308 903.5870687 748.0495707 841.8844363
462.1059821 602.2336592 492.5701908 831.1736919
518.969944 0.678526018 0.000272122 0.003747685 LIN54
4 82909973 83012926 - 6768
protein_coding lin-54 DREAM MuvB core complex component [Source:HGNC Symbol;Acc:HGNC:25397] - 781 807 861 460 659
502 6.310814237 5.257110948 5.893119628
3.286235616 4.24310386 3.462496977
ENSG00000132196 426.9188583 365.2187495 533.8779352
270.2315417 166.3224977 251.191173 442.005181
229.2484041 0.948043561 0.000274154 0.003772267
HSD17B7 1 162790702 162812817 + 4778
protein_coding hydroxysteroid 17-beta dehydrogenase 7 [Source:HGNC Symbol;Acc:HGNC:5215] - 369 394 546 269 182

256	4.22352368	3.635666465	5.293573568
2.722120531	1.659907321	2.501150677
ENSG00000186960 1080.602205	883.3844372	809.6170886
569.5958519	547.4020666	635.8276566	924.534577
584.2751917	0.661488924	0.000275283	0.003784394
LINC01551	14	28772704	28813453	+	2772
lincRNA long intergenic non-protein coding RNA 1551
[Source:HGNC Symbol;Acc:HGNC:19828]	-	934	953	828
567	599	648	18.42673385	15.15771136
13.83692435	9.889880623	9.416558184	10.91258943
ENSG00000108828 5217.897157	6202.230083	5907.85803
6772.866373	8806.867639	9857.291109	5775.99509
8479.008373	-0.553746322	0.000276277	0.003794652	VAT1
17	43014605	43025123	-	3501
protein_coding	vesicle amine transport 1 [Source:HGNC
Symbol;Acc:HGNC:16919]	-	4510	6691	6042	6742	9637
10046	70.44969932	84.26221614	79.94495566
93.11032744	119.9522354	133.9513512
ENSG00000156113 404.9365865	416.2010622	282.5837423
145.6638422	168.1502174	235.4917247	367.9071303
183.1019281	1.006458024	0.000277412	0.003806824	KCNMA1
10	76869601	77638369	-	35644
protein_coding	potassium calcium-activated channel subfamily M alpha
1 [Source:HGNC Symbol;Acc:HGNC:6284]	-	350	449	289
145	184	240	0.537002518	0.555384639
0.375589864	0.196690228	0.224952066	0.314319151
ENSG00000182179 411.8783566	399.5159417	645.3469547
130.5951689	233.9481286	308.1016731	485.5804176
224.2149902	1.114701415	0.000277835	0.003809202	UBA7
3	49805207	49813946	-	4545
protein_coding	ubiquitin like modifier activating enzyme 7
[Source:HGNC Symbol;Acc:HGNC:12471]	-	356	431	660
130	256	314	4.283618871	4.180972784
6.726861775	1.382963383	2.454509263	3.225089686
ENSG00000144659 1327.035042	1454.38634	1235.937198
1964.955002	2043.390686	1776.00009	1339.119527
1928.115259	-0.525824503	0.000278257	0.003811568
SLC25A38	3	39383324	39397351	+	2708
protein_coding	solute carrier family 25 member 38
[Source:HGNC Symbol;Acc:HGNC:26054]	-	1147	1569	1264
1956	2236	1810	23.16378209	25.54513732
21.62224927	34.92379312	35.98170486	31.20153431
ENSG00000101940 758.9668593	669.2587237	689.3478834
963.3905149	1107.598171	1118.585692	705.8578221
1063.19146	-0.591636699	0.000280144	0.003833148	WDR13
X	48590042	48608867	+	7884
protein_coding	WD repeat domain 13 [Source:HGNC
Symbol;Acc:HGNC:14352]	-	656	722	705	959	1212
1140	4.550424746	4.037611605	4.142332509
5.881298309	6.699071328	6.750007752

ENSG00000100413 1170.845216 1162.396731 1271.137941
1868.515493 1599.254785 1717.127159 1201.459962
1728.299146 -0.524358878 0.000280334 0.003833148 POLR3H
22 41525804 41544606 - 6594
protein_coding RNA polymerase III subunit H [Source:HGNC
Symbol;Acc:HGNC:30349] - 1012 1254 1300 1860 1750
1750 8.393175079 8.384603917 9.132651078
13.63845634 11.56505503 12.3889683
ENSG00000183527 1707.675433 1778.819239 1668.124098
1108.049779 1271.179089 1227.500615 1718.206257
1202.243161 0.515074763 0.000281951 0.003851823 PSMG1
21 39174769 39183851 - 2515
protein_coding proteasome assembly chaperone 1 [Source:HGNC
Symbol;Acc:HGNC:3043] - 1476 1919 1706 1103 1391
1251 32.0954213 33.64115851 31.42269957
21.20502259 24.10170344 23.22016792
ENSG00000206417 33.5518886 14.83121825 36.1785414 0
1.827719755 2.943646558 28.18721608 1.590455438
4.137893924 0.000282676 0.003858276 H1FX-AS1
3 129315392 129326225 + 3420
antisense H1FX antisense RNA 1 [Source:HGNC
Symbol;Acc:HGNC:27953] - 29 16 37 0 2
3 0.463731508 0.206266089 0.501161931 0
0.0254837 0.0409488
ENSG00000164171 1929.812075 1485.902679 1341.539427
2732.452764 2316.634789 2206.753703 1585.751394
2418.613752 -0.60957904 0.000283713 0.003868976 ITGA2
5 52989326 53094779 + 7910
protein_coding integrin subunit alpha 2 [Source:HGNC
Symbol;Acc:HGNC:6137] - 1668 1603 1372 2720 2535
2249 11.53225603 8.934926758 8.034892741
16.62622437 13.9656154 13.27269173
ENSG00000213442 1800.232367 1916.934959 1976.130599
2566.697357 2471.077109 3203.668671 1897.765975
2747.147712 -0.533416793 0.00028832 0.003928297
RPL18AP3 12 104265309 104265836 + 528
processed_pseudogene ribosomal protein L18a pseudogene 3
[Source:HGNC Symbol;Acc:HGNC:31387] - 1556 2068 2021
2555 2704 3265 161.1648702 172.6833916
177.3106602 233.9689351 223.1677128 288.6657771
ENSG00000076201 2352.103087 2642.737703 2973.484984
4171.008778 3270.704501 4206.470932 2656.108591
3882.72807 -0.547441761 0.000290372 0.003952731 PTPN23
3 47381011 47413441 + 5981
protein_coding "protein tyrosine phosphatase, non-receptor type 23
[Source:HGNC Symbol;Acc:HGNC:14406]" - 2033 2851 3041
4152 3579 4287 18.5890968 21.01635389
23.55293717 33.56485373 26.07633152 33.45998307
ENSG00000131018 1768.994402 1808.481676 1196.825261
2578.752296 2135.690534 2781.745998 1591.43378

2498.729609	-0.650947825	0.000291467	0.003964102	SYNE1
	6	152121684	152637801	-	46064
protein_coding	spectrin repeat containing nuclear envelope protein 1
[Source:HGNC Symbol;Acc:HGNC:17089]	-	1529	1951	1224
	2567	2337	2835	1.815267159	1.867366571
1.230898454	2.694420894	2.210831596	2.873012077
ENSG00000206053 2466.642293	2241.367859	2630.277739
1678.650209	1799.390099	1747.54484	2446.095964
1741.861716	0.489700249	0.000294658	0.004003944	JPT2
	16	1678256 1702280 +	4768	protein_coding
Jupiter microtubule associated homolog 2 [Source:HGNC
Symbol;Acc:HGNC:14137]	-	2132	2418	2690	1671	1969
	1781	24.45376117	22.35908401	26.13475836
16.94499401	17.99567193	17.43707793
ENSG00000106976 1243.733801	1265.288307	1936.040864
948.3218416	808.7659915	973.3657953	1481.687658
910.1512095	0.703481386	0.000297102	0.004033562	DNM1
	9	128191655	128255248	+	10775
protein_coding	dynamin 1 [Source:HGNC Symbol;Acc:HGNC:2972]	-
	1075	1365	1980	944	885	992	5.456144934
	5.585336337	8.512367545	4.23599981	3.57918865
	4.297742877
ENSG00000148834 2903.973806	2879.110243	2149.200919
3376.387404	4465.119361	4029.852138	2644.094989
3957.119635	-0.581882585	0.000297365	0.004033562	GSTO1
	10	104235356	104267459	+	1691
protein_coding	glutathione S-transferase omega 1 [Source:HGNC
Symbol;Acc:HGNC:13312]	-	2510	3106	2198	3361	4886
	4107	81.17547276	80.982616	60.21248294
96.10060491	125.9123857	113.3775665
ENSG00000177311 3772.852025	2160.723109	3070.287027
1860.478867	1769.232723	2042.890712	3001.287387
1890.867434	0.666177549	0.000298061	0.004039408	ZBTB38
	3	141324213	141449792	+	14243
protein_coding	zinc finger and BTB domain containing 38 [Source:HGNC
Symbol;Acc:HGNC:26636]	ZBTB	3261	2331	3140	1852	1936
	2082	12.52114478	7.215638335	10.21246614
6.286960396	5.923282915	6.823784243
ENSG00000162244 21061.33034	20972.26956	20805.5947
24973.8146	28677.83681	34677.13767	20946.3982
29442.9297	-0.491222962	0.000298938	0.00404771	RPL29
	3	51993522	51995942	-	2058
protein_coding	ribosomal protein L29 [Source:HGNC
Symbol;Acc:HGNC:10331]	-	18204	22625	21278	24860	31381
	35341	483.7446435	484.704638	478.9474771
584.059336	664.4770814	801.6402216
ENSG00000097021 2987.275047	3221.155214	3542.563662
1965.95958	2333.084267	2496.212282	3250.331308
2265.085376	0.521088114	0.000299647	0.004053714	ACOT7
	1	6264269 6394391 -	4124	protein_coding	acyl-

CoA thioesterase 7 [Source:HGNC Symbol;Acc:HGNC:24157] -   2582
        3475   3623   1957   2553   2544   34.23988474
37.15096596   40.69603829   22.94422461   26.97682011
28.79681698
ENSG00000130725 3254.533194   3984.036003   3790.924459
5162.527483   4728.311006   5569.379289   3676.497886
5153.405926   -0.486907182   0.000301821   0.004079527   UBE2M
        19   58555712   58558960   -   1831
protein_coding ubiquitin conjugating enzyme E2 M [Source:HGNC
Symbol;Acc:HGNC:12491] -   2813   4298   3877   5139   5174
        5676   84.01872768   103.4932499   98.08665235
135.7036378   123.1392993   144.7105114
ENSG00000155265 363.2859662   162.2164496   512.3663701
164.7508284   148.95916   98.12155195   345.956262
137.2771801   1.332936606   0.000302285   0.004080199
GOLGA7B 10   97850239   97871580   +   6458
protein_coding golgin A7 family member B [Source:HGNC
Symbol;Acc:HGNC:31668] -   314   175   524   164   163
        100   2.659048876   1.194741546   3.758683021
1.227854833   1.099887234   0.722849683
ENSG00000011677 503.2783289   523.7273946   491.8326033
233.0621475   314.3677978   331.6508456   506.2794423
293.0269303   0.788525873   0.000302405   0.004080199   GABRA3
        X   152166234   152451358   -   4193
protein_coding gamma-aminobutyric acid type A receptor alpha3 subunit
[Source:HGNC Symbol;Acc:HGNC:4077]   -   435   565   503
        232   344   338   5.673605139   5.940972518
5.557066302   2.675249792   3.575132795   3.763031673
ENSG00000115255 247.5897986   348.533629   179.9149086
494.2524852   433.1695819   571.0674323   258.6794454
499.4964998   -0.948203067   0.000303428   0.004090395   REEP6
        19   1490747 1497927 +   2449   protein_coding
receptor accessory protein 6 [Source:HGNC Symbol;Acc:HGNC:30078]
        -       214   376   184   492   474   582
4.778809308   6.769132541   3.480418702   9.713540027
8.434282714   11.09379753
ENSG00000144713 13821.06418   17191.23586   16446.56936
19607.35774   22055.09428   25494.92284   15819.62313
22385.79162   -0.500812039   0.00030856   0.004151381   RPL32
        3   12834485   12841588   -   3720
protein_coding ribosomal protein L32 [Source:HGNC
Symbol;Acc:HGNC:10336] -   11946   18546   16820   19518   24134
        25983   175.6201255   219.8068758   209.4524802
253.6842999   282.7124708   326.0561448
ENSG00000105355 3547.244498   3876.509671   3469.228781
4690.375719   4765.779261   5939.297539   3630.994316
5131.817506   -0.499013719   0.000308693   0.004151381   PLIN3
        19   4838341 4867768 -   2813   protein_coding
perilipin 3 [Source:HGNC Symbol;Acc:HGNC:16893] -   3066   4182
        3548   4669   5215   6053   59.60698169   65.546311

58.4273696	80.25190646	80.78731573	100.4493146
ENSG00000120699 1597.764074	1284.754281	1287.760514
917.1799167	954.069712	938.0420366	1390.092956
936.4305551	0.569293577	0.000308767	0.004151381	EXOSC8
13	36998816	37009613	+	3630
protein_coding	exosome component 8 [Source:HGNC
Symbol;Acc:HGNC:17035]	-	1381	1386	1317	913	1044
956	20.8056729	16.8341256	16.80666741
12.16089025	12.53292588	12.2941159
ENSG00000129910 20.82531016	25.0276808	25.42275882	0
0	1.962431039	23.75858326	0.65414368
5.198999087	0.000311286	0.004181569	CDH15	16
89171767	89195492	+	4628	protein_coding
cadherin 15 [Source:HGNC Symbol;Acc:HGNC:1754]	-	18	27
26	0	0	2	0.212703124	0.257219786
0.260245035	0	0	0.020173566
ENSG00000105974 13689.17055	9039.627526	9397.620578
6520.717239	7086.069489	7783.0015	10708.80622
7129.92941	0.586696155	0.000312771	0.004197834	CAV1
7	116524785	116561184	+	4082
protein_coding	caveolin 1 [Source:HGNC Symbol;Acc:HGNC:1527]	-
11832	9752	9611	6491	7754	7932	158.5184705
105.3306223	109.0681716	76.8846825	82.77732619
90.7101199
ENSG00000115648 1855.766528	1566.547428	2007.420148
1263.759403	844.4065267	1299.129348	1809.911368
1135.765093	0.67246752	0.000314012	0.004210411	MLPH
2	237485428	237555318	+	7514
protein_coding	melanophilin [Source:HGNC Symbol;Acc:HGNC:29643]
-	1604	1690	2053	1258	924	1324
11.67422034	9.91629577	12.65669208	8.094884693
5.358699185	8.225509915
ENSG00000100228 344.7745794	372.6343586	238.5828135
543.4768181	559.282245	563.2177082	318.6639172
555.3255904	-0.801565196	0.000314259	0.004210411	RAB36
22	23145326	23164350	+	4107
protein_coding	"RAB36, member RAS oncogene family [Source:HGNC
Symbol;Acc:HGNC:9775]"	-	298	402	244	541	612
574	3.968133493	4.315541608	2.752121349
6.369037101	6.493596771	6.524289527
ENSG00000198056 585.4226079	597.8834858	617.968599
377.7214115	406.6676454	261.0033282	600.4248976
348.4641284	0.784703588	0.000315312	0.004219687	PRIM1
12	56731596	56752373	-	2437
protein_coding	DNA primase subunit 1 [Source:HGNC
Symbol;Acc:HGNC:9369]	-	506	645	632	376	445
266	11.35506698	11.66912101	12.01334654
7.459909043	7.957251404	5.095327969
ENSG00000140319 3839.955802	3310.142524	3267.802307
2511.445555	2548.755198	2466.775816	3472.633544

2508.99219	0.468662994	0.000315503	0.004219687	SRP14
	15	40035739	40039188	-	2005
protein_coding	signal recognition particle 14 [Source:HGNC
Symbol;Acc:HGNC:11299]	-	3319	3571	3342	2500	2789
	2514	90.52897188	78.52526871	77.21372854
60.28744076	60.61676219	58.53248092
ENSG00000112379 106.4404742	159.4355962	91.91305112
222.0117871	318.0232373	243.3414488	119.2630405
261.1254911	-1.129083722	0.000316181	0.00422505
ARFGEF3 6	138161921	138344663	+	14877
protein_coding	ARFGEF family member 3 [Source:HGNC
Symbol;Acc:HGNC:21213]	-	92	172	94	221	348
	248	0.338194996	0.509738037	0.292694759
0.718254122	1.019348008	0.778184101
ENSG00000164535 888.5465669	1106.779662	896.6411476
1524.949741	1352.512619	1425.70615	963.9891256
1434.389503	-0.572666438	0.000317607	0.004240404	DAGLB
	7	6409126 6484190 -	5756	protein_coding
diacylglycerol lipase beta [Source:HGNC Symbol;Acc:HGNC:28923]	-
	768	1194	917	1518	1480	1453	7.296845445
	9.145711542	7.379908992	12.75123362	11.20468181
	11.78394929
ENSG00000232679 38.1797353	101.9646255	29.33395248
233.0621475	180.9442557	126.576802	56.49277109
180.1944017	-1.668094579	0.00031899	0.004255149
LINC01705	1	222041705	222064763	-	562
	lincRNA long intergenic non-protein coding RNA 1705
[Source:HGNC Symbol;Acc:HGNC:52493]	-	33	110	30
	232	198	129	3.211236837	8.629593281
2.472790906	19.95964836	15.35279576	10.715179
ENSG00000173852 3447.745794	2780.853423	3208.156603
2261.305578	2183.211247	2281.326083	3145.585273
2241.947636	0.488340369	0.00031991	0.004261804
DPY19L1 7	34928876	35038271	-	6278
protein_coding	dpy-19 like C-mannosyltransferase 1 [Source:HGNC
Symbol;Acc:HGNC:22205]	-	2980	3000	3281	2251	2389
	2325	25.95910203	21.06851382	24.20958667
17.33625954	16.58263097	17.28811655
ENSG00000186340 894.3313753	845.3794405	1542.965901
700.1910208	600.4059395	555.367984	1094.225572
618.6549814	0.823053852	0.000320046	0.004261804	THBS2
	6	169215780	169254044	-	6412
protein_coding	thrombospondin 2 [Source:HGNC Symbol;Acc:HGNC:11786]
	-	773	912	1578	697	657	566
6.592963827	6.270978079	11.4002911	5.255819972
4.465092432	4.120680603
ENSG00000044090 1956.422194	2398.022601	2119.866966
3109.169597	2819.257722	3233.105137	2158.10392
3053.844152	-0.500443151	0.000320585	0.004265259	CUL7
	6	43037617	43053945	-	5628

protein_coding cullin 7 [Source:HGNC Symbol;Acc:HGNC:21024] -
1691 2587 2168 3095 3085 3295 16.43176562
20.26638543 17.844634 26.58935369 23.88689373
27.33048672
ENSG00000136717 1479.753983 1770.476679 2124.755958
1045.765929 1124.961509 1320.716089 1791.662207
1163.814509 0.62281615 0.000323152 0.004295673 BIN1
2 127048027 127107355 - 6929
protein_coding bridging integrator 1 [Source:HGNC
Symbol;Acc:HGNC:1052] - 1279 1910 2173 1041 1231
1346 10.09472973 12.15337123 14.52752464
7.264092816 7.741873831 9.068188399
ENSG00000187741 2123.024675 2088.42092 2503.163945
1596.274795 1257.471191 1669.047599 2238.20318
1507.597862 0.570377935 0.000323765 0.004300085 FANCA
16 89737549 89816657 - 9736
protein_coding FA complementation group A [Source:HGNC
Symbol;Acc:HGNC:3582] - 1835 2253 2560 1589 1376
1701 10.30742578 10.20268749 12.18040856
7.891227217 6.158805181 8.155860413
ENSG00000184371 3142.307911 3394.495078 7977.857277
1600.293108 1172.482223 1539.52715 4838.220089
1437.43416 1.75103581 0.000324471 0.004305613 CSF1
1 109910242 109930992 + 5418
protein_coding colony stimulating factor 1 [Source:HGNC
Symbol;Acc:HGNC:2432] - 2716 3662 8159 1593 1283
1569 27.41482498 29.79979643 69.75902279
14.21601888 10.31920531 13.51854586
ENSG00000176619 7351.334487 9616.191135 13471.12878
6899.443229 5495.039443 6751.743989 10146.21813
6382.075554 0.66896691 0.000324745 0.004305613 LMNB2
19 2427638 2456996 - 5432 protein_coding lamin
B2 [Source:HGNC Symbol;Acc:HGNC:6638] - 6354 10374 13777
6868 6013 6881 63.97085598 84.20161867
117.4890394 61.13244161 48.23808299 59.13407835
ENSG00000141429 1789.819712 1136.442099 1671.057493
966.4042496 958.6390114 985.1403815 1532.439768
970.0612142 0.659134216 0.000329144 0.004360156 GALNT1
18 35581117 35711834 + 5126
protein_coding polypeptide N-acetylgalactosaminyltransferase 1
[Source:HGNC Symbol;Acc:HGNC:4123] - 1547 1226 1709
962 1049 1004 16.50465401 10.5449816
15.44421777 9.073977262 8.91775392 9.143261624
ENSG00000107249 718.4732006 481.0876421 804.7280965
441.0098395 351.8360528 329.6884145 668.0963131
374.1781023 0.83607797 0.000332057 0.004394942 GLIS3
9 3824127 4348392 - 11826 protein_coding GLIS
family zinc finger 3 [Source:HGNC Symbol;Acc:HGNC:28510] zf-
C2H2 621 519 823 439 385 336 2.871761959
1.934921905 3.223772723 1.794848771 1.418670221

1.326317313
ENSG00000140350 2254.918306 2305.327487 2557.920657
1409.423245 1733.592187 1783.849814 2372.72215
1642.288416 0.530801056 0.000332961 0.00440309 ANP32A
15 68778535 68820897 - 7544
protein_coding acidic nuclear phosphoprotein 32 family member A
[Source:HGNC Symbol;Acc:HGNC:13233] - 1949 2487 2616
1403 1897 1818 14.12878681 14.53476664
16.06343853 8.992018832 10.95782219 11.2496299
ENSG00000273119 28.92404189 13.90426711 29.33395248 0
0 1.962431039 24.05408716 0.65414368
5.214554028 0.000333866 0.004411248 AP005229.2 18
11390033 11390729 - 697 lincRNA novel
transcript - 25 15 30 0 0 2
1.961561565 0.948838807 1.993842883 0 0
0.133950165
ENSG00000198720 537.9871792 682.2360397 599.3904291
1040.743038 944.0172534 848.7514243 606.5378827
944.5039052 -0.637996746 0.000335772 0.004432602
ANKRD13B 17 29589769 29614761 + 3971
protein_coding ankyrin repeat domain 13B [Source:HGNC
Symbol;Acc:HGNC:26363] - 465 736 613 1036 1033
865 6.403947731 8.171690043 7.150938504
12.61423986 11.33597899 10.16862558
ENSG00000171310 296.182189 345.7527755 394.0527617
212.9705831 169.9779372 174.6563625 345.3292421
185.8682942 0.895014422 0.000336555 0.004439101 CHST11
12 104455295 104762014 + 6492
protein_coding carbohydrate sulfotransferase 11 [Source:HGNC
Symbol;Acc:HGNC:17422] - 256 373 403 212 186
178 2.156533291 2.533169665 2.875603416
1.57891433 1.248512891 1.279933856
ENSG00000138764 512.5340223 378.1960655 452.7206667
221.0072088 304.3153392 206.0552591 447.8169182
243.7926024 0.875542453 0.00033779 0.004451546 CCNG2
4 77157151 77433388 + 7471
protein_coding cyclin G2 [Source:HGNC Symbol;Acc:HGNC:1593] -
443 408 463 220 333 210 3.242796552
2.407772138 2.870811719 1.423787451 1.942334816
1.312159395
ENSG00000163171 1280.756575 1208.744288 1773.726327
815.7175163 981.4855083 909.5867865 1421.07573
902.2632704 0.655325385 0.000338128 0.004452166
CDC42EP3 2 37641882 37738468 - 10406
protein_coding CDC42 effector protein 3 [Source:HGNC
Symbol;Acc:HGNC:16943] - 1107 1304 1814 812 1074
927 5.817796313 5.524942058 8.075248894
3.772883752 4.497581662 4.1585502
ENSG00000148331 570.3821061 668.3317726 724.5486264
939.2806376 1165.171344 953.7414849 654.420835

1019.397822	-0.638908445	0.000338726	0.004456194	ASB6
	9	129634604	129642169	-	4622
protein_coding	ankyrin repeat and SOCS box containing 6 [Source:HGNC
Symbol;Acc:HGNC:17181]	-	493	721	741	935	1275
	972	5.83326479	6.877637497	7.426611789
9.780991607	12.0209514	9.817080667
ENSG00000069122 15.04050178	3.707804563	78.22387329	0
	2.741579632	0	32.32405988	0.913859877
5.104066537	0.000339897	0.00446776	ADGRF5	6
46852512	46954943	-	6650	protein_coding
adhesion G protein-coupled receptor F5 [Source:HGNC
Symbol;Acc:HGNC:19030]	-	13	4	80	0	3
	0	0.10690953	0.026519926	0.557276587	0
	0.019658854	0
ENSG00000156110 813.344058	677.601284	923.0417049
541.4676617	470.6378369	499.4386994	804.6623489
503.848066	0.675385848	0.00034029	0.004469083	ADK
	10	74151185	74709303	+	3184
protein_coding	adenosine kinase [Source:HGNC Symbol;Acc:HGNC:257]
	-	703	731	944	539	515	509
12.0747175	10.12227835	13.7341375	8.184966809
7.048436114	7.462610229
ENSG00000125520 1405.708436	1894.688132	2340.849408
1191.429771	1155.118885	1234.369123	1880.415325
1193.63926	0.656203341	0.000341104	0.004475931
SLC2A4RG	20	63739861	63743505	+	2244
	protein_coding	SLC2A4 regulator [Source:HGNC
Symbol;Acc:HGNC:15930]	Others	1215	2044	2394	1186	1264
	1258	29.61066344	40.15984212	49.42011473
25.55424492	24.54611808	26.17000613
ENSG00000138061 5.784808379	20.3929251	19.55596832	0
	0	0	15.24456727	0	6.426108756
0.000341276	1	CYP1B1	2	38066973	38109902
	-	6014	protein_coding	cytochrome P450 family 1
subfamily B member 1 [Source:HGNC Symbol;Acc:HGNC:2597] -	5
	22	20	0	0	0	0.045467523
0.161284716	0.154052598	0	0	0
ENSG00000119969 1554.956492	1181.862705	1492.120383
1102.02231	799.6273927	640.7337342	1409.646527
847.4611455	0.734012899	0.000347661	0.00455245	HELLS
	10	94501434	94613905	+	10148
protein_coding	"helicase, lymphoid specific [Source:HGNC
Symbol;Acc:HGNC:4861]"	-	1344	1275	1526	1097	875
	653	7.242917241	5.539412213	6.965888697
5.226697739	3.757389282	3.003853573
ENSG00000136068 1472.812213	1200.401727	1848.039007
1024.669786	870.9084632	985.1403815	1507.084316
960.2395437	0.6503721	0.000347744	0.00455245	FLNB
	3	58008400	58172251	+	13952
protein_coding	filamin B [Source:HGNC Symbol;Acc:HGNC:3755]	-

1273 1295 1890 1020 953 1004 4.98984033
4.09229807 6.275203185 3.534800605 2.97656267
3.359257389
ENSG00000139197 826.0706365 998.3263787 873.1739856
1408.418667 1279.403828 1283.429899 899.1903336
1323.750798 -0.557246205 0.00034801 0.00455245 PEX5
12 7188685 7218574 + 5488 protein_coding
peroxisomal biogenesis factor 5 [Source:HGNC Symbol;Acc:HGNC:9719]
- 714 1077 893 1402 1400 1308
7.115064179 8.652379478 7.537716721 12.35193869
11.11661412 11.12601592
ENSG00000044524 122.6379376 137.1887688 139.8251735
57.26095866 43.86527412 56.91050013 133.2172933
52.67891097 1.340218463 0.000348127 0.00455245 EPHA3
3 89107524 89482134 + 6674
protein_coding EPH receptor A3 [Source:HGNC Symbol;Acc:HGNC:3387]
- 106 148 143 57 48 58
0.868589101 0.977708681 0.992549765 0.412942773
0.313410565 0.405683951
ENSG00000101161 5016.585826 6036.305829 5430.692403
8422.383813 6727.836418 7927.240182 5494.528019
7692.486804 -0.485268837 0.000349271 0.004562346 PRPF6
20 63981135 64033100 + 3044
protein_coding pre-mRNA processing factor 6 [Source:HGNC
Symbol;Acc:HGNC:15860] - 4336 6512 5554 8384 7362
8079 77.90033731 94.31998038 84.52081636
133.170441 105.3925137 123.896488
ENSG00000162298 1635.943809 1863.171793 1929.196275
2761.585532 2368.724802 2551.160351 1809.437293
2560.490228 -0.500438492 0.00034948 0.004562346 SYVN1
11 65121780 65134533 - 5496
protein_coding synoviolin 1 [Source:HGNC Symbol;Acc:HGNC:20738]
- 1414 2010 1973 2749 2592 2600
14.07010693 16.12438991 16.62963883 24.18406115
20.55165832 22.08374175
ENSG00000089723 63.63289216 37.07804563 55.73450972
148.6775769 172.7195168 103.0276295 52.14848251
141.4749077 -1.44429134 0.000350711 0.004574504 OTUB2
14 94026329 94048930 + 4252
protein_coding "OTU deubiquitinase, ubiquitin aldehyde binding 2
[Source:HGNC Symbol;Acc:HGNC:20351]" - 55 40 57
148 189 105 0.707398519 0.414763655
0.620989212 1.682944043 1.936988963 1.152768443
ENSG00000099834 30.08100357 33.37024107 76.26827646
194.8881751 144.3898606 99.10276747 46.5731737
146.1269344 -1.646381066 0.000351229 0.004577358 CDHR5
11 616565 626078 - 3873 protein_coding
cadherin related family member 5 [Source:HGNC Symbol;Acc:HGNC:7521]
- 26 36 78 194 158 101
0.367130583 0.409816049 0.93293108 2.421895774

1.777739768	1.217362481
ENSG00000164941 1464.713481	1447.897682	1332.739241
1053.802555	988.7963874	806.559157	1415.116802
949.7193664	0.57524881	0.000354579	0.004617033	INTS8
8	94813311	94881746	+	7866
protein_coding	integrator complex subunit 8 [Source:HGNC
Symbol;Acc:HGNC:26048]	-	1266	1562	1363	1049	1082
822	8.801860402	8.75509868	8.026835626
6.447966354	5.994209483	4.878248406
ENSG00000006634 1004.242735	880.6035838	1062.866878
479.1838119	760.331418	546.5370443	982.5710656
595.3507581	0.722035569	0.000354878	0.004617033	DBF4
7	87876216	87909541	+	4583
protein_coding	DBF4 zinc finger [Source:HGNC Symbol;Acc:HGNC:17364]
-	868	950	1087	477	832	557
10.35772988	9.139189996	10.98707635	5.032337249
7.911012428	5.673504109
ENSG00000173465 902.4301071	1127.172587	848.7290252
1426.501075	1358.909638	1540.508366	959.4439065
1441.973026	-0.587214551	0.000359708	0.004675896	SSSCA1
11	65570430	65573942	+	2008
protein_coding	Sjogren syndrome/scleroderma autoantigen 1
[Source:HGNC Symbol;Acc:HGNC:11328]	-	780	1216	868
1420	1487	1570	21.2434773	26.69954279
20.02435207	34.1921061	32.27051214	36.49908521
ENSG00000175352 1022.754121	1363.545128	640.4579626
1519.92685	2443.661312	1565.038754	1008.919071
1842.875639	-0.86913692	0.000360661	0.004684297	NRIP3
11	8980576 9004049 -	4123	protein_coding
nuclear receptor interacting protein 3 [Source:HGNC
Symbol;Acc:HGNC:1167]	-	884	1471	655	1513	2674
1595	11.72556134	15.73016564	7.359196868
17.74299008	28.26224558	18.05898713
ENSG00000175213 399.1517781	513.530932	489.8770065
728.319211	758.5036983	727.0806999	467.5199055
737.9678697	-0.657310171	0.000363714	0.004719948	ZNF408
11	46700818	46705912	+	3078
protein_coding	zinc finger protein 408 [Source:HGNC
Symbol;Acc:HGNC:20041]	zf-C2H2 345	554	501	725	830
741	6.129784298	7.935514823	7.540003825
11.38860703	11.75081732	11.2381708
ENSG00000225663 1035.4807	1982.74849	1625.100968
2526.514228	2258.147757	2897.529429	1547.776719
2560.730471	-0.725549242	0.000364888	0.004727891	MCRIP1
17	81822361	81833302	-	3726
protein_coding	MAPK regulated corepressor interacting protein 1
[Source:HGNC Symbol;Acc:HGNC:28007]	-	895	2139	1662
2515	2471	2953	13.13635564	25.31056804
20.66286911	32.63595723	28.8993778	36.99701045
ENSG00000105372 18262.64005	20751.65519	21790.2377

24509.69946	29527.7265	29603.27222	20268.17765
27880.23273	-0.45999872	0.000364958	0.004727891	RPS19
	19	41859918	41872926	+	3799
protein_coding	ribosomal protein S19 [Source:HGNC
Symbol;Acc:HGNC:10402]	-	15785	22387	22285	24398	32311
	30170	227.2322692	259.8128122	271.7351381
310.5175493	370.6292608	370.7251525
ENSG00000130487 0	6.488657986	5.866790497	66.30216265
	22.84649694	35.3237587	4.118482828	41.4908061
	-3.29986874	0.000365254	0.004727891	KLHDC7B 22
	50545891	50551023	+	5133	protein_coding
	kelch domain containing 7B [Source:HGNC Symbol;Acc:HGNC:25145]
	-	0	7	6	66	25	36	0
	0.060125781	0.054148003	0.621690009	0.212240051
	0.327398943
ENSG00000173281 964.9060375	952.9057728	474.2322318
1387.322525	1714.40113	1206.895089	797.3480141
1436.206248	-0.849499013	0.000366546	0.0047406
PPP1R3B 8	9136255 9151574 -	5776	protein_coding
protein phosphatase 1 regulatory subunit 3B [Source:HGNC
Symbol;Acc:HGNC:14942]	-	834	1028	485	1381	1876
	1230	7.896480711	7.846931981	3.889708087
11.56026289	14.15351297	9.940860118
ENSG00000163297 2835.713067	3272.137527	2733.924372
3709.907374	4812.386114	4113.255458	2947.258322
4211.849649	-0.515041188	0.000369648	0.004776681	ANTXR2
	4	79901149	80125454	-	9864
protein_coding	ANTXR cell adhesion molecule 2 [Source:HGNC
Symbol;Acc:HGNC:21732]	-	2451	3530	2796	3693	5266
	4192	13.58892058	15.77813253	13.1306601
18.10203751	23.26410712	19.83874733
ENSG00000196365 4485.540417	5629.374278	5562.69519
9139.652664	6003.145535	8412.941864	5225.869962
7851.913354	-0.587174342	0.000370931	0.004789205	LONP1
	19	5691834 5720572 -	5085	protein_coding	"lon
peptidase 1, mitochondrial [Source:HGNC Symbol;Acc:HGNC:9479]"	-
	3877	6073	5689	9098	6569	8574	41.69649564
	52.65580806	51.82596916	86.5079841	56.29462135
	78.71156687
ENSG00000242372 2880.834573	4213.919886	3630.565519
5153.486279	4688.101171	6220.906393	3575.106659
5354.164615	-0.582335614	0.000372829	0.00480965	EIF6
	20	35278911	35284985	-	1631
protein_coding	eukaryotic translation initiation factor 6
[Source:HGNC Symbol;Acc:HGNC:6159]	-	2490	4546	3713
	5130	5130	6340	83.49108382	122.8879865
105.456522	152.0773795	137.0635581	181.4601781
ENSG00000092201 6939.456131	6850.168931	6741.920079
5413.672038	4927.532459	5111.151641	6843.84838
5150.785379	0.410037247	0.0003775	0.004865802

SUPT16H 14 21351472 21384266 - 7151
protein_coding "SPT16 homolog, facilitates chromatin remodeling
subunit [Source:HGNC Symbol;Acc:HGNC:11465]" - 5998 7390
6895 5389 5392 5209 45.87059739 45.56292727
44.66526842 36.43700079 32.85804373 34.00428248
ENSG00000128951 1890.475378 2260.833833 2235.247179
940.2852158 1464.003524 1590.550357 2128.85213
1331.613032 0.67691551 0.00037785 0.004866212 DUT
15 48331011 48343373 + 3674
protein_coding deoxyuridine triphosphatase [Source:HGNC
Symbol;Acc:HGNC:3078] - 1634 2439 2286 936 1602
1621 24.32246645 29.26891385 28.82302311
12.31793515 19.00124062 20.59633271
ENSG00000148948 28.92404189 77.86389583 62.57909863
15.06867333 6.397019142 9.812155195 56.45567879
10.42594922 2.444388557 0.000379379 0.004881793 LRRC4C
11 40114203 41459773 - 5356
protein_coding leucine rich repeat containing 4C [Source:HGNC
Symbol;Acc:HGNC:29317] - 25 84 64 15 7
10 0.255266694 0.691468938 0.553530889
0.135410365 0.05695293 0.087157641
ENSG00000279095 174.701213 227.1030295 145.691964
346.5794866 321.6786769 366.9746043 182.4987355
345.0775892 -0.917820544 0.000380112 0.004887108
AC243964.3 19 44664131 44666158 + 2028
TEC TEC - 151 245 149 345 352
374 4.071961934 5.32637932 3.403460962
8.22531163 7.563682854 8.608940126
ENSG00000144749 1640.571656 1332.955741 1636.834549
1013.619426 1072.871496 1072.468563 1536.787315
1052.986495 0.545023077 0.000382649 0.004915599 LRIG1
3 66378797 66501263 - 7032
protein_coding leucine rich repeats and immunoglobulin like domains 1
[Source:HGNC Symbol;Acc:HGNC:17360] - 1418 1438 1674
1009 1174 1093 11.02788127 9.016001638
11.0275503 6.937668123 7.275248509 7.255833957
ENSG00000123737 1733.12859 1476.633167 1385.540356
1025.674365 1089.320974 1036.163589 1531.767371
1050.386309 0.543721071 0.000383917 0.004927746 EXOSC9
4 121801317 121817021 + 4711
protein_coding exosome component 9 [Source:HGNC Symbol;Acc:HGNC:9137]
- 1498 1593 1417 1021 1192 1056
17.38975334 14.908592 13.93346727 10.47885557
11.02609548 10.46397877
ENSG00000109917 4188.201266 4645.879118 3800.702444
5468.923841 5940.089203 5919.673229 4211.594276
5776.228758 -0.455723795 0.000385989 0.004950174 ZPR1
11 116773799 116788050 - 7069
protein_coding ZPR1 zinc finger [Source:HGNC Symbol;Acc:HGNC:13051]
- 3620 5012 3887 5444 6500 6033

28.00562709   31.25986072   25.47176356   37.23585679
40.06950457   39.84018803
ENSG00000055044 3164.290183   3475.139827   3171.000264
2346.694727   2232.559681   2550.179135   3270.143425
2376.477847   0.460701007   0.000386657   0.004954588   NOP58
        2   202265716   202303666   +   3299
protein_coding NOP58 ribonucleoprotein [Source:HGNC
Symbol;Acc:HGNC:29926] -   2735   3749   3243   2336   2443
        2599   45.33876937   50.10338672   45.5372803
34.23668755   32.27005824   36.77646651
ENSG00000176485 192.0556382   208.5640067   340.2738488
132.6043253   102.3523063   100.083983   246.9644979
111.6802049   1.146329831   0.000388228   0.004970549
PLA2G16 11   63573195   63616883   -   3116
protein_coding phospholipase A2 group XVI [Source:HGNC
Symbol;Acc:HGNC:17825] -   166   225   348   132   112
        102   2.913435124   3.183603893   5.173497586
2.04822517   1.566315232   1.528089384
ENSG00000072071 461.6277086   639.5962872   600.3682275
1129.145922   864.511444   802.6342949   567.1974078
932.0972202   -0.715321694   0.00038879   0.004973584   ADGRL1
        19   14147743   14206187   -   8487
protein_coding adhesion G protein-coupled receptor L1 [Source:HGNC
Symbol;Acc:HGNC:20973] -   399   690   614   1124   946
        818   2.571067072   3.584502161   3.351325606
6.40343972   4.857306761   4.499301924
ENSG00000130508 1793.290597   1185.570509   2890.372118
864.9418492   456.0160788   579.898372   1956.411075
633.6187667   1.626649446   0.000392614   0.005018307   PXDN
        2   1631887 1744852 -   11257   protein_coding
peroxidasin [Source:HGNC Symbol;Acc:HGNC:14966] -   1550   1279
        2956   861   499   591   7.530152036   5.009355303
        12.16421869   3.698125981   1.931685755   2.450816811
ENSG00000123130 680.2934653   677.601284   688.370085
407.8587581   477.034856   428.791182   682.0882781
437.8949321   0.639025044   0.000393638   0.005027182   ACOT9
        X   23702253   23766475   -   5046
protein_coding acyl-CoA thioesterase 9 [Source:HGNC
Symbol;Acc:HGNC:17152] -   588   731   704   406   522
        437   6.372719346   6.387105485   6.462906438
3.890272327   4.507978692   4.042781099
ENSG00000065923 332.0480009   377.2691143   280.6281454
645.9437968   471.5516967   574.9922944   329.9817536
564.162596   -0.773099   0.000400709   0.005113217   SLC9A7
        X   46599252   46759172   -   10033
protein_coding solute carrier family 9 member A7 [Source:HGNC
Symbol;Acc:HGNC:17123] -   287   407   287   643   516
        586   1.564392759   1.78853546   1.325114909
3.098713164   2.241183865   2.726546066
ENSG00000125676 3732.358366   3236.913384   3941.505416

2913.276844 2113.757896 2463.832169 3636.925722
2496.955637 0.542629673 0.000406907 0.005187977 THOC2
X 123600561 123733056 - 10277
protein_coding THO complex 2 [Source:HGNC Symbol;Acc:HGNC:19073]
- 3226 3492 4031 2900 2313 2511
17.16693328 14.98103561 18.16974772 13.64372188
9.807715828 11.40581681
ENSG00000152495 585.4226079 434.7400851 588.6346465
389.7763501 222.0679502 249.2287419 536.2657798
287.0243474 0.902099567 0.000410489 0.00522929 CAMK4
5 111223653 111494886 + 13910
protein_coding calcium/calmodulin dependent protein kinase IV
[Source:HGNC Symbol;Acc:HGNC:1464] - 506 469 602
388 243 254 1.989381613 1.486550512
2.004803524 1.348670357 0.761268292 0.852418021
ENSG00000069248 881.6047969 785.1276163 760.7271678
466.124295 571.1624234 528.875165 809.1531937
522.0539611 0.631407928 0.000414537 0.005276461 NUP133
1 229440260 229508341 - 6080
protein_coding nucleoporin 133 [Source:HGNC Symbol;Acc:HGNC:18016]
- 762 847 778 464 625 539
6.854031638 6.142056244 5.927594324 3.689908677
4.479556677 4.138388149
ENSG00000021826 996.1440028 848.1602939 1035.488523
701.195599 604.9752388 564.1989237 959.9309398
623.4565872 0.622521533 0.000415316 0.005281972 CPS1
2 210477682 210679107 + 10775
protein_coding carbamoyl-phosphate synthase 1 [Source:HGNC
Symbol;Acc:HGNC:2323] - 861 915 1059 698 662
575 4.369991431 3.744016665 4.552826884
3.132126978 2.677313996 2.491131204
ENSG00000111911 841.1111382 750.8304241 496.7215954
1105.036044 1391.808593 1012.614416 696.2210526
1169.819685 -0.749546173 0.000417129 0.00530062 HINT3
6 125956781 125980244 + 3314
protein_coding histidine triad nucleotide binding protein 3
[Source:HGNC Symbol;Acc:HGNC:18468] - 727 810 508
1100 1523 1032 11.99710941 10.77622059
7.100904371 16.04875686 20.02654344 14.53694774
ENSG00000164683 223.2936034 246.5690035 263.0277739
74.33878843 148.95916 111.8585692 244.2967936
111.7188392 1.127479249 0.000418202 0.005308119 HEY1
8 79764010 79767863 - 3232
protein_coding hes related family bHLH transcription factor with YRPW
motif 1 [Source:HGNC Symbol;Acc:HGNC:4880] bHLH 193 266
269 74 163 114 3.265732961 3.628642998
3.855523759 1.107035592 2.197732599 1.646567485
ENSG00000165996 279.9847255 413.4202088 416.5421253
189.865284 216.5847909 191.3370263 369.9823532
199.2623671 0.893848073 0.000418413 0.005308119 HACD1

10	17589032	17617377	-	3857
protein_coding	3-hydroxyacyl-CoA dehydratase 1 [Source:HGNC
Symbol;Acc:HGNC:9639]	-	242	446	426	189	237
195	3.431313823	5.098227108	5.116375564
2.369263598	2.677671554	2.360103279
ENSG00000112531 4685.694787	3755.079072	4299.379636
3197.572481	3068.741468	2921.078601	4246.717831
3062.464184	0.471450864	0.000420582	0.005331208	QKI
6	163414000	163578596	+	17368
protein_coding	"QKI, KH domain containing RNA binding [Source:HGNC
Symbol;Acc:HGNC:21100]" -	4050	4051	4397	3183	3358
2977	12.75263488	10.28362877	11.72759909
8.861108303	8.425379644	8.001567257
ENSG00000162391 9.255693406	7.415609127	6.844588913
31.14192488	66.71177105	40.2298363	7.838630482
46.02784408	-2.559921949	0.000421289	0.005335743
FAM151A 1	54609182	54623556	-	2005
protein_coding	family with sequence similarity 151 member A
[Source:HGNC Symbol;Acc:HGNC:25032]	-	8	8	7
31	73	41	0.218207826	0.175917712
0.161728336	0.747564265	1.586598652	0.954587
ENSG00000052802 2097.571518	1667.585102	1767.859536
1061.839181	1456.692645	1135.266356	1844.338719
1217.932727	0.597989576	0.000421803	0.005337828	MSMO1
4	165327623	165343160	+	2892
protein_coding	methylsterol monooxygenase 1 [Source:HGNC
Symbol;Acc:HGNC:10545]	-	1813	1799	1808	1057	1594
1157	34.28421644	27.4262754	28.96026912
17.67168311	24.0186518	18.67588135
ENSG00000057704 19.66834849	64.88657986	29.33395248
132.6043253	105.0938859	134.4265262	37.96296028
124.0415791	-1.699215091	0.000423415	0.005353805	TMCC3
12	94567124	94650562	-	6515
protein_coding	transmembrane and coiled-coil domain family 3
[Source:HGNC Symbol;Acc:HGNC:29199]	-	17	70	30
132	115	137	0.142701722	0.473715481
0.213309054	0.979626958	0.769204857	0.981639856
ENSG00000006652 1830.313371	1683.343272	1573.277652
1137.182547	1186.190121	1253.012218	1695.644765
1192.128296	0.507997262	0.000426582	0.005389384	IFRD1
7	112422968	112481017	+	7489
protein_coding	interferon related developmental regulator 1
[Source:HGNC Symbol;Acc:HGNC:5456]	-	1582	1816	1609
1132	1298	1277	11.55253682	10.69118812
9.952556903	7.308425307	7.552825652	7.960000637
ENSG00000204592 3583.11031	4170.353183	3938.57202
4687.361984	6036.04449	5700.862168	3897.345171
5474.756214	-0.490209406	0.00042825	0.005405994	HLA-E
6	30489467	30494205	+	2975
protein_coding	"major histocompatibility complex, class I, E

[Source:HGNC Symbol;Acc:HGNC:4962]"	-	3097	4499	4028
	4666	6605	5810	56.93101779	66.67499335
62.71984084	75.83312984	96.74854661	91.1664824
ENSG00000171813 168.9164047	215.0526647	217.0712484
324.4787657	484.345735	327.7259835	200.3467726
378.8501614	-0.918209299	0.000431045	0.005436789	PWWP2B
	10	132397168	132417863	+	2651
protein_coding	PWWP domain containing 2B [Source:HGNC
Symbol;Acc:HGNC:25150]	-	146	232	222	323	530
	334	3.011881222	3.858444122	3.879231543
5.891067665	8.712137872	5.881427866
ENSG00000106211 5758.19826	7933.774815	6913.034802
10700.76722	8879.976429	9734.639169	6868.335959
9771.794273	-0.508464467	0.000432484	0.00545044	HSPB1
	7	76302544	76304295	+	1634
protein_coding	heat shock protein family B (small) member 1
[Source:HGNC Symbol;Acc:HGNC:5246]	-	4977	8559	7070
	10652	9717	9921	166.5751839	230.9430686
200.4332725	315.1957276	259.1425621	283.4323602
ENSG00000072864 738.1415491	735.9992058	1232.026004
518.3623626	577.5594425	472.9458804	902.0555864
522.9558952	0.786687986	0.000436011	0.005490366	NDE1
	16	15643267	15726353	+	5512
protein_coding	nudE neurodevelopment protein 1 [Source:HGNC
Symbol;Acc:HGNC:17619]	-	638	794	1260	516	632
	482	6.330036038	6.351045901	10.58921563
4.526283053	4.99650662	4.082102121
ENSG00000138092 537.9871792	466.2564238	736.2822074
311.4192488	290.607441	384.6364836	580.1752701
328.8877245	0.819200328	0.000438759	0.005519219	CENPO
	2	24793136	24822376	+	5518
protein_coding	centromere protein O [Source:HGNC
Symbol;Acc:HGNC:28152]	-	465	503	753	310	318
	392	4.608567677	4.019020736	6.321435861
2.716321769	2.51133137	3.316274005
ENSG00000145632 225.6075268	780.4928606	693.2590771
154.7050462	84.07510872	165.8254228	566.4531548
134.8685259	2.071242572	0.000439231	0.005519219	PLK2
	5	58453982	58460260	-	4768
protein_coding	polo like kinase 2 [Source:HGNC Symbol;Acc:HGNC:19699]
	-	195	842	709	154	92	169
2.236624498	7.785917592	6.888306201	1.561657138
0.840833833	1.654613234
ENSG00000186577 842.2680999	1517.419018	875.1295825
1674.631896	1952.004698	1770.112797	1078.272233
1798.916464	-0.737633516	0.000439386	0.005519219	SMIM29
	6	34246380	34249470	-	2229
protein_coding	small integral membrane protein 29 [Source:HGNC
Symbol;Acc:HGNC:1340]	-	728	1637	895	1667	2136
	1804	17.86142168	32.37968123	18.60010615

36.15986062	41.75896995	37.78091751
ENSG00000125726 131.893631	126.9923063	250.3163945
15.06867333	79.50580934	53.96685357	169.7341106
49.51377875	1.774903054	0.00044278	0.00555729	CD70
	19	6583183 6604103 -	1532	protein_coding	CD70
molecule [Source:HGNC Symbol;Acc:HGNC:11937]	-	114	137
	256	15	87	55	4.06949762	3.942718402
	7.74076095	0.473405948	2.474680209	1.675907174
ENSG00000115526 414.1922799	448.6443522	607.2128164
837.8182372	815.1630107	734.9304241	490.0164828
795.9705573	-0.699108465	0.000444089	0.005569151	CHST10
	2	100391860	100417656	-	4487
protein_coding	carbohydrate sulfotransferase 10 [Source:HGNC
Symbol;Acc:HGNC:19650]	-	358	484	621	834	892
	749	4.363366268	4.755796359	6.411180239
8.986926661	8.662981413	7.792409803
ENSG00000035403 6529.891698	5947.31852	6172.841401
4600.968257	4259.500889	4961.025666	6216.683873
4607.164937	0.432234427	0.000446044	0.005588502	VCL
	10	73995193	74121363	+	9992
protein_coding	vinculin [Source:HGNC Symbol;Acc:HGNC:12665]	-
	5644	6416	6313	4580	4661	5056	30.89080973
	28.31039233	29.26751299	22.16227141	20.32756111
	23.62113032
ENSG00000189241 1035.4807	1128.099538	1042.333112
1386.317946	1580.977588	1638.629918	1068.637783
1535.308484	-0.522606573	0.000446364	0.005588502	TSPYL1
	6	116276578	116279903	-	3326
protein_coding	TSPY like 1 [Source:HGNC Symbol;Acc:HGNC:12382] -
	895	1217	1066	1380	1730	1670	14.71619396
	16.13252294	14.8469558	20.06125314	22.66639517
	23.43906385
ENSG00000259518 118.0100909	63.03267758	80.17947013
13.05951689	11.88017841	38.26740526	87.07407954
21.06903352	2.04704933	0.000449054	0.005617585
LINC01583	15	82088594	82097694	+	2568
	lincRNA long intergenic non-protein coding RNA 1583
[Source:HGNC Symbol;Acc:HGNC:51425]	-	102	68	82
	13	13	39	2.172200279	1.167475704
1.479180895	0.244765131	0.220600723	0.708950027
ENSG00000082512 441.9593601	312.3825345	396.0083585
203.9293791	220.2402305	218.8110608	383.4500844
214.3268901	0.837919609	0.000450229	0.005627674	TRAF5
	1	211326615	211374946	+	9022
protein_coding	TNF receptor associated factor 5 [Source:HGNC
Symbol;Acc:HGNC:12035]	-	382	337	405	203	241
	223	2.31555581	1.646876514	2.079479562
1.087913664	1.164053169	1.153846604
ENSG00000146556 123.7948993	206.7101044	137.8695767
375.712255	233.9481286	365.0121732	156.1248601

324.8908523	-1.054273714	0.000453456	0.005659441	WASH2P
	2	113588550	113599043	+	2958
transcribed_unprocessed_pseudogene	"WAS protein family homolog 2, pseudogene [Source:HGNC Symbol;Acc:HGNC:33145]" -	107	223
	141	374	256	372	1.978246112	3.323844144
	2.208123698	6.113285085	3.771380866	5.870712411
ENSG00000111859 299.653074	237.2994921	412.6309316
56.25638043	165.4086378	161.9005607	316.5278326
127.855193	1.306562075	0.00045351	0.005659441	NEDD9
	6	11183298	11382348	-	10359
protein_coding	"neural precursor cell expressed, developmentally down-regulated 9 [Source:HGNC Symbol;Acc:HGNC:7733]"	-	259
	256	422	56	181	165	1.367340393
1.089572392	1.887109382	0.261379432	0.761411338
0.743553371
ENSG00000164733 56603.19302	61977.80718	44486.89454
68955.25374	86208.05768	76966.54535	54355.96491
77376.61892	-0.509463816	0.000458142	0.00571258	CTSB
	8	11842524	11869448	-	7428
protein_coding	cathepsin B [Source:HGNC Symbol;Acc:HGNC:2527]	-
	48924	66862	45497	68641	94334	78440	360.2008847
	396.8637449	283.7352681	446.799752	553.4201308
	492.9600508
ENSG00000147155 576.1669145	609.9338507	620.9019943
313.4284053	356.4053522	434.6784751	602.3342531
368.1707442	0.710362238	0.00046176	0.005753	EBP
	X	48521158	48528716	+	2409
protein_coding	"EBP, cholestenol delta-isomerase [Source:HGNC Symbol;Acc:HGNC:3133]"	-	498	658	635	312	390
	443	11.30543443	12.04267737	12.21066682
6.262085752	7.054827592	8.584459619
ENSG00000129009 16.19746346	2.780853423	27.37835565	0
	0	0	15.45222418	0	6.441584599
0.000465433	1	ISLR	15	74173671	74176872
	+	2671	protein_coding	immunoglobulin superfamily containing leucine rich repeat [Source:HGNC Symbol;Acc:HGNC:6133]
	-	14	3	28	0	0	0
0.286647963	0.049520079	0.485608857	0	0	0
ENSG00000158164 130.7366694	71.37523785	66.4902923
29.13276844	30.15737595	12.75580175	89.5340665
24.01531538	1.894857783	0.000470209	0.005853484
TMSB15A X	102513676	102516784	-	685
protein_coding	thymosin beta 15a [Source:HGNC Symbol;Acc:HGNC:30744]
	-	113	77	68	29	33	13
9.021579587	4.956032112	4.598548773	2.046956638
2.099336062	0.885928793
ENSG00000158270 164.288558	57.47097073	170.1369244
40.18312888	36.5543951	47.09834493	130.632151
41.27862297	1.660752192	0.000470927	0.005857652
COLEC12 18	316740	500722	-	7340	protein_coding

collectin subfamily member 12 [Source:HGNC Symbol;Acc:HGNC:16016]
	-	142	62	174	40	40	48
1.058003239	0.372417077	1.098134774	0.263490613
0.237477533	0.305274981
ENSG00000145730 2511.763798	1863.171793	2246.98076
1163.301581	1436.587727	1717.127159	2207.30545
1439.005489	0.616831496	0.000475335	0.005907681	PAM
	5	102753981	103031105	+	8846
protein_coding	peptidylglycine alpha-amidating monooxygenase
[Source:HGNC Symbol;Acc:HGNC:8596]	-	2171	2010	2298
	1158	1572	1750	13.42170228	10.01804736
12.03387636	6.329404345	7.743979674	9.23500531
ENSG00000197702 3739.300136	2686.304406	3587.542389
2339.662679	2369.638662	2217.547074	3337.715644
2308.949472	0.531333726	0.00048298	0.005997818	PARVA
	11	12377185	12535356	+	12849
protein_coding	parvin alpha [Source:HGNC Symbol;Acc:HGNC:14652]
	-	3232	2898	3669	2329	2593	2260
13.75614471	9.944043375	13.22759345	8.763979962
8.794107808	8.210793803
ENSG00000182492 9080.992193	5278.059796	16048.6054
3186.52212	2238.04284	3759.036655	10135.8858
3061.200538	1.727316074	0.000483836	0.006003572	BGN
	X	153494939	153509554	+	2453
protein_coding	biglycan [Source:HGNC Symbol;Acc:HGNC:1044]	-
	7849	5694	16413	3172	2449	3831	174.9892999
	102.3419936	309.9508824	62.52257367	43.50606803
	72.90555822
ENSG00000165805 16.19746346	24.10072966	9.777984162
37.16939421	119.7156439	69.66630188	16.69205909
75.51711335	-2.17623792	0.000486738	0.006034689
C12orf50	12	87980035	88034037	-	5609
	protein_coding	chromosome 12 open reading frame 50
[Source:HGNC Symbol;Acc:HGNC:26665]	-	14	26	10
	37	131	71	0.136501464	0.204372222
0.082588013	0.31894625	1.017757833	0.590906741
ENSG00000179041 1682.222276	2198.728106	1992.753172
3114.192488	2602.672931	2727.779144	1957.901185
2814.881521	-0.523213218	0.000488189	0.006047764	RRS1
	8	66429028	66430733	+	1706
protein_coding	ribosome biogenesis regulator homolog [Source:HGNC
Symbol;Acc:HGNC:17083]	-	1454	2372	2038	3100	2848
	2780	46.6101062	61.3012903	55.33852875
87.85852006	72.74774836	76.06971776
ENSG00000147274 8754.729	7362.772912	8198.83972
5947.103074	6050.666248	6152.221307	8105.44721
6049.996877	0.421847236	0.000490318	0.006069222	RBMX
	X	136848004	136880764	-	5882
protein_coding	RNA binding motif protein X-linked [Source:HGNC
Symbol;Acc:HGNC:9910]	-	7567	7943	8385	5920	6621

6270	70.35475039	59.53789836	66.03596103
48.66289064	49.05205036	49.7609378
ENSG00000198858 1086.387013	1719.494366	1647.590331
2342.676414	2062.581743	2495.231066	1484.49057
2300.163074	-0.630939774	0.000493235	0.006100385	R3HDM4
19	896503	913245	-	2957	protein_coding	R3H
domain containing 4 [Source:HGNC Symbol;Acc:HGNC:28270] -	939
1855	1685	2332	2257	2543	17.36636723
27.65836779	26.39678508	38.13102134	33.26127035
40.14588826
ENSG00000123689 276.5138405	488.5032512	613.0796069
204.9339573	223.89567	262.9657592	459.3655662
230.5984622	0.995578003	0.000498925	0.006165777	G0S2
1	209675420	209676388	+	866
protein_coding	G0/G1 switch 2 [Source:HGNC Symbol;Acc:HGNC:30229]
-	239	527	627	204	245	268
15.09297045	26.8303712	33.53915407	11.38973165
12.32840208	14.44650984
ENSG00000138600 2059.391783	1641.63047	1850.972402
1215.539649	1310.475064	1348.190124	1850.664885
1291.401612	0.518627547	0.00050061	0.006181597	SPPL2A
15	50702266	50765808	-	8144
protein_coding	signal peptide peptidase like 2A [Source:HGNC
Symbol;Acc:HGNC:30227]	-	1780	1771	1893	1210	1434
1374	11.95300084	9.587707014	10.76751052
7.183710494	7.673084556	7.875805885
ENSG00000102794 18.51138681	39.85889906	8.800185745
66.30216265	206.5323323	48.07956045	22.3901572
106.9713518	-2.252677956	0.0005011	0.006182663	ACOD1
13	76948497	76958642	+	2177
protein_coding	aconitate decarboxylase 1 [Source:HGNC
Symbol;Acc:HGNC:33904]	-	16	43	9	66	226
49	0.401935408	0.87085126	0.19150783
1.465840521	4.523854287	1.050711987
ENSG00000136213 631.7010749	813.8631017	865.3515983
1113.07267	1168.826783	1222.594537	770.3052583
1168.164663	-0.599757394	0.00050253	0.006195302	CHST12
7	2403560 2448483 +	16143	protein_coding
carbohydrate sulfotransferase 12 [Source:HGNC Symbol;Acc:HGNC:17423]
-	546	878	885	1108	1279	1246
1.849707718	2.397972659	2.539577553	3.318613917
3.452589103	3.603129168
ENSG00000108846 134.2075544	146.4582803	286.4949359
20.09156444	101.4384464	49.06077597	189.0535902
56.8635956	1.730696686	0.000503427	0.006201359	ABCC3
17	50634777	50692252	+	9614
protein_coding	ATP binding cassette subfamily C member 3 [Source:HGNC
Symbol;Acc:HGNC:54]	-	116	158	293	20	111
50	0.65985511	0.724580976	1.411776532
0.100583581	0.503126808	0.24277945

ENSG00000110104 1749.326054 2422.123331 2235.247179
3421.593424 2778.134027 3166.382481 2135.565521
3122.036644 -0.547294413 0.000508044 0.006253191 CCDC86
11 60842071 60851081 + 3384
protein_coding coiled-coil domain containing 86 [Source:HGNC
Symbol;Acc:HGNC:28359] - 1512 2613 2286 3406 3040
3227 24.43521415 34.0441906 31.29308123
48.66486307 39.14730117 44.51584759
ENSG00000255112 944.0807274 824.0595642 746.0601915
1615.361781 1322.355243 1037.144804 838.0668277
1324.953943 -0.661241967 0.000511683 0.00629292 CHMP1B
18 11851396 11854449 + 3054
protein_coding charged multivesicular body protein 1B [Source:HGNC
Symbol;Acc:HGNC:24287] - 816 889 763 1608 1447
1057 14.61220777 12.83413745 11.57331999
25.45764512 20.64705412 16.15667505
ENSG00000111275 1.156961676 12.97731597 35.20074298 0
0 0 16.44500688 0 6.535346575
0.000513251 1 ALDH2 12 111766887 111817529
+ 9720 protein_coding aldehyde dehydrogenase 2
family member [Source:HGNC Symbol;Acc:HGNC:404] - 1 14
36 0 0 0 0.005626372 0.063503217
0.171568949 0 0 0
ENSG00000078098 747.3972425 593.2487301 656.1027372
485.2112812 319.8509571 388.5613457 665.5829033
397.874528 0.742357855 0.000513577 0.006311138 FAP
2 162170684 162245151 - 7003
protein_coding fibroblast activation protein alpha [Source:HGNC
Symbol;Acc:HGNC:3590] - 646 640 671 483 350
396 5.044790138 4.029301872 4.438547272
3.334757215 2.177922972 2.639715335
ENSG00000187994 19.66834849 45.4206059 21.51156516
115.5264955 73.10879019 104.9900606 28.86683985
97.87511544 -1.753683009 0.000517433 0.006349054 RINL
19 38867834 38878279 - 4201
protein_coding Ras and Rab interactor like [Source:HGNC
Symbol;Acc:HGNC:24795] - 17 49 22 115 80
107 0.221304861 0.514253619 0.242589755
1.323568356 0.829842939 1.188987071
ENSG00000177119 2724.644746 2158.869207 2295.870681
1688.695991 1777.457462 1592.512788 2393.128211
1686.22208 0.504640991 0.000517493 0.006349054 ANO6
12 45215987 45440404 + 8235
protein_coding anoctamin 6 [Source:HGNC Symbol;Acc:HGNC:25240] -
2355 2329 2348 1681 1945 1623 15.63946962
12.46923595 13.20799649 9.869731234 10.29235127
9.200278037
ENSG00000167716 1249.51861 1494.245239 1668.124098
2342.676414 1846.910812 2359.823324 1470.629316
2183.13685 -0.569367713 0.000519917 0.006373678 WDR81

17	1716523 1738599 +	7913	protein_coding	WD
repeat domain 81 [Source:HGNC Symbol;Acc:HGNC:26600]	-	1080
1612	1706	2332	2021	2405	7.464097478
8.981685207	9.987121121	14.24913814	11.12970737
14.18795985
ENSG00000118557 42.807582	44.49365476	42.04533189
2.009156444	11.88017841	4.906077597	43.11552289
6.265137483	2.77177966	0.000521526	0.006386107	PMFBP1
16	72112157	72176878	-	5691
protein_coding	polyamine modulated factor 1 binding protein 1
[Source:HGNC Symbol;Acc:HGNC:17728]	-	37	48	43
2	13	5	0.355555869	0.371866118
0.35001151	0.016991927	0.099543605	0.041013559
ENSG00000130309 6105.286763	6574.864442	7924.078365
4636.128495	4826.094013	5329.962702	6868.076523
4930.728403	0.478196121	0.00052229	0.006386107
COLGALT1	19	17555594	17583162	+	4900
protein_coding	collagen beta(1-O)galactosyltransferase 1
[Source:HGNC Symbol;Acc:HGNC:26182]	-	5277	7093	8104
4615	5281	5432	58.89599007	63.82162206
76.6135891	45.53830293	46.96547134	51.74992409
ENSG00000177613 1228.6933	1327.394034	1189.002874
1594.265638	1806.700978	1939.863082	1248.363402
1780.276566	-0.511976005	0.000522386	0.006386107	CSTF2T
10	51695487	51699591	-	4105
protein_coding	cleavage stimulation factor subunit 2 tau variant
[Source:HGNC Symbol;Acc:HGNC:17086]	-	1062	1432	1216
1587	1977	1977	14.1483589	15.38026486
13.72217234	18.69239638	20.98708423	22.48223814
ENSG00000153044 1219.437606	1222.648555	1067.75587
757.4519794	898.3242595	613.2596997	1169.947344
756.3453129	0.628898351	0.000522602	0.006386107	CENPH
5	69189548	69210357	+	2244
protein_coding	centromere protein H [Source:HGNC
Symbol;Acc:HGNC:17268]	-	1054	1319	1092	754	983
625	25.6869459	25.91527973	22.54250847
16.24612198	19.08926746	13.0017916
ENSG00000131153 2951.409235	2308.108341	2642.01132
1788.149235	1979.420494	1822.11722	2633.842965
1863.228983	0.498932826	0.000523514	0.006392137	GINS2
16	85676198	85690073	-	3264
protein_coding	GINS complex subunit 2 [Source:HGNC
Symbol;Acc:HGNC:24575]	-	2551	2490	2702	1780	2166
1857	42.74201784	33.63435897	38.34755247
26.36762835	28.91790988	26.5587597
ENSG00000249471 336.6758476	351.3144824	333.4292599
552.5180221	510.8476715	560.2740616	340.4731966
541.2132517	-0.668324051	0.000524779	0.006402471
ZNF324B 19	58451604	58457833	+	4042
protein_coding	zinc finger protein 324B [Source:HGNC

Symbol;Acc:HGNC:33107] zf-C2H2 291 379 341 550 559
571 3.937235503 4.134060793 3.908053727
6.579116804 6.026623996 6.594560166
ENSG00000033327 201.3113316 221.5413227 228.8048294
406.8541799 318.0232373 425.8475354 217.2191612
383.5749842 -0.819192258 0.000525865 0.006410608 GAB2
11 78215297 78418348 - 6771
protein_coding GRB2 associated binding protein 2 [Source:HGNC
Symbol;Acc:HGNC:14458] - 174 239 234 405 348
434 1.405371517 1.556248856 1.600904773
2.89203421 2.239675132 2.992147176
ENSG00000163754 1298.111 1157.761975 1143.046348
856.9052234 829.7847687 803.6155104 1199.639775
830.1018342 0.530874032 0.000528157 0.00643341 GYG1
3 148991341 149027668 + 3511
protein_coding glycogenin 1 [Source:HGNC Symbol;Acc:HGNC:4699] -
1122 1249 1169 853 908 819 17.4765917
15.68431539 15.42361363 11.74679577 11.26973274
10.88927857
ENSG00000140262 3625.917892 2504.621983 2854.193577
2201.030884 2039.735246 1864.309487 2994.91115
2035.025206 0.557090327 0.000531932 0.006474236 TCF12
15 56918623 57299281 + 9446
protein_coding transcription factor 12 [Source:HGNC
Symbol;Acc:HGNC:11623] bHLH 3134 2702 2919 2191 2232
1900 18.14453169 12.61163408 14.31490959
11.21490639 10.29686092 9.38969954
ENSG00000044459 747.3972425 623.8381178 689.3478834
429.959479 479.7764356 406.2232251 686.8610812
438.6530466 0.6460668 0.000533787 0.006491648 CNTLN
9 17134982 17503923 + 11064
protein_coding centlein [Source:HGNC Symbol;Acc:HGNC:23432] -
646 673 705 428 525 414 3.193118703
2.681864645 2.95174887 1.870392784 2.067786683
1.746763908
ENSG00000118960 416.5062033 743.414815 590.5902434
1098.003997 865.4253039 980.2343039 583.5037539
981.2212015 -0.748105473 0.000536222 0.006516075 HS1BP3
2 20560448 20651089 - 9059
protein_coding HCLS1 binding protein 3 [Source:HGNC
Symbol;Acc:HGNC:24979] - 360 802 604 1093 947
999 2.173286358 3.903265263 3.088581991
5.833660067 4.555418877 5.147913779
ENSG00000000460 873.5060652 736.926157 811.5726854
448.041887 591.2673407 510.2320701 807.3349692
516.5137659 0.643423202 0.000536961 0.006519875
C1orf112 1 169662007 169854080 + 5967
protein_coding chromosome 1 open reading frame 112
[Source:HGNC Symbol;Acc:HGNC:25565] - 755 795 830
446 647 520 6.919673874 5.874150223

6.443539725	3.613932506	4.725054701	4.068116127
ENSG00000116478 3617.81916	3616.963352	3858.39255
2372.81376	2801.894384	2885.754843	3697.725021
2686.820996	0.460687294	0.000538201	0.00652974	HDAC1
	1	32292086	32333635	+	3963
protein_coding	histone deacetylase 1 [Source:HGNC
Symbol;Acc:HGNC:4852]	-	3127	3902	3946	2362	3066
	2941	43.15176079	43.41073616	46.12490284
28.81754881	33.71372003	34.64311919
ENSG00000179562 591.2074163	671.112626	637.5245673
1087.958214	911.1182978	874.2630278	633.2815365
957.7798467	-0.596172706	0.000540302	0.006550035	GCC1
	7	127580618	127593611	-	4987
protein_coding	GRIP and coiled-coil domain containing 1 [Source:HGNC
Symbol;Acc:HGNC:19095]	-	511	724	652	1083	997
	891	5.603717649	6.400783766	6.056346067
10.50002432	8.711930213	8.340351836
ENSG00000129245 1707.675433	2359.090653	2021.109326
2662.132288	2965.475302	3210.53718	2029.291804
2946.048257	-0.537333928	0.000540972	0.006552957	FXR2
	17	7591230 7614871 -	4331	protein_coding	FMR1
autosomal homolog 2 [Source:HGNC Symbol;Acc:HGNC:4024]	-	1476
	2545	2067	2650	3245	3272	18.63772445
25.90798046	22.10826943	29.58413712	32.65014506
35.26721351
ENSG00000213853 1233.321146	1212.452092	1477.453407
774.5298092	847.1481064	995.9337523	1307.742215
872.5372226	0.583888238	0.000541685	0.006556404	EMP2
	16	10528422	10580698	-	8718
protein_coding	epithelial membrane protein 2 [Source:HGNC
Symbol;Acc:HGNC:3334]	-	1066	1308	1511	771	927
	1015	6.687057426	6.614923671	8.0287892
4.276010174	4.633631229	5.434945749
ENSG00000213139 20.82531016	17.61207168	41.06753348	0
	3.65543951	0	26.50163844	1.218479837
4.403948573	0.000550122	0.006653258	CRYGS	3
186538441	186546702	-	2151	protein_coding
crystallin gamma S [Source:HGNC Symbol;Acc:HGNC:2417]	-	18
	19	42	0	4	0	0.457642983
0.389445911	0.904505758	0	0.081036034	0
ENSG00000090565 1109.526247	1466.436705	1624.123169
2116.646314	2011.40559	2088.026625	1400.028707
2072.026176	-0.564858421	0.000555603	0.006714236
RAB11FIP3	16	425619	523011	+	6317	protein_coding
	RAB11 family interacting protein 3 [Source:HGNC
Symbol;Acc:HGNC:17224]	-	959	1582	1661	2107	2201
	2128	8.302376862	11.04153772	12.18039049
16.12704787	15.1833556	15.72558399
ENSG00000168710 4814.117533	4623.632291	5505.005083
3515.019199	3655.43951	3836.552681	4980.918302

3669.003797	0.441034329	0.000558416	0.006742899	AHCYL1
	1	109984686	110023741	+	5113
protein_coding	adenosylhomocysteinase like 1 [Source:HGNC
Symbol;Acc:HGNC:344]	-	4161	4988	5630	3499	4000
	3910	44.50580244	43.01150213	51.00761976
33.08791232	34.09123986	35.69825607
ENSG00000134363 134.2075544	111.2341369	149.6031577
52.23806755	36.5543951	63.77900877	131.6816163
50.85715714	1.373700276	0.000569375	0.006869797	FST
	5	53480409	53487134	+	2969
protein_coding	follistatin [Source:HGNC Symbol;Acc:HGNC:3971]	-
	116	120	153	52	40	65	2.136694856
	1.781988949	2.387171875	0.846826349	0.587095013
	1.021995997
ENSG00000061337 195.5265232	245.6420523	455.6540619
111.5081826	106.0077458	171.7127159	298.9408792
129.7428814	1.205556884	0.000575772	0.006941498	LZTS1
	8	20246165	20303963	-	5706
protein_coding	leucine zipper tumor suppressor 1 [Source:HGNC
Symbol;Acc:HGNC:13861]	-	169	265	466	111	116
	175	1.619756196	2.047613879	3.783176516
0.940572827	0.885900241	1.431700963
ENSG00000239672 1949.480424	1940.108738	2275.336914
1537.00468	1304.991905	1502.24096	2054.975359
1448.079182	0.505248145	0.000581525	0.007005334	NME1
	17	51153536	51162428	+	3438
protein_coding	NME/NM23 nucleoside diphosphate kinase 1 [Source:HGNC
Symbol;Acc:HGNC:7849]	-	1685	2093	2327	1530	1428
	1531	26.80332952	26.84091483	31.35400092
21.51725045	18.10009827	20.78812666
ENSG00000101199 2803.31814	3463.089462	3451.628409
4757.68246	4124.249627	4628.393605	3239.345337
4503.441897	-0.474974541	0.000583123	0.007019061
ARFGAP1 20	63272785	63289793	+	6613
protein_coding	ADP ribosylation factor GTPase activating protein 1
[Source:HGNC Symbol;Acc:HGNC:15852]	-	2423	3736	3530
	4736	4513	4717	20.03778001	24.90819763
24.72741049	34.62696177	29.73893479	33.29763508
ENSG00000102241 3746.241906	3211.885703	3525.941089
2694.278791	2438.178153	2559.010075	3494.689566
2563.82234	0.446743985	0.000585627	0.007043646
HTATSF1 X	136497079	136512346	+	3136
protein_coding	HIV-1 Tat specific factor 1 [Source:HGNC
Symbol;Acc:HGNC:5276]	-	3238	3465	3606	2682	2668
	2608	56.46710248	48.71482457	53.26625014
41.35080189	37.07390809	38.82196993
ENSG00000197043 2325.492968	1941.035689	2831.704213
1354.171443	1293.111727	1899.633246	2366.077623
1515.638805	0.642614913	0.000586129	0.007044145	ANXA6
	5	151100712	151157882	-	5427

protein_coding	annexin A6 [Source:HGNC Symbol;Acc:HGNC:544]	-
	2010	2094	2896	1348	1415	1936	20.25493942
	17.01182137	24.71958593	12.00967589	11.36201128
	16.65296492
ENSG00000050405 1585.037496	1192.059167	1387.495953
829.7816114	963.2083108	997.8961833	1388.197538
930.2953685	0.57676786	0.000587103	0.007050309	LIMA1
	12	50175788	50283546	-	6317
protein_coding	LIM domain and actin binding 1 [Source:HGNC
Symbol;Acc:HGNC:24636]	-	1370	1286	1419	826	1054
	1017	11.86053837	8.975611569	10.40576406
6.322231393	7.27090268	7.515469417
ENSG00000133112 46438.12774	39484.4108	33119.98795
47119.7415	60800.0115	64180.32591	39680.84216
57366.69297	-0.531796458	0.000589079	0.007068481	TPT1
	13	45333471	45341370	-	7269
protein_coding	"tumor protein, translationally-controlled 1
[Source:HGNC Symbol;Acc:HGNC:12022]"	-	40138	42596	33872
	46905	66531	65409	301.9783254	258.3616845
215.858238	311.9936019	398.8485156	420.0576288
ENSG00000176225 616.6605732	504.2614206	628.7243816
314.4329835	338.1281546	412.1105182	583.2154585
354.8905521	0.716218834	0.000593199	0.007112335	RTTN
	18	70003031	70205945	-	19323
protein_coding	rotatin [Source:HGNC Symbol;Acc:HGNC:18654]	-
	533	544	643	313	370	420	1.508507132
	1.241247263	1.54148348	0.783196973	0.834422042
	1.014660543
ENSG00000132286 888.5465669	922.3163851	760.7271678
1279.832655	1301.336465	1167.646468	857.1967066
1249.605196	-0.543902525	0.000595342	0.007132439
TIMM10B 11	6481447 6484679 +	3041	protein_coding
translocase of inner mitochondrial membrane 10B [Source:HGNC
Symbol;Acc:HGNC:4022]	-	768	995	778	1274	1424
	1190	13.81145754	14.42582364	11.8512902
20.256025	20.40573145	18.26739321
ENSG00000254615 267.2581471	238.2264432	234.6716199
383.7488808	437.7388813	419.9602423	246.7187367
413.8160015	-0.747238229	0.00059686	0.007145029
AC027031.2	8	106270144	106272899	+	1419
	lincRNA novel transcript	-	231	257	240
	382	479	428	8.902752442	7.98517955
7.83486111	13.01613918	14.70996758	14.08015415
ENSG00000186594 308.9087674	304.9669253	300.1841138
463.1105604	524.5555696	484.7204666	304.6866022
490.7955322	-0.688130824	0.000598874	0.007163526
MIR22HG 17	1711493 1717174 -	2986	lincRNA MIR22 host
gene [Source:HGNC Symbol;Acc:HGNC:28219]	-	267	329
	307	461	574	494	4.890082327	4.85780472
	4.76267589	7.464699656	8.376848996	7.722949257

ENSG00000157193 1454.300826 1489.610483 1725.814205
1175.35652 941.2756737 1091.111658 1556.575171
1069.24795 0.542201059 0.000603898 0.007217977 LRP8
1 53242364 53328070 - 10263
protein_coding LDL receptor related protein 8 [Source:HGNC
Symbol;Acc:HGNC:6700] - 1257 1607 1765 1170 1030
1112 6.698162223 6.903598184 7.966596784
5.512044935 4.37342309 5.057972854
ENSG00000197563 637.4858833 456.0599613 605.2572196
406.8541799 237.6035681 305.1580266 566.2676881
316.5385915 0.839270529 0.000608516 0.00726749 PIGN
18 61905255 62187118 - 19003
protein_coding phosphatidylinositol glycan anchor biosynthesis class
N [Source:HGNC Symbol;Acc:HGNC:8967] - 551 492 619
405 260 311 1.585711381 1.141502567
1.508936405 1.03046696 0.596224444 0.763983988
ENSG00000119699 108.7543975 86.2064561 157.425545
49.22433288 33.81281546 43.17348286 117.4621329
42.0702104 1.48269878 0.000610282 0.00728289 TGFB3
14 75958099 75982991 - 4914
protein_coding transforming growth factor beta 3 [Source:HGNC
Symbol;Acc:HGNC:11769] - 94 93 161 49 37
44 1.046134234 0.834414331 1.517725321
0.482127767 0.328114309 0.417987756
ENSG00000188636 1345.546429 1180.008802 1372.828976
1946.872594 1702.520952 1851.553685 1299.461402
1833.649077 -0.496961653 0.000611379 0.007290285 RTL6
22 44492572 44498298 - 5495
protein_coding retrotransposon Gag like 6 [Source:HGNC
Symbol;Acc:HGNC:13343] - 1163 1273 1404 1938 1863
1887 11.5746197 10.21397204 11.8359158
17.05246993 14.77419259 16.03061704
ENSG00000109881 748.5542042 801.8127368 894.6855508
532.4264577 559.282245 531.8188116 815.0174973
541.1758381 0.591007031 0.000613367 0.007303054 CCDC34
11 27330827 27363868 - 3733
protein_coding coiled-coil domain containing 34 [Source:HGNC
Symbol;Acc:HGNC:25079] - 647 865 915 530 612
542 9.478530316 10.21626326 11.35443582
6.864661016 7.144174107 6.777777885
ENSG00000120265 1629.002039 1386.718907 1365.006589
953.3447327 964.1221707 1105.82989 1460.242512
1007.765598 0.534653941 0.000613405 0.007303054 PCMT1
6 149749443 149811420 + 2832
protein_coding protein-L-isoaspartate (D-aspartate) O-
methyltransferase [Source:HGNC Symbol;Acc:HGNC:8728] - 1408
1496 1396 949 1055 1127 27.18968139
23.2901509 22.83466397 16.20220713 16.23371093
18.57704798
ENSG00000113916 804.0883646 757.3190821 585.7012513

1030.697256 1143.238707 1082.280718 715.7028993
1085.40556 -0.60146547 0.000619052 0.00736455 BCL6
3 187721377 187745727 - 5938
protein_coding B cell CLL/lymphoma 6 [Source:HGNC
Symbol;Acc:HGNC:1001] ZBTB 695 817 599 1026 1251
1103 6.400874675 6.06618738 4.672927951
8.354267633 9.180698269 8.671242962
ENSG00000182446 5253.762969 5135.30932 4968.193753
8689.60162 6561.51392 6357.295351 5119.088681
7202.80363 -0.492651751 0.000620067 0.007370892 NPLOC4
17 81556887 81648465 - 9574
protein_coding "NPL4 homolog, ubiquitin recognition factor
[Source:HGNC Symbol;Acc:HGNC:18261]" - 4541 5540 5081
8650 7180 6479 25.93897386 25.51234032
24.58432155 43.68415112 32.68056971 31.59079772
ENSG00000172795 1000.771849 787.9084697 959.2202463
623.8430759 563.8515444 625.0342859 915.9668552
604.2429687 0.599786119 0.000622016 0.007388311 DCP2
5 112976702 113020970 + 9951
protein_coding decapping mRNA 2 [Source:HGNC Symbol;Acc:HGNC:24452]
- 865 850 981 621 617 637
4.753834892 3.766050657 4.566723706 3.017352786
2.701948304 2.98826248
ENSG00000117461 252.2176453 214.1257135 365.6966076
105.4807133 179.116536 77.51602604 277.3466555
120.7044251 1.198739081 0.000627255 0.007440307 PIK3R3
1 46040140 46133036 - 7410
protein_coding phosphoinositide-3-kinase regulatory subunit 3
[Source:HGNC Symbol;Acc:HGNC:8981] - 218 231 374
105 196 79 1.608914621 1.374446152
2.338061066 0.685128932 1.152647363 0.497685421
ENSG00000198933 233.7062585 382.8308212 312.8954932
514.3440497 563.8515444 521.0254408 309.8108576
533.0736783 -0.780808062 0.000627402 0.007440307 TBKBP1
17 47694081 47712050 + 4280
protein_coding TBK1 binding protein 1 [Source:HGNC
Symbol;Acc:HGNC:30140] - 202 413 320 512 617
531 2.581085037 4.254418815 3.463447949
5.783988336 6.282029808 5.791576375
ENSG00000183778 201.3113316 124.2114529 274.7613549
90.41203998 41.12369448 96.15912091 200.0947131
75.89828512 1.39992653 0.000627854 0.007440307
B3GALT5 21 39556442 39673137 + 14319
protein_coding "beta-1,3-galactosyltransferase 5 [Source:HGNC
Symbol;Acc:HGNC:920]" - 174 134 281 90 45
98 0.664555523 0.412597001 0.909067406
0.303900236 0.13694886 0.319491584
ENSG00000100151 440.8023984 621.9842155 487.9214097
850.8777541 698.1889463 967.4785022 516.9026745
838.8484009 -0.697170818 0.000629039 0.007448585 PICK1

22	38056311	38075701	+	4371
protein_coding	protein interacting with PRKCA 1 [Source:HGNC
Symbol;Acc:HGNC:9394]	-	381	671	499	847	764
986	4.766931178	6.768238775	5.288374409
9.369228274	7.616775406	10.53033395
ENSG00000073060 941.766804	1334.809643	1700.391446
714.2551159	764.9007174	932.1547435	1325.655964
803.7701923	0.722453874	0.000633627	0.007497097	SCARB1
12	124776856	124882668	-	7741
protein_coding	scavenger receptor class B member 1 [Source:HGNC
Symbol;Acc:HGNC:1664]	-	814	1440	1739	711	837
950	5.750717718	8.201615072	10.40650675
4.440928181	4.711801524	5.728917572
ENSG00000104221 321.6353458	317.0172902	261.0721771
666.0353612	510.8476715	403.2795785	299.908271
526.7208704	-0.812714222	0.000636953	0.007529952	BRF2
8	37843268	37849904	-	2760
protein_coding	"BRF2, RNA polymerase III transcription initiation
factor subunit [Source:HGNC Symbol;Acc:HGNC:17298]"	-	278
342	267	663	559	411	5.508462872
5.463248837	4.481306361	11.61463758	8.825947171
6.951503978
ENSG00000169710 15172.39542	18892.1912	24400.95948
12966.09111	10127.39516	15168.61072	19488.51536
12754.03233	0.611734438	0.000637388	0.007529952	FASN
17	82078333	82098332	-	9364
protein_coding	fatty acid synthase [Source:HGNC Symbol;Acc:HGNC:3594]
-	13114	20381	24955	12907	11082	15459
76.58936821	95.96172406	123.4521407	66.64462392
51.57216204	77.06656959
ENSG00000139880 211.7239867	294.7704628	501.6105875
214.9797395	113.3186248	92.23425883	336.0350123
140.177541	1.263106654	0.000637906	0.007530245	CDH24
14	23047062	23057538	-	3748
protein_coding	cadherin 24 [Source:HGNC Symbol;Acc:HGNC:14265] -
183	318	513	214	124	94	2.670214932
3.740774213	6.340452286	2.760675796	1.441719261
1.170777337
ENSG00000234741 4468.185992	4370.574629	3318.647824
4642.155964	6616.345512	7035.315275	4052.469482
6097.938917	-0.589641221	0.000639633	0.007544801	GAS5
1	173863900	173868882	-	3631
processed_transcript	growth arrest specific 5 [Source:HGNC
Symbol;Acc:HGNC:16355]	-	3862	4715	3394	4621	7240
7170	58.16754484	57.25183436	43.30001481
61.53340334	86.89022364	92.18047519
ENSG00000171222 843.4250616	1862.244842	1539.054707
2743.503124	2013.23331	2524.667532	1414.908204
2427.134655	-0.777669636	0.000640181	0.007545445	SCAND1
20	35953617	35959472	-	1996

protein_coding SCAN domain containing 1 [Source:HGNC
Symbol;Acc:HGNC:10566] -   729   2009   1574   2731   2203
        2573   19.97384632   44.37653184   36.52974553
66.1549552   48.09639858   60.17627283
ENSG00000172936 1032.009815   1241.187578   1215.403431
1618.375516   1650.430939   1669.047599   1162.866941
1645.951351   -0.50062603   0.000643101   0.007574028   MYD88
        3   38138478   38143022   +   3989
protein_coding "MYD88, innate immune signal transduction adaptor
[Source:HGNC Symbol;Acc:HGNC:7562]"   -   892   1339   1243
        1611   1806   1701   12.22912913   14.79961776
14.43475936   19.52687385   19.72932865   19.90610604
ENSG00000196510 843.4250616   854.6489519   897.618946
603.7515114   592.1812006   568.1237858   865.2309865
588.0188326   0.557341776   0.000647954   0.007625303   ANAPC7
        12   110372900   110403730   -   6009
protein_coding anaphase promoting complex subunit 7 [Source:HGNC
Symbol;Acc:HGNC:17380] -   729   922   918   601   648
        579   6.634680855   6.764920154   7.076897949
4.835857384   4.699280833   4.498030496
ENSG00000152953 59.00504546   51.90926389   94.84644637
22.10072088   16.44947779   15.69944831   68.58691857
18.08321566   1.924897338   0.000652404   0.007671773   STK32B
        4   5051442 5500998 +   5324   protein_coding
serine/threonine kinase 32B [Source:HGNC Symbol;Acc:HGNC:14217] -
        51   56   97   22   18   16   0.523873997
        0.463750017   0.84398775   0.199795568   0.147330633
        0.140290406
ENSG00000004864 1266.873035   994.6185741   1159.668922
810.6946252   610.4583981   808.521588   1140.386844
743.2248704   0.617563201   0.000653707   0.007681188
SLC25A13   7   96120220   96322147   -   3710
        protein_coding solute carrier family 25 member 13
[Source:HGNC Symbol;Acc:HGNC:10983]   -   1095   1073   1186
        807   668   824   16.14116668   12.75145581
14.80857384   10.51721717   7.846232095   10.36810383
ENSG00000187678 697.6478905   858.3567564   756.8159741
1143.210017   1150.549586   1087.186796   770.940207
1126.982133   -0.547045208   0.000655713   0.007698833   SPRY4
        5   142310427   142326455   -   6565
protein_coding sprouty RTK signaling antagonist 4 [Source:HGNC
Symbol;Acc:HGNC:15533] -   603   926   774   1138   1259
        1108   5.02316327   6.218851898   5.461459105
8.381249092   8.356984515   7.878636538
ENSG00000196369 264.9442237   324.4328993   362.7632124
194.8881751   160.8393384   163.8629918   317.3801118
173.1968351   0.875236486   0.00065761   0.007715185
SRGAP2B 1   144887265   145095528   -   7230
protein_coding SLIT-ROBO Rho GTPase activating protein 2B
[Source:HGNC Symbol;Acc:HGNC:35237]   -   229   350   371

194	176	167	1.732175525	2.134340498
2.377048638	1.297372384	1.060798674	1.078261775
ENSG00000197601 2367.143589	1984.602393	1839.238821
1509.881068	1413.74123	1433.555874	2063.661601
1452.392724	0.506415603	0.0006595	0.007727951	FAR1
11	13668670	13732346	+	7501
protein_coding	fatty acyl-CoA reductase 1 [Source:HGNC
Symbol;Acc:HGNC:26222]	-	2046	2141	1881	1503	1547
1461	14.91698925	12.58436945	11.61641412
9.688153956	8.987310494	9.092369706
ENSG00000107485 82.14427898	97.32986979	120.2692052
17.07782977	35.64053522	42.19226734	99.91445132
31.63687744	1.658380896	0.000659709	0.007727951	GATA3
10	8045378 8075203 +	3436	protein_coding	GATA
binding protein 3 [Source:HGNC Symbol;Acc:HGNC:4172]	zf-GATA 71
105	123	17	39	43	1.130055846
1.347317969	1.658266824	0.239219723	0.494618151
0.584199709
ENSG00000145431 2568.45492	1672.219858	1994.708769
1507.871911	1323.269102	1304.035425	2078.461182
1378.392146	0.592050978	0.000664169	0.00777294	PDGFC
4	156760454	156971394	-	5593
protein_coding	platelet derived growth factor C [Source:HGNC
Symbol;Acc:HGNC:8801]	-	2220	1804	2040	1501	1448
1329	21.70715303	14.22085381	16.89615185
12.97588803	11.28190245	11.09241725
ENSG00000188352 586.5795696	688.7246976	692.2812786
481.1929683	333.5588553	396.4110699	655.8618486
403.7209645	0.701176218	0.000664567	0.00777294	FOCAD
9	20658309	20995955	+	10036
protein_coding	focadhesin [Source:HGNC Symbol;Acc:HGNC:23377]	-
507	743	708	479	365	404	2.762752747
3.264089956	3.267947424	2.307682609	1.584859654
1.879172933
ENSG00000137404 828.3845598	807.3744437	1121.534783
406.8541799	588.5257611	654.4707515	919.0979289
549.9502308	0.740820111	0.000665253	0.007775013	NRM
6	30688047	30691420	-	2045
protein_coding	nurim [Source:HGNC Symbol;Acc:HGNC:8003]	-
716	871	1147	405	644	667	19.14760337
18.77840929	25.98199898	9.57553234	13.72306602
15.22574519
ENSG00000215784 144.6202095	118.649746	160.3589403
31.14192488	62.14247166	67.70387084	141.2096319
53.6627558	1.394029359	0.000667286	0.007792817	FAM72D
1	145096000	145112696	+	2397
protein_coding	family with sequence similarity 72 member D
[Source:HGNC Symbol;Acc:HGNC:33593]	-	125	128	164
31	68	69	2.85191575	2.354376388
3.169408876	0.625309283	1.236230563	1.343776657

ENSG00000173083 165.4455196	134.4079154	191.6484896
50.2289111	82.24738897	76.53481052	163.8339749
69.6703702	1.23195131	0.000669716	0.007815231	HPSE
4	83292461	83335153	-	4721
protein_coding	heparanase [Source:HGNC Symbol;Acc:HGNC:5164]	-
143	145	196	50	90	78	1.656520252
1.354153697	1.923200338	0.5120793	0.830743796
0.771270354
ENSG00000144857 248.7467603	252.1307103	455.6540619
175.8011889	134.337402	157.9756986	318.8438442
156.0380965	1.032170588	0.000683876	0.007974376	BOC
3	113211003	113287459	+	10330
protein_coding	"BOC cell adhesion associated, oncogene regulated
[Source:HGNC Symbol;Acc:HGNC:17173]"	-	215	272	466
175	147	161	1.138237399	1.160920661
2.089719768	0.819103806	0.620119818	0.727564651
ENSG00000169045 7781.724231	7821.613726	7547.625974
6006.373189	5109.390575	6165.958324	7716.987977
5760.574029	0.421885115	0.000688424	0.008015291
HNRNPH1 5	179614178	179634784	-	9480
protein_coding	heterogeneous nuclear ribonucleoprotein H1
[Source:HGNC Symbol;Acc:HGNC:5041]	-	6726	8438	7719
5979	5591	6284	38.80102857	39.24326578
37.7185648	30.49449408	25.7003923	30.94381635
ENSG00000143499 1213.652798	1007.59589	1195.847463
525.3944101	760.331418	853.6575019	1139.03205
713.1277767	0.67506418	0.000688738	0.008015291	SMYD2
1	214281102	214337131	+	4889
protein_coding	SET and MYND domain containing 2 [Source:HGNC
Symbol;Acc:HGNC:20982]	-	1049	1087	1223	523	832
870	11.73411023	9.802649282	11.58801038
5.172290014	7.415866222	8.307019907
ENSG00000197183 388.739123	406.0045997	693.2590771
157.7187809	313.453938	266.8906213	496.0009333
246.0211134	1.011203316	0.000688957	0.008015291	NOL4L
20	32443059	32585074	-	9448
protein_coding	nucleolar protein 4 like [Source:HGNC
Symbol;Acc:HGNC:16106]	-	336	438	709	157	343
272	1.9448858	2.043940193	3.476232427
0.803453939	1.582023146	1.343925069
ENSG00000141933 381.797353	625.6920201	503.5661843
677.0857216	988.7963874	884.075183	503.6851858
849.985764	-0.753699789	0.00069138	0.008037356	TPGS1
19	507497	519654	+	3642	protein_coding
tubulin polyglutamylase complex subunit 1 [Source:HGNC
Symbol;Acc:HGNC:25058]	-	330	675	515	674	1082
901	4.955285838	8.171424819	6.550428995
8.947901024	12.94630747	11.54864111
ENSG00000143416 244.1189136	194.6597396	231.7382246
59.2701151	129.7681026	109.8961382	223.5056259

99.64478529	1.163192268	0.000692744	0.008037859
SELENBP1	1	151364302	151372733	-	3504
	protein_coding	selenium binding protein 1 [Source:HGNC
Symbol;Acc:HGNC:10719]	-	211	210	237	59	142
	112	3.293161811	2.642342774	3.133189802
0.814121325	1.765968061	1.492106977
ENSG00000176974 857.3086017	1099.364053	1230.070408
587.6782599	724.6908828	732.967993	1062.247687
681.7790452	0.640184956	0.000692855	0.008037859	SHMT1
	17	18327860	18363563	-	7281
protein_coding	serine hydroxymethyltransferase 1 [Source:HGNC
Symbol;Acc:HGNC:10850]	-	741	1186	1258	585	793
	747	5.565726864	7.181705895	8.003723296
3.884776622	4.746142287	4.78933931
ENSG00000184436 750.8681275	984.4221116	846.7734284
1162.297003	1272.092949	1404.119408	860.6878892
1279.50312	-0.571240274	0.000693	0.008037859	THAP7
	22	20999104	21002196	-	2879
protein_coding	THAP domain containing 7 [Source:HGNC
Symbol;Acc:HGNC:23190]	THAP	649	1062	866	1157	1392
	1431	12.32814531	16.2636047	13.9340923
19.43089973	21.06959405	23.20299277
ENSG00000126947 1032.009815	1122.537832	913.2637207
1649.517441	1593.771626	1279.505037	1022.603789
1507.598035	-0.559952103	0.000694651	0.008050895	ARMCX1
	X	101550531	101554700	+	2141
protein_coding	armadillo repeat containing X-linked 1 [Source:HGNC
Symbol;Acc:HGNC:18073]	-	892	1211	934	1642	1744
	1304	22.78467823	24.93798927	20.20843421
37.08153486	35.49673522	28.43197051
ENSG00000160299 2139.222138	1896.542034	3270.735702
1617.370937	1170.654503	1708.296219	2435.499958
1498.773887	0.700675027	0.000697389	0.008076507	PCNT
	21	46324122	46445769	+	11660
protein_coding	pericentrin [Source:HGNC Symbol;Acc:HGNC:16068] -
	1849	2046	3345	1610	1281	1741	8.672275649
	7.736437777	13.28923641	6.676187758	4.7875043
	6.970216319
ENSG00000162852 1458.928673	1313.489767	1142.06855
1930.799343	1894.431526	1746.563625	1304.828997
1857.264831	-0.509790764	0.000700471	0.008105263	CNST
	1	246566444	246668584	+	5922
protein_coding	"consortin, connexin sorting protein [Source:HGNC
Symbol;Acc:HGNC:26486]" -	1261	1417	1168	1922	2073
	1780	11.64505104	10.54958572	9.136437665
15.69228535	15.25420213	14.03129111
ENSG00000167767 533.3593325	646.0849452	808.6392902
448.041887	285.1242818	414.0729492	662.6945226
382.4130393	0.794498781	0.000700933	0.008105263	KRT80
	12	52168996	52192000	-	4350

protein_coding keratin 80 [Source:HGNC Symbol;Acc:HGNC:27056] -
461 697 827 446 312 422 5.795706459
7.064435741 8.806811653 4.957318451 3.125531893
4.528654928
ENSG00000170006 241.8049902 300.3321696 134.9361814
507.3120021 416.7201041 401.3171475 225.6911138
441.7830846 -0.968699646 0.000704009 0.008133084
TMEM154 4 152618632 152680165 - 11587
protein_coding transmembrane protein 154 [Source:HGNC
Symbol;Acc:HGNC:26489] - 209 324 138 505 456
409 0.986438449 1.232843533 0.551709593
2.107276809 1.714953834 1.647776622
ENSG00000126561 401.4657015 355.9492381 457.6096588
174.7966106 259.5362052 246.2850954 405.0081994
226.8726371 0.835225137 0.000704403 0.008133084 STAT5A
17 42287547 42311943 + 5754
protein_coding signal transducer and activator of transcription 5A
[Source:HGNC Symbol;Acc:HGNC:11366] STAT 347 384 468
174 284 251 3.298027936 2.942356727
3.767718532 1.462111885 2.150834927 2.03633816
ENSG00000117528 1905.51588 1767.695826 1983.952986
1333.075301 1417.39667 1345.246477 1885.721564
1365.239483 0.465786717 0.000707644 0.008162704 ABCD3
1 94418455 94518666 + 7589
protein_coding ATP binding cassette subfamily D member 3 [Source:HGNC
Symbol;Acc:HGNC:67] - 1647 1907 2029 1327 1551
1371 11.86871658 11.07898816 12.38511233
8.454493343 8.906064636 8.433326951
ENSG00000136942 10266.87791 10503.28338 10920.05271
12859.60582 13495.88267 18530.25509 10563.40467
14961.91452 -0.502199699 0.000708036 0.008162704 RPL35
9 124857880 124861981 - 1535
protein_coding ribosomal protein L35 [Source:HGNC
Symbol;Acc:HGNC:10344] - 8874 11331 11168 12801 14768
18885 316.1591515 325.4571505 337.0307146
403.2150504 419.2488709 574.3209321
ENSG00000137309 10402.24243 17047.55843 13773.26849
19698.77436 18769.76802 22037.11935 13741.02312
20168.55391 -0.553512038 0.000709682 0.008175512 HMGA1
6 34236873 34246231 + 2327
protein_coding high mobility group AT-hook 1 [Source:HGNC
Symbol;Acc:HGNC:5010] HMGI/HMGY 8991 18391 14086 19609
20539 22459 211.3033231 348.4519659 280.4101673
407.4368258 384.6285426 450.5469641
ENSG00000105486 1790.976674 2081.932262 2539.342487
1174.351942 1135.013968 1758.338211 2137.417141
1355.901373 0.656948554 0.000711996 0.008195997 LIG1
19 48115445 48170603 - 6576
protein_coding DNA ligase 1 [Source:HGNC Symbol;Acc:HGNC:6598] -
1548 2246 2597 1169 1242 1792 12.87371423

15.05850666 18.29416538 8.59515916 8.230351608
12.72102882
ENSG00000210196 218.6657567 215.0526647 214.1378531
405.8496017 337.2142948 362.0685267 215.9520915
368.3774744 -0.770324543 0.000713244 0.008198165 MT-TP
MT 15956 16023 - 68 Mt_tRNA
mitochondrially encoded tRNA proline [Source:HGNC
Symbol;Acc:HGNC:7494] - 189 232 219 404 369
369 152.0014057 150.4225789 149.1892937
287.2590163 236.4699999 253.3165061
ENSG00000075213 240.6480285 270.6697331 203.3820706
452.0601999 457.8437986 341.4630008 238.2332774
417.1223331 -0.807978805 0.000713257 0.008198165 SEMA3A
7 83955777 84492724 - 9371
protein_coding semaphorin 3A [Source:HGNC Symbol;Acc:HGNC:10723]
- 208 292 208 450 501 348
1.213869809 1.37382328 1.028205335 2.321815961
2.329755715 1.733561854
ENSG00000168807 2420.363826 2027.242145 2382.89474
1757.00731 1508.782658 1606.249805 2276.83357
1624.013258 0.487357839 0.000713809 0.008198347 SNTB2
16 69187129 69309052 + 9875
protein_coding syntrophin beta 2 [Source:HGNC Symbol;Acc:HGNC:11169]
- 2092 2187 2437 1749 1651 1637
11.58562023 9.764401679 11.43196486 8.563551654
7.285654406 7.738514683
ENSG00000105248 487.0808655 792.5432254 664.902923
1058.825446 956.8112917 1076.393425 648.1756713
1030.676721 -0.667588458 0.000714999 0.008199926 YJU2
19 4247079 4269090 + 1671 protein_coding YJU2
splicing factor homolog [Source:HGNC Symbol;Acc:HGNC:25518] -
421 855 680 1054 1047 1097 13.77844981
22.55919627 18.8510228 30.49757988 27.30416059
30.6461705
ENSG00000136240 9108.759273 7177.382684 7431.267963
5713.036349 6015.939573 5680.256642 7905.803306
5803.077521 0.445919588 0.000715296 0.008199926 KDELR2
7 6445953 6484242 - 3990 protein_coding KDEL
endoplasmic reticulum protein retention receptor 2 [Source:HGNC
Symbol;Acc:HGNC:6305] - 7873 7743 7600 5687 6583
5789 107.9100934 85.55991049 88.23545964
68.91464908 71.89679934 67.72931601
ENSG00000099783 9689.554034 9870.175748 10823.25067
7983.38313 7097.949668 7881.123052 10127.66015
7654.15195 0.404052216 0.000715556 0.008199926 HNRNPM
19 8444767 8489114 + 5614 protein_coding
heterogeneous nuclear ribonucleoprotein M [Source:HGNC
Symbol;Acc:HGNC:5046] - 8375 10648 11069 7947 7767
8032 81.58439929 83.62374097 91.33525276
68.44347025 60.28919632 66.78782912

ENSG00000135677 3592.366003 2979.220967 4222.133561
2718.388669 2089.08368 2565.878583 3597.906844
2457.783644 0.549868994 0.000717 0.008210319 GNS
12 64713445 64759447 - 6284
protein_coding glucosamine (N-acetyl)-6-sulfatase [Source:HGNC
Symbol;Acc:HGNC:4422] - 3105 3214 4318 2706 2286
2615 27.02216496 22.54984983 31.83089994
20.82058043 15.85253233 19.42591807
ENSG00000179271 2384.498014 2568.581611 2679.16766
3447.712458 3239.633265 3615.779189 2544.082428
3434.374971 -0.432669056 0.000718907 0.008226
GADD45GIP1 19 12953119 12957236 - 1782
protein_coding GADD45G interacting protein 1 [Source:HGNC
Symbol;Acc:HGNC:29996] - 2061 2771 2740 3432 3545
3685 63.25065173 68.55873316 71.22710926
93.11953443 86.68962765 96.53300559
ENSG00000139737 288.0834573 431.0322805 277.6947502
566.5821172 676.2563093 492.5701908 332.2701626
578.4695391 -0.798760871 0.000727525 0.008318383 SLAIN1
13 77697854 77764242 + 4133
protein_coding SLAIN motif family member 1 [Source:HGNC
Symbol;Acc:HGNC:26387] - 249 465 284 564 740
502 3.294796944 4.960454902 3.183137438
6.598039561 7.802340731 5.670016826
ENSG00000111696 372.5416596 355.0222869 366.6744061
719.278007 496.2259134 570.0862168 364.7461175
595.1967124 -0.706266211 0.00073073 0.008348782 NT5DC3
12 103770453 103841197 - 8722
protein_coding 5'-nucleotidase domain containing 3 [Source:HGNC
Symbol;Acc:HGNC:30826] - 322 383 375 716 543
581 2.018991554 1.936050359 1.99167119
3.969155891 2.712953469 3.109611157
ENSG00000135315 362.1290045 261.4002217 261.0721771
169.7737195 115.1463446 168.7690693 294.8671344
151.2297111 0.963001572 0.000737064 0.008414857 CEP162
6 84124241 84227635 - 5474
protein_coding centrosomal protein 162 [Source:HGNC
Symbol;Acc:HGNC:21107] - 313 282 267 169 126
172 3.127045908 2.271319729 2.259482199
1.492736417 1.003054082 1.466795908
ENSG00000215375 89.08604903 100.1107232 128.0915925
302.3780448 175.4610965 205.0740436 105.7627883
227.6377283 -1.103818853 0.000739582 0.008432586 MYL5
4 673580 682033 + 4305 protein_coding myosin
light chain 5 [Source:HGNC Symbol;Acc:HGNC:7586] - 77
108 131 301 192 209 0.978165367
1.106074953 1.40961527 3.380605987 1.943509513
2.266309223
ENSG00000164715 853.8377167 788.8354209 1024.73274
1586.229013 1204.467318 1244.181279 889.1352926

1344.959203 -0.596876886 0.00073972 0.008432586 LMTK2
7 98106885 98209633 + 9139
protein_coding lemur tyrosine kinase 2 [Source:HGNC
Symbol;Acc:HGNC:17880] - 738 851 1048 1579 1318
1268 4.416237256 4.105488507 5.312085556
8.353811458 6.284566567 6.476891354
ENSG00000122203 1866.179183 2237.660054 1891.062137
2560.669888 3145.505698 2725.816713 1998.300458
2810.6641 -0.491971245 0.00074372 0.00846694
KIAA1191 5 176346061 176361968 - 3973
protein_coding KIAA1191 [Source:HGNC Symbol;Acc:HGNC:29209]
- 1613 2414 1934 2549 3442 2778
22.20294152 26.78876291 22.54967882 31.0207638
37.7529505 32.64071866
ENSG00000185115 519.4757924 620.1303132 581.7900576
949.3264198 830.6986286 810.4840191 573.7987211
863.5030225 -0.588742378 0.000743841 0.00846694 NSMCE3
15 29264992 29269829 - 4838
protein_coding "NSE3 homolog, SMC5-SMC6 complex component
[Source:HGNC Symbol;Acc:HGNC:7677]" - 449 669 595
945 909 826 5.075457432 6.096691387
5.69709626 9.444243175 8.187599993 7.970034826
ENSG00000115355 1564.212186 1342.225252 1755.148157
1051.793398 1186.190121 765.3481052 1553.861865
1001.110542 0.63394558 0.000745017 0.008474017
CCDC88A 2 55287842 55419895 - 33942
protein_coding coiled-coil domain containing 88A [Source:HGNC
Symbol;Acc:HGNC:25523] - 1352 1448 1795 1047 1298
780 2.178381676 1.880897333 2.449793509
1.491456081 1.666463712 1.072761575
ENSG00000115380 10.41265508 36.15109449 33.24514615 0
0 3.924862078 26.60296524 1.308287359
4.359450825 0.000749107 0.008514196 EFEMP1 2
55865967 55924139 - 3962 protein_coding EGF
containing fibulin extracellular matrix protein 1 [Source:HGNC
Symbol;Acc:HGNC:3218] - 9 39 34 0 0
4 0.124228932 0.433994368 0.397527248 0
0 0.047129361
ENSG00000213390 460.4707469 634.0345803 562.2340893
342.5611737 357.3192121 317.9138283 552.2464722
339.264738 0.703797459 0.000749955 0.008517506
ARHGAP19 10 97222173 97292673 - 6167
protein_coding Rho GTPase activating protein 19 [Source:HGNC
Symbol;Acc:HGNC:23724] - 398 684 575 341 391
324 3.529424015 4.890081657 4.319130757
2.673508979 2.762876082 2.452545638
ENSG00000258701 34.70885027 52.83621503 37.15633981
125.5722778 110.5770452 98.12155195 41.56713504
111.423625 -1.418744742 0.000750687 0.008519489
LINC00638 14 104821201 104823718 + 2518

lincRNA long intergenic non-protein coding RNA 638
[Source:HGNC Symbol;Acc:HGNC:28325] - 30 57 38
125 121 100 0.651568742 0.998051813
0.699085552 2.400244613 2.094055762 1.853917099
ENSG00000186017 295.0252273 267.8888797 211.2044579
493.247907 434.9973016 396.4110699 258.0395216
441.5520928 -0.776002778 0.000753783 0.008548278 ZNF566
19 36445119 36489902 - 6481
protein_coding zinc finger protein 566 [Source:HGNC
Symbol;Acc:HGNC:25919] zf-C2H2 255 289 216 491 476
404 2.151755252 1.966028365 1.54388229
3.663031785 3.200541987 2.909949013
ENSG00000160796 470.883402 769.3694469 489.8770065
1174.351942 811.5075711 936.0796056 576.7099518
973.9797061 -0.75468986 0.000756992 0.008578311 NBEAL2
3 46979683 47009703 + 9819
protein_coding neurobeachin like 2 [Source:HGNC
Symbol;Acc:HGNC:31928] - 407 830 501 1169 888
954 2.266845191 3.726874688 2.363594233
5.756366905 3.940980659 4.53552067
ENSG00000275496 129.5797077 74.15609127 84.09066379
11.05036044 23.76035681 44.15469838 95.94215425
26.32180521 1.86357748 0.000758472 0.008585464
CU633906.1 21 6228966 6267317 - 2878 lincRNA
uncharacterized LOC102724701 [Source:NCBI gene;Acc:102724701] -
112 80 86 11 26 45 2.128246588
1.225555986 1.384235929 0.184800487 0.393678009
0.729907389
ENSG00000223756 72.88858557 148.3121825 72.3570828
215.9843177 215.6709311 232.5480781 97.85261697
221.401109 -1.174069495 0.000758747 0.008585464 TSSC2
11 3380961 3408978 + 3666
transcribed_unprocessed_pseudogene tumor suppressing
subtransferable candidate 2 pseudogene [Source:HGNC
Symbol;Acc:HGNC:12384] - 63 160 74 215 236
237 0.939815929 1.924249933 0.935064814
2.835614678 2.805292432 3.017879682
ENSG00000103742 463.941632 554.3167822 824.2840648
347.5840648 264.1055046 407.2044406 614.1808263
339.6313367 0.855819742 0.00075991 0.008592267 IGDCC4
15 65381464 65423072 - 7764
protein_coding immunoglobulin superfamily DCC subclass member 4
[Source:HGNC Symbol;Acc:HGNC:13770] - 401 598 843
346 289 415 2.824577912 3.395858732
5.029728046 2.154724692 1.622074936 2.495218639
ENSG00000179364 1144.235097 1436.774268 1424.652292
1884.588745 2038.821387 1790.718323 1335.220553
1904.709485 -0.511938894 0.000760937 0.008597515 PACS2
14 105300563 105398147 + 12363
protein_coding phosphofurin acidic cluster sorting protein 2

[Source:HGNC Symbol;Acc:HGNC:23794]	-	989	1550	1457
	1876	2231	1825	4.374889973	5.527665914
5.459314808	7.336859142	7.863833303	6.891044197
ENSG00000232949 26.61011854	12.97731597	2.933395248	0
	0	0	14.17360992	0	6.314565944
0.00076642	1	AC002480.2	7	22589705
22591622	+	557	antisense	"novel transcript,
antisense to STEAP1B"	-	23	14	3	0	0
	0	2.258225741	1.108171045	0.249498831	0
	0	0
ENSG00000167625 406.0935482	348.533629	490.8548049
608.7744025	668.0315704	716.2873292	415.1606607
664.364434	-0.678533248	0.000767236	0.008656968	ZNF526
	19	42220271	42228201	+	4271
protein_coding	zinc finger protein 526 [Source:HGNC
Symbol;Acc:HGNC:29415]	zf-C2H2 351	376	502	606	731
	730	4.494405546	3.881434229	5.444733175
6.860318347	7.458412572	7.978832069
ENSG00000149968 830.6984832	466.2564238	490.8548049
1309.970002	697.2750865	1274.59896	595.9365706
1093.948016	-0.877382725	0.000767646	0.008656968	MMP3
	11	102835801	102843803	-	2048
protein_coding	matrix metallopeptidase 3 [Source:HGNC
Symbol;Acc:HGNC:7173]	-	718	503	502	1304	763
	1299	19.1729617	10.828592	11.35471455
30.78568743	16.23503329	29.6091019
ENSG00000125651 2775.55106	3658.676153	3711.722788
4953.575213	4196.444557	5413.366021	3381.983334
4854.46193	-0.521089673	0.000767897	0.008656968	GTF2F1
	19	6379569 6393981 -	3134	protein_coding
general transcription factor IIF subunit 1 [Source:HGNC
Symbol;Acc:HGNC:4652]	-	2399	3947	3796	4931	4592
	5517	41.86257789	55.52672921	56.10863035
76.07417073	63.85008577	82.17695176
ENSG00000166130 963.7490759	721.1679876	755.8381757
339.547439	456.0160788	624.0530704	813.5850797
473.2055294	0.781123161	0.000769689	0.008670771	IKBIP
	12	98613405	98645113	-	4163
protein_coding	IKBKB interacting protein [Source:HGNC
Symbol;Acc:HGNC:26430]	-	833	778	773	338	499
	636	10.94292199	8.23961926	8.601526642
3.925649362	5.223393358	7.131760342
ENSG00000198331 175.8581747	295.6974139	308.0065011
138.6317946	127.026523	117.7458623	259.8540299
127.8013933	1.025758745	0.000770529	0.008673837	HYLS1
	11	125883614	125900648	+	2999
protein_coding	"HYLS1, centriolar and ciliogenesis associated
[Source:HGNC Symbol;Acc:HGNC:26558]"	-	152	319	315
	138	139	120	2.771799646	4.689733621
4.86560158	2.224865887	2.019746816	1.867887932

ENSG00000007968 452.3720152 517.2387366 756.8159741
355.6206906 354.5776324 275.721561 575.4755753
328.6399613 0.808798371 0.000772691 0.00869178 E2F2
1 23506430 23531220 - 5457
protein_coding E2F transcription factor 2 [Source:HGNC
Symbol;Acc:HGNC:3114] E2F 391 558 774 354 388
281 3.918478934 4.508314482 6.570364491
3.136537792 3.098392049 2.403800394
ENSG00000213513 21.98227184 1.853902282 19.55596832 0
0 0 14.46404748 0 6.344456106
0.000780602 1 IMPDH1P5 10 77780337
77781876 - 1540 processed_pseudogene inosine
monophosphate dehydrogenase 1 pseudogene 5 [Source:HGNC
Symbol;Acc:HGNC:33960] - 19 2 20 0 0
0 0.674726229 0.057258931 0.601605407 0
0 0
ENSG00000105290 53.22023708 48.20145932 87.02405904
23.10529911 9.138598774 11.77458623 62.81525182
14.67282804 2.102584278 0.000784004 0.008812556 APLP1
19 35867899 35879791 + 3656
protein_coding amyloid beta precursor like protein 1 [Source:HGNC
Symbol;Acc:HGNC:597] - 46 52 89 23 10
12 0.68809176 0.627091789 1.127681032
0.304174544 0.119193456 0.153221989
ENSG00000158863 951.0224974 1266.215258 1193.891866
1669.609005 1605.651805 1646.479642 1137.043207
1640.58015 -0.528065806 0.000784626 0.008813064
FAM160B2 8 22089159 22104898 + 8278
protein_coding family with sequence similarity 160 member B2
[Source:HGNC Symbol;Acc:HGNC:16492] - 822 1366 1221
1662 1757 1678 5.430516133 7.275439527
6.832705426 9.707486916 9.249216328 9.462645497
ENSG00000175573 790.2048245 1326.467083 1098.067621
1516.913115 1676.932875 1807.398987 1071.579843
1667.081659 -0.636599388 0.000786907 0.008832195
C11orf68 11 65916808 65919117 - 1761
protein_coding chromosome 11 open reading frame 68
[Source:HGNC Symbol;Acc:HGNC:28801] - 683 1431 1123
1510 1835 1842 21.21075172 35.82731282
29.54084107 41.45899859 45.40830704 48.8288286
ENSG00000132376 882.7617586 986.2760139 941.6198748
1242.663261 1303.164185 1475.748141 936.8858824
1340.525196 -0.516462838 0.000789217 0.008851618 INPP5K
17 1494571 1516888 - 5325 protein_coding
inositol polyphosphate-5-phosphatase K [Source:HGNC
Symbol;Acc:HGNC:33882] - 763 1064 963 1237 1426
1504 7.836094028 8.809595621 8.377397654
11.23185024 11.66966828 13.18482166
ENSG00000135317 1396.452743 1095.656248 1132.290566
706.2184901 763.0729976 928.2298814 1208.133186

799.1737897 0.595631125 0.00079436 0.008902776 SNX14
6 85505496 85594156 - 7105
protein_coding sorting nexin 14 [Source:HGNC Symbol;Acc:HGNC:14977]
- 1207 1182 1158 703 835 946
9.290474606 7.334784394 7.549999675 4.784014191
5.121309126 6.21545734
ENSG00000103121 767.065591 685.9438442 685.4366897
489.2295941 468.8101171 461.1712942 712.815375
473.0703351 0.59106292 0.00079667 0.008922122 CMC2
16 80966448 81020270 - 12781
protein_coding C-X9-C motif containing 2 [Source:HGNC
Symbol;Acc:HGNC:24447] - 663 740 701 487 513
470 2.836895944 2.552706257 2.540713171
1.842321171 1.749085857 1.716639332
ENSG00000130758 474.354287 818.4978574 695.2146739
984.4866576 1071.043776 1150.965804 662.6889394
1068.832079 -0.688180364 0.000801077 0.008961515
MAP3K10 19 40191744 40215575 + 4566
protein_coding mitogen-activated protein kinase kinase kinase 10
[Source:HGNC Symbol;Acc:HGNC:6849] - 410 883 711
980 1172 1173 4.91069162 8.526263583
7.213335786 10.37746757 11.18536865 11.9924562
ENSG00000143797 623.6023432 537.6316617 485.9658128
222.0117871 432.255722 207.0364746 549.0666059
287.1013279 0.933855512 0.000801632 0.008961515 MBOAT2
2 8852690 9003813 - 8798 protein_coding
membrane bound O-acyltransferase domain containing 2 [Source:HGNC
Symbol;Acc:HGNC:25193] - 539 580 497 221 473
211 3.350422066 2.906551309 2.616826245
1.214533596 2.342803051 1.119552679
ENSG00000276550 952.1794591 818.4978574 1089.267436
663.0216265 417.633964 663.3016912 953.3149174
581.3190939 0.714071994 0.000801946 0.008961515
HERC2P2 15 22495570 22590815 + 6965
transcribed_unprocessed_pseudogene hect domain and RLD 2
pseudogene 2 [Source:HGNC Symbol;Acc:HGNC:4870] - 823 883
1114 660 457 676 6.462096322 5.589507469
7.409118244 4.581672383 2.859260187 4.530765773
ENSG00000171735 600.4631097 634.9615315 561.2562909
335.5291262 403.0122059 406.2232251 598.893644
381.5881857 0.650059815 0.0008031 0.008967856 CAMTA1
1 6785324 7769706 + 10033 protein_coding
calmodulin binding transcription activator 1 [Source:HGNC
Symbol;Acc:HGNC:18806] CG-1 519 685 574 334 441
414 2.828988997 3.010188673 2.650229818
1.609595952 1.915430396 1.92626292
ENSG00000134901 704.5896605 699.8481113 780.2831361
524.3898319 458.7576585 460.1900786 728.2403026
481.112523 0.598374921 0.000806155 0.008993762 KDELC1
13 102784281 102799007 - 2367

protein_coding KDEL motif containing 1 [Source:HGNC
Symbol;Acc:HGNC:19350] -   609   755   798   522   502
        469   14.07063662   14.06315138   15.61734086
10.66285397   9.241959413   9.249550346
ENSG00000177370 736.9845874   775.8581049   784.1943298
1005.5828   1191.67328   1121.529339   765.6790074
1106.261806   -0.530807742   0.000806597   0.008993762   TIMM22
        17   997117 1003671 +   3194   protein_coding
translocase of inner mitochondrial membrane 22 [Source:HGNC
Symbol;Acc:HGNC:17317] -   637   837   802   1001   1304
        1143   10.90684731   11.55379092   11.63166571
15.15306137   17.79103759   16.70541829
ENSG00000124194 0   0   0   17.07782977   7.310879019
        15.69944831   0   13.36271904   -6.159065582
0.000808479   1   GDAP1L1 20   44247099   44280917
        +   3385   protein_coding ganglioside induced
differentiation associated protein 1 like 1 [Source:HGNC
Symbol;Acc:HGNC:4213]   -   0   0   0   17   8
        16   0   0   0   0.242823919   0.10298878
        0.220651734
ENSG00000163378 797.1465946   677.601284   620.9019943
369.6847857   476.1209961   472.9458804   698.5499576
439.5838874   0.66722104   0.000808507   0.009008484   EOGT
        3   68975214   69013961   -   5645
protein_coding EGF domain specific O-linked N-acetylglucosamine
transferase [Source:HGNC Symbol;Acc:HGNC:28526] -   689   731
        635   368   521   482   6.674980301   5.709359483
        5.210893952   3.151991872   4.021910248   3.985925047
ENSG00000101146 2689.935896   2745.629279   2509.030736
3322.14018   3865.627281   3532.37587   2648.198637
3573.381111   -0.432373091   0.000814498   0.009068629   RAE1
        20   57351010   57379211   +   5855
protein_coding ribonucleic acid export 1 [Source:HGNC
Symbol;Acc:HGNC:9828]   -   2325   2962   2566   3307   4230
        3600   21.71654692   22.30448052   20.30169077
27.30917069   31.48270687   28.70262634
ENSG00000124243 39.33669697   83.42560268   89.95745429
201.9202226   141.648281   179.5624401   70.90658465
174.3769812   -1.292479787   0.000819273   0.009115152   BCAS4
        20   50794894   50882676   +   7583
protein_coding breast carcinoma amplified sequence 4 [Source:HGNC
Symbol;Acc:HGNC:14367] -   34   90   92   201   155
        183   0.245206836   0.523281537   0.562016709
1.281610975   0.890736482   1.12656452
ENSG00000229056 419.9770883   164.0703519   230.7604262
138.6317946   112.4047649   107.9337071   271.6026221
119.6567556   1.180548932   0.000820368   0.009120705
AC020571.1   2   196260024   196264204   +   1607
        antisense   uncharacterized LOC101927482 [Source:NCBI
gene;Acc:101927482]   -   363   177   236   138   123

110 12.35337033 4.856141664 6.802970411
4.1520677 3.335399293 3.19538244
ENSG00000101938 24.29619519 4.634755704 11.73358099 0
0 0 13.55484396 0 6.249634034
0.00082284 1 CHRDL1 X 110673856 110795819
- 3920 protein_coding chordin like 1 [Source:HGNC
Symbol;Acc:HGNC:29861] - 21 5 12 0 0
0 0.292973231 0.05623645 0.141806989 0
0 0
ENSG00000122691 550.7137576 573.7827562 763.660563
342.5611737 425.8587029 391.5049923 629.3856923
386.641623 0.703013172 0.000823726 0.009151383 TWIST1
7 19020991 19117672 - 2033
protein_coding twist family bHLH transcription factor 1 [Source:HGNC
Symbol;Acc:HGNC:12428] bHLH 476 619 781 341 466
399 12.80454901 13.42416336 17.79574242
8.10995075 9.988657819 9.161815735
ENSG00000186480 3669.882435 3023.714621 3482.917958
2028.24343 2762.598409 2350.992385 3392.171672
2380.611408 0.510534887 0.000824633 0.0091548 INSIG1
7 155297776 155310235 + 3650
protein_coding insulin induced gene 1 [Source:HGNC
Symbol;Acc:HGNC:6083] - 3172 3262 3562 2019 3023
2396 47.52641182 39.40261545 45.20677296
26.7451274 36.0914126 30.64361414
ENSG00000269974 26.61011854 24.10072966 25.42275882 0
2.741579632 2.943646558 25.37786901 1.895075397
3.73107461 0.000828384 0.009189772 AC091057.4
15 30648797 30649529 + 733
lincRNA novel transcript - 23 26 26 0
3 3 1.716005099 1.56387966 1.643129637
0 0.178351135 0.191057159
ENSG00000106399 1308.523655 1289.389037 1215.403431
751.4245101 919.3430367 955.703916 1271.105374
875.4904876 0.537632776 0.000836186 0.009269595 RPA3
7 7636518 7718607 - 3232 protein_coding
replication protein A3 [Source:HGNC Symbol;Acc:HGNC:10291] -
1131 1391 1243 748 1006 974 19.13753357
18.97534741 17.81567298 11.19003545 13.56392021
14.06804149
ENSG00000215301 8774.397349 7931.920912 8471.645478
6699.532163 6329.393511 6215.0191 8392.654579
6414.648258 0.38770723 0.000838861 0.009289554 DDX3X
X 41333284 41364472 + 15892
protein_coding DEAD-box helicase 3 X-linked [Source:HGNC
Symbol;Acc:HGNC:2745] - 7584 8557 8664 6669 6926
6334 26.09843591 23.73979332 25.25470751
20.29006216 18.99164259 18.60567963
ENSG00000184432 4802.547916 4073.023313 4391.292687
3152.366461 3166.524475 3515.695206 4422.287972

3278.195381	0.431732353	0.000839202	0.009289554	COPB2
	3	139355600	139389732	-	8177
protein_coding	coatomer protein complex subunit beta 2 [Source:HGNC
Symbol;Acc:HGNC:2232]	-	4151	4394	4491	3138	3465
	3583	27.7621725	23.69190665	25.44201551
18.55496579	18.46578773	20.45496997
ENSG00000124172 2105.67025	2530.576615	3056.597849
1537.00468	1442.984746	2076.252039	2564.281571
1685.413822	0.605791693	0.000840941	0.009299163
ATP5F1E 20	59025467	59032382	-	5126
protein_coding	ATP synthase F1 subunit epsilon [Source:HGNC
Symbol;Acc:HGNC:838]	-	1820	2730	3126	1530	1579
	2116	19.41724001	23.48107648	28.24963414
14.43158546	13.42338745	19.27006135
ENSG00000176170 3303.125584	4069.315508	3260.957718
4302.608525	5378.065379	5222.028995	3544.46627
4967.567633	-0.486852315	0.000841286	0.009299163	SPHK1
	17	76376584	76387860	+	4416
protein_coding	sphingosine kinase 1 [Source:HGNC
Symbol;Acc:HGNC:11240]	-	2855	4390	3335	4283	5885
	5322	35.35670301	43.82979239	34.98398107
46.89431822	58.07323244	56.25897836
ENSG00000104324 450.0580919	466.2564238	434.1424968
159.7279373	296.0906003	283.5712851	450.1523375
246.4632742	0.868224907	0.000842327	0.009303936	CPQ
	8	96645227	97149654	+	3457
protein_coding	carboxypeptidase Q [Source:HGNC Symbol;Acc:HGNC:16910]
	-	389	503	444	159	324	289
6.153822063	6.415087191	5.949576408	2.223816567
4.084173926	3.902514263
ENSG00000117305 726.5719323	704.482867	700.103666
904.1203998	1269.35137	1034.201158	710.3861551
1069.224309	-0.590349929	0.000843855	0.009314088	HMGCL
	1	23801885	23838620	-	5658
protein_coding	3-hydroxy-3-methylglutaryl-CoA lyase [Source:HGNC
Symbol;Acc:HGNC:5005]	-	628	760	716	900	1389
	1054	6.070038049	5.922220961	5.862090718
7.690964076	10.69788439	8.696083548
ENSG00000213186 77.51643227	52.83621503	86.04626062
13.05951689	19.19105743	27.47403455	72.13296931
19.90820295	1.85586461	0.000846203	0.00933327	TRIM59
	3	160432445	160485773	-	7865
protein_coding	tripartite motif containing 59 [Source:HGNC
Symbol;Acc:HGNC:30834]	-	67	57	88	13	21
	28	0.465876483	0.319528858	0.518306197
0.079918227	0.116353423	0.166190173
ENSG00000233608 231.3923351	239.1533943	352.0074298
171.782876	108.7493254	127.5580175	274.1843864
136.030073	1.012816982	0.000853312	0.009404889	TWIST2
	2	238848032	238910543	+	1878

protein_coding twist family bHLH transcription factor 2 [Source:HGNC Symbol;Acc:HGNC:20670] bHLH 200 258 360 171 119
130 5.824103986 6.057007006 8.87992645
4.402524069 2.761276973 3.231422914
ENSG00000148335 518.3188307 442.1556942 505.5217812
733.3421021 687.2226278 803.6155104 488.6654354
741.3934134 -0.601907356 0.000857813 0.009447684 NTMT1
9 129608884 129636131 + 3029
protein_coding N-terminal Xaa-Pro-Lys N-methyltransferase 1 [Source:HGNC Symbol;Acc:HGNC:23373] - 448 477 517
730 752 819 8.088601757 6.943094298
7.906672048 11.65265271 10.81875199 12.62207232
ENSG00000236750 21.98227184 10.19646255 15.64477466
129.5905906 38.38211485 46.11712942 15.94116968
71.3632783 -2.169069034 0.000866886 0.009536499
AC009237.9 2 95641634 95641980 - 347
processed_pseudogene pseudogene similar to part of Cdon homolog (mouse) (CDON) - 19 11 16 129 42
47 2.994462225 1.397645943 2.135959254
17.97469178 5.274464982 6.322872421
ENSG00000116285 7349.020564 6671.267361 4817.612796
8325.944304 11213.97456 8418.829157 6279.30024
9319.582672 -0.569800418 0.000867664 0.009536499 ERRFI1
1 8004404 8026308 - 4186 protein_coding ERBB receptor feedback inhibitor 1 [Source:HGNC Symbol;Acc:HGNC:18185]
- 6352 7197 4927 8288 12271 8580
82.98621907 75.80297258 54.52375957 95.73081029
127.7436526 95.68284934
ENSG00000196576 2696.877666 2404.511259 4147.820881
1943.85886 1775.629742 2224.415583 3083.069936
1981.301395 0.638045267 0.000867748 0.009536499 PLXNB2
22 50274979 50307627 - 7257
protein_coding plexin B2 [Source:HGNC Symbol;Acc:HGNC:9104] -
2331 2594 4242 1935 1943 2267 17.56628252
15.75965865 27.0779634 12.89214148 11.66740505
14.58278366
ENSG00000177963 2072.118361 2448.077963 2089.555215
2924.327204 3012.082156 3053.542697 2203.250513
2996.650686 -0.443452199 0.000870416 0.00955894 RIC8A
11 207511 215113 + 6685 protein_coding RIC8 guanine nucleotide exchange factor A [Source:HGNC Symbol;Acc:HGNC:29550] - 1791 2641 2137 2911 3296
3112 14.65172933 17.41810674 14.80831235
21.05435834 21.48544679 21.73122521
ENSG00000099139 370.2277362 125.138404 225.8714341
103.4715569 101.4384464 98.12155195 240.4125248
101.0105184 1.248564401 0.000874447 0.009596322 PCSK5
9 75890644 76362339 + 12705
protein_coding proprotein convertase subtilisin/kexin type 5 [Source:HGNC Symbol;Acc:HGNC:8747] - 320 135 231

        103   111   100   1.377431535   0.46848216
0.842247569   0.391979877   0.380721065   0.367427253
ENSG00000138035 1184.728756   1332.955741   1241.803989
858.9143798   881.8747817   919.3989417   1253.162828
886.7293678   0.499305815   0.000878939   0.009638691   PNPT1
        2   55634265   55693910   -   5134
protein_coding polyribonucleotide nucleotidyltransferase 1
[Source:HGNC Symbol;Acc:HGNC:23166]   -   1024   1438   1270
        855   965   937   10.90784116   12.34914755
11.4590948   8.052142775   8.190870256   8.51980711
ENSG00000130479 981.103501   1751.010705   1586.966829
2457.198331   2071.720342   2244.039893   1439.693678
2257.652855   -0.648177677   0.000879891   0.00964222   MAP1S
        19   17719242   17734516   +   5040
protein_coding microtubule associated protein 1S [Source:HGNC
Symbol;Acc:HGNC:15715] -   848   1889   1623   2446   2267
        2287   9.201529622   16.52476832   14.9173074
23.46535521   19.60106105   21.18271699
ENSG00000165655 152.7189412   241.0072966   267.916766
475.165499   344.5251738   406.2232251   220.5476679
408.637966   -0.886931374   0.000888608   0.009730769   ZNF503
        10   75397830   75401906   -   3347
protein_coding zinc finger protein 503 [Source:HGNC
Symbol;Acc:HGNC:23589] -   132   260   274   473   377
        414   2.15681518   3.424929164   3.792253024
6.832924859   4.908448465   5.774184606
ENSG00000254266 0   2.780853423   0   25.11445555
23.76035681   18.64309487   0.926951141
22.50596908   -4.528744541   0.000891572   1   PKIA-AS1
        8   78426103   78558503   -   2656
lincRNA PKIA antisense RNA 1 [Source:HGNC Symbol;Acc:HGNC:51659]
        -       0   3   0   25   26   19   0
        0.049799748   0   0.455106622   0.426583325
0.333942402
ENSG00000104783 1391.824896   1991.091051   1189.002874
1908.698622   2652.935224   2661.056489   1523.97294
2407.563445   -0.65947527   0.00089465   0.009789922   KCNN4
        19   43766533   43781261   -   3408
protein_coding potassium calcium-activated channel subfamily N member
4 [Source:HGNC Symbol;Acc:HGNC:6293]   -   1203   2148   1216
        1900   2903   2712   19.30459763   27.78872679
16.52861427   26.95598657   37.11983589   37.14805971
ENSG00000027847 416.5062033   507.042274   475.2100303
593.7057292   823.3877496   799.6906484   466.2528359
738.9280424   -0.663775951   0.000897215   0.00981096
B4GALT7 5   177600100   177610347   +   4927
protein_coding "beta-1,4-galactosyltransferase 7 [Source:HGNC
Symbol;Acc:HGNC:930]"   -   360   547   486   591   901
        815   3.995900368   4.894842498   4.569368283
5.799708088   7.968944945   7.721845044

ENSG00000130772 636.3289216   618.2764109   510.4107732
803.6625776   874.5639027   995.9337523   588.3387019
891.3867442   -0.599836501   0.000900764   0.009839618   MED18
        1   28329002   28335967   +   2019
protein_coding mediator complex subunit 18 [Source:HGNC
Symbol;Acc:HGNC:25944] -   550   667   522   800   957
        1015   14.89776376   14.56543545   11.97668535
19.15820802   20.65542886   23.46798269
ENSG00000055483 1898.57411   2139.403233   2072.932642
3328.16765   2473.818688   2814.12611   2036.969995
2872.037483   -0.495297748   0.000901123   0.009839618   USP36
        17   78787381   78841441   -   9235
protein_coding ubiquitin specific peptidase 36 [Source:HGNC
Symbol;Acc:HGNC:20062] -   1641   2308   2120   3313   2707
        2868   9.717765033   11.01876352   10.63411658
17.34545724   12.77350122   14.49733862
ENSG00000136861 1545.700799   1550.789259   1963.41922
1268.782294   955.8974318   1196.101718   1686.636426
1140.260481   0.565205375   0.000904759   0.009872272
CDK5RAP2   9   120388869   120580170   -   10080
        protein_coding CDK5 regulatory subunit associated protein 2
[Source:HGNC Symbol;Acc:HGNC:18672]   -   1336   1673   2008
        1263   1046   1219   7.248374749   7.317611807
9.227958488   6.058205974   4.521991588   5.645328381
ENSG00000185105 0   4.634755704   0   47.21517644
20.1049173   16.68066383   1.544918568
28.00025252   -4.121085697   0.000910005   0.009922427
MYADML2 17   81939645   81947233   -   2452
protein_coding myeloid associated differentiation marker like 2
[Source:HGNC Symbol;Acc:HGNC:34548]   -   0   5   0
        47   22   17   0   0.089904928   0
0.926784173   0.390985645   0.323649165
ENSG00000189423 46.27846703   13.90426711   60.6235018   0
        3.65543951   8.830939675   40.26874531   4.162126395
        3.270957398   0.000919206   0.010015606   USP32P3 17
        20415547   20431008   +   2935
transcribed_unprocessed_pseudogene   ubiquitin specific peptidase
32 pseudogene 3 [Source:HGNC Symbol;Acc:HGNC:43576]   -   40
        15   62   0   4   9   0.745326561
0.225329011   0.978556801   0   0.059389611   0.143146403
ENSG00000126461 3168.91803   5331.822962   5196.020783
7036.065867   6434.487397   7059.845663   4565.587258
6843.466309   -0.583614636   0.000921321   0.010031505   SCAF1
        19   49642125   49658642   +   4548
protein_coding SR-related CTD associated factor 1 [Source:HGNC
Symbol;Acc:HGNC:30403] -   2739   5752   5314   7004   7041
        7195   32.9356538   55.76123441   54.12570297
74.46066283   67.46406194   73.85099961
ENSG00000103245 524.1036391   752.6843264   685.4366897
863.9372709   1116.73677   1072.468563   654.0748851

1017.714201	-0.636810821	0.000929271	0.010110867	CIAO3
	16	729753	741329	-	8101	protein_coding
cytosolic iron-sulfur assembly component 3 [Source:HGNC
Symbol;Acc:HGNC:14179]	-	453	812	701	860	1222
	1093	3.058118307	4.419278334	4.008499572
5.132879107	6.573416816	6.298361237
ENSG00000143494 267.2581471	288.2818048	387.2081728
194.8881751	162.6670582	154.0508366	314.2493749
170.5353566	0.882868987	0.00093121	0.010124757	VASH2
	1	212950520	212992037	+	9086
protein_coding	vasohibin 2 [Source:HGNC Symbol;Acc:HGNC:25723] -
	231	311	396	194	178	157	1.390381435
	1.509112494	2.018946958	1.03235773	0.853701152
	0.806627373
ENSG00000135111 1243.733801	1046.527838	1639.767944
890.0563047	724.6908828	909.5867865	1310.009861
841.444658	0.638846725	0.000932913	0.010136058	TBX3
	12	114670254	114684164	-	5950
protein_coding	T-box 3 [Source:HGNC Symbol;Acc:HGNC:11602]	T-box
	1075	1129	1677	886	793	927	9.880665826
	8.365866581	13.05625287	7.19975922	5.807842352
	7.272919896
ENSG00000135334 1178.943948	1208.744288	1118.601388
1596.274795	2106.447017	1461.029908	1168.763208
1721.250574	-0.558700705	0.000935046	0.010152027
AKIRIN2 6	87675072	87702209	-	1999
protein_coding	akirin 2 [Source:HGNC Symbol;Acc:HGNC:21407]	-
	1019	1304	1144	1589	2305	1489	27.87764623
	28.76065386	26.51036371	38.43371095	50.24776316
	34.77186137
ENSG00000087589 19.66834849	12.05036483	6.844588913	0
	0	0	12.85443408	0	6.174739526
0.000936773	1	CASS4	20	56412112	56460387
	+	5069	protein_coding	Cas scaffold protein family
member 4 [Source:HGNC Symbol;Acc:HGNC:15878]	-	17	13
	7	0	0	0	0.183409296	0.113071986
	0.063970273	0	0	0
ENSG00000278540 2529.118223	2571.362465	3788.968863
2401.946529	1501.471779	1736.751469	2963.14985
1880.056592	0.656646826	0.000943159	0.010232835	ACACA
	17	37084988	37406818	-	13008
protein_coding	acetyl-CoA carboxylase alpha [Source:HGNC
Symbol;Acc:HGNC:84]	-	2186	2774	3875	2391	1643
	1770	9.190398481	9.402208689	13.79950901
8.887308674	5.504091347	6.351974909
ENSG00000129675 2006.171546	1476.633167	1614.345185
971.4271407	1155.118885	1297.166917	1699.049966
1141.237647	0.573533866	0.000943956	0.010234223
ARHGEF6 X	136665547	136782088	-	6434
protein_coding	Rac/Cdc42 guanine nucleotide exchange factor 6

[Source:HGNC Symbol;Acc:HGNC:685]	-	1734	1593	1651
	967	1264	1322	14.73882116	10.91612945
11.88689626	7.266857334	8.561002328	9.591718719
ENSG00000152620 1541.072952	1343.152203	1315.13887
769.5069181	1048.197279	1017.520494	1399.788008
945.0748971	0.566098839	0.000944638	0.010234356	NADK2
	5	36192592	36242279	-	4885
protein_coding	"NAD kinase 2, mitochondrial [Source:HGNC
Symbol;Acc:HGNC:26404]" -	1332	1449	1345	766	1147
	1037	14.91194762	13.07789287	12.75440408
7.581679439	10.23192734	9.909693543
ENSG00000104976 671.0377719	997.3994276	898.5967445
1252.709043	1222.744516	1409.025486	855.6779813
1294.826348	-0.596457764	0.000946113	0.010243079	SNAPC2
	19	7920316 7923250 +	2000	protein_coding	small
nuclear RNA activating complex polypeptide 2 [Source:HGNC
Symbol;Acc:HGNC:11135]	-	580	1076	919	1247	1338
	1436	15.85961757	23.7200846	21.2857017
30.14655389	29.1530982	33.51741217
ENSG00000198774 150.4050178	196.5136419	117.3358099
59.2701151	72.19493032	69.66630188	154.7514899
67.04378243	1.20684661	0.000948281	0.010259284	RASSF9
	12	85800697	85836570	-	5682
protein_coding	Ras association domain family member 9 [Source:HGNC
Symbol;Acc:HGNC:15739]	-	130	212	120	59	79
	71	1.251229098	1.645010179	0.97832347
0.502055812	0.605876991	0.583315014
ENSG00000113552 1688.007085	1497.953044	1168.469107
2092.536436	2301.099171	1956.543746	1451.476412
2116.726451	-0.544847023	0.000953446	0.010307874	GNPDA1
	5	141991749	142013041	-	3961
protein_coding	glucosamine-6-phosphate deaminase 1 [Source:HGNC
Symbol;Acc:HGNC:4417]	-	1459	1616	1195	2083	2518
	1994	20.14397446	17.98748613	13.97544092
25.42644503	27.70189515	23.49991803
ENSG00000124104 333.2049626	420.8358179	430.2313031
541.4676617	625.0801562	749.6486569	394.7573612
638.7321582	-0.693122024	0.000954861	0.010315878	SNX21
	20	45833810	45843275	+	4066
protein_coding	sorting nexin family member 21 [Source:HGNC
Symbol;Acc:HGNC:16154]	-	288	454	440	539	684
	764	3.873645079	4.922916126	5.012885189
6.409477206	7.330731704	8.771462684
ENSG00000204131 2.313923351	3.707804563	39.11193665	0
	0	0	15.04455485	0	6.406133885
0.000957438	1	NHSL2	X	71910818	72161750
	+	17706	protein_coding	NHS like 2 [Source:HGNC
Symbol;Acc:HGNC:33737]	-	2	4	40	0	0
	0	0.006177379	0.009960325	0.104650664	0
	0	0

ENSG00000068697 2652.913122   2400.803455   2445.473839
1668.604427   1949.263119   1872.159211   2499.730139
1830.008919   0.449607601   0.000959142   0.010354801
LAPTM4A 2   20032650   20052028   -   1946
protein_coding lysosomal protein transmembrane 4 alpha [Source:HGNC
Symbol;Acc:HGNC:6924]   -   2293   2590   2501   1661   2133
        1908   64.44005932   58.68010552   59.53513073
41.26938652   47.76465192   45.77006933
ENSG00000119471 1058.619933   1055.797349   1037.44412
695.1681296   776.7808958   736.8928551   1050.620467
736.2806269   0.512697944   0.00096464   0.010406815   HSDL2
        9   112379937   112472410   +   3455
protein_coding hydroxysteroid dehydrogenase like 2 [Source:HGNC
Symbol;Acc:HGNC:18572] -   915   1139   1061   692   850
        751   14.48330762   14.53481908   14.22557363
9.684099862   10.72085622   10.14700609
ENSG00000204619 1156.961676   1235.625871   1060.911282
1562.119135   1838.686073   1505.184607   1151.166276
1635.329938   -0.50658064   0.000965439   0.010408084
PPP1R11 6   30066709   30070333   +   2538
protein_coding protein phosphatase 1 regulatory inhibitor subunit 11
[Source:HGNC Symbol;Acc:HGNC:9285]   -   1000   1333   1085
        1555   2012   1534   21.54780789   23.15647714
19.80343723   29.62374714   34.54577629   28.21498201
ENSG00000162636 2269.958808   2237.660054   2175.601476
1710.796712   1697.951652   1504.203391   2227.740113
1637.650585   0.443868486   0.000974315   0.010493074
FAM102B 1   108560089   108644900   +   9355
protein_coding family with sequence similarity 102 member B
[Source:HGNC Symbol;Acc:HGNC:27637]   -   1962   2414   2225
        1703   1858   1533   11.46964362   11.37699145
11.01764258   8.801811686   8.654869334   7.649699915
ENSG00000123374 1257.617342   1227.28331   1421.718897
699.1864425   1069.216057   783.9912001   1302.206516
850.7978997   0.613634271   0.000974695   0.010493074   CDK2
        12   55966769   55972784   +   3894
protein_coding cyclin dependent kinase 2 [Source:HGNC
Symbol;Acc:HGNC:1771]   -   1087   1324   1454   696   1170
        799   15.26610727   14.99084093   17.29700517
8.642004914   13.09328172   9.578485981
ENSG00000162396 252.2176453   317.0172902   275.7391534
566.5821172   475.2071363   387.5801302   281.6580296
476.4564612   -0.757179189   0.00099412   0.010694658   PARS2
        1   54756898   54764514   -   2347
protein_coding "prolyl-tRNA synthetase 2, mitochondrial [Source:HGNC
Symbol;Acc:HGNC:30563]" -   218   342   282   564   520
        395   5.079700614   6.424613034   5.565939412
11.61895931   9.654923828   7.85651677
ENSG00000119203 2621.675157   2113.448601   2343.782804
1578.192387   1831.375194   1673.953676   2359.635521

1694.507086	0.477226491	0.000995815	0.010705361	CPSF3
2	9423568 9473101 +	3769	protein_coding
cleavage and polyadenylation specific factor 3 [Source:HGNC
Symbol;Acc:HGNC:2326]	-	2266	2280	2397	1571	2004
1706	32.87974804	26.67120685	29.46078756
20.1535364	23.17022107	21.12997218
ENSG00000176054 3.470885027	19.46597396	16.62257307	0
0	0	13.18647735	0	6.217412469
0.000996097	1	RPL23P2 21	28997613	28998033
-	421	processed_pseudogene	ribosomal protein L23
pseudogene 2 [Source:HGNC Symbol;Acc:HGNC:10324]	-	3
21	17	0	0	0	0.38970311
2.199232561	1.870549827	0	0	0
ENSG00000136960 143.4632478	123.2845017	177.9593117
51.23348932	43.86527412	85.36575019	148.2356871
60.15483788	1.302068221	0.000997198	0.010712698	ENPP2
8	119557086	119673453	-	6729
protein_coding	ectonucleotide pyrophosphatase/phosphodiesterase 2
[Source:HGNC Symbol;Acc:HGNC:3357]	-	124	133	182
51	48	87	1.007780312	0.871435144
1.252919924	0.366455179	0.31084888	0.603552093
ENSG00000117597 2929.426963	2753.044888	2244.047365
4126.807336	3654.52565	3344.963706	2642.173072
3708.765564	-0.489381302	0.00100396	0.01077777	UTP25
1	209828007	209857565	+	10376
protein_coding	"UTP25, small subunit processor component [Source:HGNC
Symbol;Acc:HGNC:28440]" -	2532	2970	2295	4108	3999
3409	13.34530338	12.62003165	10.24601961
19.14263367	16.79500118	15.33709381
ENSG00000105137 2210.953762	3096.943762	3094.731987
3819.4064	3960.668709	4145.63557	2800.876504
3975.236893	-0.504733875	0.001009397	0.010828523	SYDE1
19	15107403	15114988	+	3965
protein_coding	synapse defective Rho GTPase homolog 1 [Source:HGNC
Symbol;Acc:HGNC:25824]	-	1911	3341	3165	3802	4334
4225	26.3579851	37.15072059	36.97711113
46.36285133	47.63260276	49.74272321
ENSG00000116489 10588.51326	7964.364202	7856.610274
5919.979462	7102.518967	5650.820177	8803.162577
6224.439535	0.499893404	0.001011189	0.010840145	CAPZA1
1	112619173	112671619	+	4130
protein_coding	capping actin protein of muscle Z-line subunit alpha 1
[Source:HGNC Symbol;Acc:HGNC:1488]	-	9152	8592	8035
5893	7772	5759	121.1882942	91.72298391
90.12354893	68.99023209	82.00518978	65.09431523
ENSG00000144655 461.6277086	588.6139744	501.6105875
646.948375	832.5263483	1015.558063	517.2840902
831.6775953	-0.684396407	0.001016067	0.010884804	CSRNP1
3	39141855	39154562	-	3214
protein_coding	cysteine and serine rich nuclear protein 1

[Source:HGNC Symbol;Acc:HGNC:14300] - 399 635 513
644 911 1035 6.789248984 8.710875584
7.3939064 9.688157967 12.35182421 15.03282193
ENSG00000136770 643.2706917 647.0118963 674.6809072
424.9365879 450.5329196 434.6784751 654.9878317
436.7159942 0.584724581 0.001018669 0.010905042 DNAJC1
10 21756537 22003769 - 2501
protein_coding DnaJ heat shock protein family (Hsp40) member C1
[Source:HGNC Symbol;Acc:HGNC:20090] MYB 556 698 690
423 493 443 12.15782289 12.30483201
12.78020602 8.177638196 8.589973525 8.268677818
ENSG00000137507 64.78985384 12.97731597 54.75671131
11.05036044 2.741579632 0 44.17462704 4.597313358
3.270049473 0.001020158 0.010913336 LRRC32 11
76657524 76670747 - 4459 protein_coding
leucine rich repeat containing 32 [Source:HGNC
Symbol;Acc:HGNC:4161] - 56 14 56 11 3
0 0.686823691 0.138428184 0.581772261
0.119276924 0.029318543 0
ENSG00000186197 1.156961676 25.95463194 15.64477466 0
0 0 14.25212276 0 6.330053659
0.001021707 1 EDARADD 1 236348257 236502915
+ 3985 protein_coding EDAR associated death domain
[Source:HGNC Symbol;Acc:HGNC:14341] - 1 28 16
0 0 0 0.013723547 0.309787339
0.185991935 0 0 0
ENSG00000114166 372.5416596 443.0826453 404.8085443
225.0255217 257.7084854 256.0972506 406.8109497
246.2770859 0.724467972 0.001022757 0.010933483 KAT2B
3 20040023 20154404 + 5634
protein_coding lysine acetyltransferase 2B [Source:HGNC
Symbol;Acc:HGNC:8638] - 322 478 414 224 282
261 3.125602473 3.740632234 3.403971806
1.922349691 2.181176769 2.162567642
ENSG00000100442 833.0124065 743.414815 694.2368755
501.2845328 477.9487159 534.7624581 756.8880323
504.6652356 0.584363392 0.001030616 0.010992586 FKBP3
14 45115600 45135319 - 2262
protein_coding FK506 binding protein 3 [Source:HGNC
Symbol;Acc:HGNC:3719] - 720 802 710 499 523
545 17.40742804 15.63204245 14.54012714
10.66618622 10.07552502 11.24734295
ENSG00000110955 11636.72053 11909.46826 13542.50806
8859.37534 9660.412764 9681.653531 12362.89895
9400.480545 0.395227097 0.001031356 0.010992586
ATP5F1B 12 56638175 56646068 - 2322
protein_coding ATP synthase F1 subunit beta [Source:HGNC
Symbol;Acc:HGNC:830] - 10058 12848 13850 8819 10571
9867 236.8885822 243.953622 276.3058079
183.6362196 198.3866551 198.3667823

ENSG00000176136 2.313923351 28.73548537 22.48936357
98.44866576 56.6593124 90.27182779 17.84625743
81.79326865 -2.181423572 0.001031411 0.010992586 MC5R
18 13824149 13827323 + 1906
protein_coding melanocortin 5 receptor [Source:HGNC
Symbol;Acc:HGNC:6933] - 2 31 23 98 62
92 0.057385453 0.717088496 0.558994321
2.486018727 1.417514111 2.253258235
ENSG00000111863 506.749214 715.6062807 292.3617264
766.4931834 996.1072664 992.0088902 504.9057404
918.2031133 -0.862509484 0.001031465 0.010992586 ADTRP
6 11712054 11807046 - 4150
protein_coding androgen dependent TFPI regulating protein
[Source:HGNC Symbol;Acc:HGNC:21214] - 438 772 299
763 1090 1011 5.771925628 8.201686438
3.337532838 8.889506621 11.44555875 11.37232061
ENSG00000078902 1153.490791 1169.81234 1027.666135
1465.679626 1710.745691 1528.733779 1116.989755
1568.386365 -0.489902896 0.001031881 0.010992586 TOLLIP
11 1274371 1309654 - 5983 protein_coding toll
interacting protein [Source:HGNC Symbol;Acc:HGNC:16476] - 997
1262 1051 1459 1872 1558 9.113199302
9.299815016 8.137409451 11.79064342 13.63469537
12.15610622
ENSG00000081760 608.5618414 490.3571535 731.3932153
311.4192488 388.3904479 409.1668716 610.1040701
369.6588561 0.722381136 0.001038809 0.011058689 AACS
12 125065379 125143333 + 16094
protein_coding acetoacetyl-CoA synthetase [Source:HGNC
Symbol;Acc:HGNC:21298] - 526 529 748 310 425
417 1.787378213 1.449191016 2.152980303
0.931319965 1.150756749 1.209534036
ENSG00000198393 885.0756819 860.2106587 869.262792
1295.905906 1241.935573 1142.134865 871.5163775
1226.658781 -0.493237723 0.001044155 0.01110303 ZNF26
12 132986365 133032952 + 20863
protein_coding zinc finger protein 26 [Source:HGNC
Symbol;Acc:HGNC:13053] zf-C2H2 765 928 889 1290 1359
1164 2.005300166 1.961124549 1.973910507
2.989607461 2.838581032 2.604487384
ENSG00000155090 3223.295229 2595.463194 2303.693068
3936.942052 4567.471667 3313.564809 2707.48383
3939.326176 -0.54138932 0.001044427 0.01110303 KLF10
8 102648779 102655902 - 4003
protein_coding Kruppel like factor 10 [Source:HGNC
Symbol;Acc:HGNC:11810] zf-C2H2 2786 2800 2356 3919 4998
3377 38.06187991 30.83943403 27.26416189
47.33592737 54.40881402 39.38143221
ENSG00000100485 962.5921142 747.1226195 810.594887
581.6507906 593.0950604 461.1712942 840.1032069

545.305715	0.622649248	0.001045252	0.011104073	SOS2
	14	50117120	50231558	-	6938
protein_coding	SOS Ras/Rho guanine nucleotide exchange factor 2
[Source:HGNC Symbol;Acc:HGNC:11188]	-	832	806	829
	579	649	470	6.558186208	5.121942566
5.535064561	4.035018077	4.076326845	3.16234755
ENSG00000155368 1258.774303	1268.996112	1611.41179
821.7449856	786.8333545	1110.735968	1379.727402
906.4381027	0.606410826	0.00104611	0.011105475	DBI
	2	119366921	119372560	+	2509
protein_coding	"diazepam binding inhibitor, acyl-CoA binding protein
[Source:HGNC Symbol;Acc:HGNC:2690]"	-	1088	1369	1648
	818	861	1132	23.71499005	24.05673836
30.42699071	15.76354384	14.9541278	21.06162138
ENSG00000175455 4539.917616	3728.197488	3775.279685
3244.787657	2399.796038	2887.717274	4014.46493
2844.100323	0.497179808	0.001047454	0.011107663	CCDC14
	3	123897305	123961408	-	11423
protein_coding	coiled-coil domain containing 14 [Source:HGNC
Symbol;Acc:HGNC:25766]	-	3924	4022	3861	3230	2626
	2943	18.78639868	15.52372167	15.65748775
13.6717328	10.01781812	12.02696705
ENSG00000126368 315.8505375	418.0549645	271.8279597
530.4173012	543.7466271	585.7856651	335.2444872
553.3165311	-0.721961614	0.001047769	0.011107663	NR1D1
	17	40092787	40100725	-	2772
protein_coding	nuclear receptor subfamily 1 group D member 1
[Source:HGNC Symbol;Acc:HGNC:7962]	THR-like	273	451
	278	528	595	597	5.385972527	7.173271586
	4.64573064	9.209624284	9.353676326	10.05372822
ENSG00000145425 22241.43125	18361.0482	18491.14585
23071.14345	29164.01027	28326.71083	19697.8751
26853.95485	-0.447155159	0.001048624	0.011109016	RPS3A
	4	151099573	151104652	+	2341
protein_coding	ribosomal protein S3A [Source:HGNC
Symbol;Acc:HGNC:10421]	-	19224	19808	18911	22966	31913
	28869	449.0937973	373.0552633	374.2101273
474.3349912	594.0524841	575.6736652
ENSG00000165525 904.7440304	746.1956684	781.2609345
590.6919945	519.0724104	512.1945012	810.7335444
540.6529687	0.584062138	0.001050895	0.011125367	NEMF
	14	49782083	49853203	-	9426
protein_coding	nuclear export mediator factor [Source:HGNC
Symbol;Acc:HGNC:10663]	-	782	805	799	588	568
	522	4.537054858	3.765324437	3.92664645
3.016137297	2.62590795	2.585169976
ENSG00000134815 1081.759167	1445.116829	1187.047277
1996.096927	1613.876544	1776.981306	1237.974424
1795.651592	-0.535772822	0.001052841	0.011138254	DHX34
	19	47349281	47382704	+	5696

protein_coding DExH-box helicase 34 [Source:HGNC
Symbol;Acc:HGNC:16719] -   935   1559   1214   1987   1766
        1811   8.977105787   12.06729952   9.873046033
16.86666049   13.51074559   14.84207102
ENSG00000148737 365.5998895   363.3648472   471.2988366
253.1537119   199.2214533   248.2475264   400.0878578
233.5408972   0.777553542   0.001059072   0.011189527   TCF7L2
        10   112950250   113167678   +   6736
protein_coding transcription factor 7 like 2 [Source:HGNC
Symbol;Acc:HGNC:11641] HMG   316   392   482   252   218
        253   2.565545474   2.565771321   3.314724326
1.808838024   1.410304894   1.753333289
ENSG00000128512 1196.298373   1178.1549   1017.888151
1798.195017   1496.902479   1522.846486   1130.780475
1605.981328   -0.506207461   0.001059151   0.011189527   DOCK4
        7   111726110   112206411   -   11499
protein_coding dedicator of cytokinesis 4 [Source:HGNC
Symbol;Acc:HGNC:19192] -   1034   1271   1041   1790   1638
        1552   4.917622391   4.873258338   4.193658977
7.526519194   6.207438439   6.300538632
ENSG00000137310 2650.599199   2547.261735   2990.107557
1879.565853   1912.708723   2180.260884   2729.32283
1990.845154   0.455207355   0.001060563   0.011196701   TCF19
        6   31158542   31167159   +   3538
protein_coding transcription factor 19 [Source:HGNC
Symbol;Acc:HGNC:11629] Others 2291   2748   3058   1871   2093
        2222   35.41293916   34.24465993   40.03889731
25.56920207   25.77923334   29.31785968
ENSG00000196502 10.41265508   14.83121825   11.73358099   0
        0   0   12.32581811   0   6.11747485
0.001062433   1   SULT1A1 16   28605196   28623625
        -       8909   protein_coding sulfotransferase family 1A
member 1 [Source:HGNC Symbol;Acc:HGNC:11453]   -   9   16
        12   0   0   0   0.055246944   0.079181729
        0.062395712   0   0   0
ENSG00000096063 3536.831843   2935.654263   3500.51833
2434.093032   2567.032396   2334.311721   3324.334812
2445.145716   0.442902303   0.001063687   0.011221939   SRPK1
        6   35832966   35921342   -   6692
protein_coding SRSF protein kinase 1 [Source:HGNC
Symbol;Acc:HGNC:11305] -   3057   3167   3580   2423   2809
        2379   24.98240354   20.86536994   24.78161184
17.50647461   18.2917141   16.59527852
ENSG00000176046 947.5516124   1152.200268   860.4626062
1226.590009   1585.546887   1602.324943   986.7381622
1471.48728   -0.576331778   0.001066907   0.011248146   NUPR1
        16   28532708   28539174   -   5674
protein_coding "nuclear protein 1, transcriptional regulator
[Source:HGNC Symbol;Acc:HGNC:29990]"   -   819   1243   880
        1221   1735   1633   7.893857515   9.658635017

7.184487549	10.40465176	13.32504687	13.43516143
ENSG00000112658 1485.538792	2523.161005	2413.206491
3184.512964	3023.962334	3563.774767	2140.635429
3257.416688	-0.605084552	0.001073767	0.011312669	SRF
6	43171299	43181507	+	4202
protein_coding	serum response factor [Source:HGNC
Symbol;Acc:HGNC:11291]	SRF	1284	2722	2468	3170	3309
3632	16.71104807	28.56051477	27.20768326
36.47576681	34.31621	40.34928354
ENSG00000165891 3453.530602	2888.379755	2582.365617
4625.078134	4058.451716	3808.097431	2974.758658
4163.87576	-0.485430137	0.001077217	0.011338396	E2F7
12	77021247	77065580	-	6303
protein_coding	E2F transcription factor 7 [Source:HGNC
Symbol;Acc:HGNC:23820]	E2F	2985	3116	2641	4604	4441
3881	25.89952154	21.7963664	19.40991126
35.31744067	30.70379543	28.74368014
ENSG00000172469 575.0099528	454.206059	483.0324176
211.9660048	348.1806133	315.9513973	504.0828098
292.0326718	0.786075385	0.001077693	0.011338396	MANEA
6	95577543	95609457	+	5187
protein_coding	mannosidase endo-alpha [Source:HGNC
Symbol;Acc:HGNC:21072]	-	497	490	494	211	381
322	5.240043032	4.164988341	4.411772982
1.966832717	3.200864762	2.897915111
ENSG00000174332 45.12150535	45.4206059	51.82331606
13.05951689	5.483159264	11.77458623	47.45514244
10.10575413	2.236440457	0.001078667	0.011340842	GLIS1
1	53506237	53738106	-	3113
protein_coding	GLIS family zinc finger 1 [Source:HGNC
Symbol;Acc:HGNC:29525]	zf-C2H2 39	49	53	13	6
12	0.685141382	0.693986332	0.788677053
0.201913542	0.083990608	0.179948471
ENSG00000172985 283.4556106	359.6570426	710.8594485
268.2223853	155.3561792	220.7734919	451.3240339
214.7840188	1.072753186	0.00108098	0.011355246	SH3RF3
2	109129348	109504632	+	5803
protein_coding	SH3 domain containing ring finger 3 [Source:HGNC
Symbol;Acc:HGNC:24699]	-	245	388	727	267	170
225	2.308916496	2.947902484	5.803423929
2.224640848	1.276600319	1.8099892
ENSG00000072840 894.3313753	1004.815037	996.3765861
1391.340838	1301.336465	1357.021063	965.1743327
1349.899455	-0.483444573	0.001081523	0.011355246	EVC
4	5711197 5814305 +	7148	protein_coding	EvC
ciliary complex subunit 1 [Source:HGNC Symbol;Acc:HGNC:3497]	-
773	1084	1019	1385	1424	1383	5.914113607
6.686189733	6.603772387	9.368422017	8.681285584
9.031994659
ENSG00000204209 2721.173861	3007.029501	2922.639466

3771.186645 3904.923256 3739.412345 2883.614276
3805.174082 -0.399919271 0.001084683 0.011380612 DAXX
6 33318558 33323016 - 3084
protein_coding death domain associated protein [Source:HGNC
Symbol;Acc:HGNC:2681] - 2352 3244 2989 3754 4273
3811 41.70783634 46.37676317 44.89665667
58.85469527 60.3777773 57.68602518
ENSG00000080546 226.7644884 159.4355962 169.159126
84.38457065 69.45335068 104.9900606 185.1197369
86.27599397 1.100709403 0.001087234 0.011398975 SESN1
6 108986437 109094819 - 5121
protein_coding sestrin 1 [Source:HGNC Symbol;Acc:HGNC:21595] -
196 172 173 84 76 107 2.093129065
1.480838268 1.564925917 0.79309594 0.646721671
0.975382676
ENSG00000089820 55.53416043 143.6774268 77.24607488
247.1262426 285.1242818 145.2198969 92.15255405
225.8234738 -1.28893761 0.001087925 0.011398975
ARHGAP4 X 153907367 153934999 - 5580
protein_coding Rho GTPase activating protein 4 [Source:HGNC
Symbol;Acc:HGNC:674] - 48 155 79 246 312
148 0.470437303 1.224704905 0.655836145
2.131582395 2.436570562 1.238150828
ENSG00000161036 887.3896053 1330.174887 1201.714253
1597.279373 1718.970429 1736.751469 1139.759582
1684.333757 -0.562542882 0.001089656 0.011409297 LRWD1
7 102464929 102473168 + 5926
protein_coding leucine rich repeats and WD repeat domain containing 1
[Source:HGNC Symbol;Acc:HGNC:21769] - 767 1435 1229
1590 1881 1770 7.078291266 10.67638464
9.607108412 12.97288875 13.83202439 13.94304584
ENSG00000169403 16.19746346 18.53902282 3.911193665 0
0 0 12.88255998 0 6.179717165
0.001089954 1 PTAFR 1 28147166 28193936
- 4706 protein_coding platelet activating factor
receptor [Source:HGNC Symbol;Acc:HGNC:9582] - 14 20
4 0 0 0 0.162693734 0.187375166
0.03937409 0 0 0
ENSG00000139921 2698.034628 2444.370158 2202.002033
1438.556014 1973.023475 1718.108375 2448.135606
1709.895955 0.517357046 0.001094125 0.011441707 TMX1
14 51240162 51257546 + 4499
protein_coding thioredoxin related transmembrane protein 1
[Source:HGNC Symbol;Acc:HGNC:15487] - 2332 2637 2252
1432 2159 1751 28.34701057 25.84211737
23.18754922 15.38963222 20.91198443 18.16837933
ENSG00000206527 2768.60929 2505.548934 2681.123257
1691.709726 2087.25596 2008.548168 2651.760494
1929.171285 0.458685691 0.001094248 0.011441707 HACD2
3 123490820 123585185 - 5351

protein_coding 3-hydroxyacyl-CoA dehydratase 2 [Source:HGNC Symbol;Acc:HGNC:9640] - 2393 2703 2742 1684 2284 2047 24.45695928 22.27127357 23.73749877 15.21627514 18.60029132 17.85784
ENSG00000147408 84.45820233 115.8688926 194.5818848 14.06409511 42.03755436 63.77900877 131.6363266 39.96021941 1.71994896 0.001095478 0.011446735 CSGALNACT1 8 19404161 19758029 - 6580 protein_coding chondroitin sulfate N-acetylgalactosaminyltransferase 1 [Source:HGNC Symbol;Acc:HGNC:24290] - 73 125 199 14 46 65 0.606724705 0.837564145 1.400972591 0.102873463 0.304642532 0.461140747
ENSG00000135842 1899.731072 1727.836927 1419.7633 2587.7935 2217.937922 2334.311721 1682.443766 2380.014381 -0.500695979 0.001102347 0.011510636 FAM129A 1 184790724 184974550 - 8372 protein_coding family with sequence similarity 129 member A [Source:HGNC Symbol;Acc:HGNC:16784] - 1642 1864 1452 2576 2427 2379 10.72602107 9.816363825 8.034148458 14.87708538 12.63278644 13.26512229
ENSG00000115266 404.9365865 573.7827562 882.9519698 1100.013153 859.9421446 1333.471891 620.5571042 1097.809063 -0.821873009 0.001104345 0.011523633 APC2 19 1446302 1473244 + 12610 protein_coding "APC2, WNT signaling pathway regulator [Source:HGNC Symbol;Acc:HGNC:24036]" - 350 619 903 1095 941 1359 1.517915761 2.164260436 3.31722645 4.19855889 3.251869694 5.03095469
ENSG00000253284 13.88354011 12.05036483 10.75578258 0 0 0 12.22989584 0 6.104626452 0.001106924 1 AC092828.1 12 19147074 19154659 + 5391 sense_intronic novel transcript - 12 13 11 0 0 0 0.121732524 0.106318289 0.094520456 0 0 0
ENSG00000210140 342.460656 930.6589454 615.0352038 1766.048514 1254.729612 1990.886289 629.3849351 1670.554805 -1.407674378 0.001111988 0.01159547 MT-TC MT 5761 5826 - 66 Mt_tRNA mitochondrially encoded tRNA cysteine [Source:HGNC Symbol;Acc:HGNC:7477] - 296 1004 629 1758 1373 2029 245.2689028 670.6929407 441.4781009 1287.882232 906.5363009 1435.106552
ENSG00000114383 674.5086569 888.0191929 904.4635349 1278.828077 1167.912923 1181.383485 822.3304616 1209.374828 -0.555403497 0.001117806 0.011648189 TUSC2 3 50320027 50328251 - 2406 protein_coding "tumor suppressor 2, mitochondrial calcium regulator [Source:HGNC Symbol;Acc:HGNC:17034]" - 583 958 925 1273 1278 1204 13.25157944 17.55512168

17.80937036	25.5819707	23.14695293	23.36021845
ENSG00000163539 2199.384146	1729.690829	1838.261022
1478.739143	1334.235421	1263.805589	1922.445332
1358.926718	0.500085445	0.001119434	0.011653632	CLASP2
3	33496245	33718356	-	15211
protein_coding	cytoplasmic linker associated protein 2 [Source:HGNC
Symbol;Acc:HGNC:17078]	-	1901	1866	1880	1472	1460
1288	6.834693811	5.408636953	5.725356562
4.678980768	4.182670826	3.952793553
ENSG00000198886 54772.87965	50680.12667	61949.39645
71006.60247	70533.53306	79598.16537	55800.80093
73712.76697	-0.401628654	0.001119852	0.011653632	MT-ND4
MT	10760	12137	+	1378	protein_coding
mitochondrially encoded NADH:ubiquinone oxidoreductase core subunit 4
[Source:HGNC Symbol;Acc:HGNC:7459]	-	47342	54674	63356
70683	77182	81122	1878.849944	1749.305207
2129.810621	2480.087326	2440.76186	2748.118575
ENSG00000158301 591.2074163	675.7473817	496.7215954
842.8411283	882.7886416	926.2674504	587.8921311
883.9657401	-0.588233865	0.001135276	0.011806103
GPRASP2 X	102712176	102717733	+	3975
protein_coding	G protein-coupled receptor associated sorting protein
2 [Source:HGNC Symbol;Acc:HGNC:25169]	-	511	729	508
839	966	944	7.030374822	8.08582529
5.920099896	10.20530631	10.59006416	11.08615374
ENSG00000121931 541.4580642	360.5839938	455.6540619
592.701151	843.4926669	777.1226914	452.5653733
737.7721698	-0.706728595	0.00113619	0.011807574	LRIF1
1	110947185	110964079	-	3621
protein_coding	ligand dependent nuclear receptor interacting factor 1
[Source:HGNC Symbol;Acc:HGNC:30299]	-	468	389	466
590	923	792	7.068252265	4.736472656
5.961559017	7.878158304	11.10789521	10.2103985
ENSG00000177426 701.1187755	774.0042026	625.7909863
1058.825446	1191.67328	892.9061227	700.3046548
1047.801616	-0.581340031	0.001142745	0.011867625	TGIF1
18	3411608 3459978 +	8222	protein_coding	TGFB
induced factor homeobox 1 [Source:HGNC Symbol;Acc:HGNC:11776]
Homeobox	606	835	640	1054	1304	910
4.030787142	4.477575948	3.605827589	6.198182435
6.911283638	5.166660864
ENSG00000196230 42812.20985	39378.73837	47123.03907
33665.42538	33476.51503	32287.87788	43104.66243
33143.27276	0.379123664	0.001144552	0.011878325	TUBB
6	30720201	30725426	+	3359
protein_coding	tubulin beta class I [Source:HGNC
Symbol;Acc:HGNC:20778]	-	37004	42482	48193	33512	36632
32906	602.4671632	557.607888	664.6246028
482.3825178	475.2358825	457.3104498
ENSG00000109079 1720.402012	1713.932659	1659.323912

2331.626053	2111.016317	2460.888523	1697.886194
2301.176964	-0.438578256	0.001148989	0.011916277
TNFAIP1 17	28335602	28347009	+	3889
protein_coding	TNF alpha induced protein 1 [Source:HGNC
Symbol;Acc:HGNC:11894]	-	1487	1849	1697	2321	2310
	2508	20.91065988	20.9620101	20.21372509
28.85615179	25.8840741	30.10479158
ENSG00000179813 226.7644884	284.5740002	118.3136084
468.1334515	425.8587029	348.3315094	209.8840323
414.1078879	-0.980258727	0.001162779	0.01205112
FAM216B 13	42781550	42791549	+	3308
protein_coding	family with sequence similarity 216 member B
[Source:HGNC Symbol;Acc:HGNC:26883]	-	196	307	121
	466	466	355	3.240300466	4.091728721
1.694424901	6.811168625	6.138736804	5.009667339
ENSG00000168398 35.86581195	91.76816294	138.8473751
14.06409511	18.27719755	36.30497422	88.82711666
22.88208896	1.959497553	0.001166545	0.012081959	BDKRB2
	14	96204679	96244166	+	6845
protein_coding	bradykinin receptor B2 [Source:HGNC
Symbol;Acc:HGNC:1030]	-	31	99	142	14	20
	37	0.247675446	0.637669581	0.960986635
0.098890779	0.127325427	0.252333149
ENSG00000162086 32.39492692	36.15109449	25.42275882
121.5539649	92.29984762	63.77900877	31.32292674
92.54427375	-1.562503194	0.001168074	0.012089605	ZNF75A
	16	3305406 3318852 +	8063	protein_coding	zinc
finger protein 75a [Source:HGNC Symbol;Acc:HGNC:13146]	zf-C2H2 28
	39	26	121	101	65	0.189913608
0.213256317	0.149375422	0.725587725	0.545862565
0.376324707
ENSG00000171853 601.6200714	663.6970168	657.0805357
973.4362971	901.979699	896.8309848	640.799208
924.082327	-0.527576427	0.001172712	0.012129394
TRAPPC12	2	3379675 3485094 +	9193	protein_coding
	trafficking protein particle complex 12 [Source:HGNC
Symbol;Acc:HGNC:24284]	-	520	716	672	969	987
	914	3.093433585	3.433916418	3.386214529
5.096449596	4.678627727	4.641250098
ENSG00000272870 135.3645161	60.25182416	55.73450972
15.06867333	30.15737595	25.51160351	83.78361664
23.5792176	1.823504415	0.001174071	0.012131945
AC097534.2	4	173363780	173370446	-	2495
	antisense	uncharacterized LOC105377540 [Source:NCBI
gene;Acc:105377540]	-	117	65	57	15	33
	26	2.564543231	1.148621033	1.058295042
0.290684534	0.576370823	0.486461902
ENSG00000175414 1173.159139	1057.651252	1161.624518
1496.821551	1538.940034	1652.366935	1130.811636
1562.709506	-0.466972199	0.001174546	0.012131945	ARL10

5	176365468	176401865	+	11459
protein_coding	ADP ribosylation factor like GTPase 10 [Source:HGNC Symbol;Acc:HGNC:22042]	-	1014	1141	1188	1490	1684
1684	4.839337913	4.390084534	4.802553118
6.286960988	6.404038962	6.86027308
ENSG00000168542 148.0910945	60.25182416	195.5596832
64.29300621	7.310879019	30.4176811	134.6342006
34.00718878	1.988220911	0.001184775	0.012229347	COL3A1
2	188974320	189012746	+	6811
protein_coding	collagen type III alpha 1 chain [Source:HGNC Symbol;Acc:HGNC:2201]	-	128	65	200	64	8
31	1.027764948	0.420761926	1.360258885
0.454328844	0.05118441	0.212469624
ENSG00000171552 407.2505099	585.833121	605.2572196
933.2531683	763.9868575	819.3149588	532.7802835
838.8516615	-0.653272269	0.001186302	0.01223684	BCL2L1
20	31664452	31723989	-	3314
protein_coding	BCL2 like 1 [Source:HGNC Symbol;Acc:HGNC:992]	-
352	632	619	929	836	835	5.808779247
8.408112854	8.652479933	13.55390466	10.99290237
11.76196837
ENSG00000114450 2552.257457	1919.715813	1974.175002
1358.189756	1538.026174	1608.212236	2148.716091
1501.476055	0.516546441	0.001188986	0.012241425	GNB4
3	179396089	179451590	-	7035
protein_coding	G protein subunit beta 4 [Source:HGNC Symbol;Acc:HGNC:20731]	-	2206	2071	2019	1352	1683
1639	17.14889412	12.97926068	13.29458157
9.292098532	10.42506117	10.87579186
ENSG00000007047 742.7693958	961.2483331	741.1711995
1031.701834	1172.482223	1636.667486	815.0629761
1280.283848	-0.650962522	0.001189091	0.012241425	MARK4
19	45079288	45305283	+	6668
protein_coding	microtubule affinity regulating kinase 4 [Source:HGNC Symbol;Acc:HGNC:13538]	-	642	1037	758	1027	1283
1668	5.265433712	6.856731181	5.265941986
7.446909378	8.384741211	11.67740898
ENSG00000134056 349.4024261	361.5109449	377.4301886
220.0026306	209.2739119	228.623216	362.7811865
219.2999195	0.72662056	0.001189149	0.012241425	MRPS36
5	69217760	69230129	+	1530
protein_coding	mitochondrial ribosomal protein S36 [Source:HGNC Symbol;Acc:HGNC:16631]	-	302	390	386	219	229
233	10.79469124	11.23846854	11.68687314
6.920761778	6.522328212	7.109032932
ENSG00000143786 1848.824758	1845.559721	1626.078766
2501.399773	2527.736421	2222.453152	1773.487748
2417.196448	-0.446886792	0.001195628	0.012293068	CNIH3
1	224434660	224740549	+	6386
protein_coding	cornichon family AMPA receptor auxiliary protein 3

[Source:HGNC Symbol;Acc:HGNC:26802]	-	1598	1991	1663
	2490	2766	2265	13.68492979	13.74599887
12.06329062	18.8526172	18.87477831	16.55714027
ENSG00000145451 2.313923351	6.488657986	2.933395248
55.25180221	29.24351608	14.71823279	3.911992195
33.07118369	-3.062076171	0.001195774	0.012293068	GLRA3
	4	174636914	174829314	-	9599
protein_coding	glycine receptor alpha 3 [Source:HGNC
Symbol;Acc:HGNC:4328]	-	2	7	3	55	32
	15	0.01139459	0.032151853	0.014477638
0.277037088	0.145272224	0.07294765
ENSG00000197321 1615.118499	1163.323682	1829.460837
1091.976527	678.9978889	1078.355856	1535.967673
949.7767574	0.693627947	0.001197866	0.0123063	SVIL
	10	29457338	29736781	-	9937
protein_coding	supervillin [Source:HGNC Symbol;Acc:HGNC:11480] -
	1396	1255	1871	1087	743	1099	7.682893998
	5.568297032	8.722097828	5.289023184	3.258307902
	5.162837293
ENSG00000139350 1189.356603	998.3263787	950.4200605
683.113191	761.2452779	708.4376051	1046.034347
717.5986913	0.54289613	0.001204019	0.012361205	NEDD1
	12	96907223	96953777	+	4463
protein_coding	"neural precursor cell expressed, developmentally
down-regulated 1 [Source:HGNC Symbol;Acc:HGNC:7723]"	-	1028
	1077	972	680	833	722	12.59682049
10.63953811	10.08885392	7.366874007	8.133485791
7.551902018
ENSG00000159147 1007.71362	1065.066861	1100.023218
776.5389656	776.7808958	641.7149497	1057.601233
731.6782704	0.531629764	0.001205737	0.01237054	DONSON
	21	33559542	33588708	-	3513
protein_coding	downstream neighbor of SON [Source:HGNC
Symbol;Acc:HGNC:2993]	-	871	1149	1125	773	850
	654	13.55922033	14.42035118	14.83463375
10.63904291	10.54385376	8.690517418
ENSG00000222032 236.0201818	184.463277	240.5384104
123.5631213	74.02265007	123.6331555	220.3406231
107.0729756	1.041938355	0.001207755	0.012375476
AC112721.2	2	237428920	237434822	-	541
	lincRNA novel protein (LOC728009)	-	204	199
	246	123	81	126	20.6218496	16.21771893
	21.06397341	10.99281864	6.52448672	10.87224714
ENSG00000070495 1204.397104	1412.673539	1208.558842
1686.686835	1921.847322	1721.052021	1275.209828
1776.528726	-0.478075893	0.001207837	0.012375476	JMJD6
	17	76712832	76726799	-	5969
protein_coding	"jumonji domain containing 6, arginine demethylase and
lysine hydroxylase [Source:HGNC Symbol;Acc:HGNC:19355]" -	1041
	1524	1236	1679	2103	1754	9.537704511

11.25686211	9.592224789	13.60035779	15.35310753
13.71747085
ENSG00000139329 251.0606836	240.0803455	154.4921498
81.37083598	137.9928415	66.72265532	215.2110596
95.36211093	1.171941349	0.001209183	0.012380965	LUM
	12	91102629	91111831	-	3008
protein_coding	lumican [Source:HGNC Symbol;Acc:HGNC:6724]	-
	217	259	158	81	151	68	3.945268951
	3.796259486	2.433221867	1.301992263	2.187548614
	1.055302864
ENSG00000144134 90.24301071	139.9696223	92.89084954
242.1033515	248.5698867	174.6563625	107.7011608
221.7765335	-1.039697883	0.001215333	0.012435607	RABL2A
	2	113627229	113643396	+	6008
protein_coding	"RAB, member of RAS oncogene family like 2A
[Source:HGNC Symbol;Acc:HGNC:9799]"	-	78	151	95
	241	272	178	0.710001705	1.10810517
0.732480617	1.93949353	1.972865952	1.383044373
ENSG00000167549 86.77212568	125.138404	282.5837423
89.40746176	39.29597473	21.58674143	164.831424
50.09672597	1.720767676	0.001217686	0.012451351	CORO6
	17	29614756	29622907	-	4321
protein_coding	coronin 6 [Source:HGNC Symbol;Acc:HGNC:21356]	-
	75	135	289	89	43	22	0.949230556
	1.377474158	3.09824696	0.995879877	0.433653431
	0.237675519
ENSG00000172296 317.0074991	332.7754596	216.09345
659.0033136	488.0011745	394.4486388	288.6254696
513.817709	-0.832190229	0.001221714	0.012484187	SPTLC3
	20	13008979	13169103	+	8520
protein_coding	serine palmitoyltransferase long chain base subunit 3
[Source:HGNC Symbol;Acc:HGNC:16253]	-	274	359	221
	656	534	402	1.758756359	1.857756595
1.201586761	3.722763619	2.731242489	2.202584071
ENSG00000171302 2323.179045	2326.647364	3043.88647
3666.71051	3507.39421	3489.202387	2564.570959
3554.435702	-0.470740882	0.001223037	0.012489357	CANT1
	17	78991717	79009867	-	4421
protein_coding	calcium activated nucleotidase 1 [Source:HGNC
Symbol;Acc:HGNC:19721]	-	2008	2510	3113	3650	3838
	3556	24.83921727	25.03151667	32.61828038
39.91844048	37.83058466	37.54804012
ENSG00000140575 9071.736499	7171.820977	7856.610274
5848.654409	5994.920796	6211.094238	8033.38925
6018.223148	0.416525089	0.00122461	0.012497074	IQGAP1
	15	90388218	90502243	+	11535
protein_coding	IQ motif containing GTPase activating protein 1
[Source:HGNC Symbol;Acc:HGNC:6110]	-	7841	7737	8035
	5822	6560	6330	37.17479375	29.57256234
32.26790265	24.40370794	24.78248422	25.61722879

ENSG00000280300 19.66834849   7.415609127   9.777984162   0
        0   0   12.28731393   0   6.108911906
0.001228025   1   AC117503.4   12   123754246
123754794   -   549   TEC   novel transcript   -
        17   8   10   0   0   0   1.693445755
        0.642468147   0.843781718   0   0   0
ENSG00000172965 1278.442652   1002.034183   1302.42749
830.7861896   701.8443859   884.075183   1194.301442
805.5685862   0.567958915   0.001235632   0.012601143
MIR4435-2HG   2   111036776   111523376   -   13094
        lincRNA MIR4435-2 host gene [Source:HGNC
Symbol;Acc:HGNC:35163] -   1105   1081   1332   827   768
        901   4.615137602   3.63988209   4.71231533
3.053756395   2.555921323   3.212169767
ENSG00000198695 5445.818608   4949.919092   6077.017156
6562.909524   7256.961287   9496.203797   5490.918285
7772.024869   -0.50125809   0.00124674   0.012705947   MT-ND6
        MT   14149   14673   -   525   protein_coding
mitochondrially encoded NADH:ubiquinone oxidoreductase core subunit 6
[Source:HGNC Symbol;Acc:HGNC:7462]   -   4707   5340   6215
        6533   7941   9678   490.3199992   448.4519446
548.3833817   601.6647545   659.1351777   860.5425521
ENSG00000159259 1151.176867   1413.60049   1364.028791
818.7312509   977.8300688   938.0420366   1309.602049
911.5344521   0.523058977   0.001248234   0.012710845   CHAF1B
        21   36385378   36419015   +   5118
protein_coding chromatin assembly factor 1 subunit B [Source:HGNC
Symbol;Acc:HGNC:1911]   -   995   1525   1395   815   1070
        956   10.63206228   13.13722143   12.62630808
7.699429446   9.110497512   8.719742228
ENSG00000100418 924.4123789   1147.565512   1036.466321
1657.554066   1383.583854   1404.119408   1036.148071
1481.752443   -0.515326608   0.001248884   0.012710845   DESI1
        22   41598028   41621096   -   4972
protein_coding desumoylating isopeptidase 1 [Source:HGNC
Symbol;Acc:HGNC:24577] -   799   1238   1060   1650   1514
        1431   8.788411265   10.97800648   9.875911764
16.04552904   13.26946316   13.43552216
ENSG00000171724 532.2023708   723.0218899   330.4958647
685.1223474   970.5191898   1226.519399   528.5733751
960.7203122   -0.86177762   0.001259141   0.01280671   VAT1L
        16   77788530   77980107   +   4137
protein_coding vesicle amine transport 1 like [Source:HGNC
Symbol;Acc:HGNC:29315] -   460   780   338   682   1062
        1250   6.080888266   8.312717847   3.784718954
7.970766195   11.18658672   14.10491677
ENSG00000187091 30.08100357   61.1787753   50.84551764
100.4578222   141.648281   125.5955865   47.36843217
122.5672299   -1.366149881   0.00126348   0.012842303   PLCD1
        3   38007496   38029762   -   4314

protein_coding phospholipase C delta 1 [Source:HGNC Symbol;Acc:HGNC:9060] - 26 66 52 100 155 128 0.329600544 0.674524537 0.558374606 1.120781815 1.565705781 1.38508321
ENSG00000140612 2031.624703 1821.458992 1997.642164 1381.295055 1443.898606 1484.579081 1950.241953 1436.590914 0.440773848 0.001266584 0.012865291 SEC11A 15 84669538 84716716 - 5748 protein_coding "SEC11 homolog A, signal peptidase complex subunit [Source:HGNC Symbol;Acc:HGNC:17718]" - 1756 1965 2043 1375 1580 1513 16.70715358 15.07230776 16.46470914 11.56610565 11.97840313 12.28763223
ENSG00000176171 3086.773751 2824.420126 2023.064923 2979.579007 4417.598647 4814.824554 2644.752933 4070.667403 -0.622363827 0.001272557 0.012915774 BNIP3 10 131966455 131982013 - 5325 protein_coding BCL2 interacting protein 3 [Source:HGNC Symbol;Acc:HGNC:1084] - 2668 3047 2069 2966 4834 4907 27.40065382 25.22823107 17.99879101 26.93101682 39.55902979 43.01723398
ENSG00000171943 791.3617862 569.1480005 775.394144 410.8724928 523.6417098 404.260794 711.9679769 446.2583322 0.672817531 0.001273243 0.012915774 SRGAP2C 1 121184810 121392822 + 7311 protein_coding SLIT-ROBO Rho GTPase activating protein 2C [Source:HGNC Symbol;Acc:HGNC:30584] - 684 614 793 409 573 412 5.116512395 3.702759844 5.024569517 2.704878367 3.415359592 2.63067058
ENSG00000101463 122.6379376 109.3802346 128.0915925 41.1877071 65.79791117 22.56795695 120.0365883 43.18452507 1.472074958 0.001277782 0.01295322 SYNDIG1 20 24469199 24666616 + 2847 protein_coding synapse differentiation inducing 1 [Source:HGNC Symbol;Acc:HGNC:15885] - 106 118 131 41 72 23 2.03616567 1.827378446 2.131504649 0.696301941 1.102055908 0.377125939
ENSG00000129932 674.5086569 1047.454789 864.3737999 1233.622057 1239.193994 1447.292891 862.1124153 1306.702981 -0.598847011 0.001280461 0.012970207 DOHH 19 3490822 3500940 - 2031 protein_coding deoxyhypusine hydroxylase [Source:HGNC Symbol;Acc:HGNC:28662] - 583 1130 884 1228 1356 1475 15.69832602 24.53027845 20.16251936 29.2340954 29.09433023 33.90221961
ENSG00000022567 337.8328093 282.720098 243.4718056 729.3237892 442.3081807 390.5237767 288.0082376 520.7185822 -0.855060639 0.001281155 0.012970207 SLC45A4 8 141207166 141308305 - 8608 protein_coding solute carrier family 45 member 4 [Source:HGNC Symbol;Acc:HGNC:29196] - 292 305 249 726 484

398	1.855134089	1.562181675	1.33998379
4.077890678	2.450200934	2.158374739
ENSG00000235033 6.941770054	2.780853423	30.3117509	0
0	0	13.34479146	0	6.2320481
0.001281778	1	AL590999.1	6	39881804
39900071	-	6854	antisense	uncharacterized
LOC100505635 [Source:NCBI gene;Acc:100505635]	-	6	3
31	0	0	0	0.047874237	0.019297947
0.209517378	0	0	0
ENSG00000136270 3078.675019	3079.33169	2871.793948
3882.694828	4111.455588	3848.327267	3009.933552
3947.492561	-0.391284897	0.00128912	0.01304221	TBRG4
7	45100100	45112047	-	5803
protein_coding	transforming growth factor beta regulator 4
[Source:HGNC Symbol;Acc:HGNC:17443]	-	2661	3322	2937
3865	4499	3922	25.07766039	25.23951559
23.44519406	32.20313437	33.78485197	31.55012285
ENSG00000166592 43.96454368	113.0880392	92.89084954
17.07782977	26.50193645	30.4176811	83.31447747
24.66581577	1.757940668	0.001290818	0.013050746	RRAD
16	66921679	66925644	-	1709
protein_coding	"RRAD, Ras related glycolysis inhibitor and calcium
channel regulator [Source:HGNC Symbol;Acc:HGNC:10446]"	-	38
122	95	17	29	31	1.216007481
3.147398445	2.575040111	0.480959021	0.739459739
0.846770397
ENSG00000148541 112.2252825	118.649746	115.3802131
37.16939421	59.40089203	43.17348286	115.4184139
46.58125637	1.307361331	0.001294684	0.013081186	FAM13C
10	59246129	59363181	-	6753
protein_coding	family with sequence similarity 13 member C
[Source:HGNC Symbol;Acc:HGNC:19371]	-	97	128	118
37	65	44	0.785542519	0.835693796
0.809445687	0.264914781	0.419445177	0.304159904
ENSG00000179604 1114.154094	1229.137213	1615.322983
869.9647403	842.578807	952.7602694	1319.538097
888.4346056	0.571158063	0.001305039	0.0131771
CDC42EP4	17	73283624	73312175	-	5007
protein_coding	CDC42 effector protein 4 [Source:HGNC
Symbol;Acc:HGNC:17147]	-	963	1326	1652	866	922
971	10.51824805	11.67615605	15.28392533
8.362603716	8.024388141	9.052898984
ENSG00000142544 425.7618967	987.202965	736.2822074
1014.624004	1341.5463	1377.626589	716.4156897
1244.598965	-0.795513116	0.001306887	0.013187046	CTU1
19	51097606	51108370	-	2087
protein_coding	cytosolic thiouridylase subunit 1 [Source:HGNC
Symbol;Acc:HGNC:29590]	-	368	1065	753	1010	1468
1404	9.643175758	22.49889126	16.71379161
23.39915322	30.65223908	31.40441404

ENSG00000169299 1492.480562 1666.658151 1405.096324
995.537018 1124.961509 1137.228787 1521.411679
1085.909105 0.486550222 0.001309218 0.013201859 PGM2
4 37826633 37862937 + 3704
protein_coding phosphoglucomutase 2 [Source:HGNC
Symbol;Acc:HGNC:8906] - 1290 1798 1437 991 1231
1159 19.04642387 21.4019166 17.97166216
12.93611575 14.48257121 14.60691472
ENSG00000184220 1819.900716 1507.222555 1770.792932
1359.194334 1073.785356 1148.022158 1699.305401
1193.667283 0.509475629 0.001311495 0.013209471 CMSS1
3 99817834 100181732 + 7210
protein_coding cms1 ribosomal small subunit homolog (yeast)
[Source:HGNC Symbol;Acc:HGNC:28666] - 1573 1626 1811
1353 1175 1170 11.93131112 9.943041098
11.63551583 9.073268197 7.101681642 7.575244118
ENSG00000175581 934.825034 891.7269975 958.2424478
568.5912737 670.77315 679.0011395 928.2648264
639.4551877 0.537390579 0.001311701 0.013209471 MRPL48
11 73787316 73865133 + 4386
protein_coding mitochondrial ribosomal protein L48 [Source:HGNC
Symbol;Acc:HGNC:16653] - 808 962 980 566 734
692 10.07482349 9.670310139 10.35046603
6.239488956 7.292661076 7.365182337
ENSG00000271216 233.7062585 363.3648472 200.4486753
402.835867 664.3761309 423.8851044 265.839927
497.0323674 -0.901990207 0.001318188 0.013266052
LINC01050 13 42810366 42812562 - 760
lincRNA long intergenic non-protein coding RNA 1050
[Source:HGNC Symbol;Acc:HGNC:49044] - 202 392 205
401 727 432 14.53558416 22.74083634
12.49518598 25.51126516 41.68496261 26.53482272
ENSG00000196935 691.8630821 606.2260461 504.5439827
948.3218416 810.5937113 959.628778 600.8777036
906.1814436 -0.593401434 0.001319186 0.013267369 SRGAP1
12 63844293 64162221 + 26641
protein_coding SLIT-ROBO Rho GTPase activating protein 1 [Source:HGNC
Symbol;Acc:HGNC:17382] - 598 654 516 944 887
978 1.227567478 1.082333707 0.897225555
1.713257684 1.450880671 1.713698309
ENSG00000139278 1623.217231 1322.759278 1517.543142
900.1020869 953.1558521 1206.895089 1487.839884
1020.051009 0.544271426 0.001321477 0.013278557 GLIPR1
12 75480680 75503853 + 6273
protein_coding GLI pathogenesis related 1 [Source:HGNC
Symbol;Acc:HGNC:17001] - 1403 1427 1552 896 1043
1230 12.23142611 10.02957761 11.46090428
6.90611711 7.245487618 9.153261285
ENSG00000227827 9.255693406 11.12341369 16.62257307
118.5402302 25.58807657 39.24862078 12.33389339

61.12564252	-2.304001352	0.001322036	0.013278557
AC138969.1	16	16356224	16377507	+	9932
	unprocessed_pseudogene	polycystic kidney disease 1 (autosomal
dominant) (PKD1) pseudogene	-	8	12	17	118
	28	40	0.044050211	0.053269484	0.079289315
	0.574442433	0.122851346	0.188004964
ENSG00000240751 19.66834849	18.53902282	8.800185745	0
	0.913859877	0	15.66918568	0.304619959
5.500011328	0.001322453	1	AC026348.1	3
113360267	113361319	+	1053	processed_pseudogene
	"Rab geranylgeranyltransferase, beta subunit (RABGGTB)
pseudogene"	-	17	20	9	0	1	0
	0.882907616	0.837405063	0.395928345	0
0.041383787	0
ENSG00000104980 1582.723572	1832.582405	1661.279509
2236.191122	2233.47354	2399.071945	1692.195162
2289.578869	-0.435833586	0.001330103	0.013350808	TIMM44
	19	7926718 7943920 -	4888	protein_coding
translocase of inner mitochondrial membrane 44 [Source:HGNC
Symbol;Acc:HGNC:17316]	-	1368	1977	1699	2226	2444
	2445	15.3055737	17.83238492	16.10143701
22.0188777	21.78856368	23.35036653
ENSG00000146826 254.5315687	322.578997	401.875149
661.0124701	446.8774801	560.2740616	326.3285716
556.0546706	-0.767169854	0.001332115	0.013362229
C7orf43 7	100154420	100158715	-	3255
protein_coding	chromosome 7 open reading frame 43 [Source:HGNC
Symbol;Acc:HGNC:25604]	-	220	348	411	658	489
	571	3.696293092	4.713702934	5.849157083
9.774085127	6.546609916	8.189005281
ENSG00000196954 1257.617342	1554.497063	1067.75587
1570.155761	2199.660725	2051.721651	1293.290092
1940.512712	-0.58529718	0.001335488	0.013387285	CASP4
	11	104942866	104969436	-	7256
protein_coding	caspase 4 [Source:HGNC Symbol;Acc:HGNC:1505]	-
	1087	1677	1092	1563	2407	2091	8.192698691
	10.18989588	6.971525498	10.41508744	14.45564299
	13.45249362
ENSG00000184381 76.3594706	95.47596751	93.86864795
186.8515493	162.6670582	189.3745953	88.56802869
179.6310676	-1.017863598	0.001340383	0.013427546	PLA2G6
	22	38111495	38205690	-	6758
protein_coding	phospholipase A2 group VI [Source:HGNC
Symbol;Acc:HGNC:9039]	-	66	103	96	186	178
	193	0.534097396	0.671974813	0.65804486
1.330748463	1.147784651	1.333168849
ENSG00000153551 428.07582	438.4478896	518.2331606
201.9202226	317.1093775	294.3646558	461.5856234
271.1314186	0.767136608	0.001343113	0.013446082	CMTM7
	3	32391671	32483067	+	3751

protein_coding CKLF like MARVEL transmembrane domain containing 7 [Source:HGNC Symbol;Acc:HGNC:19178] - 370 473 530
201 347 300 5.394477334 5.559657457
6.545325685 2.590897368 4.031261848 3.733534995
ENSG00000049449 2681.837164 2778.99952 3082.998406
2157.834021 1983.989794 2215.584643 2847.94503
2119.136153 0.426650612 0.001347219 0.013478363 RCN1
11 32090904 32105755 + 4675
protein_coding reticulocalbin 1 [Source:HGNC Symbol;Acc:HGNC:9934]
- 2318 2998 3153 2148 2171 2258
27.11605644 28.27378631 31.24243042 22.21538675
20.23656545 22.54697889
ENSG00000086475 1586.194457 1615.675838 1876.395161
1315.997471 1215.433637 1086.20558 1692.755152
1205.878896 0.48947725 0.001351103 0.013508381 SEPHS1
10 13317424 13348298 - 3888
protein_coding selenophosphate synthetase 1 [Source:HGNC Symbol;Acc:HGNC:19685] - 1371 1743 1919 1310 1330
1107 19.28439024 19.76537638 22.8639454
16.29094419 14.90678482 13.29129816
ENSG00000128165 57.84808379 90.8412118 71.37928438
178.8149235 175.4610965 131.4828796 73.35619332
161.9196332 -1.138979566 0.001356603 0.013554503 ADM2
22 50481556 50486440 + 4276
protein_coding adrenomedullin 2 [Source:HGNC Symbol;Acc:HGNC:28898]
- 50 98 73 178 192 134
0.63948008 1.010467471 0.790838164 2.012720742
1.956690471 1.46289494
ENSG00000113361 9.255693406 7.415609127 19.55596832 0
0 0 12.07575695 0 6.08748101
0.001361354 1 CDH6 5 31193750 31329146
+ 12379 protein_coding cadherin 6 [Source:HGNC Symbol;Acc:HGNC:1765] - 8 8 20 0 0
0 0.035342652 0.028493013 0.074842259 0
0 0
ENSG00000144283 1200.926219 1207.817337 1442.252664
1031.701834 812.421431 779.0851225 1283.665407
874.4027958 0.554274456 0.001364186 0.013621371 PKP4
2 158456964 158682879 + 9525
protein_coding plakophilin 4 [Source:HGNC Symbol;Acc:HGNC:9026]
- 1038 1303 1475 1027 889 794
5.959736821 6.03133414 7.173473392 5.213227479
4.067198553 3.891361286
ENSG00000196967 80.9873173 77.86389583 57.69010655
191.8744404 151.7007397 132.4640951 72.18043989
158.6797584 -1.137392738 0.001365963 0.01363021
ZNF585A 19 37106734 37172741 - 10758
protein_coding zinc finger protein 585A [Source:HGNC Symbol;Acc:HGNC:26305] zf-C2H2 70 84 59 191 166
135 0.355845283 0.344256147 0.254052181

0.858426357	0.672411521	0.585798512
ENSG00000204956 38.1797353	21.31987624	31.28954932
82.37541421	90.47212786	87.32818123	30.26305362
86.7252411	-1.524659965	0.001369643	0.013653253
PCDHGA1 5	141330571	141512981	+	6252
protein_coding	"protocadherin gamma subfamily A, 1 [Source:HGNC
Symbol;Acc:HGNC:8696]"	-	33	23	32	82	99
	89	0.288662045	0.162197003	0.237101043
0.634155991	0.690040884	0.664533797
ENSG00000182768 994.9870411	936.2206523	934.7752859
1465.679626	1502.385638	1146.059727	955.3276597
1371.374997	-0.521864496	0.001370059	0.013653253	NGRN
	15	90265659	90278141	+	7694
protein_coding	"neugrin, neurite outgrowth associated [Source:HGNC
Symbol;Acc:HGNC:18077]" -	860	1010	956	1459	1644
	1168	6.112811195	5.787661859	5.755832752
9.168627451	9.311255182	7.08658004
ENSG00000130635 4723.874522	2800.319396	7959.279108
2138.747035	1559.044951	2097.838781	5161.157675
1931.876922	1.41771196	0.001372595	0.013661925	COL5A1
	9	134641774	134844843	+	11189
protein_coding	collagen type V alpha 1 chain [Source:HGNC
Symbol;Acc:HGNC:2209]	-	4083	3021	8140	2129	1706
	2138	19.95642843	11.90401346	33.70044122
9.199952903	6.6442559	8.919950881
ENSG00000020922 1839.569064	1677.781565	1544.921498
1041.747616	1198.984159	1333.471891	1687.424042
1191.401222	0.501821754	0.001372716	0.013661925	MRE11
	11	94415578	94493908	-	7689
protein_coding	"MRE11 homolog, double strand break repair nuclease
[Source:HGNC Symbol;Acc:HGNC:7230]"	-	1590	1810	1580
	1037	1312	1359	11.30894199	10.37869315
9.518963945	6.520938615	7.435712198	8.250791864
ENSG00000146476 755.4959742	742.4878638	571.034275
268.2223853	475.2071363	468.0398028	689.6727044
403.8231081	0.771411623	0.00137362	0.013662019	ARMT1
	6	151452258	151470101	+	2819
protein_coding	acidic residue methyltransferase 1 [Source:HGNC
Symbol;Acc:HGNC:17872]	-	653	801	584	267	520
	477	12.66813895	12.52770154	9.59666262
4.579493026	8.038349139	7.898949531
ENSG00000198919 777.4782461	542.2664174	790.0611203
515.3486279	395.7013269	394.4486388	703.2685946
435.1661979	0.69221981	0.001375194	0.013668787	DZIP3
	3	108589682	108694846	+	6972
protein_coding	DAZ interacting zinc finger protein 3 [Source:HGNC
Symbol;Acc:HGNC:30938]	-	672	585	808	513	433
	402	5.271164957	3.699409825	5.368543027
3.557633477	2.706382121	2.691625973
ENSG00000013619 437.3315134	437.5209385	515.2997653

211.9660048	247.6560268	350.2939405	463.3840724
269.9719907	0.779598927	0.001385696	0.013764222	MAMLD1
	X	150361422	150514178	+	5958
protein_coding	mastermind like domain containing 1 [Source:HGNC
Symbol;Acc:HGNC:2568]	-	378	472	527	211	271
	357	3.469652765	3.492813989	4.097439711
1.712313075	1.982108344	2.797137097
ENSG00000137103 240.6480285	371.7074075	273.7835565
530.4173012	535.5218882	426.828751	295.3796642
497.5893135	-0.750619678	0.001391167	0.013809587	TMEM8B
	9	35814451	35865518	+	16165
protein_coding	transmembrane protein 8B [Source:HGNC
Symbol;Acc:HGNC:21427]	-	208	401	280	528	586
	435	0.703691554	1.093711105	0.802388652
1.579281071	1.579721412	1.256202299
ENSG00000136244 35.86581195	52.83621503	55.73450972
12.05493866	10.05245865	11.77458623	48.14551223
11.29399452	2.094780046	0.001393536	0.013820299	IL6
	7	22725884	22732002	+	2069
protein_coding	interleukin 6 [Source:HGNC Symbol;Acc:HGNC:6018]
	-	31	57	57	12	11	12
0.819399918	1.214642081	1.27619436	0.280428386
0.231681199	0.270748956
ENSG00000196646 174.701213	132.5540131	106.5800274
290.3231062	273.2441033	225.6795695	137.9450845
263.0822597	-0.934139899	0.001394054	0.013820299	ZNF136
	19	12163064	12189881	+	5425
protein_coding	zinc finger protein 136 [Source:HGNC
Symbol;Acc:HGNC:12920]	zf-C2H2 151	143	109	289	299
	230	1.522200701	1.162171585	0.930741784
2.575723953	2.401762411	1.979129122
ENSG00000167969 437.3315134	507.9692252	534.8557336
262.1949159	360.0607917	286.5149317	493.3854907
302.9235464	0.703668442	0.001396387	0.013829118	ECI1
	16	2239395 2252300 -	3234	protein_coding	enoyl-
CoA delta isomerase 1 [Source:HGNC Symbol;Acc:HGNC:2703]	-
	378	548	547	261	394	292	6.392143219
	7.470927138	7.835194225	3.90212977	5.30902541
	4.214915494
ENSG00000158246 65.94681552	63.95962872	66.4902923
10.04578222	0.913859877	25.51160351	65.46557884
12.15708187	2.434074373	0.001396753	0.013829118	TENT5B
	1	27005020	27012836	-	2368
protein_coding	terminal nucleotidyltransferase 5B [Source:HGNC
Symbol;Acc:HGNC:28273]	-	57	69	68	10	1
	26	1.31639999	1.284698895	1.330238982
0.204182971	0.018402503	0.512551709
ENSG00000146938 19.66834849	6.488657986	31.28954932
1.004578222	0	0.981215519	19.14885193	0.661931247
	4.878512147	0.001399825	1	NLGN4X	X

5840637 6228863 -   7441   protein_coding neuroligin 4 X-linked
[Source:HGNC Symbol;Acc:HGNC:14287]   -   17   7   32
        1   0   1   0.124943115   0.041476366
0.199214584   0.006497853   0   0.00627357
ENSG00000173705 178.1720981   126.0653552   293.3395248
92.42119643   54.83159264   103.0276295   199.192326
83.42680621   1.256870699   0.001400147   0.013849706   SUSD5
        3   33150042   33219215   -   5008
protein_coding sushi domain containing 5 [Source:HGNC
Symbol;Acc:HGNC:29061] -   154   136   300   92   60
        105   1.681710026   1.197315338   2.774977016
0.88822854   0.522090184   0.978748286
ENSG00000147872 12651.37592   13849.577   6104.395512
23429.77787   32526.10076   20133.56124   10868.44948
25363.14662   -1.222598616   0.001400644   0.013849706   PLIN2
        9   19108375   19149290   -   5086
protein_coding perilipin 2 [Source:HGNC Symbol;Acc:HGNC:248]   -
        10935   14941   6243   23323   35592   20519   117.5809986
        129.5201289   56.861647   221.7222479   304.9542109
        188.3327602
ENSG00000197959 179.3290597   164.0703519   220.9824421
106.4852915   92.29984762   78.49724156   188.1272846
92.42746024   1.025622827   0.001403298   0.013866981   DNM3
        1   171841498   172418466   +   11329
protein_coding dynamin 3 [Source:HGNC Symbol;Acc:HGNC:29125]   -
        155   177   226   106   101   80   0.748229512
        0.688835701   0.92410074   0.452392613   0.388497649
        0.329643446
ENSG00000091317 3038.18136   2583.41283   2676.234265
2150.801973   2063.495603   1935.93822   2765.942818
2050.078599   0.431826794   0.001404916   0.013873995   CMTM6
        3   32481312   32503408   -   4413
protein_coding CKLF like MARVEL transmembrane domain containing 6
[Source:HGNC Symbol;Acc:HGNC:19177]   -   2626   2787   2737
        2141   2258   1973   32.5428442   27.84434456
28.73050937   23.45762052   22.29711162   20.87080468
ENSG00000159733 102.9695891   116.7958437   77.24607488
239.0896168   198.3075934   168.7690693   99.00383592
202.0554265   -1.028874877   0.001419924   0.014013152
ZFYVE28 4   2269582 2418663 -   9624   protein_coding zinc
finger FYVE-type containing 28 [Source:HGNC Symbol;Acc:HGNC:29334]
        -       89   126   79   238   217   172
0.505742097   0.577229993   0.380254124   1.195700908
0.982568229   0.834293516
ENSG00000179820 4030.854478   3891.340889   2818.015035
4047.445657   5596.477889   6425.980437   3580.070134
5356.634661   -0.581473396   0.001421397   0.014018647   MYADM
        19   53866223   53876437   +   3675
protein_coding myeloid associated differentiation marker [Source:HGNC
Symbol;Acc:HGNC:7544]   -   3484   4198   2882   4029   6124

6549 51.84603106 50.36386473 36.32779924
53.00796606 72.61668787 83.18857459
ENSG00000186812 570.3821061 540.4125151 394.0527617
746.401619 801.4551125 768.2917517 501.6157943
772.0494944 -0.622890704 0.001422757 0.014023005 ZNF397
18 35241030 35267133 + 8710
protein_coding zinc finger protein 397 [Source:HGNC
Symbol;Acc:HGNC:18818] zf-C2H2 493 583 403 743 877
783 3.095447745 2.951102933 2.143331501
4.124505388 4.387731422 4.19652334
ENSG00000245532 12189.74822 13641.93994 10126.0804
18505.33543 12779.41653 22318.72821 11985.92285
17867.82672 -0.575993643 0.001424413 0.014030284 NEAT1
11 65422774 65445540 + 22767
lincRNA nuclear paraspeckle assembly transcript 1 [Source:HGNC
Symbol;Acc:HGNC:30815] - 10536 14717 10356 18421 13984
22746 25.30839868 28.50016933 21.0711719
39.12087965 26.76604511 46.63857399
ENSG00000136379 499.8074439 351.3144824 439.0314889
299.3643102 213.8432113 242.3602333 430.0511384
251.8559183 0.771525038 0.001433849 0.014107775
ABHD17C 15 80679684 80755621 + 3627
protein_coding abhydrolase domain containing 17C [Source:HGNC
Symbol;Acc:HGNC:26925] - 432 379 449 298 234
247 6.513747267 4.607078501 5.734575055
3.972555057 2.811427571 3.179035909
ENSG00000169738 953.3364208 1035.404424 1152.824333
692.154395 739.3126408 761.4232431 1047.188393
730.9634263 0.518954867 0.001434126 0.014107775 DCXR
17 82035136 82037732 - 1902
protein_coding dicarbonyl and L-xylulose reductase [Source:HGNC
Symbol;Acc:HGNC:18985] - 824 1117 1179 689 809
776 23.69252851 25.89265701 28.71479686
17.51499129 18.53517141 19.04571339
ENSG00000188064 489.3947888 714.6793296 436.0980936
779.5527003 990.6241071 862.4884416 546.7240707
877.555083 -0.681960939 0.001444159 0.014197339 WNT7B
22 45920362 45977129 - 5051
protein_coding Wnt family member 7B [Source:HGNC
Symbol;Acc:HGNC:12787] - 423 771 446 776 1084
879 4.579918098 6.729936516 4.090345056
7.428233881 9.35212949 8.123768564
ENSG00000105193 19950.64714 20680.27995 21869.43938
24327.8708 27127.93046 31585.32757 20833.45549
27680.37628 -0.409949762 0.001462414 0.014367563 RPS16
19 39433207 39435948 - 2603
protein_coding ribosomal protein S16 [Source:HGNC
Symbol;Acc:HGNC:10396] - 17244 22310 22366 24217 29685
32190 362.2918453 377.8847451 398.0307347
449.8289375 496.9600559 577.288418

ENSG00000160695 1427.690708 1481.267923 1301.449692
2004.133553 1901.742405 1819.173573 1403.469441
1908.349844 -0.443308453 0.001466892 0.014402299 VPS11
11 119067692 119081978 + 5907
protein_coding "VPS11, CORVET/HOPS core subunit [Source:HGNC
Symbol;Acc:HGNC:14583]" - 1234 1598 1331 1995 2081
1854 11.42464993 11.92734447 10.43790982
16.32966012 15.35195565 14.65172622
ENSG00000173153 1023.911083 1695.393637 1409.985316
2014.179335 1957.487857 2184.185746 1376.430012
2051.95098 -0.575190525 0.001470846 0.014431849 ESRRA
11 64305572 64316743 + 3030
protein_coding estrogen related receptor alpha [Source:HGNC
Symbol;Acc:HGNC:3471] ESR-like 885 1829 1442 2005
2142 2226 15.97332599 26.61368639 22.04576063
31.99432595 30.80600884 34.29482312
ENSG00000166508 7696.109067 8438.036235 8836.364287
6105.826433 5784.733024 6962.705326 8323.503196
6284.421595 0.405511339 0.001481732 0.014526994 MCM7
7 100092728 100101940 - 4386
protein_coding minichromosome maintenance complex component 7
[Source:HGNC Symbol;Acc:HGNC:6950] - 6652 9103 9037
6078 6330 7096 82.94273004 91.50606361
95.44608313 67.00285137 62.89175015 75.52504893
ENSG00000163704 119.1670526 178.9015702 140.8029719
282.2864804 275.985683 249.2287419 146.2905316
269.1669684 -0.877113853 0.001482443 0.014526994 PRRT3
3 9945542 9952394 - 3939 protein_coding
proline rich transmembrane protein 3 [Source:HGNC
Symbol;Acc:HGNC:26591] - 103 193 144 281 302
254 1.430032661 2.160256329 1.693475692
3.449225241 3.341023727 3.01018905
ENSG00000274211 935.9819957 963.1022353 1042.333112
1348.143974 1386.325434 1323.659736 980.4724475
1352.709715 -0.464118189 0.001489308 0.014584914 SOCS7
17 38352228 38405593 + 8621
protein_coding suppressor of cytokine signaling 7 [Source:HGNC
Symbol;Acc:HGNC:29846] - 809 1039 1066 1342 1517
1349 5.131987493 5.313636732 5.727986891
7.52655236 7.668078204 7.304665621
ENSG00000077312 934.825034 1426.577806 1842.172216
871.9738967 788.6610742 949.8166228 1401.191685
870.1505313 0.688037025 0.001492355 0.014602294 SNRPA
19 40750637 40765389 + 2361
protein_coding small nuclear ribonucleoprotein polypeptide A
[Source:HGNC Symbol;Acc:HGNC:11151] - 808 1539 1884
868 863 968 18.71587287 28.73932679
36.96471543 17.77562807 15.92844596 19.13927163
ENSG00000077549 5147.322495 5090.815666 5355.401925
3808.35604 4202.841576 3998.453242 5197.846695

4003.216953	0.376707323	0.001494044	0.014602294	CAPZB
	1	19338776	19485539	-	3238
protein_coding	capping actin protein of muscle Z-line subunit beta
[Source:HGNC Symbol;Acc:HGNC:1491]	-	4449	5492	5477
	3791	4599	4075	75.14157158	74.78037559
78.35529541	56.608045	61.8935172	58.74850298
ENSG00000111077 609.7188031	790.6893231	990.5097956
450.0510435	519.9862702	535.7436736	796.9726406
501.9269958	0.667702547	0.001494773	0.014602294	TNS2
	12	53046969	53064372	+	8093
protein_coding	tensin 2 [Source:HGNC Symbol;Acc:HGNC:19737]	-
	527	853	1013	448	569	546	3.561195267
	4.647008306	5.798322418	2.676515052	3.063806433
	3.149409536
ENSG00000233117 513.690984	374.4882609	435.1202952
238.0850386	306.1430589	250.2099575	441.0998467
264.812685	0.734468301	0.001495384	0.014602294
LINC00702	10	4201141 4243912 -	6785	lincRNA long
intergenic non-protein coding RNA 702 [Source:HGNC
Symbol;Acc:HGNC:44676]	-	444	404	445	237	335
	255	3.57872091	2.625218591	3.038173804
1.688883569	2.151560452	1.75443129
ENSG00000136783 623.6023432	513.530932	452.7206667
272.2406982	391.1320275	295.3458714	529.951314
319.5728657	0.728183407	0.001495859	0.014602294
NIPSNAP3A	9	104747688	104760122	+	2585
	protein_coding	nipsnap homolog 3A [Source:HGNC
Symbol;Acc:HGNC:23619]	-	539	554	463	271	428
	301	11.40309994	9.448941828	8.297034566
5.068856074	7.215091105	5.435656247
ENSG00000177707 2035.095588	1478.48707	1868.572773
1188.416037	1287.628567	1303.05421	1794.05181
1259.699605	0.509605157	0.001499236	0.014625927
NECTIN3 3	111070071	111275563	+	6184
protein_coding	nectin cell adhesion molecule 3 [Source:HGNC
Symbol;Acc:HGNC:17664]	-	1759	1595	1911	1183	1409
	1328	15.55575417	11.37169399	14.31507613
9.249462164	9.928876527	10.02477491
ENSG00000173110 75.20250892	30.58938765	104.6244305
171.782876	107.8354655	310.0641042	70.1387757
196.5608152	-1.488014167	0.001505569	0.014669752	HSPA6
	1	161524540	161526910	+	2371
protein_coding	heat shock protein family A (Hsp70) member 6
[Source:HGNC Symbol;Acc:HGNC:5239]	-	65	33	107
	171	118	316	1.499258485	0.61364379
2.090521698	3.487111009	2.168747797	6.221592529
ENSG00000138835 2231.779072	2921.749996	2108.133385
2920.308891	4047.485397	3546.112887	2420.554151
3504.635725	-0.5337862	0.001505648	0.014669752	RGS3
	9	113444731	113597743	+	13793

protein_coding regulator of G protein signaling 3 [Source:HGNC Symbol;Acc:HGNC:9999]   -   1929   3152   2156   2907   4429
3614   7.648357933   10.0753799   7.240898773
10.19031273   13.99282948   12.23137969
ENSG00000220161 16.19746346   3.707804563   16.62257307   0
0   0   12.17594703   0   6.096675626
0.001511116   1   LINC02076   17   18411159
18414380   +   1871   lincRNA long intergenic non-protein coding RNA 2076 [Source:HGNC Symbol;Acc:HGNC:52924]   -   14
4   17   0   0   0   0.409212565
0.094258421   0.420898705   0   0   0
ENSG00000135966 1155.804714   1313.489767   1280.915925
1796.185861   1834.116774   1555.226598   1250.070135
1728.509744   -0.46718661   0.001530607   0.014903435
TGFBRAP1   2   105264391   105330034   -   6005
protein_coding transforming growth factor beta receptor associated protein 1 [Source:HGNC Symbol;Acc:HGNC:16836]   -
999   1417   1310   1788   2007   1585   9.098026327
10.40377129   10.10556825   14.39646014   14.56441209
12.32146338
ENSG00000170144 8728.118882   8683.678287   9498.333815
7439.906312   5895.310069   6917.569412   8970.043661
6750.928598   0.410107354   0.001552495   0.01508995
HNRNPA3 2   177212563   177223958   +   6033
protein_coding heterogeneous nuclear ribonucleoprotein A3 [Source:HGNC Symbol;Acc:HGNC:24941]   -   7544   9368   9714
7406   6451   7050   68.38534892   68.46167409
74.58770245   59.35421956   46.59639459   54.55088837
ENSG00000104880 121.480976   196.5136419   121.2470036
343.5657519   200.1353132   338.5193542   146.4138738
294.0734731   -1.003422877   0.00155299   0.01508995
ARHGEF18   19   7395113 7472477 +   6235   protein_coding
Rho/Rac guanine nucleotide exchange factor 18 [Source:HGNC Symbol;Acc:HGNC:17090] -   105   212   124   342   219
345   0.920974391   1.499109516   0.921271599
2.652105919   1.530616021   2.583025378
ENSG00000174705 3297.340776   3048.742302   2987.174161
5275.040244   3856.488683   3879.726164   3111.085746
4337.08503   -0.479332798   0.00155309   0.01508995
SH3PXD2B   5   172325000   172454523   -   8242
protein_coding SH3 and PX domains 2B [Source:HGNC Symbol;Acc:HGNC:29242] -   2850   3289   3055   5251   4220
3954   18.91067202   17.59402567   17.1704256
30.80424895   22.31199678   22.39494966
ENSG00000085840 1266.873035   997.3994276   1185.09168
762.4748705   712.8107044   888.9812606   1149.788048
788.0889452   0.544605789   0.001553711   0.01508995   ORC1
1   52372829   52404459   -   3157
protein_coding origin recognition complex subunit 1 [Source:HGNC Symbol;Acc:HGNC:8487]   -   1095   1076   1212   759   780

906	18.96855508	15.02697789	17.78404275
11.62434285	10.76660099	13.39675613
ENSG00000139926 2265.330961	1607.333278	1777.637521
1215.539649	1247.418733	1448.274107	1883.43392
1303.744163	0.530173934	0.001554747	0.01509042	FRMD6
14	51489100	51730727	+	9416
protein_coding	FERM domain containing 6 [Source:HGNC
Symbol;Acc:HGNC:19839]	-	1958	1734	1818	1210	1365
1476	11.37210734	8.119262849	8.943960754
6.21326872	6.31720251	7.317554126
ENSG00000174165 476.6682104	525.5812969	554.411702
745.3970407	732.0017618	807.5403725	518.8870697
761.6463917	-0.553012733	0.00155926	0.015119004
ZDHHC24 11	66520637	66546238	-	6266
protein_coding	zinc finger DHHC-type containing 24 [Source:HGNC
Symbol;Acc:HGNC:27387]	-	412	567	567	742	801
823	3.595849762	3.989574931	4.191747598
5.7255173	5.570583947	6.131341142
ENSG00000088832 4779.408682	4753.40545	5242.955107
3482.872696	3833.642186	3937.61788	4925.256413
3751.377587	0.392772992	0.00155967	0.015119004	FKBP1A
20	1368978 1393172 -	2577	protein_coding	FK506
binding protein 1A [Source:HGNC Symbol;Acc:HGNC:3711]	-	4131
5128	5362	3467	4195	4013	87.6668676
87.73392437	96.38619739	65.04900226	70.93754336
72.69436998
ENSG00000105229 542.6150259	932.5128477	808.6392902
1336.089035	1065.560617	1180.40227	761.2557213
1194.017307	-0.647939065	0.001565979	0.015170548	PIAS4
19	4007646 4039386 +	3646	protein_coding
protein inhibitor of activated STAT 4 [Source:HGNC
Symbol;Acc:HGNC:17002]	zf-MIZ	469	1006	827	1330	1166
1203	7.034786008	12.16508855	10.50730408
17.63746615	13.93607529	15.40263411
ENSG00000115257 37.02277362	63.03267758	51.82331606
128.5860124	116.0602044	120.6895089	50.62625575
121.7785752	-1.261436797	0.001574024	0.015238825	PCSK4
19	1481428 1490752 -	3792	protein_coding
proprotein convertase subtilisin/kexin type 4 [Source:HGNC
Symbol;Acc:HGNC:8746]	-	32	68	53	128	127
123	0.461504949	0.79063228	0.647455608
1.632085316	1.459466027	1.514198524
ENSG00000135052 2284.99931	2014.264829	2153.112112
1579.196965	1567.26969	1658.254228	2150.792084
1601.573628	0.425155757	0.001582295	0.015290881	GOLM1
9	86026146	86100173	-	4233
protein_coding	golgi membrane protein 1 [Source:HGNC
Symbol;Acc:HGNC:15451]	-	1975	2173	2202	1572	1715
1690	25.51605586	22.63317158	24.09747298
17.9558302	17.65527366	18.63736334

ENSG00000177873 297.3391507 262.3271729 186.7594975
468.1334515 385.6488683 418.9790268 248.808607
424.2537822 -0.771066542 0.001582639 0.015290881 ZNF619
3 40477113 40490236 + 4514
protein_coding zinc finger protein 619 [Source:HGNC
Symbol;Acc:HGNC:26910] zf-C2H2 257 283 191 466 422
427 3.113624826 2.764132382 1.960082126
4.991436821 4.073891835 4.415830107
ENSG00000143641 1576.938764 1712.078757 2145.289725
1272.800607 1219.089076 1341.321615 1811.435749
1277.7371 0.50389958 0.001583495 0.015290881 GALNT2
1 230057990 230282124 + 7743
protein_coding polypeptide N-acetylgalactosaminyltransferase 2
[Source:HGNC Symbol;Acc:HGNC:4124] - 1363 1847 2194
1267 1334 1367 9.626785814 10.51699322
13.12592202 7.911677429 7.507669881 8.241481558
ENSG00000102158 2493.252411 1800.139116 2691.87904
1646.503706 1487.76388 1694.559202 2328.423522
1609.608929 0.532479677 0.001583572 0.015290881 MAGT1
X 77826364 77895593 - 4862
protein_coding magnesium transporter 1 [Source:HGNC
Symbol;Acc:HGNC:28880] - 2155 1942 2753 1639 1628
1727 24.2396884 17.61035979 26.22972351
16.29915972 14.59143631 16.58148487
ENSG00000258768 37.02277362 38.00499677 32.26734773
146.6684204 55.74545252 107.9337071 35.76503938
103.4491934 -1.531332765 0.001584402 0.015290881
AL356019.2 14 20260480 20264308 - 3829
lincRNA novel transcript - 32 41 33
146 61 110 0.457045382 0.472098313
0.399237226 1.843608518 0.694229503 1.34107589
ENSG00000062282 68.26073887 158.5086451 84.09066379
304.3872013 215.6709311 179.5624401 103.6200159
233.2068575 -1.166025317 0.001585625 0.015293028 DGAT2
11 75759512 75801535 + 7905
protein_coding diacylglycerol O-acyltransferase 2 [Source:HGNC
Symbol;Acc:HGNC:16940] - 59 171 86 303 236
183 0.408173542 0.95373604 0.503963441
1.853283976 1.300974327 1.080675365
ENSG00000157306 1.156961676 1.853902282 2.933395248
36.16481599 4.569299387 37.28618974 1.981419735
26.00676837 -3.699354562 0.001588517 0.015311258 ZFHX2-
AS1 14 23511760 23560778 + 5097
processed_transcript ZFHX2 antisense RNA 1 [Source:HGNC
Symbol;Acc:HGNC:52658] - 1 2 3 36 5
38 0.010729515 0.017300128 0.027265224
0.341498723 0.04274782 0.348028652
ENSG00000174607 139.9923628 145.5313291 172.0925212
71.32505376 31.98509571 83.40331915 152.5387377
62.23782288 1.295876744 0.00159047 0.015317982 UGT8

4	114598455	114678224	+	4699
protein_coding	UDP glycosyltransferase 8 [Source:HGNC
Symbol;Acc:HGNC:12555]	-	121	157	176	71	35
85	1.408233392	1.473086215	1.735040747
0.730557023	0.324579582	0.844421955
ENSG00000127337 925.5693406	824.0595642	709.8816501
550.5088657	596.7505	493.5514063	819.8368516
546.936924	0.583201489	0.001591998	0.015317982	YEATS4
12	69359703	69390796	+	1714
protein_coding	YEATS domain containing 4 [Source:HGNC
Symbol;Acc:HGNC:24859]	Others	800	889	726	548	653
503	25.52547791	22.86782718	19.62132173
15.45862839	16.6020211	13.69945226
ENSG00000068885 429.2327817	363.3648472	376.4523902
173.7920324	268.674804	238.4353712	389.6833397
226.9674025	0.778350119	0.001592727	0.015317982	IFT80
3	160256986	160399880	-	7175
protein_coding	intraflagellar transport 80 [Source:HGNC
Symbol;Acc:HGNC:29262]	-	371	392	385	173	294
243	2.827787152	2.408785452	2.485657461
1.165803659	1.785599365	1.580994664
ENSG00000165271 3113.383869	3971.985639	3690.211223
5633.674669	4336.265118	4914.908537	3591.860243
4961.616108	-0.465758124	0.00159333	0.015317982	NOL6
9	33461441	33473930	-	5906
protein_coding	nucleolar protein 6 [Source:HGNC
Symbol;Acc:HGNC:19910]	-	2691	4285	3774	5608	4745
5009	24.9181025	31.98831335	29.60130849
45.91089708	35.01074658	39.59165213
ENSG00000164187 679.1365036	691.5055511	703.0370612
497.2662199	466.0685375	449.3967079	691.226372
470.9104884	0.553895547	0.001594433	0.015317982	LMBRD2
5	36098412	36151961	-	8421
protein_coding	LMBR1 domain containing 2 [Source:HGNC
Symbol;Acc:HGNC:25287]	-	587	746	719	495	510
458	3.812142677	3.905792059	3.955192985
2.842122207	2.63915627	2.538913159
ENSG00000174282 1159.275599	1456.240242	1652.479323
2009.156444	1839.599933	2328.424428	1422.665055
2059.060268	-0.53275375	0.001595226	0.015317982	ZBTB4
17	7459366 7484263 -	6126	protein_coding	zinc
finger and BTB domain containing 4 [Source:HGNC Symbol;Acc:HGNC:23847]
ZBTB	1002	1571	1690	2000	2013	2373
8.945104979	11.30662922	12.77945014	15.78535014
14.31941844	18.08284591
ENSG00000176788 1642.88558	2224.682738	1797.193489
2289.433768	3147.333418	2720.910635	1888.253935
2719.22594	-0.525861328	0.001607009	0.015421436	BASP1
5	17065598	17276843	+	2217
protein_coding	brain abundant membrane attached signal protein 1

[Source:HGNC Symbol;Acc:HGNC:957]	-	1420	2400	1838
	2279	3444	2773	35.02816316	47.72868912
38.40451366	49.70268478	67.69491502	58.38888907
ENSG00000164366 455.8429002	477.3798375	478.1434255
724.3008981	725.6047427	634.8464411	470.4553878
694.9173606	-0.562569895	0.001609167	0.015432457
CCDC127 5	196871	218215	-	9701	protein_coding
coiled-coil domain containing 127 [Source:HGNC Symbol;Acc:HGNC:30520]
	-	394	515	489	721	794	647
2.221132312	2.340586428	2.335042612	3.593519258
3.566667263	3.113392049
ENSG00000115295 475.5112487	368.9265541	285.5171375
641.9254839	515.4169709	725.1182689	376.6516468
627.4869079	-0.737633776	0.001610624	0.015436747	CLIP4
	2	29097705	29189643	+	7775
protein_coding	CAP-Gly domain containing linker protein family member
4 [Source:HGNC Symbol;Acc:HGNC:26108]	-	411	398	292
	639	564	739	2.890920421	2.256922428
1.739742246	3.973760395	3.161093225	4.437006618
ENSG00000159199 1139.607251	1283.82733	1672.035292
895.0791958	838.0095076	1023.407787	1365.156624
918.8321634	0.57170613	0.001614522	0.015464406
ATP5MC1 17	48892765	48895871	+	2615
protein_coding	ATP synthase membrane subunit c locus 1 [Source:HGNC
Symbol;Acc:HGNC:841]	-	985	1385	1710	891	917
	1043	20.59962194	23.35135241	30.29192501
16.47430975	15.28115709	18.61909857
ENSG00000160256 319.3214225	632.1806781	608.1906149
834.8045025	806.0244119	996.9149678	519.8975718
879.2479607	-0.756224972	0.001625016	0.015543835
FAM207A 21	44940010	44976989	+	1402
protein_coding	family with sequence similarity 207 member A
[Source:HGNC Symbol;Acc:HGNC:15811]	-	276	682	622
	831	882	1016	10.76603485	21.44718604
20.55156159	28.65855089	27.41442677	33.8292002
ENSG00000133789 1906.672842	1881.710816	2003.508955
1337.093614	1537.112314	1407.063055	1930.630871
1427.089661	0.435850808	0.00162639	0.015543835	SWAP70
	11	9664077 9752991 +	7190	protein_coding
switching B cell complex subunit SWAP70 [Source:HGNC
Symbol;Acc:HGNC:17070]	-	1648	2030	2049	1331	1682
	1434	12.53496223	12.44804374	13.20126423
8.950563573	10.19425983	9.310356201
ENSG00000136950 1369.842624	1597.136816	1273.093538
1958.927533	2114.671756	1816.229927	1413.357659
1963.276405	-0.473964669	0.001626408	0.015543835	ARPC5L
	9	124862130	124877733	+	3610
protein_coding	actin related protein 2/3 complex subunit 5 like
[Source:HGNC Symbol;Acc:HGNC:23366]	-	1184	1723	1302
	1950	2314	1851	17.93656242	21.04321215

16.70729874	26.11732094	27.93281792	23.93565148
ENSG00000151466 313.5366141	280.8661957	351.0296314
191.8744404	189.1689946	172.6939314	315.1441471
184.5791222	0.77155789	0.001626881	0.015543835	SCLT1
	4	128864921	129093607	-	8292
protein_coding	sodium channel and clathrin linker 1 [Source:HGNC
Symbol;Acc:HGNC:26406]	-	271	303	359	191	207
	176	1.787329857	1.611080693	2.005569013
1.113718132	1.087851587	0.990830599
ENSG00000115419 5982.648825	4244.509274	4581.963378
3599.40377	3543.948605	3566.718413	4936.373826
3570.023596	0.467246084	0.00162912	0.015555498	GLS
	2	190880827	190965552	+	9827
protein_coding	glutaminase [Source:HGNC Symbol;Acc:HGNC:4331]	-
	5171	4579	4686	3583	3878	3635	28.77718405
	20.54393562	22.0893931	17.62897527	17.19671312
	17.2675012
ENSG00000154832 1660.240005	2073.589702	1850.972402
2598.84386	2347.706025	2696.380248	1861.600703
2547.643378	-0.452111366	0.001630732	0.015561178	CXXC1
	18	50282343	50288304	-	4319
protein_coding	CXXC finger protein 1 [Source:HGNC
Symbol;Acc:HGNC:24343]	-	1435	2237	1893	2587	2569
	2748	18.17035489	22.83582667	20.30345119
28.96105919	25.92026862	29.70158052
ENSG00000155324 369.0707746	335.556313	442.9426825
238.0850386	234.8619885	220.7734919	382.5232567
231.240173	0.72613395	0.001646855	0.01570522
GRAMD2B 5	126360132	126496494	+	5051
protein_coding	GRAM domain containing 2B [Source:HGNC
Symbol;Acc:HGNC:24911]	-	319	362	453	237	257
	225	3.453886225	3.159840491	4.154543296
2.268674523	2.217248412	2.079462943
ENSG00000122870 757.8098976	609.0068995	795.9279108
534.4356141	468.8101171	385.6176992	720.9149026
462.9544768	0.638665326	0.001659598	0.015816881	BICC1
	10	58513140	58831437	+	6569
protein_coding	BicC family RNA binding protein 1 [Source:HGNC
Symbol;Acc:HGNC:19351]	-	655	657	814	532	513
	393	5.453015735	4.409608831	5.740207593
3.91573765	3.403115593	2.792796711
ENSG00000124766 736.9845874	994.6185741	880.0185745
1250.699886	1620.273563	1087.186796	870.5405787
1319.386748	-0.599392313	0.00166212	0.015831046	SOX4
	6	21592768	21598619	+	5852
protein_coding	SRY-box 4 [Source:HGNC Symbol;Acc:HGNC:11200]	HMG
	637	1073	900	1245	1773	1108	5.952917004
	8.084056915	7.124274553	10.28646732	13.20270793
	8.838559274
ENSG00000100056 614.3466498	926.9511408	742.1489979

1182.388567 1054.594299 1178.439839 761.1489295
1138.474235 -0.579586682 0.001663358 0.015832975 ESS2
22 19130279 19144684 - 6003
protein_coding ess-2 splicing factor homolog [Source:HGNC
Symbol;Acc:HGNC:16817] - 531 1000 759 1177 1154
1201 4.837499025 7.344557151 5.857008959
9.480021799 8.377145588 9.339437065
ENSG00000273391 10.41265508 10.19646255 13.68917783 0
0 0 11.43276515 0 6.008272667
0.001666877 1 AC083880.1 7 139359032
139359566 - 535 antisense "novel transcript,
antisense to LUC7L2" - 9 11 14 0 0
0 0.919990706 0.906510546 1.212206782 0
0 0
ENSG00000128944 1007.71362 1088.240639 1125.445977
821.7449856 749.3650995 704.512743 1073.800079
758.5409427 0.501807548 0.001667957 0.015863472 KNSTRN
15 40382721 40394246 + 3654
protein_coding kinetochore localized astrin (SPAG5) binding protein
[Source:HGNC Symbol;Acc:HGNC:30767] - 871 1174 1151
818 820 718 13.03599919 14.1655523
14.59181236 10.82395498 9.779213035 9.172800267
ENSG00000114841 323.9492692 359.6570426 278.6725486
621.8339194 401.1844862 574.0110789 320.7596202
532.3431615 -0.730209084 0.001668974 0.015863472 DNAH1
3 52316319 52400491 + 13872
protein_coding dynein axonemal heavy chain 1 [Source:HGNC
Symbol;Acc:HGNC:2940] - 280 388 285 619 439
585 1.103859155 1.233180371 0.951717896
2.157509841 1.379062782 1.96862421
ENSG00000231574 34.70885027 25.95463194 42.04533189 0
0 8.830939675 34.23627137 2.943646558
3.546538426 0.001670433 0.015863472 LINC02015 3
177816865 177899224 + 3005 lincRNA long
intergenic non-protein coding RNA 2015 [Source:HGNC
Symbol;Acc:HGNC:52850] - 30 28 43 0 0
9 0.545973409 0.410816154 0.662867055 0
0 0.139811878
ENSG00000177508 2033.938626 1960.501663 3050.731058
1581.206121 1637.6369 1568.963616 2348.390449
1595.935546 0.557384335 0.001671746 0.015863472 IRX3
16 54283304 54286763 - 2911
protein_coding iroquois homeobox 3 [Source:HGNC
Symbol;Acc:HGNC:14360] Homeobox 1758 2115 3120 1574
1792 1599 33.02717123 32.03333269 49.64949601
26.14350061 26.82590595 25.64202352
ENSG00000251127 28.92404189 52.83621503 29.33395248
80.36625776 101.4384464 124.614371 37.03140314
102.1396917 -1.459085294 0.001671751 0.015863472
AC091173.1 8 61759094 61765969 - 2750

lincRNA novel transcript - 25 57 30
80 111 127 0.497166695 0.913852533
0.505348542 1.4065608 1.758931322 2.155842667
ENSG00000260917 212.8809483 228.9569318 228.8048294
482.1975466 297.91832 378.7491905 223.5475698
386.2883524 -0.788048774 0.001675419 0.015888415
AL158212.3 10 112823490 112827726 + 4237
sense_overlapping uncharacterized LOC103344931
[Source:NCBI gene;Acc:103344931] - 184 247 234
480 326 386 2.374947818 2.570232715
2.558349355 5.477520226 3.352877866 4.25279919
ENSG00000182197 1410.336283 1778.819239 1699.413647
1987.055723 2267.286356 2698.342679 1629.523056
2317.561586 -0.507731671 0.00168648 0.015983393 EXT1
8 117794490 118111853 - 8325
protein_coding exostosin glycosyltransferase 1 [Source:HGNC
Symbol;Acc:HGNC:3512] - 1219 1919 1738 1978 2481
2750 8.007817671 10.1630647 9.670924343
11.48796917 12.98676912 15.4203591
ENSG00000142871 2332.434738 3868.167111 4385.425896
2056.37162 2179.555808 2622.789084 3528.675915
2286.238837 0.626468478 0.001691877 0.016024614 CYR61
1 85580761 85583962 + 2455
protein_coding cysteine rich angiogenic inducer 61 [Source:HGNC
Symbol;Acc:HGNC:2654] - 2016 4173 4485 2047 2385
2673 44.90903717 74.94296068 84.62786931
40.31508341 42.33460234 50.82688547
ENSG00000152240 904.7440304 916.7546783 759.7493694
460.0968257 673.5147297 553.405553 860.416026
562.3390361 0.612932266 0.001694408 0.016030945 HAUS1
18 46104332 46128333 + 2165
protein_coding HAUS augmin like complex subunit 1 [Source:HGNC
Symbol;Acc:HGNC:25174] - 782 989 777 458 737
564 19.75347764 20.14059743 16.62515006
10.22842568 14.8343385 12.16094261
ENSG00000167005 7364.061066 6581.3531 6997.125466
5166.545796 5626.635265 5391.779279 6980.846544
5394.98678 0.371661272 0.001694643 0.016030945 NUDT21
16 56429133 56452199 - 5954
protein_coding nudix hydrolase 21 [Source:HGNC Symbol;Acc:HGNC:13870]
- 6365 7100 7156 5143 6157 5495
58.46342986 52.57550785 55.67547839 41.76465618
45.06287758 43.08289736
ENSG00000163918 1672.966583 1446.970731 1176.291495
904.1203998 1145.980286 801.6530794 1432.07627
950.5845885 0.590535953 0.001718347 0.016245128 RFC4
3 186789880 186807058 - 2997
protein_coding replication factor C subunit 4 [Source:HGNC
Symbol;Acc:HGNC:9972] - 1446 1561 1203 900 1254
817 26.38616432 22.96413642 18.59436451

14.51967793	18.23347271	12.72569029
ENSG00000158545 3767.067216	4620.851437	4600.541548
6664.371925	5168.791467	5914.767151	4329.486734
5915.976848	-0.450159041	0.00172638	0.016310986	ZC3H18
	16	88570381	88631966	+	8648
protein_coding	zinc finger CCCH-type containing 18 [Source:HGNC
Symbol;Acc:HGNC:25091]	-	3256	4985	4705	6634	5656
	6028	20.59033573	25.41460942	25.20266128
37.09035608	28.5004894	32.53895479
ENSG00000112759 2099.885441	2130.133722	2571.609835
1731.892855	1574.580569	1670.028814	2267.209666
1658.834079	0.450978206	0.001731917	0.016353192
SLC29A1 6	44219505	44234151	+	4408
protein_coding	solute carrier family 29 member 1 (Augustine blood
group) [Source:HGNC Symbol;Acc:HGNC:11003]	-	1815	2298
	2630	1724	1723	1702	22.51799697	22.98488824
	27.63863677	18.91023353	17.03343703	18.02453235
ENSG00000187446 1390.667934	1114.195271	1023.754942
768.5023398	894.66882	739.8365017	1176.206049
801.0025538	0.553383412	0.001750089	0.016513318	CHP1
	15	41230839	41281890	+	3950
protein_coding	calcineurin like EF-hand protein 1 [Source:HGNC
Symbol;Acc:HGNC:17433]	-	1202	1202	1047	765	979
	754	16.64186845	13.41656472	12.2786902
9.364089502	10.80050827	8.910873657
ENSG00000070614 2743.156133	2872.621585	4462.671971
2420.028937	1973.937335	2356.879678	3359.48323
2250.281983	0.578368608	0.001751036	0.016513318	NDST1
	5	150485818	150558211	+	9529
protein_coding	N-deacetylase and N-sulfotransferase 1 [Source:HGNC
Symbol;Acc:HGNC:7680]	-	2371	3099	4564	2409	2160
	2402	13.6075187	14.33864813	22.18711143
12.22336244	9.877909023	11.76716144
ENSG00000229029 32.39492692	3.707804563	2.933395248	0
	0	0	13.01204224	0	6.188633012
0.001754162	1	CDCA4P1 9	14593577	14594200
	+	624	processed_pseudogene	cell division cycle
associated 4 pseudogene 1 [Source:HGNC Symbol;Acc:HGNC:49770]	-
	28	4	3	0	0	0	2.453963814
	0.282624209	0.222709694	0	0	0
ENSG00000063176 469.7264403	570.0749516	673.7031087
886.0379918	825.2154693	858.5635795	571.1681669
856.6056802	-0.583599487	0.001766918	0.016645561	SPHK2
	19	48619291	48630717	+	6009
protein_coding	sphingosine kinase 2 [Source:HGNC
Symbol;Acc:HGNC:18859]	-	406	615	689	882	903
	875	3.69503488	4.512392511	5.311527981
7.096882218	6.548534864	6.797541768
ENSG00000133398 910.5288388	1232.845017	1014.954756
1389.331681	1732.678328	1449.255322	1052.776204

1523.75511	-0.532893466	0.001768007	0.016645561	MED10
	5	6371881 6378594 -	1880	protein_coding
mediator complex subunit 10 [Source:HGNC Symbol;Acc:HGNC:28760] -
	787	1330	1038	1383	1896	1477	22.8934685
	31.19088875	25.57654986	35.56849975	43.94799652
	36.67487834
ENSG00000163376 252.2176453	271.5966843	206.3154658
406.8541799	460.5853782	348.3315094	243.3765985
405.2570225	-0.735947037	0.001769719	0.016645561	KBTBD8
	3	66998307	67011210	+	4736
protein_coding	kelch repeat and BTB domain containing 8 [Source:HGNC
Symbol;Acc:HGNC:30691]	-	218	293	211	405	504
	355	2.517326297	2.727657799	2.063826656
4.134705159	4.637430783	3.499151089
ENSG00000116885 197.8404465	201.1483976	133.958383
344.5703302	284.2104219	318.8950438	177.6490757
315.891932	-0.830819484	0.001769825	0.016645561	OSCP1
	1	36415827	36450451	-	2788
protein_coding	organic solute carrier partner 1 [Source:HGNC
Symbol;Acc:HGNC:29971]	-	171	217	137	343	311
	325	3.354270276	3.431633686	2.276303958
5.948432902	4.861006674	5.441725459
ENSG00000253710 200.1543699	260.4732706	255.2053866
432.9732137	422.2032634	339.5005697	238.611009
398.2256823	-0.737354025	0.001771446	0.016645561	ALG11
	13	52012398	52032171	+	10335
protein_coding	"ALG11, alpha-1,2-mannosyltransferase [Source:HGNC
Symbol;Acc:HGNC:32456]" -	173	281	261	431	462
	346	0.915440948	1.198753248	1.169856203
2.016359685	1.948005112	1.562829692
ENSG00000185624 10357.12092	9732.060028	12698.66803
7325.384395	6701.334481	9383.364013	10929.28299
7803.360963	0.486069215	0.001771592	0.016645561	P4HB
	17	81843159	81860694	-	5303
protein_coding	prolyl 4-hydroxylase subunit beta [Source:HGNC
Symbol;Acc:HGNC:8548]	-	8952	10499	12987	7292	7333
	9563	92.31943951	87.28915042	113.4461258
66.48539439	60.25854703	84.18186915
ENSG00000124216 86.77212568	63.03267758	109.5134226
14.06409511	44.77913399	17.66187935	86.43940862
25.50170282	1.755908354	0.001775771	0.016674574	SNAI1
	20	49982999	49988886	+	1686
protein_coding	snail family transcriptional repressor 1 [Source:HGNC
Symbol;Acc:HGNC:11128]	zf-C2H2 75	68	112	14	49
	18	2.432755179	1.778219221	3.077250906
0.40148718	1.266476418	0.498380419
ENSG00000163900 698.8048521	656.2814077	651.2137452
842.8411283	1012.556744	1031.257511	668.7666683
962.2184611	-0.525333382	0.00177824	0.016687506
TMEM41A 3	185476496	185499057	-	8668

protein_coding transmembrane protein 41A [Source:HGNC
Symbol;Acc:HGNC:30544] -   604   708   666   839   1108
        1051   3.810770097   3.601208885   3.55924417
4.679982991   5.570311157   5.660174875
ENSG00000269044 107.5974358   143.6774268   132.9805846
221.0072088   275.985683   218.8110608   128.0851491
238.6013176   -0.895812769   0.001782488   0.016717109
AC024075.2   19   16633797   16635269   -   1473
        sense_intronic "novel transcript, sense intronic C19orf42"
        -       93   155   136   220   302   223
3.452827758   4.639411656   4.276993767   7.221395824
8.934346545   7.067212532
ENSG00000112651 986.8883094   1147.565512   1187.047277
1646.503706   1389.980874   1611.155883   1107.167033
1549.213487   -0.483982761   0.001784846   0.016728961   MRPL2
        6   43054029   43059806   -   2539
protein_coding mitochondrial ribosomal protein L2 [Source:HGNC
Symbol;Acc:HGNC:14056] -   853   1238   1214   1639   1521
        1642   18.37304095   21.49769524   22.14922025
31.21170325   26.10508496   30.18953945
ENSG00000132819 495.1795972   675.7473817   805.7058949
1131.155078   971.4330497   948.8354073   658.8776246
1017.141178   -0.625204442   0.001786375   0.016733033   RBM38
        20   57391407   57409333   +   2686
protein_coding RNA binding motif protein 38 [Source:HGNC
Symbol;Acc:HGNC:15818] -   428   729   824   1126   1063
        967   8.714299327   11.96617853   14.21096792
20.26905955   17.24589962   16.8060829
ENSG00000234264 15.04050178   10.19646255   8.800185745   0
        0   0   11.34571669   0   5.99540715
0.001789415   1   DEPDC1-AS1   1   68496676
68538627   +   1247   antisense   DEPDC1 antisense RNA 1
[Source:HGNC Symbol;Acc:HGNC:50592]   -   13   11   9
        0   0   0   0.570127004   0.388919922
0.334332435   0   0   0
ENSG00000003436 1457.771711   1028.915766   1135.223961
852.8869105   793.2303736   832.0707605   1207.303813
826.0626815   0.546744946   0.001792788   0.016782826   TFPI
        2   187464230   187565760   -   11714
protein_coding tissue factor pathway inhibitor [Source:HGNC
Symbol;Acc:HGNC:11760] -   1260   1110   1161   849   868
        848   5.882474296   4.177839167   4.591234296
3.504319433   3.229037608   3.37937719
ENSG00000132254 1627.845078   2035.584705   1932.12967
2551.628684   2475.646408   2552.141566   1865.186484
2526.472219   -0.437286749   0.001796234   0.01680346   ARFIP2
        11   6474683 6481479 -   5312   protein_coding ADP
ribosylation factor interacting protein 2 [Source:HGNC
Symbol;Acc:HGNC:17160] -   1407   2196   1976   2540   2709
        2601   14.48540839   18.22670764   17.23182715

23.11941638 22.22335053 22.85747859
ENSG00000142669 4225.22404 4708.911796 4062.752419
5378.511801 5911.759547 5705.768246 4332.296085
5665.346531 -0.386975605 0.001798167 0.01680346
SH3BGRL3 1 26279176 26281522 + 1786
protein_coding SH3 domain binding glutamate rich protein like
3 [Source:HGNC Symbol;Acc:HGNC:15568] - 3652 5080 4155
5354 6469 5815 111.8263184 125.4053936
107.7685475 144.9432946 157.8389904 151.9897499
ENSG00000108946 5637.874246 4921.183607 4947.659986
3674.747136 3931.425193 4175.072035 5168.905946
3927.081455 0.396234805 0.00179829 0.01680346
PRKAR1A 17 68511780 68551319 + 6821
protein_coding protein kinase cAMP-dependent type I regulatory
subunit alpha [Source:HGNC Symbol;Acc:HGNC:9388] - 4873
5309 5060 3658 4302 4255 39.06996972
34.31615603 34.36409596 25.92966274 27.4840642
29.12041439
ENSG00000012061 1420.748938 1445.116829 1435.408075
1812.259113 1857.877131 2192.03547 1433.757947
1954.057238 -0.446598328 0.001801288 0.016821193 ERCC1
19 45407333 45478828 - 5576
protein_coding "ERCC excision repair 1, endonuclease non-catalytic
subunit [Source:HGNC Symbol;Acc:HGNC:3433]" - 1228 1559
1468 1804 2033 2234 12.04398801 12.3269975
12.1956723 15.64281772 15.88814561 18.70279181
ENSG00000066629 1146.549021 1176.300998 958.2424478
722.2917416 728.3463223 835.0144071 1093.697489
761.884157 0.52152005 0.001802931 0.016826259 EML1
14 99737693 99942060 + 8935
protein_coding echinoderm microtubule associated protein like 1
[Source:HGNC Symbol;Acc:HGNC:3330] - 991 1269 980
719 797 851 6.065600605 6.261826399
5.080821935 3.890769924 3.887070006 4.446118557
ENSG00000254470 548.3998343 746.1956684 834.062049
1264.763982 898.3242595 1132.322709 709.5525172
1098.470317 -0.629288525 0.001804904 0.016834391 AP5B1
11 65775893 65780802 - 4811
protein_coding adaptor related protein complex 5 subunit beta 1
[Source:HGNC Symbol;Acc:HGNC:25104] - 474 805 853
1259 983 1154 5.388125435 7.377249667
8.213270571 12.65294411 8.903827934 11.19738183
ENSG00000110074 709.2175072 597.8834858 775.394144
477.1746555 434.0834418 473.9270959 694.1650457
461.7283977 0.588127362 0.001807452 0.016847879
FOXRED1 11 126269055 126278131 + 5657
protein_coding FAD dependent oxidoreductase domain containing 1
[Source:HGNC Symbol;Acc:HGNC:26927] - 613 645 793
475 475 483 5.926100448 5.026983895
6.493658783 4.059837468 3.659030492 3.985721853

ENSG00000231925 2670.267548 2817.004517 3789.946661
1937.83139 2420.814815 2142.974695 3092.406242
2167.206967 0.512934265 0.001810698 0.016867845 TAPBP
6 33299694 33314387 - 5056
protein_coding TAP binding protein [Source:HGNC
Symbol;Acc:HGNC:11566] - 2308 3039 3876 1929 2649
2184 24.96453332 26.50071508 35.51232928
18.44702681 22.83144983 20.16469254
ENSG00000163597 1819.900716 1758.426314 1735.592189
1320.015784 1367.134377 1273.617744 1771.306406
1320.255968 0.423822616 0.001816678 0.016913252 SNHG16
17 76557766 76565348 + 5362
processed_transcript small nucleolar RNA host gene 16 [Source:HGNC
Symbol;Acc:HGNC:44352] - 1573 1897 1775 1314 1496
1298 16.04340791 15.59819981 15.33465478
11.84867459 12.15803479 11.30040266
ENSG00000178904 1355.959084 1182.789656 1286.782716
1030.697256 852.6312656 796.7470018 1275.177152
893.3585077 0.513276519 0.001817972 0.016914994
DPY19L3 19 32405543 32485895 + 10781
protein_coding dpy-19 like C-mannosyltransferase 3 [Source:HGNC
Symbol;Acc:HGNC:27120] - 1172 1276 1316 1026 933
812 5.945156321 5.218258465 5.65456628
4.601395159 3.771214156 3.515952661
ENSG00000161277 252.2176453 224.3221761 193.6040864
313.4284053 477.034856 364.0309577 223.3813026
384.8314063 -0.786333371 0.001822352 0.016937484 THAP8
19 36034985 36054762 - 1825
protein_coding THAP domain containing 8 [Source:HGNC
Symbol;Acc:HGNC:23191] THAP 218 242 198 312 522
371 6.53263416 5.846372127 5.025795085
8.265953193 12.46425232 9.489800371
ENSG00000103111 1529.503335 1721.348269 1694.524655
2391.900747 2297.443732 2017.379108 1648.458753
2235.574529 -0.43922485 0.001822605 0.016937484 MON1B
16 77190835 77202405 + 6942
protein_coding "MON1 homolog B, secretory trafficking associated
[Source:HGNC Symbol;Acc:HGNC:25020]" - 1322 1857 1733
2381 2514 2056 10.41457516 11.7940035
11.5642217 16.58349265 15.78117231 13.82561748
ENSG00000170909 100.6556658 292.9165605 155.4699482
377.7214115 315.2816577 447.4342769 183.0140582
380.145782 -1.050940535 0.001831791 0.017012505 OSCAR
19 54094668 54102692 - 2084
protein_coding "osteoclast associated, immunoglobulin-like receptor
[Source:HGNC Symbol;Acc:HGNC:29960]" - 87 316 159
376 345 456 2.283054352 6.685337332
3.534287425 8.723511678 7.214063789 10.21440712
ENSG00000179119 998.4579261 965.8830888 908.3747286
1223.576274 1394.550173 1353.096201 957.5719145

1323.740883	-0.467509064	0.001833195	0.017015214
SPTY2D1 11	18606401	18634791	-	6005
protein_coding	SPT2 chromatin protein domain containing 1
[Source:HGNC Symbol;Acc:HGNC:26818]	-	863	1042	929
	1218	1526	1379	7.859456177	7.650479666
7.166467869	9.806984594	11.07388782	10.72006183
ENSG00000196843 225.6075268	480.160691	422.4089158
558.5454914	661.6345512	767.3105362	376.0590445
662.4968596	-0.814824674	0.001845182	0.01711444	ARID5A
	2	96536743	96552638	+	3565
protein_coding	AT-rich interaction domain 5A [Source:HGNC
Symbol;Acc:HGNC:17361]	ARID	195	518	432	556	724
	782	2.991367631	6.406254437	5.613408765
7.54078353	8.84988505	10.23984198
ENSG00000154370 717.3162389	1196.693923	997.3543845
1384.30879	1567.26969	1436.499521	970.4548488
1462.692667	-0.590864125	0.001846125	0.01711444	TRIM11
	1	228393673	228406840	-	8493
protein_coding	tripartite motif containing 11 [Source:HGNC
Symbol;Acc:HGNC:16281]	-	620	1291	1020	1378	1715
	1464	3.992319391	6.701917481	5.563415594
7.844934285	8.799572991	8.046851532
ENSG00000173905 2263.017038	2123.645064	3110.376762
2006.142709	1366.220517	1657.273012	2499.012954
1676.545413	0.576136581	0.001850567	0.01714052	GOLIM4
	3	168008677	168095975	-	4647
protein_coding	golgi integral membrane protein 4 [Source:HGNC
Symbol;Acc:HGNC:15448]	-	1956	2291	3181	1997	1495
	1689	23.01923522	21.7363378	31.70979632
20.77813716	14.01932546	16.96692003
ENSG00000111252 2965.292775	2615.856119	3504.429524
2389.89159	1814.011857	2232.265307	3028.526139
2145.389585	0.497507005	0.001851181	0.01714052	SH2B3
	12	111405948	111451623	+	5682
protein_coding	SH2B adaptor protein 3 [Source:HGNC
Symbol;Acc:HGNC:29605]	-	2563	2822	3584	2379	1985
	2275	24.66846291	21.89725813	29.21926098
20.24391146	15.22361806	18.69072757
ENSG00000196419 11392.60162	10264.12998	11343.43943
9228.055548	8089.487635	8115.633562	11000.05701
8477.725581	0.375748717	0.001857576	0.017189325	XRCC6
	22	41621119	41664048	+	3288
protein_coding	X-ray repair cross complementing 6 [Source:HGNC
Symbol;Acc:HGNC:4055]	-	9847	11073	11601	9186	8852
	8271	163.7822533	148.4798257	163.4429054
135.081492	117.3189572	117.4281578
ENSG00000092199 10924.03214	11244.84429	12251.81415
8770.972456	8773.968683	9307.810418	11473.56353
8950.917186	0.358244071	0.001859658	0.017198179	HNRNPC
	14	21209136	21269494	-	7309

protein_coding heterogeneous nuclear ribonucleoprotein C (C1/C2) [Source:HGNC Symbol;Acc:HGNC:5035] - 9442 12131 12530
8731 9601 9486 70.64814237 73.17666265
79.4137245 57.75734786 57.24230397 60.58584846
ENSG00000198794 21.98227184 38.00499677 42.04533189
130.5951689 85.90282848 73.59116396 34.01086684
96.6963871 -1.501562281 0.001874186 0.017322057 SCAMP5
15 74957219 75021496 + 4873
protein_coding secretory carrier membrane protein 5 [Source:HGNC Symbol;Acc:HGNC:30386] - 19 41 43 130 94
75 0.213231765 0.370955149 0.40876575
1.28987658 0.840601266 0.718473721
ENSG00000067221 433.8606284 646.0849452 596.4570339
858.9143798 849.889686 833.051976 558.8008691
847.2853473 -0.598994431 0.001875608 0.017324729 STOML1
15 73978923 73994622 - 10461
protein_coding stomatin like 1 [Source:HGNC Symbol;Acc:HGNC:14560]
- 375 697 610 855 930 849
1.960436494 2.937605915 2.701214602 3.951792468
3.874077855 3.788615432
ENSG00000224431 9.255693406 15.75816939 8.800185745 0
0 0 11.27134952 0 5.988792942
0.001879336 1 AC063976.2 5 132199456
132203487 + 3556 processed_pseudogene cytoplasmic linker associated protein 2 (CLASP2) pseudogene - 8 17
9 0 0 0 0.123033378 0.210775985
0.117241999 0 0 0
ENSG00000178343 59.00504546 39.85889906 76.26827646 0
21.93263706 2.943646558 58.37740699 8.292094539
2.803183202 0.001890802 0.017443065 SHISA3 4
42397839 42402487 + 1971 protein_coding
shisa family member 3 [Source:HGNC Symbol;Acc:HGNC:25159]
- 51 43 78 0 24 3
1.41507111 0.961868692 1.833202472 0 0.530619511
0.071052713
ENSG00000274276 91.39997238 38.00499677 103.6466321
25.11445555 27.41579632 15.69944831 77.68386709
22.74323339 1.769566083 0.001891571 0.017443065 CBSL
21 6444869 6468040 - 4004 protein_coding
cystathionine-beta-synthase like [Source:HGNC Symbol;Acc:HGNC:51829]
- 79 41 106 25 30 16
1.079015629 0.451464645 1.226349483 0.301888908
0.326501953 0.18653999
ENSG00000116198 1103.741439 1325.540131 994.4209892
1674.631896 1438.415447 1777.962521 1141.234186
1630.336621 -0.514146917 0.001891843 0.017443065 CEP104
1 3812081 3857214 - 7876 protein_coding
centrosomal protein 104 [Source:HGNC Symbol;Acc:HGNC:24866] -
954 1430 1017 1667 1574 1812 6.624260152
8.005054407 5.981604595 10.23366294 8.70878599

10.73985757
ENSG00000275956 3.470885027 25.95463194 6.844588913 0
0 0 12.09003529 0 6.092708838
0.001898763 1 AC104446.1 3 52732547
52733867 + 1321 processed_pseudogene zinc finger
protein 610 (ZNF610) pseudogene - 3 28 7 0
0 0 0.124197585 0.93452123 0.245469579
0 0 0
ENSG00000007520 979.9465393 1534.104138 1343.495024
1687.691413 1820.408876 2260.720557 1285.848567
1922.940282 -0.579811727 0.001901015 0.017517064 TSR3
16 1349240 1351911 - 1243 protein_coding "TSR3,
acp transferase ribosome maturation factor [Source:HGNC
Symbol;Acc:HGNC:14175]" - 847 1655 1374 1680 1992
2304 37.26550359 58.70307179 51.20567081
65.34906371 69.83558945 86.52814272
ENSG00000072121 585.4226079 608.0799484 655.1249388
998.5507527 792.3165137 895.8497693 616.209165
895.5723452 -0.538836399 0.00190458 0.017539348
ZFYVE26 14 67727374 67816590 - 15706
protein_coding zinc finger FYVE-type containing 26 [Source:HGNC
Symbol;Acc:HGNC:20761] - 506 656 670 994 867
913 1.761893431 1.84150204 1.9761125
3.060004096 2.405537337 2.713633676
ENSG00000107815 1064.404742 1212.452092 1365.006589
1724.860807 1587.374607 1748.526056 1213.954474
1686.92049 -0.474088959 0.001910135 0.01757991 TWNK
10 100987367 100994401 + 4133
protein_coding twinkle mtDNA helicase [Source:HGNC
Symbol;Acc:HGNC:1160] - 920 1308 1396 1717 1737
1782 12.17354694 13.95327959 15.64668966
20.08658498 18.31441331 20.12743025
ENSG00000284753 82.14427898 125.138404 98.75764003
175.8011889 240.3451478 193.2994573 102.013441
203.148598 -0.991441897 0.001912057 0.017587014
EEF1AKMT4 3 184249650 184259585 + 1823
protein_coding EEF1A lysine methyltransferase 4 [Source:HGNC
Symbol;Acc:HGNC:53611] - 71 135 101 175 263
197 2.129935209 3.264984003 2.566475726
4.641438459 6.286771527 5.044586733
ENSG00000122863 476.6682104 452.3521567 599.3904291
371.6939421 265.9332243 304.176811 509.4702654
313.9346592 0.69928598 0.001921814 0.017666126 CHST3
10 71964365 72013564 + 6970
protein_coding carbohydrate sulfotransferase 3 [Source:HGNC
Symbol;Acc:HGNC:1971] - 412 488 613 370 291
310 3.232653459 3.08688892 4.074085624
2.566670756 1.819360697 2.07622756
ENSG00000188549 431.546705 461.6216681 586.6790497
278.2681675 215.6709311 370.8994664 493.2824743

288.2795216	0.775953864	0.001925751	0.017691678	CCDC9B
	15	40331452	40340967	-	7510
protein_coding	coiled-coil domain containing 9B [Source:HGNC
Symbol;Acc:HGNC:33488]	-	373	498	600	277	236
	378	2.71621165	2.923636423	3.700954699
1.783368324	1.369401073	2.349621452
ENSG00000204498 268.4151088	333.7024107	409.6975364
541.4676617	532.7803085	540.6497512	337.2716853
538.2992405	-0.67300341	0.001932889	0.01774659
NFKBIL1 6	31546870	31558829	+	1779
protein_coding	NFKB inhibitor like 1 [Source:HGNC
Symbol;Acc:HGNC:7800]	-	232	360	419	539	583
	551	7.131924706	8.921964906	10.91039642
14.64920423	14.28075618	14.45844831
ENSG00000175426 102.9695891	97.32986979	88.00185745
36.16481599	29.24351608	46.11712942	96.10043879
37.17515383	1.370956188	0.001938105	0.017768777	PCSK1
	5	96390415	96434143	-	5307
protein_coding	proprotein convertase subtilisin/kexin type 1
[Source:HGNC Symbol;Acc:HGNC:8743]	-	89	105	90
	36	32	47	0.917139993	0.872316665
0.785589875	0.327985489	0.262760142	0.413423164
ENSG00000167658 57720.818	62815.77101	62820.61484
81912.30365	71461.10083	81761.74559	61119.06795
78378.38336	-0.358819666	0.001938312	0.017768777	EEF2
	19	3976056 3985469 -	4027	protein_coding
eukaryotic translation elongation factor 2 [Source:HGNC
Symbol;Acc:HGNC:3214]	-	49890	67766	64247	81539	78197
	83327	677.5269691	741.9321314	739.0497584
979.0051307	846.1883853	965.940004
ENSG00000131370 111.0683209	68.59438442	100.7132369
256.1674466	155.3561792	177.600009	93.45864739
196.3745449	-1.07330475	0.001939818	0.017768777	SH3BP5
	3	15254853	15341368	-	4159
protein_coding	SH3 domain binding protein 5 [Source:HGNC
Symbol;Acc:HGNC:10827]	-	96	74	103	255	170
	181	1.262341981	0.784470754	1.1472307
2.964506975	1.781224249	2.031588241
ENSG00000167700 193.2125998	304.9669253	433.1646984
138.6317946	86.81668835	205.0740436	310.4480745
143.5075089	1.115262698	0.001939955	0.017768777	MFSD3
	8	144509074	144511213	+	1823
protein_coding	major facilitator superfamily domain containing 3
[Source:HGNC Symbol;Acc:HGNC:25157]	-	167	329	443
	138	95	209	5.009847605	7.956886942
11.25691828	3.660105756	2.270887054	5.351871204
ENSG00000138771 242.9619519	231.7377852	374.4967934
118.5402302	191.9105743	123.6331555	283.0655102
144.6946533	0.967304695	0.00195058	0.017855396
SHROOM3 4	76435100	76783253	+	13958

protein_coding	shroom family member 3 [Source:HGNC Symbol;Acc:HGNC:30422]	-	210	250	383	118	210	126	0.822793427	0.789679334	1.271095074	0.408752131	0.655623782	0.42139889
ENSG00000224184 151.5619795	177.974619	263.0277739	59.2701151	85.90282848	122.6519399	197.5214575	89.27496117	1.146326482	0.001951901	0.017856796	MIR3681HG	2	11848622	12578348	+	3876	lincRNA MIR3681 host gene [Source:HGNC Symbol;Acc:HGNC:52001]	-	131	192	269	59	94	125	1.848341608	2.183993886	3.2149259	0.735985842	1.056824038	1.505470606
ENSG00000171617 1133.822442	908.412118	1432.47468	808.6854687	754.8482587	751.6110879	1158.236413	771.7149385	0.585734135	0.00195616	0.017878508	ENC1	5	74627406	74641424	-	5858	protein_coding	ectodermal-neural cortex 1 [Source:HGNC Symbol;Acc:HGNC:3345]	-	980	980	1465	805	826	766	9.148953517	7.375826058	11.58485795	6.644276994	6.144538613	6.104153386
ENSG00000020181 400.3087398	679.4551862	1093.178629	476.1700772	353.6637726	325.7635525	724.3141851	385.1991341	0.91205147	0.001956613	0.017878508	ADGRA2	8	37784191	37844896	+	7061	protein_coding	adhesion G protein-coupled receptor A2 [Source:HGNC Symbol;Acc:HGNC:17849]	-	346	733	1118	474	387	332	2.679813682	4.576903134	7.334627254	3.245737152	2.38837959	2.194916019
ENSG00000108448 455.8429002	550.6089777	575.9232671	849.8731758	734.7433414	758.4795965	527.4583817	781.0320379	-0.565180138	0.001961936	0.017909123	TRIM16L 17	18697998	18736118	+	4329	protein_coding	tripartite motif containing 16 like [Source:HGNC Symbol;Acc:HGNC:32670]	-	394	594	589	846	804	773	4.977409229	6.049685768	6.302751215	9.448959635	8.093326494	8.33562069
ENSG00000090432 1493.637523	1467.363656	1485.275794	2389.89159	1914.536443	1838.797883	1482.092324	2047.741972	-0.466316369	0.001962307	0.017909123	MUL1	1	20499448	20508161	-	2438	protein_coding	mitochondrial E3 ubiquitin protein ligase 1 [Source:HGNC Symbol;Acc:HGNC:25762]	-	1291	1583	1519	2379	2095	1874	28.95924624	28.62735157	28.86200705	47.18043679	37.44630099	35.88243618
ENSG00000178252 3974.163356	5083.400056	4924.192824	6440.350981	5604.702628	6968.592619	4660.585412	6337.882076	-0.443231887	0.001963519	0.017909498	WDR6	3	49007062	49015953	+	5304	protein_coding	WD repeat domain 6 [Source:HGNC Symbol;Acc:HGNC:12758]	-	3435	5484	5036	6411	6133	7102

35.41750295 45.58562239 43.9829811 58.44178578
50.38810748 62.50621312
ENSG00000198840 4653.29986 9653.269181 9898.253367
15744.75447 15194.74818 24646.17142 8068.274136
18528.55802 -1.199370095 0.001969759 0.017955698 MT-ND3
MT 10059 10404 + 346 protein_coding
mitochondrially encoded NADH:ubiquinone oxidoreductase core subunit 3
[Source:HGNC Symbol;Acc:HGNC:7458] - 4022 10414 10123
15673 16627 25118 635.7123963 1327.013779
1355.300485 2190.167102 2094.095077 3388.870655
ENSG00000128626 964.9060375 1550.789259 1410.963115
1728.87912 1936.46908 2186.148177 1308.886137
1950.498793 -0.574697976 0.001980687 0.018044554 MRPS12
19 38930548 38933162 + 2144
protein_coding mitochondrial ribosomal protein S12 [Source:HGNC
Symbol;Acc:HGNC:10380] - 834 1673 1443 1721 2119
2228 21.27335475 34.4036973 31.17769512
38.811221 43.06899854 48.51057711
ENSG00000142541 40871.98512 38603.80721 36258.72087
44248.65695 53761.46273 52794.30103 38578.17107
50268.14023 -0.381878228 0.00198455 0.018068979 RPL13A
19 49487554 49492308 + 2369
protein_coding ribosomal protein L13a [Source:HGNC
Symbol;Acc:HGNC:10304] - 35327 41646 37082 44047 58829
53805 815.523369 775.0722571 725.104407
898.9850926 1082.143869 1060.238598
ENSG00000132481 414.1922799 602.5182416 766.5939583
213.9751613 307.0569188 446.4530614 594.4348266
322.4950472 0.882888736 0.001987639 0.018086328 TRIM47
17 75874161 75878575 - 3142
protein_coding tripartite motif containing 47 [Source:HGNC
Symbol;Acc:HGNC:19020] - 358 650 784 213 336
455 6.231198104 9.120972239 11.55878905
3.277741043 4.660061996 6.760070914
ENSG00000160145 159.6607112 130.7001109 155.4699482
63.28842799 63.05633154 83.40331915 148.6102568
69.91602623 1.087434657 0.001990143 0.018098341 KALRN
3 124080023 124726325 + 24490
protein_coding kalirin RhoGEF kinase [Source:HGNC
Symbol;Acc:HGNC:4814] - 138 141 159 63 69
85 0.308166208 0.25384247 0.300753573
0.124380695 0.122777533 0.162022816
ENSG00000072832 279.9847255 370.7804563 306.0509043
614.8018719 426.7725628 513.1757167 318.9386954
518.2500504 -0.698744297 0.001993715 0.01812005 CRMP1
4 5748084 5893058 - 5823 protein_coding
collapsin response mediator protein 1 [Source:HGNC
Symbol;Acc:HGNC:2365] - 242 400 313 612 467
523 2.272810822 3.02863655 2.490003762
5.081662859 3.494851189 4.19276899

ENSG00000125817 2293.098041 3320.338987 3982.572949
4829.007513 4186.392098 5115.076503 3198.669992
4710.158705 -0.557932812 0.002000368 0.018159282 CENPB
20 3783851 3786690 - 2840 protein_coding
centromere protein B [Source:HGNC Symbol;Acc:HGNC:1852] - 1982
3582 4073 4807 4581 5213 38.16629676
55.60850243 66.43524269 81.83837523 70.29113395
85.68709525
ENSG00000227500 2191.285414 2811.44281 3081.042809
4341.787076 3306.345036 3778.660965 2694.590344
3808.931026 -0.498901244 0.002000408 0.018159282 SCAMP4
19 1905214 1926013 + 6953 protein_coding
secretory carrier membrane protein 4 [Source:HGNC
Symbol;Acc:HGNC:30385] - 1894 3033 3151 4322 3618
3851 14.89712487 19.23242905 20.99319934
30.05479359 22.67539864 25.85516568
ENSG00000178458 143.4632478 90.8412118 126.1359957
42.19228532 59.40089203 49.06077597 120.1468184
50.21798444 1.255624265 0.002002817 0.018170364
H3F3AP6 4 139698144 139698554 - 411
processed_pseudogene "H3 histone, family 3A, pseudogene 6
[Source:HGNC Symbol;Acc:HGNC:42982]" - 124 98 129
42 65 50 16.49964408 10.51279539
14.53952921 4.940929817 6.8917598 5.679030724
ENSG00000174516 249.903722 383.7577723 236.6272167
465.1197168 413.9785245 639.7525187 290.096237
506.2835867 -0.801825021 0.002011105 0.018234719 PELI3
11 66466327 66477337 + 3926
protein_coding pellino E3 ubiquitin protein ligase family member 3
[Source:HGNC Symbol;Acc:HGNC:30010] - 216 414 242
463 453 652 3.008833589 4.64926182
2.855403756 5.702061699 5.028130079 7.752527872
ENSG00000168917 301.9669974 255.8385149 216.09345
144.659264 162.6670582 81.44088812 257.9663207
129.5890701 0.99173586 0.002012296 0.018234719
SLC35G2 3 136818647 136855892 + 4287
protein_coding solute carrier family 35 member G2 [Source:HGNC
Symbol;Acc:HGNC:28480] - 261 276 221 144 178
83 3.329520832 2.8385043 2.38803807
1.624090497 1.809360548 0.903796478
ENSG00000065802 1735.442514 1615.675838 1515.587545
2315.552802 1982.162074 2285.250945 1622.235299
2194.32194 -0.435931735 0.002029378 0.018371092 ASB1
2 238426742 238452250 + 7410
protein_coding ankyrin repeat and SOCS box containing 1 [Source:HGNC
Symbol;Acc:HGNC:16011] - 1500 1743 1550 2305 2169
2329 11.07051345 10.37082097 9.689825275
15.04021132 12.7555721 14.67227021
ENSG00000070371 325.1062309 325.3598504 328.5402678
100.4578222 189.1689946 223.7171384 326.3354497

171.1146518	0.93070056	0.002029749	0.018371092	CLTCL1
	22	19179473	19291716	-	10049
protein_coding	clathrin heavy chain like 1 [Source:HGNC
Symbol;Acc:HGNC:2093]	-	281	351	336	100	207
	228	1.529248934	1.539991161	1.548883977
0.481147651	0.897648061	1.05915138
ENSG00000088899 85.615164	234.5186386	214.1378531
311.4192488	344.5251738	436.6409062	178.0905519
364.1951096	-1.028513261	0.002032161	0.018382038	LZTS3
	20	3162617 3173592 -	5395	protein_coding
leucine zipper tumor suppressor family member 3 [Source:HGNC
Symbol;Acc:HGNC:30139]	-	74	253	219	310	377
	445	0.750127321	2.067583369	1.880421126
2.778250885	3.045148658	3.850477569
ENSG00000149636 741.6124341	637.7423849	678.5921008
412.8816493	522.7278499	435.6596906	685.9823066
457.0897299	0.584719203	0.002038	0.018423953	DSN1
	20	36751791	36773818	-	2739
protein_coding	"DSN1 homolog, MIS12 kinetochore complex component
[Source:HGNC Symbol;Acc:HGNC:16165]"	-	641	688	694
	411	572	444	12.79854825	11.07465903
11.73734564	7.255227017	9.100444267	7.567230688
ENSG00000257243 5.784808379	2.780853423	5.866790497
68.3113191	27.41579632	10.79337071	4.810817433
35.50682871	-2.890808995	0.002041998	0.018449189
AC020612.1	12	49595148	49595688	+	541
	processed_pseudogene	Protein DJ-1 (PARK7) pseudogene -
	5	3	6	68	30	11	0.50543749
	0.244488225	0.513755449	6.077330627	2.416476563
	0.949164433
ENSG00000161904 1602.391921	2359.090653	1859.772588
2693.274213	2991.977239	2563.916152	1940.418387
2749.722535	-0.502434321	0.002046841	0.018482017	LEMD2
	6	33771202	33789136	-	6788
protein_coding	LEM domain containing 2 [Source:HGNC
Symbol;Acc:HGNC:21244]	-	1385	2545	1902	2681	3274
	2613	11.15841867	16.53026862	12.97989367
19.09660639	21.01819607	17.96981524
ENSG00000167962 1317.779349	1918.788862	1940.929856
2644.04988	2312.97935	2463.832169	1725.832689
2473.620466	-0.518622756	0.002049276	0.01849308	ZNF598
	16	1997654 2009823 -	4701	protein_coding	zinc
finger protein 598 [Source:HGNC Symbol;Acc:HGNC:28079]	-	1139
	2070	1985	2632	2531	2511	13.25037549
19.4139565	19.56017409	27.07053571	23.4617548
24.93460526
ENSG00000170385 1155.804714	1368.179884	1385.540356
895.0791958	984.227088	926.2674504	1303.174985
935.1912447	0.479049248	0.002055569	0.018538929
SLC30A1 1	211571568	211578742	-	5474

protein_coding	solute carrier family 30 member 1 [Source:HGNC
Symbol;Acc:HGNC:11012]	-	999	1476	1417	891	1077
	944	9.980571446	11.88818411	11.99133437
7.869989038	8.57372418	8.050321727
ENSG00000135298 56.69112211	63.03267758	81.15726854
30.13734666	1.827719755	8.830939675	66.96035608
13.5986687	2.307463545	0.002057331	0.018543879	ADGRB3
	6	68635367	69389511	+	6490
protein_coding	adhesion G protein-coupled receptor B3 [Source:HGNC
Symbol;Acc:HGNC:945]	-	49	68	83	30	2
	9	0.412901153	0.461953406	0.592428375
0.223500127	0.013429007	0.0647357
ENSG00000197381 598.1491863	630.3267758	595.4792354
1049.784242	774.0393162	857.582364	607.9850659
893.8019741	-0.55545543	0.002060671	0.018545111	ADARB1
	21	45073853	45226560	+	10049
protein_coding	"adenosine deaminase, RNA specific B1 [Source:HGNC
Symbol;Acc:HGNC:226]"	-	517	680	609	1045	847
	874	2.813600352	2.98345866	2.807352208
5.027992957	3.67298506	4.060080292
ENSG00000139083 814.5010197	710.0445739	948.4644637
641.9254839	518.1585505	452.3403545	824.3366858
537.4747963	0.617148387	0.002060912	0.018545111	ETV6
	12	11649854	11895402	+	7075
protein_coding	ETS variant 6 [Source:HGNC Symbol;Acc:HGNC:3495]
	ETS	704	766	970	639	567	461
5.441779342	4.773492927	6.351082378	4.366923967
3.4923295	3.041728991
ENSG00000147536 861.9364484	725.8027433	996.3765861
460.0968257	617.7692771	613.2596997	861.3719259
563.7086008	0.611203776	0.002061107	0.018545111	GINS4
	8	41529206	41545046	+	5475
protein_coding	GINS complex subunit 4 [Source:HGNC
Symbol;Acc:HGNC:28226]	-	745	783	1019	458	676
	625	7.44160925	6.305384815	8.621692242
4.044665131	5.380481843	5.328953488
ENSG00000157423 16.19746346	46.34755704	15.64477466
93.42577465	82.24738897	79.47845708	26.06326505
85.05054023	-1.697755241	0.002074894	0.018658181	HYDIN
	16	70807378	71230722	-	23408
protein_coding	"HYDIN, axonemal central pair apparatus protein
[Source:HGNC Symbol;Acc:HGNC:19368]"	-	14	50	16
	93	90	81	0.032708335	0.094175873
0.031663442	0.192096679	0.167547055	0.161535041
ENSG00000134954 3724.259634	2854.082563	3460.428595
2465.234957	2641.968906	2238.1526	3346.256931
2448.452154	0.450353442	0.002077828	0.018671411	ETS1
	11	128458761	128587558	-	7149
protein_coding	"ETS proto-oncogene 1, transcription factor
[Source:HGNC Symbol;Acc:HGNC:3488]"	ETS	3219	3079	3539

2454	2891	2281	24.62466848	18.98883627
22.93177761	16.59703378	17.62225139	14.89450327
ENSG00000143924 1548.014722	1425.650855	1500.920569
1119.100139	1109.425891	1082.280718	1491.528715
1103.602249	0.434366443	0.002079927	0.018671411	EML4
2	42169350	42332548	+	8357
protein_coding	echinoderm microtubule associated protein like 4
[Source:HGNC Symbol;Acc:HGNC:1316]	-	1338	1538	1535
1114	1214	1103	8.755892563	8.114091322
8.508645571	6.445194164	6.330337754	6.161282842
ENSG00000113594 472.0403637	329.9946061	548.5449115
303.3826231	272.3302435	188.3933797	450.1932938
254.7020821	0.821253131	0.00208003	0.018671411	LIFR
5	38474963	38608354	-	11864
protein_coding	LIF receptor alpha [Source:HGNC Symbol;Acc:HGNC:6597]
-	408	356	561	302	298	192
1.880718245	1.322978596	2.19045421	1.230770339
1.094570461	0.755468093
ENSG00000246859 135.3645161	88.98730952	111.4690194
65.29758443	25.58807657	31.39889662	111.9402817
40.76151921	1.458857129	0.002081878	0.018677036
STARD4-AS1	5	111512226	111739726	+	5647
antisense	STARD4 antisense RNA 1 [Source:HGNC
Symbol;Acc:HGNC:44117]	-	117	96	114	65	28
32	1.133085773	0.749527209	0.935167746
0.556540515	0.216072174	0.264532007
ENSG00000267786 0	0	0.977798416	25.11445555
7.310879019	12.75580175	0.325932805
15.06037877	-5.369414145	0.002082919	1	AF038458.3
19	35947115	35959192	+	500
lincRNA novel transcript	-	0	0	1	25
8	13	0	0	0.092647233	2.417526375
0.697234038	1.213722446
ENSG00000076924 2023.525971	2010.557024	1799.149086
2570.71567	2570.687835	2557.047644	1944.410694
2566.150383	-0.400385326	0.002086963	0.018711673	XAB2
19	7619525 7629565 -	4673	protein_coding	XPA
binding protein 2 [Source:HGNC Symbol;Acc:HGNC:14089]	-	1749
2169	1840	2559	2813	2606	20.46862838
20.46433935	18.23998588	26.47742347	26.23206917
26.03302684
ENSG00000161692 311.2226908	443.0826453	622.8575911
294.3414191	217.4986508	259.0408971	459.0543091
256.9603223	0.838697875	0.002088282	0.018712526	DBF4B
17	44708608	44752264	+	7146
protein_coding	DBF4 zinc finger B [Source:HGNC Symbol;Acc:HGNC:17883]
-	269	478	637	293	238	264
2.058656941	2.949163449	4.129323201	1.982466353
1.451351289	1.724594318
ENSG00000086544 531.0454092	510.7500786	425.342311

746.401619	797.799673	648.5834584	489.0459329
730.9282501	-0.58039882	0.002095974	0.018770444	ITPKC
	19	40717103	40740860	+	3385
protein_coding	inositol-trisphosphate 3-kinase C [Source:HGNC
Symbol;Acc:HGNC:14897]	-	459	551	435	743	873
	661	7.415641484	7.176734563	5.952961033
10.61283366	11.23865057	9.115674777
ENSG00000279713 28.92404189	21.31987624	44.00092873	0
	8.224738897	0.981215519	31.41494895	3.068651472
	3.334338918	0.002101009	0.018804518	AC080038.3
	17	62679403	62681354	-	1952	TEC
	TEC	-	25	23	45	0	9	1
	0.700414145	0.519495728	1.067911237	0
0.200919132	0.023914771
ENSG00000169217 1844.196911	2309.035292	2172.668081
2711.356621	2916.126869	2883.792412	2108.633428
2837.091967	-0.427697192	0.00211035	0.018877074	CD2BP2
	16	30350766	30355361	-	3773
protein_coding	CD2 cytoplasmic tail binding protein 2 [Source:HGNC
Symbol;Acc:HGNC:1656]	-	1594	2491	2222	2699	3191
	2939	23.10448139	29.10857065	27.28096354
34.58735057	36.8551851	36.36292554
ENSG00000144021 3078.675019	3000.540843	2910.905885
3632.554851	4124.249627	3956.260975	2996.707249
3904.355151	-0.381835807	0.002113078	0.01889042	CIAO1
	2	96266132	96273349	+	4031
protein_coding	cytosolic iron-sulfur assembly component 1
[Source:HGNC Symbol;Acc:HGNC:14280]	-	2661	3237	2977
	3616	4513	4032	36.10162819	35.40493971
34.21121453	43.37273813	48.78778858	46.69321321
ENSG00000168916 232.5492968	98.25682093	309.9620979
131.5997471	44.77913399	65.7414398	213.5894052
80.70677363	1.405142955	0.002121719	0.018956582	ZNF608
	5	124636913	124748807	-	6922
protein_coding	zinc finger protein 608 [Source:HGNC
Symbol;Acc:HGNC:29238]	-	201	106	317	131	49
	67	1.588031728	0.675162369	2.121436922
0.915041766	0.308477209	0.451844753
ENSG00000181027 490.5517505	648.8657986	933.7974874
1315.997471	904.7212786	1160.77796	691.0716788
1127.16557	-0.704711703	0.002123886	0.018964863	FKRP
	19	46746046	46776988	+	5081
protein_coding	fukutin related protein [Source:HGNC
Symbol;Acc:HGNC:17997]	-	424	700	955	1310	990
	1183	4.563639962	6.074112105	8.706761185
12.46589077	8.490721527	10.86879971
ENSG00000184205 319.3214225	361.5109449	275.7391534
486.2158595	430.4280023	646.6210273	318.8571736
521.0882964	-0.70811174	0.002125606	0.018967242	TSPYL2
	X	53082367	53088540	+	4948

protein_coding TSPY like 2 [Source:HGNC Symbol;Acc:HGNC:24358] -
276 390 282 484 471 659 3.050521596
3.475112544 2.640109095 4.729518049 4.148105696
6.217299081
ENSG00000268001 123.7948993 135.3348666 85.06846221
197.9019097 217.4986508 247.2663109 114.7327427
220.8889572 -0.94536973 0.002126634 0.018967242 CARD8-
AS1 19 48255675 48258199 + 1969
antisense CARD8 antisense RNA 1 [Source:HGNC
Symbol;Acc:HGNC:51408] - 107 146 87 197 238
252 2.971890299 3.269197044 2.046802752
4.837508337 5.267321641 5.974490302
ENSG00000142156 28193.99908 21452.43025 40721.39284
12548.18657 13236.34646 18806.95786 30122.60739
14863.8303 1.019040674 0.002128743 0.018974982 COL6A1
21 45981737 46005050 + 5343
protein_coding collagen type VI alpha 1 chain [Source:HGNC
Symbol;Acc:HGNC:2211] - 24369 23143 41646 12491 14484
19167 249.4291728 190.9714472 361.0693103
113.0350812 118.1304721 167.4615106
ENSG00000261212 10.41265508 14.83121825 7.822387329 0
0 0 11.02208689 0 5.955919058
0.002129115 1 AC234778.2 X 141173235
141173588 - 354 lincRNA novel transcript -
9 16 8 0 0 0 1.390381435
1.992740184 1.046861386 0 0 0
ENSG00000205609 711.5314306 121.4305995 787.127725
143.6546857 55.74545252 85.36575019 540.0299183
94.92196282 2.50845686 0.002132342 0.018995987 EIF3CL
16 28379579 28403879 - 3091
protein_coding eukaryotic translation initiation factor 3 subunit C
like [Source:HGNC Symbol;Acc:HGNC:26347] - 615 131
805 143 61 87 10.88105044 1.868556562
12.0642223 2.236857144 0.859982132 1.313912013
ENSG00000132275 748.5542042 865.7723656 840.9066379
1330.061566 1158.774325 1022.426571 818.4110692
1170.420821 -0.515531448 0.002141254 0.019064266 RRP8
11 6595075 6603620 - 6922 protein_coding
ribosomal RNA processing 8 [Source:HGNC Symbol;Acc:HGNC:29030] -
647 934 860 1324 1268 1042 5.111724021
5.949072194 5.755317831 9.248208379 7.982634713
7.027197504
ENSG00000138668 6263.790512 7119.911713 7573.048733
5315.223373 4449.583743 5782.303056 6985.583653
5182.370057 0.430929064 0.002144221 0.019079574 HNRNPD
4 82352498 82374503 - 6981
protein_coding heterogeneous nuclear ribonucleoprotein D [Source:HGNC
Symbol;Acc:HGNC:5036] - 5414 7681 7745 5291 4869
5893 42.41264195 48.51031392 51.39326864
36.64555808 30.39350137 39.40622556

ENSG00000135686 1089.857899 1112.341369 1167.491309
1699.746352 1397.291753 1530.69621 1123.230192
1542.578105 -0.457391357 0.002151009 0.019128835 KLHL36
16 84648525 84667686 + 9995
protein_coding kelch like family member 36 [Source:HGNC
Symbol;Acc:HGNC:17844] - 942 1200 1194 1692 1529
1560 5.154218401 5.293371875 5.533806691
8.185001753 6.66627591 7.285977667
ENSG00000105928 1465.870443 1495.17219 1199.758657
2249.250639 1794.820799 1804.45534 1386.933763
1949.508926 -0.491206951 0.002154197 0.019146051 GSDME
7 24698351 24758113 - 8843
protein_coding gasdermin E [Source:HGNC Symbol;Acc:HGNC:2810] -
1267 1613 1227 2239 1964 1839 7.835589987
8.042085765 6.427578561 12.2420933 9.678330671
9.707963617
ENSG00000196371 417.6631649 396.7350883 443.9204809
700.1910208 551.971366 640.7337342 419.4395781
630.9653736 -0.588862869 0.002158811 0.019175914 FUT4
11 94543840 94549898 + 6059
protein_coding fucosyltransferase 4 [Source:HGNC
Symbol;Acc:HGNC:4015] - 361 428 454 697 604
653 3.258374229 3.114417095 3.471021919
5.562026351 4.344047685 5.031045727
ENSG00000120334 642.11373 440.3017919 635.5689705
318.4512964 434.9973016 268.8530523 572.6614975
340.7672168 0.747573647 0.002162091 0.019193894 CENPL
1 173799550 173824720 - 4914
protein_coding centromere protein L [Source:HGNC
Symbol;Acc:HGNC:17879] - 555 475 650 317 476
274 6.176643614 4.261793625 6.127462475
3.119071472 4.221146239 2.602923753
ENSG00000160097 41.65062033 11.12341369 16.62257307 0
0 3.924862078 23.13220236 1.308287359
4.150671079 0.002166007 0.019217496 FNDC5 1
32862268 32872482 - 3139 protein_coding
fibronectin type III domain containing 5 [Source:HGNC
Symbol;Acc:HGNC:20240] - 36 12 17 0 0
4 0.627199781 0.168548111 0.250876546 0
0 0.059485992
ENSG00000126001 1723.872897 1466.436705 1570.344256
2794.736614 1793.906939 2287.213376 1586.884619
2291.95231 -0.53045642 0.002172389 0.019262939 CEP250
20 35455164 35519280 + 16295
protein_coding centrosomal protein 250 [Source:HGNC
Symbol;Acc:HGNC:1859] - 1490 1582 1606 2782 1963
2331 5.000651812 4.280416922 4.565555557
8.254750996 5.249579687 6.677808253
ENSG00000186716 750.8681275 855.575903 968.020432
1266.773138 1127.703089 1253.993434 858.1548208

1216.156554	-0.502264907	0.002186374	0.019375705	BCR
	22	23179704	23318037	+	12389
protein_coding	"BCR, RhoGEF and GTPase activating protein
[Source:HGNC Symbol;Acc:HGNC:1014]"	-	649	923	990
	1261	1234	1278	2.86485837	3.284727951
3.701701521	4.921302379	4.340477452	4.815491679
ENSG00000178860 414.1922799	657.2083589	559.300694
838.8228154	881.8747817	783.0099845	543.5671109
834.5691939	-0.6170627	0.002188949	0.019387289	MSC
	8	71841549	71844468	-	2113
protein_coding	musculin [Source:HGNC Symbol;Acc:HGNC:7321]	bHLH
	358	709	572	835	965	798	9.265700162
	14.79383246	12.5400419	19.10681044	19.90152764
	17.629883
ENSG00000115594 318.1644608	140.8965734	201.4264737
115.5264955	116.9740643	62.79779325	220.1625027
98.43278436	1.158828766	0.002203441	0.019504344	IL1R1
	2	102064544	102179874	+	7083
protein_coding	interleukin 1 receptor type 1 [Source:HGNC
Symbol;Acc:HGNC:5993]	-	275	152	206	115	128
	64	2.123294158	0.946150676	1.34726316
0.785021977	0.787501384	0.42180213
ENSG00000285106 119.1670526	136.2618177	74.31267963
232.0575693	217.4986508	201.1491815	109.91385
216.9018005	-0.980769188	0.002216076	0.019604831
AC016831.6	7	130791264	131110161	-	11088
	lincRNA novel transcript	-	103	147	76
	231	238	205	0.508017555	0.58451825
0.317513965	1.007302656	0.935367633	0.863071309
ENSG00000168140 208.2531016	323.5059482	540.7225241
212.9705831	119.7156439	186.4309487	357.493858
173.0390586	1.048789122	0.002224549	0.019668407	VASN
	16	4371848 4383528 +	2806	protein_coding
vasorin [Source:HGNC Symbol;Acc:HGNC:18517]	-	180	349
	553	212	131	190	3.508161282	5.483675134
	9.129351326	3.652997801	2.034427542	3.160908833
ENSG00000156860 794.8326712	1712.078757	1678.879881
2079.47692	2252.664598	2444.207859	1395.26377
2258.783125	-0.694200099	0.002228033	0.019680549	FBRS
	16	30658431	30670814	+	7218
protein_coding	fibrosin [Source:HGNC Symbol;Acc:HGNC:20442]	-
	687	1847	1717	2070	2465	2491	5.205165853
	11.28194494	11.01934736	13.86611138	14.88190897
	16.11027247
ENSG00000112137 98.34174244	104.7454789	61.60130022
228.0392564	150.7868798	176.6187935	88.22950719
185.1483099	-1.069434398	0.002228497	0.019680549
PHACTR1 6	12716805	13290484	+	11198
protein_coding	phosphatase and actin regulator 1 [Source:HGNC
Symbol;Acc:HGNC:20990]	-	85	113	63	227	165

180	0.415119539	0.444909765	0.26061688
0.980136608	0.642099126	0.750374519
ENSG00000185896 4946.011164	5139.017125	6816.232759
4548.730189	3295.378718	4105.405733	5633.753683
3983.171547	0.500348837	0.002247451	0.019836482	LAMP1
13	113297241	113323672	+	5739
protein_coding	lysosomal associated membrane protein 1 [Source:HGNC Symbol;Acc:HGNC:6499]	-	4275	5544	6971	4528	3606
4184	40.73752191	42.59130576	56.26797862
38.14796802	27.38092372	34.03309821
ENSG00000139343 1005.399696	954.7596751	1070.689266
550.5088657	720.1215834	788.8972777	1010.282879
686.5092422	0.557139644	0.002252282	0.019867651	SNRPF
12	95858928	95903828	+	3043
protein_coding	small nuclear ribonucleoprotein polypeptide F [Source:HGNC Symbol;Acc:HGNC:11162]	-	869	1030	1095
548	788	804	15.61753676	14.92344984
16.66919483	8.707226114	11.28451408	12.33389174
ENSG00000276023 1175.473063	1277.338672	953.3534558
1548.05504	1649.517079	1567.001185	1135.388397
1588.191101	-0.484250173	0.002254738	0.019877845	DUSP14
17	37489831	37513501	+	2499
protein_coding	dual specificity phosphatase 14 [Source:HGNC Symbol;Acc:HGNC:17007]	-	1016	1378	975	1541	1805
1597	22.23423362	24.31178909	18.07343974
29.81519122	31.47527606	29.83215974
ENSG00000140374 1851.138681	1827.94765	1861.728184
1457.643	1392.722453	1302.072994	1846.938172
1384.146149	0.416142099	0.002259128	0.019897049	ETFA
15	76215355	76311472	-	5031
protein_coding	electron transfer flavoprotein subunit alpha [Source:HGNC Symbol;Acc:HGNC:3481]	-	1600	1972	1904
1451	1524	1327	17.39243456	17.28170356
17.53133879	13.94486492	13.20046553	12.3129649
ENSG00000204713 2037.409511	2275.665051	2427.873467
3251.819705	2879.572474	2870.055394	2246.982676
3000.482524	-0.416875158	0.002259541	0.019897049	TRIM27
6	28903002	28923989	-	7986
protein_coding	tripartite motif containing 27 [Source:HGNC Symbol;Acc:HGNC:9975]	-	1761	2455	2483	3237	3151
2925	12.05937396	13.55364632	14.40289748
19.59812891	17.19403059	17.09789322
ENSG00000123131 2269.958808	2259.906881	2458.185218
1846.414772	1407.344211	1820.154789	2329.350302
1691.304591	0.462060209	0.002260821	0.019897049	PRDX4
X	23664262	23686399	+	1904
protein_coding	peroxiredoxin 4 [Source:HGNC Symbol;Acc:HGNC:17169]
-	1962	2438	2514	1838	1540	1855
56.35426265	56.45477946	61.16469087	46.67451131
35.24620595	45.48026701

ENSG00000188177 345.931541 318.8711925 249.3385961
648.9575314 500.7952128 387.5801302 304.7137765
512.4442915 -0.750543118 0.002286427 0.020110823 ZC3H6
2 112275594 112340063 + 11803
protein_coding zinc finger CCCH-type containing 6 [Source:HGNC
Symbol;Acc:HGNC:24762] - 299 344 255 646 548
395 1.385394611 1.284990726 1.000806758
2.646313713 2.023236956 1.562250687
ENSG00000083444 3953.338046 3759.713827 5439.492589
2640.031567 2797.325085 3662.877534 4384.181487
3033.411395 0.531434106 0.002288964 0.020121561 PLOD1
1 11934205 11975538 + 5320
protein_coding "procollagen-lysine,2-oxoglutarate 5-dioxygenase 1
[Source:HGNC Symbol;Acc:HGNC:9081]" - 3417 4056 5563
2628 3061 3733 35.12594842 33.6140059
48.43952585 23.88443351 25.07323061 32.75611548
ENSG00000175643 430.3897434 432.8861828 394.0527617
154.7050462 276.8995429 286.5149317 419.1095626
239.3731736 0.807257297 0.002293754 0.020152082 RMI2
16 11249619 11381662 + 3632
protein_coding RecQ mediated genome instability 2 [Source:HGNC
Symbol;Acc:HGNC:28349] - 372 467 403 154 303
292 5.601338423 5.668980964 5.139982757
2.050104965 3.635426649 3.753038741
ENSG00000171858 10959.89795 13980.27711 15966.47034
15279.63476 20373.59211 22807.37353 13635.54847
19486.8668 -0.515076898 0.002295162 0.020152864 RPS21
20 62387116 62388520 + 1023
protein_coding ribosomal protein S21 [Source:HGNC
Symbol;Acc:HGNC:10409] - 9473 15082 16329 15210 22294
23244 506.4150645 650.0058432 739.411858
718.8773442 949.6661556 1060.672402
ENSG00000139405 581.9517229 758.2460332 679.5698992
1198.461819 893.7549601 915.4740797 673.2558851
1002.56362 -0.573392771 0.002299098 0.020175834 RITA1
12 113185526 113192368 + 2309
protein_coding RBPJ interacting and tubulin associated 1 [Source:HGNC
Symbol;Acc:HGNC:25925] - 503 818 695 1193 978
933 11.91348342 15.61936338 13.94322795
24.98145487 18.45752731 18.86269518
ENSG00000245857 10.41265508 9.269511408 12.71137941 0
0 0 10.79784863 0 5.925559174
0.002310813 1 GS1-24F4.2 8 6835554 6885276 +
1433 lincRNA uncharacterized LOC100652791 [Source:NCBI
gene;Acc:100652791] - 9 10 13 0 0
0 0.343471757 0.307671853 0.420242158 0
0 0
ENSG00000132932 79.83035562 165.9242542 160.3589403
339.547439 235.7758484 240.3978023 135.3711834
271.9070299 -1.002112145 0.002321427 0.020360102 ATP8A2

13 25372071 26025851 + 10415
protein_coding ATPase phospholipid transporting 8A2 [Source:HGNC
Symbol;Acc:HGNC:13533] - 69 179 164 338 258
245 0.362313511 0.757753088 0.729435725
1.569128977 1.079491009 1.098127698
ENSG00000119335 9656.002146 8631.769024 9834.69647
6725.651196 7903.97408 6981.348421 9374.15588
7203.657899 0.379867924 0.002350692 0.02060495 SET
9 128683424 128696400 + 4355
protein_coding SET nuclear proto-oncogene [Source:HGNC
Symbol;Acc:HGNC:10760] - 8346 9312 10058 6695 8649
7115 104.8057074 94.27331222 106.9857481
74.3299154 86.54387474 76.26631817
ENSG00000137504 1758.581747 1853.902282 1983.952986
1467.688782 1290.370147 1401.175762 1865.479005
1386.411564 0.428515682 0.002354425 0.02062515 CREBZF
11 85659708 85682908 - 5587
protein_coding CREB/ATF bZIP transcription factor [Source:HGNC
Symbol;Acc:HGNC:24905] TF_bZIP 1520 2000 2029 1461 1412
1428 14.87851644 15.78284467 16.82309245
12.64365861 11.01322782 11.93151446
ENSG00000188483 370.2277362 742.4878638 688.370085
1296.910485 919.3430367 836.9768381 600.361895
1017.743453 -0.75991315 0.002355695 0.02062515 IER5L
9 129175552 129178262 - 2711
protein_coding immediate early response 5 like [Source:HGNC
Symbol;Acc:HGNC:23679] - 320 801 704 1291 1006
853 6.45528132 13.02677633 12.02944518
23.02490999 16.1706345 14.68809759
ENSG00000267296 1.156961676 2.780853423 0.977798416
13.05951689 40.20983461 11.77458623 1.638537838
21.68131258 -3.709173022 0.002360453 1 CEBPA-DT
19 33302857 33305054 + 2198
antisense CEBPA divergent transcript [Source:HGNC
Symbol;Acc:HGNC:25710] - 1 3 1 13 44
12 0.024880954 0.060176583 0.021075349
0.285967633 0.872335579 0.254858776
ENSG00000009307 26305.83762 22618.53479 23525.82989
18118.57281 19602.29437 18958.06505 24150.06743
18892.97741 0.354128822 0.002365454 0.020698742 CSDE1
1 114716913 114758676 - 5778
protein_coding cold shock domain containing E1 [Source:HGNC
Symbol;Acc:HGNC:29905] CSD 22737 24401 24060 18036 21450
19321 215.2039989 186.1932984 192.8948094
150.9259456 161.7738632 156.0982732
ENSG00000117859 1685.693162 1604.552425 1589.900225
1251.704465 1177.051522 1217.68846 1626.71527
1215.481482 0.420359792 0.002370274 0.020719347 OSBPL9
1 51577179 51798427 + 9523
protein_coding oxysterol binding protein like 9 [Source:HGNC

Symbol;Acc:HGNC:16386]	-	1457	1731	1626	1246	1288
	1241	8.367206362	8.014145842	7.909503321
6.326237242	5.893871682	6.083367216
ENSG00000270441	6.941770054	16.68512054	16.62257307
75.34336665	60.31475191	30.4176811	13.41648789
55.35859989	-2.03490504	0.00237052	0.020719347
AC135506.1	3	49140086	49160851	-	1799
	unprocessed_pseudogene	"laminin, beta 1 (LAMB1) pseudogene"
	-	6	18	17	75	66	31
0.182395786	0.441138843	0.437744012	2.015725159
1.598716179	0.804408343
ENSG00000180758	347.0885027	311.4555833	450.7650699
230.0484128	195.5660138	234.5105092	369.7697186
220.0416453	0.749355878	0.002380543	0.020795066	GPR157
	1	9100305	9129170	-	5372	protein_coding	G
protein-coupled receptor 157 [Source:HGNC Symbol;Acc:HGNC:23687]
	-	300	336	461	229	214	239
3.054076867	2.75763785	3.975276828	2.061107743
1.735946622	2.076863399
ENSG00000178966	708.0605455	506.1153229	612.1018085
345.5749084	443.2220405	382.6740526	608.7592257
390.4903338	0.63918845	0.002382188	0.020797553	RMI1
	9	83980711	84004074	+	3507
protein_coding	RecQ mediated genome instability 1 [Source:HGNC
Symbol;Acc:HGNC:25764]	-	612	546	626	344	485
	390	9.543559138	6.864214318	8.268772116
4.742680769	6.026491807	5.191285057
ENSG00000178878	385.268238	233.5916875	391.1193665
176.8057671	227.5511095	137.3701727	336.659764
180.5756831	0.896976443	0.002388258	0.020838637	APOLD1
	12	12725917	12829975	+	7243
protein_coding	apolipoprotein L domain containing 1 [Source:HGNC
Symbol;Acc:HGNC:25268]	-	333	252	400	176	249
	140	2.514319485	1.533966989	2.558255768
1.174885108	1.498095363	0.902309617
ENSG00000167987	868.8782185	1170.739291	1058.955685
1570.155761	1425.621409	1378.607805	1032.857731
1458.128325	-0.496579436	0.002409657	0.021013368	VPS37C
	11	61130256	61161617	-	3960
protein_coding	"VPS37C, ESCRT-I subunit [Source:HGNC
Symbol;Acc:HGNC:26097]"	-	751	1263	1083	1563	1560
	1405	10.37144966	14.06183905	12.66880719
19.08380668	17.16674714	16.56254892
ENSG00000100029	1449.67298	2013.337878	1834.349829
2688.251322	2098.222279	2755.253179	1765.786895
2513.908926	-0.508977526	0.00241471	0.02104542	PES1
	22	30576625	30607083	-	4557
protein_coding	pescadillo ribosomal biogenesis factor 1 [Source:HGNC
Symbol;Acc:HGNC:8848]	-	1253	2172	1876	2676	2296
	2808	15.03719235	21.0142914	19.0702445

28.39280482	21.95591056	28.76498227
ENSG00000283360 1.156961676	0	0	7.032047554
9.138598774	28.45525006	0.385653892
14.8752988	-5.352538971	0.002425488	1	AL592295.3
	1	161403409	161470523	-	863
lincRNA novel transcript	-	1	0	0	7
	10	29	0.063370031	0	0	0.392182726
	0.504949332	1.568675949
ENSG00000255084 0	0	0	11.05036044	11.88017841
	9.812155195	0	10.91423135	-5.868110653
0.00243136	1	AP003110.1	11	78533176
78558565	+	717	antisense	uncharacterized
LOC101928896 [Source:NCBI gene;Acc:101928896]	-	0	0
	0	11	13	10	0	0	0
0.741779362	0.790101333	0.651068794
ENSG00000218428 1.156961676	0	0	27.12361199
8.224738897	8.830939675	0.385653892
14.72643019	-5.337101144	0.002436675	1	NIP7P3	6
	116137058	116137572	-	515
processed_pseudogene	"NIP7, nucleolar pre-rRNA processing protein
pseudogene 3 [Source:HGNC Symbol;Acc:HGNC:45182]"	-	1
	0	0	27	9	9	0.106190945	0
	0	2.534882024	0.761542031	0.81579552
ENSG00000148154 1052.835125	1346.860008	1391.407146
650.9666879	1096.631853	661.3392601	1263.70076
802.979267	0.654082716	0.002436812	0.021225951	UGCG
	9	111896766	111935369	+	4615
protein_coding	UDP-glucose ceramide glucosyltransferase [Source:HGNC
Symbol;Acc:HGNC:12524]	-	910	1453	1423	648	1200
	674	10.78361563	13.88122734	14.28353326
6.788979808	11.33099736	6.817642544
ENSG00000227051 1115.311055	728.5835967	1616.300782
665.030783	794.1442335	624.0530704	1153.398478
694.4093623	0.73172902	0.002443022	0.021267921
C14orf132	14	96039324	96093889	+	7775
	protein_coding	chromosome 14 open reading frame 132
[Source:HGNC Symbol;Acc:HGNC:20346]	-	964	786	1653
	662	869	636	6.780650331	4.457138263
9.848609359	4.116790894	4.870549668	3.818587563
ENSG00000053501 631.7010749	600.6643393	572.0120735
904.1203998	1000.676566	735.9116396	601.4591626
880.2362017	-0.549924459	0.002446908	0.021289634	USE1
	19	17215346	17219829	+	2431
protein_coding	unconventional SNARE in the ER 1 [Source:HGNC
Symbol;Acc:HGNC:30882]	-	546	648	585	900	1095
	750	12.28294187	11.75233074	11.1473943
17.90023642	19.62852919	14.40198454
ENSG00000133703 1281.913537	1128.099538	1176.291495
888.0471483	887.357941	828.1458984	1195.434857
867.8503292	0.461602736	0.002455241	0.021349984	KRAS

12	25204789	25250936	-	7302
protein_coding	"KRAS proto-oncogene, GTPase [Source:HGNC
Symbol;Acc:HGNC:6407]"	-	1108	1217	1203	884	971
844	8.298367128	7.34822943	7.63178724
5.853446495	5.794767278	5.395685823
ENSG00000136235 4234.479733	3695.754199	4510.584094
2834.919743	3033.100933	3431.310672	4146.939342
3099.777116	0.419798061	0.00246535	0.0214257	GPNMB
7	23235967	23275108	+	6040
protein_coding	glycoprotein nmb [Source:HGNC Symbol;Acc:HGNC:4462]
-	3660	3987	4613	2822	3319	3497
33.13895883	29.10336828	35.37927849	22.59026301
23.94577578	27.02742865
ENSG00000266017 31.23796524	38.93194792	48.88992081
75.34336665	123.3710835	105.9712761	39.68661132
101.5619087	-1.353446575	0.002474087	0.021489417
MIR4477B	9	63819574	63819654	+	81
miRNA	microRNA 4477b [Source:HGNC Symbol;Acc:HGNC:41898]
-	27	42	50	75	135	108
18.22944548	22.86115823	28.59482488	44.76900694
72.62854559	62.24217674
ENSG00000121753 141.1493244	120.5036483	159.3811418
92.42119643	36.5543951	40.2298363	140.3447049
56.40180927	1.317278632	0.002478056	0.021511668	ADGRB2
1	31727117	31764893	-	7756
protein_coding	adhesion G protein-coupled receptor B2 [Source:HGNC
Symbol;Acc:HGNC:944]	-	122	130	163	92	40
41	0.860234276	0.738991614	0.973536547
0.573523534	0.224740213	0.246769847
ENSG00000266947 4.627846703	31.51633879	25.42275882
97.44408754	46.60685375	94.19668987	20.52231477
79.41587705	-1.939410162	0.002483578	0.021547365
AC022916.1	17	35231450	35242963	-	2814
antisense	"novel transcript, antisense SLFN5"	-
4	34	26	97	51	96	0.077737507
0.532707464	0.428007827	1.666667081	0.789777361
1.592550364
ENSG00000150938 1389.510973	1444.189877	1781.548714
1189.420615	754.8482587	1135.266356	1538.416521
1026.511743	0.584235174	0.002488505	0.021577866	CRIM1
2	36355926	36551135	+	7122
protein_coding	cysteine rich transmembrane BMP regulator 1
[Source:HGNC Symbol;Acc:HGNC:2359]	-	1201	1558	1822
1184	826	1157	9.222225787	9.644938038
11.85083248	8.038054556	5.054016736	7.583635056
ENSG00000179886 304.2809207	508.8961763	595.4792354
790.6030607	680.8256087	846.7889933	469.5521108
772.7392209	-0.716890618	0.002498035	0.021648218	TIGD5
8	143597835	143603224	+	5390
protein_coding	tigger transposable element derived 5 [Source:HGNC

Symbol;Acc:HGNC:18336]	-	263	549	609	787	745
	863	2.668466138	4.49073613	5.233967038
7.059715239	6.023183651	7.47425768
ENSG00000107104 533.3593325	433.8131339	599.3904291
396.8083977	247.6560268	306.1392421	522.1876318
316.8678889	0.721156006	0.00250835	0.021724209	KANK1
	9	470291	746106	+	11908	protein_coding	KN
motif and ankyrin repeat domains 1 [Source:HGNC
Symbol;Acc:HGNC:19309]	-	461	468	613	395	271
	312	2.117175268	1.732770259	2.384647027
1.603834259	0.991719979	1.223099543
ENSG00000102271 472.0403637	281.7931468	209.2488611
99.45324398	183.6858354	184.4685177	321.0274572
155.869199	1.040018449	0.002509646	0.021724209	KLHL4
	X	87517749	87670050	+	5818
protein_coding	kelch like family member 4 [Source:HGNC
Symbol;Acc:HGNC:6355]	-	408	304	214	99	201
	188	3.83513944	2.303741918	1.703893759
0.822740155	1.505500618	1.508447391
ENSG00000163492 0	2.780853423	1.955596832	9.041203998
	31.07123583	25.51160351	1.578816752	21.87468111
	-3.749280956	0.002517169	1	CCDC141 2
178829757	179050086	-	15446	protein_coding
coiled-coil domain containing 141 [Source:HGNC Symbol;Acc:HGNC:26821]
	-	0	3	2	9	34	26	0
	0.008563261	0.005998138	0.02817265	0.09592272
	0.078578431
ENSG00000135763 587.7365313	578.4175119	683.4810929
987.5003922	797.799673	888.9812606	616.5450453
891.4271086	-0.531498233	0.00252556	0.021849597	URB2
	1	229626234	229660199	+	6001
protein_coding	URB2 ribosome biogenesis 2 homolog (S. cerevisiae)
[Source:HGNC Symbol;Acc:HGNC:28967]	-	508	624	699
	983	873	906	4.629507567	4.584531076
5.395802	7.920108069	6.339415461	7.047751891
ENSG00000149115 1877.7488	2481.448204	2466.007606
2798.754927	3126.314641	3559.849905	2275.068203
3161.639824	-0.474355673	0.002528087	0.021859089
TNKS1BP1	11	57299638	57324952	-	7149
	protein_coding	tankyrase 1 binding protein 1 [Source:HGNC
Symbol;Acc:HGNC:19081]	-	1623	2677	2522	2786	3421
	3628	12.41560638	16.50961828	16.34188842
18.84243525	20.85289588	23.69016127
ENSG00000102760 194.3695615	262.3271729	161.3367387
236.0758822	360.9746516	649.5646739	206.0111577
415.5384025	-1.011594094	0.00253275	0.02188703	RGCC
	13	41457559	41470882	+	1032
protein_coding	regulator of cell cycle [Source:HGNC
Symbol;Acc:HGNC:20369]	-	168	283	165	235	395
	662	8.902752442	12.09040075	7.406392143

11.01005229	16.67922995	29.94500073
ENSG00000109654 378.326468	265.1080263	367.6522045
100.4578222	266.8470842	120.6895089	337.0288996
162.6648051	1.048681354	0.002536606	0.021907965	TRIM2
4	153152342	153339320	+	9425
protein_coding	tripartite motif containing 2 [Source:HGNC
Symbol;Acc:HGNC:15974]	-	327	286	376	100	292
123	1.897409657	1.337884531	1.84802968
0.513002944	1.350081824	0.609213878
ENSG00000125944 7302.742097	6709.272357	7546.648176
5838.608626	5556.268055	5442.802486	7186.220877
5612.559723	0.356546175	0.002543401	0.021954247	HNRNPR
1	23303771	23344336	-	8676
protein_coding	heterogeneous nuclear ribonucleoprotein R [Source:HGNC
Symbol;Acc:HGNC:5047]	-	6312	7238	7718	5812	6080
5547	39.78708847	36.78180125	41.20858353
32.38972635	30.53814365	29.84589855
ENSG00000108479 393.3669697	671.112626	528.9889431
736.3558367	833.4402082	935.0983901	531.1561796
834.9648117	-0.651033253	0.002544938	0.021955119	GALK1
17	75751594	75765711	-	2891
protein_coding	galactokinase 1 [Source:HGNC Symbol;Acc:HGNC:4118]
-	340	724	541	733	912	953
6.431696433	11.04140735	8.668653208	12.25905799
13.74691807	15.3883071
ENSG00000113300 1087.543975	963.1022353	1203.66985
819.7358292	671.6870099	786.9348466	1084.77202
759.4525619	0.514517271	0.002550584	0.021991417	CNOT6
5	180494412	180578405	+	6586
protein_coding	CCR4-NOT transcription complex subunit 6 [Source:HGNC
Symbol;Acc:HGNC:14099]	-	940	1039	1231	816	735
802	7.805502011	6.955490778	8.658422666
5.990590713	4.863223293	5.684583861
ENSG00000067182 1854.609566	2102.325187	2018.175931
2465.234957	2767.167709	2662.037704	1991.703562
2631.480123	-0.401658694	0.002555598	0.022010869
TNFRSF1A	12	6328757 6342114 -	4005	protein_coding
TNF receptor superfamily member 1A [Source:HGNC
Symbol;Acc:HGNC:11916]	-	1603	2268	2064	2454	3028
2713	21.88898959	24.96746719	23.87314459
29.6260161	32.94670203	31.62228942
ENSG00000132612 1666.024813	1949.378249	1994.708769
2687.246744	2321.204089	2531.53604	1870.037277
2513.328958	-0.426057064	0.00255572	0.022010869	VPS4A
16	69311356	69326939	+	5670
protein_coding	vacuolar protein sorting 4 homolog A [Source:HGNC
Symbol;Acc:HGNC:13488]	-	1440	2103	2040	2675	2540
2580	13.88910132	16.35272645	16.66669793
22.81087496	19.52132336	21.24137778
ENSG00000196628 2070.9614	1399.696223	1970.263809

1382.299634 1168.826783 1222.594537 1813.640477
1257.906985 0.527538225 0.002570043 0.02212176 TCF4
18 55222331 55664787 - 27828
protein_coding transcription factor 4 [Source:HGNC
Symbol;Acc:HGNC:11634] bHLH 1790 1510 2015 1376 1279
1246 3.517756296 2.3923731 3.354250642
2.390769219 2.00284411 2.090172278
ENSG00000254527 0 3.707804563 5.866790497 38.17397244
27.41579632 19.62431039 3.191531687 28.40469305
-3.124392803 0.002573232 0.022125855 ENPP7P12
8 12205759 12206389 - 631
processed_pseudogene ectonucleotide pyrophosphatase/
phosphodiesterase 7 pseudogene 12 [Source:HGNC Symbol;Acc:HGNC:48697]
- 0 4 6 38 30 20 0
0.279488917 0.440478127 2.911759183 2.071812711
1.479608005
ENSG00000096080 476.6682104 487.5763001 494.7659986
688.1360821 727.4324624 680.9635705 486.3368364
698.8440383 -0.522951162 0.002573413 0.022125855
MRPS18A 6 43671303 43687791 - 1284
protein_coding mitochondrial ribosomal protein S18A [Source:HGNC
Symbol;Acc:HGNC:14515] - 412 526 506 685 796
694 17.54797088 18.06153589 18.2552569
25.79447923 27.01510387 25.23134968
ENSG00000181284 230.2353735 302.1860719 255.2053866
361.6481599 511.7615314 417.0165958 262.5422773
430.1420957 -0.711410503 0.002584178 0.022205922
TMEM102 17 7435443 7437679 + 2129 protein_coding
transmembrane protein 102 [Source:HGNC Symbol;Acc:HGNC:26722] -
199 326 261 360 560 425 5.111779685
6.751121073 5.678940281 8.175758524 11.46227868
9.318785268
ENSG00000182263 190.8986765 162.2164496 309.9620979
100.4578222 130.6819625 96.15912091 221.0257414
109.0996352 1.018063317 0.002606065 0.022381411 FIGN
2 163593396 163736012 - 9942
protein_coding "fidgetin, microtubule severing factor [Source:HGNC
Symbol;Acc:HGNC:13285]" - 165 175 317 100 143
98 0.907621757 0.776065269 1.477025384
0.486325966 0.626788293 0.460148862
ENSG00000133313 1604.705844 1743.595096 1835.327627
1143.210017 1280.317688 1382.532667 1727.876189
1268.686791 0.445827339 0.002625976 0.022539751 CNDP2
18 74495816 74523454 + 11848
protein_coding carnosine dipeptidase 2 [Source:HGNC
Symbol;Acc:HGNC:24437] - 1387 1881 1877 1138 1401
1409 6.402154172 6.999672294 7.338743064
4.644066533 5.152899681 5.551520955
ENSG00000261888 31.23796524 19.46597396 20.53376674
122.5585431 40.20983461 71.62873292 23.74590198

78.1323702 -1.722219257 0.002631662 0.022575877
AC144831.1 17 83104255 83106910 + 2656
lincRNA novel transcript - 27 21 21
122 44 73 0.555943179 0.348598233
0.366263533 2.220920314 0.721910242 1.283041859
ENSG00000160392 712.6883922 878.7496815 1121.534783
1473.716252 1283.973128 1249.087356 904.3242857
1335.592245 -0.561732805 0.002634598 0.022588393
C19orf47 19 40319536 40348527 - 4875
protein_coding chromosome 19 open reading frame 47
[Source:HGNC Symbol;Acc:HGNC:26723] - 616 948 1147
1467 1405 1273 6.910362101 8.573687999
10.89911547 14.5497895 12.55915158 12.18989092
ENSG00000073578 1421.905899 1472.925363 1658.346114
1112.068092 930.3093552 1214.744813 1517.725792
1085.70742 0.483684094 0.002636826 0.022594824 SDHA
5 218241 256700 + 8383 protein_coding
succinate dehydrogenase complex flavoprotein subunit A [Source:HGNC
Symbol;Acc:HGNC:10680] - 1229 1589 1696 1107 1018
1238 8.017650659 8.35715369 9.371925715
6.384830482 5.291842496 6.893935477
ENSG00000143319 2070.9614 2067.101044 2122.800361
2607.885064 2800.980524 2725.816713 2086.954268
2711.560767 -0.377755667 0.002658163 0.022764893
ISG20L2 1 156721891 156728799 - 5262
protein_coding interferon stimulated exonuclease gene 20 like 2
[Source:HGNC Symbol;Acc:HGNC:25745] - 1790 2230 2171
2596 3065 2778 18.60359601 18.68477951
19.11223318 23.85366199 25.38272431 24.64492118
ENSG00000188706 1112.997132 1157.761975 1216.38123
1787.144657 1427.449129 1567.9824 1162.380112
1594.192062 -0.455379614 0.002665547 0.022815347 ZDHHC9
X 129803288 129843909 - 5243
protein_coding zinc finger DHHC-type containing 9 [Source:HGNC
Symbol;Acc:HGNC:18475] - 962 1249 1244 1779 1562
1598 10.03436575 10.50307674 10.99114604
16.405796 12.982543 14.2279704
ENSG00000023318 1093.328784 925.0972386 963.1314399
714.2551159 718.2938636 696.6630188 993.8524873
709.7373328 0.485167241 0.002672769 0.022864361 ERP44
9 99979179 100099040 - 4854
protein_coding endoplasmic reticulum protein 44 [Source:HGNC
Symbol;Acc:HGNC:18311] - 945 998 985 711 786
710 10.64698762 9.064935687 9.400239404
7.08224661 7.056370437 6.828174519
ENSG00000239556 21.98227184 6.488657986 21.51156516 0
0.913859877 0.981215519 16.66083166 0.631691799
4.702608079 0.002681902 1 AC004951.2 7
43984995 43990207 - 793
transcribed_unprocessed_pseudogene uroplakin-like protein (UPLP)

pseudogene	-	19	7	22	0	1	1
	1.310313231	0.389187435	1.285144463	0
0.054952241	0.058867128
ENSG00000114648 1250.675571	1074.336372	1311.227676
1772.075984	1720.798149	1506.165822	1212.079873
1666.346652	-0.459484445	0.002685263	0.022958398	KLHL18
	3	47282917	47346816	+	7064
protein_coding	kelch like family member 18 [Source:HGNC
Symbol;Acc:HGNC:29120]	-	1081	1159	1341	1764	1883
	1535	8.368925776	7.233803434	8.793880164
12.0739426	11.61604343	10.14387117
ENSG00000130518 43.96454368	79.71779811	61.60130022
211.9660048	125.1988032	104.0088451	61.761214
147.0578844	-1.247097054	0.00269156	0.022990416	IQCN
	19	18257097	18274509	-	9154
protein_coding	IQ motif containing N [Source:HGNC
Symbol;Acc:HGNC:29350]	-	38	86	63	211	137
	106	0.227021716	0.414210879	0.318810119
1.114481243	0.652181172	0.540556374
ENSG00000162733 3365.601515	3001.467794	3486.829152
2723.41156	2405.279197	2293.100669	3284.63282
2473.930475	0.408891576	0.002692016	0.022990416	DDR2
	1	162631373	162787400	+	10730
protein_coding	discoidin domain receptor tyrosine kinase 2
[Source:HGNC Symbol;Acc:HGNC:2731]	-	2909	3238	3566
	2711	2632	2337	14.82650239	13.30488363
15.39515524	12.21605592	10.68918911	10.16728567
ENSG00000163508 5.784808379	4.634755704	10.75578258
63.28842799	33.81281546	19.62431039	7.058448887
38.90851795	-2.460298407	0.002697572	0.023025007	EOMES
	3	27715949	27722711	-	3656
protein_coding	eomesodermin [Source:HGNC Symbol;Acc:HGNC:3372] T-box
	5	5	11	63	37	20	0.074792583
	0.060297287	0.139376307	0.833173751	0.441015786
	0.255369981
ENSG00000124225 3956.808931	1970.698125	3627.632124
2302.493285	2018.716469	1847.628823	3185.046393
2056.279526	0.631009608	0.002726869	0.023262082	PMEPA1
	20	57648392	57711536	-	5619
protein_coding	"prostate transmembrane protein, androgen induced 1
[Source:HGNC Symbol;Acc:HGNC:14107]"	-	3420	2126	3710
	2292	2209	1883	33.28601363	16.68161855
30.58562316	19.72226535	17.1314957	15.64362237
ENSG00000134242 807.5592496	812.9361505	490.8548049
943.2989505	1226.399955	1103.867459	703.7834017
1091.188788	-0.63324346	0.002731888	0.023291899	PTPN22
	1	113813811	113871759	-	4503
protein_coding	"protein tyrosine phosphatase, non-receptor type 22
[Source:HGNC Symbol;Acc:HGNC:9652]"	-	698	877	502
	939	1342	1125	8.477117217	8.586805077

5.164213944	10.08242179	12.98700975	11.66263305
ENSG00000159753 49.74935206	99.18377207	54.75671131
221.0072088	227.5511095	75.553595	67.89661181
174.7039711	-1.360495888	0.002734038	0.023297236
CARMIL2 16	67644919	67657569	+	7699
protein_coding	capping protein regulator and myosin 1 linker 2
[Source:HGNC Symbol;Acc:HGNC:27089]	-	43	107	56
220	249	77	0.305442066	0.612750136
0.336942787	1.381623074	1.409365464	0.466876959
ENSG00000143669 1268.029997	1019.646255	1253.53757
884.0288354	875.4777626	730.0243465	1180.404607
829.8436481	0.507908375	0.002735946	0.023298033	LYST
1	235661041	235883640	-	19614
protein_coding	lysosomal trafficking regulator [Source:HGNC
Symbol;Acc:HGNC:1968]	-	1096	1100	1282	880	958
744	3.055899701	2.472637618	3.027779959
2.169290517	2.128422963	1.770731856
ENSG00000138443 1790.976674	1412.673539	1829.460837
1242.663261	1188.931701	1214.744813	1677.703683
1215.446591	0.464711949	0.002738073	0.023298033	ABI2
2	203328219	203447723	+	23331
protein_coding	abl interactor 2 [Source:HGNC Symbol;Acc:HGNC:24011]
-	1548	1524	1871	1237	1301	1238
3.628543346	2.879954134	3.714863749	2.563525031
2.429979113	2.477041751
ENSG00000100441 588.8934929	720.2410364	705.9704565
997.5461745	869.0807434	1014.576847	671.7016619
960.401255	-0.514790563	0.002738704	0.023298033	KHNYN
14	24429286	24441834	+	8015
protein_coding	KH and NYN domain containing [Source:HGNC
Symbol;Acc:HGNC:20166]	-	509	777	722	993	951
1034	3.473033468	4.274166638	4.172882218
5.990277455	5.170536259	6.022309178
ENSG00000285035 9.255693406	4.634755704	18.57816991	0
0	0	10.82287301	0	5.928838211
0.002739441	1	AC091057.7	15	30644633
30723495	+	675	transcribed_unprocessed_pseudogene
"Serine/threonine-protein kinase ULK4 (ULK4), pseudogene"
-	8	5	19	0	0	0
0.648158061	0.326587975	1.303924015	0	0	0
ENSG00000117616 709.2175072	499.6266649	614.0574053
435.9869484	363.7162312	383.6552681	607.6338592
394.4528159	0.622645531	0.002741156	0.023305925	RSRP1
1	25242237	25338213	-	6804
protein_coding	arginine and serine rich protein 1 [Source:HGNC
Symbol;Acc:HGNC:25234]	-	613	539	628	434	398
391	4.92709439	3.492676951	4.275607149
3.084087145	2.549044193	2.682615863
ENSG00000168495 2275.743616	2599.170999	2497.297155
3397.483547	2955.422844	3365.569232	2457.403923

3239.491874	-0.398303436	0.002747879	0.023350097	POLR3D
	8	22245104	22254600	+	5611
protein_coding	RNA polymerase III subunit D [Source:HGNC
Symbol;Acc:HGNC:1080]	-	1967	2804	2554	3382	3234
	3430	19.17161963	22.03290179	21.08546
29.14301978	25.11645515	28.53644621
ENSG00000233452 79.83035562	149.2391337	48.88992081
268.2223853	212.9293514	159.9381297	92.6531367
213.6966221	-1.203104701	0.002753495	0.023378925
STXBP5-AS1	6	146841901	147204614	-	6011
	antisense	STXBP5 antisense RNA 1 [Source:HGNC
Symbol;Acc:HGNC:44183]	-	69	161	50	267	233
	163	0.627764967	1.180899955	0.385323709
2.147661095	1.68914834	1.265863601
ENSG00000157985 968.3769226	1028.915766	1053.088894
1582.2107	1398.205612	1242.218848	1016.793861
1407.545053	-0.468847584	0.00275433	0.023378925	AGAP1
	2	235494089	236131800	+	13910
protein_coding	"ArfGAP with GTPase domain, ankyrin repeat and PH
domain 1 [Source:HGNC Symbol;Acc:HGNC:16922]"	-	837	1110
	1077	1575	1530	1266	3.290735988	3.518275198
	3.58666677	5.474628382	4.79317073	4.248666198
ENSG00000146109 772.8503994	833.3290756	733.3488121
1119.100139	1079.268515	1036.163589	779.8427624
1078.177414	-0.467140862	0.002758369	0.023389308	ABT1
	6	26596952	26600744	+	2949
protein_coding	activator of basal transcription 1 [Source:HGNC
Symbol;Acc:HGNC:17369]	-	668	899	750	1114	1181
	1056	12.38786325	13.44060683	11.78118421
18.26466179	17.45153862	16.7161085
ENSG00000165389 1278.442652	1030.769669	1135.223961
752.4290883	897.4103996	789.8784932	1148.145427
813.239327	0.496753211	0.002758613	0.023389308	SPTSSA
	14	34432789	34462356	-	2777
protein_coding	serine palmitoyltransferase small subunit A
[Source:HGNC Symbol;Acc:HGNC:20361]	-	1105	1112	1161
	749	982	805	21.76111334	17.65480258
19.36684139	13.04088768	15.40970078	13.53212611
ENSG00000207181 10.41265508	11.12341369	9.777984162	0
	0	0	10.43801764	0	5.876780711
0.002762441	1	SNORA14B	1	235127803
235127937	-	135	snoRNA	"small nucleolar RNA, H/ACA
box 14B [Source:HGNC Symbol;Acc:HGNC:32603]"	-	9	12
	10	0	0	0	3.645889095	3.919055696
	3.431378986	0	0	0
ENSG00000014138 369.0707746	291.9896094	351.0296314
152.6958897	229.3788292	208.9989056	337.3633384
197.0245415	0.774405275	0.002764897	0.023429594	POLA2
	11	65261762	65305589	+	5726
protein_coding	"DNA polymerase alpha 2, accessory subunit

[Source:HGNC Symbol;Acc:HGNC:30073]" - 319 315 359
152 251 213 3.046730584 2.425454702
2.904327324 1.283492871 1.91020939 1.736498033
ENSG00000077514 1696.105817 1753.791558 1585.989031
1022.66063 1401.861052 1187.270779 1678.628802
1203.93082 0.479223926 0.002788519 0.02361667 POLD3
11 74493851 74669117 + 5508
protein_coding "DNA polymerase delta 3, accessory subunit
[Source:HGNC Symbol;Acc:HGNC:20932]" - 1466 1892 1622
1018 1534 1210 14.55575548 15.14471686
13.64141352 8.936244914 12.13640402 10.25504274
ENSG00000121895 10.41265508 12.97731597 12.71137941
93.42577465 45.69299387 20.60552591 12.03378349
53.24143148 -2.141989303 0.002807849 0.023767214
TMEM156 4 38966744 39032922 - 2562
protein_coding transmembrane protein 156 [Source:HGNC
Symbol;Acc:HGNC:26260] - 9 14 13 93 50
21 0.192113594 0.240925555 0.235053479
1.755112825 0.850451354 0.382636332
ENSG00000182134 311.2226908 282.720098 260.0943787
564.5729608 427.6864226 384.6364836 284.6790558
458.965289 -0.689502528 0.002815866 0.023820173 TDRKH
1 151770107 151791416 - 5092
protein_coding tudor and KH domain containing [Source:HGNC
Symbol;Acc:HGNC:11713] - 269 305 266 562 468
392 2.889073547 2.640860145 2.419890404
5.336409358 4.005124824 3.593715625
ENSG00000136628 6529.891698 5903.751816 6334.17814
5132.390136 4729.224866 4818.749416 6255.940551
4893.454806 0.354324971 0.002817222 0.023820173 EPRS
1 219968601 220046658 - 5450
protein_coding glutamyl-prolyl-tRNA synthetase [Source:HGNC
Symbol;Acc:HGNC:3418] - 5644 6369 6478 5109 5175
4911 56.63504052 51.52389714 55.06135532
45.32529265 41.37828148 42.06486192
ENSG00000166250 6491.711962 6118.804481 4056.885629
7538.354978 8671.616377 7397.383801 5555.800691
7869.118385 -0.502336863 0.002822927 0.023854555 CLMP
11 123069865 123195281 - 5097
protein_coding CXADR like membrane protein [Source:HGNC
Symbol;Acc:HGNC:24039] - 5611 6601 4149 7504 9489
7539 60.20330699 57.09907294 37.70780539
71.18351154 81.12681214 69.04705274
ENSG00000134247 1507.521063 1087.313688 2020.131528
1134.168813 868.1668835 984.159166 1538.322093
995.4982874 0.627933699 0.002825196 0.023854555 PTGFRN
1 116910057 116990358 + 6352
protein_coding prostaglandin F2 receptor inhibitor [Source:HGNC
Symbol;Acc:HGNC:9601] - 1303 1173 2066 1129 950
1003 11.21834105 8.141819699 15.06684372

8.593788655	6.517360042	7.371170884
ENSG00000107874 828.3845598	1016.865402	995.3987877
1235.631213	1372.617536	1327.584598	946.8829163
1311.944449	-0.469806635	0.002827318	0.023854555	CUEDC2
10	102423245	102432661	-	1412
protein_coding	CUE domain containing 2 [Source:HGNC
Symbol;Acc:HGNC:28352]	-	716	1097	1018	1230	1502
1353	27.73147938	34.25357373	33.39762139
42.11837735	46.35470629	44.73105442
ENSG00000165644 86.77212568	155.7277917	158.4033434
53.24264577	45.69299387	72.60994844	133.6344203
57.18186269	1.227633813	0.00282753	0.023854555	COMTD1
10	75233969	75236030	-	1580
protein_coding	catechol-O-methyltransferase domain containing 1
[Source:HGNC Symbol;Acc:HGNC:26309]	-	75	168	162
53	50	74	2.595965337	4.687984345
4.749636609	1.621884783	1.379023018	2.186354942
ENSG00000214530 637.4858833	627.5459224	662.9473262
817.7266727	975.0884892	927.2486659	642.6597106
906.6879426	-0.496741448	0.002841348	0.023957907
STARD10 11	72754729	72793681	-	3710
protein_coding	StAR related lipid transfer domain containing 10
[Source:HGNC Symbol;Acc:HGNC:10666]	-	551	677	678
814	1067	945	8.122176112	8.045419931
8.465609666	10.60844458	12.53282881	11.89060452
ENSG00000125885 1434.632478	1395.988418	1698.435849
1249.695308	860.8560045	1039.107235	1509.685582
1049.886183	0.524426873	0.002847638	0.023997706	MCM8
20	5950652 5998977 +	7691	protein_coding
minichromosome maintenance 8 homologous recombination repair factor
[Source:HGNC Symbol;Acc:HGNC:16147]	-	1240	1506	1737
1244	942	1059	8.817258767	8.633285805
10.46211436	7.820576804	5.337362367	6.427753072
ENSG00000134318 2353.260048	2143.111038	2516.853123
1756.002732	1741.816926	1809.361418	2337.741403
1769.060359	0.402064135	0.002864303	0.024124846	ROCK2
2	11179761	11348330	-	10145
protein_coding	Rho associated coiled-coil containing protein kinase 2
[Source:HGNC Symbol;Acc:HGNC:10252]	-	2034	2312	2574
1748	1906	1844	10.96462063	10.04777118
11.75327633	8.330874525	8.187087702	8.485059678
ENSG00000172061 83.30124065	44.49365476	132.0027862
7.032047554	42.03755436	19.62431039	86.5992272
22.89797077	1.913910379	0.002865914	0.024125123	LRRC15
3	194355247	194369743	-	5940
protein_coding	leucine rich repeat containing 15 [Source:HGNC
Symbol;Acc:HGNC:20818]	-	72	48	135	7	46
20	0.662888926	0.356277791	1.052809462
0.056978736	0.337465969	0.157177214
ENSG00000158710 31807.19039	37426.57926	39583.23548

21004.72604	27897.40048	30588.4126	36272.33504
26496.84638	0.453058468	0.002873639	0.024176844	TAGLN2
	1	159918107	159925732	-	2487
protein_coding	transgelin 2 [Source:HGNC Symbol;Acc:HGNC:11554]
	-	27492	40376	40482	20909	30527	31174
604.5403077	715.7831398	754.0300103	406.4982627
534.8930304	585.1440343
ENSG00000006459 178.1720981	171.4859611	88.97965587
248.1308208	342.697454	254.1348195	146.2125717
281.6543648	-0.947597228	0.002878795	0.0242069	KDM7A
	7	140084746	140177035	-	9430
protein_coding	lysine demethylase 7A [Source:HGNC
Symbol;Acc:HGNC:22224]	-	154	185	91	247	375
	259	0.893107509	0.864955956	0.447025354
1.266445418	1.732918638	1.282136037
ENSG00000140740 1961.05004	1963.282516	2314.448851
1646.503706	1477.711422	1521.865271	2079.593803
1548.693466	0.425461769	0.002890518	0.024292111	UQCRC2
	16	21952660	21983660	+	5217
protein_coding	ubiquinol-cytochrome c reductase core protein 2
[Source:HGNC Symbol;Acc:HGNC:12586]	-	1695	2118	2367
	1639	1617	1551	17.76820591	17.89942488
21.01744294	15.19005454	13.50665419	13.87832319
ENSG00000140988 25878.91876	33251.59132	33231.45697
36762.54003	39879.93119	47536.94827	30787.32235
41393.13983	-0.427021212	0.002918587	0.024508912	RPS2
	16	1962052 1964860 -	2413	protein_coding
ribosomal protein S2 [Source:HGNC Symbol;Acc:HGNC:10404]	-
	22368	35872	33986	36595	43639	48447	506.9493201
	655.4389211	652.4469225	733.272919	788.090452
	937.2503325
ENSG00000213918 478.9821337	368.9265541	479.1212239
817.7266727	656.151392	573.0298634	442.3433039
682.3026427	-0.625866888	0.002919522	0.024508912	DNASE1
	16	3611728 3680143 +	13801	protein_coding
deoxyribonuclease 1 [Source:HGNC Symbol;Acc:HGNC:2956]	-	414
	398	490	814	718	584	1.640531214
1.271471044	1.644704876	2.851773739	2.267109444
1.975369423
ENSG00000143643 913.9997238	922.3163851	1068.733669
772.5206527	587.6119012	656.4331825	968.3499259
672.1885788	0.527205462	0.002927363	0.024561151	TTC13
	1	230906243	230978875	-	4212
protein_coding	tetratricopeptide repeat domain 13 [Source:HGNC
Symbol;Acc:HGNC:26204]	-	790	995	1093	769	643
	669	10.25730906	10.41522547	12.02082446
8.827529829	6.652443706	7.414532806
ENSG00000159217 5.784808379	13.90426711	11.73358099	0
	0	0	10.47421883	0	5.884048582
0.002927849	1	IGF2BP1 17	48997412	49055650

        +   9109   protein_coding insulin like growth factor 2
mRNA binding protein 1 [Source:HGNC Symbol;Acc:HGNC:28866]   -
        5   15   12   0   0   0   0.030018848
        0.072602991   0.061025732   0   0   0
ENSG00000198746 259.1594154   342.9719221   389.1637696
578.6370559   473.3794165   519.0630098   330.4317024
523.6931607   -0.662526095   0.002928958   0.024561151
GPATCH3 1   26890488   26900466   -   2225
protein_coding G-patch domain containing 3 [Source:HGNC
Symbol;Acc:HGNC:25720] -   224   370   398   576   518
        529   5.505702185   7.331716555   8.286201929
12.51681071   10.14514695   11.09868927
ENSG00000182004 1933.28296   2069.881898   2068.04365
1537.00468   1467.658963   1601.343728   2023.736169
1535.33579   0.398707572   0.002943773   0.024671855   SNRPE
        1   203861603   203870550   +   1474
protein_coding small nuclear ribonucleoprotein polypeptide E
[Source:HGNC Symbol;Acc:HGNC:11161]   -   1671   2233   2115
        1530   1606   1632   61.99742889   66.79211526
66.46841825   50.18745391   47.47955667   51.68549819
ENSG00000224081 18.51138681   5.561706845   7.822387329   0
        0   0   10.631827   0   5.89945596
0.002948641   1   SLC44A3-AS1   1   94613814
94855426   -   10245   transcribed_processed_pseudogene
        SLC44A3 antisense RNA 1 [Source:HGNC Symbol;Acc:HGNC:49057]
        -       16   6   8   0   0   0
0.085408822   0.025821011   0.036172663   0   0   0
ENSG00000140320 437.3315134   781.4198117   693.2590771
1123.118452   919.3430367   944.9105452   637.3368007
995.7906781   -0.642169755   0.002955887   0.024759807   BAHD1
        15   40439721   40468242   +   5196
protein_coding bromo adjacent homology domain containing 1
[Source:HGNC Symbol;Acc:HGNC:29153]   -   378   843   709
        1118   1006   963   3.97848175   7.153068602
6.320909154   10.403366   8.43698809   8.65173444
ENSG00000048028 1050.521202   841.6716359   858.5070094
524.3898319   667.1177105   683.9072171   916.8999489
625.1382532   0.551690625   0.002958708   0.024769864   USP28
        11   113797874   113875570   -   5719
protein_coding ubiquitin specific peptidase 28 [Source:HGNC
Symbol;Acc:HGNC:12625] -   908   908   878   522   730
        697   8.682813338   7.000026916   7.111756447
4.413179813   5.562389048   5.68929846
ENSG00000112787 1801.389329   3205.397045   3521.052097
4823.984622   3625.282134   4599.938355   2842.612824
4349.735037   -0.613264277   0.002960729   0.024773216   FBRSL1
        12   132489551   132585188   +   9378
protein_coding fibrosin like 1 [Source:HGNC Symbol;Acc:HGNC:29308]
        -       1557   3458   3601   4802   3967   4688
9.0797334   16.25731118   17.78751784   24.75786234

18.43361743	23.3358385
ENSG00000253719 1689.164047	1666.658151	1730.703197
2334.639788	2494.837465	1988.923858	1695.508465
2272.80037	-0.422843804	0.002966132	0.024804851
ATXN7L3B	12	74537827	74545430	+	7604
protein_coding	ataxin 7 like 3B [Source:HGNC
Symbol;Acc:HGNC:37931]	-	1460	1798	1770	2324	2730
2027	10.50039074	10.42513139	10.78285125
14.77730483	15.64512857	12.44393335
ENSG00000189057 2014.270277	1333.882692	1443.230462
1131.155078	1155.118885	1025.370218	1597.12781
1103.881394	0.532125745	0.002985386	0.024952215
FAM111B 11	59107185	59127410	+	3863
protein_coding	family with sequence similarity 111 member B
[Source:HGNC Symbol;Acc:HGNC:24200]	-	1741	1439	1476
1126	1264	1045	24.64726734	16.42366371
17.69962663	14.09337146	14.25873388	12.62808854
ENSG00000125384 57.84808379	112.161088	105.6022289
44.20144177	10.05245865	31.39889662	91.87046692
28.55093235	1.69177658	0.002987438	0.024955714	PTGER2
14	52314305	52328606	+	2473
protein_coding	prostaglandin E receptor 2 [Source:HGNC
Symbol;Acc:HGNC:9594]	-	50	121	108	44	11
32	1.105708379	2.157223844	2.023028937
0.860260093	0.193832754	0.604048622
ENSG00000048471 728.8858557	720.2410364	681.5254961
949.3264198	1013.470604	979.2530884	710.2174627
980.6833708	-0.46575957	0.002992902	0.024977054	SNX29
16	11976737	12574289	+	11298
protein_coding	sorting nexin 29 [Source:HGNC Symbol;Acc:HGNC:30542]
-	630	777	697	945	1109	998
3.049535489	3.032169021	2.857812053	4.044189103
4.277485771	4.123585528
ENSG00000001461 481.2960571	572.855805	325.6068726
710.236803	753.020539	707.4563895	459.9195782
723.5712438	-0.65368744	0.00299326	0.024977054	NIPAL3
1	24415794	24472976	+	9404
protein_coding	NIPA like domain containing 3 [Source:HGNC
Symbol;Acc:HGNC:25233]	-	416	618	333	707	824
721	2.419220327	2.897409052	1.640340731
3.635030087	3.81832762	3.579057536
ENSG00000164294 1607.019768	1154.05417	1416.829905
782.566435	1152.377305	888.0000451	1392.634614
940.9812618	0.564724502	0.002998958	0.025010951	GPX8
5	55160118	55167071	+	3868
protein_coding	glutathione peroxidase 8 (putative) [Source:HGNC
Symbol;Acc:HGNC:33100]	-	1389	1245	1449	779	1261
905	19.63859858	14.19112561	17.35339194
9.737606235	14.20650403	10.92215033
ENSG00000273449 48.59239038	39.85889906	33.24514615

7.032047554	0	11.77458623	40.56547853	6.268877929
	2.701433031	0.003002836	0.025024049	AC093788.1
	4	163529771	163530697	+	927
lincRNA novel transcript	-	42	43	34	7
	0	12	2.477788706	2.045138288	1.699032314
	0.365106464	0	0.604292978
ENSG00000104723 56.69112211	65.813531	29.33395248
150.6867333	283.296562	41.21105182	50.61286853
158.3981157	-1.646990023	0.003005567	0.025024049	TUSC3
	8	15417215	15766649	+	5190
protein_coding	tumor suppressor candidate 3 [Source:HGNC
Symbol;Acc:HGNC:30242]	-	49	71	30	150	310
	42	0.516325334	0.603149468	0.267766568
1.397414089	2.602872732	0.377770437
ENSG00000145362 646.7415767	698.9211602	485.9658128
1028.688099	775.8670359	945.8917608	610.5428499
916.815632	-0.586377304	0.003006255	0.025024049	ANK2
	4	112818109	113383740	+	18224
protein_coding	ankyrin 2 [Source:HGNC Symbol;Acc:HGNC:493]	-
	559	754	497	1024	849	964	1.677501101
	1.824154408	1.263325138	2.716798735	2.030124074
	2.469331309
ENSG00000114126 2340.53347	1941.035689	2198.09084
3299.034881	2641.055046	2801.370308	2159.886666
2913.820078	-0.432146075	0.003007649	0.025024049	TFDP2
	3	141944428	142149544	-	11897
protein_coding	transcription factor Dp-2 [Source:HGNC
Symbol;Acc:HGNC:11751]	E2F	2023	2094	2248	3284	2890
	2855	9.29936157	7.760204636	8.753088129
13.34648502	10.5856853	11.20249314
ENSG00000179262 3205.940803	3980.328199	3518.118701
4748.641256	4510.812355	4745.158252	3568.129235
4668.203954	-0.387414586	0.003008713	0.025024049	RAD23A
	19	12945855	12953642	+	2449
protein_coding	"RAD23 homolog A, nucleotide excision repair protein
[Source:HGNC Symbol;Acc:HGNC:9812]"	-	2771	4294	3598
	4727	4936	4836	61.87888128	77.30493386
68.05731788	93.32500754	87.83042083	92.18145163
ENSG00000146416 732.3567407	574.7097073	671.7475119
401.8312888	506.2783721	406.2232251	659.6046533
438.110962	0.589149912	0.003026618	0.02515928	AIG1
	6	143060496	143340304	+	7677
protein_coding	androgen induced 1 [Source:HGNC Symbol;Acc:HGNC:21607]
	-	633	620	687	400	554	414
4.509276666	3.560689524	4.145411542	2.519240719
3.14468263	2.51741512
ENSG00000188342 1846.510834	1738.96034	1752.214762
926.2211207	1388.153154	1400.194546	1779.228645
1238.189607	0.522681623	0.003047413	0.025318367	GTF2F2
	13	45120515	45284909	+	3619

protein_coding general transcription factor IIF subunit 2
[Source:HGNC Symbol;Acc:HGNC:4653] - 1596 1876 1792
922 1519 1427 24.11787371 22.85484124
22.93780614 12.31809515 18.29059311 18.40693276
ENSG00000068796 1823.371601 1534.104138 1439.319269
1020.651474 1347.029459 989.0652436 1598.931669
1118.915392 0.514318435 0.003056155 0.025377206 KIF2A
5 62306162 62537249 + 4774
protein_coding kinesin family member 2A [Source:HGNC
Symbol;Acc:HGNC:6318] - 1576 1655 1472 1016 1474
1008 18.05379519 15.28444035 14.28327675
10.28993212 13.45468909 9.856532387
ENSG00000178700 672.1947336 527.4351991 469.3432398
801.6534212 870.9084632 790.8597087 556.3243908
821.140531 -0.563110381 0.003082254 0.025580031 DHFR2
3 94047836 94063389 - 4659
protein_coding dihydrofolate reductase 2 [Source:HGNC
Symbol;Acc:HGNC:27309] - 581 569 480 798 953
806 6.81990201 5.384600831 4.772555448
8.281545598 8.913715898 8.075852294
ENSG00000104635 5570.770469 5810.129751 6115.151295
4229.274315 3872.024301 5095.452193 5832.017171
4398.916936 0.40695127 0.003094087 0.025664305
SLC39A14 8 22367249 22434129 + 6942
protein_coding solute carrier family 39 member 14
[Source:HGNC Symbol;Acc:HGNC:20858] - 4815 6268 6254
4210 4237 5193 37.93205703 39.80873126
41.73262697 29.32234525 26.5969877 34.92044337
ENSG00000087301 135.3645161 118.649746 184.8039007
77.3525231 53.91773277 73.59116396 146.2727209
68.28713994 1.100278404 0.003106574 0.0257539
TXNDC16 14 52430590 52552522 - 4739
protein_coding thioredoxin domain containing 16 [Source:HGNC
Symbol;Acc:HGNC:19965] - 117 128 189 77 59
75 1.350186825 1.190850433 1.847470665
0.785606798 0.542530178 0.738789289
ENSG00000249786 10.41265508 14.83121825 5.866790497 0
0 0 10.37022128 0 5.867806297
0.003108751 1 EAF1-AS1 3 15436171
15455940 - 4321 antisense EAF1 antisense RNA 1
[Source:HGNC Symbol;Acc:HGNC:42328] - 9 16 6
0 0 0 0.113907667 0.163256197
0.064323466 0 0 0
ENSG00000183309 1447.359056 1394.134516 1388.473751
2023.220539 1666.880416 1999.717229 1409.989108
1896.606061 -0.427690521 0.003112227 0.025786787 ZNF623
8 143636013 143656418 + 7146
protein_coding zinc finger protein 623 [Source:HGNC
Symbol;Acc:HGNC:29084] zf-C2H2 1251 1504 1420 2014 1824
2038 9.573902725 9.279376207 9.205084686

13.62691889 11.12296114 13.31334553
ENSG00000261117 16.19746346 14.83121825 1.955596832 0
0 0 10.99475952 0 5.950169243
0.003119578 1 AC009486.1 2 12715415
12716227 + 813 lincRNA novel transcript -
14 16 2 0 0 0 0.941742571
0.867687608 0.113957236 0 0 0
ENSG00000170860 1925.184228 1480.340972 1716.03622
1052.797977 1206.295038 1384.495098 1707.18714
1214.529371 0.490749523 0.003128272 0.025905683 LSM3
3 14178358 14201119 + 3815
protein_coding "LSM3 homolog, U6 small nuclear RNA and mRNA
degradation associated [Source:HGNC Symbol;Acc:HGNC:17874]" -
1664 1597 1755 1048 1320 1411 23.85357584
18.45628687 21.31007775 13.28213704 15.07780029
17.26547406
ENSG00000146757 360.9720428 329.067655 335.3848567
174.7966106 223.89567 228.623216 341.8081849
209.1051655 0.70807762 0.003134614 0.025934641 ZNF92
7 65373799 65401135 + 3512
protein_coding zinc finger protein 92 [Source:HGNC
Symbol;Acc:HGNC:13168] zf-C2H2 312 355 343 174 245
233 4.858417132 4.456642564 4.524202846
2.395498799 3.039976139 3.097044529
ENSG00000097046 783.2630545 738.7800593 786.1499266
497.2662199 564.7654042 561.2552771 769.3976801
541.0956338 0.507489669 0.003135162 0.025934641 CDC7
1 91500893 91525764 + 3557
protein_coding cell division cycle 7 [Source:HGNC
Symbol;Acc:HGNC:1745] - 677 797 804 495 618
572 10.40877249 9.878896017 10.47067403
6.728566519 7.571173659 7.506857976
ENSG00000157693 463.941632 453.2791079 467.3876429
773.525231 710.9829846 568.1237858 461.5361276
684.2106671 -0.568057499 0.00315958 0.026121888
TMEM268 9 114611206 114646422 + 4792
protein_coding transmembrane protein 268 [Source:HGNC
Symbol;Acc:HGNC:24513] - 401 489 478 770 778
579 4.576382076 4.499103745 4.620761393
7.769179084 7.074917588 5.640372548
ENSG00000033122 15.04050178 29.66243651 37.15633981
7.032047554 0.913859877 0 27.28642604 2.648635811
3.380464723 0.003161215 0.026121888 LRRC7 1
69568398 70151945 + 14652 protein_coding
leucine rich repeat containing 7 [Source:HGNC
Symbol;Acc:HGNC:18531] - 13 32 38 7 1
0 0.048522275 0.096291295 0.120140419
0.023099488 0.002974142 0
ENSG00000101544 1351.331237 1364.472079 1507.765158
2079.47692 1875.240468 1726.939314 1407.856158

1893.885567	-0.427674347	0.00316614	0.02614845	ADNP2
18	80109031	80147523	+	5893
protein_coding	ADNP homeobox 2 [Source:HGNC Symbol;Acc:HGNC:23803]
Others	1168	1472	1542	2070	2052	1760
10.83929695	11.01299208	12.12133317	16.98380993
15.1739802	13.9419096
ENSG00000149182 2052.450013	2171.846523	2042.620891
2637.017833	2750.718231	2719.92942	2088.972476
2702.555161	-0.371455176	0.003170269	0.026168413
ARFGAP2 11	47164299	47177125	-	5918
protein_coding	ADP ribosylation factor GTPase activating protein 2
[Source:HGNC Symbol;Acc:HGNC:13504]	-	1774	2343	2089
2625	3010	2772	16.39356351	17.4554595
16.35181387	21.44645736	22.16410161	21.8657461
ENSG00000189046 443.1163218	598.810437	491.8326033
808.6854687	756.6759785	696.6630188	511.2531207
754.0081554	-0.559343294	0.003173923	0.026184433	ALKBH2
12	109088188	109093631	-	2290
protein_coding	"alkB homolog 2, alpha-ketoglutarate dependent
dioxygenase [Source:HGNC Symbol;Acc:HGNC:32487]"	-	383
646	503	805	828	710	9.146564565
12.43743986	10.17501267	16.99658281	15.75627138
14.47334459
ENSG00000165487 1396.452743	1154.05417	1217.359028
680.0994563	941.2756737	976.3094419	1255.955314
865.8948573	0.535878139	0.003184663	0.026258866	MICU2
13	21492691	21604214	-	3645
protein_coding	mitochondrial calcium uptake 2 [Source:HGNC
Symbol;Acc:HGNC:31830]	-	1207	1245	1245	677	1030
995	18.10941621	15.05933439	15.82246977
8.980331169	12.31397563	12.74299709
ENSG00000102575 118.0100909	205.7831533	212.1822563
55.25180221	68.53949081	116.7646468	178.6585002
80.18531328	1.157631488	0.003188607	0.026277216	ACP5
19	11574660	11579008	-	2919
protein_coding	"acid phosphatase 5, tartrate resistant [Source:HGNC
Symbol;Acc:HGNC:124]"	-	102	222	217	55	75
119	1.911000451	3.353148887	3.443722076
0.911023985	1.119658976	1.903088138
ENSG00000234883 595.835263	543.1933685	443.9204809
721.2871634	836.1817878	762.4044586	527.6497042
773.2911366	-0.552371505	0.003191115	0.026283719
MIR155HG	21	25561909	25575168	+	1600
lincRNA MIR155 host gene [Source:HGNC Symbol;Acc:HGNC:35460]
-	515	586	454	718	915	777
17.60280829	16.14773417	13.14432613	21.69729921
24.92066971	22.66976781
ENSG00000234432 5.784808379	29.66243651	25.42275882
62.28384977	60.31475191	95.17790539	20.29000124
72.59216902	-1.826934977	0.003205787	0.026383149

AC092171.3 7 5426277 5428927 - 2651 lincRNA
uncharacterized LOC100129484 [Source:NCBI gene;Acc:100129484] -
5 32 26 62 66 97 0.103146617
0.532199189 0.454324415 1.130793174 1.084907735
1.70807935
ENSG00000153823 173.5442514 164.9973031 208.2710626
91.4166182 94.12756737 104.0088451 182.2708724
96.51767688 0.917391741 0.003208227 0.026383149 PID1
2 228850526 229271285 - 3364
protein_coding phosphotyrosine interaction domain containing 1
[Source:HGNC Symbol;Acc:HGNC:26084] - 150 178 213
91 103 106 2.438540566 2.332909938
2.933094612 1.307936386 1.334258061 1.470943237
ENSG00000213030 46.27846703 54.69011731 66.4902923
7.032047554 12.79403828 24.53038799 55.81962555
14.78549127 1.917193027 0.003208365 0.026383149 CGB8
19 49047638 49049106 - 882
protein_coding chorionic gonadotropin subunit beta 8 [Source:HGNC
Symbol;Acc:HGNC:16453] - 40 59 68 7 14
25 2.480196664 2.949289363 3.571435271
0.383734345 0.691700434 1.323175526
ENSG00000165475 439.6454368 421.7627691 295.2951217
619.824763 593.0950604 571.0674323 385.5677758
594.6624186 -0.625817586 0.003216045 0.026432082 CRYL1
13 20403666 20525873 - 4327
protein_coding crystallin lambda 1 [Source:HGNC
Symbol;Acc:HGNC:18246] - 380 455 302 617 649
582 4.802765853 4.636160468 3.233125058
6.894447761 6.536065554 6.278879165
ENSG00000143774 3251.062309 4192.60001 3569.942017
4385.988517 5222.709199 5112.132856 3671.201445
4906.943524 -0.418369694 0.00322456 0.026487826 GUK1
1 228139962 228148984 + 4725
protein_coding guanylate kinase 1 [Source:HGNC Symbol;Acc:HGNC:4693]
- 2810 4523 3651 4366 5715 5210
32.52364558 42.20449741 35.7941848 44.67691069
52.70757309 51.47329219
ENSG00000196591 4480.91257 3773.618094 3603.187164
2670.168914 3180.232373 3042.749326 3952.572609
2964.383538 0.414754146 0.003227023 0.026493827 HDAC2
6 113933028 114011308 - 15461
protein_coding histone deacetylase 2 [Source:HGNC
Symbol;Acc:HGNC:4853] - 3873 4071 3685 2658 3480
3101 13.69949725 11.60907134 11.04084639
8.312250312 9.808447267 9.362896485
ENSG00000157911 414.1922799 342.9719221 327.5624694
508.3165803 545.5743468 581.860803 361.5755571
545.2505767 -0.593942981 0.003237973 0.026569458 PEX10
1 2403964 2413797 - 3789 protein_coding
peroxisomal biogenesis factor 10 [Source:HGNC Symbol;Acc:HGNC:8851]

- 358 370 335 506 597 593
5.167174569 4.305375913 4.095648315 6.456945609
6.866071531 7.305940381
ENSG00000184990 932.5111106 1295.877695 1458.875237
814.7129381 724.6908828 940.0044677 1229.088014
826.4694295 0.573383669 0.003242446 0.026583987 SIVA1
14 104753100 104768494 + 4776
protein_coding SIVA1 apoptosis inducing factor [Source:HGNC
Symbol;Acc:HGNC:17712] - 806 1398 1492 811 793
958 9.229229306 12.90555872 14.47128047
8.21027592 7.235481992 9.363694302
ENSG00000065183 7494.797735 7660.324228 6219.775725
9080.382549 9219.018443 9330.378375 7124.965896
9209.926456 -0.3703407 0.003243222 0.026583987 WDR3
1 117929720 117966542 + 10275
protein_coding WD repeat domain 3 [Source:HGNC Symbol;Acc:HGNC:12755]
- 6478 8264 6361 9039 10088 9509
34.47893366 35.46030249 28.67781249 42.53434725
42.78404484 43.20152253
ENSG00000165383 11.56961676 1.853902282 19.55596832 0
0 0 10.99316245 0 5.950300682
0.003247533 1 LRRC18 10 48909483 48914232
- 1617 protein_coding leucine rich repeat containing
18 [Source:HGNC Symbol;Acc:HGNC:23199] - 10 2 20
0 0 0 0.338208636 0.054532315
0.57295753 0 0 0
ENSG00000223653 23.13923351 8.342560268 8.800185745 0
0 0.981215519 13.42732651 0.32707184
5.274276345 0.003250365 1 AL078459.1 1
85276715 85448124 + 2277 antisense novel
transcript - 20 9 9 0 0 1
0.480354295 0.174266311 0.183097298 0 0
0.020501376
ENSG00000205581 5353.261674 5594.150135 6174.796998
4454.299836 4310.677042 4593.069847 5707.402936
4452.682242 0.358261141 0.003256454 0.026675676 HMGN1
21 39342315 39349647 - 5102
protein_coding high mobility group nucleosome binding domain 1
[Source:HGNC Symbol;Acc:HGNC:4984] - 4627 6035 6315
4434 4717 4681 49.59681158 52.1519772
57.33705155 42.02003898 40.28877102 42.82962015
ENSG00000180089 85.615164 101.9646255 51.82331606
20.09156444 22.84649694 38.26740526 79.80103518
27.06848888 1.560214493 0.003257898 0.026675676
TMEM86B 19 55226639 55229264 - 2626
protein_coding transmembrane protein 86B [Source:HGNC
Symbol;Acc:HGNC:28448] - 74 110 53 20 25
39 1.541103159 1.846851266 0.934939705
0.368244688 0.414862218 0.693291573
ENSG00000100479 367.9138129 322.578997 425.342311

216.988896	257.7084854	213.9049832	371.9450403
229.5341215	0.695732608	0.003259999	0.026678589	POLE2
	14	49643555	49688422	-	2955
protein_coding	"DNA polymerase epsilon 2, accessory subunit
[Source:HGNC Symbol;Acc:HGNC:9178]"	-	318	348	435
	216	282	218	5.885242296	5.192251455
6.819212554	3.534251756	4.158629412	3.44385648
ENSG00000102595 645.584615	539.485564	684.4588913
506.3074239	312.5400781	360.1060956	623.1763568
392.9845325	0.665560743	0.003264083	0.026691921	UGGT2
	13	95801580	96053482	-	8105
protein_coding	UDP-glucose glycoprotein glucosyltransferase 2
[Source:HGNC Symbol;Acc:HGNC:15664]	-	558	582	700
	504	342	367	3.765094599	3.165949065
4.000805851	3.006621327	1.838788101	2.113776576
ENSG00000080166 5.784808379	0.926951141	0	23.10529911
	35.64053522	14.71823279	2.237253173	24.48802237
	-3.509026621	0.00326512	0.026691921	DCT	13
	94436808	94479682	-	7543	protein_coding
	dopachrome tautomerase [Source:HGNC Symbol;Acc:HGNC:2709]
	-	5	1	0	23	39	15
0.036251052	0.005845072	0	0.147429687	0.225309289
	0.092831034
ENSG00000179869 160.8176729	167.7781565	117.3358099
241.0987733	211.1016317	390.5237767	148.6438798
280.9080606	-0.918257704	0.003282393	0.026810613	ABCA13
	7	48171458	48647496	+	19074
protein_coding	ATP binding cassette subfamily A member 13
[Source:HGNC Symbol;Acc:HGNC:14638]	-	139	181	120
	240	231	398	0.398536163	0.418379845
0.291435145	0.608374048	0.527750677	0.974063634
ENSG00000143420 2289.627156	2805.881103	3064.420236
1785.135501	2249.923018	1880.008935	2719.976165
1971.689151	0.464298183	0.003283147	0.026810613	ENSA
	1	150600851	150629612	-	5493
protein_coding	endosulfine alpha [Source:HGNC Symbol;Acc:HGNC:3360]
	-	1979	3027	3134	1777	2462	1916
19.70293424	24.29611194	26.42967659	15.64152328
19.53156518	16.28290697
ENSG00000167566 450.0580919	807.3744437	782.2387329
1019.646895	1036.317101	1104.848675	679.8904228
1053.604224	-0.630477823	0.003285845	0.026816368
NCKAP5L 12	49791146	49828750	-	8090
protein_coding	NCK associated protein 5 like [Source:HGNC
Symbol;Acc:HGNC:29321]	-	389	871	800	1015	1134
	1126	2.629636943	4.746829048	4.580827324
6.066228109	6.108338989	6.497344655
ENSG00000169871 1117.624979	1175.374047	1318.072265
1793.172126	1409.171931	1799.549263	1203.69043
1667.297773	-0.469605688	0.003288084	0.026816368	TRIM56

7	101085439	101097967	+	11173
protein_coding	tripartite motif containing 56 [Source:HGNC
Symbol;Acc:HGNC:19028]	-	966	1268	1348	1785	1542
1834	4.728267519	5.00360955	5.58885123
7.72448685	6.014135002	7.662589645
ENSG00000135362 180.4860214	160.3625474	229.7826278
114.5219173	66.71177105	104.0088451	190.2103989
95.08084448	1.001868251	0.003289114	0.026816368	PRR5L
11	36296288	36465204	+	6616
protein_coding	proline rich 5 like [Source:HGNC
Symbol;Acc:HGNC:25878]	-	156	173	235	114	73
106	1.289507328	1.152881219	1.645412611
0.833125776	0.480823805	0.747922166
ENSG00000198642 1718.088088	1760.280216	1409.985316
2271.35136	2782.703327	1876.084073	1629.451207
2310.046253	-0.503753749	0.003298158	0.026875768	KLHL9
9	21329671	21335380	-	5710
protein_coding	kelch like family member 9 [Source:HGNC
Symbol;Acc:HGNC:18732]	-	1485	1899	1442	2261	3045
1912	14.22279853	14.66299932	11.69853848
19.14545406	23.23859068	15.6313978
ENSG00000009954 3971.849433	3626.232863	4200.621996
3199.581637	2906.07441	2995.650981	3932.901431
3033.769009	0.374478056	0.00330883	0.026948364	BAZ1B
7	73440398	73522278	-	7053
protein_coding	bromodomain adjacent to zinc finger domain 1B
[Source:HGNC Symbol;Acc:HGNC:961]	-	3433	3912	4296
3185	3180	3053	26.61917751	24.45450747
28.21583095	21.83417412	19.64770523	20.20686576
ENSG00000214078 3406.095173	3553.003723	3829.058598
2428.065563	2566.118536	3104.565904	3596.052498
2699.583334	0.413772259	0.003317907	0.0270079	CPNE1
20	35626031	35664956	-	3608
protein_coding	copine 1 [Source:HGNC Symbol;Acc:HGNC:2314]	-
2944	3833	3916	2417	2808	3164	44.62374237
46.83885267	50.27807136	32.39002909	33.91479313
40.93699706
ENSG00000234797 527.5745241	554.3167822	509.4329748
687.1315039	753.020539	843.8453467	530.4414271
761.3324632	-0.521247686	0.003326429	0.027060544
RPS3AP6 15	59768352	59769146	+	795
processed_pseudogene	ribosomal protein S3A pseudogene 6
[Source:HGNC Symbol;Acc:HGNC:18630]	-	456	598	521
684	824	860	31.36840429	33.16408452
30.35799258	41.59969913	45.16673326	50.49836981
ENSG00000188612 3444.274909	3493.67885	4122.398123
2913.276844	2731.527174	2813.144894	3686.78396
2819.316304	0.387151316	0.003327914	0.027060544	SUMO2
17	75165586	75182983	-	3372
protein_coding	small ubiquitin-like modifier 2 [Source:HGNC

Symbol;Acc:HGNC:11125]	-	2977	3769	4216	2900	2989
	2867	48.28208113	49.28020769	57.91825812
41.58260075	38.62753074	39.69046279
ENSG00000067445 112.2252825	175.1937656	74.31267963
163.7462502	292.4351608	304.176811	120.5772426
253.4527407	-1.070693356	0.003335915	0.027111183	TRO
	X	54920462	54931431	+	6716
protein_coding	trophinin [Source:HGNC Symbol;Acc:HGNC:12326]	-
	97	189	76	163	320	310	0.789870255
	1.240752259	0.524210071	1.173486596	2.076337218
	2.154750758
ENSG00000152207 124.951861	178.9015702	74.31267963
199.9110662	355.4914923	228.623216	126.0553703
261.3419248	-1.051557585	0.003340201	0.027131593
CYSLTR2 13	48653711	48711226	+	5372
protein_coding	cysteinyl leukotriene receptor 2 [Source:HGNC
Symbol;Acc:HGNC:18274]	-	108	193	76	199	389
	233	1.099467672	1.584000313	0.655360171
1.791093628	3.15552914	2.024724569
ENSG00000055130 1670.65266	1431.212561	1503.853964
1155.264955	1117.65063	1156.853097	1535.239728
1143.256228	0.424963286	0.003350086	0.027197434	CUL1
	7	148697914	148801036	+	3548
protein_coding	cullin 1 [Source:HGNC Symbol;Acc:HGNC:2551]	-
	1444	1544	1538	1150	1223	1179	22.25759803
	19.18658327	20.08053041	15.67167605	15.02108984
	15.51230124
ENSG00000144642 518.3188307	374.4882609	432.1868999
289.3185279	291.5213009	260.0221127	441.6646639
280.2873138	0.65475881	0.003358746	0.02724733	RBMS3
	3	28574791	30010391	+	18339
protein_coding	RNA binding motif single stranded interacting protein
3 [Source:HGNC Symbol;Acc:HGNC:13427]	-	448	404	442
	288	319	265	1.335971139	0.971269324
1.116475185	0.759308137	0.758007723	0.674553281
ENSG00000135124 416.5062033	338.3371664	244.449604
601.742355	524.5555696	485.7016821	333.0976579
537.3332023	-0.691327078	0.003359797	0.02724733	P2RX4
	12	121209857	121234106	+	8159
protein_coding	purinergic receptor P2X 4 [Source:HGNC
Symbol;Acc:HGNC:8535]	-	360	365	250	599	574
	495	2.413016438	1.972376817	1.419402387
3.549695547	3.065727553	2.832137286
ENSG00000095319 2916.700384	3066.354374	3176.867054
2569.711092	2060.754024	2301.931609	3053.307271
2310.798908	0.402228218	0.003370298	0.027317997	NUP188
	9	128947699	129007096	+	8041
protein_coding	nucleoporin 188 [Source:HGNC Symbol;Acc:HGNC:17859]
	-	2521	3308	3249	2558	2255	2346
17.14578985	18.13799997	18.71725275	15.38125225

12.22067183 13.61958836
ENSG00000184378 41.65062033 56.54401959 39.11193665
80.36625776 125.1988032 121.6707244 45.76885885
109.0785951 -1.251366539 0.00339792 0.027527297 ACTRT3
3 169766921 169769895 - 2005
protein_coding actin related protein T3 [Source:HGNC
Symbol;Acc:HGNC:24022] - 36 61 40 80 137
124 0.981935218 1.341372554 0.924161921
1.929198104 2.977589251 2.887043609
ENSG00000171680 232.5492968 431.0322805 419.4755205
731.3329456 549.2297863 536.7248891 361.0190326
605.7625404 -0.744418355 0.003400528 0.027533829
PLEKHG5 1 6466092 6520061 - 7053 protein_coding
pleckstrin homology and RhoGEF domain containing G5 [Source:HGNC
Symbol;Acc:HGNC:29105] - 201 465 429 728 601
547 1.558536172 2.90678578 2.817642336
4.99066837 3.713292718 3.62042436
ENSG00000107566 1802.546291 1695.393637 1934.085267
1238.644948 1406.430351 1413.931564 1810.675065
1353.002288 0.420208707 0.003404885 0.027547153 ERLIN1
10 100150094 100188334 - 5792
protein_coding ER lipid raft associated 1 [Source:HGNC
Symbol;Acc:HGNC:16947] - 1558 1829 1978 1233 1539
1441 14.71070928 13.92256039 15.8197709
10.29285228 11.57893629 11.61399042
ENSG00000122779 776.3212844 621.9842155 836.0176458
483.2021248 551.0575061 482.7580356 744.7743819
505.6725555 0.557945487 0.003407232 0.027547153 TRIM24
7 138460334 138589993 + 9223
protein_coding tripartite motif containing 24 [Source:HGNC
Symbol;Acc:HGNC:11812] - 671 671 855 481 603
492 3.978735091 3.207630021 4.294339363
2.52158774 2.849073815 2.490226956
ENSG00000148843 3060.163632 3457.527755 3310.825437
4771.746555 3672.802847 4676.473166 3276.172275
4373.674189 -0.416590312 0.003407579 0.027547153 PDCD11
10 103396626 103446292 + 7670
protein_coding programmed cell death 11 [Source:HGNC
Symbol;Acc:HGNC:13408] - 2645 3730 3386 4750 4019
4766 18.85927638 21.44111795 20.45003453
29.94328626 22.83396021 29.00712657
ENSG00000119523 620.1314582 654.4275054 589.6124449
888.0471483 957.7251515 788.8972777 621.3904695
878.2231925 -0.499129782 0.003412812 0.027574871 ALG2
9 99216426 99221956 - 2983
protein_coding "ALG2, alpha-1,3/1,6-mannosyltransferase [Source:HGNC
Symbol;Acc:HGNC:23159]" - 536 706 603 884 1048
804 9.826667223 10.43483066 9.36411017
14.32848351 15.30969811 12.58197539
ENSG00000107731 1059.776895 1066.920763 2301.737472

795.6259518 496.2259134 560.2740616 1476.145043
617.375309 1.257819535 0.003417959 0.027601872 UNC5B
10 71212570 71302864 + 6841
protein_coding unc-5 netrin receptor B [Source:HGNC
Symbol;Acc:HGNC:12568] - 916 1151 2354 792 543
571 7.322689106 7.418048888 15.94003695
5.597663759 3.458906615 3.896391199
ENSG00000173327 1721.558973 2258.97993 2685.034451
2818.846491 3070.569188 3571.624491 2221.857785
3153.680057 -0.504861656 0.003424952 0.027643738
MAP3K11 11 65597755 65615382 - 10564
protein_coding mitogen-activated protein kinase kinase kinase 11
[Source:HGNC Symbol;Acc:HGNC:6850] - 1488 2437 2746
2806 3360 3640 7.703165904 10.17094005
12.04133381 12.84282281 13.86019954 16.08492451
ENSG00000180044 86.77212568 123.2845017 170.1369244
57.26095866 46.60685375 61.81657773 126.7311839
55.22813004 1.200863017 0.003428918 0.027661141
C3orf80 3 160225496 160228213 + 2718
protein_coding chromosome 3 open reading frame 80 [Source:HGNC
Symbol;Acc:HGNC:40048] - 75 133 174 57 51
63 1.509060056 2.157427184 2.965529521
1.013973535 0.817672368 1.082024596
ENSG00000150551 41.65062033 100.1107232 49.86771922
25.11445555 18.27719755 11.77458623 63.87635425
18.38874644 1.799942189 0.003434522 0.02769174 LYPD1
2 132644853 132671579 - 2412
protein_coding LY6/PLAUR domain containing 1 [Source:HGNC
Symbol;Acc:HGNC:28431] - 36 108 51 25 20
12 0.816243827 1.97415119 0.979479449
0.5011456 0.361336048 0.232246928
ENSG00000134308 10753.95878 9026.65021 9411.309756
7234.972355 8036.483762 7432.70756 9730.63958
7568.054559 0.362481589 0.003442885 0.027744538 YWHAQ
2 9583972 9631014 - 2713 protein_coding
tyrosine 3-monooxygenase/tryptophan 5-monooxygenase activation protein
theta [Source:HGNC Symbol;Acc:HGNC:12854] - 9295 9738
9625 7202 8794 7575 187.367522 158.2537225
164.3438286 128.3525614 141.2522145 130.3403489
ENSG00000115825 2223.680341 1857.610086 1970.263809
1518.922272 1630.326021 1304.035425 2017.184745
1484.427906 0.44199233 0.0034666 0.027920925 PRKD3
2 37250502 37324808 - 10244
protein_coding protein kinase D3 [Source:HGNC Symbol;Acc:HGNC:9408]
- 1922 2004 2015 1512 1784 1329
10.2607363 8.625059612 9.111878843 7.136469891
7.588988208 6.056217265
ENSG00000147383 1036.637661 1253.237942 1077.533855
1528.968054 1472.228263 1556.207814 1122.469819
1519.13471 -0.436009945 0.003469084 0.027926218 NSDHL

X 152830967 152869729 + 1996
protein_coding NAD(P) dependent steroid dehydrogenase-like
[Source:HGNC Symbol;Acc:HGNC:13398] - 896 1352 1102
1522 1611 1586 24.54947367 29.86414686
25.57546352 36.8684884 35.17171952 37.09272006
ENSG00000105738 1898.57411 2584.339781 2629.299941
3437.666676 2864.036856 3552.981396 2370.737944
3284.894976 -0.469999046 0.003478922 0.02797742
SIPA1L3 19 37907228 38208372 + 10335
protein_coding signal induced proliferation associated 1 like 3
[Source:HGNC Symbol;Acc:HGNC:23801] - 1641 2788 2689
3422 3134 3621 8.683460095 11.89367991
12.05265644 16.00924094 13.21438966 16.35550958
ENSG00000165678 4070.191175 3563.200185 3777.235282
3099.123815 2920.696168 2805.29517 3803.542214
2941.705051 0.370542887 0.003479105 0.02797742 GHITM
10 84139440 84153245 + 2128
protein_coding growth hormone inducible transmembrane protein
[Source:HGNC Symbol;Acc:HGNC:17281] - 3518 3844 3863
3085 3196 2859 90.41051107 79.64265205
84.09216627 70.0946322 65.44760292 62.71747532
ENSG00000182749 177.0151364 211.3448601 194.5818848
277.2635893 383.8211485 310.0641042 194.3139604
323.7162806 -0.735797834 0.003490913 0.028057618 PAQR7
1 25861210 25871253 - 3297
protein_coding progestin and adipoQ receptor family member 7
[Source:HGNC Symbol;Acc:HGNC:23146] - 153 228 199
276 420 316 2.537857287 3.048946879
2.795996253 4.047541883 5.551226414 4.474187409
ENSG00000178662 3.470885027 10.19646255 17.60037149 0
0 0 10.42257302 0 5.877302771
0.003493741 1 CSRNP3 2 165469647 165689407
+ 12565 protein_coding cysteine and serine rich
nuclear protein 3 [Source:HGNC Symbol;Acc:HGNC:30729] - 3
11 18 0 0 0 0.013057303
0.038597942 0.066360931 0 0 0
ENSG00000112541 290.3973806 277.1583911 296.2729201
134.6134818 190.9967144 182.5060866 287.9428973
169.3720943 0.764714253 0.00349408 0.028068311 PDE10A
6 165327287 165988078 - 22022
protein_coding phosphodiesterase 10A [Source:HGNC
Symbol;Acc:HGNC:8772] - 251 299 303 134 209
186 0.623320881 0.598616093 0.637365169
0.294204463 0.413569141 0.394277707
ENSG00000151623 134.2075544 148.3121825 93.86864795
237.0804604 215.6709311 233.5292936 125.462795
228.7602284 -0.866587624 0.003496655 0.028074248 NR3C2
4 148078762 148444698 - 6607
protein_coding nuclear receptor subfamily 3 group C member 2
[Source:HGNC Symbol;Acc:HGNC:7979] ESR-like 116 160

96	236	236	238	0.960170581	1.067700961
0.673084178	1.727065913	1.556561534	1.681584463
ENSG00000128016 192.0556382	225.2491272	192.626288
271.2361199	364.6300911	376.7867595	203.3103511
337.5509902	-0.730958447	0.003505887	0.0281245	ZFP36
19	39406813	39409412	+	2010
protein_coding	ZFP36 ring finger protein [Source:HGNC
Symbol;Acc:HGNC:12862]	-	166	243	197	270	399
384	4.516549178	5.330208213	4.54017533
6.494846977	8.650384982	8.91828204
ENSG00000260686 101.8126275	60.25182416	46.93432398
210.9614266	119.7156439	143.2574658	69.66625853
157.9781788	-1.185552272	0.003506594	0.0281245
AC008669.1	5	122832356	122834533	+	2178
sense_overlapping	"novel transcript, overlapping SNX2"
-	88	65	48	210	131	146
2.209629755	1.315798658	1.020906145	4.66189659
2.621030158	3.129255442
ENSG00000153406 741.6124341	903.7773623	861.4404046
1081.930745	1169.740643	1218.669675	835.610067
1156.780354	-0.468522452	0.003519437	0.02821271	NMRAL1
16	4461680 4495763 -	3541	protein_coding	NmrA
like redox sensor 1 [Source:HGNC Symbol;Acc:HGNC:24987] -	641
975	881	1077	1280	1242	9.899808995
12.13983117	11.52530527	14.70587916	15.75225163
16.37350682
ENSG00000184076 366.7568512	390.2464303	447.8316746
259.1811813	190.9967144	288.4773627	401.611652
246.2184195	0.707009721	0.00352433	0.028234083	UQCR10
22	29767369	29770413	+	975
protein_coding	"ubiquinol-cytochrome c reductase, complex III subunit
X [Source:HGNC Symbol;Acc:HGNC:30863]"	-	317	421	458
258	209	294	17.78072067	19.03756671
21.76022182	12.79429343	9.341148325	14.07630766
ENSG00000092098 397.9948164	407.858502	489.8770065
646.948375	590.3534808	668.2077688	431.9101083
635.1698749	-0.555794218	0.003525797	0.028234083	RNF31
14	24146683	24160661	+	4982
protein_coding	ring finger protein 31 [Source:HGNC
Symbol;Acc:HGNC:16031]	-	344	440	501	644	646
681	3.776151693	3.893883118	4.658396582
6.250048114	5.650506678	6.38100999
ENSG00000189369 456.9998619	532.0699548	412.6309316
611.7881372	824.3016094	667.2265532	467.2335828
701.1054333	-0.585457368	0.003529575	0.028249538	GSPT2
X	51743431	51746232	+	2802
protein_coding	G1 to S phase transition 2 [Source:HGNC
Symbol;Acc:HGNC:4622]	-	395	574	422	609	902
680	7.709454993	9.031870862	6.976647425
10.50873349	14.02804028	11.32887585

```
ENSG00000184492 10.41265508     19.46597396     9.777984162
36.16481599     43.86527412     73.59116396     13.21887107
51.20708469     -1.947254223    0.003535257     0.028280208
FOXD4L1 2       113498665       113501155       +       2491
protein_coding  forkhead box D4 like 1 [Source:HGNC
Symbol;Acc:HGNC:18521]  Fork    9       21      10      36      48
        75      0.197589333     0.371688843     0.185963935
0.698763143     0.839703779     1.405508808
ENSG00000254701 2.313923351     9.269511408     20.53376674     0
        0       0       10.70573383     0       5.916177706
0.003535808     1       AC138866.2      5       70197255
70207745        -       584
unprocessed_pseudogene  "glucuronidase, beta (GUSB) pseudogene" -
        2       10      21      0       0       0       0.187288823
        0.754955078     1.665746477     0       0       0
ENSG00000168938 292.711304      204.8562021     220.9824421
76.34794487     162.6670582     125.5955865     239.5166494
121.5368632     0.976025189     0.003541404     0.028308976     PPIC
        5       123023250       123036741       -       2098
protein_coding  peptidylprolyl isomerase C [Source:HGNC
Symbol;Acc:HGNC:9256]   -       253     221     226     76      178
        128     6.594923316     4.644305159     4.990055904
1.751496706     3.697201463     2.848069098
ENSG00000072501 5352.104712     5658.109764     7707.984915
5271.021931     3187.543252     4388.977019     6239.399797
4282.514067     0.543114497     0.003542557     0.028308976     SMC1A
        X       53374149        53422728        -       10484
protein_coding  structural maintenance of chromosomes 1A [Source:HGNC
Symbol;Acc:HGNC:11111]  -       4626    6104    7883    5247    3488
        4473    24.13088939     25.66974005     34.83108235
24.19832294     14.49799888     19.91672476
ENSG00000197885 792.5187479     695.2133556     488.8992081
906.1295563     948.5865528     1124.472985     658.8771039
993.0630314     -0.592669905    0.003554336     0.028378287
NKIRAS1 3       23891660        23946591        -       3473
protein_coding  NFKB inhibitor interacting Ras like 1 [Source:HGNC
Symbol;Acc:HGNC:17899]  -       685     750     500     902     1038
        1146    10.78649884     9.521172598     6.669106869
12.55749375     13.02420334     15.40372903
ENSG00000110274 1235.63507      1777.892288     1824.571845
2354.731352     2078.117361     2388.278574     1612.699734
2273.709096     -0.494830926    0.003554943     0.028378287     CEP164
        11      117314557       117413268       +       12898
protein_coding  centrosomal protein 164 [Source:HGNC
Symbol;Acc:HGNC:29182]  -       1068    1918    1866    2344    2274
        2434    4.528387603     6.556320691     6.701804004
8.786915525     7.682926624     8.809357547
ENSG00000157470 92.55693406     128.8462086     166.2257307
31.14192488     57.57317228     71.62873292     129.2096245
53.44794336     1.273980114     0.003570174     0.028484991     FAM81A
```

15 59372693 59523549 + 5578
protein_coding family with sequence similarity 81 member A
[Source:HGNC Symbol;Acc:HGNC:28379] - 80 139 170
31 63 73 0.784343298 1.098677545
1.411798991 0.268710354 0.492176232 0.610928501
ENSG00000134030 462.7846703 685.9438442 624.8131879
854.8960669 828.8709088 933.135959 591.1805675
872.3009783 -0.559751884 0.003585663 0.028593652 CTIF
18 48539046 48863217 + 8100
protein_coding cap binding complex dependent translation initiation
factor [Source:HGNC Symbol;Acc:HGNC:23925] - 400 740
639 851 907 951 2.700658589 4.027918354
3.65441862 5.079789987 4.879562285 5.480769452
ENSG00000032444 2659.854892 3188.711925 3204.24541
3937.94663 3814.451128 4059.288604 3017.604076
3937.228788 -0.38345057 0.00359125 0.028609312 PNPLA6
19 7534004 7561764 + 7172 protein_coding
patatin like phospholipase domain containing 6 [Source:HGNC
Symbol;Acc:HGNC:16268] - 2299 3440 3277 3920 4174
4137 17.53046367 21.14716333 21.16599145
26.42694754 25.36125622 26.92720495
ENSG00000156787 495.1795972 495.9188604 568.1008798
408.8633364 310.7123583 284.5525006 519.7331124
334.7093984 0.635440711 0.00359137 0.028609312
TBC1D31 8 123041968 123152153 + 8611
protein_coding TBC1 domain family member 31 [Source:HGNC
Symbol;Acc:HGNC:30888] - 428 535 581 407 340
290 2.718221808 2.739265645 3.125539551
2.285293774 1.720615875 1.572137201
ENSG00000143971 666.4099252 499.6266649 471.2988366
323.4741875 353.6637726 381.6928371 545.7784756
352.943599 0.627627141 0.003596475 0.028621426 ETAA1
2 67397319 67412089 + 7480
protein_coding "ETAA1, ATR kinase activator [Source:HGNC
Symbol;Acc:HGNC:24648]" - 576 539 482 322 387
389 4.211294356 3.177028606 2.985024474
2.081399713 2.254592017 2.427694527
ENSG00000274762 33.5518886 21.31987624 13.68917783 0
0 4.906077597 22.85364755 1.635359199
3.811776126 0.003596635 0.028621426 AL049777.1 14
28818741 28819258 + 518 antisense
novel transcript - 29 23 14 0
0 5 3.061702233 1.957636412 1.25198963
0 0 0.450594909
ENSG00000146676 6225.610777 5612.689158 5630.16328
7742.284357 7521.066791 6972.517481 5822.821072
7411.95621 -0.348249336 0.003616086 0.028761239 PURB
7 44876293 44885361 - 9069
protein_coding purine rich element binding protein B [Source:HGNC
Symbol;Acc:HGNC:9702] Others 5381 6055 5758 7707 8230

7106 32.44877476 29.43667165 29.41133341
41.08915155 39.54567848 36.57731624
ENSG00000149089 677.979542 632.1806781 572.0120735
378.7259897 466.9823974 431.7348286 627.3907645
425.8144052 0.558348215 0.003623779 0.02878281 APIP
11 34853094 34916499 - 5337
protein_coding APAF1 interacting protein [Source:HGNC
Symbol;Acc:HGNC:17581] - 586 682 585 377 511
440 6.004752698 5.634055617 5.077630793
3.415429804 4.172365014 3.848588781
ENSG00000148291 391.0530464 515.3848343 521.1665558
648.9575314 641.5296339 897.8122003 475.8681455
729.4331219 -0.614984588 0.003624157 0.02878281 SURF2
9 133356552 133361169 + 1008
protein_coding surfeit 2 [Source:HGNC Symbol;Acc:HGNC:11475] -
338 556 533 646 702 915 18.33795408
24.31914026 24.49453124 30.98654837 30.34835655
42.37469622
ENSG00000231605 18.51138681 29.66243651 2.933395248
24.10987733 126.1126631 80.4596726 17.03573952
76.894071 -2.173757292 0.003624446 0.02878281
LINC01363 1 167175917 167195792 - 1375
antisense long intergenic non-protein coding RNA 1363
[Source:HGNC Symbol;Acc:HGNC:50598] - 16 32 3
24 138 82 0.636373369 1.026080037
0.101069708 0.84393648 4.373558964 2.783922814
ENSG00000174123 2.313923351 0.926951141 0 36.16481599
4.569299387 16.68066383 1.080291497 19.13825974
-4.193624326 0.003640091 1 TLR10 4
38772239 38782990 - 4217 protein_coding toll
like receptor 10 [Source:HGNC Symbol;Acc:HGNC:15634] - 2
1 0 36 5 17 0.025937082
0.010455152 0 0.412762388 0.051668399 0.188187753
ENSG00000107175 745.0833192 835.1829779 866.3293967
1010.605691 1321.441383 1111.717184 815.5318979
1147.921419 -0.49300761 0.003645902 0.028938163 CREB3
9 35732335 35737004 + 1903
protein_coding cAMP responsive element binding protein 3 [Source:HGNC
Symbol;Acc:HGNC:2347] TF_bZIP 644 901 886 1006 1446
1133 18.50724575 20.87468644 21.56737995
25.55997407 33.11220502 27.79311071
ENSG00000169188 1401.080589 1625.872301 1450.075051
1945.868016 1974.851195 1989.905073 1492.342647
1970.208095 -0.400415027 0.003648146 0.028940947 APEX2
X 55000357 55009057 + 3245
protein_coding apurinic/apyrimidinic endodeoxyribonuclease 2
[Source:HGNC Symbol;Acc:HGNC:17889] - 1211 1754 1483
1937 2161 2028 20.40911415 23.83136102
21.17039229 28.86131641 29.02008389 29.17422213
ENSG00000165424 1273.814805 1101.217955 1713.102825

909.1432909	960.4667312	957.666347	1362.711862
942.4254564	0.532012791	0.003652788	0.028957464
ZCCHC24 10	79382325	79445627	-	5051
protein_coding	zinc finger CCHC-type containing 24 [Source:HGNC
Symbol;Acc:HGNC:26911]	-	1101	1188	1752	905	1051
	976	11.92077973	10.36986327	16.06790255
8.663082039	9.06742444	9.020248144
ENSG00000227706 20.82531016	31.51633879	44.97872714
10.04578222	0	0.981215519	32.44012537	3.675665913
	3.157777503	0.003654016	0.028957464	AL713998.1
	6	67887646	67889339	-	1605
lincRNA novel transcript	-	18	34	46	10
	0	1	0.613327138	0.933980563	1.327655047
	0.301249392	0	0.029085129
ENSG00000020577 323.9492692	300.3321696	430.2313031
146.6684204	231.206549	229.6044316	351.5042473
202.4931337	0.795145359	0.003658664	0.028979272	SAMD4A
	14	54567097	54793315	+	12170
protein_coding	sterile alpha motif domain containing 4A [Source:HGNC
Symbol;Acc:HGNC:23023]	-	280	324	440	146	253
	234	1.258236171	1.173784553	1.674806177
0.580047413	0.905917274	0.897576172
ENSG00000184164 411.8783566	377.2691143	611.1240101
288.3139497	260.4500651	309.0828886	466.7571603
285.9489678	0.707428198	0.003665358	0.029017258	CRELD2
	22	49918167	49927540	+	5165
protein_coding	cysteine rich with EGF like domains 2 [Source:HGNC
Symbol;Acc:HGNC:28150]	-	356	407	625	287	285
	315	3.769418736	3.474225802	5.60547148
2.686660482	2.404546233	2.846992111
ENSG00000225826 4.627846703	9.269511408	16.62257307	0
	0	0	10.1733104	0	5.841849403
0.003673554	1	LINC00626	1	168786939
168792886	+	955	lincRNA long intergenic non-protein
coding RNA 626 [Source:HGNC Symbol;Acc:HGNC:44257]	-	4
	10	17	0	0	0	0.229061095
0.461668865	0.824608877	0	0	0
ENSG00000163481 546.0859109	762.8807889	660.9917293
909.1432909	996.1072664	927.2486659	656.6528097
944.1664077	-0.522825578	0.003674909	0.029077813	RNF25
	2	218663864	218672411	-	2323
protein_coding	ring finger protein 25 [Source:HGNC
Symbol;Acc:HGNC:14662]	-	472	823	676	905	1090
	945	11.11187895	15.62012782	13.48031194
18.83651631	20.44729609	18.99016047
ENSG00000099625 164.288558	291.0626582	224.8936357
503.2936892	282.3827021	430.753613	226.748284
405.4766681	-0.835787674	0.003703128	0.029285936	CBARP
	19	1228287 1238027 -	4591	protein_coding	CACN
subunit beta associated regulatory protein [Source:HGNC

Symbol;Acc:HGNC:28617]	-	142	314	230	501	309
	439	1.691514653	3.015479034	2.320721357
5.27632635	2.932984612	4.463784947
ENSG00000092203 1423.062861	1546.154503	1394.340541
1843.401037	2093.652979	1851.553685	1454.519302
1929.535901	-0.407667691	0.003705135	0.029286659	TOX4
	14	21476597	21499175	+	7091
protein_coding	TOX high mobility group box family member 4
[Source:HGNC Symbol;Acc:HGNC:20161]	HMG	1230	1668	1426
	1835	2291	1887	9.486201355	10.37104501
9.315678587	12.51208827	14.0791424	12.42254134
ENSG00000185024 684.921312	840.7446847	928.9084954
1224.580853	1157.860465	1080.318287	818.1914974
1154.253201	-0.495583509	0.003718785	0.029379362	BRF1
	14	105209286	105315589	-	13167
protein_coding	"BRF1, RNA polymerase III transcription initiation
factor subunit [Source:HGNC Symbol;Acc:HGNC:11551]"	-	592
	907	950	1219	1267	1101	2.458836118
3.037067256	3.342252259	4.476288677	4.193227034
3.903431111
ENSG00000132185 8.09873173	13.90426711	7.822387329	0
	0	0	9.941795391	0	5.807743971
0.003735337	1	FCRLA	1	161706972	161714352
	+	3275	protein_coding	Fc receptor like A
[Source:HGNC Symbol;Acc:HGNC:18504]	-	7	15	8
	0	0	0	0.116891101	0.201936076
0.113156925	0	0	0
ENSG00000101639 864.2503718	866.6993167	924.0195033
719.278007	612.2861179	456.2652166	884.9897306
595.9431138	0.570632337	0.003739684	0.02952266	CEP192
	18	12991362	13125052	+	9901
protein_coding	centrosomal protein 192 [Source:HGNC
Symbol;Acc:HGNC:25515]	-	747	935	945	716	670
	465	4.126066793	4.163576114	4.421353138
3.49651325	2.948861259	2.192400681
ENSG00000170325 534.5162942	458.8408147	503.5661843
401.8312888	236.6897083	288.4773627	498.9744311
308.9994533	0.691814963	0.003740786	0.02952266	PRDM10
	11	129899706	130002835	-	6626
protein_coding	PR/SET domain 10 [Source:HGNC Symbol;Acc:HGNC:13995]
	zf-C2H2 462	495	515	400	259	294
3.813162003	3.293727951	3.600462179	2.918836553
1.703361905	2.071294895
ENSG00000146005 6.941770054	12.05036483	10.75578258	0
	0	0	9.915972488	0	5.804271849
0.003743387	1	PSD2	5	139795821	139844466
	+	4513	protein_coding	pleckstrin and Sec7 domain
containing 2 [Source:HGNC Symbol;Acc:HGNC:19092]	-	6
	13	11	0	0	0	0.072707737
0.127002414	0.112909324	0	0	0

ENSG00000171792 693.0200438 532.996906 772.4607488
405.8496017 519.0724104 359.1248801 666.1592328
428.0156307 0.637342122 0.003754677 0.029617003 RHNO1
12 2876258 2889523 + 2643 protein_coding RAD9-
HUS1-RAD1 interacting nuclear orphan 1 [Source:HGNC
Symbol;Acc:HGNC:28206] - 599 575 790 404 568
366 12.39436758 9.591899937 13.84625686
7.390697354 9.365042882 6.464425848
ENSG00000178802 608.5618414 817.5709062 654.1471404
933.2531683 1071.957636 969.4409332 693.4266294
991.5505792 -0.515158359 0.003757985 0.029617562 MPI
15 74890005 74902219 + 9090
protein_coding mannose phosphate isomerase [Source:HGNC
Symbol;Acc:HGNC:7216] - 526 882 669 929 1173
988 3.164583604 4.277979114 3.409295854
4.941434548 5.623319074 5.073867213
ENSG00000166794 2216.738571 1885.41862 2352.582989
1550.064197 1444.812466 1765.20672 2151.58006
1586.694461 0.439313266 0.003758623 0.029617562 PPIB
15 64155812 64163205 - 1911
protein_coding peptidylprolyl isomerase B [Source:HGNC
Symbol;Acc:HGNC:9255] - 1916 2034 2406 1543 1581
1799 54.8314247 46.92715435 58.3226692
39.03969855 36.05203472 43.9457127
ENSG00000166188 220.9796801 307.7477788 315.8288884
461.1014039 444.1359004 426.828751 281.5187824
444.0220184 -0.655425146 0.00376457 0.02964914 ZNF319
16 57994668 58000453 - 4274
protein_coding zinc finger protein 319 [Source:HGNC
Symbol;Acc:HGNC:13644] zf-C2H2 191 332 323 459 486
435 2.443957009 3.424818209 3.500825472
5.192534422 4.955190429 4.751172242
ENSG00000136450 3579.639425 2667.765383 3010.641323
2445.143392 2343.136726 2039.947065 3086.015377
2276.075728 0.438857715 0.003772456 0.029695953 SRSF1
17 58003360 58007346 - 3340
protein_coding serine and arginine rich splicing factor 1
[Source:HGNC Symbol;Acc:HGNC:10780] - 3094 2878 3079
2434 2564 2079 50.66039309 37.99078617
42.70371695 35.235085 33.45262112 29.05721979
ENSG00000123146 1767.83744 1941.035689 2894.283312
1270.791451 1264.78207 1838.797883 2201.052147
1458.123802 0.594355046 0.003774437 0.029696254 ADGRE5
19 14380501 14408725 + 5382
protein_coding adhesion G protein-coupled receptor E5 [Source:HGNC
Symbol;Acc:HGNC:1711] - 1528 2094 2960 1265 1384
1874 15.52652881 17.15406068 25.47712826
11.36444022 11.20600971 16.2544369
ENSG00000153944 1487.852715 1210.59819 1713.102825
1113.07267 1026.264642 1009.67077 1470.51791

1049.669361	0.486274332	0.003782587	0.029745067	MSI2
	17	57255851	57684685	+	21479
protein_coding	musashi RNA binding protein 2 [Source:HGNC
Symbol;Acc:HGNC:18585]	-	1286	1306	1752	1108	1123
	1029	3.274323788	2.680791741	3.778526737
2.494174983	2.278370223	2.236389026
ENSG00000116096 422.2910116	590.4678767	555.3895004
644.9392185	840.7510872	881.1315365	522.7161296
788.9406141	-0.592838871	0.003792465	0.029807408	SPR
	2	72887360	72892158	+	1480
protein_coding	sepiapterin reductase [Source:HGNC
Symbol;Acc:HGNC:11257]	-	365	637	568	642	920
	898	13.48732621	18.976306	17.77825275
20.97367476	27.08848457	28.32439597
ENSG00000270083 18.51138681	8.342560268	3.911193665	0
	0	0	10.25504691	0	5.847550684
0.003797912	1	AL021878.2	22	42089630
42090028	-	399	sense_intronic	"novel transcript,
sense intronic to NDUFA6"	-	16	9	4	0
	0	0	2.193015997	0.994497216	0.464397156
	0	0	0
ENSG00000143294 1563.055224	2125.498966	1892.039935
2512.450133	2729.699454	2349.029954	1860.198042
2530.39318	-0.44342313	0.003808428	0.029917492	PRCC
	1	156750610	156800817	+	4108
protein_coding	proline rich mitotic checkpoint control factor
[Source:HGNC Symbol;Acc:HGNC:9343]	-	1351	2293	1935
	2501	2987	2394	17.98538036	24.6097713
21.81991177	29.43638492	31.68570579	27.20443728
ENSG00000160209 2552.257457	3322.192889	2813.126043
4028.35867	3813.537268	3661.896319	2895.858796
3834.597419	-0.404733231	0.003810549	0.029918782	PDXK
	21	43719094	43762307	+	11912
protein_coding	pyridoxal kinase [Source:HGNC Symbol;Acc:HGNC:8819]
	-	2206	3584	2877	4010	4173	3732
10.12780979	13.26530605	11.18813332	16.27649557
15.26589594	14.62523948
ENSG00000103740 10.41265508	8.342560268	10.75578258	0
	0	0	9.836999309	0	5.790667618
0.003820374	1	ACSBG1	15	78167468	78245688
	-	8230	protein_coding	acyl-CoA synthetase bubblegum
family member 1 [Source:HGNC Symbol;Acc:HGNC:29567]	-	9
	9	11	0	0	0	0.059804985
0.048214385	0.061914919	0	0	0
ENSG00000100578 842.2680999	685.0168931	833.0842506
514.3440497	636.9603346	383.6552681	786.7897479
511.6532174	0.619992442	0.003823463	0.030001686
KIAA0586	14	58427385	58551289	+	10184
	protein_coding	KIAA0586 [Source:HGNC Symbol;Acc:HGNC:19960]
	-	728	739	852	512	697	391

3.909378331	3.199337126	3.87546358	2.430819921
2.98244872	1.792273991
ENSG00000169084 208.2531016	278.0853423	329.5180662
131.5997471	158.0977588	171.7127159	271.95217
153.8034073	0.82331551	0.003825033	0.030001686	DHRSX
	X	2219516 2502805 -	3620	protein_coding
dehydrogenase/reductase X-linked [Source:HGNC Symbol;Acc:HGNC:18399]
	-	180	300	337	131	173	175
2.719309546	3.653815739	4.312447154	1.749701409
2.082553324	2.256708756
ENSG00000138413 1756.267824	1531.323285	1568.38866
1157.274112	1251.988032	1230.444261	1618.659923
1213.235468	0.415494587	0.003836884	0.030079206	IDH1
	2	208236227	208266074	-	4721
protein_coding	"isocitrate dehydrogenase (NADP(+)) 1, cytosolic
[Source:HGNC Symbol;Acc:HGNC:5382]"	-	1518	1652	1604
	1152	1370	1254	17.5845996	15.42801316
15.73884359	11.79830707	12.64576668	12.39965415
ENSG00000100503 1800.232367	1686.124125	2064.132457
1417.459871	1147.808006	1473.78571	1850.162983
1346.351196	0.458820673	0.003841999	0.030092405	NIN
	14	50719763	50831121	-	13683
protein_coding	ninein [Source:HGNC Symbol;Acc:HGNC:14906]	-
	1556	1819	2111	1411	1256	1502	6.219034677
	5.861183658	7.146762701	4.985938339	4.000063726
	5.124301111
ENSG00000257242 21.98227184	21.31987624	27.37835565
115.5264955	77.67808958	33.36132766	23.56016791
75.52197092	-1.679714008	0.003842505	0.030092405
LINC01619	12	91984976	92142914	-	3642
	processed_transcript	long intergenic non-protein coding RNA
1619 [Source:HGNC Symbol;Acc:HGNC:27409]	-	19	23
	28	115	85	34	0.285304336	0.278433735
	0.356139829	1.526719017	1.017038942	0.435797778
ENSG00000135924 480.1390954	618.2764109	565.1674845
852.8869105	726.5186025	820.2961743	554.5276636
799.9005624	-0.527304182	0.003855815	0.030166687	DNAJB2
	2	219279267	219286900	+	5886
protein_coding	DnaJ heat shock protein family (Hsp40) member B2
[Source:HGNC Symbol;Acc:HGNC:5228]	-	415	667	578
	849	795	836	3.855871495	4.996196768
4.548938197	6.974107686	5.885799566	6.630282843
ENSG00000188191 341.3036943	486.6493489	299.2063153
610.783559	497.1397733	701.5690964	375.7197862
603.1641429	-0.681659463	0.00385651	0.030166687
PRKAR1B 7	549197	727650	-	4491	protein_coding
protein kinase cAMP-dependent type I regulatory subunit beta
[Source:HGNC Symbol;Acc:HGNC:9390]	-	295	525	306
	608	544	715	3.592308895	5.154068738
3.156318546	6.545785062	5.278547602	7.432056842

ENSG00000160226 170.0733663	272.5236354	262.0499755
360.6435817	399.3567664	405.2420095	234.8823258
388.4141192	-0.723219645	0.00385791	0.030166687
CFAP410 21	44328944	44339402	-	6063
protein_coding	cilia and flagella associated protein 410 [Source:HGNC
Symbol;Acc:HGNC:1260]	-	147	294	268	359	437
413	1.325941853	2.137931175	2.047621503
2.862912646	3.140888117	3.179863804
ENSG00000266903 1.156961676	29.66243651	25.42275882	0
0	1.962431039	18.74738567	0.65414368
4.857201396	0.003867556	1	AC243964.2	19
44632199	44718759	-	1418	antisense	"novel
transcript, antisense to CEACAM16, CEACAM19, PVR & IGSF23"	-
1	32	26	0	0	2	0.038567233
0.994964775	0.849375193	0	0	0.065841513
ENSG00000184481 157.3467879	251.2037592	219.0268452
387.7671937	360.0607917	308.1016731	209.1924641
351.9765528	-0.748104114	0.003880498	0.030327797	FOXO4
X	71096197	71103535	+	3447
protein_coding	forkhead box O4 [Source:HGNC Symbol;Acc:HGNC:7139]
Fork	136	271	224	386	394	314
2.15770634	3.466266624	3.010295925	5.414361361
4.980965529	4.25240285
ENSG00000102032 10.41265508	12.05036483	5.866790497
21.09614266	66.71177105	41.21105182	9.443270136
43.00632184	-2.189053659	0.003909465	0.030538576	RENBP
X	153935263	153944691	-	2057
protein_coding	renin binding protein [Source:HGNC
Symbol;Acc:HGNC:9959]	-	9	13	6	21	73
42	0.239278088	0.278639716	0.135119931
0.49361258	1.546490178	0.953149522
ENSG00000197530 1033.166776	1293.096841	1282.871522
1733.902011	1542.595473	1637.648702	1203.045047
1638.048729	-0.444523942	0.003917045	0.030582157	MIB2
1	1615415 1630610 +	8767	protein_coding
mindbomb E3 ubiquitin protein ligase 2 [Source:HGNC
Symbol;Acc:HGNC:30577]	-	893	1395	1312	1726	1688
1669	5.570512654	7.015476255	6.932426668
9.518992865	8.390349147	8.88692195
ENSG00000138594 2775.55106	1967.917272	2010.353544
1470.702517	1812.184137	1517.940409	2251.273959
1600.275688	0.491774588	0.003927831	0.030650719	TMOD3
15	51829628	51947295	+	10632
protein_coding	tropomodulin 3 [Source:HGNC Symbol;Acc:HGNC:11873]
-	2399	2123	2056	1464	1983	1547
12.33985319	8.803776004	8.957990513	6.657747578
8.127675277	6.792370726
ENSG00000129355 220.9796801	303.1130231	379.3857855
122.5585431	154.4423193	213.9049832	301.1594962
163.6352819	0.88111068	0.003933285	0.030677619	CDKN2D

19	10566462	10569059	-	1503
protein_coding	cyclin dependent kinase inhibitor 2D [Source:HGNC
Symbol;Acc:HGNC:1790]	-	191	327	388	122	169
218	6.949748675	9.592299495	11.9584585
3.924660249	4.899889902	6.770855554
ENSG00000070047 1441.574248	2061.539337	2146.267523
2873.093715	2226.162661	2968.176946	1883.127036
2689.144441	-0.513393898	0.003956395	0.030842124	PHRF1
11	576486	612222	+	5769	protein_coding	PHD
and ring finger domains 1 [Source:HGNC Symbol;Acc:HGNC:24351]	-
1246	2224	2195	2860	2436	3025	11.81169478
16.99684062	17.6252969	23.96992696	18.40074228
24.47771511
ENSG00000120656 830.6984832	928.8050431	800.8169028
1324.034097	1135.013968	1084.243149	853.440143
1181.097071	-0.468376453	0.00396455	0.030889947	TAF12
1	28589323	28643085	-	2337
protein_coding	TATA-box binding protein associated factor 12
[Source:HGNC Symbol;Acc:HGNC:11545]	-	718	1002	819
1318	1242	1105	16.80197927	18.90353245
16.23407863	27.26829064	23.15908951	22.07240221
ENSG00000169972 491.7087122	723.0218899	578.8566624
959.372202	901.0658391	784.9724156	597.8624215
881.8034856	-0.559393708	0.003967982	0.030900937	PUSL1
1	1308567 1311677 +	2489	protein_coding
pseudouridylate synthase like 1 [Source:HGNC Symbol;Acc:HGNC:26914]
-	425	780	592	955	986	800
9.338104855	13.81667888	11.01791115	18.55152823
17.26277524	15.00414064
ENSG00000172995 9.255693406	21.31987624	7.822387329	0
0	0.981215519	12.79931899	0.32707184
5.211345548	0.00399387	1	ARPP21	3	35638945
35794496	+	10092	protein_coding	cAMP regulated
phosphoprotein 21 [Source:HGNC Symbol;Acc:HGNC:16968]	-	8
23	8	0	0	1	0.043351832
0.10048114	0.036721059	0	0	0.004625608
ENSG00000075399 409.5644332	724.8757921	701.0814644
854.8960669	866.3391638	1186.289563	611.8405632
969.1749313	-0.662147249	0.00399468	0.031093014	VPS9D1
16	89707134	89720986	-	3428
protein_coding	VPS9 domain containing 1 [Source:HGNC
Symbol;Acc:HGNC:13526]	-	354	782	717	851	948
1209	5.647511989	10.05772826	9.689041102
12.00300435	12.05108423	16.46385466
ENSG00000102393 870.0351801	810.1552971	874.151784
621.8339194	443.2220405	666.2453377	851.4474204
577.1004326	0.56148878	0.003999309	0.031100911	GLA
X	101397803	101408012	-	2275
protein_coding	galactosidase alpha [Source:HGNC Symbol;Acc:HGNC:4296]
-	752	874	894	619	485	679

18.07719956	16.93807258	18.20365406	13.15559407
9.290068908	13.93267187
ENSG00000145860 2026.996856	1692.612783	2077.821634
1464.675048	1562.70039	1135.266356	1932.477091
1387.547265	0.477598832	0.003999764	0.031100911	RNF145
5	159157409	159210053	-	4614
protein_coding	ring finger protein 145 [Source:HGNC
Symbol;Acc:HGNC:20853]	-	1752	1826	2125	1458	1710
1157	20.76592229	17.44846156	21.33456538
15.27851519	16.15017074	11.705819
ENSG00000105732 768.2225527	885.2383395	794.9501123
1199.466397	1230.055395	983.1779505	816.1370015
1137.566581	-0.478725405	0.004003536	0.031114414	ZNF574
19	42068477	42081549	+	4666
protein_coding	zinc finger protein 574 [Source:HGNC
Symbol;Acc:HGNC:26166]	zf-C2H2 664	955	813	1194	1346
1002	7.782480795	9.023865116	8.071388783
12.37259533	12.5706844	10.02464548
ENSG00000159399 1043.579431	756.3921309	559.300694
593.7057292	443.2220405	427.8099665	786.4240855
488.2459121	0.687060504	0.004018409	0.031214137	HK2
2	74833981	74893359	+	6064
protein_coding	hexokinase 2 [Source:HGNC Symbol;Acc:HGNC:4923] -
902	816	572	591	485	436	8.134709674
5.932871255	4.369575945	4.712262821	3.485307844
3.356397064
ENSG00000167130 396.8378548	497.7727626	529.9667416
235.071304	302.4876194	354.2188025	474.8591197
297.259242	0.676353043	0.004023319	0.031225134	DOLPP1
9	129081100	129090438	+	2249
protein_coding	dolichyldiphosphatase 1 [Source:HGNC
Symbol;Acc:HGNC:29565]	-	343	537	542	234	331
361	8.340640016	10.52734336	11.16380615
5.030690722	6.413530082	7.493138885
ENSG00000135045 765.9086293	875.041877	1058.955685
653.9804225	643.3573537	576.9547254	899.9687303
624.7641672	0.527093778	0.00402391	0.031225134
C9orf40 9	74946581	74952886	-	2327
protein_coding	chromosome 9 open reading frame 40 [Source:HGNC
Symbol;Acc:HGNC:23433]	-	662	944	1083	651	704
588	15.55809141	17.88584937	21.55929371
13.52651199	13.18362598	11.79578854
ENSG00000131652 603.9339947	629.3998246	551.4783067
759.4611358	1113.995191	774.1790449	594.9373754
882.5451238	-0.569332956	0.004028974	0.031248566	THOC6
16	3024027 3027755 +	1860	protein_coding	THO
complex 6 [Source:HGNC Symbol;Acc:HGNC:28369]	-	522	679
564	756	1219	789	15.348017	16.09499285
14.04651591	19.65214988	28.55941841	19.80204736
ENSG00000004478 3098.343368	3620.671156	3138.732916

4543.707298	3740.428478	4811.880907	3285.915813
4365.338895	-0.409564465	0.004036168	0.031288492	FKBP4
	12	2794953 2805423 +	5180	protein_coding	FK506
binding protein 4 [Source:HGNC Symbol;Acc:HGNC:3720]	-	2678
	3906	3210	4523	4093	4904	28.27323648
33.24577315	28.70633366	42.21803781	34.43266067
44.19434866
ENSG00000119711 291.5543423	309.601681	381.3413823
196.8973315	208.3600521	201.1491815	327.4991352
202.1355217	0.69661101	0.004061467	0.031463724
ALDH6A1 14	74056850	74084493	-	6680
protein_coding	aldehyde dehydrogenase 6 family member A1 [Source:HGNC
Symbol;Acc:HGNC:7179]	-	252	334	390	196	228
	205	2.063092931	2.204468829	2.704522509
1.418668172	1.487363029	1.432595011
ENSG00000206560 4626.689741	3815.330896	4006.040111
3427.620894	3187.543252	2839.637713	4149.353583
3151.60062	0.396607082	0.004062889	0.031463724
ANKRD28 3	15667236	15859771	-	11276
protein_coding	ankyrin repeat domain 28 [Source:HGNC
Symbol;Acc:HGNC:29024]	-	3999	4116	4097	3412	3488
	2894	19.39505653	16.09363906	16.83113303
14.63036536	13.47969317	11.98090144
ENSG00000204946 215.1948717	298.4782674	351.0296314
533.4310359	384.7350084	497.4762684	288.2342568
471.8807709	-0.709064994	0.004065496	0.03146797	ZNF783
	7	149262171	149297302	+	6726
protein_coding	zinc finger family member 783 [Source:HGNC
Symbol;Acc:HGNC:27222]	zf-C2H2 186	322	359	531	421
	507	1.512344718	2.110731379	2.472521299
3.817146907	2.727619776	3.518820652
ENSG00000116337 2900.502921	3481.628485	3458.472998
4427.176224	3845.522364	4726.515157	3280.201468
4333.071249	-0.401294009	0.004069145	0.03148027	AMPD2
	1	109616104	109632051	+	6823
protein_coding	adenosine monophosphate deaminase 2 [Source:HGNC
Symbol;Acc:HGNC:469]	-	2507	3756	3537	4407	4208
	4817	20.09433672	24.2708044	24.0138694
31.22977791	26.87564882	32.9569726
ENSG00000175221 1867.336145	2657.568921	2395.60612
3585.339674	2991.063379	2950.515067	2306.837062
3175.639373	-0.460605995	0.004077509	0.031529025	MED16
	19	867630	893218	-	4222	protein_coding
mediator complex subunit 16 [Source:HGNC Symbol;Acc:HGNC:17556] -
	1614	2867	2450	3569	3273	3007	20.90643652
	29.9394227	26.88130269	40.87234311	33.78207907
	33.2476715
ENSG00000081059 78.67339395	77.86389583	126.1359957
44.20144177	41.12369448	24.53038799	94.22442849
36.61850808	1.363928032	0.004110711	0.031769689	TCF7

5 134114711 134151865 + 10063
protein_coding transcription factor 7 [Source:HGNC
Symbol;Acc:HGNC:11639] HMG 68 84 129 44 45
25 0.369552507 0.368032161 0.593833499
0.211410435 0.194869396 0.115973449
ENSG00000183751 1203.240143 1545.227552 1324.916854
2124.68294 1642.2062 1853.516116 1357.794849
1873.468419 -0.46377763 0.004113057 0.031771755 TBL3
16 1972037 1982933 + 7139 protein_coding
transducin beta like 3 [Source:HGNC Symbol;Acc:HGNC:11587] -
1040 1667 1355 2115 1797 1889 7.966923923
10.29513808 8.792337877 14.32432633 10.96905699
12.35209468
ENSG00000144647 629.3871516 1050.235643 873.1739856
1265.76856 1211.778197 1263.805589 850.9322599
1247.117449 -0.550213932 0.00412101 0.031817114
POMGNT2 3 43079232 43106076 - 2668
protein_coding "protein O-linked mannose N-
acetylglucosaminyltransferase 2 (beta 1,4-) [Source:HGNC
Symbol;Acc:HGNC:25902]" - 544 1133 893 1260 1326
1288 11.15084521 18.72311232 15.50486858
22.83420714 21.65789763 22.53596054
ENSG00000115561 1404.551474 1169.81234 1294.605103
1005.5828 981.4855083 804.596726 1289.656306
930.5550115 0.47037502 0.004128952 0.031859081 CHMP3
2 86503431 86563479 - 7018
protein_coding charged multivesicular body protein 3 [Source:HGNC
Symbol;Acc:HGNC:29865] - 1214 1262 1324 1001 1074
820 9.460193848 7.928297698 8.739308635
6.896391852 6.668827982 5.454394228
ENSG00000175198 542.6150259 450.4982545 491.8326033
232.0575693 385.6488683 310.0641042 494.9819612
309.2568472 0.677178062 0.004130614 0.031859081 PCCA
13 100089015 100530437 + 7288
protein_coding propionyl-CoA carboxylase subunit alpha [Source:HGNC
Symbol;Acc:HGNC:8653] - 469 486 503 231 422
316 3.519323516 2.940098383 3.197143113
1.532515347 2.52326396 2.024066395
ENSG00000125107 6069.420951 5670.160129 6380.134665
5069.101708 4683.531872 4431.169286 6039.905248
4727.934289 0.35330823 0.004137656 0.031897299 CNOT1
16 58519951 58629886 - 10852
protein_coding CCR4-NOT transcription complex subunit 1 [Source:HGNC
Symbol;Acc:HGNC:7877] - 5246 6117 6525 5046 5125
4516 26.43706348 24.85207487 27.85307745
22.4821933 20.57987262 19.42630415
ENSG00000279039 4.627846703 4.634755704 21.51156516 0
0 0 10.25805585 0 5.853184765
0.004139611 1 AC011447.6 19 20207192
20209789 - 2598 TEC TEC - 4 5

22	0	0	0	0.084200672	0.084852534
0.392270808	0	0	0
ENSG00000109606 5191.287039	4916.548851	5122.685902
3916.850488	3946.046951	4196.658777	5076.840597
4019.852072	0.336742382	0.004180477	0.032211166	DHX15
4	24517441	24584550	-	8162
protein_coding	DEAH-box helicase 15 [Source:HGNC
Symbol;Acc:HGNC:2738]	-	4487	5304	5239	3899	4318
4277	30.06451428	28.65107245	29.73406345
23.09712162	23.05391276	24.46181603
ENSG00000004799 133.0505927	108.4532835	168.1813276
51.23348932	86.81668835	43.17348286	136.5617346
60.40788684	1.174467914	0.004193929	0.032298535	PDK4
7	95583499	95596491	-	4519
protein_coding	pyruvate dehydrogenase kinase 4 [Source:HGNC
Symbol;Acc:HGNC:8812]	-	115	117	172	51	95
44	1.391714691	1.141504107	1.763147158
0.54566871	0.916093627	0.45452353
ENSG00000153531 18.51138681	41.71280134	39.11193665
99.45324398	66.71177105	102.046414	33.1120416
89.40380969	-1.425369039	0.004199954	0.032309202
ADPRHL1 13	113399610	113453524	-	10924
protein_coding	ADP-ribosylhydrolase like 1 [Source:HGNC
Symbol;Acc:HGNC:21303]	-	16	45	40	99	73
104	0.08010009	0.181620464	0.169621444
0.438182188	0.29120563	0.444424184
ENSG00000127220 596.9922247	754.5382286	831.1286537
1045.765929	955.8974318	1113.679615	727.5530357
1038.447659	-0.512232716	0.004201494	0.032309202	ABHD8
19	17292131	17310236	-	2210
protein_coding	abhydrolase domain containing 8 [Source:HGNC
Symbol;Acc:HGNC:23759]	-	516	814	850	1041	1046
1135	12.76886045	16.23925454	17.8167755
22.77506747	20.6251924	23.9745036
ENSG00000196449 1058.619933	1322.759278	964.1092383
1292.892172	2015.97489	1582.700633	1115.162817
1630.522565	-0.548012314	0.004201655	0.032309202	YRDC
1	37802944	37808185	-	1826
protein_coding	yrdC N6-threonylcarbamoyltransferase domain containing
[Source:HGNC Symbol;Acc:HGNC:28905]	-	915	1427	986
1287	2206	1613	27.40406782	34.45538904
25.01373805	34.07838383	52.64575189	41.23629426
ENSG00000251191 17.35442514	5.561706845	6.844588913	0
0	0	9.920240298	0	5.799502836
0.004204752	1	LINC00589	8	29673922
29748109	-	2074	lincRNA long intergenic non-protein
coding RNA 589 [Source:HGNC Symbol;Acc:HGNC:32299]	-	15
6	7	0	0	0	0.395527988
0.127548823	0.156347789	0	0	0
ENSG00000135597 1151.176867	1237.479773	1019.843748

1694.723461	1454.864925	1475.748141	1136.166796
1541.778842	-0.44025411	0.004208942	0.032348971	REPS1
6	138903493	138988261	-	9602
protein_coding	RALBP1 associated Eps domain containing 1 [Source:HGNC
Symbol;Acc:HGNC:15578]	-	995	1335	1043	1687	1592
1504	5.66703757	6.129901868	5.031819602
8.494828148	7.225035071	7.311932448
ENSG00000164610 549.556796	619.2033621	560.2784925
782.566435	826.1293292	805.5779415	576.3462168
804.7579019	-0.481203788	0.004212562	0.032360524	RP9
7	33094797	33109401	-	1898
protein_coding	"RP9, pre-mRNA splicing factor [Source:HGNC
Symbol;Acc:HGNC:10288]" -	475	668	573	779	904
821	13.68649094	15.51723053	13.98494844
19.84460533	20.75538626	20.19263452
ENSG00000132661 495.1795972	668.3317726	500.6327891
760.4657141	821.5600298	836.9768381	554.7147196
806.334194	-0.53870201	0.004221209	0.032410656	NXT1
20	23350736	23354777	+	1123
protein_coding	nuclear transport factor 2 like export factor 1
[Source:HGNC Symbol;Acc:HGNC:15913]	-	428	721	512
757	899	853	20.84292787	28.30671462
21.11993905	32.5924749	34.88498441	35.45808777
ENSG00000152465 599.306148	558.9515379	601.3460259
476.1700772	365.543951	308.1016731	586.5345706
383.2719004	0.614017875	0.004232166	0.032478478	NMT2
10	15102584	15168693	-	4700
protein_coding	N-myristoyltransferase 2 [Source:HGNC
Symbol;Acc:HGNC:7858]	-	518	603	615	474	400
314	6.027352824	5.656573208	6.061494475
4.876202134	3.70869169	3.118730345
ENSG00000198804 122005.0796	109268.0735	137942.9116
166903.6395	153453.5229	151958.8851	123072.0216
157438.6825	-0.355287956	0.004239386	0.03250504	MT-CO1
MT	5904	7445	+	1542	protein_coding
mitochondrially encoded cytochrome c oxidase I [Source:HGNC
Symbol;Acc:HGNC:7419]	-	105453	117879	141075	166143	167918
154868	3739.979988	3370.435552	4238.07015
5209.534169	4745.38526	4688.385908
ENSG00000105619 216.3518334	284.5740002	254.2275882
329.5016568	384.7350084	534.7624581	251.7178073
416.3330411	-0.724641774	0.00423988	0.03250504	TFPT
19	54107013	54115675	-	1240
protein_coding	TCF3 fusion partner [Source:HGNC
Symbol;Acc:HGNC:13630]	-	187	307	260	328	421
545	8.247353962	10.9156763	9.713016324
12.78949437	14.79513759	20.51733044
ENSG00000143393 2008.485469	2220.974933	2149.200919
2824.87396	2938.973366	2539.385764	2126.22044
2767.744363	-0.380255518	0.004251557	0.032568263	PI4KB

1	151291797	151327715	-	5456
protein_coding	phosphatidylinositol 4-kinase beta [Source:HGNC
Symbol;Acc:HGNC:8984]	-	1736	2396	2198	2812	3216
2588	17.40083432	19.36183033	18.66189675
24.91966336	25.68622463	22.1429738
ENSG00000230630 183.9569064	145.5313291	172.0925212
84.38457065	93.2137075	87.32818123	167.1935856
88.30881979	0.919573601	0.004252388	0.032568263	DNM3OS
1	172138397	172144840	-	3195
antisense	DNM3 opposite strand/antisense RNA [Source:HGNC
Symbol;Acc:HGNC:41228]	-	159	157	176	84	102
89	2.721579184	2.166520226	2.551786063
1.271187577	1.391194676	1.300364725
ENSG00000106635 1251.832533	1407.111832	1290.693909
1872.533806	1643.12006	1724.976883	1316.546091
1746.876916	-0.407608712	0.004263492	0.032636955	BCL7B
7	73536356	73558002	-	3542
protein_coding	BCL tumor suppressor 7B [Source:HGNC
Symbol;Acc:HGNC:1005]	-	1082	1518	1320	1864	1798
1758	16.70603614	18.89544711	17.2634595
25.44477223	22.12074392	23.16948335
ENSG00000006625 636.3289216	849.087245	705.9704565
347.5840648	444.1359004	604.42876	730.4622077
465.3829084	0.650870068	0.004269162	0.032650149	GGCT
7	30496621	30504844	-	1597
protein_coding	gamma-glutamylcyclotransferase [Source:HGNC
Symbol;Acc:HGNC:21705]	-	550	916	722	346	486
616	18.8344302	25.28858419	20.94279961
10.47544303	13.26141759	18.00619014
ENSG00000197046 6135.367766	6906.71295	6761.476048
7587.579311	8970.448557	8843.695477	6601.185588
8467.241115	-0.35910332	0.004270317	0.032650149
SIGLEC15	18	45825512	45844080	+	3598
protein_coding	sialic acid binding Ig like lectin 15
[Source:HGNC Symbol;Acc:HGNC:27596]	-	5303	7451	6915
7553	9816	9013	80.60373766	91.30348663
89.02940712	101.4984809	118.8863486	116.9376193
ENSG00000078018 193.2125998	74.15609127	171.1147228
52.23806755	8.224738897	76.53481052	146.161138
45.66587232	1.679665635	0.004271623	0.032650149	MAP2
2	209424058	209734118	+	11559
protein_coding	microtubule associated protein 2 [Source:HGNC
Symbol;Acc:HGNC:6839]	-	167	80	175	52	9
78	0.790116116	0.305143189	0.701326486
0.217512538	0.033929764	0.315007123
ENSG00000139112 171.230328	190.0249839	149.6031577
275.2544328	291.5213009	277.683992	170.2861565
281.4865752	-0.724919112	0.004276347	0.032655686
GABARAPL1	12	10212458	10223130	+	4086
protein_coding	GABA type A receptor associated protein like 1

[Source:HGNC Symbol;Acc:HGNC:4068]	-	148	205	153
	274	319	283	1.980879538	2.212022075
1.734584752	3.242301648	3.402130109	3.233211457
ENSG00000172244 297.3391507	192.8058373	274.7613549
161.7370937	138.9067014	131.4828796	254.968781
144.0422249	0.822754954	0.00427662	0.032655686
C5orf34 5	43486701	43515145	-	3011
protein_coding	chromosome 5 open reading frame 34 [Source:HGNC
Symbol;Acc:HGNC:24738]	-	257	208	281	161	152
	134	4.66785203	3.045695891	4.323127261
2.585332091	2.1998417	2.077495438
ENSG00000224592 11.56961676	1.853902282	17.60037149	0
	0	0	10.34129684	0	5.861840273
0.004285944	1	AL139158.2	1	38047314
38119025	+	684	lincRNA novel transcript	-
	10	2	18	0	0	0	0.799537082
	0.128916306	1.219042535	0	0	0
ENSG00000069849 3875.821614	3669.799567	4017.773692
3198.577059	2321.204089	3085.922809	3854.464957
2868.567985	0.426334836	0.004311908	0.032908703	ATP1B3
	3	141876124	141926514	+	2584
protein_coding	ATPase Na+/K+ transporting subunit beta 3 [Source:HGNC
Symbol;Acc:HGNC:806]	-	3350	3959	4109	3184	2540
	3145	70.90012657	67.5502484	73.66243786
59.57743016	42.83510197	56.81646068
ENSG00000259298 4.627846703	12.97731597	11.73358099	0
	0	0	9.779581223	0	5.785374154
0.004325105	1	AC012170.2	15	50497195
50498744	-	667	antisense	"novel transcript,
antisense to USP8"	-	4	14	12	0	0
	0	0.327966036	0.925414201	0.833408389	0
	0	0
ENSG00000197903 258.0024537	212.2718113	236.6272167
403.8404453	297.91832	447.4342769	235.6338272
383.0643474	-0.701570028	0.004336805	0.033082202
HIST1H2BK	6	27146418	27146798	-	381
	protein_coding	histone cluster 1 H2B family member k
[Source:HGNC Symbol;Acc:HGNC:13954]	-	223	229	242
	402	326	456	32.00918379	26.49991401
29.42339934	51.0155172	37.28646592	55.8709303
ENSG00000135439 167.759443	232.6647364	254.2275882
306.3963577	395.7013269	382.6740526	218.2172558
361.5905791	-0.726834188	0.004339037	0.033082722	AGAP2
	12	57723761	57742157	-	6728
protein_coding	"ArfGAP with GTPase domain, ankyrin repeat and PH
domain 2 [Source:HGNC Symbol;Acc:HGNC:16921]"	-	145	251
	260	305	433	390	1.17862794	1.644832569
	1.790151641	2.191871416	2.804532721	2.705980484
ENSG00000182534 2703.819436	2706.697331	2487.519171
1634.448767	2256.320037	1960.468608	2632.678646

1950.412471	0.432501792	0.004354628	0.033185041	MXRA7
	17	76672551	76711016	-	9257
protein_coding	matrix remodeling associated 7 [Source:HGNC
Symbol;Acc:HGNC:7541]	-	2337	2920	2544	1627	2469
	1998	13.80648614	13.90741921	12.7306125
8.498034809	11.62276412	10.07560785
ENSG00000121073 1486.695753	1378.376346	1349.361814
1023.665208	1123.133789	1004.764692	1404.811305
1050.52123	0.418865496	0.004361528	0.033194992
SLC35B1 17	49700934	49709014	-	5928
protein_coding	solute carrier family 35 member B1 [Source:HGNC
Symbol;Acc:HGNC:20798]	-	1285	1487	1380	1019	1229
	1024	11.85467482	11.05953154	10.78383781
8.31126645	9.034461794	8.063763788
ENSG00000184060 23.13923351	25.0276808	23.46716199
69.31589732	62.14247166	67.70387084	23.87802544
66.38741328	-1.474235722	0.004361643	0.033194992	ADAP2
	17	30906344	30959322	+	3750
protein_coding	ArfGAP with dual PH domains 2 [Source:HGNC
Symbol;Acc:HGNC:16487]	-	20	27	24	69	68
	69	0.291671128	0.317443511	0.296471144
0.889649706	0.790198576	0.858942039
ENSG00000267751 1.156961676	7.415609127	2.933395248
39.17855066	18.27719755	25.51160351	3.835322017
27.6557839	-2.823763495	0.004362448	0.033194992
AC009005.1	19	567210	572228	-	865	antisense
	uncharacterized LOC105372233 [Source:NCBI gene;Acc:105372233]
	-	1	8	3	39	20	26
0.06322351	0.40776302	0.160659941	2.179965979
1.007563638	1.403147337
ENSG00000115806 2538.373917	2213.559324	2071.954844
3297.025725	2831.1379	2885.754843	2274.629362
3004.639489	-0.401816031	0.004366149	0.033206626
GORASP2 2	170928464	170967129	+	5340
protein_coding	golgi reassembly stacking protein 2 [Source:HGNC
Symbol;Acc:HGNC:17500]	-	2194	2388	2119	3282	3098
	2941	22.46932774	19.7163729	18.38197434
29.71656016	25.28126227	25.70986542
ENSG00000092345 16.19746346	14.83121825	0.977798416	0
	0	0	10.66882671	0	5.906709332
0.004381681	1	DAZL	3	16586792	16670306
	-	3631	protein_coding	deleted in azoospermia like
[Source:HGNC Symbol;Acc:HGNC:2685]	-	14	16	1
	0	0	0	0.210861115	0.19427982
0.012757812	0	0	0
ENSG00000250548 6.941770054	19.46597396	21.51156516	0
	0	1.962431039	15.97310306	0.65414368
4.625884758	0.004396626	1	LINC01303	14
61556313	61570653	-	5148	lincRNA long
intergenic non-protein coding RNA 1303 [Source:HGNC

Symbol;Acc:HGNC:50470] -   6   21   22   0   0
        2   0.06373932   0.179851769   0.197964172   0
        0   0.018135832
ENSG00000184557 659.4681552   1106.779662   1351.317411
535.4401923   563.8515444   813.4276656   1039.188409
637.5731341   0.705584946   0.004401548   0.03345921   SOCS3
        17   78356778   78360077   -   2734
protein_coding suppressor of cytokine signaling 3 [Source:HGNC
Symbol;Acc:HGNC:19391] -   570   1194   1382   533   617
        829   11.40173803   19.25483381   23.41596114
9.426053823   9.834340738   14.1547452
ENSG00000171476 5.784808379   4.634755704   19.55596832   0
        0   0   9.991844135   0   5.814772251
0.004402414   1   HOPX   4   56647988   56681899
        -       7753   protein_coding HOP homeobox [Source:HGNC
Symbol;Acc:HGNC:24961] -   5   5   20   0   0
        0   0.035269145   0.028433752   0.119498559   0
        0   0
ENSG00000174744 1517.933719   1852.048379   1819.682852
2476.285317   2270.027936   2157.692927   1729.888317
2301.335393   -0.411286639   0.004409619   0.0335039   BRMS1
        11   66337333   66345125   -   2514
protein_coding "BRMS1, transcriptional repressor and anoikis
regulator [Source:HGNC Symbol;Acc:HGNC:17262]" -   1312   1998
        1861   2465   2484   2199   28.54061153   35.04000573
        34.29126888   47.40813455   43.0571139   40.83250198
ENSG00000197774 98.34174244   164.9973031   228.8048294
474.1609208   195.5660138   330.6696301   164.0479583
333.4655215   -1.020599845   0.004414403   0.033523583   EME2
        16   1773207 1781708 +   6909   protein_coding
essential meiotic structure-specific endonuclease subunit 2
[Source:HGNC Symbol;Acc:HGNC:27289]   -   85   178   234
        472   214   337   0.672819308   1.135896516
1.568928386   3.30314792   1.349761941   2.276988011
ENSG00000145555 4156.963301   4122.151723   5695.675774
3342.231745   3200.337291   3753.149362   4658.263599
3431.906132   0.440893988   0.004424666   0.033584838   MYO10
        5   16661914   16936276   -   13963
protein_coding myosin X [Source:HGNC Symbol;Acc:HGNC:7593]   -
        3593   4447   5825   3327   3502   3825   14.07256269
        14.04178598   19.32500645   11.52060481   10.92939196
        12.78788545
ENSG00000214655 939.4528807   1268.069161   1060.911282
1792.167548   1218.175217   1712.221081   1089.477774
1574.187949   -0.530166447   0.004432564   0.033628093   ZSWIM8
        10   73785582   73801797   +   7953
protein_coding zinc finger SWIM-type containing 8 [Source:HGNC
Symbol;Acc:HGNC:23528] -   812   1368   1085   1784   1333
        1745   5.58367021   7.583838446   6.319769106
10.84588722   7.303949549   10.24260641

ENSG00000151612 473.1973254 319.7981436 458.5874572
320.4604528 198.3075934 224.698354 417.1943087
247.8221334 0.751352057 0.004452648 0.033763705 ZNF827
4 145757627 145938635 - 8433
protein_coding zinc finger protein 827 [Source:HGNC
Symbol;Acc:HGNC:27193] zf-C2H2 409 345 469 319 217
229 2.652381074 1.803727608 2.576280808
1.828983549 1.121337203 1.267650167
ENSG00000213347 163.1315963 168.7051076 393.0749633
138.6317946 39.29597473 121.6707244 241.6372224
99.86616459 1.276997521 0.004456181 0.033773746 MXD3
5 177301461 177312757 - 5154
protein_coding MAX dimerization protein 3 [Source:HGNC
Symbol;Acc:HGNC:14008] bHLH 141 182 402 138 43
124 1.496130275 1.556900764 3.613134218
1.294600853 0.363565479 1.123112619
ENSG00000164096 1659.083043 1662.023396 1347.406217
1723.856229 2588.051173 2263.664203 1556.170885
2191.857202 -0.494444926 0.004460031 0.033786172 C4orf3
4 119296419 119304445 - 3148
protein_coding chromosome 4 open reading frame 3 [Source:HGNC
Symbol;Acc:HGNC:19225] - 1434 1793 1378 1716 2832
2307 24.91203127 25.11189714 20.27761858
26.35625959 39.20280326 34.21045944
ENSG00000171475 1517.933719 1561.912672 1484.297996
2017.19307 1843.255373 2116.481875 1521.381462
1992.310106 -0.388923002 0.004463572 0.033796247 WIPF2
17 40219304 40284136 + 8123
protein_coding WAS/WASL interacting protein family member 2
[Source:HGNC Symbol;Acc:HGNC:30923] - 1312 1685 1518
2008 2017 2157 8.833078591 9.145709656
8.656807776 11.95221706 10.82051777 12.39594749
ENSG00000058600 1435.78944 1686.124125 1525.365529
2034.2709 1956.573998 2122.369169 1549.093031
2037.738022 -0.395110563 0.004471341 0.033838314 POLR3E
16 22297375 22335103 + 9488
protein_coding RNA polymerase III subunit E [Source:HGNC
Symbol;Acc:HGNC:30347] - 1241 1819 1560 2025 2141
2163 7.153059181 8.452632377 7.61644619
10.31933159 9.833329433 10.64211332
ENSG00000118263 802.9314029 769.3694469 593.5236386
1121.109296 1038.144821 927.2486659 721.9414962
1028.834261 -0.511496595 0.004487022 0.033940183 KLF7
2 207074137 207167267 - 9262
protein_coding Kruppel like factor 7 [Source:HGNC
Symbol;Acc:HGNC:6350] zf-C2H2 694 830 607 1116 1136
945 4.097787247 3.95100222 3.035892367
5.825867921 5.344808537 4.762917595
ENSG00000240527 84.45820233 86.2064561 66.4902923
146.6684204 189.1689946 133.4453106 79.05165024

156.4275752	-0.985701384	0.004507285	0.034076592
AL365273.1	10	95833508	95873758	+	587
	sense_intronic	"novel transcript, sense intronic to ENTPD1"
	-	73	93	68	146	207	136
6.801104871	6.985199356	5.366279232	12.02585522
15.36706195	10.81550601
ENSG00000132406 429.2327817	335.556313	339.2960504
221.0072088	222.9818101	252.1723885	368.0283817
232.0538025	0.664285124	0.004528965	0.034223574
TMEM128 4	4235542 4248212 -	1737	protein_coding
transmembrane protein 128 [Source:HGNC Symbol;Acc:HGNC:28201]	-
	371	362	347	220	244	257	11.68069822
	9.188459598	9.254055763	6.123843436	6.121369646
	6.906839129
ENSG00000134321 5.784808379	36.15109449	10.75578258
60.27469332	152.6145995	22.56795695	17.56389515
78.48574993	-2.150133516	0.004532703	0.034234893	RSAD2
	2	6865806 6898239 +	4834	protein_coding
radical S-adenosyl methionine domain containing 2 [Source:HGNC
Symbol;Acc:HGNC:30908]	-	5	39	11	60	167
	23	0.056566339	0.355706596	0.105411622
0.600130668	1.505457234	0.222109547
ENSG00000152894 4052.83675	2766.022204	3621.765333
2726.425295	2496.665185	2436.358135	3480.208096
2553.149538	0.446618042	0.004537984	0.034257861	PTPRK
	6	127968779	128520674	-	9000
protein_coding	"protein tyrosine phosphatase, receptor type K
[Source:HGNC Symbol;Acc:HGNC:9674]"	-	3503	2984	3704
	2714	2732	2483	21.28591584	14.61807775
19.06474165	14.58037018	13.2280791	12.87894374
ENSG00000254122 62.47593049	27.80853423	42.04533189
1.004578222	5.483159264	17.66187935	44.1099322
8.049872279	2.4521286	0.004543134	0.034269055
PCDHGB7 5	141417645	141512979	+	4966
protein_coding	"protocadherin gamma subfamily B, 7 [Source:HGNC
Symbol;Acc:HGNC:8714]"	-	54	30	43	1	6
	18	0.594677843	0.266347422	0.401110653
0.009736312	0.052650577	0.169204468
ENSG00000004975 1299.267962	1841.851917	1698.435849
2061.394512	2250.836878	2325.480781	1613.185243
2212.570724	-0.455172986	0.004543951	0.034269055	DVL2
	17	7225341 7234545 -	5350	protein_coding
dishevelled segment polarity protein 2 [Source:HGNC
Symbol;Acc:HGNC:3086]	-	1123	1987	1737	2052	2463
	2370	11.47943959	16.37487687	15.04002272
18.5449126	20.0617691	20.67952695
ENSG00000133935 592.364378	717.460183	709.8816501
380.7351461	524.5555696	458.2276476	673.2354037
454.5061211	0.56691656	0.004548117	0.034283563	ERG28
	14	75649791	75661189	-	2633

protein_coding	ergosterol biosynthesis 28 homolog [Source:HGNC
Symbol;Acc:HGNC:1187]	-	512	774	726	379	574
	467	10.63442015	12.96056873	12.77286192
6.95968474	9.499913065	8.279651501
ENSG00000135898 0	0	0	17.07782977	9.138598774
	3.924862078	0	10.04709688	-5.747998498
0.004554614	1	GPR55	2	230907318	230961066
	-	4130	protein_coding	G protein-coupled receptor 55
[Source:HGNC Symbol;Acc:HGNC:4511]	-	0	0	0
	17	10	4	0	0	0	0.199021542
	0.105513626	0.045212235
ENSG00000135744 20.82531016	2.780853423	6.844588913	0
	0	0	10.15025083	0	5.831243265
0.004560575	1	AGT	1	230702523	230714297
	-	2291	protein_coding	angiotensinogen [Source:HGNC
Symbol;Acc:HGNC:333]	-	18	3	7	0	0
	0	0.429677021	0.057733797	0.141538767	0
	0	0
ENSG00000204568 1963.363964	2207.070666	2107.155587
2649.072771	2744.321212	2687.549308	2092.530072
2693.647764	-0.364072131	0.004569452	0.034427407
MRPS18B 6	30617709	30626395	+	1888
protein_coding	mitochondrial ribosomal protein S18B [Source:HGNC
Symbol;Acc:HGNC:14516]	-	1697	2381	2155	2637	3003
	2739	49.15577697	55.60212163	52.87467858
67.53196027	69.31256009	67.72298282
ENSG00000211683 2.313923351	20.3929251	7.822387329	0
	0	0	10.17641193	0	5.844280291
0.004571212	1	AP000346.1	22	23630618
23638941	-	1087	processed_transcript	novel
transcript	-	2	22	8	0	0	0
	0.100622514	0.892333289	0.340928179	0	0
	0
ENSG00000125637 593.5213396	727.6566456	712.8150454
914.166182	901.979699	1032.238726	677.9976769
949.4615358	-0.484906045	0.004571959	0.034429328	PSD4
	2	113157325	113209396	+	13442
protein_coding	pleckstrin and Sec7 domain containing 4 [Source:HGNC
Symbol;Acc:HGNC:19096]	-	513	785	729	910	987
	1052	2.087123686	2.574777609	2.512268732
3.273246542	3.199719141	3.653405553
ENSG00000254545 0	1.853902282	7.822387329	20.09156444
	42.95141424	20.60552591	3.22542987	27.88283486
	-3.090665768	0.004588234	0.034534876	AL354919.2
	1	31789130	31791322	+	537
antisense	novel transcript	-	0	2	8
	20	47	21	0	0.164206244	0.69010974
	1.800764525	3.814013009	1.825538703
ENSG00000156642 1797.918444	1629.580106	1741.458979
1228.599166	1477.711422	1038.12602	1722.985843

1248.145536	0.464719478	0.00460453	0.034640478	NPTN
	15	73560014	73634134	-	4032
protein_coding	neuroplastin [Source:HGNC Symbol;Acc:HGNC:17867]
	-	1554	1758	1781	1223	1617	1058
21.07779633	19.22349306	20.461895	14.665847
17.47624378	12.24929743
ENSG00000235374 62.47593049	59.32487301	63.55689705
139.6363729	114.2324847	126.576802	61.78590018
126.8152198	-1.037317183	0.004607174	0.034643317	SSR4P1
	21	45070952	45074165	-	2636
transcribed_processed_pseudogene	signal sequence receptor
subunit 4 pseudogene 1 [Source:HGNC Symbol;Acc:HGNC:23131]	-
	54	64	65	139	125	129	1.120322522
	1.070455274	1.142274302	2.549591548	2.066441927
	2.284495675
ENSG00000231298 128.422746	40.7858502	72.3570828
32.1465031	17.36333767	29.43646558	80.521893
26.31543545	1.612018476	0.004624977	0.03476009	MANCR
	10	4650185 4678154 -	1920	lincRNA mitotically
associated long non coding RNA [Source:HGNC Symbol;Acc:HGNC:44678]
	-	111	44	74	32	19	30
3.16166945	1.010381547	1.785389379	0.805842125
0.431231989	0.729400509
ENSG00000171451 658.3111935	390.2464303	458.5874572
790.6030607	699.1028062	812.4464501	502.3816937
767.3841057	-0.612913941	0.004627349	0.034760825	DSEL
	18	67506582	67516980	-	9531
protein_coding	dermatan sulfate epimerase like [Source:HGNC
Symbol;Acc:HGNC:18144]	-	569	421	469	787	765
	828	3.264889668	1.947500529	2.279485474
3.992431554	3.497691997	4.055439278
ENSG00000163605 2647.128314	1948.451298	1995.686567
1333.075301	1934.64136	1281.467468	2197.088726
1516.39471	0.534320872	0.00463331	0.034788509	PPP4R2
	3	72996785	73069199	+	5964
protein_coding	protein phosphatase 4 regulatory subunit 2
[Source:HGNC Symbol;Acc:HGNC:18296]	-	2288	2102	2041
	1327	2117	1306	20.98036783	15.53921354
15.85286735	10.75807344	15.46827274	10.22236957
ENSG00000114857 2243.348689	2114.375552	2250.891954
1922.762717	1450.295625	1538.545935	2202.872065
1637.201426	0.428291599	0.00464892	0.034872998	NKTR
	3	42600614	42648741	+	17356
protein_coding	natural killer cell triggering receptor [Source:HGNC
Symbol;Acc:HGNC:7833]	-	1939	2281	2302	1914	1587
	1568	6.109742126	5.794415071	6.144097992
5.332041347	3.984610573	4.217377267
ENSG00000090615 3990.36082	3809.769189	4498.850513
5985.277047	4490.707438	5948.128479	4099.660174
5474.704321	-0.417187771	0.004649125	0.034872998	GOLGA3

	12	132768909	132828858	-	10549
protein_coding	golgin A3 [Source:HGNC Symbol;Acc:HGNC:4426]	-
	3449	4110	4601	5958	4914	6062	17.88037467
	17.17767919	20.20428085	27.30803325	20.29936523
	26.82567604
ENSG00000150636 222.1366417	175.1937656	193.6040864
72.32963199	140.7344211	88.30939675	196.9781646
100.4578166	0.968747041	0.004654781	0.034898304
CCDC102B	18	68715209	69055189	+	5805
	protein_coding	coiled-coil domain containing 102B
[Source:HGNC Symbol;Acc:HGNC:26295]	-	192	189	198
	72	154	90	1.808813195	1.435468075
1.580030324	0.599696465	1.156051268	0.723746241
ENSG00000259956 1755.110862	2369.287116	2767.169518
3333.190541	3155.558157	3065.317283	2297.189165
3184.68866	-0.470812282	0.004663422	0.034945948	RBM15B
	3	51391268	51397908	+	6641
protein_coding	RNA binding motif protein 15B [Source:HGNC
Symbol;Acc:HGNC:24303]	-	1517	2556	2830	3318	3453
	3124	12.4924268	16.96919839	19.74037557
24.15706222	22.65800644	21.95955731
ENSG00000143061 75.20250892	93.62206523	231.7382246
42.19228532	63.97019142	51.02320701	133.5209329
52.39522792	1.349923408	0.004675888	0.035022195	IGSF3
	1	116574399	116667755	-	7400
protein_coding	immunoglobulin superfamily member 3 [Source:HGNC
Symbol;Acc:HGNC:5950]	-	65	101	237	42	70
	52	0.480370523	0.60176041	1.483607712
0.274421913	0.41221607	0.328033094
ENSG00000169221 1384.883126	1617.529741	1831.416433
2143.769926	2228.904241	2099.801212	1611.276433
2157.491793	-0.42071448	0.004681825	0.035049493
TBC1D10B	16	30357102	30370264	-	6699
	protein_coding	TBC1 domain family member 10B [Source:HGNC
Symbol;Acc:HGNC:24510]	-	1197	1745	1873	2134	2439
	2140	9.771897105	11.48469356	12.95180375
15.40230268	15.86574319	14.91247853
ENSG00000138796 1644.042541	1411.746588	1386.518154
998.5507527	1246.504873	979.2530884	1480.769094
1074.769571	0.461655842	0.004688569	0.035082795	HADH
	4	107989714	108035175	+	12086
protein_coding	hydroxyacyl-CoA dehydrogenase [Source:HGNC
Symbol;Acc:HGNC:4799]	-	1421	1523	1418	994	1364
	998	6.429929345	5.555859716	5.434956804
3.976536847	4.918020992	3.854730208
ENSG00000078618 5814.889382	4973.092871	5181.353807
4365.896953	4084.953652	4058.307389	5323.11202
4169.719331	0.352184054	0.004693061	0.035089297	NRDC
	1	51789191	51878937	-	6773
protein_coding	nardilysin convertase [Source:HGNC

Symbol;Acc:HGNC:7995] - 5026 5365 5299 4346 4470
4136 40.58224995 34.92388976 36.24226234
31.02486232 28.75974594 28.5066045
ENSG00000163545 93.71389573 139.0426711 172.0925212
68.3113191 42.95141424 71.62873292 134.949696
60.96382209 1.14958782 0.004694028 0.035089297 NUAK2
1 205302059 205321791 - 3441
protein_coding NUAK family kinase 2 [Source:HGNC
Symbol;Acc:HGNC:29558] - 81 150 176 68 47
73 1.287345321 1.921943181 2.369356719
0.955488483 0.595212143 0.990339778
ENSG00000178425 797.1465946 802.739688 807.6614917
576.6278994 588.5257611 580.8795875 802.5159248
582.0110827 0.463479907 0.004723673 0.035275482 NT5DC1
6 116100849 116249497 + 7309
protein_coding 5'-nucleotidase domain containing 1 [Source:HGNC
Symbol;Acc:HGNC:21556] - 689 866 826 574 644
592 5.155324094 5.223888373 5.235094688
3.79712721 3.839604599 3.781027018
ENSG00000166450 249.903722 161.2894985 343.2072441
181.8286582 40.20983461 95.17790539 251.4668215
105.7387994 1.251479919 0.004725329 0.035275482 PRTG
15 55611546 55743090 - 14566
protein_coding protogenin [Source:HGNC Symbol;Acc:HGNC:26373] -
216 174 351 181 44 97 0.810976292
0.526675239 1.116270035 0.600813228 0.131634876
0.310869035
ENSG00000079974 577.3238762 551.5359288 320.7178805
732.3375239 705.4998254 832.0707605 483.1925618
756.6360366 -0.647651607 0.004725858 0.035275482 RABL2B
22 50767501 50783663 - 5870
protein_coding "RAB, member of RAS oncogene family like 2B
[Source:HGNC Symbol;Acc:HGNC:9800]" - 499 595 328
729 772 848 4.648974426 4.469025394
2.588440571 6.004690723 5.731097499 6.7437861
ENSG00000109919 2375.24232 2346.113337 2343.782804
1986.051145 1789.33764 1610.174667 2355.046154
1795.187817 0.391646883 0.004728492 0.03527792 MTCH2
11 47617315 47642623 - 2897
protein_coding mitochondrial carrier 2 [Source:HGNC
Symbol;Acc:HGNC:17587] - 2053 2531 2397 1977 1958
1641 38.75566265 38.51923097 38.32851512
32.99585532 29.45254241 26.44271972
ENSG00000173068 684.921312 450.4982545 653.169342
428.9549008 297.91832 406.2232251 596.1963028
377.6988153 0.658297711 0.004741977 0.035361266 BNC2
9 16409503 16870843 - 15323
protein_coding basonuclin 2 [Source:HGNC Symbol;Acc:HGNC:30988]
zf-C2H2 592 486 668 427 326 414
2.112869227 1.398383934 2.019459355 1.347365088

0.927112414	1.261254055
ENSG00000146147 16.19746346	12.05036483	1.955596832	0
	0	0	10.06780837	0	5.822353335
0.004743918	1	MLIP	6	53929982	54266280
	+	7293	protein_coding	muscular LMNA interacting
protein [Source:HGNC Symbol;Acc:HGNC:21355]	-	14	13
	2	0	0	0	0.104982409	0.078590689
	0.012703583	0	0	0
ENSG00000118418 617.8175348	844.4524893	673.7031087
439.000683	486.1734548	529.8563805	711.9910443
485.0101728	0.554571854	0.004756025	0.035448733	HMGN3
	6	79201245	79234738	-	1014
protein_coding	high mobility group nucleosomal binding domain 3
[Source:HGNC Symbol;Acc:HGNC:12312]	Others	534	911	689
	437	532	540	28.80036652	39.61086988
31.47630339	20.83745613	22.86295045	24.86004101
ENSG00000174343 35.86581195	65.813531	21.51156516
102.4669786	95.04142725	120.6895089	41.06363603
106.0659716	-1.365308544	0.004763242	0.035470539	CHRNA9
	4	40335329	40355217	+	3168
protein_coding	cholinergic receptor nicotinic alpha 9 subunit
[Source:HGNC Symbol;Acc:HGNC:14079]	-	31	71	22
	102	104	123	0.535144706	0.988114185
0.32169178	1.556740469	1.430562262	1.812449749
ENSG00000166166 897.8022604	1300.512451	1170.424704
1562.119135	1574.580569	1540.508366	1122.913138
1559.069357	-0.472526009	0.004763591	0.035470539
TRMT61A 14	103529184	103537073	+	3229
protein_coding	tRNA methyltransferase 61A [Source:HGNC
Symbol;Acc:HGNC:23790]	-	776	1403	1197	1555	1723
	1570	13.14281483	19.1568273	17.17230372
23.2843203	23.25283073	22.69748006
ENSG00000008311 410.7213949	321.6520459	494.7659986
121.5539649	286.9520015	260.0221127	409.0464798
222.842693	0.875117259	0.0047759	0.035544881	AASS
	7	122075647	122144280	-	4591
protein_coding	aminoadipate-semialdehyde synthase [Source:HGNC
Symbol;Acc:HGNC:17366]	-	355	347	506	121	314
	265	4.228786633	3.332392436	5.105586986
1.274322332	2.98044391	2.694539888
ENSG00000138073 654.8403085	900.0695578	877.0851793
1211.521336	1091.148694	1122.510554	810.6650152
1141.726861	-0.492889659	0.004786642	0.035607495	PREB
	2	27130756	27134675	-	3221
protein_coding	prolactin regulatory element binding [Source:HGNC
Symbol;Acc:HGNC:9356]	Others	566	971	897	1206	1194
	1144	9.609934312	13.29114705	12.90042963
18.10330213	16.15370694	16.57987819
ENSG00000185238 760.1238209	802.739688	697.1702707
542.4722399	440.4804609	584.8044496	753.3445932

522.5857168	0.528101787	0.004790591	0.035619532	PRMT3
	11	20387530	20509294	+	5467
protein_coding	protein arginine methyltransferase 3 [Source:HGNC
Symbol;Acc:HGNC:30163]	-	657	866	713	540	482
	596	6.572203592	6.983976608	6.041474013
4.775797484	3.841992937	5.089126212
ENSG00000173166 236.0201818	249.3498569	220.9824421
149.6821551	122.4572236	142.2762503	235.4508269
138.138543	0.770020098	0.004792945	0.035619715	RAPH1
	2	203394345	203535410	-	11697
protein_coding	Ras association (RalGDS/AF-6) and pleckstrin homology
domains 1 [Source:HGNC Symbol;Acc:HGNC:14436]	-	204	269
	226	149	134	145	0.953784785	1.013938813
	0.895027553	0.615903958	0.499216471	0.578681433
ENSG00000173818 466.2555553	733.2183524	541.7003226
734.3466803	892.8411002	1003.783476	580.3914101
876.990419	-0.594381297	0.004829411	0.035857411	ENDOV
	17	80415165	80438086	+	7253
protein_coding	endonuclease V [Source:HGNC Symbol;Acc:HGNC:26640]
	-	403	791	554	731	977	1023
3.038659807	4.80831337	3.538299109	4.873050544
5.869964625	6.584214822
ENSG00000124333 2573.082767	1868.7335	1865.639378
1423.487341	1704.348671	1401.175762	2102.485215
1509.670591	0.477199868	0.004829621	0.035857411	VAMP7
	X	155881293	155943769	+	2667
protein_coding	vesicle associated membrane protein 7 [Source:HGNC
Symbol;Acc:HGNC:11486]	-	2224	2016	1908	1417	1865
	1428	45.60437204	33.32740277	33.14040492
25.68905041	30.47294433	24.99488987
ENSG00000072210 885.0756819	950.1249194	877.0851793
709.2322247	640.6157741	618.1657773	904.0952602
656.004592	0.46307361	0.004839007	0.035909657
ALDH3A2 17	19648136	19677598	+	6457
protein_coding	aldehyde dehydrogenase 3 family member A2 [Source:HGNC
Symbol;Acc:HGNC:403]	-	765	1025	897	706	701
	630	6.479259311	6.998855659	6.435230577
5.286583926	4.730922452	4.554658279
ENSG00000151640 548.3998343	725.8027433	626.7687848
790.6030607	925.7400558	1013.595632	633.6571208
909.9795827	-0.521268838	0.004851677	0.035986213	DPYSL4
	10	132186900	132205776	+	3176
protein_coding	dihydropyrimidinase like 4 [Source:HGNC
Symbol;Acc:HGNC:3016]	-	474	783	641	787	1013
	1033	8.161924266	10.86964164	9.349319287
11.98106585	13.89912784	15.18328918
ENSG00000165684 672.1947336	1015.011499	1066.778072
1304.94711	1268.43751	1409.025486	917.9947683
1327.470035	-0.531008875	0.004908116	0.036387182	SNAPC4
	9	136375577	136400168	-	5045

protein_coding	small nuclear RNA activating complex polypeptide 4
[Source:HGNC Symbol;Acc:HGNC:11137]	MYB	581	1095	1091
	1299	1388	1436	6.298101777	9.569448435
10.01765419	12.44942224	11.98910858	13.28737846
ENSG00000100505 219.8227184	206.7101044	114.4024147
363.6573164	274.1579632	321.8386904	180.3117458
319.8846567	-0.828058077	0.004911412	0.036390388	TRIM9
	14	50975262	51096061	-	8945
protein_coding	tripartite motif containing 9 [Source:HGNC
Symbol;Acc:HGNC:16288]	-	190	223	117	362	300
	328	1.161630399	1.099153826	0.605909794
1.956723416	1.461502315	1.711746839
ENSG00000181929 1469.341328	1423.796952	1353.273008
1756.002732	2001.353132	1813.28628	1415.470429
1856.880715	-0.391877463	0.004915551	0.036390388	PRKAG1
	12	49002274	49018825	-	4184
protein_coding	protein kinase AMP-activated non-catalytic subunit
gamma 1 [Source:HGNC Symbol;Acc:HGNC:9385]	-	1270	1536
	1384	1748	2190	1848	16.59994916	16.18577496
	15.32310826	20.19998137	22.80925165	20.61846486
ENSG00000231079 2.313923351	12.97731597	0	8.036625776
	75.85036983	41.21105182	5.097079774	41.69934914
	-3.015554474	0.004916212	0.036390388	AC105402.3
	2	148866470	148888823	-	4156
antisense	novel transcript	-	2	14	0
	8	83	42	0.026317775	0.148520518	0
	0.093071275	0.870284305	0.471758558
ENSG00000163870 770.536476	1100.291004	894.6855508
1330.061566	1261.126631	1271.655313	921.837677
1287.614503	-0.481117829	0.00491807	0.036390388	TPRA1
	3	127571266	127598267	-	5622
protein_coding	transmembrane protein adipocyte associated 1
[Source:HGNC Symbol;Acc:HGNC:30413]	-	666	1187	915
	1324	1380	1296	6.478554262	9.308802917
7.539329229	11.38671263	10.69662678	10.76118744
ENSG00000180901 940.6098424	1011.303695	1177.269293
1418.464449	1567.26969	1294.22327	1043.060943
1426.65247	-0.451542123	0.004923567	0.036413435	KCTD2
	17	75032575	75065889	+	5279
protein_coding	potassium channel tetramerization domain containing 2
[Source:HGNC Symbol;Acc:HGNC:21294]	-	813	1091	1204
	1412	1715	1319	8.422356037	9.111860172
10.56518925	12.93255253	14.1569944	11.66377597
ENSG00000184313 0	0	0	17.07782977	5.483159264
	6.868508636	0	9.809832558	-5.712914616
0.004924068	1	MROH7	1	54641754	54710266
	+	9088	protein_coding	maestro heat like repeat
family member 7 [Source:HGNC Symbol;Acc:HGNC:24802]	-	0
	0	0	17	6	7	0	0	0
	0.090444429	0.02877011	0.035956363

ENSG00000164307 771.6934377 742.4878638 1058.955685
568.5912737 626.9078759 573.0298634 857.7123287
589.509671 0.541056806 0.004927343 0.036423741 ERAP1
5 96760810 96808100 - 7945
protein_coding endoplasmic reticulum aminopeptidase 1 [Source:HGNC
Symbol;Acc:HGNC:18173] - 667 801 1083 566 686
584 4.591204581 4.445008262 6.31447155
3.444480624 3.762606591 3.431349706
ENSG00000164587 11626.30788 14928.54812 14597.55255
16145.58118 17160.46078 21714.29945 13717.46952
18340.1138 -0.418916263 0.004939236 0.036494008 RPS14
5 150442635 150449756 - 5099
protein_coding ribosomal protein S14 [Source:HGNC
Symbol;Acc:HGNC:10387] - 10049 16105 14929 16072 18778
22130 107.7786022 139.2546401 135.6276266
152.4004075 160.4807408 202.601398
ENSG00000114416 4756.269449 4283.441222 4354.136347
3521.046668 3676.458287 3339.076413 4464.615673
3512.193789 0.345988843 0.004950078 0.036556446 FXR1
3 180868141 180982753 + 12054
protein_coding FMR1 autosomal homolog 1 [Source:HGNC
Symbol;Acc:HGNC:4023] - 4111 4621 4453 3505 4023
3403 18.65138137 16.90202499 17.11291384
14.05911721 14.54378491 13.17882824
ENSG00000205531 3069.419326 3078.404739 2865.927158
2298.474972 2532.30572 2135.12497 3004.583741
2321.968554 0.371679626 0.00496638 0.036643471 NAP1L4
11 2944431 2992377 - 5200 protein_coding
nucleosome assembly protein 1 like 4 [Source:HGNC
Symbol;Acc:HGNC:7640] - 2653 3321 2931 2288 2771
2176 27.90156857 28.15784993 26.1104845
21.2742321 23.22158075 19.53446778
ENSG00000169100 6650.215712 9344.594451 8576.269908
10578.20868 10593.4637 11212.34974 8190.360024
10794.67404 -0.398153209 0.004966656 0.036643471
SLC25A6 X 1386152 1392724 - 2164 protein_coding solute
carrier family 25 member 6 [Source:HGNC Symbol;Acc:HGNC:10992] -
5748 10081 8771 10530 11592 11427 145.2627347
205.390483 187.7562101 235.2731305 233.431636
246.5023176
ENSG00000284977 23.13923351 1.853902282 5.866790497 0
0 0 10.2866421 0 5.849883493
0.004977444 1 AL160272.1 9 117759455
117879988 + 17450 lincRNA novel transcript -
20 2 6 0 0 0 0.062680042
0.005053224 0.015927891 0 0 0
ENSG00000070950 1892.789301 1516.492066 1495.053778
1237.64037 1267.52365 1099.942597 1634.778382
1201.702206 0.443424164 0.004980112 0.036725024 RAD18
3 8775402 8963773 - 6983

protein_coding "RAD18, E3 ubiquitin protein ligase [Source:HGNC Symbol;Acc:HGNC:18278]" -   1636   1636   1529   1232   1387
        1121   12.81256171   10.32940285   10.14303442
8.530409548   8.655517061   7.493929557
ENSG00000167685 562.2833744   728.5835967   807.6614917
1140.196282   993.3656868   898.7934158   699.5094876
1010.785128   -0.530001382   0.004998398   0.036842097   ZNF444
        19   56132599   56160893   +   7239
protein_coding zinc finger protein 444 [Source:HGNC Symbol;Acc:HGNC:16052] zf-C2H2 486   786   826   1135   1087
        916   3.671575011   4.787159827   5.285717236
7.580860437   6.543491841   5.906945078
ENSG00000154845 2246.819574   2046.708119   2018.175931
1707.782977   1657.741818   1400.194546   2103.901208
1588.573114   0.405104816   0.005013974   0.036939091   PPP4R1
        18   9546791 9615240 -   8131   protein_coding
protein phosphatase 4 regulatory subunit 1 [Source:HGNC Symbol;Acc:HGNC:9320]   -   1942   2208   2064   1700   1814
        1427   13.0617082   11.97261634   11.75894036
10.10895299   9.721917233   8.192681055
ENSG00000170222 171.230328   167.7781565   149.6031577
239.0896168   303.4014793   267.8718368   162.8705474
270.1209777   -0.730734524   0.005026255   0.037011738   ADPRM
        17   10697594   10711233   +   1620
protein_coding "ADP-ribose/CDP-alcohol diphosphatase, manganese dependent [Source:HGNC Symbol;Acc:HGNC:30925]" -   148   181
        153   238   332   273   4.99621839   4.926035284
        4.375008207   7.103349101   8.930621161   7.86671956
ENSG00000136444 1258.774303   1591.575109   1380.651364
1747.966106   1832.289054   2154.749281   1410.333592
1911.668147   -0.438288716   0.005040264   0.037097024   RSAD1
        17   50478800   50485975   +   4198
protein_coding radical S-adenosyl methionine domain containing 1 [Source:HGNC Symbol;Acc:HGNC:25634]   -   1088   1717   1412
        1740   2005   2196   14.17363269   18.03274407
15.58097814   20.0404759   20.81280142   24.41945333
ENSG00000119333 1272.657843   1780.673142   2135.511741
1217.548805   1212.692057   1194.139287   1729.614242
1208.126717   0.518229481   0.005053779   0.037178596   WDR34
        9   128633661   128656787   -   2604
protein_coding WD repeat domain 34 [Source:HGNC Symbol;Acc:HGNC:28296] -   1100   1921   2184   1212   1327
        1217   23.10183183   32.5252275   38.85206529
22.50416257   22.20693087   21.81703027
ENSG00000183814 831.8554448   610.8608018   672.7253103
468.1334515   524.5555696   457.2464321   705.1471857
483.3118177   0.543745441   0.005065372   0.037245955   LIN9
        1   226231149   226309869   -   4442
protein_coding lin-9 DREAM MuvB core complex component [Source:HGNC Symbol;Acc:HGNC:30830] -   719   659   688   466   574

        466   8.852074267   6.540949835   7.174841968
5.072342596   5.631083093   4.897262668
ENSG00000177733 3052.064901   3846.847235   4084.263984
4714.485596   4699.06749   5036.579261   3661.058706
4816.710782   -0.39548458   0.005082615   0.037354774
HNRNPA0 5   137745651   137754376   -   8726
protein_coding heterogeneous nuclear ribonucleoprotein A0
[Source:HGNC Symbol;Acc:HGNC:5030]   -   2638   4150   4177
        4693   5142   5133   16.5331001   20.96847499
22.17439209   26.00378473   25.67884355   27.46009855
ENSG00000160714 2313.923351   2384.118334   2093.466409
3096.11008   2865.864576   2798.426662   2263.836032
2920.133772   -0.367250656   0.005089983   0.037390951   UBE2Q1
        1   154548577   154559028   -   4161
protein_coding ubiquitin conjugating enzyme E2 Q1 [Source:HGNC
Symbol;Acc:HGNC:15698] -   2000   2572   2141   3082   3136
        2852   26.28615065   27.25255385   23.83534307
35.81262334   32.84255501   31.99615863
ENSG00000114503 4114.155719   3599.35128   3101.576576
4867.181486   4827.921732   4473.361553   3605.027858
4722.82159   -0.389885868   0.00509787   0.037413623   NCBP2
        3   196935402   196942597   -   5114
protein_coding nuclear cap binding protein subunit 2 [Source:HGNC
Symbol;Acc:HGNC:7659]   -   3556   3883   3172   4845   5283
        4559   38.02732193   33.47654463   28.73259893
45.80725571   45.01720059   41.61547963
ENSG00000138468 548.3998343   419.9088668   420.4533189
279.2727457   339.0420145   282.5900696   462.9206733
300.30161   0.622779485   0.005099795   0.037413623   SENP7
        3   101324205   101513241   -   6396
protein_coding SUMO specific peptidase 7 [Source:HGNC
Symbol;Acc:HGNC:30402] -   474   453   430   278   371
        288   4.052887972   3.122652844   3.114314418
2.1015395   2.527691408   2.101987207
ENSG00000114023 1492.480562   1455.313291   1202.692052
1414.446137   2216.110203   2426.54598   1383.495302
2019.034106   -0.545611   0.005100412   0.037413623
FAM162A 3   122384176   122412334   +   3793
protein_coding family with sequence similarity 162 member A
[Source:HGNC Symbol;Acc:HGNC:17865]   -   1290   1570   1230
        1408   2425   2473   18.59951331   18.24949149
15.02189509   17.9482053   27.86040966   30.43598136
ENSG00000101574 395.6808931   279.9392445   332.4514615
189.865284   233.9481286   194.2806729   336.0238664
206.0313618   0.703858931   0.005106125   0.037437572   METTL4
        18   2537525 2571509 -   5724   protein_coding
methyltransferase like 4 [Source:HGNC Symbol;Acc:HGNC:24726]   -
        342   302   340   189   256   198   3.267542114
        2.326169065   2.751577489   1.596479681   1.948942104
        1.614773453

ENSG00000164418 135.3645161 42.63975248 73.33488121
40.18312888 20.1049173 2.943646558 83.77971658
21.07723091 1.990124801 0.005111686 0.037446974 GRIK2
6 101181257 102070083 + 7585
protein_coding glutamate ionotropic receptor kainate type subunit 2
[Source:HGNC Symbol;Acc:HGNC:4580] - 117 46 75
40 22 3 0.843577503 0.267384486
0.458044987 0.25497971 0.126393777 0.018463401
ENSG00000171951 57.84808379 63.03267758 92.89084954
6.027469332 29.24351608 28.45525006 71.25720363
21.24207849 1.743594114 0.005112307 0.037446974 SCG2
2 223596940 223602503 - 2719
protein_coding secretogranin II [Source:HGNC Symbol;Acc:HGNC:10575]
- 50 68 95 6 32 29
1.005670034 1.102639797 1.618515465 0.106694801
0.512860638 0.497891631
ENSG00000169504 15443.12445 11975.28179 12248.88076
9313.444696 11251.44281 9646.329772 13222.429
10070.40576 0.392743305 0.005120199 0.037469917 CLIC4
1 24745357 24844324 + 4495
protein_coding chloride intracellular channel 4 [Source:HGNC
Symbol;Acc:HGNC:13518] - 13348 12919 12527 9271 12312
9831 162.3982013 126.7164974 129.098096
99.72363523 119.3596423 102.097248
ENSG00000108344 4821.059303 5930.633399 6038.883018
7875.893261 6565.169359 7489.61806 5596.858573
7310.226893 -0.385082018 0.005120341 0.037469917 PSMD3
17 39980797 39997960 + 4964
protein_coding "proteasome 26S subunit, non-ATPase 3 [Source:HGNC
Symbol;Acc:HGNC:9560]" - 4167 6398 6176 7840 7184
7633 45.90779571 56.82591284 57.63389491
76.3634439 63.06568955 71.78100348
ENSG00000154065 203.6252549 203.0022998 169.159126
266.2132288 396.6151868 294.3646558 191.9288936
319.0643572 -0.73429263 0.005133766 0.037550186
ANKRD29 18 23598926 23662885 - 6391
protein_coding ankyrin repeat domain 29 [Source:HGNC
Symbol;Acc:HGNC:27110] - 176 219 173 265 434
300 1.506047131 1.510807928 1.253948619
2.004833326 2.959235373 2.191283018
ENSG00000135541 949.8655358 687.7977465 563.2118877
541.4676617 429.5141424 459.2088631 733.6250567
476.7302224 0.621106235 0.005140375 0.037580538 AHI1
6 135283532 135497776 - 8705
protein_coding Abelson helper integration site 1 [Source:HGNC
Symbol;Acc:HGNC:21575] - 821 742 576 539 470
468 5.15785459 3.758106539 3.065181275
2.993789123 2.352814458 2.509707529
ENSG00000134014 1812.958946 1794.577409 1581.100039
2228.154496 2434.522713 2128.256462 1729.545464

2263.644557	-0.388471359	0.005162476	0.037724077	ELP3
	8	28089673	28191156	+	5390
protein_coding	elongator acetyltransferase complex subunit 3
[Source:HGNC Symbol;Acc:HGNC:20696]	-	1567	1936	1617
	2218	2664	2169	15.89918798	15.83618424
13.89708489	19.89637662	21.53793456	18.78524323
ENSG00000132182 6.941770054	7.415609127	13.68917783	0
	0	0	9.348852336	0	5.718573539
0.005182059	1	NUP210	3	13316235	13420309
	-	8182	protein_coding	nucleoporin 210 [Source:HGNC
Symbol;Acc:HGNC:30052]	-	6	8	14	0	0
	0	0.040103889	0.043108655	0.079263093	0
	0	0
ENSG00000059758 1340.918582	1281.973428	891.7521555
1641.480815	1840.513793	1501.259745	1171.548055
1661.084784	-0.504169137	0.005186196	0.037877981	CDK17
	12	96278261	96400560	-	6547
protein_coding	cyclin dependent kinase 17 [Source:HGNC
Symbol;Acc:HGNC:8750]	-	1159	1383	912	1634	2014
	1530	9.681347476	9.313518834	6.452900271
12.06732273	13.40527486	10.90925581
ENSG00000213707 37.02277362	64.88657986	84.09066379
9.041203998	26.50193645	19.62431039	62.00000576
18.38915028	1.751969991	0.00518962	0.037877981
HMGB1P10	22	26560526	26561088	+	563
	processed_pseudogene	high mobility group box 1 pseudogene
10 [Source:HGNC Symbol;Acc:HGNC:4994]	-	32	70	86
	9	29	20	3.108395676	5.481805258
7.076076382	0.772921399	2.244647768	1.65831732
ENSG00000108578 2346.318278	2231.171396	2094.444207
1666.59527	1862.44643	1552.282952	2223.977961
1693.774884	0.392611708	0.005190971	0.037877981	BLMH
	17	30248195	30292056	-	5127
protein_coding	bleomycin hydrolase [Source:HGNC Symbol;Acc:HGNC:1059]
	-	2028	2407	2142	1659	2038	1582
21.63213308	20.69887447	19.35345936	15.64531405
17.32205687	14.40420181
ENSG00000187257 1409.179321	1119.756978	1365.006589
910.1478692	1049.111139	864.4508727	1297.980963
941.236627	0.463000865	0.005198366	0.037911324	RSBN1L
	7	77696443	77783022	+	7206
protein_coding	round spermatid basic protein 1 like [Source:HGNC
Symbol;Acc:HGNC:24765]	-	1218	1208	1396	906	1148
	881	9.243740463	7.391058411	8.974156033
6.079042174	6.942345573	5.707260378
ENSG00000111412 1384.883126	1305.147206	1206.603246
1668.604427	2053.443145	1564.057538	1298.877859
1762.035036	-0.44040482	0.0052005	0.037911324
C12orf49	12	116710185	116738070	-	8664
	protein_coding	chromosome 12 open reading frame 49

[Source:HGNC Symbol;Acc:HGNC:26128]	-	1197	1408	1234
	1661	2247	1594	7.555625428	7.165032574
6.597800384	9.269416686	11.30168573	8.588472102
ENSG00000151611 232.5492968	279.9392445	156.4477466
312.423827	436.8250214	383.6552681	222.9787626
377.6347055	-0.760222036	0.00520425	0.037920576	MMAA
	4	145599042	145660035	+	8882
protein_coding	methylmalonic aciduria (cobalamin deficiency) cblA
type [Source:HGNC Symbol;Acc:HGNC:18871]	-	201	302
	160	311	478	391	1.237599147	1.499098371
	0.834471809	1.692976137	2.345177536	2.055000938
ENSG00000134871 3996.145628	4411.360479	11638.73455
2607.885064	1436.587727	2385.334928	6682.080218
2143.26924	1.640521877	0.005235149	0.038127548	COL4A2
	13	110305812	110513209	+	19293
protein_coding	collagen type IV alpha 2 chain [Source:HGNC
Symbol;Acc:HGNC:2203]	-	3454	4759	11903	2596	1572
	2431	9.790779766	10.87551667	28.57979604
6.505881375	3.550678702	5.88208411
ENSG00000164011 312.3796524	251.2037592	226.8492325
582.6553688	350.0083331	375.805544	263.4775481
436.1564153	-0.727980096	0.005240063	0.038145163	ZNF691
	1	42846573	42852477	+	2036
protein_coding	zinc finger protein 691 [Source:HGNC
Symbol;Acc:HGNC:28028]	zf-C2H2 270	271	232	580	383
	383	7.252382533	5.868477924	5.278526024
13.77372591	8.197465509	8.781466242
ENSG00000181163 21006.95315	18364.756	19428.85453
14478.98591	16816.84946	15322.66155	19600.18789
15539.49898	0.334868139	0.005260587	0.038276341	NPM1
	5	171387116	171411137	+	3828
protein_coding	nucleophosmin 1 [Source:HGNC Symbol;Acc:HGNC:7910]
	-	18157	19812	19870	14413	18402	15616
259.3981517	228.1867108	240.452 182.0470618	209.4844038
	190.4337445
ENSG00000164484 622.4453815	484.7954467	264.9833708
559.5500697	983.3132281	817.3525277	457.4080663
786.7386085	-0.783784421	0.005263799	0.038281493
TMEM200A	6	130365734	130443063	+	6137
	protein_coding	transmembrane protein 200A [Source:HGNC
Symbol;Acc:HGNC:21075]	-	538	523	271	557	1076
	833	4.794252078	3.757331587	2.045576018
4.388340201	7.640376248	6.336288075
ENSG00000121068 260.316377	403.2237463	753.8825789
300.3688884	212.0154916	245.3038799	472.4742341
252.5627533	0.90495711	0.005295108	0.038490877	TBX2
	17	61399896	61409466	+	6029
protein_coding	T-box 2 [Source:HGNC Symbol;Acc:HGNC:11597]	T-box
	225	435	771	299	232	250	2.040948034
	3.181104464	5.92395226	2.397878209	1.67687735

1.935712081
ENSG00000172301 1685.693162 1860.39094 2069.021449
1314.992893 1479.539142 1435.518305 1871.70185
1410.01678 0.408821259 0.005303181 0.038531242 COPRS
17 31851864 31859337 - 1432
protein_coding coordinator of PRMT5 and differentiation stimulator
[Source:HGNC Symbol;Acc:HGNC:28848] - 1457 2007 2116
1309 1619 1463 55.64309091 61.79286229
68.45025986 44.19751431 49.26771591 47.69219862
ENSG00000137154 21083.31262 24183.22831 22234.15819
23825.58169 31772.16636 33463.37408 22500.23304
29687.04071 -0.399881967 0.005309089 0.038555843 RPS6
9 19375715 19380254 - 2714
protein_coding ribosomal protein S6 [Source:HGNC
Symbol;Acc:HGNC:10429] - 18223 26089 22739 23717 34767
34104 367.2017519 423.8200979 388.1181692
422.5237511 558.2335986 586.5992619
ENSG00000254064 52.06327541 35.22414335 70.40148596
166.7599849 89.55826799 110.8773537 52.56296824
122.3985355 -1.219659085 0.005313314 0.038568203
AC105206.2 8 22254576 22275162 - 515
antisense uncharacterized LOC100507071 [Source:NCBI
gene;Acc:100507071] - 45 38 72 166 98
113 4.778592504 3.253196718 6.476311407
15.58483022 8.292346564 10.24276598
ENSG00000198816 511.3770607 1001.107232 1065.800274
1292.892172 1293.111727 1426.687365 859.4281888
1337.563755 -0.636953235 0.005322607 0.038617319 ZNF358
19 7515292 7521026 + 2069 protein_coding zinc
finger protein 358 [Source:HGNC Symbol;Acc:HGNC:16838] zf-C2H2 442
1080 1090 1287 1415 1454 11.68305689
23.014271 24.40441845 30.07594436 29.80262697
32.80574854
ENSG00000183935 74.04554725 75.08304241 136.8917783
218.9980524 180.9442557 165.8254228 95.34012264
188.5892436 -0.982341827 0.005341497 0.038735988 HTR7P1
12 13000420 13004830 + 4411
transcribed_processed_pseudogene 5-hydroxytryptamine receptor 7
pseudogene 1 [Source:HGNC Symbol;Acc:HGNC:30411] - 64
81 140 218 198 169 0.793483004
0.809621288 1.470257602 2.389574925 1.95608053
1.788527749
ENSG00000235944 141.1493244 188.1710816 186.7594975
322.4696093 254.9669058 282.5900696 172.0266345
286.6755282 -0.734441564 0.005362833 0.038872274
ZNF815P 7 5823160 5854365 + 2302
transcribed_unprocessed_pseudogene "zinc finger protein 815,
pseudogene [Source:HGNC Symbol;Acc:HGNC:22029]" - 122 203
191 321 279 288 2.89833929 3.887985858
3.843532022 6.742189107 5.281502403 5.840273751

ENSG00000278384 245.2758753 169.6320588 210.2266595
279.2727457 280.5549824 581.860803 208.3781978
380.5628437 -0.870046432 0.005367779 0.038881649
AL354822.1 GL000218.1 51867 54893 - 3027
protein_coding - 212 183 215 278 307
593 3.830170903 2.665462806 3.290246947
4.440517556 4.419616154 9.14509683
ENSG00000153214 767.065591 621.9842155 675.6587056
451.0556217 486.1734548 509.2508546 688.2361707
482.159977 0.512575161 0.005369213 0.038881649
TMEM87B 2 112055223 112119318 + 5367
protein_coding transmembrane protein 87B [Source:HGNC
Symbol;Acc:HGNC:25913] - 663 671 691 449 532
519 6.755797849 5.512198935 5.964154811
4.044976121 4.319551286 4.514210415
ENSG00000164970 594.6783013 696.1403068 673.7031087
1074.898698 915.6875972 794.7845708 654.8405723
928.4569552 -0.502984524 0.005379162 0.038935252
FAM219A 9 34398184 34458570 - 3782
protein_coding family with sequence similarity 219 member A
[Source:HGNC Symbol;Acc:HGNC:19920] - 514 751 689
1070 1002 810 7.432523777 8.7549238
8.439178117 13.67928726 11.54528863 9.997917073
ENSG00000171051 18.51138681 68.59438442 20.53376674
5.02289111 10.05245865 0 35.87984599 5.025116587
2.830812761 0.00539691 0.039036806 FPR1 19
51745172 51804110 - 2594 protein_coding
formyl peptide receptor 1 [Source:HGNC Symbol;Acc:HGNC:3826]
- 16 74 21 5 11 0
0.337322044 1.257753996 0.375017711 0.093196853
0.184791211 0
ENSG00000213693 54.37719876 132.5540131 72.3570828
183.8378146 168.1502174 186.4309487 86.42943157
179.4729936 -1.049154236 0.0053983 0.039036806
SEC14L1P1 11 43897456 43899636 + 2181
processed_pseudogene SEC14 like 1 pseudogene 1 [Source:HGNC
Symbol;Acc:HGNC:44189] - 47 143 74 183 184
190 1.178519859 2.890775264 1.571732053
4.056921839 3.67638305 4.066717187
ENSG00000136807 1034.323738 1154.981121 1046.244305
1349.148552 1569.09741 1401.175762 1078.516388
1439.807241 -0.416685511 0.005401007 0.03903792 CDK9
9 127785679 127790787 + 3000
protein_coding cyclin dependent kinase 9 [Source:HGNC
Symbol;Acc:HGNC:1780] - 894 1246 1070 1343 1717
1428 16.29712426 18.31178774 16.52208982
21.64491947 24.94064256 22.2204571
ENSG00000254272 13.88354011 9.269511408 4.888992081 0
0 0 9.347347866 0 5.715325467
0.005401473 1 AC087854.1 8 22492631

22493044	+	414	processed_pseudogene	basic
transcription factor 3-like (BTF3L) pseudogene -	12	10
	5	0	0	0	1.585169172	1.064960787
	0.559463965	0	0	0
ENSG00000102870 490.5517505	597.8834858	792.0167171
846.8594412	912.9460175	1020.46414	626.8173178
926.756533	-0.563228631	0.005409167	0.039076658	ZNF629
	16	30778449	30787202	-	6087
protein_coding	zinc finger protein 629 [Source:HGNC
Symbol;Acc:HGNC:29008]	zf-C2H2 424	645	810	843	999
	1040	3.809406053	4.671865926	6.164305769
6.696154867	7.151889309	7.975833392
ENSG00000155329 410.7213949	383.7577723	302.1397106
169.7737195	275.0718231	215.8674143	365.5396259
220.2376523	0.72948123	0.005411479	0.039076658
ZCCHC10 5	132996985	133026604	-	2559
protein_coding	zinc finger CCHC-type containing 10 [Source:HGNC
Symbol;Acc:HGNC:25954]	-	355	414	309	169	301
	220	7.586697707	7.132865144	5.593590246
3.193137611	5.125719161	4.013270481
ENSG00000164237 212.8809483	199.2944953	166.2257307
322.4696093	329.9034157	279.646423	192.8003915
310.6731494	-0.68932803	0.00541959	0.03911675	CMBL
	5	10275875	10308026	-	3324
protein_coding	carboxymethylenebutenolidase homolog [Source:HGNC
Symbol;Acc:HGNC:25090]	-	184	215	170	321	361
	285	3.027272534	2.851749688	2.369138018
4.66922964	4.732654325	4.002486546
ENSG00000083817 195.5265232	189.0980327	164.2701339
240.0941951	380.165709	300.251949	182.9648966
306.8372843	-0.747076876	0.005428964	0.039165912	ZNF416
	19	57571566	57578927	-	2561
protein_coding	zinc finger protein 416 [Source:HGNC
Symbol;Acc:HGNC:20645]	zf-C2H2 169	204	168	239	416
	306	3.608874993	3.512000321	3.038800289
4.512212445	7.078518149	5.577735089
ENSG00000243789 40.49365865	5.561706845	4.888992081	0
	0	1.962431039	16.98145253	0.65414368
4.704601617	0.005434743	1	JMJD7	15	41828085
	41837581	+	1971	protein_coding	jumonji domain
containing 7 [Source:HGNC Symbol;Acc:HGNC:34397]	-	35
	6	5	0	0	2	0.971127232
0.134214236	0.117512979	0	0	0.047368475
ENSG00000233429 115.6961676	62.10572644	122.224802
20.09156444	36.5543951	55.92928461	100.0088987
37.52508138	1.412324614	0.005444719	0.039261044
HOTAIRM1	7	27095647	27100265	+	1496
	antisense	"HOXA transcript antisense RNA, myeloid-
specific 1 [Source:HGNC Symbol;Acc:HGNC:37117]" -	100	67
	125	20	40	57	3.655637462	1.974591063

3.870623021	0.646397426	1.165163833	1.778645091
ENSG00000081026 726.5719323	561.7323914	710.8594485
386.7626155	495.3120536	475.8895269	666.3879241
452.654732	0.556988512	0.005455519	0.039320379	MAGI3
1	113390749	113685923	+	8180
protein_coding	"membrane associated guanylate kinase, WW and PDZ
domain containing 3 [Source:HGNC Symbol;Acc:HGNC:29647]"	-
628	606	727	385	542	485	4.19856666
3.266278998	4.117025557	2.275666636	2.887384233
2.767798507
ENSG00000261008 37.02277362	36.15109449	43.02313031
9.041203998	10.96631853	9.812155195	38.73233281
9.939892574	1.961288359	0.005465044	0.039370468
LINC01572	16	72283301	72665009	-	4752
lincRNA long intergenic non-protein coding RNA 1572
[Source:HGNC Symbol;Acc:HGNC:51385]	-	32	39	44
9	12	10	0.368271626	0.361844631
0.428922373	0.091572969	0.110043251	0.098235759
ENSG00000035928 2060.548744	2002.214464	1909.640307
1591.251904	1509.696517	1521.865271	1990.801172
1540.937897	0.369478745	0.005471428	0.039397886	RFC1
4	39287456	39366375	-	6719
protein_coding	replication factor C subunit 1 [Source:HGNC
Symbol;Acc:HGNC:9969]	-	1781	2160	1953	1584	1652
1551	14.49619396	14.17369451	13.46480468
11.39860627	10.71430487	10.77589107
ENSG00000111276 550.7137576	659.9892123	679.5698992
412.8816493	480.6902955	410.1480871	630.0909564
434.573344	0.536335142	0.00547591	0.039411599	CDKN1B
12	12715058	12722371	+	2811
protein_coding	cyclin dependent kinase inhibitor 1B [Source:HGNC
Symbol;Acc:HGNC:1785]	-	476	712	695	411	526
418	9.260636123	11.16742658	11.45318867
7.069394094	8.154240124	6.94163017
ENSG00000090863 3091.401598	2320.158706	4143.909688
2487.335678	1689.726913	2302.912824	3185.156664
2159.991805	0.560431989	0.005478568	0.03941217	GLG1
16	74447427	74607144	-	14111
protein_coding	golgi glycoprotein 1 [Source:HGNC
Symbol;Acc:HGNC:4316]	-	2672	2503	4238	2476	1849
2347	10.35555488	7.820544935	13.91251406
8.483871169	5.710021152	7.764282589
ENSG00000205726 1153.490791	1178.1549	1216.38123
934.2577465	867.2530237	860.5260106	1182.67564
887.3455936	0.414685898	0.005481373	0.039413808	ITSN1
21	33642400	33899861	+	21748
protein_coding	intersectin 1 [Source:HGNC Symbol;Acc:HGNC:6183]
-	997	1271	1244	930	949	877
2.507093591	2.576678206	2.649741525	2.067591989
1.901540089	1.882462376

ENSG00000259207 109.9113592 88.06035838 129.0693909
45.20601999 31.98509571 63.77900877 109.0137028
46.99004149 1.215184922 0.005484672 0.039418982 ITGB3
17 47253846 47311816 + 4394
protein_coding integrin subunit beta 3 [Source:HGNC
Symbol;Acc:HGNC:6156] - 95 95 132 45 35
65 1.182383241 0.95322958 1.391606134
0.495169262 0.347109572 0.690556695
ENSG00000101773 2103.356326 1608.260229 1538.076909
1142.205438 1414.65509 1233.387908 1749.897821
1263.416146 0.469211432 0.00550803 0.039551541 RBBP8
18 22798261 23026488 + 5091
protein_coding "RB binding protein 8, endonuclease [Source:HGNC
Symbol;Acc:HGNC:9891]" - 1818 1735 1573 1137 1548
1257 19.52924683 15.02554869 14.31291465
10.79837945 13.25032275 11.52598942
ENSG00000141551 1970.305734 2901.357071 2942.195434
3843.516277 3195.767991 3764.923948 2604.619413
3601.402739 -0.466981109 0.00550829 0.039551541 CSNK1D
17 82239023 82273731 - 11024
protein_coding casein kinase 1 delta [Source:HGNC
Symbol;Acc:HGNC:2452] - 1703 3130 3009 3826 3497
3837 8.448316123 12.51811944 12.64402771
16.78058039 13.82340479 16.24795211
ENSG00000133639 1874.277915 1616.60279 1131.312767
2253.268952 2421.728675 1928.088496 1540.731157
2201.028708 -0.51507077 0.005522128 0.039632285 BTG1
12 92140278 92145897 - 4854
protein_coding BTG anti-proliferation factor 1 [Source:HGNC
Symbol;Acc:HGNC:1130] - 1620 1744 1157 2243 2650
1965 18.25197878 15.8409297 11.04170253
22.34244606 23.79056191 18.89769427
ENSG00000014164 468.5694787 830.5482222 780.2831361
942.2943723 1022.609203 1191.195641 693.1336123
1052.033072 -0.600599346 0.005524938 0.03963385 ZC3H3
8 143437655 143541453 - 3731
protein_coding zinc finger CCCH-type containing 3 [Source:HGNC
Symbol;Acc:HGNC:28972] - 405 896 798 938 1119
1214 5.936418187 10.58806792 9.907865403
12.15566732 13.06963428 15.18935994
ENSG00000075275 657.1542318 627.5459224 1050.155499
530.4173012 429.5141424 548.4994754 778.2852177
502.8103063 0.630866441 0.005542058 0.03973801 CELSR1
22 46360834 46537170 - 12806
protein_coding cadherin EGF LAG seven-pass G-type receptor 1
[Source:HGNC Symbol;Acc:HGNC:1850] - 568 677 1074
528 470 559 2.425657902 2.33082211
3.885019828 1.993524794 1.599347951 2.037719241
ENSG00000115568 736.9845874 859.2837076 883.9297682
1359.194334 986.0548077 1138.210003 826.7326877

1161.153048	-0.489252721	0.005546105	0.039748389	ZNF142
	2	218637916	218659655	-	6994
protein_coding	zinc finger protein 142 [Source:HGNC
Symbol;Acc:HGNC:12927]	zf-C2H2 637	927	904	1353	1079
	1160	4.980907965	5.843702043	5.987496304
9.353483514	6.722865372	7.74244978
ENSG00000101298 122.6379376	138.11572	136.8917783
269.2269635	213.8432113	203.1116125	132.5484786
228.7272624	-0.785852673	0.005556408	0.039803566	SNPH
	20	1266280 1309328 +	5709	protein_coding
syntaphilin [Source:HGNC Symbol;Acc:HGNC:15931] -	106	149
	140	268	234	207	1.015407893	1.150694887
	1.135979381	2.269739248	1.786135541	1.692607802
ENSG00000131791 973.0047693	720.2410364	697.1702707
561.5592261	551.0575061	542.6121823	796.8053588
551.7429715	0.529217884	0.005573945	0.039910494	PRKAB2
	1	147155106	147172550	-	5460
protein_coding	protein kinase AMP-activated non-catalytic subunit
beta 2 [Source:HGNC Symbol;Acc:HGNC:9379]	-	841	777
	713	559	603	553	8.423606399	6.274257436
	6.049219493	4.950173053	4.81263879	4.728011502
ENSG00000141456 2091.78671	2733.578914	2544.231479
3893.745189	2849.415098	3268.428895	2456.532368
3337.196394	-0.441560653	0.005587782	0.039990838	PELP1
	17	4669774 4704337 -	6577
protein_coding	"proline, glutamate and leucine rich protein 1
[Source:HGNC Symbol;Acc:HGNC:30134]"	-	1808	2949	2602
	3876	3118	3331	15.03367983	19.76882645
18.32660022	28.49424427	20.65888446	23.64246891
ENSG00000006062 521.7897157	977.0065025	1260.382158
691.1498168	424.0309831	517.1005788	919.7261255
544.0937929	0.758477069	0.00559769	0.040043
MAP3K14 17	45263119	45317145	-	5784
protein_coding	mitogen-activated protein kinase kinase kinase 14
[Source:HGNC Symbol;Acc:HGNC:6853]	-	451	1054	1289
	688	464	527	4.264253065	8.034267447
10.32350301	5.751238402	3.495813813	4.253323022
ENSG00000181544 268.4151088	228.0299806	243.4718056
165.7554066	118.8017841	150.1259745	246.638965
144.8943884	0.767603163	0.005612211	0.040128099	FANCB
	X	14835961	14873069	-	3593
protein_coding	FA complementation group B [Source:HGNC
Symbol;Acc:HGNC:3583]	-	232	246	249	165	130
	153	3.531225731	3.018643651	3.210292363
2.220383255	1.576684263	1.987834618
ENSG00000154473 4330.507552	3864.459306	4031.46287
3022.77587	3152.816577	3419.536085	4075.476576
3198.376178	0.349482358	0.005621312	0.040174377	BUB3
	10	123154277	123170467	+	8300
protein_coding	"BUB3, mitotic checkpoint protein [Source:HGNC

Symbol;Acc:HGNC:1151]" -   3743   4169   4123   3009   3450
        3485   24.66246304   22.14561578   23.01111687
17.52852256   18.11338426   19.60066138
ENSG00000144560 1367.528701   1501.660848   1477.453407
1774.08514   2072.634202   1854.497332   1448.880985
1900.405558   -0.391231947   0.005633272   0.040241043   VGLL4
        3   11556070   11771350   -   7236
protein_coding vestigial like family member 4 [Source:HGNC
Symbol;Acc:HGNC:28966] -   1182   1620   1511   1766   2268
        1890   8.933335221   9.87075595   9.673159791
11.8003084   13.6585026   12.19296373
ENSG00000175471 575.0099528   538.5586128   564.1896861
450.0510435   369.1993905   254.1348195   559.2527506
357.7950845   0.644343496   0.0056394   0.040265999   MCTP1
        5   94703741   95284575   -   12089
protein_coding multiple C2 and transmembrane domain containing 1
[Source:HGNC Symbol;Acc:HGNC:26183]   -   497   581   577
        448   404   259   2.248333461   2.11894514
2.210995667   1.791797197   1.456295761   1.000127623
ENSG00000139291 671.0377719   561.7323914   644.3691563
416.8999621   486.1734548   390.5237767   625.7131065
431.1990646   0.536243162   0.005651365   0.040332594   TMEM19
        12   71686087   71705046   +   6488
protein_coding transmembrane protein 19 [Source:HGNC
Symbol;Acc:HGNC:25605] -   580   606   659   415   532
        398   4.888908004   4.118089119   4.705188525
3.092704826   3.573216978   2.863638988
ENSG00000115963 3333.206588   3748.590414   1577.188845
5348.374454   8052.01938   5156.287555   2886.328616
6185.560463   -1.099703867   0.005665433   0.040414128   RND3
        2   150468195   150539011   -   3594
protein_coding Rho family GTPase 3 [Source:HGNC Symbol;Acc:HGNC:671]
        -       2881   4044   1613   5324   8811   5255
43.83892523   49.60974927   20.79020398   71.62443193
106.8330743   68.25597637
ENSG00000268902 3.470885027   0   9.777984162   19.08698622
        10.05245865   79.47845708   4.41628973   36.20596732
        -3.036344894   0.005671599   0.04043924   CSAG2   X
        152708261   152714549   -   1083   protein_coding
        CSAG family member 2 [Source:HGNC Symbol;Acc:HGNC:16847]
        -       3   0   10   19   11   81
0.151491237   0   0.427734223   0.848254868   0.442611635
        3.491424042
ENSG00000144597 1014.65539   1167.958437   920.1083096
1432.528545   1560.872671   1254.974649   1034.240712
1416.125288   -0.453255974   0.005681052   0.040487758   EAF1
        3   15427355   15450635   +   5152
protein_coding ELL associated factor 1 [Source:HGNC
Symbol;Acc:HGNC:20907] -   877   1260   941   1426   1708
        1279   9.309330561   10.78272797   8.46089343

13.38273529	14.44676505	11.58886026
ENSG00000172339 233.7062585	183.5363259	264.0055724
110.5036044	159.9254785	105.9712761	227.0827189
125.4667864	0.854278425	0.005712682	0.040694209	ALG14
	1	94974407	95072945	-	9633
protein_coding	"ALG14, UDP-N-acetylglucosaminyltransferase subunit
[Source:HGNC Symbol;Acc:HGNC:28287]"	-	202	198	270
	110	175	108	1.146791649	0.906228232
1.298388498	0.552118553	0.791653409	0.523369284
ENSG00000167895 5.784808379	5.561706845	16.62257307	0
	0	0	9.323029433	0	5.714838671
0.005730769	1	TMC8	17	78130770	78142968
	+	5459	protein_coding	transmembrane channel like 8
[Source:HGNC Symbol;Acc:HGNC:20474]	-	5	6	17
	0	0	0	0.050090068	0.04845874
0.144257461	0	0	0
ENSG00000103599 109.9113592	95.47596751	87.02405904
196.8973315	131.5958223	228.623216	97.47046191
185.7054566	-0.930674042	0.005747863	0.0408929	IQCH
	15	67254800	67502260	+	7108
protein_coding	IQ motif containing H [Source:HGNC
Symbol;Acc:HGNC:25721]	-	95	103	89	196	144
	233	0.730921773	0.638886577	0.58002277
1.333244709	0.882823064	1.530222339
ENSG00000249839 24.29619519	28.73548537	15.64477466
1.004578222	1.827719755	5.887293117	22.89215174
2.906530365	2.97928433	0.005748245	0.0408929
AC011330.1	15	43663654	43684339	-	3235
	unprocessed_pseudogene	histidine acid phosphatase domain
containing 2A (HISPPD2A) pseudogene	-	21	31	16
	1	2	6	0.355009294	0.422494799
0.229112167	0.014946067	0.026941037	0.08658108
ENSG00000072682 389.8960847	291.9896094	483.0324176
265.2086506	163.5809181	256.0972506	388.3060372
228.2956064	0.766843966	0.0057486	0.0408929	P4HA2
	5	132191838	132295315	-	8502
protein_coding	prolyl 4-hydroxylase subunit alpha 2 [Source:HGNC
Symbol;Acc:HGNC:8547]	-	337	315	494	264	179
	261	2.167721639	1.633516069	2.691586269
1.50135724	0.91746716	1.433063526
ENSG00000123119 252.2176453	195.5866907	256.183185
123.5631213	125.1988032	157.9756986	234.662507
135.5792077	0.790857434	0.005757131	0.040930483	NECAB1
	8	90791550	90959408	+	6408
protein_coding	N-terminal EF-hand calcium binding protein 1
[Source:HGNC Symbol;Acc:HGNC:20983]	-	218	211	262
	123	137	161	1.86049584	1.451756938
1.894005536	0.928076604	0.93165831	1.172868733
ENSG00000236104 253.374607	410.6393554	496.7215954
500.2799546	685.3949081	703.5315275	386.9118526

629.7354634	-0.70121416	0.005759238	0.040930483	ZBTB22
	6	33314406	33317942	-	2728
protein_coding	zinc finger and BTB domain containing 22 [Source:HGNC
Symbol;Acc:HGNC:13085]	-	219	443	508	498	750
	717	4.390302668	7.159675156	8.626245266
8.826452599	11.98051522	12.26932938
ENSG00000176396 657.1542318	789.762372	650.2359467
916.1753385	1116.73677	905.6619245	699.0508502
979.5246777	-0.486400624	0.005774285	0.041009034	EID2
	19	39538705	39540333	-	1629
protein_coding	EP300 interacting inhibitor of differentiation 2
[Source:HGNC Symbol;Acc:HGNC:28292]	-	568	852	665
	912	1222	923	19.06873855	23.05963711
18.91050021	27.06917193	32.68953323	26.45005945
ENSG00000172572 183.9569064	64.88657986	115.3802131
63.28842799	52.09001301	22.56795695	121.4078998
45.98213265	1.398129219	0.00577776	0.041009034	PDE3A
	12	20369245	20684381	+	8398
protein_coding	phosphodiesterase 3A [Source:HGNC
Symbol;Acc:HGNC:8778]	-	159	70	118	63	57
	23	1.035418611	0.367498971	0.650891489
0.362715317	0.295772358	0.127849196
ENSG00000143167 10.41265508	25.95463194	35.20074298
6.027469332	0	0	23.85601	2.009156444
3.59112016	0.005778338	0.041009034	GPA33	1
167052836	167166479	-	3200	protein_coding
glycoprotein A33 [Source:HGNC Symbol;Acc:HGNC:4445]	-	9
	28	36	6	0	0	0.153810946
0.385782045	0.521140684	0.090657239	0	0
ENSG00000099377 264.9442237	185.3902282	116.3580115
315.4375617	341.7835942	345.3878629	188.8974878
334.2030062	-0.825999283	0.005789535	0.041069432	HSD3B7
	16	30985207	30989152	+	2495
protein_coding	"hydroxy-delta-5-steroid dehydrogenase, 3 beta- and
steroid delta-isomerase 7 [Source:HGNC Symbol;Acc:HGNC:18324]"	-
	229	200	119	314	374	352	5.019490598
	3.534218563	2.209422982	6.084996246	6.532202657
	6.585945755
ENSG00000138698 1293.483153	903.7773623	931.8418906
701.195599	792.3165137	692.7381567	1043.034135
728.7500898	0.516200329	0.005825207	0.041303311
RAP1GDS1	4	98261384	98443861	+	5424
	protein_coding	Rap1 GTPase-GDP dissociation stimulator 1
[Source:HGNC Symbol;Acc:HGNC:9859]	-	1118	975	953
	698	867	706	11.27241153	7.925358069
8.139086716	6.222099592	6.965591706	6.076185948
ENSG00000108953 16637.1089	17305.25085	18537.10237
12380.42201	15426.86859	13456.38963	17493.15404
13754.56008	0.346867894	0.005838319	0.041377089	YWHAE
	17	1344272 1400378 -	3847	protein_coding

tyrosine 3-monooxygenase/tryptophan 5-monooxygenase activation protein epsilon [Source:HGNC Symbol;Acc:HGNC:12851]	-	14380	18669
	18958	12324	16881	13714	204.423779	213.9601173
	228.2825885	154.8926178	191.2205581	166.4132854
ENSG00000085063 4752.798564	4717.254356	3484.873555
4814.943418	7867.419685	5666.519625	4318.308825
6116.294243	-0.502333173	0.005848915	0.041432975	CD59
	11	33703010	33736491	-	10358
protein_coding	CD59 molecule (CD59 blood group) [Source:HGNC Symbol;Acc:HGNC:1689]	-	4108	5089	3564	4793	8609
	5775	21.68948504	21.66159851	15.93911648
22.37343872	36.21891189	26.02688048
ENSG00000234616 953.3364208	1044.673936	1309.272079
1526.958897	1395.464033	1624.8929	1102.427479
1515.771944	-0.458816334	0.005872038	0.041577512	JRK
	8	142657460	142681968	-	11229
protein_coding	Jrk helix-turn-helix protein [Source:HGNC Symbol;Acc:HGNC:6199]	-	824	1127	1339	1520	1527
	1656	4.013107954	4.425035836	5.523850943
6.544910659	5.92593049	6.884387168
ENSG00000260804 623.6023432	536.7047106	694.2368755
384.753459	476.1209961	405.2420095	618.1813097
422.0388216	0.550006455	0.005880209	0.04161609
LINC01963	2	216217045	216220192	+	3148
	lincRNA long intergenic non-protein coding RNA 1963
[Source:HGNC Symbol;Acc:HGNC:25283]	-	539	579	710
	383	521	413	9.363727235	8.10919601
10.4478296	5.882545117	7.212097634	6.124369201
ENSG00000127990 1152.333829	979.7873559	1012.999159
701.195599	868.1668835	697.6442343	1048.373448
755.6689056	0.471515506	0.005891376	0.041654229	SGCE
	7	94524204	94656572	-	20884
protein_coding	sarcoglycan epsilon [Source:HGNC Symbol;Acc:HGNC:10808]	-	996	1057	1036	698	950
	711	2.608196853	2.231491622	2.297992075
1.616005947	1.982296063	1.589285613
ENSG00000067141 1084.07309	1031.69662	1370.873379
720.2825852	818.8184502	935.0983901	1162.214363
824.7331418	0.494927372	0.005892255	0.041654229	NEO1
	15	73051710	73305206	+	8117
protein_coding	neogenin 1 [Source:HGNC Symbol;Acc:HGNC:7754]	-
	937	1113	1402	717	896	953	6.313043148
	6.045518804	8.001196263	4.270953334	4.810287804
	5.480792882
ENSG00000143819 575.0099528	487.5763001	711.837247
391.7855066	368.2855306	427.8099665	591.4745
395.9603346	0.579024451	0.005893772	0.041654229	EPHX1
	1	225810092	225845563	+	2508
protein_coding	epoxide hydrolase 1 [Source:HGNC Symbol;Acc:HGNC:3401]
	-	497	526	728	390	403	436

10.83736173	9.246815024	13.44640856	7.518622696
7.002225807	8.115307733
ENSG00000112996 1734.285552	1778.819239	1313.183273
2149.797395	2247.181439	2093.913919	1608.762688
2163.630917	-0.42768422	0.005907213	0.04172993	MRPS30
5	44808925	44820428	+	6202
protein_coding	mitochondrial ribosomal protein S30 [Source:HGNC
Symbol;Acc:HGNC:8769]	-	1499	1919	1343	2140	2459
2134	13.21796458	13.64197253	10.03105719
16.68334873	17.27767755	16.06233536
ENSG00000149084 1778.250096	1485.902679	1554.699482
1140.196282	1321.441383	1142.134865	1606.284085
1201.25751	0.418577482	0.005916112	0.041773487
HSD17B12	11	43680558	43856617	+	7838
protein_coding	hydroxysteroid 17-beta dehydrogenase 12
[Source:HGNC Symbol;Acc:HGNC:18646]	-	1537	1603	1590
1135	1446	1164	10.7241609	9.017003146
9.397110224	7.0015117	8.039362867	6.932561916
ENSG00000172115 4151.178492	4342.766095	4036.351862
3485.88643	3240.547125	3219.368119	4176.765483
3315.267225	0.333332173	0.005933023	0.041873556	CYCS
7	25120091	25125361	-	4457
protein_coding	"cytochrome c, somatic [Source:HGNC
Symbol;Acc:HGNC:19986]" -	3588	4685	4128	3470	3546
3281	44.0255219	46.34479005	42.90417055
37.64333193	34.67006812	34.36446857
ENSG00000171453 963.7490759	1078.971128	899.5745429
1321.020362	1355.254198	1258.899511	980.7649156
1311.724691	-0.419293049	0.005952187	0.041989424	POLR1C
6	43509702	43562419	+	5550
protein_coding	RNA polymerase I and III subunit C [Source:HGNC
Symbol;Acc:HGNC:20194]	-	833	1164	920	1315	1483
1283	8.208177342	9.246853034	7.678869731
11.45602588	11.6441225	10.79144767
ENSG00000226419 273.0429555	342.9719221	312.8954932
431.9686355	436.8250214	535.7436736	309.6367903
468.1791102	-0.595202147	0.005957554	0.041995908
SLC16A1-AS1	1	112956415	113047055	+	10138
antisense	SLC16A1 antisense RNA 1 [Source:HGNC
Symbol;Acc:HGNC:49445]	-	236	370	320	430	478
546	1.273076287	1.609101335	1.46217767
2.050772028	2.054632755	2.51412225
ENSG00000162772 77.51643227	127.9192574	71.37928438
156.7142026	231.206549	160.9193452	92.27165803
182.9466989	-0.985453225	0.0059586	0.041995908	ATF3
1	212565334	212620777	+	4040
protein_coding	activating transcription factor 3 [Source:HGNC
Symbol;Acc:HGNC:785]	TF_bZIP 67	138	73	156	253
164	0.906960035	1.506023259	0.837035641
1.867000567	2.728963668	1.894996965

ENSG00000169064 21.98227184 3.707804563 10.75578258 0
0 0.981215519 12.14861966 0.32707184
5.129221278 0.005963694 1 ZBBX 3 167240287
167381346 - 3972 protein_coding zinc finger B-
box domain containing [Source:HGNC Symbol;Acc:HGNC:26245] -
19 4 11 0 0 1 0.261600804
0.044400178 0.128287961 0 0 0.011752677
ENSG00000143333 54.37719876 103.8185278 51.82331606
106.4852915 184.5996952 166.8066383 70.00634753
152.6305417 -1.12150163 0.005965739 0.042026848 RGS16
1 182598623 182604408 - 2427
protein_coding regulator of G protein signaling 16 [Source:HGNC
Symbol;Acc:HGNC:9997] - 47 112 53 106 202
170 1.059065436 2.034614824 1.011599367
2.111724728 3.626938494 3.269830051
ENSG00000137962 1847.667796 854.6489519 1246.692981
867.9555838 809.6798514 875.2442434 1316.336576
850.9598929 0.628543044 0.005992736 0.042197582
ARHGAP29 1 94148988 94275068 - 15524
protein_coding Rho GTPase activating protein 29 [Source:HGNC
Symbol;Acc:HGNC:30207] - 1597 922 1275 864 886
892 5.625951641 2.618552255 3.804600026
2.690985297 2.487073875 2.682299423
ENSG00000126767 903.5870687 1038.185278 1030.599531
1319.011206 1304.078045 1323.659736 990.7906257
1315.582995 -0.408488076 0.006000318 0.042231522 ELK1
X 47635521 47650604 - 3398
protein_coding "ELK1, ETS transcription factor [Source:HGNC
Symbol;Acc:HGNC:3321]" ETS 781 1120 1054 1313 1427
1349 12.56962647 14.53210764 14.36877328
18.68282596 18.30034159 18.5325257
ENSG00000147614 24.29619519 19.46597396 12.71137941
103.4715569 31.98509571 52.98563805 18.82451619
62.81409688 -1.741214104 0.006011805 0.042292897
ATP6V0D2 8 85987323 86154228 + 3010
protein_coding ATPase H+ transporting V0 subunit d2
[Source:HGNC Symbol;Acc:HGNC:18266] - 21 21 13
103 35 54 0.381546533 0.307600302
0.200068775 1.654519712 0.506710783 0.837477793
ENSG00000171227 8.09873173 15.75816939 3.911193665 0
0 0 9.256031596 0 5.704697311
0.006018759 1 TMEM37 2 119429901 119438520
+ 2292 protein_coding transmembrane protein 37
[Source:HGNC Symbol;Acc:HGNC:18216] - 7 17 4
0 0 0 0.167023715 0.327015446
0.080844008 0 0 0
ENSG00000198917 688.3921971 861.1376098 913.2637207
1217.548805 1068.302197 1120.548123 820.9311759
1135.466375 -0.466965053 0.00602717 0.042381486 SPOUT1
9 128819651 128829821 - 4593

protein_coding SPOUT domain containing methyltransferase 1
[Source:HGNC Symbol;Acc:HGNC:26933]   -   595   929   934
        1212   1169   1142   7.08459834   8.917707564
9.42004303   12.75872835   11.09115216   11.60688534
ENSG00000022267 2083.687978   1951.232151   1885.195346
1676.641053   1459.434224   1349.171339   1973.371825
1495.082205   0.400362025   0.006034092   0.042388798   FHL1
        X   136146702   136211359   +   5997
protein_coding four and a half LIM domains 1 [Source:HGNC
Symbol;Acc:HGNC:3702]   -   1801   2105   1928   1669   1597
        1375   16.42382757   15.47576083   14.89276843
13.45623318   11.60458102   10.70322574
ENSG00000139572 11.56961676   24.10072966   10.75578258
74.33878843   49.34843338   37.28618974   15.47537633
53.65780385   -1.786460252   0.006034928   0.042388798   GPR84
        12   54362445   54364487   -   2043
protein_coding G protein-coupled receptor 84 [Source:HGNC
Symbol;Acc:HGNC:4535]   -   10   26   11   74   54
        38   0.267686424   0.561098282   0.249417415
1.751316219   1.151818344   0.868282935
ENSG00000198900 4079.446869   3989.59771   4507.650699
3477.849805   3360.262769   2998.594627   4192.231759
3278.9024   0.35452473   0.006036529   0.042388798   TOP1
        20   41028818   41124487   +   3738
protein_coding DNA topoisomerase I [Source:HGNC
Symbol;Acc:HGNC:11986] -   3526   4304   4610   3462   3677
        3056   51.58669723   50.76529609   57.1299816
44.7805046   42.86599713   38.16454497
ENSG00000166037 2221.366417   1863.171793   1876.395161
1591.251904   1580.977588   1246.14371   1986.97779
1472.791067   0.431636208   0.006084414   0.042705434   CEP57
        11   95789965   95832693   +   7101
protein_coding centrosomal protein 57 [Source:HGNC
Symbol;Acc:HGNC:30794] -   1920   2010   1919   1584   1730
        1270   14.78687593   12.47988268   12.51866212
10.78541551   10.61659348   8.348918933
ENSG00000189159 2456.229638   2738.21367   3425.227852
1776.094297   2125.638075   2383.372497   2873.22372
2095.034956   0.455842268   0.006094707   0.042758044   JPT1
        17   75135248   75168281   -   3414
protein_coding Jupiter microtubule associated homolog 1 [Source:HGNC
Symbol;Acc:HGNC:14569] -   2123   2954   3503   1768   2326
        2429   34.00800769   38.14880446   47.53123256
25.03917182   29.68963041   33.21314747
ENSG00000172716 1841.882988   1683.343272   1680.835477
1289.878437   1274.834529   1418.837641   1735.353912
1327.850202   0.385944892   0.006098836   0.04276739   SLFN11
        17   35350305   35373701   -   5982
protein_coding schlafen family member 11 [Source:HGNC
Symbol;Acc:HGNC:26633] -   1592   1816   1719   1284   1395

1446 14.5543015 13.38453826 13.31165103
10.37814733 10.16216862 11.28412582
ENSG00000259469 15.04050178 4.634755704 7.822387329 0
0 0 9.165881606 0 5.685769156
0.00610609 1 AC084757.2 15 48729080
48729844 - 540 lincRNA novel transcript -
13 5 8 0 0 0 1.316571062
0.408234968 0.686275797 0 0 0
ENSG00000115268 22184.74013 19059.96936 19873.75281
23826.58627 26572.30366 26174.9052 20372.82077
25524.59837 -0.325295311 0.006106224 0.042799562 RPS15
19 1438358 1440494 + 2137 protein_coding
ribosomal protein S15 [Source:HGNC Symbol;Acc:HGNC:10388] -
19175 20562 20325 23718 29077 26676 490.7107399
424.2235663 440.583763 536.6297665 592.9303379
582.7230838
ENSG00000133110 118.0100909 114.9419415 27.37835565
224.0209435 271.4163836 130.5016641 86.77679601
208.6463304 -1.267631101 0.006113971 0.042817394 POSTN
13 37562583 37598844 - 5464
protein_coding periostin [Source:HGNC Symbol;Acc:HGNC:16953] -
102 124 28 223 297 133 1.020902327
1.000564183 0.2373831 1.973310328 2.368668892
1.136284248
ENSG00000141424 2642.500467 2351.675044 2200.046436
1832.350677 1748.213945 1944.76916 2398.073983
1841.777927 0.380560595 0.00611437 0.042817394
SLC39A6 18 36108532 36129385 - 3682
protein_coding solute carrier family 39 member 6 [Source:HGNC
Symbol;Acc:HGNC:18607] - 2284 2537 2250 1824 1913
1982 33.92399794 30.37880184 28.30747874
23.95202666 22.64069653 25.12846163
ENSG00000165819 1268.029997 1104.92576 1339.58383
899.0975087 778.6086156 1017.520494 1237.513196
898.4088727 0.462045318 0.006141278 0.042986135 METTL3
14 21498133 21511375 - 5894
protein_coding methyltransferase like 3 [Source:HGNC
Symbol;Acc:HGNC:17563] - 1096 1192 1370 895 852
1037 10.16939544 8.91661637 10.76745069
7.341995607 6.299238633 8.213242782
ENSG00000104450 606.2479181 527.4351991 592.5458402
494.2524852 296.0906003 318.8950438 575.4096525
369.7460431 0.638456442 0.006146341 0.043001882 SPAG1
8 100157906 100259278 + 5055
protein_coding sperm associated antigen 1 [Source:HGNC
Symbol;Acc:HGNC:11212] - 524 569 606 492 324
325 5.66897889 4.96278047 5.553335605
4.705926711 2.793074038 3.001291905
ENSG00000155561 3305.439508 3038.54584 3415.449868
2888.162388 2302.013031 2205.772488 3253.145072

2465.315969 0.400109507 0.006151522 0.043018445 NUP205
7 135557919 135648757 + 7619
protein_coding nucleoporin 205 [Source:HGNC Symbol;Acc:HGNC:18658]
- 2857 3278 3493 2875 2519 2248
20.50722893 18.96902171 21.23748416 18.24488339
14.40750542 13.77350177
ENSG00000231942 0 0 0 24.10987733 10.96631853
0 0 11.69206529 -5.966794994 0.006163285
1 HNRNPA1P36 8 81807772 81808726
+ 955 processed_pseudogene heterogeneous nuclear
ribonucleoprotein A1 pseudogene 36 [Source:HGNC
Symbol;Acc:HGNC:48765] - 0 0 0 24 12
0 0 0 0 1.21509179 0.547565998
0
ENSG00000182810 484.7669421 549.6820265 445.8760778
782.566435 777.6947557 589.7105272 493.4416821
716.6572393 -0.538181671 0.006187728 0.043251849 DDX28
16 68021274 68023442 - 2169
protein_coding DEAD-box helicase 28 [Source:HGNC
Symbol;Acc:HGNC:17330] - 419 593 456 779 851
601 10.56450575 12.05394205 9.738851562
17.36517331 17.09734227 12.93483687
ENSG00000216867 13.88354011 4.634755704 8.800185745 0
0 0 9.106160519 0 5.676832568
0.006188344 1 RPL22P12 2 110633947
110634333 + 387 processed_pseudogene ribosomal
protein L22 pseudogene 12 [Source:HGNC Symbol;Acc:HGNC:36345] -
12 5 9 0 0 0 1.69576237
0.569630188 1.077293403 0 0 0
ENSG00000040531 738.1415491 704.482867 766.5939583
1070.880385 922.9984762 1000.83983 736.4061248
998.2395636 -0.438798351 0.006196392 0.043292618 CTNS
17 3636468 3661542 + 3398
protein_coding "cystinosin, lysosomal cystine transporter
[Source:HGNC Symbol;Acc:HGNC:2518]" - 638 760 784
1066 1010 1020 10.26814557 9.861073043
10.68796798 15.16823493 12.95258935 14.01273255
ENSG00000133030 6509.066388 6687.952481 8360.176458
6140.986671 4866.303847 5365.28646 7185.731776
5457.52566 0.397006172 0.006201867 0.043293566 MPRIP
17 17042545 17217679 + 18153
protein_coding myosin phosphatase Rho interacting protein
[Source:HGNC Symbol;Acc:HGNC:30321] - 5626 7215 8550
6113 5325 5468 16.94907623 17.52354167
21.81826252 16.28197954 12.78291209 14.06132137
ENSG00000161682 202.4682933 386.5386257 432.1868999
468.1334515 615.0276975 620.1282083 340.3979396
567.7631191 -0.736112787 0.006207626 0.043293566
FAM171A2 17 44353215 44363875 - 3592
protein_coding family with sequence similarity 171 member A2

[Source:HGNC Symbol;Acc:HGNC:30480]	-	175	417	442
466	673	632	2.664381647	5.118393662
5.700177731	6.272646384	8.16464552	8.213472098
ENSG00000188779 18.51138681	29.66243651	31.28954932
98.44866576	49.34843338	72.60994844	26.48779088
73.46901586	-1.465909711	0.00620794	0.043293566	SKOR1
15	67819704	67834561	+	4479
protein_coding	SKI family transcriptional corepressor 1 [Source:HGNC
Symbol;Acc:HGNC:21326]	-	16	32	32	98	54
74	0.195359094	0.31499443	0.330956848
1.057903928	0.525377289	0.771252692
ENSG00000275395 3.470885027	43.56670362	27.37835565
101.4624004	68.53949081	86.34696571	24.80531477
85.44961898	-1.772516469	0.006208479	0.043293566	FCGBP
19	39863323	39934626	-	12961
protein_coding	Fc fragment of IgG binding protein [Source:HGNC
Symbol;Acc:HGNC:13572]	-	3	47	28	101	75
88	0.01265836	0.159879693	0.100074165
0.376776736	0.252162993	0.316949592
ENSG00000111731 894.3313753	812.0091994	740.193401
479.1838119	568.4208438	661.3392601	815.5113252
569.6479719	0.517114925	0.006210688	0.043293566	C2CD5
12	22448583	22544546	-	6759
protein_coding	C2 calcium dependent domain containing 5 [Source:HGNC
Symbol;Acc:HGNC:29062]	-	773	876	757	477	622
674	6.254487951	5.71420238	5.188190198
3.412220981	4.010204648	4.655040737
ENSG00000127948 861.9364484	1223.575506	1096.112025
1573.169496	1256.557331	1640.592349	1060.541326
1490.106392	-0.489643025	0.006227968	0.04339423	POR
7	75899200	75986855	+	6112
protein_coding	cytochrome p450 oxidoreductase [Source:HGNC
Symbol;Acc:HGNC:9208]	-	745	1320	1121	1566	1375
1672	6.666035772	9.521920334	8.496199916
12.38824052	9.803427701	12.77023718
ENSG00000180776 2468.956216	1815.897285	2025.998318
1599.288529	1650.430939	1433.555874	2103.617273
1561.091781	0.429769484	0.006235261	0.043425252
ZDHHC20 13	21372573	21459370	-	5574
protein_coding	zinc finger DHHC-type containing 20 [Source:HGNC
Symbol;Acc:HGNC:20749]	-	2134	1959	2072	1592	1806
1461	20.93737172	15.4953514	17.21968658
13.80947969	14.11917689	12.23571316
ENSG00000185909 116.8531292	145.5313291	190.6706912
228.0392564	238.517428	332.6320611	151.0183832
266.3962485	-0.816895784	0.00624064	0.043442926
KLHDC8B 3	49171611	49176486	+	2158
protein_coding	kelch domain containing 8B [Source:HGNC
Symbol;Acc:HGNC:28557]	-	101	157	195	227	261
339	2.559556061	3.207614515	4.185868944

5.085991539 5.270449601 7.333212899
ENSG00000266074 790.2048245 847.2333427 1857.816991
640.9209057 316.1955176 403.2795785 1165.085053
453.4653339 1.361648651 0.006246151 0.043461498 BAHCC1
17 81395475 81466332 + 13473
protein_coding BAH domain and coiled-coil containing 1 [Source:HGNC
Symbol;Acc:HGNC:29279] - 683 914 1900 638 346
411 2.772369464 2.990996081 6.532685445
2.289589293 1.119103842 1.424044458
ENSG00000210077 108.7543975 163.1434008 81.15726854
306.3963577 179.116536 200.167966 117.6850223
228.5602866 -0.955584756 0.00627641 0.043652177 MT-TV
MT 1602 1670 + 69 Mt_tRNA
mitochondrially encoded tRNA valine [Source:HGNC
Symbol;Acc:HGNC:7500] - 94 176 83 305 196
204 74.50295108 112.4598591 55.72261093
213.7233462 123.7843038 138.0152615
ENSG00000133422 963.7490759 1018.719304 1109.801202
1416.455293 1311.388924 1365.852003 1030.756527
1364.565407 -0.404337261 0.006295073 0.043762063 MORC2
22 30925130 30968298 - 6258
protein_coding MORC family CW-type zinc finger 2 [Source:HGNC
Symbol;Acc:HGNC:23573] - 833 1099 1135 1410 1435
1392 7.279543664 7.742765238 8.401614656
10.89393477 9.992518017 10.38364214
ENSG00000166503 1962.207002 1707.444001 1832.394232
1285.860124 1470.400543 1439.443167 1834.015078
1398.567945 0.390644856 0.006305568 0.043815101 HDGFL3
15 83112738 83208018 - 16253
protein_coding HDGF like 3 [Source:HGNC Symbol;Acc:HGNC:24937] -
1696 1842 1874 1280 1609 1467 5.706726056
4.996777928 5.3411959 3.807830874 4.314009593
4.213496275
ENSG00000120889 3618.976122 4670.906799 4114.575735
5230.838802 6442.712136 4837.392511 4134.819552
5503.647816 -0.412427143 0.006317767 0.043864014
TNFRSF10B 8 23020133 23069179 - 5158
protein_coding TNF receptor superfamily member 10b
[Source:HGNC Symbol;Acc:HGNC:11905] - 3128 5039 4208
5207 7050 4930 33.16500899 43.07219244
37.79173661 48.80984813 59.56160292 44.61815597
ENSG00000197632 492.8656739 456.0599613 147.6475608
866.9510056 1111.253611 920.3801573 365.5243987
966.1949246 -1.402722686 0.006318346 0.043864014
SERPINB2 18 63871692 63903890 + 2748
protein_coding serpin family B member 2 [Source:HGNC
Symbol;Acc:HGNC:8584] - 426 492 151 863 1216
938 8.477886215 7.893731178 2.545438888
15.18431777 19.28303743 15.93426905
ENSG00000135776 403.7796248 469.9642284 407.7419395

315.4375617	197.3937335	296.3270869	427.1619309
269.7194607	0.664630017	0.006334039	0.043952995	ABCB10
1	229516582	229558695	-	4256
protein_coding	ATP binding cassette subfamily B member 10
[Source:HGNC Symbol;Acc:HGNC:41]	-	349	507	417
314	216	302	4.484546385	5.252188422
4.53875658	3.567214669	2.211621125	3.312465468
ENSG00000175575 549.556796	721.1679876	652.1915436
944.3035287	1009.815165	779.0851225	640.972109
911.0679386	-0.506485211	0.006342596	0.043992408	PAAF1
11	73876699	73931124	+	7937
protein_coding	proteasomal ATPase associated factor 1 [Source:HGNC
Symbol;Acc:HGNC:25687]	-	475	778	667	940	1105
794	3.272894016	4.32172546	3.892888003
5.726281447	6.066867296	4.669927712
ENSG00000136689 9.255693406	5.561706845	0	23.10529911
54.83159264	20.60552591	4.939133417	32.84747255
-2.752267489	0.006348701	0.044014782	IL1RN	2
113107214	113134016	+	3256	protein_coding
interleukin 1 receptor antagonist [Source:HGNC
Symbol;Acc:HGNC:6000]	-	8	6	0	23	60
21	0.134369377	0.08124578	0	0.341542424
0.803018317	0.301079325
ENSG00000090013 985.7313477	1193.913069	753.8825789
1172.342785	1443.898606	1636.667486	977.842332
1417.636293	-0.53570559	0.006364556	0.044104701	BLVRB
19	40447765	40465840	-	1689
protein_coding	biliverdin reductase B [Source:HGNC
Symbol;Acc:HGNC:1063]	-	852	1288	771	1167	1580
1668	27.58701163	33.62173892	21.14594919
33.40738045	40.76486751	46.10122149
ENSG00000184900 4502.894842	4693.153626	5205.798768
3603.422082	3788.863052	3973.922854	4800.615745
3788.735996	0.341578277	0.006382402	0.044208325	SUMO3
21	44805617	44818779	-	2732
protein_coding	small ubiquitin-like modifier 3 [Source:HGNC
Symbol;Acc:HGNC:11124]	-	3892	5063	5324	3587	4146
4050	77.90885995	81.70736223	90.27340163
63.48218965	66.13132138	69.20227374
ENSG00000204628 24067.11678	27432.19206	24617.05293
29608.93852	30534.80008	36931.97094	25372.12059
32358.56985	-0.350880413	0.006389778	0.044239376	RACK1
5	181236909	181248096	-	5990
protein_coding	receptor for activated C kinase 1 [Source:HGNC
Symbol;Acc:HGNC:4399]	-	20802	29594	25176	29474	33413
37639	189.9209974	217.826546	194.6983914
237.9104253	243.0788909	293.3305455
ENSG00000250954 0	0.926951141	0	24.10987733
2.741579632	10.79337071	0.308983714
12.54827589	-5.105635072	0.006393416	1	AC016687.3

4	33776347	34039893	-	2680
lincRNA uncharacterized LOC101928622 [Source:NCBI gene;Acc:101928622]
-	0	1	0	24	3	11	0
0.01645126	0	0.432989798	0.048780366
0.191603716
ENSG00000095015 395.6808931	412.4932577	618.9463974
328.4970786	272.3302435	307.1204576	475.7068494
302.6492599	0.653331293	0.00639907	0.044264386	MAP3K1
5	56815574	56896152	+	7716
protein_coding	mitogen-activated protein kinase kinase kinase 1
[Source:HGNC Symbol;Acc:HGNC:6848]	-	342	445	633
327	298	313	2.423977587	2.5427388
3.800265568	2.049069789	1.682994291	1.893643208
ENSG00000167723 151.5619795	243.78815	97.77984162
241.0987733	304.3153392	385.6176992	164.3766571
310.3439372	-0.915573761	0.006399182	0.044264386	TRPV3
17	3510502 3557995 -	9780	protein_coding
transient receptor potential cation channel subfamily V member 3
[Source:HGNC Symbol;Acc:HGNC:18084]	-	131	263	100
240	333	393	0.732532932	1.185634564
0.473656609	1.186516012	1.483761085	1.875857014
ENSG00000148484 1585.037496	1573.963037	1704.302639
1227.594587	1175.223802	1318.753658	1621.101057
1240.524016	0.386163733	0.006407377	0.044301026	RSU1
10	16590611	16817528	-	4191
protein_coding	Ras suppressor protein 1 [Source:HGNC
Symbol;Acc:HGNC:10464]	-	1370	1698	1743	1222	1286
1344	17.87712262	17.86298292	19.26558416
14.0979109	13.37155471	14.97020142
ENSG00000110330 3009.257319	2283.08066	2754.458138
1808.2408	2313.89321	1827.023297	2682.265372
1983.052436	0.435266524	0.006426205	0.044398844	BIRC2
11	102347211	102378670	+	4532
protein_coding	baculoviral IAP repeat containing 2 [Source:HGNC
Symbol;Acc:HGNC:590]	-	2601	2463	2817	1800	2532
1862	31.3866644	23.96119473	28.7938277
19.2036517	24.34626797	19.17943509
ENSG00000259807 75.20250892	43.56670362	114.4024147
44.20144177	14.62175804	5.887293117	77.72387574
21.57016431	1.851952144	0.006427333	0.044398844
AC009093.1	16	29217170	29220031	-	2099
lincRNA novel transcript	-	65	47	117
44	16	6	1.693540671	0.987232348
2.582116774	1.01354131	0.332174387	0.133439636
ENSG00000125755 2702.662474	3357.417032	3394.916101
4291.558164	3808.967969	4191.752699	3151.665203
4097.426278	-0.378238975	0.006442257	0.044481838	SYMPK
19	45815410	45863290	-	10628
protein_coding	symplekin [Source:HGNC Symbol;Acc:HGNC:22935]	-
2336	3622	3472	4272	4168	4272	12.02031934

15.02556661	15.13319494	19.43483755	17.08971272
18.76401339
ENSG00000228526 168.9164047	118.649746	173.0703197
37.16939421	55.74545252	110.8773537	153.5454901
67.93073348	1.175903434	0.006451085	0.044522683
MIR34AHG	1	9148011 9198906 -	8702	lincRNA MIR34A
host gene [Source:HGNC Symbol;Acc:HGNC:51913]	-	146	128
177	37	61	113	0.917547359	0.648522202
0.942229383	0.20558142	0.305470555	0.6061853
ENSG00000138650 150.4050178	152.0199871	123.2026004
300.3688884	219.3263706	211.9425522	141.8758685
243.8792704	-0.781508839	0.006458156	0.044551365	PCDH10
4	133149315	133208606	+	10161
protein_coding	protocadherin 10 [Source:HGNC Symbol;Acc:HGNC:13404]
-	130	164	126	299	240	216
0.69968347	0.711608873	0.574429255	1.422774109
1.029279654	0.992346485
ENSG00000272721 3.470885027	8.342560268	15.64477466	0
0	0	9.152739984	0	5.689637473
0.006462651	1	AC131235.3	3	184134019
184135238	-	386	antisense	"novel transcript,
antisense to EIF2B5"	-	3	9	16	0	0
0	0.425038884	1.027990646	1.920149899	0
0	0
ENSG00000238260 0	0	0	12.05493866	6.397019142
8.830939675	0	9.09429916	-5.604095451
0.006476896	1	AL513320.1	1	3623190 3624743 -
622	antisense	novel transcript	-	0
0	0	12	7	9	0	0	0
0.932807604	0.490417832	0.675457706
ENSG00000227036 122.6379376	100.1107232	155.4699482
36.16481599	79.50580934	53.96685357	126.0728697
56.5458263	1.154322645	0.006493417	0.044774411
LINC00511	17	72290091	72640472	-	27605
processed_transcript	long intergenic non-protein coding RNA
511 [Source:HGNC Symbol;Acc:HGNC:43564] -	106	108	159
36	87	55	0.209996872	0.172492399
0.266815975	0.063054483	0.137337804	0.093008143
ENSG00000103888 683.7643503	200.2214464	847.7512268
244.112508	116.0602044	146.2011124	577.2456745
168.7912749	1.774051907	0.006511574	0.044863207	CEMIP
15	80779343	80951776	+	7621
protein_coding	cell migration inducing hyaluronidase 1 [Source:HGNC
Symbol;Acc:HGNC:29213]	-	591	216	867	243	127
149	4.241019135	1.249613613	5.269987579
1.54168458	0.726190155	0.912683801
ENSG00000101457 1052.835125	1161.469779	1070.689266
1340.107348	1487.76388	1503.222176	1094.998057
1443.697801	-0.39864098	0.006512164	0.044863207
DNTTIP1 20	45791937	45811427	+	1883

protein_coding deoxynucleotidyltransferase terminal interacting
protein 1 [Source:HGNC Symbol;Acc:HGNC:16160]   -   910   1253
        1095   1334   1628   1532   26.42930757   29.33828404
        26.93805622   34.25363976   37.67581696   37.97995809
ENSG00000181873 190.8986765   288.2818048   345.1628409
526.3989883   393.8736072   423.8851044   274.7811074
448.0525666   -0.703078501   0.006517041   0.044876577   IBA57
        1   228165815   228182257   +   10219
protein_coding "IBA57, iron-sulfur cluster assembly [Source:HGNC
Symbol;Acc:HGNC:27302]" -   165   311   353   524   431
        432   0.883019426   1.341794316   1.60017972
2.479271593   1.837923661   1.973428443
ENSG00000206897 5.784808379   11.12341369   9.777984162   0
        0   0   8.895402077   0   5.647841006
0.006519692   1   SNORA9B 12   123616708   123616840
        +   133   snoRNA "small nucleolar RNA, H/ACA box 9B
[Source:HGNC Symbol;Acc:HGNC:52223]"   -   5   12   10
        0   0   0   2.055952497   3.977988864
3.48297867   0   0   0
ENSG00000260742 11.56961676   9.269511408   5.866790497   0
        0   0   8.901972888   0   5.645748317
0.006519817   1   AC009962.1   2   181887851
181891663   -   3813   antisense   "novel transcript,
antisense to SSFA2"   -   10   10   6   0   0
        0   0.143426007   0.115629102   0.072893181   0
        0   0
ENSG00000173209 573.8529912   397.6620394   523.1221526
257.1720248   357.3192121   347.3502939   498.2123944
320.6138436   0.634525026   0.006525426   0.044914089   AHSA2P
        2   61177418   61191203   +   9083
transcribed_unitary_pseudogene "activator of HSP90 ATPase homolog 2,
pseudogene [Source:HGNC Symbol;Acc:HGNC:20437]" -   496   429
        535   256   391   354   2.986393798   2.082389359
        2.728518631   1.362736435   1.875884267   1.81936562
ENSG00000183763 569.2251445   516.3117855   543.6559194
367.6756293   388.3904479   383.6552681   543.0642831
379.9071151   0.514981206   0.006529057   0.044918853   TRAIP
        3   49828599   49856574   -   3263
protein_coding TRAF interacting protein [Source:HGNC
Symbol;Acc:HGNC:30764] -   492   557   556   366   425
        391   8.245988821   7.526136302   7.893328431
5.42331997   5.675844047   5.593784348
ENSG00000106034 385.268238   344.8258244   322.6734773
158.7233591   286.9520015   179.5624401   350.9225132
208.4126002   0.749960575   0.006547847   0.04502786   CPED1
        7   120988677   121297444   +   7705
protein_coding cadherin like and PC-esterase domain containing 1
[Source:HGNC Symbol;Acc:HGNC:26159]   -   333   372   330
        158   314   183   2.363558213   2.128649979
1.984009524   0.991483886   1.775888123   1.10872664

ENSG00000203485 2826.457374 3503.875312 3448.695014
3920.868801 4067.590314 4858.979252 3259.6759
4282.479456 -0.393438631 0.006557205 0.04507194 INF2
14 104689606 104722535 + 9488
protein_coding "inverted formin, FH2 and WH2 domain containing
[Source:HGNC Symbol;Acc:HGNC:23791]" - 2443 3780 3527
3903 4451 4952 14.0813244 17.56511841
17.22000366 19.88955616 20.44285348 24.36419102
ENSG00000003756 1634.786848 1582.305597 1752.214762
1285.860124 1217.261357 1314.828796 1656.435736
1272.650092 0.380331877 0.006580623 0.045212575 RBM5
3 50088908 50119021 + 10368
protein_coding RNA binding motif protein 5 [Source:HGNC
Symbol;Acc:HGNC:9902] - 1413 1707 1792 1280 1332
1340 7.453184739 7.25892803 8.006550968
5.969200925 5.598450389 6.03331285
ENSG00000214866 13.88354011 11.12341369 0 55.25180221
61.22861179 20.60552591 8.335651266 45.6953133
-2.464050197 0.006583949 0.045215109 DCDC2C 2
3703592 3847411 + 2242 protein_coding doublecortin
domain containing 2C [Source:HGNC Symbol;Acc:HGNC:32696] -
12 12 0 55 67 21 0.292711881
0.23598239 0 1.186119095 1.302260273
0.437249904
ENSG00000108588 3867.722882 3490.897996 3960.083585
2891.176123 2847.587378 3161.476404 3772.901488
2966.746635 0.346731315 0.006587318 0.045217927 CCDC47
17 63745250 63776351 - 4537
protein_coding coiled-coil domain containing 47 [Source:HGNC
Symbol;Acc:HGNC:24856] - 3343 3766 4050 2878 3116
3222 40.29603454 36.5970007 41.35125547
30.67066743 29.92865965 33.15147015
ENSG00000277791 3371.386323 3138.656563 3108.421165
2746.516859 2407.106917 2359.823324 3206.154684
2504.482367 0.356281982 0.006592831 0.045225711 PSMB3
17 38752736 38764231 + 1179
protein_coding proteasome subunit beta 3 [Source:HGNC
Symbol;Acc:HGNC:9540] - 2914 3386 3179 2734 2634
2405 135.1669316 126.6213987 124.9048145
112.1207313 97.35551607 95.22419533
ENSG00000198805 1011.184505 1212.452092 991.487594
1369.240117 1487.76388 1437.480736 1071.708064
1431.494911 -0.417244223 0.006594368 0.045225711 PNP
14 20468954 20477094 + 4139
protein_coding purine nucleoside phosphorylase [Source:HGNC
Symbol;Acc:HGNC:7892] - 874 1308 1014 1363 1628
1465 11.54810487 13.93305256 11.34867044
15.92214761 17.14026657 16.52297456
ENSG00000169564 2090.629748 2928.238654 2871.793948
3670.728823 3062.344449 4035.739432 2630.220783

3589.604235	-0.448175259	0.006602323	0.045259963	PCBP1
	2	70087454	70089203	+	1750
protein_coding	poly(rC) binding protein 1 [Source:HGNC
Symbol;Acc:HGNC:8647]	-	1807	3159	2937	3654	3351
	4113	56.46961368	79.58762321	77.74426349
100.9559014	83.44397358	109.7151741
ENSG00000159202 4085.231677	4649.586922	4086.219581
5441.800229	5527.938398	5180.817943	4273.679394
5383.518857	-0.332955721	0.006637615	0.0454815	UBE2Z
	17	48908369	48929056	+	5753
protein_coding	ubiquitin conjugating enzyme E2 Z [Source:HGNC
Symbol;Acc:HGNC:25847]	-	3531	5016	4179	5417	6049
	5280	33.56588144	38.44121553	33.64964237
45.52664826	45.81923229	42.84356334
ENSG00000075391 2504.822028	2313.670048	1850.972402
3067.98189	2907.90213	2830.806774	2223.154826
2935.563598	-0.401292755	0.006649499	0.045542512	RASAL2
	1	178094141	178484147	+	16044
protein_coding	RAS protein activator like 2 [Source:HGNC
Symbol;Acc:HGNC:9874]	-	2165	2496	1893	3054	3182
	2885	7.37972129	6.859080276	5.465632366
9.203597043	8.642633959	8.394197826
ENSG00000156675 48.59239038	62.10572644	56.71230814
7.032047554	0	25.51160351	55.80347498	10.84788369
	2.367965339	0.006656228	0.045547229	RAB11FIP1
	8	37858618	37899467	-	9602
protein_coding	RAB11 family interacting protein 1 [Source:HGNC
Symbol;Acc:HGNC:30265]	-	42	67	58	7	0
	26	0.239211636	0.307643015	0.279813554
0.03524825	0	0.126403088
ENSG00000080031 126.1088227	101.9646255	60.6235018
236.0758822	125.1988032	225.6795695	96.23231665
195.6514183	-1.0254423	0.006656955	0.045547229	PTPRH
	19	55181248	55209506	-	4344
protein_coding	"protein tyrosine phosphatase, receptor type H
[Source:HGNC Symbol;Acc:HGNC:9672]"	-	109	110	62
	235	137	230	1.372244169	1.116443698
0.661156586	2.615647781	1.374324689	2.4716334
ENSG00000049541 1587.351419	1367.252933	1519.498739
918.1844949	1222.744516	1149.003373	1491.367697
1096.644128	0.443012437	0.006659126	0.045547229	RFC2
	7	74231499	74254458	-	2211
protein_coding	replication factor C subunit 2 [Source:HGNC
Symbol;Acc:HGNC:9970]	-	1372	1475	1554	914	1338
	1171	33.93595549	29.41285864	32.55852544
19.98750888	26.37096174	24.72374117
ENSG00000149925 187.4277915	439.3748408	398.9417538
515.3486279	504.4506523	749.6486569	341.9147953
589.815979	-0.784342109	0.006666476	0.045573613	ALDOA
	16	30064164	30070457	+	3916

protein_coding "aldolase, fructose-bisphosphate A [Source:HGNC
Symbol;Acc:HGNC:414]"   -   162   474   408   513   552
764   2.262387769   5.336661006   4.826362476
6.333968489   6.142638994   9.107448231
ENSG00000166004 710.3744689   630.3267758   707.9260533
572.6095866   339.9558744   448.4154924   682.875766
453.6603178   0.590487695   0.006668946   0.045573613   CEP295
11   93661639   93730358   +   9495
protein_coding centrosomal protein 295 [Source:HGNC
Symbol;Acc:HGNC:29366] -   614   680   724   570   372
457   3.536454826   3.157533025   3.532206236
2.902559312   1.707287138   2.246814753
ENSG00000134874 1647.513426   1694.466685   1854.883595
1205.493866   1501.471779   1171.57133   1732.287902
1292.845658   0.421985938   0.006676739   0.045592654   DZIP1
13   95578202   95644703   -   8470
protein_coding DAZ interacting zinc finger protein 1 [Source:HGNC
Symbol;Acc:HGNC:20908] -   1424   1828   1897   1200   1643
1194   9.194355499   9.515393198   10.37495869
6.850133765   8.453036628   6.58062211
ENSG00000154767 739.2985108   926.0241897   744.1045947
1213.530492   1086.579394   1019.482925   803.1424317
1106.530937   -0.461618258   0.006677697   0.045592654   XPC
3   14145147   14178783   -   4798
protein_coding "XPC complex subunit, DNA damage recognition and
repair factor [Source:HGNC Symbol;Acc:HGNC:12816]"   -   639
999   761   1208   1189   1039   7.283419546
9.17992647   7.347284705   12.17328829   10.79891714
10.1088404
ENSG00000099840 26.61011854   40.7858502   43.02313031
3.013734666   10.96631853   10.79337071   36.80636635
8.25780797   2.153574822   0.006687024   0.045635955   IZUMO4
19   2096429 2099593 +   2770   protein_coding IZUMO
family member 4 [Source:HGNC Symbol;Acc:HGNC:26950]   -   23
44   44   3   12   11   0.45409088
0.700336668   0.735826396   0.052365192   0.188781779
0.185378324
ENSG00000198786 31045.90961   30916.6014   37294.20939
43288.28017   36057.25532   51754.21257   33085.57347
43699.91602   -0.401413231   0.006697442   0.045686659   MT-ND5
MT   12337   14148   +   1812   protein_coding
mitochondrially encoded NADH:ubiquinone oxidoreductase core subunit 5
[Source:HGNC Symbol;Acc:HGNC:7461]   -   26834   33353   38141
43091   39456   52745   809.8823509   811.5413781
975.0712195   1149.819305   948.8847334   1358.842555
ENSG00000140563 341.3036943   406.0045997   493.7882002
266.2132288   308.8846386   145.2198969   413.6988314
240.1059214   0.784949436   0.006702314   0.045699501   MCTP2
15   94231538   94483952   +   12978
protein_coding multiple C2 and transmembrane domain containing 2

[Source:HGNC Symbol;Acc:HGNC:25636]	-	295	438	505
	265	338	148	1.243108279	1.48799098
1.802544786	0.987277684	1.134925955	0.532353338
ENSG00000237548 13.88354011	12.05036483	1.955596832	0
	0	0	9.296500591	0	5.707917209
0.006726048	1	TTLL11-IT1	9	121884636
121963719	-	1052	lincRNA TTLL11 intronic transcript 1
[Source:HGNC Symbol;Acc:HGNC:24214]	-	12	13	2
	0	0	0	0.623821328	0.544830699
0.088067712	0	0	0
ENSG00000169255 864.2503718	676.6743328	637.5245673
420.918275	582.1287419	475.8895269	726.1497573
492.978848	0.557426933	0.006755154	0.04603925
B3GALNT1	3	161083883	161105384	-	4183
	protein_coding	"beta-1,3-N-acetylgalactosaminyltransferase 1
(globoside blood group) [Source:HGNC Symbol;Acc:HGNC:918]"	-
	747	730	652	419	637	485	9.76624129
	7.694297132	7.220415452	4.84314392	6.636057883
	5.412524932
ENSG00000173581 565.7542594	974.225649	688.370085
1103.026888	1060.991318	1095.03652	742.7833311
1086.351575	-0.547174196	0.006764385	0.046081618
CCDC106 19	55641062	55653161	+	2709
protein_coding	coiled-coil domain containing 106 [Source:HGNC
Symbol;Acc:HGNC:30181]	-	489	1051	704	1098	1161
	1116	9.871759511	17.1051808	12.03832628
19.59722377	18.67591172	19.23097155
ENSG00000260219 27.76708022	50.98231275	21.51156516
82.37541421	95.95528713	78.49724156	33.42031937
85.6093143	-1.353020076	0.006769998	0.046094259
AC106782.2	16	30355441	30357104	+	992
	lincRNA uncharacterized LOC101928707 [Source:NCBI
gene;Acc:101928707]	-	24	55	22	82	105
	80	1.323104914	2.444471484	1.027338265
3.99671699	4.612498359	3.764647787
ENSG00000172216 2139.222138	4870.201294	3885.770906
5915.961149	6940.765769	7415.045681	3631.731446
6757.257533	-0.895632546	0.006772271	0.046094259	CEBPB
	20	50190734	50192689	+	1956
protein_coding	CCAAT enhancer binding protein beta [Source:HGNC
Symbol;Acc:HGNC:1834]	C/EBP	1849	5254	3974	5889	7595
	7557	51.69669431	118.428213	94.11556811
145.5706832	169.2066883	180.3543442
ENSG00000198546 236.0201818	221.5413227	225.8714341
419.9136968	286.0381416	372.8618974	227.8109795
359.6045786	-0.658385291	0.006777057	0.046106305	ZNF511
	10	133308475	133313162	+	2728
protein_coding	zinc finger protein 511 [Source:HGNC
Symbol;Acc:HGNC:28445]	-	204	239	231	418	313
	380	4.089597006	3.862668989	3.922564285

7.408548567	4.999868351	6.50257345
ENSG00000273230 255.6885303	420.8358179	387.2081728
556.536335	501.7090727	579.898372	354.577507
546.0479266	-0.620701418	0.006780335	0.046108088
AC102953.2	7	1464497 1467522 -	3026	lincRNA novel
transcript	-	221	454	396	554	549	591
3.994091987	6.614863505	6.062178473	8.852013295
7.906094817	9.117265314
ENSG00000100207 956.8073058	1027.061864	1009.087965
1475.725408	1192.58714	1332.490675	997.6523785
1333.601075	-0.418270477	0.006802639	0.046239194	TCF20
22	42160013	42343616	-	7548
protein_coding	transcription factor 20 [Source:HGNC
Symbol;Acc:HGNC:11631]	-	827	1108	1032	1469	1305
1358	5.991952071	6.47204945	6.333594599
9.410032444	7.534201271	8.398735693
ENSG00000160948 1332.81985	1393.207565	1337.628233
2011.1656	1676.019015	1660.216659	1354.551883
1782.467092	-0.39583746	0.006816802	0.046314871	VPS28
8	144423601	144428563	-	3769
protein_coding	"VPS28, ESCRT-I subunit [Source:HGNC
Symbol;Acc:HGNC:18178]" -	1152	1503	1368	2002	1834
1692	16.71556476	17.5819403	16.81366599
25.68260972	21.20468336	20.95657264
ENSG00000123104 467.412517	445.8634987	610.1462117
266.2132288	394.787467	323.8011214	507.8074091
328.2672724	0.628933946	0.006832738	0.04640252	ITPR2
12	26336515	26833198	-	12750
protein_coding	"inositol 1,4,5-trisphosphate receptor type 2
[Source:HGNC Symbol;Acc:HGNC:6181]"	-	404	481	624
265	432	330	1.732869641	1.663293344
2.267132281	1.004932532	1.476495609	1.20823049
ENSG00000236591 9.255693406	10.19646255	6.844588913	0
0	0	8.765581623	0	5.624716329
0.006852297	1	AL357992.1	6	149027700
149032573	-	130	antisense	novel transcript
-	8	11	7	0	0	0
3.365436088	3.730639557	2.494348571	0	0	0
ENSG00000050438 145.7771711	136.2618177	195.5596832
83.37999243	31.07123583	99.10276747	159.1995574
71.18466524	1.163454045	0.00685981	0.046565679	SLC4A8
12	51391317	51515763	+	17980
protein_coding	solute carrier family 4 member 8 [Source:HGNC
Symbol;Acc:HGNC:11034]	-	126	147	200	83	34
101	0.383244182	0.360463757	0.515279381
0.223197652	0.082403912	0.262227191
ENSG00000272764 17.35442514	0.926951141	10.75578258	0
0	0	9.679052951	0	5.763755358
0.006896314	1	AL596094.1	10	5813985 5814441 +
276	lincRNA novel transcript	-	15	1

11	0	0	0	2.972192197	0.159744118
1.846231085	0	0	0
ENSG00000153914 2052.450013	1928.058373	1993.730971
1628.421298	1472.228263	1545.414443	1991.413119
1548.688001	0.362721257	0.006916198	0.046927619	SREK1
5	66139971	66183615	+	18240
protein_coding	splicing regulatory glutamic acid and lysine rich
protein 1 [Source:HGNC Symbol;Acc:HGNC:17882]	-	1774	2080
2039	1621	1611	1575	5.31892044	5.027735926
5.1783911	4.296941067	3.848835097	4.030897548
ENSG00000182095 2966.449737	4200.015619	5592.029142
6996.887316	4836.146471	6768.424653	4252.831499
6200.486147	-0.543703801	0.006940284	0.047070153	TNRC18
7	5306790 5425414 -	12572	protein_coding
trinucleotide repeat containing 18 [Source:HGNC
Symbol;Acc:HGNC:11962]	-	2564	4531	5719	6965	5292
6898	11.15342782	15.88999087	21.07260274
26.78662297	18.34315606	25.61325973
ENSG00000214160 1649.82735	1759.353265	1806.971473
2902.226483	1940.12452	2261.701772	1738.717363
2368.017592	-0.445338341	0.006948456	0.047094793	ALG3
3	184242301	184249548	-	3464
protein_coding	"ALG3, alpha-1,3- mannosyltransferase [Source:HGNC
Symbol;Acc:HGNC:23056]" -	1426	1898	1848	2889	2123
2305	22.51315466	24.15751638	24.7130609
40.32467492	26.70734451	31.06269141
ENSG00000116497 1132.665481	984.4221116	1061.88908
831.7907678	823.3877496	661.3392601	1059.658891
772.1725925	0.456170348	0.006952046	0.047094793
S100PBP 1	32816767	32858879	+	9194
protein_coding	S100P binding protein [Source:HGNC
Symbol;Acc:HGNC:25768]	-	979	1062	1086	828	901
674	5.82335016	5.092768972	5.47176934
4.354387292	4.270501604	3.422168843
ENSG00000131724 2450.444829	2182.969937	2269.470124
1645.499128	2047.046125	1399.213331	2300.96163
1697.252861	0.438661787	0.006953159	0.047094793
IL13RA1 X	118727572	118794539	+	4372
protein_coding	interleukin 13 receptor subunit alpha 1 [Source:HGNC
Symbol;Acc:HGNC:5974]	-	2118	2355	2321	1638	2240
1426	26.49357195	23.74896657	24.59220345
18.11485911	22.32679901	15.22598537
ENSG00000225806 24.29619519	4.634755704	0.977798416	0
0	0	9.969583103	0	5.804665344
0.006966748	1	AL121917.1	20	58863528
58888809	-	1521	antisense	uncharacterized
LOC101927932 [Source:NCBI gene;Acc:101927932]	-	21	5
1	0	0	0	0.755065789	0.144935492
0.030456027	0	0	0
ENSG00000116754 5691.094483	5000.901405	5269.355665

4556.766815	4085.867512	3967.054345	5320.450517
4203.229557	0.339965601	0.006973837	0.047198822	SRSF11
	1	70205682	70253052	+	8473
protein_coding	serine and arginine rich splicing factor 11
[Source:HGNC Symbol;Acc:HGNC:10782]	-	4919	5395	5389
	4536	4471	4043	31.74931275	28.07295959
29.46276033	25.88433763	22.99461069	22.27473627
ENSG00000198732 1392.981858	1479.414021	1813.816062
1011.61027	1006.159725	1352.114986	1562.070647
1123.294993	0.476041604	0.006974694	0.047198822	SMOC1
	14	69854131	70032366	+	4369
protein_coding	SPARC related modular calcium binding 1 [Source:HGNC
Symbol;Acc:HGNC:20318]	-	1204	1596	1855	1007	1101
	1378	15.07089885	16.10589266	19.66818683
11.14419345	10.98155578	14.72357282
ENSG00000170775 31.23796524	43.56670362	81.15726854
18.082408	18.27719755	5.887293117	51.98731247
14.08229955	1.884810316	0.006979198	0.047202846	GPR37
	7	124745997	124765627	-	3021
protein_coding	G protein-coupled receptor 37 [Source:HGNC
Symbol;Acc:HGNC:4494]	-	27	47	83	18	20
	6	0.488773613	0.685932042	1.272711074
0.288086559	0.288494719	0.092714265
ENSG00000128524 596.9922247	1197.620874	921.086108
1247.686152	1583.719168	1280.486253	905.2330689
1370.630524	-0.597450431	0.006981464	0.047202846
ATP6V1F 7	128862826	128865844	+	789
protein_coding	ATPase H+ transporting V1 subunit F [Source:HGNC
Symbol;Acc:HGNC:16832]	-	516	1292	942	1242	1733
	1305	35.76575615	72.19705265	55.3065229
76.11071628	95.71503385	77.21106525
ENSG00000185043 905.9009921	1218.94075	1026.688337
1179.374833	1504.213358	1792.680754	1050.510026
1492.089648	-0.505726401	0.007010745	0.047379864	CIB1
	15	90229975	90234047	-	1472
protein_coding	calcium and integrin binding 1 [Source:HGNC
Symbol;Acc:HGNC:16920]	-	783	1315	1050	1174	1646
	1827	29.09033113	39.3869091	33.04334044
38.56217342	48.72822801	57.93977084
ENSG00000101955 1903.201957	1902.103741	1572.299853
1151.246642	1473.142122	1372.720512	1792.535184
1332.369759	0.427685312	0.007019865	0.047420535	SRPX
	X	38149336	38220924	-	1987
protein_coding	sushi repeat containing protein X-linked [Source:HGNC
Symbol;Acc:HGNC:11309]	-	1645	2052	1608	1146	1612
	1399	45.27544712	45.53165613	37.4878586
27.88611198	35.35295888	32.86744033
ENSG00000160752 1412.650206	2011.483976	1992.753172
1169.32905	1085.665534	1556.207814	1805.629118
1270.4008	0.507801827	0.007046235	0.047577649	FDPS

1	155308748	155320666	+	3718
protein_coding	farnesyl diphosphate synthase [Source:HGNC
Symbol;Acc:HGNC:3631]	-	1221	2170	2038	1164	1188
1586	17.95977912	25.73263776	25.39201992
15.13717429	13.92405253	19.91314396
ENSG00000101972 5856.540002	4979.581529	5486.426913
4371.924422	4599.456763	3697.220077	5440.849481
4222.867088	0.365444379	0.007058821	0.047641587	STAG2
X	123960212	124422664	+	8187
protein_coding	stromal antigen 2 [Source:HGNC Symbol;Acc:HGNC:11355]
-	5062	5372	5611	4352	5033	3768
33.81365079	28.9297827	31.74811422	25.70190493
26.78926126	21.48484078
ENSG00000159055 532.2023708	598.810437	618.9463974
348.588643	340.8697343	486.6828977	583.3197351
392.0470917	0.573923676	0.007064871	0.047661375	MIS18A
21	32268219	32279069	-	1723
protein_coding	MIS18 kinetochore protein A [Source:HGNC
Symbol;Acc:HGNC:1286]	-	460	646	633	347	373
496	14.60048448	16.53031763	17.01848469
9.737453883	9.433701975	13.43824129
ENSG00000228300 1020.440198	1391.353662	1178.247091
1574.174074	1495.074759	1830.948159	1196.680317
1633.398998	-0.448075171	0.007072615	0.047692574
C19orf24	19	1275438 1279249 +	2193	protein_coding
chromosome 19 open reading frame 24 [Source:HGNC
Symbol;Acc:HGNC:26073]	-	882	1501	1205	1567	1636
1866	21.99503545	30.17699692	25.45369706
34.54868973	32.50897417	39.72089665
ENSG00000088833 1192.827488	1635.141812	1397.273937
2098.563906	1754.610965	1858.422194	1408.414412
1903.865688	-0.434112868	0.007123752	0.048016224	NSFL1C
20	1442162 1473842 -	6531	protein_coding	NSFL1
cofactor [Source:HGNC Symbol;Acc:HGNC:15912]	-	1031	1764
1429	2089	1920	1894	8.633237615	11.90838467
10.13572925	15.46535782	12.81091479	13.53774492
ENSG00000235865 53.22023708	17.61207168	45.95652556
10.04578222	0	9.812155195	38.92961144	6.619312472
2.562356546	0.007126933	0.048016491	GSN-AS1 9
121280768	121285530	-	4763	antisense
GSN antisense RNA 1 [Source:HGNC Symbol;Acc:HGNC:23372] -
46	19	47	10	0	10	0.528167851
0.175876161	0.457108958	0.10151276	0
0.098008886
ENSG00000153147 3352.874936	2967.170602	2944.151031
2469.25327	2524.994841	2246.002324	3088.065523
2413.416812	0.355370152	0.007130333	0.048018236
SMARCA5 4	143513463	143557486	+	8385
protein_coding	"SWI/SNF related, matrix associated, actin dependent
regulator of chromatin, subfamily a, member 5 [Source:HGNC

Symbol;Acc:HGNC:11101]" - 2898 3201 3011 2458 2763
2289 18.90122826 16.83125753 16.63451505
14.1735953 14.35940404 12.74350112
ENSG00000253471 0 0 0 5.02289111 9.138598774
12.75580175 0 8.972430546 -5.585609877
0.007143462 1 AC012574.1 8 23707141
23789487 + 252 lincRNA novel transcript -
0 0 0 5 10 13 0 0
0 0.959335863 1.729251085 2.408179457
ENSG00000175745 2556.885303 1821.458992 3300.069655
2019.202226 1390.894733 1834.873021 2559.471317
1748.323327 0.549941277 0.007161339 0.048205811 NR2F1
5 93583224 93594615 + 4314
protein_coding nuclear receptor subfamily 2 group F member 1
[Source:HGNC Symbol;Acc:HGNC:7975] RXR-like 2210 1965
3375 2010 1522 1870 28.01604625 20.08243509
36.24065949 22.52771448 15.37422064 20.23520002
ENSG00000197756 11186.66244 13119.1395 13309.79204
13525.64118 16342.55619 20127.67395 12538.53133
16665.29044 -0.410434116 0.007168988 0.048217827 RPL37A
2 216498189 216579180 + 6841
protein_coding ribosomal protein L37a [Source:HGNC
Symbol;Acc:HGNC:10348] - 9669 14153 13612 13464 17883
20513 77.29593991 91.21428837 92.17322983
95.1602839 113.9145985 139.9766596
ENSG00000144868 152.7189412 160.3625474 209.2488611
271.2361199 256.7946256 332.6320611 174.1101165
286.8876022 -0.719366962 0.007169433 0.048217827
TMEM108 3 133038391 133397792 + 8425
protein_coding transmembrane protein 108 [Source:HGNC
Symbol;Acc:HGNC:28451] - 132 173 214 270 281
339 0.856838031 0.905336753 1.176647346
1.549512454 1.453432972 1.878346995
ENSG00000108592 5360.203444 5521.847946 5497.182696
7175.70224 6079.909764 7482.749551 5459.744695
6912.787185 -0.340350562 0.007192785 0.048353605 FTSJ3
17 63819433 63830012 - 5212
protein_coding FtsJ RNA methyltransferase homolog 3 [Source:HGNC
Symbol;Acc:HGNC:17136] - 4633 5957 5622 7143 6653
7626 48.61302047 50.39148432 49.96764599
66.2639712 55.62521648 68.30278777
ENSG00000137825 173.5442514 241.0072966 261.0721771
377.7214115 335.386575 358.1436646 225.2079084
357.0838837 -0.662723208 0.007201377 0.048390091 ITPKA
15 41493393 41503551 + 2092
protein_coding inositol-trisphosphate 3-kinase A [Source:HGNC
Symbol;Acc:HGNC:6178] - 150 260 267 376 367
365 3.921247832 5.479559231 5.912239749
8.690152169 7.644744617 8.144739906
ENSG00000159063 1623.217231 1406.184881 1712.125027

1034.715569	1121.30607	1347.208908	1580.509046
1167.743516	0.436498163	0.007215301	0.048462347	ALG8
	11	78100936	78139660	-	3448
protein_coding	"ALG8, alpha-1,3-glucosyltransferase [Source:HGNC
Symbol;Acc:HGNC:23161]" -	1403	1517	1751	1030	1227
	1373	22.25282367	19.39779126	23.52455109
14.44345804	15.50728981	18.58871273
ENSG00000259146 26.61011854	17.61207168	9.777984162	0
	1.827719755	2.943646558	18.00005813	1.590455438
	3.49138423	0.007255194	1	AC005476.2	14
	71292729	71321814	-	973	antisense
	"novel transcript, antisense to SIPA1L1"	-	23
	19	10	0	2	3	1.292735599
0.860943633	0.47609061	0	0.089572718	0.143931036
ENSG00000117318 2677.209318	3295.311306	3581.675598
2258.291843	2371.466382	2618.864221	3184.732074
2416.207482	0.398712277	0.007256373	0.048716811	ID3
	1	23557918	23559794	-	1797
protein_coding	"inhibitor of DNA binding 3, HLH protein [Source:HGNC
Symbol;Acc:HGNC:5362]"	bHLH	2314	3555	3663	2248	2595
	2669	70.42226517	87.22188856	94.42593576
60.4852453	62.92857289	69.33404412
ENSG00000231672 189.7417148	237.2994921	72.3570828
220.0026306	549.2297863	281.6088541	166.4660966
350.2804237	-1.073926557	0.007286695	0.048878745	DIRC3
	2	217284019	217756593	-	4665
lincRNA disrupted in renal carcinoma 3 [Source:HGNC
Symbol;Acc:HGNC:17805]	-	164	256	74	219	601
	287	1.922591034	2.41948133	0.734822638
2.269831837	5.614116514	2.871946097
ENSG00000174233 733.5137024	812.9361505	1084.378444
635.8980145	574.8178629	627.9779325	876.9427655
612.8979366	0.517507402	0.007286888	0.048878745	ADCY6
	12	48766194	48789037	-	8410
protein_coding	adenylate cyclase 6 [Source:HGNC Symbol;Acc:HGNC:237]
	-	634	877	1109	633	629	640
4.122759251	4.597667451	6.108548215	3.639225197
3.259216778	3.552466687
ENSG00000140718 2145.006947	1903.030692	2043.59869
1766.048514	1458.520364	1384.495098	2030.545443
1536.354659	0.402293898	0.007306075	0.048967432	FTO
	16	53701692	54158512	+	21379
protein_coding	"FTO, alpha-ketoglutarate dependent dioxygenase
[Source:HGNC Symbol;Acc:HGNC:24678]"	-	1854	2053	2090
	1758	1596	1411	4.742606097	4.233850513
4.528572809	3.975874799	3.253150066	3.080957179
ENSG00000080493 68.26073887	54.69011731	84.09066379
29.13276844	22.84649694	27.47403455	69.01383999
26.48443331	1.382448204	0.007306516	0.048967432	SLC4A4
	4	71062667	71572087	+	9622

protein_coding solute carrier family 4 member 4 [Source:HGNC
Symbol;Acc:HGNC:11030] - 59 59 86 29 25
28 0.335336921 0.270346416 0.414033569
0.145724932 0.113222634 0.135843454
ENSG00000143756 2864.637109 2570.435514 2198.09084
3418.57969 3935.080632 2881.829981 2544.387821
3411.830101 -0.423538782 0.007317689 0.049020823 FBXO28
1 224114087 224162047 + 6044
protein_coding F-box protein 28 [Source:HGNC Symbol;Acc:HGNC:29046]
- 2476 2773 2248 3403 4306 2937
22.40375927 20.22829935 17.22956477 27.22317092
31.04617975 22.68430755
ENSG00000116761 173.5442514 330.9215573 172.0925212
463.1105604 378.3379893 327.7259835 225.5194433
389.7248444 -0.786884089 0.007343448 0.049153016 CTH
1 70411218 70439851 + 2414
protein_coding cystathionine gamma-lyase [Source:HGNC
Symbol;Acc:HGNC:2501] - 150 357 176 461 414
334 3.398198204 6.520259917 3.377363907
9.233468589 7.473459289 6.458850569
ENSG00000113593 821.4427898 772.1503003 746.0601915
476.1700772 653.4098124 521.0254408 779.8844272
550.2017768 0.502513101 0.007344006 0.049153016 PPWD1
5 65563236 65587549 + 3777
protein_coding peptidylprolyl isomerase domain and WD repeat
containing 1 [Source:HGNC Symbol;Acc:HGNC:28954] - 710
833 763 474 715 531 10.28030682
9.723709476 9.35793467 6.067818383 8.249310579
6.562866531
ENSG00000130244 193.2125998 341.1180198 139.8251735
405.8496017 318.0232373 485.7016821 224.7185977
403.1915071 -0.84157328 0.007347069 0.049153016 FAM98C
19 38403135 38409088 + 4598
protein_coding family with sequence similarity 98 member C
[Source:HGNC Symbol;Acc:HGNC:27119] - 167 368 143
404 348 495 1.986287991 3.528684337
1.440686632 4.248284712 3.29813839 5.025534605
ENSG00000138798 49.74935206 26.88158308 57.69010655
7.032047554 16.44947779 12.75580175 44.77368056
12.07910903 1.88528818 0.007352412 0.049167243 EGF
4 109912884 110012266 + 6127
protein_coding epidermal growth factor [Source:HGNC
Symbol;Acc:HGNC:3229] - 43 29 59 7 18
13 0.383809118 0.20868156 0.446073668
0.055239708 0.128021592 0.099047041
ENSG00000120327 121.480976 110.3071858 59.64570339
205.9385355 205.6184724 152.0884055 97.1446217
187.8818045 -0.953715583 0.007357704 0.049181119
PCDHB14 5 141222932 141227759 + 4828
protein_coding protocadherin beta 14 [Source:HGNC

Symbol;Acc:HGNC:8685] - 105 119 61 205 225
155 1.189369371 1.086709986 0.585281813
2.052994643 2.030831329 1.498685387
ENSG00000100387 1066.718665 835.1829779 804.7280965
630.8751234 691.7919272 611.2972686 902.2099131
644.6547731 0.483934311 0.007374994 0.049275148 RBX1
22 40951347 40973309 + 3092
protein_coding ring-box 1 [Source:HGNC Symbol;Acc:HGNC:9928] -
922 901 823 628 757 623 16.30745349
12.84751885 12.32999231 9.820223566 10.66878571
9.405775253
ENSG00000070404 216.3518334 342.044971 225.8714341
337.5382826 474.2932764 457.2464321 261.4227462
423.025997 -0.692928558 0.007382975 0.049289167 FSTL3
19 676365 683399 + 3067 protein_coding
follistatin like 3 [Source:HGNC Symbol;Acc:HGNC:3973] - 187
369 231 336 519 466 3.334437207
5.304525581 3.488997512 5.296960299 7.374153602
7.092807555
ENSG00000130770 642.11373 892.6539486 936.7308827
386.7626155 540.0911876 681.944786 823.8328538
536.2661964 0.619906096 0.007383541 0.049289167
ATP5IF1 1 28236109 28246906 + 2225
protein_coding ATP synthase inhibitory factor subunit 1 [Source:HGNC
Symbol;Acc:HGNC:871] - 555 963 958 385 591
695 13.64136032 19.08227849 19.94517952
8.366271049 11.57486843 14.58145376
ENSG00000101974 1948.323462 1695.393637 1802.082481
1132.159656 1523.404416 1402.156977 1815.266527
1352.573683 0.423989887 0.007392526 0.049321817 ATP11C
X 139726346 139945276 - 7407
protein_coding ATPase phospholipid transporting 11C [Source:HGNC
Symbol;Acc:HGNC:13554] - 1684 1829 1843 1127 1667
1429 12.43353025 10.8869272 11.5261813
7.356695624 9.80735403 9.006082479
ENSG00000164024 2657.540969 2231.171396 2205.913227
1986.051145 1665.966557 1755.394564 2364.875197
1802.470755 0.391585975 0.007396488 0.049321817 METAP1
4 98995620 99062813 + 4117
protein_coding methionyl aminopeptidase 1 [Source:HGNC
Symbol;Acc:HGNC:15789] - 2297 2407 2256 1977 1823
1789 30.51229264 25.77681064 25.38403653
23.21811825 19.29587155 20.28502323
ENSG00000178927 1361.743892 1554.497063 1471.586616
1721.847073 2071.720342 1951.637668 1462.609191
1915.068361 -0.388648718 0.007398111 0.049321817 CYBC1
17 82442589 82450829 - 7234
protein_coding cytochrome b-245 chaperone 1 [Source:HGNC
Symbol;Acc:HGNC:28672] - 1177 1677 1505 1714 2267
1989 8.898005527 10.22088534 9.637412573

11.45601384	13.65625487	12.83519037
ENSG00000103496 720.787124	915.8277272	743.1267963
1030.697256	946.758833	1467.898417	793.2472158
1148.451502	-0.533198671	0.007406869	0.049358676	STX4
	16	31032889	31042975	+	4078
protein_coding	syntaxin 4 [Source:HGNC Symbol;Acc:HGNC:11439]	-
	623	988	760	1026	1036	1496	8.354789994
	10.68178128	8.633140853	12.16469868	11.0705993
	17.12499321
ENSG00000151023 65.94681552	113.0880392	17.60037149
167.7645631	202.8768928	129.5204486	65.5450754
166.7206348	-1.345690341	0.007410171	0.049359166	ENKUR
	10	24981979	25062279	-	4076
protein_coding	"enkurin, TRPC channel interacting protein
[Source:HGNC Symbol;Acc:HGNC:28388]"	-	57	122	18
	167	222	132	0.764778012	1.319652587
0.204569454	1.980995606	2.373435297	1.51177024
ENSG00000258565 15.04050178	0	17.60037149	0	0
	0	10.88029109	0	5.934502442	0.007414869
	1	BLZF2P	14	68867857	68869951	-
	1130	unprocessed_pseudogene	basic leucine zipper nuclear
factor 2 pseudogene [Source:HGNC Symbol;Acc:HGNC:20049] -	13
	0	18	0	0	0	0.629157853	0
	0.737898313	0	0	0
ENSG00000159593 2795.219409	2349.821142	2015.242536
1820.295738	1734.506047	1833.891806	2386.761029
1796.231197	0.409746566	0.007419033	0.049396674	NAE1
	16	66802875	66873256	-	4810
protein_coding	NEDD8 activating enzyme E1 subunit 1 [Source:HGNC
Symbol;Acc:HGNC:621]	-	2416	2535	2061	1812	1898
	1869	27.4692351	23.23629306	19.84885098
18.21437751	17.1952989	18.13887136
ENSG00000120899 69.41770054	113.0880392	53.77891289
177.8103453	134.337402	165.8254228	78.76155087
159.32439	-1.013518705	0.007435219	0.049482886	PTK2B
	8	27311482	27459391	+	7933
protein_coding	protein tyrosine kinase 2 beta [Source:HGNC
Symbol;Acc:HGNC:9612]	-	60	122	55	177	147
	169	0.413626646	0.678041591	0.321164616
1.078790289	0.807492465	0.994478243
ENSG00000173597 3.470885027	4.634755704	0.977798416
17.07782977	47.52071363	7.849724156	3.027813049
24.14942252	-2.997127724	0.007463995	0.049652782
SULT1B1 4	69721162	69787961	-	7356
protein_coding	sulfotransferase family 1B member 1 [Source:HGNC
Symbol;Acc:HGNC:17845]	-	3	5	1	17	52
	8	0.022303563	0.029968309	0.006297392
0.111739936	0.308049296	0.050768497
ENSG00000146535 4924.028892	4512.398154	6049.638801
4110.734085	3870.196581	3960.185837	5162.021949

3980.372167 0.375067319 0.007473838 0.049696637 GNA12
7 2728112 2844324 - 6120 protein_coding G
protein subunit alpha 12 [Source:HGNC Symbol;Acc:HGNC:4380] -
4256 4868 6187 4092 4235 4036 38.03162743
35.06978516 46.83075394 32.32848995 30.15508731
30.78546879
ENSG00000189212 357.5011578 303.1130231 244.449604
231.0529911 150.7868798 153.069621 301.6879283
178.303164 0.75859955 0.007478664 0.0497071
DPY19L2P1 7 35079989 35186041 - 8272
transcribed_unprocessed_pseudogene DPY19L2 pseudogene 1
[Source:HGNC Symbol;Acc:HGNC:22305] - 309 327 250
230 165 156 2.042879105 1.742894843
1.400012582 1.344369115 0.869224615 0.880359608
ENSG00000186907 236.0201818 569.1480005 446.8538762
729.3237892 710.0691248 616.2033462 417.3406862
685.1987534 -0.713175289 0.007483922 0.049720432
RTN4RL2 11 57460549 57477534 + 2387
protein_coding reticulon 4 receptor like 2 [Source:HGNC
Symbol;Acc:HGNC:23053] - 204 614 457 726 777
628 4.67382515 11.34096239 8.868828092
14.70569039 14.18493002 12.28155226
ENSG00000174015 0 15.75816939 0.977798416 26.11903377
77.67808958 19.62431039 5.578655937 41.14047791
-2.858382505 0.007490394 0.049741816 SPERT 13
45702311 45714559 + 2125 protein_coding
spermatid associated [Source:HGNC Symbol;Acc:HGNC:30720]
- 0 17 1 26 85 20 0
0.352715013 0.021799349 0.591582925 1.743085094
0.439356542
ENSG00000114107 447.7441685 326.2868016 484.010216
168.7691413 297.0044602 291.4210093 419.347062
252.3982036 0.731252017 0.007493923 0.049743641 CEP70
3 138494344 138594538 - 4420
protein_coding centrosomal protein 70 [Source:HGNC
Symbol;Acc:HGNC:29972] - 387 352 495 168 325
297 4.788322669 3.511190171 5.187825809
1.837757606 3.204200541 3.136752233
ENSG00000147465 18.51138681 4.634755704 3.911193665 0
0 0 9.01911206 0 5.660909064
0.007497782 1 STAR 8 38143649 38151265
- 3767 protein_coding steroidogenic acute regulatory
protein [Source:HGNC Symbol;Acc:HGNC:11359] - 16 5
4 0 0 0 0.232283882 0.058520542
0.049188868 0 0 0
ENSG00000141568 1716.931127 2018.899585 2273.381318
3025.789605 2547.841338 2437.33935 2003.070676
2670.323431 -0.414378554 0.00749924 0.049757328 FOXK2
17 82519713 82644662 + 9991
protein_coding forkhead box K2 [Source:HGNC Symbol;Acc:HGNC:6036]

Fork	1484	2178	2325	3012	2788	2484
8.12305988	9.611316404	10.77994274	14.57629755
12.16024733	11.60616307
ENSG00000080189 2000.386737	2707.624282	2622.455352
3608.444974	2989.235659	3163.438835	2443.488791
3253.706489	-0.412639345	0.007507038	0.049787461
SLC35C2 20	46345980	46364458	-	7708
protein_coding	solute carrier family 35 member C2 [Source:HGNC
Symbol;Acc:HGNC:17117]	-	1729	2921	2682	3592	3271
3224	12.26727214	16.7079747	16.11831071
22.53179745	18.49257701	19.52537407
ENSG00000130956 284.6125722	324.4328993	385.252576
507.3120021	483.4318752	477.851958	331.4326825
489.5319451	-0.561559138	0.007513	0.049805388	HABP4
9	96450201	96491336	+	4056
protein_coding	hyaluronan binding protein 4 [Source:HGNC
Symbol;Acc:HGNC:17062]	-	246	350	394	505	529
487	3.316896145	3.804556657	4.499877916
6.019974454	5.683506008	5.605018505
ENSG00000099194 14149.64129	12981.02378	16446.56936
10455.65013	10788.11585	12658.66142	14525.74481
11300.80913	0.362188205	0.00752267	0.049847882	SCD
10	100347124	100364834	+	5362
protein_coding	stearoyl-CoA desaturase [Source:HGNC
Symbol;Acc:HGNC:10571]	-	12230	14004	16820	10408	11805
12901	124.7367315	115.148756	145.3120527
93.85160204	95.93957262	112.3162517
ENSG00000125148 18389.90584	15346.60309	14225.98916
17879.4832	25228.92964	20415.1701	15987.49936
21174.52764	-0.405460742	0.007535086	0.049908515	MT2A
16	56608199	56609497	+	1299
protein_coding	metallothionein 2A [Source:HGNC Symbol;Acc:HGNC:7406]
-	15895	16556	14549	17798	27607	20806
669.1848403	561.92742	518.8316349	662.4655029
926.122983	747.6967259
ENSG00000143093 1504.050178	1615.675838	1598.70041
2111.623423	1877.982048	2084.101763	1572.808809
2024.569078	-0.363957586	0.007540678	0.049911791	STRIP1
1	110031577	110074641	+	7352
protein_coding	striatin interacting protein 1 [Source:HGNC
Symbol;Acc:HGNC:25916]	-	1300	1743	1635	2102	2055
2124	9.670135658	10.45263648	10.30183796
13.82383145	12.18049466	13.48636936
ENSG00000268942 52.06327541	41.71280134	18.57816991
5.02289111	9.138598774	11.77458623	37.45141555
8.645358706	2.111484308	0.007543479	0.049911791
CKS1BP3 5	62512246	62512482	-	237
processed_pseudogene	CDC28 protein kinase regulatory subunit 1B
pseudogene 3 [Source:HGNC Symbol;Acc:HGNC:24233]	-	45
45	19	5	10	12	10.38386135

8.371400616 3.713707637 1.020053323 1.838697357
2.363626965
ENSG00000105185 1458.928673 1691.685832 1503.853964
925.2165425 1238.280134 1253.012218 1551.48949
1138.836298 0.446115406 0.007546552 0.049911791 PDCD5
19 32581068 32587452 + 2253
protein_coding programmed cell death 5 [Source:HGNC
Symbol;Acc:HGNC:8764] - 1261 1825 1538 921 1355
1277 30.60896238 35.71376487 31.62260182
19.76512908 26.20817025 26.45914104
ENSG00000052795 805.2453263 747.1226195 701.0814644
902.1112434 1157.860465 1024.389002 751.1498034
1028.120237 -0.453495283 0.00754864 0.049911791 FNIP2
4 158769138 158908049 + 10868
protein_coding folliculin interacting protein 2 [Source:HGNC
Symbol;Acc:HGNC:29280] - 696 806 717 898 1267
1044 3.502307891 3.269786301 3.056131109
3.995102474 5.08025583 4.484323186
ENSG00000241560 21.98227184 0 10.75578258 0 0
0 10.91268481 0 5.936891929 0.007549192
1 ZBTB20-AS1 3 114351811 114388978
+ 2035 antisense ZBTB20 antisense RNA 1
[Source:HGNC Symbol;Acc:HGNC:40640] - 19 0 11
0 0 0 0.510603633 0 0.250397926
0 0 0
ENSG00000166848 2313.923351 2976.440113 2254.803148
3069.991047 3685.596886 3242.917292 2515.055537
3332.835075 -0.405967135 0.007552685 0.049916946
TERF2IP 16 75647786 75761872 + 3379
protein_coding TERF2 interacting protein [Source:HGNC
Symbol;Acc:HGNC:19246] - 2000 3211 2306 3056 4033
3305 32.36953917 41.8973034 31.61357183
43.72868068 52.01141007 45.65930618
ENSG00000171094 11.56961676 65.813531 13.68917783
74.33878843 105.0938859 107.9337071 30.35744186
95.78879382 -1.64871734 0.007574206 0.050037552 ALK
2 29192774 29921566 - 7362
protein_coding ALK receptor tyrosine kinase [Source:HGNC
Symbol;Acc:HGNC:427] - 10 71 14 74 115
110 0.074284619 0.42520317 0.088091637
0.486000956 0.68070764 0.697497906
ENSG00000005175 1323.564157 1182.789656 1134.246163
953.3447327 954.069712 791.8409242 1213.533325
899.7517896 0.431167857 0.007591565 0.050115693 RPAP3
12 47661249 47706061 - 5381
protein_coding RNA polymerase II associated protein 3 [Source:HGNC
Symbol;Acc:HGNC:26151] - 1144 1276 1160 949 1044
807 11.62673423 10.4549423 9.986135462
8.527160489 8.454659163 7.000943593
ENSG00000105518 274.1999171 325.3598504 246.4052009

388.7717719	413.9785245	479.814389	281.9883228
427.5215618	-0.599771081	0.007594072	0.050115693
TMEM205 19	11342776	11346518	-	1606
protein_coding	transmembrane protein 205 [Source:HGNC
Symbol;Acc:HGNC:29631]	-	237	351	252	387	453
489	8.070445663	9.635972092	7.2687119
11.65109224	12.29168038	14.2137723
ENSG00000161547 6376.015795	5762.855243	5374.957894
4456.308993	4767.60698	4695.116261	5837.942977
4639.677411	0.331282133	0.00759669	0.050115693	SRSF2
17	76734115	76737374	-	3017
protein_coding	serine and arginine rich splicing factor 2
[Source:HGNC Symbol;Acc:HGNC:10783]	-	5511	6217	5497
4436	5217	4785	99.89639446	90.85305078
84.40202813	71.0914617	75.35362062	74.0376572
ENSG00000119640 114.5392059	136.2618177	143.7363672
42.19228532	68.53949081	80.4596726	131.5124636
63.73048291	1.045137786	0.007599148	0.050115693	ACYP1
14	75053221	75069483	-	1841
protein_coding	acylphosphatase 1 [Source:HGNC Symbol;Acc:HGNC:179]
-	99	147	147	42	75	82
2.940871975	3.520444518	3.698843888	1.103053859
1.77527678	2.079247077
ENSG00000066651 629.3871516	659.9892123	548.5449115
473.1563426	388.3904479	424.8663199	612.6404251
428.8043701	0.515056757	0.007608325	0.050154576	TRMT11
6	125986430	126203817	+	5771
protein_coding	tRNA methyltransferase 11 homolog [Source:HGNC
Symbol;Acc:HGNC:21080]	-	544	712	561	471	425
433	5.155164619	5.4395488	4.503127491
3.946126919	3.209197561	3.502538017
ENSG00000183010 864.2503718	1312.562815	1121.534783
1511.890224	1381.756135	1714.183513	1099.449324
1535.94329	-0.481367378	0.007619876	0.050189335	PYCR1
17	81932384	81942412	-	2886
protein_coding	pyrroline-5-carboxylate reductase 1 [Source:HGNC
Symbol;Acc:HGNC:9721]	-	747	1416	1147	1505	1512
1747	14.15529706	21.63220971	18.41066802
25.21397917	22.83042847	28.25807764
ENSG00000161526 1624.374193	1901.17679	1825.549643
2187.971368	2401.623758	2302.912824	1783.700209
2297.50265	-0.36487453	0.007620164	0.050189335
SAP30BP 17	75667116	75708062	+	8268
protein_coding	SAP30 binding protein [Source:HGNC
Symbol;Acc:HGNC:30785]	-	1404	2051	1867	2178	2628
2347	9.286698639	10.93702363	10.46035216
12.73674999	13.85107531	13.25130523
ENSG00000162614 327.4201542	230.8108341	220.9824421
139.6363729	94.12756737	193.2994573	259.7378101
142.3544659	0.867252099	0.007624296	0.050194923	NEXN

1	77888513	77943895	+	4436
protein_coding	nexilin F-actin binding protein [Source:HGNC
Symbol;Acc:HGNC:29557]	-	283	249	226	139	103
197	3.488908749	2.474809461	2.360039965
1.515041326	1.011822389	2.073102257
ENSG00000171056 70.57466222	79.71779811	111.4690194
55.25180221	23.76035681	18.64309487	87.25382659
32.5517513	1.425685389	0.007645685	0.050314069	SOX7
8	10723768	10730512	-	3216
protein_coding	SRY-box 7 [Source:HGNC Symbol;Acc:HGNC:18196]	HMG
61	86	114	55	26	19	1.037309864
1.179006961	1.642068489	0.82689024	0.352302647
0.275793227
ENSG00000257219 57.84808379	44.49365476	10.75578258
82.37541421	105.0938859	122.6519399	37.69917371
103.3737467	-1.460262714	0.007655796	0.050358933
LINC02407	12	76259839	76305131	+	1201
lincRNA long intergenic non-protein coding RNA 2407
[Source:HGNC Symbol;Acc:HGNC:53336]	-	50	48	11
82	115	125	2.276783365	1.762106641
0.424279583	3.301201711	4.172664151	4.858621207
ENSG00000152642 1187.042679	975.1526002	1174.335898
885.0334136	823.3877496	746.7050103	1112.177059
818.3753912	0.442192957	0.007661065	0.050371913	GPD1L
3	32105689	32168713	+	4802
protein_coding	glycerol-3-phosphate dehydrogenase 1 like [Source:HGNC
Symbol;Acc:HGNC:28956]	-	1026	1052	1201	881	901
761	11.68476326	9.65889716	11.58572745
8.8706403	8.176383121	7.397901369
ENSG00000065548 2436.561289	2525.014908	2083.688425
1811.254534	1899.000825	1726.939314	2348.421541
1812.398225	0.373689231	0.007673906	0.050434655	ZC3H15
2	186486156	186509363	+	3125
protein_coding	zinc finger CCCH-type containing 15 [Source:HGNC
Symbol;Acc:HGNC:29528]	-	2106	2724	2131	1803	2078
1760	36.85556369	38.43182778	31.58900044
27.89632034	28.97704661	26.29109546
ENSG00000255458 19.66834849	2.780853423	4.888992081	0
0	0	9.11273133	0	5.675255406
0.007683737	1	AC108471.2	4	39639140
39666644	+	2301	lincRNA novel transcript	-
17	3	5	0	0	0	0.404042468
0.05748289	0.100659749	0	0	0
ENSG00000105339 151.5619795	150.1660848	251.294193
72.32963199	97.78300688	116.7646468	184.3407524
95.6257619	0.947167588	0.007689903	0.050476849	DENND3
8	141117278	141195808	+	14670
protein_coding	DENN domain containing 3 [Source:HGNC
Symbol;Acc:HGNC:29134]	-	131	162	257	72	107
119	0.488355288	0.486876551	0.811531656

0.237303202	0.317842715	0.37867173
ENSG00000148225 78.67339395	85.27950496	107.5578258
164.7508284	143.4760008	194.2806729	90.50357489
167.5025007	-0.886493343	0.007690963	0.050476849	WDR31
9	113313222	113340298	-	4933
protein_coding	WD repeat domain 31 [Source:HGNC
Symbol;Acc:HGNC:21421]	-	68	92	110	164	157
198	0.753863142	0.822262851	1.032961239
1.607436957	1.386906344	1.873700232
ENSG00000198938 31749.3423	41972.34766	52026.69813
54188.95845	50394.80294	68184.66645	41916.12936
57589.47595	-0.458273719	0.007693218	0.050476849	MT-CO3
MT	9207	9990	+	784	protein_coding
mitochondrially encoded cytochrome c oxidase III [Source:HGNC
Symbol;Acc:HGNC:7422]	-	27442	45280	53208	53942	55145
69490	1914.231286	2546.386443	3143.86094
3326.688972	3065.128428	4137.636028
ENSG00000130396 1513.305872	1319.978425	1456.91964
1052.797977	1058.249738	1154.890666	1430.067979
1088.646127	0.393271072	0.007693534	0.050476849	AFDN
6	167826922	167972023	+	13720
protein_coding	"afadin, adherens junction formation factor
[Source:HGNC Symbol;Acc:HGNC:7137]"	-	1308	1424	1490
1048	1158	1177	5.213727701	4.576040251
5.030771742	3.693247289	3.678011186	4.004685242
ENSG00000263680 11.56961676	8.342560268	5.866790497	0
0	0	8.592989174	0	5.594481407
0.00770414	1	AC007639.1	17	72425939
72428852	+	833	lincRNA novel transcript	-
10	9	6	0	0	0	0.656522646
0.476355809	0.333663503	0	0	0
ENSG00000260536 13.88354011	11.12341369	1.955596832	0
0	0	8.987516877	0	5.658860764
0.007795914	1	AL035071.2	20	32843128
32854257	-	459	antisense	"novel transcript,
antisense to MAPRE1"	-	12	12	2	0	0
0	1.42976043	1.15266344	0.201845823	0
0	0
ENSG00000111817 966.0629992	852.7950496	1041.355313
699.1864425	691.7919272	719.2309758	953.404454
703.4031152	0.438568512	0.007804199	0.051180949	DSE
6	116254173	116444860	+	19193
protein_coding	dermatan sulfate epimerase [Source:HGNC
Symbol;Acc:HGNC:21144]	-	835	920	1065	696	757
733	2.379240396	2.113386467	2.570450236
1.753345862	1.718745658	1.782818562
ENSG00000135912 651.3694234	768.4424958	632.6355753
1008.596535	872.7361829	929.2110969	684.1491648
936.8479383	-0.4528511	0.007825168	0.051296457	TTLL4
2	218710845	218755416	+	7685

protein_coding tubulin tyrosine ligase like 4 [Source:HGNC Symbol;Acc:HGNC:28976] - 563 829 647 1004 955
947 4.006445467 4.756030343 3.899984353
6.316711724 5.415244844 5.752440602
ENSG00000243156 461.6277086 661.8431146 738.2378042
1154.260377 685.3949081 1005.745907 620.5695425
948.4670642 -0.61069314 0.007830641 0.051296823 MICAL3
22 17787649 18024559 - 22354
protein_coding "microtubule associated monooxygenase, calponin and LIM domain containing 3 [Source:HGNC Symbol;Acc:HGNC:24694]" -
399 714 755 1149 750 1025 0.976140567
1.408240802 1.564566982 2.48522663 1.462058044
2.140497153
ENSG00000273033 349.4024261 247.4959546 258.1387819
546.4905528 374.6825497 417.9978113 285.0123875
446.3903046 -0.648690783 0.007832499 0.051296823
LINC02035 3 122886941 122892416 + 5476
lincRNA long intergenic non-protein coding RNA 2035 [Source:HGNC Symbol;Acc:HGNC:52875] - 302 267 264
544 410 426 3.016047773 2.14971942
2.2332788 4.803266427 3.262714064 3.6315514
ENSG00000151320 64.78985384 75.08304241 50.84551764
132.6043253 170.8917971 93.21547435 63.57280463
132.2371989 -1.056916921 0.007839189 0.051296823 AKAP6
14 32329273 32837681 + 19748
protein_coding A-kinase anchoring protein 6 [Source:HGNC Symbol;Acc:HGNC:376] - 56 81 52 132 187
95 0.155081367 0.180840566 0.121978329
0.32318562 0.412645474 0.224567303
ENSG00000259431 34.70885027 69.52133556 38.13413823
164.7508284 72.19493032 104.9900606 47.45477469
113.9786064 -1.258883594 0.007845926 0.051296823 THTPA
14 23555988 23560271 + 2894
protein_coding thiamine triphosphatase [Source:HGNC Symbol;Acc:HGNC:18987] - 30 75 39 164 79
107 0.566914338 1.142606511 0.624264353
2.739974606 1.189562219 1.725962226
ENSG00000171148 2687.621973 3172.026804 3131.888327
3533.101607 3980.773626 3975.885285 2997.179035
3829.920173 -0.353484337 0.007846654 0.051296823 TADA3
3 9779860 9793011 - 4234 protein_coding
transcriptional adaptor 3 [Source:HGNC Symbol;Acc:HGNC:19422] -
2323 3422 3203 3517 4356 4052 30.00496116
35.6338797 35.04358598 40.16268427 44.83277439
44.67500593
ENSG00000134686 5610.107166 7308.082794 6676.407586
7753.334718 8698.118313 8681.794916 6531.532515
8377.749316 -0.35898511 0.007851744 0.051296823 PHC2
1 33323623 33431052 - 4975
protein_coding polyhomeotic homolog 2 [Source:HGNC

Symbol;Acc:HGNC:3183]	-	4849	7884	6828	7718	9518
	8848	53.303265	69.86947637	63.57741752
75.00891883	83.37027099	83.02293163
ENSG00000105705 625.9162666	929.7319943	803.7502981
1345.130239	875.4777626	1232.406692	786.4661863
1151.004898	-0.548256777	0.007851843	0.051296823	SUGP1
	19	19276018	19320844	-	5356
protein_coding	SURP and G-patch domain containing 1 [Source:HGNC
Symbol;Acc:HGNC:18643]	-	541	1003	822	1339	958
	1256	5.523971249	8.256468392	7.109412362
12.08763187	7.794415236	10.94699972
ENSG00000106105 7836.10143	7379.458032	6374.267875
9332.531683	9535.213961	8462.983855	7196.609112
9110.243166	-0.340266444	0.007852068	0.051296823	GARS
	7	30594681	30634033	+	4152
protein_coding	glycyl-tRNA synthetase [Source:HGNC
Symbol;Acc:HGNC:4162]	-	6773	7961	6519	9290	10434
	8625	89.21100738	84.53649493	72.73209411
108.1831408	109.5095729	96.97232196
ENSG00000117122 579.6377995	699.8481113	862.4182031
377.7214115	509.0199517	546.5370443	713.968038
477.7594692	0.579949108	0.007855643	0.051298254	MFAP2
	1	16974502	16980835	-	2915
protein_coding	microfibril associated protein 2 [Source:HGNC
Symbol;Acc:HGNC:7033]	-	501	755	882	376	557
	557	9.399264683	11.41937541	14.01627087
6.236637508	8.326744403	8.919955174
ENSG00000159714 500.9644056	957.5405285	754.8603773
1112.068092	1156.032745	1025.370218	737.7884371
1097.823685	-0.572007552	0.007871512	0.051379935	ZDHHC1
	16	67394152	67416833	-	3540
protein_coding	zinc finger DHHC-type containing 1 [Source:HGNC
Symbol;Acc:HGNC:17916]	-	433	1033	772	1107	1265
	1045	6.689279569	12.86562882	10.10221237
15.1197836	15.57205257	13.78031244
ENSG00000100297 3397.996442	4032.237463	4494.939319
3051.908639	2785.444906	3294.921714	3975.057741
3044.091753	0.385237752	0.007874899	0.051380104	MCM5
	22	35400063	35425430	+	7338
protein_coding	minichromosome maintenance complex component 5
[Source:HGNC Symbol;Acc:HGNC:6948]	-	2937	4350	4597
	3038	3048	3358	21.88874954	26.13638432
29.02012322	20.01756644	18.10072011	21.3623497
ENSG00000010361 547.2428726	680.3821374	640.4579626
843.8457065	806.0244119	929.2110969	622.6943242
859.6937384	-0.464289377	0.007885609	0.051414177	FUZ
	19	49806869	49817376	-	2950
protein_coding	fuzzy planar cell polarity protein [Source:HGNC
Symbol;Acc:HGNC:26219]	-	473	734	655	840	882
	947	8.768672248	10.97003471	10.28541311

13.76760783	13.02882248	14.98559526
ENSG00000134250 10071.35139	9027.577161	11318.01667
13913.40838	11837.22699	12939.28906	10138.98174
12896.64147	-0.347077258	0.007886848	0.051414177	NOTCH2
1	119911553	120069626	-	16807
protein_coding	notch 2 [Source:HGNC Symbol;Acc:HGNC:7882]	-
8705	9739	11575	13850	12953	13187	28.32521977
25.54807155	31.9031272	39.84380352	33.58449043
36.62704162
ENSG00000180573 238.3341052	282.720098	199.4708769
453.0647781	323.5063966	363.0497422	240.1750267
379.873639	-0.660551456	0.007894092	0.051439467
HIST1H2AC	6	26124145	26139116	+	1668
protein_coding	histone cluster 1 H2A family member c
[Source:HGNC Symbol;Acc:HGNC:4733]	-	206	305	204
451	354	370	6.754075123	8.061906389
5.665478254	13.07319418	9.248383143	10.3550384
ENSG00000116171 1797.918444	1651.826933	1517.543142
1325.038675	1326.010682	1086.20558	1655.76284
1245.751646	0.410152955	0.007905686	0.051493063	SCP2
1	52927229	53051703	+	8305
protein_coding	sterol carrier protein 2 [Source:HGNC
Symbol;Acc:HGNC:10606]	-	1554	1782	1552	1319	1451
1107	10.23307343	9.460237094	8.656743229
7.679030194	7.613535435	6.222344038
ENSG00000108523 1136.136366	1811.262529	1479.409004
2042.307525	1796.648519	2379.447635	1475.602633
2072.801226	-0.489497515	0.007918411	0.051553085	RNF167
17	4940008 4945222 +	2744	protein_coding	ring
finger protein 167 [Source:HGNC Symbol;Acc:HGNC:24544]	-	982
1954	1513	2033	1966	2425	19.57140903
31.3960065	25.54213975	35.82238425	31.2218048
41.25472265
ENSG00000144152 112.2252825	99.18377207	308.9842995
34.15565955	96.86914701	80.4596726	173.4644514
70.49482638	1.298286151	0.007921646	0.051553085	FBLN7
2	112138385	112188216	+	6578
protein_coding	fibulin 7 [Source:HGNC Symbol;Acc:HGNC:26740]	-
97	107	316	34	106	82	0.80644096
0.717172894	2.225336387	0.249911513	0.702215795
0.581923665
ENSG00000277342 0	7.415609127	2.933395248	53.24264577
15.53561792	13.73701727	3.449668125	27.50509365
-2.963025456	0.007931673	0.051579025	AC048344.4
12	32109076	32109602	+	527
sense_intronic	"novel transcript, sense intronic to BICD1"	-
0	8	3	53	17	14	0
0.669288449	0.263701801	4.862576769	1.405713786
1.240119271
ENSG00000167680 350.5593877	598.810437	651.2137452

802.6579994	831.6124884	789.8784932	533.5278566
808.0496603	-0.597268506	0.00793238	0.051579025	SEMA6B
	19	4542593 4559808 -	4412	protein_coding
semaphorin 6B [Source:HGNC Symbol;Acc:HGNC:10739]	-	303
	646	666	799	910	805	3.755794637
6.455516154	6.992640178	8.756135872	8.988029441
8.517387626
ENSG00000134070 566.9112211	624.7650689	600.3682275
306.3963577	509.9338116	371.8806819	597.3481725
396.0702837	0.592248197	0.00793887	0.051599279	IRAK2
	3	10164865	10243743	+	3483
protein_coding	interleukin 1 receptor associated kinase 2
[Source:HGNC Symbol;Acc:HGNC:6113]	-	490	674	614
	305	558	379	7.693736679	8.531794377
8.16614999	4.233968098	6.981348568	5.079626396
ENSG00000133739 749.7111659	701.7020136	567.1230814
461.1014039	539.1773277	368.9370353	672.8454203
456.4052556	0.559157753	0.007961736	0.051725904	LRRCC1
	8	85107147	85146076	+	5065
protein_coding	leucine rich repeat and coiled-coil centrosomal
protein 1 [Source:HGNC Symbol;Acc:HGNC:29373]	-	648	757
	580	459	590	376	6.996651926	6.589468523
	5.304579953	4.381617398	5.076111577	3.465408458
ENSG00000158373 146.9341328	92.69511408	50.84551764
176.8057671	181.8581156	228.623216	96.82492151
195.7623662	-1.019885642	0.007990926	0.051878297
HIST1H2BD	6	26158146	26171349	+	871
	protein_coding	histone cluster 1 H2B family member d
[Source:HGNC Symbol;Acc:HGNC:4747]	-	127	100	52
	176	199	233	7.974074313	5.061926128
2.765589034	9.770026221	9.956197868	12.48773867
ENSG00000228335 0	3.707804563	5.866790497	26.11903377
	23.76035681	21.58674143	3.191531687	23.822044
	-2.872683105	0.007991979	0.051878297	AC073063.1
	7	99442890	99443496	-	607
processed_pseudogene	cytokine induced protein 29 kDa (CIP29)
pseudogene	-	0	4	6	26	26	22
	0	0.290539549	0.457894066	2.071027537
1.866565587	1.691920785
ENSG00000197747 3281.143312	3862.605404	3945.416609
1454.629265	2793.669645	3184.044361	3696.388442
2477.447757	0.577243403	0.007995808	0.051881116
S100A10 1	151982915	151994390	-	1543
protein_coding	S100 calcium binding protein A10 [Source:HGNC
Symbol;Acc:HGNC:10487]	-	2836	4167	4035	1448	3057
	3245	100.5159573	119.0670332	121.1379079
45.37366417	86.33524194	98.17362128
ENSG00000272405 480.1390954	391.1733814	596.4570339
802.6579994	552.8852258	879.1691054	489.2565036
744.9041102	-0.60631082	0.00801402	0.05197723

AL365181.3	1	156641666	156644887	-	3222
	antisense	"novel transcript, antisense to BCAN"	-
	415	422	610	799	605	896	7.043966362
	5.774586256	8.770144615	11.99009046	8.182545639
	12.98160856
ENSG00000057149 0	0	0	18.082408	0
14.71823279	0	10.93354693	-5.869095804	0.008016589
	1	SERPINB3	18	63655197	63661963
	-	1777	protein_coding	serpin family B member 3
[Source:HGNC Symbol;Acc:HGNC:10569]	-	0	0	0
	18	0	15	0	0	0	0.489763362
	0	0.394048671
ENSG00000150403 1552.642569	1484.975728	1527.321126
1148.232908	1217.261357	1168.627684	1521.646474
1178.040649	0.369050583	0.008024543	0.052023405	TMCO3
	13	113490995	113554590	+	10407
protein_coding	transmembrane and coiled-coil domains 3 [Source:HGNC
Symbol;Acc:HGNC:20329]	-	1342	1602	1562	1143	1332
	1191	7.052152156	6.786891638	6.952771085
5.310334671	5.577470321	5.34234884
ENSG00000267198 17.35442514	0	14.66697624	0	0
	0	10.67380046	0	5.906100205	0.008031882
	1	AC091132.4	17	45545804	45563230
	+	2278	lincRNA novel transcript	-	15
	0	15	0	0	0	0.360107571	0
	0.305028202	0	0	0
ENSG00000113649 2561.51315	2441.589305	2774.991905
2139.751613	2035.165947	1920.238772	2592.69812
2031.718777	0.351762192	0.008063052	0.052233665	TCERG1
	5	146447311	146511961	+	8714
protein_coding	transcription elongation regulator 1 [Source:HGNC
Symbol;Acc:HGNC:15630]	-	2214	2634	2838	2130	2227
	1957	13.89487914	13.3269931	15.0868055
11.81852462	11.13682151	10.48381397
ENSG00000167333 386.4251997	417.1280134	346.1406393
504.2982675	595.8366401	551.4431219	383.2312841
550.5260098	-0.522697988	0.008063809	0.052233665	TRIM68
	11	4598672 4608259 -	4282	protein_coding
tripartite motif containing 68 [Source:HGNC Symbol;Acc:HGNC:21161]
	-	334	450	354	502	652	562
4.265741328	4.633400154	3.829649737	5.668371042
6.635284221	6.126828
ENSG00000122140 910.5288388	1311.635864	1065.800274
1459.652157	1523.404416	1491.44759	1095.988326
1491.501387	-0.443697663	0.008083175	0.052312168	MRPS2
	9	135499984	135504673	+	2464
protein_coding	mitochondrial ribosomal protein S2 [Source:HGNC
Symbol;Acc:HGNC:14495]	-	787	1415	1090	1453	1667
	1520	17.46741914	25.31918338	20.49218416
28.51189791	29.48176595	28.79711099

ENSG00000166685 1014.65539 1157.761975 973.8872225
1518.922272 1267.52365 1406.081839 1048.768196
1397.509254 -0.413710752 0.008085496 0.052312168 COG1
17 73192632 73208507 + 5317
protein_coding component of oligomeric golgi complex 1 [Source:HGNC
Symbol;Acc:HGNC:6545] - 877 1249 996 1512 1387
1433 9.020438415 10.35689888 8.677510221
13.74948233 11.36758993 12.58130138
ENSG00000110075 3329.735703 2752.117937 3061.486841
2263.314734 2559.721517 2286.23216 3047.78016
2369.756137 0.362671881 0.008086194 0.052312168 PPP6R3
11 68460731 68615334 + 8305
protein_coding protein phosphatase 6 regulatory subunit 3
[Source:HGNC Symbol;Acc:HGNC:1173] - 2878 2969 3131
2253 2801 2330 18.95159931 15.76175305
17.46408702 13.11664521 14.69711423 13.09671329
ENSG00000108559 1859.237413 1839.998015 2251.869752
1471.707095 1550.820212 1537.564719 1983.701727
1520.030675 0.384171213 0.008090316 0.05231669 NUP88
17 5360963 5420160 - 5294 protein_coding
nucleoporin 88 [Source:HGNC Symbol;Acc:HGNC:8067] - 1607
1985 2303 1465 1697 1567 16.6007096
16.53143417 20.15173562 13.37996275 13.96871649
13.81755161
ENSG00000026508 12451.22155 11904.8335 13365.52655
7851.783383 10001.2825 11130.90885 12573.86054
9661.324912 0.380109497 0.008099136 0.052349207 CD44
11 35138870 35232402 + 10004
protein_coding CD44 molecule (Indian blood group) [Source:HGNC
Symbol;Acc:HGNC:1681] - 10762 12843 13669 7816 10944
11344 58.83205485 56.6013458 63.29443336
37.77566202 47.67173948 52.93447018
ENSG00000267481 0 3.707804563 11.73358099 33.15108133
29.24351608 33.36132766 5.147128519 31.91864169
-2.613678009 0.008102193 0.052349207 AC011477.2
19 19788755 19790531 - 921
sense_intronic "novel transcript, sense intronic to ZNF506" -
0 4 12 33 32 34 0
0.191484806 0.603565033 1.732429324 1.514080429
1.723317597
ENSG00000071082 6227.9247 9326.055428 7631.716638
7976.351083 11372.07231 13147.30675 7728.565589
10831.91005 -0.486917186 0.008121633 0.05245264 RPL31
2 101001715 101024032 + 5791
protein_coding ribosomal protein L31 [Source:HGNC
Symbol;Acc:HGNC:10334] - 5383 10061 7805 7940 12444
13399 50.83531601 76.59872522 62.43409175
66.29307344 93.64078273 108.0102218
ENSG00000185650 3541.459689 3878.363573 3541.585863
4667.27042 4355.456176 4674.510735 3653.803042

4565.745777	-0.321309164	0.008144787	0.052579963
ZFP36L1 14	68787660	68796253	-	5160
protein_coding	ZFP36 ring finger protein like 1 [Source:HGNC
Symbol;Acc:HGNC:1107]	Others	3061	4184	3622	4646	4766
4764	32.44205384	35.74999062	32.51630587
43.53421526	40.24972654	43.09908866
ENSG00000151849 266.1011854	269.742782	358.8520187
176.8057671	144.3898606	219.7922764	298.2319954
180.3293014	0.726859299	0.008157349	0.052638833	CENPJ
13	24882284	24922889	-	5812
protein_coding	centromere protein J [Source:HGNC
Symbol;Acc:HGNC:17272]	-	230	291	367	176	158
224	2.16419776	2.207503198	2.925114795
1.464159126	1.184650055	1.799154455
ENSG00000273102 28.92404189	11.12341369	8.800185745	0
0	2.943646558	16.28254711	0.981215519
4.059374208	0.008170554	1	AP000569.1	21
33967101	33968573	-	591	lincRNA novel
transcript	-	25	12	9	0	0	3
2.313381406	0.895215768	0.705435781	0	0
0.236962602
ENSG00000091140 2162.361372	2118.083357	1760.037149
1359.194334	1601.082505	1615.080745	2013.493959
1525.119195	0.400513758	0.008170838	0.052703632	DLD
7	107890970	107931730	+	5299
protein_coding	dihydrolipoamide dehydrogenase [Source:HGNC
Symbol;Acc:HGNC:2898]	-	1869	2285	1800	1353	1752
1646	19.28901694	19.01193159	15.7355179
12.34539794	14.40783679	14.50046559
ENSG00000234678 61.31896881	61.1787753	39.11193665
18.082408	12.79403828	22.56795695	53.86989359
17.81480108	1.597635745	0.008181396	0.052729997	ELF3-
AS1	1	201995696	202010463	-	5569
antisense	ELF3 antisense RNA 1 [Source:HGNC
Symbol;Acc:HGNC:40211]	-	53	66	40	18	14
23	0.520467199	0.522517302	0.332724843
0.156277517	0.109549252	0.192795394
ENSG00000076321 814.5010197	798.1049323	692.2812786
1102.02231	998.848846	998.8773988	768.2957435
1033.249518	-0.427646873	0.008183966	0.052729997	KLHL20
1	173714941	173786702	+	4254
protein_coding	kelch like family member 20 [Source:HGNC
Symbol;Acc:HGNC:25056]	-	704	861	708	1097	1093
1018	9.050444017	8.923590323	7.709713293
12.46838944	11.19647395	11.17111
ENSG00000203364 8.09873173	0	0	25.11445555
33.81281546	17.66187935	2.699577243
25.52971679	-3.30020255	0.008188533	0.052729997
AL390760.1	9	93147040	93148556	+	410
lincRNA novel transcript	-	7	0	0

25	37	18	0.933703305	0	0
2.948202896	3.932570029	2.049437527
ENSG00000166741 1088.700937	1304.220255	1445.186059
952.3401545	954.9835719	945.8917608	1279.369084
951.071829	0.428350833	0.008188723	0.052729997	NNMT
11	114257787	114313285	+	2919
protein_coding	nicotinamide N-methyltransferase [Source:HGNC
Symbol;Acc:HGNC:7861]	-	941	1407	1478	948	1045
964	17.62991592	21.25171389	23.45539737
15.70274069	15.60058174	15.41661315
ENSG00000092010 1919.39942	1548.935356	1908.662508
888.0471483	1548.078632	1312.866365	1792.332428
1249.664049	0.51979368	0.008192416	0.052731562	PSME1
14	24136158	24138967	+	1781
protein_coding	proteasome activator subunit 1 [Source:HGNC
Symbol;Acc:HGNC:9568]	-	1659	1671	1952	884	1694
1338	50.94213933	41.36628201	50.77130772
23.99880197	41.44842995	35.07019897
ENSG00000115687 503.2783289	612.7147041	645.3469547
424.9365879	362.8023713	433.6972596	587.1133292
407.1454063	0.529226706	0.008197818	0.052744125	PASK
2	241106099	241150264	-	10686
protein_coding	PAS domain containing serine/threonine kinase
[Source:HGNC Symbol;Acc:HGNC:17270]	-	435	661	660
423	397	442	2.22622369	2.727220468
2.861088037	1.913931605	1.618951858	1.930870446
ENSG00000274386 10.41265508	5.561706845	14.66697624
52.23806755	15.53561792	60.83536221	10.21377939
42.86968256	-2.070772642	0.008232434	0.052944563
TMEM269 1	42785007	42816619	+	4373
protein_coding	transmembrane protein 269 [Source:HGNC
Symbol;Acc:HGNC:52381]	-	9	6	15	52	17
62	0.112553174	0.060493085	0.158896466
0.574943387	0.169405709	0.66184798
ENSG00000130349 275.3568788	380.9769189	303.117509
368.6802075	535.5218882	594.6166048	319.8171022
499.6062335	-0.642596134	0.008248344	0.053024573
C6orf203	6	107028213	107051342	+	2036
protein_coding	chromosome 6 open reading frame 203
[Source:HGNC Symbol;Acc:HGNC:17971]	-	238	411	310
367	586	606	6.392840899	8.900163936
7.053202877	8.715443806	12.54233626	13.89443484
ENSG00000164330 78.67339395	74.15609127	132.0027862
46.21059821	41.12369448	36.30497422	94.94409047
41.21308897	1.204873921	0.008256412	0.053043415	EBF1
5	158695916	159099761	-	6178
protein_coding	early B cell factor 1 [Source:HGNC
Symbol;Acc:HGNC:3126]	COE	68	80	135	46	45
37	0.601943489	0.57092103	1.012251247
0.360007165	0.317411902	0.27957598

ENSG00000283689 49.74935206   58.39792187   39.11193665
94.43035287   108.7493254   109.8961382   49.08640353
104.3586055   -1.087782983   0.008258215   0.053043415
AC018553.2   16   54285604   54289568   +   1064
        antisense   "novel transcript, antisense to IRX3"   -
        43   63   40   94   119   112   2.210148935
        2.610555192   1.741489335   4.271569158   4.873757665
        4.913856058
ENSG00000181333 9.255693406   1.853902282   15.64477466   0
        0   0   8.918123449   0   5.64856094
0.008269563   1   HEPHL1 11   94021361   94113751
        +   5345   protein_coding hephaestin like 1 [Source:HGNC
Symbol;Acc:HGNC:30477] -   8   2   16   0   0
        0   0.08185345   0.016497428   0.138667514   0
        0   0
ENSG00000112378 1080.602205   995.5455253   823.3062664
1196.452662   1368.962096   1350.152555   966.4846656
1305.189105   -0.434035023   0.008272851   0.053115106   PERP
        6   138088505   138107511   -   4290
protein_coding "PERP, TP53 apoptosis effector [Source:HGNC
Symbol;Acc:HGNC:17637]" -   934   1074   842   1191   1498
        1376   11.90650495   11.03776001   9.09195453
13.4231884   15.21644214   14.97294322
ENSG00000010327 38.1797353   37.07804563   58.66790497
16.07325155   8.224738897   14.71823279   44.6418953
13.00540775   1.783073935   0.008279069   0.053132714   STAB1
        3   52495338   52524495   +   10588
protein_coding stabilin 1 [Source:HGNC Symbol;Acc:HGNC:18628] -
        33   40   60   16   9   15   0.170449103
        0.166563568   0.262506326   0.073064643   0.037041381
        0.066133782
ENSG00000153993 77.51643227   42.63975248   44.97872714
6.027469332   10.05245865   29.43646558   55.04497063
15.17213119   1.857722741   0.008298025   0.05323202   SEMA3D
        7   84995553   85186855   -   8062
protein_coding semaphorin 3D [Source:HGNC Symbol;Acc:HGNC:10726]
        -       67   46   46   6   11   30
0.454492501   0.251564292   0.264312373   0.035984019
0.059457753   0.173709871
ENSG00000128609 1966.834849   1670.365956   1496.031577
1227.594587   1358.909638   1277.542606   1711.07746
1288.01561   0.40919701   0.008326674   0.0533934   NDUFA5
        7   123536997   123558255   -   9851
protein_coding NADH:ubiquinone oxidoreductase subunit A5 [Source:HGNC
Symbol;Acc:HGNC:7688]   -   1700   1802   1530   1222   1487
        1302   9.437638   8.065075281   7.194714543
5.997801705   6.577929994   6.169879767
ENSG00000140939 681.450427   859.2837076   758.7715709
1068.871228   901.0658391   1231.425477   766.5019018
1067.120848   -0.476472102   0.008340782   0.053442703   NOL3

16	67170154	67175735	+	2992
protein_coding	nucleolar protein 3 [Source:HGNC Symbol;Acc:HGNC:7869]
-	589	927	776	1064	986	1255
10.76585233	13.66004415	12.01441386	17.19417154
14.36064424	19.58069815
ENSG00000138356 1099.113592	1079.898079	887.8409619
1357.185178	1275.748389	1462.99234	1022.284211
1365.308635	-0.417618243	0.008341355	0.053442703	AOX1
2	200585868	200677064	+	5556
protein_coding	aldehyde oxidase 1 [Source:HGNC Symbol;Acc:HGNC:553]
-	950	1165	908	1351	1396	1491
9.350957453	9.244802684	7.570526208	11.75694072
10.94918463	12.52741435
ENSG00000166847 2119.55379	2246.002614	2124.755958
2777.658784	2631.916447	2761.140472	2163.437454
2723.571901	-0.332024271	0.008349957	0.053475406	DCTN5
16	23641392	23677455	+	12740
protein_coding	dynactin subunit 5 [Source:HGNC Symbol;Acc:HGNC:24594]
-	1832	2423	2173	2765	2880	2814
7.864131267	8.385287241	7.901194525	10.49365844
9.851030359	10.31099796
ENSG00000164654 1227.536338	983.4951604	1056.022289
828.7770332	705.4998254	859.5447951	1089.017929
797.9405512	0.448334615	0.008355044	0.053485574	MIOS
7	7566872 7608929 +	6714	protein_coding
meiosis regulator for oocyte development [Source:HGNC
Symbol;Acc:HGNC:21905]	-	1061	1061	1080	825	772
876	8.642288495	6.967356055	7.45152005
5.941195291	5.010655692	6.090722389
ENSG00000229751 0	1.853902282	0	27.12361199
8.224738897	7.849724156	0.617967427
14.39935835	-4.469579264	0.008359956	1	AC044781.1
10	13991815	14007526	+	570
antisense	novel transcript	-	0	2	0
27	9	8	0	0.154699567	0
2.290288144	0.688059906	0.655180808
ENSG00000141576 386.4251997	443.0826453	632.6355753
367.6756293	288.7797213	277.683992	487.3811401
311.3797808	0.647436618	0.008365068	0.05352733	RNF157
17	76142453	76240373	-	7805
protein_coding	ring finger protein 157 [Source:HGNC
Symbol;Acc:HGNC:29402]	-	334	478	647	366	316
283	2.34028243	2.700156567	3.840023031
2.267302122	1.764301377	1.692620373
ENSG00000138430 3426.920483	2982.928771	2915.794877
2281.397142	2579.826434	2432.433273	3108.548044
2431.21895	0.354233722	0.00837579	0.053573518	OLA1
2	174072447	174248698	-	6100
protein_coding	Obg like ATPase 1 [Source:HGNC Symbol;Acc:HGNC:28833]
-	2962	3218	2982	2271	2823	2479

26.55522172	23.25895309	22.64541375	18.0006636
20.16692304	18.97110936
ENSG00000162434 3923.257042	3129.387052	3695.100215
2824.87396	2858.553697	2711.09848	3582.581436
2798.175379	0.356257205	0.008392822	0.053660005	JAK1
	1	64833229	64966504	-	6317
protein_coding	Janus kinase 1 [Source:HGNC Symbol;Acc:HGNC:6190]
	-	3391	3376	3779	2812	3128	2763
29.35699681	23.56272524	27.71203832	21.52314126
21.57816279	20.41813373
ENSG00000164332 1335.133774	1114.195271	1032.555127
881.0151007	839.8372273	860.5260106	1160.628058
860.4594462	0.431164566	0.008402818	0.053701454	UBLCP1
	5	159263081	159286040	+	2676
protein_coding	ubiquitin like domain containing CTD phosphatase 1
[Source:HGNC Symbol;Acc:HGNC:28110]	-	1154	1202	1056
	877	919	877	23.58383417	19.80397259
18.28017146	15.84581936	14.96538865	15.29887584
ENSG00000115649 851.5237933	1256.018796	1052.111096
1458.647578	1362.565077	1511.0719	1053.217895
1444.094852	-0.454368086	0.008419932	0.05378834	CNPPD1
	2	219171897	219178106	-	2760
protein_coding	cyclin Pas1/PHO80 domain containing 1 [Source:HGNC
Symbol;Acc:HGNC:25220]	-	736	1355	1076	1452	1491
	1540	14.58355638	21.64532799	18.05949679
25.43658185	23.54112206	26.04699787
ENSG00000113273 679.1365036	621.0572644	650.2359467
533.4310359	445.9636202	384.6364836	650.1432383
454.6770466	0.515840063	0.008429449	0.053826646	ARSB
	5	78777209	78986087	-	9053
protein_coding	arylsulfatase B [Source:HGNC Symbol;Acc:HGNC:714]
	-	587	670	665	531	488	392
3.546012757	3.262993738	3.402762051	2.835980349
2.349015591	2.021340988
ENSG00000216906 8.09873173	10.19646255	6.844588913	0
	0	0	8.379927731	0	5.560275282
0.008452695	1	AL355312.1	6	149904243
149906418	+	2176	processed_pseudogene	multimerin 2
(MMRN2) pseudogene	-	7	11	7	0	0
	0	0.175927553	0.222878282	0.149018986	0
	0	0
ENSG00000123572 6.941770054	18.53902282	15.64477466	0
	1.827719755	0	13.70852251	0.609239918
4.451377042	0.008456296	1	NRK	X	105822543
	105958610	+	9573	protein_coding	Nik related
kinase [Source:HGNC Symbol;Acc:HGNC:25391]	-	6	20
	16	0	2	0	0.034276613	0.092111933
	0.077423782	0	0.009104174	0
ENSG00000137996 1214.80976	1361.691226	1336.650435
965.3996714	1041.80026	979.2530884	1304.383807

995.48434	0.390120713	0.008476404	0.054103875	RTCA
	1	100266207	100292769	+	3037
protein_coding	RNA 3'-terminal phosphate cyclase [Source:HGNC
Symbol;Acc:HGNC:17981]	-	1050	1469	1367	961	1140
	998	18.90772251	21.32607646	20.85096592
15.29959069	16.35756509	15.34022696
ENSG00000103126 1086.387013	1446.04378	1372.828976
1593.26106	2016.888749	1681.8034	1301.753257
1763.984403	-0.437898149	0.008481642	0.054114714	AXIN1
	16	287440	352673	-	7487	protein_coding	axin 1
[Source:HGNC Symbol;Acc:HGNC:903]	-	939	1560	1404
	1586	2207	1714	6.858868426	9.186513619
8.68683816	10.24227816	12.84556165	10.68683294
ENSG00000052749 4432.32018	4877.616903	5094.329748
6847.205161	5289.42097	6372.013583	4801.422277
6169.546572	-0.361525882	0.008511113	0.054280092	RRP12
	10	97356358	97426076	-	8529
protein_coding	ribosomal RNA processing 12 homolog [Source:HGNC
Symbol;Acc:HGNC:29100]	-	3831	5262	5210	6816	5788
	6494	24.56454647	27.2011138	28.2971088
38.63960551	29.57256573	35.54350121
ENSG00000174990 0	0	0	15.06867333	2.741579632
	8.830939675	0	8.880397546	-5.568955838
0.008518118	1	CA5A	16	87881546	87936580
	-	8140	protein_coding	carbonic anhydrase 5A
[Source:HGNC Symbol;Acc:HGNC:1377]	-	0	0	0
	15	3	9	0	0	0	0.089098024
	0.016060366	0.051613599
ENSG00000115641 407.2505099	275.3044888	190.6706912
454.0693564	483.4318752	480.7956045	291.0752299
472.765612	-0.702130024	0.00853651	0.054419361	FHL2
	2	105357712	105438513	-	6626
protein_coding	four and a half LIM domains 2 [Source:HGNC
Symbol;Acc:HGNC:3703]	-	352	297	195	452	529
	490	2.905266288	1.976236771	1.363281796
3.298285304	3.479067366	3.452158158
ENSG00000214063 605.0909564	859.2837076	1074.600459
520.371519	532.7803085	646.6210273	846.3250411
566.5909516	0.579798874	0.008543818	0.054443245	TSPAN4
	11	842808	867116	+	5841	protein_coding
tetraspanin 4 [Source:HGNC Symbol;Acc:HGNC:11859]	-	523
	927	1099	518	583	659	4.896764245
6.99723542	8.715914112	4.287891327	4.34950612
5.266768679
ENSG00000133195 448.9011302	617.3494598	407.7419395
715.2596941	857.200565	617.1845617	491.3308432
729.8816069	-0.570394307	0.008549056	0.054453927
SLC39A11	17	72645949	73092712	-	5583
	protein_coding	solute carrier family 39 member 11
[Source:HGNC Symbol;Acc:HGNC:14463]	-	388	666	417

712	938	629	3.800658165	5.259452768
3.459958446	6.166142858	7.321394494	5.25931343
ENSG00000024422 3600.464735	3629.940668	3822.214009
4043.427344	4704.550649	5651.801392	3684.20647
4799.926462	-0.381645569	0.008573804	0.054588814	EHD2
19	47713343	47743134	+	3923
protein_coding	EH domain containing 2 [Source:HGNC
Symbol;Acc:HGNC:3243]	-	3112	3916	3909	4025	5148
5760	43.38264159	44.01070576	46.15830134
49.60766586	57.1845658	68.54096445
ENSG00000197713 1278.442652	1129.02649	1138.157356
967.4088278	860.8560045	836.9768381	1181.875499
888.4138901	0.411510076	0.00860221	0.054732588	RPE
2	210002565	210022259	+	3522
protein_coding	ribulose-5-phosphate-3-epimerase [Source:HGNC
Symbol;Acc:HGNC:10293]	-	1105	1218	1164	963	942
853	17.15803855	15.24726311	15.30967899
13.22020385	11.65521123	11.30591498
ENSG00000154380 1727.343782	1342.225252	1855.861394
1365.221804	1081.096235	1158.815528	1641.810143
1201.711189	0.45012519	0.008603546	0.054732588	ENAH
1	225486835	225653142	-	15044
protein_coding	"ENAH, actin regulator [Source:HGNC
Symbol;Acc:HGNC:18271]" -	1493	1448	1898	1359	1183
1181	5.427392069	4.243646456	5.844338192
4.367745737	3.426731033	3.664650894
ENSG00000060688 1812.958946	1511.857311	1714.080624
1389.331681	1293.111727	1122.510554	1679.632293
1268.317987	0.404924828	0.008615473	0.054763951
SNRNP40 1	31259568	31296782	-	3881
protein_coding	small nuclear ribonucleoprotein U5 subunit 40
[Source:HGNC Symbol;Acc:HGNC:30857]	-	1567	1631	1753
1383	1415	1144	22.08106756	18.52867127
20.92380814	17.22978086	15.88807916	13.76031632
ENSG00000168701 799.4605179	996.4724764	693.2590771
1188.416037	1129.530808	1112.698399	829.7306905
1143.548415	-0.462337293	0.008615641	0.054763951
TMEM208 16	67227103	67229278	+	1787
protein_coding	transmembrane protein 208 [Source:HGNC
Symbol;Acc:HGNC:25015]	-	691	1075	709	1183	1236
1134	21.14697285	26.52270835	18.37909567
32.00821154	30.14064321	29.62337511
ENSG00000183828 27.76708022	42.63975248	61.60130022
137.6272164	116.0602044	63.77900877	44.00271097
105.8221432	-1.261965068	0.008620368	0.054771222	NUDT14
14	105172938	105181323	-	1916
protein_coding	nudix hydrolase 14 [Source:HGNC Symbol;Acc:HGNC:20141]
-	24	46	63	137	127	65
0.685031354	1.058513216	1.523166925	3.457214127
2.888463034	1.583667075

ENSG00000013374 1026.225006	1111.414418	1024.73274
1248.69073	1423.793689	1492.428805	1054.124055
1388.304408	-0.397159736	0.008643696	0.054896626	NUB1
	7	151341699	151378449	+	5411
protein_coding	negative regulator of ubiquitin like proteins 1
[Source:HGNC Symbol;Acc:HGNC:17623]	-	887	1199	1048
	1243	1558	1521	8.964803995	9.769573557
8.971936776	11.10694986	12.54724901	13.12192998
ENSG00000187531 601.6200714	799.0318834	846.7734284
1021.656052	1082.923955	1005.745907	749.1417944
1036.775305	-0.467792189	0.008657051	0.054958614	SIRT7
	17	81911939	81921323	-	6152
protein_coding	sirtuin 7 [Source:HGNC Symbol;Acc:HGNC:14935]	-
	520	862	866	1017	1185	1025	4.622551194
	6.177672726	6.52084716	7.992926928	8.393838738
	7.777742745
ENSG00000090861 7016.972563	7831.810189	7077.304936
11004.14984	8094.970794	9164.552952	7308.695896
9421.22453	-0.366201308	0.008670011	0.055018043	AARS
	16	70252295	70289543	-	4498
protein_coding	alanyl-tRNA synthetase [Source:HGNC
Symbol;Acc:HGNC:20]	-	6065	8449	7238	10954	8858
	9340	73.7404981	82.81706152	74.54209312
117.748261	85.81729526	96.9334033
ENSG00000109756 795.9896329	943.6362614	1083.400645
768.5023398	498.9674931	680.9635705	941.0088465
649.4778011	0.535888684	0.008683717	0.055082151
RAPGEF2 4	159103013	159360174	+	13275
protein_coding	Rap guanine nucleotide exchange factor 2 [Source:HGNC
Symbol;Acc:HGNC:16854]	-	688	1018	1108	765	546
	694	2.834318302	3.381015846	3.866408051
2.786301584	1.792324786	2.440455969
ENSG00000266094 573.8529912	429.1783782	578.8566624
398.8175541	357.3192121	308.1016731	527.2960106
354.7461464	0.571236985	0.008695223	0.055132255	RASSF5
	1	206507530	206589448	+	6976
protein_coding	Ras association domain family member 5 [Source:HGNC
Symbol;Acc:HGNC:17609]	-	496	463	592	397	391
	314	3.888390893	2.926230126	3.931132577
2.751599687	2.442468004	2.101208805
ENSG00000185432 42.807582	26.88158308	83.11286537
20.09156444	2.741579632	14.71823279	50.93401015
12.51712562	2.030442154	0.008711643	0.055213467
METTL7A 12	50923472	50932517	+	4083
protein_coding	methyltransferase like 7A [Source:HGNC
Symbol;Acc:HGNC:24550]	-	37	29	85	20	3
	15	0.495583749	0.313150115	0.964366247
0.236838244	0.032018462	0.171497548
ENSG00000111554 301.9669974	361.5109449	407.7419395
286.3047933	207.4461922	155.0320521	357.0732939

216.2610125	0.724554732	0.008744489	0.055398676	MDM1
	12	68272443	68332381	-	6549
protein_coding	Mdm1 nuclear protein [Source:HGNC
Symbol;Acc:HGNC:29917]	-	261	390	417	285	227
	158	2.179516844	2.625569837	2.949602688
2.104122818	1.51046082	1.126232699
ENSG00000179409 1962.207002	2381.337481	2334.982618
2950.446238	2665.729262	2979.951533	2226.1757
2865.375678	-0.36370818	0.008766462	0.055514877	GEMIN4
	17	744414	753999	-	5751	protein_coding	gem
nuclear organelle associated protein 4 [Source:HGNC
Symbol;Acc:HGNC:15717]	-	1696	2569	2388	2937	2917
	3037	16.12787665	19.69494148	19.23505403
24.69231425	22.10302217	24.65173328
ENSG00000119801 809.873173	710.971525	585.7012513
783.5710132	1172.482223	1076.393425	702.1819831
1010.815554	-0.526491426	0.008775886	0.055532171	YPEL5
	2	30146941	30160533	+	3421
protein_coding	yippee like 5 [Source:HGNC Symbol;Acc:HGNC:18329]
	-	700	767	599	780	1283	1097
11.19024715	9.884990306	8.111033666	11.02408987
16.34301502	14.96923441
ENSG00000114626 400.3087398	548.7550754	470.3210382
663.0216265	624.1662963	741.7989327	473.1282845
676.3289518	-0.514084685	0.008776459	0.055532171	ABTB1
	3	127672935	127680920	+	6149
protein_coding	ankyrin repeat and BTB domain containing 1
[Source:HGNC Symbol;Acc:HGNC:18275]	-	346	592	481
	660	683	756	3.07727507	4.24474076
3.623623263	5.189680947	4.840328181	5.739358304
ENSG00000041802 2311.609428	2423.977233	2263.603333
3070.995625	2873.175455	2851.4123	2333.063332
2931.861126	-0.329490438	0.008782902	0.055549947	LSG1
	3	194640788	194672477	-	4818
protein_coding	large 60S subunit nuclear export GTPase 1 [Source:HGNC
Symbol;Acc:HGNC:25652]	-	1998	2615	2315	3057	3144
	2906	22.67897389	23.92978824	22.25802652
30.67819895	28.43638198	28.15625243
ENSG00000172238 0	0	3.911193665	22.10072088
9.138598774	22.56795695	1.303731222
17.93575887	-3.757991952	0.008783641	1	ATOH1	4
	93828753	93830964	+	2212	protein_coding
	atonal bHLH transcription factor 1 [Source:HGNC
Symbol;Acc:HGNC:797]	bHLH	0	0	4	22	10
	23	0	0	0.083767841	0.480882281
0.197003288	0.48538768
ENSG00000178691 3159.662336	2914.334387	2925.572861
2399.937372	2543.272039	2098.819996	2999.856528
2347.343136	0.353650797	0.008791566	0.055581746	SUZ12
	17	31937018	32001045	+	6547

protein_coding "SUZ12, polycomb repressive complex 2 subunit
[Source:HGNC Symbol;Acc:HGNC:17101]"   -   2731   3144   2992
        2389   2783   2139   22.81256252   21.17259813
21.17004124   17.64310527   18.52377355   15.25156744
ENSG00000149428 2841.497876   2442.516256   4441.160406
2399.937372   1597.427066   2567.841014   3241.724846
2188.401818   0.567058593   0.008802057   0.05562507   HYOU1
        11   119044189   119057202   -   7694
protein_coding hypoxia up-regulated 1 [Source:HGNC
Symbol;Acc:HGNC:16931] -   2456   2635   4542   2389   1748
        2617   17.4570515   15.09949406   27.34622632
15.01292048   9.900288356   15.87806504
ENSG00000158352 16.19746346   4.634755704   4.888992081   0
        0   0   8.573737082   0   5.588444939
0.008802807   1   SHROOM4 X   50591647   50814302
        -       10333   protein_coding shroom family member 4
[Source:HGNC Symbol;Acc:HGNC:29215]   -   14   5   5
        0   0   0   0.074096265   0.021334258
0.022415376   0   0   0
ENSG00000230309 13.88354011   5.561706845   5.866790497   0
        0   0   8.437345817   0   5.566421111
0.008808061   1   AL121718.1   6   78604467
78606036   -   387   lincRNA novel transcript   -
        12   6   6   0   0   0   1.69576237
        0.683556226   0.718195602   0   0   0
ENSG00000160172 129.5797077   152.9469382   112.4468179
240.0941951   296.0906003   163.8629918   131.6578213
233.3492624   -0.825659818   0.008816054   0.055690506
FAM86C2P   11   67791648   67805336   -   3402
        transcribed_unprocessed_pseudogene   "family with sequence
similarity 86 member C2, pseudogene [Source:HGNC
Symbol;Acc:HGNC:42392]" -   112   165   115   239   324
        167   1.800439059   2.138373644   1.565907077
3.396759574   4.150202605   2.291543985
ENSG00000125166 4433.477141   3876.509671   4021.684886
3419.584268   3298.120298   3084.941593   4110.557233
3267.54872   0.330958251   0.008821636   0.055702747   GOT2
        16   58707131   58734357   -   3766
protein_coding glutamic-oxaloacetic transaminase 2 [Source:HGNC
Symbol;Acc:HGNC:4433]   -   3832   4182   4113   3404   3609
        3144   55.64676187   48.95957856   50.59188367
43.7029197   41.76044945   38.9716019
ENSG00000252657 15.04050178   5.561706845   4.888992081   0
        0   0   8.497066903   0   5.576087734
0.008827696   1   RF00156 17   29777619   29777744
        +   126   snoRNA   -   13   6   5
        0   0   0   5.64244741   2.099494123
1.838238743   0   0   0
ENSG00000232774 3.470885027   9.269511408   12.71137941   0
        0   0   8.483925282   0   5.580305571

0.008829428	1	FLJ22447	14	61570540
61658696	+	2653	lincRNA uncharacterized LOC400221
[Source:NCBI gene;Acc:400221]	-	3	10	13	0
	0	0	0.061841315	0.16618687	0.226990958
	0	0	0
ENSG00000227440 3.470885027	9.269511408	12.71137941	0
	0	0	8.483925282	0	5.580305571
0.008829428	1	ATP5MC1P4	3	172828736
172829176	-	441	processed_pseudogene	ATP synthase
membrane subunit c locus 1 pseudogene 4 [Source:HGNC
Symbol;Acc:HGNC:39507]	-	3	10	13	0	0
	0	0.3720295	0.999759106	1.36554878	0
	0	0
ENSG00000171155 600.4631097	640.5232383	572.0120735
373.7030986	428.6002825	475.8895269	604.3328072
426.0643027	0.504262462	0.008855661	0.055874461
C1GALT1C1	X	120625793	120630150	-	1785
	protein_coding	C1GALT1 specific chaperone 1 [Source:HGNC
Symbol;Acc:HGNC:24338]	-	519	691	585	372	469
	485	15.90097849	17.06765222	15.18168938
10.07641245	11.4496766	12.68380492
ENSG00000105875 277.6708022	460.694717	447.8316746
331.5108133	158.0977588	188.3933797	395.3990646
226.0006506	0.80903474	0.008856141	0.055874461	WDR91
	7	135183839	135211534	-	6032
protein_coding	WD repeat domain 91 [Source:HGNC
Symbol;Acc:HGNC:24997]	-	240	497	458	330	173
	192	2.175928505	3.63269565	3.517277233
2.645171431	1.249808195	1.485887508
ENSG00000198324 146.9341328	263.254124	283.5615407
365.6664728	352.7499127	426.828751	231.2499325
381.7483788	-0.72038482	0.008862967	0.055892769	PHETA1
	12	111360651	111369121	-	3361
protein_coding	PH domain containing endocytic trafficking adaptor 1
[Source:HGNC Symbol;Acc:HGNC:26509]	-	127	284	290
	364	386	435	2.066473885	3.72549329
3.996979688	5.236415355	5.004692401	6.041806058
ENSG00000206503 2149.634793	2996.833038	3017.485912
1395.35915	2244.439859	2115.50066	2721.317915
1918.433223	0.504493055	0.008866355	0.055892769	HLA-A
	6	29941260	29945884	+	2710
protein_coding	"major histocompatibility complex, class I, A
[Source:HGNC Symbol;Acc:HGNC:4931]"	-	1858	3233	3086
	1389	2456	2156	37.49480778	52.59813818
52.75080441	24.78187553	39.49277667	37.13859771
ENSG00000272872 28.92404189	38.00499677	32.26734773
103.4715569	40.20983461	114.8022158	33.06546213
86.16120242	-1.378451926	0.008885259	0.055988851
AP000525.1	22	15823197	15823890	+	694
	sense_intronic	"novel transcript, sense intronic to novel

transcript"	-	25	41	33	103	44	117
	1.970040938	2.604703804	2.20270798	7.175942841
	2.762814991	7.869958226
ENSG00000185728 3647.900164	3148.853025	3008.685727
4282.51696	4606.767642	3741.374776	3268.479639
4210.219793	-0.365560261	0.008893975	0.056020683	YTHDF3
	8	63168553	63212786	+	6163
protein_coding	YTH N6-methyladenosine RNA binding protein 3
[Source:HGNC Symbol;Acc:HGNC:26465]	-	3153	3397	3077
	4263	5041	3813	27.97863456	24.30173815
23.12798432	33.44447488	35.64372854	28.88156173
ENSG00000120256 143.4632478	71.37523785	91.91305112
24.10987733	54.83159264	48.07956045	102.2505123
42.34034348	1.267542029	0.008912937	0.056111523	LRP11
	6	149818798	149864026	-	5652
protein_coding	LDL receptor related protein 11 [Source:HGNC
Symbol;Acc:HGNC:16936]	-	124	77	94	24	60
	49	1.199814883	0.60065145	0.770421078
0.205310096	0.462602201	0.404706298
ENSG00000047644 1094.485745	983.4951604	1194.869665
879.0059443	812.421431	734.9304241	1090.95019
808.7859331	0.431679977	0.008915738	0.056111523	WWC3
	X	10015562	10144478	+	7114
protein_coding	WWC family member 3 [Source:HGNC
Symbol;Acc:HGNC:29237]	-	946	1061	1222	875	889
	749	7.272303383	6.575601427	7.957191332
5.946965358	5.445609533	4.914892154
ENSG00000177700 367.9138129	799.9588346	756.8159741
297.3551537	332.6449954	511.2132856	641.5628738
380.4044782	0.755241509	0.008929467	0.05617317	POLR2L
	11	837356	842545	-	1142	protein_coding	RNA
polymerase II subunit L [Source:HGNC Symbol;Acc:HGNC:9199]	-
	318	863	774	296	364	521	15.22845095
	33.31797897	31.39621631	12.53218576	13.88973236
	21.29696196
ENSG00000233184 409.5644332	269.742782	203.3820706
167.7645631	152.6145995	189.3745953	294.2297619
169.9179193	0.790508447	0.008932883	0.05617317
AC093157.1	1	101025878	101087268	+	2840
	antisense	uncharacterized LOC102606465 [Source:NCBI
gene;Acc:102606465]	-	354	291	208	167	167
	193	6.816785597	4.517608656	3.392715561
2.843147215	2.562457841	3.17237855
ENSG00000120306 554.1846427	424.5436225	378.4079871
625.8522323	725.6047427	618.1657773	452.3787507
656.5409174	-0.539034677	0.008939807	0.056193596	CYSTM1
	5	140174642	140282052	+	2371
protein_coding	cysteine rich transmembrane module containing 1
[Source:HGNC Symbol;Acc:HGNC:30239]	-	479	458	387
	623	794	630	11.04838176	8.516632

7.561045767	12.70450385	14.59309959	12.40380789
ENSG00000102900 2990.745932	2911.553533	3024.330501
2721.402403	2070.806482	2001.67966	2975.543322
2264.629515	0.393993598	0.008944055	0.05619719	NUP93
16	56730105	56850286	+	11136
protein_coding	nucleoporin 93 [Source:HGNC Symbol;Acc:HGNC:28958]
-	2585	3141	3093	2709	2266	2040
12.69480511	12.43576974	12.8662846	11.76199524
8.867256698	8.551592171
ENSG00000063245 3800.619105	5423.591125	6128.840473
7331.411864	6073.512745	7491.580491	5117.683567
6965.5017	-0.4444827	0.008950749	0.056216146	EPN1
19	55675226	55709858	+	17616
protein_coding	epsin 1 [Source:HGNC Symbol;Acc:HGNC:21604]	-
3285	5851	6268	7298	6646	7635	10.19818263
14.6439 16.48254014	20.03077598	16.44037173
20.23241738
ENSG00000198948 168.9164047	141.8235245	108.5356242
253.1537119	243.0867274	211.9425522	139.7585178
236.0609972	-0.758240261	0.008957747	0.056236995	MFAP3L
4	169986597	170033031	-	8741
protein_coding	microfibril associated protein 3 like [Source:HGNC
Symbol;Acc:HGNC:29083]	-	146	153	111	252	266
216	0.913453509	0.771728019	0.588253222
1.393928947	1.326108669	1.153555958
ENSG00000110917 4425.37841	4068.388557	5294.778424
3552.188593	3691.993905	3516.676422	4596.181797
3586.952973	0.357661283	0.008969841	0.056289804	MLEC
12	120686869	120701864	+	6723
protein_coding	malectin [Source:HGNC Symbol;Acc:HGNC:28973]	-
3825	4389	5415	3536	4040	3584	31.11451537
28.78302451	37.31107873	25.4302343	26.18646356
24.88576098
ENSG00000259354 45.12150535	27.80853423	31.28954932
6.027469332	4.569299387	13.73701727	34.73986297
8.111261997	2.099265443	0.008974773	0.056297645
AC025580.2	15	45448427	45513767	+	3886
antisense	"novel transcript, antisense to SLC30A4"
-	39	30	32	6	5	14
0.548853608	0.340370895	0.381460556	0.074653414
0.056069387	0.16817881
ENSG00000172006 93.71389573	86.2064561	62.57909863
153.700468	156.270039	142.2762503	80.83315016
150.7489191	-0.900866851	0.008981826	0.056318776	ZNF554
19	2819874 2835773 +	4685	protein_coding	zinc
finger protein 554 [Source:HGNC Symbol;Acc:HGNC:26629]	zf-C2H2 81
93	64	153	171	145	0.94551873
0.875200005	0.632809273	1.579003352	1.590541895
1.444789055
ENSG00000100139 1240.262916	1833.509357	1945.818848

2472.267004	2101.877718	2340.199014	1673.19704
2304.781245	-0.461301536	0.009031682	0.056608171
MICALL1 22	37905657	37942822	+	5968
protein_coding	MICAL like 1 [Source:HGNC Symbol;Acc:HGNC:29804]
	-	1072	1978	1990	2461	2300	2385
9.823374104	14.61273238	15.4463801	19.93811129
16.7941342	18.65544465
ENSG00000162878 31.23796524	48.20145932	128.0915925
28.12819022	12.79403828	23.54917247	69.1770057
21.49046699	1.691021255	0.009051343	0.056686043	PKDCC
	2	42048020	42058528	+	3925
protein_coding	"protein kinase domain containing, cytoplasmic
[Source:HGNC Symbol;Acc:HGNC:25123]"	-	27	52	131
	28	14	24	0.376200021	0.584114034
1.546087576	0.34492096	0.15543434	0.28544183
ENSG00000111961 1043.579431	834.2560268	997.3543845
789.5984825	524.5555696	704.512743	958.3966142
672.8889317	0.510329415	0.009051523	0.056686043	SASH1
	6	148272304	148552050	+	11143
protein_coding	SAM and SH3 domain containing 1 [Source:HGNC
Symbol;Acc:HGNC:19182]	-	902	900	1020	786	574
	718	4.426893966	3.561019377	4.240338207
3.410528099	2.244751961	3.007934324
ENSG00000151233 753.1820509	653.5005543	772.4607488
586.6736817	515.4169709	446.4530614	726.381118
516.181238	0.492661745	0.009056602	0.056694627	GXYLT1
	12	42081845	42144879	-	7497
protein_coding	glucoside xylosyltransferase 1 [Source:HGNC
Symbol;Acc:HGNC:27482]	-	651	705	790	584	564
	455	4.748847141	4.146059822	4.881373468
3.766400968	3.278311302	2.833152303
ENSG00000073417 1293.483153	1313.489767	1522.432134
1128.141343	1050.938859	924.3050193	1376.468351
1034.461741	0.41228194	0.009079508	0.05681475	PDE8A
	15	84980440	85139145	+	7886
protein_coding	phosphodiesterase 8A [Source:HGNC
Symbol;Acc:HGNC:8793]	-	1118	1417	1557	1123	1150
	942	7.7531778	7.922222497	9.146065255
6.885321123	6.354767494	5.576223417
ENSG00000279145 35.86581195	26.88158308	33.24514615
95.43493109	63.05633154	72.60994844	31.99751373
77.03373702	-1.269315101	0.009095538	0.056891769
AC011912.1	15	55171378	55176739	-	5362
	lincRNA TEC	-	31	29	34	95	69
	74	0.316176507	0.238454293	0.293734232
0.856639335	0.560764973	0.644244836
ENSG00000227165 5.784808379	10.19646255	8.800185745	0
	0	0	8.260485558	0	5.540774389
0.009125402	1	WDR11-AS1	10	120761812
120851345	-	4685	antisense	WDR11 antisense RNA 1

[Source:HGNC Symbol;Acc:HGNC:27437]	-	5	11	9
	0	0	0	0.058365354	0.10351828
0.088988804	0	0	0
ENSG00000095383 145.7771711	268.8158308	178.9371102
311.4192488	281.4688422	412.1105182	197.8433707
334.9995364	-0.757026836	0.009148018	0.057196629	TBC1D2
	9	98198999	98255721	-	3681
protein_coding	TBC1 domain family member 2 [Source:HGNC
Symbol;Acc:HGNC:18026]	-	126	290	183	310	308
	420	1.871972396	3.473490684	2.30296707
4.071899897	3.646225272	5.326347643
ENSG00000102226 2672.581471	2948.631579	2837.571004
3547.165702	3313.655916	3792.397983	2819.594685
3551.0732	-0.33254684	0.009164928	0.057278925	USP11
	X	47232690	47248328	+	5269
protein_coding	ubiquitin specific peptidase 11 [Source:HGNC
Symbol;Acc:HGNC:12609]	-	2310	3181	2902	3531	3626
	3865	23.97609739	26.61763274	25.51359547
32.40191926	29.98873862	34.24264753
ENSG00000275963 0	0.926951141	0.977798416	21.09614266
	7.310879019	10.79337071	0.634916519	13.06679747
	-4.310239479	0.009168067	1	AC008115.4	12
	12648939	12649713	+	775	lincRNA novel
transcript	-	0	1	1	21	8	11
	0	0.056889518	0.059772408	1.310143326
0.449828411	0.66257801
ENSG00000129315 2199.384146	1721.348269	1588.922426
2824.87396	2319.376369	2263.664203	1836.551613
2469.304844	-0.427548113	0.009176962	0.057330697	CCNT1
	12	48688458	48716998	-	7381
protein_coding	cyclin T1 [Source:HGNC Symbol;Acc:HGNC:1599]	-
	1901	1857	1625	2812	2538	2307	14.0851548
	11.09253113	10.19860134	18.42049632	14.98425
	14.59077717
ENSG00000130741 4604.707469	4423.410844	4507.650699
3879.681093	3488.203152	3510.789129	4511.923004
3626.224458	0.315279435	0.009182683	0.057343002	EIF2S3
	X	24054716	24077971	+	3472
protein_coding	eukaryotic translation initiation factor 2 subunit
gamma [Source:HGNC Symbol;Acc:HGNC:3267]	-	3980	4772
	4610	3862	3817	3578	62.68997091	60.59749569
	61.50687535	53.78160633	47.90722785	48.10682065
ENSG00000122707 321.6353458	144.604378	277.6947502
123.5631213	73.10879019	177.600009	247.978158
124.7573068	0.990688159	0.009207432	0.057474074	RECK
	9	36036433	36124451	+	6045
protein_coding	reversion inducing cysteine rich protein with kazal
motifs [Source:HGNC Symbol;Acc:HGNC:11345]	-	278	156
	284	123	80	181	2.515030195	1.137790363
	2.176328707	0.983807259	0.576703092	1.397746153

ENSG00000115073 944.0807274 1062.286007 885.885365
1215.539649 1201.725739 1457.105046 964.0840333
1291.456811 -0.421469151 0.009214707 0.057494289 ACTR1B
2 97655963 97664107 - 2892
protein_coding ARP1 actin related protein 1 homolog B [Source:HGNC
Symbol;Acc:HGNC:168] - 816 1146 906 1210 1315
1485 15.43073393 17.47110151 14.51217026
20.2296467 19.81463433 23.97034037
ENSG00000181104 3284.614197 2483.302106 3154.377691
2480.30363 2248.095298 2049.75922 2974.097998
2259.38605 0.396291462 0.009218192 0.057494289 F2R
5 76716043 76735781 + 4066
protein_coding coagulation factor II thrombin receptor [Source:HGNC
Symbol;Acc:HGNC:3537] - 2839 2679 3226 2469 2460
2089 38.18499438 29.04954251 36.75356277
29.35992434 26.36491227 23.98375072
ENSG00000079999 2605.477694 2692.793064 2540.320285
3509.996308 3172.921494 3154.607895 2612.863681
3279.175232 -0.327618691 0.009251243 0.057676896 KEAP1
19 10486120 10503741 - 3761
protein_coding kelch like ECH associated protein 1 [Source:HGNC
Symbol;Acc:HGNC:23177] - 2252 2905 2598 3494 3472
3215 32.7461137 34.05467667 31.99913725
44.91803857 40.22860574 39.90466596
ENSG00000212694 69.41770054 36.15109449 70.40148596
4.018312888 12.79403828 31.39889662 58.65676033
16.07041593 1.866385852 0.009259745 0.05770637
LINC01089 12 121795267 121803906 - 4132
lincRNA long intergenic non-protein coding RNA 1089
[Source:HGNC Symbol;Acc:HGNC:27886] - 60 39 72
4 14 32 0.794119116 0.41613884
0.807187893 0.046805932 0.147647576 0.361522808
ENSG00000158555 266.1011854 388.392528 324.6290742
495.2570635 410.323085 586.7668806 326.3742625
497.4490097 -0.606149068 0.009279725 0.057771049 GDPD5
11 75434640 75525903 - 9867
protein_coding glycerophosphodiester phosphodiesterase domain
containing 5 [Source:HGNC Symbol;Acc:HGNC:28804] - 230
419 332 493 449 598 1.274786397
1.872245747 1.558674432 2.415811296 1.982986742
2.829189852
ENSG00000232119 1758.581747 1529.469382 1878.350757
1329.056988 1334.235421 1309.922718 1722.133962
1324.405042 0.378668424 0.009279853 0.057771049 MCTS1
X 120594010 120621074 + 2837
protein_coding "MCTS1, re-initiation and release factor [Source:HGNC
Symbol;Acc:HGNC:23357]" - 1520 1650 1921 1323 1460
1335 29.30076538 25.64239385 31.36681951
22.54767285 22.42601549 21.96685917
ENSG00000174804 194.3695615 138.11572 229.7826278

118.5402302	78.59194946	107.9337071	187.4226364
101.6886289	0.882569042	0.009283322	0.057771049	FZD4
	11	86945679	86955391	-	7383
protein_coding	frizzled class receptor 4 [Source:HGNC
Symbol;Acc:HGNC:4042]	-	168	149	235	118	86
	110	1.244431873	0.889789667	1.474475123
0.772770181	0.507603	0.695513962
ENSG00000072364 3920.943119	3166.465097	3000.863339
5333.305781	4325.2988	3807.116216	3362.757185
4488.573599	-0.416824808	0.00928524	0.057771049	AFF4
	5	132875379	132963634	-	11297
protein_coding	AF4/FMR2 family member 4 [Source:HGNC
Symbol;Acc:HGNC:17869]	AF-4	3389	3416	3069	5309	4733
	3880	16.40601683	13.33179697	12.58450726
22.72222275	18.25710753	16.0329941
ENSG00000104756 1735.442514	1546.154503	1572.299853
1157.274112	1385.411574	1154.890666	1617.965623
1232.525451	0.392053281	0.009293023	0.057795952	KCTD9
	8	25427847	25458476	-	4383
protein_coding	potassium channel tetramerization domain containing 9
[Source:HGNC Symbol;Acc:HGNC:22401]	-	1500	1668	1608
	1152	1516	1177	18.71606312	16.77870868
16.99483802	12.70814686	15.07253595	12.53577037
ENSG00000065665 198.9974082	164.0703519	181.8705054
77.3525231	120.6295038	105.9712761	181.6460885
101.3177677	0.840318827	0.009296843	0.057796195
SEC61A2 10	12129637	12169961	+	3916
protein_coding	Sec61 translocon alpha 2 subunit [Source:HGNC
Symbol;Acc:HGNC:17702]	-	172	177	186	77	132
	108	2.402041335	1.992803793	2.200253482
0.950712619	1.468891933	1.287440326
ENSG00000258673 12.72657843	11.12341369	1.955596832	0
	0	0	8.601862985	0	5.595908916
0.009349019	1	LINC01397	1	110082688
110109719	+	579	antisense	long intergenic non-
protein coding RNA 1397 [Source:HGNC Symbol;Acc:HGNC:50677]	-
	11	12	2	0	0	0	1.038983939
	0.913769463	0.160012492	0	0	0
ENSG00000113387 4655.613783	3603.986036	3323.536817
2516.468446	3381.281546	2693.436601	3861.045545
2863.728865	0.430715842	0.009353716	0.05812612	SUB1
	5	32531633	32604079	+	9324
protein_coding	"SUB1 homolog, transcriptional regulator [Source:HGNC
Symbol;Acc:HGNC:19985]" PC4	4024	3888	3399	2505	3700
	2745	23.60208771	18.38475935	16.88695537
12.98992614	17.29251085	13.74314472
ENSG00000129484 537.9871792	670.1856748	784.1943298
531.4218795	434.9973016	392.4862078	664.1223946
452.968463	0.55302304	0.009359385	0.058137718	PARP2
	14	20343582	20357905	+	4096

protein_coding poly(ADP-ribose) polymerase 2 [Source:HGNC Symbol;Acc:HGNC:272]   -   465   723   802   529   476
        400   6.208514756   7.782377751   9.070200264
6.244489513   5.064138823   4.558753179
ENSG00000105854 1180.100909   914.900776   1084.378444
875.9922096   702.7582457   738.8552862   1059.793376
772.5352472   0.455805852   0.009377762   0.058228207   PON2
        7   95404863   95435329   -   3843
protein_coding paraoxonase 2 [Source:HGNC Symbol;Acc:HGNC:9205]
        -       1020   987   1109   872   769   753
14.51524933   11.32350109   13.36791322   10.97102784
8.719961211   9.146830423
ENSG00000152217 254.5315687   248.4229057   466.4098445
177.8103453   164.4947779   220.7734919   323.1214396
187.6928717   0.784573774   0.009385567   0.058253007   SETBP1
        18   44680173   45068510   +   14025
protein_coding SET binding protein 1 [Source:HGNC Symbol;Acc:HGNC:15573] -   220   268   477   177   180
        225   0.857856258   0.842492187   1.575498394
0.61019917   0.55927864   0.748903196
ENSG00000108556 34.70885027   40.7858502   28.35615407   0
        13.70789816   3.924862078   34.61695151   5.877586746
        2.546289531   0.009407   0.058362343   CHRNE   17
        4897774 4934438 -   2864   protein_coding cholinergic receptor nicotinic epsilon subunit [Source:HGNC Symbol;Acc:HGNC:1966]
        -       30   44   29   0   15   4
0.572852686   0.677350757   0.469058964   0   0.228232161
        0.065197811
ENSG00000196204 841.1111382   920.4624829   833.0842506
1235.631213   1026.264642   1195.120503   864.8859572
1152.338786   -0.413527357   0.009411091   0.058364037
RNF216P1   7   4973988 5040675 +   5510
transcribed_unprocessed_pseudogene   ring finger protein 216 pseudogene 1 [Source:HGNC Symbol;Acc:HGNC:33610]   -   727
        993   852   1230   1123   1218   7.215684317
7.945689826   7.162925789   10.79331194   8.881508896
10.31909772
ENSG00000247315 882.7617586   948.2710171   1090.245234
1333.075301   1195.32872   1356.039848   973.7593366
1294.814623   -0.410570394   0.009415858   0.058369922   ZCCHC3
        20   296968 300321 +   3354   protein_coding zinc finger CCHC-type containing 3 [Source:HGNC Symbol;Acc:HGNC:16230]
        -       763   1023   1115   1327   1308   1382
12.44102585   13.44765422   15.39977108   19.1297406
16.99430011   19.23494818
ENSG00000166797 1168.531292   1041.893082   934.7752859
653.9804225   891.0133805   726.0994844   1048.399887
757.0310958   0.468951873   0.009467778   0.058667984   CIAO2A
        15   64072559   64094018   -   2427
protein_coding cytosolic iron-sulfur assembly component 2A

[Source:HGNC Symbol;Acc:HGNC:26235]	-	1010	1124	956
	651	975	740	22.75864021	20.41881305
18.24696217	12.96917734	17.50626253	14.23337787
ENSG00000125952 770.536476	916.7546783	917.1749144
1148.232908	1076.526936	1259.880727	868.1553562
1161.546857	-0.4192512	0.009480591	0.05872358	MAX
	14	65006174	65102695	-	4430
protein_coding	MYC associated factor X [Source:HGNC
Symbol;Acc:HGNC:6913]	bHLH	666	989	938	1143	1178
	1284	8.22176796	9.842978202	9.808476772
12.47509095	11.58777788	13.53029711
ENSG00000114054 742.7693958	749.9034729	742.1489979
618.8201848	526.3832894	459.2088631	744.9406222
534.8041124	0.478309205	0.009496125	0.058782873	PCCB
	3	136250306	136337896	+	7532
protein_coding	propionyl-CoA carboxylase subunit beta [Source:HGNC
Symbol;Acc:HGNC:8654]	-	642	809	759	616	576
	468	4.661432819	4.735568993	4.66803303
3.954318234	3.332504694	2.900558157
ENSG00000111145 4819.902341	3691.119443	3270.735702
5612.578526	5650.395622	4517.516252	3927.252495
5260.163467	-0.421884292	0.009497854	0.058782873	ELK3
	12	96194382	96269835	+	4648
protein_coding	"ELK3, ETS transcription factor [Source:HGNC
Symbol;Acc:HGNC:3325]"	ETS	4166	3982	3345	5587	6183
	4604	49.01712771	37.77192288	33.33745623
58.1184159	57.96845491	46.23972381
ENSG00000101265 108.7543975	92.69511408	111.4690194
38.17397244	25.58807657	70.6475174	104.306177
44.8031888	1.22041329	0.009535568	0.058992404	RASSF2
	20	4780023 4823645 -	5759	protein_coding	Ras
association domain family member 2 [Source:HGNC Symbol;Acc:HGNC:9883]
	-	94	100	114	38	28	72
0.89263824	0.765573478	0.916980771	0.319034562
0.211870041	0.58362173
ENSG00000133597 345.931541	506.1153229	542.678121
841.8365501	591.2673407	658.3956136	464.9083283
697.1665014	-0.582862739	0.009542225	0.059009707	ADCK2
	7	140673153	140696261	+	2913
protein_coding	aarF domain containing kinase 2 [Source:HGNC
Symbol;Acc:HGNC:19039]	-	299	546	555	838	647
	671	5.613392583	8.263920224	8.825817732
13.90928323	9.678819567	10.75296102
ENSG00000100294 414.1922799	530.2160526	547.5671131
790.6030607	563.8515444	829.127114	497.3251485
727.860573	-0.548133189	0.009550961	0.059039847	MCAT
	22	43132206	43143394	-	2388
protein_coding	malonyl-CoA-acyl carrier protein transacylase
[Source:HGNC Symbol;Acc:HGNC:29622]	-	358	572	560
	787	617	845	8.198670202	10.56077194

10.8631596	15.93461689	11.2592494	16.51841688
ENSG00000137331 2698.034628	3342.585814	2777.9253
3645.614368	4623.21712	3393.043266	2939.515247
3887.291585	-0.403085592	0.009600673	0.059323159	IER3
	6	30743199	30744554	-	1356
protein_coding	immediate early response 3 [Source:HGNC
Symbol;Acc:HGNC:5392]	-	2332	3606	2841	3629	5059
	3458	94.05103286	117.246528	97.05412533
129.3982775	162.5786779	119.0450482
ENSG00000067365 258.0024537	311.4555833	236.6272167
398.8175541	362.8023713	455.284001	268.6950846
405.6346422	-0.593287544	0.00961209	0.059348188
METTL22 16	8621683 8649654 +	8254	protein_coding
methyltransferase like 22 [Source:HGNC Symbol;Acc:HGNC:28368]	-
	223	336	242	397	397	464	1.47752593
	1.794769873	1.358167573	2.325558446	2.095967962
	2.624215835
ENSG00000136111 1609.333691	1680.562418	2087.599619
1305.951689	1273.920669	1474.766926	1792.498576
1351.546428	0.407649195	0.009612489	0.059348188	TBC1D4
	13	75283503	75482114	-	7977
protein_coding	TBC1 domain family member 4 [Source:HGNC
Symbol;Acc:HGNC:19165]	-	1391	1813	2135	1300	1394
	1503	9.536351508	10.02056409	12.3982601
7.879614609	7.615208165	8.795599064
ENSG00000106538 3.470885027	1.853902282	21.51156516	0
	0	0	8.945450821	0	5.655534203
0.009617267	1	RARRES2 7	150338317	150341674
	-	1921	protein_coding	retinoic acid receptor
responder 2 [Source:HGNC Symbol;Acc:HGNC:9868]	-	3	2
	22	0	0	0	0.085406043	0.045902526
	0.530515127	0	0	0
ENSG00000253500 1.156961676	2.780853423	0.977798416	0
	61.22861179	8.830939675	1.638537838	23.35318382
	-3.821542493	0.009635734	0.059467687	AF121898.1
	8	87540835	87755718	-	2910
antisense	"novel transcript, antisense to CNBD1"	-	1
	3	1	0	67	9	0.018793243
0.045452966	0.015918768	0	1.003322176	0.144376183
ENSG00000110066 1220.594568	1055.797349	1030.599531
1451.615531	1580.977588	1328.565813	1102.330483
1453.719644	-0.399843445	0.009659386	0.059589605	KMT5B
	11	68154863	68213828	-	8632
protein_coding	lysine methyltransferase 5B [Source:HGNC
Symbol;Acc:HGNC:24283]	-	1055	1139	1054	1445	1730
	1354	6.683989219	5.817632058	5.656289573
8.093896226	8.733599435	7.322396951
ENSG00000266129 17.35442514	24.10072966	15.64477466	0
	0	4.906077597	19.03330982	1.635359199
3.549799781	0.009675564	1	SRP68P1 17	16787235

16788141	-	269	processed_pseudogene	signal recognition particle 68 pseudogene 1 [Source:HGNC Symbol;Acc:HGNC:30427]	-	15	26	16	0	0
5	3.049535489	4.261426733	2.755308033	0
0	0.867688337
ENSG00000099949 253.374607	318.8711925	290.4061296
392.7900848	393.8736072	508.2696391	287.550643
431.6444437	-0.584719715	0.009699042	0.059808466	LZTR1
22	20982013	20999038	+	8935
protein_coding	leucine zipper like transcription regulator 1 [Source:HGNC Symbol;Acc:HGNC:6742]	Others	219	344	297
391	431	518	1.340430406	1.697453334
1.539800117	2.115842893	2.102041622	2.706333035
ENSG00000164087 506.749214	573.7827562	806.6836933
460.0968257	382.9072886	428.791182	629.0718878
423.9317654	0.570330205	0.009702688	0.059808466	POC1A
3	52075253	52154690	-	2247
protein_coding	POC1 centriolar protein A [Source:HGNC Symbol;Acc:HGNC:24488]	-	438	619	825	458	419
437	10.66020977	12.14567161	17.00800332
9.855158697	8.125863979	9.078715366
ENSG00000084731 588.8934929	602.5182416	531.9223384
728.319211	857.200565	768.2917517	574.444691
784.6038426	-0.450095608	0.009738081	0.060002438	KIF3C
2	25926596	25982749	-	6403
protein_coding	kinesin family member 3C [Source:HGNC Symbol;Acc:HGNC:6321]	-	509	650	544	725	938
783	4.347393916	4.475729311	3.935662545
5.474641954	6.383780331	5.708530109
ENSG00000215712 369.0707746	378.1960655	260.0943787
471.1471861	553.7990857	476.8707425	335.7870729
500.6056714	-0.576797738	0.009751953	0.060039691
TMEM242 6	157289386	157323601	-	4644
protein_coding	transmembrane protein 242 [Source:HGNC Symbol;Acc:HGNC:17206]	-	319	408	266	469	606
486	3.756584695	3.873485281	2.653333751
4.882945175	5.686421011	4.885287128
ENSG00000130939 2038.566473	1943.816542	2102.266595
1772.075984	1251.074172	1554.245383	2028.216537
1525.798513	0.41088176	0.009751982	0.060039691	UBE4B
1	10032832	10181239	+	9128
protein_coding	ubiquitination factor E4B [Source:HGNC Symbol;Acc:HGNC:12500]	-	1762	2097	2150	1764	1369
1584	10.55662235	10.12877111	10.9110183
9.343813595	6.535614302	8.10075657
ENSG00000147439 240.6480285	408.7854531	395.0305601
561.5592261	499.8813529	534.7624581	348.1546806
532.0676791	-0.60959344	0.009771655	0.060136593	BIN3
8	22620418	22669148	-	5080
protein_coding	bridging integrator 3 [Source:HGNC

Symbol;Acc:HGNC:1054] - 208 441 404 559 547
545 2.239207476 3.827443912 3.684004132
5.320461588 4.692261548 5.008167272
ENSG00000068120 2231.779072 2687.231357 2608.766174
3190.540433 2997.460398 3427.38581 2509.258868
3205.12888 -0.352741252 0.009776341 0.060141215 COASY
17 42561467 42566277 + 4032
protein_coding Coenzyme A synthase [Source:HGNC
Symbol;Acc:HGNC:29932] - 1929 2899 2668 3176 3280
3493 26.16413715 31.70017428 30.65263103
38.08563376 35.44964725 40.44120598
ENSG00000177822 220.9796801 116.7958437 118.3136084
80.36625776 81.33352909 67.70387084 152.0297107
76.4678859 0.988378903 0.009784456 0.060166923
AC098864.1 4 181874438 182145249 - 5551
antisense uncharacterized LOC90768 [Source:NCBI
gene;Acc:90768] - 191 126 121 80 89 69
1.881728023 1.00076769 1.009756364 0.696818987
0.698678497 0.580261691
ENSG00000150990 1737.756437 2585.266732 2376.050151
3327.163071 2632.830307 3102.603473 2233.02444
3020.865617 -0.435377476 0.009804311 0.060250554 DHX37
12 124946825 124989122 - 6618
protein_coding DEAH-box helicase 37 [Source:HGNC
Symbol;Acc:HGNC:17210] - 1502 2789 2430 3312 2881
3162 12.41188899 18.58042781 17.00912476
24.19718148 18.97033906 22.30391691
ENSG00000186298 4816.431456 3614.182498 3946.394408
3053.917795 3543.034745 2855.337162 4125.669454
3150.763234 0.388582014 0.009805938 0.060250554 PPP1CC
12 110719680 110742939 - 6906
protein_coding protein phosphatase 1 catalytic subunit gamma
[Source:HGNC Symbol;Acc:HGNC:9283] - 4163 3899 4036
3040 3877 2910 32.96662968 24.89204739
27.07241753 21.28375378 24.46402009 19.67036645
ENSG00000078967 325.1062309 363.3648472 328.5402678
526.3989883 474.2932764 457.2464321 339.003782
485.9795656 -0.518945708 0.009820871 0.060318063 UBE2D4
7 43926438 43956136 + 4713
protein_coding ubiquitin conjugating enzyme E2 D4 (putative)
[Source:HGNC Symbol;Acc:HGNC:21647] - 281 392 336
524 519 466 3.260645563 3.667098582
3.302511157 5.375700489 4.798754317 4.615667467
ENSG00000162595 37.02277362 23.17377852 28.35615407
12.05493866 1.827719755 1.962431039 29.51756874
5.281696486 2.490084552 0.009831808 0.060360983 DIRAS3
1 68045896 68051631 - 2039
protein_coding DIRAS family GTPase 3 [Source:HGNC
Symbol;Acc:HGNC:687] - 32 25 29 12 2
2 0.858276982 0.540575976 0.65884496

0.284554355	0.042743627	0.045788752
ENSG00000064933 815.6579814	559.8784891	568.1008798
303.3826231	486.1734548	475.8895269	647.8791167
421.8152016	0.617686433	0.009841186	0.060394306	PMS1
	2	189784085	189877629	+	5454
protein_coding	"PMS1 homolog 1, mismatch repair system component
[Source:HGNC Symbol;Acc:HGNC:9121]"	HMG	705	604	581
	302	532	485	7.069174401	4.882651898
4.934730671	2.677275266	4.250647553	4.15119028
ENSG00000124783 8309.298755	6662.9248	6989.303079
5920.984041	6092.703803	5283.845572	7320.508878
5765.844472	0.344243644	0.009849122	0.060418749	SSR1
	6	7268306 7347446 -	11349	protein_coding	signal
sequence receptor subunit 1 [Source:HGNC Symbol;Acc:HGNC:11323] -
	7182	7188	7148	5894	6667	5385	34.60847936
	27.92443729	29.17624543	25.110407	25.59949846
	22.15002126
ENSG00000213753 822.5997514	1025.207962	1046.244305
1395.35915	1207.208898	1274.59896	964.6840062
1292.389003	-0.421029767	0.009860699	0.060465508
CENPBD1P1	19	58573503	58599801	+	4969
	transcribed_processed_pseudogene	CENPB DNA-binding
domains containing 1 pseudogene 1 [Source:HGNC Symbol;Acc:HGNC:28421]
	-	711	1106	1070	1389	1321	1299
7.825197666	9.813413261	9.975099508	13.51557309
11.58490345	12.20355015
ENSG00000165476 2448.130906	1865.952647	1760.037149
1549.059618	1480.453001	1534.621072	2024.7069
1521.377897	0.411835303	0.009870639	0.060502187	REEP3
	10	63521363	63625123	+	5248
protein_coding	receptor accessory protein 3 [Source:HGNC
Symbol;Acc:HGNC:23711]	-	2116	2013	1800	1542	1620
	1564	22.05040394	16.91156918	15.88843547
14.2066527	13.45178093	13.91198043
ENSG00000129083 3927.884889	2870.767683	2978.373976
2122.673783	2799.152805	2371.597911	3259.008849
2431.141499	0.422337068	0.009888567	0.060577239	COPB1
	11	14443440	14500027	-	5131
protein_coding	coatomer protein complex subunit beta 1 [Source:HGNC
Symbol;Acc:HGNC:2231]	-	3395	3097	3046	2113	3063
	2417	36.18532492	26.61173246	27.49985096
19.91125796	26.013787	21.98976922
ENSG00000175322 323.9492692	221.5413227	360.8076156
181.8286582	163.5809181	208.0176901	302.0994025
184.4757555	0.711148848	0.009893451	0.060577239	ZNF519
	18	14057457	14132490	-	15787
protein_coding	zinc finger protein 519 [Source:HGNC
Symbol;Acc:HGNC:30574]	zf-C2H2 280	239	369	181	179
	212	0.96995846	0.667470767	1.082752544
0.554345061	0.494096776	0.62687693

ENSG00000160688 671.0377719 850.9411473 776.3719424
1203.48471 937.6202342 1010.651985 766.1169539
1050.585643 -0.454565942 0.009897694 0.060577239 FLAD1
1 154983338 154993111 + 4019
protein_coding flavin adenine dinucleotide synthetase 1 [Source:HGNC
Symbol;Acc:HGNC:24671] - 580 918 794 1198 1026
1030 7.892320261 10.07067621 9.151766945
14.4125235 11.12469088 11.96369284
ENSG00000105063 3024.29782 3717.074075 3862.303744
5199.696877 3941.477651 4749.083114 3534.558546
4630.085881 -0.389191896 0.009898948 0.060577239 PPP6R1
19 55229780 55258995 - 6583
protein_coding protein phosphatase 6 regulatory subunit 1
[Source:HGNC Symbol;Acc:HGNC:29195] - 2614 4010 3950
5176 4313 4840 21.71583039 26.85681302
27.79557716 38.01645607 28.55053171 34.32160133
ENSG00000154957 273.0429555 297.5513162 249.3385961
474.1609208 423.1171232 339.5005697 273.3109559
412.2595379 -0.592740922 0.009902696 0.060577239 ZNF18
17 11977439 11997510 - 3012
protein_coding zinc finger protein 18 [Source:HGNC
Symbol;Acc:HGNC:12969] zf-C2H2 236 321 255 472 463
346 4.285009096 4.698768221 3.921820106
7.57684229 6.698608886 5.362498295
ENSG00000234129 27.76708022 24.10072966 23.46716199 0
6.397019142 4.906077597 25.11165729 3.767698913
2.726258928 0.00991905 0.060653005 AC073529.1
X 10847877 11111141 - 3080
lincRNA novel transcript - 24 26 24 0
7 5 0.426142881 0.372183049 0.360963244
0 0.099038926 0.075781871
ENSG00000131023 1355.959084 1103.998809 1245.715182
1637.462502 1647.689359 1540.508366 1235.224358
1608.553409 -0.381574508 0.009928009 0.060683513 LATS1
6 149658153 149718256 - 8643
protein_coding large tumor suppressor kinase 1 [Source:HGNC
Symbol;Acc:HGNC:6514] - 1172 1191 1274 1630 1803
1570 7.415796633 6.075488546 6.828217885
9.118519011 9.090542707 8.479713423
ENSG00000065883 2770.923213 2691.866113 2934.373047
4209.18275 3293.550998 3321.414533 2799.054124
3608.049427 -0.366191878 0.009938056 0.060720646 CDK13
7 39949646 40099580 + 25093
protein_coding cyclin dependent kinase 13 [Source:HGNC
Symbol;Acc:HGNC:1733] - 2395 2904 3001 4190 3604
3385 5.219725252 5.102440903 5.540077813
8.073514932 6.258795958 6.297267214
ENSG00000083457 275.3568788 354.0953358 326.584671
194.8881751 173.6333767 239.4165868 318.6789619
202.6460462 0.654569532 0.009953279 0.060789364 ITGAE

17   3714628 3801243 -   6657   protein_coding
integrin subunit alpha E [Source:HGNC Symbol;Acc:HGNC:6147]   -
238   382   334   194   190   244   1.955208663
2.529989763   2.324183243   1.409043463   1.243751569
1.711028743
ENSG00000232677 1.156961676   0.926951141   0.977798416
6.027469332   21.01877718   14.71823279   1.020570411
13.9214931   -3.777918516   0.009977913   1   LINC00665
19   36313067   36331718   -   7201
lincRNA long intergenic non-protein coding RNA 665 [Source:HGNC
Symbol;Acc:HGNC:44323] -   1   1   1   6   23
15   0.007594547   0.006122674   0.006432942
0.040286511   0.139185381   0.097239896
ENSG00000011009 2015.427239   2792.903787   2528.586704
2970.537803   2963.647582   3962.148268   2445.639244
3298.777884   -0.431312084   0.00999048   0.060992202   LYPLA2
1   23790970   23795539   +   2196
protein_coding lysophospholipase II [Source:HGNC
Symbol;Acc:HGNC:6738]   -   1742   3013   2586   2957   3243
4038   43.38209566   60.49239145   54.55048806
65.10587878   64.35365392   85.83808391
ENSG00000189343 1840.726026   1757.499363   1798.171287
2131.714987   2424.470255   2280.344867   1798.798892
2278.84337   -0.341477355   0.009998257   0.06101531
RPS2P46 17   19445970   19446852   -   883
processed_pseudogene   ribosomal protein S2 pseudogene 46
[Source:HGNC Symbol;Acc:HGNC:35839]   -   1591   1896   1839
2122   2653   2324   98.53810109   94.66982785
96.47693137   116.1945859   130.9287869   122.8630963
ENSG00000100280 1034.323738   1294.950744   1600.656007
904.1203998   901.979699   1027.332649   1309.97683
944.4775826   0.47255747   0.010019662   0.06112154   AP1B1
22   29327680   29423179   -   6721
protein_coding adaptor related protein complex 1 subunit beta 1
[Source:HGNC Symbol;Acc:HGNC:554]   -   894   1397   1637
900   987   1047   7.274419397   9.164240304
11.28280909   6.4745536   6.399438283   7.272082917
ENSG00000147394 133.0505927   193.7327884   73.33488121
257.1720248   244.9144471   237.4541557   133.3727541
246.5135426   -0.885188902   0.010056802   0.061323625   ZNF185
X   152914442   152973480   +   4812
protein_coding zinc finger protein 185 with LIM domain [Source:HGNC
Symbol;Acc:HGNC:12976] -   115   209   75   256   268
242   1.306973959   1.914937595   0.722001501
2.572264139   2.426988805   2.34766315
ENSG00000185338 45.12150535   12.97731597   47.91212239
6.027469332   7.310879019   10.79337071   35.33698124
8.043906355   2.132769746   0.010063716   0.061341321   SOCS1
16   11254405   11256200   -   1796
protein_coding suppressor of cytokine signaling 1 [Source:HGNC

Symbol;Acc:HGNC:19383] -   39   14   49   6   8
        11   1.187552963   0.343681109   1.263840311
0.161527375   0.194107472   0.285912003
ENSG00000244509 498.6504822   455.1330102   523.1221526
236.0758822   365.543951   372.8618974   492.3018817
324.8272435   0.599159299   0.010071202   0.061362478
APOBEC3C   22   39014083   39020352   +   2818
        protein_coding apolipoprotein B mRNA editing enzyme catalytic
subunit 3C [Source:HGNC Symbol;Acc:HGNC:17353] -   431   491
        535   235   400   380   8.364326828   7.682002803
        8.794582941   4.032070249   6.185539724   6.294897222
ENSG00000264340 0   0   0   12.05493866   11.88017841
        1.962431039   0   8.632516037   -5.529982082
0.010080306   1   AC009704.1   18   74561381
74562100   -   331   antisense   "novel transcript,
antisense to CNDP1"   -   0   0   0   12   13
        2   0   0   0   1.752889214   1.711488385
        0.282064245
ENSG00000013016 188.5847531   193.7327884   160.3589403
316.4421399   255.8807657   281.6088541   180.8921606
284.6439199   -0.653893781   0.010080361   0.061393816   EHD3
        2   31234337   31269447   +   4636
protein_coding EH domain containing 3 [Source:HGNC
Symbol;Acc:HGNC:3244]   -   163   209   164   315   280
        287   1.922821147   1.987635829   1.638712915
3.285249387   2.631923136   2.889911247
ENSG00000169933 10.41265508   10.19646255   3.911193665   0
        0   0   8.173437098   0   5.522955293
0.010104407   1   FRMPD4 X   12138466   12724523
        +   8478   protein_coding FERM and PDZ domain containing
4 [Source:HGNC Symbol;Acc:HGNC:29007]   -   9   11   4
        0   0   0   0.058055559   0.0572049
0.021855917   0   0   0
ENSG00000163159 1397.609704   1564.693526   1587.944628
1870.524649   2002.266991   1941.825513   1516.749286
1938.205718   -0.353433528   0.010131501   0.061659572   VPS72
        1   151169987   151195321   -   4545
protein_coding vacuolar protein sorting 72 homolog [Source:HGNC
Symbol;Acc:HGNC:11644] -   1208   1688   1624   1862   2191
        1979   14.53542583   16.37466835   16.55215685
19.80829091   21.00714764   20.32628181
ENSG00000188486 4122.254451   6450.652989   8283.908182
4256.397927   4167.201041   4861.922899   6285.605207
4428.507289   0.505416013   0.010132063   0.061659572   H2AFX
        11   119093854   119095467   -   1614
protein_coding H2A histone family member X [Source:HGNC
Symbol;Acc:HGNC:4739]   -   3563   6959   8472   4237   4560
        4955   120.7277216   190.0978758   243.155934
126.9276239   123.1175345   143.3131904
ENSG00000170425 328.5771159   357.8031404   417.5199237

178.8149235   264.1055046   262.9657592   367.9667267
235.2953958   0.645002953   0.010139887   0.061682629
ADORA2B 17   15944917   15975746   +   2551
protein_coding adenosine A2b receptor [Source:HGNC
Symbol;Acc:HGNC:264]   -   284   386   427   178   289
        268   6.088391825   6.671305119   7.753894224
3.373733396   4.936805098   4.904224824
ENSG00000169057 2208.639839   2357.236751   2388.761531
3439.675832   2733.354893   2779.783567   2318.212707
2984.271431   -0.364129877   0.010175887   0.061877   MECP2
        X   154021573   154137103   -   18366
protein_coding methyl-CpG binding protein 2 [Source:HGNC
Symbol;Acc:HGNC:6990]   MBD   1909   2543   2443   3424   2991
        2833   5.684418722   6.104719843   6.161853134
9.01405892   7.096765105   7.200754929
ENSG00000143507 1553.79953   1759.353265   1254.515368
1940.845125   2730.613314   1738.7139   1522.556055
2136.724113   -0.489034754   0.010181381   0.061885797   DUSP10
        1   221701424   221742176   -   2914
protein_coding dual specificity phosphatase 10 [Source:HGNC
Symbol;Acc:HGNC:3065]   -   1343   1898   1283   1932   2988
        1772   25.20467942   28.71710252   20.39574459
32.05669839   44.6837531   28.38704629
ENSG00000141002 3696.492554   3565.981039   4449.960592
3306.066929   2809.205263   3006.444352   3904.144728
3040.572181   0.360787444   0.010196667   0.061954075   TCF25
        16   89873570   89913627   +   7967
protein_coding transcription factor 25 [Source:HGNC
Symbol;Acc:HGNC:29181] Others 3195   3847   4551   3291   3074
        3064   21.9316223   21.28929731   26.46150092
19.97258516   16.81386839   17.9531219
ENSG00000261373 87.92908735   64.88657986   127.1137941
165.7554066   177.2888162   177.600009   93.30982044
173.5480773   -0.895631741   0.010208201   0.061999509
VPS9D1-AS1   16   89711856   89718165   +   1753
        antisense   VPS9D1 antisense RNA 1 [Source:HGNC
Symbol;Acc:HGNC:48915] -   76   70   130   165   194
        181   2.370971802   1.760556965   3.435293851
4.550962371   4.822568572   4.819951793
ENSG00000146223 9999.619763   8971.960092   8258.485423
10885.60961   11658.11046   11284.95969   9076.688426
11276.22659   -0.31315163   0.010223969   0.062048202   RPL7L1
        6   42879618   42889925   +   7809
protein_coding ribosomal protein L7 like 1 [Source:HGNC
Symbol;Acc:HGNC:21370] -   8643   9679   8446   10836   12757
        11501   60.52904236   54.64733972   50.10235157
67.09262593   71.18880953   68.75213933
ENSG00000173511 1394.138819   1975.332881   1730.703197
1860.478867   2602.672931   2525.648747   1700.058299
2329.600182   -0.454109129   0.010228275   0.062048202   VEGFB

11	64234538	64238793	+	1545
protein_coding	vascular endothelial growth factor B [Source:HGNC
Symbol;Acc:HGNC:12681]	-	1205	2131	1770	1852	2848
2574	42.65336272	60.81194918	53.06977403
57.95804331	80.32858169	77.77250628
ENSG00000126457 7185.888968	7821.613726	8542.046964
6071.670774	6312.030173	6561.388179	7849.849886
6315.029709	0.313954449	0.010228395	0.062048202	PRMT1
19	49675786	49689029	+	4796
protein_coding	protein arginine methyltransferase 1 [Source:HGNC
Symbol;Acc:HGNC:5187]	-	6211	8438	8736	6044	6907
6687	70.8234482	77.57009165	84.37929777
60.93214933	62.75796886	65.08758901
ENSG00000240849 665.2529635	737.8531081	842.8622347
918.1844949	1098.459573	1030.276295	748.6561021
1015.640121	-0.439614534	0.010234201	0.062058796
TMEM189 20	50118254	50153734	-	7931
protein_coding	transmembrane protein 189 [Source:HGNC
Symbol;Acc:HGNC:16735]	-	575	796	862	914	1202
1050	3.96492163	4.425059104	5.034794763
5.572107191	6.604426564	6.180269094
ENSG00000100321 11.56961676	18.53902282	0	0	0
0	10.03621319	0	5.820321298	0.010252547
1	SYNGR1	22	39349925	39385588	+
6953	protein_coding	synaptogyrin 1 [Source:HGNC
Symbol;Acc:HGNC:11498]	-	10	20	0	0	0
0	0.078654302	0.126821161	0	0	0
0
ENSG00000181789 4111.841795	3714.293221	4168.354648
3066.977312	3225.925367	3320.433318	3998.163222
3204.445332	0.319148923	0.010268067	0.062223448	COPG1
3	129249606	129277773	+	4425
protein_coding	coatomer protein complex subunit gamma 1 [Source:HGNC
Symbol;Acc:HGNC:2236]	-	3554	4007	4263	3053	3530
3384	43.92369439	39.92454959	44.62770087
33.3591323	34.7632225	35.69958069
ENSG00000148308 1336.290735	1389.49976	1409.985316
1908.698622	1548.992492	1934.957004	1378.591937
1797.549373	-0.382527609	0.01027006	0.062223448	GTF3C5
9	133030675	133058503	+	3592
protein_coding	general transcription factor IIIC subunit 5
[Source:HGNC Symbol;Acc:HGNC:4668]	-	1155	1499	1442
1900	1695	1972	17.58491887	18.39921367
18.59650744	25.57516766	20.56326026	25.6281123
ENSG00000157445 62.47593049	18.53902282	27.37835565
14.06409511	3.65543951	0	36.13110299	5.906511539
2.615613012	0.010273565	0.062223448	CACNA2D3
3	54122547	55074557	+	4670
protein_coding	calcium voltage-gated channel auxiliary subunit
alpha2delta 3 [Source:HGNC Symbol;Acc:HGNC:15460]	-	54

20	28	14	4	0	0.632370486
0.1888196	0.27774331	0.144948048	0.037325163	0
ENSG00000145781 682.6073887	637.7423849	571.034275
444.0235741	470.6378369	448.4154924	630.4613495
454.3589678	0.472001386	0.010282502	0.062252916
COMMD10 5	116084991	116412762	+	2464
protein_coding	COMM domain containing 10 [Source:HGNC
Symbol;Acc:HGNC:30201]	-	590	688	584	442	515
457	13.09501562	12.31067008	10.97929867
8.673268325	9.108044069	8.658078765
ENSG00000223496 2154.26264	2726.163305	2464.052009
2836.928899	3406.869623	3252.729447	2448.159318
3165.509323	-0.370462285	0.010300952	0.062339925	EXOSC6
16	70246778	70251930	-	5153
protein_coding	exosome component 6 [Source:HGNC
Symbol;Acc:HGNC:19055]	-	1862	2941	2520	2824	3728
3315	19.76124247	25.16337212	22.65389348
26.49755281	31.52639837	30.03097456
ENSG00000240445 190.8986765	133.4809643	183.8261022
297.3551537	286.9520015	238.4353712	169.4019143
274.2475088	-0.696814969	0.010320816	0.06239409	FOXO3B
17	18670946	18672873	-	1928
processed_pseudogene	forkhead box O3B pseudogene [Source:HGNC
Symbol;Acc:HGNC:3822]	-	165	144	188	296	314
243	4.680277755	3.292982483	4.517033126
7.42311003	7.097104766	5.883628999
ENSG00000123908 3636.330547	3273.064478	3200.334216
4817.957153	3682.855306	4503.779234	3369.909747
4334.863898	-0.363332417	0.010323318	0.06239409	AGO2
8	140520156	140635619	-	15833
protein_coding	"argonaute 2, RISC catalytic component [Source:HGNC
Symbol;Acc:HGNC:3263]"	-	3143	3531	3273	4796	4030
4590	10.85615117	9.832602078	9.576024518
14.64593759	11.09175919	13.53304449
ENSG00000102007 3610.87739	3279.553136	3701.944804
2335.644366	2817.430002	3060.411205	3530.791777
2737.828525	0.366824327	0.01032531	0.06239409	PLP2
X	49171926	49175239	+	1279
protein_coding	proteolipid protein 2 [Source:HGNC
Symbol;Acc:HGNC:9087]	-	3121	3538	3786	2325	3083
3119	133.449803	121.9610745	137.1236993
87.89286663	105.0416604	113.838946
ENSG00000204237 380.6403913	420.8358179	400.8973506
534.4356141	616.8554173	537.7061047	400.7911866
562.9990453	-0.49001753	0.010326228	0.06239409	OXLD1
17	81665036	81666635	-	1600
protein_coding	oxidoreductase like domain containing 1 [Source:HGNC
Symbol;Acc:HGNC:27901]	-	329	454	410	532	675
548	11.24528918	12.5103606	11.87042668
16.07655039	18.3841006	15.98845915

ENSG00000125388 104.1265508 135.3348666 125.1581973
171.782876 232.1204089 217.8298453 121.5398715
207.2443767 -0.768496668 0.010333862 0.062415545 GRK4
4 2963608 3040747 + 4686 protein_coding G
protein-coupled receptor kinase 4 [Source:HGNC Symbol;Acc:HGNC:4543]
- 90 146 128 171 254 222
1.050352172 1.373676692 1.265348461 1.764391848
2.362055132 2.211549814
ENSG00000187079 4809.489686 4039.653072 4763.833884
3899.772658 3422.405241 3417.573654 4537.65888
3579.917184 0.341939559 0.010341341 0.062436052 TEAD1
11 12674591 12944483 + 10279
protein_coding TEA domain transcription factor 1 [Source:HGNC
Symbol;Acc:HGNC:11714] TEA 4157 4358 4872 3882 3745
3483 22.11688049 18.69262605 21.9562855
18.26021478 15.87667496 15.81789339
ENSG00000133835 1299.267962 1400.623174 1568.38866
961.3813585 1003.418145 1228.48183 1422.759932
1064.427111 0.418912095 0.010353096 0.062458417
HSD17B4 5 119452443 119637199 + 10664
protein_coding hydroxysteroid 17-beta dehydrogenase 4 [Source:HGNC
Symbol;Acc:HGNC:5213] - 1123 1511 1604 957 1098
1252 5.759096194 6.247097525 6.967655717
4.339033647 4.486842258 5.480626778
ENSG00000269834 12.72657843 34.29719221 16.62257307
34.15565955 80.41966921 78.49724156 21.21544791
64.35752344 -1.593960598 0.010353217 0.062458417
ZNF528-AS1 19 52388842 52397766 - 4160
processed_transcript ZNF528 antisense RNA 1 [Source:HGNC
Symbol;Acc:HGNC:51305] - 11 37 17 34 88
80 0.144608582 0.39214109 0.18930324
0.39517258 0.921823848 0.897723703
ENSG00000265688 112.2252825 261.4002217 219.0268452
293.3368408 318.0232373 423.8851044 197.5507832
345.0817275 -0.80151529 0.010392735 0.062663088 MAFG-
DT 17 81927829 81930753 + 1895
antisense MAFG divergent transcript [Source:HGNC
Symbol;Acc:HGNC:43649] - 97 282 224 292 348
432 2.799350203 6.561057623 5.475720345
7.450318748 8.002554258 10.64193418
ENSG00000115966 1239.105955 857.4298053 1028.643934
732.3375239 827.9570489 690.7757257 1041.726565
750.3567662 0.472317891 0.010395342 0.062663088 ATF2
2 175072250 175168382 - 5751
protein_coding activating transcription factor 2 [Source:HGNC
Symbol;Acc:HGNC:784] TF_bZIP 1071 925 1052 729 906
704 10.18452588 7.091405553 8.473734022
6.128940105 6.865045624 5.714461714
ENSG00000047188 896.6452987 880.6035838 855.5736141
745.3970407 583.9564617 603.4475445 877.6074989

644.2670156	0.44616188	0.010400744	0.06267094	YTHDC2
	5	113513683	113595285	+	10816
protein_coding	YTH domain containing 2 [Source:HGNC
Symbol;Acc:HGNC:24721]	-	775	950	875	742	639
	615	3.918589195	3.872495169	3.747518886
3.31694632	2.574499295	2.654327295
ENSG00000244045 328.5771159	425.4705736	322.6734773
588.6828381	492.5704739	490.6077597	358.9070556
523.9536906	-0.544637259	0.010416059	0.062738491
TMEM199 17	28357581	28363683	+	4620
protein_coding	transmembrane protein 199 [Source:HGNC
Symbol;Acc:HGNC:18085]	-	284	459	330	586	539
	500	3.361793841	4.380308193	3.308829737
6.132772535	5.083998191	5.052124735
ENSG00000076770 1388.354011	1102.144906	1175.313696
1006.587378	757.5898384	926.2674504	1221.937538
896.8148891	0.446082281	0.010422092	0.062748197	MBNL3
	X	132369317	132489968	-	12199
protein_coding	muscleblind like splicing regulator 3 [Source:HGNC
Symbol;Acc:HGNC:20564]	-	1200	1189	1202	1002	829
	944	5.379621585	4.297259509	4.564389442
3.971409833	2.961344256	3.612383075
ENSG00000100625 397.9948164	216.906567	274.7613549
170.7782977	168.1502174	194.2806729	296.5542461
177.736396	0.736606779	0.01042588	0.062748197	SIX4
	14	60709528	60724348	-	6658
protein_coding	SIX homeobox 4 [Source:HGNC Symbol;Acc:HGNC:10890]
	Homeobox	344	234	281	170	184	198
	2.825591429	1.549551535	1.955082034	1.234543357
	1.204294298	1.388249211
ENSG00000232716 0	0.926951141	0.977798416	45.20601999
	0	5.887293117	0.634916519
17.03110437	-4.699038942	0.010431745	1	AC016831.2
	7	130840204	130843725	-	1723
processed_pseudogene	zinc finger protein 131 (ZNF131) pseudogene
	-	0	1	1	45	0	6	0
	0.025588727	0.026885442	1.262782204	0
0.16255937
ENSG00000279267 89.08604903	116.7958437	55.73450972
136.6226382	224.8095298	152.0884055	87.2054675
171.1735245	-0.972983782	0.01044037	0.062806215
AL078621.3	2	113605867	113607908	-	2042
	TEC	TEC	-	77	126	57	136	246
	155	2.062194861	2.72050022	1.293068624
3.220211429	5.249742081	3.543414812
ENSG00000105698 1307.366694	1896.542034	1631.945557
1935.822234	2197.833005	2366.691833	1611.951428
2166.782357	-0.426148972	0.010457465	0.062806215	USF2
	19	35268978	35279821	+	4952
protein_coding	"upstream transcription factor 2, c-fos interacting

[Source:HGNC Symbol;Acc:HGNC:12594]"   bHLH   1130   2046   1669
        1927   2405   2412   12.47936595   18.21624889
15.61270509   18.81491649   21.16377045   22.73749954
ENSG00000163006 522.9466774   439.3748408   460.543054
256.1674466   328.075696   372.8618974   474.2881907
319.0350133   0.571147665   0.010458347   0.062806215
CCDC138 2   108786757   108885477   +   5796
protein_coding coiled-coil domain containing 138 [Source:HGNC
Symbol;Acc:HGNC:26531] -   452   474   471   255   359
        380   4.264859915   3.60565295   3.764393251
2.127223   2.69913539   3.060562521
ENSG00000172071 226.7644884   202.0753487   266.9389676
138.6317946   131.5958223   154.0508366   231.9262683
141.4261512   0.713768716   0.010458817   0.062806215
EIF2AK3 2   88556741   88627576   -   5434
protein_coding eukaryotic translation initiation factor 2 alpha
kinase 3 [Source:HGNC Symbol;Acc:HGNC:3255]   -   196   218
        273   138   144   157   1.972564214   1.768767776
        2.327263021   1.227893411   1.154785856   1.348733219
ENSG00000079785 3874.664652   3025.568524   2999.885541
2478.294474   2679.437161   2500.137144   3300.039572
2552.622926   0.370109239   0.010458997   0.062806215   DDX1
        2   15591178   15631111   +   3833
protein_coding DEAD-box helicase 1 [Source:HGNC Symbol;Acc:HGNC:2734]
        -       3349   3264   3068   2467   2932   2548
47.78273903   37.54441042   37.07822981   31.11942377
33.33371704   31.03177661
ENSG00000224877 458.1568236   585.833121   704.0148596
385.7580373   466.0685375   294.3646558   582.6682681
382.0637435   0.609198362   0.01046017   0.062806215
NDUFAF8 17   81239239   81241281   +   1376
protein_coding NADH:ubiquinone oxidoreductase complex assembly factor
8 [Source:HGNC Symbol;Acc:HGNC:33551]   -   396   632   720
        384   510   300   15.7387945   20.2503532
24.23910156   13.49317046   16.15140621   10.17768152
ENSG00000226864 9.255693406   17.61207168   13.68917783
85.38914887   24.67421669   35.3237587   13.51898097
48.46237475   -1.83406458   0.01047614   0.062877407   ATE1-
AS1   10   121928312   121951965   +   1172
transcribed_unitary_pseudogene ATE1 antisense RNA 1 [Source:HGNC
Symbol;Acc:HGNC:49496] -   8   19   14   85   27
        36   0.373299225   0.714759518   0.553353778
3.506650885   1.003909931   1.433906802
ENSG00000197982 779.7921694   1015.011499   965.0870368
1254.718199   1132.272388   1323.659736   919.9635685
1236.883441   -0.426100896   0.010486771   0.062916513
C1orf122   1   37806979   37809454   +   2231
        protein_coding chromosome 1 open reading frame 122
[Source:HGNC Symbol;Acc:HGNC:24789]   -   674   1095   987
        1249   1239   1349   16.52171168   21.63956403

20.49368413 27.06849343 24.20083406 28.22659001
ENSG00000140527 0 0 0 14.06409511 8.224738897
2.943646558 0 8.410826854 -5.49165944
0.010530887 1 WDR93 15 89690797 89743638
+ 4068 protein_coding WD repeat domain 93
[Source:HGNC Symbol;Acc:HGNC:26924] - 0 0 0
14 9 3 0 0 0 0.166398079
0.096409574 0.034425983
ENSG00000140511 208.2531016 290.1357071 421.4311174
180.82408 174.5472366 191.3370263 306.606642
182.2361143 0.752080725 0.010550725 0.063224458 HAPLN3
15 88877288 88895626 - 3524
protein_coding hyaluronan and proteoglycan link protein 3
[Source:HGNC Symbol;Acc:HGNC:21446] - 180 313 431
180 191 195 2.793388353 3.915997409
5.665572824 2.46966372 2.36187041 2.583120984
ENSG00000103194 3800.619105 3780.106752 3643.276899
4736.586317 4689.928891 4412.526191 3741.334252
4613.0138 -0.302191332 0.010551563 0.063224458 USP10
16 84699978 84779922 + 5083
protein_coding ubiquitin specific peptidase 10 [Source:HGNC
Symbol;Acc:HGNC:12608] - 3285 4078 3726 4715 5132
4497 35.34353437 35.37211837 33.95667802
44.85003681 43.99720983 41.29988227
ENSG00000196653 84.45820233 103.8185278 56.71230814
183.8378146 195.5660138 105.9712761 81.66301275
161.7917015 -0.98627256 0.010553917 0.063224458 ZNF502
3 44712643 44723831 + 3273
protein_coding zinc finger protein 502 [Source:HGNC
Symbol;Acc:HGNC:23718] zf-C2H2 73 112 58 183 214
108 1.219752081 1.508710717 0.820889015
2.703375048 2.849222503 1.540365511
ENSG00000013523 413.0353182 432.8861828 464.4542477
636.9025928 629.6494555 556.3491995 436.7919162
607.6337493 -0.475898911 0.010554642 0.063224458 ANGEL1
14 76786178 76826246 - 6414
protein_coding angel homolog 1 [Source:HGNC Symbol;Acc:HGNC:19961]
- 357 467 475 634 689 567
3.04392518 3.21012455 3.430576512 4.779269478
4.681110188 4.126673785
ENSG00000204580 682.6073887 767.5155446 869.262792
976.4500318 996.1072664 1175.496192 773.1285751
1049.351164 -0.440087349 0.010562296 0.063245522 DDR1
6 30876421 30900156 + 9122
protein_coding discoidin domain receptor tyrosine kinase 1
[Source:HGNC Symbol;Acc:HGNC:2730] - 590 828 889
972 1090 1198 3.537175893 4.001973669
4.51454669 5.152018496 5.207089324 6.130738413
ENSG00000261645 3.470885027 6.488657986 0 25.11445555
21.01877718 24.53038799 3.319847671 23.55454024

-2.820503228 0.010572087 0.063265762 DISC1FP1
11 90251204 90915052 + 3175
processed_transcript DISC1 fusion partner 1 [Source:HGNC
Symbol;Acc:HGNC:33625] - 3 7 0 25 23
25 0.051674019 0.097204925 0 0.380712815
0.315676828 0.36757191
ENSG00000269486 20.82531016 27.80853423 16.62257307
61.27927154 72.19493032 45.1359139 21.75213915
59.53670525 -1.451169125 0.010573953 0.063265762
ERVK9-11 19 38935297 38938632 - 3336
lincRNA "endogenous retrovirus group K9, member 11
[Source:HGNC Symbol;Acc:HGNC:51160]" - 18 30 17
61 79 46 0.295080952 0.396487199
0.236061594 0.884107367 1.031952356 0.643691576
ENSG00000066557 1311.99454 937.1476034 885.885365
621.8339194 871.8223231 690.7757257 1045.00917
728.1439894 0.520061894 0.010602223 0.063387504 LRRC40
1 70144805 70205620 - 2884
protein_coding leucine rich repeat containing 40 [Source:HGNC
Symbol;Acc:HGNC:26004] - 1134 1011 906 619 954
704 21.50366627 15.45574193 14.55242593
10.37759241 14.41490274 11.39523901
ENSG00000259865 232.5492968 179.8285213 186.7594975
103.4715569 124.2849433 124.614371 199.7124385
117.4569571 0.764058489 0.010602593 0.063387504
AL390728.6 1 247187281 247188526 - 1246
lincRNA novel transcript - 201 194 191
103 136 127 8.822115267 6.864638087
7.100971682 3.99687346 4.756411974 4.758079723
ENSG00000105877 113.3822442 174.2668145 100.7132369
304.3872013 236.6897083 164.8442073 129.4540985
235.3070389 -0.860169347 0.010609402 0.063403412 DNAH11
7 21543215 21901839 + 15998
protein_coding dynein axonemal heavy chain 11 [Source:HGNC
Symbol;Acc:HGNC:2942] - 98 188 103 303 259
168 0.335007937 0.518114939 0.298245561
0.915752583 0.705492936 0.490218419
ENSG00000166106 5.784808379 32.44328993 27.37835565
1.004578222 0 5.887293117 21.86881799 2.297290446
3.262880108 0.010617429 0.06342659 ADAMTS15
11 130448974 130476641 + 5673
protein_coding ADAM metallopeptidase with thrombospondin type 1 motif
15 [Source:HGNC Symbol;Acc:HGNC:16305] - 5 35 28
1 0 6 0.048200543 0.272012723
0.228637627 0.00852292 0 0.04937243
ENSG00000125484 1905.51588 1471.998412 1693.546857
2326.603162 2203.316164 2060.552591 1690.353716
2196.823973 -0.37860252 0.010628328 0.063466892 GTF3C4
9 132670035 132694955 + 9043
protein_coding general transcription factor IIIC subunit 4

[Source:HGNC Symbol;Acc:HGNC:4667]	-	1647	1588	1732
	2316	2411	2100	9.960377099	7.742334403
8.872332573	12.383039	11.61831848	10.84058701
ENSG00000172819 91.39997238	95.47596751	123.2026004
43.19686355	64.8840513	35.3237587	103.3595134
47.80155785	1.1112569	0.010659546	0.063628458	RARG
	12	53210567	53232980	-	5127
protein_coding	retinoic acid receptor gamma [Source:HGNC
Symbol;Acc:HGNC:9866]	THR-like	79	103	126	43
	71	36	0.842671851	0.885743278	1.138438786
	0.405514469	0.603467143	0.327782089
ENSG00000198455 482.4530188	550.6089777	460.543054
640.9209057	740.2265007	682.9260016	497.8683501
688.0244693	-0.466459704	0.010669342	0.063662071	ZXDB
	X	57591652	57597545	+	5894
protein_coding	zinc finger X-linked duplicated B [Source:HGNC
Symbol;Acc:HGNC:13199]	zf-C2H2 417	594	471	638	810
	696	3.869195163	4.443347419	3.701802389
5.233735416	5.988712785	5.512456101
ENSG00000077684 957.9642675	885.2383395	1122.512582
750.4199319	654.3236722	779.0851225	988.5717296
727.9429088	0.441753672	0.010679436	0.063697437	JADE1
	4	128809623	128875224	+	9129
protein_coding	jade family PHD finger 1 [Source:HGNC
Symbol;Acc:HGNC:30027]	-	828	955	1148	747	716
	794	4.960230317	4.612263625	5.825338101
3.956385588	3.41781391	4.06016171
ENSG00000122965 628.2301899	793.4701766	684.4588913
1160.287846	892.8411002	871.3193813	702.0530859
974.8161093	-0.472648759	0.010684632	0.063703576	RBM19
	12	113816738	113966371	-	5912
protein_coding	RNA binding motif protein 19 [Source:HGNC
Symbol;Acc:HGNC:29098]	-	543	856	700	1155	977
	888	5.022964594	6.38371217	5.484866614
9.446018142	7.201429876	7.011720181
ENSG00000125347 629.3871516	660.9161634	704.0148596
375.712255	510.8476715	518.0817943	664.7727249
468.2139069	0.505543915	0.010705289	0.063801856	IRF1
	5	132481609	132490798	-	7799
protein_coding	interferon regulatory factor 1 [Source:HGNC
Symbol;Acc:HGNC:6116]	IRF	544	713	720	374	559
	528	3.814649958	4.030737979	4.2765744
2.318643067	3.1234279	3.160392613
ENSG00000166987 411.8783566	691.5055511	849.7068236
1209.512179	870.9084632	911.5492176	651.0302438
997.3232867	-0.61397237	0.010720377	0.063866874	MBD6
	12	57520710	57530148	+	5646
protein_coding	methyl-CpG binding domain protein 6 [Source:HGNC
Symbol;Acc:HGNC:20445]	-	356	746	869	1204	953
	929	3.44829043	5.82548263	7.129865848

10.31066863	7.355473321	7.68105502
ENSG00000186638 418.8201266	343.8988733	423.3867142
297.3551537	240.3451478	257.0784661	395.3685714
264.9262559	0.57747262	0.010741629	0.063966833	KIF24
	9	34252381	34311371	-	6131
protein_coding	kinesin family member 24 [Source:HGNC
Symbol;Acc:HGNC:19916]	-	362	371	433	296	263
	262	3.229029161	2.667943029	3.271591235
2.33432656	1.869317321	1.994876485
ENSG00000182919 535.6732559	496.8458115	346.1406393
672.0628305	630.5633154	691.7569412	459.5532356
664.7943624	-0.533421089	0.010745524	0.063966833
C11orf54	11	93741591	93764749	+	6269
	protein_coding	chromosome 11 open reading frame 54
[Source:HGNC Symbol;Acc:HGNC:30204]	-	463	536	354
	669	690	705	4.039033302	3.769645214
2.615817543	5.159754808	4.796334004	5.249728976
ENSG00000156026 476.6682104	463.4755704	515.2997653
324.4787657	384.7350084	298.2895179	485.1478487
335.8344307	0.530218091	0.010754739	0.063996768	MCU
	10	72692131	72887694	+	4533
protein_coding	mitochondrial calcium uniporter [Source:HGNC
Symbol;Acc:HGNC:23526]	-	412	500	527	323	421
	304	4.970570176	4.863156472	5.38551639
3.445228409	4.047202871	3.130645554
ENSG00000104946 1058.619933	1190.205265	1175.313696
1416.455293	1407.344211	1642.55478	1141.379631
1488.784761	-0.382916492	0.010771473	0.064071401
TBC1D17 19	49877425	49888749	+	4745
protein_coding	TBC1 domain family member 17 [Source:HGNC
Symbol;Acc:HGNC:25699]	-	915	1284	1202	1410	1540
	1674	10.54580144	11.93061318	11.73466529
14.36759616	14.14305082	16.4689258
ENSG00000104327 15.04050178	5.561706845	3.911193665	0
	0	0	8.171134098	0	5.519413863
0.0107776	1	CALB1	8	90058608	90095475
	-	7138	protein_coding	calbindin 1 [Source:HGNC
Symbol;Acc:HGNC:1434]	-	13	6	4	0	0
	0	0.099600501	0.037060277	0.025958877	0
	0	0
ENSG00000259642 9.255693406	6.488657986	16.62257307
53.24264577	47.52071363	21.58674143	10.78897482
40.78336694	-1.91813709	0.010803438	0.0642299	ST20-
AS1	15	79922771	79926993	+	4223
antisense	ST20 antisense RNA 1 [Source:HGNC
Symbol;Acc:HGNC:27521]	-	8	7	17	53	52
	22	0.103600921	0.073082083	0.186479156
0.606814577	0.536587881	0.243191077
ENSG00000163257 1903.201957	1740.814243	1600.656007
2478.294474	2133.862814	2140.031048	1748.224069

2250.729445	-0.364714936	0.010806522	0.0642299	DCAF16
	4	17800655	17810758	-	4816
protein_coding	DDB1 and CUL4 associated factor 16 [Source:HGNC
Symbol;Acc:HGNC:25987]	-	1645	1878	1637	2467	2335
	2181	18.67988236	17.19265972	15.74579732
24.76759787	21.12802998	21.14049846
ENSG00000207975 0	0	0	0	22.84649694
7.849724156	0	10.2320737	-5.776662048	0.010811476
	1	MIR181B1	1	198858873	198858982
	-	110	miRNA	microRNA 181b-1 [Source:HGNC
Symbol;Acc:HGNC:31550]	-	0	0	0	0	25
	8	0	0	0	0	9.90389258
3.395027822
ENSG00000167173 760.1238209	891.7269975	880.0185745
1237.64037	1007.987445	1141.153649	843.9564643
1128.927154	-0.418905923	0.010820981	0.064281984
C15orf39	15	75195643	75212169	+	5812
	protein_coding	chromosome 15 open reading frame 39
[Source:HGNC Symbol;Acc:HGNC:24497]	-	657	962	900
	1232	1103	1163	6.182077948	7.297656619
7.173306036	10.24911388	8.270057032	9.341145675
ENSG00000048991 1073.660435	1047.454789	1294.605103
985.4912358	752.1066791	755.53595	1138.573442
831.0446216	0.454611552	0.010823695	0.064281984	R3HDM1
	2	135531455	135725270	+	7898
protein_coding	R3H domain containing 1 [Source:HGNC
Symbol;Acc:HGNC:9757]	-	928	1130	1324	981	823
	770	6.425775666	6.308052106	7.765569511
6.00555425	4.540893367	4.551134093
ENSG00000125630 3016.199089	3088.601201	2694.812435
4008.267106	3724.89286	3389.118404	2933.204242
3707.426123	-0.337954645	0.010860306	0.064474367	POLR1B
	2	112541915	112577150	+	6329
protein_coding	RNA polymerase I subunit B [Source:HGNC
Symbol;Acc:HGNC:20454]	-	2607	3332	2756	3990	4076
	3454	22.52685939	23.21153464	20.17188917
30.48168821	28.06452379	25.47611927
ENSG00000146143 1455.457788	1182.789656	1293.627305
988.5049705	1071.043776	909.5867865	1310.624916
989.7118444	0.404524402	0.010865685	0.064481258	PRIM2
	6	57314805	57646849	+	4667
protein_coding	DNA primase subunit 2 [Source:HGNC
Symbol;Acc:HGNC:9370]	-	1258	1276	1323	984	1172
	927	14.74136002	12.05443422	13.13180724
10.19432592	10.94330261	9.272310559
ENSG00000256006 0	17.61207168	11.73358099	0	0
	0	9.781884223	0	5.786887374	0.010871734
	1	AC084117.1	11	18405609	18406731
	+	270	sense_intronic	novel transcript	-
	0	19	12	0	0	0	0

3.102585759	2.058827392	0	0	0
ENSG00000153113 4539.917616	3690.192492	4347.291758
3400.497282	3149.161138	3401.874206	4192.467288
3317.177542	0.337699154	0.01090941	0.064715617	CAST
	5	96525267	96779595	+	12448
protein_coding	calpastatin [Source:HGNC Symbol;Acc:HGNC:1515]	-
	3924	3981	4446	3385	3446	3467	17.2394788
	14.10024166	16.54521193	13.1480186	12.06352674
	13.0017047
ENSG00000139636 240.6480285	266.0349774	395.0305601
192.8790186	188.2551347	174.6563625	300.5711887
185.2635053	0.699007204	0.010916456	0.064732297	LMBR1L
	12	49096551	49110900	-	6047
protein_coding	limb development membrane protein 1 like [Source:HGNC
Symbol;Acc:HGNC:18268]	-	208	287	404	192	206
	178	1.881126836	2.092550203	3.09488027
1.535191215	1.484519305	1.374124458
ENSG00000189266 2264.173999	1959.574712	2349.649594
1489.789503	1845.083093	1720.070806	2191.132768
1684.981134	0.378625164	0.010926299	0.06476554	PNRC2
	1	23956839	23963462	+	2779
protein_coding	proline rich nuclear receptor coactivator 2
[Source:HGNC Symbol;Acc:HGNC:23158]	-	1957	2114	2403
	1483	2019	1753	38.51208147	33.53902198
40.05600936	25.80202673	31.65966899	29.44688804
ENSG00000277310 0	0.926951141	0	12.05493866
17.36333767	2.943646558	0.308983714
10.78730763	-4.890302455	0.01095778	1	AC090246.1
	18	50256036	50256461	-	426
sense_intronic	"novel transcript, sense intronic to CCDC11"	-
	0	1	0	12	19	3	0
0.103496189	0	1.36198669	1.943580797	0.328743891
ENSG00000250786 83.30124065	59.32487301	95.82424478
5.02289111	29.24351608	43.17348286	79.48345282
25.81329668	1.620384416	0.010991199	0.065124985	SNHG18
	5	9546200 9550609 +	1951	lincRNA small
nucleolar RNA host gene 18 [Source:HGNC Symbol;Acc:HGNC:49007]	-
	72	64	98	5	32	44	2.018226665
	1.44629426	2.326865402	0.123912167	0.714745297
	1.052789253
ENSG00000285077 10.41265508	5.561706845	7.822387329	0
	0	0	7.932249752	0	5.478845828
0.011003208	1	ARHGAP11B	15	30624494
30638805	+	2337	protein_coding	Rho GTPase activating
protein 11B [Source:HGNC Symbol;Acc:HGNC:15782] -	9	6
	8	0	0	0	0.210609768	0.113194805
	0.158574639	0	0	0
ENSG00000173227 18.51138681	2.780853423	3.911193665	0
	0	0	8.401144633	0	5.557770757
0.011019432	1	SYT12	11	67006778	67050863

+   10830   protein_coding synaptotagmin 12 [Source:HGNC
Symbol;Acc:HGNC:18381] -   16   3   4   0   0
0   0.080795326   0.012213124   0.017109369   0
0   0
ENSG00000176894 93.71389573   107.5263323   171.1147228
77.3525231   38.38211485   58.87293117   124.118317
58.20252304   1.095496601   0.011059638   0.065505107   PXMP2
12   132687606   132704991   +   1408
protein_coding peroxisomal membrane protein 2 [Source:HGNC
Symbol;Acc:HGNC:9716]   -   81   116   175   77   42
60   3.146132991   3.632363411   5.757551743
2.644169472   1.299885901   1.989274115
ENSG00000099814 813.344058   1154.981121   1063.844677
1259.74109   1310.475064   1581.719417   1010.723285
1383.978524   -0.452540673   0.011068692   0.065533341
CEP170B 14   104865280   104896770   +   7199
protein_coding centrosomal protein 170B [Source:HGNC
Symbol;Acc:HGNC:20362] -   703   1246   1088   1254   1434
1612   5.340450134   7.630971415   7.000985491
8.422219958   8.680316798   10.45295064
ENSG00000120942 1377.941356   1351.494763   1315.13887
1776.094297   1658.655678   1704.371357   1348.191663
1713.040444   -0.345571195   0.01108201   0.065586794   UBIAD1
1   11273206   11296049   +   5082
protein_coding UbiA prenyltransferase domain containing 1
[Source:HGNC Symbol;Acc:HGNC:30791]   -   1191   1458   1345
1768   1815   1737   12.81656999   12.64901831
12.25998897   16.82088402   15.56325977   15.95552848
ENSG00000123975 1033.166776   1106.779662   1166.51351
781.5618567   903.8074188   820.2961743   1102.153316
835.2218166   0.400147572   0.011091763   0.065619111   CKS2
9   89311198   89316703   +   613
protein_coding CDC28 protein kinase regulatory subunit 2 [Source:HGNC
Symbol;Acc:HGNC:2000]   -   893   1194   1193   778   989
836   79.66832697   85.87718701   90.15346535
61.36494354   70.30632782   63.66369464
ENSG00000117152 876.9769502   531.1430037   823.3062664
503.2936892   316.1955176   601.4851134   743.8087401
473.6581068   0.650953174   0.011118471   0.065751671   RGS4
1   163068775   163076802   +   4836
protein_coding regulator of G protein signaling 4 [Source:HGNC
Symbol;Acc:HGNC:10000] -   758   573   842   501   346
613   8.571910466   5.223989409   8.065443535
5.009018667   3.117801089   5.917254085
ENSG00000245025 5.784808379   18.53902282   12.71137941
1.004578222   0   0.981215519   12.3450702   0.661931247
4.246653784   0.01116988   1   AC107959.1   8
22984596   23019335   -   2268   antisense
"novel transcript, antisense to RHOBTB2"   -   5
20   13   1   0   1   0.120565116

0.388795208	0.265523374	0.021318575	0	0.02058273
ENSG00000179046 519.4757924	558.9515379	205.3376674
772.5206527	1348.857179	900.7558469	427.9216659
1007.377893	-1.235414925	0.011193919	0.066172251	TRIML2
	4	188091273	188109603	-	3050
protein_coding	tripartite motif family like 2 [Source:HGNC
Symbol;Acc:HGNC:26378]	-	449	603	210	769	1476
	918	8.050840347	8.716686583	3.189494894
12.19067398	21.08847212	14.05040613
ENSG00000164163 4865.023846	4392.821456	4280.801466
3532.097029	3912.234135	3344.963706	4512.882256
3596.431623	0.327276137	0.011203804	0.066205083	ABCE1
	4	145097932	145129179	+	6265
protein_coding	ATP binding cassette subfamily E member 1 [Source:HGNC
Symbol;Acc:HGNC:69]	-	4205	4739	4378	3516	4281
	3409	36.70621783	33.35028821	32.37107617
27.13494887	29.77712405	25.4010671
ENSG00000247626 386.4251997	543.1933685	451.7428683
664.0262048	654.3236722	632.8840101	460.4538122
650.4112957	-0.496886861	0.011223874	0.066298054	MARS2
	2	197705369	197708387	+	3019
protein_coding	"methionyl-tRNA synthetase 2, mitochondrial
[Source:HGNC Symbol;Acc:HGNC:25133]"	-	334	586	462
	661	716	645	6.050316121	8.557924702
7.08894029	10.58618704	10.33495303	9.97338622
ENSG00000233461 54.37719876	81.57170039	68.44588913
9.041203998	40.20983461	20.60552591	68.13159609
23.2855215	1.545527388	0.011235079	0.066338608
AL445524.1	1	231522388	231528556	-	1021
	antisense	novel transcript	-	47	88
	70	9	44	21	2.517484635	3.800063799
	3.175958023	0.426204454	1.877956517	0.96015111
ENSG00000130762 19.66834849	9.269511408	15.64477466	0
	0	2.943646558	14.86087818	0.981215519
3.929165938	0.011250383	1	ARHGEF16	1
3454426 3481113 +	5563	protein_coding	Rho guanine nucleotide
exchange factor 16 [Source:HGNC Symbol;Acc:HGNC:15515]	-	17
	10	16	0	0	3	0.167122366
0.079254677	0.133233482	0	0	0.025174348
ENSG00000272948 0	0	7.822387329	39.17855066
24.67421669	10.79337071	2.607462443
24.88204602	-3.237774789	0.011259656	0.066458056
AP001412.1	21	37267784	37268497	+	714
	antisense	"novel transcript, antisense to DSCR3"	-
	0	0	8	39	27	11	0	0
	0.519032116	2.640995199	1.647874564	0.719184815
ENSG00000173915 1465.870443	1466.436705	1492.120383
639.9163274	1245.591013	1152.928235	1474.809177
1012.811859	0.54182403	0.011264944	0.066463602	ATP5MD
	10	103389041	103396492	-	924

protein_coding ATP synthase membrane subunit DAPIT [Source:HGNC Symbol;Acc:HGNC:30889] -   1267   1582   1526   637   1363
        1175   74.9893098   75.48635687   76.50415421
33.33256062   64.28097899   59.36246564
ENSG00000258982 9.255693406   6.488657986   7.822387329   0
        0   0   7.855579574   0   5.465592174
0.011277   1   AL133523.1   14   100207407
100238555   -   331   antisense   "novel transcript, antisense to YY1"   -   8   7   8   0   0
        0   1.321772482   0.932403734   1.11960402   0
        0   0
ENSG00000110880 6417.666415   4856.297027   5425.803411
3921.873379   4518.123234   4572.464321   5566.588951
4337.486978   0.359694015   0.011315335   0.066735159   CORO1C
        12   108645109   108731596   -   6532
protein_coding coronin 1C [Source:HGNC Symbol;Acc:HGNC:2254]   -
        5547   5239   5549   3904   4944   4660   46.44154963
        35.36194793   39.35238012   28.89780455   32.98305536
        33.3031855
ENSG00000225222 0   0   0.977798416   20.09156444   0
        18.64309487   0.325932805   12.9115531   -5.147136247
        0.011321159   1   CHCHD4P5   1   246178778
        246179204   +   427   processed_pseudogene
coiled-coil-helix-coiled-coil-helix domain containing 4 pseudogene 5 [Source:HGNC Symbol;Acc:HGNC:44493]   -   0   0   1
        20   0   19   0   0   0.108486221
2.264661709   0   2.077168662
ENSG00000196535 509.0631373   761.0268866   812.5504838
976.4500318   811.5075711   1286.373546   694.2135026
1024.77705   -0.560664781   0.011336733   0.066835569   MYO18A
        17   29071124   29180412   -   15047
protein_coding myosin XVIIIA [Source:HGNC Symbol;Acc:HGNC:31104]
        -       440   821   831   972   888   1311
1.599180437   2.405620932   2.558312299   3.123327755
2.571707921   4.067230696
ENSG00000259953 4.627846703   7.415609127   11.73358099   0
        0   0   7.925678941   0   5.481198169
0.011346258   1   AL138756.1   9   112032555
112037730   -   4836   sense_overlapping   "novel transcript, overlapping SUSD1" -   4   8   12   0
        0   0   0.045234356   0.07293528   0.114946939
        0   0   0
ENSG00000250362 9.255693406   7.415609127   6.844588913   0
        0   0   7.838630482   0   5.462619892
0.011363096   1   AC008592.1   5   95861786
95874519   +   850   processed_transcript   novel transcript   -   8   8   7   0   0   0
        0.514713755   0.414958838   0.381488605   0   0
        0
ENSG00000075643 204.7822166   136.2618177   241.5162088

110.5036044	112.4047649	105.9712761	194.1867477
109.6265485	0.823930282	0.011395319	0.067155067	MOCOS
	18	36187519	36272157	+	6281
protein_coding	molybdenum cofactor sulfurase [Source:HGNC
Symbol;Acc:HGNC:18234]	-	177	147	247	110	123
	108	1.541129685	1.031864091	1.821673815
0.846769308	0.853365175	0.802677331
ENSG00000169213 1232.164185	972.3717467	1366.962186
826.7678767	595.8366401	1037.144804	1190.499372
819.9164403	0.538157074	0.011401169	0.067163648	RAB3B
	1	51907956	51990764	-	12844
protein_coding	"RAB3B, member RAS oncogene family [Source:HGNC
Symbol;Acc:HGNC:9778]"	-	1065	1049	1398	823	652
	1057	4.534652624	3.600884151	5.042075335
3.098137973	2.212105811	3.841675927
ENSG00000116288 2459.700523	2905.991827	3173.933659
2090.52728	2026.027348	2454.020014	2846.542003
2190.191547	0.378473632	0.011416752	0.06722954	PARK7
	1	7954291 7985505 +	2397	protein_coding
Parkinsonism associated deglycase [Source:HGNC Symbol;Acc:HGNC:16369]
	-	2126	3135	3246	2081	2217	2501
48.50538308	57.66382794	62.73110494	41.97640706
40.30475233	48.70703505
ENSG00000279349 5.784808379	12.05036483	5.866790497	0
	0	0	7.900654569	0	5.476733519
0.011427142	1	AC092692.1	3	112525548
112528142	-	2595	TEC	TEC	-	5	13
	6	0	0	0	0.105372517	0.220871636
	0.107106627	0	0	0
ENSG00000134545 0	0	0	0	24.67421669
5.887293117	0	10.18716994	-5.770504688	0.01146573
	1	KLRC1	12	10442264	10454685	-
	2698	protein_coding	killer cell lectin like receptor C1
[Source:HGNC Symbol;Acc:HGNC:6374]	-	0	0	0
	0	27	6	0	0	0	0
0.436094306	0.10381386
ENSG00000198887 1699.576702	1529.469382	1494.07598
1261.750247	1140.497127	1260.861943	1574.374021
1221.036439	0.36649005	0.0114687	0.067509442	SMC5
	9	70258962	70354888	+	6637
protein_coding	structural maintenance of chromosomes 5 [Source:HGNC
Symbol;Acc:HGNC:20465]	-	1469	1650	1528	1256	1248
	1285	12.10443969	10.96089669	10.66483136
9.149956687	8.194101995	9.038104239
ENSG00000090905 1780.564019	1609.187181	1829.460837
1496.821551	1070.129916	1354.077417	1739.737345
1307.009628	0.412781418	0.011495385	0.067630833	TNRC6A
	16	24610209	24827632	+	12924
protein_coding	trinucleotide repeat containing 6A [Source:HGNC
Symbol;Acc:HGNC:11969]	-	1539	1736	1871	1490	1171

1380	6.512329756	5.922249903	6.706243123
5.574302535	3.948376364	4.984575435
ENSG00000234745 1867.336145	2671.473188	2466.985404
1014.624004	1877.982048	1898.65203	2335.264912
1597.086028	0.548226388	0.011498171	0.067630833	HLA-B
6	31269491	31357188	-	3051
protein_coding	"major histocompatibility complex, class I, B
[Source:HGNC Symbol;Acc:HGNC:4932]"	-	1614	2882	2523
1010	2055	1935	28.93050639	41.64719217
38.30694328	16.00591044	29.35135913	29.60634513
ENSG00000006007 1433.475516	1283.82733	1292.649506
907.1341345	1095.717993	1053.825468	1336.650784
1018.892532	0.391099424	0.011503701	0.067637339	GDE1
16	19501689	19522145	-	4416
protein_coding	glycerophosphodiester phosphodiesterase 1 [Source:HGNC
Symbol;Acc:HGNC:29644]	-	1239	1385	1322	903	1199
1074	15.34394222	13.82785022	13.86771304
9.886894548	11.83174269	11.35327748
ENSG00000004776 115.6961676	52.83621503	192.626288
22.10072088	73.10879019	44.15469838	120.3862235
46.45473648	1.370939306	0.011524008	0.067730685	HSPB6
19	35754569	35758079	-	1721
protein_coding	heat shock protein family B (small) member 6
[Source:HGNC Symbol;Acc:HGNC:26511]	-	100	57	197
22	80	45	3.17770694	1.460252449
5.302587108	0.618077632	2.02566542	1.220612124
ENSG00000127837 2202.855031	2805.881103	2423.962274
2997.661415	3018.479175	3597.136094	2477.566136
3204.425561	-0.37078799	0.011532323	0.067753507	AAMP
2	218264123	218270257	-	3140
protein_coding	angio associated migratory cell protein [Source:HGNC
Symbol;Acc:HGNC:18]	-	1904	3027	2479	2984	3303
3666	33.16133521	42.50272067	36.5720525
45.94839937	45.83925212	54.50154935
ENSG00000142784 1225.222415	1300.512451	1546.877094
1811.254534	1795.734659	1685.728262	1357.53732
1764.239152	-0.377731686	0.01155473	0.06785907	WDTC1
1	27234516	27308633	+	6024
protein_coding	WD and tetratricopeptide repeats 1 [Source:HGNC
Symbol;Acc:HGNC:29175]	-	1059	1403	1582	1803	1965
1718	9.614035239	10.26849192	12.16533217
14.47144772	14.21465061	13.31325444
ENSG00000172986 541.4580642	378.1960655	475.2100303
323.4741875	341.7835942	272.7779144	464.95472
312.6785654	0.571078061	0.011569692	0.067895731	GXYLT2
3	72888073	72998138	+	3667
protein_coding	glucoside xylosyltransferase 2 [Source:HGNC
Symbol;Acc:HGNC:33383]	-	468	408	486	322	374
278	6.979585888	4.905499221	6.139426651
4.245669444	4.444462948	3.538994778

ENSG00000271303 104.1265508	223.3952249	115.3802131
199.9110662	207.4461922	458.2276476	147.6339963
288.528302	-0.964101534	0.011572115	0.067895731	SRXN1
	20	646615	653370	-	2698	protein_coding
sulfiredoxin 1 [Source:HGNC Symbol;Acc:HGNC:16132]	-	90
	241	118	199	227	467	1.824295878
3.938302356	2.026014353	3.566254622	3.666422502
8.0801788
ENSG00000168056 2010.799392	1433.993415	3423.272255
1317.002049	880.9609218	1173.533761	2289.355021
1123.832244	1.026584937	0.011574297	0.067895731	LTBP3
	11	65538805	65558930	-	10298
protein_coding	latent transforming growth factor beta binding protein
3 [Source:HGNC Symbol;Acc:HGNC:6716]	-	1738	1547	3501
	1311	964	1196	9.229785271	6.623253599
15.74859009	6.155325456	4.079272749	5.421560743
ENSG00000131149 2754.72575	2698.354771	3445.761619
2421.033515	2131.121234	2344.123876	2966.280713
2298.759542	0.367969802	0.011591342	0.067969636	GSE1
	16	85169525	85676204	+	13387
protein_coding	Gse1 coiled-coil protein [Source:HGNC
Symbol;Acc:HGNC:28979]	-	2381	2911	3524	2410	2332
	2389	9.726819231	9.587224563	12.19424994
8.704322944	7.591085455	8.330650644
ENSG00000148700 514.8479457	559.8784891	721.6152311
413.8862275	362.8023713	465.0961562	598.7805553
413.9282517	0.533489892	0.011605063	0.068014548	ADD3
	10	109996368	110135565	+	6541
protein_coding	adducin 3 [Source:HGNC Symbol;Acc:HGNC:245]	-
	445	604	738	412	397	474	3.720579378
	4.0712404	5.226544693	3.045469703	2.644873805
	3.382830428
ENSG00000091409 735.8276258	581.1983653	694.2368755
470.1426079	439.566601	529.8563805	670.4209555
479.8551965	0.481997261	0.011613418	0.068014548	ITGA6
	2	172427354	172506282	+	6533
protein_coding	integrin subunit alpha 6 [Source:HGNC
Symbol;Acc:HGNC:6142]	-	636	627	710	468	481
	540	5.324013772	4.231446367	5.034404957
3.463653278	3.20841853	3.8585767
ENSG00000144730 769.3795144	575.6366585	474.2322318
698.1818643	1071.957636	910.5680021	606.4161349
893.5691675	-0.560672969	0.011618238	0.068014548	IL17RD
	3	57089982	57170306	-	9561
protein_coding	interleukin 17 receptor D [Source:HGNC
Symbol;Acc:HGNC:17616]	-	665	621	485	695	1173
	928	3.803759411	2.863665187	2.349853981
3.514655016	5.346299591	4.530964858
ENSG00000260941 149.2480562	123.2845017	94.84644637
160.7325155	228.4649694	253.153604	122.4596681

214.1170296 -0.808409172 0.011618551 0.068014548
LINC00622 1 119597702 119599271 - 1570
sense_overlapping long intergenic non-protein coding RNA
622 [Source:HGNC Symbol;Acc:HGNC:44251] - 129 133 97
160 250 258 4.493500255 3.734959927
2.862032345 4.927442292 6.939033018 7.671249171
ENSG00000163346 704.5896605 687.7977465 861.4404046
828.7770332 991.537967 1454.1614 751.2759372
1091.492133 -0.538748567 0.011621247 0.068014548 PBXIP1
1 154944076 154956123 - 3851
protein_coding PBX homeobox interacting protein 1 [Source:HGNC
Symbol;Acc:HGNC:21199] - 609 742 881 825 1085
1482 8.648454138 8.495018806 10.59753466
10.35813689 12.2776378 17.96473109
ENSG00000198483 290.3973806 266.0349774 274.7613549
111.5081826 137.0789816 234.5105092 277.064571
161.0325578 0.782721994 0.011646241 0.068134746
ANKRD35 1 145866560 145885866 - 3363
protein_coding ankyrin repeat domain 35 [Source:HGNC
Symbol;Acc:HGNC:26323] - 251 287 281 111 150
239 4.081704563 3.762608111 3.870632228
1.595869328 1.94367205 3.317546887
ENSG00000152422 326.2631926 297.5513162 253.2497898
178.8149235 198.3075934 189.3745953 292.3547662
188.8323707 0.629619965 0.011655798 0.068164569 XRCC4
5 83077498 83353787 + 2676
protein_coding X-ray repair cross complementing 4 [Source:HGNC
Symbol;Acc:HGNC:12831] - 282 321 259 178 217
193 5.763120655 5.288748087 4.483489023
3.216141216 3.533720716 3.366799358
ENSG00000134215 192.0556382 200.2214464 274.7613549
141.6455293 111.490905 143.2574658 222.3461465
132.1313001 0.752174897 0.011664738 0.068180634 VAV3
1 107571160 107965144 - 7271
protein_coding vav guanine nucleotide exchange factor 3 [Source:HGNC
Symbol;Acc:HGNC:12659] - 166 216 281 141 122
146 1.248557812 1.309765554 1.790253911
0.937618536 0.731179967 0.937356396
ENSG00000213676 961.4351525 1196.693923 1326.872451
883.0242572 596.7505 957.666347 1161.667175
812.480368 0.516624828 0.011667465 0.068180634 ATF6B
6 32115264 32128253 - 3629
protein_coding activating transcription factor 6 beta [Source:HGNC
Symbol;Acc:HGNC:2349] TF_bZIP 831 1291 1357 879 653
976 12.52301118 15.68459222 17.32189235
11.71124653 7.841241158 12.55477359
ENSG00000166169 322.7923075 337.4102153 265.9611692
575.6233212 415.8062442 399.3547164 308.7212307
463.5947606 -0.586350206 0.011686168 0.068263831 POLL
10 101578882 101588270 - 5420

protein_coding DNA polymerase lambda [Source:HGNC Symbol;Acc:HGNC:9184] - 279 364 272 573 455 407 2.815137613 2.960983962 2.324727608 5.111596357 3.658227481 3.505428865
ENSG00000204469 8224.840553 11811.21144 13825.09181 17415.36806 12784.89969 15977.1323 11287.04793 15392.46668 -0.447440992 0.011721226 0.06844247 PRRC2A 6 31620720 31637771 + 9115 protein_coding proline rich coiled-coil 2A [Source:HGNC Symbol;Acc:HGNC:13918] - 7109 12742 14139 17336 13990 16283 42.65270254 61.63322396 71.85623818 91.958831 66.88359975 83.39188403
ENSG00000272843 16.19746346 1.853902282 13.68917783 0 0 0.981215519 10.58018119 0.32707184 4.930952703 0.011722844 1 AC211476.2 7 72924418 72925125 - 708 antisense "novel transcript, antisense to POM121" - 14 2 14 0 0 1 1.081407783 0.124546262 0.916003712 0 0 0.065934509
ENSG00000147457 2530.275185 2658.495872 2438.62925 3100.128393 3164.696755 3198.762593 2542.466769 3154.529247 -0.311149896 0.011748002 0.068533119 CHMP7 8 23243637 23262000 + 4698 protein_coding charged multivesicular body protein 7 [Source:HGNC Symbol;Acc:HGNC:28439] - 2187 2868 2494 3086 3463 3260 25.45836351 26.91535377 24.5915494 31.76026561 32.1216671 32.39295916
ENSG00000253106 38.1797353 28.73548537 29.33395248 82.37541421 74.02265007 66.72265532 32.08305772 74.3735732 -1.216164249 0.011748208 0.068533119 AC090198.1 8 124488510 124491643 + 2355 antisense "novel transcript, antisense to TATDN1" - 33 31 30 82 81 68 0.766333377 0.580369713 0.590109762 1.683542783 1.498831132 1.347919751
ENSG00000178685 290.3973806 632.1806781 535.8335321 680.0994563 714.6384241 880.150321 486.1371969 758.2960671 -0.639539581 0.011751998 0.068533119 PARP10 8 143977153 144012772 - 6033 protein_coding poly(ADP-ribose) polymerase family member 10 [Source:HGNC Symbol;Acc:HGNC:25895] - 251 682 548 677 782 897 2.27528136 4.984080031 4.207747678 5.425709782 5.648485594 6.940730052
ENSG00000107890 716.1592773 663.6970168 640.4579626 499.2753763 541.0050474 407.2044406 673.4380856 482.4949548 0.480421686 0.011754683 0.068533119 ANKRD26 10 26991914 27100498 - 7941 protein_coding ankyrin repeat domain 26 [Source:HGNC Symbol;Acc:HGNC:29186] - 619 716 655 497 592 415 4.262949282 3.975317168 3.820925411

3.026093963	3.248666338	2.439601752
ENSG00000118762 1298.111	1189.278314	1132.290566
967.4088278	868.1668835	935.0983901	1206.55996
923.5580338	0.385444123	0.011787316	0.068697179	PKD2
	4	88007668	88077777	+	5498
protein_coding	"polycystin 2, transient receptor potential cation
channel [Source:HGNC Symbol;Acc:HGNC:9009]"	-	1122	1283
	1158	963	950	953	11.16047899	10.28859042
	9.756774771	8.468817384	7.529696433	8.091596185
ENSG00000179627 97.18478076	114.9419415	113.4246163
193.8835969	200.1353132	160.9193452	108.5171128
184.9794184	-0.768240748	0.011796492	0.068724457	ZBTB42
	14	104800596	104804712	+	3905
protein_coding	zinc finger and BTB domain containing 42 [Source:HGNC
Symbol;Acc:HGNC:32550]	ZBTB	84	124	116	193	219
	164	1.176394433	1.400021177	1.376066451
2.389667556	2.443890113	1.960509024
ENSG00000183850 26.61011854	21.31987624	25.42275882	0
	5.483159264	5.887293117	24.45091787	3.790150794
	2.681653818	0.011801316	0.068726369	ZNF730	19
	23075210	23147219	+	3291	protein_coding
	zinc finger protein 730 [Source:HGNC Symbol;Acc:HGNC:32470]
	zf-C2H2 23	23	26	0	6	6
0.382203506	0.308129949	0.365972052	0	0.079447817
	0.085107808
ENSG00000173898 919.7845322	1276.411721	1467.675423
1819.29116	1506.041078	1723.014452	1221.290559
1682.78223	-0.46158636	0.011823014	0.068806189	SPTBN2
	11	66685248	66729226	-	9642
protein_coding	"spectrin beta, non-erythrocytic 2 [Source:HGNC
Symbol;Acc:HGNC:11276]" -	795	1377	1501	1811	1648
	1756	4.509150328	6.296522666	7.21134081
9.08139445	7.44815452	8.501653886
ENSG00000222031 0	0	0	14.06409511	15.53561792
	0	0	9.866571008	-5.72287438	0.011825198
	1	AC023469.1	2	151001220	151048774
	-	469	lincRNA novel transcript	-	0
	0	0	14	17	0	0	0	0
	1.443299328	1.579554723	0
ENSG00000060491 1166.217369	1949.378249	1904.751315
2188.975946	2101.877718	2777.821136	1673.448978
2356.224933	-0.492963089	0.011826292	0.068806189	OGFR
	20	62804835	62814000	+	4702
protein_coding	opioid growth factor receptor [Source:HGNC
Symbol;Acc:HGNC:15768]	-	1008	2103	1948	2179	2300
	2831	11.72391389	19.71925967	19.19149396
22.40659281	21.31590662	28.1062743
ENSG00000167112 1266.873035	1642.557422	1545.899296
1922.762717	2035.165947	1841.74153	1485.109917
1933.223398	-0.379874435	0.011828525	0.068806189	TRUB2

9	128305161	128322742	-	6125
protein_coding	TruB pseudouridine synthase family member 2
[Source:HGNC Symbol;Acc:HGNC:17170]	-	1095	1772	1581
1914	2227	1877	9.776935248	12.75532658
11.95716529	15.10904647	15.84428777	14.30553866
ENSG00000149231 1674.123545	1294.950744	1202.692052
1048.779664	1106.684312	929.2110969	1390.58878
1028.225024	0.434793256	0.011865337	0.068989182	CCDC82
11	96352769	96389956	-	17313
protein_coding	coiled-coil domain containing 82 [Source:HGNC
Symbol;Acc:HGNC:26282]	-	1447	1397	1230	1044	1211
947	4.570786277	3.557607525	3.291055742
2.915609698	3.048108429	2.553428408
ENSG00000125741 1237.948993	1152.200268	1307.316482
1580.201543	1554.475651	1592.512788	1232.488581
1575.729994	-0.354536898	0.01186901	0.068989182	OPA3
19	45527427	45602212	-	9999
protein_coding	"OPA3, outer mitochondrial membrane lipid metabolism
regulator [Source:HGNC Symbol;Acc:HGNC:8142]"	-	1070	1243
1337	1573	1701	1623	5.852237222	5.480857595
6.19408691	7.606298604	7.413210684	7.577186682
ENSG00000139517 481.2960571	370.7804563	496.7215954
547.495131	732.0017618	653.489536	449.5993696
644.3288096	-0.520207493	0.011878001	0.069015206	LNX2
13	27545911	27620404	-	4692
protein_coding	ligand of numb-protein X 2 [Source:HGNC
Symbol;Acc:HGNC:20421]	-	416	400	508	545	801
666	4.848752761	3.75868513	5.015429899
5.616163146	7.439317777	6.626165235
ENSG00000080839 1195.141411	940.855408	1051.133297
723.2963199	736.5710612	892.9061227	1062.376705
784.2578346	0.437370632	0.011888742	0.069051369	RBL1
20	36996349	37095995	-	5954
protein_coding	RB transcriptional corepressor like 1 [Source:HGNC
Symbol;Acc:HGNC:9893]	-	1033	1015	1075	720	806
910	9.488251853	7.516076122	8.363770161
5.846889452	5.899087109	7.134747334
ENSG00000235636 69.41770054	76.00999355	58.66790497
144.659264	129.7681026	111.8585692	68.03186635
128.7619786	-0.920154882	0.011900614	0.069094071	NUS1P1
X	47512602	47513483	-	882
processed_pseudogene	NUS1 pseudogene 1 [Source:HGNC
Symbol;Acc:HGNC:38472]	-	60	82	60	144	142
114	3.720294995	4.099012335	3.151266416
7.893963672	7.01581869	6.033680398
ENSG00000234664 65.94681552	52.83621503	71.37928438
25.11445555	28.3296562	22.56795695	63.38743831
25.33735623	1.32155303	0.011922262	0.069193477
HMGN2P5 15	29730713	29730985	+	273
processed_pseudogene	high mobility group nucleosomal binding domain

2 pseudogene 5 [Source:HGNC Symbol;Acc:HGNC:33568]	-	57
	57	73	25	31	23	11.41844387
9.205474231	12.38690107	4.427703983	4.948318491
3.932884794
ENSG00000135823 1209.024951	1076.190275	998.3321829
1461.661313	1444.812466	1358.983494	1094.515803
1421.819091	-0.377976476	0.011952555	0.069342962	STX6
	1	180972712	181023121	-	6477
protein_coding	syntaxin 6 [Source:HGNC Symbol;Acc:HGNC:11441]	-
	1045	1161	1021	1455	1581	1385	8.823423124
	7.903005436	7.302209704	10.86151266	10.6369366
	9.982099906
ENSG00000226291 0	2.780853423	1.955596832	34.15565955
	10.96631853	6.868508636	1.578816752	17.33016224
	-3.416511651	0.011964655	1	AC091729.2	7
	1080863 1082178 +	302	antisense	novel
transcript	-	0	3	2	34	12	7
	0	0.43797394	0.306778916	5.44343687
1.731541484	1.082024596
ENSG00000179532 411.8783566	406.0045997	436.0980936
765.4886052	458.7576585	619.1469928	417.9936833
614.4644188	-0.555297646	0.011970297	0.069419549	DNHD1
	11	6497260 6593758 +	19629	protein_coding	dynein
heavy chain domain 1 [Source:HGNC Symbol;Acc:HGNC:26532]	-
	356	438	446	762	502	631	0.991851229
	0.983806966	1.052541285	1.876972946	1.114459113
	1.500642424
ENSG00000142459 453.5289769	629.3998246	650.2359467
742.3833061	827.0431891	862.4884416	577.7215828
810.6383122	-0.487457258	0.011977752	0.069436442	EVI5L
	19	7830233 7864976 +	6252	protein_coding
ecotropic viral integration site 5 like [Source:HGNC
Symbol;Acc:HGNC:30464]	-	392	679	665	739	905
	879	3.428955195	4.788337603	4.927256054
5.715137527	6.307949497	6.563204577
ENSG00000179950 4939.069394	5773.051705	5711.320549
7199.812117	6161.243294	7249.220258	5474.480549
6870.09189	-0.32741594	0.011995304	0.069511834	PUF60
	8	143816344	143829859	-	5424
protein_coding	poly(U) binding splicing factor 60 [Source:HGNC
Symbol;Acc:HGNC:17042]	-	4269	6228	5841	7167	6742
	7388	43.04286656	50.62474877	49.88500053
63.88794811	54.16611221	63.58479007
ENSG00000261716 121.480976	126.9923063	147.6475608
81.37083598	59.40089203	70.6475174	132.040281
70.47308181	0.907324535	0.012013326	0.069578854
HIST2H2BC	1	149844498	149850937	-	6440
	transcribed_unprocessed_pseudogene	histone cluster 2 H2B
family member c (pseudogene) [Source:NCBI gene;Acc:337873]	-
	105	137	151	81	65	72	0.891657659

0.937926179	1.086159327	0.608135517	0.439831254
0.521906451
ENSG00000141522 4421.907525	8574.298053	7187.796157
9776.555257	8441.323688	9924.994979	6728.000578
9380.957975	-0.479337593	0.012015973	0.069578854
ARHGDIA 17	81867721	81871406	-	2903
protein_coding	Rho GDP dissociation inhibitor alpha [Source:HGNC
Symbol;Acc:HGNC:678]	-	3822	9250	7351	9732	9237
10115	72.00097204	140.4845792	117.3010346
162.0900219	138.6572254	162.6540521
ENSG00000177946 534.5162942	552.4628799	558.3228956
692.154395	761.2452779	760.4420276	548.4340233
737.9472335	-0.428110593	0.012069731	0.069851811
CENPBD1 16	89969773	89972832	-	3060
protein_coding	CENPB DNA-binding domain containing 1 [Source:HGNC
Symbol;Acc:HGNC:28272]	-	462	596	571	689	833
775	8.256866481	8.587342628	8.644047358
10.8867691	11.86266245	11.82296249
ENSG00000183291 4564.213811	3803.280531	3615.898543
2919.304313	3554.001063	2754.271963	3994.464295
3075.859113	0.376692566	0.012076106	0.069851811
SELENOF 1	86862445	86914424	-	2201
protein_coding	selenoprotein F [Source:HGNC Symbol;Acc:HGNC:17705]
-	3945	4103	3698	2906	3889	2807
98.02157529	82.18932853	77.83041032	63.83763421
76.99747764	59.53445824
ENSG00000168300 550.7137576	569.1480005	455.6540619
349.5932213	427.6864226	301.2331645	525.17194
359.5042695	0.546140471	0.012076819	0.069851811	PCMTD1
8	51817575	51899186	-	7323
protein_coding	protein-L-isoaspartate (D-aspartate) O-
methyltransferase domain containing 1 [Source:HGNC
Symbol;Acc:HGNC:30483]	-	476	614	466	348	468
307	3.554779208	3.696692233	2.947808986
2.297689959	2.784937266	1.95702051
ENSG00000089060 188.5847531	230.8108341	196.5374816
353.6115342	241.2590076	383.6552681	205.311023
326.17527	-0.666315323	0.012082073	0.069855767	SLC8B1
12	113298759	113359493	-	6768
protein_coding	solute carrier family 8 member B1 [Source:HGNC
Symbol;Acc:HGNC:26175]	-	163	249	201	352	264
391	1.317109757	1.62208256	1.375745697
2.514684645	1.699817024	2.696885096
ENSG00000198959 19.66834849	75.08304241	113.4246163
35.16023777	14.62175804	13.73701727	69.39200239
21.17300436	1.717709958	0.012105802	0.0699665	TGM2
20	38127387	38166578	-	8036
protein_coding	transglutaminase 2 [Source:HGNC Symbol;Acc:HGNC:11778]
-	17	81	116	35	16	14
0.1156921	0.444405115	0.668683361	0.210585921

0.086763818	0.081326886
ENSG00000212242 0	0	0	20.09156444	3.65543951
	1.962431039	0	8.569811663	-5.517538082
0.012118667	1	RNA5SP219	6	136630243
136630355	+	113	rRNA_pseudogene "RNA, 5S ribosomal
pseudogene 219 [Source:HGNC Symbol;Acc:HGNC:43119]"	-	0
	0	0	20	4	2	0	0	0
	8.557615485	1.542553181	0.826223585
ENSG00000165801 1470.49829	1509.076457	1812.838264
1173.347363	993.3656868	1393.326038	1597.471004
1186.679696	0.429227132	0.012126037	0.070032589
ARHGEF40	14	21070270	21090240	+	8189
	protein_coding	Rho guanine nucleotide exchange factor 40
[Source:HGNC Symbol;Acc:HGNC:25516]	-	1271	1628	1854
	1168	1087	1420	8.488078594	8.765112354
10.48772556	6.896253036	5.784386059	8.09475128
ENSG00000073150 48.59239038	38.93194792	24.4449604
113.5173391	51.17615314	104.9900606	37.32309957
89.8945176	-1.270521003	0.012126399	0.070032589	PANX2
	22	50170731	50180294	+	3102
protein_coding	pannexin 2 [Source:HGNC Symbol;Acc:HGNC:8600]	-
	42	42	25	113	56	107	0.740461035
	0.596954809	0.373336689	1.761318377	0.786692177
	1.610230394
ENSG00000186141 2814.887757	2446.224061	2208.846622
3298.030303	3202.16501	3012.331645	2489.986147
3170.842319	-0.349059458	0.012134488	0.070052839	POLR3C
	1	145824088	145842505	+	2793
protein_coding	RNA polymerase III subunit C [Source:HGNC
Symbol;Acc:HGNC:30076]	-	2433	2639	2259	3283	3504
	3070	47.63935644	41.65838338	37.46689912
56.83307618	54.67033808	51.31135408
ENSG00000136160 19.66834849	25.95463194	75.29047804
9.041203998	6.397019142	14.71823279	40.30448616
10.05215198	2.007624495	0.012163096	0.070191488	EDNRB
	13	77895481	77975529	-	5607
protein_coding	endothelin receptor type B [Source:HGNC
Symbol;Acc:HGNC:3180]	-	17	28	77	9	7
	15	0.165810901	0.220171668	0.636154531
0.077609193	0.054403405	0.124883982
ENSG00000188171 8.09873173	3.707804563	11.73358099	0
	0	0	7.846705762	0	5.464185453
0.012180324	1	ZNF626	19	20620061	20661596
	-	6713	protein_coding	zinc finger protein 626
[Source:HGNC Symbol;Acc:HGNC:30461]	zf-C2H2 7	4	12
	0	0	0	0.05702642	0.026271042
0.082807001	0	0	0
ENSG00000163714 5444.661646	5054.664571	4828.368579
3990.184698	4426.737246	3929.768155	5109.231599
4115.563367	0.311851726	0.012193186	0.070320371	U2SURP

3	142964497	143060546	+	11118
protein_coding	U2 snRNP associated SURP domain containing
[Source:HGNC Symbol;Acc:HGNC:30855]	-	4706	5453	4938
3972	4844	4005	23.14834604	21.62433626
20.57438544	17.27363691	18.98611305	16.81596855
ENSG00000092108 1231.007223	1183.716607	1001.265578
745.3970407	950.4142725	844.8265623	1138.663136
846.8792918	0.426560581	0.012196266	0.070320371	SCFD1
14	30622112	30735812	+	6571
protein_coding	sec1 family domain containing 1 [Source:HGNC
Symbol;Acc:HGNC:20726]	-	1064	1277	1024	742	1040
861	8.855332516	8.568274827	7.218898661
5.459761284	6.897003873	6.116707599
ENSG00000048828 3800.619105	3250.817651	3958.127989
2936.382143	2978.26934	2842.58136	3669.854915
2919.077614	0.330066059	0.012201222	0.070320371
FAM120A 9	93451722	93566107	+	7204
protein_coding	family with sequence similarity 120A [Source:HGNC
Symbol;Acc:HGNC:13247]	-	3285	3507	4048	2923	3259
2897	24.93769922	21.46327647	26.02970556
19.61807216	19.71375042	18.77244441
ENSG00000147044 565.7542594	483.8684955	420.4533189
274.2498546	376.5102695	345.3878629	490.025358
332.049329	0.560160295	0.01220383	0.070320371	CASK
X	41514934	41923645	-	19939
protein_coding	calcium/calmodulin dependent serine protein kinase
[Source:HGNC Symbol;Acc:HGNC:1497]	-	489	522	430
273	412	352	1.341220548	1.154253201
0.999004715	0.662003812	0.900435151	0.824110269
ENSG00000150712 829.5415215	883.3844372	849.7068236
1358.189756	1084.751674	1001.821045	854.2109275
1148.254159	-0.42643163	0.012213279	0.070348304	MTMR12
5	32226994	32313009	-	5545
protein_coding	myotubularin related protein 12 [Source:HGNC
Symbol;Acc:HGNC:18191]	-	717	953	869	1352	1187
1021	7.071512574	7.577488887	7.259733557
11.78898344	9.328413015	8.595481847
ENSG00000185386 209.4100633	186.3171793	199.4708769
223.0163653	324.4202565	420.9414579	198.3993732
322.7926932	-0.70294777	0.012226866	0.070400035	MAPK11
22	50263713	50270767	-	2688
protein_coding	mitogen-activated protein kinase 11 [Source:HGNC
Symbol;Acc:HGNC:6873]	-	181	201	204	222	355
429	3.682510749	3.296861865	3.515631595
3.993235529	5.755163769	7.450305196
ENSG00000241749 223.2936034	350.3875312	133.958383
351.6023777	388.3904479	474.9083114	235.8798392
404.9670457	-0.778766438	0.012252016	0.070518284
RPSAP52 12	65758020	65826997	-	2035
transcribed_processed_pseudogene	ribosomal protein SA

pseudogene 52 [Source:HGNC Symbol;Acc:HGNC:35752]	-	193
	378	137	350	425	484	5.186657951
8.189574618	3.118592351	8.315815539	9.100874263
11.10265855
ENSG00000136826 138.8354011	190.0249839	167.2035292
283.2910586	242.1728675	262.9657592	165.354638
262.8098951	-0.666187285	0.012265322	0.070568301	KLF4
	9	107484852	107490482	-	3659
protein_coding	Kruppel like factor 4 [Source:HGNC
Symbol;Acc:HGNC:6348]	zf-C2H2 120	205	171	282	265
	268	1.793550252	2.470161847	2.164891607
3.72638665	3.156036827	3.419152098
ENSG00000196663 638.642845	752.6843264	772.4607488
1116.086405	838.9233675	996.9149678	721.26264
983.9749133	-0.447165926	0.012278419	0.07060602	TECPR2
	14	102362963	102502481	+	9384
protein_coding	tectonin beta-propeller repeat containing 2
[Source:HGNC Symbol;Acc:HGNC:19957]	-	552	812	790
	1111	918	1016	3.216960967	3.815065407
3.899792933	5.724364455	4.26297985	5.054192101
ENSG00000078487 41.65062033	35.22414335	36.1785414
86.39372709	71.28107044	90.27182779	37.68443503
82.64887511	-1.134400484	0.012284714	0.07060602	ZCWPW1
	7	100400826	100428992	-	3318
protein_coding	zinc finger CW-type and PWWP domain containing 1
[Source:HGNC Symbol;Acc:HGNC:23486]	-	36	38	37
	86	78	92	0.593363506	0.504941624
0.516568356	1.253208368	1.024417099	1.294367147
ENSG00000196810 550.7137576	606.2260461	650.2359467
756.4474012	785.0056347	913.5116486	602.3919168
818.3215615	-0.441390323	0.012291931	0.07060602	CTBP1-
DT	4	1249300 1288291 +	7820	antisense	CTBP1
divergent transcript [Source:HGNC Symbol;Acc:HGNC:28307]	-
	476	654	665	753	859	931	3.328855261
	3.687270113	3.939284507	4.655747724	4.786796982
	5.557621472
ENSG00000162929 395.6808931	359.6570426	417.5199237
228.0392564	321.6786769	223.7171384	390.9526198
257.8116906	0.599622948	0.012294179	0.07060602
KIAA1841	2	61065871	61138034	+	7992
	protein_coding	KIAA1841 [Source:HGNC Symbol;Acc:HGNC:29387]
	-	342	388	427	227	352	228
2.340266649	2.140475239	2.474998019	1.373319537
1.919312916	1.331758286
ENSG00000196776 3655.998895	3209.10485	3083.976205
2343.680992	2938.059506	2476.587971	3316.359983
2586.10949	0.358478121	0.012299651	0.07060602	CD47
	3	108043094	108091862	-	8225
protein_coding	CD47 molecule [Source:HGNC Symbol;Acc:HGNC:1682]
	-	3160	3462	3154	2333	3215	2524

21.01095965	18.55774124	17.76348764	13.71450221
17.03349112	14.32515995
ENSG00000114853 410.7213949	366.1457006	488.8992081
632.8842799	487.0873147	729.043131	421.9221012
616.3382418	-0.546473329	0.012304167	0.07060602	ZBTB47
	3	42653684	42665854	+	3781
protein_coding	zinc finger and BTB domain containing 47 [Source:HGNC
Symbol;Acc:HGNC:26955]	ZBTB	355	395	500	630	533
	743	5.134715534	4.606004694	6.125841882
8.056289955	6.142980397	9.173354401
ENSG00000095303 25.45315687	25.0276808	29.33395248
1.004578222	6.397019142	7.849724156	26.60493005
5.08377384	2.383206212	0.012304209	0.07060602	PTGS1
	9	122370530	122395703	+	5791
protein_coding	prostaglandin-endoperoxide synthase 1 [Source:HGNC
Symbol;Acc:HGNC:9604]	-	22	27	30	1	7
	8	0.207760905	0.205562626	0.239977291
0.008349254	0.052674822	0.064488527
ENSG00000186814 380.6403913	305.8938765	254.2275882
460.0968257	526.3832894	414.0729492	313.5872853
466.8510214	-0.575897715	0.01231102	0.070618597
ZSCAN30 18	35251058	35290245	-	8271
protein_coding	zinc finger and SCAN domain containing 30 [Source:HGNC
Symbol;Acc:HGNC:33517]	zf-C2H2 329	330	260	458	576
	422	2.175367269	1.759097361	1.456189124
2.677371732	3.03475098	2.381773539
ENSG00000107902 175.8581747	172.4129122	198.4930785
76.34794487	104.180026	129.5204486	182.2547218
103.3494732	0.818157033	0.012331384	0.070708878	LHPP
	10	124461834	124617888	+	4064
protein_coding	phospholysine phosphohistidine inorganic pyrophosphate
phosphatase [Source:HGNC Symbol;Acc:HGNC:30042] -	152	186
	203	76	114	132	2.045429906	2.017870089
	2.31390111	0.904192935	1.222389891	1.516234128
ENSG00000079313 783.2630545	1147.565512	1423.674494
1584.219856	1500.557919	1645.498426	1118.167687
1576.758734	-0.494957579	0.012342766	0.070747604	REXO1
	19	1815246 1848463 -	5561	protein_coding	RNA
exonuclease 1 homolog [Source:HGNC Symbol;Acc:HGNC:24616]	-
	677	1238	1456	1577	1642	1677	6.657796037
	9.815257724	12.12860733	13.71134362	12.86704606
	14.07752163
ENSG00000051180 3.470885027	6.488657986	13.68917783	0
	0	0	7.882906946	0	5.473829751
0.012344394	1	RAD51	15	40694774	40732339
	+	3046	protein_coding	RAD51 recombinase [Source:HGNC
Symbol;Acc:HGNC:9817]	-	3	7	14	0	0
	0	0.053862446	0.101321614	0.212912222	0
	0	0
ENSG00000105835 6799.463768	7582.460332	4054.930032

7541.368713 10952.61063 7741.790449 6145.618044
8745.256597 -0.508998872 0.0123823 0.07094761 NAMPT
7 106248285 106286326 - 9049
protein_coding nicotinamide phosphoribosyltransferase [Source:HGNC
Symbol;Acc:HGNC:30092] - 5877 8180 4147 7507 11985
7890 35.51810733 39.85535423 21.22931118
40.11132831 57.7159765 40.70262801
ENSG00000149761 322.7923075 486.6493489 501.6105875
595.7148857 556.5406653 809.5028036 437.0174147
653.9194515 -0.579799439 0.012388593 0.070957069 NUDT22
11 64225941 64230686 + 3721
protein_coding nudix hydrolase 22 [Source:HGNC Symbol;Acc:HGNC:28189]
- 279 525 513 593 609 825
4.100522941 6.22061884 6.38645933 7.705418652
7.132080236 10.34999916
ENSG00000119559 536.8302175 661.8431146 847.7512268
951.3355763 867.2530237 1070.506132 682.1415196
963.0315772 -0.496502524 0.012413008 0.071070281
C19orf25 19 1461143 1479556 - 5111 protein_coding
chromosome 19 open reading frame 25 [Source:HGNC
Symbol;Acc:HGNC:26711] - 464 714 867 947 949
1091 4.964857778 6.159228111 7.858066003
8.95870662 8.091311653 9.964715539
ENSG00000275966 0 5.561706845 23.46716199 0 0
0 9.676289611 0 5.770040158 0.012427221
1 AC110285.6 17 81345476 81345966
- 491 lincRNA novel transcript - 0
6 24 0 0 0 0 0.538770386
2.264290818 0 0 0
ENSG00000064607 2563.827073 2224.682738 2546.187076
2241.214013 1656.827958 1670.028814 2444.898962
1856.023595 0.397587006 0.012433188 0.071159169 SUGP2
19 18990888 19034023 - 8468
protein_coding SURP and G-patch domain containing 2 [Source:HGNC
Symbol;Acc:HGNC:18641] - 2216 2400 2604 2231 1813
1702 14.3114494 12.49580819 14.24500436
12.73854828 9.329869142 9.382633279
ENSG00000185774 145.7771711 90.8412118 88.00185745
213.9751613 256.7946256 131.4828796 108.2067468
200.7508888 -0.895362056 0.012466079 0.071291007 KCNIP4
4 20728616 21948799 - 4256
protein_coding potassium voltage-gated channel interacting protein 4
[Source:HGNC Symbol;Acc:HGNC:30083] - 126 98 90
213 281 134 1.619062592 1.015215908
0.979587751 2.419798486 2.87715526 1.469769446
ENSG00000105426 826.0706365 907.4851669 1388.473751
846.8594412 616.8554173 679.0011395 1040.676518
714.238666 0.543721278 0.012473262 0.071291007 PTPRS
19 5158495 5340803 - 8565
protein_coding "protein tyrosine phosphatase, receptor type S

[Source:HGNC Symbol;Acc:HGNC:9681]"	-	714	979	1420
843	675	692	4.558957643	5.039521269
7.680039132	4.758843511	3.434274485	3.771592496
ENSG00000269973 127.2657843	160.3625474	126.1359957
245.1170862	234.8619885	197.2243194	137.9214425
225.7344647	-0.709518642	0.012475061	0.071291007
AC010969.2	2	9936360 9939590 +	3231	lincRNA novel
transcript	-	110	173	129	244	257	201
1.861874654	2.360712519	1.849503715	3.651355218
3.46620914	2.904056993
ENSG00000203724 127.2657843	131.627062	175.0259165
88.40288354	77.67808958	70.6475174	144.6395876
78.90949684	0.87509655	0.012476257	0.071291007
C1orf53 1	197902647	197907367	+	611
protein_coding	chromosome 1 open reading frame 53 [Source:HGNC
Symbol;Acc:HGNC:30003]	-	110	142	179	88	85
72	9.845690683	10.24663089	13.57107581
6.963742094	6.062284493	5.500945244
ENSG00000188786 942.9237657	1033.550522	996.3765861
1506.867333	1192.58714	1224.556968	990.9502913
1308.003814	-0.400009299	0.01247954	0.071291007	MTF1
1	37809567	37859620	-	8024
protein_coding	metal regulatory transcription factor 1 [Source:HGNC
Symbol;Acc:HGNC:7428]	zf-C2H2 815	1115	1019	1500	1305
1248	5.554710144	6.126577129	5.882822161
9.038608081	7.087257128	7.26055302
ENSG00000140688 741.6124341	858.3567564	785.1721282
969.4179843	1039.97254	1151.94702	795.0471062
1053.779182	-0.405979234	0.012489249	0.071319819
C16orf58	16	31489471	31509309	-	3630
protein_coding	chromosome 16 open reading frame 58
[Source:HGNC Symbol;Acc:HGNC:25848]	-	641	926	803
965	1138	1174	9.657086406	11.24704207
10.24734543	12.85351488	13.6613694	15.09758585
ENSG00000165526 1053.992087	1103.071858	1149.890937
1522.940585	1293.111727	1463.973555	1102.318294
1426.675289	-0.371735563	0.012499579	0.071352155	RPUSD4
11	126202094	126211692	-	4083
protein_coding	RNA pseudouridylate synthase domain containing 4
[Source:HGNC Symbol;Acc:HGNC:25898]	-	911	1190	1176
1516	1415	1492	12.20207555	12.84995301
13.34229066	17.95233889	15.10204144	17.05828944
ENSG00000161980 563.4403361	670.1856748	616.0130022
371.6939421	453.2744992	489.6265442	616.5463377
438.1983285	0.492953943	0.012506905	0.071367323	POLR3K
16	46407	53628	-	1889	protein_coding	RNA
polymerase III subunit K [Source:HGNC Symbol;Acc:HGNC:14121]	-
487	723	630	370	496	499	14.09911056
16.87486462	15.44937971	9.470458005	11.4421679
12.33146355

ENSG00000050820 2873.892802 4659.783385 4612.275129
5289.104339 5983.040617 5164.137279 4048.650439
5478.760745 -0.436115301 0.012530355 0.071474455 BCAR1
16 75228187 75268053 - 9178
protein_coding "BCAR1, Cas family scaffold protein [Source:HGNC
Symbol;Acc:HGNC:971]" - 2484 5027 4717 5265 6547
5263 14.80124512 24.14875747 23.80785554
27.7364924 31.08514413 26.76895098
ENSG00000198857 155.0328645 234.5186386 151.5587545
299.3643102 278.7272626 289.4585782 180.3700859
289.1833837 -0.678947033 0.01255028 0.071561406
HSD3BP5 1 119601340 119609250 + 1132
transcribed_unprocessed_pseudogene "hydroxy-delta-5-steroid
dehydrogenase, 3 beta, pseudogene 5 [Source:HGNC
Symbol;Acc:HGNC:5223]" - 134 253 155 298 305
295 6.473707669 9.853897769 6.342897993
12.72831907 11.74118714 12.16526643
ENSG00000148498 1242.57684 1162.396731 1142.06855
868.960162 866.3391638 985.1403815 1182.347373
906.8132358 0.38259963 0.012564746 0.071617177 PARD3
10 34109560 34815325 - 6513
protein_coding par-3 family cell polarity regulator [Source:HGNC
Symbol;Acc:HGNC:16051] - 1074 1254 1168 865 948
1004 9.018159577 8.488880428 8.30738275
6.421496435 6.342870679 7.196124533
ENSG00000189067 5.784808379 0 23.46716199 0 0
0 9.750656789 0 5.778761973 0.01257615
1 LITAF 16 11547722 11636381 -
5060 protein_coding lipopolysaccharide induced TNF factor
[Source:HGNC Symbol;Acc:HGNC:16841] zf-LITAF-like 5 0
24 0 0 0 0.054039858 0
0.219716757 0 0 0
ENSG00000280070 8.09873173 9.269511408 11.73358099 0
0.913859877 0 9.700608044 0.304619959
4.80996999 0.012577324 1 AL049833.3 14
96992864 96996601 + 3738 TEC TEC -
7 10 12 0 1 0 0.102412615
0.117949108 0.148711449 0 0.011657872 0
ENSG00000141503 1433.475516 2567.65466 2138.445136
2564.688201 2797.325085 3178.157068 2046.525104
2846.723451 -0.475538266 0.012588829 0.071722691 MINK1
17 4833388 4898061 + 7817 protein_coding
misshapen like kinase 1 [Source:HGNC Symbol;Acc:HGNC:17565] -
1239 2770 2187 2553 3061 3239 8.668139803
15.62333032 12.96018279 15.79108311 17.06403823
19.34269001
ENSG00000115317 327.4201542 440.3017919 356.8964219
478.1792337 547.4020666 593.6353893 374.8727893
539.7388965 -0.52471616 0.012592641 0.071722691 HTRA2
2 74529377 74533348 + 3001

protein_coding HtrA serine peptidase 2 [Source:HGNC
Symbol;Acc:HGNC:14348] -   283   475   365   476   599
        605   5.157213999   6.978491794   5.634161931
7.669060675   8.698000428   9.410992235
ENSG00000112276 362.1290045   294.7704628   261.0721771
209.9568484   207.4461922   171.7127159   305.9905481
196.3719188   0.638615562   0.012613603   0.071815327   BVES
        6   105096822   105137174   -   5808
protein_coding blood vessel epicardial substance [Source:HGNC
Symbol;Acc:HGNC:1152]   -   313   318   267   209   227
        175   2.947219233   2.413984461   2.129546411
1.739886406   1.703169406   1.406557455
ENSG00000123427 423.4479733   337.4102153   331.4736631
216.988896   261.3639249   249.2287419   364.1106172
242.5271876   0.584740382   0.012633606   0.071902436
EEF1AKMT3   12   57771492   57782541   +   3172
        protein_coding EEF1A lysine methyltransferase 3 [Source:HGNC
Symbol;Acc:HGNC:24936] -   366   364   339   216   286
        254   6.310192665   5.059436657   4.950726964
3.292469716   3.929085253   3.738062632
ENSG00000125485 857.3086017   875.9688281   864.3737999
1237.64037   1159.688184   1013.595632   865.8837432
1136.974729   -0.392865355   0.012640034   0.071905266   DDX31
        9   132592997   132670401   -   5546
protein_coding DEAD-box helicase 31 [Source:HGNC
Symbol;Acc:HGNC:16715] -   741   945   884   1232   1269
        1033   7.306898178   7.512524498   7.383713815
10.74068696   9.971037615   8.694938051
ENSG00000163655 4898.575735   5042.614206   4667.03184
3579.312205   4138.871385   4060.26982   4869.407261
3926.151136   0.310574422   0.012643511   0.071905266   GMPS
        3   155870536   155944026   +   8885
protein_coding guanine monophosphate synthase [Source:HGNC
Symbol;Acc:HGNC:4378]   -   4234   5440   4773   3563   4529
        4138   26.06082346   26.99450856   24.88493198
19.38918733   22.2128092   21.74097867
ENSG00000174365 166.6024813   190.0249839   259.1165803
366.671051   337.2142948   272.7779144   205.2480152
325.5544201   -0.664187502   0.012703703   0.072220716   SNHG11
        20   38446578   38450921   +   2813
processed_transcript   small nucleolar RNA host gene 11 [Source:HGNC
Symbol;Acc:HGNC:25046] -   144   205   265   365   369
        278   2.799545128   3.213054461   4.363938259
6.273708686   5.716302877   4.613399876
ENSG00000006194 693.0200438   910.2660203   850.6846221
1216.544227   1215.433637   929.2110969   817.9902287
1120.39632   -0.453114099   0.012715799   0.072262608   ZNF263
        16   3263800 3301401 +   6945   protein_coding zinc
finger protein 263 [Source:HGNC Symbol;Acc:HGNC:13056] zf-C2H2 599
        982   870   1211   1330   947   4.716819802

6.234091836	5.802958415	8.430883916	8.345223813
6.365371639
ENSG00000258857 0	0	1.955596832	9.041203998
17.36333767	11.77458623	0.651865611
12.72637597	-4.258729205	0.012737417	1	AL359397.1
	14	50397013	50398345	+	501
lincRNA novel transcript	-	0	0	2	9
	19	12	0	0	0.184924616	0.86857235
	1.652625588	1.118122935
ENSG00000126458 450.0580919	706.3367693	517.2553621
681.1040345	892.8411002	837.9580536	557.8834078
803.9677295	-0.526106122	0.012744164	0.072396892	RRAS
	19	49635292	49640201	-	1160
protein_coding	RAS related [Source:HGNC Symbol;Acc:HGNC:10447] -
	389	762	529	678	977	854	18.33945075
	28.96215944	21.1251664	28.26004969	36.70245985
	34.36733983
ENSG00000118939 74.04554725	100.1107232	132.9805846
23.10529911	60.31475191	50.04199149	102.3789517
44.4873475	1.201658198	0.012759472	0.072456927	UCHL3
	13	75549480	75606020	+	1905
protein_coding	ubiquitin C-terminal hydrolase L3 [Source:HGNC
Symbol;Acc:HGNC:12515]	-	64	108	136	23	66
	51	1.837298442	2.499555208	3.307092818
0.58375965	1.509758743	1.249744494
ENSG00000134668 588.8934929	1043.746985	539.7447257
936.2669029	1571.838989	903.6994934	724.1284011
1137.268462	-0.650764427	0.012772477	0.072503846	SPOCD1
	1	31790422	31816051	-	7085
protein_coding	SPOC domain containing 1 [Source:HGNC
Symbol;Acc:HGNC:26338]	-	509	1126	552	932	1720
	921	3.928915066	7.007006073	3.609122965
6.360295218	10.57906267	6.068282792
ENSG00000158042 1384.883126	1563.766575	1282.871522
1725.865385	1713.48727	2078.21447	1410.507074
1839.189042	-0.382631794	0.012782742	0.072535182	MRPL17
	11	6680782 6683401 -	2276	protein_coding
mitochondrial ribosomal protein L17 [Source:HGNC
Symbol;Acc:HGNC:14053]	-	1197	1687	1312	1718	1875
	2118	28.76183599	32.67960382	26.70324455
36.49657567	35.89943488	43.44099198
ENSG00000207750 15.04050178	3.707804563	4.888992081	0
	0	0	7.879099476	0	5.466368529
0.012797374	1	MIR553	1	100281241	100281308
	+	68	miRNA	microRNA 553 [Source:HGNC
Symbol;Acc:HGNC:32809]	-	13	4	5	0	0
	0	10.45512314	2.59349274	3.406148258	0
	0	0
ENSG00000167977 817.9719047	1345.006105	1105.890009
1643.489971	1509.696517	1373.701727	1089.622673

1508.962739 -0.468733946 0.012802134 0.072618265 KCTD5
16 2682475 2709030 + 3420 protein_coding
potassium channel tetramerization domain containing 5 [Source:HGNC
Symbol;Acc:HGNC:21423] - 707 1451 1131 1636 1652
1400 11.30545434 18.70575597 15.31930118
23.12908274 21.04953637 19.10944023
ENSG00000182118 325.1062309 383.7577723 351.0296314
414.8908057 621.4247166 514.1569322 353.2978782
516.8241515 -0.548567592 0.012816944 0.072675304 FAM89A
1 231018958 231040246 - 2394
protein_coding family with sequence similarity 89 member A
[Source:HGNC Symbol;Acc:HGNC:25057] - 281 414 359
413 680 524 6.419140575 7.624478656
6.946607459 8.341172871 12.37779724 10.21770069
ENSG00000103326 1877.7488 2727.090256 3576.786606
4227.265158 3098.898844 4293.799113 2727.208554
3873.321039 -0.505752265 0.012896944 0.073101811 CAPN15
16 527717 554636 + 7987 protein_coding
calpain 15 [Source:HGNC Symbol;Acc:HGNC:11182] Others 1623 2942
3658 4208 3391 4376 11.11295481 16.24025866
21.21594948 25.47377234 18.5013195 25.57641468
ENSG00000225200 27.76708022 45.4206059 32.26734773
75.34336665 83.16124884 80.4596726 35.15167795
79.6547627 -1.176140735 0.01290754 0.073134751
AC246787.1 14 105978520 105979146 - 627
processed_pseudogene ribosomal protein S8 (RPS8) pseudogene
- 24 49 33 75 91 82
2.093333452 3.445581264 2.438085069 5.78355592
6.324591051 6.105093891
ENSG00000099326 592.364378 786.0545674 656.1027372
1063.848337 733.8294816 1081.299502 678.1738942
959.659107 -0.499807016 0.012922614 0.073193035 MZF1
19 58561931 58573575 - 6691
protein_coding myeloid zinc finger 1 [Source:HGNC
Symbol;Acc:HGNC:13108] zf-C2H2 512 848 671 1059 803
1102 4.184789755 5.587773328 4.645515849
7.652549487 5.229776306 7.688411161
ENSG00000231628 6.941770054 16.68512054 5.866790497 0
0.913859877 0 9.831227029 0.304619959
4.830909756 0.01295479 1 AL133406.2 6
105279016 105281755 + 1198 antisense novel
transcript - 6 18 6 0 1 0
0.273898179 0.662444723 0.232004756 0
0.036374898 0
ENSG00000081692 126.1088227 210.417909 159.3811418
205.9385355 277.8134027 357.1624491 165.3026245
280.3047958 -0.759659463 0.012955779 0.073353699 JMJD4
1 227730425 227735411 - 4315
protein_coding jumonji domain containing 4 [Source:HGNC
Symbol;Acc:HGNC:25724] - 109 227 163 205 304

364	1.381466668	2.319417957	1.749884
2.297070252	3.070091939	3.937917555
ENSG00000142227 4347.861977	5761.928292	5571.495375
5985.277047	6479.266531	7992.000406	5227.095215
6818.847995	-0.383327641	0.012980547	0.073466723	EMP3
19	48321509	48330553	+	1667
protein_coding	epithelial membrane protein 3 [Source:HGNC
Symbol;Acc:HGNC:3335]	-	3758	6216	5698	5958	7090
8145	123.2866037	164.4028583	158.3395115
172.8089039	185.3400318	228.0875208
ENSG00000183647 455.8429002	373.5613098	428.2757063
215.9843177	268.674804	344.4066473	419.2266388
276.3552563	0.600492026	0.01301055	0.073609281	ZNF530
19	57599885	57612722	+	5019
protein_coding	zinc finger protein 530 [Source:HGNC
Symbol;Acc:HGNC:29297]	zf-C2H2 394	403	438	215	294
351	4.293127028	3.540151178	4.042586959
2.071202114	2.552635075	3.264644955
ENSG00000139178 185.1138681	152.0199871	146.6697624
223.0163653	324.4202565	235.4917247	161.2678725
260.9761155	-0.696618802	0.013028409	0.073683053	C1RL
12	7089587 7109273 -	5014	protein_coding
complement C1r subcomponent like [Source:HGNC Symbol;Acc:HGNC:21265]
-	160	164	150	222	355	240
1.745140373	1.442093689	1.385828171	2.140769267
3.085337098	2.234461869
ENSG00000253626 463.941632	359.6570426	355.9186235
200.9156444	327.1618361	227.6420005	393.1724327
251.9064937	0.640353186	0.013045171	0.073750567
EIF5AL1 10	79512601	79516440	+	3840
protein_coding	eukaryotic translation initiation factor 5A-like 1
[Source:HGNC Symbol;Acc:HGNC:17419]	-	401	388	364
200	358	232	5.710943466	4.454864092
4.391092796	2.51825664	4.062659269	2.820348634
ENSG00000152104 5762.826107	4096.197091	5807.144794
3813.378931	3358.43505	4585.220122	5222.055997
3919.011368	0.414047404	0.013102396	0.074046704	PTPN14
1	214348696	214552449	-	18756
protein_coding	"protein tyrosine phosphatase, non-receptor type 14
[Source:HGNC Symbol;Acc:HGNC:9647]"	-	4981	4419	5939
3796	3675	4673	14.52349135	10.38766022
14.66815724	9.785594069	8.538384678	11.63058589
ENSG00000231058 0	0	0	24.10987733	5.483159264
0	0	9.864345531	-5.720963891	0.013111817
1	MSANTD2P1	21	23101223	23103074
+	1368	processed_pseudogene	Myb/SANT DNA binding
domain containing 2 pseudogene 1 [Source:HGNC Symbol;Acc:HGNC:39637]
-	0	0	0	24	6	0	0
0	0	0.848254868	0.191127752	0
ENSG00000170027 9875.824864	9515.153461	9355.575246

12026.81047	12576.53963	10663.85027	9582.184524
11755.73346	-0.294983599	0.013133462	0.074194841	YWHAG
7	76326794	76359031	-	3750
protein_coding	tyrosine 3-monooxygenase/tryptophan 5-monooxygenase
activation protein gamma [Source:HGNC Symbol;Acc:HGNC:12852]	-
8536	10265	9568	11972	13762	10868	124.4852373
120.6873202	118.1931629	154.3606707	159.9222471
135.2895954
ENSG00000217416 21.98227184	14.83121825	14.66697624
1.004578222	3.65543951	1.962431039	17.16015544
2.207482924	2.946407932	0.013149637	1	ISCA1P1 5
62776877	62777263	-	387
processed_pseudogene	iron-sulfur cluster assembly 1 pseudogene 1
[Source:HGNC Symbol;Acc:HGNC:33263]	-	19	16	15
1	4	2	2.684957086	1.822816603
1.795489004	0.124936764	0.450409585	0.241248747
ENSG00000092607 12.72657843	19.46597396	10.75578258	0
0	2.943646558	14.31611166	0.981215519
3.878087425	0.013157526	1	TBX15	1	118883046
118989556	-	4349	protein_coding	T-box 15
[Source:HGNC Symbol;Acc:HGNC:11594]	T-box	11	21	11
0	0	3	0.138324144	0.212894207
0.117167114	0	0	0.032201632
ENSG00000100028 2857.695339	2857.790367	2900.150102
2232.172809	2422.642535	2299.969178	2871.878603
2318.261507	0.308888677	0.01317156	0.074382577	SNRPD3
22	24555503	24609980	+	2544
protein_coding	small nuclear ribonucleoprotein D3 polypeptide
[Source:HGNC Symbol;Acc:HGNC:11160]	-	2470	3083	2966
2222	2651	2344	53.09755935	53.43063993
54.00780109	42.23068871	45.40997037	43.01169289
ENSG00000087302 2031.624703	1754.71851	1936.040864
1592.256482	1485.022301	1412.950348	1907.461359
1496.743044	0.349594637	0.013184236	0.074426666	RTRAF
14	51989475	52010691	+	8323
protein_coding	"RNA transcription, translation and transport factor
[Source:HGNC Symbol;Acc:HGNC:23169]"	-	1756	1893	1980
1585	1625	1440	11.53823366	10.02777723
11.02015623	9.207687862	8.508089865	8.076601091
ENSG00000181577 60.16200714	114.9419415	74.31267963
143.6546857	230.2926891	117.7458623	83.13887608
163.8977457	-0.976572739	0.013208205	0.074534449
C6orf223	6	44000580	44005958	+	3917
protein_coding	chromosome 6 open reading frame 223
[Source:HGNC Symbol;Acc:HGNC:28692]	-	52	124	76
143	252	120	0.726013146	1.395732115
0.898798785	1.765158395	2.803532319	1.430124051
ENSG00000124160 1558.427377	1343.152203	1248.648577
2002.124396	1641.29234	1767.169151	1383.409386
1803.528629	-0.382942795	0.013263769	0.074816872	NCOA5

20	46060985	46089952	-	3220
protein_coding	nuclear receptor coactivator 5 [Source:HGNC
Symbol;Acc:HGNC:15909]	-	1347	1449	1277	1993	1796
1801	22.87738794	19.84021946	18.37119815
29.92627369	24.30575178	26.10981995
ENSG00000149658 2146.163908	2449.931865	2416.139886
2968.528646	2905.16055	2873.980257	2337.411887
2915.889818	-0.318722196	0.013268041	0.074816872	YTHDF1
20	63195429	63216234	-	3301
protein_coding	YTH N6-methyladenosine RNA binding protein 1
[Source:HGNC Symbol;Acc:HGNC:15867]	-	1855	2643	2471
2955	3179	2929	30.73216119	35.30088528
34.6760545	43.28258368	41.9665822	41.42093358
ENSG00000214826 273.0429555	404.1506974	503.5661843
326.4879222	206.5323323	173.6751469	393.5866124
235.5651338	0.742163462	0.013278511	0.074847029	DDX12P
12	9418673 9448229 -	2947	unprocessed_pseudogene
"DEAD/H-box helicase 12, pseudogene [Source:HGNC
Symbol;Acc:HGNC:2737]"	-	236	436	515	325	226
177	4.379520664	6.522893854	8.095236648
5.33217558	3.341849604	2.803749224
ENSG00000188933 204.7822166	176.1207168	211.2044579
139.6363729	106.0077458	107.9337071	197.3691304
117.8592753	0.744042447	0.013286637	0.074847029
USP32P1 17	16786489	16804455	+	9299
transcribed_unprocessed_pseudogene	ubiquitin specific peptidase
32 pseudogene 1 [Source:HGNC Symbol;Acc:HGNC:43570]	-	177
190	216	139	116	110	1.040954463
0.90084757	1.076019048	0.722736135	0.543601116
0.552207719
ENSG00000245556 69.41770054	110.3071858	102.6688337
144.659264	182.7719755	177.600009	94.13124
168.3437495	-0.835659217	0.013288077	0.074847029
SCAMP1-AS1	5	78342365	78360507	-	1792
lincRNA SCAMP1 antisense RNA 1 [Source:HGNC
Symbol;Acc:HGNC:50544]	-	60	119	105	144	200
181	1.831082693	2.927810163	2.714274393
3.885310245	4.863518678	4.715053288
ENSG00000241157 15.04050178	5.561706845	2.933395248	0
0	0	7.845201293	0	5.460443684
0.013307204	1	AC104763.1	17	60245288
60245647	-	360	processed_pseudogene	ribosomal
protein L32 (RPL32) pseudogene	-	13	6	3	0
0	0	1.974856593	0.734822943	0.386030136
0	0	0
ENSG00000166411 858.4655634	775.8581049	817.4394759
629.8705452	562.9376845	642.6961653	817.2543814
611.8347983	0.417539192	0.013312017	0.074933961	IDH3A
15	78131498	78171949	+	6832
protein_coding	isocitrate dehydrogenase 3 (NAD(+)) alpha [Source:HGNC

Symbol;Acc:HGNC:5384]	-	742	837	836	627	616
	655	5.939511949	5.4014649	5.668405041
4.437321537	3.929085253	4.475478531
ENSG00000229848 2.313923351	18.53902282	12.71137941
27.12361199	53.91773277	49.06077597	11.18810853
43.36737358	-1.93989382	0.013315731	0.074933961
AC139149.1	17	81514047	81527776	+	957
	antisense	"novel transcript, antisense to ACTG1"	-
	2	20	13	27	59	50	0.114291194
	0.921408079	0.629265425	1.364121465	2.68657316
	2.438956769
ENSG00000110719 693.0200438	1241.187578	1374.784573
678.0902999	497.1397733	944.9105452	1102.997398
706.7135395	0.643152205	0.01331993	0.074933961	TCIRG1
	11	68039016	68050895	+	4384
protein_coding	"T cell immune regulator 1, ATPase H+ transporting V0 subunit a3 [Source:HGNC Symbol;Acc:HGNC:11647]"	-	599	1339
	1406	675	544	963	7.472243048	13.4661668
	14.85652476	7.444481309	5.407380767	10.25419985
ENSG00000040633 1372.156547	1394.134516	1751.236963
1914.726091	1901.742405	2035.040987	1505.842676
1950.503161	-0.373022601	0.013327869	0.074933961	PHF23
	17	7235028 7239722 -	2783	protein_coding	PHD finger protein 23 [Source:HGNC Symbol;Acc:HGNC:28428]	-	1186
	1504	1791	1906	2081	2074	23.305917
23.82695737	29.8115691	33.11394373	32.58498096
34.78897087
ENSG00000265972 2788.277638	3882.071378	1770.792932
4885.263894	6049.752388	4307.53613	2813.713983
5080.850804	-0.852568427	0.01332802	0.074933961	TXNIP
	1	145992435	145996600	-	3625
protein_coding	thioredoxin interacting protein [Source:HGNC Symbol;Acc:HGNC:16952]	-	2410	4188	1811	4863	6620
	4390	36.3583147	50.93691286	23.14263976
64.86306626	79.58085051	56.53306674
ENSG00000106479 165.4455196	188.1710816	185.7816991
332.5153915	211.1016317	320.8574749	179.7994334
288.158166	-0.67899306	0.013338222	0.07496375	ZNF862
	7	149838367	149867479	+	8430
protein_coding	zinc finger protein 862 [Source:HGNC Symbol;Acc:HGNC:34519]	-	143	203	190	331	231
	327	0.927690642	1.061701476	1.044067272
1.898460807	1.194106337	1.810782188
ENSG00000022840 2030.467741	2661.276725	2383.872539
3097.114658	3081.535507	2862.20567	2358.539002
3013.618612	-0.353178857	0.013360353	0.075060534	RNF10
	12	120533480	120577594	+	5911
protein_coding	ring finger protein 10 [Source:HGNC Symbol;Acc:HGNC:10055]	-	1755	2871	2438	3083	3372
	2917	16.23719006	21.41441383	19.10623863

25.21818242	24.85908872	23.03676572
ENSG00000049249 136.5214777	41.71280134	161.3367387
61.27927154	47.52071363	27.47403455	113.1903392
45.42467324	1.315967621	0.013386721	0.075181046
TNFRSF9 1	7915894 7943165 -	6331	protein_coding	TNF
receptor superfamily member 9 [Source:HGNC Symbol;Acc:HGNC:11924]
-	118	45	165	61	52	28
1.019305591	0.31338208	1.207296903	0.465863557
0.357923017	0.206458018
ENSG00000173546 885.0756819	641.4501895	1605.544999
664.0262048	633.304895	704.512743	1044.023624
667.2812809	0.645906689	0.013407166	0.075268216	CSPG4
15	75674322	75712848	-	8290
protein_coding	chondroitin sulfate proteoglycan 4 [Source:HGNC
Symbol;Acc:HGNC:2466]	-	765	692	1642	661	693
718	5.04663177	3.680319492	9.175317007
3.855210938	3.642816557	4.043113652
ENSG00000278902 13.88354011	28.73548537	10.75578258
80.36625776	47.52071363	36.30497422	17.79160268
54.73064854	-1.614738023	0.013415768	0.075277666
AC010947.1	18	73921939	73922631	-	693
TEC	TEC	-	12	31	11	80	52
37	0.946984181	1.972252055	0.735295497
5.581590475	3.269856598	2.492381536
ENSG00000164342 39.33669697	52.83621503	32.26734773	0
17.36333767	8.830939675	41.48008658	8.731425782
2.24085344	0.013418698	0.075277666	TLR3	4
186069152	186088069	+	6724	protein_coding
toll like receptor 3 [Source:HGNC Symbol;Acc:HGNC:11849]
-	34	57	33	0	19	9
0.276532338	0.37374992	0.227346719	0	0.123135845
0.062482851
ENSG00000213999 0	1.853902282	8.800185745	40.18312888
22.84649694	12.75580175	3.551362676	25.26180919
-2.81120179	0.013426113	0.075285775	MEF2B	19
19145568	19170289	-	1848	protein_coding
myocyte enhancer factor 2B [Source:HGNC Symbol;Acc:HGNC:6995]
SRF	0	2	9	40	25	13	0
0.047715776	0.225602027	1.046548214	0.589517416
0.328388108
ENSG00000105556 684.921312	760.0999355	710.8594485
1020.651474	954.069712	876.2254589	718.6268987
950.3155482	-0.402733407	0.013429993	0.075285775	MIER2
19	301444	344815	-	7939	protein_coding	MIER
family member 2 [Source:HGNC Symbol;Acc:HGNC:29210]	-	592
820	727	1016	1044	893	4.078031889
4.553884468	4.242003912	6.18769819	5.73051026
5.250875157
ENSG00000205730 1598.921036	1655.534738	1823.594046
2366.786291	1867.929589	2328.424428	1692.683273

2187.713436	-0.369797054	0.013449138	0.075365457
ITPRIPL2	16	19113932	19121629	+	7440
	protein_coding	ITPRIP like 2 [Source:HGNC
Symbol;Acc:HGNC:27257]	-	1382	1786	1865	2356	2044
	2373	10.1585055	10.58382078	11.61203554
15.31100037	11.97199574	14.889182
ENSG00000183826 607.4048798	768.4424958	876.1073809
985.4912358	944.9311132	1171.57133	750.6515855
1033.997893	-0.46103937	0.013512976	0.075695441	BTBD9
	6	38168451	38640148	-	9537
protein_coding	BTB domain containing 9 [Source:HGNC
Symbol;Acc:HGNC:21228]	-	525	829	896	981	1034
	1194	3.010524969	3.832451839	4.352098167
4.973457845	4.724625111	5.844381805
ENSG00000101911 1101.427515	1167.031486	957.2646494
871.9738967	830.6986286	720.2121913	1075.241217
807.6282389	0.412895319	0.013550596	0.075878377	PRPS2
	X	12791355	12824222	+	2758
protein_coding	phosphoribosyl pyrophosphate synthetase 2 [Source:HGNC
Symbol;Acc:HGNC:9465]	-	952	1259	979	868	909
	734	18.87719227	20.12636879	16.44337214
15.21691728	14.36243973	12.42361069
ENSG00000204709 0	0	0.977798416	26.11903377
10.96631853	0	0.325932805	12.3617841	-5.085226334
	0.013559329	1	LINC01556	6	28943877
	28944537	+	466	lincRNA long intergenic non-
protein coding RNA 1556 [Source:HGNC Symbol;Acc:HGNC:21195]	-
	0	0	1	26	12	0	0	0
	0.099406902	2.697668916	1.122157786	0
ENSG00000144559 467.412517	449.5713033	445.8760778
815.7175163	560.1961049	569.1050013	454.2866327
648.3395408	-0.512971017	0.013563066	0.075881518	TAMM41
	3	11790442	11846919	-	5925
protein_coding	TAM41 mitochondrial translocator assembly and
maintenance homolog [Source:HGNC Symbol;Acc:HGNC:25187] -	404
	485	456	812	613	580	3.728959986
3.609003821	3.565159331	6.626266384	4.508485919
4.569678799
ENSG00000137522 653.6833468	970.5178445	812.5504838
1030.697256	1105.770452	1196.101718	812.2505584
1110.856475	-0.450606593	0.01356449	0.075881518	RNF121
	11	71928701	71997597	+	3567
protein_coding	ring finger protein 121 [Source:HGNC
Symbol;Acc:HGNC:21070]	-	565	1047	831	1026	1210
	1219	8.662436244	12.94128884	10.79196108
13.9073847	14.78226075	15.95315674
ENSG00000163466 5715.390678	5011.097867	5119.752507
3940.960365	4770.34856	3855.195776	5282.080351
4188.8349	0.334344747	0.013568336	0.075881518	ARPC2
	2	218217094	218254356	+	6562

protein_coding actin related protein 2/3 complex subunit 2
[Source:HGNC Symbol;Acc:HGNC:705]   -   4940   5406   5236
        3923   5220   3929   41.1704331   36.32233615
36.96288556   28.90568719   34.66513331   27.95064528
ENSG00000100601 414.1922799   352.2414335   283.5615407
498.2707981   551.0575061   471.9646649   349.998418
507.0976564   -0.53637816   0.013571013   0.075881518   ALKBH1
        14   77672404   77708020   -   2844
protein_coding "alkB homolog 1, histone H2A dioxygenase [Source:HGNC
Symbol;Acc:HGNC:17911]" -   358   380   290   496   603
        481   6.884115486   5.890985619   4.723575503
8.4324408   9.239454217   7.895170626
ENSG00000230910 12.72657843   14.83121825   20.53376674   0
        0   3.924862078   16.03052114   1.308287359
3.624777966   0.013632577   1   AL391807.1   6
68628117   68635161   -   5924   processed_transcript
        uncharacterized LOC101928307 [Source:NCBI gene;Acc:101928307]
        -       11   16   21   0   0   4
0.101548228   0.119080018   0.164212684   0   0
0.031520346
ENSG00000189043 3114.540831   3249.8907   2912.861482
2056.37162   2824.740881   2297.025531   3092.431004
2392.712678   0.369915653   0.013644575   0.076263229   NDUFA4
        7   10931951   10940256   -   3556
protein_coding "NDUFA4, mitochondrial complex associated [Source:HGNC
Symbol;Acc:HGNC:7687]" -   2692   3506   2979   2047   3091
        2341   41.40073163   43.46944721   38.80710152
27.83282615   37.87876846   30.73163718
ENSG00000198604 1752.796939   1924.350568   1866.617176
1356.1806   1656.827958   1235.350339   1847.921561
1416.119632   0.38388813   0.013649257   0.076263229   BAZ1A
        14   34752731   34875647   -   7698
protein_coding bromodomain adjacent to zinc finger domain 1A
[Source:HGNC Symbol;Acc:HGNC:960]   -   1515   2076   1909
        1350   1813   1259   10.76290331   11.89004232
11.48763101   8.479242935   10.26309845   7.634733098
ENSG00000127564 424.604935   845.3794405   1019.843748
339.547439   523.6417098   550.4619064   763.2760412
471.2170184   0.69649996   0.01366078   0.076299723   PKMYT1
        16   2968024 2980539 -   5034
protein_coding "protein kinase, membrane associated tyrosine/
threonine 1 [Source:HGNC Symbol;Acc:HGNC:29650]"   -   367
        912   1043   338   573   561   3.987012211
7.987586698   9.597841044   3.246420002   4.960209371
5.202303509
ENSG00000197562 575.0099528   641.4501895   863.3960015
931.2440118   976.916209   964.5348556   693.2853813
957.5650255   -0.465249282   0.013669747   0.07632192   RAB40C
        16   589357 629272 +   4180
protein_coding "RAB40C, member RAS oncogene family [Source:HGNC

Symbol;Acc:HGNC:18285]" - 497 692 883 927 1069
983 6.502417035 7.29900684 9.785586891
10.72271268 11.14448544 10.97800115
ENSG00000104356 779.7921694 946.4171148 971.9316257
643.9346403 663.462271 706.475174 899.3803033
671.2906951 0.422658413 0.013690316 0.07640886 POP1
8 98117297 98159834 + 4852
protein_coding "POP1 homolog, ribonuclease P/MRP subunit [Source:HGNC
Symbol;Acc:HGNC:30129]" - 674 1021 994 641 726
720 7.596854649 9.277669721 9.49004011
6.387610907 6.520402815 6.927200214
ENSG00000115211 842.2680999 751.7573752 657.0805357
1048.779664 954.069712 994.9525367 750.3686703
999.2673042 -0.41384941 0.01370846 0.076475078 EIF2B4
2 27364352 27370486 - 5341
protein_coding eukaryotic translation initiation factor 2B subunit
delta [Source:HGNC Symbol;Acc:HGNC:3260] - 728 811
672 1044 1044 1014 7.454242449 6.69471717
5.828397334 9.451029901 8.517978086 8.862605394
ENSG00000133243 2634.401736 3270.283625 3348.959575
3947.992413 3682.855306 4111.293027 3084.548312
3914.046915 -0.343240841 0.013713881 0.076475078 BTBD2
19 1985438 2034881 - 5944 protein_coding BTB
domain containing 2 [Source:HGNC Symbol;Acc:HGNC:15504] - 2277
3528 3425 3930 4030 4190 20.94975472
26.16879552 26.69219143 31.96796316 29.54505774
32.90646709
ENSG00000175806 585.4226079 641.4501895 840.9066379
446.0327306 482.5180153 534.7624581 689.2598118
487.771068 0.499355895 0.013717189 0.076475078 MSRA
8 10054268 10428891 + 4162
protein_coding methionine sulfoxide reductase A [Source:HGNC
Symbol;Acc:HGNC:7377] - 506 692 860 444 528
545 6.64879823 7.330573905 9.571914952
5.15800918 5.528285258 6.112803879
ENSG00000198796 263.7872621 184.463277 196.5374816
124.5676995 60.31475191 158.9569142 214.9293402
114.6131219 0.907409326 0.013723207 0.076480741 ALPK2
18 58481247 58628957 - 9431
protein_coding alpha kinase 2 [Source:HGNC Symbol;Acc:HGNC:20565]
- 228 199 201 124 66 162
1.322122861 0.930313428 0.987280975 0.635718949
0.304961341 0.801868781
ENSG00000113648 3459.31541 3503.875312 3752.790321
3073.004781 2707.766817 2857.299593 3571.993681
2879.357064 0.31111859 0.013728815 0.076484108 H2AFY
5 135333900 135399914 - 17730
protein_coding H2A histone family member Y [Source:HGNC
Symbol;Acc:HGNC:4740] - 2990 3780 3838 3059 2963
2912 9.222680538 9.399765565 10.027639

8.342034044	7.282517109	7.667056627
ENSG00000183955 778.6352078	926.0241897	1101.978815
611.7881372	768.5561569	652.5083204	935.5460708
677.6175382	0.46561815	0.013736307	0.076497969	KMT5A
12	123383773	123409358	+	4579
protein_coding	lysine methyltransferase 5A [Source:HGNC
Symbol;Acc:HGNC:29489]	-	673	999	1127	609	841
665	8.037835863	9.618975148	11.40133557
6.430546242	8.003573729	6.779490205
ENSG00000122390 590.0504546	813.8631017	787.127725
1068.871228	933.9647947	980.2343039	730.3470938
994.3567756	-0.443942754	0.013742758	0.076506022	NAA60
16	3443611 3486963 +	7397
protein_coding	"N(alpha)-acetyltransferase 60, NatF catalytic subunit
[Source:HGNC Symbol;Acc:HGNC:25875]"	-	510	878	805
1064	1022	999	3.770589642	5.233266545
5.041302032	6.954841321	6.020795479	6.304576304
ENSG00000178297 46.27846703	62.10572644	87.02405904
229.0438346	96.86914701	96.15912091	65.13608417
140.6907008	-1.107732583	0.013759112	0.076569184
TMPRSS9 19	2360238 2426239 +	4328	protein_coding
transmembrane serine protease 9 [Source:HGNC Symbol;Acc:HGNC:30079]
-	40	67	89	228	106	98
0.50543749	0.682529628	0.952588228	2.547116513
1.067277149	1.057024027
ENSG00000279968 21.98227184	12.05036483	19.55596832	0
0.913859877	4.906077597	17.86286833	1.939979158
3.203558006	0.01376074	1	GVQW2	6
138725211	138773652	-	2993	protein_coding	GVQW
motif containing 2 [Source:HGNC Symbol;Acc:HGNC:51715]	-	19
13	20	0	1	5	0.347169526
0.1915008	0.309546384	0	0.014559682	0.077984685
ENSG00000096433 2758.196635	2748.410133	3020.419308
2304.502441	1476.797562	2480.512833	2842.342025
2087.270945	0.445703703	0.013795411	0.076743251	ITPR3
6	33620365	33696574	+	9870
protein_coding	"inositol 1,4,5-trisphosphate receptor type 3
[Source:HGNC Symbol;Acc:HGNC:6182]"	-	2384	2965	3089
2294	1616	2528	13.20942189	13.24468101
14.49783696	11.23770112	7.134816393	11.95655188
ENSG00000119950 853.8377167	864.8454144	463.4764493
964.3950931	1156.032745	1075.412209	727.3865268
1065.280016	-0.550928071	0.013819032	0.076846687	MXI1
10	110207605	110287365	+	4607
protein_coding	"MAX interactor 1, dimerization protein [Source:HGNC
Symbol;Acc:HGNC:7534]"	bHLH	738	933	474	960	1265
1096	8.760580049	8.928888289	4.766093799
10.07521302	11.96550165	11.10550668
ENSG00000240184 1519.09068	1120.683929	1868.572773
1076.907854	1079.268515	1120.548123	1502.782461

1092.241497	0.46016208	0.01384545	0.0769656
PCDHGC3 5	141475947	141512979	+	5432
protein_coding	"protocadherin gamma subfamily C, 3 [Source:HGNC
Symbol;Acc:HGNC:8716]"	-	1313	1209	1911	1072	1181
	1142	13.21903272	9.812970597	16.29683924
9.541930315	9.474334942	9.814142927
ENSG00000053254 624.7593049	671.112626	672.7253103
910.1478692	867.2530237	823.2398208	656.1990804
866.8802379	-0.401299894	0.013859881	0.077002702	FOXN3
	14	89124871	89619149	-	11033
protein_coding	forkhead box N3 [Source:HGNC Symbol;Acc:HGNC:1928]
	Fork	540	724	688	906	949	839
2.676670142	2.893202995	2.888665642	3.970414022
3.748272805	3.549885771
ENSG00000135506 1147.705982	1119.756978	1554.699482
818.7312509	719.2077235	1151.94702	1274.054147
896.6286648	0.507184279	0.013862199	0.077002702	OS9
	12	57693955	57721557	+	5169
protein_coding	"OS9, endoplasmic reticulum lectin [Source:HGNC
Symbol;Acc:HGNC:16994]" -	992	1208	1590	815	787
	1174	10.49542073	10.3037274	14.24928418
7.623462934	6.634784142	10.60248339
ENSG00000234841 2.313923351	1.853902282	0.977798416
19.08698622	9.138598774	17.66187935	1.715208016
15.29582145	-3.16639379	0.013868883	1	AC017007.2
	4	112826307	112827607	+	1301
processed_pseudogene	"TAF4b RNA polymerase II, TATA box binding
protein (TBP)-associated factor, 105kDa (TAF4B) pseudogene"	-
	2	2	1	19	10	18	0.084071232
	0.067777673	0.035606162	0.706118388	0.334951017
	0.645864247
ENSG00000082153 7958.739367	6361.66568	6488.67029
5011.84075	5899.879369	5571.34172	6936.358446
5494.353946	0.336024739	0.013869712	0.077016448	BZW1
	2	200810594	200827338	+	8085
protein_coding	basic leucine zipper and W2 domains 1 [Source:HGNC
Symbol;Acc:HGNC:18380]	-	6879	6863	6636	4989	6456
	5678	46.53074413	37.4255277	38.0214617
29.83559452	34.7970234	32.78395914
ENSG00000205097 276.5138405	379.1230166	248.3607977
344.5703302	562.0238246	493.5514063	301.3325516
466.715187	-0.630697771	0.013888555	0.077093077	FRG2
	4	190024351	190027257	-	2103
protein_coding	FSHD region gene 2 [Source:HGNC Symbol;Acc:HGNC:19136]
	-	239	409	254	343	615	503
6.215174706	8.574681417	5.594958889	7.885987128
12.74366777	11.16541188
ENSG00000085644 396.8378548	468.1103261	483.0324176
625.8522323	666.2038506	572.0486479	449.3268662
621.3682436	-0.466895108	0.013927852	0.07728315	ZNF213

16 3129777 3142805 + 6443 protein_coding zinc
finger protein 213 [Source:HGNC Symbol;Acc:HGNC:13005] zf-C2H2 343
505 494 623 729 583 2.911392115
3.455709324 3.551740875 4.675210093 4.930579829
4.224024799
ENSG00000272335 203.6252549 172.4129122 135.9139798
308.4055142 243.0867274 263.9469747 170.6507157
271.8130721 -0.672949932 0.013933919 0.077288761
AC093297.2 5 44826076 44828592 + 2517
lincRNA novel transcript - 176 186 139
307 266 269 3.824055309 3.258094574
2.558197325 5.897342845 4.605290376 4.989018338
ENSG00000236045 27.76708022 8.342560268 2.933395248
72.32963199 27.41579632 50.04199149 13.01434524
49.92913993 -1.955775149 0.013952281 0.077362538
AL031283.3 1 15334166 15335464 - 727
antisense novel transcript - 24 9
3 72 30 51 1.805392124 0.545810714
0.191156601 4.788497908 1.79823084 3.274777525
ENSG00000172845 2722.330823 1859.463989 2170.712484
1688.695991 1790.2515 1641.573564 2250.835765
1706.840352 0.398556152 0.013991493 0.077551835 SP3
2 173906459 173965702 - 10704
protein_coding Sp3 transcription factor [Source:HGNC
Symbol;Acc:HGNC:11208] zf-C2H2 2353 2006 2220 1681 1959
1673 12.02182881 8.262639146 9.607476477
7.593164865 7.975298252 7.296185656
ENSG00000240857 306.5948441 366.1457006 346.1406393
531.4218795 417.633964 510.2320701 339.6270613
486.4293045 -0.516980624 0.01400081 0.07757535 RDH14
2 18554723 18560680 - 1587
protein_coding retinol dehydrogenase 14 [Source:HGNC
Symbol;Acc:HGNC:19979] - 265 395 354 529 457
520 9.131952838 10.97372637 10.33305619
16.1168425 12.54867498 15.29580903
ENSG00000272072 12.72657843 6.488657986 15.64477466
80.36625776 16.44947779 35.3237587 11.62000369
44.04649809 -1.924251169 0.014008211 0.077588231
AC004492.1 7 107192559 107193300 - 742
antisense "novel transcript, antisense to HBP1" -
11 7 16 80 18 36 0.810743532
0.415937515 0.998891996 5.212994878 1.057127079
2.264877051
ENSG00000082438 414.1922799 261.4002217 421.4311174
254.1582902 220.2402305 232.5480781 365.6745397
235.6488662 0.633116865 0.014014379 0.077594283 COBLL1
2 164653624 164843679 - 17178
protein_coding cordon-bleu WH2 repeat protein like 1 [Source:HGNC
Symbol;Acc:HGNC:23571] - 358 282 431 253 241
237 1.139738296 0.723786482 1.162270266

0.712113369	0.611368477	0.644053261
ENSG00000166913 11942.15842	10253.93352	10105.54663
8488.685976	9119.407717	8644.508727	10767.21286
8750.867473	0.299036807	0.014079701	0.077927729	YWHAB
	20	44885676	44908532	+	7933
protein_coding	tyrosine 3-monooxygenase/tryptophan 5-monooxygenase
activation protein beta [Source:HGNC Symbol;Acc:HGNC:12849]	-
	10322	11062	10335	8450	9979	8810	71.15757074
	61.47947608	60.34975099	51.50157032	54.8161041
	51.84232734
ENSG00000142534 14768.61579	21559.02963	19071.95811
19735.94375	23477.97411	31688.3552	18466.53451
24967.42435	-0.435083765	0.014094339	0.077980515	RPS11
	19	49496365	49499689	+	1390
protein_coding	ribosomal protein S11 [Source:HGNC
Symbol;Acc:HGNC:10384]	-	12765	23258	19505	19646	25691
	32295	502.2277803	737.7199428	650.0303138
683.3773116	805.4244452	1084.592319
ENSG00000174842 595.835263	555.2437334	541.7003226
321.465031	504.4506523	323.8011214	564.259773
383.2389349	0.557024549	0.014109712	0.078037324	GLMN
	1	92246402	92298987	-	2910
protein_coding	"glomulin, FKBP associated protein [Source:HGNC
Symbol;Acc:HGNC:14373]" -	515	599	554	320	552
	330	9.678520021	9.075442121	8.818997745
5.316896494	8.266176735	5.293793383
ENSG00000259039 0	0	0	15.06867333	4.569299387
	3.924862078	0	7.854278265	-5.392104452
0.014117666	1	AL161804.1	14	57578409
57600404	+	1430	antisense	"novel transcript,
antisense to SLC35F4"	-	0	0	0	15	5
	4	0	0	0	0.507173365	0.152367578
	0.130577993
ENSG00000168282 9.255693406	45.4206059	32.26734773
67.30674088	73.10879019	94.19668987	28.98121568
78.20407365	-1.422389724	0.014135628	0.078147526	MGAT2
	14	49620795	49623481	+	2687
protein_coding	"mannosyl (alpha-1,6-)-glycoprotein beta-1,2-N-
acetylglucosaminyltransferase [Source:HGNC Symbol;Acc:HGNC:7045]"
	-	8	49	33	67	80	96
0.16282348	0.804011705	0.568916762	1.205614195
1.297420986	1.667821632
ENSG00000165632 365.5998895	478.3067887	572.9898719
724.3008981	664.3761309	632.8840101	472.29885
673.8536797	-0.511360811	0.014142786	0.078147526	TAF3
	10	7818504 8016627 +	4872	protein_coding	TATA-
box binding protein associated factor 3 [Source:HGNC
Symbol;Acc:HGNC:17303]	-	316	516	586	721	727
	645	3.547108849	4.669564515	5.571765016
7.155322316	6.502580375	6.180142241

ENSG00000101335 852.680755 1192.986118 1050.155499
830.7861896 716.4661439 736.8928551 1031.940791
761.3817295 0.439623092 0.014144973 0.078147526 MYL9
20 36541484 36551447 + 2821
protein_coding myosin light chain 9 [Source:HGNC
Symbol;Acc:HGNC:15754] - 737 1287 1074 827 784
751 14.28759445 20.11450821 17.6361446
14.17436591 12.11076492 12.42747467
ENSG00000166317 13.88354011 9.269511408 0.977798416 0
0 0 8.043616644 0 5.498028297
0.014165205 1 SYNPO2L 10 73644881 73663803
- 5147 protein_coding synaptopodin 2 like
[Source:HGNC Symbol;Acc:HGNC:23532] - 12 10 1
0 0 0 0.127503407 0.085660339
0.00900012 0 0 0
ENSG00000236453 111.0683209 124.2114529 29.33395248
259.1811813 157.1838989 156.0132676 88.20457541
190.7927826 -1.113634343 0.014171179 0.078264025
AC003092.1 7 94022833 94066661 + 1064
lincRNA novel transcript - 96 134 30
258 172 159 4.934285994 5.552609456
1.306117001 11.72409407 7.044422843 6.975920654
ENSG00000134851 1318.93631 1376.522444 1702.347043
956.3584674 1089.320974 1249.087356 1465.935266
1098.255599 0.416789981 0.014181173 0.078290931
TMEM165 4 55395957 55453397 + 7045
protein_coding transmembrane protein 165 [Source:HGNC
Symbol;Acc:HGNC:30760] - 1140 1485 1741 952 1192
1273 8.849496598 9.293502373 11.44775245
6.533669577 7.373163351 8.435162277
ENSG00000162631 242.9619519 88.98730952 94.84644637
27.12361199 55.74545252 95.17790539 142.2652359
59.34898997 1.25826874 0.014207513 0.078408031 NTNG1
1 107140007 107483458 + 9138
protein_coding netrin G1 [Source:HGNC Symbol;Acc:HGNC:23319] -
210 96 97 27 61 97 1.256790397
0.463184521 0.491725846 0.142861046 0.290895685
0.495526194
ENSG00000267500 35.86581195 23.17377852 44.97872714
6.027469332 6.397019142 14.71823279 34.67277254
9.047573755 1.938744003 0.014222185 0.078460678
ZNF887P 19 11648364 11652877 + 1407
transcribed_unprocessed_pseudogene "zinc finger protein 887,
pseudogene [Source:HGNC Symbol;Acc:HGNC:38700]" - 31 25
46 6 7 15 1.204931364 0.783393329
1.514489233 0.206185618 0.216801629 0.497671989
ENSG00000224078 113.3822442 111.2341369 131.0249878
264.2040724 170.8917971 172.6939314 118.547123
202.5966003 -0.772297513 0.014256663 0.078622513 SNHG14
15 24978583 25419462 + 43324

processed_transcript   small nucleolar RNA host gene 14 [Source:HGNC Symbol;Acc:HGNC:37462] -   98   120   134   263   187
        176   0.123706421   0.122119961   0.143277735
0.293513728   0.188092577   0.189640092
ENSG00000189157 8.09873173   8.342560268   5.866790497   0
        0   0   7.436027498   0   5.387192528
0.014260085   1   FAM47E 4   76214040   76283780
        +   9508   protein_coding family with sequence similarity 47 member E [Source:HGNC Symbol;Acc:HGNC:34343]   -
        7   9   6   0   0   0   0.040262763
        0.041733739   0.029232404   0   0   0
ENSG00000138944 1036.637661   1068.774665   1605.544999
818.7312509   997.0211263   841.8829157   1236.985775
885.878431   0.481758044   0.01427095   0.078672925
SHISAL1 22   44243667   44312851   -   6751
protein_coding shisa like 1 [Source:HGNC Symbol;Acc:HGNC:29335]
        -       896   1153   1642   815   1091   858
7.258294985   7.53000314   11.26697941   5.837013762
7.042311649   5.932875238
ENSG00000177410 1034.323738   1004.815037   881.9741714
1000.559909   1398.205612   1647.460857   973.7043154
1348.742126   -0.470349029   0.0142851   0.078722542   ZFAS1
        20   49278178   49295738   +   2243
antisense   ZNFX1 antisense RNA 1 [Source:HGNC Symbol;Acc:HGNC:33101] -   894   1084   902   996   1530
        1679   21.79731287   21.3075721   18.62857865
21.46996227   29.72492414   34.94358496
ENSG00000108468 2263.017038   2411.926868   2724.146387
1889.611636   1988.559093   1991.867505   2466.363431
1956.679411   0.334141582   0.01429289   0.078733233   CBX1
        17   48070052   48101521   -   3087
protein_coding chromobox 1 [Source:HGNC Symbol;Acc:HGNC:1551] -
        1956   2602   2786   1881   2176   2030   34.65189053
        37.1624742   41.80680112   29.46140013   30.71714581
        30.6976722
ENSG00000104081 13.88354011   18.53902282   44.97872714
64.29300621   91.38598774   54.94806909   25.80043002
70.20902101   -1.440163212   0.014299832   0.078733233   BMF
        15   40087890   40108892   -   5447
protein_coding Bcl2 modifying factor [Source:HGNC Symbol;Acc:HGNC:24132] -   12   20   46   64   100
        56   0.120481006   0.161884988   0.391203663
0.568098726   0.800020697   0.479928662
ENSG00000183735 1072.503473   869.4801701   916.1971159
671.0582523   710.9829846   762.4044586   952.7269198
714.8152319   0.413792693   0.014302492   0.078733233   TBK1
        12   64451880   64502108   +   4762
protein_coding TANK binding kinase 1 [Source:HGNC Symbol;Acc:HGNC:11584] -   927   938   937   668   778
        777   10.64596553   8.684551707   9.114915684

6.782476347	7.119488677	7.616889646
ENSG00000147041 105.2835125	50.98231275	75.29047804
40.18312888	31.98509571	24.53038799	77.18543443
32.23287086	1.257436055	0.014338089	0.078900783	SYTL5
X	38006582	38128819	+	4791
protein_coding	synaptotagmin like 5 [Source:HGNC
Symbol;Acc:HGNC:15589]	-	91	55	77	40	35
25	1.038747363	0.506139785	0.744503957
0.403677959	0.318346787	0.243590235
ENSG00000162825 160.8176729	144.604378	162.3145371
309.4100924	210.1877718	231.5668626	155.912196
250.3882423	-0.683361108	0.014347511	0.078924215	NBPF20
1	145289900	145405778	-	18995
protein_coding	NBPF member 20 [Source:HGNC Symbol;Acc:HGNC:32000]
-	139	156	166	308	230	236
0.40019367	0.362092274	0.404828655	0.783993812
0.52765145	0.579987643
ENSG00000132622 5.784808379	20.3929251	31.28954932
56.25638043	49.34843338	69.66630188	19.15576093
58.42370523	-1.598845481	0.014373323	0.079012466
HSPA12B 20	3732667 3753111 +	3151	protein_coding	heat
shock protein family A (Hsp70) member 12B [Source:HGNC
Symbol;Acc:HGNC:16193]	-	5	22	32	56	54
71	0.086779334	0.307828081	0.470439772
0.859292142	0.74679939	1.051855256
ENSG00000004897 4227.537963	3212.812654	3980.617352
3190.540433	2981.92478	2767.027765	3806.989323
2979.830993	0.353203294	0.014373891	0.079012466	CDC27
17	47117703	47189422	-	6824
protein_coding	cell division cycle 27 [Source:HGNC
Symbol;Acc:HGNC:1728]	-	3654	3466	4071	3176	3263
2820	29.2835846	22.39357843	27.63532269
22.50311772	20.83707013	19.29106152
ENSG00000140675 3.470885027	10.19646255	5.866790497
68.3113191	21.93263706	8.830939675	6.511379358
33.02496528	-2.32997264	0.014382658	0.07903224	SLC5A2
16	31483002	31490860	+	4468
protein_coding	solute carrier family 5 member 2 [Source:HGNC
Symbol;Acc:HGNC:11037]	-	3	11	6	68	24
9	0.036720011	0.108545914	0.062207184
0.735862997	0.234075886	0.094031937
ENSG00000137252 9.255693406	2.780853423	10.75578258	0
0	0	7.597443135	0	5.416677039
0.014414757	1	HCRTR2	6	55106460	55282620
+	1964	protein_coding	hypocretin receptor 2
[Source:HGNC Symbol;Acc:HGNC:4849]	-	8	3	11
0	0	0	0.222763081	0.067346298
0.25944999	0	0	0
ENSG00000078124 1074.817397	882.4574861	968.9982304
738.3649932	780.4363353	688.8132947	975.4243711

735.8715411	0.405867912	0.01444808	0.079363203	ACER3
	11	76860867	77026797	+	8442
protein_coding	alkaline ceramidase 3 [Source:HGNC
Symbol;Acc:HGNC:16066]	-	929	952	991	735	854
	702	6.018178695	4.971936331	5.43789431
4.20962304	4.408299782	3.881841513
ENSG00000137857 9.255693406	26.88158308	29.33395248
92.42119643	29.24351608	74.57237948	21.82374299
65.41236399	-1.574539773	0.014470426	0.0794574	DUOX1
	15	45129933	45165576	+	8784
protein_coding	dual oxidase 1 [Source:HGNC Symbol;Acc:HGNC:3062]
	-	8	29	30	92	32	76
0.049807228	0.14555919	0.158209072	0.506403521
0.158750919	0.403893906
ENSG00000233077 6.941770054	7.415609127	7.822387329	0
	0	0	7.393255503	0	5.379395446
0.014485959	1	LINC01271	20	50310711
50321342	-	2250	lincRNA long intergenic non-protein
coding RNA 1271 [Source:HGNC Symbol;Acc:HGNC:50327]	-	6
	8	8	0	0	0	0.145835564
0.156762228	0.164706191	0	0	0
ENSG00000008226 5.784808379	18.53902282	12.71137941	0
	0	1.962431039	12.3450702	0.65414368
4.253193383	0.014526721	1	DLEC1	3	38039205
	38124025	+	8338	protein_coding	"DLEC1, cilia
and flagella associated protein [Source:HGNC Symbol;Acc:HGNC:2899]"
	-	5	20	13	0	0	2
0.032794637	0.105755281	0.072224396	0	0
0.011197321
ENSG00000279140 0	18.53902282	8.800185745	0	0
	0	9.113069521	0	5.684948787	0.014538494
	1	AL590326.1	14	104815449	104815753
	-	305	TEC	novel transcript	-	0
	20	9	0	0	0	0	2.891106661
	1.366926383	0	0	0
ENSG00000033170 1019.283236	915.8277272	1074.600459
592.701151	893.7549601	695.6818033	1003.237141
727.3793048	0.463245707	0.014543248	0.079804431	FUT8
	14	65410592	65744121	+	6700
protein_coding	fucosyltransferase 8 [Source:HGNC
Symbol;Acc:HGNC:4019]	-	881	988	1099	590	978
	709	7.191108119	6.501537919	7.59845587
4.257733018	6.360959784	4.939892161
ENSG00000258770 9.255693406	3.707804563	4.888992081
47.21517644	38.38211485	5.887293117	5.950830017
30.49486147	-2.370336169	0.014544067	0.079804431
LINC02330	14	87634379	87655294	-	555
	lincRNA long intergenic non-protein coding RNA 2330
[Source:HGNC Symbol;Acc:HGNC:53250]	-	8	4	5
	47	42	6	0.788300345	0.317761273

0.417329877	4.094549175	3.297728557	0.504666298
ENSG00000115221 3.470885027	9.269511408	9.777984162	0
	0	0	7.506126866	0	5.403631751
0.014549766	1	ITGB6	2	160099666	160200313
	-	4687	protein_coding	integrin subunit beta 6
[Source:HGNC Symbol;Acc:HGNC:6161]	-	3	10	10
	0	0	0	0.035004269	0.094067371
0.098834257	0	0	0
ENSG00000139651 638.642845	697.9942091	748.9935868
1106.040622	810.5937113	915.4740797	695.2102136
944.0361378	-0.44066635	0.014550496	0.079811064	ZNF740
	12	53180700	53195141	+	9063
protein_coding	zinc finger protein 740 [Source:HGNC
Symbol;Acc:HGNC:27465]	zf-C2H2 552	753	766	1101	887
	933	3.330901656	3.663168991	3.915247721
5.873764843	4.264913601	4.805689416
ENSG00000267011 2.313923351	2.780853423	2.933395248
22.10072088	3.65543951	34.34254318	2.676057341
20.03290119	-2.898675609	0.014573318	1	AC011498.1
	19	4457962 4471493 -	837	lincRNA novel
transcript	-	2	3	3	22	4	35
	0.130677029	0.158026439	0.166034467	1.270862132
	0.208253894	1.952039593
ENSG00000099968 1176.630024	1272.703916	1127.401574
1368.235538	1668.708136	1583.681848	1192.245171
1540.208508	-0.369461108	0.014579631	0.079915682
BCL2L13 22	17628855	17730855	+	7662
protein_coding	BCL2 like 13 [Source:HGNC Symbol;Acc:HGNC:17164]
	-	1017	1373	1153	1362	1826	1614
7.258945204	7.900641352	6.970912243	8.594807941
10.3852564	9.83348407
ENSG00000163629 581.9517229	482.0145932	624.8131879
449.0464652	399.3567664	335.5757077	562.9265014
394.6596464	0.512064	0.0145933	0.079915682	PTPN13
	4	86594315	86815171	+	9496
protein_coding	"protein tyrosine phosphatase, non-receptor type 13
[Source:HGNC Symbol;Acc:HGNC:9646]"	-	503	520	639
	447	437	342	2.896823212	2.414329804
3.117185217	2.275977863	2.005392234	1.681246665
ENSG00000160062 217.508795	119.5766972	199.4708769
40.18312888	104.180026	118.7270779	178.852123
87.69674425	1.025903301	0.014594653	0.079915682	ZBTB8A
	1	32539427	32605939	+	7075
protein_coding	zinc finger and BTB domain containing 8A [Source:HGNC
Symbol;Acc:HGNC:24172]	ZBTB	188	129	204	40	114
	121	1.453202438	0.803891107	1.335691552
0.273359873	0.702161487	0.798371384
ENSG00000172315 1109.526247	949.1979682	870.2405904
1393.349994	1534.370734	1056.769114	976.3216019
1328.163281	-0.444674524	0.014598978	0.079915682	TP53RK

20	46684365	46689779	-	3520
protein_coding	TP53 regulating kinase [Source:HGNC
Symbol;Acc:HGNC:16197]	-	959	1024	890	1387	1679
1077	14.89946439	12.82600046	11.71250526
19.05175615	20.78579455	14.28298814
ENSG00000112851 2444.660021	2236.733103	2237.202776
1891.620792	1913.622583	1706.333788	2306.198633
1837.192388	0.327773812	0.014600578	0.079915682	ERBIN
5	65926475	66082549	+	10216
protein_coding	erbb2 interacting protein [Source:HGNC
Symbol;Acc:HGNC:15842]	-	2113	2413	2288	1883	2094
1739	11.31132096	10.41382789	10.37474884
8.911907133	8.932116746	7.94629591
ENSG00000145012 3320.480009	2422.123331	3214.023394
2229.159075	2213.368623	2466.775816	2985.542245
2303.101171	0.374154808	0.014600935	0.079915682	LPP
3	188153284	188890671	+	25535
protein_coding	LIM domain containing preferred translocation partner
in lipoma [Source:HGNC Symbol;Acc:HGNC:6679]	-	2870	2613
3287	2219	2422	2514	6.146682027	4.511671862
5.963020435	4.201676934	4.133299489	4.595951605
ENSG00000127580 475.5112487	602.5182416	604.2794212
817.7266727	711.8968445	766.3293207	560.7696372
765.3176126	-0.447404739	0.014642372	0.080113795	WDR24
16	684622	690444	-	3651	protein_coding	WD
repeat domain 24 [Source:HGNC Symbol;Acc:HGNC:20852]	-	411
650	618	814	779	781	6.156369837
7.8493823	7.841138012	10.77987658	9.297886116
9.985854567
ENSG00000135637 105.2835125	116.7958437	142.7585688
214.9797395	186.427415	201.1491815	121.6126417
200.852112	-0.722266335	0.014684394	0.080301744
CCDC142 2	74471986	74483408	-	4897
protein_coding	coiled-coil domain containing 142 [Source:HGNC
Symbol;Acc:HGNC:25889]	-	91	126	146	214	204
205	1.016262735	1.13442137	1.381100262
2.112928912	1.815342859	1.954203527
ENSG00000204899 2869.264956	2819.78537	2506.097341
1742.943215	2529.564141	1969.299548	2731.715889
2080.602301	0.39250977	0.01468723	0.080301744	MZT1
13	72708357	72727687	-	2299
protein_coding	mitotic spindle organizing protein 1 [Source:HGNC
Symbol;Acc:HGNC:33830]	-	2480	3042	2563	1735	2768
2007	58.99394274	58.33835735	51.64307465
36.48898008	52.46693715	40.75251698
ENSG00000072858 2.313923351	18.53902282	2.933395248	0
0	0	7.928780472	0	5.484343532
0.01474011	1	SIDT1	3	113532296	113629578
+	8060	protein_coding	SID1 transmembrane family
member 1 [Source:HGNC Symbol;Acc:HGNC:25967]	-	2	20

3 0 0 0 0.013570307 0.10940292
0.017242041 0 0 0
ENSG00000100097 8450.448079 11256.89465 12638.04453
6384.094601 7381.24623 10072.17731 10781.79575
7945.839379 0.440404004 0.01474202 0.080572489 LGALS1
22 37675608 37679806 + 1099
protein_coding galectin 1 [Source:HGNC Symbol;Acc:HGNC:6561] -
7304 12144 12925 6355 8077 10265 363.460973
487.1896171 544.7977624 279.5883551 320.2661125
436.0208901
ENSG00000122299 1347.860352 1073.409421 1156.735526
1032.706412 798.7135329 839.9204847 1192.668433
890.4468099 0.421323933 0.014759696 0.08064027 ZC3H7A
16 11750586 11797267 - 7076
protein_coding zinc finger CCCH-type containing 7A [Source:HGNC
Symbol;Acc:HGNC:30959] - 1165 1158 1183 1028 874
856 9.003944593 7.215304986 7.744606854
7.024355888 5.38247729 5.647184492
ENSG00000124659 572.6960295 654.4275054 532.9001368
651.9712661 861.7698644 935.0983901 586.6745572
816.2798402 -0.476386661 0.014796045 0.080781087 TBCC
6 42744481 42746096 - 1616
protein_coding tubulin folding cofactor C [Source:HGNC
Symbol;Acc:HGNC:11580] - 495 706 545 649 943
953 16.75168721 19.26181922 15.62275426
19.41800269 25.42897964 27.52945286
ENSG00000053372 3564.598923 3926.565033 3669.677456
4407.08466 4791.367337 4501.816803 3720.28047
4566.756267 -0.295674371 0.014797604 0.080781087 MRTO4
1 19251539 19260128 + 2822
protein_coding "MRT4 homolog, ribosome maturation factor [Source:HGNC
Symbol;Acc:HGNC:18477]" - 3081 4236 3753 4387 5243
4588 59.70757071 66.18093522 61.60614175
75.16433881 80.96204065 75.89487249
ENSG00000163964 890.8604903 937.1476034 1035.488523
731.3329456 784.0917748 634.8464411 954.4988721
716.7570539 0.413338508 0.014801323 0.080781087 PIGX
3 196639775 196736007 + 4446
protein_coding phosphatidylinositol glycan anchor biosynthesis class
X [Source:HGNC Symbol;Acc:HGNC:26046] - 770 1011 1059
728 858 647 9.471439283 10.02572193
11.03389781 7.917045437 8.409621068 6.793301004
ENSG00000109452 541.4580642 286.4279025 356.8964219
308.4055142 217.4986508 206.0552591 394.9274629
243.9864747 0.693538822 0.014807149 0.08078404 INPP4B
4 142023160 142847432 - 14153
protein_coding inositol polyphosphate-4-phosphatase type II B
[Source:HGNC Symbol;Acc:HGNC:6075] - 468 309 365
307 238 210 1.808389843 0.96259573
1.194666852 1.048796152 0.732802679 0.692654761

ENSG00000184588 252.2176453 107.5263323 188.7150943
84.38457065 110.5770452 98.12155195 182.8196907
97.69438925 0.90105481 0.014818018 0.080814498 PDE4B
1 65792514 66374579 + 8644
protein_coding phosphodiesterase 4B [Source:HGNC
Symbol;Acc:HGNC:8781] - 218 116 193 84 121
100 1.379229216 0.591666784 1.034296385
0.469857046 0.609999122 0.540046651
ENSG00000134186 2444.660021 2402.657357 2733.924372
2162.856912 1824.978175 2015.416677 2527.080583
2001.083921 0.336871818 0.01483891 0.080899579
PRPF38B 1 108692323 108701803 + 5817
protein_coding pre-mRNA processing factor 38B [Source:HGNC
Symbol;Acc:HGNC:25512] - 2113 2592 2796 2153 1997
2054 19.86530082 19.64580782 22.26591563
17.89559665 14.96020686 16.48342329
ENSG00000282849 12.72657843 8.342560268 1.955596832 0
0 0 7.674911844 0 5.430375352
0.014865933 1 AL359834.1 1 200478020
200483604 - 638 lincRNA novel transcript -
11 9 2 0 0 0 0.942902352
0.621950453 0.145215098 0 0 0
ENSG00000176912 31.23796524 32.44328993 58.66790497
5.02289111 19.19105743 9.812155195 40.78305338
11.34203458 1.841542276 0.0148682 0.081024336 TYMSOS
18 641320 658340 - 976
bidirectional_promoter_lncRNA TYMS opposite strand [Source:HGNC
Symbol;Acc:HGNC:29553] - 27 35 60 5 21
10 1.512894553 1.581073955 2.847763298
0.247697374 0.937622617 0.478295416
ENSG00000148110 1834.941218 1736.179487 1727.769801
1465.679626 1386.325434 1358.983494 1766.296835
1403.662851 0.33141892 0.014872394 0.081024336
MFSD14B 9 94374551 94461042 + 3428
protein_coding major facilitator superfamily domain containing 14B
[Source:HGNC Symbol;Acc:HGNC:23376] - 1586 1873 1767
1459 1517 1385 25.30212998 24.08967396
23.87801343 20.57859382 19.28427719 18.86057791
ENSG00000100983 1379.098317 1424.723903 1532.210118
1188.416037 1135.927828 1071.487347 1445.344113
1131.943737 0.35280107 0.014879643 0.081034952 GSS
20 34928433 34956027 - 6174
protein_coding glutathione synthetase [Source:HGNC
Symbol;Acc:HGNC:4624] - 1192 1537 1567 1183 1243
1092 10.55855151 10.97592676 11.75722494
9.264443476 8.773302446 8.256615282
ENSG00000250990 6.941770054 9.269511408 5.866790497 0
0 0 7.35935732 0 5.373076698
0.01493829 1 AC098851.1 7 77246340
77258123 - 570 antisense novel transcript

- 6 10 6 0 0 0
0.575666699 0.773497835 0.487617014 0 0 0
ENSG00000115226 220.9796801 243.78815 149.6031577
236.0758822 438.6527412 337.5381387 204.7903293
337.422254 -0.721187281 0.014965791 0.081472869 FNDC4
2 27491883 27495245 - 1982
protein_coding fibronectin type III domain containing 4 [Source:HGNC
Symbol;Acc:HGNC:20239] - 191 263 153 235 480
344 5.270167638 5.85040668 3.575940109
5.732782019 10.55349199 8.102160242
ENSG00000173409 694.1770054 659.0622611 619.9241958
375.712255 507.192232 526.912734 657.7211541
469.9390737 0.48439394 0.014972918 0.081472869 ARV1
1 230978981 231000595 + 1987
protein_coding "ARV1 homolog, fatty acid homeostasis modulator
[Source:HGNC Symbol;Acc:HGNC:29561]" - 600 711 634
374 555 537 16.5138409 15.77631945
14.78066067 9.100703212 12.17176934 12.61602249
ENSG00000232098 489.3947888 611.787753 535.8335321
1029.692678 608.6306784 729.043131 545.6720246
789.1221623 -0.531186437 0.014976043 0.081472869
AC012313.1 19 58404238 58408484 - 3417
lincRNA novel transcript - 423 660 548
1025 666 743 6.770022333 8.51594631
7.429131326 14.50374325 8.493522627 10.15055692
ENSG00000224715 2.313923351 2.780853423 0 30.13734666
11.88017841 7.849724156 1.698258925 16.62241641
-3.295057699 0.015000027 1 Z82186.1 22
47345569 47373541 + 4364 lincRNA
uncharacterized LOC339685 [Source:NCBI gene;Acc:339685] - 2
3 0 30 13 8 0.025063399
0.030308921 0 0.332382178 0.129812708 0.085575862
ENSG00000228748 11.56961676 6.488657986 18.57816991 0
0 1.962431039 12.21214822 0.65414368
4.234023162 0.015005305 1 AL450306.1 10
77782866 77793176 + 3532 antisense novel
transcript - 10 7 19 0 0 2
0.15483674 0.087379852 0.249192726 0 0
0.026433541
ENSG00000090857 1276.128728 1238.406724 1340.561629
1812.259113 1445.726326 1708.296219 1285.03236
1655.427219 -0.365219702 0.015010676 0.081632234 PDPR
16 70113626 70162537 + 10385
protein_coding pyruvate dehydrogenase phosphatase regulatory subunit
[Source:HGNC Symbol;Acc:HGNC:30264] - 1103 1336 1371
1804 1582 1741 5.808496397 5.671969871
6.115520266 8.399070929 6.638325997 7.825972295
ENSG00000173988 21.98227184 56.54401959 22.48936357
2.009156444 12.79403828 7.849724156 33.671885
7.550972961 2.152865956 0.015059742 0.081869944 LRRC63

13	46211943	46277366	+	2677
protein_coding	leucine rich repeat containing 63 [Source:HGNC
Symbol;Acc:HGNC:34296]	-	19	61	23	2	14
8	0.388150315	1.004651465	0.397998945
0.036122919	0.227896818	0.139504318
ENSG00000180185 882.7617586	862.9915121	778.3275393
952.3401545	1333.321561	1103.867459	841.36027
1129.843058	-0.425824549	0.015087754	0.081967878	FAHD1
16	1826941 1840207 +	3039	protein_coding
fumarylacetoacetate hydrolase domain containing 1 [Source:HGNC
Symbol;Acc:HGNC:14169]	-	763	931	796	948	1459
1125	13.7305695	13.50681461	12.13346449
15.0826917	20.92103613	17.28095973
ENSG00000171408 127.2657843	104.7454789	141.7807703
78.35710132	57.57317228	62.79779325	124.5973445
66.24268895	0.911877707	0.015088481	0.081967878	PDE7B
6	135851696	136195574	+	5946
protein_coding	phosphodiesterase 7B [Source:HGNC
Symbol;Acc:HGNC:8792]	-	110	113	145	78	63
64	1.011725026	0.837890944	1.129654283
0.634265245	0.461715275	0.50245955
ENSG00000086717 46.27846703	16.68512054	48.88992081
17.07782977	6.397019142	3.924862078	37.28450279
9.133236998	2.031327303	0.015094563	0.081971786	PPEF1
X	18675909	18827921	+	3513
protein_coding	protein phosphatase with EF-hand domain 1 [Source:HGNC
Symbol;Acc:HGNC:9243]	-	40	18	50	17	7
4	0.622696686	0.225906285	0.659317055
0.233976364	0.086831737	0.053153012
ENSG00000164111 12984.58089	9827.535995	10394.97496
8035.621198	8378.267356	9716.977289	11069.03061
8710.288614	0.345629783	0.015109393	0.081998999	ANXA5
4	121667955	121697113	-	3034
protein_coding	annexin A5 [Source:HGNC Symbol;Acc:HGNC:543]	-
11223	10602	10631	7999	9168	9903	202.2963743
154.065778	162.3158751	127.4739187	131.6793354
152.3692179
ENSG00000232059 0	0	1.955596832	18.082408
10.96631853	7.849724156	0.651865611
12.29948356	-4.208632448	0.015109439	1	AL451007.1
1	244694432	244694720	-	289
processed_pseudogene	ribosomal protein L37 (RPL37) pseudogene
-	0	0	2	18	12	8	0
0	0.32057866	3.011451539	1.809430894
1.292225123
ENSG00000160785 1411.493244	1412.673539	1580.122241
2130.710409	1893.517666	1657.273012	1468.096341
1893.833696	-0.367219735	0.015110367	0.081998999
SLC25A44	1	156193932	156212796	+	7293
protein_coding	solute carrier family 25 member 44

[Source:HGNC Symbol;Acc:HGNC:29036]	-	1220	1524	1616
2121	2072	1689	9.148467084	9.213246936
10.26449526	14.06163017	12.38061263	10.81108973
ENSG00000100519 2272.272731	1987.383246	1922.351686
1520.931428	1646.775499	1703.390142	2060.669221
1623.699023	0.343450836	0.015115666	0.081998999	PSMC6
14	52707172	52728587	+	7482
protein_coding	"proteasome 26S subunit, ATPase 6 [Source:HGNC
Symbol;Acc:HGNC:9553]"	-	1964	2144	1966	1514	1802
1736	14.35550558	12.63400473	12.17217718
9.783841035	10.4953199	10.83123685
ENSG00000088543 200.1543699	158.5086451	151.5587545
233.0621475	286.9520015	277.683992	170.0739232
265.8993803	-0.646904359	0.015152623	0.082170327
C3orf18 3	50558025	50571027	-	3248
protein_coding	chromosome 3 open reading frame 18 [Source:HGNC
Symbol;Acc:HGNC:24837]	-	173	171	155	232	314
283	2.912894767	2.321207942	2.210640557
3.453609107	4.21281342	4.06739594
ENSG00000131759 1136.136366	1233.771968	1209.536641
1428.510232	1522.490556	1603.306159	1193.148325
1518.102315	-0.347251277	0.015169854	0.082234593	RARA
17	40309192	40357643	+	4731
protein_coding	retinoic acid receptor alpha [Source:HGNC
Symbol;Acc:HGNC:9864]	THR-like	982	1331	1237	1422
1666	1634	11.35149997	12.40392311	12.1120933
14.53275208	15.34548598	16.12297349
ENSG00000163072 91.39997238	38.93194792	110.491221
26.11903377	42.03755436	28.45525006	80.27438044
32.20394607	1.314488302	0.015175409	0.082235547
NOSTRIN 2	168786539	168865514	+	5248
protein_coding	nitric oxide synthase trafficking [Source:HGNC
Symbol;Acc:HGNC:20203]	-	79	42	113	26	46
29	0.823242869	0.352849431	0.997440671
0.239541485	0.38196415	0.257958716
ENSG00000169083 310.0657291	420.8358179	599.3904291
294.3414191	283.296562	283.5712851	443.4306587
287.0697554	0.628514898	0.015191935	0.082295928	AR
X	67544032	67730619	+	12775
protein_coding	androgen receptor [Source:HGNC Symbol;Acc:HGNC:644]
ESR-like	268	454	613	293	310	289
1.147277821	1.56685534	2.22280836	1.108939691
1.057448883	1.056046326
ENSG00000154240 285.7695339	276.23144	317.7844853
147.6729986	223.89567	193.2994573	293.2618197
188.2893753	0.638393378	0.015204551	0.082335093	CEP112
17	65635538	66192084	-	7849
protein_coding	centrosomal protein 112 [Source:HGNC
Symbol;Acc:HGNC:28514]	-	247	298	325	147	245
197	1.720985998	1.673924604	1.91810107

0.905532876	1.360223748	1.171650097
ENSG00000260454 18.51138681	10.19646255	18.57816991
75.34336665	41.12369448	29.43646558	15.76200642
48.63450891	-1.629105188	0.015213946	0.082356798
AL355607.2	9	90997054	91001871	+	1395
	lincRNA novel transcript	-	16	11	19
	75	45	30	0.627249737	0.347658167
0.630930975	2.599490725	1.405713786	1.003906076
ENSG00000148672 4290.013894	3734.686146	3559.186235
2524.505072	3385.850846	3102.603473	3861.295425
3004.319797	0.361756143	0.015252094	0.082534076	GLUD1
	10	87050486	87094866	-	3519
protein_coding	glutamate dehydrogenase 1 [Source:HGNC
Symbol;Acc:HGNC:4335]	-	3708	4029	3640	2513	3705
	3162	57.62556166	50.4791413	47.91644313
34.52823972	45.88043673	41.94581476
ENSG00000172893 2192.442375	3007.956452	4200.621996
2232.172809	2159.45089	2386.316143	3133.673608
2259.313281	0.472282361	0.015264684	0.082572971	DHCR7
	11	71428193	71452868	-	4160
protein_coding	7-dehydrocholesterol reductase [Source:HGNC
Symbol;Acc:HGNC:2860]	-	1895	3245	4296	2222	2363
	2432	24.91211479	34.39183341	47.83804223
25.82569041	24.75306537	27.29080057
ENSG00000066739 1361.743892	1137.36905	1020.821546
1003.573644	736.5710612	852.6762864	1173.311496
864.2736638	0.440821338	0.01527998	0.082626478	ATG2B
	14	96279202	96363870	-	14778
protein_coding	autophagy related 2B [Source:HGNC
Symbol;Acc:HGNC:20187]	-	1177	1227	1044	999	806
	869	4.355675462	3.660689204	3.272557547
3.268519215	2.376719762	2.745049309
ENSG00000171872 9.255693406	10.19646255	2.933395248	0
	0	0	7.461850401	0	5.391846914
0.015306654	1	KLF17	1	44118850	44135140
	+	3131	protein_coding	Kruppel like factor 17
[Source:HGNC Symbol;Acc:HGNC:18830]	zf-C2H2 8	11	3
	0	0	0	0.139733852	0.154897203
0.044385452	0	0	0
ENSG00000223478 17.35442514	18.53902282	22.48936357
5.02289111	0	2.943646558	19.46093717	2.655512556
	2.887801488	0.015307662	1	AL441992.1	9
	128724445	128733194	+	544	antisense
	uncharacterized LOC100506100 [Source:NCBI gene;Acc:100506100]
	-	15	20	23	5	0	3
1.507950453	1.620932962	1.958535249	0.444398231	0
	0.257435474
ENSG00000104979 1198.612296	1841.851917	1820.660651
2298.474972	1902.656265	2401.034376	1620.374955
2200.721871	-0.440892565	0.015325172	0.082841551

C19orf53	19	13774168	13778462	+	1174
	protein_coding	chromosome 19 open reading frame 53
[Source:HGNC Symbol;Acc:HGNC:24991]	-	1036	1987	1862
	2288	2082	2447	48.25989484	74.62145763
73.47067596	94.22998884	77.28073181	97.29979119
ENSG00000167552 9283.460486	6803.821374	6901.301221
8442.475378	11544.79183	10039.7972	7662.861027
10009.02147	-0.385532851	0.015338938	0.082886652	TUBA1A
	12	49184796	49189324	-	3421
protein_coding	tubulin alpha 1a [Source:HGNC Symbol;Acc:HGNC:20766]
	-	8024	7340	7058	8404	12633	10232
128.2722045	94.59690853	95.57207949	118.7775016
160.9207395	139.6218837
ENSG00000224080 33.5518886	16.68512054	9.777984162
94.43035287	31.98509571	54.94806909	20.00499776
60.45450589	-1.603627258	0.015389528	0.083130643
UBE2FP1 3	37143512	37143958	-	447
processed_pseudogene	UBE2F pseudogene 1 [Source:HGNC
Symbol;Acc:HGNC:44535]	-	29	18	10	94	35
	56	3.548012878	1.775411137	1.036322513
10.16767245	3.412079323	5.848258217
ENSG00000197714 146.9341328	186.3171793	228.8048294
409.8679146	332.6449954	201.1491815	187.3520472
314.5540305	-0.746079623	0.015395788	0.083131216	ZNF460
	19	57280051	57293569	+	3505
protein_coding	zinc finger protein 460 [Source:HGNC
Symbol;Acc:HGNC:21628]	zf-C2H2 127	201	234	408	364
	205	1.98157453	2.528377944	3.092646567
5.628249705	4.525556165	2.730309465
ENSG00000116521 3612.034352	4344.619997	4334.580379
4603.981992	5297.645709	5573.304151	4097.078243
5158.310617	-0.332123485	0.01540051	0.083131216	SCAMP3
	1	155255979	155262430	-	2442
protein_coding	secretory carrier membrane protein 3 [Source:HGNC
Symbol;Acc:HGNC:10565]	-	3122	4687	4433	4583	5797
	5680	69.91686582	84.62199346	84.09197015
90.74138718	103.4466041	108.5797186
ENSG00000116815 893.1744137	609.9338507	557.3450972
377.7214115	493.4843338	530.837596	686.8177872
467.3477804	0.554095214	0.015410938	0.083158138	CD58
	1	116514535	116571039	-	1783
protein_coding	CD58 molecule [Source:HGNC Symbol;Acc:HGNC:1688]
	-	772	658	570	376	540	541
23.67885346	16.27078508	14.80900802	10.19618527
13.19778394	14.16419698
ENSG00000227120 0	0	0	6.027469332	15.53561792
	1.962431039	0	7.841839429	-5.392678805
0.015422672	1	AC009238.1	2	95436133
95436556	+	424	processed_pseudogene	pseudogene
similar to part of Cdon homolog (mouse) (CDON)	-	0	0

0	6	17	2	0	0	0
0.684205578	1.747196144	0.22019638
ENSG00000113732 1609.333691	1197.620874	1021.799345
1752.988997	1864.27415	1567.9824	1276.251303
1728.415182	-0.438387117	0.01543582	0.083263014
ATP6V0E1	5	172983757	173035445	+	1623
protein_coding	ATPase H+ transporting V0 subunit e1
[Source:HGNC Symbol;Acc:HGNC:863]	-	1391	1292	1045
1745	2040	1598	46.87090325	35.09764297
29.82635801	51.98500953	54.77346876	45.96256859
ENSG00000272733 3.470885027	1.853902282	0	9.041203998
19.19105743	20.60552591	1.774929103	16.27926244
-3.227324453	0.015437114	1	AP000345.2	22
23580880	23583859	-	1081	lincRNA novel
transcript	-	3	2	0	9	21	21
0.151771516	0.081571465	0	0.402548332
0.84654919	0.906858727
ENSG00000134343 17.35442514	18.53902282	3.911193665
41.1877071	63.05633154	32.38011214	13.26821387
45.5413836	-1.782937704	0.015441686	0.083265272	ANO3
11	26309599	26663288	+	7700
protein_coding	anoctamin 3 [Source:HGNC Symbol;Acc:HGNC:14004] -
15	20	4	41	69	33	0.10653572
0.114517861	0.024064216	0.257450861	0.390496336
0.20006414
ENSG00000126698 4198.613921	3887.633085	3960.083585
3238.760188	3519.274388	2950.515067	4015.44353
3236.183214	0.311094192	0.01551686	0.083641129	DNAJC8
1	28199456	28233025	-	2888
protein_coding	DnaJ heat shock protein family (Hsp40) member C8
[Source:HGNC Symbol;Acc:HGNC:15470]	-	3629	4194	4050
3224	3851	3007	68.72021223	64.02730103
64.96213506	53.97579662	58.10786615	48.60514858
ENSG00000100099 1217.123683	1287.535135	1310.249878
1683.6731	1694.296213	1475.748141	1271.636232
1617.905818	-0.34727731	0.015536371	0.083696744	HPS4
22	26443423	26483837	-	10782
protein_coding	"HPS4, biogenesis of lysosomal organelles complex 3
subunit 2 [Source:HGNC Symbol;Acc:HGNC:15844]"	-	1052	1389
1340	1676	1854	1504	5.335942304	5.679850127
5.75715506	7.515811916	7.493228911	6.511702408
ENSG00000109519 990.3591944	1027.061864	978.7762146
1176.361098	1281.231548	1405.100624	998.7324243
1287.564423	-0.366427738	0.015538128	0.083696744	GRPEL1
4	7058906 7068197 -	4177	protein_coding	"GrpE
like 1, mitochondrial [Source:HGNC Symbol;Acc:HGNC:19696]"	-
856	1108	1001	1171	1402	1432	11.20737754
11.69524282	11.10125447	13.5548163	14.62655795
16.00385392
ENSG00000099331 4240.264541	4178.695743	4942.770994

5503.0795	5323.233786	5674.369349	4453.910426
5500.227545	-0.304350836	0.015553444	0.083749738	MYO9B
	19	17075781	17214537	+	9948
protein_coding	myosin IXB [Source:HGNC Symbol;Acc:HGNC:7609]	-
	3665	4508	5055	5478	5825	5783	20.14804514
	19.97938376	23.5389908	26.62486828	25.51636176
	27.13710103
ENSG00000104774 536.8302175	624.7650689	840.9066379
368.6802075	348.1806133	599.5226824	667.5006415
438.7945011	0.605977469	0.015569557	0.083806977	MAN2B1
	19	12646511	12666742	-	5374
protein_coding	mannosidase alpha class 2B member 1 [Source:HGNC
Symbol;Acc:HGNC:6826]	-	464	674	860	367	381
	611	4.721880927	5.529631525	7.413157802
3.301943355	3.089483722	5.307494881
ENSG00000152684 880.4478352	803.6666391	612.1018085
959.372202	1152.377305	1013.595632	765.4054276
1041.781713	-0.445545875	0.015609969	0.083994932	PELO
	5	52787940	52804046	+	4349
protein_coding	pelota mRNA surveillance and ribosome rescue factor
[Source:HGNC Symbol;Acc:HGNC:8829]	-	761	867	626
	955	1261	1033	9.569515756	8.789489421
6.667873951	10.61732669	12.63526273	11.08809529
ENSG00000175054 1896.260186	1657.38864	1686.702268
1507.871911	1221.830656	1347.208908	1746.783698
1358.970492	0.362084948	0.015675806	0.084319512	ATR
	3	142449235	142578826	-	12496
protein_coding	ATR serine/threonine kinase [Source:HGNC
Symbol;Acc:HGNC:882]	-	1639	1788	1725	1501	1337
	1373	7.173030043	6.308563166	6.394705357
5.807789834	4.662501542	5.129151848
ENSG00000262406 0	0	1.955596832	12.05493866
5.483159264	19.62431039	0.651865611
12.38746944	-4.21874501	0.015700848	1	MMP12	11
	102862736	102875034	-	1874	protein_coding
	matrix metallopeptidase 12 [Source:HGNC Symbol;Acc:HGNC:7158]
	-	0	0	2	12	6	20	0
	0	0.049438224	0.3096085	0.139521219
0.498203122
ENSG00000112739 5429.621144	5313.283939	5143.219669
4520.601999	3993.567664	4461.586967	5295.374917
4325.25221	0.2919764	0.015754138	0.084674487	PRPF4B
	6	4021267 4064983 +	7859	protein_coding	pre-
mRNA processing factor 4B [Source:HGNC Symbol;Acc:HGNC:17346]	-
	4693	5732	5260	4500	4370	4547	32.65712723
	32.15680195	31.00422723	27.68512199	24.23107858
	27.00870126
ENSG00000119927 948.7085741	751.7573752	876.1073809
664.0262048	666.2038506	591.6729582	858.8577767
640.6343379	0.42221437	0.015759804	0.084674487	GPAM

10	112149864	112215377	-	9808
protein_coding	"glycerol-3-phosphate acyltransferase, mitochondrial
[Source:HGNC Symbol;Acc:HGNC:24865]"	-	820	811	896
661	729	603	4.572230411	3.645644821
4.231847494	3.258533715	3.238960628	2.870006569
ENSG00000103512 1500.579293	1646.265226	2414.18429
1447.597218	1209.950478	1408.04427	1853.67627
1355.197322	0.452301196	0.015760741	0.084674487	NOMO1
16	14833681	14896160	+	5819
protein_coding	NODAL modulator 1 [Source:HGNC Symbol;Acc:HGNC:30060]
-	1297	1776	2469	1441	1324	1435
12.18951235	13.4563899	19.65509687	11.97338204
9.915125729	11.51196816
ENSG00000013441 1187.042679	823.1326131	715.7484406
637.907171	634.2187549	660.3580446	908.6412443
644.1613235	0.495262192	0.015763956	0.084674487	CLK1
2	200853009	200864744	-	5170
protein_coding	CDC like kinase 1 [Source:HGNC Symbol;Acc:HGNC:2068]
-	1026	888	732	635	694	673
10.85304317	7.572798144	6.558778944	5.938604441
5.849618256	6.076738629
ENSG00000117758 2264.173999	1989.237148	1483.320197
2407.973998	3126.314641	2244.039893	1912.243782
2592.776177	-0.439675508	0.01581731	0.084931232	STX12
1	27773183	27824452	+	3846
protein_coding	syntaxin 12 [Source:HGNC Symbol;Acc:HGNC:11430] -
1957	2146	1517	2397	3421	2287	27.82763245
24.6010926	18.27169161	30.13422111	38.76166216
27.75894271
ENSG00000047634 686.0782737	564.5132448	576.9010655
427.9503226	458.7576585	446.4530614	609.1641947
444.3870141	0.454066968	0.015882213	0.085249786	SCML1
X	17737449	17754988	+	5497
protein_coding	Scm polycomb group protein like 1 [Source:HGNC
Symbol;Acc:HGNC:10580]	-	593	609	590	426	502
455	5.899614973	4.884560731	4.971972644
3.747011954	3.979573937	3.863951758
ENSG00000111358 1451.986903	1322.759278	1366.962186
1000.559909	1143.238707	1093.074089	1380.569456
1078.957568	0.35520913	0.015961541	0.085645519	GTF2H3
12	123633739	123662606	+	3996
protein_coding	general transcription factor IIH subunit 3
[Source:HGNC Symbol;Acc:HGNC:4657]	-	1255	1427	1398
996	1251	1114	17.1756412	15.74462972
16.20631021	12.05133268	13.64238897	13.01384852
ENSG00000178965 42.807582	52.83621503	15.64477466
4.018312888	8.224738897	15.69944831	37.09619056
9.314166699	1.99310199	0.0159674	0.085646891	ERICH3
1	74568111	74673738	-	8779
protein_coding	glutamate rich 3 [Source:HGNC Symbol;Acc:HGNC:25346]

- 37 57 16 4 9 16
0.230489628 0.286262042 0.084426229 0.022030084
0.044674125 0.085078724
ENSG00000156011 2805.632064 2041.146412 2372.138958
2110.618844 1809.442557 1517.940409 2406.305811
1812.66727 0.408394128 0.015992898 0.085725373 PSD3
8 18527301 19084730 - 15213
protein_coding pleckstrin and Sec7 domain containing 3 [Source:HGNC
Symbol;Acc:HGNC:19093] - 2425 2202 2426 2101 1980
1547 8.717492661 6.381700337 7.387174993
6.677477043 5.671643473 4.747024621
ENSG00000158615 2634.401736 2547.261735 2353.560788
3357.300418 3472.667534 2722.873067 2511.741419
3184.28034 -0.342434757 0.015993247 0.085725373
PPP1R15B 1 204403387 204411791 - 5227
protein_coding protein phosphatase 1 regulatory subunit 15B
[Source:HGNC Symbol;Acc:HGNC:14951] - 2277 2748 2407
3342 3800 2775 23.82348231 23.17918631
21.33172842 30.91399711 31.68032982 24.78315101
ENSG00000101158 3429.234407 3712.439319 3629.587721
2775.649627 2933.490207 3052.561481 3590.420482
2920.567105 0.298001224 0.016007813 0.085773373 NELFCD
20 58981208 58995133 + 6298
protein_coding negative elongation factor complex member C/D
[Source:HGNC Symbol;Acc:HGNC:15934] - 2964 4005 3712
2763 3210 3111 25.7377309 28.03714722
27.30283642 21.21189385 22.21063493 23.05915511
ENSG00000185122 2555.728342 2865.205976 2830.726415
3515.019199 3189.370972 3515.695206 2750.553578
3406.695126 -0.308372667 0.016043144 0.085932568 HSF1
8 144291591 144314722 + 5307
protein_coding heat shock transcription factor 1 [Source:HGNC
Symbol;Acc:HGNC:5224] HSF 2209 3091 2895 3499 3490
3583 22.76362072 25.67934106 25.26980766
31.87836738 28.65727802 31.51691906
ENSG00000110841 5493.254036 4025.748805 4325.780193
4040.413609 3436.113139 3234.086352 4614.927678
3570.204367 0.370096922 0.016065417 0.08599705
PPFIBP1 12 27523431 27695564 + 11690
protein_coding PPFIA binding protein 1 [Source:HGNC
Symbol;Acc:HGNC:9249] - 4748 4343 4424 4022 3760
3296 22.21216607 16.3798257 17.5308536
16.63522853 14.01625311 13.16190427
ENSG00000271122 923.2554172 891.7269975 885.885365
1240.654104 1164.257484 1076.393425 900.2892599
1160.435004 -0.366330122 0.016066434 0.08599705
AC018647.2 7 35695214 35699413 + 4200
antisense uncharacterized LOC101930085 [Source:NCBI
gene;Acc:101930085] - 798 962 906 1235 1274
1097 10.39078392 10.09856673 9.992665805

14.21735749	13.2183953	12.19279784
ENSG00000186501 1239.105955	1497.026092	1378.695767
1689.700569	1646.775499	1956.543746	1371.609271
1764.339938	-0.362733905	0.016081819	0.086049269
TMEM222 1	27322145	27336400	+	4476
protein_coding	transmembrane protein 222 [Source:HGNC
Symbol;Acc:HGNC:25363]	-	1071	1615	1410	1682	1802
	1994	13.08561401	15.9080302	14.5925601
18.16925542	17.54378541	20.7960624
ENSG00000133028 2699.191589	2493.498569	2496.319356
3177.480916	3310.000476	3002.51949	2563.003172
3163.333627	-0.303827686	0.016095292	0.086091226	SCO1
	17	10672474	10698375	-	10325
protein_coding	"SCO1, cytochrome c oxidase assembly protein
[Source:HGNC Symbol;Acc:HGNC:10603]"	-	2333	2690	2553
	3163	3622	3060	12.35718052	11.48672378
11.45415907	14.81188556	15.28681407	13.83494389
ENSG00000108106 4089.859524	5768.416949	6616.761882
4295.576477	4079.470493	4143.673139	5491.679452
4172.906703	0.396423814	0.016101117	0.086092262	UBE2S
	19	55399745	55407777	-	2788
protein_coding	ubiquitin conjugating enzyme E2 S [Source:HGNC
Symbol;Acc:HGNC:17895]	-	3535	6223	6767	4276	4464
	4223	69.34120132	98.4103983	112.4361232
74.15597402	69.77342055	70.70894343
ENSG00000187624 47.4354287	42.63975248	29.33395248
68.3113191	95.95528713	93.21547435	39.80304456
85.82736019	-1.111314331	0.016111509	0.086117706
C17orf97	17	410327	431062	+	2710	protein_coding
	chromosome 17 open reading frame 97 [Source:HGNC
Symbol;Acc:HGNC:33800]	-	41	46	30	68	105
	95	0.827388116	0.748380562	0.512807561
1.213223568	1.688412683	1.636440994
ENSG00000125266 349.4024261	426.3975248	265.9611692
532.4264577	562.9376845	439.5845527	347.2537067
511.649565	-0.5589363	0.016134327	0.086209527	EFNB2
	13	106489731	106535662	-	5019
protein_coding	ephrin B2 [Source:HGNC Symbol;Acc:HGNC:3227]	-
	302	460	272	530	616	448	3.290670971
	4.040867349	2.510464961	5.105754049	5.348378253
	4.166840284
ENSG00000182359 98.34174244	78.79084697	50.84551764
149.6821551	140.7344211	133.4453106	75.99270235
141.2872956	-0.897712821	0.016150446	0.086265501	KBTBD3
	11	106051098	106077765	-	5516
protein_coding	kelch repeat and BTB domain containing 3 [Source:HGNC
Symbol;Acc:HGNC:22934]	-	85	85	52	149	154
	136	0.842731798	0.679404824	0.436698341
1.306060297	1.216620307	1.150961209
ENSG00000266852 9.255693406	5.561706845	6.844588913	0

0	0	7.220663055	0	5.343463753
0.016216683	1	MIR4482 10	104268336	104268405
-	70	miRNA	microRNA 4482 [Source:HGNC
Symbol;Acc:HGNC:41792]	-	8	6	7	0	0
0	6.250095592	3.779089421	4.632361631	0
0	0
ENSG00000183853 4464.715107	4932.30702	6453.469547
4546.721033	3670.061268	3959.204621	5283.497225
4058.662307	0.380672147	0.016219496	0.086604065
KIRREL1 1	157993273	158100262	+	7872
protein_coding	kirre like nephrin family adhesion molecule 1
[Source:HGNC Symbol;Acc:HGNC:15734]	-	3859	5321	6600
4526	4016	4035	26.80923403	29.801775
38.83839782	27.79909647	22.23142066	23.92789474
ENSG00000099804 932.5111106	1608.260229	1363.050992
1554.082509	1843.255373	2015.416677	1301.274111
1804.25152	-0.470687372	0.016225712	0.086607002	CDC34
19	531712	542092	+	1942	protein_coding	cell
division cycle 34 [Source:HGNC Symbol;Acc:HGNC:1734]	-	806
1735	1394	1547	2017	2054	22.69763088
39.38983953	33.25186464	38.51609991	45.26007511
49.37387913
ENSG00000198783 386.4251997	494.0649581	529.9667416
608.7744025	629.6494555	728.0619154	470.1522998
655.4952578	-0.47821898	0.016282273	0.08687857	ZNF830
17	34961530	34963775	+	2246
protein_coding	zinc finger protein 830 [Source:HGNC
Symbol;Acc:HGNC:28291]	-	334	533	542	606	689
742	8.132637742	10.46288411	11.17871774
13.04560092	13.3680502	15.4219819
ENSG00000284490 0	0	0.977798416	15.06867333
5.483159264	7.849724156	0.325932805
9.467185584	-4.699787799	0.016286924	1	AC011471.4
19	6199405 6205121 +	216	unprocessed_pseudogene
acyl-CoA synthetase bubblegum family member 2 (ACSBG2)
pseudogene	-	0	0	1	15	6	8
0	0	0.214461187	3.35767552	1.21047576
1.728949354
ENSG00000104427 698.8048521	420.8358179	419.4755205
290.3231062	368.2855306	357.1624491	513.0387302
338.5903619	0.597666978	0.016298688	0.086893622
ZC2HC1A 8	78666047	78719765	+	4181
protein_coding	zinc finger C2HC-type containing 1A [Source:HGNC
Symbol;Acc:HGNC:24277]	-	604	454	429	289	403
364	7.900443723	4.78750944	4.753128773
3.342095777	4.20033062	4.064126824
ENSG00000121552 17.35442514	38.00499677	13.68917783
78.35710132	46.60685375	62.79779325	23.01619991
62.58724944	-1.43659458	0.016300583	0.086893622	CSTA
3	122325244	122341972	+	1373

protein_coding cystatin A [Source:HGNC Symbol;Acc:HGNC:2481]   -
        15   41   14   78   51   64   0.59746908
        1.316580073   0.472345687   2.746788889   1.618669698
        2.175982872
ENSG00000165501 474.354287   329.9946061   517.2553621
206.9431137   338.1281546   308.1016731   440.5347518
284.3909805   0.630145212   0.016302146   0.086893622   LRR1
        14   49598697   49614672   +   2359
protein_coding leucine rich repeat protein 1 [Source:HGNC
Symbol;Acc:HGNC:19742] -   410   356   529   206   370
        314   9.504967332   6.653589683   10.38795805
4.222216475   6.8349034   6.213663681
ENSG00000278356 4.627846703   4.634755704   12.71137941   0
        0   0   7.324660606   0   5.366817295
0.016315998   1   AC005911.1   12   2885819 2886329 +
        456   sense_intronic novel transcript   -   4
        5   13   0   0   0   0.479722249
0.483436147   1.320629412   0   0   0
ENSG00000167207 4.627846703   4.634755704   12.71137941   0
        0   0   7.324660606   0   5.366817295
0.016315998   1   NOD2   16   50693588   50734041
        +   6873   protein_coding nucleotide binding
oligomerization domain containing 2 [Source:HGNC
Symbol;Acc:HGNC:5331]   -   4   5   13   0   0
        0   0.031827928   0.032074332   0.087619236   0
        0   0
ENSG00000128596 92.55693406   61.1787753   106.5800274
34.15565955   24.67421669   54.94806909   86.77191224
37.92598178   1.194631259   0.016332102   0.087022949
CCDC136 7   128790757   128822132   +   6188
protein_coding coiled-coil domain containing 136 [Source:HGNC
Symbol;Acc:HGNC:22225] -   80   66   109   34   27
        56   0.707024388   0.470248684   0.815978374
0.265662239   0.190139373   0.422458213
ENSG00000163877 926.7263022   908.412118   973.8872225
1237.64037   1231.883115   1131.341494   936.3418809
1200.288326   -0.358290838   0.016350549   0.087090883   SNIP1
        1   37534449   37554344   -   5769
protein_coding Smad nuclear interacting protein 1 [Source:HGNC
Symbol;Acc:HGNC:30587] -   801   980   996   1232   1348
        1153   7.593232359   7.489615019   7.997629025
10.325507   10.18234836   9.329853066
ENSG00000177731 4696.107442   4400.237066   4971.127148
4230.278893   2996.546538   3758.05544   4689.157218
3661.626957   0.356952373   0.01635834   0.087102035   FLII
        17   18244836   18258916   -   6465
protein_coding "FLII, actin remodeling protein [Source:HGNC
Symbol;Acc:HGNC:3750]" -   4059   4747   5084   4211   3279
        3830   34.33564696   32.37312771   36.42834731
31.49328249   22.10199545   27.6551667

ENSG00000165312 236.0201818 342.044971 304.0953074
361.6481599 478.8625758 467.0585873 294.0534867
435.856441 -0.566329086 0.016367123 0.087109621 OTUD1
10 23439458 23442390 + 2933
protein_coding OTU deubiquitinase 1 [Source:HGNC
Symbol;Acc:HGNC:27346] - 204 369 311 360 524
476 3.803757461 5.546873494 4.911914311
5.934602761 7.785344266 7.576016739
ENSG00000078304 2762.824482 2563.946856 2212.757816
1965.95958 2153.967731 1785.812245 2513.176384
1968.579852 0.352049228 0.016375543 0.087109621
PPP2R5C 14 101761798 101927989 + 14289
protein_coding protein phosphatase 2 regulatory subunit B'gamma
[Source:HGNC Symbol;Acc:HGNC:9311] - 2388 2766 2263
1957 2357 1820 9.13960021 8.53462213
7.33643668 6.622015698 7.188136971 5.945872437
ENSG00000130818 325.1062309 310.5286322 335.3848567
552.5180221 438.6527412 401.3171475 323.6732399
464.1626369 -0.519963375 0.016376859 0.087109621 ZNF426
19 9523224 9538645 - 7545 protein_coding zinc
finger protein 426 [Source:HGNC Symbol;Acc:HGNC:20725] zf-C2H2 281
335 343 550 480 409 2.036769057
1.957580007 2.105897998 3.524558001 2.772302337
2.530521897
ENSG00000123178 959.1212292 846.3063916 792.9945155
460.0968257 621.4247166 760.4420276 866.1407121
613.9878566 0.495853586 0.016438056 0.087369389 SPRYD7
13 49912706 49936490 - 3340
protein_coding SPRY domain containing 7 [Source:HGNC
Symbol;Acc:HGNC:14297] - 829 913 811 458 680
775 13.57384159 12.05197629 11.24803977
6.630102273 8.87199 10.83181594
ENSG00000147010 2117.239867 1930.839226 1981.019591
1650.522019 1562.70039 1617.043176 2009.699561
1610.088528 0.319682585 0.016442614 0.087369389
SH3KBP1 X 19533975 19887601 - 5780
protein_coding SH3 domain containing kinase binding protein 1
[Source:HGNC Symbol;Acc:HGNC:13867] - 1830 2083 2026
1643 1710 1648 17.31481932 15.88895699
16.23730911 13.74393022 12.89219512 13.30991876
ENSG00000172461 53.22023708 51.90926389 92.89084954
35.16023777 25.58807657 17.66187935 66.0067835
26.13673123 1.338074172 0.016442842 0.087369389 FUT9
6 96015984 96215612 + 13719
protein_coding fucosyltransferase 9 [Source:HGNC
Symbol;Acc:HGNC:4020] - 46 56 95 35 28
18 0.183370761 0.179969756 0.320777283
0.123352173 0.088939395 0.061248589
ENSG00000154654 263.7872621 188.1710816 263.0277739
187.8561275 105.0938859 131.4828796 238.3287059

141.477631	0.752692091	0.016460064	0.087430505	NCAM2
	21	20998315	21543329	+	8380
protein_coding	neural cell adhesion molecule 2 [Source:HGNC
Symbol;Acc:HGNC:7657]	-	228	203	269	187	115
	134	1.487940418	1.068036211	1.486999139
1.078943752	0.598015471	0.746460473
ENSG00000089597 6527.577774	7086.541472	12737.77997
5119.330619	4115.111028	5536.017961	8783.966405
4923.486536	0.835227397	0.01647103	0.087450348	GANAB
	11	62624826	62646726	-	5030
protein_coding	glucosidase II alpha subunit [Source:HGNC
Symbol;Acc:HGNC:4138]	-	5642	7645	13027	5096	4503
	5642	61.34226524	67.01059323	119.9717196
48.98494793	39.01149194	52.36138586
ENSG00000165029 483.6099804	510.7500786	254.2275882
743.3878843	705.4998254	491.5889753	416.1958824
646.8255616	-0.636542665	0.01647524	0.087450348	ABCA1
	9	104781002	104928237	-	11350
protein_coding	ATP binding cassette subfamily A member 1 [Source:HGNC
Symbol;Acc:HGNC:29]	-	418	551	260	740	772
	501	2.014072654	2.140374141	1.061157731
3.152369193	2.964012539	2.060572503
ENSG00000172137 69.41770054	179.8285213	34.22294457
262.1949159	591.2673407	204.092828	94.48972214
352.5183616	-1.898584256	0.016486421	0.087479318	CALB2
	16	71358713	71390438	+	1703
protein_coding	calbindin 2 [Source:HGNC Symbol;Acc:HGNC:1435]	-
	60	194	35	261	647	208	1.926776386
	5.022512658	0.952041439	7.410151307	16.55572601
	5.701573442
ENSG00000146374 42.807582	60.25182416	33.24514615
84.38457065	97.78300688	102.046414	45.43485077
94.73799719	-1.058368128	0.01655875	0.08783262	RSPO3
	6	127118604	127197765	+	3179
protein_coding	R-spondin 3 [Source:HGNC Symbol;Acc:HGNC:20866] -
	37	65	34	84	107	104	0.636510993
	0.901481434	0.495439747	1.277585502	1.466735649
	1.527175145
ENSG00000165609 1843.039949	1711.151806	1676.924284
1471.707095	1333.321561	1344.265262	1743.705346
1383.097973	0.334129914	0.016574408	0.087885182	NUDT5
	10	12165325	12196144	-	4969
protein_coding	nudix hydrolase 5 [Source:HGNC Symbol;Acc:HGNC:8052]
	-	1593	1846	1715	1465	1459	1370
17.5324049	16.3793498	15.98812678	14.25508609
12.7951356	12.87056482
ENSG00000165512 1034.323738	735.0722547	616.0130022
481.1929683	664.3761309	491.5889753	795.1363317
545.7193582	0.541677254	0.016622806	0.088085468	ZNF22
	10	45000475	45005326	+	2551

protein_coding zinc finger protein 22 [Source:HGNC
Symbol;Acc:HGNC:13012] zf-C2H2 894 793 630 479 727
501 19.16557145 13.70555689 11.44017181
9.078754476 12.41888341 9.167972525
ENSG00000110090 1627.845078 1674.07376 1623.145371
1383.304212 1129.530808 1344.265262 1641.68807
1285.700094 0.352910268 0.016623705 0.088085468 CPT1A
11 68754620 68844410 - 6565
protein_coding carnitine palmitoyltransferase 1A [Source:HGNC
Symbol;Acc:HGNC:2328] - 1407 1806 1660 1377 1236
1370 11.7207143 12.12877595 11.71320687
10.1414587 8.204315218 9.74163543
ENSG00000124593 4.627846703 25.95463194 35.20074298
51.23348932 42.95141424 112.8397847 21.92774054
69.00822943 -1.644491891 0.01665074 0.088198152
AL365205.1 6 41780349 41790141 + 4479
protein_coding novel transcript - 4 28
36 51 47 115 0.048839774 0.275620126
0.372326454 0.55054184 0.457272826 1.198568373
ENSG00000202111 49.74935206 74.15609127 59.64570339
84.38457065 116.0602044 164.8442073 61.18371557
121.7629941 -0.990283936 0.016657285 0.088202255
VTRNA1-2 5 140718925 140719013 + 89
misc_RNA vault RNA 1-2 [Source:HGNC
Symbol;Acc:HGNC:12655] - 43 80 61 84 127
168 26.42245468 39.6309003 31.74989433
45.63420572 62.18309184 88.11813786
ENSG00000156256 2301.196773 1809.408627 1996.664366
1497.826129 1754.610965 1489.485159 2035.756589
1580.640751 0.364488138 0.016745826 0.08864039 USP16
21 29024629 29054488 + 5694
protein_coding ubiquitin specific peptidase 16 [Source:HGNC
Symbol;Acc:HGNC:12614] - 1989 1952 2042 1491 1920
1518 19.10345999 15.11458783 16.61271944
12.66080725 14.69407877 12.44515599
ENSG00000111669 14887.78284 16665.65456 17833.08751
18508.34916 19921.23147 22760.27519 16462.17497
20396.61861 -0.309133269 0.016778307 0.088751554 TPI1
12 6867119 6870948 + 2700 protein_coding
triosephosphate isomerase 1 [Source:HGNC Symbol;Acc:HGNC:12009] -
12868 17979 18238 18424 21799 23196 260.6405604
293.5862598 312.9074497 329.9296735 351.8288145
401.0470921
ENSG00000139629 34.70885027 15.75816939 13.68917783
64.29300621 48.4345735 59.85414669 21.38539916
57.52724213 -1.437334576 0.016778439 0.088751554 GALNT6
12 51351247 51392867 - 7347
protein_coding polypeptide N-acetylgalactosaminyltransferase 6
[Source:HGNC Symbol;Acc:HGNC:4128] - 30 17 14
64 53 61 0.223308846 0.102017068

0.088271489	0.421183307	0.314357935	0.387583992
ENSG00000233223 48.59239038	52.83621503	69.42368755
103.4715569	73.10879019	181.5248711	56.95076432
119.3684061	-1.065693289	0.016793416	0.088800051
AC016876.1	17	7581964 7584072 -	1058	antisense
	uncharacterized LOC100996842 [Source:NCBI gene;Acc:100996842]
	-	42	57	71	103	80	185
2.170992562	2.375325581	3.108673685	4.707092941
3.295056889	8.162667318
ENSG00000171603 2623.989081	2603.805755	4023.640483
2303.497863	1665.052697	2667.924997	3083.811773
2212.158519	0.479492756	0.016818645	0.088894713	CLSTN1
	1	9729026 9824526 -	5683	protein_coding
calsyntenin 1 [Source:HGNC Symbol;Acc:HGNC:17447]	-	2268
	2809	4115	2293	1822	2719	21.82529422
21.7925495	33.54243905	19.50866788	13.9710586
22.33456958
ENSG00000181638 230.2353735	210.417909	316.8066868
443.0189959	280.5549824	463.1337252	252.4866564
395.5692345	-0.646945699	0.016822948	0.088894713	ZFP41
	8	143246821	143262705	+	6910
protein_coding	ZFP41 zinc finger protein [Source:HGNC
Symbol;Acc:HGNC:26786]	zf-C2H2 199	227	324	441	307
	472	1.574960774	1.448377494	2.172048001
3.085757254	1.936060506	3.18867302
ENSG00000198598 696.4909288	939.0015057	852.6402189
1027.683521	1146.894146	1128.397847	829.3775511
1100.991838	-0.407827702	0.01685334	0.089024535	MMP17
	12	131828393	131851783	+	4003
protein_coding	matrix metallopeptidase 17 [Source:HGNC
Symbol;Acc:HGNC:7163]	-	602	1013	872	1023	1255
	1150	8.224426313	11.15726667	10.09098012
12.35638012	13.66207715	13.41091118
ENSG00000130559 1145.392059	1344.079154	1481.3646
2058.380777	1755.524825	1462.011124	1323.611938
1758.638908	-0.409502119	0.016865547	0.089058242
CAMSAP1 9	135808487	135907228	-	8642
protein_coding	calmodulin regulated spectrin associated protein 1
[Source:HGNC Symbol;Acc:HGNC:19946]	-	990	1450	1515
	2049	1921	1490	6.264921669	7.397546406
8.12083762	11.46380824	9.686607457	8.048557337
ENSG00000146731 13318.94281	11222.59746	11482.2868
10263.77569	9732.607694	9442.236944	12007.94236
9812.873444	0.291163053	0.016873913	0.089071653	CCT6A
	7	56051630	56063989	+	3901
protein_coding	chaperonin containing TCP1 subunit 6A [Source:HGNC
Symbol;Acc:HGNC:1620]	-	11512	12107	11743	10217	10650
	9623	161.3873696	136.8341662	139.4458412
126.6335143	118.9685738	115.1544091
ENSG00000141252 1174.316101	1280.119526	1260.382158

1730.888277 1602.910225 1417.856426 1238.272595
1583.884976 -0.35484732 0.016889628 0.089123832 VPS53
17 508668 721717 - 20899
protein_coding "VPS53, GARP complex subunit [Source:HGNC
Symbol;Acc:HGNC:25608]" - 1015 1381 1289 1723 1754
1445 2.656043901 2.913413515 2.857129118
3.986217468 3.657317641 3.227664436
ENSG00000118412 1247.204686 994.6185741 1024.73274
927.2256989 781.3501952 741.7989327 1088.852
816.7916089 0.414309286 0.01692331 0.089228983
CASP8AP2 6 89829894 89874436 + 8171
protein_coding caspase 8 associated protein 2 [Source:HGNC
Symbol;Acc:HGNC:1510] - 1078 1073 1048 923 855
756 7.215032025 5.78973211 5.941396389
5.461698308 4.559838929 4.31909365
ENSG00000102981 64.78985384 248.4229057 115.3802131
287.3093715 231.206549 304.176811 142.8643242
274.2309105 -0.936532686 0.016924667 0.089228983 PARD6A
16 67660946 67662778 + 1488
protein_coding par-6 family cell polarity regulator alpha
[Source:HGNC Symbol;Acc:HGNC:15943] - 56 268 118
286 253 310 2.058163199 7.940828577
3.673512584 9.293179343 7.409283078 9.725340116
ENSG00000267100 584.2656462 641.4501895 667.8363182
799.6442647 1019.867623 750.6298724 631.1840513
856.7139201 -0.440653125 0.016927066 0.089228983 ILF3-
DT 19 10651862 10653844 - 1983
lincRNA ILF3 divergent tranacript [Source:HGNC Symbol;Acc:HGNC:27115]
- 505 692 683 796 1116 765
13.92718603 15.38570277 15.95513361 19.40848204
24.52449527 18.00879925
ENSG00000183508 38.1797353 75.08304241 30.3117509
114.5219173 108.7493254 87.32818123 47.8581762
103.5331413 -1.109242709 0.016947266 0.089304673 TENT5C
1 117605934 117628372 + 5751
protein_coding terminal nucleotidyltransferase 5C [Source:HGNC
Symbol;Acc:HGNC:24712] - 33 81 31 114 119
89 0.313808921 0.620977135 0.249701288
0.958435078 0.901700253 0.722424847
ENSG00000189306 856.15164 969.5908933 804.7280965
1051.793398 1346.115599 1088.168011 876.8235433
1162.02567 -0.406290585 0.016966626 0.089375883 RRP7A
22 42509968 42519802 - 4082
protein_coding ribosomal RNA processing 7 homolog A [Source:HGNC
Symbol;Acc:HGNC:24286] - 740 1046 823 1047 1473
1109 9.914103126 11.29776774 9.339621809
12.40151942 15.72491636 12.68249155
ENSG00000157191 2035.095588 2259.906881 2379.961345
2560.669888 2786.358766 3040.786895 2224.987938
2795.938516 -0.329305238 0.016973054 0.089378947 NECAP2

1 16440672 16460078 + 5559
protein_coding NECAP endocytosis associated 2 [Source:HGNC
Symbol;Acc:HGNC:25528] - 1759 2438 2434 2549 3049
3099 17.30469217 19.33619358 20.28272749
22.17044335 23.9011803 26.02381351
ENSG00000172331 516.0049074 474.5989841 372.5411966
613.7972937 927.5677756 498.4574839 454.381696
679.940851 -0.5824862 0.016997037 0.089423626 BPGM
7 134646808 134679813 + 2539
protein_coding bisphosphoglycerate mutase [Source:HGNC
Symbol;Acc:HGNC:1093] - 446 512 381 611 1015
508 9.606537238 8.890807723 6.951279171
11.63535734 17.42055308 9.340003678
ENSG00000121774 6836.486542 7830.883238 8086.392902
6112.858481 6191.400669 6311.178221 7584.58756
6205.145791 0.289719447 0.016999808 0.089423626
KHDRBS1 1 32013829 32060850 + 3588
protein_coding "KH RNA binding domain containing, signal transduction
associated 1 [Source:HGNC Symbol;Acc:HGNC:18116]" - 5909
8448 8270 6085 6775 6432 90.06504459
103.8091007 106.771546 81.99915267 82.28401277
83.6834617
ENSG00000100307 71.73162389 119.5766972 100.7132369
157.7187809 168.1502174 182.5060866 97.34051931
169.4583616 -0.79637902 0.01700266 0.089423626 CBX7
22 39120167 39152674 - 7461
protein_coding chromobox 7 [Source:HGNC Symbol;Acc:HGNC:1557] -
62 129 103 157 184 186 0.454453406
0.762301244 0.63950308 1.017428336 1.07468053
1.163756019
ENSG00000070610 836.4832915 792.5432254 1026.688337
553.5226003 551.971366 794.7845708 885.2382846
633.426179 0.483118717 0.017004938 0.089423626 GBA2
9 35736866 35749228 - 4803
protein_coding glucosylceramidase beta 2 [Source:HGNC
Symbol;Acc:HGNC:18986] - 723 855 1050 551 604
810 8.232285497 7.848514881 10.12696172
5.546770903 5.480030173 7.872605115
ENSG00000137868 17.35442514 13.90426711 92.89084954
16.07325155 0 5.887293117 41.38318059 7.320181556
2.507614557 0.01703263 0.089538449 STRA6 15
74179466 74212267 - 7690 protein_coding
stimulated by retinoic acid 6 [Source:HGNC
Symbol;Acc:HGNC:30650] - 15 15 95 16 0
6 0.106674258 0.086000084 0.572268342
0.100599277 0 0.0364226
ENSG00000213672 734.6706641 892.6539486 949.4422621
596.7194639 508.1060918 750.6298724 858.9222916
618.4851427 0.474641776 0.017059915 0.089647209
NCKIPSD 3 48673844 48686364 - 3431

protein_coding	NCK interacting protein with SH3 domain [Source:HGNC Symbol;Acc:HGNC:15486]	-	635	963	971	594	556
	765	10.1215662	12.37483814	13.10994796
8.370799572	7.061755409	10.4084666
ENSG00000197933 222.1366417	159.4355962	135.9139798
107.4898698	92.29984762	98.12155195	172.4954059
99.30375644	0.795063622	0.017071378	0.089647209	ZNF823
	19	11721265	11739009	-	2459
protein_coding	zinc finger protein 823 [Source:HGNC Symbol;Acc:HGNC:30936]	zf-C2H2 192	172	139	107	101
	100	4.270093776	3.083925487	2.618537075
2.103906646	1.78986981	1.898399047
ENSG00000136451 2643.657429	2056.904582	2386.805934
1725.865385	2026.027348	1784.83103	2362.455981
1845.574588	0.355730824	0.017072263	0.089647209	VEZF1
	17	57971547	57988259	-	4913
protein_coding	vascular endothelial zinc finger 1 [Source:HGNC Symbol;Acc:HGNC:12949]	zf-C2H2 2285	2219	2441	1718	2217
	1819	25.43514121	19.91335775	23.015662
16.90743054	19.66425633	17.28351102
ENSG00000160201 93.71389573	259.5463194	321.6956789
93.42577465	70.36721056	165.8254228	224.985298
109.8728027	1.036439021	0.017076776	0.089647209	U2AF1
	21	43092956	43107587	-	11112
protein_coding	U2 small nuclear RNA auxiliary factor 1 [Source:HGNC Symbol;Acc:HGNC:12453]	-	81	280	329	93	77
	169	0.398646081	1.110963413	1.371532556
0.404661542	0.301965335	0.709970833
ENSG00000119242 673.3516953	717.460183	743.1267963
1054.807133	950.4142725	824.2210364	711.3128915
943.1474807	-0.406626455	0.017084123	0.08965499	CCDC92
	12	123918660	123972831	-	8223
protein_coding	coiled-coil domain containing 92 [Source:HGNC Symbol;Acc:HGNC:29563]	-	582	774	760	1050	1040
	840	3.870681236	4.149966858	4.281399538
6.173909019	5.511396382	4.768645427
ENSG00000102144 35276.91845	38200.58347	30721.44844
35275.76427	47563.66504	50908.4048	34732.98345
44582.61137	-0.360179195	0.01709132	0.089661978	PGK1
	X	77910739	78129296	+	5512
protein_coding	phosphoglycerate kinase 1 [Source:HGNC Symbol;Acc:HGNC:8896]	-	30491	41211	31419	35115	52047
	51883	302.5221455	329.6384794	264.0496555
308.024088	411.4765507	439.4018762
ENSG00000180104 1317.779349	1384.865004	1189.002874
1613.352625	1538.026174	1827.023297	1297.215742
1659.467365	-0.355209185	0.017098752	0.089668025	EXOC3
	5	443158	471937	+	8182	protein_coding
exocyst complex component 3 [Source:HGNC Symbol;Acc:HGNC:30378] -
	1139	1494	1216	1606	1683	1862	7.6130549

8.050541262	6.884565807	9.49046042	8.963615905
10.62346612
ENSG00000108848 3351.717975	3164.611195	3241.40175
2753.548907	2270.027936	2776.83992	3252.576973
2600.138921	0.323056525	0.017110016	0.089668025	LUC7L3
17	50719544	50756213	+	8151
protein_coding	LUC7 like 3 pre-mRNA splicing factor [Source:HGNC
Symbol;Acc:HGNC:24309]	-	2897	3414	3315	2741	2484
2830	19.43713785	18.46658467	18.83974826
16.25920695	13.28003734	16.20770705
ENSG00000144034 760.1238209	684.0899419	574.9454687
513.3394715	519.0724104	430.753613	673.0530772
487.7218316	0.463962677	0.017110069	0.089668025	TPRKB
2	73729104	73737400	-	2047
protein_coding	TP53RK binding protein [Source:HGNC
Symbol;Acc:HGNC:24259]	-	657	738	588	511	568
439	17.55263167	15.89543718	13.30644181
12.06991673	12.09174809	10.01135158
ENSG00000176222 91.39997238	76.93694469	58.66790497
150.6867333	156.270039	109.8961382	75.66827401
138.9509702	-0.879309695	0.017133039	0.089757633	ZNF404
19	43872363	43901385	-	2479
protein_coding	zinc finger protein 404 [Source:HGNC
Symbol;Acc:HGNC:19417]	zf-C2H2 79	83	60	150	171
112	1.742790875	1.476167106	1.121184743
2.925606746	3.005925283	2.109053185
ENSG00000213033 3.470885027	0	1.955596832	30.13734666
1.827719755	22.56795695	1.808827286	18.17767445
-3.361867058	0.01714867	1	AURKAP1 1
220266706	220267917	-	1212
transcribed_processed_pseudogene	aurora kinase A pseudogene 1
[Source:HGNC Symbol;Acc:HGNC:18611]	-	3	0	2
30	2	23	0.135367169	0	0.076441611
1.196795235	0.071909451	0.885872565
ENSG00000088986 3584.267271	3204.470094	3100.598778
2466.239535	2841.190359	2634.56367	3296.445381
2647.331188	0.316082601	0.017180228	0.089958777	DYNLL1
12	120469850	120498493	+	2957
protein_coding	dynein light chain LC8-type 1 [Source:HGNC
Symbol;Acc:HGNC:15476]	-	3098	3457	3171	2455	3109
2685	57.2960657	51.54446224	49.67608635
40.14222015	45.81714202	42.38761698
ENSG00000108984 429.2327817	305.8938765	357.8742203
265.2086506	181.8581156	267.8718368	364.3336262
238.3128677	0.612058686	0.017183203	0.089958777	MAP2K6
17	69414698	69553861	+	16655
protein_coding	mitogen-activated protein kinase kinase 6 [Source:HGNC
Symbol;Acc:HGNC:6846]	-	371	330	366	264	199
273	1.21821512	0.873581163	1.0179792
0.766408842	0.520675373	0.765180768

ENSG00000142686 1014.65539 1053.016496 1114.690194
1431.523966 1235.538554 1409.025486 1060.78736
1358.696002 -0.356719595 0.017225958 0.090129036
C1orf216 1 35713875 35719472 - 3629
protein_coding chromosome 1 open reading frame 216
[Source:HGNC Symbol;Acc:HGNC:26800] - 877 1136 1140
1425 1352 1436 13.21622239 13.80146922
14.55192135 18.98580922 16.23485152 18.47198246
ENSG00000163820 1634.786848 1406.184881 1318.072265
2083.495232 1686.071474 1851.553685 1453.014664
1873.706797 -0.367166015 0.017227516 0.090129036 FYCO1
3 45917899 45995824 - 8895
protein_coding FYVE and coiled-coil domain containing 1 [Source:HGNC
Symbol;Acc:HGNC:14673] - 1413 1517 1348 2074 1845
1887 8.687422077 7.519233757 7.020150061
11.2736362 9.038763347 9.903118677
ENSG00000222808 3.470885027 9.269511408 8.800185745 0
0 0 7.18019406 0 5.339576876
0.017290836 1 RNU4-47P 17 77152561
77152674 - 114 snRNA "RNA, U4 small nuclear 47,
pseudogene [Source:HGNC Symbol;Acc:HGNC:46983]" - 3 10
9 0 0 0 1.439166748 3.867489174
3.657127604 0 0 0
ENSG00000250267 2.313923351 13.90426711 5.866790497 0
0 0 7.36166032 0 5.376905585
0.01731411 1 AC021237.1 8 109298598
109316513 + 1643 antisense novel transcript
- 2 15 6 0 0 0
0.066571316 0.402520176 0.169167193 0 0 0
ENSG00000178764 119.1670526 179.8285213 118.3136084
290.3231062 212.9293514 194.2806729 139.1030608
232.5110435 -0.73882781 0.017327478 0.090620992 ZHX2
8 122781394 122974512 + 4716
protein_coding zinc fingers and homeoboxes 2 [Source:HGNC
Symbol;Acc:HGNC:18513] Homeobox 103 194 121 289
233 198 1.194422954 1.813685126 1.188540622
2.962956413 2.152983603 1.959915871
ENSG00000163913 756.6529359 704.482867 999.3099813
557.5409132 602.2336592 634.8464411 820.1485948
598.2070045 0.455326225 0.017333668 0.090622359 IFT122
3 129440036 129520507 + 9636
protein_coding intraflagellar transport 122 [Source:HGNC
Symbol;Acc:HGNC:13556] - 654 760 1022 555 659
647 3.711723955 3.477368846 4.913110821
2.784821789 2.980212425 3.134393552
ENSG00000162607 4338.606284 3436.207879 3747.901329
2725.420716 3476.322974 2801.370308 3840.905164
3001.037999 0.355634862 0.017351022 0.090635578 USP1
1 62436297 62451804 + 4263
protein_coding ubiquitin specific peptidase 1 [Source:HGNC

Symbol;Acc:HGNC:12607]	-	3750	3707	3833	2713	3804
	2855	48.10726287	38.339038	41.65104887
30.77057966	38.88514953	31.26344381
ENSG00000177303 399.1517781	634.0345803	809.6170886
1106.040622	658.8929716	1044.013313	614.2678157
936.3156356	-0.606796824	0.017352583	0.090635578
CASKIN2 17	75500261	75515583	-	5823
protein_coding	CASK interacting protein 2 [Source:HGNC
Symbol;Acc:HGNC:18200]	-	345	684	828	1101	721
	1064	3.240164188	5.178968501	6.586974808
9.142011123	5.395691022	8.52983978
ENSG00000132471 1910.143727	3134.948758	2730.013178
3178.485494	3305.431177	3945.467604	2591.701888
3476.461425	-0.423243785	0.017353983	0.090635578	WBP2
	17	75845699	75856507	-	5399
protein_coding	WW domain binding protein 2 [Source:HGNC
Symbol;Acc:HGNC:12738]	-	1651	3382	2792	3164	3617
	4021	16.72354944	27.61812773	23.95546152
28.33507483	29.19401179	34.76696509
ENSG00000152049 219.8227184	205.7831533	247.3829993
133.6089035	175.4610965	87.32818123	224.3296236
132.1327271	0.762556495	0.017373402	0.09070601	KCNE4
	2	223051814	223198399	+	4124
protein_coding	potassium voltage-gated channel subfamily E regulatory
subunit 4 [Source:HGNC Symbol;Acc:HGNC:6244]	-	190	222
	253	133	192	89	2.51958873	2.373385451
	2.841870739	1.559316236	2.028809033	1.007435814
ENSG00000169914 603.9339947	663.6970168	576.9010655
441.0098395	460.5853782	464.1149407	614.8440257
455.2367195	0.433775348	0.017393787	0.090762213	OTUD3
	1	19882513	19912945	+	6767
protein_coding	OTU deubiquitinase 3 [Source:HGNC
Symbol;Acc:HGNC:29038]	-	522	716	590	439	504
	473	4.218606712	4.664990931	4.038855272
3.136675273	3.245584777	3.262954367
ENSG00000211584 297.3391507	322.578997	234.6716199
469.1380297	356.4053522	428.791182	284.8632559
418.1115213	-0.553275711	0.017401403	0.090762213
SLC48A1 12	47753916	47782753	+	4921
protein_coding	solute carrier family 48 member 1 [Source:HGNC
Symbol;Acc:HGNC:26035]	-	257	348	240	467	390
	437	2.856106983	3.117883164	2.259229408
4.588436566	3.453582538	4.145473161
ENSG00000163320 4073.66206	3330.535449	3589.497986
2387.882434	3410.525063	2663.01892	3664.565165
2820.475472	0.377359299	0.017401978	0.090762213	CGGBP1
	3	88051944	88149885	-	6490
protein_coding	CGG triplet repeat binding protein 1 [Source:HGNC
Symbol;Acc:HGNC:1888]	-	3521	3593	3671	2377	3732
	2714	29.66989716	24.40880278	26.20246464

17.70866007	25.05852685	19.52140998
ENSG00000106804 469.7264403	453.2791079	466.4098445
327.4925004	367.3716707	295.3458714	463.1384642
330.0700142	0.488175237	0.017408807	0.090766862	C5
	9	120952335	121050276	-	6533
protein_coding	complement C5 [Source:HGNC Symbol;Acc:HGNC:1331]
	-	406	489	477	326	402	301
3.398662879	3.300123243	3.382269246	2.412715745
2.681464135	2.150799235
ENSG00000109084 1759.738709	1698.17449	1893.017734
1465.679626	1374.445256	1426.687365	1783.643644
1422.270749	0.326703779	0.017429024	0.090841288	TMEM97
	17	28319095	28328685	+	3034
protein_coding	transmembrane protein 97 [Source:HGNC
Symbol;Acc:HGNC:28106]	-	1521	1832	1936	1459	1504
	1454	27.41626886	26.62219443	29.5591698
23.2509623	21.6018456	22.37148772
ENSG00000237424 166.6024813	139.9696223	181.8705054
276.2590111	238.517428	242.3602333	162.814203
252.3788908	-0.632781504	0.017471373	0.091030976	FOXD2-
AS1	1	47432133	47434641	-	2509
lincRNA FOXD2 adjacent opposite strand RNA 1 [Source:HGNC
Symbol;Acc:HGNC:44256]	-	144	151	186	275	261
	247	3.138748683	2.65344594	3.434114242
5.299479897	4.533132818	4.595601132
ENSG00000100897 802.9314029	824.0595642	1023.754942
705.2139119	601.3197993	670.1701998	883.5819696
658.9013037	0.423846383	0.017480544	0.091047725	DCAF11
	14	24114195	24125242	+	6350
protein_coding	DDB1 and CUL4 associated factor 11 [Source:HGNC
Symbol;Acc:HGNC:20258]	-	694	889	1047	702	658
	683	5.976961493	6.172512721	7.637925398
5.345207921	4.515551149	5.021032289
ENSG00000276900 35.86581195	28.73548537	43.02313031
57.26095866	134.337402	60.83536221	35.87480921
84.14457428	-1.232050063	0.017489789	0.09106485
AC023157.3	12	31729117	31731204	+	2088
	antisense	"novel transcript, antisense to AMN1"	-
	31	31	44	57	147	62	0.811943692
	0.654583656	0.97616816	1.319913825	3.067929943
	1.38614043
ENSG00000154144 901.2731454	1221.721604	1043.31091
1606.320577	1420.13825	1202.970227	1055.43522
1409.809684	-0.416924439	0.017522904	0.091206208	TBRG1
	11	124622836	124635398	+	6627
protein_coding	transforming growth factor beta regulator 1
[Source:HGNC Symbol;Acc:HGNC:29551]	-	779	1318	1067
	1599	1554	1226	6.428582176	8.768643177
7.458472703	11.66628844	10.21862923	8.636137243
ENSG00000112837 139.9923628	26.88158308	92.89084954

32.1465031	27.41579632	39.24862078	86.58826513
32.9369734	1.391684086	0.017546275	0.091282661	TBX18
	6	84687351	84764519	-	8469
protein_coding	T-box 18 [Source:HGNC Symbol;Acc:HGNC:11595]	T-box
	121	29	95	32	30	40	0.781354199
	0.150973187	0.519629655	0.182691803	0.154364603
	0.220482383
ENSG00000115053 17267.65301	18527.8994	19180.49373
15998.91276	13305.79982	15613.10135	18325.34871
14972.60464	0.291568568	0.017549535	0.091282661	NCL
	2	231453531	231483641	-	6929
protein_coding	nucleolin [Source:HGNC Symbol;Acc:HGNC:7667]	-
	14925	19988	19616	15926	14560	15912	117.7981558
	127.1840755	131.1421645	111.1315487	91.56919819
	107.2013476
ENSG00000279894 23.13923351	0	3.911193665	0	0
	0	9.01680906	0	5.659880302	0.01754971
	1	Z73965.1	X	103596608	103598904
	+	2297	TEC	TEC	-	20	0	4
	0	0	0	0.476171845	0	0.08066803
	0	0	0
ENSG00000121690 305.4378824	305.8938765	234.6716199
162.741672	201.049173	187.4121642	282.0011262
183.7343364	0.617230452	0.017564785	0.091330909	DEPDC7
	11	33015864	33033582	+	3326
protein_coding	DEP domain containing 7 [Source:HGNC
Symbol;Acc:HGNC:29899]	-	264	330	240	162	220
	191	4.340866151	4.37447212	3.342654214
2.355016673	2.882431755	2.680755207
ENSG00000130023 554.1846427	473.672033	371.5633981
322.4696093	348.1806133	293.3834403	466.4733579
321.3445543	0.536522778	0.017606585	0.091517123	ERMARD
	6	169751622	169781584	+	4251
protein_coding	ER membrane associated RNA degradation [Source:HGNC
Symbol;Acc:HGNC:21056]	-	479	511	380	321	381
	299	6.162247271	5.299852136	4.140901952
3.651027835	3.905642325	3.28341758
ENSG00000108518 12487.08737	15318.79455	16131.71827
10882.59588	11425.99005	12849.99844	14645.86673
11719.52812	0.321650326	0.017624866	0.091581006	PFN1
	17	4945652 4949061 -	1987	protein_coding
profilin 1 [Source:HGNC Symbol;Acc:HGNC:8881]	-	10793	16526
	16498	10833	12503	13096	297.0564746	366.6940299
	384.6235641	263.6040585	274.2047425	307.6711927
ENSG00000196422 307.7518057	450.4982545	746.0601915
246.1216644	278.7272626	391.5049923	501.4367506
305.4513064	0.716097533	0.017636615	0.09161092
PPP1R26 9	135479079	135488893	+	5276
protein_coding	protein phosphatase 1 regulatory subunit 26
[Source:HGNC Symbol;Acc:HGNC:29089]	-	266	486	763

245 305 399 2.757220904 4.061303453
6.699188637 2.245238672 2.519147809 3.530320582
ENSG00000236719 32.39492692 6.488657986 4.888992081 0
0 2.943646558 14.590859 0.981215519
3.898225447 0.017646915 1 OVAAL 1 180552749
180566520 + 1604 lincRNA ovarian adenocarcinoma
amplified long non-coding RNA [Source:HGNC Symbol;Acc:HGNC:49422]
- 28 7 5 0 0 3
0.954659239 0.192409998 0.1444003 0 0
0.087309787
ENSG00000141524 93.71389573 172.4129122 344.1850425
68.3113191 80.41966921 144.2386814 203.4372835
97.65655656 1.060436637 0.017653599 0.091667989 TMC6
17 78110458 78132407 - 8669
protein_coding transmembrane channel like 6 [Source:HGNC
Symbol;Acc:HGNC:18021] - 81 186 352 68 88
147 0.510988032 0.945971167 1.880945085
0.379263568 0.442356351 0.791579189
ENSG00000103485 458.1568236 472.7450818 672.7253103
335.5291262 376.5102695 401.3171475 534.5424052
371.1188477 0.526806601 0.017664556 0.09169374 QPRT
16 29679008 29698699 + 3642
protein_coding quinolinate phosphoribosyltransferase [Source:HGNC
Symbol;Acc:HGNC:9755] - 396 510 688 334 412
409 5.946343006 6.173965419 8.750864367
4.434123059 4.929647575 5.242390916
ENSG00000197070 171.230328 271.5966843 244.449604
346.5794866 326.2479762 369.9182508 229.0922054
347.5819046 -0.598863886 0.017691638 0.091790528 ARRDC1
9 137605654 137615360 + 3821
protein_coding arrestin domain containing 1 [Source:HGNC
Symbol;Acc:HGNC:28633] - 148 293 250 345 357
377 2.11826061 3.380839397 3.030856864
4.365593296 4.071456285 4.605855921
ENSG00000081870 576.1669145 678.5282351 589.6124449
154.7050462 392.0458874 541.6309667 614.7691982
362.7939668 0.760735303 0.017695211 0.091790528 HSPB11
1 53916574 53945929 - 3236
protein_coding heat shock protein family B (small) member 11
[Source:HGNC Symbol;Acc:HGNC:25019] - 498 732 603
154 429 552 8.416190217 9.973245876
8.631996488 2.300983076 5.777066636 7.96299789
ENSG00000151470 148.0910945 109.3802346 204.359869
72.32963199 100.5245865 78.49724156 153.9437327
83.78382002 0.876446767 0.017708522 0.091828419
C4orf33 4 129093317 129116640 + 7015
protein_coding chromosome 4 open reading frame 33 [Source:HGNC
Symbol;Acc:HGNC:27025] - 128 118 209 72 110
80 0.997876987 0.741631709 1.380133401
0.496256305 0.683319174 0.532363593

ENSG00000253414 0 7.415609127 18.57816991 0 0
0 8.664593011 0 5.611091202 0.0177192
1 AC124067.2 8 37516410 37517021
- 510 sense_intronic novel transcript -
0 8 19 0 0 0 0
0.691598064 1.725781784 0 0 0
ENSG00000075651 252.2176453 229.8838829 238.5828135
190.8698622 120.6295038 138.3513882 240.2281139
149.9502514 0.680578196 0.017724212 0.091878612 PLD1
3 171600405 171810950 - 12859
protein_coding phospholipase D1 [Source:HGNC Symbol;Acc:HGNC:9067]
- 218 248 244 190 132 141
0.927137207 0.850312263 0.878992331 0.714410158
0.447327227 0.511867967
ENSG00000072195 643.2706917 1183.716607 767.5717567
1438.556014 968.6914701 1365.852003 864.8530184
1257.699829 -0.539108694 0.017740039 0.091929484 SPEG
2 219434846 219498287 + 17910
protein_coding SPEG complex locus [Source:HGNC Symbol;Acc:HGNC:16901]
- 556 1277 785 1432 1060 1392
1.697750701 3.143614399 2.030376259 3.865882488
2.579104131 3.628187187
ENSG00000132604 1153.490791 1210.59819 1184.113882
1553.077931 1580.977588 1367.814434 1182.734288
1500.623318 -0.343345909 0.017797329 0.092195112 TERF2
16 69355561 69408571 - 5621
protein_coding telomeric repeat binding factor 2 [Source:HGNC
Symbol;Acc:HGNC:11729] MYB 997 1306 1211 1546 1730
1394 9.700101658 10.243858 9.980056815
13.29833046 13.41192498 11.57697843
ENSG00000099866 5.784808379 16.68512054 3.911193665
56.25638043 21.01877718 29.43646558 8.793707526
35.57054107 -2.004618764 0.01782732 0.09231919
MADCAM1 19 489176 505342 + 2651 protein_coding
mucosal vascular addressin cell adhesion molecule 1 [Source:HGNC
Symbol;Acc:HGNC:6765] - 5 18 4 56 23
30 0.103146617 0.299362044 0.069896064
1.021361577 0.378073908 0.528271964
ENSG00000172638 1317.779349 1255.091845 1689.635663
776.5389656 1237.366274 1074.430994 1420.835619
1029.445411 0.464633368 0.017835318 0.092329326 EFEMP2
11 65866441 65873592 - 5397
protein_coding EGF containing fibulin extracellular matrix protein 2
[Source:HGNC Symbol;Acc:HGNC:3219] - 1139 1354 1728
773 1354 1095 11.5416 11.06114802 14.83179711
6.92513577 10.93263488 9.471259523
ENSG00000143157 3038.18136 2640.8838 2815.08164
3814.383509 3353.86575 3367.531663 2831.382267
3511.926974 -0.310925357 0.017848411 0.092365828 POGK
1 166839447 166856344 + 8448

protein_coding pogo transposable element derived with KRAB domain
[Source:HGNC Symbol;Acc:HGNC:18800]   -   2626   2849   2879
        3797   3670   3432   16.99947579   14.86868299
15.78665854   21.73141014   18.93087801   18.96441323
ENSG00000158623 986.8883094   841.6716359   949.4422621
690.1452385   784.0917748   611.2972686   926.0007358
695.178094   0.412963838   0.017909085   0.092648455   COPG2
        7   130506238   130668748   -   4139
protein_coding coatomer protein complex subunit gamma 2 [Source:HGNC
Symbol;Acc:HGNC:2237]   -   853   908   971   687   858
        623   11.27063324   9.672180221   10.86741518
8.025323118   9.033383733   7.026493617
ENSG00000198648 951.0224974   891.7269975   877.0851793
674.071987   744.7958001   665.2641222   906.6115581
694.7106364   0.383600067   0.017921794   0.092682836   STK39
        2   167954020   168248141   -   4247
protein_coding serine/threonine kinase 39 [Source:HGNC
Symbol;Acc:HGNC:17717] -   822   962   897   671   815
        678   10.58483931   9.986809576   9.783914253
7.639087343   8.362457922   7.452353867
ENSG00000231084 9.255693406   31.51633879   27.37835565   0
        4.569299387   5.887293117   22.71679595   3.485530835
        2.701772929   0.017961057   0.092854473   RPL22P24
        1   185171335   185171710   -   376
processed_pseudogene   ribosomal protein L22 pseudogene 24
[Source:HGNC Symbol;Acc:HGNC:53758]   -   8   34   28
        0   5   6   1.163581626   3.986805329
3.449631002   0   0.579483077   0.744919668
ENSG00000058453 549.556796   620.1303132   597.4348323
1082.935323   601.3197993   840.9017002   589.0406472
841.718941   -0.514183584   0.017973435   0.092887054   CROCC
        1   16740273   16972979   +   8871
protein_coding "ciliary rootlet coiled-coil, rootletin [Source:HGNC
Symbol;Acc:HGNC:21299]" -   475   669   611   1078   658
        857   2.928301184   3.324968203   3.19059064
5.875534735   3.23230186   4.509768808
ENSG00000240342 6763.597956   5633.082083   5421.892218
7049.125384   7354.744293   7641.706466   5939.524086
7348.525381   -0.307295535   0.017986005   0.092894103   RPS2P5
        12   118246084   118246962   +   879
processed_pseudogene   ribosomal protein S2 pseudogene 5 [Source:HGNC
Symbol;Acc:HGNC:31386] -   5846   6077   5545   7017   8048
        7788   363.717878   304.8135853   292.2234954
385.9791256   398.9860307   413.6024509
ENSG00000131165 4535.289769   6069.67607   5323.134578
6134.959202   7030.324037   6879.302007   5309.366806
6681.528415   -0.331452768   0.017986953   0.092894103   CHMP1A
        16   89644431   89657845   -   3208
protein_coding charged multivesicular body protein 1A [Source:HGNC
Symbol;Acc:HGNC:8740]   -   3920   6548   5444   6107   7693

7011	66.82614676	89.99290456	78.61152344
92.04385019	104.5008855	102.0214856
ENSG00000140497 1124.566749	1400.623174	1239.848392
1862.488024	1503.299498	1528.733779	1255.012771
1631.5071	-0.377857845	0.018010126	0.092982369	SCAMP2
15	74843730	74873365	-	5000
protein_coding	secretory carrier membrane protein 2 [Source:HGNC
Symbol;Acc:HGNC:10564]	-	972	1511	1268	1854	1645
1558	10.6314126	13.3238096	11.7476691
17.92837559	14.3368749	14.5459967
ENSG00000107938 1003.085773	872.2610235	916.1971159
758.4565576	688.1364877	689.7945102	930.5146374
712.1291852	0.385557716	0.018068458	0.093252031	EDRF1
10	125719515	125764143	+	7741
protein_coding	erythroid differentiation regulatory factor 1
[Source:HGNC Symbol;Acc:HGNC:24640]	-	867	941	937
755	753	703	6.125150198	5.359527627
5.607186214	4.715753553	4.238932554	4.239399004
ENSG00000011304 10979.5663	12987.51243	15118.71911
11167.89609	8590.282848	10764.91546	13028.59928
10174.3648	0.356842811	0.018077041	0.093264842	PTBP1
19	797075	812327	+	6883	protein_coding
polypyrimidine tract binding protein 1 [Source:HGNC
Symbol;Acc:HGNC:9583]	-	9490	14011	15462	11117	9400
10971	75.40205038	89.74811205	104.0615655
78.09281042	59.51256678	74.40711765
ENSG00000062822 1644.042541	2284.007611	2371.161159
1483.762034	1387.239294	1842.722746	2099.737104
1571.241358	0.418831793	0.018097965	0.093314454	POLD1
19	50384204	50418016	+	4932
protein_coding	"DNA polymerase delta 1, catalytic subunit
[Source:HGNC Symbol;Acc:HGNC:9175]"	-	1421	2464	2425
1477	1518	1878	15.75671656	22.02680939
22.77671727	14.47966932	13.41242484	17.77536617
ENSG00000127423 282.2986489	377.2691143	282.5837423
161.7370937	220.2402305	229.6044316	314.0505018
203.8605853	0.623816076	0.018104107	0.093314454	AUNIP
1	25831913	25859458	-	2408
protein_coding	aurora kinase A and ninein interacting protein
[Source:HGNC Symbol;Acc:HGNC:28363]	-	244	407	289
161	241	234	5.541509173	7.4519835
5.559603453	3.232738757	4.361332098	4.536338047
ENSG00000174720 851.5237933	995.5455253	951.3978589
737.360415	749.3650995	640.7337342	932.8223925
709.1530829	0.395895359	0.01810497	0.093314454	LARP7
4	112636964	112657592	+	4083
protein_coding	La ribonucleoprotein domain family member 7
[Source:HGNC Symbol;Acc:HGNC:24912]	-	736	1074	973
734	820	653	9.858098362	11.59735255
11.03915716	8.691963551	8.751713061	7.465859921

ENSG00000285781 6.941770054 9.269511408 4.888992081 0
0 0 7.033424515 0 5.307600793
0.018122161 1 AL117340.1 10 31362799
31364049 - 1251 unprocessed_pseudogene putative
UPF0607 protein FLJ37424 [Source:NCBI gene;Acc:100505502] -
6 10 5 0 0 0 0.26229418
0.352433066 0.185146348 0 0 0
ENSG00000112576 1337.447697 2049.488972 1991.775374
1455.633844 1193.501 1347.208908 1792.904014
1332.114584 0.429313863 0.01819415 0.093708595 CCND3
6 41934933 42050357 - 5802
protein_coding cyclin D3 [Source:HGNC Symbol;Acc:HGNC:1585] -
1156 2211 2037 1449 1306 1373 10.89619388
16.80138084 16.2635654 12.07513174 9.808984544
11.04685996
ENSG00000170035 1644.042541 1354.275617 1659.323912
904.1203998 1234.624694 1335.434322 1552.547357
1158.059805 0.42252588 0.018199082 0.093708595 UBE2E3
2 180967248 181076585 + 2973
protein_coding ubiquitin conjugating enzyme E2 E3 [Source:HGNC
Symbol;Acc:HGNC:12479] - 1421 1461 1697 900 1351
1361 26.13929568 21.66652512 26.44170094
14.63689026 19.80245511 21.37023273
ENSG00000092330 519.4757924 650.7197009 619.9241958
714.2551159 993.3656868 756.5171655 596.706563
821.3793227 -0.460591482 0.018199831 0.093708595 TINF2
14 24239643 24242674 - 2712
protein_coding TERF1 interacting nuclear factor 2 [Source:HGNC
Symbol;Acc:HGNC:11824] - 449 702 634 711 1087
771 9.054226791 11.41251562 10.82934098
12.67596794 17.46620112 13.27121633
ENSG00000105971 19.66834849 6.488657986 0 0 0
0 8.719002158 0 5.612863597 0.018224263
1 CAV2 7 116287380 116508541 +
6926 protein_coding caveolin 2 [Source:HGNC
Symbol;Acc:HGNC:1528] - 17 7 0 0 0
0 0.134233572 0.044560444 0 0 0
0
ENSG00000047056 665.2529635 809.228346 678.5921008
1255.722778 816.0768705 907.6243555 717.6911368
993.1413345 -0.467863192 0.018237209 0.093869435 WDR37
10 1049538 1132384 + 6000 protein_coding WD
repeat domain 37 [Source:HGNC Symbol;Acc:HGNC:31406] - 575
873 694 1250 893 925 5.240965575
6.415004292 5.358098287 10.07302656 6.485729121
7.196751686
ENSG00000188529 5494.410998 4288.075978 4213.333375
3792.282788 3831.814466 3461.728353 4665.27345
3695.275202 0.336009233 0.018295406 0.094137283 SRSF10
1 23964804 23980927 - 10277

protein_coding	serine and arginine rich splicing factor 10
[Source:HGNC Symbol;Acc:HGNC:16713]	-	4749	4626	4309
	3775	4193	3528	25.27147122	19.8460111
19.42283377	17.7603621	17.77940012	16.02537702
ENSG00000122367 2.313923351	0.926951141	7.822387329
23.10529911	34.72667534	9.812155195	3.687753941
22.54804321	-2.609855782	0.018321778	0.09424126	LDB3
	10	86668449	86736068	+	6676
protein_coding	LIM domain binding 3 [Source:HGNC
Symbol;Acc:HGNC:15710]	-	2	1	8	23	38
	10	0.016383564	0.006604161	0.055510625
0.166576113	0.248042367	0.069924554
ENSG00000187147 989.2022327	1890.980327	1530.254521
1932.808499	2017.802609	2174.373591	1470.145694
2041.661567	-0.472967205	0.018329578	0.094249667	RNF220
	1	44405194	44651724	+	9687
protein_coding	ring finger protein 220 [Source:HGNC
Symbol;Acc:HGNC:25552]	-	855	2040	1565	1924	2208
	2216	4.826935857	9.284848583	7.483891765
9.603222349	9.93272398	10.67889932
ENSG00000099381 843.4250616	1122.537832	1425.630091
1643.489971	1361.651217	1646.479642	1130.530995
1550.540277	-0.454949713	0.018379342	0.094456175	SETD1A
	16	30957294	30984664	+	6832
protein_coding	SET domain containing 1A [Source:HGNC
Symbol;Acc:HGNC:29010]	-	729	1211	1458	1636	1490
	1678	5.835450418	7.815022693	9.885806877
11.57808299	9.503793875	11.46542439
ENSG00000131016 27.76708022	64.88657986	31.28954932
115.5264955	84.07510872	77.51602604	41.31440313
92.37254343	-1.154815541	0.018382097	0.094456175	AKAP12
	6	151239999	151358557	+	8741
protein_coding	A-kinase anchoring protein 12 [Source:HGNC
Symbol;Acc:HGNC:370]	-	24	70	32	115	92
	79	0.150156741	0.353078179	0.169586514
0.636118369	0.458654126	0.421902411
ENSG00000226652 0	0	0.977798416	4.018312888
12.79403828	10.79337071	0.325932805
9.201907295	-4.661072474	0.01841221	1	PSMD10P2
	3	186760693	186762050	+	1087
transcribed_processed_pseudogene	"proteasome 26S subunit, non-
ATPase, 10 pseudogene 2 [Source:HGNC Symbol;Acc:HGNC:30151]"	-
	0	0	1	4	14	11	0	0
	0.042616022	0.177922824	0.56125095	0.472399225
ENSG00000030066 3461.629334	3008.883403	3732.256554
2844.965525	2706.852957	2633.582454	3400.923097
2728.466979	0.317758101	0.018422488	0.094631911	NUP160
	11	47778087	47848555	-	8576
protein_coding	nucleoporin 160 [Source:HGNC Symbol;Acc:HGNC:18017]
	-	2992	3246	3817	2832	2962	2684

19.07969946	16.68774678	20.61768231	15.96649882
15.05077556	14.60978332
ENSG00000144381 16802.55442	15706.26013	16007.53787
14102.26908	13026.15869	13338.64377	16172.11747
13489.02385	0.261707807	0.01845646	0.094774571	HSPD1
2	197486581	197516737	-	4245
protein_coding	heat shock protein family D (Hsp60) member 1
[Source:HGNC Symbol;Acc:HGNC:5261]	-	14523	16944	16371
14038	14254	13594	187.0998139	175.9836035
178.648745	159.8927456	146.3247051	149.4911927
ENSG00000273489 0	0.926951141	0	13.05951689
7.310879019	6.868508636	0.308983714
9.079634847	-4.640002989	0.018491339	1	AC008264.2
7	131493964	131497694	-	3731
antisense	"novel transcript, antisense to MKLN1"	-	0
1	0	13	8	7	0	0.01181704
0	0.168468737	0.093437957	0.0875828
ENSG00000214279 104.1265508	152.0199871	131.0249878
188.8607057	204.7046125	237.4541557	129.0571752
210.3398247	-0.702276711	0.018492733	0.094928947	SCART1
10	133453928	133523558	+	8015
protein_coding	scavenger receptor family member expressed on T cells
1 [Source:HGNC Symbol;Acc:HGNC:32411]	-	90	164	134
188	224	242	0.614092362	0.902140706
0.774468445	1.134110938	1.217876048	1.409476616
ENSG00000233765 0	0	0	22.10072088	0
4.906077597	0	9.002266161	-5.588361275	0.018514417
1	AL591479.1	9	66253424	66254464
-	635	unprocessed_pseudogene	CDRT15 protein
pseudogene	-	0	0	0	22	0	5
0	0	0	1.675136386	0	0.36757191
ENSG00000188766 411.8783566	415.2741111	286.4949359
437.9961048	544.6604869	645.6398118	371.2158012
542.7654679	-0.548618397	0.018534906	0.095113496	SPRED3
19	38388421	38399587	+	5864
protein_coding	sprouty related EVH1 domain containing 3 [Source:HGNC
Symbol;Acc:HGNC:31041]	-	356	448	293	436	596
658	3.320096823	3.368356192	2.314600883
3.594957365	4.429053189	5.238150447
ENSG00000162642 728.8858557	808.3013948	678.5921008
1045.765929	894.66882	966.4972867	738.5931171
968.9773453	-0.391273871	0.018569313	0.095258084
C1orf52 1	85249953	85259672	-	3420
protein_coding	chromosome 1 open reading frame 52 [Source:HGNC
Symbol;Acc:HGNC:24871]	-	630	872	694	1041	979
985	10.07416724	11.24150186	9.400172433
14.71722196	12.47427125	13.44485616
ENSG00000230417 24.29619519	7.415609127	18.57816991	0
4.569299387	0	16.76332474	1.523099796
3.430698387	0.018584065	1	LINC00856	10

78179185	78551355	+	9354	lincRNA long
intergenic non-protein coding RNA 856 [Source:HGNC
Symbol;Acc:HGNC:45111]	-	21	8	19	0	5
	0	0.122776894	0.037707399	0.094093298	0
	0.023293312	0
ENSG00000189129 5.784808379	3.707804563	11.73358099	0
	0	0	7.075397979	0	5.315916392
0.018607487	1	PLAC9	10	80131682	80145359
	+	1240	protein_coding	placenta specific 9
[Source:HGNC Symbol;Acc:HGNC:19255]	-	5	4	12
	0	0	0	0.220517486	0.142223795
0.448293061	0	0	0
ENSG00000167291 2245.662613	2704.843429	3717.589578
3930.914583	3596.038618	3967.054345	2889.365207
3831.335849	-0.406785258	0.018615725	0.095464138
TBC1D16 17	79932343	80035848	-	11555
protein_coding	TBC1 domain family member 16 [Source:HGNC
Symbol;Acc:HGNC:28356]	-	1941	2918	3802	3913	3935
	4043	9.186504631	11.13395074	15.2420934
16.37348456	14.83998236	16.33352146
ENSG00000259589 5.784808379	9.269511408	5.866790497	0
	0	0	6.973703428	0	5.296023504
0.018628882	1	AC073167.1	15	63675146
63713758	+	580	antisense	"novel transcript,
antisense to HERC1"	-	5	10	6	0	0
	0	0.471451176	0.760161665	0.479209824	0
	0	0
ENSG00000235106 804.0883646	799.0318834	807.6614917
922.2028078	1102.115012	1108.773537	803.5939133
1044.363786	-0.378259755	0.018632286	0.095489218	BRD3OS
	9	134025439	134034666	+	5994
protein_coding	BRD3 opposite strand [Source:HGNC
Symbol;Acc:HGNC:24742]	-	695	862	826	918	1206
	1130	6.341073377	6.340514283	6.383601447
7.405035742	8.767770368	8.800507973
ENSG00000156467 1305.05277	1822.385943	2112.044579
1304.94711	1205.381178	1384.495098	1746.494431
1298.274462	0.428490365	0.018633109	0.095489218	UQCRB
	8	96225920	96235634	-	9715
protein_coding	ubiquinol-cytochrome c reductase binding protein
[Source:HGNC Symbol;Acc:HGNC:12582]	-	1128	1966	2160
	1299	1319	1411	6.34981405	8.922255724
10.29943502	6.464985611	5.916441686	6.780008599
ENSG00000228857 2.313923351	2.780853423	1.955596832
31.14192488	7.310879019	12.75580175	2.350124535
17.06953522	-2.856719699	0.018648605	1	AC104653.1
	2	113831049	113843356	-	671
lincRNA novel transcript	-	2	3	2	31
	8	13	0.163005474	0.197120909	0.138073372
	2.233779959	0.519548463	0.904413149

ENSG00000112149 240.6480285 221.5413227 273.7835565
117.535652 188.2551347 154.0508366 245.3243026
153.2805411 0.677388556 0.018669866 0.095619765 CD83
6 14117256 14136918 + 2515
protein_coding CD83 molecule [Source:HGNC Symbol;Acc:HGNC:1703]
- 208 239 280 117 206 157
4.522931999 4.189805568 5.15730122 2.249308834
3.569339258 2.914121794
ENSG00000188157 3018.513012 2873.548537 6292.132808
2069.431137 1715.31499 2376.503988 4061.398119
2053.750038 0.983764816 0.018671093 0.095619765 AGRN
1 1020123 1056118 + 10758 protein_coding agrin
[Source:HGNC Symbol;Acc:HGNC:329] - 2609 3100 6435
2060 1877 2422 13.26286203 12.70469115
27.7089116 9.258420397 7.603110991 10.50965923
ENSG00000120647 399.1517781 435.6670362 480.0990223
346.5794866 299.7460398 281.6088541 438.3059456
309.3114602 0.503628614 0.01869476 0.095702305 CCDC77
12 389273 442645 + 3614 protein_coding
coiled-coil domain containing 77 [Source:HGNC Symbol;Acc:HGNC:28203]
- 345 470 491 345 328 287
5.220662996 5.733814884 6.293551635 4.615642497
3.954980014 3.707146802
ENSG00000154727 3821.444415 3237.840335 3088.865197
2484.321943 2955.422844 2613.958144 3382.716649
2684.567643 0.333136932 0.018702484 0.095702305 GABPA
21 25734570 25772460 + 5429
protein_coding GA binding protein transcription factor subunit alpha
[Source:HGNC Symbol;Acc:HGNC:4071] ETS 3303 3493 3159
2473 3234 2664 33.27234762 28.36695384
26.95455957 22.02447126 25.95845089 22.90658853
ENSG00000136937 2628.616927 2190.385546 2290.003891
2063.403668 1836.858354 1714.183513 2369.668788
1871.481845 0.340250518 0.018705991 0.095702305 NCBP1
9 97633626 97673748 + 5548
protein_coding nuclear cap binding protein subunit 1 [Source:HGNC
Symbol;Acc:HGNC:7658] - 2272 2363 2342 2054 2010
1747 22.39580036 18.77851421 19.55477819
17.90050171 15.78767592 14.69949749
ENSG00000163191 3644.429279 4079.511971 3922.927246
4437.222007 4948.551236 4860.941683 3882.289498
4748.904975 -0.290575559 0.018714381 0.095713196
S100A11 1 152032506 152047907 - 680
protein_coding S100 calcium binding protein A11 [Source:HGNC
Symbol;Acc:HGNC:10488] - 3150 4401 4012 4417 5415
4954 253.3356761 285.3490387 273.3093362
314.0651175 347.0149186 340.0894231
ENSG00000182484 211.7239867 266.0349774 259.1165803
459.0922475 359.1469318 293.3834403 245.6251815
370.5408732 -0.591741074 0.018749421 0.095860335 WASH6P

X 156020826 156025710 + 4618
transcribed_unprocessed_pseudogene "WAS protein family homolog 6, pseudogene [Source:HGNC Symbol;Acc:HGNC:31685]" - 183 287
265 457 393 299 2.16716448 2.740071693
2.6582413 4.784796679 3.708490916 3.022479024
ENSG00000106346 868.8782185 912.1199226 886.8631635
1161.292425 1214.519777 1048.91939 889.2871015
1141.577197 -0.360234495 0.018783387 0.096001885 USP42
7 6104884 6161564 + 7072 protein_coding
ubiquitin specific peptidase 42 [Source:HGNC Symbol;Acc:HGNC:20068]
- 751 984 907 1156 1329 1069
5.807542514 6.134607827 5.941108597 7.903451609
8.189197151 7.056372342
ENSG00000145725 2553.414418 1988.310197 2102.266595
1809.245378 1764.663423 1648.442073 2214.663737
1740.783625 0.346899191 0.018811989 0.096101292
PPIP5K2 5 103120149 103212799 + 17270
protein_coding diphosphoinositol pentakisphosphate kinase 2
[Source:HGNC Symbol;Acc:HGNC:29035] - 2207 2145 2150
1801 1931 1680 6.988833729 5.476069066
5.766981764 5.0422293 4.872462821 4.541120017
ENSG00000164002 2.313923351 8.342560268 7.822387329
21.09614266 24.67421669 35.3237587 6.159623649
27.03137268 -2.118578708 0.018825603 0.096101292 EXO5
1 40508741 40516556 + 3092
protein_coding exonuclease 5 [Source:HGNC Symbol;Acc:HGNC:26115]
- 2 9 8 21 27 36
0.035374086 0.128332597 0.119854117 0.328383272
0.380524721 0.543511893
ENSG00000166938 990.3591944 823.1326131 869.262792
695.1681296 699.1028062 648.5834584 894.2515332
680.9514647 0.392457105 0.018827497 0.096101292 DIS3L
15 66293217 66333898 + 5436
protein_coding DIS3 like exosome 3'-5' exoribonuclease [Source:HGNC Symbol;Acc:HGNC:28698] - 856 888 889 692 765
661 8.61170272 7.202238117 7.575734897
6.154997245 6.132542757 5.676335379
ENSG00000267311 2.313923351 6.488657986 12.71137941 0
0 0 7.171320249 0 5.33791652
0.018828077 1 AC007673.1 18 54879457
54880809 + 1100 lincRNA "novel transcript, sense intronic to RAB27B" - 2 7 13 0 0
0 0.099433339 0.28056876 0.54746092 0
0 0
ENSG00000108262 2462.014446 3058.938765 2785.747688
3231.72814 3580.503 3556.906258 2768.900299
3456.379133 -0.319663591 0.018835661 0.096101292 GIT1
17 29573469 29594054 - 5635
protein_coding GIT ArfGAP 1 [Source:HGNC Symbol;Acc:HGNC:4272] -
2128 3300 2849 3217 3918 3625 20.65248978

25.81986561	23.42076005	27.60313167	30.29905678
30.0303315
ENSG00000104518 203.6252549	335.556313	466.4098445
239.0896168	134.337402	217.8298453	335.1971375
197.0856214	0.768333318	0.018836302	0.096101292	GSDMD
8	143553207	143563062	+	5116
protein_coding	gasdermin D [Source:HGNC Symbol;Acc:HGNC:25697] -
176	362	477	238	147	222	1.881381394
3.119693964	4.319070559	2.249301318	1.252118397
2.025668965
ENSG00000135046 10227.54121	8538.146958	7300.242975
5885.823803	7588.692422	6864.583774	8688.643716	6779.7
0.357754294	0.018840555	0.096101292	ANXA1	9
73151757	73170393	+	4561	protein_coding
annexin A1 [Source:HGNC Symbol;Acc:HGNC:533]	-	8840
9211	7466	5859	8304	6996	105.9953725
89.03908083	75.82813405	62.1104452	79.33884358
71.60374948
ENSG00000083845 27666.42455	30616.26923	30272.63896
33538.84852	33346.74693	43260.81104	29518.44425
36715.46883	-0.31475074	0.018863909	0.096157998	RPS5
19	58386400	58394806	+	3617
protein_coding	ribosomal protein S5 [Source:HGNC
Symbol;Acc:HGNC:10426]	-	23913	33029	30960	33386	36490
44089	361.5599085	402.6065853	396.5106887
446.2899394	439.6265903	569.0203201
ENSG00000253540 89.08604903	207.6370555	92.89084954
248.1308208	201.049173	249.2287419	129.871318
232.8029119	-0.83836951	0.018866128	0.096157998
FAM86HP 3	130099092	130111472	-	1301
transcribed_unprocessed_pseudogene	"family with sequence
similarity 86 member H, pseudogene [Source:HGNC
Symbol;Acc:HGNC:42359]" -	77	224	95	247	220
254	3.236742433	7.591099426	3.382585357
9.17953904	7.368922381	9.113862159
ENSG00000174326 0	0	0	8.036625776	3.65543951
9.812155195	0	7.168073494	-5.260510128
0.018868134	1	SLC16A11	17	7041630 7043923 -
2041	protein_coding	solute carrier family 16 member 11
[Source:HGNC Symbol;Acc:HGNC:23093]	-	0	0	0
8	4	10	0	0	0	0.189517011
0.085403483	0.228719415
ENSG00000153006 1440.417286	951.9788217	889.7965587
1486.775769	1688.813053	1337.396753	1094.064222
1504.328525	-0.460518962	0.018870543	0.096157998
SREK1IP1	5	64718144	64768685	-	7537
protein_coding	SREK1 interacting protein 1 [Source:HGNC
Symbol;Acc:HGNC:26716]	-	1245	1027	910	1480	1848
1363	9.033697606	6.007667473	5.593006613
9.494332054	10.68469303	8.441961679

ENSG00000145416 170.0733663   177.0476679   166.2257307
108.494448   104.180026   100.083983   171.1155883
104.252819   0.715074599   0.018899466   0.096243605   1-Mar
        4   163524298   164384050   -   10421
protein_coding membrane associated ring-CH-type finger 1 [Source:HGNC
Symbol;Acc:HGNC:26077] -   147   191   170   108   114
        102   0.771440884   0.808086645   0.755687052
0.501089816   0.47670977   0.456916469
ENSG00000176438 864.2503718   879.6766327   1385.540356
906.1295563   381.0795689   728.0619154   1043.155787
671.7570135   0.635645212   0.018899935   0.096243605   SYNE3
        14   95407266   95475836   -   18164
protein_coding spectrin repeat containing nuclear envelope family
member 3 [Source:HGNC Symbol;Acc:HGNC:19861]   -   747   949
        1417   902   417   742   2.249074395   2.303502443
        3.613772534   2.401022671   1.000421829   1.906946232
ENSG00000221962 2.313923351   9.269511408   9.777984162   0
        0   0   7.120472974   0   5.328159302
0.018903363   1   TMEM14EP   3   152339695
152340990   -   1296   transcribed_processed_pseudogene
        "transmembrane protein 14E, pseudogene [Source:HGNC
Symbol;Acc:HGNC:34386]" -   2   10   10   0   0
        0   0.084395581   0.340195807   0.357435311   0
        0   0
ENSG00000145414 568.0681828   616.4225087   537.7891289
867.9555838   707.3275451   730.0243465   574.0932734
768.4358251   -0.420207173   0.018908523   0.096255276   NAF1
        4   163110073   163166921   -   4808
protein_coding nuclear assembly factor 1 ribonucleoprotein
[Source:HGNC Symbol;Acc:HGNC:25126]   -   491   665   550
        864   774   744   5.584852992   6.098052293
5.299082565   8.688613925   7.015119919   7.22361369
ENSG00000117280 1434.632478   1369.106835   1228.114811
1740.934059   1803.045538   1567.001185   1343.951375
1703.66026   -0.342476821   0.018950922   0.096438997   RAB29
        1   205767986   205775460   -   3882
protein_coding "RAB29, member RAS oncogene family [Source:HGNC
Symbol;Acc:HGNC:9789]" -   1240   1477   1256   1733   1973
        1597   17.46871128   16.77486069   14.98775427
21.58461209   22.1477775   19.20416465
ENSG00000182324 150.4050178   174.2668145   181.8705054
391.7855066   198.3075934   243.3414488   168.8474459
277.8115163   -0.716753635   0.018959018   0.096448091   KCNJ14
        19   48455509   48466980   +   4340
protein_coding potassium voltage-gated channel subfamily J member 14
[Source:HGNC Symbol;Acc:HGNC:6260]   -   130   188   186
        390   217   248   1.638129893   1.909862396
1.985297842   4.34486307   2.178856368   2.66752186
ENSG00000000419 2051.293051   1715.786562   1656.390517
1454.629265   1433.846148   1381.551451   1807.823377

1423.342288   0.344513428   0.018999327   0.096620995   DPM1
20   50934867   50958555   -   1207
protein_coding "dolichyl-phosphate mannosyltransferase subunit 1, catalytic [Source:HGNC Symbol;Acc:HGNC:3005]"   -   1773   1851
1694   1448   1569   1408   80.33340555   67.61345157
65.01425521   58.00460962   56.64665519   54.45545869
ENSG00000237550 3511.378686   4067.461606   3569.942017
3662.692197   5442.94943   5440.840055   3716.26077
4848.827228   -0.383757948   0.01901207   0.096653646   RPL9P9
15   82372196   82372912   -   717
transcribed_processed_pseudogene   ribosomal protein L9 pseudogene 9 [Source:HGNC Symbol;Acc:HGNC:17251]   -   3035
4388   3651   3646   5956   5545   231.4910754
269.824525   235.8821801   245.8661412   361.9879645
361.0176465
ENSG00000124608 644.4276534   833.3290756   630.6799784
934.2577465   891.9272404   988.0840281   702.8122358
938.0896716   -0.41578693   0.019107238   0.097105169   AARS2
6   44299654   44313326   -   4298
protein_coding "alanyl-tRNA synthetase 2, mitochondrial [Source:HGNC Symbol;Acc:HGNC:21022]" -   557   899   645   930   976
1007   7.087343739   9.222045031   6.951775831
10.46207319   9.895597091   10.93727408
ENSG00000153179 463.941632   513.530932   591.5680418
318.4512964   462.413098   294.3646558   523.0135353
358.4096834   0.544939646   0.019114281   0.097108678   RASSF3
12   64507001   64697567   +   4226
protein_coding Ras association domain family member 3 [Source:HGNC Symbol;Acc:HGNC:14271] -   401   554   605   317   506
300   5.189309728   5.779818889   6.631752927
3.626861622   5.217706209   3.313887782
ENSG00000110429 757.8098976   654.4275054   629.70218
442.0144177   562.0238246   495.5138373   680.6465277
499.8506932   0.444390729   0.019148239   0.097248881   FBXO3
11   33740939   33774543   -   12446
protein_coding F-box protein 3 [Source:HGNC Symbol;Acc:HGNC:13582]
-       655   706   644   440   615   505
2.878102231   2.500972189   2.396947526   1.709322842
2.153296908   1.894120556
ENSG00000230733 726.5719323   796.25103   947.4866653
1200.470975   1003.418145   1061.675192   823.4365425
1088.521438   -0.401953885   0.019169807   0.097326085
AC092171.2   7   5475804 5479811 +   3251   lincRNA novel transcript   -   628   859   969   1195   1098   1082
10.56421879   11.64957689   13.80731597   17.77264852
14.71783631   15.53661225
ENSG00000243402 0   0   0.977798416   26.11903377   0
7.849724156   0.325932805   11.32291931   -4.9573739
0.019192308   1   AC022973.1   8   129864478
129865052   +   575   processed_pseudogene

ribosomal protein L15 (RPL15) pseudogene	-	0	0
	1	26	0	8	0	0	0.080562811
	2.186284721	0	0.649483583
ENSG00000112983 806.402288	1063.212959	1106.867807
761.4702923	700.930526	768.2917517	992.1610179
743.56419	0.417031383	0.019207054	0.097459005	BRD8
	5	138139766	138178986	-	7046
protein_coding	bromodomain containing 8 [Source:HGNC
Symbol;Acc:HGNC:19874]	-	697	1147	1132	758	767
	783	5.409845372	7.177194853	7.442284085
5.201490184	4.743635634	5.187584202
ENSG00000136098 207.09614	249.3498569	194.5818848
149.6821551	126.1126631	135.4077417	217.0092939
137.06752	0.663933364	0.019208738	0.097459005	NEK3
	13	52132639	52159861	-	4272
protein_coding	NIMA related kinase 3 [Source:HGNC
Symbol;Acc:HGNC:7746]	-	179	269	199	149	138
	138	2.291482262	2.77622713	2.157865086
1.686383098	1.407688103	1.507974085
ENSG00000183230 10.41265508	5.561706845	4.888992081	0
	0	0	6.954451336	0	5.288350146
0.019270773	1	CTNNA3	10	65912518	67696169
	-	11136	protein_coding	catenin alpha 3 [Source:HGNC
Symbol;Acc:HGNC:2511]	-	9	6	5	0	0
	0	0.044198548	0.023755052	0.020799037	0
	0	0
ENSG00000162836 290.3973806	297.5513162	281.6059439
355.6206906	451.4467794	428.791182	289.8515469
411.952884	-0.507453875	0.019289463	0.097836106	ACP6
	1	147629652	147670496	-	11960
protein_coding	"acid phosphatase 6, lysophosphatidic [Source:HGNC
Symbol;Acc:HGNC:29609]" -	251	321	288	354	494
	437	1.147723449	1.183335274	1.115485075
1.431110931	1.799924825	1.705675036
ENSG00000234323 12.72657843	12.05036483	14.66697624
3.013734666	0	0	13.14797317	1.004578222
3.734697526	0.019336126	1	LINC01505	9
105993310	106740875	+	10944	lincRNA long
intergenic non-protein coding RNA 1505 [Source:HGNC
Symbol;Acc:HGNC:51186]	-	11	13	15	3	0
	0	0.054968174	0.052372249	0.063491799
0.013253982	0	0
ENSG00000168374 7050.524452	5900.044011	6455.425144
5364.447706	5303.128869	5241.653305	6468.664536
5303.076626	0.286498845	0.01934205	0.098070289	ARF4
	3	57571363	57598220	-	2595
protein_coding	ADP ribosylation factor 4 [Source:HGNC
Symbol;Acc:HGNC:655]	-	6094	6365	6602	5340	5803
	5342	128.428024	108.142151	117.8529923
99.49588316	97.44819654	96.09760351

ENSG00000088305 108.7543975 158.5086451 278.6725486
124.5676995 86.81668835 76.53481052 181.9785304
95.97306613 0.925203695 0.019358298 0.098120126 DNMT3B
20 32762385 32809356 + 4630
protein_coding DNA methyltransferase 3 beta [Source:HGNC
Symbol;Acc:HGNC:2979] - 94 171 285 124 95
78 1.110303159 1.628354945 2.851453704
1.294916935 0.894131123 0.786429231
ENSG00000128928 956.8073058 1027.988815 1181.180487
813.7083598 674.4285895 892.9061227 1055.325536
793.681024 0.411660456 0.019396563 0.098247432 IVD
15 40405795 40435947 + 6533
protein_coding isovaleryl-CoA dehydrogenase [Source:HGNC
Symbol;Acc:HGNC:6186] - 827 1109 1208 810 738
910 6.922892121 7.484328582 8.565579138
5.99478452 4.92268789 6.50241629
ENSG00000128578 621.2884199 729.5105478 405.7863427
841.8365501 892.8411002 767.3105362 585.5284368
833.9960622 -0.51033818 0.019399595 0.098247432 STRIP2
7 129434433 129488399 + 5876
protein_coding striatin interacting protein 2 [Source:HGNC
Symbol;Acc:HGNC:22209] - 537 787 415 838 977
782 4.99789596 5.905095195 3.271664529
6.895463247 7.245550276 6.212565803
ENSG00000179632 2136.908215 3138.656563 2951.973418
3455.749084 3349.296451 3866.970362 2742.512732
3557.338632 -0.374834156 0.019402695 0.098247432 MAF1
8 144104499 144107611 + 2377
protein_coding "MAF1 homolog, negative regulator of RNA polymerase
III [Source:HGNC Symbol;Acc:HGNC:24966]" - 1847 3386
3019 3440 3665 3941 42.49447093 62.80463992
58.83508525 69.9729973 67.18980722 77.39685061
ENSG00000156509 84.45820233 50.05536161 128.0915925
40.18312888 30.15737595 46.11712942 87.53505215
38.81921142 1.173644233 0.019433799 0.098372344 FBXO43
8 100133360 100145800 - 3950
protein_coding F-box protein 43 [Source:HGNC Symbol;Acc:HGNC:28521]
- 73 54 131 40 33 47
1.010695838 0.602740844 1.536302212 0.489625595
0.364062077 0.555452337
ENSG00000058866 409.5644332 297.5513162 280.6281454
509.3211586 451.4467794 457.2464321 329.247965
472.6714567 -0.523452292 0.019452227 0.098423044 DGKG
3 186105668 186362237 - 10978
protein_coding diacylglycerol kinase gamma [Source:HGNC
Symbol;Acc:HGNC:2853] - 354 321 287 507 494
466 1.763497094 1.289186544 1.211047357
2.232985739 1.960931036 1.98156684
ENSG00000154920 437.3315134 470.8911796 552.4561051
433.9777919 276.8995429 279.646423 486.8929327

330.1745859	0.561401834	0.019456691	0.098423044	EME1
	17	50373220	50381483	+	3294
protein_coding	essential meiotic structure-specific endonuclease 1
[Source:HGNC Symbol;Acc:HGNC:24965]	-	378	508	565
	432	303	285	6.275710738	6.799454554
7.945611177	6.341052786	4.008460713	4.038939064
ENSG00000124920 476.6682104	469.9642284	786.1499266
403.8404453	339.0420145	429.7723975	577.5941218
390.8849524	0.564027075	0.01946823	0.098448834	MYRF
	11	61752642	61788518	+	10680
protein_coding	myelin regulatory factor [Source:HGNC
Symbol;Acc:HGNC:1181]	NDT80/PhoG	412	507	804	402
	371	438	2.10969987	2.093006922	3.487283475
	1.819935585	1.513774742	1.914471447
ENSG00000267260 38.1797353	94.54901637	22.48936357
232.0575693	82.24738897	74.57237948	51.73937175
129.6257792	-1.320815894	0.0194927	0.098539983
AC020928.1	19	36773153	36777078	+	2050
	lincRNA uncharacterized LOC728485 [Source:NCBI
gene;Acc:728485]	-	33	102	23	231	90
	76	0.88034883	2.193715322	0.519728378
5.448278952	1.913142177	1.730636134
ENSG00000178605 1600.077998	2146.818842	2069.021449
2606.880486	2254.492318	2615.920575	1938.639429
2492.431126	-0.361901609	0.019502826	0.098558578	GTPBP6
	X	304529	318819	-	2964	protein_coding	GTP
binding protein 6 (putative) [Source:HGNC Symbol;Acc:HGNC:30189]
	-	1383	2316	2116	2595	2467	2666
25.5175335	34.45040356	33.07043594	42.33118045
36.27016639	41.98827004
ENSG00000134853 503.2783289	211.3448601	386.2303744
206.9431137	234.8619885	233.5292936	366.9511878
225.1114653	0.703002809	0.019509972	0.098562107	PDGFRA
	4	54229097	54298247	+	9732
protein_coding	platelet derived growth factor receptor alpha
[Source:HGNC Symbol;Acc:HGNC:8803]	-	435	228	395
	206	257	238	2.444454002	1.032920043
1.880171439	1.023449308	1.150772886	1.141618223
ENSG00000255248 1792.133636	1574.889988	1012.999159
1829.336942	2185.952827	1979.111703	1460.007594
1998.133824	-0.453194567	0.019537338	0.098667752
MIR100HG	11	122028327	122556721	-	19385
	processed_transcript	mir-100-let-7a-2-mir-125b-1 cluster
host gene [Source:HGNC Symbol;Acc:HGNC:39522]	-	1549	1699
	1036	1821	2392	2017	4.369988813	3.86421722
	2.475690818	4.541981458	5.377172485	4.857201592
ENSG00000131469 8311.612678	14105.41551	12631.19994
13340.79879	14569.66803	19567.39989	11682.74271
15825.95557	-0.437810798	0.01956206	0.098710925	RPL27
	17	42998273	43002959	+	2026

protein_coding ribosomal protein L27 [Source:HGNC
Symbol;Acc:HGNC:10328] - 7184 15217 12918 13280 15943
19942 193.9195503 331.1490836 295.3644993
316.9274458 342.9171478 459.4891986
ENSG00000165934 2391.439784 2209.85152 2165.823492
1869.520071 1903.570125 1648.442073 2255.704932
1807.177423 0.319598566 0.019562398 0.098710925 CPSF2
14 92121937 92172145 + 13595
protein_coding cleavage and polyadenylation specific factor 2
[Source:HGNC Symbol;Acc:HGNC:2325] - 2067 2384 2215
1861 2083 1680 8.314879838 7.73145081
7.547393169 6.618634179 6.676804434 5.768675446
ENSG00000111832 1342.075544 893.5808998 962.1536415
453.0647781 951.3281324 742.7801482 1065.936695
715.7243529 0.573604046 0.019565258 0.098710925 RWDD1
6 116571367 116597675 + 5909
protein_coding RWD domain containing 1 [Source:HGNC
Symbol;Acc:HGNC:20993] - 1160 964 984 451 1041
757 10.73590629 7.192783723 7.714069801
3.690317803 7.677067114 5.980368225
ENSG00000261596 0 3.707804563 0.977798416 25.11445555
9.138598774 11.77458623 1.56186766 15.34254685
-3.252002859 0.019689352 1 AC005632.2 16
21626742 21627569 + 828 sense_intronic
"novel transcript, sense intronic to METTL9" - 0
4 1 25 10 12 0 0.212992157
0.055946397 1.459858922 0.526293809 0.676545399
ENSG00000159167 2869.264956 3414.888003 1613.367387
3759.131707 6274.561918 4347.765967 2632.506782
4793.819864 -0.864767125 0.019692865 0.099298197 STC1
8 23841915 23854807 - 4149
protein_coding stanniocalcin 1 [Source:HGNC Symbol;Acc:HGNC:11373]
- 2480 3684 1650 3742 6866 4431
32.68909963 39.14805093 18.42226245 43.60753769
72.11389646 49.8544984
ENSG00000105722 976.4756543 1949.378249 1948.752243
2299.47955 2264.544776 2326.461997 1624.868716
2296.828774 -0.498584288 0.019694651 0.099298197 ERF
19 42247572 42255157 - 3295
protein_coding ETS2 repressor factor [Source:HGNC
Symbol;Acc:HGNC:3444] ETS 844 2103 1993 2289 2478
2371 14.00818086 28.139593 28.01910996
33.58857585 32.77211581 33.59094106
ENSG00000265496 2.313923351 12.05036483 6.844588913 0
0 0 7.069625699 0 5.318285034
0.019699335 1 AC100778.3 18 49487339
49491878 + 582 antisense microRNA 1539 -
2 13 7 0 0 0 0.187932428
0.984814253 0.557156897 0 0 0
ENSG00000262777 10.41265508 6.488657986 3.911193665 0

0	0	6.937502244	0	5.28500083
0.019701118	1	AC032044.1	17	1424473 1426484 +
691	antisense	"novel transcript, antisense to CRK"
-	9	7	4	0	0	0
0.712293817	0.446636232	0.268154074	0	0	0
ENSG00000170889 12120.33051	14210.16099	13456.4618
14613.5994	15485.35562	20085.48168	13262.31777
16728.14557	-0.334894339	0.019750063	0.099544744	RPS9
19	54200742	54249003	+	4371
protein_coding	ribosomal protein S9 [Source:HGNC
Symbol;Acc:HGNC:10442]	-	10476	15330	13762	14547	16945
20470	131.07184	154.630552	145.8489151
160.9140067	168.9348943	218.6165679
ENSG00000265681 214.03791	329.067655	307.0287027
478.1792337	377.4241294	402.298363	283.3780892
419.3005753	-0.562924539	0.019764418	0.099569843	RPL17
18	49488453	49492523	-	2466
protein_coding	ribosomal protein L17 [Source:HGNC
Symbol;Acc:HGNC:10307]	-	185	355	314	476	413
410	4.102734079	6.347010821	5.898465337
9.332867432	7.298196917	7.76134199
ENSG00000132801 165.4455196	200.2214464	143.7363672
367.6756293	243.0867274	212.9237677	169.8011111
274.5620415	-0.692245286	0.019768069	0.099569843	ZSWIM3
20	45857617	45879122	+	2773
protein_coding	zinc finger SWIM-type containing 3 [Source:HGNC
Symbol;Acc:HGNC:16157]	-	143	216	147	366	266
217	2.820206314	3.434296913	2.455669527
6.381641926	4.180135549	3.653052385
ENSG00000156795 639.7998067	608.0799484	464.4542477
403.8404453	398.4429066	429.7723975	570.7780009
410.6852498	0.474441232	0.019792989	0.099662523	WDYHV1
8	123416725	123470028	+	4930
protein_coding	WDYHV motif containing 1 [Source:HGNC
Symbol;Acc:HGNC:25490]	-	553	656	475	402	436
438	6.134411774	5.866659439	4.463228752
3.94257851	3.853879823	4.147374251
ENSG00000188501 133.0505927	72.30218899	72.3570828
66.30216265	16.44947779	30.4176811	92.56995483
37.72310718	1.296035992	0.019823493	0.099751995	LCTL
15	66547179	66565979	-	3518
protein_coding	lactase like [Source:HGNC Symbol;Acc:HGNC:15583]
-	115	78	74	66	18	31
1.787708553	0.977535922	0.97440239	0.907087781
0.222964267	0.411350372
ENSG00000009830 864.2503718	795.3240788	966.0648352
695.1681296	604.9752388	689.7945102	875.2130953
663.3126262	0.400120682	0.019833511	0.099751995	POMT2
14	77274956	77320884	-	10008
protein_coding	protein O-mannosyltransferase 2 [Source:HGNC

Symbol;Acc:HGNC:19743] -   747   858   988   692   662
        703   4.081953169   3.779844635   4.5731148
3.343181957   2.882499831   3.279095492
ENSG00000060138 4659.084668   5106.573835   4275.912474
5344.356141   5745.437049   6215.0191   4680.523659
5768.270764   -0.301432688   0.019835465   0.099751995   YBX3
        12   10699089   10723312   -   8610
protein_coding Y-box binding protein 3 [Source:HGNC
Symbol;Acc:HGNC:2428]   CSD   4027   5509   4373   5320   6287
        6334   25.57838918   28.21003201   23.5276625
29.87512268   31.81990705   34.34163306
ENSG00000247271 60.16200714   70.4482867   76.26827646
36.16481599   21.93263706   33.36132766   68.95952343
30.48626024   1.180433289   0.019836859   0.099751995   ZBED5-
AS1   11   10858259   10906802   +   1995
antisense   ZBED5 antisense RNA 1 [Source:HGNC
Symbol;Acc:HGNC:48646] -   52   76   78   36   24
        34   1.425460398   1.679595298   1.811148908
0.872490722   0.524236119   0.795576695
ENSG00000085185 245.2758753   337.4102153   391.1193665
495.2570635   382.9072886   573.0298634   324.601819
483.7314052   -0.573678088   0.019851968   0.099795142   BCORL1
        X   129981107   130058083   +   7996
protein_coding BCL6 corepressor like 1 [Source:HGNC
Symbol;Acc:HGNC:25657] -   212   364   400   493   419
        584   1.449965898   2.007070169   2.317339485
2.981091803   2.283493792   3.409463908
ENSG00000109689 389.8960847   411.5663065   368.6300029
669.0490959   522.7278499   457.2464321   390.030798
549.6744593   -0.494652913   0.01987111   0.099858534   STIM2
        4   26857678   27025381   +   6909
protein_coding stromal interaction molecule 2 [Source:HGNC
Symbol;Acc:HGNC:19205] -   337   444   377   666   572
        466   2.667530667   2.83335985   2.527717955
4.6607977   3.607774909   3.148594698
ENSG00000089127 19.66834849   26.88158308   9.777984162
27.12361199   87.73054823   51.02320701   18.77597191
55.29245575   -1.558610534   0.020032173   0.100634842   OAS1
        12   112906777   112933222   +   4947
protein_coding 2'-5'-oligoadenylate synthetase 1 [Source:HGNC
Symbol;Acc:HGNC:8086]   -   17   29   10   27   96
        52   0.187932428   0.258458039   0.093639815
0.263890083   0.845644679   0.490690296
ENSG00000092470 1293.483153   1224.502457   1092.200831
737.360415   1044.54184   935.0983901   1203.39548
905.6668816   0.409487201   0.020047506   0.100670355   WDR76
        15   43826963   43868419   +   4078
protein_coding WD repeat domain 76 [Source:HGNC
Symbol;Acc:HGNC:25773] -   1118   1321   1117   734   1143
        953   14.99302603   14.28201728   12.68844517

8.702620691	12.21399131	10.90917014
ENSG00000181915 1256.46038	1243.04148	1051.133297
1460.656735	1643.12006	1434.537089	1183.545052
1512.771295	-0.3544087	0.020053295	0.100670355	ADO
	10	62804857	62808483	+	3627
protein_coding	2-aminoethanethiol dioxygenase [Source:HGNC
Symbol;Acc:HGNC:23506]	-	1086	1341	1075	1454	1798
	1462	16.37483688	16.30103501	13.72977324
19.38286931	21.60233664	18.81680364
ENSG00000244398 2026.996856	1673.146809	1955.596832
2140.756191	2570.687835	2436.358135	1885.246832
2382.60072	-0.338205639	0.020058998	0.100670355
AC116533.1	11	16974693	16975013	-	321
	processed_pseudogene	ribosomal protein L36a (RPL36A)
pseudogene	-	1752	1805	2000	2131	2813	2483
	298.4858736	247.9168995	288.6206624	320.9812277
	381.8768201	361.0918805
ENSG00000157796 726.5719323	788.8354209	597.4348323
512.3348932	524.5555696	529.8563805	704.2807285
522.2489478	0.431399731	0.020099985	0.100815006	WDR19
	4	39182404	39285810	+	8873
protein_coding	WD repeat domain 19 [Source:HGNC
Symbol;Acc:HGNC:18340]	-	628	851	611	510	574
	540	3.870649755	4.228565251	3.189871472
2.779079119	2.819032019	2.840987443
ENSG00000198838 108.7543975	57.47097073	81.15726854
58.26553688	12.79403828	25.51160351	82.46087893
32.19039289	1.358797867	0.02010101	0.100815006	RYR3
	15	33310945	33866121	+	20370
protein_coding	ryanodine receptor 3 [Source:HGNC
Symbol;Acc:HGNC:10485]	-	94	62	83	58	14
	26	0.252366403	0.13419447	0.188751112
0.137669641	0.029949916	0.059583822
ENSG00000145723 232.5492968	189.0980327	131.0249878
73.33421021	114.2324847	124.614371	184.2241058
104.0603553	0.822021352	0.020117499	0.100864616	GIN1
	5	103086000	103120151	-	4182
protein_coding	gypsy retrotransposon integrase 1 [Source:HGNC
Symbol;Acc:HGNC:25959]	-	201	204	134	73	125
	127	2.628492497	2.150701297	1.484305257
0.843995339	1.302520545	1.417639248
ENSG00000228775 0	0	0	0	5.483159264
20.60552591	0	8.696228391	-5.540178405	0.0201406
	1	WEE2-AS1	7	141704338	141738346
	-	3504	antisense	WEE2 antisense RNA 1
[Source:HGNC Symbol;Acc:HGNC:48669]	-	0	0	0
	0	6	21	0	0	0	0
0.074618369	0.279770058
ENSG00000236274 8.09873173	6.488657986	5.866790497	0
	0	0	6.818060071	0	5.261349058

0.020171891 1 AC004865.1 1 35509742
35511438 + 1003 processed_pseudogene zinc finger
protein pseudogene - 7 7 6 0 0
0 0.381673335 0.307702528 0.277110367 0
0 0
ENSG00000285692 9.255693406 0.926951141 11.73358099 0
0 0 7.305408514 0 5.359771455
0.020172072 1 AC023509.6 12 53531752
53621218 + 867 antisense - 8
1 12 0 0 0 0.504621328
0.050852799 0.641157319 0 0 0
ENSG00000174574 1544.543837 1556.350965 1566.433063
1925.776452 2051.615425 1821.136004 1555.775955
1932.842627 -0.313140139 0.020189458 0.101185862
AKIRIN1 1 38991223 39006059 + 2741
protein_coding akirin 1 [Source:HGNC Symbol;Acc:HGNC:25744] -
1335 1679 1602 1917 2245 1856 26.63587345
27.00695486 27.07421865 33.81538169 35.69159099
31.60930683
ENSG00000172809 4993.446592 4211.139033 4343.380565
4683.343671 5936.433764 6674.227963 4515.98873
5764.668466 -0.352371374 0.02019481 0.101185862 RPL38
17 74203582 74210655 + 3829
protein_coding ribosomal protein L38 [Source:HGNC
Symbol;Acc:HGNC:10349] - 4316 4543 4442 4662 6496
6802 61.64399583 52.31079598 53.73975024
58.86919801 73.92975171 82.92725637
ENSG00000166402 499.8074439 446.7904499 549.5227099
419.9136968 355.4914923 232.5480781 498.7068679
335.9844224 0.569730926 0.020219651 0.101277132 TUB
11 8019244 8106112 + 6722 protein_coding tubby
bipartite transcription factor [Source:HGNC Symbol;Acc:HGNC:12406]
Tub 432 482 562 418 389 237
3.514632749 3.161422123 3.872935491 3.006623102
2.521794487 1.645871306
ENSG00000147454 3016.199089 2752.117937 3217.934588
2647.063615 2157.623171 2373.560342 2995.417204
2392.749042 0.324156063 0.020253533 0.101413611
SLC25A37 8 23528805 23575463 + 8147
protein_coding solute carrier family 25 member 37
[Source:HGNC Symbol;Acc:HGNC:29786] - 2607 2969 3291
2635 2361 2419 17.49999915 16.06743084
18.71253483 15.63810482 12.6286483 13.86066885
ENSG00000164742 52.06327541 71.37523785 54.75671131
124.5676995 124.2849433 89.29061227 59.39840819
112.7144184 -0.92214205 0.020275902 0.101492375 ADCY1
7 45574140 45723116 + 13812
protein_coding adenylate cyclase 1 [Source:HGNC Symbol;Acc:HGNC:232]
- 45 77 56 124 136 91
0.178176596 0.245792209 0.187816574 0.434076557

0.429082633	0.307560713
ENSG00000258944 0	0.926951141	0	17.07782977
15.53561792	0	0.308983714	10.87114923	-4.900772607
	0.020295708	1	AC004846.2	14	73272182
	73274081	-	777	antisense	"novel
transcript, antisense to PAPLN" -	0	1	0	17
	17	0	0	0.056743084	0	1.057862249
	0.953424923	0
ENSG00000101040 1858.080451	1827.020699	2428.851266
1579.196965	1636.72304	1547.376874	2037.984139
1587.765627	0.360249304	0.020312643	0.101643	ZMYND8
	20	47209214	47356889	-	7625
protein_coding	zinc finger MYND-type containing 8 [Source:HGNC
Symbol;Acc:HGNC:9397]	-	1606	1971	2484	1572	1791
	1577	11.51861879	11.39674246	15.09086727
9.968134979	10.23562427	9.65467994
ENSG00000224855 8.09873173	3.707804563	8.800185745	0
	0	0	6.868907346	0	5.271592075
0.020330098	1	OPA1-AS1	3	193618609
193627337	-	497	antisense	OPA1 antisense RNA 1
[Source:HGNC Symbol;Acc:HGNC:40421]	-	7	4	9
	0	0	0	0.77025826	0.354844077
0.838858243	0	0	0
ENSG00000130803 1148.862944	1142.003806	1048.199902
1556.091666	1436.587727	1271.655313	1113.022217
1421.444902	-0.352958839	0.020330154	0.101697336	ZNF317
	19	9140380 9163424 +	5065	protein_coding	zinc
finger protein 317 [Source:HGNC Symbol;Acc:HGNC:13507]	zf-C2H2 993
	1232	1072	1549	1572	1296	10.72172124
10.72420769	9.804327085	14.78676547	13.5248261
11.94459937
ENSG00000267365 8.09873173	18.53902282	9.777984162
2.009156444	0	0.981215519	12.13857957	0.996790655
	3.629153907	0.020362455	1	KCNJ2-AS1	17
	70166961	70169402	-	2442	antisense
	KCNJ2 antisense RNA 1 [Source:HGNC Symbol;Acc:HGNC:43720]
	-	7	20	10	2	0	1
0.156764273	0.361092355	0.189695398	0.039599122	0
	0.019116148
ENSG00000263884 0	0	3.911193665	26.11903377
11.88017841	7.849724156	1.303731222
15.28297878	-3.529132934	0.020365315	1	AP000845.1
	18	268148	270278	+	2131	lincRNA novel
transcript	-	0	0	4	26	13	8
	0	0	0.086951884	0.589917276	0.265838881
	0.175247799
ENSG00000156427 21.98227184	12.97731597	8.800185745	0
	3.65543951	0	14.58659119	1.218479837
3.549182399	0.020382312	1	FGF18	5	171419656
	171457623	+	1986	protein_coding	fibroblast

growth factor 18 [Source:HGNC Symbol;Acc:HGNC:3674]	-	19
	14	9	0	4	0	0.523201607
0.310801245	0.209925754	0	0.087768635	0
ENSG00000131748 1571.153956	2011.483976	2064.132457
2372.81376	2279.166534	2539.385764	1882.256796
2397.12202	-0.348285221	0.020397136	0.10198326	STARD3
	17	39637065	39663484	+	5160
protein_coding	StAR related lipid transfer domain containing 3
[Source:HGNC Symbol;Acc:HGNC:17579]	-	1358	2170	2111
	2362	2494	2588	14.39278312	18.54146263
18.95138644	22.13254766	21.06227822	23.4131909
ENSG00000145354 1597.764074	1198.547825	1266.248949
898.0929305	1017.126044	1155.871882	1354.186949
1023.696952	0.402962811	0.020400655	0.10198326	CISD2
	4	102868974	102892807	+	6352
protein_coding	CDGSH iron sulfur domain 2 [Source:HGNC
Symbol;Acc:HGNC:24212]	-	1381	1293	1295	894	1113
	1178	11.88989178	8.97474243	9.444125177
6.805001823	7.635601817	8.657267498
ENSG00000086827 1742.384284	1391.353662	1479.409004
1279.832655	1279.403828	904.680709	1537.71565
1154.639064	0.412866598	0.020420343	0.102048312	ZW10
	11	113733187	113773811	-	2992
protein_coding	zw10 kinetochore protein [Source:HGNC
Symbol;Acc:HGNC:13194]	-	1506	1501	1513	1274	1400
	922	27.52695009	22.11836706	23.42501052
20.58775803	20.39036708	14.38518222
ENSG00000044115 9171.235203	7906.893231	8744.451236
7286.205844	6539.581283	7371.872198	8607.526557
7065.886442	0.284682384	0.02043688	0.102097577	CTNNA1
	5	138610967	138935034	+	8830
protein_coding	catenin alpha 1 [Source:HGNC Symbol;Acc:HGNC:2509]
	-	7927	8530	8943	7253	7156	7513
49.0956334	42.59143626	46.91643269	39.71533136
35.31573311	39.71903798
ENSG00000226026 0	12.97731597	11.73358099	0	0
	0	8.236965655	0	5.538816308	0.020463067
	1	AC092802.1	1	95163219	95233982
	-	2505	antisense	uncharacterized LOC101928118
[Source:NCBI gene;Acc:101928118]	-	0	14	12
	0	0	0	0	0.246407693	0.221909539
	0	0	0
ENSG00000256185 11.56961676	8.342560268	5.866790497
1.004578222	0	0	8.592989174	0.334859407
4.632450814	0.020505873	1	AC055720.2	12
26335864	26336950	-	172
3prime_overlapping_ncRNA	novel transcript	-	10
	9	6	1	0	0	3.179554444
2.307002263	1.615940104	0.281107718	0	0
ENSG00000188878 197.8404465	253.9846126	246.4052009

147.6729986	123.3710835	171.7127159	232.74342
147.5855993	0.658929404	0.020524488	0.10250175	FBF1
	17	75909574	75941140	-	6730
protein_coding	Fas binding factor 1 [Source:HGNC
Symbol;Acc:HGNC:24674]	-	171	274	252	147	135
	175	1.389555056	1.795020681	1.73455443
1.056096217	0.87413257	1.213861173
ENSG00000114554 5368.302175	4910.987144	9858.163632
3787.259897	2960.906003	4322.254363	6712.484317
3690.140088	0.86320426	0.020549743	0.102594355	PLXNA1
	3	126988594	127037392	+	10944
protein_coding	plexin A1 [Source:HGNC Symbol;Acc:HGNC:9099]	-
	4640	5298	10082	3770	3240	4405	23.18657539
	21.34370588	42.67495428	16.65583778	12.90112323
	18.78952772
ENSG00000215421 562.2833744	460.694717	461.5208524
756.4474012	619.5969969	639.7525187	494.8329813
671.9323056	-0.442165573	0.020574205	0.102682951	ZNF407
	18	74597870	75065671	+	12190
protein_coding	zinc finger protein 407 [Source:HGNC
Symbol;Acc:HGNC:19904]	zf-C2H2 486	497	472	753	678
	652	2.180355333	1.797573434	1.793662584
2.986706087	2.42373194	2.496835474
ENSG00000163344 716.1592773	776.785056	784.1943298
848.8685976	1023.523063	1130.360278	759.046221
1000.917313	-0.398847423	0.020594823	0.102752302	PMVK
	1	154924734	154936991	-	1280
protein_coding	phosphomevalonate kinase [Source:HGNC
Symbol;Acc:HGNC:9141]	-	619	838	802	845	1120
	1152	26.4469377	28.86476373	29.02464085
31.91890291	38.12998643	42.0134693
ENSG00000234754 23.13923351	0	2.933395248	0	0
	0	8.690876254	0	5.606504128	0.020628865
	1	C1orf140	1	221330080	221336296
	-	4340	lincRNA	uncharacterized LOC400804 [Source:NCBI
gene;Acc:400804]	-	20	0	3	0	0
	0	0.252019984	0	0.032020933	0	0
	0
ENSG00000135316 7698.42299	7094.884032	8126.482637
6488.570736	6249.887702	6235.624626	7639.929886
6324.694355	0.272542652	0.020650392	0.1029829
SYNCRIP 6	85607785	85643792	-	8205
protein_coding	synaptotagmin binding cytoplasmic RNA interacting
protein [Source:HGNC Symbol;Acc:HGNC:16918]	-	6654	7654
	8311	6459	6839	6355	44.35054121	41.1285909
	46.92206888	38.06167667	36.32223936	36.15621876
ENSG00000033800 945.2376891	819.4248085	824.2840648
723.2963199	556.5406653	664.2829067	862.9821875
648.039964	0.413162242	0.020654515	0.1029829	PIAS1
	15	68054179	68198603	+	10776

protein_coding protein inhibitor of activated STAT 1 [Source:HGNC
Symbol;Acc:HGNC:2752]   zf-MIZ 817   884   843   720   609
        677   4.146285344   3.61683453   3.623868648
3.230547494   2.462738545   2.932764035
ENSG00000274425 8.09873173   8.342560268   0.977798416
48.21975466   33.81281546   6.868508636   5.806363471
29.63369292   -2.356130365   0.020699428   0.103173187
AC114271.1   19   10333436   10336248   +   2813
        antisense   "novel transcript, antisense to ICAM3" -
        7   9   1   48   37   7   0.136088999
        0.141060928   0.016467692   0.825035663   0.573179421
        0.116164745
ENSG00000186566 1779.407057   2076.370555   2137.467338
2959.487442   2152.140011   2597.27748   1997.748317
2569.634978   -0.362744031   0.020757164   0.103426744
GPATCH8 17   44395284   44503430   -   9734
protein_coding G-patch domain containing 8 [Source:HGNC
Symbol;Acc:HGNC:29066] -   1538   2240   2186   2946   2355
        2647   8.640914468   10.14590133   10.40306403
14.63331148   10.54285339   12.69429642
ENSG00000181404 167.759443   304.0399742   297.2507185
397.8129759   405.7537856   370.8994664   256.3500452
391.4887426   -0.608283281   0.020763831   0.103426744   WASHC1
        9   14521   73865   -   2270   protein_coding WASH
complex subunit 1 [Source:HGNC Symbol;Acc:HGNC:24361]   -   145
        328   304   396   444   378   3.493307834
6.370623576   6.203691348   8.434717571   8.523455747
7.773417227
ENSG00000188021 2354.41701   2275.665051   2350.627392
2557.656153   3267.962922   2895.566998   2326.903151
2907.062024   -0.321331868   0.020799835   0.103572336   UBQLN2
        X   56563639   56567868   +   4230
protein_coding ubiquilin 2 [Source:HGNC Symbol;Acc:HGNC:12509] -
        2035   2455   2404   2546   3576   2951   26.30987344
        25.58851525   26.32670771   29.10175957   36.83967078
        32.56678432
ENSG00000237115 17.35442514   12.05036483   31.28954932
4.018312888   5.483159264   0.981215519   20.23144643
3.494229224   2.528742838   0.020925376   1   AL139805.1
        6   131825981   131828413   -   2433
processed_pseudogene   T-cell activation leucine repeat-rich protein
(TA-LRRP) pseudogene   -   15   13   32   4   6
        1   0.337166069   0.235578255   0.609270745
0.079491208   0.107465172   0.019186861
ENSG00000150787 902.4301071   793.4701766   692.2812786
600.7377768   641.5296339   538.6873202   796.0605208
593.651577   0.422483731   0.02092902   0.104181678   PTS
        11   112226365   112269955   +   3130
protein_coding 6-pyruvoyltetrahydropterin synthase [Source:HGNC
Symbol;Acc:HGNC:9689]   -   780   856   708   598   702

549	13.62840333	12.05766976	10.47831321
9.237576818	9.773528244	8.187928522
ENSG00000225163 32.39492692	18.53902282	9.777984162	0
0	6.868508636	20.2373113	2.289502879
3.147843597	0.020932013	1	LINC00618	14
96931367	96945394	+	700	sense_overlapping
long intergenic non-protein coding RNA 618 [Source:HGNC
Symbol;Acc:HGNC:20110]	-	28	20	10	0	0
7	2.187533457	1.259696474	0.661765947	0
0	0.466816326
ENSG00000198712 42100.67842	39908.02747	54809.51242
59006.91561	54849.86984	56740.74984	45606.07277
56865.8451	-0.318328877	0.020993904	0.104470639	MT-CO2
MT	7586	8269	+	684	protein_coding
mitochondrially encoded cytochrome c oxidase II [Source:HGNC
Symbol;Acc:HGNC:7421]	-	36389	43053	56054	58738	60020
57827	2909.435489	2775.116857	3796.233902
4152.066204	3823.829216	3946.577143
ENSG00000077238 1102.584477	1046.527838	1283.84932
725.3054763	936.7063744	937.0608211	1144.320545
866.3575572	0.40126317	0.021031206	0.104622206	IL4R
16	27313668	27364778	+	5894
protein_coding	interleukin 4 receptor [Source:HGNC
Symbol;Acc:HGNC:6015]	-	953	1129	1313	722	1025
955	8.842549138	8.445352249	10.31946186
5.922816568	7.578309389	7.563786748
ENSG00000103540 1242.57684	1000.180281	1308.294281
915.1707603	1014.384464	702.5503119	1183.683801
877.368512	0.431550383	0.021044199	0.104652789	CCP110
16	19523811	19553408	+	10142
protein_coding	centriolar coiled-coil protein 110 [Source:HGNC
Symbol;Acc:HGNC:24342]	-	1074	1079	1338	911	1110
716	5.791291001	4.690636692	6.111319131
4.34306158	4.769336557	3.295607268
ENSG00000135926 2462.014446	2672.400139	2135.511741
2634.004098	3330.105393	3277.259835	2423.308775
3080.456442	-0.346222224	0.021071409	0.104753852	TMBIM1
2	218274192	218292586	-	5098
protein_coding	transmembrane BAX inhibitor motif containing 1
[Source:HGNC Symbol;Acc:HGNC:23410]	-	2128	2883	2184
2622	3644	3340	22.82792858	24.93324297
19.84518988	24.86761143	31.14849982	30.58388637
ENSG00000116525 111.0683209	304.9669253	283.5615407
163.7462502	85.90282848	126.576802	233.198929
125.4086269	0.897995813	0.021078227	0.104753852	TRIM62
1	33145402	33182059	-	3646
protein_coding	tripartite motif containing 62 [Source:HGNC
Symbol;Acc:HGNC:25574]	-	96	329	290	163	94
129	1.439956198	3.978443471	3.684544358
2.161584197	1.12349149	1.651654032

ENSG00000126012 2003.857622 2054.123728 2251.869752
2713.365778 2382.4327 2736.610084 2103.283701
2610.802854 -0.31162914 0.021142301 0.105038137 KDM5C
X 53191321 53225422 - 8727
protein_coding lysine demethylase 5C [Source:HGNC
Symbol;Acc:HGNC:11114] ARID 1732 2216 2303 2701 2607
2789 10.85369528 11.19537739 12.22450881
14.96445225 13.01771182 14.91865168
ENSG00000213371 0 0 0 32.1465031 0 0
0 10.71550103 -5.840040276 0.021153366 1
NAP1L1P3 3 120805866 120806745 +
880 processed_pseudogene nucleosome assembly protein 1
like 1 pseudogene 3 [Source:HGNC Symbol;Acc:HGNC:38007] - 0
0 0 32 0 0 0 0 0
1.758201 0 0
ENSG00000235505 15.04050178 0 9.777984162 0 0
0 8.272828649 0 5.537708372 0.021181266
1 CASP17P 11 104901549 104919073 -
4307 transcribed_unprocessed_pseudogene "caspase 17,
pseudogene [Source:HGNC Symbol;Acc:HGNC:53657]" - 13 0
10 0 0 0 0.165068116 0
0.107554252 0 0 0
ENSG00000134900 2541.844802 2389.680041 2235.247179
2199.021728 1707.090251 1730.864176 2388.924007
1878.992052 0.346419229 0.021184637 0.105214276 TPP2
13 102597003 102679958 + 6752
protein_coding tripeptidyl peptidase 2 [Source:HGNC
Symbol;Acc:HGNC:12016] - 2197 2578 2286 2189 1868
1764 17.79476824 16.83388815 15.68361773
15.67525247 12.05599436 12.19585305
ENSG00000198618 284.6125722 196.5136419 218.0490468
137.6272164 134.337402 168.7690693 233.0584203
146.9112292 0.66431266 0.021239523 0.105452612
PPIAP22 21 18857779 18858276 + 498
processed_pseudogene peptidylprolyl isomerase A pseudogene 22
[Source:HGNC Symbol;Acc:HGNC:17236] - 246 212 223
137 147 172 27.01472041 18.76897156
20.7433061 13.30124953 12.86312795 16.12297349
ENSG00000148344 32.39492692 52.83621503 113.4246163
25.11445555 15.53561792 33.36132766 66.21858607
24.67046704 1.428375766 0.021250933 0.105475005 PTGES
9 129738331 129753047 - 1896
protein_coding prostaglandin E synthase [Source:HGNC
Symbol;Acc:HGNC:9599] - 28 57 116 25 17
34 0.80763366 1.325471764 2.834145302
0.637533327 0.390723188 0.837117883
ENSG00000178397 828.3845598 722.0949387 802.7724997
1050.78882 1058.249738 933.135959 784.4173327
1014.058172 -0.370957777 0.021270858 0.105520802
FAM220A 7 6329409 6348981 - 2395 protein_coding family

with sequence similarity 220 member A [Source:HGNC
Symbol;Acc:HGNC:22422]	-	716	779	821	1046	1158
	951	16.34941498	14.34055297	15.87961962
21.11676483	21.06985949	18.53621401
ENSG00000275591 34.70885027	76.00999355	97.77984162
14.06409511	46.60685375	13.73701727	69.49956181
24.80265538	1.484458197	0.021273966	0.105520802	XKR5
	8	6808517 6835644 -	5031	protein_coding	XK
related 5 [Source:HGNC Symbol;Acc:HGNC:20782]	-	30	82
	100	14	51	14	0.326108148	0.718610391
	0.920763592	0.134547284	0.441747862	0.129903171
ENSG00000146197 67.10377719	92.69511408	185.7816991
67.30674088	33.81281546	58.87293117	115.1935301
53.33082917	1.11426012	0.021283677	0.105534728	SCUBE3
	6	35214419	35253079	+	7356
protein_coding	"signal peptide, CUB domain and EGF like domain
containing 3 [Source:HGNC Symbol;Acc:HGNC:13655]"	-	58
	100	190	67	37	60	0.431202218
0.599366185	1.1965045	0.440386806	0.219188922
0.380763724
ENSG00000072954 55.53416043	78.79084697	45.95652556
178.8149235	90.47212786	93.21547435	60.09384432
120.8341752	-1.005234075	0.021309221	0.105627129
TMEM38A 19	16661127	16690029	+	3005
protein_coding	transmembrane protein 38A [Source:HGNC
Symbol;Acc:HGNC:28462]	-	48	85	47	178	99
	95	0.873557454	1.247120469	0.724529107
2.86402459	1.435652449	1.475792044
ENSG00000135414 281.1416872	304.0399742	286.4949359
387.7671937	366.4578108	488.6453287	290.5588658
414.2901111	-0.511228692	0.021335623	0.105723716	GDF11
	12	55743280	55757278	+	8657
protein_coding	growth differentiation factor 11 [Source:HGNC
Symbol;Acc:HGNC:4216]	-	243	328	293	386	401
	498	1.535089032	1.670476553	1.567843315
2.155862725	2.018531601	2.685393671
ENSG00000112167 432.7036667	433.8131339	346.1406393
588.6828381	543.7466271	529.8563805	404.2191467
554.0952819	-0.455276424	0.02134453	0.10573358	SAYSD1
	6	39104064	39115189	-	2925
protein_coding	SAYSVFN motif domain containing 1 [Source:HGNC
Symbol;Acc:HGNC:21025]	-	374	468	354	586	595
	540	6.992628316	7.054300253	5.606345359
9.686635593	8.864407103	8.618147549
ENSG00000171490 5576.555277	6178.129354	5488.38251
6639.257469	7599.658741	6765.481007	5747.689047
7001.465739	-0.284649266	0.021355484	0.105753575	RSL1D1
	16	11833850	11851585	-	6167
protein_coding	ribosomal L1 domain containing 1 [Source:HGNC
Symbol;Acc:HGNC:24534]	-	4820	6665	5613	6609	8316

6895 42.74327576 47.64969919 42.16222772
51.8158969 58.76234653 52.19229065
ENSG00000165629 2689.935896 2379.483579 2366.272167
1781.117188 2056.184724 2111.575798 2478.563881
1982.959237 0.321526873 0.021413442 0.105967178
ATP5F1C 10 7788129 7807815 + 2326 protein_coding ATP
synthase F1 subunit gamma [Source:HGNC Symbol;Acc:HGNC:833] -
2325 2567 2420 1773 2250 2152 54.66482468
48.6575364 48.19567991 36.8553247 42.15328312
43.18954138
ENSG00000235897 19.66834849 48.20145932 43.02313031
71.32505376 88.64440811 86.34696571 36.96431271
82.10547586 -1.14480918 0.021422416 0.105967178
TM4SF19-AS1 3 196318330 196325570 + 2136
antisense TM4SF19 antisense RNA 1 [Source:HGNC
Symbol;Acc:HGNC:41085] - 17 52 44 71 97
88 0.435253614 1.073336883 0.954231797
1.607157047 1.978923855 1.923213326
ENSG00000101361 4358.274632 5076.911398 4850.857943
6025.460176 5247.383416 6339.633471 4762.014658
5870.825688 -0.301789433 0.021423176 0.105967178 NOP56
20 2652145 2658393 + 5398 protein_coding NOP56
ribonucleoprotein [Source:HGNC Symbol;Acc:HGNC:15911] - 3767
5477 4961 5998 5742 6461 38.16431333
44.73462681 42.57344582 53.7247988 46.3541803
55.8744031
ENSG00000167487 244.1189136 261.4002217 391.1193665
420.918275 387.476588 529.8563805 298.8795006
446.0837479 -0.576652538 0.021429124 0.105967178 KLHL26
19 18636965 18671714 + 4797
protein_coding kelch like family member 26 [Source:HGNC
Symbol;Acc:HGNC:25623] - 211 282 400 419 424
540 2.405511567 2.591870793 3.862715557
4.223237653 3.851720241 5.254968017
ENSG00000108389 1920.556382 1593.429011 1854.883595
1491.79866 1466.745103 1265.76802 1789.622996
1408.103928 0.345562354 0.021433278 0.105967178 MTMR4
17 58489529 58517905 - 7475
protein_coding myotubularin related protein 4 [Source:HGNC
Symbol;Acc:HGNC:7452] - 1660 1719 1897 1485 1605
1290 12.14483458 10.13908205 11.75597326
9.605422519 9.356694234 8.056094448
ENSG00000198055 792.5187479 1004.815037 924.0195033
1346.134818 1155.118885 1067.562485 907.1177626
1189.605396 -0.390312608 0.021451495 0.106009867 GRK6
5 177403204 177442901 + 4355
protein_coding G protein-coupled receptor kinase 6 [Source:HGNC
Symbol;Acc:HGNC:4545] - 685 1084 945 1340 1264
1088 8.60195418 10.97425585 10.05185245
14.87708538 12.64787347 11.66236882

ENSG00000158417 3842.269725 3620.671156 3618.831938
3045.881169 2971.872321 3125.17143 3693.924273
3047.64164 0.277384003 0.021455781 0.106009867 EIF5B
2 99337353 99401326 + 7883
protein_coding eukaryotic translation initiation factor 5B
[Source:HGNC Symbol;Acc:HGNC:30793] - 3321 3906 3701
3032 3252 3185 23.03944758 21.84613788
21.74853533 18.59682854 17.97701613 18.8609666
ENSG00000141644 1686.850123 1902.103741 1908.662508
2177.925585 2524.080981 2172.41116 1832.538791
2291.472576 -0.322253005 0.021489957 0.106089673 MBD1
18 50266882 50281774 - 6734
protein_coding methyl-CpG binding domain protein 1 [Source:HGNC
Symbol;Acc:HGNC:6916] MBD 1458 2052 1952 2168 2762
2214 11.84074763 13.43501644 13.42793274
15.56637119 17.8734817 15.34795582
ENSG00000178971 329.7340776 357.8031404 419.4755205
441.0098395 530.0387289 580.8795875 369.0042462
517.3093853 -0.486848735 0.021491902 0.106089673 CTC1
17 8224821 8248095 - 8566 protein_coding CST
telomere replication complex component 1 [Source:HGNC
Symbol;Acc:HGNC:26169] - 285 386 429 439 580
592 1.819539561 1.986749867 2.319966308
2.4779222 2.950587656 3.226188008
ENSG00000064102 1079.445243 1062.286007 961.1758431
737.360415 900.1519793 743.7613638 1034.302365
793.7579193 0.38141095 0.021492753 0.106089673 INTS13
12 26905181 26938326 - 3345
protein_coding integrator complex subunit 13 [Source:HGNC
Symbol;Acc:HGNC:20174] - 933 1146 983 734 985
758 15.2538768 15.10505996 13.61318829
10.60965237 12.83212868 10.57837892
ENSG00000119280 905.9009921 1018.719304 1186.069479
796.6305301 811.5075711 775.1602604 1036.896592
794.4327872 0.384732553 0.02160142 0.106565728
C1orf198 1 230837119 230869589 - 5161
protein_coding chromosome 1 open reading frame 198
[Source:HGNC Symbol;Acc:HGNC:25900] - 783 1099 1213
793 888 790 8.297029147 9.388534172
10.88753082 7.429174249 7.497866516 7.145609324
ENSG00000110852 16.19746346 4.634755704 0 42.19228532
41.12369448 18.64309487 6.944073055 33.98635823
-2.313846157 0.021603139 0.106565728 CLEC2B 12
9852984 9870136 - 2638 protein_coding C-type lectin
domain family 2 member B [Source:HGNC Symbol;Acc:HGNC:2053] -
14 5 0 42 45 19 0.290233779
0.083565915 0 0.769796116 0.743355091
0.336221008
ENSG00000081148 0 0 0 20.09156444 5.483159264
0 0 8.524907902 -5.510518922 0.021605687

1	IMPG2	3	101222546	101320560	-
8337	protein_coding	interphotoreceptor matrix proteoglycan
2 [Source:HGNC Symbol;Acc:HGNC:18362]	-	0	0	0
20	6	0	0	0	0	0.11599023
0.031361733	0
ENSG00000241409 0	0	0	20.09156444	5.483159264
0	0	8.524907902	-5.510518922	0.021605687
1	AC064852.1	2	232760146	232767949
-	851	3prime_overlapping_ncRNA	novel
transcript	-	0	0	0	20	6	0
0	0	0	1.13632262	0.307241791	0
ENSG00000111011 1220.594568	1263.434405	1339.58383
1058.825446	698.1889463	1081.299502	1274.537601
946.1046316	0.430417899	0.021620459	0.106597677	RSRC2
12	122503458	122527000	-	8741
protein_coding	arginine and serine rich coiled-coil 2 [Source:HGNC
Symbol;Acc:HGNC:30559]	-	1055	1363	1370	1054	764
1102	6.60064008	6.874936538	7.260422646
5.830163137	3.808823395	5.885271602
ENSG00000142208 2222.523379	2451.785768	2949.040023
1843.401037	2122.896495	2035.040987	2541.11639
2000.446173	0.345292901	0.021623562	0.106597677	AKT1
14	104769349	104795751	-	11162
protein_coding	AKT serine/threonine kinase 1 [Source:HGNC
Symbol;Acc:HGNC:391]	-	1921	2645	3016	1835	2323
2074	9.41195971	10.44762597	12.51675567
7.94868464	9.069133385	8.673867221
ENSG00000163682 6249.906972	7857.764821	7264.064434
7141.54658	9568.112917	10918.9663	7123.912076
9209.541933	-0.370383898	0.021632213	0.106605946	RPL9
4	39452521	39458949	-	5022
protein_coding	ribosomal protein L9 [Source:HGNC
Symbol;Acc:HGNC:10369]	-	5402	8477	7429	7109	10470
11128	58.82644233	74.42167369	68.52611422
68.44362803	90.85076133	103.4395076
ENSG00000132467 1878.905761	1445.116829	1298.516297
1910.707778	1954.746278	2141.012263	1540.846296
2002.15544	-0.378496928	0.021682738	0.106820501	UTP3
4	70688479	70690551	+	2073
protein_coding	"UTP3, small subunit processome component [Source:HGNC
Symbol;Acc:HGNC:24477]" -	1624	1559	1328	1902	2139
2182	42.84315406	33.1574231	29.67571754
44.36213376	44.96453228	49.13619018
ENSG00000243051 0	0	0	0	26.50193645
0.981215519	0	9.161050655	-5.61767475	0.021687822
1	RN7SL269P	1	169957942	169958246
-	305	misc_RNA	"RNA, 7SL, cytoplasmic 269,
pseudogene [Source:HGNC Symbol;Acc:HGNC:46285]" -	0	0
0	0	29	1	0	0	0	0
4.143398994	0.153054533

ENSG00000128602 156.1898262	185.3902282	204.359869
365.6664728	257.7084854	229.6044316	181.9799745
284.3264633	-0.642172572	0.021710132	0.106920999	SMO
	7	129188872	129213545	+	4311
protein_coding	"smoothened, frizzled class receptor [Source:HGNC
Symbol;Acc:HGNC:11119]" -	135	200	209	364	282
	234	1.712578385	2.045436167	2.245798146
4.082484808	2.850556695	2.53386732
ENSG00000143751 1366.371739	1296.804646	1094.156428
1594.265638	1820.408876	1434.537089	1252.444271
1616.403868	-0.368480099	0.021725971	0.106964547	SDE2
	1	225982702	225999331	-	3975
protein_coding	SDE2 telomere maintenance homolog [Source:HGNC
Symbol;Acc:HGNC:26643]	-	1181	1399	1119	1587	1992
	1462	16.2482831	15.51724222	13.04053501
19.30372003	21.83789627	17.16944573
ENSG00000243155 8.09873173	27.80853423	5.866790497
41.1877071	40.20983461	54.94806909	13.92468548
45.44853693	-1.696193368	0.021740178	0.107000034
AL162431.2	1	180944042	180976482	-	2424
	antisense	novel transcript	-	7	30
	6	41	44	56	0.157928364	0.545660601
	0.114662417	0.817810077	0.791003962	1.078453557
ENSG00000155252 907.0579538	1019.646255	869.262792
1285.860124	1270.26523	1060.693977	931.9890002
1205.606443	-0.371158156	0.021760041	0.107063326	PI4K2A
	10	97640686	97676434	+	4189
protein_coding	phosphatidylinositol 4-kinase type 2 alpha
[Source:HGNC Symbol;Acc:HGNC:30031]	-	784	1100	889
	1280	1390	1081	10.2352962	11.5775398
9.830913082	14.77409291	14.45982502	12.04651344
ENSG00000279329 12.72657843	8.342560268	23.46716199
23.10529911	52.09001301	62.79779325	14.84543356
45.99770179	-1.631975545	0.021785904	0.107156086
AC020910.5	19	34675717	34677581	-	1865
	TEC	novel transcript	-	11	9	24
	23	57	64	0.322558553	0.212763748
0.596121604	0.596279964	1.33184786	1.601943423
ENSG00000156140 93.71389573	110.3071858	147.6475608
86.39372709	53.00387289	34.34254318	117.2228808
57.91338106	1.019228575	0.021794854	0.107165626
ADAMTS3 4	72280969	72569386	-	7172
protein_coding	ADAM metallopeptidase with thrombospondin type 1 motif
3 [Source:HGNC Symbol;Acc:HGNC:219]	-	81	119	151
	86	58	35	0.617645741	0.731544313
0.975302017	0.57977487	0.352408448	0.227810533
ENSG00000254999 2272.272731	2550.042588	3037.041881
2081.486076	1855.135551	2228.340445	2619.785733
2054.987357	0.350658338	0.021820271	0.107256104	BRK1
	3	10115592	10127190	+	1233

protein_coding "BRICK1, SCAR/WAVE actin nucleating complex subunit [Source:HGNC Symbol;Acc:HGNC:23057]"   -   1964   2751   3106
        2072   2030   2271   87.11102413   98.36972828
116.6919321   81.25084588   71.74498664   85.98052517
ENSG00000266066 34.70885027   11.12341369   21.51156516
79.36167954   24.67421669   86.34696571   22.44794304
63.46095398   -1.506208625   0.021829973   0.107269304
POLRMTP1   17   62136972   62140639   -   3668
        processed_pseudogene   RNA polymerase mitochondrial pseudogene 1 [Source:HGNC Symbol;Acc:HGNC:28865]   -   30
        12   22   79   27   88   0.447287375
0.144240054   0.277840665   1.041355418   0.320769476
1.119951926
ENSG00000105516 112.2252825   136.2618177   215.1156516
290.3231062   222.9818101   246.2850954   154.5342506
253.1966705   -0.710137023   0.021867482   0.107419088   DBP
        19   48630030   48637438   -   6544
protein_coding D-box binding PAR bZIP transcription factor [Source:HGNC Symbol;Acc:HGNC:2697]   TF_bZIP 97   147   220
        289   244   251   0.810630904   0.990394003
1.557334289   2.135284603   1.624819541   1.790508828
ENSG00000105298 551.8707193   910.2660203   846.7734284
929.2348554   988.7963874   1339.359184   769.6367227
1085.796809   -0.495366592   0.021876914   0.107430899   CACTIN
        19   3610641 3626815 -   5952
protein_coding "cactin, spliceosome C complex subunit [Source:HGNC Symbol;Acc:HGNC:29938]" -   477   982   866   925   1082
        1365   4.382785027   7.274154536   6.739961648
7.514152878   7.921782896   10.70571714
ENSG00000165916 4768.996027   4930.453118   5549.98381
4023.335779   4021.897321   4433.131717   5083.144319
4159.454939   0.289419839   0.021904429   0.107531474   PSMC3
        11   47418769   47426473   -   2276
protein_coding "proteasome 26S subunit, ATPase 3 [Source:HGNC Symbol;Acc:HGNC:9549]" -   4122   5319   5676   4005   4401
        4518   99.04451791   103.0366406   115.5240976
85.08078322   84.26315355   92.66591208
ENSG00000166086 1183.571794   1112.341369   1447.141656
1012.614848   879.1332021   985.1403815   1247.68494
958.9628105   0.379951979   0.021937331   0.107658421   JAM3
        11   134068925   134152001   +   5451
protein_coding junctional adhesion molecule 3 [Source:HGNC Symbol;Acc:HGNC:15532] -   1023   1200   1480   1008   962
        1004   10.26346875   9.705971729   12.57731648
8.940989124   7.690551553   8.598121278
ENSG00000111642 650.2124618   799.9588346   750.9491836
519.3669408   475.2071363   630.921579   733.7068266
541.8318854   0.438226781   0.021977658   0.107802558   CHD4
        12   6570082 6614524 -   12713   protein_coding
chromodomain helicase DNA binding protein 4 [Source:HGNC

Symbol;Acc:HGNC:1919]	-	562	863	768	517	520
	643	2.417591841	2.992931015	2.798437609
1.966272533	1.782435792	2.361070537
ENSG00000213096 360.9720428	294.7704628	306.0509043
161.7370937	280.5549824	174.6563625	320.5978033
205.6494795	0.63863814	0.021980806	0.107802558	ZNF254
	19	24033405	24129961	+	7602
protein_coding	zinc finger protein 254 [Source:HGNC
Symbol;Acc:HGNC:13047]	zf-C2H2 312	318	313	161	307
	178	2.244509467	1.844306992	1.907299646
1.02399828	1.75982348	1.093045329
ENSG00000274204 15.04050178	5.561706845	0.977798416	0
	0	0	7.193335682	0	5.334915412
0.02199171	1	AL138689.1	13	106506046
106506713	-	668	sense_intronic	"novel transcript,
sense intronic to EFNB2"	-	13	6	1	0
	0	0	1.064293972	0.396012364	0.069346731
	0	0	0
ENSG00000185453 144.6202095	346.6797267	348.0962362
479.1838119	434.0834418	431.7348286	279.7987241
448.3340274	-0.67748011	0.022051731	0.108115717	ZSWIM9
	19	48170692	48197620	+	3751
protein_coding	zinc finger SWIM-type containing 9 [Source:HGNC
Symbol;Acc:HGNC:34495]	-	125	374	356	477	475
	440	1.822458559	4.396008222	4.396482913
6.148547484	5.518297919	5.475851326
ENSG00000149136 9580.799637	10887.9681	11905.67352
9267.234098	7988.049188	9078.205986	10791.48042
8777.829758	0.298064432	0.022074312	0.10819173	SSRP1
	11	57325985	57335877	-	6323
protein_coding	structure specific recognition protein 1 [Source:HGNC
Symbol;Acc:HGNC:11327]	HMG	8281	11746	12176	9225	8741
	9252	71.62329812	81.90318161	89.20391463
70.54145439	60.24160528	68.30594091
ENSG00000032219 278.8277638	298.4782674	287.4727344
154.7050462	237.6035681	175.637578	288.2595885
189.3153974	0.605769123	0.022094776	0.108240336	ARID4A
	14	58298385	58373887	+	7568
protein_coding	AT-rich interaction domain 4A [Source:HGNC
Symbol;Acc:HGNC:9885]	ARID	241	322	294	154	260
	179	1.741528684	1.875895779	1.799569661
0.983877013	1.497100041	1.104124237
ENSG00000205918 3.470885027	4.634755704	5.866790497
46.21059821	10.05245865	14.71823279	4.657477076
23.66042989	-2.337569405	0.02209839	0.108240336	PDPK2P
	16	2616121 2643296 -	4426
transcribed_unprocessed_pseudogene	"3-phosphoinositide dependent
protein kinase 2, pseudogene [Source:HGNC Symbol;Acc:HGNC:49897]"
	-	3	5	6	46	11	15
0.037068461	0.049807249	0.062797492	0.50251339

0.108302847	0.158207069
ENSG00000143248 24.29619519	31.51633879	30.3117509
53.24264577	74.02265007	67.70387084	28.70809496
64.98972223	-1.176635514	0.02210826	0.108246237	RGS5
	1	163111121	163321791	-	6821
protein_coding	regulator of G protein signaling 5 [Source:HGNC
Symbol;Acc:HGNC:10001]	-	21	34	31	53	81
	69	0.168370483	0.219768187	0.210531023
0.375689482	0.51748238	0.472222936
ENSG00000269713 1189.356603	1219.867701	972.9094241
1544.036727	1578.236008	1266.749236	1127.377909
1463.007324	-0.376156341	0.022122528	0.108246237	NBPF9
	1	149054027	149103561	-	9046
protein_coding	NBPF member 9 [Source:HGNC Symbol;Acc:HGNC:31991]
	-	1028	1316	995	1537	1727	1291
6.214858485	6.414063628	5.095290541	8.215206805
8.319444941	6.662169758
ENSG00000118246 1365.214777	1179.081851	1125.445977
1570.155761	1520.662836	1543.452012	1223.247535
1544.75687	-0.337197505	0.022134207	0.108246237
FASTKD2 2	206765357	206792509	+	6079
protein_coding	FAST kinase domains 2 [Source:HGNC
Symbol;Acc:HGNC:29160]	-	1180	1272	1151	1563	1664
	1573	10.61560075	9.225479028	8.77092982
12.43162929	11.92833359	12.07932357
ENSG00000166925 430.3897434	858.3567564	639.4801642
847.8640194	1005.245865	915.4740797	642.7422213
922.8613214	-0.520471385	0.02213457	0.108246237
TSC22D4 7	100463359	100479279	-	3715
protein_coding	TSC22 domain family member 4 [Source:HGNC
Symbol;Acc:HGNC:21696]	TSC22	372	926	654	844	1100
	933	5.476194119	10.98970732	8.154951566
10.98461513	12.90305251	11.72381243
ENSG00000067177 338.989771	507.042274	335.3848567
562.5638043	580.3010222	534.7624581	393.8056339
559.2090949	-0.50460531	0.022134999	0.108246237	PHKA1
	X	72578814	72714319	-	6350
protein_coding	phosphorylase kinase regulatory subunit alpha 1
[Source:HGNC Symbol;Acc:HGNC:8925]	-	293	547	343
	560	635	545	2.523414579	3.797935274
2.502204787	4.263983527	4.357712735	4.006533818
ENSG00000228485 2.313923351	8.342560268	9.777984162	0
	0	0	6.81148926	0	5.264014799
0.022228435	1	GRK5-IT1	10	119208531
119211760	+	400	sense_intronic	GRK5 intronic
transcript 1 [Source:HGNC Symbol;Acc:HGNC:49478]	-	2
	9	10	0	0	0	0.273441682
0.992010973	1.158090408	0	0	0
ENSG00000115310 8051.296301	8827.355714	7764.697223
10447.61351	9521.506063	9752.301048	8214.449746

9907.140207	-0.270243322	0.022254392	0.108795295	RTN4
	2	54972187	55112621	-	6644
protein_coding	reticulon 4 [Source:HGNC Symbol;Acc:HGNC:14085] -
	6959	9523	7941	10400	10419	9939	57.28117598
	63.1943307	55.36662209	75.68414899	68.3368588
	69.83274322
ENSG00000166272 1071.346512	926.9511408	1147.935341
1379.285899	1299.508746	1333.471891	1048.744331
1337.422178	-0.350998029	0.022275224	0.108862326	WBP1L
	10	102743901	102834516	+	7880
protein_coding	WW domain binding protein 1 like [Source:HGNC
Symbol;Acc:HGNC:23510]	-	926	1000	1174	1373	1422
	1359	6.426573545	5.595098551	6.901513395
8.424527189	7.863791256	8.050804396
ENSG00000163590 323.9492692	323.5059482	305.0731058
484.206703	378.3379893	471.9646649	317.5094411
444.8364524	-0.486186254	0.022287796	0.108888954	PPM1L
	3	160755602	161078907	+	12447
protein_coding	"protein phosphatase, Mg2+/Mn2+ dependent 1L
[Source:HGNC Symbol;Acc:HGNC:16381]"	-	280	349	312
	482	414	481	1.230234932	1.236216954
1.161160785	1.872335041	1.449419999	1.803958003
ENSG00000133065 1173.159139	1303.293304	1352.29521
1724.860807	1643.12006	1470.842064	1276.249218
1612.940977	-0.33740798	0.022318699	0.108969688
SLC41A1 1	205789093	205813748	-	6357
protein_coding	solute carrier family 41 member 1 [Source:HGNC
Symbol;Acc:HGNC:19429]	-	1014	1406	1383	1717	1798
	1499	8.723292928	9.75140215	10.07795522
13.059282	12.32525955	11.00767142
ENSG00000166012 2577.710613	2635.322093	2216.669009
2009.156444	2029.682788	1956.543746	2476.567239
1998.460993	0.309376112	0.022324508	0.108969688	TAF1D
	11	93729948	93784391	-	8420
protein_coding	"TATA-box binding protein associated factor, RNA
polymerase I subunit D [Source:HGNC Symbol;Acc:HGNC:28759]"	-
	2228	2843	2267	2000	2221	1994	14.47097548
	14.88670993	12.47216605	11.48468587	11.49463181
	11.05500894
ENSG00000108423 197.8404465	223.3952249	219.0268452
139.6363729	156.270039	107.9337071	213.4208389
134.613373	0.664883214	0.022325706	0.108969688	TUBD1
	17	59859482	59892945	-	3330
protein_coding	tubulin delta 1 [Source:HGNC Symbol;Acc:HGNC:16811]
	-	171	241	224	139	171	110
2.808319979	3.190852779	3.116063079	2.018235232
2.237744378	1.54203591
ENSG00000099795 1290.012268	1862.244842	1893.995532
2111.623423	1952.918558	2664.981351	1682.084214
2243.174444	-0.414660362	0.022341945	0.109014143	NDUFB7

19	14566078	14572062	-	531
protein_coding	NADH:ubiquinone oxidoreductase subunit B7 [Source:HGNC
Symbol;Acc:HGNC:7702]	-	1115	2009	1937	2102	2137
2716	114.8352074	166.8089596	168.9808753
191.3988866	175.3753694	238.7708362
ENSG00000125734 857.3086017	799.9588346	961.1758431
1310.97458	1029.006222	1082.280718	872.8144264
1140.75384	-0.38606207	0.0223723	0.109127427	GPR108
19	6729914 6737603 -	3213	protein_coding	G
protein-coupled receptor 108 [Source:HGNC Symbol;Acc:HGNC:17829]
-	741	863	983	1305	1126	1103
12.6125295	11.84224463	14.17246027	19.63816943
15.27166057	16.02547174
ENSG00000127054 2392.596745	2804.954152	2775.969703
3541.138233	2806.463684	3771.792457	2657.8402
3373.131458	-0.343496188	0.022386089	0.109159853	INTS11
1	1311585 1324691 -	8220	protein_coding
integrator complex subunit 11 [Source:HGNC Symbol;Acc:HGNC:26052]
-	2068	3026	2839	3525	3071	3844
13.75857418	16.2304688	15.9991176	20.73425905
16.28045719	21.8301941
ENSG00000278959 0	3.707804563	0.977798416	9.041203998
23.76035681	11.77458623	1.56186766	14.85871568
-3.208860031	0.022388357	1	AC006378.2	7
93954044	93956519	-	2476	TEC	tec
-	0	4	1	9	26	12	0
0.07122678	0.018709053	0.17574909	0.457595037
0.226243777
ENSG00000267787 86.77212568	64.88657986	82.13506696
158.7233591	153.5284594	104.0088451	77.9312575
138.7535545	-0.83418435	0.022402174	0.109203453
AC027097.2	18	57639455	57738603	+	2670
antisense	"novel transcript, antisense to ATP8B1" -
75	70	84	158	168	106	1.536189226
1.155901259	1.457372199	2.861192264	2.741931609
1.853278296
ENSG00000164062 2595.065039	2942.142921	2893.305513
2094.545593	2238.9567	2469.719463	2810.171158
2267.740585	0.309595571	0.022419245	0.109241451	APEH
3	49674002	49683963	+	4669
protein_coding	acylaminoacyl-peptide hydrolase [Source:HGNC
Symbol;Acc:HGNC:586]	-	2243	3174	2959	2085	2450
2517	26.27242206	29.97208851	29.35780267
21.5915292	22.86655858	25.16548921
ENSG00000100416 894.3313753	1016.865402	1208.558842
784.5755914	606.8029586	900.7558469	1039.91854
764.044799	0.445476699	0.022424261	0.109241451	TRMU
22	46330875	46357340	+	9219
protein_coding	tRNA 5-methylaminomethyl-2-thiouridylate
methyltransferase [Source:HGNC Symbol;Acc:HGNC:25481]	-	773

1097 1236 781 664 918 4.585539002
5.246344084 6.210650804 4.096080049 3.138649806
4.648415087
ENSG00000253390 19.66834849 12.97731597 15.64477466 0
0 4.906077597 16.09681304 1.635359199
3.305456755 0.022431077 1 AC104561.1 8
23458601 23484971 + 630 lincRNA novel
transcript - 17 14 16 0 0 5
1.475717015 0.979763924 1.176472795 0 0
0.370489147
ENSG00000234383 3.470885027 7.415609127 2.933395248
71.32505376 12.79403828 1.962431039 4.606629801
28.69384103 -2.630137012 0.022439073 0.109278786
CTBP2P8 1 68161761 68163090 - 1330
processed_pseudogene C-terminal binding protein 2 pseudogene 8
[Source:HGNC Symbol;Acc:HGNC:45200] - 3 8 3
71 14 2 0.12335715 0.265199258
0.10448936 2.581118385 0.458706604 0.070197944
ENSG00000064763 217.508795 235.4455898 219.0268452
133.6089035 166.3224977 134.4265262 223.9937433
144.7859758 0.629141899 0.022458253 0.109337362 FAR2
12 29149103 29340980 + 5411
protein_coding fatty acyl-CoA reductase 2 [Source:HGNC
Symbol;Acc:HGNC:25531] - 188 254 224 133 182
137 1.900093744 2.069617751 1.917665876
1.188434699 1.465724853 1.181922687
ENSG00000131446 1774.779211 2500.914178 2979.351774
2948.437082 3283.49854 3359.681939 2418.348388
3197.205853 -0.402378216 0.022577021 0.109880586 MGAT1
5 180784782 180815652 - 11576
protein_coding "mannosyl (alpha-1,3-)-glycoprotein beta-1,2-N-
acetylglucosaminyltransferase [Source:HGNC Symbol;Acc:HGNC:7044]"
- 1534 2698 3047 2935 3593 3424
7.247054949 10.27584122 12.19316335 12.25888029
13.52562358 13.80769781
ENSG00000143537 1329.348965 1810.335578 2202.002033
1247.686152 1062.819037 1576.81334 1780.562192
1295.772843 0.459090157 0.022611265 0.110012223 ADAM15
1 155050566 155062775 + 6584
protein_coding ADAM metallopeptidase domain 15 [Source:HGNC
Symbol;Acc:HGNC:193] - 1149 1953 2252 1242 1163
1607 9.543878882 13.07815195 15.84459051
9.120801207 7.697478602 11.39389179
ENSG00000131238 4598.922661 4682.957164 4853.791338
3741.049299 3810.795689 4141.710708 4711.890387
3897.851898 0.273672441 0.02263538 0.110065042 PPT1
1 40071461 40097727 - 5088
protein_coding palmitoyl-protein thioesterase 1 [Source:HGNC
Symbol;Acc:HGNC:9325] - 3975 5052 4964 3724 4170
4221 42.72526284 43.77742344 45.19466025

35.38863294 35.71474471 38.72703833
ENSG00000163399 9861.941324 10598.75934 12490.39697
9560.570939 7404.092727 9300.941909 10983.69921
8755.201858 0.327246576 0.022636521 0.110065042 ATP1A1
1 116372668 116410261 + 7261
protein_coding ATPase Na+/K+ transporting subunit alpha 1
[Source:HGNC Symbol;Acc:HGNC:799] - 8524 11434 12774
9517 8102 9479 64.20098881 69.42816854
81.49536906 63.37308499 48.62441617 60.94135725
ENSG00000157657 406.0935482 445.8634987 717.7040375
455.0739346 272.3302435 291.4210093 523.2203615
339.6083958 0.624653705 0.022648827 0.110089862 ZNF618
9 113876282 114056591 + 10384
protein_coding zinc finger protein 618 [Source:HGNC
Symbol;Acc:HGNC:29416] zf-C2H2 351 481 734 453 298
297 1.848575317 2.042275629 3.274415868
2.109282449 1.250576267 1.335173812
ENSG00000248926 0 0 0 11.05036044 13.70789816
0 0 8.252752868 -5.465359099 0.022654535
1 AC114801.2 4 77958000 77958780
- 781 processed_pseudogene FUS interacting
protein (serine/arginine-rich) 1 (FUSIP1) pseudogene - 0
0 0 11 15 0 0 0 0
0.680993345 0.836948669 0
ENSG00000182327 6.941770054 24.10072966 43.02313031
60.27469332 76.7642297 63.77900877 24.68854334
66.9393106 -1.431161635 0.022657611 0.110097554 GLTPD2
17 4788959 4790390 + 1023 protein_coding
glycolipid transfer protein domain containing 2 [Source:HGNC
Symbol;Acc:HGNC:33756] - 6 26 44 60 84
65 0.320752706 1.120551115 1.992413605
2.835808064 3.578180545 2.966086135
ENSG00000132768 1707.675433 2168.138718 2083.688425
2797.750348 2240.784419 2541.348195 1986.500859
2526.627654 -0.346437232 0.022692853 0.110233758 DPH2
1 43970000 43973369 + 2930
protein_coding DPH2 homolog [Source:HGNC Symbol;Acc:HGNC:3004] -
1476 2339 2131 2785 2452 2590 27.54948279
35.19626342 33.69134006 45.95775395 36.46795777
41.2646513
ENSG00000130921 363.2859662 329.9946061 412.6309316
234.0667257 256.7946256 280.6276386 368.637168
257.1629966 0.519321288 0.022715736 0.110309864
C12orf65 12 123232916 123257959 + 7090
protein_coding chromosome 12 open reading frame 65
[Source:HGNC Symbol;Acc:HGNC:26784] - 314 356 422
233 281 286 2.422022234 2.213796624
2.757202551 1.588952455 1.727104765 1.883067265
ENSG00000279118 46.27846703 50.05536161 59.64570339
26.11903377 13.70789816 21.58674143 51.99317734

20.47122445	1.347951449	0.022788194	0.110626586
AC093535.2	5	126496279	126498604	+	2326
	TEC	tec	-	40	54	61	26	15
	22	0.940470102	1.023571081	1.214849783
0.540461614	0.281021887	0.441528769
ENSG00000223343 2.313923351	5.561706845	18.57816991	0
	0	0.981215519	8.817933368	0.32707184
4.674472718	0.022857029	1	AC137630.1	3
48985049	48989988	-	782	antisense	novel
transcript	-	2	6	19	0	0	1
	0.139867868	0.338281662	1.125509859	0	0
	0.059695182
ENSG00000144824 517.161869	568.2210493	490.8548049
301.3734666	468.8101171	335.5757077	525.4125744
368.5864305	0.510820756	0.022870775	0.110992234	PHLDB2
	3	111732497	111976517	+	12309
protein_coding	pleckstrin homology like domain family B member 2
[Source:HGNC Symbol;Acc:HGNC:29573]	-	447	613	502
	300	513	342	1.986001006	2.19569322
1.88922377	1.178418901	1.816156173	1.297028055
ENSG00000010610 3.470885027	2.780853423	20.53376674	0
	0	0.981215519	8.92850173	0.32707184
4.691399273	0.022936612	1	CD4	12	6786858
6820808 +	4583	protein_coding	CD4 molecule [Source:HGNC
Symbol;Acc:HGNC:1678]	-	3	3	21	0	0
	1	0.035798606	0.0288606	0.212261825	0
	0	0.010185824
ENSG00000198743 7237.952243	4932.30702	5094.329748
8416.356344	6520.390225	7437.613638	5754.863004
7458.120069	-0.374221157	0.022946756	0.111268902	SLC5A3
	21	34073570	34106262	+	11576
protein_coding	solute carrier family 5 member 3 [Source:HGNC
Symbol;Acc:HGNC:11038]	-	6256	5321	5210	8378	7135
	7580	29.55513413	20.26603082	20.8488287
34.99315129	26.85926086	30.56727495
ENSG00000119900 1349.017314	1490.537434	1739.503382
1235.631213	1179.793102	1178.439839	1526.35271
1197.954718	0.349918207	0.02295255	0.111268902	OGFRL1
	6	71288803	71308950	+	9075
protein_coding	opioid growth factor receptor like 1 [Source:HGNC
Symbol;Acc:HGNC:21378]	-	1166	1608	1779	1230	1291
	1201	7.02662262	7.81220028	9.080960157
6.553294635	6.199236519	6.17792184
ENSG00000167635 3269.573696	2584.339781	2726.101984
2285.415455	2369.638662	2221.471936	2860.005154
2292.175351	0.318902509	0.022956245	0.111268902	ZNF146
	19	36214602	36238774	+	4146
protein_coding	zinc finger protein 146 [Source:HGNC
Symbol;Acc:HGNC:12931]	zf-C2H2 2826	2788	2788	2275	2593
	2264	37.27670978	29.64813842	31.15056495

26.53098168	27.25409822	25.49136906
ENSG00000272070 47.4354287	15.75816939	32.26734773
73.33421021	82.24738897	65.7414398	31.82031528
73.77434633	-1.221342581	0.022956899	0.111268902
AC005618.1	5	141326210	141329357	+	3148
	lincRNA novel transcript	-	41	17	33
	73	90	67	0.712268677	0.238093838
0.485603348	1.121216171	1.245851799	0.993541735
ENSG00000186312 171.230328	196.5136419	219.0268452
380.7351461	249.4837465	268.8530523	195.5902717
299.6906483	-0.614066116	0.022977078	0.111331406	CA5BP1
	X	15674932	15703724	+	2409
transcribed_unprocessed_pseudogene	carbonic anhydrase 5B
pseudogene 1 [Source:HGNC Symbol;Acc:HGNC:29544]	-	148
	212	224	379	273	274	3.359847983
3.880011554	4.307384829	7.606828526	4.938379314
5.309575475
ENSG00000105656 492.8656739	725.8027433	702.0592628
752.4290883	964.1221707	917.4365107	640.24256
877.9959232	-0.454550524	0.023002379	0.111418684	ELL
	19	18442663	18522127	-	4929
protein_coding	elongation factor for RNA polymerase II [Source:HGNC
Symbol;Acc:HGNC:23114]	-	426	783	718	749	1055
	935	4.726563465	7.003851057	6.74789136
7.347239824	9.327220401	8.855209259
ENSG00000118680 4207.869615	4055.411241	3892.615495
2983.597319	3648.128631	3258.61674	4051.96545
3296.780897	0.297386891	0.02302392	0.111481684	MYL12B
	18	3261479 3278431 +	1932	protein_coding	myosin
light chain 12B [Source:HGNC Symbol;Acc:HGNC:29827]	-	3637
	4375	3981	2970	3992	3321	102.9510764
99.84007377	95.45254479	74.32767425	90.04135217
80.24311683
ENSG00000184517 492.8656739	393.9542349	484.010216
258.1766031	406.6676454	268.8530523	456.9433749
311.2324336	0.552611098	0.02302997	0.111481684	ZFP1
	16	75148492	75172236	+	3761
protein_coding	ZFP1 zinc finger protein [Source:HGNC
Symbol;Acc:HGNC:23328]	zf-C2H2 426	425	495	257	445
	274	6.194424706	4.982181613	6.096833309
3.303931286	5.156028097	3.400895326
ENSG00000174132 173.5442514	233.5916875	181.8705054
232.0575693	321.6786769	349.3127249	196.3354814
301.0163237	-0.615447681	0.023046874	0.111528194
FAM174A 5	100535305	100586741	+	1416
protein_coding	family with sequence similarity 174 member A
[Source:HGNC Symbol;Acc:HGNC:24943]	-	150	252	186
	231	352	356	5.793255978	7.846414476
6.084881804	7.887691985	10.83273222	11.73634265
ENSG00000170004 2763.981443	2968.097553	3960.083585

2735.466499	2237.12898	2535.460902	3230.720861
2502.68546	0.368643847	0.023063555	0.111573595	CHD3
	17	7884806 7912760 +	9758	protein_coding
chromodomain helicase DNA binding protein 3 [Source:HGNC
Symbol;Acc:HGNC:1918]	-	2389	3202	4050	2723	2448
	2584	13.3890588	14.46753267	19.22634209
13.49236384	10.93224101	12.36168667
ENSG00000177548 520.6327541	774.0042026	799.8391044
885.0334136	894.66882	1096.998951	698.158687
958.9003948	-0.456586472	0.02317876	0.112095447	RABEP2
	16	28904421	28936526	-	4566
protein_coding	"rabaptin, RAB GTPase binding effector protein 2
[Source:HGNC Symbol;Acc:HGNC:24817]"	-	450	835	818
	881	979	1118	5.389783485	8.062774736
8.298887022	9.329131564	9.343409479	11.43015006
ENSG00000183684 2306.981581	3073.769983	2851.260182
2114.637157	2260.889337	2164.561436	2744.003915
2180.02931	0.332282788	0.023190147	0.112115045	ALYREF
	17	81887844	81891586	-	1574
protein_coding	Aly/REF export factor [Source:HGNC
Symbol;Acc:HGNC:19071]	-	1994	3316	2916	2105	2474
	2206	69.2811581	92.88460784	85.81935525
64.66191891	68.49416332	65.42546469
ENSG00000197361 12.72657843	13.90426711	12.71137941
35.16023777	46.60685375	33.36132766	13.11407499
38.37613973	-1.548914764	0.023208278	0.112167227	FBXL22
	15	63597353	63602428	+	3614
protein_coding	F-box and leucine rich repeat protein 22 [Source:HGNC
Symbol;Acc:HGNC:27537]	-	11	15	13	35	51
	34	0.166455922	0.182994092	0.166631713
0.468253587	0.614951161	0.439174186
ENSG00000177337 53.22023708	52.83621503	52.80111447
78.35710132	106.9216057	117.7458623	52.95252219
101.0081898	-0.932147624	0.023225159	0.112213338
DLGAP1-AS1	18	3593732 3598352 +	2105	antisense
	DLGAP1 antisense RNA 1 [Source:HGNC Symbol;Acc:HGNC:31676]
	-	46	57	54	78	117	120
1.195089537	1.193869105	1.188349302	1.791610995
2.422101615	2.661185704
ENSG00000105821 1213.652798	998.3263787	1134.246163
919.1890732	890.0995206	776.1414759	1115.408446
861.8100232	0.37163793	0.023235165	0.112225853	DNAJC2
	7	103312474	103344873	-	6564
protein_coding	DnaJ heat shock protein family (Hsp40) member C2
[Source:HGNC Symbol;Acc:HGNC:13192]	MYB	1049	1077	1160
	915	974	791	8.739802699	7.234043049
8.186379482	6.739904426	6.466197752	5.62540697
ENSG00000111912 465.0985936	527.4351991	383.2969791
505.3028457	798.7135329	647.6022428	458.6102573
650.5395405	-0.504648677	0.023242431	0.112225853	NCOA7

6	125781161	125932030	+	7820
protein_coding	nuclear receptor coactivator 7 [Source:HGNC Symbol;Acc:HGNC:21081]	-	402	569	392	503	874
660	2.811344149	3.208037759	2.322104552
3.110014748	4.870384822	3.939882032
ENSG00000012983 1498.26537	1265.288307	1101.978815
1047.775086	1020.781483	892.9061227	1288.510831
987.1542305	0.383791305	0.023282144	0.112382109	MAP4K5
14	50418501	50561126	-	7620
protein_coding	mitogen-activated protein kinase kinase kinase kinase 5 [Source:HGNC Symbol;Acc:HGNC:6867]	-	1295	1365	1127
1043	1117	910	9.294146415	7.897900135
6.851275011	6.618057766	6.387880742	5.574840633
ENSG00000051108 1684.5362	1852.975331	1669.101896
1977.009941	2385.17428	2139.049832	1735.537809
2167.078018	-0.320338467	0.023309021	0.112476325
HERPUD1 16	56932048	56944863	+	5155
protein_coding	homocysteine inducible ER protein with ubiquitin like domain 1 [Source:HGNC Symbol;Acc:HGNC:13744]	-	1456	1999
1707	1968	2610	2180	15.44640501	17.09692799
15.33936238	18.45855249	22.06329823	19.74121416
ENSG00000176076 3.470885027	9.269511408	16.62257307
38.17397244	38.38211485	27.47403455	9.787656503
34.67670728	-1.814318504	0.023341636	0.112598168	KCNE5
X	109623700	109625172	-	1473
protein_coding	potassium voltage-gated channel subfamily E regulatory subunit 5 [Source:HGNC Symbol;Acc:HGNC:6241]	-	3	10
17	38	42	28	0.111381541	0.299316881
0.534624221	1.247332006	1.242525016	0.887363009
ENSG00000226479 809.873173	769.3694469	851.6624205
1068.871228	1071.043776	968.4597177	810.3016801
1036.124907	-0.354791131	0.023363795	0.112669508
TMEM185B	2	120221278	120223408	-	2131
protein_coding	transmembrane protein 185B [Source:HGNC Symbol;Acc:HGNC:18896]	-	700	830	871	1064	1172
987	17.9642588	17.17230528	18.93377279
24.14123006	23.96639759	21.62119725
ENSG00000183690 19.66834849	8.342560268	4.888992081	0
1.827719755	0	10.96663361	0.609239918
4.126905295	0.023371813	1	EFHC2	X	44147882
44343672	-	3319	protein_coding	EF-hand domain containing 2 [Source:HGNC Symbol;Acc:HGNC:26233]	-	17
9	5	0	2	0	0.28011501
0.119555405	0.069785502	0	0.026259191	0
ENSG00000128342 59.00504546	104.7454789	162.3145371
37.16939421	60.31475191	57.89171565	108.6883538
51.79195392	1.070654978	0.023385617	0.112739176	LIF
22	30240447	30246851	-	3969
protein_coding	"LIF, interleukin 6 family cytokine [Source:HGNC Symbol;Acc:HGNC:6596]"	-	51	113	166	37	66

59	0.702722388	1.2552531	1.937445278
0.45073558	0.72463855	0.693932054
ENSG00000167081 285.7695339	320.7250947	373.518995
193.8835969	252.2253262	219.7922764	326.6712079
221.9670665	0.557628506	0.023437391	0.112933157	PBX3
9	125747345	125967377	+	4529
protein_coding	PBX homeobox 3 [Source:HGNC Symbol;Acc:HGNC:8634]
Homeobox	247	346	382	193	276	224
2.982561073	3.368276506	3.907180709	2.060422125
2.655616505	2.308828813
ENSG00000265511 9.255693406	17.61207168	5.866790497
22.10072088	25.58807657	67.70387084	10.91151853
38.46422277	-1.812090921	0.02344063	0.112933157
AC020558.2	17	17507351	17508308	+	795
antisense	novel transcript	-	8	19
6	22	28	69	0.550322882	1.053708371
0.349612199	1.338002019	1.534791907	4.051613392
ENSG00000150779 474.354287	754.5382286	865.3515983
820.7404074	1062.819037	1088.168011	698.0813713
990.5758186	-0.503818047	0.023448267	0.112934362	TIMM8B
11	112084800	112086798	-	1166
protein_coding	translocase of inner mitochondrial membrane 8 homolog
B [Source:HGNC Symbol;Acc:HGNC:11818]	-	410	814	885
817	1163	1109	19.23003254	30.7793761
35.15986315	33.87854285	43.46500782	44.39959734
ENSG00000175387 2371.771435	2003.141415	1903.773516
1703.764665	1696.123932	1607.231021	2092.895456
1669.039873	0.326068558	0.023484782	0.113074604	SMAD2
18	47808957	47931146	-	36366
protein_coding	SMAD family member 2 [Source:HGNC
Symbol;Acc:HGNC:6768]	MH1	2050	2161	1947	1696	1856
1638	3.082854581	2.61995113	2.480121019
2.254922032	2.224032018	2.102637467
ENSG00000140848 920.9414939	1129.953441	1154.779929
729.3237892	762.1591378	938.0420366	1068.558288
809.8416545	0.400550849	0.023495034	0.113088344	CPNE2
16	57092537	57148367	+	7781
protein_coding	copine 2 [Source:HGNC Symbol;Acc:HGNC:2315]	-
796	1219	1181	726	834	956	5.594642822
6.907203451	7.030997412	4.51130741	4.670778076
5.735463401
ENSG00000092208 274.1999171	352.2414335	323.6512757
195.8927533	186.427415	256.0972506	316.6975421
212.8058063	0.574863137	0.023571981	0.113412384	GEMIN2
14	39114223	39136973	+	2757
protein_coding	gem nuclear organelle associated protein 2
[Source:HGNC Symbol;Acc:HGNC:10884]	-	237	380	331
195	204	261	4.701173643	6.076881792
5.561522307	3.419787037	3.224422916	4.419262277
ENSG00000132507 9004.632722	12581.50783	12640.00013

8792.068599	9139.512634	9299.960694	11408.71356
9077.180642	0.329934035	0.023577194	0.113412384	EIF5A
	17	7306999 7312463 +	2815	protein_coding
eukaryotic translation initiation factor 5A [Source:HGNC
Symbol;Acc:HGNC:3300]	-	7783	13573	12927	8752	10001
	9478	151.2040222	212.5844079	212.7266032
150.324624	154.8187747	157.1753156
ENSG00000135336 1025.068045	674.8204305	729.4376185
632.8842799	593.0950604	542.6121823	809.7753646
589.5305075	0.456850089	0.023593943	0.113457255	ORC3
	6	87590067	87667453	+	3689
protein_coding	origin recognition complex subunit 3 [Source:HGNC
Symbol;Acc:HGNC:8489]	-	886	728	746	630	649
	553	13.13468855	8.700749837	9.367692537
8.257205834	7.666455856	6.997815886
ENSG00000260368 12.72657843	1.853902282	5.866790497	0
	0	0	6.815757071	0	5.257183072
0.023598111	1	AC027373.1	8	100913247
100914388	-	1142	sense_overlapping	"novel
transcript, overlapping YWHAZ"	-	11	2	6	0
	0	0	0.526770316	0.07721432	0.243381522
	0	0	0
ENSG00000103226 201.3113316	43.56670362	32.26734773
14.06409511	22.84649694	55.92928461	92.38179431
30.94662555	1.574373978	0.023605279	0.113476069	NOMO3
	16	16232495	16294814	+	8274
protein_coding	NODAL modulator 3 [Source:HGNC Symbol;Acc:HGNC:25242]
	-	174	47	33	14	25	57
1.150081042	0.250447268	0.18475699	0.081811383
0.131668864	0.321592102
ENSG00000168303 945.2376891	1415.454392	1378.695767
1630.430454	1453.037205	1920.238772	1246.462616
1667.902144	-0.419323139	0.023647123	0.113641488	MPLKIP
	7	40126023	40134652	-	7661
protein_coding	M-phase specific PLK1 interacting protein [Source:HGNC
Symbol;Acc:HGNC:16002]	-	817	1527	1410	1623	1590
	1957	5.832185206	8.787949097	8.525818953
10.24316749	9.044202127	11.92480811
ENSG00000196968 658.3111935	482.9415444	644.3691563
483.2021248	269.5886638	459.2088631	595.207298
403.9998839	0.559234002	0.023659808	0.113666714	FUT11
	10	73772291	73780251	+	4910
protein_coding	fucosyltransferase 11 [Source:HGNC
Symbol;Acc:HGNC:19233]	-	569	521	659	481	295
	468	6.33760966	4.678322851	6.217365204
4.73657917	2.618177713	4.449491657
ENSG00000109738 432.7036667	377.2691143	336.3626552
251.1445555	296.0906003	255.1160351	382.1118121
267.450397	0.513477494	0.023684157	0.113747944	GLRB
	4	157076057	157172090	+	3459

protein_coding	glycine receptor beta [Source:HGNC
Symbol;Acc:HGNC:4329]	-	374	407	344	250	324
	260	5.913107206	5.187735261	4.606916453
3.494545208	4.081812449	3.50888247
ENSG00000128510 341.3036943	223.3952249	471.2988366
254.1582902	138.9067014	235.4917247	345.3325853
209.5189054	0.721457424	0.023697049	0.113774116	CPA4
	7	130293134	130324180	+	3958
protein_coding	carboxypeptidase A4 [Source:HGNC
Symbol;Acc:HGNC:15740]	-	295	241	482	253	152
	240	4.076063478	2.684572955	5.641228667
3.090622399	1.673502617	2.830619458
ENSG00000187522 861.9364484	543.1933685	694.2368755
485.2112812	569.3347036	443.5094148	699.7888975
499.3517999	0.485470938	0.023767587	0.114076957	HSPA14
	10	14838164	14871741	+	2644
protein_coding	heat shock protein family A (Hsp70) member 14
[Source:HGNC Symbol;Acc:HGNC:29526]	-	745	586	710
	483	623	452	15.40953504	9.771699953
12.43939772	8.832566104	10.26798424	7.980369862
ENSG00000156475 0	4.634755704	19.55596832	0	0
	0	8.063574676	0	5.507107156	0.023785836
	1	PPP2R2B 5	146581146	147084784	-
	13206	protein_coding	protein phosphatase 2 regulatory
subunit Bbeta [Source:HGNC Symbol;Acc:HGNC:9305]	-	0
	5	20	0	0	0	0	0.016692934
	0.070155409	0	0	0
ENSG00000162366 0	13.90426711	9.777984162	0	0
	0	7.894083758	0	5.477669748	0.023803773
	1	PDZK1IP1	1	47183593	47191044
	-	1438	protein_coding	PDZK1 interacting protein 1
[Source:HGNC Symbol;Acc:HGNC:16887]	-	0	15	10
	0	0	0	0	0.459903094	0.322139195
	0	0	0
ENSG00000101181 1081.759167	1649.973031	1705.280438
2089.522702	1869.757309	1922.201203	1479.004212
1960.493738	-0.405791303	0.023826185	0.114319674	MTG2
	20	62183029	62203568	+	5209
protein_coding	mitochondrial ribosome associated GTPase 2
[Source:HGNC Symbol;Acc:HGNC:16239]	-	935	1780	1744
	2080	2046	1959	9.816393658	15.06605688
15.50938507	19.30679539	17.11629921	17.55602192
ENSG00000196544 74.04554725	118.649746	93.86864795
224.0209435	113.3186248	173.6751469	95.52131374
170.3382384	-0.830986228	0.023833113	0.114319674	BORCS6
	17	8188333 8190907 -	2575	protein_coding	BLOC-1
related complex subunit 6 [Source:HGNC Symbol;Acc:HGNC:25939]	-
	64	128	96	223	124	177	1.35924409
	2.191627263	1.727016375	4.187249565	2.098471375
	3.208795714

ENSG00000134996 448.9011302 468.1103261 431.2091015
294.3414191 354.5776324 327.7259835 449.4068526
325.548345 0.464837847 0.023842666 0.114329622 OSTF1
9 75088543 75147265 + 1319
protein_coding osteoclast stimulating factor 1 [Source:HGNC
Symbol;Acc:HGNC:8510] - 388 505 441 293 388
334 16.08724377 16.88031476 15.48803244
10.74048867 12.81874557 11.8208228
ENSG00000101104 511.3770607 566.3671471 696.1924723
405.8496017 343.6113139 508.2696391 591.3122267
419.2435182 0.497020457 0.023861268 0.114382942
PABPC1L 20 44910062 44959035 + 4984
protein_coding poly(A) binding protein cytoplasmic 1 like
[Source:HGNC Symbol;Acc:HGNC:15797] - 442 611 712
404 376 518 4.849968841 5.405017875
6.617659473 3.919264267 3.287520041 4.851742709
ENSG00000143162 2021.212047 2076.370555 1802.082481
1171.338207 1537.112314 1796.605616 1966.555028
1501.685379 0.388963021 0.023882292 0.114447838 CREG1
1 167529117 167553805 - 3286
protein_coding cellular repressor of E1A stimulated genes 1
[Source:HGNC Symbol;Acc:HGNC:2351] - 1747 2240 1843
1166 1682 1831 29.07502244 30.05483979
25.98126137 17.15663879 22.30576026 26.01158527
ENSG00000272333 1222.908491 1802.919969 2374.094554
2929.350095 2053.443145 2527.611178 1799.974338
2503.468139 -0.475376051 0.023926372 0.114623145 KMT2B
19 35718019 35738878 + 10279
protein_coding lysine methyltransferase 2B [Source:HGNC
Symbol;Acc:HGNC:15840] - 1057 1945 2428 2916 2247
2576 5.623657127 8.34262452 10.94208974
13.71632826 9.526004978 11.69879224
ENSG00000100997 860.7794867 952.9057728 1066.778072
804.6671558 711.8968445 693.7193723 960.1544439
736.7611242 0.382647753 0.023955965 0.114728961 ABHD12
20 25294743 25390983 - 3429
protein_coding abhydrolase domain containing 12 [Source:HGNC
Symbol;Acc:HGNC:15868] - 744 1028 1091 801 779
707 11.86588577 13.21781252 14.7387184
11.29448018 9.899848996 9.624938529
ENSG00000168389 198.9974082 302.1860719 334.4070583
164.7508284 223.89567 127.5580175 278.5301795
172.068172 0.695407014 0.023970829 0.114730374 MFSD2A
1 39955112 39969968 + 4007
protein_coding major facilitator superfamily domain containing 2A
[Source:HGNC Symbol;Acc:HGNC:25897] - 172 326 342
164 245 130 2.347490358 3.587006929
3.953750132 1.978908537 2.664436287 1.514502678
ENSG00000165775 2141.536062 1801.993018 1541.010304
2264.319312 2519.511682 2214.603427 1828.179794

2332.811474	-0.352202094	0.023971271	0.114730374	FUNDC2
X	155025980	155060303	+	10252
protein_coding	FUN14 domain containing 2 [Source:HGNC
Symbol;Acc:HGNC:24925]	-	1851	1944	1576	2254	2757
2257	9.873986611	8.360295364	7.121148977
10.63032471	11.71889779	10.27706249
ENSG00000184110 178.1720981	267.8888797	363.7410108
1536.000101	267.7609441	669.1889843	269.9339962
824.3166766	-1.61019618	0.024055093	0.11507444	EIF3C
16	28688558	28735730	+	6982
protein_coding	eukaryotic translation initiation factor 3 subunit C
[Source:HGNC Symbol;Acc:HGNC:3279]	-	154	289	372
1529	293	682	1.206245175	1.824954144
2.468115908	10.58836387	1.828716459	4.559850101
ENSG00000275023 2894.718113	3793.084068	4337.513774
4488.455496	4407.546189	5278.939495	3675.105318
4724.980393	-0.362225935	0.024058214	0.11507444	MLLT6
17	38705542	38729803	+	13315
protein_coding	"MLLT6, PHD finger containing [Source:HGNC
Symbol;Acc:HGNC:7138]"	-	2502	4092	4436	4468	4823
5380	10.27639638	13.54966046	15.43308764
16.22457055	15.78464027	18.86197395
ENSG00000267277 1.156961676	2.780853423	6.844588913
8.036625776	26.50193645	27.47403455	3.59413467
20.67086559	-2.510983469	0.024097808	0.115218319
AC024575.1	19	11368123	11374935	-	682
antisense	"novel transcript, antisense to SWSAP1" -
1	3	7	8	29	28	0.080188177
0.193941539	0.475462338	0.567161613	1.852986354
1.916547964
ENSG00000268201 20.82531016	3.707804563	0	0	0
0	8.177704909	0	5.519269021	0.0241015
1	AC020915.2	19	58266635	58267685
+	550	sense_intronic	"novel transcript, sense
intronic to ZNF544"	-	18	4	0	0	0
0	1.789800101	0.320650011	0	0	0
0
ENSG00000065308 2485.153679	2646.445507	3338.203793
2352.722196	1758.266404	2402.996807	2823.26766
2171.328469	0.379100945	0.024103368	0.115218319	TRAM2
6	52497402	52576915	-	6908
protein_coding	translocation associated membrane protein 2
[Source:HGNC Symbol;Acc:HGNC:16855]	-	2148	2855	3414
2342	1924	2449	17.00500096	18.22165173
22.89357645	16.39214467	12.13699957	16.54940911
ENSG00000196361 0	14.83121825	8.800185745	0	0
0	7.877134666	0	5.474673725	0.024114393
1	ELAVL3	19	11451326	11481046	-
4761	protein_coding	ELAV like RNA binding protein 3
[Source:HGNC Symbol;Acc:HGNC:3314]	-	0	16	9

0 0 0 0 0.148168457 0.087568273
0 0 0
ENSG00000226276 30.08100357 0 0 0 0 0
10.02700119 0 5.813578856 0.024127685 1
AC093382.1 2 215939308 215941719 -
379 lincRNA novel transcript - 26 0
0 0 0 0 3.751706457 0 0
0 0 0
ENSG00000224945 15.04050178 20.3929251 33.24514615
50.2289111 68.53949081 52.00442253 22.89285768
56.92427481 -1.31029817 0.024158752 0.115446966
AL353150.1 9 89088604 89109934 - 1038
sense_intronic novel transcript - 13 22
34 50 75 53 0.684921362 0.934456922
1.517343887 2.329023482 3.148636369 2.383551566
ENSG00000271447 5.784808379 32.44328993 40.08973506 0
10.05245865 0 26.10594446 3.350819551
2.947750418 0.02417772 0.115501501 MMP28 17
35756249 35795707 - 2858 protein_coding matrix
metallopeptidase 28 [Source:HGNC Symbol;Acc:HGNC:14366] - 5
35 41 0 11 0 0.095675886
0.539932883 0.664544531 0 0.167721624 0
ENSG00000107551 372.5416596 154.8008405 171.1147228
73.33421021 144.3898606 157.9756986 232.8190743
125.2332565 0.891604973 0.024191239 0.115529982 RASSF4
10 44959407 44995891 + 9481
protein_coding Ras association domain family member 4 [Source:HGNC
Symbol;Acc:HGNC:20793] - 322 167 175 73 158
161 1.857361495 0.776598026 0.855039854
0.372280193 0.726208852 0.792716258
ENSG00000160113 956.8073058 1470.144509 1999.597761
876.9967878 919.3430367 1274.59896 1475.516525
1023.646261 0.528062062 0.02421844 0.115623765 NR2F6
19 17231883 17245940 - 2676
protein_coding nuclear receptor subfamily 2 group F member 6
[Source:HGNC Symbol;Acc:HGNC:7977] RXR-like 827 1586
2045 873 1006 1299 16.9010666 26.13069927
35.40052143 15.77354652 16.38213383 22.66047858
ENSG00000273176 21.98227184 2.780853423 0 0 0
0 8.254375087 0 5.532366388 0.024277802
1 AL008635.1 22 35298838 35299541
- 385 antisense "novel transcript, antisense
to TOM1" - 19 3 0 0 0 0
2.698904915 0.343553584 0 0 0 0
ENSG00000168476 634.0149983 969.5908933 1357.184202
621.8339194 657.9791117 764.3668897 986.9300311
681.3933069 0.535222342 0.024279482 0.115879001 REEP4
8 22138020 22141951 - 2266
protein_coding receptor accessory protein 4 [Source:HGNC
Symbol;Acc:HGNC:26176] - 548 1046 1388 619 720

779	13.22559945	20.35193641	28.37474821
13.20784489	13.84621875	16.04809875
ENSG00000264522 939.4528807	836.109929	1022.777143
1185.402302	1077.440795	1365.852003	932.7799843
1209.565034	-0.374909992	0.024300275	0.115942043	OTUD7B
1	149937812	150010676	-	12177
protein_coding	OTU deubiquitinase 7B [Source:HGNC
Symbol;Acc:HGNC:16683]	-	812	902	1046	1180	1179
1392	3.646787319	3.265879747	3.979182283
4.685359484	4.219219278	5.336358095
ENSG00000135930 1276.128728	1319.978425	1293.627305
1561.114557	1529.801435	1767.169151	1296.578152
1619.361714	-0.320562704	0.024316565	0.115953283	EIF4E2
2	232550052	232583644	+	7177
protein_coding	eukaryotic translation initiation factor 4E family
member 2 [Source:HGNC Symbol;Acc:HGNC:3293]	-	1103	1424
1323	1554	1674	1801	8.404797977	8.747843423
8.539242634	10.46909847	10.16415093	11.71431242
ENSG00000220205 458.1568236	629.3998246	510.4107732
622.8384977	672.6008698	943.9293297	532.6558072
746.4562324	-0.485887722	0.024317801	0.115953283	VAMP2
17	8159149 8163546 -	3268	protein_coding
vesicle associated membrane protein 2 [Source:HGNC
Symbol;Acc:HGNC:12643]	-	396	679	522	620	736
962	6.626860841	9.160552845	7.399304686
9.172988692	9.814187801	13.74165561
ENSG00000124209 3590.05208	3051.523156	2971.529387
4416.125864	3946.046951	3597.136094	3204.368207
3986.436303	-0.315286024	0.024396545	0.116292478	RAB22A
20	58309696	58367507	+	9285
protein_coding	"RAB22A, member RAS oncogene family [Source:HGNC
Symbol;Acc:HGNC:9764]"	-	3103	3292	3039	4396	4318
3666	18.27656521	15.6319039	15.16181691
22.89164446	20.26559353	18.43132633
ENSG00000153714 133.0505927	146.4582803	85.06846221
204.9339573	210.1877718	182.5060866	121.5257784
199.2092719	-0.713458941	0.024438343	0.116452604
LURAP1L 9	12775021	12822131	+	2132
protein_coding	leucine rich adaptor protein 1 like [Source:HGNC
Symbol;Acc:HGNC:31452]	-	115	158	87	204	230
186	2.949886815	3.267411585	1.890316426
4.626410698	4.701097229	4.072600214
ENSG00000135047 649.0555001	958.4674796	801.7947013
912.1570256	1100.287292	1248.106141	803.1058937
1086.850153	-0.435596431	0.024445372	0.116452604	CTSL
9	87725519	87731393	+	2366
protein_coding	cathepsin L [Source:HGNC Symbol;Acc:HGNC:2537]	-
561	1034	820	908	1204	1272	12.96709921
19.26813837	16.05467683	18.55548561	22.17534291
25.0968033

ENSG00000268218 83.30124065 80.64474925 95.82424478
141.6455293 121.5433637 182.5060866 86.59007823
148.5649932 -0.778188749 0.024454439 0.116459505
AC137932.3 16 89268104 89273044 + 3660
antisense "novel transcript, antisense to ANKRD11"
- 72 87 98 141 133 186
1.075836127 1.048026165 1.240359125 1.862684256
1.58354042 2.372345261
ENSG00000131899 2095.257595 2656.64197 2821.926229
1893.629949 1952.004698 2133.162539 2524.608598
1992.932395 0.341550047 0.0244646 0.11647161 LLGL1
17 18225587 18244875 + 6638
protein_coding "LLGL1, scribble cell polarity complex component
[Source:HGNC Symbol;Acc:HGNC:6628]" - 1811 2866 2886
1885 2136 2174 14.92024364 19.03587726
20.14009591 13.7301513 14.02240796 15.28862145
ENSG00000163449 65.94681552 47.27450818 51.82331606 0
14.62175804 29.43646558 55.01487992 14.68607454
1.903223386 0.024498932 0.116576373 TMEM169 2
216081866 216102783 + 3628 protein_coding
transmembrane protein 169 [Source:HGNC Symbol;Acc:HGNC:25130]
- 57 51 53 0 16 30
0.85921587 0.619778998 0.676723171 0 0.192181378
0.386011295
ENSG00000283787 43.96454368 12.97731597 44.00092873 0
1.827719755 16.68066383 33.64759613 6.169461195
2.447815033 0.024501857 0.116576373 PRR33 11
1888577 1891895 - 3319 protein_coding proline rich
33 [Source:HGNC Symbol;Acc:HGNC:35118] - 38 14 45
0 2 17 0.626139435 0.185975074
0.628069519 0 0.026259191 0.239104475
ENSG00000205710 10.41265508 12.97731597 13.68917783 0
0 2.943646558 12.35971629 0.981215519
3.665264276 0.02456113 1 C17orf107 17
4899418 4902932 + 3408 protein_coding chromosome 17 open
reading frame 107 [Source:HGNC Symbol;Acc:HGNC:37238] - 9
14 14 0 0 3 0.144423424
0.181118331 0.190296546 0 0 0.041092986
ENSG00000112249 553.027681 468.1103261 453.6984651
253.1537119 381.9934288 391.5049923 491.6121574
342.2173777 0.521478728 0.024593571 0.116976331 ASCC3
6 100508194 100881372 - 11731
protein_coding activating signal cointegrator 1 complex subunit 3
[Source:HGNC Symbol;Acc:HGNC:18697] - 478 505 464
252 418 399 2.228371393 1.897974186
1.832252832 1.038644014 1.552743946 1.58775649
ENSG00000284820 23.13923351 0 1.955596832 0 0
0 8.364943449 0 5.551118115 0.024599708
1 AC068946.2 2 219209772 219222738
- 5245 protein_coding novel transcript -

20	0	2	0	0	0	0.208535125
0	0.017663915	0	0	0
ENSG00000140022 188.5847531	159.4355962	168.1813276
129.5905906	106.0077458	53.96685357	172.0672256
96.52173	0.833509841	0.024617695	0.11705465	STON2
14	81260656	81436465	-	7812
protein_coding	stonin 2 [Source:HGNC Symbol;Acc:HGNC:30652]	-
163	172	172	129	116	55	1.141090481
0.970733842	1.019926012	0.798415009	0.6470746
0.328659727
ENSG00000279494 0	0	4.888992081	31.14192488
11.88017841	6.868508636	1.629664027
16.63020398	-3.331548879	0.024632703	1	AL117328.2
22	49851425	49853364	+	1940
lincRNA novel transcript	-	0	0	5	31
13	7	0	0	0.119390764	0.772611522
0.292011678	0.16843888
ENSG00000137269 409.5644332	295.6974139	279.650347
173.7920324	213.8432113	260.0221127	328.3040647
215.8857855	0.603191367	0.024646764	0.117156432	LRRC1
6	53794497	53924121	+	4897
protein_coding	leucine rich repeat containing 1 [Source:HGNC
Symbol;Acc:HGNC:14307]	-	354	319	286	173	234
265	3.953373718	2.872066802	2.705442978
1.708115429	2.082305044	2.526165535
ENSG00000128789 1206.711028	865.7723656	1003.221175
693.1589732	805.110552	823.2398208	1025.234856
773.8364487	0.404920325	0.024663618	0.117200102	PSMG2
18	12658043	12725740	+	3356
protein_coding	proteasome assembly chaperone 2 [Source:HGNC
Symbol;Acc:HGNC:24929]	-	1043	934	1026	690	881
839	16.99640492	12.27040457	14.16210678
9.940960658	11.43964517	11.67040814
ENSG00000262094 4.627846703	0	19.55596832	0	0
0	8.061271675	0	5.504342234	0.024667647
1	AC139099.1	17	83220527	83227721
-	2625	lincRNA novel transcript	-	4
0	20	0	0	0	0.083334608	0
0.352941839	0	0	0
ENSG00000151247 1515.619795	1272.703916	1256.470965
954.3493109	1131.358528	1073.449778	1348.264892
1053.052539	0.355849551	0.024686444	0.117272117	EIF4E
4	98871684	98930637	-	14067
protein_coding	eukaryotic translation initiation factor 4E
[Source:HGNC Symbol;Acc:HGNC:3287]	-	1310	1373	1285
950	1238	1094	5.092892637	4.303313716
4.231595007	3.26530185	3.835109381	3.630461791
ENSG00000100664 8879.680861	8208.152352	7466.468706
9806.692603	10246.19695	9568.813746	8184.767306
9873.901098	-0.270775773	0.024699168	0.117283401	EIF5

14	103333544	103345025	+	8505
protein_coding	eukaryotic translation initiation factor 5
[Source:HGNC Symbol;Acc:HGNC:3299]	-	7675	8855	7636
9762	11212	9752	49.35132065	45.90375422
41.59049197	55.49651374	57.44700199	53.52607651
ENSG00000165246 207.09614	212.2718113	264.9833708
404.8450235	265.0193645	352.2563715	228.1171073
340.7069198	-0.577616525	0.024704164	0.117283401	NLGN4Y
Y	14522573	14845650	+	8589
protein_coding	neuroligin 4 Y-linked [Source:HGNC
Symbol;Acc:HGNC:15529]	-	179	229	271	403	290
359	1.139738296	1.175511379	1.461602052
2.268629943	1.471343222	1.951182453
ENSG00000231365 217.508795	234.5186386	208.2710626
331.5108133	296.0906003	321.8386904	220.0994988
316.4800346	-0.523443572	0.024726546	0.117319502
AL359915.2	1	119140396	119275973	+	5462
antisense	uncharacterized LOC101929147 [Source:NCBI
gene;Acc:101929147]	-	188	253	213	330	324
328	1.882352114	2.042221215	1.806468377
2.921214587	2.584948602	2.803291007
ENSG00000139579 1263.40215	1360.764275	1525.365529
1052.797977	987.8825275	1203.951442	1383.177318
1081.543982	0.35530491	0.024727117	0.117319502	NABP2
12	56222015	56229854	+	2218
protein_coding	nucleic acid binding protein 2 [Source:HGNC
Symbol;Acc:HGNC:28412]	-	1092	1468	1560	1048	1081
1227	26.92500603	29.18088585	32.58108271
22.84551524	21.23844665	25.82432964
ENSG00000164346 1420.748938	1522.053773	1486.253593
1090.971949	1295.853306	1132.322709	1476.352101
1173.049322	0.331720079	0.024764315	0.117459534	NSA2
5	74766991	74780113	+	4962
protein_coding	"NSA2, ribosome biogenesis homolog [Source:HGNC
Symbol;Acc:HGNC:30728]" -	1228	1642	1520	1086	1418
1154	13.53431623	14.58983401	14.19022507
10.58215898	12.45311701	10.85663119
ENSG00000185716 601.6200714	532.996906	421.4311174
736.3558367	657.9791117	711.3812516	518.6826982
701.9054	-0.437196639	0.024792162	0.117510163	MOSMO
16	22007638	22087534	+	6122
protein_coding	modulator of smoothened [Source:HGNC
Symbol;Acc:HGNC:27087]	-	520	575	431	733	720
725	4.645203356	4.141030959	3.261267336
5.789110855	5.125046014	5.52828873
ENSG00000006606 1.156961676	4.634755704	7.822387329
39.17855066	20.1049173	10.79337071	4.538034903
23.35894623	-2.347492524	0.024796474	0.117510163	CCL26
7	75769533	75789896	-	706
protein_coding	C-C motif chemokine ligand 26 [Source:HGNC

Symbol;Acc:HGNC:10625]	-	1	5	8	39	22
	11	0.077462233	0.312247709	0.524913499
2.67092149	1.357927481	0.727334218
ENSG00000226380 94.87085741	50.98231275	65.51249388
167.7645631	93.2137075	135.4077417	70.45522135
132.1286708	-0.910582345	0.02479805	0.117510163	LINC-
PINT	7	130876809	130913310	-	6619
lincRNA "long intergenic non-protein coding RNA, p53 induced
transcript [Source:NCBI gene;Acc:378805]"	-	82	55
	67	167	102	138	0.67751074	0.366356808
	0.468905015	1.21990302	0.671531499	0.973268665
ENSG00000238250 8.09873173	0	15.64477466	0	0
	0	7.91450213	0	5.476393249	0.024801323
	1	ST6GAL2-IT1	2	106822923	106825031
	-	388	sense_intronic	ST6GAL2 intronic transcript 1
[Source:HGNC Symbol;Acc:HGNC:41380]	-	7	0	16
	0	0	0	0.986645245	0	1.910252219
	0	0	0
ENSG00000181754 85.615164	126.9923063	106.5800274
176.8057671	163.5809181	183.4873021	106.3958326
174.6246624	-0.711941446	0.024816611	0.117556851	AMIGO1
	1	109504175	109509738	-	5144
protein_coding	adhesion molecule with Ig like domain 1 [Source:HGNC
Symbol;Acc:HGNC:20824]	-	74	137	109	176	179
	187	0.786729568	1.174231064	0.981585182
1.654294875	1.516389152	1.697018913
ENSG00000128262 8.09873173	2.780853423	8.800185745	0
	0	0	6.559923633	0	5.204808641
0.024822991	1	POM121L9P	22	24251828
24265525	+	5137	transcribed_unprocessed_pseudogene
	"POM121 transmembrane nucleoporin like 9, pseudogene
[Source:HGNC Symbol;Acc:HGNC:30080]"	-	7	3	9
	0	0	0	0.074521774	0.025748127
0.081158759	0	0	0
ENSG00000257605 3.470885027	10.19646255	13.68917783
44.20144177	27.41579632	26.49281903	9.118841801
32.70335237	-1.830356893	0.024823282	0.117556851
AC073611.1	12	53298655	53300314	-	647
	antisense	"novel transcript, antisense to PFDN5 and
C12orf10"	-	3	11	14	44	30	27
	0.253578067	0.749587546	1.002365732	3.288134791
	2.020577775	1.948074311
ENSG00000251288 0	0	0	14.06409511	10.05245865
	0	0	8.038851253	-5.426794997	0.024831085
	1	AC018797.3	4	102751401	102752641
	+	1241	processed_pseudogene	WD repeat domain 12
(WDR12) pseudogene	-	0	0	0	14	11
	0	0	0	0	0.545453171	0.386259791
	0
ENSG00000168528 441.9593601	233.5916875	407.7419395

276.2590111	229.3788292	186.4309487	361.0976624
230.6895963	0.645294932	0.02483718	0.117586239
SERINC2 1	31409565	31434680	+	3549
protein_coding	serine incorporator 2 [Source:HGNC
Symbol;Acc:HGNC:23231]	-	382	252	417	275	251
190	5.886431253	3.130606621	5.442927022
3.746518755	3.081955189	2.499157561
ENSG00000174915 566.9112211	622.9111666	832.1064522
935.2623247	794.1442335	1015.558063	673.97628
914.9882069	-0.440253032	0.024898988	0.117823054	PTDSS2
11	448268	491399	+	6113	protein_coding
phosphatidylserine synthase 2 [Source:HGNC Symbol;Acc:HGNC:15463]
-	490	672	851	931	869	1035
4.3836553	4.846730093	6.448780874	7.363707033
6.194752768	7.90372807
ENSG00000230555 5.784808379	21.31987624	13.68917783
47.21517644	46.60685375	31.39889662	13.59795415
41.74030894	-1.606658087	0.024902616	0.117823054
AL450326.1	10	43420738	43422100	+	760
lincRNA novel transcript	-	5	23	14
47	51	32	0.359791687	1.334283765
0.853329774	2.990098411	2.924254599	1.965542423
ENSG00000236682 8.09873173	5.561706845	10.75578258	0
0	0.981215519	8.138740384	0.32707184
4.555476219	0.024918575	1	AC068282.1	2
127389130	127400580	+	2690	lincRNA novel
transcript	-	7	6	11	0	0	1
0.142311656	0.098340617	0.189427427	0	0
0.017353767
ENSG00000034053 738.1415491	1101.217955	987.5764003
1406.409511	1174.309942	1180.40227	942.3119683
1253.707241	-0.410820074	0.024926164	0.117897981	APBA2
15	28884483	29118315	+	6869
protein_coding	amyloid beta precursor protein binding family A member
2 [Source:HGNC Symbol;Acc:HGNC:579]	-	638	1188	1010
1400	1285	1203	5.079510648	7.625299079
6.811304772	9.854525912	8.152075797	8.175571985
ENSG00000033178 2406.480285	1958.647761	1777.637521
1657.554066	1642.2062	1532.658641	2047.588522
1610.806302	0.345670415	0.024986927	0.118148826	UBA6
4	67612652	67701179	-	11432
protein_coding	ubiquitin like modifier activating enzyme 6
[Source:HGNC Symbol;Acc:HGNC:25581]	-	2080	2113	1818
1650	1797	1562	9.950292143	8.149129873
7.366719249	6.978513853	6.849903591	6.37829864
ENSG00000215861 4.627846703	24.10072966	21.51156516
43.19686355	56.6593124	48.07956045	16.74671384
49.31191213	-1.546712571	0.025018118	0.11825973
AC245297.1	1	149197992	149321732	-	933
unprocessed_pseudogene	phosphodiesterase 4D interacting

protein-like [Source:NCBI gene;Acc:653513]	-	4	26
	22	43	62	49	0.234462321	1.228642863
	1.092303922	2.228373722	2.895800532	2.451661302
ENSG00000182307 3131.895256	3403.764589	3000.863339
3924.887113	3901.267817	3733.525052	3178.841062
3853.226661	-0.277485583	0.025047347	0.118361296
C8orf33 8	145052378	145066685	+	4440
protein_coding	chromosome 8 open reading frame 33 [Source:HGNC
Symbol;Acc:HGNC:26104]	-	2707	3672	3069	3907	4269
	3805	33.34264115	36.46310604	32.01963479
42.54628624	41.89881907	40.00531799
ENSG00000199133 8.09873173	7.415609127	3.911193665	0
	0	0	6.475178174	0	5.186881495
0.025061448	1	MIRLET7D	9	94178834
94178920	+	87	miRNA	microRNA let-7d [Source:HGNC
Symbol;Acc:HGNC:31481]	-	7	8	4	0	0
	0	4.400210977	4.054195547	2.12982144	0
	0	0
ENSG00000198931 2121.867713	3310.142524	3707.811594
4087.628785	3452.562617	4769.68864	3046.607277
4103.293347	-0.429182326	0.025088845	0.118520757	APRT
	16	88809339	88811944	-	1803
protein_coding	adenine phosphoribosyltransferase [Source:HGNC
Symbol;Acc:HGNC:626]	-	1834	3571	3792	4069	3778
	4861	55.62862397	87.32288617	97.42604163
109.1171916	91.31136281	125.8565812
ENSG00000269416 6.941770054	3.707804563	8.800185745	0
	0	0	6.483253454	0	5.18875103
0.025129937	1	LINC01224	19	23399233
23416075	-	3296	lincRNA	long intergenic non-protein
coding RNA 1224 [Source:HGNC Symbol;Acc:HGNC:49676]	-	6
	4	9	0	0	0	0.09955401
0.053506525	0.126490457	0	0	0
ENSG00000122970 472.0403637	510.7500786	469.3432398
335.5291262	426.7725628	222.7359229	484.0445607
328.3458706	0.559448676	0.025148277	0.118764819	IFT81
	12	110124335	110218797	+	7005
protein_coding	intraflagellar transport 81 [Source:HGNC
Symbol;Acc:HGNC:14313]	-	408	551	480	334	467
	227	3.185273557	3.467986652	3.174209255
2.305364194	2.905141824	1.512738128
ENSG00000198482 75.20250892	116.7958437	71.37928438
225.0255217	151.7007397	105.9712761	87.79254568
160.8991792	-0.871544694	0.025160467	0.118785687	ZNF808
	19	52527652	52564464	+	5008
protein_coding	zinc finger protein 808 [Source:HGNC
Symbol;Acc:HGNC:33230]	zf-C2H2 65	126	73	224	166
	108	0.709812673	1.109277446	0.675244407
2.162643402	1.444449509	1.006712523
ENSG00000089818 822.5997514	705.4098182	607.2128164

1173.347363	753.020539	997.8961833	711.7407954
974.7546952	-0.453989715	0.025185079	0.118865176	NECAP1
	12	8076939 8097859 +	8763	protein_coding	NECAP
endocytosis associated 1 [Source:HGNC Symbol;Acc:HGNC:24539]	-
	711	761	621	1168	824	1017	4.437225517
	3.828827522	3.282775959	6.444529968	4.097632425
	5.417690324
ENSG00000143793 555.3416043	794.3971277	646.3247531
824.7587203	875.4777626	969.4409332	665.354495
889.892472	-0.418402162	0.025232864	0.119053948
C1orf35 1	228100726	228105411	-	4308
protein_coding	chromosome 1 open reading frame 35 [Source:HGNC
Symbol;Acc:HGNC:19032]	-	480	857	661	821	958
	988	6.093407959	8.770797523	7.10768579
9.214434325	9.690549676	10.70600115
ENSG00000184599 43.96454368	54.69011731	8.800185745
76.34794487	74.93650995	115.7834313	35.81828224
89.02262871	-1.313996842	0.025261935	0.119154333
FAM19A3 1	112720419	112727235	+	1282
protein_coding	"family with sequence similarity 19 member A3, C-C
motif chemokine like [Source:HGNC Symbol;Acc:HGNC:21590]"	-
	38	59	9	76	82	118	1.621027133
	2.029074273	0.325204795	2.866333923	2.787304558
	4.29674933
ENSG00000198700 4532.975845	3852.408941	4901.70346
3858.584951	3463.528935	3415.611223	4429.029416
3579.241703	0.307289331	0.025288786	0.11924419	IPO9
	1	201829141	201884294	+	11915
protein_coding	importin 9 [Source:HGNC Symbol;Acc:HGNC:19425]	-
	3918	4156	5013	3841	3790	3481	17.98312229
	15.37855217	19.48974306	15.58660311	13.86129355
	13.63816726
ENSG00000161509 34.70885027	19.46597396	50.84551764
99.45324398	83.16124884	54.94806909	35.00678062
79.18752064	-1.178974091	0.025310131	0.11925717	GRIN2C
	17	74842023	74861504	-	8574
protein_coding	glutamate ionotropic receptor NMDA type subunit 2C
[Source:HGNC Symbol;Acc:HGNC:4587]	-	30	21	52
	99	91	56	0.191351772	0.107986577
0.280945655	0.558281108	0.462505084	0.304895197
ENSG00000162231 2024.682933	2144.96494	2060.221263
2658.113975	2532.30572	2412.808962	2076.623045
2534.409553	-0.287272111	0.02532093	0.11925717	NXF1
	11	62792123	62806302	-	6194
protein_coding	nuclear RNA export factor 1 [Source:HGNC
Symbol;Acc:HGNC:8071]	-	1750	2314	2107	2646	2771
	2459	15.45117674	16.47123303	15.75780749
20.65474584	19.49503066	18.53247247
ENSG00000224032 418.8201266	367.9996029	232.716023
512.3348932	478.8625758	490.6077597	339.8452509

493.9350762	-0.540608519	0.025322427	0.11925717
EPB41L4A-AS1	5	112160526	112164276	+	3344
	lincRNA EPB41L4A antisense RNA 1 [Source:HGNC
Symbol;Acc:HGNC:30749]	-	362	397	238	510	524
	500	5.920208669	5.234295007	3.296955946
7.374033798	6.828473305	6.979909174
ENSG00000123349 2364.829665	2357.236751	2587.254609
2706.33373	2999.288118	3327.301827	2436.440342
3010.974558	-0.305428213	0.02532348	0.11925717	PFDN5
	12	53295291	53299450	+	3380
protein_coding	prefoldin subunit 5 [Source:HGNC Symbol;Acc:HGNC:8869]
	-	2044	2543	2646	2694	3282	3391
33.07188156	33.17138599	36.26399076	38.5373731
42.31364851	46.83355503
ENSG00000272752 1.156961676	0	0	23.10529911	0
	7.849724156	0.385653892	10.31834109	-4.82329766
	0.025330743	1	STAG3L5P-PVRIG2P-PILRB	7
100336104	100367831	+	3816	processed_transcript
	STAG3L5P-PVRIG2P-PILRB readthrough [Source:HGNC
Symbol;Acc:HGNC:48898]	-	1	0	0	23	0
	8	0.014331325	0	0	0.291420894	0
	0.097865058
ENSG00000141699 994.9870411	1009.449792	1121.534783
1140.196282	1495.988619	1385.476313	1041.990539
1340.553738	-0.36354624	0.025336297	0.11925717
RETREG3 17	42579513	42610623	-	5145
protein_coding	reticulophagy regulator family member 3 [Source:HGNC
Symbol;Acc:HGNC:27258]	-	860	1089	1147	1135	1637
	1412	9.141296274	9.332037142	10.32715023
10.66624853	13.86506462	12.81136349
ENSG00000162923 3852.68238	3969.204785	3910.215866
4708.458127	4673.479413	4650.961562	3910.70101
4677.633034	-0.258305685	0.025338346	0.11925717	WDR26
	1	224385143	224437033	-	10976
protein_coding	WD repeat domain 26 [Source:HGNC
Symbol;Acc:HGNC:21208]	-	3330	4282	3999	4687	5114
	4740	16.59185134	17.20031984	16.87756392
20.64676771	20.30370165	20.15952426
ENSG00000113739 669.8808102	645.157994	572.0120735
741.3787279	845.3203866	882.112752	629.0169592
822.9372888	-0.388153337	0.025361599	0.119329873	STC2
	5	173314713	173329503	-	6128
protein_coding	stanniocalcin 2 [Source:HGNC Symbol;Acc:HGNC:11374]
	-	579	696	585	738	925	899
5.167191056	5.00754016	4.422212066	5.822893161
6.577813773	6.848366134
ENSG00000233892 13.88354011	0.926951141	5.866790497	0
	0	0	6.892427249	0	5.272789315
0.02537568	1	PAIP1P1 6	30186798	30188614
	-	1203	processed_pseudogene	poly(A) binding

protein interacting protein 1 pseudogene 1 [Source:HGNC
Symbol;Acc:HGNC:18240]	-	12	1	6	0	0
	0	0.545519565	0.036649523	0.23104048	0
	0	0
ENSG00000115541 673.3516953	1067.847714	912.2859223
619.824763	604.061379	720.2121913	884.4951106
648.0327778	0.44987293	0.025408576	0.119514122	HSPE1
	2	197499994	197503457	+	1977
protein_coding	heat shock protein family E (Hsp10) member 1
[Source:HGNC Symbol;Acc:HGNC:5269]	-	582	1152	933
	617	661	734	16.09944957	25.69092656
21.8613728	15.08966892	14.56979321	17.33147107
ENSG00000198160 1593.136227	1395.988418	1200.736455
1019.646895	1257.471191	897.8122003	1396.620367
1058.310096	0.399533492	0.025431067	0.119583118	MIER1
	1	66924895	66988619	+	8273
protein_coding	MIER1 transcriptional regulator [Source:HGNC
Symbol;Acc:HGNC:29657]	Others	1377	1506	1228	1015	1376
	915	9.102603562	8.02593994	6.876030561
5.932042234	7.247930284	5.163023545
ENSG00000198910 1.156961676	6.488657986	12.71137941	0
	0	0	6.785666357	0	5.258747904
0.025438524	1	L1CAM	X	153861514	153886174
	-	7270	protein_coding	L1 cell adhesion molecule
[Source:HGNC Symbol;Acc:HGNC:6470]	-	1	7	13
	0	0	0	0.007522467	0.042451944
0.082834527	0	0	0
ENSG00000141101 1196.298373	1474.779265	1522.432134
1717.82876	1710.745691	1878.046504	1397.836591
1768.873651	-0.339021814	0.025467162	0.119716023	NOB1
	16	69741867	69754940	-	2119
protein_coding	NIN1 (RPN12) binding protein 1 homolog [Source:HGNC
Symbol;Acc:HGNC:29540]	-	1034	1591	1557	1710	1872
	1914	26.68604996	33.10344414	34.03769259
39.0181227	38.4975849	42.1654765
ENSG00000127586 1165.060407	1480.340972	1639.767944
1090.971949	988.7963874	1203.951442	1428.389774
1094.57326	0.384702856	0.025477487	0.119727742	CHTF18
	16	788046	800737	+	5916	protein_coding
chromosome transmission fidelity factor 18 [Source:HGNC
Symbol;Acc:HGNC:18435]	-	1007	1597	1677	1086	1082
	1227	9.308849693	11.90174686	13.1312888
8.875705351	7.969988471	9.681941032
ENSG00000023171 286.9264956	289.2087559	389.1637696
564.5729608	413.9785245	411.1293027	321.7663404
463.2269293	-0.524860336	0.025487763	0.119739222
GRAMD1B 11	123358428	123627774	+	14863
protein_coding	GRAM domain containing 1B [Source:HGNC
Symbol;Acc:HGNC:29214]	-	248	312	398	562	453
	419	0.912514797	0.925512043	1.240449391

1.828230939	1.328159772	1.315993005
ENSG00000155792 37.02277362	17.61207168	23.46716199
79.36167954	47.52071363	58.87293117	26.03400243
61.91844144	-1.256254407	0.025507452	0.119794907	DEPTOR
8	119873717	120050913	+	2622
protein_coding	DEP domain containing MTOR interacting protein
[Source:HGNC Symbol;Acc:HGNC:22953]	-	32	19	24
79	52	60	0.667439651	0.319488236
0.424014795	1.456785535	0.864229833	1.068229578
ENSG00000170581 974.161731	1015.93845	818.4172743
1174.351942	1369.875956	1095.03652	936.1724852
1213.088139	-0.37409751	0.025523345	0.119832731	STAT2
12	56341597	56360155	-	6109
protein_coding	signal transducer and activator of transcription 2
[Source:HGNC Symbol;Acc:HGNC:11363]	STAT	842	1096	837
1169	1499	1116	7.537662347	7.909961815
6.346843486	9.252212578	10.69276705	8.52786085
ENSG00000153443 348.2454644	448.6443522	476.1878287
757.4519794	469.723977	588.7293117	424.3592151
605.301756	-0.510886104	0.025534887	0.119850113	UBALD1
16	4608883 4615027 -	3619	protein_coding	UBA
like domain containing 1 [Source:HGNC Symbol;Acc:HGNC:29576]	-
301	484	487	754	514	600	4.548546357
5.896451579	6.233656022	10.07358324	6.189180287
7.739425127
ENSG00000159176 4145.393684	4855.370076	4160.532261
5317.232529	4936.671058	5915.748367	4387.098674
5389.883985	-0.296818199	0.025543972	0.119855957	CSRP1
1	201483530	201509456	-	10318
protein_coding	cysteine and glycine rich protein 1 [Source:HGNC
Symbol;Acc:HGNC:2469]	-	3583	5238	4255	5293	5402
6029	18.9909197	22.3822596	19.10321646
24.80319267	22.81485191	27.27694928
ENSG00000197355 539.1441409	788.8354209	867.3071951
458.0876692	513.5892511	598.5414669	731.7622523
523.4061291	0.484392991	0.025556711	0.119862567	UAP1L1
9	137077501	137084539	+	5283
protein_coding	UDP-N-acetylglucosamine pyrophosphorylase 1 like 1
[Source:HGNC Symbol;Acc:HGNC:28082]	-	466	851	887
456	562	610	4.823919133	7.102036621
7.777597515	4.173356149	4.635689111	5.390080609
ENSG00000183864 802.9314029	779.5659095	894.6855508
1073.894119	1192.58714	940.0044677	825.7276211
1068.828576	-0.372407873	0.025561063	0.119862567	TOB2
22	41433492	41447023	-	4565
protein_coding	"transducer of ERBB2, 2 [Source:HGNC
Symbol;Acc:HGNC:11980]" -	694	841	915	1069	1305
958	8.314064728	8.122489749	9.285018385
11.32239078	12.45742633	9.796495945
ENSG00000129103 1733.12859	1642.557422	1744.392374

1227.594587	1520.662836	1329.547029	1706.692795
1359.268151	0.328055478	0.025589503	0.119946711	SUMF2
	7	56064002	56080670	+	4101
protein_coding	sulfatase modifying factor 2 [Source:HGNC
Symbol;Acc:HGNC:20415]	-	1498	1772	1784	1222	1664
	1355	19.97637844	19.05056701	20.15150732
14.40730178	17.68162397	15.42394833
ENSG00000175906 160.8176729	237.2994921	224.8936357
339.547439	300.6598997	288.4773627	207.6702669
309.5615671	-0.573532787	0.0255947	0.119946711	ARL4D
	17	43398959	43401137	+	1612
protein_coding	ADP ribosylation factor like GTPase 4D [Source:HGNC
Symbol;Acc:HGNC:656]	-	139	256	230	338	329
	294	4.715681615	7.001786851	6.609448978
10.13801383	8.89384299	8.513895764
ENSG00000172270 14740.84871	14160.10563	19149.20418
11747.53773	11991.66931	14496.47808	16016.71951
12745.22837	0.32964247	0.02560892	0.119976574	BSG
	19	571277	583493	+	3797	protein_coding
basigin (Ok blood group) [Source:HGNC Symbol;Acc:HGNC:1116]	-
	12741	15276	19584	11694	13122	14774	183.509111
	177.3793302	238.9259157	148.9099469	150.5975942
	181.6366709
ENSG00000183506 179.3290597	260.4732706	270.8501613
159.7279373	116.9740643	170.7315004	236.8841639
149.1445007	0.669800538	0.025624439	0.120012499
PI4KAP2 22	21473000	21517533	-	7474
transcribed_unitary_pseudogene	phosphatidylinositol 4-kinase alpha
pseudogene 2 [Source:HGNC Symbol;Acc:HGNC:33577]	-	155
	281	277	159	128	174	1.134157365
1.657628421	1.716837265	1.028596986	0.746303492
1.086781384
ENSG00000132357 264.9442237	177.0476679	173.0703197
277.2635893	307.0569188	343.4254318	205.0207371
309.2486466	-0.595708605	0.025634208	0.12002148	CARD6
	5	40841184	40860175	+	4399
protein_coding	caspase recruitment domain family member 6
[Source:HGNC Symbol;Acc:HGNC:16394]	-	229	191	177
	276	336	350	2.846926357	1.914314828
1.86389636	3.033586176	3.328464376	3.714155807
ENSG00000230177 28.92404189	23.17377852	25.42275882
70.32047554	53.00387289	53.96685357	25.84019308
59.09706733	-1.195249736	0.02568744	0.120199454
AL080317.1	6	111277932	111278742	+	557
	antisense	novel transcript	-	25	25
	26	70	58	55	2.454593197	1.978876866
	2.162323203	6.076367907	4.537654195	4.609496931
ENSG00000041988 185.1138681	385.6116746	359.8298171
364.6618946	591.2673407	494.5326218	310.18512
483.4872857	-0.638516586	0.025687945	0.120199454	THAP3

1	6624866 6635586 +	4072	protein_coding	THAP domain containing 3 [Source:HGNC Symbol;Acc:HGNC:20855] THAP	160	416	368	363	647	504	2.148854084	4.504219218	4.186417191	4.310226297	6.923968909	5.777883794
ENSG00000214756 107.5974358	137.1887688	126.1359957	161.7370937	191.9105743	244.3226643	123.6407335	199.3234441	-0.687438945	0.025700198	0.120219988	CSKMT	11	62665309	62668496	+	3185	protein_coding	citrate synthase lysine methyltransferase [Source:HGNC Symbol;Acc:HGNC:33113]	-	93	148	129	161	210	249	1.596865083	2.048737122	1.876215543	2.444092599	2.873217188	3.649521666
ENSG00000101474 2319.70816	2400.803455	2627.344344	2163.86149	1693.382353	2011.491815	2449.28532	1956.245219	0.324567781	0.025726626	0.1203068	APMAP	20	24962925	24992979	-	2454	protein_coding	adipocyte plasma membrane associated protein [Source:HGNC Symbol;Acc:HGNC:13238]	-	2005	2590	2687	2154	1853	2050	44.68219827	46.53279761	50.72190588	42.43970506	32.9048154	38.99647381
ENSG00000173198 12.72657843	0.926951141	6.844588913	0	0	0	6.832706162	0	5.260819111	0.025753566	1	CYSLTR1 X	78271464	78327691	-	5571	protein_coding	cysteinyl leukotriene receptor 1 [Source:HGNC Symbol;Acc:HGNC:17451]	-	11	1	7	0	0	0	0.107982714	0.007914087	0.058205944	0	0	0
ENSG00000213614 504.4352906	578.4175119	676.636504	422.9274315	427.6864226	448.4154924	586.4964355	433.0097822	0.438452424	0.02575395	0.120373053	HEXA	15	72340919	72376476	-	12611	protein_coding	hexosaminidase subunit alpha [Source:HGNC Symbol;Acc:HGNC:4878]	-	436	624	692	421	468	457	1.890739409	2.181569343	2.541903298	1.614112447	1.617167203	1.691658558
ENSG00000171621 329.7340776	420.8358179	402.8529475	494.2524852	507.192232	579.898372	384.474281	527.1143631	-0.453950307	0.025756542	0.120373053	SPSB1	1	9292880 9369532 +	3403	protein_coding	splA/ryanodine receptor domain and SOCS box containing 1 [Source:HGNC Symbol;Acc:HGNC:30628]	-	285	454	412	492	555	591	4.580128088	5.882038486	5.608383756	6.99043771	7.10705427	8.107212706
ENSG00000075624 200702.7697	182752.1252	195007.2271	154867.7879	174916.436	155381.3648	192820.7074	161721.8629	0.253740417	0.025778443	0.120438589	ACTB	7	5527147 5563784 -	3519	protein_coding	actin beta [Source:HGNC Symbol;Acc:HGNC:132]	-	173474	197154	199435	154162	191404	158356	2695.937617	2470.132694

2625.334021   2118.162552   2370.22918   2100.686731
ENSG00000267655 2.313923351   6.488657986   0   22.10072088
        10.05245865   26.49281903   2.934193779   19.54866619
        -2.721743105   0.025794931   1   AC125437.1   18
        79117207   79117920   +   714   sense_intronic
        "novel transcript, sense intronic to ATP9B"   -   2
        7   0   22   11   27   0.153188617
0.43224879   0   1.489792164   0.671356304   1.765271819
ENSG00000099624 1714.617203   2372.994921   2379.961345
2717.384091   2439.092013   3236.048783   2155.857823
2797.508296   -0.375359541   0.025820735   0.120581129
ATP5F1D 19   1241746 1244826 +   2250   protein_coding ATP
synthase F1 subunit delta [Source:HGNC Symbol;Acc:HGNC:837]   -
        1482   2560   2434   2705   2669   3298   36.02138426
        50.16391291   50.11185871   58.12807861   51.69215685
        68.42489963
ENSG00000174032 568.0681828   596.9565347   643.3913578
754.4382447   802.3689724   787.9160621   602.8053584
781.5744264   -0.374392817   0.025824728   0.120581129
SLC25A30   13   45393316   45418455   -   6794
        protein_coding solute carrier family 25 member 30
[Source:HGNC Symbol;Acc:HGNC:27371]   -   491   644   658
        751   878   803   3.952306916   4.17921085
4.48644974   5.344604967   5.63154516   5.517419921
ENSG00000180999 0   0   0   0   10.05245865
13.73701727   0   7.929825308   -5.407863513   0.025862452
        1   C1orf105   1   172420688   172468831
        +   1725   protein_coding chromosome 1 open reading
frame 105 [Source:HGNC Symbol;Acc:HGNC:29591]   -   0   0
        0   0   11   14   0   0   0   0
        0.277883131   0.378865424
ENSG00000155189 3373.700246   2846.666954   3020.419308
2555.646997   2567.946256   2421.639902   3080.262169
2515.077718   0.292170137   0.025873834   0.120773528   AGPAT5
        8   6708357 6759666 +   4684   protein_coding 1-
acylglycerol-3-phosphate O-acyltransferase 5 [Source:HGNC
Symbol;Acc:HGNC:20886] -   2916   3071   3089   2544   2810
        2468   34.0459413   28.90659169   30.54945576
26.26040605   26.14255505   24.59655618
ENSG00000263001 1712.30328   1612.894985   1980.041793
1477.734565   1400.033332   1353.096201   1768.413353
1410.288033   0.326521452   0.025884047   0.12078432   GTF2I
        7   74657667   74760692   +   10438
protein_coding general transcription factor IIi [Source:HGNC
Symbol;Acc:HGNC:4659]   GTF2I   1480   1740   2025   1471   1532
        1379   7.75423816   7.349637406   8.986905828
6.813913196   6.395876519   6.167270674
ENSG00000008441 2440.032174   2352.601995   3808.524831
2231.168231   1756.438684   2401.034376   2867.053
2129.547097   0.429240457   0.025934407   0.120982384   NFIX

19	12995608	13098796	+	6302
protein_coding	nuclear factor I X [Source:HGNC Symbol;Acc:HGNC:7788]
CTF/NFI 2109	2538	3895	2221	1922	2447
18.30176159	17.75608343	28.63067051	17.04007007
13.29026321	18.12598459
ENSG00000132563 166.6024813	348.533629	291.383928
351.6023777	411.2369448	481.7768201	268.8400128
414.8720475	-0.623396277	0.026010326	0.121299528	REEP2
5	138439017	138446969	+	3284
protein_coding	receptor accessory protein 2 [Source:HGNC
Symbol;Acc:HGNC:17975]	-	144	376	298	350	450
491	2.398026932	5.047991959	4.203543746
5.153070835	5.971287244	6.979501092
ENSG00000132326 497.4935206	650.7197009	677.6143024
857.9098016	746.6235198	831.089545	608.6091746
811.8742888	-0.414451454	0.026056933	0.12147982	PER2
2	238244038	238290102	-	6877
protein_coding	period circadian regulator 2 [Source:HGNC
Symbol;Acc:HGNC:8846]	-	430	702	693	854	817
847	3.419512093	4.500616891	4.668062543
6.004267919	5.177041304	5.749504548
ENSG00000105220 6727.732144	7570.409967	8508.801817
4680.329936	5798.440922	7247.257827	7602.314643
5908.676228	0.363655425	0.026098047	0.121630336	GPI
19	34359480	34402413	+	10680
protein_coding	glucose-6-phosphate isomerase [Source:HGNC
Symbol;Acc:HGNC:4458]	-	5815	8167	8702	4659	6345
7386	29.77646782	33.71516278	37.74420498
21.09223854	25.88922032	32.28375824
ENSG00000214226 82.14427898	101.9646255	86.04626062
190.8698622	123.3710835	146.2011124	90.0517217
153.480686	-0.767165209	0.026105131	0.121630336
C17orf67	17	56791913	56838773	-	2410
protein_coding	chromosome 17 open reading frame 67
[Source:HGNC Symbol;Acc:HGNC:27900]	-	71	110	88
190	135	149	1.61115016	2.012378184
1.691484745	3.811867313	2.441042404	2.88612583
ENSG00000117054 1427.690708	1515.565115	1345.450621
1045.765929	1031.747802	1288.335977	1429.568815
1121.949903	0.349726487	0.026138148	0.121747064	ACADM
1	75724347	75787575	+	4783
protein_coding	acyl-CoA dehydrogenase medium chain [Source:HGNC
Symbol;Acc:HGNC:89]	-	1234	1635	1376	1041	1129
1313	14.10943072	15.07132149	13.32663518
10.52329064	10.28613355	12.81475717
ENSG00000137221 798.3035562	1027.988815	934.7752859
1159.283268	1032.661661	1467.898417	920.3558858
1219.947782	-0.405763001	0.026174022	0.121877027	TJAP1
6	43477523	43506556	+	6741
protein_coding	tight junction associated protein 1 [Source:HGNC

Symbol;Acc:HGNC:17949]	-	690	1109	956	1154	1130
	1496	5.597827049	7.253392468	6.56955603
8.277185688	7.304873744	10.35984606
ENSG00000170037 592.364378	719.3140853	896.6411476
487.2204377	523.6417098	601.4851134	736.106537
537.449087	0.454508099	0.026190599	0.121917078	CNTROB
	17	7932101 7949918 +	6234
protein_coding	"centrobin, centriole duplication and spindle assembly protein [Source:HGNC Symbol;Acc:HGNC:29616]"	-	512	776
	917	485	573	613	4.491566932	5.488186754
	6.81404494	3.761630708	4.005404872	4.590285652
ENSG00000147606 2.313923351	7.415609127	9.777984162	0
	0	0	6.502505547	0	5.196880162
0.026216212	1	SLC26A7 8	91209494	91398152
	+	6273	protein_coding	solute carrier family 26
member 7 [Source:HGNC Symbol;Acc:HGNC:14467]	-	2	8
	10	0	0	0	0.017436103	0.056227485
	0.073846033	0	0	0
ENSG00000089692 63.63289216	18.53902282	31.28954932
19.08698622	0	4.906077597	37.8204881	7.997687939
	2.246497597	0.026246543	0.122061469	LAG3	12
	6772512 6778455 +	3056	protein_coding	lymphocyte
activating 3 [Source:HGNC Symbol;Acc:HGNC:6476] -	55	20
	32	19	0	5	0.984246893	0.28854304
	0.485064045	0.300608646	0	0.076377017
ENSG00000240583 304.2809207	181.6824236	296.2729201
165.7554066	37.46825497	30.4176811	260.7454215
77.88044757	1.743960865	0.026249262	0.122061469	AQP1
	7	30911694	30925516	+	3435
protein_coding	aquaporin 1 (Colton blood group) [Source:HGNC
Symbol;Acc:HGNC:633]	-	263	196	303	165	41
	31	4.18720014	2.515725709	4.086187989
2.322514421	0.520134562	0.421289843
ENSG00000165895 527.5745241	463.4755704	455.6540619
359.6390035	344.5251738	361.0873112	482.2347188
355.0838295	0.441019094	0.026252419	0.122061469
ARHGAP42	11	100687653	100993941	+	8948
	protein_coding	Rho GTPase activating protein 42 [Source:HGNC
Symbol;Acc:HGNC:26545]	-	456	500	466	358	377
	368	2.786978254	2.463644199	2.412472642
1.93445338	1.836005477	1.919852568
ENSG00000070423 1184.728756	1852.048379	1784.482109
2082.490654	2041.562966	2197.922764	1607.086415
2107.325461	-0.390213121	0.026257187	0.122061469	RNF126
	19	647526	663277	-	3496	protein_coding	ring
finger protein 126 [Source:HGNC Symbol;Acc:HGNC:21151]	-	1024
	1998	1825	2073	2234	2240	16.01855163
25.19753272	24.18209376	28.67009253	27.84648241
29.91042818
ENSG00000128340 1469.341328	2007.776171	1879.328556

1817.282004	2374.207962	2964.252084	1785.482018
2385.24735	-0.417466131	0.026261541	0.122061469	RAC2
22	37225261	37244448	-	2532
protein_coding	Rac family small GTPase 2 [Source:HGNC
Symbol;Acc:HGNC:9802]	-	1270	2166	1922	1809	2598
3021	27.43056369	37.71626764	35.16350338
34.54427497	44.71302404	55.69716112
ENSG00000061918 343.6176177	264.1810751	333.4292599
238.0850386	226.6372496	165.8254228	313.7426509
210.1825703	0.577033815	0.026281359	0.122116455
GUCY1B1 4	155758992	155807591	+	3724
protein_coding	guanylate cyclase 1 soluble subunit beta 1
[Source:HGNC Symbol;Acc:HGNC:4687]	-	297	285	341
237	248	169	4.36155637	3.374186983
4.241770452	3.077087813	2.90202137	2.118473658
ENSG00000232085 13.88354011	5.561706845	0.977798416	0
0	0	6.80768179	0	5.255618479
0.026316304	1	AL606534.2	1	243164638
243169445	+	488	antisense	"novel transcript,
antisense to CEP170"	-	12	6	1	0	0
0	1.344795158	0.542082499	0.094925443	0
0	0
ENSG00000008056 15.04050178	0	5.866790497	31.14192488
36.5543951	27.47403455	6.969097427	31.72345151
-2.210175688	0.026336378	0.122280435	SYN1	X
47571898	47619943	-	3822	protein_coding
synapsin I [Source:HGNC Symbol;Acc:HGNC:11494]	-	13
0	6	31	40	28	0.18601475	0
0.072721533	0.392168067	0.45606622	0.341989982
ENSG00000154529 1.156961676	3.707804563	15.64477466	0
0	0	6.836513633	0	5.268957435
0.026336909	1	CNTNAP3B	9	41890314
42129510	-	13220	protein_coding	contactin associated
protein like 3B [Source:HGNC Symbol;Acc:HGNC:32035]	-	1
4	16	0	0	0	0.004136788
0.013340205	0.056064891	0	0	0
ENSG00000131381 1439.260325	1301.439402	1333.71704
1719.837916	1962.971017	1486.541512	1358.138922
1723.116815	-0.343803848	0.026338624	0.122280435	RBSN
3	15070073	15099163	-	7921
protein_coding	"rabenosyn, RAB effector [Source:HGNC
Symbol;Acc:HGNC:20759]" -	1244	1404	1364	1712	2148
1515	8.588851221	7.814857305	7.976948952
10.45020869	11.81715308	8.928503134
ENSG00000058063 2220.209456	1743.595096	1849.994603
1426.501075	1646.775499	1529.714995	1937.933052
1534.330523	0.336315461	0.026340685	0.122280435	ATP11B
3	182793500	182921635	+	9196
protein_coding	ATPase phospholipid transporting 11B (putative)
[Source:HGNC Symbol;Acc:HGNC:13553]	-	1919	1881	1892

1420   1802   1559   11.4122355   9.018281573
9.530696179   7.466044915   8.53914566   7.913947929
ENSG00000067369 1668.338736   1477.560119   1604.567201
1402.391198   1014.384464   1277.542606   1583.488685
1231.439423   0.36286098   0.026348646   0.122280435
TP53BP1 15   43403061   43510728   -   12991
protein_coding tumor protein p53 binding protein 1 [Source:HGNC
Symbol;Acc:HGNC:11999] -   1442   1594   1641   1396   1110
1302   6.070401134   5.4097811   5.851516771
5.195699821   3.723393993   4.678584065
ENSG00000260651 0   8.342560268   14.66697624   0   0
0   7.669845503   0   5.435470036   0.026424142
1   AF213884.3   4   102500841   102501319
-       479   antisense   "novel transcript, antisense
NFKB1" -   0   9   15   0   0   0   0
0.828401648   1.450635166   0   0   0
ENSG00000141867 3625.917892   4108.247456   4884.103089
6121.899685   4902.858242   4908.040028   4206.089479
5310.932652   -0.336275933   0.026488798   0.122893552   BRD4
19   15235519   15332545   -   12093
protein_coding bromodomain containing 4 [Source:HGNC
Symbol;Acc:HGNC:13575] -   3134   4432   4995   6094   5365
5002   14.17293032   16.15844844   19.13391743
24.3651794   19.33277832   19.30881717
ENSG00000100324 833.0124065   810.1552971   936.7308827
1279.832655   949.5004126   1131.341494   859.9661954
1120.224854   -0.381091369   0.026496983   0.12289423   TAB1
22   39399741   39437060   +   4456
protein_coding TGF-beta activated kinase 1 (MAP3K7) binding protein 1
[Source:HGNC Symbol;Acc:HGNC:18157]   -   720   874   958
1274   1039   1153   8.83653551   8.647691905
9.959161676   13.82373699   10.1608248   12.07897718
ENSG00000270270 0   0   0   9.041203998   14.62175804
0   0   7.887654012   -5.400402153   0.026543581
1   BNIP3P10   19   19870723   19871299
-       577   processed_pseudogene   BCL2 interacting
protein 3 pseudogene 10 [Source:HGNC Symbol;Acc:HGNC:49690]   -
0   0   0   9   16   0   0   0
0   0.754167673   1.208377882   0
ENSG00000146083 703.4326988   1074.336372   948.4644637
1146.223751   1185.276261   1279.505037   908.7445116
1203.66835   -0.404405689   0.026595682   0.123314587   RNF44
5   176526697   176538025   -   5660
protein_coding ring finger protein 44 [Source:HGNC
Symbol;Acc:HGNC:19180] -   608   1159   970   1141   1297
1304   5.874648154   9.028195663   7.938852972
9.746987963   9.985783424   10.75492029
ENSG00000267264 3.470885027   12.05036483   3.911193665   0
0   0   6.477481174   0   5.19143249
0.02660748   1   AC006504.3   19   27803538

27804060	+	523	processed_pseudogene	vomeronasal receptor pseudogene	-	3	13	4	0	0
	0	0.313699827	1.095911846	0.354291521	0
	0	0
ENSG00000234840 118.0100909	169.6320588	106.5800274
93.42577465	66.71177105	56.91050013	131.4073924
72.34934861	0.862845343	0.026634221	0.123455832
LINC01239	9	22646200	22824213	+	2568
	lincRNA long intergenic non-protein coding RNA 1239
[Source:HGNC Symbol;Acc:HGNC:49796]	-	102	183	109
	93	73	58	2.172200279	3.141883144
1.966228263	1.751012094	1.238757904	1.054335938
ENSG00000162419 609.7188031	556.1706845	539.7447257
826.7678767	905.6351385	590.6917427	568.5447378
774.3649193	-0.446353411	0.026662755	0.12355063	GMEB1
	1	28668732	28719353	+	6459
protein_coding	glucocorticoid modulatory element binding protein 1
[Source:HGNC Symbol;Acc:HGNC:4370]	SAND	527	600	552
	823	991	602	4.462107648	4.095622534
3.958915653	6.160780945	6.686009167	4.350881375
ENSG00000106400 1757.424785	1518.345969	1508.742956
1868.515493	1958.401717	2126.294031	1594.837903
1984.403747	-0.31572743	0.026676663	0.123577622	ZNHIT1
	7	101217668	101224190	+	5510
protein_coding	zinc finger HIT-type containing 1 [Source:HGNC
Symbol;Acc:HGNC:21688]	-	1519	1638	1543	1860	2143
	2167	15.07651235	13.10678745	12.97229401
16.32159367	16.94841813	18.35918289
ENSG00000270177 5.784808379	8.342560268	4.888992081	0
	0	0	6.338786909	0	5.15791598
0.026707839	1	AC104109.2	5	134226410
134227827	+	1418	lincRNA novel transcript	-
	5	9	5	0	0	0	0.192836165
	0.279833843	0.163341383	0	0	0
ENSG00000260231 18.51138681	23.17377852	19.55596832
65.29758443	30.15737595	60.83536221	20.41371122
52.0967742	-1.348441177	0.026721824	0.123749326	KDM7A-
DT	7	140177184	140179640	+	2457
bidirectional_promoter_lncRNA	KDM7A divergent transcript
[Source:HGNC Symbol;Acc:HGNC:48959]	-	16	25	20
	65	33	62	0.356130803	0.448609855
0.377074614	1.279114484	0.585284983	1.177965494
ENSG00000185989 2806.789025	3475.139827	3413.494271
3892.74061	4027.38048	3979.810147	3231.807708
3966.643746	-0.295274605	0.026730361	0.123751373	RASA3
	13	113977783	114132611	-	4763
protein_coding	RAS p21 protein activator 3 [Source:HGNC
Symbol;Acc:HGNC:20331]	-	2426	3749	3491	3875	4407
	4056	27.8551132	34.70314356	33.95249728
39.33619442	40.32005044	39.75240429

ENSG00000239305 319.3214225 322.578997 308.0065011
329.5016568 561.1099647 481.7768201 316.6356402
457.4628139 -0.531410592 0.026754335 0.123824864 RNF103
2 86603393 86623866 - 7599
protein_coding ring finger protein 103 [Source:HGNC
Symbol;Acc:HGNC:12859] - 276 348 315 328 614
491 1.986311469 2.019095019 1.920244656
2.086981579 3.521036478 3.016276034
ENSG00000239900 298.4961123 262.3271729 271.8279597
161.7370937 187.3412749 213.9049832 277.550415
187.6611173 0.563840744 0.026817483 0.12406076 ADSL
22 40346500 40390463 + 7111
protein_coding adenylosuccinate lyase [Source:HGNC
Symbol;Acc:HGNC:291] - 258 283 278 161 205
218 1.984192209 1.754646825 1.810992172
1.094703266 1.256266504 1.431106159
ENSG00000081853 15.04050178 7.415609127 2.933395248
45.20601999 38.38211485 13.73701727 8.46316872
32.44171737 -1.952276325 0.026821535 0.12406076
PCDHGA2 5 141338760 141512979 + 6724
protein_coding "protocadherin gamma subfamily A, 2 [Source:HGNC
Symbol;Acc:HGNC:8700]" - 13 8 3 45 42
14 0.105732953 0.052456129 0.020667884
0.323583245 0.272195025 0.097195547
ENSG00000185933 3.470885027 1.853902282 14.66697624 0
0 0 6.663921184 0 5.230400651
0.026841395 1 CALHM1 10 103453387 103458888
- 3053 protein_coding calcium homeostasis modulator
1 [Source:HGNC Symbol;Acc:HGNC:23494] - 3 2 15
0 0 0 0.053738948 0.028882657
0.227597198 0 0 0
ENSG00000017483 17.35442514 28.73548537 6.844588913
53.24264577 63.05633154 34.34254318 17.64483314
50.21384016 -1.506584865 0.026850207 0.124109932
SLC38A5 X 48458537 48470256 - 4014
protein_coding solute carrier family 38 member 5 [Source:HGNC
Symbol;Acc:HGNC:18070] - 15 31 7 53 69
35 0.204365981 0.340500915 0.080783586
0.638410054 0.749083654 0.407039646
ENSG00000197603 712.6883922 574.7097073 711.837247
500.2799546 406.6676454 561.2552771 666.4117822
489.4009591 0.445311358 0.026853132 0.124109932
CPLANE1 5 37106228 37249428 - 15146
protein_coding ciliogenesis and planar polarity effector 1
[Source:HGNC Symbol;Acc:HGNC:25801] - 616 620 728
498 445 572 2.224218622 1.804794235
2.226567587 1.589763812 1.280326269 1.762966712
ENSG00000147894 421.13405 492.2110558 349.0740346
543.4768181 722.863163 506.307208 420.8063801
590.8823964 -0.4898353 0.026856522 0.124109932

C9orf72 9 27546545 27573866 - 6332
protein_coding chromosome 9 open reading frame 72 [Source:HGNC
Symbol;Acc:HGNC:28337] - 364 531 357 541 791
516 3.143802031 3.697324536 2.611738949
4.131022643 5.443699895 3.804125458
ENSG00000225234 0 3.707804563 0.977798416 56.25638043
3.65543951 0 1.56186766
19.97060665 -3.642401386 0.026899599 1 TRAPPC12-AS1
2 3481242 3482409 - 908 antisense
TRAPPC12 antisense RNA 1 [Source:HGNC Symbol;Acc:HGNC:41046] -
0 4 1 56 4 0 0
0.194226329 0.051017199 2.981970859 0.191969724 0
ENSG00000169826 1336.290735 1303.293304 1070.689266
1597.279373 1784.768341 1382.532667 1236.757768
1588.19346 -0.361187929 0.026914549 0.124340497
CSGALNACT2 10 43138486 43185308 + 3730
protein_coding chondroitin sulfate N-
acetylgalactosaminyltransferase 2 [Source:HGNC Symbol;Acc:HGNC:24292]
- 1155 1406 1095 1590 1953 1409
16.93432402 16.61921273 13.59902409 20.61054657
22.81665676 17.63389283
ENSG00000154217 56.69112211 70.4482867 66.4902923
121.5539649 106.9216057 115.7834313 64.5432337
114.7530006 -0.828072419 0.026969164 0.124529489
PITPNC1 17 67377281 67697261 + 7819
protein_coding phosphatidylinositol transfer protein cytoplasmic 1
[Source:HGNC Symbol;Acc:HGNC:21045] - 49 76 68
121 117 118 0.342720103 0.428544906
0.402865572 0.748230442 0.652068538 0.70449324
ENSG00000137075 695.3339671 583.9792187 754.8603773
517.3577843 523.6417098 488.6453287 678.0578544
509.8816076 0.410855071 0.026978504 0.124529489 RNF38
9 36336396 36487548 - 6138
protein_coding ring finger protein 38 [Source:HGNC
Symbol;Acc:HGNC:18052] - 601 630 772 515 573
498 5.354788236 4.525302581 5.82630039
4.056780981 4.068050501 3.787463834
ENSG00000280355 38.1797353 40.7858502 41.06753348
98.44866576 76.7642297 66.72265532 40.01103966
80.6451836 -1.01014763 0.026979896 0.124529489
AL132656.4 10 79681973 79684094 - 2122
TEC novel transcript - 33 44 42
98 84 68 0.850478371 0.91420008
0.916867052 2.232964983 1.725013524 1.495924135
ENSG00000165507 325.1062309 114.9419415 67.46809072
107.4898698 68.53949081 59.85414669 169.1720877
78.62783575 1.103025749 0.026992958 0.124552173 DEPP1
10 44970981 44978810 - 2979
protein_coding "DEPP1, autophagy regulator [Source:HGNC
Symbol;Acc:HGNC:23355]" - 281 124 69 107 75

        61   5.158584269   1.83520735   1.072953852
1.736658759   1.097107939   0.955884386
ENSG00000212443 4.627846703   5.561706845   8.800185745   0
        0   0   6.329913098   0   5.156143851
0.027016551   1   SNORA53 12   98599635   98599883
        +   249   snoRNA "small nucleolar RNA, H/ACA box 53
[Source:HGNC Symbol;Acc:HGNC:32646]"   -   4   6   9
        0   0   0   0.878527493   1.062394616
1.674347578   0   0   0
ENSG00000187144 3.470885027   3.707804563   5.866790497
50.2289111   2.741579632   19.62431039   4.348493363
24.19826704   -2.471153026   0.027029339   0.124682407
SPATA21 1   16387117   16437424   -   4506
protein_coding spermatogenesis associated 21 [Source:HGNC
Symbol;Acc:HGNC:28026] -   3   4   6   50   3
        20   0.036410344   0.039138372   0.061682578
0.536512733   0.029012735   0.207197659
ENSG00000250764 0   0.926951141   4.888992081   18.082408
        12.79403828   15.69944831   1.938647741   15.5252982
        -2.97803725   0.02706638   1   AC025178.1   5
        32103445   32121941   -   834   antisense
        novel transcript   -   0   1   5   18
        14   16   0   0.05286496   0.277719522
1.043536565   0.731510531   0.895570888
ENSG00000100564 441.9593601   494.0649581   388.1859712
589.6874163   716.4661439   513.1757167   441.4034298
606.4430923   -0.458403036   0.027080645   0.124871918   PIGH
        14   67581955   67600287   -   2915
protein_coding phosphatidylinositol glycan anchor biosynthesis class
H [Source:HGNC Symbol;Acc:HGNC:8964]   -   382   533   397
        587   784   523   7.166704809   8.061625289
6.308911038   9.736452706   11.72022911   8.37546958
ENSG00000106991 1508.678025   1802.919969   2979.351774
1873.538384   1319.613663   1238.293986   2096.983256
1477.148678   0.505877832   0.027086759   0.124871918   ENG
        9   127815012   127854756   -   3935
protein_coding endoglin [Source:HGNC Symbol;Acc:HGNC:3349]   -
        1304   1945   3047   1865   1444   1262   18.12289472
        21.79258893   35.86990061   22.91581544   15.99119997
        14.97133934
ENSG00000268941 782.1060928   959.3944308   808.6392902
1100.013153   840.7510872   1643.535995   850.0466046
1194.766745   -0.490578519   0.027095981   0.124876775
LINC01711   20   58634772   58635738   +   967
        lincRNA long intergenic non-protein coding RNA 1711
[Source:HGNC Symbol;Acc:HGNC:28663]   -   676   1035   827
        1095   920   1675   38.23093633   47.18976707
39.61699141   54.75059732   41.45910772   80.86011844
ENSG00000270673 28.92404189   14.83121825   11.73358099   0
        5.483159264   2.943646558   18.49628038   2.808935274

	2.703896256	0.027104189	1	YTHDF3-AS1	8
	63167725	63168442	-	718	lincRNA YTHDF3
antisense RNA 1 (head to head) [Source:HGNC Symbol;Acc:HGNC:48728]
	-	25	16	12	0	6	3
1.904189987	0.982493071	0.774210858	0	0.364154268
	0.195048604
ENSG00000259030 0	0	3.911193665	12.05493866
20.1049173	9.812155195	1.303731222
13.99067039	-3.403633716	0.027207047	1	FPGT-TNNI3K
	1	74198235	74544393	+	9896
protein_coding	FPGT-TNNI3K readthrough [Source:HGNC
Symbol;Acc:HGNC:42952]	-	0	0	4	12	22
	10	0	0	0.018724178	0.058630389
0.096877203	0.047172224
ENSG00000127152 135.3645161	265.1080263	47.91212239
353.6115342	653.4098124	332.6320611	149.4615549
446.5511359	-1.578669537	0.027221199	0.125416053	BCL11B
	14	99169287	99271524	-	7855
protein_coding	B cell CLL/lymphoma 11B [Source:HGNC
Symbol;Acc:HGNC:13222]	zf-C2H2 117	286	49	352	715
	339	0.814581205	1.605291114	0.288969726
2.166694548	3.966600389	2.014649705
ENSG00000140807 32.39492692	6.488657986	79.20167171
18.082408	5.483159264	4.906077597	39.36175221
9.490548286	2.055800833	0.02724548	0.125490102	NKD1
	16	50548330	50649249	+	17425
protein_coding	naked cuticle homolog 1 [Source:HGNC
Symbol;Acc:HGNC:17045]	-	28	7	81	18	6
	5	0.087877958	0.017711658	0.215335031
0.049946026	0.015005037	0.013395017
ENSG00000171084 155.0328645	126.0653552	113.4246163
202.9248008	187.3412749	226.660785	131.507612
205.6422869	-0.646725235	0.027272629	0.125577315
FAM86JP 3	125916620	125930024	+	2519
transcribed_unprocessed_pseudogene	"family with sequence
similarity 86 member J, pseudogene [Source:HGNC
Symbol;Acc:HGNC:44097]" -	134	136	116	202	205
	231	2.909185027	2.380371264	2.133203451
3.87725548	3.546372016	4.2808484
ENSG00000219790 4.627846703	7.415609127	6.844588913	0
	0	0	6.296014914	0	5.148773124
0.027280661	1	OSTCP6	6	56975606	56976062
	-	457	processed_pseudogene
oligosaccharyltransferase complex subunit pseudogene 6 [Source:HGNC
Symbol;Acc:HGNC:42868]	-	4	8	7	0	0
	0	0.478672529	0.771805279	0.70955211	0
	0	0
ENSG00000149357 2427.305596	2720.601598	2478.718985
2837.933477	3104.382004	3444.066473	2542.208726
3128.793985	-0.299377651	0.02728897	0.125601951

LAMTOR1 11	72085895	72103387	-	2778
protein_coding	"late endosomal/lysosomal adaptor, MAPK and MTOR
activator 1 [Source:HGNC Symbol;Acc:HGNC:26068]"	-	2098
	2935	2535	2825	3397	3510	41.3017026
46.5811088	42.27155052	49.16855298	53.28707762
58.98219232
ENSG00000116641 2715.389053	2061.539337	2270.447922
1920.753561	1894.431526	1809.361418	2349.125437
1874.848835	0.324898596	0.027297055	0.125601951	DOCK7
	1	62454298	62688368	-	24872
protein_coding	dedicator of cytokinesis 7 [Source:HGNC
Symbol;Acc:HGNC:19190]	-	2347	2224	2322	1912	2073
	1844	5.160563107	3.942375905	4.324679844
3.716878762	3.632011298	3.460957319
ENSG00000213063 0	0	0	11.05036044	2.741579632
	5.887293117	0	6.559744397	-5.132095389
0.027308194	1	RPL29P7 1	168938467	168938923
	+	457	processed_pseudogene	ribosomal protein L29
pseudogene 7 [Source:HGNC Symbol;Acc:HGNC:36859]	-	0
	0	0	11	3	6	0	0	0
	1.163798255	0.286064293	0.612887955
ENSG00000176533 80.9873173	197.440593	199.4708769
211.9660048	294.2628805	305.1580266	159.2995957
270.462304	-0.760466227	0.027311002	0.125601951	GNG7
	19	2511219 2702709 -	4561	protein_coding	G
protein subunit gamma 7 [Source:HGNC Symbol;Acc:HGNC:4410]	-
	70	213	204	211	322	311	0.839329873
	2.058986453	2.071917941	2.236781693	3.076482133
	3.183071196
ENSG00000102893 919.7845322	604.3721438	734.3266105
669.0490959	494.3981937	446.4530614	752.8277622
536.6334503	0.487754468	0.02731122	0.125601951	PHKB
	16	47461123	47701523	+	11822
protein_coding	phosphorylase kinase regulatory subunit beta
[Source:HGNC Symbol;Acc:HGNC:8927]	-	795	652	751
	666	541	455	3.677654159	2.4315914
2.942736919	2.723858172	1.994182532	1.796662393
ENSG00000124357 594.6783013	722.0949387	534.8557336
718.2734287	830.6986286	931.173528	617.2096579
826.7151951	-0.42125842	0.027325769	0.125601951	NAGK
	2	71064344	71079805	+	8894
protein_coding	N-acetylglucosamine kinase [Source:HGNC
Symbol;Acc:HGNC:17174]	-	514	779	547	715	909
	949	3.160535746	3.861662284	2.849001363
3.886960553	4.453745083	4.98098373
ENSG00000008394 2234.092996	1597.136816	1467.675423
1328.05241	1396.377893	1328.565813	1766.301745
1350.998705	0.386015281	0.027327277	0.125601951	MGST1
	12	16347142	16609259	+	7440
protein_coding	microsomal glutathione S-transferase 1 [Source:HGNC

Symbol;Acc:HGNC:7061]	-	1931	1723	1501	1322	1528
	1354	14.19397549	10.21048331	9.345665065
8.591316847	8.949711102	8.495555172
ENSG00000241015 112.2252825	122.3575506	139.8251735
269.2269635	167.2363576	172.6939314	124.8026689
203.0524175	-0.700679768	0.027347527	0.125605095	TPM3P9
	19	53431984	53444670	+	2920
transcribed_processed_pseudogene	tropomyosin 3 pseudogene 9
[Source:HGNC Symbol;Acc:HGNC:44142]	-	97	132	143
	268	183	176	1.816701587	1.993081407
2.268588059	4.437651153	2.731032296	2.813687442
ENSG00000269293 77.51643227	52.83621503	31.28954932	0
	5.483159264	33.36132766	53.88073221	12.94816231
	2.056730099	0.027351954	0.125605095	ZSCAN16-AS1
	6	28121795	28137293	-	2163
antisense	ZSCAN16 antisense RNA 1 [Source:HGNC
Symbol;Acc:HGNC:48982]	-	67	57	32	0	6
	34	1.693998401	1.161855971	0.685323958	0
	0.120879688	0.733784331
ENSG00000186918 613.1896881	579.344463	556.3672988
667.0399394	842.578807	786.9348466	582.96715
765.5178643	-0.393632276	0.027352611	0.125605095	ZNF395
	8	28345585	28402701	-	5819
protein_coding	zinc finger protein 395 [Source:HGNC
Symbol;Acc:HGNC:18737]	-	530	625	569	664	922
	802	4.981065185	4.73549757	4.52966793
5.51722809	6.904641935	6.433866525
ENSG00000111196 684.921312	595.1026324	559.300694
523.3852537	462.413098	333.6132766	613.1082128
439.8038761	0.478789803	0.02737293	0.125660654	MAGOHB
	12	10604190	10613623	-	4856
protein_coding	"mago homolog B, exon junction complex subunit
[Source:HGNC Symbol;Acc:HGNC:25504]"	-	592	642	572
	521	506	340	6.667111855	5.828949704
5.45657095	5.187525705	4.540779745	3.268483334
ENSG00000130475 119.1670526	154.8008405	117.3358099
233.0621475	161.7531983	230.5856471	130.4345677
208.4669976	-0.674534316	0.027417755	0.125828646	FCHO1
	19	17747718	17788568	+	5694
protein_coding	FCH domain only 1 [Source:HGNC Symbol;Acc:HGNC:29002]
	-	103	167	120	232	177	235
0.98926917	1.293102545	0.976261672	1.970025005
1.354610387	1.926621646
ENSG00000169733 733.5137024	1294.950744	1094.156428
1281.841811	1374.445256	1598.400081	1040.873625
1418.229049	-0.445278549	0.027433962	0.125837697	RFNG
	17	82047902	82051831	-	3865
protein_coding	RFNG O-fucosylpeptide 3-beta-N-
acetylglucosaminyltransferase [Source:HGNC Symbol;Acc:HGNC:9974]
	-	634	1397	1119	1276	1504	1629

8.970868124	15.93605668	13.41167572	15.96255448
16.95730907	19.67513051
ENSG00000106511 381.797353	277.1583911	352.0074298
123.5631213	211.1016317	294.3646558	336.9877246
209.6764696	0.683518042	0.02743619	0.125837697	MEOX2
	7	15611212	15686812	-	2500
protein_coding	mesenchyme homeobox 2 [Source:HGNC
Symbol;Acc:HGNC:7014]	Homeobox	330	299	360	123
	231	300	7.218860409	5.273089439	6.670600749
	2.378845953	4.026526567	5.601795907
ENSG00000138398 1689.164047	1714.859611	1672.035292
1272.800607	1485.936161	1332.490675	1692.01965
1363.742481	0.3110085	0.027464663	0.125930506	PPIG
	2	169584340	169641406	+	7368
protein_coding	peptidylprolyl isomerase G [Source:HGNC
Symbol;Acc:HGNC:14650]	-	1460	1850	1710	1267	1626
	1358	10.83672247	11.07021535	10.75100216
8.314348308	9.616776476	8.603916532
ENSG00000213041 0	0	0	9.041203998	6.397019142
	3.924862078	0	6.454361739	-5.109844222
0.027483074	1	AC093422.1	1	205202191
205202742	+	552	processed_pseudogene	ribosomal
protein L17 (RPL17) pseudogene	-	0	0	0	9
	7	4	0	0	0	0.788323818
0.552608499	0.3382727
ENSG00000176715 1107.212324	1535.95804	1324.916854
1813.263691	1545.337053	1737.732685	1322.695739
1698.777809	-0.360201761	0.027498295	0.126046907	ACSF3
	16	89088375	89164121	+	14618
protein_coding	acyl-CoA synthetase family member 3 [Source:HGNC
Symbol;Acc:HGNC:27288]	-	957	1657	1355	1805	1691
	1771	3.580294019	4.997680735	4.293918464
5.97022179	5.040971566	5.655573352
ENSG00000198585 688.3921971	675.7473817	954.3312542
639.9163274	357.3192121	611.2972686	772.823611
536.1776027	0.528208462	0.027510166	0.126056215	NUDT16
	3	131381671	131388830	+	7029
protein_coding	nudix hydrolase 16 [Source:HGNC Symbol;Acc:HGNC:26442]
	-	595	729	976	637	391	623
4.629329944	4.572649812	6.43218801	4.381745058
2.42405133	4.137524126
ENSG00000179588 465.0985936	761.9538378	789.0833218
826.7678767	1014.384464	953.7414849	672.0452511
931.6312752	-0.470000138	0.027516817	0.126056215	ZFPM1
	16	88453317	88537016	+	6110
protein_coding	"zinc finger protein, FOG family member 1 [Source:HGNC
Symbol;Acc:HGNC:19762]" zf-C2H2 402	822	807	823	1110
	972	3.598152413	5.931500417	6.118356524
6.512681526	7.916630338	7.426276079
ENSG00000175348 955.6503441	800.8857857	696.1924723

491.2387506	659.8068315	658.3956136	817.5762007
603.1470652	0.437897681	0.027564612	0.126237337	TMEM9B
	11	8947294 8965011 -	3064	protein_coding	TMEM9
domain family member B [Source:HGNC Symbol;Acc:HGNC:1168]	-
	826	864	712	489	722	671	14.74300453
	12.4325135	10.7644957	7.716516953	10.26850064
	10.22303376
ENSG00000177200 2340.53347	1643.484373	2219.602405
1753.993576	1387.239294	1641.573564	2067.873416
1594.268811	0.37503246	0.027604982	0.126384356	CHD9
	16	53055033	53329150	+	16504
protein_coding	chromodomain helicase DNA binding protein 9
[Source:HGNC Symbol;Acc:HGNC:25701]	-	2023	1773	2270
	1746	1518	1673	6.703496401	4.736455688
6.371462011	5.115124879	4.00812405	4.732087449
ENSG00000176476 397.9948164	494.0649581	489.8770065
697.1772861	564.7654042	602.466329	460.6455937
621.4696731	-0.430727258	0.02761935	0.126412275	SGF29
	16	28553915	28591790	+	2124
protein_coding	SAGA complex associated factor 29 [Source:HGNC
Symbol;Acc:HGNC:25156]	-	344	533	501	694	618
	614	8.857244695	11.06385957	10.92661571
15.79814787	12.67922067	13.49459623
ENSG00000122912 587.7365313	466.2564238	524.0999511
728.319211	707.3275451	649.5646739	526.0309687
695.0704767	-0.403014589	0.027676855	0.126637556
SLC25A16	10	68477999	68527474	-	7815
	protein_coding	solute carrier family 25 member 16
[Source:HGNC Symbol;Acc:HGNC:10986]	-	508	503	536
	725	774	662	3.554916815	2.837742344
3.177153979	4.485493593	4.315892076	3.954349424
ENSG00000161653 20.82531016	96.40291865	86.04626062
159.7279373	127.9403828	135.4077417	67.75816314
141.0253539	-1.051224559	0.027704958	0.126728215	NAGS
	17	44004546	44009063	+	2822
protein_coding	N-acetylglutamate synthase [Source:HGNC
Symbol;Acc:HGNC:17996]	-	18	104	88	159	140
	138	0.348827093	1.624838825	1.444535165
2.724214696	2.161870244	2.282801309
ENSG00000122218 7151.180118	6442.310429	7199.529738
5526.184799	5354.305022	6289.59148	6931.006762
5723.360434	0.276171777	0.027733845	0.126822401	COPA
	1	160288686	160343400	-	7723
protein_coding	coatomer protein complex subunit alpha [Source:HGNC
Symbol;Acc:HGNC:2230]	-	6181	6950	7363	5501	5859
	6410	43.76908034	39.67644273	44.16428679
34.43949912	33.05948325	38.74521102
ENSG00000172236 0	0	0	0	29.24351608	0
	0	9.747838692	-5.706890069	0.02774237	1
	TPSAB1	16	1240696 1242554 +	1512	protein_coding

tryptase alpha/beta 1 [Source:HGNC Symbol;Acc:HGNC:12019]
- 0 0 0 0 32 0 0
0 0 0 0.922267246 0
ENSG00000226107 11.56961676 2.780853423 4.888992081 0
0 0 6.413154087 0 5.169688733
0.027749098 1 AC004383.1 X 134522702
134522990 + 289 processed_pseudogene ribosomal
protein L36a (RPL36A) pseudogene - 10 3 5
0 0 0 1.89232998 0.457675189
0.801446649 0 0 0
ENSG00000150768 2468.956216 2473.105644 2321.29344
2048.334995 1814.011857 2067.4211 2421.118433
1976.589317 0.292744109 0.027750719 0.126861616 DLAT
11 112024814 112064390 + 4831
protein_coding dihydrolipoamide S-acetyltransferase [Source:HGNC
Symbol;Acc:HGNC:2896] - 2134 2668 2374 2039 1985
2107 24.15750568 24.3490906 22.7638719
20.40710527 17.90531935 20.35980124
ENSG00000010256 4694.95048 4269.536955 5235.13272
3834.475073 3626.195994 4141.710708 4733.206718
3867.460592 0.291442037 0.027775951 0.126939001 UQCRC1
3 48599002 48610976 - 3236
protein_coding ubiquinol-cytochrome c reductase core protein 1
[Source:HGNC Symbol;Acc:HGNC:12585] - 4058 4606 5354
3817 3968 4221 68.58012028 62.75515096
76.64296716 57.0315091 53.4344998 60.89096756
ENSG00000162769 645.584615 553.3898311 826.2396617
485.2112812 359.1469318 581.860803 675.0713693
475.4063387 0.506299082 0.027850055 0.127214431 FLVCR1
1 212858255 212899363 + 6442
protein_coding feline leukemia virus subgroup C cellular receptor 1
[Source:HGNC Symbol;Acc:HGNC:24682] - 558 597 845
483 393 593 4.737052426 4.08589845
6.076289318 3.625163735 2.658461821 4.297145002
ENSG00000069702 377.1695063 322.578997 279.650347
230.0484128 233.0342687 215.8674143 326.4662834
226.3166986 0.527489986 0.027852862 0.127214431 TGFBR3
1 91680343 91906335 - 8705
protein_coding transforming growth factor beta receptor 3
[Source:HGNC Symbol;Acc:HGNC:11774] - 326 348 286
229 255 220 2.048064064 1.762562096
1.521947647 1.271943802 1.276526993 1.179777043
ENSG00000250497 10.41265508 10.19646255 0.977798416
12.05493866 40.20983461 40.2298363 7.195638682
30.83153652 -2.105443242 0.027861362 0.127215243
AC007126.1 4 13777377 13782157 - 4781
lincRNA novel transcript - 9 11 1
12 44 41 0.102948134 0.101439687
0.009689106 0.121356689 0.401044468 0.400323559
ENSG00000150753 10509.83986 9653.269181 10085.99066

8668.505478	8129.697469	8649.414804	10083.03324
8482.53925	0.249331571	0.027881624	0.127251963	CCT5
	5	10249921	10266412	+	5839
protein_coding	chaperonin containing TCP1 subunit 5 [Source:HGNC
Symbol;Acc:HGNC:1618]	-	9084	10414	10315	8629	8896
	8815	85.08115228	78.63448667	81.83389318
71.45345123	66.39186932	70.47415499
ENSG00000142185 23.13923351	55.61706845	50.84551764
25.11445555	0	1.962431039	43.20060654	9.025628863
	2.267685751	0.027886052	0.127251963	TRPM2	21
	44350163	44443081	+	6705	protein_coding
	transient receptor potential cation channel subfamily M member
2 [Source:HGNC Symbol;Acc:HGNC:12339]	-	20	60	52
	25	0	2	0.163127029	0.394535808
0.359258471	0.18027788	0	0.013924424
ENSG00000146122 142.3062861	278.0853423	247.3829993
144.659264	155.3561792	101.0651985	222.5915426
133.6935472	0.737157343	0.027933508	0.127398778	DAAM2
	6	39792298	39904877	+	12955
protein_coding	dishevelled associated activator of morphogenesis 2
[Source:HGNC Symbol;Acc:HGNC:18143]	-	123	300	253
	144	170	103	0.519233144	1.020981318
0.904660357	0.537435427	0.571834168	0.371146905
ENSG00000152558 8831.088471	7440.636808	7691.362342
5766.278994	7427.853084	6215.0191	7987.695873
6469.717059	0.303911234	0.027934893	0.127398778
TMEM123 11	102396332	102470384	-	4647
protein_coding	transmembrane protein 123 [Source:HGNC
Symbol;Acc:HGNC:30138]	-	7633	8027	7866	5740	8128
	6334	89.82915257	76.1578278	78.41221561
59.72283792	76.2201186	63.6284615
ENSG00000178607 220.9796801	294.7704628	260.0943787
337.5382826	350.9221929	422.9038889	258.6148405
370.4547881	-0.516929663	0.027952565	0.127441354	ERN1
	17	64039142	64130819	-	10864
protein_coding	endoplasmic reticulum to nucleus signaling 1
[Source:HGNC Symbol;Acc:HGNC:3449]	-	191	318	266
	336	384	431	0.961475723	1.290539557
1.134212255	1.495377139	1.540281379	1.851968302
ENSG00000132153 4428.849295	5494.966363	5197.97638
6450.396764	6131.085918	5851.969358	5040.597346
6144.484013	-0.285482937	0.027994574	0.127594827	DHX30
	3	47802909	47850195	+	7744
protein_coding	DExH-box helicase 30 [Source:HGNC
Symbol;Acc:HGNC:16716]	-	3828	5928	5316	6421	6709
	5964	27.03343903	33.75023558	31.79963124
40.09022947	37.75296325	35.95160854
ENSG00000108379 297.3391507	249.3498569	454.6762635
257.1720248	173.6333767	214.8861988	333.7884237
215.2305334	0.633857119	0.028015905	0.127653989	WNT3

17	46762506	46833154	-	4334
protein_coding	Wnt family member 3 [Source:HGNC
Symbol;Acc:HGNC:12782]	-	257	269	465	256	190
219	3.242940116	2.736511837	4.970115733
2.855961015	1.910395523	2.358854991
ENSG00000285560 4.627846703	0	0	21.09614266
18.27719755	5.887293117	1.542615568
15.08687778	-3.356914721	0.028016668	1	AC103739.3
15	90630535	90631755	-	888
antisense	"novel transcript, antisense to CRTC3"	-	4
0	0	21	20	6	0.246343858	0
0	1.143424637	0.981466832	0.315416436
ENSG00000257954 0	3.707804563	2.933395248	32.1465031
8.224738897	9.812155195	2.213733271	16.72779907
-2.885502813	0.028022594	1	AC125611.2	12
49389516	49390162	+	647
processed_pseudogene	FGFR1 oncogene partner 2 (FGFR1OP2) pseudogene
-	0	4	3	32	9	10	0
0.27257729	0.214792657	2.391370757	0.606173333
0.721509004
ENSG00000271032 31.23796524	3.707804563	14.66697624	0
0	4.906077597	16.53758202	1.635359199
3.340849846	0.028027104	1	AC020907.4	19
35014961	35025335	-	526	antisense	novel
transcript	-	27	4	15	0	0	5
2.807195977	0.33528043	1.321015674	0	0
0.443741754
ENSG00000100124 1063.24778	1208.744288	1252.559771
1375.267586	1663.224977	1423.743719	1174.850613
1487.412094	-0.340076973	0.028052197	0.127781268
ANKRD54 22	37830855	37849327	-	4487
protein_coding	ankyrin repeat domain 54 [Source:HGNC
Symbol;Acc:HGNC:25185]	-	919	1304	1281	1369	1820
1451	11.20093184	12.8131373	13.22499498
14.75192158	17.67558988	15.09584329
ENSG00000166136 97.18478076	144.604378	203.3820706
79.36167954	83.16124884	89.29061227	148.3904098
83.93784689	0.823929857	0.028100925	0.127938546	NDUFB8
10	100523740	100530000	-	1999
protein_coding	NADH:ubiquinone oxidoreductase subunit B8 [Source:HGNC
Symbol;Acc:HGNC:7703]	-	84	156	208	79	91
91	2.29805916	3.440691719	4.82006613
1.910801237	1.98375117	2.12507682
ENSG00000259498 46.27846703	29.66243651	40.08973506
12.05493866	7.310879019	19.62431039	38.67687953
12.99670936	1.574269656	0.028103463	0.127938546	TPM1-
AS	15	63046034	63049387	-	3354
antisense	TPM1 antisense RNA [Source:HGNC Symbol;Acc:HGNC:53635]
-	40	32	41	12	8	20
0.652216296	0.420649985	0.566269609	0.172989365

0.103940673	0.278363939
ENSG00000162227 430.3897434	854.6489519	756.8159741
984.4866576	955.8974318	941.9668987	680.6182231
960.7836627	-0.495853032	0.028115417	0.12795486	TAF6L
	11	62771303	62787342	+	3149
protein_coding	TATA-box binding protein associated factor 6 like
[Source:HGNC Symbol;Acc:HGNC:17305]	-	372	922	774
	980	1046	960	6.460483059	12.90898863
11.38598889	15.04716321	14.47496831	14.23130113
ENSG00000199753 12.72657843	24.10072966	23.46716199	0
	0	7.849724156	20.09815669	2.616574719
2.948585447	0.028125527	1	SNORD104	17
64146083	64146152	+	70	snoRNA	"small
nucleolar RNA, C/D box 104 [Source:HGNC Symbol;Acc:HGNC:32768]" -
	11	26	24	0	0	8	8.593881439
	16.37605416	15.88238274	0	0	5.335043721
ENSG00000134852 1299.267962	1132.734294	1291.671708
950.330998	918.4291768	1069.524916	1241.224655
979.4283637	0.341564738	0.028186559	0.128240161	CLOCK
	4	55427903	55547138	-	11762
protein_coding	clock circadian regulator [Source:HGNC
Symbol;Acc:HGNC:2082]	bHLH	1123	1222	1321	946	1005
	1090	5.221476094	4.580617087	5.202643866
3.888760331	3.723432494	4.326048247
ENSG00000135378 322.7923075	239.1533943	264.0055724
469.1380297	403.0122059	320.8574749	275.3170914
397.6692368	-0.531902621	0.028194883	0.128240161	PRRG4
	11	32829943	32858123	+	5530
protein_coding	proline rich and Gla domain 4 [Source:HGNC
Symbol;Acc:HGNC:30799]	-	279	258	270	467	441
	327	2.759140301	2.056972723	2.261731719
4.083127729	3.475138004	2.760378634
ENSG00000164828 6135.367766	5524.628799	6005.637872
4863.163173	4479.741119	5266.183693	5888.544813
4869.695995	0.274048986	0.028219342	0.128301228	SUN1
	7	816615	896435	+	11811	protein_coding	Sad1
and UNC84 domain containing 1 [Source:HGNC Symbol;Acc:HGNC:18587]
	-	5303	5960	6142	4841	4902	5367
24.55441945	22.24813178	24.08937866	19.81753481
18.0861128	21.21245635
ENSG00000178401 245.2758753	213.1987624	394.0527617
173.7920324	116.9740643	227.6420005	284.1757998
172.8026991	0.718696177	0.028225095	0.128301228
DNAJC22 12	49346917	49357546	+	5109
protein_coding	DnaJ heat shock protein family (Hsp40) member C22
[Source:HGNC Symbol;Acc:HGNC:25802]	-	212	230	403
	173	128	232	2.269314411	1.984841772
3.654025714	1.637236496	1.091773792	2.119815767
ENSG00000119906 1774.779211	1378.376346	1451.05285
1236.635791	1295.853306	1060.693977	1534.736136

1197.727691	0.357044633	0.028285186	0.128536161	SLF2
	10	100912569	100965136	+	9208
protein_coding	SMC5-SMC6 complex localization factor 2 [Source:HGNC
Symbol;Acc:HGNC:17814]	-	1534	1487	1484	1231	1418
	1081	9.110763259	7.119993807	7.465708798
6.463890024	6.710726172	5.480326324
ENSG00000133884 1707.675433	1694.466685	1532.210118
2124.68294	2087.25596	1876.084073	1644.784079
2029.340991	-0.303238904	0.028329148	0.128697676	DPF2
	11	65333754	65353249	+	7715
protein_coding	double PHD fingers 2 [Source:HGNC
Symbol;Acc:HGNC:9964]	Others	1476	1828	1567	2115	2284
	1912	10.46273293	10.44658203	9.408827837
13.25487565	12.90086311	11.56905787
ENSG00000274100 0	0	0.977798416	14.06409511
4.569299387	5.887293117	0.325932805
8.173562537	-4.487682348	0.028332408	1	AL627230.1
	9	67721884	67722094	-	211
unprocessed_pseudogene	family with sequence similarity 27-like
(FAM27L) pseudogene	-	0	0	1	14	5
	6	0	0	0.219543205	3.208091872
1.03263335	1.327439788
ENSG00000109046 1465.870443	1276.411721	1361.095395
1066.862072	1187.103981	998.8773988	1367.79252
1084.28115	0.334590969	0.028343724	0.128725643	WSB1
	17	27294076	27315926	+	8602
protein_coding	WD repeat and SOCS box containing 1 [Source:HGNC
Symbol;Acc:HGNC:19221]	-	1267	1377	1392	1062	1299
	1018	8.055117677	7.057785579	7.496218776
5.969339711	6.580642692	5.52451778
ENSG00000103249 519.4757924	605.299095	686.4144881
911.1524474	802.3689724	702.5503119	603.7297918
805.3572439	-0.414885476	0.028364761	0.128782924	CLCN7
	16	1444934 1475580 -	8119	protein_coding
chloride voltage-gated channel 7 [Source:HGNC Symbol;Acc:HGNC:2025]
	-	449	653	702	907	878	716
3.024395006	3.546047901	4.005318223	5.401395299
4.712491417	4.116769172
ENSG00000077348 1170.845216	1732.471682	1527.321126
1879.565853	1769.232723	2083.120548	1476.879341
1910.639708	-0.370727031	0.028392506	0.128837471	EXOSC5
	19	41386374	41397479	-	1488
protein_coding	exosome component 5 [Source:HGNC
Symbol;Acc:HGNC:24662]	-	1012	1869	1562	1871	1936
	2123	37.19394924	55.37839034	48.62734454
60.79558934	56.69712269	66.60289376
ENSG00000230882 39.33669697	45.4206059	43.02313031
53.24264577	120.6295038	89.29061227	42.59347773
87.72092062	-1.042366333	0.028393631	0.128837471
AC005077.4	7	76071469	76074963	-	3495

processed_pseudogene hypothetical protein LOC285908
(LOC285908) pseudogene - 34 49 44 53 132
91 0.532018151 0.618134321 0.583187158
0.733212577 1.645831419 1.215458816
ENSG00000130723 3544.930574 3836.650772 4508.628497
5327.278311 4467.860941 4903.133951 3963.403281
4899.424401 -0.305667657 0.028410464 0.128863287 PRRC2B
9 131373636 131500197 + 12390
protein_coding proline rich coiled-coil 2B [Source:HGNC
Symbol;Acc:HGNC:28121] - 3064 4139 4611 5303 4889
4997 13.52421815 14.72848504 17.23956375
20.69433796 17.19520384 18.82712816
ENSG00000104848 46.27846703 41.71280134 42.04533189
14.06409511 20.1049173 15.69944831 43.34553342
16.62282024 1.380446962 0.02841618 0.128863287 KCNA7
19 49067418 49072941 - 4372
protein_coding potassium voltage-gated channel subfamily A member 7
[Source:HGNC Symbol;Acc:HGNC:6226] - 40 45 43
14 22 16 0.500350745 0.453801909
0.455607388 0.154827856 0.219281062 0.170838545
ENSG00000172831 1048.207278 1457.167193 1247.670779
1580.201543 1529.801435 1692.596771 1251.015084
1600.866583 -0.354956598 0.028446665 0.128963277 CES2
16 66934444 66945096 + 5343
protein_coding carboxylesterase 2 [Source:HGNC Symbol;Acc:HGNC:1864]
- 906 1572 1276 1573 1674 1725
9.273373162 12.9718323 11.06287374 14.23458352
13.65302474 15.07127385
ENSG00000102302 1172.002177 1423.796952 1260.382158
1623.398407 1520.662836 1685.728262 1285.393763
1609.929835 -0.324212435 0.028476446 0.129060013 FGD1
X 54445454 54496166 - 4275
protein_coding "FYVE, RhoGEF and PH domain containing 1 [Source:HGNC
Symbol;Acc:HGNC:3663]" - 1013 1536 1289 1616 1664
1718 12.95889703 15.84123565 13.96751846
18.2770649 16.96195086 18.76001046
ENSG00000197121 600.4631097 474.5989841 613.0796069
397.8129759 343.6113139 478.8331735 562.7139002
406.7524878 0.468082016 0.028488474 0.129062145 PGAP1
2 196833004 196927796 - 12761
protein_coding post-GPI attachment to proteins 1 [Source:HGNC
Symbol;Acc:HGNC:25712] - 519 512 627 396 376
488 2.224218056 1.768964878 2.276068288
1.500416024 1.283990274 1.785176451
ENSG00000164197 133.0505927 128.8462086 157.425545
212.9705831 229.3788292 199.1867505 139.7741154
213.8453876 -0.613409291 0.028493801 0.129062145 RNF180
5 64165844 64372869 + 5434
protein_coding ring finger protein 180 [Source:HGNC
Symbol;Acc:HGNC:27752] - 115 139 161 212 251

203	1.15737186	1.127792297	1.372488448
1.886329008	2.012855901	1.743903461
ENSG00000164125 308.9087674	107.5263323	300.1841138
53.24264577	13.70789816	0	238.8730712	22.31684798
3.420635742	0.028512136	0.129106939	FAM198B 4
158124474	158173318	-	7880	protein_coding
family with sequence similarity 198 member B [Source:HGNC
Symbol;Acc:HGNC:25312]	-	267	116	307	53	15
0	1.853018506	0.649031432	1.804739874
0.325200248	0.082951385	0
ENSG00000144043 1752.796939	1866.879598	2072.932642
1673.627318	1337.890861	1519.90284	1897.536393
1510.473673	0.329524604	0.028547619	0.129195118	TEX261
2	70985938	70994945	-	4260
protein_coding	testis expressed 261 [Source:HGNC
Symbol;Acc:HGNC:30712]	-	1515	2014	2120	1666	1464
1549	19.44902105	20.8441325	23.05306727
18.90891521	14.97580151	16.97414292
ENSG00000184752 537.9871792	543.1933685	603.3016228
283.2910586	390.2181677	508.2696391	561.4940568
393.9262885	0.511290325	0.028548512	0.129195118
NDUFA12 12	94897055	95003770	-	1815
protein_coding	NADH:ubiquinone oxidoreductase subunit A12
[Source:HGNC Symbol;Acc:HGNC:23987]	-	465	586	617
282	427	518	14.0110614	14.23491718
15.74747728	7.512313362	10.25202941	13.32291221
ENSG00000138379 10.41265508	8.342560268	0.977798416	0
0	0	6.577671255	0	5.208381804
0.028549477	1	MSTN	2	190055697	190062729
-	2822	protein_coding	myostatin [Source:HGNC
Symbol;Acc:HGNC:4223]	-	9	9	1	0	0
0	0.174413546	0.140611052	0.016415172	0
0	0
ENSG00000257027 124.951861	150.1660848	115.3802131
251.1445555	216.5847909	156.9944831	130.166053
208.2412765	-0.677044516	0.028572564	0.129265695
AC010186.3	12	9658567 9662085 +	2508	sense_intronic
novel transcript	-	108	162	118	250
237	160	2.355000133	2.847878391	2.179500289
4.819629933	4.117934283	2.978094581
ENSG00000091947 1103.741439	1052.089545	1017.888151
1178.370254	1532.543014	1326.603382	1057.906378
1345.838884	-0.347735033	0.028615058	0.129419645
TMEM101 17	44011188	44023946	-	3029
protein_coding	transmembrane protein 101 [Source:HGNC
Symbol;Acc:HGNC:28653]	-	954	1135	1041	1173	1677
1352	17.22438856	16.52077993	15.92039768
18.72405703	24.1263924	20.83643685
ENSG00000139890 1.156961676	11.12341369	0	37.16939421
20.1049173	16.68066383	4.093458455	24.65165845

-2.567132778 0.028627453 0.129437405 REM2 14
22883165 22887686 + 2305 protein_coding
RRAD and GEM like GTPase 2 [Source:HGNC Symbol;Acc:HGNC:20248]
- 1 12 0 37 22 17
0.023725959 0.229532546 0 0.776125604 0.415920521
0.344289698
ENSG00000144741 651.3694234 748.0495707 679.5698992
812.7037816 1005.245865 878.1878899 692.9962978
898.7125122 -0.374793465 0.028676583 0.129613608
SLC25A26 3 66133610 66388116 + 5850
protein_coding solute carrier family 25 member 26
[Source:HGNC Symbol;Acc:HGNC:20661] - 563 807 695
809 1100 895 5.263168104 6.082072974
5.503403989 6.686423375 8.193989759 7.141890793
ENSG00000049246 193.2125998 237.2994921 281.6059439
137.6272164 165.4086378 161.9005607 237.3726786
154.978805 0.615892815 0.02868338 0.129613608 PER3
1 7784320 7845177 + 8099 protein_coding period
circadian regulator 3 [Source:HGNC Symbol;Acc:HGNC:8847] -
167 256 288 137 181 165 1.127664179
1.393614076 1.647265279 0.8178815 0.973880732
0.951039557
ENSG00000136448 3534.517919 3509.437019 3547.452654
4666.265841 4114.197168 4014.15269 3530.469197
4264.8719 -0.272602558 0.028838567 0.130276351 NMT1
17 45051610 45109016 + 6669
protein_coding N-myristoyltransferase 1 [Source:HGNC
Symbol;Acc:HGNC:7857] - 3055 3786 3628 4645 4502
4091 25.05216191 25.0295966 25.20049183
33.67644328 29.41733803 28.63616116
ENSG00000221978 4027.383593 4129.567332 4461.694173
3491.9139 2694.972778 3850.289698 4206.215033
3345.725459 0.330372948 0.028867709 0.130369475 CCNL2
1 1385711 1399328 - 6850 protein_coding cyclin
L2 [Source:HGNC Symbol;Acc:HGNC:20570] - 3481 4455 4563
3476 2949 3924 27.79125535 28.67418579
30.85761478 24.53524578 18.76043045 26.74141988
ENSG00000280424 0 1.853902282 4.888992081 32.1465031
18.27719755 2.943646558 2.247631454 17.78911574
-2.961414666 0.028890134 1 BX537318.2 22
46006616 46010777 + 4162 TEC dynein
heavy chain -like pseudogene [Source:NCBI gene;Acc:730668] -
0 2 5 32 20 3 0
0.02118663 0.055650668 0.371748409 0.209404745
0.033648462
ENSG00000197712 1473.969175 1074.336372 1108.823404
908.1387127 882.7886416 1014.576847 1219.042984
935.1680671 0.381741327 0.028910606 0.13052464
FAM114A1 4 38867677 38945739 + 4871
protein_coding family with sequence similarity 114 member A1

[Source:HGNC Symbol;Acc:HGNC:25087]	-	1274	1159	1134
	904	966	1034	14.30362156	10.49057431
10.78443459	8.973286153	8.642066316	9.909424772
ENSG00000176919 10.41265508	20.3929251	1.955596832	0
	0	1.962431039	10.92039234	0.65414368
4.073169353	0.028917675	1	C8G	9	136945185
	136946975	+	1152	protein_coding	complement C8
gamma chain [Source:HGNC Symbol;Acc:HGNC:1354]	-	9	22
	2	0	0	2	0.427252628	0.841984622
	0.080422945	0	0	0.081044501
ENSG00000125648 470.883402	765.6616423	721.6152311
752.4290883	974.1746293	977.2906574	652.7200918
901.298125	-0.464381027	0.028935038	0.130596374
SLC25A23	19	6436079 6465203 -	4859	protein_coding
	solute carrier family 25 member 23 [Source:HGNC
Symbol;Acc:HGNC:19375]	-	407	826	738	749	1066
	996	4.580809411	7.494921806	7.035774612
7.453086045	9.560242387	9.568821985
ENSG00000067560 14742.00567	11384.81391	12366.21657
11096.57104	10959.92151	9101.755159	12831.01205
10386.08257	0.304889694	0.028952206	0.130635294	RHOA
	3	49359145	49412998	-	2582
protein_coding	ras homolog family member A [Source:HGNC
Symbol;Acc:HGNC:667]	-	12742	12282	12647	11046	11993
	9276	269.8833396	209.723363	226.8996032
206.8473767	202.4091744	167.7067481
ENSG00000229647 19.66834849	25.95463194	19.55596832
58.26553688	63.97019142	36.30497422	21.72631625
52.84690084	-1.280576766	0.02897657	0.130703526
MYOSLID 2	207239650	207245887	+	1204
lincRNA "myocardin-induced smooth muscle lncRNA, inducer of
differentiation [Source:HGNC Symbol;Acc:HGNC:51821]"	-	17
	28	20	58	70	37	0.772177508
1.025334339	0.769495288	2.329178235	2.533553916
1.434568442
ENSG00000196781 701.1187755	889.8730952	833.0842506
887.04257	1326.924542	1024.389002	808.0253738
1079.452038	-0.417515487	0.028991122	0.130703526	TLE1
	9	81583683	81689305	-	4906
protein_coding	transducin like enhancer of split 1 [Source:HGNC
Symbol;Acc:HGNC:11837]	-	606	960	852	883	1452
	1044	6.755224598	8.627354569	8.0447862
8.702306518	12.89726639	9.933881856
ENSG00000126858 1489.009677	1283.82733	1100.023218
1016.633161	1083.837815	891.9249072	1290.953408
997.4652942	0.371477466	0.028992978	0.130703526	RHOT1
	17	32142454	32253374	+	6446
protein_coding	ras homolog family member T1 [Source:HGNC
Symbol;Acc:HGNC:21168]	-	1287	1385	1125	1012	1186
	909	10.91900233	9.473128539	8.084714296

7.590867797	8.017758772	6.582935773
ENSG00000237788 2.313923351	0.926951141	4.888992081
18.082408	10.05245865	21.58674143	2.709955524
16.57386936	-2.613021388	0.029018345	1	AL162615.1
	20	49829125	49831085	+	1653
processed_pseudogene	cytokine-like nuclear factor n-pac (N-PAC)
pseudogene	-	2	1	5	18	11	22
	0.066168586	0.026672339	0.140119832	0.526503022
	0.289986933	0.621292145
ENSG00000182568 504.4352906	426.3975248	407.7419395
564.5729608	721.0354433	536.7248891	446.191585
607.4444311	-0.446371094	0.029029519	0.13079949	SATB1
	3	18345387	18445588	-	13409
protein_coding	SATB homeobox 1 [Source:HGNC Symbol;Acc:HGNC:10541]
	CUT	436	460	417	562	789	547
1.778217219	1.512500054	1.440595719	2.026474491
2.564125101	1.904307033
ENSG00000160741 918.6275705	1110.487467	1087.311839
1421.478184	1209.036618	1327.584598	1038.808959
1319.366467	-0.34414276	0.029031378	0.13079949	CRTC2
	1	153947669	153958625	-	3612
protein_coding	CREB regulated transcription coactivator 2
[Source:HGNC Symbol;Acc:HGNC:27301]	-	794	1198	1112
	1415	1323	1353	12.02174394	14.62322069
14.26131266	18.94130576	15.96138967	17.48622615
ENSG00000129451 10.41265508	12.05036483	0	0	0
	0	7.487673304	0	5.396699794	0.029071351
	1	KLK10	19	51012739	51020175	-
	3853	protein_coding	kallikrein related peptidase 10
[Source:HGNC Symbol;Acc:HGNC:6358]	-	9	13	0
	0	0	0	0.127743324	0.148757305	0
	0	0	0
ENSG00000259515 1.156961676	0.926951141	7.822387329
42.19228532	10.05245865	9.812155195	3.302100049
20.68563306	-2.636439049	0.029109784	0.131114106
AL117209.1	14	102933574	102937177	+	828
	antisense	"novel transcript, antisense to CDC42BPB"
	-	1	1	8	42	11	10
0.066048715	0.053248039	0.447571172	2.452562989
0.578923189	0.563787833
ENSG00000187838 17.35442514	9.269511408	24.4449604	0
	0	5.887293117	17.02296565	1.962431039
3.12273137	0.029146829	1	PLSCR3	17	7389727
7394843 -	2834	protein_coding	phospholipid scramblase 3
[Source:HGNC Symbol;Acc:HGNC:16495]	-	15	10	25
	0	0	6	0.289458379	0.155572959
0.40864164	0	0	0.098831967
ENSG00000184838 12.72657843	0	9.777984162	0	0
	0	7.501520865	0	5.396838812	0.029219719
	1	PRR16	5	120464278	120687332	+

2274	protein_coding	proline rich 16 [Source:HGNC
Symbol;Acc:HGNC:29654]	-	11	0	10	0	0
0	0.264543404	0	0.203709834	0	0
0
ENSG00000255495 59.00504546	157.5816939	106.5800274
174.7966106	262.2777848	147.1823279	107.7222556
194.7522411	-0.850781467	0.029279285	0.131838714
AC145124.1	8	12194467	12196280	+	1361
antisense	novel transcript	-	51	170
109	174	287	150	2.049305774	5.507122717
3.70997368	6.181478166	9.189298715	5.144926439
ENSG00000236552 2301.196773	1890.053376	1960.485824
2268.337625	3085.190946	2455.982445	2050.578658
2603.170339	-0.344732903	0.02930082	0.131862789
RPL13AP5	10	96750288	96750899	+	612
processed_pseudogene	ribosomal protein L13a pseudogene 5
[Source:HGNC Symbol;Acc:HGNC:23736]	-	1989	2039	2005
2258	3376	2503	177.7370934	146.8925471
151.762828	178.3913253	240.386245	190.921775
ENSG00000106785 269.5720704	297.5513162	340.2738488
206.9431137	175.4610965	237.4541557	302.4657452
206.6194553	0.550953504	0.029301884	0.131862789	TRIM14
9	98069275	98119212	-	5469
protein_coding	tripartite motif containing 14 [Source:HGNC
Symbol;Acc:HGNC:16283]	-	233	321	348	206	192
242	2.329929126	2.587802136	2.947635487
1.821212043	1.529860752	2.0656345
ENSG00000116001 2000.386737	1633.28791	1553.721683
1299.924219	1451.209485	1358.002279	1729.13211
1369.711995	0.335564863	0.029314941	0.131864646	TIA1
2	70209444	70248660	-	6607
protein_coding	TIA1 cytotoxic granule associated RNA binding protein
[Source:HGNC Symbol;Acc:HGNC:11802]	-	1729	1762	1589
1294	1588	1384	14.31150805	11.75805684
11.14094541	9.469590219	10.47381236	9.778625618
ENSG00000197694 4016.970938	3931.199788	5154.95325
3389.446921	2927.093187	3992.565949	4367.707992
3436.368686	0.346122016	0.029319548	0.131864646	SPTAN1
9	128552558	128633662	+	16236
protein_coding	"spectrin alpha, non-erythrocytic 1 [Source:HGNC
Symbol;Acc:HGNC:11273]" -	3472	4241	5272	3374	3203
4069	11.69486968	11.51657096	15.04176553
10.04771371	8.596793478	11.69916007
ENSG00000102780 1483.224868	1314.416718	1555.67728
1219.557962	986.9686676	1218.669675	1451.106289
1141.732101	0.346016202	0.029392555	0.132154116	DGKH
13	42040036	42256578	+	19637
protein_coding	diacylglycerol kinase eta [Source:HGNC
Symbol;Acc:HGNC:2854]	-	1282	1418	1591	1214	1080
1242	3.570323741	3.183721342	3.753163597

2.989129723	2.396664335	2.952517576
ENSG00000011243	780.9491311	1121.61088	952.3756573
1332.070722	1121.30607	1274.59896	951.645223
1242.658584	-0.383868983	0.029403921	0.132166345	AKAP8L
	19	15380048	15419141	-	5081
protein_coding	A-kinase anchoring protein 8 like [Source:HGNC
Symbol;Acc:HGNC:29857]	-	675	1210	974	1326	1227
	1299	7.265228713	10.49953664	8.879984706
12.61814593	10.5233488	11.93454845
ENSG00000120594	795.9896329	257.6924172	419.4755205
377.7214115	227.5511095	281.6088541	491.0525235
295.627125	0.730889754	0.029425733	0.13219798	PLXDC2
	10	19816239	20289856	+	12567
protein_coding	plexin domain containing 2 [Source:HGNC
Symbol;Acc:HGNC:21013]	-	688	278	429	376	249
	287	2.993998207	0.97532002	1.581350473
1.446629931	0.863428401	1.066096009
ENSG00000221819	6.941770054	2.780853423	19.55596832
29.13276844	26.50193645	50.04199149	9.7595306
35.22556546	-1.850183883	0.029428254	0.13219798	GAS8-
AS1	16	90028908	90029901	-	994
antisense	GAS8 antisense RNA 1 [Source:HGNC
Symbol;Acc:HGNC:1197]	-	6	3	20	29	29
	51	0.330110683	0.133066529	0.932064715
1.410629072	1.271364883	2.395134065
ENSG00000150593	771.6934377	714.6793296	774.4163456
460.0968257	620.5108568	620.1282083	753.596371
566.9119636	0.410138397	0.029464604	0.132307464	PDCD4
	10	110871795	110900006	+	4547
protein_coding	programmed cell death 4 [Source:HGNC
Symbol;Acc:HGNC:8763]	-	667	771	792	458	679
	632	8.022238927	7.47589825	8.068683554
4.870143301	6.507338789	6.488408132
ENSG00000136867	4.627846703	4.634755704	0	0
52.09001301	23.54917247	3.087534136
25.21306183	-3.0372966	0.029473366	0.132307464
SLC31A2	9	113150942	113164137	+	2504
protein_coding	solute carrier family 31 member 2 [Source:HGNC
Symbol;Acc:HGNC:11017]	-	4	5	0	0	57
	24	0.08736156	0.088037893	0	0
0.99197135	0.447427788
ENSG00000215859	63.63289216	43.56670362	29.33395248
10.04578222	25.58807657	12.75580175	45.51118276
16.12988685	1.490015064	0.029478591	0.132307464
PDZK1P1	1	147993862	148014956	-	3674
transcribed_unprocessed_pseudogene	PDZ domain containing 1
pseudogene 1 [Source:HGNC Symbol;Acc:HGNC:31974]	-	55
	47	30	10	28	13	0.818687671
0.56401761	0.378254896	0.131601871	0.332106578
0.165177252

ENSG00000272068 86.77212568 101.9646255 117.3358099
127.5814342 166.3224977 218.8110608 102.024187
170.9049976 -0.742977902 0.029494615 0.132340529
AL365181.2 1 156637783 156641004 - 3222
lincRNA novel transcript - 75 110 120
127 182 223 1.273005969 1.505223906
1.725274351 1.905809122 2.461526126 3.230913737
ENSG00000196814 345.931541 267.8888797 330.4958647
409.8679146 438.6527412 455.284001 314.7720951
434.6015523 -0.466600079 0.029545182 0.132512174 MVB12B
9 126326849 126507041 + 7884
protein_coding multivesicular body subunit 12B [Source:HGNC
Symbol;Acc:HGNC:23368] - 299 289 338 408 480
464 2.074050304 1.616163094 1.985969345
2.502158196 2.653097556 2.747371576
ENSG00000104897 1742.384284 3149.779977 2983.262968
3767.168333 2998.374258 3843.42119 2625.142409
3536.32126 -0.429338444 0.029550995 0.132512174 SF3A2
19 2236504 2248679 + 3134 protein_coding
splicing factor 3a subunit 2 [Source:HGNC Symbol;Acc:HGNC:10766]
- 1506 3398 3051 3750 3281 3917
26.27971751 47.80335087 45.09679431 57.85401343
45.62110876 58.34459308
ENSG00000174780 5136.90984 4578.211685 5085.529562
3764.154598 4414.857068 4032.795785 4933.550362
4070.602484 0.277216413 0.029558874 0.132512174 SRP72
4 56467336 56503681 + 6426
protein_coding signal recognition particle 72 [Source:HGNC
Symbol;Acc:HGNC:11303] - 4440 4939 5201 3747 4831
4110 37.78652564 33.88693292 37.49286157
28.19318807 32.76083135 29.85706657
ENSG00000261103 3.470885027 13.90426711 1.955596832 0
0 0 6.443582991 0 5.18408332
0.02959452 1 AC009063.3 16 83710179
83717898 - 566 antisense "novel transcript,
antisense to CDH13" - 3 15 2 0 0
0 0.289867508 1.168446376 0.16368769 0
0 0
ENSG00000118046 1165.060407 2141.257135 2287.070495
2444.138814 2318.462509 2930.890757 1864.462679
2564.49736 -0.459287504 0.029614774 0.132699354 STK11
19 1177558 1228435 + 8639 protein_coding
serine/threonine kinase 11 [Source:HGNC Symbol;Acc:HGNC:11389] -
1007 2310 2339 2433 2537 2987 6.374714063
11.7891492 12.54206952 13.61695027 12.79721867
16.14052974
ENSG00000225855 173.5442514 99.18377207 130.0471893
97.44408754 45.69299387 73.59116396 134.2584043
72.24274846 0.893871985 0.029617989 0.132699354 RUSC1-
AS1 1 155316863 155324176 - 3516

antisense	RUSC1 antisense RNA 1 [Source:HGNC
Symbol;Acc:HGNC:26680]	-	150	107	133	97	50
	75	2.333120155	1.341741551	1.752286965
1.333902493	0.619697488	0.995768613
ENSG00000204438 418.8201266	457.9138636	447.8316746
557.5409132	538.2634678	676.0574929	441.5218883
590.6206246	-0.419159109	0.029665599	0.132873723	GPANK1
	6	31661229	31666283	-	3528
protein_coding	G-patch domain and ankyrin repeats 1 [Source:HGNC
Symbol;Acc:HGNC:13920]	-	362	494	458	555	589
	689	5.611444951	6.17351248	6.013666744
7.606162913	7.275206352	9.116679374
ENSG00000180353 196.6834849	309.601681	293.3395248
355.6206906	340.8697343	487.6641132	266.5415636
394.7181793	-0.56427857	0.029696268	0.13297213	HCLS1
	3	121631399	121660927	-	5093
protein_coding	hematopoietic cell-specific Lyn substrate 1
[Source:HGNC Symbol;Acc:HGNC:4844]	-	170	334	300
	354	373	497	1.825450067	2.891390492
2.728663832	3.360708174	3.19149195	4.555423401
ENSG00000272168 76.3594706	37.07804563	47.91212239
12.05493866	29.24351608	21.58674143	53.78321287
20.96173206	1.353478201	0.029717412	0.133006593	CASC15
	6	21664772	22368328	+	13289
lincRNA cancer susceptibility 15 [Source:HGNC Symbol;Acc:HGNC:28245]
	-	66	40	49	12	32	22
0.27161037	0.132709388	0.170807224	0.043660646
0.104934011	0.077281655
ENSG00000079931 144.6202095	129.7731597	117.3358099
84.38457065	48.4345735	87.32818123	130.576393
73.3824418	0.832295392	0.029722292	0.133006593	MOXD1
	6	132296055	132401545	-	5293
protein_coding	monooxygenase DBH like 1 [Source:HGNC
Symbol;Acc:HGNC:21063]	-	125	140	120	84	53
	89	1.291525043	1.16616526	1.050223684
0.767323693	0.436347582	0.78493582
ENSG00000123500 477.8251721	511.6770297	350.051833
261.1903377	362.8023713	312.0265352	446.5180116
312.0064148	0.516453807	0.029730067	0.133006593
COL10A1 6	116118923	116158747	-	3816
protein_coding	collagen type X alpha 1 chain [Source:HGNC
Symbol;Acc:HGNC:2185]	-	413	552	358	260	397
	318	5.91883725	6.377708562	4.345873857
3.294323152	4.533574308	3.890136046
ENSG00000285517 224.4505651	195.5866907	121.2470036
174.7966106	346.3528935	374.8243284	180.4280865
298.6579442	-0.728752153	0.029765889	0.133091149
LINC00941	12	30795458	30879268	+	18914
	lincRNA long intergenic non-protein coding RNA 941
[Source:NCBI gene;Acc:100287314]	-	194	211	124

174	379	382	0.56093567	0.4918504
0.303697178	0.444802357	0.873201399	0.942813981
ENSG00000144362 42.807582	23.17377852	17.60037149
110.5036044	42.03755436	52.00442253	27.86057734
68.18186044	-1.296724913	0.029766379	0.133091149
PHOSPHO2	2	169694454	169701708	+	1488
protein_coding	"phosphatase, orphan 2 [Source:HGNC
Symbol;Acc:HGNC:28316]" -	37	25	18	110	46
53	1.359857828	0.740748934	0.560366326
3.574299747	1.347142378	1.662719439
ENSG00000244462 2444.660021	2440.662354	2741.746759
3249.810548	3108.951303	2916.172524	2542.356378
3091.644792	-0.282123133	0.029803729	0.133219184	RBM12
20	35648925	35664956	-	6702
protein_coding	RNA binding motif protein 12 [Source:HGNC
Symbol;Acc:HGNC:9898]	-	2113	2633	2804	3235	3402
2972	17.24208518	17.32129641	19.38099375
23.33839995	22.12017118	20.70095672
ENSG00000151445 831.8554448	766.5885935	705.9704565
1022.66063	1039.97254	890.9436917	768.1381649
984.5256207	-0.358606002	0.02982705	0.133284452
VIPAS39 14	77426675	77457952	-	3695
protein_coding	"VPS33B interacting protein, apical-basolateral
polarity regulator, spe-39 homolog [Source:HGNC
Symbol;Acc:HGNC:20347]" -	719	827	722	1018	1138
908	10.64165464	9.867906477	9.051597017
13.32093017	13.42104761	11.47142689
ENSG00000178695 2288.470195	2066.174093	1947.774445
2538.569167	2977.355481	2338.236583	2100.806244
2618.053744	-0.317918912	0.029841703	0.133310962	KCTD12
13	76880166	76886390	-	6225
protein_coding	potassium channel tetramerization domain containing 12
[Source:HGNC Symbol;Acc:HGNC:14678]	-	1978	2229	1992
2527	3258	2383	17.37727381	15.787184
14.82355722	19.62759566	22.8071134	17.87025388
ENSG00000124570 2534.903031	2618.636973	2899.172304
1975.000784	1931.899781	2517.799023	2684.237436
2141.566529	0.326007907	0.029863936	0.133339374
SERPINB6	6	2948159 2972165 -	13201	protein_coding
serpin family B member 6 [Source:HGNC Symbol;Acc:HGNC:8950]
-	2191	2825	2965	1966	2114	2566
9.076747604	9.43507983	10.40447863	7.20075275
6.978414304	9.07393903
ENSG00000269378 525.2606008	474.5989841	611.1240101
402.835867	365.543951	421.9226734	536.9945317
396.7674971	0.436783019	0.029865508	0.133339374
AC022149.1	19	14621634	14622242	-	609
processed_pseudogene	integrin beta 1 pseudogene 1	-
454	512	625	401	400	430	40.76930171
37.06693072	47.54065714	31.83671843	28.62208693

32.96075862
ENSG00000255135 68.26073887 64.88657986 83.11286537
24.10987733 51.17615314 5.887293117 72.08672803
27.05777453 1.410251584 0.029882058 0.133358921
AP002360.1 11 76441338 76444656 - 754
lincRNA novel transcript - 59 70 85
24 56 6 4.27932606 4.093178197
5.222158338 1.539008833 3.236497522 0.371471877
ENSG00000120800 2141.536062 2074.516653 2243.069567
1932.808499 1587.374607 1709.277435 2153.040761
1743.153514 0.304827515 0.029887333 0.133358921 UTP20
12 101280109 101386616 + 10024
protein_coding "UTP20, small subunit processome component
[Source:HGNC Symbol;Acc:HGNC:17897]" - 1851 2238 2294
1924 1737 1742 10.09857449 9.843577891
10.60119471 9.280368605 7.551224084 8.112470462
ENSG00000171940 1851.138681 1362.618177 1994.708769
1414.446137 1496.902479 940.0044677 1736.155209
1283.784361 0.43516044 0.030004376 0.133842107 ZNF217
20 53567065 53609907 - 6181
protein_coding zinc finger protein 217 [Source:HGNC
Symbol;Acc:HGNC:13009] zf-C2H2 1600 1470 2040 1408 1638
958 14.15650191 10.4855822 15.2888169
11.01400141 11.5481855 7.235237662
ENSG00000279926 16.19746346 17.61207168 4.888992081
49.22433288 29.24351608 39.24862078 12.89950907
39.23882325 -1.606492859 0.030022524 0.133874911
AL138831.3 6 3982673 3984130 + 1458 TEC TEC
- 14 19 5 49 32 40
0.525128059 0.574552918 0.158860138 1.624949141
0.956425292 1.280703225
ENSG00000188167 64.78985384 123.2845017 138.8473751
302.3780448 101.4384464 208.0176901 108.9739102
203.9447271 -0.900576113 0.030029245 0.133874911 TMPPE
3 33090421 33096801 - 3966
protein_coding transmembrane protein with metallophosphoesterase
domain [Source:HGNC Symbol;Acc:HGNC:33865] - 56 133
142 301 111 212 0.772200414 1.478539356
1.658586363 3.669568526 1.219632157 2.495336889
ENSG00000198815 2995.373778 2829.054882 2526.631107
3735.021829 3579.58914 3007.425567 2783.686589
3440.678845 -0.305857072 0.030071494 0.133994643 FOXJ3
1 42176539 42335877 - 6893
protein_coding forkhead box J3 [Source:HGNC Symbol;Acc:HGNC:29178]
Fork 2589 3052 2584 3718 3917 3065
20.54085348 19.52136621 17.36547578 26.07968391
24.7630361 20.75717449
ENSG00000166278 30.08100357 29.66243651 38.13413823
8.036625776 0 14.71823279 32.62585944 7.584952856
2.111054386 0.030074766 0.133994643 C2 6

31897785 31945672 + 5536 protein_coding
complement C2 [Source:HGNC Symbol;Acc:HGNC:1248] -
26 32 39 8 0 15 0.256845511
0.254851888 0.326340505 0.069870704 0
0.126485637
ENSG00000104671 596.9922247 371.7074075 470.3210382
306.3963577 396.6151868 298.2895179 479.6735568
333.7670208 0.52136833 0.030082398 0.133994643 DCTN6
8 30156297 30183640 + 1984
protein_coding dynactin subunit 6 [Source:HGNC Symbol;Acc:HGNC:16964]
- 516 401 481 305 434 304
14.22337782 8.911209682 11.23067512 7.432918793
9.532496609 7.152830795
ENSG00000212766 5.784808379 16.68512054 0 0 0
0 7.489976305 0 5.399730957 0.030086473
1 EWSAT1 15 69072926 69095820 +
3576 lincRNA Ewing sarcoma associated transcript 1
[Source:HGNC Symbol;Acc:HGNC:26596] - 5 18 0
0 0 0 0.076465795 0.221926392 0
0 0 0
ENSG00000198934 157.3467879 210.417909 154.4921498
287.3093715 266.8470842 230.5856471 174.0856155
261.5807009 -0.585997214 0.030106448 0.134028486 MAGEE1
X 76427724 76431353 + 3630
protein_coding MAGE family member E1 [Source:HGNC
Symbol;Acc:HGNC:24934] - 136 227 158 286 292
235 2.048929409 2.757104265 2.016289636
3.809435499 3.505377738 3.02208916
ENSG00000238251 0 0 0 10.04578222 0
12.75580175 0 7.600527991 -5.34453452 0.030106629
1 AL133477.1 9 96407284 96407797
+ 514 processed_pseudogene NSA2 ribosome
biogenesis homolog (S. cerevisiae) (NSA2) pseudogene - 0
0 0 10 0 13 0 0 0
0.940671741 0 1.180663858
ENSG00000077150 1244.890763 1917.86191 1562.521869
1728.87912 2524.080981 2022.285186 1575.091514
2091.748429 -0.408859266 0.030107531 0.134028486 NFKB2
10 102394110 102402529 + 4484
protein_coding nuclear factor kappa B subunit 2 [Source:HGNC
Symbol;Acc:HGNC:7795] RHD 1076 2069 1598 1721 2762
2061 13.12324933 20.34364856 16.50872856
18.55737239 26.84211101 21.45647741
ENSG00000104517 3414.193905 3066.354374 3652.077084
3038.849122 2290.132853 2784.689644 3377.541788
2704.557206 0.320669851 0.030116347 0.134028703 UBR5
8 102253012 102412841 - 13085
protein_coding ubiquitin protein ligase E3 component n-recognin 5
[Source:HGNC Symbol;Acc:HGNC:16806] - 2951 3308 3735
3025 2506 2838 12.33360954 11.14617178

13.22267535	11.17771079	8.345760883	10.12475913
ENSG00000187189 883.9187202	1073.409421	824.2840648
1321.020362	1159.688184	1105.82989	927.2040687
1195.512812	-0.366172072	0.0301274	0.134038874	TSPYL4
	6	116249961	116254140	-	4180
protein_coding	TSPY like 4 [Source:HGNC Symbol;Acc:HGNC:21559] -
	764	1158	843	1315	1269	1127	9.995667233
	12.21423399	9.342298697	15.21075207	13.22951546
	12.58617222
ENSG00000114993 301.9669974	474.5989841	425.342311
504.2982675	547.4020666	630.921579	400.6360975
560.873971	-0.483640777	0.030143868	0.134073119	RTKN
	2	74425836	74442422	-	5190
protein_coding	rhotekin [Source:HGNC Symbol;Acc:HGNC:10466]	-
	261	512	435	502	599	643	2.750222699
	4.349472217	3.88261524	4.676679152	5.029421828
	5.783485498
ENSG00000230993 16.19746346	0.926951141	2.933395248	0
	0	0	6.685936616	0	5.227426157
0.03014685	1	RPL12P15	2	121658475
121658973	-	499	processed_pseudogene	ribosomal
protein L12 pseudogene 15 [Source:HGNC Symbol;Acc:HGNC:36619]	-
	14	1	3	0	0	0	1.534342104
	0.088355464	0.278498695	0	0	0
ENSG00000086666 1242.57684	967.736991	886.8631635
729.3237892	875.4777626	745.7237948	1032.392331
783.5084488	0.396963217	0.030194664	0.134203534	ZFAND6
	15	80059568	80138393	+	5153
protein_coding	zinc finger AN1-type containing 6 [Source:HGNC
Symbol;Acc:HGNC:30164]	-	1074	1044	907	726	958
	760	11.39826767	8.932526518	8.153603725
6.812047925	8.101472541	6.884929311
ENSG00000106477 1001.928811	1030.769669	944.55327
873.9830532	762.1591378	676.0574929	992.4172499
770.7332279	0.364839119	0.030202197	0.134203534	CEP41
	7	130393771	130442433	-	12566
protein_coding	centrosomal protein 41 [Source:HGNC
Symbol;Acc:HGNC:12370]	-	866	1112	966	870	834
	689	3.768908113	3.901590542	3.561086532
3.347521798	2.892195147	2.55957702
ENSG00000139266 400.3087398	474.5989841	477.1656271
489.2295941	717.3800038	644.6585963	450.691117
617.0893981	-0.45297816	0.030205165	0.134203534	9-Mar
	12	57755098	57760407	+	4005
protein_coding	membrane associated ring-CH-type finger 9 [Source:HGNC
Symbol;Acc:HGNC:25139]	-	346	512	488	487	785
	657	4.724635307	5.636394709	5.644425658
5.879327563	8.541334575	7.65788579
ENSG00000187172 92.55693406	226.1760784	131.0249878
48.21975466	94.12756737	94.19668987	149.9193334

78.84800397	0.928369724	0.030208305	0.134203534	BAGE2
	21	10413477	10516431	+	4080
transcribed_unprocessed_pseudogene	BAGE family member 2
[Source:HGNC Symbol;Acc:HGNC:15723]	-	80	244	134
	48	103	96	1.072320322	2.636717619
1.521412889	0.568829735	1.100108852	1.098391354
ENSG00000164292 2993.059855	2414.707722	2601.921585
2243.22317	2240.784419	1992.84872	2669.896387
2158.952103	0.306093997	0.030229924	0.134260561
RHOBTB3 5	95713522	95824383	+	8833
protein_coding	Rho related BTB domain containing 3 [Source:HGNC
Symbol;Acc:HGNC:18757]	-	2587	2605	2661	2233	2452
	2031	16.017064	13.00269738	13.95529752
12.22310969	12.09680927	10.73365739
ENSG00000106733 82.14427898	97.32986979	68.44588913
130.5951689	141.648281	145.2198969	82.64001263
139.1544489	-0.751215451	0.030263719	0.134323173	NMRK1
	9	75060573	75088217	-	3865
protein_coding	nicotinamide riboside kinase 1 [Source:HGNC
Symbol;Acc:HGNC:26057]	-	71	105	70	130	155
	148	1.004624033	1.197770903	0.838978821
1.626279062	1.747595017	1.787550225
ENSG00000167770 2280.371463	2792.903787	2814.103842
3499.950526	3016.651455	3231.142706	2629.126364
3249.248229	-0.305110884	0.030267388	0.134323173	OTUB1
	11	63985853	64001811	+	5336
protein_coding	"OTU deubiquitinase, ubiquitin aldehyde binding 1
[Source:HGNC Symbol;Acc:HGNC:23077]"	-	1971	3013	2878
	3484	3301	3293	20.200658	24.89529453
24.98488901	31.56919749	26.95803924	28.80858621
ENSG00000233834 3.470885027	10.19646255	4.888992081	0
	0	0	6.185446552	0	5.124553797
0.030269857	1	AC005083.1	7	20217577
20221700	+	901	processed_transcript
uncharacterized LOC100506098 [Source:NCBI gene;Acc:100506098]	-
	3	11	5	0	0	0	0.18209213
	0.538272078	0.257067793	0	0	0
ENSG00000144596 3.470885027	10.19646255	4.888992081	0
	0	0	6.185446552	0	5.124553797
0.030269857	1	GRIP2	3	14489111	14556075
	-	11885	protein_coding	glutamate receptor interacting
protein 2 [Source:HGNC Symbol;Acc:HGNC:23841]	-	3	11
	5	0	0	0	0.013804376	0.040806322
	0.019488269	0	0	0
ENSG00000272734 45.12150535	64.88657986	65.51249388
6.027469332	17.36333767	40.2298363	58.5068597
21.2068811	1.464992255	0.03027353	0.134323173	ADIRF-
AS1	10	86965345	86971311	-	5748
processed_transcript	ADIRF antisense RNA 1 [Source:HGNC
Symbol;Acc:HGNC:45127]	-	39	70	67	6	19

41	0.37105865	0.536926994	0.539958645
0.050470279	0.144044088	0.332976154
ENSG00000085832 1708.832395	1357.983421	1547.854893
1011.61027	1428.362988	1111.717184	1538.22357
1183.896814	0.377029653	0.030279169	0.134323173	EPS15
1	51354263	51519328	-	7440
protein_coding	epidermal growth factor receptor pathway substrate 15
[Source:HGNC Symbol;Acc:HGNC:3419]	-	1477	1465	1583
1007	1563	1133	10.85681087	8.681577512
9.856221052	6.544217901	9.154711029	7.108909904
ENSG00000272913 180.4860214	192.8058373	135.9139798
284.2956368	239.4312879	242.3602333	169.7352795
255.362386	-0.589232318	0.030302412	0.134384889
AC009237.14	2	95525109	95526702	-	1594
lincRNA novel transcript	-	156	208	139
283	262	247	5.35218349	5.753193431
4.039512338	8.584190264	7.162614408	7.233603037
ENSG00000105058 2610.10554	3067.281325	2656.678297
3078.027672	3469.925955	3702.126155	2778.021721
3416.693261	-0.298374348	0.030310812	0.134384889	FAM32A
19	16185380	16192046	+	1921
protein_coding	family with sequence similarity 32 member A
[Source:HGNC Symbol;Acc:HGNC:24563]	-	2256	3309	2717
3064	3797	3773	64.22534461	75.94572988
65.51861818	77.1192172	86.13344746	91.68651725
ENSG00000111052 41.65062033	5.561706845	33.24514615
2.009156444	11.88017841	1.962431039	26.81915777
5.283921963	2.330445549	0.030327178	0.134384889	LIN7A
12	80792520	80937925	-	6795
protein_coding	"lin-7 homolog A, crumbs cell polarity complex
component [Source:HGNC Symbol;Acc:HGNC:17787]"	-	36	6
34	2	13	2	0.289739531	0.038931017
0.231788514	0.014231208	0.083370516	0.013739995
ENSG00000197136 2352.103087	3212.812654	3713.678385
4638.137651	3511.049649	3909.16263	3092.864708
4019.449977	-0.37767097	0.030333667	0.134384889	PCNX3
11	65615773	65637439	+	7972
protein_coding	pecanex 3 [Source:HGNC Symbol;Acc:HGNC:18760]	-
2033	3466	3798	4617	3842	3984	13.9464862
19.16881325	22.06937967	28.00230625	21.00142038
23.32910488
ENSG00000061455 0	0	0	0	0	28.45525006
0	9.485083355	-5.664906732	0.030338556	1
PRDM6	5	123089121	123194266	+	9350
protein_coding	PR/SET domain 6 [Source:HGNC
Symbol;Acc:HGNC:9350]	zf-C2H2 0	0	0	0	0
29	0	0	0	0	0	0.144787951
ENSG00000270956 2.313923351	1.853902282	3.911193665
25.11445555	15.53561792	7.849724156	2.693006433
16.16659921	-2.583993727	0.030345105	1	AC009948.4

2	178541125	178541799	+	675
antisense	"novel transcript, antisense to TTN"	-	2
2	4	25	17	8	0.162039515
0.13063519	0.274510319	1.790760277	1.097498022
0.553263793
ENSG00000182578 284.6125722	337.4102153	365.6966076
261.1903377	188.2551347	231.5668626	329.2397984
227.0041117	0.537972083	0.030349329	0.134384889	CSF1R
5	150053291	150113372	-	5151
protein_coding	colony stimulating factor 1 receptor [Source:HGNC
Symbol;Acc:HGNC:2433]	-	246	364	374	260	206
236	2.611790092	3.115615041	3.363430887
2.440523616	1.742746697	2.138781845
ENSG00000100749 1069.032588	1008.522841	924.9973017
737.360415	841.6649471	778.1039069	1000.85091
785.7097563	0.348651222	0.03035122	0.134384889	VRK1
14	96797304	96931722	+	2744
protein_coding	vaccinia related kinase 1 [Source:HGNC
Symbol;Acc:HGNC:12718]	-	924	1088	946	734	921
793	18.41546023	17.48150208	15.97016801
12.9334137	14.62628801	13.49071961
ENSG00000142657 2993.059855	3262.868016	3653.054883
2649.072771	2781.789467	2694.417816	3302.994251
2708.426685	0.286473255	0.030354616	0.134384889	PGD
1	10398592	10420144	+	2966
protein_coding	phosphogluconate dehydrogenase [Source:HGNC
Symbol;Acc:HGNC:8891]	-	2587	3520	3736	2637	3044
2746	47.70017746	52.32454672	58.34963943
42.9873031	44.72312059	43.21907046
ENSG00000237070 0	8.342560268	13.68917783	0	0
0	7.343912698	0	5.372895919	0.03036352
1	AC005550.2	7	15667947	15681980
+	3743	antisense	uncharacterized LOC105375166
[Source:NCBI gene;Acc:105375166]	-	0	9	14
0	0	0	0	0.106012394	0.173264929
0	0	0
ENSG00000116030 2018.898124	2077.297507	1899.862323
1673.627318	1632.153741	1609.193452	1998.685984
1638.324837	0.286851697	0.030426715	0.134665083	SUMO1
2	202206180	202238608	-	2164
protein_coding	small ubiquitin-like modifier 1 [Source:HGNC
Symbol;Acc:HGNC:12502]	-	1745	2241	1943	1666	1786
1640	44.09942101	45.65817602	41.59278489
37.22365009	35.96522618	35.37794704
ENSG00000198952 3704.591286	4314.957561	4972.104946
5901.897054	5060.042141	5152.362693	4330.551264
5371.433963	-0.31052746	0.03047537	0.134841384	SMG5
1	156249224	156282825	-	5304
protein_coding	"SMG5, nonsense mediated mRNA decay factor
[Source:HGNC Symbol;Acc:HGNC:24644]"	-	3202	4655	5085

5875 5537 5251 33.015093 38.69457918
44.41093306 53.5556842 45.49143178 46.21516828
ENSG00000204362 1.156961676 10.19646255 7.822387329 0
0 0 6.391937185 0 5.173285279
0.030516367 1 AL590644.1 1 17189783
17197617 + 4676 lincRNA novel transcript -
1 11 8 0 0 0 0.011695538
0.103717524 0.079253407 0 0 0
ENSG00000146021 23.13923351 0.926951141 0 0 0
0 8.022061552 0 5.49065407 0.030536386
1 KLHL3 5 137617500 137736090 -
11854 protein_coding kelch like family member 3
[Source:HGNC Symbol;Acc:HGNC:6354] - 20 1 0
0 0 0 0.092269844 0.003719367 0
0 0 0
ENSG00000283839 2.313923351 11.12341369 19.55596832
41.1877071 34.72667534 35.3237587 10.99776845
37.07938038 -1.741144333 0.03059258 0.135320824
AC096667.1 2 184593577 184599008 - 3465
antisense proline-rich protein 18-like [Source:NCBI
gene;Acc:105373780] - 2 12 20 41 38
36 0.031566139 0.152690482 0.267380181
0.572113024 0.477902118 0.485003975
ENSG00000162408 1600.077998 1438.628171 1460.830834
2121.669205 1777.457462 1717.127159 1499.845667
1872.084609 -0.320008211 0.030644321 0.135489947 NOL9
1 6521347 6554535 - 7293 protein_coding
nucleolar protein 9 [Source:HGNC Symbol;Acc:HGNC:26265] - 1383
1552 1494 2112 1945 1750 10.37076228
9.38251919 9.489576686 14.00196271 11.62176233
11.20154353
ENSG00000183496 224.4505651 185.3902282 266.9389676
162.741672 159.9254785 105.9712761 225.5932536
142.8794755 0.658418099 0.030655094 0.135489947 MEX3B
15 82041778 82046141 - 4364
protein_coding mex-3 RNA binding family member B [Source:HGNC
Symbol;Acc:HGNC:25297] - 194 200 273 162 175
108 2.431149695 2.02059471 2.897879756
1.794863761 1.747478755 1.155274133
ENSG00000285589 75.20250892 56.54401959 69.42368755
35.16023777 16.44947779 38.26740526 67.05673869
29.95904027 1.163885364 0.030657403 0.135489947
AC010422.8 19 12668071 12681834 - 5025
protein_coding novel transcript - 65 61
71 35 18 39 0.707411317 0.535214323
0.654522738 0.336769843 0.156097173 0.362305208
ENSG00000179841 97.18478076 57.47097073 100.7132369
31.14192488 31.98509571 58.87293117 85.12299612
40.66665059 1.064890225 0.030697285 0.135626992 AKAP5
14 64465499 64474503 + 7512

protein_coding A-kinase anchoring protein 5 [Source:HGNC Symbol;Acc:HGNC:375]   -   84   62   103   31   35
        60   0.611530919   0.363889956   0.635161406
0.1995296   0.203035072   0.37285649
ENSG00000238039 1.156961676   0   0   0   7.310879019
        21.58674143   0.385653892   9.632540149   -4.726090024
        0.030734451   1   AC244197.2   X   149511509
        149526264   -   655   lincRNA novel transcript
        -       1   0   0   0   8   22
0.083493643   0   0   0   0.532239723   1.567932697
ENSG00000269343 478.9821337   449.5713033   419.4755205
653.9804225   529.124869   610.3160531   449.3429859
597.8071149   -0.412025901   0.030772464   0.135894125
ZNF587B 19   57819741   57846238   +   4380
protein_coding zinc finger protein 587B [Source:HGNC Symbol;Acc:HGNC:37142] zf-C2H2 414   485   429   651   579
        622   5.169171526   4.88204284   4.537176118
7.186345525   5.760538068   6.629218139
ENSG00000175137 912.8427621   1276.411721   1250.604174
1610.33889   1299.508746   1567.001185   1146.619552
1492.28294   -0.37918897   0.030778097   0.135894125
SH3BP5L 1   248810446   248826633   -   6442
protein_coding SH3 binding domain protein 5 like [Source:HGNC Symbol;Acc:HGNC:29360] -   789   1377   1279   1603   1422
        1597   6.698090258   9.424258235   9.197129038
12.03134051   9.619167199   11.57258106
ENSG00000137713 1954.10827   1771.40363   1634.878952
1364.217226   1543.509333   1404.119408   1786.796951
1437.281989   0.313591905   0.030784415   0.135894125
PPP2R1B 11   111726908   111766427   -   6720
protein_coding protein phosphatase 2 scaffold subunit Abeta [Source:HGNC Symbol;Acc:HGNC:9303]   -   1689   1911   1672
        1358   1689   1431   13.74532742   12.53791646
11.52575692   9.770835764   10.95264406   9.940686933
ENSG00000087266 1498.26537   1217.086848   1635.85675
1150.242064   715.552284   1304.035425   1450.402989
1056.609925   0.457200222   0.030815761   0.135993229   SH3BP2
        4   2793023 2841098 +   13968   protein_coding SH3 domain binding protein 2 [Source:HGNC Symbol;Acc:HGNC:10825]   -
        1295   1313   1673   1145   783   1329   5.070260286
        4.14442665   5.548354102   3.963441722   2.44279
4.441572857
ENSG00000185761 76.3594706   99.18377207   85.06846221
150.6867333   102.3523063   201.1491815   86.87056829
151.3960737   -0.799099943   0.030828299   0.136009294
ADAMTSL5   19   1505018 1513604 -   3625   protein_coding
        ADAMTS like 5 [Source:HGNC Symbol;Acc:HGNC:27912]   -
        66   107   87   150   112   205   0.995704884
        1.301396771   1.111766792   2.000711482   1.346382969
        2.639926807

ENSG00000164403 246.4328369	532.0699548	611.1240101
315.4375617	233.0342687	335.5757077	463.208934
294.6825127	0.654326519	0.030842698	0.13603356
SHROOM1 5	132822141	132830898	-	4420
protein_coding	shroom family member 1 [Source:HGNC
Symbol;Acc:HGNC:24084]	-	213	574	625	314	255
	342	2.635433407	5.725633972	6.550285112
3.434856478	2.51406504	3.612017723
ENSG00000142327 618.9744965	975.1526002	981.7096098
1004.578222	1211.778197	1262.824374	858.6122355
1159.726931	-0.432705301	0.030869291	0.136111579
RNPEPL1 2	240565804	240581372	+	7867
protein_coding	arginyl aminopeptidase like 1 [Source:HGNC
Symbol;Acc:HGNC:10079]	-	535	1052	1004	1000	1326
	1287	3.719112748	5.8957702	5.911899171
6.145993071	7.345019813	7.636870611
ENSG00000197111 3942.925391	3383.371664	3847.636768
3073.004781	3002.029697	3162.457619	3724.644608
3079.164033	0.274417679	0.030885977	0.136145886	PCBP2
	12	53452102	53481162	+	7926
protein_coding	poly(rC) binding protein 2 [Source:HGNC
Symbol;Acc:HGNC:8648]	-	3408	3650	3935	3059	3285
	3223	23.51474269	20.30358624	22.99816177
18.66064391	18.06092144	18.98245038
ENSG00000101367 4441.575873	3736.540049	4192.799609
3586.344253	3529.326847	2949.533852	4123.63851
3355.068317	0.297397321	0.030945488	0.136333051	MAPRE1
	20	32819893	32850405	+	2623
protein_coding	microtubule associated protein RP/EB family member 1
[Source:HGNC Symbol;Acc:HGNC:6890]	-	3839	4031	4288
	3570	3862	3006	80.04137383	67.75611018
75.72842804	65.80685595	64.16121458	53.49789838
ENSG00000171723 502.1213673	535.7777594	487.9214097
427.9503226	346.3528935	357.1624491	508.6068454
377.1552217	0.431936381	0.030951544	0.136333051	GPHN
	14	66507407	67181803	+	6007
protein_coding	gephyrin [Source:HGNC Symbol;Acc:HGNC:15465]	-
	434	578	499	426	379	364	3.951179959
	4.242327229	3.848091317	3.428887084	2.749414228
	2.82871887
ENSG00000111737 1339.76162	1856.683135	1699.413647
1968.973315	2171.331069	2051.721651	1631.952801
2064.008678	-0.338264693	0.030955192	0.136333051	RAB35
	12	120095095	120117502	-	4022
protein_coding	"RAB35, member RAS oncogene family [Source:HGNC
Symbol;Acc:HGNC:9774]"	-	1158	2003	1738	1960	2376
	2091	15.7456722	21.95699187	20.01751496
23.56216656	25.74322591	24.26934204
ENSG00000250131 2.313923351	6.488657986	9.777984162	0
	0	0	6.193521833	0	5.12646323

0.031015864 1 AC078881.1 4 177444979
177677126 + 2161 antisense novel transcript
 - 2 7 10 0 0 0
0.050613916 0.14281612 0.214361945 0 0 0
ENSG00000013364 2752.411827 3243.402042 2628.322143
3044.876591 3578.67528 4276.137234 2874.712004
3633.229702 -0.337722548 0.031061017 0.136759724 MVP
 16 29820394 29848039 + 6141
protein_coding major vault protein [Source:HGNC Symbol;Acc:HGNC:7531]
 - 2379 3499 2688 3031 3916 4358
21.18605315 25.12110872 20.27648276 23.86426459
27.7883131 33.12791967
ENSG00000284631 2.313923351 2.780853423 1.955596832
14.06409511 11.88017841 17.66187935 2.350124535
14.53538429 -2.626299871 0.031071846 1 SCYGR4 2
 227616998 227618655 + 1658 protein_coding
 small cysteine and glycine repeat containing 4 [Source:HGNC
Symbol;Acc:HGNC:34223] - 2 3 2 14 13
 18 0.065969043 0.079775712 0.05587891
0.408267422 0.341678321 0.506796976
ENSG00000122641 652.5263851 553.3898311 559.300694
329.5016568 509.0199517 439.5845527 588.4056367
426.0353871 0.46466793 0.031080205 0.136804808 INHBA
 7 41667168 41705834 - 8302
protein_coding inhibin subunit beta A [Source:HGNC
Symbol;Acc:HGNC:6066] - 564 597 572 328 557
 448 3.715276048 3.170483958 3.191653641
1.910259337 2.92368826 2.519076293
ENSG00000155093 536.8302175 601.5912904 616.0130022
444.0235741 406.6676454 473.9270959 584.8115034
441.5394385 0.406180758 0.031138167 0.137020485 PTPRN2
 7 157539056 158587788 - 4833
protein_coding "protein tyrosine phosphatase, receptor type N2
[Source:HGNC Symbol;Acc:HGNC:9677]" - 464 649 630
 442 445 483 5.250442397 5.920547362
6.038460227 4.421877333 4.012377751 4.665265575
ENSG00000119314 2501.351143 2017.972634 2101.288796
1597.279373 2064.409463 1524.808917 2206.870858
1728.832584 0.351667709 0.03116308 0.13709065 PTBP3
 9 112217716 112333667 - 8239
protein_coding polypyrimidine tract binding protein 3 [Source:HGNC
Symbol;Acc:HGNC:10253] - 2162 2177 2149 1590 2259
 1554 14.35079298 11.6497843 12.08271046
9.330906507 11.94814064 8.804861875
ENSG00000220091 0 0 0.977798416 3.013734666
9.138598774 11.77458623 0.325932805
7.975639891 -4.454322068 0.031183155 1 LAP3P1 6
 82924829 82925122 - 294
processed_pseudogene leucine aminopeptidase 3 pseudogene 1
[Source:HGNC Symbol;Acc:HGNC:42364] - 0 0 1

3	10	12	0	0	0.157563321
0.49337273	1.482215216	1.905372757
ENSG00000129493 451.2150535	321.6520459	316.8066868
275.2544328	249.4837465	231.5668626	363.2245954
252.1016807	0.525446192	0.031192151	0.137179059
HEATR5A 14	31291791	31420582	-	10243
protein_coding	HEAT repeat containing 5A [Source:HGNC
Symbol;Acc:HGNC:20276]	-	390	347	324	274	273
236	2.08224653	1.493606724	1.465278891
1.29337543	1.161432761	1.075550648
ENSG00000242125 1184.728756	1251.38404	1358.162
1127.136765	892.8411002	967.4785022	1264.758265
995.8187892	0.345388865	0.031227112	0.137293314	SNHG3
1	28505980	28510892	+	4088
processed_transcript	small nucleolar RNA host gene 3 [Source:HGNC
Symbol;Acc:HGNC:10118]	-	1024	1350	1389	1122	977
986	13.69883965	14.55984794	15.73960447
13.27037472	10.41459233	11.25931744
ENSG00000249669 11.56961676	13.90426711	24.4449604	0
0	5.887293117	16.63961476	1.962431039
3.091488599	0.031252137	1	CARMN	5	149406689
149432835	+	17988	lincRNA cardiac mesoderm
enhancer-associated non-coding RNA [Source:HGNC
Symbol;Acc:HGNC:42872]	-	10	15	25	0	0
6	0.030402678	0.036765658	0.064381277	0
0	0.015570925
ENSG00000120948 3215.196497	2656.64197	2863.971561
2526.514228	2409.848497	2160.636574	2911.936676
2365.666433	0.299462628	0.031256772	0.13737112	TARDBP
1	11012344	11026420	+	6071
protein_coding	TAR DNA binding protein [Source:HGNC
Symbol;Acc:HGNC:11571]	-	2779	2866	2929	2515	2637
2202	25.03358375	20.81372974	22.34918007
20.02990885	18.9281642	16.93179952
ENSG00000005073 32.39492692	31.51633879	31.28954932	0
12.79403828	9.812155195	31.73360501	7.535397826
2.067877863	0.031262781	0.13737112	HOXA11	7
27181510	27185223	-	2307	protein_coding
homeobox A11 [Source:HGNC Symbol;Acc:HGNC:5101] Homeobox
28	34	32	0	14	10	0.663750941
0.649778415	0.64254691	0	0.26444724
0.202347779
ENSG00000197646 264.9442237	324.4328993	158.4033434
347.5840648	393.8736072	387.5801302	249.2601555
376.3459341	-0.594374957	0.031280438	0.137409213
PDCD1LG2	9	5510570 5571254 +	2365	protein_coding
programmed cell death 1 ligand 2 [Source:HGNC
Symbol;Acc:HGNC:18731]	-	229	350	162	346	431
395	5.295403401	6.524854885	3.17311875
7.073692394	7.941539911	7.796720871

ENSG00000128591 4287.69997 3868.167111 4949.615583
3043.872013 2474.732548 4385.052157 4368.494221
3301.218906 0.404212338 0.031310238 0.137500606 FLNC
7 128830377 128859274 + 9280
protein_coding filamin C [Source:HGNC Symbol;Acc:HGNC:3756] -
3706 4173 5062 3030 2708 4469 21.83997573
19.82596643 25.26833467 15.78686404 12.71625656
22.48062671
ENSG00000089159 2017.741162 3114.555833 2747.613549
3396.478969 3344.727151 3297.865361 2626.636848
3346.35716 -0.34888077 0.031321735 0.13751159 PXN
12 120210439 120265771 - 7460
protein_coding paxillin [Source:HGNC Symbol;Acc:HGNC:9718] -
1744 3360 2810 3381 3660 3361 12.78504809
19.85794977 17.44897612 21.91328867 21.37966302
21.03176502
ENSG00000106355 1635.943809 1319.051473 1476.475608
1205.493866 1211.778197 1116.623261 1477.156964
1177.965108 0.32600412 0.031346608 0.137581281 LSM5
7 32485332 32495283 - 4701
protein_coding "LSM5 homolog, U6 small nuclear RNA and mRNA
degradation associated [Source:HGNC Symbol;Acc:HGNC:17162]" -
1414 1423 1510 1200 1326 1138 16.44954429
13.34592276 14.87952789 12.34218953 12.2916977
11.30051007
ENSG00000107282 173.5442514 188.1710816 179.9149086
257.1720248 265.9332243 261.9845437 180.5434138
261.6965976 -0.535083766 0.031441386 0.137957654 APBA1
9 69427530 69672306 - 11384
protein_coding amyloid beta precursor protein binding family A member
1 [Source:HGNC Symbol;Acc:HGNC:578] - 150 203 184
256 291 267 0.720594735 0.786203746
0.748730271 1.087292256 1.11392692 1.094869632
ENSG00000157326 149.2480562 202.0753487 152.5365529
220.0026306 231.206549 320.8574749 167.9533193
257.3555515 -0.614146298 0.031450639 0.137958656 DHRS4
14 23953586 23969279 + 2305
protein_coding dehydrogenase/reductase 4 [Source:HGNC
Symbol;Acc:HGNC:16985] - 129 218 156 219 253
327 3.060648763 4.169841256 3.135134119
4.593824521 4.783085996 6.622513599
ENSG00000226284 25.45315687 4.634755704 22.48936357 0
3.65543951 3.924862078 17.52575871 2.526767196
2.783612234 0.031451602 1 ARPC3P1 20
49134480 49135012 + 533 processed_pseudogene
actin related protein 2/3 complex subunit 3 pseudogene 1
[Source:HGNC Symbol;Acc:HGNC:16092] - 22 5 23
0 4 4 2.257304693 0.413596403
1.998955301 0 0.327032851 0.350331201
ENSG00000162039 0 0 2.933395248 31.14192488

1.827719755	6.868508636	0.977798416
13.27938442	-3.739981548	0.031457868	1	MEIOB	16
	1833987 1884294 -	2303	protein_coding	meiosis
specific with OB domains [Source:HGNC Symbol;Acc:HGNC:28569]	-
	0	0	3	31	2	7	0	0
	0.060343399	0.650832111	0.037843793	0.141889461
ENSG00000238269 0	0	0	28.12819022	0	0
	0	9.376063406	-5.64733261	0.031463326	1
	PAGE2B	X	55075063	55078909	+	507
	protein_coding	PAGE family member 2B [Source:HGNC
Symbol;Acc:HGNC:31805]	-	0	0	0	28	0
	0	0	0	0	2.670246094	0	0
ENSG00000077254 2367.143589	2108.813845	2193.201847
1770.066827	1866.10187	1839.779099	2223.053094
1825.315932	0.284105048	0.031547891	0.138318853	USP33
	1	77695987	77759852	-	6918
protein_coding	ubiquitin specific peptidase 33 [Source:HGNC
Symbol;Acc:HGNC:20059]	-	2046	2275	2243	1762	2042
	1875	16.17408736	14.49889155	15.01935117
12.31477731	12.86274849	12.65222045
ENSG00000135723 2623.989081	2487.009911	2933.395248
2329.616897	1951.090838	2245.021109	2681.464747
2175.242948	0.301979797	0.03155085	0.138318853	FHOD1
	16	67229387	67247658	-	5407
protein_coding	formin homology 2 domain containing 1 [Source:HGNC
Symbol;Acc:HGNC:17905]	-	2268	2683	3000	2319	2135
	2288	22.93936509	21.87752864	25.70202496
20.73698414	17.20679987	19.75357412
ENSG00000155876 1601.234959	1822.385943	1617.27858
2047.330416	2148.484572	1984.01778	1680.299827
2059.944256	-0.293670658	0.031627261	0.138614089	RRAGA
	9	19049395	19050983	+	1589
protein_coding	Ras related GTP binding A [Source:HGNC
Symbol;Acc:HGNC:16963]	-	1384	1966	1654	2038	2351
	2022	47.63288711	54.5498517	48.21854083
62.01282255	64.47440303	59.40230398
ENSG00000259933 6.941770054	8.342560268	14.66697624	0
	0	1.962431039	9.983768855	0.65414368
3.944078143	0.031704095	1	AC009065.2	16
2091436 2095433 +	691	sense_overlapping
uncharacterized LOC105371049 [Source:NCBI gene;Acc:105371049]	-
	6	9	15	0	0	2	0.474862545
	0.574246584	1.005577778	0	0	0.135113264
ENSG00000258858 0	0	1.955596832	15.06867333
2.741579632	13.73701727	0.651865611
10.51575675	-3.983991159	0.031727494	1	AL583722.4
	14	104690091	104691284	-	716
antisense	"novel transcript, antisense to INF2"	-	0
	0	2	15	3	14	0	0
0.129395576	1.012930045	0.182585729	0.912769352

ENSG00000182667 1442.73121	1354.275617	1335.672636
921.1982296	1138.669407	1200.02658	1377.559821
1086.631406	0.341909911	0.031728749	0.139019026	NTM
	11	131370478	132336822	+	6956
protein_coding	neurotrimin [Source:HGNC Symbol;Acc:HGNC:17941] -
	1247	1461	1366	917	1246	1223	9.803961405
	9.260290279	9.096903376	6.373984145	7.805793658
	8.207538329
ENSG00000094804 2326.64993	2097.690432	1972.219405
1848.423929	1722.625869	1617.043176	2132.186589
1729.364325	0.301856145	0.031746494	0.139056919	CDC6
	17	40287633	40304657	+	5557
protein_coding	cell division cycle 6 [Source:HGNC
Symbol;Acc:HGNC:1744]	-	2011	2263	2017	1840	1885
	1648	19.79093838	17.95469843	16.81388053
16.00953223	14.78187602	13.84404003
ENSG00000176678 1222.908491	1348.71391	1772.748529
1139.191704	1094.804133	1135.266356	1448.123643
1123.087398	0.367139224	0.031815611	0.139319747	FOXL1
	16	86576368	86582160	+	3742
protein_coding	forkhead box L1 [Source:HGNC Symbol;Acc:HGNC:3817]
	Fork	1057	1455	1813	1134	1198	1157
15.44777435	17.14325038	22.44380448	14.65245809
13.95120218	14.43363144
ENSG00000100083 1092.171822	1571.182184	1348.384016
1875.547541	1577.322148	1700.446495	1337.246007
1717.772061	-0.360434743	0.031831514	0.139349473	GGA1
	22	37608475	37633564	+	9369
protein_coding	"golgi associated, gamma adaptin ear containing, ARF
binding protein 1 [Source:HGNC Symbol;Acc:HGNC:17842]"	-	944
	1695	1379	1867	1726	1733	5.510277467
7.976464223	6.818258821	9.63501279	8.027977566
8.634781644
ENSG00000141556 2282.685386	2454.566621	2772.05851
1906.689465	1843.255373	2278.382436	2503.103506
2009.442425	0.317169475	0.03187349	0.139493283	TBCD
	17	82752064	82945922	+	13546
protein_coding	tubulin folding cofactor D [Source:HGNC
Symbol;Acc:HGNC:11581]	-	1973	2648	2835	1898	2017
	2322	7.965457536	8.618682207	9.694924867
6.774642048	6.488636193	8.001974811
ENSG00000245904 0	0.926951141	0	11.05036044
6.397019142	5.887293117	0.308983714
7.778224234	-4.416842119	0.031973037	1	AC025164.1
	12	92145573	92189660	+	7589
antisense	uncharacterized LOC101928617 [Source:NCBI
gene;Acc:101928617]	-	0	1	0	11	7
	6	0	0.005809642	0	0.070082462
0.040195005	0.036907339
ENSG00000183604 98.34174244	59.32487301	59.64570339

151.6913115	112.4047649	120.6895089	72.43743961
128.2618618	-0.82820708	0.031985341	0.139907658	SMG1P5
	16	30267553	30335374	-	4791
transcribed_unprocessed_pseudogene	SMG1 pseudogene 5 [Source:HGNC
Symbol;Acc:HGNC:49862]	-	85	64	61	151	123
	123	0.970258526	0.588962659	0.589801836
1.523884294	1.118761566	1.198463954
ENSG00000157224 1458.928673	1308.855011	1104.91221
1726.869964	1724.453589	1462.99234	1290.898631
1638.105297	-0.34413876	0.031986476	0.139907658	CLDN12
	7	90383721	90513402	+	6741
protein_coding	claudin 12 [Source:HGNC Symbol;Acc:HGNC:2034]	-
	1261	1412	1130	1719	1887	1491	10.23023175
	9.235157948	7.765270202	12.32970728	12.1984927
	10.32522091
ENSG00000149782 1060.933857	1440.482073	1510.698553
851.8823323	1010.729024	1177.458623	1337.371494
1013.35666	0.400777166	0.032003532	0.139942219	PLCB3
	11	64251523	64269150	+	4964
protein_coding	phospholipase C beta 3 [Source:HGNC
Symbol;Acc:HGNC:9056]	-	917	1554	1545	848	1106
	1200	10.10257947	13.8023552	14.41780564
8.259719443	9.70916657	11.28484268
ENSG00000039523 1467.027405	1839.071063	2274.359116
1249.695308	1379.014555	1630.780193	1860.152528
1419.830019	0.390094518	0.03202939	0.140015238	RIPOR1
	16	67518418	67546788	+	6729
protein_coding	RHO family interacting cell polarization regulator 1
[Source:HGNC Symbol;Acc:HGNC:25836]	-	1268	1984	2326
	1244	1509	1662	10.30536641	12.99945358
16.012592	8.938632219	9.772311662	11.52992619
ENSG00000144579 1042.42247	1047.454789	1116.645791
1300.928798	1270.26523	1413.931564	1068.841017
1328.375197	-0.313459161	0.032064423	0.140128313	CTDSP1
	2	218398256	218405941	+	4727
protein_coding	CTD small phosphatase 1 [Source:HGNC
Symbol;Acc:HGNC:21614]	-	901	1130	1142	1295	1390
	1441	10.42398797	10.53966481	11.19136235
13.24601927	12.81409076	14.23063941
ENSG00000120217 329.7340776	335.556313	182.8483038
453.0647781	498.0536332	327.7259835	282.7128981
426.2814649	-0.593283574	0.032094244	0.140197928	CD274
	9	5450503 5470566 +	4108	protein_coding	CD274
molecule [Source:HGNC Symbol;Acc:HGNC:17635]	-	285	362
	187	451	545	334	3.794103185	3.885188491
	2.108694316	5.30820056	5.781288804	3.795439453
ENSG00000268912 155.0328645	156.6547428	121.2470036
259.1811813	264.1055046	159.9381297	144.311537
227.7416052	-0.658871942	0.032098695	0.140197928
AC012313.5	19	58428632	58431148	-	2517

lincRNA novel transcript	-	134	169	124
258	289	163	2.911496655	2.960311737
2.282132866	4.95607314	5.003492175	3.023085461
ENSG00000173376 16.19746346	0	5.866790497	0	0
0	7.354751319	0	5.366708897	0.032149278
1	NDNF	4	121035613	121073021	-
3556	protein_coding	neuron derived neurotrophic factor
[Source:HGNC Symbol;Acc:HGNC:26256]	-	14	0	6
0	0	0	0.215308411	0	0.078161332
0	0	0
ENSG00000103415 565.7542594	668.3317726	682.5032945
929.2348554	700.0166661	897.8122003	638.8631088
842.3545739	-0.397918775	0.032155159	0.140404432	HMOX2
16	4474690 4510347 +	3793	protein_coding	heme
oxygenase 2 [Source:HGNC Symbol;Acc:HGNC:5014]	-	489	721
698	925	766	915	7.050513186	8.38081743
8.524620139	11.79125703	8.800442803	11.26119003
ENSG00000184675 389.8960847	528.3621503	446.8538762
799.6442647	497.1397733	610.3160531	455.0373704
635.7000304	-0.48095405	0.032197149	0.140547633	AMER1
X	64185117	64205744	-	8443
protein_coding	APC membrane recruitment protein 1 [Source:HGNC
Symbol;Acc:HGNC:26837]	-	337	570	457	796	544
622	2.182869759	2.976542064	2.507389868
4.558453143	2.80776469	3.439059037
ENSG00000225311 4.627846703	5.561706845	7.822387329	0
0	0	6.003980292	0	5.079765078
0.032316244	1	AL024474.2	6	144311699
144333301	-	501	antisense	"novel transcript,
antisense to UTRN"	-	4	6	8	0	0
0	0.436633425	0.528016486	0.739698464	0
0	0
ENSG00000197134 134.2075544	113.0880392	88.97965587
90.41203998	43.86527412	38.26740526	112.0917498
57.51490645	0.96326309	0.032329807	0.141071866	ZNF257
19	22052452	22091480	+	5029
protein_coding	zinc finger protein 257 [Source:HGNC
Symbol;Acc:HGNC:13498]	zf-C2H2 116	122	91	90	48
39	1.261452978	1.069577241	0.838228094
0.865290808	0.41592804	0.362017035
ENSG00000099954 0	14.83121825	6.844588913	0	0
0	7.225269056	0	5.350253705	0.032340148
1	CECR2	22	17359949	17558149	+
10759	protein_coding	"CECR2, histone acetyl-lysine reader
[Source:HGNC Symbol;Acc:HGNC:1840]"	-	0	16	7
0	0	0	0	0.065566505	0.030138983
0	0	0
ENSG00000256628 38.1797353	46.34755704	67.46809072
93.42577465	113.3186248	83.40331915	50.66512769
96.7159062	-0.930606809	0.032343871	0.141071866

ZBTB11-AS1 3 101676475 101679217 + 2743
antisense ZBTB11 antisense RNA 1 [Source:HGNC
Symbol;Acc:HGNC:48573] - 33 50 69 93 124
85 0.65793478 0.803670736 1.165267782
1.639299693 1.969946698 1.446569
ENSG00000185596 244.1189136 286.4279025 222.9380389
419.9136968 405.7537856 272.7779144 251.1616183
366.1484656 -0.543429321 0.032353846 0.141071866 WASH3P
15 101961603 101976543 + 2351
transcribed_unprocessed_pseudogene "WAS protein family homolog 3,
pseudogene [Source:HGNC Symbol;Acc:HGNC:24362]" - 211 309
228 418 444 278 4.908225856 5.794818104
4.492464704 8.596563374 8.229793511 5.519988877
ENSG00000151388 731.1997791 554.3167822 631.6577768
511.330315 456.0160788 474.9083114 639.0581127
480.7515684 0.409908796 0.032358295 0.141071866
ADAMTS12 5 33523535 33892192 - 9778
protein_coding ADAM metallopeptidase with thrombospondin type
1 motif 12 [Source:HGNC Symbol;Acc:HGNC:14605] - 632 598
646 509 499 484 3.534774864 2.696404908
3.060447549 2.516917416 2.223868536 2.310688296
ENSG00000010319 27.76708022 29.66243651 56.71230814
7.032047554 18.27719755 12.75580175 38.04727495
12.68834895 1.581916925 0.032363383 0.141071866 SEMA3G
3 52433053 52445085 - 5011
protein_coding semaphorin 3G [Source:HGNC Symbol;Acc:HGNC:30400]
- 24 32 58 7 20 13
0.261927774 0.281552594 0.536174366 0.067542146
0.173925872 0.121105812
ENSG00000180855 40.49365865 63.03267758 66.4902923
65.29758443 115.1463446 151.10719 56.67220951
110.5170397 -0.9607576 0.032401732 0.141198768 ZNF443
19 12429707 12441112 - 2753
protein_coding zinc finger protein 443 [Source:HGNC
Symbol;Acc:HGNC:20878] zf-C2H2 35 68 68 65 126
154 0.695274891 1.089022015 1.144208467
1.141585284 1.994448982 2.611322707
ENSG00000112357 86.77212568 134.4079154 80.17947013
141.6455293 214.7570712 153.069621 100.4531704
169.8240738 -0.75606588 0.03248274 0.141489052 PEX7
6 136822564 136913937 + 1835
protein_coding peroxisomal biogenesis factor 7 [Source:HGNC
Symbol;Acc:HGNC:8860] - 75 145 82 141 235
156 2.23521811 3.483901692 2.070047159
3.715217644 5.580722032 3.968574757
ENSG00000270872 163.1315963 138.11572 185.7816991
105.4807133 103.2661661 94.19668987 162.3430051
100.9811898 0.684576411 0.032486856 0.141489052
SRGAP2D 1 143975087 144068350 + 1021
unprocessed_pseudogene SLIT-ROBO Rho GTPase activating protein 2D

(pseudogene) [Source:HGNC Symbol;Acc:HGNC:43932]	-	141
	149	190	105	113	96	7.552453905
6.434198933	8.620457492	4.972385295	4.822933782
4.389262219
ENSG00000237818 0	0	0	13.05951689	9.138598774
	0	0	7.399371887	-5.307181005	0.032504319
	1	RPS3AP29	7	97898347	97899115
	-	769	processed_pseudogene	ribosomal protein S3a
pseudogene 29 [Source:HGNC Symbol;Acc:HGNC:35531]	-	0
	0	0	13	10	0	0	0	0
	0.817369125	0.566672657	0
ENSG00000270874 0	0	0	7.032047554	6.397019142
	4.906077597	0	6.111714764	-5.031388565
0.03250784	1	RPAP2P1 10	86854696	86856236
	-	1168	processed_pseudogene	RNA polymerase II
associated protein 2 pseudogene 1 [Source:HGNC Symbol;Acc:HGNC:45125]
	-	0	0	0	7	7	5	0
	0	0	0.289771997	0.261164291	0.199835756
ENSG00000230943 9.255693406	0.926951141	8.800185745	0
	0	0	6.327610097	0	5.151614901
0.032538091	1	LINC02541	6	113616927
113650090	-	2145	lincRNA long intergenic non-protein
coding RNA 2541 [Source:HGNC Symbol;Acc:HGNC:53574]	-	8
	1	9	0	0	0	0.203965824
0.020554488	0.194364824	0	0	0
ENSG00000238113 151.5619795	176.1207168	167.2035292
237.0804604	236.6897083	254.1348195	164.9620751
242.6349961	-0.555560547	0.032555297	0.141746748
LINC01410	9	62801461	62813486	+	3038
	lincRNA long intergenic non-protein coding RNA 1410
[Source:HGNC Symbol;Acc:HGNC:50702]	-	131	190	171
	236	259	259	2.358186989	2.757400115
2.607418825	3.755998844	3.715100719	3.979770517
ENSG00000271869 1.156961676	1.853902282	1.955596832
21.09614266	8.224738897	8.830939675	1.65548693
12.71727374	-2.929735688	0.032617534	1	AC026979.2
	8	30155830	30156232	-	403
lincRNA novel transcript	-	1	2	2	21
	9	9	0.135703068	0.218805839	0.229893877
	2.519506395	0.973186467	1.042517849
ENSG00000166479 3419.978713	2353.528947	2896.238909
2292.447503	2198.746865	2386.316143	2889.915523
2292.503504	0.333735165	0.032632289	0.142031212	TMX3
	18	68673688	68715298	-	7637
protein_coding	thioredoxin related transmembrane protein 3
[Source:HGNC Symbol;Acc:HGNC:24718]	-	2956	2539	2962
	2282	2406	2432	21.16783063	14.65797134
17.9665512	14.44754534	13.7287637	14.86574969
ENSG00000047849 7729.660955	8475.114281	9098.414262
7603.652562	6000.403955	7136.380473	8434.3965

6913.478997	0.286999562	0.032639213	0.142031212	MAP4
	3	47850690	48089272	-	14943
protein_coding	microtubule associated protein 4 [Source:HGNC
Symbol;Acc:HGNC:6862]	-	6681	9143	9305	7569	6566
	7273	24.45109922	26.9764552	28.84569697
24.49074099	19.14792332	22.72070626
ENSG00000236863 10.41265508	10.19646255	8.800185745	0
	0	1.962431039	9.803101125	0.65414368
3.91623764	0.032650813	1	RPL23AP23	1
235295865	235296335	+	471	processed_pseudogene
	ribosomal protein L23a pseudogene 23 [Source:HGNC
Symbol;Acc:HGNC:36272]	-	9	11	9	0	0
	2	1.045000059	1.0296882	0.885164643	0
	0	0.198223493
ENSG00000169976 1296.954038	1749.156803	1674.968687
2039.293791	1784.768341	2172.41116	1573.693176
1998.82443	-0.344295736	0.032666913	0.142111297	SF3B5
	6	144094881	144095573	-	693
protein_coding	splicing factor 3b subunit 5 [Source:HGNC
Symbol;Acc:HGNC:21083]	-	1121	1887	1713	2030	1953
	2214	88.46410554	120.0528912	114.5055624
141.6328583	122.808268	149.1387222
ENSG00000103313 18.51138681	3.707804563	0	0	0
	0	7.406397125	0	5.376402033	0.032685239
	1	MEFV	16	3242028 3256627 -	4819
protein_coding	"MEFV, pyrin innate immunity regulator [Source:HGNC
Symbol;Acc:HGNC:6998]"	-	16	4	0	0	0
	0	0.181575718	0.036596287	0	0	0
	0
ENSG00000115738 79.83035562	112.161088	106.5800274
169.7737195	162.6670582	151.10719	99.52382368
161.1826559	-0.692987796	0.032689093	0.14213972	ID2
	2	8678845 8684453 +	2205	protein_coding
inhibitor of DNA binding 2 [Source:HGNC Symbol;Acc:HGNC:5361]	bHLH
	69	121	109	169	178	154	1.711335698
	2.419417037	2.289920262	3.705777391	3.51779078
	3.260304496
ENSG00000164967 170.0733663	237.2994921	205.3376674
253.1537119	373.7686899	284.5525006	204.2368419
303.8249675	-0.571902189	0.032701115	0.14213972	RPP25L
	9	34610486	34612104	-	1092
protein_coding	ribonuclease P/MRP subunit p25 like [Source:HGNC
Symbol;Acc:HGNC:19909]	-	147	256	210	252	409
	290	7.361891443	10.33597107	8.908387752
11.15781404	16.32146986	12.39713685
ENSG00000175183 255.6885303	298.4782674	276.7169518
153.700468	208.3600521	204.092828	276.9612498
188.7177827	0.553507485	0.032708366	0.14213972	CSRP2
	12	76858715	76879060	-	5873
protein_coding	cysteine and glycine rich protein 2 [Source:HGNC

Symbol;Acc:HGNC:2470]   -   221   322   283   153   228
        208   2.057912881   2.417295975   2.232178344
1.259599984   1.691739322   1.65329126
ENSG00000140455 1296.954038   983.4951604   1188.025076
964.3950931   904.7212786   835.0144071   1156.158092
901.3769263   0.358567658   0.032710639   0.14213972   USP3
        15   63504511   63594640   +   7764
protein_coding ubiquitin specific peptidase 3 [Source:HGNC
Symbol;Acc:HGNC:12626] -   1121   1061   1215   960   990
        851   7.89613925   6.025093837   7.249252166
5.978426892   5.556588882   5.116701353
ENSG00000076928 4130.353182   5710.945979   4827.390781
6329.847377   5510.575061   6363.182644   4889.563314
6067.868361   -0.31121392   0.032732452   0.142178123
ARHGEF1 19   41883161   41930150   +   8636
protein_coding Rho guanine nucleotide exchange factor 1 [Source:HGNC
Symbol;Acc:HGNC:681]   -   3570   6161   4937   6301   6030
        6485   22.60738317   31.45375742   26.48213221
35.27752127   30.42729017   35.05446817
ENSG00000131711 3998.459551   2886.525853   3514.207508
3133.279474   2320.290229   2724.835498   3466.397637
2726.135067   0.34644186   0.032738078   0.142178123   MAP1B
        5   72107234   72209570   +   12630
protein_coding microtubule associated protein 1B [Source:HGNC
Symbol;Acc:HGNC:6836]   -   3456   3114   3594   3119   2539
        2777   14.96459942   10.87049237   13.18187467
11.94024507   8.760279204   10.26404542
ENSG00000232814 0   19.46597396   7.822387329   0
0.913859877   0   9.096120429   0.304619959   4.721184348
        0.032822147   1   COL4A2-AS1   13   110502575
        110508179   -   540   antisense   COL4A2
antisense RNA 1 [Source:HGNC Symbol;Acc:HGNC:40156]   -   0
        21   8   0   1   0   0   1.714586867
        0.686275797   0   0.080698384   0
ENSG00000197951 208.2531016   408.7854531   321.6956789
468.1334515   529.124869   390.5237767   312.9114112
462.5940324   -0.561907232   0.032826123   0.142497871   ZNF71
        19   56595264   56626481   +   6170
protein_coding zinc finger protein 71 [Source:HGNC
Symbol;Acc:HGNC:13141] zf-C2H2 180   441   329   466   579
        398   1.595445795   3.151282832   2.470092345
3.65175783   4.089328483   3.011230106
ENSG00000255717 1389.510973   1593.429011   1476.475608
1136.177969   1146.894146   1299.129348   1486.471864
1194.067154   0.316361072   0.032830346   0.142497871   SNHG1
        11   62851988   62855914   -   3756
processed_transcript   small nucleolar RNA host gene 1 [Source:HGNC
Symbol;Acc:HGNC:32688] -   1201   1719   1510   1131   1255
        1324   17.48687222   20.17828497   18.62317908
14.55922433   14.56051513   16.45539976

ENSG00000264364 2082.531016 2290.496269 2231.335986
2791.722879 2450.058331 2798.426662 2201.454424
2680.069291 -0.2835174 0.032843707 0.1425154 DYNLL2
17 58083415 58095536 + 6805
protein_coding dynein light chain LC8-type 2 [Source:HGNC
Symbol;Acc:HGNC:24596] - 1800 2471 2282 2779 2681
2852 14.46568781 16.00952969 15.53423842
19.74520439 17.16829955 19.56444027
ENSG00000186834 1187.042679 1217.086848 1601.633806
1791.16297 1484.108441 1877.065289 1335.254444
1717.445567 -0.362749792 0.03295692 0.142966069 HEXIM1
17 45148502 45152101 + 3600
protein_coding hexamethylene bisacetamide inducible 1 [Source:HGNC
Symbol;Acc:HGNC:24953] - 1026 1313 1638 1783 1624
1913 15.58617588 16.0803754 21.07724542
23.94694181 19.65812634 24.80610086
ENSG00000236562 1.156961676 0 1.955596832 10.04578222
17.36333767 5.887293117 1.037519503 11.09880434
-3.435317128 0.032971633 1 AC116049.1 4
66094142 66094526 + 385
processed_pseudogene interferon induced transmembrane protein 3
(1-8U) (IFITM3) pseudogene - 1 0 2 10
19 6 0.142047627 0 0.240642163
1.255857857 2.150559532 0.727505962
ENSG00000178381 598.1491863 489.4302024 365.6966076
829.7816114 651.5820926 558.3116306 484.4253321
679.8917782 -0.489951234 0.033005191 0.143112168
ZFAND2A 7 1152071 1160759 - 2281 protein_coding zinc
finger AN1-type containing 2A [Source:HGNC Symbol;Acc:HGNC:28073]
- 517 528 374 826 713 569
12.39538358 10.20569524 7.595367164 17.50878374
13.62143437 11.64482636
ENSG00000015676 2739.685248 3332.389351 3706.833796
4125.802758 3846.436224 4105.405733 3259.636132
4025.881572 -0.304267432 0.033014799 0.143112168 NUDCD3
7 44379121 44490880 - 10113
protein_coding NudC domain containing 3 [Source:HGNC
Symbol;Acc:HGNC:22208] - 2368 3595 3791 4107 4209
4184 12.80549596 15.67302569 17.36505779
19.63567847 18.13666855 19.31335416
ENSG00000176402 15.04050178 4.634755704 5.866790497
24.10987733 46.60685375 19.62431039 8.514015995
30.11368049 -1.839515269 0.033022646 0.143112168 GJC3
7 99923269 99929620 - 1116
protein_coding gap junction protein gamma 3 [Source:HGNC
Symbol;Acc:HGNC:17495] - 13 5 6 24 51
20 0.637050514 0.197533049 0.249051701
1.03979629 1.991427863 0.836588397
ENSG00000103642 1091.01486 1326.467083 1190.958471
806.6763123 1095.717993 890.9436917 1202.813471

931.1126657	0.369433015	0.033028045	0.143112168	LACTB
	15	63121800	63142061	+	3972
protein_coding	lactamase beta [Source:HGNC Symbol;Acc:HGNC:16468]
	-	943	1431	1218	803	1199	908
12.98366094	15.88416362	14.204976	9.774791938
13.15432419	10.6714306
ENSG00000189366 0	12.05036483	7.822387329	15.06867333
	48.4345735	26.49281903	6.62425072	29.99868862
	-2.160880617	0.033061763	0.143166576	ALG1L	3
	125929275	125937039	-	843	protein_coding
	"ALG1, chitobiosyldiphosphodolichol beta-mannosyltransferase
like [Source:HGNC Symbol;Acc:HGNC:33721]"	-	0	13
	8	15	53	27	0	0.679907349
0.439607272	0.860329671	2.739724496	1.495141256
ENSG00000244486 698.8048521	1167.958437	2174.623678
535.4401923	498.0536332	828.1458984	1347.128989
620.5465747	1.118457336	0.033065863	0.143166576	SCARF2
	22	20424815	20437826	-	3341
protein_coding	scavenger receptor class F member 2 [Source:HGNC
Symbol;Acc:HGNC:19869]	-	604	1260	2224	533	545
	844	9.886786951	16.627541	30.83619356
7.713508277	7.108510748	11.79266623
ENSG00000181191 634.0149983	758.2460332	675.6587056
786.5847478	985.1409479	905.6619245	689.306579
892.4625401	-0.372277499	0.033068698	0.143166576	PJA1
	X	69160851	69165793	-	3117
protein_coding	praja ring finger ubiquitin ligase 1 [Source:HGNC
Symbol;Acc:HGNC:16648]	-	548	818	691	783	1078
	923	9.614760463	11.57045558	10.26936762
12.14580142	15.07094748	13.82327457
ENSG00000227666 0	0	0.977798416	30.13734666	0
	0.981215519	0.325932805	10.37285406	-4.830881976
	0.033094628	1	CYCSP24 9	94038030
94038345	+	316	processed_pseudogene	"cytochrome c,
somatic pseudogene 24 [Source:HGNC Symbol;Acc:HGNC:24398]"	-
	0	0	1	30	0	1	0	0
	0.146593723	4.590239952	0	0.147726685
ENSG00000166454 2021.212047	1913.227155	1913.5515
2443.134236	2671.212422	2099.801212	1949.330234
2404.715956	-0.30311265	0.033135071	0.143413316	ATMIN
	16	81035847	81047358	+	6262
protein_coding	ATM interactor [Source:HGNC Symbol;Acc:HGNC:29034]
	-	1747	2064	1957	2432	2923	2140
15.25718999	14.53217395	14.47705479	18.7781033
20.34109602	15.95316092
ENSG00000110987 401.4657015	411.5663065	412.6309316
474.1609208	614.1138376	550.4619064	408.5543132
546.2455549	-0.419243675	0.033244327	0.143812718	BCL7A
	12	122019422	122062044	+	6565
protein_coding	BCL tumor suppressor 7A [Source:HGNC

Symbol;Acc:HGNC:1004] - 347 444 422 472 672
561 2.890609709 2.981825316 2.977694758
3.476229852 4.460598565 3.989093049
ENSG00000184489 30.08100357 27.80853423 58.66790497
103.4715569 63.97019142 77.51602604 38.85248092
81.65259144 -1.06913958 0.033246166 0.143812718 PTP4A3
8 141391993 141432454 + 2877
protein_coding "protein tyrosine phosphatase type IVA, member 3
[Source:HGNC Symbol;Acc:HGNC:9636]" - 26 30 60
103 70 79 0.49422897 0.459743239
0.966081675 1.731006024 1.060270739 1.281838364
ENSG00000171574 374.8555829 471.8181307 486.9436112
736.3558367 491.656614 608.3536221 444.5391083
612.1220243 -0.460098014 0.033294787 0.143962579 ZNF584
19 58401504 58418327 + 3696
protein_coding zinc finger protein 584 [Source:HGNC
Symbol;Acc:HGNC:27318] zf-C2H2 324 509 498 733 538
620 4.794107414 6.071832435 6.241656094
9.588998012 6.343207391 7.83079334
ENSG00000162885 340.1467327 388.392528 420.4533189
225.0255217 329.9034157 251.191173 382.9975265
268.7067035 0.511117245 0.033299645 0.143962579
B3GALNT2 1 235449923 235504481 - 5702
protein_coding "beta-1,3-N-acetylgalactosaminyltransferase 2
[Source:HGNC Symbol;Acc:HGNC:28596]" - 294 419 430
224 361 256 2.819777431 3.239819149
3.493362858 1.89942444 2.758916691 2.095843201
ENSG00000163694 83.30124065 62.10572644 128.0915925
51.23348932 36.5543951 50.04199149 91.16618654
45.94329197 0.989684031 0.033310998 0.143970942 RBM47
4 40423267 40630875 - 8544
protein_coding RNA binding motif protein 47 [Source:HGNC
Symbol;Acc:HGNC:30358] - 72 67 131 51 40
51 0.460856768 0.345738323 0.710252076
0.288609188 0.204012769 0.278647385
ENSG00000255142 2.313923351 6.488657986 3.911193665
20.09156444 12.79403828 26.49281903 4.237925001
19.79280725 -2.208905505 0.033324273 0.143987609
AP006621.2 11 781645 782105 + 284 lincRNA novel
transcript - 2 7 4 20 14 27
0.38512913 1.086709986 0.6524453 3.404966725
2.14816825 4.438042532
ENSG00000178814 98.34174244 252.1307103 162.3145371
292.3322626 264.1055046 275.721561 170.9289966
277.3864427 -0.694809012 0.033344935 0.144036174 OPLAH
8 144051266 144063965 - 4452
protein_coding "5-oxoprolinase, ATP-hydrolysing [Source:HGNC
Symbol;Acc:HGNC:8149]" - 85 272 166 291 289
281 1.044139397 2.693690573 1.727250743
3.160378145 2.828793757 2.946437275

ENSG00000096654 317.0074991   243.78815   298.2285169
188.8607057   187.3412749   215.8674143   286.3413887
197.356465   0.536064681   0.033357787   0.144050985   ZNF184
        6   27450743   27473118   -   3122
protein_coding zinc finger protein 184 [Source:HGNC
Symbol;Acc:HGNC:12975] zf-C2H2 274   263   305   188   205
        220   4.799681032   3.714127495   4.52552946
2.911562834   2.861406505   3.289544895
ENSG00000142408 65.94681552   75.08304241   86.04626062
161.7370937   102.3523063   128.5392331   75.69203952
130.876211   -0.787821075   0.03339363   0.144165046   CACNG8
        19   53963040   53990215   +   8747
protein_coding calcium voltage-gated channel auxiliary subunit gamma
8 [Source:HGNC Symbol;Acc:HGNC:13628]   -   57   81   88
        161   112   131   0.356377635   0.40828164
0.466043013   0.889954833   0.557978537   0.699130429
ENSG00000249700 28.92404189   27.80853423   3.911193665
125.5722778   59.40089203   12.75580175   20.21458993
65.90965718   -1.707462586   0.033420243   0.144239203
SRD5A3-AS1   4   55363971   55395847   -   5148
        processed_transcript   SRD5A3 antisense RNA 1 [Source:HGNC
Symbol;Acc:HGNC:44138] -   25   30   4   125   65
        13   0.265580499   0.256931099   0.035993486
1.17401242   0.550216254   0.11788291
ENSG00000185267 9.255693406   20.3929251   13.68917783
38.17397244   23.76035681   62.79779325   14.44593211
41.57737417   -1.517050321   0.033468396   0.144406257   CDNF
        10   14819250   14838575   -   1733
protein_coding cerebral dopamine neurotrophic factor [Source:HGNC
Symbol;Acc:HGNC:24913] -   8   22   14   38   26
        64   0.252456256   0.559703569   0.374224252
1.060196217   0.653782638   1.723961041
ENSG00000181856 2.313923351   4.634755704   0.977798416
15.06867333   19.19105743   12.75580175   2.642159157
15.67184417   -2.559733015   0.033503203   1   SLC2A4 17
        7281667 7288257 +   4046   protein_coding solute carrier
family 2 member 4 [Source:HGNC Symbol;Acc:HGNC:11009]   -   2
        5   1   15   21   13   0.027033285
0.054485142   0.011449238   0.179253068   0.226178862
0.149990416
ENSG00000248916 0   0.926951141   0   25.11445555   0
        3.924862078   0.308983714   9.679772543   -4.731016163
        0.033506211   1   NUP210P3   3   129323046
        129325426   -   388   unprocessed_pseudogene
nucleoporin 210 pseudogene 3 [Source:HGNC Symbol;Acc:HGNC:42712]
        -       0   1   0   25   0   4   0
        0.113632414   0   3.115369039   0   0.481253944
ENSG00000280890 11.56961676   2.780853423   3.911193665   0
        0   0   6.087221281   0   5.094046066
0.033545596   1   ELDR   7   55235965   55255635

- 2941 lincRNA EGFR long non-coding downstream RNA
[Source:HGNC Symbol;Acc:HGNC:49511] - 10 3 4
0 0 0 0.185951501 0.044973863
0.063003898 0 0 0
ENSG00000251870 8.09873173 0.926951141 9.777984162 0
0 0 6.267889011 0 5.138645252
0.033549191 1 RNU2-69P 18 58926197
58926296 - 100 snRNA "RNA, U2 small nuclear 69,
pseudogene [Source:HGNC Symbol;Acc:HGNC:48562]" - 7 1
10 0 0 0 3.82818355 0.440893766
4.632361631 0 0 0
ENSG00000184831 214.03791 306.8208276 300.1841138
205.9385355 164.4947779 182.5060866 273.6809505
184.3131334 0.572360566 0.033566582 0.144778934 APOO
X 23833353 23907934 - 2353
protein_coding apolipoprotein O [Source:HGNC Symbol;Acc:HGNC:28727]
- 185 331 307 205 180 186
4.299762958 6.202117997 6.04392274 4.212434397
3.333566903 3.690090801
ENSG00000163638 34.70885027 66.74048214 65.51249388
86.39372709 100.5245865 130.5016641 55.6539421
105.8066592 -0.922190829 0.033573711 0.144778934
ADAMTS9 3 64515654 64688000 - 10680
protein_coding ADAM metallopeptidase with thrombospondin type 1 motif
9 [Source:HGNC Symbol;Acc:HGNC:13202] - 30 72 67
86 110 133 0.153618923 0.297231752
0.290606956 0.389339454 0.448828091 0.581334937
ENSG00000102931 32.39492692 112.161088 108.5356242
205.9385355 103.2661661 186.4309487 84.36387972
165.2118835 -0.964263054 0.033598759 0.144846089 ARL2BP
16 57245098 57253635 + 2155
protein_coding ADP ribosylation factor like GTPase 2 binding protein
[Source:HGNC Symbol;Acc:HGNC:17146] - 28 121 111
205 113 190 0.710567712 2.475552003
2.386042418 4.599470133 2.285018743 4.115781989
ENSG00000113161 1639.414694 1440.482073 1980.041793
1496.821551 1270.26523 1179.421054 1686.646187
1315.502612 0.358607231 0.033609872 0.144853149 HMGCR
5 75336329 75362104 + 6215
protein_coding 3-hydroxy-3-methylglutaryl-CoA reductase [Source:HGNC
Symbol;Acc:HGNC:5006] - 1417 1554 2025 1490 1390
1202 12.46876472 11.02411765 15.09337458
11.59167916 9.74613146 9.028370448
ENSG00000101162 3.470885027 16.68512054 26.40055724
59.2701151 39.29597473 40.2298363 15.51885427
46.26530871 -1.564980453 0.033624289 0.144874437 TUBB1
20 59019254 59026654 + 3496
protein_coding tubulin beta 1 class VI [Source:HGNC
Symbol;Acc:HGNC:16257] - 3 18 27 59 43
41 0.04692935 0.227004799 0.357762483

0.815984303	0.535988695	0.547467659
ENSG00000159363 1725.029858	1609.187181	2101.288796
1603.306842	1269.35137	1410.006701	1811.835278
1427.554971	0.34412923	0.033642205	0.144910787
ATP13A2 1	16985958	17011928	-	5253
protein_coding	ATPase cation transporting 13A2 [Source:HGNC
Symbol;Acc:HGNC:30213]	-	1491	1736	2149	1596	1389
	1437	15.52261748	14.57056115	18.95097115
14.69016598	11.52267845	12.77013249
ENSG00000186222 433.8606284	543.1933685	455.6540619
766.4931834	667.1177105	517.1005788	477.569353
650.2371576	-0.444462585	0.03374095	0.145295185
BLOC1S4 4	6716055 6717671 +	1617	protein_coding
biogenesis of lysosomal organelles complex 1 subunit 4 [Source:HGNC
Symbol;Acc:HGNC:24206]	-	375	586	466	763	730
	527	12.68282385	15.97796826	13.34991045
22.81475107	19.67303832	15.21411277
ENSG00000273456 11.56961676	12.97731597	9.777984162
51.23348932	21.93263706	30.4176811	11.44163896
34.52793583	-1.59164757	0.033763076	0.145332306
AC064836.3	2	202374932	202375604	-	673
	lincRNA novel transcript	-	10	14	10
	51	24	31	0.812605296	0.917163852
0.68831525	3.664007284	1.554013457	2.150268364
ENSG00000037757 549.556796	647.9388475	544.6337178
884.0288354	760.331418	656.4331825	580.7097871
766.9311453	-0.400683188	0.033768585	0.145332306	MRI1
	19	13764532	13774282	+	3456
protein_coding	methylthioribose-1-phosphate isomerase 1 [Source:HGNC
Symbol;Acc:HGNC:28469]	-	475	699	557	880	832
	669	7.516481425	8.917382589	7.46593006
12.31147691	10.49078992	9.036461857
ENSG00000270165 11.56961676	3.707804563	2.933395248	0
	0	0	6.07027219	0	5.090191421
0.033812527	1	AC010530.1	16	67738588
67739922	-	569	sense_intronic	novel transcript
	-	10	4	3	0	0	0
0.961130693	0.309942893	0.244236993	0	0	0
ENSG00000115363 804.0883646	916.7546783	815.4838791
458.0876692	822.4738897	557.3304151	845.4423073
612.630658	0.464248901	0.033830761	0.145535453	EVA1A
	2	75469302	75569722	-	2744
protein_coding	"eva-1 homolog A, regulator of programmed cell death
[Source:HGNC Symbol;Acc:HGNC:25816]"	-	695	989	834
	456	900	568	13.85145547	15.89081393
14.07940817	8.034927309	14.29278958	9.662961841
ENSG00000155367 20.82531016	10.19646255	3.911193665
25.11445555	52.09001301	34.34254318	11.64432213
37.18233725	-1.688520154	0.033834827	0.145535453	PPM1J
	1	112709994	112715477	-	2874

protein_coding "protein phosphatase, Mg2+/Mn2+ dependent 1J
[Source:HGNC Symbol;Acc:HGNC:20785]" - 18 11 4
25 57 35 0.342515677 0.168748484
0.064472674 0.42058566 0.86426453 0.568495873
ENSG00000218052 10.41265508 2.780853423 4.888992081 0
0 0 6.027500195 0 5.080545494
0.03388097 1 ADAMTS7P4 15 85255369
85330334 - 5516 transcribed_unprocessed_pseudogene
ADAMTS7 pseudogene 4 [Source:HGNC Symbol;Acc:HGNC:49410]
- 9 3 5 0 0 0
0.089230426 0.023978994 0.041990225 0 0 0
ENSG00000267672 2.313923351 0 1.955596832 11.05036044
13.70789816 11.77458623 1.423173395 12.17761495
-3.127139623 0.033898537 1 AC010632.2 19
37091341 37092564 + 1224 sense_intronic
novel transcript - 2 0 2 11
15 12 0.089360027 0 0.075692184
0.434522714 0.534033423 0.457663064
ENSG00000116213 1147.705982 1353.348666 1287.760514
1612.348046 1589.202327 1490.466374 1262.938387
1564.005582 -0.307973039 0.033968706 0.146017849 WRAP73
1 3630767 3652761 - 7604 protein_coding "WD
repeat containing, antisense to TP73 [Source:HGNC
Symbol;Acc:HGNC:12759]" - 992 1460 1317 1605 1739
1519 7.134512065 8.465345845 8.023172368
10.20549666 9.965889593 9.325276151
ENSG00000227376 1.156961676 9.269511408 0 27.12361199
13.70789816 22.56795695 3.475491028 21.1331557
-2.580266234 0.033974867 0.146017849 FTH1P16 11
77734475 77735026 - 552
processed_pseudogene ferritin heavy chain 1 pseudogene 16
[Source:HGNC Symbol;Acc:HGNC:3986] - 1 10 0
27 15 23 0.099073073 0.79872059 0
2.364971453 1.184161069 1.945068023
ENSG00000088386 6.941770054 9.269511408 1.955596832 0
0 0 6.055626098 0 5.091203566
0.033982655 1 SLC15A1 13 98683801 98752654
- 4192 protein_coding solute carrier family 15
member 1 [Source:HGNC Symbol;Acc:HGNC:10920] - 6 10
2 0 0 0 0.078275291 0.10517504
0.022100962 0 0 0
ENSG00000157021 106.4404742 70.4482867 46.93432398
3.013734666 46.60685375 29.43646558 74.60769495
26.35235133 1.496362328 0.033998181 0.146017849
FAM92A1P1 15 41163162 41164374 + 890
unprocessed_pseudogene family with sequence similarity 92
member A1 pseudogene 1 [Source:HGNC Symbol;Acc:HGNC:32278] -
92 76 48 3 51 30 5.65317635
3.764935528 2.49835234 0.162979306 2.497116287
1.573538176

ENSG00000104529 1903.201957 2876.32939 3381.226923
4121.784445 2768.995429 4118.161535 2720.252757
3669.647136 -0.431480596 0.034004993 0.146017849 EEF1D
8 143579697 143599541 - 9390
protein_coding eukaryotic translation elongation factor 1 delta
[Source:HGNC Symbol;Acc:HGNC:3211] - 1645 3103 3458
4103 3030 4197 9.580651057 14.56968429
17.05932537 21.12696638 14.06162895 20.86504918
ENSG00000117501 0 8.342560268 8.800185745 42.19228532
31.98509571 8.830939675 5.714248671 27.66944024
-2.257025768 0.034012452 0.146017849 MROH9 1
170935471 171064765 + 3831 protein_coding
maestro heat like repeat family member 9 [Source:HGNC
Symbol;Acc:HGNC:26287] - 0 9 9 42 35
9 0 0.103577236 0.108826037 0.530076261
0.398120454 0.109667109
ENSG00000144476 64.78985384 5.561706845 3.911193665
9.041203998 0 0.981215519 24.75425145 3.340806506
2.894767716 0.034015696 0.146017849 ACKR3 2
236567787 236582358 + 2258 protein_coding
atypical chemokine receptor 3 [Source:HGNC
Symbol;Acc:HGNC:23692] - 56 6 4 9 0
1 1.356309495 0.117155119 0.082061322
0.192716894 0 0.020673885
ENSG00000224870 268.4151088 450.4982545 418.4977221
462.1059821 539.1773277 614.2409152 379.1370284
538.508075 -0.504431599 0.034015874 0.146017849
AL391244.1 1 1399522 1402046 + 2525
processed_transcript uncharacterized LOC148413 [Source:NCBI
gene;Acc:148413] - 232 486 428 460 590
626 5.024829328 8.48611367 7.85208229
8.808412929 10.18237827 11.57334732
ENSG00000162461 17.35442514 7.415609127 16.62257307
36.16481599 47.52071363 32.38011214 13.79753578
38.68854725 -1.494494914 0.034024668 0.146017849
SLC25A34 1 15736405 15741396 + 3195
protein_coding solute carrier family 25 member 34
[Source:HGNC Symbol;Acc:HGNC:27653] - 15 8 17
36 52 33 0.256752753 0.110395935
0.246479336 0.544794676 0.709236502 0.482157707
ENSG00000121057 1604.705844 1605.479376 1804.038078
2290.438346 1944.693819 1953.600099 1671.407766
2062.910755 -0.303416641 0.034032943 0.146017849 AKAP1
17 57085092 57121349 + 5399
protein_coding A-kinase anchoring protein 1 [Source:HGNC
Symbol;Acc:HGNC:367] - 1387 1732 1845 2280 2128
1991 14.04940223 14.14387854 15.83016709
20.41844836 17.17579682 17.21487876
ENSG00000269907 2.313923351 12.05036483 3.911193665 0
0 0 6.091827282 0 5.103647423

0.03403824	1	AL158827.2	9	96910076
96910405	+	330	processed_pseudogene	"prothymosin,
alpha (PTMA) pseudogene"	-	2	13	4	0
0	0	0.331444463	1.736854229	0.56149838
0	0	0
ENSG00000236609 236.0201818	390.2464303	351.0296314
293.3368408	572.0762833	634.8464411	325.7654145
500.0865217	-0.617237239	0.034101716	0.146271868	ZNF853
7	6615617 6624290 +	3857	protein_coding	zinc
finger protein 853 [Source:HGNC Symbol;Acc:HGNC:21767]	zf-C2H2 204
421	359	292	626	647	2.892512479
4.812452046	4.311687388	3.660449579	7.072668323
7.83070165
ENSG00000133083 594.6783013	366.1457006	487.9214097
330.506235	383.8211485	311.0453197	482.9151372
341.7909011	0.496975086	0.034113961	0.146283342	DCLK1
13	35768652	36131306	-	14375
protein_coding	doublecortin like kinase 1 [Source:HGNC
Symbol;Acc:HGNC:2700]	-	514	395	499	329	420
317	1.95546469	1.211499391	1.608033707
1.106596421	1.273209982	1.02943148
ENSG00000135870 1136.136366	879.6766327	980.7318114
836.8136589	804.1966921	669.1889843	998.8482699
770.0664451	0.374597484	0.034143605	0.146343954	RC3H1
1	173931214	174022297	-	11508
protein_coding	ring finger and CCCH-type domains 1 [Source:HGNC
Symbol;Acc:HGNC:29434]	-	982	949	1003	833	880
682	4.666662007	3.635802778	4.037416333
3.499825287	3.332279464	2.766499253
ENSG00000128973 518.3188307	640.5232383	660.9917293
518.3623626	374.6825497	451.359139	606.6112661
448.1346838	0.438094493	0.034147242	0.146343954	CLN6
15	68206992	68257211	-	7345
protein_coding	"CLN6, transmembrane ER protein [Source:HGNC
Symbol;Acc:HGNC:2077]"	-	448	691	676	516	410
460	3.335653468	4.147822902	4.263412475
3.39671507	2.432487708	2.923560378
ENSG00000152102 4047.051942	4086.000629	4540.895845
5305.177591	5055.472842	4814.824554	4224.649472
5058.491662	-0.259804171	0.034176464	0.14642814
FAM168B 2	131047876	131093460	-	5435
protein_coding	family with sequence similarity 168 member B
[Source:HGNC Symbol;Acc:HGNC:27016]	-	3498	4408	4644
5281	5532	4907	35.19775544	35.75822851
39.58176157	46.98052175	44.35486081	42.14659999
ENSG00000257086 1.156961676	8.342560268	7.822387329
41.1877071	12.79403828	21.58674143	5.773969758
25.18949561	-2.106643072	0.034274717	0.146807955
AP001453.3	11	64246939	64249494	-	853
lincRNA novel transcript	-	1	9	8

41 14 22 0.064112938 0.465186857
0.434453611 2.323999563 0.715216627 1.203981144
ENSG00000169252 3.470885027 4.634755704 9.777984162 0
0 0 5.961208298 0 5.07008405
0.034276556 1 ADRB2 5 148825245 148828687
+ 3443 protein_coding adrenoceptor beta 2
[Source:HGNC Symbol;Acc:HGNC:286] - 3 5 10
0 0 0 0.04765176 0.064027558
0.13454434 0 0 0
ENSG00000249690 0 0 0 3.013734666 5.483159264
9.812155195 0 6.103016375 -5.029465935
0.034278484 1 AC110813.1 4 150579089
150581545 + 2209 antisense uncharacterized
LOC729558 [Source:NCBI gene;Acc:729558] - 0 0 0
3 6 10 0 0 0 0.065663912
0.118362501 0.211324729
ENSG00000186073 468.5694787 345.7527755 362.7632124
213.9751613 318.9370972 285.5337162 392.3618222
272.8153249 0.5224575 0.034288228 0.146820539
C15orf41 15 36579611 36810248 + 9293
protein_coding chromosome 15 open reading frame 41
[Source:HGNC Symbol;Acc:HGNC:26929] - 405 373 371
213 349 291 2.383382789 1.769647849
1.849355607 1.108217191 1.636545512 1.461783608
ENSG00000128965 97.18478076 154.8008405 123.2026004
117.535652 300.6598997 226.660785 125.0627406
214.9521122 -0.780240342 0.034309899 0.146820539 CHAC1
15 40952962 40956519 + 2058
protein_coding ChaC glutathione specific gamma-
glutamylcyclotransferase 1 [Source:HGNC Symbol;Acc:HGNC:28680] -
84 167 126 117 329 231 2.232176997
3.577709373 2.836139774 2.748790922 6.966411516
5.239775083
ENSG00000165724 748.5542042 908.412118 753.8825789
955.3538891 1007.987445 1119.566908 803.6163004
1027.636081 -0.354203862 0.03431997 0.146820539
ZMYND19 9 137582079 137590490 - 2372
protein_coding zinc finger MYND-type containing 19 [Source:HGNC
Symbol;Acc:HGNC:21146] - 647 980 771 951 1103
1141 14.91709683 18.21567835 15.05712824
19.3850555 20.26373165 22.45520352
ENSG00000059804 992.6731178 926.0241897 589.6124449
1113.07267 1409.171931 932.1547435 836.1032508
1151.466448 -0.462378049 0.034330398 0.146820539 SLC2A3
12 7919230 7936275 - 6159 protein_coding solute
carrier family 2 member 3 [Source:HGNC Symbol;Acc:HGNC:11007] -
858 999 603 1108 1542 950 7.618540779
7.151369898 4.535337009 8.698227709 10.91020139
7.200446652
ENSG00000135916 618.9744965 1027.061864 1277.98253

685.1223474 686.3087679 719.2309758 974.6729635
696.8873637 0.484893168 0.034337898 0.146820539 ITM2C
2 230864639 230879248 + 2611
protein_coding integral membrane protein 2C [Source:HGNC
Symbol;Acc:HGNC:6175] - 535 1108 1307 682 751
733 11.2057679 18.70970098 23.18842073
12.6292837 12.53405693 13.10518447
ENSG00000140395 715.0023156 654.4275054 635.5689705
424.9365879 562.9376845 527.8939495 668.3329305
505.256074 0.402764055 0.034339815 0.146820539 WDR61
15 78277835 78299794 - 7279
protein_coding WD repeat domain 61 [Source:HGNC
Symbol;Acc:HGNC:30300] - 618 706 650 423 616
538 4.643136683 4.276287933 4.136605386
2.809764134 3.687801958 3.450297886
ENSG00000166734 2483.996718 2083.786165 2013.286939
1779.108031 1933.727501 1552.282952 2193.68994
1755.039495 0.321401864 0.034344884 0.146820539 CASC4
15 44288729 44415758 + 7095
protein_coding cancer susceptibility 4 [Source:HGNC
Symbol;Acc:HGNC:24892] - 2147 2248 2059 1771 2116
1582 16.54909913 13.9694036 13.44331585
12.06889136 12.99636385 10.40878685
ENSG00000168404 422.2910116 422.6897202 385.252576
543.4768181 527.2971493 548.4994754 410.0777693
539.7578143 -0.396522751 0.03437852 0.14692324 MLKL
16 74671855 74700960 - 5692
protein_coding mixed lineage kinase domain like pseudokinase
[Source:HGNC Symbol;Acc:HGNC:26617] - 365 456 394
541 577 559 3.506894378 3.53210747
3.206518768 4.595508674 4.417428405 4.584510295
ENSG00000076513 1492.480562 1249.530138 1414.874308
936.2669029 1141.410987 1198.064149 1385.628336
1091.914013 0.343188133 0.034388972 0.146926833
ANKRD13A 12 109999186 110039763 + 7350
protein_coding ankyrin repeat domain 13A [Source:HGNC
Symbol;Acc:HGNC:21268] - 1290 1348 1447 932 1249
1221 9.598361088 8.08605165 9.119765007
6.130978452 7.40514722 7.754867122
ENSG00000182871 702.2757372 619.2033621 1961.463623
618.8201848 309.7984984 435.6596906 1094.314241
454.759458 1.267042968 0.034402348 0.146942915
COL18A1 21 45405137 45513720 + 8525
protein_coding collagen type XVIII alpha 1 chain [Source:HGNC
Symbol;Acc:HGNC:2195] - 607 668 2006 616 339
444 3.893937855 3.454745285 10.9003137
3.493715535 1.732861721 2.431277989
ENSG00000075303 760.1238209 831.4751733 662.9473262
463.1105604 712.8107044 471.9646649 751.5154401
549.2953099 0.451658881 0.034457687 0.147138175

SLC25A40	7	87833568	87876357	-	5081
protein_coding	solute carrier family 25 member 40
[Source:HGNC Symbol;Acc:HGNC:29680]	-	657	897	678
461	780	481	7.071489281	7.783540797
6.18134459	4.386851638	6.689659385	4.419182299
ENSG00000112679 320.4783842	490.3571535	349.0740346
538.453927	560.1961049	504.344777	386.6365241
534.331603	-0.465283539	0.034514145	0.147329822	DUSP22
6	291630	351355	+	5686	protein_coding	dual
specificity phosphatase 22 [Source:HGNC Symbol;Acc:HGNC:16077]	-
277	529	357	536	613	514	2.664204923
4.101878335	2.90846483	4.557840791	4.697991394
4.21990136
ENSG00000267796 16.19746346	29.66243651	33.24514615
27.12361199	74.02265007	93.21547435	26.36834871
64.78724547	-1.292751278	0.034521844	0.147329822	LIN37
19	35748361	35754519	+	2820
protein_coding	lin-37 DREAM MuvB core complex component [Source:HGNC
Symbol;Acc:HGNC:33234]	-	14	32	34	27	81
95	0.271502379	0.500304982	0.558511686
0.462930582	1.251683445	1.572608189
ENSG00000267470 3.470885027	7.415609127	6.844588913	0
0	0	5.910361022	0	5.058335747
0.034550889	1	ZNF571-AS1	19	37548914
37587348	+	4560	antisense	ZNF571 antisense RNA 1
[Source:HGNC Symbol;Acc:HGNC:44324]	-	3	8	7
0	0	0	0.035979169	0.077349783
0.071110815	0	0	0
ENSG00000124549 84.45820233	109.3802346	143.7363672
43.19686355	56.6593124	81.44088812	112.5249347
60.43235469	0.898155326	0.034563481	0.14746635
BTN2A3P 6	26421391	26432383	+	4177
transcribed_unprocessed_pseudogene	"butyrophilin subfamily 2
member A3, pseudogene [Source:HGNC Symbol;Acc:HGNC:13229]"	-
73	118	147	43	62	83	0.955769346
1.24552225	1.630254153	0.497743041	0.646823533
0.927597678
ENSG00000146463 2686.465011	2137.549331	2264.581132
2080.481498	1867.01573	1761.281857	2362.865158
1902.926362	0.311991572	0.034582039	0.147504361	ZMYM4
1	35268967	35422058	+	7647
protein_coding	zinc finger MYM-type containing 4 [Source:HGNC
Symbol;Acc:HGNC:13055]	-	2322	2306	2316	2071	2043
1795	16.60603076	13.29542335	14.02974962
13.09453935	11.64222194	10.95769981
ENSG00000214842 0	0	0.977798416	15.06867333
5.483159264	2.943646558	0.325932805
7.831826384	-4.426230879	0.034590745	1	RAD51AP2
2	17510584	17518439	-	3724
protein_coding	RAD51 associated protein 2 [Source:HGNC

Symbol;Acc:HGNC:34417]	-	0	0	1	15	6
	3	0	0	0.01243921	0.194752393
0.070210194	0.037606041
ENSG00000088387 1861.551336	1848.340575	1354.250806
2347.699305	2015.06103	2059.571375	1688.047572
2140.777237	-0.342907443	0.034593976	0.147514118	DOCK9
	13	98793429	99086625	-	14816
protein_coding	dedicator of cytokinesis 9 [Source:HGNC
Symbol;Acc:HGNC:14132]	-	1609	1994	1385	2337	2205
	2099	5.939088372	5.933734942	4.330332653
7.62656471	6.485391861	6.613441329
ENSG00000184260 41.65062033	30.58938765	11.73358099	0
	10.96631853	6.868508636	27.99119632	5.944942388
	2.22583057	0.034657048	0.147692509	HIST2H2AC
	1	149886975	149887364	+	390
protein_coding	histone cluster 2 H2A family member c [Source:HGNC
Symbol;Acc:HGNC:4738]	-	36	33	12	0	12
	7	5.048154132	3.730639557	1.42534204	0
	1.340834688	0.837875456
ENSG00000065882 1330.505927	1310.708913	1685.724469
958.3676238	1240.107854	1170.590115	1442.313103
1123.021864	0.360932381	0.034660794	0.147692509	TBC1D1
	4	37891087	38139175	+	9032
protein_coding	TBC1 domain family member 1 [Source:HGNC
Symbol;Acc:HGNC:11578]	-	1150	1414	1724	954	1357
	1193	6.963196069	6.902389115	8.842107454
5.1069977	6.547183549	6.165986231
ENSG00000275700 2763.981443	3366.686544	3075.176019
3675.751714	3381.281546	4310.479777	3068.614669
3789.171013	-0.304001324	0.034668604	0.147692509	AATF
	17	36948875	37056871	+	4714
protein_coding	apoptosis antagonizing transcription factor
[Source:HGNC Symbol;Acc:HGNC:19235]	-	2389	3632	3145
	3659	3700	4393	27.71540851	33.96958331
30.90534012	37.52960969	34.20351532	43.5028451
ENSG00000261819 3.470885027	32.44328993	17.60037149
66.30216265	48.4345735	41.21105182	17.83818215
51.98259599	-1.530398395	0.034674456	0.147692509
AC138932.3	16	14988259	14990160	-	1902
	processed_pseudogene	uncharacterized protein KIAA2013
[Source:NCBI gene;Acc:102724984]	-	3	35	18
	66	53	42	0.086259206	0.811318707
0.438393845	1.677778557	1.214294295	1.030824694
ENSG00000144736 1679.908353	1409.892685	1570.344256
1837.373568	1846.910812	2062.515022	1553.381765
1915.599801	-0.302759511	0.034698122	0.147706495	SHQ1
	3	72749277	72861914	-	5841
protein_coding	"SHQ1, H/ACA ribonucleoprotein assembly factor
[Source:HGNC Symbol;Acc:HGNC:25543]"	-	1452	1521	1606
	1829	2021	2102	13.59484069	11.48090083

12.73681352	15.14006416	15.07779051	16.79931375
ENSG00000110092 8490.941738	8582.640613	6302.888591
8806.132694	9370.719183	10939.57183	7792.156981
9705.474568	-0.316817061	0.03471236	0.147706495	CCND1
	11	69641087	69654474	+	4830
protein_coding	cyclin D1 [Source:HGNC Symbol;Acc:HGNC:1582]	-
	7339	9259	6446	8766	10254	11149	83.09683252
	84.5183308	61.82236662	87.75170269	92.51342938
	107.7543522
ENSG00000247596 947.5516124	1379.303298	1240.82619
1376.272164	1565.44197	1661.197874	1189.227033
1534.304003	-0.366793349	0.034712801	0.147706495	TWF2
	3	52228610	52239260	-	1824
protein_coding	twinfilin actin binding protein 2 [Source:HGNC
Symbol;Acc:HGNC:9621]	-	819	1488	1269	1370	1713
	1693	24.55578264	35.96764931	32.22843701
36.31591155	40.9252298	43.32894952
ENSG00000106367 853.8377167	1478.48707	1231.048206
1406.409511	1553.561792	1801.511694	1187.790997
1587.160999	-0.417283439	0.034723196	0.147706495	AP1S1
	7	101154400	101161596	+	1756
protein_coding	adaptor related protein complex 1 subunit sigma 1
[Source:HGNC Symbol;Acc:HGNC:559]	-	738	1595	1259
	1400	1700	1836	22.98405028	40.04701346
33.21266113	38.54825654	42.18742398	48.80835841
ENSG00000198833 1103.741439	1153.127219	1258.426562
640.9209057	1085.665534	907.6243555	1171.765073
878.0702652	0.415982063	0.034728532	0.147706495	UBE2J1
	6	89326625	89352848	-	4290
protein_coding	ubiquitin conjugating enzyme E2 J1 [Source:HGNC
Symbol;Acc:HGNC:17598]	-	954	1244	1287	638	1188
	925	12.16146223	12.78489148	13.89708489
7.190591268	12.06751219	10.06538697
ENSG00000061676 6988.048521	5336.457718	5155.931048
4725.535956	5023.487746	4349.728398	5826.812429
4699.584033	0.309931332	0.034735713	0.147706495	NCKAP1
	2	182909115	183038858	-	22520
protein_coding	NCK associated protein 1 [Source:HGNC
Symbol;Acc:HGNC:7666]	-	6040	5757	5273	4704	5497
	4433	14.6677421	11.27098317	10.84655545
10.09950628	10.63692136	9.189150849
ENSG00000077380 2327.806892	2077.297507	2162.890097
1750.979841	1825.892035	1834.873021	2189.331498
1803.914966	0.279083856	0.034749188	0.147722702
DYNC1I2 2	171687409	171748420	+	4695
protein_coding	dynein cytoplasmic 1 intermediate chain 2 [Source:HGNC
Symbol;Acc:HGNC:2964]	-	2012	2241	2212	1743	1998
	1870	23.43619444	21.04457783	21.8248859
17.94994024	18.54464333	18.5931103
ENSG00000232859 5.784808379	50.05536161	12.71137941

65.29758443 86.81668835 43.17348286 22.85051646
65.09591855 -1.500160664 0.034772179 0.147779344 LYRM9
17 27878314 27894752 - 5380
protein_coding LYR motif containing 9 [Source:HGNC
Symbol;Acc:HGNC:27314] - 5 54 13 65 95
44 0.050825591 0.442532776 0.111934389
0.584160648 0.769484591 0.381782868
ENSG00000285618 2.313923351 0 1.955596832 9.041203998
22.84649694 5.887293117 1.423173395 12.59166468
-3.175809449 0.034778298 1 AC074132.1 5
91132303 91142470 - 687 antisense
"novel transcript, antisense to ADGRV1" - 2 0
2 9 25 6 0.159209131 0
0.134857689 0.633413024 1.585776105 0.407699848
ENSG00000163431 10.41265508 1.853902282 5.866790497 0
0 0 6.044449287 0 5.084492982
0.034800397 1 LMOD1 1 201896452 201946588
- 3971 protein_coding leiomodin 1 [Source:HGNC
Symbol;Acc:HGNC:6647] - 9 2 6 0 0
0 0.123947375 0.022205679 0.069992873 0
0 0
ENSG00000213414 0 0 0 27.12361199 0 0
0 9.041203998 -5.594847411 0.03486326 1
AC008677.1 5 157986146 157987706 -
1561 processed_pseudogene cell division cycle 37 homolog
(S. cerevisiae) (CDC37) pseudogene - 0 0 0
27 0 0 0 0 0 0.836299963
0 0
ENSG00000221990 57.84808379 127.9192574 91.91305112
120.5493866 171.805657 194.2806729 92.56013078
162.2119055 -0.80555819 0.034876504 0.148142754 EXOC3-
AS1 5 441498 443160 - 1663 antisense EXOC3
antisense RNA 1 [Source:HGNC Symbol;Acc:HGNC:25175] - 50
138 94 120 188 198 1.644267481
3.658649409 2.618412467 3.488913589 4.926337909
5.55800556
ENSG00000176946 1167.374331 1426.577806 1319.050063
1449.606374 1705.262531 1750.488487 1304.334067
1635.119131 -0.32567573 0.03487707 0.148142754 THAP4
2 241584405 241637449 - 3147
protein_coding THAP domain containing 4 [Source:HGNC
Symbol;Acc:HGNC:23187] THAP 1009 1539 1349 1443 1866
1784 17.5343284 21.56134431 19.85718411
22.17026094 25.83886865 26.4633087
ENSG00000242641 8.09873173 11.12341369 0.977798416
30.13734666 33.81281546 17.66187935 6.733314612
27.20401383 -2.014992873 0.03488988 0.148155998
LINC00971 3 84638406 84881679 - 8031
lincRNA long intergenic non-protein coding RNA 971
[Source:HGNC Symbol;Acc:HGNC:48737] - 7 12 1

30	37	18	0.047667582	0.065878785
0.005768101	0.180614597	0.200766245	0.104628239
ENSG00000101442 391.0530464	397.6620394	405.7863427
532.4264577	597.6643598	465.0961562	398.1671428
531.7289912	-0.417456629	0.034905477	0.14818107	ACTR5
20	38748442	38772520	+	2565
protein_coding	ARP5 actin related protein 5 homolog [Source:HGNC
Symbol;Acc:HGNC:14671]	-	338	429	415	530	654
474	7.206494235	7.374012691	7.494854101
9.990557338	11.11089329	8.626547303
ENSG00000275835 816.8149431	741.5609127	807.6614917
630.8751234	523.6417098	669.1889843	788.6791158
607.9019392	0.375698936	0.034915842	0.14818392
TUBGCP5 15	22983192	23039673	-	6478
protein_coding	tubulin gamma complex associated protein 5
[Source:HGNC Symbol;Acc:HGNC:18600]	-	706	800	826
628	573	682	5.96016757	5.444813409
5.90665438	4.687269414	3.854537508	4.914614604
ENSG00000119787 992.6731178	918.6085806	939.6642779
661.0124701	794.1442335	784.9724156	950.3153254
746.7097064	0.34737932	0.034936226	0.148215589	ATL2
2	38294880	38377285	-	4884
protein_coding	atlastin GTPase 2 [Source:HGNC Symbol;Acc:HGNC:24047]
-	858	991	961	658	869	800
9.607410454	8.946063102	9.114863898	6.514055506
7.753587975	7.646459059
ENSG00000125503 1132.665481	1951.232151	1979.063994
2184.957633	1997.697692	2611.995713	1687.653875
2264.883679	-0.423714293	0.034942695	0.148215589
PPP1R12C	19	55090913	55117559	-	7726
protein_coding	protein phosphatase 1 regulatory subunit 12C
[Source:HGNC Symbol;Acc:HGNC:14947]	-	979	2105	2024
2175	2186	2662	6.929831914	12.0124434
12.13551636	13.61149331	12.32974377	16.08419698
ENSG00000114127 1323.564157	916.7546783	1247.670779
820.7404074	950.4142725	907.6243555	1162.663205
892.9263451	0.380046523	0.034953837	0.148221723	XRN1
3	142306607	142448062	-	11982
protein_coding	5'-3' exoribonuclease 1 [Source:HGNC
Symbol;Acc:HGNC:30654]	-	1144	989	1276	817	1040
925	5.221453587	3.63915819	4.933144251
3.296810296	3.782357908	3.603781515
ENSG00000102290 473.1973254	413.4202088	615.0352038
425.9411661	344.5251738	295.3458714	500.5509127
355.2707371	0.494843665	0.034982578	0.148302459
PCDH11X X	91779261	92623230	+	11630
protein_coding	protocadherin 11 X-linked [Source:HGNC
Symbol;Acc:HGNC:8656]	-	409	446	629	424	377
301	1.923261359	1.690787786	2.505378733
1.762736342	1.412603354	1.208183267

ENSG00000260563 93.71389573 86.2064561 73.33488121
134.6134818 132.5096822 149.144759 84.41841101
138.7559743 -0.718036635 0.035011411 0.148383542
AC132872.1 17 82293716 82294910 + 1195
lincRNA novel transcript - 81 93 75
134 145 152 3.70690816 3.431223449
2.907339936 5.42173279 5.287601227 5.937747405
ENSG00000231948 8.09873173 4.634755704 4.888992081 0
0 0 5.874159838 0 5.045285904
0.035029844 1 HS1BP3-IT1 2 20590775
20592548 - 600 sense_intronic HS1BP3 intronic
transcript 1 [Source:HGNC Symbol;Acc:HGNC:41474] - 7
5 5 0 0 0 0.638030592
0.367411471 0.386030136 0 0 0
ENSG00000284431 8.09873173 4.634755704 4.888992081 0
0 0 5.874159838 0 5.045285904
0.035029844 1 AL022238.4 22 40346529
40410054 + 4563 protein_coding novel transcript
- 7 5 5 0 0 0
0.083896199 0.048311831 0.050760044 0 0 0
ENSG00000243679 68.26073887 120.5036483 118.3136084
159.7279373 145.3037205 208.9989056 102.3593318
171.3435212 -0.739451326 0.035030346 0.148422646
AC018638.5 7 128653969 128654722 + 754
processed_pseudogene novel pseudogene - 59
130 121 159 159 213 4.27932606
7.601616652 7.433895987 10.19593352 9.189341179
13.18725164
ENSG00000119402 2474.741024 2269.176393 2219.602405
2875.102871 2720.560855 2793.520584 2321.173274
2796.39477 -0.268905343 0.035041095 0.14842705 FBXW2
9 120751978 120793412 - 11263
protein_coding F-box and WD repeat domain containing 2 [Source:HGNC
Symbol;Acc:HGNC:13608] - 2139 2448 2270 2862 2977
2847 10.38607401 9.582774915 9.336287759
12.28617683 11.5181664 11.79992967
ENSG00000115091 7049.36749 6054.844852 5594.962537
4471.377666 5862.411114 4751.045545 6233.058293
5028.278108 0.309667477 0.03505737 0.148435314 ACTR3
2 113889960 113962596 + 12049
protein_coding ARP3 actin related protein 3 homolog [Source:HGNC
Symbol;Acc:HGNC:170] - 6093 6532 5722 4451 6415
4842 27.65507792 23.90171863 21.99881588
17.86108373 23.20086912 18.7594377
ENSG00000165650 1397.609704 1265.288307 1375.762372
1842.396459 1774.715882 1437.480736 1346.220128
1684.864359 -0.323948465 0.035062466 0.148435314 PDZD8
10 117277274 117375467 - 9881
protein_coding PDZ domain containing 8 [Source:HGNC
Symbol;Acc:HGNC:26974] - 1208 1365 1407 1834 1942

1465	6.685913411	6.090678983	6.596227928
8.974280682	8.564596834	6.921221708
ENSG00000133393 1021.59716	1053.943447	1004.198973
761.4702923	931.2232151	716.2873292	1026.57986
802.9936122	0.354086117	0.035102077	0.148561864	FOPNL
16	15865720	15888625	-	2391
protein_coding	FGFR1OP N-terminal like [Source:HGNC
Symbol;Acc:HGNC:26435]	-	883	1137	1027	758	1019
730	20.19648727	20.96596452	19.89726221
15.32818897	18.57176611	14.25244323
ENSG00000141098 778.6352078	787.9084697	1089.267436
1198.461819	1175.223802	1075.412209	885.270371
1149.699277	-0.3767048	0.03512547	0.148619725	GFOD2
16	67674531	67719421	-	8674
protein_coding	glucose-fructose oxidoreductase domain containing 2
[Source:HGNC Symbol;Acc:HGNC:28159]	-	673	850	1114
1193	1286	1096	4.24316929	4.320494592
5.949332323	6.650009142	6.460708529	5.898440083
ENSG00000108433 1332.81985	1256.945747	1281.893724
1643.489971	1741.816926	1417.856426	1290.553107
1601.054441	-0.311303832	0.035153157	0.148695715	GOSR2
17	46923075	46975524	+	14366
protein_coding	golgi SNAP receptor complex member 2 [Source:HGNC
Symbol;Acc:HGNC:4431]	-	1152	1356	1311	1636	1906
1445	4.385421382	4.16157557	4.227360503
5.506157802	5.78156792	4.695458655
ENSG00000199691 0	2.780853423	5.866790497	24.10987733
12.79403828	15.69944831	2.882547973	17.53445464
-2.581091737	0.035164816	1	RN7SKP173	20
38761528	38761842	+	315	misc_RNA
"RNA, 7SK small nuclear pseudogene 173 [Source:HGNC
Symbol;Acc:HGNC:45897]" -	0	3	6	24	14
16	0	0.419898825	0.882354596	3.683849714
1.936761216	2.371130542
ENSG00000010404 8655.230296	6940.083192	5275.222455
8682.569573	10380.53435	7821.268906	6956.845314
8961.457609	-0.365468055	0.035207338	0.148883701	IDS
X	149476990	149521096	-	9292
protein_coding	iduronate 2-sulfatase [Source:HGNC
Symbol;Acc:HGNC:5389]	-	7481	7487	5395	8643	11359
7971	44.02964322	35.52487758	26.89581468
44.97348355	53.27083401	40.0451241
ENSG00000127415 387.5821614	380.0499677	415.5643269
580.6462123	466.0685375	531.8188116	394.3988187
526.1778538	-0.415509791	0.03523779	0.148971267	IDUA
4	986997	1004506 +	4000
protein_coding	"iduronidase, alpha-L- [Source:HGNC
Symbol;Acc:HGNC:5391]"	-	335	410	425	578	510
542	4.580148176	4.519161099	4.921884233
6.986651223	5.556083738	6.325361211

ENSG00000105711 94.87085741 152.0199871 130.0471893
40.18312888 98.69686676 62.79779325 125.6460113
67.22592963 0.901721659 0.035254888 0.14900234 SCN1B
19 35030466 35040449 + 7993
protein_coding sodium voltage-gated channel beta subunit 1
[Source:HGNC Symbol;Acc:HGNC:10586] - 82 164 133
40 108 64 0.561046364 0.904623766
0.770804575 0.241964356 0.588806425 0.373780118
ENSG00000020129 1575.781802 2332.20907 2208.846622
2856.015885 2195.091426 2854.355946 2038.945832
2635.154419 -0.369447544 0.035279379 0.149061024 NCDN
1 35557473 35567274 + 4917
protein_coding neurochondrin [Source:HGNC Symbol;Acc:HGNC:17597]
- 1362 2516 2259 2843 2402 2909
15.14856909 22.56027486 21.28229596 27.95618256
21.28782996 27.6178298
ENSG00000269896 24.29619519 29.66243651 36.1785414
72.32963199 67.62563093 52.00442253 30.04572437
63.98656182 -1.08802728 0.035292492 0.149061024
AL513477.1 1 2350414 2352820 - 2407
transcribed_processed_pseudogene small nuclear
ribonucleoprotein polypeptide N pseudogene [Source:NCBI
gene;Acc:100129534] - 21 32 37 72 74
53 0.477131311 0.586148754 0.712078855
1.446297457 1.339720575 1.027888045
ENSG00000139233 821.4427898 988.1299161 842.8622347
1177.365676 1089.320974 1080.318287 884.1449802
1115.668312 -0.334933277 0.035317853 0.149061024 LLPH
12 66116555 66130768 - 7993
protein_coding "LLP homolog, long-term synaptic facilitation factor
[Source:HGNC Symbol;Acc:HGNC:28229]" - 710 1066 862
1172 1192 1101 4.857840469 5.880054477
4.995740931 7.089555639 6.498678319 6.430186093
ENSG00000138336 163.1315963 126.0653552 141.7807703
115.5264955 63.97019142 71.62873292 143.6592406
83.70847329 0.779560318 0.035327173 0.149061024 TET1
10 68560656 68694482 + 9288
protein_coding tet methylcytosine dioxygenase 1 [Source:HGNC
Symbol;Acc:HGNC:29484] - 141 136 145 115 70
73 0.830216994 0.64558088 0.723183071
0.598655325 0.328423656 0.366899136
ENSG00000186020 426.9188583 482.0145932 341.2516472
591.6965728 532.7803085 561.2552771 416.7283663
561.9107195 -0.430958407 0.035333848 0.149061024 ZNF529
19 36534774 36605276 - 8173
protein_coding zinc finger protein 529 [Source:HGNC
Symbol;Acc:HGNC:29328] zf-C2H2 369 520 349 589 583
572 2.46910512 2.805148149 1.978091532
3.484456221 3.108462651 3.267085993
ENSG00000007255 160.8176729 380.9769189 204.359869

332.5153915   425.8587029   406.2232251   248.7181536
388.1991065   -0.639891212   0.035334637   0.149061024
TRAPPC6A   19   45162928   45178237   -   933
        protein_coding trafficking protein particle complex 6A
[Source:HGNC Symbol;Acc:HGNC:23069]   -   139   411   209
        331   466   414   8.147565663   19.42200833
10.37688726   17.15329539   21.76521045   20.71403631
ENSG00000164615 657.1542318   788.8354209   600.3682275
937.2714811   1007.987445   750.6298724   682.1192934
898.6295994   -0.397458602   0.035337028   0.149061024   CAMLG
        5   134738501   134752160   +   2221
protein_coding calcium modulating ligand [Source:HGNC
Symbol;Acc:HGNC:1471]   -   568   851   614   933   1103
        765   13.98603111   16.89331809   12.80625863
20.31114009   21.64140994   16.07899545
ENSG00000229051 8.09873173   2.780853423   6.844588913   0
        0   0   5.908058022   0   5.053240353
0.035377642   1   LINC01788   1   70706453
70786468   +   2086   lincRNA long intergenic non-protein
coding RNA 1788 [Source:HGNC Symbol;Acc:HGNC:52577]   -   7
        3   7   0   0   0   0.183517907
0.063407541   0.155448377   0   0   0
ENSG00000176731 439.6454368   490.3571535   429.2535047
351.6023777   330.8172756   330.6696301   453.085365
337.6964278   0.424515157   0.035386997   0.149230627
C8orf59 8   85214076   85220421   -   2514
protein_coding chromosome 8 open reading frame 59 [Source:HGNC
Symbol;Acc:HGNC:32235] -   380   529   439   350   362
        337   8.266335658   9.277358874   8.089127908
6.731378131   6.274828998   6.257641277
ENSG00000280020 38.1797353   34.29719221   26.40055724
67.30674088   62.14247166   73.59116396   32.95916158
67.6801255   -1.039871765   0.035408461   0.149279961
AC116407.3   17   32178946   32180074   -   1129
        TEC   TEC   -   33   37   27   67   68
        75   1.598507619   1.444913139   1.107827848
2.869340427   2.624663118   3.101082765
ENSG00000266242 0   4.634755704   16.62257307   0   0
        0   7.08577626   0   5.320772943   0.035485454
        1   GRAMD4P7   18   14485806   14487501
        +   1232   processed_pseudogene   GRAM domain containing
4 pseudogene 7 [Source:HGNC Symbol;Acc:HGNC:49142]   -   0
        5   17   0   0   0   0   0.178934158
        0.639205745   0   0   0
ENSG00000213190 2129.966445   1630.507057   1877.372959
1588.238169   1554.475651   1353.096201   1879.282154
1498.603341   0.32608933   0.035529105   0.14974729   MLLT11
        1   151057758   151068497   +   4075
protein_coding "MLLT11, transcription factor 7 cofactor [Source:HGNC
Symbol;Acc:HGNC:16997]" -   1841   1759   1920   1581   1701

1379 24.70704966 19.03146341 21.82609652
18.75881815 18.19010887 15.79729357
ENSG00000161677 271.8859938 704.482867 489.8770065
650.9666879 644.2712136 916.4552952 488.7486224
737.2310655 -0.591216484 0.035555554 0.149817463 JOSD2
19 50505998 50511353 - 1514
protein_coding Josephin domain containing 2 [Source:HGNC
Symbol;Acc:HGNC:28853] - 235 760 501 648 705
934 8.488612326 22.13205165 15.32901702
20.69428125 20.29185917 28.79831229
ENSG00000251361 48.59239038 21.31987624 86.04626062
19.08698622 13.70789816 25.51160351 51.98617575
19.43549596 1.420290788 0.035584933 0.149899939
AC012625.1 5 93019663 93068669 - 625
lincRNA novel transcript - 42 23 88
19 15 26 3.675056208 1.622489058
6.522365177 1.469856036 1.045851056 1.941955914
ENSG00000154451 89.08604903 146.4582803 126.1359957
63.28842799 90.47212786 41.21105182 120.5601083
64.99053589 0.891751743 0.035595223 0.149901974 GBP5
1 89258950 89272804 - 8143
protein_coding guanylate binding protein 5 [Source:HGNC
Symbol;Acc:HGNC:19895] - 77 158 129 63 99
42 0.517131512 0.855473597 0.733850731
0.374073834 0.529796833 0.240774723
ENSG00000161956 447.7441685 824.9865154 855.5736141
931.2440118 962.2944509 1065.600054 709.434766
986.3795056 -0.47410827 0.03561194 0.149931073 SENP3
17 7561875 7571969 + 3668 protein_coding SUMO
specific peptidase 3 [Source:HGNC Symbol;Acc:HGNC:17862] -
387 890 875 927 1053 1086 5.770007143
10.69780402 11.05048099 12.21944901 12.51000957
13.82122491
ENSG00000187140 26.61011854 1.853902282 9.777984162 0
1.827719755 1.962431039 12.74733499 1.263383598
3.319223942 0.035657934 1 FOXD3 1
63323041 63325126 + 2086 protein_coding
forkhead box D3 [Source:HGNC Symbol;Acc:HGNC:3804] Fork 23
2 10 0 2 2 0.60298741
0.042271694 0.22206911 0 0.041780563 0.044757078
ENSG00000273669 27.76708022 20.3929251 4.888992081
87.39830532 36.5543951 30.4176811 17.68299913
51.45679384 -1.546297523 0.035658475 0.150044316
AC015819.1 18 75073543 75074205 + 663
lincRNA novel transcript - 24 22 5
87 40 31 1.979668287 1.462995905
0.349348539 6.344639354 2.629087623 2.182700768
ENSG00000246922 45.12150535 61.1787753 51.82331606
129.5905906 62.14247166 110.8773537 52.70786557
100.8701387 -0.933186481 0.035667904 0.150044316 UBAP1L

15 65092770 65115197 - 8536
protein_coding ubiquitin associated protein 1 like [Source:HGNC
Symbol;Acc:HGNC:40028] - 39 66 53 129 68
113 0.249864705 0.340897242 0.287623203
0.730695647 0.34714675 0.617973814
ENSG00000185495 143.4632478 136.2618177 125.1581973
65.29758443 84.07510872 93.21547435 134.9610876
80.8627225 0.738090234 0.035668283 0.150044316
AC138393.1 1 223992743 224010612 + 4349
transcribed_unprocessed_pseudogene septin 7 (SEPT7)
pseudogene - 124 147 128 65 92 95
1.559290347 1.490259452 1.363399146 0.722645272
0.921843117 1.019718347
ENSG00000108691 85.615164 107.5263323 123.2026004
36.16481599 84.07510872 43.17348286 105.4480323
54.47113586 0.951476725 0.035726427 0.150247562 CCL2
17 34255218 34257203 + 1986
protein_coding C-C motif chemokine ligand 2 [Source:HGNC
Symbol;Acc:HGNC:10618] - 74 116 126 36 92
44 2.037732576 2.575210314 2.938960552
0.876444607 2.018678609 1.034235565
ENSG00000136158 874.6630268 748.0495707 760.7271678
860.9235363 1111.253611 1096.017735 794.4799218
1022.731627 -0.365130259 0.035814305 0.150535561 SPRY2
13 80335976 80340951 - 3038
protein_coding sprouty RTK signaling antagonist 2 [Source:HGNC
Symbol;Acc:HGNC:11270] - 756 807 778 857 1216
1117 13.60907911 11.71169417 11.86299325
13.63936868 17.44232616 17.16372073
ENSG00000106868 49.74935206 71.37523785 126.1359957
68.3113191 10.96631853 14.71823279 82.4201952
31.33195681 1.399890441 0.035814603 0.150535561 SUSD1
9 112040785 112175408 - 4183
protein_coding sushi domain containing 1 [Source:HGNC
Symbol;Acc:HGNC:25413] - 43 77 129 68 12
15 0.562179887 0.811590245 1.428579131
0.785999491 0.125012079 0.167397678
ENSG00000102763 871.1921418 739.7070104 840.9066379
640.9209057 604.9752388 669.1889843 817.2685967
638.3617096 0.356092387 0.035880911 0.15077281 VWA8
13 41566837 41961120 - 7633
protein_coding von Willebrand factor A domain containing 8
[Source:HGNC Symbol;Acc:HGNC:29071] - 753 798 860
638 662 682 5.395036988 4.60937017
5.219220494 4.041351571 3.779386651 4.170951579
ENSG00000250519 13.88354011 0.926951141 3.911193665 0
0 0 6.240561638 0 5.128578586
0.035892316 1 AP002784.1 11 94238150
94279206 + 723 lincRNA novel transcript -
12 1 4 0 0 0 0.907690231

0.060981157	0.256285567	0	0	0
ENSG00000179362 27.76708022	11.12341369	25.42275882	0
7.310879019	4.906077597	21.43775091	4.072318872
2.385353275	0.035896781	0.150798046	HMGN2P46
15	45511136	45586290	+	3091
transcribed_processed_pseudogene	high mobility group
nucleosomal binding domain 2 pseudogene 46 [Source:HGNC
Symbol;Acc:HGNC:26817]	-	24	12	26	0	8
5	0.424626359	0.171165487	0.3896519	0
0.112784542	0.075512185
ENSG00000116977 780.9491311	628.4728735	523.1221526
806.6763123	1107.598171	725.1182689	644.1813857
879.7975842	-0.450897326	0.035914087	0.150829298	LGALS8
1	236518000	236552981	+	9710
protein_coding	galectin 8 [Source:HGNC Symbol;Acc:HGNC:6569]	-
675	678	535	803	1212	739	3.801712368
3.078537314	2.552331074	3.998503973	5.439287163
3.552803961
ENSG00000055950 911.6858005	1090.094542	977.7984162
1131.155078	1357.081918	1265.76802	993.1929194
1251.335005	-0.332972187	0.03594848	0.150932275	MRPL43
10	100969458	100987515	-	3573
protein_coding	mitochondrial ribosomal protein L43 [Source:HGNC
Symbol;Acc:HGNC:14517]	-	788	1176	1000	1126	1485
1290	12.06112765	14.51136492	12.96490801
15.23724992	18.11140054	16.85398992
ENSG00000172663 496.3365589	507.9692252	304.0953074
566.5821172	664.3761309	590.6917427	436.1336972
607.2166636	-0.4780811	0.035958651	0.150933526
TMEM134 11	67461710	67469272	-	5892
protein_coding	transmembrane protein 134 [Source:HGNC
Symbol;Acc:HGNC:26142]	-	429	548	311	564	727
602	3.981890076	4.10064127	2.445119598
4.628258232	5.376879088	4.769576171
ENSG00000082641 3569.22677	4032.237463	4762.856085
4874.213533	4958.603695	5308.37596	4121.440106
5047.064396	-0.292103144	0.036006105	0.151072833	NFE2L1
17	48048329	48061487	+	5825
protein_coding	"nuclear factor, erythroid 2 like 1 [Source:HGNC
Symbol;Acc:HGNC:7781]"	TF_bZIP 3085	4350	4871	4852	5426
5410	28.96369406	32.92511384	38.73688156
40.27412178	40.59218764	43.35581667
ENSG00000103044 15.04050178	15.75816939	37.15633981
79.36167954	21.93263706	78.49724156	22.65167033
59.93051939	-1.399597097	0.036011605	0.151072833	HAS3
16	69105564	69118719	+	5289
protein_coding	hyaluronan synthase 3 [Source:HGNC
Symbol;Acc:HGNC:4820]	-	13	17	38	79	24
80	0.134420188	0.141712876	0.332822352
0.722195438	0.197740793	0.706093894

ENSG00000168785 1440.417286 1123.464783 1271.137941
888.0471483 925.7400558 1165.684037 1278.340003
993.1570804 0.363722433 0.036053083 0.151205344 TSPAN5
4 98470367 98658629 - 7430
protein_coding tetraspanin 5 [Source:HGNC Symbol;Acc:HGNC:17753]
- 1245 1212 1300 884 1013 1188
9.163792578 7.191968293 8.105074187 5.752606501
5.94126918 7.464034923
ENSG00000146729 2548.786572 1986.456295 1960.485824
1640.476237 1966.626456 1512.053116 2165.242897
1706.385269 0.34300837 0.036082465 0.151287064
NIPSNAP2 7 55951793 56000181 + 5651
protein_coding nipsnap homolog 2 [Source:HGNC
Symbol;Acc:HGNC:4179] - 2203 2143 2005 1633 2152
1541 21.31983811 16.71979013 16.43582564
13.97211315 16.59493507 12.72985237
ENSG00000165943 413.0353182 476.4528864 323.6512757
487.2204377 785.9194946 467.0585873 404.3798268
580.0661732 -0.520886069 0.036109375 0.151358382 MOAP1
14 93182196 93184928 - 2537
protein_coding modulator of apoptosis 1 [Source:HGNC
Symbol;Acc:HGNC:16658] - 357 514 331 485 860
476 7.695599569 8.932573733 6.043798581
9.24320293 14.77190758 8.758556207
ENSG00000170903 1210.181913 1105.852711 941.6198748
1336.089035 1561.786531 1245.162494 1085.884833
1381.012687 -0.347457837 0.036155931 0.151511986
MSANTD4 11 105995623 106022403 - 5105
protein_coding Myb/SANT DNA binding domain containing 4 with coiled-
coils [Source:HGNC Symbol;Acc:HGNC:29383] - 1046 1193
963 1330 1709 1269 11.2054848 10.3033548
8.738421647 12.59670942 14.58830767 11.60411199
ENSG00000084073 3053.221862 2423.977233 2738.813364
2280.392564 2227.990381 2214.603427 2738.67082
2240.995458 0.289009902 0.036193259 0.151626841
ZMPSTE24 1 40258107 40294184 + 3572
protein_coding zinc metallopeptidase STE24 [Source:HGNC
Symbol;Acc:HGNC:12877] - 2639 2615 2801 2270 2438
2257 40.40384094 32.2770772 36.32487382
30.72667901 29.74273138 29.49620512
ENSG00000234756 5.784808379 12.97731597 10.75578258
9.041203998 49.34843338 44.15469838 9.839302309
34.18144525 -1.790270133 0.036235928 0.151673676
AC024597.1 10 62289521 62304033 + 706
lincRNA uncharacterized LOC105378325 [Source:NCBI
gene;Acc:105378325] - 5 14 11 9 54
45 0.387311165 0.874293587 0.721756062
0.616366498 3.333094727 2.975458166
ENSG00000134278 812.1870964 835.1829779 765.6161599
1109.054357 1075.613076 885.0563986 804.3287447

1023.241277	-0.347318372	0.036240559	0.151673676	SPIRE1
	18	12446512	12658134	-	6532
protein_coding	spire type actin nucleation factor 1 [Source:HGNC
Symbol;Acc:HGNC:30622]	-	702	901	783	1104	1177
	902	5.877405416	6.081526071	5.552876848
8.17192014	7.852155373	6.446238911
ENSG00000130811 3843.426687	5721.142441	5460.026356
6167.105705	5643.998603	7276.694292	5008.198495
6362.599533	-0.345058579	0.036243194	0.151673676	EIF3G
	19	10115017	10119918	-	3486
protein_coding	eukaryotic translation initiation factor 3 subunit G
[Source:HGNC Symbol;Acc:HGNC:3274]	-	3322	6172	5584
	6139	6176	7416	52.11550592	78.06070919
74.20283233	85.14741488	77.20376894	99.30894637
ENSG00000276337 23.13923351	5.561706845	40.08973506
6.027469332	4.569299387	4.906077597	22.93022514
5.167615439	2.149753659	0.036244694	0.151673676
AC105429.1	16	87470370	87474370	-	4001
	sense_intronic	"novel transcript, sense intronic to ZCCHC14"
	-	20	6	41	6	5	5
0.273373339	0.066117535	0.474698393	0.072507664
0.054457795	0.058337456
ENSG00000185485 163.1315963	198.3675441	242.4940072
118.5402302	116.9740643	152.0884055	201.3310492
129.2009	0.641381323	0.036254047	0.151673676	SDHAP1
	3	195959748	195990318	-	3353
transcribed_unprocessed_pseudogene	succinate dehydrogenase
complex flavoprotein subunit A pseudogene 1 [Source:HGNC
Symbol;Acc:HGNC:32455]	-	141	214	248	118	128
	155	2.299748117	2.813935755	3.426262107
1.701569414	1.663546764	2.157963927
ENSG00000126524 2254.918306	2117.156406	1708.213833
1540.018414	1676.932875	1661.197874	2026.762848
1626.049721	0.317490158	0.036291883	0.151746468	SBDS
	7	66987677	66995601	-	1732
protein_coding	"SBDS, ribosome maturation factor [Source:HGNC
Symbol;Acc:HGNC:19440]"	-	1949	2284	1747	1533	1835
	1693	61.54016611	58.14095618	46.72480237
42.79524171	46.16860779	45.63048725
ENSG00000100632 2179.715797	2729.87111	2860.060367
2051.348729	2131.121234	2109.613367	2589.882425
2097.36111	0.304665883	0.036297003	0.151746468	ERH
	14	69380123	69398627	-	1446
protein_coding	"ERH, mRNA splicing and mitosis factor [Source:HGNC
Symbol;Acc:HGNC:3447]"	-	1884	2945	2925	2042	2332
	2150	71.25368315	89.79475382	93.70441059
68.2792373	70.27791216	69.40906639
ENSG00000187514 17571.93393	19109.09777	20954.22006
15603.10894	16621.28345	16325.46381	19211.75059
16183.2854	0.24751383	0.036301225	0.151746468	PTMA

2	231706895	231713541	+	3378
protein_coding	prothymosin alpha [Source:HGNC Symbol;Acc:HGNC:9623]
-	15188	20615	21430	15532	18188	16638
245.8870497	269.0652748	293.8765831	222.3150956
234.6301931	229.9256964
ENSG00000168874 24.29619519	31.51633879	26.40055724
8.036625776	0	10.79337071	27.40436374	6.276665497
2.134138582	0.036376881	0.151985064	ATOH8	2
85751344	85788066	+	6076	protein_coding
atonal bHLH transcription factor 8 [Source:HGNC
Symbol;Acc:HGNC:24126]	bHLH	21	34	27	8	0
11	0.189014988	0.246714747	0.205848855
0.063660997	0	0.084512501
ENSG00000106636 4450.831566	4482.735717	4526.228868
6019.432706	4950.378956	5206.329546	4486.598717
5392.04707	-0.265134616	0.036378187	0.151985064	YKT6
7	44200968	44214294	+	3707
protein_coding	YKT6 v-SNARE homolog [Source:HGNC
Symbol;Acc:HGNC:16959]	-	3847	4836	4629	5992	5417
5306	56.75371736	57.51719049	57.84516318
78.15386046	63.67879657	66.81757279
ENSG00000132953 1324.721119	1069.701617	1096.112025
1046.770507	804.1966921	864.4508727	1163.511587
905.1393574	0.36199264	0.036618282	0.15290855	XPO4
13	20777329	20903048	-	10500
protein_coding	exportin 4 [Source:HGNC Symbol;Acc:HGNC:17796]	-
1145	1154	1121	1042	880	881	5.963632878
4.845632435	4.945597513	4.798214252	3.652178293
3.916811265
ENSG00000102858 1050.521202	1745.448998	1720.925212
2195.003415	1841.427653	1923.182418	1505.631804
1986.537829	-0.399067113	0.036628923	0.15290855	MGRN1
16	4616493 4690974 +	10219	protein_coding
mahogunin ring finger 1 [Source:HGNC Symbol;Acc:HGNC:20254]	-
908	1883	1760	2185	2015	1960	4.859282658
8.124111566	7.978233165	10.33818403	8.592612938
8.953517938
ENSG00000260293 57.84808379	12.05036483	50.84551764
2.009156444	17.36333767	14.71823279	40.24798875
11.36357564	1.817830871	0.036629235	0.15290855
AC106820.4	16	2476558 2482173 +	5616	sense_intronic
"novel transcript, intronic to TBC1D24"	-	50	13
52	2	19	15	0.486897582	0.102058742
0.428922373	0.017218849	0.14742974	0.124683848
ENSG00000285095 8.09873173	19.46597396	4.888992081
21.09614266	72.19493032	18.64309487	10.81789926
37.31138928	-1.781321725	0.036684547	0.153089669
AC025887.2	18	32470288	32745567	+	5012
antisense	novel transcript	-	7	21
5	21	79	19	0.076380358	0.184732025

0.046212706	0.202586009	0.686870124	0.176965487
ENSG00000280401 12.72657843	24.10072966	0.977798416	0
0	2.943646558	12.60170217	0.981215519
3.692142147	0.036687464	1	AC022532.1	10
70434829	70436556	+	1728	TEC	TEC	-
11	26	1	0	0	3	0.348131771
0.663381824	0.026807648	0	0	0.081044501
ENSG00000187535 357.5011578	272.5236354	272.8057581
395.8038195	406.6676454	440.5657682	300.9435171
414.3457444	-0.463039158	0.036692651	0.153089669	IFT140
16	1510427 1612110 -	8168	protein_coding
intraflagellar transport 140 [Source:HGNC Symbol;Acc:HGNC:29077]
-	309	294	279	394	445	449
2.068890298	1.586958462	1.582307658	2.332285484
2.374121165	2.566118146
ENSG00000119431 61.31896881	79.71779811	56.71230814
81.37083598	118.8017841	156.9944831	65.91635835
119.0557011	-0.851812665	0.036716179	0.153146039	HDHD3
9	113373419	113376999	-	2105
protein_coding	haloacid dehalogenase like hydrolase domain containing
3 [Source:HGNC Symbol;Acc:HGNC:28171]	-	53	86	58
81	130	160	1.376950989	1.801276193
1.276375176	1.86051911	2.691224017	3.548247605
ENSG00000223901 39.33669697	36.15109449	50.84551764
11.05036044	25.58807657	9.812155195	42.11110304
15.48353073	1.439266014	0.036726357	0.153146704
AP001469.1	21	46220269	46225364	+	1117
antisense	novel transcript	-	34	39
52	11	28	10	1.6646405	1.539378412
2.15651571	0.476146645	1.092354132	0.417919718
ENSG00000159625 0	0	0.977798416	15.06867333
11.88017841	0	0.325932805	8.982950579	-4.625345544
0.036734499	1	DRC7	16	57694793
57731805	+	5151	protein_coding	dynein regulatory
complex subunit 7 [Source:HGNC Symbol;Acc:HGNC:25289]	-	0
0	1	15	13	0	0	0
0.008993131	0.140799439	0.10997916	0
ENSG00000122729 1907.829803	2030.022998	1848.039007
1557.096244	1619.359703	1592.512788	1928.630603
1589.656245	0.278910892	0.036753846	0.153219539	ACO1
9	32384603	32454769	+	7555
protein_coding	aconitase 1 [Source:HGNC Symbol;Acc:HGNC:117]	-
1649	2190	1890	1550	1772	1623	11.93660712
12.78037521	11.58856848	9.919697897	10.22086958
10.02836395
ENSG00000169375 2131.123407	1615.675838	1909.640307
1603.306842	1510.610377	1414.912779	1885.479851
1509.61 0.32032043	0.036769093	0.153241308	SIN3A	15
75369379	75455842	-	12453	protein_coding
SIN3 transcription regulator family member A [Source:HGNC

Symbol;Acc:HGNC:19353] -   1842   1743   1953   1596   1653
        1442   8.089288983   6.171025727   7.264917904
6.196694923   5.784388622   5.405517879
ENSG00000281183 55.53416043   33.37024107   43.02313031
16.07325155   0.913859877   22.56795695   43.97584394
13.18502279   1.741189024   0.036796355   0.153249442   NPTN-
IT1   15   73567012   73569294   -   2283
sense_intronic NPTN intronic transcript 1 [Source:HGNC
Symbol;Acc:HGNC:45091] -   48   36   44   16   1
        23   1.149820477   0.695233271   0.892789802
0.338856084   0.01908766   0.4702924
ENSG00000253910 2.313923351   12.05036483   18.57816991
81.37083598   13.70789816   25.51160351   10.98081936
40.19677922   -1.860244721   0.036802631   0.153249442
PCDHGB2 5   141360042   141512979   +   4769
protein_coding "protocadherin gamma subfamily B, 2 [Source:HGNC
Symbol;Acc:HGNC:8709]" -   2   13   19   81   15
        26   0.022934928   0.120184923   0.18455624
0.821218857   0.137063726   0.254502505
ENSG00000162813 1520.247642   1336.663545   1199.758657
939.2806376   1137.755547   1124.472985   1352.223281
1067.169723   0.340950991   0.036813509   0.153249442   BPNT1
        1   220057482   220090462   -   3113
protein_coding "3'(2'), 5'-bisphosphate nucleotidase 1 [Source:HGNC
Symbol;Acc:HGNC:1096]" -   1314   1442   1227   935   1245
        1146   23.08399424   20.42302635   18.2586178
14.52224324   17.42805125   17.18507899
ENSG00000174521 15.04050178   13.90426711   29.33395248
72.32963199   22.84649694   58.87293117   19.42624046
51.3496867   -1.399089377   0.036816128   0.153249442   TTC9B
        19   40216058   40218399   -   2342
protein_coding tetratricopeptide repeat domain 9B [Source:HGNC
Symbol;Acc:HGNC:26395] -   13   15   30   72   25
        60   0.303564634   0.282382856   0.59338535
1.486438078   0.465170019   1.195942764
ENSG00000114541 38.1797353   37.07804563   74.31267963
34.15565955   4.569299387   11.77458623   49.85682019
16.83318172   1.572107812   0.036821168   0.153249442   FRMD4B
        3   69169990   69542583   -   9993
protein_coding FERM domain containing 4B [Source:HGNC
Symbol;Acc:HGNC:24886] -   33   40   76   34   5
        12   0.180597929   0.176481043   0.352306098
0.164506948   0.021803826   0.056057199
ENSG00000141985 3082.145904   4320.519267   4708.099374
5376.502644   4578.437986   5380.985909   4036.921515
5111.975513   -0.340300182   0.036883304   0.153466269   SH3GL1
        19   4360370 4400547 -   3685   protein_coding "SH3
domain containing GRB2 like 1, endophilin A2 [Source:HGNC
Symbol;Acc:HGNC:10830]" -   2664   4661   4815   5352   5010
        5484   39.53588284   55.76677998   60.52868726

70.22307276 59.24597233 69.47139021
ENSG00000149256 214.03791 202.0753487 220.9824421
123.5631213 143.4760008 157.9756986 212.3652336
141.6716069 0.583610869 0.036894867 0.153469469 TENM4
11 78652831 79440948 - 16330
protein_coding teneurin transmembrane protein 4 [Source:HGNC
Symbol;Acc:HGNC:29945] - 185 218 226 123 157
161 0.619555557 0.588578328 0.641098425
0.364183398 0.418959522 0.460241448
ENSG00000167315 1541.072952 1356.129519 1294.605103
973.4362971 1291.284007 1027.332649 1397.269191
1097.350984 0.347924695 0.036904152 0.153469469 ACAA2
18 49782167 49813960 - 5072
protein_coding acetyl-CoA acyltransferase 2 [Source:HGNC
Symbol;Acc:HGNC:83] - 1332 1463 1324 969 1413
1047 14.36215775 12.71742073 12.09236356
9.237314894 12.14007905 9.636370127
ENSG00000133250 365.5998895 501.4805672 491.8326033
653.9804225 512.6753912 679.982355 452.97102
615.5460563 -0.44083728 0.036969701 0.153633235 ZNF414
19 8509678 8514164 - 2758 protein_coding zinc
finger protein 414 [Source:HGNC Symbol;Acc:HGNC:20630] - 316
541 503 651 561 693 6.265958779
8.64842376 8.448433287 11.41268796 8.86394795
11.72964879
ENSG00000115840 570.3821061 556.1706845 440.9870857
359.6390035 372.85483 429.7723975 522.5132921
387.422077 0.431203661 0.036972789 0.153633235
SLC25A12 2 171784370 171999859 - 5169
protein_coding solute carrier family 25 member 12
[Source:HGNC Symbol;Acc:HGNC:10982] - 493 600 451
358 408 438 5.21597018 5.117745395
4.041778092 3.348711326 3.439633964 3.955611348
ENSG00000105281 4170.846841 5079.692252 5615.496304
6567.932416 5192.551823 6795.898688 4955.345132
6185.460976 -0.319669792 0.036973682 0.153633235 SLC1A5
19 46774883 46788594 - 3332
protein_coding solute carrier family 1 member 5 [Source:HGNC
Symbol;Acc:HGNC:10943] - 3605 5480 5743 6538 5682
6926 59.16910349 72.51193987 79.8428957
94.87267369 74.31129581 97.03390969
ENSG00000270038 30.08100357 41.71280134 38.13413823
90.41203998 48.4345735 87.32818123 36.64264771
75.39159824 -1.037066859 0.036995896 0.153683768
AL133467.4 14 95644508 95645232 + 725
lincRNA novel transcript - 26 45 39
90 53 89 1.961236893 2.736581995
2.491891084 6.002134447 3.185638276 5.730572824
ENSG00000173674 2759.353597 2683.523553 2434.718056
1947.877173 2451.886051 1984.01778 2625.865069

2127.927001 0.303066742 0.037072833 0.153958465 EIF1AX
X 20124518 20141844 - 4427
protein_coding eukaryotic translation initiation factor 1A X-linked
[Source:HGNC Symbol;Acc:HGNC:3250] - 2385 2895 2490
1939 2683 2022 29.46276991 28.83188281
26.0550722 21.17724708 26.41008193 21.3214956
ENSG00000107140 303.123959 377.2691143 311.9176948
456.0785128 375.5964096 547.5182599 330.770256
459.7310608 -0.473763279 0.037092005 0.153958465 TESK1
9 35605305 35610041 + 3298
protein_coding testis associated actin remodelling kinase 1
[Source:HGNC Symbol;Acc:HGNC:11731] - 262 407 319
454 411 558 4.344555532 5.440987346
4.480665132 6.655894324 5.430624422 7.898226491
ENSG00000164930 2592.751115 1864.098744 2084.666223
1799.199596 1539.853893 1839.779099 2180.505361
1726.277529 0.336635315 0.037092237 0.153958465 FZD6
8 103298433 103332866 + 4065
protein_coding frizzled class receptor 6 [Source:HGNC
Symbol;Acc:HGNC:4044] - 2241 2011 2132 1791 1685
1875 30.14921573 21.81149724 24.29568265
21.30277853 18.06333569 21.53211834
ENSG00000116954 1013.498428 855.575903 733.3488121
951.3355763 1342.46016 1120.548123 867.474381
1138.11462 -0.392680263 0.037114305 0.154008215 RRAGC
1 38838198 38859823 - 5167
protein_coding Ras related GTP binding C [Source:HGNC
Symbol;Acc:HGNC:19902] - 876 923 750 947 1469
1142 9.271721059 7.875845671 6.723962112
8.861612064 12.38916201 10.31748101
ENSG00000148180 3185.115493 2060.612386 2286.092697
1759.016467 1976.678915 2116.481875 2510.606859
1950.725752 0.363488065 0.037124372 0.154008215 GSN
9 121207794 121332843 + 11018
protein_coding gelsolin [Source:HGNC Symbol;Acc:HGNC:4620] -
2753 2223 2338 1751 2163 2157 13.66463879
8.895505912 9.829788976 7.683951138 8.554849017
9.13888922
ENSG00000175416 599.306148 1059.505154 992.4653924
1155.264955 1231.883115 1180.40227 883.7588981
1189.183447 -0.427080402 0.037138509 0.15402375 CLTB
5 176392455 176416569 - 2039
protein_coding clathrin light chain B [Source:HGNC
Symbol;Acc:HGNC:2091] - 518 1143 1015 1150 1348
1203 13.89335864 24.71513361 23.05957359
27.26979236 28.80920435 27.54193426
ENSG00000073536 1235.63507 1464.582803 1515.587545
1887.602479 1716.22885 1633.72384 1405.268472
1745.851723 -0.312535976 0.037148268 0.15402375 NLE1
17 35128753 35142315 - 5463

protein_coding notchless homolog 1 [Source:HGNC
Symbol;Acc:HGNC:19889] -   1068   1580   1550   1879   1878
        1665   10.6914046   12.75145799   13.14325559
16.63017411   14.98038535   14.22751569
ENSG00000214389 156.1898262   129.7731597   118.3136084
179.8195017   222.0679502   212.9237677   134.7588648
204.9370732   -0.606854879   0.037159783   0.15402972
RPS3AP26   7   98385801   98386584   -   784
        processed_pseudogene   ribosomal protein S3a pseudogene 26
[Source:HGNC Symbol;Acc:HGNC:36513]   -   135   140   121
        179   243   217   9.416996707   7.873102961
7.149435681   11.03921482   13.50668616   12.92080901
ENSG00000162889 3327.421779   3369.467397   3324.514615
3839.497965   4278.691946   3839.496328   3340.46793
3985.895413   -0.254903719   0.037183959   0.15408815
MAPKAPK2   1   206684944   206734283   +   4195
        protein_coding mitogen-activated protein kinase-activated
protein kinase 2 [Source:HGNC Symbol;Acc:HGNC:6887]   -   2876
        3635   3400   3822   4682   3913   37.49312409
38.20378638   37.5447665   44.05142219   48.63602152
43.54355857
ENSG00000241360 122.6379376   251.2037592   213.1600547
302.3780448   238.517428   377.767975   195.6672505
306.2211493   -0.64298371   0.037221847   0.154203353   PDXP
        22   37658727   37666934   +   2203
protein_coding pyridoxal phosphatase [Source:HGNC
Symbol;Acc:HGNC:30259] -   106   271   218   301   261
        385   2.631395216   5.423613733   4.583998346
6.606222776   5.162791756   8.158160932
ENSG00000156486 0   0   4.888992081   28.12819022
15.53561792   2.943646558   1.629664027
15.53581823   -3.235128659   0.037249755   1   KCNS2   8
        98427022   98432848   +   5348   protein_coding
        potassium voltage-gated channel modifier subfamily S member 2
[Source:HGNC Symbol;Acc:HGNC:6301]   -   0   0   5
        28   17   3   0   0   0.04330929
0.253144123   0.13852116   0.026186406
ENSG00000226144 16.19746346   4.634755704   12.71137941   0
        2.741579632   0   11.18119952   0.913859877
3.578790058   0.03726506   1   RPS27AP3   20
37049254   37049707   +   454   processed_pseudogene
        ribosomal protein S27a pseudogene 3 [Source:HGNC
Symbol;Acc:HGNC:16538] -   14   5   13   0   3
        0   1.686424471   0.485565821   1.326447163   0
        0.287954586   0
ENSG00000278829 0   0   3.911193665   36.16481599   0
        13.73701727   1.303731222   16.63394442   -3.654863725
        0.037272662   1   AC099811.5   17   42272069
        42275571   -   819   sense_intronic "novel
transcript, sense intronic to STAT5B"   -   0   0   4

36	0	14	0	0	0.226244768
2.125297912	0	0.797976625
ENSG00000277103 0	0	4.888992081	26.11903377
17.36333767	2.943646558	1.629664027
15.47533933	-3.229692284	0.037281144	1	AL445670.1
9	97151894	97152694	-	801
processed_pseudogene	sorting nexin 18 (SNX18) pseudogene	-
0	0	5	26	19	3	0	0
0.289161151	1.569430356	1.033664694	0.174837575
ENSG00000189149 32.39492692	29.66243651	18.57816991
62.28384977	44.77913399	71.62873292	26.87851111
59.56390556	-1.149694243	0.037285794	0.154426425	CRYM-
AS1	16	21300849	21339037	+	2746
transcribed_unitary_pseudogene	CRYM antisense RNA 1 [Source:HGNC
Symbol;Acc:HGNC:34405]	-	28	32	19	62	49
73	0.557637808	0.513787345	0.320520288
1.091672507	0.777596227	1.240990232
ENSG00000233387 0	0	0	18.082408	0.913859877
0.981215519	0	6.659161131	-5.153121089
0.037417531	1	AL121748.1	10	33096257
33116672	-	552	lincRNA novel transcript	-
0	0	0	18	1	1	0	0
0	1.576647636	0.078944071	0.084568175
ENSG00000070476 1018.126275	1116.049174	1182.158285
1521.936006	1400.947192	1231.425477	1105.444578
1384.769558	-0.324629912	0.037431926	0.154989666	ZXDC
3	126437601	126475919	-	7357
protein_coding	ZXD family zinc finger C [Source:HGNC
Symbol;Acc:HGNC:28160]	zf-C2H2 880	1204	1209	1515	1533
1255	6.541489202	7.215387984	7.612512182
9.956646616	9.080295804	7.963225344
ENSG00000116266 2031.624703	1798.285213	1513.631948
1138.187126	1743.644646	1179.421054	1781.180621
1353.750942	0.395300023	0.037520008	0.155312309	STXBP3
1	108746674	108809526	+	5895
protein_coding	syntaxin binding protein 3 [Source:HGNC
Symbol;Acc:HGNC:11446]	-	1756	1940	1548	1133	1908
1202	16.29053754	14.50948101	12.16436947
9.292815547	14.10435267	9.518460107
ENSG00000240625 0	0	2.933395248	11.05036044
6.397019142	16.68066383	0.977798416
11.37601447	-3.51699027	0.037608042	1	RN7SL403P
6	43036196	43036500	-	305
misc_RNA	"RNA, 7SL, cytoplasmic 403, pseudogene [Source:HGNC
Symbol;Acc:HGNC:46419]" -	0	0	3	11	7
17	0	0	0.455642128	1.743789516
1.000130792	2.60192706
ENSG00000105607 403.7796248	456.0599613	420.4533189
256.1674466	348.1806133	327.7259835	426.7643017
310.6913478	0.457848111	0.037640301	0.155768078	GCDH

19	12891026	12914207	+	4136
protein_coding	glutaryl-CoA dehydrogenase [Source:HGNC
Symbol;Acc:HGNC:4189]	-	349	492	430	255	381
334	4.614658949	5.24467439	4.816043282
2.980992386	4.014237312	3.769744989
ENSG00000165175 1436.946401	1771.40363	1562.521869
1973.996206	1991.300673	1895.708384	1590.290633
1953.668421	-0.296440607	0.037659594	0.155802705
MID1IP1 X	38801432	38806537	+	3790
protein_coding	MID1 interacting protein 1 [Source:HGNC
Symbol;Acc:HGNC:20715]	-	1242	1911	1598	1965	2179
1932	17.92161315	22.23081758	19.53169891
25.0682814	25.05397375	23.79654726
ENSG00000162337 490.5517505	414.34716	659.0361325
402.835867	394.787467	325.7635525	521.311681
374.4622955	0.477262099	0.037669052	0.155802705	LRP5
11	68312609	68449275	+	6164
protein_coding	LDL receptor related protein 5 [Source:HGNC
Symbol;Acc:HGNC:6697]	-	424	447	674	401	432
332	3.761819378	3.197266601	5.065236437
3.145451253	3.054075116	2.514325439
ENSG00000179922 190.8986765	190.951935	278.6725486
292.3322626	287.8658614	390.5237767	220.1743867
323.5739669	-0.55459286	0.037688541	0.155841149	ZNF784
19	55620742	55624601	-	2058
protein_coding	zinc finger protein 784 [Source:HGNC
Symbol;Acc:HGNC:33111]	zf-C2H2 165	206	285	291	315
398	4.384633387	4.41322234	6.415078061
6.836736395	6.669968473	9.027837588
ENSG00000187049 313.5366141	275.3044888	320.7178805
285.3002151	143.4760008	164.8442073	303.1863278
197.8734744	0.616446446	0.037706758	0.155849596
TMEM216 11	61391687	61398863	+	2364
protein_coding	transmembrane protein 216 [Source:HGNC
Symbol;Acc:HGNC:25018]	-	271	297	328	284	157
168	6.26926361	5.539147565	6.427303786
5.80860821	2.894081639	3.317476425
ENSG00000269335 373.6986213	479.2337398	431.2091015
459.0922475	615.9415574	678.019924	428.0471542
584.3512429	-0.448329386	0.037710974	0.155849596	IKBKG
X	154541199	154565046	+	3529
protein_coding	inhibitor of nuclear factor kappa B kinase subunit
gamma [Source:HGNC Symbol;Acc:HGNC:5961]	-	323	517
441	457	674	691	5.005478228	6.459112409
5.78881122	6.261317955	8.322749741	9.140552025
ENSG00000169018 2131.123407	1598.990718	1706.258236
2643.045302	2508.545364	1836.835452	1812.12412
2329.475373	-0.362793873	0.037735179	0.155907481	FEM1B
15	68277803	68295865	+	7569
protein_coding	fem-1 homolog B [Source:HGNC Symbol;Acc:HGNC:3649]

- 1842 1725 1745 2631 2745 1872
13.30901251 10.04811396 10.67970808 16.80674301
15.80383334 11.54551673
ENSG00000130340 1955.265232 1986.456295 1569.366458
2012.170179 2794.583505 2193.016686 1837.029328
2333.25679 -0.345238744 0.037769806 0.156008383 SNX9
6 157700387 157945077 + 4637
protein_coding sorting nexin 9 [Source:HGNC Symbol;Acc:HGNC:14973]
- 1690 2143 1605 2003 3058 2235
19.93169907 20.37600475 16.03394526 20.88550929
28.73816162 22.50020461
ENSG00000071553 2577.710613 2780.853423 3571.897614
2301.488707 2345.878305 2523.686316 2976.82055
2390.351109 0.316753121 0.037862176 0.156347674
ATP6AP1 X 154428632 154436516 + 4884
protein_coding ATPase H+ transporting accessory protein 1
[Source:HGNC Symbol;Acc:HGNC:868] - 2228 3000 3653
2291 2567 2572 24.94791433 27.08192665
34.64786454 22.68039691 22.9038669 24.58336587
ENSG00000233538 0 12.97731597 12.71137941 0 0
0.981215519 8.562898461 0.32707184 4.633529385
0.037925694 1 AC017104.3 2 231452195
231453153 + 568 lincRNA novel transcript
- 0 14 13 0 0 1 0
1.086709986 1.060223613 0 0 0.082185973
ENSG00000160213 1428.84767 4071.169411 1457.897438
3657.669306 7780.602996 4839.354942 2319.30484
5425.875748 -1.226089757 0.037942303 0.156636237 CSTB
21 43772511 43776445 - 3935
protein_coding cystatin B [Source:HGNC Symbol;Acc:HGNC:2482] -
1235 4392 1491 3641 8514 4932 17.16393786
49.20979465 17.55235373 44.73806114 94.28606411
58.50922789
ENSG00000230002 42.807582 67.66743328 60.6235018
128.5860124 137.9928415 60.83536221 57.03283903
109.138072 -0.933707979 0.037955121 0.15664685 ALMS1-
IT1 2 73456764 73459482 + 2105
sense_intronic ALMS1 intronic transcript 1 [Source:HGNC
Symbol;Acc:HGNC:41305] - 37 73 62 128 151
62 0.961267671 1.528990257 1.364401051
2.940079581 3.125960204 1.374945947
ENSG00000127804 1384.883126 1288.462086 1409.007518
1728.87912 1772.888162 1517.940409 1360.784243
1673.235897 -0.298404129 0.037976284 0.156691891
METTL16 17 2405562 2511891 - 8934 protein_coding
methyltransferase like 16 [Source:HGNC Symbol;Acc:HGNC:28484] -
1197 1390 1441 1721 1940 1547 7.327282148
6.85966347 7.471718279 9.313997964 9.462684919
8.083331717
ENSG00000171462 50.90631373 41.71280134 91.91305112

120.5493866	148.95916	80.4596726	61.51072206
116.6560731	-0.923142471	0.038038652	0.156906872	DLK2
	6	43450352	43456632	-	2236
protein_coding	delta like non-canonical Notch ligand 2 [Source:HGNC
Symbol;Acc:HGNC:21113]	-	44	45	94	120	163
	82	1.076156889	0.887308563	1.947414997
2.594840474	3.176686833	1.711938224
ENSG00000183426 267.2581471	208.5640067	198.4930785
94.43035287	155.3561792	176.6187935	224.7717441
142.1351085	0.659437309	0.03806537	0.156974726	NPIPA1
	16	14922802	14952060	+	7277
protein_coding	nuclear pore complex interacting protein family member
A1 [Source:HGNC Symbol;Acc:HGNC:7909]	-	231	225	203
	94	170	180	1.736018375	1.3632142
1.292248744	0.624563637	1.018017267	1.154692024
ENSG00000227954 37.02277362	23.17377852	19.55596832	0
	10.96631853	6.868508636	26.58417349	5.944942388
	2.151172047	0.038091007	0.157038088	TARID	6
	133502252	133892802	-	7862	antisense
	TCF21 antisense RNA inducing promoter demethylation
[Source:HGNC Symbol;Acc:HGNC:50506]	-	32	25	20
	0	12	7	0.222593076	0.140197712
0.117841812	0	0.066513041	0.041563397
ENSG00000233901 3.470885027	11.12341369	2.933395248	0
	0	0	5.842564655	0	5.042410329
0.038100017	1	LINC01503	9	129332300
129359538	+	1594	lincRNA long intergenic non-protein
coding RNA 1503 [Source:HGNC Symbol;Acc:HGNC:51184]	-	3
	12	3	0	0	0	0.102926606
0.331915006	0.08718372	0	0	0
ENSG00000129292 2375.24232	2098.617383	2130.622749
1961.941268	1777.457462	1661.197874	2201.494151
1800.198868	0.290126455	0.038188885	0.15739916
PHF20L1 8	132775358	132848807	+	20571
protein_coding	PHD finger protein 20 like 1 [Source:HGNC
Symbol;Acc:HGNC:24280]	-	2053	2264	2179	1953	1945
	1693	5.457933727	4.852381925	4.906866946
4.590373837	4.120242706	3.841913564
ENSG00000196504 4714.618829	3747.663462	3826.125202
3515.019199	3568.622821	2827.863127	4096.135831
3303.835049	0.309857631	0.038211016	0.157424365
PRPF40A 2	152651593	152717997	-	9536
protein_coding	pre-mRNA processing factor 40 homolog A [Source:HGNC
Symbol;Acc:HGNC:16463]	-	4075	4043	3913	3499	3905
	2882	23.36985853	18.69267507	19.00842184
17.74103353	17.84487021	14.10827024
ENSG00000153814 752.0250892	608.0799484	624.8131879
296.3505755	522.7278499	577.935941	661.6394085
465.6714554	0.505846091	0.038215596	0.157424365	JAZF1
	7	27830573	28180743	-	4206

protein_coding	JAZF zinc finger 1 [Source:HGNC Symbol;Acc:HGNC:28917]
	-	650	656	639	295	572	589
8.451597404	6.876517127	7.037753406	3.391204377
5.926323549	6.537204369
ENSG00000138757 5173.932614	4297.345489	4199.644197
5762.260682	5622.979826	5154.325124	4556.9741
5513.188544	-0.275037845	0.038272018	0.157614314	G3BP2
	4	75642782	75724525	-	7183
protein_coding	G3BP stress granule assembly factor 2 [Source:HGNC Symbol;Acc:HGNC:30291]	-	4472	4636	4295	5736	6153
	5253	34.04792434	28.45584711	27.69872366
38.61041705	37.32842331	34.13874646
ENSG00000111335 4.627846703	25.95463194	8.800185745
5.02289111	115.1463446	31.39889662	13.1275548
50.52271076	-1.936934737	0.038321575	0.157748155	OAS2
	12	112978395	113011723	+	6264
protein_coding	2'-5'-oligoadenylate synthetase 2 [Source:HGNC Symbol;Acc:HGNC:8087]	-	4	28	9	5	126
	32	0.034922309	0.19707895	0.06655692
0.038593971	0.876551412	0.238475773
ENSG00000168653 2791.748524	2991.271332	3161.222279
2201.030884	2307.49619	2784.689644	2981.414045
2431.07224	0.294548362	0.038325155	0.157748155	NDUFS5
	1	39026318	39034636	+	548
protein_coding	NADH:ubiquinone oxidoreductase subunit S5 [Source:HGNC Symbol;Acc:HGNC:7712]	-	2413	3227	3233	2191	2525
	2838	240.8083135	259.6285004	273.2924298
193.3138791	200.7887711	241.7563379
ENSG00000168175 3161.97626	3162.757293	2785.747688
4050.459391	3402.300324	3584.380293	3036.82708
3679.046669	-0.27675608	0.038345216	0.157788242
MAPK1IP1L	14	55051631	55070192	+	6988
	protein_coding	mitogen-activated protein kinase 1 interacting protein 1 like [Source:HGNC Symbol;Acc:HGNC:19840]	-	2733
	3412	2849	4032	3723	3653	21.38855516
21.52732583	18.88608799	27.89772851	23.21660634
24.40297706
ENSG00000100941 2600.849847	2940.289019	3117.221351
2534.550854	1985.817514	2480.512833	2886.120072
2333.627067	0.306910265	0.038380889	0.157892531	PNN
	14	39175183	39183218	+	4201
protein_coding	"pinin, desmosome associated protein [Source:HGNC Symbol;Acc:HGNC:9162]"	-	2248	3172	3188	2523	2173
	2528	29.26431333	33.29005059	35.1534608
29.03793879	22.54060884	28.09120855
ENSG00000151090 62.47593049	106.5993812	131.0249878
20.09156444	57.57317228	65.7414398	100.0334332
47.80205884	1.06575136	0.038393931	0.157902014	THRB
	3	24117153	24495756	-	10968
protein_coding	thyroid hormone receptor beta [Source:HGNC

Symbol;Acc:HGNC:11799] THR-like   54   115   134   20
        63   67   0.269253298   0.462279204   0.565952278
        0.088166534   0.250306257   0.285163146
ENSG00000140829 2916.700384   3399.129833   3267.802307
4173.017934   3259.738183   4440.000226   3194.544175
3957.585448   -0.308734176   0.038403852   0.157902014   DHX38
        16   72093562   72112912   +   6361
protein_coding DEAH-box helicase 38 [Source:HGNC
Symbol;Acc:HGNC:17211] -   2521   3667   3342   4154   3567
        4525   21.6741544   25.41671811   24.33792261
31.57492394   24.43634857   33.20773264
ENSG00000105641 0   15.75816939   4.888992081   0   0
        0   6.882387158   0   5.280321391   0.038439751
        1   SLC5A5 19   17871973   17895174   +
        3652   protein_coding solute carrier family 5 member 5
[Source:HGNC Symbol;Acc:HGNC:11040]   -   0   17   5
        0   0   0   0   0.205235324   0.063422257
        0   0   0
ENSG00000136144 1595.450151   1372.81464   1311.227676
1241.658682   1176.137662   965.5160712   1426.497489
1127.770805   0.338586187   0.038440486   0.15801014   RCBTB1
        13   49531946   49585583   -   4534
protein_coding RCC1 and BTB domain containing protein 1 [Source:HGNC
Symbol;Acc:HGNC:18243] -   1379   1481   1341   1236   1287
        984   16.63326333   14.40149244   13.7009196
13.18069078   12.36959923   10.13117036
ENSG00000226210 192.0556382   329.9946061   315.8288884
359.6390035   385.6488683   471.9646649   279.2930442
405.7508455   -0.536524599   0.038459947   0.158047639   WASH8P
        12   14522   32015   -   1817   unprocessed_pseudogene
        "WAS protein family homolog 8, pseudogene [Source:HGNC
Symbol;Acc:HGNC:53913]" -   166   356   323   358   422
        481   4.996292706   8.638314839   8.234743021
9.52641103   10.1208298   12.35765837
ENSG00000163121 0   0   0.977798416   0   13.70789816
        12.75580175   0.325932805   8.821233305   -4.600276392
        0.038479605   1   NEURL3 2   96497643
96508109   -   2497   protein_coding neuralized E3
ubiquitin protein ligase 3 [Source:HGNC Symbol;Acc:HGNC:25162] -
        0   0   1   0   15   13   0   0
        0.018551709   0   0.261776896   0.243036133
ENSG00000221843 85.615164   75.08304241   51.82331606
139.6363729   124.2849433   104.0088451   70.84050749
122.6433871   -0.793839246   0.038506441   0.158196174
C2orf16 2   27537386   27582721   +   16402
protein_coding chromosome 2 open reading frame 16 [Source:HGNC
Symbol;Acc:HGNC:25275] -   74   81   53   139   136
        106   0.246734355   0.217731954   0.149686115
0.409750233   0.361327236   0.301685956
ENSG00000156976 13153.49729   12766.89806   8544.980359

13538.7007	17867.78832	12871.58518	11488.45857
14759.35807	-0.361507414	0.038533486	0.158235962	EIF4A2
	3	186783205	186789900	+	6133
protein_coding	eukaryotic translation initiation factor 4A2
[Source:HGNC Symbol;Acc:HGNC:3284]	-	11369	13773	8739
	13477	19552	13118	101.378069	99.01238931
66.00718783	106.2481753	138.9238536	99.84830521
ENSG00000244306 745.0833192	646.0849452	770.5051519
847.8640194	915.6875972	987.1028126	720.5578054
916.8848097	-0.348038458	0.038536828	0.158235962
DUXAP10 14	19268853	19337730	-	12311
transcribed_processed_pseudogene	double homeobox A pseudogene
10 [Source:NCBI gene;Acc:503639]	-	644	697	788
	844	1002	1006	2.860798364	2.496165663
2.965072671	3.314746585	3.546769686	3.814614763
ENSG00000186468 4003.087398	3828.308212	3674.566448
4294.571899	4296.969144	5429.065469	3835.320686
4673.535504	-0.28521293	0.038569891	0.158329194	RPS23
	5	82273358	82278577	-	4204
protein_coding	ribosomal protein S23 [Source:HGNC
Symbol;Acc:HGNC:10410]	-	3460	4130	3758	4275	4702
	5533	45.00990582	43.31330287	41.40916986
49.16710396	48.73921332	61.43898024
ENSG00000108384 746.2402808	590.4678767	640.4579626
474.1609208	495.3120536	541.6309667	659.0553734
503.7013137	0.386978966	0.038585343	0.158350105	RAD51C
	17	58692573	58735611	+	6068
protein_coding	RAD51 paralog C [Source:HGNC Symbol;Acc:HGNC:9820]
	-	645	637	655	472	542	552
5.813114205	4.628367317	5.000324437	3.760950721
3.892353828	4.246582263
ENSG00000240661 1.156961676	12.97731597	3.911193665	0
	0	0	6.015157104	0	5.086198519
0.038627052	1	AC063952.1	3	120349449
120423826	+	4265	transcribed_unitary_pseudogene	novel
V-set domain-containing pseudogene	-	1	14	4
	0	0	0	0.012822588	0.1447248
0.043445361	0	0	0
ENSG00000261465 0	0	0	4.018312888	17.36333767
	0	0	7.127216853	-5.255088795	0.038644384
	1	AC099518.4	16	19062499	19067691
	-	696	antisense	uncharacterized LOC102723385
[Source:NCBI gene;Acc:102723385]	-	0	0	0
	4	19	0	0	0	0	0.277876595
	1.189605488	0
ENSG00000101849 836.4832915	869.4801701	948.4644637
1192.43435	1082.923955	1045.975744	884.8093084
1107.111349	-0.32300972	0.038686629	0.158703748	TBL1X
	X	9463295 9741037 +	9092	protein_coding
transducin beta like 1 X-linked [Source:HGNC Symbol;Acc:HGNC:11585]

- 723 938 970 1187 1185 1066
4.348841535 4.548596044 4.942136804 6.31237089
5.679596999 5.473231446
ENSG00000116906 1767.83744 1646.265226 1555.67728
1205.493866 1338.80472 1471.823279 1656.593316
1338.707289 0.307101003 0.038692279 0.158703748 GNPAT
1 231241207 231277973 + 3261
protein_coding glyceronephosphate O-acyltransferase [Source:HGNC
Symbol;Acc:HGNC:4416] - 1528 1776 1591 1200 1465
1500 25.62520027 24.01187758 22.6006972
17.79228243 19.57696767 21.47269207
ENSG00000280913 0 0 0 26.11903377 0 0
0 8.706344591 -5.540382146 0.038696119 1
CTSLP2 10 46753605 46759740 + 1476
transcribed_unprocessed_pseudogene cathepsin L pseudogene
2 [Source:NCBI gene;Acc:1517] - 0 0 0 26
0 0 0 0 0 0.851703059 0
0
ENSG00000263017 0 0 0 26.11903377 0 0
0 8.706344591 -5.540382146 0.038696119 1
AC036164.1 17 959034 959366 + 333
processed_pseudogene chromosome 19 open reading frame 70 (C19orf70)
pseudogene - 0 0 0 26 0 0
0 0 0 3.775116261 0 0
ENSG00000271524 6.941770054 0 13.68917783 0 0
0 6.876982627 0 5.27368853 0.038715404
1 BNIP3P17 19 20221869 20222463
+ 595 processed_pseudogene BCL2 interacting
protein 3 pseudogene 17 [Source:HGNC Symbol;Acc:HGNC:49697] -
6 0 14 0 0 0 0.551479023
0 1.089967443 0 0 0
ENSG00000272419 231.3923351 164.9973031 190.6706912
128.5860124 74.93650995 155.0320521 195.6867765
119.5181915 0.711379112 0.038741256 0.158862011
LINC01145 1 145164099 145216058 - 6056
transcribed_unprocessed_pseudogene long intergenic non-
protein coding RNA 1145 [Source:HGNC Symbol;Acc:HGNC:49462] -
200 178 195 128 82 158 1.806087729
1.295889866 1.491595968 1.021939815 0.590046969
1.217915777
ENSG00000188785 522.9466774 494.0649581 404.8085443
650.9666879 570.2485635 652.5083204 473.9400599
624.5745239 -0.398577123 0.038762331 0.15889224 ZNF548
19 57389850 57402992 + 6161
protein_coding zinc finger protein 548 [Source:HGNC
Symbol;Acc:HGNC:26561] zf-C2H2 452 533 414 648 624
665 4.012194135 3.814257055 3.112802654
5.085398769 4.413589915 5.038676457
ENSG00000161204 1489.009677 1362.618177 1309.272079
1796.185861 1479.539142 1915.332694 1386.966644

1730.352566	-0.319241263	0.038769416	0.15889224	ABCF3
	3	184186023	184194012	+	5207
protein_coding	ATP binding cassette subfamily F member 3 [Source:HGNC
Symbol;Acc:HGNC:72]	-	1287	1470	1339	1788	1619
	1952	13.51716708	12.44697207	11.91229542
16.602793	13.54933151	17.50000898
ENSG00000219992 4.627846703	5.561706845	6.844588913	0
	0	0	5.678047487	0	4.99911281
0.038782119	1	AL391422.1	6	3754099 3754840 +
	436	processed_pseudogene	FUN14 domain containing 1
(FUNDC1) pseudogene	-	4	6	7	0	0
	0	0.501727857	0.60673454	0.743727785	0
	0	0
ENSG00000257403 0	1.853902282	0	0	31.07123583
	4.906077597	0.617967427	11.99243781	-4.226191284
	0.038792703	1	AC008740.1	16	21347139
	21352138	+	255	unprocessed_pseudogene	BTG3
associated nuclear protein (BNAP) pseudogene	-	0	2
	0	0	34	5	0	0.345799032	0
	0	5.810283647	0.915326129
ENSG00000173559 569.2251445	633.1076292	429.2535047
771.5160745	647.9266531	755.53595	543.8620928
724.9928925	-0.414522688	0.03879813	0.158967305	NABP1
	2	191678068	191696659	+	13806
protein_coding	nucleic acid binding protein 1 [Source:HGNC
Symbol;Acc:HGNC:26232]	-	492	683	439	768	709
	770	1.948910729	2.181156324	1.472987655
2.689642555	2.23788087	2.603567801
ENSG00000184787 4535.289769	4308.468903	3850.570163
5588.468649	4448.669883	5436.915193	4231.442945
5158.017909	-0.285689236	0.03883569	0.159078561	UBE2G2
	21	44768580	44802019	-	7388
protein_coding	ubiquitin conjugating enzyme E2 G2 [Source:HGNC
Symbol;Acc:HGNC:12483]	-	3920	4648	3938	5563	4868
	5541	29.01709242	27.73787525	24.69171644
36.40687391	28.71324526	35.01122442
ENSG00000223949 4.627846703	11.12341369	0.977798416
13.05951689	46.60685375	15.69944831	5.576352936
25.12193965	-2.16527462	0.038894903	0.159278431	ROR1-
AS1	1	64094442	64171297	-	3544
antisense	ROR1 antisense RNA 1 [Source:HGNC
Symbol;Acc:HGNC:40508]	-	4	12	1	13	51
	16	0.061724985	0.149286828	0.013070998
0.17735803	0.627097487	0.210752291
ENSG00000100077 285.7695339	234.5186386	303.117509
157.7187809	180.0303959	223.7171384	274.4685605
187.1554384	0.551839097	0.03892112	0.159343108	GRK3
	22	25564849	25729294	+	9303
protein_coding	G protein-coupled receptor kinase 3 [Source:HGNC
Symbol;Acc:HGNC:290]	-	247	253	310	157	197

228 1.452006783 1.19903389 1.543622601
0.815976869 0.922787712 1.144083868
ENSG00000143727 1833.784256 1670.365956 1290.693909
1166.315316 1324.182962 1283.429899 1598.281374
1257.976059 0.344956788 0.038938815 0.159372868 ACP1
2 264140 278283 + 4160 protein_coding acid
phosphatase 1 [Source:HGNC Symbol;Acc:HGNC:122] - 1585 1802
1320 1161 1449 1308 20.83678203 19.09833091
14.69883979 13.49398135 15.17866768 14.67778254
ENSG00000254944 10.41265508 3.707804563 1.955596832
39.17855066 23.76035681 7.849724156 5.358685492
23.59621054 -2.157446961 0.038960998 0.15942098
ATP5PBP5 11 122960327 122961086 + 760
processed_pseudogene ATP synthase peripheral stalk-membrane
subunit b pseudogene 5 [Source:HGNC Symbol;Acc:HGNC:39746] -
9 4 2 39 26 8 0.647625037
0.23204935 0.121904253 2.48114549 1.490796462
0.491385606
ENSG00000184304 159.6607112 239.1533943 253.2497898
155.7096244 147.1314403 119.7082934 217.3546318
140.849786 0.62763 0.03899867 0.159532423 PRKD1 14
29576479 30191898 - 5187 protein_coding
protein kinase D1 [Source:HGNC Symbol;Acc:HGNC:9407] -
138 258 259 155 161 122 1.454981767
2.192993861 2.313055066 1.444829721 1.352596396
1.097967837
ENSG00000254978 2.313923351 7.415609127 10.75578258
17.07782977 26.50193645 33.36132766 6.828438352
25.64703129 -1.896887272 0.039032639 0.159619178
ALG1L9P 11 71800541 71804640 - 549
transcribed_unprocessed_pseudogene "asparagine-linked
glycosylation 1-like 9, pseudogene [Source:HGNC
Symbol;Acc:HGNC:44378]" - 2 8 11 17 29
34 0.199228912 0.642468147 0.92815989
1.497193019 2.30188833 2.891030067
ENSG00000138678 59.00504546 38.93194792 32.26734773
132.6043253 60.31475191 71.62873292 43.40144704
88.18260338 -1.0258638 0.039042284 0.159619178 GPAT3
4 83535914 83605875 + 3468
protein_coding glycerol-3-phosphate acyltransferase 3 [Source:HGNC
Symbol;Acc:HGNC:28157] - 51 42 33 132 66
73 0.804240242 0.533954388 0.440795657
1.840331496 0.829322493 0.98262952
ENSG00000270021 3.470885027 0.926951141 3.911193665
24.10987733 8.224738897 14.71823279 2.769676611
15.68428301 -2.511425703 0.039053191 1 AC026691.1
5 135399280 135401296 + 2017
antisense "novel transcript, antisense to H2AFY" - 3
1 4 24 9 15 0.081341105
0.021858888 0.091866368 0.575316143 0.194444297

0.347161372
ENSG00000167670 1760.89567 2084.713116 2353.560788
1714.815025 1368.962096 1810.342633 2066.389858
1631.373252 0.341522363 0.039057484 0.159619178 CHAF1A
19 4402662 4445018 + 4299 protein_coding
chromatin assembly factor 1 subunit A [Source:HGNC
Symbol;Acc:HGNC:1910] - 1522 2249 2407 1707 1498
1845 19.36163016 23.06513327 25.93648394
19.19849975 15.18458636 20.03433637
ENSG00000114391 9918.632446 10402.2457 9954.965675
9815.733807 12785.81354 14990.02949 10091.94794
12530.52561 -0.312243991 0.039061644 0.159619178 RPL24
3 101681091 101686782 - 2682
protein_coding ribosomal protein L24 [Source:HGNC
Symbol;Acc:HGNC:10325] - 8573 11222 10181 9771 13991
15277 174.8110023 184.4783684 175.8466583
176.1495168 227.3257229 265.904288
ENSG00000128203 102.9695891 112.161088 124.1803989
210.9614266 176.3749563 145.2198969 113.103692
177.5187599 -0.649241777 0.03911968 0.159813616 ASPHD2
22 26429273 26445015 + 3357
protein_coding aspartate beta-hydroxylase domain containing 2
[Source:HGNC Symbol;Acc:HGNC:30437] - 89 121 127
210 193 148 1.449884403 1.589161324
1.752487123 3.024608512 2.505327846 2.058052314
ENSG00000155506 5510.608461 6245.796787 6945.30215
8970.883523 6659.296927 7444.482146 6233.902466
7691.554199 -0.302984293 0.039151262 0.159875269 LARP1
5 154712902 154817607 + 8492
protein_coding La ribonucleoprotein domain family member 1
[Source:HGNC Symbol;Acc:HGNC:29531] - 4763 6738 7103
8930 7287 7587 30.67363948 34.98283318
38.74666117 50.84434886 37.39360893 41.70672942
ENSG00000074964 444.2732835 482.0145932 647.3025515
356.6252688 387.476588 411.1293027 524.5301427
385.0770532 0.446432314 0.039155688 0.159875269
ARHGEF10L 1 17539835 17697874 + 7817
protein_coding Rho guanine nucleotide exchange factor 10 like
[Source:HGNC Symbol;Acc:HGNC:25540] - 384 520 662
355 424 419 2.686493692 2.932899555
3.923018293 2.195783198 2.36365639 2.502188057
ENSG00000068784 466.2555553 438.4478896 463.4764493
345.5749084 362.8023713 318.8950438 456.0599647
342.4241078 0.41303632 0.03921661 0.160081259 SRBD1
2 45388680 45612165 - 5045
protein_coding S1 RNA binding domain 1 [Source:HGNC
Symbol;Acc:HGNC:25521] - 403 473 474 344 397
325 4.368562851 4.133652155 4.352308054
3.296844689 3.429161459 3.007240948
ENSG00000238078 16.19746346 27.80853423 8.800185745

40.18312888	41.12369448	55.92928461	17.60206114
45.74536932	-1.374187447	0.039268966	0.160252186
LINC01352	1	220829255	220832429	+	962
	lincRNA long intergenic non-protein coding RNA 1352
[Source:HGNC Symbol;Acc:HGNC:50578]	-	14	30	9
	40	45	57	0.795880156	1.374928584
0.433381026	2.010416944	2.038431113	2.765959517
ENSG00000136891 2103.356326	1831.655454	1451.05285
2245.232326	2511.286943	2059.571375	1795.354877
2272.030215	-0.340195754	0.039284505	0.160272813	TEX10
	9	100302077	100352939	-	7261
protein_coding	testis expressed 10 [Source:HGNC
Symbol;Acc:HGNC:25988]	-	1818	1976	1484	2235	2748
	2099	13.69279653	11.99843109	9.467600414
14.88271987	16.49221126	13.49466282
ENSG00000150625 41.65062033	63.95962872	38.13413823
5.02289111	29.24351608	17.66187935	47.91479576
17.30942885	1.465503744	0.039339803	0.160455595	GPM6A
	4	175632934	176002664	-	7040
protein_coding	glycoprotein M6A [Source:HGNC Symbol;Acc:HGNC:4460]
	-	36	69	39	5	32	18
0.279656266	0.432125992	0.256622306	0.034339863
0.198077852	0.119356447
ENSG00000103018 3539.145766	3608.620791	3875.992922
3170.448869	3210.389749	2806.276386	3674.586493
3062.371668	0.262963222	0.039429859	0.16078001	CYB5B
	16	69424525	69466266	+	6903
protein_coding	cytochrome b5 type B [Source:HGNC
Symbol;Acc:HGNC:24374]	-	3059	3893	3964	3156	3513
	2860	24.23462569	24.86454339	26.60101623
22.10549975	22.17679971	19.34078938
ENSG00000134262 998.4579261	786.9815186	776.3719424
1185.402302	1059.163598	1022.426571	853.9371291
1088.99749	-0.351626562	0.039454625	0.160838093	AP4B1
	1	113894748	113905201	-	5369
protein_coding	adaptor related protein complex 4 subunit beta 1
[Source:HGNC Symbol;Acc:HGNC:572]	-	863	849	794
	1180	1159	1042	8.79047017	6.971853365
6.850614891	10.62648956	9.40694554	9.059836305
ENSG00000188283 230.2353735	268.8158308	191.6484896
289.3185279	381.9934288	316.9326128	230.2332313
329.4148565	-0.516864198	0.039469381	0.160855351	ZNF383
	19	37217926	37248738	+	8417
protein_coding	zinc finger protein 383 [Source:HGNC
Symbol;Acc:HGNC:18609]	zf-C2H2 199	290	196	288	418
	323	1.292975995	1.519058953	1.078701295
1.654384213	2.164101133	1.791394477
ENSG00000147100 666.4099252	777.7120072	447.8316746
683.113191	965.9498904	963.5536401	630.6512023
870.8722405	-0.465768664	0.039491544	0.160902781

SLC16A2 X	74421461	74533917	+	4324
protein_coding	solute carrier family 16 member 2 [Source:HGNC
Symbol;Acc:HGNC:10923]	-	576	839	458	680	1057
	982	7.28503279	8.554807343	4.906618009
7.603690725	10.65241064	10.60161035
ENSG00000279901 8.09873173	0.926951141	8.800185745	0
	0	0	5.941956205	0	5.061292669
0.039521185	1	AC092117.2	16	2683106 2684764 +
	1659	TEC	tec	-	7	1	9	0
	0	0	0.230752474	0.026575875	0.251303524
	0	0	0
ENSG00000100938 940.6098424	1061.359056	912.2859223
1068.871228	1286.714707	1314.828796	971.4182736
1223.471577	-0.33267994	0.039523581	0.160990405	GMPR2
	14	24232422	24239242	+	3640
protein_coding	guanosine monophosphate reductase 2 [Source:HGNC
Symbol;Acc:HGNC:4377]	-	813	1145	933	1064	1408
	1340	12.21473009	13.86877368	11.87360825
14.13323111	16.8562075	17.1849966
ENSG00000116729 2020.055086	1904.884594	2442.540444
1749.975263	1474.055982	1862.347056	2122.493375
1695.459434	0.324277934	0.039548503	0.161013479	WLS
	1	68098473	68233120	-	4469
protein_coding	wntless Wnt ligand secretion mediator [Source:HGNC
Symbol;Acc:HGNC:30238]	-	1746	2055	2498	1742	1613
	1898	21.36626436	20.27381268	25.89312901
18.84686035	15.72832992	19.82585334
ENSG00000181788 787.8909012	946.4171148	851.6624205
1246.681574	1040.8864	999.8586143	861.9901455
1095.808863	-0.345555313	0.039551997	0.161013479	SIAH2
	3	150741127	150763477	-	2617
protein_coding	siah E3 ubiquitin protein ligase 2 [Source:HGNC
Symbol;Acc:HGNC:10858]	-	681	1021	871	1241	1139
	1019	14.23108793	17.20109037	15.41760405
22.92816378	18.96612459	18.17676101
ENSG00000040341 1173.159139	985.3490627	1115.667993
729.3237892	965.9498904	866.4133037	1091.392065
853.8956611	0.353331873	0.039571307	0.161013479	STAU2
	8	73420369	73747708	-	8998
protein_coding	staufen double-stranded RNA binding protein 2
[Source:HGNC Symbol;Acc:HGNC:11371]	-	1014	1063	1141
	726	1057	883	6.16292211	5.208602723
5.874110493	3.901142805	5.119029075	4.58100484
ENSG00000105499 54.37719876	101.9646255	79.20167171
150.6867333	200.1353132	80.4596726	78.51449865
143.760573	-0.870074206	0.039584932	0.161013479
PLA2G4C 19	48047843	48110817	-	6613
protein_coding	phospholipase A2 group IVC [Source:HGNC
Symbol;Acc:HGNC:9037]	-	47	110	81	150	219
	82	0.388681659	0.73337841	0.567399504

1.096715428 1.443125796 0.578843773
ENSG00000245694 646.7415767 558.0245868 533.8779352
383.7488808 487.0873147 439.5845527 579.5480329
436.8069161 0.406851073 0.039593876 0.161013479 CRNDE
16 54845189 54929189 - 5846
lincRNA colorectal neoplasia differentially expressed [Source:HGNC Symbol;Acc:HGNC:37078] - 559 602 546 382 533
448 5.229349994 4.540165019 4.32649581
3.159408399 3.973077126 3.577381352
ENSG00000100344 264.9442237 246.5690035 349.0740346
259.1811813 170.8917971 120.6895089 286.8624206
183.5874958 0.64456715 0.039600404 0.161013479 PNPLA3
22 43923739 43964488 + 3232
protein_coding patatin like phospholipase domain containing 3 [Source:HGNC Symbol;Acc:HGNC:18590] - 229 266 357
258 187 123 3.874885224 3.628642998
5.116810341 3.859664633 2.521325128 1.776559655
ENSG00000080854 107.5974358 53.76316617 86.04626062
188.8607057 96.86914701 152.0884055 82.46895421
145.9394194 -0.826127412 0.039602975 0.161013479 IGSF9B
11 133908564 133956985 - 5753
protein_coding immunoglobulin superfamily member 9B [Source:HGNC Symbol;Acc:HGNC:32326] - 93 58 88 188 106
155 0.884063148 0.444495714 0.708583041
1.580027667 0.802915957 1.257718242
ENSG00000188906 65.94681552 64.88657986 29.33395248
87.39830532 106.0077458 109.8961382 53.38911595
101.1007298 -0.92323155 0.039618744 0.161034763 LRRK2
12 40196744 40369285 + 13491
protein_coding leucine rich repeat kinase 2 [Source:HGNC Symbol;Acc:HGNC:18618] - 57 70 30 87 116
112 0.23106035 0.228764092 0.103010043
0.311800155 0.374690295 0.387543017
ENSG00000166226 8385.658226 8897.804001 8360.176458
6836.154801 7071.447731 7774.170561 8547.879562
7227.257698 0.242150323 0.039648603 0.161106026 CCT2
12 69585334 69601570 + 4322
protein_coding chaperonin containing TCP1 subunit 2 [Source:HGNC Symbol;Acc:HGNC:1615] - 7248 9599 8550 6805 7738
7923 91.71241612 97.9208528 91.63973148
76.12802859 78.01939182 85.5757924
ENSG00000116711 397.9948164 434.7400851 431.2091015
249.1353991 365.543951 300.251949 421.3146677
304.9770997 0.465809149 0.039663781 0.161106026
PLA2G4A 1 186828953 186988981 + 2875
protein_coding phospholipase A2 group IVA [Source:HGNC Symbol;Acc:HGNC:9035] - 344 469 441 248 400
306 6.543578342 7.192319171 7.105639928
4.170758545 6.062904675 4.968549413
ENSG00000114698 411.8783566 239.1533943 315.8288884

191.8744404	199.2214533	260.0221127	322.2868798
217.0393354	0.568778994	0.039667893	0.161106026	PLSCR4
	3	146192339	146251179	-	4227
protein_coding	phospholipid scramblase 4 [Source:HGNC
Symbol;Acc:HGNC:16497]	-	356	258	323	191	218
	265	4.605878346	2.691047825	3.53975114
2.184752957	2.247412766	2.926575024
ENSG00000164331 220.9796801	231.7377852	179.9149086
277.2635893	380.165709	261.9845437	210.8774579
306.4712807	-0.540093945	0.039678956	0.161108154	ANKRA2
	5	73552335	73565686	-	2659
protein_coding	ankyrin repeat family A member 2 [Source:HGNC
Symbol;Acc:HGNC:13208]	-	191	250	184	276	416
	267	3.928346092	4.145296783	3.205545469
5.018708382	6.817632561	4.68747495
ENSG00000196507 334.3619243	414.34716	468.3654413
213.9751613	277.8134027	355.200018	405.6915085
282.3295274	0.523668536	0.039742307	0.161322528	TCEAL3
	X	103607451	103629690	+	2305
protein_coding	transcription elongation factor A like 3 [Source:HGNC
Symbol;Acc:HGNC:28247]	-	289	447	479	213	304
	362	6.856802268	8.550087345	9.626469507
4.467966315	5.747265386	7.33134533
ENSG00000284770 299.653074	318.8711925	277.6947502
243.1079297	181.8581156	205.0740436	298.7396722
210.013363	0.50916613	0.039791633	0.161479875	TBCE
	1	235367360	235452443	+	12444
protein_coding	tubulin folding cofactor E [Source:HGNC
Symbol;Acc:HGNC:11582]	-	259	344	284	242	199
	209	1.138241653	1.218799867	1.057208858
0.940278661	0.696869844	0.784029348
ENSG00000154723 3607.406505	2959.754993	2871.793948
2451.170862	2440.005873	2801.370308	3146.318482
2564.182348	0.294892155	0.039825321	0.161545105	ATP5PF
	21	25716503	25735673	-	2043
protein_coding	ATP synthase peripheral stalk subunit F6 [Source:HGNC
Symbol;Acc:HGNC:847]	-	3118	3193	2937	2440	2670
	2855	83.46462702	68.90718523	66.59444988
57.74610234	56.95101813	65.23546791
ENSG00000112695 1797.918444	1578.597793	1623.145371
1170.333629	1464.917383	1389.401176	1666.553869
1341.550729	0.312477757	0.039837159	0.161545105	COX7A2
	6	75237675	75250323	-	1922
protein_coding	cytochrome c oxidase subunit 7A2 [Source:HGNC
Symbol;Acc:HGNC:2288]	-	1554	1703	1660	1165	1603
	1416	44.2173126	39.0656651	40.00895061
29.30716157	36.3445032	34.39187914
ENSG00000011590 0	13.90426711	9.777984162	44.20144177
	18.27719755	33.36132766	7.894083758	31.94665566
	-1.998227213	0.039839409	0.161545105	ZBTB32	19

35704527 35717038 + 2770 protein_coding
zinc finger and BTB domain containing 32 [Source:HGNC
Symbol;Acc:HGNC:16763] ZBTB 0 15 10 44 20
34 0 0.238751137 0.167233272 0.768022819
0.314636299 0.572987548
ENSG00000254538 5.784808379 8.342560268 2.933395248 0
0 0 5.686921298 0 5.001073413
0.03984318 1 AC027018.1 8 73670441
73732897 + 707 lincRNA novel transcript -
5 9 3 0 0 0 0.386763341
0.561250904 0.196564143 0 0 0
ENSG00000235522 5.784808379 8.342560268 2.933395248 0
0 0 5.686921298 0 5.001073413
0.03984318 1 AC010978.1 2 105846534
105857177 - 2166 antisense novel transcript
- 5 9 3 0 0 0
0.126242697 0.183196856 0.064160133 0 0 0
ENSG00000142552 2148.477832 2622.344777 3669.677456
1852.442241 1859.704851 2638.488532 2813.500022
2116.878541 0.410699784 0.039856512 0.161571601 RCN3
19 49527618 49546962 + 2093
protein_coding reticulocalbin 3 [Source:HGNC Symbol;Acc:HGNC:21145]
- 1857 2829 3753 1844 2035 2689
48.52185416 59.59333318 83.06379934 42.59836249
42.36954332 59.97463447
ENSG00000162723 100.6556658 262.3271729 44.97872714
247.1262426 624.1662963 381.6928371 135.9871886
417.661792 -1.618239046 0.039875752 0.161606741 SLAMF9
1 159951492 159954254 - 1156
protein_coding SLAM family member 9 [Source:HGNC
Symbol;Acc:HGNC:18430] - 87 283 46 246 683
389 4.115817707 10.79350655 1.843327293
10.28912609 25.74669376 15.70861165
ENSG00000003249 92.55693406 152.0199871 176.0037149
170.7782977 243.0867274 257.0784661 140.1935454
223.6478304 -0.671310875 0.039922589 0.161724331 DBNDD1
16 90004865 90020128 - 4791
protein_coding dysbindin domain containing 1 [Source:HGNC
Symbol;Acc:HGNC:28455] - 80 164 180 170 266
262 0.913184495 1.509216815 1.74039886
1.715631324 2.419435583 2.552825658
ENSG00000159314 39.33669697 63.95962872 46.93432398
135.61806 58.48703215 99.10276747 50.07688322
97.7359532 -0.960498962 0.03993531 0.161724331
ARHGAP27 17 45393902 45434421 - 10426
protein_coding Rho GTPase activating protein 27 [Source:HGNC
Symbol;Acc:HGNC:31813] - 34 69 48 135 64
101 0.178342935 0.29178659 0.213268136
0.626061885 0.267498192 0.45221992
ENSG00000204922 224.4505651 332.7754596 260.0943787

284.2956368	364.6300911	581.860803	272.4401345
410.262177	-0.589344915	0.039949126	0.161724331	UQCC3
	11	62670273	62673687	+	2398
protein_coding	ubiquinol-cytochrome c reductase complex assembly
factor 3 [Source:HGNC Symbol;Acc:HGNC:34399]	-	194	359
	266	283	399	593	4.424327468	6.60053636
	5.138482877	5.706088107	7.250739706	11.54387327
ENSG00000227214 15.04050178	11.12341369	1.955596832
59.2701151	28.3296562	13.73701727	9.373170769
33.77892952	-1.85674064	0.039951756	0.161724331	HCG15
	6	28986203	28987484	+	1032
antisense	HLA complex group 15 [Source:HGNC
Symbol;Acc:HGNC:18361]	-	13	12	2	59	31
	14	0.688903463	0.51266717	0.08977445
2.764225893	1.309002857	0.633277961
ENSG00000143147 1277.28569	1171.666242	1373.806775
1379.285899	1658.655678	1729.882961	1274.252902
1589.274846	-0.318920822	0.039957663	0.161724331	GPR161
	1	168079543	168137667	-	11224
protein_coding	G protein-coupled receptor 161 [Source:HGNC
Symbol;Acc:HGNC:23694]	-	1104	1264	1405	1373	1815
	1763	5.379180633	4.965161439	5.798706425
5.914582524	7.046728987	7.332476675
ENSG00000160323 13.88354011	31.51633879	4.888992081
56.25638043	67.62563093	23.54917247	16.76295699
49.14372794	-1.54723674	0.039984217	0.161741913
ADAMTS13	9	133414358	133459402	+	5708
	protein_coding	ADAM metallopeptidase with thrombospondin type
1 motif 13 [Source:HGNC Symbol;Acc:HGNC:1366]	-	12	34
	5	56	74	24	0.114971976	0.262620673
	0.0405778	0.474356962	0.564945239	0.196278763
ENSG00000278730 386.4251997	303.1130231	498.6771922
268.2223853	253.139186	312.0265352	396.071805
277.7960355	0.511723961	0.039997616	0.161741913
AC005332.6	17	68126666	68129586	+	2921
	lincRNA novel transcript	-	334	327	510
	267	277	318	6.253305159	4.935715899
8.08799874	4.419579199	4.132442409	5.082081189
ENSG00000258077 20.82531016	15.75816939	5.866790497
67.30674088	41.12369448	17.66187935	14.15009002
42.03077157	-1.576081968	0.040004068	0.161741913
AC078923.1	12	75563202	75984015	-	1385
	lincRNA novel transcript	-	18	17	6
	67	45	18	0.710750943	0.541169243
0.200679926	2.338978586	1.415863344	0.606692697
ENSG00000204685 76.3594706	51.90926389	71.37928438
125.5722778	127.9403828	93.21547435	66.54933962
115.576045	-0.79896319	0.040004329	0.161741913
STARD7-AS1	2	96208416	96242621	+	2349
	processed_transcript	STARD7 antisense RNA 1 [Source:HGNC

Symbol;Acc:HGNC:40827] - 66 56 73 125 140
95 1.536581611 1.051087735 1.439601529
2.572931433 2.59718937 1.887933203
ENSG00000269845 21.98227184 0 9.777984162 0
0.913859877 0.981215519 10.586752 0.631691799
4.050135404 0.040024519 1 AC092364.2 19
21750747 21753438 + 2692
transcribed_processed_pseudogene cyclin Y like 1 pseudogene
[Source:NCBI gene;Acc:641367] - 19 0 10 0
1 1 0.385987516 0 0.172078812 0
0.01618764 0.017340874
ENSG00000256128 28.92404189 39.85889906 68.44588913
99.45324398 95.95528713 74.57237948 45.74294336
89.99363686 -0.97269142 0.040060316 0.161907532
LINC00944 12 126729787 126772411 - 1853
lincRNA long intergenic non-protein coding RNA 944
[Source:HGNC Symbol;Acc:HGNC:48640] - 25 43 70
99 105 76 0.737835084 1.023120989
1.749947729 2.583217605 2.46929216 1.914627131
ENSG00000251322 21.98227184 17.61207168 33.24514615
59.2701151 42.95141424 62.79779325 24.27982989
55.00644086 -1.179006878 0.04007215 0.161907532 SHANK3
22 50674415 50733298 + 7455
protein_coding SH3 and multiple ankyrin repeat domains 3 [Source:HGNC
Symbol;Acc:HGNC:14294] - 19 19 34 59 47
64 0.139380066 0.112367291 0.211268002
0.382653403 0.274731722 0.400754458
ENSG00000163795 261.4733387 486.6493489 368.6300029
622.8384977 492.5704739 476.8707425 372.2508968
530.7599047 -0.509624834 0.040078424 0.161907532 ZNF513
2 27377231 27380790 - 2365
protein_coding zinc finger protein 513 [Source:HGNC
Symbol;Acc:HGNC:26498] zf-C2H2 226 525 377 620 539
486 5.226031304 9.787282327 7.384356596
12.67540256 9.93153135 9.592927451
ENSG00000167548 2083.687978 2612.148315 3685.322231
5927.01151 2919.782308 5377.061047 2793.719508
4741.284955 -0.76298634 0.040087657 0.161907532 KMT2D
12 49018975 49059774 - 20485
protein_coding lysine methyltransferase 2D [Source:HGNC
Symbol;Acc:HGNC:7133] - 1801 2818 3769 5900 3195
5480 4.808088548 6.065114142 8.523002679
13.92570721 6.796627869 12.4879349
ENSG00000250651 1.156961676 4.634755704 1.955596832
20.09156444 21.93263706 4.906077597 2.582438071
15.64342637 -2.580028548 0.040090874 1 PABPC1P7
4 102896725 102898237 + 1513
processed_pseudogene poly(A) binding protein cytoplasmic 1
pseudogene 7 [Source:HGNC Symbol;Acc:HGNC:37987] - 1
5 2 20 24 5 0.036145629

0.145701839	0.061234126	0.639134534	0.691243263
0.154268449
ENSG00000249348 67.10377719	53.76316617	60.6235018
167.7645631	78.59194946	89.29061227	60.49681505
111.8823749	-0.887446244	0.040123341	0.162008851	UGDH-
AS1	4	39528019	39594707	+	2787
antisense	UGDH antisense RNA 1 [Source:HGNC
Symbol;Acc:HGNC:40601]	-	58	58	62	167	86
91	1.138113926	0.91753995	1.030521784
2.897214959	1.344683514	1.524229839
ENSG00000158669 2324.336007	2220.047982	2486.541372
3092.091767	2663.901543	2744.459808	2343.641787
2833.484373	-0.273762883	0.040187485	0.162225001	GPAT4
8	41577187	41625001	+	7939
protein_coding	glycerol-3-phosphate acyltransferase 4 [Source:HGNC
Symbol;Acc:HGNC:20880]	-	2009	2395	2543	3078	2915
2797	13.83913187	13.30067476	14.83826128
18.74580219	16.00041897	16.44647012
ENSG00000197721 5.784808379	0	14.66697624	0	0
0	6.81726154	0	5.261641015	0.040188424
1	CR1L	1	207645113	207738416	+
2768	protein_coding	complement C3b/C4b receptor 1 like
[Source:HGNC Symbol;Acc:HGNC:2335]	-	5	0	15
0	0	0	0.098786735	0	0.251031158
0	0	0
ENSG00000270926 5.784808379	0	14.66697624	0	0
0	6.81726154	0	5.261641015	0.040188424
1	AC008013.3	12	31105105	31106830
-	675	unprocessed_pseudogene	WAS protein family
homolog 4 pseudogene	-	5	0	15	0	0
0	0.405098788	0	1.029413696	0	0
0
ENSG00000246089 106.4404742	138.11572	147.6475608
55.25180221	47.52071363	112.8397847	130.734585
71.87076686	0.865115186	0.040224374	0.162331045
AC016065.1	8	6403551 6407142 -	2934	antisense
uncharacterized LOC100287015 [Source:NCBI gene;Acc:100287015]
-	92	149	151	55	52	115
1.714835362	2.239031053	2.384071596	0.906366398
0.772328092	1.829716341
ENSG00000235436 0	7.415609127	12.71137941	0	0
0	6.708996179	0	5.242462726	0.040251384
1	DPY19L2P4	7	90119400	90125600
+	2018	transcribed_unprocessed_pseudogene
DPY19L2 pseudogene 4 [Source:HGNC Symbol;Acc:HGNC:22176]	-
0	8	13	0	0	0	0
0.174784446	0.298417746	0	0	0
ENSG00000232973 0	6.488657986	13.68917783	0	0
0	6.725945271	0	5.245953183	0.040268504
1	CYP1B1-AS1	2	38073447	38231651

+ 7447 antisense CYP1B1 antisense RNA 1
[Source:HGNC Symbol;Acc:HGNC:28543] - 0 7 14
0 0 0 0 0.041442948 0.087086159
0 0 0
ENSG00000107672 971.8478076 953.8327239 784.1943298
689.1406603 808.7659915 574.0110789 903.2916204
690.6392436 0.386722923 0.040287406 0.162535193
NSMCE4A 10 121957088 121975217 - 3546
protein_coding "NSE4 homolog A, SMC5-SMC6 complex component
[Source:HGNC Symbol;Acc:HGNC:25935]" - 840 1029 802
686 885 585 12.95493587 12.79412535
10.47702772 9.35376815 10.8758482 7.701284559
ENSG00000145916 503.2783289 499.6266649 464.4542477
594.7103074 553.7990857 814.4088812 489.1197472
654.3060914 -0.419706005 0.040296224 0.162535193 RMND5B
5 178130996 178150565 + 7701
protein_coding required for meiotic nuclear division 5 homolog B
[Source:HGNC Symbol;Acc:HGNC:26181] - 435 539 475
592 606 830 3.089134703 3.0858556
2.85725461 3.716856548 3.429131175 5.031262826
ENSG00000204301 13.88354011 32.44328993 44.00092873
3.013734666 17.36333767 2.943646558 30.10925292
7.773572965 1.947555708 0.040334623 0.162647159 NOTCH4
6 32194843 32224067 - 9748
protein_coding notch 4 [Source:HGNC Symbol;Acc:HGNC:7884] -
12 35 45 3 19 3 0.067322532
0.158302029 0.213845172 0.014880138 0.084936953
0.014366526
ENSG00000104957 661.7820785 794.3971277 690.3256818
742.3833061 1031.747802 1030.276295 715.5016293
934.8024677 -0.385448607 0.040360707 0.162647545
CCDC130 19 13731760 13763296 + 3307
protein_coding coiled-coil domain containing 130 [Source:HGNC
Symbol;Acc:HGNC:28118] - 572 857 706 739 1129
1050 9.45924658 11.42564128 9.889468738
10.80466883 14.87710214 14.82180653
ENSG00000178741 1089.857899 1543.37365 1542.965901
1069.875806 997.9349861 1183.345916 1392.065816
1083.718903 0.362019123 0.040371132 0.162647545 COX5A
15 74919791 74938168 - 1411
protein_coding cytochrome c oxidase subunit 5A [Source:HGNC
Symbol;Acc:HGNC:2267] - 942 1665 1578 1065 1092
1206 36.51056904 52.0260893 51.80628387
36.49419687 33.72517581 39.89939678
ENSG00000087152 1571.153956 1810.335578 1976.130599
2232.172809 2067.151043 2275.43879 1785.873378
2191.587547 -0.294885356 0.040377727 0.162647545
ATXN7L3 17 44191805 44200113 - 5301
protein_coding ataxin 7 like 3 [Source:HGNC Symbol;Acc:HGNC:25416]
- 1358 1953 2021 2222 2262 2319

14.009953	16.24345453	17.6608241	20.26690664
18.5948806	20.42156308
ENSG00000096060 688.3921971	592.321779	798.861306
569.5958519	312.5400781	587.7480962	693.1917607
489.961342	0.501125682	0.040379287	0.162647545	FKBP5
6	35573585	35728583	-	10628
protein_coding	FK506 binding protein 5 [Source:HGNC
Symbol;Acc:HGNC:3721]	-	595	639	817	567	342
599	3.061682365	2.650838505	3.561008141
2.579483354	1.402274892	2.631002813
ENSG00000170442 1.156961676	38.00499677	36.1785414
8.036625776	4.569299387	0	25.11349995	4.201975054
2.584286444	0.040387917	0.162647545	KRT86	12
52249300	52309163	+	2394	protein_coding
keratin 86 [Source:HGNC Symbol;Acc:HGNC:6463]	-	1
41	37	8	5	0	0.022843917
0.75508122	0.715945616	0.161572356	0.091013215	0
ENSG00000160193 835.3263299	1063.212959	1055.044491
1516.913115	1138.669407	1153.909451	984.5279265
1269.830658	-0.36628564	0.04041185	0.162701068	WDR4
21	42843094	42879568	-	2738
protein_coding	WD repeat domain 4 [Source:HGNC Symbol;Acc:HGNC:12756]
-	722	1147	1079	1510	1246	1176
14.42110259	18.46987397	18.25536231	26.6651923
19.83093524	20.05025562
ENSG00000115677 12371.3912	11534.98	13330.32581
10716.84047	10049.71707	10764.91546	12412.23233
10510.491	0.239936433	0.040456845	0.162839332	HDLBP
2	241227264	241317061	-	12182
protein_coding	high density lipoprotein binding protein [Source:HGNC
Symbol;Acc:HGNC:4857]	-	10693	12444	13633	10668	10997
10971	48.00380738	45.03761305	51.84122978
42.34144043	39.33817678	42.04105982
ENSG00000273415 27.76708022	36.15109449	14.66697624
41.1877071	71.28107044	66.72265532	26.19505032
59.73047762	-1.189220329	0.040515146	0.163031072
AP000904.1	11	128096042	128183554	+	1959
lincRNA novel transcript	-	24	39	15
41	78	68	0.669994933	0.87773644
0.35469844	1.011930387	1.735077046	1.620393575
ENSG00000213590 9.255693406	7.415609127	0.977798416	0
0	0	5.88303365	0	5.047356128
0.040518326	1	AL807752.1	9	136935840
136937161	+	1322	processed_pseudogene	ribosomal
protein L30 (RPL30) pseudogene	-	8	8	1	0
0	0	0.330943034	0.266804094	0.035040557
0	0	0
ENSG00000152409 566.9112211	530.2160526	564.1896861
985.4912358	598.5782197	665.2641222	553.7723199
749.7778592	-0.436925702	0.040534474	0.163065924	JMY

5 79236189 79327215 + 9042
protein_coding "junction mediating and regulatory protein, p53
cofactor [Source:HGNC Symbol;Acc:HGNC:28916]" - 490 572
577 981 655 678 2.963645748 2.789108981
2.956063549 5.245727435 3.156715153 3.500348028
ENSG00000198829 0 0 0 0 12.79403828
7.849724156 0 6.881254147 -5.204008151 0.040563974
1 SUCNR1 3 151873643 151884619 +
4181 protein_coding succinate receptor 1 [Source:HGNC
Symbol;Acc:HGNC:4542] - 0 0 0 0 14
8 0 0 0 0 0.145917193
0.089321469
ENSG00000241494 384.1112763 431.9592316 478.1434255
509.3211586 766.7284372 502.382346 431.4046445
592.8106472 -0.458455069 0.040578041 0.163198239
AL355032.1 14 101677943 101678380 + 438
processed_pseudogene ribosomal protein L26-like 1 (RPL26L1)
pseudogene - 332 466 489 507 839 512
41.45325958 46.90787554 51.71746205 55.96739141
83.47308185 54.56848372
ENSG00000133858 1219.437606 865.7723656 1067.75587
781.5618567 939.447954 683.9072171 1050.988614
801.6390093 0.389781112 0.040600105 0.163225852 ZFC3H1
12 71609472 71667725 - 9670
protein_coding zinc finger C3H1-type containing [Source:HGNC
Symbol;Acc:HGNC:28328] - 1054 934 1092 778 1028
697 5.960859007 4.258477531 5.231167426
3.890042439 4.632604645 3.364746421
ENSG00000130669 1159.275599 2584.339781 2430.806863
2692.269635 2802.808244 2994.669765 2058.140747
2829.915881 -0.458833053 0.040606261 0.163225852 PAK4
19 39125770 39182816 + 7115
protein_coding p21 (RAC1) activated kinase 4 [Source:HGNC
Symbol;Acc:HGNC:16059] - 1002 2788 2486 2680 3067
3052 7.701716529 17.2763432 16.18559524
18.21214528 18.78440613 20.02422243
ENSG00000139438 54.37719876 54.69011731 121.2470036
149.6821551 124.2849433 138.3513882 76.77143989
137.4394956 -0.838038771 0.040642441 0.163328339
FAM222A 12 109714228 109770507 + 3722
protein_coding family with sequence similarity 222 member A
[Source:HGNC Symbol;Acc:HGNC:25915] - 47 59 124
149 136 141 0.690583507 0.698891246
1.543290818 1.935579956 1.592286222 1.768433689
ENSG00000224273 5.784808379 6.488657986 0 27.12361199
5.483159264 33.36132766 4.091155455 21.98936631
-2.429307626 0.040678533 0.163430417 AC005077.2
7 76108434 76132187 - 690
transcribed_unprocessed_pseudogene pseudogene similar to part of
RAB guanine nucleotide exchange factor (GEF) 1 RABGEF1 - 5

7	0	27	6	34	0.396292293
0.447283531	0	1.891977163	0.378931542	2.300254358
ENSG00000011422 4607.021393	4936.014825	3616.876341
4662.247528	5957.452541	5750.90416	4386.63752
5456.868076	-0.315024053	0.040713675	0.163522616	PLAUR
19	43646095	43670547	-	4457
protein_coding	"plasminogen activator, urokinase receptor
[Source:HGNC Symbol;Acc:HGNC:9053]"	-	3982	5325	3699
4641	6519	5861	48.85998557	52.67577525
38.44537957	50.34660042	63.73778174	61.38681813
ENSG00000180481 12.72657843	14.83121825	1.955596832
59.2701151	30.15737595	14.71823279	9.83779784
34.71524128	-1.820546199	0.04072722	0.163522616
GLIPR1L2	12	75391070	75432688	+	3790
protein_coding	GLIPR1 like 2 [Source:HGNC
Symbol;Acc:HGNC:28592]	-	11	16	2	59	33
15	0.158726042	0.186129294	0.02444518
0.752686312	0.379431452	0.184755802
ENSG00000279412 23.13923351	47.27450818	31.28954932
96.43950931	45.69299387	75.553595	33.901097
72.56203273	-1.091720954	0.040742908	0.163522616
AC020763.3	16	70713987	70716890	+	2904
TEC	novel transcript	-	20	51	32
96	50	77	0.376641435	0.774296903
0.510453072	1.598364545	0.750294892	1.23777056
ENSG00000178440 2.313923351	0.926951141	10.75578258
35.16023777	9.138598774	23.54917247	4.665552357
22.616003	-2.270920498	0.040744269	0.163522616
LINC00843	10	49972763	49982053	+	711
lincRNA long intergenic non-protein coding RNA 843
[Source:HGNC Symbol;Acc:HGNC:45009]	-	2	1	11
35	10	24	0.153834983	0.062010375
0.716680421	2.380124419	0.612899119	1.57575131
ENSG00000271367 3.470885027	5.561706845	7.822387329	0
0	0	5.618326401	0	4.984755262
0.040811665	1	AL034374.1	6	53350158
53350705	+	548	lincRNA novel transcript	-
3	6	8	0	0	0	0.299388703
0.4827304	0.676257172	0	0	0
ENSG00000198517 404.9365865	814.7900528	908.3747286
988.5049705	916.601457	1113.679615	709.3671226
1006.262014	-0.50303991	0.04089356	0.164078703	MAFK
7	1530714 1543043 +	4458	protein_coding	MAF
bZIP transcription factor K [Source:HGNC Symbol;Acc:HGNC:6782]
TF_bZIP 350	879	929	984	1003	1135	4.29361098
8.693262004	9.653351179	10.6722564	9.804364903
11.88507244
ENSG00000148219 42.807582	47.27450818	57.69010655
100.4578222	81.33352909	88.30939675	49.25739891
90.03358268	-0.868210596	0.040989822	0.164421783	ASTN2

9	116425225	117415070	-	8290
protein_coding	astrotactin 2 [Source:HGNC Symbol;Acc:HGNC:17021]
-	37	51	59	100	89	90
0.244085458	0.271237419	0.329685568	0.583239174
0.46783647	0.506796976
ENSG00000117481 1058.619933	1075.263323	936.7308827
1402.391198	1156.032745	1275.580175	1023.538046
1278.001373	-0.32023118	0.04101638	0.164485153	NSUN4
1	46340177	46365152	+	8468
protein_coding	NOP2/Sun RNA methyltransferase family member 4
[Source:HGNC Symbol;Acc:HGNC:31802]	-	915	1160	958
1396	1265	1300	5.90928529	6.039640627
5.240673645	7.970871089	6.509809412	7.166523656
ENSG00000182013 244.1189136	287.3548537	288.4505328
412.8816493	354.5776324	357.1624491	273.3081
374.8739103	-0.454610373	0.041051327	0.164582122	PNMA8A
19	46466491	46471563	-	3696
protein_coding	PNMA family member 8A [Source:HGNC
Symbol;Acc:HGNC:25578]	-	211	310	295	411	388
364	3.122088471	3.697972603	3.697366562
5.37664145	4.574655144	4.597433509
ENSG00000108823 3.470885027	6.488657986	6.844588913	0
0	0	5.601377309	0	4.980609161
0.041070072	1	SGCA	17	50164214	50175931
+	2452	protein_coding	sarcoglycan alpha [Source:HGNC
Symbol;Acc:HGNC:10805]	-	3	7	7	0	0
0	0.066910689	0.125866899	0.132245234	0
0	0
ENSG00000105699 6.941770054	21.31987624	25.42275882
46.21059821	60.31475191	34.34254318	17.8948017
46.95596443	-1.38339932	0.041075394	0.164635435	LSR
19	35248330	35267964	+	2916
protein_coding	lipolysis stimulated lipoprotein receptor [Source:HGNC
Symbol;Acc:HGNC:29572]	-	6	23	26	46	66
35	0.112527441	0.347755714	0.413036359
0.762731229	0.986313582	0.560307661
ENSG00000259015 0	6.488657986	5.866790497	16.07325155
30.15737595	15.69944831	4.118482828	20.64335861
-2.303452571	0.041095123	0.164649417	AL442663.4
14	72960595	72961993	+	289
processed_pseudogene	chromosome 17 open reading frame 98 (C17orf98)
pseudogene	-	0	7	6	16	33	16
0	1.067908775	0.961735979	2.676845813
4.975934957	2.584450245
ENSG00000168803 411.8783566	278.0853423	311.9176948
272.2406982	231.206549	180.5436556	333.9604645
227.9969676	0.549402138	0.041108125	0.164649417	ADAL
15	43330657	43354555	+	5735
protein_coding	adenosine deaminase like [Source:HGNC
Symbol;Acc:HGNC:31853]	-	356	300	319	271	253

184 3.394777292 2.306331818 2.576675432
2.284741578 1.922408582 1.497719336
ENSG00000132405 440.8023984 633.1076292 627.7465832
735.3512585 751.1928192 756.5171655 567.2188703
747.6870811 -0.397140705 0.041111194 0.164649417
TBC1D14 4 6909242 7033118 + 6241 protein_coding TBC1
domain family member 14 [Source:HGNC Symbol;Acc:HGNC:29246] -
381 683 642 732 822 771 3.338608585
4.825035123 4.76522379 5.670979991 5.739528711
5.766950601
ENSG00000168906 6364.446178 6217.061302 5697.631371
6907.479855 7431.508523 7247.257827 6093.046284
7195.415402 -0.239990332 0.041215381 0.16498304 MAT2A
2 85539165 85545280 + 4487
protein_coding methionine adenosyltransferase 2A [Source:HGNC
Symbol;Acc:HGNC:6904] - 5501 6707 5827 6876 8132
7386 67.0471448 65.90315327 60.15772504
74.09365434 78.97686642 76.84210788
ENSG00000160877 1677.59443 2554.677344 3227.712572
3216.659467 3144.591838 3452.897413 2486.661449
3271.382906 -0.395253066 0.041216081 0.16498304 NACC1
19 13118103 13141141 + 4474
protein_coding nucleus accumbens associated 1 [Source:HGNC
Symbol;Acc:HGNC:20967] - 1450 2756 3301 3202 3441
3519 17.72420381 27.15921365 34.17842142
34.60402079 33.51562254 36.71718037
ENSG00000206989 2.313923351 0 0 25.11445555
2.741579632 3.924862078 0.771307784
10.59363242 -3.861455928 0.041223429 1 SNORD63 5
138561043 138561110 - 68 snoRNA "small
nucleolar RNA, C/D box 63 [Source:HGNC Symbol;Acc:HGNC:10220]" -
2 0 0 25 3 4 1.608480483
0 0 17.77592923 1.922520324 2.745978386
ENSG00000241837 500.9644056 468.1103261 633.6133737
260.1857595 412.1508047 459.2088631 534.2293685
377.1818091 0.501906977 0.041237069 0.165020503 ATP5PO
21 33903453 33915980 - 3336
protein_coding ATP synthase peripheral stalk subunit OSCP
[Source:HGNC Symbol;Acc:HGNC:850] - 433 505 648
259 451 468 7.098336233 6.674201191
8.998112521 3.75383292 5.891272313 6.548862121
ENSG00000134452 1103.741439 1174.447095 1019.843748
1424.491919 1324.182962 1315.810012 1099.344094
1354.828298 -0.301328004 0.04124703 0.165020503 FBH1
10 5890203 5937594 + 6676 protein_coding F-box
DNA helicase 1 [Source:HGNC Symbol;Acc:HGNC:13620] - 954
1267 1043 1418 1449 1341 7.814959999
8.367471559 7.237197695 10.26977951 9.458247084
9.376882753
ENSG00000257167 39.33669697 152.0199871 158.4033434

69.31589732	26.50193645	66.72265532	116.5866758
54.18016303	1.10987749	0.041290572	0.165151484	TMPO-
AS1	12	98512973	98516422	-	3357
antisense	TMPO antisense RNA 1 [Source:HGNC
Symbol;Acc:HGNC:44158]	-	34	164	162	69	29
	68	0.553888424	2.153904605	2.235456015
0.99379994	0.376448226	0.945591604
ENSG00000066926 698.8048521	627.5459224	855.5736141
514.3440497	674.4285895	403.2795785	727.3081295
530.6840726	0.454234009	0.041324925	0.165214599	FECH
	18	57548283	57586772	-	4473
protein_coding	ferrochelatase [Source:HGNC Symbol;Acc:HGNC:3647]
	-	604	677	875	512	738	411
7.384698235	6.673040005	9.06174028	5.534422105
7.189787612	4.289325057
ENSG00000189171 408.4074715	333.7024107	488.8992081
249.1353991	284.2104219	342.4442163	410.3363634
291.9300124	0.490872843	0.041327967	0.165214599
S100A13 1	153618787	153631360	-	2216
protein_coding	S100 calcium binding protein A13 [Source:HGNC
Symbol;Acc:HGNC:10490]	-	353	360	500	248	311
	349	8.71163482	7.162534101	10.45207949
5.411069864	6.115743054	7.351935813
ENSG00000165804 267.2581471	511.6770297	425.342311
482.1975466	595.8366401	633.8652256	401.4258293
570.6331374	-0.505587322	0.041369322	0.165336685	ZNF219
	14	21090046	21104722	-	6679
protein_coding	zinc finger protein 219 [Source:HGNC
Symbol;Acc:HGNC:13011]	zf-C2H2 231	552	435	480	652
	646	1.891451672	3.643859241	3.017034451
3.474809582	4.253973205	4.515097265
ENSG00000114062 2414.579017	2117.156406	2004.486753
2911.267687	2747.976651	2360.80454	2178.740725
2673.349626	-0.295454152	0.041385705	0.165358932	UBE3A
	15	25333728	25439056	-	12813
protein_coding	ubiquitin protein ligase E3A [Source:HGNC
Symbol;Acc:HGNC:12496]	-	2087	2284	2050	2898	3007
	2406	8.907715456	7.859216117	7.411489381
10.93575499	10.22683384	8.765785369
ENSG00000137275 1810.645022	1745.448998	1677.902082
1949.886329	2494.837465	2007.566953	1744.665368
2150.763582	-0.302199422	0.041405009	0.165392832	RIPK1
	6	3063991 3115187 +	4231	protein_coding
receptor interacting serine/threonine kinase 1 [Source:HGNC
Symbol;Acc:HGNC:10019]	-	1565	1883	1716	1941	2730
	2046	20.22860943	19.62190879	18.78783399
22.18113303	28.11759813	22.57400619
ENSG00000214485 5953.724783	3240.621188	3491.718144
2931.359252	3637.162312	2979.951533	4228.688039
3182.824366	0.409510048	0.041436439	0.165475141	RPL7P1

5	150094302	150095048	+	747
processed_pseudogene	ribosomal protein L7 pseudogene 1 [Source:HGNC
Symbol;Acc:HGNC:10367]	-	5146	3496	3571	2918	3980
3037	376.7418732	206.3406433	221.4479704
188.8712707	232.1780012	189.7886453
ENSG00000182544 392.2100081	518.1656877	483.0324176
608.7744025	541.9189073	706.475174	464.4693711
619.0561613	-0.413111461	0.041469861	0.165546598	MFSD5
12	53251251	53254405	+	2581
protein_coding	major facilitator superfamily domain containing 5
[Source:HGNC Symbol;Acc:HGNC:28156]	-	339	559	494
606	593	720	7.183008931	9.548997097
8.866279139	11.35235167	10.01210249	13.0223849
ENSG00000153707 102.9695891	67.66743328	135.9139798
63.28842799	37.46825497	61.81657773	102.1836674
54.1910869	0.915888099	0.041475991	0.165546598	PTPRD
9	8314246 10612723	-	11165	protein_coding
"protein tyrosine phosphatase, receptor type D [Source:HGNC
Symbol;Acc:HGNC:9668]"	-	89	73	139	63	41
63	0.435939269	0.288269099	0.57671139
0.272824293	0.160023486	0.263407331
ENSG00000144278 222.1366417	243.78815	218.0490468
137.6272164	197.3937335	110.8773537	227.9912795
148.6327679	0.616335568	0.041507884	0.165630649
GALNT13 2	153871913	154453849	+	6520
protein_coding	polypeptide N-acetylgalactosaminyltransferase 13
[Source:HGNC Symbol;Acc:HGNC:23242]	-	192	263	223
137	216	113	1.610454079	1.778451847
1.584381355	1.015954335	1.443659434	0.809052834
ENSG00000237418 9.255693406	2.780853423	4.888992081	0
0	0	5.641846303	0	4.985543294
0.041521964	1	AC069257.2	3	196298061
196298215	-	155	processed_pseudogene	ribosomal
protein L39 (RPL39) pseudogene	-	8	3	5	0
0	0	2.822623816	0.853342772	1.494310204
0	0	0
ENSG00000283265 3.470885027	12.97731597	11.73358099	0
0	1.962431039	9.393927331	0.65414368
3.85798171	0.041535962	1	AL356234.3	6
137693068	137700415	+	1160	lincRNA novel
transcript	-	3	14	12	0	0	2
0.141435353	0.532113166	0.479209824	0	0
0.080485573
ENSG00000106153 6426.922109	8139.557968	7945.58993
8270.692502	9151.392812	9880.840281	7504.023335
9100.975198	-0.278223825	0.041550547	0.165757623	CHCHD2
7	56101569	56106576	-	1132
protein_coding	coiled-coil-helix-coiled-coil-helix domain containing
2 [Source:HGNC Symbol;Acc:HGNC:21645]	-	5555	8781	8126
8233	10014	10070	268.3690008	342.0042542

332.5315425	351.6518488	385.4958952	415.2685864
ENSG00000264978 0	0	1.955596832	20.09156444
6.397019142	2.943646558	0.651865611
9.81074338	-3.885159902	0.041593132	1	RN7SL630P
	12	93831076	93831372	+	297
misc_RNA	"RNA, 7SL, cytoplasmic 630, pseudogene [Source:HGNC
Symbol;Acc:HGNC:46646]" -	0	0	2	20	7
	3	0	0	0.311943544	3.255927777
1.027070342	0.471531642
ENSG00000130985 5745.471682	5824.034018	7020.592628
5467.919262	4498.018317	5326.03784	6196.699443
5097.32514	0.281881378	0.041593882	0.165887208	UBA1
	X	47190861	47215128	+	6437
protein_coding	ubiquitin like modifier activating enzyme 1
[Source:HGNC Symbol;Acc:HGNC:12469]	-	4966	6283	7180
	5443	4922	5428	42.1908154	43.03457403
51.67058647	40.8842506	33.32089806	39.36428484
ENSG00000268379 0	1.853902282	0	9.041203998
12.79403828	5.887293117	0.617967427
9.240845133	-3.841384922	0.041647359	1	AC025588.1
	19	57175233	57177921	+	2072
processed_pseudogene	pentatricopeptide repeat domain 3 pseudogene
	-	0	2	0	9	14	6	0
	0.042557313	0	0.21001677	0.29444005
0.135178473
ENSG00000106665 952.1794591	1205.036483	1457.897438
1728.87912	1489.5916	1437.480736	1205.037794
1551.983819	-0.364318374	0.041749963	0.166466269	CLIP2
	7	74289475	74405943	+	7315
protein_coding	CAP-Gly domain containing linker protein 2
[Source:HGNC Symbol;Acc:HGNC:2586]	-	823	1300	1491
	1721	1630	1465	6.15290511	7.835432611
9.44203854	11.37542827	9.71028265	9.349089774
ENSG00000085872 1935.596883	2946.777677	2871.793948
3302.048616	2902.418971	3704.088586	2584.722836
3302.852057	-0.353203181	0.041859786	0.16686064	CHERP
	19	16517889	16542530	-	6113
protein_coding	calcium homeostasis endoplasmic reticulum protein
[Source:HGNC Symbol;Acc:HGNC:16930]	-	1673	3179	2937
	3287	3176	3775	14.96705167	22.9282068
22.2562508	25.99839422	22.64043129	28.82760721
ENSG00000091010 9.255693406	0	10.75578258	0	0
	0	6.670491995	0	5.228420716	0.041876947
	1	POU4F3	5	146338839	146341722	+
	2569	protein_coding	POU class 4 homeobox 3 [Source:HGNC
Symbol;Acc:HGNC:9220]	Pou	8	0	11	0	0
	0	0.170302332	0	0.198349467	0	0
	0
ENSG00000168884 985.7313477	1192.059167	1205.625447
1492.803238	1281.231548	1450.236538	1127.805321

1408.090441	-0.319472322	0.041882246	0.166906647	TNIP2
	4	2741648 2756376 -	2686	protein_coding
TNFAIP3 interacting protein 2 [Source:HGNC Symbol;Acc:HGNC:19118]
	-	852	1286	1233	1486	1402	1478
17.3471566	21.10906116	21.26471292	26.74939831
22.74576789	25.68706363
ENSG00000187801 83.30124065	84.35255382	32.26734773
82.37541421	187.3412749	115.7834313	66.64038073
128.5000401	-0.949638515	0.041914579	0.166991967	ZFP69B
	1	40450102	40463718	+	2386
protein_coding	ZFP69 zinc finger protein B [Source:HGNC
Symbol;Acc:HGNC:28053]	zf-C2H2 72	91	33	82	205
	118	1.650276707	1.681531127	0.640687066
1.661669428	3.744053272	2.308647377
ENSG00000141367 19656.77887	16863.09515	17288.4538
15909.5053	14366.79113	15207.85934	17936.10927
15161.38526	0.242426807	0.041927914	0.167001571	CLTC
	17	59619689	59696956	+	11836
protein_coding	clathrin heavy chain [Source:HGNC
Symbol;Acc:HGNC:2092]	-	16990	18192	17681	15837	15721
	15499	78.50243629	67.7656251	69.19971781
64.69477052	57.88070456	61.12864337
ENSG00000277481 5.784808379	9.269511408	1.955596832	0
	0	0	5.669972206	0	4.996953633
0.041944472	1	PKD1L3	16	71929538	71999978
	-	5199	protein_coding	"polycystin 1 like 3,
transient receptor potential channel interacting [Source:HGNC
Symbol;Acc:HGNC:21716]" -	5	10	2	0	0
	0	0.052595053	0.084803571	0.017820202	0
	0	0
ENSG00000088205 4440.418911	4552.257053	4228.000351
3446.70788	3815.364988	3860.101854	4406.892105
3707.391574	0.24931711	0.041948642	0.167017286	DDX18
	2	117814650	117832379	+	7851
protein_coding	DEAD-box helicase 18 [Source:HGNC
Symbol;Acc:HGNC:2741]	-	3838	4911	4324	3431	4175
	3934	26.73466249	27.5790254	25.51309603
21.12987643	23.17341825	23.39135683
ENSG00000133433 129.5797077	123.2845017	224.8936357
85.38914887	95.95528713	105.9712761	159.252615
95.77190403	0.734210554	0.041953711	0.167017286	GSTT2B
	22	23957414	23961186	-	1099
protein_coding	glutathione S-transferase theta 2B (gene/pseudogene)
[Source:HGNC Symbol;Acc:HGNC:33437]	-	112	133	230
	85	105	108	5.573333649	5.335657038
9.694660375	3.73957674	4.163419811	4.587457976
ENSG00000054611 822.5997514	957.5405285	919.1305112
1119.100139	1163.343624	1075.412209	899.7569304
1119.285324	-0.314466537	0.041978311	0.167064668
TBC1D22A	22	46762617	47175699	+	5315

protein_coding TBC1 domain family member 22A [Source:HGNC
Symbol;Acc:HGNC:1309] - 711 1033 940 1114 1273
1096 7.315786868 8.569017122 8.192699781
10.13405223 10.43719344 9.626165434
ENSG00000247950 1.156961676 20.3929251 11.73358099
43.19686355 54.83159264 17.66187935 11.09448926
38.56344518 -1.783340601 0.04198747 0.167064668
SEC24B-AS1 4 109347475 109433817 - 2584
antisense SEC24B antisense RNA 1 [Source:HGNC
Symbol;Acc:HGNC:44003] - 1 22 12 43 60
18 0.021164217 0.375373949 0.215125153
0.804594691 1.011852802 0.325181651
ENSG00000037897 329.7340776 376.3421632 386.2303744
548.4997092 477.034856 435.6596906 364.102205
487.064752 -0.418841142 0.042064931 0.167329326 METTL1
12 57768471 57772793 - 2621
protein_coding methyltransferase like 1 [Source:HGNC
Symbol;Acc:HGNC:7030] - 285 406 395 546 522
444 5.946652378 6.829563865 6.981239391
10.07225792 8.678847951 7.907914862
ENSG00000196584 675.6656186 581.1983653 624.8131879
474.1609208 554.7129456 386.5989147 627.225724
471.8242604 0.40986767 0.042136253 0.167569432 XRCC2
7 152644779 152676165 - 4853
protein_coding X-ray repair cross complementing 2 [Source:HGNC
Symbol;Acc:HGNC:12829] - 584 627 639 472 607
394 6.581081491 5.696278408 6.099482964
4.702544607 5.450508202 3.789936787
ENSG00000238793 8.09873173 3.707804563 4.888992081 0
0 0 5.565176125 0 4.966824433
0.042172847 1 SNORD124 17 40027542
40027645 - 104 snoRNA "small nucleolar RNA, C/D box
124 [Source:HGNC Symbol;Acc:HGNC:33563]" - 7 4
5 0 0 0 3.680945721 1.695745253
2.227096938 0 0 0
ENSG00000236031 8.09873173 3.707804563 4.888992081 0
0 0 5.565176125 0 4.966824433
0.042172847 1 AL591721.1 1 243545532
243548329 + 751 antisense novel transcript
- 7 4 5 0 0 0
0.509744814 0.234830235 0.308412892 0 0 0
ENSG00000243943 741.6124341 540.4125151 608.1906149
501.2845328 508.1060918 410.1480871 630.0718547
473.1795706 0.411997085 0.042184947 0.167719452 ZNF512
2 27582969 27623215 + 4398
protein_coding zinc finger protein 512 [Source:HGNC
Symbol;Acc:HGNC:29380] zf-C2H2 641 583 622 499 556
418 7.970719339 5.844498987 6.551452785
5.485882951 5.509068397 4.436771807
ENSG00000196109 0 0.926951141 0 9.041203998

6.397019142	5.887293117	0.308983714
7.108505419	-4.287193981	0.042202432	1	ZNF676	19
	22179091	22215775	-	3088	protein_coding
	zinc finger protein 676 [Source:HGNC Symbol;Acc:HGNC:20429]
	zf-C2H2 0	1	0	9	7	6	0
	0.014277648	0	0.140917988	0.098782348
0.090702654
ENSG00000280169 0	0.926951141	0	12.05493866	0
	13.73701727	0.308983714	8.597318646	-4.560317391
	0.042204715	1	AL136526.1	13	100537365
	100539567	+	2203	antisense	"novel
transcript, antisense to GGACT" -	0	1	0	12
	0	14	0	0.020013335	0	0.263371008
	0	0.296660398
ENSG00000068078 9.255693406	4.634755704	2.933395248	0
	0	0	5.607948119	0	4.977230131
0.042208593	1	FGFR3	4	1793293 1808872 +	4848
	protein_coding	fibroblast growth factor receptor 3
[Source:HGNC Symbol;Acc:HGNC:3690]	-	8	5	3
	0	0	0	0.09024478	0.045471717
0.028665604	0	0	0
ENSG00000145545 735.8276258	655.3544566	738.2378042
513.3394715	592.1812006	555.367984	709.8066288
553.629552	0.357939189	0.042208758	0.167750617	SRD5A1
	5	6633343 6674386 +	7715	protein_coding
steroid 5 alpha-reductase 1 [Source:HGNC Symbol;Acc:HGNC:11284] -
	636	707	755	511	648	566	4.508332077
	4.040335611	4.533289736	3.202478231	3.660139796
	3.424731565
ENSG00000259330 432.7036667	372.6343586	492.8104017
252.1491337	350.0083331	335.5757077	432.7161424
312.5777248	0.468497296	0.042214733	0.167750617	INAFM2
	15	40323664	40326715	+	3052
protein_coding	InaF motif containing 2 [Source:HGNC
Symbol;Acc:HGNC:35165]	-	374	402	504	251	383
	342	6.701650664	5.807316312	7.649771501
3.976403146	5.468558249	5.231034841
ENSG00000112303 3.470885027	8.342560268	4.888992081	0
	0	0	5.567479125	0	4.972266564
0.04221832	1	VNN2	6	132743870	132763459
	-	2633	protein_coding	vanin 2 [Source:HGNC
Symbol;Acc:HGNC:12706]	-	3	9	5	0	0
	0	0.062311056	0.150704288	0.087967369	0
	0	0
ENSG00000279675 3.470885027	8.342560268	4.888992081	0
	0	0	5.567479125	0	4.972266564
0.04221832	1	AC080100.1	11	40107244
40112599	-	5356	TEC	TEC	-	3	9
	5	0	0	0	0.030632003	0.074085958
	0.043244601	0	0	0

ENSG00000111639 1217.123683   1162.396731   1211.492238
756.4474012   893.7549601   1140.172434   1197.004217
930.1249316   0.36381536   0.042247187   0.167835953   MRPL51
        12   6491886 6493841 -   1551   protein_coding
mitochondrial ribosomal protein L51 [Source:HGNC
Symbol;Acc:HGNC:14044] -   1052   1254   1239   753   978
        1162   37.09357184   35.64673   37.00513257
23.47385377   27.47803388   34.97360221
ENSG00000145779 446.5872068   353.1683847   316.8066868
236.0758822   326.2479762   215.8674143   372.1874261
259.3970909   0.519046705   0.042344689   0.168179596
TNFAIP8 5   119268692   119399688   +   9591
protein_coding TNF alpha induced protein 8 [Source:HGNC
Symbol;Acc:HGNC:17260] -   386   381   324   235   357
        220   2.200990289   1.751439107   1.564889134
1.184691269   1.622045091   1.07079128
ENSG00000261061 238.3341052   223.3952249   293.3395248
203.9293791   155.3561792   154.0508366   251.6896183
171.1121316   0.557378926   0.04237617   0.168260914
AC092718.4   16   81030770   81031485   +   716
        sense_intronic novel transcript   -   206   241
        300   203   170   157   15.73435378   14.84013932
        19.40933644   13.70831995   10.34652465   10.2360563
ENSG00000196372 576.1669145   748.0495707   677.6143024
875.9922096   723.7770229   1027.332649   667.2769292
875.7006271   -0.391095102   0.042434296   0.168402422   ASB13
        10   5638867 5666595 -   3003   protein_coding
ankyrin repeat and SOCS box containing 13 [Source:HGNC
Symbol;Acc:HGNC:19765] -   498   807   693   872   792
        1047   9.069194653   11.84819411   10.6900653
14.03984681   11.49286875   16.27561415
ENSG00000277152 13.88354011   15.75816939   35.20074298
10.04578222   0   0   21.61415083   3.348594073
2.703143401   0.042436851   0.168402422   AC110048.2   15
        66860303   66867023   +   6721   lincRNA novel
transcript   -   12   17   36   10   0   0
        0.097643213   0.11151903   0.248125307   0.071939484
        0   0
ENSG00000142409 736.9845874   1314.416718   1382.60696
1523.945163   1372.617536   1746.563625   1144.669422
1547.708774   -0.434232482   0.042450277   0.168402422   ZNF787
        19   56087366   56121280   -   3671
protein_coding zinc finger protein 787 [Source:HGNC
Symbol;Acc:HGNC:26998] zf-C2H2 637   1418   1414   1517   1502
        1780   9.489640509   17.03043748   17.84298378
19.98031877   17.82970452   22.63506019
ENSG00000117682 975.3186926   892.6539486   768.5495551
1245.676995   994.2795466   1109.754753   878.8407321
1116.570431   -0.345679237   0.042455873   0.168402422   DHDDS
        1   26432282   26471294   +   5596

protein_coding dehydrodolichyl diphosphate synthase subunit
[Source:HGNC Symbol;Acc:HGNC:20603]   -   843   963   786
        1240   1088   1131   8.238432382   7.587217592
6.506497931   10.71384097   8.472465075   9.43476169
ENSG00000207955 1.156961676   0   0   7.032047554
8.224738897   5.887293117   0.385653892
7.048026523   -4.275596506   0.042475186   1   AL359091.1
        9   128392007   128393510   -   1504
lincRNA novel transcript   -   1   0   0   7
        9   6   0.036361926   0   0   0.2250357
        0.260767384   0.186229917
ENSG00000259138 0   0   0   13.05951689   7.310879019
        0   0   6.790131969   -5.182946051   0.042486209
        1   AL049780.2   14   75127153   75136930
        +   367   antisense   "novel transcript, antisense
to NEK9 and TMED10"   -   0   0   0   13   8
        0   0   0   0   1.712688985   0.949910133
        0
ENSG00000151287 537.9871792   510.7500786   494.7659986
397.8129759   371.9409701   410.1480871   514.5010855
393.3006777   0.387417918   0.042512319   0.168582574   TEX30
        13   102765990   102773811   -   2154
protein_coding testis expressed 30 [Source:HGNC
Symbol;Acc:HGNC:25188] -   465   551   506   396   407
        418   11.80597792   11.27820172   10.88196372
8.888954915   8.233932606   9.058924052
ENSG00000127507 23.13923351   3.707804563   2.933395248
2.009156444   0   0   9.926811109   0.669718815
3.908134609   0.042559973   1   ADGRE2 19   14732393
        14778541   -   8622   protein_coding adhesion G
protein-coupled receptor E2 [Source:HGNC Symbol;Acc:HGNC:3337] -
        20   4   3   2   0   0   0.126857658
        0.020454362   0.016118169   0.011215618   0   0
ENSG00000064042 0   0.926951141   2.933395248   13.05951689
        18.27719755   3.924862078   1.28678213   11.75385884
        -3.164989729   0.042568931   1   LIMCH1 4
41359607   41700044   +   10862   protein_coding LIM
and calponin homology domains 1 [Source:HGNC Symbol;Acc:HGNC:29191]
        -       0   1   3   13   20   4   0
        0.004059048   0.012794223   0.057867507   0.08023776
        0.017190806
ENSG00000085788 2083.687978   1960.501663   2337.916013
1739.929481   1777.457462   1744.601194   2127.368551
1753.996045   0.27839678   0.042588922   0.168758176   DDHD2
        8   38225218   38275558   +   10686
protein_coding DDHD domain containing 2 [Source:HGNC
Symbol;Acc:HGNC:29106] -   1801   2115   2391   1732   1945
        1778   9.217077851   8.726280317   10.36494166
7.83671286   7.931640717   7.767166637
ENSG00000179051 5886.621006   6557.25237   8277.063593

6025.460176 5145.94497 5655.726254 6906.97899
5609.0438 0.300444444 0.042591255 0.168758176 RCC2
1 17406760 17439724 - 4126
protein_coding regulator of chromosome condensation 2 [Source:HGNC
Symbol;Acc:HGNC:30297] - 5088 7074 8465 5998 5631
5764 67.43922825 75.59094763 95.03863599
70.2875579 59.47232286 65.21399177
ENSG00000176783 1381.412241 1274.557819 1277.004732
1032.706412 1105.770452 1068.543701 1310.991597
1069.006855 0.294027751 0.042599011 0.168758176 RUFY1
5 179550558 179610026 + 5911
protein_coding RUN and FYVE domain containing 1 [Source:HGNC
Symbol;Acc:HGNC:19760] - 1194 1375 1306 1028 1210
1089 11.04684042 10.25594532 10.23492521
8.40878739 8.920372881 8.600287236
ENSG00000118363 2242.191728 2235.806152 2241.11397
1941.849703 1810.356417 1857.440978 2239.70395
1869.882366 0.260420939 0.042600759 0.168758176 SPCS2
11 74949247 74979031 + 2982
protein_coding signal peptidase complex subunit 2 [Source:HGNC
Symbol;Acc:HGNC:28962] - 1938 2412 2292 1933 1981
1893 35.54191684 35.66182975 35.6048721
31.341908 28.94912451 29.63391362
ENSG00000241369 0 0 24.4449604 0 0 0
8.148320135 0 5.521095169 0.042616337 1
LINC01192 3 163127923 163361563 -
3079 lincRNA long intergenic non-protein coding RNA 1192
[Source:HGNC Symbol;Acc:HGNC:37197] - 0 0 25
0 0 0 0 0 0.376125498 0
0 0
ENSG00000131668 0 0 0 17.07782977 3.65543951
0 0 6.911089761 -5.207522534 0.042626989
1 BARX1 9 93951622 93955372 -
1777 protein_coding BARX homeobox 1 [Source:HGNC
Symbol;Acc:HGNC:955] Homeobox 0 0 0 17
4 0 0 0 0 0.462554287
0.098091452 0
ENSG00000165716 26.61011854 14.83121825 16.62257307
106.4852915 18.27719755 35.3237587 19.35463662
53.36208259 -1.466406088 0.042644741 0.168888643 FAM69B
9 136712570 136724050 + 2704
protein_coding family with sequence similarity 69 member B
[Source:HGNC Symbol;Acc:HGNC:28290] - 23 16 17
106 20 36 0.465174459 0.260883885
0.291235753 1.895397897 0.322316031 0.621501025
ENSG00000145388 780.9491311 798.1049323 765.6161599
1001.564487 994.2795466 921.3613728 781.5567411
972.4018023 -0.315182659 0.042662448 0.168915008
METTL14 4 118685368 118715433 + 6862
protein_coding methyltransferase like 14 [Source:HGNC

Symbol;Acc:HGNC:29330]	-	675	861	783	997	1088
	939	5.379572587	5.532053809	5.285833805
7.024989203	6.909343422	6.387941266
ENSG00000011198 1228.6933	956.6135774	968.020432
1232.617478	1448.467906	1293.242055	1051.109103
1324.775813	-0.334786826	0.042694216	0.168997016	ABHD5
	3	43690108	43734371	+	7549
protein_coding	abhydrolase domain containing 5 [Source:HGNC
Symbol;Acc:HGNC:21396]	-	1062	1032	990	1227	1585
	1318	7.693603562	6.027319728	6.075027176
7.858802124	9.149522699	8.150270461
ENSG00000227619 10.41265508	4.634755704	1.955596832	0
	0	0	5.667669206	0	4.991754421
0.042716747	1	AL391056.1	9	129575409
129584556	+	2449	lincRNA novel transcript (FLJ20378)
	-	9	5	2	0	0	0
0.200977962	0.09001506	0.037830638	0	0	0
ENSG00000177738 105.2835125	167.7781565	138.8473751
154.7050462	221.1540903	275.721561	137.3030147
217.1935658	-0.659480297	0.04272692	0.169002426
AC025171.1	5	43014414	43067419	-	2784
	sense_overlapping	uncharacterized LOC648987 [Source:NCBI
gene;Acc:648987]	-	91	181	142	154	242
	281	1.78758571	2.866442946	2.36277066
2.674562225	3.787954317	4.711759608
ENSG00000110497 905.9009921	937.1476034	1111.756799
1187.411458	1230.055395	1263.805589	984.9351316
1227.090814	-0.316837114	0.042736338	0.169002426	AMBRA1
	11	46396414	46594125	-	7673
protein_coding	autophagy and beclin 1 regulator 1 [Source:HGNC
Symbol;Acc:HGNC:25990]	-	783	1011	1137	1182	1346
	1288	5.580733406	5.809247976	6.864323178
7.44823713	7.64431297	7.836041018
ENSG00000139835 16.19746346	15.75816939	38.13413823
7.032047554	0	7.849724156	23.36325703	4.96059057
	2.244508701	0.042737464	0.169002426	GRTP1	13
	113324164	113364148	-	2916	protein_coding
	growth hormone regulated TBC protein 1 [Source:HGNC
Symbol;Acc:HGNC:20310]	-	14	17	39	7	0
	8	0.26256403	0.257036832	0.619554539
0.116067796	0	0.128070323
ENSG00000132386 215.1948717	217.8335181	332.4514615
226.0301	119.7156439	141.2950348	255.1599504
162.3469262	0.653888713	0.042742508	0.169002426
SERPINF1	17	1761959 1777574 +	3069	protein_coding
	serpin family F member 1 [Source:HGNC Symbol;Acc:HGNC:8824]
	-	186	235	340	225	131	144
3.314444632	3.376019386	5.131974437	3.54476008
1.860085918	2.19034053
ENSG00000138777 1250.675571	1261.580503	1187.047277

777.5435438	1219.089076	840.9017002	1233.101117
945.8447735	0.382127886	0.042750859	0.169002426	PPA2
	4	105369077	105474081	-	4654
protein_coding	pyrophosphatase (inorganic) 2 [Source:HGNC
Symbol;Acc:HGNC:28883]	-	1081	1361	1214	774	1334
	857	12.7026411	12.89334799	12.08355612
8.041106205	12.49073655	8.596080597
ENSG00000240098 0	0	0	12.05493866	8.224738897
	0	0	6.75989252	-5.17674503	0.042801864
	1	RN7SL351P	11	126245035	126245282
	+	248	misc_RNA	"RNA, 7SL, cytoplasmic 351,
pseudogene [Source:HGNC Symbol;Acc:HGNC:46367]" -	0	0
	0	12	9	0	0	0	0
2.339541653	1.581428009	0
ENSG00000136999 961.4351525	997.3994276	803.7502981
1038.733882	1173.396083	1278.523822	920.8616261
1163.551262	-0.337635576	0.042834239	0.16925984	NOV
	8	119416306	119424353	+	2673
protein_coding	nephroblastoma overexpressed [Source:HGNC
Symbol;Acc:HGNC:7885]	-	831	1076	822	1034	1284
	1303	17.00187339	17.74791216	14.24542185
18.70349623	20.93267173	22.75576776
ENSG00000083642 2046.665204	1850.194477	1776.659722
1846.414772	1306.819625	1322.67852	1891.173135
1491.970972	0.342083377	0.042838119	0.16925984	PDS5B
	13	32586427	32778019	+	13212
protein_coding	PDS5 cohesin associated factor B [Source:HGNC
Symbol;Acc:HGNC:20418]	-	1769	1996	1817	1838	1430
	1348	7.322408958	6.660792889	6.370724405
6.726329816	4.716567675	4.762855032
ENSG00000196696 123.7948993	107.5263323	101.6910353
43.19686355	42.03755436	94.19668987	111.004089
59.81036926	0.892207567	0.042947171	0.169608599
PDXDC2P-NPIPB14P	16	69976297	70065948	-
	6114	processed_transcript	nuclear pore complex-
interacting protein [Source:NCBI gene;Acc:283970]	-	107
	116	104	43	46	96	0.957090611
0.836501093	0.787971229	0.340051142	0.327861933
0.73297951
ENSG00000089737 3521.791341	3693.900296	3572.875413
4553.75308	4253.103869	4003.359319	3596.189017
4270.07209	-0.247709813	0.042948576	0.169608599	DDX24
	14	94048291	94081245	-	8636
protein_coding	DEAD-box helicase 24 [Source:HGNC
Symbol;Acc:HGNC:13266]	-	3044	3985	3654	4533	4654
	4080	19.2764354	20.34462317	19.60010352
25.37898809	23.48401467	22.05431459
ENSG00000272146 0	13.90426711	5.866790497	0	0
	0	6.590352537	0	5.217668955	0.042969327
	1	ARF4-AS1	3	57597715	57600927

+ 597 antisense ARF4 antisense RNA 1
[Source:HGNC Symbol;Acc:HGNC:51593] - 0 15 6
0 0 0 0 1.107773281 0.465563983
0 0 0
ENSG00000171763 433.8606284 505.1883718 466.4098445
647.9529532 550.1436462 630.921579 468.4862816
609.6727261 -0.379085624 0.042982608 0.169671412
SPATA5L1 15 45402331 45421419 + 3406
protein_coding spermatogenesis associated 5 like 1
[Source:HGNC Symbol;Acc:HGNC:28762] - 375 545 477
645 602 643 6.021176207 7.054818037
6.487482379 9.156221442 7.70212292 8.812768565
ENSG00000223656 9.255693406 25.95463194 6.844588913
23.10529911 80.41966921 25.51160351 14.01830475
43.01219061 -1.611870988 0.04298668 0.169671412
HMGB3P10 1 99698242 99699178 + 572
processed_pseudogene high mobility group box 3 pseudogene
10 [Source:HGNC Symbol;Acc:HGNC:39302] - 8 28 7
23 88 26 0.764871838 2.158221231
0.566897402 1.944164567 6.704173439 2.121892389
ENSG00000136695 71.73162389 260.4732706 18.57816991
369.6847857 1156.946605 231.5668626 116.9276881
586.0660844 -2.324945605 0.043005362 0.169701334 IL36RN
2 113058638 113065382 + 3299
protein_coding interleukin 36 receptor antagonist [Source:HGNC
Symbol;Acc:HGNC:15561] - 62 281 19 368 1266
236 1.027789287 3.755415222 0.266792577
5.393450778 16.7228382 3.339455982
ENSG00000120992 3050.907939 2458.274426 2620.499755
1976.005363 2302.926891 2351.9736 2709.89404
2210.301951 0.293597771 0.043023278 0.169704948 LYPLA1
8 54046367 54102017 - 4694
protein_coding lysophospholipase I [Source:HGNC Symbol;Acc:HGNC:6737]
- 2637 2652 2680 1967 2520 2397
30.72286817 24.90946457 26.44808089 20.26107533
23.39462312 23.83806417
ENSG00000171105 185.1138681 241.0072966 223.9158373
260.1857595 325.3341164 339.5005697 216.6790007
308.3401485 -0.507701835 0.04302848 0.169704948 INSR
19 7112255 7294034 - 10451 protein_coding
insulin receptor [Source:HGNC Symbol;Acc:HGNC:6091] - 160
260 229 259 356 346 0.837253261
1.096855603 1.015032833 1.198238123 1.484399324
1.545483194
ENSG00000158092 557.6555277 501.4805672 592.5458402
653.9804225 748.4512396 717.2685447 550.560645
706.5667356 -0.360356943 0.043044428 0.169715141 NCK1
3 136862208 136949823 + 3727
protein_coding NCK adaptor protein 1 [Source:HGNC
Symbol;Acc:HGNC:7664] - 482 541 606 651 819

731 7.072653115 6.399879992 7.532093235
8.445450335 9.575977275 9.155962811
ENSG00000166167 584.2656462 539.485564 465.4320461
692.154395 651.5820926 706.475174 529.7277521
683.4038872 -0.368039215 0.043053269 0.169715141 BTRC
10 101354033 101557321 + 6613
protein_coding beta-transducin repeat containing E3 ubiquitin protein
ligase [Source:HGNC Symbol;Acc:HGNC:1144] - 505 582
476 689 713 720 4.176260381 3.880238495
3.334347704 5.037579532 4.698395857 5.082530688
ENSG00000221867 1867.336145 2064.320191 2236.224978
2658.113975 2343.136726 2485.418911 2055.960438
2495.556537 -0.279188252 0.043090907 0.169819722 MAGEA3
X 152698752 152702347 + 1788
protein_coding MAGE family member A3 [Source:HGNC
Symbol;Acc:HGNC:6801] - 1614 2227 2287 2646 2564
2533 49.36631711 54.91445282 59.25173966
71.55229068 62.4897956 66.13231279
ENSG00000123815 494.0226355 676.6743328 613.0796069
610.783559 839.8372273 948.8354073 594.5921918
799.8187312 -0.4270115 0.043142679 0.16993664 COQ8B
19 40691529 40718207 - 5488
protein_coding coenzyme Q8B [Source:HGNC Symbol;Acc:HGNC:19041]
- 427 730 627 608 919 967
4.255087401 5.864658328 5.2924394 5.356618206
7.297263126 8.225426144
ENSG00000160293 629.3871516 749.9034729 785.1721282
456.0785128 554.7129456 633.8652256 721.4875842
548.2188947 0.396673245 0.043142808 0.16993664 VAV2
9 133761894 133992604 - 5682
protein_coding vav guanine nucleotide exchange factor 2 [Source:HGNC
Symbol;Acc:HGNC:12658] - 544 809 803 454 607
646 5.235912534 6.277420917 6.546614555
3.863276924 4.6552827 5.30734506
ENSG00000177600 9411.883232 9536.473337 10085.99066
10006.60367 11394.91881 13890.08689 9678.115744
11763.86979 -0.281553923 0.043176333 0.170024885 RPLP2
11 809647 812880 + 1955 protein_coding
ribosomal protein lateral stalk subunit P2 [Source:HGNC
Symbol;Acc:HGNC:10377] - 8135 10288 10315 9961 12469
14156 227.5650214 232.0161157 244.4133515
246.3527388 277.9351412 338.0180002
ENSG00000082258 888.5465669 667.4048214 688.370085
616.8110283 545.5743468 561.2552771 748.1071578
574.5468841 0.379995905 0.043229413 0.170190068 CCNT2
2 134918235 134959342 + 9081
protein_coding cyclin T2 [Source:HGNC Symbol;Acc:HGNC:1600] -
768 720 704 614 597 572 4.625112034
3.495688926 3.591215272 3.269158009 2.864832621
2.940413371

ENSG00000184500 1015.812351 1028.915766 1015.932554
623.8430759 855.3728453 897.8122003 1020.220224
792.3427072 0.364442238 0.043246832 0.170214808 PROS1
3 93873033 93980003 - 5444
protein_coding protein S [Source:HGNC Symbol;Acc:HGNC:9456] -
878 1110 1039 621 936 915 8.82005132
8.989567965 8.840969388 5.515370605 7.492320206
7.846012819
ENSG00000261693 0 0 0 18.082408 2.741579632
0 0 6.94132921 -5.213621073 0.043285872
1 AC134682.1 8 142403652 142407028
+ 3377 antisense "novel transcript, antisense
to TSNARE1" - 0 0 0 18 3 0
0 0 0 0.257716759 0.038712284 0
ENSG00000118156 6.941770054 3.707804563 5.866790497 0
0 0 5.505455038 0 4.952144964
0.043292821 1 ZNF541 19 47520685 47555856
- 4655 protein_coding zinc finger protein 541
[Source:HGNC Symbol;Acc:HGNC:25294] zf-C2H2 6 4 6
0 0 0 0.0704898 0.037885608
0.059708206 0 0 0
ENSG00000063322 1883.533608 1740.814243 1738.525584
2119.660048 2378.777261 2009.529384 1787.624478
2169.322231 -0.279526546 0.043302288 0.170389207 MED29
19 39391303 39400637 + 3835
protein_coding mediator complex subunit 29 [Source:HGNC
Symbol;Acc:HGNC:23074] - 1628 1878 1778 2110 2603
2048 23.21580488 21.59057346 21.47676396
26.6022459 29.57790417 24.92933076
ENSG00000144199 0 0 1.955596832 5.02289111
10.05245865 12.75580175 0.651865611
9.277050505 -3.805701314 0.043318771 1 FAHD2B 2
97083583 97094882 - 2662 protein_coding
fumarylacetoacetate hydrolase domain containing 2B
[Source:HGNC Symbol;Acc:HGNC:25318] - 0 0 2
5 11 13 0 0 0.034803619
0.090816167 0.180070774 0.22797191
ENSG00000115233 3526.419188 2791.049885 2700.679225
2418.01978 2414.417796 2531.53604 3006.049433
2454.657872 0.291987959 0.043336453 0.17047423 PSMD14
2 161308038 161411717 + 7698
protein_coding "proteasome 26S subunit, non-ATPase 14 [Source:HGNC
Symbol;Acc:HGNC:16889]" - 3048 3011 2762 2407 2642
2580 21.6536827 17.24514327 16.62065839
15.11817611 14.95593277 15.64544193
ENSG00000273472 0 21.31987624 5.866790497 33.15108133
39.29597473 33.36132766 9.062222245 35.26946124
-1.942537147 0.043363551 0.17047423 AC096733.2
4 140756528 140757921 + 1394
lincRNA novel transcript - 0 23 6 33

43 34 0 0.727443086 0.199384288
1.144596418 1.344201202 1.138576404
ENSG00000106052 5182.031346 4236.166714 4354.136347
4256.397927 3780.638313 3108.490766 4590.778135
3715.175668 0.305153429 0.043364833 0.17047423
TAX1BP1 7 27739331 27844564 + 9165
protein_coding Tax1 binding protein 1 [Source:HGNC
Symbol;Acc:HGNC:11575] - 4479 4570 4453 4237 4137
3168 26.72657489 21.98455548 22.50726279
22.35255701 19.67033015 16.13610605
ENSG00000164924 24554.19764 21526.58634 22072.82145
18581.68337 20721.77272 18441.94569 22717.86848
19248.46726 0.239033061 0.043371435 0.17047423 YWHAZ
8 100916525 100953388 - 8555
protein_coding tyrosine 3-monooxygenase/tryptophan 5-monooxygenase
activation protein zeta [Source:HGNC Symbol;Acc:HGNC:12855] -
21223 23223 22574 18497 22675 18795 135.6692652
119.6829447 122.2337013 104.5400008 115.501036
102.5577187
ENSG00000232888 26.61011854 13.90426711 37.15633981
59.2701151 49.34843338 65.7414398 25.89024182
58.11999609 -1.168376084 0.043389343 0.17047423
RPS11P5 12 132825701 132826184 + 484
processed_pseudogene ribosomal protein S11 pseudogene 5
[Source:HGNC Symbol;Acc:HGNC:36312] - 23 15 38
59 54 67 2.598825905 1.366406299
3.63697814 5.89396926 4.861910903 6.462126821
ENSG00000198832 201.3113316 223.3952249 267.916766
199.9110662 493.4843338 376.7867595 230.8744408
356.7273865 -0.627796559 0.043390804 0.17047423
SELENOM 22 31104772 31120069 - 3187
protein_coding selenoprotein M [Source:HGNC Symbol;Acc:HGNC:30397]
- 174 241 274 199 540 384
2.985808139 3.334025653 3.982639118 3.019063373
7.383636263 5.624646031
ENSG00000108506 1047.050317 830.5482222 817.4394759
757.4519794 658.8929716 686.8508636 898.3460049
701.0652716 0.357104076 0.043442023 0.170631603 INTS2
17 61865367 61928016 - 8627
protein_coding integrator complex subunit 2 [Source:HGNC
Symbol;Acc:HGNC:29241] - 905 896 836 754 721
700 5.736982088 4.579121527 4.488993073
4.225837224 3.641950715 3.787775912
ENSG00000144158 75.20250892 69.52133556 115.3802131
37.16939421 29.24351608 64.76022428 86.70135253
43.72437819 0.989229667 0.043501255 0.170820365
AL078621.1 2 113657908 113658675 + 768
processed_pseudogene small nuclear ribonucleoprotein
polypeptide A' (SNRPA1) pseudogene - 65 75 118
37 32 66 4.628570141 4.305603182

7.117430631	2.329387392	1.81571364	4.011702798
ENSG00000229567 8.09873173	5.561706845	2.933395248	0
0	0	5.531277941	0	4.958424494
0.043517212	1	AL139421.1	1	93278961
93279129	-	169	processed_pseudogene	ribosomal
protein L39 (RPL39) pseudogene	-	7	6	3	0
0	0	2.265197367	1.56530331	0.822312716
0	0	0
ENSG00000230601 2.313923351	12.97731597	1.955596832	0
0	0	5.748945385	0	5.020211153
0.043533972	1	TEX48	9	114666434	114682066
-	708	protein_coding	testis expressed 48
[Source:HGNC Symbol;Acc:HGNC:52393]	-	2	14	2
0	0	0	0.154486826	0.871823831
0.130857673	0	0	0
ENSG00000090266 598.1491863	755.4651798	814.5060807
355.6206906	523.6417098	676.0574929	722.7068156
518.4399644	0.479596021	0.043536354	0.17091429	NDUFB2
7	140690777	140722790	+	4447
protein_coding	NADH:ubiquinone oxidoreductase subunit B2 [Source:HGNC
Symbol;Acc:HGNC:7697]	-	517	815	833	354	573
689	6.357964906	8.08024329	8.677214389
3.848906394	5.614952546	7.232661307
ENSG00000185722 2422.677749	2426.758087	2939.262039
2279.387986	1909.967144	2167.505083	2596.232625
2118.953404	0.29329788	0.043628107	0.171215951	ANKFY1
17	4163907 4263995 -	14138	protein_coding
ankyrin repeat and FYVE domain containing 1 [Source:HGNC
Symbol;Acc:HGNC:20763]	-	2094	2618	3006	2269	2090
2209	8.099970044	8.164237366	9.849256658
7.759750098	6.441943427	7.293798721
ENSG00000092140 800.6174796	769.3694469	798.861306
639.9163274	656.151392	579.898372	789.6160775
625.3220305	0.336329326	0.043642239	0.171215951	G2E3
14	30559123	30620063	+	9353
protein_coding	G2/M-phase specific E3 ubiquitin protein ligase
[Source:HGNC Symbol;Acc:HGNC:20338]	-	692	830	817
637	718	591	4.046223544	3.912560949
4.046444406	3.292984712	3.345277177	2.949732154
ENSG00000136731 2625.146042	2182.969937	3430.116844
2368.795448	1754.610965	2267.589065	2746.077608
2130.331826	0.366412547	0.043646795	0.171215951	UGGT1
2	128091200	128195677	+	11349
protein_coding	UDP-glucose glycoprotein glucosyltransferase 1
[Source:HGNC Symbol;Acc:HGNC:15663]	-	2269	2355	3508
2358	1920	2311	10.93381226	9.148866142
14.31872817	10.04586693	7.372286943	9.505793712
ENSG00000219294 16.19746346	0	3.911193665	0	0
0	6.702885708	0	5.232086079	0.043662489
1	PIP5K1P1	6	7986537 7988192 +	1656

processed_pseudogene phosphatidylinositol-4-phosphate 5-kinase type 1 pseudogene 1 [Source:HGNC Symbol;Acc:HGNC:28372] - 14 0 4 0 0 0 0.462341008 0 0.111892793 0 0 0
ENSG00000174891 1248.361648 851.8680984 1086.33404 873.9830532 882.7886416 679.0011395 1062.187929 811.9242781 0.386819233 0.043670389 0.171264556 RSRC1 3 158105855 158545730 + 5117 protein_coding arginine and serine rich coiled-coil 1 [Source:HGNC Symbol;Acc:HGNC:24152] - 1079 919 1111 870 966 692 11.53189662 7.918338299 10.05775605 8.220629064 8.226598598 6.313013431
ENSG00000154978 2244.505651 2728.017208 2579.432222 2773.640471 3321.880654 3096.716179 2517.31836 3064.079102 -0.283336797 0.043688582 0.171291961 VOPP1 7 55436056 55572988 - 5364 protein_coding "VOPP1, WBP1/VOPP1 family member [Source:HGNC Symbol;Acc:HGNC:34518]" - 1940 2943 2638 2761 3635 3156 19.77915225 24.18997675 22.78182324 24.88736137 29.53073414 27.46592698
ENSG00000108785 111.0683209 130.7001109 152.5365529 207.947692 137.9928415 285.5337162 131.4349949 210.4914165 -0.677554167 0.04374745 0.17142509 HSD17B1P1 17 42546764 42548706 + 955 unprocessed_pseudogene hydroxysteroid 17-beta dehydrogenase 1 pseudogene 1 [Source:HGNC Symbol;Acc:HGNC:5216] - 96 141 156 207 151 291 5.49746628 6.509530992 7.566999105 10.48016669 6.890205477 14.22445557
ENSG00000235427 2.313923351 5.561706845 8.800185745 0 0 0 5.558605314 0 4.970211452 0.043750088 1 AC006159.1 7 116542718 116551969 - 386 antisense novel transcript - 2 6 9 0 0 0 0.283359256 0.685327097 1.080084318 0 0 0
ENSG00000172037 2268.801846 2050.415924 3144.599706 1768.057671 1233.710835 2470.700678 2487.939159 1824.156394 0.447914699 0.043762085 0.17142509 LAMB2 3 49121114 49133118 - 6865 protein_coding laminin subunit beta 2 [Source:HGNC Symbol;Acc:HGNC:6487] - 1961 2212 3216 1760 1350 2518 15.62182487 14.2062201 21.70091042 12.39576524 8.569427812 17.12226523
ENSG00000129219 691.8630821 715.6062807 798.861306 586.6736817 489.8288943 630.921579 735.4435563 569.141385 0.370425381 0.043766558 0.17142509 PLD2 17 4807096 4823434 + 4888 protein_coding phospholipase D2 [Source:HGNC Symbol;Acc:HGNC:9068] - 598 772 817 584 536 643 6.690594351 6.963379443 7.742715738 5.776740601 4.7785066 6.140812138

ENSG00000118402 70.57466222 38.93194792 60.6235018
136.6226382 107.8354655 70.6475174 56.71003731
105.035207 -0.892501098 0.043771804 0.17142509 ELOVL4
6 79914812 79947580 - 3042
protein_coding ELOVL fatty acid elongase 4 [Source:HGNC
Symbol;Acc:HGNC:14415] - 61 42 62 136 118
72 1.09664317 0.608729065 0.944136822
2.161627791 1.690368517 1.104890711
ENSG00000234072 175.8581747 177.0476679 155.4699482
240.0941951 239.4312879 249.2287419 169.4585969
242.918075 -0.519761558 0.043778606 0.17142509
AC074117.1 2 27356246 27367622 + 3412
antisense uncharacterized LOC105374363 [Source:NCBI
gene;Acc:105374363] - 152 191 159 239 262
254 2.436291658 2.468074715 2.158691382
3.386804241 3.346192077 3.475127394
ENSG00000197124 8.09873173 4.634755704 7.822387329
1.004578222 0 0 6.851958255 0.334859407
4.306174161 0.043817694 1 ZNF682 19 19997058
20039506 - 4968 protein_coding zinc finger
protein 682 [Source:HGNC Symbol;Acc:HGNC:28857] zf-C2H2 7 5
8 1 0 0 0.077056835 0.044373366
0.074595195 0.009732393 0 0
ENSG00000130304 601.6200714 787.9084697 823.3062664
914.166182 860.8560045 1100.923813 737.6116025
958.6486665 -0.377093021 0.043855166 0.171680901
SLC27A1 19 17468769 17506168 + 8249
protein_coding solute carrier family 27 member 1 [Source:HGNC
Symbol;Acc:HGNC:10995] - 520 850 842 910 942
1122 3.447440289 4.543092507 4.728389494
5.333856227 4.976318823 6.34947166
ENSG00000176692 411.8783566 485.7223978 705.9704565
420.918275 286.9520015 420.9414579 534.5237369
376.2705781 0.507722682 0.04389429 0.171702686 FOXC2
16 86566829 86569728 + 2900
protein_coding forkhead box C2 [Source:HGNC Symbol;Acc:HGNC:3801]
Fork 356 524 722 419 314 429
6.713464748 7.966494251 11.5329831 6.985817593
4.718351031 6.905662195
ENSG00000012174 802.9314029 724.8757921 716.726239
595.7148857 603.1475191 572.0486479 748.1778114
590.3036842 0.34141677 0.043894701 0.171702686 MBTPS2
X 21839636 21885424 + 8927
protein_coding "membrane bound transcription factor peptidase, site 2
[Source:HGNC Symbol;Acc:HGNC:15455]" - 694 782 733
593 660 583 4.251563289 3.862203706
3.803653048 3.211813913 3.221788289 3.048660444
ENSG00000159228 1361.743892 1515.565115 1279.938127
1764.039358 1571.838989 1761.281857 1385.749045
1699.053401 -0.293777264 0.043895562 0.171702686 CBR1

21 36069941 36073166 + 2450
protein_coding carbonyl reductase 1 [Source:HGNC
Symbol;Acc:HGNC:1548] - 1177 1635 1309 1756 1720
1795 26.27272326 29.42291049 24.75004643
34.65450052 30.59292206 34.20144099
ENSG00000187800 2.313923351 6.488657986 7.822387329 0
0 0 5.541656222 0 4.966025667
0.04389857 1 PEAR1 1 156893698 156916434
+ 5530 protein_coding platelet endothelial
aggregation receptor 1 [Source:HGNC Symbol;Acc:HGNC:33631] -
2 7 8 0 0 0 0.019778784
0.055809337 0.067014273 0 0 0
ENSG00000279108 94.87085741 82.49865154 61.60130022
135.61806 137.9928415 120.6895089 79.65693639
131.4334701 -0.72458255 0.043905658 0.171702686
AC008537.3 19 40840159 40842039 + 1881
TEC TEC - 82 89 63 135 151
123 2.384074209 2.086100221 1.55150868
3.470133552 3.498217028 3.052546945
ENSG00000170468 448.9011302 563.5862936 509.4329748
583.659947 820.6461699 619.1469928 507.3067995
674.4843699 -0.410532846 0.043973419 0.171903369 RIOX1
14 73490964 73493392 + 2429
protein_coding ribosomal oxygenase 1 [Source:HGNC
Symbol;Acc:HGNC:20968] - 388 608 521 581 898
631 8.735724387 11.03595758 9.936024742
11.56511176 16.11044066 12.1268465
ENSG00000073584 365.5998895 310.5286322 358.8520187
278.2681675 171.805657 270.8154834 344.9935135
240.2964359 0.522232951 0.043979465 0.171903369
SMARCE1 17 40624962 40648864 - 18680
protein_coding "SWI/SNF related, matrix associated, actin dependent
regulator of chromatin, subfamily e, member 1 [Source:HGNC
Symbol;Acc:HGNC:11109]" HMG 316 335 367 277 188
276 0.925134599 0.790682075 0.91010531
0.716975167 0.438570661 0.689728618
ENSG00000143947 9138.840276 9400.211519 9575.579889
6151.032453 8548.245293 8321.688821 9371.543895
7673.655522 0.288336383 0.043996589 0.171926346 RPS27A
2 55231903 55235853 + 3130
protein_coding ribosomal protein S27a [Source:HGNC
Symbol;Acc:HGNC:10417] - 7899 10141 9793 6123 9354
8481 138.0137922 142.8467629 144.9351995
94.58475394 130.2301755 126.4878357
ENSG00000227678 15.04050178 1.853902282 0.977798416 0
0 0 5.957400827 0 5.060715918
0.044017999 1 AL355581.1 6 130133410
130146179 - 2906 antisense "novel transcript,
antisense to L3MBTL3" - 13 2 1 0 0
0 0.244648442 0.030343687 0.01594068 0

0	0
ENSG00000260366 2.313923351	12.97731597	5.866790497	0
	0	0.981215519	7.052676607	0.32707184
4.353810868	0.044024017	1	AC026803.1	19
48926171	48926444	+	274	sense_overlapping
	"novel transcript, overlapping NUCB1"	-	2	14
	6	0	0	1	0.399184937	2.252741869
	1.014385759	0	0	0.170370922
ENSG00000224805 0	0.926951141	0	7.032047554
9.138598774	4.906077597	0.308983714
7.025574642	-4.271253034	0.044040113	1	LINC00853
	1	47179250	47180339	+	661
antisense	long intergenic non-protein coding RNA 853
[Source:HGNC Symbol;Acc:HGNC:43716]	-	0	1	0
	7	10	5	0	0.066701024	0
0.512032818	0.659260626	0.353113711
ENSG00000110717 2035.095588	1883.564718	2297.826278
1845.410194	1562.70039	1660.216659	2072.162195
1689.442414	0.294700371	0.04409838	0.172280076	NDUFS8
	11	68030617	68036644	+	4523
protein_coding	NADH:ubiquinone oxidoreductase core subunit S8
[Source:HGNC Symbol;Acc:HGNC:7715]	-	1759	2032	2350
	1837	1710	1692	21.26835812	19.80756427
24.06820657	19.63739089	16.47510232	17.46303831
ENSG00000253948 9.255693406	12.05036483	15.64477466
4.018312888	0	0	12.3169443	1.339437629
3.221303069	0.044101024	1	AC104986.2	8
98996763	99013044	-	380	lincRNA
uncharacterized LOC105375666 [Source:NCBI gene;Acc:105375666]	-
	8	13	16	4	0	0	1.151333399
	1.508320778	1.950468055	0.508952921	0	0
ENSG00000109794 0	0	0	0	6.397019142
13.73701727	0	6.711345471	-5.166792955	0.04412584
	1	FAM149A 4	186104419	186172667	+
	5218	protein_coding	family with sequence similarity 149
member A [Source:HGNC Symbol;Acc:HGNC:24527]	-	0	0
	0	0	7	14	0	0	0	0
	0.058459159	0.125247768
ENSG00000175711 264.9442237	255.8385149	274.7613549
177.8103453	149.8730199	221.7547074	265.1813645
183.1460242	0.534516708	0.044137933	0.172390542
B3GNTL1 17	82942155	83051810	-	6891
protein_coding	"UDP-GlcNAc:betaGal beta-1,3-N-
acetylglucosaminyltransferase like 1 [Source:HGNC
Symbol;Acc:HGNC:21727]" -	229	276	281	177	164
	226	1.81738921	1.765878383	1.888976373
1.24191603	1.037098953	1.530989545
ENSG00000234718 12.72657843	3.707804563	0.977798416	0
	0	0	5.804060471	0	5.024580422
0.044157483	1	AC007161.1	7	7738524 7742574 -

1496	transcribed_processed_pseudogene	ribosomal protein L23a (RPL23A) pseudogene	-	11	4	1
0	0	0	0.402120121	0.117886034
0.030964984	0	0	0
ENSG00000256481 12.72657843	3.707804563	0.977798416	0
0	0	5.804060471	0	5.024580422
0.044157483	1	AP006333.2	11	64081690
64087175	+	732	antisense	"novel transcript, antisense to MACROD1"	-	11	4	1	0	0
0	0.821819263	0.240925555	0.063283629	0
0	0
ENSG00000222287 2.313923351	3.707804563	23.46716199	0
0	1.962431039	9.829629968	0.65414368
3.921827271	0.044179446	1	RNU6-1043P	16
30701337	30701435	-	99	snRNA	"RNA, U6 small nuclear 1043, pseudogene [Source:HGNC Symbol;Acc:HGNC:48006]"	-
2	4	24	0	0	2	1.104814877
1.781388953	11.22996759	0	0	0.943063284
ENSG00000126785 315.8505375	176.1207168	290.4061296
154.7050462	212.9293514	139.3326038	260.7924613
168.9890005	0.623716712	0.044316222	0.172992426	RHOJ
14	63204114	63293219	+	3793
protein_coding	ras homolog family member J [Source:HGNC Symbol;Acc:HGNC:688]	-	273	190	297	154	233
142	3.936176073	2.208537187	3.627238082
1.963084955	2.676897093	1.747638234
ENSG00000012963 2239.877804	1882.637767	2017.198133
1408.418667	1924.588902	1584.663064	2046.571235
1639.223544	0.319683487	0.044324857	0.172992426	UBR7
14	93207056	93229215	+	3694
protein_coding	ubiquitin protein ligase E3 component n-recognin 7 (putative) [Source:HGNC Symbol;Acc:HGNC:20344]	-	1936	2031
2063	1402	2106	1615	28.66178109	24.24080234
25.87049823	18.35068748	24.84391722	20.40899745
ENSG00000105825 401.4657015	827.7673688	359.8298171
762.4748705	2108.274737	824.2210364	529.6876291
1231.656881	-1.217225215	0.044328277	0.172992426	TFPI2
7	93885396	93890991	-	2682
protein_coding	tissue factor pathway inhibitor 2 [Source:HGNC Symbol;Acc:HGNC:11761]	-	347	893	368	759	2307
840	7.075634878	14.68001987	6.356111411
13.68309111	37.48412856	14.62064554
ENSG00000172575 8.09873173	5.561706845	10.75578258
51.23348932	30.15737595	6.868508636	8.138740384
29.4197913	-1.854863785	0.044337301	0.172992426
RASGRP1 15	38488103	38565575	-	6097
protein_coding	RAS guanyl releasing protein 1 [Source:HGNC Symbol;Acc:HGNC:9878]	-	7	6	11	51	33
7	0.062787987	0.043387938	0.083575493
0.404441021	0.235861112	0.053595445

ENSG00000147475 1675.280506 1939.181787 2033.820706
2295.461237 2303.840751 2246.002324 1882.761
2281.768104 -0.276916052 0.044374048 0.173030934 ERLIN2
8 37736601 37759101 + 9415
protein_coding ER lipid raft associated 2 [Source:HGNC
Symbol;Acc:HGNC:1356] - 1448 2092 2080 2285 2521
2289 8.41090931 9.796598598 10.23400127
11.73456775 11.66839491 11.34936345
ENSG00000121039 248.7467603 218.7604692 249.3385961
318.4512964 301.5737595 372.8618974 238.9486085
330.9623178 -0.470358738 0.044379119 0.173030934 RDH10
8 73294612 73325281 + 5321
protein_coding retinol dehydrogenase 10 [Source:HGNC
Symbol;Acc:HGNC:19975] - 215 236 255 317 330
380 2.209733571 1.955476954 2.219981612
2.880495624 2.702584482 3.333775676
ENSG00000116120 2702.662474 2373.921872 2503.163945
2208.062932 1876.154328 2170.448729 2526.582764
2084.888663 0.277137183 0.044381127 0.173030934 FARSB
2 222570536 222656337 - 3369
protein_coding phenylalanyl-tRNA synthetase subunit beta [Source:HGNC
Symbol;Acc:HGNC:17800] - 2336 2561 2560 2198 2053
2212 37.91984384 33.5152548 35.19989842
31.54480838 26.55501409 30.64997661
ENSG00000238137 0 0 0 0 4.569299387
15.69944831 0 6.756249233 -5.176062668 0.044389894
1 ARPC3P2 1 211442274 211442806 +
533 processed_pseudogene actin related protein 2/3
complex subunit 3 pseudogene 2 [Source:HGNC Symbol;Acc:HGNC:39431]
- 0 0 0 0 5 16 0
0 0 0 0.408791063 1.401324805
ENSG00000100583 98.34174244 53.76316617 55.73450972
89.40746176 164.4947779 117.7458623 69.27980611
123.8827007 -0.843958988 0.044405671 0.17308248 SAMD15
14 77376689 77391497 + 2616
protein_coding sterile alpha motif domain containing 15 [Source:HGNC
Symbol;Acc:HGNC:18631] - 85 58 57 89 180
120 1.776952827 0.977516759 1.009344851
1.644952961 2.998426194 2.141359292
ENSG00000065057 319.3214225 332.7754596 327.5624694
430.9640572 426.7725628 438.6033372 326.5531172
432.1133191 -0.403817255 0.044424346 0.173111133 NTHL1
16 2039815 2047866 - 2430 protein_coding nth
like DNA glycosylase 1 [Source:HGNC Symbol;Acc:HGNC:8028] -
276 359 335 429 467 447 6.211514755
6.513615717 6.386177558 8.535957323 8.374698961
8.587115124
ENSG00000258539 18.51138681 0 1.955596832 0 0
0 6.822327881 0 5.256832008 0.044445238
1 AC068896.1 10 124617080 124791727

- 3422 protein_coding novel transcript (METTL10-
FAM53B readthrough) - 16 0 2 0 0
0 0.255702333 0 0.027074001 0 0
0
ENSG00000084693 631.7010749 784.2006652 930.8640922
583.659947 533.6941684 664.2829067 782.2552774
593.8790074 0.398405195 0.044455008 0.17318647 AGBL5
2 27042364 27070622 + 5283
protein_coding ATP/GTP binding protein like 5 [Source:HGNC
Symbol;Acc:HGNC:26147] - 546 846 952 581 584
677 5.652059756 7.060309026 8.347545472
5.317368252 4.817157368 5.982105856
ENSG00000164258 776.3212844 756.3921309 782.2387329
532.4264577 530.9525888 719.2309758 771.6507161
594.2033407 0.377090531 0.044479998 0.173196988 NDUFS4
5 53560633 53683340 + 1176
protein_coding NADH:ubiquinone oxidoreductase subunit S4 [Source:HGNC
Symbol;Acc:HGNC:7711] - 671 816 800 530 581
733 31.20397427 30.59262865 31.51266416
21.79062889 21.52917601 29.09662982
ENSG00000176410 53.22023708 81.57170039 102.6688337
110.5036044 171.805657 126.576802 79.15359039
136.2953545 -0.781512412 0.044480367 0.173196988
DNAJC30 7 73680969 73683453 - 2485
protein_coding DnaJ heat shock protein family (Hsp40) member C30
[Source:HGNC Symbol;Acc:HGNC:16410] - 46 88 105
110 188 129 1.012339427 1.561313939
1.957335901 2.140264798 3.296780661 2.423312113
ENSG00000233966 172.3872897 185.3902282 232.716023
96.43950931 143.4760008 142.2762503 196.8311803
127.3972535 0.627551395 0.044547443 0.173413997
UBE2SP1 17 15704232 15704900 - 669
processed_pseudogene ubiquitin conjugating enzyme E2 S pseudogene 1
[Source:HGNC Symbol;Acc:HGNC:30864] - 149 200 238
96 157 145 12.18021245 13.18068059
16.47985154 6.938192286 10.22662032 10.11784263
ENSG00000166455 0 0 0 0 24.67421669 0
0 8.224738897 -5.461878964 0.044577398 1
C16orf46 16 81053497 81077267 -
2376 protein_coding chromosome 16 open reading frame 46
[Source:HGNC Symbol;Acc:HGNC:26525] - 0 0 0
0 27 0 0 0 0 0
0.495194629 0
ENSG00000259130 0 0 0 0 24.67421669 0
0 8.224738897 -5.461878964 0.044577398 1
AL133371.3 14 20870278 20873592 -
583 lincRNA novel transcript - 0 0
0 0 27 0 0 0 0 0
2.018151696 0
ENSG00000259736 16.19746346 4.634755704 11.73358099 0

0	2.943646558	10.85526672	0.981215519
3.473258319	0.044598469	1	CRTC3-AS1	15
90620007	90717141	-	957	antisense	CRTC3
antisense RNA 1 [Source:HGNC Symbol;Acc:HGNC:51433]	-	14
5	12	0	0	3	0.800038359
0.23035202	0.580860393	0	0	0.146337406
ENSG00000167114 752.0250892	793.4701766	822.328468
1263.759403	913.8598774	883.0939675	789.2745779
1020.237749	-0.369846573	0.044601571	0.173580504
SLC27A4 9	128340646	128361470	+	3110
protein_coding	solute carrier family 27 member 4 [Source:HGNC
Symbol;Acc:HGNC:10998]	-	650	856	841	1258	1000
900	11.43003816	12.13521105	12.52673997
19.5578661	14.01193806	13.50915412
ENSG00000002586 4405.710061	6303.267758	5736.743308
3415.565955	5037.195644	4499.854372	5481.907042
4317.538657	0.34456106	0.044628755	0.17364209	CD99
X	2691133 2741309 +	4912	protein_coding	CD99
molecule (Xg blood group) [Source:HGNC Symbol;Acc:HGNC:7082]	-
3808	6800	5867	3400	5512	4586	42.39682108
61.03578191	55.3299383	33.46738466	48.90006636
43.58346232
ENSG00000151718 1166.217369	1230.064164	1492.120383
1095.99484	981.4855083	1033.219942	1296.133972
1036.900097	0.32236686	0.04465192	0.173688014	WWC2
4	183099293	183320777	+	9966
protein_coding	WW and C2 domain containing 2 [Source:HGNC
Symbol;Acc:HGNC:24148]	-	1008	1327	1526	1091	1074
1053	5.531391042	5.870620381	7.093100391
5.293038882	4.696150389	4.932345884
ENSG00000182372 1126.880672	1047.454789	1055.044491
1493.807816	1292.197867	1213.763598	1076.459984
1333.256427	-0.30880902	0.044676676	0.173740105	CLN8
8	1755778 1801711 +	9459	protein_coding	"CLN8,
transmembrane ER and ERGIC protein [Source:HGNC
Symbol;Acc:HGNC:2079]"	-	974	1130	1079	1487	1414
1237	5.631297144	5.267046784	5.284193044
7.600933966	6.514225402	6.104786919
ENSG00000278374 2.313923351	2.780853423	16.62257307	0
0.913859877	0	7.239116616	0.304619959
4.389291011	0.04468167	1	RF00015 8	123454981
123455107	-	127	snRNA	-	2
3	17	0	1	0	0.861233645
1.041481337	6.200799034	0	0.343126987	0
ENSG00000113578 423.4479733	333.7024107	405.7863427
256.1674466	235.7758484	340.4817853	387.6455756
277.4750267	0.482013782	0.044743119	0.173944141	FGF1
5	142592178	142698070	-	5275
protein_coding	fibroblast growth factor 1 [Source:HGNC
Symbol;Acc:HGNC:3665]	-	366	360	415	255	258

347	3.794489314	3.008943236	3.644417208
2.337324078	2.131355234	3.07081071
ENSG00000198856 2094.100633	1748.229852	1902.795718
1395.35915	1655.000238	1626.855331	1915.042068
1559.071573	0.296220191	0.044751901	0.173944141	OSTC
4	108650584	108667820	+	1617
protein_coding	oligosaccharyltransferase complex non-catalytic
subunit [Source:HGNC Symbol;Acc:HGNC:24448]	-	1810	1886
1946	1389	1811	1658	61.21576311	51.42397293
55.74876768	41.53301341	48.80530466	47.86527321
ENSG00000118503 452.3720152	558.9515379	381.3413823
700.1910208	705.4998254	492.5701908	464.2216451
632.753679	-0.446512252	0.044772606	0.173980383
TNFAIP3 6	137867188	137883312	+	5051
protein_coding	TNF alpha induced protein 3 [Source:HGNC
Symbol;Acc:HGNC:11896]	-	391	603	390	697	772
502	4.233446752	5.263491205	3.576759129
6.67200904	6.660372662	4.639512877
ENSG00000177674 893.1744137	1251.38404	1082.422847
1200.470975	1506.041078	1406.081839	1075.660433
1370.864631	-0.34923194	0.044822364	0.174129472	AGTRAP
1	11736084	11754802	+	2435
protein_coding	angiotensin II receptor associated protein
[Source:HGNC Symbol;Acc:HGNC:13539]	-	772	1350	1107
1195	1648	1433	17.33856087	24.44380221
21.05964816	23.72849296	29.49285662	27.47218869
ENSG00000137312 2166.989219	2071.7358	2458.185218
1531.981789	1912.708723	1972.243194	2232.303412
1805.644569	0.305919731	0.04486714	0.17425914	FLOT1
6	30727709	30742733	-	3186
protein_coding	flotillin 1 [Source:HGNC Symbol;Acc:HGNC:3757]	-
1873	2235	2514	1525	2093	2010	32.15042503
30.92898828	36.55291005	23.1433002	28.62740978
29.45074747
ENSG00000137871 529.8884475	355.9492381	321.6956789
309.4100924	290.607441	249.2287419	402.5111215
283.0820918	0.506180731	0.044903925	0.174315045
ZNF280D 15	56630181	56734086	-	12113
protein_coding	zinc finger protein 280D [Source:HGNC
Symbol;Acc:HGNC:25953]	-	458	384	329	308	318
254	2.067799726	1.397698391	1.25819118
1.229419836	1.14402101	0.978876799
ENSG00000089053 3078.675019	2921.749996	3100.598778
2695.28337	2396.140599	2540.36698	3033.674598
2543.930316	0.254031404	0.04490434	0.174315045	ANAPC5
12	121308245	121399896	-	12304
protein_coding	anaphase promoting complex subunit 5 [Source:HGNC
Symbol;Acc:HGNC:15713]	-	2661	3152	3171	2683	2622
2589	11.82750839	11.29467775	11.93857179
10.54327579	9.286348173	9.822719984

ENSG00000169612 202.4682933 364.2917984 307.0287027
389.7763501 356.4053522 520.0442253 291.2629314
422.0753092 -0.532833264 0.044958766 0.174482016 RAMAC
15 82986207 82991057 + 1568
protein_coding RNA guanine-7 methyltransferase activating subunit
[Source:HGNC Symbol;Acc:HGNC:31022] - 175 393 314
388 390 530 6.103608977 11.05046237
9.276540512 11.96428869 10.83869877 15.77886815
ENSG00000154358 4.627846703 5.561706845 6.844588913
12.05493866 34.72667534 18.64309487 5.678047487
21.80823629 -1.939670944 0.044976326 0.174487119 OBSCN
1 228208130 228378874 + 32290
protein_coding "obscurin, cytoskeletal calmodulin and titin-
interacting RhoGEF [Source:HGNC Symbol;Acc:HGNC:15719]" - 4
6 7 12 38 19 0.006774647
0.008192513 0.010042283 0.017968607 0.051283086
0.027468288
ENSG00000130052 677.979542 656.2814077 967.0426336
569.5958519 571.1624234 604.42876 767.1011944
581.7290118 0.399384615 0.044982909 0.174487119 STARD8
X 68647666 68725842 + 5558
protein_coding StAR related lipid transfer domain containing 8
[Source:HGNC Symbol;Acc:HGNC:19161] - 586 708 989
567 625 616 5.765988692 5.616278988
8.242903299 4.932484543 4.900270708 5.173782953
ENSG00000219085 11.56961676 4.634755704 0.977798416 0
0 0 5.727390293 0 5.006264878
0.045001434 1 NPM1P37 6 69705287 69706160
- 874 processed_pseudogene nucleophosmin 1
pseudogene 37 [Source:HGNC Symbol;Acc:HGNC:45216] - 10
5 1 0 0 0 0.625724673
0.252227555 0.053001849 0 0 0
ENSG00000196172 83.30124065 50.05536161 75.29047804
43.19686355 11.88017841 38.26740526 69.54902677
31.11481574 1.162107968 0.045006147 0.17453297 ZNF681
19 23739195 23758891 - 6497
protein_coding zinc finger protein 681 [Source:HGNC
Symbol;Acc:HGNC:26457] zf-C2H2 72 54 77 43 13
39 0.606058215 0.366450105 0.549010075
0.32000503 0.087194498 0.280219127
ENSG00000127884 1674.123545 1905.811546 2070.977045
1415.450715 1552.647932 1634.705055 1883.637379
1534.267901 0.296251464 0.045056819 0.174685161 ECHS1
10 133362480 133373689 - 1617
protein_coding "enoyl-CoA hydratase, short chain 1 [Source:HGNC
Symbol;Acc:HGNC:3151]" - 1447 2056 2118 1409 1699
1666 48.93878962 56.05921969 60.67620244
42.13104096 45.78697549 48.09622748
ENSG00000117595 5.784808379 5.561706845 4.888992081 0
0 0 5.411835768 0 4.928738927

0.04508317	1	IRF6	1	209785623	209806175
	-	4965	protein_coding	interferon regulatory factor 6
[Source:HGNC Symbol;Acc:HGNC:6121]	IRF	5	6	5
	0	0	0	0.055073853	0.053280213
0.046650167	0	0	0
ENSG00000127920 675.6656186	696.1403068	502.5883859
692.154395	892.8411002	878.1878899	624.7981038
821.0611284	-0.394538331	0.045088244	0.174722911	GNG11
	7	93921699	93928610	+	3055
protein_coding	G protein subunit gamma 11 [Source:HGNC
Symbol;Acc:HGNC:4403]	-	584	751	514	689	977
	895	10.45433338	10.83833774	7.793891583
10.90458705	13.93612223	13.67596109
ENSG00000130270 38.1797353	57.47097073	107.5578258
31.14192488	33.81281546	29.43646558	67.73617727
31.46373531	1.108209462	0.045089415	0.174722911	ATP8B3
	19	1782075 1812276 -	7014	protein_coding	ATPase
phospholipid transporting 8B3 [Source:HGNC Symbol;Acc:HGNC:13535]
	-	33	62	110	31	37	30
0.257301839	0.389726454	0.726489563	0.213696372
0.229876492	0.19966481
ENSG00000179918 1435.78944	1504.441702	1493.098181
1847.41935	1750.955525	1744.601194	1477.776441
1780.992023	-0.269062071	0.045117523	0.174761251	SEPHS2
	16	30443631	30446181	-	2551
protein_coding	selenophosphate synthetase 2 [Source:HGNC
Symbol;Acc:HGNC:19686]	-	1241	1623	1527	1839	1916
	1778	26.60455724	28.05059121	27.72879738
34.85559391	32.72982203	32.53623782
ENSG00000157538 2149.634793	1823.312894	1838.261022
2479.299052	2421.728675	2163.58022	1937.06957
2354.869316	-0.282172053	0.045131281	0.174761251	VPS26C
	21	37223420	37267919	-	9301
protein_coding	VPS26 endosomal protein sorting factor C [Source:HGNC
Symbol;Acc:HGNC:3044]	-	1858	1967	1880	2468	2650
	2205	10.92473165	9.324137591	9.363337132
12.82970668	12.41580341	11.06687451
ENSG00000280241 5.784808379	9.269511408	2.933395248
38.17397244	15.53561792	15.69944831	5.995905012
23.13634622	-1.943101145	0.045142032	0.174761251
AC079298.3	4	154142122	154298819	+	1376
	antisense	"novel transcript, antisense to DCHS2"	-
	5	10	3	38	17	16	0.198722153
	0.320416981	0.100996256	1.33526166	0.538380207
	0.542809681
ENSG00000109111 5266.489548	5862.965966	5915.680418
7844.751336	5639.429304	7390.515293	5681.711977
6958.231977	-0.292243125	0.045145037	0.174761251	SUPT6H
	17	28662091	28702684	+	10620
protein_coding	"SPT6 homolog, histone chaperone [Source:HGNC

Symbol;Acc:HGNC:11470]" -	4552	6325	6050	7809	6171
	7532	23.44080108	26.25850347	26.38963076
35.55266188	25.32151157	33.10791492
ENSG00000085978 926.7263022	935.2937011	854.5958157
1277.823498	1214.519777	946.8729763	905.5386064
1146.405417	-0.340359606	0.045184485	0.174869677
ATG16L1 2	233210051	233295674	+	6047
protein_coding	autophagy related 16 like 1 [Source:HGNC
Symbol;Acc:HGNC:21498]	-	801	1009	874	1272	1329
	965	7.244147095	7.356735731	6.695359791
10.1706418	9.577311436	7.449607312
ENSG00000173599 673.3516953	1022.427108	1237.892795
524.3898319	779.5224754	813.4276656	977.8905328
705.779991	0.470949909	0.045245755	0.175062481	PC
	11	66848233	66958376	-	5927
protein_coding	pyruvate carboxylase [Source:HGNC
Symbol;Acc:HGNC:8636]	-	582	1103	1266	522	853
	829	5.37010491	8.204923632	9.894668171
4.258305273	6.271518413	6.529285201
ENSG00000267064 12.72657843	0	11.73358099	0	0
	0.981215519	8.153386476	0.32707184	4.555305243
	0.045259638	1	UXT-AS1 X	47658833
47660377	+	1545	antisense	UXT antisense RNA 1
[Source:HGNC Symbol;Acc:HGNC:49239]	-	11	0	12
	0	0	1	0.389366797	0	0.359795078
	0	0	0.030214649
ENSG00000083812 295.0252273	346.6797267	469.3432398
451.0556217	512.6753912	565.1801392	370.3493979
509.6370507	-0.459548749	0.045260754	0.175076202	ZNF324
	19	58467045	58475436	+	5664
protein_coding	zinc finger protein 324 [Source:HGNC
Symbol;Acc:HGNC:14096]	zf-C2H2 255	374	480	449	561
	576	2.462133791	2.911268863	3.925730196
3.832871971	4.316166745	4.747284667
ENSG00000173875 798.3035562	594.1756813	572.9898719
965.3996714	843.4926669	760.4420276	655.1563698
856.4447886	-0.387617221	0.045282673	0.175116675	ZNF791
	19	12610918	12633840	+	6642
protein_coding	zinc finger protein 791 [Source:HGNC
Symbol;Acc:HGNC:26895]	zf-C2H2 690	641	586	961	923
	775	5.681263496	4.254936824	4.086967654
6.995612304	6.055659221	5.44689329
ENSG00000099889 40.49365865	86.2064561	75.29047804
133.6089035	104.180026	119.7082934	67.3301976
119.165741	-0.818374861	0.045309403	0.175134329	ARVCF
	22	19969896	20016808	-	5621
protein_coding	"ARVCF, delta catenin family member [Source:HGNC
Symbol;Acc:HGNC:728]"	-	35	93	77	133	114
	122	0.340525133	0.729463089	0.634570086
1.144034897	0.883791588	1.013193235

ENSG00000113742 443.1163218 461.6216681 449.7872714
692.154395 597.6643598 495.5138373 451.5084205
595.110864 -0.39814052 0.045310151 0.175134329 CPEB4
5 173888280 173961976 + 10272
protein_coding cytoplasmic polyadenylation element binding protein 4
[Source:HGNC Symbol;Acc:HGNC:21747] - 383 498 460
689 654 505 2.039099772 2.137510664
2.074461011 3.243137991 2.774478319 2.294998485
ENSG00000282855 0 0 1.955596832 12.05493866
7.310879019 7.849724156 0.651865611
9.07184728 -3.772391159 0.045323212 1 AC093591.3
4 127809170 127809477 + 308
processed_pseudogene INTS3 and NABP interacting protein (INIP)
pseudogene - 0 0 2 12 8 8
0 0 0.300802703 1.883786785 1.131873438
1.212509936
ENSG00000100304 1548.014722 2235.806152 2038.709698
2351.717618 2397.968318 2507.005652 1940.843524
2418.897196 -0.317067252 0.04535085 0.175247332 TTLL12
22 43166622 43187133 - 4622
protein_coding tubulin tyrosine ligase like 12 [Source:HGNC
Symbol;Acc:HGNC:28974] - 1338 2412 2085 2341 2624
2555 15.83145698 23.00812988 20.89674167
24.48909235 24.73958939 25.80518632
ENSG00000160219 174.701213 124.2114529 176.0037149
129.5905906 84.9889686 74.57237948 158.3054603
96.38397957 0.715703323 0.045408012 0.175379964 GAB3
X 154675249 154751583 - 5272
protein_coding GRB2 associated binding protein 3 [Source:HGNC
Symbol;Acc:HGNC:17515] - 151 134 180 129 93
76 1.566376859 1.120632864 1.581610572
1.183083848 0.768716397 0.672952214
ENSG00000182612 56.69112211 120.5036483 118.3136084
114.5219173 173.6333767 221.7547074 98.50279293
169.9700005 -0.78353557 0.045416994 0.175379964
TSPAN10 17 81637171 81648749 + 4745
protein_coding tetraspanin 10 [Source:HGNC Symbol;Acc:HGNC:29942]
- 49 130 121 114 190 226
0.564747837 1.207928125 1.181276623 1.161635434
1.744921854 2.223403363
ENSG00000177732 1041.265508 1619.383643 1832.394232
1614.357203 1964.798736 2416.733824 1497.681128
1998.629921 -0.415704212 0.045422888 0.175379964 SOX12
20 325595 330224 + 4630 protein_coding SRY-
box 12 [Source:HGNC Symbol;Acc:HGNC:11198] HMG 900 1747
1874 1607 2150 2463 10.63056216 16.63588356
18.74955874 16.78170576 20.2355991 24.83301533
ENSG00000213654 219.8227184 490.3571535 500.6327891
514.3440497 595.8366401 667.2265532 403.6042203
592.469081 -0.551866672 0.045431063 0.175379964 GPSM3

6	32190766	32195523	-	1854
protein_coding	G protein signaling modulator 3 [Source:HGNC Symbol;Acc:HGNC:13945]	-	190	529	512	512	652
680	5.604522072	12.57997854	12.79271389
13.35246498	15.32485816	17.12163438
ENSG00000273523 9.255693406	0.926951141	6.844588913	0
0	0	5.675744487	0	4.994019347
0.045432927	1	AL139082.1	13	52128891
52132723	+	3433	antisense	uncharacterized
LOC101929657 [Source:NCBI gene;Acc:101929657]	-	8	1
7	0	0	0	0.127441506	0.012842813
0.094455378	0	0	0
ENSG00000276740 35.86581195	21.31987624	12.71137941
5.02289111	6.397019142	7.849724156	23.29902253
6.423211469	1.854700402	0.045454765	0.175427165
AL445649.1	13	107465984	107466674	-	691
sense_intronic	"novel transcript, sense intronic to FAM155A"
-	31	23	13	5	7	8
2.453456482	1.467519047	0.871500741	0.349859099
0.441447021	0.540453054
ENSG00000163632 35.86581195	22.24682738	15.64477466
89.40746176	32.89895559	51.02320701	24.58580466
57.77654145	-1.237210049	0.045482124	0.175488446
C3orf49 3	63819362	63848636	+	1581
protein_coding	chromosome 3 open reading frame 49 [Source:HGNC Symbol;Acc:HGNC:25190]	-	31	24	16	89	36
52	1.072320322	0.669288449	0.468803201
2.721819701	0.992268555	1.535385764
ENSG00000260541 2.313923351	1.853902282	2.933395248
13.05951689	17.36333767	9.812155195	2.367073627
13.41166992	-2.50426314	0.045556697	1	AL031722.1
16	1841020 1843547 -	220	sense_overlapping
"novel transcript, overlapping C16orf73"	-	2
2	3	13	19	10	0.497166695
0.400812514	0.631685677	2.857076625	3.763479181
2.121892389
ENSG00000198846 275.3568788	141.8235245	160.3589403
352.6069559	305.2291991	227.6420005	192.5131145
295.1593852	-0.619698271	0.045574829	0.175754605	TOX
8	58805418	59119208	-	4131
protein_coding	thymocyte selection associated high mobility group box [Source:HGNC Symbol;Acc:HGNC:18988]	HMG	238	153	164
351	334	232	3.150768354	1.632939873
1.839039718	4.108214754	3.523301994	2.621674837
ENSG00000187742 1160.432561	1214.305995	1053.088894
1255.722778	1587.374607	1416.87521	1142.60915
1419.990865	-0.31374401	0.045581256	0.175754605
SECISBP2	9	89318506	89359662	+	9813
protein_coding	SECIS binding protein 2 [Source:HGNC Symbol;Acc:HGNC:30972]	-	1003	1310	1077	1250	1737

1444 5.589768821 5.885772273 5.084126645
6.158989031 7.713591176 6.869283339
ENSG00000129028 303.123959 168.7051076 258.1387819
189.865284 172.7195168 88.30939675 243.3226162
150.2980658 0.693523308 0.045589067 0.175754605 THAP10
15 70881342 70892785 - 2399
protein_coding THAP domain containing 10 [Source:HGNC
Symbol;Acc:HGNC:23193] THAP 262 182 264 189 189
90 5.97263199 3.344838073 5.097721845
3.809191203 3.43312925 1.751290925
ENSG00000108819 2021.212047 2745.629279 3559.186235
3563.238954 3150.074997 4078.912914 2775.34252
3597.408955 -0.373919577 0.045597093 0.175754605
PPP1R9B 17 50133735 50150630 - 4298
protein_coding protein phosphatase 1 regulatory subunit 9B
[Source:HGNC Symbol;Acc:HGNC:9298] - 1747 2962 3640
3547 3447 4157 22.22906555 30.38453546
39.23172717 39.90212215 34.94889669 45.15019696
ENSG00000116793 1531.817259 1282.900379 1476.475608
1209.512179 760.331418 1291.279624 1430.397749
1087.041074 0.396152806 0.045642555 0.17588549 PHTF1
1 113696831 113759489 - 7170
protein_coding putative homeodomain transcription factor 1
[Source:HGNC Symbol;Acc:HGNC:8939] - 1324 1384 1510
1204 832 1316 10.09865515 8.510418017
9.755740674 8.119112287 5.05664853 8.568065334
ENSG00000138821 1349.017314 1119.756978 1142.06855
947.3172634 907.4628583 1041.069666 1203.614281
965.2832626 0.317894009 0.045667417 0.175936947
SLC39A8 4 102251041 102431258 - 6480
protein_coding solute carrier family 39 member 8 [Source:HGNC
Symbol;Acc:HGNC:20862] - 1166 1208 1168 943 993
1061 9.840524734 8.219130696 8.349688866
7.03619559 6.677791275 7.643396935
ENSG00000169926 539.1441409 520.01959 548.5449115
569.5958519 778.6086156 744.7425793 535.9028808
697.6490156 -0.380939764 0.045718474 0.176051916 KLF13
15 31326855 31435665 + 9409
protein_coding Kruppel like factor 13 [Source:HGNC
Symbol;Acc:HGNC:13672] zf-C2H2 466 561 561 567 852
759 2.708551895 2.628774605 2.761988389
2.913672982 3.945978585 3.765688076
ENSG00000160305 966.0629992 1024.281011 1146.957542
791.607639 626.9078759 986.1215971 1045.767184
801.545704 0.384245481 0.045720292 0.176051916 DIP2A
21 46458899 46569852 + 13557
protein_coding disco interacting protein 2 homolog A [Source:HGNC
Symbol;Acc:HGNC:17217] - 835 1105 1173 788 686
1005 3.368352948 3.593624041 4.008084527
2.810372181 2.205053431 3.460576877

ENSG00000255434 0 0 0 10.04578222 0
9.812155195 0 6.619312472 -5.144992207 0.045725127
1 AP001922.6 11 75596144 75597270
- 546 sense_intronic novel transcript -
0 0 0 10 0 10 0 0
0 0.885540797 0 0.854974955
ENSG00000116209 4052.83675 3981.25515 3855.459155
2884.144075 3514.705089 3499.014542 3963.183685
3299.287902 0.264383724 0.045735404 0.176065758 TMEM59
1 54026681 54053504 - 9149
protein_coding transmembrane protein 59 [Source:HGNC
Symbol;Acc:HGNC:1239] - 3503 4295 3943 2871 3846
3566 20.93925484 20.69776723 19.96436978
15.172627 18.31868311 18.19507068
ENSG00000169228 30.08100357 57.47097073 82.13506696
69.31589732 102.3523063 160.9193452 56.56234709
110.8625163 -0.966857476 0.045780019 0.176193139 RAB24
5 177301198 177303744 - 2065
protein_coding "RAB24, member RAS oncogene family [Source:HGNC
Symbol;Acc:HGNC:9765]" - 26 62 84 69 112
164 0.688569853 1.323748837 1.884350494
1.615586633 2.363505212 3.707403263
ENSG00000237849 0 0 0 13.05951689 0
6.868508636 0 6.642675174 -5.149888522 0.045795673
1 NFYAP1 13 63975451 63976422 -
972 processed_pseudogene nuclear transcription factor Y
subunit alpha pseudogene 1 [Source:HGNC Symbol;Acc:HGNC:34078] -
0 0 0 13 0 7 0 0
0 0.646663434 0 0.336184597
ENSG00000243547 143.4632478 101.0376744 147.6475608
67.30674088 86.81668835 82.42210364 130.716161
78.84851096 0.72750107 0.045870562 0.176497175
HNRNPKP4 3 96349554 96350939 + 1386
processed_pseudogene heterogeneous nuclear
ribonucleoprotein K pseudogene 4 [Source:HGNC Symbol;Acc:HGNC:42377]
- 124 109 151 67 95 84
4.8927516 3.467346354 5.046800911 2.337291012
2.986888239 2.829189852
ENSG00000000003 1861.551336 1167.958437 1423.674494
1141.20086 1127.703089 1195.120503 1484.394756
1154.674817 0.361637304 0.045964337 0.176813491 TSPAN6
X 100627109 100639991 - 4535
protein_coding tetraspanin 6 [Source:HGNC Symbol;Acc:HGNC:11858]
- 1609 1260 1456 1136 1234 1218
19.4032047 12.24974961 14.87258773 12.11162056
11.85759099 12.53764685
ENSG00000179954 254.5315687 204.8562021 442.9426825
212.9705831 144.3898606 220.7734919 300.7768178
192.7113119 0.643131121 0.045989924 0.176867414 SSC5D
19 55488404 55519098 + 7314

protein_coding scavenger receptor cysteine rich family member with 5 domains [Source:HGNC Symbol;Acc:HGNC:26641]   -   220   221
        453   212   158   225   1.644986877   1.332205664
        2.869100108   1.401464565   0.941370812   1.436063348
ENSG00000121440 261.4733387   328.1407039   400.8973506
249.1353991   258.6223453   176.6187935   330.1704644
228.1255126   0.534151736   0.046030991   0.176980824   PDZRN3
        3   73382433   73624940   -   6136
protein_coding PDZ domain containing ring finger 3 [Source:HGNC
Symbol;Acc:HGNC:17704] -   226   354   410   248   283
        180   2.01427054   2.54361788   3.09528727
1.954193419   2.009831656   1.369409038
ENSG00000273382 18.51138681   16.68512054   15.64477466
17.07782977   44.77913399   72.60994844   16.947094
44.82230407   -1.404923361   0.046067244   0.177075675
AL356488.3   1   109087971   109090858   -   2888
        antisense   "novel transcript, antisense to TMEM167B"
        -       16   18   16   17   49   74
0.302982473   0.274795283   0.256640534   0.284611831
0.739362618   1.196136014
ENSG00000265867 0   0   0   14.06409511   0
5.887293117   0   6.650462742   -5.15152246   0.046072071
        1   MIR4516 16   2133119 2133204 +   86   miRNA
        microRNA 4516 [Source:HGNC Symbol;Acc:HGNC:41617]   -
        0   0   0   14   0   6   0   0
        0   7.871016103   0   3.256858085
ENSG00000206647 5.784808379   0   13.68917783   0   0
        0   6.491328735   0   5.190814819   0.046193462
        1   RF00410 2   10155072   10155206   -
        135   snoRNA   -   5   0   14   0
        0   0   2.025493942   0   4.803930581   0
        0   0
ENSG00000232815 20.82531016   63.95962872   45.95652556
94.43035287   69.45335068   99.10276747   43.58048815
87.66215701   -1.000972319   0.046197179   0.177530486
DUX4L50 9   63817748   63818462   -   715
unprocessed_pseudogene double homeobox 4 like 50 (pseudogene)
[Source:HGNC Symbol;Acc:HGNC:51788]   -   18   69   47
        94   76   101   1.376769309   4.254778999
3.045048904   6.356572845   4.631974376   6.594188655
ENSG00000133812 1191.670526   1157.761975   1169.446906
1089.967371   910.2044379   789.8784932   1172.959802
930.0167673   0.33489158   0.046258449   0.177721263   SBF2
        11   9778667 10294207   -   16767   protein_coding
        SET binding factor 2 [Source:HGNC Symbol;Acc:HGNC:2135] -
        1030   1249   1196   1085   996   805   3.359514912
        3.284286476   3.304290876   3.12878406   2.588585844
        2.241230644
ENSG00000184995 27.76708022   30.58938765   14.66697624
58.26553688   37.46825497   69.66630188   24.34114804

55.13336458	-1.179555348	0.046321395	0.177918383	IFNE
	9	21480839	21482313	-	1475
protein_coding	interferon epsilon [Source:HGNC Symbol;Acc:HGNC:18163]
	-	24	33	15	58	41	71
0.889844118	0.986406391	0.471087624	1.901241081
1.211296421	2.247048076
ENSG00000100906 2058.234821	2827.20098	2044.576488
2546.605793	3604.263357	2700.30511	2310.004096
2950.39142	-0.352858355	0.046344636	0.177962936	NFKBIA
	14	35401511	35404749	-	2373
protein_coding	NFKB inhibitor alpha [Source:HGNC
Symbol;Acc:HGNC:7797]	-	1779	3050	2091	2535	3944
	2752	40.99896777	56.66776172	40.81866064
51.65132204	72.42654458	54.13731681
ENSG00000266088 13.88354011	12.05036483	5.866790497	0
	0	2.943646558	10.60023181	0.981215519
3.440743718	0.046354347	1	AC004585.1	17
40517026	40527002	+	1198	lincRNA novel
transcript	-	12	13	6	0	0	3
	0.547796358	0.4784323	0.232004756	0	0
	0.116898913
ENSG00000140332 558.8124894	656.2814077	804.7280965
831.7907678	918.4291768	859.5447951	673.2739979
869.9215799	-0.368969505	0.046368937	0.178011539	TLE3
	15	70047790	70098176	-	12634
protein_coding	transducin like enhancer of split 3 [Source:HGNC
Symbol;Acc:HGNC:11839]	-	483	708	823	828	1005
	876	2.090744538	2.470736	3.017598245
3.16876973	3.466440794	3.23675084
ENSG00000204390 31.23796524	29.66243651	31.28954932
71.32505376	53.91773277	60.83536221	30.72998369
62.02604958	-1.013068229	0.046399899	0.178085678	HSPA1L
	6	31809619	31815065	-	2544
protein_coding	heat shock protein family A (Hsp70) member 1 like
[Source:HGNC Symbol;Acc:HGNC:5234]	-	27	32	32
	71	59	62	0.580418665	0.554583353
0.582686998	1.349405445	1.010633064	1.137681297
ENSG00000186106 378.326468	489.4302024	396.986157
548.4997092	583.0426018	531.8188116	421.5809424
554.4537075	-0.39431672	0.046422047	0.178125961
ANKRD46 8	100509752	100559784	-	5820
protein_coding	ankyrin repeat domain 46 [Source:HGNC
Symbol;Acc:HGNC:27229]	-	327	528	406	546	638
	542	3.072695191	3.999860968	3.231510004
4.535977321	4.777011555	4.347327293
ENSG00000090006 1468.184366	2276.592002	2065.110255
2603.866751	2190.522126	2589.427756	1936.628874
2461.272211	-0.345181347	0.046469151	0.178261959	LTBP4
	19	40592883	40629818	+	8108
protein_coding	latent transforming growth factor beta binding protein

4 [Source:HGNC Symbol;Acc:HGNC:6717]   -   1269   2456   2112
        2592   2397   2639   8.55938566   13.35514416
12.06653646   15.45690272   12.88287793   15.19398475
ENSG00000263815 2.313923351   17.61207168   0   0   0
        0   6.641998343   0   5.228088844   0.046482281
        1   RN7SL426P   17   21229334   21229628
        -       295   misc_RNA   "RNA, 7SL, cytoplasmic 426,
pseudogene [Source:HGNC Symbol;Acc:HGNC:46442]" -   2   19
        0   0   0   0   0.370768383   2.839654763
        0   0   0   0
ENSG00000153317 3386.426825   2893.941462   3624.698729
3152.366461   2405.279197   2447.151506   3301.689005
2668.265721   0.307323267   0.046485423   0.178279643   ASAP1
        8   130052104   130443660   -   8280
protein_coding "ArfGAP with SH3 domain, ankyrin repeat and PH domain
1 [Source:HGNC Symbol;Acc:HGNC:2720]"   -   2927   3122   3707
        3138   2632   2494   19.33245903   16.62403788
20.73932919   18.32414919   13.85205304   14.06086855
ENSG00000240135 0   0   0   15.06867333   0
4.906077597   0   6.658250309   -5.153158626   0.046521611
        1   PSMD12P 3   72876737   72878102   -
        1366   unprocessed_pseudogene "proteasome 26S subunit, non-
ATPase, 12 pseudogene [Source:HGNC Symbol;Acc:HGNC:30153]"   -
        0   0   0   15   0   5   0   0
        0   0.530935514   0   0.170869812
ENSG00000115896 13.88354011   14.83121825   18.57816991
31.14192488   70.36721056   23.54917247   15.76430942
41.68610264   -1.403247275   0.046562271   0.178529581   PLCL1
        2   197804702   198572581   +   8013
protein_coding phospholipase C like 1 (inactive) [Source:HGNC
Symbol;Acc:HGNC:9063]   -   12   16   19   31   77
        24   0.081899418   0.088035695   0.109840099
0.187054331   0.418749383   0.139817694
ENSG00000124224 481.2960571   435.6670362   355.9186235
293.3368408   294.2628805   348.3315094   424.2939056
311.9770769   0.443005876   0.046676551   0.178860739
PPP4R1L 20   58228940   58309439   -   9251
transcribed_unprocessed_pseudogene   protein phosphatase 4
regulatory subunit 1 like (pseudogene) [Source:HGNC
Symbol;Acc:HGNC:15755] -   416   470   364   292   322
        355   2.459231213   2.239974813   1.822699853
1.526143555   1.516791159   1.791371696
ENSG00000025796 2232.936034   2004.995318   2357.471981
1915.730669   1773.802022   1769.131582   2198.467778
1819.554758   0.27285176   0.046682554   0.178860739   SEC63
        6   107867756   107958189   -   8587
protein_coding "SEC63 homolog, protein translocation regulator
[Source:HGNC Symbol;Acc:HGNC:21082]"   -   1930   2163   2411
        1907   1941   1803   12.29166057   11.10577868
13.00643286   10.73767974   9.850146057   9.801675032

ENSG00000199906 0 0 0.977798416 0 22.84649694
2.943646558 0.325932805 8.596714498 -4.564460808
0.046682609 1 RNU5B-2P 3 40498891
40499003 - 113 snRNA "RNA, U5B small nuclear 2, pseudogene [Source:HGNC Symbol;Acc:HGNC:34182]" -
0 0 1 0 25 3 0 0
0.409943507 0 9.640957379 1.239335378
ENSG00000104951 30.08100357 71.37523785 60.6235018
102.4669786 133.4235421 72.60994844 54.02658107
102.8334897 -0.923937139 0.046683741 0.178860739 IL4I1
19 49889654 49929539 - 2563
protein_coding interleukin 4 induced 1 [Source:HGNC Symbol;Acc:HGNC:19094] - 26 77 62 102 146
74 0.554778286 1.324573545 1.120586895
1.924211395 2.482349042 1.347811474
ENSG00000138079 8.09873173 0.926951141 7.822387329 0
0 0 5.6160234 0 4.979553444
0.046696803 1 SLC3A1 2 44275458 44321494
+ 6541 protein_coding solute carrier family 3 member 1 [Source:HGNC Symbol;Acc:HGNC:11025] - 7 1 8
0 0 0 0.058525968 0.006740464
0.056656311 0 0 0
ENSG00000260953 4.627846703 5.561706845 5.866790497 0
0 0 5.352114682 0 4.913680816
0.046699344 1 AC009093.4 16 29262273
29264479 - 561 lincRNA novel transcript -
4 6 6 0 0 0 0.389934663
0.471544135 0.495439747 0 0 0
ENSG00000251333 38.1797353 14.83121825 20.53376674 0
2.741579632 12.75580175 24.51490676 5.165793795
2.244627461 0.046717825 0.178946479 RTN3P1 4
145375543 145376253 + 711
processed_pseudogene reticulon 3 pseudogene 1 [Source:HGNC Symbol;Acc:HGNC:19212] - 33 16 21 0 3
13 2.538277218 0.992165999 1.368208077 0
0.183869736 0.85353196
ENSG00000119401 558.8124894 516.3117855 647.3025515
768.5023398 709.1552649 725.1182689 574.1422754
734.2586245 -0.354797619 0.046785623 0.179120643 TRIM32
9 116687302 116701300 + 3780
protein_coding tripartite motif containing 32 [Source:HGNC Symbol;Acc:HGNC:16380] - 483 557 662 765 776
739 6.9879541 6.496767924 8.112760317
9.785225802 8.945992282 9.126382661
ENSG00000275160 37.02277362 33.37024107 40.08973506
24.10987733 9.138598774 4.906077597 36.82758325
12.71818457 1.537026365 0.04679492 0.179120643
AL354718.1 9 41479960 41480548 + 589
processed_pseudogene ribosomal protein L7a (RPL7A) pseudogene - 32 36 41 24 10 5

2.971182964 2.69476665 3.224564124 1.970140339
0.739849361 0.396278714
ENSG00000119013 350.5593877 431.0322805 400.8973506
179.8195017 164.4947779 415.0541647 394.1630063
253.1228148 0.639785034 0.046798446 0.179120643 NDUFB3
2 201071433 201085750 + 1103
protein_coding NADH:ubiquinone oxidoreductase subunit B3 [Source:HGNC
Symbol;Acc:HGNC:7698] - 303 465 410 179 180
423 15.02317855 18.58708985 17.21911395
7.846549792 7.111407909 17.90238492
ENSG00000152760 30.08100357 6.488657986 2.933395248 0
0 3.924862078 13.1676856 1.308287359
3.332805194 0.046814799 1 TCTEX1D1 1
66752459 66778787 + 3100 protein_coding Tctex1
domain containing 1 [Source:HGNC Symbol;Acc:HGNC:26882] - 26
7 3 0 0 4 0.45867637
0.099556657 0.044829306 0 0 0.060234365
ENSG00000133895 1172.002177 1623.091448 1571.322055
1777.098875 1559.958811 2287.213376 1455.471893
1874.75702 -0.364557799 0.046901646 0.179470008 MEN1
11 64803510 64811294 - 4246
protein_coding menin 1 [Source:HGNC Symbol;Acc:HGNC:7010] -
1013 1751 1607 1769 1707 2331 13.04740575
18.18193556 17.53227777 20.14415524 17.51911361
25.62762258
ENSG00000272047 426.9188583 412.4932577 470.3210382
616.8110283 557.4545252 526.912734 436.5777181
567.0594292 -0.377110371 0.046913204 0.179470008 GTF2H5
6 158168350 158199344 + 7565
protein_coding general transcription factor IIH subunit 5
[Source:HGNC Symbol;Acc:HGNC:21157] - 369 445 481
614 610 537 2.667547408 2.59349274
2.94536146 3.924286038 3.513819919 3.313686277
ENSG00000285584 61.31896881 83.42560268 55.73450972
74.33878843 134.337402 147.1823279 66.8263604
118.6195061 -0.826906356 0.046938638 0.179502308
AL158800.1 14 97180525 97217778 + 1320
lincRNA novel transcript - 53 90 57
74 147 150 2.195819569 3.006093858
2.000337977 2.710559875 4.852907364 5.304730972
ENSG00000176124 166.6024813 139.9696223 147.6475608
98.44866576 77.67808958 110.8773537 151.4065548
95.66803635 0.662261953 0.046945132 0.179502308 DLEU1
13 50082171 50723236 + 12394
processed_transcript deleted in lymphocytic leukemia 1 [Source:HGNC
Symbol;Acc:HGNC:13747] - 144 151 151 98 85
113 0.635397809 0.537154741 0.564375187
0.382310125 0.298858789 0.425611141
ENSG00000184208 388.739123 520.9465412 457.6096588
655.989579 543.7466271 600.5038979 455.7651077

600.0800347	-0.395400105	0.046995454	0.179649787
C22orf46	22	41688939	41698136	+	5341
	protein_coding	chromosome 22 open reading frame 46
[Source:HGNC Symbol;Acc:HGNC:26294]	-	336	562	468
	653	595	612	3.440419592	4.639249136
4.059062429	5.911420044	4.85459479	5.349028108
ENSG00000088280 84.45820233	88.98730952	211.2044579
106.4852915	51.17615314	35.3237587	128.2166566
64.32840112	0.997180915	0.047016542	0.179655785	ASAP3
	1	23428563	23484568	-	4988
protein_coding	"ArfGAP with SH3 domain, ankyrin repeat and PH domain
3 [Source:HGNC Symbol;Acc:HGNC:14987]"	-	73	96	216
	106	56	36	0.800370601	0.848552556
2.005994612	1.027497176	0.489237998	0.336916354
ENSG00000121864 1608.176729	1249.530138	1241.803989
777.5435438	1246.504873	1104.848675	1366.503619
1042.965697	0.389020476	0.047028875	0.179655785	ZNF639
	3	179322991	179338583	+	6859
protein_coding	zinc finger protein 639 [Source:HGNC
Symbol;Acc:HGNC:30950]	zf-C2H2 1390	1348	1270	774	1364
	1126	11.08277994	8.664889871	8.57719678
5.4560881	8.66586991	7.663437565
ENSG00000141994 867.7212568	1477.560119	1356.206403
1795.181283	1432.018428	1626.855331	1233.82926
1618.018347	-0.390094659	0.047032279	0.179655785	DUS3L
	19	5784832 5791238 -	3642	protein_coding
dihydrouridine synthase 3 like [Source:HGNC Symbol;Acc:HGNC:26920]
	-	750	1594	1387	1787	1567	1658
11.26201327	19.29666839	17.64164081	23.72388595
18.74941202	21.25155046
ENSG00000103522 42.807582	43.56670362	151.5587545
47.21517644	17.36333767	37.28618974	79.31101338
33.95490128	1.227346561	0.047080473	0.17979495	IL21R
	16	27402162	27452042	+	5467
protein_coding	interleukin 21 receptor [Source:HGNC
Symbol;Acc:HGNC:6006]	-	37	47	155	47	19
	38	0.370124099	0.379037991	1.313363916
0.415671263	0.151447854	0.32447449
ENSG00000167447 926.7263022	983.4951604	875.1295825
1157.274112	1172.482223	1098.961382	928.4503484
1142.905905	-0.299742121	0.047135496	0.179960122	SMG8
	17	59209400	59215247	+	3752
protein_coding	"SMG8, nonsense mediated mRNA decay factor
[Source:HGNC Symbol;Acc:HGNC:25551]"	-	801	1061	895
	1152	1283	1120	11.67520189	12.46770484
11.05000975	14.84536452	14.90124051	13.93481569
ENSG00000100156 1.156961676	0	2.933395248	18.082408
	7.310879019	8.830939675	1.363452308	11.40807556
	-3.068235707	0.047152629	1	SLC16A8 22
38078134	38084093	-	2203	protein_coding	solute

carrier family 16 member 8 [Source:HGNC Symbol;Acc:HGNC:16270]	-
	1	0	3	18	8	9	0.024824483
	0	0.063082546	0.395056512	0.158246491
0.190710256
ENSG00000279236 10.41265508	0	8.800185745	0	0
	0	6.404280276	0	5.16883812	0.047164902
	1	AC064801.2	18	62551203	62552505
	+	1303	TEC	novel transcript	-	9
	0	9	0	0	0	0.377739853	0
	0.319963582	0	0	0
ENSG00000229097 34.70885027	36.15109449	22.48936357
14.06409511	2.741579632	12.75580175	31.11643611
9.853825498	1.663871718	0.047176897	0.180073213
CALM2P2 10	70163685	70164124	+	440
processed_pseudogene	calmodulin 2 pseudogene 2 [Source:HGNC
Symbol;Acc:HGNC:1447]	-	30	39	23	14	3
	13	3.728750211	3.907922015	2.421461762
1.538425875	0.297116777	1.379230053
ENSG00000106633 0	0.926951141	0	11.05036044	0
	13.73701727	0.308983714	8.262459238	-4.503017
	0.047180863	1	GCK	7	44144271
44198170	-	4122	protein_coding	glucokinase
[Source:HGNC Symbol;Acc:HGNC:4195]	-	0	1	0
	11	0	14	0	0.010696113	0
0.129028579	0	0.158549941
ENSG00000229191 5.784808379	7.415609127	2.933395248	0
	0	0	5.377937585	0	4.920103021
0.047207897	1	AL358473.1	1	201023949
201028792	+	3508	antisense	"novel transcript,
antisense KIF21B"	-	5	8	3	0	0
	0	0.077948028	0.100545899	0.039615407	0
	0	0
ENSG00000126878 20.82531016	18.53902282	25.42275882	0
	6.397019142	7.849724156	21.59569727	4.748914433
	2.180084276	0.04722163	0.180155084	AIF1L	9
	131096476	131123152	+	5299	protein_coding
	allograft inflammatory factor 1 like [Source:HGNC
Symbol;Acc:HGNC:28904]	-	18	20	26	0	7
	8	0.185769024	0.166406403	0.227290814	0
	0.057565558	0.070476139
ENSG00000160211 1777.093134	2356.3098	2244.047365
1713.810447	1698.865512	1775.018875	2125.816766
1729.231611	0.298387259	0.047221916	0.180155084	G6PD
	X	154531390	154547572	-	3995
protein_coding	glucose-6-phosphate dehydrogenase [Source:HGNC
Symbol;Acc:HGNC:4057]	-	1536	2542	2295	1706	1859
	1809	21.02660445	28.05386615	26.61143916
20.64730911	20.27781721	21.13819106
ENSG00000117335 3093.715521	2600.09795	3021.397106
2222.127027	2428.125694	2563.916152	2905.070192

2404.722958 0.272459488 0.047234822 0.180159362 CD46
1 207752057 207795513 + 8708
protein_coding CD46 molecule [Source:HGNC Symbol;Acc:HGNC:6953]
- 2674 2805 3090 2212 2657 2613
16.79336376 14.20196386 16.43775544 12.28196679
13.29632836 14.00770623
ENSG00000237781 6.941770054 7.415609127 1.955596832 0
0 0 5.437658671 0 4.935120551
0.047243619 1 AL356356.1 1 150548562
150557724 - 665 antisense "novel transcript,
antisense to ADAMTSL4" - 6 8 2 0 0
0 0.493428599 0.530398515 0.139319147 0
0 0
ENSG00000143353 664.0960019 517.2387366 446.8538762
646.948375 801.4551125 702.5503119 542.7295382
716.9845998 -0.403189135 0.047268702 0.180229755
LYPLAL1 1 219173844 219212865 + 2966
protein_coding lysophospholipase like 1 [Source:HGNC
Symbol;Acc:HGNC:20440] - 574 558 457 644 877
716 10.58364973 8.294629849 7.13752281
10.49822647 12.88507778 11.26906571
ENSG00000151694 1047.050317 899.1426066 1121.534783
789.5984825 892.8411002 748.6674414 1022.575902
810.369008 0.335102341 0.047276857 0.180229755 ADAM17
2 9488486 9556732 - 10750 protein_coding ADAM
metallopeptidase domain 17 [Source:HGNC Symbol;Acc:HGNC:195] -
905 970 1147 786 977 763 4.603994834
3.978297235 4.942622131 3.535210661 3.960451481
3.313310292
ENSG00000127870 2313.923351 1854.829233 1656.390517
2408.978576 2393.399019 2387.297359 1941.714367
2396.558318 -0.304162794 0.047336618 0.180393263 RNF6
13 26132115 26222493 - 4621
protein_coding ring finger protein 6 [Source:HGNC
Symbol;Acc:HGNC:10069] - 2000 2001 1694 2398 2619
2433 23.66948125 19.09172096 16.9816503
25.09079527 24.69779194 24.57831898
ENSG00000285533 24.29619519 52.83621503 45.95652556
88.40288354 56.6593124 100.083983 41.02964526
81.71539297 -0.98769037 0.047343349 0.180393263
AP001362.2 11 65663086 65671716 + 824
lincRNA uncharacterized LOC105369347 [Source:NCBI
gene;Acc:105369347] - 21 57 47 88 62
102 1.393756147 3.049871924 2.642245105
5.163648567 3.278861524 5.778551603
ENSG00000004848 3.470885027 0 0.977798416 13.05951689
6.397019142 15.69944831 1.482894481 11.71866145
-3.030099593 0.047353831 1 ARX X
25003694 25016420 - 3097 protein_coding
aristaless related homeobox [Source:HGNC Symbol;Acc:HGNC:18060]

Homeobox 3 0 1 13 7 16
0.052975463 0 0.014957577 0.202956686 0.098495283
0.241170849
ENSG00000145868 1233.321146 1096.5832 1057.977886
1481.752877 1538.026174 1203.951442 1129.294077
1407.910165 -0.318634431 0.047370141 0.180450374 FBXO38
5 148383935 148442836 + 8722
protein_coding F-box protein 38 [Source:HGNC Symbol;Acc:HGNC:28844]
- 1066 1183 1082 1475 1683 1227
6.683990671 5.980019777 5.746635273 8.176682877
8.408656883 6.567113408
ENSG00000137100 937.1389573 1067.847714 1064.822475
772.5206527 785.0056347 899.7746314 1023.269716
819.1003063 0.321549413 0.04739357 0.180494645 DCTN3
9 34613545 34620523 - 3740
protein_coding dynactin subunit 3 [Source:HGNC Symbol;Acc:HGNC:2713]
- 810 1152 1089 769 859 917
11.84426538 13.58047107 13.4883471 9.941592418
10.00875733 11.44573718
ENSG00000213024 2949.095311 3099.724615 3262.913315
3565.24811 3820.848147 3641.290793 3103.91108
3675.795683 -0.24388468 0.047446286 0.180640456 NUP62
19 49906825 49929763 - 6020
protein_coding nucleoporin 62 [Source:HGNC Symbol;Acc:HGNC:8066]
- 2549 3344 3337 3549 4181 3711
23.15624079 24.49084307 25.67805775 28.5043226
30.26511121 28.77666751
ENSG00000144802 800.6174796 948.2710171 780.2831361
772.5206527 632.3910352 539.6685357 843.0572109
648.1934079 0.379709703 0.04745549 0.180640456 NFKBIZ
3 101827991 101861022 + 5902
protein_coding NFKB inhibitor zeta [Source:HGNC
Symbol;Acc:HGNC:29805] - 692 1023 798 769 692
550 6.412119419 7.642059004 6.263342226
6.29982305 5.109348039 4.350202966
ENSG00000104856 671.0377719 1130.880392 750.9491836
1046.770507 1459.434224 960.6099936 850.9557825
1155.604908 -0.440866419 0.047488442 0.180694909 RELB
19 45001430 45038198 + 3035
protein_coding "RELB proto-oncogene, NF-kB subunit [Source:HGNC
Symbol;Acc:HGNC:9956]" RHD 580 1220 768 1042 1597
979 10.45114831 17.7229125 11.72208808
16.60008226 22.93004032 15.05809498
ENSG00000170296 223.2936034 183.5363259 260.0943787
337.5382826 345.4390337 263.9469747 222.3081027
315.6414303 -0.506325234 0.047493435 0.180694909
GABARAP 17 7240014 7242770 - 2266 protein_coding GABA
type A receptor-associated protein [Source:HGNC Symbol;Acc:HGNC:4067]
- 193 198 266 336 378 269
4.657920976 3.852469798 5.437811977 7.1693633

7.269264845	5.541641287
ENSG00000139324 1401.080589	1101.217955	1230.070408
1014.624004	1083.837815	866.4133037	1244.122984
988.2917075	0.331431562	0.047545549	0.180848172	TMTC3
12	88142296	88199887	+	7327
protein_coding	transmembrane and tetratricopeptide repeat containing
3 [Source:HGNC Symbol;Acc:HGNC:26899]	-	1211	1188	1258
1010	1186	883	9.038839282	7.148652842
7.953474727	6.664942373	7.053701794	5.625751542
ENSG00000167555 94.87085741	140.8965734	64.53469547
182.8332364	216.5847909	112.8397847	100.1007088
170.752604	-0.769583709	0.047608326	0.18104191	ZNF528
19	52397849	52418412	+	8530
protein_coding	zinc finger protein 528 [Source:HGNC
Symbol;Acc:HGNC:29384]	zf-C2H2 82	152	66	182	237
115	0.525726095	0.785648914	0.358424229
1.031629074	1.210759576	0.62935378
ENSG00000110455 240.6480285	224.3221761	289.4283312
167.7645631	92.29984762	219.7922764	251.4661786
159.952229	0.653899918	0.047624292	0.181057585	ACCS
11	44065925	44084222	+	5826
protein_coding	1-aminocyclopropane-1-carboxylate synthase homolog
(inactive) [Source:HGNC Symbol;Acc:HGNC:23989]	-	208	242
296	167	101	224	1.952484377	1.831381588
2.353551395	1.385948866	0.75545655	1.794831049
ENSG00000215251 426.9188583	451.4252056	428.2757063
513.3394715	700.930526	515.1381477	435.5399234
576.4693817	-0.404723109	0.047676093	0.181209456
FASTKD5 20	3146519 3159897 -	2874	protein_coding	FAST
kinase domains 5 [Source:HGNC Symbol;Acc:HGNC:25790]	-	369
487	438	511	767	525	7.021571379
7.4709556	7.05975781	8.596770894	11.62966482
8.527438097
ENSG00000243244 96.02781908	87.13340724	162.3145371
32.1465031	85.90282848	64.76022428	115.1585878
60.93651862	0.916787531	0.047717122	0.18132032	STON1
2	48529383	48598513	+	7709
protein_coding	stonin 1 [Source:HGNC Symbol;Acc:HGNC:17003]	-
83	94	166	32	94	66	0.588809434
0.537605578	0.997499067	0.20070267	0.531359446
0.399661143
ENSG00000227057 3240.649654	4225.970251	3746.923531
4574.849223	4155.320863	4880.565994	3737.847812
4536.912027	-0.279187057	0.047806858	0.181616163	WDR46
6	33279108	33289527	-	3115
protein_coding	WD repeat domain 46 [Source:HGNC
Symbol;Acc:HGNC:13923]	-	2801	4559	3832	4554	4547
4974	49.17561167	64.52759802	56.98622719
70.68645335	63.61001543	74.54075131
ENSG00000254554 15.04050178	19.46597396	2.933395248

75.34336665	7.310879019	45.1359139	12.479957
42.59671986	-1.770070131	0.047824148	0.18163671
AC080023.1	11	10302657	10303704	-	803
	antisense	novel transcript	-	13	21
	3	75	8	46	0.885365347	1.153022302
	0.173064569	4.515927225	0.434143236	2.67416575
ENSG00000006282 652.5263851	745.2687172	854.5958157
529.412723	513.5892511	679.982355	750.7969727
574.3281097	0.387243741	0.0478405	0.181653683
SPATA20 17	50543058	50555852	+	5337
protein_coding	spermatogenesis associated 20 [Source:HGNC
Symbol;Acc:HGNC:26125]	-	564	804	874	527	562
	693	5.779318296	6.641907208	7.586067202
4.774354129	4.588785005	6.06152733
ENSG00000013288 591.2074163	605.299095	773.4385472
565.577539	524.5555696	374.8243284	656.6483528
488.3191457	0.42755953	0.047859793	0.181681813	MAN2B2
	4	6575175 6623362 +	8898	protein_coding
mannosidase alpha class 2B member 2 [Source:HGNC
Symbol;Acc:HGNC:29623]	-	511	653	791	563	574
	382	3.140676547	3.235599338	4.118001855
3.059265788	2.811111609	2.004088968
ENSG00000236536 0	0	0	24.10987733	0	0
	0	8.036625776	-5.424868665	0.047886284	1
	AC003986.3	7	19119933	19121916	+
	692	antisense	novel transcript	-	0
	0	0	24	0	0	0	0	0
	1.676896907	0	0
ENSG00000242516 0	0	0	24.10987733	0	0
	0	8.036625776	-5.424868665	0.047886284	1
	LINC00960	3	75672391	75679303	+
	3181	lincRNA long intergenic non-protein coding RNA 960
[Source:HGNC Symbol;Acc:HGNC:48710]	-	0	0	0
	24	0	0	0	0	0	0.364794926
	0	0
ENSG00000154736 52.06327541	79.71779811	49.86771922
88.40288354	144.3898606	92.23425883	60.54959758
108.3423343	-0.837906082	0.047909172	0.181824108
ADAMTS5 21	26917912	26966513	-	9056
protein_coding	ADAM metallopeptidase with thrombospondin type 1 motif
5 [Source:HGNC Symbol;Acc:HGNC:221]	-	45	86	51
	88	158	94	0.271750788	0.418693285
0.260877256	0.469837281	0.760289987	0.484548748
ENSG00000064115 1347.860352	1269.923063	1387.495953
1032.706412	1218.175217	988.0840281	1335.093123
1079.655219	0.306032798	0.047944515	0.18191308	TM7SF3
	12	26973195	27014434	-	3888
protein_coding	transmembrane 7 superfamily member 3 [Source:HGNC
Symbol;Acc:HGNC:23049]	-	1165	1370	1419	1028	1333
	1007	16.38680863	15.53560852	16.90669021

12.78403865	14.94040915	12.09063888
ENSG00000166164 1574.624841	1518.345969	1585.989031
1403.395776	1279.403828	1139.191218	1559.65328
1273.996941	0.291852812	0.047967694	0.181955867	BRD7
	16	50313487	50368934	-	8492
protein_coding	bromodomain containing 7 [Source:HGNC
Symbol;Acc:HGNC:14310]	-	1361	1638	1622	1397	1400
	1161	8.76481699	8.50428625	8.847963455
7.954037554	7.184170784	6.382168558
ENSG00000143434 121.480976	241.9342478	224.8936357
258.1766031	310.7123583	315.9513973	196.1029531
294.9467862	-0.585982367	0.048005617	0.182054543	SEMA6C
	1	151131685	151146664	-	5809
protein_coding	semaphorin 6C [Source:HGNC Symbol;Acc:HGNC:10740]
	-	105	261	230	257	340	322
0.98851357	1.980948061	1.834124936	2.139109238
2.550563488	2.58762019
ENSG00000259899 0	0	0.977798416	19.08698622	0
	5.887293117	0.325932805	8.324759778	-4.513433136
	0.048050347	1	AC010333.2	16	12560756
	12611044	-	698	antisense	"novel
transcript, antisense to a novel protein"	-	0	0
	1	19	0	6	0	0	0.066366212
	1.316131837	0	0.401274778
ENSG00000156239 315.8505375	406.0045997	438.0536904
623.8430759	494.3981937	454.3027855	386.6362759
524.1813517	-0.437649599	0.048052628	0.182187631	N6AMT1
	21	28872191	28885371	-	4865
protein_coding	N-6 adenine-specific DNA methyltransferase 1
[Source:HGNC Symbol;Acc:HGNC:16021]	-	273	438	448
	621	541	463	3.0688419	3.969403277
4.265771862	6.171773396	4.845884049	4.4426713
ENSG00000100557 5.784808379	8.342560268	17.60037149	0
	0	2.943646558	10.57591338	0.981215519
3.440429553	0.04807583	1	CCDC198 14	57469301
	57493867	-	4107	protein_coding	coiled-coil
domain containing 198 [Source:HGNC Symbol;Acc:HGNC:20189]	-
	5	9	18	0	0	3	0.066579421
	0.096616603	0.203025345	0	0	0.034099074
ENSG00000164574 1595.450151	1302.366353	2162.890097
1633.444189	965.9498904	1159.796744	1686.9022
1253.063608	0.429154119	0.048128728	0.182406597
GALNT10 5	154190730	154420984	+	10496
protein_coding	polypeptide N-acetylgalactosaminyltransferase 10
[Source:HGNC Symbol;Acc:HGNC:19873]	-	1379	1405	2212
	1626	1057	1182	7.185138714	5.9018268
9.762560907	7.490277982	4.388435939	5.257020739
ENSG00000170855 499.8074439	538.5586128	508.4551764
285.3002151	476.1209961	370.8994664	515.6070777
377.4402258	0.449427889	0.048134246	0.182406597	TRIAP1

12	120443961	120446412	-	1442
protein_coding	TP53 regulated inhibitor of apoptosis 1 [Source:HGNC
Symbol;Acc:HGNC:26937]	-	432	581	520	284	521
378	16.38374573	17.76416629	16.70477149
9.522572682	15.7445793	12.23693281
ENSG00000172336 462.7846703	629.3998246	573.9676703
777.5435438	657.9791117	729.043131	555.3840551
721.5219289	-0.37615989	0.048149535	0.182419315	POP7
7	100706053	100707495	+	988
protein_coding	"POP7 homolog, ribonuclease P/MRP subunit [Source:HGNC
Symbol;Acc:HGNC:19949]" -	400	679	587	774	720
743	22.1410269	30.30029018	27.52222953
37.87784239	31.75661103	35.10572165
ENSG00000185201 1029.695891	1491.464386	1568.38866
944.3035287	1075.613076	1152.928235	1363.182979
1057.614947	0.366788916	0.048179355	0.182487068	IFITM2
11	307631	315272	+	1543	protein_coding
interferon induced transmembrane protein 2 [Source:HGNC
Symbol;Acc:HGNC:5413]	-	890	1609	1604	940	1177
1175	31.54414739	45.97524751	48.15494528
29.45527922	33.24062145	35.54822959
ENSG00000153187 17828.77942	19424.26116	21483.209
17149.15483	14619.01646	17507.82851	19578.74986
16425.33327	0.253419011	0.048270562	0.18278724	HNRNPU
1	244840638	244864560	-	22512
protein_coding	heterogeneous nuclear ribonucleoprotein U [Source:HGNC
Symbol;Acc:HGNC:5048]	-	15410	20955	21971	17071	15997
17843	37.43546839	41.04001804	45.21038442
36.66452802	30.96585405	36.99983874
ENSG00000207110 1.156961676	2.780853423	1.955596832	0
26.50193645	19.62431039	1.964470644	15.37541561
-2.957740409	0.048291287	1	RNU1-106P	8
127999131	127999294	+	164	snRNA	"RNA,
U1 small nuclear 106, pseudogene [Source:HGNC Symbol;Acc:HGNC:48448]"
-	1	3	2	0	29	20
0.333465466	0.806512986	0.56492215	0	7.705711544
5.692882019
ENSG00000232434 55.53416043	133.4809643	227.827031
162.741672	285.1242818	290.4397938	138.9473852
246.1019158	-0.822152263	0.048311515	0.18289701	AJM1
9	136844415	136848801	+	4387
protein_coding	apical junction component 1 homolog [Source:HGNC
Symbol;Acc:HGNC:37284]	-	48	144	233	162	312
296	0.598367939	1.447200872	2.460315159
1.785453716	3.099171127	3.149706687
ENSG00000149476 610.8757648	712.8254273	700.103666
683.113191	864.511444	1131.341494	674.6016194
892.9887097	-0.404203794	0.048401755	0.183193272	TKFC
11	61333210	61353295	+	8136
protein_coding	triokinase and FMN cyclase [Source:HGNC

Symbol;Acc:HGNC:24552]	-	528	769	716	680	946
	1153	3.54909558	4.167248106	4.076660433
4.041096201	5.066858711	6.615526344
ENSG00000225137 144.6202095	136.2618177	122.224802
96.43950931	85.90282848	66.72265532	134.3689431
83.02166437	0.694200992	0.048425611	0.183238197
DYNC1I2P1	10	50264978	50266815	+	1838
	processed_pseudogene	dynein cytoplasmic 1 intermediate
chain 2 pseudogene 1 [Source:HGNC Symbol;Acc:HGNC:45012]	-
	125	147	125	96	94	68	3.719282946
	3.526190619	3.150409162	2.525381197	2.228645251
	1.727068016
ENSG00000102904 85.615164	29.66243651	37.15633981
80.36625776	106.0077458	108.9149227	50.81131344
98.42964207	-0.961947371	0.048493691	0.183419051
TSNAXIP1	16	67806765	67832148	+	3852
	protein_coding	translin associated factor X interacting
protein 1 [Source:HGNC Symbol;Acc:HGNC:18586]	-	74	32
	38	80	116	111	1.050606671	0.366266887
	0.456982715	1.004164642	1.312291478	1.345187231
ENSG00000272391 2381.027129	2654.788067	3186.645038
3626.527382	2974.613901	3514.713991	2740.820078
3371.951758	-0.298660661	0.048497403	0.183419051
POM121C 7	75416787	75486271	-	8767
protein_coding	POM121 transmembrane nucleoporin C [Source:HGNC
Symbol;Acc:HGNC:34005]	-	2058	2864	3259	3610	3255
	3582	12.83775481	14.40309964	17.22010557
19.90936515	16.17925739	19.07307036
ENSG00000145337 1330.505927	1482.194874	1498.964972
1199.466397	1089.320974	1237.31277	1437.221924
1175.366714	0.290631657	0.04853593	0.183519356	PYURF
	4	88520985	88523813	-	1361
protein_coding	PIGY upstream reading frame [Source:HGNC
Symbol;Acc:HGNC:44317]	-	1150	1599	1533	1194	1192
	1261	46.20983607	51.79934838	52.17788671
42.41772948	38.1660072	43.2516816
ENSG00000198612 2442.346097	2063.39324	1796.21569
1475.725408	1888.948367	1676.897323	2100.651676
1680.523699	0.321399079	0.048552144	0.183530328	COPS8
	2	237085312	237100466	+	4743
protein_coding	COP9 signalosome subunit 8 [Source:HGNC
Symbol;Acc:HGNC:24335]	-	2111	2226	1837	1469	2067
	1709	24.34051828	20.69216788	17.94148918
14.97510539	18.99091761	16.82034789
ENSG00000129696 518.3188307	491.2841046	468.3654413
715.2596941	630.5633154	563.2177082	492.6561256
636.3469059	-0.369486106	0.048562843	0.183530328	TTI2
	8	33473386	33513601	-	4222
protein_coding	TELO2 interacting protein 2 [Source:HGNC
Symbol;Acc:HGNC:26262]	-	448	530	479	712	690

574	5.803025751	5.534668306	5.255568975
8.153854944	7.121794854	6.34657913
ENSG00000252777 2.313923351	0	0	28.12819022	0
6.868508636	0.771307784	11.66556628	-3.991504468
0.0486159	1	RF00422 1	28689665
28689794	+	130	scaRNA		-	2	0
0	28	0	7	0.841359022	0	0
10.41395977	0	2.513626368
ENSG00000226469 9.255693406	10.19646255	26.40055724	0
3.65543951	3.924862078	15.28423773	2.526767196
2.591008989	0.048662954	1	ADAM1B	12
111927018	111929017	+	2000	unitary_pseudogene
ADAM metallopeptidase domain 1B (pseudogene) [Source:HGNC
Symbol;Acc:HGNC:44366]	-	8	11	27	0	4
4	0.218753346	0.242491571	0.62536882	0
0.087154255	0.093363265
ENSG00000251669 86.77212568	184.463277	86.04626062
187.8561275	229.3788292	170.7315004	119.0938878
195.988819	-0.715821739	0.048671021	0.183893697
FAM86EP 4	3941760 3955419 -	3476
transcribed_unprocessed_pseudogene	"family with sequence
similarity 86 member E, pseudogene [Source:HGNC
Symbol;Acc:HGNC:28017]" -	75	199	88	187	251
174	1.179984244	2.524104125	1.17274978
2.601135973	3.146679795	2.336767567
ENSG00000117448 1402.237551	1204.109532	1338.606032
1660.567801	1410.085791	1808.380202	1314.984372
1626.344598	-0.306763114	0.048709392	0.183974144	AKR1A1
1	45550543	45570049	+	3121
protein_coding	aldo-keto reductase family 1 member A1 [Source:HGNC
Symbol;Acc:HGNC:380]	-	1212	1299	1369	1653	1543
1843	21.23750841	18.35056077	20.31945874
25.60827361	21.544219	27.56624441
ENSG00000113391 1303.895809	978.8604047	907.3969302
697.1772861	909.290578	850.7138554	1063.384381
819.0605732	0.375605575	0.048716382	0.183974144
FAM172A 5	93618069	94111699	-	5071
protein_coding	family with sequence similarity 172 member A
[Source:HGNC Symbol;Acc:HGNC:25365]	-	1127	1056	928
694	995	867	12.15416193	9.181301847
8.47728573	6.61709053	8.550432206	7.981261176
ENSG00000188770 0	0	0	13.05951689	6.397019142
0	0	6.485512009	-5.116569584	0.048727534
1	OPTC	1	203494143	203508864	+
1359	protein_coding	opticin [Source:HGNC
Symbol;Acc:HGNC:8158]	-	0	0	0	13	7
0	0	0	0	0.462514244	0.224459081
0
ENSG00000176986 1507.521063	1828.874601	1801.104683
2476.285317	1881.637488	2017.379108	1712.500116

2125.100638 -0.310892992 0.048732551 0.183989753 SEC24C
10 73744384 73772161 + 5341
protein_coding "SEC24 homolog C, COPII coat complex component
[Source:HGNC Symbol;Acc:HGNC:10705]" - 1303 1973 1842
2465 2059 2056 13.34186526 16.28690133
15.97605341 22.31493171 16.79934567 17.96993756
ENSG00000279345 13.88354011 23.17377852 11.73358099 0
0 6.868508636 16.26363321 2.289502879
2.835295801 0.048746491 1 Z98885.3 22
49805452 49807208 + 1757 antisense "novel
transcript, antisense to BRD1" - 12 25 12 0
0 7 0.373511689 0.627338881 0.316382126
0 0 0.1859826
ENSG00000130684 42.807582 90.8412118 63.55689705
104.4761351 118.8017841 122.6519399 65.73523029
115.309953 -0.80615061 0.048788116 0.184154058 ZNF337
20 25674215 25696841 - 3823
protein_coding zinc finger protein 337 [Source:HGNC
Symbol;Acc:HGNC:15809] zf-C2H2 37 98 65 104 130
125 0.529288111 1.130201126 0.787610531
1.315316469 1.481827506 1.526341635
ENSG00000184349 569.2251445 401.369844 459.5652556
291.3276844 380.165709 377.767975 476.7200813
349.7537895 0.445260035 0.048812309 0.184184251 EFNA5
5 107376889 107670895 - 5703
protein_coding ephrin A5 [Source:HGNC Symbol;Acc:HGNC:3225] -
492 433 470 290 416 385 4.717983785
3.347483791 3.817657315 2.458645094 3.17869279
3.151399006
ENSG00000139613 1244.890763 1746.375949 1742.436778
1214.53507 1209.950478 1334.453106 1577.901163
1252.979552 0.333312635 0.048820212 0.184184251
SMARCC2 12 56162983 56189567 - 8555
protein_coding "SWI/SNF related, matrix associated, actin dependent
regulator of chromatin subfamily c member 2 [Source:HGNC
Symbol;Acc:HGNC:11105]" MYB 1076 1884 1782 1209 1324
1360 6.878392753 9.70945476 9.649174082
6.832938368 6.744139873 7.421042697
ENSG00000177105 961.4351525 1051.162594 1135.223961
1342.116505 1128.616949 1464.954771 1049.273902
1311.896075 -0.321715169 0.048839184 0.184210364 RHOG
11 3826978 3840983 - 1782 protein_coding ras
homolog family member G [Source:HGNC Symbol;Acc:HGNC:672] -
831 1134 1161 1336 1235 1493 25.50281009
28.056876 30.1805379 36.24932925 30.20075886
39.11093008
ENSG00000177465 0 0 0 16.07325155 3.65543951
0 0 6.576230354 -5.135900328 0.048872834
1 ACOT4 14 73591706 73596496 +
2362 protein_coding acyl-CoA thioesterase 4 [Source:HGNC

Symbol;Acc:HGNC:19748]	-	0	0	0	16	4
	0	0	0	0	0.327522625	0.073796998
	0
ENSG00000152942 894.3313753	738.7800593	718.6818359
597.7240421	625.994016	626.0155014	783.9310902
616.5778532	0.345602347	0.04892374	0.184483773	RAD17
	5	69369293	69414801	+	4159
protein_coding	RAD17 checkpoint clamp loader component [Source:HGNC
Symbol;Acc:HGNC:9807]	-	773	797	735	595	685
	638	10.16448282	8.448962042	8.186549168
6.917182943	7.177285943	7.161067942
ENSG00000121101 3.470885027	5.561706845	6.844588913	0
	0	0	5.292393595	0	4.898451791
0.048926738	1	TEX14	17	58556678	58692055
	-	5325	protein_coding	"testis expressed 14,
intercellular bridge forming factor [Source:HGNC
Symbol;Acc:HGNC:11737]" -	3	6	7	0	0
	0	0.03081033	0.049678171	0.060894895	0
	0	0
ENSG00000155275 137.6784394	226.1760784	214.1378531
221.0072088	398.4429066	260.0221127	192.6641236
293.1574094	-0.60416273	0.048937447	0.184489952	TRMT44
	4	8436140 8493531 +	7724	protein_coding	tRNA
methyltransferase 44 homolog [Source:HGNC Symbol;Acc:HGNC:26653]
	-	119	244	219	220	436	265
0.842557229	1.392776785	1.313422058	1.377151223
2.459817132	1.601583717
ENSG00000197479 3.470885027	12.97731597	6.844588913
40.18312888	16.44947779	23.54917247	7.764263304
26.72725971	-1.769273856	0.049003549	0.184693603
PCDHB11 5	141199582	141203779	+	4198
protein_coding	protocadherin beta 11 [Source:HGNC
Symbol;Acc:HGNC:8682]	-	3	14	7	40	18
	24	0.039081708	0.147034605	0.077242809
0.460700595	0.186848093	0.266879271
ENSG00000175048 153.8759029	194.6597396	165.2479323
273.2452764	195.5660138	283.5712851	171.2611916
250.7941918	-0.548481553	0.04907267	0.184877018
ZDHHC14 6	157381133	157678146	+	11121
protein_coding	zinc finger DHHC-type containing 14 [Source:HGNC
Symbol;Acc:HGNC:20341]	-	133	210	169	272	214
	289	0.654037294	0.832548249	0.703955684
1.182568427	0.838549164	1.213109595
ENSG00000171791 284.6125722	263.254124	356.8964219
222.0117871	203.7907527	219.7922764	301.587706
215.198272	0.48725397	0.049076401	0.184877018	BCL2
	18	63123346	63320128	-	11765
protein_coding	"BCL2, apoptosis regulator [Source:HGNC
Symbol;Acc:HGNC:990]"	-	246	284	365	221	223
	224	1.143504527	1.064290943	1.437154267

0.908241953	0.825983799	0.888796064
ENSG00000062582 55.53416043	87.13340724	74.31267963
90.41203998	106.0077458	185.4497332	72.3267491
127.2898396	-0.812654491	0.049112814	0.18496861	MRPS24
	7	43866558	43869893	-	2613
protein_coding	mitochondrial ribosomal protein S24 [Source:HGNC
Symbol;Acc:HGNC:14510]	-	48	94	76	90	116
	189	1.004607787	1.586070187	1.347338247
1.665345379	1.934537609	3.376513032
ENSG00000163071 0	0	0	11.05036044	3.65543951
	1.962431039	0	5.556076997	-4.892756617
0.049127838	1	SPATA18 4	52051331	52097292
	+	4941	protein_coding	spermatogenesis associated 18
[Source:HGNC Symbol;Acc:HGNC:29579]	-	0	0	0
	11	4	2	0	0	0	0.107641328
	0.035277982	0.018895621
ENSG00000102081 2415.735979	1939.181787	2033.820706
1847.41935	1810.356417	1542.470797	2129.57949
1733.415521	0.296531156	0.049173988	0.185111849	FMR1
	X	147911951	147951125	+	8919
protein_coding	fragile X mental retardation 1 [Source:HGNC
Symbol;Acc:HGNC:3775]	-	2088	2092	2080	1839	1981
	1572	12.80292034	10.34140327	10.80313061
9.969348588	9.678920203	8.227775129
ENSG00000196943 779.7921694	889.8730952	828.1952585
1190.425193	965.9498904	978.2718729	832.6201744
1044.882319	-0.326989238	0.049184981	0.185111849	NOP9
	14	24299862	24309124	+	6033
protein_coding	NOP9 nucleolar protein [Source:HGNC
Symbol;Acc:HGNC:19826]	-	674	960	847	1185	1057
	997	6.109719668	7.015713827	6.503580808
9.496995703	7.634845618	7.714501518
ENSG00000119537 3139.993988	2675.180992	2293.915084
3383.419452	3691.080045	2938.740481	2703.030022
3337.746659	-0.304656158	0.049187174	0.185111849	KDSR
	18	63327726	63367510	-	9313
protein_coding	3-ketodihydrosphingosine reductase [Source:HGNC
Symbol;Acc:HGNC:4021]	-	2714	2886	2346	3368	4039
	2995	15.93730754	13.66283054	11.66919402
17.48572711	18.89917507	15.01250827
ENSG00000143702 4991.132669	4777.50618	3877.948518
5742.169117	6106.411701	4809.918476	4548.862456
5552.833098	-0.287853008	0.049252547	0.185312255	CEP170
	1	243124428	243255348	-	10939
protein_coding	centrosomal protein 170 [Source:HGNC
Symbol;Acc:HGNC:28920]	-	4314	5154	3966	5716	6682
	4902	21.5673721	20.77307312	16.79490468
25.26479707	26.61873709	20.91903856
ENSG00000160993 208.2531016	268.8158308	302.1397106
334.5245479	381.9934288	362.0685267	259.7362144

359.5288345	-0.467602783	0.04931636	0.185501942	ALKBH4
	7	102456238	102464876	-	2312
protein_coding	"alkB homolog 4, lysine demethylase [Source:HGNC
Symbol;Acc:HGNC:21900]" -	180	290	309	333	418
	369	4.257742456	5.530241872	6.191175364
6.963981685	7.878563682	7.450485473
ENSG00000169621 114.5392059	129.7731597	118.3136084
69.31589732	89.55826799	59.85414669	120.8753247
72.90943733	0.728802778	0.049327232	0.185501942	APLF
	2	68467561	68655862	+	7135
protein_coding	aprataxin and PNKP like factor [Source:HGNC
Symbol;Acc:HGNC:28724]	-	99	140	121	69	98
	61	0.758815039	0.865103395	0.785586205
0.467580434	0.598536578	0.399100152
ENSG00000185504 1298.111	1697.247539	1762.970544
1838.378146	1983.989794	2081.158117	1586.109694
1967.842019	-0.310546589	0.049347677	0.185533187
FAAP100 17	81539885	81553961	-	5354
protein_coding	FA core complex associated protein 100 [Source:HGNC
Symbol;Acc:HGNC:26171]	-	1122	1831	1803	1830	2171
	2121	11.46064876	15.07800682	15.59982821
16.52623558	17.6701426	18.49304121
ENSG00000128915 1441.574248	1001.107232	991.487594
830.7861896	996.1072664	822.2586053	1144.723025
883.0506871	0.37336555	0.04936009	0.185534225	ICE2
	15	60419609	60479160	-	16010
protein_coding	interactor of little elongation complex ELL subunit 2
[Source:HGNC Symbol;Acc:HGNC:29885]	-	1246	1080	1014
	827	1090	838	4.256194078	2.97417406
2.93392548	2.497556917	2.966837528	2.443423365
ENSG00000140682 1119.938902	1050.235643	987.5764003
750.4199319	869.9946033	910.5680021	1052.583648
843.6608457	0.318780604	0.049385294	0.18558333
TGFB1I1 16	31471585	31477960	+	5403
protein_coding	transforming growth factor beta 1 induced transcript 1
[Source:HGNC Symbol;Acc:HGNC:11767]	-	968	1133	1010
	747	952	928	9.797947375	9.245468011
8.65942115	6.684775872	7.678220478	8.01787063
ENSG00000041982 7973.779869	5628.447327	7455.712923
9405.865893	6540.495143	11128.94642	7019.313373
9025.102486	-0.362667212	0.049399944	0.18559276	TNC
	9	115019578	115118257	-	9636
protein_coding	tenascin C [Source:HGNC Symbol;Acc:HGNC:5318]	-
	6892	6072	7625	9363	7157	11342	39.114987
	27.78234689	36.65603719	46.98069623	32.36628274
	54.94635496
ENSG00000105520 931.354149	1346.860008	1234.9594
1609.334312	1325.096822	1523.827702	1171.057852
1486.086279	-0.342746554	0.049554334	0.186127052	PLPPR2
	19	11355386	11365698	+	3273

protein_coding phospholipid phosphatase related 2 [Source:HGNC
Symbol;Acc:HGNC:29566] - 805 1453 1263 1602 1450
1553 13.45069075 19.57282743 17.87556596
23.66561107 19.30547958 22.14988554
ENSG00000242791 2.313923351 0.926951141 5.866790497
26.11903377 18.27719755 4.906077597 3.03588833
16.43410297 -2.435684652 0.049567365 1 AC117395.1
3 149976755 149979355 + 721
lincRNA novel transcript - 2 1 6 26
20 5 0.151701349 0.061150314 0.385494727
1.743569646 1.208796875 0.323728381
ENSG00000158292 119.1670526 217.8335181 220.9824421
252.1491337 319.8509571 264.9281903 185.9943376
278.9760937 -0.582308428 0.049569236 0.186137291 GPR153
1 6247346 6260975 - 4082 protein_coding G
protein-coupled receptor 153 [Source:HGNC Symbol;Acc:HGNC:23618]
- 103 235 226 251 350 270
1.379935976 2.538217417 2.564707812 2.973048114
3.736402394 3.087712099
ENSG00000260966 219.8227184 153.8738894 123.2026004
380.7351461 211.1016317 193.2994573 165.6330694
261.7120784 -0.661617035 0.049645195 0.186376742
AP001486.2 11 103050687 103055799 - 5113
sense_overlapping "novel transcript, overlapping to
DCUN1D5" - 190 166 126 379 231 197
2.032228422 1.431417272 1.141555966 3.583972212
1.968769102 1.798607787
ENSG00000110048 2244.505651 2195.020302 2138.445136
2660.123132 2727.871734 2445.189075 2192.65703
2611.061314 -0.252072934 0.049684366 0.186478001 OSBP
11 59574398 59616144 - 5492
protein_coding oxysterol binding protein [Source:HGNC
Symbol;Acc:HGNC:8503] - 1940 2368 2187 2648 2985
2492 19.31816691 19.01013178 18.44678603
23.31249031 23.68494631 21.18183327
ENSG00000258458 0 0 8.800185745 19.08698622
15.53561792 21.58674143 2.933395248
18.73644852 -2.661448844 0.049734626 1 AL160314.2
14 22701476 22766562 - 2513
processed_transcript "novel transcript, antisense to OXA1L" -
0 0 9 19 17 22 0 0
0.165902327 0.365563081 0.29479155 0.408673266
ENSG00000204574 5377.557869 6272.67837 5869.723892
7453.970407 6527.701104 6781.180455 5839.98671
6920.950656 -0.244838928 0.049773616 0.186767126 ABCF1
6 30571376 30597179 + 4523
protein_coding ATP binding cassette subfamily F member 1 [Source:HGNC
Symbol;Acc:HGNC:70] - 4648 6767 6003 7420 7143
6911 56.19973198 65.96347807 61.48146556
79.31923811 68.81968178 71.32804833

ENSG00000260572 10.41265508 10.19646255 12.71137941
53.24264577 21.93263706 21.58674143 11.10683235
32.25400808 -1.536081258 0.049821443 0.186900712
AC069224.1 3 160753428 160755142 - 1715
antisense "novel transcript, antisense to PPM1L" -
9 11 13 53 24 22 0.286994185
0.282789004 0.351141115 1.494214552 0.609825689
0.598831438
ENSG00000248713 3.470885027 13.90426711 19.55596832 0
0 3.924862078 12.31037349 1.308287359
3.244584241 0.049824219 1 C4orf54 4 99636529
99654648 - 10086 protein_coding chromosome 4
open reading frame 54 [Source:HGNC Symbol;Acc:HGNC:27741] -
3 15 20 0 0 4 0.016266608
0.065570161 0.09185726 0 0 0.018513437
ENSG00000183648 201.3113316 164.9973031 263.0277739
62.28384977 158.0977588 156.9944831 209.7788029
125.7920306 0.736665064 0.049884275 0.187090507 NDUFB1
14 92116122 92121917 - 2263
protein_coding NADH:ubiquinone oxidoreductase subunit B1 [Source:HGNC
Symbol;Acc:HGNC:7695] - 174 178 269 62 173
160 4.204936164 3.467922683 5.50643075
1.324671986 3.331349108 3.300513128
ENSG00000076356 517.161869 571.9288539 706.9482549
580.6462123 328.075696 385.6176992 598.6796593
431.4465358 0.473663602 0.049916008 0.187159551 PLXNA2
1 208022242 208244320 - 13781
protein_coding plexin A2 [Source:HGNC Symbol;Acc:HGNC:9100] -
447 617 723 578 359 393 1.773868833
1.973960188 2.430300747 2.027908344 1.13519982
1.331244583
ENSG00000182963 2389.12586 2135.695429 3107.443367
1977.009941 2010.49173 2150.824419 2544.088218
2046.108697 0.314298306 0.04992717 0.187159551 GJC1
17 44798448 44830816 - 8660
protein_coding gap junction protein gamma 1 [Source:HGNC
Symbol;Acc:HGNC:4280] - 2065 2304 3178 1968 2200
2192 13.04057907 11.73001428 16.99959037
10.98774112 11.07040187 11.81595133
ENSG00000233589 12.72657843 9.269511408 8.800185745 0
0 2.943646558 10.2654252 0.981215519
3.394390195 0.049960652 1 AL138789.1 1
68479129 68483539 + 960 antisense novel
transcript - 11 10 9 0 0 3
0.626637188 0.459264339 0.434283903 0 0
0.145880102
ENSG00000108349 3216.353458 3792.157117 3887.726503
4756.677881 4156.234722 4171.147173 3632.079026
4361.353259 -0.263705579 0.049972757 0.187284514 CASC3
17 40140318 40172183 + 6380

protein_coding "CASC3, exon junction complex subunit [Source:HGNC Symbol;Acc:HGNC:17040]" - 2780 4091 3976 4735 4548 4251 23.82971399 28.27110338 28.86876151 35.88397299 31.06407135 31.10401567
