# Supplementary material for: Multiomic screening of invasive GBM cells reveals targetable transsulfuration pathway alterations
Source: J Clin Invest. 2024 Feb 1;134(3):e170397. doi: 10.1172/JCI170397 (PMC10849762; doi:10.1172/JCI170397)
Supplement: Supplemental table 15 [file jci-134-170397-s077.pdf]

SUPPLEMENTAL TABLE 13. Results of bulk RNA-seq comparing GBM43 knockdown vs. GBM43 control cells in the core fractions after invading 3D hydrogels.

gene_id CTHKD_CORE1 CTHKD_CORE2 CTHKD_CORE3 NT_CORE1
NT_CORE2 NT_CORE3 CTH_KD_Core Wt_Core
log2FoldChange pvalue padj gene_name gene_chr
gene_start gene_end gene_strand gene_length
gene_biotype gene_description tf_family
CTHKD_CORE1_count CTHKD_CORE2_count CTHKD_CORE3_count
NT_CORE1_count NT_CORE2_count NT_CORE3_count
CTHKD_CORE1_fpkm CTHKD_CORE2_fpkm CTHKD_CORE3_fpkm
NT_CORE1_fpkm NT_CORE2_fpkm NT_CORE3_fpkm
ENSG00000251562 8418.163108 6151.503808 7290.64312
3726.095814 3439.975321 3772.086973 7286.770012
3646.052703 0.998740712 4.92E-14 9.03E-11 MALAT1
11 65497688 65506516 + 8829
lincRNA metastasis associated lung adenocarcinoma transcript 1
[Source:HGNC Symbol;Acc:HGNC:29665] - 8752 7070 7836
3381 2838 4086 47.92885 34.89526451
41.43133681 21.27872969 19.758239 21.51879081
ENSG00000245532 17718.3481 12167.27429 21162.96177
6395.307309 8206.001735 8008.530223 17016.19472
7536.613089 1.174889579 2.42E-12 2.22E-09 NEAT1
11 65422774 65445540 + 22767
lincRNA nuclear paraspeckle assembly transcript 1 [Source:HGNC
Symbol;Acc:HGNC:30815] - 18421 13984 22746 5803 6770
8675 39.12087965 26.76604511 46.63857399
14.16311582 18.27806516 17.71718394
ENSG00000248527 2322.882073 2289.194699 2012.463128
3827.486177 3753.912463 3700.079439 2208.179967
3760.492693 -0.768019881 3.06E-10 1.87E-07
MTATP6P1 1 633696 634376 + 681
unprocessed_pseudogene MT-ATP6 pseudogene 1 [Source:HGNC
Symbol;Acc:HGNC:44575] - 2415 2631 2163 3473 3097
4008 171.4633244 168.357448 148.270736
283.3803387 279.5383862 273.6601868
ENSG00000179813 448.2248638 405.4597984 330.2933011
109.1048464 88.48421369 144.015068 394.6593211
113.8680427 1.788974628 6.31E-09 1 FAM216B 13
42781550 42791549 + 3308 protein_coding
family with sequence similarity 216 member B [Source:HGNC
Symbol;Acc:HGNC:26883] - 466 466 355 99 73
156 6.811168625 6.138736804 5.009667339
1.662959748 1.356452903 2.192755028
ENSG00000172893 2137.243879 2056.01181 2262.741714
3492.457154 4946.631179 3393.585833 2151.999134

3944.224722	-0.873728204	2.36E-08	1.08E-05	DHCR7
	11	71428193	71452868	-	4160
protein_coding	7-dehydrocholesterol reductase [Source:HGNC
Symbol;Acc:HGNC:2860]	-	2222	2363	2432	3169	4081
	3676	25.82569041	24.75306537	27.29080057
42.3292869	60.30045989	41.08782858
ENSG00000185567 2601.820293	2379.683581	3317.81947
1740.167196	1203.627729	1283.211183	2766.441115
1409.002036	0.973307642	4.16E-08	1	AHNAK2	14
	104937244	104978357	-	18771	protein_coding
	AHNAK nucleoprotein 2 [Source:HGNC Symbol;Acc:HGNC:20125]
	-	2705	2735	3566	1579	993	1390
6.967565759	6.34933905	8.868291604	4.674194327
3.251690476	3.443169548
ENSG00000137310 1799.632447	1821.088751	2067.356944
3192.694343	3152.704655	2861.837889	1896.026047
3069.078963	-0.694647523	8.78E-08	3.17E-05	TCF19
	6	31158542	31167159	+	3538
protein_coding	transcription factor 19 [Source:HGNC
Symbol;Acc:HGNC:11629]	Others	1871	2093	2222	2897	2601
	3100	25.56920207	25.77923334	29.31785968
45.49908996	45.18870377	40.74130007
ENSG00000188486 4075.383579	3967.589443	4610.150161
6392.001101	6237.531009	6536.06847	4217.707728
6388.533527	-0.599112744	1.03E-07	3.17E-05	H2AFX
	11	119093854	119095467	-	1614
protein_coding	H2A histone family member X [Source:HGNC
Symbol;Acc:HGNC:4739]	-	4237	4560	4955	5800	5146
	7080	126.9276239	123.1175345	143.3131904
199.6808914	195.9808386	203.9673932
ENSG00000116133 5156.509646	3934.526198	4950.677902
7583.337858	9215.691461	6849.947464	4680.571249
7882.992261	-0.751974055	1.57E-07	4.12E-05	DHCR24
	1	54849627	54887271	-	6290
protein_coding	24-dehydrocholesterol reductase [Source:HGNC
Symbol;Acc:HGNC:2859]	-	5361	4522	5321	6881	7603
	7420	41.20940825	31.32842129	39.49013781
60.78731433	74.29878387	54.85096568
ENSG00000134824 6015.446992	6123.661075	7994.958695
13860.72377	14472.62344	8374.106934	6711.355587
12235.81805	-0.86630761	9.92E-07	0.000227623	FADS2
	11	61792980	61867354	+	11026
protein_coding	fatty acid desaturase 2 [Source:HGNC
Symbol;Acc:HGNC:3575]	-	6254	7038	8593	12577	11940
	9071	27.42465073	27.81569221	36.38085149
63.38275559	66.56312437	38.25323751
ENSG00000137857 88.49074565	27.84273294	70.71067855
5.510345777	0	1.846347025	62.34805238	2.452230934
	4.656022391	4.07E-06	1	DUOX1	15
45129933	45165576	+	8784	protein_coding	dual

oxidase 1 [Source:HGNC Symbol;Acc:HGNC:3062]	-	92	32
	76	5	0	2	0.506403521	0.158750919
	0.403893906	0.03162931	0	0.010586897
ENSG00000115758 5353.690112	4725.43383	4933.930637
6449.308698	7483.582676	7960.5252	5004.351526
7297.805524	-0.544415778	6.17E-06	0.001258086	ODC1
	2	10439968	10448504	-	2968
protein_coding	ornithine decarboxylase 1 [Source:HGNC
Symbol;Acc:HGNC:8109]	-	5566	5431	5303	5852	6174
	8623	90.67352966	79.73968284	83.40724307
109.5601112	127.8645306	135.0907173
ENSG00000167615 2322.882073	1444.341771	2527.906758
1110.885709	1218.173079	1127.194859	2098.376868
1152.084549	0.864992883	8.38E-06	1	LENG8	19
	54448887	54462037	+	6469	protein_coding
	leukocyte receptor cluster member 8 [Source:HGNC
Symbol;Acc:HGNC:15500]	-	2415	1660	2717	1008	1005
	1221	18.05016601	11.18225868	19.60643
8.658355724	9.549404081	8.776261011
ENSG00000105011 732.9342194	656.0443948	876.4402526
1671.838909	1421.807982	1083.805704	755.1396223
1392.484198	-0.882485311	8.73E-06	1	ASF1B	19
	14119509	14136956	-	2304	protein_coding
	anti-silencing function 1B histone chaperone [Source:HGNC
Symbol;Acc:HGNC:20996]	-	762	754	942	1517	1173
	1174	15.99092967	14.26091755	19.08597998
36.5860185	31.2941311	23.69281374
ENSG00000165029 711.7733889	671.7059321	466.1322363
307.4772944	178.1805399	301.8777387	616.5371857
262.5118576	1.229816883	9.39E-06	1	ABCA1	9
	104781002	104928237	-	11350	protein_coding
	ATP binding cassette subfamily A member 1 [Source:HGNC
Symbol;Acc:HGNC:29]	-	740	772	501	279	147
	327	3.152369193	2.964012539	2.060572503
1.365904656	0.796102217	1.339623968
ENSG00000196230 32233.71596	31872.96853	30615.86301
42975.18672	44387.56035	39229.33525	31574.1825
42197.36077	-0.418376976	1.66E-05	0.003040965	TUBB
	6	30720201	30725426	+	3359
protein_coding	tubulin beta class I [Source:HGNC
Symbol;Acc:HGNC:20778]	-	33512	36632	32906	38995	36620
	42494	482.3825178	475.2358825	457.3104498
645.0761191	670.1248088	588.2319746
ENSG00000114270 2227.658336	1518.29903	2551.16685
1261.869183	1263.021242	1072.727622	2099.041405
1199.206016	0.80786716	1.84E-05	1	COL7A1	3
	48564073	48595267	-	11071	protein_coding
	collagen type VII alpha 1 chain [Source:HGNC
Symbol;Acc:HGNC:2214]	-	2316	1745	2742	1145	1042
	1162	10.11469801	6.868583437	11.56183149

5.746860823	5.785331224	4.88034243
ENSG00000141030 3347.25864	3386.372393	3328.984314
4626.486314	4797.54134	4438.618249	3354.205116
4620.881968	-0.462051879	2.65E-05	0.004416978	COPS3
	17	17246820	17281293	-	2788
protein_coding	COP9 signalosome subunit 3 [Source:HGNC
Symbol;Acc:HGNC:2239]	-	3480	3892	3578	4198	3958
	4808	60.35144752	60.83291953	59.90921136
83.6684471	87.26304846	80.18676772
ENSG00000254527 36.55052538	26.10256213	18.6080733	0
	0	0	27.0870536	0	7.101184645
2.87E-05	1	ENPP7P12	8	12205759
12206389	-	631	processed_pseudogene	ectonucleotide
pyrophosphatase/phosphodiesterase 7 pseudogene 12 [Source:HGNC
Symbol;Acc:HGNC:48697]	-	38	30	20	0	0
	0	2.911759183	2.071812711	1.479608005	0
	0	0
ENSG00000179698 38.47423724	43.50427021	6.512825656	0
	0	0	29.49711104	0	7.224634276
2.90E-05	1	WDR97	8	144107726	144118315
	+	8967	protein_coding	WD repeat domain 97
[Source:HGNC Symbol;Acc:HGNC:26959]	-	40	50	7
	0	0	0	0.215682068	0.242986101
0.036441555	0	0	0
ENSG00000146670 915.6868463	897.9281372	805.7295741
1580.367169	1206.051954	1461.383671	873.1148525
1415.934264	-0.697875686	3.27E-05	1	CDCA5	11
	65066300	65084164	-	3925	protein_coding
	cell division cycle associated 5 [Source:HGNC
Symbol;Acc:HGNC:14626]	-	952	1032	866	1434	995
	1583	11.72731266	11.45773132	10.29969269
20.30119178	15.58227196	18.75306556
ENSG00000111863 733.8960753	948.3930906	940.6381055
466.1752527	504.2388068	529.9015963	874.3090905
500.1052186	0.805781529	3.86E-05	1	ADTRP	6
	11712054	11807046	-	4150	protein_coding
	androgen dependent TFPI regulating protein [Source:HGNC
Symbol;Acc:HGNC:21214]	-	763	1090	1011	423	416
	574	8.889506621	11.44555875	11.37232061
5.663753118	6.16158713	6.431241436
ENSG00000187778 1202.319914	1152.863161	1166.726196
1622.245797	1852.107925	1808.496911	1173.969757
1760.950211	-0.584995272	3.93E-05	1	MCRS1	12
	49556544	49568153	-	3269	protein_coding
	microspherule protein 1 [Source:HGNC Symbol;Acc:HGNC:6960]
	-	1250	1325	1254	1472	1528	1959
18.48827145	17.66280017	17.9072399	25.02101556
28.7313342	27.86445374
ENSG00000240342 6749.343067	7002.447333	7245.983744
8726.183573	9689.627455	10114.28901	6999.258048

9510.033344	-0.442295515	3.94E-05	0.006028478	RPS2P5
	12	118246084	118246962	+	879
processed_pseudogene	ribosomal protein S2 pseudogene 5 [Source:HGNC
Symbol;Acc:HGNC:31386]	-	7017	8048	7788	7918	7994
	10956	385.9791256	398.9860307	413.6024509
500.5398579	559.0139286	579.5543457
ENSG00000089157 36136.92733	40262.332	42767.86528
47242.39849	61490.46795	59038.79249	39722.37487
55923.88631	-0.493507862	4.64E-05	0.006369658	RPLP0
	12	120196686	120201235	-	4001
protein_coding	ribosomal protein lateral stalk subunit P0
[Source:HGNC Symbol;Acc:HGNC:10371]	-	37570	46274	45967
	42867	50730	63952	454.0188248	503.9959988
536.319571	595.3420838	779.3699758	743.2186475
ENSG00000231595 30.77938979	20.0119643	25.12089896	0
	0	0	25.30408435	0	7.00284443
4.71E-05	1	AC005224.1	17	14210488
14217922	+	1537	lincRNA novel transcript	-
	32	23	27	0	0	0	1.006647287
	0.652097547	0.820041691	0	0	0
ENSG00000160408 15.3896949	25.23247672	36.28574294	0
	0	0	25.63597152	0	7.022149988
4.81E-05	1	ST6GALNAC6	9	127885321
127905408	-	5307	protein_coding	"ST6 N-
acetylgalactosaminide alpha-2,6-sialyltransferase 6 [Source:HGNC
Symbol;Acc:HGNC:23364]" -	16	29	39	0	0
	0	0.145771328	0.238126379	0.343053264	0
	0	0
ENSG00000142230 2960.592555	2741.639109	2910.302665
4066.635183	3858.154139	3946.566767	2870.844776
3957.118697	-0.463070535	4.86E-05	0.006369658	SAE1
	19	47113274	47210636	+	3396
protein_coding	SUMO1 activating enzyme subunit 1 [Source:HGNC
Symbol;Acc:HGNC:30660]	-	3078	3151	3128	3690	3183
	4275	43.8230046	40.43331222	42.99768747
60.37688852	57.61244642	58.53282101
ENSG00000129003 1268.687973	1182.446064	1136.022875
830.9601432	721.2069472	560.3663222	1195.718971
704.1778042	0.764864413	4.88E-05	1	VPS13C	15
	61852389	62060473	-	19703	protein_coding
	vacuolar protein sorting 13 homolog C [Source:HGNC
Symbol;Acc:HGNC:23594]	-	1319	1359	1221	754	595
	607	3.236783523	3.005700455	2.892872829
2.126429713	1.856230775	1.432475991
ENSG00000229163 0	0	0	18.73517564	14.5453502
	38.77328753	0	24.01793779	-7.151429952
5.22E-05	1	NAP1L1P2	Y	2929001 2931120 -
	1089	processed_pseudogene	nucleosome assembly protein 1
like 1 pseudogene 2 [Source:HGNC Symbol;Acc:HGNC:38005] -	0
	0	0	17	12	42	0	0	0

0.867427298 0.677330647 1.793297854
ENSG00000073756 202.9516014 220.1316073 150.7253938
465.0731836 424.2393807 356.3449759 191.2695342
415.2191801 -1.117343433 5.27E-05 1 PTGS2 1
186671791 186680427 - 5269 protein_coding
prostaglandin-endoperoxide synthase 2 [Source:HGNC
Symbol;Acc:HGNC:9605] - 211 253 162 422 350
386 1.936223439 2.092429915 1.435267503
4.45037181 4.083073577 3.406356705
ENSG00000101057 1224.4426 1164.174271 1397.466305
2399.204551 2094.530428 1622.939035 1262.027725
2038.891338 -0.691663815 5.29E-05 1 MYBL2 20
43667019 43716496 + 2777 protein_coding
MYB proto-oncogene like 2 [Source:HGNC Symbol;Acc:HGNC:7548]
MYB 1273 1338 1502 2177 1728 1758
22.16428574 20.99610961 25.24876201 43.56067421
38.24857139 29.43567567
ENSG00000010256 3671.404088 3452.498884 3927.233871
4672.773219 5390.26436 5303.631831 3683.712281
5122.223137 -0.475662462 6.42E-05 0.00786335 UQCRC1
3 48599002 48610976 - 3236
protein_coding ubiquinol-cytochrome c reductase core protein 1
[Source:HGNC Symbol;Acc:HGNC:12585] - 3817 3968 4221
4240 4447 5745 57.0315091 53.4344998
60.89096756 72.80637149 84.47067735 82.54913717
ENSG00000196460 852.2043548 773.5059244 1009.487977
511.3600881 564.8444326 427.4293364 878.3994186
501.2112857 0.81061857 6.73E-05 1 RFX8 2
101397361 101474703 - 2854 protein_coding
"RFX family member 8, lacking RFX DNA binding domain
[Source:HGNC Symbol;Acc:HGNC:37253]" RFX 886 889 1085
464 466 463 15.01000959 13.57395453
17.74687152 9.033916153 10.03643111 7.543241992
ENSG00000185813 653.1001771 660.3948218 760.1397945
1088.844326 1133.325203 1071.804448 691.2115978
1097.991326 -0.667530015 6.81E-05 1 PCYT2 17
81900965 81911464 - 6897 protein_coding
"phosphate cytidylyltransferase 2, ethanolamine [Source:HGNC
Symbol;Acc:HGNC:8756]" - 679 759 817 988 935
1161 4.760041782 4.795569039 5.529780165
7.959921087 8.332949408 7.827138378
ENSG00000254995 23.08454234 26.10256213 21.3992843 0
0 0 23.52879626 0 6.898341234
8.17E-05 1 STX16-NPEPL1 20 58651434
58715410 + 3859 protein_coding STX16-NPEPL1
readthrough (NMD candidate) [Source:HGNC Symbol;Acc:HGNC:41993] -
24 30 23 0 0 0 0.300702944
0.338770101 0.278226885 0 0 0
ENSG00000251050 19.23711862 22.62222051 26.98170629 0
0 0 22.94701514 0 6.862170183

0.000101052	1	AC112184.1	5	80019609
80019920	+	312	processed_pseudogene	ring-box 1
(RBX1) pseudogene	-	20	26	29	0	0
	0	3.099392788	3.631427279	4.338997898	0
	0	0
ENSG00000124766 1197.510634	1542.661422	1030.887261
692.0994296	700.6010344	843.7805906	1257.019772
745.4936849	0.75324673	0.000105682	1	SOX4	6
	21592768	21598619	+	5852	protein_coding
	SRY-box 4 [Source:HGNC Symbol;Acc:HGNC:11200]	HMG	1245
	1773	1108	628	578	914	10.28646732
13.20270793	8.838559274	5.963035158	6.071148854
7.262278447
ENSG00000150672 3.847423724	31.32307455	41.86816493	0
	0	0	25.6795544	0	7.024952204
0.000105795	1	DLG2	11	83455012	85627922
	-	16709	protein_coding	discs large MAGUK scaffold
protein 2 [Source:HGNC Symbol;Acc:HGNC:2901]	-	4	36
	45	0	0	0	0.011574727	0.093888119
	0.125721076	0	0	0
ENSG00000099250 6226.093441	6094.078171	6748.217784
5159.887786	4401.180547	4321.375213	6356.129799
4627.481182	0.457973969	0.000111898	0.012840275	NRP1
	10	33177492	33336262	-	11522
protein_coding	neuropilin 1 [Source:HGNC Symbol;Acc:HGNC:8004] -
	6473	7004	7253	4682	3631	4681	27.16307624
	26.48968929	29.38568659	22.57956558	19.37071887
	18.89042759
ENSG00000198482 215.4557285	144.4341771	100.4835958
40.77655875	35.15126297	51.69771671	153.4578338
42.54184615	1.846828916	0.00011429	1	ZNF808	19
	52527652	52564464	+	5008	protein_coding
	zinc finger protein 808 [Source:HGNC Symbol;Acc:HGNC:33230]
	zf-C2H2 224	166	108	37	29	56
2.162643402	1.444449509	1.006712523	0.410534299
0.355943477	0.51994179
ENSG00000079459 3219.331801	2959.16046	3096.383398
4525.095952	5511.475612	3712.080695	3091.625219
4582.884086	-0.567551648	0.000120366	0.012999581	FDFT1
	8	11795573	11839309	+	5202
protein_coding	farnesyl-diphosphate farnesyltransferase 1
[Source:HGNC Symbol;Acc:HGNC:3629]	-	3347	3401	3328
	4106	4547	4021	31.10903797	28.49015958
29.86475839	43.85919344	53.72815985	35.9413796
ENSG00000167900 933.000253	1257.273409	1220.689609
2378.265237	2296.953218	1317.368603	1136.987757
1997.529019	-0.812267602	0.000131135	1	TK1	17
	78174075	78187233	-	1861	protein_coding
	thymidine kinase 1 [Source:HGNC Symbol;Acc:HGNC:11830]	-
	970	1445	1312	2158	1895	1427	25.20151084

33.83608223	32.91042553	64.43429942	62.5907566
35.65402939
ENSG00000279425 31.74124572	6.960683234	56.75462358
1.102069155	0	0	31.81885084	0.367356385
6.371191191	0.000139208	1	AC092279.2	19
24078481	24080669	+	2189	TEC	TEC	-
33	8	61	1	0	0	0.728902425
0.159258574	1.30085865	0.025384364	0	0
ENSG00000186871 319.3361691	401.1093714	345.1797598
786.877377	584.2382329	576.0602719	355.2084334
649.0586273	-0.869107472	0.00014297	1	ERCC6L	X
72204657	72239047	-	4364	protein_coding
"ERCC excision repair 6 like, spindle assembly checkpoint
helicase [Source:HGNC Symbol;Acc:HGNC:20794]"	-	332	461
371	714	482	624	3.67836277	4.60335832
3.968580586	9.091290044	6.789060151	6.648610114
ENSG00000167513 3303.975123	3243.678387	3800.698972
4161.413131	5110.266369	5661.823154	3449.450827
4977.834218	-0.52932382	0.000148836	0.015066501	CDT1
16	88803213	88809258	+	3336
protein_coding	chromatin licensing and DNA replication factor 1
[Source:HGNC Symbol;Acc:HGNC:24576]	-	3435	3728	4085
3776	4216	6133	49.78539027	48.69770107
57.16261061	62.89527021	77.68227225	85.48264288
ENSG00000109971 89632.46864	82091.6878	83199.48695
103935.04	112222.2252	110225.9942	84974.5478
108794.4198	-0.35650451	0.000155917	0.015066501	HSPA8
11	123057489	123063230	-	4849
protein_coding	heat shock protein family A (Hsp70) member 8
[Source:HGNC Symbol;Acc:HGNC:5241]	-	93187	94349	89423
94309	92584	119399	929.1896485	847.8982035
860.8809297	1080.719529	1173.629767	1144.931551
ENSG00000106133 7.694847448	26.10256213	34.42493561	0
0	0	22.74078173	0	6.849525207
0.000159348	1	NSUN5P2 7	72947581	72954790
-	2576	transcribed_unprocessed_pseudogene	NOP2/
Sun RNA methyltransferase family member 5 pseudogene 2 [Source:HGNC
Symbol;Acc:HGNC:16609]	-	8	30	37	0	0
0	0.150156918	0.507497601	0.670504815	0
0	0
ENSG00000254266 24.04639827	22.62222051	17.67766964	0
0	0	21.44876281	0	6.764679253
0.00016848	1	PKIA-AS1	8	78426103
78558503	-	2656	lincRNA PKIA antisense RNA 1
[Source:HGNC Symbol;Acc:HGNC:51659]	-	25	26	19
0	0	0	0.455106622	0.426583325
0.333942402	0	0	0
ENSG00000119333 1165.769388	1154.603331	1132.301261
1818.414106	1471.504595	1824.190861	1150.891327
1704.703187	-0.56724229	0.000193753	1	WDR34	9

128633661	128656787	-	2604	protein_coding
WD repeat domain 34 [Source:HGNC Symbol;Acc:HGNC:28296] -
1212	1327	1217	1650	1214	1976	22.50416257
22.20693087	21.81703027	35.20910681	28.65662675
35.28393217
ENSG00000072840 1332.170464	1239.001616	1286.748269
924.6360214	655.7528713	857.6281933	1285.97345
812.672362	0.661262211	0.00019564	1	EVC	4
5711197 5814305 +	7148	protein_coding	EvC ciliary
complex subunit 1 [Source:HGNC Symbol;Acc:HGNC:3497]	-	1385
1424	1383	839	541	929	9.368422017
8.681285584	9.031994659	6.522130145	4.652218259
6.043133498
ENSG00000186603 39.43609317	12.18119566	13.95605498	0
0	0	21.85778127	0	6.791134924
0.000199687	1	HPDL	1	45326905	45328533
+	1629	protein_coding	4-hydroxyphenylpyruvate
dioxygenase like [Source:HGNC Symbol;Acc:HGNC:28242]	-	41
14	15	0	0	0	1.216925492
0.374511837	0.429849287	0	0	0
ENSG00000159720 1670.743752	1974.223782	1706.360322
2258.139699	2849.676526	2670.740972	1783.775952
2592.852399	-0.539389244	0.000200799	1	ATP6V0D1
16	67438014	67481237	-	8212
protein_coding	ATPase H+ transporting V0 subunit d1 [Source:HGNC
Symbol;Acc:HGNC:13724]	-	1737	2269	1834	2049	2351
2893	10.22709039	12.04048977	10.42548881
13.86452711	17.59750303	16.38062662
ENSG00000099797 2397.906836	2279.623759	2471.152135
3187.183997	3723.60965	3066.782409	2382.894243
3325.858686	-0.480733685	0.000204397	0.018763635	TECR
19	14517085	14565980	+	5918
protein_coding	"trans-2,3-enoyl-CoA reductase [Source:HGNC
Symbol;Acc:HGNC:4551]"	-	2493	2620	2656	2892	3072
3322	20.36800693	19.29234094	20.95072931
27.1540974	31.90756119	26.10091218
ENSG00000179532 732.9342194	436.7828729	587.0847127
320.7021242	235.1498282	333.2656381	585.6006017
296.3725302	0.980699097	0.000206404	1	DNHD1	11
6497260 6593758 +	19629	protein_coding	dynein heavy
chain domain 1 [Source:HGNC Symbol;Acc:HGNC:26532]	-	762
502	631	291	194	361	1.876972946
1.114459113	1.500642424	0.82377168	0.607506481
0.85514556
ENSG00000219470 31.74124572	20.0119643	12.09524765	0
0	0	21.28281922	0	6.753176427
0.000207302	1	AL355802.1	6	43538822
43539703	-	882	processed_pseudogene	40S ribosomal
protein S2 (RPS2) pseudogene	-	33	23	13	0
0	0	1.809033342	1.136364999	0.688051273

0 0 0
ENSG00000248323 1143.646702 1927.23917 1184.403866
790.1835844 825.4486236 878.8611841 1418.429913
831.4977974 0.77061159 0.000223771 1 LUCAT1 5
91054834 91314547 - 16538 antisense
lung cancer associated transcript 1 [Source:HGNC
Symbol;Acc:HGNC:48498] - 1189 2215 1273 717 681
952 3.476162607 5.836457679 3.593283241
2.409063301 2.531112709 2.676609277
ENSG00000254910 21.16083048 25.23247672 14.88645864 0
0 0 20.42658862 0 6.69445621
0.000248671 1 AC136475.2 11 310139 311141 -
392 antisense novel transcript - 22
29 16 0 0 0 2.713550012
3.223818095 1.905372757 0 0 0
ENSG00000071054 8827.913734 7832.508809 7776.313834
6325.876952 6381.772398 5731.061167 8145.578792
6146.236839 0.406423765 0.000255204 0.021487853 MAP4K4
2 101696850 101894689 + 11961
protein_coding mitogen-activated protein kinase kinase kinase kinase
4 [Source:HGNC Symbol;Acc:HGNC:6866] - 9178 9002 8358
5740 5265 6208 37.1006723 32.79669764
32.61977133 26.66591225 27.05691301 24.133218
ENSG00000184009 122007.5774 127926.0467 130604.4841
157820.7113 160482.485 159034.1779 126846.0361
159112.4581 -0.326966765 0.000262595 0.021487853 ACTG1
17 81509971 81523847 - 2993
protein_coding actin gamma 1 [Source:HGNC Symbol;Acc:HGNC:144] -
126846 147027 140374 143204 132399 172269 2049.138326
2140.666323 2189.40444 2658.645762 2719.101054
2676.279294
ENSG00000187144 48.09279655 2.610256213 18.6080733 0
0 0 23.10370869 0 6.870775843
0.000284082 1 SPATA21 1 16387117 16437424
- 4506 protein_coding spermatogenesis associated 21
[Source:HGNC Symbol;Acc:HGNC:28026] - 50 3 20
0 0 0 0.536512733 0.029012735
0.207197659 0 0 0
ENSG00000168003 17709.6914 15000.27237 15635.43359
19557.31923 21005.90991 24404.09181 16115.13245
21655.77365 -0.426415511 0.000288782 0.021487853 SLC3A2
11 62856102 62888875 + 5178
protein_coding solute carrier family 3 member 2 [Source:HGNC
Symbol;Acc:HGNC:11026] - 18412 17240 16805 17746 17330
26435 171.9254369 145.0887747 151.5034444
190.4366244 205.7234869 237.3822745
ENSG00000100297 2922.118318 2652.020312 3124.295508
3882.589635 3816.942314 4085.042794 2899.478046
3928.191581 -0.438276297 0.000291908 0.021487853 MCM5
22 35400063 35425430 + 7338

protein_coding minichromosome maintenance complex component 5
[Source:HGNC Symbol;Acc:HGNC:6948] - 3038 3048 3358
3523 3149 4425 20.01756644 18.10072011
21.3623497 26.67761369 26.3780273 28.03926245
ENSG00000167553 9584.894352 9135.896745 8803.47948
11243.30952 12575.66736 11810.15875 9174.756859
11876.37854 -0.37232495 0.000295247 0.021487853 TUBA1C
12 49188736 49274603 + 4631
protein_coding tubulin alpha 1c [Source:HGNC Symbol;Acc:HGNC:20768]
- 9965 10500 9462 10202 10375 12793
104.0408133 98.80367895 95.37931489 122.4116021
137.70848 128.4483817
ENSG00000244313 1849.648955 2291.804955 2397.650245
3086.895704 3100.583817 3034.471336 2179.701385
3073.983619 -0.495814139 0.000304294 0.021487853
AC024293.1 11 46428653 46429150 - 498
processed_pseudogene ribosomal protein S10 (RPS10)
pseudogene - 1923 2634 2577 2801 2558 3287
186.7029405 230.4862519 241.5633877 312.5329489
315.7319687 306.9031737
ENSG00000196123 496.3176604 402.8495422 489.3923279
251.2717674 276.3616537 224.3311636 462.8531768
250.6548616 0.88590099 0.000310326 1 KIAA0895L
16 67175602 67184040 - 4780
protein_coding KIAA0895 like [Source:HGNC Symbol;Acc:HGNC:34408]
- 516 463 526 228 228 243
5.219429328 4.220964428 5.136932787 2.650446205
2.931934816 2.363792744
ENSG00000141499 899.3352954 759.5845579 834.5720877
1084.436049 1301.808843 1503.849652 831.1639803
1296.698181 -0.642292935 0.000316801 1 WRAP53 17
7686071 7703502 + 5087 protein_coding WD repeat
containing antisense to TP53 [Source:HGNC Symbol;Acc:HGNC:25522]
- 935 873 897 984 1074 1629
8.886916297 7.478441553 8.23145752 10.74843921
12.97746613 14.88985144
ENSG00000169908 1779.433472 2885.2032 2536.280391
4061.124838 4003.607641 3099.093482 2400.305688
3721.27532 -0.632173768 0.000321556 0.021865841 TM4SF1
3 149369022 149377865 - 3668
protein_coding transmembrane 4 L six family member 1 [Source:HGNC
Symbol;Acc:HGNC:11853] - 1850 3316 2726 3685 3303
3357 24.38617117 39.39524381 34.69305626
55.82390446 55.35114471 42.55523885
ENSG00000101096 46.16908469 58.29572208 66.05866023
103.5945006 220.604478 194.7896112 56.84115567
172.9961966 -1.605161356 0.000322574 1 NFATC2 20
51386957 51562831 - 8286 protein_coding
nuclear factor of activated T cells 2 [Source:HGNC
Symbol;Acc:HGNC:7776] RHD 48 67 71 94 182

211 0.280089949 0.352361517 0.399999507
0.630369175 1.350124503 1.184045917
ENSG00000120658 195.256754 163.576056 217.7144577
321.8041934 438.7847309 387.7328753 192.1824225
382.7739332 -0.994124002 0.0003252 1 ENOX1 13
43213518 43786908 - 3101 protein_coding
ecto-NOX disulfide-thiol exchanger 1 [Source:HGNC
Symbol;Acc:HGNC:25474] - 203 188 234 292 362
420 3.165158685 2.641889694 3.522574014
5.232305928 7.175532974 6.297650316
ENSG00000171848 752.171338 1249.44264 993.6711144
2639.455627 1630.291334 1243.514722 998.4283643
1837.753894 -0.879682836 0.000333079 1 RRM2 2
10120698 10131419 + 5323 protein_coding
ribonucleotide reductase regulatory subunit M2 [Source:HGNC
Symbol;Acc:HGNC:10452] - 782 1436 1068 2395 1345
1347 7.103158463 11.75591863 9.366143823
25.00121387 15.53149204 11.76636043
ENSG00000174371 495.3558044 798.7384011 777.8174641
1459.139562 1056.962114 1057.956846 690.6372232
1191.352841 -0.786147897 0.000355231 1 EXO1 1
241847967 241895148 + 4140 protein_coding
exonuclease 1 [Source:HGNC Symbol;Acc:HGNC:3511] -
515 918 836 1324 872 1146 6.014618758
9.662754326 9.426532564 17.77050164 12.94683174
12.87108904
ENSG00000117410 2894.224496 3022.676694 2937.284371
3619.195106 4128.455231 4111.814826 2951.395187
3953.155054 -0.421602511 0.000372266 0.024410026
ATP6V0B 1 43974487 43978295 + 3535
protein_coding ATPase H+ transporting V0 subunit b [Source:HGNC
Symbol;Acc:HGNC:861] - 3009 3474 3157 3284 3406
4454 41.15607842 42.82515995 41.68993323
51.62092399 59.2246613 58.58572554
ENSG00000251093 9.618559309 33.06324536 16.74726597 0
0 0 19.80969021 0 6.650885519
0.000377159 1 AC093281.2 5 91226475
91227071 - 597 lincRNA novel transcript -
10 38 18 0 0 0 0.809891583
2.7737535 1.407486409 0 0 0
ENSG00000130193 850.280643 866.6050626 1034.608876
1142.845714 1532.110221 1641.402506 917.1648604
1438.786147 -0.649875492 0.000378445 1 THEM6 8
142727203 142736927 + 3055 protein_coding
thioesterase superfamily member 6 [Source:HGNC
Symbol;Acc:HGNC:29656] - 884 996 1112 1037 1264
1778 13.99079093 14.20714201 16.99180864
18.86164582 25.43215838 27.06148097
ENSG00000133424 15.3896949 9.570939447 34.42493561 0
0 0 19.79518998 0 6.64856299

0.000390682	1	LARGE1	22	33162226	33922841
	-	10687	protein_coding	LARGE xylosyl- and
glucuronyltransferase 1 [Source:HGNC Symbol;Acc:HGNC:6511]	-
	16	11	37	0	0	0	0.072387802
	0.044853411	0.161618827	0	0	0
ENSG00000119725 32.70310165	20.8820497	6.512825656	0
	0	0	20.032659	0	6.665881168
0.000397739	1	ZNF410	14	73886617	73932511
	+	6486	protein_coding	zinc finger protein 410
[Source:HGNC Symbol;Acc:HGNC:20144]	zf-C2H2 34	24	7
	0	0	0	0.253456357	0.161247465
0.05038104	0	0	0
ENSG00000207751 25.0082542	17.40170808	14.88645864	0
	0	0	19.09880698	0	6.597025424
0.00039967	1	AP000553.1	22	21652270
21670237	+	1093	processed_transcript	novel
transcript	-	26	20	16	0	0	0
	1.150149785	0.797385679	0.683354182	0	0
	0
ENSG00000255142 19.23711862	12.18119566	25.12089896	0
	0	0	18.84640441	0	6.577720518
0.000453753	1	AP006621.2	11	781645	782105	+
	284	lincRNA novel transcript	-	20	14
	27	0	0	0	3.404966725	2.14816825
	4.438042532	0	0	0
ENSG00000097021 1882.352057	2221.328037	2366.946924
2826.807384	3156.340992	3013.238346	2156.875673
2998.795574	-0.475284209	0.000469891	0.02905492	ACOT7
	1	6264269 6394391 -	4124	protein_coding	acyl-
CoA thioesterase 7 [Source:HGNC Symbol;Acc:HGNC:24157]	-	1957
	2553	2544	2565	2604	3264	22.94422461
26.97682011	28.79681698	34.56055885	38.81232716
36.80124503
ENSG00000088325 1714.989125	1797.596445	1900.814688
3036.200523	2478.770096	2241.465289	1804.466753
2585.478636	-0.518610025	0.000485404	1	TPX2	20
	31739271	31801805	+	3605	protein_coding
	"TPX2, microtubule nucleation factor [Source:HGNC
Symbol;Acc:HGNC:1249]"	-	1783	2066	2043	2755	2045
	2428	23.9137283	24.97374344	26.4550833
42.46473105	34.8686717	31.31658747
ENSG00000182481 3725.268021	3608.244171	3289.90736
5351.647819	4890.874003	4231.827382	3541.139851
4824.783068	-0.446056652	0.000488327	0.02905492	KPNA2
	17	68035519	68046842	+	3140
protein_coding	karyopherin subunit alpha 2 [Source:HGNC
Symbol;Acc:HGNC:6395]	-	3873	4147	3536	4856	4035
	4584	59.63744999	57.55233985	52.56887029
85.93321765	78.98802431	67.88064792
ENSG00000197912 4976.642587	4105.933023	4785.06605

3004.240518   3247.249431   3778.549188   4622.54722
3343.346379   0.467027879   0.000490579   0.02905492   SPG7
        16   89490719   89557768   +   36008
protein_coding "SPG7, paraplegin matrix AAA peptidase subunit
[Source:HGNC Symbol;Acc:HGNC:11237]"   -   5174   4719   5143
        2726   2679   4093   6.947501368   5.710966007
6.667508227   4.206674367   4.573209257   5.285350133
ENSG00000130584 243.3495505   251.4546818   241.9049529
135.5545061   87.27210117   105.2417805   245.5697284
109.3561292   1.166360556   0.000522608   1   ZBTB46 20
        63743666   63831244   -   6084   protein_coding
        zinc finger and BTB domain containing 46 [Source:HGNC
Symbol;Acc:HGNC:16094] -   253   289   260   123   72
        114   2.010631732   2.069985175   1.994941562
1.123383264   0.727429068   0.87125777
ENSG00000090615 5730.737637   4275.599676   5640.107018
3860.548251   3499.368835   3891.176356   5215.481444
3750.364481   0.475541356   0.000524808   0.030110856   GOLGA3
        12   132768909   132828858   -   10549
protein_coding golgin A3 [Source:HGNC Symbol;Acc:HGNC:4426]   -
        5958   4914   6062   3503   2887   4215   27.30803325
        20.29936523   26.82567604   18.45189135   16.82220231
        18.57878482
ENSG00000125971 2780.725496   2967.861314   2866.573692
3505.681983   4224.212119   3846.864028   2871.720168
3858.919377   -0.426110887   0.000543821   0.030256217
DYNLRB1 20   34516409   34540958   +   2460
protein_coding dynein light chain roadblock-type 1 [Source:HGNC
Symbol;Acc:HGNC:15468] -   2891   3411   3081   3181   3485
        4167   56.82169715   60.42340707   58.46589833
71.85228861   87.07932131   78.76248528
ENSG00000188177 621.3589314   476.8068015   367.5094478
270.0069431   225.452928   275.1057068   488.5583936
256.8551926   0.926467583   0.000579781   1   ZC3H6   2
        112275594   112340063   +   11803   protein_coding
        zinc finger CCCH-type containing 6 [Source:HGNC
Symbol;Acc:HGNC:24762] -   646   548   395   245   186
        298   2.646313713   2.023236956   1.562250687
1.153415334   0.968652263   1.173964259
ENSG00000112715 3837.805164   5628.58248   5914.5761
3560.785441   3832.699777   3069.55193   5126.987915
3487.679049   0.55614878   0.000585378   0.031530171   VEGFA
        6   43770184   43786487   +   14431
protein_coding vascular endothelial growth factor A [Source:HGNC
Symbol;Acc:HGNC:12680] -   3990   6469   6357   3231   3162
        3325   13.36834625   19.53436608   20.56372657
12.44092222   13.46830055   10.71337338
ENSG00000166803 219.3031523   160.0957144   196.3151734
524.584918   332.1188295   320.3412089   191.90468
392.3483188   -1.031985418   0.000587937   1   PCLAF   15

64364311	64387687	-	3734	protein_coding
PCNA clamp associated factor [Source:HGNC
Symbol;Acc:HGNC:28961]	-	228	184	211	476	274
347	2.952308588	2.147346393	2.637874791
7.083447554	4.510488807	4.321019033
ENSG00000120279 8.656703379	22.62222051	10.23444032
24.24552142	139.3929394	90.47100425	13.83778807
84.70315501	-2.609028105	0.000599424	1	MYCT1	6
152697895	152724567	+	3030	protein_coding
MYC target 1 [Source:HGNC Symbol;Acc:HGNC:23172]	-
9	26	11	22	115	98	0.143615428
0.373929146	0.169471273	0.403452207	2.332934642
1.503884438
ENSG00000113575 2576.812039	2788.623721	2293.445035
3453.884733	3511.48996	3367.736974	2552.960265
3444.370556	-0.43190731	0.000601065	0.031530171	PPP2CA
5	134194334	134226142	-	5661
protein_coding	protein phosphatase 2 catalytic subunit alpha
[Source:HGNC Symbol;Acc:HGNC:9299]	-	2679	3205	2465
3134	2897	3648	22.88130421	24.67138194
20.326837	30.76223467	31.45594237	29.96351045
ENSG00000258458 18.27526269	14.79145187	20.46888063	0
0	0	17.8451984	0	6.499142424
0.000613949	1	AL160314.2	14	22701476
22766562	-	2513	processed_transcript	"novel
transcript, antisense to OXA1L" -	19	17	22	0
0	0	0.365563081	0.29479155	0.408673266
0	0	0
ENSG00000140365 584.808406	642.9931137	632.6744923
867.3284253	905.4480497	1094.883786	620.1586707
955.8867537	-0.62474632	0.000627548	1	COMMD4	15
75335891	75343224	+	6779	protein_coding
COMM domain containing 4 [Source:HGNC Symbol;Acc:HGNC:26027]
-	608	739	680	787	747	1186
4.336498114	4.750478996	4.682624301	6.450912365
6.77333145	8.134859814
ENSG00000225806 0	0	0	19.8372448	21.81802529
9.231735127	0	16.96233507	-6.645410704
0.000630072	1	AL121917.1	20	58863528
58888809	-	1521	antisense	uncharacterized
LOC101927932 [Source:NCBI gene;Acc:101927932]	-	0	0
0	18	18	10	0	0	0
0.657590204	0.727429068	0.305704481
ENSG00000209082 69.25362703	95.70939447	73.50188955
197.2703788	226.6650405	161.5553647	79.48830368
195.1635947	-1.29374168	0.000633478	1	MT-TL1	MT
3230	3304	+	75	Mt_tRNA mitochondrially
encoded tRNA leucine 1 (UUA/G) [Source:HGNC Symbol;Acc:HGNC:7490]
-	72	110	79	179	187	175
46.41650639	63.91312012	49.17131962	132.6184083

153.2596055	108.4945202
ENSG00000108518 10419.7853	10878.67781	12184.5664
14268.48936	14348.98797	14203.02449	11161.00984
14273.50061	-0.354862241	0.000633596	0.032313373	PFN1
	17	4945652 4949061 -	1987	protein_coding
profilin 1 [Source:HGNC Symbol;Acc:HGNC:8881]	-	10833	12503
	13096	12947	11838	15385	263.6040585	274.2047425
	307.6711927	362.0623155	366.2080011	360.0233358
ENSG00000117318 2162.252133	2257.871624	2483.247382
2759.581165	3226.643518	3648.381722	2301.123713
3211.535469	-0.481162835	0.000661357	0.032817595	ID3
	1	23557918	23559794	-	1797
protein_coding	"inhibitor of DNA binding 3, HLH protein [Source:HGNC
Symbol;Acc:HGNC:5362]"	bHLH	2248	2595	2669	2504	2662
	3952	60.4852453	62.92857289	69.33404412
77.42804452	91.05574127	102.2586081
ENSG00000242071 129.8505507	203.5999846	260.5130263
523.4828488	638.7832961	240.0251133	197.9878538
467.4304194	-1.237628339	0.000662158	1	RPL7AP6 14
	69885340	69886140	-	801
processed_pseudogene	ribosomal protein L7a pseudogene 6
[Source:HGNC Symbol;Acc:HGNC:19785]	-	135	234	280
	475	527	260	8.148965308	12.73039676
16.31817368	32.95134431	40.44133274	15.09287065
ENSG00000160796 1124.409583	772.635839	887.6050966
567.565615	608.4804832	566.8285368	928.2168396
580.9582117	0.675991052	0.000670311	1	NBEAL2	3
	46979683	47009703	+	9819	protein_coding
	neurobeachin like 2 [Source:HGNC Symbol;Acc:HGNC:31928] -
	1169	888	954	515	502	614	5.756366905
	3.940980659	4.53552067	2.914419155	3.14256173
	2.907583055
ENSG00000104969 4692.895087	4183.370624	4304.977759
4967.025683	6237.531009	7131.515386	4393.747823
6112.024026	-0.476390661	0.000679546	0.032832791	SGTA
	19	2754714 2783371 -	3801	protein_coding	small
glutamine rich tetratricopeptide repeat containing alpha [Source:HGNC
Symbol;Acc:HGNC:10819]	-	4879	4808	4627	4507	5146
	7725	62.06320011	55.12202797	56.82607573
65.88730321	83.21838293	94.4999626
ENSG00000083223 2366.16559	2747.729707	2106.433898
1364.361614	1718.775548	1850.962893	2406.776398
1644.700019	0.549103585	0.00068757	1	TUT7	9
	86287733	86354454	-	6707	protein_coding
	terminal uridylyl transferase 7 [Source:HGNC
Symbol;Acc:HGNC:25817]	-	2460	3158	2264	1238	1418
	2005	17.73405362	20.51834921	15.75774804
10.25662275	12.99556856	13.90007325
ENSG00000188807 1665.934472	1548.75202	1383.51025
1897.763086	2220.59013	2443.640288	1532.732247

2187.331168	-0.513364192	0.000699769	1	TMEM201 1
	9588922 9614873 +	6966	protein_coding	transmembrane
protein 201 [Source:HGNC Symbol;Acc:HGNC:33719] -	1732	1780
	1487	1722	1832	2647	12.02169303	11.13512585
	9.964913524	13.7360455	16.1655081	17.66857357
ENSG00000227775 29.81753386	18.27179349	6.512825656	0
	0	0	18.20071767	0	6.52747843
0.000713462	1	AL031282.1	1	1724512 1737251 +
	2759	transcribed_processed_pseudogene	ribosomal
protein S7 (RPS7) pseudogene	-	31	21	7	0
	0	0	0.543264354	0.331685275	0.118438357
	0	0	0
ENSG00000143702 5497.968501	5813.910671	4560.838767
3784.50548	3955.123141	4079.503753	5290.90598
3939.710791	0.425399602	0.000721628	0.032853751	CEP170
	1	243124428	243255348	-	10939
protein_coding	centrosomal protein 170 [Source:HGNC
Symbol;Acc:HGNC:28920]	-	5716	6682	4902	3434	3263
	4419	25.26479707	26.61873709	20.91903856
17.44354348	18.33524898	18.78353798
ENSG00000073111 4711.17035	3843.167231	5163.740342
6110.973467	6250.864247	6171.414933	4572.692641
6177.750882	-0.434121472	0.000733082	0.032853751	MCM2
	3	127598223	127622436	+	4902
protein_coding	minichromosome maintenance complex component 2
[Source:HGNC Symbol;Acc:HGNC:6944]	-	4898	4417	5550
	5545	5157	6685	48.31107378	39.26564087
52.85252156	62.85506607	64.66528332	63.41019997
ENSG00000125166 3274.157589	3140.138224	2925.189123
3888.09998	4078.758617	4335.222816	3113.161645
4100.693804	-0.397631917	0.000739964	0.032853751	GOT2
	16	58707131	58734357	-	3766
protein_coding	glutamic-oxaloacetic transaminase 2 [Source:HGNC
Symbol;Acc:HGNC:4433]	-	3404	3609	3144	3528	3365
	4696	43.7029197	41.76044945	38.9716019
52.05474273	54.9227296	57.98007739
ENSG00000136930 3505.003012	3372.451027	3594.149359
4177.944168	4631.481925	5048.835941	3490.534466
4619.420678	-0.404456991	0.00076221	0.032853751	PSMB7
	9	124353466	124415444	-	1787
protein_coding	proteasome subunit beta 7 [Source:HGNC
Symbol;Acc:HGNC:9544]	-	3644	3876	3863	3791	3821
	5469	98.595032	94.51871607	100.9127849
117.8803117	131.4316153	142.3031092
ENSG00000173821 3422.283402	2979.172424	3807.211798
2389.285929	2735.737949	2211.923737	3402.889208
2445.649205	0.476873738	0.000775355	0.032853751	RNF213
	17	80260866	80398786	+	28570
protein_coding	ring finger protein 213 [Source:HGNC
Symbol;Acc:HGNC:14539]	-	3558	3424	4092	2168	2257

2396	6.021392258	5.222544069	6.686077718
4.216587152	4.855888197	3.899488029
ENSG00000142657 2536.41409	2648.539971	2554.888465
3086.895704	3769.669926	3534.83138	2579.947508
3463.799003	-0.424906887	0.000787345	0.032853751	PGD
1	10398592	10420144	+	2966
protein_coding	phosphogluconate dehydrogenase [Source:HGNC
Symbol;Acc:HGNC:8891]	-	2637	3044	2746	2801	3110
3829	42.9873031	44.72312059	43.21907046
52.47518831	64.45203029	60.02680634
ENSG00000234393 0	0	0	7.714484088	8.484787614
35.08059348	0	17.0932884	-6.661985457
0.000797834	1	AL592546.1	10	113710681
113719332	-	692	antisense	novel transcript
-	0	0	0	7	7	38	0
0	0	0.56208758	0.621783661	2.553339244
ENSG00000188897 25.97011014	9.570939447	16.74726597	0
0	0	17.42943852	0	6.464627698
0.000806086	1	AC099489.1	16	11359845
11527245	-	12027	protein_coding	vitellogenin
[Source:NCBI gene;Acc:400499]	-	27	11	18	0
0	0	0.108544462	0.039856024	0.069865252
0	0	0
ENSG00000205138 664.6424483	675.1862737	650.352162
864.0222178	1024.235076	1129.96438	663.393628
1006.073891	-0.601185126	0.000820781	1	SDHAF1	19
35995199	35996315	+	1117	protein_coding
succinate dehydrogenase complex assembly factor 1 [Source:HGNC
Symbol;Acc:HGNC:33867]	-	691	776	699	784	845
1224	29.91066651	30.27381453	29.21258832
39.00092731	46.49977977	50.95176852
ENSG00000181019 277.0145081	399.3692005	351.6925854
671.1601157	553.93542	541.902852	342.692098
588.9994625	-0.780629878	0.000878986	1	NQO1	16
69706996	69726951	-	3254	protein_coding
NAD(P)H quinone dehydrogenase 1 [Source:HGNC
Symbol;Acc:HGNC:2874]	-	288	459	378	609	457
587	4.279333718	6.146865844	5.422758791
10.39948392	8.632687336	8.38786768
ENSG00000079385 19.23711862	17.40170808	13.95605498	0
0	0	16.86496056	0	6.417777761
0.000884845	1	CEACAM1 19	42507304	42561234
-	5524	protein_coding	carcinoembryonic antigen
related cell adhesion molecule 1 [Source:HGNC Symbol;Acc:HGNC:1814]
-	20	20	15	0	0	0
0.175056218	0.157773814	0.126760407	0	0	0
ENSG00000161203 7012.891593	7268.693467	7938.204071
9174.725719	10379.31948	9113.568918	7406.596377
9555.871371	-0.367471181	0.000885502	0.036128494	AP2M1
3	184174689	184184091	+	4756

protein_coding adaptor related protein complex 2 subunit mu 1
[Source:HGNC Symbol;Acc:HGNC:564]   -   7291   8354   8532
        8325   8563   9872   74.12188729   76.54401218
83.74425756   97.26451821   110.6703946   96.51488974
ENSG00000174282 1923.711862   1751.481919   2207.847897
1340.116093   1486.049945   1354.295543   1961.013893
1393.487194   0.493074017   0.00088887   1   ZBTB4   17
        7459366 7484263 -   6126   protein_coding zinc finger
and BTB domain containing 4 [Source:HGNC Symbol;Acc:HGNC:23847] ZBTB
        2000   2013   2373   1216   1226   1467   15.78535014
        14.31941844   18.08284591   11.02982511   12.30157838
        11.13484407
ENSG00000163754 820.4631091   790.0375471   762.0006018
1149.458129   1147.870553   1115.193603   790.8337526
1137.507428   -0.524364091   0.000896353   1   GYG1   3
        148991341   149027668   +   3511   protein_coding
        glycogenin 1 [Source:HGNC Symbol;Acc:HGNC:4699] -   853
        908   819   1043   947   1208   11.74679577
11.26973274   10.88927857   16.50690009   16.5793122
15.99805269
ENSG00000143195 247.1969743   135.7333231   205.61921
577.4842374   336.9672795   331.4192911   196.1831691
415.2902693   -1.082525192   0.000904516   1   ILDR2   1
        166895711   166975482   -   13884   protein_coding
        immunoglobulin like domain containing receptor 2 [Source:HGNC
Symbol;Acc:HGNC:18131] -   257   156   221   524   278
        359   0.894993198   0.489630644   0.743059694
2.097146286   1.23077184   1.202294504
ENSG00000092847 1839.06854   1485.235785   1665.422561
1255.256768   1098.17394   1154.890064   1663.242295
1169.440257   0.507905777   0.000934601   1   AGO1   1
        35869808   35930528   +   13886   protein_coding
        "argonaute 1, RISC catalytic component [Source:HGNC
Symbol;Acc:HGNC:3262]" -   1912   1707   1790   1139   906
        1251   6.657511779   5.356917499   6.01758046
4.557835081   4.010498857   4.18900778
ENSG00000115268 22813.29897   25299.4733   24819.44817
28271.38004   31971.89184   31948.26576   24310.74015
30730.51255   -0.338072076   0.00095375   0.037601102   RPS15
        19   1438358 1440494 +   2137   protein_coding
ribosomal protein S15 [Source:HGNC Symbol;Acc:HGNC:10388]   -
        23718   29077   26676   25653   26377   34607   536.6297665
        592.9303379   582.7230838   667.0304848   758.6967739
        752.9921504
ENSG00000198899 61331.78158   59122.30322   64470.46117
67685.78132   86366.65312   87186.35289   61641.51532
80412.92911   -0.383531366   0.000962555   0.037601102   MT-
ATP6   MT   8527   9207   +   681   protein_coding
mitochondrially encoded ATP synthase membrane subunit 6 [Source:HGNC
Symbol;Acc:HGNC:7414]   -   63764   67950   69293   61417   71253

94442 4527.199758 4348.114249 4749.941798
5011.336096 6431.368625 6448.357126
ENSG00000199691 23.08454234 12.18119566 14.88645864 0
0 0 16.71739888 0 6.404673554
0.000978451 1 RN7SKP173 20 38761528
38761842 + 315 misc_RNA "RNA, 7SK small
nuclear pseudogene 173 [Source:HGNC Symbol;Acc:HGNC:45897]" -
24 14 16 0 0 0 3.683849714
1.936761216 2.371130542 0 0 0
ENSG00000147421 679.0702872 358.4751865 445.6633556
263.3945281 266.6647536 252.026369 494.4029431
260.6952169 0.923097353 0.000993898 1 HMBOX1 8
28890394 29064764 + 6634 protein_coding
homeobox containing 1 [Source:HGNC Symbol;Acc:HGNC:26137]
Homeobox 706 412 479 239 220 273
5.145533978 2.706327475 3.370591196 2.001863575
2.038424234 1.913454757
ENSG00000267519 886.8311683 683.0170423 962.0373898
418.7862791 616.9652708 494.8210028 843.9618668
510.1908509 0.726672401 0.000998865 1 AC020916.1
19 13823880 13842928 - 19049
antisense novel transcript - 922 785 1034
380 509 536 2.340237616 1.795792166
2.533928713 1.108468761 1.642453038 1.308349058
ENSG00000149968 1254.260134 663.8751634 1208.594361
2007.970001 1492.110508 1659.865976 1042.243219
1719.982162 -0.723139373 0.001044038 1 MMP3 11
102835801 102843803 - 2048 protein_coding
matrix metallopeptidase 3 [Source:HGNC Symbol;Acc:HGNC:7173]
- 1304 763 1299 1822 1231 1798
30.78568743 16.23503329 29.6091019 49.43453621
36.94668355 40.82166865
ENSG00000137965 1925.635574 2149.981034 1944.54366
1603.510621 1363.626581 1392.145657 2006.720089
1453.094286 0.465821899 0.00106783 1 IFI44 1
78649796 78664078 + 2038 protein_coding
interferon induced protein 44 [Source:HGNC
Symbol;Acc:HGNC:16938] - 2002 2471 2090 1455 1125
1508 47.49644555 52.83566324 47.87272426
39.67079075 33.93092529 34.40552427
ENSG00000233927 1025.338422 1249.44264 1353.737333
1839.35342 1506.655858 1969.129103 1209.506132
1771.712794 -0.551079967 0.001075353 1 RPS28 19
8321158 8323340 + 2119 protein_coding ribosomal
protein S28 [Source:HGNC Symbol;Acc:HGNC:10418] - 1066 1436
1455 1669 1243 2133 24.32357825 29.53126705
32.05369295 43.7660572 36.05682916 46.80485637
ENSG00000105447 6714.716254 5874.816649 6387.221162
7226.267452 8399.939738 9907.498139 6325.584688
8511.23511 -0.428348395 0.001095645 0.041908438 GRWD1

19	48445773	48457022	+	5763
protein_coding	glutamate rich WD repeat containing 1 [Source:HGNC
Symbol;Acc:HGNC:21270]	-	6981	6752	6865	6557	6930
10732	58.56932716	51.05548567	55.60808737
63.22205492	73.91489686	86.58906748
ENSG00000104549 1485.105557	1501.767408	1598.433497
2216.261072	2215.74168	1868.50319	1528.435487
2100.168647	-0.458034898	0.001100564	1	SQLE	8
124998497	125022283	+	4305	protein_coding
squalene epoxidase [Source:HGNC Symbol;Acc:HGNC:11279]	-
1544	1726	1718	2011	1828	2024	17.34104865
17.47134072	18.62927868	25.9567885	26.10059428
21.86091676
ENSG00000215914 19.23711862	16.53162268	13.02565131	0
0	0	16.26479754	0	6.365463758
0.001117954	1	MMP23A	1	1699942 1701782 +	1017
unprocessed_pseudogene	matrix metallopeptidase 23A
(pseudogene) [Source:HGNC Symbol;Acc:HGNC:7170] -	20	19
14	0	0	0	0.950846165	0.81412529
0.642618344	0	0	0
ENSG00000160752 1119.600304	1033.66146	1475.620213
1575.958892	2123.621129	1751.260154	1209.627326
1816.946725	-0.586779239	0.001135234	1	FDPS	1
155308748	155320666	+	3718	protein_coding
farnesyl diphosphate synthase [Source:HGNC
Symbol;Acc:HGNC:3631]	-	1164	1188	1586	1430	1752
1897	15.13717429	13.92405253	19.91314396
21.37168162	28.96490288	23.72405726
ENSG00000130827 748.3239143	835.2819881	1239.297682
578.5863066	469.0875438	636.9897238	940.9678615
561.5545247	0.743769875	0.00115196	1	PLXNA3	X
154458281	154477779	+	11710	protein_coding
plexin A3 [Source:HGNC Symbol;Acc:HGNC:9101]	-	778
960	1332	525	387	690	3.212357847
3.572505744	5.309985872	2.491233596	2.031427981
2.739827458
ENSG00000104894 179.8670591	130.5128106	60.47623824
36.36828213	14.5453502	45.23550212	123.6187027
32.04971148	1.939976527	0.001172861	1	CD37	19
49335171	49343335	+	4161	protein_coding
CD37 molecule [Source:HGNC Symbol;Acc:HGNC:1666]	-
187	150	65	33	12	49	2.172926854
1.570913026	0.729225214	0.440684999	0.177268223
0.547557059
ENSG00000230648 27.893822	18.27179349	4.652018326	0
0	0	16.93921127	0	6.423918528
0.001206072	1	AL138831.1	6	4018843 4021215 -
209	antisense	novel transcript	-	29
21	5	0	0	0	6.708924867
4.378563036	1.116785468	0	0	0

ENSG00000268218 135.6216863 115.7213588 173.0550817
24.24552142 75.15097601 30.46472592 141.4660423
43.28707445 1.71532948 0.001206508 1 AC137932.3
16 89268104 89273044 + 3660
antisense "novel transcript, antisense to ANKRD11" -
141 133 186 22 62 33 1.862684256
1.58354042 2.372345261 0.334005516 1.041257073
0.41924112
ENSG00000101440 18.27526269 29.58290374 3.721614661 0
0 0 17.19326036 0 6.446247125
0.001210081 1 ASIP 20 34194569 34269344
+ 778 protein_coding agouti signaling protein
[Source:HGNC Symbol;Acc:HGNC:745] - 19 34 4
0 0 0 1.180796944 1.904398882
0.240008394 0 0 0
ENSG00000127580 782.9507278 677.7965299 726.6452625
952.1877503 1067.871127 1241.668375 729.1308401
1087.242417 -0.577144943 0.001217205 1 WDR24 16
684622 690444 - 3651 protein_coding WD repeat
domain 24 [Source:HGNC Symbol;Acc:HGNC:20852] - 814 779
781 864 881 1345 10.77987658 9.297886116
9.985854567 13.14964272 14.83240023 17.12937312
ENSG00000196670 455.9197113 530.7520966 457.7586033
273.3131505 306.6644666 281.5679214 481.4768037
287.1818462 0.746173004 0.001248835 1 ZFP62 5
180847611 180861285 - 4197 protein_coding
ZFP62 zinc finger protein [Source:HGNC Symbol;Acc:HGNC:23241]
zf-C2H2 474 610 492 248 253 305
5.460602819 6.333582961 5.47232862 3.283407269
3.70534723 3.37902876
ENSG00000198464 253.9299658 355.8649303 319.1284572
168.6165808 168.4836398 143.0918945 309.6411178
160.0640383 0.953461942 0.001322894 1 ZNF480 19
52297177 52325922 + 4836 protein_coding
zinc finger protein 480 [Source:HGNC Symbol;Acc:HGNC:23305]
zf-C2H2 264 409 343 153 139 155
2.63948289 3.685493194 3.310959464 1.757993165
1.766753125 1.490309343
ENSG00000255741 50.01650841 3.480341617 14.88645864 0
1.212112516 0 22.79443622 0.404037505
5.889387411 0.001337678 1 AP000808.2 11
68941503 68942852 - 569 antisense novel
transcript - 52 4 16 0 1 0
4.418677381 0.306341844 1.312664536 0
0.108027691 0
ENSG00000240694 9.618559309 14.79145187 23.26009163 0
0 0 15.89003427 0 6.332045333
0.001387652 1 PNMA2 8 26504686 26514092
- 5579 protein_coding PNMA family member 2
[Source:HGNC Symbol;Acc:HGNC:9159] - 10 17 25

0 0 0 0.086665222 0.132785654
0.209184588 0 0 0
ENSG00000083845 32112.52211 31749.4164 41020.56719
41334.20574 49554.796 47192.62997 34960.83524
46027.21057 -0.39674749 0.001411621 0.052892589 RPS5
19 58386400 58394806 + 3617
protein_coding ribosomal protein S5 [Source:HGNC
Symbol;Acc:HGNC:10426] - 33386 36490 44089 37506 40883
51120 446.2899394 439.6265903 569.0203201
576.1880995 694.7708813 657.1633798
ENSG00000280424 30.77938979 17.40170808 2.791210996 0
0 0 16.99076962 0 6.428191704
0.001443095 1 BX537318.2 22 46006616
46010777 + 4162 TEC dynein heavy chain -like
pseudogene [Source:NCBI gene;Acc:730668] - 32 20
3 0 0 0 0.371748409 0.209404745
0.033648462 0 0 0
ENSG00000119777 1656.315913 1375.605024 1391.883883
2164.463821 2189.075204 1815.8823 1474.601607
2056.473775 -0.479601975 0.001444259 1 TMEM214 2
27032910 27041695 + 4910 protein_coding
transmembrane protein 214 [Source:HGNC Symbol;Acc:HGNC:25983]
- 1722 1581 1496 1964 1806 1967
16.95715038 14.0316575 14.22316137 22.22654888
22.60911766 18.62747057
ENSG00000167766 386.6660842 356.7350157 345.1797598
212.699347 221.8165905 123.7052507 362.8602866
186.0737294 0.966580661 0.001451679 1 ZNF83 19
52594060 52690496 - 8028 protein_coding
zinc finger protein 83 [Source:HGNC Symbol;Acc:HGNC:13158]
zf-C2H2 402 410 371 193 183 134
2.421140016 2.225538393 2.157310124 1.335863208
1.401170826 0.776119245
ENSG00000163931 6780.122457 6006.199545 6718.444866
7660.482699 9412.053689 8250.401683 6501.588956
8440.979357 -0.376567387 0.00145485 0.053422088 TKT
3 53224707 53256052 - 9507
protein_coding transketolase [Source:HGNC Symbol;Acc:HGNC:11834]
- 7049 6903 7221 6951 7765 8937
35.84967585 31.64120228 35.45682851 40.62710142
50.20480983 43.70984658
ENSG00000213018 15.3896949 23.49230591 8.373632987 0
0 0 15.75187793 0 6.319804564
0.001502493 1 AL590762.1 X 71420083
71420514 - 432 processed_pseudogene "pseudogene
similar to part of poly(A) binding protein, nuclear 1 (PABPN1)" -
16 27 9 0 0 0 1.790760277
2.72357046 0.972534012 0 0 0
ENSG00000144893 22.12268641 21.75213511 4.652018326 0
0 0 16.17561328 0 6.357751758

0.001520807 1 MED12L 3 151085697 151437072
+ 12619 protein_coding mediator complex subunit 12
like [Source:HGNC Symbol;Acc:HGNC:16050] - 23 25
5 0 0 0 0.088126011 0.086332371
0.018496566 0 0 0
ENSG00000166682 15.3896949 28.71281834 4.652018326 0
0 0 16.25151052 0 6.36509284
0.001543171 1 TMPRSS5 11 113687546 113706373
- 2666 protein_coding transmembrane serine protease
5 [Source:HGNC Symbol;Acc:HGNC:14908] - 16 33 5
0 0 0 0.290175709 0.539401801
0.087549949 0 0 0
ENSG00000105568 8283.503277 8460.710471 8707.647903
9822.742382 10798.71041 11523.97496 8483.953884
10715.14258 -0.336908133 0.001543715 0.055421692
PPP2R1A 19 52190039 52229533 + 7437
protein_coding protein phosphatase 2 scaffold subunit Aalpha
[Source:HGNC Symbol;Acc:HGNC:9302] - 8612 9724 9359
8913 8909 12483 55.98961177 56.9778118
58.74591893 66.59447028 73.63402449 78.04627991
ENSG00000269486 58.67321179 68.73674694 42.7985686
8.816553243 15.75746271 8.308561615 56.73617577
10.96085919 2.381147924 0.001556123 1 ERVK9-11
19 38935297 38938632 - 3336
lincRNA "endogenous retrovirus group K9, member 11 [Source:HGNC
Symbol;Acc:HGNC:51160]" - 61 79 46 8 13
9 0.884107367 1.031952356 0.643691576
0.133252691 0.239532623 0.125443304
ENSG00000285103 15.3896949 12.18119566 18.6080733 0
0 0 15.39298795 0 6.285852777
0.001565535 1 AL451123.1 9 27529085
27543902 + 9804 antisense novel transcript
- 16 14 20 0 0 0
0.07890743 0.06222764 0.095229769 0 0 0
ENSG00000110958 7287.982389 6765.784103 6158.34186
8089.187601 9141.752598 8657.521202 6737.369451
8629.487134 -0.357071422 0.001569678 0.055421692 PTGES3
12 56663341 56688408 - 3399
protein_coding prostaglandin E synthase 3 [Source:HGNC
Symbol;Acc:HGNC:16049] - 7577 7776 6619 7340 7542
9378 107.7822733 99.69277502 90.9048914
119.9932839 136.390061 128.2891956
ENSG00000203880 1892.932472 1596.606717 1596.572689
1310.360226 1183.021816 1162.275453 1695.370626
1218.552498 0.476428937 0.001577195 1 PCMTD2 20
64255695 64287821 + 5997 protein_coding
protein-L-isoaspartate (D-aspartate) O-methyltransferase
domain containing 2 [Source:HGNC Symbol;Acc:HGNC:15882] - 1968
1835 1716 1189 976 1259 15.86690647
13.33400512 13.35762572 11.01691121 10.00375689

9.761638025
ENSG00000106089 1642.84993	1474.79476	1378.858232
829.858074	886.0542494	1261.978192	1498.834307
992.6301718	0.593319299	0.001578049	1	STX1A	7
	73699206	73719672	-	3693	protein_coding
	syntaxin 1A [Source:HGNC Symbol;Acc:HGNC:11433] -	1708
	1695	1482	753	731	1367	22.36195531
20.00087486	18.73332777	11.32994267	12.16705383
17.2115596
ENSG00000205534 185.6381947	275.8170731	102.3444032
62.81794186	8.484787614	76.62340156	187.9332237
49.30871034	1.923915847	0.001593696	1	SMG1P2	16
	29527568	29594966	-	4221
unprocessed_pseudogene	SMG1 pseudogene 2 [Source:HGNC
Symbol;Acc:HGNC:49859]	-	193	317	110	57	7
	83	2.210768018	3.272672204	1.216531528
0.750363235	0.101936577	0.914310608
ENSG00000257954 30.77938979	7.830768638	9.304036652	0
	0	0	15.97139836	0	6.338348174
0.001611042	1	AC125611.2	12	49389516
49390162	+	647	processed_pseudogene	FGFR1 oncogene
partner 2 (FGFR1OP2) pseudogene -	32	9	10	0
	0	0	2.391370757	0.606173333	0.721509004
	0	0	0
ENSG00000080854 180.828915	92.22905285	144.2125681
41.87862791	53.33295072	54.46723725	139.0901787
49.89293862	1.477895335	0.001629871	1	IGSF9B	11
	133908564	133956985	-	5753	protein_coding
	immunoglobulin superfamily member 9B [Source:HGNC
Symbol;Acc:HGNC:32326]	-	188	106	155	38	44
	59	1.580027667	0.802915957	1.257718242
0.367029749	0.470116682	0.476857542
ENSG00000173915 612.702228	1185.926406	1093.224307
1552.81544	1447.262344	1643.248853	963.9509802
1547.775546	-0.682960738	0.001645194	1	ATP5MD	10
	103389041	103396492	-	924	protein_coding
	ATP synthase membrane subunit DAPIT [Source:HGNC
Symbol;Acc:HGNC:30889]	-	637	1363	1175	1409	1194
	1780	33.33256062	64.28097899	59.36246564
84.73270393	79.42911356	89.57339791
ENSG00000204396 8.656703379	20.8820497	16.74726597	0
	0	0	15.42867302	0	6.289975925
0.001650499	1	VWA7	6	31765590	31777294
	-	3638	protein_coding	von Willebrand factor A domain
containing 7 [Source:HGNC Symbol;Acc:HGNC:13939]	-	9
	24	18	0	0	0	0.119613729
0.287479675	0.230970145	0	0	0
ENSG00000139318 1675.553032	2488.444256	2164.118925
1350.034715	1311.505743	1663.55867	2109.372071
1441.699709	0.548657119	0.001653209	1	DUSP6	12

89347232	89353271	-	4632	protein_coding
dual specificity phosphatase 6 [Source:HGNC
Symbol;Acc:HGNC:3072]	-	1742	2860	2326	1225	1082
1802	18.18363966	26.9064301	23.441597
14.69533807	14.35840074	18.08911226
ENSG00000243709 7.694847448	14.79145187	24.19049529	0
0	0	15.55893154	0	6.301738806
0.001656571	1	LEFTY1	1	225886282	225911382
-	1891	protein_coding	left-right determination
factor 1 [Source:HGNC Symbol;Acc:HGNC:6552]	-	8	17
26	0	0	0	0.20455009	0.391756301
0.64184159	0	0	0
ENSG00000279494 29.81753386	11.31111026	6.512825656	0
0	0	15.88048992	0	6.330345457
0.001666087	1	AL117328.2	22	49851425
49853364	+	1940	lincRNA novel transcript	-
31	13	7	0	0	0	0.772611522
0.292011678	0.16843888	0	0	0
ENSG00000150995 6262.643966	7201.696891	7512.079193
6169.383132	4667.8453	4734.033773	6992.140017
5190.420735	0.429940695	0.001675591	0.057177438	ITPR1
3	4493345 4847840 +	32454
protein_coding	"inositol 1,4,5-trisphosphate receptor type 1
[Source:HGNC Symbol;Acc:HGNC:6180]"	-	6511	8277	8074
5598	3851	5128	9.700199806	11.11381904
11.61359158	9.584659875	7.293779787	7.347012907
ENSG00000225224 9.618559309	34.80341617	4.652018326	0
0	0	16.35799794	0	6.374976407
0.001677035	1	RPS27AP12	7	157192368
157192832	+	465	processed_pseudogene	ribosomal
protein S27a pseudogene 12 [Source:HGNC Symbol;Acc:HGNC:35732]	-
10	40	5	0	0	0	1.03979629
3.748570095	0.501953038	0	0	0
ENSG00000052802 1016.681719	1386.916134	1076.477041
1806.291346	1940.592139	1407.839607	1160.024965
1718.24103	-0.565939444	0.001679436	1	MSMO1	4
165327623	165343160	+	2892	protein_coding
methylsterol monooxygenase 1 [Source:HGNC
Symbol;Acc:HGNC:10545]	-	1057	1594	1157	1639	1601
1525	17.67168311	24.0186518	18.67588135
31.49145368	34.02831179	24.51898981
ENSG00000198886 67986.86277	67154.93167	75476.20613
78382.46454	93765.38792	95356.43848	70206.00019
89168.09698	-0.344938854	0.001681689	0.057177438	MT-ND4
MT	10760	12137	+	1378	protein_coding
mitochondrially encoded NADH:ubiquinone oxidoreductase core subunit 4
[Source:HGNC Symbol;Acc:HGNC:7459]	-	70683	77182	81122
71123	77357	103292	2480.087326	2440.76186
2748.118575	2867.958701	3450.624977	3485.366777
ENSG00000180610 28.85567793	7.830768638	10.23444032	0

0	0	15.64029563	0	6.308163906
0.001745183	1	ZBTB12BP	4	39770081
39771371	-	1122	processed_pseudogene	"zinc finger
and BTB domain containing 12B, pseudogene [Source:HGNC
Symbol;Acc:HGNC:37702]" -	30	9	11	0	0
0	1.292794853	0.34954915	0.457663064	0
0	0
ENSG00000272129 48.09279655	6.960683234	8.373632987
1.102069155	0	0	21.14237092	0.367356385
5.780896479	0.001749913	1	AL359715.3	6
80355424	80356859	+	1436	lincRNA novel
transcript	-	50	8	9	1	0	0
1.683514188	0.242769512	0.292572906	0.038695245
0	0
ENSG00000243243 13.46598303	13.05128106	18.6080733	0
0	0	15.04177913	0	6.252685675
0.001789024	1	AC073130.2	7	116237929
116327896	-	917	antisense	novel transcript
-	14	15	20	0	0	0
0.738175992	0.712821058	1.018138115	0	0	0
ENSG00000150637 23.08454234	9.570939447	13.02565131	0
0	0	15.22704437	0	6.269793633
0.001792257	1	CD226	18	69831158	69961803
-	13638	protein_coding	CD226 molecule [Source:HGNC
Symbol;Acc:HGNC:16961]	-	24	11	14	0	0
0	0.085086718	0.035147998	0.047920726	0
0	0
ENSG00000212127 63.48249144	31.32307455	71.64108222
6.612414933	10.90901265	12.92442918	55.48221607
10.14861892	2.445984373	0.001820118	1	TAS2R14 12
10937406	11171573	-	2350	protein_coding
taste 2 receptor member 14 [Source:HGNC Symbol;Acc:HGNC:14920]
-	66	36	77	6	9	14
1.357929708	0.667564504	1.529568386	0.141871589
0.235408428	0.277007286
ENSG00000275993 1012.834295	1099.787951	852.2497573
504.7476732	584.2382329	787.4670064	988.2906679
625.4843041	0.65878515	0.00182434	1	SIK1B	21
6111134 6123739 +	4806	protein_coding	salt inducible
kinase 1B (putative) [Source:HGNC Symbol;Acc:HGNC:52389]	-
1053	1264	916	458	482	853	10.5936549
11.46098397	8.897289934	5.295338841	6.164681335
8.252704272
ENSG00000150991 16905.57984	16569.03635	15936.88438
17682.6996	23748.92053	22988.86681	16470.50019
21473.49565	-0.382676073	0.001842221	0.060811209	UBC
12	124911604	124917368	-	3898
protein_coding	ubiquitin C [Source:HGNC Symbol;Acc:HGNC:12468] -
17576	19043	17129	16045	19593	24902	218.0115113
212.8884649	205.1333207	228.7230482	308.9629932

297.0457972
ENSG00000181856 14.42783896 18.27179349 12.09524765 0
0 0 14.9316267 0 6.242402668
0.001869267 1 SLC2A4 17 7281667 7288257 + 4046
protein_coding solute carrier family 2 member 4 [Source:HGNC
Symbol;Acc:HGNC:11009] - 15 21 13 0 0
0 0.179253068 0.226178862 0.149990416 0
0 0
ENSG00000276180 130.8124066 135.7333231 88.38834819
286.5379804 233.9377156 219.715296 118.3113593
246.7303307 -1.059745922 0.001878953 1 HIST1H4I
6 27138588 27139881 + 1294
protein_coding histone cluster 1 H4 family member i [Source:HGNC
Symbol;Acc:HGNC:4793] - 136 156 95 260 193
238 5.081662859 5.253502216 3.427167769
11.16480431 9.16791109 8.552118282
ENSG00000213658 10.58041524 13.05128106 21.3992843 0
0 0 15.01032687 0 6.249766697
0.001887724 1 LAT 16 28984826 28990783
+ 4430 protein_coding linker for activation of T
cells [Source:HGNC Symbol;Acc:HGNC:18874] - 11 15
23 0 0 0 0.120057743 0.14755235
0.242365135 0 0 0
ENSG00000213442 2457.541904 2352.710933 3037.767967
2921.585331 3947.850466 4223.518821 2616.006934
3697.651539 -0.499410362 0.001899428 0.060811209
RPL18AP3 12 104265309 104265836 + 528
processed_pseudogene ribosomal protein L18a pseudogene 3
[Source:HGNC Symbol;Acc:HGNC:31387] - 2555 2704 3265
2651 3257 4575 233.9689351 223.1677128
288.6657771 278.9894938 379.1675796 402.8915826
ENSG00000259974 0 0 0.930403665 7.714484088
14.5453502 32.31107295 0.310134555
18.19030241 -5.789057324 0.001912077 1 LINC00261
20 22547671 22578642 - 5552
lincRNA long intergenic non-protein coding RNA 261 [Source:HGNC
Symbol;Acc:HGNC:16189] - 0 0 1 7 12
35 0 0 0.008408075 0.070058466
0.132855381 0.293122803
ENSG00000185989 3727.191732 3834.466377 3773.717266
2984.403273 2838.767513 3058.473848 3778.458458
2960.548211 0.351769658 0.001918573 0.060811209 RASA3
13 113977783 114132611 - 4763
protein_coding RAS p21 protein activator 3 [Source:HGNC
Symbol;Acc:HGNC:20331] - 3875 4407 4056 2708 2342
3313 39.33619442 40.32005044 39.75240429
31.59221833 30.22412032 32.34237233
ENSG00000103018 3035.617318 3056.610025 2660.954482
3786.709618 4088.455517 3562.526586 2917.727275
3812.563907 -0.385669345 0.001921051 0.060811209 CYB5B

16	69424525	69466266	+	6903
protein_coding	cytochrome b5 type B [Source:HGNC
Symbol;Acc:HGNC:24374]	-	3156	3513	2860	3436	3373
3859	22.10549975	22.17679971	19.34078938
27.65841734	30.03487494	25.99368929
ENSG00000226419 413.5980503	415.9008232	508.0004012
224.8221077	259.3920785	295.4155241	445.8330916
259.8765701	0.777638813	0.001927318	1	SLC16A1-AS1
1	112956415	113047055	+	10138
antisense	SLC16A1 antisense RNA 1 [Source:HGNC
Symbol;Acc:HGNC:49445]	-	430	478	546	204	214
320	2.050772028	2.054632755	2.51412225
1.118123883	1.297504422	1.467670988
ENSG00000197702 2240.162463	2256.131453	2102.712283
3188.286067	2841.191738	2675.35684	2199.668733
2901.611548	-0.399388501	0.001979733	0.061606617	PARVA
11	12377185	12535356	+	12849
protein_coding	parvin alpha [Source:HGNC Symbol;Acc:HGNC:14652]
-	2329	2593	2260	2893	2344	2898
8.763979962	8.794107808	8.210793803	12.51097477
11.21335673	10.48721255
ENSG00000126091 187.5619065	187.9384473	174.9158891
84.85932497	79.99942608	87.70148371	183.472081
84.18674492	1.12317073	0.002000341	1	ST3GAL3 1
43705824	43931165	+	19475	protein_coding
"ST3 beta-galactoside alpha-2,3-sialyltransferase 3
[Source:HGNC Symbol;Acc:HGNC:10866]"	-	195	216	188
77	66	95	0.484125949	0.483320129
0.450636556	0.219697595	0.20831178	0.226817812
ENSG00000198804 159805.6299	146103.0009	144089.7548
153933.7134	219105.0948	230517.3493	149999.4619
201185.3858	-0.423572067	0.002014645	0.06164813	MT-CO1
MT	5904	7445	+	1542	protein_coding
mitochondrially encoded cytochrome c oxidase I [Source:HGNC
Symbol;Acc:HGNC:7419]	-	166143	167918	154868	139677	180763
249701	5209.534169	4745.38526	4688.385908
5033.297127	7205.639441	7529.513669
ENSG00000085733 4819.86007	4436.565476	5086.516838
3978.469651	3389.066596	3713.927042	4780.980795
3693.821096	0.372024477	0.00204947	0.061685696	CTTN
11	70398404	70436584	+	6949
protein_coding	cortactin [Source:HGNC Symbol;Acc:HGNC:3338]	-
5011	5099	5467	3610	2796	4023	34.86609487
31.9757911	36.72592966	28.86668638	24.73216958
26.91898863
ENSG00000270021 23.08454234	7.830768638	13.95605498	0
0	0	14.95712199	0	6.243878419
0.002050139	1	AC026691.1	5	135399280
135401296	+	2017	antisense	"novel transcript,
antisense to H2AFY"	-	24	9	15	0	0

0	0.575316143	0.194444297	0.347161372	0
0	0
ENSG00000263624 26.93196607	13.05128106	31.63372462	0
2.424225033	0	23.87232392	0.808075011
4.984904034	0.002051574	1	AC055811.1	17
17167946	17185554	-	1231	antisense	novel
transcript	-	28	15	34	0	2	0
1.099768294	0.530996678	1.289338348	0
0.099866379	0
ENSG00000253522 114.4608558	200.9897284	159.0990267
78.24691003	60.60562582	26.77203187	158.1832036
55.20818924	1.524055608	0.002065293	1	MIR3142HG
5	160438594	160487426	+	2377
lincRNA MIR3142 host gene [Source:HGNC Symbol;Acc:HGNC:51944]	-
119	231	171	71	50	29	2.420577523
4.234882801	3.358249544	1.659744395	1.29296921
0.567283085
ENSG00000205609 137.5453981	53.07520966	80.94511887
1413.954726	104.2416764	673.9166643	90.52190889
730.7043557	-3.01334227	0.002075185	1	EIF3CL	16
28379579	28403879	-	3091	protein_coding
eukaryotic translation initiation factor 3 subunit C like
[Source:HGNC Symbol;Acc:HGNC:26347]	-	143	61	87
1283	86	730	2.236857144	0.859982132
1.313912013	23.06426902	1.710199623	10.98132824
ENSG00000243423 0	0	0	6.612414933	13.33323768
22.15616431	0	14.03393897	-6.375573989
0.00211976	1	AC004223.1	17	35076737
35077007	+	271	processed_pseudogene	ribosomal
protein L37 (RPL37) pseudogene	-	0	0	0	6
11	24	0	0	0	1.230251783
2.495001175	4.117873196
ENSG00000141552 1143.646702	1310.348619	1224.411223
1502.120259	1933.319464	1691.253875	1226.135515
1708.897866	-0.478545625	0.002122059	1	ANAPC11 17
81890790	81900991	+	4027	protein_coding
anaphase promoting complex subunit 11 [Source:HGNC
Symbol;Acc:HGNC:14452]	-	1189	1506	1316	1363	1595
1832	14.27583243	16.29678515	15.25528394
18.80729211	24.34593275	21.15314069
ENSG00000186812 714.6589567	763.0648995	728.5060698
494.8290508	526.0568321	451.4318477	735.4099754
490.7725769	0.58439813	0.002128193	1	ZNF397	18
35241030	35267133	+	8710	protein_coding
zinc finger protein 397 [Source:HGNC Symbol;Acc:HGNC:18818]
zf-C2H2 743	877	783	449	434	489
4.124505388	4.387731422	4.19652334	2.864443297
3.062802089	2.610488127
ENSG00000167693 8.656703379	7.830768638	28.84251362	0
0	0	15.10999521	0	6.259045242

0.002170448	1	NXN	17	799313	979770	-	5880
	protein_coding	nucleoredoxin [Source:HGNC
Symbol;Acc:HGNC:18008]	-	9	9	31	0	0
	0	0.074005909	0.066699685	0.246110648	0
	0	0
ENSG00000042753 4448.583681	4642.775717	4838.099059
5189.643653	6105.410745	7155.517897	4643.152819
6150.190765	-0.4057009	0.002262018	0.066058804	AP2S1
	19	46838136	46850992	-	1704
protein_coding	adaptor related protein complex 2 subunit sigma 1
[Source:HGNC Symbol;Acc:HGNC:565]	-	4625	5336	5200
	4709	5037	7751	131.2330925	136.4598307
142.4556862	153.5575391	181.6978216	211.5042821
ENSG00000188229 9929.238775	8886.182234	11391.86248
10997.5481	15364.73826	14215.02575	10069.0945
13525.7707	-0.425784822	0.002299861	0.066058804	TUBB4B
	9	137241213	137243707	+	2111
protein_coding	tubulin beta 4B class IVb [Source:HGNC
Symbol;Acc:HGNC:20771]	-	10323	10213	12244	9979	12676
	15398	236.4388893	210.8257706	270.7578915
262.6702171	369.0977157	339.1619317
ENSG00000100075 1901.589175	2124.748557	2756.78606
2806.970139	3946.638353	2993.851702	2261.041264
3249.153398	-0.522799907	0.002311381	0.066058804
SLC25A1 22	19175575	19178830	-	2096
protein_coding	solute carrier family 25 member 1 [Source:HGNC
Symbol;Acc:HGNC:10979]	-	1977	2442	2963	2547	3256
	3243	45.60543552	50.77067987	65.99125824
67.52268607	95.48617092	71.94269266
ENSG00000271383 898.3734395	669.9657613	813.1728034
545.5242319	378.1791051	562.2126693	793.8373347
495.3053354	0.678953854	0.002321708	1	NBPF19	1
	149390623	149556361	+	18785	protein_coding
	NBPF member 19 [Source:HGNC Symbol;Acc:HGNC:31999]	-
	934	770	874	495	312	609	2.404013451
	1.786233062	2.171932225	1.464219017	1.02091775
	1.507429852
ENSG00000250651 19.23711862	20.8820497	4.652018326	0
	0	0	14.92372888	0	6.241630595
0.002328454	1	PABPC1P7	4	102896725
102898237	+	1513	processed_pseudogene	poly(A)
binding protein cytoplasmic 1 pseudogene 7 [Source:HGNC
Symbol;Acc:HGNC:37987]	-	20	24	5	0	0
	0	0.639134534	0.691243263	0.154268449	0
	0	0
ENSG00000143384 19369.85474	24095.2751	20022.28687
15695.66891	17058.05944	17185.79811	21162.47224
16646.50882	0.346305783	0.002338683	0.066058804	MCL1
	1	150574551	150579738	-	4371
protein_coding	"MCL1, BCL2 family apoptosis regulator [Source:HGNC

Symbol;Acc:HGNC:6943]" - 20138 27693 21520 14242 14073
18616 222.7597627 276.0881693 229.8304124
181.0515381 197.9033936 198.032551
ENSG00000223478 4.809279655 0 2.791210996 31.96000551
21.81802529 39.69646105 2.533496883 31.15816395
-3.636102245 0.002427021 1 AL441992.1 9
128724445 128733194 + 544 antisense
uncharacterized LOC100506100 [Source:NCBI gene;Acc:100506100]
- 5 0 3 29 18 43
0.444398231 0 0.257435474 2.96217793 2.033859581
3.675365835
ENSG00000187678 1094.592049 1095.437524 1030.887261
804.5104835 726.0553973 757.925454 1073.638945
762.8304449 0.49295583 0.002434175 1 SPRY4 5
142310427 142326455 - 6565 protein_coding
sprouty RTK signaling antagonist 4 [Source:HGNC
Symbol;Acc:HGNC:15533] - 1138 1259 1108 730 599
821 8.381249092 8.356984515 7.878636538
6.178743596 5.608406089 5.814862434
ENSG00000238133 0 0 0 18.73517564 9.69690013
12.00125567 0 13.47777715 -6.31566883
0.002435435 1 MAP3K20-AS1 2 173166446
173282036 - 3049 antisense MAP3K20 antisense RNA
1 [Source:HGNC Symbol;Acc:HGNC:27935] - 0 0 0
17 8 13 0 0 0 0.309815785
0.16127978 0.198251712
ENSG00000130164 2497.939853 2519.767331 2513.0203
4085.370359 3792.700064 2643.96894 2510.242494
3507.346454 -0.482149777 0.002443818 0.067982573 LDLR
19 11089362 11133816 + 7718
protein_coding low density lipoprotein receptor [Source:HGNC
Symbol;Acc:HGNC:6547] - 2597 2896 2701 3707 3129
2864 16.26928218 16.35130355 16.33675687
26.68884967 24.92000638 17.25437599
ENSG00000131389 2049.714989 1755.832346 2230.177585
1589.183722 1472.716707 1327.523511 2011.908307
1463.141314 0.459716197 0.002476125 1 SLC6A6 3
14402576 14489349 + 8254 protein_coding
solute carrier family 6 member 6 [Source:HGNC
Symbol;Acc:HGNC:11052] - 2131 2018 2397 1442 1215
1438 12.48303539 10.65406385 13.55656327
9.707621604 9.048137122 8.10075392
ENSG00000279349 0 0 0 13.22482987 15.75746271
11.07808215 0 13.35345824 -6.301281408
0.002484066 1 AC092692.1 3 112525548
112528142 - 2595 TEC TEC - 0 0
0 12 13 12 0 0 0
0.256954322 0.307930956 0.215018042
ENSG00000188517 0 0 0 8.816553243 6.060562582
26.77203187 0 13.88304923 -6.361767713

0.002492198	1	COL25A1 4	108808725	109302752
	-	8744	protein_coding	collagen type XXV alpha 1
chain [Source:HGNC Symbol;Acc:HGNC:18603]	-	0	0
	0	8	5	29	0	0	0
0.050838401	0.035148534	0.154212248
ENSG00000164683 71.17733889	141.8239209	106.0660178
177.433134	317.5734793	226.1775106	106.3557592
240.394708	-1.17440372	0.002498536	1	HEY1	8
	79764010	79767863	-	3232	protein_coding
	hes related family bHLH transcription factor with YRPW motif 1
[Source:HGNC Symbol;Acc:HGNC:4880]	bHLH	74	163	114
	161	262	245	1.107035592	2.197732599
1.646567485	2.768003071	4.9828441	3.524729151
ENSG00000125740 95.22373716	56.55555128	96.76198118
330.6207466	229.0892656	97.85639235	82.84708987
219.1888015	-1.403021525	0.002521747	1	FOSB	19
	45467995	45475179	+	5553	protein_coding
	"FosB proto-oncogene, AP-1 transcription factor subunit
[Source:HGNC Symbol;Acc:HGNC:3797]"	TF_bZIP 99	65	104
	300	189	106	0.862002921	0.51008703
0.874282331	3.001965003	2.09209543	0.887583479
ENSG00000197301 168.3247879	112.2410171	133.0477241
57.30759608	27.87858788	63.69897238	137.8711764
49.62838545	1.468341184	0.00253731	1	AC090673.1
	12	65851340	65882167	-	6033
antisense	uncharacterized LOC100129940 [Source:NCBI
gene;Acc:100129940]	-	175	129	143	52	23
	69	1.402509914	0.931783429	1.106493197
0.47894105	0.234337542	0.531798103
ENSG00000215021 4449.545537	4636.685119	4915.322563
5453.038181	6282.379172	6012.629088	4667.184406
5916.01548	-0.342027637	0.00257198	0.070027852	PHB2
	12	6965327 6970825 -	2499	protein_coding
prohibitin 2 [Source:HGNC Symbol;Acc:HGNC:30306]	-	4626
	5329	5283	4948	5183	6513	89.50361752
92.92617514	98.68710076	110.0209723	127.4859466
121.1841553
ENSG00000168028 28232.39529	38204.58001	34689.17025
42230.18797	42683.33015	44757.29824	33708.71518
43223.60545	-0.358687971	0.00264869	0.070027852	RPSA
	3	39406689	39412542	+	3936
protein_coding	ribosomal protein SA [Source:HGNC
Symbol;Acc:HGNC:6502]	-	29352	43909	37284	38319	35214
	48482	360.5652142	486.1351842	442.1946108
540.967433	549.9302763	572.7386026
ENSG00000244480 24.04639827	16.53162268	3.721614661	0
	0	0	14.76654521	0	6.225927136
0.002656293	1	AC005154.2	7	30523143
30524535	-	1393	transcribed_processed_pseudogene
	"pseudogene similar golgi autoantigen, golgin subfamily a, 8A

GOLGA8A"	-	25	19	4	0	0	0
	0.867740982	0.59437575	0.134046325	0	0
	0
ENSG00000142546 3763.742258	3418.565553	4326.377043
3882.589635	6347.833248	6224.035823	3836.228285
5484.819568	-0.515819263	0.002661785	0.070027852	NOSIP
	19	49555711	49590262	-	4248
protein_coding	nitric oxide synthase interacting protein [Source:HGNC
Symbol;Acc:HGNC:17946]	-	3913	3929	4650	3523	5237
	6742	44.53757394	40.30473949	51.09924468
46.08294004	75.77839909	73.79641394
ENSG00000267786 24.04639827	6.960683234	12.09524765	0
	0	0	14.36744305	0	6.185751461
0.002662626	1	AF038458.3	19	35947115
35959192	+	500	lincRNA novel transcript	-
	25	8	13	0	0	0	2.417526375
	0.697234038	1.213722446	0	0	0
ENSG00000277159 23.08454234	17.40170808	3.721614661	0
	0	0	14.73595503	0	6.2230218
0.002669447	1	AL139384.2	13	112602828
112606417	-	3590	lincRNA novel transcript	-
	24	20	4	0	0	0	0.323234724
	0.242769512	0.052012961	0	0	0
ENSG00000165288 1221.557032	884.8768561	971.3414265
768.1422013	481.208669	705.3045637	1025.925105
651.5518113	0.653848125	0.002671729	1	BRWD3	X
	80670854	80809688	-	12694	protein_coding
	bromodomain and WD repeat domain containing 3 [Source:HGNC
Symbol;Acc:HGNC:17342]	-	1270	1017	1044	697	397
	764	4.837338106	3.491250868	3.839264565
3.051028945	1.922380591	2.798503683
ENSG00000105738 3291.470996	2726.847657	3368.991672
2471.941116	2067.863953	2465.796453	3129.103441
2335.200507	0.421775936	0.002685025	0.070027852
SIPA1L3 19	37907228	38208372	+	10335
protein_coding	signal induced proliferation associated 1 like 3
[Source:HGNC Symbol;Acc:HGNC:23801]	-	3422	3134	3621
	2243	1706	2671	16.00924094	13.21438966
16.35550958	12.05954261	10.14649174	12.01695473
ENSG00000116221 2639.332675	2547.610064	3048.932811
3412.006105	3484.823484	3857.018936	2745.29185
3584.616175	-0.385094052	0.002708049	0.070027852	MRPL37
	1	54184041	54225464	+	2549
protein_coding	mitochondrial ribosomal protein L37 [Source:HGNC
Symbol;Acc:HGNC:14034]	-	2744	2928	3277	3096	2875
	4178	52.0493713	50.05642561	60.01400937
67.49057997	69.32906988	76.21309846
ENSG00000254941 15.3896949	23.49230591	13.95605498	0
	1.212112516	0	17.61268526	0.404037505
5.519062685	0.00271859	1	AP000866.4	11

124807822	124808269	-	448	processed_pseudogene
	ring finger protein 181 (RNF181) pseudogene	-	16
	27	15	0	1	0	1.726804553
2.626300086	1.56300109	0	0.137204813	0
ENSG00000087494 7.694847448	60.9059783	69.78027489
136.6565753	209.6954653	143.0918945	46.12703354
163.1479784	-1.820155256	0.002741598	1	PTHLH	12
	27958084	27972705	-	3377	protein_coding
	parathyroid hormone like hormone [Source:HGNC
Symbol;Acc:HGNC:9607]	-	8	70	75	124	173
	155	0.114540782	0.90328662	1.036755239
2.040340584	3.148925622	2.134182997
ENSG00000123384 1966.995379	1165.914442	1966.873348
1363.259545	1031.507751	742.2315042	1699.927723
1045.666267	0.70146355	0.002752434	1	LRP1	12
	57128493	57213351	+	20839	protein_coding
	LDL receptor related protein 1 [Source:HGNC
Symbol;Acc:HGNC:6692]	-	2045	1340	2114	1237	851
	804	4.744797194	2.802118655	4.735590538
3.298411748	2.510152145	1.793949412
ENSG00000280537 8.656703379	10.44102485	23.26009163	0
	0	0	14.11927329	0	6.161394968
0.002826539	1	AC068946.1	2	219075329
219170827	-	4650	protein_coding	"novel protein,
SLC23A3-NHEJ1 readthrough"	-	9	12	25	0
	0	0	0.093581666	0.112457103	0.250976519
	0	0	0
ENSG00000161800 968.5889225	983.1965068	1096.945921
1649.797526	1307.869405	1322.907644	1016.243784
1426.858191	-0.489538366	0.002861134	1	RACGAP1 12
	49976923	50033136	-	4487	protein_coding
	Rac GTPase activating protein 1 [Source:HGNC
Symbol;Acc:HGNC:9804]	-	1007	1130	1179	1497	1079
	1433	10.85112128	10.9744047	12.26602291
18.53863588	14.78130353	14.84981827
ENSG00000130726 18206.00906	15830.33384	17044.99515
17943.88999	23459.22564	25969.79409	17027.11268
22457.63657	-0.399424672	0.002861838	0.070591725	TRIM28
	19	58544091	58550722	+	4034
protein_coding	tripartite motif containing 28 [Source:HGNC
Symbol;Acc:HGNC:16384]	-	18928	18194	18320	16282	19354
	28131	226.8663323	196.539974	211.9998781
224.2765672	294.9050456	324.2502316
ENSG00000112514 2346.928472	2451.030584	2926.119527
3446.170249	3362.40012	3305.884349	2574.692861
3371.484906	-0.38894851	0.002863642	0.070591725	CUTA
	6	33416442	33418317	-	1876
protein_coding	cutA divalent cation tolerance homolog [Source:HGNC
Symbol;Acc:HGNC:21101]	-	2440	2817	3145	3127	2774
	3581	62.88661358	65.43537727	78.25892025

92.62049354	90.89102119	88.75697762
ENSG00000133119 1522.617939	1551.362276	1306.286746
1951.764474	1909.077213	1994.054787	1460.088987
1951.632158	-0.418678103	0.002868106	1	RFC3	13
	33818049	33966558	+	2874	protein_coding
	replication factor C subunit 3 [Source:HGNC
Symbol;Acc:HGNC:9971]	-	1583	1783	1404	1771	1575
	2160	26.631484	27.034801	22.80480588
34.24079512	33.68535701	34.94604288
ENSG00000214279 180.828915	194.8991306	225.157687
36.36828213	86.05998866	116.3198626	200.2952442
79.58271113	1.32857716	0.002870889	1	SCART1	10
	133453928	133523558	+	8015	protein_coding
	scavenger receptor family member expressed on T cells 1
[Source:HGNC Symbol;Acc:HGNC:32411]	-	188	224	242
	33	71	126	1.134110938	1.217876048
1.409476616	0.228782318	0.544505389	0.730967447
ENSG00000214282 24.04639827	4.350427021	14.88645864	0
	0	0	14.42776131	0	6.19165134
0.002878833	1	KRT8P14 X	45632292	45633733
	-	1442	processed_pseudogene	keratin 8 pseudogene
14 [Source:HGNC Symbol;Acc:HGNC:33366]	-	25	5	16
	0	0	0	0.838254638	0.151099609
0.51796541	0	0	0
ENSG00000112972 595.3888213	650.8238824	561.9638138
1239.8278	1003.629164	661.9154086	602.7255058
968.4574573	-0.683276842	0.002883707	1	HMGCS1	5
	43289395	43313512	-	4702	protein_coding
	3-hydroxy-3-methylglutaryl-CoA synthase 1 [Source:HGNC
Symbol;Acc:HGNC:5007]	-	619	748	604	1125	828
	717	6.365158766	6.932303543	5.99653468
13.29480407	10.82418166	7.090347964
ENSG00000165916 3852.233003	3829.245864	4203.563759
4618.77183	5116.326931	5413.489479	3961.680876
5049.529413	-0.350160266	0.002888004	0.070591725	PSMC3
	11	47418769	47426473	-	2276
protein_coding	"proteasome 26S subunit, ATPase 3 [Source:HGNC
Symbol;Acc:HGNC:9549]"	-	4005	4401	4518	4191	4221
	5864	85.08078322	84.26315355	92.66591208
102.3192732	113.996221	119.7988701
ENSG00000111911 1058.041524	1325.140071	960.1765825
806.7146218	787.8731356	686.8410935	1114.452726
760.4762836	0.552129069	0.002896931	1	HINT3	6
	125956781	125980244	+	3314	protein_coding
	histidine triad nucleotide binding protein 3 [Source:HGNC
Symbol;Acc:HGNC:18468]	-	1100	1523	1032	732	650
	744	16.04875686	20.02654344	14.53694774
12.273562	12.05613806	10.43882098
ENSG00000130635 2047.791277	1484.3657	1989.203036
1138.437438	1576.958384	913.0186041	1840.453338

1209.471475	0.606571904	0.002913346	1	COL5A1	9
	134641774	134844843	+	11189	protein_coding
	collagen type V alpha 1 chain [Source:HGNC
Symbol;Acc:HGNC:2209]	-	2129	1706	2138	1033	1301
	989	9.199952903	6.6442559	8.919950881
5.130044015	7.147157998	4.109945244
ENSG00000122026 8042.077439	9985.100099	14016.53122
15096.14329	14368.38177	15098.5028	10681.23625
14854.34262	-0.475808407	0.00291586	0.070591725	RPL21
	13	27251309	27256691	+	2249
protein_coding	ribosomal protein L21 [Source:HGNC
Symbol;Acc:HGNC:10313]	-	8361	11476	15065	13698	11854
	16355	179.7504492	222.3615445	312.6984413
338.4384911	323.9834514	338.1365453
ENSG00000110921 353.9629826	527.271755	458.6890069
721.8552968	637.5711836	745.9241983	446.6412482
701.7835596	-0.651797215	0.002924576	1	MVK	12
	109573255	109598117	+	6480	protein_coding
	mevalonate kinase [Source:HGNC Symbol;Acc:HGNC:7530]	-
	368	606	493	655	526	808	2.745832425
	4.075268391	3.551550131	5.616662622	4.98951231
	5.79785531
ENSG00000172803 87.52888972	67.86666153	63.26744923
7.714484088	1.212112516	28.61837889	72.88766683
12.51499183	2.519373042	0.002942505	1	SNX32	11
	65833641	65856896	+	3966	protein_coding
	sorting nexin 32 [Source:HGNC Symbol;Acc:HGNC:26423]	-
	91	78	68	7	1	31	1.109404438
	0.857038813	0.800391078	0.098074787	0.015498678
	0.363446091
ENSG00000202523 0	0	0	25.34759057	25.45436284
	0	0	16.93398447	-6.642286666	0.002979652
	1	RF00019 9	83992932	83993033	+
	102	misc_RNA		-	0	0	0
	23	21	0	0	0	0	12.52967216
	12.65512628	0
ENSG00000249626 33.66495758	10.44102485	1.86080733	0
	0	0	15.32226325	0	6.278622403
0.00299332	1	AC024560.2	3	197634315
197635811	+	1497	processed_pseudogene	"protein
phosphatase 4, regulatory subunit 2 (PPP4R2) pseudogene"	-
	35	12	2	0	0	0	1.130439855
	0.34931565	0.062366911	0	0	0
ENSG00000172531 3442.482377	3724.835616	4276.135245
4572.484926	5186.629457	5061.76037	3814.484413
4940.291584	-0.373080938	0.003002231	0.070591725	PPP1CA
	11	67398183	67421183	-	2711
protein_coding	protein phosphatase 1 catalytic subunit alpha
[Source:HGNC Symbol;Acc:HGNC:9281]	-	3579	4281	4596
	4149	4279	5483	63.83125706	68.81360465

79.14008972 85.04053053 97.01974506 94.04154304
ENSG00000269997 15.3896949 22.62222051 4.652018326 0
0 0 14.22131124 0 6.172360792
0.003012657 1 AC068790.3 12 123966077
123966629 - 553 sense_intronic "novel transcript,
sense intronic to CCDC92" - 16 26 5 0
0 0 1.398930271 2.048834197 0.422076244
0 0 0
ENSG00000231704 20.19897455 3.480341617 19.53847697 0
0 0 14.40593105 0 6.189550553
0.003026865 1 AC004895.1 7 6081103 6093085 +
820 lincRNA novel transcript - 21 4
21 0 0 0 1.238245216 0.212571353
1.195505224 0 0 0
ENSG00000147162 5193.060171 4467.888551 5041.857462
3851.731698 4155.121706 3058.473848 4900.935395
3688.442417 0.410411673 0.003071492 0.070591725 OGT
X 71533083 71575897 + 9812
protein_coding O-linked N-acetylglucosamine (GlcNAc) transferase
[Source:HGNC Symbol;Acc:HGNC:8127] - 5399 5135 5419
3495 3428 3313 26.60461658 22.80560018
25.78146829 19.79254697 21.47487447 15.69982872
ENSG00000233328 15.3896949 24.36239132 15.81686231
54.00138862 75.15097601 79.39292209 18.52298284
69.51509557 -1.906419368 0.003083612 1 PFN1P1 1
171670517 171670939 - 423
processed_pseudogene profilin 1 pseudogene 1 [Source:HGNC
Symbol;Acc:HGNC:42989] - 16 28 17 49 62
86 1.82886156 2.884537981 1.87609398
6.43676652 9.009458358 9.453423236
ENSG00000146701 5675.911849 5015.17227 5139.549847
6092.238291 7252.069185 6774.247236 5276.877988
6706.184904 -0.345805513 0.0030837 0.070591725 MDH2
7 76048051 76067508 + 3641
protein_coding malate dehydrogenase 2 [Source:HGNC
Symbol;Acc:HGNC:6971] - 5901 5764 5524 5528 5983
7338 78.36211555 68.98614723 70.82376771
84.36443437 101.0056538 93.71045501
ENSG00000198763 101563.3296 89093.26505 89124.29749
93244.96917 139926.2689 143320.8415 93260.29738
125497.3599 -0.428327357 0.003085536 0.070591725 MT-ND2
MT 4470 5511 + 1042 protein_coding
mitochondrially encoded NADH:ubiquinone oxidoreductase core subunit 2
[Source:HGNC Symbol;Acc:HGNC:7456] - 105591 102396 95791
84609 115440 155248 4899.597455 4282.268265
4291.439793 4511.914766 6809.825123 6927.703839
ENSG00000218428 25.97011014 7.830768638 8.373632987 0
0 0 14.05817059 0 6.154348036
0.003110509 1 NIP7P3 6 116137058 116137572
- 515 processed_pseudogene "NIP7, nucleolar pre-

rRNA processing protein pseudogene 3 [Source:HGNC
Symbol;Acc:HGNC:45182]" - 27 9 9 0 0
0 2.534882024 0.761542031 0.81579552 0
0 0
ENSG00000151611 299.1371945 415.9008232 363.7878331
234.7407301 187.87744 186.4810496 359.6086169
203.0330732 0.825345454 0.003115536 1 MMAA 4
145599042 145660035 + 8882 protein_coding
methylmalonic aciduria (cobalamin deficiency) cblA type
[Source:HGNC Symbol;Acc:HGNC:18871] - 311 478 391
213 155 202 1.692976137 2.345177536
2.055000938 1.332541914 1.072675322 1.057478676
ENSG00000283360 6.732991517 8.700854042 26.98170629 0
0 0 14.13851728 0 6.163310877
0.003115656 1 AL592295.3 1 161403409
161470523 - 863 lincRNA novel transcript -
7 10 29 0 0 0 0.392182726
0.504949332 1.568675949 0 0 0
ENSG00000221962 0 0 0 27.55172889 7.272675098
5.539041076 0 13.45448169 -6.312138716
0.00312 1 TMEM14EP 3 152339695 152340990
- 1296
transcribed_processed_pseudogene "transmembrane protein 14E,
pseudogene [Source:HGNC Symbol;Acc:HGNC:34386]" - 0 0
0 25 6 6 0 0 0
1.07188218 0.284572945 0.215266905
ENSG00000147155 300.0990505 339.3333077 412.1688237
505.8497423 601.2078081 573.2907514 350.5337273
560.1161006 -0.67599326 0.003131041 1 EBP X
48521158 48528716 + 2409 protein_coding
"EBP, cholestenol delta-isomerase [Source:HGNC
Symbol;Acc:HGNC:3133]" - 312 390 443 459 496
621 6.262085752 7.054827592 8.584459619
10.58736606 12.65587675 11.98631863
ENSG00000116649 5865.397467 4776.768869 4638.062271
6231.099005 6515.104775 7011.502829 5093.409536
6585.902203 -0.370902284 0.003133009 0.070591725 SRM
1 11054584 11060024 - 1898
protein_coding spermidine synthase [Source:HGNC
Symbol;Acc:HGNC:11296] - 6098 5490 4985 5654 5375
7595 155.3432648 126.0476445 122.6069222
165.5280656 174.0722812 186.0641007
ENSG00000242299 394.3609317 462.0153497 548.9381625
791.2856536 706.661597 664.6849292 468.4381479
720.8773933 -0.621513707 0.003143226 1 AC073861.1
3 101576489 101576947 - 459
processed_pseudogene ribosomal protein S18 (RPS18) pseudogene
- 410 531 590 718 583 720
43.18892434 50.41275517 60.00471287 86.92081752
78.07342457 72.93749255

ENSG00000161996 1962.186099 1302.51785 1831.964817
1248.644353 904.2359372 1269.36358 1698.889589
1140.747957 0.573752651 0.00314452 1 WDR90 16
649311 667833 + 10534 protein_coding WD repeat
domain 90 [Source:HGNC Symbol;Acc:HGNC:26960] - 2040 1497
1969 1133 746 1375 9.363496875 6.192800422
8.725663044 5.97652361 4.353042163 6.06932512
ENSG00000139990 1252.336422 1149.382819 1090.433096
825.4497974 890.9026995 790.2365269 1164.050779
835.5296746 0.478830361 0.003182382 1 DCAF5 14
69050881 69153150 - 8295 protein_coding
DDB1 and CUL4 associated factor 5 [Source:HGNC
Symbol;Acc:HGNC:20224] - 1302 1321 1172 749 735
856 7.58919672 6.939769166 6.595644768
5.017385507 5.446510045 4.798310993
ENSG00000122705 3817.60619 4044.156959 3743.944349
4898.697396 5008.448917 4639.870075 3868.569166
4849.005463 -0.325716494 0.00321322 0.070591725 CLTA
9 36190856 36304781 + 2515
protein_coding clathrin light chain A [Source:HGNC
Symbol;Acc:HGNC:2090] - 3969 4648 4024 4445 4132
5026 76.30347659 80.53538288 74.69061209
98.2077632 100.9879796 92.92135047
ENSG00000196419 8835.608582 7701.995998 7695.368715
10085.03484 9989.019247 10017.35579 8077.657765
10030.46996 -0.3124239 0.003221445 0.070591725 XRCC6
22 41621119 41664048 + 3288
protein_coding X-ray repair cross complementing 6 [Source:HGNC
Symbol;Acc:HGNC:4055] - 9186 8852 8271 9151 8241
10851 135.081492 117.3189572 117.4281578
154.6495961 154.0619766 153.4507349
ENSG00000065427 7549.607202 6898.037085 6674.715894
8404.379379 8946.602483 8854.157161 7040.786727
8735.046341 -0.311106968 0.003233897 0.070591725 KARS
16 75627474 75648643 - 4065
protein_coding lysyl-tRNA synthetase [Source:HGNC
Symbol;Acc:HGNC:6215] - 7849 7928 7174 7626 7381
9591 93.358743 84.98879844 82.38475571
104.2433344 111.6097192 109.7070051
ENSG00000171161 1003.215736 893.5777102 984.3670778
1265.17539 1267.869692 1424.45673 960.3868413
1319.167271 -0.458488938 0.003258148 1 ZNF672 1
248838210 248849517 + 3741 protein_coding
zinc finger protein 672 [Source:HGNC Symbol;Acc:HGNC:26179]
zf-C2H2 1043 1027 1058 1148 1046 1543
13.48024597 11.96303389 13.20212971 17.05164269
17.1866541 19.1782615
ENSG00000169727 3221.255513 3748.327921 3620.200661
3970.755167 4860.57119 5032.218818 3529.928032
4621.181725 -0.388638019 0.003259189 0.070591725 GPS1

17	82050691	82057470	+	6155
protein_coding	G protein pathway suppressor 1 [Source:HGNC
Symbol;Acc:HGNC:4549]	-	3349	4308	3891	3603	4010
5451	26.30802869	30.50044917	29.51067949
32.52731747	40.04641794	41.17931736
ENSG00000008988 11245.05769	14924.57494	13809.0512
16289.68419	16776.84934	18208.67437	13326.22794
17091.73596	-0.35902572	0.003268135	0.070591725	RPS20
8	56067295	56074581	-	3002
protein_coding	ribosomal protein S20 [Source:HGNC
Symbol;Acc:HGNC:10405]	-	11691	17153	14842	14781	13841
19724	188.2964747	248.9934928	230.795733
273.5931204	283.4028027	305.5028908
ENSG00000267523 84.64332192	13.92136647	53.96341258
7.714484088	4.848450065	11.07808215	50.84270032
7.880338769	2.677953825	0.003280019	1	AC008735.2
19	55661901	55674715	-	561
antisense	"novel transcript, antisense to U2AF2A"	-	88
16	58	7	4	12	7.584396469
1.242841422	4.826265041	0.693341543	0.438272772
0.994602171
ENSG00000176155 727.1630838	656.9144802	915.5172065
569.7697534	373.330655	500.3600439	766.5315902
481.1534841	0.670898319	0.003284214	1	CCDC57	17
82101460	82212830	-	12397	protein_coding
coiled-coil domain containing 57 [Source:HGNC
Symbol;Acc:HGNC:27564]	-	756	755	984	517	308
542	2.948535838	2.653926849	3.705309868
2.31731987	1.527149223	2.032889176
ENSG00000279632 38.47423724	9.570939447	6.512825656
1.102069155	0	0	18.18600078	0.367356385
5.563778169	0.003289936	1	AP003108.4	11
61426448	61427325	-	878	TEC	novel
transcript	-	40	11	7	1	0	0
2.202757517	0.545954899	0.372177025	0.06328744
0	0
ENSG00000207088 26.93196607	4.350427021	11.16484398	0
0	0	14.14907902	0	6.163411953
0.003316762	1	SNORA7B 3	129397210	129397348
-	139	snoRNA	"small nucleolar RNA, H/ACA box 7B
[Source:HGNC Symbol;Acc:HGNC:32593]"	-	28	5	12
0	0	0	9.739674603	1.567522567
4.030068998	0	0	0
ENSG00000184208 628.0919229	517.7008155	569.4070431
360.3766138	318.7855918	410.8122132	571.7332605
363.3248063	0.65252596	0.003347218	1	C22orf46
22	41688939	41698136	+	5341
protein_coding	chromosome 22 open reading frame 46 [Source:HGNC
Symbol;Acc:HGNC:26294]	-	653	595	612	327	263
445	5.911420044	4.85459479	5.349028108

3.402022788	3.026777736	3.874078809
ENSG00000136527 5018.964248	5436.293606	5429.83579
6626.741832	6502.98365	6519.451347	5295.031214
6549.725609	-0.306757494	0.003349835	0.071515084	TRA2B
	3	185915906	185938136	-	11769
protein_coding	transformer 2 beta homolog [Source:HGNC
Symbol;Acc:HGNC:10781]	-	5218	6248	5836	6013	5365
	7062	21.43708492	23.1344967	23.14844146
28.38988836	28.02060601	27.90096141
ENSG00000092439 1735.188099	1937.680195	2093.408247
1626.654073	1179.385478	1336.755246	1922.09218
1380.931599	0.476897201	0.003353804	1	TRPM7	15
	50552473	50686815	-	12398	protein_coding
	transient receptor potential cation channel subfamily M member
7 [Source:HGNC Symbol;Acc:HGNC:17994]	-	1804	2227	2250
	1476	973	1448	7.035356638	7.827574013
8.471823943	6.615257733	4.824014099	5.430601659
ENSG00000132002 7303.372084	5776.496999	6505.382427
7445.579214	8862.966719	9043.407731	6528.41717
8450.651221	-0.372431934	0.003421992	0.071828215	DNAJB1
	19	14514770	14529770	-	3672
protein_coding	DnaJ heat shock protein family (Hsp40) member B1
[Source:HGNC Symbol;Acc:HGNC:5270]	-	7593	6639	6992
	6756	7312	9796	99.97972637	78.78773107
88.88833737	102.2348613	122.3998457	124.0443884
ENSG00000126457 5813.457247	6009.679887	6221.609309
6689.559773	8267.819474	7919.905566	6014.915481
7625.761604	-0.342314032	0.003442747	0.071828215	PRMT1
	19	49675786	49689029	+	4796
protein_coding	protein arginine methyltransferase 1 [Source:HGNC
Symbol;Acc:HGNC:5187]	-	6044	6907	6687	6070	6821
	8579	60.93214933	62.75796886	65.08758901
70.32691394	87.42109366	83.17417686
ENSG00000008710 2651.836802	1902.006694	2508.368281
1464.649908	1644.836685	1927.586295	2354.070592
1679.024296	0.486965217	0.003489967	1	PKD1	16
	2088710 2135898 -	18532	protein_coding	"polycystin 1,
transient receptor potential channel interacting [Source:HGNC
Symbol;Acc:HGNC:9008]"	-	2757	2186	2696	1329	1357
	2088	7.19309326	5.1402763	6.791154833
3.984875278	4.500957543	5.238889291
ENSG00000184967 1299.467363	1239.001616	1316.521186
1449.220939	1856.956375	2058.676933	1284.996722
1788.284749	-0.477182012	0.003506924	1	NOC4L	12
	132144448	132152473	+	2029	protein_coding
	nucleolar complex associated 4 homolog [Source:HGNC
Symbol;Acc:HGNC:28461]	-	1351	1424	1415	1315	1532
	2230	32.19396877	30.58345458	32.55520457
36.01270549	46.41133688	51.10387523
ENSG00000196739 1240.794151	863.124721	1163.004581

778.0608237   746.66131   705.3045637   1088.974484
743.3422325   0.55081888   0.003511869   1   COL27A1 9
        114155560   114312511   +   13988   protein_coding
        collagen type XXVII alpha 1 chain [Source:HGNC
Symbol;Acc:HGNC:22986] -   1290   992   1250   706   616
        764   4.458977728   3.090399652   4.171578545
2.804536658   2.706901475   2.539620085
ENSG00000146826 632.9012026   425.4717627   531.2604928
276.619358   232.7256031   401.580478   529.8778194
303.6418131   0.800585769   0.003514334   1   C7orf43 7
        100154420   100158715   -   3255   protein_coding
        chromosome 7 open reading frame 43 [Source:HGNC
Symbol;Acc:HGNC:25604] -   658   489   571   251   192
        435   9.774085127   6.546609916   8.189005281
4.284841605   3.625747832   6.213971859
ENSG00000157106 3675.251512   2834.738247   2978.222132
2567.821132   2185.438867   2360.554672   3162.737297
2371.271557   0.415286764   0.003549739   0.073228322   SMG1
        16   18804853   18926454   -   25988
protein_coding "SMG1, nonsense mediated mRNA decay associated PI3K
related kinase [Source:HGNC Symbol;Acc:HGNC:30045]"   -   3821
        3258   3201   2330   1803   2557   7.108948959
5.463070683   5.749880938   4.981901156   4.26452072
4.574976716
ENSG00000229751 25.97011014   7.830768638   7.443229322   0
        0   0   13.74803603   0   6.122158721
0.003555649   1   AC044781.1   10   13991815
14007526   +   570   antisense   novel transcript
        -       27   9   8   0   0   0
2.290288144   0.688059906   0.655180808   0   0   0
ENSG00000257557 10.58041524   6.960683234   23.26009163   0
        0   0   13.6003967   0   6.107072995
0.0035635   1   PPP1R12A-AS1   12   79934901
79942712   +   3234   bidirectional_promoter_lncRNA
PPP1R12A antisense RNA 1 [Source:HGNC Symbol;Acc:HGNC:51903]   -
        11   8   25   0   0   0   0.164457577
        0.10779747   0.360866053   0   0   0
ENSG00000171612 578.0754145   841.3725859   788.0519044
1085.538118   1056.962114   1088.421572   735.8333016
1076.973935   -0.549210855   0.003572517   1   SLC25A33
        1   9539482 9585179 +   3854   protein_coding solute
carrier family 25 member 33 [Source:HGNC Symbol;Acc:HGNC:29681] -
        601   967   847   985   872   1179   7.539872087
        10.93385629   10.25930015   14.2015767   13.90759819
        14.22437237
ENSG00000279123 7.694847448   13.92136647   18.6080733   0
        0   0   13.40809574   0   6.087148953
0.003587302   1   AC027281.2   16   70778433
70779145   +   713   TEC   novel transcript   -
        8   16   20   0   0   0   0.542502412

0.977887851	1.309442708	0	0	0
ENSG00000173281 1328.323041	1632.280218	1144.396508
1013.903623	807.2669359	1009.028649	1368.333256
943.3997361	0.536076803	0.003604972	1	PPP1R3B 8
9136255 9151574 -	5776	protein_coding	protein
phosphatase 1 regulatory subunit 3B [Source:HGNC
Symbol;Acc:HGNC:14942]	-	1381	1876	1230	920	666
1093	11.56026289	14.15351297	9.940860118
8.850599451	7.087521752	8.79881113
ENSG00000173825 28.85567793	20.8820497	3.721614661
1.102069155	0	0	17.81978076	0.367356385
5.535327538	0.003619603	1	TIGD3	11	65354767
65357613	+	2012	protein_coding	tigger
transposable element derived 3 [Source:HGNC Symbol;Acc:HGNC:18334]
-	30	24	4	1	0	0
0.720932318	0.519806688	0.092806427	0.027617481	0
0
ENSG00000117419 2541.22337	2544.999807	2524.185144
2802.561862	3361.188008	4117.353867	2536.802774
3427.034579	-0.43428174	0.003651343	0.073349648	ERI3
1	44221070	44355260	-	2785
protein_coding	ERI1 exoribonuclease family member 3 [Source:HGNC
Symbol;Acc:HGNC:17276]	-	2642	2925	2713	2543	2773
4460	45.86789718	45.76771903	45.47478245
50.73798367	61.20290413	74.46302538
ENSG00000253406 22.12268641	13.05128106	5.582421991	0
0	0	13.58546316	0	6.105496578
0.003655212	1	AC012613.2	5	149216523
149276805	-	4533	antisense	"novel transcript,
antisense to ABLIM3"	-	23	15	6	0	0
0	0.245325862	0.144199627	0.061789057	0
0	0
ENSG00000108829 6844.566805	6496.057628	7109.214406
7620.80821	8700.543642	9621.31435	6816.612946
8647.5554	-0.343365943	0.00366759	0.073349648	LRRC59
17	50375059	50397553	-	4570
protein_coding	leucine rich repeat containing 59 [Source:HGNC
Symbol;Acc:HGNC:28817]	-	7116	7466	7641	6915	7178
10422	75.2871671	71.19186714	78.05128104
84.07909492	96.54607312	106.0390643
ENSG00000111640 40654.76463	42664.6378	53496.34994
57251.39055	68687.99207	52580.27059	45605.25079
59506.55107	-0.383824811	0.003675473	0.073349648	GAPDH
12	6533927 6538374 +	2981	protein_coding
glyceraldehyde-3-phosphate dehydrogenase [Source:HGNC
Symbol;Acc:HGNC:4141]	-	42267	49035	57498	51949	56668
56956	685.5524138	716.8079302	900.4027201
968.3386345	1168.485344	888.3999459
ENSG00000197479 38.47423724	15.66153728	22.32968796	0
0	3.692694051	25.48848749	1.230898017

4.284323969	0.003709825	1	PCDHB11 5	141199582
	141203779	+	4198	protein_coding	protocadherin
beta 11 [Source:HGNC Symbol;Acc:HGNC:8682]	-	40	18
	24	0	0	4	0.460700595	0.186848093
	0.266879271	0	0	0.044304575
ENSG00000178035 10281.27805	9224.645456	10273.51727
10935.83223	13102.9363	13647.27404	9926.480258
12562.01419	-0.33978165	0.003739736	0.073829632	IMPDH2
	3	49024325	49029408	-	2773
protein_coding	inosine monophosphate dehydrogenase 2 [Source:HGNC
Symbol;Acc:HGNC:6053]	-	10689	10602	11042	9923	10810
	14783	186.3753294	166.6082597	185.8848131
198.840646	239.6200666	247.8812774
ENSG00000112576 1393.729244	1136.331538	1277.444232
1769.923064	1641.200347	1710.640519	1269.168338
1707.254643	-0.428103206	0.00376302	1	CCND3	6
	41934933	42050357	-	5802	protein_coding
	cyclin D3 [Source:HGNC Symbol;Acc:HGNC:1585]	-	1449
	1306	1373	1606	1354	1853	12.07513174
9.808984544	11.04685996	15.38083312	14.3445953
14.85007725
ENSG00000166432 159.6680845	94.83930906	123.7436875
28.65379804	72.72675098	19.38664377	126.0836937
40.25573093	1.655634581	0.003769246	1	ZMAT1	X
	101882288	101932031	-	7514	protein_coding
	zinc finger matrin-type 1 [Source:HGNC Symbol;Acc:HGNC:29377]
	-	166	109	133	26	60	21
1.068164435	0.632140921	0.826278564	0.192271184
0.490825842	0.129950849
ENSG00000108106 4112.895961	3884.061245	3929.094678
4701.427017	4910.267804	5341.481945	3975.350628
4984.392255	-0.32652083	0.003780678	0.07384388	UBE2S
	19	55399745	55407777	-	2788
protein_coding	ubiquitin conjugating enzyme E2 S [Source:HGNC
Symbol;Acc:HGNC:17895]	-	4276	4464	4223	4266	4051
	5786	74.15597402	69.77342055	70.70894343
85.02372447	89.31344349	96.49763686
ENSG00000228589 66.36805924	72.21708855	87.45794453
234.7407301	210.9075778	98.77956586	75.34769744
181.4759579	-1.266123894	0.003795949	1	SPCS2P4 1
	28095742	28096422	-	681
processed_pseudogene	signal peptidase complex subunit 2 pseudogene
4 [Source:HGNC Symbol;Acc:HGNC:45237]	-	69	83	94
	213	174	107	4.898952125	5.311162365
6.443573363	17.37979042	15.70541789	7.3057984
ENSG00000008083 941.6569564	762.1948141	585.2239054
406.6635183	494.5419067	540.9796785	763.0252253
480.7283678	0.665882056	0.003817552	1	JARID2	6
	15246296	15522040	+	6015	protein_coding
	jumonji and AT-rich interaction domain containing 2

[Source:HGNC Symbol;Acc:HGNC:6196]	ARID	979	876	629
	369	408	586	7.869520601	6.346394607
4.881587178	3.408809866	4.169383963	4.529945765
ENSG00000159199 857.0136345	797.8683157	970.4110228
1223.296763	1199.991391	1203.818261	875.0976577
1209.035471	-0.466478514	0.003823124	1	ATP5MC1 17
	48892765	48895871	+	2615	protein_coding
	ATP synthase membrane subunit c locus 1 [Source:HGNC
Symbol;Acc:HGNC:841]	-	891	917	1043	1110	990
	1304	16.47430975	15.28115709	18.61909857
23.58649069	23.27077578	23.1865918
ENSG00000172500 1525.503506	1693.186197	1690.54346
2186.505204	2111.500003	2093.757527	1636.411054
2130.587578	-0.380578914	0.003884763	1	FIBP	11
	65883741	65888539	-	3353	protein_coding
	FGF1 intracellular binding protein [Source:HGNC
Symbol;Acc:HGNC:3705]	-	1586	1946	1817	1984	1742
	2268	22.87024653	25.2911094	25.29690616
32.87911794	31.93463505	31.45143859
ENSG00000168439 11518.22477	10011.20266	10109.76623
12484.23939	13010.81575	13647.27404	10546.39789
13047.44306	-0.307088727	0.003904025	0.075231367	STIP1
	11	64185272	64204543	+	4657
protein_coding	stress induced phosphoprotein 1 [Source:HGNC
Symbol;Acc:HGNC:11387]	-	11975	11506	10866	11328	10734
	14783	124.3284446	107.6655416	108.920468
135.1633808	141.6780965	147.6003397
ENSG00000204387 366.4671097	442.0033854	532.1908965
170.8207191	316.3613668	261.2581041	446.8871305
249.4800633	0.841712834	0.00392774	1	C6orf48 6
	31834608	31839766	+	1801	protein_coding
	chromosome 6 open reading frame 48 [Source:HGNC
Symbol;Acc:HGNC:19078]	-	381	508	572	155	261
	283	10.22851248	12.29160505	14.82614871
4.782225259	8.907875832	7.306404983
ENSG00000178982 3928.219622	3312.415134	3357.826828
3924.468262	4787.844439	5323.018474	3532.820528
4678.443725	-0.405437637	0.003933666	0.075231367	EIF3K
	19	38619082	38636955	+	2798
protein_coding	eukaryotic translation initiation factor 3 subunit K
[Source:HGNC Symbol;Acc:HGNC:24656]	-	4084	3807	3609
	3561	3950	5766	70.57310732	59.29168114
60.21229875	70.71903196	86.77542426	95.82039262
ENSG00000265692 0	36.54358698	14.88645864	0	0
	0	17.14334854	0	6.442488849	0.003986497
	1	LINC01970	17	82290046	82292814
	-	1810	lincRNA long intergenic non-protein coding RNA
1970 [Source:HGNC Symbol;Acc:HGNC:52796]	-	0	42
	16	0	0	0	0	1.011181961
0.412655315	0	0	0

ENSG00000182871 592.5032535 294.958952 413.0992273
210.4952087 231.5134906 271.4130127 433.5204776
237.8072373 0.864906389 0.00399759 1 COL18A1 21
45405137 45513720 + 8525 protein_coding
collagen type XVIII alpha 1 chain [Source:HGNC
Symbol;Acc:HGNC:2195] - 616 339 444 191 191
294 3.493715535 1.732861721 2.431277989
1.244947459 1.377166151 1.603555371
ENSG00000146083 1097.477617 1128.500769 1213.246379
880.5532552 823.0243986 797.621915 1146.408255
833.7331896 0.459670385 0.004047336 1 RNF44 5
176526697 176538025 - 5660 protein_coding
ring finger protein 44 [Source:HGNC Symbol;Acc:HGNC:19180]
- 1141 1297 1304 799 679 864
9.746987963 9.985783424 10.75492029 7.844086818
7.373958741 7.097874717
ENSG00000263990 25.0082542 2.610256213 13.95605498 0
0 0 13.85818847 0 6.133409229
0.004053622 1 AC004253.1 17 31873926
31886666 + 488 lincRNA novel transcript -
26 3 15 0 0 0 2.576052694
0.267892176 1.434886247 0 0 0
ENSG00000275481 14.42783896 26.10256213 1.86080733 0
0 0 14.13040281 0 6.163318933
0.004053687 1 AC025031.4 12 46388856
46392126 + 3271 lincRNA novel transcript -
15 30 2 0 0 0 0.221723605
0.399667937 0.028542729 0 0 0
ENSG00000049130 468.4238384 476.8068015 370.3006587
600.6276897 816.963836 628.6811622 438.5104329
682.090896 -0.636538532 0.004054832 1 KITLG 12
88492793 88580851 - 6205 protein_coding
KIT ligand [Source:HGNC Symbol;Acc:HGNC:6343] - 487
548 398 545 674 681 3.794795631
3.848552102 2.994244925 4.880527454 6.67675547
5.103126619
ENSG00000173295 291.4423471 274.0769023 290.2859435
159.8000275 163.6351897 161.5553647 285.2683977
161.6635273 0.819306027 0.004087685 1 FAM86B3P
8 8228595 8244865 + 4870
transcribed_unprocessed_pseudogene "family with sequence
similarity 86 member B3, pseudogene [Source:HGNC
Symbol;Acc:HGNC:44371]" - 303 315 312 145 135
175 3.008256639 2.818643761 2.99069186
1.65444024 1.703931641 1.670860167
ENSG00000196440 337.6114318 336.7230514 390.7695394
217.1076236 231.5134906 170.7870999 355.0346742
206.4694047 0.783873969 0.00410257 1 ARMCX4 X
101418287 101533459 + 13351 protein_coding
armadillo repeat containing X-linked 4 [Source:HGNC

Symbol;Acc:HGNC:28615] - 351 387 420 197 191
185 1.271143371 1.263152444 1.468525629
0.819906773 0.879360455 0.644301215
ENSG00000185105 45.20722875 19.14187889 15.81686231
4.408276622 0 0 26.72198999 1.469425541
4.198722121 0.00416481 1 MYADML2 17 81939645
81947233 - 2452 protein_coding myeloid
associated differentiation marker like 2 [Source:HGNC
Symbol;Acc:HGNC:34548] - 47 22 17 4 0
0 0.926784173 0.390985645 0.323649165
0.09064661 0 0
ENSG00000272159 11.54227117 22.62222051 5.582421991 0
0 0 13.24897122 0 6.070412692
0.004185483 1 AC087623.3 8 38408048
38408742 - 695 antisense "novel transcript,
antisense to LETM2" - 12 26 6 0 0
0 0.834829252 1.630223469 0.4030069 0
0 0
ENSG00000105131 16.35155083 13.05128106 9.304036652 0
0 0 12.90228951 0 6.031285479
0.004216457 1 EPHX3 19 15226919 15233435
- 2034 protein_coding epoxide hydrolase 3
[Source:HGNC Symbol;Acc:HGNC:23760] - 17 15 10
0 0 0 0.40410962 0.321365246
0.229506551 0 0 0
ENSG00000148082 1709.217989 1728.859698 1746.36768
1287.216774 1334.53588 1339.524767 1728.148456
1320.425807 0.388192492 0.00423049 1 SHC3 9
89005771 89178767 - 9929 protein_coding
SHC adaptor protein 3 [Source:HGNC Symbol;Acc:HGNC:18181]
- 1777 1987 1877 1168 1101 1451
8.65332736 8.720692119 8.8247985 6.536561862
6.815993514 6.795054117
ENSG00000123908 4613.061045 3506.444179 4270.552823
3533.233712 2770.889212 3000.313916 4130.019349
3101.478947 0.413032662 0.004243448 0.080319286 AGO2
8 140520156 140635619 - 15833
protein_coding "argonaute 2, RISC catalytic component [Source:HGNC
Symbol;Acc:HGNC:3263]" - 4796 4030 4590 3206 2286
3250 14.64593759 11.09175919 13.53304449
11.25154988 8.874836779 9.544455717
ENSG00000253661 25.97011014 0.870085404 16.74726597 0
0 0 14.52915384 0 6.201625326
0.00432605 1 ZFHX4-AS1 8 76491200
76683278 - 2251 antisense ZFHX4 antisense RNA 1
[Source:HGNC Symbol;Acc:HGNC:44165] - 27 1 18
0 0 0 0.579948575 0.019359008
0.373287155 0 0 0
ENSG00000025770 877.212609 1078.905901 1197.429517
1332.401609 1581.806834 1495.541091 1051.182676

1469.916511	-0.483490039	0.004422677	1	NCAPH2	22
	50508216	50523472	+	3159	protein_coding
	non-SMC condensin II complex subunit H2 [Source:HGNC
Symbol;Acc:HGNC:25071]	-	912	1240	1287	1209	1305
	1620	13.95874678	17.10529849	19.01844289
21.26614245	25.39266283	23.84494949
ENSG00000089159 3252.034903	3184.51258	3127.086719
2665.905287	2423.01292	2480.567229	3187.878067
2523.161812	0.337328885	0.00448682	0.084059205	PXN
	12	120210439	120265771	-	7460
protein_coding	paxillin [Source:HGNC Symbol;Acc:HGNC:9718]	-
	3381	3660	3361	2419	1999	2687	21.91328867
	21.37966302	21.03176502	18.0181038	16.47105157
	16.74788064
ENSG00000112343 419.3691859	550.7640609	512.6524195
328.4166083	270.3010911	327.726597	494.2618888
308.8147655	0.677900344	0.004502823	1	TRIM38	6
	25962802	25991226	+	9413	protein_coding
	tripartite motif containing 38 [Source:HGNC
Symbol;Acc:HGNC:10059]	-	436	633	551	298	223
	355	2.239544246	2.93044955	2.732559178
1.759139373	1.456210521	1.753603132
ENSG00000166436 509.7836434	281.907671	457.7586033
254.5779749	184.2411025	253.872716	416.4833059
230.8972645	0.849256149	0.004511457	1	TRIM66	11
	8612037 8682694 -	11752	protein_coding	tripartite
motif containing 66 [Source:HGNC Symbol;Acc:HGNC:29005] -	530
	324	492	231	152	275	2.180546253
1.201411612	1.954336557	1.092225322	0.795022034
1.088057706
ENSG00000272004 0	0	0	0	31.51492542
14.7707762	0	15.42856721	-6.509152191	0.004519627
	1	FO704657.1	1	1659325 1662602 +	400
	antisense	"novel transcript, antisense to SLC35E2B"
	-	0	0	0	0	26	16	0
	0	0	0	3.995404154	1.85990606
ENSG00000275479 15.3896949	19.14187889	4.652018326	0
	0	0	13.06119737	0	6.04943207
0.004525433	1	AC087741.2	17	80149627
80149798	+	172	lincRNA novel transcript	-
	16	22	5	0	0	0	4.497723488
	5.573818615	1.357024202	0	0	0
ENSG00000258727 12.5041271	25.23247672	2.791210996	0
	0	0	13.50927161	0	6.098559872
0.004526117	1	AL135999.1	14	23561097
23568073	+	3243	antisense	uncharacterized
LOC102724814 [Source:NCBI gene;Acc:102724814]	-	13	29
	3	0	0	0	0.193819567	0.389681373
	0.043183749	0	0	0
ENSG00000061337 106.7660083	100.9299069	162.8206414

229.2303843	208.4833528	300.9545651	123.5055189
246.2227674	-0.99694559	0.004588857	1	LZTS1	8
	20246165	20303963	-	5706	protein_coding
	leucine zipper tumor suppressor 1 [Source:HGNC
Symbol;Acc:HGNC:13861]	-	111	116	175	208	172
	326	0.940572827	0.885900241	1.431700963
2.025553	1.852866118	2.656543005
ENSG00000279700 45.20722875	26.10256213	22.32968796
6.612414933	0	0	31.21315962	2.204138311
3.835021449	0.004615648	1	AC131212.2	12
132565071	132566425	+	1355	TEC	novel
transcript	-	47	30	24	6	0	0
	1.677103168	0.964807248	0.826833344	0.246050357
	0	0
ENSG00000135047 873.3651853	1047.582827	1183.473462
753.8153023	675.1466716	739.4619837	1034.807158
722.8079859	0.517478047	0.004625818	1	CTSL	9
	87725519	87731393	+	2366	protein_coding
	cathepsin L [Source:HGNC Symbol;Acc:HGNC:2537]	-	908
	1204	1272	684	557	801	18.55548561
22.17534291	25.0968033	16.06398926	14.47064253
15.74159715
ENSG00000133318 3124.108064	3124.476687	3238.735159
4514.075261	4067.849605	3575.451015	3162.43997
4052.458627	-0.357541903	0.004687334	0.08596425	RTN3
	11	63681446	63759891	+	5792
protein_coding	reticulon 3 [Source:HGNC Symbol;Acc:HGNC:10469] -
	3248	3591	3481	4096	3356	3873	27.11369359
	27.01751801	28.05572564	39.29555603	35.61564052
	31.09209328
ENSG00000198830 4953.558044	5308.391051	5309.813717
8574.098029	6821.769242	5467.033542	5190.587604
6954.300271	-0.421835476	0.004725285	0.08596425	HMGN2
	1	26472450	26475972	+	2509
protein_coding	high mobility group nucleosomal binding domain 2
[Source:HGNC Symbol;Acc:HGNC:4986]	-	5150	6101	5707
	7780	5628	5922	99.24480533	105.9641507
106.1825735	172.3022622	137.8798454	109.7485421
ENSG00000166741 911.8394225	909.2392474	896.9091332
1226.60297	1189.082378	1242.591548	905.9959344
1219.425632	-0.428771267	0.004733257	1	NNMT	11
	114257787	114313285	+	2919	protein_coding
	nicotinamide N-methyltransferase [Source:HGNC
Symbol;Acc:HGNC:7861]	-	948	1045	964	1113	981
	1346	15.70274069	15.60058174	15.41661315
21.18717789	20.65771458	21.44084924
ENSG00000169710 12414.6745	9642.28645	14383.11026
19804.18272	17164.72534	13015.82336	12146.6904
16661.57714	-0.455924246	0.004752846	0.08596425	FASN
	17	82078333	82098332	-	9364

protein_coding	fatty acid synthase [Source:HGNC Symbol;Acc:HGNC:3594]
	-	12907	11082	15459	17970	14161	14099
66.64462392	51.57216204	77.06656959	106.6347403
92.95652454	70.00965277
ENSG00000100201 6747.419356	6681.385819	6930.576902
5740.678231	5312.689159	5570.428976	6786.460692
5541.265455	0.292375798	0.004775792	0.08596425	DDX17
	22	38483440	38507660	-	9995
protein_coding	DEAD-box helicase 17 [Source:HGNC
Symbol;Acc:HGNC:2740]	-	7015	7679	7449	5209	4383
	6034	33.93486247	33.4796159	34.79054336
28.95900278	26.95479495	28.07071827
ENSG00000156471 3570.409216	3194.953604	3095.452994
4174.637961	4126.031006	4085.042794	3286.938605
4128.570587	-0.3289417	0.004853787	0.086519931	PTDSS1
	8	96261715	96336995	+	6295
protein_coding	phosphatidylserine synthase 1 [Source:HGNC
Symbol;Acc:HGNC:9587]	-	3712	3672	3327	3788	3404
	4425	28.51106562	25.41941408	24.67192875
33.43692103	33.23848168	32.68500522
ENSG00000279861 32.70310165	1.740170808	7.443229322	0
	0	0	13.96216726	0	6.143979455
0.004898636	1	AC073548.1	19	47487638
47489519	-	1882	TEC	TEC	-	34	2
	8	0	0	0	0.873495183	0.046309381
	0.198434145	0	0	0
ENSG00000169064 0	0	0.930403665	16.53103733
4.848450065	24.00251133	0.310134555
15.12733291	-5.523408875	0.00493754	1	ZBBX	3
	167240287	167381346	-	3972	protein_coding
	zinc finger B-box domain containing [Source:HGNC
Symbol;Acc:HGNC:26245]	-	0	0	1	15	4
	26	0	0	0.011752677	0.209842795
0.061901064	0.304365292
ENSG00000073605 93.3000253	100.0598215	78.15390788
42.98069706	27.87858788	15.69394972	90.50458489
28.85107822	1.654522029	0.005001625	1	GSDMB	17
	39904595	39919854	-	5905	protein_coding
	gasdermin B [Source:HGNC Symbol;Acc:HGNC:23690] -	97
	115	84	39	23	17	0.794242365
0.848665478	0.664057093	0.366992128	0.239417171
0.133862841
ENSG00000005483 1462.982871	1350.372547	1497.019497
1218.888486	802.4184858	1016.414038	1436.791639
1012.57367	0.504290512	0.005018109	1	KMT2E	7
	105014179	105114361	+	9231	protein_coding
	lysine methyltransferase 2E [Source:HGNC
Symbol;Acc:HGNC:18541]	-	1521	1552	1609	1106	662
	1101	7.966758999	7.326584514	8.136794148
6.657611056	4.40815238	5.545868736

ENSG00000100519 1456.249879	1567.893898	1615.180763
2117.074848	1964.834389	1946.972938	1546.441514
2009.627392	-0.37785447	0.005034207	1	PSMC6	14
	52707172	52728587	+	7482	protein_coding
	"proteasome 26S subunit, ATPase 6 [Source:HGNC
Symbol;Acc:HGNC:9553]"	-	1514	1802	1736	1921	1621
	2109	9.783841035	10.4953199	10.83123685
14.26664007	13.31719231	13.10659543
ENSG00000187889 0	0	0	29.7558672	15.75746271
	0	0	15.17110997	-6.484202447	0.005035202
	1	FYB2	1	56718804	56819696	-
	4633	protein_coding	FYN binding protein 2 [Source:HGNC
Symbol;Acc:HGNC:27295]	-	0	0	0	27	13
	0	0	0	0	0.323827336	0.172475897
	0
ENSG00000169045 5750.936611	4864.647495	5846.656632
4884.370497	3818.154426	3976.108319	5487.413579
4226.211081	0.376705106	0.005084654	0.089763698
HNRNPH1 5	179614178	179634784	-	9480
protein_coding	heterogeneous nuclear ribonucleoprotein H1
[Source:HGNC Symbol;Acc:HGNC:5041]	-	5979	5591	6284
	4432	3150	4307	30.49449408	25.7003923
30.94381635	25.97786515	20.42441267	21.12504061
ENSG00000101412 1092.668338	1232.911018	1467.24658
1660.818217	1567.261484	2049.445198	1264.275312
1759.174966	-0.477082771	0.005112013	1	E2F1	20
	33675683	33686404	-	2503	protein_coding
	E2F transcription factor 1 [Source:HGNC Symbol;Acc:HGNC:3113]
	E2F	1136	1417	1577	1507	1293	2220
21.94414672	24.66991189	29.41148004	33.45526285
31.75301989	41.24042602
ENSG00000105254 1902.551031	1850.671655	1751.950102
2323.16178	2282.407868	2439.947594	1835.057596
2348.505747	-0.35610937	0.005131426	1	TBCB	19
	36114289	36125947	+	3326	protein_coding
	tubulin folding cofactor B [Source:HGNC Symbol;Acc:HGNC:1989]
	-	1978	2127	1883	2108	1883	2643
28.75446283	27.86787429	26.42859714	35.21765262
34.79969482	36.94927628
ENSG00000237149 60.59692365	65.25640532	41.86816493
147.6772668	115.150689	135.7065064	55.90716463
132.8448207	-1.248677923	0.005153159	1	ZNF503-AS2
	10	75401519	75408982	+	3348
antisense	ZNF503 antisense RNA 2 [Source:HGNC
Symbol;Acc:HGNC:23525]	-	63	75	45	134	95
	147	0.909821754	0.976190129	0.627441298
2.223982639	1.744156763	2.041563552
ENSG00000172428 338.5732877	406.3298838	347.0405671
544.4221628	621.8137209	508.6686055	363.9812462
558.3014964	-0.616129557	0.005188028	1	COPS9	2

240126563 240136807 - 2039 protein_coding
COP9 signalosome subunit 9 [Source:HGNC Symbol;Acc:HGNC:21314]
- 352 467 373 494 513 551
8.346927748 9.980636819 8.539602228 13.46237757
15.46491365 12.56508385
ENSG00000130881 771.4084566 789.1674616 998.3231327
1184.724342 1506.655858 1033.031161 852.9663503
1241.470454 -0.540748621 0.005196236 1 LRP3 19
33177603 33208867 + 5095 protein_coding
LDL receptor related protein 3 [Source:HGNC
Symbol;Acc:HGNC:6695] - 802 907 1073 1075 1243
1119 7.610819048 7.757498432 9.83108768
11.72401376 14.99596094 10.21214368
ENSG00000177971 750.2476261 844.8529275 802.0079594
855.2056646 1410.898969 1284.134356 799.036171
1183.412997 -0.566432635 0.005202692 1 IMP3 15
75639085 75648706 - 2565 protein_coding
"IMP3, U3 small nucleolar ribonucleoprotein [Source:HGNC
Symbol;Acc:HGNC:14497]" - 780 971 862 776 1164
1391 14.70308438 16.4964486 15.68794045
16.81072313 27.89413966 25.21568547
ENSG00000262943 37.51238131 5.220512425 7.443229322 0
1.212112516 0 16.72537435 0.404037505
5.442723096 0.005252442 1 ALOX12P2 17
6853861 6954107 + 5655 transcribed_unprocessed_pseudogene
arachidonate 12-lipoxygenase pseudogene 2 [Source:HGNC
Symbol;Acc:HGNC:432] - 39 6 8 0 1
0 0.333451914 0.046235679 0.066039445 0
0.01086963 0
ENSG00000184661 151.9732371 250.5845964 199.1063844
520.1766414 418.1788181 227.1006841 200.5547393
388.4853812 -0.951984079 0.005266297 1 CDCA2 8
25458997 25507920 + 3908 protein_coding
cell division cycle associated 2 [Source:HGNC
Symbol;Acc:HGNC:14623] - 158 288 214 472 345
246 1.954806383 3.211415731 2.556261353
6.711189273 5.426401202 2.926924839
ENSG00000105835 7220.652474 10427.97357 7340.884918
6087.830015 6427.832674 6427.133996 8329.836987
6314.265561 0.399742559 0.00530892 0.092830263 NAMPT
7 106248285 106286326 - 9049
protein_coding nicotinamide phosphoribosyltransferase [Source:HGNC
Symbol;Acc:HGNC:30092] - 7507 11985 7890 5524 5303
6962 40.11132831 57.7159765 40.70262801
33.92072495 36.02204788 35.77374845
ENSG00000122432 34.62681351 7.830768638 53.96341258
1.102069155 0 6.462214589 32.14033158 2.521427915
3.620732227 0.005385611 1 SPATA1 1
84506300 84566194 + 4280 protein_coding
spermatogenesis associated 1 [Source:HGNC Symbol;Acc:HGNC:14682]

- 36 9 58 1 0 7
0.40668668 0.091634146 0.632601563 0.012982797 0
0.076047561
ENSG00000011332 73.10105075 95.70939447 140.4909534
173.0248574 212.1196904 264.0276246 103.1004662
216.3907241 -1.070269504 0.005394512 1 DPF1 19
38211006 38229714 - 4800 protein_coding
double PHD fingers 1 [Source:HGNC Symbol;Acc:HGNC:20225]
- 76 110 151 157 175 286
0.765550019 0.998642502 1.468526357 1.817483424
2.241011946 2.770485069
ENSG00000280388 5.771135586 10.44102485 21.3992843 0
0 0 12.53714825 0 5.990104713
0.005432149 1 AC006330.1 7 76043977
76045963 - 1987 TEC TEC - 6 12
23 0 0 0 0.146000586 0.263173391
0.540351056 0 0 0
ENSG00000265511 21.16083048 24.36239132 64.1978529
8.816553243 0 0 36.57369157 2.938851081
3.64660045 0.005441768 1 AC020558.2 17
17507351 17508308 + 795 antisense novel
transcript - 22 28 69 8 0 0
1.338002019 1.534791907 4.051613392 0.559158462
0 0
ENSG00000137959 612.702228 1121.540086 833.641684
501.4414657 527.2689446 606.5249979 855.9613327
545.0784694 0.650952804 0.005462078 1 IFI44L 1
78619922 78646145 + 10240 protein_coding
interferon induced protein 44 like [Source:HGNC
Symbol;Acc:HGNC:17817] - 637 1289 896 455 435
657 3.007742775 5.485441129 4.084642849
2.469013609 2.611179097 2.983296585
ENSG00000202078 21.16083048 1.740170808 16.74726597 0
0 0 13.21608909 0 6.065027297
0.005469012 1 RF00019 1 160326104 160326216
- 113 misc_RNA - 22 2
18 0 0 0 9.413377034 0.77127659
7.436012265 0 0 0
ENSG00000285744 69.25362703 75.69743017 40.93776127
5.510345777 0 23.07933782 61.96293949 9.529894532
2.675844734 0.005477744 1 AC083837.2 8
78805293 78956082 + 2921
bidirectional_promoter_lncRNA novel transcript - 72
87 44 5 0 25 1.191796638
1.297915125 0.703181045 0.095115324 0 0.397960044
ENSG00000128585 1525.503506 1203.328114 1321.173205
1044.761559 869.0846742 1020.106732 1350.001608
977.9843217 0.464410398 0.005606722 1 MKLN1 7
131110096 131496636 + 12878 protein_coding
muskelin 1 [Source:HGNC Symbol;Acc:HGNC:7109] - 1586

	1383	1420	948	717	1105	5.95464642
4.679854568	5.147376784	4.090458212	3.422300141
3.989742577
ENSG00000180998 22.12268641	20.8820497	40.93776127
85.96139412	90.90843872	78.46974858	27.98083246
85.11319381	-1.605024149	0.00565584	1	GPR137C 14
	52553148	52637713	+	6193	protein_coding
	G protein-coupled receptor 137C [Source:HGNC
Symbol;Acc:HGNC:25445]	-	23	24	44	78	75
	85	0.179567598	0.168876321	0.331663464
0.699850966	0.744402021	0.638188338
ENSG00000271122 1187.892075	1108.488805	1020.652821
718.5490893	892.114812	774.5425772	1105.6779
795.0688262	0.476237426	0.005657311	1	AC018647.2
	7	35695214	35699413	+	4200
antisense	uncharacterized LOC101930085 [Source:NCBI
gene;Acc:101930085]	-	1235	1274	1097	652	736
	839	14.21735749	13.2183953	12.19279784
8.62601778	10.77149252	9.288459431
ENSG00000261783 39.43609317	20.0119643	1.86080733	0
	1.212112516	0.923173513	20.43628827	0.71176201
	4.846562116	0.005695101	1	AC009054.2	16
	75379818	75381260	-	374	sense_intronic
	"novel transcript, sense intronic to CFDP1"	-	41
	23	2	0	1	1	5.300458896
2.679876816	0.249634399	0	0.164352289	0.124325271
ENSG00000233968 7.694847448	5.220512425	7.443229322
66.12414933	32.72703794	20.30981728	6.786196398
39.72033485	-2.54964773	0.005719731	1	AL157895.1
	10	19710328	19728550	-	2151
antisense	uncharacterized LOC101928834 [Source:NCBI
gene;Acc:101928834]	-	8	6	8	60	27
	22	0.1798253	0.121554051	0.173618345
1.549968541	0.771561794	0.475568728
ENSG00000285531 43.28351689	21.75213511	37.21614661
4.408276622	4.848450065	6.462214589	34.08393287
5.239647092	2.694829658	0.005756959	1	Z83840.1
	22	41676745	41677125	-	381
processed_pseudogene	"LLP homolog, long-term synaptic facilitation
(LLPH) pseudogene"	-	45	25	40	4	4
	7	5.710692223	2.859391559	4.900958798
0.583373986	0.645330774	0.85428756
ENSG00000198498 800.2641345	823.1007924	834.5720877
613.8525196	590.2987954	540.0565049	819.3123382
581.4026067	0.495410933	0.005776788	1	TMA16	4
	163494442	163520539	+	3513	protein_coding
	translation machinery associated 16 homolog [Source:HGNC
Symbol;Acc:HGNC:25638]	-	832	946	897	557	487
	585	11.45107853	11.73468901	11.91956288
8.810267383	8.52114924	7.742990643

ENSG00000099341 5989.476882 6030.561937 5749.894651
6469.145942 7999.942608 7777.736845 5923.311157
7415.608465 -0.324155876 0.005801891 0.100493139 PSMD8
19 38374536 38383824 + 3105
protein_coding "proteasome 26S subunit, non-ATPase 8 [Source:HGNC
Symbol;Acc:HGNC:9566]" - 6227 6931 6180 5870 6600
8425 96.96577607 97.27313033 92.91223484
105.0481819 130.6561002 126.1651252
ENSG00000258857 8.656703379 16.53162268 11.16484398 0
0 0 12.11772335 0 5.941419334
0.005828868 1 AL359397.1 14 50397013
50398345 + 501 lincRNA novel transcript -
9 19 12 0 0 0 0.86857235
1.652625588 1.118122935 0 0 0
ENSG00000214460 0 0 0 0 23.03013781
20.30981728 0 14.4466517 -6.415718597 0.005835549
1 TPT1P6 6 85427453 85427966 -
514 processed_pseudogene "tumor protein,
translationally-controlled 1 pseudogene 6 [Source:HGNC
Symbol;Acc:HGNC:49297]" - 0 0 0 0 19
22 0 0 0 0 2.272154413
1.990171854
ENSG00000183160 600.1981009 587.3076479 695.9419416
1182.520204 1107.87084 644.3751119 627.8158968
978.2553852 -0.638951596 0.005840341 1 TMEM119 12
108589846 108598320 - 3189 protein_coding
transmembrane protein 119 [Source:HGNC Symbol;Acc:HGNC:27884]
- 624 675 748 1073 914 698
9.460874617 9.223756966 10.94947041 18.69636794
17.6172873 10.17728465
ENSG00000221978 3343.411216 2565.881857 3650.903982
2152.341061 2364.831519 2603.349306 3186.732352
2373.507295 0.424730038 0.005859761 0.100546933 CCNL2
1 1385711 1399328 - 6850 protein_coding cyclin
L2 [Source:HGNC Symbol;Acc:HGNC:20570] - 3476 2949 3924
1953 1951 2820 24.53524578 18.76043045
26.74141988 15.84249999 17.50709378 19.14209887
ENSG00000221038 0 0 0 15.42896818 0
27.69520538 0 14.37472452 -6.411549563 0.005928422
1 RNU6ATAC7P 17 31563768 31563894
- 127 snRNA "RNA, U6atac small nuclear 7,
pseudogene [Source:HGNC Symbol;Acc:HGNC:46906]" - 0 0
0 14 0 30 0 0 0
6.125426857 0 10.9836972
ENSG00000104833 42.32166096 6.09059783 0 0 0
0 16.1374196 0 6.353272785 0.006055967
1 TUBB4A 19 6494319 6502848 - 3466
protein_coding tubulin beta 4A class IVa [Source:HGNC
Symbol;Acc:HGNC:20774] - 44 7 0 0 0
0 0.61379781 0.088009201 0 0 0

0
ENSG00000011304 10692.95238 8178.8028 10207.45861
11710.58685 12496.88004 12388.98854 9693.071265
12198.81848 -0.331775678 0.006118399 0.103132857 PTBP1
19 797075 812327 + 6883 protein_coding
polypyrimidine tract binding protein 1 [Source:HGNC
Symbol;Acc:HGNC:9583] - 11117 9400 10971 10626 10310
13420 78.09281042 59.51256678 74.40711765
85.783564 92.07214392 90.65792287
ENSG00000164032 3447.291657 3553.428791 3964.450017
5177.520892 4706.632901 4116.430693 3655.056822
4666.861495 -0.352366501 0.006133078 0.103132857 H2AFZ
4 99948086 99950388 - 2303
protein_coding H2A histone family member Z [Source:HGNC
Symbol;Acc:HGNC:4741] - 3584 4084 4261 4698 3883
4459 75.2445899 77.2770248 86.37014169
113.352504 103.638427 90.0273678
ENSG00000278389 26.93196607 24.36239132 7.443229322
2.204138311 0 0 19.57919557 0.73471277
4.756454953 0.006134389 1 AC099518.6 16
19111035 19111484 - 450 lincRNA novel
transcript - 28 28 8 2 0 0
3.008477266 2.711465702 0.82989569 0.246961654
0 0
ENSG00000075624 148281.634 166537.8267 147335.0028
204114.2283 180231.4343 177990.6228 154051.4878
187445.4284 -0.283054059 0.006178984 0.103132857 ACTB
7 5527147 5563784 - 3519 protein_coding actin
beta [Source:HGNC Symbol;Acc:HGNC:132] - 154162 191404 158356
185210 148692 192803 2118.162552 2370.22918
2100.686731 2924.537595 2597.261611 2547.566554
ENSG00000243406 46.16908469 30.45298915 48.38099059
2.204138311 0 12.92442918 41.66768814 5.04285583
3.008856692 0.006193036 1 MRPS31P5 13
52167709 52194465 - 3873
transcribed_unprocessed_pseudogene mitochondrial ribosomal
protein S31 pseudogene 5 [Source:HGNC Symbol;Acc:HGNC:39253] -
48 35 52 2 0 14 0.599231944
0.393803113 0.626760881 0.028694228 0
0.168078265
ENSG00000213553 1211.938473 1377.345195 1709.151533
1532.978195 2293.316881 2328.243599 1432.811734
2051.512892 -0.517909272 0.006226033 1 RPLP0P6 2
38481851 38482804 + 954
processed_pseudogene ribosomal protein lateral stalk subunit P0
pseudogene 6 [Source:HGNC Symbol;Acc:HGNC:36404] - 1260
1583 1837 1391 1892 2522 63.85918725
72.30879728 89.88905556 81.01973138 121.9046067
122.9214644
ENSG00000277791 2629.714115 2291.804955 2237.620815

2852.154974	2917.554827	3550.52533	2386.379962
3106.745044	-0.381016197	0.006383332	0.104696918	PSMB3
	17	38752736	38764231	+	1179
protein_coding	proteasome subunit beta 3 [Source:HGNC
Symbol;Acc:HGNC:9540]	-	2734	2634	2405	2588	2407
	3846	112.1207313	97.35551607	95.22419533
121.9726643	125.490152	151.6793619
ENSG00000171314 7043.670982	7791.614795	8487.142234
9383.016789	9930.837846	9427.447912	7774.14267
9580.434182	-0.301346569	0.00638674	0.104696918	PGAM1
	10	97426160	97433441	+	2568
protein_coding	phosphoglycerate mutase 1 [Source:HGNC
Symbol;Acc:HGNC:8888]	-	7323	8955	9122	8514	8193
	10212	137.8780813	151.9599593	165.8215935
184.2258929	196.1079933	184.9042123
ENSG00000183929 0	0	0	20.93931395	21.81802529
	0	0	14.25244642	-6.393432747	0.00644628
	1	DUSP5P1 1	228650241	228651379	+
	1139	processed_pseudogene	dual specificity phosphatase 5
pseudogene 1 [Source:HGNC Symbol;Acc:HGNC:32020]	-	0
	0	0	19	18	0	0	0	0
	0.92691929	0.971395621	0
ENSG00000070950 1185.006507	1206.808456	1042.982509
1556.121647	1471.504595	1509.388693	1144.93249
1512.338312	-0.401513441	0.006558111	1	RAD18	3
	8775402 8963773 -	6983	protein_coding	"RAD18, E3
ubiquitin protein ligase [Source:HGNC Symbol;Acc:HGNC:18278]"	-
	1232	1387	1121	1412	1214	1635	8.530409548
	8.655517061	7.493929557	11.23581807	10.68621739
	10.88696265
ENSG00000151914 6570.437864	4416.553512	6209.514061
4948.290508	4012.092429	3785.934576	5732.168479
4248.772504	0.432032642	0.006573207	0.106279634	DST
	6	56457987	56954628	-	42341
protein_coding	dystonin [Source:HGNC Symbol;Acc:HGNC:1090]	-
	6831	5076	6674	4490	3310	4101	7.80053502
	5.224191645	7.358192194	5.892468557	4.8052307
	4.503598611
ENSG00000167522 14917.42363	11150.14446	12300.86686
9336.729885	9487.204665	11185.17028	12789.47832
10003.03494	0.354374701	0.006599062	0.106279634
ANKRD11 16	89267619	89490561	-	19538
protein_coding	ankyrin repeat domain 11 [Source:HGNC
Symbol;Acc:HGNC:21316]	-	15509	12815	13221	8472	7827
	12116	38.37999441	28.58229538	31.58858962
24.09449817	24.62422602	28.83435078
ENSG00000160062 38.47423724	99.18973608	112.5788435
271.1090122	175.7563149	148.6309355	83.41427227
198.4987542	-1.249187189	0.006635559	1	ZBTB8A	1
	32539427	32605939	+	7075	protein_coding

zinc finger and BTB domain containing 8A [Source:HGNC
Symbol;Acc:HGNC:24172] ZBTB 40 114 121 246 145
161 0.273359873 0.702161487 0.798371384
1.932060433 1.259763202 1.058109101
ENSG00000033050 592.5032535 677.7965299 668.9602353
824.3477283 837.5697488 1150.274197 646.4200062
937.3972246 -0.537001305 0.006642555 1 ABCF2 7
151212490 151227230 - 4177 protein_coding
ATP binding cassette subfamily F member 2 [Source:HGNC
Symbol;Acc:HGNC:71] - 616 779 719 748 691
1246 7.130458448 8.127024709 8.035454586
9.950597656 10.16859458 13.87025946
ENSG00000227436 25.97011014 0 19.53847697 0 0
0 15.16952903 0 6.264039799 0.006661033
1 FCF1P1 7 23619333 23622826 +
571 processed_pseudogene FCF1 pseudogene 1 [Source:HGNC
Symbol;Acc:HGNC:44613] - 27 0 21 0 0
0 2.286277132 0 1.716837625 0 0
0
ENSG00000271550 26.93196607 10.44102485 17.67766964 0
0 1.846347025 18.35022019 0.615449008
4.786427338 0.006705223 1 BNIP3P11 7
64678954 64687393 - 563 processed_pseudogene
BCL2 interacting protein 3 pseudogene 11 [Source:HGNC
Symbol;Acc:HGNC:49724] - 28 12 19 0 0
2 2.404644351 0.928819766 1.575401454 0
0 0.165178158
ENSG00000106086 796.4167108 736.9623374 674.5426573
598.4235514 455.7543061 436.6610715 735.9739018
496.9463097 0.566900983 0.006706638 1 PLEKHA8 7
30027404 30130483 + 10886 protein_coding
pleckstrin homology domain containing A8 [Source:HGNC
Symbol;Acc:HGNC:30037] - 828 847 725 543 376
473 3.677589267 3.390577519 3.108964138
2.771682905 2.123082522 2.020337053
ENSG00000120437 516.5166349 421.1213357 529.3996855
873.9408402 938.1750876 516.0539936 489.012552
776.0566405 -0.665327036 0.006748619 1 ACAT2 6
159760328 159779055 + 4299 protein_coding
acetyl-CoA acetyltransferase 2 [Source:HGNC
Symbol;Acc:HGNC:94] - 537 484 569 793 774
559 6.03959834 4.906101335 6.178611055
10.24985651 11.06676979 6.046100765
ENSG00000224543 3.847423724 10.44102485 42.7985686
1.102069155 0 0.923173513 19.02900572 0.675080889
4.777728422 0.006748754 1 SNRPGP15 19
14489388 14489609 + 222
processed_pseudogene small nuclear ribonucleoprotein polypeptide G
pseudogene 15 [Source:HGNC Symbol;Acc:HGNC:49371] - 4
12 46 1 0 1 0.871180676

2.355520398	9.67277071	0.250298974	0	0.209448881
ENSG00000106399 719.4682363	875.3059167	906.2131699
1272.889875	1164.840128	1039.493375	833.662441
1159.074459	-0.474889141	0.006752474	1	RPA3	7
7636518 7718607 -	3232	protein_coding	replication
protein A3 [Source:HGNC Symbol;Acc:HGNC:10291]	-	748	1006
974	1155	961	1126	11.19003545	13.56392021
14.06804149	19.85741333	18.27676786	16.19936745
ENSG00000203588 13.46598303	11.31111026	10.23444032	0
0	0	11.6705112	0	5.886540439
0.00681291	1	IGBP1-AS1	X	70163842
70165206	-	542	antisense	IGBP1 antisense RNA 1
[Source:HGNC Symbol;Acc:HGNC:40295]	-	14	13	11
0	0	0	1.248906614	1.045207852
0.947413207	0	0	0
ENSG00000134057 841.6239396	912.7195891	988.0886924
1471.262322	1138.173653	1177.046229	914.1440737
1262.160735	-0.465318828	0.006845881	1	CCNB1	5
69167010	69178245	+	2558	protein_coding
cyclin B1 [Source:HGNC Symbol;Acc:HGNC:1579]	-	875
1049	1062	1335	939	1275	16.53898028
17.87037005	19.3807247	28.99965086	22.56380887
23.17611637
ENSG00000002822 3147.192606	2797.324575	2897.277013
3157.42813	3799.972739	4926.977037	2947.264731
3961.459302	-0.427027694	0.006885752	0.109932521	MAD1L1
7	1815793 2233243 -	7230	protein_coding
mitotic arrest deficient 1 like 1 [Source:HGNC Symbol;Acc:HGNC:6762]
-	3272	3215	3114	2865	3135	5337
21.88145587	19.3776576	20.10603095	22.01903961
26.65303122	34.32337013
ENSG00000138629 2002.584048	1528.740055	1752.880505
1919.804469	2584.223885	2721.515516	1761.401536
2408.514623	-0.451737643	0.006933665	1	UBL7	15
74445977	74461182	-	1885	protein_coding
ubiquitin like 7 [Source:HGNC Symbol;Acc:HGNC:28221]	-
2082	1757	1884	1742	2132	2948	53.40360649
40.6180439	46.65686776	51.35099224	69.52215186
72.71887354
ENSG00000134278 1061.888948	1024.090521	839.224106
731.7739192	635.1469585	714.5362989	975.0678582
693.8190589	0.490528195	0.006936551	1	SPIRE1	18
12446512	12658134	-	6532	protein_coding
spire type actin nucleation factor 1 [Source:HGNC
Symbol;Acc:HGNC:30622]	-	1104	1177	902	664	524
774	8.17192014	7.852155373	6.446238911
5.648510585	4.930971258	5.509672728
ENSG00000100739 18.27526269	9.570939447	25.12089896
37.47035128	135.7566018	42.46598159	17.65570036
71.8976449	-2.024707017	0.006942041	1	BDKRB1	14

96255824	96268967	+	2758	protein_coding
bradykinin receptor B1 [Source:HGNC Symbol;Acc:HGNC:1029]
-	19	11	27	34	112	46
0.333089203	0.173802901	0.456999303	0.685009665
2.496152537	0.775522831
ENSG00000102977 1246.565287	1271.194776	1119.275609
1528.569919	1656.95781	1584.165748	1212.345224
1589.897825	-0.390974429	0.006945586	1	ACD	16
67657512	67660815	-	3223	protein_coding
"ACD, shelterin complex subunit and telomerase recruitment
factor [Source:HGNC Symbol;Acc:HGNC:25070]"	-	1296	1461
1203	1387	1367	1716	19.44222266	19.75370247
17.42414023	23.91267708	26.07087271	24.75642878
ENSG00000267672 10.58041524	13.05128106	11.16484398	0
0	0	11.59884676	0	5.877940186
0.006999542	1	AC010632.2	19	37091341
37092564	+	1224	sense_intronic	novel transcript
-	11	15	12	0	0	0
0.434522714	0.534033423	0.457663064	0	0	0
ENSG00000163479 4789.08068	5356.245749	5374.01157
5934.642402	6858.132617	6427.133996	5173.112666
6406.636338	-0.3084335	0.007013043	0.110999536	SSR2
1	156009048	156020959	-	6651
protein_coding	signal sequence receptor subunit 2 [Source:HGNC
Symbol;Acc:HGNC:11324]	-	4979	6156	5776	5385	5658
6962	36.19565124	40.33390407	40.540236
44.98946238	52.29056754	48.67187637
ENSG00000204681 266.4340929	274.0769023	277.2602922
195.0662405	103.0295639	141.2455474	272.5904291
146.4471173	0.895340856	0.007026294	1	GABBR1	6
29555629	29633976	-	8470	protein_coding
gamma-aminobutyric acid type B receptor subunit 1 [Source:HGNC
Symbol;Acc:HGNC:4070]	-	277	315	298	177	85
153	1.581239211	1.620636968	1.642399823
1.16118629	0.616854696	0.839922158
ENSG00000198133 20.19897455	20.0119643	23.26009163
58.40966524	58.18140078	88.62465722	21.15701016
68.40524108	-1.694913314	0.007047309	1	TMEM229B
14	67447084	67533739	-	6377
protein_coding	transmembrane protein 229B [Source:HGNC
Symbol;Acc:HGNC:20130]	-	21	23	25	53	48
96	0.159222374	0.157170132	0.183007811
0.461818681	0.462670895	0.699980327
ENSG00000237452 0	0	0	0	27.87858788
13.84760269	0	13.90873019	-6.359628595	0.00704843
1	BHMG1	19	45733251	45764534	+
2749	protein_coding	basic helix-loop-helix and HMG-box
containing 1 [Source:HGNC Symbol;Acc:HGNC:44318]	HMG	0
0	0	0	23	15	0	0	0
0	0.514280972	0.253715814

ENSG00000236829 32.70310165 33.06324536 35.35533928
84.85932497 129.6960392 66.46849292 33.70722876
93.67461904 -1.472274875 0.007058427 1 Z97634.1
16 382097 392960 + 2291
transcribed_processed_pseudogene uncharacterized LOC100134368
[Source:NCBI gene;Acc:100134368] - 34 38 38
77 107 72 0.717554751 0.722798271
0.774291592 1.8675734 2.870820565 1.461296773
ENSG00000005379 292.404203 304.5298915 387.9783284
145.4731285 219.3923655 200.3286523 328.304141
188.3980487 0.801477325 0.007091498 1 TSPOAP1 17
58301228 58328760 - 11354 protein_coding
TSPO associated protein 1 [Source:HGNC Symbol;Acc:HGNC:16831]
- 304 350 417 132 181 217
1.294571108 1.343314653 1.71448307 0.646006793
0.979889367 0.888672748
ENSG00000049541 879.1363209 1164.174271 1089.502692
1483.385083 1436.353332 1341.371114 1044.271095
1420.369843 -0.443295745 0.007099143 1 RFC2 7
74231499 74254458 - 2211 protein_coding
replication factor C subunit 2 [Source:HGNC
Symbol;Acc:HGNC:9970] - 914 1338 1171 1346 1185
1453 19.98750888 26.37096174 24.72374117
33.82737991 32.94404845 30.55680128
ENSG00000213366 132.7361185 174.8871663 210.2712283
54.00138862 94.54477627 89.54783073 172.6315044
79.36466521 1.121193059 0.007106201 1 GSTM2 1
109668022 109709551 + 9116 protein_coding
glutathione S-transferase mu 2 [Source:HGNC
Symbol;Acc:HGNC:4634] - 138 201 226 49 78
97 0.731940851 0.960838372 1.157311206
0.298678394 0.525941749 0.494764392
ENSG00000213592 20.19897455 8.700854042 6.512825656 0
0 0 11.80421808 0 5.902465191
0.007113034 1 PPIAP42 11 57718044 57718530
- 487 processed_pseudogene peptidylprolyl
isomerase A pseudogene 42 [Source:HGNC Symbol;Acc:HGNC:53666] -
21 10 7 0 0 0 2.084930344
0.894807543 0.670988558 0 0 0
ENSG00000164078 16.35155083 3.480341617 26.05130263
1.102069155 0 0 15.29439836 0.367356385
5.314166413 0.007153242 1 MST1R 3 49887002
49903873 - 5252 protein_coding macrophage
stimulating 1 receptor [Source:HGNC Symbol;Acc:HGNC:7381] -
17 4 28 1 0 0 0.156503992
0.033188977 0.248873898 0.01058004 0 0
ENSG00000224982 0 0 0 0 26.66647536
14.7707762 0 13.81241719 -6.349841819 0.007202277
1 TMEM233 12 119593459 119643066 +
3146 protein_coding transmembrane protein 233 [Source:HGNC

Symbol;Acc:HGNC:37219]	-	0	0	0	0	22
	16	0	0	0	0	0.429844449
0.236478838
ENSG00000101493 347.2299911	321.0615142	301.4507875
224.8221077	119.9991391	195.7127847	323.2474309
180.1780105	0.841245401	0.007260048	1	ZNF516	18
	76358190	76495190	-	8217	protein_coding
	zinc finger protein 516 [Source:HGNC Symbol;Acc:HGNC:28990]
	zf-C2H2 361	369	324	204	99	212
2.124198664	1.956913715	1.840677735	1.379522932
0.740575376	1.199647331
ENSG00000171488 612.702228	649.953797	659.6561986
946.6774045	927.266075	802.2377826	640.7707412
892.0604207	-0.476724529	0.007268361	1	LRRC8C	1
	89633072	89769903	+	11724	protein_coding
	leucine rich repeat containing 8 VRAC subunit C [Source:HGNC
Symbol;Acc:HGNC:25075]	-	637	747	709	859	765
	869	2.627028831	2.776536518	2.823036292
4.071265244	4.010818279	3.446473827
ENSG00000180044 54.82578806	44.37435562	58.61543091
141.0648519	116.3628016	108.9344745	52.60519153
122.1207093	-1.215287088	0.007350673	1	C3orf80 3
	160225496	160228213	+	2718	protein_coding
	chromosome 3 open reading frame 80 [Source:HGNC
Symbol;Acc:HGNC:40048]	-	57	51	63	128	96
	118	1.013973535	0.817672368	1.082024596
2.61681223	2.171046577	2.018661838
ENSG00000133624 100.0330168	122.682042	120.0220728
46.28690453	54.54506323	43.3891551	114.2457105
48.07370762	1.251019574	0.007395675	1	ZNF767P 7
	149547154	149624752	-	4012
transcribed_unprocessed_pseudogene	"zinc finger family member
767, pseudogene [Source:HGNC Symbol;Acc:HGNC:21884]"	-	104
	141	129	42	45	47	1.253353654
1.531499241	1.500979711	0.581701803	0.689443926
0.544713265
ENSG00000183023 18.27526269	24.36239132	26.05130263
94.77794737	46.06027562	79.39292209	22.89631888
73.41038169	-1.681482259	0.007436654	1	SLC8A1	2
	40097270	40611053	-	21922	protein_coding
	solute carrier family 8 member A1 [Source:HGNC
Symbol;Acc:HGNC:11068]	-	19	28	28	86	38
	86	0.041905849	0.055659135	0.059624382
0.217986863	0.106549345	0.182410274
ENSG00000279267 130.8124066	214.0410094	144.2125681
14.32689902	92.12055124	65.5453194	163.0219947
57.33092322	1.509438611	0.007488552	1	AL078621.3
	2	113605867	113607908	-	2042	TEC
	TEC	-	136	246	155	13	76	71
	3.220211429	5.249742081	3.543414812	0.353752614

2.287732357	1.616715601
ENSG00000258102 26.93196607	41.7640994	81.87552254
135.5545061	141.8171644	113.5503421	50.19052934
130.3073375	-1.375570384	0.007590974	1	MAP1LC3B2
12	116548105	116576448	+	1767
protein_coding	microtubule associated protein 1 light chain 3 beta 2
[Source:HGNC Symbol;Acc:HGNC:34390]	-	28	48	88
123	117	123	0.766165687	1.183758977
2.324835124	3.86794781	4.07002121	3.236678627
ENSG00000116237 3468.452487	3268.040778	3563.446038
3968.551029	4180.576069	4745.111855	3433.313101
4298.079651	-0.324361149	0.007604673	0.119334874	ICMT
1	6221193 6235972 -	4910	protein_coding
isoprenylcysteine carboxyl methyltransferase [Source:HGNC
Symbol;Acc:HGNC:5350]	-	3606	3756	3830	3601	3449
5140	35.50957274	33.33517115	36.41357489
40.75244528	43.17765605	48.67574923
ENSG00000173698 12.5041271	9.570939447	12.09524765	0
0	0	11.39010473	0	5.851351756
0.007647326	1	ADGRG2	X	18989309	19122637
-	10155	protein_coding	adhesion G protein-coupled
receptor G2 [Source:HGNC Symbol;Acc:HGNC:4516]	-	13	11
13	0	0	0	0.061896293	0.047203191
0.059759845	0	0	0
ENSG00000244560 119.2701354	68.73674694	173.0550817
27.55172889	38.78760052	63.69897238	120.353988
43.3461006	1.468126736	0.007663734	1	AC004890.2
7	149285281	149297312	+	4288
transcribed_unprocessed_pseudogene	AI894139 pseudogene
[Source:NCBI gene;Acc:155060]	-	124	79	186	25
32	69	1.398196224	0.802843531	2.024902905
0.32396439	0.458714599	0.748213142
ENSG00000119943 102.9185846	94.83930906	89.31875186
14.32689902	25.45436284	53.54406374	95.69221518
31.10844187	1.612689987	0.007731882	1	PYROXD2 10
98383565	98415184	-	3867	protein_coding
pyridine nucleotide-disulphide oxidoreductase domain 2
[Source:HGNC Symbol;Acc:HGNC:23517]	-	107	109	96
13	21	58	1.337860471	1.228318304
1.158892352	0.186801872	0.333804727	0.697404651
ENSG00000223745 401.0939232	318.451258	378.6742917
167.5145116	101.8174514	294.4923506	366.0731576
187.9414379	0.957600995	0.007734162	1	CCDC18-AS1
1	93262186	93346025	-	4786
processed_transcript	CCDC18 antisense RNA 1 [Source:HGNC
Symbol;Acc:HGNC:52262]	-	417	366	407	152	84
319	4.212739232	3.332475681	3.969791987
1.764748971	1.078832328	3.099195744
ENSG00000067560 10624.66061	10434.93425	8630.424398
12322.23523	13254.45037	11379.95989	9896.673088

12318.88183 -0.315766437 0.00774073 0.120440516 RHOA
3 49359145 49412998 - 2582
protein_coding ras homolog family member A [Source:HGNC
Symbol;Acc:HGNC:667] - 11046 11993 9276 11181 10935
12327 206.8473767 202.4091744 167.7067481
240.6226211 260.321423 221.9893687
ENSG00000181227 39.43609317 26.10256213 28.84251362
5.510345777 1.212112516 6.462214589 31.46038964
4.394890961 2.820062784 0.00775341 1 DLSTP1 1
75743423 75744776 - 1354
processed_pseudogene dihydrolipoamide S-succinyltransferase
pseudogene 1 [Source:HGNC Symbol;Acc:HGNC:2912] - 41 30
31 5 1 7 1.464085397 0.965519808
1.068781838 0.205193398 0.045397161 0.240386677
ENSG00000258754 10.58041524 17.40170808 6.512825656 0
0 0 11.49831633 0 5.865758881
0.007763839 1 LINC01579 15 93718542
94070820 - 6795 lincRNA long intergenic non-protein
coding RNA 1579 [Source:HGNC Symbol;Acc:HGNC:27519] - 11
20 7 0 0 0 0.078271641
0.128262332 0.048089982 0 0 0
ENSG00000060558 6.732991517 21.75213511 6.512825656 0
0 0 11.66598409 0 5.887142118
0.007800959 1 GNA15 19 3136193 3163769 + 2613
protein_coding G protein subunit alpha 15 [Source:HGNC
Symbol;Acc:HGNC:4383] - 7 25 7 0 0
0 0.129526863 0.416926209 0.125056038 0
0 0
ENSG00000130402 12241.54043 11895.80765 14545.9309
17460.08163 15353.82924 15151.12369 12894.42633
15988.34485 -0.310269431 0.00780744 0.120457649 ACTN4
19 38647649 38731583 + 6098
protein_coding actinin alpha 4 [Source:HGNC Symbol;Acc:HGNC:166]
- 12727 13672 15634 15843 12667 16412
100.9113092 97.70194903 119.6819684 144.3650434
127.683186 125.1425806
ENSG00000102978 3356.877199 3717.874932 3255.482424
3875.97722 4462.998285 4666.642107 3443.411519
4335.20587 -0.332295766 0.007962212 0.121821849 POLR2C
16 57462387 57472010 + 3940
protein_coding RNA polymerase II subunit C [Source:HGNC
Symbol;Acc:HGNC:9189] - 3490 4273 3499 3517 3682
5055 42.82825912 47.26016883 41.45660719
49.60074392 57.44271025 59.65625085
ENSG00000069020 1164.807532 879.6563437 1000.18394
717.4470202 762.4187728 709.9204313 1014.882605
729.9287414 0.475497585 0.00806379 1 MAST4 5
66596361 67169595 + 14675 protein_coding
microtubule associated serine/threonine kinase family member 4
[Source:HGNC Symbol;Acc:HGNC:19037] - 1211 1011 1075

651	629	769	3.989948129	3.002144856
3.419608518	2.464988641	2.634631595	2.436571993
ENSG00000132341 14033.47803	15361.35781	13601.57118
15838.9379	18552.59417	18056.35074	14332.13568
17482.6276	-0.286645932	0.008118367	0.123176705	RAN
12	130871879	130877678	+	4825
protein_coding	"RAN, member RAS oncogene family [Source:HGNC
Symbol;Acc:HGNC:9846]"	-	14590	17655	14619	14372	15306
19559	146.2039785	159.4516442	141.4380904
165.5129329	194.9897361	188.4865421
ENSG00000207971 8.656703379	6.960683234	18.6080733	0
0	0	11.40848664	0	5.853629285
0.0081479	1	MIR125B1	11	122099757
122099844	-	88	miRNA	microRNA 125b-1 [Source:HGNC
Symbol;Acc:HGNC:31506]	-	9	8	20	0	0
0	4.944940312	3.961557032	10.60946194	0
0	0
ENSG00000130706 7516.9041	6152.373893	6180.671548
6811.88945	9196.297661	10004.43136	6616.649847
8670.872823	-0.390189657	0.008184944	0.123176705	ADRM1
20	62302093	62308862	+	2142
protein_coding	adhesion regulating molecule 1 [Source:HGNC
Symbol;Acc:HGNC:15759]	-	7815	7071	6643	6181	7587
10837	176.4049357	143.8533462	144.7740827
160.3434858	217.7198256	235.2451211
ENSG00000085840 730.0486516	678.6666153	842.9457207
1158.274682	849.6908739	1200.125567	750.5536625
1069.363708	-0.511585638	0.008196324	1	ORC1	1
52372829	52404459	-	3157	protein_coding
origin recognition complex subunit 1 [Source:HGNC
Symbol;Acc:HGNC:8487]	-	759	780	906	1051	701
1300	11.62434285	10.76660099	13.39675613
18.49865606	13.64868455	19.14695817
ENSG00000184076 248.1588302	181.8478495	273.5386776
382.4179969	403.6334679	365.576711	234.5151191
383.8760586	-0.711214604	0.008225365	1	UQCR10	22
29767369	29770413	+	975	protein_coding
"ubiquinol-cytochrome c reductase, complex III subunit X
[Source:HGNC Symbol;Acc:HGNC:30863]"	-	258	209	294
347	333	396	12.79429343	9.341148325
14.07630766	19.77592939	20.99360289	18.88519999
ENSG00000130717 1092.668338	1344.28195	1204.872746
1439.302317	1615.745984	1831.576249	1213.941011
1628.87485	-0.42434299	0.008242578	1	UCK1	9
131523801	131531268	-	2688	protein_coding
uridine-cytidine kinase 1 [Source:HGNC Symbol;Acc:HGNC:14859]
-	1136	1545	1295	1306	1333	1984
20.43385388	25.04712119	22.48984902	26.99764959
30.48233595	34.31969515
ENSG00000114626 634.8249144	594.2683311	703.3851709

349.3559223	487.2692316	458.8172358	644.1594721
431.8141299	0.57700011	0.00825032	1	ABTB1	3
	127672935	127680920	+	6149	protein_coding
	ankyrin repeat and BTB domain containing 1 [Source:HGNC
Symbol;Acc:HGNC:18275]	-	660	683	756	317	402
	497	5.189680947	4.840328181	5.739358304
2.864618635	4.01854578	3.758226182
ENSG00000177363 334.725864	305.3999769	358.2054111
157.5958892	255.7557409	164.3248853	332.777084
192.5588385	0.791483061	0.008395829	1	LRRN4CL 11
	62686402	62689899	-	2585	protein_coding
	LRRN4 C-terminal like [Source:HGNC Symbol;Acc:HGNC:33724]
	-	348	351	385	143	211	178
6.509084552	5.917049014	6.952583572	3.07388442
5.01729074	3.201772521
ENSG00000133265 3319.364818	2813.856197	3258.273635
3451.680595	4236.333244	4335.222816	3130.498217
4007.745552	-0.356568254	0.00840718	0.123753357	HSPBP1
	19	55262231	55280381	-	2159
protein_coding	HSPA (Hsp70) binding protein 1 [Source:HGNC
Symbol;Acc:HGNC:24989]	-	3451	3234	3502	3132	3495
	4696	77.28470142	65.2748633	75.71981344
80.60855847	99.50431125	101.1361609
ENSG00000104853 4165.798037	4098.102254	4404.530951
5241.440903	5379.355347	4879.895188	4222.810414
5166.897146	-0.290958955	0.008418361	0.123753357	CLPTM1
	19	44954585	44993341	+	5229
protein_coding	"CLPTM1, transmembrane protein [Source:HGNC
Symbol;Acc:HGNC:2087]"	-	4331	4710	4734	4756	4438
	5286	40.04707106	39.25191621	42.26254514
50.54000118	52.16942094	47.00451058
ENSG00000012963 1348.522015	1832.399861	1502.601919
2183.198997	2009.682552	2008.825564	1561.174599
2067.235704	-0.404776518	0.008422856	1	UBR7	14
	93207056	93229215	+	3694	protein_coding
	ubiquitin protein ligase E3 component n-recognin 7 (putative)
[Source:HGNC Symbol;Acc:HGNC:20344]	-	1402	2106	1615
	1981	1658	2176	18.35068748	24.84391722
20.40899745	29.79885851	27.58893877	27.39006217
ENSG00000214485 2806.695607	3462.939909	2825.635931
3976.265513	3757.548801	3774.856494	3031.757149
3836.223602	-0.339381522	0.008425474	0.123753357	RPL7P1
	5	150094302	150095048	+	747
processed_pseudogene	ribosomal protein L7 pseudogene 1 [Source:HGNC
Symbol;Acc:HGNC:10367]	-	2918	3980	3037	3608	3100
	4089	188.8712707	232.1780012	189.7886453
268.3848339	255.087074	254.5232891
ENSG00000128185 1203.28177	1265.974263	1190.916691
1265.17539	1886.047075	1921.12408	1220.057575
1690.782182	-0.470841542	0.008460228	1	DGCR6L	22

20314276 20320080 - 1154 protein_coding
DiGeorge syndrome critical region gene 6 like [Source:HGNC
Symbol;Acc:HGNC:18551] - 1251 1455 1280 1148 1556
2081 52.41465329 54.9434318 51.77858724
55.27746559 82.88026749 83.84888455
ENSG00000085382 547.2960247 525.5315842 388.908732
317.3959168 327.2703794 296.3386976 487.245447
313.6683312 0.635956042 0.008473099 1 HACE1 6
104728093 104859919 - 9238 protein_coding
HECT domain and ankyrin repeat containing E3 ubiquitin protein
ligase 1 [Source:HGNC Symbol;Acc:HGNC:21033] - 569 604
418 288 270 321 2.978074274 2.849164854
2.112245335 1.732313834 1.796524592 1.615690207
ENSG00000279488 7.694847448 0 36.28574294 0 0
0 14.6601968 0 6.215192836 0.008549944
1 AC004623.1 19 1372238 1374364 - 2127
TEC TEC - 8 0 39 0 0
0 0.181854358 0 0.855939666 0 0
0
ENSG00000067225 49989.57644 52453.96868 61308.01911
64284.7959 70930.40023 64502.13333 54583.85475
66572.44316 -0.286439849 0.008588005 0.124186412 PKM
15 72199029 72231822 - 8571
protein_coding pyruvate kinase M1/2 [Source:HGNC
Symbol;Acc:HGNC:9021] - 51972 60286 65894 58331 58518
69870 293.1832476 306.5092404 358.8892189
378.1638148 419.6675019 379.0445584
ENSG00000134250 13321.70464 11270.21624 12269.23313
10961.17982 8472.666489 10009.9704 12287.05134
9814.605569 0.324026102 0.008590237 0.124186412 NOTCH2
1 119911553 120069626 - 16807
protein_coding notch 2 [Source:HGNC Symbol;Acc:HGNC:7882] -
13850 12953 13187 9946 6990 10843 39.84380352
33.58449043 36.62704162 32.88291414 25.56432534
29.99786013
ENSG00000264885 292.404203 100.9299069 155.3774121
52.89931946 64.24196336 108.9344745 182.9038407
75.35858578 1.274986598 0.00859337 1 AC026271.3
17 18667629 18669461 - 1833
sense_intronic novel transcript - 304 116 167
48 53 118 8.018854532 2.757745103
4.253045628 1.455093216 1.777300098 2.99330217
ENSG00000127957 151.9732371 91.35896745 81.87552254
24.24552142 65.45407588 12.92442918 108.4025757
34.20800883 1.6735325 0.008607177 1 PMS2P3 7
75502930 75528148 - 1727
transcribed_unprocessed_pseudogene "PMS1 homolog 2, mismatch
repair system component pseudogene 3 [Source:HGNC
Symbol;Acc:HGNC:9128]" - 158 105 88 22 54
14 4.423499331 2.649448971 2.378681914

0.707851875	1.921979639	0.376935218
ENSG00000095564 1968.919091	1548.75202	1717.525166
1389.709205	1123.628303	1389.376137	1745.065425
1300.904548	0.423072896	0.008609739	1	BTAF1	10
	91923769	92030325	+	7582	protein_coding
	B-TFIID TATA-box binding protein associated factor 1
[Source:HGNC Symbol;Acc:HGNC:17307]	-	2047	1780	1846
	1261	927	1505	13.05374964	10.23045195
11.36564148	9.241518774	7.515247958	9.229618242
ENSG00000116883 43.28351689	70.47691774	81.87552254
24.24552142	8.484787614	21.23299079	65.21198572
17.98776661	1.852213813	0.008672625	1	AL591845.1
	1	36323734	36329221	+	1838
antisense	"novel transcript, antisense to EVA1B"	-	45
	81	88	22	7	23	1.183772436
1.920428355	2.235029197	0.665103476	0.23409918
0.581853093
ENSG00000138834 1146.53227	855.2939524	1379.788635
675.5683923	753.9339851	888.0929192	1127.204953
772.5317656	0.544287629	0.008740798	1	MAPK8IP3
	16	1706183 1770317 +	10285	protein_coding
mitogen-activated protein kinase 8 interacting protein 3 [Source:HGNC
Symbol;Acc:HGNC:6884]	-	1192	983	1483	613	622
	962	5.603678053	4.164931083	6.73105115
3.311831421	3.717349963	4.349124039
ENSG00000268575 180.828915	207.0803262	169.3334671
111.3089847	44.8481631	100.6259129	185.7475694
85.59435356	1.114429333	0.008758535	1	AL031282.2
	1	1702736 1737688 -	5079	processed_transcript
	"novel transcript, readthrough between SLC35E2 and CDK11A"
	-	188	238	182	101	37	109
1.789702534	2.042007543	1.672781478	1.104982003
0.447786371	0.997882263
ENSG00000185689 17.31340676	7.830768638	8.373632987	0
	0	0	11.17260279	0	5.823164387
0.008787323	1	C6orf201	6	4079206 4130951 +
	3322	protein_coding	chromosome 6 open reading frame 201
[Source:HGNC Symbol;Acc:HGNC:21620]	-	18	9	9
	0	0	0	0.261983593	0.118059647
0.126470407	0	0	0
ENSG00000177990 179.8670591	84.39828421	183.289522
70.53242595	58.18140078	65.5453194	149.1849551
64.75304871	1.202737347	0.008788519	1	DPY19L2 12
	63558913	63668939	-	5646	protein_coding
	dpy-19 like 2 [Source:HGNC Symbol;Acc:HGNC:19414]	-
	187	97	197	64	48	71	1.601407836
	0.748668323	1.628813605	0.629870319	0.52257391
	0.584720733
ENSG00000280035 10.58041524	7.830768638	14.88645864	0
	0	0	11.09921417	0	5.813969042

0.008812508	1	AC011676.5	8	141224288
141226947	-	2660	TEC	TEC	-	11	9
	16	0	0	0	0.19994579	0.147441408
	0.280791775	0	0	0
ENSG00000131462 1647.65921	1660.122951	1915.701147
2168.872098	2558.769522	2072.524536	1741.161103
2266.722052	-0.380165261	0.008814614	1	TUBG1	17
	42609676	42615234	+	2411	protein_coding
	tubulin gamma 1 [Source:HGNC Symbol;Acc:HGNC:12417]	-
	1713	1908	2059	1968	2111	2245	34.35273894
	34.48575653	39.86623037	45.35654106	53.81934192
	43.29623709
ENSG00000149806 5606.658221	5729.512387	7595.815523
7862.161355	7821.762068	8409.187527	6310.662044
8031.036983	-0.34786052	0.008829227	0.126644221	FAU
	11	65120628	65122473	-	1846
protein_coding	"FAU, ubiquitin like and ribosomal protein S30 fusion
[Source:HGNC Symbol;Acc:HGNC:3597]"	-	5829	6585	8164
	7134	6453	9109	152.6734695	155.4471201
206.4511637	214.7402488	214.8707643	229.4404699
ENSG00000213999 38.47423724	21.75213511	12.09524765	0
	4.848450065	0	24.10720666	1.616150022
3.961948501	0.008833317	1	MEF2B	19	19145568
	19170289	-	1848	protein_coding	myocyte
enhancer factor 2B [Source:HGNC Symbol;Acc:HGNC:6995]	SRF	40
	25	13	0	4	0	1.046548214
0.589517416	0.328388108	0	0.133047091	0
ENSG00000053900 544.4104569	730.0016542	654.0737766
542.2180245	345.4520671	345.2668938	642.8286292
410.9789951	0.645894542	0.008837082	1	ANAPC4	4
	25377213	25418498	+	7494	protein_coding
	anaphase promoting complex subunit 4 [Source:HGNC
Symbol;Acc:HGNC:19990]	-	566	839	703	492	285
	374	3.651774561	4.878730965	4.379128328
3.648072474	2.337644852	2.32053932
ENSG00000275882 366.4671097	54.81538047	31.63372462
25.34759057	44.8481631	50.7745432	150.9720716
40.32343229	1.902819971	0.008849654	1	IKBKGP1 X
	154639978	154648275	-	1073
unprocessed_pseudogene	inhibitor of nuclear factor kappa B kinase
subunit gamma pseudogene 1 [Source:HGNC Symbol;Acc:HGNC:24455]	-
	381	63	34	23	37	55	17.16826745
	2.558582501	1.479194321	1.191077876	2.119577801
	2.383383814
ENSG00000285756 17.31340676	18.27179349	0.930403665	0
	0	0	12.17186797	0	5.947574799
0.008918013	1	BX890604.2	X	3817528 3937855 -
	14235	lincRNA novel transcript	-	18	21
	1	0	0	0	0.061138707	0.064286595
	0.003279356	0	0	0

ENSG00000142513 50.97836434 10.44102485 38.14655027 0
8.484787614 0 33.18864649 2.828262538
3.59762167 0.008958455 1 ACP4 19 50790415
50795224 + 1347 protein_coding acid
phosphatase 4 [Source:HGNC Symbol;Acc:HGNC:14376] - 53
12 41 0 7 0 1.902433524
0.388214943 1.420896017 0 0.319431547 0
ENSG00000129682 7.694847448 39.15384319 35.35533928
52.89931946 119.9991391 106.164954 27.40134331
93.02113751 -1.760698604 0.008969113 1 FGF13 X
138614731 139222777 - 20681 protein_coding
fibroblast growth factor 13 [Source:HGNC Symbol;Acc:HGNC:3670]
- 8 45 38 48 99 115
0.018703362 0.094819918 0.085774481 0.128967935
0.294246307 0.25855761
ENSG00000253311 0 0 0 15.42896818 8.484787614
7.385388102 0 10.43304796 -5.945530081
0.008979076 1 LINC01847 5 159698586
159937766 - 7553 antisense long intergenic non-
protein coding RNA 1847 [Source:HGNC Symbol;Acc:HGNC:52662] -
0 0 0 14 7 8 0 0
0 0.102996056 0.056967337 0.049249465
ENSG00000117632 4419.728003 5915.710663 5403.784487
7047.732249 6986.616544 6009.859568 5246.407718
6681.402787 -0.3486018 0.009012441 0.127724303 STMN1
1 25884181 25906991 - 6261
protein_coding stathmin 1 [Source:HGNC Symbol;Acc:HGNC:6510] -
4595 6799 5808 6395 5764 6510 35.48485447
47.32165611 43.3040923 56.75562214 56.58842786
48.34686332
ENSG00000185278 1124.409583 877.0460875 803.8687667
715.2428819 665.4497715 580.6761395 935.1081458
653.7895976 0.516689837 0.009014186 1 ZBTB37 1
173868082 173903549 + 19464 protein_coding
zinc finger and BTB domain containing 37 [Source:HGNC
Symbol;Acc:HGNC:28365] ZBTB 1169 1008 864 649 549
629 2.903913206 2.256768618 2.072180977
1.852783372 1.73375453 1.502621393
ENSG00000204248 108.6897202 44.37435562 56.75462358 0
15.75746271 24.00251133 69.93956646 13.25332468
2.39212078 0.0090346 1 COL11A2 6
33162681 33192499 - 6740 protein_coding
collagen type XI alpha 2 chain [Source:HGNC Symbol;Acc:HGNC:2187]
- 113 51 61 0 13 26
0.810624571 0.329737907 0.422489553 0 0.118557987
0.179367795
ENSG00000168939 58.67321179 92.22905285 108.8572288
120.1255379 193.9380026 248.3336749 86.58649782
187.4657385 -1.114916311 0.009042724 1 SPRY3 X
155767812 155782459 + 9019 protein_coding

sprouty RTK signaling antagonist 3 [Source:HGNC Symbol;Acc:HGNC:11271] - 61 106 117 109 160 269 0.327018758 0.512160494 0.605582771 0.671552785 1.090458033 1.386835376
ENSG00000136271 6367.486263 5419.761983 5199.095681 5886.151359 7595.097027 8680.60054 5662.114642 7387.282975 -0.383856531 0.00904366 0.127724303 DDX56 7 44565417 44575051 - 4068 protein_coding DEAD-box helicase 56 [Source:HGNC Symbol;Acc:HGNC:18193] - 6620 6229 5588 5341 6266 9403 78.68252016 66.72613724 64.12413046 72.95476744 94.67968547 107.4772412
ENSG00000252743 0 0 2.791210996 7.714484088 25.45436284 24.00251133 0.930403665 19.05711942 -4.362883033 0.009155585 1 RNU6-850P 6 31756951 31757053 - 103 snRNA "RNA, U6 small nuclear 850, pseudogene [Source:HGNC Symbol;Acc:HGNC:47813]" - 0 0 3 7 21 26 0 0 1.359659201 3.776355392 12.53226098 11.73727125
ENSG00000239306 1882.352057 1505.247749 1870.111367 2054.256906 2432.70982 2387.326704 1752.570391 2291.431143 -0.386973746 0.009159678 1 RBM14 11 66616582 66627347 + 3937 protein_coding RNA binding motif protein 14 [Source:HGNC Symbol;Acc:HGNC:14219] - 1957 1730 2010 1864 2007 2586 24.03403157 19.1486996 23.83288835 26.30828493 31.3349725 30.54176449
ENSG00000163481 870.4796175 948.3930906 879.2314636 994.0663782 1307.869405 1434.611639 899.3680572 1245.515807 -0.470022286 0.009162733 1 RNF25 2 218663864 218672411 - 2323 protein_coding ring finger protein 25 [Source:HGNC Symbol;Acc:HGNC:14662] - 905 1090 945 902 1079 1554 18.83651631 20.44729609 18.99016047 21.5759224 28.55088634 31.10518744
ENSG00000144554 401.0939232 415.0307378 365.6486404 922.4318831 512.7235944 481.8965736 393.9244338 639.0173504 -0.697543995 0.009214089 1 FANCD2 3 10026414 10101930 + 8378 protein_coding FA complementation group D2 [Source:HGNC Symbol;Acc:HGNC:3585] - 417 477 393 837 423 522 2.406561228 2.481056308 2.189768631 5.551331288 3.103468713 2.897084517
ENSG00000239264 396.2846435 395.8888589 544.2861441 300.8648794 190.3016651 312.0326473 445.4865489 267.7330639 0.732627843 0.009216584 1 TXNDC5 6 7881517 7910814 - 4387 protein_coding thioredoxin domain containing 5 [Source:HGNC Symbol;Acc:HGNC:21073] - 412 455 585 273 157 338 4.540783526

4.51962456	6.224927067	3.457857217	2.199780653
3.582449557
ENSG00000143368 5657.636586	5310.131222	5542.414634
6050.359663	6923.586693	7357.692896	5503.394147
6777.213084	-0.300493841	0.009228026	0.128426108	SF3B4
1	149923317	149928344	-	2245
protein_coding	splicing factor 3b subunit 4 [Source:HGNC
Symbol;Acc:HGNC:10771]	-	5882	6103	5957	5490	5712
7970	126.6805357	118.4637899	123.8674767
135.8839124	156.3936853	165.0718407
ENSG00000279948 35.58866945	13.05128106	13.02565131
3.306207466	0	0	20.55520061	1.102069155
4.236232456	0.009231923	1	AC008895.1	19
46946535	46949156	+	2622	TEC	TEC	-
37	15	14	3	0	0	0.682291959
0.249297067	0.249253568	0.063577085	0	0
ENSG00000170889 13992.11823	14743.59717	19045.36303
17481.02094	21299.24114	22451.57983	15927.02614
20410.61397	-0.35786694	0.00923325	0.128426108	RPS9
19	54200742	54249003	+	4371
protein_coding	ribosomal protein S9 [Source:HGNC
Symbol;Acc:HGNC:10442]	-	14547	16945	20470	15862	17572
24320	160.9140067	168.9348943	218.6165679
201.6458009	247.1085363	258.7103373
ENSG00000147854 1549.549905	1741.910979	1634.71924
1439.302317	1201.203504	988.7188321	1642.060041
1209.741551	0.441495715	0.00925557	1	UHRF2	9
6413151 6507054 +	9919	protein_coding	ubiquitin like
with PHD and ring finger domains 2 [Source:HGNC
Symbol;Acc:HGNC:12557]	-	1611	2002	1757	1306	991
1071	7.852878293	8.795383503	8.268941264
7.31622967	6.141198348	5.020565053
ENSG00000260300 0	0	0	4.408276622	0
36.003767	0	13.47068121	-6.319142918	0.00926013
1	AC009119.2	16	83908132	83951445
+	3055	protein_coding	novel protein	-	0
0	0	4	0	39	0	0	0
0.072754661	0	0.59358704
ENSG00000017483 50.97836434	60.03589289	32.56412828
5.510345777	3.636337549	19.38664377	47.85946184
9.511109031	2.310759765	0.009282135	1	SLC38A5 X
48458537	48470256	-	4014	protein_coding
solute carrier family 38 member 5 [Source:HGNC
Symbol;Acc:HGNC:18070]	-	53	69	35	5	3
21	0.638410054	0.749083654	0.407039646
0.06921571	0.045940027	0.243261256
ENSG00000164300 819.5012532	826.581134	653.143373
609.4442429	504.2388068	476.3575326	766.4085867
530.0135274	0.532522643	0.009285068	1	SERINC5 5
80111651	80256079	-	9570	protein_coding

serine incorporator 5 [Source:HGNC Symbol;Acc:HGNC:18825]
- 852 950 702 553 416 516
4.304561068 4.325838139 3.424295303 3.210888592
2.671952621 2.507083404
ENSG00000109084 1403.347803 1308.608448 1352.806929
1708.207191 1744.229911 1740.182071 1354.92106
1730.873058 -0.353378537 0.009353678 1 TMEM97 17
28319095 28328685 + 3034 protein_coding
transmembrane protein 97 [Source:HGNC Symbol;Acc:HGNC:28106]
- 1459 1504 1454 1550 1439 1885
23.2509623 21.6018456 22.37148772 28.38756655
29.15362597 28.88862
ENSG00000225282 29.81753386 12.18119566 0 0 0
0 13.99957651 0 6.148675513 0.009436989
1 AP000350.2 22 23926900 23929574
+ 1763 processed_pseudogene kelch-like 5
(Drosophila) (KLHL5) pseudogene - 31 14 0 0
0 0 0.85017944 0.346046389 0 0
0 0
ENSG00000167548 5674.949993 2779.922867 5098.612085
3283.064014 2831.494838 3351.119851 4517.828315
3155.226234 0.517650739 0.009468354 0.130417851 KMT2D
12 49018975 49059774 - 20485
protein_coding lysine methyltransferase 2D [Source:HGNC
Symbol;Acc:HGNC:7133] - 5900 3195 5480 2979 2336
3630 13.92570721 6.796627869 12.4879349
8.08065525 7.009454651 8.239515496
ENSG00000099960 21.16083048 3.480341617 9.304036652 0
0 0 11.31506958 0 5.840937952
0.009504261 1 SLC7A4 22 21028718 21032840
- 2447 protein_coding solute carrier family 7 member
4 [Source:HGNC Symbol;Acc:HGNC:11062] - 22 4 10
0 0 0 0.434700288 0.071233555
0.190770873 0 0 0
ENSG00000175866 2001.622192 1625.319535 1965.942945
1887.844463 3009.675378 2796.29257 1864.294891
2564.604137 -0.460196756 0.009508868 1 BAIAP2 17
81035122 81117432 + 10288 protein_coding
BAI1 associated protein 2 [Source:HGNC Symbol;Acc:HGNC:947]
- 2081 1868 2113 1713 2483 3029
9.78007851 7.912332222 9.587703109 9.252060224
14.83519039 13.68987037
ENSG00000146830 1251.374566 974.4956528 1411.42236
741.6925416 728.4796223 1045.032416 1212.43086
838.4015268 0.530916499 0.009531339 1 GIGYF1 7
100679507 100694037 - 6917 protein_coding
GRB10 interacting GYF protein 1 [Source:HGNC
Symbol;Acc:HGNC:9126] - 1301 1120 1517 673 601
1132 9.094121189 7.056004429 10.23796972
5.406414413 5.340772226 7.609562165

ENSG00000213736 0 0 0 0 0 49.85136969
0 16.61712323 -6.621170699 0.009552872 1
AC026310.1 12 24967127 24967504 -
378 processed_pseudogene brain protein I3 (BR13)
pseudogene - 0 0 0 0 0 54
0 0 0 0 0 6.642521643
ENSG00000149600 594.4269653 723.0409709 441.941741
846.3891114 959.9931129 792.0828739 586.4698924
866.1550327 -0.561792494 0.009627576 1 COMMD7 20
32702691 32743997 - 2584 protein_coding
COMM domain containing 7 [Source:HGNC Symbol;Acc:HGNC:16223]
- 618 831 475 768 792 858
11.56370975 14.01416131 8.581182455 16.51508276
18.83996243 15.43923568
ENSG00000267128 11.54227117 3.480341617 18.6080733 0
0 0 11.2102287 0 5.827920253
0.009628683 1 RNF157-AS1 17 76140556
76154650 + 2283 antisense RNF157 antisense RNA 1
[Source:HGNC Symbol;Acc:HGNC:44127] - 12 4 20
0 0 0 0.254142063 0.076350639
0.408949913 0 0 0
ENSG00000092621 7660.220634 5806.079903 7369.727432
7842.32411 8604.786753 10336.77382 6945.342656
8927.961562 -0.362472312 0.009636515 0.130417851 PHGDH
1 119648411 119744226 + 9217
protein_coding phosphoglycerate dehydrogenase [Source:HGNC
Symbol;Acc:HGNC:8923] - 7964 6673 7921 7116 7099
11197 41.7775416 31.54932959 40.11774021
42.90010899 47.34290999 56.48629748
ENSG00000224945 48.09279655 65.25640532 49.31139426
12.12276071 21.81802529 2.769520538 54.22019871
12.23676885 2.164588244 0.009638083 1 AL353150.1
9 89088604 89109934 - 1038
sense_intronic novel transcript - 50 75 53
11 18 3 2.329023482 3.148636369
2.383551566 0.588853655 1.065914848 0.134386276
ENSG00000106153 7918.959879 8713.035238 9369.164908
9366.485752 11149.01092 11871.0882 8667.053342
10795.52829 -0.316863618 0.00965983 0.130417851 CHCHD2
7 56101569 56106576 - 1132
protein_coding coiled-coil-helix-coiled-coil-helix domain containing
2 [Source:HGNC Symbol;Acc:HGNC:21645] - 8233 10014 10070
8499 9198 12859 351.6518488 385.4958952
415.2685864 417.1895736 499.4526694 528.1919617
ENSG00000198888 48197.63884 41786.72162 43506.60579
41276.89815 63975.29861 72778.38388 44496.98875
59343.52688 -0.41540074 0.009660582 0.130417851 MT-ND1
MT 3307 4262 + 956 protein_coding
mitochondrially encoded NADH:ubiquinone oxidoreductase core subunit 1
[Source:HGNC Symbol;Acc:HGNC:7455] - 50109 48026 46761

37454 52780 78835 2534.306048 2189.158074
2283.347092 2176.969565 3393.585955 3834.353929
ENSG00000079462 1038.804405 1176.355467 1065.312197
1355.545061 1505.443745 1432.765292 1093.49069
1431.251366 -0.388056068 0.009670649 1 PAFAH1B3
19 42297033 42303546 - 1419
protein_coding platelet activating factor acetylhydrolase 1b
catalytic subunit 3 [Source:HGNC Symbol;Acc:HGNC:8576] - 1080
1352 1145 1230 1242 1552 36.79955581
41.51957448 37.6677021 48.16535434 53.80053081
50.85578233
ENSG00000275091 0 0 0 0 26.66647536
12.00125567 0 12.88924367 -6.249571588 0.009691477
1 AC022098.4 19 14006422 14006773
+ 352 antisense "novel transcript, antisense
to RFX1" - 0 0 0 0 22 13
0 0 0 0 3.841734764 1.717242811
ENSG00000176095 2083.379946 1992.495576 2122.25076
2445.491456 2483.618546 2945.846679 2066.042094
2624.98556 -0.345879761 0.009696166 1 IP6K1 3
49724294 49786542 - 5200 protein_coding
inositol hexakisphosphate kinase 1 [Source:HGNC
Symbol;Acc:HGNC:18360] - 2166 2290 2281 2219 2049
3191 20.13985434 19.19069647 20.47707767
23.71188075 24.2206601 28.53346268
ENSG00000135604 0 0 0 8.816553243 9.69690013
12.00125567 0 10.17156968 -5.910070226
0.009726482 1 STX11 6 144150526 144188370
+ 1926 protein_coding syntaxin 11 [Source:HGNC
Symbol;Acc:HGNC:11429] - 0 0 0 8 8
13 0 0 0 0.230805284 0.255317783
0.313847077
ENSG00000214029 277.976364 190.5487035 307.9636132
153.1876126 163.6351897 96.01004532 258.8295603
137.6109492 0.91387465 0.009799025 1 ZNF891 12
133106817 133130473 - 15455 protein_coding
zinc finger protein 891 [Source:HGNC Symbol;Acc:HGNC:38709]
zf-C2H2 289 219 331 139 135 104
0.904128272 0.617495367 0.999781325 0.49975579
0.536923137 0.31289264
ENSG00000279865 20.19897455 2.610256213 11.16484398 0
0 0 11.32469158 0 5.842147554
0.009825263 1 AC006511.3 12 8080381 8082163 -
1783 TEC TEC - 21 3 12 0
0 0 0.569467794 0.073321022 0.314178122
0 0 0
ENSG00000116521 4408.185732 5043.885088 5284.692818
5459.650596 6244.803684 6768.708195 4912.254546
6157.720825 -0.326080311 0.00983943 0.131652932 SCAMP3
1 155255979 155262430 - 2442

protein_coding secretory carrier membrane protein 3 [Source:HGNC
Symbol;Acc:HGNC:10565] -   4583   5797   5680   4954   5152
        7332   90.74138718   103.4466041   108.5797186
112.7255561   129.6813596   139.6071993
ENSG00000118600 438.6063045   406.3298838   363.7878331
300.8648794   241.2103907   219.715296   402.9080071
253.9301887   0.666690848   0.009888365   1   RXYLT1 12
        63779803   63809558   +   7804   protein_coding
        ribitol xylosyltransferase 1 [Source:HGNC
Symbol;Acc:HGNC:13530] -   456   467   391   273   199
        238   2.825197403   2.607703546   2.338867033
1.943826193   1.567412031   1.418047291
ENSG00000167552 8083.437244   10991.78891   9519.890302
12173.45589   14938.07465   10229.68569   9531.705486
12447.07208   -0.384826321   0.009895482   0.131652932   TUBA1A
        12   49184796   49189324   -   3421
protein_coding tubulin alpha 1a [Source:HGNC Symbol;Acc:HGNC:20766]
        -       8404   12633   10232   11046   12324   11081
118.7775016   160.9207395   139.6218837   179.4171726
221.4348517   150.6110717
ENSG00000169403 0   0   0   8.816553243   30.30281291
        0   0   13.03978872   -6.264172501   0.009967173
        1   PTAFR   1   28147166   28193936   -
        4706   protein_coding platelet activating factor receptor
[Source:HGNC Symbol;Acc:HGNC:9582]   -   0   0   0
        8   25   0   0   0   0   0.094460471
        0.326539291   0
ENSG00000115760 2951.935852   1967.263099   2327.86997
2015.684485   1596.352184   1742.028419   2415.68964
1784.688363   0.436492627   0.009987366   1   BIRC6   2
        32357028   32618899   +   17858   protein_coding
        baculoviral IAP repeat containing 6 [Source:HGNC
Symbol;Acc:HGNC:13516] -   3069   2261   2502   1829   1317
        1887   8.30931621   5.517296727   6.540342964
5.691056936   4.533152365   4.913263993
ENSG00000223653 0   0   0.930403665   11.02069155
13.33323768   12.92442918   0.310134555
12.42611947   -5.236478049   0.010002796   1   AL078459.1
        1   85276715   85448124   +   2277
antisense   novel transcript   -   0   0   1
        10   11   14   0   0   0.020501376
0.244033255   0.296945682   0.285888064
ENSG00000069188 13.46598303   5.220512425   13.95605498   0
        0   0   10.88085015   0   5.784933836
0.010058606   1   SDK2   17   73334384   73644089
        -       11607   protein_coding sidekick cell adhesion
molecule 2 [Source:HGNC Symbol;Acc:HGNC:19308] -   14   6
        15   0   0   0   0.058318892   0.0225263
        0.060327775   0   0   0
ENSG00000167723 230.8454234   289.7384396   365.6486404

197.2703788	179.3926524	98.77956586	295.4108345
158.4808657	0.901294731	0.01011869	1	TRPV3	17
	3510502 3557995 -	9780	protein_coding	transient
receptor potential cation channel subfamily V member 3 [Source:HGNC
Symbol;Acc:HGNC:18084]	-	240	333	393	179	148
	107	1.186516012	1.483761085	1.875857014
1.017012334	0.930186904	0.508716637
ENSG00000131504 3794.521648	3450.758713	3687.189725
4558.158027	4287.24197	4503.240395	3644.156695
4449.546797	-0.288212516	0.010170056	0.134285642	DIAPH1
	5	141515016	141619055	-	7375
protein_coding	diaphanous related formin 1 [Source:HGNC
Symbol;Acc:HGNC:2876]	-	3945	3966	3963	4136	3537
	4878	25.86343471	23.43415418	25.08465218
31.16237497	29.47951915	30.75465004
ENSG00000165280 11326.81544	10021.64369	10227.92749
12401.58421	12951.42224	12510.84744	10525.46221
12621.28463	-0.261984838	0.010250926	0.134285642	VCP
	9	35056064	35073249	-	5438
protein_coding	valosin containing protein [Source:HGNC
Symbol;Acc:HGNC:12666]	-	11776	11518	10993	11253	10685
	13552	104.7031651	92.29888798	94.36763271
114.984992	120.7765677	115.876457
ENSG00000198576 54.82578806	41.7640994	53.03300892
241.353145	139.3929394	41.54280807	49.87429879
140.7629642	-1.495603993	0.010270361	1	ARC	8
	142611044	142614472	-	2991	protein_coding
	activity regulated cytoskeleton associated protein
[Source:HGNC Symbol;Acc:HGNC:648]	-	57	48	57
	219	115	45	0.921424295	0.699332034
0.889619878	4.068550823	2.363354051	0.699563463
ENSG00000136877 1134.028143	1308.608448	1291.400287
1353.340923	1833.926237	1842.654331	1244.678959
1676.640497	-0.429794238	0.010278291	1	FPGS	9
	127794597	127814327	+	3931	protein_coding
	folylpolyglutamate synthase [Source:HGNC Symbol;Acc:HGNC:3824]
	-	1179	1504	1388	1228	1513	1996
14.50146831	16.67260227	16.48285576	17.35830706
23.65828419	23.60959359
ENSG00000111145 5373.889086	5379.738054	4283.578475
4066.635183	3809.669639	4114.584346	5012.401872
3996.963056	0.326508585	0.010312786	0.134285642	ELK3
	12	96194382	96269835	+	4648
protein_coding	"ELK3, ETS transcription factor [Source:HGNC
Symbol;Acc:HGNC:3325]"	ETS	5587	6183	4604	3690	3143
	4457	58.1184159	57.96845491	46.23972381
44.11357862	41.56479299	44.58692615
ENSG00000131002 858.9373463	596.0085019	751.7661615
562.0552693	458.1785312	488.3587882	735.5706699
502.8641962	0.548229657	0.010332645	1	TXLNGY	Y

19567313 19606274 + 11377
transcribed_unprocessed_pseudogene "taxilin gamma pseudogene, Y-linked [Source:HGNC Symbol;Acc:HGNC:18473]" - 893 685
808 510 378 529 3.795114797 2.623743714
3.315351947 2.490889498 2.042261743 2.162016142
ENSG00000279530 10.58041524 13.05128106 8.373632987 0
0 0 10.6684431 0 5.757364599
0.010376204 1 AC092881.1 12 70321542
70324274 - 2733 TEC TEC - 11 15
9 0 0 0 0.194605123 0.239171939
0.153726562 0 0 0
ENSG00000232729 23.08454234 0 17.67766964 0 0
0 13.58740399 0 6.105112946 0.01039108
1 AC211433.1 7 74688939 74729001
- 2847 processed_transcript uncharacterized
LOC101926943 [Source:NCBI gene;Acc:101926943] - 24 0
19 0 0 0 0.40759138 0
0.311538819 0 0 0
ENSG00000230373 11.54227117 23.49230591 20.46888063
2.204138311 1.212112516 0.923173513 18.50115257
1.44647478 3.686938497 0.01046518 1 GOLGA6L5P
15 84507885 84516814 - 3899
transcribed_unprocessed_pseudogene "golgin A6 family-like 5, pseudogene [Source:HGNC Symbol;Acc:HGNC:30472]" - 12 27
22 2 1 1 0.14880901 0.30176518
0.263399825 0.028502884 0.015765005 0.011925533
ENSG00000270923 12.5041271 16.53162268 3.721614661 0
0 0 10.91912148 0 5.791066069
0.010513975 1 TAS2R6P 7 141787815 141788640
+ 826 processed_pseudogene taste 2 receptor
member 6 pseudogene [Source:HGNC Symbol;Acc:HGNC:20616] - 13
19 4 0 0 0 0.760964718
1.002379443 0.226061175 0 0 0
ENSG00000170345 101.9567287 160.0957144 159.0990267
544.4221628 133.3323768 243.7178074 140.3838233
307.157449 -1.129471827 0.010521147 1 FOS 14
75278774 75282230 + 3238 protein_coding
"Fos proto-oncogene, AP-1 transcription factor subunit
[Source:HGNC Symbol;Acc:HGNC:3796]" TF_bZIP 106 184 171
494 110 264 1.582815292 2.47627901
2.465274604 8.477389706 2.088157253 3.791037676
ENSG00000196689 142.3546778 87.00854042 93.04036652
46.28690453 54.54506323 19.38664377 107.4678616
40.07287051 1.429603262 0.010521671 1 TRPV1 17
3565444 3609411 - 5519 protein_coding transient
receptor potential cation channel subfamily V member 1 [Source:HGNC
Symbol;Acc:HGNC:12716] - 148 100 100 42 45
21 1.296589612 0.789583753 0.84583498
0.42286422 0.501186633 0.176925291
ENSG00000108107 28205.46332 29720.37724 30192.52934

30125.06036	38505.17831	40552.24289	29372.78997
36394.16052	-0.309241381	0.01052622	0.134686665	RPL28
	19	55385345	55403250	+	6933
protein_coding	ribosomal protein L28 [Source:HGNC
Symbol;Acc:HGNC:10330]	-	29324	34158	32451	27335	31767
	43927	204.5046687	214.6989061	218.500744
219.0836267	281.6452058	294.605847
ENSG00000166226 6545.42961	6732.720858	7371.588239
8310.703501	8896.90587	7859.899287	6883.246236
8355.836219	-0.279574712	0.010542191	0.134686665	CCT2
	12	69585334	69601570	+	4322
protein_coding	chaperonin containing TCP1 subunit 2 [Source:HGNC
Symbol;Acc:HGNC:1615]	-	6805	7738	7923	7541	7340
	8514	76.12802859	78.01939182	85.5757924
96.95187709	104.3899423	91.59671561
ENSG00000100401 6147.221255	4230.355235	4933.000233
5319.687813	6799.951216	8666.752938	5103.525574
6928.797322	-0.441336227	0.01056366	0.134686665
RANGAP1 22	41245611	41286251	-	5348
protein_coding	Ran GTPase activating protein 1 [Source:HGNC
Symbol;Acc:HGNC:9854]	-	6391	4862	5302	4827	5610
	9388	57.78014607	39.61705183	46.28010767
50.15311867	64.47907861	81.62302773
ENSG00000185022 567.4949993	586.4375625	664.3082169
836.470489	792.7215857	877.0148371	606.0802596
835.4023039	-0.463316557	0.01058187	1	MAFF	22
	38200767	38216511	+	5615	protein_coding
	MAF bZIP transcription factor F [Source:HGNC
Symbol;Acc:HGNC:6780]	TF_bZIP 590	674	714	759	654
	950	5.080465044	5.230807451	5.936008129
7.511108905	7.159378908	7.866922337
ENSG00000107186 1190.777643	814.3999384	941.5685092
781.3670312	684.8435717	587.1383541	982.2486967
684.4496523	0.521458145	0.01062813	1	MPDZ	9
	13105704	13279590	-	11273	protein_coding
	multiple PDZ domain crumbs cell polarity complex component
[Source:HGNC Symbol;Acc:HGNC:7208]	-	1238	936	1012
	709	565	636	5.309851241	3.618219747
4.190704528	3.494771391	3.080748892	2.623304032
ENSG00000261423 1.923711862	54.81538047	0	0
1.212112516	0	18.91303078	0.404037505	5.622808348
	0.010650917	1	TMEM202-AS1	15	72407778
	72475168	-	2713	lincRNA TMEM202 antisense RNA
1 [Source:HGNC Symbol;Acc:HGNC:53265]	-	2	63	0
	0	1	0	0.035643588	1.011927395	0
	0	0.022656748	0
ENSG00000183691 0	9.570939447	0	7.714484088
44.8481631	56.31358428	3.190313149
36.29207716	-3.493320484	0.010656746	1	NOG	17
	56593699	56595590	+	1892	protein_coding

noggin [Source:HGNC Symbol;Acc:HGNC:7866]	-	0
11	0	7	37	61	0	0.253355392
0	0.20558383	1.202065	1.49913147
ENSG00000169976 1952.56754	1699.276794	2059.913715
2061.97139	2671.495986	2772.290059	1903.91935
2501.919145	-0.394303883	0.010674352	1	SF3B5	6
144094881	144095573	-	693	protein_coding
splicing factor 3b subunit 5 [Source:HGNC
Symbol;Acc:HGNC:21083]	-	2030	1953	2214	1871	2204
3003	141.6328583	122.808268	149.1387222
150.0211867	195.4905263	201.4898232
ENSG00000278384 267.3959488	267.1162191	551.7293735
139.9627827	231.5134906	210.4835609	362.0805138
193.9866114	0.900394848	0.010693164	1	AL354822.1
GL000218.1	51867	54893	-	3027	protein_coding
-	278	307	593	127	191	228
4.440517556	4.419616154	9.14509683	2.331327806
3.878540283	3.502300807
ENSG00000108465 317.4124572	239.2734862	349.8317781
185.1476181	189.0895525	162.4785382	302.1725738
178.9052363	0.756757717	0.010715783	1	CDK5RAP3
17	47967810	47981774	+	6070
protein_coding	CDK5 regulatory subunit associated protein 3
[Source:HGNC Symbol;Acc:HGNC:18673]	-	330	275	376
168	156	176	2.628611874	1.974252063
2.891646432	1.537916068	1.579731461	1.348202086
ENSG00000214078 2324.805785	2443.199815	2943.797197
3270.941253	3643.610224	2982.77362	2570.600932
3299.108366	-0.359678511	0.010739037	0.135147702	CPNE1
20	35626031	35664956	-	3608
protein_coding	copine 1 [Source:HGNC Symbol;Acc:HGNC:2314]	-
2417	2808	3164	2968	3006	3231	32.39002909
33.91479313	40.93699706	45.70980951	51.2117725
41.63911086
ENSG00000213593 2607.591429	2359.671616	2580.939767
2972.280512	3261.794781	3127.711861	2516.067604
3120.595718	-0.310672732	0.010747039	0.135147702	TMX2
11	57712600	57740973	+	2246
protein_coding	thioredoxin related transmembrane protein 2
[Source:HGNC Symbol;Acc:HGNC:30739]	-	2711	2712	2774
2697	2691	3388	58.36076582	52.61850818
57.65576523	66.72417891	73.64636331	70.13982337
ENSG00000187726 20.19897455	20.0119643	0	0	0
0	13.40364628	0	6.086559901	0.010771603
1	DNAJB13 11	73950319	73970366	+
3475	protein_coding	DnaJ heat shock protein family (Hsp40)
member B13 [Source:HGNC Symbol;Acc:HGNC:30718]	-	21	23
0	0	0	0	0.292190238	0.288424152
0	0	0	0
ENSG00000105364 1434.127193	1417.369124	1703.569111

1768.820994	1901.804538	2377.171795	1518.355143
2015.932443	-0.409469796	0.010799354	1	MRPL4	19
	10251901	10260045	+	4070	protein_coding
	mitochondrial ribosomal protein L4 [Source:HGNC
Symbol;Acc:HGNC:14276]	-	1491	1629	1831	1605	1569
	2575	17.7126871	17.44155785	21.00099981
21.91253744	23.69604656	29.41804732
ENSG00000275956 0	0	0	25.34759057	0
12.00125567	0	12.44961541	-6.202194825	0.010855044
	1	AC104446.1	3	52732547	52733867
	+	1321	processed_pseudogene	zinc finger protein
610 (ZNF610) pseudogene -	0	0	0	23	0
	13	0	0	0	0.967469009	0
0.457584761
ENSG00000134253 362.619686	270.5965607	231.6705126
152.0855434	162.4230772	184.6347025	288.2955864
166.3811077	0.791858609	0.0108564	1	TRIM45	1
	117111060	117122587	-	4368	protein_coding
	tripartite motif containing 45 [Source:HGNC
Symbol;Acc:HGNC:19018]	-	377	311	249	138	134
	200	4.173111004	3.102675505	2.661109548
1.75553099	1.885686661	2.129013347
ENSG00000151240 622.3207873	780.4666076	693.1507306
486.0124975	450.9058561	531.7479433	698.6460418
489.5554323	0.512541263	0.010912686	1	DIP2C	10
	274190	689668	-	8563	protein_coding	disco
interacting protein 2 homolog C [Source:HGNC Symbol;Acc:HGNC:29150]
	-	647	897	745	441	372	576
3.653251347	4.564835132	4.061405612	2.861703859
2.670326441	3.12771777
ENSG00000124788 534.7918976	448.9640686	405.655998
252.3738366	264.2405286	363.730364	463.1373214
293.4482431	0.656371828	0.010916025	1	ATXN1	6
	16299112	16761491	-	17812	protein_coding
	ataxin 1 [Source:HGNC Symbol;Acc:HGNC:10548]	-	556
	516	436	229	218	394	1.509257427
1.262396009	1.142667404	0.714389133	0.752300183
1.028524292
ENSG00000203786 0	0	0	8.816553243	15.75746271
	5.539041076	0	10.03768568	-5.887970839
0.010954849	1	KPRP	1	152759561	152762052
	+	2492	protein_coding	keratinocyte proline rich
protein [Source:HGNC Symbol;Acc:HGNC:31823]	-	0	0
	0	8	13	6	0	0	0
0.178383217	0.320658439	0.111952612
ENSG00000169228 66.36805924	97.44956528	152.5862011
26.44965973	26.66647536	60.00627833	105.4679419
37.70747114	1.477060735	0.01096849	1	RAB24	5
	177301198	177303744	-	2065	protein_coding
	"RAB24, member RAS oncogene family [Source:HGNC

Symbol;Acc:HGNC:9765]"	-	69	112	164	24	22
	65	1.615586633	2.363505212	3.707403263
0.645807716	0.654862294	1.463606464
ENSG00000280300 0	0	0	7.714484088	10.90901265
	11.07808215	0	9.900526296	-5.870648489
0.011003258	1	AC117503.4	12	123754246
123754794	-	549	TEC	novel transcript	-
	0	0	0	7	9	12	0	0
	0	0.708496549	1.007668135	1.016342109
ENSG00000143933 5408.5159	6100.168769	5380.524396
7023.486727	6747.830378	6690.238447	5629.736355
6820.518518	-0.276732905	0.011107693	0.13873282	CALM2
	2	47160082	47176601	-	6466
protein_coding	calmodulin 2 [Source:HGNC Symbol;Acc:HGNC:1445] -
	5623	7011	5783	6373	5567	7247	42.04686299
	47.25011443	41.75067755	54.76716518	52.92158968
	52.11390047
ENSG00000075240 403.979491	401.9794568	366.5790441
254.5779749	267.8768661	240.0251133	390.8459973
254.1599848	0.621565276	0.011139169	1	GRAMD4	22
	46576012	46679790	+	4885	protein_coding
	GRAM domain containing 4 [Source:HGNC Symbol;Acc:HGNC:29113]
	-	420	462	394	231	221	260
4.157056611	4.121316855	3.765110179	2.627601224
2.780834007	2.474798237
ENSG00000121653 657.9094568	526.4016696	834.5720877
656.8332166	1368.475031	1145.658329	672.9610713
1056.988859	-0.651386008	0.011139862	1	MAPK8IP1
	11	45885651	45906465	+	3498
protein_coding	mitogen-activated protein kinase 8 interacting protein
1 [Source:HGNC Symbol;Acc:HGNC:6882]	-	684	605	897
	596	1129	1241	9.454477074	7.536924542
11.97067593	9.46756942	19.83907855	16.4961651
ENSG00000213918 782.9507278	624.7213202	543.3557405
398.9490343	489.6934566	431.1220304	650.3425962
439.9215071	0.564250547	0.011190202	1	DNASE1	16
	3611728 3680143 +	13801	protein_coding
deoxyribonuclease 1 [Source:HGNC Symbol;Acc:HGNC:2956]	-	814
	718	584	362	404	467	2.851773739
2.267109444	1.975369423	1.457505017	1.799360446
1.573393468
ENSG00000237649 541.5248891	683.8871277	476.3666766
1165.989166	832.7212987	622.2189476	567.2595645
873.6431376	-0.622173977	0.011282534	1	KIFC1	6
	33391536	33409924	+	3345	protein_coding
	kinesin family member C1 [Source:HGNC Symbol;Acc:HGNC:6389]
	-	563	786	512	1058	687	674
8.137921369	10.23964786	7.145290245	17.57525315
12.62431944	9.369033516
ENSG00000278900 13.46598303	9.570939447	8.373632987	0

0	0	10.47018516	0	5.729831957
0.011304236	1	AC139792.2	5	34105750
34106336	-	587	TEC	TEC	-	14	11
9	0	0	0	1.153164199	0.816607157
0.715732015	0	0	0
ENSG00000196704 1664.972616	1900.266523	1488.645864
2046.542422	2081.19719	2452.872023	1684.628335
2193.537212	-0.381048515	0.011348259	1	AMZ2	17
68247574	68257164	+	4214	protein_coding
archaelysin family metallopeptidase 2 [Source:HGNC
Symbol;Acc:HGNC:28041]	-	1731	2184	1600	1857	1717
2657	19.86112081	22.58482348	17.72439774
24.48665239	25.04512042	29.31757476
ENSG00000226797 5.771135586	5.220512425	21.3992843	0
0	0	10.79697744	0	5.77414574
0.011393896	1	AC005828.1	17	63381231
63414312	-	524	antisense	"novel transcript,
antisense to TANC2"	-	6	6	23	0	0
0	0.553631994	0.498974741	2.049002956	0
0	0
ENSG00000250374 51.94022027	0	0	0	0	0
17.31340676	0	6.454734987	0.011416179	1
TRIM75P 4	165053864	165060554	+	1840
protein_coding	"tripartite motif containing 75, pseudogene
[Source:HGNC Symbol;Acc:HGNC:32686]"	-	54	0	0
0	0	0	1.418982872	0	0	0
0	0
ENSG00000072832 588.6558297	406.3298838	486.6011169
309.6814327	283.6343288	365.576711	493.8622768
319.6308242	0.626049875	0.011480359	1	CRMP1	4
5748084 5893058 -	5823	protein_coding	collapsin
response mediator protein 1 [Source:HGNC Symbol;Acc:HGNC:2365]	-
612	467	523	281	234	396	5.081662859
3.494851189	4.19276899	2.681461547	2.47011076
3.162127768
ENSG00000116285 7971.861956	10676.818	7982.863447
7118.264675	6864.19318	7193.368011	8877.181133
7058.608622	0.330750045	0.011520727	0.142919285	ERRFI1
1	8004404 8026308 -	4186	protein_coding	ERBB
receptor feedback inhibitor 1 [Source:HGNC Symbol;Acc:HGNC:18185]
-	8288	12271	8580	6459	5663	7792
95.73081029	127.7436526	95.68284934	85.73893886
83.15621201	86.55272348
ENSG00000222009 388.5897961	323.6717704	423.3336677
293.1503953	204.8470153	197.5591317	378.5317447
231.8521808	0.707494217	0.011605235	1	BTBD19	1
44808482	44815585	+	3520	protein_coding
BTB domain containing 19 [Source:HGNC Symbol;Acc:HGNC:27145]
-	404	372	455	266	169	214
5.549321905	4.60531005	6.034131481	4.199049718

2.951150796	2.826845858
ENSG00000182054 958.0085072	1026.700777	1074.616233
1423.873349	1618.170209	1119.809471	1019.775173
1387.284343	-0.443136154	0.011622735	1	IDH2	15
	90083045	90102504	-	2814	protein_coding
	"isocitrate dehydrogenase (NADP(+)) 2, mitochondrial
[Source:HGNC Symbol;Acc:HGNC:5383]"	-	996	1180	1155
	1292	1335	1213	17.11340632	18.27328013
19.16037157	25.51234999	29.16114234	20.04323073
ENSG00000257022 34.62681351	6.09059783	0	0	0
	0	13.57247045	0	6.103563794	0.011636082
	1	AC008250.1	12	21827210	21887957
	+	522	antisense	novel transcript	-
	36	7	0	0	0	0	3.334519137
	0.584367608	0	0	0	0
ENSG00000175063 516.5166349	589.0478187	641.978529
1276.196082	1020.598739	515.1308201	582.5143275
937.3085469	-0.685288343	0.011683126	1	UBE2C	20
	45812576	45816957	+	1286	protein_coding
	ubiquitin conjugating enzyme E2 C [Source:HGNC
Symbol;Acc:HGNC:15937]	-	537	677	690	1158	842
	558	20.189917	22.94068057	25.04691016
50.03566019	40.24560711	20.17549731
ENSG00000273291 4.809279655	16.53162268	10.23444032	0
	0	0	10.52511422	0	5.738461881
0.011828196	1	AC092042.3	3	42809446
43055871	+	1654	protein_coding	novel protein	-
	5	19	11	0	0	0	0.146162417
	0.500583688	0.310458258	0	0	0
ENSG00000071282 209.6845929	166.1863122	169.3334671
262.292459	316.3613668	323.1107295	181.7347907
300.5881851	-0.72659899	0.011831605	1	LMCD1	3
	8501707 8574673 +	9130	protein_coding	LIM and
cysteine rich domains 1 [Source:HGNC Symbol;Acc:HGNC:6633]	-
	218	191	182	238	261	350	1.154481379
	0.911635413	0.930564855	1.448499078	1.757183392
	1.782494855
ENSG00000094804 1769.814913	1640.110987	1533.30524
2380.469376	1779.381174	2311.626476	1647.743713
2157.159009	-0.389099775	0.011874301	1	CDC6	17
	40287633	40304657	+	5557	protein_coding
	cell division cycle 6 [Source:HGNC Symbol;Acc:HGNC:1744]
	-	1840	1885	1648	2160	1468	2504
16.00953223	14.78187602	13.84404003	21.59858988
16.23801802	20.95197397
ENSG00000285373 24.04639827	6.09059783	2.791210996	0
	0	0	10.97606903	0	5.797160426
0.011883669	1	LINC02478	2	160257718
160271880	-	614	lincRNA long intergenic non-protein
coding RNA 2478 [Source:HGNC Symbol;Acc:HGNC:53446]	-	25

7	3	0	0	0	1.968669686
0.496807641	0.228086153	0	0	0
ENSG00000006007 868.5559056	1043.2324	999.2535364
1233.215385	1185.446041	1482.616661	970.3472806
1300.426029	-0.422855305	0.011942762	1	GDE1	16
19501689	19522145	-	4416	protein_coding
glycerophosphodiester phosphodiesterase 1 [Source:HGNC
Symbol;Acc:HGNC:29644]	-	903	1199	1074	1119	978
1606	9.886894548	11.83174269	11.35327748
14.08033752	13.61310362	16.91015134
ENSG00000104728 51.94022027	23.49230591	23.26009163
6.612414933	7.272675098	3.692694051	32.89753927
5.85926136	2.498183759	0.012003728	1	ARHGEF10
8	1823976 1958641 +	10792	protein_coding	Rho
guanine nucleotide exchange factor 10 [Source:HGNC
Symbol;Acc:HGNC:14103]	-	54	27	25	6	6
4	0.241931846	0.109023577	0.108139438
0.030893091	0.034174068	0.017234118
ENSG00000100412 1076.316787	1043.2324	1079.268252
1266.27746	1506.655858	1393.992004	1066.272479
1388.975107	-0.381308279	0.012073206	1	ACO2	22
41469125	41528989	+	4041	protein_coding
aconitase 2 [Source:HGNC Symbol;Acc:HGNC:118]	-	1119
1199	1160	1149	1243	1510	13.38882461
12.92971435	13.40032016	15.79949559	18.90730537
17.37477203
ENSG00000179528 42.32166096	44.37435562	60.47623824
88.16553243	133.3323768	118.1662096	49.05741827
113.221373	-1.206386992	0.012076424	1	LBX2	2
74497517	74503316	-	2389	protein_coding
ladybird homeobox 2 [Source:HGNC Symbol;Acc:HGNC:15525]
Homeobox	44	51	65	80	110	128
0.890507832	0.930277729	1.270115578	1.860740802
2.830244112	2.491293174
ENSG00000234091 28.85567793	1.740170808	3.721614661	0
0	0	11.43915447	0	5.856354566
0.012093304	1	AL157392.2	10	13729383
13756200	+	474	antisense	novel transcript
-	30	2	4	0	0	0
3.060159968	0.183869736	0.393937827	0	0	0
ENSG00000088986 2361.35631	2705.095522	2498.133841
2694.559085	3687.246275	3347.427157	2521.528558
3243.077506	-0.362893145	0.012093739	0.149020833	DYNLL1
12	120469850	120498493	+	2957
protein_coding	dynein light chain LC8-type 1 [Source:HGNC
Symbol;Acc:HGNC:15476]	-	2455	3109	2685	2445	3042
3626	40.14222015	45.81714202	42.38761698
45.94514036	63.23466839	57.017411
ENSG00000158079 249.1206861	281.907671	210.2712283
167.5145116	122.4233641	132.0138123	247.0998618

140.6505627	0.813066953	0.012143928	1	PTPDC1	9
	94030794	94109856	+	6404	protein_coding
	protein tyrosine phosphatase domain containing 1 [Source:HGNC
Symbol;Acc:HGNC:30184]	-	259	324	226	152	101
	143	1.955463245	2.204714126	1.647415515
1.318877042	0.969432133	1.038282974
ENSG00000170962 26.93196607	27.84273294	21.3992843
108.0027772	59.3935133	55.39041076	25.39132777
74.26223376	-1.547443761	0.01221908	1	PDGFD	11
	103907186	104164379	-	4266	protein_coding
	platelet derived growth factor D [Source:HGNC
Symbol;Acc:HGNC:30620]	-	28	32	23	98	49
	60	0.317349923	0.32687953	0.251682501
1.276489563	0.70602908	0.653975408
ENSG00000173209 246.2351183	340.2033931	329.3628975
168.6165808	190.3016651	193.8664377	305.2671363
184.2615612	0.728414468	0.012229479	1	AHSA2P	2
	61177418	61191203	+	9083
transcribed_unitary_pseudogene	"activator of HSP90 ATPase homolog 2,
pseudogene [Source:HGNC Symbol;Acc:HGNC:20437]" -	256	391
	354	153	157	210	1.362736435	1.875884267
	1.81936562	0.935996361	1.062472501	1.075031027
ENSG00000114302 2744.174971	2672.902362	2918.676298
3510.09026	3133.310855	3691.770877	2778.584544
3445.057331	-0.310494679	0.012289493	0.14903889
PRKAR2A 3	48744597	48847846	-	6644
protein_coding	protein kinase cAMP-dependent type II regulatory
subunit alpha [Source:HGNC Symbol;Acc:HGNC:9391]	-	2853
	3072	3137	3185	2585	3999	20.76219972
20.14884636	22.04098154	26.63740148	23.91543495
27.98677127
ENSG00000130522 4753.492011	5931.372201	5704.304871
5115.805019	8913.875445	8087.923145	5463.056361
7372.534536	-0.43239768	0.012334218	0.14903889	JUND
	19	18279760	18281622	-	1863
protein_coding	"JunD proto-oncogene, AP-1 transcription factor
subunit [Source:HGNC Symbol;Acc:HGNC:6206]"	TF_bZIP 4942	6817
	6131	4642	7354	8761	128.2599607	159.4553286
	153.6259201	138.4536231	242.6376163	218.6612586
ENSG00000263887 132.7361185	57.42563668	13.95605498
15.42896818	15.75746271	18.46347025	68.03927004
16.54996705	2.037412901	0.012338068	1	AC053481.2
	17	62282702	62297026	-	1500
transcribed_unprocessed_pseudogene	ubiquitin specific peptidase
32 (USP32) pseudogene	-	138	66	15	14	13
	20	4.448248529	1.917393604	0.466816326
0.518619474	0.532720554	0.619968687
ENSG00000134909 1290.810659	1058.893937	1154.630949
943.371197	855.7514365	828.0866409	1168.111848
875.7364248	0.415622205	0.012389108	1	ARHGAP32

11 128965060 129279324 - 12841
protein_coding Rho GTPase activating protein 32 [Source:HGNC
Symbol;Acc:HGNC:17399] - 1342 1217 1241 856 706
897 5.053065018 4.130002647 4.511479324
3.704136329 3.379505949 3.248064278
ENSG00000101680 7.694847448 2.610256213 3.721614661
4.408276622 15.75746271 87.70148371 4.675572774
35.95574101 -2.952087697 0.012414963 1 LAMA1 18
6941744 7117814 - 15872 protein_coding laminin
subunit alpha 1 [Source:HGNC Symbol;Acc:HGNC:6481] - 8
3 4 4 13 95 0.024370226
0.008236604 0.011764524 0.014003622 0.050345314
0.278306256
ENSG00000285399 57.71135586 8.700854042 56.75462358
13.22482987 0 2.769520538 41.05561116 5.331450134
2.942610188 0.012451697 1 AC104162.2 3
63863155 63905838 + 5642
bidirectional_promoter_lncRNA novel transcript - 60
10 61 12 0 3 0.514184979
0.077237021 0.504711022 0.118184414 0 0.024724026
ENSG00000162601 1505.304532 1250.312726 1212.315976
1069.007081 1032.719864 844.7037641 1322.644411
982.1435696 0.429988906 0.012454083 1 MYSM1 1
58654739 58700092 - 8938 protein_coding
"Myb like, SWIRM and MPN domains 1 [Source:HGNC
Symbol;Acc:HGNC:29401]" MYB 1565 1437 1303 970 852
915 8.465940426 7.006078766 6.805344285
6.030362613 5.859311736 4.76005271
ENSG00000142945 605.9692365 580.3469646 783.3998861
1313.666433 848.4787614 772.6962302 656.5720291
978.2804749 -0.575083002 0.012460807 1 KIF2C 1
44739818 44767767 + 3646 protein_coding
kinesin family member 2C [Source:HGNC Symbol;Acc:HGNC:6393]
- 630 667 842 1192 700 837
8.354589227 7.972008762 10.78056352 18.16651554
11.80126971 10.67431001
ENSG00000067715 25.0082542 0 13.95605498 0 0
0 12.98810306 0 6.039946592 0.012506358
1 SYT1 12 78863993 79452008 +
6494 protein_coding synaptotagmin 1 [Source:HGNC
Symbol;Acc:HGNC:11509] - 26 0 15 0 0
0 0.1935808 0 0.107826376 0 0
0
ENSG00000227953 26.93196607 2.610256213 3.721614661 0
0 0 11.08794565 0 5.811431752
0.012567444 1 LINC01341 1 246776013
246792385 + 3984 processed_transcript long
intergenic non-protein coding RNA 1341 [Source:HGNC
Symbol;Acc:HGNC:49457] - 28 3 4 0 0
0 0.339812944 0.032814102 0.046869109 0

0	0
ENSG00000219992 0	0	0	0	4.848450065
32.31107295	0	12.38650767	-6.197594429	0.012625591
1	AL391422.1	6	3754099 3754840 +	436
processed_pseudogene	FUN14 domain containing 1 (FUNDC1)
pseudogene	-	0	0	0	0	4	35
0	0	0	0	0.563924369	3.732609639
ENSG00000116539 2928.85131	2306.596407	2838.661582
2381.571445	1984.228189	1840.807984	2691.369766
2068.869206	0.379587438	0.012632179	0.14903889	ASH1L
1	155335268	155562807	-	13296
protein_coding	ASH1 like histone lysine methyltransferase
[Source:HGNC Symbol;Acc:HGNC:19088]	Others	3045	2651	3051
2161	1637	1994	11.07305627	8.688550287
10.71191794	9.031207155	7.567893873	6.973248879
ENSG00000267044 3.847423724	18.27179349	9.304036652	0
0	0	10.47441795	0	5.731695034
0.012636047	1	AC005757.1	19	45135993
45136977	-	472	antisense	"novel transcript,
antisense to PPP1R37"	-	4	21	10	0	0
0	0.409750233	1.93881287	0.989017639	0
0	0
ENSG00000123136 3272.233877	2756.430561	2725.152335
3280.859876	3653.307124	4186.59188	2917.938924
3706.919627	-0.34558892	0.012645182	0.14903889	DDX39A
19	14408819	14419383	-	3840
protein_coding	DExD-box helicase 39A [Source:HGNC
Symbol;Acc:HGNC:17821]	-	3402	3168	2929	2977	3014
4535	42.83554545	35.95113007	35.6069015
43.07840887	48.24578574	54.91324207
ENSG00000127824 1723.645828	1580.94518	1257.905755
1801.883069	2078.772965	2131.607641	1520.832254
2004.087892	-0.398211739	0.012658296	1	TUBA4A	2
219249711	219278170	-	4079	protein_coding
tubulin alpha 4a [Source:HGNC Symbol;Acc:HGNC:12407]	-
1792	1817	1352	1635	1715	2309	21.24151637
19.41153234	15.47280393	22.27286554	25.84388377
26.32093094
ENSG00000264853 18.27526269	12.18119566	1.86080733	0
0	0	10.77242189	0	5.770909103
0.012666203	1	AC011933.2	17	75370947
75373736	-	2790	sense_intronic	"novel transcript,
sense intronic to GRB2" -	19	14	2	0	0
0	0.329268825	0.218666589	0.033463536	0
0	0
ENSG00000168496 2643.180098	2916.526275	3347.592387
3718.38133	3359.975895	4283.525099	2969.099587
3787.294108	-0.351433475	0.012666476	0.14903889	FEN1
11	61792637	61797244	+	2478
protein_coding	flap structure-specific endonuclease 1 [Source:HGNC

Symbol;Acc:HGNC:3650]	-	2748	3352	3598	3374	2772
	4640	53.61874477	58.94694548	67.78067552
75.6581678	68.76054086	87.0658204
ENSG00000177600 9581.046928	10849.09491	13170.79428
11489.07095	15705.34187	16311.5528	11200.31204
14501.98854	-0.372732077	0.012675616	0.14903889	RPLP2
	11	809647	812880	+	1955	protein_coding
ribosomal protein lateral stalk subunit P2 [Source:HGNC
Symbol;Acc:HGNC:10377]	-	9961	12469	14156	10425	12957
	17669	246.3527388	277.9351412	338.0180002
296.3066139	407.3850217	420.2388769
ENSG00000270392 171.2103557	46.98461183	92.10996285
20.93931395	33.93915046	50.7745432	103.4349768
35.2176692	1.54947235	0.012680999	1	PFN1P2	1
	120432204	120434052	-	1849
transcribed_processed_pseudogene	profilin 1 pseudogene 2
[Source:HGNC Symbol;Acc:HGNC:24298]	-	178	54	99
	19	28	55	4.654620819	1.272668944
2.499449228	0.570990304	0.930825946	1.383110239
ENSG00000242615 11.54227117	8.700854042	10.23444032	0
	0	0	10.15918851	0	5.686349324
0.01274374	1	AC022415.1	19	12141806
12143322	-	1517	transcribed_processed_pseudogene
	ribosomal protein L17 (RPL17) pseudogene	-	12
	10	11	0	0	0	0.382469565
0.287258585	0.338495688	0	0	0
ENSG00000279381 17.31340676	13.05128106	1.86080733	0
	0	0	10.74183172	0	5.766925404
0.012751144	1	CR392039.1	21	9088188 9088391 +
	204	processed_pseudogene	"ankyrin repeat domain 20
family, member A2 (ANKRD20A2) pseudogene"	-	18	15
	2	0	0	0	4.266223014	3.204200541
	0.457663064	0	0	0
ENSG00000188986 1993.927345	2332.698969	2272.976154
2414.63352	2825.434276	3275.419623	2199.867489
2838.495806	-0.36792184	0.012765252	0.14903889	NELFB
	9	137255173	137273546	+	2698
protein_coding	negative elongation factor complex member B
[Source:HGNC Symbol;Acc:HGNC:24324]	-	2073	2681	2443
	2191	2331	3548	37.14997906	43.30254946
42.26954349	45.1245076	53.10650102	61.14665216
ENSG00000198393 1240.794151	1182.446064	1082.989866
918.0236065	752.7218726	943.48333	1168.743361
871.409603	0.422729135	0.012789294	1	ZNF26	12
	132986365	133032952	+	20863	protein_coding
	zinc finger protein 26 [Source:HGNC Symbol;Acc:HGNC:13053]
	zf-C2H2 1290	1359	1164	833	621	1022
2.989607461	2.838581032	2.604487384	2.21860653
1.829625491	2.277745283
ENSG00000179950 6893.621457	5866.115795	6873.822278

```
6736.948747	8559.93859	9752.404988	6544.519844
8349.764109	-0.351598219	0.012793583	0.14903889	PUF60
	8	143816344	143829859	-	5424
protein_coding	poly(U) binding splicing factor 60 [Source:HGNC
Symbol;Acc:HGNC:17042]	-	7167	6742	7388	6113	7062
	10564	63.88794811	54.16611221	63.58479007
62.62485864	80.03047464	90.56069145
ENSG00000065923 618.4733636	448.9640686	545.2165478
425.398694	349.0884047	268.6434922	537.5513267
347.710197	0.629452949	0.012863491	1	SLC9A7	X
	46599252	46759172	-	10033	protein_coding
	solute carrier family 9 member A7 [Source:HGNC
Symbol;Acc:HGNC:17123]	-	643	516	586	386	288
	291	3.098713164	2.241183865	2.726546066
2.137807203	1.764448698	1.348631176
ENSG00000103888 233.7309912	110.5008463	138.6301461
87.06346328	69.09041343	71.08436048	160.9539946
75.74607906	1.08673402	0.012892635	1	CEMIP	15
	80779343	80951776	+	7621	protein_coding
	cell migration inducing hyaluronidase 1 [Source:HGNC
Symbol;Acc:HGNC:29213]	-	243	127	149	79	57
	77	1.54168458	0.726190155	0.912683801
0.57600622	0.459737843	0.469796505
ENSG00000166508 5846.160348	5507.640609	6602.144408
6992.628791	7397.522687	7432.469951	5985.315122
7274.207143	-0.28141304	0.012906987	0.14903889	MCM7
	7	100092728	100101940	-	4386
protein_coding	minichromosome maintenance complex component 7
[Source:HGNC Symbol;Acc:HGNC:6950]	-	6078	6330	7096
	6345	6103	8051	67.00285137	62.89175015
75.52504893	80.38500493	85.53071505	85.35170821
ENSG00000166262 94.26188123	155.7452874	129.3261095
54.00138862	43.63605059	71.08436048	126.444426
56.24059989	1.165628903	0.012928899	1	FAM227B 15
	49326962	49620931	-	6020	protein_coding
	family with sequence similarity 227 member B [Source:HGNC
Symbol;Acc:HGNC:26543]	-	98	179	139	49	36
	77	0.78710161	1.29573186	1.077864938
0.452284425	0.367581266	0.594737403
ENSG00000132646 2855.750259	2881.722859	2807.958262
3891.406188	3381.79392	3287.420879	2848.477126
3520.206996	-0.305364897	0.012983369	0.14903889	PCNA
	20	5114953 5126626 -	1471	protein_coding
proliferating cell nuclear antigen [Source:HGNC Symbol;Acc:HGNC:8729]
	-	2969	3312	3018	3531	2790	3561
97.58852218	98.11519089	95.77509657	133.381958
116.5839836	112.5616159
ENSG00000116668 272.2052285	296.6991228	280.9819069
115.7172613	173.3320898	202.1749993	283.2954194
163.7414501	0.789563705	0.012983754	1	SWT1	1
```

185157080	185291765	+	4054	protein_coding
"SWT1, RNA endoribonuclease homolog [Source:HGNC
Symbol;Acc:HGNC:16785]" -	283	341	302	105	143
219	3.375234159	3.665466312	3.477516781
1.439188229	2.168201564	2.511836625
ENSG00000156697 2717.243005	2425.798107	3016.368683
3396.577137	3391.490821	3372.352842	2719.803265
3386.806933	-0.316510533	0.012988139	0.14903889	UTP14A
X	129906121	129929761	+	4087
protein_coding	"UTP14A, small subunit processome component
[Source:HGNC Symbol;Acc:HGNC:10665]"	-	2825	2788	3242
3082	2798	3653	33.42066067	29.726702
37.03005939	41.90251019	42.08142449	41.56004916
ENSG00000233554 0	0	0	0	21.81802529
13.84760269	0	11.88854266	-6.133735653	0.013008873
1	B4GALT1-AS1	9	33166975	33179983
+	843	antisense	B4GALT1 antisense RNA 1
[Source:HGNC Symbol;Acc:HGNC:49910]	-	0	0	0
0	18	15	0	0	0	0
1.31247878	0.827360347
ENSG00000145757 0	33.93333077	5.582421991	0	0
0	13.17191759	0	6.062625243	0.013017237
1	SPATA9	5	95652181	95698711	-
2811	protein_coding	spermatogenesis associated 9
[Source:HGNC Symbol;Acc:HGNC:22988]	-	0	39	6
0	0	0	0	0.604591948	0.099640624
0	0	0
ENSG00000196747 17.31340676	4.350427021	9.304036652	0
0	0	10.32262348	0	5.708675866
0.013115455	1	HIST1H2AI	6	27808199
27808701	+	503	protein_coding	histone cluster 1 H2A
family member i [Source:HGNC Symbol;Acc:HGNC:4725]	-	18
5	10	0	0	0	1.730237564
0.43317224	0.928064266	0	0	0
ENSG00000267493 48.09279655	10.44102485	32.56412828
3.306207466	0	7.385388102	30.36598323	3.563865189
3.057712436	0.013136652	1	CIRBP-AS1	19
1267814 1270241 -	994	antisense	CIRBP
antisense RNA 1 [Source:HGNC Symbol;Acc:HGNC:28588]	-	50
12	35	3	0	8	2.432119089
0.52608202	1.643719456	0.167705349	0	0.374226571
ENSG00000137776 1831.373693	1489.586212	1836.616835
1291.62505	1427.868544	1242.591548	1719.192247
1320.695047	0.380680145	0.013187525	1	SLTM	15
58879045	58933653	-	8157	protein_coding
SAFB like transcription modulator [Source:HGNC
Symbol;Acc:HGNC:20709]	-	1904	1712	1974	1172	1178
1346	11.28593899	9.146014715	11.29698942
7.983791617	8.876917596	7.67265403
ENSG00000267016 7.694847448	8.700854042	13.95605498	0

0	0	10.11725216	0	5.680584488
0.013212438	1	AC111170.1	17	77469162
77472770	-	1132	antisense	"novel transcript,
antisense to SEPT9"	-	8	10	15	0	0
0	0.341699841	0.384956955	0.61857287	0
0	0
ENSG00000108298 28055.41379	28787.64568	31948.20106
32428.3849	37093.06722	36819.85238	29597.08684
35447.1015	-0.260215697	0.013236812	0.150948991	RPL19
17	39200283	39204732	+	1761
protein_coding	ribosomal protein L19 [Source:HGNC
Symbol;Acc:HGNC:10312]	-	29168	33086	34338	29425	30602
39884	800.84508	818.7352843	910.2520719
928.4727439	1068.1637	1053.101836
ENSG00000269321 16.35155083	8.700854042	5.582421991	0
0	0	10.21160895	0	5.693533345
0.013261444	1	AC011466.2	19	48145908
48147413	+	359	antisense	"novel transcript,
antisense to LIG1"	-	17	10	6	0	0
0	2.289579296	1.213847559	0.780194416	0
0	0
ENSG00000155275 211.6083048	379.3572363	246.5569713
166.4124425	147.877727	161.5553647	279.1741708
158.6151781	0.815887304	0.013289341	1	TRMT44	4
8436140 8493531 +	7724	protein_coding	tRNA
methyltransferase 44 homolog [Source:HGNC Symbol;Acc:HGNC:26653]
-	220	436	265	151	122	175
1.377151223	2.459817132	1.601583717	1.086292362
0.970878594	1.053481229
ENSG00000033867 2067.028396	1969.873355	1925.005183
1837.149282	1441.201782	1067.188581	1987.302311
1448.513215	0.456849326	0.013299298	1	SLC4A7	3
27372721	27484420	-	9616	protein_coding
solute carrier family 4 member 7 [Source:HGNC
Symbol;Acc:HGNC:11033]	-	2149	2264	2069	1667	1189
1156	10.80545794	10.25983947	10.04412414
9.632814316	7.600370439	5.589775908
ENSG00000235947 7.694847448	6.960683234	15.81686231	0
0	0	10.15746433	0	5.6861333
0.013316926	1	EGOT	3	4749192 4751590 -	1464
lincRNA eosinophil granule ontogeny transcript [Source:HGNC
Symbol;Acc:HGNC:37129]	-	8	8	17	0	0
0	0.264210533	0.238126379	0.542068138	0
0	0
ENSG00000270083 0	0	0	17.63310649	0
17.54029674	0	11.72446774	-6.116771561	0.013337331
1	AL021878.2	22	42089630	42090028
-	399	sense_intronic	"novel transcript, sense
intronic to NDUFA6"	-	0	0	0	16	0
19	0	0	0	2.228225452	0

2.214173881
ENSG00000038219 2366.16559 1517.428945 1713.803551
1538.488541 1267.869692 1285.980703 1865.799362
1364.112979 0.451655084 0.013345756 1 BOD1L1 4
13568738 13627723 - 15860 protein_coding
biorientation of chromosomes in cell division 1 like 1
[Source:HGNC Symbol;Acc:HGNC:31792] - 2460 1744 1842
1396 1046 1393 7.499514353 4.791835442
5.421662495 4.890961891 4.053926419 4.083936226
ENSG00000184990 780.06516 689.9777256 891.3267113
902.5946383 1069.083239 1334.908899 787.1231989
1102.195592 -0.48652321 0.013349961 1 SIVA1 14
104753100 104768494 + 4776 protein_coding
SIVA1 apoptosis inducing factor [Source:HGNC
Symbol;Acc:HGNC:17712] - 811 793 958 819 882
1446 8.21027592 7.235481992 9.363694302
9.528655535 11.35145749 14.07780655
ENSG00000163453 61.55877958 62.64614911 82.8059262
7.714484088 37.57548801 1.846347025 69.0036183
15.71210637 2.151632617 0.01339748 1 IGFBP7 4
57030773 57110385 - 1930 protein_coding
insulin like growth factor binding protein 7 [Source:HGNC
Symbol;Acc:HGNC:5476] - 64 72 89 7 31
2 1.603333554 1.625675217 2.15267632
0.201536065 0.987305929 0.048184095
ENSG00000184216 11636.53305 8818.315572 11744.48547
12540.44492 13501.72132 13991.61776 10733.11136
13344.59467 -0.314261701 0.013418559 0.152077004 IRAK1
X 154010500 154019980 - 4001
protein_coding interleukin 1 receptor associated kinase 1
[Source:HGNC Symbol;Acc:HGNC:6112] - 12098 10135 12623
11379 11139 15156 146.1996205 110.3859499
147.2787423 158.0329291 171.1295517 176.1355676
ENSG00000245648 1.923711862 30.45298915 1.86080733 0
0 0 11.41250278 0 5.856123472
0.013441382 1 AC022075.1 12 10363769
10398506 + 2830 antisense uncharacterized
LOC101928100 [Source:NCBI gene;Acc:101928100] - 2 35
2 0 0 0 0.034169984 0.538939738
0.032990553 0 0 0
ENSG00000196091 0 0 0 9.918622399 4.848450065
13.84760269 0 9.538225052 -5.81891277
0.013521584 1 MYBPC1 12 101568353 101686018
+ 7385 protein_coding "myosin binding protein C,
slow type [Source:HGNC Symbol;Acc:HGNC:7549]" - 0 0
0 9 4 15 0 0 0
0.067717989 0.033293301 0.094443436
ENSG00000154122 2568.155336 2513.676733 2464.639309
2175.484513 1989.076639 1905.43013 2515.490459
2023.330427 0.314270691 0.013524443 1 ANKH 5

14704800	14871785	-	12702	protein_coding
	ANKH inorganic pyrophosphate transport regulator [Source:HGNC
Symbol;Acc:HGNC:15492]	-	2670	2889	2649	1974	1641
	2064	10.16343162	9.911377809	9.735446752
8.635491948	7.941157924	7.55559382
ENSG00000158859 242.3876946	227.9623759	157.2382194
139.9627827	112.726464	55.39041076	209.1960966
102.6932192	1.029950424	0.013622776	1	ADAMTS4 1
	161184308	161199056	-	10334	protein_coding
	ADAM metallopeptidase with thrombospondin type 1 motif 4
[Source:HGNC Symbol;Acc:HGNC:220]	-	252	262	169
	127	93	60	1.179052925	1.104819757
0.763421318	0.682884582	0.553174117	0.269968946
ENSG00000104313 160.6299405	109.6307609	154.4470084
284.3338421	350.3005172	161.5553647	141.5692366
265.3965747	-0.905264325	0.013631937	1	EYA1	8
	71197433	71592025	-	9418	protein_coding
	EYA transcriptional coactivator and phosphatase 1 [Source:HGNC
Symbol;Acc:HGNC:3519]	-	167	126	166	258	289
	175	0.857351677	0.583002553	0.822802188
1.522204717	1.886194685	0.863993312
ENSG00000174373 959.9322191	658.654651	788.9823081
633.6897644	541.8142948	504.052738	802.5230594
559.8522657	0.519557619	0.013636855	1	RALGAPA1
	14	35538352	35809304	-	14258
protein_coding	Ral GTPase activating protein catalytic alpha subunit
1 [Source:HGNC Symbol;Acc:HGNC:17770]	-	998	757	848
	575	447	546	3.384333458	2.313640441
2.77640794	2.240893815	1.927064597	1.780594594
ENSG00000237854 200.0660336	174.0170808	220.5056687
121.2276071	110.302239	96.01004532	198.196261
109.1799638	0.861123048	0.013637066	1	LINC00674
	17	68101908	68115518	+	734
transcribed_unprocessed_pseudogene	long intergenic non-protein
coding RNA 674 [Source:HGNC Symbol;Acc:HGNC:44355]	-	208
	200	237	110	91	104	13.70151188
11.87387666	15.0729522	8.32738548	7.620661875
6.588223101
ENSG00000153914 1559.168464	1401.707586	1465.385773
1158.274682	1198.779279	1088.421572	1475.420608
1148.491844	0.361663487	0.01366611	1	SREK1	5
	66139971	66183615	+	18240	protein_coding
	splicing regulatory glutamic acid and lysine rich protein 1
[Source:HGNC Symbol;Acc:HGNC:17882]	-	1621	1611	1575
	1051	989	1179	4.296941067	3.848835097
4.030897548	3.201768486	3.332873405	3.005522539
ENSG00000241685 467.4619824	509.8700469	612.2056117
730.67185	722.4190597	771.7730566	529.8458803
741.6213221	-0.485301199	0.013708359	1	ARPC1A	7
	99325910	99366262	+	2053	protein_coding

```
	actin related protein 2/3 complex subunit 1A [Source:HGNC
Symbol;Acc:HGNC:703]	-	486	586	658	663	596
	836	11.44586282	12.43847863	14.9617702
17.94471737	17.84451179	18.93426043
ENSG00000152234 3872.431978	4085.050973	3739.29233
5780.35272	5025.418493	4055.501241	3898.925094
4953.757485	-0.345185812	0.013746245	0.154835001
ATP5F1A 18	46080248	46104334	-	10908
protein_coding	ATP synthase F1 subunit alpha [Source:HGNC
Symbol;Acc:HGNC:823]	-	4026	4695	4019	5245	4146
	4393	17.84554673	18.75638182	17.19962241
26.71852055	23.36315707	18.72608939
ENSG00000089685 720.4300923	579.4768792	757.3485835
1639.878903	878.7815743	713.6131253	685.7518517
1077.424534	-0.651622034	0.013755778	1	BIRC5	17
	78214186	78225636	+	3815	protein_coding
	baculoviral IAP repeat containing 5 [Source:HGNC
Symbol;Acc:HGNC:593]	-	749	666	814	1488	725
	773	9.49267237	7.607435601	9.96037979
21.67306994	11.6812905	9.421411431
ENSG00000225137 92.33816937	81.788028	63.26744923
136.6565753	151.5140645	182.7883555	79.13121553
156.9863318	-0.989382051	0.013763536	1	DYNC1I2P1
	10	50264978	50266815	+	1838
processed_pseudogene	dynein cytoplasmic 1 intermediate chain 2
pseudogene 1 [Source:HGNC Symbol;Acc:HGNC:45012]	-	96
	94	68	124	125	198	2.525381197
2.228645251	1.727068016	3.748765045	4.180342507
5.00899619
ENSG00000272072 76.94847448	15.66153728	33.49453195
2.204138311	0	14.7707762	42.0348479	5.658304838
	2.860999483	0.013796973	1	AC004492.1	7
	107192559	107193300	-	742	antisense
	"novel transcript, antisense to HBP1"	-	80	18
	36	2	0	16	5.212994878	1.057127079
	2.264877051	0.149774588	0	1.002644776
ENSG00000172247 0	0	0	45.18483537	0	0
	0	15.06161179	-6.475208128	0.013820462	1
	C1QTNF4 11	47589664	47594659	-	1664
	protein_coding	C1q and TNF related 4 [Source:HGNC
Symbol;Acc:HGNC:14346]	-	0	0	0	41	0
	0	0	0	0	1.369123354	0	0
ENSG00000041353 1002.25388	1273.805032	1168.587003
1974.907927	1517.56487	1257.362324	1148.215305
1583.278374	-0.46296114	0.013832265	1	RAB27B	18
	54717860	54895516	+	7432	protein_coding
	"RAB27B, member RAS oncogene family [Source:HGNC
Symbol;Acc:HGNC:9767]"	-	1042	1464	1256	1792	1252
	1362	6.778962547	8.58408429	7.889145652
13.39813496	10.35490188	8.521232689
```

ENSG00000261315 17.31340676 0 20.46888063 0 0
0 12.5940958 0 5.995690563 0.013843036
1 LARP4P 15 36617089 36619265 +
2177 transcribed_processed_pseudogene La
ribonucleoprotein domain family member 4 pseudogene [Source:HGNC
Symbol;Acc:HGNC:21438] - 18 0 22 0 0
0 0.399774688 0 0.471748239 0 0
0
ENSG00000159792 1130.180719 1128.500769 1122.997224
1184.724342 1558.776696 1840.807984 1127.226237
1528.103007 -0.439439198 0.013853337 1 PSKH1 16
67893272 67929678 + 3977 protein_coding
protein serine kinase H1 [Source:HGNC Symbol;Acc:HGNC:9529]
- 1175 1297 1207 1075 1286 1994
14.28510682 14.21160025 14.16764659 15.01982653
19.87617161 23.31312977
ENSG00000248428 0 0 0 15.42896818 0
19.38664377 0 11.60520398 -6.102394039 0.013890485
1 AC010395.1 5 110970951 111008899
- 697 lincRNA novel transcript - 0
0 0 14 0 21 0 0 0
1.116110776 0 1.400933546
ENSG00000230160 0 0 0 5.510345777 14.5453502
8.308561615 0 9.454752529 -5.802673244
0.013991975 1 AC004854.1 7 44919669
44920329 + 349 processed_pseudogene mitochondrial
ribosomal protein S23 (MRPS23) pseudogene - 0 0
0 5 12 9 0 0 0
0.796079831 2.113504512 1.199079838
ENSG00000115361 3.847423724 4.350427021 23.26009163 0
0 0 10.48598079 0 5.732003701
0.014020886 1 ACADL 2 210187939 210225491
- 4383 protein_coding acyl-CoA dehydrogenase long
chain [Source:HGNC Symbol;Acc:HGNC:88] - 4 5 25
0 0 0 0.04412551 0.04971153
0.266265301 0 0 0
ENSG00000126247 5730.737637 6311.599522 6216.957291
6576.04665 8608.423091 7391.850316 6086.431483
7525.440019 -0.306042141 0.014022147 0.156181498 CAPNS1
19 36139575 36150353 + 3478
protein_coding calpain small subunit 1 [Source:HGNC
Symbol;Acc:HGNC:1481] - 5958 7254 6682 5967 7102
8007 82.82703933 90.88800512 89.68564368
95.33195599 125.515815 107.0462034
ENSG00000145107 174.0959235 207.0803262 110.7180362
73.83863341 88.48421369 81.23926912 163.964762
81.18737207 1.014850776 0.014029916 1 TM4SF19 3
196319342 196338503 - 1899 protein_coding
transmembrane 4 L six family member 19 [Source:HGNC
Symbol;Acc:HGNC:25167] - 181 238 119 67 73

88	4.608449434	5.461483049	2.925283978
1.960477587	2.362899528	2.154709496
ENSG00000277310 11.54227117	16.53162268	2.791210996	0
0	0	10.28836828	0	5.705318155
0.014056046	1	AC090246.1	18	50256036
50256461	-	426	sense_intronic	"novel transcript,
sense intronic to CCDC11"	-	12	19	3	0
0	0	1.36198669	1.943580797	0.328743891
0	0	0
ENSG00000228022 30.77938979	55.68546587	4.652018326	0
0	7.385388102	30.37229133	2.461796034
3.574535235	0.014074315	1	HCG20	6	30766825
30792250	+	1371	lincRNA HLA complex group 20
[Source:HGNC Symbol;Acc:HGNC:31334]	-	32	64	5
0	0	8	1.128531641	2.034234975
0.170246654	0	0	0.271321088
ENSG00000141469 448.2248638	626.4614911	570.3374468
279.9255655	439.9968434	352.6522819	548.3412672
357.5248969	0.618110768	0.014094142	1	SLC14A1 18
45687025	45752520	+	7182	protein_coding
solute carrier family 14 member 1 (Kidd blood group)
[Source:HGNC Symbol;Acc:HGNC:10918]	-	466	720	613
254	363	382	3.137196576	4.368634321
3.984383285	1.965171058	3.106766292	2.473141586
ENSG00000150990 3185.666843	2506.71605	2941.936389
3176.163306	3878.760052	3918.871562	2878.106427
3657.93164	-0.346094866	0.014102117	0.156181498	DHX37
12	124946825	124989122	-	6618
protein_coding	DEAH-box helicase 37 [Source:HGNC
Symbol;Acc:HGNC:17210]	-	3312	2881	3162	2882	3200
4245	24.19718148	18.97033906	22.30391691
24.19798802	29.72148986	29.82510284
ENSG00000197157 3793.559792	3287.182657	3919.790641
4857.920837	4481.179973	4235.520076	3666.844363
4524.873629	-0.303325342	0.014120985	0.156181498	SND1
7	127652180	128092609	+	6500
protein_coding	staphylococcal nuclease and tudor domain containing 1
[Source:HGNC Symbol;Acc:HGNC:30646]	-	3944	3778	4213
4408	3697	4588	29.33761237	25.32836725
30.25687969	37.68254903	34.96096842	32.82018847
ENSG00000204577 21.16083048	40.0239286	41.86816493
11.02069155	0	0	34.35097467	3.673563851
3.232693323	0.014132259	1	LILRB3	19	54216278
54223506	-	3736	protein_coding	leukocyte
immunoglobulin like receptor B3 [Source:HGNC Symbol;Acc:HGNC:6607]
-	22	46	45	10	0	0
0.28471938	0.536549213	0.562278765	0.14873226	0
0
ENSG00000163395 229.8835675	180.1076787	386.1175211
122.3296763	44.8481631	191.0969171	265.3695891

119.4249188	1.147314841	0.014151581	1	IGFN1	1
	201190825	201228952	+	12116	protein_coding
	immunoglobulin-like and fibronectin type III domain containing
1 [Source:HGNC Symbol;Acc:HGNC:24607]	-	239	207	415
	111	37	207	0.953761643	0.744508531
1.598949943	0.509067953	0.187711042	0.794405238
ENSG00000163482 865.6703379	492.4683388	790.8431154
251.2717674	442.4210685	594.5237422	716.327264
429.405526	0.736893252	0.014227236	1	STK36	2
	218672026	218702716	+	6458	protein_coding
	serine/threonine kinase 36 [Source:HGNC Symbol;Acc:HGNC:17209]
	-	900	566	850	228	365	644
6.73822774	3.819240335	6.144222309	1.961773438
3.47409895	4.636805136
ENSG00000129038 1588.985998	1545.271678	1737.063643
2180.994859	2374.528419	1763.261409	1623.773773
2106.261562	-0.374787048	0.014254729	1	LOXL1	15
	73925989	73952137	+	3637	protein_coding
	lysyl oxidase like 1 [Source:HGNC Symbol;Acc:HGNC:6665] -
	1652	1776	1867	1979	1959	1910	21.96180132
	21.27934511	23.96332361	30.23531773	33.108423
	24.41861819
ENSG00000133935 364.5433978	499.429022	434.4985116
731.7739192	607.2683707	558.5199752	432.8236438
632.520755	-0.546538428	0.014255911	1	ERG28	14
	75649791	75661189	-	2633	protein_coding
	ergosterol biosynthesis 28 homolog [Source:HGNC
Symbol;Acc:HGNC:1187]	-	379	574	467	664	501
	605	6.95968474	9.499913065	8.279651501
14.01294005	11.69591563	10.6840407
ENSG00000229647 55.78764399	60.9059783	34.42493561
18.73517564	13.33323768	1.846347025	50.37285263
11.30492012	2.168137919	0.01427341	1	MYOSLID 2
	207239650	207245887	+	1204
lincRNA "myocardin-induced smooth muscle lncRNA, inducer of
differentiation [Source:HGNC Symbol;Acc:HGNC:51821]"	-	58
	70	37	17	11	2	2.329178235
2.533553916	1.434568442	0.784575023	0.56158249
0.077238624
ENSG00000278829 34.62681351	0	13.02565131	0	0
	0.923173513	15.88415494	0.307724504	5.368391076
	0.014275319	1	AC099811.5	17	42272069
	42275571	-	819	sense_intronic	"novel
transcript, sense intronic to STAT5B"	-	36	0	14
	0	0	1	2.125297912	0	0.797976625
	0	0	0.056773689
ENSG00000128591 2914.423471	2356.191275	4157.97398
4037.981385	4136.940018	4514.318477	3142.862908
4229.746627	-0.428655522	0.014288087	0.157083399	FLNC
	7	128830377	128859274	+	9280

protein_coding filamin C [Source:HGNC Symbol;Acc:HGNC:3756] -
3030 2708 4469 3664 3413 4890 15.78686404
12.71625656 22.48062671 21.93913661 22.60662198
24.50145645
ENSG00000179943 598.274389 711.7298607 750.8357578
763.7339247 990.2959258 1159.505932 686.9466692
971.1785942 -0.499935142 0.014293088 1 FIZ1 19
55591371 55601970 - 6790 protein_coding
FLT3 interacting zinc finger 1 [Source:HGNC
Symbol;Acc:HGNC:25917] zf-C2H2 622 818 807 693 817
1256 4.429164669 5.249792368 5.548170467
5.67120706 7.396046662 8.601038333
ENSG00000168209 563.6475755 729.1315688 692.2203269
221.5159002 562.4202076 423.7366423 661.6664904
402.5575834 0.717686551 0.014299769 1 DDIT4 10
72273920 72276036 + 2058 protein_coding
DNA damage inducible transcript 4 [Source:HGNC
Symbol;Acc:HGNC:24944] - 586 838 744 201 464
459 13.76744855 17.74423359 16.87615871
5.427036352 13.85861948 10.37046746
ENSG00000243716 1090.744626 719.5606293 905.2827662
689.8952913 614.5410458 621.2957741 905.1960071
641.9107037 0.49554306 0.01434982 1 NPIPB5 16
22479121 22536521 + 10005 protein_coding
nuclear pore complex interacting protein family member B5
[Source:HGNC Symbol;Acc:HGNC:37233] - 1134 827 973
626 507 673 5.480209713 3.602027418
4.539852921 3.476716542 3.114857811 3.127728082
ENSG00000205755 2.885567793 25.23247672 3.721614661 0
0 0 10.61321973 0 5.751227697
0.014431683 1 CRLF2 X 1187549 1212750 - 2010
protein_coding cytokine receptor like factor 2 [Source:HGNC
Symbol;Acc:HGNC:14281] - 3 29 4 0 0
0 0.072164966 0.628724723 0.092898771 0
0 0
ENSG00000068784 330.8784402 345.4239055 302.3811912
217.1076236 220.604478 180.9420085 326.2278456
206.2180367 0.663079815 0.014468101 1 SRBD1 2
45388680 45612165 - 5045 protein_coding
S1 RNA binding domain 1 [Source:HGNC Symbol;Acc:HGNC:25521]
- 344 397 325 197 182 196
3.296844689 3.429161459 3.007240948 2.169786982
2.217469104 1.80644989
ENSG00000136002 0 12.18119566 25.12089896 0 0
0 12.43403154 0 5.978417153 0.014552305
1 ARHGEF4 2 130836916 131047263 +
10611 protein_coding Rho guanine nucleotide exchange factor
4 [Source:HGNC Symbol;Acc:HGNC:684] - 0 14 27
0 0 0 0 0.057495032 0.11878278
0 0 0

ENSG00000215067 91.37631344 64.38631991 59.54583457
19.8372448 20.60591278 34.15741997 71.76948931
24.86685918 1.523143594 0.014600641 1 ALOX12-AS1
17 6876635 7012349 - 3217 antisense ALOX12
antisense RNA 1 [Source:HGNC Symbol;Acc:HGNC:51342] - 95
74 64 18 17 37 1.427820986
1.002395842 0.928698938 0.310909139 0.324821839
0.534788034
ENSG00000111669 17721.23367 18966.99173 21581.64342
20505.09871 25987.69235 25199.86738 19423.28961
23897.55281 -0.299072617 0.014617135 0.158877008 TPI1
12 6867119 6870948 + 2700 protein_coding
triosephosphate isomerase 1 [Source:HGNC Symbol;Acc:HGNC:12009] -
18424 21799 23196 18606 21440 27297 329.9296735
351.8288145 401.0470921 382.9140449 488.0995161
470.0912567
ENSG00000168282 64.44434737 69.60683234 89.31875186
11.02069155 38.78760052 24.00251133 74.45664386
24.60360114 1.601547849 0.014639907 1 MGAT2 14
49620795 49623481 + 2687 protein_coding
"mannosyl (alpha-1,6-)-glycoprotein beta-1,2-N-
acetylglucosaminyltransferase [Source:HGNC Symbol;Acc:HGNC:7045]"
- 67 80 96 10 32 26
1.205614195 1.297420986 1.667821632 0.206797068
0.732031336 0.449921451
ENSG00000146535 3935.914469 3684.811687 3755.109193
4693.712533 4519.967573 4473.698843 3791.945116
4562.45965 -0.266886184 0.014683603 0.158877008 GNA12
7 2728112 2844324 - 6120 protein_coding G
protein subunit alpha 12 [Source:HGNC Symbol;Acc:HGNC:4380] -
4092 4235 4036 4259 3729 4846 32.32848995
30.15508731 30.78546879 38.66947373 37.45314754
36.81823843
ENSG00000232716 43.28351689 0 5.582421991 1.102069155
0 0 16.28864629 0.367356385 5.404560309
0.014723518 1 AC016831.2 7 130840204
130843725 - 1723 processed_pseudogene zinc
finger protein 131 (ZNF131) pseudogene - 45 0 6
1 0 0 1.262782204 0 0.16255937
0.032249781 0 0
ENSG00000110092 8431.629091 8921.855735 10373.07046
8397.766964 6242.379459 7485.090841 9242.185096
7375.079088 0.325479059 0.014793435 0.158877008 CCND1
11 69641087 69654474 + 4830
protein_coding cyclin D1 [Source:HGNC Symbol;Acc:HGNC:1582] -
8766 10254 11149 7620 5150 8108 87.75170269
92.51342938 107.7543522 87.66371764 65.54015415
78.05444272
ENSG00000106628 4737.14046 4669.748365 5053.952709
5250.257456 6264.197484 6099.407399 4820.280511

5871.287446	-0.284570345	0.014836319	0.158877008	POLD2
	7	44114681	44124358	-	3695
protein_coding	"DNA polymerase delta 2, accessory subunit
[Source:HGNC Symbol;Acc:HGNC:9176]"	-	4925	5367	5432
	4764	5168	6607	64.445561	63.2959249
68.62642166	71.64227258	85.97168177	83.14207942
ENSG00000279031 24.04639827	13.05128106	0	0	0
	0	12.36589311	0	5.969837021	0.01484786
	1	AC004232.3	16	3292879 3293403 +	525
	TEC	TEC	-	25	15	0	0	0
	0	2.302406071	1.245060782	0	0	0
	0
ENSG00000110108 4309.114571	4096.362083	4886.48005
5102.58019	5443.597311	5692.287879	4430.652234
5412.821793	-0.288976984	0.014883903	0.158877008
TMEM109 11	60913874	60923443	+	2421
protein_coding	transmembrane protein 109 [Source:HGNC
Symbol;Acc:HGNC:28771]	-	4480	4708	5252	4630	4491
	6166	89.47144286	84.74230301	101.26887
106.2669572	114.0238303	118.4240063
ENSG00000232097 6.732991517	16.53162268	6.512825656	0
	0	0	9.925813285	0	5.65386963
0.014890483	1	AC079781.3	7	97938439
97938794	-	356	processed_pseudogene	novel
pseudogene	-	7	19	7	0	0	0
	0.950712619	2.325745561	0.917897269	0	0
	0
ENSG00000271032 0	0	4.652018326	20.93931395
20.60591278	21.23299079	1.550672775
20.92607251	-3.759120716	0.014934965	1	AC020907.4
	19	35014961	35025335	-	526
antisense	novel transcript	-	0	0	5
	19	17	23	0	0	0.443741754
2.007150327	1.986600486	2.033167271
ENSG00000186480 1941.987125	2630.268177	2229.247182
3717.279261	3576.944036	2126.0686	2267.167494
3140.097299	-0.469388273	0.014974905	0.158924425	INSIG1
	7	155297776	155310235	+	3650
protein_coding	insulin induced gene 1 [Source:HGNC
Symbol;Acc:HGNC:6083]	-	2019	3023	2396	3373	2951
	2303	26.7451274	36.0914126	30.64361414
51.34941738	49.69625989	29.33810723
ENSG00000276916 53.86393213	37.41367238	34.42493561
12.12276071	3.636337549	12.92442918	41.90084671
9.561175812	2.12042461	0.015139652	1	AL442125.2
	13	113511747	113514473	+	418
sense_intronic	"novel transcript, sense intronic"	-	56
	43	37	11	3	14	6.477582631
4.482814536	4.132106231	1.462272953	0.441156145
1.55733761

ENSG00000176563 36.55052538	35.67350157	33.49453195
6.612414933	0	10.15490864	35.23951963	5.589107858
	2.63328868	0.015140205	1	CNTD1	17
42798792	42811587	+	3378	protein_coding	cyclin
N-terminal domain containing 1 [Source:HGNC Symbol;Acc:HGNC:26847]
	-	38	41	36	6	0	11
0.543907651	0.528911256	0.497495196	0.098696931	0
	0.151413312
ENSG00000154529 0	0	0	1.102069155	19.39380026
	9.231735127	0	9.909201515	-5.869994003
0.015175529	1	CNTNAP3B	9	41890314
42129510	-	13220	protein_coding	contactin associated
protein like 3B [Source:HGNC Symbol;Acc:HGNC:32035]	-	0
	0	0	1	16	10	0	0	0
	0.004203205	0.074393654	0.035172202
ENSG00000129195 258.7392454	213.170924	234.4617236
580.7904449	374.5427675	252.9495425	235.4572977
402.7609183	-0.773728407	0.015195445	1	PIMREG	17
	6444415 6451469 +	2823	protein_coding	PICALM
interacting mitotic regulator [Source:HGNC Symbol;Acc:HGNC:25483]
	-	269	245	252	527	309	274
4.607258907	3.781932767	4.16711704	10.37317682
6.728139097	4.513055796
ENSG00000166275 287.5949234	394.1486881	331.2237048
415.4800716	504.2388068	624.9884681	337.6557721
514.9024488	-0.609129474	0.015213538	1	BORCS7	10
	102854223	102864961	+	2708	protein_coding
	BLOC-1 related complex subunit 7 [Source:HGNC
Symbol;Acc:HGNC:23516]	-	299	453	356	377	416
	677	5.338555288	7.289674554	6.136876362
7.735791108	9.442609523	11.62441287
ENSG00000133142 833.9290921	937.9520658	925.7516469
705.3242595	512.7235944	719.1521664	899.2109349
645.7333401	0.476679463	0.015219623	1	TCEAL4	X
	103576231	103587736	+	2518	protein_coding
	transcription elongation factor A like 4 [Source:HGNC
Symbol;Acc:HGNC:26121]	-	867	1078	995	640	423
	779	16.64809664	18.65613316	18.44647514
14.1233035	10.32599717	14.38509552
ENSG00000253167 0.961855931	0	40.0073576	0	0
	0	13.65640451	0	6.113044443	0.015260207
	1	WASHC5-AS1	8	125040684	125044989
	+	587	antisense	WASHC5 antisense RNA 1
[Source:HGNC Symbol;Acc:HGNC:43440]	-	1	0	43
	0	0	0	0.082368871	0	3.419608518
	0	0	0
ENSG00000235079 16.35155083	14.79145187	5.582421991
1.102069155	0	0	12.24180823	0.367356385
4.99385105	0.015283256	1	ZRANB2-AS1	1
71048855	71067184	+	967	antisense	ZRANB2

antisense RNA 1 [Source:HGNC Symbol;Acc:HGNC:43594]	-	17
	17	6	1	0	0	0.850009273
0.766092208	0.289648185	0.057462639	0	0
ENSG00000279583 18.27526269	6.960683234	4.652018326	0
	0	0	9.962654749	0	5.657662039
0.015349836	1	AC009086.3	16	29595130
29596137	-	1008	TEC	novel transcript	-
	19	8	5	0	0	0	0.91136907
	0.345850217	0.231555717	0	0	0
ENSG00000141543 4153.29391	3792.702277	3733.709908
4571.382857	4808.450352	4712.800782	3893.235365
4697.544664	-0.270964678	0.015437511	0.162253262	EIF4A3
	17	80135214	80147183	-	4967
protein_coding	eukaryotic translation initiation factor 4A3
[Source:HGNC Symbol;Acc:HGNC:18683]	-	4318	4359	4013
	4148	3967	5105	42.0329329	38.24294305
37.71560126	46.40412963	49.09252847	47.78951297
ENSG00000206053 1607.261261	1713.198161	1657.048928
2011.276209	2119.984791	2055.907413	1659.16945
2062.389471	-0.313715225	0.015462927	1	JPT2	16
	1678256 1702280 +	4768	protein_coding	Jupiter
microtubule associated homolog 2 [Source:HGNC Symbol;Acc:HGNC:14137]
	-	1671	1969	1781	1825	1749	2227
16.94499401	17.99567193	17.43707793	21.26858835
22.54763121	21.71775795
ENSG00000163041 2228.620192	2483.223744	2453.474465
3011.955002	3031.493403	2779.675447	2388.439467
2941.041284	-0.299981341	0.015465316	0.162253262	H3F3A
	1	226061851	226072001	+	3146
protein_coding	H3 histone family member 3A [Source:HGNC
Symbol;Acc:HGNC:4764]	-	2317	2854	2637	2733	2501
	3011	35.6097178	39.53246073	39.12888272
48.27174038	48.86549851	44.50236131
ENSG00000197172 1776.547904	1738.430638	1673.796194
1996.94931	2187.863092	2299.62522	1729.591579
2161.479207	-0.321777122	0.015479451	1	MAGEA6	X
	152766136	152769747	-	1863	protein_coding
	MAGE family member A6 [Source:HGNC Symbol;Acc:HGNC:6804]
	-	1847	1998	1799	1812	1805	2491
47.93527873	46.73489021	45.07796939	54.04523158
59.55410627	62.17157804
ENSG00000183688 522.2877705	553.3743171	417.7512457
732.8759884	819.388061	595.4469157	497.8044444
715.903655	-0.523112551	0.015482377	1	RFLNB	17
	439978	445939	-	3621	protein_coding	refilin B
[Source:HGNC Symbol;Acc:HGNC:28705]	-	543	636	449
	665	676	645	7.250576202	7.65397763
5.788470869	10.20481566	11.47533919	8.282514559
ENSG00000137343 533.8300417	482.027314	615.9272264
362.5807521	421.8151557	332.3424646	543.928194

372.2461241	0.548170432	0.015552737	1	ATAT1	6
	30626842	30646823	+	4035	protein_coding
	alpha tubulin acetyltransferase 1 [Source:HGNC
Symbol;Acc:HGNC:21186]	-	555	554	662	329	348
	360	6.650444302	5.983080187	7.658795725
4.530690571	5.301308343	4.148489353
ENSG00000258858 14.42783896	2.610256213	13.02565131	0
	0	0	10.02124883	0	5.665909222
0.015640076	1	AL583722.4	14	104690091
104691284	-	716	antisense	"novel transcript,
antisense to INF2"	-	15	3	14	0	0
	0	1.012930045	0.182585729	0.912769352	0
	0	0
ENSG00000248487 130.8124066	163.576056	198.1759807
239.1490067	298.179679	291.72283	164.1881478
276.3505053	-0.750929739	0.015657725	1	ABHD14A 3
	51971426	51981199	+	2378	protein_coding
	abhydrolase domain containing 14A [Source:HGNC
Symbol;Acc:HGNC:24538]	-	136	188	213	217	246
	316	2.765210992	3.445121927	4.18132369
5.070606715	6.358733402	6.178830056
ENSG00000163166 822.386821	1015.389667	784.3302898
624.8732111	667.8739965	624.9884681	874.0355925
639.2452252	0.451842	0.015673376	1	IWS1	2
	127436207	127526886	-	4150	protein_coding
	"IWS1, SUPT6H interacting protein [Source:HGNC
Symbol;Acc:HGNC:25467]" -	855	1167	843	567	551
	677	9.961373736	12.25409822	9.482558131
7.591839286	8.161140645	7.585279534
ENSG00000204392 1311.009634	1269.454605	1199.290324
1478.976807	1656.95781	1678.329446	1259.918188
1604.754688	-0.349137021	0.015685635	1	LSM2	6
	31797396	31806984	-	1077	protein_coding
	"LSM2 homolog, U6 small nuclear RNA and mRNA degradation
associated [Source:HGNC Symbol;Acc:HGNC:13940]" -	1363	1459
	1289	1342	1367	1818	61.19012904	59.03345293
	55.87058901	69.2386922	78.01896263	78.48907189
ENSG00000144848 1355.255007	1562.673386	1389.092672
2011.276209	1682.412173	1809.420085	1435.673688
1834.369489	-0.353515251	0.015696073	1	ATG3	3
	112532509	112562046	-	6111	protein_coding
	autophagy related 3 [Source:HGNC Symbol;Acc:HGNC:20962] -
	1409	1796	1493	1825	1388	1960	11.14807613
	12.80715443	11.40495457	16.59444105	13.96125767
	14.91333611
ENSG00000110427 1332.170464	1296.427252	1197.429517
1117.498124	907.8722747	866.8599285	1275.342411
964.0767756	0.403946067	0.015738223	1	KIAA1549L
	11	33376466	33674102	+	13012
protein_coding	KIAA1549 like [Source:HGNC Symbol;Acc:HGNC:24836]

	-	1385	1490	1287	1014	749	939
5.146440253	4.990003055	4.617219574	4.330179943
3.538222365	3.355463784
ENSG00000205143 29.81753386	6.09059783	27.91210996
4.408276622	0	0	21.27341388	1.469425541
3.867178331	0.015791054	1	ARID3C	9	34621379
	34628107	-	1411	protein_coding	AT-rich
interaction domain 3C [Source:HGNC Symbol;Acc:HGNC:21209]	ARID
	31	7	30	4	0	0	1.062272397
	0.216187024	0.992522308	0.15752338	0	0
ENSG00000259488 0	32.19315996	27.91210996	2.204138311
	0	0	20.03508997	0.73471277	4.78581263
	0.015793843	1	AC023355.1	15	48312353
	48331856	-	4710	antisense	"novel
transcript, antisense to DUT"	-	0	37	30	2
	0	0	0	0.342325629	0.297335239
0.023595063	0	0
ENSG00000233913 2861.521395	4555.767177	4041.673522
4899.799465	4796.329227	5191.004662	3819.654031
4962.377785	-0.377516151	0.015824361	0.165076859
RPL10P9 5	168616352	168616996	+	645
processed_pseudogene	ribosomal protein L10 pseudogene 9
[Source:HGNC Symbol;Acc:HGNC:35579]	-	2975	5236	4344
	4446	3957	5623	223.0121229	353.7516881
314.3953672	383.0202958	377.097537	405.3585959
ENSG00000223519 11.54227117	13.92136647	10.23444032	0
	1.212112516	0	11.89935932	0.404037505
4.953098456	0.015830652	1	KIF28P	1	246771837
	246839611	-	3525	transcribed_unitary_pseudogene
	"kinesin family member 28, pseudogene [Source:HGNC
Symbol;Acc:HGNC:49205]" -	12	16	11	0	1
	0	0.16459754	0.19779689	0.14567318	0
	0.017437661	0
ENSG00000188981 30.77938979	0	6.512825656	0	0
	0	12.43073848	0	5.976491815	0.015884406
	1	MSANTD1 4	3244369 3271738 +	7553
protein_coding	Myb/SANT DNA binding domain containing 1 [Source:HGNC
Symbol;Acc:HGNC:33741]	-	32	0	7	0	0
	0	0.204847991	0	0.043263793	0	0
	0
ENSG00000198712 56497.49367	52222.52596	53802.45275
49768.34099	78359.43784	81097.1004	54174.15746
69741.62641	-0.364423771	0.015930406	0.165244215	MT-CO2
	MT	7586	8269	+	684	protein_coding
mitochondrially encoded cytochrome c oxidase II [Source:HGNC
Symbol;Acc:HGNC:7421]	-	58738	60020	57827	45159	64647
	87846	4152.066204	3823.829216	3946.577143
3668.599126	5809.511749	5971.685225
ENSG00000271869 20.19897455	7.830768638	8.373632987	0
	0	0.923173513	12.13445872	0.307724504

4.980312634	0.015945155	1	AC026979.2	8
30155830	30156232	-	403	lincRNA novel
transcript	-	21	9	9	0	0	1
	2.519506395	0.973186467	1.042517849	0	0
	0.115378788
ENSG00000130176 4.809279655	7.830768638	16.74726597	0
	0	0	9.795771422	0	5.634075855
0.016042273	1	CNN1	19	11538717	11550323
	+	2887	protein_coding	calponin 1 [Source:HGNC
Symbol;Acc:HGNC:2155]	-	5	9	18	0	0
	0	0.083738357	0.135848336	0.291052784	0
	0	0
ENSG00000105220 4481.286782	5520.69189	6871.961471
7247.206766	7375.704662	6881.335364	5624.646714
7168.082264	-0.349749579	0.016093086	0.165993853	GPI
	19	34359480	34402413	+	10680
protein_coding	glucose-6-phosphate isomerase [Source:HGNC
Symbol;Acc:HGNC:4458]	-	4659	6345	7386	6576	6085
	7454	21.09223854	25.88922032	32.28375824
34.21390109	35.02165698	32.45257437
ENSG00000232640 43.28351689	0	4.652018326	1.102069155
	0	0	15.97851174	0.367356385	5.376812448
	0.016102169	1	AL354892.2	6	169725091
	169725854	-	403	antisense	novel
transcript	-	45	0	5	1	0	0
	5.398942276	0	0.579176583	0.137881817	0
	0
ENSG00000120008 1121.524015	1166.784527	982.5062704
923.5339522	734.5401849	784.6974858	1090.271604
814.2572076	0.421088901	0.016173455	1	WDR11	10
	120851175	120909524	+	10837	protein_coding
	WD repeat domain 11 [Source:HGNC Symbol;Acc:HGNC:13831] -
	1166	1341	1056	838	606	850	5.202243707
	5.392352845	4.548842298	4.296818299	3.437248341
	3.647042888
ENSG00000262001 134.6598303	94.83930906	85.5971372
312.9876401	138.1808269	175.4029674	105.0320922
208.8571448	-0.992384054	0.016175938	1	DLGAP1-AS2
	18	3603000 3608336 +	3171	antisense	DLGAP1
antisense RNA 2 [Source:HGNC Symbol;Acc:HGNC:28146]	-	140
	109	92	284	114	190	2.134681125
1.497920808	1.354370922	4.976616117	2.209815266
2.786046605
ENSG00000285532 9.618559309	0	26.98170629	0	0
	0	12.20008853	0	5.95005148	0.016193263
	1	AC091614.2	1	90841587	90849472
	+	754	lincRNA novel transcript	-	10
	0	29	0	0	0	0.64125368	0
	1.795447406	0	0	0
ENSG00000113240 188.5237625	261.0256213	213.992843

143.2689902	133.3323768	95.08687181	221.1807422
123.8960796	0.838667338	0.016195766	1	CLK4	5
178602664	178630615	-	7924	protein_coding
CDC like kinase 4 [Source:HGNC Symbol;Acc:HGNC:13659]	-
196	300	230	130	110	103	1.195949443
1.649815523	1.354969143	0.91161388	0.853287883
0.604399054
ENSG00000161958 10.58041524	13.92136647	4.652018326	0
0	0	9.717933345	0	5.622889254
0.016201863	1	FGF11	17	7438273 7444937 +	5565
protein_coding	fibroblast growth factor 11 [Source:HGNC
Symbol;Acc:HGNC:3667]	-	11	16	5	0	0
0	0.095571573	0.125289135	0.041942168	0
0	0
ENSG00000164096 1650.544778	2464.081865	2146.441256
1595.796137	1597.564296	1575.857186	2087.022633
1589.739207	0.392899372	0.016214553	1	C4orf3	4
119296419	119304445	-	3148	protein_coding
chromosome 4 open reading frame 3 [Source:HGNC
Symbol;Acc:HGNC:19225]	-	1716	2832	2307	1448	1318
1707	26.35625959	39.20280326	34.21045944
25.55911911	25.73522957	25.21330721
ENSG00000095380 780.06516	723.0409709	714.5500149
937.8608513	973.3263506	1013.644517	739.2187153
974.9439063	-0.399599671	0.016231913	1	NANS	9
98056739	98083075	+	4922	protein_coding
N-acetylneuraminate synthase [Source:HGNC
Symbol;Acc:HGNC:19237]	-	811	831	768	851	803
1098	7.966736651	7.357292326	7.283928038
9.60726996	10.02816096	10.37269836
ENSG00000167767 428.9877452	271.4666461	392.6303467
706.4263286	515.1478194	466.2026239	364.3615793
562.5922573	-0.626901885	0.016281506	1	KRT80	12
52168996	52192000	-	4350	protein_coding
keratin 80 [Source:HGNC Symbol;Acc:HGNC:27056]	-	446
312	422	641	425	505	4.957318451
3.125531893	4.528654928	8.188056226	6.005470435
5.39800322
ENSG00000143319 2496.977997	2666.811764	2584.661382
2923.789469	3202.401268	3379.73823	2582.817048
3168.642989	-0.295019077	0.016310769	0.166758629
ISG20L2 1	156721891	156728799	-	5262
protein_coding	interferon stimulated exonuclease gene 20 like 2
[Source:HGNC Symbol;Acc:HGNC:25745]	-	2596	3065	2778
2653	2642	3661	23.85366199	25.38272431
24.64492118	28.01550465	30.86237399	32.3504185
ENSG00000153214 431.873313	462.8854351	482.8795022
358.1724755	293.3312289	278.7984008	459.2127501
310.1007018	0.566957185	0.016333489	1	TMEM87B 2
112055223	112119318	+	5367	protein_coding

transmembrane protein 87B [Source:HGNC Symbol;Acc:HGNC:25913]
- 449 532 519 325 242 302
4.044976121 4.319551286 4.514210415 3.364835283
2.77160369 2.616413407
ENSG00000121644 2476.779022 2759.040817 2514.881107
3138.692955 3299.370269 3047.395766 2583.566982
3161.819663 -0.291134241 0.016348885 0.166758629 DESI2
1 244652935 244709033 + 5104
protein_coding desumoylating isopeptidase 2 [Source:HGNC
Symbol;Acc:HGNC:24264] - 2575 3171 2703 2848 2722
3301 24.39314426 27.07348567 24.72187555
31.00568731 32.78119758 30.07224679
ENSG00000180596 35.58866945 2.610256213 0 0 0
0 12.73297522 0 6.011232644 0.016406076
1 HIST1H2BC 6 26114873 26123926
- 834 protein_coding histone cluster 1 H2B family
member c [Source:HGNC Symbol;Acc:HGNC:4757] - 37 3
0 0 0 0 2.145047383 0.156752257
0 0 0 0
ENSG00000218980 35.58866945 2.610256213 0 0 0
0 12.73297522 0 6.011232644 0.016406076
1 FTH1P15 6 57004520 57004799 +
280 processed_pseudogene ferritin heavy chain 1
pseudogene 15 [Source:HGNC Symbol;Acc:HGNC:3985] - 37
3 0 0 0 0 6.389176847
0.466897793 0 0 0 0
ENSG00000196243 13.46598303 14.79145187 1.86080733 0
0 0 10.03941408 0 5.669734217
0.016419749 1 LINC00615 12 90918023
90948669 + 1406 lincRNA long intergenic non-protein
coding RNA 615 [Source:HGNC Symbol;Acc:HGNC:26343] - 14
17 2 0 0 0 0.481441952
0.52689272 0.06640346 0 0 0
ENSG00000125968 1609.184972 2118.657959 2031.071201
1812.903761 2705.435136 3572.681494 1919.638044
2697.006797 -0.49077332 0.016425023 1 ID1 20
31605283 31606515 + 1233 protein_coding
"inhibitor of DNA binding 1, HLH protein [Source:HGNC
Symbol;Acc:HGNC:5360]" bHLH 1673 2435 2183 1645 2232
3870 65.60456812 86.05864161 82.64882714
74.13356227 111.2700989 145.9415339
ENSG00000143669 846.4332192 833.5418173 692.2203269
672.2621848 550.2990824 432.9683775 790.7317878
551.8432149 0.519929948 0.016427298 1 LYST 1
235661041 235883640 - 19614 protein_coding
lysosomal trafficking regulator [Source:HGNC
Symbol;Acc:HGNC:1968] - 880 958 744 610 454
469 2.169290517 2.128422963 1.770731856
1.728127207 1.422777675 1.111828212
ENSG00000127564 325.1073047 498.5589366 521.9564562

814.4291059	684.8435717	537.2869844	448.5408992
678.8532207	-0.596879186	0.016447665	1	PKMYT1	16
	2968024	2980539	-	5034	protein_coding	"protein
kinase, membrane associated tyrosine/threonine 1 [Source:HGNC
Symbol;Acc:HGNC:29650]"	-	338	573	561	739	565
	582	3.246420002	4.960209371	5.202303509
8.157240576	6.898943636	5.375771389
ENSG00000141371	21.16083048	14.79145187	0	0	0
	0	11.98409412	0	5.924779547	0.016471099
	1	C17orf64	17	60392429	60431421
	+	1184	protein_coding	chromosome 17 open reading
frame 64 [Source:HGNC Symbol;Acc:HGNC:26990]	-	22	17
	0	0	0	0	0.898405072	0.625685106
	0	0	0	0
ENSG00000184613	9.618559309	14.79145187	4.652018326	0
	0	0	9.687343169	0	5.618483712
0.016486685	1	NELL2	12	44508275	44921848
	-	6424	protein_coding	neural EGFL like 2
[Source:HGNC Symbol;Acc:HGNC:7751]	-	10	17	5
	0	0	0	0.075265454	0.115319297
0.036333774	0	0	0
ENSG00000141627	1012.834295	1201.587943	930.4036652
1507.630605	1313.929968	1317.368603	1048.275301
1379.643058	-0.395995803	0.016554046	1	DYM	18
	49041474	49461347	-	7256	protein_coding
	dymeclin [Source:HGNC Symbol;Acc:HGNC:21317]	-	1053
	1381	1000	1368	1084	1427	7.016690387
8.293827574	6.433521576	10.47612971	9.182889711
9.144452686
ENSG00000279652	25.97011014	25.23247672	22.32968796	0
	0	6.462214589	24.51075827	2.15407153
3.450045219	0.016616645	1	Z82217.1	22
35992321	36000469	+	8149	lincRNA	novel
transcript	-	27	29	24	0	0	7
	0.160199318	0.155078745	0.137484253	0	0
	0.039941534
ENSG00000202566	14.42783896	15.66153728	0.930403665	0
	0	0	10.33992664	0	5.712281203
0.016624258	1	MIR421	X	74218377	74218461
	-	85	miRNA	microRNA 421 [Source:HGNC
Symbol;Acc:HGNC:32793]	-	15	18	1	0	0
	0	8.532446028	9.228097557	0.549195677	0
	0	0
ENSG00000141076	4737.14046	4561.857774	4649.227115
5305.360914	6038.744556	5419.951693	4649.40845
5588.019055	-0.265171421	0.016683527	0.168606066	UTP4
	16	69131291	69231130	+	5681
protein_coding	"UTP4, small subunit processome component [Source:HGNC
Symbol;Acc:HGNC:1983]"	-	4925	5243	4997	4814	4982
	5871	41.91627317	40.21736996	41.06110155

47.08616719	53.90465789	48.0527569
ENSG00000102225 1128.257007	1376.47511	1247.671315
1549.509233	1638.776122	1611.860953	1250.801144
1600.048769	-0.355017165	0.016687317	1	CDK16	X
	47217860	47229997	+	6524	protein_coding
	cyclin dependent kinase 16 [Source:HGNC Symbol;Acc:HGNC:8749]
	-	1173	1582	1341	1406	1352	1746
8.693312193	10.56698582	9.595350898	11.97521755
12.73825972	12.44403733
ENSG00000245556 138.5072541	174.0170808	168.4030634
102.4924315	67.87830091	78.46974858	160.3091328
82.94682698	0.950366143	0.016710874	1	SCAMP1-AS1
	5	78342365	78360507	-	1792
lincRNA SCAMP1 antisense RNA 1 [Source:HGNC Symbol;Acc:HGNC:50544]
	-	144	200	181	93	56	85
3.885310245	4.863518678	4.715053288	2.883745879
1.920867382	2.205524764
ENSG00000136240 5470.074679	5727.772216	5386.106818
7427.946108	6399.954086	6228.65169	5527.984571
6685.517295	-0.274205107	0.016713673	0.168606066	KDELR2
	7	6445953 6484242 -	3990	protein_coding	KDEL
endoplasmic reticulum protein retention receptor 2 [Source:HGNC
Symbol;Acc:HGNC:6305]	-	5687	6583	5789	6740	5280
	6747	68.91464908	71.89679934	67.72931601
93.86399715	81.34079019	78.62647987
ENSG00000236234 8.656703379	6.960683234	13.02565131	0
	0	0	9.547679308	0	5.5967777
0.016760165	1	AC091132.2	17	45452844
45464065	+	769	antisense	"novel transcript,
antisense to PLEKHM1"	-	9	8	14	0	0
	0	0.565870933	0.453338126	0.849860671	0
	0	0
ENSG00000257093 866.6321938	588.1777333	614.066419
488.2166359	501.8145818	444.0464596	689.6254487
478.0258924	0.528898374	0.016788098	1	KIAA1147
	7	141656728	141702153	-	7356
protein_coding	KIAA1147 [Source:HGNC Symbol;Acc:HGNC:29472]	-
	901	676	660	443	414	481	5.922216595
	4.00464085	4.188400963	3.346370702	3.459441419
	3.040425553
ENSG00000165646 4.809279655	14.79145187	9.304036652	0
	0	0	9.634922726	0	5.610921069
0.016788671	1	SLC18A2 10	117241073	117279430
	+	4145	protein_coding	solute carrier family 18
member A2 [Source:HGNC Symbol;Acc:HGNC:10935]	-	5	17
	10	0	0	0	0.058323917	0.178724045
	0.11262155	0	0	0
ENSG00000049759 1154.227117	1102.398207	1023.444032
1459.139562	1283.627155	1480.770314	1093.356452
1407.845677	-0.3651439	0.016835525	1	NEDD4L	18

58044367	58401540	+	17455	protein_coding
"neural precursor cell expressed, developmentally down-regulated 4-like, E3 ubiquitin protein ligase [Source:HGNC Symbol;Acc:HGNC:7728]"	-	1200	1267	1100	1324	1059
1604	3.324012202	3.163117752	2.941838775
4.214831097	3.729266906	4.272829161
ENSG00000188157 1981.423218	1633.150304	2253.437677
1515.345089	1666.65471	1253.66963	1956.003733
1478.556476	0.404242308	0.016846752	1	AGRN	1
1020123 1056118 +	10758	protein_coding	agrin
[Source:HGNC Symbol;Acc:HGNC:329]	-	2060	1877	2422
1375	1375	1358	9.258420397	7.603110991
10.50965923	7.102041437	7.85630831	5.869474878
ENSG00000104695 2276.712989	2695.524582	2245.064044
2857.66532	3002.402703	3100.016656	2405.767205
2986.694893	-0.311989061	0.016853378	0.168766452	PPP2CB
8	30774457	30814314	-	4516
protein_coding	protein phosphatase 2 catalytic subunit beta
[Source:HGNC Symbol;Acc:HGNC:9300]	-	2367	3098	2413
2593	2477	3358	25.34227161	29.89414095
24.94304237	31.90513798	33.7147104	34.57464875
ENSG00000169981 351.0774148	481.1572285	392.6303467
555.4428543	690.9041343	540.0565049	408.28833
595.4678312	-0.543210972	0.016897351	1	ZNF35	3
44648727	44660791	+	2663	protein_coding
zinc finger protein 35 [Source:HGNC Symbol;Acc:HGNC:13099]
zf-C2H2 365	553	422	504	570	585
6.6270907	9.049249503	7.397539969	10.51650454
13.15682352	10.21446719
ENSG00000084207 4542.845562	4974.278256	5589.86522
6093.34036	6568.437726	5790.144272	5035.663013
6150.640786	-0.288400061	0.016913414	0.168766452	GSTP1
11	67583595	67586656	+	1911
protein_coding	glutathione S-transferase pi 1 [Source:HGNC Symbol;Acc:HGNC:4638]	-	4723	5717	6008	5529	5419
6272	119.4974052	130.3665291	146.762558
160.7673846	174.3033862	152.6076767
ENSG00000180611 126.9649829	195.769216	120.0220728
185.1476181	306.6644666	301.8777387	147.5854239
264.5632745	-0.841360592	0.017039866	1	MB21D2	3
192796815	192918161	-	3368	protein_coding
Mab-21 domain containing 2 [Source:HGNC Symbol;Acc:HGNC:30438]
-	132	225	129	168	253	327
1.894973168	2.911179826	1.787984145	2.771719279
4.617381925	4.514469133
ENSG00000267278 25.97011014	6.09059783	41.86816493	0
6.060562582	0	24.64295763	2.020187527
3.658874238	0.017040108	1	MAP3K14-AS1	17
45247925	45268630	+	3775	antisense
MAP3K14 antisense RNA 1 [Source:HGNC Symbol;Acc:HGNC:44359]	-

27	7	45	0	5	0	0.345818342
0.080805269	0.556469792	0	0.081414247	0
ENSG00000101115 11.54227117	10.44102485	6.512825656	0
0	0	0	9.498707226	0	5.589571897
0.017053352	1	SALL4	20	51782331	51802520
-	5772	protein_coding	spalt like transcription
factor 4 [Source:HGNC Symbol;Acc:HGNC:15924]	zf-C2H2 12	12
7	0	0	0	0.100520847	0.090596938
0.056613206	0	0	0
ENSG00000130653 55.78764399	40.0239286	66.98906389
24.24552142	7.272675098	14.7707762	54.26687883
15.42965757	1.810532478	0.017077757	1	PNPLA7	9
137459953	137550534	-	9976	protein_coding
patatin like phospholipase domain containing 7 [Source:HGNC
Symbol;Acc:HGNC:24768]	-	58	46	72	22	6
16	0.281107718	0.200937035	0.336916354
0.122540115	0.03696938	0.074575223
ENSG00000169777 0	0	0	0	6.060562582
27.69520538	0	11.25192265	-6.05865721	0.017159431
1	TAS2R1	5	9628997 9712378 -	1727
protein_coding	taste 2 receptor member 1 [Source:HGNC
Symbol;Acc:HGNC:14909]	-	0	0	0	0	5
30	0	0	0	0	0.177961078
0.807718324
ENSG00000213139 0	3.480341617	0	5.510345777
10.90901265	41.54280807	1.160113872
19.32072217	-4.036024532	0.017160342	1	CRYGS	3
186538441	186546702	-	2151	protein_coding
crystallin gamma S [Source:HGNC Symbol;Acc:HGNC:2417]	-
0	4	0	5	9	45	0
0.081036034	0	0.129164045	0.257187265	0.972754215
ENSG00000178404 37.51238131	47.85469723	31.63372462	0
14.5453502	0	39.00026772	4.848450065
3.039971504	0.017225551	1	CEP295NL	17
78870910	78903217	-	2758	protein_coding	CEP295
N-terminal like [Source:HGNC Symbol;Acc:HGNC:44659]	-	39
55	34	0	12	0	0.683709417
0.869014505	0.575480604	0	0.267444915	0
ENSG00000228672 435.7207367	297.5692083	315.4068425
180.7393415	207.2712403	258.4885836	349.5655958
215.4997218	0.696068123	0.017230927	1	PROB1	5
139390592	139395713	-	5122	protein_coding
proline rich basic protein 1 [Source:HGNC
Symbol;Acc:HGNC:41906]	-	453	342	339	164	171
280	4.27621807	2.909679335	3.08962777
1.779165373	2.052125403	2.541847407
ENSG00000183495 7335.113329	4860.297068	6711.001637
5555.530612	4682.39065	4399.844962	6302.137345
4879.255408	0.369171125	0.017248264	0.170233168	EP400
12	131949920	132081102	+	18705

protein_coding E1A binding protein p400 [Source:HGNC
Symbol;Acc:HGNC:11958] -   7626   5586   7213   5041   3863
        4766   19.71243639   13.01373074   18.00131599
14.97514474   12.69446363   11.84751708
ENSG00000137817 594.4269653   612.5401246   558.2421991
415.4800716   461.8148687   373.8852727   588.4030963
417.060071   0.497701349   0.017301577   1   PARP6   15
        72241181   72272999   -   7696   protein_coding
        poly(ADP-ribose) polymerase family member 6 [Source:HGNC
Symbol;Acc:HGNC:26921] -   618   704   600   377   381
        405   3.882617722   3.986265288   3.639420418
2.722001341   3.043037308   2.446926827
ENSG00000160013 0   7.830768638   13.95605498   29.7558672
        50.90872568   43.3891551   7.262274539   41.35124933
        -2.506386839   0.017311122   1   PTGIR   19
46620468   46625118   -   3546   protein_coding
prostaglandin I2 receptor [Source:HGNC Symbol;Acc:HGNC:9602]   -
        0   9   15   27   42   47   0
0.110601846   0.197468835   0.423094205   0.72804449
0.616297129
ENSG00000099800 1846.763387   2022.948565   2272.04575
2059.767251   2759.9802   3375.122363   2047.252568
2731.623271   -0.416372726   0.017325176   0.170233168   TIMM13
        19   2425624 2427894 -   1973   protein_coding
translocase of inner mitochondrial membrane 13 [Source:HGNC
Symbol;Acc:HGNC:11816] -   1920   2325   2442   1869   2277
        3656   47.0517044   51.35165793   57.77828013
52.63737945   70.93871308   86.16087881
ENSG00000206989 24.04639827   2.610256213   3.721614661   0
        0   0   10.12608972   0   5.680529117
0.017330196   1   SNORD63 5   138561043   138561110
        -       68   snoRNA "small nucleolar RNA, C/D box 63
[Source:HGNC Symbol;Acc:HGNC:10220]"   -   25   3   4
        0   0   0   17.77592923   1.922520324
2.745978386   0   0   0
ENSG00000110107 8935.641598   7537.549857   8109.398346
8658.957354   10584.16649   10874.98398   8194.1966
10039.36928   -0.293074732   0.017338563   0.170233168   PRPF19
        11   60890730   60906588   -   2739
protein_coding pre-mRNA processing factor 19 [Source:HGNC
Symbol;Acc:HGNC:17896] -   9290   8663   8716   7857   8732
        11780   163.9928443   137.827183   148.5495105
159.3957599   195.9607329   199.9789466
ENSG00000223959 747.3620583   487.2478264   569.4070431
503.645604   326.0582669   354.4986289   601.3389759
394.7341666   0.606951097   0.01735838   1   AFG3L1P 16
        89972586   90002161   +   9532
transcribed_unitary_pseudogene "AFG3 like matrix AAA peptidase
subunit 1, pseudogene [Source:HGNC Symbol;Acc:HGNC:314]"   -
        777   560   612   457   269   384   3.941288278

        2.560133374   2.99718413   2.664061277   1.734664963
        1.873174379
ENSG00000179071 139.46911   95.70939447   117.2308618
28.65379804   54.54506323   69.23801345   117.4697888
50.81229158   1.205807034   0.017411605   1   CCDC89 11
        85684866   85686277   -   1412   protein_coding
        coiled-coil domain containing 89 [Source:HGNC
Symbol;Acc:HGNC:26762] -   145   110   126   26   45
        75   4.965174565   3.394818703   4.165641432
1.023176825   1.958958236   2.469776107
ENSG00000162458 1074.393075   997.1178733   858.762583
678.8745997   856.963549   562.2126693   976.7578437
699.3502727   0.483325906   0.017412902   1   FBLIM1 1
        15756607   15786594   +   4716   protein_coding
        filamin binding LIM protein 1 [Source:HGNC
Symbol;Acc:HGNC:24686] -   1117   1146   923   616   707
        609   11.45198032   10.58935283   9.136375498
7.25803335   9.214949883   6.004467719
ENSG00000141576 352.0392707   274.9469877   263.3042372
370.2952362   442.4210685   554.8272812   296.7634986
455.8478619   -0.620491257   0.01742243   1   RNF157 17
        76142453   76240373   -   7805   protein_coding
        ring finger protein 157 [Source:HGNC Symbol;Acc:HGNC:29402]
        -       366   316   283   336   365   601
2.267302122   1.764301377   1.692620373   2.392094947
2.874533122   3.580408527
ENSG00000148719 933.9621089   1043.2324   1146.257316
1265.17539   1253.324342   1628.478076   1041.150608
1382.325936   -0.409534237   0.017493347   1   DNAJB12 10
        72332830   72355230   -   5649   protein_coding
        DnaJ heat shock protein family (Hsp40) member B12 [Source:HGNC
Symbol;Acc:HGNC:14891] -   971   1199   1232   1148   1034
        1764   8.310915595   9.249243352   10.18087649
11.29229869   11.2511347   14.51971274
ENSG00000143774 4199.462995   4972.538085   4847.403096
4666.160804   6395.105636   6825.944953   4673.134725
5962.403798   -0.351549485   0.017551704   0.170965309   GUK1
        1   228139962   228148984   +   4725
protein_coding guanylate kinase 1 [Source:HGNC Symbol;Acc:HGNC:4693]
        -       4366   5715   5210   4234   5276   7394
44.67691069   52.70757309   51.47329219   49.79217352
68.63574218   72.76267411
ENSG00000101294 3533.85869   2649.410056   3187.562957
3758.05582   4295.726758   3722.235603   3123.610568
3925.339394   -0.329576858   0.017667251   0.170965309   HM13
        20   31514410   31577923   +   10805
protein_coding histocompatibility minor 13 [Source:HGNC
Symbol;Acc:HGNC:16435] -   3674   3045   3426   3410   3544
        4032   16.4405218   12.28064348   14.80159862
17.53644879   20.16119649   17.35109031

ENSG00000225630 4342.779528   3375.931368   3326.193103
3861.650321   5301.780146   5040.52738   3681.634667
4734.652615   -0.362985146   0.017692488   0.170965309
MTND2P28   1   629640 630683 +   1044
unprocessed_pseudogene MT-ND2 pseudogene 28 [Source:HGNC
Symbol;Acc:HGNC:42129] -   4515   3880   3575   3504   4374
        5460   209.1021376   161.9533086   159.8532916
186.4986285   257.5287028   243.1773728
ENSG00000051341 345.3062792   254.064938   328.4324938
636.9959718   519.9962695   325.88025   309.2679037
494.2908304   -0.675763129   0.017715783   1   POLQ   3
        121431427   121546641   -   9400   protein_coding
        DNA polymerase theta [Source:HGNC Symbol;Acc:HGNC:9186] -
        359   292   353   578   429   353   1.846578656
        1.353672467   1.753044286   3.416740759   2.805283768
        1.746135211
ENSG00000140632 1206.167337   857.0341232   1211.385572
837.5725581   850.9029864   690.5337875   1091.529011
793.0031107   0.461374757   0.01771945   1   GLYR1   16
        4803203 4847342 -   5470   protein_coding glyoxylate
reductase 1 homolog [Source:HGNC Symbol;Acc:HGNC:24434] -   1254
        985   1302   760   702   748   11.08438053
7.847069551   11.11142332   7.720373469   7.888549336
6.358362582
ENSG00000262370 12.5041271   11.31111026   4.652018326   0
        0   0   9.489085228   0   5.588149063
0.017722214   1   AC108134.3   16   3076911 3087100 -
        1326   lincRNA uncharacterized LOC105371058 [Source:NCBI
gene;Acc:105371058]   -   13   13   5   0   0
        0   0.474024779   0.427226739   0.176024255   0
        0   0
ENSG00000227001 60.59692365   65.25640532   58.61543091
35.26621297   9.69690013   7.385388102   61.48958662
17.4495004   1.820752114   0.017727359   1   NBPF2P 1
        21424625   21427967   -   661
unprocessed_pseudogene "NBPF member 2, pseudogene [Source:HGNC
Symbol;Acc:HGNC:31987]" -   63   75   63   32   8
        8   4.608295359   4.944454692   4.449232755
2.690051302   0.743936535   0.562755238
ENSG00000204709 25.0082542   10.44102485   0   0   0
        0   11.81642635   0   5.904079284   0.01774878
        1   LINC01556   6   28943877   28944537
        +   466   lincRNA long intergenic non-protein coding RNA
1556 [Source:HGNC Symbol;Acc:HGNC:21195]   -   26   12
        0   0   0   0   2.697668916   1.122157786
        0   0   0   0
ENSG00000100997 770.4466007   677.7965299   657.7953913
911.4111915   1069.083239   857.6281933   702.0128406
946.0408747   -0.429885013   0.017768698   1   ABHD12 20
        25294743   25390983   -   3429   protein_coding

abhydrolase domain containing 12 [Source:HGNC
Symbol;Acc:HGNC:15868]	-	801	779	707	827	882
929	11.29448018	9.899848996	9.624938529
13.40139685	15.81060396	12.59735149
ENSG00000203279 27.893822	7.830768638	0	0	0
0	11.90819688	0	5.915008736	0.017800842
1	AL590705.1	9	97200475	97238700
-	806	lincRNA novel transcript	-	29
9	0	0	0	0	1.739659178
0.486593233	0	0	0	0
ENSG00000135898 16.35155083	8.700854042	3.721614661	0
0	0	9.591339843	0	5.603133132
0.017821356	1	GPR55	2	230907318	230961066
-	4130	protein_coding	G protein-coupled receptor 55
[Source:HGNC Symbol;Acc:HGNC:4511]	-	17	10	4
0	0	0	0.199021542	0.105513626
0.045212235	0	0	0
ENSG00000067064 909.9157107	1058.893937	944.3597202
1431.587833	1292.111942	1113.347256	971.0564559
1279.015677	-0.396770615	0.017912084	1	IDI1	10
1039152 1049170 -	4345	protein_coding	isopentenyl-
diphosphate delta isomerase 1 [Source:HGNC Symbol;Acc:HGNC:5387]
-	946	1217	1015	1299	1066	1206
10.52695029	12.20560736	10.90491531	16.61236306
15.08046677	12.90590741
ENSG00000279759 163.5155083	111.3709317	194.454366
98.08415483	47.27238814	79.39292209	156.4469353
74.91648835	1.059713529	0.017922826	1	AC118344.2
19	40355842	40360606	+	4765	TEC
TEC	-	170	128	209	89	39	86
1.724992586	1.170592298	2.047525961	1.037860887
0.503093912	0.839202105
ENSG00000280432 31.74124572	0	4.652018326	0	0
0	12.13108802	0	5.941250488	0.017996032
1	AP000962.2	21	16525919	16527019
+	1101	TEC	TEC	-	33	0	5
0	0	0	1.449198372	0	0.211996515
0	0	0
ENSG00000225916 10.58041524	2.610256213	15.81686231	0
0	0	9.66917792	0	5.614492187
0.01800214	1	AC007879.2	2	207226949
207228308	+	551	lincRNA novel transcript	-
11	3	17	0	0	0	0.96525554
0.237262036	1.440268155	0	0	0
ENSG00000131153 1712.103557	1884.604986	1727.759606
2468.634908	2048.470153	2162.072367	1774.822716
2226.392476	-0.326993784	0.018032061	1	GINS2	16
85676198	85690073	-	3264	protein_coding
GINS complex subunit 2 [Source:HGNC Symbol;Acc:HGNC:24575]
-	1780	2166	1857	2240	1690	2342

26.36762835   28.91790988   26.5587597   38.13378484
31.82613603   33.3632046
ENSG00000228000 8.656703379   23.49230591   22.32968796   0
        3.636337549   0   18.15956575   1.212112516
3.973638965   0.018053825   1   RPL7AP65   17
18311904   18312651   +   748   processed_pseudogene
        ribosomal protein L7a pseudogene 65 [Source:HGNC
Symbol;Acc:HGNC:37031] -   9   27   24   0   3
        0   0.581757684   1.572971175   1.497806392   0
        0.246528434   0
ENSG00000166073 2460.427471   2725.107486   2974.500518
3140.897093   3512.702072   3411.12613   2720.011825
3354.908432   -0.302587902   0.018055295   0.173557704   GPR176
        15   39799032   39920892   -   5119
protein_coding G protein-coupled receptor 176 [Source:HGNC
Symbol;Acc:HGNC:32370] -   2558   3132   3197   2850   2898
        3695   24.16109579   26.66215333   29.15436204
30.93654245   34.79850704   33.56296587
ENSG00000130377 0.961855931   0   2.791210996   50.69518115
        15.75746271   0   1.251022309
22.15088129   -4.152590233   0.018179657   1   ACSBG2 19
        6135247 6193094 +   3447   protein_coding acyl-CoA
synthetase bubblegum family member 2 [Source:HGNC
Symbol;Acc:HGNC:24174] -   1   0   3   46   13
        0   0.014026843   0   0.040628053   0.741529771
        0.231819214   0
ENSG00000114942 5666.293289   7340.04047   6437.462959
6811.88945   8893.269532   8646.44312   6481.265573
8117.200701   -0.32464698   0.018294137   0.174825425   EEF1B2
        2   206159585   206162928   +   1915
protein_coding eukaryotic translation elongation factor 1 beta 2
[Source:HGNC Symbol;Acc:HGNC:3208]   -   5891   8436   6919
        6181   7337   9366   148.7378368   191.9669172
168.663298   179.3502593   235.5033563   227.4135791
ENSG00000182551 807.958982   824.8409632   936.9164908
1376.484375   1023.022964   1054.264152   856.5721454
1151.257163   -0.426521922   0.018379555   1   ADI1   2
        3497361 3519736 -   5560   protein_coding acireductone
dioxygenase 1 [Source:HGNC Symbol;Acc:HGNC:30576]   -   840
        948   1007   1249   844   1142   7.304755952
7.430056966   8.454748918   12.48244584   9.330716951
9.550416909
ENSG00000273888 4.809279655   13.92136647   27.91210996
2.204138311   0   0   15.54758536   0.73471277
4.42129855   0.018388237   1   FRMD6-AS1   14
51649516   51651744   -   2229   antisense   FRMD6
antisense RNA 1 [Source:HGNC Symbol;Acc:HGNC:20129]   -   5
        16   30   2   0   0   0.10845789
0.312801273   0.628285768   0.049857669   0   0
ENSG00000271147 94.26188123   81.788028   86.52754086

42.98069706 3.636337549 34.15741997 87.5258167
26.92481819 1.694139356 0.018390778 1 ARMCX5-GPRASP2
X 102599512 102714671 + 2711
processed_transcript ARMCX5-GPRASP2 readthrough [Source:HGNC
Symbol;Acc:HGNC:42000] - 98 94 93 39 3
37 1.747824306 1.5109738 1.601398682
0.799368689 0.068020387 0.634604613
ENSG00000162366 0 0 0 6.612414933 26.66647536
0 0 11.09296343 -6.030680188 0.0183922
1 PDZK1IP1 1 47183593 47191044
- 1438 protein_coding PDZK1 interacting protein 1
[Source:HGNC Symbol;Acc:HGNC:16887] - 0 0 0
6 22 0 0 0 0 0.231848563
0.940396827 0
ENSG00000254604 5.771135586 13.92136647 8.373632987 0
0 0 9.355378347 0 5.568342184
0.018488146 1 AP002336.2 11 70282367
70363368 - 539 antisense novel transcript
- 6 16 9 0 0 0
0.538224796 1.293569643 0.779470673 0 0 0
ENSG00000072195 1377.377693 922.2905285 1295.121902
844.1849731 856.963549 939.790636 1198.263375
880.3130527 0.444244547 0.018512903 1 SPEG 2
219434846 219498287 + 17910 protein_coding
SPEG complex locus [Source:HGNC Symbol;Acc:HGNC:16901] -
1432 1060 1392 766 707 1018 3.865882488
2.579104131 3.628187187 2.376540542 2.426449115
2.642915088
ENSG00000283317 2.885567793 16.53162268 9.304036652 0
0 0 9.573742375 0 5.60199171
0.018520555 1 AL831711.1 1 161433444
161440996 - 569 lincRNA novel transcript -
3 19 10 0 0 0 0.254923695
1.45512376 0.820415335 0 0 0
ENSG00000235488 35.58866945 0 1.86080733 0 0
0 12.48315893 0 5.982514794 0.018534895
1 JARID2-AS1 6 15247815 15248634
- 455 antisense JARID2 antisense RNA 1
[Source:HGNC Symbol;Acc:HGNC:40314] - 37 0 2
0 0 0 3.931801137 0 0.205193989
0 0 0
ENSG00000167645 1011.872439 1155.473417 1343.502893
1330.197471 1563.625146 1753.106501 1170.282916
1548.976372 -0.404721286 0.018581468 1 YIF1B 19
38305104 38317273 - 3193 protein_coding
"Yip1 interacting factor homolog B, membrane trafficking
protein [Source:HGNC Symbol;Acc:HGNC:30511]" - 1052 1328
1444 1207 1290 1899 15.93008297 18.12415444
21.11126759 21.00488921 24.83351253 27.65394306
ENSG00000123143 6159.725382 5940.94314 6484.913546

6458.125251 8250.849898 7998.375314 6195.194023
7569.116821 -0.28899356 0.018657585 0.174825425 PKN1
19 14433053 14471867 + 4606
protein_coding protein kinase N1 [Source:HGNC Symbol;Acc:HGNC:9405]
- 6404 6828 6970 5860 6807 8664
67.22465872 64.59935423 70.64068148 70.69451609
90.84042913 87.4632333
ENSG00000109066 1451.4406 1285.116142 1329.546838
1513.14095 1773.320611 1925.739948 1355.36786
1737.400503 -0.358644121 0.018664874 1 TMEM104 17
74776483 74839779 + 4983 protein_coding
transmembrane protein 104 [Source:HGNC Symbol;Acc:HGNC:25984]
- 1509 1477 1429 1373 1463 2086
14.6419719 12.91659986 13.38712682 15.31058178
18.04682467 19.46500121
ENSG00000213303 13.46598303 1.740170808 13.95605498 0
0 0 9.720736273 0 5.621952259
0.018679472 1 AC008481.1 19 11523436
11523831 + 396 processed_pseudogene ribosomal
protein S12 (RPS12) pseudogene - 14 2 15 0
0 0 1.709362083 0.220086502 1.768243657
0 0 0
ENSG00000135318 10505.39048 12879.87424 11833.80422
9463.467838 10151.44232 9765.329418 11739.68964
9793.413193 0.26157761 0.0186868 0.174825425 NT5E
6 85449584 85495791 + 4533
protein_coding 5'-nucleotidase ecto [Source:HGNC
Symbol;Acc:HGNC:8021] - 10922 14803 12719 8587 8375
10578 116.4977854 142.3058055 130.9825026
105.2610717 113.5655103 108.5047778
ENSG00000225082 0 0 0 14.32689902 18.18168774
0 0 10.83619559 -5.997727049 0.018700162
1 DAP3P1 1 155586644 155602197 +
278 unprocessed_pseudogene death associated protein 3
pseudogene 1 [Source:HGNC Symbol;Acc:HGNC:17624] - 0
0 0 13 15 0 0 0 0
2.598427477 3.316605551 0
ENSG00000237276 12.5041271 6.960683234 8.373632987 0
0 0 9.279481108 0 5.555458673
0.018717544 1 ANO7L1 1 16215909 16228027
- 1990 transcribed_unprocessed_pseudogene
anoctamin 7 like 1 [Source:HGNC Symbol;Acc:HGNC:32248] - 13
8 9 0 0 0 0.315857717
0.175184432 0.211122961 0 0 0
ENSG00000203325 1.923711862 2.610256213 26.05130263 0
0 0 10.19509023 0 5.691371903
0.018722538 1 AL445248.1 1 32052291
32073474 - 624 antisense novel transcript
- 2 3 28 0 0 0
0.154969639 0.20950542 2.09468864 0 0 0

ENSG00000167658 78428.77075 68038.06836 77527.74621
79433.83852 92708.42581 95836.4887 74664.86177
89326.25101 -0.258665446 0.018779356 0.174825425 EEF2
19 3976056 3985469 - 4027 protein_coding
eukaryotic translation elongation factor 2 [Source:HGNC
Symbol;Acc:HGNC:3214] - 81539 78197 83327 72077 76485
103812 979.0051307 846.1883853 965.940004
994.5511322 1167.459979 1198.662577
ENSG00000285805 22.12268641 2.610256213 4.652018326 0
0 0 9.794986983 0 5.632603333
0.018820757 1 AC012378.2 15 55588666
55589811 + 663 antisense "novel transcript,
antisense to PYGO1" - 23 3 5 0 0
0 1.67731845 0.197181572 0.352048511 0
0 0
ENSG00000167799 50.01650841 68.73674694 51.17220159
90.36967074 112.726464 163.4017118 56.64181898
122.1659488 -1.109778663 0.018840372 1 NUDT8 11
67627938 67629930 - 1475 protein_coding
nudix hydrolase 8 [Source:HGNC Symbol;Acc:HGNC:8055] -
52 79 55 82 93 177 1.704560969
2.333961397 1.740671044 3.089113573 3.875594121
5.57971818
ENSG00000177156 1545.702481 1379.085366 1550.052506
1647.593387 2229.074917 1891.582528 1491.613451
1922.750277 -0.366111215 0.018859855 1 TALDO1 11
747329 765024 + 3005 protein_coding transaldolase
1 [Source:HGNC Symbol;Acc:HGNC:11559] - 1607 1585 1666
1495 1839 2049 25.85667144 22.98494072
25.88073206 27.64450131 37.6170395 31.70505422
ENSG00000130723 5100.722002 4253.847541 4649.227115
4115.126226 3486.035597 3826.55421 4667.932219
3809.238678 0.293091343 0.018868326 0.174825425 PRRC2B
9 131373636 131500197 + 12390
protein_coding proline rich coiled-coil 2B [Source:HGNC
Symbol;Acc:HGNC:28121] - 5303 4889 4997 3734 2876
4145 20.69433796 17.19520384 18.82712816
16.74615285 14.26806028 15.55550972
ENSG00000149948 4461.087808 4600.141532 4301.256144
3345.881956 3071.493116 4243.828638 4454.161828
3553.73457 0.325349002 0.018905428 0.174825425 HMGA2
12 65824131 65966295 + 15403
protein_coding high mobility group AT-hook 2 [Source:HGNC
Symbol;Acc:HGNC:5009] HMGI/HMGY 4638 5287 4623 3036
2534 4597 14.55883571 14.95762334 14.0108542
10.9523798 10.11226996 13.87714757
ENSG00000177283 5807.686111 4867.257751 6393.733987
4393.949723 4559.967286 4890.97327 5689.559283
4614.963426 0.301826014 0.018924257 0.174825425 FZD8
10 35638249 35642278 - 4030

protein_coding	frizzled class receptor 8 [Source:HGNC
Symbol;Acc:HGNC:4046]	-	6038	5594	6872	3987	3762
	5298	72.44180769	60.48894551	79.60202951
54.97348039	57.38007417	61.1276818
ENSG00000167106 861.8229141	938.8221512	1032.748068
597.3214822	709.085822	785.6206593	944.4643779
697.3426545	0.437105839	0.018941442	1	FAM102A 9
	127940579	127980513	-	6100	protein_coding
	family with sequence similarity 102 member A [Source:HGNC
Symbol;Acc:HGNC:31419]	-	896	1079	1110	542	585
	851	7.10197912	7.708150888	8.49452658
4.937208809	5.894858588	6.486803513
ENSG00000155761 8.656703379	6.960683234	12.09524765	0
	0	0	9.237544753	0	5.549144475
0.018998679	1	SPAG17	1	117953861	118185223
	-	9552	protein_coding	sperm associated antigen 17
[Source:HGNC Symbol;Acc:HGNC:26620]	-	9	8	13
	0	0	0	0.045556402	0.036496757
0.063532373	0	0	0
ENSG00000261024 0	0	0	3.306207466	12.12112516
	11.07808215	0	8.835138261	-5.706301585
0.019012816	1	GS1-279B7.1	1	185321157
185335039	-	1205	transcribed_processed_pseudogene
	microtubule associated protein 1 light chain 3 beta pseudogene
[Source:NCBI gene;Acc:100288079]	-	0	0	0
	3	10	12	0	0	0	0.138339516
	0.510105861	0.463047152
ENSG00000142541 42366.86819	51186.25425	50060.36921
53172.63261	55541.41972	64646.1484	47871.16388
57786.73358	-0.271595408	0.019027001	0.174825425	RPL13A
	19	49487554	49492308	+	2369
protein_coding	ribosomal protein L13a [Source:HGNC
Symbol;Acc:HGNC:10304]	-	44047	58829	53805	48248	45822
	70026	898.9850926	1082.143869	1060.238598
1131.686925	1188.93015	1374.438389
ENSG00000171320 207.7608811	352.3845887	294.9379619
676.6704614	319.9977043	427.4293364	285.0278106
474.6991674	-0.735629268	0.01907623	1	ESCO2	8
	27771949	27812640	+	7973	protein_coding
	establishment of sister chromatid cohesion N-acetyltransferase
2 [Source:HGNC Symbol;Acc:HGNC:27230]	-	216	405	317
	614	264	463	1.309885105	2.213562847
1.85602377	4.279161236	2.03530511	2.700164636
ENSG00000232109 0	11.31111026	23.26009163	0	0
	0	11.52373396	0	5.868736913	0.019100102
	1	VN1R54P 10	42660728	42661776	+
	1049	processed_pseudogene	vomeronasal 1 receptor 54
pseudogene [Source:HGNC Symbol;Acc:HGNC:37374]	-	0	13
	25	0	0	0	0	0.540040663
1.112526991	0	0	0

ENSG00000105699 44.24537282 57.42563668 32.56412828 0
18.18168774 0 44.74504593 6.060562582
2.911568258 0.019108496 1 LSR 19 35248330
35267964 + 2916 protein_coding lipolysis
stimulated lipoprotein receptor [Source:HGNC Symbol;Acc:HGNC:29572]
- 46 66 35 0 15 0
0.762731229 0.986313582 0.560307661 0 0.316192162
0
ENSG00000274012 241.4258387 163.576056 446.5937593
101.3903623 86.05998866 225.2543371 283.865218
137.5682294 1.041054426 0.019113508 1 RN7SL2 14
49862550 49862849 - 300 misc_RNA
"RNA, 7SL, cytoplasmic 2 [Source:HGNC Symbol;Acc:HGNC:23134]"
- 251 188 480 92 71 244
40.45327467 27.30833314 74.69061209 17.04035414
14.54736897 37.81808989
ENSG00000184313 16.35155083 5.220512425 6.512825656 0
0 0 9.361629636 0 5.567785294
0.019123515 1 MROH7 1 54641754 54710266
+ 9088 protein_coding maestro heat like repeat
family member 7 [Source:HGNC Symbol;Acc:HGNC:24802] - 17
6 7 0 0 0 0.090444429
0.02877011 0.035956363 0 0 0
ENSG00000167483 10.58041524 0 24.19049529 0 0
0 11.59030351 0 5.876022103 0.01913248
1 FAM129C 19 17523301 17553839 +
6628 protein_coding family with sequence similarity 129
member C [Source:HGNC Symbol;Acc:HGNC:24130] - 11 0
26 0 0 0 0.080243784 0
0.183120466 0 0 0
ENSG00000143315 1034.956982 854.423867 902.4915552
792.3877227 581.8140078 671.1471438 930.6241346
681.7829581 0.448369894 0.019136333 1 PIGM 1
160024953 160031991 - 7039 protein_coding
phosphatidylinositol glycan anchor biosynthesis class M
[Source:HGNC Symbol;Acc:HGNC:18858] - 1076 982 970
719 480 727 7.390988433 6.079377619
6.432900068 5.675837706 4.191578773 4.802357244
ENSG00000095319 2460.427471 1962.042587 2182.726999
2518.22802 2785.434562 2980.927273 2201.732352
2761.529952 -0.327152834 0.019222638 0.174825425 NUP188
9 128947699 129007096 + 8041
protein_coding nucleoporin 188 [Source:HGNC Symbol;Acc:HGNC:17859]
- 2558 2255 2346 2285 2298 3229
15.38125225 12.22067183 13.61958836 15.79022018
17.56658423 18.67192099
ENSG00000087365 12008.7713 10766.43679 13154.04702
13758.23134 14168.3832 15058.80634 11976.41837
14328.47363 -0.258756149 0.019234179 0.174825425 SF3B2
11 66050729 66069308 + 9795

protein_coding splicing factor 3b subunit 2 [Source:HGNC Symbol;Acc:HGNC:10769] - 12485 12374 14138 12484 11689 16312 61.62902866 55.05088044 67.37977755 70.82088725 73.35340505 77.43437379
ENSG00000096384 54807.5128 46687.91271 51664.38512 60953.24085 60231.08305 58775.68803 51053.27021 59986.67064 -0.232643972 0.019259507 0.174825425 HSP90AB1 6 44246166 44253888 + 2980 protein_coding heat shock protein 90 alpha family class B member 1 [Source:HGNC Symbol;Acc:HGNC:5258] - 56981 53659 55529 55308 49691 63667 924.5172507 784.6661331 869.8605282 1031.296951 1024.964522 993.411402
ENSG00000117399 1073.431219 1017.999923 1257.905755 1707.105122 1713.927098 1132.7339 1116.445632 1517.92204 -0.442517615 0.019299837 1 CDC20 1 43358955 43363203 + 2038 protein_coding cell division cycle 20 [Source:HGNC Symbol;Acc:HGNC:1723] - 1116 1170 1352 1549 1414 1227 26.47654008 25.01729097 30.96838431 42.23371469 42.64740299 27.9944153
ENSG00000136270 3717.573173 3914.514234 3649.043175 4037.981385 4755.117401 4980.521101 3760.376861 4591.206629 -0.288071326 0.019329826 0.174825425 TBRG4 7 45100100 45112047 - 5803 protein_coding transforming growth factor beta regulator 4 [Source:HGNC Symbol;Acc:HGNC:17443] - 3865 4499 3922 3664 3923 5395 32.20313437 33.78485197 31.55012285 35.08447144 41.5540251 43.22847318
ENSG00000231028 0 26.97264753 15.81686231 0 1.212112516 0 14.26316995 0.404037505 5.215381195 0.019347536 1 LINC00271 6 135497801 135716055 + 3825 lincRNA long intergenic non-protein coding RNA 271 [Source:HGNC Symbol;Acc:HGNC:32526] - 0 31 17 0 1 0 0 0.353174104 0.207473922 0 0.016070002 0
ENSG00000162063 261.6248132 316.7110871 293.0771545 509.1559498 351.5126297 452.3550212 290.4710183 437.6745336 -0.591819205 0.019366339 1 CCNF 16 2429394 2458854 + 6309 protein_coding cyclin F [Source:HGNC Symbol;Acc:HGNC:1591] - 272 364 315 462 290 490 2.084536928 2.514197869 2.330751982 4.06905436 2.825431812 3.61132497
ENSG00000197140 20.19897455 67.86666153 33.49453195 6.612414933 15.75746271 4.615867564 40.52005601 8.995248403 2.186082604 0.019407718 1 ADAM32 8 39106990 39284911 + 10044 protein_coding ADAM metallopeptidase domain 32 [Source:HGNC Symbol;Acc:HGNC:15479] - 21 78 36 6 13 5 0.101091306 0.338412578 0.167317679 0.033193771 0.079558028 0.023146979

ENSG00000269304 22.12268641 1.740170808 5.582421991 0
0 0 9.81509307 0 5.635503554
0.019454356 1 FKBP1AP1 19 57826461
57826778 - 318 processed_pseudogene FK506 binding
protein 1A pseudogene 1 [Source:HGNC Symbol;Acc:HGNC:3714] -
23 2 6 0 0 0 3.497050731
0.274069983 0.88078552 0 0 0
ENSG00000121068 287.5949234 201.8598138 232.6009163
100.2882931 168.4836398 144.015068 240.6852178
137.595667 0.806968196 0.019463598 1 TBX2 17
61399896 61409466 + 6029 protein_coding
T-box 2 [Source:HGNC Symbol;Acc:HGNC:11597] T-box 299
232 250 91 139 156 2.397878209
1.67687735 1.935712081 0.838702914 1.417153444
1.20312384
ENSG00000099834 186.6000506 137.4734939 93.97077018
27.55172889 3.636337549 96.01004532 139.3481049
42.39937059 1.707130508 0.019464757 1 CDHR5 11
616565 626078 - 3873 protein_coding cadherin
related family member 5 [Source:HGNC Symbol;Acc:HGNC:7521] -
194 158 101 25 3 104 2.421895774
1.777739768 1.217362481 0.358677848 0.047612515
1.248581398
ENSG00000177674 1149.417837 1433.900746 1333.268452
1450.323009 1728.472448 1931.278989 1305.529012
1703.358148 -0.383890035 0.019479833 1 AGTRAP 1
11736084 11754802 + 2435 protein_coding
angiotensin II receptor associated protein [Source:HGNC
Symbol;Acc:HGNC:13539] - 1195 1648 1433 1316 1426
2092 23.72849296 29.49285662 27.47218869
30.03094284 35.99713362 39.94787965
ENSG00000152520 1181.159083 1225.950335 1016.931206
978.63741 774.5398979 829.0098144 1141.346875
860.7290408 0.407063526 0.019527586 1 PAN3 13
28138506 28295335 + 6352 protein_coding
poly(A) specific ribonuclease subunit PAN3 [Source:HGNC
Symbol;Acc:HGNC:29991] - 1228 1409 1093 888 639
898 9.347362683 9.666273999 8.032592
7.768094854 6.183547894 6.573502999
ENSG00000105290 22.12268641 8.700854042 11.16484398
52.89931946 41.21182555 51.69771671 13.99612815
48.60295391 -1.79984582 0.019548533 1 APLP1 19
35867899 35879791 + 3656 protein_coding
amyloid beta precursor like protein 1 [Source:HGNC
Symbol;Acc:HGNC:597] - 23 10 12 48 34
56 0.304174544 0.119193456 0.153221989
0.729536615 0.571636683 0.712217857
ENSG00000255348 16.35155083 6.960683234 4.652018326 0
0 0 9.321417462 0 5.56176095
0.019569225 1 AP001775.2 11 134178824

134186166	+	334	antisense	novel transcript
	-	17	8	5	0	0	0
2.460954992	1.043763529	0.698826835	0	0	0
ENSG00000136827 1843.87782	1832.399861	1730.550817
2190.913481	2246.044493	2199.922481	1802.276166
2212.293485	-0.295665897	0.019627201	1	TOR1A	9
	129812944	129824134	-	3766	protein_coding
	torsin family 1 member A [Source:HGNC Symbol;Acc:HGNC:3098]
	-	1917	2106	1860	1988	1853	2383
24.6117794	24.36894058	23.05571868	29.3324344
30.24422525	29.422173
ENSG00000092607 0	0	2.791210996	0	26.66647536
	34.15741997	0.930403665	20.27463178	-4.451713849
	0.019670712	1	TBX15	1	118883046
118989556	-	4349	protein_coding	T-box 15 [Source:HGNC
Symbol;Acc:HGNC:11594]	T-box	0	0	3	0	22
	37	0	0	0.032201632	0	0.310942892
	0.395588205
ENSG00000005194 2126.663463	1943.770793	2059.913715
2223.975556	2766.040762	2666.125105	2043.449324
2552.047141	-0.320705479	0.019678731	1	CIAPIN1 16
	57428169	57447528	-	3509	protein_coding
	cytokine induced apoptosis inhibitor 1 [Source:HGNC
Symbol;Acc:HGNC:28050]	-	2211	2234	2214	2018	2282
	2888	30.46537939	27.7433179	29.45372883
31.95581052	39.97418629	38.26879952
ENSG00000183808 1036.880694	783.0768638	915.5172065
686.5890838	716.3584971	618.5262535	911.8249213
673.8246115	0.436717756	0.019719881	1	RBM12B	8
	93729356	93741017	-	7618	protein_coding
	RNA binding motif protein 12B [Source:HGNC
Symbol;Acc:HGNC:32310]	-	1078	900	984	623	591
	670	6.841936025	5.148256054	6.029761937
4.544217627	4.768632702	4.089449528
ENSG00000141034 556.914584	502.0392782	614.066419
438.6235239	324.8461544	397.887784	557.6734271
387.1191541	0.525748409	0.019762409	1	GID4	17
	18039292	18068404	+	5210	protein_coding
	GID complex subunit 4 homolog [Source:HGNC
Symbol;Acc:HGNC:28453]	-	579	577	660	398	268
	431	5.37331198	4.826104123	5.913604124
4.244801562	3.161873065	3.846542763
ENSG00000110841 3868.584554	3271.52112	3066.61048
3132.08054	2538.163609	2358.708325	3402.238718
2676.317491	0.346351692	0.019772799	0.17712177
PPFIBP1 12	27523431	27695564	+	11690
protein_coding	PPFIA binding protein 1 [Source:HGNC
Symbol;Acc:HGNC:9249]	-	4022	3760	3296	2842	2094
	2555	16.63522853	14.01625311	13.16190427
13.50895037	11.010563	10.16266036

ENSG00000147324 846.4332192	845.7230129	870.8578306
1127.416746	1063.022677	1110.577736	854.3380209
1100.339053	-0.365212659	0.019775984	1	MFHAS1	8
	8783354 8893645 -	6883	protein_coding	malignant
fibrous histiocytoma amplified sequence 1 [Source:HGNC
Symbol;Acc:HGNC:16982]	-	880	972	936	1023	877
	1203	6.181674298	6.15385265	6.348105197
8.2586661	7.831936976	8.126786976
ENSG00000104904 11857.75992	13529.82804	12792.11999
14288.3266	15495.64641	15296.98511	12726.56932
15026.98604	-0.239681784	0.019776668	0.17712177	OAZ1
	19	2269509 2273490 +	3436	protein_coding
ornithine decarboxylase antizyme 1 [Source:HGNC Symbol;Acc:HGNC:8095]
	-	12328	15550	13749	12965	12784	16570
173.4765142	197.2131346	186.7944604	209.6676413
228.697263	224.2334358
ENSG00000110717 1766.929345	1487.846041	1574.243002
1891.150671	2227.862805	1972.821797	1609.672796
2030.611757	-0.335049368	0.019806074	1	NDUFS8	11
	68030617	68036644	+	4523	protein_coding
	NADH:ubiquinone oxidoreductase core subunit S8 [Source:HGNC
Symbol;Acc:HGNC:7715]	-	1837	1710	1692	1716	1838
	2137	19.63739089	16.47510232	17.46303831
21.08155974	24.97849567	21.9689324
ENSG00000102595 484.7753892	297.5692083	341.4581451
273.3131505	249.6951784	154.1699766	374.6009142
225.7261018	0.73241114	0.019822185	1	UGGT2	13
	95801580	96053482	-	8105	protein_coding
	UDP-glucose glycoprotein glucosyltransferase 2 [Source:HGNC
Symbol;Acc:HGNC:15664]	-	504	342	367	248	206
	167	3.006621327	1.838788101	2.113776576
1.700241864	1.562289671	0.958063887
ENSG00000112319 368.3908216	284.5179272	388.908732
559.851131	475.1481064	485.5892677	347.2724936
506.862835	-0.545955128	0.019995734	1	EYA4	6
	133240598	133532120	+	8968	protein_coding
	EYA transcriptional coactivator and phosphatase 4 [Source:HGNC
Symbol;Acc:HGNC:3522]	-	383	327	418	508	392
	526	2.064925516	1.588951901	2.175838806
3.147604491	2.686815392	2.727226214
ENSG00000142507 1853.496379	1640.110987	1673.796194
2024.501039	2568.466422	1991.285267	1722.467853
2194.750909	-0.349193913	0.020041542	1	PSMB6	17
	4796144 4798503 +	1448	protein_coding	proteasome
subunit beta 6 [Source:HGNC Symbol;Acc:HGNC:9543]	-	1927
	1885	1799	1837	2119	2157	64.34493541
56.72851178	57.99741503	70.49407855	89.95177861
69.26480268
ENSG00000107165 37.51238131	19.14187889	6.512825656	0
	0	4.615867564	21.05569529	1.538622521

3.709172136	0.02004754	1	TYRP1	9	12685439
	12710290	+	3741	protein_coding	tyrosinase
related protein 1 [Source:HGNC Symbol;Acc:HGNC:12450]	-	39
	22	7	0	0	5	0.504055218
0.256267523	0.087348684	0	0	0.062146019
ENSG00000272308 34.62681351	0	1.86080733	0	0
	0	12.16254028	0	5.944966861	0.020133843
	1	AC104113.1	5	60866457	60866935
	-	479	lincRNA novel transcript	-	36
	0	2	0	0	0	3.633860104	0
	0.194912871	0	0	0
ENSG00000185650 4468.782655	4146.827037	4432.443061
4173.535892	3269.067456	2905.227045	4349.350918
3449.276798	0.334688481	0.020145161	0.179546192
ZFP36L1 14	68787660	68796253	-	5160
protein_coding	ZFP36 ring finger protein like 1 [Source:HGNC
Symbol;Acc:HGNC:1107]	Others	4646	4766	4764	3787	2697
	3147	43.53421526	40.24972654	43.09908866
40.78097898	32.12762375	28.35816071
ENSG00000096746 1974.690226	1921.148573	2448.822447
1665.226494	1494.534733	1800.18835	2114.887082
1653.316525	0.354715227	0.020157374	1	HNRNPH3 10
	68331174	68343191	+	5373	protein_coding
	heterogeneous nuclear ribonucleoprotein H3 [Source:HGNC
Symbol;Acc:HGNC:5043]	-	2053	2208	2632	1511	1233
	1950	18.47452688	17.90774189	22.86731005
15.62642628	14.105666	16.87519457
ENSG00000234740 6.732991517	27.84273294	0	0	0
	0	11.52524148	0	5.869697933	0.020166214
	1	AL162386.2	9	13274510	13279187
	-	457	sense_intronic	novel transcript	-
	7	32	0	0	0	0	0.740598889
	3.051352462	0	0	0	0
ENSG00000167524 130.8124066	82.6581134	126.5348985
74.94070257	12.12112516	38.77328753	113.3351395
41.94503842	1.431108131	0.020178849	1	SGK494	17
	28607964	28614200	-	4663	protein_coding
	uncharacterized serine/threonine-protein kinase SgK494
[Source:NCBI gene;Acc:124923]	-	136	95	136	68
	10	42	1.410180514	0.887803367	1.361505903
	0.810318102	0.131820193	0.418807927
ENSG00000166337 252.0062539	225.3521197	482.8795022
110.2069155	232.7256031	179.0956615	320.0792919
174.0093934	0.879927617	0.020277829	1	TAF10	11
	6606296 6612667 -	6103	protein_coding	TATA-box
binding protein associated factor 10 [Source:HGNC
Symbol;Acc:HGNC:11543]	-	262	259	519	100	192
	194	2.075673964	1.849332457	3.969812764
0.910476359	1.933771783	1.478050859
ENSG00000164171 2616.248132	2205.6665	2092.477843

2038.827938	1349.081231	1856.501934	2304.797492
1748.137034	0.39828809	0.020296185	1	ITGA2	5
	52989326	53094779	+	7910	protein_coding
	integrin subunit alpha 2 [Source:HGNC Symbol;Acc:HGNC:6137]
	-	2720	2535	2249	1850	1113	2011
16.62622437	13.9656154	13.27269173	12.99592776
8.649002866	11.82133719
ENSG00000272325 203.9134574	235.7931445	291.2163472
109.1048464	104.2416764	192.9432642	243.640983
135.429929	0.843579035	0.0203378	1	NUDT3	6
	34279679	34392674	-	9905	protein_coding
	nudix hydrolase 3 [Source:HGNC Symbol;Acc:HGNC:8050]	-
	212	271	313	99	86	209	1.034862375
	1.192266685	1.475149015	0.555383225	0.533692785
	0.981121571
ENSG00000122642 1646.697354	1671.434062	1906.39711
1548.407163	1366.050806	1066.265407	1741.509508
1326.907792	0.392931638	0.020355575	1	FKBP9	7
	32957404	33006931	+	5170	protein_coding
	FK506 binding protein 9 [Source:HGNC Symbol;Acc:HGNC:3725]
	-	1712	1921	2049	1405	1127	1155
16.01085166	16.19181076	18.50109577	15.10072591
13.3992575	10.38777321
ENSG00000250251 294.3279149	215.7811803	452.1761813
238.0469376	157.5746271	156.0163237	320.7617588
183.8792961	0.802788383	0.020492312	1	PKD1P6	16
	15125242	15154564	-	6015
transcribed_unprocessed_pseudogene	"polycystin 1, transient
receptor potential channel interacting pseudogene 6 [Source:HGNC
Symbol;Acc:HGNC:30070]" -	306	248	486	216	130
	169	2.459727583	1.79669619	3.77178278
1.995400897	1.328480184	1.306417806
ENSG00000185267 36.55052538	22.62222051	59.54583457
13.22482987	9.69690013	5.539041076	39.57286015
9.486923691	2.06643041	0.020498509	1	CDNF	10
	14819250	14838575	-	1733	protein_coding
	cerebral dopamine neurotrophic factor [Source:HGNC
Symbol;Acc:HGNC:24913]	-	38	26	64	12	8
	6	1.060196217	0.653782638	1.723961041
0.384764262	0.283751904	0.160984368
ENSG00000135473 552.1053044	455.9247518	473.5754656
383.5200661	344.2399546	289.876483	493.8685073
339.2121679	0.542713484	0.020564147	1	PAN2	12
	56316223	56334053	-	7288	protein_coding
	poly(A) specific ribonuclease subunit PAN2 [Source:HGNC
Symbol;Acc:HGNC:20074]	-	574	524	509	348	284
	314	3.808068439	3.133152406	3.260284162
2.653279024	2.39528578	2.003329112
ENSG00000105197 2373.860438	2117.787874	2070.148155
2180.994859	3079.977904	3239.415856	2187.265489

2833.462873	-0.373638074	0.020592551	0.182490169	TIMM50
	19	39480412	39493785	+	6911
protein_coding	translocase of inner mitochondrial membrane 50
[Source:HGNC Symbol;Acc:HGNC:23656]	-	2468	2434	2225
	1979	2541	3509	17.26654635	15.3475225
15.02917558	15.9117133	22.60014015	23.60877718
ENSG00000248124 196.2186099	171.4068246	137.6997424
123.4317454	71.51463846	61.85262535	168.4417257
85.59966974	0.977854745	0.020631052	1	RRN3P1	16
	21796106	21820410	-	5253
transcribed_unprocessed_pseudogene	"RRN3 homolog, RNA polymerase
I transcription factor pseudogene 1 [Source:HGNC
Symbol;Acc:HGNC:30548]" -	204	197	148	112	59
	67	1.877690388	1.634245972	1.315225893
1.184738947	0.690385992	0.593059709
ENSG00000261136 0	22.62222051	11.16484398	0	0
	0	11.26235483	0	5.836367925	0.02065128
	1	AC023908.3	15	39782571	39785617
	+	3047	antisense	uncharacterized LOC105370941
[Source:NCBI gene;Acc:105370941]	-	0	26	12
	0	0	0	0	0.371842898	0.183846272
	0	0	0
ENSG00000013275 6518.497644	6157.594406	5519.154542
6950.750163	7493.279576	7320.765956	6065.082197
7254.931898	-0.258432944	0.020674267	0.182490169	PSMC4
	19	39971005	39981441	+	2387
protein_coding	"proteasome 26S subunit, ATPase 4 [Source:HGNC
Symbol;Acc:HGNC:9551]"	-	6777	7077	5932	6307	6182
	7930	137.2733661	129.1978761	116.0098217
146.8190656	159.1929908	154.4727174
ENSG00000112137 218.3412963	143.5640917	167.4726597
89.26760159	83.63576363	112.6271686	176.4593493
95.17684459	0.888018977	0.02071248	1	PHACTR1 6
	12716805	13290484	+	11198	protein_coding
	phosphatase and actin regulator 1 [Source:HGNC
Symbol;Acc:HGNC:20990]	-	227	165	180	81	69
	122	0.980136608	0.642099126	0.750374519
0.401935716	0.378752918	0.506582736
ENSG00000128039 1137.875566	1478.275102	1341.642085
1453.629216	1824.229337	1891.582528	1319.264251
1723.147027	-0.385256985	0.0207764	1	SRD5A3	4
	55346109	55373096	+	4420	protein_coding
	steroid 5 alpha-reductase 3 [Source:HGNC
Symbol;Acc:HGNC:25812]	-	1183	1699	1442	1319	1505
	2049	12.94087648	16.75057452	15.22961858
16.58191062	20.92963193	21.55513301
ENSG00000121380 27.893822	0	6.512825656	0	0
	0	11.46888255	0	5.860292573	0.020797488
	1	BCL2L14 12	12049844	12211084	+
	5112	protein_coding	BCL2 like 14 [Source:HGNC

Symbol;Acc:HGNC:16657]	-	29	0	7	0	0
0	0.274288986	0	0.063922423	0	0
0
ENSG00000179195 1986.232497	1851.54174	1685.891441
1582.571307	1487.262058	1268.440406	1841.221893
1446.091257	0.3489773	0.02082531	1	ZNF664	12
123971845	124015439	+	6340	protein_coding
zinc finger protein 664 [Source:HGNC Symbol;Acc:HGNC:25406]
zf-C2H2 2065	2128	1812	1436	1227	1374
15.74823963	14.62651846	13.34181675	12.58569566
11.89604683	10.07693583
ENSG00000143226 0	0	0	11.02069155	20.60591278
0	0	10.54220144	-5.957679807	0.020828764
1	FCGR2A	1	161505430	161524013	+
5314	protein_coding	Fc fragment of IgG receptor IIa
[Source:HGNC Symbol;Acc:HGNC:3616]	-	0	0	0
10	17	0	0	0	0	0.104566
0.196641298	0
ENSG00000204178 1276.38282	1185.926406	1188.12548
1404.036104	1681.20006	1548.161981	1216.811569
1544.466048	-0.343897558	0.020841144	1	MACO1	1
25430858	25500209	+	3979	protein_coding
macoilin 1 [Source:HGNC Symbol;Acc:HGNC:25572]	-	1327
1363	1277	1274	1387	1677	16.12494345
14.92727434	14.98176546	17.79129384	21.42643324
19.59702477
ENSG00000149781 538.6393213	704.7691774	530.3300892
373.6014437	374.5427675	479.1270531	591.246196
409.0904214	0.530429878	0.020861475	1	FERMT3	11
64206678	64223886	+	3066	protein_coding
fermitin family member 3 [Source:HGNC Symbol;Acc:HGNC:23151]
-	560	810	570	339	309	519
8.831146574	11.51254832	8.678581395	6.143835674
6.194891282	7.87093318
ENSG00000267395 0	23.49230591	10.23444032	0	0
0	11.24224874	0	5.833844593	0.020916466
1	DM1-AS	19	45767796	45772504	+
1027	antisense	DM1 locus antisense RNA [Source:HGNC
Symbol;Acc:HGNC:53125]	-	0	27	11	0	0
0	0	1.145649891	0.499998012	0	0
0
ENSG00000207331 6.732991517	13.92136647	6.512825656	0
0	0	9.05572788	0	5.521360747
0.020947665	1	RNU6-1263P	3	98908459
98908564	+	106	snRNA	"RNA, U6 small nuclear 1263,
pseudogene [Source:HGNC Symbol;Acc:HGNC:48226]" -	7	16
7	0	0	0	3.192959363	6.577679601
3.08274932	0	0	0
ENSG00000215712 451.1104316	527.271755	452.1761813
355.9683372	351.5126297	271.4130127	476.8527893

326.2979932	0.548939205	0.020948701	1	TMEM242 6
	157289386	157323601	-	4644	protein_coding
	transmembrane protein 242 [Source:HGNC Symbol;Acc:HGNC:17206]
	-	469	606	486	323	290	294
4.882945175	5.686421011	4.885287128	3.864758446
3.838425776	2.943649772
ENSG00000090013 1122.485871	1374.734939	1551.913314
1782.045824	1659.382035	1785.417574	1349.711375
1742.281811	-0.368348571	0.020972487	1	BLVRB	19
	40447765	40465840	-	1689	protein_coding
	biliverdin reductase B [Source:HGNC Symbol;Acc:HGNC:1063]
	-	1167	1580	1668	1617	1369	1934
33.40738045	40.76486751	46.10122149	53.19764586
49.82200016	53.24242629
ENSG00000168374 5136.310671	5049.105601	4970.216379
6129.708642	6267.833822	5609.202263	5051.877551
6002.248243	-0.248530499	0.020981612	0.183486337	ARF4
	3	57571363	57598220	-	2595
protein_coding	ADP ribosylation factor 4 [Source:HGNC
Symbol;Acc:HGNC:655]	-	5340	5803	5342	5562	5171
	6076	99.49588316	97.44819654	96.09760351
119.0983284	122.4854595	108.8708017
ENSG00000125743 3899.363944	4398.281718	4734.824252
4890.982912	5599.959825	5351.636853	4344.156638
5280.859863	-0.281619981	0.020989763	0.183486337	SNRPD2
	19	45687454	45692569	-	1608
protein_coding	small nuclear ribonucleoprotein D2 polypeptide
[Source:HGNC Symbol;Acc:HGNC:11159]	-	4054	5055	5089
	4438	4620	5797	121.8986558	136.9915291
147.7380772	153.3604228	176.6051205	167.6286603
ENSG00000259972 143.3165337	66.99657613	107.9268252
56.20552693	50.90872568	24.92568484	106.0799783
44.01331248	1.27303927	0.021069994	1	AC009120.2
	16	74305127	74335346	-	3297
processed_transcript	"novel transcript, antisense to PSMD7"	-
	149	77	116	51	42	27	2.185086016
	1.017724843	1.642423226	0.85953442	0.783028742
	0.380781495
ENSG00000165118 276.0526522	283.6478418	315.4068425
230.3324535	173.3320898	111.703995	291.7024455
171.7895128	0.76607532	0.021085347	1	C9orf64 9
	83938311	83956986	-	2729	protein_coding
	chromosome 9 open reading frame 64 [Source:HGNC
Symbol;Acc:HGNC:28144]	-	287	326	339	209	143
	121	5.084866761	5.205622396	5.798854319
4.255541147	3.220919435	2.061640099
ENSG00000147123 781.0270159	1130.24094	1027.165646
1109.78364	1343.020668	1539.853419	979.4778675
1330.885909	-0.442384453	0.021118442	1	NDUFB11 X
	47142216	47145504	-	1418	protein_coding

NADH:ubiquinone oxidoreductase subunit B11 [Source:HGNC
Symbol;Acc:HGNC:20372] - 812 1299 1104 1007 1108
1668 27.68732604 39.92009057 36.34451505
39.46074528 48.02981234 54.69540388
ENSG00000105640 26983.90629 26514.11252 36185.25935
30904.22326 40093.0457 41892.69083 29894.42605
37629.9866 -0.332021633 0.0211211 0.183486337 RPL18A
19 17859876 17864153 + 3527
protein_coding ribosomal protein L18a [Source:HGNC
Symbol;Acc:HGNC:10311] - 28054 30473 38892 28042 33077
45379 384.5834132 376.5029209 514.7553313
441.7896823 576.4584555 598.2469315
ENSG00000276809 0 0 2.791210996 13.22482987
48.48450065 0 0.930403665 20.56977684 -4.468013034
0.021122457 1 AL138955.1 13 95744726
95745765 + 1040 sense_intronic "novel
transcript, sense intronic to DNAJC3" - 0 0 3
12 40 0 0 0 0.134658555
0.641150448 2.36414447 0
ENSG00000198920 920.4961259 789.1674616 744.3229322
684.3849455 576.9655578 534.5174639 817.9955066
598.6226557 0.450757486 0.021183169 1 KIAA0753
17 6578148 6640927 - 6299 protein_coding
KIAA0753 [Source:HGNC Symbol;Acc:HGNC:29110] - 957 907
800 621 476 579 7.345841373 6.274718925
5.92876743 5.478126232 4.644967766 4.274034008
ENSG00000166250 7217.766906 8256.240401 7014.313232
6349.020404 6420.559999 6049.556029 7496.106846
6273.045477 0.257104131 0.021186875 0.183486337 CLMP
11 123069865 123195281 - 5097
protein_coding CXADR like membrane protein [Source:HGNC
Symbol;Acc:HGNC:24039] - 7504 9489 7539 5761 5297
6553 71.18351154 81.12681214 69.04705274
62.80515406 63.87967524 59.78008834
ENSG00000278920 19.23711862 1.740170808 7.443229322 0
0 0 9.47350625 0 5.584484916
0.021229862 1 AC005005.4 22 31051630
31068327 + 16698 sense_overlapping "novel
transcript, sense overlapping SMTN" - 20 2 8
0 0 0 0.057911759 0.005219443
0.022365137 0 0 0
ENSG00000269713 1478.372566 1502.637493 1201.151132
897.0842925 1066.659014 1201.971914 1394.05373
1055.238407 0.40126921 0.021238688 1 NBPF9 1
149054027 149103561 - 9046 protein_coding
NBPF member 9 [Source:HGNC Symbol;Acc:HGNC:31991] -
1537 1727 1291 814 880 1302 8.215206805
8.319444941 6.662169758 5.000113528 5.979618116
6.692454372
ENSG00000148219 96.18559309 77.43760098 83.73632987

51.7972503 30.30281291 18.46347025 85.78650798
33.52117782 1.359368035 0.02126347 1 ASTN2 9
116425225 117415070 - 8290 protein_coding
astrotactin 2 [Source:HGNC Symbol;Acc:HGNC:17021] -
100 89 90 47 25 20 0.583239174
0.46783647 0.506796976 0.315032508 0.185367178
0.112177688
ENSG00000178201 57.71135586 47.85469723 66.98906389
27.55172889 18.18168774 5.539041076 57.51837233
17.09081924 1.758877825 0.021329859 1 VN1R1 19
57454790 57457142 - 2353 protein_coding
vomeronasal 1 receptor 1 [Source:HGNC Symbol;Acc:HGNC:13548]
- 60 55 72 25 15 6
1.232907628 1.018589887 1.428422246 0.590377945
0.39184715 0.118566047
ENSG00000167325 1754.425218 2383.163922 2113.877127
3635.726144 2646.041623 2139.916203 2083.822089
2807.22799 -0.429540982 0.021358335 0.18410283 RRM1
11 4094707 4138876 + 4177 protein_coding
ribonucleotide reductase catalytic subunit M1 [Source:HGNC
Symbol;Acc:HGNC:10451] - 1824 2739 2272 3299 2183
2318 21.11356527 28.57499445 25.39158946
43.8863926 32.12451803 25.8035806
ENSG00000112159 2937.508013 1763.663114 2483.247382
1974.907927 1593.927959 1811.266432 2394.80617
1793.367439 0.416883382 0.021391097 1 MDN1 6
89642499 89819723 - 18602 protein_coding
midasin AAA ATPase 1 [Source:HGNC Symbol;Acc:HGNC:18302]
- 3054 2027 2669 1792 1315 1962
7.937991128 4.748459152 6.697843097 5.352915761
4.345237041 4.904224935
ENSG00000231942 23.08454234 10.44102485 0 0 0
0 11.17518906 0 5.823631852 0.021427703
1 HNRNPA1P36 8 81807772 81808726
+ 955 processed_pseudogene heterogeneous nuclear
ribonucleoprotein A1 pseudogene 36 [Source:HGNC
Symbol;Acc:HGNC:48765] - 24 12 0 0 0
0 1.21509179 0.547565998 0 0 0
0
ENSG00000166913 8127.682616 8682.582249 8196.85629
10609.61976 10319.92596 8900.315836 8335.707052
9943.287186 -0.254270761 0.021465078 0.184158335 YWHAB
20 44885676 44908532 + 7933
protein_coding tyrosine 3-monooxygenase/tryptophan 5-monooxygenase
activation protein beta [Source:HGNC Symbol;Acc:HGNC:12849] -
8450 9979 8810 9627 8514 9641 51.50157032
54.8161041 51.84232734 67.43192553 65.96955458
56.50874299
ENSG00000110066 1389.88182 1505.247749 1259.766563
1099.865017 1126.052528 1025.645773 1384.965377

1083.854439	0.354142254	0.021470371	1	KMT5B	11
	68154863	68213828	-	8632	protein_coding
	lysine methyltransferase 5B [Source:HGNC
Symbol;Acc:HGNC:24283]	-	1445	1730	1354	998	929
	1111	8.093896226	8.733599435	7.322396951
6.424378992	6.615331966	5.984579566
ENSG00000236896 0	17.40170808	15.81686231	0	0
	0	11.0728568	0	5.811584977	0.021510882
	1	AL354726.1	9	97986551	97987656
	-	402	antisense	novel transcript	-
	0	20	17	0	0	0	0
2.168016286	1.974098889	0	0	0
ENSG00000112312 576.1517026	692.5879818	576.8502724
998.4746548	837.5697488	708.9972578	615.1966523
848.3472205	-0.4628029	0.021519291	1	GMNN	6
	24774931	24786099	+	2476	protein_coding
	"geminin, DNA replication inhibitor [Source:HGNC
Symbol;Acc:HGNC:17493]" -	599	796	620	906	691
	768	11.69707834	14.00944805	11.68926179
20.33244476	17.15436977	14.42253488
ENSG00000144711 1262.916837	1199.847772	1284.887462
1001.780862	899.3874871	1026.568946	1249.217357
975.9124318	0.355633734	0.021580221	1	IQSEC1	3
	12897220	13283281	-	8550	protein_coding
	IQ motif and Sec7 domain 1 [Source:HGNC Symbol;Acc:HGNC:29112]
	-	1313	1379	1381	909	742	1112
7.425057614	7.028404517	7.54003913	5.907582729
5.33439475	6.047413856
ENSG00000147133 1788.090176	1549.622105	1858.016119
1383.09679	1306.657293	1429.072598	1731.909467
1372.942227	0.334670546	0.021598995	1	TAF1	X
	71366239	71532374	+	10860	protein_coding
	TATA-box binding protein associated factor 1 [Source:HGNC
Symbol;Acc:HGNC:11535]	-	1859	1781	1997	1255	1078
	1548	8.276577404	7.146488381	8.584090259
6.421344117	6.101495507	6.627842037
ENSG00000129255 2164.175845	2025.558821	1828.243202
2257.03763	2573.314872	2655.047023	2005.992623
2495.133175	-0.314948811	0.021692736	1	MPDU1	17
	7583529 7592789 +	3270	protein_coding	mannose-P-
dolichol utilization defect 1 [Source:HGNC Symbol;Acc:HGNC:7207]
	-	2250	2328	1965	2048	2123	2876
33.26871158	31.02371635	28.05180672	34.80120192
39.90704785	40.89518218
ENSG00000235286 8.656703379	8.700854042	9.304036652	0
	0	0	8.887198024	0	5.493573515
0.021723174	1	AP000925.1	11	111670956
111672404	-	1449	processed_pseudogene	keratin 8
(KRT8) pseudogene	-	9	10	10	0	0
	0	0.300313835	0.300739319	0.322164476	0

0	0
ENSG00000126001 2675.8832	1707.977649	2168.770944
1535.182333	1461.807695	1917.431386	2184.210597
1638.140471	0.414335577	0.021780769	1	CEP250	20
35455164	35519280	+	16295	protein_coding
centrosomal protein 250 [Source:HGNC Symbol;Acc:HGNC:1859]
-	2782	1963	2331	1393	1206	2077
8.254750996	5.249579687	6.677808253	4.750166093
4.549255232	5.926702802
ENSG00000166669 15.3896949	6.09059783	5.582421991	0
0	0	9.020904905	0	5.514430929
0.021789817	1	ATF7IP2 16	10326434	10483638
+	6391	protein_coding	activating transcription
factor 7 interacting protein 2 [Source:HGNC Symbol;Acc:HGNC:20397]
-	16	7	6	0	0	0
0.12104654	0.047729603	0.04382566	0	0	0
ENSG00000214425 376.085669	190.5487035	315.4068425
214.9034853	143.0292769	151.4004561	294.0137383
169.7777394	0.791729881	0.02183683	1	LRRC37A4P
17	45506741	45550335	-	6924
transcribed_unprocessed_pseudogene	"leucine rich repeat
containing 37 member A4, pseudogene [Source:HGNC
Symbol;Acc:HGNC:25479]" -	391	219	339	195	118
164	2.730366298	1.378306021	2.285539202
1.564910829	1.047544083	1.101330856
ENSG00000175166 13439.05107	11920.17004	11742.62466
14142.85347	14431.41162	15186.20428	12367.28192
14586.82313	-0.23819801	0.021851674	0.186367697	PSMD2
3	184298709	184309054	+	5453
protein_coding	"proteasome 26S subunit, non-ATPase 2 [Source:HGNC
Symbol;Acc:HGNC:9559]"	-	13972	13700	12621	12833	11906
16450	123.8865891	109.4822382	108.0449082
130.7689812	134.2077949	140.2689102
ENSG00000267405 14.42783896	3.480341617	9.304036652	0
0	0	9.070739078	0	5.522156096
0.021859805	1	AC005180.1	17	44794747
44797783	-	587	lincRNA novel transcript	-
15	4	10	0	0	0	1.235533071
0.296948057	0.795257795	0	0	0
ENSG00000185860 0	26.10256213	7.443229322	0	0
0	11.18193048	0	5.82624182	0.021925691
1	CCDC190 1	162824458	162868815	-
4316	protein_coding	coiled-coil domain containing 190
[Source:HGNC Symbol;Acc:HGNC:28736]	-	0	30	8
0	0	0	0	0.302899402	0.086527586
0	0	0
ENSG00000075407 814.6919735	616.8905516	666.1690243
569.7697534	456.9664186	475.4343591	699.2505165
500.7235104	0.481513234	0.02192924	1	ZNF37A	10
38094334	38150293	+	13190	protein_coding

zinc finger protein 37A [Source:HGNC Symbol;Acc:HGNC:13102]
zf-C2H2 847 709 716 517 377 515
3.104844336 2.342394488 2.534044648 2.177999578
1.756887346 1.815488288
ENSG00000147439 537.6774654 475.9367161 507.0699975
318.4979859 313.9371417 418.1976013 506.8947263
350.2109096 0.531712698 0.021957576 1 BIN3 8
22620418 22669148 - 5080 protein_coding
bridging integrator 3 [Source:HGNC Symbol;Acc:HGNC:1054]
- 559 547 545 289 259 453
5.320461588 4.692261548 5.008167272 3.161157789
3.133887571 4.146345695
ENSG00000233276 5753.822179 6447.332845 6796.598774
7009.159828 7444.795075 8593.82223 6332.5846
7682.592378 -0.278915146 0.021962535 0.186367697 GPX1
3 49357171 49358600 - 1430
polymorphic_pseudogene glutathione peroxidase 1 [Source:HGNC
Symbol;Acc:HGNC:4553] - 5982 7410 7305 6360 6142
9309 202.2607381 225.8087508 238.46806
247.1343547 264.0104606 302.6899565
ENSG00000111215 7.694847448 5.220512425 13.95605498 0
0 0 8.957138284 0 5.504543031
0.022021653 1 PRR4 12 10845849 10849475
- 1310 protein_coding proline rich 4 [Source:HGNC
Symbol;Acc:HGNC:18020] - 8 6 15 0 0
0 0.295270397 0.199589896 0.53452251 0
0 0
ENSG00000161547 4266.79291 4539.235554 4451.981538
4667.262873 5678.747139 5737.523382 4419.336667
5361.177798 -0.278743611 0.02210035 0.186367697 SRSF2
17 76734115 76737374 - 3017
protein_coding serine and arginine rich splicing factor 2
[Source:HGNC Symbol;Acc:HGNC:10783] - 4436 5217 4785
4235 4685 6215 71.0914617 75.35362062
74.0376572 77.99919996 95.45125552 95.78485385
ENSG00000135926 2521.986251 3170.591213 3107.548242
3580.622686 3830.275552 3448.05307 2933.375235
3619.650436 -0.303011831 0.022128626 0.186367697 TMBIM1
2 218274192 218292586 - 5098
protein_coding transmembrane BAX inhibitor motif containing 1
[Source:HGNC Symbol;Acc:HGNC:23410] - 2622 3644 3340
3249 3160 3735 24.86761143 31.14849982
30.58388637 35.41293513 38.10084536 34.06605107
ENSG00000197124 0.961855931 0 0 48.49104284 0
0 0.320618644 16.16368095 -5.61524539
0.022158596 1 ZNF682 19 19997058 20039506
- 4968 protein_coding zinc finger protein 682
[Source:HGNC Symbol;Acc:HGNC:28857] zf-C2H2 1 0 0
44 0 0 0.009732393 0 0
0.492133731 0 0

ENSG00000243927 1237.908583 1315.569131 1411.42236
1713.717537 1878.7744 1470.615406 1321.633358
1687.702448 -0.352108659 0.022194249 1 MRPS6 21
34073224 34143034 + 4299 protein_coding
mitochondrial ribosomal protein S6 [Source:HGNC
Symbol;Acc:HGNC:14051] - 1287 1512 1517 1555 1550
1593 14.47479155 15.32649839 16.47267657
20.09902507 22.16213588 17.22976479
ENSG00000146197 64.44434737 32.19315996 55.82421991 0
14.5453502 17.54029674 50.82057575 10.69521565
2.243275234 0.022227477 1 SCUBE3 6
35214419 35253079 + 7356
protein_coding "signal peptide, CUB domain and EGF like domain
containing 3 [Source:HGNC Symbol;Acc:HGNC:13655]" - 67
37 60 0 12 19 0.440386806
0.219188922 0.380763724 0 0.100273664 0.12009997
ENSG00000246225 0 0 0 17.63310649 0
12.92442918 0 10.18584522 -5.91337538 0.022242866
1 AC006299.1 11 22829380 22945393
+ 2421 antisense "novel transcript, antisense
to SVIP" - 0 0 0 16 0 14
0 0 0 0.367229226 0 0.268883569
ENSG00000183696 1228.290024 1031.051204 1098.806729
1320.278848 1506.655858 1462.306844 1119.382652
1429.747183 -0.353216851 0.022257879 1 UPP1 7
48088628 48108733 + 4577 protein_coding
uridine phosphorylase 1 [Source:HGNC Symbol;Acc:HGNC:12576]
- 1277 1185 1181 1198 1243 1584
13.48997676 11.28225823 12.04522789 14.54413675
16.69312235 16.09182433
ENSG00000130518 202.9516014 119.2017004 98.62278851
22.04138311 103.0295639 46.15867564 140.2586968
57.07654088 1.301080801 0.022287498 1 IQCN 19
18257097 18274509 - 9154 protein_coding
IQ motif containing N [Source:HGNC Symbol;Acc:HGNC:29350]
- 211 137 106 20 85 50
1.114481243 0.652181172 0.540556374 0.121403479
0.570762429 0.2539745
ENSG00000197771 3225.102936 3410.734785 3165.233269
4184.556583 3966.032153 3659.459804 3267.023663
3936.682847 -0.268793747 0.022366025 0.186789966 MCMBP
10 119829404 119892556 - 6000
protein_coding minichromosome maintenance complex binding protein
[Source:HGNC Symbol;Acc:HGNC:25782] - 3353 3920 3402
3797 3272 3964 27.01988645 28.47038987
26.46848566 35.16425254 33.52041639 30.71944842
ENSG00000149485 5233.45812 4541.84581 5491.242432
10895.05567 8593.877741 5167.002151 5088.848787
8218.645187 -0.691509001 0.02238224 0.186789966 FADS1
11 61799625 61829318 - 10230

protein_coding	fatty acid desaturase 1 [Source:HGNC
Symbol;Acc:HGNC:3574]	-	5441	5220	5902	9886	7090
	5597	25.71605279	22.23583625	26.93206211
53.69786467	42.60082029	25.43962419
ENSG00000172296 630.9774907	464.6256059	374.0222734
469.4814602	192.7258901	169.8639263	489.8751233
277.3570922	0.821208016	0.022386786	1	SPTLC3	20
	13008979	13169103	+	8520	protein_coding
	serine palmitoyltransferase long chain base subunit 3
[Source:HGNC Symbol;Acc:HGNC:16253]	-	656	534	402
	426	159	184	3.722763619	2.731242489
2.202584071	2.77831861	1.147109535	1.004174633
ENSG00000204580 934.9239649	948.3930906	1114.623591
627.0773494	823.0243986	786.5438328	999.3135488
745.548527	0.422651191	0.022396599	1	DDR1	6
	30876421	30900156	+	9122	protein_coding
	discoidin domain receptor tyrosine kinase 1 [Source:HGNC
Symbol;Acc:HGNC:2730]	-	972	1090	1198	569	679
	852	5.152018496	5.207089324	6.130738413
3.466045361	4.575378916	4.342907156
ENSG00000177000 1128.257007	856.1640378	815.9640144
577.4842374	766.0551103	689.610614	933.4616864
677.7166539	0.461968181	0.02240556	1	MTHFR	1
	11785723	11806920	-	11978	protein_coding
	methylenetetrahydrofolate reductase [Source:HGNC
Symbol;Acc:HGNC:7436]	-	1173	984	877	524	632
	747	4.734944794	3.57988757	3.417915491
2.43085482	3.243247782	2.899795097
ENSG00000137100 739.6672109	747.4033622	853.180161
1005.08707	1035.144089	1003.489608	780.083578
1014.573589	-0.379101227	0.02253093	1	DCTN3	9
	34613545	34620523	-	3740	protein_coding
	dynactin subunit 3 [Source:HGNC Symbol;Acc:HGNC:2713]	-
	769	859	917	912	854	1087	9.941592418
	10.00875733	11.44573718	13.54987472	14.03568551
	13.514157
ENSG00000032389 218.3412963	203.5999846	200.9671917
273.3131505	315.1492542	376.6547932	207.6361575
321.7057327	-0.632697585	0.022541077	1	EIPR1	2
	3188925 3377882 -	5775	protein_coding	EARP complex
and GARP complex interacting protein 1 [Source:HGNC
Symbol;Acc:HGNC:12383]	-	227	234	216	248	260
	408	1.900531557	1.765722563	1.746014309
2.386226893	2.767379501	3.285028885
ENSG00000204959 23.08454234	12.18119566	11.16484398
1.102069155	2.424225033	0	15.47686066	1.175431396
	3.771538086	0.022541959	1	ARHGEF34P	7
	144272445	144286966	-	4532
unprocessed_pseudogene	"Rho guanine nucleotide exchange factor 34,
pseudogene [Source:HGNC Symbol;Acc:HGNC:38086]" -	24	14

12	1	2	0	0.256048689	0.134616016
0.123605382	0.012260894	0.027126106	0
ENSG00000132394 1200.396202	1187.666577	1175.099829
1259.665045	1624.230772	1731.87351	1187.720869
1538.589775	-0.373686643	0.022570969	1	EEFSEC	3
128153454	128408646	+	2421	protein_coding
"eukaryotic elongation factor, selenocysteine-tRNA specific
[Source:HGNC Symbol;Acc:HGNC:24614]"	-	1248	1365	1263
1143	1340	1876	24.92418765	24.56950799
24.35311934	26.23393781	34.02180642	36.03039827
ENSG00000260306 22.12268641	4.350427021	1.86080733	0
0	0	9.444640254	0	5.580213536
0.022582393	1	AC092375.2	16	21794095
21795759	-	1665	lincRNA novel transcript	-
23	5	2	0	0	0	0.667905185
0.130862244	0.056074033	0	0	0
ENSG00000132613 692.5362703	853.5537816	726.6452625
531.1973329	623.0258334	512.3612996	757.5784381
555.5281553	0.448607869	0.022684933	1	MTSS1L	16
70661204	70686066	-	4999	protein_coding
"MTSS1L, I-BAR domain containing [Source:HGNC
Symbol;Acc:HGNC:25094]" -	720	981	781	482	514
555	6.963868733	8.551542695	7.293129631
5.357669814	6.320149369	5.162271771
ENSG00000258976 82.71961006	96.57947987	78.15390788
3.306207466	42.42393807	35.08059348	85.81766594
26.93691301	1.672155666	0.022689647	1	AC013451.2
14	74614693	74616647	-	717
lincRNA novel transcript	-	86	111	84	3
35	38	5.79936592	6.746249841	5.468977873
0.232495281	3.000518086	2.464310679
ENSG00000180089 19.23711862	21.75213511	36.28574294
41.87862791	65.45407588	109.857648	25.75833222
72.39678393	-1.49326888	0.022745783	1	TMEM86B 19
55226639	55229264	-	2626	protein_coding
transmembrane protein 86B [Source:HGNC Symbol;Acc:HGNC:28448]
-	20	25	39	38	54	119
0.368244688	0.414862218	0.693291573	0.804083071
1.263998033	2.107090833
ENSG00000252777 26.93196607	0	6.512825656	0	0
0	11.14826391	0	5.819383232	0.022796876
1	RF00422 1	28689665	28689794	+
130	scaRNA	-	28	0	7	0
0	0	10.41395977	0	2.513626368	0
0	0
ENSG00000203943 40.3979491	26.10256213	11.16484398
5.510345777	4.848450065	0	25.88845174	3.452931947
2.93035246	0.022944796	1	SAMD13	1
84298366	84389957	+	3455	protein_coding
sterile alpha motif domain containing 13 [Source:HGNC

Symbol;Acc:HGNC:24582]	-	42	30	12	5	4
	0	0.587763286	0.378383161	0.162135916
0.080414432	0.071163828	0
ENSG00000105928 2153.595429	1708.847734	1711.01234
1294.931258	1558.776696	1496.464264	1857.818501
1450.057406	0.357400535	0.022967187	1	GSDME	7
	24698351	24758113	-	8843	protein_coding
	gasdermin E [Source:HGNC Symbol;Acc:HGNC:2810]	-	2239
	1964	1839	1175	1286	1621	12.2420933
9.678330671	9.707963617	7.383296092	8.938995194
8.52343018
ENSG00000214026 840.6620836	503.7794491	766.6526201
936.7587821	1541.807121	729.3070751	703.6980509
1069.290993	-0.602999444	0.022981043	1	MRPL23	11
	1947278 1984522 +	2975	protein_coding	mitochondrial
ribosomal protein L23 [Source:HGNC Symbol;Acc:HGNC:10322]	-
	874	579	824	850	1272	790	14.2044911
	8.481061088	12.92963537	15.87610634	26.2813398
	12.34727552
ENSG00000236526 0	11.31111026	21.3992843	0	0
	0	10.90346485	0	5.788958972	0.022987026
	1	AL035448.1	20	11909404	11918677
	-	1831	antisense	novel transcript	-
	0	13	23	0	0	0	0
0.309395224	0.586388612	0	0	0
ENSG00000268379 8.656703379	12.18119566	5.582421991	0
	0	0	8.806773676	0	5.48086395
0.023066237	1	AC025588.1	19	57175233
57177921	+	2072	processed_pseudogene
pentatricopeptide repeat domain 3 pseudogene	-	9	14
	6	0	0	0	0.21001677	0.29444005
	0.135178473	0	0	0
ENSG00000197776 86.56703379	88.74871123	67.91946756
29.7558672	39.99971304	32.31107295	81.07840419
34.02221773	1.254671207	0.0230815	1	KLHDC1	14
	49693105	49753152	+	3006	protein_coding
	kelch domain containing 1 [Source:HGNC Symbol;Acc:HGNC:19836]
	-	90	102	73	27	33	35
1.447620584	1.478665	1.133652421	0.499099152
0.674795727	0.541389821
ENSG00000156381 565.5712874	544.6734631	628.022474
694.3035679	806.0548233	841.0110701	579.4224082
780.4564871	-0.430043874	0.023096401	1	ANKRD9	14
	102501760	102509799	-	7215	protein_coding
	ankyrin repeat domain 9 [Source:HGNC Symbol;Acc:HGNC:20096]
	-	588	626	675	630	665	911
3.940417209	3.780912228	4.367304501	4.85194934
5.665427288	5.871013239
ENSG00000230989 3442.482377	4071.129606	3613.687836
4259.497286	4504.210111	4668.488454	3709.09994

4477.398617	-0.271562177	0.023153213	0.192144665	HSBP1
	16	83807843	83819737	+	9525
protein_coding	heat shock factor binding protein 1 [Source:HGNC
Symbol;Acc:HGNC:5203]	-	3579	4679	3884	3865	3716
	5057	18.16761553	21.40654896	19.03532397
22.54740457	23.98049156	24.68646967
ENSG00000263766 36.55052538	2.610256213	7.443229322
2.204138311	0	0	15.5346703	0.73471277
4.417210564	0.023182795	1	AC025682.1	17
47603860	47649420	-	1242	antisense	"novel
transcript, antisense to NPEPPS"	-	38	3	8
	2	0	0	1.479323707	0.105258762
0.300686844	0.08947886	0	0
ENSG00000217416 0.961855931	3.480341617	1.86080733
12.12276071	14.5453502	27.69520538	2.101001626
18.12110543	-3.102772346	0.023223361	1	ISCA1P1 5
	62776877	62777263	-	387
processed_pseudogene	iron-sulfur cluster assembly 1 pseudogene 1
[Source:HGNC Symbol;Acc:HGNC:33263]	-	1	4	2
	11	12	30	0.124936764	0.450409585
0.241248747	1.579405928	1.905976937	3.604469109
ENSG00000226284 0	3.480341617	3.721614661	44.08276622
	4.848450065	18.46347025	2.400652093	22.46489551
	-3.219879986	0.023242441	1	ARPC3P1 20
49134480	49135012	+	533	processed_pseudogene
	actin related protein 2/3 complex subunit 3 pseudogene 1
[Source:HGNC Symbol;Acc:HGNC:16092]	-	0	4	4
	40	4	20	0	0.327032851	0.350331201
	4.170084218	0.461296482	1.744752401
ENSG00000204673 2974.058538	2233.509233	2904.720243
2791.541171	3728.4581	3942.874073	2704.096005
3487.624448	-0.367342181	0.023318239	0.192144665	AKT1S1
	19	49869033	49878459	-	5737
protein_coding	AKT1 substrate 1 [Source:HGNC Symbol;Acc:HGNC:28426]
	-	3092	2567	3122	2533	3076	4271
26.05888635	19.49842878	25.40353091	24.53366233
32.95708875	34.61590893
ENSG00000100029 2573.926471	1997.716088	2612.573492
2432.266626	3164.82578	3825.631037	2394.738684
3140.907814	-0.391701218	0.02336712	0.192144665	PES1
	22	30576625	30607083	-	4557
protein_coding	pescadillo ribosomal biogenesis factor 1 [Source:HGNC
Symbol;Acc:HGNC:8848]	-	2676	2296	2808	2207	2611
	4144	28.39280482	21.95591056	28.76498227
26.91134155	35.21885264	42.28357863
ENSG00000234899 0	0	0	0	10.90901265
19.38664377	0	10.09855214	-5.900924669	0.023393357
	1	SOX9-AS1	17	72034107	72237203
	-	10700	processed_transcript	SOX9 antisense RNA 1
[Source:HGNC Symbol;Acc:HGNC:49321]	-	0	0	0

0 9 21 0 0 0 0
0.051701851 0.091257073
ENSG00000182472 8.656703379 0 24.19049529 0 0
0 10.94906622 0 5.79395089 0.023494543
1 CAPN12 19 38730187 38769904 -
5966 protein_coding calpain 12 [Source:HGNC
Symbol;Acc:HGNC:13249] - 9 0 26 0 0
0 0.072939113 0 0.203439901 0 0
0
ENSG00000163655 3427.092682 3940.616796 3850.010367
4767.551166 4355.120271 4373.07293 3739.239948
4498.581456 -0.266633439 0.023539046 0.192144665 GMPS
3 155870536 155944026 + 8885
protein_coding guanine monophosphate synthase [Source:HGNC
Symbol;Acc:HGNC:4378] - 3563 4529 4138 4326 3593
4737 19.38918733 22.2128092 21.74097867
27.05460058 24.85691031 24.79002534
ENSG00000277142 215.4557285 208.820497 122.8132838
96.98208568 94.54477627 106.164954 182.3631698
99.2306053 0.877258974 0.023564027 1 LINC00235
16 525155 527407 - 2253 lincRNA long
intergenic non-protein coding RNA 235 [Source:HGNC
Symbol;Acc:HGNC:14138] - 224 240 132 88 78
115 4.807154087 4.642037534 2.735009098
2.170368732 2.128044823 2.373382123
ENSG00000279342 195.256754 67.86666153 60.47623824
42.98069706 24.24225033 56.31358428 107.8665513
41.17884389 1.383810409 0.023649216 1 AP000866.6
11 124789240 124792818 - 3579
sense_intronic "novel transcript, sense intronic to MSANTD2" -
203 78 65 39 20 61 2.742430031
0.949711074 0.847808359 0.605501122 0.343491233
0.792499788
ENSG00000232491 0 0 0 0 0 37.85011402
0 12.61670467 -6.224163289 0.023761645 1
SAPCD2P3 7 66556216 66557065 +
850 processed_pseudogene suppressor APC domain
containing 2 pseudogene 3 [Source:HGNC Symbol;Acc:HGNC:51279] -
0 0 0 0 0 41 0 0
0 0 0 2.242827896
ENSG00000273682 0 0 0 12.12276071 6.060562582
6.462214589 0 8.215179293 -5.601097903
0.023772958 1 AC109583.2 3 46918807
46918935 - 129 processed_pseudogene biliverdin
reductase B (flavin reductase (NADPH) (BLVRB) pseudogene -
0 0 0 11 5 7 0 0
0 4.738217785 2.382471171 2.523128376
ENSG00000145425 22089.98331 27767.03551 26859.82341
28670.32908 32734.31062 30435.18437 25572.28074
30613.27469 -0.259543586 0.023786561 0.192144665 RPS3A

4	151099573	151104652	+	2341
protein_coding	ribosomal protein S3A [Source:HGNC
Symbol;Acc:HGNC:10421]	-	22966	31913	28869	26015	27006
32968	474.3349912	594.0524841	575.6736652
617.4964429	709.0979173	654.8204078
ENSG00000085224 3214.522521	2499.755366	2560.470887
2262.547976	2254.52928	2119.606385	2758.249591
2212.227881	0.318265222	0.023816999	0.192144665	ATRX
X	77504878	77786233	-	19266
protein_coding	"ATRX, chromatin remodeler [Source:HGNC
Symbol;Acc:HGNC:886]"	-	3342	2873	2752	2053	1860
2296	8.387182751	6.498343553	6.668112363
5.921196	5.934289763	5.541295954
ENSG00000232712 0	18.27179349	13.95605498	0	0
0	10.74261616	0	5.767999407	0.023877895
1	KIZ-AS1 20	21154023	21218289	-
5451	antisense	KIZ antisense RNA 1 [Source:HGNC
Symbol;Acc:HGNC:51231]	-	0	21	15	0	0
0	0	0.167881063	0.128457987	0	0
0
ENSG00000198356 1968.919091	1922.018658	2304.609879
2252.629354	2666.647536	2856.298848	2065.182542
2591.858579	-0.32794476	0.023878854	1	ASNA1	19
12737139	12748323	+	1652	protein_coding
"arsA arsenite transporter, ATP-binding, homolog 1 (bacterial)
[Source:HGNC Symbol;Acc:HGNC:752]"	-	2047	2209	2477
2044	2200	3094	59.91133764	58.26989971
69.99419119	68.75161306	81.85778673	87.08458459
ENSG00000198952 5650.903594	4817.662883	4885.549646
5531.285091	6467.832387	6614.538219	5118.038708
6204.551899	-0.277845836	0.023901065	0.192144665	SMG5
1	156249224	156282825	-	5304
protein_coding	"SMG5, nonsense mediated mRNA decay factor
[Source:HGNC Symbol;Acc:HGNC:24644]"	-	5875	5537	5251
5019	5336	7165	53.5556842	45.49143178
46.21516828	52.58062256	61.83860241	62.81215554
ENSG00000100767 10.58041524	21.75213511	26.05130263	0
0	4.615867564	19.46128432	1.538622521
3.596122805	0.023901726	1	PAPLN	14	73237497
73274640	+	7699	protein_coding	"papilin,
proteoglycan like sulfated glycoprotein [Source:HGNC
Symbol;Acc:HGNC:19262]" -	11	25	28	0	0
5	0.069081154	0.141502557	0.16977344	0
0	0.030197202
ENSG00000230366 34.62681351	0	0.930403665	0	0
0	11.85240573	0	5.907713058	0.023908801
1	DSCR9	21	37208503	37221736	+
1901	lincRNA Down syndrome critical region 9 [Source:HGNC
Symbol;Acc:HGNC:16301]	-	36	0	1	0	0
0	0.915633345	0	0.024556356	0	0

0
ENSG00000257038 13.46598303 10.44102485 2.791210996 0
0 0 8.899406293 0 5.495468341
0.023913193 1 AP002761.3 11 73307235
73309361 - 1695 antisense novel transcript
- 14 12 3 0 0 0
0.399355389 0.308510636 0.082622359 0 0 0
ENSG00000051108 1892.932472 2270.922905 2028.27999
3477.028185 2158.772392 2495.338005 2064.045122
2710.379527 -0.39299425 0.023943935 0.192144665
HERPUD1 16 56932048 56944863 + 5155
protein_coding homocysteine inducible ER protein with ubiquitin like
domain 1 [Source:HGNC Symbol;Acc:HGNC:13744] - 1968 2610
2180 3155 1781 2703 18.45855249 22.06329823
19.74121416 34.00812886 21.23648377 24.38082483
ENSG00000137168 2181.489251 2583.283565 2150.16287
2788.234963 2727.253162 2991.082181 2304.978562
2835.523435 -0.298902266 0.023965756 0.192144665 PPIL1
6 36854827 36875024 - 1810
protein_coding peptidylprolyl isomerase like 1 [Source:HGNC
Symbol;Acc:HGNC:9260] - 2268 2969 2311 2530 2250
3240 60.58508086 71.48093432 59.60290212
77.67012247 76.4101942 83.23336512
ENSG00000156486 26.93196607 14.79145187 2.791210996
2.204138311 0 0 14.83820964 0.73471277
4.35232232 0.02403504 1 KCNS2 8 98427022
98432848 + 5348 protein_coding potassium
voltage-gated channel modifier subfamily S member 2 [Source:HGNC
Symbol;Acc:HGNC:6301] - 28 17 3 2 0
0 0.253144123 0.13852116 0.026186406
0.020780244 0 0
ENSG00000259363 0 19.14187889 13.02565131 0 0
0 10.72251007 0 5.765356724 0.024088696
1 AC090825.1 15 99807023 99877148
+ 1291 lincRNA ubiquitin conjugating enzyme E2 Q2
pseudogene [Source:NCBI gene;Acc:400464] - 0 22
14 0 0 0 0 0.742600156
0.506229943 0 0 0
ENSG00000160271 191.4093303 154.0051166 211.201632
54.00138862 129.6960392 106.164954 185.5386929
96.62079394 0.941823535 0.024088719 1 RALGDS 9
133097720 133149334 - 7584 protein_coding
ral guanine nucleotide dissociation stimulator [Source:HGNC
Symbol;Acc:HGNC:9842] - 199 177 227 49 107
115 1.26869132 1.017029475 1.397248232
0.35901269 0.867227046 0.705067237
ENSG00000276692 0 0 0 13.22482987 16.96957523
0 0 10.0648017 -5.891132385 0.024089305
1 CR769767.2 9 39873931 39874390
- 460 processed_pseudogene novel pseudogene

-	0	0	0	12	14	0	0
0	0	1.449557536	1.870757798	0
ENSG00000185825 6182.809924	6606.558474	7275.756662
7000.343275	8924.784458	8483.964582	6688.37502
8136.364105	-0.282702612	0.024196896	0.192685051	BCAP31
X	153700492	153724746	-	3533
protein_coding	B cell receptor associated protein 31 [Source:HGNC
Symbol;Acc:HGNC:16695]	-	6428	7593	7820	6352	7363
9190	87.96976811	93.65443759	103.3258892
99.90308413	128.1027707	120.9491699
ENSG00000010292 1699.59943	1745.391321	1444.916892
2858.767389	1923.622563	1750.33698	1629.969214
2177.575644	-0.417643331	0.024237035	1	NCAPD2	12
6493356 6531955 +	6487	protein_coding	non-SMC
condensin I complex subunit D2 [Source:HGNC Symbol;Acc:HGNC:24305]
-	1767	2006	1553	2594	1587	1896
13.17024543	13.47552296	11.17567063	22.21969624
15.03766442	13.59018764
ENSG00000163191 4248.517647	4711.512464	4609.219757
4612.159415	6170.864821	5925.850778	4523.083289
5569.625005	-0.300234374	0.024243054	0.192685051
S100A11 1	152032506	152047907	-	680
protein_coding	S100 calcium binding protein A11 [Source:HGNC
Symbol;Acc:HGNC:10488]	-	4417	5415	4954	4185	5091
6419	314.0651175	347.0149186	340.0894231
341.9783348	460.1946278	438.9241544
ENSG00000147471 969.5507784	1066.724706	1058.799371
1237.623662	1539.382896	1221.358557	1031.691618
1332.788372	-0.368742395	0.024346677	1	PLPBP	8
37762593	37779767	+	3428	protein_coding
pyridoxal phosphate binding protein [Source:HGNC
Symbol;Acc:HGNC:9457]	-	1008	1226	1138	1123	1270
1323	14.21742465	15.58505196	15.4969947
18.20333605	22.77247678	17.94527215
ENSG00000282855 11.54227117	6.960683234	7.443229322	0
0	0	8.648727909	0	5.453962192
0.024374265	1	AC093591.3	4	127809170
127809477	+	308	processed_pseudogene	INTS3 and NABP
interacting protein (INIP) pseudogene	-	12	8	8
0	0	0	1.883786785	1.131873438
1.212509936	0	0	0
ENSG00000224533 0	0	0	5.510345777	15.75746271
3.692694051	0	8.320167513	-5.616349738
0.024376911	1	TMLHE-AS1	X	155466540
155611616	+	1845	antisense	TMLHE antisense RNA 1
[Source:HGNC Symbol;Acc:HGNC:44261]	-	0	0	0
5	13	4	0	0	0	0.150586375
0.433106141	0.100807917
ENSG00000248449 5.771135586	26.97264753	0	0	0
0	10.91459437	0	5.791225238	0.024439294

1	PCDHGB8P	5	141426286	141429158
+	2873	transcribed_unitary_pseudogene	"protocadherin
gamma subfamily B, 8 pseudogene [Source:HGNC Symbol;Acc:HGNC:8715]"
-	6	31	0	0	0	0
0.100975693	0.47020221	0	0	0	0
ENSG00000114854 11.54227117	4.350427021	10.23444032	0
0	0	8.70904617	0	5.463689606
0.024445905	1	TNNC1	3	52451102	52454070
-	1168	protein_coding	"troponin C1, slow skeletal
and cardiac type [Source:HGNC Symbol;Acc:HGNC:11943]"	-	12
5	11	0	0	0	0.496751995
0.186545922	0.439638663	0	0	0
ENSG00000273489 12.5041271	6.960683234	6.512825656	0
0	0	8.659211998	0	5.455652878
0.024503637	1	AC008264.2	7	131493964
131497694	-	3731	antisense	"novel transcript,
antisense to MKLN1"	-	13	8	7	0	0
0	0.168468737	0.093437957	0.0875828	0
0	0
ENSG00000197989 516.5166349	333.2427098	494.9747499
316.2938476	313.9371417	264.0276246	448.2446982
298.0862047	0.588958235	0.02454872	1	SNHG12	1
28578538	28583132	-	4266	antisense
small nucleolar RNA host gene 12 [Source:HGNC
Symbol;Acc:HGNC:30062]	-	537	383	532	287	259
286	6.086318158	3.912339376	5.821525673
3.738290863	3.731867994	3.117282778
ENSG00000279953 52.9020762	6.960683234	10.23444032	0
6.060562582	0	23.36573325	2.020187527
3.578340814	0.024589414	1	AC117503.3	12
123649068	123650485	+	1418	TEC	novel
transcript	-	55	8	11	0	5	0
1.875373069	0.245851212	0.36212832	0
0.21674103	0
ENSG00000114503 4660.191985	4596.661191	4241.71031
4138.269679	3336.945757	3644.689028	4499.521162
3706.634821	0.279571235	0.024648036	0.195059461	NCBP2
3	196935402	196942597	-	5114
protein_coding	nuclear cap binding protein subunit 2 [Source:HGNC
Symbol;Acc:HGNC:7659]	-	4845	5283	4559	3755	2753
3948	45.80725571	45.01720059	41.61547963
40.80010317	33.08970138	35.89611422
ENSG00000135617 256.8155336	306.2700623	349.8317781
438.6235239	421.8151557	468.9721445	304.3057913
443.1369413	-0.54244337	0.024712748	1	PRADC1	2
73228006	73233238	-	1407	protein_coding
protease associated domain containing 1 [Source:HGNC
Symbol;Acc:HGNC:16047]	-	267	352	376	398	348
508	9.175260015	10.90202475	12.47497785
15.71813514	15.20311241	16.78806465

ENSG00000125703 510.7454993	587.3076479	404.7255944
403.3573109	340.6036171	260.3349306	500.9262472
334.7652862	0.582871449	0.024713654	1	ATG4C	1
62784135	62865513	+	3110	protein_coding
autophagy related 4C cysteine peptidase [Source:HGNC
Symbol;Acc:HGNC:16040]	-	531	675	435	366	281
282	8.255347299	9.458058188	6.529424489
6.539322259	5.553839067	4.216185763
ENSG00000169738 662.7187364	703.899092	721.9932442
996.2705165	943.0235377	813.3158647	696.2036909
917.5366396	-0.397643366	0.024728649	1	DCXR	17
82035136	82037732	-	1902	protein_coding
dicarbonyl and L-xylulose reductase [Source:HGNC
Symbol;Acc:HGNC:18985]	-	689	809	776	904	778
881	17.51499129	18.53517141	19.04571339
26.41009488	25.14296232	21.53755572
ENSG00000123575 2432.533649	2344.880164	2087.825825
1904.375501	1957.561714	1676.483099	2288.413213
1846.140105	0.310262961	0.024759063	1	FAM199X X
104166620	104195902	+	7457	protein_coding
"family with sequence similarity 199, X-linked [Source:HGNC
Symbol;Acc:HGNC:25195]" -	2529	2695	2244	1728	1615
1816	16.39781199	15.74900875	14.04768452
12.87631637	13.31238116	11.32355305
ENSG00000243402 25.0082542	0	7.443229322	0	0
0	10.81716118	0	5.775907793	0.024769491
1	AC022973.1	8	129864478	129865052
+	575	processed_pseudogene	ribosomal protein L15
(RPL15) pseudogene	-	26	0	8	0	0
0	2.186284721	0	0.649483583	0	0
0
ENSG00000134153 1049.384821	1325.140071	1259.766563
1690.574084	1795.138637	1286.903877	1211.430485
1590.872199	-0.392262921	0.024809818	1	EMC7	15
34084017	34101948	-	1538	protein_coding
ER membrane protein complex subunit 7 [Source:HGNC
Symbol;Acc:HGNC:24301]	-	1091	1523	1354	1534	1481
1394	34.29806599	43.15212286	41.09683386
55.42185628	59.18969243	42.14416527
ENSG00000249915 2058.371692	1863.722936	1730.550817
1486.691291	1442.413894	1601.706045	1884.215148
1510.27041	0.318750343	0.024814677	1	PDCD6	5
271621	353856	+	9493	protein_coding	programmed
cell death 6 [Source:HGNC Symbol;Acc:HGNC:8765] -	2140	2142
1860	1349	1190	1735	10.89962381	9.832740629
9.146511804	7.896243137	7.705322859	8.498201343
ENSG00000102900 2605.667717	1971.613526	1898.023477
2705.579777	2849.676526	2622.73595	2158.434907
2725.997417	-0.336831547	0.02488071	0.196055725	NUP93
16	56730105	56850286	+	11136

protein_coding nucleoporin 93 [Source:HGNC Symbol;Acc:HGNC:28958]
        -       2709   2266   2040   2455   2351   2841
11.76199524   8.867256698   8.551592171   12.24995005
12.9768943   11.86241271
ENSG00000247679 40.3979491   19.14187889   16.74726597
47.38897368   78.78731356   84.93196317   25.42903132
70.3694168   -1.470998816   0.024890314   1   AC139795.2
        5   177611253   177619754   +   1751
antisense   novel transcript   -   42   22   18
        43   65   92   1.159749946   0.547513879
0.479879718   1.364565394   2.281784211   2.443051935
ENSG00000187240 939.7332445   858.774294   1020.652821
858.5118721   604.8441456   599.1396098   939.7201197
687.4985425   0.451019993   0.024899302   1   DYNC2H1 11
        103109410   103479863   +   15012   protein_coding
        dynein cytoplasmic 2 heavy chain 1 [Source:HGNC
Symbol;Acc:HGNC:2962]   -   977   987   1097   779   499
        649   3.146713653   2.865082914   3.411254391
2.883440178   2.043192803   2.010190236
ENSG00000228261 15.3896949   11.31111026   0.930403665   0
        0   0   9.210402938   0   5.545003503
0.024918374   1   AL162742.1   10   104323369
104327004   +   954   lincRNA uncharacterized LOC101927472
[Source:NCBI gene;Acc:101927472]   -   16   13   1
        0   0   0   0.810910314   0.593818297
0.048932529   0   0   0
ENSG00000260194 0.961855931   17.40170808   9.304036652   0
        0   0   9.222533556   0   5.548241239
0.024938838   1   AC007496.1   16   53998313
53999969   -   739   antisense   "novel transcript,
antisense to FTO"   -   1   20   10   0   0
        0   0.065426965   1.17935392   0.631686503   0
        0   0
ENSG00000279344 23.08454234   4.350427021   0.930403665   0
        0   0   9.455124343   0   5.581816736
0.024944977   1   AC007342.6   16   53478957
53481550   -   2594   TEC   novel transcript   -
        24   5   1   0   0   0   0.447344896
        0.083996005   0.017996003   0   0   0
ENSG00000251323 2.885567793   0.870085404   25.12089896   0
        0   0   9.625517386   0   5.608249357
0.024998133   1   AP003086.1   11   78423982
78429836   -   1642   lincRNA uncharacterized LOC101928865
[Source:NCBI gene;Acc:101928865]   -   3   1   27
        0   0   0   0.088338357   0.026539054
0.767602971   0   0   0
ENSG00000106261 1995.851057   1670.563976   1871.041771
1531.876126   1432.716994   1479.847141   1845.818935
1481.480087   0.316994783   0.025001081   1   ZKSCAN1 7
        100015572   100041689   +   10319   protein_coding

zinc finger with KRAB and SCAN domains 1 [Source:HGNC
Symbol;Acc:HGNC:13101]	zf-C2H2	2075	1920	2011	1390	1182
1603	9.722584024	8.108158205	9.097467106
7.484955651	7.040884567	7.223154894
ENSG00000239288	6.732991517	1.740170808	18.6080733	0
0	0	9.027078543	0	5.51547432
0.025150938	1	AC063923.1	3	109241507
109243125	+	1619	processed_pseudogene	bromodomain
containing 7 (BRD7) pseudogene	-	7	2	20	0
0	0	0.209051076	0.053832152	0.576672422
0	0	0
ENSG00000273893	78.87218634	47.85469723	40.93776127
101.3903623	128.4839267	122.7820772	55.88821495
117.5521221	-1.073727587	0.02520129	1	AL133520.1
20	46681676	46682375	-	700
sense_intronic	"novel transcript, sense intronic to SLC13A3"	-
82	55	44	92	106	133	5.663918935
3.423917149	2.934274046	7.303008918	9.307974513
8.834553785
ENSG00000197081	2649.91309	1868.943448	2415.327915
2032.215523	1728.472448	1618.323168	2311.394818
1793.003713	0.366397326	0.025207431	1	IGF2R	6
159969099	160113507	+	17843	protein_coding
insulin like growth factor 2 receptor [Source:HGNC
Symbol;Acc:HGNC:5467]	-	2755	2148	2596	1844	1426
1753	7.465432004	5.245960296	6.791768095
5.742553962	4.912459809	4.568199466
ENSG00000259563	16.35155083	5.220512425	4.652018326	0
0	0	8.741360526	0	5.468861267
0.025214522	1	AC025430.1	15	44516650
44517483	-	498	antisense	"novel transcript,
antisense to CTDSPL2"	-	17	6	5	0	0
0	1.650520015	0.525025631	0.46869109	0
0	0
ENSG00000147481	16.35155083	5.220512425	4.652018326	0
0	0	8.741360526	0	5.468861267
0.025214522	1	SNTG1	8	49909789	50796691
+	10599	protein_coding	syntrophin gamma 1
[Source:HGNC Symbol;Acc:HGNC:13740]	-	17	6	5
0	0	0	0.077550615	0.024668626
0.022021716	0	0	0
ENSG00000130066	355.8866945	491.5982534	508.9308049
502.5435349	848.4787614	645.2982854	452.1385842
665.4401939	-0.556681564	0.02524277	1	SAT1	X
23783173	23786226	+	2498	protein_coding
spermidine/spermine N1-acetyltransferase 1 [Source:HGNC
Symbol;Acc:HGNC:10540]	-	370	565	547	456	700
699	7.161607355	9.856315835	10.2221189
10.14342103	17.22475154	13.01115228
ENSG00000238121	0	0	1.86080733	0	38.78760052

10.15490864	0.62026911	16.31416972	-4.72055407
0.025254161	1	LINC00426	13	30340270
30377145	-	3972	lincRNA long intergenic non-protein coding RNA 426 [Source:HGNC Symbol;Acc:HGNC:42761]	-
0	0	2	0	32	11	0	0
0.023505354	0	0.495208509	0.128769931
ENSG00000229119 257.7773895	188.8085327	261.4434299
250.1696983	545.4506323	368.3462316	236.009784
387.9888541	-0.716674138	0.025260884	1	AC026403.1
5	166382305	166382599	+	295
processed_pseudogene	60S acidic ribosomal protein (RPLP0) pseudogene	-	268	217	281	227	450	399
43.92522498	32.05503944	44.46623304	42.75785251
93.76437389	62.89004389
ENSG00000260279 173.1340676	124.4222128	148.8645864
72.73656426	16.96957523	98.77956586	148.8069556
62.82856845	1.237573684	0.025300113	1	AC137932.1
16	89296128	89298317	+	1576
antisense	uncharacterized LOC105371414 [Source:NCBI gene;Acc:105371414]	-	180	143	160	66	14
107	5.522268368	3.954015997	4.739252036
2.327018125	0.546033367	3.1568837
ENSG00000143549 14471.12248	15015.06382	15480.05618
17908.62378	18925.92483	16044.75565	14988.74749
17626.43475	-0.233772338	0.025336634	0.198795128	TPM3
1	154155304	154194648	-	8389
protein_coding	tropomyosin 3 [Source:HGNC Symbol;Acc:HGNC:12012]
-	15045	17257	16638	16250	15614	17380
86.71280083	89.64244686	92.58421772	107.6354212
114.4066689	96.33200418
ENSG00000273319 0	0	0	8.816553243	6.060562582
9.231735127	0	8.036283651	-5.570401981
0.025343548	1	AC058791.1	7	130936464
130939661	+	3198	lincRNA novel transcript	-
0	0	0	8	5	10	0	0
0	0.139002807	0.096103434	0.145396033
ENSG00000275131 330.8784402	200.9897284	244.6961639
144.3710594	122.4233641	190.1737436	258.8547775
152.3227224	0.762069122	0.025433567	1	AC241952.1
1	120489860	120701289	-	9444
transcribed_unprocessed_pseudogene	phosphodiesterase 4D interacting protein-like [Source:NCBI gene;Acc:100996724]	-
344	231	263	131	101	206	1.761179739
1.065895428	1.300007345	0.77077454	0.657374352
1.014243563
ENSG00000118985 1616.87982	2070.803262	1991.994247
2168.872098	2678.768661	2326.397252	1893.225776
2391.346004	-0.336595276	0.025445238	1	ELL2	5
95885098	95962071	-	7328	protein_coding
elongation factor for RNA polymerase II 2 [Source:HGNC

Symbol;Acc:HGNC:17064]	-	1681	2380	2141	1968	2210
	2520	11.09132597	14.15305173	13.63883397
14.92284668	18.53762845	15.98991291
ENSG00000118263 1073.431219	988.4170192	879.2314636
732.8759884	766.0551103	756.0791069	980.3599006
751.6700685	0.38318431	0.025481035	1	KLF7	2
	207074137	207167267	-	9262	protein_coding
	Kruppel like factor 7 [Source:HGNC Symbol;Acc:HGNC:6350]
	zf-C2H2 1116	1136	945	665	632	819
5.825867921	5.344808537	4.762917595	3.989595931
4.194301655	4.111593239
ENSG00000123892 9.618559309	10.44102485	5.582421991	0
	0	0	8.547335384	0	5.437486046
0.025564647	1	RAB38	11	88113242	88175467
	-	1465	protein_coding	"RAB38, member RAS oncogene
family [Source:HGNC Symbol;Acc:HGNC:9776]"	-	10	12
	6	0	0	0	0.33003773	0.356945753
	0.191187574	0	0	0
ENSG00000151287 380.8949487	354.1247595	388.908732
492.6249125	524.8447196	544.6723725	374.6428134
520.7140015	-0.475336704	0.025590763	1	TEX30	13
	102765990	102773811	-	2154	protein_coding
	testis expressed 30 [Source:HGNC Symbol;Acc:HGNC:25188] -
	396	407	418	447	433	590	8.888954915
	8.233932606	9.058924052	11.53118309	12.35633168
	12.73612553
ENSG00000199473 0	0	0	0	0	36.92694051
	0	12.30898017	-6.18856777	0.025605692	1
	RF00092 7	17373201	17373326	-	126
	snoRNA	-	0	0	0	0	0
	40	0	0	0	0	0	14.76115921
ENSG00000233762 282.7856437	335.852966	468.9234473
543.3200936	406.057693	722.8448605	362.5206857
557.407549	-0.621897251	0.025633412	1	AC007969.1
	2	171517270	171517716	-	447
processed_pseudogene	ribosomal protein S15 (RPS15) pseudogene
	-	294	386	504	493	335	783
31.80101808	37.63036053	52.63432395	61.28461185
46.06643923	81.44890632
ENSG00000141503 2455.618192	2663.331422	3013.577472
1744.575473	2117.560566	2506.416087	2710.842362
2122.850709	0.352346893	0.025678051	0.200616599	MINK1
	17	4833388 4898061 +	7817	protein_coding
misshapen like kinase 1 [Source:HGNC Symbol;Acc:HGNC:17565]	-
	2553	3061	3239	1583	1747	2715	15.79108311
	17.06403823	19.34269001	11.2525991	13.73726111
	16.1495617
ENSG00000237382 0	5.220512425	26.98170629	0	0
	0	10.73407291	0	5.765973482	0.025927575
	1	RPL21P121	17	18312760	18313533

+ 465 processed_pseudogene ribosomal protein L21
pseudogene 121 [Source:HGNC Symbol;Acc:HGNC:36429] - 0
6 29 0 0 0 0 0.562285514
2.911327622 0 0 0
ENSG00000279897 31.74124572 0 1.86080733 0 0
0 11.20068435 0 5.826080096 0.025949518
1 BIRC6-AS2 2 32557273 32574818
- 1066 transcribed_unprocessed_pseudogene BIRC6
antisense RNA 2 [Source:HGNC Symbol;Acc:HGNC:50490] - 33
0 2 0 0 0 1.496779932 0
0.0875828 0 0 0
ENSG00000251667 31.74124572 0 1.86080733 0 0
0 11.20068435 0 5.826080096 0.025949518
1 BRCC3P1 5 176308063 176309013 +
951 processed_pseudogene BRCA1/BRCA2-containing complex
subunit 3 pseudogene 1 [Source:HGNC Symbol;Acc:HGNC:51444] -
33 0 2 0 0 0 1.677778557
0 0.09817378 0 0 0
ENSG00000125835 4366.825926 4509.65265 4589.68128
5153.275371 5322.386059 5379.332059 4488.719952
5284.99783 -0.235611168 0.025965313 0.201151678 SNRPB
20 2461634 2470853 - 1278 protein_coding small
nuclear ribonucleoprotein polypeptides B and B1 [Source:HGNC
Symbol;Acc:HGNC:11153] - 4540 5183 4933 4676 4391
5827 171.7616548 176.7294609 180.1881795
203.3085731 211.1932062 212.0045503
ENSG00000080824 36418.75111 31434.44548 31767.70274
42751.46668 38182.75638 36757.99976 33206.96645
39230.74094 -0.240497356 0.025965658 0.201151678
HSP90AA1 14 102080738 102139699 - 5248
protein_coding heat shock protein 90 alpha family class A
member 1 [Source:HGNC Symbol;Acc:HGNC:5253] - 37863 36128
34144 38792 31501 39817 348.8368945 299.9913218
303.7152557 410.7337482 368.9588012 352.7814386
ENSG00000281189 0 24.36239132 7.443229322 0 0
0 10.60187355 0 5.749381872 0.026008345
1 GHET1 7 148987527 148989432 +
1906 lincRNA gastric carcinoma proliferation enhancing
transcript 1 [Source:HGNC Symbol;Acc:HGNC:49425] - 0
28 8 0 0 0 0 0.640167663
0.195935499 0 0 0
ENSG00000085552 107.7278643 38.28375779 42.7985686
13.22482987 19.39380026 28.61837889 62.93673022
20.41233634 1.618889202 0.026008402 1 IGSF9 1
159927039 159945604 - 6628 protein_coding
immunoglobulin superfamily member 9 [Source:HGNC
Symbol;Acc:HGNC:18132] - 112 44 46 12 16
31 0.817027622 0.289286905 0.323982362
0.100602967 0.148383238 0.217475437
ENSG00000138336 110.6134321 60.9059783 67.91946756

30.85793635	39.99971304	26.77203187	79.8129593
32.54322709	1.296887519	0.026078329	1	TET1	10
	68560656	68694482	+	9288	protein_coding
	tet methylcytosine dioxygenase 1 [Source:HGNC
Symbol;Acc:HGNC:29484]	-	115	70	73	28	33
	29	0.598655325	0.328423656	0.366899136
0.16751275	0.218393191	0.145180006
ENSG00000187522 464.5764146	542.0632068	420.5424567
661.2414933	641.2075211	648.9909794	475.7273594
650.4799979	-0.451087163	0.026080736	1	HSPA14	10
	14838164	14871741	+	2644	protein_coding
	heat shock protein family A (Hsp70) member 14 [Source:HGNC
Symbol;Acc:HGNC:29526]	-	483	623	452	600	529
	703	8.832566104	10.26798424	7.980369862
12.60961548	12.29820085	12.36302912
ENSG00000253471 4.809279655	8.700854042	12.09524765	0
	0	0	8.535127115	0	5.435513783
0.026087099	1	AC012574.1	8	23707141
23789487	+	252	lincRNA novel transcript	-
	5	10	13	0	0	0	0.959335863
	1.729251085	2.408179457	0	0	0
ENSG00000063177 20364.41377	20955.13688	24467.75559
22748.91151	27637.37748	29007.95812	21929.10208
26464.74904	-0.27124712	0.026156534	0.201749092	RPL18
	19	48615328	48619536	-	4209
protein_coding	ribosomal protein L18 [Source:HGNC
Symbol;Acc:HGNC:10310]	-	21172	24084	26298	20642	22801
	31422	243.2115391	249.3493788	291.6687035
272.511536	332.9832049	347.1250191
ENSG00000118473 2.885567793	6.09059783	1.86080733
30.85793635	32.72703794	7.385388102	3.612324318
23.65678746	-2.702292464	0.02618419	1	SGIP1	1
	66533383	66748299	+	10477	protein_coding
	SH3 domain GRB2 like endophilin interacting protein 1
[Source:HGNC Symbol;Acc:HGNC:25412]	-	3	7	2
	28	27	8	0.013844763	0.029115194
0.008911259	0.148502283	0.158406931	0.035504554
ENSG00000143575 2007.393328	1952.471647	2018.975953
2084.012773	2637.556835	2736.286292	1992.946976
2485.951967	-0.319067287	0.026277624	1	HAX1	1
	154272511	154275875	+	2155	protein_coding
	HCLS1 associated protein X-1 [Source:HGNC
Symbol;Acc:HGNC:16915]	-	2087	2244	2170	1891	2176
	2964	46.82484959	45.37683238	47.00656271
48.7591693	62.06674596	63.95315037
ENSG00000163354 0	4.350427021	27.91210996	0	0
	0	10.75417899	0	5.768616295	0.02628403
	1	DCST2	1	155018520	155033781	-
	3071	protein_coding	DC-STAMP domain containing 2
[Source:HGNC Symbol;Acc:HGNC:26562]	-	0	5	30

0 0 0 0 0.07094941 0.456023763
0 0 0
ENSG00000052344 10.58041524 20.8820497 0 0 0
0 10.48748831 0 5.733151275 0.026300911
1 PRSS8 16 31131433 31135762 -
3010 protein_coding serine protease 8 [Source:HGNC
Symbol;Acc:HGNC:9491] - 11 24 0 0 0
0 0.17669628 0.347458823 0 0 0
0
ENSG00000103150 581.9228382 509.8700469 624.3008593
346.0497148 351.5126297 496.6673498 572.0312482
398.0765648 0.52117342 0.026331306 1 MLYCD 16
83899126 83927026 + 16161 protein_coding
malonyl-CoA decarboxylase [Source:HGNC Symbol;Acc:HGNC:7150]
- 605 586 671 314 290 538
1.810040785 1.580112408 1.93820775 1.079626315
1.103004103 1.54790771
ENSG00000241697 0.961855931 12.18119566 13.95605498 0
0 0 9.033035523 0 5.517806468
0.026366489 1 TMEFF1 9 100473113 100577636
+ 2611 protein_coding transmembrane protein with EGF
like and two follistatin like domains 1 [Source:HGNC
Symbol;Acc:HGNC:11866] - 1 14 15 0 0
0 0.018518011 0.233657519 0.268182493 0
0 0
ENSG00000271425 155.8206608 110.5008463 162.8206414
105.7986389 52.1208382 52.62089023 143.0473829
70.18012245 1.027736415 0.026391889 1 NBPF10 1
146064699 146144804 - 13249 protein_coding
NBPF member 10 [Source:HGNC Symbol;Acc:HGNC:31992] -
162 127 175 96 43 57 0.591198238
0.41771418 0.6165964 0.40262448 0.199495322
0.20004273
ENSG00000105426 810.8445498 587.3076479 643.8393363
474.991806 482.4207815 521.5930347 680.6638447
493.0018741 0.464634777 0.026423675 1 PTPRS 19
5158495 5340803 - 8565 protein_coding "protein
tyrosine phosphatase, receptor type S [Source:HGNC
Symbol;Acc:HGNC:9681]" - 843 675 692 431 398
565 4.758843511 3.434274485 3.771592496
2.796159535 2.856295035 3.067270648
ENSG00000004142 4066.726876 4114.633877 3933.746696
4206.597966 4981.782442 5598.124181 4038.36915
4928.834863 -0.287651643 0.026463676 0.201749092
POLDIP2 17 28347177 28357522 - 2121
protein_coding DNA polymerase delta interacting protein 2
[Source:HGNC Symbol;Acc:HGNC:23781] - 4228 4729 4228
3817 4110 6064 96.38190959 97.15994119
93.05513552 99.99851141 119.1100792 132.9381229
ENSG00000232352 16.35155083 6.960683234 2.791210996 0

0	0	8.701148352	0	5.462415062
0.026508719	1	SEMA3B-AS1	3	50266641
50267371	-	338	antisense	SEMA3B antisense RNA 1
(head to head) [Source:HGNC Symbol;Acc:HGNC:49096]	-	17
8	3	0	0	0	2.431831264
1.031411298	0.414334017	0	0	0
ENSG00000076108 1817.907709	1322.529814	1751.950102
1368.769891	1175.749141	1237.052507	1630.795875
1260.523846	0.371256447	0.026543128	1	BAZ2A	12
56595596	56636816	-	10918	protein_coding
bromodomain adjacent to zinc finger domain 2A [Source:HGNC
Symbol;Acc:HGNC:962]	MBD	1890	1520	1883	1242	970
1340	8.369893475	6.066791865	8.051063757
6.321069269	5.461048135	5.706800972
ENSG00000160410 2474.85531	2562.401515	2352.990869
2803.663931	2995.130028	3116.633779	2463.415898
2971.809246	-0.270758862	0.026620421	0.201749092	SHKBP1
19	40576851	40591399	+	3936
protein_coding	SH3KBP1 binding protein 1 [Source:HGNC
Symbol;Acc:HGNC:19214]	-	2573	2945	2529	2544	2471
3376	31.60719188	32.60534554	29.99437214
35.91485033	38.58913253	39.88213198
ENSG00000105372 23467.361	28113.3295	28070.27858
26732.8915	34245.81492	36204.09565	26550.32303
32394.26736	-0.287016575	0.026621236	0.201749092	RPS19
19	41859918	41872926	+	3799
protein_coding	ribosomal protein S19 [Source:HGNC
Symbol;Acc:HGNC:10402]	-	24398	32311	30170	24257	28253
39217	310.5175493	370.6292608	370.7251525
354.7969177	457.1330657	479.994314
ENSG00000270231 1023.414711	993.6375316	973.2022338
821.0415208	717.5706097	779.1584447	996.751492
772.5901917	0.367216124	0.026654248	1	NBPF8	1
120436353	120467739	+	5330
unprocessed_pseudogene	NBPF member 8 [Source:HGNC
Symbol;Acc:HGNC:31990]	-	1064	1142	1046	745	592
844	9.651962711	9.336787887	9.16116091
7.766781856	6.827187933	7.362855134
ENSG00000105676 2146.862438	2207.406671	2159.466907
2098.339672	3041.190303	3228.337774	2171.245338
2789.28925	-0.361524131	0.026659191	0.201749092	ARMC6
19	19033575	19060311	+	4052
protein_coding	armadillo repeat containing 6 [Source:HGNC
Symbol;Acc:HGNC:25049]	-	2232	2537	2321	1904	2509
3497	26.63336065	27.28409973	26.73940502
26.11016107	38.06085892	40.12889617
ENSG00000131467 7066.755525	5846.103831	6235.565364
6709.397018	8487.211839	8087.923145	6382.80824
7761.510667	-0.282186175	0.026702086	0.201749092	PSME3
17	42824385	42843758	+	5152

protein_coding	proteasome activator subunit 3 [Source:HGNC Symbol;Acc:HGNC:9570]	-	7347	6719	6702	6088	7002	8761	68.95017964	56.83127304	60.72599018	65.66150504	83.53983483	79.06947298
ENSG00000256407 0	0	0	19.8372448	0	9.231735127	0	9.689659975	-5.840639722	0.026722656	1	AL357673.1	1	54132686	54200073	-	3952	protein_coding	novel transcript	-	0	0	0	18	0	10	0	0	0	0.253085703	0	0.117656001
ENSG00000066136 922.4198378	1052.803339	1352.806929	1365.463684	1580.594721	1422.610383	1109.343369	1456.222929	-0.392285514	0.026779731	1	NFYC	1	40691648	40771603	+	5990	protein_coding	nuclear transcription factor Y subunit gamma [Source:HGNC Symbol;Acc:HGNC:7806]	NF-YC	959	1210	1454	1239	1304	1541	7.740927523	8.802725225	11.33140129	11.49361188	13.38129451	11.96208363
ENSG00000102466 299.1371945	196.6393014	247.4873749	98.08415483	89.69632621	214.176255	247.7546236	133.9855787	0.882089369	0.026795516	1	FGF14	13	101710804	102402457	-	13759	protein_coding	fibroblast growth factor 14 [Source:HGNC Symbol;Acc:HGNC:3671]	-	311	226	266	89	74	232	1.092885678	0.715781	0.902486682	0.359430709	0.330591901	0.78402901
ENSG00000164031 1063.81266	1329.490498	1129.51005	991.8622399	905.4480497	805.9304766	1174.271069	901.0802554	0.38272802	0.026807782	1	DNAJB14 4	99896248	99946726	-	13078	protein_coding	DnaJ heat shock protein family (Hsp40) member B14 [Source:HGNC Symbol;Acc:HGNC:25881]	-	1106	1528	1214	900	747	873	4.088980227	5.091439868	4.333346224	3.823958937	3.510966042	3.103872898
ENSG00000251474 401.0939232	324.5418558	294.0075582	187.3517564	166.0594147	283.4142684	339.8811124	212.2751465	0.676135995	0.026887559	1	RPL32P3 3	129382922	129399655	-	4504	transcribed_unprocessed_pseudogene	ribosomal protein L32 pseudogene 3 [Source:HGNC Symbol;Acc:HGNC:27024]	-	417	373	316	170	137	307	4.4765031	3.608851799	3.275176707	2.097309786	1.869689743	3.169355908
ENSG00000066427 735.8197872	438.5230437	463.3410253	369.1931671	373.330655	365.576711	545.8946187	369.3668444	0.563214714	0.02692035	1	ATXN3	14	92044496	92106625	-	9665	protein_coding	ataxin 3 [Source:HGNC Symbol;Acc:HGNC:7106]	-	765	504	498	335	308	396	3.827020541	2.272413056	2.405323643	1.925994277	1.958827617

1.905128815
ENSG00000166507 25.0082542	4.350427021	3.721614661	0
	0	0.923173513	11.0267653	0.307724504
4.841664749	0.02693028	1	NDST2	10	73801911
	73811798	-	4654	protein_coding	N-deacetylase
and N-sulfotransferase 2 [Source:HGNC Symbol;Acc:HGNC:7681]	-
	26	5	4	0	0	1	0.270114679
	0.046816854	0.04012173	0	0	0.009990901
ENSG00000260641 6.732991517	13.05128106	5.582421991	0
	0	0	8.455564857	0	5.422410798
0.026977029	1	AC114811.2	4	98658904
98664550	+	5647	antisense	"novel transcript,
antisense TSPAN5"	-	7	15	6	0	0
	0	0.059935132	0.11575295	0.049599751	0
	0	0
ENSG00000101928 727.1630838	777.8563514	786.1910971
1115.293985	900.5995996	970.2553619	763.7368441
995.3829823	-0.382231617	0.027052425	1	MOSPD1	X
	134887626	134915267	-	2675	protein_coding
	motile sperm domain containing 1 [Source:HGNC
Symbol;Acc:HGNC:25235]	-	756	894	845	1012	743
	1051	13.66467244	14.56372032	14.74616056
21.0217453	17.07310014	18.26879691
ENSG00000233421 0	0	0	11.02069155	18.18168774
	0	0	9.734126433	-5.842682675	0.027053866
	1	LINC01783	1	16533886	16536172
	-	2068	lincRNA long intergenic non-protein coding RNA
1783 [Source:HGNC Symbol;Acc:HGNC:52573]	-	0	0
	0	10	15	0	0	0	0
0.268696191	0.445849296	0
ENSG00000024048 1739.997379	1345.152035	1443.986488
1283.910566	1123.628303	1127.194859	1509.711967
1178.244576	0.357498994	0.027056021	1	UBR2	6
	42564062	42693504	+	8840	protein_coding
	ubiquitin protein ligase E3 component n-recognin 2
[Source:HGNC Symbol;Acc:HGNC:21289]	-	1809	1546	1552
	1165	927	1221	9.894355683	7.621067748
8.195689335	7.322943848	6.445770364	6.422356616
ENSG00000239521 211.6083048	120.9418712	106.0660178
49.59311199	112.726464	29.54155241	146.2053979
63.95370947	1.198779504	0.02708496	1	CASTOR3 7
	100200653	100272232	-	4874	protein_coding
	CASTOR family member 3 [Source:HGNC Symbol;Acc:HGNC:29954]
	-	220	139	114	45	93	32
2.182420199	1.242761736	1.091855993	0.513025595
1.172856243	0.305277975
ENSG00000145020 29.81753386	0	2.791210996	0	0
	0	10.86958162	0	5.782783416	0.027088157
	1	AMT	3	49416775	49422753	-
	5746	protein_coding	aminomethyltransferase [Source:HGNC

Symbol;Acc:HGNC:473]	-	31	0	3	0	0
	0	0.260853873	0	0.024372589	0	0
	0
ENSG00000173812 11734.64236	13966.61091	12660.93308
15244.92263	14140.50462	15966.2859	12787.39545
15117.23771	-0.241499967	0.027157785	0.203449352	EIF1
	17	41688885	41692668	+	3623
protein_coding	eukaryotic translation initiation factor 1
[Source:HGNC Symbol;Acc:HGNC:3249]	-	12200	16052	13608
	13833	11666	17295	162.8143625	193.0720531
175.3363665	212.1583292	197.9251571	221.9643618
ENSG00000205177 7.694847448	0.870085404	18.6080733	0
	0	0	9.057668719	0	5.520242762
0.027199002	1	C11orf91	11	33698261
33700801	-	891	protein_coding	chromosome 11 open
reading frame 91 [Source:HGNC Symbol;Acc:HGNC:34444]	-	8
	1	20	0	0	0	0.434123704
0.048908112	1.047848093	0	0	0
ENSG00000128805 1279.268388	1299.907594	1907.327514
1142.845714	1093.32549	1154.890064	1495.501165
1130.353756	0.403678014	0.027211646	1	ARHGAP22
	10	48446034	48656265	-	6783
protein_coding	Rho GTPase activating protein 22 [Source:HGNC
Symbol;Acc:HGNC:30320]	-	1330	1494	2050	1037	902
	1251	9.480495587	9.598146582	14.10841025
8.495109535	8.173951955	8.575639396
ENSG00000274554 24.04639827	0	7.443229322	0	0
	0	10.49654253	0	5.732499106	0.027225774
	1	AC083806.2	12	116948738	116951422
	-	2685	antisense	"novel transcript, antisense
to FBXW8"	-	25	0	8	0	0	0
	0.450191131	0	0.139088663	0	0	0
ENSG00000183396 24.04639827	0	7.443229322	0	0
	0	10.49654253	0	5.732499106	0.027225774
	1	TMEM89	3	48620759	48621855	-
	662	protein_coding	transmembrane protein 89 [Source:HGNC
Symbol;Acc:HGNC:32372]	-	25	0	8	0	0
	0	1.825926265	0	0.56412849	0	0
	0
ENSG00000174547 942.6188123	926.6409555	932.2644725
1041.455352	1317.566305	1242.591548	933.8414135
1200.537735	-0.362361492	0.027227582	1	MRPL11	11
	66435075	66466738	-	2539	protein_coding
	mitochondrial ribosomal protein L11 [Source:HGNC
Symbol;Acc:HGNC:14042]	-	980	1065	1002	945	1087
	1346	18.66227528	18.2787084	18.42260568
20.68145795	26.31565617	24.64979871
ENSG00000066322 1815.983998	2210.016927	2181.796595
2510.513536	2547.860509	2563.652845	2069.26584
2540.67563	-0.295969936	0.027238126	1	ELOVL1	1

43363397	43368074	-	2507	protein_coding
ELOVL fatty acid elongase 1 [Source:HGNC
Symbol;Acc:HGNC:14418]	-	1888	2540	2345	2278	2102
2777	36.41236374	44.15073932	43.66510903
50.49070438	51.53778364	51.50537623
ENSG00000070018 957.0466513	802.2187427	747.1141431
682.1808072	579.3897828	610.2176919	835.4598457
623.9294273	0.420921547	0.02730622	1	LRP6	12
12116025	12267012	-	10337	protein_coding
LDL receptor related protein 6 [Source:HGNC
Symbol;Acc:HGNC:6698]	-	995	922	803	619	478
661	4.654036457	3.886825135	3.626327846
3.327424242	2.84237085	2.973294732
ENSG00000101132 686.7651347	801.3486573	549.8685661
748.3049565	1021.810851	1044.109243	679.3274527
938.0750169	-0.465534075	0.027310382	1	PFDN4	20
54207847	54228052	+	1975	protein_coding
prefoldin subunit 4 [Source:HGNC Symbol;Acc:HGNC:8868]	-
714	921	591	679	843	1131	17.47963374
20.32128319	13.96903536	19.10357809	26.23661696
26.62726271
ENSG00000105193 23293.26508	25828.48522	29949.69398
28647.18563	33540.36544	32632.33733	26357.1481
31606.62946	-0.262027246	0.027313076	0.203449352	RPS16
19	39433207	39435948	-	2603
protein_coding	ribosomal protein S16 [Source:HGNC
Symbol;Acc:HGNC:10396]	-	24217	29685	32190	25994	27671
35348	449.8289375	496.9600559	577.288418
554.8952282	653.4284604	631.4248887
ENSG00000010322 2488.321293	1645.331499	1860.80733
1503.222328	1478.77727	1637.709812	1998.153374
1539.903136	0.375379708	0.027324547	1	NISCH	3
52455118	52493071	+	9161	protein_coding
nischarin [Source:HGNC Symbol;Acc:HGNC:18006]	-	2587
1891	2000	1364	1220	1774	13.65383851
8.995125841	10.19138359	8.273390643	8.185859905
9.004129872
ENSG00000168268 4608.251765	4101.582596	4619.454198
6838.339109	5939.35133	4215.210259	4443.096186
5664.300233	-0.350127543	0.027330071	0.203449352	NT5DC2
3	52524385	52535054	-	4719
protein_coding	5'-nucleotidase domain containing 2 [Source:HGNC
Symbol;Acc:HGNC:25717]	-	4791	4714	4965	6205	4900
4566	49.08823421	43.53095536	49.11513152
73.06406856	63.8253879	44.99009891
ENSG00000147804 0	0	0	36.36828213	0	0
0	12.12276071	-6.162006419	0.027370141	1
SLC39A4 8	144409742	144416895	-	5033
protein_coding	solute carrier family 39 member 4 [Source:HGNC
Symbol;Acc:HGNC:17129]	-	0	0	0	33	0

0 0 0 0 0.364333456 0 0
ENSG00000079246 9844.595453 9181.141186 8978.395369
12927.27119 11442.34215 9543.767775 9334.710669
11304.46037 -0.276117254 0.02741973 0.203449352 XRCC5
2 216107464 216206303 + 5463
protein_coding X-ray repair cross complementing 5 [Source:HGNC
Symbol;Acc:HGNC:12833] - 10235 10552 9650 11730 9440
10338 90.58532837 84.17094048 82.45977561
119.3105521 106.2155626 87.99061344
ENSG00000081307 1383.148829 1207.678541 1149.048527
937.8608513 1033.931976 956.4077592 1246.625299
976.0668623 0.35313024 0.027431399 1 UBA5 3
132654446 132678097 + 6827 protein_coding
ubiquitin like modifier activating enzyme 5 [Source:HGNC
Symbol;Acc:HGNC:23230] - 1438 1388 1235 851 853
1036 10.18427692 8.85968255 8.444677927
6.926465907 7.680093168 7.056037345
ENSG00000266412 3073.129699 3401.163845 3461.101635
4065.533114 4358.756609 3608.685261 3311.798393
4010.991661 -0.276002115 0.027500028 0.203449352 NCOA4
10 46005088 46030714 - 4079
protein_coding nuclear receptor coactivator 4 [Source:HGNC
Symbol;Acc:HGNC:7671] - 3195 3909 3720 3689 3596
3909 37.87201161 41.76096858 42.57309956
50.25357859 54.18927467 44.55977438
ENSG00000226555 0 39.15384319 0 0 0 0
13.05128106 0 6.049207729 0.027516533 1
AGKP1 Y 14639096 14640358 + 1263
processed_pseudogene acylglycerol kinase pseudogene 1
[Source:HGNC Symbol;Acc:HGNC:37661] - 0 45 0
0 0 0 0 1.552629241 0 0
0 0
ENSG00000168010 202.9516014 214.0410094 248.4177786
96.98208568 145.453502 151.4004561 221.8034632
131.2786812 0.755988071 0.027572073 1 ATG16L2 11
72814308 72843674 + 8617 protein_coding
autophagy related 16 like 2 [Source:HGNC
Symbol;Acc:HGNC:25464] - 211 246 267 88 120
164 1.183934235 1.244049359 1.446442601
0.567464402 0.855997534 0.884950081
ENSG00000165637 2132.434599 2284.844272 2111.085916
2811.378415 2916.342714 2323.627732 2176.121596
2683.782954 -0.302004489 0.027591987 0.203449352 VDAC2
10 75210154 75231448 + 2827
protein_coding voltage dependent anion channel 2 [Source:HGNC
Symbol;Acc:HGNC:12672] - 2217 2626 2269 2551 2406
2517 37.91762273 40.47878897 37.46750063
50.14142748 52.31390926 41.39886411
ENSG00000123144 3875.317546 3471.640763 4431.512657
4032.47104 4975.721879 5806.761395 3926.156989

4938.318105	-0.331134727	0.027703579	0.203455086	TRIR
	19	12730640	12734775	-	1402
protein_coding	telomerase RNA component interacting RNase
[Source:HGNC Symbol;Acc:HGNC:28424]	-	4029	3990	4763
	3659	4105	6290	138.9474146	124.0176449
158.5910242	145.019512	179.975135	208.6092924
ENSG00000242125 1079.202355	850.0734399	917.3780139
763.7339247	728.4796223	670.2239702	948.8846028
720.8125057	0.396765568	0.02775885	1	SNHG3	1
	28505980	28510892	+	4088
processed_transcript	small nucleolar RNA host gene 3 [Source:HGNC
Symbol;Acc:HGNC:10118]	-	1122	977	986	693	601
	726	13.27037472	10.41459233	11.25931744
9.419641863	9.036722478	8.25765533
ENSG00000231663 0	12.18119566	18.6080733	0	0
	0	10.26308965	0	5.701751151	0.02783987
	1	AL355472.1	1	234372807	234373593
	-	575	antisense	uncharacterized LOC101927765
[Source:NCBI gene;Acc:101927765]	-	0	14	20
	0	0	0	0	1.061008318	1.623708958
	0	0	0
ENSG00000104804 9.618559309	0	21.3992843	0	0
	0	10.3392812	0	5.711214759	0.027952851
	1	TULP2	19	48880965	48898733	-
	2240	protein_coding	tubby like protein 2 [Source:HGNC
Symbol;Acc:HGNC:12424]	Tub	10	0	23	0	0
	0	0.215850569	0	0.479320334	0	0
	0
ENSG00000153922 2007.393328	1792.375933	1470.968195
1531.876126	1237.566879	1327.523511	1756.912485
1365.655505	0.363309947	0.028002348	1	CHD1	5
	98853985	98928957	-	10437	protein_coding
	chromodomain helicase DNA binding protein 1 [Source:HGNC
Symbol;Acc:HGNC:1915]	-	2087	2060	1581	1390	1021
	1438	9.668252455	8.601023508	7.071348191
7.40033126	6.01308605	6.406402497
ENSG00000170677 472.2712621	495.078595	431.7073006
536.7076787	690.9041343	698.8423491	466.3523859
642.1513874	-0.461565629	0.028016675	1	SOCS6	18
	70288901	70330200	+	5984	protein_coding
	suppressor of cytokine signaling 6 [Source:HGNC
Symbol;Acc:HGNC:16833]	-	491	569	464	487	570
	757	3.967264204	4.143613881	3.619698781
4.522196401	5.855050308	5.882139403
ENSG00000129654 0	0	0	0	8.484787614
20.30981728	0	9.598201631	-5.828302574	0.028019472
	1	FOXJ1	17	76136333	76141299	-
	2641	protein_coding	forkhead box J1 [Source:HGNC
Symbol;Acc:HGNC:3816]	Fork	0	0	0	0	7
	22	0	0	0	0	0.162920974

0.387333712
ENSG00000196993 3.847423724	18.27179349	3.721614661	0
	0	0	8.613610625	0	5.44982674
0.028088991	1	NPIPB9	16	28751787	28772807
	+	2123	protein_coding	nuclear pore complex
interacting protein family member B9 [Source:HGNC
Symbol;Acc:HGNC:41987]	-	4	21	4	0	0
	0	0.091098497	0.431050247	0.087954089	0
	0	0
ENSG00000128886 10.58041524	7.830768638	6.512825656	0
	0	0	8.308003178	0	5.396148083
0.028093117	1	ELL3	15	43772600	43777543
	-	2880	protein_coding	elongation factor for RNA
polymerase II 3 [Source:HGNC Symbol;Acc:HGNC:23113]	-	11
	9	7	0	0	0	0.184672154
0.136178523	0.113462301	0	0	0
ENSG00000131473 7206.224635	6189.787566	7205.045983
9325.709193	8930.84502	6959.805112	6867.019395
8405.453109	-0.291513331	0.028144356	0.205868677	ACLY
	17	41866908	41930542	-	5217
protein_coding	ATP citrate lyase [Source:HGNC Symbol;Acc:HGNC:115]
	-	7492	7114	7744	8462	7368	7539
69.43495342	59.42259613	69.29318814	90.12893264
86.81127618	67.19298345
ENSG00000132837 0	2.610256213	9.304036652	23.14345226
	12.12112516	48.00502266	3.971430955	27.75653336
	-2.807192795	0.028157783	1	DMGDH	5
78997606	79236038	-	5254	protein_coding
dimethylglycine dehydrogenase [Source:HGNC Symbol;Acc:HGNC:24475]
	-	0	3	10	21	10	52	0
	0.024882258	0.0888497	0.222096273	0.116992303
	0.460197541
ENSG00000117054 1001.292024	982.3264214	1221.620012
909.2070532	778.1762355	758.8486275	1068.412819
815.4106387	0.390005284	0.028176645	1	ACADM	1
	75724347	75787575	+	4783	protein_coding
	acyl-CoA dehydrogenase medium chain [Source:HGNC
Symbol;Acc:HGNC:89]	-	1041	1129	1313	825	642
	822	10.52329064	10.28613355	12.81475717
9.584415025	8.250533032	7.991024364
ENSG00000197299 252.9681098	271.4666461	311.6852278
386.8262736	399.9971304	422.8134688	278.7066613
403.2122909	-0.53301122	0.02824843	1	BLM	15
	90717327	90816165	+	6674	protein_coding
	Bloom syndrome RecQ like helicase [Source:HGNC
Symbol;Acc:HGNC:1058]	-	263	312	335	351	330
	458	1.905332444	2.037168675	2.343174544
2.922354906	3.039310691	3.190878691
ENSG00000185347 243.3495505	353.2546741	425.194475
414.3780024	561.208095	559.4431487	340.5995665

511.6764154	-0.58696422	0.028249531	1	TEDC1	14
	105489855	105499575	+	6698	protein_coding
	tubulin epsilon and delta complex 1 [Source:HGNC
Symbol;Acc:HGNC:20127]	-	253	406	457	376	463
	606	1.82631882	2.641432324	3.185056148
3.119282763	4.248965531	4.206864259
ENSG00000224738 25.97011014	17.40170808	24.19049529
62.81794186	103.0295639	29.54155241	22.52077117
65.12968605	-1.529606298	0.028250779	1	AC099850.1
	17	59106598	59118267	+	1338
antisense	novel transcript	-	27	20	26
	57	85	32	0.97568329	0.65137709
0.907116926	2.367177291	3.904902301	1.112051456
ENSG00000230536 21.16083048	1.740170808	3.721614661	0
	0	0	8.874205317	0	5.490067554
0.028318725	1	AL360268.1	9	128090969
128094457	-	765	antisense	novel transcript
	-	22	2	4	0	0	0
1.390472686	0.11392713	0.244086968	0	0	0
ENSG00000104852 4765.996138	3636.086904	5720.121734
3495.763361	3631.489099	4008.419392	4707.401592
3711.890617	0.342571772	0.028362776	0.206643082
SNRNP70 19	49085419	49108605	+	4684
protein_coding	small nuclear ribonucleoprotein U1 subunit 70
[Source:HGNC Symbol;Acc:HGNC:11150]	-	4955	4179	6148
	3172	2996	4342	51.14792138	38.87891016
61.27213428	37.62949031	39.31626764	43.10264791
ENSG00000257097 9.618559309	8.700854042	6.512825656	0
	0	0	8.277413003	0	5.390997138
0.028364778	1	CLIP1-AS1	12	122395542
122400857	+	1809	lincRNA CLIP1 antisense RNA 1
[Source:HGNC Symbol;Acc:HGNC:48586]	-	10	10	7
	0	0	0	0.267277653	0.240890698
0.1806365	0	0	0
ENSG00000087111 761.7898973	838.7623297	832.7112803
1031.536729	1013.326064	1066.265407	811.0878358
1037.042733	-0.354607562	0.028381285	1	PIGS	17
	28553383	28571872	-	5990	protein_coding
	phosphatidylinositol glycan anchor biosynthesis class S
[Source:HGNC Symbol;Acc:HGNC:14937]	-	792	964	895
	936	836	1155	6.392924503	7.013080262
6.97496847	8.682825439	8.578805376	8.965740815
ENSG00000172172 532.8681857	666.4854197	798.2863447
853.0015263	1006.053389	857.6281933	665.8799834
905.5610361	-0.443003217	0.028385033	1	MRPL13	8
	120380761	120445402	-	4209	protein_coding
	mitochondrial ribosomal protein L13 [Source:HGNC
Symbol;Acc:HGNC:14278]	-	554	766	858	774	830
	929	6.364027615	7.930643752	9.515999224
10.21819246	12.12122539	10.26284586

ENSG00000147813 199.1041777 170.5367392 218.6448613
91.4717399 140.6050519 108.9344745 196.0952594
113.6704221 0.787944308 0.028466716 1 NAPRT 8
143574785 143578649 - 3079 protein_coding
nicotinate phosphoribosyltransferase [Source:HGNC
Symbol;Acc:HGNC:30450] - 207 196 235 83 116
118 3.25058759 2.773990569 3.562904726
1.497891813 2.315771264 1.781982097
ENSG00000172336 744.4764906 626.4614911 691.2899232
829.858074 870.2967867 1019.183558 687.4093016
906.4461396 -0.399875861 0.028466858 1 POP7 7
100706053 100707495 + 988 protein_coding
"POP7 homolog, ribonuclease P/MRP subunit [Source:HGNC
Symbol;Acc:HGNC:19949]" - 774 720 743 753 718
1104 37.87784239 31.75661103 35.10572165
42.34967436 44.66988762 51.95688994
ENSG00000091483 1766.929345 1943.770793 1774.27979
2216.261072 2310.286456 2151.917458 1828.326643
2226.154995 -0.283743847 0.028632648 1 FH 1
241497603 241519761 - 2142 protein_coding
fumarate hydratase [Source:HGNC Symbol;Acc:HGNC:3700] -
1837 2234 1907 2011 1906 2331 41.46588189
45.44878735 41.56016493 52.16805532 54.69539839
50.60038546
ENSG00000270157 87.52888972 13.92136647 26.05130263
13.22482987 9.69690013 0 42.5005196 7.640576665
2.488338739 0.028641727 1 AC004918.3 7
141662922 141663846 - 925 sense_intronic
novel transcript - 91 16 28 12
8 0 4.756646488 0.753766527 1.413065634
0.720861045 0.531613027 0
ENSG00000236942 16.35155083 4.350427021 4.652018326 0
0 0 8.451332058 0 5.420069445
0.028749424 1 AC096533.1 1 205625483
205626153 + 353 processed_pseudogene GABA(A)
receptor-associated protein (GABARAP) pseudogene - 17
5 5 0 0 0 2.328495658
0.617239764 0.661212926 0 0 0
ENSG00000269693 16.35155083 23.49230591 20.46888063 0
0 5.539041076 20.10424579 1.846347025
3.386278985 0.028754994 1 AC010422.6 19
12525720 12580975 - 5857 protein_coding novel
transcript zf-C2H2 17 27 22 0 0 6
0.140337881 0.200884828 0.175345043 0 0
0.047632902
ENSG00000175104 211.6083048 356.7350157 328.4324938
415.4800716 476.3602189 451.4318477 298.9252715
447.7573794 -0.582164271 0.028789328 1 TRAF6 11
36487027 36510272 - 4871 protein_coding
TNF receptor associated factor 6 [Source:HGNC

Symbol;Acc:HGNC:12036] -   220   410   353   377   393
        489   2.183764329   3.667957753   3.383004782
4.300661532   4.959315991   4.667902193
ENSG00000136802 2565.269768   2526.728014   2565.122905
3121.059848   2899.373139   3135.097249   2552.373562
3051.843412   -0.258018739   0.028790896   0.208480881   LRRC8A
        9   128882112   128918039   +   5230
protein_coding leucine rich repeat containing 8 VRAC subunit A
[Source:HGNC Symbol;Acc:HGNC:19027]   -   2667   2904   2757
        2832   2392   3396   24.65599557   24.19655408
24.60827169   30.08871244   28.11297761   30.1923565
ENSG00000124257 4.809279655   0   0   37.47035128
10.90901265   9.231735127   1.603093218
19.20369969   -3.598700427   0.028899359   1   NEURL2 20
        45888625   45891287   -   1301   protein_coding
        neuralized E3 ubiquitin protein ligase 2 [Source:HGNC
Symbol;Acc:HGNC:16156] -   5   0   0   34   9
        10   0.185820628   0   0   1.452157306
0.425218913   0.35739932
ENSG00000052749 6556.010025   5036.05432   6042.041402
5756.107199   7832.67108   8694.448143   5878.035249
7427.742141   -0.337740415   0.02891694   0.208480881   RRP12
        10   97356358   97426076   -   8529
protein_coding ribosomal RNA processing 12 homolog [Source:HGNC
Symbol;Acc:HGNC:29100] -   6816   5788   6494   5223   6462
        9418   38.63960551   29.57256573   35.54350121
34.02780654   46.5710682   51.34422345
ENSG00000019582 0   0   0.930403665   6.612414933
14.5453502   7.385388102   0.310134555
9.51438441   -4.849393201   0.028956694   1   CD74   5
        150401637   150412929   -   3184   protein_coding
        CD74 molecule [Source:HGNC Symbol;Acc:HGNC:1697]   -
        0   0   1   6   12   8   0   0
        0.014661317   0.1047105   0.231662398   0.11682827
ENSG00000106263 17365.34698   15150.79714   16564.90686
16323.84833   20778.03275   22427.57732   16360.35033
19843.1528   -0.27849127   0.028961817   0.208480881   EIF3B
        7   2354086 2380745 +   8998   protein_coding
eukaryotic translation initiation factor 3 subunit B [Source:HGNC
Symbol;Acc:HGNC:3280]   -   18054   17413   17804   14812   17142
        24294   97.01271653   84.3307978   92.36716893
91.47022728   117.1016089   125.5405585
ENSG00000112695 1120.56216   1394.746903   1317.45159
1675.145116   1573.322046   1568.471798   1277.586884
1605.64632   -0.32948168   0.028964979   1   COX7A2 6
        75237675   75250323   -   1922   protein_coding
        cytochrome c oxidase subunit 7A2 [Source:HGNC
Symbol;Acc:HGNC:2288]   -   1165   1603   1416   1520   1298
        1699   29.30716157   36.3445032   34.39187914
43.94426938   41.51152319   41.10276269

ENSG00000203706 7.694847448 11.31111026 14.88645864
26.44965973 38.78760052 55.39041076 11.29747212
40.20922367 -1.832515956 0.029013813 1 SERTAD4-AS1
1 210231456 210234047 - 1004
antisense SERTAD4 antisense RNA 1 [Source:HGNC
Symbol;Acc:HGNC:32019] - 8 13 16 24 32
60 0.385263167 0.564245673 0.743930399
1.328279814 1.959131672 2.778744114
ENSG00000185201 904.1445751 1024.090521 1093.224307
1137.335368 1635.139784 1214.896343 1007.153134
1329.123832 -0.399498129 0.029030879 1 IFITM2 11
307631 315272 + 1543 protein_coding interferon
induced transmembrane protein 2 [Source:HGNC Symbol;Acc:HGNC:5413]
- 940 1177 1175 1032 1349 1316
29.45527922 33.24062145 35.54822959 37.16428782
53.7394706 39.65710264
ENSG00000239254 15.3896949 6.09059783 3.721614661 0
0 0 8.400635795 0 5.411646337
0.029046708 1 AC009220.2 7 139172516
139174266 - 1751 processed_pseudogene Male-specific
lethal-3 homolog 1 (Msl3l1) pseudogene - 16 7 4
0 0 0 0.441809503 0.174208961
0.106639937 0 0 0
ENSG00000254088 0 0 0 3.306207466 0
25.84885836 0 9.718355274 -5.848413615 0.029080014
1 SLC2A3P4 8 86503591 86505061
+ 1471 processed_pseudogene solute carrier family
2 member 3 pseudogene 4 [Source:HGNC Symbol;Acc:HGNC:31076] -
0 0 0 3 0 28 0 0
0 0.113323669 0 0.885067466
ENSG00000110321 16403.49105 17884.60548 16422.55509
20042.22966 20050.76524 18735.80644 16903.55054
19609.60045 -0.214178877 0.029193564 0.208480881 EIF4G2
11 10797050 10809110 - 8655
protein_coding eukaryotic translation initiation factor 4 gamma 2
[Source:HGNC Symbol;Acc:HGNC:3297] - 17054 20555 17651
18186 16542 20295 95.27092962 103.4925306
95.20248368 116.7567932 117.4811812 109.0317547
ENSG00000166920 0 0 0 0 18.18168774
10.15490864 0 9.445532128 -5.801514344 0.02919753
1 C15orf48 15 45430529 45448761
+ 1405 protein_coding chromosome 15 open reading
frame 48 [Source:HGNC Symbol;Acc:HGNC:29898] - 0 0
0 0 15 11 0 0 0 0
0.65623939 0.364038553
ENSG00000160957 567.4949993 525.5315842 797.3559411
742.7946108 887.2663619 990.5651792 630.1275082
873.5420506 -0.471755476 0.0292011 1 RECQL4 8
144511288 144517845 - 4236 protein_coding
RecQ like helicase 4 [Source:HGNC Symbol;Acc:HGNC:9949] -

590	604	857	674	732	1073	6.734374698
6.21354696	9.444324622	8.841297182	10.62190688
11.77808783
ENSG00000151665 225.0742878	233.1828883	167.4726597
131.1462295	134.5444893	106.164954	208.576612
123.9518909	0.752481616	0.029235111	1	PIGF	2
46580937	46617119	-	4978	protein_coding
phosphatidylinositol glycan anchor biosynthesis class F
[Source:HGNC Symbol;Acc:HGNC:8962]	-	234	268	180
119	111	115	2.272805029	2.346056676
1.687965822	1.328324285	1.370614894	1.074172343
ENSG00000033122 6.732991517	0.870085404	0	19.8372448
26.66647536	17.54029674	2.534358974	21.34800563
-3.084333664	0.029237883	1	LRRC7	1
69568398	70151945	+	14652	protein_coding
leucine rich repeat containing 7 [Source:HGNC Symbol;Acc:HGNC:18531]
-	7	1	0	18	22	19
0.023099488	0.002974142	0	0.068263357	0.092293928
0.06029589
ENSG00000162772 150.0495252	220.1316073	152.5862011
314.0897093	326.0582669	213.2530814	174.2557779
284.4670192	-0.704935406	0.029256168	1	ATF3	1
212565334	212620777	+	4040	protein_coding
activating transcription factor 3 [Source:HGNC
Symbol;Acc:HGNC:785]	TF_bZIP 156	253	164	285	269
231	1.867000567	2.728963668	1.894996965
3.91990497	4.092778818	2.658652846
ENSG00000257310 26.93196607	0.870085404	0.930403665	0
0	0	9.577485045	0	5.600014521
0.029277577	1	AL589743.1	14	19244962
19247673	+	2712	lincRNA novel transcript	-
28	1	1	0	0	0	0.499194237
0.016068262	0.017212991	0	0	0
ENSG00000182752 303.9464742	341.0734785	280.0515032
177.433134	183.02899	239.1019398	308.357152
199.8546879	0.624055915	0.029281595	1	PAPPA	9
116153804	116402322	+	11573	protein_coding
pappalysin 1 [Source:HGNC Symbol;Acc:HGNC:8602] -	316
392	301	161	151	259	1.320207957
1.476041988	1.214133881	0.7730222	0.802007361
1.040602414
ENSG00000132485 3369.381326	3342.868123	3129.87793
2437.776972	3042.402416	2600.579785	3280.709126
2693.586391	0.284777242	0.029292367	0.208480881	ZRANB2
1	71063291	71081297	-	4993
protein_coding	zinc finger RANBP2-type containing 2 [Source:HGNC
Symbol;Acc:HGNC:13058]	-	3503	3842	3364	2212	2510
2817	33.92187018	33.53160891	31.45143439
24.6170269	30.90007373	26.23350376
ENSG00000236552 2171.870692	2937.408325	2328.800374

2868.686012 3146.644092 3245.878071 2479.359797
3087.069392 -0.316151163 0.029296333 0.208480881
RPL13AP5 10 96750288 96750899 + 612
processed_pseudogene ribosomal protein L13a pseudogene 5
[Source:HGNC Symbol;Acc:HGNC:23736] - 2258 3376 2503
2603 2596 3516 178.3913253 240.386245
190.921775 236.3386713 260.7357764 267.1335665
ENSG00000140199 1267.726117 1307.738363 1203.011939
914.717399 913.9328373 1123.502165 1259.49214
984.0508004 0.35523855 0.029310833 1 SLC12A6 15
34229996 34338060 - 10056 protein_coding
solute carrier family 12 member 6 [Source:HGNC
Symbol;Acc:HGNC:10914] - 1318 1503 1293 830 754
1217 6.337111698 6.513168498 6.002322086
4.58632547 4.608859207 5.627251579
ENSG00000204070 735.8197872 692.5879818 640.1177217
792.3877227 865.4483366 1109.654562 689.5084969
922.4968739 -0.420863194 0.02938973 1 SYS1 20
45361937 45376798 + 6117 protein_coding
"SYS1, golgi trafficking protein [Source:HGNC
Symbol;Acc:HGNC:16162]" - 765 796 688 719 714
1202 6.046780044 5.670654467 5.25044355
6.531342425 7.174755263 9.136860733
ENSG00000132434 1163.845676 1324.269985 1630.997625
1594.694068 1744.229911 1965.436409 1373.037762
1768.120129 -0.365088908 0.02941845 1 LANCL2 7
55365448 55433742 + 5235 protein_coding
LanC like 2 [Source:HGNC Symbol;Acc:HGNC:6509] - 1210
1522 1753 1447 1439 2129 11.1755756
12.66941506 15.63188192 15.35903354 16.8962944
18.90993315
ENSG00000159685 264.510381 210.5606678 242.8353566
322.9062625 305.4523541 469.895318 239.3021352
366.0846449 -0.615299368 0.029420155 1 CHCHD6 3
126704220 126960406 + 2763 protein_coding
coiled-coil-helix-coiled-coil-helix domain containing 6
[Source:HGNC Symbol;Acc:HGNC:28184] - 275 242 261
293 252 509 4.812303677 3.816744415
4.409665616 5.892488981 5.60617972 8.565799715
ENSG00000232888 56.74949993 46.98461183 62.33704557
13.22482987 31.51492542 10.15490864 55.35705244
18.29822131 1.607019343 0.029472034 1 RPS11P5 12
132825701 132826184 + 484
processed_pseudogene ribosomal protein S11 pseudogene 5
[Source:HGNC Symbol;Acc:HGNC:36312] - 59 54 67
12 26 11 5.89396926 4.861910903
6.462126821 1.37767865 3.301986904 1.056764807
ENSG00000091164 1925.635574 2134.319497 2007.811109
2216.261072 2630.28416 2583.039489 2022.588727
2476.52824 -0.292041415 0.029529523 1 TXNL1 18

56597208	56651600	-	11298	protein_coding
thioredoxin like 1 [Source:HGNC Symbol;Acc:HGNC:12436]	-
2002	2453	2158	2011	2170	2798	8.567689506
9.461381961	8.91653063	9.890597849	11.80607461
11.51535041
ENSG00000075415 6182.809924	6404.698661	7366.936221
7452.191629	8059.336121	8183.010017	6651.481602
7898.179256	-0.247872149	0.029544769	0.20943705
SLC25A3 12	98593591	98606379	+	8896
protein_coding	solute carrier family 25 member 3 [Source:HGNC
Symbol;Acc:HGNC:10989]	-	6428	7361	7918	6762	6649
8864	34.93673457	36.05791754	41.54959157
42.23693894	45.94189648	46.33039376
ENSG00000271716 20.19897455	0	10.23444032	0	0
0	10.14447162	0	5.683368511	0.029545519
1	AC022001.2	3	11607184	11610748
+	580	antisense	"novel transcript, antisense
to VGLL4"	-	21	0	11	0	0	0
1.750622547	0	0.885341307	0	0	0
ENSG00000234751 20.19897455	0	10.23444032	0	0
0	10.14447162	0	5.683368511	0.029545519
1	AP002381.1	11	72940498	72941112
+	615	processed_pseudogene	ribosomal protein L15
(RPL15) pseudogene	-	21	0	11	0	0
0	1.650993622	0	0.834956029	0	0
0
ENSG00000176349 35.58866945	18.27179349	14.88645864	0
7.272675098	0	22.91564053	2.424225033
3.284775426	0.029572848	1	AC104129.1	7
1838586 1849931 +	4764	antisense	"novel transcript,
antisense to MAD1L1"	-	37	21	16	0	6
0	0.375518371	0.192090612	0.156781302	0
0.07741531	0
ENSG00000138688 1472.60143	1350.372547	1399.327112
1355.545061	901.8117121	941.636983	1407.433697
1066.331252	0.400432149	0.029597468	1	KIAA1109
4	122152333	122362758	+	20470
protein_coding	KIAA1109 [Source:HGNC Symbol;Acc:HGNC:26953]	-
1531	1552	1504	1230	744	1020	3.616250981
3.303942435	3.429857126	3.338868481	2.2340992
2.316932317
ENSG00000167085 2467.160463	2133.449411	2548.375639
2692.354947	2866.646101	3203.412089	2382.995171
2920.804379	-0.293946332	0.02966436	0.209476018	PHB
17	49404049	49414905	-	3838
protein_coding	prohibitin [Source:HGNC Symbol;Acc:HGNC:8912]	-
2565	2452	2739	2443	2365	3470	32.31347134
27.84031169	33.31448452	35.36963192	37.87682216
42.03930451
ENSG00000100347 1067.660083	1261.623836	1169.517407

1249.746422	1589.079509	1652.480588	1166.267109
1497.102173	-0.360310458	0.029774771	1	SAMM50	22
	43955421	44010531	+	6435	protein_coding
	SAMM50 sorting and assembly machinery component [Source:HGNC
Symbol;Acc:HGNC:24276]	-	1110	1450	1257	1134	1311
	1790	8.340184229	9.819243926	9.118696522
9.792115941	12.52280162	12.93407866
ENSG00000196372 838.7383718	689.1076402	974.1326375
1033.740868	1053.325777	1217.665863	833.9928831
1101.577503	-0.402133618	0.029804169	1	ASB13	10
	5638867 5666595 -	3003	protein_coding	ankyrin repeat
and SOCS box containing 13 [Source:HGNC Symbol;Acc:HGNC:19765]	-
	872	792	1047	938	869	1319	14.03984681
	11.49286875	16.27561415	17.35639598	17.78737268
	20.4230444
ENSG00000005022 7457.269033	6815.378971	6590.049161
8997.292585	8115.093297	7632.798603	6954.232388
8248.394828	-0.246175106	0.029847341	0.209960607
SLC25A5 X	119468400	119471319	+	1500
protein_coding	solute carrier family 25 member 5 [Source:HGNC
Symbol;Acc:HGNC:10991]	-	7753	7833	7083	8164	6695
	8268	249.9077598	227.559759	220.4306689
302.4292418	274.3510853	256.2950551
ENSG00000179632 3308.784402	3188.863007	3666.720845
3389.964722	4530.876586	4712.800782	3388.122751
4211.21403	-0.313875342	0.029979679	0.210086606	MAF1
	8	144104499	144107611	+	2377
protein_coding	"MAF1 homolog, negative regulator of RNA polymerase
III [Source:HGNC Symbol;Acc:HGNC:24966]"	-	3440	3665
	3941	3076	3738	5105	69.9729973	67.18980722
	77.39685061	71.90667265	96.6623781	99.86138448
ENSG00000283752 4.809279655	10.44102485	9.304036652	0
	0	0	8.184780386	0	5.375293965
0.030027742	1	AC241585.3	1	145139986
145148375	+	686	unprocessed_pseudogene
phosphodiesterase 4D interacting protein (PDE4DIP) pseudogene	-
	5	12	10	0	0	0	0.352409093
	0.762282111	0.68049027	0	0	0
ENSG00000249456 31.74124572	22.62222051	61.4066419
14.32689902	0	8.308561615	38.59003604	7.545153545
	2.345463994	0.030032549	1	AL731577.2	10
	124917143	124942881	+	555
sense_overlapping	"novel transcript, sense overlapping ZRANB1"
	-	33	26	66	13	0	9
2.874896229	2.041451011	5.551329277	1.301554664	0
	0.75401597
ENSG00000074800 86276.55329	85170.04997	119783.8895
105254.2168	140622.0215	115919.2053	97076.83091
120598.4812	-0.313007584	0.030218445	0.210954623	ENO1
	1	8861000 8879250 -	5276	protein_coding

enolase 1 [Source:HGNC Symbol;Acc:HGNC:3350]	-	89698	97887
	128744	95506	116014	125566	822.0139528	808.4977758
	1139.116774	1005.860869	1351.614911	1106.619429
ENSG00000240270 0	0	0	12.12276071	0
15.69394972	0	9.272236809	-5.77876616	0.030293343
	1	RPL12P37	17	80976454	80976948
	+	495	processed_pseudogene	ribosomal protein L12
pseudogene 37 [Source:HGNC Symbol;Acc:HGNC:35565]	-	0
	0	0	11	0	17	0	0	0
	1.234808271	0	1.596889041
ENSG00000182544 582.8846942	515.9606447	669.8906389
560.9532001	938.1750876	1034.877508	589.5786593
844.6685985	-0.519232953	0.03029337	1	MFSD5	12
	53251251	53254405	+	2581	protein_coding
	major facilitator superfamily domain containing 5 [Source:HGNC
Symbol;Acc:HGNC:28156]	-	606	593	720	509	774
	1121	11.35235167	10.01210249	13.0223849
10.95826558	18.43318222	20.19522175
ENSG00000108179 2701.85331	3025.286951	2838.661582
3004.240518	3585.428823	3900.408091	2855.267281
3496.692477	-0.292485155	0.030387756	0.211333031	PPIF
	10	79347469	79355337	+	2674
protein_coding	peptidylprolyl isomerase F [Source:HGNC
Symbol;Acc:HGNC:9259]	-	2809	3477	3051	2726	2958
	4225	50.79156011	56.66330285	53.26314919
56.64694489	67.99611926	73.46768047
ENSG00000023697 493.4320926	408.0700546	441.0113373
742.7946108	557.5717575	563.1358428	447.5044948
621.1674037	-0.473218386	0.030427371	1	DERA	12
	15911172	16037282	+	2844	protein_coding
	deoxyribose-phosphate aldolase [Source:HGNC
Symbol;Acc:HGNC:24269]	-	513	469	474	674	460
	610	8.721455908	7.186242169	7.780272093
13.16868315	9.942042145	9.97312497
ENSG00000241278 0	8.700854042	21.3992843	0	0
	0	10.03337945	0	5.668859247	0.030495827
	1	ENPP7P4 3	125848223	125909372	+
	1086	transcribed_unprocessed_pseudogene	ectonucleotide
pyrophosphatase/phosphodiesterase 7 pseudogene 4 [Source:HGNC
Symbol;Acc:HGNC:48687]	-	0	10	23	0	0
	0	0	0.401262683	0.98865336	0	0
	0
ENSG00000168280 252.9681098	419.3811648	276.3298886
260.0883207	149.0898395	165.2480588	316.2263877
191.4754063	0.724313615	0.030507303	1	KIF5C	2
	148875250	149026759	+	9215	protein_coding
	kinesin family member 5C [Source:HGNC Symbol;Acc:HGNC:6325]
	-	263	482	297	236	123	179
1.379944518	2.279346216	1.504551803	1.423078008
0.82045947	0.903209942

ENSG00000111696 688.6888466 472.4563745 540.5645295
486.0124975 253.3315159 389.5792224 567.2365835
376.3077453 0.59078159 0.030509255 1 NT5DC3 12
103770453 103841197 - 8722 protein_coding
5'-nucleotidase domain containing 3 [Source:HGNC
Symbol;Acc:HGNC:30826] - 716 543 581 441 209
422 3.969155891 2.712953469 3.109611157
2.809535673 1.47291459 2.249714393
ENSG00000160211 1640.926218 1617.488766 1683.10023
1868.007218 2557.557409 1850.03972 1647.171738
2091.868116 -0.344247025 0.030552685 1 G6PD X
154531390 154547572 - 3995 protein_coding
glucose-6-phosphate dehydrogenase [Source:HGNC
Symbol;Acc:HGNC:4057] - 1706 1859 1809 1695 2110
2004 20.64730911 20.27781721 21.13819106
23.57571987 32.46482243 23.324479
ENSG00000223791 0 0 0 0 35.15126297 0
0 11.71708766 -6.110070486 0.030563963 1
UBE2E1-AS1 3 23804024 23806905 -
778 antisense UBE2E1 antisense RNA 1 [Source:HGNC
Symbol;Acc:HGNC:40598] - 0 0 0 0 29
0 0 0 0 0 2.291214563 0
ENSG00000089327 2923.080174 2772.962183 3703.936991
3387.760584 4135.727906 4194.900442 3133.32645
3906.129644 -0.318146098 0.030602589 0.212023978 FXYD5
19 35154730 35169883 + 2993
protein_coding FXYD domain containing ion transport regulator 5
[Source:HGNC Symbol;Acc:HGNC:4029] - 3039 3187 3981
3074 3412 4544 49.09363617 46.40170561
62.09140635 57.07017312 70.07283135 70.59316018
ENSG00000254094 12.5041271 17.40170808 0 0 0
0 9.968611729 0 5.659683821 0.030607682
1 AC078852.2 4 1356581 1358075 + 527
sense_intronic novel transcript - 13 20
0 0 0 0 1.192707509 1.653780924
0 0 0 0
ENSG00000115464 2383.478997 1651.422097 2014.323935
1742.371335 1543.019233 1432.765292 2016.408343
1572.71862 0.358539129 0.030633054 1 USP34 2
61187463 61470769 - 18125 protein_coding
ubiquitin specific peptidase 34 [Source:HGNC
Symbol;Acc:HGNC:20066] - 2478 1898 2165 1581 1273
1552 6.610350738 4.563276564 5.576040523
4.846920521 4.317156064 3.981481662
ENSG00000104133 1098.439473 962.3144571 979.7150594
895.9822234 767.2672228 636.0665503 1013.489663
766.4386655 0.403741282 0.030638398 1 SPG11 15
44562696 44663678 - 11853 protein_coding
"SPG11, spatacsin vesicle trafficking associated [Source:HGNC
Symbol;Acc:HGNC:11226]" - 1142 1106 1053 813 633

689	4.65842423	4.066169143	4.147115421
3.811310267	3.282636437	2.70285007
ENSG00000117448 1589.947854	1342.541779	1714.733955
1895.558947	2175.741967	1783.571227	1549.074529
1951.624047	-0.333025109	0.030640618	1	AKR1A1	1
45550543	45570049	+	3121	protein_coding
aldo-keto reductase family 1 member A1 [Source:HGNC
Symbol;Acc:HGNC:380]	-	1653	1543	1843	1720	1795
1932	25.60827361	21.544219	27.56624441
30.62292861	35.35233016	28.78355101
ENSG00000185697 436.6825926	377.6170654	367.5094478
920.2277448	507.8751443	398.8109575	393.9363686
608.9712822	-0.627958766	0.03066696	1	MYBL1	8
66562175	66614247	-	7239	protein_coding
MYB proto-oncogene like 1 [Source:HGNC Symbol;Acc:HGNC:7547]
MYB	454	434	395	835	419	432
3.032344175	2.612580919	2.547208849	6.409437877
3.557810451	2.774828767
ENSG00000119681 1709.217989	1231.170847	1586.338249
1325.789194	1090.901265	1062.572713	1508.909028
1159.754391	0.3796244	0.030682115	1	LTBP2	14
74498170	74612378	-	9091	protein_coding
latent transforming growth factor beta binding protein 2
[Source:HGNC Symbol;Acc:HGNC:6715]	-	1777	1415	1705
1203	900	1151	9.450983099	6.782712045
8.755052635	7.353024503	6.085247013	5.887008786
ENSG00000240793 0	0	0	9.918622399	18.18168774
0	0	9.366770048	-5.787065179	0.0306999
1	UBA52P8 16	48334471	48334854	-
384	processed_pseudogene	ubiquitin A-52 residue
ribosomal protein fusion product 1 pseudogene 8 [Source:HGNC
Symbol;Acc:HGNC:35625]	-	0	0	0	9	15
0	0	0	0	1.302336848	2.401084227
0
ENSG00000180673 1.923711862	0	0.930403665	9.918622399
9.69690013	19.38664377	0.951371842	13.0007221
-3.792744304	0.030738905	1	EXOC5P1 4
62816826	62818794	+	1969	processed_pseudogene
exocyst complex component 5 pseudogene 1 [Source:HGNC
Symbol;Acc:HGNC:43870]	-	2	0	1	9	8
21	0.04911176	0	0.023708295	0.253985449
0.249742026	0.495911976
ENSG00000165525 565.5712874	494.2085096	485.6707132
411.071795	357.5731923	340.6510262	515.1501701
369.7653378	0.478619754	0.030755889	1	NEMF	14
49782083	49853203	-	9426	protein_coding
nuclear export mediator factor [Source:HGNC
Symbol;Acc:HGNC:10663]	-	588	568	522	373	295
369	3.016137297	2.62590795	2.585169976
2.198839044	1.923720357	1.820245428

ENSG00000140961 837.7765159	703.0290066	967.6198118
792.3877227	1293.324055	1418.917689	836.1417781
1168.209822	-0.482940868	0.030768713	1	OSGIN1	16
	83948282	83966332	+	3460	protein_coding
	oxidative stress induced growth inhibitor 1 [Source:HGNC
Symbol;Acc:HGNC:30093]	-	871	808	1040	719	1067
	1537	12.17147672	10.17639275	14.03147337
11.54688486	18.95551904	20.65517062
ENSG00000150593 440.5300164	590.7879895	588.0151164
443.0318005	306.6644666	376.6547932	539.7777074
375.4503534	0.523375129	0.030812063	1	PDCD4	10
	110871795	110900006	+	4547	protein_coding
	programmed cell death 4 [Source:HGNC Symbol;Acc:HGNC:8763]
	-	458	679	632	402	253	408
4.870143301	6.507338789	6.488408132	4.912619667
3.420132466	4.172210647
ENSG00000157021 2.885567793	44.37435562	27.91210996
89.26760159	58.18140078	91.39417776	25.05734445
79.61439338	-1.665377562	0.030884511	1	FAM92A1P1
	15	41163162	41164374	+	890
unprocessed_pseudogene	family with sequence similarity 92 member A1
pseudogene 1 [Source:HGNC Symbol;Acc:HGNC:32278]	-	3
	51	30	81	48	99	0.162979306
2.497116287	1.573538176	5.057164212	3.315114942
5.172210672
ENSG00000189007 316.4506013	310.6204893	321.9196682
166.4124425	199.9985652	252.026369	316.3302529
206.1457922	0.616047383	0.030985542	1	ADAT2	6
	143422832	143450673	-	7175	protein_coding
	"adenosine deaminase, tRNA specific 2 [Source:HGNC
Symbol;Acc:HGNC:21172]" -	329	357	346	151	165
	273	2.217048578	2.1682278	2.251128204
1.16941076	1.41354422	1.769178935
ENSG00000273456 49.05465248	20.8820497	28.84251362	0
	13.33323768	0	32.92640527	4.44441256
2.919746964	0.031004024	1	AC064836.3	2
202374932	202375604	-	673	lincRNA novel
transcript	-	51	24	31	0	11	0
	3.664007284	1.554013457	2.150268364	0
1.004673579	0
ENSG00000111261 115.4227117	96.57947987	117.2308618
67.22621848	43.63605059	51.69771671	109.7443511
54.18666193	1.01720534	0.031029823	1	MANSC1	12
	12326056	12350541	-	6015	protein_coding
	MANSC domain containing 1 [Source:HGNC Symbol;Acc:HGNC:25505]
	-	120	111	126	61	36	56
0.964599052	0.80416644	0.97786961	0.563515994
0.36788682	0.432895841
ENSG00000071539 836.8146599	919.6802723	685.7075012
1215.582278	1098.17394	923.1735127	814.0674778

1078.976577	-0.405793831	0.031050464	1	TRIP13	5
	892643	919357	+	3560	protein_coding	thyroid
hormone receptor interactor 13 [Source:HGNC Symbol;Acc:HGNC:12307]
	-	870	1057	737	1103	906	1000
11.8159997	12.93848978	9.664146964	17.21621026
15.64319863	13.06113806
ENSG00000174444 35462.66632	41654.46864	40986.14226
44706.53736	46942.69353	45861.41377	39367.75907
45836.88155	-0.219479953	0.03108027	0.214261239	RPL4
	15	66498015	66524532	-	5637
protein_coding	ribosomal protein L4 [Source:HGNC
Symbol;Acc:HGNC:10353]	-	36869	47874	44052	40566	38728
	49678	316.2383534	370.0924951	364.8073935
399.8767881	422.3032221	409.7765356
ENSG00000251414 32.70310165	39.15384319	8.373632987
5.510345777	0	6.462214589	26.74352594	3.990853455
	2.724270878	0.031141327	1	AC138956.2	5
	176354206	176356168	+	528	antisense
	novel transcript	-	34	45	9	5
	0	7	3.113480937	3.713959718	0.795709646
	0.526196706	0	0.616446137
ENSG00000119684 650.2146093	750.8837039	548.9381625
434.2152472	494.5419067	492.9746558	650.0121585
473.9106032	0.455991094	0.031183181	1	MLH3	14
	75013764	75051532	-	8363	protein_coding
	mutL homolog 3 [Source:HGNC Symbol;Acc:HGNC:7128]	-
	676	863	590	394	408	534	3.908281309
	4.496838563	3.293335311	2.617858501	2.998785668
	2.968999869
ENSG00000176731 336.6495758	314.9709163	313.5460352
487.1145667	424.2393807	443.1232861	321.7221758
451.4924112	-0.489088913	0.031200877	1	C8orf59 8
	85214076	85220421	-	2514	protein_coding
	chromosome 8 open reading frame 59 [Source:HGNC
Symbol;Acc:HGNC:32235]	-	350	362	337	442	350
	480	6.731378131	6.274828998	6.257641277
9.769425821	8.557563515	8.877833222
ENSG00000143819 375.1238131	350.6444179	405.655998
582.9945832	507.8751443	479.1270531	377.1414097
523.3322602	-0.472451258	0.031226413	1	EPHX1	1
	225810092	225845563	+	2508	protein_coding
	epoxide hydrolase 1 [Source:HGNC Symbol;Acc:HGNC:3401]	-
	390	403	436	529	419	519	7.518622696
	7.002225807	8.115307733	11.72033927	10.26913471
	9.622121662
ENSG00000119979 398.2083554	555.9845733	366.5790441
338.3352307	319.9977043	204.0213463	440.2573243
287.4514271	0.617187256	0.031281635	1	FAM45A	10
	119104086	119137984	+	6291	protein_coding
	family with sequence similarity 45 member A [Source:HGNC

Symbol;Acc:HGNC:31793]	-	414	639	394	307	264
	221	3.181865901	4.426289045	2.923631096
2.711631897	2.579476656	1.63344158
ENSG00000163682 6837.833813	9109.794182	10353.53199
9637.594764	11718.70381	11394.73067	8767.053327
10917.00975	-0.31638058	0.031316551	0.214261239	RPL9
	4	39452521	39458949	-	5022
protein_coding	ribosomal protein L9 [Source:HGNC
Symbol;Acc:HGNC:10369]	-	7109	10470	11128	8745	9668
	12343	68.44362803	90.85076133	103.4395076
96.75984168	118.3333865	114.2812649
ENSG00000047410 4660.191985	4006.743287	4752.501922
4338.846265	3436.338984	2927.383209	4473.145731
3567.522819	0.326545602	0.031339325	0.214261239	TPR
	1	186311652	186375693	-	12350
protein_coding	"translocated promoter region, nuclear basket protein
[Source:HGNC Symbol;Acc:HGNC:12017]"	-	4845	4605	5108
	3937	2835	3171	18.96828386	16.24879931
19.30767442	17.71374958	14.11020963	11.93878971
ENSG00000055130 1106.134321	1064.114449	1096.945921
1437.098179	1381.808269	1262.901365	1089.064897
1360.602604	-0.320884246	0.031385372	1	CUL1	7
	148697914	148801036	+	3548	protein_coding
	cullin 1 [Source:HGNC Symbol;Acc:HGNC:2551]	-	1150
	1223	1179	1304	1140	1368	15.67167605
15.02108984	15.51230124	20.42236453	19.75006823
17.92806856
ENSG00000090006 2493.130573	2085.594714	2455.335272
1860.292734	2018.16734	1862.040975	2344.686853
1913.50035	0.293249285	0.031386212	1	LTBP4	19
	40592883	40629818	+	8108	protein_coding
	latent transforming growth factor beta binding protein 4
[Source:HGNC Symbol;Acc:HGNC:6717]	-	2592	2397	2639
	1688	1665	2017	15.45690272	12.88287793
15.19398475	11.56833205	12.62257204	11.56706501
ENSG00000242372 4934.320926	4463.538124	5898.759237
5206.17469	6535.710688	7353.077029	5098.872762
6364.987469	-0.320135726	0.031392306	0.214261239	EIF6
	20	35278911	35284985	-	1631
protein_coding	eukaryotic translation initiation factor 6
[Source:HGNC Symbol;Acc:HGNC:6159]	-	5130	5130	6340
	4724	5392	7965	152.0773795	137.0635581
181.4601781	160.9414729	203.209161	227.0716089
ENSG00000063169 589.6176857	548.1538047	694.0811342
498.1352582	359.9974173	455.1245418	610.6175415
437.7524058	0.479237591	0.031526268	1	BICRA	19
	47608196	47703277	+	7507	protein_coding
	BRD4 interacting chromatin remodeling complex associated
protein [Source:HGNC Symbol;Acc:HGNC:4332]	-	613	630
	746	452	297	493	3.948164827	3.657065437

4.638936711	3.345677399	2.431853416	3.053595603
ENSG00000197785 2658.569793	2096.035739	2543.723621
2211.852795	3378.157583	4105.352611	2432.776384
3231.787663	-0.410060586	0.031565216	0.214643467	ATAD3A
1	1512151 1534687 +	3069
protein_coding	"ATPase family, AAA domain containing 3A [Source:HGNC
Symbol;Acc:HGNC:25567]" -	2764	2409	2734	2007	2787
4447	43.54540827	34.20570212	41.58604868
36.3381261	55.8196926	67.37538489
ENSG00000225871 25.0082542	5.220512425	0	0	0
0	10.07625554	0	5.673841152	0.031586275
1	AC245100.2	1	148435105	148436926
+	1679	processed_pseudogene	"family with sequence
similarity 91, member A1 (FAM91A1) pseudogene"	-	26	6
0	0	0	0	0.748727644	0.155725291
0	0	0	0
ENSG00000134780 512.6692112	486.377741	380.5350991
219.3117619	370.90643	330.4961176	459.8606837
306.9047698	0.58366095	0.031607384	1	DAGLA	11
61680433	61747001	+	5757	protein_coding
diacylglycerol lipase alpha [Source:HGNC Symbol;Acc:HGNC:1165]
-	533	559	409	199	306	358
4.476434107	4.23130349	3.316447406	1.92074137
3.267176203	2.891464172
ENSG00000122435 980.1311936	960.5742863	975.0630411
843.0829039	784.236798	503.1295644	971.9228403
710.1497555	0.454037411	0.031627525	1	TRMT13	1
100133150	100150497	+	4133	protein_coding
tRNA methyltransferase 13 homolog [Source:HGNC
Symbol;Acc:HGNC:25502]	-	1019	1104	1048	765	647
545	11.92092609	11.64024887	11.83700724
10.28508946	9.622462684	6.131434809
ENSG00000163872 1292.734371	1188.536662	1225.341627
904.7987766	1092.113377	921.3271657	1235.537553
972.7464398	0.345526315	0.031643257	1	YEATS2	3
183697818	183812625	+	10212	protein_coding
YEATS domain containing 2 [Source:HGNC Symbol;Acc:HGNC:25489]
-	1344	1366	1317	821	901	998
6.363406673	5.829059534	6.020339804	4.467292556
5.42327148	4.544130062
ENSG00000233030 8.656703379	6.09059783	9.304036652	0
0	0	8.01711262	0	5.344643305
0.031758964	1	AC243772.2	1	149785659
149793020	-	3543	antisense	"novel transcript,
antisense to FCGR1A"	-	9	7	10	0	0
0	0.122820984	0.086096498	0.13175736	0
0	0
ENSG00000227170 25.97011014	4.350427021	0	0	0
0	10.10684572	0	5.678128041	0.031856
1	AF178030.1	8	115509602	115511325

+ 402 antisense novel transcript -
27 5 0 0 0 0 3.247423488
0.542004072 0 0 0 0
ENSG00000129214 0 0 0 0 14.5453502
12.92442918 0 9.156593125 -5.7578859 0.03188742
1 SHBG 17 7613946 7633383 + 1972
protein_coding sex hormone binding globulin [Source:HGNC
Symbol;Acc:HGNC:10839] - 0 0 0 0 12
14 0 0 0 0 0.374043141
0.330105031
ENSG00000176945 46.16908469 51.33503885 53.96341258
82.65518666 87.27210117 146.7845885 50.48917871
105.5706255 -1.066301168 0.031896875 1 MUC20 3
195720882 195741123 + 4249 protein_coding
"mucin 20, cell surface associated [Source:HGNC
Symbol;Acc:HGNC:23282]" - 48 59 58 75 72
159 0.546205065 0.605095437 0.637216919
0.980813819 1.041581183 1.739968602
ENSG00000273056 0 0 0 14.32689902 13.33323768
0 0 9.220045567 -5.764929452 0.031907744
1 AL354694.1 9 7786105 7786688 - 584
lincRNA novel transcript - 0 0 0
13 11 0 0 0 0 1.236922669
1.157783079 0
ENSG00000248309 12.5041271 3.480341617 8.373632987 0
0 0 8.119367235 0 5.362360224
0.031931234 1 MEF2C-AS1 5 88883328
89466398 + 7865 antisense MEF2C antisense RNA 1
[Source:HGNC Symbol;Acc:HGNC:48908] - 13 4 9
0 0 0 0.079918227 0.022162557
0.05341827 0 0 0
ENSG00000236778 40.3979491 65.25640532 38.14655027
23.14345226 0 0 47.9336349 7.714484088
2.639641939 0.031946594 1 INTS6-AS1 13
51452367 51552364 + 9866 antisense INTS6
antisense RNA 1 [Source:HGNC Symbol;Acc:HGNC:42691] - 42
75 41 21 0 0 0.205830342
0.331267439 0.193994216 0.118274257 0 0
ENSG00000165283 3802.216495 3812.714241 3631.365505
4065.533114 4815.723027 4514.318477 3748.765414
4465.19154 -0.252227379 0.031955789 0.216497521 STOML2
9 35099776 35103195 - 1806
protein_coding stomatin like 2 [Source:HGNC Symbol;Acc:HGNC:14559]
- 3953 4382 3903 3689 3973 4890
105.8303628 105.7336501 100.8850564 113.5018533
135.2222566 125.8989567
ENSG00000158555 474.194974 390.6683465 556.3813918
556.5449235 661.8134339 767.1571891 473.7482374
661.8385155 -0.483222663 0.032047489 1 GDPD5 11
75434640 75525903 - 9867 protein_coding

glycerophosphodiester phosphodiesterase domain containing 5 [Source:HGNC Symbol;Acc:HGNC:28804] - 493 449 598 505 546 831 2.415811296 1.982986742 2.829189852 2.843926012 3.401377815 3.916038147
ENSG00000277589 0 0 37.21614661 0 0 0 12.4053822 0 5.9744852 0.032065609 1 AC244093.4 17 36998598 37000034 + 710 sense_intronic "novel transcript, sense intronic AATF" - 0 0 40 0 0 0 0 0 2.62995113 0 0 0
ENSG00000284693 54.82578806 13.92136647 12.09524765 0 1.212112516 9.231735127 26.94746739 3.481282548 2.917727388 0.032096589 1 AL928921.2 1 9425094 9440564 - 2623 lincRNA uncharacterized LOC100506022 [Source:NCBI gene;Acc:100506022] - 57 16 13 0 1 10 1.0506977 0.265815493 0.231361503 0 0.023434143 0.177268973
ENSG00000254732 8.656703379 7.830768638 7.443229322 0 0 0 7.976900446 0 5.337614202 0.032099813 1 AP001931.1 11 57742163 57793243 + 580 protein_coding "novel protein, C11orf31-CTNND1 readthrough" - 9 9 8 0 0 0 0.750266806 0.676196804 0.643884587 0 0 0
ENSG00000171735 321.2598809 383.7076633 385.1871174 490.4207742 504.2388068 516.0539936 363.3848872 503.5711915 -0.470551367 0.032109133 1 CAMTA1 1 6785324 7769706 + 10033 protein_coding calmodulin binding transcription activator 1 [Source:HGNC Symbol;Acc:HGNC:18806] CG-1 334 441 414 445 416 559 1.609595952 1.915430396 1.92626292 2.46457048 2.54864812 2.590669509
ENSG00000117280 1666.896328 1716.678503 1485.854653 1330.197471 1323.626868 1284.134356 1623.143161 1312.652898 0.306477034 0.032110901 1 RAB29 1 205767986 205775460 - 3882 protein_coding "RAB29, member RAS oncogene family [Source:HGNC Symbol;Acc:HGNC:9789]" - 1733 1973 1597 1207 1092 1391 21.58461209 22.1477775 19.20416465 17.27681897 17.29077532 16.66105957
ENSG00000214264 0 0 0 0 4.848450065 23.07933782 0 9.309262628 -5.785414721 0.032240991 1 KCTD9P4 11 112180773 112183191 + 1166 processed_pseudogene potassium channel tetramerization domain containing 9 pseudogene 4 [Source:HGNC Symbol;Acc:HGNC:49913] - 0 0 0 0 4 25 0 0 0 0 0.210867088 0.996947931
ENSG00000127603 9038.560183 7023.329383 8761.611315 8117.841399 5675.110801 6095.714705 8274.500294

6629.555635	0.319709997	0.032259158	0.216792031	MACF1
	1	39081316	39487177	+	40739
protein_coding	microtubule-actin crosslinking factor 1 [Source:HGNC
Symbol;Acc:HGNC:13664]	-	9397	8072	9417	7366	4682
	6603	11.15270151	8.63434478	10.79066579
10.0469304	7.064288142	7.536365469
ENSG00000235128 10.58041524	7.830768638	5.582421991	0
	0	0	7.997868623	0	5.341272506
0.032267533	1	AC013474.1	2	126679622
126680420	+	799	processed_pseudogene	novel
pseudogene	-	11	9	6	0	0	0
	0.665651818	0.490856253	0.350550432	0	0
	0
ENSG00000172354 4105.201113	4655.826998	5033.483829
4466.686287	6403.590424	6377.282626	4598.170647
5749.186445	-0.322307733	0.032284193	0.216792031	GNB2
	7	100673531	100679174	+	2857
protein_coding	G protein subunit beta 2 [Source:HGNC
Symbol;Acc:HGNC:4398]	-	4268	5351	5410	4053	5283
	6908	72.22962945	81.61750384	88.39609105
78.82761867	113.6626378	112.4276432
ENSG00000247400 61.55877958	33.06324536	47.45058692
24.24552142	0	6.462214589	47.35753729	10.235912
	2.20718773	0.03228547	1	DNAJC3-DT	13
	95648733	95676925	-	3010	lincRNA DNAJC3
divergent transcript [Source:HGNC Symbol;Acc:HGNC:39808]	-
	64	38	51	22	0	7	1.028051083
	0.550143136	0.790951249	0.406132953	0
0.108134073
ENSG00000075826 129.8505507	74.82734476	160.9598341
61.7158727	26.66647536	75.70022804	121.8792432
54.69419204	1.150389039	0.032343401	1	SEC31B	10
	100486642	100519864	-	7938	protein_coding
	"SEC31 homolog B, COPII coat complex component [Source:HGNC
Symbol;Acc:HGNC:23197]" -	135	86	173	56	22
	82	0.822287883	0.472112995	1.01737496
0.392002626	0.170356593	0.480323434
ENSG00000168610 2880.758513	3017.456182	2709.335473
2387.081791	2326.043919	2469.489147	2869.183389
2394.204952	0.260977248	0.032353495	0.216792031	STAT3
	17	42313324	42388568	-	8812
protein_coding	signal transducer and activator of transcription 3
[Source:HGNC Symbol;Acc:HGNC:11364]	STAT	2995	3468	2912
	2166	1919	2675	16.4332535	17.14996342
15.42634067	13.65827987	13.38590833	14.11498159
ENSG00000187957 605.9692365	628.2016619	704.3155745
458.4607687	472.7238814	513.2844731	646.1621576
481.4897077	0.423914767	0.032428725	1	DNER	2
	229357629	229714558	-	3935	protein_coding
	delta/notch like EGF repeat containing [Source:HGNC

Symbol;Acc:HGNC:24456]	-	630	722	757	416	390
	556	7.740999319	7.995599987	8.980430964
5.874361077	6.092102904	6.569935002
ENSG00000254503 0	0	2.791210996	0	36.36337549
	16.61712323	0.930403665	17.66016624	-4.249763559
	0.03251119	1	AC010319.1	19	17417031
	17418288	-	1258	processed_pseudogene	high-
mobility group box 3 (HMGB3) pseudogene -	0	0	3
	0	30	18	0	0	0.111323448	0
	1.465844743	0.665308209
ENSG00000275981 12.5041271	2.610256213	9.304036652	0
	0	0	8.139473322	0	5.365834306
0.032575656	1	AC018816.2	3	4750333 4750685 +
	353	misc_RNA		-	13	3	10
	0	0	0	1.780614327	0.370343859
1.322425851	0	0	0
ENSG00000130560 1276.38282	1285.116142	1280.235443
1272.889875	1729.684561	1981.130358	1280.578135
1661.234931	-0.375846049	0.032682161	1	UBAC1	9
	135932969	135961380	-	2979	protein_coding
	UBA domain containing 1 [Source:HGNC Symbol;Acc:HGNC:30221]
	-	1327	1477	1376	1155	1427	2146
21.5378147	21.60571235	21.56224451	21.54386032
29.44427262	33.49579057
ENSG00000155657 770.4466007	386.3179195	646.6305473
427.6028323	340.6036171	440.3537656	601.1316892
402.853405	0.57620101	0.032789398	1	TTN	2
	178525989	178830802	-	118976	protein_coding
	titin [Source:HGNC Symbol;Acc:HGNC:12403]	-	801
	444	695	388	281	477	0.325517521
0.162623088	0.272691422	0.181210937	0.14517583
0.18641894
ENSG00000080644 6.732991517	6.09059783	9.304036652
52.89931946	15.75746271	25.84885836	7.375875333
31.50188018	-2.095768669	0.032789485	1	CHRNA3	15
	78593052	78621295	-	4765	protein_coding
	cholinergic receptor nicotinic alpha 3 subunit [Source:HGNC
Symbol;Acc:HGNC:1957]	-	7	7	10	48	13
	28	0.071029106	0.064016766	0.097967749
0.559745197	0.167697971	0.273228592
ENSG00000259950 0	0	0	34.16414382	0	0
	0	11.38804794	-6.071796448	0.03279706	1
	AC034105.3	16	31894542	31894983	-
	442	processed_pseudogene	"protein tyrosine phosphatase,
receptor type, f polypeptide (PTPRF), interacting protein (liprin),
alpha 1 (PPFIA1) pseudogene"	-	0	0	0	31
	0	0	0	0	0	3.897189001	0
	0
ENSG00000273033 523.2496264	356.7350157	396.3519614
296.4566028	209.6954653	336.9583321	425.4455345

281.0368001	0.595965582	0.03283967	1	LINC02035
	3	122886941	122892416	+	5476
lincRNA long intergenic non-protein coding RNA 2035 [Source:HGNC
Symbol;Acc:HGNC:52875]	-	544	410	426	269	173
	365	4.803266427	3.262714064	3.6315514
2.729611783	1.941914139	3.099277355
ENSG00000254859 15.3896949	2.610256213	6.512825656	0
	0	0	8.170925588	0	5.371230022
0.032882711	1	AC067930.4	8	143541973
143549729	-	498	antisense	novel transcript
	-	16	3	7	0	0	0
1.553430602	0.262512815	0.656167526	0	0	0
ENSG00000122034 3534.820546	4376.529583	3559.724423
4303.580052	4848.450065	4722.032518	3823.691518
4624.687545	-0.274259312	0.032921507	0.219795949	GTF3A
	13	27424544	27435823	+	3001
protein_coding	general transcription factor IIIA [Source:HGNC
Symbol;Acc:HGNC:4662]	zf-C2H2 3675	5030	3826	3905	4000
	5115	59.20965962	73.03997021	59.51480378
72.30479289	81.92969839	79.25207845
ENSG00000124103 0	0	0	0	13.33323768
13.84760269	0	9.060280123	-5.743048964	0.032939426
	1	FAM209A 20	56517187	56526142	+
	1137	protein_coding	family with sequence similarity 209
member A [Source:HGNC Symbol;Acc:HGNC:16100]	-	0	0
	0	0	11	15	0	0	0	0
	0.594674862	0.613425482
ENSG00000234832 0	0	0	20.93931395	0
6.462214589	0	9.133842847	-5.754877759	0.032990227
	1	AL096677.1	20	23346941	23351486
	-	814	antisense	novel transcript	-
	0	0	0	19	0	7	0	0
	0	1.297003774	0	0.399856954
ENSG00000272752 22.12268641	0	7.443229322	0	0
	0	9.855305244	0	5.641555312	0.032995429
	1	STAG3L5P-PVRIG2P-PILRB	7	100336104
100367831	+	3816	processed_transcript	STAG3L5P-
PVRIG2P-PILRB readthrough [Source:HGNC Symbol;Acc:HGNC:48898]	-
	23	0	8	0	0	0	0.291420894
	0	0.097865058	0	0	0
ENSG00000160953 1482.21999	1453.042625	1400.257516
1082.231911	1252.112229	1152.120544	1445.173377
1162.154895	0.314690884	0.033104229	1	MUM1	19
	1354711 1378431 +	9876	protein_coding	melanoma
associated antigen (mutated) 1 [Source:HGNC Symbol;Acc:HGNC:29641]
	-	1541	1670	1505	982	1033	1248
7.54436643	7.368752803	7.11379678	5.525129354
6.429343072	5.875766411
ENSG00000198431 3428.054538	2593.72459	3268.508076
2995.423964	4804.814015	4159.819848	3096.762401

3986.685942	-0.364441828	0.033219417	0.220981339	TXNRD1
	12	104215779	104350305	+	6708
protein_coding	thioredoxin reductase 1 [Source:HGNC
Symbol;Acc:HGNC:12437]	-	3564	2981	3513	2718	3964
	4506	25.68892069	19.36544673	24.44731293
22.51481807	36.32352201	31.23411116
ENSG00000161956 891.640448	916.1999307	1010.41838
523.4828488	726.0553973	831.779335	939.4195864
693.772527	0.436613357	0.033309425	1	SENP3	17
	7561875 7571969 +	3668	protein_coding	SUMO specific
peptidase 3 [Source:HGNC Symbol;Acc:HGNC:17862] -	927	1053
	1086	475	599	901	12.21944901	12.51000957
	13.82122491	7.195754306	10.03794601	11.42158779
ENSG00000159167 3599.264894	5974.006386	4122.61864
3805.444794	3506.64151	3391.739486	4565.29664
3567.94193	0.355795191	0.033352926	0.221068491	STC1
	8	23841915	23854807	-	4149
protein_coding	stanniocalcin 1 [Source:HGNC Symbol;Acc:HGNC:11373]
	-	3742	6866	4431	3453	2893	3674
43.60753769	72.11389646	49.8544984	46.24504295
42.86001898	41.17434842
ENSG00000272668 162.5536523	93.09913825	230.740109
66.12414933	119.9991391	53.54406374	162.1309665
79.88911739	1.024653676	0.033354839	1	AL590560.1
	1	159854870	159867685	+	7134
antisense	uncharacterized LOC107985216 [Source:NCBI
gene;Acc:107985216]	-	169	107	248	60	99
	58	1.145393769	0.653595827	1.622798553
0.467337024	0.853000822	0.378029687
ENSG00000130414 875.2888972	658.654651	735.0188955
497.0331891	643.6317462	533.5942904	756.3208146
558.0864085	0.438926227	0.033466347	1	NDUFA10 2
	239892450	240025402	-	9517	protein_coding
	NADH:ubiquinone oxidoreductase subunit A10 [Source:HGNC
Symbol;Acc:HGNC:7684]	-	910	757	790	451	531
	578	4.623198489	3.466206305	3.875012054
2.633228314	3.429586903	2.823961602
ENSG00000174977 146.2021015	177.4974225	137.6997424
158.6979584	310.3008042	307.4167797	153.7997555
258.8051808	-0.750690982	0.03349575	1	AC026271.1
	17	18650195	18651542	+	1348
processed_pseudogene	poly(A) binding protein interacting protein 1
(PAIP1) pseudogene	-	152	204	148	144	256
	333	5.451988263	6.594758146	5.1252831
5.935873588	11.67340177	11.48643765
ENSG00000123485 325.1073047	263.6358775	304.2419985
657.9352858	402.4213554	318.4948619	297.6617269
459.6171677	-0.626311134	0.033525062	1	HJURP	2
	233833416	233854566	-	3821	protein_coding
	Holliday junction recognition protein [Source:HGNC

Symbol;Acc:HGNC:25444]	-	338	303	327	597	332
	345	4.277016041	3.455605755	3.994999698
8.681791208	5.340825717	4.198296197
ENSG00000139151 12.5041271	16.53162268	0	0	0
	0	9.678583261	0	5.617047229	0.033526429
	1	PLCZ1	12	18683169	18738100	-
	3431	protein_coding	phospholipase C zeta 1 [Source:HGNC
Symbol;Acc:HGNC:19218]	-	13	19	0	0	0
	0	0.183199317	0.24131898	0	0	0
	0
ENSG00000169918 0	8.700854042	20.46888063	0	0
	0	9.723244892	0	5.62358298	0.033543983
	1	OTUD7A	15	31475398	31870789	-
	14331	protein_coding	OTU deubiquitinase 7A [Source:HGNC
Symbol;Acc:HGNC:20718]	-	0	10	22	0	0
	0	0	0.030407597	0.071662544	0	0
	0
ENSG00000103723 0	9.570939447	19.53847697	0	0
	0	9.703138805	0	5.620664667	0.033548501
	1	AP3B2	15	82659281	82709914	-
	9108	protein_coding	adaptor related protein complex 3
subunit beta 2 [Source:HGNC Symbol;Acc:HGNC:567]	-	0
	11	21	0	0	0	0	0.052629381
	0.107632223	0	0	0
ENSG00000238061 0	10.44102485	18.6080733	0	0
	0	9.683032718	0	5.617740593	0.033594849
	1	AL356273.2	1	185280844	185281816
	+	973	processed_pseudogene	"proteasome (prosome,
macropain) 26S subunit, non-ATPase, 7 (Mov34 homolog) (PSMD7)
pseudogene"	-	0	12	20	0	0	0
	0	0.537436309	0.959540238	0	0	0
ENSG00000258388 20.19897455	24.36239132	14.88645864	0
	6.060562582	0	19.8159415	2.020187527
3.343000103	0.033630929	1	PPT2-EGFL8	6
32153845	32171978	+	5512	protein_coding	PPT2-
EGFL8 readthrough (NMD candidate) [Source:HGNC Symbol;Acc:HGNC:48343]
	-	21	28	16	0	5	0
0.184209194	0.221364217	0.135505465	0	0.055758124
	0
ENSG00000182173 655.023889	860.5144648	826.1984547
899.2884308	1072.719577	1104.115521	780.5789362
1025.37451	-0.39342207	0.033672006	1	TSEN54	17
	75516060	75524739	+	3440	protein_coding
	tRNA splicing endonuclease subunit 54 [Source:HGNC
Symbol;Acc:HGNC:27561]	-	681	989	888	816	885
	1196	9.571717797	12.52842411	12.05037492
13.18086038	15.8136524	16.16604395
ENSG00000116194 0	6.960683234	22.32968796	0	0
	0	9.763457066	0	5.629402824	0.033686096
	1	ANGPTL1 1	178849705	178871052	-

3403	protein_coding	angiopoietin like 1 [Source:HGNC Symbol;Acc:HGNC:489]	-	0	8	24	0	0	0	0	0.102444026	0.329226912	0	0	0
ENSG00000177105 1285.039524	1074.555474	1389.092672	1486.691291	1555.140358	1682.945314	1249.562557	1574.925654	-0.334296468	0.033732339	1	RHOG	11	3826978 3840983 -	1782	protein_coding	ras homolog family member G [Source:HGNC Symbol;Acc:HGNC:672]	-	1336	1235	1493	1349	1283	1823	36.24932925	30.20075886	39.11093008	42.06455449	44.25540473	47.56746279
ENSG00000242808 0	28.71281834	1.86080733	0	0	0	10.19120856	0	5.692665937	0.033782958	1	SOX2-OT 3	180989762	181836880	+	31629	sense_overlapping	SOX2 overlapping transcript [Source:HGNC Symbol;Acc:HGNC:20209]	-	0	33	2	0	0	0	0	0.045466034	0.002951825	0	0	0
ENSG00000160951 9.618559309	0	19.53847697	0	0	0	9.719012093	0	5.621927177	0.033788583	1	PTGER1	19	14472466	14475362	-	1421	protein_coding	prostaglandin E receptor 1 [Source:HGNC Symbol;Acc:HGNC:9593]	-	10	0	21	0	0	0	0.340257055	0	0.689876343	0	0	0
ENSG00000128652 0	12.18119566	16.74726597	0	0	0	9.642820544	0	5.611874741	0.033798298	1	HOXD3	2	176136612	176173102	+	3548	protein_coding	homeobox D3 [Source:HGNC Symbol;Acc:HGNC:5137]	Homeobox	0	14	18	0	0	0	0	0.171950333	0.236829026	0	0	0
ENSG00000207975 0	21.75213511	7.443229322	0	0	0	9.731788143	0	5.625819041	0.033814129	1	MIR181B1	1	198858873	198858982	-	110	miRNA	microRNA 181b-1 [Source:HGNC Symbol;Acc:HGNC:31550]	-	0	25	8	0	0	0	0	9.90389258	3.395027822	0	0	0
ENSG00000252821 36.55052538	0	0	0	0	0	12.18350846	0	5.947654465	0.033849295	1	RNU6-388P	7	33001950	33002054	-	105	snRNA	"RNA, U6 small nuclear 388, pseudogene [Source:HGNC Symbol;Acc:HGNC:47351]"	-	38	0	0	0	0	0	17.49828614	0	0	0	0	0
ENSG00000126067 4881.41885	4516.613333	3986.779705	5678.962358	5162.387207	5149.461854	4461.603963	5330.270473	-0.2566465	0.03393278	0.223171528	PSMB2

1	35599544	35641844	-	4843
protein_coding	proteasome subunit beta 2 [Source:HGNC
Symbol;Acc:HGNC:9539]	-	5075	5191	4285	5153	4259
5578	50.66672043	46.70841794	41.30307568
59.12317075	54.05557996	53.55438779
ENSG00000106636 5763.440738	4713.252635	4936.721847
5371.485064	6316.318322	7099.204313	5137.805074
6262.3359	-0.285746727	0.034017304	0.223171528	YKT6
7	44200968	44214294	+	3707
protein_coding	YKT6 v-SNARE homolog [Source:HGNC
Symbol;Acc:HGNC:16959]	-	5992	5417	5306	4874	5211
7690	78.15386046	63.67879657	66.81757279
73.05921179	86.40638728	96.45722688
ENSG00000185624 7013.853448	6380.336269	8897.45025
10006.78793	9094.48021	8207.935702	7430.546656
9103.067948	-0.292849189	0.034034874	0.223171528	P4HB
17	81843159	81860694	-	5303
protein_coding	prolyl 4-hydroxylase subunit beta [Source:HGNC
Symbol;Acc:HGNC:8548]	-	7292	7333	9563	9080	7503
8891	66.48539439	60.25854703	84.18186915
95.14287377	86.96823966	77.95787658
ENSG00000104472 1602.451981	1772.363968	1697.986689
2229.485901	1831.502012	2183.305358	1690.934213
2081.43109	-0.300016568	0.034206252	1	CHRAC1	8
140511298	140517137	+	3301	protein_coding
chromatin accessibility complex subunit 1 [Source:HGNC
Symbol;Acc:HGNC:13544]	-	1666	2037	1825	2023	1511
2365	24.40229591	26.89082351	25.80853663
34.05355073	28.13625557	33.31322199
ENSG00000252026 0	0	0.930403665	19.8372448	0
12.92442918	0.310134555	10.92055799	-5.052141864
0.03421756	1	RNU6-1262P	16	68284223
68284318	-	96	snRNA	"RNA, U6 small nuclear
1262, pseudogene [Source:HGNC Symbol;Acc:HGNC:48225]"	-	0
0	1	18	0	14	0	0
0.486267006	10.41869479	0	6.78090751
ENSG00000121966 29.81753386	196.6393014	43.72897226
16.53103733	25.45436284	47.08184915	90.06193583
29.68908311	1.597601888	0.034285733	1	CXCR4	2
136114349	136118165	-	2224	protein_coding
C-X-C motif chemokine receptor 4 [Source:HGNC
Symbol;Acc:HGNC:2561]	-	31	226	47	15	21
51	0.673950698	4.428251251	0.986527307
0.374773194	0.580405972	1.066268087
ENSG00000065243 1860.22937	1888.085327	1702.638707
1595.796137	1413.323194	1429.072598	1816.984468
1479.39731	0.296595277	0.034304572	1	PKN2	1
88684222	88836255	+	6897	protein_coding
protein kinase N2 [Source:HGNC Symbol;Acc:HGNC:9406]	-
1934	2170	1830	1448	1166	1548	13.55805715

13.71065193	12.3861661	11.66595722	10.39167809
10.4361845
ENSG00000147536 440.5300164	588.1777333	581.5022907
976.4332717	599.9956956	691.456961	536.7366801
755.9619761	-0.493957808	0.03432505	1	GINS4	8
41529206	41545046	+	5475	protein_coding
GINS complex subunit 4 [Source:HGNC Symbol;Acc:HGNC:28226]
-	458	676	625	886	495	749
4.044665131	5.380481843	5.328953488	8.992110643
5.557358782	6.36104858
ENSG00000042088 819.5012532	1171.134954	927.6124542
1278.40022	1270.293917	1229.667119	972.7495538
1259.453752	-0.372187364	0.034430834	1	TDP1	14
89954939	90044768	+	6227	protein_coding
tyrosyl-DNA phosphodiesterase 1 [Source:HGNC
Symbol;Acc:HGNC:18884]	-	852	1346	997	1160	1048
1332	6.615488907	9.419433663	7.47415893
10.35121114	10.3449829	9.946181435
ENSG00000092853 764.6754651	794.3879741	784.3302898
1635.470627	847.2666489	849.3196317	781.131243
1110.685636	-0.507619479	0.034436557	1	CLSPN	1
35720218	35769967	-	5411	protein_coding
claspin [Source:HGNC Symbol;Acc:HGNC:19715]	-	795
913	843	1484	699	920	7.103801396
7.352784564	7.272706754	15.23941903	7.940484494
7.905717867
ENSG00000048140 690.6125584	679.5367007	758.2789871
794.5918611	1033.931976	951.7918916	709.4760821
926.7719097	-0.385340143	0.034694494	1	TSPAN17 5
176647387	176659054	+	5215	protein_coding
tetraspanin 17 [Source:HGNC Symbol;Acc:HGNC:13594]	-
718	781	815	721	853	1031	6.656889499
6.526123962	7.295403745	7.682330653	10.05407403
9.192536663
ENSG00000238140 24.04639827	5.220512425	0	0	0
0	9.7556369	0	5.627200373	0.034749916
1	AC104170.2	1	51461721	51463416
+	678	antisense	novel transcript	-
25	6	0	0	0	0	1.782836559
0.385638295	0	0	0	0
ENSG00000162086 116.3845676	87.87862583	60.47623824
42.98069706	49.69661317	12.92442918	88.24647724
35.2005798	1.333105305	0.034759955	1	ZNF75A	16
3305406 3318852 +	8063	protein_coding	zinc finger
protein 75a [Source:HGNC Symbol;Acc:HGNC:13146] zf-C2H2 121	101
65	39	41	14	0.725587725	0.545862565
0.376324707	0.268769505	0.312560834	0.080735101
ENSG00000113739 709.849677	804.8289989	836.432895
570.8718225	363.6337549	711.7667783	783.703857
548.7574519	0.512364574	0.03485692	1	STC2	5

173314713	173329503	-	6128	protein_coding
stanniocalcin 2 [Source:HGNC Symbol;Acc:HGNC:11374]	-
738	925	899	518	300	771	5.822893161
6.577813773	6.848366134	4.697026893	3.009191721
5.850145122
ENSG00000254856 18.27526269	1.740170808	4.652018326	0
0	0	8.222483941	0	5.380076835
0.034896124	1	NDUFA3P2	11	68488609
68488840	-	232	processed_pseudogene
NADH:ubiquinone oxidoreductase subunit A3 pseudogene 2 [Source:HGNC
Symbol;Acc:HGNC:45051]	-	19	2	5	0	0
0	3.959741476	0.375664891	1.006069667	0
0	0
ENSG00000210140 1690.942727	1194.62726	1887.789037
1571.550616	2060.591278	2906.150218	1591.119674
2179.430704	-0.454496306	0.034971781	1	MT-TC	MT
5761	5826	-	66	Mt_tRNA mitochondrially
encoded tRNA cysteine [Source:HGNC Symbol;Acc:HGNC:7477]	-
1758	1373	2029	1426	1700	3148	1287.882232
906.5363009	1435.106552	1200.570405	1583.260387
2217.797075
ENSG00000134545 0	23.49230591	5.582421991	0	0
0	9.691575969	0	5.619967582	0.035041912
1	KLRC1	12	10442264	10454685	-
2698	protein_coding	killer cell lectin like receptor C1
[Source:HGNC Symbol;Acc:HGNC:6374]	-	0	27	6
0	0	0	0	0.436094306	0.10381386
0	0	0
ENSG00000067066 973.3982021	1366.034085	1053.216949
981.9436175	804.8427108	783.7743123	1130.883079
856.8535469	0.400793785	0.03504928	1	SP100	2
230415942	230544090	+	12618	protein_coding
SP100 nuclear antigen [Source:HGNC Symbol;Acc:HGNC:11206]
SAND	1012	1570	1132	891	664	849
3.877851785	5.422102548	4.187954355	3.92373099
3.234632282	3.128586632
ENSG00000254064 159.6680845	85.26836962	105.1356142
28.65379804	50.90872568	80.31609561	116.6906894
53.29287311	1.126000854	0.035080146	1	AC105206.2
8	22254576	22275162	-	515
antisense	uncharacterized LOC100507071 [Source:NCBI
gene;Acc:100507071]	-	166	98	113	26	42
87	15.58483022	8.292346564	10.24276598
2.805292577	5.012904391	7.854943069
ENSG00000168137 2901.919344	2386.644264	2578.148556
2454.308009	1799.987087	2044.829331	2622.237388
2099.708142	0.320362556	0.035109412	1	SETD5	3
9397615 9479240 +	10808	protein_coding	SET domain
containing 5 [Source:HGNC Symbol;Acc:HGNC:25566]	-	3017
2743	2771	2227	1485	2215	13.49681175

```
11.05959107	11.96843114	11.44951063	8.445560509
9.529265181
ENSG00000075426	954.1610835	728.2614834	1029.956857
1247.542284	1121.204078	1157.659585	904.1264747
1175.468649	-0.378987628	0.035168212	1	FOSL2	2
	28392448	28417312	+	7157	protein_coding
	"FOS like 2, AP-1 transcription factor subunit [Source:HGNC
Symbol;Acc:HGNC:3798]"	TF_bZIP	992	837	1107	1132	925
	1254	6.701651987	5.096277155	7.220422976
8.788756928	7.944344628	8.146996644
ENSG00000255248	1751.53965	2081.244287	1876.624193
1616.735451	1512.71642	1526.005817	1903.136043
1551.819229	0.29457056	0.0351925	1	MIR100HG
	11	122028327	122556721	-	19385
processed_transcript	mir-100-let-7a-2-mir-125b-1 cluster host gene
[Source:HGNC Symbol;Acc:HGNC:39522]	-	1821	2392	2017
	1467	1248	1653	4.541981458	5.377172485
4.857201592	4.205100233	3.957274169	3.964953208
ENSG00000212128	7.694847448	12.18119566	3.721614661	0
	0	0	7.865885923	0	5.318024223
0.035221061	1	TAS2R13	12	10907926	10909562
	-	1637	protein_coding	taste 2 receptor member 13
[Source:HGNC Symbol;Acc:HGNC:14919]	-	8	14	4
	0	0	0	0.236288467	0.372681602
0.114066298	0	0	0
ENSG00000243970	216.4175845	149.6546895	194.454366
98.08415483	86.05998866	137.5528534	186.8422133
107.2323323	0.797583316	0.035252756	1	PPIEL	1
	39522280	39559671	-	5416
transcribed_unprocessed_pseudogene	peptidylprolyl isomerase E
like pseudogene [Source:HGNC Symbol;Acc:HGNC:33195]	-	225
	172	209	89	71	149	2.008653746
1.383911725	1.80141455	0.913110621	0.805799611
1.279200531
ENSG00000272320	0	0	0	9.918622399	0
16.61712323	0	8.845248543	-5.711178331	0.035297051
	1	AL445309.1	6	3311662	3313650	+	1989
	antisense	"novel transcript, antisense to SLC22A23"
	-	0	0	0	9	0	18	0
	0	0	0.251431548	0	0.420793226
ENSG00000256746	12.5041271	11.31111026	0.930403665	0
	0	0	8.248547008	0	5.386083837
0.035335448	1	AC018410.1	11	47270657
47272110	-	536	antisense	uncharacterized
LOC101928943 [Source:NCBI gene;Acc:101928943]	-	13	13
	1	0	0	0	1.172680704	1.05690794
	0.087092598	0	0	0
ENSG00000160789	14542.29982	16561.20558	23318.70706
23608.52545	24656.79281	19907.31363	18140.73749
22724.21063	-0.324956899	0.035409875	0.230814014	LMNA
```

1	156082573	156140089	+	6985
protein_coding	lamin A/C [Source:HGNC Symbol;Acc:HGNC:6636]	-
15119	19034	25063	21422	20342	21564	104.6544918
118.7468922	167.4991778	170.4141482	179.0088901
143.5469373
ENSG00000198727 23903.08174	26411.44245	31110.83776
28081.82415	33026.42973	37541.77407	27141.78731
32883.34265	-0.276867443	0.035507531	0.230814014	MT-CYB
MT	14747	15887	+	1141	protein_coding
mitochondrially encoded cytochrome b [Source:HGNC
Symbol;Acc:HGNC:7427]	-	24851	30355	33438	25481	27247
40666	1053.075336	1159.319633	1368.04595
1240.91738	1467.845709	1657.207271
ENSG00000166016 139.46911	110.5008463	103.2748068
39.6744896	88.48421369	42.46598159	117.7482544
56.87489496	1.054445794	0.035525487	1	ABTB2	11
34150988	34358008	-	4973	protein_coding
ankyrin repeat and BTB domain containing 2 [Source:HGNC
Symbol;Acc:HGNC:23842]	-	145	127	111	36	73
46	1.409778099	1.112868525	1.04195882
0.40225003	0.90230167	0.430100939
ENSG00000236478 5.771135586	0	23.26009163	0	0
0	9.677075738	0	5.615847071	0.035532457
1	AC012513.2	2	216174896	216176032
-	1137	processed_pseudogene	heterogeneous nuclear
ribonucleoprotein A3 (HNRNPA3) pseudogene	-	6	0
25	0	0	0	0.255147902	0
1.02642112	0	0	0
ENSG00000142102 142.3546778	158.3555436	163.7510451
104.6965698	89.69632621	66.46849292	154.8204221
86.9537963	0.834571781	0.035544108	1	PGGHG	11
289135	296107	+	5260	protein_coding	protein-
glucosylgalactosylhydroxylysine glucosidase [Source:HGNC
Symbol;Acc:HGNC:26210]	-	148	182	176	95	74
72	1.360433093	1.507801745	1.561970975
1.003575163	0.864755506	0.636469754
ENSG00000180846 2.885567793	8.700854042	12.09524765	0
0	0	7.893889828	0	5.323017434
0.03555126	1	CSNK1G2-AS1	19	1952531 1954586 -
1346	antisense	CSNK1G2 antisense RNA 1 [Source:HGNC
Symbol;Acc:HGNC:28604]	-	3	10	13	0	0
0	0.10776492	0.323752804	0.450862722	0
0	0
ENSG00000168517 45.20722875	50.46495345	43.72897226
12.12276071	21.81802529	12.92442918	46.46705149
15.62173839	1.578137693	0.035563713	1	HEXIM2	17
45160700	45170040	+	2038	protein_coding
hexamethylene bisacetamide inducible 2 [Source:HGNC
Symbol;Acc:HGNC:28591]	-	47	58	47	11	18
14	1.115051419	1.240173399	1.076563656

0.299916631	0.542894805	0.319414682
ENSG00000081985 0	18.27179349	10.23444032	0	0
	0	9.502077935	0	5.591146931	0.035576793
	1	IL12RB2 1	67307364	67397090	+
	4423	protein_coding	interleukin 12 receptor subunit beta 2
[Source:HGNC Symbol;Acc:HGNC:5972]	-	0	21	11
	0	0	0	0	0.20690022	0.11609721
	0	0	0
ENSG00000185085 1744.806659	1796.72636	1858.946523
1916.498261	2099.378878	2818.448734	1800.159847
2278.108625	-0.340271633	0.035577203	1	INTS5	11
	62646848	62653302	-	3285	protein_coding
	integrator complex subunit 5 [Source:HGNC
Symbol;Acc:HGNC:29352]	-	1814	2065	1998	1739	1732
	3053	26.69949981	27.39323226	28.39266418
29.41550114	32.4085704	43.2137991
ENSG00000099956 3437.673097	3362.010002	3537.394735
3961.938614	3955.123141	4253.983547	3445.692611
4057.0151	-0.235796033	0.035577541	0.230814014
SMARCB1 22	23786931	23838008	+	16525
protein_coding	"SWI/SNF related, matrix associated, actin dependent
regulator of chromatin, subfamily b, member 1 [Source:HGNC
Symbol;Acc:HGNC:11103]" -	3574	3864	3802	3595	3263
	4608	10.45717309	10.18953223	10.74030662
12.08841804	12.13732457	12.96588067
ENSG00000069011 136.5835422	159.225629	118.1612655
213.8014162	253.3315159	201.2518258	137.9901456
222.7949193	-0.689812337	0.035581761	1	PITX1	5
	135027735	135034813	-	3511	protein_coding
	paired like homeodomain 1 [Source:HGNC Symbol;Acc:HGNC:9004]
	Homeobox	142	183	127	194	209	218
	1.955504103	2.271322787	1.688569449	3.070315069
	3.659003432	2.887065801
ENSG00000107796 9.618559309	29.58290374	32.56412828
65.02208017	79.99942608	50.7745432	23.92186378
65.26534982	-1.444655862	0.035606349	1	ACTA2	10
	88935074	88991339	-	2976	protein_coding
	"actin, alpha 2, smooth muscle, aorta [Source:HGNC
Symbol;Acc:HGNC:130]"	-	10	34	35	59	66
	55	0.16246817	0.497856966	0.549011136
1.101618266	1.363196206	0.859331597
ENSG00000233559 45.20722875	18.27179349	22.32968796
4.408276622	8.484787614	6.462214589	28.6029034
6.451759608	2.150443233	0.035685688	1	LINC00513
	7	130853720	130928649	+	1616
lincRNA long intergenic non-protein coding RNA 513 [Source:HGNC
Symbol;Acc:HGNC:43566]	-	47	21	24	4	7
	7	1.406234401	0.566286927	0.693291573
0.137540525	0.266258845	0.201413094
ENSG00000154118 12.5041271	29.58290374	29.77291729	0

8.484787614	0	23.95331604	2.828262538
3.122335756	0.035785141	1	JPH3	16	87601835
87698156	+	5941	protein_coding	junctophilin 3
[Source:HGNC Symbol;Acc:HGNC:14203]	-	13	34	32
0	7	0	0.105799841	0.249389384
0.251441212	0	0.072424557	0
ENSG00000165322 763.7136092	972.7554819	815.9640144
818.8373825	490.9055691	516.9771671	850.8110352
608.9067062	0.482917425	0.035810004	1	ARHGAP12
10	31805404	31928876	-	5938
protein_coding	Rho GTPase activating protein 12 [Source:HGNC
Symbol;Acc:HGNC:16348]	-	794	1118	877	743	405
560	6.465193471	8.204652809	6.894542228
6.952814844	4.192394959	4.385093439
ENSG00000128578 806.0352701	850.0734399	727.5756662
510.258019	610.9047082	673.9166643	794.5614588
598.3597972	0.408634755	0.035863153	1	STRIP2	7
129434433	129488399	+	5876	protein_coding
striatin interacting protein 2 [Source:HGNC
Symbol;Acc:HGNC:22209]	-	838	977	782	463	504
730	6.895463247	7.245550276	6.212565803
4.378357782	5.272251384	5.776597276
ENSG00000280287 345.3062792	304.5298915	300.5203839
179.6372723	201.2106777	250.1800219	316.7855182
210.3426573	0.5890883	0.035895735	1	AC131212.3
12	132550729	132554947	+	4219	TEC
novel transcript	-	359	350	323	163
166	271	4.114207009	3.615073376	3.573872319
2.146792763	2.41849906	2.986694372
ENSG00000259349 8.656703379	12.18119566	2.791210996	0
0	0	7.876370011	0	5.319873936
0.035949481	1	AC011921.1	17	60526293
60550798	+	718	antisense	"novel transcript,
antisense to APPBP2"	-	9	14	3	0	0
0	0.606065108	0.849693291	0.195048604	0
0	0
ENSG00000212242 19.23711862	3.480341617	1.86080733	0
0	0	8.192755855	0	5.37500225
0.0360235	1	RNA5SP219	6	136630243
136630355	+	113	rRNA_pseudogene "RNA, 5S ribosomal
pseudogene 219 [Source:HGNC Symbol;Acc:HGNC:43119]"	-	20
4	2	0	0	0	8.557615485
1.542553181	0.826223585	0	0	0
ENSG00000269534 15.3896949	0	13.02565131	0	0
0	9.471782069	0	5.584501331	0.036066334
1	AC011466.3	19	48118432	48127706
+	1009	antisense	"novel transcript, antisense
to LIG1"	-	16	0	14	0	0	0
0.766708067	0	0.647713435	0	0	0
ENSG00000136942 12312.71777	12849.42125	17570.67322

15722.11857	17751.3878	18876.12881	14244.27075
17449.8784	-0.292864226	0.036088997	0.232611482	RPL35
	9	124857880	124861981	-	1535
protein_coding	ribosomal protein L35 [Source:HGNC
Symbol;Acc:HGNC:10344]	-	12801	14768	18885	14266	14645
	20447	403.2150504	419.2488709	574.3209321
516.4233654	586.4464429	619.3729513
ENSG00000156931 907.9919988	1066.724706	962.9677935
851.8994571	729.6917348	688.6874405	979.228166
756.7595442	0.372318159	0.036089966	1	VPS8	3
	184812143	185052614	+	10366	protein_coding
	"VPS8, CORVET complex subunit [Source:HGNC
Symbol;Acc:HGNC:29122]" -	944	1226	1035	773	602
	746	4.403135052	5.153922259	4.66095791
4.143623935	3.569707625	3.346251979
ENSG00000151835 5536.442739	4330.415057	4808.326142
4439.134558	3825.427101	3870.866539	4891.727979
4045.142733	0.274081926	0.036124506	0.232611482	SACS
	13	23328826	23433740	-	15918
protein_coding	sacsin molecular chaperone [Source:HGNC
Symbol;Acc:HGNC:10519]	-	5756	4977	5168	4028	3156
	4193	17.48370626	13.6250385	15.155841
14.0608963	12.18697315	12.24806211
ENSG00000231292 9.618559309	6.09059783	7.443229322	0
	0	0	7.717462154	0	5.289626998
0.036136568	1	IGKV1OR2-108	2	113406396
113406872	+	353	IG_V_gene	immunoglobulin kappa
variable 1/OR2-108 (non-functional) [Source:HGNC
Symbol;Acc:HGNC:5767]	-	10	7	8	0	0
	0	1.369703328	0.86413567	1.057940681	0
	0	0
ENSG00000095906 1482.21999	1185.056321	1379.788635
1471.262322	1730.896673	1915.585039	1349.021649
1705.914678	-0.339135075	0.036146691	1	NUBP2	16
	1782901 1789191 +	4983	protein_coding	nucleotide
binding protein 2 [Source:HGNC Symbol;Acc:HGNC:8042]	-	1541
	1362	1483	1335	1428	2075	14.95247097
11.91090657	13.89300844	14.88683662	17.61508246
19.36235739
ENSG00000279716 41.35980503	18.27179349	43.72897226
12.12276071	2.424225033	10.15490864	34.45352359
8.233964794	2.055047535	0.036150804	1	AC006128.1
	19	15398696	15400356	+	1661	TEC
	TEC	-	43	21	47	11	2	11
	1.251699387	0.550945018	1.320913143	0.36798922
	0.074012951	0.307931467
ENSG00000101310 1639.002506	1586.165692	1503.532323
2012.378278	2193.923655	1672.790405	1576.233507
1959.697446	-0.313566571	0.036180886	1	SEC23B	20
	18507482	18561415	+	4385	protein_coding

"Sec23 homolog B, coat complex II component [Source:HGNC
Symbol;Acc:HGNC:10702]" - 1704 1823 1616 1826 1810
1812 18.78889369 18.11655716 17.20353893
23.13892717 25.3720955 19.21408085
ENSG00000117114 615.5877958 566.4255982 649.4217583
1043.65949 866.6604492 612.9872125 610.4783841
841.1023839 -0.461591057 0.036197319 1 ADGRL2 1
81306160 81992436 + 7754 protein_coding
adhesion G protein-coupled receptor L2 [Source:HGNC
Symbol;Acc:HGNC:18582] - 640 651 698 947 715
664 3.990758008 3.658590393 4.202189776
6.786349558 5.667970815 3.981743693
ENSG00000235618 8.656703379 6.09059783 8.373632987 0
0 0 7.706978065 0 5.287732549
0.036215229 1 FAM21EP 10 50021182 50067803
- 2549
transcribed_unprocessed_pseudogene "family with sequence
similarity 21 member E, pseudogene [Source:HGNC
Symbol;Acc:HGNC:45010]" - 9 7 9 0 0
0 0.170715868 0.119670416 0.16482334 0
0 0
ENSG00000198363 13596.79544 14999.40228 13309.42443
12519.50561 12288.39669 11150.08969 13968.54072
11985.99733 0.220930273 0.036234686 0.232611482 ASPH
8 61500556 61714640 - 20047
protein_coding aspartate beta-hydroxylase [Source:HGNC
Symbol;Acc:HGNC:757] - 14136 17239 14305 11360 10138
12078 34.09403186 37.4732428 33.31075741
31.48770331 31.08495598 28.01409861
ENSG00000233270 22.12268641 26.97264753 31.63372462
44.08276622 98.18111382 60.92945184 26.90968619
67.73111063 -1.329657042 0.036281938 1 SNRPEP4 19
5576660 5576938 - 279 processed_pseudogene small
nuclear ribonucleoprotein polypeptide E pseudogene 4 [Source:HGNC
Symbol;Acc:HGNC:43446] - 23 31 34 40 81
66 3.985885779 4.841903039 5.6888011
7.966504975 17.84547761 10.99944444
ENSG00000266445 13.46598303 0 14.88645864 0 0
0 9.450813892 0 5.581380149 0.036288678
1 NARF-AS1 17 82476597 82477924
- 838 antisense NARF antisense RNA 1
[Source:HGNC Symbol;Acc:HGNC:53622] - 14 0 16
0 0 0 0.807765376 0 0.891296087
0 0 0
ENSG00000204427 103.8804405 28.71281834 43.72897226
25.34759057 16.96957523 17.54029674 58.77407705
19.95248751 1.557759466 0.036347588 1 ABHD16A 6
31686949 31703444 - 4153 protein_coding
abhydrolase domain containing 16A [Source:HGNC
Symbol;Acc:HGNC:13921] - 108 33 47 23 14

19 1.257369846 0.346266603 0.528301645
0.307735748 0.207211314 0.212727036
ENSG00000187672 13.46598303 14.79145187 0 0 0
0 9.419144968 0 5.577694641 0.036356203
1 ERC2 3 55508308 56468363 -
7933 protein_coding ELKS/RAB6-interacting/CAST family
member 2 [Source:HGNC Symbol;Acc:HGNC:31922] - 14 17
0 0 0 0 0.085328045 0.093383482
0 0 0 0
ENSG00000222031 13.46598303 14.79145187 0 0 0
0 9.419144968 0 5.577694641 0.036356203
1 AC023469.1 2 151001220 151048774
- 469 lincRNA novel transcript - 14
17 0 0 0 0 1.443299328
1.579554723 0 0 0 0
ENSG00000274100 13.46598303 4.350427021 5.582421991 0
0 0 7.799610682 0 5.304423642
0.036423528 1 AL627230.1 9 67721884
67722094 - 211 unprocessed_pseudogene family with
sequence similarity 27-like (FAM27L) pseudogene - 14 5
6 0 0 0 3.208091872 1.03263335
1.327439788 0 0 0
ENSG00000271216 385.7042283 632.5520889 401.9343834
244.6593525 358.7853048 343.4205467 473.3969002
315.6217347 0.585304383 0.036425981 1 LINC01050
13 42810366 42812562 - 760
lincRNA long intergenic non-protein coding RNA 1050 [Source:HGNC
Symbol;Acc:HGNC:49044] - 401 727 432 222 296
372 25.51126516 41.68496261 26.53482272
16.23122977 23.94007348 22.75937679
ENSG00000230185 21.16083048 21.75213511 29.77291729
8.816553243 0 0 24.22862762 2.938851081
3.0508296 0.036475417 1 C9orf147 9
112433816 112487204 - 1718 antisense
chromosome 9 open reading frame 147 [Source:HGNC
Symbol;Acc:HGNC:31438] - 22 25 32 8 0
0 0.619156929 0.634125835 0.869506544
0.258749114 0 0
ENSG00000239665 465.5382706 241.013657 410.3080163
224.8221077 261.8163035 234.4860722 372.286648
240.3748278 0.630968968 0.036561235 1 AL157392.3
10 13631143 13668445 + 15012
processed_transcript novel transcript - 484 277
441 204 216 254 1.558863263 0.80408102
1.371342923 0.755098583 0.884428147 0.786730847
ENSG00000112759 1658.239625 1499.157152 1583.547038
1877.925841 2064.227615 1838.038464 1580.314605
1926.73064 -0.285749748 0.036565149 1 SLC29A1 6
44219505 44234151 + 4408 protein_coding
solute carrier family 29 member 1 (Augustine blood group)

[Source:HGNC Symbol;Acc:HGNC:11003]	-	1724	1723	1702
	1704	1703	1991	18.91023353	17.03343703
18.02453235	21.48028544	23.74763812	21.00200185
ENSG00000284738 24.04639827	11.31111026	10.23444032	0
	3.636337549	0	15.19731628	1.212112516
3.709528012	0.036582527	1	AL358472.5	1
153923337	153935240	+	1267	antisense
uncharacterized LOC101928059 [Source:NCBI gene;Acc:101928059]	-
	25	13	11	0	3	0	0.954035665
	0.447121275	0.40528647	0	0.145543227	0
ENSG00000114857 1840.992252	1380.825537	1458.872947
1422.77128	1147.870553	1049.648284	1560.230245
1206.763372	0.370758967	0.036625121	1	NKTR	3
	42600614	42648741	+	17356	protein_coding
	natural killer cell triggering receptor [Source:HGNC
Symbol;Acc:HGNC:7833]	-	1914	1587	1568	1291	947
	1137	5.332041347	3.984610573	4.217377267
4.133221163	3.353881375	3.046083761
ENSG00000100220 1654.392201	1536.570824	1574.243002
1728.044436	1997.561427	2132.530814	1588.402009
1952.712226	-0.298206524	0.036662164	1	RTCB	22
	32387582	32412255	-	2948	protein_coding
	"RNA 2',3'-cyclic phosphate and 5'-OH ligase [Source:HGNC
Symbol;Acc:HGNC:26935]" -	1720	1766	1692	1568	1648
	2310	28.20994141	26.10488701	26.79285016
29.5549768	34.36189357	36.43472692
ENSG00000273391 0	0	0	13.22482987	0
12.92442918	0	8.716419681	-5.689146057	0.036663471
	1	AC083880.1	7	139359032	139359566
	-	535	antisense	"novel transcript, antisense
to LUC7L2"	-	0	0	0	12	0	14
	0	0	0	1.246348535	0	1.216760974
ENSG00000117152 481.8898214	301.0495499	570.3374468
1041.455352	712.7221596	391.4255694	451.0922727
715.201027	-0.664502101	0.036710377	1	RGS4	1
	163068775	163076802	+	4836	protein_coding
	regulator of G protein signaling 4 [Source:HGNC
Symbol;Acc:HGNC:10000]	-	501	346	613	945	588
	424	5.009018667	3.117801089	5.917254085
10.85819308	7.473747034	4.07671717
ENSG00000268043 690.6125584	600.3589289	640.1177217
466.1752527	385.4517802	557.5968017	643.696403
469.7412782	0.452783498	0.03671215	1	NBPF12	1
	146938744	146996202	+	7272	protein_coding
	NBPF member 12 [Source:HGNC Symbol;Acc:HGNC:24297]	-
	718	690	688	423	318	604	4.773883215
	4.134793437	4.416524092	3.232202343	2.687946435
	3.862016159
ENSG00000115685 1074.393075	1049.322998	1155.561352
1472.364392	1303.020955	1308.136868	1093.092475

1361.174071	-0.316436146	0.036734026	1	PPP1R7	2
	241149576	241183652	+	3781	protein_coding
	protein phosphatase 1 regulatory subunit 7 [Source:HGNC
Symbol;Acc:HGNC:9295]	-	1117	1206	1242	1336	1075
	1417	14.28392997	13.89950161	15.33419403
19.63413733	17.47628615	17.42585881
ENSG00000207475 6.732991517	6.09059783	10.23444032	0
	0	0	7.686009888	0	5.283937231
0.036755899	1	SNORA80E	1	155919909
155920045	-	137	snoRNA	"small nucleolar RNA, H/ACA
box 80E [Source:HGNC Symbol;Acc:HGNC:32635]"	-	7	7
	11	0	0	0	2.470464908	2.226568551
	3.748160278	0	0	0
ENSG00000273165 8.656703379	7.830768638	6.512825656	0
	0	0	7.666765891	0	5.280420658
0.036763132	1	AL121652.1	2	31852976
31853423	-	448	lincRNA novel transcript	-
	9	9	7	0	0	0	0.971327561
	0.875433362	0.729400509	0	0	0
ENSG00000160803 2937.508013	2451.030584	3007.064646
3092.40605	3570.883473	3520.983778	2798.534414
3394.757767	-0.278769234	0.036788777	0.235345624	UBQLN4
	1	156035301	156053794	-	3878
protein_coding	ubiquilin 4 [Source:HGNC Symbol;Acc:HGNC:1237]	-
	3054	2817	3232	2806	2946	3814	38.07697549
	31.65465904	38.90537298	40.20609603	46.69520625
	45.73028438
ENSG00000170385 857.0136345	937.0819804	878.3010599
975.3312025	1586.655284	1027.49212	890.7988916
1196.492869	-0.424736564	0.036831835	1	SLC30A1 1
	211571568	211578742	-	5474	protein_coding
	solute carrier family 30 member 1 [Source:HGNC
Symbol;Acc:HGNC:11012]	-	891	1077	944	885	1309
	1113	7.869989038	8.57372418	8.050321727
8.983602375	14.69881127	9.454126072
ENSG00000164211 366.4671097	557.7247441	547.0773551
1006.189139	621.8137209	537.2869844	490.4230696
721.7632814	-0.55678949	0.036864961	1	STARD4	5
	111496033	111512590	-	5684	protein_coding
	StAR related lipid transfer domain containing 4 [Source:HGNC
Symbol;Acc:HGNC:18058]	-	381	641	588	913	513
	582	3.240948447	4.914310104	4.829134402
8.925421854	5.547670468	4.761019207
ENSG00000197933 102.9185846	87.87862583	93.04036652
191.760033	162.4230772	136.6296799	94.61252565
163.6042634	-0.789612794	0.037009969	1	ZNF823	19
	11721265	11739009	-	2459	protein_coding
	zinc finger protein 823 [Source:HGNC Symbol;Acc:HGNC:30936]
	zf-C2H2 107	101	100	174	134	148
2.103906646	1.78986981	1.898399047	3.93190271

3.34960526	2.798557309
ENSG00000235109 93.3000253	69.60683234	66.05866023
122.3296763	173.3320898	125.5515977	76.32183929
140.4044546	-0.878799231	0.037010185	1	ZSCAN31 6
28324693	28356271	-	4957	protein_coding
zinc finger and SCAN domain containing 31 [Source:HGNC
Symbol;Acc:HGNC:14097]	zf-C2H2 97	80	71	111	143
136	0.946137012	0.703282265	0.668629395
1.244274221	1.773227585	1.275707203
ENSG00000151725 456.8815672	775.2460952	597.3191531
889.3698084	831.5091862	790.2365269	609.8156051
837.0385072	-0.456212487	0.037022254	1	CENPU	4
184694618	184734133	-	2953	protein_coding
centromere protein U [Source:HGNC Symbol;Acc:HGNC:21348]
-	475	891	642	807	686	856
7.777345262	13.1483984	10.1488683	15.18525647
14.27933653	13.47849295
ENSG00000105669 2778.801785	2019.468223	2842.383197
2665.905287	3387.854483	3648.381722	2546.884402
3234.047164	-0.344888428	0.037027044	0.235872734	COPE
19	18899514	18919397	-	2885
protein_coding	coatomer protein complex subunit epsilon [Source:HGNC
Symbol;Acc:HGNC:2234]	-	2889	2321	3055	2419	2795
3952	48.41756462	35.05806329	49.43237
46.59100671	59.5502179	63.69452989
ENSG00000274756 0	10.44102485	17.67766964	0	0
0	9.372898163	0	5.570805521	0.037031488
1	AC243732.1	17	36574462	36575325
-	864	processed_pseudogene	ribosomal protein S2
(RPS2) pseudogene	-	0	12	19	0	0
0	0	0.60523788	1.026563679	0	0
0
ENSG00000065802 2217.077921	1887.215242	2166.910136
1679.553393	1700.59386	1770.646797	2090.4011
1716.93135	0.283673294	0.03707735	1	ASB1	2
238426742	238452250	+	7410	protein_coding
ankyrin repeat and SOCS box containing 1 [Source:HGNC
Symbol;Acc:HGNC:16011]	-	2305	2169	2329	1524	1403
1918	15.04021132	12.7555721	14.67227021
11.42822554	11.63822699	12.0354245
ENSG00000159433 716.5826686	408.94014	489.3923279
461.7669761	338.179392	275.1057068	538.3050455
358.3506917	0.587485584	0.037125106	1	STARD9	15
42575659	42720981	+	20706	protein_coding
StAR related lipid transfer domain containing 9 [Source:HGNC
Symbol;Acc:HGNC:19162]	-	745	470	526	419	279
298	1.739647589	0.989145651	1.185865871
1.124423353	0.828238384	0.669192512
ENSG00000138356 1299.467363	1214.639224	1387.231865
2208.546587	1362.414468	1536.160725	1300.446151

1702.373927	-0.388649179	0.037170402	1	AOX1	2
	200585868	200677064	+	5556	protein_coding
	aldehyde oxidase 1 [Source:HGNC Symbol;Acc:HGNC:553]	-
	1351	1396	1491	2004	1124	1664	11.75694072
	10.94918463	12.52741435	20.0422984	12.43516163
	13.92586251
ENSG00000275468 31.74124572	4.350427021	0	0	0
	0.923173513	12.03055758	0.307724504	4.967540765
	0.037189181	1	AC004678.1	19	2013728
2014656 -	929	processed_pseudogene	novel pseudogene
	-	33	5	0	0	0	1
1.717510664	0.234537822	0	0	0	0.050051293
ENSG00000084623 8986.619963	8683.452334	8560.644123
9433.71197	10490.83383	10506.63775	8743.57214
10143.72785	-0.214319079	0.037247611	0.235872734	EIF3I
	1	32221928	32231604	+	1725
protein_coding	eukaryotic translation initiation factor 3 subunit I
[Source:HGNC Symbol;Acc:HGNC:3272]	-	9343	9980	9201
	8560	8655	11381	261.8776686	252.1157861
248.9957688	275.7380557	308.4077856	306.7766793
ENSG00000149547 3794.521648	3797.92279	3142.903581
3957.530337	4345.423371	4618.637084	3578.449339
4307.196931	-0.267507101	0.037256587	0.235872734	EI24
	11	125569216	125584687	+	3558
protein_coding	"EI24, autophagy associated transmembrane protein
[Source:HGNC Symbol;Acc:HGNC:13276]"	-	3945	4365	3378
	3591	3585	5003	53.60956463	53.46097833
44.31999853	56.08174328	61.93420631	65.38160496
ENSG00000275708 28.85567793	18.27179349	2.791210996	0
	0	3.692694051	16.6395608	1.230898017
3.691038164	0.037338292	1	MIR3648-1	21
8208473 8208652 +	180	miRNA	microRNA 3648-1 [Source:HGNC
Symbol;Acc:HGNC:38941]	-	30	21	3	0	0
	4	8.058421249	5.083998191	0.778027209	0
	0	1.033281144
ENSG00000264187 0	0	0	0	32.72703794	0
	0	10.90901265	-6.00690402	0.037362604	1
	AC055811.2	17	17202649	17237185	-
	1693	protein_coding	novel transcript	-	0
	0	0	0	27	0	0	0	0
	0	0.980289083	0
ENSG00000225216 0	13.05128106	14.88645864	0	0
	0	9.312579902	0	5.56170657	0.037482817
	1	AC007362.1	2	205756469	205764006
	-	1623	antisense	novel transcript	-
	0	15	16	0	0	0	0
0.402746094	0.460200937	0	0	0
ENSG00000076242 598.274389	790.9076325	833.641684
1247.542284	951.5083253	817.9317323	740.9412352
1005.660781	-0.440099233	0.037543519	1	MLH1	3

36993332 37050918 + 3532 protein_coding
mutL homolog 1 [Source:HGNC Symbol;Acc:HGNC:7127] -
622 909 896 1132 785 886 8.514730493
11.21506477 11.84222615 17.8089279 13.66143506
11.66390692
ENSG00000256616 4.809279655 9.570939447 0.930403665
31.96000551 21.81802529 24.92568484 5.103540922
26.23457188 -2.356917038 0.037571934 1 AP002414.2
18 12073232 12076654 - 3423
transcribed_processed_pseudogene G protein-coupled receptor 125
(GPR125) pseudogene - 5 11 1 29 18
27 0.070625953 0.140037511 0.013637637
0.47076389 0.32323097 0.366764999
ENSG00000272078 0 0 0 0 0 32.31107295
0 10.77035765 -5.996070587 0.037611741 1
AL139423.1 1 10639241 10654333 +
7923 antisense "novel transcript, antisense to CASZ1"
- 0 0 0 0 0 35 0
0 0 0 0 0.205404241
ENSG00000143418 3883.012393 3650.008271 3569.02846
4028.062763 4768.450639 4383.227838 3700.683041
4393.24708 -0.247421538 0.037644222 0.23652823 CERS2
1 150960583 150975004 - 4644
protein_coding ceramide synthase 2 [Source:HGNC
Symbol;Acc:HGNC:14076] - 4037 4195 3836 3655 3934
4748 42.03080954 39.36392103 38.5595914
43.73279294 52.07023104 47.53894258
ENSG00000153774 930.1146852 807.4392551 923.8908395
739.4884033 647.2680837 674.8398378 887.14826
687.1987749 0.368184561 0.037647237 1 CFDP1 16
75293698 75433485 - 3317 protein_coding
craniofacial development protein 1 [Source:HGNC
Symbol;Acc:HGNC:1873] - 967 928 993 671 534
731 14.09555625 12.19161115 13.97493552
11.24058961 9.8956231 10.24714599
ENSG00000229932 6.732991517 6.09059783 17.67766964
27.55172889 31.51492542 52.62089023 10.16708633
37.22918151 -1.875698323 0.037698015 1 YWHAZP3 10
23136924 23137661 + 738
processed_pseudogene tyrosine 3-monooxygenase/tryptophan 5-
monooxygenase activation protein zeta pseudogene 3 [Source:HGNC
Symbol;Acc:HGNC:31101] - 7 7 19 25 26
57 0.458609339 0.413333186 1.201830648
1.882329682 2.165530707 3.591282026
ENSG00000113328 551.1434484 947.5230052 707.1067855
1019.413969 1279.990817 793.9292209 735.2577464
1031.111336 -0.486659141 0.037725793 1 CCNG1 5
163437569 163446151 + 3096 protein_coding
cyclin G1 [Source:HGNC Symbol;Acc:HGNC:1592] - 573
1089 760 925 1056 860 8.948595689

15.32800119	11.45931549	16.60171004	20.96574631
12.91601431
ENSG00000092820 2075.685099	2395.345118	2719.569913
3211.429519	2867.858214	2745.518027	2396.86671
2941.60192	-0.295291481	0.037744186	0.23652823	EZR
	6	158765741	158819412	-	3342
protein_coding	ezrin [Source:HGNC Symbol;Acc:HGNC:12691]	-
	2158	2753	2923	2914	2366	2974	31.22095701
	35.89701724	40.82896827	48.45015218	43.51667002
	41.37762285
ENSG00000131043 3145.268894	2666.811764	2836.800775
2771.703926	3934.517228	4157.973501	2882.960478
3621.398218	-0.329189284	0.037746608	0.23652823	AAR2
	20	36236459	36270918	+	3101
protein_coding	AAR2 splicing factor homolog [Source:HGNC
Symbol;Acc:HGNC:15886]	-	3270	3065	3049	2515	3246
	4504	50.98556108	43.07123358	45.89883833
45.06592263	64.34193379	67.53480244
ENSG00000267508 117.3464236	100.9299069	117.2308618
60.61380355	72.72675098	27.69520538	111.8357308
53.67858664	1.064608426	0.037810559	1	ZNF285	19
	44382298	44401608	-	6866	protein_coding
	zinc finger protein 285 [Source:HGNC Symbol;Acc:HGNC:13079]
	zf-C2H2 122	116	126	55	60	30
0.859126763	0.736228776	0.856668468	0.445113672
0.537149049	0.203164804
ENSG00000090889 724.277516	811.7896822	667.0994279
1469.058184	911.5086123	728.3839015	734.3888754
1036.316899	-0.496287733	0.037818245	1	KIF4A	X
	70290090	70420832	+	4533	protein_coding
	kinesin family member 4A [Source:HGNC Symbol;Acc:HGNC:13339]
	-	753	933	717	1333	752	789
8.031755394	8.969216815	7.38379231	16.34016637
10.19716582	8.093237819
ENSG00000232850 6.732991517	9.570939447	6.512825656	0
	0	0	7.60558554	0	5.269236842
0.037875096	1	PTGES2-AS1	9	128128529
128130628	+	2100	lincRNA	PTGES2 antisense RNA 1 (head
to head) [Source:NCBI gene;Acc:389791]	-	7	11	7
	0	0	0	0.161168425	0.228261143
0.155605442	0	0	0
ENSG00000268182 4.809279655	6.960683234	11.16484398	0
	0	0	7.644935624	0	5.27645934
0.037876516	1	SMIM17	19	56643145	56655766
	+	1076	protein_coding	small integral membrane
protein 17 [Source:HGNC Symbol;Acc:HGNC:27114]	-	5	8
	12	0	0	0	0.224677172	0.323993512
	0.520613002	0	0	0
ENSG00000095066 360.6959741	341.0734785	398.2127687
218.2096928	317.5734793	201.2518258	366.6607404

245.6783326	0.579800633	0.037903188	1	HOOK2	19
	12763003	12872740	-	5950	protein_coding
	hook microtubule tethering protein 2 [Source:HGNC
Symbol;Acc:HGNC:19885]	-	375	392	428	198	262
	218	3.047302153	2.870963684	3.357939283
1.849099445	2.706647417	1.703611433
ENSG00000100280 865.6703379	858.774294	974.1326375
1054.680182	1021.810851	1443.843374	899.5257564
1173.444802	-0.38446898	0.037937133	1	AP1B1	22
	29327680	29423179	-	6721	protein_coding
	adaptor related protein complex 1 subunit beta 1 [Source:HGNC
Symbol;Acc:HGNC:554]	-	900	987	1047	957	843
	1564	6.4745536	6.399438283	7.272082917
7.912069364	7.709763204	10.8201647
ENSG00000239672 1471.639574	1242.481957	1424.448011
1559.427855	1812.108212	1751.260154	1379.523181
1707.59874	-0.307939831	0.037975933	1	NME1	17
	51153536	51162428	+	3438	protein_coding
	NME/NM23 nucleoside diphosphate kinase 1 [Source:HGNC
Symbol;Acc:HGNC:7849]	-	1530	1428	1531	1415	1495
	1897	21.51725045	18.10009827	20.78812666
22.86981288	26.72899812	25.65620852
ENSG00000273416 13.46598303	0	4.652018326	41.87862791
	39.99971304	16.61712323	6.039333786	32.83182139
	-2.448354678	0.03799812	1	AL732292.2	1
	235104180	235104609	-	430	lincRNA novel
transcript	-	14	0	5	38	33	18
	1.574203221	0	0.542809681	4.910516613
4.717292919	1.946413319
ENSG00000184995 55.78764399	35.67350157	66.05866023
12.12276071	16.96957523	25.84885836	52.50660193
18.31373143	1.513681882	0.038028994	1	IFNE	9
	21480839	21482313	-	1475	protein_coding
	interferon epsilon [Source:HGNC Symbol;Acc:HGNC:18163]	-
	58	41	71	11	14	28	1.901241081
	1.211296421	2.247048076	0.414393284	0.583422771
	0.882667283
ENSG00000115738 162.5536523	154.875202	143.2821644
224.8221077	192.7258901	322.1875559	153.5703396
246.5785179	-0.68517672	0.038083542	1	ID2	2
	8678845 8684453 +	2205	protein_coding	inhibitor of
DNA binding 2 [Source:HGNC Symbol;Acc:HGNC:5361]	bHLH	169
	178	154	204	159	349	3.705777391
3.51779078	3.260304496	5.140834435	4.432368816
7.359492233
ENSG00000102032 20.19897455	63.51623451	39.07695394
16.53103733	1.212112516	12.00125567	40.930721
9.914801838	2.037765024	0.038132811	1	RENBP	X
	153935263	153944691	-	2057	protein_coding
	renin binding protein [Source:HGNC Symbol;Acc:HGNC:9959]

- 21 73 42 15 1 13
0.49361258 1.546490178 0.953149522 0.405199603
0.029882234 0.293859732
ENSG00000187961 567.4949993 423.7315919 701.5243636
348.2538531 379.3912176 454.2013683 564.2503182
393.948813 0.517184847 0.038139327 1 KLHL17 1
960587 965715 + 3395 protein_coding kelch like
family member 17 [Source:HGNC Symbol;Acc:HGNC:24023] - 590
487 754 316 313 492 8.402595352
6.250975264 10.36758496 5.172009902 5.666983121
6.738393089
ENSG00000186326 12.5041271 9.570939447 50.24179792
6.612414933 3.636337549 0 24.10562149 3.416250827
2.836844796 0.038249439 1 RGS9BP 19
32675407 32678300 + 2894 protein_coding
regulator of G protein signaling 9 binding protein [Source:HGNC
Symbol;Acc:HGNC:30304] - 13 11 54 6 3
0 0.217193109 0.165635246 0.871046357
0.11520326 0.063719167 0
ENSG00000203280 0 15.66153728 12.09524765 0 0
0 9.252261641 0 5.552547875 0.038265882
1 AL022323.1 22 25102433 25112692
- 957 antisense uncharacterized LOC100128531
[Source:NCBI gene;Acc:100128531] - 0 18 13
0 0 0 0 0.819632489 0.63412876
0 0 0
ENSG00000204625 23.08454234 5.220512425 0 0 0
0 9.435018256 0 5.579001901 0.038271038
1 HCG9 6 29975112 29978410 +
700 lincRNA HLA complex group 9 [Source:HGNC
Symbol;Acc:HGNC:21243] - 24 6 0 0 0
0 1.657732371 0.373518234 0 0 0
0
ENSG00000231058 23.08454234 5.220512425 0 0 0
0 9.435018256 0 5.579001901 0.038271038
1 MSANTD2P1 21 23101223 23103074
+ 1368 processed_pseudogene Myb/SANT DNA binding
domain containing 2 pseudogene 1 [Source:HGNC Symbol;Acc:HGNC:39637]
- 24 6 0 0 0 0
0.848254868 0.191127752 0 0 0 0
ENSG00000115128 1577.443727 1811.517812 1831.034413
1817.312037 2204.832667 2506.416087 1739.99865
2176.186931 -0.322956674 0.038326048 1 SF3B6 2
24067584 24076443 - 1197 protein_coding
splicing factor 3b subunit 6 [Source:HGNC
Symbol;Acc:HGNC:30096] - 1640 2082 1968 1649 1819
2715 66.2446659 75.79580547 76.74975177
76.54882854 93.40839479 105.464598
ENSG00000140988 35199.11779 37969.65696 45075.26637
41087.34225 47849.35369 52626.42927 39414.68037

47187.7084	-0.259694064	0.03840714	0.239012647	RPS2
16	1962052 1964860 -	2413	protein_coding
ribosomal protein S2 [Source:HGNC Symbol;Acc:HGNC:10404]	-
36595	43639	48447	37282	39476	57006	733.272919
788.090452	937.2503325	858.5269326	1005.59517
1098.485338
ENSG00000157353 394.3609317	472.4563745	471.7146583
155.3917509	368.482205	330.4961176	446.1773215
284.7900245	0.647904915	0.038411371	1	FUK	16
70454421	70480274	+	7405	protein_coding
fucokinase [Source:HGNC Symbol;Acc:HGNC:29500]	-	410
543	507	141	304	358	2.677071745
3.195459845	3.196163093	1.058049761	2.523456839
2.247962085
ENSG00000277452 10.58041524	1.740170808	11.16484398	0
0	0	7.828476677	0	5.309640903
0.038422234	1	RN7SL473P	1	150566562
150566860	-	299	misc_RNA	"RNA, 7SL, cytoplasmic
473, pseudogene [Source:HGNC Symbol;Acc:HGNC:46489]"	-	11
2	12	0	0	0	1.778781948
0.291485802	1.873510337	0	0	0
ENSG00000177954 7837.202125	11831.42133	11288.58767
10984.32327	13344.14669	14216.8721	10319.07037
12848.44735	-0.316268718	0.03844462	0.239012647	RPS27
1	153990755	153992155	+	1035
protein_coding	ribosomal protein S27 [Source:HGNC
Symbol;Acc:HGNC:10416]	-	8148	13598	12133	9967	11009
15400	380.6377759	572.5234568	547.235022
535.1014799	653.8150031	691.8491141
ENSG00000171790 53.86393213	52.20512425	36.28574294
27.55172889	9.69690013	6.462214589	47.45159978
14.5702812	1.707641167	0.038463875	1	SLFNL1	1
41015597	41023237	-	4310	protein_coding
schlafen like 1 [Source:HGNC Symbol;Acc:HGNC:26313]	-
56	60	39	25	8	7	0.628220311
0.606642144	0.422409204	0.322310744	0.114093283
0.075518227
ENSG00000184786 95.22373716	40.894014	46.52018326
19.8372448	21.81802529	26.77203187	60.87931147
22.80910065	1.413244632	0.038470312	1	TCTE3	6
169740114	169751587	-	2626	protein_coding
t-complex-associated-testis-expressed 3 [Source:HGNC
Symbol;Acc:HGNC:11695]	-	99	47	50	18	18
29	1.822811204	0.779940969	0.888835349
0.380881455	0.421332678	0.513492724
ENSG00000268163 16.35155083	4.350427021	2.791210996	0
0	0	7.831062948	0	5.310070029
0.038473518	1	AC004076.1	19	57437970
57477536	-	1028	protein_coding	novel transcript
-	17	5	3	0	0	0

0.79957098	0.211951009	0.136230445	0	0	0
ENSG00000260751 0	2.610256213	6.512825656	31.96000551
	14.5453502	17.54029674	3.04102729	21.34855081
	-2.809118841	0.038533615	1	AC008870.2	16
	23568673	23569696	+	1024	sense_intronic
	novel transcript	-	0	3	7	29
	12	19	0	0.127667365	0.319112723
1.573657025	0.720325268	0.862749393
ENSG00000210082 222960.1285	189874.3873	188540.7203
213135.7664	286779.7608	420740.9443	200458.4121
306885.4905	-0.614400267	0.038533629	0.239012647	MT-
RNR2	MT	1671	3229	+	1559	Mt_rRNA
mitochondrially encoded 16S RNA [Source:HGNC Symbol;Acc:HGNC:7471]
	-	231802	218225	202644	193396	236595	455755
7189.062844	6099.81951	6067.833706	6893.081538
9328.39242	13593.03217
ENSG00000124217 585.7702619	627.3315765	740.6013175
662.3435624	924.8418499	1077.343489	651.2343853
888.1763006	-0.448190537	0.038574685	1	MOCS3	20
	50958826	50963931	+	5106	protein_coding
	molybdenum cofactor synthesis 3 [Source:HGNC
Symbol;Acc:HGNC:15765]	-	609	721	796	601	763
	1167	5.766837298	6.153370314	7.277434296
6.540421013	9.185252251	10.62725407
ENSG00000100299 182.7526269	58.29572208	86.52754086
58.40966524	58.18140078	12.92442918	109.1919633
43.17183173	1.343863304	0.038598041	1	ARSA	22
	50622754	50628173	-	2932	protein_coding
	arylsulfatase A [Source:HGNC Symbol;Acc:HGNC:713]	-
	190	67	93	53	48	14	3.133219722
	0.995793838	1.480692984	1.00443988	1.006293417
	0.222021528
ENSG00000254858 252.0062539	244.4939986	186.080733
349.3559223	352.7247422	306.4936062	227.5269952
336.1914236	-0.562626192	0.038843877	1	MPV17L2 19
	18193182	18196948	+	2135	protein_coding
	MPV17 mitochondrial inner membrane protein like 2 [Source:HGNC
Symbol;Acc:HGNC:28177]	-	262	281	200	317	291
	332	5.933413678	5.735443928	4.372986656
8.250370018	8.378040778	7.230548149
ENSG00000042062 25.97011014	12.18119566	28.84251362
3.306207466	2.424225033	6.462214589	22.33127314
4.064215696	2.441458987	0.038950609	1	RIPOR3	20
	50586108	50691528	-	6009	protein_coding
	RIPOR family member 3 [Source:HGNC Symbol;Acc:HGNC:16168]
	-	27	14	31	3	2	7
0.217251496	0.101527672	0.240827194	0.027741574
0.020458564	0.054166011
ENSG00000197980 39.43609317	29.58290374	29.77291729
15.42896818	3.636337549	5.539041076	32.93063807

8.201448934 2.005164592 0.038979921 1 LEKR1 3
156825481 157046129 + 5602 protein_coding
"leucine, glutamate and lysine rich 1 [Source:HGNC
Symbol;Acc:HGNC:33765]" - 41 34 32 14 3
6 0.353868552 0.264480959 0.266656951
0.138866335 0.032917399 0.049801126
ENSG00000064687 50.01650841 64.38631991 60.47623824
28.65379804 15.75746271 0 58.29302219 14.80375358
1.986353066 0.039059452 1 ABCA7 19
1040101 1065572 + 8367 protein_coding ATP binding cassette
subfamily A member 7 [Source:HGNC Symbol;Acc:HGNC:37] - 52
74 65 26 13 0 0.300493299
0.385407843 0.362651621 0.172669497 0.095503864 0
ENSG00000108666 586.7321179 646.4734554 530.3300892
476.0938751 435.1483934 392.3487429 587.8452208
434.5303371 0.436733646 0.039083454 1 C17orf75
17 32324565 32350023 - 5740
protein_coding chromosome 17 open reading frame 75 [Source:HGNC
Symbol;Acc:HGNC:30173] - 610 743 570 432 359
425 5.138296476 5.640732687 4.635632501
4.181998744 3.844411931 3.442770364
ENSG00000232059 17.31340676 10.44102485 7.443229322 0
0 1.846347025 11.73255364 0.615449008
4.160459301 0.039092761 1 AL451007.1 1
244694432 244694720 - 289 processed_pseudogene
ribosomal protein L37 (RPL37) pseudogene - 18
12 8 0 0 2 3.011451539
1.809430894 1.292225123 0 0 0.321783055
ENSG00000134871 2496.977997 1367.774255 2261.81131
1578.163031 1612.109647 1430.918945 2042.187854
1540.397207 0.406783683 0.039178541 1 COL4A2 13
110305812 110513209 + 19293 protein_coding
collagen type IV alpha 2 chain [Source:HGNC
Symbol;Acc:HGNC:2203] - 2596 1572 2431 1432 1330
1550 6.505881375 3.550678702 5.88208411
4.12434795 4.237397801 3.735622237
ENSG00000285781 0 0 0 0 10.90901265
14.7707762 0 8.559929617 -5.661805897 0.039243802
1 AL117340.1 10 31362799 31364049
- 1251 unprocessed_pseudogene putative UPF0607
protein FLJ37424 [Source:NCBI gene;Acc:100505502] - 0
0 0 0 9 16 0 0 0
0 0.442214074 0.594694184
ENSG00000166133 398.2083554 310.6204893 380.5350991
337.2331616 633.934846 648.0678059 363.1213146
539.7452712 -0.572379789 0.039273259 1 RPUSD2 15
40569300 40574943 + 2482 protein_coding
RNA pseudouridylate synthase domain containing 2 [Source:HGNC
Symbol;Acc:HGNC:24180] - 414 357 409 306 523
702 8.064914739 6.267942976 7.692501094

6.850648628   12.95231124   13.15122939
ENSG00000242020 5.771135586   11.31111026   5.582421991   0
        0   0   7.554889277   0   5.259894227
0.039290984   1   RN7SL68P   5   141479316
141479625   +   310   misc_RNA   "RNA, 7SL, cytoplasmic
68, pseudogene [Source:HGNC Symbol;Acc:HGNC:46084]"   -   6
        13   6   0   0   0   0.935816661
1.827427921   0.903515469   0   0   0
ENSG00000084636 442.4537282   218.3914365   485.6707132
324.0083317   208.4833528   154.1699766   382.1719593
228.8872204   0.74025701   0.039313154   1   COL16A1 1
        31652247   31704319   -   9269   protein_coding
        collagen type XVI alpha 1 chain [Source:HGNC
Symbol;Acc:HGNC:2193]   -   460   251   522   294   172
        167   2.3995299   1.180047358   2.628958053
1.762489311   1.140625102   0.837750329
ENSG00000140265 639.6341941   517.7008155   597.3191531
355.9683372   512.7235944   398.8109575   584.8847209
422.500963   0.469978812   0.039405954   1   ZSCAN29 15
        43358172   43371025   -   6634   protein_coding
        zinc finger and SCAN domain containing 29 [Source:HGNC
Symbol;Acc:HGNC:26673] zf-C2H2 665   595   642   323   423
        432   4.846714016   3.908409824   4.517577344
2.705447426   3.919333868   3.027884451
ENSG00000131437 680.9939991   588.1777333   543.3557405
425.398694   339.3915046   530.8247698   604.1758243
431.8716561   0.482456784   0.039454818   1   KIF3A   5
        132692628   132737638   -   6860   protein_coding
        kinesin family member 3A [Source:HGNC Symbol;Acc:HGNC:6319]
        -       708   676   584   386   280   575
4.99011275   4.294189226   3.97406318   3.126620943
2.508888009   3.897397903
ENSG00000115307 2527.757387   2002.066515   2526.045951
2625.128728   2900.585252   3124.942341   2351.956618
2883.552107   -0.294306047   0.039456747   0.243914435   AUP1
        2   74526645   74529939   -   2634
protein_coding "AUP1, lipid droplet regulating VLDL assembly factor
[Source:HGNC Symbol;Acc:HGNC:891]"   -   2628   2301   2715
        2382   2393   3385   48.24038962   38.06794611
48.11717251   50.25022725   55.84371322   59.75495457
ENSG00000270872 100.9948727   98.31965068   89.31875186
39.6744896   49.69661317   53.54406374   96.21109176
47.63838883   1.013076037   0.039486132   1   SRGAP2D 1
        143975087   144068350   +   1021
unprocessed_pseudogene SLIT-ROBO Rho GTPase activating protein 2D
(pseudogene) [Source:HGNC Symbol;Acc:HGNC:43932]   -   105
        113   96   36   41   58   4.972385295
4.822933782   4.389262219   1.959245249   2.468342806
2.641394502
ENSG00000139697 2095.884074   1874.163961   1690.54346

1810.699622	1208.476179	1395.838351	1886.863831
1471.671384	0.358335266	0.039528545	1	SBNO1	12
	123289109	123364843	-	11124	protein_coding
	strawberry notch homolog 1 [Source:HGNC Symbol;Acc:HGNC:22973]
	-	2179	2154	1817	1643	997	1512
9.471035545	8.438073743	7.625002369	8.207079246
5.509111197	6.320069136
ENSG00000127948 1506.266388	1196.367431	1555.634928
1497.711982	2098.166766	1846.347025	1419.422916
1814.075258	-0.353937166	0.03955953	1	POR	7
	75899200	75986855	+	6112	protein_coding
	cytochrome p450 oxidoreductase [Source:HGNC
Symbol;Acc:HGNC:9208]	-	1566	1375	1672	1359	1731
	2000	12.38824052	9.803427701	12.77023718
12.35515377	17.4084892	15.2152001
ENSG00000257243 65.4062033	26.10256213	10.23444032
12.12276071	6.060562582	0	33.91440192	6.061107764
	2.496508388	0.039663543	1	AC020612.1	12
	49595148	49595688	+	541
processed_pseudogene	Protein DJ-1 (PARK7) pseudogene -	68
	30	11	11	5	0	6.077330627
2.416476563	0.949164433	1.129815331	0.568093865	0
ENSG00000207034 21.16083048	2.610256213	0.930403665	0
	0	0	8.23383012	0	5.382082312
0.039704579	1	RF00019 18	2778941 2779057 +	117
	misc_RNA		-	22	3	1	0
	0	0	9.091552178	1.11736224	0.398988312
	0	0	0
ENSG00000130224 8.656703379	8.700854042	10.23444032	0
	0	0.923173513	9.197332579	0.307724504
4.581253048	0.039739456	1	LRCH2	X	115110616
	115234072	-	4929	protein_coding	leucine rich
repeats and calponin homology domain containing 2 [Source:HGNC
Symbol;Acc:HGNC:29292]	-	9	10	11	0	0
	1	0.088284591	0.088409672	0.104178932	0
	0	0.009433486
ENSG00000184319 268.3578047	380.2273217	348.9013744
512.4621573	489.6934566	412.6585602	332.4955003
471.6047247	-0.503117732	0.039739606	1	RPL23AP82
	22	50756948	50801309	+	4250
transcribed_unprocessed_pseudogene	ribosomal protein L23a
pseudogene 82 [Source:HGNC Symbol;Acc:HGNC:33730]	-	279
	437	375	465	404	447	3.174069922
4.480754036	4.118967578	6.079614841	5.843052591
4.890458875
ENSG00000131143 7469.77316	7328.72936	8336.41684
8089.187601	9793.869132	9439.449168	7711.639787
9107.501967	-0.240021186	0.039805537	0.245244849	COX4I1
	16	85798633	85807044	+	4792
protein_coding	cytochrome c oxidase subunit 4I1 [Source:HGNC

Symbol;Acc:HGNC:2265]	-	7766	8423	8960	7340	8080
	10225	78.35772047	76.59644067	87.28452164
85.11209766	103.6434621	99.21504311
ENSG00000256188 17.31340676	0	10.23444032	0	0
	0	9.182615691	0	5.539667227	0.03982798
	1	TAS2R30 12	11132958	11134644	-
	1687	protein_coding	taste 2 receptor member 30
[Source:HGNC Symbol;Acc:HGNC:19112]	-	18	0	11
	0	0	0	0.515891817	0	0.304385275
	0	0	0
ENSG00000158636 824.3105328	634.2922597	565.6854284
547.7283702	471.5117688	463.4331034	674.7627403
494.2244142	0.449165878	0.03985233	1	EMSY	11
	76444923	76553025	+	11783	protein_coding
	"EMSY, BRCA2 interacting transcriptional repressor
[Source:HGNC Symbol;Acc:HGNC:18071]"	-	857	729	608
	497	389	502	3.516625822	2.696064316
2.408761147	2.343756852	2.029275835	1.980974374
ENSG00000114841 595.3888213	381.9674925	544.2861441
185.1476181	220.604478	516.9771671	507.2141526
307.5764211	0.718741829	0.039863008	1	DNAH1	3
	52316319	52400491	+	13872	protein_coding
	dynein axonemal heavy chain 1 [Source:HGNC
Symbol;Acc:HGNC:2940]	-	619	439	585	168	182
	560	2.157509841	1.379062782	1.96862421
0.672949144	0.806454126	1.877067823
ENSG00000261616 15.3896949	0	12.09524765	0	0
	0	9.161647514	0	5.536445078	0.039865047
	1	AC036108.3	15	99139317	99145370
	+	2677	antisense	"novel transcript, antisense
to TTC23"	-	16	0	13	0	0	0
	0.288983354	0	0.226694517	0	0	0
ENSG00000144034 491.5083807	494.2085096	408.447209
582.9945832	569.6928827	739.4619837	464.7213664
630.7164832	-0.441519358	0.039894979	1	TPRKB	2
	73729104	73737400	-	2047	protein_coding
	TP53RK binding protein [Source:HGNC Symbol;Acc:HGNC:24259]
	-	511	568	439	529	470	801
12.06991673	12.09174809	10.01135158	14.359849
14.11326108	18.1947332
ENSG00000227372 629.0537788	555.1144879	659.6561986
393.4386885	471.5117688	500.3600439	614.6081551
455.1035004	0.432851731	0.039915775	1	TP73-AS1
	1	3735511 3747373 -	9701
transcribed_unitary_pseudogene	TP73 antisense RNA 1 [Source:HGNC
Symbol;Acc:HGNC:29052]	-	654	638	709	357	389
	542	3.259586123	2.865911478	3.411738736
2.044860826	2.464793029	2.597848378
ENSG00000142208 1765.005633	2021.208394	1929.657202
2522.636297	2260.589843	2154.686979	1905.29041

2312.637706 -0.279261781 0.039928007 1 AKT1 14
104769349 104795751 - 11162 protein_coding
AKT serine/threonine kinase 1 [Source:HGNC
Symbol;Acc:HGNC:391] - 1835 2323 2074 2289 1865
2334 7.94868464 9.069133385 8.673867221
11.39503906 10.27032479 9.722766404
ENSG00000176014 3677.175224 5009.951758 5147.923479
5823.333417 5382.991685 5668.285368 4611.683487
5624.870157 -0.286477529 0.039944805 0.245279808 TUBB6
18 12307669 12344320 + 4117
protein_coding tubulin beta 6 class V [Source:HGNC
Symbol;Acc:HGNC:20776] - 3823 5758 5533 5284 4441
6140 44.89775725 60.94658715 62.73730214
71.31715101 66.30515069 69.34553806
ENSG00000153904 451.1104316 416.7709086 481.9490986
862.9201487 539.3900698 511.4381261 449.9434796
637.9161148 -0.50340696 0.039981513 1 DDAH1 1
85318481 85578363 - 4568 protein_coding
dimethylarginine dimethylaminohydrolase 1 [Source:HGNC
Symbol;Acc:HGNC:2715] - 469 479 518 783 445
554 4.964185069 4.569492995 5.293582676
9.524621155 5.987992889 5.639163514
ENSG00000100605 827.1961006 838.7623297 777.8174641
958.8001652 1043.628877 1081.959357 814.5919648
1028.129466 -0.336021592 0.040050373 1 ITPK1 14
92936914 93116320 - 6783 protein_coding
inositol-tetrakisphosphate 1-kinase [Source:HGNC
Symbol;Acc:HGNC:6177] - 860 964 836 870 861
1172 6.130245267 6.193181596 5.753478522
7.127044643 7.802408684 8.034092224
ENSG00000280062 0 0 0.930403665 0 0
38.77328753 0.310134555 12.92442918 -5.297719116
0.04007149 1 AL031719.1 16 1530715 1533301 +
2587 TEC TEC - 0 0 1 0
0 42 0 0 0.018044698 0 0
0.754890361
ENSG00000073067 20.19897455 0 7.443229322 0 0
0 9.214067957 0 5.54449341 0.040156516
1 CYP2W1 7 983199 989640 + 2773
protein_coding cytochrome P450 family 2 subfamily W member 1
[Source:HGNC Symbol;Acc:HGNC:20243] - 21 0 8
0 0 0 0.366159783 0 0.134674742
0 0 0
ENSG00000175793 12.5041271 0 14.88645864 0 0
0 9.130195248 0 5.531602567 0.040237877
1 SFN 1 26863138 26864457 +
1320 protein_coding stratifin [Source:HGNC
Symbol;Acc:HGNC:10773] - 13 0 16 0 0
0 0.476179437 0 0.56583797 0 0
0

ENSG00000220091 2.885567793 8.700854042 11.16484398 0
0 0 7.583755273 0 5.26523267
0.040244395 1 LAP3P1 6 82924829 82925122
- 294 processed_pseudogene leucine aminopeptidase
3 pseudogene 1 [Source:HGNC Symbol;Acc:HGNC:42364] - 3
10 12 0 0 0 0.49337273
1.482215216 1.905372757 0 0 0
ENSG00000092758 12.5041271 0 21.3992843 1.102069155
0 0 11.30113713 0.367356385 4.877584128
0.040245071 1 COL9A3 20 62816244
62841159 + 4841 protein_coding collagen type IX alpha
3 chain [Source:HGNC Symbol;Acc:HGNC:2219] - 13 0
23 1 0 0 0.129840293 0
0.22178838 0.011478284 0 0
ENSG00000135446 2668.188352 2517.157074 2824.705528
3124.366056 3139.371417 3223.721906 2670.016985
3162.48646 -0.244334603 0.040263797 0.246414441 CDK4
12 57747727 57756013 - 3168
protein_coding cyclin dependent kinase 4 [Source:HGNC
Symbol;Acc:HGNC:1773] - 2774 2893 3036 2835 2590
3492 42.33723588 39.7943906 44.73656453
49.72558876 50.25299514 51.25309313
ENSG00000277957 0 0 0 19.8372448 6.060562582
0 0 8.63260246 -5.67096173 0.040342059
1 SENP3-EIF4A1 17 7563287 7578715 + 3519
protein_coding SENP3-EIF4A1 readthrough (NMD candidate)
[Source:HGNC Symbol;Acc:HGNC:49182] - 0 0 0
18 5 0 0 0 0 0.284226968
0.087336965 0
ENSG00000138698 671.3754398 754.3640455 656.8649876
980.8415483 972.1142381 763.464495 694.201491
905.4734271 -0.382374446 0.040368357 1 RAP1GDS1
4 98261384 98443861 + 5424
protein_coding Rap1 GTPase-GDP dissociation stimulator 1 [Source:HGNC
Symbol;Acc:HGNC:9859] - 698 867 706 890 802
827 6.222099592 6.965591706 6.076185948
9.117638507 9.088705842 7.089520242
ENSG00000252906 26.93196607 0 1.86080733 0 0
0 9.597591132 0 5.603186554 0.040425793
1 SCARNA3 1 175968398 175968540 -
143 scaRNA small Cajal body-specific RNA 3 [Source:HGNC
Symbol;Acc:HGNC:32577] - 28 0 2 0 0
0 9.467236152 0 0.652889966 0 0
0
ENSG00000119227 225.0742878 308.0102331 348.9013744
405.5614492 466.6633188 403.4268251 293.9952985
425.2171977 -0.53159826 0.040495872 1 PIGZ 3
196946343 196969060 - 3211 protein_coding
phosphatidylinositol glycan anchor biosynthesis class Z
[Source:HGNC Symbol;Acc:HGNC:30596] - 234 354 375

368	385	437	3.523520222	4.804205258
5.451763378	6.368241971	7.370005028	6.328082748
ENSG00000164045 695.4218381	636.9025159	756.4181798
1040.353283	790.2973606	892.7087868	696.2475113
907.7864767	-0.383127696	0.040513952	1	CDC25A	3
48157146	48188402	-	4117	protein_coding
cell division cycle 25A [Source:HGNC Symbol;Acc:HGNC:1725]
-	723	732	813	944	652	967
8.490996205	7.747985723	9.218403514	12.74098989
9.734509851	10.92135754
ENSG00000146411 3.847423724	2.610256213	16.74726597	0
0	0	7.73498197	0	5.292856694
0.040620266	1	SLC2A12 6	133988697	134052636
-	4468	protein_coding	solute carrier family 2 member
12 [Source:HGNC Symbol;Acc:HGNC:18067]	-	4	3	18
0	0	0	0.043286059	0.029259486
0.188063873	0	0	0
ENSG00000130045 0	8.700854042	18.6080733	0	0
0	9.102975782	0	5.52853156	0.040705298
1	NXNL2	9	88534033	88584274	+
2498	protein_coding	nucleoredoxin like 2 [Source:HGNC
Symbol;Acc:HGNC:30482]	-	0	10	20	0	0
0	0	0.174448068	0.373752062	0	0
0
ENSG00000187676 457.8434231	437.6529583	411.23842
865.124287	509.0872568	496.6673498	435.5782672
623.6262979	-0.517504309	0.040709335	1	B3GLCT	13
31199936	31332276	+	4566	protein_coding
beta 3-glucosyltransferase [Source:HGNC Symbol;Acc:HGNC:20207]
-	476	503	442	785	420	538
5.04048425	4.800546443	4.518896537	9.553132321
5.654064304	5.478698315
ENSG00000182359 143.3165337	133.9931523	126.5348985
89.26760159	41.21182555	81.23926912	134.6148615
70.57289875	0.928366838	0.040753182	1	KBTBD3	11
106051098	106077765	-	5516	protein_coding
kelch repeat and BTB domain containing 3 [Source:HGNC
Symbol;Acc:HGNC:22934]	-	149	154	136	81	34
88	1.306060297	1.216620307	1.150961209
0.815967395	0.378880296	0.741804447
ENSG00000079616 689.6507025	633.4221743	808.5207851
965.4125801	1138.173653	748.6937188	710.5312206
950.7599839	-0.419306667	0.040760918	1	KIF22	16
29790719	29805385	+	4312	protein_coding
kinesin family member 22 [Source:HGNC Symbol;Acc:HGNC:6391]
-	717	728	869	876	939	811
8.039732888	7.357177345	9.407777989	11.28853016
13.38548773	8.745267942
ENSG00000126500 58.67321179	24.36239132	25.12089896
5.510345777	10.90901265	12.92442918	36.05216736

9.781262534	1.877566771	0.040766478	1	FLRT1	11
	64103188	64119173	+	3949	protein_coding
	fibronectin leucine rich transmembrane protein 1 [Source:HGNC
Symbol;Acc:HGNC:3760]	-	61	28	27	5	9
	14	0.746868113	0.308979379	0.319170443
0.070354991	0.140088581	0.164843535
ENSG00000261485 75.02476261	22.62222051	26.98170629
1.102069155	16.96957523	12.00125567	41.54289647
10.02430002	2.052628149	0.04079639	1	PAN3-AS1
	13	28136843	28138193	-	1351
antisense	PAN3 antisense RNA 1 [Source:HGNC
Symbol;Acc:HGNC:39932]	-	78	26	29	1	14
	13	2.791518242	0.838641977	1.002048367
0.041129809	0.636971567	0.447423738
ENSG00000071127 8093.055803	7652.40113	8255.471721
9646.411317	8974.481071	9098.798141	8000.309552
9239.896843	-0.207848017	0.040935726	0.249326009	WDR1
	4	10074339	10116949	-	7827
protein_coding	WD repeat domain 1 [Source:HGNC Symbol;Acc:HGNC:12754]
	-	8414	8795	8873	8753	7404	9856
51.97666262	48.96650506	52.92016426	62.14034188
58.14581156	58.55127804
ENSG00000226752 509.7836434	507.2597907	430.776897
423.1945557	298.179679	301.8777387	482.606777
341.0839911	0.500977895	0.040974938	1	CUTALP	9
	120824828	120854385	+	7224
transcribed_unitary_pseudogene	"cutA divalent cation tolerance
homolog-like, pseudogene [Source:HGNC Symbol;Acc:HGNC:27367]"	-
	530	583	463	384	246	327	3.547311679
	3.516814126	2.991915265	2.953694203	2.093171101
	2.104752497
ENSG00000119878 438.6063045	527.271755	424.2640713
342.7435073	329.6946044	332.3424646	463.3807103
334.9268588	0.468618874	0.04104048	1	CRIPT	2
	46616416	46625742	+	2561	protein_coding
	CXXC repeat containing interactor of PDZ3 domain [Source:HGNC
Symbol;Acc:HGNC:14312]	-	456	606	456	311	272
	360	8.60907479	10.31149519	8.311918956
6.747810135	6.528398943	6.536179047
ENSG00000279110 41.35980503	17.40170808	22.32968796	0
	10.90901265	0	27.03040036	3.636337549
2.927197549	0.041049485	1	AL022323.4	22
25052122	25065241	-	13120	antisense	novel
transcript	-	43	20	24	0	9	0
	0.158465906	0.066428548	0.08539323	0
0.042165382	0
ENSG00000232871 2.885567793	3.480341617	16.74726597	0
	0	0	7.704391794	0	5.287312255
0.041059575	1	SEC1P	19	48638071	48682245
	+	2634	transcribed_unitary_pseudogene	"secretory

blood group 1, pseudogene [Source:HGNC Symbol;Acc:HGNC:44149]" -
3 4 18 0 0 0 0.055068938
0.066176351 0.319008879 0 0 0
ENSG00000167986 7525.560804 6661.373855 7488.819101
8274.335219 8576.908165 8318.716523 7225.251253
8389.986636 -0.215642368 0.04109758 0.249326009 DDB1
11 61299451 61342596 - 13950
protein_coding damage specific DNA binding protein 1 [Source:HGNC
Symbol;Acc:HGNC:2717] - 7824 7656 8049 7508 7076
9011 27.11788725 23.91587721 26.93480003
29.90625968 31.17891348 30.03514965
ENSG00000176454 508.8217875 488.1179118 532.1908965
246.8634908 378.1791051 436.6610715 509.7101986
353.9012225 0.525305084 0.041107024 1 LPCAT4 15
34358618 34367278 - 4799 protein_coding
lysophosphatidylcholine acyltransferase 4 [Source:HGNC
Symbol;Acc:HGNC:30059] - 529 561 572 224 312
473 5.329741414 5.094138038 5.564053724
2.593637711 3.996236704 4.582910848
ENSG00000114200 13.46598303 3.480341617 14.88645864
65.02208017 23.03013781 29.54155241 10.61092776
39.19792346 -1.888702748 0.041155142 1 BCHE 3
165772904 165837472 - 3082 protein_coding
butyrylcholinesterase [Source:HGNC Symbol;Acc:HGNC:983] -
14 4 16 59 19 32 0.219632506
0.056556947 0.242344621 1.063730032 0.378938147
0.48277899
ENSG00000230836 0 25.23247672 19.53847697 2.204138311
0 0 14.92365123 0.73471277 4.358945827
0.041162221 1 LINC01293 2 74940258
74942670 + 1266 lincRNA long intergenic non-
protein coding RNA 1293 [Source:HGNC Symbol;Acc:HGNC:50362] -
0 29 21 2 0 0 0
0.998212238 0.774339877 0.087782579 0 0
ENSG00000143158 623.2826433 1159.823844 854.1105646
1044.761559 1213.324629 1338.601593 879.0723506
1198.895927 -0.447432231 0.041196236 1 MPC2 1
167916729 167937040 - 2552 protein_coding
mitochondrial pyruvate carrier 2 [Source:HGNC
Symbol;Acc:HGNC:24515] - 648 1333 918 948 1001
1450 12.27709319 22.76187726 16.79221735
20.64142666 24.11019748 26.41912017
ENSG00000120802 2066.06654 2274.403247 2285.071402
3090.201912 2574.526985 2413.175562 2208.513729
2692.63482 -0.285708988 0.041269505 0.249326009 TMPO
12 98515512 98550379 + 8593
protein_coding thymopoietin [Source:HGNC Symbol;Acc:HGNC:11875]
- 2148 2614 2456 2804 2124 2614
12.08622519 13.25620981 13.34226575 18.13198041
15.19347308 14.14463645

ENSG00000035687 2549.880073 3079.232246 2619.086318
2460.920424 2310.286456 1973.74497 2749.399545
2248.317283 0.2907484 0.041282738 0.249326009 ADSS
1 244408494 244452134 - 3666
protein_coding adenylosuccinate synthase [Source:HGNC
Symbol;Acc:HGNC:292] - 2651 3539 2815 2233 1906
2138 34.96378843 42.06749965 35.84527977
33.8460745 31.9578678 27.1172883
ENSG00000177030 1712.103557 1636.630645 1462.594562
1782.045824 1941.804251 2194.38344 1603.776255
1972.744505 -0.299093479 0.041288364 1 DEAF1 11
644233 706715 - 4452 protein_coding "DEAF1,
transcription factor [Source:HGNC Symbol;Acc:HGNC:14677]" SAND
1780 1881 1572 1617 1602 2377 19.33152267
18.41162995 16.48327187 20.18212575 22.11845136
24.82590243
ENSG00000151320 126.9649829 162.7059706 88.38834819
71.6344951 79.99942608 38.77328753 126.0197672
63.46906957 0.994189125 0.041335709 1 AKAP6 14
32329273 32837681 + 19748 protein_coding
A-kinase anchoring protein 6 [Source:HGNC Symbol;Acc:HGNC:376]
- 132 187 95 65 66 42
0.32318562 0.412645474 0.224567303 0.182895189
0.205432039 0.098891096
ENSG00000143248 50.97836434 70.47691774 64.1978529
28.65379804 31.51492542 11.07808215 61.88437833
23.74893521 1.389764475 0.041383773 1 RGS5 1
163111121 163321791 - 6821 protein_coding
regulator of G protein signaling 5 [Source:HGNC
Symbol;Acc:HGNC:10001] - 53 81 69 26 26
12 0.375689482 0.51748238 0.472222936
0.211805553 0.2343002 0.081802055
ENSG00000111275 0 0 0 0 31.51492542 0
0 10.50497514 -5.952418974 0.041389803 1
ALDH2 12 111766887 111817529 + 9720
protein_coding aldehyde dehydrogenase 2 family member
[Source:HGNC Symbol;Acc:HGNC:404] - 0 0 0
0 26 0 0 0 0 0
0.164419924 0
ENSG00000245904 10.58041524 6.09059783 5.582421991 0
0 0 7.417811687 0 5.232430776
0.041463 1 AC025164.1 12 92145573
92189660 + 7589 antisense uncharacterized
LOC101928617 [Source:NCBI gene;Acc:101928617] - 11 7
6 0 0 0 0.070082462 0.040195005
0.036907339 0 0 0
ENSG00000285560 20.19897455 17.40170808 5.582421991
3.306207466 0 0 14.39436821 1.102069155
3.719861087 0.041486587 1 AC103739.3 15
90630535 90631755 - 888 antisense "novel

transcript, antisense to CRTC3" - 21 20 6 3
0 0 1.143424637 0.981466832 0.315416436
0.18772423 0 0
ENSG00000154832 2488.321293 2235.249404 2556.749272
2556.800441 2943.00919 3400.048047 2426.773323
2966.619226 -0.29015653 0.041570155 0.250238705 CXXC1
18 50282343 50288304 - 4319
protein_coding CXXC finger protein 1 [Source:HGNC
Symbol;Acc:HGNC:24343] - 2587 2569 2748 2320 2428
3683 28.96105919 25.92026862 29.70158052
29.84810917 34.55515446 39.65057895
ENSG00000270580 48.09279655 13.05128106 35.35533928
11.02069155 7.272675098 0 32.1664723 6.097788884
2.413358798 0.041591544 1 PKD1P6-NPIPP1 16
15104723 15131601 - 3043
processed_transcript PKD1P6-NPIPP1 readthrough [Source:NCBI
gene;Acc:105369154] - 50 15 38 10 6
0 0.794454937 0.21480674 0.582945132
0.182603918 0.121198336 0
ENSG00000178896 1688.057159 1891.565669 2109.225109
1763.310649 2441.194608 3280.958664 1896.282646
2495.15464 -0.396340605 0.041630038 1 EXOSC4 8
144078626 144080647 + 1407 protein_coding
exosome component 4 [Source:HGNC Symbol;Acc:HGNC:18189] -
1755 2174 2267 1600 2014 3554 60.30929335
67.33239152 75.21482658 63.18848296 87.98582873
117.4503578
ENSG00000131737 0 3.480341617 1.86080733 8.816553243
1.212112516 45.23550212 1.780382982 18.42138929
-3.365683915 0.041687489 1 KRT34 17
41377650 41382403 - 1751 protein_coding
keratin 34 [Source:HGNC Symbol;Acc:HGNC:6452] - 0 4
2 8 1 49 0 0.099547978
0.053319969 0.253872631 0.035104372 1.301190704
ENSG00000166592 16.35155083 25.23247672 28.84251362
102.4924315 38.78760052 46.15867564 23.47551372
62.4795692 -1.411295801 0.041731176 1 RRAD 16
66921679 66925644 - 1709 protein_coding
"RRAD, Ras related glycolysis inhibitor and calcium channel
regulator [Source:HGNC Symbol;Acc:HGNC:10446]" - 17 29
31 93 32 50 0.480959021 0.739459739
0.846770397 3.023799072 1.150946869 1.360375995
ENSG00000228634 39.43609317 0 14.88645864 3.306207466
0 0 18.10751727 1.102069155 4.047854792
0.041736817 1 AL136115.1 1 31933020
31933975 + 517 lincRNA novel transcript
- 41 0 16 3 0 0
3.834374521 0 1.44469269 0.322435429 0 0
ENSG00000104419 36200.40982 48558.59633 49890.10533
27459.15508 43015.44898 37405.14439 44883.03716

35959.91615	0.319797919	0.04174234	0.250454041	NDRG1
	8	133237171	133302022	-	9823
protein_coding	N-myc downstream regulated 1 [Source:HGNC
Symbol;Acc:HGNC:7679]	-	37636	55809	53622	24916	35488
	40518	185.2509878	247.5817877	254.8266824
140.9438796	222.0673656	191.793937
ENSG00000102034 1141.72299	1185.926406	1237.436875
1376.484375	1764.835824	1344.140635	1188.36209
1495.153611	-0.330653495	0.041780654	1	ELF4	X
	130064874	130110716	-	4316	protein_coding
	E74 like ETS transcription factor 4 [Source:HGNC
Symbol;Acc:HGNC:3319]	ETS	1187	1363	1330	1249	1456
	1456	13.29751532	13.76172951	14.38521114
16.08025924	20.73611053	15.68595472
ENSG00000222365 7.694847448	0	19.53847697	0	0
	0	9.077774806	0	5.523503018	0.041790819
	1	SNORD12B	20	49280319	49280409
	+	91	snoRNA	"small nucleolar RNA, C/D box 12B
[Source:HGNC Symbol;Acc:HGNC:33573]"	-	8	0	21
	0	0	0	4.250595823	0	10.77268444
	0	0	0
ENSG00000248916 24.04639827	0	3.721614661	0	0
	0	9.256004311	0	5.550925854	0.041871969
	1	NUP210P3	3	129323046	129325426
	-	388	unprocessed_pseudogene	nucleoporin 210
pseudogene 3 [Source:HGNC Symbol;Acc:HGNC:42712]	-	25
	0	4	0	0	0	3.115369039	0
	0.481253944	0	0	0
ENSG00000080947 92.33816937	78.30768638	60.47623824
13.22482987	59.3935133	7.385388102	77.040698
26.66791042	1.541683391	0.041878069	1	CROCCP3 1
	16467436	16499257	-	6813
transcribed_unprocessed_pseudogene	"ciliary rootlet coiled-coil,
rootletin pseudogene 3 [Source:HGNC Symbol;Acc:HGNC:29405]"	-
	96	90	65	12	49	8	0.68129321
	0.575655579	0.445370045	0.097871197	0.442084259
	0.054598739
ENSG00000116688 4132.133079	4095.491998	3856.523192
4409.378691	4801.177677	4959.28811	4028.049423
4723.281493	-0.229784846	0.041950676	0.250533572	MFN2
	1	11980181	12013514	+	4973
protein_coding	mitofusin 2 [Source:HGNC Symbol;Acc:HGNC:16877] -
	4296	4707	4145	4001	3961	5372	41.76832216
	41.24623737	38.90918298	44.70562139	48.95913581
	50.22830964
ENSG00000136982 789.6837193	689.9777256	734.0884918
1228.807108	924.8418499	788.3901799	737.9166456
980.6797127	-0.409973168	0.041975017	1	DSCC1	8
	119833976	119856010	-	2603	protein_coding
	DNA replication and sister chromatid cohesion 1 [Source:HGNC

Symbol;Acc:HGNC:24453]	-	821	793	789	1115	763
	854	15.25001271	13.27570572	14.14975339
23.8019612	18.01763273	15.25508812
ENSG00000229539 22.12268641	8.700854042	13.02565131	0
	3.636337549	0	14.61639726	1.212112516
3.651908859	0.042005249	1	AL353194.1	20
3888239 3888868 -	441	antisense	novel transcript
	-	23	10	14	0	3	0
2.52168284	0.988143477	1.481956589	0	0.418148001
	0
ENSG00000126107 1195.586922	1274.675117	1183.473462
1937.437575	1530.898108	1237.052507	1217.911834
1568.46273	-0.364415263	0.04201397	1	HECTD3	1
	45002540	45011329	-	5077	protein_coding
	HECT domain E3 ubiquitin protein ligase 3 [Source:HGNC
Symbol;Acc:HGNC:26117]	-	1243	1465	1272	1758	1263
	1340	11.83764146	12.57445176	11.69569364
19.24082772	15.29126967	12.27237601
ENSG00000154277 5955.811924	4659.30734	6018.78131
6302.7335	6462.983937	7203.52292	5544.633525
6656.413452	-0.263845247	0.042028508	0.250533572	UCHL1
	4	41256413	41268455	+	2282
protein_coding	ubiquitin C-terminal hydrolase L1 [Source:HGNC
Symbol;Acc:HGNC:12513]	-	6192	5355	6469	5719	5332
	7803	131.1947705	102.2592099	132.3328138
139.2568285	143.6222946	158.9926269
ENSG00000144426 336.6495758	219.2615219	201.8975953
191.760033	133.3323768	142.168721	252.6028977
155.7537103	0.697185713	0.042060941	1	NBEAL1	2
	203014879	203226378	+	16376	protein_coding
	neurobeachin like 1 [Source:HGNC Symbol;Acc:HGNC:20681] -
	350	252	217	174	110	154	1.033383282
	0.6705811	0.618582942	0.59040967	0.412887957
	0.437264187
ENSG00000269881 21.16083048	6.09059783	0	0	0
	0	9.083809437	0	5.524391366	0.042094953
	1	AC004754.1	16	249547	269943	+	2242
	protein_coding	family with sequence similarity 234 member A
[Source:NCBI gene;Acc:83986]	-	22	7	0	0
	0	0	0.474447638	0.136057043	0	0
	0	0
ENSG00000134318 1681.324167	1658.38278	1715.664359
1570.448546	1333.323768	1143.811982	1685.123769
1349.194766	0.321253963	0.042288147	1	ROCK2	2
	11179761	11348330	-	10145	protein_coding
	Rho associated coiled-coil containing protein kinase 2
[Source:HGNC Symbol;Acc:HGNC:10252]	-	1748	1906	1844
	1425	1100	1239	8.330874525	8.187087702
8.485059678	7.805035031	6.66481339	5.678717615
ENSG00000163961 1082.087922	908.369162	912.7259956

856.3077338	671.510334	714.5362989	967.7276933
747.4514555	0.37238469	0.042289525	1	RNF168	3
	196468783	196503768	-	5347	protein_coding
	ring finger protein 168 [Source:HGNC Symbol;Acc:HGNC:26661]
	-	1125	1044	981	777	554	774
10.17287141	8.508419854	8.564556113	8.074634599
6.368643528	6.730724193
ENSG00000221230 33.66495758	0	0	0	0	0
	11.22165253	0	5.828982673	0.042291615	1
	MIR548L 11	94466495	94466580	-	86
	miRNA	microRNA 548l [Source:HGNC Symbol;Acc:HGNC:35292]
	-	35	0	0	0	0	0
19.67754026	0	0	0	0	0
ENSG00000202399 33.66495758	0	0	0	0	0
	11.22165253	0	5.828982673	0.042291615	1
	RF00019 8	97772313	97772425	+	113
	misc_RNA		-	35	0	0	0
	0	0	14.9758271	0	0	0	0
	0
ENSG00000214097 33.66495758	0	0	0	0	0
	11.22165253	0	5.828982673	0.042291615	1
	SMCO1	3	196506877	196515366	-	1868
	protein_coding	single-pass membrane protein with coiled-coil
domains 1 [Source:HGNC Symbol;Acc:HGNC:27407]	-	35	0
	0	0	0	0	0.905925301	0	0
	0	0	0
ENSG00000210154 12.5041271	12.18119566	18.6080733	0
	3.636337549	0	14.43113202	1.212112516
3.634484259	0.042307504	1	MT-TD	MT	7518	7585
	+	68	Mt_tRNA mitochondrially encoded tRNA aspartic
acid [Source:HGNC Symbol;Acc:HGNC:7478] -	13	14	20
	0	3	0	9.243483197	8.971761514
13.72989193	0	2.711812774	0
ENSG00000164749 8.656703379	6.960683234	31.63372462
77.14484088	18.18168774	64.62214589	15.75037041
53.31622484	-1.762152732	0.04246823	1	HNF4G	8
	75407914	75566843	+	4806	protein_coding
	hepatocyte nuclear factor 4 gamma [Source:HGNC
Symbol;Acc:HGNC:5026]	RXR-like	9	8	34	70
	15	70	0.090544059	0.072537873	0.330248753
	0.809331264	0.19184693	0.677244196
ENSG00000143512 14.42783896	4.350427021	3.721614661	0
	0	0	7.499960215	0	5.247832544
0.042472324	1	HHIPL2	1	222522258	222548103
	-	3289	protein_coding	HHIP like 2 [Source:HGNC
Symbol;Acc:HGNC:25842]	-	15	5	4	0	0
	0	0.220510159	0.066246773	0.056773041	0
	0	0
ENSG00000259039 14.42783896	4.350427021	3.721614661	0
	0	0	7.499960215	0	5.247832544

0.042472324 1 AL161804.1 14 57578409
57600404 + 1430 antisense "novel transcript,
antisense to SLC35F4" - 15 5 4 0 0
0 0.507173365 0.152367578 0.130577993 0
0 0
ENSG00000141741 204.8753133 354.1247595 340.5277415
308.5793635 597.5714705 480.0502266 299.8426048
462.0670202 -0.6230212 0.042577979 1 MIEN1 17
39728496 39730787 - 2292 protein_coding
migration and invasion enhancer 1 [Source:HGNC
Symbol;Acc:HGNC:28230] - 213 407 366 280 493
520 4.493308183 7.738172266 7.454396822
6.788213009 13.22146763 10.5492054
ENSG00000100991 2933.660589 2812.116027 2718.63951
2905.054294 3581.792486 3729.620991 2821.472042
3405.489257 -0.271557812 0.042594663 0.251427816
TRPC4AP 20 35002404 35092871 - 3226
protein_coding transient receptor potential cation channel subfamily
C member 4 associated protein [Source:HGNC Symbol;Acc:HGNC:16181]
- 3050 3232 2922 2636 2955 4040
45.71268098 43.65817595 42.28261944 45.40389248
56.30415984 58.23016493
ENSG00000132507 8418.163108 8701.724128 8818.365939
9510.856811 9452.053402 11453.81377 8646.084391
10138.908 -0.22993316 0.042627239 0.251427816 EIF5A
17 7306999 7312463 + 2815 protein_coding
eukaryotic translation initiation factor 5A [Source:HGNC
Symbol;Acc:HGNC:3300] - 8752 10001 9478 8630 7798
12407 150.324624 154.8187747 157.1753156
170.3509031 170.2755108 204.9365407
ENSG00000230683 0 0 0.930403665 0 0
37.85011402 0.310134555 12.61670467 -5.26299291
0.042631691 1 DDTP1 3 38515448 38515830
- 383 processed_pseudogene D-dopachrome
tautomerase pseudogene 1 [Source:HGNC Symbol;Acc:HGNC:44546] -
0 0 1 0 0 41 0 0
0.121884158 0 0 4.977555382
ENSG00000198625 1421.623066 1274.675117 1277.444232
1066.802942 1123.628303 1021.953079 1324.580805
1070.794775 0.307118268 0.042669569 1 MDM4 1
204516379 204558120 + 11008 protein_coding
"MDM4, p53 regulator [Source:HGNC Symbol;Acc:HGNC:6974]"
- 1478 1465 1373 968 927 1107
6.491831362 5.799463261 5.822481968 4.88628709
5.176290881 4.675953871
ENSG00000231210 0 14.79145187 35.35533928 0 0
2.769520538 16.71559705 0.923173513 4.112471786
0.042752234 1 LINC01510 7 116563594
116663829 - 1691 lincRNA long intergenic non-
protein coding RNA 1510 [Source:HGNC Symbol;Acc:HGNC:51196] -

0	17	38	0	0	3	0
0.438090577	1.049025451	0	0	0.082491398
ENSG00000159377 5785.563425	6427.320881	5855.960669
6619.027347	7094.494558	7339.229426	6022.948325
7017.583777	-0.220511385	0.042791319	0.251427816	PSMB4
1	151399534	151401944	+	2158
protein_coding	proteasome subunit beta 4 [Source:HGNC
Symbol;Acc:HGNC:9541]	-	6015	7387	6294	6006	5853
7950	134.7675732	149.167859	136.1511563
154.6485781	166.7149106	171.2957968
ENSG00000128165 171.2103557	167.0563976	124.6740911
110.2069155	84.84787614	65.5453194	154.3136148
86.8667037	0.830898829	0.042795312	1	ADM2	22
50481556	50486440	+	4276	protein_coding
adrenomedullin 2 [Source:HGNC Symbol;Acc:HGNC:28898]	-
178	192	134	100	70	71	2.012720742
1.956690471	1.46289494	1.299494205	1.006254194
0.772061098
ENSG00000175741 159.6680845	97.44956528	99.55319218
217.1076236	232.7256031	155.0931501	118.8902807
201.6421256	-0.761631923	0.042796304	1	RWDD4P2 7
39854494	39855056	-	563
processed_pseudogene	RWD domain containing 4 pseudogene 2
[Source:HGNC Symbol;Acc:HGNC:23754]	-	166	112	107
197	192	168	14.2561058	8.668984482
8.871997661	19.44329542	20.96236091	13.87496528
ENSG00000110955 8482.607455	9197.672808	9180.292964
10066.29967	10873.86138	10070.89985	8953.524409
10337.0203	-0.207205717	0.042808673	0.251427816
ATP5F1B 12	56638175	56646068	-	2322
protein_coding	ATP synthase F1 subunit beta [Source:HGNC
Symbol;Acc:HGNC:830]	-	8819	10571	9867	9134	8971
10909	183.6362196	198.3866551	198.3667823
218.5802083	237.479432	218.4508528
ENSG00000111186 2265.170717	2317.907517	2559.540483
2988.811549	2721.192599	2805.524305	2380.872906
2838.509485	-0.253680862	0.042863239	0.251427816	WNT5B
12	1529891 1647243 +	4305	protein_coding	Wnt
family member 5B [Source:HGNC Symbol;Acc:HGNC:16265]	-	2355
2664	2751	2712	2245	3039	26.44959169
26.96619449	29.83070178	35.00487838	32.05461387
32.82377768
ENSG00000227615 66.36805924	82.6581134	62.33704557
120.1255379	95.75688879	180.9420085	70.45440607
132.2748117	-0.910650229	0.042892223	1	AP001324.1
11	74745716	74746114	-	399
processed_pseudogene	ribosomal protein S12 (RPS12) pseudogene
-	69	95	67	109	79	196
8.361369416	10.37550651	7.838770379	15.17978589
12.17030762	22.84095161

ENSG00000114346 1026.300278   1239.001616   1180.682251
2014.582416   1364.838693   1182.58527   1148.661382
1520.668793   -0.404318964   0.042912839   1   ECT2   3
        172750682   172821474   +   5499   protein_coding
        epithelial cell transforming 2 [Source:HGNC
Symbol;Acc:HGNC:3155]   -   1067   1424   1269   1828   1126
        1281   9.381708099   11.28456617   10.77268444
18.4716 12.58641453   10.83169514
ENSG00000198938 51884.43263   47980.85962   64653.75069
51726.71788   73805.53112   77844.76011   54839.68098
67792.33637   -0.305912525   0.043007782   0.251472252   MT-CO3
        MT   9207   9990   +   784   protein_coding
mitochondrially encoded cytochrome c oxidase III [Source:HGNC
Symbol;Acc:HGNC:7422]   -   53942   55145   69490   46936   60890
        84323   3326.688972   3065.128428   4137.636028
3326.611283   4773.943465   5001.04779
ENSG00000177182 15.3896949   11.31111026   0   0   0
        0   8.900268383   0   5.495608779   0.043090707
        1   CLVS1   8   61057158   61501645   +
        7393   protein_coding clavesin 1 [Source:HGNC
Symbol;Acc:HGNC:23139] -   16   13   0   0   0
        0   0.104640665   0.076626898   0   0   0
        0
ENSG00000280042 11.54227117   6.960683234   8.373632987
1.102069155   0   0   8.958862464   0.367356385
4.542930785   0.043098808   1   AC022336.3   3
124791119   124793104   -   1986   TEC   TEC   -
        12   8   9   1   0   0   0.292148202
        0.17553727   0.211548184   0.027979039   0   0
ENSG00000204394 7107.153474   5156.996191   6598.422794
6412.940415   7854.489106   9024.94426   6287.524153
7764.124594   -0.304506539   0.043254333   0.251521614   VARS
        6   31777518   31795953   -   6081
protein_coding valyl-tRNA synthetase [Source:HGNC
Symbol;Acc:HGNC:12651] -   7389   5927   7092   5819   6480
        9776   58.75054229   42.4735461   54.44271305
53.17229401   65.50091437   74.75103454
ENSG00000111247 165.4392201   304.5298915   240.0441456
465.0731836   355.1489673   282.4910949   236.6710857
367.5710819   -0.633672224   0.043265335   1   RAD51AP1
        12   4538798 4560048 +   2558   protein_coding RAD51
associated protein 1 [Source:HGNC Symbol;Acc:HGNC:16956]   -
        172   350   258   422   293   306   3.251090981
        5.962468559   4.708311649   9.166930832   7.040677315
        5.562267928
ENSG00000143786 2395.021268   2406.656228   2107.364302
1571.550616   1961.198051   2071.601363   2303.013933
1868.116677   0.30173277   0.043347428   1   CNIH3   1
        224434660   224740549   +   6386   protein_coding
        cornichon family AMPA receptor auxiliary protein 3

[Source:HGNC Symbol;Acc:HGNC:26802]	-	2490	2766	2265
	1426	1618	2244	18.8526172	18.87477831
16.55714027	12.40802486	15.57388499	16.33898058
ENSG00000214842 14.42783896	5.220512425	2.791210996	0
	0	0	7.479854128	0	5.244079412
0.043351451	1	RAD51AP2	2	17510584
17518439	-	3724	protein_coding	RAD51 associated
protein 2 [Source:HGNC Symbol;Acc:HGNC:34417]	-	15	6
	3	0	0	0	0.194752393	0.070210194
	0.037606041	0	0	0
ENSG00000061987 1294.658083	1003.208471	1082.059463
898.1863617	967.265788	764.3876685	1126.642006
876.6132727	0.362611564	0.043415963	1	MON2	12
	62466817	62600479	+	16017	protein_coding
	"MON2 homolog, regulator of endosome-to-Golgi trafficking
[Source:HGNC Symbol;Acc:HGNC:29177]"	-	1346	1153	1163
	815	798	828	4.063171006	3.136943737
3.389569748	2.827407963	3.062450488	2.403699534
ENSG00000186416 1245.603431	1185.926406	1121.136417
1409.54645	1301.808843	1759.568715	1184.222084
1490.308003	-0.332468491	0.043435948	1	NKRF	X
	119588337	119606443	-	4552	protein_coding
	NFKB repressing factor [Source:HGNC Symbol;Acc:HGNC:19374]
	Others	1295	1363	1205	1279	1074	1906
13.75525771	13.04824793	12.35750598	15.6127834
14.50271753	19.46935935
ENSG00000034152 1923.711862	1513.078518	2144.580448
1998.051379	2263.014068	2858.145195	1860.456943
2373.070214	-0.351631047	0.043478599	1	MAP2K3	17
	21284672	21315240	+	3783	protein_coding
	mitogen-activated protein kinase kinase 3 [Source:HGNC
Symbol;Acc:HGNC:6843]	-	2000	1739	2305	1813	1867
	3096	25.56200237	20.03188593	28.44334207
26.63014348	30.33579193	38.05358949
ENSG00000227279 4.809279655	8.700854042	8.373632987	0
	0	0	7.294588895	0	5.209056254
0.043571531	1	AC110015.1	18	27954519
27963687	+	440	antisense	"novel transcript,
antisense to CDH2"	-	5	10	9	0	0
	0	0.549437812	0.990389258	0.954851575	0
	0	0
ENSG00000280417 5.771135586	15.66153728	0	41.87862791
	32.72703794	29.54155241	7.144224287	34.71573942
	-2.274975303	0.043580904	1	AC096887.2	3
	53046166	53048122	+	1957	TEC	TEC
	-	6	18	0	38	27	32
0.148238715	0.400811595	0	1.078958684	0.848047735
	0.760309069
ENSG00000137409 6152.030534	6597.85762	6100.656833
6519.841123	7848.428543	7812.817438	6283.514996

7393.695702	-0.234717783	0.043586755	0.251521614	MTCH1
	6	36968141	36986298	-	3126
protein_coding	mitochondrial carrier 1 [Source:HGNC
Symbol;Acc:HGNC:17586]	-	6396	7583	6557	5916	6475
	8463	98.92833456	105.7086874	97.91793495
105.1601593	127.3204483	125.8827974
ENSG00000158483 308.7557538	416.7709086	435.4289153
430.9090398	580.6018953	625.9116416	386.9851926
545.8075256	-0.496196781	0.043626717	1	FAM86C1 11
	71787510	71801236	+	2972	protein_coding
	family with sequence similarity 86 member C1 [Source:HGNC
Symbol;Acc:HGNC:25561]	-	321	479	468	391	479
	678	5.222247418	7.023366084	7.350943484
7.310380731	9.906815353	10.60747231
ENSG00000149269 1644.773642	1848.931484	1706.360322
2205.24038	2309.074344	1848.193372	1733.355149
2120.836032	-0.290462833	0.043669068	1	PAK1	11
	77321707	77474635	-	6299	protein_coding
	p21 (RAC1) activated kinase 1 [Source:HGNC
Symbol;Acc:HGNC:8590]	-	1710	2125	1834	2001	1905
	2002	13.12579807	14.70096771	13.59169933
17.65174008	18.5896294	14.77826612
ENSG00000076248 1490.876693	1376.47511	1662.63135
1760.004441	1693.321185	2186.998052	1509.994384
1880.107893	-0.316939019	0.043670759	1	UNG	12
	109097574	109110992	+	2567	protein_coding
	uracil DNA glycosylase [Source:HGNC Symbol;Acc:HGNC:12572]
	-	1550	1582	1787	1597	1397	2369
29.19490363	26.8558689	32.49710844	34.56934024
33.45167723	42.91115559
ENSG00000178031 1407.195227	1232.911018	1322.103608
1081.129841	1092.113377	1036.723855	1320.736618
1069.989024	0.303824136	0.043707799	1	ADAMTSL1
	9	17906563	18910950	+	13652
protein_coding	ADAMTS like 1 [Source:HGNC Symbol;Acc:HGNC:14632]
	-	1463	1417	1421	981	901	1123
5.181425558	4.52305812	4.858965709	3.99286633
4.056727831	3.824850764
ENSG00000159335 2861.521395	2680.73313	3596.94057
3545.356473	3903.002303	3652.99759	3046.398365
3700.452122	-0.280580053	0.043726992	0.251521614	PTMS
	12	6765516 6770952 +	1475	protein_coding
parathymosin [Source:HGNC Symbol;Acc:HGNC:9629] -	2975	3081
	3866	3217	3220	3957	97.52055545	91.02449449
	122.3533501	121.1911996	134.1872373	124.7398013
ENSG00000260877 77.91033041	26.10256213	79.08431154
22.04138311	0	25.84885836	61.03240136	15.96341382
	1.924621519	0.043749899	1	AP005233.2	11
	69371463	69372512	+	372	lincRNA novel
transcript	-	81	30	85	20	0	28

10.52793744	3.514284464	10.66650206	2.987439366
0	3.499823231
ENSG00000148303 15260.8062	17689.70635	19104.90886
19107.67502	21620.45095	20302.43189	17351.80714
20343.51929	-0.229450317	0.043809289	0.251521614	RPL7A
9	133348214	133351426	+	2242
protein_coding	ribosomal protein L7a [Source:HGNC
Symbol;Acc:HGNC:10364]	-	15866	20331	20534	17338	17837
21992	342.1630103	395.1679644	427.5471199
429.7099738	489.0278179	456.1000677
ENSG00000115234 2235.353184	2283.104101	2185.51821
2314.345226	2887.252014	2919.997821	2234.658498
2707.198354	-0.276814992	0.043828661	0.251521614	SNX17
2	27370496	27377533	+	3765
protein_coding	sorting nexin 17 [Source:HGNC Symbol;Acc:HGNC:14979]
-	2324	2624	2349	2100	2382	3163
29.84505336	30.37088504	29.12487513	30.99319565
38.8887637	39.06296725
ENSG00000167770 3351.106063	2872.151919	3063.819269
3157.42813	4032.698342	4077.657406	3095.692417
3755.927959	-0.279053007	0.04383819	0.251521614	OTUB1
11	63985853	64001811	+	5336
protein_coding	"OTU deubiquitinase, ubiquitin aldehyde binding 1
[Source:HGNC Symbol;Acc:HGNC:23077]"	-	3484	3301	3293
2865	3327	4417	31.56919749	26.95803924
28.80858621	29.83464325	38.32519208	38.48952899
ENSG00000080603 387.6279402	356.7350157	326.5716865
250.1696983	161.2109647	296.3386976	356.9782141
235.9064535	0.594989114	0.04385818	1	SRCAP	16
30698209	30741409	+	13674	protein_coding
Snf2 related CREBBP activator protein [Source:HGNC
Symbol;Acc:HGNC:16974]	-	403	410	351	227	133
321	1.424986294	1.306612711	1.198277975
0.922448917	0.597865407	1.09154206
ENSG00000149346 329.9165843	323.6717704	240.9745493
214.9034853	192.7258901	191.0969171	298.1876347
199.5754308	0.579493339	0.043877181	1	SLX4IP	20
10435303	10636829	+	6492	protein_coding
SLX4 interacting protein [Source:HGNC Symbol;Acc:HGNC:16225]
-	343	372	259	195	159	207
2.554564222	2.497025782	1.862375667	1.669045376
1.505448743	1.482596097
ENSG00000284770 232.7691353	173.1469954	194.454366
112.4110539	130.9081518	129.2442918	200.1234989
124.1878325	0.687909198	0.043877676	1	TBCE	1
235367360	235452443	+	12444	protein_coding
tubulin folding cofactor E [Source:HGNC Symbol;Acc:HGNC:11582]
-	242	199	209	102	108	140
0.940278661	0.696869844	0.784029348	0.455462067
0.533471365	0.523117262

ENSG00000115350 453.9959994 328.8922828 416.820842
435.3173164 593.935133 656.3763676 399.9030414
561.8762723 -0.491502129 0.043903237 1 POLE4 2
74958492 74970128 + 2151 protein_coding
"DNA polymerase epsilon 4, accessory subunit [Source:HGNC
Symbol;Acc:HGNC:18755]" - 472 378 448 395 490
711 10.60969269 7.657905225 9.722627329
10.20395956 14.00241773 15.3695166
ENSG00000163661 1639.002506 1924.628914 1331.407645
709.7325361 1472.716707 1349.679676 1631.679688
1177.376306 0.470875339 0.044071958 1 PTX3 3
157436789 157443628 + 1940 protein_coding
pentraxin 3 [Source:HGNC Symbol;Acc:HGNC:9692] - 1704
2212 1431 644 1215 1462 42.46871075
49.68691016 34.43371968 18.44574417 38.49655866
35.04101366
ENSG00000058673 16.35155083 0 10.23444032 0 0
0 8.861997048 0 5.488401137 0.044090371
1 ZC3H11A 1 203795654 203854999 +
12798 protein_coding zinc finger CCCH-type containing 11A
[Source:HGNC Symbol;Acc:HGNC:29093] - 17 0 11
0 0 0 0.06422558 0 0.040123297
0 0 0
ENSG00000278931 17.31340676 0 9.304036652 0 0
0 8.872481136 0 5.490068199 0.044122592
1 CR381670.1 21 8857260 8880976 + 1693
unprocessed_pseudogene "ankyrin repeat domain 20 family,
member A3 (ANKRD20A3) pseudogene" - 18 0 10
0 0 0 0.514063494 0 0.275733211
0 0 0
ENSG00000006062 661.7568805 403.7196276 490.3227316
393.4386885 336.9672795 367.4230581 518.5997465
365.9430087 0.502270228 0.044189822 1 MAP3K14 17
45263119 45317145 - 5784 protein_coding
mitogen-activated protein kinase kinase kinase 14 [Source:HGNC
Symbol;Acc:HGNC:6853] - 688 464 527 357 278
398 5.751238402 3.495813813 4.253323022
3.429667164 2.95436311 3.199527195
ENSG00000260923 0 0 0 9.918622399 1.212112516
10.15490864 0 7.095214518 -5.3924831
0.044199214 1 LINC02193 16 90173217
90222678 + 3723 lincRNA long intergenic non-protein
coding RNA 2193 [Source:HGNC Symbol;Acc:HGNC:53055] - 0
0 0 9 1 11 0 0 0
0.134326444 0.016510276 0.137382263
ENSG00000241288 105.8041524 92.22905285 59.54583457
20.93931395 47.27238814 46.15867564 85.85967994
38.12345924 1.170622688 0.044200087 1 AC092902.2
3 125827238 125916384 - 6732
processed_transcript uncharacterized LOC101927056 [Source:NCBI

gene;Acc:101927056]	-	110	106	64	19	39
	50	0.790041299	0.686152035	0.443794487
0.156827254	0.356096627	0.345347976
ENSG00000182450 18.27526269	0	8.373632987	0	0
	0	8.882965225	0	5.491734267	0.044213307
	1	KCNK4	11	64291302	64300031	+
	2632	protein_coding	potassium two pore domain channel
subfamily K member 4 [Source:HGNC Symbol;Acc:HGNC:6279] -	19
	0	9	0	0	0	0.349034963	0
	0.159625643	0	0	0
ENSG00000179271 3301.089555	3084.452758	3428.537506
3269.839184	4117.546218	4567.862541	3271.35994
3985.082648	-0.284945711	0.044217957	0.25225284
GADD45GIP1	19	12953119	12957236	-	1782
	protein_coding	GADD45G interacting protein 1 [Source:HGNC
Symbol;Acc:HGNC:29996]	-	3432	3545	3685	2967	3397
	4948	93.11953443	86.68962765	96.53300559
92.51707426	117.1750661	129.1079571
ENSG00000226952 5.771135586	10.44102485	5.582421991	0
	0	0	7.264860809	0	5.203343008
0.044277616	1	AC093019.1	1	100099239
100099490	+	252	processed_pseudogene	novel
pseudogene	-	6	12	6	0	0	0
	1.151203036	2.075101303	1.111467442	0	0
	0
ENSG00000077549 3646.395834	4001.522774	3791.394936
4229.741419	4535.725036	4626.022472	3813.104515
4463.829642	-0.227311929	0.044341601	0.25225284	CAPZB
	1	19338776	19485539	-	3238
protein_coding	capping actin protein of muscle Z-line subunit beta
[Source:HGNC Symbol;Acc:HGNC:1491]	-	3791	4599	4075
	3838	3742	5011	56.608045	61.8935172
58.74850298	65.86279695	71.03531309	71.95791589
ENSG00000184900 3450.177224	3607.374086	3768.134844
4449.05318	4227.848457	4017.651127	3608.562051
4231.517588	-0.229623382	0.044377815	0.25225284	SUMO3
	21	44805617	44818779	-	2732
protein_coding	small ubiquitin-like modifier 3 [Source:HGNC
Symbol;Acc:HGNC:11124]	-	3587	4146	4050	4037	3488
	4352	63.48218965	66.13132138	69.20227374
82.1088743	78.47713532	74.06946535
ENSG00000147127 59.63506772	114.8512734	91.17955919
22.04138311	69.09041343	11.07808215	88.55530009
34.06995956	1.387940724	0.044379543	1	RAB41	X
	70282093	70285002	+	1344	protein_coding
	"RAB41, member RAS oncogene family [Source:HGNC
Symbol;Acc:HGNC:18293]" -	62	132	98	20	57
	12	2.230455881	4.279896437	3.40386904
0.826880539	2.606891447	0.415157603
ENSG00000134330 461.6908469	513.3503885	485.6707132

366.9890288	402.4213554	283.4142684	486.9039829
350.9415509	0.474236836	0.044538372	1	IAH1	2
	9473658 9496543 +	5908	protein_coding	isoamyl
acetate hydrolyzing esterase 1 (putative) [Source:HGNC
Symbol;Acc:HGNC:27696]	-	480	590	522	333	332
	307	3.928275761	4.351811973	4.124545057
3.131956998	3.454179937	2.416177896
ENSG00000184752 271.2433725	371.5264676	481.9490986
535.6056095	585.4503454	485.5892677	374.9063129
535.5484075	-0.513750556	0.044543306	1	NDUFA12 12
	94897055	95003770	-	1815	protein_coding
	NADH:ubiquinone oxidoreductase subunit A12 [Source:HGNC
Symbol;Acc:HGNC:23987]	-	282	427	518	486	483
	526	7.512313362	10.25202941	13.32291221
14.87892942	16.35753513	13.47535245
ENSG00000185787 3808.949487	3778.780911	4090.054512
4420.399382	4614.51235	4577.094276	3892.59497
4537.335336	-0.221125012	0.044588407	0.252667638
MORF4L1 15	78810487	78898133	+	6584
protein_coding	mortality factor 4 like 1 [Source:HGNC
Symbol;Acc:HGNC:16989]	-	3960	4343	4396	4011	3807
	4958	29.08081544	28.74475457	31.16835612
33.8512635	35.54188152	35.01448301
ENSG00000127980 510.7454993	403.7196276	391.699943
347.151784	279.9979913	300.0313916	435.3883566
309.060389	0.493987512	0.04477734	1	PEX1	7
	92487020	92528531	-	7849	protein_coding
	peroxisomal biogenesis factor 1 [Source:HGNC
Symbol;Acc:HGNC:8850]	-	531	464	421	315	231
	325	3.27100651	2.576097222	2.50388168
2.230017486	1.809026843	1.925307267
ENSG00000077152 454.9578553	558.5948295	540.5645295
665.6497699	726.0553973	653.606847	518.0390714
681.7706714	-0.395576116	0.044806111	1	UBE2T	1
	202331657	202341980	-	1652	protein_coding
	ubiquitin conjugating enzyme E2 T [Source:HGNC
Symbol;Acc:HGNC:25009]	-	473	642	581	604	599
	708	13.8437043	16.9349369	16.41769281
20.31603439	22.28764284	19.92756493
ENSG00000100418 1587.062286	1317.309302	1331.407645
1291.62505	1865.441163	2476.874535	1411.926411
1877.980249	-0.412093949	0.044873736	1	DESI1	22
	41598028	41621096	-	4972	protein_coding
	desumoylating isopeptidase 1 [Source:HGNC
Symbol;Acc:HGNC:24577]	-	1650	1514	1431	1172	1539
	2683	16.04552904	13.26946316	13.43552216
13.09810704	19.02632277	25.09115024
ENSG00000168135 10.58041524	0	15.81686231	0	0
	0	8.799092516	0	5.478371305	0.044925765
	1	KCNJ4	22	38426327	38455199	-

2065 protein_coding potassium voltage-gated channel
subfamily J member 4 [Source:HGNC Symbol;Acc:HGNC:6265] - 11
0 17 0 0 0 0.257557289 0
0.384303997 0 0 0
ENSG00000198040 1386.996252 1267.714434 1285.817865
1243.134007 796.3579232 1007.182302 1313.509517
1015.558078 0.370673728 0.044927743 1 ZNF84 12
133037292 133063304 + 9520 protein_coding
zinc finger protein 84 [Source:HGNC Symbol;Acc:HGNC:13159]
zf-C2H2 1442 1457 1382 1128 657 1091
7.323682841 6.669314554 6.776682373 6.58391469
4.242049983 5.328669936
ENSG00000148200 165.4392201 97.44956528 145.1429718
46.28690453 94.54477627 73.85388102 136.0105857
71.56185394 0.927126521 0.044928542 1 NR6A1 9
124517275 124771310 - 7124 protein_coding
nuclear receptor subfamily 6 group A member 1 [Source:HGNC
Symbol;Acc:HGNC:7985] GCNF-like 172 112 156 42
78 80 1.167362539 0.685098016 1.02222553
0.32759512 0.67300463 0.522152179
ENSG00000267169 104.8422965 110.5008463 120.9524765
55.10345777 20.60591278 80.31609561 112.0985398
52.00848872 1.101241649 0.045058556 1 AC022098.1
19 14137179 14171267 + 3186
antisense uncharacterized LOC100507373 [Source:NCBI
gene;Acc:100507373] - 109 127 130 50 17
87 1.654176866 1.73706691 1.904774712
0.87203974 0.327982378 1.269709881
ENSG00000134453 1667.858184 1322.529814 1438.404066
1285.012635 1027.871414 1210.280475 1476.264022
1174.388175 0.32946755 0.04507681 1 RBM17 10
6088987 6117457 + 6419 protein_coding RNA binding
motif protein 17 [Source:HGNC Symbol;Acc:HGNC:16944] - 1734
1520 1546 1166 848 1311 13.06119562
10.31893341 11.24315375 10.09353326 8.12037035
9.496560386
ENSG00000232060 46.16908469 6.960683234 19.53847697 0
3.636337549 7.385388102 24.2227483 3.67390855
2.703095449 0.045093695 1 SLC4A1APP1 9
30558880 30559481 - 602
processed_pseudogene solute carrier family 4 member 1 adaptor
protein pseudogene 1 [Source:HGNC Symbol;Acc:HGNC:49811] -
48 8 21 0 3 8 3.855191561
0.579098038 1.628429043 0 0.306317722
0.61790899
ENSG00000186141 3157.773021 3048.779256 2856.339252
3170.65296 3939.365678 3730.544165 3020.963843
3613.520934 -0.258362673 0.045175315 0.255205778 POLR3C
1 145824088 145842505 + 2793
protein_coding RNA polymerase III subunit C [Source:HGNC

Symbol;Acc:HGNC:30076]	-	3283	3504	3070	2877	3250
	4041	56.83307618	54.67033808	51.31135408
57.23754129	71.52531605	67.27426055
ENSG00000239494 0	5.220512425	21.3992843	0	0
	0	8.873265575	0	5.491384531	0.045268705
	1	RN7SL333P	1	169859756	169860052
	+	297	misc_RNA	"RNA, 7SL, cytoplasmic 333,
pseudogene [Source:HGNC Symbol;Acc:HGNC:46349]" -	0	6
	23	0	0	0	0	0.880346007
3.615075922	0	0	0
ENSG00000172482 0	0	0	0	9.69690013
14.7707762	0	8.155892111	-5.592366746	0.045282008
	1	AGXT	2	240868479	240880502	+
	4459	protein_coding	alanine--glyoxylate and serine--
pyruvate aminotransferase [Source:HGNC Symbol;Acc:HGNC:341]	-
	0	0	0	0	8	16	0	0
	0	0	0.110280792	0.166845128
ENSG00000213549 28.85567793	0	4.652018326	0	0
	0.923173513	11.16923208	0.307724504	4.860072632
	0.045329678	1	AC005077.1	7	76113026
	76113400	-	375	processed_pseudogene	"GrpE-
like 1, mitochondrial (E. coli) (GRPEL1) pseudogene"	-	30
	0	5	0	0	1	3.868042199	0
	0.622421767	0	0	0.123993737
ENSG00000008300 744.4764906	681.2768715	678.2642719
459.5628378	465.4512063	642.5287649	701.3392113
522.5142696	0.423058713	0.045336649	1	CELSR3	3
	48636469	48662915	-	13873	protein_coding
	cadherin EGF LAG seven-pass G-type receptor 3 [Source:HGNC
Symbol;Acc:HGNC:3230]	-	774	783	729	417	384
	696	2.69756421	2.45951782	2.453031798
1.670235508	1.701406933	2.332758988
ENSG00000144560 1698.637574	1973.353697	1758.462927
1607.918898	1378.171931	1456.767803	1810.151399
1480.952877	0.289616152	0.045339696	1	VGLL4	3
	11556070	11771350	-	7236	protein_coding
	vestigial like family member 4 [Source:HGNC
Symbol;Acc:HGNC:28966]	-	1766	2268	1890	1459	1137
	1578	11.8003084	13.6585026	12.19296373
11.20388848	9.658490716	10.14003511
ENSG00000233509 2.885567793	15.66153728	3.721614661	0
	0	0	7.422906577	0	5.235228073
0.045369985	1	ZNF197-AS1	3	44617128
44624797	-	623	antisense	ZNF197 antisense RNA 1
[Source:HGNC Symbol;Acc:HGNC:40620]	-	3	18	4
	0	0	0	0.23282758	1.259050229
0.299721557	0	0	0
ENSG00000256742 0	0	0	6.612414933	18.18168774
	0	0	8.264700892	-5.606070239	0.04537536
	1	AC145422.1	12	121580792	121593504

+	544	lincRNA novel transcript	-	0
0	0	6	15	0	0	0	0
0.612864399	1.694882984	0
ENSG00000143621 5029.544663	4767.19793	4849.263903
5329.606436	6326.015223	5524.2703	4882.002165
5726.630653	-0.230081093	0.045389834	0.255631088	ILF2
1	153661788	153671048	-	2281
protein_coding	interleukin enhancer binding factor 2 [Source:HGNC
Symbol;Acc:HGNC:6037]	-	5229	5479	5212	4836	5219
5984	110.8395038	104.6729859	106.6657908
117.80753	140.6401665	121.9824404
ENSG00000142347 63.48249144	82.6581134	122.8132838
28.65379804	66.6661884	18.46347025	89.65129622
37.9278189	1.248874442	0.045393101	1	MYO1F	19
8520790 8577577 -	8549	protein_coding	myosin IF
[Source:HGNC Symbol;Acc:HGNC:7600]	-	66	95	132
26	55	20	0.3732758	0.484246941
0.720783191	0.168993529	0.395452871	0.108779159
ENSG00000214629 22.12268641	0	4.652018326	0	0
0	8.924901579	0	5.498393563	0.045429086
1	RPSAP6	10	74371535	74372416	+
882	processed_pseudogene	ribosomal protein SA
pseudogene 6 [Source:HGNC Symbol;Acc:HGNC:31458]	-	23
0	5	0	0	0	1.26084142	0
0.264635105	0	0	0
ENSG00000134744 1412.004507	1168.524698	1489.576268
1320.278848	959.9931129	809.6231707	1356.701824
1029.965044	0.397891781	0.045520332	1	TUT4	1
52408282	52553487	-	16199	protein_coding
terminal uridylyl transferase 4 [Source:HGNC
Symbol;Acc:HGNC:28981]	-	1468	1343	1601	1198	792
877	4.381663952	3.61282067	4.613697989
4.109421193	3.005275815	2.517343068
ENSG00000236700 0	0	0	7.714484088	16.96957523
0	0	8.228019772	-5.5998204	0.045530581
1	LINC01010	6	134437716	134504581
+	2583	lincRNA long intergenic non-protein coding RNA
1010 [Source:HGNC Symbol;Acc:HGNC:48978]	-	0	0
0	7	14	0	0	0	0
0.150586375	0.33315857	0
ENSG00000173868 0	0	0	4.408276622	0
20.30981728	0	8.239364634	-5.610049966	0.045647345
1	PHOSPHO1	17	49223362	49230766
-	2375	protein_coding	phosphoethanolamine/
phosphocholine phosphatase [Source:HGNC Symbol;Acc:HGNC:16815]	-
0	0	0	4	0	22	0	0
0	0.093585469	0	0.430715088
ENSG00000187535 378.9712368	387.1880049	417.7512457
307.4772944	181.8168774	312.0326473	394.6368291
267.1089397	0.561070542	0.045710302	1	IFT140	16

1510427 1612110 - 8168 protein_coding intraflagellar transport 140 [Source:HGNC Symbol;Acc:HGNC:29077] - 394 445 449 279 150 338 2.332285484 2.374121165 2.566118146 1.898018835 1.128815308 1.924119271
ENSG00000133195 684.8414228 816.1401092 585.2239054 465.0731836 419.3909306 630.5275092 695.4018125 504.9972078 0.460214594 0.045711199 1 SLC39A11 17 72645949 73092712 - 5583 protein_coding solute carrier family 39 member 11 [Source:HGNC Symbol;Acc:HGNC:14463] - 712 938 629 422 346 683 6.166142858 7.321394494 5.25931343 4.200073271 3.809393454 5.68832097
ENSG00000185245 89.45260158 58.29572208 67.91946756 34.16414382 9.69690013 40.61963456 71.88926374 28.16022617 1.344151183 0.04578938 1 GP1BA 17 4932297 4935030 + 2501 protein_coding glycoprotein Ib platelet subunit alpha [Source:HGNC Symbol;Acc:HGNC:4439] - 93 67 73 31 8 44 1.797920455 1.167400053 1.362558647 0.688747516 0.196618173 0.818031454
ENSG00000144199 4.809279655 9.570939447 12.09524765 0 1.212112516 0 8.825155583 0.404037505 4.522071376 0.045792708 1 FAHD2B 2 97083583 97094882 - 2662 protein_coding fumarylacetoacetate hydrolase domain containing 2B [Source:HGNC Symbol;Acc:HGNC:25318] - 5 11 13 0 1 0 0.090816167 0.180070774 0.22797191 0 0.023090818 0
ENSG00000283296 0 13.05128106 13.02565131 0 0 0 8.692310792 0 5.462347507 0.04582538 1 MIR4256 1 112461770 112461833 - 64 miRNA microRNA 4256 [Source:HGNC Symbol;Acc:HGNC:38388] - 0 15 14 0 0 0 0 10.21338922 10.21160712 0 0 0
ENSG00000104237 57.71135586 75.69743017 88.38834819 38.57242044 30.30281291 31.38789943 73.93237807 33.42104426 1.145743515 0.045845053 1 RP1 8 54509422 54871720 + 15273 protein_coding "RP1, axonemal microtubule associated [Source:HGNC Symbol;Acc:HGNC:10263]" - 60 87 95 35 25 34 0.189945109 0.248229561 0.290365684 0.127337329 0.100615066 0.103510781
ENSG00000116670 902.2208632 991.0272754 904.3523626 1081.129841 1259.384904 1151.19737 932.5335004 1163.904039 -0.31936512 0.045866033 1 MAD2L2 1 11674480 11691650 - 3324 protein_coding mitotic arrest deficient 2 like 2 [Source:HGNC Symbol;Acc:HGNC:6764] - 938 1139 972 981 1039

1247	13.64404175	14.93211434	13.65058569
16.39910082	19.21329684	17.44361354
ENSG00000065328 911.8394225	736.092252	802.0079594
1234.317454	1049.689439	889.9392663	816.6465446
1057.982053	-0.373254882	0.045871997	1	MCM10	10
13161554	13211104	+	5454	protein_coding
minichromosome maintenance 10 replication initiation factor
[Source:HGNC Symbol;Acc:HGNC:18043]	-	948	846	862
1120	866	964	8.404162094	6.759488401
7.377991797	11.4107695	9.760006763	8.218506792
ENSG00000008853 524.2114824	421.9914211	596.3887494
867.3284253	558.78387	696.9960021	514.1972176
707.7027658	-0.461458607	0.045890272	1	RHOBTB2 8
22987417	23020199	+	6580	protein_coding
Rho related BTB domain containing 2 [Source:HGNC
Symbol;Acc:HGNC:18756]	-	545	485	641	787	461
755	4.004716943	3.21199191	4.547557214
6.646008347	4.306479577	5.335216851
ENSG00000108528 1549.549905	1360.813572	1524.931607
1574.856823	1945.440589	1942.357071	1478.431695
1820.884828	-0.300770372	0.045912954	1	SLC25A11
17	4937130 4940251 -	2669	protein_coding	solute
carrier family 25 member 11 [Source:HGNC Symbol;Acc:HGNC:10981] -
1611	1564	1639	1429	1605	2104	29.18422622
25.53564151	28.66661512	29.75059793	36.96356266
36.65457428
ENSG00000030304 9.618559309	16.53162268	0	0	0
0	8.71672733	0	5.466256156	0.045945378
1	MUSK	9	110668771	110801620	+
4246	protein_coding	muscle associated receptor tyrosine
kinase [Source:HGNC Symbol;Acc:HGNC:7525]	-	10	19
0	0	0	0	0.113873122	0.194998921
0	0	0	0
ENSG00000230555 45.20722875	44.37435562	29.77291729
18.73517564	10.90901265	8.308561615	39.78483389
12.65091663	1.656502241	0.045958416	1	AL450326.1
10	43420738	43422100	+	760
lincRNA novel transcript	-	47	51	32	17
9	9	2.990098411	2.924254599	1.965542423
1.24293201	0.727907639	0.550630084
ENSG00000185404 633.8630585	523.7914134	592.6671347
374.7035128	500.6024692	418.1976013	583.4405355
431.1678611	0.436853175	0.045958475	1	SP140L	2
230327184	230403732	+	5650	protein_coding
SP140 nuclear body protein like [Source:HGNC
Symbol;Acc:HGNC:25105]	SAND	659	602	637	340	413
453	5.639468605	4.643085074	5.263044237
3.343817088	4.493129791	3.728041793
ENSG00000199133 0	0	0	7.714484088	0
16.61712323	0	8.110535772	-5.586485648	0.045972529

1	MIRLET7D	9	94178834	94178920
+	87	miRNA	microRNA let-7d [Source:HGNC
Symbol;Acc:HGNC:31481]	-	0	0	0	7	0
18	0	0	0	4.470857534	0
9.620203759
ENSG00000230433 23.08454234	0	3.721614661	0	0
0	8.935385668	0	5.500059736	0.046099459
1	AL031770.1	6	524171	525581	+	851
antisense	novel transcript	-	24	0
4	0	0	0	1.363587144	0
0.21942013	0	0	0
ENSG00000137145 1169.616812	1206.808456	950.8725458
1012.801554	784.236798	779.1584447	1109.099271
858.7322655	0.369333687	0.046124448	1	DENND4C 9
19230435	19373545	+	8745	protein_coding
DENN domain containing 4C [Source:HGNC Symbol;Acc:HGNC:26079]
-	1216	1387	1022	919	647	844
6.723183697	6.911546671	5.455532129	5.839393488
4.547700203	4.487594953
ENSG00000163156 1761.15821	1673.174232	1618.902377
1741.269266	2169.681404	2301.471567	1684.411606
2070.807412	-0.298194256	0.046154208	1	SCNM1	1
151156664	151170297	+	3265	protein_coding
sodium channel modifier 1 [Source:HGNC Symbol;Acc:HGNC:23136]
-	1831	1923	1740	1580	1790	2493
27.11479811	25.66579354	24.87780724	26.88969926
33.6990149	35.50341353
ENSG00000184434 17.31340676	13.05128106	3.721614661	0
0	1.846347025	11.36210083	0.615449008
4.117650415	0.046168101	1	LRRC19	9	26993136
27005693	-	3609	protein_coding	leucine rich
repeat containing 19 [Source:HGNC Symbol;Acc:HGNC:23379]	-
18	15	4	0	0	2	0.241149763
0.181118568	0.051739133	0	0	0.02576761
ENSG00000259863 56.74949993	11.31111026	46.52018326
17.63310649	6.060562582	6.462214589	38.19359781
10.05196122	1.926193387	0.046205324	1	SH3RF3-AS1
2	109127327	109128930	-	1604
lincRNA SH3RF3 antisense RNA 1 [Source:HGNC Symbol;Acc:HGNC:44168]
-	59	13	50	16	5	7
1.778479503	0.353181207	1.45516311	0.554278027
0.191607719	0.202919925
ENSG00000057608 5464.303544	5714.720935	5574.978762
6498.90181	6642.376589	6195.417444	5584.667747
6445.565281	-0.206713229	0.046338437	0.259520159	GDI2
10	5765223 5842132 -	4033	protein_coding	GDP
dissociation inhibitor 2 [Source:HGNC Symbol;Acc:HGNC:4227]	-
5681	6568	5992	5897	5480	6711	68.10794612
70.96815583	69.35689122	81.24842472	83.52177141
77.37310667

ENSG00000144713 18773.50406 20998.64115 24174.67843
21932.27826 26084.66135 28235.26189 21315.60788
25417.4005 -0.253928301 0.046363079 0.259520159 RPL32
3 12834485 12841588 - 3720
protein_coding ribosomal protein L32 [Source:HGNC
Symbol;Acc:HGNC:10336] - 19518 24134 25983 19901 21520
30585 253.6842999 282.7124708 326.0561448
297.2651541 355.587665 382.2931912
ENSG00000178502 443.4155842 359.345272 279.1210996
179.6372723 216.9681404 310.1863003 360.6273186
235.5972377 0.612100234 0.04641409 1 KLHL11 17
41853545 41865431 - 2383 protein_coding
kelch like family member 11 [Source:HGNC
Symbol;Acc:HGNC:19008] - 461 413 300 163 179
336 9.35358505 7.552393452 5.876831627
3.800805149 4.617175142 6.556110325
ENSG00000183340 616.5496517 929.2512117 566.6158321
546.6263011 478.7844439 508.6686055 704.1388985
511.3597835 0.461790072 0.046432262 1 JRKL 11
96389989 96507574 + 3511 protein_coding
JRK like [Source:HGNC Symbol;Acc:HGNC:6200] - 641
1068 609 496 395 551 8.827310773
13.25558873 8.09715586 7.849877702 6.915341415
7.297125029
ENSG00000023909 811.8064057 1062.374279 982.5062704
895.9822234 1344.232781 1590.627962 952.2289849
1276.947655 -0.423582572 0.046481448 1 GCLM 1
93885205 93909456 - 5420 protein_coding
glutamate-cysteine ligase modifier subunit [Source:HGNC
Symbol;Acc:HGNC:4312] - 844 1221 1056 813 1109
1723 7.529122731 9.816913745 9.095166786
8.33495583 12.5770741 14.78144899
ENSG00000160813 319.3361691 446.3538124 374.0222734
296.4566028 206.0591278 277.8752273 379.904085
260.1303193 0.545597372 0.046546151 1 PPP1R35 7
100435282 100436565 - 1187 protein_coding
protein phosphatase 1 regulatory subunit 35 [Source:HGNC
Symbol;Acc:HGNC:28320] - 332 513 402 269 170
301 13.52348368 18.83324881 15.80961777
12.5925477 8.803301228 11.79089562
ENSG00000277067 32.70310165 0.870085404 8.373632987
2.204138311 0 0.923173513 13.98227335 1.042437275
3.731309192 0.046596667 1 CU634019.1 21
7048891 7087229 + 3058 lincRNA uncharacterized
LOC102724843 [Source:NCBI gene;Acc:102724843] - 34 1
9 2 0 1 0.537579442 0.014250205
0.137388716 0.036341643 0 0.015205249
ENSG00000130714 148.1258134 180.1076787 195.3847697
275.5172889 220.604478 301.8777387 174.5394206
265.9998352 -0.608762395 0.046601347 1 POMT1 9

131502902	131523806	+	6067	protein_coding
protein O-mannosyltransferase 1 [Source:HGNC
Symbol;Acc:HGNC:9202]	-	154	207	210	250	182
327	1.22729211	1.486808202	1.615813884
2.289697223	1.843931372	2.506136812
ENSG00000271967 0	21.75213511	4.652018326	0	0
0	8.801384477	0	5.481020114	0.046657363
1	AL583856.2	6	70596438	70596980
+	543	lincRNA novel transcript	-	0
25	5	0	0	0	0	2.006313414
0.429849287	0	0	0
ENSG00000206739 18.27526269	7.830768638	0	0	0
0	8.702010442	0	5.462688545	0.046660974
1	RF00019 22	19045256	19045367	+
112	misc_RNA		-	19	9	0
0	0	0	8.202321628	3.501733448	0
0	0	0
ENSG00000269343 626.168211	503.7794491	578.7110797
477.1959443	270.3010911	451.4318477	569.5529133
399.6429611	0.509460897	0.04666275	1	ZNF587B 19
57819741	57846238	+	4380	protein_coding
zinc finger protein 587B [Source:HGNC Symbol;Acc:HGNC:37142]
zf-C2H2 651	579	622	433	223	489
7.186345525	5.760538068	6.629218139	5.493205288
3.129522748	5.191176161
ENSG00000136932 200.0660336	198.3794722	227.0184943
301.9669486	356.3610798	263.1044511	208.488 307.1441598
-0.557811726	0.046720827	1	TRMO	9
97904489	97922570	-	4203	protein_coding	tRNA
methyltransferase O [Source:HGNC Symbol;Acc:HGNC:30967] -	208
228	244	274	294	285	2.392793176
2.363926966	2.710044812	3.622456813	4.299671741
3.152945676
ENSG00000164897 885.8693124	1044.97257	930.4036652
1010.597416	1271.50603	1361.680931	953.748516
1214.594792	-0.348910103	0.046748799	1	TMUB1	7
151081080	151083546	-	2005	protein_coding
transmembrane and ubiquitin like domain containing 1
[Source:HGNC Symbol;Acc:HGNC:21709]	-	921	1201	1000
917	1049	1475	22.20989318	26.10280796
23.28260975	25.41364754	32.15943954	34.20650173
ENSG00000149716 582.8846942	346.2939909	495.9051535
283.2317729	231.5134906	430.1988569	475.0279462
314.9813735	0.590119002	0.046833959	1	LTO1	11
69653076	69675416	-	7872	protein_coding
"LTO1, ABCE1 maturation factor [Source:HGNC
Symbol;Acc:HGNC:17589]" -	606	398	533	257	191
466	3.722106156	2.203213502	3.160735538
1.814095231	1.491405162	2.752528658
ENSG00000264349 6.732991517	12.18119566	2.791210996	0

0	0	7.235132724	0	5.197589723
0.046923071	1	MIR4258 1	154975693	154975783
+	91	miRNA	microRNA 4258 [Source:HGNC
Symbol;Acc:HGNC:38281]	-	7	14	3	0	0
0	3.719271346	6.704173439	1.538954919	0
0	0
ENSG00000118655 1176.349804	983.1965068	1201.151132
1416.158865	1326.051093	1421.68721	1120.232481
1387.965722	-0.309576948	0.04694123	1	DCLRE1B 1
113905141	113914086	+	3940	protein_coding
DNA cross-link repair 1B [Source:HGNC Symbol;Acc:HGNC:17641]
-	1223	1130	1291	1285	1094	1540
15.00829825	12.49800861	15.29593595	18.1225351
17.06744297	18.17420896
ENSG00000197757 11.54227117	11.31111026	3.721614661	0
0	0.923173513	8.858332029	0.307724504
4.527239922	0.046994292	1	HOXC6	12	53990624
54030823	+	3540	protein_coding	homeobox C6
[Source:HGNC Symbol;Acc:HGNC:5128]	Homeobox	12	13
4	0	0	1	0.163900093	0.160028999
0.052747607	0	0	0.01313493
ENSG00000183458 180.828915	140.0837501	321.9196682
168.6165808	81.21153859	108.011301	214.2774444
119.2798068	0.844309243	0.047004846	1	AC138932.1
16	14911551	14935708	+	6830
transcribed_unprocessed_pseudogene	polycystic kidney disease 1
(autosomal dominant) (PKD1) pseudogene	-	188	161	346
153	67	117	1.330878356	1.027220718
2.364838194	1.244751822	0.602977989	0.796519067
ENSG00000137492 1864.076794	2591.114334	1919.422761
2578.841824	2610.89036	2673.510493	2124.871296
2621.080892	-0.30259947	0.047058897	0.262074246	THAP12
11	76349956	76380971	-	7727
protein_coding	THAP domain containing 12 [Source:HGNC
Symbol;Acc:HGNC:9440]	THAP	1938	2978	2063	2340	2154
2896	12.1267403	16.79470496	12.46333738
16.82739886	17.1349226	17.42684078
ENSG00000102710 1787.12832	1506.117835	1209.524765
1319.176779	1136.96154	1059.803193	1500.92364
1171.980504	0.357065674	0.047078576	1	SUPT20H 13
37009312	37059713	-	10933	protein_coding
"SPT20 homolog, SAGA complex component [Source:HGNC
Symbol;Acc:HGNC:20596]" -	1858	1731	1300	1197	938
1148	8.216891986	6.899479324	5.550729198
6.083686776	5.273644501	4.882402261
ENSG00000105671 4445.698113	3415.955297	3841.636734
3921.162055	4899.358791	5587.969273	3901.096714
4802.830039	-0.300258595	0.047104848	0.262074246	DDX49
19	18919675	18928633	+	2780
protein_coding	DEAD-box helicase 49 [Source:HGNC

Symbol;Acc:HGNC:18684] - 4622 3926 4129 3558 4042
6053 80.38710002 61.54093597 69.33397871
71.11696126 89.37146426 101.2411095
ENSG00000260051 10.58041524 3.480341617 7.443229322 0
0 0 7.167995393 0 5.182640239
0.047107282 1 AL031600.1 16 1451760 1452653 +
570 antisense "novel transcript, antisense to CLCN7"
- 11 4 8 0 0 0
0.933080355 0.305804402 0.655180808 0 0 0
ENSG00000256588 16.35155083 9.570939447 0 0 0
0 8.640830091 0 5.452743137 0.047111522
1 AC027544.2 11 18507608 18508820
- 630 sense_intronic novel transcript -
17 11 0 0 0 0 1.304696774
0.760870478 0 0 0 0
ENSG00000279901 0 0 0 12.12276071 12.12112516
0 0 8.081295291 -5.57460031 0.047196555
1 AC092117.2 16 2683106 2684764 + 1659
TEC tec - 0 0 0 11 10
0 0 0 0 0.368432848 0.370510887
0
ENSG00000135269 8.656703379 5.220512425 21.3992843
2.204138311 0 0 11.75883337 0.73471277
4.015272945 0.04722506 1 TES 7 116210493
116258783 + 4280 protein_coding testin LIM
domain protein [Source:HGNC Symbol;Acc:HGNC:14620] - 9
6 23 2 0 0 0.10167167
0.061089431 0.25085924 0.025965594 0 0
ENSG00000174715 8.656703379 6.09059783 6.512825656 0
0 0 7.086708955 0 5.166698837
0.047288958 1 PPIAP72 3 138643874 138644694
- 504 processed_pseudogene peptidylprolyl
isomerase A pseudogene 72 [Source:HGNC Symbol;Acc:HGNC:53696] -
9 7 7 0 0 0 0.863402277
0.60523788 0.648356008 0 0 0
ENSG00000198805 1311.009634 1416.499038 1363.04137
1315.870572 1847.259475 2046.675678 1363.51668
1736.601908 -0.349189877 0.047305245 1 PNP 14
20468954 20477094 + 4139 protein_coding
purine nucleoside phosphorylase [Source:HGNC
Symbol;Acc:HGNC:7892] - 1363 1628 1465 1194 1524
2217 15.92214761 17.14026657 16.52297456
16.02953574 22.63272783 24.90584522
ENSG00000122490 764.6754651 867.475148 767.5830238
869.5325636 1019.386626 1197.356046 799.9112123
1028.758412 -0.363421094 0.04738455 1 PQLC1 18
79902420 79951664 - 5225 protein_coding
PQ loop repeat containing 1 [Source:HGNC
Symbol;Acc:HGNC:26188] - 795 997 825 789 841
1297 7.35668313 8.315099708 7.370784088

8.39078807	9.893661815	11.54209646
ENSG00000250571 527.0970502	522.0512425	673.6122536
244.6593525	545.4506323	376.6547932	574.2535154
388.9215927	0.563212362	0.047385441	1	GLI4	8
	143267433	143276931	+	3057	protein_coding
	GLI family zinc finger 4 [Source:HGNC Symbol;Acc:HGNC:4320]
	zf-C2H2 548	600	724	222	450	408
8.667350038	8.552919991	11.05577428	4.035241946
9.048246745	6.205770956
ENSG00000135624 9266.520039	7641.09002	8657.406105
8717.367019	10902.95208	10745.73969	8521.672054
10122.0196	-0.248344359	0.047518076	0.263418741	CCT7
	2	73233420	73253021	+	3591
protein_coding	chaperonin containing TCP1 subunit 7 [Source:HGNC
Symbol;Acc:HGNC:1622]	-	9634	8782	9305	7910	8995
	11640	129.715673	106.5704072	120.9614567
122.397662	153.968941	150.7192045
ENSG00000158164 27.893822	28.71281834	12.09524765
71.6344951	81.21153859	30.46472592	22.90062933
61.10358654	-1.412589117	0.047567023	1	TMSB15A X
	102513676	102516784	-	685	protein_coding
	thymosin beta 15a [Source:HGNC Symbol;Acc:HGNC:30744]	-
	29	33	13	65	67	33	2.046956638
	2.099336062	0.885928793	5.27272145	6.012174696
	2.240032846
ENSG00000162813 899.3352954	1083.256328	1066.2426
1358.851269	1168.476466	1281.364836	1016.278075
1269.56419	-0.321056677	0.047573815	1	BPNT1	1
	220057482	220090462	-	3113	protein_coding
	"3'(2'), 5'-bisphosphate nucleotidase 1 [Source:HGNC
Symbol;Acc:HGNC:1096]"	-	935	1245	1146	1233	964
	1388	14.52224324	17.42805125	17.18507899
22.00878154	19.03466656	20.7320078
ENSG00000284954 12.5041271	13.05128106	14.88645864
3.306207466	0	0	13.48062227	1.102069155
3.625191655	0.04757785	1	AL662884.3	6
32152802	32154365	-	1060
bidirectional_promoter_lncRNA	uncharacterized LOC100507547
[Source:NCBI gene;Acc:100507547]	-	13	15	16
	3	0	0	0.592978167	0.616657463
0.704628416	0.157263318	0	0
ENSG00000105755 153.896949	275.8170731	235.3921273
330.6207466	368.482205	305.5704327	221.7020498
334.8911281	-0.593664274	0.047665185	1	ETHE1	19
	43506719	43527244	-	1604	protein_coding
	"ETHE1, persulfide dioxygenase [Source:HGNC
Symbol;Acc:HGNC:23287]" -	160	317	253	300	304
	331	4.822995261	8.612187887	7.363125334
10.39271301	11.64974931	9.59521362
ENSG00000175334 2845.169844	2789.493806	3053.584829

3343.677818	3381.79392	3462.823846	2896.082826
3396.098528	-0.229862258	0.047720835	0.263418741	BANF1
	11	66002079	66004149	+	1645
protein_coding	barrier to autointegration factor 1 [Source:HGNC
Symbol;Acc:HGNC:17397]	-	2958	3206	3282	3034	2790
	3751	86.94277223	84.92903969	93.13624197
102.4853333	104.2523039	106.0259518
ENSG00000229927 0	0.870085404	8.373632987	28.65379804
	24.24225033	12.00125567	3.081239464	21.63243468
	-2.81128813	0.047759377	1	RHEBP1	10
46634911	46635466	-	556	processed_pseudogene
	RHEB pseudogene 1 [Source:HGNC Symbol;Acc:HGNC:10010]	-
	0	1	9	26	20	13	0
0.078376128	0.755637937	2.598427477	2.211070368
1.087175305
ENSG00000235636 138.5072541	123.5521274	106.0660178
70.53242595	75.15097601	57.23675779	122.7084664
67.64005325	0.861198931	0.04776987	1	NUS1P1	X
	47512602	47513483	-	882
processed_pseudogene	NUS1 pseudogene 1 [Source:HGNC
Symbol;Acc:HGNC:38472]	-	144	142	114	64	62
	62	7.893963672	7.01581869	6.033680398
4.032027008	4.320862682	3.268542396
ENSG00000130449 494.3939485	573.3862814	577.7806761
450.7462846	346.6641797	418.1976013	548.520302
405.2026885	0.436330742	0.047787225	1	ZSWIM6	5
	61332273	61546170	+	5501	protein_coding
	zinc finger SWIM-type containing 6 [Source:HGNC
Symbol;Acc:HGNC:29316]	-	514	659	621	409	286
	453	4.517755159	5.220383008	5.269822544
4.131366339	3.195742279	3.829019475
ENSG00000235527 14.42783896	11.31111026	0	0	0
	0	8.57964974	0	5.442731682	0.047794009
	1	HIPK1-AS1	1	113924000	113929492
	-	3087	antisense	HIPK1 antisense RNA 1
[Source:HGNC Symbol;Acc:HGNC:50576]	-	15	13	0
	0	0	0	0.234939395	0.18351236	0
	0	0	0
ENSG00000175315 0	0	0	0	0	29.54155241
	0	9.847184136	-5.866885458	0.047803152	1
	CST6	11	66011841	66013505	+	759
	protein_coding	cystatin E/M [Source:HGNC
Symbol;Acc:HGNC:2478]	-	0	0	0	0	0
	32	0	0	0	0	0	1.960375294
ENSG00000236088 799.3022786	662.1349926	762.0006018
592.9132056	579.3897828	543.749199	741.1459577
572.0173958	0.373804324	0.047810625	1	COX10-AS1
	17	13756478	14069495	-	6456
processed_transcript	COX10 antisense RNA 1 [Source:HGNC
Symbol;Acc:HGNC:38873]	-	831	761	819	538	478

589	6.223557674	5.136647137	5.921972903
4.630531017	4.551051343	4.242118453
ENSG00000172725 2515.253259	2411.876741	2323.217952
2432.266626	3169.67423	3213.566998	2416.782651
2938.502618	-0.282103892	0.047884836	0.263418741	CORO1B
11	67435510	67443821	-	5327
protein_coding	coronin 1B [Source:HGNC Symbol;Acc:HGNC:2253]	-
2615	2772	2497	2207	2615	3481	23.73505339
22.67613986	21.88174141	23.02139731	30.17424113
30.38451753
ENSG00000164733 66022.75296	82078.63652	72980.8635
68300.73591	59731.69269	61807.38985	73694.08433
63279.93948	0.219806215	0.047920403	0.263418741	CTSB
8	11842524	11869448	-	7428
protein_coding	cathepsin B [Source:HGNC Symbol;Acc:HGNC:2527]	-
68641	94334	78440	61975	49279	66951	446.799752
553.4201308	492.9600508	463.6141515	407.7907322
419.0985818
ENSG00000231584 12.5041271	25.23247672	9.304036652
4.408276622	0	0	15.68021349	1.469425541
3.426908362	0.047936896	1	FAHD2CP 2	96010551
96023380	+	2140
transcribed_unprocessed_pseudogene	"fumarylacetoacetate hydrolase
domain containing 2C, pseudogene [Source:HGNC Symbol;Acc:HGNC:44135]"
-	13	29	10	4	0	0
0.293718158	0.590531165	0.21813847	0.103862378	0
0
ENSG00000078124 706.9641092	743.0529352	653.143373
933.4525746	972.1142381	788.3901799	701.0534725
897.9856642	-0.356358562	0.047982221	1	ACER3	11
76860867	77026797	+	8442	protein_coding
alkaline ceramidase 3 [Source:HGNC Symbol;Acc:HGNC:16066]
-	735	854	702	847	802	854
4.20962304	4.408299782	3.881841513	5.575067194
5.839509653	4.703742523
ENSG00000172175 721.3919482	669.0956759	680.1250793
1233.215385	865.4483366	698.8423491	690.2042344
932.5020236	-0.433594763	0.047983314	1	MALT1	18
58671386	58754477	+	11527	protein_coding
MALT1 paracaspase [Source:HGNC Symbol;Acc:HGNC:6819]	-
750	769	731	1119	714	757	3.145909223
2.907158058	2.96037767	5.394185	3.807406779
3.053589155
ENSG00000114745 974.360058	968.4050549	1122.06682
1118.600193	1557.564583	1227.820772	1021.610644
1301.328516	-0.348651594	0.04803102	1	GORASP1 3
39096659	39108363	-	6256	protein_coding
golgi reassembly stacking protein 1 [Source:HGNC
Symbol;Acc:HGNC:16769]	-	1013	1113	1206	1015	1285
1330	7.829137524	7.752772178	8.999048731

9.015324134	12.62565005	9.885210437
ENSG00000070759 13.46598303	105.2803339	68.84987122
29.7558672	9.69690013	21.23299079	62.53206272
20.22858604	1.626220394	0.048045822	1	TESK2	1
	45343883	45491166	-	3322	protein_coding
	testis associated actin remodelling kinase 2 [Source:HGNC
Symbol;Acc:HGNC:11732]	-	14	121	74	27	8
	23	0.203765017	1.587246361	1.039867793
0.451623133	0.148025903	0.321928352
ENSG00000162408 2031.439726	1692.316111	1628.206414
1369.87196	1334.53588	1614.630474	1783.987417
1439.679438	0.308679362	0.048070372	1	NOL9	1
	6521347 6554535 -	7293	protein_coding	nucleolar
protein 9 [Source:HGNC Symbol;Acc:HGNC:26265]	-	2112	1945
	1750	1243	1101	1749	14.00196271	11.62176233
	11.20154353	9.470588324	9.279583107	11.1510205
ENSG00000197283 488.6228129	304.5298915	380.5350991
306.3752252	189.0895525	287.1069625	391.2292678
260.8572467	0.582937513	0.048086864	1	SYNGAP1 6
	33419661	33457541	+	12129	protein_coding
	synaptic Ras GTPase activating protein 1 [Source:HGNC
Symbol;Acc:HGNC:11497]	-	508	350	409	278	156
	311	2.0250695	1.25748162	1.574143599
1.273596461	0.790582074	1.192247474
ENSG00000232956 621.3589314	551.6341463	494.9747499
598.4235514	701.8131469	970.2553619	555.9892759
756.8306867	-0.446028987	0.048099698	1	SNHG15	7
	44983023	44986961	-	3647	lincRNA small
nucleolar RNA host gene 15 [Source:HGNC Symbol;Acc:HGNC:27797]	-
	646	634	532	543	579	1051	8.56442028
	7.575513776	6.809604749	8.273249275	9.758659405
	13.39978934
ENSG00000140406 699.2692618	686.4973839	735.9492992
835.3684198	810.9032734	1102.269174	707.2386483
916.1802891	-0.374468838	0.048159807	1	TLNRD1	15
	81000944	81005788	+	4845	protein_coding
	talin rod domain containing 1 [Source:HGNC
Symbol;Acc:HGNC:13519]	-	727	789	791	758	669
	1194	7.255073991	7.096460987	7.621294397
8.693356064	8.487498227	11.45886396
ENSG00000215146 6.732991517	19.14187889	0	0	0
	0	8.624956803	0	5.451344561	0.04820188
	1	BX322639.1	10	42331866	42367974
	-	7208	transcribed_unprocessed_pseudogene	zinc
finger protein 91 pseudogene [Source:NCBI gene;Acc:441666]	-
	7	22	0	0	0	0	0.046955285
	0.133004551	0	0	0	0
ENSG00000113070 695.4218381	904.018735	662.4474096
618.2607962	482.4207815	594.5237422	753.9626609
565.06844	0.415690909	0.04820283	1	HBEGF	5

140332843	140346631	-	3018	protein_coding
heparin binding EGF like growth factor [Source:HGNC
Symbol;Acc:HGNC:3059]	-	723	1039	712	561	398
644	11.58297925	15.00219858	11.01302928
10.32893797	8.10608581	9.921964071
ENSG00000167695 1283.115812	1439.121259	1583.547038
1752.289957	1592.715846	1970.97545	1435.26137
1771.993751	-0.30448745	0.048234478	1	FAM57A	17
732412	742972	+	2740	protein_coding	family with
sequence similarity 57 member A [Source:HGNC Symbol;Acc:HGNC:29646]
-	1334	1654	1702	1590	1314	2135
23.54000134	26.30531702	28.99713088	32.24471964
29.4776028	36.23083429
ENSG00000112118 6366.524407	5430.203008	5895.968026
7351.903336	6722.376015	6593.305228	5897.565147
6889.19486	-0.224242051	0.048286153	0.264636945	MCM3
6	52264009	52284881	-	3650
protein_coding	minichromosome maintenance complex component 3
[Source:HGNC Symbol;Acc:HGNC:6945]	-	6619	6241	6337
6671	5546	7142	87.68003876	74.51091831
81.04698781	101.55706	93.39730849	90.98252795
ENSG00000112357 135.6216863	204.47007	145.1429718
124.5338146	90.90843872	41.54280807	161.7449093
85.66168712	0.920910537	0.048323962	1	PEX7	6
136822564	136913937	+	1835	protein_coding
peroxisomal biogenesis factor 7 [Source:HGNC
Symbol;Acc:HGNC:8860]	-	141	235	156	113	75
45	3.715217644	5.580722032	3.968574757
3.421798397	2.512306113	1.140269383
ENSG00000135686 1627.460235	1330.360583	1451.429718
1227.705039	1215.748854	1136.426594	1469.750179
1193.293496	0.300696582	0.048486305	1	KLHL36	16
84648525	84667686	+	9995	protein_coding
kelch like family member 36 [Source:HGNC
Symbol;Acc:HGNC:17844]	-	1692	1529	1560	1114	1003
1231	8.185001753	6.66627591	7.285977667
6.193190459	6.168300099	5.726724262
ENSG00000122986 10.58041524	6.960683234	3.721614661	0
0	0	7.087571045	0	5.166858776
0.048544721	1	HVCN1	12	110627841	110704950
-	3688	protein_coding	hydrogen voltage gated channel
1 [Source:HGNC Symbol;Acc:HGNC:28240]	-	11	8	4
0	0	0	0.144212528	0.094527391
0.050630838	0	0	0
ENSG00000214535 21.16083048	6.09059783	0.930403665
1.102069155	0	0	9.393943992	0.367356385
4.610754648	0.048561503	1	RPS15AP1	20
21166206	21166596	-	391	processed_pseudogene
ribosomal protein S15a pseudogene 1 [Source:HGNC
Symbol;Acc:HGNC:16598]	-	22	7	1	1	0

0	2.720490038	0.780153175	0.119390365
0.142113484	0	0
ENSG00000279368 0	18.27179349	7.443229322	0	0
0	8.57167427	0	5.442647396	0.048568479
1	AL031123.3	6	6685148 6686984 +	1837
TEC	TEC	-	0	21	8	0	0
0	0	0.498159866	0.203295079	0	0
0
ENSG00000169933 0	0	0	2.204138311	6.060562582
12.00125567	0	6.755318853	-5.32146049
0.048580044	1	FRMPD4	X	12138466	12724523
+	8478	protein_coding	FERM and PDZ domain containing
4 [Source:HGNC Symbol;Acc:HGNC:29007]	-	0	0	0
2	5	13	0	0	0	0.013108368
0.036251331	0.071298593
ENSG00000174748 30542.77323	35976.29129	35600.03544
37649.98856	39729.41195	40284.52257	34039.69999
39221.30769	-0.204412665	0.048606916	0.265141844	RPL15
3	23916545	23924374	+	7222
protein_coding	ribosomal protein L15 [Source:HGNC
Symbol;Acc:HGNC:10306]	-	31754	41348	38263	34163	32777
43637	212.5896774	249.4914237	247.3247447
262.851561	278.9710116	280.949601
ENSG00000182095 6699.326559	4604.491959	6417.924482
4587.913894	4648.4515	5261.165849	5907.247667
4832.510414	0.289492813	0.048667103	0.265141844	TNRC18
7	5306790 5425414 -	12572	protein_coding
trinucleotide repeat containing 18 [Source:HGNC
Symbol;Acc:HGNC:11962]	-	6965	5292	6898	4163	3835
5699	26.78662297	18.34315606	25.61325973
18.39984151	18.75030585	21.07780114
ENSG00000237685 5.771135586	0	1.86080733	12.12276071
20.60591278	22.15616431	2.543980972	18.29494593
-2.858243887	0.048774739	1	AL139039.3	6
10511036	10514546	-	1475	antisense
"novel transcript, antisense to GCNT2"	-	6	0
2	11	17	24	0.196680112	0
0.063297129	0.414393284	0.708441936	0.756571957
ENSG00000174903 3237.607064	2935.668154	2691.657803
3008.648794	3833.911889	3914.255694	2954.977674
3585.605459	-0.279189806	0.048815423	0.265163069	RAB1B
11	66268533	66277492	+	2007
protein_coding	"RAB1B, member RAS oncogene family [Source:HGNC
Symbol;Acc:HGNC:18370]" -	3366	3374	2893	2730	3163
4240	81.09012234	73.25821011	67.28946835
75.58355561	96.87220375	98.23121194
ENSG00000280063 62.52063551	31.32307455	66.05866023
14.32689902	2.424225033	31.38789943	53.3007901
16.04634116	1.717691614	0.048823457	1	AC012676.5
16	4346694 4348648 -	1955	TEC	tec	-

65	36	71	13	2	34	1.607562295
0.802443266	1.695343177	0.369495058	0.062882615
0.808654809
ENSG00000109790 1186.930219	1379.085366	1091.363499
953.2898194	1113.931402	772.6962302	1219.126361
946.6391507	0.366161748	0.048891449	1	KLHL5	4
39045039	39126857	+	5144	protein_coding
kelch like family member 5 [Source:HGNC Symbol;Acc:HGNC:6356]
-	1234	1585	1173	865	919	837
11.59886293	13.42724472	10.64493682	9.343878685
10.98150621	7.56581149
ENSG00000172301 1259.069414	1408.668269	1361.180562
1762.20858	1517.56487	1630.324423	1342.972748
1636.699291	-0.285384161	0.048951282	1	COPRS	17
31851864	31859337	-	1432	protein_coding
coordinator of PRMT5 and differentiation stimulator
[Source:HGNC Symbol;Acc:HGNC:28848]	-	1309	1619	1463
1599	1252	1766	44.19751431	49.26771591
47.69219862	62.04652874	53.74136228	57.34277413
ENSG00000227071 0	0	0	0	7.272675098
16.61712323	0	7.963266109	-5.558875474	0.048988989
1	FOCAD-AS1	9	20683304	20684688
-	423	lincRNA FOCAD antisense RNA 1 [Source:HGNC
Symbol;Acc:HGNC:49789]	-	0	0	0	0	6
18	0	0	0	0	0.871883067
1.978623468
ENSG00000174197 3147.192606	2487.574171	2565.122905
2583.2501	1972.107064	2097.450221	2733.296561
2217.602462	0.301503919	0.049012574	0.265448629	MGA
15	41621224	41773081	+	17651
protein_coding	"MGA, MAX dimerization protein [Source:HGNC
Symbol;Acc:HGNC:14010]" T-box	3272	2859	2757	2344	1627
2272	8.962830772	7.058354037	7.291443032
7.379048011	5.665856856	5.98508097
ENSG00000230021 1.923711862	4.350427021	8.373632987
11.02069155	24.24225033	36.003767	4.882590623
23.75556963	-2.283425904	0.0490204	1	AL669831.3
1	586071	827796	-	5495
transcribed_processed_pseudogene	uncharacterized LOC101928626
[Source:NCBI gene;Acc:101928626]	-	2	5	9
10	20	39	0.017598008	0.039651617
0.076457633	0.101121696	0.223722498	0.330010629
ENSG00000255670 18.27526269	0	7.443229322	0	0
0	8.57283067	0	5.440430252	0.049082173
1	AC007619.1	12	12485353	12491581
+	412	lincRNA novel transcript	-	19
0	8	0	0	0	2.229757336	0
0.906439467	0	0	0
ENSG00000268564 0	0	0.930403665	5.510345777
24.24225033	0	0.310134555	9.917532034	-4.906506707

0.049154246	1	AC003956.1	19	15124160
15126174	+	356	antisense	"novel
transcript, antisense to ILVBL" -	0	0	1	5
20	0	0	0	0.131128181	0.780426576
3.453244731	0
ENSG00000233013 0	28.71281834	21.3992843	2.204138311
0	1.846347025	16.70403421	1.350161779
3.608372573	0.049249885	1	FAM157B 9	138217068
138253217	+	2073
transcribed_unprocessed_pseudogene	family with sequence
similarity 157 member B [Source:HGNC Symbol;Acc:HGNC:34080]	-
0	33	23	2	0	2	0
0.693702461	0.517934177	0.053609621	0	0.044860252
ENSG00000273748 201.9897455	228.8324613	255.8610079
174.1269266	143.0292769	123.7052507	228.8944049
146.9538181	0.640500926	0.049253563	1	AL592183.1
GL000219.1	54224	83311	-	372	protein_coding
-	210	263	275	158	118	134
27.29465262	30.80856047	34.50927138	23.60077099
19.49783665	16.74915403
ENSG00000261499 35.58866945	8.700854042	17.67766964
6.612414933	2.424225033	0	20.65573104	3.012213322
2.793009307	0.049312933	1	AC233699.1	17
36274191	36320547	+	1168
unprocessed_pseudogene	aminopeptidase puromycin sensitive (NPEPPS)
pseudogene	-	37	10	19	6	2	0
1.531651984	0.373091844	0.759375872	0.285443693
0.105253007	0
ENSG00000258366 85.60517785	103.5401631	135.8389351
67.22621848	42.42393807	58.1599313	108.328092
55.93669595	0.952003884	0.049351671	1	RTEL1	20
63657810	63696253	+	6440	protein_coding
regulator of telomere elongation helicase 1 [Source:HGNC
Symbol;Acc:HGNC:15888]	-	89	119	146	61	35
63	0.668198284	0.805229527	1.058310303
0.526327439	0.334063892	0.45486833
ENSG00000147394 246.2351183	233.1828883	225.157687
147.6772668	133.3323768	178.172488	234.8585645
153.0607105	0.615735113	0.049360718	1	ZNF185	X
152914442	152973480	+	4812	protein_coding
zinc finger protein 185 with LIM domain [Source:HGNC
Symbol;Acc:HGNC:12976]	-	256	268	242	134	110
193	2.572264139	2.426988805	2.34766315
1.547359492	1.405123272	1.864930744
ENSG00000122783 483.8135333	391.5384319	584.2935017
599.5256205	675.1466716	691.456961	486.548489
655.3764177	-0.430212881	0.049426906	1	CYREN	7
135092363	135170795	-	5321	protein_coding
cell cycle regulator of NHEJ [Source:HGNC
Symbol;Acc:HGNC:22432]	-	503	450	628	544	557

749	4.570628703	3.685342475	5.509502959
5.680907062	6.434418383	6.545149591
ENSG00000031691 358.7722622	386.3179195	331.2237048
473.8897368	610.9047082	410.8122132	358.7712955
498.5355527	-0.473176647	0.049427801	1	CENPQ	6
49463378	49493107	+	1733	protein_coding
centromere protein Q [Source:HGNC Symbol;Acc:HGNC:21347]
-	373	444	356	430	504	445
10.40666287	11.16459581	9.589533289	13.78738606
17.87636996	11.93967393
ENSG00000179941 621.3589314	797.8683157	746.1837395
555.4428543	566.0565451	540.0565049	721.8036622
553.8519681	0.382586967	0.049501482	1	BBS10	12
76344474	76348442	-	3595	protein_coding
Bardet-Biedl syndrome 10 [Source:HGNC Symbol;Acc:HGNC:26291]
-	646	917	802	504	467	585
8.688300629	11.11550091	10.41409438	7.790111708
7.984823965	7.566377226
ENSG00000118495 760.8280414	939.6922366	950.8725458
681.078738	707.8737095	684.0715729	883.7976079
691.0080068	0.35533486	0.049514042	1	PLAGL1	6
143940300	144064599	-	8733	protein_coding
PLAG1 like zinc finger 1 [Source:HGNC Symbol;Acc:HGNC:9046]
zf-C2H2 791	1080	1022	618	584	741
4.379396227	5.389132892	5.463028567	3.932213217
4.110519825	3.945352085
ENSG00000126267 1728.455108	1769.753712	2243.203237
1887.844463	2390.285882	3075.090971	1913.804019
2451.073772	-0.357384657	0.04952558	1	COX6B1	19
35648223	35658861	+	936	protein_coding
cytochrome c oxidase subunit 6B1 [Source:HGNC
Symbol;Acc:HGNC:2280]	-	1797	2034	2411	1713	1972
3331	92.826814	94.69644983	120.2451027
101.693585	129.5025804	165.474014
ENSG00000263142 209.6845929	113.981188	129.3261095
76.04277172	103.0295639	73.85388102	150.9972968
84.30873888	0.842058953	0.049594343	1	LRRC37A17P
17	46978481	47054569	+	5074
transcribed_unprocessed_pseudogene	"leucine rich repeat
containing 37 member A17, pseudogene [Source:HGNC
Symbol;Acc:HGNC:48365]" -	218	131	139	69	85
80	2.07733839	1.125069705	1.278822807
0.755632574	1.029712116	0.733112361
ENSG00000238086 10.58041524	0	14.88645864	0	0
0	8.488957961	0	5.426580497	0.049640161
1	PPP1R26P1	13	48316863	48320475
-	3613	processed_pseudogene	protein phosphatase 1
regulatory subunit 26 pseudogene 1 [Source:HGNC
Symbol;Acc:HGNC:42015]	-	11	0	16	0	0
0	0.147206145	0	0.206727407	0	0

0
ENSG00000269190 1790.975743 1766.273371 1978.038192
1469.058184 1504.231633 1613.7073 1845.095769
1528.999039 0.270809219 0.049683939 1 FBXO17 19
38941401 38975910 - 4960 protein_coding
F-box protein 17 [Source:HGNC Symbol;Acc:HGNC:18754] -
1862 2030 2126 1333 1241 1748 18.15094399
17.83499365 20.00910299 14.93346253 15.37933175
16.38667234
ENSG00000167680 768.5228888 791.7777179 748.9749505
943.371197 1066.659014 899.1710014 769.7585191
969.7337376 -0.332552463 0.049751105 1 SEMA6B 19
4542593 4559808 - 4412 protein_coding semaphorin 6B
[Source:HGNC Symbol;Acc:HGNC:10739] - 799 910 805
856 880 974 8.756135872 8.988029441
8.517387626 10.780783 12.26011457 10.26489405
ENSG00000099204 19.23711862 18.27179349 12.09524765
41.87862791 31.51492542 65.5453194 16.53471992
46.31295758 -1.488680128 0.04977008 1 ABLIM1 10
114431113 114685003 - 10039 protein_coding
actin binding LIM protein 1 [Source:HGNC Symbol;Acc:HGNC:78]
- 20 21 13 38 26 71
0.096325386 0.091156457 0.060450366 0.21033192
0.159195304 0.328850807
ENSG00000271367 0 0 0 8.816553243 0
14.7707762 0 7.862443149 -5.541313466 0.049820494
1 AL034374.1 6 53350158 53350705
+ 548 lincRNA novel transcript - 0
0 0 8 0 16 0 0 0
0.811187915 0 1.357595664
ENSG00000119599 766.599177 701.2888358 638.2569143
932.3505055 844.8424239 898.2478279 702.048309
891.8135857 -0.345432505 0.0498617 1 DCAF4 14
72926332 72959703 + 5581 protein_coding
DDB1 and CUL4 associated factor 4 [Source:HGNC
Symbol;Acc:HGNC:20229] - 797 806 686 846 697
973 6.904742951 6.293346111 5.737968094
8.423069501 7.676585932 8.106471046
ENSG00000148834 3232.797784 4251.237285 3821.167853
3729.402022 5061.781868 5112.534913 3768.400974
4634.572935 -0.298442803 0.049904939 0.269486672 GSTO1
10 104235356 104267459 + 1691
protein_coding glutathione S-transferase omega 1 [Source:HGNC
Symbol;Acc:HGNC:13312] - 3361 4886 4107 3384 4176
5538 96.10060491 125.9123857 113.3775665
111.1984645 151.7973684 152.2791212
