# Supplementary material for: Multiomic screening of invasive GBM cells reveals targetable transsulfuration pathway alterations
Source: J Clin Invest. 2024 Feb 1;134(3):e170397. doi: 10.1172/JCI170397 (PMC10849762; doi:10.1172/JCI170397)
Supplement: Supplemental table 18 [file jci-134-170397-s080.pdf]

Pharmacologic Inhibitor	Target	Concentration
Metformin (Cayman Chemical)	Complex 1	500 μM
Deguelin (Cayman Chemical)	Complex 1	0.5 μM
Cystathionine-y-lyase-IN-1 (Med Chem Express)	CTH (CSE)	10 – 40 μM
Entacapone (Sigma)	COMT	0.5 μM
Desipramine (Sigma)	SMPD1 (ASM)	0.5 μM
5'-Deoxy-5'-methylthioadenosine (Cayman Chemical)	SMS (MTA)	50 μM

Supplemental Table 16. Drug concentrations used for invasion assays. Shown are the concentrations used and vendors for the six drugs used to target the products of the metabolic genes that emerged from a CRISPR screen of metabolic genes whose targeting slowed GBM invasion in hydrogel devices. These concentrations represent the highest concentrations which did not affect GBM43 cell viability after 48 hours in culture.
