# Supplementary material for: Multiomic screening of invasive GBM cells reveals targetable transsulfuration pathway alterations
Source: J Clin Invest. 2024 Feb 1;134(3):e170397. doi: 10.1172/JCI170397 (PMC10849762; doi:10.1172/JCI170397)
Supplement: Supplemental table 19 [file jci-134-170397-s081.pdf]

Additional Cell Culture Reagents	Concentration
N-Acetyl-L-cysteine (Sigma-Aldrich, A9165)	1 – 8 mM
L-Glutathione (Sigma-Aldrich, G6013)	100 µM
MnTBAP (Sigma-Aldrich, 475870)	30 µM
L-Cysteine (Sigma-Aldrich, C7477-25G)	50 µM
Erastin (MedChemExpress, HY-15763)	1 nM – 5 µM
Hydrogen peroxide solution (Sigma-Aldrich, H1009)	0.1 – 100 µM
Sodium Hydrogen Sulfide, NaHS (Cayman Chemical, 207683-19-0)	0.01 – 1000 µM
L-Methionine (Sigma, M5308)	0.201 mM
L-Glutamine (Gibco, 25030081)	4 mM
LIVE/DEAD – Ethidium homodimer (ThermoFisher, R37601)	4 µM
LIVE/DEAD – Calcien AM (ThermoFisher, R37601)	1 µM

Supplemental Table 19. Concentrations used for additional cell culture reagents. Shown are the concentrations used and vendors for reagents used for cell culture experiments.
