# Supplementary material for: Multiomic screening of invasive GBM cells reveals targetable transsulfuration pathway alterations
Source: J Clin Invest. 2024 Feb 1;134(3):e170397. doi: 10.1172/JCI170397 (PMC10849762; doi:10.1172/JCI170397)
Supplement: Supplemental table 7 [file jci-134-170397-s087.pdf]

SUPPLEMENTAL TABLE 5. Results of bulk RNA-sequencing comparing invasive vs. core cells in 3D hydrogels. Shown are the results of bulk RNA-seq comparing edge vs. core fractions after GBM43 cells invaded 3D hydrogels.

gene_id	NT_INV1	NT_INV2	NT_INV3	NT_CORE1	NT_CORE2	NT_CORE3	WT_Invasive	Wt_Core	log2FoldChange	pvalue	padj
ENSG00000141753	3110.963562	2740.829989	3411.912992	589.4660736	681.4706214	546.0505157	3087.902181	605.6624036	2.3510759	1.75E-50	3.09E-46
ENSG00000134871	6106.706252	6013.542065	8497.813961	1483.506885	1518.184131	1337.09052	6872.687426	1446.260512	2.248631173	1.23E-45	1.09E-41
ENSG00000114270	3686.160675	4517.015262	4357.018704	1186.183927	1189.434485	1002.386571	4186.731547	1126.001661	1.895226338	3.45E-38	2.04E-34
ENSG00000084636	1758.252372	1560.246455	2176.184369	304.5747375	196.3365944	144.0607206	1831.561065	214.9906842	3.092903409	5.43E-38	2.40E-34
ENSG00000184371	5901.667534	5021.462499	5686.909151	1705.204143	1600.371543	1229.26064	5536.679728				

gene_id	gene_name	gene_chr	gene_start	gene_end	gene_strand	gene_length	gene_biotype	gene_description	tf_family
ENSG00000141753	IGFBP4	17	40443461	40457731	+	2200	protein_coding	insulin like growth factor binding protein 4 [Source:HGNC Symbol;Acc:HGNC:5473]	-
ENSG00000134871	COL4A2	13	110305812	110513209	+	19293	protein_coding	collagen type IV alpha 2 chain [Source:HGNC Symbol;Acc:HGNC:2203]	-
ENSG00000114270	COL7A1	3	48564073	48595267	-	11071	protein_coding	collagen type VII alpha 1 chain [Source:HGNC Symbol;Acc:HGNC:2214]	-
ENSG00000084636	COL16A1	1	31652247	31704319	-	9269	protein_coding	collagen type XVI alpha 1 chain [Source:HGNC Symbol;Acc:HGNC:2193]	-

gene_id	NT_INV1_count	NT_INV2_count	NT_INV3_count	NT_CORE1_count	NT_CORE2_count	NT_CORE3_count	NT_INV1_fpkm	NT_INV2_fpkm	NT_INV3_fpkm	NT_CORE1_fpkm	NT_CORE2_fpkm	NT_CORE3_fpkm
ENSG00000141753	3429	3097	2935	569	597	633	76.32617308	66.70293832	83.00270986	14.37148445	16.68011385	13.37864245
ENSG00000134871	6731	6795	7310	1432	1330	1550	17.08474534	16.68845521	23.57352125	4.12434795	4.237397801	3.735622237
ENSG00000114270	4063	5104	3748	1145	1042	1162	17.97167909	21.8449098	21.06296849	5.746860823	5.785331224	4.88034243
ENSG00000084636	1938	1763	1872	294	172	167	10.23881213	9.012512311	12.56550099	1.762489311	1.140625102	0.837750329

1511.612108	1.873599345	3.62E-34	1.28E-30	CSF1
	1	109910242	109930992	+	5418
protein_coding	colony stimulating factor 1 [Source:HGNC
Symbol;Acc:HGNC:2432]	-	6505	5674	4892	1646	1402
	1425	58.79453823	49.62228647	56.17645042
16.88118284	15.90583134	12.22944876
ENSG00000157168 1046.060364	1000.929518	1106.692051
243.4525963	256.8356613	235.5004594	1051.227311
245.2629057	2.100295425	6.07E-31	1.79E-27	NRG1
	8	31639386	32767959	+	11328
protein_coding	neuregulin 1 [Source:HGNC Symbol;Acc:HGNC:7997] -
	1153	1131	952	235	225	273	4.984306614
	4.73081326	5.228661835	1.152727531	1.220890285
	1.120573699
ENSG00000103888 731.2442786	452.2325232	700.9824649
81.8415111	65.06503421	66.42320649	628.1530889
71.10991726	3.142767227	1.73E-30	4.38E-27	CEMIP
	15	80779343	80951776	+	7621
protein_coding	cell migration inducing hyaluronidase 1 [Source:HGNC
Symbol;Acc:HGNC:29213]	-	806	511	603	79	57
	77	5.179069795	3.177132882	4.922800059
0.57600622	0.459737843	0.469796505
ENSG00000164104 4482.727023	4224.081866	4772.028223
1322.931768	1049.03099	733.2431885	4492.945704
1035.068649	2.118808483	1.03E-29	2.27E-26	HMGB2
	4	173331695	173335125	-	2775
protein_coding	high mobility group box 2 [Source:HGNC
Symbol;Acc:HGNC:5000]	HMG	4941	4773	4105	1277	919
	850	87.19278508	81.49948938	92.03584852
25.57054317	20.35634882	14.24252388
ENSG00000182871 1147.672472	1191.203476	2473.782231
197.8699825	218.0249392	253.6158793	1604.219393
223.170267	2.843690409	7.48E-27	1.47E-23
COL18A1 21	45405137	45513720	+	8525
protein_coding	collagen type XVIII alpha 1 chain [Source:HGNC
Symbol;Acc:HGNC:2195]	-	1265	1346	2128	191	191
	294	7.266492362	7.481300737	15.53045153
1.244947459	1.377166151	1.603555371
ENSG00000133216 1535.068634	1346.962623	1773.962258
455.8261378	381.2582706	326.0775591	1551.997838
387.7206558	2.001752736	5.64E-26	9.98E-23	EPHB2
	1	22710839	22921500	+	12516
protein_coding	EPH receptor B2 [Source:HGNC Symbol;Acc:HGNC:3393]
	-	1692	1522	1526	440	334	378
6.620083812	5.762030314	7.58570284	1.953435904
1.640318838	1.404291488
ENSG00000166033 4646.939448	4546.220102	7188.848363
1303.248367	1305.866651	706.5013781	5460.669304
1105.205465	2.305532345	6.65E-25	1.07E-21	HTRA1
	10	122458551	122514908	+	2279

protein_coding HtrA serine peptidase 1 [Source:HGNC
Symbol;Acc:HGNC:9476]   -   5122   5137   6184   1258   1144
819 110.0585869 106.8050239 168.8231512
30.6724424 30.85524928 16.70977472
ENSG00000116962 858.2594138 638.9665006 1047.404977
177.1506126 135.8375276 144.0607206 848.210297
152.3496202 2.475977465 9.38E-25   1.38E-21   NID1
1   235975830   236065162   -   5864
protein_coding nidogen 1 [Source:HGNC Symbol;Acc:HGNC:7821]   -
946 722 901 171 119 167 7.899977572
5.834043685 9.559554994 1.620369994 1.247384548
1.32419983
ENSG00000197594 3680.717169 3043.498332 3200.339512
970.7024797 699.7344907 446.8470255 3308.185004
705.7613319 2.230032293 3.13E-24   3.87E-21   ENPP1
6   131808016   131895155   +   9959
protein_coding ectonucleotide pyrophosphatase/phosphodiesterase 1
[Source:HGNC Symbol;Acc:HGNC:3356] -   4057   3439   2753
937 613 518 19.94885432 16.36224474
17.1987675 5.228003891 3.783485748 2.418494174
ENSG00000186193 1011.584827 967.2997024 993.9303607
297.322958 234.0058248 254.4785184 990.9382967
261.9357671 1.919151493 3.18E-24   3.87E-21   SAPCD2
9   137062124   137070588   -   3848
protein_coding suppressor APC domain containing 2 [Source:HGNC
Symbol;Acc:HGNC:28055] -   1115   1093   855 287 205
295 14.18954559 13.45896115 13.82413283
4.144373395 3.27465957 3.564658834
ENSG00000085117 2050.387183 2209.832897 3206.15197
722.0700409 647.2258666 645.2540059 2488.790684
671.5166378 1.889433538 3.28E-24   3.87E-21   CD82
11 44564427   44620363   +   4054
protein_coding CD82 molecule [Source:HGNC Symbol;Acc:HGNC:6210]
-   2260   2497   2758   697 567 748
27.29941737 29.18510357 42.32698791 9.553468531
8.596995012 8.579241076
ENSG00000111206 2575.685492 1881.499696 2398.220274
731.3937574 570.7459141 467.5503625 2285.135154
589.896678 1.954580622 6.21E-24   6.74E-21   FOXM1
12 2857681 2877155 -   4899   protein_coding
forkhead box M1 [Source:HGNC Symbol;Acc:HGNC:3818] Fork   2839
2126   2063   706 500 542 28.37831706
20.56280048 26.19983976 8.007727858 6.273500329
5.144259464
ENSG00000186185 1671.156279 1466.436969 1604.238477
482.7613186 397.2391562 287.2588021 1580.610575
389.0864256 2.024241687 6.47E-24   6.74E-21   KIF18B
17 44924709   44947711   -   4655
protein_coding kinesin family member 18B [Source:HGNC
Symbol;Acc:HGNC:27102] -   1842   1657   1380   466 348

333	19.3775392	16.86666674	18.44447286
5.562605681	4.595226459	3.326255199
ENSG00000189403 11226.32345	11212.00359	13055.94372
5324.878064	4723.493185	4963.625067	11831.42359
5003.998772	1.241332431	3.59E-23	3.52E-20	HMGB1
13	30456704	30617597	-	7723
protein_coding	high mobility group box 1 [Source:HGNC
Symbol;Acc:HGNC:4983]	HMG	12374	12669	11231	5140	4138
5754	78.46080514	77.72893945	90.47721813
36.98189216	32.9345559	34.64294791
ENSG00000187498 3329.611045	2784.194752	4044.30845
984.1700702	679.1876378	451.1602207	3386.038082
704.8393095	2.265072381	5.31E-23	4.94E-20	COL4A1
13	110148963	110307233	-	14738
protein_coding	collagen type IV alpha 1 chain [Source:HGNC
Symbol;Acc:HGNC:2202]	-	3670	3146	3479	950	595
523	12.19428092	10.11455096	14.68664925
3.581765069	2.481565677	1.650038793
ENSG00000072571 1421.662264	1071.72913	1185.741483
306.6466745	195.1951026	131.9837739	1226.377626
211.2751837	2.539851587	1.82E-22	1.61E-19	HMMR
5	163460203	163491945	+	3936
protein_coding	hyaluronan mediated motility receptor [Source:HGNC
Symbol;Acc:HGNC:5012]	-	1567	1211	1020	296	171
153	19.49586865	14.57858906	16.12322552
4.178771893	2.670474165	1.807454441
ENSG00000166851 2517.621431	2088.588561	2744.642785
568.7467037	610.6981281	270.8686602	2450.284259
483.4378307	2.343825006	2.05E-22	1.73E-19	PLK1
16	23677656	23690367	+	4760
protein_coding	polo like kinase 1 [Source:HGNC Symbol;Acc:HGNC:9077]
-	2775	2360	2361	549	535	314
28.5485936	23.49262201	30.8599971	6.408810575
6.908665878	3.067282053
ENSG00000115163 430.9442088	420.3726977	490.5714763
37.29486582	74.19696883	28.46708849	447.2961276
46.65297438	3.275462311	4.17E-22	3.35E-19	CENPA
2	26764289	26801067	+	1983
protein_coding	centromere protein A [Source:HGNC
Symbol;Acc:HGNC:1851]	-	475	475	422	36	65
33	11.73004232	11.35003854	13.24026213
1.008769238	2.014828116	0.773788452
ENSG00000117650 1049.689368	818.6205166	1035.78006
269.3518087	210.0344964	163.0387796	968.0299814
214.1416949	2.178483743	1.10E-21	8.48E-19	NEK2
1	211658657	211675630	-	3095
protein_coding	NIMA related kinase 2 [Source:HGNC
Symbol;Acc:HGNC:7745]	-	1157	925	891	260	184
189	18.30633431	14.16144338	17.91116758
4.667934337	3.654302793	2.839436554

ENSG00000113721 3916.60242	2837.294461	5448.598363
1050.472054	981.6829722	589.1824679	4067.498415
873.7791647	2.219488168	1.27E-21	9.36E-19	PDGFRB
	5	150113837	150155872	-	7137
protein_coding	platelet derived growth factor receptor beta
[Source:HGNC Symbol;Acc:HGNC:8804]	-	4317	3206	4687
	1014	860	683	29.62068076	21.28502978
40.85884665	7.894675832	7.406791418	4.449754235
ENSG00000115884 1595.854449	1374.397473	1786.749666
481.7253501	431.483911	288.1214411	1585.667196
400.4435674	1.987200186	1.48E-21	1.05E-18	SDC1
	2	20200797	20225433	-	3595
protein_coding	syndecan 1 [Source:HGNC Symbol;Acc:HGNC:10658]	-
	1759	1553	1537	465	378	334	23.9604863
	20.46911198	26.60001119	7.187305445	6.463090918
	4.319948707
ENSG00000143228 1883.453005	1319.527773	1619.350868
459.9700117	280.8069897	219.1103175	1607.443882
319.9624397	2.330186944	1.64E-21	1.12E-18	NUF2
	1	163266576	163355764	+	3362
protein_coding	"NUF2, NDC80 kinetochore complex component
[Source:HGNC Symbol;Acc:HGNC:14621]"	-	2076	1491	1393
	444	246	254	30.2383663	21.01388616
25.77865498	7.33833113	4.497640699	3.512910018
ENSG00000138778 1799.078666	980.5746297	1210.153808
300.4308635	148.3939377	123.3573835	1329.935701
190.7273949	2.803360796	3.37E-21	2.21E-18	CENPE
	4	103105806	103198409	-	9444
protein_coding	centromere protein E [Source:HGNC
Symbol;Acc:HGNC:1856]	-	1983	1108	1041	290	130
	143	10.2824219	5.559173428	6.858065011
1.706294784	0.846125403	0.704062279
ENSG00000157456 1290.110874	1046.949266	1571.688711
387.4522171	310.4857773	255.3411574	1302.916283
317.7597173	2.036480713	4.47E-21	2.83E-18	CCNB2
	15	59105078	59125045	+	2801
protein_coding	cyclin B2 [Source:HGNC Symbol;Acc:HGNC:1580]	-
	1422	1183	1352	374	272	296	24.86080408
	20.0123494	30.03104394	7.419429919	5.969021668
	4.913711119
ENSG00000248527 1362.690951	1194.743457	819.5566132
3597.918583	3535.200192	3457.457293	1125.663674
3530.192023	-1.647642964	1.81E-20	1.11E-17
MTATP6P1	1	633696	634376	+	681
unprocessed_pseudogene	MT-ATP6 pseudogene 1 [Source:HGNC
Symbol;Acc:HGNC:44575]	-	1502	1350	705	3473	3097
	4008	108.0069008	93.93189791	64.4093404
283.3803387	279.5383862	273.6601868
ENSG00000198763 40008.86035	41950.54025	26412.97278
87652.25839	131773.8166	133922.9865	36124.12446

117783.0205	-1.705060657	5.45E-20	3.21E-17	MT-ND2
	MT	4470	5511	+	1042	protein_coding
mitochondrially encoded NADH:ubiquinone oxidoreductase core subunit 2
[Source:HGNC Symbol;Acc:HGNC:7456]	-	44099	47402	22721
	84609	115440	155248	2072.476935	2155.536533
1356.646096	4511.914766	6809.825123	6927.703839
ENSG00000004399 3573.661555	2935.528923	4194.269873
1212.083139	1392.62003	1058.458109	3567.820117
1221.053759	1.547308653	5.80E-20	3.31E-17	PLXND1
	3	129555175	129606818	-	9738
protein_coding	plexin D1 [Source:HGNC Symbol;Acc:HGNC:9107]	-
	3939	3317	3608	1170	1220	1227	19.80819461
	16.13994841	23.05173431	6.676181503	7.700827951
	5.858761387
ENSG00000088325 6920.510368	5523.254751	6614.577488
2854.093203	2334.350789	2094.487602	6352.780869
2427.643865	1.388000406	1.56E-19	8.62E-17	TPX2
	20	31739271	31801805	+	3605
protein_coding	"TPX2, microtubule nucleation factor [Source:HGNC
Symbol;Acc:HGNC:1249]"	-	7628	6241	5690	2755	2045
	2428	103.617736	82.03049918	98.20053276
42.46473105	34.8686717	31.31658747
ENSG00000134222 822.8766261	777.0257444	897.4435537
243.4525963	202.0440536	143.1980815	832.4486414
196.2315771	2.087542652	1.66E-19	8.91E-17	PSRC1
	1	109279556	109283186	-	2855
protein_coding	proline and serine rich coiled-coil 1 [Source:HGNC
Symbol;Acc:HGNC:24472]	-	907	878	772	235	177
	166	15.55714351	14.5718551	16.82356515
4.573764437	3.810785587	2.703541208
ENSG00000132967 1252.913584	1395.637356	1708.862725
483.7972871	469.1531414	485.6657825	1452.471222
479.538737	1.597902766	1.76E-19	9.14E-17
HMGB1P5 3	22381819	22382929	+	1111
transcribed_processed_pseudogene	high mobility group box 1
pseudogene 5 [Source:HGNC Symbol;Acc:HGNC:4997] -	1381	1577
	1470	467	411	563	60.870701	67.25801956
	82.32090238	23.35688192	22.73919695	23.56271629
ENSG00000163359 26821.96756	21721.32104	35328.12124
9530.910153	8483.567267	5007.619658	27957.13661
7674.032359	1.865262061	4.04E-19	2.04E-16	COL6A3
	2	237324003	237414375	-	19633
protein_coding	collagen type VI alpha 3 chain [Source:HGNC
Symbol;Acc:HGNC:2213]	-	29564	24544	30390	9200	7432
	5805	73.74035271	59.23591708	96.30545338
26.03833465	23.26839323	13.74822324
ENSG00000112984 1600.390704	1062.879179	1653.063126
401.955776	219.166431	169.0772529	1438.77767
263.39982	2.450713662	4.36E-19	2.14E-16	KIF20A
	5	138178719	138187715	+	3958

protein_coding kinesin family member 20A [Source:HGNC
Symbol;Acc:HGNC:9787] - 1764 1201 1422 388 192
196 21.8248604 14.3778404 22.35273416
5.447133 2.981760787 2.302561823
ENSG00000087586 2479.51689 2023.09892 2486.569639
801.8396151 422.3519764 368.3468723 2329.728483
530.8461546 2.13438139 5.08E-19 2.43E-16 AURKA
20 56369389 56392337 - 2928
protein_coding aurora kinase A [Source:HGNC Symbol;Acc:HGNC:11393]
- 2733 2286 2139 774 370 427
45.70852878 36.99402575 45.4513261 14.68865167
7.767441872 6.78090751
ENSG00000164176 3274.268736 2229.302791 4322.143954
945.8392358 940.5892664 639.2155325 3275.238494
841.8813449 1.960406584 6.67E-19 3.10E-16 EDIL3
5 83940554 84384793 - 5738
protein_coding EGF like repeats and discoidin domains 3 [Source:HGNC
Symbol;Acc:HGNC:3173] - 3609 2519 3718 913 824
741 30.80030532 20.8014718 40.31398112
8.841425204 8.827018321 6.004663604
ENSG00000176619 11945.77347 11759.81559 12717.65865
6256.213741 5489.434202 5911.665377 12141.08257
5885.771107 1.044479994 1.15E-18 5.21E-16 LMNB2
19 2427638 2456996 - 5432 protein_coding lamin
B2 [Source:HGNC Symbol;Acc:HGNC:6638] - 13167 13288 10940
6039 4809 6853 118.7013772 115.9114394
125.3038478 61.77564833 54.4179749 58.66134126
ENSG00000138182 1866.215237 1161.998636 1413.589846
483.7972871 369.8433523 405.4403513 1480.60124
419.6936636 1.818614175 1.50E-18 6.62E-16 KIF20B
10 89701610 89774939 + 6851
protein_coding kinesin family member 20B [Source:HGNC
Symbol;Acc:HGNC:7212] - 2057 1313 1216 467 324
470 14.70310327 9.08107368 11.04298428
3.787694617 2.906955629 3.189884134
ENSG00000188157 3824.970073 3635.560089 6355.34185
1424.45668 1569.551264 1171.463823 4605.290671
1388.490589 1.729888013 1.06E-17 4.59E-15 AGRN
1 1020123 1056118 + 10758 protein_coding agrin
[Source:HGNC Symbol;Acc:HGNC:329] - 4216 4108 5467
1375 1375 1358 19.1910062 18.09361461
31.61727092 7.102041437 7.85630831 5.869474878
ENSG00000147872 5794.61192 6414.44487 7910.755678
13809.46004 20228.37669 21515.08043 6706.604156
18517.63905 -1.465424621 1.83E-17 7.72E-15 PLIN2
9 19108375 19149290 - 5086
protein_coding perilipin 2 [Source:HGNC Symbol;Acc:HGNC:248] -
6387 7248 6805 13330 17721 24941 61.49633641
67.52557168 83.24508868 145.6350258 214.1702926
228.0176811

ENSG00000137801 1335.473422 1552.281499 1489.151804
440.2866103 142.6864785 218.2476785 1458.968908
267.0735891 2.449131895 2.92E-17 1.20E-14 THBS1
15 39581079 39599466 + 9158
protein_coding thrombospondin 1 [Source:HGNC Symbol;Acc:HGNC:11785]
- 1472 1754 1281 425 125 253
7.871107691 9.075182987 8.702726539 2.578697116
0.838989903 1.284549665
ENSG00000225630 1668.434526 1700.075689 1072.979793
3630.033606 4992.885256 4710.009187 1480.496669
4444.30935 -1.584936362 3.01E-17 1.21E-14
MTND2P28 1 629640 630683 + 1044
unprocessed_pseudogene MT-ND2 pseudogene 28 [Source:HGNC
Symbol;Acc:HGNC:42129] - 1839 1921 923 3504 4374
5460 86.26009143 87.18731572 55.00574472
186.4986285 257.5287028 243.1773728
ENSG00000050165 1424.384017 1182.353525 1608.888443
528.3439324 353.8624667 425.2810493 1405.208662
435.8291495 1.687883266 3.21E-17 1.26E-14 DKK3
11 11963106 12009769 - 5059
protein_coding dickkopf WNT signaling pathway inhibitor 3
[Source:HGNC Symbol;Acc:HGNC:2893] - 1570 1336 1384
510 310 493 15.19720272 12.5131952
17.02073293 5.601670256 3.76655553 4.531200275
ENSG00000138448 1571.358673 1281.472982 2035.522879
512.804405 481.7095515 574.5176042 1629.451511
523.0105202 1.637894927 5.35E-17 2.06E-14 ITGAV
2 186590065 186680901 + 8211
protein_coding integrin subunit alpha V [Source:HGNC
Symbol;Acc:HGNC:6150] - 1732 1448 1751 495 422
666 10.329529 8.356009991 13.26773964
3.349817835 3.159102804 3.771457301
ENSG00000198888 19209.2247 21190.32395 13212.88009
38801.16401 60247.93869 68006.14913 17870.80958
55685.08394 -1.639627615 9.72E-17 3.66E-14 MT-ND1
MT 3307 4262 + 956 protein_coding
mitochondrially encoded NADH:ubiquinone oxidoreductase core subunit 1
[Source:HGNC Symbol;Acc:HGNC:7455] - 21173 23944 11366
37454 52780 78835 1084.559015 1186.766428
739.7016817 2176.969565 3393.585955 3834.353929
ENSG00000117399 3971.944729 3485.995908 4286.106713
1604.715199 1614.069445 1058.458109 3914.68245
1425.747584 1.457906658 1.22E-16 4.50E-14 CDC20
1 43358955 43363203 + 2038
protein_coding cell division cycle 20 [Source:HGNC
Symbol;Acc:HGNC:1723] - 4378 3939 3687 1549 1414
1227 105.1962506 91.58160322 112.557854
42.23371469 42.64740299 27.9944153
ENSG00000142945 3306.02252 2378.866972 3225.914328
1234.874446 799.0442797 722.0288809 2970.26794

918.6492022	1.693265725	1.71E-16	6.17E-14	KIF2C
	1	44739818	44767767	+	3646
protein_coding	kinesin family member 2C [Source:HGNC
Symbol;Acc:HGNC:6393]	-	3644	2688	2775	1192	700
	837	48.9429776	34.93325394	47.35361837
18.16651554	11.80126971	10.67431001
ENSG00000119403 1827.203445	1670.870849	1756.524883
772.8324972	517.0957982	518.4460662	1751.533059
602.7914539	1.539162425	2.50E-16	8.86E-14	PHF19
	9	120855652	120894896	-	6733
protein_coding	PHD finger protein 19 [Source:HGNC
Symbol;Acc:HGNC:24566]	-	2014	1888	1511	746	453
	601	14.64804061	13.28678221	13.96248426
6.156618693	4.135584965	4.150466145
ENSG00000075218 1250.191831	1131.908801	1490.314295
419.5672404	392.6731889	224.2861518	1290.804976
345.5088604	1.903696679	2.68E-16	9.30E-14	GTSE1
	22	46296741	46330810	+	4242
protein_coding	G2 and S-phase expressed 1 [Source:HGNC
Symbol;Acc:HGNC:13698]	-	1378	1279	1282	405	344
	260	15.90769432	14.28651565	18.80287278
5.305134545	4.984655384	2.849926777
ENSG00000110492 1738.292851	2146.113246	2393.570307
790.4439616	874.3827404	690.9738753	2092.658801
785.2668591	1.414605968	2.95E-16	1.00E-13	MDK
	11	46380756	46383837	+	2196
protein_coding	midkine [Source:HGNC Symbol;Acc:HGNC:6972]	-
	1916	2425	2059	763	766	801	42.72596226
	52.32459168	58.33522234	19.30653096	21.44093864
	16.96020895
ENSG00000121068 493.5445255	560.2019319	610.3081162
94.27313304	158.6673641	134.5716911	554.6848579
129.1707294	2.103078238	4.44E-16	1.48E-13	TBX2
	17	61399896	61409466	+	6029
protein_coding	T-box 2 [Source:HGNC Symbol;Acc:HGNC:11597]	T-box
	544	633	525	91	139	156	4.418575696
	4.97490586	5.417773769	0.838702914	1.417153444
	1.20312384
ENSG00000134057 3276.083238	2509.846254	3384.013193
1383.017941	1071.860827	1099.864783	3056.647562
1184.914517	1.366971916	4.72E-16	1.54E-13	CCNB1
	5	69167010	69178245	+	2558
protein_coding	cyclin B1 [Source:HGNC Symbol;Acc:HGNC:1579]	-
	3611	2836	2911	1335	939	1275	69.128261
	52.53299086	70.80248683	28.99965086	22.56380887
	23.17611637
ENSG00000197461 1300.090634	1388.557395	1879.748998
381.2364061	560.4724876	297.6104706	1522.799009
413.1064548	1.884147948	5.46E-16	1.75E-13	PDGFA
	7	497258	520296	-	4151	protein_coding

platelet derived growth factor subunit A [Source:HGNC
Symbol;Acc:HGNC:8799]   -   1433   1569   1617   368   491
        345   16.90527113   17.91004393   24.23617798
4.926144295   7.270698218   3.8645362
ENSG00000137573 242.2360079   217.7088077   261.5606212
32.11502334   31.96177119   13.80222472   240.5018123
25.95967309   3.226439608   6.94E-16   2.19E-13   SULF1
        8   69466624   69660915   +   9404
protein_coding sulfatase 1 [Source:HGNC Symbol;Acc:HGNC:20391] -
        267   246   225   31   28   16   1.390360191
        1.239506839   1.488595646   0.183172856   0.183017564
        0.079111274
ENSG00000166949 1730.127592   1766.450326   2441.232465
773.8684657   659.7822767   500.3306463   1979.270128
644.6604629   1.618877831   7.20E-16   2.23E-13   SMAD3
        15   67063763   67195195   +   9113
protein_coding SMAD family member 3 [Source:HGNC
Symbol;Acc:HGNC:6769]   MH1   1907   1996   2100   747   578
        580   10.24750187   10.37828535   14.33721411
4.55482059   3.89864623   2.959358924
ENSG00000204262 2719.938396   2279.747514   4579.054609
1095.018699   859.5433466   712.5398514   3192.913506
889.0339657   1.844531002   1.29E-15   3.93E-13   COL5A2
        2   189031896   189225312   -   7453
protein_coding collagen type V alpha 2 chain [Source:HGNC
Symbol;Acc:HGNC:2210]   -   2998   2576   3939   1057   753
        826   19.69832011   16.37725753   32.88226357
7.880538765   6.210280482   5.153234958
ENSG00000068489 1675.692534   1151.378694   1468.226954
490.0130981   228.2983656   200.9948975   1431.766061
306.4354538   2.224988492   1.46E-15   4.38E-13   PRR11
        17   59155499   59204705   +   4080
protein_coding proline rich 11 [Source:HGNC Symbol;Acc:HGNC:25619]
        -       1847   1301   1263   473   200   233
22.16845445   15.10927321   19.25972289   6.441885797
3.013125305   2.655380588
ENSG00000136696 44.45529733   52.21471403   34.8747495
156.4312427   466.8701577   896.281968   43.84825362
506.5277895   -3.527112195   3.44E-15   1.01E-12   IL36B
        2   113022091   113052867   -   1869
protein_coding interleukin 36 beta [Source:HGNC
Symbol;Acc:HGNC:15564] -   49   59   30   151   409
        1039   1.283853479   1.495784922   0.998662632
4.48931097   13.45121043   25.84861418
ENSG00000162591 329.3321007   246.0286526   297.5978624
19.6834014   21.68834474   54.34625985   290.9862052
31.906002   3.171161689   4.53E-15   1.31E-12   MEGF6
        1   3489920 3611495 -   7039   protein_coding
multiple EGF like domains 6 [Source:HGNC Symbol;Acc:HGNC:3232] -
        363   278   256   19   19   63   2.525366083

1.871372622 2.262746213 0.149987366 0.16591666
0.416160256
ENSG00000106003 809.2678617 1081.464077 938.1307615
242.4166278 364.1358932 267.418104 942.9542334
291.3235417 1.696514257 7.73E-15 2.21E-12 LFNG
7 2512529 2529177 + 3418 protein_coding LFNG
O-fucosylpeptide 3-beta-N-acetylglucosaminyltransferase [Source:HGNC
Symbol;Acc:HGNC:6560] - 892 1222 807 234 319
310 12.77972415 16.94047519 14.68954429
3.804134317 5.7367508 4.217165584
ENSG00000169679 1770.046635 1276.163011 1819.299432
636.0846559 450.8892721 333.8413105 1621.836359
473.6050795 1.776805015 1.03E-14 2.89E-12 BUB1
2 110637698 110678114 - 6032
protein_coding BUB1 mitotic checkpoint serine/threonine kinase
[Source:HGNC Symbol;Acc:HGNC:1148] - 1951 1442 1565
614 395 387 15.83888553 11.32740343
16.14209339 5.656126083 4.025159766 2.983188185
ENSG00000111665 659.5714523 469.0474312 498.7089178
153.3233372 141.5449867 148.3739158 542.4426004
147.7474132 1.876356778 1.16E-14 3.20E-12 CDCA3
12 6844793 6852066 - 5653 protein_coding cell
division cycle associated 3 [Source:HGNC Symbol;Acc:HGNC:14624] -
727 530 429 148 124 172 6.297731038
4.442457943 4.721556091 1.454771464 1.348310945
1.414752531
ENSG00000110031 755.7400547 678.7912824 640.5328991
1508.370129 1641.465249 1649.365855 691.6880787
1599.733744 -1.208982003 1.22E-14 3.33E-12 LPXN
11 58526871 58578220 - 2467
protein_coding leupaxin [Source:HGNC Symbol;Acc:HGNC:14061] -
833 767 551 1456 1438 1912 16.5350128
14.73169285 13.8959837 32.79474582 35.8291988
36.0370935
ENSG00000164932 720.357267 744.2809238 933.4807949
299.394895 235.1473166 233.7751813 799.3729952
256.1057976 1.641661574 1.67E-14 4.48E-12 CTHRC1
8 103371515 103382997 + 1776
protein_coding collagen triple helix repeat containing 1 [Source:HGNC
Symbol;Acc:HGNC:18831] - 794 841 803 289 206
271 21.89304776 22.4377588 28.13062818
9.042050432 7.129705958 7.095080831
ENSG00000101447 1569.544171 1185.893505 1408.93988
463.0779172 391.5316971 190.643229 1388.125852
348.4176144 1.996731093 2.08E-14 5.49E-12 FAM83D
20 38926312 38953106 + 2475
protein_coding family with sequence similarity 83 member D
[Source:HGNC Symbol;Acc:HGNC:16122] - 1730 1340 1212
447 343 221 34.2294234 25.65405585
30.46732064 10.03562359 8.518561771 4.151911508

ENSG00000140945 2644.636566 2400.99185 3618.836506
1261.809627 849.2699202 728.0673542 2888.154974
946.3823004 1.609754258 2.62E-14 6.80E-12 CDH13
16 82626803 83800640 + 11187
protein_coding cadherin 13 [Source:HGNC Symbol;Acc:HGNC:1753] -
2915 2713 3113 1218 744 844 12.76008609
11.49112626 17.31300001 6.049865142 4.087960188
3.508001955
ENSG00000161888 671.3657148 630.116549 763.757014
175.0786756 189.4876435 77.63751407 688.4130926
147.4012777 2.228880598 3.19E-14 8.17E-12 SPC24
19 11131520 11155808 - 2839
protein_coding "SPC24, NDC80 kinetochore complex component
[Source:HGNC Symbol;Acc:HGNC:26913]" - 740 712 657
169 166 90 12.76424145 11.88340892
14.39815431 3.307755161 3.594099166 1.474036152
ENSG00000140416 10641.14658 9646.447169 16528.30628
5151.871325 4439.26172 3225.40739 12271.96668
4272.180145 1.522375767 3.51E-14 8.87E-12 TPM1
15 63042632 63071915 + 12876
protein_coding tropomyosin 1 [Source:HGNC Symbol;Acc:HGNC:12010]
- 11729 10900 14218 4973 3889 3739
44.60758398 40.11178333 68.70121037 21.46097926
18.56540105 13.50223043
ENSG00000266074 790.2155914 889.4201289 1274.090848
346.0134773 280.8069897 290.7093583 984.5755228
305.8432751 1.685629445 4.02E-14 1.00E-11 BAHCC1
17 81395475 81466332 + 13473
protein_coding BAH domain and coiled-coil containing 1 [Source:HGNC
Symbol;Acc:HGNC:29279] - 871 1005 1096 334 246
337 3.165793104 3.534501683 5.061195241
1.377508225 1.122323761 1.163045243
ENSG00000087245 19669.20094 17745.92281 26207.21176
10419.77112 9294.026465 8379.675686 21207.44517
9364.491091 1.179266728 4.98E-14 1.22E-11 MMP2
16 55389700 55506691 + 4933
protein_coding matrix metallopeptidase 2 [Source:HGNC
Symbol;Acc:HGNC:7166] - 21680 20052 22544 10058 8142
9714 215.2171351 192.6074233 284.3326331
113.2954737 101.4535721 91.56257585
ENSG00000089685 3275.175987 3254.127178 3786.235304
1541.52112 827.5815754 666.819982 3438.512823
1011.974226 1.764992359 5.47E-14 1.32E-11 BIRC5
17 78214186 78225636 + 3815
protein_coding baculoviral IAP repeat containing 5 [Source:HGNC
Symbol;Acc:HGNC:593] - 3610 3677 3257 1488 725
773 46.33843297 45.6694234 53.11657491
21.67306994 11.6812905 9.421411431
ENSG00000198830 13985.27364 14740.47927 16773.59202
8059.83489 6424.316009 5108.548426 15166.44831

6530.899775	1.21562334	5.59E-14	1.34E-11	HMGN2
	1	26472450	26475972	+	2509
protein_coding	high mobility group nucleosomal binding domain 2
[Source:HGNC Symbol;Acc:HGNC:4986]	-	15415	16656	14429
	7780	5628	5922	300.8649187	314.5549267
357.8017157	172.3022622	137.8798454	109.7485421
ENSG00000168003 9129.666471	11372.18772	9004.66032
18384.29691	19782.05338	22803.86316	9835.504836
20323.40448	-1.047024663	6.26E-14	1.48E-11	SLC3A2
	11	62856102	62888875	+	5178
protein_coding	solute carrier family 3 member 2 [Source:HGNC
Symbol;Acc:HGNC:11026]	-	10063	12850	7746	17746	17330
	26435	95.16869413	117.5892235	93.07269579
190.4366244	205.7234869	237.3822745
ENSG00000115963 2585.665253	2781.539766	2055.285237
5194.346033	5043.110897	5587.313096	2474.163419
5274.923342	-1.091719828	6.56E-14	1.53E-11	RND3
	2	150468195	150539011	-	3594
protein_coding	Rho family GTPase 3 [Source:HGNC Symbol;Acc:HGNC:671]
	-	2850	3143	1768	5014	4418	6477
38.83251072	41.43742399	30.60631435	77.52080974
75.5605306	83.7966858
ENSG00000134508 692.232487	562.8569174	568.4584168
138.8197783	216.8834474	154.4123891	607.8492737
170.0385383	1.840906595	7.00E-14	1.60E-11
CABLES1 18	23134564	23260467	+	6654
protein_coding	Cdk5 and Abl enzyme substrate 1 [Source:HGNC
Symbol;Acc:HGNC:25097]	-	763	636	489	134	190
	179	5.615267176	4.528983724	4.572280959
1.119010201	1.755165867	1.250838536
ENSG00000114554 7554.678794	5830.348069	6577.377755
3492.249796	2522.69694	2370.532096	6654.134873
2795.159611	1.251457994	7.09E-14	1.60E-11	PLXNA1
	3	126988594	127037392	+	10944
protein_coding	plexin A1 [Source:HGNC Symbol;Acc:HGNC:9099]	-
	8327	6588	5658	3371	2210	2748	37.25985529
	28.52357435	32.16575172	17.11570182	12.41262256
	11.67539714
ENSG00000162745 811.0823636	525.687121	614.9580828
200.977888	146.110954	96.61557307	650.5758558
147.9014717	2.140368034	7.15E-14	1.60E-11
OLFML2B 1	161983192	162023854	-	3415
protein_coding	olfactomedin like 2B [Source:HGNC
Symbol;Acc:HGNC:24558]	-	894	594	529	194	128
	112	12.81963011	8.241802017	9.63766465
3.156625537	2.303915899	1.524959581
ENSG00000142949 1841.719461	1608.036193	2135.497161
823.5949534	803.610247	653.0177573	1861.750938
760.0743193	1.292835771	7.35E-14	1.62E-11	PTPRF
	1	43525187	43623666	+	10712

protein_coding "protein tyrosine phosphatase, receptor type F
[Source:HGNC Symbol;Acc:HGNC:9670]" - 2030 1817 1837
795 704 757 9.280132082 8.037311567
10.66953369 4.123904584 4.039703172 3.285915066
ENSG00000143369 916.3234756 919.5099641 1250.841015
388.4881856 373.2678278 292.4346363 1028.891485
351.3968832 1.550559299 9.94E-14 2.17E-11 ECM1
1 150508062 150513789 + 3360
protein_coding extracellular matrix protein 1 [Source:HGNC
Symbol;Acc:HGNC:3153] - 1010 1039 1076 375 327
339 14.72010066 14.65219571 19.92415174
6.20160404 5.982129846 4.69128091
ENSG00000198899 37829.64353 35687.42955 25339.99298
63626.07705 81334.71723 81469.35671 32952.35535
75476.717 -1.195604648 1.10E-13 2.38E-11 MT-
ATP6 MT 8527 9207 + 681 protein_coding
mitochondrially encoded ATP synthase membrane subunit 6 [Source:HGNC
Symbol;Acc:HGNC:7414] - 41697 40325 21798 61417 71253
94442 2998.37799 2805.78058 1991.481989
5011.336096 6431.368625 6448.357126
ENSG00000080986 1374.485213 1049.604252 1425.214763
474.4735707 308.2027936 188.0553119 1283.101409
323.5772254 1.989124564 1.28E-13 2.73E-11 NDC80
18 2571511 2616635 + 3600
protein_coding "NDC80, kinetochore complex component [Source:HGNC
Symbol;Acc:HGNC:16909]" - 1515 1186 1226 458 270
218 20.60814093 15.61020581 21.1882367
7.069277352 4.610081716 2.815691119
ENSG00000089597 8483.703783 8278.244662 11531.91717
4426.693379 4605.919527 3966.41433 9431.288537
4333.009079 1.122043858 1.42E-13 2.98E-11 GANAB
11 62624826 62646726 - 5030
protein_coding glucosidase II alpha subunit [Source:HGNC
Symbol;Acc:HGNC:4138] - 9351 9354 9920 4273 4035
4598 91.03717142 88.11621245 122.701687
47.20379889 49.3086275 42.50421503
ENSG00000076382 2351.594504 1969.114216 2150.609552
1006.961377 609.5566362 527.0724567 2157.106091
714.5301567 1.594622255 1.43E-13 2.98E-11 SPAG5
17 28577565 28599279 - 5472
protein_coding sperm associated antigen 5 [Source:HGNC
Symbol;Acc:HGNC:13452] - 2592 2225 1850 972 534
611 23.19623992 19.26683453 21.0345142
9.870342431 5.99849814 5.191897856
ENSG00000087086 37268.96243 49303.07998 38204.12558
76173.72746 93037.29146 110768.0292 41592.056
93326.34939 -1.165969982 1.51E-13 3.11E-11 FTL
19 48965301 48966878 + 878
protein_coding ferritin light chain [Source:HGNC
Symbol;Acc:HGNC:3999] - 41079 55710 32864 73529 81505

        128406 2291.152677   3006.526827   2328.803004
4653.462166   5706.070012   6800.202094
ENSG00000117298 2615.604535   2805.434635   3879.234636
1360.226634   1296.734717   1019.639352   3100.091269
1225.533567   1.339041669   1.56E-13   3.16E-11   ECE1
        1   21217247   21345572   -   7333
protein_coding endothelin converting enzyme 1 [Source:HGNC
Symbol;Acc:HGNC:3146]   -   2883   3170   3337   1313   1136
        1182   19.25269988   20.48349301   28.31270528
9.949358612   9.52234707   7.494916688
ENSG00000148606 1399.888241   1395.637356   1389.177522
2642.75563   2871.99344   2711.274519   1394.90104
2742.007863   -0.974943564   1.58E-13   3.18E-11   POLR3A
        10   77969251   78029545   -   8917
protein_coding RNA polymerase III subunit A [Source:HGNC
Symbol;Acc:HGNC:30074] -   1543   1577   1195   2551   2516
        3143   8.473753825   8.379910254   8.337886621
15.8965813   17.34359927   16.38915764
ENSG00000225614 639.611931   551.3519804   1054.379926
128.4600934   206.6100209   71.59904076   748.447946
135.556385   2.469925548   1.75E-13   3.47E-11   ZNF469
        16   88427471   88440757   +   13287
protein_coding zinc finger protein 469 [Source:HGNC
Symbol;Acc:HGNC:23216] zf-C2H2 705   623   907   124   181
        83   2.598309406   2.221710934   4.247048286
0.51856929   0.837334528   0.290457219
ENSG00000128606 787.4938385   698.2611758   778.8694054
266.2439032   123.2811174   108.6925197   754.8748066
166.0725134   2.185808194   1.78E-13   3.50E-11   LRRC17
        7   102912991   102944949   +   2674
protein_coding leucine rich repeat containing 17 [Source:HGNC
Symbol;Acc:HGNC:16895] -   868   789   670   257   108
        126   15.89596895   13.98112251   15.58907141
5.340522684   2.48261693   2.190988814
ENSG00000197632 117.9426256   217.7088077   127.8740815
499.3368145   655.2163094   945.4523936   154.5085049
700.0018392   -2.177939428   1.91E-13   3.71E-11
SERPINB2   18   63871692   63903890   +   2748
        protein_coding serpin family B member 2 [Source:HGNC
Symbol;Acc:HGNC:8584]   -   130   246   110   482   574
        1096   2.316622699   4.241747568   2.490478537
9.746357861   12.83933481   18.54491486
ENSG00000125844 3203.503161   3191.292522   3510.724783
1787.045654   1303.583668   1406.964283   3301.840155
1499.197868   1.138881113   1.95E-13   3.75E-11   RRBP1
        20   17613678   17682295   -   6980
protein_coding ribosome binding protein 1 [Source:HGNC
Symbol;Acc:HGNC:10448] -   3531   3606   3020   1725   1142
        1631   24.77256511   24.47917294   26.91896557
13.73237708   10.05675897   10.86499564

ENSG00000134690 1623.979229 1515.111702 1784.424683
702.3866395 577.5948651 369.2095114 1641.171871
549.7303387 1.579328921 2.18E-13 4.14E-11 CDCA8
1 37692418 37709719 + 2469
protein_coding cell division cycle associated 8 [Source:HGNC
Symbol;Acc:HGNC:14629] - 1790 1712 1535 678 506
428 35.50263803 32.85557796 38.68068322
15.25880938 12.59728013 8.06034623
ENSG00000091986 1155.837731 971.7246781 1440.327154
423.7111144 450.8892721 288.1214411 1189.296521
387.5739426 1.619016693 2.36E-13 4.44E-11 CCDC80
3 112596794 112649530 - 13071
protein_coding coiled-coil domain containing 80 [Source:HGNC
Symbol;Acc:HGNC:30649] - 1274 1098 1239 409 395
334 4.772976509 3.980338124 5.897518859
1.738707538 1.857529164 1.188142881
ENSG00000142627 5523.343881 4472.765504 5479.985638
2500.827947 1499.920262 1186.991326 5158.698341
1729.246512 1.577145529 2.42E-13 4.50E-11 EPHA2
1 16124337 16156087 - 4159
protein_coding EPH receptor A2 [Source:HGNC Symbol;Acc:HGNC:3386]
- 6088 5054 4714 2414 1314 1376
71.68270856 57.58014551 70.51922072 32.25227759
19.42020478 15.38369042
ENSG00000075702 1330.029916 1219.523321 1246.191049
578.0704202 404.0881072 514.132871 1265.248095
498.7637994 1.342068063 2.80E-13 5.16E-11 WDR62
19 36054881 36105106 + 9812
protein_coding WD repeat domain 62 [Source:HGNC
Symbol;Acc:HGNC:24502] - 1466 1378 1072 558 354
596 7.31652927 6.65454121 6.797419803
3.160011791 2.217650398 2.824357959
ENSG00000101255 2326.191477 3347.936664 3003.878423
5483.381244 7327.236045 7423.871624 2892.668855
6744.829637 -1.221461923 3.13E-13 5.71E-11 TRIB3
20 362835 397559 + 3434 protein_coding
tribbles pseudokinase 3 [Source:HGNC Symbol;Acc:HGNC:16228] -
2564 3783 2584 5293 6419 8606 36.56338653
52.19903704 46.81651319 85.64729414 114.8985228
116.5284766
ENSG00000075426 2493.125655 2068.233673 2796.95491
1172.716336 1055.879941 1081.749363 2452.771412
1103.448547 1.151934049 3.21E-13 5.80E-11 FOSL2
2 28392448 28417312 + 7157
protein_coding "FOS like 2, AP-1 transcription factor subunit
[Source:HGNC Symbol;Acc:HGNC:3798]" TF_bZIP 2748 2337 2406
1132 925 1254 18.80244793 15.47227358
20.91565417 8.788756928 7.944344628 8.146996644
ENSG00000006062 950.7990123 830.1254536 854.4313627
369.8407527 317.3347282 343.33034 878.4519429

343.5019403   1.354353645   3.90E-13   6.98E-11
MAP3K14 17   45263119   45317145   -   5784
protein_coding mitogen-activated protein kinase kinase kinase 14
[Source:HGNC Symbol;Acc:HGNC:6853]   -   1048   938   735
        357   278   398   8.872820218   7.684241315
7.906165504   3.429667164   2.95436311   3.199527195
ENSG00000122566 22669.47989   21951.41978   24623.89813
13728.65449   10537.11107   10569.91622   23081.59926
11611.89393   0.991129514   4.84E-13   8.56E-11
HNRNPA2B1   7   26173057   26201529   -   9267
        protein_coding heterogeneous nuclear ribonucleoprotein A2/B1
[Source:HGNC Symbol;Acc:HGNC:5033]   -   24987   24804   21182
        13252   9231   12253   132.0394289   126.8262059
142.2114771   79.46105152   61.22896921   61.48006084
ENSG00000144057 498.0807803   416.8327171   534.746159
103.5968495   127.8470848   45.7198694   483.2198855
92.38793455   2.394573883   5.05E-13   8.85E-11
ST6GAL2 2   106801600   106887108   -   7708
protein_coding "ST6 beta-galactoside alpha-2,6-sialyltransferase 2
[Source:HGNC Symbol;Acc:HGNC:10861]"   -   549   471   460
        100   112   53   3.48786223   2.895380479
3.712983098   0.720892219   0.893148508   0.319716597
ENSG00000118515 458.1617378   545.1570143   383.6222445
1027.680747   1359.516767   1719.239617   462.3136655
1368.812377   -1.565041156   5.27E-13   9.13E-11   SGK1
        6   134169246   134318112   -   9992
protein_coding serum/glucocorticoid regulated kinase 1 [Source:HGNC
Symbol;Acc:HGNC:10810] -   505   616   330   992   1191
        1993   2.474956877   2.921155847   2.054794342
5.5165974   7.326671103   9.274401465
ENSG00000164611 1456.1378   1308.022836   1818.13694
675.4514587   598.141718   478.7646701   1527.432526
584.1192823   1.387178851   6.34E-13   1.09E-10   PTTG1
        5   160421822   160428744   +   2013
protein_coding pituitary tumor-transforming 1 [Source:HGNC
Symbol;Acc:HGNC:9690]   -   1605   1478   1564   652   524
        555   39.04450717   34.79021399   48.33924027
17.9976526   16.00054856   12.81976979
ENSG00000091136 4457.323996   3176.247605   4723.203574
1617.146821   1519.325623   758.2597208   4118.925058
1298.244055   1.666431895   7.11E-13   1.21E-10   LAMB1
        7   107923799   108003255   -   8140
protein_coding laminin subunit beta 1 [Source:HGNC
Symbol;Acc:HGNC:6486]   -   4913   3589   4063   1561   1331
        879   29.55636647   20.89178242   31.05483771
10.65590995   10.05080879   5.021060893
ENSG00000118193 1566.822418   969.0696927   1183.4165
468.2577597   186.063168   244.1268498   1239.769537
299.4825925   2.049376009   7.39E-13   1.25E-10   KIF14
        1   200551497   200620734   -   7277

protein_coding	kinesin family member 14 [Source:HGNC
Symbol;Acc:HGNC:19181]	-	1727	1095	1018	452	163
	283	11.62167286	7.129977922	8.703666659
3.451422322	1.376837194	1.808277501
ENSG00000095752 631.4466723	938.0948623	1676.312959
199.9419195	276.2410224	283.8082459	1081.951498
253.3303959	2.093142852	9.13E-13	1.52E-10	IL11
	19	55364389	55370463	-	2671
protein_coding	interleukin 11 [Source:HGNC Symbol;Acc:HGNC:5966]
	-	696	1060	1442	193	242	329
12.76039256	18.80435399	33.58908853	4.015091664
5.56914901	5.727340825
ENSG00000101160 2908.646597	3692.199779	3098.040247
1715.563828	1288.744274	1313.799266	3232.962208
1439.369123	1.167599656	9.37E-13	1.55E-10	CTSZ
	20	58995185	59007247	-	2038
protein_coding	cathepsin Z [Source:HGNC Symbol;Acc:HGNC:2547]	-
	3206	4172	2665	1656	1129	1523	77.03498847
	96.99884453	81.35792814	45.15108556	34.05156858
	34.74775429
ENSG00000162723 101.6121082	88.49951531	86.02438209
275.5676196	496.5489453	444.2591083	92.0453352
405.4585577	-2.137828322	1.07E-12	1.75E-10	SLAMF9
	1	159951492	159954254	-	1156
protein_coding	SLAM family member 9 [Source:HGNC
Symbol;Acc:HGNC:18430]	-	112	100	74	266	435
	515	4.744482752	4.098912116	3.982728779
12.78603374	23.13016778	20.71478419
ENSG00000145934 722.1717689	469.9324263	789.3318303
202.0138565	69.63100152	76.77487503	660.4786752
116.139911	2.507962199	1.10E-12	1.79E-10	TENM2
	5	167284799	168264157	+	11042
protein_coding	teneurin transmembrane protein 2 [Source:HGNC
Symbol;Acc:HGNC:29943]	-	796	531	679	195	61
	89	3.530156901	2.278626895	3.825858276
0.981293478	0.339570108	0.374777303
ENSG00000137807 2520.343183	1507.146746	2155.259519
867.1056302	563.8969631	409.7535465	2060.916483
613.58538	1.748724694	1.39E-12	2.23E-10	KIF23
	15	69414246	69448427	+	6576
protein_coding	kinesin family member 23 [Source:HGNC
Symbol;Acc:HGNC:6392]	-	2778	1703	1854	837	494
	475	20.6870765	12.27098102	17.5410171
7.072544637	4.617559546	3.358635107
ENSG00000183087 2603.810272	2969.158739	3258.464095
1430.672491	1496.495787	1165.42535	2943.811035
1364.197876	1.110080056	1.41E-12	2.24E-10	GAS6
	13	113820549	113864067	-	6006
protein_coding	growth arrest specific 6 [Source:HGNC
Symbol;Acc:HGNC:4168]	-	2870	3355	2803	1381	1311

1351 23.40050621 26.46876252 29.03652342
12.77674992 13.41728745 10.45926193
ENSG00000029993 2346.150998 2042.568813 2430.77004
1155.104872 1079.851269 1111.07909 2273.163284
1115.345077 1.026854389 1.47E-12 2.32E-10 HMGB3
X 150980509 150990775 + 3967
protein_coding high mobility group box 3 [Source:HGNC
Symbol;Acc:HGNC:5004] HMG 2586 2308 2091 1115 946
1288 31.92236093 27.56766895 32.79432369
15.61797454 14.65805329 15.09679232
ENSG00000162413 3950.170706 5334.750783 4334.931362
7985.245159 12273.32014 15334.27167 4539.95095
11864.27899 -1.385886178 1.73E-12 2.69E-10 KLHL21
1 6590724 6614607 - 6901 protein_coding kelch
like family member 21 [Source:HGNC Symbol;Acc:HGNC:29041] -
4354 6028 3729 7708 10752 17776 30.89620093
41.38925956 33.61918667 62.0642801 95.7689197
119.7713742
ENSG00000135451 571.5681086 446.9225523 491.7339679
149.1794633 173.5067579 150.0991939 503.4082096
157.5951383 1.676927244 1.74E-12 2.69E-10 TROAP
12 49323236 49331731 + 3954
protein_coding trophinin associated protein [Source:HGNC
Symbol;Acc:HGNC:12327] - 630 505 423 144 152
174 7.802478274 6.05175244 6.655957635
2.023661507 2.362948646 2.046178898
ENSG00000173545 1005.23407 1063.764174 877.6811957
1823.304551 2438.226545 2740.604247 982.22648
2334.045114 -1.248324146 1.96E-12 3.02E-10 ZNF622
5 16451519 16465792 - 1699
protein_coding zinc finger protein 622 [Source:HGNC
Symbol;Acc:HGNC:30958] - 1108 1202 755 1760 2136
3177 31.9355989 33.52258724 27.64778983
57.56139793 77.27788539 86.94705051
ENSG00000283632 78.02358307 127.439302 211.5734803
9.323716454 9.131934625 8.626390453 139.0121218
9.027347177 3.94385693 2.21E-12 3.36E-10
EXOC3L2 19 45212621 45245431 - 2964
protein_coding exocyst complex component 3 like 2 [Source:HGNC
Symbol;Acc:HGNC:30162] - 86 144 182 9 8
10 1.420852303 2.302028699 3.820322579
0.168723802 0.165904875 0.156874668
ENSG00000179046 242.2360079 281.4284587 173.2112558
690.9909861 651.7918339 860.0511281 232.2919075
734.2779827 -1.657685388 2.65E-12 4.01E-10 TRIML2
4 188091273 188109603 - 3050
protein_coding tripartite motif family like 2 [Source:HGNC
Symbol;Acc:HGNC:26378] - 267 318 149 667 571
997 4.286867946 4.940304541 3.039437908
12.15172795 11.5075701 15.19939624

ENSG00000168487 1036.987854 1037.214319 1608.888443
354.3012253 479.4265678 444.2591083 1227.696872
425.9956338 1.526259727 2.74E-12 4.11E-10 BMP1
8 22164736 22212326 + 7471
protein_coding bone morphogenetic protein 1 [Source:HGNC
Symbol;Acc:HGNC:1067] - 1143 1172 1384 342 420
515 7.491972551 7.433191406 11.52561744
2.543662066 3.455555831 3.205232301
ENSG00000122952 1995.044874 1771.760297 2077.372578
998.6736291 674.6216705 610.7484441 1948.05925
761.3479145 1.355751051 3.05E-12 4.54E-10 ZWINT
10 56357228 56361275 - 2378
protein_coding ZW10 interacting kinetochore protein [Source:HGNC
Symbol;Acc:HGNC:13195] - 2199 2002 1787 964 591
708 45.28371648 39.89134355 46.75408358
22.52564458 15.27646927 13.84370785
ENSG00000170312 2329.820481 1890.349647 2251.746326
1011.105251 518.23729 445.9843864 2157.305485
658.4423091 1.71258991 3.18E-12 4.69E-10 CDK1
10 60778331 60794852 + 3931
protein_coding cyclin dependent kinase 1 [Source:HGNC
Symbol;Acc:HGNC:1722] - 2568 2136 1937 976 454
517 31.99047283 25.74688216 30.6572661
13.7961789 7.099048925 6.115310564
ENSG00000185950 942.6337537 1081.464077 992.767869
2211.792737 1850.358253 2325.674866 1005.6219
2129.275285 -1.082533082 3.29E-12 4.81E-10 IRS2
13 109752698 109786568 - 8138
protein_coding insulin receptor substrate 2 [Source:HGNC
Symbol;Acc:HGNC:6126] - 1039 1222 854 2135 1621
2696 6.252109067 7.115082845 6.52900546
14.57780839 12.24370027 15.40398973
ENSG00000198826 2067.624951 1611.576174 1877.424015
879.5372522 555.9065203 439.083274 1852.20838
624.8423488 1.568470123 3.46E-12 5.02E-10
ARHGAP11A 15 32615144 32639949 + 6428
protein_coding Rho GTPase activating protein 11A [Source:HGNC
Symbol;Acc:HGNC:15783] - 2279 1821 1615 849 487
509 17.36189561 13.42333777 15.63160259
7.339117922 4.656937971 3.681907998
ENSG00000196878 1537.790387 1333.687696 1902.998831
707.566482 587.8682915 416.6546589 1591.492305
570.6964775 1.480354854 3.68E-12 5.30E-10 LAMB3
1 209614870 209652466 - 4688
protein_coding laminin subunit beta 3 [Source:HGNC
Symbol;Acc:HGNC:6490] - 1695 1507 1637 683 515
483 17.70560549 15.23183022 21.72540709
8.095527349 6.752537213 4.790607012
ENSG00000173546 1189.406016 1024.824387 1621.675852
383.3083431 495.4074534 510.6823148 1278.635418

463.1327038	1.463897036	4.00E-12	5.70E-10	CSPG4
	15	75674322	75712848	-	8290
protein_coding	chondroitin sulfate proteoglycan 4 [Source:HGNC
Symbol;Acc:HGNC:2466]	-	1311	1158	1395	370	434
	592	7.744205069	6.618818463	10.46951404
2.48004315	3.21797421	3.320459552
ENSG00000117519 4570.730367	4346.211197	5275.387107
2687.302276	1957.658485	2010.811615	4730.776224
2218.590792	1.092302641	4.66E-12	6.59E-10	CNN3
	1	94896949	94927278	-	2838
protein_coding	calponin 3 [Source:HGNC Symbol;Acc:HGNC:2157]	-
	5038	4911	4538	2594	1715	2331	86.93095598
	81.99436065	99.48530759	50.78899559	37.14489144
	38.1909886
ENSG00000103187 5004.396328	5114.38699	6577.377755
2815.762369	2794.371995	2828.593429	5565.387024
2812.909265	0.984113566	5.08E-12	7.14E-10	COTL1
	16	84565594	84618077	-	6762
protein_coding	coactosin like F-actin binding protein 1 [Source:HGNC
Symbol;Acc:HGNC:18304]	-	5516	5779	5658	2718	2448
	3279	39.94641179	40.49523922	52.05885637
22.33501917	22.25274582	22.54744148
ENSG00000177508 2548.467963	2983.318661	4322.143954
1389.233752	1296.734717	1394.024697	3284.643526
1359.997722	1.271505831	6.19E-12	8.61E-10	IRX3
	16	54283304	54286763	-	2911
protein_coding	iroquois homeobox 3 [Source:HGNC
Symbol;Acc:HGNC:14360]	Homeobox	2809	3371	3718	1341
	1136	1616	47.25396087	54.87101425	79.46465946
	25.59756274	23.98741706	25.81250595
ENSG00000136205 4375.671409	3695.739759	4932.45207
2373.403822	1916.56478	1758.921013	4334.62108
2016.296538	1.104183359	7.56E-12	1.04E-09	TNS3
	7	47275154	47582558	-	11797
protein_coding	tensin 3 [Source:HGNC Symbol;Acc:HGNC:21616]	-
	4823	4176	4243	2291	1679	2039	20.02047449
	16.77317783	22.37733167	10.79109593	8.74835659
	8.036679784
ENSG00000166401 1289.203623	1088.544038	1224.103707
2278.09472	2332.067805	2506.829066	1200.617123
2372.33053	-0.982876484	8.43E-12	1.16E-09
SERPINB8	18	63969925	64019779	+	5868
	protein_coding	serpin family B member 8 [Source:HGNC
Symbol;Acc:HGNC:8952]	-	1421	1230	1053	2199	2043
	2906	11.85857914	9.932108314	11.16464999
20.82318549	21.40058384	23.02695557
ENSG00000105894 632.3539233	569.9368786	869.5437541
243.4525963	93.60232991	94.89029498	690.6115187
143.9817404	2.262297514	1.04E-11	1.41E-09	PTN
	7	137227341	137343865	-	1713

protein_coding pleiotrophin [Source:HGNC Symbol;Acc:HGNC:9630] -
        697   644   748   235   82   110   19.92526466
        17.81373327   27.16758794   7.622940728   2.942414483
        2.985838684
ENSG00000087303 271.2680388   226.5587592   327.8226453
55.94229872   61.64055872   66.42320649   275.2164811
61.33535464   2.16302673   1.44E-11   1.94E-09   NID2
        14   52004803   52069228   -   6392
protein_coding nidogen 2 [Source:HGNC Symbol;Acc:HGNC:13389]   -
        299   256   282   54   54   77   2.290673046
        1.897709099   2.744853618   0.469428051   0.519283297
        0.560125026
ENSG00000150995 2466.815376   2564.715954   2135.497161
5799.351634   4395.88503   4423.613024   2389.009497
4872.949896   -1.028008308   1.69E-11   2.27E-09   ITPR1
        3   4493345 4847840 +   32454
protein_coding "inositol 1,4,5-trisphosphate receptor type 1
[Source:HGNC Symbol;Acc:HGNC:6180]"   -   2719   2898   1837
        5598   3851   5128   4.102699036   4.231132154
3.52166281   9.584659875   7.293779787   7.347012907
ENSG00000184226 347.47712   326.5632115   202.2735471
697.2067971   997.6638578   1086.062558   292.1046262
926.9777376   -1.663386101   1.70E-11   2.27E-09   PCDH9
        13   66302834   67230445   -   30944
protein_coding protocadherin 9 [Source:HGNC Symbol;Acc:HGNC:8661]
        -       383   369   174   673   874   1259
0.60610938   0.565036307   0.349848199   1.208511133
1.736130395   1.891822106
ENSG00000198886 47129.87318   44775.44478   32982.21309
73681.18726   88302.38335   89103.71226   41629.17702
83695.76096   -1.007522657   1.89E-11   2.49E-09   MT-ND4
        MT   10760   12137   +   1378   protein_coding
mitochondrially encoded NADH:ubiquinone oxidoreductase core subunit 4
[Source:HGNC Symbol;Acc:HGNC:7459]   -   51948   50594   28372
        71123   77357   103292 1846.070566   1739.707516
1280.995429   2867.958701   3450.624977   3485.366777
ENSG00000166147 2976.690419   1924.864458   3795.535237
1158.212777   1120.944975   741.0069399   2899.030038
1006.721564   1.52625259   1.90E-11   2.49E-09   FBN1
        15   48408306   48645849   -   16057
protein_coding fibrillin 1 [Source:HGNC Symbol;Acc:HGNC:3603] -
        3281   2175   3265   1118   982   859   10.00623058
        6.418318946   12.6510224   3.86891724   3.759191419
        2.487481014
ENSG00000169607 899.0857073   775.2557541   1068.329826
390.5601226   267.1090878   197.5443414   914.2237626
285.0711839   1.682383981   1.94E-11   2.52E-09   CKAP2L
        2   112736607   112764677   -   5219
protein_coding cytoskeleton associated protein 2 like [Source:HGNC
Symbol;Acc:HGNC:26877] -   991   876   919   377   234

229 9.298545942 7.953224655 10.95557209
4.013895827 2.755979106 2.040230349
ENSG00000119630 1709.26082 1587.681305 2656.29342
756.2570013 641.5184074 311.4126953 1984.411848
569.729368 1.801411402 2.09E-11 2.70E-09 PGF
14 74941834 74955784 - 5962
protein_coding placental growth factor [Source:HGNC
Symbol;Acc:HGNC:8893] - 1884 1794 2285 730 562
361 15.4745351 14.25794411 23.84520604
6.803665164 5.794176282 2.815439817
ENSG00000122642 2605.624774 2533.741123 3225.914328
1455.535735 1286.46129 996.3480973 2788.426742
1246.115041 1.16234913 2.35E-11 3.02E-09 FKBP9
7 32957404 33006931 + 5170
protein_coding FK506 binding protein 9 [Source:HGNC
Symbol;Acc:HGNC:3725] - 2872 2863 2775 1405 1127
1155 27.2033617 26.23960214 33.39483415
15.10072591 13.3992575 10.38777321
ENSG00000164442 1328.215414 1719.545583 1487.989312
3040.567533 2955.322343 4477.959284 1511.91677
3491.283053 -1.207666257 2.71E-11 3.44E-09 CITED2
6 139371807 139374620 - 2535
protein_coding Cbp/p300 interacting transactivator with Glu/Asp rich
carboxy-terminal domain 2 [Source:HGNC Symbol;Acc:HGNC:1987] -
1464 1943 1280 2935 2589 5191 28.28080691
36.31794593 31.41512938 64.33424158 62.77712854
95.21471753
ENSG00000112742 1360.876449 887.6501386 1217.128757
476.5455077 307.0613018 194.0937852 1155.218448
325.9001982 1.827231912 3.02E-11 3.81E-09 TTK
6 80003887 80042527 + 4579
protein_coding TTK protein kinase [Source:HGNC Symbol;Acc:HGNC:12401]
- 1500 1003 1047 460 269 225
16.0416597 10.37902912 14.22600263 5.582120815
3.611012541 2.284772131
ENSG00000162511 3925.67493 3085.978099 3802.510187
1871.99507 1552.428886 1062.771304 3604.721072
1495.731753 1.26960103 3.88E-11 4.87E-09 LAPTM5
1 30732469 30757820 - 3274
protein_coding lysosomal protein transmembrane 5 [Source:HGNC
Symbol;Acc:HGNC:29612] - 4327 3487 3271 1807 1360
1232 64.71976083 50.46609642 62.15967222
30.6684284 25.53333795 17.49697821
ENSG00000188610 506.246039 444.2675669 442.9093186
112.9205659 180.3557089 108.6925197 464.4743082
133.9895982 1.798135228 3.95E-11 4.91E-09 FAM72B
1 121167646 121185539 - 3033
protein_coding family with sequence similarity 72 member B
[Source:HGNC Symbol;Acc:HGNC:24805] - 558 502 381
109 158 126 9.00928804 7.842558152

7.815547591   1.996945127   3.202078959   1.931653178
ENSG00000132205 131.5513901   159.2991276   266.2105878
10.35968495   18.26386925   31.05500563   185.6870352
19.89285328   3.207140069   4.36E-11   5.40E-09
EMILIN2 18   2847030 2915993 +   6435   protein_coding
elastin microfibril interfacer 2 [Source:HGNC Symbol;Acc:HGNC:19881]
        -       145   180   229   10   16   36
1.103438504   1.325410463   2.21408239   0.086350229
0.152833582   0.260126722
ENSG00000179218 16494.72981   15905.13289   18384.80544
10497.46876   7514.440705   7431.635375   16928.22272
8481.181613   0.997085423   4.94E-11   6.07E-09   CALR
        19   12938578   12944489   +   2714
protein_coding calreticulin [Source:HGNC Symbol;Acc:HGNC:1455] -
        18181   17972   15815   10133   6583   8615   328.0474053
        313.7711485   362.5485726   207.4628038   149.0944139
        147.5966351
ENSG00000168056 2039.500172   1842.559909   3287.526386
891.9688741   1007.937284   932.5128079   2389.862155
944.1396554   1.339325045   5.10E-11   6.22E-09   LTBP3
        11   65538805   65558930   -   10298
protein_coding latent transforming growth factor beta binding protein
3 [Source:HGNC Symbol;Acc:HGNC:6716]   -   2248   2082   2828
        861   883   1081   10.6898621   9.579752271
17.08572312   4.645819233   5.27054076   4.880943996
ENSG00000121064 564.3101008   631.8865393   774.2194388
216.5174154   195.1951026   272.5939383   656.8053597
228.1021521   1.522300066   5.18E-11   6.25E-09   SCPEP1
        17   56978105   57006768   +   3853
protein_coding serine carboxypeptidase 1 [Source:HGNC
Symbol;Acc:HGNC:29507] -   622   714   666   209   171
        316   7.905331008   8.780629321   10.75429801
3.014111547   2.7280006   3.81345909
ENSG00000198804 99462.83065   94057.28487   71200.28857
144700.9715   206339.4873   215401.8322   88240.1347
188814.097   -1.097447897   5.19E-11   6.25E-09   MT-CO1
        MT   5904   7445   +   1542   protein_coding
mitochondrially encoded cytochrome c oxidase I [Source:HGNC
Symbol;Acc:HGNC:7419]   -   109631 106280 61248   139677 180763
        249701 3481.590474   3265.830291   2471.236926
5033.297127   7205.639441   7529.513669
ENSG00000130821 4972.642545   5486.969949   5436.973447
8653.444838   9070.294067   9508.007557   5298.86198
9077.248821   -0.776668103   6.25E-11   7.47E-09   SLC6A8
        X   153688099   153696593   +   5498
protein_coding solute carrier family 6 member 8 [Source:HGNC
Symbol;Acc:HGNC:11055] -   5481   6200   4677   8353   7946
        11022   48.81842345   53.43347202   52.92604979
84.42086341   88.83644797   93.21519004
ENSG00000237649 2027.705909   1936.369395   2341.258183

1096.054668 784.204886 581.4187165 2101.777829
820.5594234 1.357557699 6.88E-11 8.17E-09 KIFC1
6 33391536 33409924 + 3345
protein_coding kinesin family member C1 [Source:HGNC
Symbol;Acc:HGNC:6389] - 2235 2188 2014 1058 687
674 32.71975845 30.99400055 37.46020854
17.57525315 12.62431944 9.369033516
ENSG00000011028 4734.03554 4614.364728 7630.59519
941.6953619 1327.554996 628.863864 5659.665153
966.038074 2.550939335 7.82E-11 9.22E-09 MRC2
17 62627401 62693597 + 6860
protein_coding mannose receptor C type 2 [Source:HGNC
Symbol;Acc:HGNC:16875] - 5218 5214 6564 909 1163
729 37.2484875 36.01416516 59.5321138
7.36294932 10.42084555 4.941222732
ENSG00000142892 1154.023229 956.6797605 1105.529559
538.7036173 410.9370581 427.0063274 1072.077516
458.8823343 1.224077931 8.24E-11 9.66E-09 PIGK
1 77088990 77219430 - 5513
protein_coding phosphatidylinositol glycan anchor biosynthesis class
K [Source:HGNC Symbol;Acc:HGNC:8965] - 1272 1081 951
520 360 495 11.29868244 9.291035988
10.73246229 5.241159722 4.013856746 4.174920641
ENSG00000137831 2501.290913 1681.490791 1985.535738
1056.687865 727.1302945 675.4463725 2056.105814
819.7548439 1.327048303 8.69E-11 1.01E-08 UACA
15 70654554 70763593 - 11031
protein_coding uveal autoantigen with coiled-coil domains and ankyrin
repeats [Source:HGNC Symbol;Acc:HGNC:15947] - 2757 1900
1708 1020 637 783 12.23913049 8.161409274
9.633405209 5.138038224 3.549538638 3.300486005
ENSG00000235385 75.30183018 53.98470434 36.03724115
293.1790841 275.0995306 236.3630984 55.10792522
268.2139044 -2.274736666 8.79E-11 1.02E-08
LINC02154 X 13266048 13303452 - 1557
lincRNA long intergenic non-protein coding RNA 2154
[Source:HGNC Symbol;Acc:HGNC:53015] - 83 61 31
283 241 274 2.610466735 1.856383345
1.238739333 10.09973239 9.514276974 8.182631028
ENSG00000166833 1690.20855 1134.563786 1564.713761
695.1348601 450.8892721 353.6820086 1463.162032
499.9020469 1.550121745 9.69E-11 1.11E-08 NAV2
11 19350724 20121598 + 14673
protein_coding neuron navigator 2 [Source:HGNC Symbol;Acc:HGNC:15997]
- 1863 1282 1346 671 395 410
6.217597738 4.139954313 5.707330061 2.541064251
1.654723895 1.299259668
ENSG00000154229 3046.548744 2451.436574 3344.488477
1602.643262 1092.40768 927.3369737 2947.491265
1207.462638 1.28770793 1.09E-10 1.24E-08 PRKCA

17	66302636	66810743	+	11152
protein_coding	protein kinase C alpha [Source:HGNC
Symbol;Acc:HGNC:9393]	-	3358	2770	2877	1547	957
1075	14.74540191	11.76937631	16.0506989
7.708140046	5.274806555	4.482153458
ENSG00000143401 4029.10154	2847.914403	3438.6503
1908.253968	1302.442176	1331.914686	3438.555414
1514.20361	1.183301965	1.10E-10	1.25E-08	ANP32E
1	150218417	150236156	-	3605
protein_coding	acidic nuclear phosphoprotein 32 family member E
[Source:HGNC Symbol;Acc:HGNC:16673]	-	4441	3218	2958
1842	1141	1544	60.32595248	42.29677077
51.05047028	28.39202707	19.45484323	19.91466683
ENSG00000105472 1787.284403	2404.531831	2598.168838
1169.608431	978.2584967	908.3589147	2263.328357
1018.741947	1.151616133	1.22E-10	1.37E-08
CLEC11A 19	50723329	50725718	+	1469
protein_coding	C-type lectin domain containing 11A [Source:HGNC
Symbol;Acc:HGNC:10576]	-	1970	2717	2235	1129	857
1053	65.67092203	87.63836839	94.65914519
42.70553725	35.85967807	33.33017497
ENSG00000158109 975.2947884	1108.898927	999.7428189
1752.858693	2295.540066	2518.906012	1027.978845
2189.101591	-1.090559364	1.33E-10	1.49E-08	TPRG1L
1	3625002 3630127 +	2414	protein_coding	tumor
protein p63 regulated 1 like [Source:HGNC Symbol;Acc:HGNC:27007]
-	1075	1253	860	1692	2011	2920
21.80719877	24.59462732	22.16501513	38.94710098
51.20615483	56.24405235
ENSG00000101057 3622.653107	3980.708199	4237.282064
2255.303413	1972.497879	1516.519442	3946.881123
1914.773578	1.043934238	1.39E-10	1.54E-08	MYBL2
20	43667019	43716496	+	2777
protein_coding	MYB proto-oncogene like 2 [Source:HGNC
Symbol;Acc:HGNC:7548]	MYB	3993	4498	3645	2177	1728
1758	70.41288097	76.74852049	81.66359593
43.56067421	38.24857139	29.43567567
ENSG00000166825 1099.588171	1087.659043	1656.550601
555.2791133	478.285076	319.1764468	1281.265938
450.9135453	1.507406541	1.47E-10	1.62E-08	ANPEP
15	89784889	89815401	-	4871
protein_coding	"alanyl aminopeptidase, membrane [Source:HGNC
Symbol;Acc:HGNC:500]"	-	1212	1229	1425	536	419
370	12.18465323	11.95529217	18.20134918
6.114468384	5.287413233	3.531950535
ENSG00000166710 11441.34193	13251.03243	16657.34285
7480.728502	7120.626024	6922.678338	13783.23907
7174.677621	0.941830414	1.53E-10	1.69E-08	B2M
15	44711487	44718877	+	6079
protein_coding	beta-2-microglobulin [Source:HGNC Symbol;Acc:HGNC:914]

- 12611 14973 14329 7221 6238 8025
101.5888551 116.7086706 146.6528765 66.00506229
63.07548335 61.38240719
ENSG00000196611 789.3083404 949.5997993 683.5450901
2129.951226 1703.105808 1595.019595 807.4844099
1809.358876 -1.162750705 1.57E-10 1.71E-08 MMP1
11 102789920 102798160 - 1970
protein_coding matrix metallopeptidase 1 [Source:HGNC
Symbol;Acc:HGNC:7155] - 870 1073 588 2056 1492
1849 21.62627444 25.80833199 18.57025839
57.99211231 46.55324481 43.64170438
ENSG00000182628 1472.468318 1158.458655 1385.690047
701.350671 476.0020923 471.8635578 1338.87234
549.7387737 1.28436632 1.68E-10 1.82E-08 SKA2
17 59109951 59155269 - 3148
protein_coding spindle and kinetochore associated complex subunit 2
[Source:HGNC Symbol;Acc:HGNC:28006] - 1623 1309 1192
677 417 547 25.24715694 19.70295492
23.55854033 11.94994726 8.142329842 8.079483917
ENSG00000165304 2116.616504 1731.935515 2021.572979
1053.579959 779.6389186 613.3363612 1956.708332
815.518413 1.263338989 1.70E-10 1.84E-08 MELK
9 36572862 36677683 + 3087
protein_coding maternal embryonic leucine zipper kinase [Source:HGNC
Symbol;Acc:HGNC:16870] - 2333 1957 1739 1017 683
711 37.00895249 30.03866565 35.04852932
18.30612262 13.59976595 10.70937163
ENSG00000159261 13.60876449 18.58489822 25.5748163
98.41700702 163.2333314 222.5608737 19.25615967
161.4037374 -3.079504635 1.81E-10 1.94E-08 CLDN14
21 36460621 36576569 - 2853
protein_coding claudin 14 [Source:HGNC Symbol;Acc:HGNC:2035] -
15 21 22 95 143 258 0.257464983
0.348773889 0.479764109 1.85026476 3.080928545
4.204834941
ENSG00000124762 968.9440317 1285.012962 1195.041416
1948.656739 3036.368263 3002.846517 1149.666137
2662.623839 -1.211803196 1.91E-10 2.04E-08 CDKN1A
6 36676460 36687339 + 3050
protein_coding cyclin dependent kinase inhibitor 1A [Source:HGNC
Symbol;Acc:HGNC:1784] - 1068 1452 1028 1881 2660
3481 17.14747178 22.55761696 20.97008167
34.26896594 53.60794477 53.06830324
ENSG00000090889 2916.811856 2114.253421 2843.454576
1380.946004 858.4018548 680.6222067 2624.839951
973.3233551 1.431643586 2.04E-10 2.16E-08 KIF4A
X 70290090 70420832 + 4533
protein_coding kinesin family member 4A [Source:HGNC
Symbol;Acc:HGNC:13339] - 3215 2389 2446 1333 752
789 34.73153211 24.97220385 33.57202828

16.34016637	10.19716582	8.093237819
ENSG00000128039 601.5073904	863.7552694	566.1334335
1366.442445	1717.945201	1767.547404	677.1320311
1617.311683	-1.255003306	2.11E-10	2.22E-08	SRD5A3
4	55346109	55373096	+	4420
protein_coding	steroid 5 alpha-reductase 3 [Source:HGNC
Symbol;Acc:HGNC:25812]	-	663	976	487	1319	1505
2049	7.345475975	10.46294613	6.855095958
16.58191062	20.92963193	21.55513301
ENSG00000171604 1245.655576	1155.80367	1626.325818
661.9838682	554.7650285	477.9020311	1342.595021
564.8836426	1.249003171	2.24E-10	2.35E-08	CXXC5
5	139647299	139683882	+	3852
protein_coding	CXXC finger protein 5 [Source:HGNC
Symbol;Acc:HGNC:26943]	-	1373	1306	1399	639	486
554	17.45472225	16.06509653	22.59634946
9.217786043	7.755277654	6.687356939
ENSG00000101439 1922.464797	1933.71441	2679.543253
1064.975613	1067.294859	920.4358613	2178.574153
1017.568778	1.098096006	2.35E-10	2.44E-08	CST3
20	23626706	23638473	-	3695
protein_coding	cystatin C [Source:HGNC Symbol;Acc:HGNC:2475]	-
2119	2185	2305	1028	935	1067	28.08310977
28.01969731	38.81175968	15.4593317	15.55408716
13.42706202
ENSG00000145147 2173.773314	1796.540161	2559.806613
1155.104872	964.5605948	870.4027967	2176.706696
996.6894211	1.126843682	2.36E-10	2.44E-08	SLIT2
4	20253260	20620561	+	9712
protein_coding	slit guidance ligand 2 [Source:HGNC
Symbol;Acc:HGNC:11086]	-	2396	2030	2202	1115	845
1009	12.08110958	9.904072367	14.10637704
6.379376545	5.348049218	4.830738299
ENSG00000123689 710.3775064	517.7221646	656.8077822
263.1359977	200.9025618	226.8740689	628.3024844
230.3042095	1.44702249	2.38E-10	2.45E-08	G0S2
1	209675420	209676388	+	866
protein_coding	G0/G1 switch 2 [Source:HGNC Symbol;Acc:HGNC:30229]
-	783	585	565	254	176	263
44.27642562	32.00843311	40.59171516	16.29775813
12.49229226	14.12111125
ENSG00000141526 3749.668242	4127.617394	6480.890948
2175.533839	2217.918622	1671.79447	4786.058862
2021.748977	1.243237496	2.43E-10	2.49E-08
SLC16A3 17	82228397	82261129	+	8995
protein_coding	solute carrier family 16 member 3 [Source:HGNC
Symbol;Acc:HGNC:10924]	-	4133	4664	5575	2100	1943
1938	22.50053897	24.56879264	38.56120091
12.9726939	13.27758203	10.01805988
ENSG00000175063 2416.916573	2408.956807	2800.442385

1199.651517 961.1361193 481.3525873 2542.105255
880.7134079 1.530288537 2.73E-10 2.78E-08 UBE2C
20 45812576 45816957 + 1286
protein_coding ubiquitin conjugating enzyme E2 C [Source:HGNC
Symbol;Acc:HGNC:15937] - 2664 2722 2409 1158 842
558 101.4429653 100.2936861 116.5474238
50.03566019 40.24560711 20.17549731
ENSG00000212907 10841.64904 9192.444655 7106.311456
16778.54574 18002.46762 17958.41964 9046.801718
17579.811 -0.958265959 2.75E-10 2.78E-08 MT-
ND4L MT 10470 10766 + 297 protein_coding
mitochondrially encoded NADH:ubiquinone oxidoreductase core subunit 4L
[Source:HGNC Symbol;Acc:HGNC:7460] - 11950 10387 6113
16196 15771 20818 1970.335306 1657.143521
1280.574334 3030.144661 3263.999944 3259.219222
ENSG00000001036 1811.780179 1693.880723 1902.998831
1017.321062 655.2163094 640.9408106 1802.886578
771.159394 1.225362845 2.85E-10 2.87E-08 FUCA2
6 143494811 143511690 - 2793
protein_coding alpha-L-fucosidase 2 [Source:HGNC
Symbol;Acc:HGNC:4008] - 1997 1914 1637 982 574
743 35.01352315 32.4711327 36.46570299
19.53676244 12.6324712 12.36940747
ENSG00000107731 1430.734773 1192.973466 2195.946727
590.5020421 639.2354238 657.3309525 1606.551655
629.0228061 1.351732457 2.91E-10 2.90E-08 UNC5B
10 71212570 71302864 + 6841
protein_coding unc-5 netrin receptor B [Source:HGNC
Symbol;Acc:HGNC:12568] - 1577 1348 1889 570 560
762 11.28861825 9.336771762 17.17984393
4.629854138 5.031712247 5.179244327
ENSG00000203805 440.9239695 322.1382357 485.9215097
134.6759043 106.15874 56.93417699 416.327905
99.25627378 2.07262119 3.22E-10 3.20E-08 PLPP4
10 120456954 120589855 + 1785
protein_coding phospholipid phosphatase 4 [Source:HGNC
Symbol;Acc:HGNC:23531] - 486 364 418 130 93
66 13.33296479 9.66250216 14.56950873
4.046850637 3.202521753 1.719240896
ENSG00000099901 5136.854969 6173.726188 6042.631596
3281.948192 3328.590171 3301.319626 5784.404251
3303.952663 0.807917121 3.29E-10 3.25E-08 RANBP1
22 20115938 20127357 + 4622
protein_coding RAN binding protein 1 [Source:HGNC
Symbol;Acc:HGNC:9847] - 5662 6976 5198 3168 2916
3827 59.98858354 71.5159598 69.97021053
38.08616771 38.77974408 38.49989448
ENSG00000137812 1606.741461 1014.204445 1261.30344
592.5739791 283.0899734 233.7751813 1294.083115
369.8130446 1.807920633 3.31E-10 3.25E-08 KNL1

15	40594020	40664342	+	10250
protein_coding	kinetochore scaffold 1 [Source:HGNC
Symbol;Acc:HGNC:24054]	-	1771	1146	1085	572	248
271	8.461032814	5.297697948	6.585863412
3.100874624	1.487219858	1.229352542
ENSG00000221955 384.6744096	423.9126783	320.8476954
821.5230165	875.5242322	911.8094709	376.4782611
869.6189065	-1.206105702	3.39E-10	3.31E-08
SLC12A8 3	125082636	125212864	-	5586
protein_coding	solute carrier family 12 member 8 [Source:HGNC
Symbol;Acc:HGNC:15595]	-	424	479	276	793	767
1057	3.717008967	4.063132854	3.07407881
7.888315997	8.439987293	8.79842779
ENSG00000162063 1037.895105	845.1703712	1171.791583
478.6174446	331.0326302	422.6931322	1018.285686
410.781069	1.308124045	3.45E-10	3.35E-08	CCNF
16	2429394 2458854 +	6309	protein_coding	cyclin
F [Source:HGNC Symbol;Acc:HGNC:1591]	-	1144	955	1008
462	290	490	8.879615909	7.172478994
9.940468452	4.06905436	2.825431812	3.61132497
ENSG00000181019 1077.814148	1205.363399	1519.376586
630.9048134	521.6617655	506.3691196	1267.518044
552.9785661	1.196234425	3.63E-10	3.51E-08	NQO1
16	69706996	69726951	-	3254
protein_coding	NAD(P)H quinone dehydrogenase 1 [Source:HGNC
Symbol;Acc:HGNC:2874]	-	1188	1362	1307	609	457
587	17.87835578	19.83288985	24.98992114
10.39948392	8.632687336	8.38786768
ENSG00000137804 248.5867647	238.9486913	265.0480962
65.26601518	68.48950969	61.24737221	250.8611841
65.00096569	1.949438747	3.65E-10	3.51E-08	NUSAP1
15	41332694	41381050	+	2875
protein_coding	nucleolar and spindle associated protein 1
[Source:HGNC Symbol;Acc:HGNC:18538]	-	274	270	228
63	60	71	4.667038648	4.449921563
4.93405339	1.21762833	1.282805347	1.148289828
ENSG00000168140 355.6423787	301.7833472	635.8829325
134.6759043	78.76293614	66.42320649	431.1028861
93.28734899	2.208504174	3.81E-10	3.64E-08	VASN
16	4371848 4383528 +	2806	protein_coding
vasorin [Source:HGNC Symbol;Acc:HGNC:18517]	-	392	341
547	130	69	77	6.841118038	5.758284962
12.12848363	2.574350815	1.511502202	1.275951235
ENSG00000162576 3867.610868	4540.025136	7365.547094
2380.655601	2264.719787	1700.261558	5257.727699
2115.212316	1.313627719	3.86E-10	3.67E-08	MXRA8
1	1352689 1361777 -	3622	protein_coding	matrix
remodeling associated 8 [Source:HGNC Symbol;Acc:HGNC:7542]	-
4263	5130	6336	2298	1984	1971	57.63623059
67.11125494	108.8362499	35.25442389	33.66980352

25.30283576
ENSG00000136153 3049.270497 2027.523896 2751.617735
1387.161815 1086.70022 926.4743346 2609.470709
1133.445457 1.203343345 3.91E-10 3.69E-08 LMO7
13 75620434 75859870 + 14367
protein_coding LIM domain 7 [Source:HGNC Symbol;Acc:HGNC:6646] -
3361 2291 2367 1339 952 1074 11.45594986
7.55588672 10.25035751 5.178768872 4.073035701
3.475915481
ENSG00000101384 1885.267507 2067.348678 2500.519539
1105.378384 898.3540688 1118.842842 2151.045241
1040.858432 1.046169692 4.00E-10 3.77E-08 JAG1
20 10637684 10673999 - 9298
protein_coding jagged 1 [Source:HGNC Symbol;Acc:HGNC:6188] -
2078 2336 2151 1067 787 1297 10.94421673
11.90446102 14.39321177 6.376566911 5.202745122
6.486067326
ENSG00000170961 573.3826105 742.5109335 594.0332331
1534.269341 1453.119097 1195.617717 636.642259
1394.335385 -1.129939304 4.47E-10 4.18E-08 HAS2
8 121612116 121641390 - 4190
protein_coding hyaluronan synthase 2 [Source:HGNC
Symbol;Acc:HGNC:4819] - 632 839 511 1481 1273
1386 7.386381569 9.487993504 7.587762412
19.6405244 18.67504861 15.38084606
ENSG00000185215 927.2104872 692.0662097 1297.340681
356.3731622 373.2678278 336.4292277 972.2057928
355.3567392 1.451298123 4.57E-10 4.25E-08
TNFAIP2 14 103123442 103137439 + 5029
protein_coding TNF alpha induced protein 2 [Source:HGNC
Symbol;Acc:HGNC:11895] - 1022 782 1116 344 327
390 9.951715313 7.368032931 13.80668465
3.800921065 3.99680976 3.605902582
ENSG00000259781 1362.690951 1321.297764 1599.58851
782.1562137 597.0002261 602.1220536 1427.859075
660.4261645 1.112069761 4.67E-10 4.32E-08
HMGB1P6 15 71164770 71165415 - 646
processed_pseudogene high mobility group box 1 pseudogene 6
[Source:HGNC Symbol;Acc:HGNC:4998] - 1502 1493 1376
755 523 698 113.8586678 109.5099878
132.5234589 64.94212231 49.76414319 50.24049651
ENSG00000013810 2396.957052 2264.702597 2383.107882
1364.370508 1012.503252 828.1334835 2348.255844
1068.335748 1.1368083 4.96E-10 4.57E-08 TACC3
4 1721490 1745176 + 6528 protein_coding
transforming acidic coiled-coil containing protein 3 [Source:HGNC
Symbol;Acc:HGNC:11524] - 2642 2559 2050 1317 887
960 19.81898236 18.57447643 19.53802054
11.2103113 8.352006705 6.837889926
ENSG00000173207 2068.532202 2567.370939 2497.032064

1292.888682	1263.631454	1018.776712	2377.645069
1191.765616	0.996988834	5.70E-10	5.23E-08	CKS1B
	1	154974653	154979249	+	2338
protein_coding	CDC28 protein kinase regulatory subunit 1B
[Source:HGNC Symbol;Acc:HGNC:19083]	-	2280	2901	2148
	1248	1107	1181	47.75501917	58.79354713
57.16057868	29.66074958	29.10385207	23.48747922
ENSG00000123485 1192.127769	1154.918675	1250.841015
618.4731915	378.975287	297.6104706	1199.29582
431.6863163	1.475124387	5.84E-10	5.33E-08	HJURP
	2	233833416	233854566	-	3821
protein_coding	Holliday junction recognition protein [Source:HGNC
Symbol;Acc:HGNC:25444]	-	1314	1305	1076	597	332
	345	16.84019093	16.18303282	17.52032186
8.681791208	5.340825717	4.198296197
ENSG00000140961 275.8042937	491.17231	315.0352371
744.8613478	1217.971781	1325.876213	360.6706136
1096.236447	-1.602944233	5.89E-10	5.34E-08	OSGIN1
	16	83948282	83966332	+	3460
protein_coding	oxidative stress induced growth inhibitor 1
[Source:HGNC Symbol;Acc:HGNC:30093]	-	304	555	271
	719	1067	1537	4.302552401	7.600520333
4.8730407	11.54688486	18.95551904	20.65517062
ENSG00000142192 10641.14658	9008.365664	13612.77722
5920.559948	5757.684781	4224.343405	11087.42982
5300.862711	1.064686539	6.55E-10	5.92E-08	APP
	21	25880550	26171128	-	6316
protein_coding	amyloid beta precursor protein [Source:HGNC
Symbol;Acc:HGNC:620]	-	11729	10179	11710	5715	5044
	4897	90.93845019	76.36413449	115.3510681
50.27894508	49.08856276	36.05113987
ENSG00000277443 4338.474119	3718.749633	5963.582164
2054.325525	2481.603234	2067.745792	4673.601972
2201.22485	1.086113572	6.67E-10	5.98E-08	MARCKS
	6	113857362	113863471	+	4275
protein_coding	myristoylated alanine rich protein kinase C substrate
[Source:HGNC Symbol;Acc:HGNC:6759]	-	4782	4202	5130
	1983	2174	2397	54.77749101	46.5743036
74.66001841	25.77499791	31.25869053	26.07131477
ENSG00000154146 444.5529733	584.9817962	723.0698062
217.5533839	162.0918396	211.3465661	584.2015253
196.9972632	1.565593398	6.69E-10	5.98E-08	NRGN
	11	124739846	124747210	+	1309
protein_coding	neurogranin [Source:HGNC Symbol;Acc:HGNC:8000]	-
	490	661	622	210	142	245	18.33095609
	23.92700023	29.56361819	8.914391262	6.668007168
	8.702768997
ENSG00000169919 941.7265027	945.1748235	1283.390781
454.7901693	386.9657298	504.6438415	1056.764036
448.7999135	1.233297713	8.40E-10	7.47E-08	GUSB

7 65960684 65982314 - 3992
protein_coding glucuronidase beta [Source:HGNC Symbol;Acc:HGNC:4696]
- 1038 1068 1104 439 339 585
12.73313972 12.67672768 17.20621667 6.11063061
5.219832004 6.813909351
ENSG00000169744 468.1414984 248.683638 609.1456245
99.45297551 119.856642 45.7198694 441.9902537
88.34316229 2.32780828 8.68E-10 7.67E-08 LDB2
4 16501541 16898809 - 5586
protein_coding LIM domain binding 2 [Source:HGNC
Symbol;Acc:HGNC:6533] - 516 281 524 96 105
53 4.52352978 2.383591507 5.836294553
0.954953765 1.155408952 0.441169984
ENSG00000117632 10323.60874 10381.87814 12293.3492
6625.018525 6579.558898 5615.780185 10999.61203
6273.452536 0.810153833 8.88E-10 7.82E-08 STMN1
1 25884181 25906991 - 6261
protein_coding stathmin 1 [Source:HGNC Symbol;Acc:HGNC:6510] -
11379 11731 10575 6395 5764 6510 88.99980953
88.78053787 105.0856752 56.75562214 56.58842786
48.34686332
ENSG00000130635 4477.283517 4568.34498 8067.69205
1070.155455 1485.080868 853.1500158 5704.440183
1136.12878 2.328174624 8.97E-10 7.86E-08 COL5A1
9 134641774 134844843 + 11189
protein_coding collagen type V alpha 1 chain [Source:HGNC
Symbol;Acc:HGNC:2209] - 4935 5162 6940 1033 1301
989 21.59854854 21.86015149 38.59002352
5.130044015 7.147157998 4.109945244
ENSG00000136271 3226.184435 3955.928334 3436.325317
5533.107731 7152.587795 8111.394943 3539.479362
6932.36349 -0.969835747 9.08E-10 7.91E-08 DDX56
7 44565417 44575051 - 4068
protein_coding DEAD-box helicase 56 [Source:HGNC
Symbol;Acc:HGNC:18193] - 3556 4470 2956 5341 6266
9403 42.80647749 52.06585682 45.20956539
72.95476744 94.67968547 107.4772412
ENSG00000100242 1432.549275 1559.36146 2145.959586
747.9692533 850.411412 566.7538527 1712.62344
721.711506 1.247314795 9.34E-10 8.10E-08 SUN2
22 38734725 38794143 - 6489
protein_coding Sad1 and UNC84 domain containing 2 [Source:HGNC
Symbol;Acc:HGNC:14210] - 1579 1762 1846 722 745
657 11.91606982 12.86632658 17.69949067
6.18260452 7.057093294 4.707806601
ENSG00000129195 1004.326819 1106.243941 1243.866065
545.9553968 352.7209749 236.3630984 1118.145609
378.34649 1.564607771 9.40E-10 8.11E-08 PIMREG
17 6444415 6451469 + 2823 protein_coding PICALM
interacting mitotic regulator [Source:HGNC Symbol;Acc:HGNC:25483]

	-	1107	1250	1070	527	309	274
19.20283836	20.98097062	23.58195173	10.37317682
6.728139097	4.513055796
ENSG00000160213 1466.117561	2056.728736	1806.512024
2728.741016	5156.118588	6135.08889	1776.452774
4673.316164	-1.395572726	9.47E-10	8.13E-08	CSTB
	21	43772511	43776445	-	3935
protein_coding	cystatin B [Source:HGNC Symbol;Acc:HGNC:2482]	-
	1616	2324	1554	2634	4517	7112	20.11061275
	27.98451779	24.57045078	37.19487278	70.55904825
	84.03844917
ENSG00000156970 1414.404256	921.2799544	1113.667001
561.4949242	296.7878753	276.0444945	1149.783737
378.109098	1.605135091	1.02E-09	8.71E-08	BUB1B
	15	40161023	40221136	+	4476
protein_coding	BUB1 mitotic checkpoint serine/threonine kinase B
[Source:HGNC Symbol;Acc:HGNC:1149]	-	1559	1041	958
	542	260	320	17.05629587	11.02013951
13.31626036	6.728546411	3.570513096	3.324228883
ENSG00000138180 1675.692534	1214.21335	1562.388777
762.4728122	362.9944014	355.4072867	1484.098221
493.6248334	1.588395404	1.04E-09	8.87E-08	CEP55
	10	93496632	93529092	+	3271
protein_coding	centrosomal protein 55 [Source:HGNC
Symbol;Acc:HGNC:1161]	-	1847	1372	1344	736	318
	412	27.65126694	19.87467374	25.56380942
12.50285844	5.975770858	5.856628682
ENSG00000135476 1046.060364	840.7453955	889.3061122
380.2004376	357.2869422	171.66517	925.3706238
303.05085	1.613465641	1.09E-09	9.24E-08	ESPL1
	12	53268299	53293643	+	8890
protein_coding	"extra spindle pole bodies like 1, separase
[Source:HGNC Symbol;Acc:HGNC:16856]"	-	1153	950	765
	367	313	199	6.351206448	5.063470512
5.353853963	2.293909854	2.164162846	1.040836068
ENSG00000176945 5.443505796	8.849951531	0	77.69763712
	82.18741163	137.1596082	4.764485776	99.01488565
	-4.323659086	1.12E-09	9.45E-08	MUC20	3
	195720882	195741123	+	4249	protein_coding
	"mucin 20, cell surface associated [Source:HGNC
Symbol;Acc:HGNC:23282]" -	6	10	0	75	72
	159	0.069150162	0.111516649	0	0.980813819
	1.041581183	1.739968602
ENSG00000111424 304.8363246	196.468924	189.4861389
61.1221412	43.37668947	37.09347895	230.2637958
47.19743654	2.290178512	1.18E-09	9.86E-08	VDR
	12	47841537	47943048	-	5674
protein_coding	vitamin D receptor [Source:HGNC Symbol;Acc:HGNC:12679]
	THR-like	336	222	163	59	38	43
	2.89987067	1.853916133	1.787331542	0.577796257

0.411662802   0.3523791
ENSG00000117906 2631.935052   2045.223799   2497.032064
1367.478413   1181.444042   1048.969079   2391.396972
1199.297178   0.995927139   1.28E-09   1.07E-07   RCN2
15   76931619   76954392   +   10019
protein_coding reticulocalbin 2 [Source:HGNC Symbol;Acc:HGNC:9935]
-       2901   2311   2148   1320   1035   1216
14.17921004   10.92954317   13.33879958   7.320851513
6.349848057   5.643391978
ENSG00000228253 992.5325568   1015.089441   666.1077154
1732.139323   2115.184358   2003.910502   891.2432376
1950.411394   -1.128410656   1.34E-09   1.11E-07   MT-
ATP8   MT   8366   8572   +   207   protein_coding
mitochondrially encoded ATP synthase membrane subunit 8 [Source:HGNC
Symbol;Acc:HGNC:7415]   -   1094   1147   573   1672   1853
2323   258.8067864   262.5545285   172.2229893
448.8259629   550.2403492   521.8069779
ENSG00000179820 3000.278944   3336.431727   3522.349699
5167.410853   6641.199456   5959.973164   3286.353457
5922.861158   -0.849924015   1.35E-09   1.11E-07   MYADM
19   53866223   53876437   +   3675
protein_coding myeloid associated differentiation marker [Source:HGNC
Symbol;Acc:HGNC:7544]   -   3307   3770   3030   4988   5818
6909   44.06619329   48.60830168   51.29701945
75.41906518   97.31140291   87.41558482
ENSG00000150991 10653.84809   11516.44193   11720.24081
16622.1145   22365.24939   21481.43751   11296.84361
20156.26713   -0.835336924   1.36E-09   1.12E-07   UBC
12   124911604   124917368   -   3898
protein_coding ubiquitin C [Source:HGNC Symbol;Acc:HGNC:12468] -
11743   13013   10082   16045   19593   24902   147.5250973
158.1838115   160.9206229   228.7230482   308.9629932
297.0457972
ENSG00000244486 1410.775252   1497.411799   1875.099031
675.4514587   862.9678221   731.5179104   1594.428694
756.6457304   1.075291146   1.39E-09   1.14E-07   SCARF2
22   20424815   20437826   -   3341
protein_coding scavenger receptor class F member 2 [Source:HGNC
Symbol;Acc:HGNC:19869] -   1555   1692   1613   652   756
848   22.79200866   23.99663379   30.03756602
10.84384157   13.90889665   11.80185827
ENSG00000120802 5286.551379   4196.647016   4713.90364
2904.85566   2424.528643   2254.938464   4732.367345
2528.107589   0.904664532   1.41E-09   1.15E-07   TMPO
12   98515512   98550379   +   8593
protein_coding thymopoietin [Source:HGNC Symbol;Acc:HGNC:11875]
-       5827   4742   4055   2804   2124   2614
33.20694248   26.14828312   29.35978652   18.13198041
15.19347308   14.14463645
ENSG00000132002 4173.354444   4894.023197   4553.479793

6999.003152 8346.588248 8450.412088 4540.285811
7932.001162 -0.804921451 1.42E-09 1.15E-07 DNAJB1
19 14514770 14529770 - 3672
protein_coding DnaJ heat shock protein family (Hsp40) member B1
[Source:HGNC Symbol;Acc:HGNC:5270] - 4600 5530 3917
6756 7312 9796 61.34565992 71.3590237
66.36784951 102.2348613 122.3998457 124.0443884
ENSG00000179750 361.9931354 338.0681485 559.1584836
149.1794633 54.79160775 70.73640171 419.7399225
91.56915758 2.195347105 1.49E-09 1.20E-07
APOBEC3B 22 38982347 38992804 + 1896
protein_coding apolipoprotein B mRNA editing enzyme catalytic
subunit 3B [Source:HGNC Symbol;Acc:HGNC:17352] - 399 382
481 144 48 82 10.30536186 9.546660332
15.78387344 4.2202308 1.556145727 2.010974379
ENSG00000035499 616.9306569 543.387024 531.258684
247.5964703 162.0918396 216.5224004 563.8587883
208.7369034 1.432324551 1.50E-09 1.21E-07
DEPDC1B 5 60596912 60700190 - 3806
protein_coding DEP domain containing 1B [Source:HGNC
Symbol;Acc:HGNC:24902] - 680 614 457 239 142
251 8.749209429 7.644094159 7.470579001
3.4893229 2.293331945 3.06645048
ENSG00000110237 1842.626712 1531.92661 1625.163327
938.5874564 713.4323926 582.2813556 1666.572216
744.7670682 1.162874733 1.53E-09 1.22E-07
ARHGEF17 11 73308289 73369388 + 9908
protein_coding Rho guanine nucleotide exchange factor 17
[Source:HGNC Symbol;Acc:HGNC:21726] - 2031 1731 1398
906 625 675 10.03812522 8.278230424
8.778655777 5.081059065 3.877406907 3.167734635
ENSG00000163535 1402.609993 1011.54946 1118.316967
570.8186407 422.3519764 274.3192164 1177.49214
422.4966112 1.48038074 1.54E-09 1.22E-07 SGO2
2 200510008 200583782 + 6022
protein_coding shugoshin 2 [Source:HGNC Symbol;Acc:HGNC:30812] -
1546 1143 962 551 370 318 12.5717988
8.993565874 9.938965143 5.084203103 3.776663866
2.455372497
ENSG00000162490 160.583421 219.478798 549.8585504
66.30198367 19.40536108 37.09347895 309.9735898
40.9336079 2.916295467 1.55E-09 1.23E-07 DRAXIN
1 11691729 11725857 + 7346
protein_coding dorsal inhibitory axon guidance protein [Source:HGNC
Symbol;Acc:HGNC:25054] - 177 248 473 64 17
43 1.179915825 1.599658204 4.006056437
0.4841067 0.142247734 0.272175199
ENSG00000196154 7608.206601 8887.121328 9032.56012
5165.338916 4045.447039 2987.319014 8509.296016
4066.034989 1.065618791 1.59E-09 1.25E-07 S100A4

1	153543613	153550136	-	1025
protein_coding	S100 calcium binding protein A4 [Source:HGNC
Symbol;Acc:HGNC:10494]	-	8386	10042	7770	4986	3544
3463	400.644953	464.2188726	471.6327992
270.2965188	212.5285152	157.0940167
ENSG00000163430	5294.716637	4736.494059	7051.674348
3072.682556	2934.77549	2215.257068	5694.295015
2740.905038	1.05493922	1.65E-09	1.29E-07	FSTL1
3	120392293	120451253	-	8727
protein_coding	follistatin like 1 [Source:HGNC Symbol;Acc:HGNC:3972]
-	5836	5352	6066	2966	2571	2568
32.74756334	29.05879289	43.24583397	18.88505328
18.1085827	13.68236153
ENSG00000168615	4655.104706	3833.799003	5034.751336
2705.949709	1800.132613	1558.788755	4507.885015
2021.623692	1.157053574	1.75E-09	1.37E-07	ADAM9
8	38996869	39105144	+	6913
protein_coding	ADAM metallopeptidase domain 9 [Source:HGNC
Symbol;Acc:HGNC:216]	-	5131	4332	4331	2612	1577
1807	36.34662927	29.69260712	38.97880078
20.99513441	14.02208181	12.15409464
ENSG00000164308	1130.434704	823.0454924	1195.041416
503.4806885	442.8988293	420.9678541	1049.507204
455.7824573	1.203014878	1.76E-09	1.37E-07	ERAP2
5	96875939	96919716	+	7422
protein_coding	endoplasmic reticulum aminopeptidase 2 [Source:HGNC
Symbol;Acc:HGNC:29499]	-	1246	930	1028	486	388
488	8.221021346	5.937292424	8.617454743
3.638541753	3.21335077	3.057242513
ENSG00000146670	2565.705732	2388.601918	2510.981964
1485.578822	1135.784369	1365.557609	2488.429871
1328.9736	0.904447263	1.97E-09	1.53E-07	CDCA5
11	65066300	65084164	-	3925
protein_coding	cell division cycle associated 5 [Source:HGNC
Symbol;Acc:HGNC:14626]	-	2828	2699	2160	1434	995
1583	35.28323746	32.58289466	34.23898933
20.30119178	15.58227196	18.75306556
ENSG00000112655	2582.9435	2871.809272	3459.57515
1538.413215	1596.947068	1632.113074	2971.442641
1589.157785	0.902397625	2.03E-09	1.57E-07	PTK7
6	43076268	43161719	+	9024
protein_coding	protein tyrosine kinase 7 (inactive) [Source:HGNC
Symbol;Acc:HGNC:9618]	-	2847	3245	2976	1485	1399
1892	15.44959375	17.03891967	20.51826747
9.144067234	9.529409458	9.748842713
ENSG00000131043	1586.781939	1803.620122	1691.425351
2605.460765	3705.282474	3885.32626	1693.942471
3398.689833	-1.004676657	2.04E-09	1.57E-07	AAR2
20	36236459	36270918	+	3101
protein_coding	AAR2 splicing factor homolog [Source:HGNC

Symbol;Acc:HGNC:15886]	-	1749	2038	1455	2515	3246
	4504	27.61955816	31.14073467	29.19228388
45.06592263	64.34193379	67.53480244
ENSG00000146918 2184.660326	1685.915767	2106.43487
1135.42147	643.8013911	644.3913668	1992.336987
807.8714094	1.30237454	2.35E-09	1.80E-07	NCAPG2
	7	158631311	158704829	-	6988
protein_coding	non-SMC condensin II complex subunit G2 [Source:HGNC
Symbol;Acc:HGNC:21904]	-	2408	1905	1812	1096	564
	747	16.87455271	12.91720418	16.13288892
8.715046356	4.961049586	4.970484498
ENSG00000163808 1437.08553	943.4048332	1095.067134
571.8546092	276.2410224	376.9732628	1158.519166
408.3562981	1.503956758	2.63E-09	2.01E-07	KIF15
	3	44761717	44873376	+	5285
protein_coding	kinesin family member 15 [Source:HGNC
Symbol;Acc:HGNC:17273]	-	1584	1066	942	552	242
	437	14.67705322	9.557375599	11.08952024
5.803715696	2.814606813	3.844744315
ENSG00000124172 2348.872751	3421.391262	3397.963093
1628.542474	1220.254764	1526.87111	3056.075702
1458.556116	1.066604559	2.67E-09	2.02E-07
ATP5F1E 20	59025467	59032382	-	5126
protein_coding	ATP synthase F1 subunit epsilon [Source:HGNC
Symbol;Acc:HGNC:838]	-	2589	3866	2923	1572	1069
	1770	24.73330381	35.73630851	35.47783096
17.04064321	12.8187732	16.05556831
ENSG00000110811 1136.78546	1107.128937	1246.191049
635.0486874	543.3501102	571.929687	1163.368482
583.4428282	0.995096784	2.91E-09	2.20E-07	P3H3
	12	6828410 6839851 +	4601	protein_coding	prolyl
3-hydroxylase 3 [Source:HGNC Symbol;Acc:HGNC:19318]	-	1253
	1251	1072	613	476	663	13.3360594
12.88343045	14.49604067	7.403213684	6.35919408
6.700270147
ENSG00000179403 1181.240758	959.334746	1053.217435
508.660531	555.9065203	472.7261968	1064.597646
512.4310827	1.055789413	3.01E-09	2.27E-07	VWA1
	1	1434861 1442882 +	5065	protein_coding	von
Willebrand factor A domain containing 1 [Source:HGNC
Symbol;Acc:HGNC:30910]	-	1302	1084	906	491	487
	548	12.58810098	10.1408947	11.12898596
5.386592054	5.910127794	5.030742946
ENSG00000122691 662.2932052	790.3006717	778.8694054
257.9561552	320.7592037	359.7204819	743.8210941
312.8119469	1.248334079	3.03E-09	2.27E-07	TWIST1
	7	19020991	19117672	-	2033
protein_coding	twist family bHLH transcription factor 1 [Source:HGNC
Symbol;Acc:HGNC:12428]	bHLH	730	893	670	249	281
	417	17.58385788	20.81327973	20.50426805

6.805718976	8.496035169	9.537393347
ENSG00000268858 256.7520234	323.0232309	277.8355043
556.3150818	762.5165412	1022.227269	285.8702529
780.3529639	-1.449208334	3.21E-09	2.39E-07
AL118506.1	20	63861212	63864293	-	3082
antisense	"novel transcript, antisense to ABHD16B"
-	283	365	239	537	668	1185
4.496581659	5.611599539	4.824719987	9.681746227
13.32266747	17.87790948
ENSG00000164109 2280.828928	2131.068329	2286.621075
1357.118728	888.0806423	820.3697321	2232.839444
1021.856368	1.127993978	3.25E-09	2.42E-07	MAD2L1
4	120055608	120067074	-	5748
protein_coding	mitotic arrest deficient 2 like 1 [Source:HGNC
Symbol;Acc:HGNC:6763]	-	2514	2408	1967	1310	778
951	21.41791533	19.85025837	21.29092093
12.66387397	8.319748493	7.692983051
ENSG00000177283 1949.682326	2596.575779	2549.344188
4130.406389	4294.292258	4570.261662	2365.200764
4331.653436	-0.873256029	3.45E-09	2.55E-07	FZD8
10	35638249	35642278	-	4030
protein_coding	frizzled class receptor 8 [Source:HGNC
Symbol;Acc:HGNC:4046]	-	2149	2934	2193	3987	3762
5298	26.11319747	34.49701394	33.85637311
54.97348039	57.38007417	61.1276818
ENSG00000136108 3882.126883	2794.814694	3743.223113
1944.512865	1761.321891	1349.167467	3473.38823
1685.000741	1.043941045	3.50E-09	2.58E-07	CKAP2
13	52455429	52476628	+	4547
protein_coding	cytoskeleton associated protein 2 [Source:HGNC
Symbol;Acc:HGNC:1990]	-	4279	3158	3220	1877	1543
1564	46.08355941	32.90891866	44.05931736
22.93777889	20.85875255	15.99347415
ENSG00000154734 3392.211362	3523.165705	2004.135604
6803.205106	5575.046089	7060.700586	2973.17089
6479.650593	-1.123462763	3.69E-09	2.71E-07
ADAMTS1 21	26835747	26845409	-	7063
protein_coding	ADAM metallopeptidase with thrombospondin type 1 motif
1 [Source:HGNC Symbol;Acc:HGNC:217]	-	3739	3981	1724
6567	4884	8185	25.92357796	26.70726478
15.18640235	51.66421722	42.5043921	53.8840829
ENSG00000124731 369.2511432	461.9674699	267.3730795
842.2423864	849.2699202	1142.134096	366.1972309
944.5488008	-1.365173602	3.89E-09	2.85E-07	TREM1
6	41267926	41286719	-	4329
protein_coding	triggering receptor expressed on myeloid cells 1
[Source:HGNC Symbol;Acc:HGNC:17760]	-	407	522	230
813	744	1324	4.604002035	5.713593753
3.30557562	10.43554183	10.56410502	14.22104195
ENSG00000186141 1837.183206	1994.779075	1914.623747

2980.48136	3709.848442	3485.924382	1915.528676
3392.084728	-0.824360196	3.92E-09	2.85E-07	POLR3C
1	145824088	145842505	+	2793
protein_coding	RNA polymerase III subunit C [Source:HGNC
Symbol;Acc:HGNC:30076]	-	2025	2254	1647	2877	3250
4041	35.50444886	38.2392545	36.68846232
57.23754129	71.52531605	67.27426055
ENSG00000134369 3994.626003	3085.093104	5034.751336
2147.56269	1985.054289	1616.585571	4038.156814
1916.40085	1.075278793	3.93E-09	2.85E-07	NAV1
1	201622885	201826969	+	22453
protein_coding	neuron navigator 1 [Source:HGNC Symbol;Acc:HGNC:15989]
-	4403	3486	4331	2073	1739	1874
9.602912964	7.356639035	12.00108893	5.130231576
4.760719194	3.880844382
ENSG00000010292 4866.494182	3595.735307	4933.614562
2687.302276	1811.547531	1635.56363	4465.28135
2044.804479	1.126862115	4.02E-09	2.91E-07	NCAPD2
12	6493356 6531955 +	6487	protein_coding	non-
SMC condensin I complex subunit D2 [Source:HGNC
Symbol;Acc:HGNC:24305]	-	5364	4063	4244	2594	1587
1896	40.4924034	29.67764019	40.70411568
22.21969624	15.03766442	13.59018764
ENSG00000103043 1794.542411	1801.850132	1664.688043
2821.97818	3031.802296	3247.836005	1753.693528
3033.87216	-0.790633305	4.15E-09	2.98E-07	VAC14
16	70687439	70801161	-	8276
protein_coding	"Vac14, PIKFYVE complex component [Source:HGNC
Symbol;Acc:HGNC:25507]" -	1978	2036	1432	2724	2656
3765	11.70400474	11.65691776	10.76538045
18.28936659	19.72672312	21.15317278
ENSG00000169499 1310.070395	1018.629421	1338.027889
573.9265462	641.5184074	488.2536996	1222.242568
567.8995511	1.106624741	4.18E-09	3.00E-07
PLEKHA2 8	38901235	38973909	+	6325
protein_coding	pleckstrin homology domain containing A2 [Source:HGNC
Symbol;Acc:HGNC:14336]	-	1444	1151	1151	554	562
566	11.17983379	8.622659461	11.32196063
4.866999241	5.461640948	4.160896561
ENSG00000154096 513.5040467	492.0573051	594.0332331
222.7332264	73.055477	120.7694663	533.198195
138.8527232	1.939622437	4.28E-09	3.06E-07	THY1
11	119417378	119424985	-	5925
protein_coding	Thy-1 cell surface antigen [Source:HGNC
Symbol;Acc:HGNC:11801]	-	566	556	511	215	64
140	4.677962759	4.446444519	5.365860676
2.016332493	0.66395551	1.098678685
ENSG00000172985 451.8109811	420.3726977	403.3846025
174.0427071	151.8184131	157.8629453	425.1894271
161.2413552	1.399091552	4.38E-09	3.11E-07	SH3RF3

2 109129348 109504632 + 5803
protein_coding SH3 domain containing ring finger 3 [Source:HGNC
Symbol;Acc:HGNC:24699] - 498 475 347 168 133
183 4.202478069 3.878532901 3.720349587
1.608676638 1.408790553 1.46632263
ENSG00000196576 3051.99225 3070.048186 3960.609051
1939.333022 1732.784595 1338.815798 3360.883162
1670.311139 1.008917541 4.83E-09 3.42E-07 PLXNB2
22 50274979 50307627 - 7257
protein_coding plexin B2 [Source:HGNC Symbol;Acc:HGNC:9104] -
3364 3469 3407 1872 1518 1552 22.70008836
22.65028222 29.20934761 14.33378101 12.85766211
9.944102953
ENSG00000001617 811.0823636 741.6259383 1004.392786
373.9846267 399.5221399 260.5169917 852.3670291
344.6745861 1.307712812 4.86E-09 3.42E-07 SEMA3F
3 50155045 50189075 + 4826
protein_coding semaphorin 3F [Source:HGNC Symbol;Acc:HGNC:10728]
- 894 838 864 361 350 302
9.071495402 8.227788926 11.13866831 4.156539653
4.457877057 2.909716277
ENSG00000196262 25551.81621 28013.63658 32812.48931
18651.57678 15528.85483 14122.26381 28792.64736
16100.89847 0.838553601 5.46E-09 3.83E-07 PPIA
7 44796680 44824564 + 4432
protein_coding peptidylprolyl isomerase A [Source:HGNC
Symbol;Acc:HGNC:9253] - 28164 31654 28226 18004 13604
16371 311.1883053 338.419428 396.2382821
225.7258495 188.6749449 171.7538476
ENSG00000076706 2741.712419 2490.376361 2493.544589
1544.629026 946.2967256 767.7487503 2575.211123
1086.224834 1.245908365 5.79E-09 4.05E-07 MCAM
11 119308529 119321521 - 7422
protein_coding melanoma cell adhesion molecule [Source:HGNC
Symbol;Acc:HGNC:6934] - 3022 2814 2145 1491 829
890 19.93894583 17.96509772 17.98097317
11.16268674 6.865638629 5.575708682
ENSG00000145431 2044.036426 2111.598435 2183.159318
1282.528997 1184.868518 1109.353812 2112.931393
1192.250442 0.825738267 6.48E-09 4.50E-07 PDGFC
4 156760454 156971394 - 5593
protein_coding platelet derived growth factor C [Source:HGNC
Symbol;Acc:HGNC:8801] - 2253 2386 1878 1238 1038
1286 19.72627376 20.21399067 20.89092237
12.29951167 11.40774735 10.69121756
ENSG00000148082 583.3623711 706.2261322 478.9465598
1210.011202 1256.782503 1251.689255 589.5116877
1239.49432 -1.070465747 6.49E-09 4.50E-07 SHC3
9 89005771 89178767 - 9929
protein_coding SHC adaptor protein 3 [Source:HGNC

```
Symbol;Acc:HGNC:18181]  -       643     798     412     1168    1101
        1451    3.171276766     3.808235713     2.581657064
6.536561862     6.815993514     6.795054117
ENSG00000105447 4168.818189     4954.202867     4097.783066
6792.845421     7910.538369     9257.842234     4406.934707
7987.075341     -0.857859106    6.86E-09        4.74E-07        GRWD1
        19      48445773        48457022        +       5763
protein_coding  glutamate rich WD repeat containing 1 [Source:HGNC
Symbol;Acc:HGNC:21270]  -       4595    5598    3525    6557    6930
        10732   39.04501371     46.02679297     38.05549264
63.22205492     73.91489686     86.58906748
ENSG00000123384 2758.042937     1665.560878     3638.598864
1281.493028     971.4095458     693.5617924     2687.400893
982.1547888     1.452331725     6.89E-09        4.74E-07        LRP1
        12      57128493        57213351        +       20839
protein_coding  LDL receptor related protein 1 [Source:HGNC
Symbol;Acc:HGNC:6692]   -       3040    1882    3130    1237    851
        804     7.143735932     4.279265035     9.344892494
3.298411748     2.510152145     1.793949412
ENSG00000204388 2223.672118     3297.491941     2181.996827
4440.160969     5254.286885     6488.770899     2567.720295
5394.406251     -1.07077696     7.40E-09        5.07E-07        HSPA1B
        6       31827735        31830255        +       2521
protein_coding  heat shock protein family A (Hsp70) member 1B
[Source:HGNC Symbol;Acc:HGNC:5233]      -       2451    3726    1877
        4286    4603    7522    47.61010608     70.03198653
46.32317022     94.46944516     112.2316866     138.7367452
ENSG00000134243 1927.001052     1547.856523     1792.562124
999.7095976     583.3023242     789.3147264     1755.806566
790.7755494     1.150145667     7.76E-09        5.30E-07        SORT1
        1       109309568       109397951       -       9160
protein_coding  sortilin 1 [Source:HGNC Symbol;Acc:HGNC:11186]  -
        2124    1749    1542    965     511     915     11.35501526
        9.047337191     10.47359429     5.853880914     3.429041859
        4.644688987
ENSG00000084234 5747.434869     5408.205381     7294.635103
3725.342708     3031.802296     2349.828759     6150.091784
3035.657921     1.018697909     7.97E-09        5.42E-07        APLP2
        11      130068147       130144811       +       7463
protein_coding  amyloid beta precursor like protein 2 [Source:HGNC
Symbol;Acc:HGNC:598]    -       6335    6111    6275    3596    2656
        2724    41.56826147     38.79942441     52.31269993
26.77430985     21.87570153     16.97167395
ENSG00000150687 4300.369579     4168.327171     4456.992986
2849.949329     2132.306735     2174.713033     4308.563245
2385.656366     0.852806371     8.77E-09        5.94E-07        PRSS23
        11      86791059        86952910        +       11725
protein_coding  serine protease 23 [Source:HGNC Symbol;Acc:HGNC:14370]
        -       4740    4710    3834    2751    1868    2521
19.79676254     19.03419423     20.34445704     13.03736375
```

9.792901375	9.997490783
ENSG00000140545 498.9880313	527.4571113	702.1449565
255.8842182	203.1855454	217.3850394	576.1966997
225.4849344	1.352077027	8.87E-09	5.99E-07	MFGE8
15	88898683	88913411	-	6837
protein_coding	milk fat globule-EGF factor 8 protein [Source:HGNC
Symbol;Acc:HGNC:7036]	-	550	596	604	247	178
252	3.93936111	4.130542743	5.49639841
2.007443898	1.600301391	1.713823048
ENSG00000204389 1297.368881	2193.902985	1163.654142
3056.10706	3481.550076	4691.893767	1551.642002
3743.183634	-1.270020187	9.16E-09	6.16E-07	HSPA1A
6	31815464	31817946	+	2483
protein_coding	heat shock protein family A (Hsp70) member 1A
[Source:HGNC Symbol;Acc:HGNC:5232]	-	1430	2479	1001
2950	3050	5439	28.20252556	47.30709152
25.08211788	66.01723641	75.5040904	101.8528903
ENSG00000099998 29.93928188	58.40968011	66.26202404	0
0	0	51.53699534	0	8.119211766
1.00E-08	6.73E-07	GGT5	22	24219654
24245142	-	6054	protein_coding	gamma-
glutamyltransferase 5 [Source:HGNC Symbol;Acc:HGNC:4260]	-
33	66	57	0	0	0	0.266931733
0.516568548	0.585786401	0	0	0
ENSG00000087074 2821.550504	3825.834047	2873.679359
5381.856331	5731.430469	7948.356163	3173.68797
6353.880988	-1.001467037	1.03E-08	6.88E-07
PPP1R15A	19	48872392	48876057	+	3093
protein_coding	protein phosphatase 1 regulatory subunit 15A
[Source:HGNC Symbol;Acc:HGNC:14375]	-	3110	4323	2472
5195	5021	9214	49.23899188	66.22649279
49.72506884	93.32922845	99.78325379	138.5157973
ENSG00000075223 1347.267684	1056.684213	2070.397628
687.8830806	589.0097833	433.0448007	1491.449842
569.9792216	1.387812407	1.06E-08	7.06E-07	SEMA3C
7	80742538	80922359	-	6268
protein_coding	semaphorin 3C [Source:HGNC Symbol;Acc:HGNC:10725]
-	1485	1194	1781	664	516	502
11.6018207	9.026134065	17.67835205	5.886418498
5.060204564	3.723966345
ENSG00000123473 1490.613337	1332.802701	1471.714429
854.6740083	646.0843747	671.9958163	1431.710155
724.2513998	0.98307878	1.09E-08	7.20E-07	STIL
1	47250139	47314147	-	7291
protein_coding	"STIL, centriolar assembly protein [Source:HGNC
Symbol;Acc:HGNC:10879]" -	1643	1506	1266	825	566
779	11.03517307	9.787331865	10.80322582
6.28751297	4.771739133	4.967997602
ENSG00000121621 853.723159	474.3574021	635.8829325
291.1071471	194.0536108	186.3300338	654.6544978

223.8302639	1.548954692	1.12E-08	7.41E-07	KIF18A
11	28020620	28108308	-	4668
protein_coding	kinesin family member 18A [Source:HGNC
Symbol;Acc:HGNC:29441]	-	941	536	547	281	170
216	9.871597961	5.4407702	7.290600912
3.344933717	2.238542964	2.15156228
ENSG00000100065 275.8042937	286.7384296	304.5728123
102.560881	90.17785443	60.38473317	289.0385118
84.37448953	1.780434277	1.20E-08	7.91E-07	CARD10
22	37490362	37519542	-	5652
protein_coding	caspase recruitment domain family member 10
[Source:HGNC Symbol;Acc:HGNC:16422]	-	304	324	262
99	79	70	2.633905044	2.716247239
2.884071247	0.973296328	0.859156536	0.575873249
ENSG00000066056 797.4735991	1061.109189	1394.98998
498.300846	407.5125827	245.8521279	1084.524256
383.8885189	1.499347009	1.24E-08	8.11E-07	TIE1
1	43300993	43323108	+	6694
protein_coding	tyrosine kinase with immunoglobulin like and EGF like
domains 1 [Source:HGNC Symbol;Acc:HGNC:11809]	-	879	1199
1200	481	357	285	6.430309114	8.487111659
11.15327434	3.992743506	3.278157898	1.979658004
ENSG00000164105 678.6237226	693.8362	789.3318303
319.0782964	335.5985975	353.6820086	720.597251
336.1196342	1.09909893	1.36E-08	8.87E-07	SAP30
4	173369969	173377532	+	1320
protein_coding	Sin3A associated protein 30 [Source:HGNC
Symbol;Acc:HGNC:10532]	-	748	784	679	308	294
410	27.7495759	28.14288217	32.00388415
12.96548685	13.6905457	14.44245236
ENSG00000143127 2493.125655	2090.358552	2445.882431
1350.902917	1183.727026	810.8807026	2343.122213
1115.170215	1.071956554	1.36E-08	8.87E-07	ITGA10
1	145891208	145910189	-	6320
protein_coding	integrin subunit alpha 10 [Source:HGNC
Symbol;Acc:HGNC:6135]	-	2748	2362	2104	1304	1037
940	21.29258226	17.708805	20.71264224
11.46496034	10.08576949	6.915789938
ENSG00000117984 1878.916751	2402.761841	3077.115397
1124.025817	1313.857094	1117.980203	2452.93133
1185.287705	1.049091731	1.38E-08	8.93E-07	CTSD
11	1752752 1764573 -	3126	protein_coding
cathepsin D [Source:HGNC Symbol;Acc:HGNC:2529]	-	2071	2715
2647	1085	1151	1296	32.44291052	41.15354968
52.68315971	19.28647276	22.63256155	19.27733728
ENSG00000128590 505.3387881	461.9674699	445.2343019
943.7672989	1029.625629	872.9907138	470.8468533
948.7945472	-1.009736186	1.41E-08	9.13E-07	DNAJB9
7	108569568	108574850	+	2837
protein_coding	DnaJ heat shock protein family (Hsp40) member B9

[Source:HGNC Symbol;Acc:HGNC:6968]	-	557	522	383
	911	902	1012	9.614452163	8.718416411
8.399361723	17.84313186	19.54314985	16.5864023
ENSG00000110203 9.07250966	6.194966072	2.3249833
62.15810969	69.63100152	127.6705787	5.86415301
86.4865633	-3.855846662	1.55E-08	1.00E-06	FOLR3
	11	72114869	72139892	+	1396
protein_coding	folate receptor 3 [Source:HGNC Symbol;Acc:HGNC:3795]
	-	10	7	2	60	61	148
0.350786818	0.237595966	0.089135648	2.388239493
2.685911984	4.929550446
ENSG00000205336 153.3254133	160.1841227	313.8727455
50.76245625	18.26386925	36.2308399	209.1274271
35.0857218	2.56852321	1.58E-08	1.01E-06	ADGRG1
	16	57610652	57665580	+	8659
protein_coding	adhesion G protein-coupled receptor G1 [Source:HGNC
Symbol;Acc:HGNC:4512]	-	169	181	270	49	16
	42	0.955757354	0.990460764	1.940005098
0.31444188	0.113579409	0.225534284
ENSG00000100109 1498.778596	1694.765718	1487.989312
2384.799475	3126.546117	3066.681806	1560.511209
2859.342466	-0.873448079	1.59E-08	1.02E-06	TFIP11
	22	26491225	26512505	-	5436
protein_coding	tuftelin interacting protein 11 [Source:HGNC
Symbol;Acc:HGNC:17165]	-	1652	1915	1280	2302	2739
	3555	14.88193072	16.69228423	14.64999135
23.53086623	30.97133633	30.40823236
ENSG00000184661 1151.301476	821.2755021	1126.454409
488.9771296	393.8146807	212.2092051	1033.010462
365.0003385	1.502696614	1.61E-08	1.02E-06	CDCA2
	8	25458997	25507920	+	3908
protein_coding	cell division cycle associated 2 [Source:HGNC
Symbol;Acc:HGNC:14623]	-	1269	928	969	472	345
	246	15.90141421	11.2517445	15.42680779
6.711189273	5.426401202	2.926924839
ENSG00000160049 1841.719461	1918.669492	1385.690047
3056.10706	3011.255443	3611.007044	1715.359667
3226.123182	-0.910749405	1.73E-08	1.10E-06	DFFA
	1	10456522	10472526	-	6622
protein_coding	DNA fragmentation factor subunit alpha [Source:HGNC
Symbol;Acc:HGNC:2772]	-	2030	2168	1192	2950	2638
	4186	15.01189593	15.51302678	11.19937858
24.75397131	24.48685305	29.39280718
ENSG00000093009 1295.554379	1269.968045	1418.239813
778.0123397	681.4706214	644.3913668	1327.920746
701.2914426	0.921126069	1.83E-08	1.15E-06	CDC45
	22	19479459	19520612	+	2846
protein_coding	cell division cycle 45 [Source:HGNC
Symbol;Acc:HGNC:1739]	-	1428	1435	1220	751	597
	747	24.57095266	23.89150159	26.67053832

14.66280588	12.89397416	12.20440818
ENSG00000168461 4943.610514	4081.597646	5596.234803
3008.452509	2542.102301	2134.168998	4873.814321
2561.574603	0.928079152	1.96E-08	1.23E-06	RAB31
18	9708165 9862551 +	5586
protein_coding	"RAB31, member RAS oncogene family [Source:HGNC Symbol;Acc:HGNC:9771]"	-	5449	4612	4814	2904	2227
2474	47.76882514	39.12143783	53.61817171
28.88735139	24.50567367	20.59348189
ENSG00000130592 445.4602243	478.7823778	867.2187708
187.5102976	171.2237742	52.62098176	597.153791
137.1183512	2.126135274	2.17E-08	1.36E-06	LSP1
11	1852970 1892267 +	5399	protein_coding
lymphocyte specific protein 1 [Source:HGNC Symbol;Acc:HGNC:6707]
-	491	541	746	181	150	61
4.453452743	4.747996372	8.596711386	1.862847447
1.707753924	0.525348535
ENSG00000166963 896.3639544	1085.004058	799.7942551
1574.672112	1845.792286	2063.432596	927.0540891
1827.965665	-0.978873579	2.25E-08	1.41E-06	MAP1A
15	43510958	43531620	+	10629
protein_coding	microtubule associated protein 1A [Source:HGNC Symbol;Acc:HGNC:6835]	-	988	1226	688	1520	1617
2392	4.55190533	5.46543211	4.027197029
7.946268299	9.351148914	10.46404952
ENSG00000082781 4528.089571	4186.912069	5209.125083
2623.072229	2633.421648	1634.700991	4641.375575
2297.064956	1.015188583	2.30E-08	1.43E-06	ITGB5
3	124761948	124901418	-	6447
protein_coding	integrin subunit beta 5 [Source:HGNC Symbol;Acc:HGNC:6160]	-	4991	4731	4481	2532	2307
1895	37.91041889	34.7713633	43.24382691
21.82318201	21.99567452	13.6672948
ENSG00000167693 54.43505796	26.54985459	62.77454909	0
0	0	47.91982055	0	8.013842704
2.33E-08	1.44E-06	NXN	17	799313	979770	-
5880	protein_coding	nucleoredoxin [Source:HGNC Symbol;Acc:HGNC:18008]	-	60	30	54	0	0
0	0.499692243	0.241752164	0.571377692	0
0	0
ENSG00000119865 967.1295297	877.0301967	1012.530227
509.6964995	472.5776169	364.8963162	952.2299845
449.0568108	1.085547415	2.34E-08	1.44E-06	CNRIP1
2	68284171	68320051	-	3548
protein_coding	cannabinoid receptor interacting protein 1 [Source:HGNC Symbol;Acc:HGNC:24546]	-	1066	991	871
492	414	423	14.7130353	13.23477262
15.27359922	7.705370666	7.1723932	5.543547515
ENSG00000137563 1749.179862	1577.946358	1753.037408
1025.60881	612.9811117	773.7872236	1693.387876

804.1257151	1.073902126	2.51E-08	1.55E-06	GGH
	8	63015079	63039171	-	3130
protein_coding	gamma-glutamyl hydrolase [Source:HGNC
Symbol;Acc:HGNC:4248]	-	1928	1783	1508	990	537
	897	30.16416971	26.99189939	29.97532155
17.57530622	10.54574604	13.3253653
ENSG00000160113 1625.793731	1754.060393	1915.786239
890.9329056	1053.596957	974.7821212	1765.213455
973.1039947	0.859062058	2.62E-08	1.61E-06	NR2F6
	19	17231883	17245940	-	2676
protein_coding	nuclear receptor subfamily 2 group F member 6
[Source:HGNC Symbol;Acc:HGNC:7977]	RXR-like	1792	1982
	1648	860	923	1130	32.79295702	35.09489779
	38.31580416	17.85765325	21.20132249	19.63465852
ENSG00000210140 626.0031665	1082.349072	805.6067134
1477.291074	1940.536108	2715.587715	837.9863174
2044.471632	-1.286871014	2.92E-08	1.79E-06	MT-TC
	MT	5761	5826	-	66	Mt_tRNA
mitochondrially encoded tRNA cysteine [Source:HGNC
Symbol;Acc:HGNC:7477]	-	690	1223	693	1426	1700
	3148	511.9574164	878.0292064	653.275161
1200.570405	1583.260387	2217.797075
ENSG00000109586 757.5545566	544.2720192	656.8077822
292.1431156	300.2123508	215.6597613	652.8781193
269.3384092	1.279413491	2.94E-08	1.79E-06	GALNT7
	4	173168753	173323967	+	6865
protein_coding	polypeptide N-acetylgalactosaminyltransferase 7
[Source:HGNC Symbol;Acc:HGNC:4129]	-	835	615	565
	282	263	250	5.956273308	4.244836969
5.120528089	2.282551633	2.354846305	1.693286653
ENSG00000273812 304.8363246	340.723134	569.6209084
146.0715578	113.007691	67.28584553	405.0601223
108.7883648	1.898626169	2.94E-08	1.79E-06
BX640514.2	20	62596732	62603115	-	625
	lincRNA novel transcript	-	336	385	490
	141	99	78	26.32618589	29.18818922
48.77787869	12.53577357	9.736492585	5.802906907
ENSG00000113368 4524.460567	3762.999391	4158.232632
2642.75563	2036.421421	1561.376672	4148.564197
2080.184575	0.996292522	3.08E-08	1.86E-06	LMNB1
	5	126776623	126837020	+	3633
protein_coding	lamin B1 [Source:HGNC Symbol;Acc:HGNC:6637]	-
	4987	4252	3577	2551	1784	1810	67.22064168
	55.45673523	61.257658	39.01729025	30.1840014
	23.16563425
ENSG00000168393 1457.045051	1827.514991	1785.587174
993.4937866	882.3731832	849.6994596	1690.049072
908.5221431	0.895508271	3.77E-08	2.27E-06	DTYMK
	2	241675742	241686991	-	2451
protein_coding	deoxythymidylate kinase [Source:HGNC

Symbol;Acc:HGNC:3061]	-	1606	2065	1536	959	773
985	32.08713291	39.92116307	38.99013609
21.74139165	19.38579174	18.68632669
ENSG00000120738 380.1381547	302.6683424	349.9099866
1367.478413	775.0729513	650.4298401	344.2388279
930.9937349	-1.435181749	3.78E-08	2.27E-06	EGR1
5	138465490	138469315	+	3138
protein_coding	early growth response 1 [Source:HGNC
Symbol;Acc:HGNC:3238]	zf-C2H2 419	342	301	1320	679
754	6.538675236	5.164159027	5.967884412
23.37399978	13.30038447	11.17247585
ENSG00000128595 7017.586222	6099.386595	8132.791583
4561.369283	3885.638183	3551.484949	7083.2548
3999.497472	0.82454342	3.95E-08	2.37E-06	CALU
7	128739292	128771807	+	4537
protein_coding	calumenin [Source:HGNC Symbol;Acc:HGNC:1458]	-
7735	6892	6996	4403	3404	4117	83.48726275
71.97852294	95.93738314	53.92522301	46.1177523
42.19326234
ENSG00000213949 448.1819772	247.7986429	466.1591516
138.8197783	116.4321665	104.3793245	387.3799239
119.8770898	1.69217015	4.11E-08	2.46E-06	ITGA1
5	52787896	52959210	+	18566
protein_coding	integrin subunit alpha 1 [Source:HGNC
Symbol;Acc:HGNC:6134]	-	494	280	401	134	102
121	1.30297861	0.714605124	1.343794543
0.401049977	0.337698542	0.303038664
ENSG00000132128 2536.673701	3167.397653	2822.529726
4247.470829	5378.709494	5328.521383	2842.20036
4984.900569	-0.81053286	4.27E-08	2.54E-06	LRRC41
1	46261196	46303608	-	8606
protein_coding	leucine rich repeat containing 41 [Source:HGNC
Symbol;Acc:HGNC:16917]	-	2796	3579	2428	4100	4712
6177	15.90979226	19.70546999	17.55311456
26.47247572	33.65513215	33.37392439
ENSG00000130584 288.5058072	296.4733763	365.0223781
127.4241249	82.18741163	98.34085116	316.6671872
102.6507959	1.623281686	4.43E-08	2.63E-06	ZBTB46
20	63743666	63831244	-	6084
protein_coding	zinc finger and BTB domain containing 46 [Source:HGNC
Symbol;Acc:HGNC:16094]	-	318	335	314	123	72
114	2.559567565	2.60904784	3.211051635
1.123383264	0.727429068	0.87125777
ENSG00000129355 371.0656451	428.3376541	611.4706078
140.8917153	110.7247073	203.5828147	470.2913023
151.7330791	1.62650701	4.57E-08	2.70E-06	CDKN2D
19	10566462	10569059	-	1503
protein_coding	cyclin dependent kinase inhibitor 2D [Source:HGNC
Symbol;Acc:HGNC:1790]	-	409	484	526	136	97
236	13.32579141	15.25853443	21.77376895

5.027961823	3.96698094	7.301028446
ENSG00000137809 202.3169654	155.7591469	288.2979292
39.36680281	60.49906689	17.25278091	215.4580138
39.0395502	2.475087501	4.79E-08	2.82E-06	ITGA11
15	68296533	68432162	-	10864
protein_coding	integrin subunit alpha 11 [Source:HGNC
Symbol;Acc:HGNC:6136]	-	223	176	248	38	53
20	1.005179886	0.767625427	1.420262991
0.194359549	0.299870313	0.085599506
ENSG00000104341 3780.514775	3526.705685	3666.498664
2477.000671	1867.480631	1903.844373	3657.906375
2082.775225	0.81255772	5.20E-08	3.06E-06
LAPTM4B 8	97775057	97853013	+	3197
protein_coding	lysosomal protein transmembrane 4 beta [Source:HGNC
Symbol;Acc:HGNC:13646]	-	4167	3985	3154	2391	1636
2207	63.82775182	59.0625414	61.37986082
41.55745885	31.45487932	32.09894178
ENSG00000185728 2564.798481	2352.317117	2405.195224
3787.500817	3867.374314	4156.19492	2440.770274
3937.02335	-0.68984237	5.22E-08	3.06E-06	YTHDF3
8	63168553	63212786	+	6163
protein_coding	YTH N6-methyladenosine RNA binding protein 3
[Source:HGNC Symbol;Acc:HGNC:26465]	-	2827	2658	2069
3656	3388	4818	22.462719	20.43568735
20.88695685	32.96294934	33.79080936	36.35010302
ENSG00000160957 1538.697638	1709.810636	1422.889779
698.2427656	835.5720182	925.6116956	1557.132685
819.8088265	0.925325778	5.24E-08	3.06E-06	RECQL4
8	144511288	144517845	-	4236
protein_coding	RecQ like helicase 4 [Source:HGNC
Symbol;Acc:HGNC:9949]	-	1696	1932	1224	674	732
1073	19.60643257	21.61113675	17.97762483
8.841297182	10.62190688	11.77808783
ENSG00000121060 2351.594504	2312.492335	2272.671176
3403.156506	4277.16988	3938.809881	2312.252671
3873.045422	-0.744028611	5.55E-08	3.23E-06	TRIM25
17	56836387	56914080	-	9501
protein_coding	tripartite motif containing 25 [Source:HGNC
Symbol;Acc:HGNC:12932]	-	2592	2613	1955	3285	3747
4566	13.35962792	13.03156374	12.80219065
19.21224426	24.24162536	22.34588746
ENSG00000130706 4180.612451	5009.072567	4347.718771
6403.321267	8660.4985	9348.419334	4512.467929
8137.413034	-0.850646785	5.69E-08	3.30E-06	ADRM1
20	62302093	62308862	+	2142
protein_coding	adhesion regulating molecule 1 [Source:HGNC
Symbol;Acc:HGNC:15759]	-	4608	5660	3740	6181	7587
10837	105.3468823	125.2054996	108.6323019
160.3434858	217.7198256	235.2451211
ENSG00000143799 8227.85901	7592.373419	7878.205911

5503.064645 4479.213934 4293.354528 7899.479447
4758.544369 0.731319126 6.05E-08 3.50E-06 PARP1
1 226360691 226408079 - 7169
protein_coding poly(ADP-ribose) polymerase 1 [Source:HGNC
Symbol;Acc:HGNC:270] Others 9069 8579 6777 5312 3924
4977 61.948316 56.70280304 58.81468182
41.17290684 33.64478664 32.28048703
ENSG00000129315 1378.114217 1344.307638 1208.991316
2191.073367 2128.88226 2398.136546 1310.471057
2239.364057 -0.772761774 6.11E-08 3.52E-06 CCNT1
12 48688458 48716998 - 7381
protein_coding cyclin T1 [Source:HGNC Symbol;Acc:HGNC:1599] -
1519 1519 1040 2115 1865 2780 10.07792802
9.751445758 8.766474636 15.92235161 15.53141381
17.51300246
ENSG00000161800 2578.407245 2138.14829 2572.594021
1550.844837 1231.669683 1236.161752 2429.716519
1339.558757 0.85893344 6.15E-08 3.53E-06
RACGAP1 12 49976923 50033136 - 4487
protein_coding Rac GTPase activating protein 1 [Source:HGNC
Symbol;Acc:HGNC:9804] - 2842 2416 2213 1497 1079
1433 31.01677843 25.51333909 30.68542841
18.53863588 14.78130353 14.84981827
ENSG00000105671 2092.120728 2750.564936 2494.707081
3685.975905 4613.909969 5221.554141 2445.797581
4507.146672 -0.882072887 6.41E-08 3.67E-06 DDX49
19 18919675 18928633 + 2780
protein_coding DEAD-box helicase 49 [Source:HGNC
Symbol;Acc:HGNC:18684] - 2306 3108 2146 3558 4042
6053 40.62030599 52.97398632 48.02769769
71.11696126 89.37146426 101.2411095
ENSG00000109971 66121.35765 71464.24361 64555.4863
97701.15279 105683.8794 102998.2394 67380.36252
102127.7572 -0.599964183 6.53E-08 3.73E-06 HSPA8
11 123057489 123063230 - 4849
protein_coding heat shock protein family A (Hsp70) member 8
[Source:HGNC Symbol;Acc:HGNC:5241] - 72881 80751 55532
94309 92584 119399 736.0220451 789.0820532
712.5215065 1080.719529 1173.629767 1144.931551
ENSG00000136695 29.93928188 50.44472373 43.01219105
178.1865811 586.7267997 1051.556996 41.13206555
605.4901257 -3.880254408 6.56E-08 3.73E-06 IL36RN
2 113058638 113065382 + 3299
protein_coding interleukin 36 receptor antagonist [Source:HGNC
Symbol;Acc:HGNC:15561] - 33 57 37 172 514
1219 0.489846837 0.818689049 0.697792432
2.897064571 9.576970808 17.18115707
ENSG00000104824 4684.136737 4608.169762 5203.312625
2786.755251 3151.658938 3038.214717 4831.873042
2992.209635 0.691236646 7.20E-08 4.08E-06 HNRNPL

19	38836388	38852347	-	6821
protein_coding	heterogeneous nuclear ribonucleoprotein L [Source:HGNC Symbol;Acc:HGNC:5045]	-	5163	5207	4476	2690	2761
3522	37.06660065	36.17145419	40.82713219
21.91372837	24.88087889	24.00890318
ENSG00000170017 4083.536598	2772.689815	4279.131763
2238.727917	1578.683198	1224.084805	3711.786059
1680.49864	1.143402502	7.35E-08	4.16E-06	ALCAM
3	105366909	105576900	+	7179
protein_coding	activated leukocyte cell adhesion molecule
[Source:HGNC Symbol;Acc:HGNC:400]	-	4501	3133	3681
2161	1383	1419	30.70250022	20.67868332
31.9013242	16.72641459	11.84146913	9.190718412
ENSG00000132561 217.7402318	102.6594378	187.1611556
43.51067679	11.41491828	11.21430759	169.1869417
22.04663422	2.942979593	7.47E-08	4.21E-06	MATN2
8	97868840	98036716	+	6933
protein_coding	matrilin 2 [Source:HGNC Symbol;Acc:HGNC:6908]	-
240	116	161	42	10	13	1.69519134
0.792799249	1.444812607	0.336620169	0.08865968
0.087187288
ENSG00000146648 2683.648357	2155.848193	2953.891282
1638.902159	918.9009217	1004.111849	2597.795944
1187.304976	1.129408722	7.51E-08	4.22E-06	EGFR
7	55019021	55211628	+	12521
protein_coding	epidermal growth factor receptor [Source:HGNC Symbol;Acc:HGNC:3236]	-	2958	2436	2541	1582	805
1164	11.56878734	9.218594441	12.62619511
7.020685315	3.951884335	4.322599341
ENSG00000185269 98.89035529	182.3090015	272.0230461
40.4027713	35.38624667	18.11541995	184.4074676
31.30147931	2.564166742	7.77E-08	4.35E-06	NOTUM
17	81952507	81961840	-	3019
protein_coding	"notum, palmitoleoyl-protein carboxylesterase
[Source:HGNC Symbol;Acc:HGNC:27106]"	-	109	206	234
39	31	21	1.768039928	3.233184947
4.822359586	0.717816666	0.631169408	0.323435138
ENSG00000117758 1398.073739	1302.712865	1486.82682
2069.865053	2706.477125	2801.851619	1395.871141
2526.064599	-0.856182987	7.87E-08	4.38E-06	STX12
1	27773183	27824452	+	3846
protein_coding	syntaxin 12 [Source:HGNC Symbol;Acc:HGNC:11430] -
1541	1472	1279	1998	2371	3248	19.62104087
18.13530947	20.69036305	28.8667737	37.89392876
39.26790745
ENSG00000154839 891.8276996	702.6861516	875.3562124
426.8190199	277.3825142	171.66517	823.2900212
291.9555681	1.497371856	7.88E-08	4.38E-06	SKA1
18	50374995	50394173	+	2950
protein_coding	spindle and kinetochore associated complex subunit 1

[Source:HGNC Symbol;Acc:HGNC:28109]	-	983	794	753
	412	243	199	16.31774663	12.75336905
15.88107171	7.76045605	5.06327619	3.136621237
ENSG00000175745 3296.042759	2781.539766	3292.176352
2074.008927	1493.071311	1488.914992	3123.252959
1685.331743	0.890008254	8.49E-08	4.70E-06	NR2F1
	5	93583224	93594615	+	4314
protein_coding	nuclear receptor subfamily 2 group F member 1
[Source:HGNC Symbol;Acc:HGNC:7975]	RXR-like	3633	3143
	2832	2002	1308	1726	41.23955218	34.52158132
	40.84321823	25.78671237	18.63695529	18.60337193
ENSG00000186854 184.1719461	172.5740549	172.0487642
49.72648776	7.990442797	15.52750281	176.2649217
24.41481112	2.851604551	8.50E-08	4.70E-06
TRABD2A 2	84821650	84907008	-	8771
protein_coding	TraB domain containing 2A [Source:HGNC
Symbol;Acc:HGNC:27013]	-	203	195	148	48	7
	18	1.133380172	1.053445182	1.049831141
0.304091422	0.049056469	0.095423296
ENSG00000107438 1156.744982	1137.218772	1535.65147
715.85423	630.1034892	546.0505157	1276.538408
630.6694116	1.01715787	8.55E-08	4.71E-06	PDLIM1
	10	95237572	95291024	-	2239
protein_coding	PDZ and LIM domain 1 [Source:HGNC
Symbol;Acc:HGNC:2067]	-	1275	1285	1321	691	552
	633	27.88590701	27.19414913	36.70756451
17.14888932	15.15417661	13.1456067
ENSG00000130766 476.3067571	585.8667914	549.8585504
845.3502918	1283.036815	1394.887336	537.344033
1174.424814	-1.128439338	9.18E-08	5.04E-06	SESN2
	1	28259527	28282491	+	3453
protein_coding	sestrin 2 [Source:HGNC Symbol;Acc:HGNC:20746]	-
	525	662	473	816	1124	1617	7.445457837
	9.084224363	8.522586327	13.13123652	20.00861801
	21.77431291
ENSG00000150093 10702.83965	9232.269437	12508.41015
7129.535182	5954.021376	5252.609147	10814.50641
6112.055235	0.823225092	9.51E-08	5.21E-06	ITGB1
	10	32900319	33005792	-	6011
protein_coding	integrin subunit beta 1 [Source:HGNC
Symbol;Acc:HGNC:6153]	-	11797	10432	10760	6882	5216
	6089	96.10667119	82.23321906	111.3710694
63.61799592	53.33818274	47.1010148
ENSG00000135111 1389.001229	1281.472982	1596.101035
727.2498834	843.562461	575.3802432	1422.191749
715.3975292	0.992244761	1.01E-07	5.49E-06	TBX3
	12	114670254	114684164	-	5950
protein_coding	T-box 3 [Source:HGNC Symbol;Acc:HGNC:11602]	T-box
	1531	1448	1373	702	739	667	12.60047475
	11.53129379	14.35689149	6.555898031	7.63439863

5.212425807
ENSG00000138080 2660.059832 3162.087682 3549.087007
1783.937748 1929.12119 1589.84376 3123.74484
1767.634233 0.821571485 1.10E-07 6.00E-06
EMILIN1 2 27078567 27086408 + 3958
protein_coding elastin microfibril interfacer 1 [Source:HGNC
Symbol;Acc:HGNC:19880] - 2932 3573 3053 1722 1690
1843 36.27578837 42.77437447 47.99078579
24.17516244 26.24570693 21.65112979
ENSG00000069275 16341.4044 13295.28219 15527.40097
10111.05251 8966.41831 7372.97592 15054.69585
8816.81558 0.771969346 1.12E-07 6.09E-06 NUCKS1
1 205712819 205750276 - 6546
protein_coding nuclear casein kinase and cyclin dependent kinase
substrate 1 [Source:HGNC Symbol;Acc:HGNC:29923] - 18012 15023
13357 9760 7855 8547 134.7456088 108.7444515
126.9520656 82.84873093 73.75942944 60.71118658
ENSG00000137845 1752.808866 1359.352555 2092.48497
1035.968495 684.8950969 653.8803963 1734.88213
791.5813294 1.131834036 1.15E-07 6.19E-06 ADAM10
15 58588807 58749978 - 16168
protein_coding ADAM metallopeptidase domain 10 [Source:HGNC
Symbol;Acc:HGNC:188] - 1932 1536 1800 1000 600
758 5.85166567 4.501542513 6.926646933
3.436811739 2.281089419 2.179936902
ENSG00000121152 1729.220341 1287.667948 1615.863393
931.3356769 460.0212068 436.4953569 1544.250561
609.2840802 1.342013532 1.16E-07 6.26E-06 NCAPH
2 96335787 96373845 + 3278
protein_coding non-SMC condensin I complex subunit H [Source:HGNC
Symbol;Acc:HGNC:1112] - 1906 1455 1390 899 403
506 28.47361645 21.03199573 26.38230262
15.23922166 7.556896204 7.177489829
ENSG00000108387 40.82629347 29.20484005 49.98714095 0
0 0 40.00609149 0 7.753743451
1.17E-07 6.28E-06 4-Sep 17 58520250
58540818 - 8012 protein_coding septin 4 [Source:HGNC
Symbol;Acc:HGNC:9165] - 45 33 43 0 0
0 0.275042785 0.195163878 0.333913795 0
0 0
ENSG00000000971 555.2375912 341.6081291 463.8341683
180.2585181 186.063168 146.6486377 453.5599629
170.9901079 1.408953368 1.22E-07 6.55E-06 CFH
1 196651878 196747504 + 8145
protein_coding complement factor H [Source:HGNC Symbol;Acc:HGNC:4883]
- 612 386 399 174 163 170
3.679501777 2.245549624 3.047815362 1.187053255
1.230109793 0.970485053
ENSG00000117122 752.1110508 752.2458802 1181.091516
370.8767212 393.8146807 426.1436884 895.1494824

396.9450301 1.171413191 1.25E-07 6.66E-06 MFAP2
1 16974502 16980835 - 2915
protein_coding microfibril associated protein 2 [Source:HGNC
Symbol;Acc:HGNC:7033] - 829 850 1016 358 345
494 13.92658567 13.81677888 21.68512827
6.824274871 7.274914544 7.879876446
ENSG00000173706 2147.463036 1798.310151 2059.935204
1079.479172 1273.90488 1027.403103 2001.902797
1126.929052 0.829534143 1.25E-07 6.68E-06 HEG1
3 124965710 125055958 - 9925
protein_coding heart development protein with EGF like domains 1
[Source:HGNC Symbol;Acc:HGNC:29227] - 2367 2032 1772
1042 1116 1191 11.67875173 9.701069792
11.10810685 5.833769253 6.911638886 5.57971818
ENSG00000145632 510.7822938 528.3421064 441.746827
203.049825 126.7055929 228.599347 493.6237424
186.118255 1.404169425 1.27E-07 6.76E-06 PLK2
5 58453982 58460260 - 4768
protein_coding polo like kinase 2 [Source:HGNC Symbol;Acc:HGNC:19699]
- 563 597 380 196 111 265
5.782302816 5.932865806 4.958544287 2.284188119
1.430981741 2.584286419
ENSG00000157613 1585.874689 1593.876271 2243.608884
935.4795509 1026.201154 770.3366674 1807.786615
910.6724573 0.989368153 1.28E-07 6.80E-06
CREB3L1 11 46277661 46321422 + 4037
protein_coding cAMP responsive element binding protein 3 like 1
[Source:HGNC Symbol;Acc:HGNC:18856] TF_bZIP 1748 1801 1930
903 899 893 21.20368591 21.1388523
29.74441324 12.42913899 13.68826179 10.28546019
ENSG00000092969 311.1870813 276.1184878 270.8605544
95.30910153 41.09370581 13.80222472 286.0553745
50.06834402 2.520528928 1.36E-07 7.19E-06 TGFB2
1 218346235 218444619 + 5151
protein_coding transforming growth factor beta 2 [Source:HGNC
Symbol;Acc:HGNC:11768] - 343 312 233 92 36
16 3.260853244 2.870050147 2.81430536
0.99244928 0.429594103 0.144430678
ENSG00000166803 805.6388578 762.865822 1067.167335
493.1210036 312.7687609 299.3357487 878.5573381
368.4085044 1.253573868 1.37E-07 7.19E-06 PCLAF
15 64364311 64387687 - 3734
protein_coding PCNA clamp associated factor [Source:HGNC
Symbol;Acc:HGNC:28961] - 888 862 918 476 274
347 11.64574659 10.93854085 15.29590629
7.083447554 4.510488807 4.321019033
ENSG00000168385 6661.943843 5354.220676 6612.252505
4160.449476 3451.871288 3055.467498 6209.472341
3555.929421 0.804324279 1.42E-07 7.46E-06 2-Sep
2 241315100 241354027 + 7378

protein_coding septin 2 [Source:HGNC Symbol;Acc:HGNC:7729] -
7343 6050 5688 4016 3024 3542 48.73753509
38.85466462 47.96536829 30.24594074 25.19361545
22.32240195
ENSG00000198712 36848.90523 37926.46729 30131.78357
46783.30126 73794.02222 75779.38957 34969.05203
65452.23768 -0.904344816 1.43E-07 7.49E-06 MT-CO2
MT 7586 8269 + 684 protein_coding
mitochondrially encoded cytochrome c oxidase II [Source:HGNC
Symbol;Acc:HGNC:7421] - 40616 42855 25920 45159 64647
87846 2907.834817 2968.737775 2357.684792
3668.599126 5809.511749 5971.685225
ENSG00000124496 956.2425181 738.9709529 863.7312959
464.1138857 407.5125827 315.7258906 852.981589
395.7841196 1.109118941 1.45E-07 7.57E-06 TRERF1
6 42224931 42452051 - 7732
protein_coding transcriptional regulating factor 1 [Source:HGNC
Symbol;Acc:HGNC:18273] zf-C2H2 1054 835 743 448 357
366 6.675402377 5.117066617 5.978659432
3.219572523 2.838074104 2.201001093
ENSG00000204525 1683.857793 2191.247999 3418.887942
910.616307 1293.310241 987.7217068 2431.331245
1063.882752 1.19226346 1.47E-07 7.67E-06 HLA-C
6 31268749 31272130 - 2507
protein_coding "major histocompatibility complex, class I, C
[Source:HGNC Symbol;Acc:HGNC:4933]" - 1856 2476 2941
879 1133 1145 36.25369873 46.79751016
72.98734016 19.48258523 27.77940479 21.23646229
ENSG00000145040 177.8211893 217.7088077 189.4861389
41.4387398 41.09370581 6.038473317 195.0053786
29.52363964 2.737911513 1.51E-07 7.82E-06 UCN2
3 48561727 48563773 - 1481
protein_coding urocortin 2 [Source:HGNC Symbol;Acc:HGNC:18414] -
196 246 163 40 36 7 6.480816075
7.870575501 6.847615913 1.500779803 1.494152076
0.21977283
ENSG00000114346 3623.560358 2416.921763 2978.303607
1893.750409 1285.319799 1105.040617 3006.261909
1428.036941 1.074245438 1.57E-07 8.10E-06 ECT2
3 172750682 172821474 + 5499
protein_coding epithelial cell transforming 2 [Source:HGNC
Symbol;Acc:HGNC:3155] - 3994 2731 2562 1828 1126
1281 35.56747414 23.53230244 28.98693204
18.4716 12.58641453 10.83169514
ENSG00000075275 843.7433984 772.6007687 820.7191048
468.2577597 382.3997624 344.1929791 812.354424
398.2835004 1.029066872 1.60E-07 8.26E-06 CELSR1
22 46360834 46537170 - 12806
protein_coding cadherin EGF LAG seven-pass G-type receptor 1
[Source:HGNC Symbol;Acc:HGNC:1850] - 930 873 706

452 335 399 3.556297911 3.230183445
3.43003104 1.961268174 1.607972695 1.448739884
ENSG00000070614 3629.003864 2969.158739 3576.986807
2249.087602 1830.952892 1743.393511 3391.71647
1941.144668 0.805137754 1.63E-07 8.36E-06 NDST1
5 150485818 150558211 + 9529
protein_coding N-deacetylase and N-sulfotransferase 1 [Source:HGNC
Symbol;Acc:HGNC:7680] - 4000 3355 3077 2171 1604
2021 20.55612964 16.68290353 20.09032748
12.65973282 10.34676052 9.861659532
ENSG00000150990 1997.766627 2204.522926 1918.111222
2985.661202 3652.77385 3661.902747 2040.133592
3433.445933 -0.750770812 1.64E-07 8.43E-06 DHX37
12 124946825 124989122 - 6618
protein_coding DEAH-box helicase 37 [Source:HGNC
Symbol;Acc:HGNC:17210] - 2202 2491 1650 2882 3200
4245 16.29368197 17.83501199 15.51186541
24.19798802 29.72148986 29.82510284
ENSG00000113140 8515.457567 8862.341463 14675.29459
5596.301809 5906.078719 4676.366264 10684.36454
5392.915598 0.986304079 1.74E-07 8.88E-06 SPARC
5 151661096 151687165 - 4831
protein_coding secreted protein acidic and cysteine rich [Source:HGNC
Symbol;Acc:HGNC:11219] - 9386 10014 12624 5402 5174
5421 95.14198234 98.21933523 162.5798786
62.13403905 65.83195419 52.17631314
ENSG00000100234 7374.135851 7012.701593 5734.571309
4559.297346 2442.792512 2386.059599 6707.136251
3129.383153 1.099990063 1.83E-07 9.35E-06 TIMP3
22 32801701 32863043 + 4603
protein_coding TIMP metallopeptidase inhibitor 3 [Source:HGNC
Symbol;Acc:HGNC:11822] - 8128 7924 4933 4401 2140
2766 86.47118361 81.56990055 66.67714367
53.12787401 28.57723187 27.94101761
ENSG00000130770 833.7636377 1030.134358 926.505845
485.8692241 406.3710908 515.8581491 930.1346136
469.3661547 0.985576989 1.87E-07 9.50E-06
ATP5IF1 1 28236109 28246906 + 2225
protein_coding ATP synthase inhibitory factor subunit 1 [Source:HGNC
Symbol;Acc:HGNC:871] - 919 1164 797 469 356
598 20.22619452 24.78845196 22.28615531
11.71264205 9.834840995 12.4968969
ENSG00000128567 2375.183029 1819.550035 1760.012358
1226.586698 788.7708533 954.9414231 1984.915141
990.0996581 1.003406015 1.88E-07 9.51E-06 PODXL
7 131500262 131558217 - 6874
protein_coding podocalyxin like [Source:HGNC Symbol;Acc:HGNC:9171]
- 2618 2056 1514 1184 691 1107
18.6504278 14.17228977 13.70323779 9.570931726
6.178967057 7.488056475

ENSG00000149503 2582.9435 2323.112277 2534.231797
1409.953122 1189.434485 1626.0746 2480.095858
1408.487402 0.815343842 1.89E-07 9.54E-06 INCENP
11 62123973 62153163 + 4473
protein_coding inner centromere protein [Source:HGNC
Symbol;Acc:HGNC:6058] - 2847 2625 2180 1361 1042
1885 31.16859692 27.80717374 30.32246071
16.90718367 14.31911513 19.59491909
ENSG00000128487 901.8074602 666.4013503 780.0318971
439.2506418 222.5909065 215.6597613 782.7469025
292.5004366 1.420705961 1.95E-07 9.81E-06 SPECC1
17 20009344 20319026 + 13676
protein_coding sperm antigen with calponin homology and coiled-coil
domains 1 [Source:HGNC Symbol;Acc:HGNC:30615] - 994 753
671 424 195 250 3.559229365 2.608929388
3.052602634 1.722736313 0.876441391 0.849986317
ENSG00000113552 1289.203623 1293.862914 1109.017034
1908.253968 2185.956851 2254.938464 1230.694524
2116.383094 -0.781615943 1.97E-07 9.91E-06 GNPDA1
5 141991749 142013041 - 3961
protein_coding glucosamine-6-phosphate deaminase 1 [Source:HGNC
Symbol;Acc:HGNC:4417] - 1421 1462 954 1842 1915
2614 17.56782186 17.48916081 14.98478027
25.8402569 29.71743326 30.68539788
ENSG00000183856 1894.340017 1524.846649 1477.526887
969.6665112 513.6713227 354.5446476 1632.237851
612.6274938 1.414717532 1.98E-07 9.91E-06 IQGAP3
1 156525405 156572604 - 6742
protein_coding IQ motif containing GTPase activating protein 3
[Source:HGNC Symbol;Acc:HGNC:20669] - 2088 1723 1271
936 450 411 15.16597828 12.10940962
11.72907191 7.714346541 4.102712889 2.834549802
ENSG00000075239 1424.384017 1392.982371 1691.425351
896.1127481 832.1475427 653.0177573 1502.930579
793.7593494 0.921473599 2.09E-07 1.05E-05 ACAT1
11 108121516 108147776 + 5584
protein_coding acetyl-CoA acetyltransferase 1 [Source:HGNC
Symbol;Acc:HGNC:93] - 1570 1574 1455 865 729
757 13.76838262 13.35628751 16.2115459
8.607613172 8.024712443 6.303496093
ENSG00000108175 2160.16455 1792.115185 2430.77004
1188.255864 1118.661992 1273.255231 2127.683258
1193.391029 0.833335992 2.11E-07 1.05E-05 ZMIZ1
10 79069035 79316528 + 11048
protein_coding zinc finger MIZ-type containing 1 [Source:HGNC
Symbol;Acc:HGNC:16493] zf-MIZ 2381 2025 2091 1147 980
1476 10.55369195 8.684959605 11.7754419
5.768883863 5.452425878 6.212032369
ENSG00000129474 684.0672283 664.63136 732.3697394
355.3371938 367.5603687 288.9840802 693.6894426

337.2938809	1.041563223	2.11E-07	1.05E-05	AJUBA
	14	22971174	22982642	-	5979
protein_coding	ajuba LIM protein [Source:HGNC Symbol;Acc:HGNC:20250]
	-	754	751	630	343	322	335
6.175490757	5.95165604	6.555696548	3.187701232
3.310355829	2.605237206
ENSG00000119326 1696.559306	1392.982371	1515.889111
959.3068263	688.3195724	542.5999595	1535.143596
730.0754527	1.073086172	2.13E-07	1.06E-05
CTNNAL1 9	108942569	109013529	-	3131
protein_coding	catenin alpha like 1 [Source:HGNC
Symbol;Acc:HGNC:2512]	-	1870	1574	1304	926	603
	629	29.24739715	23.82034796	25.91202598
16.43387437	11.83808911	9.341112358
ENSG00000168036 11207.27118	9522.547848	11663.27872
7554.282265	5524.820448	4935.157978	10797.69925
6004.753564	0.846586752	2.14E-07	1.06E-05	CTNNB1
	3	41194741	41260096	+	11927
protein_coding	catenin beta 1 [Source:HGNC Symbol;Acc:HGNC:2514]
	-	12353	10760	10033	7292	4840	5721
50.71890932	42.74718226	52.33671255	33.97249821
24.94373607	22.30343458
ENSG00000081803 449.0892282	398.2478189	490.5714763
181.2944866	166.6578069	70.73640171	445.9695078
139.5628984	1.680339311	2.17E-07	1.07E-05	CADPS2
	7	122318425	122886759	-	6351
protein_coding	calcium dependent secretion activator 2 [Source:HGNC
Symbol;Acc:HGNC:16018]	-	495	450	422	175	146
	82	3.816732911	3.357351728	4.134063896
1.531115594	1.413051867	0.600347571
ENSG00000143815 2440.505098	1881.499696	2087.835003
1252.48591	1275.046372	980.8205945	2136.613266
1169.450959	0.87028244	2.20E-07	1.08E-05	LBR
	1	225401502	225428925	-	5257
protein_coding	lamin B receptor [Source:HGNC Symbol;Acc:HGNC:6518]
	-	2690	2126	1796	1209	1117	1137
25.05780277	19.16248042	21.25568972	12.77910291
13.06058278	10.05665394
ENSG00000137834 434.5732127	362.8480128	323.1726787
151.2514003	155.2428886	100.0661293	373.5313014
135.5201394	1.467115802	2.22E-07	1.09E-05	SMAD6
	15	66702228	66782848	+	8563
protein_coding	SMAD family member 6 [Source:HGNC
Symbol;Acc:HGNC:6772]	MH1	479	410	278	146	136
	116	2.739291519	2.268738043	2.019880602
0.947412162	0.976248376	0.629887606
ENSG00000168398 78.02358307	97.34946684	198.7860721
25.89921237	12.55641011	7.763751407	124.7197073
15.40645796	3.022290111	2.26E-07	1.10E-05	BDKRB2
	14	96204679	96244166	+	6845

protein_coding	bradykinin receptor B2 [Source:HGNC
Symbol;Acc:HGNC:1030]	-	86	110	171	25	11
	9	0.615252918	0.761457509	1.55428088
0.202945114	0.098779448	0.06113643
ENSG00000163507 1823.574442	1369.972497	1253.165999
888.8609686	566.1799468	643.5287278	1482.237646
699.5232144	1.083594615	2.28E-07	1.11E-05	CIP2A
	3	108549869	108589644	-	4579
protein_coding	cell proliferation regulating inhibitor of protein
phosphatase 2A [Source:HGNC Symbol;Acc:HGNC:29302]	-	2010
	1548	1078	858	496	746	21.49582399
16.01868103	14.64721188	10.41186882	6.658223867
7.575288932
ENSG00000171310 492.6372745	405.3277801	496.3839345
184.4023921	183.7801843	226.0114299	464.7829964
198.0646688	1.228195862	2.32E-07	1.13E-05	CHST11
	12	104455295	104762014	+	6492
protein_coding	carbohydrate sulfotransferase 11 [Source:HGNC
Symbol;Acc:HGNC:17422]	-	543	458	427	178	161
	262	4.095906197	3.3428232	4.092193964
1.523538856	1.524385205	1.876522596
ENSG00000236509 0	0	0	18.64743291	55.93309958
	40.54403513	0	38.37485587	-7.737154907
2.34E-07	1.13E-05	RPL21P133	X	134607157
	134607632	+	476	processed_pseudogene
ribosomal protein L21 pseudogene 133 [Source:HGNC
Symbol;Acc:HGNC:36003]	-	0	0	0	18	49
	47	0	0	0	2.101249369	6.32756314
	4.591154665
ENSG00000101665 937.1902479	1051.374242	1291.528223
620.5451284	495.4074534	540.8746814	1093.364238
552.2757544	0.984277231	2.46E-07	1.19E-05	SMAD7
	18	48919853	48952052	-	5033
protein_coding	SMAD family member 7 [Source:HGNC
Symbol;Acc:HGNC:6773]	MH1	1033	1188	1111	599	434
	627	10.05083341	11.18448396	13.73390298
6.613204242	5.300418478	5.792574506
ENSG00000060656 1232.954063	1038.099315	1508.914162
594.6459161	728.2717864	577.1055213	1259.98918
633.3410746	0.992443135	2.52E-07	1.21E-05	PTPRU
	1	29236516	29326813	+	6682
protein_coding	"protein tyrosine phosphatase, receptor type U
[Source:HGNC Symbol;Acc:HGNC:9683]"	-	1359	1173	1298
	574	638	669	9.959594782	8.317982104
12.08579067	4.773286089	5.868965649	4.655332064
ENSG00000108963 815.6186184	758.4408462	575.4333667
1128.169691	1633.474806	1658.854884	716.4976104
1473.499794	-1.039169382	2.52E-07	1.21E-05	DPH1
	17	2030110 2043430 +	5757	protein_coding
diphthamide biosynthesis 1 [Source:HGNC Symbol;Acc:HGNC:3003]	-

899	857	495	1089	1431	1923	7.647018588
7.053603338	5.349532325	10.51099172	15.27885342
15.53152403
ENSG00000167004 5004.396328	4227.621846	6239.092685
3297.487719	2220.201606	1582.942648	5157.036953
2366.877324	1.123701267	2.55E-07	1.22E-05	PDIA3
15	43746392	43773279	+	4240
protein_coding	protein disulfide isomerase family A member 3
[Source:HGNC Symbol;Acc:HGNC:4606]	-	5516	4777	5367
3183	1945	1835	63.70698974	53.38457941
78.75399347	41.71409498	28.19688345	20.12339399
ENSG00000258791 40.82629347	70.79961225	12.78740815
270.3877772	181.4972007	226.8740689	41.47110462
226.2530156	-2.435311201	2.62E-07	1.25E-05
LINC00520	14	55781132	55796731	-	3482
lincRNA long intergenic non-protein coding RNA 520
[Source:HGNC Symbol;Acc:HGNC:19843]	-	45	80	11
261	159	263	0.632866971	1.088648456
0.196548967	4.165084189	2.806827467	3.512028244
ENSG00000224051 1137.692711	1621.311121	1271.765865
1873.031039	3175.630266	4012.1342	1343.589899
3020.265168	-1.168630009	2.65E-07	1.26E-05	CPTP
1	1324756 1328897 +	2556	protein_coding
ceramide-1-phosphate transfer protein [Source:HGNC
Symbol;Acc:HGNC:28116]	-	1254	1832	1094	1808	2782
4651	24.02510921	33.9618282	26.62951882
39.30516469	66.90269867	84.60898949
ENSG00000138166 995.2543097	987.6545909	842.8064462
1438.960239	1770.453825	2028.927034	941.9051156
1746.1137	-0.89018887	2.83E-07	1.34E-05	DUSP5
10	110497838	110511544	+	2615
protein_coding	dual specificity phosphatase 5 [Source:HGNC
Symbol;Acc:HGNC:3071]	-	1097	1116	725	1389	1551
2352	20.54298826	20.22175956	17.2493669
29.51498699	36.45754872	41.82121466
ENSG00000071575 1946.053322	1727.510539	2777.192552
1073.263361	1228.245207	955.8040622	2150.252138
1085.770877	0.985689601	2.90E-07	1.38E-05	TRIB2
2	12716889	12742734	+	4588
protein_coding	tribbles pseudokinase 2 [Source:HGNC
Symbol;Acc:HGNC:30809]	-	2145	1952	2389	1036	1076
1108	22.8945742	20.15964336	32.39661144
12.54724534	14.41571615	11.22916257
ENSG00000179776 331.1466026	133.6342681	225.5233801
59.05020421	63.92354238	43.13195226	230.1014169
55.36856628	2.058938659	2.91E-07	1.38E-05	CDH5
16	66366622	66404786	+	6082
protein_coding	cadherin 5 [Source:HGNC Symbol;Acc:HGNC:1764]	-
365	151	194	57	56	50	2.938834518
1.1764053	1.984550528	0.520763436	0.565964214

0.38225626
ENSG00000186340 1057.854626 906.2350368 1277.578323
639.1925614 456.5967313 411.4788246 1080.555995
502.4227057 1.104744549 2.94E-07 1.39E-05 THBS2
6 169215780 169254044 - 6412
protein_coding thrombospondin 2 [Source:HGNC Symbol;Acc:HGNC:11786]
- 1166 1024 1099 617 400 477
8.904995827 7.567159425 10.66377628 5.346920095
3.834544992 3.459042384
ENSG00000153044 1252.006333 1208.903379 1311.290581
723.1060094 741.9696883 708.2266562 1257.400098
724.434118 0.795495655 3.02E-07 1.42E-05 CENPH
5 69189548 69210357 + 2244
protein_coding centromere protein H [Source:HGNC
Symbol;Acc:HGNC:17268] - 1380 1366 1128 698 650
821 30.11514214 28.84392035 31.27469577
17.28401417 17.80483135 17.0118413
ENSG00000100526 504.4315371 443.3825717 489.4089846
164.7189907 239.7132839 106.1046026 479.0743645
170.1789591 1.497916353 3.09E-07 1.45E-05 CDKN3
14 54396849 54420218 + 1836
protein_coding cyclin dependent kinase inhibitor 3 [Source:HGNC
Symbol;Acc:HGNC:1791] - 556 501 421 159 210
123 14.82964649 12.92979055 14.2664614
4.812120469 7.030625711 3.115038744
ENSG00000067445 83.46708887 71.6846074 32.5497662
279.7114936 204.3270372 245.8521279 62.56715416
243.2968863 -1.95106369 3.11E-07 1.45E-05 TRO
X 54920462 54931431 + 6716
protein_coding trophinin [Source:HGNC Symbol;Acc:HGNC:12326] -
92 81 28 270 179 285 0.670819724
0.571479653 0.259390574 2.233907161 1.638285939
1.973173121
ENSG00000105426 990.7180548 734.5459771 1156.679192
446.5024213 454.3137476 487.3910606 960.6477412
462.7357432 1.052488439 3.19E-07 1.49E-05 PTPRS
19 5158495 5340803 - 8565
protein_coding "protein tyrosine phosphatase, receptor type S
[Source:HGNC Symbol;Acc:HGNC:9681]" - 1092 830 995
431 398 565 6.243440175 4.5917387
7.227740642 2.796159535 2.856295035 3.067270648
ENSG00000168268 9185.00878 9016.33062 11007.63343
6428.184511 5593.309958 3938.809881 9736.324277
5320.10145 0.872057539 3.28E-07 1.53E-05 NT5DC2
3 52524385 52535054 - 4719
protein_coding 5'-nucleotidase domain containing 2 [Source:HGNC
Symbol;Acc:HGNC:25717] - 10124 10188 9469 6205 4900
4566 105.0584146 102.2975894 124.8420771
73.06406856 63.8253879 44.99009891
ENSG00000175264 534.370819 406.2127753 504.5213761

244.4885648	119.856642	158.7255843	481.7016568
174.3569304	1.465069354	3.30E-07	1.53E-05	CHST1
	11	45648877	45665622	-	3478
protein_coding	carbohydrate sulfotransferase 1 [Source:HGNC
Symbol;Acc:HGNC:1969]	-	589	459	434	236	105
	184	8.293052231	6.253304095	7.763668772
3.770461139	1.855697068	2.459910258
ENSG00000130522 3120.943323	3910.793582	3759.497996
4808.965753	8394.530904	7557.580676	3597.0783
6920.359111	-0.944054936	3.31E-07	1.53E-05	JUND
	19	18279760	18281622	-	1863
protein_coding	"JunD proto-oncogene, AP-1 transcription factor
subunit [Source:HGNC Symbol;Acc:HGNC:6206]"	TF_bZIP 3440	4419
	3234	4642	7354	8761	90.42203383	112.3925662
	108.0025494	138.4536231	242.6376163	218.6612586
ENSG00000090530 517.1330506	445.152562	631.2329659
244.4885648	188.3461516	94.02765594	531.1728595
175.6207908	1.599393117	3.39E-07	1.56E-05	P3H2
	3	189956728	190122437	-	5605
protein_coding	prolyl 3-hydroxylase 2 [Source:HGNC
Symbol;Acc:HGNC:19317]	-	570	503	543	236	165
	109	4.979983712	4.252250187	6.02741451
2.339636724	1.809487917	0.90423622
ENSG00000156113 474.4922552	352.2280709	495.2214429
210.3016045	166.6578069	141.4728034	440.6472563
172.8107383	1.351122436	3.54E-07	1.63E-05	KCNMA1
	10	76869601	77638369	-	35644
protein_coding	potassium calcium-activated channel subfamily M alpha
1 [Source:HGNC Symbol;Acc:HGNC:6284]	-	523	398	426
	203	146	164	0.718528398	0.529082111
0.743583956	0.316462057	0.251775682	0.213938246
ENSG00000169045 6544.001218	6568.434026	6141.443386
4591.412369	3595.699259	3715.386368	6417.959543
3967.499332	0.69395985	3.56E-07	1.64E-05
HNRNPH1 5	179614178	179634784	-	9480
protein_coding	heterogeneous nuclear ribonucleoprotein H1
[Source:HGNC Symbol;Acc:HGNC:5041]	-	7213	7422	5283
	4432	3150	4307	37.25943615	37.0970225
34.67201804	25.97786515	20.42441267	21.12504061
ENSG00000069020 361.0858845	340.723134	352.2349699
674.4154902	717.9983599	663.3694258	351.3479961
685.261092	-0.96352682	3.63E-07	1.66E-05	MAST4
	5	66596361	67169595	+	14675
protein_coding	microtubule associated serine/threonine kinase family
member 4 [Source:HGNC Symbol;Acc:HGNC:19037]	-	398	385
	303	651	629	769	1.328108774	1.24310857
	1.284610197	2.464988641	2.634631595	2.436571993
ENSG00000143977 1019.750086	1584.141324	1374.06513
695.1348601	671.197195	669.4078991	1325.985513
678.5799847	0.966437886	3.64E-07	1.66E-05	SNRPG

2	70281362	70293771	-	1795
protein_coding	small nuclear ribonucleoprotein polypeptide G
[Source:HGNC Symbol;Acc:HGNC:11163]	-	1124	1790	1182
671	588	776	30.66412255	47.2514368
40.96942514	20.77160766	20.13539869	20.10149168
ENSG00000122861 4108.032374	4559.495029	5245.162324
2681.086465	2192.805802	3080.484031	4637.563242
2651.458766	0.805926348	3.82E-07	1.74E-05	PLAU
10	73909177	73917497	+	2950
protein_coding	"plasminogen activator, urokinase [Source:HGNC
Symbol;Acc:HGNC:9052]"	-	4528	5152	4512	2588	1921
3571	75.16455416	82.75233923	95.15988787
48.74771907	40.02696939	56.28580119
ENSG00000064666 4212.366235	4028.497937	4746.453407
2962.869895	2526.121416	2510.279622	4329.10586
2666.423644	0.699010502	3.84E-07	1.75E-05	CNN2
19	1026581 1039068 +	3645	protein_coding
calponin 2 [Source:HGNC Symbol;Acc:HGNC:2156]	-	4643	4552
4083	2860	2213	2910	62.37776854	59.17403191
69.69292527	43.59940315	37.31910686	37.12158186
ENSG00000168077 386.4889115	331.8731824	670.757682
123.2802509	34.24475485	161.3135015	463.0399253
106.2795024	2.116622737	3.91E-07	1.77E-05	SCARA3
8	27633868	27676776	+	4031
protein_coding	scavenger receptor class A member 3 [Source:HGNC
Symbol;Acc:HGNC:19000]	-	426	375	577	119	30
187	5.175180295	4.408033744	8.905736918
1.640386577	0.457462835	2.157048085
ENSG00000029534 224.0909886	195.5839288	226.6858717
81.8415111	55.93309958	39.68139608	215.4535964
59.15200225	1.868628009	3.99E-07	1.81E-05	ANK1
8	41653220	41896762	-	10797
protein_coding	ankyrin 1 [Source:HGNC Symbol;Acc:HGNC:492]	-
247	221	195	79	49	46	1.12026956
0.969874661	1.123668889	0.406570659	0.278958975
0.198100581
ENSG00000144724 2950.380141	2241.692723	2987.60354
1735.247229	1437.138212	1124.881315	2726.558801
1432.422252	0.928934968	4.08E-07	1.84E-05	PTPRG
3	61561569	62297613	+	14629
protein_coding	"protein tyrosine phosphatase, receptor type G
[Source:HGNC Symbol;Acc:HGNC:9671]"	-	3252	2533	2570
1675	1259	1304	10.88590602	8.204403112
10.93013007	6.362271751	5.290033842	4.144708289
ENSG00000163453 585.1768731	371.6979643	767.2444889
7.251779464	35.38624667	1.725278091	574.7064421
14.78776808	5.292256116	4.15E-07	1.87E-05	IGFBP7
4	57030773	57110385	-	1930
protein_coding	insulin like growth factor binding protein 7
[Source:HGNC Symbol;Acc:HGNC:5476]	-	645	420	660

7	31	2	16.36556823	10.31141871
21.27617105	0.201536065	0.987305929	0.048184095
ENSG00000100911 1050.596619	1309.792827	1479.85187
629.8688449	746.5356556	461.5118892	1280.080439
612.6387966	1.064259321	4.24E-07	1.90E-05	PSME2
14	24143362	24147570	-	3582
protein_coding	proteasome activator subunit 2 [Source:HGNC
Symbol;Acc:HGNC:9569]	-	1158	1480	1273	608	654
535	15.83112075	19.57774082	22.11106538
9.431701368	11.22275616	6.944791612
ENSG00000143641 1496.964094	1745.210442	2122.709753
1027.680747	1025.059662	831.5840396	1788.294763
961.4414828	0.89540124	4.36E-07	1.95E-05	GALNT2
1	230057990	230282124	+	7743
protein_coding	polypeptide N-acetylgalactosaminyltransferase 2
[Source:HGNC Symbol;Acc:HGNC:4124]	-	1650	1972	1826
992	898	964	10.43526227	12.06768852
14.67230548	7.118925639	7.128767285	5.788936594
ENSG00000108821 34404.77113	32846.59511	64901.90881
12187.13337	10482.31946	6158.380144	44051.09168
9609.277659	2.196692226	4.45E-07	1.99E-05	COL1A1
17	50183289	50201632	-	9819
protein_coding	collagen type I alpha 1 chain [Source:HGNC
Symbol;Acc:HGNC:2197]	-	37922	37115	55830	11764	9183
7139	189.1266184	179.1053859	353.7587694
66.5732562	57.48634335	33.80657237
ENSG00000149782 1359.061947	1274.39302	1493.80177
861.9257878	744.252672	627.1385859	1375.752246
744.4390152	0.886408133	4.51E-07	2.01E-05	PLCB3
11	64251523	64269150	+	4964
protein_coding	phospholipase C beta 3 [Source:HGNC
Symbol;Acc:HGNC:9056]	-	1498	1440	1285	832	652
727	14.77776391	13.74539296	16.10564794
9.313300095	8.07352479	6.809789009
ENSG00000136824 2502.198164	1685.915767	2343.583166
1377.838098	950.8626929	929.9248908	2177.232366
1086.208561	1.003186622	4.55E-07	2.02E-05	SMC2
9	104094260	104141417	+	6468
protein_coding	structural maintenance of chromosomes 2 [Source:HGNC
Symbol;Acc:HGNC:14011]	-	2758	1905	2016	1330	833
1078	20.88107889	13.95569308	19.39221257
11.42598563	7.916301937	7.749608583
ENSG00000020181 753.0183018	643.3914763	1056.70491
392.6320596	376.6923033	223.4235127	817.7048959
330.9159585	1.306236979	4.55E-07	2.02E-05	ADGRA2
8	37784191	37844896	+	7061
protein_coding	adhesion G protein-coupled receptor A2 [Source:HGNC
Symbol;Acc:HGNC:17849]	-	830	727	909	379	330
259	5.756262153	4.878593583	8.009483634
2.98253152	2.872731844	1.705550452

ENSG00000176890 1331.844418 1484.136872 1717.000167
886.7890316 792.1953288 515.8581491 1510.993819
731.6141698 1.047234729 4.67E-07 2.07E-05 TYMS
18 657604 673578 + 2470 protein_coding
thymidylate synthetase [Source:HGNC Symbol;Acc:HGNC:12441] -
1468 1677 1477 856 694 598 29.10434205
32.17085107 37.2040645 19.25700996 17.2706975
11.25732615
ENSG00000134247 1452.508797 1585.911314 1822.786907
966.5586057 883.514675 597.8088584 1620.402339
815.960713 0.990589627 4.72E-07 2.08E-05 PTGFRN
1 116910057 116990358 + 6352
protein_coding prostaglandin F2 receptor inhibitor [Source:HGNC
Symbol;Acc:HGNC:9601] - 1601 1792 1568 933 774
693 12.34268161 13.36761585 15.35827415
8.16174831 7.489931252 5.072870354
ENSG00000117525 741.2240392 627.4615636 662.6202404
370.8767212 292.221908 198.4069804 677.1019477
287.1685365 1.239637922 4.77E-07 2.10E-05 F3
1 94529225 94541800 - 2875
protein_coding "coagulation factor III, tissue factor [Source:HGNC
Symbol;Acc:HGNC:3541]" - 817 709 570 358 256
230 13.91595101 11.6851644 12.33513348
6.919221304 5.473302815 3.71981212
ENSG00000182481 7623.629867 6570.204017 7920.055611
5030.663011 4605.919527 3954.337384 7371.296498
4530.30664 0.702389394 4.88E-07 2.14E-05 KPNA2
17 68035519 68046842 + 3140
protein_coding karyopherin subunit alpha 2 [Source:HGNC
Symbol;Acc:HGNC:6395] - 8403 7424 6813 4856 4035
4584 131.0489058 112.0301083 134.9943486
85.93321765 78.98802431 67.88064792
ENSG00000173905 2550.282465 2312.492335 2623.743654
1695.880426 1354.9508 1197.342995 2495.506151
1416.058074 0.817697224 4.91E-07 2.15E-05 GOLIM4
3 168008677 168095975 - 4647
protein_coding golgi integral membrane protein 4 [Source:HGNC
Symbol;Acc:HGNC:15448] - 2811 2613 2257 1637 1187
1388 29.62216909 26.64361675 30.21800114
19.5743816 15.70093106 13.88825915
ENSG00000184557 1134.063707 1315.987793 1585.63861
550.0992708 815.0251653 396.8139608 1345.230037
587.312799 1.197171738 4.96E-07 2.16E-05 SOCS3
17 78356778 78360077 - 2734
protein_coding suppressor of cytokine signaling 3 [Source:HGNC
Symbol;Acc:HGNC:19391] - 1250 1487 1364 531 714
460 22.38928302 25.77145266 31.04007105
10.79215203 16.05266201 7.823306397
ENSG00000171603 3058.343006 3119.607915 4187.294923
2097.836202 2060.39275 1749.431984 3455.081948

1969.220312	0.810989915	4.97E-07	2.17E-05	CLSTN1
	1	9729026 9824526 -	5683	protein_coding
calsyntenin 1 [Source:HGNC Symbol;Acc:HGNC:17447]	-	3371
	3525	3602	2025	1805	2028	29.0475682
29.39056305	39.43418768	19.79973671	19.52301601
16.59286244
ENSG00000064225 202.3169654	194.6989337	145.3114562
306.6466745	600.4247016	611.6110831	180.7757851
506.2274864	-1.483730682	5.02E-07	2.18E-05
ST3GAL6 3	98732236	98821201	+	6288
protein_coding	"ST3 beta-galactoside alpha-2,3-sialyltransferase 6
[Source:HGNC Symbol;Acc:HGNC:18080]"	-	223	220	125
	296	526	709	1.736684841	1.657816999
1.236813812	2.615719811	5.141863831	5.242817257
ENSG00000198492 3008.444203	3447.941117	3180.577154
4492.995362	5588.743991	5541.593227	3212.320825
5207.777527	-0.697030161	5.03E-07	2.18E-05	YTHDF2
	1	28736621	28769775	+	3859
protein_coding	YTH N6-methyladenosine RNA binding protein 2
[Source:HGNC Symbol;Acc:HGNC:31675]	-	3316	3896	2736
	4337	4896	6424	42.07929227	47.8377352
44.11112774	62.44917239	77.98552331	77.40370905
ENSG00000157224 1056.040124	940.7498478	867.2187708
1620.254726	1610.64497	1640.739464	954.669581
1623.87972	-0.76573995	5.20E-07	2.25E-05	CLDN12
	7	90383721	90513402	+	6741
protein_coding	claudin 12 [Source:HGNC Symbol;Acc:HGNC:2034]	-
	1164	1063	746	1564	1411	1902	8.455851293
	7.471974451	6.88527589	12.89212374	12.86619256
	13.11949757
ENSG00000118705 3974.666482	3997.523107	4576.729626
2759.82007	2158.561047	1441.469845	4182.973071
2119.950321	0.980865152	5.21E-07	2.25E-05	RPN2
	20	37178410	37241623	+	2819
protein_coding	ribophorin II [Source:HGNC Symbol;Acc:HGNC:10382]
	-	4381	4517	3937	2664	1891	1671
76.10389085	75.9244152	86.8914782	52.51110874
41.23289358	27.56210559
ENSG00000111725 755.7400547	690.2962194	775.3819305
1132.313565	1389.195555	1553.612921	740.4727349
1358.374013	-0.876123129	5.44E-07	2.34E-05	PRKAB1
	12	119667753	119681630	+	6246
protein_coding	protein kinase AMP-activated non-catalytic subunit
beta 1 [Source:HGNC Symbol;Acc:HGNC:9378]	-	833	780
	667	1093	1217	1801	6.530880016	5.917238355
	6.644016474	9.72367032	11.97666656	13.40734396
ENSG00000177706 1991.41587	2109.828445	4026.871075
941.6953619	1309.291127	1330.189408	2709.371797
1193.725299	1.181817179	5.57E-07	2.39E-05	FAM20C
	7	192969	260745	+	4629

protein_coding "FAM20C, golgi associated secretory pathway kinase
[Source:HGNC Symbol;Acc:HGNC:22140]" - 2195 2384 3464
909 1147 1542 23.22073848 24.40312874
46.55834661 10.91160776 15.23083093 15.48917231
ENSG00000111799 2395.14255 2805.434635 2946.916332
1750.786756 1544.438444 1143.859374 2715.831173
1479.694858 0.876568484 5.63E-07 2.41E-05
COL12A1 6 75084326 75206051 - 12481
protein_coding collagen type XII alpha 1 chain [Source:HGNC
Symbol;Acc:HGNC:2188] - 2640 3170 2535 1690 1353
1326 10.3581746 12.03472913 12.63675097
7.524010017 6.663398299 4.93997964
ENSG00000170801 490.8227726 484.9773439 782.3568804
152.2873688 303.6368263 167.3519748 586.0523323
207.7587233 1.497837199 5.77E-07 2.47E-05 HTRA3
4 8269765 8307111 + 3321 protein_coding HtrA
serine peptidase 3 [Source:HGNC Symbol;Acc:HGNC:30406] - 541
548 673 147 266 194 7.977321093
7.818764343 12.60819843 2.459577451 4.923343317
2.716213307
ENSG00000248323 347.47712 466.3924457 355.7224449
742.7894108 777.355935 821.2323711 389.8640035
780.459239 -1.000449066 5.80E-07 2.47E-05 LUCAT1
5 91054834 91314547 - 16538
antisense lung cancer associated transcript 1 [Source:HGNC
Symbol;Acc:HGNC:48498] - 383 527 306 717 681
952 1.134082033 1.509920455 1.151185433
2.409063301 2.531112709 2.676609277
ENSG00000146576 1403.517244 1694.765718 1584.476119
2298.81409 2701.911157 3378.95714 1560.919694
2793.227463 -0.83990422 5.98E-07 2.54E-05
C7orf26 7 6590017 6608726 + 2728 protein_coding
chromosome 7 open reading frame 26 [Source:HGNC
Symbol;Acc:HGNC:21702] - 1547 1915 1363 2219 2367
3917 27.76992017 33.26219101 31.0855343
45.19859968 53.33364332 66.76367336
ENSG00000038427 577.9188653 300.0133569 476.6215765
208.2296675 133.5545439 107.8298807 451.5179329
149.871364 1.592339339 6.10E-07 2.59E-05 VCAN
5 83471465 83582303 + 14831
protein_coding versican [Source:HGNC Symbol;Acc:HGNC:2464] -
637 339 410 201 117 125 2.103282852
1.083067949 1.71996761 0.753074021 0.484911839
0.391895788
ENSG00000152952 5092.399672 4636.489607 7413.209251
3481.890111 3061.481083 2282.542914 5714.032844
2941.971369 0.957736334 6.16E-07 2.61E-05 PLOD2
3 146069440 146163653 - 7646
protein_coding "procollagen-lysine,2-oxoglutarate 5-dioxygenase 2
[Source:HGNC Symbol;Acc:HGNC:9082]" - 5613 5239 6377

	3361	2682	2646	35.94921671	32.46687923
51.89063316	24.42565746	21.56114598	16.09113077
ENSG00000230882 8.165258694	24.77986429	11.6249165
93.23716454	110.7247073	105.2419635	14.85667983
103.0679451	-2.78550078	6.30E-07	2.66E-05
AC005077.4	7	76071469	76074963	-	3495
	processed_pseudogene	hypothetical protein LOC285908
(LOC285908) pseudogene	-	9	28	10	90	97
	122	0.126102592	0.379609692	0.178016258
1.430893705	1.705972061	1.623093987
ENSG00000162390 234.0707492	267.2685362	202.2735471
400.9198075	579.8778487	883.3423824	234.5376108
621.3800129	-1.40543803	6.57E-07	2.77E-05	ACOT11
	1	54542257	54639192	+	9768
protein_coding	acyl-CoA thioesterase 11 [Source:HGNC
Symbol;Acc:HGNC:18156]	-	258	302	174	387	508
	1024	1.29342943	1.464966632	1.108282419
2.201493248	3.196726061	4.874446676
ENSG00000254139 19.05227029	21.23988367	4.6499666
78.73360561	86.75337894	198.4069804	14.98070685
121.2979883	-3.000117187	6.79E-07	2.85E-05
AC104051.2	8	57746149	57750199	+	2461
	lincRNA uncharacterized LOC286178 [Source:NCBI
gene;Acc:286178]	-	21	24	4	76	76
	230	0.417865354	0.462089467	0.101124229
1.715987114	1.898232212	4.345574907
ENSG00000140391 3887.570389	3857.693872	4417.46827
2713.201488	2610.591811	2283.405553	4054.244177
2535.732951	0.677104489	6.79E-07	2.85E-05	TSPAN3
	15	77041404	77083984	-	8506
protein_coding	tetraspanin 3 [Source:HGNC Symbol;Acc:HGNC:17752]
	-	4285	4359	3800	2619	2287	2647
24.66914692	24.28219439	27.79489673	17.10890299
16.52677621	14.4697018
ENSG00000109685 4804.801116	4034.692903	4347.718771
3077.862398	2479.320251	2226.471376	4395.737596
2594.551342	0.760844086	6.88E-07	2.87E-05	NSD2
	4	1871424 1982207 +	19776	protein_coding
nuclear receptor binding SET domain protein 2 [Source:HGNC
Symbol;Acc:HGNC:12766]	HMG	5296	4559	3740	2971	2172
	2581	13.11409141	10.92339352	11.76630212
8.347880856	6.751009633	6.068489003
ENSG00000233608 280.3405485	237.178701	315.0352371
119.1363769	84.47039528	86.26390453	277.5181622
96.62355891	1.521536154	6.89E-07	2.87E-05	TWIST2
	2	238848032	238910543	+	1878
protein_coding	twist family bHLH transcription factor 2 [Source:HGNC
Symbol;Acc:HGNC:20670]	bHLH	309	268	271	115	74
	100	8.057337864	6.761851782	8.978019608
3.402626626	2.422052162	2.475913285

ENSG00000136869 501.7097842 362.8480128 539.3961256
235.1648483 108.4417237 85.40126548 467.9846408
143.0026125 1.711607665 6.98E-07 2.90E-05 TLR4
9 117704175 117724730 + 13272
protein_coding toll like receptor 4 [Source:HGNC
Symbol;Acc:HGNC:11850] - 553 410 464 227 95
99 2.040409993 1.463773648 2.175146206
0.950389277 0.439981679 0.346840529
ENSG00000109881 968.0367807 817.7355215 942.7807281
551.1352393 437.1913702 434.7700788 909.5176767
474.3655628 0.939108607 7.56E-07 3.14E-05 CCDC34
11 27330827 27363868 - 3733
protein_coding coiled-coil domain containing 34 [Source:HGNC
Symbol;Acc:HGNC:25079] - 1067 924 811 532 383
504 13.99700485 11.72844464 13.51667
7.918915085 6.306496285 6.277743465
ENSG00000144736 1161.281236 1245.18818 1050.892452
1866.815228 1852.641237 1999.597307 1152.453956
1906.351257 -0.725650528 7.64E-07 3.17E-05 SHQ1
3 72749277 72861914 - 5841
protein_coding "SHQ1, H/ACA ribonucleoprotein assembly factor
[Source:HGNC Symbol;Acc:HGNC:25543]" - 1280 1407 904
1802 1623 2318 10.73127803 11.41388078
9.629152634 17.14271575 17.07963848 18.45258623
ENSG00000167889 374.6946489 321.2532406 444.0718103
105.6687865 130.1300684 183.7421166 380.0065666
139.8469905 1.437796875 7.70E-07 3.18E-05 MGAT5B
17 76868456 76950393 + 6386
protein_coding "alpha-1,6-mannosylglycoprotein 6-beta-N-
acetylglucosaminyltransferase B [Source:HGNC Symbol;Acc:HGNC:24140]"
- 413 363 382 102 114 213
3.167012817 2.69342044 3.721699425 0.88753053
1.09729474 1.550892541
ENSG00000133030 7434.921666 7046.331409 7176.060955
5042.058665 4631.032347 4843.718239 7219.104677
4838.936417 0.577089297 7.75E-07 3.20E-05 MPRIP
17 17042545 17217679 + 18153
protein_coding myosin phosphatase Rho interacting protein
[Source:HGNC Symbol;Acc:HGNC:30321] - 8195 7962 6173
4867 4057 5615 22.10697061 20.78261567
21.15703068 14.89789751 13.73738153 14.38243338
ENSG00000117385 1453.416047 1508.031741 1821.624415
1017.321062 823.0156081 660.7815087 1594.357401
833.7060596 0.93568955 7.89E-07 3.25E-05 P3H1
1 42746335 42767084 - 6766
protein_coding prolyl 3-hydroxylase 1 [Source:HGNC
Symbol;Acc:HGNC:19316] - 1602 1704 1567 982 721
766 11.59469161 11.93339559 14.40933206
8.064761676 6.550140738 5.264144406
ENSG00000149948 1961.476588 2096.553518 1493.80177

3145.200351	2892.540293	3965.551691	1850.610625
3334.430778	-0.8491275	8.15E-07	3.34E-05	HMGA2
	12	65824131	65966295	+	15403
protein_coding	high mobility group AT-hook 2 [Source:HGNC
Symbol;Acc:HGNC:5009]	HMGI/HMGY	2162	2369	1285	3036
	2534	4597	6.873517738	7.287627838	5.190445782
	10.9523798	10.11226996	13.87714757
ENSG00000115414 26922.67242	19873.45116	48314.31546
8138.568496	6062.463099	3940.535159	31703.47968
6047.188918	2.39031807	8.16E-07	3.34E-05	FN1
	2	215360440	215436172	-	17315
protein_coding	fibronectin 1 [Source:HGNC Symbol;Acc:HGNC:3778]
	-	29675	22456	41561	7856	5311	4568
83.92607548	61.45204566	149.3380029	25.21105516
18.85389854	12.26689414
ENSG00000123146 2145.648535	2369.132025	2967.841182
1604.715199	1327.554996	1073.985611	2494.207247
1335.418602	0.901386197	8.20E-07	3.35E-05	ADGRE5
	19	14380501	14408725	+	5382
protein_coding	adhesion G protein-coupled receptor E5 [Source:HGNC
Symbol;Acc:HGNC:1711]	-	2365	2677	2553	1549	1163
	1245	21.51870517	23.56845526	29.51304146
15.99262552	13.28260879	10.75614569
ENSG00000077312 1361.7837	1516.881692	1617.025885
783.1921821	777.355935	975.6447602	1498.563759
845.3976258	0.824580668	8.62E-07	3.51E-05	SNRPA
	19	40750637	40765389	+	2361
protein_coding	small nuclear ribonucleoprotein polypeptide A
[Source:HGNC Symbol;Acc:HGNC:11151]	-	1501	1714	1391
	756	681	1131	31.13245641	34.39863991
36.65540223	17.79253595	17.72958153	22.27397029
ENSG00000184678 67.13657148	58.40968011	68.58700734
197.8699825	167.7992987	278.6324116	64.71108631
214.767231	-1.734027466	8.68E-07	3.53E-05
HIST2H2BE	1	149884459	149886652	-	2194
	protein_coding	histone cluster 2 H2B family member e
[Source:HGNC Symbol;Acc:HGNC:4760]	-	74	66	59
	191	147	323	1.6516719	1.425390149
1.673101294	4.837364216	4.118395699	6.845369843
ENSG00000126787 1634.866241	1008.009479	1464.739479
356.3731622	243.1377594	106.1046026	1369.205066
235.2051747	2.54234296	8.76E-07	3.55E-05	DLGAP5
	14	55148112	55191678	-	3667
protein_coding	DLG associated protein 5 [Source:HGNC
Symbol;Acc:HGNC:16864]	-	1802	1139	1260	344	213
	123	24.06426272	14.71767657	21.37797091
5.212662132	3.570393257	1.559643069
ENSG00000115306 3292.413756	2512.50124	3352.625918
2035.678092	1608.361986	1482.876519	3052.513638
1708.972199	0.836873676	8.93E-07	3.62E-05	SPTBN1

2	54456285	54671445	+	18504
protein_coding	"spectrin beta, non-erythrocytic 1 [Source:HGNC
Symbol;Acc:HGNC:11275]" -	3629	2839	2884	1965	1409
1719	9.603953132	7.269862781	9.696979621
5.900773961	4.680505216	4.319577547
ENSG00000132780 5641.286506	5346.25572	5023.126419
3805.112282	2777.249618	3162.43474	5336.889549
3248.265547	0.716315564	8.97E-07	3.62E-05	NASP
1	45583846	45618904	+	8304
protein_coding	nuclear autoantigenic sperm protein [Source:HGNC
Symbol;Acc:HGNC:7644]	-	6218	6041	4321	3673	2433
3666	36.66840849	34.47052803	32.37455238
24.57794859	18.00951961	20.52750366
ENSG00000232112 535.2780699	873.4902161	782.3568804
342.9055718	327.6081547	346.7808962	730.3750555
339.0982076	1.106398034	9.22E-07	3.71E-05	TMA7
3	48440257	48444208	+	1139
protein_coding	translation machinery associated 7 homolog
[Source:HGNC Symbol;Acc:HGNC:26932]	-	590	987	673
331	287	402	25.36629105	41.06008739
36.7619201	16.14790974	15.48836351	16.41093582
ENSG00000218336 1639.402496	1323.952749	2180.834335
1016.285093	831.0060509	700.4629048	1714.72986
849.2513497	1.013548145	9.57E-07	3.85E-05	TENM3
4	182143987	182803024	+	11983
protein_coding	teneurin transmembrane protein 3 [Source:HGNC
Symbol;Acc:HGNC:29944]	-	1807	1496	1876	981	728
812	7.384503094	5.915513844	9.740340102
4.548995338	3.734334184	3.150804725
ENSG00000149968 951.7062633	890.305124	924.1808617
1887.534598	1405.17644	1551.025003	922.064083
1614.578681	-0.808358373	9.75E-07	3.91E-05	MMP3
11	102835801	102843803	-	2048
protein_coding	matrix metallopeptidase 3 [Source:HGNC
Symbol;Acc:HGNC:7173]	-	1049	1006	795	1822	1231
1798	25.08269628	23.27525615	24.15149521
49.43453621	36.94668355	40.82166865
ENSG00000104723 23.58852512	42.47976735	34.8747495
105.6687865	158.6673641	188.0553119	33.64768065
150.7971542	-2.165243726	9.84E-07	3.94E-05	TUSC3
8	15417215	15766649	+	5190
protein_coding	tumor suppressor candidate 3 [Source:HGNC
Symbol;Acc:HGNC:30242]	-	26	48	30	102	139
218	0.24532097	0.438228199	0.359634
1.09205587	1.646246265	1.953080545
ENSG00000145781 794.7518462	603.5666944	739.3446893
306.6466745	400.6636317	339.0171448	712.55441
348.775817	1.031277544	1.01E-06	4.02E-05
COMMD10 5	116084991	116412762	+	2464
protein_coding	COMM domain containing 10 [Source:HGNC

Symbol;Acc:HGNC:30201]	-	876	682	636	296	351
	393	17.40973202	13.11505487	16.05917604
6.675181076	8.756161702	7.416224448
ENSG00000198642 1175.797252	1244.303185	1105.529559
1754.93063	2019.299044	2043.591898	1175.209999
1939.273858	-0.722257287	1.04E-06	4.14E-05	KLHL9
	9	21329671	21335380	-	5710
protein_coding	kelch like family member 9 [Source:HGNC
Symbol;Acc:HGNC:18732]	-	1296	1406	951	1694	1769
	2369	11.1146957	11.66744207	10.36218294
16.4850148	19.043163	19.29123229
ENSG00000164985 2628.306048	2316.917311	2454.019873
1692.772521	1434.855228	1297.409124	2466.414411
1475.012291	0.742038149	1.09E-06	4.33E-05	PSIP1
	9	15464066	15511019	-	6768
protein_coding	PC4 and SFRS1 interacting protein 1 [Source:HGNC
Symbol;Acc:HGNC:9527]	-	2897	2618	2111	1634	1257
	1504	20.96123315	18.32887178	19.40594204
13.41540369	11.41621891	10.33281144
ENSG00000143870 3476.585702	3061.198235	3879.234636
2424.166278	1418.874342	1235.299113	3472.339524
1692.779911	1.036634001	1.10E-06	4.34E-05	PDIA6
	2	10783391	10837977	-	4101
protein_coding	protein disulfide isomerase family A member 6
[Source:HGNC Symbol;Acc:HGNC:30168]	-	3832	3459	3337
	2340	1243	1432	45.75772403	39.96568247
50.62596144	31.70575736	18.63068056	16.23619531
ENSG00000175216 7841.370099	5494.934906	7313.234969
4601.772054	3800.026296	3030.450966	6883.179991
3810.749772	0.8531361	1.11E-06	4.39E-05	CKAP5
	11	46743048	46846308	-	8538
protein_coding	cytoskeleton associated protein 5 [Source:HGNC
Symbol;Acc:HGNC:28959]	-	8643	6209	6291	4442	3329
	3513	49.57206906	34.45814944	45.84272037
28.90909175	23.96652149	19.131676
ENSG00000187244 85.2815908	119.4743457	227.8483634
22.79130689	36.5277385	12.07694663	144.2014333
23.79866401	2.609383169	1.15E-06	4.53E-05	BCAM
	19	44809059	44821421	+	5009
protein_coding	basal cell adhesion molecule (Lutheran blood group)
[Source:HGNC Symbol;Acc:HGNC:6722]	-	94	135	196
	22	32	14	0.918978827	1.277053753
2.434511813	0.244052743	0.392686804	0.129959497
ENSG00000176692 547.0723325	509.7572082	780.0318971
260.0280922	302.4953345	272.5939383	612.2871459
278.372455	1.136227784	1.16E-06	4.56E-05	FOXC2
	16	86566829	86569728	+	2900
protein_coding	forkhead box C2 [Source:HGNC Symbol;Acc:HGNC:3801]
	Fork	603	576	671	251	265	316
10.18234945	9.411328364	14.39565297	4.809365318

5.616881172	5.066640646
ENSG00000155368 1192.127769	1701.845679	1446.139612
882.6451577	674.6216705	761.710277	1446.704354
772.9923684	0.90403304	1.18E-06	4.64E-05	DBI
2	119366921	119372560	+	2509
protein_coding	"diazepam binding inhibitor, acyl-CoA binding protein
[Source:HGNC Symbol;Acc:HGNC:2690]"	-	1314	1923	1244
852	591	883	25.64622142	36.31659006
30.84796828	18.86909092	14.4788537	16.36405989
ENSG00000172137 56.24955989	68.14462679	29.06229125
129.4960619	275.0995306	236.3630984	51.15215931
213.652897	-2.054233367	1.21E-06	4.74E-05	CALB2
16	71358713	71390438	+	1703
protein_coding	calbindin 2 [Source:HGNC Symbol;Acc:HGNC:1435]	-
62	77	25	125	241	274	1.782812724
2.142409661	0.91333943	4.078565194	8.698607897
7.481125373
ENSG00000174840 2021.355152	2227.5328	1814.649465
3059.214965	3167.639823	3564.424535	2021.179139
3263.759775	-0.691074155	1.22E-06	4.76E-05	PDE12
3	57556276	57566844	+	8947
protein_coding	phosphodiesterase 12 [Source:HGNC
Symbol;Acc:HGNC:25386]	-	2228	2517	1561	2953	2775
4132	12.19456836	13.33006352	10.8550621
18.33994603	19.06482883	21.47404672
ENSG00000274012 275.8042937	878.800187	381.2972612
95.30910153	81.0459198	210.483927	511.9672473
128.9463161	1.985454239	1.27E-06	4.96E-05	RN7SL2
14	49862550	49862849	-	300
misc_RNA	"RNA, 7SL, cytoplasmic 2 [Source:HGNC
Symbol;Acc:HGNC:23134]"	-	304	993	328	92	71
244	49.62277103	156.8391336	68.02357233
17.04035414	14.54736897	37.81808989
ENSG00000188643 6137.552785	8640.20768	8233.928356
11028.9206	13639.68585	14179.19799	7670.56294
12949.26815	-0.755546131	1.28E-06	4.99E-05
S100A16 1	153606886	153613145	-	1701
protein_coding	S100 calcium binding protein A16 [Source:HGNC
Symbol;Acc:HGNC:20441]	-	6765	9763	7083	10646	11949
16437	194.756594	271.9602405	259.0715806
347.7716628	431.7920159	449.313285
ENSG00000198840 7963.848979	12545.69129	8480.376586
13355.70584	21259.14381	21002.67284	9663.305619
18539.17416	-0.939926377	1.28E-06	4.99E-05	MT-ND3
MT	10059	10404	+	346	protein_coding
mitochondrially encoded NADH:ubiquinone oxidoreductase core subunit 3
[Source:HGNC Symbol;Acc:HGNC:7458]	-	8778	14176	7295
12892	18624	24347	1242.362006	1941.350923
1311.765015	2070.409452	3308.599687	3271.902662
ENSG00000128708 1560.471661	1570.866397	1527.514028

1066.011581   687.1780806   703.9134609   1552.950695
819.0343743   0.923237303   1.34E-06   5.19E-05   HAT1
        2   171922448   171983682   +   6790
protein_coding histone acetyltransferase 1 [Source:HGNC
Symbol;Acc:HGNC:4821]   -   1720   1775   1314   1029   602
        816   12.40473115   12.38668302   12.04016497
8.42088321   5.449718593   5.587935733
ENSG00000111247 781.1430817   699.146171   712.6073814
437.1787048   334.4571057   263.9675479   730.9655447
345.2011195   1.083895253   1.35E-06   5.23E-05
RAD51AP1   12   4538798 4560048 +   2558   protein_coding
        RAD51 associated protein 1 [Source:HGNC Symbol;Acc:HGNC:16956]
        -       861   790   613   422   293   306
16.48281161   14.63366106   14.90962708   9.166930832
7.040677315   5.562267928
ENSG00000143384 10721.89192   10837.65065   10683.29826
14754.2633   16064.2145   16058.88847   10747.61361
15625.78876   -0.539910569   1.35E-06   5.23E-05   MCL1
        1   150574551   150579738   -   4371
protein_coding "MCL1, BCL2 family apoptosis regulator [Source:HGNC
Symbol;Acc:HGNC:6943]" -   11818   12246   9190   14242   14073
        18616   132.4011821   132.7516383   130.8101825
181.0515381   197.9033936   198.032551
ENSG00000135862 5987.856375   4764.813904   7398.09686
3807.184219   3505.521404   3138.280847   6050.255713
3483.662157   0.796266214   1.37E-06   5.29E-05   LAMC1
        1   183023460   183145592   +   8406
protein_coding laminin subunit gamma 1 [Source:HGNC
Symbol;Acc:HGNC:6492]   -   6600   5384   6364   3675   3071
        3638   38.44883927   30.34884072   47.10289844
24.29293574   22.45627877   20.12353749
ENSG00000109917 3842.207841   3809.904134   3191.039579
5092.821121   5970.002261   6242.918771   3614.383851
5768.580718   -0.674237072   1.38E-06   5.31E-05   ZPR1
        11   116773799   116788050   -   7069
protein_coding ZPR1 zinc finger [Source:HGNC Symbol;Acc:HGNC:13051]
        -       4235   4305   2745   4916   5230   7237
29.33756849   28.85636449   24.15968201   38.64256412
45.47692248   47.60270249
ENSG00000148834 1803.61492   2655.870455   2683.030728
3505.717387   4766.869874   4777.295033   2380.838701
4349.960765   -0.869821472   1.39E-06   5.34E-05   GSTO1
        10   104235356   104267459   +   1691
protein_coding glutathione S-transferase omega 1 [Source:HGNC
Symbol;Acc:HGNC:13312] -   1988   3001   2308   3384   4176
        5538   57.57069284   84.0908667   84.91786048
111.1984645   151.7973684   152.2791212
ENSG00000164919 995.2543097   1410.682274   1179.929025
621.5810969   592.4342588   708.2266562   1195.288536
640.7473373   0.898875068   1.42E-06   5.44E-05   COX6C

8	99873200	99894062	-	3066
protein_coding	cytochrome c oxidase subunit 6C [Source:HGNC
Symbol;Acc:HGNC:2285]	-	1097	1594	1015	600	519
821	17.52117231	24.63443508	20.59684678
10.87404544	10.40501157	12.45093669
ENSG00000275832 2404.21506	2277.092529	2796.95491
1627.506505	1495.354295	1480.288602	2492.754166
1534.383134	0.699729884	1.44E-06	5.50E-05
ARHGAP23	17	38419280	38512392	+	8133
protein_coding	Rho GTPase activating protein 23 [Source:HGNC
Symbol;Acc:HGNC:29293]	-	2650	2573	2406	1571	1310
1716	15.95599109	14.99047708	18.4056728
10.73340351	9.900745192	9.810644285
ENSG00000133112 29424.87058	36395.42567	42229.83417
53202.16206	64777.37827	58498.14158	36016.71014
58825.89397	-0.707816522	1.46E-06	5.55E-05	TPT1
13	45333471	45341370	-	7269
protein_coding	"tumor protein, translationally-controlled 1
[Source:HGNC Symbol;Acc:HGNC:12022]"	-	32433	41125	36327
51355	56748	67813	218.4948157	268.0758446
310.9293448	392.5727121	479.8696148	433.7797828
ENSG00000124920 571.5681086	496.4822809	763.757014
293.1790841	281.9484816	157.8629453	610.6024678
244.3301703	1.323156796	1.47E-06	5.58E-05	MYRF
11	61752642	61788518	+	10680
protein_coding	myelin regulatory factor [Source:HGNC
Symbol;Acc:HGNC:1181]	NDT80/PhoG	630	561	657	283
247	183	2.888670327	2.488960758	3.827374539
1.472404806	1.421585748	0.796729422
ENSG00000128951 2101.193237	2442.586623	2973.65364
1594.355514	1421.157326	1115.392286	2505.811167
1376.968375	0.863924895	1.54E-06	5.84E-05	DUT
15	48331011	48343373	+	3674
protein_coding	deoxyuridine triphosphatase [Source:HGNC
Symbol;Acc:HGNC:3078]	-	2316	2760	2558	1539	1245
1293	30.86939277	35.59560435	43.31798382
23.27616952	20.82943835	16.36403467
ENSG00000142185 74.39457921	87.61452016	156.9363727
10.35968495	17.12237742	0	106.3151574	9.160687457
3.567370168	1.56E-06	5.90E-05	TRPM2	21
44350163	44443081	+	6705	protein_coding
transient receptor potential cation channel subfamily M member
2 [Source:HGNC Symbol;Acc:HGNC:12339]	-	82	99	135
10	15	0	0.598885439	0.699621026
1.252684873	0.082873038	0.137511759	0
ENSG00000198157 577.9188653	465.5074505	428.9594188
268.3158402	144.9694622	117.3189102	490.7952449
176.8680708	1.474470234	1.59E-06	6.00E-05	HMGN5
X	81113701	81201942	-	2416
protein_coding	high mobility group nucleosome binding domain 5

[Source:HGNC Symbol;Acc:HGNC:8013]	-	637	526	369
259	127	136	12.91133608	10.31609315
9.502465091	5.956825497	3.231127914	2.617417469
ENSG00000163156 1015.213831	1111.553912	1245.028557
1636.830222	2043.270372	2150.55914	1123.9321
1943.553245	-0.790908141	1.59E-06	6.01E-05	SCNM1
1	151156664	151170297	+	3265
protein_coding	sodium channel modifier 1 [Source:HGNC
Symbol;Acc:HGNC:23136]	-	1119	1256	1071	1580	1790
2493	16.78323148	18.22774291	20.40859615
26.88969926	33.6990149	35.50341353
ENSG00000179772 48.0843012	10.61994184	40.68720775	0
0	0	33.13048359	0	7.481805536
1.65E-06	6.20E-05	FOXS1	20	31844301
31845619	-	1319	protein_coding	forkhead box S1
[Source:HGNC Symbol;Acc:HGNC:3735]	Fork	53	12	35
0	0	0	1.967703951	0.43108498
1.650935459	0	0	0
ENSG00000114315 382.8599076	212.3988367	415.009519
80.8055426	115.2906746	135.4343301	336.7560878
110.5101825	1.604465011	1.65E-06	6.20E-05	HES1
3	194136145	194138732	+	2062
protein_coding	hes family bHLH transcription factor 1 [Source:HGNC
Symbol;Acc:HGNC:5192]	bHLH	422	240	357	78	101
157	10.02195558	5.515044507	10.77175338
2.101928725	3.010787283	3.540315851
ENSG00000167703 68.04382245	92.03949592	162.748831
11.39565344	18.26386925	21.56597613	107.6107165
17.07516628	2.648266592	1.77E-06	6.63E-05
SLC43A2 17	1569267 1628886 -	10606	protein_coding	solute
carrier family 43 member 2 [Source:HGNC Symbol;Acc:HGNC:23087]	-
75	104	140	11	16	25	0.346288703
0.464630973	0.821264895	0.057630595	0.092729031
0.109602233
ENSG00000176136 17.23776835	11.50493699	5.812458249
46.61858227	97.02680539	139.7475253	11.51838786
94.46430433	-3.021928092	1.80E-06	6.72E-05	MC5R
18	13824149	13827323	+	1906
protein_coding	melanocortin 5 receptor [Source:HGNC
Symbol;Acc:HGNC:6933]	-	19	13	5	45	85
162	0.48815685	0.323181801	0.163212702
1.311902806	2.74121683	3.952056423
ENSG00000197256 3160.862365	2948.80385	3788.560287
2094.728297	1950.809534	1256.865089	3299.408834
1767.46764	0.900939043	1.81E-06	6.75E-05	KANK2
19	11164267	11197791	-	7638
protein_coding	KN motif and ankyrin repeat domains 2 [Source:HGNC
Symbol;Acc:HGNC:29300]	-	3484	3332	3259	2022	1709
1457	22.33711992	20.67053796	26.5467618
14.7100294	13.75339034	8.869740539

ENSG00000158402 220.4619847 170.8040646 301.0853373
65.26601518 86.75337894 37.09347895 230.7837955
63.03762436 1.87790191 1.82E-06 6.77E-05 CDC25C
5 138285265 138338355 - 3338
protein_coding cell division cycle 25C [Source:HGNC
Symbol;Acc:HGNC:1727] - 243 193 259 63 76
43 3.564910449 2.739664722 4.827477723
1.048736204 1.399505534 0.598981131
ENSG00000105373 4525.367818 5418.825323 5696.209084
7040.441891 9042.898263 10165.33851 5213.467408
8749.559555 -0.74716208 1.84E-06 6.85E-05 NOP53
19 47745522 47757058 + 5341
protein_coding NOP53 ribosome biogenesis factor [Source:HGNC
Symbol;Acc:HGNC:4333] - 4988 6123 4900 6796 7922
11784 45.73330108 54.32104578 57.07952477
70.70381305 91.17160921 102.5890892
ENSG00000187601 114.3136217 115.9343651 58.12458249
315.9703909 230.5813493 339.8797838 96.12418976
295.4771747 -1.615076107 1.96E-06 7.25E-05 MAGEH1
X 55452105 55453566 + 1462
protein_coding MAGE family member H1 [Source:HGNC
Symbol;Acc:HGNC:24092] - 126 131 50 305 202
394 4.220382913 4.245710363 2.127793502
11.59216383 8.492808999 12.53083084
ENSG00000092841 4808.43012 6822.427635 8093.266867
3329.602743 4222.378272 3319.435046 6574.708207
3623.805354 0.859420001 1.96E-06 7.25E-05 MYL6
12 56158161 56163496 + 4609
protein_coding myosin light chain 6 [Source:HGNC
Symbol;Acc:HGNC:7587] - 5300 7709 6962 3214 3699
3848 56.31159712 79.25337731 93.97972231
38.74817103 49.33157523 38.82034345
ENSG00000167549 156.9544171 175.2290403 94.16182364
43.51067679 25.11282022 23.29125422 142.1150937
30.63825041 2.218024511 1.97E-06 7.25E-05 CORO6
17 29614756 29622907 - 4321
protein_coding coronin 6 [Source:HGNC Symbol;Acc:HGNC:21356] -
173 198 81 42 22 27 1.960606871
2.171237668 1.166292812 0.540103595 0.312957796
0.290543067
ENSG00000164877 1316.421152 1439.887114 1145.054275
661.9838682 803.610247 432.1821617 1300.45418
632.5920923 1.04184188 1.97E-06 7.25E-05
MICALL2 7 1428465 1459502 - 8843 protein_coding MICAL
like 2 [Source:HGNC Symbol;Acc:HGNC:29672] - 1451 1627
985 639 704 501 8.035195929 8.717949897
6.930163041 4.015256343 4.893509033 2.634323578
ENSG00000120279 10.88701159 2.654985459 3.48747495
22.79130689 131.2715602 84.53862644 5.676490667
79.53383119 -3.794748898 1.99E-06 7.33E-05 MYCT1

6	152697895	152724567	+	3030
protein_coding	MYC target 1 [Source:HGNC Symbol;Acc:HGNC:23172]
-	12	3	3	22	115	98
0.19393996	0.046914281	0.061600675	0.403452207
2.332934642	1.503884438
ENSG00000226674 101.6121082	132.749273	87.18687374
15.53952742	15.98088559	27.60444945	107.1827516
19.70828749	2.434168278	2.03E-06	7.43E-05	TEX41
2	144667967	145262988	+	15056
lincRNA testis expressed 41 [Source:HGNC Symbol;Acc:HGNC:48667] -
112	150	75	15	14	32	0.364281487
0.472071839	0.309926352	0.055359696	0.057156521
0.098826039
ENSG00000105968 3964.686721	3838.223979	4702.278724
2896.567912	2496.442628	2422.290439	4168.396475
2605.100326	0.677979498	2.04E-06	7.49E-05	H2AFV
7	44826791	44848083	-	4749
protein_coding	H2A histone family member V [Source:HGNC
Symbol;Acc:HGNC:20664]	-	4370	4337	4045	2796	2187
2808	45.06173933	43.27267006	52.99357311
32.71500878	28.30700839	27.49324182
ENSG00000120708 29736.96491	24710.83467	52539.97261
9104.091133	10591.90267	5268.13665	35662.59073
8321.376819	2.099541131	2.05E-06	7.51E-05	TGFBI
5	136028895	136063818	+	7810
protein_coding	transforming growth factor beta induced [Source:HGNC
Symbol;Acc:HGNC:11771]	-	32777	27922	45196	8788	9279
6107	205.5165736	169.4033248	360.0441946
62.52461958	73.02936107	36.35866296
ENSG00000111252 3292.413756	2513.386235	3721.135771
2169.318028	1471.382967	1400.06317	3175.645254
1680.254722	0.918264641	2.23E-06	8.14E-05	SH2B3
12	111405948	111451623	+	5682
protein_coding	SH2B adaptor protein 3 [Source:HGNC
Symbol;Acc:HGNC:29605]	-	3629	2840	3201	2094	1289
1623	31.27623174	23.68337281	35.05026383
20.47799778	13.94437483	13.28153615
ENSG00000170540 3560.052791	2765.609853	3663.011189
2307.101838	1752.189956	1602.783346	3329.557944
1887.35838	0.818984203	2.24E-06	8.15E-05
ARL6IP1 16	18791667	18801678	-	3666
protein_coding	ADP ribosylation factor like GTPase 6 interacting
protein 1 [Source:HGNC Symbol;Acc:HGNC:697]	-	3924	3125
3151	2227	1535	1858	52.41616244	40.39094386
53.47647709	33.75513118	25.73731746	23.56591284
ENSG00000164032 6020.51741	7587.063448	7560.845691
4866.979989	4432.412769	3846.507503	7056.142183
4381.966754	0.687387715	2.28E-06	8.30E-05	H2AFZ
4	99948086	99950388	-	2303
protein_coding	H2A histone family member Z [Source:HGNC

Symbol;Acc:HGNC:4741]	-	6636	8573	6504	4698	3883
	4459	141.1045841	176.3864935	175.7087711
113.352504	103.638427	90.0273678
ENSG00000243742 108.8701159	144.25421	33.71225785
330.4739499	313.9102527	420.9678541	95.61219457
355.1173522	-1.886188595	2.29E-06	8.31E-05
RPLP0P2 11	61615036	61639449	+	3909
transcribed_processed_pseudogene	ribosomal protein lateral
stalk subunit P0 pseudogene 2 [Source:HGNC Symbol;Acc:HGNC:17960]
	-	120	163	29	319	275	488
1.50329516	1.975824539	0.461571701	4.534579875
4.324285741	5.804772047
ENSG00000079616 1543.233893	1526.616639	1841.386773
907.5084015	1071.860827	699.6002657	1637.079102
892.9898313	0.875272193	2.41E-06	8.72E-05	KIF22
	16	29790719	29805385	+	4312
protein_coding	kinesin family member 22 [Source:HGNC
Symbol;Acc:HGNC:6391]	-	1701	1725	1584	876	939
	811	19.31764786	18.95556737	22.85510768
11.28853016	13.38548773	8.745267942
ENSG00000270872 244.0505098	123.0143263	151.1239145
37.29486582	46.80116496	50.03306463	172.7295835
44.70969847	1.949140186	2.42E-06	8.73E-05
SRGAP2D 1	143975087	144068350	+	1021
unprocessed_pseudogene	SLIT-ROBO Rho GTPase activating protein 2D
(pseudogene) [Source:HGNC Symbol;Acc:HGNC:43932]	-	269
	139	130	36	41	58	12.90194605
6.450828544	7.921810637	1.959245249	2.468342806
2.641394502
ENSG00000102038 4436.457224	3136.422823	3981.533901
2572.309773	1785.293219	1145.584652	3851.471316
1834.395881	1.070499647	2.44E-06	8.79E-05
SMARCA1 X	129446501	129523500	-	4363
protein_coding	"SWI/SNF related, matrix associated, actin dependent
regulator of chromatin, subfamily a, member 1 [Source:HGNC
Symbol;Acc:HGNC:11097]" -	4890	3544	3425	2483	1564
	1328	54.8848308	38.48885053	48.84073708
31.62303511	22.03428162	14.15284923
ENSG00000210135 493.5445255	525.687121	475.4590848
687.8830806	1103.822598	1764.959487	498.2302438
1185.555055	-1.251179976	2.46E-06	8.86E-05	MT-TN
	MT	5657	5729	-	73	Mt_tRNA
mitochondrially encoded tRNA asparagine [Source:HGNC
Symbol;Acc:HGNC:7493]	-	544	594	409	664	967
	2046	364.9259297	385.5582725	348.5838759
505.4256321	814.2372639	1303.20815
ENSG00000134014 1270.151352	1400.062332	1371.740147
1893.750409	2311.520952	2487.851007	1347.317944
2231.040789	-0.727908043	2.49E-06	8.93E-05	ELP3
	8	28089673	28191156	+	5390

protein_coding elongator acetyltransferase complex subunit 3
[Source:HGNC Symbol;Acc:HGNC:20696]   -   1400   1582   1180
        1828   2025   2884   12.71943892   13.90734264
13.62072074   18.84514441   23.09317372   24.87926288
ENSG00000171848 3549.165779   3197.487488   3732.760688
2481.144545   1535.306509   1161.974794   3493.137985
1726.141949   1.017264416   2.53E-06   9.08E-05   RRM2
        2   10120698   10131419   +   5323
protein_coding ribonucleotide reductase regulatory subunit M2
[Source:HGNC Symbol;Acc:HGNC:10452]   -   3912   3613   3211
        2395   1345   1347   35.98910641   32.16162148
37.53104751   25.00121387   15.53149204   11.76636043
ENSG00000178343 69.85832438   44.24975766   61.61205744
5.179842475   5.707459141   0   58.57337983   3.629100538
        4.061754817   2.54E-06   9.09E-05   SHISA3 4
        42397839   42402487   +   1971   protein_coding
        shisa family member 3 [Source:HGNC Symbol;Acc:HGNC:25159]
        -       77   50   53   5   5   0
1.913078471   1.202014816   1.673000582   0.140959848
0.155930381   0
ENSG00000128564 63.50756762   65.48964133   89.51185704
165.7549592   202.0440536   412.3414636   72.83635533
260.0468255   -1.841679545   2.56E-06   9.12E-05   VGF
        7   101162509   101165593   -   2704
protein_coding VGF nerve growth factor inducible [Source:HGNC
Symbol;Acc:HGNC:12684] -   70   74   77   160   177
        478   1.267710351   1.296735717   1.771702853
3.287951018   4.023592031   8.219629222
ENSG00000182158 1691.115801   1085.889053   1677.475451
954.1269838   432.6254029   420.9678541   1484.826768
602.5734136   1.301180652   2.59E-06   9.21E-05
CREB3L2 7   137874979   138002067   -   8788
protein_coding cAMP responsive element binding protein 3 like 2
[Source:HGNC Symbol;Acc:HGNC:23720]   TF_bZIP 1864   1227   1443
        921   379   488   10.38686635   6.615778484
10.21605281   5.823467091   2.650919391   2.582027075
ENSG00000125944 6607.508785   6714.458227   6756.401469
4954.001343   3548.898094   4076.832128   6692.789494
4193.243855   0.674441944   2.62E-06   9.30E-05   HNRNPR
        1   23303771   23344336   -   8676
protein_coding heterogeneous nuclear ribonucleoprotein R [Source:HGNC
Symbol;Acc:HGNC:5047]   -   7283   7587   5812   4782   3109
        4726   41.10734711   41.43591958   41.67857951
30.6268317   22.02665446   25.32825046
ENSG00000161011 7110.12582   8056.995874   8803.549265
11209.17911   13138.57094   12318.48557   7990.223653
12222.07854   -0.613287062   2.63E-06   9.33E-05   SQSTM1
        5   179806398   179838078   +   7654
protein_coding sequestosome 1 [Source:HGNC Symbol;Acc:HGNC:11280]
        -       7837   9104   7573   10820   11510   14280

50.14066302	56.35990235	61.55826141	78.55084233
92.43452758	86.75025652
ENSG00000108107 18769.20798	21485.91233	23772.95424
28318.19881	36261.77091	37893.14534	21342.69152
34157.70502	-0.678523984	2.66E-06	9.40E-05	RPL28
	19	55385345	55403250	+	6933
protein_coding	ribosomal protein L28 [Source:HGNC
Symbol;Acc:HGNC:10330]	-	20688	24278	20450	27335	31767
	43927	146.1254935	165.9274151	183.518123
219.0836267	281.6452058	294.605847
ENSG00000125740 56.24955989	49.55972857	30.2247829
310.7905485	215.7419555	91.4397388	45.34469045
205.9907476	-2.17639692	2.70E-06	9.54E-05	FOSB
	19	45467995	45475179	+	5553
protein_coding	"FosB proto-oncogene, AP-1 transcription factor
subunit [Source:HGNC Symbol;Acc:HGNC:3797]"	TF_bZIP 62	56
	26	300	189	106	0.54675492	0.477844723
	0.291308073	3.001965003	2.09209543	0.887583479
ENSG00000106991 2165.608056	1984.159133	2349.395624
1304.284335	1327.554996	1435.431371	2166.387605
1355.756901	0.675550107	2.73E-06	9.60E-05	ENG
	9	127815012	127854756	-	3935
protein_coding	endoglin [Source:HGNC Symbol;Acc:HGNC:3349]	-
	2387	2242	2021	1259	1163	1664	29.70546574
	26.99711226	31.9542349	17.77841489	18.16696327
	19.66253929
ENSG00000102054 2786.167717	2582.415857	2931.803941
1915.505747	1708.813267	1416.453312	2766.795838
1680.257442	0.719848666	2.79E-06	9.82E-05	RBBP7
	X	16839283	16870414	-	7392
protein_coding	"RB binding protein 7, chromatin remodeling factor
[Source:HGNC Symbol;Acc:HGNC:9890]"	-	3071	2918	2522
	1849	1497	1642	20.34447756	18.70465793
21.22706602	13.89911015	12.44821849	10.32861793
ENSG00000105880 30.84653284	24.77986429	30.2247829	0
	0	0	28.61706001	0	7.271518142
2.87E-06	0.000100621	DLX5	7	97020392
97024950	-	1748	protein_coding	distal-less homeobox 5
[Source:HGNC Symbol;Acc:HGNC:2918]	Homeobox	34	28
	26	0	0	0	0.952502605	0.759002216
	0.925419755	0	0	0
ENSG00000163376 246.7722627	229.2137447	144.1489646
504.516657	418.9275009	533.11093	206.7116573
485.5183626	-1.2289648	2.89E-06	0.000101302	KBTBD8
	3	66998307	67011210	+	4736
protein_coding	kelch repeat and BTB domain containing 8 [Source:HGNC
Symbol;Acc:HGNC:30691]	-	272	259	124	487	367
	618	2.812457017	2.591281003	1.628984073
5.713856263	4.763231954	6.067472261
ENSG00000184867 623.2814136	585.8667914	548.6960587

949.9831098	985.1074477	1182.678131	585.9480879
1039.25623	-0.826744918	2.95E-06	0.00010325	ARMCX2
X	101655281	101659891	-	3589
protein_coding	armadillo repeat containing X-linked 2 [Source:HGNC
Symbol;Acc:HGNC:16869]	-	687	662	472	917	863
1371	9.373719689	8.739990729	8.182299779
14.19737066	14.78034929	17.76212878
ENSG00000166845 788.4010894	574.3618544	730.0447561
417.4953034	291.0804162	269.1433821	697.6025666
325.9063673	1.098370139	2.97E-06	0.000103773
C18orf54	18	54357917	54385218	+	6165
protein_coding	chromosome 18 open reading frame 54
[Source:HGNC Symbol;Acc:HGNC:13796]	-	869	649	628
403	255	312	6.902642468	4.988133368
6.337725272	3.632319221	2.542461936	2.353165818
ENSG00000005884 10139.4368	9988.055298	14895.00551
7910.655427	6339.845614	5557.12073	11674.16587
6602.54059	0.822165867	3.01E-06	0.000104861	ITGA3
17	50055968	50090481	+	9802
protein_coding	integrin subunit alpha 3 [Source:HGNC
Symbol;Acc:HGNC:6139]	-	11176	11286	12813	7636	5554
6442	55.83421036	54.55716424	81.32853974
43.28757581	34.82880209	30.55885237
ENSG00000240694 23.58852512	28.3198449	33.71225785	0
0	0	28.54020929	0	7.267000502
3.02E-06	0.000105014	PNMA2	8	26504686
26514092	-	5579	protein_coding	PNMA family member 2
[Source:HGNC Symbol;Acc:HGNC:9159]	-	26	32	29
0	0	0	0.22821578	0.271781604
0.323406305	0	0	0
ENSG00000179300 235.8852512	230.0987398	213.8984636
91.16522755	29.67878753	15.52750281	226.6274848
45.45717263	2.322450486	3.07E-06	0.000106487	RTL3
X	78656069	78659328	-	2648
protein_coding	retrotransposon Gag like 3 [Source:HGNC
Symbol;Acc:HGNC:22997]	-	260	260	184	88	26
18	4.808216902	4.652451002	4.323213553
1.846616599	0.603535371	0.316071649
ENSG00000179115 5573.242684	6981.726763	6112.381095
8194.510795	10750.57004	12515.16727	6222.450181
10486.74937	-0.753058879	3.08E-06	0.000106487	FARSA
19	12922479	12934037	-	2435
protein_coding	phenylalanyl-tRNA synthetase subunit alpha
[Source:HGNC Symbol;Acc:HGNC:3592]	-	6143	7889	5258
7910	9418	14508	123.5407499	153.5145102
134.3471515	180.5051352	237.7426399	277.0381634
ENSG00000198938 36929.65057	43347.94759	32729.9524
48624.21728	69505.43742	72740.31221	37669.18352
63623.3223	-0.756152343	3.11E-06	0.000107333	MT-CO3
MT	9207	9990	+	784	protein_coding

mitochondrially encoded cytochrome c oxidase III [Source:HGNC
Symbol;Acc:HGNC:7422]	-	40705	48981	28155	46936	60890
	84323	2542.496595	2960.315681	2234.324849
3326.611283	4773.943465	5001.04779
ENSG00000078053 465.4197455	351.3430758	499.8714095
218.5893524	196.3365944	149.2365548	438.8780769
188.0541672	1.223806456	3.13E-06	0.000107828	AMPH
	7	38383704	38631567	-	5636
protein_coding	amphiphysin [Source:HGNC Symbol;Acc:HGNC:471]	-
	513	397	430	211	172	173	4.457332831
	3.337689736	4.746836988	2.080288242	1.875879005
	1.427269998
ENSG00000240342 5478.888584	5899.377691	6435.553774
8202.798543	9125.085674	9451.07338	5937.940016
8926.319199	-0.588291024	3.20E-06	0.000110011	RPS2P5
	12	118246084	118246962	+	879
processed_pseudogene	ribosomal protein S2 pseudogene 5 [Source:HGNC
Symbol;Acc:HGNC:31386]	-	6039	6666	5536	7918	7994
	10956	336.4378416	359.3377756	391.8447686
500.5398579	559.0139286	579.5543457
ENSG00000083223 838.2998926	996.5045424	695.1700066
1282.528997	1618.635412	1729.591286	843.3248139
1543.585232	-0.87123528	3.27E-06	0.000112325	TUT7
	9	86287733	86354454	-	6707
protein_coding	terminal uridylyl transferase 7 [Source:HGNC
Symbol;Acc:HGNC:25817]	-	924	1126	598	1238	1418
	2005	6.746404056	7.954932978	5.54727536
10.25662275	12.99556856	13.90007325
ENSG00000165805 4.53625483	4.424975766	17.43737475
40.4027713	97.02680539	115.5936321	8.799535115
84.34106959	-3.296618021	3.29E-06	0.000112651
C12orf50	12	87980035	88034037	-	5609
	protein_coding	chromosome 12 open reading frame 50
[Source:HGNC Symbol;Acc:HGNC:26665]	-	5	5	15
	39	85	134	0.043652915	0.042238745
0.166384423	0.386359158	0.931495682	1.110837101
ENSG00000155090 1989.601368	1940.794371	2187.809285
2805.402684	3443.880846	3453.144098	2039.401675
3234.142543	-0.665597642	3.44E-06	0.000117585	KLF10
	8	102648779	102655902	-	4003
protein_coding	Kruppel like factor 10 [Source:HGNC
Symbol;Acc:HGNC:11810]	zf-C2H2 2193	2193	1882	2708	3017
	4003	26.82759399	25.95849337	29.25101063
37.5902413	46.32730965	46.4976515
ENSG00000176393 1332.751669	1269.08305	1598.426019
859.8538508	837.8550019	776.3751407	1400.086912
824.6946645	0.763254443	3.47E-06	0.00011861	RNPEP
	1	201982372	202006147	+	3479
protein_coding	arginyl aminopeptidase [Source:HGNC
Symbol;Acc:HGNC:10078]	-	1469	1434	1375	830	734

900 20.67740578 19.53085085 24.58980677
13.25670852 12.96847745 12.02871122
ENSG00000144893 39.01179154 30.97483036 16.2748831 0
0 0 28.753835 0 7.281136132
3.52E-06 0.000119801 MED12L 3 151085697
151437072 + 12619 protein_coding mediator complex
subunit 12 like [Source:HGNC Symbol;Acc:HGNC:16050] - 43
35 14 0 0 0 0.166867669
0.131422446 0.069025561 0 0 0
ENSG00000183963 970.7585336 1056.684213 900.9310287
1404.773279 1700.822824 2106.564549 976.1245917
1737.386884 -0.831802648 3.53E-06 0.000119801 SMTN
22 31064105 31104757 + 10242
protein_coding smoothelin [Source:HGNC Symbol;Acc:HGNC:11126] -
1070 1194 775 1356 1490 2442 5.115966473
5.523902394 4.707862581 7.356766326 8.942292205
11.08643477
ENSG00000131171 1655.733013 1503.606765 2100.622411
1090.874825 953.1456765 995.4854582 1753.32073
1013.168653 0.7904343 3.53E-06 0.000119801
SH3BGRL X 81201943 81298547 + 2304
protein_coding SH3 domain binding glutamate rich protein like
[Source:HGNC Symbol;Acc:HGNC:10823] - 1825 1699 1807
1053 835 1154 38.78904414 34.9411621
48.79580919 25.39556855 22.27672589 23.28918829
ENSG00000179598 185.0791971 147.7941906 90.67434869
257.9561552 504.5393881 471.8635578 141.1825788
411.4530337 -1.539389018 3.54E-06 0.000119907 PLD6
17 17200995 17206315 - 2560
protein_coding phospholipase D family member 6 [Source:HGNC
Symbol;Acc:HGNC:30447] - 204 167 78 249 442
547 3.902284112 3.091028054 1.89566453
5.404697921 10.61279228 9.935240379
ENSG00000255248 851.0014061 817.7355215 978.8179692
1519.765782 1424.581802 1425.942342 882.5182989
1456.763308 -0.723941715 3.56E-06 0.000120407
MIR100HG 11 122028327 122556721 - 19385
processed_transcript mir-100-let-7a-2-mir-125b-1 cluster
host gene [Source:HGNC Symbol;Acc:HGNC:39522] - 938 924
842 1467 1248 1653 2.369549124 2.258565067
2.702421782 4.205100233 3.957274169 3.964953208
ENSG00000101224 1886.174758 1792.115185 2019.247996
1340.543232 1013.644743 937.6886422 1899.179313
1097.292206 0.791575171 3.68E-06 0.000124365 CDC25B
20 3786772 3806121 + 4314 protein_coding cell
division cycle 25B [Source:HGNC Symbol;Acc:HGNC:1726] - 2079
2025 1737 1294 888 1087 23.59951252
22.24187151 25.05108406 16.66733557 12.65261185
11.71602855
ENSG00000136167 3140.902844 3018.718467 2093.647461

5180.878443	4295.433749	4602.179307	2751.089591
4692.8305	-0.769933821	3.70E-06	0.000124629	LCP1
	13	46125920	46211871	-	5061
protein_coding	lymphocyte cytosolic protein 1 [Source:HGNC
Symbol;Acc:HGNC:6528]	-	3462	3411	1801	5001	3763
	5335	33.498041	31.93536049	22.14033675
54.9076126	45.70305605	49.01501102
ENSG00000196422 605.1363943	592.0617574	874.1937207
394.7039966	297.9293672	268.2807431	690.4639575
320.3047023	1.107651923	3.75E-06	0.000126239
PPP1R26 9	135479079	135488893	+	5276
protein_coding	protein phosphatase 1 regulatory subunit 26
[Source:HGNC Symbol;Acc:HGNC:29089]	-	667	669	752
	381	261	311	6.190842147	6.008246909
8.867881893	4.012658796	3.040766561	2.740858532
ENSG00000164535 717.6355141	931.0149011	710.2823981
1121.95388	1724.794152	1626.0746	786.3109378
1490.940878	-0.922437954	3.80E-06	0.00012746	DAGLB
	7	6409126 6484190 -	5756	protein_coding
diacylglycerol lipase beta [Source:HGNC Symbol;Acc:HGNC:28923]	-
	791	1052	611	1083	1511	1885	6.72952455
	8.660069859	6.60430728	10.45489595	16.13581995
	15.22725384
ENSG00000210082 103109.0723	112828.9171	69184.52805
200352.163	270071.2591	393152.0581	95040.83913
287858.4934	-1.59873815	3.97E-06	0.000132822	MT-
RNR2	MT	1671	3229	+	1559	Mt_rRNA
mitochondrially encoded 16S RNA [Source:HGNC Symbol;Acc:HGNC:7471]
	-	113650	127491	59514	193396	236595	455755
3569.866772	3874.894238	2375.088911	6893.081538
9328.39242	13593.03217
ENSG00000180340 1046.967615	1156.688665	1297.340681
753.1490958	630.1034892	605.5726098	1166.998987
662.9417316	0.815633499	3.97E-06	0.000132822	FZD2
	17	44557459	44559570	+	2112
protein_coding	frizzled class receptor 2 [Source:HGNC
Symbol;Acc:HGNC:4040]	-	1154	1307	1116	727	552
	702	26.75719468	29.3229807	32.87586038
19.12725028	16.06543628	15.4551853
ENSG00000115216 2441.412349	2664.720406	2657.455912
3279.876255	5285.107164	4878.223801	2587.862889
4481.069073	-0.792117902	4.10E-06	0.000136734	NRBP1
	2	27427790	27442259	+	5329
protein_coding	nuclear receptor binding protein 1 [Source:HGNC
Symbol;Acc:HGNC:7993]	-	2691	3011	2286	3166	4630
	5655	24.72843666	26.77265713	26.68931039
33.01241028	53.4050875	49.34213159
ENSG00000111716 476.3067571	753.1308753	731.2072478
231.0209744	305.91981	347.6435352	653.5482934
294.8614399	1.146828731	4.18E-06	0.000139231	LDHB

12	21635342	21757857	-	2983
protein_coding	lactate dehydrogenase B [Source:HGNC
Symbol;Acc:HGNC:6541]	-	525	851	629	223	268
403	8.61856048	13.51769825	13.11910593
4.153972847	5.522413231	6.281781279
ENSG00000102595 547.9795835	423.0276832	518.4712759
256.9201867	235.1473166	144.0607206	496.4928475
212.0427413	1.230034944	4.29E-06	0.000142799	UGGT2
13	95801580	96053482	-	8105
protein_coding	UDP-glucose glycoprotein glucosyltransferase 2
[Source:HGNC Symbol;Acc:HGNC:15664]	-	604	478	446
248	206	167	3.64932551	2.794482011
3.423644685	1.700241864	1.562289671	0.958063887
ENSG00000142156 26517.13123	30917.30567	51178.69489
12966.18168	13119.16558	8346.032763	36204.37726
11477.12668	1.657413861	4.40E-06	0.000145959	COL6A1
21	45981737	46005050	+	5343
protein_coding	collagen type VI alpha 1 chain [Source:HGNC
Symbol;Acc:HGNC:2211]	-	29228	34935	44025	12516	11493
9675	267.8814296	309.8146957	512.6500889
130.1644609	132.219525	84.19703879
ENSG00000158301 438.2022166	487.6323294	417.3345023
746.9332848	858.4018548	810.0180635	447.7230161
805.1177344	-0.845621371	4.43E-06	0.000146597
GPRASP2 X	102712176	102717733	+	3975
protein_coding	G protein-coupled receptor associated sorting protein
2 [Source:HGNC Symbol;Acc:HGNC:25169]	-	483	551	359
721	752	939	5.950297519	6.568117397
5.619066375	10.07883129	11.62861703	10.98397352
ENSG00000141456 2136.576025	2367.362035	2087.835003
3031.243816	3494.106486	4160.508115	2197.257688
3561.952806	-0.697030205	4.47E-06	0.000147885	PELP1
17	4669774 4704337 -	6577
protein_coding	"proline, glutamate and leucine rich protein 1
[Source:HGNC Symbol;Acc:HGNC:30134]"	-	2355	2675	1796
2926	3061	4823	17.53443406	19.27180468
16.9896854	24.72057246	28.60769375	34.09733514
ENSG00000102317 3490.194466	4032.922913	4218.682197
2748.424417	2465.622349	2311.010002	3913.933192
2508.352256	0.64187206	4.48E-06	0.000147932	RBM3
X	48574449	48579066	+	3731
protein_coding	RNA binding motif protein 3 [Source:HGNC
Symbol;Acc:HGNC:9900]	-	3847	4557	3629	2653	2160
2679	50.49235428	57.87356296	60.5157703
39.51154796	35.58572861	33.38708345
ENSG00000176171 2025.891407	2485.06639	2669.080828
3767.817416	3810.299722	3723.150119	2393.346208
3767.089086	-0.654780694	4.49E-06	0.000147932	BNIP3
10	131966455	131982013	-	5325
protein_coding	BCL2 interacting protein 3 [Source:HGNC

Symbol;Acc:HGNC:1084]	-	2233	2808	2296	3637	3338
	4316	20.53514598	24.98641404	26.82619754
37.95209309	38.53133714	37.68711059
ENSG00000136891 1445.250789	1393.867366	1205.503841
2035.678092	2082.081095	2439.54322	1348.207332
2185.767469	-0.696845617	4.57E-06	0.000150049	TEX10
	9	100302077	100352939	-	7261
protein_coding	testis expressed 10 [Source:HGNC
Symbol;Acc:HGNC:25988]	-	1593	1575	1037	1965	1824
	2828	10.74355528	10.27804612	8.885649255
15.0375873	15.44101189	18.10981386
ENSG00000165996 430.9442088	388.5128722	425.4719439
219.6253209	190.6291353	140.6101644	414.9763416
183.6215402	1.178503265	4.57E-06	0.000150049	HACD1
	10	17589032	17617377	-	3857
protein_coding	3-hydroxyacyl-CoA dehydratase 1 [Source:HGNC
Symbol;Acc:HGNC:9639]	-	475	439	366	212	167
	163	6.030768452	5.39313538	5.903890488
3.054205576	2.661424757	1.965029089
ENSG00000164692 15423.26642	12104.07871	26574.55912
5643.95636	5142.420686	3047.703747	18033.96808
4611.360264	1.967480822	4.60E-06	0.000150754	COL1A2
	7	94394561	94431232	+	11156
protein_coding	collagen type I alpha 2 chain [Source:HGNC
Symbol;Acc:HGNC:2198]	-	17000	13677	22860	5448	4505
	3533	74.62238053	58.09099057	127.4895438
27.13567549	24.8218216	14.72536776
ENSG00000164294 1312.792148	1368.202507	1394.98998
928.2277714	752.2431148	654.7430354	1358.661545
778.4046405	0.804151856	4.64E-06	0.000151782	GPX8
	5	55160118	55167071	+	3868
protein_coding	glutathione peroxidase 8 (putative) [Source:HGNC
Symbol;Acc:HGNC:33100]	-	1447	1546	1200	896	659
	759	18.31937907	18.93866949	19.3019696
12.8716312	10.47240211	9.124022101
ENSG00000144741 544.3505796	584.0968011	464.99666
930.2997084	933.7403154	936.8260032	531.1480135
933.622009	-0.812414282	4.67E-06	0.000152379
SLC25A26	3	66133610	66388116	+	5850
	protein_coding	solute carrier family 25 member 26
[Source:HGNC Symbol;Acc:HGNC:20661]	-	600	660	400
	898	818	1086	5.022547675	5.345822201
4.254132103	8.529675596	8.594978562	8.631871714
ENSG00000197321 1222.974302	1238.993214	1599.58851
910.616307	609.5566362	542.5999595	1353.852009
687.5909676	0.977444274	4.70E-06	0.00015324	SVIL
	10	29457338	29736781	-	9937
protein_coding	supervillin [Source:HGNC Symbol;Acc:HGNC:11480] -
	1348	1400	1376	879	534	629	6.642985216
	6.675736508	8.615291782	4.915250193	3.303188268

2.943244721
ENSG00000242802 1439.807283 1711.580626 1338.027889
2042.929872 2695.062206 3464.358406 1496.471933
2734.116828 -0.869464843 4.83E-06 0.000157187 AP5Z1
7 4775615 4794395 + 8396 protein_coding
adaptor related protein complex 5 subunit zeta 1 [Source:HGNC
Symbol;Acc:HGNC:22197] - 1587 1934 1151 1972 2361
4016 9.256209601 10.91466676 8.529228322
13.05108218 17.28506103 22.24089667
ENSG00000204713 2005.931886 2025.753905 1862.311623
2623.072229 3658.481309 3328.061437 1964.665805
3203.204992 -0.704962159 4.87E-06 0.000157865 TRIM27
6 28903002 28923989 - 7986
protein_coding tripartite motif containing 27 [Source:HGNC
Symbol;Acc:HGNC:9975] - 2211 2289 1602 2532 3205
3858 13.55776557 13.58134957 12.48073185
17.61758758 24.66869004 22.46280234
ENSG00000127152 41.73354444 90.26950562 74.39946559
121.2083139 287.6559407 411.4788246 68.80083855
273.4476931 -1.9925284 4.87E-06 0.000157865 BCL11B
14 99169287 99271524 - 7855
protein_coding B cell CLL/lymphoma 11B [Source:HGNC
Symbol;Acc:HGNC:13222] zf-C2H2 46 102 64 117 252
477 0.286774364 0.615290803 0.506921407
0.827659522 1.971976393 2.823600225
ENSG00000104142 1064.205383 1046.064271 893.9560788
1362.298571 1878.895549 2610.345751 1001.408578
1950.51329 -0.961887563 5.01E-06 0.000161889 VPS18
15 40894430 40903975 + 4049
protein_coding "VPS18, CORVET/HOPS core subunit [Source:HGNC
Symbol;Acc:HGNC:15972]" - 1173 1182 769 1315 1646
3026 14.18661944 13.83235545 11.81640614
18.04637675 24.98788015 34.74978845
ENSG00000185085 1011.584827 1370.857492 1210.153808
1801.549213 1977.063846 2633.637005 1197.532042
2137.416688 -0.836246449 5.02E-06 0.000161889 INTS5
11 62646848 62653302 - 3285
protein_coding integrator complex subunit 5 [Source:HGNC
Symbol;Acc:HGNC:29352] - 1115 1549 1041 1739 1732
3053 16.62142204 22.3430514 19.71615402
29.41550114 32.4085704 43.2137991
ENSG00000077152 1000.697815 1100.048975 1153.191717
625.7249709 683.7536051 610.7484441 1084.646169
640.0756734 0.761114051 5.03E-06 0.000162176 UBE2T
1 202331657 202341980 - 1652
protein_coding ubiquitin conjugating enzyme E2 T [Source:HGNC
Symbol;Acc:HGNC:25009] - 1103 1243 992 604 599
708 32.69596449 35.65229788 37.36013835
20.31603439 22.28764284 19.92756493
ENSG00000171724 364.7148883 431.8776347 291.7854041

660.9478997	940.5892664	655.6056744	362.7926424
752.3809469	-1.049654558	5.13E-06	0.00016492	VAT1L
	16	77788530	77980107	+	4137
protein_coding	vesicle amine transport 1 like [Source:HGNC
Symbol;Acc:HGNC:29315]	-	402	488	251	638	824
	760	4.7584906	5.589342746	3.774809568
8.569336588	12.24303387	8.541990607
ENSG00000052749 3990.996999	4671.889413	3921.084335
5410.863449	7376.320194	8124.334528	4194.656916
6970.506057	-0.732676954	5.26E-06	0.00016878	RRP12
	10	97356358	97426076	-	8529
protein_coding	ribosomal RNA processing 12 homolog [Source:HGNC
Symbol;Acc:HGNC:29100]	-	4399	5279	3373	5223	6462
	9418	25.25716091	29.32783393	24.60509654
34.02780654	46.5710682	51.34422345
ENSG00000171320 1183.962511	899.1550756	886.9811289
636.0846559	301.3538426	399.401878	990.032905
445.6134588	1.151579254	5.32E-06	0.000170498	ESCO2
	8	27771949	27812640	+	7973
protein_coding	establishment of sister chromatid cohesion N-
acetyltransferase 2 [Source:HGNC Symbol;Acc:HGNC:27230] -	1305
	1016	763	614	264	463	8.015256614
6.038073353	5.954010833	4.279161236	2.03530511
2.700164636
ENSG00000100335 1744.643608	1833.709957	1549.601369
2474.928734	2628.85568	3288.380041	1709.318311
2797.388152	-0.71061795	5.40E-06	0.000172715	MIEF1
	22	39499432	39518132	+	7555
protein_coding	mitochondrial elongation factor 1 [Source:HGNC
Symbol;Acc:HGNC:25979]	-	1923	2072	1333	2389	2303
	3812	12.46446089	12.99516276	10.97747679
17.57088858	18.7372922	23.46115784
ENSG00000106799 1755.530619	1923.094468	2150.609552
1343.651138	920.0424135	1098.139505	1943.078213
1120.611019	0.79344982	5.43E-06	0.000173255	TGFBR1
	9	99104038	99154192	+	6844
protein_coding	transforming growth factor beta receptor 1
[Source:HGNC Symbol;Acc:HGNC:11772]	-	1935	2173	1850
	1297	806	1273	13.84521334	15.04444484
16.8177764	10.53033091	7.238897065	8.648671882
ENSG00000168528 648.6844407	472.5874118	432.4468938
226.8771004	279.6654979	173.3904481	517.9062487
226.6443488	1.195749483	5.49E-06	0.000174917
SERINC2 1	31409565	31434680	+	3549
protein_coding	serine incorporator 2 [Source:HGNC
Symbol;Acc:HGNC:23231]	-	715	534	372	219	245
	201	9.865718647	7.129543095	6.521444268
3.428863204	4.243336228	2.63342574
ENSG00000156976 8719.589034	8302.139531	8340.877588
11629.78232	12105.52084	12328.83724	8454.202051

12021.38013	-0.507859569	5.56E-06	0.000176785	EIF4A2
	3	186783205	186789900	+	6133
protein_coding	eukaryotic translation initiation factor 4A2
[Source:HGNC Symbol;Acc:HGNC:3284]	-	9611	9381	7175
	11226	10605	14292	76.74044197	72.47740112
72.78732975	101.7101083	106.2882039	108.3555251
ENSG00000040275 1400.795491	1063.764174	1150.866733
815.3072055	472.5776169	501.1932853	1205.142133
596.3593692	1.015182721	5.58E-06	0.000177258	SPDL1
	5	169583634	169604778	+	4777
protein_coding	spindle apparatus coiled-coil protein 1 [Source:HGNC
Symbol;Acc:HGNC:26010]	-	1544	1202	990	787	414
	581	15.82780672	11.92272885	12.89397429
9.154434776	5.327119756	5.655251313
ENSG00000186472 259.4737763	200.8938998	54.63710754
988.3139441	1195.141944	1782.212268	171.6682612
1321.889385	-2.943368059	5.67E-06	0.000179476	PCLO
	7	82754013	83162930	-	22873
protein_coding	piccolo presynaptic cytomatrix protein [Source:HGNC
Symbol;Acc:HGNC:13406]	-	286	227	47	954	1047
	2066	0.612310331	0.470250394	0.12784436
2.317593629	2.813655435	4.199892799
ENSG00000184613 12.70151352	4.424975766	88.34936539	0
	0	0	35.15861823	0	7.563420804
5.67E-06	0.000179476	NELL2	12	44508275
44921848	-	6424	protein_coding	neural EGFL like 2
[Source:HGNC Symbol;Acc:HGNC:7751]	-	14	5	76
	0	0	0	0.10672132	0.03688 0.736062863
	0	0	0
ENSG00000239264 569.7536066	554.0069659	503.3588844
282.8193991	179.214217	291.5719973	542.3731523
251.2018711	1.10832508	5.73E-06	0.000181001	TXNDC5
	6	7881517 7910814 -	4387	protein_coding
thioredoxin domain containing 5 [Source:HGNC Symbol;Acc:HGNC:21073]
	-	628	626	433	273	157	338
7.010043176	6.761345671	6.140830478	3.457857217
2.199780653	3.582449557
ENSG00000157227 15906.83119	18162.75553	27596.38928
12497.92392	12444.54391	9364.809476	20555.32533
11435.7591	0.845943955	5.75E-06	0.000181198	MMP14
	14	22836557	22849027	+	4464
protein_coding	matrix metallopeptidase 14 [Source:HGNC
Symbol;Acc:HGNC:7160]	-	17533	20523	23739	12064	10902
	10856	192.3360667	217.8427446	330.8606214
150.1686188	150.1168186	113.077622
ENSG00000231925 2662.781585	2606.310726	3495.612391
1810.872929	1385.771079	1900.393817	2921.568234
1699.012608	0.78111376	5.77E-06	0.000181498	TAPBP
	6	33299694	33314387	-	5056
protein_coding	TAP binding protein [Source:HGNC

Symbol;Acc:HGNC:11566] - 2935 2945 3007 1748 1214
2203 28.42691454 27.59971991 37.00268251
19.21084229 14.75906963 20.25995377
ENSG00000178896 910.8799698 1436.347134 1182.254008
1657.549592 2298.964542 3065.819167 1176.493704
2340.777767 -0.992783582 5.86E-06 0.000184046 EXOSC4
8 144078626 144080647 + 1407
protein_coding exosome component 4 [Source:HGNC
Symbol;Acc:HGNC:18189] - 1004 1623 1017 1600 2014
3554 34.94365259 54.65763841 44.97111983
63.18848296 87.98582873 117.4503578
ENSG00000214063 949.8917614 955.7947654 1329.890447
617.437223 603.8491771 549.5010718 1078.525658
590.2624906 0.869013926 5.95E-06 0.000186696 TSPAN4
11 842808 867116 + 5841 protein_coding
tetraspanin 4 [Source:HGNC Symbol;Acc:HGNC:11859] - 1047
1080 1144 596 529 637 8.777850078
8.761187807 12.18556484 5.669843834 5.566930841
5.07087896
ENSG00000167601 10785.39948 9111.910097 9790.504675
4200.852247 2199.654753 1696.811002 9895.938085
2699.106001 1.874418195 6.18E-06 0.00019345 AXL
19 41219203 41261766 + 5154
protein_coding AXL receptor tyrosine kinase [Source:HGNC
Symbol;Acc:HGNC:905] - 11888 10296 8422 4055 1927
1967 112.9517765 94.65652582 101.6664524
43.71781903 22.9818328 17.74561127
ENSG00000151348 1907.948781 1906.27956 2822.529726
1230.730572 1260.206978 1314.661905 2212.252689
1268.533152 0.80152878 6.20E-06 0.000193861 EXT2
11 44095549 44245429 + 5286
protein_coding exostosin glycosyltransferase 2 [Source:HGNC
Symbol;Acc:HGNC:3513] - 2103 2154 2428 1188 1104
1524 19.48232561 19.30834193 28.57777221
12.48824256 12.83776066 13.40567932
ENSG00000270647 2973.968666 3847.958926 4031.521042
2341.288798 2313.803936 2097.075519 3617.816211
2250.722751 0.684697068 6.24E-06 0.000194554 TAF15
17 35713791 35864615 + 5909
protein_coding TATA-box binding protein associated factor 15
[Source:HGNC Symbol;Acc:HGNC:11547] - 3278 4348 3468
2260 2027 2431 27.16587155 34.8659888
36.51505385 21.25232716 21.08565609 19.12942813
ENSG00000197299 761.1835605 607.106675 734.6947227
363.6249417 376.6923033 395.0886827 700.9949861
378.4686426 0.888482162 6.27E-06 0.000195311 BLM
15 90717327 90816165 + 6674
protein_coding Bloom syndrome RecQ like helicase [Source:HGNC
Symbol;Acc:HGNC:1058] - 839 686 632 351 330
458 6.156082652 4.870396899 5.891660627

2.922354906	3.039310691	3.190878691
ENSG00000152377 1860.771731	1415.992245	2553.994155
1217.262982	918.9009217	683.2101239	1943.586044
939.7913423	1.04834614	6.38E-06	0.000198229	SPOCK1
5	136975298	137598379	-	6222
protein_coding	"SPARC (osteonectin), cwcv and kazal like domains
proteoglycan 1 [Source:HGNC Symbol;Acc:HGNC:11251]"	-	2051
1600	2197	1175	805	792	16.14225996
12.18474421	21.96882841	10.4934888	7.952674985
5.918698166
ENSG00000162244 17233.2321	21877.96518	22269.85254
29672.20963	32501.69682	30522.75734	20460.34994
30898.88793	-0.594754223	6.40E-06	0.000198606	RPL29
3	51993522	51995942	-	2058
protein_coding	ribosomal protein L29 [Source:HGNC
Symbol;Acc:HGNC:10331]	-	18995	24721	19157	28642	28473
35383	451.9835314	569.1766891	579.1472192
773.3391801	850.4234319	799.4297391
ENSG00000108379 352.0133748	361.9630176	427.7969272
210.3016045	124.4226093	95.75293403	380.5911065
143.4923826	1.408722051	6.56E-06	0.000203108	WNT3
17	46762506	46833154	-	4334
protein_coding	Wnt family member 3 [Source:HGNC
Symbol;Acc:HGNC:12782]	-	388	409	368	203	109
111	4.384009657	4.47157832	5.28281933
2.60267041	1.545912651	1.190872016
ENSG00000101161 4715.890521	5386.080502	5304.449398
6782.485736	8362.569133	8282.197474	5135.473474
7809.084114	-0.604723143	6.63E-06	0.00020503	PRPF6
20	63981135	64033100	+	3044
protein_coding	pre-mRNA processing factor 6 [Source:HGNC
Symbol;Acc:HGNC:15860]	-	5198	6086	4563	6547	7326
9601	83.62195383	94.73571577	93.26370565
119.5115108	147.9345539	146.6570145
ENSG00000106070 664.1077071	616.8416217	873.0312291
372.9486582	406.3710908	316.5885296	717.9935193
365.3027595	0.975042064	6.69E-06	0.00020608	GRB10
7	50590063	50793462	-	9795
protein_coding	growth factor receptor bound protein 10 [Source:HGNC
Symbol;Acc:HGNC:4564]	-	732	697	751	360	356
367	3.65961437	3.371745438	4.770263214
2.04225564	2.234050149	1.742178469
ENSG00000176994 1310.070395	1228.373273	955.5681362
1957.980455	1777.302776	2168.67456	1164.670601
1967.985931	-0.75619286	6.69E-06	0.00020608	SMCR8
17	18315310	18328055	+	8279
protein_coding	"Smith-Magenis syndrome chromosome region, candidate 8
[Source:HGNC Symbol;Acc:HGNC:17921]"	-	1444	1388	822
1890	1557	2514	8.541182355	7.943977846
6.177329703	12.68516046	11.56000682	14.11947045

ENSG00000114251 2963.988906 2691.270261 5648.546927
1078.443203 994.2393823 489.9789777 3767.935364
854.2205211 2.141301143 6.77E-06 0.000208134 WNT5A
3 55465715 55490539 - 7397
protein_coding Wnt family member 5A [Source:HGNC
Symbol;Acc:HGNC:12784] - 3267 3041 4859 1041 871
568 21.62829076 19.47992329 40.8693873
7.820007228 7.237855302 3.570456408
ENSG00000244586 51.71330506 55.75469465 106.9492318
11.39565344 4.565967313 0 71.4724105 5.320540252
3.771463398 6.90E-06 0.000211972 WNT5A-AS1
3 55487699 55488308 + 500
antisense WNT5A antisense RNA 1 [Source:HGNC
Symbol;Acc:HGNC:40616] - 57 63 92 11 4
0 5.582561741 5.970311431 11.44786949
1.222460188 0.49174205 0
ENSG00000076716 319.35234 489.4023197 447.5592852
118.1004084 127.8470848 230.3246251 418.771315
158.7573728 1.394913871 7.00E-06 0.000214499 GPC4
X 133300103 133415490 - 4960
protein_coding glypican 4 [Source:HGNC Symbol;Acc:HGNC:4452] -
352 553 385 114 112 267 3.475278956
5.282869658 4.829319067 1.277130329 1.387981592
2.502998579
ENSG00000138587 401.9121779 254.8786041 305.7353039
160.5751167 87.89487077 104.3793245 320.8420286
117.6164373 1.447586356 7.01E-06 0.000214499 MNS1
15 56421544 56465137 - 2504
protein_coding meiosis specific nuclear structural 1 [Source:HGNC
Symbol;Acc:HGNC:29636] - 443 288 263 155 77
121 8.663593868 5.44985069 6.534739364
3.439611698 1.890182599 2.246891307
ENSG00000181588 1679.321538 1931.944419 2142.472111
942.7313304 1156.331222 1287.057456 1917.912689
1128.706669 0.76406773 7.46E-06 0.000227894 MEX3D
19 1554669 1568058 - 2850 protein_coding mex-3
RNA binding family member D [Source:HGNC Symbol;Acc:HGNC:16734] -
1851 2183 1843 910 1013 1492 31.80462229
36.29404025 40.23345436 17.74224516 21.848013
24.34192843
ENSG00000092964 2034.963917 1780.610248 2567.944055
1479.363011 1017.069219 908.3589147 2127.839406
1134.930381 0.906690242 7.62E-06 0.0002321 DPYSL2
8 26514022 26658178 + 7305
protein_coding dihydropyrimidinase like 2 [Source:HGNC
Symbol;Acc:HGNC:3014] - 2243 2012 2209 1428 891
1053 15.03618764 13.05071173 18.81405209
10.86225592 7.497299219 6.702536212
ENSG00000132394 697.6759928 939.8648526 802.1192384
1184.11199 1529.59905 1618.310849 813.220028

1444.007296	-0.828396727	7.62E-06	0.0002321	EEFSEC
	3	128153454	128408646	+	2421
protein_coding	"eukaryotic elongation factor, selenocysteine-tRNA
specific [Source:HGNC Symbol;Acc:HGNC:24614]"	-	769	1062
	690	1143	1340	1876	15.55464966	20.78529382
	17.73213985	26.23393781	34.02180642	36.03039827
ENSG00000094916 5912.554545	5101.997058	5881.045257
4107.615082	3522.643782	3573.050926	5631.86562
3734.436597	0.592647155	7.64E-06	0.000232287	CBX5
	12	54230940	54280133	-	15040
protein_coding	chromobox 5 [Source:HGNC Symbol;Acc:HGNC:1555]	-
	6517	5765	5059	3965	3086	4142	21.2191786
	18.16259572	20.92780547	14.64898044	12.61233349
	12.80540376
ENSG00000160695 1187.591514	1141.643748	1162.49165
1752.858693	1843.509302	1790.838658	1163.908971
1795.735551	-0.625537572	7.66E-06	0.000232293	VPS11
	11	119067692	119081978	+	5907
protein_coding	"VPS11, CORVET/HOPS core subunit [Source:HGNC
Symbol;Acc:HGNC:14583]" -	1309	1290	1000	1692	1615
	2076	10.85178946	10.3478275	10.53270391
15.91642149	16.80555719	16.34148036
ENSG00000024526 2199.176342	1282.357977	1676.312959
615.365286	326.4666629	233.7751813	1719.282426
391.8690434	2.133753187	7.71E-06	0.000233428	DEPDC1
	1	68474152	68497221	-	5904
protein_coding	DEP domain containing 1 [Source:HGNC
Symbol;Acc:HGNC:22949]	-	2424	1449	1442	594	286
	271	20.10550335	11.62916353	15.1958766
5.590519155	2.977604722	2.134292608
ENSG00000066279 4165.189185	2153.193208	2836.479626
854.6740083	495.4074534	232.0499032	3051.620673
527.3771216	2.533030718	7.77E-06	0.0002348	ASPM
	1	197084128	197146694	-	10887
protein_coding	abnormal spindle microtubule assembly [Source:HGNC
Symbol;Acc:HGNC:19048]	-	4591	2433	2440	825	434
	269	20.650366	10.58913114	13.94403455
4.210733633	2.450354202	1.148881074
ENSG00000213347 290.3203091	290.2784102	417.3345023
69.40988916	167.7992987	43.99459131	332.6444072
93.73459307	1.833729453	7.78E-06	0.000234885	MXD3
	5	177301461	177312757	-	5154
protein_coding	MAX dimerization protein 3 [Source:HGNC
Symbol;Acc:HGNC:14008]	bHLH	320	328	359	67	147
	51	3.040424669	3.015475959	4.333680411
0.722341276	1.753154863	0.460104817
ENSG00000136295 11910.39068	11566.00166	14009.18687
8932.120363	8516.67053	7530.838865	12495.19307
8326.543253	0.585570522	8.02E-06	0.000241755	TTYH3
	7	2631951 2664802 +	6129	protein_coding	tweety

family member 3 [Source:HGNC Symbol;Acc:HGNC:22222]	-	13128
	13069	12051	8622	7461	8730	104.8908561
101.0367057	122.3320664	78.16825928	74.82638753
66.23013503
ENSG00000122966 1417.126009	861.9852791	1083.442218
733.4656944	336.7400893	344.1929791	1120.851169
471.4662543	1.249697215	8.18E-06	0.000245981	CIT
	12	119685790	119877291	-	13230
protein_coding	citron rho-interacting serine/threonine kinase
[Source:HGNC Symbol;Acc:HGNC:1985]	-	1562	974	932
	708	295	399	5.781624325	3.488394182
4.382913653	2.973619918	1.370596227	1.402310125
ENSG00000156467 1667.527275	2227.5328	2302.895958
1106.414353	1065.011876	1402.651088	2065.985345
1191.359105	0.793201419	8.20E-06	0.000246319	UQCRB
	8	96225920	96235634	-	9715
protein_coding	ubiquinol-cytochrome c reductase binding protein
[Source:HGNC Symbol;Acc:HGNC:12582]	-	1838	2517	1981
	1068	933	1626	9.264700526	12.27628187
12.68669553	6.108583172	5.903182352	7.782314085
ENSG00000148848 1763.695878	1427.497182	1792.562124
1107.450321	918.9009217	645.2540059	1661.251728
890.5350829	0.900229752	8.27E-06	0.000248035	ADAM12
	10	126012381	126388455	-	9860
protein_coding	ADAM metallopeptidase domain 12 [Source:HGNC
Symbol;Acc:HGNC:190]	-	1944	1613	1542	1069	805
	748	9.654905541	7.751466836	9.730032825
6.024386601	5.018412146	3.527408045
ENSG00000269378 536.1853209	498.2522712	646.3453573
242.4166278	292.221908	308.8247782	560.2609831
281.154438	0.993099282	8.31E-06	0.000248763
AC022149.1	19	14621634	14622242	-	609
	processed_pseudogene	integrin beta 1 pseudogene 1	-
	591	563	556	234	256	358	47.5224554
	43.80438053	56.80209392	21.35062577	25.83866271
	27.33359481
ENSG00000196950 822.8766261	521.2621452	785.8443553
372.9486582	311.6272691	141.4728034	709.9943756
275.3495769	1.368702637	8.43E-06	0.000251407
SLC39A10	2	195575977	195737702	+	6917
	protein_coding	solute carrier family 39 member 10
[Source:HGNC Symbol;Acc:HGNC:20861]	-	907	589	676
	360	273	164	6.42122954	4.034818096
6.080450634	2.891989879	2.426008016	1.102445402
ENSG00000150764 1613.092218	1039.869305	1551.926353
902.3285591	647.2258666	647.841923	1401.629292
732.4654495	0.936073374	8.43E-06	0.000251407	DIXDC1
	11	111927144	112022584	+	8509
protein_coding	DIX domain containing 1 [Source:HGNC
Symbol;Acc:HGNC:23695]	-	1778	1175	1335	871	567

751	10.23250385	6.543133537	9.76134334
5.687896367	4.095924054	4.103859005
ENSG00000143322 4996.23107	4806.408677	3651.386272
7714.857382	6320.440253	7425.596902	4484.67534
7153.631512	-0.673431788	8.44E-06	0.000251407	ABL2
1	179099327	179229684	-	13257
protein_coding	"ABL proto-oncogene 2, non-receptor tyrosine kinase
[Source:HGNC Symbol;Acc:HGNC:77]"	-	5507	5431	3141
7447	5537	8608	20.34222735	19.41158452
14.74108759	31.21390766	25.67300039	30.19173147
ENSG00000140450 72.58007728	146.0242003	181.3486974
234.1288798	552.4820448	454.6107769	133.3176583
413.7405672	-1.636860653	8.67E-06	0.000257899	ARRDC4
15	97960698	97973838	+	4072
protein_coding	arrestin domain containing 4 [Source:HGNC
Symbol;Acc:HGNC:28087]	-	80	165	156	226	484
527	0.962079368	1.920006132	2.383546757
3.083988241	7.306088902	6.017746154
ENSG00000152818 1226.603306	572.5918641	932.3183032
545.9553968	238.5717921	226.8740689	910.5044911
337.1337526	1.433735901	8.82E-06	0.000261794	UTRN
6	144285701	144853034	+	14604
protein_coding	utrophin [Source:HGNC Symbol;Acc:HGNC:12635]	-
1352	647	802	527	209	263	4.533499278
2.099224552	3.416720006	2.00516832	0.879674134
0.837365266
ENSG00000137507 52.62055603	29.20484005	41.8496994
3.107905485	0	0	41.22503183	1.035968495
5.343108154	8.85E-06	0.000262341	LRRC32	11
76657524	76670747	-	4459	protein_coding
leucine rich repeat containing 32 [Source:HGNC Symbol;Acc:HGNC:4161]
-	58	33	36	3	0	0
0.636970332	0.350673468	0.502310059	0.037384866	0
0
ENSG00000070081 995.2543097	723.0410401	867.2187708
536.6316804	351.5794831	216.5224004	861.8380402
368.2445213	1.228264637	9.20E-06	0.000272131	NUCB2
11	17208153	17349980	+	9025
protein_coding	nucleobindin 2 [Source:HGNC Symbol;Acc:HGNC:8044]
-	1097	817	746	518	308	251
5.952345074	4.289446809	5.142786125	3.189294272
2.097736168	1.293175682
ENSG00000147416 3531.928011	3846.188935	3838.547428
5262.719954	5601.300401	5381.142364	3738.888125
5415.05424	-0.534401697	9.27E-06	0.000273822
ATP6V1B2	8	20197367	20226819	+	7816
protein_coding	ATPase H+ transporting V1 subunit B2
[Source:HGNC Symbol;Acc:HGNC:854]	-	3893	4346	3302
5080	4907	6238	24.39093993	26.34702673
26.2844785	36.11529821	38.59036333	37.11007549

ENSG00000170558 1922.464797   1754.945389   2727.205411
1399.593437   851.5529038   1116.254925   2134.871865
1122.467088   0.926608414   9.32E-06   0.000274777   CDH2
        18   27950966   28177446   -   4950
protein_coding cadherin 2 [Source:HGNC Symbol;Acc:HGNC:1759]   -
        2119   1983   2346   1351   746   1294   20.96304861
        18.98208685   29.48693656   15.16569068   9.263625483
        12.15514364
ENSG00000164949 1330.937167   1520.421673   1213.641282
2024.282439   2111.759882   2347.240842   1355.000041
2161.094388   -0.673084676   9.39E-06   0.000276439   GEM
        8   94249253   94262350   -   2648
protein_coding GTP binding protein overexpressed in skeletal muscle
[Source:HGNC Symbol;Acc:HGNC:4234]   -   1467   1718   1044
        1954   1850   2721   27.12943921   30.7419647
24.52953777   41.00328221   42.94386292   47.77949763
ENSG00000181222 4880.102946   4474.535494   4749.940881
7116.067591   6407.193632   6870.057357   4701.526441
6797.77286   -0.532023182   9.47E-06   0.00027817   POLR2A
        17   7484366 7514616 +   8355   protein_coding RNA
polymerase II subunit A [Source:HGNC Symbol;Acc:HGNC:9187]   -
        5379   5056   4086   6869   5613   7964   31.5270818
        28.67391885   30.42697339   45.68347225   41.29485526
        44.32163932
ENSG00000108582 2550.282465   1983.274138   2622.581162
1628.542474   1319.564553   924.7490565   2385.379255
1290.952028   0.886241008   9.56E-06   0.000280555   CPD
        17   30378905   30469989   +   12532
protein_coding carboxypeptidase D [Source:HGNC Symbol;Acc:HGNC:2301]
        -       2811   2241   2256   1572   1156   1072
10.98421798   8.47320885   11.20019427   6.970183299
5.670022837   3.977456304
ENSG00000143319 1754.623368   2164.698145   1798.374582
2748.424417   3015.82141   3158.121545   1905.898698
2974.122457   -0.641804568   9.64E-06   0.000282219
ISG20L2 1   156721891   156728799   -   5262
protein_coding interferon stimulated exonuclease gene 20 like 2
[Source:HGNC Symbol;Acc:HGNC:25745]   -   1934   2446   1547
        2653   2642   3661   17.998417   22.02581818
18.29137344   28.01550465   30.86237399   32.3504185
ENSG00000108861 2619.233539   3129.342861   2708.605544
3732.594487   4754.313464   4872.185328   2819.060648
4453.031093   -0.659503712   9.67E-06   0.000282219   DUSP3
        17   43766121   43778988   -   5012
protein_coding dual specificity phosphatase 3 [Source:HGNC
Symbol;Acc:HGNC:3069]   -   2887   3536   2330   3603   4165
        5648   28.20748755   33.42932711   28.92355728
39.94525919   51.08004881   52.39799195
ENSG00000135916 840.1143945   1144.298733   1225.266199
504.516657   691.7440479   406.3029903   1069.893109

534.1878984	1.003356521	9.67E-06	0.000282219	ITM2C
	2	230864639	230879248	+	2611
protein_coding	integral membrane protein 2C [Source:HGNC
Symbol;Acc:HGNC:6175]	-	926	1293	1054	487	606
	471	17.36731968	23.46486683	25.11542812
10.36416058	14.26635782	8.387741806
ENSG00000135636 29.03203091	22.12487883	54.63710754	0
	0	0.862639045	35.26467243	0.287546348
6.608212879	9.82E-06	0.000286079	DYSF	2
71453722	71686768	+	8386	protein_coding
dysferlin [Source:HGNC Symbol;Acc:HGNC:3097]	-	32	25
	47	0	0	1	0.186863209	0.141257525
	0.348698313	0	0	0.005544676
ENSG00000137135 226.8127415	242.488672	280.1604876
106.704755	107.3002318	76.77487503	249.8206337
96.92662062	1.368286856	1.01E-05	0.00029415
ARHGEF39	9	35658875	35675866	-	6496
	protein_coding	Rho guanine nucleotide exchange factor 39
[Source:HGNC Symbol;Acc:HGNC:25909]	-	250	274	241
	103	94	89	1.884615141	1.99862349
2.308223578	0.881055471	0.889465684	0.637052183
ENSG00000168874 72.58007728	84.9595347	53.4746159
8.287747959	10.27342645	0	70.33807596	6.187058138
	3.544850405	1.02E-05	0.000297634	ATOH8	2
	85751344	85788066	+	6076	protein_coding
	atonal bHLH transcription factor 8 [Source:HGNC
Symbol;Acc:HGNC:24126]	bHLH	80	96	46	8	9
	0	0.644764185	0.748651861	0.471028205
0.07316178	0.091048355	0
ENSG00000139289 6566.682492	6787.027829	6797.088677
8549.847988	10627.28892	11063.34576	6716.932999
10080.16089	-0.585702094	1.04E-05	0.000301637	PHLDA1
	12	76025447	76033932	-	8069
protein_coding	pleckstrin homology like domain family A member 1
[Source:HGNC Symbol;Acc:HGNC:8933]	-	7238	7669	5847
	8253	9310	12825	43.9265957	45.03451222
45.08376994	56.83347004	70.92140419	73.90412449
ENSG00000125148 13498.98712	13587.33059	13960.36222
19978.65242	17744.49047	22997.09431	13682.22664
20240.07907	-0.565001403	1.04E-05	0.000301637	MT2A
	16	56608199	56609497	+	1299
protein_coding	metallothionein 2A [Source:HGNC Symbol;Acc:HGNC:7406]
	-	14879	15353	12009	19285	15545	26659
560.9101208	560.0290297	575.1810117	824.9403294
735.5783452	954.257807
ENSG00000136848 1251.099082	1160.228646	1335.702906
850.5301343	699.7344907	683.2101239	1249.010211
744.4915829	0.746343924	1.05E-05	0.000303339	DAB2IP
	9	121567057	121785530	+	9127
protein_coding	DAB2 interacting protein [Source:HGNC

Symbol;Acc:HGNC:17294] - 1379 1311 1149 821 613
792 7.398861524 6.806143195 7.832471527
4.998355602 4.128381129 4.034857016
ENSG00000006625 830.1346339 791.1856669 683.5450901
405.0636815 372.126336 481.3525873 768.2884636
419.5142016 0.872083092 1.06E-05 0.000304494 GGCT
7 30496621 30504844 - 1597
protein_coding gamma-glutamylcyclotransferase [Source:HGNC
Symbol;Acc:HGNC:21705] - 915 894 588 391 326
558 28.05723447 26.52522298 22.90758235
13.60454072 12.54758205 16.24651818
ENSG00000114268 746.667545 654.0114182 1276.415832
318.0423279 519.3787818 206.1707318 892.3649316
347.8639472 1.360630953 1.06E-05 0.000305711 PFKFB4
3 48517684 48562015 - 6107
protein_coding "6-phosphofructo-2-kinase/fructose-2,6-biphosphatase 4
[Source:HGNC Symbol;Acc:HGNC:8875]" - 823 739 1098
307 455 239 6.599341441 5.733805531
11.18616618 2.79333163 4.579634695 1.819705045
ENSG00000096746 2185.567577 2479.756419 2660.943387
1565.348396 1407.459424 1682.146138 2442.089128
1551.651319 0.653557895 1.06E-05 0.000306261
HNRNPH3 10 68331174 68343191 + 5373
protein_coding heterogeneous nuclear ribonucleoprotein H3
[Source:HGNC Symbol;Acc:HGNC:5043] - 2409 2802 2289
1511 1233 1950 21.9557685 24.71028368
26.50548764 15.62642628 14.105666 16.87519457
ENSG00000170275 8194.290725 7568.478549 8647.775384
5988.933869 5379.850986 4304.568836 8136.848219
5224.45123 0.639325579 1.07E-05 0.000307287 CRTAP
3 33113979 33147773 + 6695
protein_coding cartilage associated protein [Source:HGNC
Symbol;Acc:HGNC:2379] - 9032 8552 7439 5781 4713
4990 66.06356884 60.52621994 69.13067923
47.98046269 43.27072966 34.65620328
ENSG00000182473 1652.104009 1757.600374 1823.949399
2368.223979 2870.851948 2860.511074 1744.551261
2699.862334 -0.630286086 1.08E-05 0.000308639 EXOC7
17 76081017 76121576 - 10086
protein_coding exocyst complex component 7 [Source:HGNC
Symbol;Acc:HGNC:23214] - 1821 1986 1569 2286 2515
3316 8.841372034 9.330109079 9.678561775
12.59416288 15.32732569 15.28715173
ENSG00000213085 205.0387183 196.468924 181.3486974
49.72648776 87.89487077 33.64292277 194.2854466
57.08809376 1.776063741 1.13E-05 0.000322941 CFAP45
1 159872364 159900163 - 2625
protein_coding cilia and flagella associated protein 45 [Source:HGNC
Symbol;Acc:HGNC:17229] - 226 222 156 48 77
39 4.21607002 4.007283863 3.697448531

1.016070806	1.803054182	0.690822251
ENSG00000231663 26.31027801	23.89486913	23.249833	0
	0	0	24.48499338	0	7.047196772
1.16E-05	0.000331705	AL355472.1	1	234372807
	234373593	-	575	antisense
uncharacterized LOC101927765 [Source:NCBI gene;Acc:101927765]	-
	29	27	20	0	0	0	2.469783226
	2.224960782	2.164058505	0	0	0
ENSG00000210144 19.05227029	87.61452016	49.98714095
154.3593057	184.9216762	351.0940914	52.21797713
230.1250244	-2.139876601	1.17E-05	0.000333932	MT-TY
	MT	5826	5891	-	66	Mt_tRNA
mitochondrially encoded tRNA tyrosine [Source:HGNC
Symbol;Acc:HGNC:7502]	-	21	99	43	149	162
	407	15.58131267	71.07513608	40.535111
125.4452948	150.8754016	286.7355176
ENSG00000132669 1371.763461	1200.053428	1191.553941
858.8178823	703.1589662	662.5067868	1254.456943
741.4945451	0.759156153	1.20E-05	0.00034066	RIN2
	20	19757606	20002459	+	8273
protein_coding	Ras and Rab interactor 2 [Source:HGNC
Symbol;Acc:HGNC:18750]	-	1512	1356	1025	829	616
	768	8.949884906	7.766459932	7.708461145
5.568055428	4.576832809	4.31647484
ENSG00000131389 2101.193237	2000.974041	3168.952238
1493.86657	1386.912571	1240.474947	2423.706505
1373.751363	0.818688546	1.20E-05	0.000341143	SLC6A6
	3	14402576	14489349	+	8254
protein_coding	solute carrier family 6 member 6 [Source:HGNC
Symbol;Acc:HGNC:11052]	-	2316	2261	2726	1442	1215
	1438	13.74050752	12.97963676	20.54793738
9.707621604	9.048137122	8.10075392
ENSG00000146938 18.14501932	19.46989337	37.1997328	0
	0	0	24.93821516	0	7.070600751
1.22E-05	0.000345432	NLGN4X	X	5840637 6228863 -
	7441	protein_coding	neuroligin 4 X-linked [Source:HGNC
Symbol;Acc:HGNC:14287]	-	20	22	32	0	0
	0	0.131621663	0.140093446	0.26756267	0
	0	0
ENSG00000166503 1911.577785	1658.480917	2059.935204
1268.025438	1118.661992	1145.584652	1876.664635
1177.424027	0.672106906	1.22E-05	0.000345438	HDGFL3
	15	83112738	83208018	-	16253
protein_coding	HDGF like 3 [Source:HGNC Symbol;Acc:HGNC:24937] -
	2107	1874	1772	1224	980	1328	6.348332771
	5.463393631	6.783237588	4.184657576	3.70629429
	3.799229754
ENSG00000135597 899.9929583	878.800187	845.1314295
1218.29895	1555.853362	1551.887642	874.6415249
1442.013318	-0.721165992	1.22E-05	0.00034653	REPS1

6	138903493	138988261	-	9602
protein_coding	RALBP1 associated Eps domain containing 1 [Source:HGNC
Symbol;Acc:HGNC:15578]	-	992	993	727	1176	1363
1799	5.05916279	4.90020205	4.710636098
6.80546279	8.725323029	8.711651224
ENSG00000147862 332.0538535	284.9684393	363.8598864
179.2225496	106.15874	105.2419635	326.9607264
130.2077511	1.328357764	1.23E-05	0.000347646	NFIB
9	14081843	14398983	-	12765
protein_coding	nuclear factor I B [Source:HGNC Symbol;Acc:HGNC:7785]
CTF/NFI 366	322	313	173	93	122
1.404070613	1.195257544	1.525563766	0.753073434
0.447826191	0.444395886
ENSG00000052795 704.0267496	700.0311661	527.7712091
1120.917911	1047.889498	1171.463823	643.9430416
1113.423744	-0.788786479	1.24E-05	0.000349347	FNIP2
4	158769138	158908049	+	10868
protein_coding	folliculin interacting protein 2 [Source:HGNC
Symbol;Acc:HGNC:29280]	-	776	791	454	1082	918
1358	3.49655831	3.448683146	2.599040636
5.532095576	5.192068477	5.810067237
ENSG00000126522 412.7991895	488.5173245	549.8585504
279.7114936	203.1855454	212.2092051	483.7250215
231.7020814	1.061179245	1.24E-05	0.000350614	ASL
7	66075798	66093558	+	3195
protein_coding	argininosuccinate lyase [Source:HGNC
Symbol;Acc:HGNC:746]	-	455	552	473	270	178
246	6.973795657	8.18643195	9.210795176
4.695749764	3.424494713	3.580100867
ENSG00000148180 2435.968844	2136.3783	3223.589345
1706.240111	1530.740542	1238.749669	2598.645496
1491.910107	0.800523799	1.26E-05	0.000354085	GSN
9	121207794	121332843	+	11018
protein_coding	gelsolin [Source:HGNC Symbol;Acc:HGNC:4620]	-
2685	2414	2773	1647	1341	1436	11.93356507
10.38151985	15.6586367	8.306209386	7.481236258
6.060140457
ENSG00000131724 2086.677222	1735.475495	2120.384769
1194.471675	1319.564553	1252.551894	1980.845829
1255.529374	0.657643892	1.26E-05	0.000354087
IL13RA1 X	118727572	118794539	+	4372
protein_coding	interleukin 13 receptor subunit alpha 1 [Source:HGNC
Symbol;Acc:HGNC:5974]	-	2300	1961	1824	1153	1156
1452	25.76180961	21.25317808	25.9568225
14.65416906	16.25268211	15.44249542
ENSG00000073050 780.2358307	900.9250659	918.3684034
558.3870188	468.0116496	451.1602207	866.5097667
492.5196297	0.815075638	1.29E-05	0.00036097	XRCC1
19	43543040	43580473	-	3459
protein_coding	X-ray repair cross complementing 1 [Source:HGNC

Symbol;Acc:HGNC:12828] -   860   1018   790   539   410
        523   12.17521314   13.94516499   14.20964984
8.658651234   7.285857199   7.030434153
ENSG00000244038 6425.151341   7141.025891   7115.611389
4785.138478   4939.235141   3683.468723   6893.92954
4469.280781   0.625549679   1.31E-05   0.000366065   DDOST
        1   20651767   20661544   -   2721
protein_coding dolichyl-diphosphooligosaccharide--protein
glycosyltransferase non-catalytic subunit [Source:HGNC
Symbol;Acc:HGNC:2728]   -   7082   8069   6121   4619   4327
        4270   127.4547614   140.5133586   139.9589528
94.32601   97.74751237   72.96764862
ENSG00000119574 664.1077071   858.4452985   735.8572144
1000.745566   1554.71187   1685.596694   752.8034067
1413.68471   -0.909187771   1.31E-05   0.000366275   ZBTB45
        19   58513530   58538911   -   3418
protein_coding zinc finger and BTB domain containing 45 [Source:HGNC
Symbol;Acc:HGNC:23715] ZBTB   732   970   633   966   1362
        1954   10.48739694   13.44702204   11.52228195
15.7042468   24.49358805   26.58174694
ENSG00000138696 228.6272434   249.5686332   122.0616232
387.4522171   448.6062885   686.66068   200.0858333
507.5730619   -1.340455974   1.34E-05   0.000373779   BMPR1B
        4   94757968   95158448   +   6312
protein_coding bone morphogenetic protein receptor type 1B
[Source:HGNC Symbol;Acc:HGNC:1077]   -   252   282   105
        374   393   796   1.955069651   2.116940048
1.034973322   3.292430799   3.827127407   5.863772274
ENSG00000125484 1521.45987   1301.82787   1356.627755
2014.958723   2126.599276   2381.746404   1393.305165
2174.434801   -0.642283806   1.34E-05   0.000374116   GTF3C4
        9   132670035   132694955   +   9043
protein_coding general transcription factor IIIC subunit 4
[Source:HGNC Symbol;Acc:HGNC:4667]   -   1677   1471   1167
        1945   1863   2761   9.081324936   7.707731592
8.029068661   11.95140926   12.66332299   14.19661791
ENSG00000153214 657.7569503   484.0923488   581.2458249
336.6897608   276.2410224   260.5169917   574.3650413
291.1492583   0.98063296   1.36E-05   0.000378395
TMEM87B 2   112055223   112119318   +   5367
protein_coding transmembrane protein 87B [Source:HGNC
Symbol;Acc:HGNC:25913] -   725   547   500   325   242
        302   6.61507991   4.829277615   5.796225266
3.364835283   2.77160369   2.616413407
ENSG00000115268 17403.79528   19991.15551   21638.61957
26575.6998   30109.12995   29853.34944   19677.85679
28846.05973   -0.551856096   1.37E-05   0.000379909   RPS15
        19   1438358 1440494 +   2137   protein_coding
ribosomal protein S15 [Source:HGNC Symbol;Acc:HGNC:10388]   -
        19183   22589   18614   25653   26377   34607   439.5827971

500.8629696 541.9285535 667.0304848 758.6967739
752.9921504
ENSG00000229689 395.5614212 485.8623391 224.3608884
836.0265754 652.9333257 984.2711507 368.5948829
824.4103506 -1.159303064 1.39E-05 0.000384834
AC009237.3 2 95525345 95532405 + 4978
transcribed_unprocessed_pseudogene Ankyrin repeat domain-containing protein pseudogene - 436 549 193 807
572 1141 4.289041817 5.225693011 2.412177506
9.008047884 7.062988461 10.65765777
ENSG00000183853 5155.90724 4963.052819 5550.897628
3906.637194 3398.221172 3197.802941 5223.285896
3500.887103 0.577241473 1.39E-05 0.000385014
KIRREL1 1 157993273 158100262 + 7872
protein_coding kirre like nephrin family adhesion molecule 1 [Source:HGNC Symbol;Acc:HGNC:15734] - 5683 5608 4775
3771 2977 3707 35.35259143 33.75587425
37.73941267 26.61849461 23.24561868 21.89618828
ENSG00000119227 156.9544171 228.3287495 163.9113226
381.2364061 439.4743538 376.9732628 183.0648298
399.2280076 -1.122641157 1.40E-05 0.000387535 PIGZ
3 196946343 196969060 - 3211
protein_coding phosphatidylinositol glycan anchor biosynthesis class Z [Source:HGNC Symbol;Acc:HGNC:30596] - 173 258 141
368 385 437 2.638362594 3.80720131
2.732031194 6.368241971 7.370005028 6.328082748
ENSG00000100714 3408.541879 3089.51808 3375.875751
2343.360735 2216.77713 1793.426575 3291.311903
2117.854814 0.636443985 1.42E-05 0.00039163 MTHFD1
14 64388031 64463457 + 9377
protein_coding "methylenetetrahydrofolate dehydrogenase, cyclohydrolase and formyltetrahydrofolate synthetase 1 [Source:HGNC Symbol;Acc:HGNC:7432]" - 3757 3491 2904 2262 1942
2079 19.62031441 17.64056024 19.26812888
13.40419472 12.73012505 10.30911992
ENSG00000136960 160.583421 190.2739579 174.3737475
74.58973163 50.22564044 46.58250844 175.0770421
57.13262684 1.617294332 1.45E-05 0.000399312 ENPP2
8 119557086 119673453 - 6729
protein_coding ectonucleotide pyrophosphatase/phosphodiesterase 2 [Source:HGNC Symbol;Acc:HGNC:3357] - 177 215 150
72 44 54 1.288105461 1.513959901
1.386907758 0.594557705 0.401929153 0.373142099
ENSG00000173334 299.3928188 384.0878965 275.510521
554.2431448 611.8396199 695.2870705 319.6637454
620.4566117 -0.95562333 1.45E-05 0.000399614 TRIB1
8 125430321 125438405 + 4138
protein_coding tribbles pseudokinase 1 [Source:HGNC Symbol;Acc:HGNC:16891] - 330 434 237 535 536
806 3.905279639 4.969648632 3.563401072

7.184149137 7.961990656 9.056816604
ENSG00000106105 5228.487317 5787.868301 6210.030394
7885.792183 8400.238364 8796.330345 5742.128671
8360.786964 -0.542249488 1.45E-05 0.000399614 GARS
7 30594681 30634033 + 4152
protein_coding glycyl-tRNA synthetase [Source:HGNC
Symbol;Acc:HGNC:4162] - 5763 6540 5342 7612 7359
10197 67.97042075 74.6357402 80.04853451
101.8716824 108.9453801 114.194738
ENSG00000183876 178.7284403 219.478798 261.5606212
102.560881 63.92354238 58.65945508 219.9226198
75.04795948 1.551448243 1.48E-05 0.00040457 ARSI
5 150296343 150339307 - 3269
protein_coding arylsulfatase family member I [Source:HGNC
Symbol;Acc:HGNC:32521] - 197 248 225 99 56
68 2.951073248 3.594704548 4.282273922
1.682799281 1.052980835 0.967219426
ENSG00000145833 3654.406891 2724.900076 3173.602204
2312.281681 1767.02935 1484.601797 3184.303057
1854.637609 0.780152791 1.48E-05 0.000405873 DDX46
5 134758771 134855133 + 9222
protein_coding DEAD-box helicase 46 [Source:HGNC
Symbol;Acc:HGNC:18681] - 4028 3079 2730 2232 1548
1721 21.38912544 15.82016511 18.41808088
13.44872509 10.31794477 8.677343118
ENSG00000149639 2849.675284 2031.063876 2637.693554
1752.858693 1303.583668 915.260027 2506.144238
1323.900796 0.921145451 1.53E-05 0.000417618 SOGA1
20 36777442 36863686 - 15128
protein_coding "suppressor of glucose, autophagy associated 1
[Source:HGNC Symbol;Acc:HGNC:16111]" - 3141 2295 2269
1692 1142 1061 10.16752161 7.18832352
9.331679764 6.214853369 4.640149233 3.26110578
ENSG00000145794 86.18884177 26.54985459 52.31212425
7.251779464 0 0 55.0169402 2.417259821
4.525538801 1.54E-05 0.000420138 MEGF10 5
127290831 127465737 + 9007 protein_coding
multiple EGF like domains 10 [Source:HGNC Symbol;Acc:HGNC:29634]
- 95 30 45 7 0 0
0.516502141 0.157821996 0.310841644 0.043184701 0
0
ENSG00000255921 63.50756762 54.86969949 20.9248497
143.9996208 142.6864785 270.0060212 46.43403894
185.5640402 -1.991438867 1.60E-05 0.000436266
AC026310.2 12 24949163 24960158 + 728
antisense "novel transcript, antisense to BCAT1" -
70 62 18 139 125 313 4.708638445
4.035401499 1.538324555 10.60951337 10.55421638
19.99143533
ENSG00000135506 1038.802356 1217.753331 1671.662993

664.0558052	751.1016229	759.1223598	1309.406226
724.7599293	0.852284384	1.64E-05	0.000447365	OS9
	12	57693955	57721557	+	5169
protein_coding	"OS9, endoplasmic reticulum lectin [Source:HGNC
Symbol;Acc:HGNC:16994]"	-	1145	1376	1438	641	658
	880	10.84744953	12.61357932	17.30849076
6.89070315	7.824682452	7.916025018
ENSG00000152767 955.3352672	803.575599	1115.991984
624.6890024	489.6999943	409.7535465	958.30095
508.0475144	0.915663513	1.66E-05	0.000452124	FARP1
	13	98142562	98455176	+	36228
protein_coding	"FERM, ARH/RhoGEF and pleckstrin domain protein 1
[Source:HGNC Symbol;Acc:HGNC:3591]"	-	1053	908	960
	603	429	475	1.423353245	1.187593824
1.648669944	0.924879166	0.72788085	0.609649566
ENSG00000135124 221.3692357	292.0484005	263.8856045
546.9913653	485.134027	483.0778654	259.1010803
505.0677525	-0.962957551	1.69E-05	0.000458717	P2RX4
	12	121209857	121234106	+	8159
protein_coding	purinergic receptor P2X 4 [Source:HGNC
Symbol;Acc:HGNC:8535]	-	244	330	227	528	425
	560	1.4644737	1.916476276	1.73099483
3.595911818	3.201838019	3.191406403
ENSG00000198585 703.1194986	723.9260353	734.6947227
453.7542008	312.7687609	403.7150732	720.5800855
390.079345	0.88422043	1.69E-05	0.000458717	NUDT16
	3	131381671	131388830	+	7029
protein_coding	nudix hydrolase 16 [Source:HGNC Symbol;Acc:HGNC:26442]
	-	775	818	632	438	274	468
5.399292342	5.514246817	5.59410201	3.462522553
2.396096913	3.095874364
ENSG00000182810 418.2426953	497.3672761	420.8219773
680.6313012	788.7708533	1066.22186	445.4773162
845.2080048	-0.924333958	1.70E-05	0.000459397	DDX28
	16	68021274	68023442	-	2169
protein_coding	DEAD-box helicase 28 [Source:HGNC
Symbol;Acc:HGNC:17330]	-	461	562	362	657	691
	1236	10.40806646	12.27730951	10.38378925
16.83130776	19.58239721	26.49658702
ENSG00000116786 2405.122311	2918.714015	2578.406479
3430.091687	4323.971045	4955.861315	2634.080935
4236.641349	-0.685713765	1.71E-05	0.0004609
PLEKHM2 1	15684332	15734769	+	4122
protein_coding	pleckstrin homology and RUN domain containing M2
[Source:HGNC Symbol;Acc:HGNC:29131]	-	2651	3298	2218
	3311	3788	5745	31.4941886	37.91133734
33.47806907	44.63373565	56.48710834	64.80567876
ENSG00000134910 4392.001926	3732.024561	4722.041082
3189.746996	2421.104168	1944.388408	4282.022523
2518.41319	0.765929105	1.75E-05	0.000472879	STT3A

11	125591712	125625215	+	7393
protein_coding	"STT3A, catalytic subunit of the oligosaccharyltransferase complex [Source:HGNC Symbol;Acc:HGNC:6172]"
-	4841	4217	4062	3079	2121	2254
32.0658724	27.0277153	34.18425028	23.14200731
17.63466941	14.17634336
ENSG00000142694 295.7638149	515.0671791	385.9472278
137.7838098	213.4589719	145.7859987	398.9260739
165.6762601	1.27033408	1.76E-05	0.000472879	EVA1B
1	36322031	36324154	-	1211
protein_coding	eva-1 homolog B [Source:HGNC Symbol;Acc:HGNC:25558]
-	326	582	332	133	187	169
13.18263236	22.77221536	17.05692686	6.102665155
9.49171793	6.488937327
ENSG00000104147 174.1921855	133.6342681	124.3866065
52.83439324	39.95221399	33.64292277	144.07102
42.14317666	1.776998672	1.78E-05	0.000478786	OIP5
15	41309268	41332621	-	1236
protein_coding	Opa interacting protein 5 [Source:HGNC Symbol;Acc:HGNC:20300]	-	192	151	107	51	35
39	7.606965411	5.788751644	5.386071986
2.292787203	1.740591802	1.467158907
ENSG00000165389 1036.080603	1034.559334	1322.915498
744.8613478	601.5661934	615.9242783	1131.185145
654.1172732	0.789471282	1.78E-05	0.000478914	SPTSSA
14	34432789	34462356	-	2777
protein_coding	serine palmitoyltransferase small subunit A [Source:HGNC Symbol;Acc:HGNC:20361]	-	1142	1169	1138
719	527	714	20.13811923	19.94642518
25.49606918	14.38682809	11.66492889	11.95510377
ENSG00000109674 381.0454057	323.908226	495.2214429
225.8411319	103.8757564	71.59904076	400.0583582
133.7719763	1.581692157	1.83E-05	0.000490896	NEIL3
4	177309836	177362943	+	2959
protein_coding	nei like DNA glycosylase 3 [Source:HGNC Symbol;Acc:HGNC:24573]	-	420	366	426	218	91
83	6.950771453	5.860876379	8.957183688
4.093771254	1.890356815	1.30425991
ENSG00000228716 2034.963917	1675.295825	2068.072645
1429.636523	730.55477	804.8422292	1926.110796
988.3445074	0.962519519	1.86E-05	0.000497417	DHFR
5	80626228	80654983	-	4409
protein_coding	dihydrofolate reductase [Source:HGNC Symbol;Acc:HGNC:2861]	-	2243	1893	1779	1380	640
933	24.91253135	20.34402852	25.10398668
17.39206025	8.922513944	9.839489419
ENSG00000185697 1412.589754	1109.783922	1163.654142
865.0336932	478.285076	372.6600676	1228.675939
571.9929456	1.103884048	1.86E-05	0.000497485	MYBL1
8	66562175	66614247	-	7239

protein_coding MYB proto-oncogene like 1 [Source:HGNC
Symbol;Acc:HGNC:7547]   MYB   1557   1254   1001   835   419
        432   10.53267587   8.208152199   8.603246123
6.409437877   3.557810451   2.774828767
ENSG00000102760 150.6036604   255.7635993   141.8239813
250.7043758   784.204886   552.951628   182.7304136
529.2869632   -1.53206583   1.86E-05   0.000497485   RGCC
        13   41457559   41470882   +   1032
protein_coding regulator of cell cycle [Source:HGNC
Symbol;Acc:HGNC:20369] -   166   289   122   242   687
        641   7.876931601   13.2691953   7.355072873
13.03009891   40.91894237   28.88080873
ENSG00000081923 470.8632513   261.0735702   406.8720775
179.2225496   114.1491828   168.2146138   379.6029663
153.8621154   1.300391728   1.89E-05   0.000502251   ATP8B1
        18   57646426   57803315   -   7028
protein_coding ATPase phospholipid transporting 8B1 [Source:HGNC
Symbol;Acc:HGNC:3706]   -   519   295   350   173   100
        195   3.616298644   1.988917202   3.098440339
1.367811951   0.874612354   1.290131196
ENSG00000140534 817.4331203   594.7167429   688.1950567
443.3945158   324.1836792   246.7147669   700.1149733
338.097654   1.05154475   1.91E-05   0.000508503   TICRR
        15   89575482   89631056   +   8402
protein_coding TOPBP1 interacting checkpoint and replication
regulator [Source:HGNC Symbol;Acc:HGNC:28704]   -   901   672
        592   428   284   286   5.251347975   3.789771598
        4.383750982   2.830565021   2.077700877   1.582757478
ENSG00000117640 938.0974988   961.1047363   926.505845
1395.449563   1563.843805   1438.881928   941.9026934
1466.058432   -0.637937173   1.94E-05   0.000515776   MTFR1L
        1   25818640   25832942   +   5817
protein_coding mitochondrial fission regulator 1 like [Source:HGNC
Symbol;Acc:HGNC:28836] -   1034   1086   797   1347   1370
        1668   8.70462685   8.846209133   8.524444827
12.86709702   14.4766763   13.33300373
ENSG00000180758 416.4281934   399.1328141   476.6215765
214.4454784   240.8547757   163.0387796   430.727528
206.1130112   1.065350372   1.95E-05   0.000517651   GPR157
        1   9100305 9129170 -   5372   protein_coding G
protein-coupled receptor 157 [Source:HGNC Symbol;Acc:HGNC:23687]
        -       459   451   410   207   211   189
4.184131884   3.978020151   4.748480942   2.141146509
2.414314327   1.635900248
ENSG00000179604 1464.303059   1307.137841   1889.048931
1076.371266   751.1016229   747.9080523   1553.49661
858.4603138   0.855241169   1.96E-05   0.000518555
CDC42EP4   17   73283624   73312175   -   5007
        protein_coding CDC42 effector protein 4 [Source:HGNC
Symbol;Acc:HGNC:17147] -   1614   1477   1625   1039   658

867	15.78536479	13.97749497	20.19215264
11.53054937	8.077847732	8.051420781
ENSG00000197183 392.8396683	425.6826687	425.4719439
204.0857935	136.9790194	229.461986	414.6647603
190.1755996	1.121259806	2.01E-05	0.000532395	NOL4L
20	32443059	32585074	-	9448
protein_coding	nucleolar protein 4 like [Source:HGNC
Symbol;Acc:HGNC:16106]	-	433	481	366	197	120
266	2.244278223	2.412301754	2.410172059
1.158612968	0.780708165	1.309099841
ENSG00000164904 1393.537484	1323.067754	1512.401637
1018.35703	720.2813436	736.6937447	1409.668958
825.1107062	0.77253755	2.03E-05	0.000536386
ALDH7A1 5	126531200	126595418	-	17970
protein_coding	aldehyde dehydrogenase 7 family member A1 [Source:HGNC
Symbol;Acc:HGNC:877]	-	1536	1495	1301	983	631
854	4.185735892	3.942026653	4.504390834
3.039607339	2.158383649	2.20973814
ENSG00000130764 2735.361662	3387.761446	2663.26837
3831.011494	4785.133744	6173.045008	2928.797159
4929.730082	-0.751210733	2.07E-05	0.000544583	LRRC47
1	3778558 3796504 -	4516	protein_coding
leucine rich repeat containing 47 [Source:HGNC Symbol;Acc:HGNC:29207]
-	3015	3828	2291	3698	4192	7156
32.69354896	40.1646916	31.56298018	45.50142702
57.05775777	73.67962669
ENSG00000051825 591.5276298	435.4176153	468.4841349
250.7043758	263.6846123	250.1653231	498.47646
254.8514371	0.968535134	2.08E-05	0.000546846
MPHOSPH9	12	123152320	123244014	-	10304
protein_coding	M-phase phosphoprotein 9 [Source:HGNC
Symbol;Acc:HGNC:7215]	-	652	492	403	242	231
290	3.098635051	2.262484922	2.433358196
1.305033198	1.378013557	1.308648965
ENSG00000198853 688.6034832	693.8362	698.6574816
936.5155194	1292.168749	1375.909277	693.6990549
1201.531182	-0.792822581	2.09E-05	0.000549103	RUSC2
9	35490127	35561898	+	5682
protein_coding	RUN and SH3 domain containing 2 [Source:HGNC
Symbol;Acc:HGNC:23625]	-	759	784	601	904	1132
1595	6.54137776	6.537945171	6.580821169
8.840549185	12.24595214	13.05240305
ENSG00000138942 300.3000697	342.4931243	333.6351035
587.3941366	667.7727195	539.1494033	325.4760992
598.1054198	-0.877302717	2.10E-05	0.000551741	RNF185
22	31160183	31207019	+	3447
protein_coding	ring finger protein 185 [Source:HGNC
Symbol;Acc:HGNC:26783]	-	331	387	287	567	585
625	4.702354797	5.319810011	5.180211121
9.140160441	10.43186463	8.430818737

ENSG00000123416 15526.69303 13191.73775 17594.31112
11137.69729 10185.53158 7780.141549 15437.58064
9701.123474 0.670264577 2.11E-05 0.000552073 TUBA1B
12 49127782 49131397 - 3574
protein_coding tubulin alpha 1b [Source:HGNC Symbol;Acc:HGNC:18809]
- 17114 14906 15135 10751 8923 9019
234.4907216 197.6209622 263.472155 167.1499909
153.4630075 117.3369667
ENSG00000205581 5138.669471 5768.398408 5972.882097
4034.061319 3563.737488 3956.925301 5626.649992
3851.574702 0.546586951 2.11E-05 0.000552758 HMGN1
21 39342315 39349647 - 5102
protein_coding high mobility group nucleosome binding domain 1
[Source:HGNC Symbol;Acc:HGNC:4984] - 5664 6518 5138
3894 3122 4587 54.36400878 60.53413524
62.65568643 42.40992814 37.61315855 41.80414101
ENSG00000213442 1640.309746 2033.718862 2440.069973
2746.35248 3717.838884 3946.573632 2038.03286
3470.254999 -0.768462785 2.14E-05 0.000558146
RPL18AP3 12 104265309 104265836 + 528
processed_pseudogene ribosomal protein L18a pseudogene 3
[Source:HGNC Symbol;Acc:HGNC:31387] - 1808 2298 2099
2651 3257 4575 167.6846031 206.2255842
247.3348779 278.9894938 379.1675796 402.8915826
ENSG00000214866 5.443505796 10.61994184 6.974949899
40.4027713 79.90442797 61.24737221 7.679465844
60.5181905 -2.972622619 2.17E-05 0.000566673 DCDC2C
2 3703592 3847411 + 2242 protein_coding
doublecortin domain containing 2C [Source:HGNC Symbol;Acc:HGNC:32696]
- 6 12 6 39 70 71
0.131052203 0.253613331 0.166503163 0.966587206
1.919153852 1.472494762
ENSG00000146676 4830.204143 4540.025136 4089.645624
6320.443787 6342.128597 6768.265949 4486.624968
6476.946111 -0.529532706 2.24E-05 0.000583299 PURB
7 44876293 44885361 - 9069
protein_coding purine rich element binding protein B [Source:HGNC
Symbol;Acc:HGNC:9702] Others 5324 5130 3518 6101 5556
7846 28.74797963 26.80306157 24.13477641
37.38123683 37.6573882 40.22721068
ENSG00000154920 465.4197455 494.7122906 365.0223781
228.9490374 224.8738902 129.3958568 441.7181381
194.4062614 1.188175064 2.24E-05 0.000583894 EME1
17 50373220 50381483 + 3294
protein_coding essential meiotic structure-specific endonuclease 1
[Source:HGNC Symbol;Acc:HGNC:24965] - 513 559 314
221 197 150 7.626450466 8.041085017
5.930794824 3.728041365 3.67612264 2.117379394
ENSG00000085662 4683.229486 5716.183694 6869.163159
3424.911844 3800.026296 3771.457906 5756.192113

3665.465349	0.650865396	2.25E-05	0.00058488	AKR1B1
	7	134442350	134459284	-	3660
protein_coding	aldo-keto reductase family 1 member B [Source:HGNC
Symbol;Acc:HGNC:381]	-	5162	6459	5909	3306	3329
	4372	69.0662058	83.6200918	100.4476432
50.19191981	55.90878701	55.54309627
ENSG00000127824 1231.139561	1081.464077	1290.365731
1693.808489	1957.658485	1991.833556	1200.98979
1881.100177	-0.647942607	2.26E-05	0.000586193	TUBA4A
	2	219249711	219278170	-	4079
protein_coding	tubulin alpha 4a [Source:HGNC Symbol;Acc:HGNC:12407]
	-	1357	1222	1110	1635	1715	2309
16.29126567	14.19527928	16.930747	22.27286554
25.84388377	26.32093094
ENSG00000129173 489.0082707	348.6880903	390.5971944
242.4166278	141.5449867	134.5716911	409.4311851
172.8444352	1.245177861	2.28E-05	0.000589315	E2F8
	11	19224063	19241620	-	4140
protein_coding	E2F transcription factor 8 [Source:HGNC
Symbol;Acc:HGNC:24727]	E2F	539	394	336	234	124
	156	6.375541949	4.509436975	5.049469844
3.140707994	1.84106323	1.752085419
ENSG00000143742 4958.126529	4903.758143	4992.901636
3713.947054	3386.806254	3225.40739	4951.595436
3442.053566	0.524719622	2.28E-05	0.000589325	SRP9
	1	225777813	225790466	+	1728
protein_coding	signal recognition particle 9 [Source:HGNC
Symbol;Acc:HGNC:11304]	-	5465	5541	4295	3585	2967
	3739	154.8727863	151.939556	154.6415794
115.2809284	105.5409911	100.6103698
ENSG00000112893 603.3218924	574.3618544	707.9574148
271.4237457	337.8815811	383.0117361	628.5470538
330.7723543	0.924353004	2.29E-05	0.000590094	MAN2A1
	5	109689366	109869625	+	11815
protein_coding	mannosidase alpha class 2A member 1 [Source:HGNC
Symbol;Acc:HGNC:6824]	-	665	649	609	262	296
	444	2.756237282	2.602779705	3.206936889
1.232195473	1.53994548	1.74735144
ENSG00000122545 6741.781928	5953.362395	7770.094188
5157.051168	3752.083639	2832.043986	6821.74617
3913.726264	0.801700164	2.31E-05	0.00059513	7-Sep
	7	35800932	35907105	+	10906
protein_coding	septin 7 [Source:HGNC Symbol;Acc:HGNC:1717]	-
	7431	6727	6684	4978	3287	3283	33.36648448
	29.22687453	38.13096484	25.36304794	18.52599621
	13.99704657
ENSG00000145860 1645.753252	1498.296794	2349.395624
1085.694983	1060.445908	1055.870191	1831.148557
1067.337027	0.777926738	2.34E-05	0.000601667	RNF145
	5	159157409	159210053	-	4614

protein_coding	ring finger protein 145 [Source:HGNC
Symbol;Acc:HGNC:20853]	-	1814	1693	2021	1048	929
	1224	19.25255515	17.38624554	27.25182365
12.62105723	12.37614771	12.33487764
ENSG00000165480 938.0974988	754.0158705	856.756346
588.4301051	281.9484816	320.0390858	849.6232384
396.8058908	1.098419058	2.34E-05	0.000601667	SKA3
	13	21153595	21176602	-	3048
protein_coding	spindle and kinetochore associated complex subunit 3
[Source:HGNC Symbol;Acc:HGNC:20262]	-	1034	852	737
	568	247	371	16.61247191	13.24497287
15.04386307	10.35488826	4.981146912	5.659655087
ENSG00000123700 31.75378381	39.82478189	20.9248497
1.035968495	0	0	30.8344718	0.345322832
6.419805767	2.34E-05	0.000601667	KCNJ2	17
70168673	70180048	+	5577	protein_coding
potassium voltage-gated channel subfamily J member 2 [Source:HGNC
Symbol;Acc:HGNC:6263]	-	35	45	18	1	0
	0	0.307323721	0.382329941	0.200806935
0.009963488	0	0
ENSG00000216775 329.3321007	246.0286526	262.7231129
98.41700702	122.1396256	31.91764468	279.3612887
84.15809244	1.738254766	2.35E-05	0.000603638
AL109918.1	6	52664366	52669155	+	3821
	transcribed_unprocessed_pseudogene	uncharacterized
LOC730101 [Source:NCBI gene;Acc:730101] -	363	278	226
	95	107	37	4.652198864	3.447420018
3.679918904	1.381524564	1.721290216	0.450252056
ENSG00000100632 2264.498411	2793.044703	2969.003674
1666.873308	1817.25499	1686.459334	2675.515596
1723.529211	0.634311909	2.37E-05	0.000607282	ERH
	14	69380123	69398627	-	1446
protein_coding	"ERH, mRNA splicing and mitosis factor [Source:HGNC
Symbol;Acc:HGNC:3447]"	-	2496	3156	2554	1609	1592
	1955	84.52885216	103.4177637	109.8903222
61.83007806	67.67404419	62.86508208
ENSG00000175352 880.940688	772.6007687	826.5315631
1164.428588	1342.39439	2067.745792	826.6910066
1524.856257	-0.884029679	2.38E-05	0.000607282	NRIP3
	11	8980576 9004049 -	4123	protein_coding
nuclear receptor interacting protein 3 [Source:HGNC
Symbol;Acc:HGNC:1167]	-	971	873	711	1124	1176
	2397	11.53279517	10.03292001	10.72909554
15.14833916	17.53239906	27.03246923
ENSG00000001461 423.6862011	370.8129692	394.0846693
669.2356477	679.1876378	715.1277685	396.1946132
687.8503513	-0.796120128	2.39E-05	0.000609078	NIPAL3
	1	24415794	24472976	+	9404
protein_coding	NIPA like domain containing 3 [Source:HGNC
Symbol;Acc:HGNC:25233]	-	467	419	339	646	595

829	2.431828499	2.111192544	2.242817439
3.817085968	3.88912324	4.098952902
ENSG00000141429 1499.685847	1244.303185	1610.050935
986.2420071	861.8263303	775.5125017	1451.346656
874.5269464	0.730795266	2.44E-05	0.000621051	GALNT1
18	35581117	35711834	+	5126
protein_coding	polypeptide N-acetylgalactosaminyltransferase 1
[Source:HGNC Symbol;Acc:HGNC:4123]	-	1653	1406	1385
952	755	899	15.79148366	12.99670196
16.81039886	10.31977884	9.053483407	8.15477735
ENSG00000122483 662.2932052	440.7275863	320.8476954
236.2008168	194.0536108	182.8794776	474.6228289
204.3779684	1.217441322	2.45E-05	0.000623521	CCDC18
1	93179919	93278730	+	5633
protein_coding	coiled-coil domain containing 18 [Source:HGNC
Symbol;Acc:HGNC:30370]	-	730	498	276	228	170
212	6.346171326	4.18905471	3.048429653
2.249091579	1.855053889	1.749955995
ENSG00000221866 328.4248497	188.5039676	440.5843353
142.9636523	97.02680539	108.6925197	319.1710509
116.2276591	1.455179463	2.48E-05	0.000630646	PLXNA4
7	132123332	132648688	-	16372
protein_coding	plexin A4 [Source:HGNC Symbol;Acc:HGNC:9102]	-
362	213	379	138	85	126	1.082768264
0.616459157	1.440271346	0.46837035	0.319127735
0.357849016
ENSG00000164465 1083.257653	997.3895376	1232.241149
756.2570013	600.4247016	584.8692727	1104.296113
647.1836585	0.770581793	2.50E-05	0.000635395	DCBLD1
6	117453817	117569858	+	6819
protein_coding	"discoidin, CUB and LCCL domain containing 1
[Source:HGNC Symbol;Acc:HGNC:21479]"	-	1194	1127	1060
730	526	678	8.574569403	7.83122436
9.671459587	5.948592419	4.741463524	4.623171685
ENSG00000167526 50101.12009	69211.04595	74994.66132
85556.49412	113704.001	114486.8662	64768.94245
104582.4538	-0.691283472	2.52E-05	0.000638212	RPL13
16	89560657	89566828	+	6172
protein_coding	ribosomal protein L13 [Source:HGNC
Symbol;Acc:HGNC:10303]	-	55223	78205	64512	82586	99610
132717	438.1499457	600.3922032	650.3115019
743.5198339	992.0290339	999.8426465
ENSG00000164761 652.3134445	668.1713406	1024.155144
385.3802801	408.6540745	429.5942445	781.5466429
407.8761997	0.936457207	2.53E-05	0.000639433
TNFRSF11B	8	118923557	118952200	-	2827
protein_coding	TNF receptor superfamily member 11b
[Source:HGNC Symbol;Acc:HGNC:11909]	-	719	755	881
372	358	498	12.45465682	12.65457558
19.38906857	7.311882016	7.784031385	8.190955234

ENSG00000100664 6970.409172 7096.776133 6525.065631
9226.335416 10109.05163 9593.408823 6864.083645
9642.931956 -0.490278641 2.53E-05 0.000639433 EIF5
14 103333544 103345025 + 8505
protein_coding eukaryotic translation initiation factor 5
[Source:HGNC Symbol;Acc:HGNC:3299] - 7683 8019 5613
8906 8856 11121 44.23695231 44.67579982
41.06081553 58.18625642 64.00452076 60.79957464
ENSG00000146374 34.47553671 29.20484005 33.71225785
90.12925906 120.9981338 150.0991939 32.46421154
120.4088622 -1.893407944 2.55E-05 0.000643804 RSPO3
6 127118604 127197765 + 3179
protein_coding R-spondin 3 [Source:HGNC Symbol;Acc:HGNC:20866] -
38 33 29 87 106 174 0.585358262
0.491869454 0.567563315 1.520690274 2.049569726
2.545011438
ENSG00000167900 3031.125477 3151.46774 4111.732966
2235.620012 2163.127014 1230.985918 3431.442061
1876.577648 0.871098852 2.59E-05 0.000653146 TK1
17 78174075 78187233 - 1861
protein_coding thymidine kinase 1 [Source:HGNC Symbol;Acc:HGNC:11830]
- 3341 3561 3537 2158 1895 1427
87.91415093 90.66758359 118.2484708 64.43429942
62.5907566 35.65402939
ENSG00000163481 501.7097842 727.4660159 697.4949899
934.4435824 1231.669683 1340.541076 642.2235967
1168.88478 -0.864715342 2.63E-05 0.000661233 RNF25
2 218663864 218672411 - 2323
protein_coding ring finger protein 25 [Source:HGNC
Symbol;Acc:HGNC:14662] - 553 822 600 902 1079
1554 11.65747801 16.76675617 16.06974137
21.5759224 28.55088634 31.10518744
ENSG00000005893 4769.418328 3471.835986 5028.938877
3176.279405 2518.130973 1908.157568 4423.39773
2534.189315 0.803769178 2.64E-05 0.000662856 LAMP2
X 120427827 120469365 - 5526
protein_coding lysosomal associated membrane protein 2 [Source:HGNC
Symbol;Acc:HGNC:6501] - 5257 3923 4326 3066 2206
2212 46.58603836 33.63828675 48.70600187
30.82998501 24.53815965 18.61252355
ENSG00000115648 1666.620025 1749.635418 2322.658316
1390.26972 833.2890346 891.9687728 1912.971253
1038.509176 0.880904348 2.65E-05 0.000664615 MLPH
2 237485428 237555318 + 7514
protein_coding melanophilin [Source:HGNC Symbol;Acc:HGNC:29643]
- 1837 1977 1998 1342 730 1034
11.97199837 12.46699885 16.54364262 9.924151117
5.971714405 6.398532293
ENSG00000258807 3.629003864 7.079961225 3.48747495
47.65455077 25.11282022 84.53862644 4.73214668

52.43533247	-3.460531141	2.65E-05	0.000664615
AL359237.1	14	87710419	87872291	-	1845
lincRNA novel transcript	-	4	8	3
46	22	98	0.106167674	0.205456581
0.101165337	1.385394646	0.732948855	2.469793955
ENSG00000126777 5439.876792	3929.37848	4362.831162
3460.134773	2372.020019	2193.691092	4577.362145
2675.281961	0.775004152	2.67E-05	0.000668593	KTN1
14	55559072	55701526	+	17575
protein_coding	kinectin 1 [Source:HGNC Symbol;Acc:HGNC:6467]	-
5996	4440	3753	3340	2078	2543	16.70686541
11.97054924	13.28587241	10.55998197	7.267709669
6.727938991
ENSG00000158480 458.1617378	516.8371694	460.3466934
752.1131273	818.4496408	862.6390453	478.4485335
811.0672711	-0.761241525	2.70E-05	0.000674173	SPATA2
20	49903391	49915508	-	4270
protein_coding	spermatogenesis associated 2 [Source:HGNC
Symbol;Acc:HGNC:14681]	-	505	584	396	726	717
1000	5.791515015	6.480543243	5.769978003
9.447584595	10.32140075	10.88937974
ENSG00000008283 775.6995759	746.0509141	762.5945223
388.4881856	431.483911	496.017451	761.4483374
438.6631826	0.794587842	2.70E-05	0.000674173	CYB561
17	63432304	63446378	-	5320
protein_coding	cytochrome b561 [Source:HGNC Symbol;Acc:HGNC:2571]
-	855	843	656	375	378	575
7.87015283	7.508313248	7.671831465	3.916802552
4.367445837	5.025592032
ENSG00000234883 312.0943323	358.423037	213.8984636
473.4376022	660.9237685	722.0288809	294.8052776
618.7967505	-1.067527286	2.73E-05	0.000680545
MIR155HG	21	25561909	25575168	+	1600
lincRNA MIR155 host gene [Source:HGNC Symbol;Acc:HGNC:35460]
-	344	405	184	457	579	837
10.52851556	11.99392921	7.154918431	15.87114506
22.24364428	24.32408394
ENSG00000117054 1319.142905	1143.413738	1256.653474
854.6740083	732.8377537	709.0892952	1239.736705
765.5336857	0.695655885	2.75E-05	0.000683998	ACADM
1	75724347	75787575	+	4783
protein_coding	acyl-CoA dehydrogenase medium chain [Source:HGNC
Symbol;Acc:HGNC:89]	-	1454	1292	1081	825	642
822	14.88650368	12.79936941	14.06151646
9.584415025	8.250533032	7.991024364
ENSG00000133142 960.778773	1055.799218	1143.891783
663.0198367	482.8510433	671.9958163	1053.489925
605.9555654	0.796298959	2.85E-05	0.000706891	TCEAL4
X	103576231	103587736	+	2518
protein_coding	transcription elongation factor A like 4 [Source:HGNC

Symbol;Acc:HGNC:26121]	-	1059	1193	984	640	423
	779	20.59533772	22.44973189	24.31342935
14.1233035	10.32599717	14.38509552
ENSG00000174276 356.5496296	619.4966072	365.0223781
807.0194575	755.6675903	1339.678437	447.0228716
967.4551617	-1.113580731	2.89E-05	0.000716145	ZNHIT2
	11	65116403	65117708	-	1306
protein_coding	zinc finger HIT-type containing 2 [Source:HGNC
Symbol;Acc:HGNC:1177]	-	393	700	314	779	662
	1553	14.73594721	25.39693479	14.95868158
33.14410716	31.15746908	55.29161775
ENSG00000116171 1496.056843	1350.502604	1679.800434
967.5945742	989.673415	907.4962756	1508.786627
954.9214216	0.659720825	2.92E-05	0.000722226	SCP2
	1	52927229	53051703	+	8305
protein_coding	sterol carrier protein 2 [Source:HGNC
Symbol;Acc:HGNC:10606]	-	1649	1526	1445	934	867
	1052	9.723210823	8.706454559	10.82517827
6.249126025	6.416922895	5.889889148
ENSG00000162852 1056.947375	1015.089441	1027.642619
1511.478034	1714.520726	1532.909583	1033.226478
1586.302781	-0.618204695	2.93E-05	0.000723718	CNST
	1	246566444	246668584	+	5922
protein_coding	"consortin, connexin sorting protein [Source:HGNC
Symbol;Acc:HGNC:26486]" -	1165	1147	884	1459	1502
	1777	9.633546674	9.177437925	9.287326392
13.68985766	15.5900996	13.95243612
ENSG00000164161 111.5918688	29.20484005	82.53690714
9.323716454	10.27342645	0	74.44453867	6.532380969
	3.540441302	2.93E-05	0.000723718	HHIP	4
	144646021	144745271	+	12074	protein_coding
	hedgehog interacting protein [Source:HGNC
Symbol;Acc:HGNC:14866]	-	123	33	71	9	9
	0	0.498864527	0.129505797	0.365859237
0.04141936	0.045818271	0
ENSG00000065183 5485.23934	5632.99415	4688.328824
7648.555398	7584.071706	7473.042049	5268.854105
7568.556384	-0.522275909	2.93E-05	0.000723718	WDR3
	1	117929720	117966542	+	10275
protein_coding	WD repeat domain 3 [Source:HGNC Symbol;Acc:HGNC:12755]
	-	6046	6365	4033	7383	6644	8663
28.81475928	29.35235952	24.42042613	39.92666919
39.74615789	39.20283746
ENSG00000072864 840.1143945	685.8712437	971.8430193
500.372783	477.1435842	419.242576	832.6095525
465.5863144	0.838350166	2.94E-05	0.000724003	NDE1
	16	15643267	15726353	+	5512
protein_coding	nudE neurodevelopment protein 1 [Source:HGNC
Symbol;Acc:HGNC:17619]	-	926	775	836	483	418
	486	8.226790944	6.662219456	9.436347271

4.869114255	4.661379191	4.099756645
ENSG00000171658 4.53625483	5.309970919	3.48747495
35.22292883	38.81072216	66.42320649	4.444566899
46.81895249	-3.388746975	3.01E-05	0.000739546
NMRAL2P 3	185959943	185980872	+	2628
transcribed_unprocessed_pseudogene	"NmrA like redox sensor 2,
pseudogene [Source:HGNC Symbol;Acc:HGNC:52332]" -	5	6
	3	34	34	77	0.093169406	0.108181333
	0.07102361	0.718895226	0.795244943	1.362374112
ENSG00000148773 8504.570555	5434.755235	7228.373079
3087.186115	1662.012102	1280.156343	7055.899623
2009.784853	1.811860275	3.04E-05	0.000745851	MKI67
	10	128096659	128126385	-	12807
protein_coding	marker of proliferation Ki-67 [Source:HGNC
Symbol;Acc:HGNC:7107]	-	9374	6141	6218	2980	1456
	1484	35.84315441	22.72051278	30.207178
12.92947522	6.988135633	5.387874977
ENSG00000156509 121.5716294	115.9343651	105.7867401
33.15099184	36.5277385	25.01653231	114.4309115
31.56508755	1.863886113	3.12E-05	0.00076579	FBXO43
	8	100133360	100145800	-	3950
protein_coding	F-box protein 43 [Source:HGNC Symbol;Acc:HGNC:28521]
	-	134	131	91	32	32	29
1.661255326	1.571450266	1.433346345	0.450157952
0.497966633	0.341375163
ENSG00000130508 1789.098905	1189.433486	2613.281229
580.1423571	485.134027	219.1103175	1863.937873
428.1289005	2.122670969	3.16E-05	0.000773361	PXDN
	2	1631887 1744852 -	11257	protein_coding
peroxidasin [Source:HGNC Symbol;Acc:HGNC:14966] -	1972	1344
	2248	560	425	254	8.578531061	5.657219679
	12.42454483	2.764250549	2.320671262	1.049160832
ENSG00000124795 5883.522514	4919.688056	5197.500167
4216.391774	2703.052649	2640.538118	5333.570246
3186.660847	0.743164836	3.17E-05	0.000775752	DEK
	6	18223868	18264823	-	4361
protein_coding	DEK proto-oncogene [Source:HGNC Symbol;Acc:HGNC:2768]
	-	6485	5559	4471	4070	2368	3061
72.82031903	60.40001246	63.786009	51.85854961
33.37666745	32.63685192
ENSG00000169855 1813.594681	1354.042584	1889.048931
1165.464557	952.0041847	763.4355551	1685.562065
960.3014322	0.811931205	3.22E-05	0.000787186	ROBO1
	3	78597240	79767815	-	10875
protein_coding	roundabout guidance receptor 1 [Source:HGNC
Symbol;Acc:HGNC:10249]	-	1999	1530	1625	1125	834
	885	9.001444581	6.666357591	9.296745587
5.7482454	4.713941029	3.783946812
ENSG00000129038 2848.768033	2927.563967	4104.758016
2050.181651	2236.182491	1647.640576	3293.696672

1978.001573 0.735698286 3.23E-05 0.000787889 LOXL1
15 73925989 73952137 + 3637
protein_coding lysyl oxidase like 1 [Source:HGNC
Symbol;Acc:HGNC:6665] - 3140 3308 3531 1979 1959
1910 42.27805803 43.09715886 60.40338305
30.23531773 33.108423 24.41861819
ENSG00000105825 424.5934521 436.3026105 402.2221109
546.9913653 901.7785443 1078.298807 421.0393911
842.3562387 -1.00071947 3.26E-05 0.000793583 TFPI2
7 93885396 93890991 - 2682
protein_coding tissue factor pathway inhibitor 2 [Source:HGNC
Symbol;Acc:HGNC:11761] - 468 493 346 528 790
1250 8.545072722 8.709928434 8.026462332
10.93924106 18.10571492 21.67116494
ENSG00000163932 598.7856375 566.396898 791.6568136
382.2723746 355.0039586 241.5389327 652.279783
326.2717553 1.000279002 3.27E-05 0.000796117 PRKCD
3 53156009 53192717 + 3396
protein_coding protein kinase C delta [Source:HGNC
Symbol;Acc:HGNC:9399] - 660 640 681 369 311
280 9.517106681 8.929738338 12.47631344
6.037688852 5.629114306 3.833728628
ENSG00000171135 909.0654679 1400.062332 970.6805277
1571.564207 2012.450093 2191.103175 1093.269443
1925.039158 -0.815866407 3.29E-05 0.000798388 JAGN1
3 9890572 9894349 + 1781 protein_coding
jagunal homolog 1 [Source:HGNC Symbol;Acc:HGNC:26926] - 1002
1582 835 1517 1763 2540 27.55069035
42.0890381 29.16952806 47.32969491 60.84652118
66.31332668
ENSG00000150403 1389.001229 1266.428064 1710.025217
882.6451577 965.7020866 798.8037559 1455.151503
882.3836667 0.721616797 3.30E-05 0.000799764 TMCO3
13 113490995 113554590 + 10407
protein_coding transmembrane and coiled-coil domains 3 [Source:HGNC
Symbol;Acc:HGNC:20329] - 1531 1431 1471 852 846
926 7.204076562 6.515391546 8.794151939
4.549106286 4.996802322 4.137294637
ENSG00000133835 1536.883136 1217.753331 1397.314963
993.4937866 740.8281965 673.7210944 1383.98381
802.6810258 0.786315599 3.32E-05 0.000805397
HSD17B4 5 119452443 119637199 + 10664
protein_coding hydroxysteroid 17-beta dehydrogenase 4 [Source:HGNC
Symbol;Acc:HGNC:5213] - 1694 1376 1202 959 649
781 7.77896743 6.113990201 7.012795552
4.997013404 3.740864009 3.405351259
ENSG00000142856 954.4280162 809.7705651 848.6189044
550.0992708 533.0766838 470.1382797 870.9391619
517.7714114 0.751116309 3.33E-05 0.000805574
ITGB3BP 1 63440770 63593721 - 5459

protein_coding integrin subunit beta 3 binding protein [Source:HGNC Symbol;Acc:HGNC:6157] - 1052 915 730 531 467 545 9.436942939 7.942083351 8.31987138 5.404972273 5.258370059 4.642099298
ENSG00000140937 280.3405485 131.8642778 219.7109218 97.38103852 35.38624667 27.60444945 210.6385827 53.45724488 1.980652748 3.39E-05 0.000820037 CDH11 16 64943753 65126112 - 13381 protein_coding cadherin 11 [Source:HGNC Symbol;Acc:HGNC:1750] - 309 149 189 94 31 32 1.130833309 0.52762351 0.87877983 0.390347432 0.142403441 0.111196835
ENSG00000205542 20069.29862 23058.54871 28245.05962 17515.11934 13826.89051 15098.77121 23790.96898 15480.26036 0.619909906 3.42E-05 0.000825766 TMSB4X X 12975108 12977227 + 1705 protein_coding thymosin beta 4 X-linked [Source:HGNC Symbol;Acc:HGNC:11881] - 22121 26055 24297 16907 12113 17503 635.34418 724.0909758 886.6150866 551.0033166 436.6914552 477.3304365
ENSG00000162627 1220.252549 1192.973466 1803.024549 901.2925906 794.4783124 650.4298401 1405.416855 782.0669144 0.845393979 3.44E-05 0.00082875 SNX7 1 98661701 98760500 + 2067 protein_coding sorting nexin 7 [Source:HGNC Symbol;Acc:HGNC:14971] - 1345 1348 1551 870 696 754 31.86474822 30.90123639 46.6850865 23.387878 20.69741574 16.96140747
ENSG00000074410 2651.894574 3166.512658 3857.147294 5071.065783 5871.833964 4499.52526 3225.184842 5147.475002 -0.674694518 3.52E-05 0.000847415 CA12 15 63321378 63382161 - 7313 protein_coding carbonic anhydrase 12 [Source:HGNC Symbol;Acc:HGNC:1371] - 2923 3578 3318 4895 5144 5216 19.57320413 23.18308372 28.22849048 37.19368138 43.23672063 33.16446742
ENSG00000100162 479.935761 492.9423003 474.2965932 287.9992416 255.6941695 223.4235127 482.3915515 255.7056413 0.917004959 3.54E-05 0.000851838 CENPM 22 41938721 41947164 - 1778 protein_coding centromere protein M [Source:HGNC Symbol;Acc:HGNC:18352] - 529 557 408 278 224 259 14.56976674 14.84396355 14.2769439 8.68810544 7.743969288 6.773279943
ENSG00000005022 10933.28139 10302.22858 12699.05878 8457.646792 7642.28779 7132.299626 11311.52292 7744.078069 0.546575788 3.55E-05 0.000853222 SLC25A5 X 119468400 119471319 + 1500 protein_coding solute carrier family 25 member 5 [Source:HGNC Symbol;Acc:HGNC:10991] - 12051 11641 10924 8164 6695

8268	393.4236932	367.7269596	453.1033561
302.4292418	274.3510853	256.2950551
ENSG00000178913 2038.592921	1993.009085	2231.983968
2740.136669	3470.135158	3241.797532	2087.861991
3150.689786	-0.593869659	3.57E-05	0.00085508	TAF7
5	141260225	141320821	-	2723
protein_coding	TATA-box binding protein associated factor 7
[Source:HGNC Symbol;Acc:HGNC:11541]	-	2247	2252	1920
2645	3040	3758	40.40955935	39.18746639
43.86927266	53.97468031	68.62356919	64.1711988
ENSG00000214160 1685.672295	1900.084594	1587.963594
2497.720041	2501.008596	2753.543833	1724.573494
2584.090823	-0.583177365	3.58E-05	0.00085508	ALG3
3	184242301	184249548	-	3464
protein_coding	"ALG3, alpha-1,3- mannosyltransferase [Source:HGNC
Symbol;Acc:HGNC:23056]" -	1858	2147	1366	2411	2191
3192	26.26615543	29.36842132	24.53463846
38.67509335	38.8787107	42.8465657
ENSG00000143499 1134.063707	1077.039101	1016.017702
764.5447492	575.3118814	568.4791308	1075.706837
636.1119205	0.758460192	3.58E-05	0.00085508	SMYD2
1	214281102	214337131	+	4889
protein_coding	SET and MYND domain containing 2 [Source:HGNC
Symbol;Acc:HGNC:20982]	-	1250	1217	874	738	504
659	12.52041313	11.79497383	11.12239314
8.387805826	6.336622854	6.267529625
ENSG00000131408 1976.899855	2703.660193	2347.070641
3009.488478	4179.001583	4335.623842	2342.543563
3841.371301	-0.713594073	3.58E-05	0.00085508	NR1H2
19	50329653	50382982	+	3753
protein_coding	nuclear receptor subfamily 1 group H member 2
[Source:HGNC Symbol;Acc:HGNC:7965]	THR-like	2179	3055
2019	2905	3661	5026	28.431996	38.57083946
33.47068504	43.01100753	59.96095271	62.26943683
ENSG00000182446 5103.286684	5242.711287	4637.179191
6254.141804	7838.624384	8411.59333	4994.392387
7501.453173	-0.586805316	3.59E-05	0.00085508	NPLOC4
17	81556887	81648465	-	9574
protein_coding	"NPL4 homolog, ubiquitin recognition factor
[Source:HGNC Symbol;Acc:HGNC:18261]"	-	5625	5924	3989
6037	6867	9751	28.77118749	29.31892669
25.92253442	35.03803938	44.08805953	47.35728011
ENSG00000166965 578.8261163	707.1111273	782.3568804
420.6032089	373.2678278	350.2314524	689.4313747
381.3674964	0.853931851	3.61E-05	0.000859684	RCCD1
15	90954870	90963125	+	4969
protein_coding	RCC1 domain containing 1 [Source:HGNC
Symbol;Acc:HGNC:30457]	-	638	799	673	406	327
406	6.287534275	7.619109644	8.426610382
4.54013828	4.045070695	3.799164119

ENSG00000122565 7286.132508 6954.291913 6758.726452
5496.848834 4478.072442 4188.112565 6999.716958
4721.01128 0.568349707 3.63E-05 0.000862405 CBX3
7 26201162 26213356 + 4641
protein_coding chromobox 3 [Source:HGNC Symbol;Acc:HGNC:1553] -
8031 7858 5814 5306 3923 4855 84.73966466
80.22817199 77.94177746 63.52837124 51.95820031
48.64169318
ENSG00000136270 2555.725971 3244.392231 2677.21827
3795.788565 4478.072442 4653.937649 2825.778824
4309.266219 -0.608682852 3.64E-05 0.000864554 TBRG4
7 45100100 45112047 - 5803
protein_coding transforming growth factor beta regulator 4
[Source:HGNC Symbol;Acc:HGNC:17443] - 2817 3666 2303
3664 3923 5395 23.77184884 29.93410867
24.69154207 35.08447144 41.5540251 43.22847318
ENSG00000149273 25603.52951 35628.13487 35733.83083
42832.11742 51934.45371 56534.77511 32321.83174
50433.78208 -0.641912398 3.69E-05 0.000875326 RPS3
11 75399486 75422280 + 5894
protein_coding ribosomal protein S3 [Source:HGNC
Symbol;Acc:HGNC:10420] - 28221 40258 30739 41345 45497
65537 234.47198 323.644704 324.4788918
389.7848081 474.4822709 517.0201198
ENSG00000150556 126.1078843 130.0942875 172.0487642
25.89921237 61.64055872 21.56597613 142.750312
36.36858241 1.982396574 3.72E-05 0.000881357 LYPD6B
2 149038107 149215262 + 3771
protein_coding LY6/PLAUR domain containing 6B [Source:HGNC
Symbol;Acc:HGNC:27018] - 139 147 148 25 54
25 1.805040503 1.847086538 2.441810909
0.368379556 0.880206533 0.308258098
ENSG00000138092 712.1920083 645.1614666 684.7075818
402.9917445 424.6349601 334.7039496 680.6870189
387.4435514 0.814403099 3.74E-05 0.000883507 CENPO
2 24793136 24822376 + 5518
protein_coding centromere protein O [Source:HGNC
Symbol;Acc:HGNC:28152] - 785 729 589 389 372
388 6.966532125 6.259970304 6.641106506
3.91723791 4.143893678 3.269497786
ENSG00000067955 3852.187602 3907.253601 4173.345023
2829.22996 2794.371995 2780.285643 3977.595409
2801.295866 0.505673665 3.76E-05 0.000887476 CBFB
16 67029116 67101058 + 4129
protein_coding core-binding factor subunit beta [Source:HGNC
Symbol;Acc:HGNC:1539] CBF 4246 4415 3590 2731 2448
3223 50.35745699 50.66549218 54.09491121
36.75266711 36.44298068 36.29496992
ENSG00000196449 918.1379776 1157.57366 995.0928523
1652.369749 1656.304643 1512.206246 1023.601497

```
1606.960213	-0.650193501	3.79E-05	0.000893116	YRDC
	1	37802944	37808185	-	1826
protein_coding	yrdC N6-threonylcarbamoyltransferase domain containing
[Source:HGNC Symbol;Acc:HGNC:28905]	-	1012	1308	856
	1595	1451	1753	27.13991123	33.94168602
29.16619923	48.53689138	48.84431231	44.63876401
ENSG00000227051 966.2222788	896.5000901	1246.191049
643.3364353	626.6790137	520.1713443	1036.304473
596.7289311	0.796163483	3.83E-05	0.000901391
C14orf132	14	96039324	96093889	+	7775
	protein_coding	chromosome 14 open reading frame 132
[Source:HGNC Symbol;Acc:HGNC:20346]	-	1065	1013	1072
	621	549	603	6.707765842	6.173557372
8.578300078	4.438162976	4.340295584	3.606184419
ENSG00000166670 53.52780699	40.70977704	39.5247161
290.0711786	141.5449867	86.26390453	44.58743338
172.6266899	-1.950019127	3.83E-05	0.000901901	MMP10
	11	102770503	102780628	-	1758
protein_coding	matrix metallopeptidase 10 [Source:HGNC
Symbol;Acc:HGNC:7156]	-	59	46	34	280	124
	100	1.643470165	1.23983931	1.203280539
8.85016167	4.335609654	2.644917605
ENSG00000142798 1429.827522	1272.62303	2122.709753
932.3716454	999.9468415	692.6991534	1608.386768
875.0058801	0.878309732	3.85E-05	0.000904528	HSPG2
	1	21822245	21937297	-	18104
protein_coding	heparan sulfate proteoglycan 2 [Source:HGNC
Symbol;Acc:HGNC:5273]	-	1576	1438	1826	900	876
	803	4.262951148	3.763663488	6.27527957
2.762358318	2.974246269	2.062395833
ENSG00000019549 1090.515661	868.1802452	1274.090848
600.861727	695.1685234	554.6769061	1077.595585
616.9023855	0.804731212	3.87E-05	0.000908747	SNAI2
	8	48917604	48921740	-	3392
protein_coding	snail family transcriptional repressor 2 [Source:HGNC
Symbol;Acc:HGNC:11094]	zf-C2H2 1202	981	1096	580	609
	643	17.35310952	13.7037556	20.10303169
9.501325436	11.03592675	8.814265895
ENSG00000106397 1886.174758	2055.843741	2286.621075
1272.169312	1512.476672	1196.480356	2076.213191
1327.042113	0.646041629	3.92E-05	0.000917777	PLOD3
	7	101205977	101218420	-	5671
protein_coding	"procollagen-lysine,2-oxoglutarate 5-dioxygenase 3
[Source:HGNC Symbol;Acc:HGNC:9083]"	-	2079	2323	1967
	1228	1325	1387	17.95244172	19.40957399
21.58000591	12.0323585	14.36162529	11.37228754
ENSG00000165424 1555.028156	1314.217802	1915.786239
889.8969371	1103.822598	715.9904076	1595.010732
903.2366475	0.820897677	3.97E-05	0.000929706
ZCCHC24 10	79382325	79445627	-	5051
```

protein_coding zinc finger CCHC-type containing 24 [Source:HGNC
Symbol;Acc:HGNC:26911] -   1714   1485   1648   859   967
        830   16.61736398   13.93078296   20.29956285
9.449913625   11.76783216   7.640675261
ENSG00000142230 5036.150112   5088.72213   5757.821142
3822.723746   3633.368489   3687.781919   5294.231128
3714.624718   0.511002963   3.99E-05   0.000932508   SAE1
        19   47113274   47210636   +   3396
protein_coding SUMO1 activating enzyme subunit 1 [Source:HGNC
Symbol;Acc:HGNC:30660] -   5551   5750   4953   3690   3183
        4275   80.04463513   80.22811788   90.74182155
60.37688852   57.61244642   58.53282101
ENSG00000198440 88.0033437   56.6396898   58.12458249
225.8411319   165.5163151   179.4289214   67.58920533
190.2621228   -1.490981623   4.03E-05   0.000940736   ZNF583
        19   56397966   56436035   +   8060
protein_coding zinc finger protein 583 [Source:HGNC
Symbol;Acc:HGNC:26427] zf-C2H2 97   64   50   218   145
        208   0.589339263   0.376245551   0.385959566
1.502911804   1.10580951   1.199939394
ENSG00000204619 1022.471839   1149.608704   1019.505177
1456.571704   1769.312334   1766.684765   1063.861907
1664.189601   -0.64526414   4.04E-05   0.000940736
PPP1R11 6   30066709   30070333   +   2538
protein_coding protein phosphatase 1 regulatory inhibitor subunit 11
[Source:HGNC Symbol;Acc:HGNC:9285]   -   1127   1299   877
        1406   1550   2048   21.7450786   24.25179978
21.49882983   30.78263172   37.53940983   37.52056354
ENSG00000189326 0.907250966   3.539980612   2.3249833
21.75533839   20.54685291   84.53862644   2.257404959
42.28027258   -4.230450857   4.04E-05   0.000940736
SPANXN4 X   143025918   143034702   +   625
protein_coding SPANX family member N4 [Source:HGNC
Symbol;Acc:HGNC:33177] -   1   4   2   21   18
        98   0.078351744   0.303253914   0.199093382
1.867030106   1.770271379   7.290831755
ENSG00000140511 342.9408651   308.8633084   418.496994
165.7549592   190.6291353   151.824472   356.7670558
169.4028555   1.07493996   4.08E-05   0.000947439   HAPLN3
        15   88877288   88895626   -   3524
protein_coding hyaluronan and proteoglycan link protein 3
[Source:HGNC Symbol;Acc:HGNC:21446]   -   378   349   360
        160   167   176   5.252724023   4.692626276
6.355847197   2.522877285   2.912915803   2.322243662
ENSG00000100836 635.9829272   1080.579082   828.8565464
306.6466745   392.6731889   540.8746814   848.4728518
413.3981816   1.03601478   4.10E-05   0.000951073   PABPN1
        14   23321289   23326185   +   3876
protein_coding poly(A) binding protein nuclear 1 [Source:HGNC
Symbol;Acc:HGNC:8565]   -   701   1221   713   296   344

627	8.85651644	14.92651207	11.44491596
4.243458765	5.455342657	7.521678919
ENSG00000144152 137.9021468	157.5291373	165.0738143
50.76245625	18.26386925	1.725278091	153.5016995
23.58386786	2.710819276	4.12E-05	0.000956017	FBLN7
2	112138385	112188216	+	6578
protein_coding	fibulin 7 [Source:HGNC Symbol;Acc:HGNC:26740]	-
152	178	142	49	16	2	1.131562124
1.282190557	1.343078268	0.413917944	0.149511113
0.014137322
ENSG00000132481 509.8750429	538.0770531	789.3318303
379.1644691	260.2601368	251.8906012	612.4279754
297.1050691	1.042722254	4.14E-05	0.000957757	TRIM47
17	75874161	75878575	-	3142
protein_coding	tripartite motif containing 47 [Source:HGNC
Symbol;Acc:HGNC:19020]	-	562	608	679	366	228
292	8.759086564	9.16903941	13.44529824
6.472721905	4.460422794	4.321233048
ENSG00000205544 64.41481858	110.6243941	155.7738811
4.14387398	19.40536108	31.05500563	110.2710313
18.20141356	2.5872261	4.17E-05	0.000965061
TMEM256 17	7402975 7404137 -	707	protein_coding
transmembrane protein 256 [Source:HGNC Symbol;Acc:HGNC:28618]	-
71	125	134	4	17	36	4.917763265
8.377550222	11.79212926	0.314378343	1.478008283
2.367631477
ENSG00000141756 4665.991718	6018.852036	6215.842852
3568.911465	4096.814171	3439.341874	5633.562202
3701.68917	0.605922148	4.19E-05	0.000966842	FKBP10
17	41812680	41823217	+	4273
protein_coding	FK506 binding protein 10 [Source:HGNC
Symbol;Acc:HGNC:18169]	-	5143	6801	5347	3445	3589
3987	58.94029634	75.41649122	77.85457493
44.79900591	51.62831198	43.38547543
ENSG00000234741 3235.256945	4194.877026	2915.529058
5814.891162	5303.371034	4973.114096	3448.554343
5363.792097	-0.636849912	4.21E-05	0.000971452	GAS5
1	173863900	173868882	-	3631
processed_transcript	growth arrest specific 5 [Source:HGNC
Symbol;Acc:HGNC:16355]	-	3566	4740	2508	5613	4646
5765	48.09321092	61.85553016	42.97423257
85.89756188	78.65028791	73.82510628
ENSG00000166734 2385.16279	1872.649744	2329.633266
1591.247608	1294.451733	1256.00245	2195.815267
1380.567264	0.66944449	4.23E-05	0.000975392	CASC4
15	44288729	44415758	+	7095
protein_coding	cancer susceptibility 4 [Source:HGNC
Symbol;Acc:HGNC:24892]	-	2629	2116	2004	1536	1134
1456	18.14541352	14.13154691	17.5732531
12.02959094	9.824444757	9.542012767

ENSG00000173465 689.5107341 1015.089441 888.1436205
1044.256243 1872.046598 2171.262477 864.2479318
1695.855106 -0.972802782 4.30E-05 0.000988017 SSSCA1
11 65570430 65573942 + 2008
protein_coding Sjogren syndrome/scleroderma autoantigen 1
[Source:HGNC Symbol;Acc:HGNC:11328] - 760 1147 764
1008 1640 2517 18.53440153 27.06612918
23.67208419 27.89387609 50.2027491 58.28415778
ENSG00000081059 88.91059467 58.40968011 166.2363059
27.97114936 15.98088559 3.450556181 104.5188602
15.80086371 2.735497414 4.30E-05 0.000988017 TCF7
5 134114711 134151865 + 10063
protein_coding transcription factor 7 [Source:HGNC
Symbol;Acc:HGNC:11639] HMG 98 66 143 27 14
4 0.476899961 0.310772731 0.884128543
0.149089938 0.085516107 0.01848262
ENSG00000023902 1917.021291 2294.792432 2327.308283
1456.571704 1506.769213 1322.425656 2179.707335
1428.588858 0.609710298 4.34E-05 0.0009949
PLEKHO1 1 150149183 150164720 + 6565
protein_coding pleckstrin homology domain containing O1 [Source:HGNC
Symbol;Acc:HGNC:24310] - 2113 2593 2002 1406 1320
1533 15.76135134 18.71518943 18.97300798
11.90042945 12.35909188 10.85771512
ENSG00000236938 1.814501932 4.424975766 1.16249165
60.0861727 39.95221399 20.70333709 2.467323116
40.24724126 -3.99387745 4.40E-05 0.001008025
AC003092.2 7 94071759 94077157 - 446
lincRNA novel transcript - 2 5 1
58 35 24 0.219595694 0.531204306
0.139499287 7.226120152 4.823702842 2.502115776
ENSG00000251361 73.48732824 55.75469465 56.96209084
10.35968495 0 8.626390453 62.06803791 6.328691801
3.271115085 4.42E-05 0.001011586 AC012625.1
5 93019663 93068669 - 625
lincRNA novel transcript - 81 63 49 10
0 10 6.346491242 4.776249145 4.877787869
0.889061955 0 0.743962424
ENSG00000164077 650.4989426 816.8505263 691.6825317
1068.083518 1119.803483 1552.750281 719.6773335
1246.879094 -0.793349521 4.47E-05 0.001020262 MON1A
3 49907160 49930173 - 6446
protein_coding "MON1 homolog A, secretory trafficking associated
[Source:HGNC Symbol;Acc:HGNC:28207]" - 717 923 595
1031 981 1800 5.447002041 6.784812349
5.742929847 8.887516249 9.35461819 12.98414097
ENSG00000177565 3371.34459 2755.874907 3124.777555
2290.526342 1965.648928 1683.871416 3083.999017
1980.015562 0.639584377 4.47E-05 0.001020262
TBL1XR1 3 177019340 177228000 - 11603

```
protein_coding	transducin beta like 1 X-linked receptor 1
[Source:HGNC Symbol;Acc:HGNC:29529]	-	3716	3114	2688
	2211	1722	1952	15.6831789	12.71670969
14.4133794	10.58840377	9.122423185	7.822409353
ENSG00000081320 2656.430828	1843.444904	2372.645457
1642.010064	1246.509076	882.4797433	2290.840397
1256.999628	0.866401589	4.49E-05	0.00102358	STK17B
	2	196133566	196176503	-	6580
protein_coding	serine/threonine kinase 17b [Source:HGNC
Symbol;Acc:HGNC:11396]	-	2928	2083	2041	1585	1092
	1023	21.7908345	14.9999502	19.29851793
13.38490881	10.20103188	7.229042171
ENSG00000178773 3.629003864	14.15992245	1.16249165
30.04308635	78.76293614	89.71446071	6.317139321
66.1734944	-3.358994427	4.50E-05	0.001024554	CPNE7
	16	89575768	89597246	+	3048
protein_coding	copine 7 [Source:HGNC Symbol;Acc:HGNC:2320]	-
	4	16	1	29	69	104	0.064264882
	0.248731885	0.020412297	0.528682675	1.391494481
	1.586534041
ENSG00000173039 2517.621431	2899.244122	3241.02672
3598.954551	5355.879658	4955.861315	2885.964091
4636.898508	-0.684340463	4.51E-05	0.001024554	RELA
	11	65653596	65663094	-	5464
protein_coding	"RELA proto-oncogene, NF-kB subunit [Source:HGNC
Symbol;Acc:HGNC:9955]"	RHD	2775	3276	2788	3474	4692
	5745	24.8702975	28.4092418	31.74599367
35.32898555	52.78307324	48.88891066
ENSG00000074696 2680.926604	2699.235217	3024.803273
1936.225117	2013.591585	1728.728647	2801.655032
1892.84845	0.565854154	4.53E-05	0.001029582	HACD3
	15	65530418	65578352	+	7097
protein_coding	3-hydroxyacyl-CoA dehydratase 3 [Source:HGNC
Symbol;Acc:HGNC:24175]	-	2955	3050	2602	1869	1764
	2004	20.38972477	20.36345546	22.81073786
14.63344366	15.27816288	13.12967361
ENSG00000035862 10141.2513	8144.610394	12948.99449
5159.123105	3863.949838	2424.878356	10411.61873
3815.983766	1.448121648	4.60E-05	0.001043146	TIMP2
	17	78852977	78925387	-	5633
protein_coding	TIMP metallopeptidase inhibitor 2 [Source:HGNC
Symbol;Acc:HGNC:11821]	-	11178	9203	11139	4980	3385
	2811	97.17466175	77.41339456	123.0306446
49.12489501	36.93739656	23.20342595
ENSG00000259807 51.71330506	51.32971888	58.12458249
4.14387398	5.707459141	10.35166854	53.72253548
6.734333888	2.977811496	4.61E-05	0.001043146
AC009093.1	16	29217170	29220031	-	2099
	lincRNA	novel transcript	-	57	58	50
	4	5	12	1.329814612	1.309308526
```

1.482055312 0.105891133 0.146421525 0.26582745
ENSG00000168939 71.67282631 42.47976735 49.98714095
112.9205659 182.6386925 232.0499032 54.71324487
175.8697205 -1.684621077 4.63E-05 0.001048396 SPRY3
X 155767812 155782459 + 9019
protein_coding sprouty RTK signaling antagonist 3 [Source:HGNC
Symbol;Acc:HGNC:11271] - 79 48 43 109 160
269 0.42894083 0.252179217 0.296631259
0.671552785 1.090458033 1.386835376
ENSG00000140525 2172.866064 1762.02535 2156.422011
1546.700963 978.2584967 827.2708444 2030.437808
1117.410101 0.861917788 4.72E-05 0.001065642 FANCI
15 89243949 89317261 + 7766
protein_coding FA complementation group I [Source:HGNC
Symbol;Acc:HGNC:25568] - 2395 1991 1855 1493 857
959 15.10208169 12.14787501 14.86118273
10.68253846 6.783140237 5.741855239
ENSG00000140612 1643.031499 1777.955263 2350.558116
1319.823863 1155.18973 958.3919793 1923.848293
1144.468524 0.749217047 4.72E-05 0.001065642 SEC11A
15 84669538 84716716 - 5748
protein_coding "SEC11 homolog A, signal peptidase complex subunit
[Source:HGNC Symbol;Acc:HGNC:17718]" - 1811 2009 2022
1274 1012 1111 15.42873694 16.56111672
21.88624409 12.31585911 10.8220893 8.987280935
ENSG00000106333 1799.985917 2024.86891 3010.853373
715.85423 725.9888027 264.8301869 2278.5694
568.8910732 2.002387646 4.74E-05 0.001067805 PCOLCE
7 100602177 100608175 + 3532
protein_coding procollagen C-endopeptidase enhancer [Source:HGNC
Symbol;Acc:HGNC:8738] - 1984 2288 2590 691 636
307 27.5074072 30.69458501 45.62321812
10.87099751 11.06837286 4.041556911
ENSG00000186416 910.8799698 889.4201289 769.5694722
1325.003705 1225.962223 1644.19002 856.6231903
1398.385316 -0.707043023 4.87E-05 0.001096182 NKRF
X 119588337 119606443 - 4552
protein_coding NFKB repressing factor [Source:HGNC
Symbol;Acc:HGNC:19374] Others 1004 1005 662 1279 1074
1906 10.80090492 10.46141063 9.048208147
15.6127834 14.50271753 19.46935935
ENSG00000136068 1417.126009 1214.21335 1613.53841
913.7242125 909.7689871 600.3967755 1414.959256
807.963325 0.809126646 4.95E-05 0.001111229 FLNB
3 58008400 58172251 + 13952
protein_coding filamin B [Source:HGNC Symbol;Acc:HGNC:3755] -
1562 1372 1388 882 797 696 5.482431896
4.659551161 6.189560968 3.512725078 3.511310329
2.319550276
ENSG00000116977 535.2780699 463.7374602 463.8341683

833.9546384	786.4878696	804.8422292	487.6165662
808.4282458	-0.728958014	4.95E-05	0.001111229	LGALS8
	1	236518000	236552981	+	9710
protein_coding	galectin 8 [Source:HGNC Symbol;Acc:HGNC:6569]	-
	590	524	399	805	689	933	2.97551035
	2.557045747	2.556586624	4.606686882	4.361615246
	4.467796998
ENSG00000114023 1066.927136	1223.063302	1406.614896
1706.240111	2055.826783	2102.251353	1232.201778
1954.772749	-0.666581067	4.96E-05	0.001111229
FAM162A 3	122384176	122412334	+	3793
protein_coding	family with sequence similarity 162 member A
[Source:HGNC Symbol;Acc:HGNC:17865]	-	1176	1382	1210
	1647	1801	2437	15.18284515	17.26440602
19.84766286	24.128082	29.18624544	29.87471044
ENSG00000167658 56230.50762	55801.59939	64778.6847
74669.50121	87307.00248	89552.28457	58936.93057
83842.92942	-0.508540129	5.04E-05	0.001127522	EEF2
	19	3976056 3985469 -	4027	protein_coding
eukaryotic translation elongation factor 2 [Source:HGNC
Symbol;Acc:HGNC:3214]	-	61979	63053	55724	72077	76485
	103812	753.6880315	741.9088743	860.9293241
994.5511322	1167.459979	1198.662577
ENSG00000151503 3546.444026	3113.412949	3170.114729
2572.309773	1795.566646	1794.289214	3276.657235
2054.055211	0.673904962	5.11E-05	0.001142526	NCAPD3
	11	134150119	134225454	-	9114
protein_coding	non-SMC condensin II complex subunit D3 [Source:HGNC
Symbol;Acc:HGNC:28952]	-	3909	3518	2727	2483	1573
	2080	21.00319332	18.28998089	18.61585383
15.13839172	10.60881946	10.61170892
ENSG00000143756 2103.91499	2000.089046	1668.175518
2827.158023	2762.410224	3073.582918	1924.059851
2887.717055	-0.585378061	5.12E-05	0.001143019	FBXO28
	1	224114087	224162047	+	6044
protein_coding	F-box protein 28 [Source:HGNC Symbol;Acc:HGNC:29046]
	-	2319	2260	1435	2729	2420	3563
18.78905668	17.71782567	14.7718297	25.089449
24.6115106	27.41084254
ENSG00000010404 5944.308329	5872.827836	5790.370908
8903.113245	7564.666345	8254.593024	5869.169024
8240.790872	-0.489649541	5.15E-05	0.001148024	IDS
	X	149476990	149521096	-	9292
protein_coding	iduronate 2-sulfatase [Source:HGNC
Symbol;Acc:HGNC:5389]	-	6552	6636	4981	8594	6627
	9569	34.529745	33.83947504	33.35140907
51.39231626	43.83844387	47.88377391
ENSG00000152234 6901.458098	6482.589497	8498.976452
5433.654756	4732.62512	3789.573326	7294.341349
4651.951067	0.648971882	5.16E-05	0.001148024

ATP5F1A 18	46080248	46104334	-	10908
protein_coding	ATP synthase F1 subunit alpha [Source:HGNC
Symbol;Acc:HGNC:823]	-	7607	7325	7311	5245	4146
	4393	34.15049245	31.81917686	41.70023489
26.71852055	23.36315707	18.72608939
ENSG00000166130 920.8597305	659.3213891	795.1442885
511.7684365	430.3424192	365.7589552	791.775136
435.9566036	0.861706403	5.16E-05	0.001148024	IKBIP
	12	98613405	98645113	-	4163
protein_coding	IKBKB interacting protein [Source:HGNC
Symbol;Acc:HGNC:26430]	-	1015	745	684	494	377
	424	11.9395598	8.479618285	10.22248631
6.59375159	5.566501104	4.735768493
ENSG00000010165 2202.805345	2508.961259	2105.272378
3002.236698	3487.257535	3740.4029	2272.346327
3409.965711	-0.585416614	5.22E-05	0.001160138
METTL13 1	171781664	171814023	+	3827
protein_coding	methyltransferase like 13 [Source:HGNC
Symbol;Acc:HGNC:24248]	-	2428	2835	1811	2898	3055
	4336	31.06840113	35.10112548	29.44196789
42.07769706	49.06819839	52.6819485
ENSG00000158321 949.8917614	945.1748235	756.7820641
595.6818846	463.4456822	319.1764468	883.9495497
459.4346712	0.946059384	5.23E-05	0.001160138	AUTS2
	7	69598296	70793495	+	24197
protein_coding	"AUTS2, activator of transcription and developmental
regulator [Source:HGNC Symbol;Acc:HGNC:14262]"	-	1047	1068
	651	575	406	370	2.11891649	2.091395499
	1.673887671	1.320439063	1.031363765	0.711002647
ENSG00000124813 1100.495422	946.0598187	1163.654142
726.2139149	597.0002261	634.0396983	1070.069794
652.4179464	0.71320401	5.31E-05	0.001177005	RUNX2
	6	45328157	45664349	+	8192
protein_coding	runt related transcription factor 2 [Source:HGNC
Symbol;Acc:HGNC:10472]	Runt	1213	1069	1001	701	523
	735	7.251027309	6.183212929	7.602404625
4.754886098	3.924272034	4.171847394
ENSG00000138411 609.6726491	650.4714375	452.2092518
1039.0764	908.6274952	992.0349021	570.7844462
979.9129326	-0.778156216	5.35E-05	0.001184421	HECW2
	2	196189099	196593692	-	23683
protein_coding	"HECT, C2 and WW domain containing E3 ubiquitin
protein ligase 2 [Source:HGNC Symbol;Acc:HGNC:29853]"	-	672
	735	389	1003	796	1150	1.389508608
1.470540754	1.021926669	2.353294402	2.065968583
2.257834701
ENSG00000083845 25465.62736	30269.48922	33112.41216
38855.03437	46667.61041	44098.10799	29615.84291
43206.91759	-0.54492582	5.37E-05	0.001187215	RPS5
	19	58386400	58394806	+	3617

protein_coding	ribosomal protein S5 [Source:HGNC
Symbol;Acc:HGNC:10426]	-	28069	34203	28484	37506	40883
	51120	380.020579	448.0661468	489.9585209
576.1880995	694.7708813	657.1633798
ENSG00000130487 1.814501932	1.769990306	6.974949899
38.33083431	30.82027936	60.38473317	3.519814046
43.17861561	-3.669003967	5.38E-05	0.001187215
KLHDC7B 22	50545891	50551023	+	5133
protein_coding	kelch domain containing 7B [Source:HGNC
Symbol;Acc:HGNC:25145]	-	2	2	6	37	27
	70	0.019080397	0.018462273	0.07272552
0.400536873	0.323325427	0.634100059
ENSG00000162772 141.5311507	90.26950562	73.23697394
295.251021	307.0613018	199.2696195	101.6792101
267.1939808	-1.388925056	5.49E-05	0.001210663	ATF3
	1	212565334	212620777	+	4040
protein_coding	activating transcription factor 3 [Source:HGNC
Symbol;Acc:HGNC:785]	TF_bZIP 156	102	63	285	269
	231	1.890914607	1.196314172	0.970210635
3.91990497	4.092778818	2.658652846
ENSG00000236453 55.34230892	69.02962194	27.8997996
152.2873688	254.5526777	124.2200225	50.75724349
177.020023	-1.79259661	5.50E-05	0.001211947
AC003092.1	7	94022833	94066661	+	1064
	lincRNA novel transcript	-	61	78	24
	147	223	144	2.807481419	3.473596876
1.403383805	7.676933002	12.88280981	6.292915241
ENSG00000113070 792.0300933	896.5000901	1052.054943
581.1783256	454.3137476	555.5395452	913.5283755
530.3438728	0.78299066	5.55E-05	0.001222148	HBEGF
	5	140332843	140346631	-	3018
protein_coding	heparin binding EGF like growth factor [Source:HGNC
Symbol;Acc:HGNC:3059]	-	873	1013	905	561	398
	644	14.165232	15.9043766	18.65675852
10.32893797	8.10608581	9.921964071
ENSG00000151693 575.1971124	428.3376541	570.7834001
337.7257293	186.063168	222.5608737	524.7727222
248.783257	1.076032398	5.58E-05	0.001225859	ASAP2
	2	9206765 9405683 +	7257
protein_coding	"ArfGAP with SH3 domain, ankyrin repeat and PH domain
2 [Source:HGNC Symbol;Acc:HGNC:2721]"	-	634	484	491
	326	163	258	4.278197389	3.160200805
4.209506802	2.496160581	1.380631702	1.65307897
ENSG00000229056 268.5462859	247.7986429	266.2105878
141.9276838	59.35757507	94.89029498	260.8518389
98.72518462	1.3997431	5.70E-05	0.001251055
AC020571.1	2	196260024	196264204	+	1607
	antisense	uncharacterized LOC101927482 [Source:NCBI
gene;Acc:101927482]	-	296	280	229	137	52
	110	9.019958052	8.2559793	8.865973976

4.737145608   1.989000201   3.182788839
ENSG00000144366 300.3000697   189.3889628   225.5233801
533.5237749   461.1626986   438.220635   238.4041375
477.6357028   -1.001855008   5.71E-05   0.001251788   GULP1
        2   188291669   188595931   +   11979
protein_coding "GULP, engulfment adaptor PTB domain containing 1
[Source:HGNC Symbol;Acc:HGNC:18649]"   -   331   214   194
        515   404   508   1.353119374   0.846485746
1.007599658   2.388904056   2.073042283   1.97185132
ENSG00000196235 4262.265038   4898.448173   4537.20491
5807.639382   6722.245376   8098.455357   4565.972707
6876.113372   -0.590799149   5.73E-05   0.001253569   SUPT5H
        19   39436156   39476670   +   8568
protein_coding "SPT5 homolog, DSIF elongation factor subunit
[Source:HGNC Symbol;Acc:HGNC:11469]"   -   4698   5535   3903
        5606   5889   9388   26.85110966   30.61009012
28.34170283   36.35680235   42.24832124   50.94770685
ENSG00000152661 5962.453348   5674.588922   9070.922344
4564.477189   4423.280834   3547.171754   6902.654871
4178.309926   0.724139454   5.74E-05   0.001254944   GJA1
        6   121435577   121449727   +   3413
protein_coding gap junction protein alpha 1 [Source:HGNC
Symbol;Acc:HGNC:4274]   -   6572   6412   7803   4406   3875
        4112   94.29527904   89.01919574   142.2434133
71.73320713   69.78832562   56.0206103
ENSG00000100442 719.450016   669.9413309   803.2817301
404.027713   436.0498784   453.7481378   730.8910257
431.2752431   0.760002739   5.75E-05   0.001256279   FKBP3
        14   45115600   45135319   -   2262
protein_coding FK506 binding protein 3 [Source:HGNC
Symbol;Acc:HGNC:3719]   -   793   757   691   390   382
        526   17.16758753   15.85731742   19.00606864
9.580409   10.38049641   10.81245123
ENSG00000153774 892.7349505   1133.678791   1175.279058
695.1348601   609.5566362   630.5891421   1067.230933
645.0935461   0.725760681   5.78E-05   0.001261353   CFDP1
        16   75293698   75433485   -   3317
protein_coding craniofacial development protein 1 [Source:HGNC
Symbol;Acc:HGNC:1873]   -   984   1281   1011   671   534
        731   14.52707941   18.29911553   18.96323953
11.24058961   9.8956231   10.24714599
ENSG00000078177 411.8919386   284.0834442   328.9851369
607.077538   614.1226036   643.5287278   341.6535065
621.5762898   -0.86334193   5.80E-05   0.001262444   N4BP2
        4   40056826   40158252   +   9835
protein_coding NEDD4 binding protein 2 [Source:HGNC
Symbol;Acc:HGNC:29851] -   454   321   283   586   538
        746   2.260529465   1.546525584   1.790271582
3.310817907   3.362445638   3.526918965
ENSG00000108055 3451.182675   2739.944994   3070.140447

2395.15916	1575.258723	1697.673641	3087.089372
1889.363841	0.708340524	5.91E-05	0.0012851	SMC3
	10	110567691	110604636	+	4275
protein_coding	structural maintenance of chromosomes 3 [Source:HGNC
Symbol;Acc:HGNC:2468]	-	3804	3096	2641	2312	1380
	1968	43.57456625	34.31557447	38.43608355
30.05133393	19.84222306	21.40523466
ENSG00000178999 1659.362017	1762.910345	1990.185705
716.8901985	574.1703896	237.2257375	1804.152689
509.4287752	1.824989804	5.92E-05	0.001287552	AURKB
	17	8204733 8210600 -	2241	protein_coding	aurora
kinase B [Source:HGNC Symbol;Acc:HGNC:11390]	-	1829	1992
	1712	692	503	275	39.96690632	42.11859916
	47.53010245	17.15837999	13.79664497	5.705869774
ENSG00000144559 389.2106644	364.6180031	428.9594188
554.2431448	773.9314595	819.507093	394.2626954
715.8938991	-0.861798816	6.01E-05	0.001305061	TAMM41
	3	11790442	11846919	-	5925
protein_coding	TAM41 mitochondrial translocator assembly and
maintenance homolog [Source:HGNC Symbol;Acc:HGNC:25187] -	429
	412	369	535	678	950	3.545664352
3.294847377	3.874760449	5.017385507	7.033778686
7.45531965
ENSG00000168542 113.4063707	115.0493699	254.5856713
34.18696033	60.49906689	12.07694663	161.013804
35.58765795	2.186895072	6.12E-05	0.001326927	COL3A1
	2	188974320	189012746	+	6811
protein_coding	collagen type III alpha 1 chain [Source:HGNC
Symbol;Acc:HGNC:2201]	-	125	130	219	33	53
	14	0.898727056	0.904396583	2.000507027
0.269224825	0.478313182	0.095575851
ENSG00000154133 700.3977457	762.865822	749.8071142
1136.457439	1041.040547	1671.79447	737.6902273
1283.097485	-0.799576129	6.14E-05	0.001328433	ROBO4
	11	124883691	124898500	-	7033
protein_coding	roundabout guidance receptor 4 [Source:HGNC
Symbol;Acc:HGNC:17985]	-	772	862	645	1097	912
	1938	5.375332909	5.807551761	5.705923488
8.667184744	7.970793925	12.81280373
ENSG00000141543 2618.326288	3320.501815	3122.452572
4297.197317	4528.298082	4403.772326	3020.426891
4409.755908	-0.545996994	6.16E-05	0.001331451	EIF4A3
	17	80135214	80147183	-	4967
protein_coding	eukaryotic translation initiation factor 4A3
[Source:HGNC Symbol;Acc:HGNC:18683]	-	2886	3752	2686
	4148	3967	5105	28.45318256	35.79275358
33.64485763	46.40412963	49.09252847	47.78951297
ENSG00000125648 594.2493827	631.8865393	530.0961924
997.6376606	924.6083808	917.8479442	585.4107048
946.6979952	-0.692419709	6.16E-05	0.001331451

SLC25A23	19	6436079 6465203 -	4859	protein_coding
	solute carrier family 25 member 23 [Source:HGNC
Symbol;Acc:HGNC:19375]	-	655	714	456	963	810
	1064	6.601202941	6.962701127	5.83881601
11.01263973	10.24673442	10.18182778
ENSG00000083857 2778.002458	2193.902985	3352.625918
1950.728676	1566.126788	1597.607512	2774.843787
1704.820992	0.702362507	6.24E-05	0.001344392	FAT1
	4	186587783	186726722	-	16177
protein_coding	FAT atypical cadherin 1 [Source:HGNC
Symbol;Acc:HGNC:3595]	-	3062	2479	2884	1883	1372
	1852	9.269064076	7.261142872	11.0918533
6.467916107	5.21318919	5.323215094
ENSG00000128342 108.8701159	130.9792827	125.5490982
32.11502334	34.24475485	4.313195226	121.7994989
23.5576578	2.384629368	6.24E-05	0.001344392	LIF
	22	30240447	30246851	-	3969
protein_coding	"LIF, interleukin 6 family cytokine [Source:HGNC
Symbol;Acc:HGNC:6596]"	-	120	148	108	31	30
	5	1.480569609	1.76688001	1.692970939
0.434002907	0.464608891	0.058576029
ENSG00000129460 661.3859542	750.4758898	670.757682
978.9902277	1244.226093	1140.408818	694.2065087
1121.208379	-0.691116405	6.45E-05	0.001387002	NGDN
	14	23469688	23509862	+	4654
protein_coding	neuroguidin [Source:HGNC Symbol;Acc:HGNC:20271] -
	729	848	577	945	1090	1322	7.670608775
	8.633679329	7.713585199	11.28281515	14.396187
	13.20797062
ENSG00000129116 3074.673524	2062.923702	2734.180361
1762.18241	1526.174574	797.9411169	2623.925862
1362.099367	0.946560131	6.45E-05	0.001387002	PALLD
	4	168497066	168928457	+	10114
protein_coding	"palladin, cytoskeletal associated protein
[Source:HGNC Symbol;Acc:HGNC:17068]"	-	3389	2331	2352
	1701	1337	925	16.40881819	10.92058152
14.46842358	9.345303452	8.125607086	4.252553652
ENSG00000117597 2024.076905	2264.702597	2073.885103
3258.120916	2938.199966	2989.044292	2120.888202
3061.788391	-0.529580091	6.48E-05	0.001391585	UTP25
	1	209828007	209857565	+	10376
protein_coding	"UTP25, small subunit processor component [Source:HGNC
Symbol;Acc:HGNC:28440]" -	2231	2559	1784	3145	2574
	3465	10.52927069	11.68602372	10.69723985
16.84235164	15.24845841	15.52759854
ENSG00000033327 178.7284403	153.9891566	210.4109886
348.0854143	432.6254029	333.8413105	181.0428619
371.5173759	-1.038500685	6.50E-05	0.001394457	GAB2
	11	78215297	78418348	-	6771
protein_coding	GRB2 associated binding protein 2 [Source:HGNC

Symbol;Acc:HGNC:14458] -   197   174   181   336   379
        387   1.424761253   1.217651128   1.663154548
2.757391975   3.440596604   2.657597272
ENSG00000115946 983.4600471   1180.583534   1034.617568
1511.478034   1828.669909   1619.173488   1066.220383
1653.107144   -0.632189582   6.52E-05   0.00139675   PNO1
        2   68157844   68176238   +   2674
protein_coding partner of NOB1 homolog [Source:HGNC
Symbol;Acc:HGNC:32790] -   1084   1334   890   1459   1602
        1877   19.85164786   23.63855187   20.70787097
30.31837586   36.82548447   32.63877781
ENSG00000143036 13.60876449   21.23988367   25.5748163   0
        0   0   20.14115482   0   6.763697208
6.54E-05   0.001398556   SLC44A3 1   94820342
94895246   +   2869   protein_coding solute carrier family
44 member 3 [Source:HGNC Symbol;Acc:HGNC:28689] -   15   24
        22   0   0   0   0.256029138   0.396375802
        0.477088534   0   0   0
ENSG00000134291 1993.230372   2066.463683   2190.134268
1579.851955   1152.906746   832.4466787   2083.276108
1188.401793   0.810390847   6.55E-05   0.001399056
TMEM106C   12   47963569   47968878   +   3475
        protein_coding transmembrane protein 106C [Source:HGNC
Symbol;Acc:HGNC:28775] -   2197   2335   1884   1525   1010
        965   30.96021241   31.83893386   33.73128889
24.38524248   17.86544857   12.91229747
ENSG00000120437 1319.142905   1176.158558   1536.813961
821.5230165   883.514675   482.2152263   1344.038475
729.0843059   0.883519138   6.58E-05   0.001403644   ACAT2
        6   159760328   159779055   +   4299
protein_coding acetyl-CoA acetyltransferase 2 [Source:HGNC
Symbol;Acc:HGNC:94]   -   1454   1329   1322   793   774
        559   16.56249061   14.64819041   19.13246188
10.24985651   11.06676979   6.046100765
ENSG00000112029 1069.648889   755.7858608   947.4306947
661.9838682   366.4188768   314.0006125   924.2884814
447.4677859   1.04715495   6.59E-05   0.001404053   FBXO5
        6   152970519   152983579   -   3833
protein_coding F-box protein 5 [Source:HGNC Symbol;Acc:HGNC:13584]
        -       1179   854   815   639   321   364
15.06272924   10.55712083   13.22895795   9.263478173
5.147704082   4.415639224
ENSG00000183955 1064.205383   871.7202258   869.5437541
625.7249709   517.0957982   543.4625985   935.1564543
562.0944559   0.734655148   6.62E-05   0.001408238   KMT5A
        12   123383773   123409358   +   4579
protein_coding lysine methyltransferase 5A [Source:HGNC
Symbol;Acc:HGNC:29489] -   1173   985   748   604   453
        630   12.54457788   10.19276538   10.16337151
7.329567331   6.080998814   6.397361967

ENSG00000132376 826.50563	1002.699508	778.8694054
1194.471675	1451.977605	1678.695582	869.3581813
1441.714954	-0.729472825	6.63E-05	0.001408238	INPP5K
	17	1494571 1516888 -	5325	protein_coding
inositol polyphosphate-5-phosphatase K [Source:HGNC
Symbol;Acc:HGNC:33882]	-	911	1133	670	1153	1272
	1946	8.377751002	10.08176891	7.828202243
12.03155439	14.68300205	16.99238119
ENSG00000079974 373.787398	377.0079352	338.2850701
495.1929406	722.5643272	851.4247377	363.0268011
689.7273352	-0.925994972	6.63E-05	0.001408238	RABL2B
	22	50767501	50783663	-	5870
protein_coding	"RAB, member of RAS oncogene family like 2B
[Source:HGNC Symbol;Acc:HGNC:9800]"	-	412	426	291
	478	633	987	3.437065419	3.438728901
3.084336365	4.524825539	6.628465023	7.81825929
ENSG00000139343 832.8563868	1163.768626	1005.555277
624.6890024	523.9447491	621.9627516	1000.726763
590.1988344	0.761173475	6.67E-05	0.001412564	SNRPF
	12	95858928	95903828	+	3043
protein_coding	small nuclear ribonucleoprotein polypeptide F
[Source:HGNC Symbol;Acc:HGNC:11162]	-	918	1315	865
	603	459	721	14.77302431	20.47624142
17.68564901	11.01101625	9.271672726	11.01702489
ENSG00000163041 3734.244976	3812.55912	4480.242819
2831.301897	2854.871062	2597.406165	4009.015638
2761.193041	0.537854396	6.67E-05	0.001412564	H3F3A
	1	226061851	226072001	+	3146
protein_coding	H3 histone family member 3A [Source:HGNC
Symbol;Acc:HGNC:4764]	-	4116	4308	3854	2733	2501
	3011	64.06861435	64.88486676	76.21840192
48.27174038	48.86549851	44.50236131
ENSG00000101150 6085.83948	7600.338375	7047.024382
8517.732965	10938.91619	12300.37015	6911.067412
10585.6731	-0.615209178	6.68E-05	0.001412564
TPD52L2 20	63865228	63891545	+	4694
protein_coding	tumor protein D52 like 2 [Source:HGNC
Symbol;Acc:HGNC:12007]	-	6708	8588	6062	8222	9583
	14259	69.98075961	86.6912752	80.3488552
97.32993444	125.4890302	141.2462745
ENSG00000111885 605.1363943	521.2621452	696.3324983
387.4522171	318.4762201	267.418104	607.5770126
324.4488471	0.905328715	6.72E-05	0.001420271	MAN1A1
	6	119177209	119349761	-	5014
protein_coding	mannosidase alpha class 1A member 1 [Source:HGNC
Symbol;Acc:HGNC:6821]	-	667	589	599	374	279
	310	6.514336491	5.566182044	7.432746813
4.144759315	3.42032389	2.874804939
ENSG00000102226 2288.086936	2246.117699	2715.580494
2965.977801	4537.430017	4076.832128	2416.595043

3860.079982	-0.675918988	6.77E-05	0.001429041	USP11
	X	47232690	47248328	+	5269
protein_coding	ubiquitin specific peptidase 11 [Source:HGNC
Symbol;Acc:HGNC:12609]	-	2522	2538	2336	2863	3975
	4726	23.43935017	22.82390022	27.58363431
30.19292534	46.37204991	41.70580774
ENSG00000165672 2435.061593	2632.860581	2614.443721
1944.512865	1659.729118	1525.145832	2560.788631
1709.795938	0.582991055	6.82E-05	0.001437055	PRDX3
	10	119167703	119178833	-	3417
protein_coding	peroxiredoxin 3 [Source:HGNC Symbol;Acc:HGNC:9354]
	-	2684	2975	2249	1877	1454	1768
38.46504247	41.25422492	40.94975646	30.52328962
26.15572653	24.05848635
ENSG00000145414 441.8312204	407.0977704	464.99666
681.6672696	698.5929988	869.5401576	437.9752169
749.9334754	-0.777418503	6.91E-05	0.001454859	NAF1
	4	163110073	163166921	-	4808
protein_coding	nuclear assembly factor 1 ribonucleoprotein
[Source:HGNC Symbol;Acc:HGNC:25126]	-	487	460	400
	658	612	1008	4.960131447	4.533355879
5.176096673	7.604549274	7.824098753	9.748259715
ENSG00000196365 4712.261517	5345.370725	4727.85354
6338.055252	7040.721596	8849.813965	4928.495261
7409.530271	-0.588320013	6.92E-05	0.00145527	LONP1
	19	5691834 5720572 -	5085	protein_coding	"lon
peptidase 1, mitochondrial [Source:HGNC Symbol;Acc:HGNC:9479]"	-
	5194	6040	4067	6118	6168	10259	50.01953748
	56.28237587	49.76111027	66.85448675	74.55911905
	93.8091262
ENSG00000083720 1005.23407	882.3401677	856.756346
673.3795217	370.9848442	340.7424229	914.7768613
461.7022629	0.987223365	6.93E-05	0.00145527	OXCT1
	5	41730065	41870519	-	4194
protein_coding	3-oxoacid CoA-transferase 1 [Source:HGNC
Symbol;Acc:HGNC:8527]	-	1108	997	737	650	325
	395	12.93719183	11.26401378	10.93316515
8.61186026	4.763238143	4.379249486
ENSG00000100353 5451.671055	6121.511474	6264.667501
7447.57751	8959.569359	9531.298811	5945.95001
8646.14856	-0.540287558	7.06E-05	0.001482087	EIF3D
	22	36510850	36529436	-	4070
protein_coding	eukaryotic translation initiation factor 3 subunit D
[Source:HGNC Symbol;Acc:HGNC:3278]	-	6009	6917	5389
	7189	7849	11049	72.29969719	80.52853666
82.3797787	98.149054	118.5406434	126.2291281
ENSG00000127948 878.2189351	1171.733583	1062.517368
1407.881185	1975.922355	1725.278091	1037.489962
1703.02721	-0.71489018	7.07E-05	0.001482087	POR
	7	75899200	75986855	+	6112

protein_coding	cytochrome p450 oxidoreductase [Source:HGNC Symbol;Acc:HGNC:9208]	-	968	1324	914	1359	1731	2000	7.755694528	10.26434121	9.303999894	12.35515377	17.4084892	15.2152001
ENSG00000179409 1909.763283	2200.982946	1965.77338	2569.201867	3204.167562	3557.523423	2025.506536	3110.297617	-0.618836009	7.13E-05	0.001492174	GEMIN4	17	744414	753999	-	5751	protein_coding	gem nuclear organelle associated protein 4 [Source:HGNC Symbol;Acc:HGNC:15717]	-	2105	2487	1691	2480	2807	4124	17.92410239	20.49079736	18.2939331	23.96185065	30.00173739	33.34312551
ENSG00000079308 511.6895448	434.5326202	770.7319639	258.9921237	262.5431205	324.352281	572.318043	281.9625084	1.018438085	7.14E-05	0.001492174	TNS1	2	217799588	218033982	-	16281	protein_coding	tensin 1 [Source:HGNC Symbol;Acc:HGNC:11973]	-	564	491	663	250	230	376	1.696393936	1.428982324	2.533607283	0.853239546	0.868348623	1.073835573
ENSG00000203760 410.9846876	390.2828625	437.0968604	258.9921237	111.8661992	168.2146138	412.7881368	179.6909789	1.198469475	7.14E-05	0.001492174	CENPW	6	126340174	126348875	+	795	protein_coding	centromere protein W [Source:HGNC Symbol;Acc:HGNC:21488]	-	453	441	376	250	98	195	27.90356911	26.28438995	29.42575149	17.47370195	7.57715737	11.40508433
ENSG00000124788 531.6490661	450.4625329	625.4205076	237.2367853	248.8452185	339.8797838	535.8440355	275.3205959	0.957614055	7.21E-05	0.001503171	ATXN1	6	16299112	16761491	-	17812	protein_coding	ataxin 1 [Source:HGNC Symbol;Acc:HGNC:10548]	-	586	509	538	229	218	394	1.611067041	1.354040133	1.879214851	0.714389133	0.752300183	1.028524292
ENSG00000170037 757.5545566	726.5810207	714.9323647	453.7542008	436.0498784	450.2975816	733.0226473	446.7005536	0.714547367	7.24E-05	0.00150773	CNTROB	17	7932101 7949918 +	6234	protein_coding	"centrobin, centriole duplication and spindle assembly protein [Source:HGNC Symbol;Acc:HGNC:29616]"	-	835	821	615	438	382	522	6.559162056	6.240261654	6.137834365	3.904085824	3.766551632	3.893451088
ENSG00000215030 2561.169477	2826.674519	3236.376753	3778.177101	4657.286659	4429.651497	2874.74025	4288.371752	-0.577328157	7.25E-05	0.00150773	RPL13P12	17	17383377	17384012	-	636	processed_pseudogene	ribosomal protein L13 pseudogene 12 [Source:HGNC Symbol;Acc:HGNC:35701]	-	2823	3194	2784

3647 4080 5135 217.3614117 237.9601516
272.3447212 318.6329551 394.321455 375.4173592
ENSG00000198805 842.8361474 1142.528743 840.4814629
1236.946383 1739.633546 1912.470763 941.9487843
1629.683564 -0.790541976 7.31E-05 0.001519236 PNP
14 20468954 20477094 + 4139
protein_coding purine nucleoside phosphorylase [Source:HGNC
Symbol;Acc:HGNC:7892] - 929 1291 723 1194 1524
2217 10.99129771 14.77941543 10.8680022
16.02953574 22.63272783 24.90584522
ENSG00000106976 1283.760117 1085.889053 1221.778724
820.487048 722.5643272 745.3201351 1197.142631
762.7905034 0.650034117 7.36E-05 0.001528069 DNM1
9 128191655 128255248 + 10775
protein_coding dynamin 1 [Source:HGNC Symbol;Acc:HGNC:2972] -
1415 1227 1051 792 633 864 6.430842075
5.395773672 6.068652695 4.084321743 3.611052407
3.728442775
ENSG00000168916 176.9139384 98.234462 284.8104542
45.58261378 42.23519764 74.18695789 186.6529515
54.00158977 1.781459919 7.39E-05 0.001532339 ZNF608
5 124636913 124748807 - 6922
protein_coding zinc finger protein 608 [Source:HGNC
Symbol;Acc:HGNC:29238] - 195 111 245 44 37
86 1.379531749 0.759832429 2.202121799
0.353210109 0.328562118 0.577694023
ENSG00000168778 246.7722627 200.0089046 274.3480294
108.776692 105.0172482 100.0661293 240.3763989
104.6200231 1.199422996 7.63E-05 0.001579514 TCTN2
12 123671113 123708403 + 5106
protein_coding tectonic family member 2 [Source:HGNC
Symbol;Acc:HGNC:25774] - 272 226 236 105 92
116 2.608655784 2.097268671 2.875663328
1.142669229 1.107527139 1.056350876
ENSG00000166450 247.6795137 194.6989337 281.3229793
68.37392066 133.5545439 64.6979284 241.2338089
88.87546432 1.445018309 7.63E-05 0.001579514 PRTG
15 55611546 55743090 - 14566
protein_coding protogenin [Source:HGNC Symbol;Acc:HGNC:26373] -
273 220 242 66 117 75 0.91780628
0.715663414 1.033669988 0.251776779 0.493733865
0.239415341
ENSG00000153885 960.778773 948.7148041 1145.054275
674.4154902 614.1226036 611.6110831 1018.182617
633.3830589 0.684207294 7.74E-05 0.001600006 KCTD15
19 33795933 33815763 + 6388
protein_coding potassium channel tetramerization domain containing 15
[Source:HGNC Symbol;Acc:HGNC:23297] - 1059 1072 985
651 538 709 8.118199809 7.951632841
9.593524072 5.662759597 5.176839832 5.160744351

ENSG00000124201 1451.601546 1477.941906 1431.027221
1945.548833 2112.901374 2579.290745 1453.523557
2212.580318 -0.60652348 7.90E-05 0.001631014 ZNFX1
20 49237946 49278426 - 9340
protein_coding zinc finger NFX1-type containing 1 [Source:HGNC
Symbol;Acc:HGNC:29271] - 1600 1670 1231 1878 1851
2990 8.388837659 8.472196807 8.200078752
11.17276734 12.18167203 14.88522248
ENSG00000104881 1301.905136 1619.54113 1633.300768
2604.424796 1972.497879 2587.917136 1518.249011
2388.279937 -0.654181668 8.00E-05 0.001648715
PPP1R13L 19 45379634 45406349 - 5032
protein_coding protein phosphatase 1 regulatory subunit 13
like [Source:HGNC Symbol;Acc:HGNC:18838] - 1435 1830
1405 2514 1728 3000 13.96496824 17.2320481
17.37170871 27.7611009 21.1081643 27.72117538
ENSG00000184164 554.3303402 573.4768592 591.7082498
398.8478705 229.4398575 232.0499032 573.1718164
286.7792104 0.999320531 8.01E-05 0.00164925 CRELD2
22 49918167 49927540 + 5165
protein_coding cysteine rich with EGF like domains 2 [Source:HGNC
Symbol;Acc:HGNC:28150] - 611 648 509 385 201
269 5.792947171 5.944716125 6.131324519
4.141927066 2.392065634 2.421658907
ENSG00000168175 2410.565817 2314.262325 2209.896626
3298.523688 3080.886444 3561.836618 2311.574923
3313.748917 -0.51964047 8.02E-05 0.001650209
MAPK1IP1L 14 55051631 55070192 + 6988
protein_coding mitogen-activated protein kinase 1 interacting
protein 1 like [Source:HGNC Symbol;Acc:HGNC:19840] - 2657
2615 1901 3184 2699 4129 18.61947116
17.73149026 16.92528799 25.31816387 23.74090928
27.47407027
ENSG00000072682 347.47712 293.8183908 601.008183
163.6830222 219.166431 149.2365548 414.1012313
177.3620027 1.223202607 8.03E-05 0.0016506 P4HA2
5 132191838 132295315 - 8502
protein_coding prolyl 4-hydroxylase subunit alpha 2 [Source:HGNC
Symbol;Acc:HGNC:8547] - 383 332 517 158 192
173 2.206004311 1.850305432 3.783347988
1.032637827 1.388121524 0.946141344
ENSG00000106049 973.4802865 928.3599156 1013.692719
665.0917737 522.8032573 304.511583 971.8443069
497.4688713 0.967589428 8.05E-05 0.0016506 HIBADH
7 27525442 27662995 - 2323
protein_coding 3-hydroxyisobutyrate dehydrogenase [Source:HGNC
Symbol;Acc:HGNC:4907] - 1073 1049 872 642 458
353 22.61930183 21.39699175 23.35469079
15.35669865 12.1189119 7.065721472
ENSG00000138758 4300.369579 3307.226887 4243.094522

3039.531564	2320.652887	1974.580775	3950.230329
2444.921742	0.692272034	8.05E-05	0.0016506	11-Sep
	4	76949703	77040384	+	9787
protein_coding	septin 11 [Source:HGNC Symbol;Acc:HGNC:25589]	-
	4740	3737	3650	2934	2033	2289	23.71687349
	18.09255703	23.20331964	16.65798876	12.76836093
	10.87494884
ENSG00000115361 30.84653284	8.849951531	20.9248497	0
	0	0	20.20711136	0	6.769438563
8.10E-05	0.001658585	ACADL	2	210187939
210225491	-	4383	protein_coding	acyl-CoA dehydrogenase
long chain [Source:HGNC Symbol;Acc:HGNC:88]	-	34	10
	18	0	0	0	0.379870991	0.108107287
	0.255509988	0	0	0
ENSG00000236552 1940.609816	2043.453809	2079.697562
2696.625992	2963.312786	3033.038883	2021.253729
2897.65922	-0.519848111	8.12E-05	0.00166114
RPL13AP5	10	96750288	96750899	+	612
	processed_pseudogene	ribosomal protein L13a pseudogene 5
[Source:HGNC Symbol;Acc:HGNC:23736]	-	2139	2309	1789
	2603	2596	3516	171.1543912	178.7717747
181.8719675	236.3386713	260.7357764	267.1335665
ENSG00000165119 13479.0276	12949.24908	15133.3163
11007.16526	9125.085674	7799.119608	13853.86433
9310.456847	0.573395511	8.24E-05	0.001682333	HNRNPK
	9	83968083	83980616	-	5114
protein_coding	heterogeneous nuclear ribonucleoprotein K [Source:HGNC
Symbol;Acc:HGNC:5044]	-	14857	14632	13018	10625	7994
	9041	142.2653325	135.5718148	158.376372
115.4463638	96.08393493	82.20282894
ENSG00000171943 686.7889812	512.4121937	702.1449565
427.8549884	277.3825142	221.6982346	633.7820438
308.9785791	1.037174051	8.25E-05	0.001682333
SRGAP2C 1	121184810	121392822	+	7311
protein_coding	SLIT-ROBO Rho GTPase activating protein 2C
[Source:HGNC Symbol;Acc:HGNC:30584]	-	757	579	604
	413	243	257	5.070464882	3.752564974
5.140045949	3.138956602	2.043039907	1.634509155
ENSG00000253500 1.814501932	0	0	13.46759043
14.83939377	75.91223598	0.604833977
34.73974006	-5.780349687	8.26E-05	0.001683632
AF121898.1	8	87540835	87755718	-	2910
	antisense	"novel transcript, antisense to CNBD1"	-
	2	0	0	13	13	88	0.033656247
	0	0	0.248234652	0.274598224	1.406114547
ENSG00000183751 1149.486974	1367.317512	1360.11523
1717.635765	2006.742634	2383.471682	1292.306572
2035.950027	-0.656346088	8.27E-05	0.001683632	TBL3
	16	1972037 1982933 +	7139	protein_coding
transducin beta like 3 [Source:HGNC Symbol;Acc:HGNC:11587]	-

1267 1545 1170 1658 1758 2763 8.69096331
10.25457209 10.19659868 12.90503503 15.13661793
17.99593936
ENSG00000170456 473.5850042 273.4635023 409.1970608
219.6253209 158.6673641 111.2804368 385.4151891
163.1910406 1.241575659 8.31E-05 0.001690392
DENND5B 12 31382223 31591097 - 11310
protein_coding DENN domain containing 5B [Source:HGNC
Symbol;Acc:HGNC:28338] - 522 309 352 212 139
129 2.260146454 1.294560392 1.936363578
1.041562414 0.755439267 0.530344566
ENSG00000188612 3570.032551 4074.517685 4738.315965
2791.935094 2894.823276 2712.999797 4127.622067
2799.919389 0.559725891 8.39E-05 0.001704665 SUMO2
17 75165586 75182983 - 3372
protein_coding small ubiquitin-like modifier 2 [Source:HGNC
Symbol;Acc:HGNC:11125] - 3935 4604 4076 2695 2536
3145 57.14600229 64.6955173 75.20616722
44.41025299 46.22841927 43.36747152
ENSG00000198732 1536.883136 1655.825931 2145.959586
1349.866949 779.6389186 833.3093177 1779.556218
987.6050617 0.849146443 8.44E-05 0.001710683 SMOC1
14 69854131 70032366 + 4369
protein_coding SPARC related modular calcium binding 1 [Source:HGNC
Symbol;Acc:HGNC:20318] - 1694 1871 1846 1303 683
966 18.98716152 20.29168835 26.28793659
16.57198054 9.609173151 10.28078081
ENSG00000105323 9966.151861 9734.061689 11286.63143
7597.792942 7418.555391 7369.525364 10328.94833
7461.957899 0.468963376 8.44E-05 0.001710683
HNRNPUL1 19 41262496 41307598 + 6543
protein_coding heterogeneous nuclear ribonucleoprotein U like
1 [Source:HGNC Symbol;Acc:HGNC:17011] - 10985 10999 9709
7334 6499 8543 82.21514451 79.65310732
92.32183487 62.28393302 61.05440129 60.71059709
ENSG00000177946 439.1094675 505.3322324 520.7962592
775.9404027 768.2240004 870.4027967 488.412653
804.8557332 -0.72174185 8.50E-05 0.001720537
CENPBD1 16 89969773 89972832 - 3060
protein_coding CENPB DNA-binding domain containing 1 [Source:HGNC
Symbol;Acc:HGNC:28272] - 484 571 448 749 673
1009 7.745556366 8.841808868 9.108847561
13.60104993 13.51888887 15.33206875
ENSG00000127528 848.2796532 810.6555603 820.7191048
580.1423571 412.07855 469.2756406 826.5514394
487.1655159 0.762376631 8.54E-05 0.00172733 KLF2
19 16324817 16327874 + 2018
protein_coding Kruppel like factor 2 [Source:HGNC
Symbol;Acc:HGNC:6347] zf-C2H2 935 916 706 560 361
544 22.68919735 21.50803589 21.76658944

15.41980596	10.9959663	12.53455026
ENSG00000178381 362.9003864	459.3124845	373.1598196
671.3075847	714.5738844	660.7815087	398.4575635
682.2209926	-0.774722211	8.65E-05	0.001747489
ZFAND2A 7	1152071 1160759 -	2281	protein_coding	zinc
finger AN1-type containing 2A [Source:HGNC Symbol;Acc:HGNC:28073]
	-	400	519	321	648	626	766
8.587433552	10.78123502	8.755613732	15.78562437
16.86927461	15.61473084
ENSG00000104415 2076.697461	1382.362429	2111.084836
373.9846267	456.5967313	89.71446071	1856.714909
306.7652729	2.598369437	8.71E-05	0.001754571	WISP1
	8	133191039	133231690	+	5190
protein_coding	WNT1 inducible signaling pathway protein 1
[Source:HGNC Symbol;Acc:HGNC:12769]	-	2289	1562	1816
	361	400	104	21.5976808	14.26067599
21.7698448	3.865021265	4.737399323	0.931744847
ENSG00000151623 123.3861314	114.1643748	82.53690714
397.811902	220.3079228	206.1707318	106.6958044
274.7635189	-1.361262036	8.71E-05	0.001754571	NR3C2
	4	148078762	148444698	-	6607
protein_coding	nuclear receptor subfamily 3 group C member 2
[Source:HGNC Symbol;Acc:HGNC:7979]	ESR-like	136	129
	71	384	193	239	1.00800639	0.925149342
	0.668591558	3.229527308	1.795561821	1.681994658
ENSG00000228526 118.8498765	193.8139385	242.9607548
19.6834014	87.89487077	17.25278091	185.20819
41.61035103	2.164300867	8.82E-05	0.001774048
MIR34AHG	1	9148011 9198906 -	8702	lincRNA MIR34A
host gene [Source:HGNC Symbol;Acc:HGNC:51913]	-	131	219
	209	19	77	20	0.737192487	1.192481024
	1.494287122	0.121323957	0.543899934	0.106866586
ENSG00000069399 389.2106644	389.3978674	716.0948563
143.9996208	289.9389244	200.9948975	498.2344627
211.6444809	1.235060144	8.85E-05	0.001778389	BCL3
	19	44747705	44760044	+	3242
protein_coding	B cell CLL/lymphoma 3 [Source:HGNC
Symbol;Acc:HGNC:998]	-	429	440	616	139	254
	233	6.479969552	6.430816343	11.8215534
2.382395354	4.81579583	3.341749784
ENSG00000211459 22400.9336	30353.56376	17439.69973
46647.58939	60275.33449	96296.39662	23398.0657
67739.7735	-1.533606893	8.95E-05	0.001796359	MT-
RNR1	MT	648	1601	+	954	Mt_rRNA
mitochondrially encoded 12S RNA [Source:HGNC Symbol;Acc:HGNC:7470]
	-	24691	34298	15002	45028	52804	111630
1267.415425	1703.518531	978.3801504	2622.686171
3402.24675	5440.810102
ENSG00000167657 1544.141144	1809.815088	1615.863393
2045.001809	2656.251484	3858.58445	1656.606542

2853.279248	-0.78470685	9.04E-05	0.001813247	DAPK3
	19	3958453 3971123 -	3549	protein_coding	death
associated protein kinase 3 [Source:HGNC Symbol;Acc:HGNC:2676]	-
	1702	2045	1390	1974	2327	4473	23.48454984
	27.30321279	24.36776218	30.90673957	40.3030343
	58.60354893
ENSG00000172638 1240.21207	1223.948297	1510.076653
601.8976955	988.5319232	698.7376267	1324.745673
763.0557485	0.796352205	9.09E-05	0.001821688	EFEMP2
	11	65866441	65873592	-	5397
protein_coding	EGF containing fibulin extracellular matrix protein 2
[Source:HGNC Symbol;Acc:HGNC:3219]	-	1367	1383	1299
	581	866	810	12.40351511	12.14216703
14.97488789	5.981853298	9.863086323	6.978524683
ENSG00000263465 1036.080603	1202.708413	979.9804609
1516.657877	1727.077136	1676.107665	1072.923159
1639.947559	-0.611450091	9.11E-05	0.001822994	SRSF8
	11	95067197	95071224	+	4028
protein_coding	serine and arginine rich splicing factor 8
[Source:HGNC Symbol;Acc:HGNC:16988]	-	1142	1359	843
	1464	1513	1943	13.88370335	15.98661204
13.0210186	20.19592078	23.08855888	22.42922961
ENSG00000182718 16401.28296	17152.97606	24976.1331
12295.91007	13865.70124	11456.70916	19510.13071
12539.44015	0.637707004	9.12E-05	0.001822994	ANXA2
	15	60347134	60402883	-	8824
protein_coding	annexin A2 [Source:HGNC Symbol;Acc:HGNC:537]	-
	18078	19382	21485	11869	12147	13281	100.3260159
	104.078142	151.4874675	74.74130459	84.61568844
	69.98360263
ENSG00000087995 909.9727189	1097.39399	935.8057782
1380.946004	1739.633546	1488.914992	981.0574956
1536.498181	-0.646572725	9.16E-05	0.001827896
METTL2A 17	62423867	62450822	+	3843
protein_coding	methyltransferase like 2A [Source:HGNC
Symbol;Acc:HGNC:25755]	-	1003	1240	805	1333	1524
	1726	12.78083512	15.28895286	13.03263831
19.27399796	24.37597202	20.88341048
ENSG00000184640 6208.31836	6127.70644	7416.696726
4419.441599	4855.906237	4596.140833	6584.240509
4623.829557	0.509780417	9.32E-05	0.001858711	9-Sep
	17	77280569	77500596	+	12844
protein_coding	septin 9 [Source:HGNC Symbol;Acc:HGNC:7323]	-
	6843	6924	6380	4266	4254	5328	26.09005092
	25.5436646	30.90489187	18.45578821	20.35844246
	19.28834376
ENSG00000197043 2391.513546	2184.168038	3281.713928
1711.419954	1753.331448	1362.969692	2619.131837
1609.240364	0.70262802	9.36E-05	0.001863955	ANXA6
	5	151100712	151157882	-	5427

protein_coding	annexin A6 [Source:HGNC Symbol;Acc:HGNC:544]	-
	2636	2468	2823	1652	1536	1580	23.78560859
	21.54823854	32.36368036	16.91462076	17.39717589
	13.53718249
ENSG00000224321 14.51601546	28.3198449	15.11239145	0
	0	0	19.31608393	0	6.706246754
9.41E-05	0.001872126	RPL12P14	1	15792796
	15793285	+	490	processed_pseudogene
ribosomal protein L12 pseudogene 14 [Source:HGNC
Symbol;Acc:HGNC:36495]	-	16	32	13	0	0
	0	1.599015178	3.094427693	1.650646665	0
	0	0
ENSG00000165801 1691.115801	1579.716348	2218.034068
1079.479172	1299.0177	1007.562405	1829.622072
1128.686426	0.696877518	9.43E-05	0.001873498
ARHGEF40	14	21070270	21090240	+	8189
	protein_coding	Rho guanine nucleotide exchange factor 40
[Source:HGNC Symbol;Acc:HGNC:25516]	-	1864	1785	1908
	1042	1138	1168	11.14663347	10.32841763
14.4962058	7.070479892	8.541983951	6.63197667
ENSG00000149177 796.5663481	610.6466557	681.2201068
343.9415403	479.4265678	304.511583	696.1443702
375.959897	0.890938793	9.46E-05	0.001878651	PTPRJ
	11	47980558	48170841	+	9620
protein_coding	"protein tyrosine phosphatase, receptor type J
[Source:HGNC Symbol;Acc:HGNC:9673]"	-	878	690	586
	332	420	353	4.469388708	3.398603181
3.789914309	1.917675215	2.683623452	1.706202805
ENSG00000131174 1542.326642	1836.364943	2138.984636
1320.859831	1099.256631	797.9411169	1839.225407
1072.685859	0.778221615	9.55E-05	0.001894537	COX7B
	X	77899438	77907373	+	3024
protein_coding	cytochrome c oxidase subunit 7B [Source:HGNC
Symbol;Acc:HGNC:2291]	-	1700	2075	1840	1275	963
	925	27.52934118	32.51342755	37.85671127
23.42828193	19.57455332	14.22299194
ENSG00000142173 32765.36864	37224.66613	66339.91098
16321.68364	19471.5676	11951.86397	45443.31525
15915.0384	1.513682791	9.67E-05	0.001914573	COL6A2
	21	46098097	46132849	+	5386
protein_coding	collagen type VI alpha 2 chain [Source:HGNC
Symbol;Acc:HGNC:2212]	-	36115	42062	57067	15755	17058
	13855	328.3597782	370.0411405	659.212661
162.5414397	194.6745239	119.6110215
ENSG00000197019 545.2578306	731.0059965	942.7807281
1044.256243	1402.893457	1576.041536	739.6815184
1341.063745	-0.859955357	9.72E-05	0.001922541
SERTAD1 19	40421592	40426025	-	2114
protein_coding	SERTA domain containing 1 [Source:HGNC
Symbol;Acc:HGNC:17932]	-	601	826	811	1008	1229

	1827	13.92188919	18.51405311	23.8683676
26.49522383	35.73503898	40.18505643
ENSG00000140795 15.42326642	38.93978674	6.974949899	0
	0	0	20.44600102	0	6.790471523
9.73E-05	0.001922541	MYLK3	16	46703369
46790407	-	9522	protein_coding	myosin light chain
kinase 3 [Source:HGNC Symbol;Acc:HGNC:29826]	-	17	44
	6	0	0	0	0.087427775	0.21895302
	0.039203958	0	0	0
ENSG00000127580 463.6052436	708.8811176	594.0332331
895.0767796	1005.654301	1160.249516	588.8398648
1020.326865	-0.793425767	9.75E-05	0.001924013	WDR24
	16	684622	690444	-	3651	protein_coding	WD
repeat domain 24 [Source:HGNC Symbol;Acc:HGNC:20852]	-	511
	801	511	864	881	1345	6.853899796
10.39554168	8.707949741	13.14964272	14.83240023
17.12937312
ENSG00000131747 12303.23035	8156.115331	11707.45341
4541.685882	2765.8347	1403.513727	10722.26636
2903.678103	1.884729788	9.76E-05	0.001924013	TOP2A
	17	40388516	40417950	-	6437
protein_coding	DNA topoisomerase II alpha [Source:HGNC
Symbol;Acc:HGNC:11989]	-	13561	9216	10071	4384	2423
	1627	103.1660708	67.83993104	97.34102912
37.8441783	23.1375444	11.75262995
ENSG00000156876 871.8681783	864.6402646	660.2952571
506.588594	406.3710908	481.3525873	798.9345667
464.7707574	0.781846987	9.77E-05	0.001925003	SASS6
	1	100083563	100132955	-	3972
protein_coding	SAS-6 centriolar assembly protein [Source:HGNC
Symbol;Acc:HGNC:25403]	-	961	977	568	489	356
	558	11.8479396	11.65498623	8.897048182
6.840875128	5.509194666	6.532147416
ENSG00000196739 1254.728086	1026.594378	1035.78006
731.3937574	703.1589662	659.0562306	1105.700841
697.8696514	0.664575399	9.92E-05	0.001949732
COL27A1 9	114155560	114312511	+	13988
protein_coding	collagen type XXVII alpha 1 chain [Source:HGNC
Symbol;Acc:HGNC:22986]	-	1383	1160	891	706	616
	764	4.84167061	3.929423213	3.9630443
2.804536658	2.706901475	2.539620085
ENSG00000142546 2460.46462	3561.220496	3185.227121
3649.717008	5977.992704	5815.912443	3068.970746
5147.874052	-0.746277429	9.92E-05	0.001949732	NOSIP
	19	49555711	49590262	-	4248
protein_coding	nitric oxide synthase interacting protein [Source:HGNC
Symbol;Acc:HGNC:17946]	-	2712	4024	2740	3523	5237
	6742	31.26323108	44.88486309	40.13034574
46.08294004	75.77839909	73.79641394
ENSG00000102978 2610.161029	3029.338409	2819.042251

3643.501197 4202.972911 4360.640374 2819.513896
4069.038161 -0.529299516 0.00010165 0.001995653 POLR2C
16 57462387 57472010 + 3940
protein_coding RNA polymerase II subunit C [Source:HGNC
Symbol;Acc:HGNC:9189] - 2877 3423 2425 3517 3682
5055 35.75792619 41.16585293 38.2932624
49.60074392 57.44271025 59.65625085
ENSG00000183255 9366.458973 10031.42006 11051.80812
7305.649826 7738.173103 6045.374429 10149.89572
7029.732453 0.530019787 0.000102265 0.002005507
PTTG1IP 21 44849585 44873903 - 3537
protein_coding PTTG1 interacting protein [Source:HGNC
Symbol;Acc:HGNC:13524] - 10324 11335 9507 7052 6779
7008 142.9359984 151.8493389 167.2304201
110.7871238 117.8088548 92.12766234
ENSG00000111110 204.1314673 159.2991276 166.2363059
88.05732207 37.66923033 21.56597613 176.5556336
49.09750951 1.850906489 0.000102399 0.002005902 PPM1H
12 62643982 62935037 - 7063
protein_coding "protein phosphatase, Mg2+/Mn2+ dependent 1H
[Source:HGNC Symbol;Acc:HGNC:18583]" - 225 180 143
85 33 25 1.55999065 1.207562839
1.259660984 0.668716075 0.287191838 0.164581805
ENSG00000161381 265.824533 106.1994184 116.249165
64.23004668 5.707459141 8.626390453 162.7577055
26.18796543 2.63828709 0.000103629 0.002027761 PLXDC1
17 39063303 39154394 - 9277
protein_coding plexin domain containing 1 [Source:HGNC
Symbol;Acc:HGNC:20945] - 293 120 100 62 5
10 1.546638253 0.612914831 0.67065519
0.371360901 0.033129113 0.050121431
ENSG00000178202 3876.683378 2647.905498 3581.636773
2600.280922 1763.604875 1479.425963 3368.741883
1947.770586 0.790547253 0.00010548 0.002061688 KDELC2
11 108472105 108498432 - 4993
protein_coding KDEL motif containing 2 [Source:HGNC
Symbol;Acc:HGNC:28496] - 4273 2992 3081 2510 1545
1715 41.90829674 28.39399254 38.39166779
27.93342564 19.0201649 15.97105394
ENSG00000189320 318.4450891 377.0079352 146.4739479
640.2285298 632.3864728 574.5176042 280.6423241
615.7108689 -1.129786481 0.000105694 0.002063606
FAM180A 7 135728348 135748846 - 2526
protein_coding family with sequence similarity 180 member A
[Source:HGNC Symbol;Acc:HGNC:33773] - 351 426 126
618 554 666 6.804597696 7.99102876
3.103444946 13.59462313 13.48105184 12.25947581
ENSG00000265763 7.258007728 12.38993214 41.8496994 0
0 0 20.49921309 0 6.785352663
0.000106021 0.002067707 ZNF488 10 47365496

47384273	-	3496	protein_coding	zinc finger protein
488 [Source:HGNC Symbol;Acc:HGNC:23535] zf-C2H2 8	14	36
	0	0	0	0.11205913	0.189750554
0.640675215	0	0	0
ENSG00000136143 919.9524795	831.8954439	924.1808617
613.293349	489.6999943	545.1878766	892.009595
549.39374	0.698601279	0.000106414	0.002071511	SUCLA2
	13	47745736	48037968	-	8928
protein_coding	succinate-CoA ligase ADP-forming beta subunit
[Source:HGNC Symbol;Acc:HGNC:11448]	-	1014	940	795
	592	429	632	5.561762723	4.988846171
5.540127934	3.684508551	2.953591781	3.29150042
ENSG00000259863 54.43505796	56.6396898	72.07448229
16.57549592	5.707459141	6.038473317	61.04974335
9.440476125	2.695002142	0.000106451	0.002071511
SH3RF3-AS1	2	109127327	109128930	-	1604
	lincRNA SH3RF3 antisense RNA 1 [Source:HGNC
Symbol;Acc:HGNC:44168]	-	60	64	62	16	5
	7	1.83178952	1.890610436	2.404884217
0.554278027	0.191607719	0.202919925
ENSG00000112559 1650.289507	1308.022836	2027.385437
265.2079347	504.5393881	97.47821212	1661.89926
289.0751783	2.524303276	0.000107105	0.002081958	MDFI
	6	41636882	41654246	+	2523
protein_coding	MyoD family inhibitor [Source:HGNC
Symbol;Acc:HGNC:6967]	-	1819	1478	1744	256	442
	113	35.30564354	27.75770938	43.00669577
5.638125757	10.76842975	2.08253453
ENSG00000087253 284.8768033	334.5281679	466.1591516
159.5391482	204.3270372	127.6705787	361.8547076
163.8455881	1.144376246	0.000107861	0.002094356	LPCAT2
	16	55508998	55586670	+	7439
protein_coding	lysophosphatidylcholine acyltransferase 2 [Source:HGNC
Symbol;Acc:HGNC:26032]	-	314	378	401	154	179
	148	2.067015689	2.407707258	3.35379614
1.150318769	1.479060138	0.925077621
ENSG00000185896 5025.263101	5061.287281	6437.878757
4040.27713	3760.074082	3411.737424	5508.143046
3737.362879	0.559418707	0.000108163	0.002097914	LAMP1
	13	113297241	113323672	+	5739
protein_coding	lysosomal associated membrane protein 1 [Source:HGNC
Symbol;Acc:HGNC:6499]	-	5539	5719	5538	3900	3294
	3955	47.26327632	47.21829625	60.0376346
37.76073385	35.28049991	32.04359848
ENSG00000072778 4757.624066	5745.388534	5412.561122
3395.904726	4000.928858	3698.133587	5305.191241
3698.32239	0.520596233	0.000108487	0.002101887	ACADVL
	17	7217125 7225273 +	4865	protein_coding	acyl-
CoA dehydrogenase very long chain [Source:HGNC Symbol;Acc:HGNC:92]
	-	5244	6492	4656	3278	3505	4287

52.78475644   63.22984357   59.54385846   37.44019899
44.28458079   40.97336731
ENSG00000110697 1528.717878   1333.687696   1755.362391
1054.615928   1010.220268   879.0291871   1539.255988
981.288461   0.649390584   0.000108854   0.002106687
PITPNM1 11   67491768   67506263   -   6449
protein_coding phosphatidylinositol transfer protein membrane
associated 1 [Source:HGNC Symbol;Acc:HGNC:9003] -   1685   1507
        1510   1018   885   1019   12.79487984   11.07254149
        14.56771435   8.771370275   8.435255738   7.34704712
ENSG00000124225 2931.327871   3072.703172   5196.337675
1797.405339   2185.956851   2468.010309   3733.456239
2150.457499   0.795252037   0.00010955   0.002116514   PMEPA1
        20   57648392   57711536   -   5619
protein_coding "prostate transmembrane protein, androgen induced 1
[Source:HGNC Symbol;Acc:HGNC:14107]"   -   3231   3472   4470
        1735   1915   2861   28.15831153   29.27838553
49.49431724   17.15744007   20.9487014   23.67499216
ENSG00000158483 302.1145717   258.4185847   261.5606212
405.0636815   546.7745857   584.8692727   274.0312592
512.2358466   -0.902451796   0.000109601   0.002116514
FAM86C1 11   71787510   71801236   +   2972
protein_coding family with sequence similarity 86 member C1
[Source:HGNC Symbol;Acc:HGNC:25561]   -   333   292   225
        391   479   678   5.486862942   4.655437357
4.71021314   7.310380731   9.906815353   10.60747231
ENSG00000188549 522.5765564   284.9684393   396.4096526
202.0138565   157.5258723   201.8575366   401.3182161
187.1324218   1.09931444   0.000110178   0.002125336   CCDC9B
        15   40331452   40340967   -   7510
protein_coding coiled-coil domain containing 9B [Source:HGNC
Symbol;Acc:HGNC:33488] -   576   322   341   195   138
        234   3.755875865   2.031619513   2.825018451
1.442801941   1.129500713   1.448795
ENSG00000170185 767.5343172   707.9961225   661.4577488
1081.551109   1122.086467   1095.551588   712.3293962
1099.729721   -0.625834101   0.000111442   0.002145716   USP38
        4   143184917   143223830   +   7787
protein_coding ubiquitin specific peptidase 38 [Source:HGNC
Symbol;Acc:HGNC:20067] -   846   800   569   1044   983
        1270   5.320211185   4.867951617   4.546204194
7.449761472   7.75944579   7.58341048
ENSG00000179833 743.0385411   710.651108   564.9709418
1078.443203   1050.172482   1102.4527   672.8868636
1077.022795   -0.677415093   0.000111516   0.002145716
SERTAD2 2   64631621   64751005   -   6274
protein_coding SERTA domain containing 2 [Source:HGNC
Symbol;Acc:HGNC:30784] -   819   803   486   1041   920
        1278   6.392460762   6.06453451   4.819462457
9.219731186   9.013442098   9.471469336

ENSG00000125304 2987.577431 2456.746545 3156.164829
2215.936611 1690.549398 1469.936933 2866.829602
1792.14098 0.67785671 0.000111598 0.002145716 TM9SF2
13 99446311 99564006 + 5524
protein_coding transmembrane 9 superfamily member 2 [Source:HGNC
Symbol;Acc:HGNC:11865] - 3293 2776 2715 2139 1481
1704 29.19219453 23.81180036 30.57898111
21.51637765 16.47967903 14.34322921
ENSG00000168159 2261.776658 2718.70511 2567.944055
2820.942212 4838.78386 4973.114096 2516.141941
4210.946722 -0.743023584 0.000111928 0.002148175 RNF187
1 228487061 228495766 + 3285
protein_coding ring finger protein 187 [Source:HGNC
Symbol;Acc:HGNC:27146] - 2493 3072 2209 2723 4239
5765 37.16341269 44.31107419 41.83764096
46.06004003 79.31866625 81.60090134
ENSG00000145386 2818.828751 2083.27859 2781.842518
1095.018699 574.1703896 335.5665886 2561.31662
668.2518924 1.938675515 0.000111969 0.002148175 CCNA2
4 121816444 121823933 - 2798
protein_coding cyclin A2 [Source:HGNC Symbol;Acc:HGNC:1578] -
3107 2354 2393 1057 503 389 54.37787432
39.86439608 53.21105076 20.99129929 11.0501363
6.464469776
ENSG00000126062 1258.35709 1800.965137 1292.690715
1707.27608 3016.962902 3020.099298 1450.67098
2581.446093 -0.831197725 0.000113091 0.002165774
TMEM115 3 50354749 50359610 - 2197
protein_coding transmembrane protein 115 [Source:HGNC
Symbol;Acc:HGNC:30055] - 1387 2035 1112 1648 2643
3501 30.9154155 43.88951659 31.49064651
41.68110213 73.94596253 74.09571138
ENSG00000148841 2126.596264 2397.45187 2120.384769
2655.187252 3648.207883 4188.112565 2214.810968
3497.169233 -0.659068885 0.000113131 0.002165774 ITPRIP
10 104309698 104338448 - 7279
protein_coding "inositol 1,4,5-trisphosphate receptor interacting
protein [Source:HGNC Symbol;Acc:HGNC:29370]" - 2344 2709
1824 2563 3196 4855 15.76937829 17.63452339
15.59049704 19.56540898 26.98872769 31.01333947
ENSG00000101146 2147.463036 2239.922733 2326.145791
2807.474621 3431.324435 3618.770795 2237.843853
3285.856617 -0.554432186 0.000114384 0.002187395 RAE1
20 57351010 57379211 + 5855
protein_coding ribonucleic acid export 1 [Source:HGNC
Symbol;Acc:HGNC:9828] - 2367 2531 2001 2710 3006
4195 19.79703004 20.48291141 21.26312224
25.71902112 31.55799747 33.31471359
ENSG00000196123 478.1212591 407.0977704 421.9844689
236.2008168 260.2601368 209.621288 435.7344995

235.3607472	0.890335418	0.000114859	0.002194098
KIAA0895L	16	67175602	67184040	-	4780
	protein_coding	KIAA0895 like [Source:HGNC
Symbol;Acc:HGNC:34408]	-	527	460	363	228	228
	243	5.398976065	4.559911102	4.724823341
2.650446205	2.931934816	2.363792744
ENSG00000171222 1159.466735	1727.510539	1604.238477
1857.491511	2625.431205	3465.221045	1497.071917
2649.381254	-0.823956953	0.000115912	0.002210511	SCAND1
	20	35953617	35959472	-	1996
protein_coding	SCAN domain containing 1 [Source:HGNC
Symbol;Acc:HGNC:10566]	-	1278	1952	1380	1793	2300
	4017	31.35443653	46.33889968	43.01554167
49.91508285	70.82957881	93.57768841
ENSG00000170373 7.258007728	26.54985459	24.41232465	0
	0	0	19.40672899	0	6.710404485
0.000115968	0.002210511	CST1	20	23747553
23751268	-	872	protein_coding	cystatin SN
[Source:HGNC Symbol;Acc:HGNC:2473]	-	8	30	21
	0	0	0	0.449264586	1.630163672
1.498337525	0	0	0
ENSG00000138600 1660.269268	1689.455747	1800.699566
1226.586698	1196.283436	1007.562405	1716.808194
1143.477513	0.58672232	0.000116872	0.002225341	SPPL2A
	15	50702266	50765808	-	8144
protein_coding	signal peptide peptidase like 2A [Source:HGNC
Symbol;Acc:HGNC:30227]	-	1830	1909	1549	1184	1048
	1168	11.00378277	11.10694456	11.83369848
8.078411676	7.909897903	6.668621924
ENSG00000162512 2654.616326	2300.102403	2172.696894
1795.333402	1450.836114	1470.799572	2375.805208
1572.323029	0.595822749	0.000117246	0.002230078	SDC3
	1	30869467	30908761	-	7067
protein_coding	syndecan 3 [Source:HGNC Symbol;Acc:HGNC:10660]	-
	2926	2599	1869	1733	1271	1705	20.27532918
	17.42599676	16.45436234	13.62622372	11.05497639
	11.21812591
ENSG00000012048 2461.371871	1919.554487	1833.249332
1605.751167	1043.323531	948.9029498	2071.391897
1199.325883	0.78888869	0.000117666	0.002235653	BRCA1
	17	43044295	43170245	-	9187
protein_coding	"BRCA1, DNA repair associated [Source:HGNC
Symbol;Acc:HGNC:1100]"	-	2713	2169	1577	1550	914
	1100	14.46121427	11.18696493	10.6798419
9.374972996	6.115329181	5.567368744
ENSG00000204628 20435.82801	23283.33748	24635.52304
28994.68624	34081.52151	32539.60743	22784.89618
31871.93839	-0.484241653	0.000117873	0.002237191	RACK1
	5	181236909	181248096	-	5990
protein_coding	receptor for activated C kinase 1 [Source:HGNC

Symbol;Acc:HGNC:4399]   -   22525   26309   21192   27988   29857
37721   184.1478535   208.115276   220.1161811
259.6313231   306.3844403   292.8110037
ENSG00000142731 989.8108039   739.855948   673.0826653
562.5308927   310.4857773   203.5828147   800.9164724
358.8664949   1.159807471   0.000118295   0.002242783   PLK4
4   127880861   127899195   +   4482
protein_coding polo like kinase 4 [Source:HGNC Symbol;Acc:HGNC:11397]
-       1091   836   579   543   272   236
11.9201462   8.838139784   8.037362535   6.731936659
3.730305598   2.448336848
ENSG00000170004 3050.177748   2788.619727   3758.335504
1978.699825   1857.207204   2396.411268   3199.044326
2077.439432   0.621990677   0.000118592   0.00224601   CHD3
17   7884806 7912760 +   9758   protein_coding
chromodomain helicase DNA binding protein 3 [Source:HGNC
Symbol;Acc:HGNC:1918]   -   3362   3151   3233   1910   1627
2778   16.87196162   15.30079619   20.61349999
10.87638562   10.24882551   13.23739248
ENSG00000091317 2637.378558   2489.491366   2430.77004
1882.354755   1717.945201   1666.618635   2519.213321
1755.639531   0.521238004   0.000119189   0.002254908   CMTM6
3   32481312   32503408   -   4413
protein_coding CKLF like MARVEL transmembrane domain containing 6
[Source:HGNC Symbol;Acc:HGNC:19177]   -   2907   2813   2091
1817   1505   1932   32.25817457   30.20384588
29.47996421   22.87878955   20.96283098   20.35655171
ENSG00000174442 1815.409183   1397.407347   1401.96493
1185.147958   565.0384549   697.8749876   1538.260486
816.0204669   0.914678677   0.000120729   0.002281591   ZWILCH
15   66504959   66550128   +   4849
protein_coding zwilch kinetochore protein [Source:HGNC
Symbol;Acc:HGNC:25468] -   2001   1579   1206   1144   495
809   20.20801186   15.42966108   15.47397783
13.10949264   6.274807038   7.757599518
ENSG00000260231 13.60876449   15.0449176   16.2748831
75.62570013   74.19696883   59.52209412   14.9761884
69.78158769   -2.22173385   0.000121986   0.002302887   KDM7A-
DT   7   140177184   140179640   +   2457
bidirectional_promoter_lncRNA   KDM7A divergent transcript
[Source:HGNC Symbol;Acc:HGNC:48959]   -   15   17   14
73   65   69   0.298961171   0.327846239
0.354511009   1.650934135   1.626131117   1.305794853
ENSG00000013563 257.6592743   234.5237156   291.7854041
139.8557468   118.7151501   93.16501689   261.322798
117.2453046   1.157639562   0.000122216   0.002303346
DNASE1L1   X   154401238   154412112   -   4130
protein_coding deoxyribonuclease 1 like 1 [Source:HGNC
Symbol;Acc:HGNC:2957]   -   284   265   251   135   104
108   3.367417557   3.040340769   3.78120755

1.8163342 1.547856331 1.215919216
ENSG00000136827 1343.638681 1571.751392 1341.515364
2059.505368 2115.184358 2055.668845 1418.968479
2076.78619 -0.54907947 0.000122377 0.002303346 TOR1A
9 129812944 129824134 - 3766
protein_coding torsin family 1 member A [Source:HGNC
Symbol;Acc:HGNC:3098] - 1481 1776 1154 1988 1853
2383 19.25765608 22.34544905 19.06480378
29.3324344 30.24422525 29.422173
ENSG00000111412 1131.341955 1161.998636 1092.742151
1470.039294 1733.926087 2033.24023 1128.694247
1745.735204 -0.629409876 0.000122401 0.002303346
C12orf49 12 116710185 116738070 - 8664
protein_coding chromosome 12 open reading frame 49
[Source:HGNC Symbol;Acc:HGNC:26128] - 1247 1313 940
1419 1519 2357 7.048175239 7.180798221
6.750194031 9.100725087 10.77672226 12.64946498
ENSG00000185262 1710.168071 1970.884206 2302.895958
1035.968495 1406.317932 1321.563017 1994.649412
1254.616482 0.668507324 0.000123545 0.002322411 UBALD2
17 76265202 76271299 + 1797
protein_coding UBA like domain containing 2 [Source:HGNC
Symbol;Acc:HGNC:28438] - 1885 2227 1981 1000 1232
1532 51.36791769 58.7216947 68.58722707
30.92174302 42.14150009 39.64073572
ENSG00000118523 1280.131113 1220.408316 1698.4003
1082.587077 429.2009274 477.9020311 1399.646577
663.2300119 1.077277678 0.000124256 0.002333299 CTGF
6 131948176 131951373 - 2339
protein_coding connective tissue growth factor [Source:HGNC
Symbol;Acc:HGNC:2500] - 1411 1379 1461 1045 376
554 29.54101924 27.93575963 38.86215152
24.82550618 9.881092919 11.01312481
ENSG00000164574 2163.793554 1653.170946 1919.273714
1319.823863 1255.641011 1218.046332 1912.079405
1264.503735 0.596635728 0.00012449 0.002335202
GALNT10 5 154190730 154420984 + 10496
protein_coding polypeptide N-acetylgalactosaminyltransferase 10
[Source:HGNC Symbol;Acc:HGNC:19873] - 2385 1868 1651
1274 1100 1412 11.12738834 8.432949327
9.786560784 6.744622541 6.441933293 6.255209977
ENSG00000272899 64.41481858 86.72952501 52.31212425
132.6039673 214.6004637 212.2092051 67.81882261
186.4712121 -1.455241803 0.000125062 0.002343447
ATP6V1FNB 7 128866308 128872044 + 3902
protein_coding ATP6V1F neighbor [Source:HGNC
Symbol;Acc:HGNC:52392] - 71 98 45 128 188
246 0.891045266 1.190050117 0.717516835
1.822782071 2.961542329 2.931425492
ENSG00000076924 1608.555963 1675.295825 2044.822812

2203.504989	2979.293672	3279.75365	1776.224867
2820.85077	-0.667981599	0.000125745	0.002353762	XAB2
	19	7619525 7629565 -	4673	protein_coding	XPA
binding protein 2 [Source:HGNC Symbol;Acc:HGNC:14089]	-	1773
	1893	1759	2127	2610	3802	18.57982581
19.19469757	23.41946151	25.29203374	34.33144527
37.83095891
ENSG00000164604 448.1819772	309.7483036	391.759686
216.5174154	187.2046598	103.5166854	383.2299889
169.0795869	1.183526916	0.000126332	0.002361097	GPR85
	7	113078331	113087778	-	7396
protein_coding	G protein-coupled receptor 85 [Source:HGNC
Symbol;Acc:HGNC:4536]	-	494	350	337	209	164
	120	3.270835705	2.242319959	2.834913715
1.570223336	1.362995135	0.754423767
ENSG00000162851 1221.1598	1260.233098	1061.354876
1867.851196	1561.560821	1989.245638	1180.915925
1806.219219	-0.612933394	0.000126404	0.002361097	TFB2M
	1	246540560	246566324	-	1848
protein_coding	"transcription factor B2, mitochondrial [Source:HGNC
Symbol;Acc:HGNC:18559]" -	1346	1424	913	1803	1368
	2306	35.66742663	36.51190252	30.73800361
54.21329496	45.50210525	58.02142011
ENSG00000143420 2772.558952	3298.376936	3270.089011
2105.087982	1950.809534	2330.8507	3113.674966
2128.916072	0.547992605	0.000126594	0.002362162	ENSA
	1	150600851	150629612	-	5493
protein_coding	endosulfine alpha [Source:HGNC Symbol;Acc:HGNC:3360]
	-	3056	3727	2813	2032	1709	2702
27.24409804	32.14964891	31.86155589	20.55541022
19.12404795	22.87213806
ENSG00000136122 427.315205	347.8030952	370.8348363
220.6612894	173.5067579	81.9507093	381.9843788
158.7062522	1.270780226	0.000126747	0.002362523	BORA
	13	72727749	72756198	+	3189
protein_coding	"bora, aurora kinase A activator [Source:HGNC
Symbol;Acc:HGNC:24724]" -	471	393	319	213	152
	95	7.232610398	5.839349531	6.223619178
3.711394569	2.929789572	1.385160518
ENSG00000119537 2204.619847	2396.566875	2200.596693
3291.271908	3610.538653	2925.209003	2267.261138
3275.673188	-0.530393888	0.000128058	0.002384431	KDSR
	18	63327726	63367510	-	9313
protein_coding	3-ketodihydrosphingosine reductase [Source:HGNC
Symbol;Acc:HGNC:4021]	-	2430	2708	1893	3177	3163
	3391	12.77748425	13.77797835	12.64642747
18.95569253	20.8764644	16.93047742
ENSG00000198695 4759.438568	6035.666944	4161.720107
7410.282644	6820.413673	8405.554857	4985.608539
7545.417058	-0.597683222	0.000128854	0.002396733	MT-ND6

MT 14149 14673 - 525 protein_coding
mitochondrially encoded NADH:ubiquinone oxidoreductase core subunit 6
[Source:HGNC Symbol;Acc:HGNC:7462] - 5246 6820 3580
7153 5975 9744 489.3252948 615.533242
424.2585173 757.0785912 699.5616065 862.9964118
ENSG00000112186 443.6457224 310.6332987 344.0975284
217.5533839 138.1205112 70.73640171 366.1255165
142.1367656 1.368286157 0.00013027 0.002420525 CAP2
6 17393216 17557792 + 3637
protein_coding cyclase associated actin cytoskeleton regulatory
protein 2 [Source:HGNC Symbol;Acc:HGNC:20039] - 489 351
296 210 121 82 6.584067 4.572884752
5.063551794 3.208396525 2.044981716 1.048338582
ENSG00000111602 1870.751492 1655.825931 1553.088844
1144.745187 1206.556862 1001.523932 1693.222089
1117.60866 0.600321027 0.00013352 0.002478309
TIMELESS 12 56416373 56449403 - 5818
protein_coding timeless circadian regulator [Source:HGNC
Symbol;Acc:HGNC:11813] - 2062 1871 1336 1105 1057
1161 17.35575967 15.23794885 14.28695207
10.5535994 11.1673115 9.278751013
ENSG00000266028 1327.308163 1121.288859 1300.828156
887.8250001 760.2335576 772.0619455 1249.808393
806.7068344 0.631383917 0.000133927 0.002483264 SRGAP2
1 206203345 206464443 + 15675
protein_coding SLIT-ROBO Rho GTPase activating protein 2 [Source:HGNC
Symbol;Acc:HGNC:19751] - 1463 1267 1119 857 666
895 4.570518384 3.829971182 4.441497108
3.037982837 2.611644379 2.65488983
ENSG00000182149 2363.388766 2393.026894 2674.893286
3292.307877 3853.676412 3470.396879 2477.102982
3538.793723 -0.514782537 0.000134438 0.002490136 IST1
16 71845996 71931199 + 7806
protein_coding "IST1, ESCRT-III associated factor [Source:HGNC
Symbol;Acc:HGNC:28977]" - 2605 2704 2301 3178 3376
4023 16.34210002 16.41362781 18.33981364
22.62233293 26.58405649 23.96362439
ENSG00000065534 4007.327517 3290.411979 3122.452572
2827.158023 1670.002545 1585.530565 3473.397356
2027.563711 0.776867205 0.000135169 0.002501042 MYLK
3 123610049 123884331 - 22167
protein_coding myosin light chain kinase [Source:HGNC
Symbol;Acc:HGNC:7590] - 4417 3718 2686 2729 1463
1838 9.757738194 7.947470142 7.538864432
6.840827795 4.056810906 3.855401428
ENSG00000151718 1229.325059 1130.138811 1132.266867
855.7099768 704.300458 646.1166449 1163.910245
735.3756932 0.663045399 0.000135469 0.002503982 WWC2
4 183099293 183320777 + 9966
protein_coding WW and C2 domain containing 2 [Source:HGNC

Symbol;Acc:HGNC:24148]	-	1355	1277	974	826	617
	749	6.658050669	6.071506374	6.080578795
4.605440843	3.805499256	3.494555586
ENSG00000139880 272.1752898	253.9936089	319.6852037
103.5968495	78.76293614	167.3519748	281.9513675
116.5705868	1.267945692	0.000137263	0.002534485	CDH24
	14	23047062	23057538	-	3748
protein_coding	cadherin 24 [Source:HGNC Symbol;Acc:HGNC:14265] -
	300	287	275	100	69	194	3.919677681
	3.628346506	4.564991342	1.482560624	1.13161024
	2.406762111
ENSG00000173846 400.097676	583.2118059	614.9580828
615.365286	1281.895323	1341.403715	532.7558549
1079.554775	-1.019729874	0.000138008	0.002545444	PLK3
	1	44800225	44805990	+	3479
protein_coding	polo like kinase 3 [Source:HGNC Symbol;Acc:HGNC:2154]
	-	441	659	529	594	1123	1555
6.207444486	8.975474692	9.460369296	9.487331155
19.84141714	20.78293995
ENSG00000198890 1437.992781	1406.257298	1211.316299
1800.513244	2052.402307	2252.350547	1351.85546
2035.088699	-0.589835358	0.000138144	0.002545444	PRMT6
	1	107056679	107067636	+	4335
protein_coding	protein arginine methyltransferase 6 [Source:HGNC
Symbol;Acc:HGNC:18241]	-	1585	1589	1042	1738	1798
	2611	17.90477419	17.36845694	14.95496716
22.2778212	25.49458493	28.00585192
ENSG00000186642 89.81784563	69.9146171	159.261356
278.6755251	229.4398575	290.7093583	106.3312729
266.2749136	-1.331692805	0.000138858	0.002555942	PDE2A
	11	72576141	72674591	-	7182
protein_coding	phosphodiesterase 2A [Source:HGNC
Symbol;Acc:HGNC:8777]	-	99	79	137	269	201
	337	0.675022855	0.52120447	1.18681223
2.081224467	1.72027555	2.181802918
ENSG00000103995 703.1194986	470.8174215	502.1963928
335.6537923	214.6004637	307.0995001	558.7111043
285.7845854	0.966206331	0.000140148	0.002577005	CEP152
	15	48712928	48811146	-	8168
protein_coding	centrosomal protein 152 [Source:HGNC
Symbol;Acc:HGNC:29298]	-	775	532	432	324	188
	356	4.646379269	3.086187757	3.290598265
2.204150905	1.414781852	2.026587161
ENSG00000136717 1422.569515	1811.585078	2039.010354
1192.399738	1136.925861	1016.188795	1757.721649
1115.171465	0.656274821	0.000141416	0.002597623	BIN1
	2	127048027	127107355	-	6929
protein_coding	bridging integrator 1 [Source:HGNC
Symbol;Acc:HGNC:1052]	-	1568	2047	1754	1151	996
	1178	11.08164365	13.99824925	15.74946749

9.230321028	8.835601847	7.905070496
ENSG00000148331 678.6237226	736.3159674	618.4455577
865.0336932	1288.744274	1256.00245	677.7950826
1136.593472	-0.745247102	0.000141877	0.002603381	ASB6
	9	129634604	129642169	-	4622
protein_coding	ankyrin repeat and SOCS box containing 6 [Source:HGNC
Symbol;Acc:HGNC:17181]	-	748	832	532	835	1129
	1456	7.925019514	8.529426399	7.161245094
10.03849433	15.01451683	14.64746443
ENSG00000145649 19.05227029	14.15992245	20.9248497	0
	0	0	18.04568081	0	6.605670376
0.000143419	0.002628959	GZMA	5	55102648
55110252	+	894	protein_coding	granzyme A
[Source:HGNC Symbol;Acc:HGNC:4708]	-	21	16	18
	0	0	0	1.150298251	0.848025486
1.252684873	0	0	0
ENSG00000231500 25533.67119	36767.12364	38090.2014
43196.77833	55392.03245	55453.02575	33463.66541
51347.27884	-0.617720225	0.000144392	0.00264404	RPS18
	6	33272010	33276510	+	1898
protein_coding	ribosomal protein S18 [Source:HGNC
Symbol;Acc:HGNC:10401]	-	28144	41545	32766	41697	48526
	64283	726.1365502	1037.16773	1074.073658
1220.732888	1571.540747	1574.82009
ENSG00000173848 974.3875375	938.0948623	977.6554776
728.2858519	471.436125	383.8743751	963.3792924
527.8654507	0.868745418	0.000144614	0.002645381	NET1
	10	5412551 5458463 +	4175	protein_coding
neuroepithelial cell transforming 1 [Source:HGNC
Symbol;Acc:HGNC:14592]	-	1074	1060	841	703	413
	445	12.59727137	12.03028251	12.5327496
9.356445427	6.080522951	4.956037106
ENSG00000181894 91.63234756	64.60464618	75.56195724
250.7043758	159.8088559	179.4289214	77.26631699
196.6473844	-1.34770108	0.000145133	0.002652126	ZNF329
	19	58126252	58155110	-	4309
protein_coding	zinc finger protein 329 [Source:HGNC
Symbol;Acc:HGNC:14209]	zf-C2H2 101	73	65	242	140
	208	1.147819407	0.802736123	0.938520383
3.120692057	1.997095816	2.244490952
ENSG00000204116 697.6759928	631.8865393	512.6588176
1145.781155	1019.352203	885.0676605	614.0737833
1016.733673	-0.72579387	0.000146504	0.00267373	CHIC1
	X	73563200	73687102	+	6939
protein_coding	cysteine rich hydrophobic domain 1 [Source:HGNC
Symbol;Acc:HGNC:1934]	-	769	714	441	1106	893
	1026	5.426978935	4.875596595	3.954108195
8.856666329	7.910463511	6.875139132
ENSG00000041357 3105.520057	3122.2629	3503.749833
2144.454784	2260.15382	2436.092664	3243.844263

2280.233756	0.508068958	0.000146618	0.00267373	PSMA4
	15	78540405	78552419	+	6996
protein_coding	proteasome subunit alpha 4 [Source:HGNC
Symbol;Acc:HGNC:9533]	-	3423	3528	3014	2070	1980
	2824	23.95994307	23.89489995	26.80404225
16.44116502	17.39653478	18.76920638
ENSG00000105640 17809.33646	23634.68056	27175.5673
29050.62853	37757.1252	39145.69724	22873.19477
35317.81699	-0.626796959	0.000147664	0.002690039	RPL18A
	19	17859876	17864153	+	3527
protein_coding	ribosomal protein L18a [Source:HGNC
Symbol;Acc:HGNC:10311]	-	19630	26706	23377	28042	33077
	45379	272.5483289	358.781322	412.3729445
441.7896823	576.4584555	598.2469315
ENSG00000102265 3473.863949	5479.004993	5699.696559
6206.487253	9415.024599	8712.654357	4884.1885
8111.388736	-0.731967361	0.000148237	0.0026977	TIMP1
	X	47582313	47586789	+	1541
protein_coding	TIMP metallopeptidase inhibitor 1 [Source:HGNC
Symbol;Acc:HGNC:11820]	-	3829	6191	4903	5991	8248
	10100	121.6778175	190.3639055	197.9548292
216.0273432	328.9980878	304.7542376
ENSG00000087266 1199.385777	1208.903379	1275.25334
873.3214412	733.9792455	812.6059807	1227.847499
806.6355558	0.605580059	0.000148833	0.002704406	SH3BP2
	4	2793023 2841098 +	13968	protein_coding	SH3
domain binding protein 2 [Source:HGNC Symbol;Acc:HGNC:10825]	-
	1322	1366	1097	843	643	942	4.634745723
	4.633860056	4.886290103	3.35355468	2.829593875
	3.135795226
ENSG00000120868 381.0454057	274.3484975	442.9093186
180.2585181	181.4972007	179.4289214	366.1010739
180.3948801	1.019631129	0.000148911	0.002704406	APAF1
	12	98645141	98735433	+	7908
protein_coding	apoptotic peptidase activating factor 1 [Source:HGNC
Symbol;Acc:HGNC:576]	-	420	310	381	174	159
	208	2.600826091	1.857468571	2.997541204
1.222628827	1.235884325	1.223003479
ENSG00000107815 1036.987854	1168.193602	973.005511
1309.464178	1808.123056	2089.311768	1059.395656
1735.633	-0.712152543	0.000149167	0.002706268	TWNK
	10	100987367	100994401	+	4133
protein_coding	twinkle mtDNA helicase [Source:HGNC
Symbol;Acc:HGNC:1160]	-	1143	1320	837	1264	1584
	2422	13.54283255	15.13334618	12.59989423
16.99392559	23.55793028	27.2483213
ENSG00000160299 2357.03801	1866.454778	1829.761857
1533.233372	1101.539614	1169.738545	2017.751548
1268.170511	0.670229904	0.000149652	0.002712286	PCNT
	21	46324122	46445769	+	11660

protein_coding pericentrin [Source:HGNC Symbol;Acc:HGNC:16068] -
        2598   2109   1574   1480   965   1356   10.91111869
        8.570466667   8.39871848   7.053021515   5.087168504
        5.407445578
ENSG00000108106 5878.98626   6815.347674   7007.499666
4419.441599   4624.183396   4991.229516   6567.277866
4678.284837   0.489106917   0.0001521   0.002753827   UBE2S
        19   55399745   55407777   -   2788
protein_coding ubiquitin conjugating enzyme E2 S [Source:HGNC
Symbol;Acc:HGNC:17895] -   6480   7701   6028   4266   4051
        5786   113.8179921   130.8822628   134.5201432
85.02372447   89.31344349   96.49763686
ENSG00000187514 20080.18563   20545.16248   25140.04442
15766.40452   15792.53944   15647.40964   21921.79751
15735.4512   0.478276818   0.000152675   0.002758116   PTMA
        2   231706895   231713541   +   3378
protein_coding prothymosin alpha [Source:HGNC Symbol;Acc:HGNC:9623]
        -       22133   23215   21626   15219   13835   18139
320.8553774   325.6383036   398.3120086   250.3447657
251.7484924   249.6805508
ENSG00000188501 124.2933823   74.33959286   75.56195724
6.215810969   30.82027936   18.11541995   91.39831081
18.38383676   2.321036068   0.000152785   0.002758116   LCTL
        15   66547179   66565979   -   3518
protein_coding lactase like [Source:HGNC Symbol;Acc:HGNC:15583]
        -       137   84   65   6   27   21
1.907011955   1.131383633   1.149540742   0.094769253
0.471753672   0.277558465
ENSG00000272129 25.40302705   17.69990306   27.8997996
1.035968495   0   0   23.66757657   0.345322832
6.034923652   0.000152804   0.002758116   AL359715.3   6
        80355424   80356859   +   1436   lincRNA novel
transcript   -   28   20   24   1   0   0
        0.954843674   0.659936268   1.039833125   0.038695245
        0   0
ENSG00000197081 2700.886126   2198.32796   2700.468103
1910.325905   1627.767347   1512.206246   2533.227396
1683.433166   0.589585227   0.000153449   0.002766937   IGF2R
        6   159969099   160113507   +   17843
protein_coding insulin like growth factor 2 receptor [Source:HGNC
Symbol;Acc:HGNC:5467]   -   2977   2484   2323   1844   1426
        1753   8.17033084   6.596448207   8.100058975
5.742553962   4.912459809   4.568199466
ENSG00000131018 1284.667368   1557.591469   1069.492318
2444.885648   1841.226319   1975.443414   1303.917052
2087.185127   -0.677943965   0.000153907   0.002772355   SYNE1
        6   152121684   152637801   -   46064
protein_coding spectrin repeat containing nuclear envelope protein 1
[Source:HGNC Symbol;Acc:HGNC:17089]   -   1416   1760   920
        2360   1613   2290   1.505325052   1.810412173

1.242604799	2.846835672	2.152385611	2.311558309
ENSG00000090863 2932.235122	2568.255934	3865.284736
2306.06587	1890.310467	1529.459027	3121.925264
1908.611788	0.709849228	0.000154407	0.002778535	GLG1
	16	74447427	74607144	-	14111
protein_coding	golgi glycoprotein 1 [Source:HGNC
Symbol;Acc:HGNC:4316]	-	3232	2902	3325	2226	1656
	1773	11.21610958	9.744645781	14.66022732
8.765554852	7.213564191	5.842274545
ENSG00000099783 9323.818177	9013.675635	9690.530394
7158.5423	6338.704122	7041.722527	9342.674735
6846.322983	0.448365421	0.000154694	0.002780868	HNRNPM
	19	8444767 8489114 +	5614	protein_coding
heterogeneous nuclear ribonucleoprotein M [Source:HGNC
Symbol;Acc:HGNC:5046]	-	10277	10185	8336	6910	5553
	8163	89.64428998	85.96369327	92.38301767
68.3939494	60.79986646	67.6096062
ENSG00000169826 914.5089737	846.9403615	846.2939211
1279.421091	1261.34847	1347.442189	869.2477521
1296.070583	-0.576322669	0.000157319	0.002825187
CSGALNACT2	10	43138486	43185308	+	3730
	protein_coding	chondroitin sulfate N-
acetylgalactosaminyltransferase 2 [Source:HGNC Symbol;Acc:HGNC:24292]
	-	1008	957	728	1235	1105	1562
13.23367253	12.15708762	12.14309504	18.39798114
18.20961679	19.47167068
ENSG00000102794 0	5.309970919	16.2748831	62.15810969
	42.23519764	95.75293403	7.194951339	66.71541379
	-3.252007866	0.00015873	0.002847647	ACOD1	13
	76948497	76958642	+	2177	protein_coding
	aconitate decarboxylase 1 [Source:HGNC Symbol;Acc:HGNC:33904]
	-	0	6	14	60	37	111	0
	0.130592809	0.40010728	1.531457204	1.04469774
	2.370803545
ENSG00000083937 1670.249028	1369.972497	1438.002171
2085.40458	2255.587852	2209.218595	1492.741232
2183.403676	-0.548437159	0.000161063	0.002886573	CHMP2B
	3	87227271	87255548	+	3106
protein_coding	charged multivesicular body protein 2B [Source:HGNC
Symbol;Acc:HGNC:24537]	-	1841	1548	1237	2013	1976
	2561	29.02558761	23.61543478	24.77850471
36.01259087	39.10505032	38.3388556
ENSG00000168404 326.6103478	330.9881873	248.7732131
492.0850351	547.9160775	574.5176042	302.123916
538.1729056	-0.831102747	0.000161828	0.002897349	MLKL
	16	74671855	74700960	-	5692
protein_coding	mixed lineage kinase domain like pseudokinase
[Source:HGNC Symbol;Acc:HGNC:26617]	-	360	374	214
	475	480	666	3.097178907	3.113387315
2.339137377	4.637039142	5.183507201	5.440519308

ENSG00000072062 1342.73143 1375.282468 1770.474783
1137.493407 784.204886 733.2431885 1496.162893
884.9804939 0.757340615 0.000162015 0.002897767 PRKACA
19 14091688 14118084 - 6144
protein_coding protein kinase cAMP-activated catalytic subunit alpha
[Source:HGNC Symbol;Acc:HGNC:9380] - 1480 1554 1523
1098 687 850 11.79612027 11.98467464
15.42252713 9.93031847 6.873103601 6.432780562
ENSG00000099139 339.3118613 185.8489822 556.8335003
101.5249125 191.7706271 100.0661293 360.6647812
131.1205563 1.460858954 0.000162193 0.002898008 PCSK5
9 75890644 76362339 + 12705
protein_coding proprotein convertase subtilisin/kexin type 5
[Source:HGNC Symbol;Acc:HGNC:8747] - 374 210 479
98 168 116 1.441536411 0.783197092
2.345674198 0.428611135 0.812796776 0.424535818
ENSG00000182578 375.6018999 309.7483036 318.5227121
203.049825 136.9790194 151.824472 334.6243052
163.9511055 1.029351165 0.000163089 0.002911082 CSF1R
5 150053291 150113372 - 5151
protein_coding colony stimulating factor 1 receptor [Source:HGNC
Symbol;Acc:HGNC:2433] - 414 350 274 196 120
176 3.935840359 3.219607536 3.309526474
2.114348467 1.431980343 1.588737461
ENSG00000213853 1398.073739 1329.26272 1487.989312
1053.579959 866.3922976 684.9354019 1405.10859
868.3025529 0.695002874 0.000163816 0.002921107 EMP2
16 10528422 10580698 - 8718
protein_coding epithelial membrane protein 2 [Source:HGNC
Symbol;Acc:HGNC:3334] - 1541 1502 1280 1017 759
794 8.655944389 8.163558492 9.134819106
6.482106048 5.35145985 4.234817079
ENSG00000083457 283.0623014 347.8030952 337.1225785
175.0786756 144.9694622 168.2146138 322.6626583
162.7542505 0.986033259 0.000164875 0.00293704 ITGAE
17 3714628 3801243 - 6657 protein_coding
integrin subunit alpha E [Source:HGNC Symbol;Acc:HGNC:6147] -
312 393 290 169 127 195 2.295116423
2.797308946 2.710355683 1.410652982 1.172661115
1.362031252
ENSG00000109089 1387.186727 1291.207928 1474.039412
926.1558344 834.4305264 987.7217068 1384.144689
916.1026892 0.594396537 0.000165705 0.002948844 CDR2L
17 74987632 75005800 + 3536
protein_coding cerebellar degeneration related protein 2 like
[Source:HGNC Symbol;Acc:HGNC:29999] - 1529 1459 1268
894 731 1145 21.17502407 19.55102254
22.31073325 14.04873777 12.70727653 15.05650763
ENSG00000103512 1555.028156 1669.985854 2627.231129
953.0910153 1104.96409 1331.914686 1950.748379

1129.98993 0.786545233 0.00016613 0.002953446 NOMO1
16 14833681 14896160 + 5819
protein_coding NODAL modulator 1 [Source:HGNC Symbol;Acc:HGNC:30060]
- 1714 1887 2260 920 968 1544
14.42418035 15.36561629 24.16389437 8.785197186
10.22526001 12.33757929
ENSG00000115355 1810.872928 1108.013932 1192.716433
983.1341017 603.8491771 671.9958163 1370.534431
752.9930317 0.864263214 0.000167575 0.002976144
CCDC88A 2 55287842 55419895 - 33942
protein_coding coiled-coil domain containing 88A [Source:HGNC
Symbol;Acc:HGNC:25523] - 1996 1252 1026 949 529
779 2.879730137 1.747806462 1.880688107
1.553605775 0.958000207 1.067163706
ENSG00000276043 5358.224205 5319.705865 5842.683032
4476.419866 2849.163603 3472.984796 5506.871034
3599.522755 0.613242149 0.000168233 0.002984825 UHRF1
19 4903080 4962154 + 5145 protein_coding
ubiquitin like with PHD and ring finger domains 1 [Source:HGNC
Symbol;Acc:HGNC:12556] - 5906 6011 5026 4321 2496
4026 56.21299787 55.35894305 60.77765671
46.66711259 29.81992605 36.3847512
ENSG00000151388 670.4584639 554.0069659 805.6067134
462.0419487 223.7323983 328.6654763 676.6907144
338.1466078 0.999262632 0.000169453 0.003003454
ADAMTS12 5 33523535 33892192 - 9778
protein_coding ADAM metallopeptidase with thrombospondin type
1 motif 12 [Source:HGNC Symbol;Acc:HGNC:14605] - 739 626
693 446 196 381 3.701034121 3.033547091
4.409507121 2.534526693 1.232121111 1.811782084
ENSG00000176438 847.3724022 784.9907008 926.505845
637.1206244 414.3615336 377.8359018 852.956316
476.4393533 0.840424881 0.000171919 0.003044115 SYNE3
14 95407266 95475836 - 18164
protein_coding spectrin repeat containing nuclear envelope family
member 3 [Source:HGNC Symbol;Acc:HGNC:19861] - 934 887
797 615 363 438 2.51804836 2.313867933
2.7299436 1.881376289 1.228407592 1.121227227
ENSG00000217801 38.10454057 36.28480128 48.8246493
3.107905485 6.848950969 0 41.07133038 3.318952151
3.680722717 0.000172225 0.003046482 AL390719.1
1 1059734 1069355 + 2171
transcribed_unprocessed_pseudogene uncharacterized LOC100288175
[Source:NCBI gene;Acc:100288175] - 42 41 42
3 6 0 0.947366777 0.894850477
1.203639173 0.076784485 0.169878645 0
ENSG00000116459 4617.000166 4720.564147 5436.973447
3876.594108 3045.500198 3284.929484 4924.84592
3402.341263 0.533316726 0.000172416 0.003046821 ATP5PB
1 111448864 111462773 + 3383

protein_coding ATP synthase peripheral stalk-membrane subunit b
[Source:HGNC Symbol;Acc:HGNC:840]   -   5089   5334   4677
        3742   2668   3808   73.66465117   74.70977946
86.01460885   61.46301058   48.47649234   52.339065
ENSG00000100029 1461.581306   2120.448387   1844.874248
2286.382468   2980.435163   3574.776204   1808.96798
2947.197945   -0.704421029   0.000172953   0.003053252   PES1
        22   30576625   30607083   -   4557
protein_coding pescadillo ribosomal biogenesis factor 1 [Source:HGNC
Symbol;Acc:HGNC:8848]   -   1611   2396   1587   2207   2611
        4144   17.31191836   24.91347027   21.66729742
26.91134155   35.21885264   42.28357863
ENSG00000140264 1455.230549   2622.240639   2541.206747
823.5949534   1164.321665   1557.926116   2206.225978
1181.947578   0.899675326   0.000173221   0.00305495   SERF2
        15   43777087   43802589   +   7347
protein_coding small EDRK-rich factor 2 [Source:HGNC
Symbol;Acc:HGNC:10757] -   1604   2963   2186   795   1020
        1806   10.69111516   19.10944406   18.51172817
6.01269442   8.533702374   11.42980245
ENSG00000125630 2383.348288   2376.211986   1940.198564
3194.926838   3006.689475   3596.34218   2233.252946
3265.986164   -0.548135727   0.000173552   0.00305676   POLR1B
        2   112541915   112577150   +   6329
protein_coding RNA polymerase I subunit B [Source:HGNC
Symbol;Acc:HGNC:20454] -   2627   2685   1669   3084   2634
        4169   20.32608141   20.10183182   16.4069588
27.07642469   25.58161951   30.62864735
ENSG00000260966 84.37433984   83.18954439   112.76169
199.9419195   214.6004637   226.0114299   93.44185809
213.5179377   -1.196230981   0.00017367   0.00305676
AP001486.2   11   103050687   103055799   -   5113
        sense_overlapping   "novel transcript, overlapping to
DCUN1D5"   -   93   94   97   193   188   262
        0.890708998   0.871121037   1.180328213   2.097459385
        2.260109167   2.382629512
ENSG00000162521 4803.893865   4222.311876   4933.614562
3587.558898   3268.091104   2840.670376   4653.273434
3232.106793   0.52587615   0.000174522   0.003066568   RBBP4
        1   32651142   32686211   +   9470
protein_coding "RB binding protein 4, chromatin remodeling factor
[Source:HGNC Symbol;Acc:HGNC:9887]"   -   5295   4771   4244
        3463   2863   3293   27.3807077   23.87183909
27.88253415   20.31957201   18.58312419   16.16861314
ENSG00000107249 591.5276298   543.387024   784.6818637
397.811902   311.6272691   365.7589552   639.8655058
358.3993754   0.834253488   0.000174686   0.003066568   GLIS3
        9   3824127 4348392 -   11826   protein_coding GLIS
family zinc finger 3 [Source:HGNC Symbol;Acc:HGNC:28510]   zf-
C2H2   652   614   675   384   273   424   2.699842345

2.460123657	3.55118048	1.804286058	1.418966468
1.667089822
ENSG00000173744 1706.539067	1556.706474	1589.126085
2387.907381	2163.127014	2377.433209	1617.457209
2309.489201	-0.513950013	0.000174747	0.003066568	AGFG1
2	227472152	227561214	+	9321
protein_coding	ArfGAP with FG repeats 1 [Source:HGNC
Symbol;Acc:HGNC:5175]	-	1881	1759	1367	2305	1895
2756	9.88223031	8.94189925	9.124579369
13.74106726	12.49666324	13.74825958
ENSG00000104695 1819.945438	2058.498726	2059.935204
2686.266307	2827.475258	2896.741914	1979.459789
2803.494493	-0.502361478	0.000175179	0.003071098	PPP2CB
8	30774457	30814314	-	4516
protein_coding	protein phosphatase 2 catalytic subunit beta
[Source:HGNC Symbol;Acc:HGNC:9300]	-	2006	2326	1772
2593	2477	3358	21.75232478	24.4051914
24.41274591	31.90513798	33.7147104	34.57464875
ENSG00000180891 1355.432943	1308.022836	1338.027889
924.0838975	848.1284283	929.0622518	1333.82789
900.4248592	0.566493827	0.000175628	0.003075929	CUEDC1
17	57861243	57955323	-	5577
protein_coding	CUE domain containing 1 [Source:HGNC
Symbol;Acc:HGNC:31350]	-	1494	1478	1151	892	743
1077	13.11833256	12.55741452	12.84048789
8.887431236	8.189087838	8.979374335
ENSG00000237973 137.9021468	143.3692148	77.88694054
186.4743291	335.5985975	376.1106237	119.7194341
299.3945168	-1.318924419	0.000176253	0.003083818
MTCO1P12	1	631074	632616	+	1543
unprocessed_pseudogene	MT-CO1 pseudogene 12 [Source:HGNC
Symbol;Acc:HGNC:52014]	-	152	162	67	180	294
436	4.823989407	4.974798896	2.701566879
6.482143225	11.711938	13.13867534
ENSG00000196659 189.6154519	201.7788949	211.5734803
342.9055718	326.4666629	577.9681603	200.9892757
415.7801317	-1.051136923	0.000176518	0.003085401	TTC30B
2	177548998	177553014	-	4017
protein_coding	tetratricopeptide repeat domain 30B [Source:HGNC
Symbol;Acc:HGNC:26425]	-	209	228	182	331	286
670	2.547845787	2.689425114	2.818878796
4.578658003	4.376345103	7.755396192
ENSG00000072501 5915.276298	5248.021258	5746.196225
4607.987865	3306.901826	3524.743139	5636.497927
3813.210944	0.563751956	0.00017679	0.003087101	SMC1A
X	53374149	53422728	-	10484
protein_coding	structural maintenance of chromosomes 1A [Source:HGNC
Symbol;Acc:HGNC:11111]	-	6520	5930	4943	4448	2897
4086	30.45434526	26.80119274	29.33394307
23.57489733	16.98512875	18.12184319

ENSG00000064393 1956.033083 1695.650713 1521.70157
2374.43979 2539.819318 2704.373407 1724.461789
2539.544172 -0.558045427 0.000178088 0.003103797 HIPK2
7 139561570 139777778 - 15402
protein_coding homeodomain interacting protein kinase 2 [Source:HGNC
Symbol;Acc:HGNC:14402] - 2156 1916 1309 2292 2225
3135 6.854887331 5.89447088 5.287731252
8.268934235 8.879740137 9.464364203
ENSG00000102241 3433.944906 3085.978099 3163.139779
2458.353238 2277.276197 2080.685377 3227.687595
2272.104938 0.506756351 0.000178097 0.003103797
HTATSF1 X 136497079 136512346 + 3136
protein_coding HIV-1 Tat specific factor 1 [Source:HGNC
Symbol;Acc:HGNC:5276] - 3785 3487 2721 2373 1995
2412 59.10422314 52.68686214 53.98328818
42.04687539 39.1033717 35.76286206
ENSG00000279095 170.5631816 206.2038707 106.9492318
323.2221704 386.9657298 332.1160324 161.2387614
347.4346442 -1.102858344 0.00017949 0.003125002
AC243964.3 19 44664131 44666158 + 2028
TEC TEC - 188 233 92 312 339
385 4.539610399 5.443953553 2.82245303
8.548672647 10.27493558 8.827216877
ENSG00000143479 626.9104175 459.3124845 394.0846693
729.3218204 867.5337894 1050.694357 493.4358571
882.5166557 -0.837974597 0.000181823 0.003162512 DYRK3
1 206635536 206684419 + 4336
protein_coding dual specificity tyrosine phosphorylation regulated
kinase 3 [Source:HGNC Symbol;Acc:HGNC:3094] - 691 519
339 704 760 1218 7.804003534 5.671586044
4.864265498 9.021846409 10.77386871 13.06137904
ENSG00000186594 303.0218226 282.3134538 141.8239813
406.09965 675.7631623 504.6438415 242.3864193
528.8355513 -1.121713105 0.00018456 0.003206962
MIR22HG 17 1711493 1717174 - 2986 lincRNA MIR22 host
gene [Source:HGNC Symbol;Acc:HGNC:28219] - 334 319
122 392 592 585 5.477537342 5.062060373
2.542007771 7.294714636 12.1865076 9.109553291
ENSG00000177000 257.6592743 445.152562 309.2227789
542.8474913 721.4228354 644.3913668 337.3448717
636.2205645 -0.914170087 0.000184882 0.003209396 MTHFR
1 11785723 11806920 - 11978
protein_coding methylenetetrahydrofolate reductase [Source:HGNC
Symbol;Acc:HGNC:7436] - 284 503 266 524 632
747 1.161081525 1.989803164 1.381669512
2.43085482 3.243247782 2.899795097
ENSG00000101463 156.9544171 123.8993214 146.4739479
39.36680281 34.24475485 0.862639045 142.4425621
24.82473223 2.53221037 0.000185376 0.003214816
SYNDIG1 20 24469199 24666616 + 2847

protein_coding	synapse differentiation inducing 1 [Source:HGNC Symbol;Acc:HGNC:15885]	-	173	140	126	38	30	1	2.975687492	2.33005949	2.75353071	0.741665663	0.647710814	0.016332157
ENSG00000131899 2545.746211	2698.350222	3021.315798	1914.469779	2052.402307	1767.547404	2755.13741	1911.473163	0.527495933	0.000185966	0.003221895	LLGL1	17	18225587	18244875	+	6638	protein_coding	"LLGL1, scribble cell polarity complex component [Source:HGNC Symbol;Acc:HGNC:6628]"	-	2806	3049	2599	1848	1798	2049	20.70041738	21.76439589	24.35992114	15.4695173	16.64944647	14.3527701
ENSG00000245532 4277.688305	5623.259203	3005.040915	6011.725176	7727.899677	7483.393718	4301.996141	7074.339523	-0.717238787	0.000188146	0.003256189	NEAT1	11	65422774	65445540	+	22767	lincRNA nuclear paraspeckle assembly transcript 1 [Source:HGNC Symbol;Acc:HGNC:30815]	-	4715	6354	2585	5803	6770	8675	10.14155553	13.22415235	7.064177229	14.16311582	18.27806516	17.71718394
ENSG00000131116 422.7789501	723.0410401	757.9445557	265.2079347	404.0881072	288.1214411	634.588182	319.139161	0.992240629	0.000188314	0.003256189	ZNF428	19	43607219	43619874	-	1803	protein_coding	zinc finger protein 428 [Source:HGNC Symbol;Acc:HGNC:20804]	-	466	817	652	256	354	334	12.656653	21.47102466	22.49876687	7.889623563	12.06854448	8.613541653
ENSG00000163590 301.2073207	292.0484005	217.3859385	522.1281214	554.7650285	422.6931322	270.2138866	499.862094	-0.884286615	0.000188903	0.003261425	PPM1L	3	160755602	161078907	+	12447	protein_coding	"protein phosphatase, Mg2+/Mn2+ dependent 1L [Source:HGNC Symbol;Acc:HGNC:16381]"	-	332	330	187	504	486	490	1.306177137	1.25624889	0.934724796	2.249976026	2.400042542	1.830469128
ENSG00000168913 0	0.884995153	0	40.4027713	18.26386925	12.07694663	0.294998384	23.58119573	-6.072247533	0.000188985	0.003261425	ENHO	9	34521040	34523041	-	1082	protein_coding	energy homeostasis associated [Source:HGNC Symbol;Acc:HGNC:24838]	-	0	1	0	39	16	14	0	0.043792444	0	2.002854451	0.908950184	0.601633199
ENSG00000008441 3364.993833	2524.006177	3638.598864	2323.677334	1961.082961	1932.311461	3175.866291	2072.357252	0.615625441	0.000189286	0.003263436	NFIX	19	12995608	13098796	+	6302	protein_coding	nuclear factor I X [Source:HGNC Symbol;Acc:HGNC:7788]	CTF/NFI 3709	2852	3130	2243	1718	2240

28.82087209	21.44359337	30.90101788	19.77711407
16.75683992	16.52725156
ENSG00000174233 806.5461088	738.9709529	1005.555277
466.1858227	578.7363569	481.3525873	850.3574463
508.7582556	0.74087207	0.000191295	0.003294855	ADCY6
	12	48766194	48789037	-	8410
protein_coding	adenylate cyclase 6 [Source:HGNC Symbol;Acc:HGNC:237]
	-	889	835	865	450	507	558
5.176478907	4.704537347	6.399218779	2.973230379
3.705606707	3.085099826
ENSG00000063177 13172.37678	17557.41884	19168.32482
21384.46167	26027.15517	27105.84408	16632.70681
24839.15364	-0.578667858	0.000193609	0.003331465	RPL18
	19	48615328	48619536	-	4209
protein_coding	ribosomal protein L18 [Source:HGNC
Symbol;Acc:HGNC:10310]	-	14519	19839	16489	20642	22801
	31422	168.9220966	223.3404015	243.7374363
272.511536	332.9832049	347.1250191
ENSG00000091137 110.6846178	74.33959286	87.18687374
21.75533839	15.98088559	0	90.73702815	12.57874133
	2.868036452	0.000194299	0.0033401	SLC26A4 7
	107660635	107717809	+	5800	protein_coding
	solute carrier family 26 member 4 [Source:HGNC
Symbol;Acc:HGNC:8818]	-	122	84	75	21	14
	0	1.030055252	0.686242693	0.80452606
0.201188589	0.148370446	0
ENSG00000002586 4064.484328	5263.066176	6814.526052
3573.055339	3347.995532	3447.105625	5380.692185
3456.052165	0.63835796	0.000194757	0.003344154	CD99
	X	2691133 2741309 +	4912	protein_coding	CD99
molecule (Xg blood group) [Source:HGNC Symbol;Acc:HGNC:7082]	-
	4480	5947	5862	3449	2933	3996	44.6630461
	57.3675128	74.2496315	39.01637169	36.70295786
	37.82667251
ENSG00000162231 1504.222102	1627.506087	1406.614896
2238.727917	1927.979698	2895.879275	1512.781028
2354.19563	-0.638311588	0.000194913	0.003344154	NXF1
	11	62792123	62806302	-	6194
protein_coding	nuclear RNA export factor 1 [Source:HGNC
Symbol;Acc:HGNC:8071]	-	1658	1839	1210	2161	1689
	3357	13.1081683	14.06814931	12.15404992
19.38633037	16.76122704	25.20061609
ENSG00000076201 2208.248851	2381.521957	2229.658984
2869.632731	3260.100661	3807.688746	2273.143264
3312.474046	-0.543401668	0.000196288	0.003364476	PTPN23
	3	47381011	47413441	+	5981
protein_coding	"protein tyrosine phosphatase, non-receptor type 23
[Source:HGNC Symbol;Acc:HGNC:14406]"	-	2434	2691	1918
	2770	2856	4414	19.92853873	21.31897578
19.95177998	25.73463484	29.35159869	34.31543784

ENSG00000198483 264.9172821 347.8030952 311.5477622
143.9996208 180.3557089 119.9068273 308.0893798
148.0873856 1.059931103 0.00019758 0.003383346
ANKRD35 1 145866560 145885866 - 3363
protein_coding ankyrin repeat domain 35 [Source:HGNC
Symbol;Acc:HGNC:26323] - 292 393 268 139 158
139 4.251915918 5.537224399 4.958094195
2.296677293 2.887869605 1.921847624
ENSG00000083444 3701.583941 4578.079927 5833.383099
2923.503093 3434.748911 2680.219514 4704.348989
3012.823839 0.642834945 0.000198759 0.003400246 PLOD1
1 11934205 11975538 + 5320
protein_coding "procollagen-lysine,2-oxoglutarate 5-dioxygenase 1
[Source:HGNC Symbol;Acc:HGNC:9081]" - 4080 5173 5018
2822 3009 3107 37.55581701 46.07414523
58.68483276 29.4752448 34.76625535 27.1556773
ENSG00000188641 1858.049978 1346.962623 1990.185705
1365.406476 740.8281965 658.1935915 1731.732769
921.4760881 0.910268204 0.000203058 0.003470245 DPYD
1 97077743 97995000 - 7131
protein_coding dihydropyrimidine dehydrogenase [Source:HGNC
Symbol;Acc:HGNC:3012] - 2048 1522 1712 1318 649
763 14.06397868 10.11324799 14.93688958
10.27015546 5.59424678 4.975137862
ENSG00000120658 103.4266101 157.5291373 224.3608884
302.5028005 413.2200418 362.308399 161.7722119
359.3437471 -1.155241759 0.000203243 0.003470245 ENOX1
13 43213518 43786908 - 3101
protein_coding ecto-NOX disulfide-thiol exchanger 1 [Source:HGNC
Symbol;Acc:HGNC:25474] - 114 178 193 292 362
420 1.800245644 2.719848269 3.872241092
5.232305928 7.175532974 6.297650316
ENSG00000086475 1589.503692 1563.786436 1859.98664
1168.572462 1101.539614 1131.782427 1671.092256
1133.964835 0.558809431 0.000204499 0.003488314 SEPHS1
10 13317424 13348298 - 3888
protein_coding selenophosphate synthetase 1 [Source:HGNC
Symbol;Acc:HGNC:19685] - 1752 1767 1600 1128 965
1312 22.06665622 21.53459627 25.60357284
16.12110798 15.2562718 15.69056553
ENSG00000124784 1892.525515 1838.134933 1569.363727
2622.036261 2360.605101 2694.884377 1766.674725
2559.175246 -0.53433122 0.000204785 0.003489825 RIOK1
6 7389496 7418037 + 3237 protein_coding RIO
kinase 1 [Source:HGNC Symbol;Acc:HGNC:18656] - 2086 2077
1350 2531 2068 3124 31.55733268 30.40326591
25.9476431 43.44716962 39.26948405 44.8744712
ENSG00000146950 409.1701857 248.683638 281.3229793
178.1865811 127.8470848 119.9068273 313.0589343
141.9801644 1.142052857 0.000205349 0.003496076

SHROOM2 X	9786456 9949443 +	8191	protein_coding	shroom family member 2 [Source:HGNC Symbol;Acc:HGNC:630]	-	451
281	242	172	112	139	2.696300545
1.625533166	1.838168361	1.166819194	0.840482077
0.789057937
ENSG00000148411 535.2780699	498.2522712	611.4706078
356.3731622	310.4857773	271.7312993	548.3336497
312.8634129	0.809681221	0.00020579	0.003500214	NACC2
9	136006537	136095285	-	7212
protein_coding	NACC family member 2 [Source:HGNC Symbol;Acc:HGNC:23846]	-	590	563	526	344	272
315	4.006129437	3.698955594	4.537711416
2.650420416	2.318251483	2.030887441
ENSG00000116830 2092.120728	1662.020898	1655.388109
1443.104113	856.1188711	714.2651295	1803.176578
1004.496038	0.844647852	0.000208928	0.003550167	TTF2
1	117060303	117107453	+	10181
protein_coding	transcription termination factor 2 [Source:HGNC Symbol;Acc:HGNC:12398]	-	2306	1878	1424	1393	750
828	11.09168556	8.740405695	8.702146663
7.602785235	4.528122696	3.78155932
ENSG00000058799 470.8632513	492.0573051	548.6960587
671.3075847	886.9391505	973.0568431	503.8722051
843.7678594	-0.744959038	0.000209951	0.003564131	YIPF1
1	53851719	53889834	-	2072
protein_coding	Yip1 domain family member 1 [Source:HGNC Symbol;Acc:HGNC:25231]	-	519	556	472	648	777
1128	12.26609405	12.71485703	14.17291212
17.37790019	23.05040858	25.31339329
ENSG00000242299 244.0505098	443.3825717	402.2221109
743.8253793	665.4897358	621.1001126	363.2183975
676.8050759	-0.898375948	0.000210472	0.003567495
AC073861.1	3	101576489	101576947	-	459
processed_pseudogene	ribosomal protein S18 (RPS18) pseudogene	-	269	501	346	718	583	720
28.69910003	51.7191622	46.89972108	86.92081752
78.07342457	72.93749255
ENSG00000188766 224.0909886	232.7537253	265.0480962
344.9775088	454.3137476	613.3363612	240.6309367
470.8758725	-0.970760333	0.000210694	0.003567495	SPRED3
19	38388421	38399587	+	5864
protein_coding	sprouty related EVH1 domain containing 3 [Source:HGNC Symbol;Acc:HGNC:31041]	-	247	263	228	333	398
711	2.062679133	2.125143337	2.419066081
3.155457357	4.171924791	5.637760951
ENSG00000025039 33.56828574	26.54985459	34.8747495
105.6687865	90.17785443	113.0057149	31.66429661
102.9507853	-1.704729535	0.000210754	0.003567495	RRAGD
6	89364636	89412270	-	5072
protein_coding	Ras related GTP binding D [Source:HGNC

Symbol;Acc:HGNC:19903] - 37 30 30 102 79
131 0.357232664 0.280264732 0.368000879
1.117462532 0.957403932 1.200944863
ENSG00000102158 2531.230195 2167.35313 2512.144455
1976.627888 1188.292993 1133.507705 2403.575927
1432.809529 0.746450868 0.000211062 0.003569291 MAGT1
X 77826364 77895593 - 4862
protein_coding magnesium transporter 1 [Source:HGNC
Symbol;Acc:HGNC:28880] - 2790 2449 2161 1908 1041
1314 28.10075136 23.867134 27.6532805
21.80597247 13.16082563 12.56641589
ENSG00000122863 537.9998228 537.1920579 652.1578156
298.3589265 222.5909065 393.3634046 575.7832321
304.7710792 0.914335704 0.000211519 0.003571669 CHST3
10 71964365 72013564 + 6970
protein_coding carbohydrate sulfotransferase 3 [Source:HGNC
Symbol;Acc:HGNC:1971] - 593 607 561 288 195
456 4.166300577 4.126504792 5.007684161
2.295999311 1.71968615 3.042027128
ENSG00000109332 4795.728606 5330.325807 5006.851536
6364.990433 7112.635581 7323.805494 5044.301983
6933.810503 -0.459010989 0.000211607 0.003571669 UBE2D3
4 102794383 102868896 - 10069
protein_coding ubiquitin conjugating enzyme E2 D3 [Source:HGNC
Symbol;Acc:HGNC:12476] - 5286 6023 4307 6144 6231
8490 25.70807164 28.34346639 26.61309459
33.90602749 38.03809604 39.20598483
ENSG00000132357 163.3051739 142.4842197 188.3236473
337.7257293 300.2123508 326.9401982 164.7043469
321.6260928 -0.968079459 0.0002129 0.003590073 CARD6
5 40841184 40860175 + 4399
protein_coding caspase recruitment domain family member 6
[Source:HGNC Symbol;Acc:HGNC:16394] - 180 161 162
326 263 379 2.003767031 1.734196698
2.291225843 4.117898917 3.67493064 4.006049084
ENSG00000146834 2161.071801 2519.581201 2014.598029
2612.712544 3661.905785 4545.24513 2231.750344
3606.621153 -0.692467766 0.000214431 0.003612447 MEPCE
7 100428790 100434126 + 3420
protein_coding methylphosphate capping enzyme [Source:HGNC
Symbol;Acc:HGNC:20247] - 2382 2847 1733 2522 3208
5269 34.10706388 39.44462231 31.52675728
40.97613763 57.65747426 71.63629408
ENSG00000112578 1428.920271 1920.439482 1723.975117
1876.138944 3189.328168 3684.331362 1691.111623
2916.599492 -0.786514487 0.000214953 0.00361779 BYSL
6 41921188 41933046 + 2248
protein_coding bystin like [Source:HGNC Symbol;Acc:HGNC:1157] -
1575 2170 1483 1811 2794 4271 34.30938511
45.73933728 41.04419013 44.76454629 76.39720235

88.34140105
ENSG00000088305 137.9021468 125.6693117 149.9614228
60.0861727 46.80116496 43.13195226 137.8442938
50.00642997 1.46348735 0.0002166 0.003642055 DNMT3B
20 32762385 32809356 + 4630
protein_coding DNA methyltransferase 3 beta [Source:HGNC
Symbol;Acc:HGNC:2979] - 152 142 129 58 41
50 1.607649169 1.453228124 1.73346695
0.696079825 0.544314904 0.502134465
ENSG00000260454 27.21752898 6.194966072 4.6499666
62.15810969 86.75337894 81.08807026 12.68748722
76.66651963 -2.580812978 0.00021693 0.003644143
AL355607.2 9 90997054 91001871 + 1395
lincRNA novel transcript - 30 7 4
60 76 94 1.053114835 0.237766286
0.178399088 2.389951493 3.348780984 3.133175083
ENSG00000140350 2139.297778 2001.859036 2801.604876
1576.744049 1382.346604 1586.393204 2314.253897
1515.161286 0.610192297 0.000217473 0.003649783 ANP32A
15 68778535 68820897 - 7544
protein_coding acidic nuclear phosphoprotein 32 family member A
[Source:HGNC Symbol;Acc:HGNC:13233] - 2358 2262 2410
1522 1211 1839 15.30632056 14.20749009
19.87568977 11.21050086 9.867106678 11.33472708
ENSG00000171033 674.0874677 712.4210983 642.8578824
872.2854727 1140.350336 1262.040923 676.4554828
1091.558911 -0.690335632 0.00022068 0.003700106 PKIA
8 78516139 78605267 + 4216
protein_coding cAMP-dependent protein kinase inhibitor alpha
[Source:HGNC Symbol;Acc:HGNC:9017] - 743 805 553
842 999 1463 8.630121204 9.047356823
8.160774466 11.09745859 14.56505893 16.13521446
ENSG00000259431 37.19728961 43.3647625 23.249833
95.30910153 126.7055929 117.3189102 34.6039617
113.1112015 -1.701079313 0.000220904 0.003700358 THTPA
14 23555988 23560271 + 2894
protein_coding thiamine triphosphatase [Source:HGNC
Symbol;Acc:HGNC:18987] - 41 49 20 92 111
136 0.6937676 0.802276358 0.429970159
1.76644998 2.357609171 2.185100416
ENSG00000144659 1133.156457 1353.157589 1059.029893
1506.298192 1833.235876 2472.323504 1181.781313
1937.285857 -0.713125411 0.000222968 0.003731391
SLC25A38 3 39383324 39397351 + 2708
protein_coding solute carrier family 25 member 38
[Source:HGNC Symbol;Acc:HGNC:26054] - 1249 1529 911
1454 1606 2866 22.5861632 26.75378707
20.93035351 29.83512008 36.45392042 49.21058685
ENSG00000232679 30.84653284 38.05479158 20.9248497
139.8557468 77.62144432 100.9287683 29.94205804

106.1353198	-1.819225231	0.000224664	0.003756225
LINC01705	1	222041705	222064763	-	562
lincRNA long intergenic non-protein coding RNA 1705
[Source:HGNC Symbol;Acc:HGNC:52493]	-	34	43	18
135	68	117	2.962588175	3.625422125
1.992705118	13.34779403	7.437379756	9.68011606
ENSG00000182667 1451.601546	1279.702991	1611.213427
1052.543991	933.7403154	686.66068	1447.505988
890.9816621	0.700623609	0.000225402	0.003765005	NTM
11	131370478	132336822	+	6956
protein_coding	neurotrimin [Source:HGNC Symbol;Acc:HGNC:17941] -
1600	1446	1386	1016	818	796	11.26390795
9.849975731	12.39682594	8.116077366	7.228381913
5.32089284
ENSG00000139926 1956.940334	1614.231159	2186.646793
1254.557847	1399.468981	961.8425355	1919.272762
1205.289788	0.671631393	0.000226279	0.003776091	FRMD6
14	51489100	51730727	+	9416
protein_coding	FERM domain containing 6 [Source:HGNC
Symbol;Acc:HGNC:19839]	-	2157	1824	1881	1211	1226
1115	11.21792104	9.178777132	12.42879979
7.146439755	8.003342091	5.506040933
ENSG00000152217 305.7435755	216.8238125	332.4726119
161.6110852	123.2811174	83.67598739	285.0133333
122.8560633	1.215739033	0.000226531	0.00377672	SETBP1
18	44680173	45068510	+	14025
protein_coding	SET binding protein 1 [Source:HGNC
Symbol;Acc:HGNC:15573]	-	337	245	286	156	108
97	1.1766728	0.827731828	1.268732339
0.618064461	0.473334593	0.321588035
ENSG00000081692 176.0066874	184.0789918	203.4360387
354.3012253	315.0517446	393.3634046	187.8405727
354.2387915	-0.917372521	0.000228748	0.003809764	JMJD4
1	227730425	227735411	-	4315
protein_coding	jumonji domain containing 4 [Source:HGNC
Symbol;Acc:HGNC:25724]	-	194	208	175	342	276
456	2.201656762	2.284067718	2.523272155
4.404101806	3.931657177	4.913772673
ENSG00000144554 1411.682503	974.3796636	1016.017702
867.1056302	482.8510433	450.2975816	1134.026623
600.0847517	0.918846211	0.000228943	0.003809764	FANCD2
3	10026414	10101930	+	8378
protein_coding	FA complementation group D2 [Source:HGNC
Symbol;Acc:HGNC:3585]	-	1556	1101	874	837	423
522	9.09489983	6.226921686	6.490496547
5.551331288	3.103468713	2.897084517
ENSG00000263528 451.8109811	284.0834442	394.0846693
183.3664236	178.0727252	208.758649	376.6596982
190.0659326	0.985183341	0.0002292	0.003810447	IKBKE
1	206470476	206496889	+	3791

protein_coding	inhibitor of nuclear factor kappa B kinase subunit epsilon [Source:HGNC Symbol;Acc:HGNC:14552]	-	498	321
	339	177	156	242	6.432862104	4.012154873
	5.563559799	2.594367681	2.529403843	2.968196165
ENSG00000178719 3078.302528	3849.728916	3805.997662
4660.822259	5181.231408	5559.708647	3578.009702
5133.920771	-0.521126403	0.00022984	0.003817505	GRINA
	8	143990058	143993415	+	2527
protein_coding	glutamate ionotropic receptor NMDA type subunit associated protein 1 [Source:HGNC Symbol;Acc:HGNC:4589] -	3393
	4350	3274	4499	4539	6445	65.75174774
81.56624244	80.60839608	98.92881224	110.408447
118.5901717
ENSG00000004700 1329.122665	1057.569208	1249.678524
918.904055	583.3023242	361.44576	1212.123466
621.2173797	0.96530806	0.00023347	0.003871528	RECQL
	12	21468911	21501669	-	3778
protein_coding	RecQ like helicase [Source:HGNC Symbol;Acc:HGNC:9948]
	-	1465	1195	1075	887	511	419
18.98909882	14.98761031	17.70326447	13.04588993
8.313928912	5.156833239
ENSG00000271334 15.42326642	8.849951531	27.8997996	0
	0	0	17.39100585	0	6.549881974
0.00023353	0.003871528	LINC02104	5	39520431
	39524708	+	599	lincRNA long intergenic non-protein coding RNA 2104 [Source:HGNC Symbol;Acc:HGNC:52959]	-
	17	10	24	0	0	0	1.38979512
	0.791042138	2.492821983	0	0	0
ENSG00000265415 225.9054905	139.8292342	156.9363727
92.20119605	26.25431205	38.81875704	174.2236992
52.42475504	1.73265788	0.000233811	0.003872557
AC099850.3	17	59202677	59203829	-	321
	antisense	"novel transcript, antisense to PRR11"	-
	249	158	135	89	23	45	37.98595052
	23.32268225	26.1658943	15.40625273	4.404231754
	6.51836236
ENSG00000002822 2304.417454	2568.255934	2429.607548
2968.049738	3578.576881	4603.904585	2434.093645
3716.843735	-0.610972603	0.0002353	0.003893566	MAD1L1
	7	1815793 2233243 -	7230	protein_coding
mitotic arrest deficient 1 like 1 [Source:HGNC Symbol;Acc:HGNC:6762]
	-	2540	2902	2090	2865	3135	5337
17.20378882	19.01890686	17.98518193	22.01903961
26.65303122	34.32337013
ENSG00000162618 3.629003864	0.884995153	1.16249165
19.6834014	29.67878753	43.13195226	1.892163556
30.8313804	-4.009757419	0.000236399	0.003908089	ADGRL4
	1	78889764	79006718	-	3677
protein_coding	adhesion G protein-coupled receptor L4 [Source:HGNC Symbol;Acc:HGNC:20822]	-	4	1	1	19	26

50	0.053271515	0.012886436	0.016920501
0.287125665	0.434637384	0.632277013
ENSG00000156642 1627.608233	1551.396503	2157.584502
1268.025438	1152.906746	901.4578023	1778.86308
1107.463329	0.683707506	0.000238135	0.003933111	NPTN
15	73560014	73634134	-	4032
protein_coding	neuroplastin [Source:HGNC Symbol;Acc:HGNC:17867]
-	1794	1753	1856	1224	1010	1045
21.78866385	20.60097777	28.63942505	16.86836299
15.39742901	12.05110263
ENSG00000077097 4812.966375	3448.826112	4218.682197
3279.876255	2515.847989	2146.245945	4160.158228
2647.323396	0.652286407	0.000238845	0.003941173	TOP2B
3	25597905	25664907	-	6044
protein_coding	DNA topoisomerase II beta [Source:HGNC
Symbol;Acc:HGNC:11990]	-	5305	3897	3629	3166	2204
2488	42.98229654	30.55148967	37.35677349
29.10707055	22.41478073	19.14066131
ENSG00000178401 317.5378381	294.703386	263.8856045
165.7549592	92.46083808	31.91764468	292.0422762
96.71114731	1.598743231	0.000239568	0.003949411
DNAJC22 12	49346917	49357546	+	5109
protein_coding	DnaJ heat shock protein family (Hsp40) member C22
[Source:HGNC Symbol;Acc:HGNC:25802]	-	350	333	227
160	81	37	3.354755126	3.088408732
2.76437401	1.740187816	0.974532835	0.336741653
ENSG00000168569 777.5140778	936.324872	696.3324983
1067.04755	1462.251032	1351.755384	803.3904827
1293.684655	-0.686341609	0.000241771	0.003982011
TMEM223 11	62771629	62792021	-	2280
protein_coding	transmembrane protein 223 [Source:HGNC
Symbol;Acc:HGNC:28464]	-	857	1058	599	1030	1281
1567	18.40664594	21.98757134	16.34552304
25.10235235	34.53517356	31.95693855
ENSG00000101938 33.56828574	3.539980612	18.5998664	0
0	0	18.56937758	0	6.647600468
0.000242042	0.003982771	CHRDL1	X	110673856
110795819	-	3920	protein_coding	chordin like 1
[Source:HGNC Symbol;Acc:HGNC:29861]	-	37	4	16
0	0	0	0.462215325	0.048350433
0.253945641	0	0	0
ENSG00000203666 330.2393516	324.7932212	392.9221777
161.6110852	180.3557089	46.58250844	349.3182502
129.5164342	1.436036296	0.00024336	0.003996996	EFCAB2
1	244969705	245127164	+	8607
protein_coding	EF-hand calcium binding domain 2 [Source:HGNC
Symbol;Acc:HGNC:28166]	-	364	367	338	156	158
54	2.070991251	2.020415549	2.443271583
1.007128391	1.128372892	0.291724548
ENSG00000143368 3863.074613	4942.69793	4531.392451

5687.467037 6520.201323 6875.233191 4445.721665
6360.967183 -0.516907217 0.000243382 0.003996996 SF3B4
1 149923317 149928344 - 2245
protein_coding splicing factor 3b subunit 4 [Source:HGNC
Symbol;Acc:HGNC:10771] - 4258 5585 3898 5490 5712
7970 92.87909933 117.8781396 108.0270051
135.8839124 156.3936853 165.0718407
ENSG00000132153 4342.103123 4969.247785 4189.619906
5624.272959 6422.033025 7233.228395 4500.323605
6426.51146 -0.513972919 0.000243584 0.003996996 DHX30
3 47802909 47850195 + 7744
protein_coding DExH-box helicase 30 [Source:HGNC
Symbol;Acc:HGNC:16716] - 4786 5615 3604 5429 5626
8385 30.26467632 34.35665368 28.95518104
38.9552989 44.65619789 50.34643696
ENSG00000149781 196.8734596 229.2137447 184.8361723
351.1933198 352.7209749 447.7096645 203.6411255
383.8746531 -0.914336509 0.000243949 0.003999063 FERMT3
11 64206678 64223886 + 3066
protein_coding fermitin family member 3 [Source:HGNC
Symbol;Acc:HGNC:23151] - 217 259 159 339 309
519 3.465901906 4.002709338 3.226501122
6.143835674 6.194891282 7.87093318
ENSG00000082497 658.6642013 435.4176153 445.2343019
343.9415403 221.4494147 163.0387796 513.1053728
242.8099115 1.081388675 0.000244162 0.003999063
SERTAD4 1 210232799 210246631 + 5467
protein_coding SERTA domain containing 4 [Source:HGNC
Symbol;Acc:HGNC:25236] - 726 492 383 332 194
189 6.50303708 4.26424815 4.358695666
3.374434895 2.181222738 1.607473227
ENSG00000125845 16.33051739 20.35488852 15.11239145
48.69051926 78.76293614 99.20349021 17.26593245
75.5523152 -2.127289603 0.000245342 0.004014661 BMP2
20 6767664 6780280 + 3601 protein_coding bone
morphogenetic protein 2 [Source:HGNC Symbol;Acc:HGNC:1069] -
18 23 13 47 69 115 0.244781204
0.302643364 0.22460896 0.725248401 1.177804826
1.484929165
ENSG00000172037 2236.373631 2165.58314 2598.168838
1630.614411 1648.3142 966.1557307 2333.375203
1415.028114 0.722237406 0.000248385 0.00406071 LAMB2
3 49121114 49133118 - 6865
protein_coding laminin subunit beta 2 [Source:HGNC
Symbol;Acc:HGNC:6487] - 2465 2447 2235 1574 1444
1120 17.58348947 16.88961962 20.25554032
12.74019954 12.92927021 7.585924207
ENSG00000135334 1001.605066 931.8998962 1067.167335
1230.730572 2255.587852 1574.316258 1000.224099
1686.878227 -0.753880817 0.000250163 0.004085989

AKIRIN2 6 87675072 87702209 - 1999
protein_coding akirin 2 [Source:HGNC Symbol;Acc:HGNC:21407] -
1104 1053 918 1188 1976 1825 27.04487402
24.95985269 28.57174291 33.02293656 60.76052341
42.45033216
ENSG00000046774 33.56828574 316.8282648 249.9357047
23.82727538 61.64055872 46.58250844 200.1107518
44.01678085 2.185478059 0.000250511 0.004087909 MAGEC2
X 142202345 142205290 - 1991
protein_coding MAGE family member C2 [Source:HGNC
Symbol;Acc:HGNC:13574] - 37 358 215 23 54
54 0.910037204 8.519972783 6.718526686
0.641901839 1.66713151 1.261111593
ENSG00000189171 421.8716992 492.0573051 625.4205076
328.4020129 269.3920714 188.9179509 513.116504
262.2373451 0.96918923 0.000250891 0.004090331
S100A13 1 153618787 153631360 - 2216
protein_coding S100 calcium binding protein A13 [Source:HGNC
Symbol;Acc:HGNC:10490] - 465 556 538 317 236
219 10.27571098 11.88862084 15.10495258
7.948799634 6.546205085 4.595210144
ENSG00000130589 542.5360777 652.2414278 783.519372
904.400496 1283.036815 1107.628534 659.4322925
1098.355282 -0.736991457 0.000253018 0.004121215 HELZ2
20 63558086 63574239 - 12066
protein_coding helicase with zinc finger 2 [Source:HGNC
Symbol;Acc:HGNC:30021] - 598 737 674 873 1124
1284 2.426981951 2.894213785 3.475389
4.020341698 5.725986905 4.948034521
ENSG00000110841 4014.585524 3163.857672 4148.932698
2944.222463 2390.283888 2204.042761 3775.791965
2512.849704 0.587423038 0.000254571 0.004142685
PPFIBP1 12 27523431 27695564 + 11690
protein_coding PPFIA binding protein 1 [Source:HGNC
Symbol;Acc:HGNC:9249] - 4425 3575 3569 2842 2094
2555 18.5364877 14.49065364 18.99498187
13.50895037 11.010563 10.16266036
ENSG00000074855 886.3841938 935.4398768 1086.929693
640.2285298 636.9524401 596.0835803 969.5845878
624.4215168 0.634466378 0.000256628 0.004169673 ANO8
19 17323223 17334829 - 4979
protein_coding anoctamin 8 [Source:HGNC Symbol;Acc:HGNC:29329] -
977 1057 935 618 558 691 9.609064775
10.05910408 11.68359062 6.896970882 6.888734278
6.453078366
ENSG00000089818 555.2375912 566.396898 570.7834001
727.2498834 981.6829722 1057.59547 564.1392964
922.1761084 -0.709446603 0.0002567 0.004169673 NECAP1
12 8076939 8097859 + 8763 protein_coding NECAP
endocytosis associated 1 [Source:HGNC Symbol;Acc:HGNC:24539] -

612	640	491	702	860	1226	3.420009355
3.460617528	3.486065373	4.451397157	6.032439844
6.50532018
ENSG00000241468 698.5832438	1277.933001	1319.428023
524.2000584	684.8950969	605.5726098	1098.648089
604.889255	0.860729647	0.000257436	0.004177783	ATP5MF
7	99448475	99466331	-	2275
protein_coding	ATP synthase membrane subunit f [Source:HGNC
Symbol;Acc:HGNC:848]	-	770	1444	1135	506	600
702	16.57440733	30.07545685	31.03997102
12.35893817	16.21127637	14.34784675
ENSG00000130816 8001.046269	7330.414853	6933.1002
6016.905018	4370.77221	5023.147161	7421.520441
5136.941463	0.530802626	0.000257912	0.004181666	DNMT1
19	10133345	10231286	-	9796
protein_coding	DNA methyltransferase 1 [Source:HGNC
Symbol;Acc:HGNC:2976]	-	8819	8283	5964	5808	3829
5823	44.08585315	40.06501648	37.87875577
32.94502754	24.02613705	27.63942677
ENSG00000103154 0.907250966	0.884995153	3.48747495
42.47470829	17.12237742	29.32972754	1.759907023
29.64227108	-4.13590648	0.000258931	0.004193007	NECAB2
16	83968632	84002776	+	2520
protein_coding	N-terminal EF-hand calcium binding protein 2
[Source:HGNC Symbol;Acc:HGNC:23746]	-	1	1	3
41	15	34	0.019432476	0.018802946
0.074067479	0.904056056	0.365879501	0.627349266
ENSG00000129810 632.3539233	381.432911	387.1097194
303.538769	101.5927727	172.5278091	466.9655179
192.5531169	1.27765872	0.000259085	0.004193007	SGO1
3	20160593	20186292	-	3828
protein_coding	shugoshin 1 [Source:HGNC Symbol;Acc:HGNC:25088] -
697	431	333	293	89	200	8.916399781
5.334967546	5.412266225	4.253120965	1.429109275
2.429344383
ENSG00000100243 2836.06652	3370.061543	4048.958417
2107.159919	2247.59741	2503.378509	3418.36216
2286.045279	0.579872734	0.000262196	0.00423948	CYB5R3
22	42617840	42649568	-	5973
protein_coding	cytochrome b5 reductase 3 [Source:HGNC
Symbol;Acc:HGNC:2873]	-	3126	3808	3483	2034	1969
2902	25.62861532	30.20861858	36.2800441
18.92214985	20.2628515	22.59102372
ENSG00000165891 2578.407245	2508.076264	2290.10855
3609.314236	3250.968727	3397.935199	2458.86402
3419.406054	-0.475516181	0.000263276	0.004253049	E2F7
12	77021247	77065580	-	6303
protein_coding	E2F transcription factor 7 [Source:HGNC
Symbol;Acc:HGNC:23820]	E2F	2842	2834	1970	3484	2848
3939	22.08032442	21.30487447	19.4457978

30.7144599	27.77410276	29.05826579
ENSG00000189159 2556.633222	2954.113821	3084.090347
1852.311669	2229.33354	1827.069498	2864.945797
1969.571569	0.540831199	0.000264786	0.004273539	JPT1
17	75135248	75168281	-	3414
protein_coding	Jupiter microtubule associated homolog 1 [Source:HGNC
Symbol;Acc:HGNC:14569]	-	2818	3338	2653	1788	1953
2118	40.42091642	46.32860852	48.34823004
29.10154467	35.16301344	28.84652193
ENSG00000123992 922.6742324	1172.618578	1069.492318
1276.313186	1806.981564	2086.723851	1054.928376
1723.339533	-0.70840469	0.00026504	0.004273744	DNPEP
2	219373527	219400022	-	4753
protein_coding	aspartyl aminopeptidase [Source:HGNC
Symbol;Acc:HGNC:2981]	-	1017	1325	920	1232	1583
2419	10.47808271	13.20913883	12.04278297
14.40306555	20.47200886	23.66459478
ENSG00000132467 1115.918688	1303.597861	976.4929859
1573.636144	1596.947068	2321.361671	1132.003178
1830.648294	-0.693464249	0.000266201	0.004288545	UTP3
4	70688479	70690551	+	2073
protein_coding	"UTP3, small subunit processome component [Source:HGNC
Symbol;Acc:HGNC:24477]" -	1230	1473	840	1519	1399
2691	29.05591075	33.66897426	25.21081181
40.71650718	41.48258126	60.35946946
ENSG00000204356 1758.252372	2165.58314	2115.734803
2606.496733	2958.746819	3278.891011	2013.190105
2948.044854	-0.550628756	0.000266625	0.004291469	NELFE
6	31952087	31959110	-	2719
protein_coding	negative elongation factor complex member E
[Source:HGNC Symbol;Acc:HGNC:13974]	-	1938	2447	1820
2516	2592	3801	34.90384318	42.64333897
41.64559075	51.41779789	58.59669883	65.00094643
ENSG00000213694 485.3792668	265.4985459	596.3582164
282.8193991	114.1491828	150.0991939	449.0786764
182.3559253	1.29887195	0.000267187	0.004296608	S1PR3
9	88990863	89005010	+	13578
protein_coding	sphingosine-1-phosphate receptor 3 [Source:HGNC
Symbol;Acc:HGNC:3167]	-	535	300	513	273	100
174	1.929508345	1.04691613	2.350652369
1.117220475	0.452701106	0.595860315
ENSG00000112992 1408.96075	973.4946684	1183.4165
759.3649068	816.1666571	546.9131547	1188.623973
707.4815729	0.749716125	0.000271753	0.004366069	NNT
5	43602692	43707405	+	8245
protein_coding	nicotinamide nucleotide transhydrogenase [Source:HGNC
Symbol;Acc:HGNC:7863]	-	1553	1100	1018	733	715
634	9.223791541	6.32162116	7.681817135
4.939981907	5.330436106	3.575441001
ENSG00000234323 14.51601546	23.89486913	11.6249165	0

0	0	16.67860036	0	6.494904691
0.000272688	0.004377113	LINC01505	9	105993310
106740875	+	10944	lincRNA long intergenic non-protein coding RNA 1505 [Source:HGNC Symbol;Acc:HGNC:51186]	-
16	27	10	0	0	0	0.071593333
0.116899895	0.056850038	0	0	0
ENSG00000144749 1342.73143	1362.892536	2348.233133
959.3068263	1090.124696	892.8314119	1684.619033
980.7543113	0.779951009	0.000272942	0.004377212	LRIG1
3	66378797	66501263	-	7032
protein_coding	leucine rich repeats and immunoglobulin like domains 1 [Source:HGNC Symbol;Acc:HGNC:17360]	-	1480	1540	2020
926	955	1035	10.30650781	10.37691596
17.87225507	7.317187238	8.347796813	6.843724304
ENSG00000107551 220.4619847	219.478798	576.5958583
155.3952742	120.9981338	117.3189102	338.845547
131.2374394	1.366139539	0.000273894	0.004388502	RASSF4
10	44959407	44995891	+	9481
protein_coding	Ras association domain family member 4 [Source:HGNC Symbol;Acc:HGNC:20793]	-	243	248	496	150	106
136	1.25510717	1.239435626	3.254875464
0.879122016	0.687225204	0.666984559
ENSG00000259974 0	0	0	7.251779464	13.69790194
30.19236658	0	17.04734933	-6.570455713
0.00027442	0.004392959	LINC00261	20	22547671
22578642	-	5552	lincRNA long intergenic non-protein coding RNA 261 [Source:HGNC Symbol;Acc:HGNC:16189]	-
0	0	0	7	12	35	0	0
0	0.070058466	0.132855381	0.293122803
ENSG00000159111 1314.60665	1463.781983	1596.101035
1767.362252	2487.310694	2467.147669	1458.163223
2240.606872	-0.620174502	0.000276747	0.004426195	MRPL10
17	47823272	47831534	-	2764
protein_coding	mitochondrial ribosomal protein L10 [Source:HGNC Symbol;Acc:HGNC:14055]	-	1449	1654	1373	1706	2179
2860	25.67196017	28.35462496	30.90575412
34.29675506	48.45811896	48.11262058
ENSG00000145247 880.940688	1302.712865	991.6053774
1282.528997	2066.100209	2034.102869	1058.419644
1794.244025	-0.761223379	0.000278573	0.004451375	OCIAD2
4	48885019	48906937	-	2483
protein_coding	OCIA domain containing 2 [Source:HGNC Symbol;Acc:HGNC:28685]	-	971	1472	853	1238	1810
2358	19.15010651	28.09037463	21.37367288
27.70486057	44.80734545	44.15685148
ENSG00000168476 1084.164904	1117.748878	1229.916166
730.3577889	654.0748175	840.2104301	1143.943316
741.5476788	0.623939407	0.000279525	0.004462561	REEP4
8	22138020	22141951	-	2266
protein_coding	receptor accessory protein 4 [Source:HGNC

Symbol;Acc:HGNC:26176]	-	1195	1263	1058	705	573
	974	25.82478314	26.41009028	29.04909513
17.28786072	15.54325874	19.98619266
ENSG00000137055 1336.380673	1239.87821	1095.067134
1877.174913	1986.195781	1605.371263	1223.775339
1822.913986	-0.573876922	0.000282075	0.004499216	PLAA
	9	26904083	26947463	-	4825
protein_coding	phospholipase A2 activating protein [Source:HGNC
Symbol;Acc:HGNC:9043]	-	1473	1401	942	1812	1740
	1861	14.94975628	13.75837867	12.14675947
20.86761999	22.16661053	17.93412009
ENSG00000261499 41.73354444	35.39980612	33.71225785
6.215810969	2.282983656	0	36.94853614	2.832931542
	3.7361436	0.00028408	0.004527103	AC233699.1
	17	36274191	36320547	+	1168
unprocessed_pseudogene	aminopeptidase puromycin sensitive (NPEPPS)
pseudogene	-	46	40	29	6	2	0
	1.928606706	1.622720002	1.544763509	0.285443693
	0.105253007	0
ENSG00000059122 588.8058769	664.63136	717.257348
829.8107644	1246.509076	1159.386877	656.898195
1078.568906	-0.715919491	0.000286786	0.004566121
FLYWCH1 16	2911937 2951208 +	8419	protein_coding
FLYWCH-type zinc finger 1 [Source:HGNC Symbol;Acc:HGNC:25404]	-
	649	751	617	801	1092	1344	3.774964491
	4.226743255	4.559649934	5.286692497	7.972774652
	7.422834495
ENSG00000112667 391.9324173	686.7562388	717.257348
235.1648483	352.7209749	325.2149201	598.648668
304.3669144	0.97529761	0.000287413	0.004571984	DNPH1
	6	43225629	43229484	-	1250
protein_coding	2'-deoxynucleoside 5'-phosphate N-hydrolase 1
[Source:HGNC Symbol;Acc:HGNC:21218]	-	432	776	617
	227	309	377	16.92397665	29.41562965
30.71015424	10.09085319	15.19482934	14.02369169
ENSG00000181467 1992.323121	1762.910345	1675.150467
1252.48591	1243.084601	1267.216758	1810.127978
1254.262423	0.529505005	0.000288299	0.004581959	RAP2B
	3	153162270	153170620	+	8351
protein_coding	"RAP2B, member of RAS oncogene family [Source:HGNC
Symbol;Acc:HGNC:9862]"	-	2196	1992	1441	1209	1089
	1469	12.87723246	11.30257223	10.73574886
8.044514908	8.015613283	8.179265963
ENSG00000103042 465.4197455	451.3475281	418.496994
692.0269546	719.1398517	703.9134609	445.0880892
705.0267558	-0.662838839	0.000289179	0.004591823
SLC38A7 16	58665109	58685104	-	6991
protein_coding	solute carrier family 38 member 7 [Source:HGNC
Symbol;Acc:HGNC:25582]	-	513	510	360	668	630
	816	3.593409789	3.456665179	3.20383429

5.309445949	5.539219914	5.427275586
ENSG00000049860 3034.754481	2592.150803	2793.467435
2207.648863	1876.612566	1585.530565	2806.790906
1889.930664	0.570969236	0.000291615	0.004625164	HEXB
	5	74640023	74722647	+	5067
protein_coding	hexosaminidase subunit beta [Source:HGNC
Symbol;Acc:HGNC:4879]	-	3345	2929	2403	2131	1644
	1838	32.32763257	27.39018138	29.50595754
23.36924002	19.94335726	16.86652525
ENSG00000256663 281.2477995	298.2433666	280.1604876
171.9707702	136.9790194	107.8298807	286.5505512
138.9265567	1.046773987	0.000291802	0.004625164
AC112777.1	12	20551590	20553012	+	1423
	processed_pseudogene	ubiquitin-like with PHD and ring
finger domains 1 (UHRF1) pseudogene	-	310	337	241
	166	120	125	10.66806068	11.22151364
10.53704874	6.482092611	5.183507201	4.084473955
ENSG00000137404 984.3672981	901.810061	995.0928523
710.6743875	541.0671266	573.6549651	960.4234038
608.4654931	0.658218771	0.000292243	0.004628009	NRM
	6	30688047	30691420	-	2045
protein_coding	nurim [Source:HGNC Symbol;Acc:HGNC:8003]	-
	1085	1019	856	686	474	665	25.98155316
	23.6106157	26.04277741	18.63986862	14.24729411
	15.1202632
ENSG00000187123 477.2140081	374.3529498	416.1720107
277.6395566	208.8930046	182.8794776	422.5796562
223.1373463	0.922450054	0.000293126	0.00463784	LYPD6
	2	149329985	149474148	+	5033
protein_coding	LY6/PLAUR domain containing 6 [Source:HGNC
Symbol;Acc:HGNC:28751]	-	526	423	358	268	183
	212	5.117849345	3.982354138	4.425506091
2.958829277	2.234969082	1.958573836
ENSG00000138668 5900.760283	7031.286492	7249.297929
4825.541249	4894.716959	4897.20186	6727.114901
4872.486689	0.465213069	0.000293513	0.004639808	HNRNPD
	4	82352498	82374503	-	6981
protein_coding	heterogeneous nuclear ribonucleoprotein D [Source:HGNC
Symbol;Acc:HGNC:5036]	-	6504	7945	6236	4658	4288
	5677	45.62381296	53.92655839	55.5770275
37.07608677	37.75587146	37.81222856
ENSG00000105011 1909.763283	1987.699114	2229.658984
1571.564207	1338.969914	1012.738239	2042.373794
1307.757453	0.643573048	0.000294543	0.004651942	ASF1B
	19	14119509	14136956	-	2304
protein_coding	anti-silencing function 1B histone chaperone
[Source:HGNC Symbol;Acc:HGNC:20996]	-	2105	2246	1918
	1517	1173	1174	44.74023995	46.19061217
51.79322747	36.5860185	31.2941311	23.69281374
ENSG00000120742 4137.064405	4933.847979	4060.583333

5706.11447	6101.273822	6556.056744	4377.165239
6121.148345	-0.483702128	0.00029665	0.004681042	SERP1
	3	150541994	150603228	-	5747
protein_coding	stress associated endoplasmic reticulum protein 1
[Source:HGNC Symbol;Acc:HGNC:10759]	-	4560	5575	3493
	5508	5345	7600	38.85548454	45.96530174
37.81501135	53.25553821	57.16811502	61.48984712
ENSG00000134775 735.7805334	715.9610789	892.7935871
517.9842475	458.8797149	236.3630984	781.5117331
404.4090203	0.95201766	0.000297105	0.004684032	FHOD3
	18	36297714	36780055	+	7965
protein_coding	formin homology 2 domain containing 3 [Source:HGNC
Symbol;Acc:HGNC:26178]	-	811	809	768	500	402
	274	4.98613184	4.812704339	5.999047304
3.488158958	3.102327433	1.599542563
ENSG00000116584 3521.94825	3666.534919	4765.053273
4700.189061	6725.669852	7065.013781	3984.512147
6163.624231	-0.629717649	0.000298292	0.004698566
ARHGEF2 1	155946851	156007070	-	9135
protein_coding	Rho/Rac guanine nucleotide exchange factor 2
[Source:HGNC Symbol;Acc:HGNC:682]	-	3882	4143	4099
	4537	5892	8190	20.81017167	21.48982221
27.91747997	27.59766072	39.6461981	41.68754962
ENSG00000143167 39.01179154	23.89486913	22.08734135
3.107905485	0	0	28.33133401	1.035968495
4.798701157	0.000300688	0.004732092	GPA33	1
167052836	167166479	-	3200	protein_coding
glycoprotein A33 [Source:HGNC Symbol;Acc:HGNC:4445]	-	43
	27	19	3	0	0	0.658032223
0.39979764	0.369411549	0.052093474	0	0
ENSG00000121058 694.046989	657.5513988	613.7955911
951.0190783	1033.050104	994.6228192	655.1313263
992.897334	-0.599175528	0.000301771	0.004744918	COIL
	17	56938187	56961054	-	3030
protein_coding	coilin [Source:HGNC Symbol;Acc:HGNC:2184]	-
	765	743	528	918	905	1153	12.36367243
	11.61910365	10.84171884	16.83496029	18.35918131
	17.69366079
ENSG00000086062 1944.23882	1742.555456	2270.346192
1467.967357	1336.686931	1111.941729	1985.71349
1305.532006	0.605058907	0.000302822	0.00475528
B4GALT1 9	33104082	33167356	-	4901
protein_coding	"beta-1,4-galactosyltransferase 1 [Source:HGNC
Symbol;Acc:HGNC:924]"	-	2143	1969	1953	1417	1171
	1289	21.41243966	19.0365154	24.79273209
16.06560894	14.68654204	12.22923338
ENSG00000145730 1801.800418	1753.175398	2925.991483
1297.032556	1501.061754	1016.188795	2160.322433
1271.427702	0.764769681	0.000302968	0.00475528	PAM
	5	102753981	103031105	+	8846

protein_coding peptidylglycine alpha-amidating monooxygenase
[Source:HGNC Symbol;Acc:HGNC:8596]   -   1986   1981   2517
        1252   1315   1178   10.99413316   10.61118732
17.70284746   7.86446959   9.1374745   6.191977557
ENSG00000159055 554.3303402   512.4121937   526.6087174
378.1285006   253.4111859   255.3411574   531.1170838
295.626948   0.845647185   0.000303626   0.004761385   MIS18A
        21   32268219   32279069   -   1723
protein_coding MIS18 kinetochore protein A [Source:HGNC
Symbol;Acc:HGNC:1286]   -   611   579   453   365   222
        296   17.365393   15.92281052   16.35760705
11.77116997   7.919815369   7.987988882
ENSG00000140632 1130.434704   1106.243941   1160.166667
787.3360561   801.3272634   645.2540059   1132.281771
744.6391085   0.605461567   0.000305995   0.004793412   GLYR1
        16   4803203 4847342 -   5470   protein_coding
glyoxylate reductase 1 homolog [Source:HGNC Symbol;Acc:HGNC:24434]
        -       1246   1250   998   760   702   748
11.15473865   10.82802195   11.35141657   7.720373469
7.888549336   6.358362582
ENSG00000112096 2026.798658   2770.034829   3377.038243
3309.919341   4681.257987   5648.560468   2724.62391
4546.579266   -0.739214854   0.00030621   0.004793412   SOD2
        6   159669057   159745186   -   16493
protein_coding superoxide dismutase 2 [Source:HGNC
Symbol;Acc:HGNC:11180] -   2234   3130   2905   3195   4101
        6548   6.633033541   8.992306875   10.95855583
10.7642369   15.28401554   18.46035421
ENSG00000198786 29527.38994   34543.13082   20674.91399
40897.96424   40473.87575   47214.82287   28248.47825
42862.22095   -0.601484467   0.000309149   0.004835136   MT-ND5
        MT   12337   14148   +   1812   protein_coding
mitochondrially encoded NADH:ubiquinone oxidoreductase core subunit 5
[Source:HGNC Symbol;Acc:HGNC:7461]   -   32546   39032   17785
        39478   35457   54733   879.5653461   1020.678702
610.6642878   1210.623202   1202.793726   1404.501081
ENSG00000186432 3962.872219   3589.540341   3288.688878
5499.956739   4898.141435   4749.690583   3613.700479
5049.262919   -0.48230877   0.000310856   0.004857538   KPNA4
        3   160494995   160565588   -   9413
protein_coding karyopherin subunit alpha 4 [Source:HGNC
Symbol;Acc:HGNC:6397]   -   4368   4056   2829   5309   4291
        5506   22.72392015   20.41720684   18.69871384
31.33983534   28.02062487   27.19813759
ENSG00000135074 2336.171237   2111.598435   2723.717936
1509.406097   1599.230051   1780.486989   2390.495869
1629.707713   0.551926228   0.000312453   0.004878187   ADAM19
        5   157395534   157575775   -   7704
protein_coding ADAM metallopeptidase domain 19 [Source:HGNC
Symbol;Acc:HGNC:197]   -   2575   2386   2343   1457   1401

2064	16.36777487	14.67508435	18.92181801
10.5088531	11.17813168	12.45731473
ENSG00000092203 1081.443151	1088.544038	1354.302772
1643.046033	1743.058022	1826.206859	1174.763321
1737.436971	-0.56565047	0.000317196	0.004947866	TOX4
14	21476597	21499175	+	7091
protein_coding	TOX high mobility group box family member 4
[Source:HGNC Symbol;Acc:HGNC:20161]	HMG	1192	1230	1165
1586	1527	2117	8.231850103	8.219096261
10.22175075	12.42818591	13.23667519	13.8817555
ENSG00000248774 18.14501932	15.0449176	15.11239145	0
0	0	16.10077612	0	6.442467413
0.000318687	0.004966749	AC097534.1	4	173322206
173329694	-	521	antisense	novel
transcript	-	20	17	13	0	0	0
1.879840301	1.546100209	1.552431605	0	0
0
ENSG00000197557 185.0791971	222.1337834	240.6357715
392.6320596	408.6540745	373.5227066	215.949584
391.6029469	-0.859803364	0.000322314	0.005018848	TTC30A
2	177612992	177618966	-	5975
protein_coding	tetratricopeptide repeat domain 30A [Source:HGNC
Symbol;Acc:HGNC:25853]	-	204	251	207	379	358
433	1.671940975	1.990500324	2.155456598
3.524628463	3.682921628	3.369620602
ENSG00000111328 534.370819	359.3080322	517.3087842
257.9561552	277.3825142	233.7751813	470.3292118
256.3712836	0.875716566	0.000324601	0.005050017
CDK2AP1 12	123260971	123272334	-	3117
protein_coding	cyclin dependent kinase 2 associated protein 1
[Source:HGNC Symbol;Acc:HGNC:14002]	-	589	406	445
249	243	271	9.253524434	6.171854401
8.882394447	4.438892101	4.792000244	4.042625459
ENSG00000174080 223.1837376	268.1535314	212.7359719
109.8126605	107.3002318	118.1815492	234.6910803
111.7648138	1.070269508	0.000326031	0.005067808	CTSF
11	66563463	66568841	-	2896
protein_coding	cathepsin F [Source:HGNC Symbol;Acc:HGNC:2531]	-
246	303	183	106	94	137	4.15973087
4.957588912	3.931509947	2.033852021	1.995155071
2.199647188
ENSG00000104907 1189.406016	1609.806184	1156.679192
1793.261465	1951.951026	2505.103787	1318.630464
2083.438759	-0.65978596	0.000331901	0.005154516	TRMT1
19	13104902	13117567	-	3364
protein_coding	tRNA methyltransferase 1 [Source:HGNC
Symbol;Acc:HGNC:25980]	-	1311	1819	995	1731	1710
2904	19.08426279	25.62141747	18.4023777
28.59256548	31.24550034	40.13947085
ENSG00000125520 1849.88472	1987.699114	2304.05845

1108.48629   1655.163151   1116.254925   2047.214095
1293.301455   0.663089562   0.000333063   0.00516803
SLC2A4RG   20   63739861   63743505   +   2244
        protein_coding SLC2A4 regulator [Source:HGNC
Symbol;Acc:HGNC:15930] Others 2039   2246   1982   1070   1450
        1294   44.49621364   47.42565527   54.95252395
26.49555181   39.71846993   26.81281686
ENSG00000247679 8.165258694   7.964956378   22.08734135
44.54664528   74.19696883   79.36279217   12.73918547
66.03546876   -2.39548499   0.000334548   0.00518652
AC139795.2   5   177611253   177619754   +   1751
        antisense   novel transcript   -   9   9
        19   43   65   92   0.251701061   0.243547011
        0.675109628   1.364565394   2.281784211   2.443051935
ENSG00000151445 577.0116144   660.2063842   557.9959919
780.0842767   1014.786235   1104.177978   598.4046635
966.3494966   -0.691214785   0.000336829   0.005217316
VIPAS39 14   77426675   77457952   -   3695
protein_coding "VPS33B interacting protein, apical-basolateral
polarity regulator, spe-39 homolog [Source:HGNC
Symbol;Acc:HGNC:20347]" -   636   746   480   753   889
        1280   8.428908832   9.566450432   8.082275335
11.32381009   14.78885934   16.10744084
ENSG00000148840 2245.446141   2472.676458   1846.03674
3183.531185   2915.370129   3498.001329   2188.053113
3198.967548   -0.547632705   0.000337475   0.005222749   PPRC1
        10   102132994   102150331   +   6095
protein_coding "peroxisome proliferator-activated receptor gamma,
coactivator-related 1 [Source:HGNC Symbol;Acc:HGNC:30025]"   -
        2475   2794   1588   3073   2554   4055   19.88520978
        21.72096584   16.21002314   28.01566231   25.75695642
        30.93486084
ENSG00000140807 88.0033437   46.01974796   61.61205744
2.07193699   0   18.11541995   65.21171637   6.72911898
        3.234459683   0.000339535   0.0052464   NKD1   16
        50548330   50649249   +   17425   protein_coding
        naked cuticle homolog 1 [Source:HGNC Symbol;Acc:HGNC:17045]
        -       97   52   53   2   0   21
0.272601117   0.141402471   0.189238688   0.006377776   0
        0.056037342
ENSG00000160014 5683.020051   5718.838679   6286.754843
4437.053064   4443.827687   4246.77202   5896.204524
4375.884257   0.430144955   0.000339597   0.0052464   CALM3
        19   46601074   46610793   +   3667
protein_coding calmodulin 3 [Source:HGNC Symbol;Acc:HGNC:1449] -
        6264   6462   5408   4283   3893   4923   83.65068904
        83.49923268   91.75560848   64.90067416   65.2560608
        62.42376284
ENSG00000196155 26.31027801   41.5947722   34.8747495   0
        5.707459141   0   34.25993324   1.90248638

4.252078009	0.000340872	0.005261505	PLEKHG4 16
67277510	67289499	+	7328	protein_coding
pleckstrin homology and RhoGEF domain containing G4 [Source:HGNC
Symbol;Acc:HGNC:24501]	-	29	47	30	0	5
	0	0.193794399	0.303905695	0.254708032	0
	0.041940336	0
ENSG00000144567 1735.571098	1814.240064	1868.124081
2238.727917	2741.863371	2771.659252	1805.978414
2584.083514	-0.517101661	0.00034452	0.00530997
RETREG2 2	219176225	219185479	+	5689
protein_coding	reticulophagy regulator family member 2 [Source:HGNC
Symbol;Acc:HGNC:28450]	-	1913	2050	1607	2161	2402
	3213	16.46674347	17.07435741	17.57465424
21.10721222	25.95281252	26.26067046
ENSG00000119950 488.1010197	441.6125814	667.270207
992.4578181	864.1093139	807.4301464	532.327936
887.9990928	-0.739991747	0.000344612	0.00530997	MXI1
	10	110207605	110287365	+	4607
protein_coding	"MAX interactor 1, dimerization protein [Source:HGNC
Symbol;Acc:HGNC:7534]"	bHLH	538	499	574	958	757
	936	5.718639859	5.132261472	7.751763723
11.55471773	10.10008497	9.446885566
ENSG00000158792 520.7620545	675.2513018	676.5701402
658.8759628	1308.149635	1634.700991	624.1944988
1200.57553	-0.944384186	0.000349291	0.005377386
SPATA2L 16	89696343	89701705	-	2482
protein_coding	spermatogenesis associated 2 like [Source:HGNC
Symbol;Acc:HGNC:28393]	-	574	763	582	636	1146
	1895	11.32501534	14.56629837	14.58908498
14.23860303	28.38116383	35.50082578
ENSG00000139737 304.8363246	323.908226	305.7353039
486.9051926	583.3023242	500.3306463	311.4932848
523.512721	-0.748172343	0.000350963	0.005398417	SLAIN1
	13	77697854	77764242	+	4133
protein_coding	SLAIN motif family member 1 [Source:HGNC
Symbol;Acc:HGNC:26387]	-	336	366	263	470	511
	580	3.981095133	4.196064168	3.959106549
6.318943851	7.599812105	6.525196678
ENSG00000278981 0	0.884995153	0	8.287747959
18.26386925	38.81875704	0.294998384
21.79012475	-5.962907164	0.000352271	0.005413826
AC079298.1	4	154235980	154237598	+	1619
	antisense	"novel transcript, antisense to DCHS2"	-
	0	1	0	8	16	45	0
0.029267093	0	0.274571327	0.607463928	1.292399208
ENSG00000163719 992.5325568	1316.872788	992.767869
1425.492649	1744.199513	2058.256762	1100.724405
1742.649641	-0.662667332	0.000352589	0.005414011	MTMR14
	3	9649433 9702393 +	6019	protein_coding
myotubularin related protein 14 [Source:HGNC Symbol;Acc:HGNC:26190]

- 1094 1488 854 1376 1528 2386
8.900648742 11.71399485 8.827553818 12.70299521
15.60437473 18.43219745
ENSG00000100567 1578.616681 1571.751392 1607.725952
1229.694603 1026.201154 979.9579554 1586.031342
1078.617904 0.556447736 0.000353003 0.005415667 PSMA3
14 58244831 58272012 + 3119
protein_coding proteasome subunit alpha 3 [Source:HGNC
Symbol;Acc:HGNC:9532] - 1740 1776 1383 1187 899
1136 27.31885903 26.9807506 27.58758295
21.14693293 17.71706087 16.93534213
ENSG00000108468 2562.983979 2575.335896 2364.508016
1955.908518 1731.643103 1415.590673 2500.94263
1701.047432 0.556708738 0.000354672 0.005436568 CBX1
17 48070052 48101521 - 3087
protein_coding chromobox 1 [Source:HGNC Symbol;Acc:HGNC:1551] -
2825 2910 2034 1888 1517 1641 44.81366943
44.66659022 40.99408202 33.98422764 30.20621516
24.71741047
ENSG00000241749 98.89035529 232.7537253 136.011523
258.9921237 353.8624667 492.5668949 155.8852012
368.4738284 -1.240532256 0.000355342 0.005442115
RPSAP52 12 65758020 65826997 - 2035
transcribed_processed_pseudogene ribosomal protein SA
pseudogene 52 [Source:HGNC Symbol;Acc:HGNC:35752] - 109
263 117 250 310 571 2.622954566
6.123754559 3.577077049 6.826335651 9.36363854
13.04676118
ENSG00000179119 905.436464 838.09041 838.1564796
1522.873688 1179.161059 1215.458415 860.5611179
1305.831054 -0.601421686 0.00035642 0.005453906
SPTY2D1 11 18606401 18634791 - 6005
protein_coding SPT2 chromatin protein domain containing 1
[Source:HGNC Symbol;Acc:HGNC:26818] - 998 947 721
1470 1033 1409 8.13853458 7.472456716
7.470146166 13.60242583 10.57388712 10.91010674
ENSG00000144460 0 0 0 9.323716454 34.24475485
6.901112362 0 16.82319455 -6.544026722
0.000358631 0.005482999 NYAP2 2 225399710
225654018 + 5461 protein_coding neuronal tyrosine-
phosphorylated phosphoinositide-3-kinase adaptor 2 [Source:HGNC
Symbol;Acc:HGNC:29291] - 0 0 0 9 30
8 0 0 0 0.091576149 0.337673079
0.068115952
ENSG00000103742 438.2022166 523.9171306 481.2715431
319.0782964 205.4685291 91.4397388 481.1302968
205.3288548 1.231115926 0.000364856 0.005564677 IGDCC4
15 65381464 65423072 - 7764
protein_coding immunoglobulin superfamily DCC subclass member 4
[Source:HGNC Symbol;Acc:HGNC:13770] - 483 592 414

308	180	106	3.046423575	3.612955569
3.317581962	2.204333158	1.4250639	0.634821105
ENSG00000134242 621.4669117	565.5119028	430.1219105
1003.853472	834.4305264	820.3697321	539.033575
886.21791	-0.715571048	0.000364864	0.005564677	PTPN22
1	113813811	113871759	-	4503
protein_coding	"protein tyrosine phosphatase, non-receptor type 22
[Source:HGNC Symbol;Acc:HGNC:9652]"	-	685	639	370
969	731	951	7.44933162	6.723963574
5.11218573	11.9573206	9.978443215	9.819957046
ENSG00000164283 88.0033437	96.46447169	68.58700734
192.6901401	187.2046598	194.9564242	84.35160758
191.6170747	-1.180445106	0.000364917	0.005564677	ESM1
5	54977864	55022671	-	2695
protein_coding	endothelial cell specific molecule 1 [Source:HGNC
Symbol;Acc:HGNC:3466]	-	97	109	59	186	164
226	1.762550821	1.916435333	1.362072074
3.835007506	3.740523941	3.899246471
ENSG00000100097 9271.197621	12746.58519	18945.12642
7097.420159	9659.30385	7985.449642	13654.30308
8247.391217	0.727273537	0.000367718	0.005602553	LGALS1
22	37675608	37679806	+	1099
protein_coding	galectin 1 [Source:HGNC Symbol;Acc:HGNC:6561]	-
10219	14403	16297	6851	8462	9257	455.3437609
620.9858568	922.607158	346.3923712	473.2849437
391.6549226
ENSG00000223756 58.06406182	91.15450077	10.46242485
186.4743291	162.0918396	243.2642108	53.22699581
197.2767932	-1.881284822	0.000368189	0.005604897	TSSC2
11	3380961 3408978 +	3666
transcribed_unprocessed_pseudogene	tumor suppressing
subtransferable candidate 2 pseudogene [Source:HGNC
Symbol;Acc:HGNC:12384]	-	64	103	9	180	142
282	0.854901732	1.331285509	0.152741445
2.728299781	2.380911452	3.576742423
ENSG00000099821 2708.144133	3173.592619	2884.141783
3326.494837	4681.257987	5410.472092	2921.959512
4472.741639	-0.614341495	0.000371667	0.005652976	POLRMT
19	617224	633604	-	5124	protein_coding	RNA
polymerase mitochondrial [Source:HGNC Symbol;Acc:HGNC:9200]	-
2985	3586	2481	3211	4101	6272	28.52751208
33.16099896	30.12482202	34.82115947	49.19579786
56.91515812
ENSG00000189060 4569.823116	4536.485155	6300.704742
3948.075934	3328.590171	2957.989286	5135.671004
3411.551797	0.589983716	0.00037443	0.005689256	H1F0
22	37805093	37807436	+	2344
protein_coding	H1 histone family member 0 [Source:HGNC
Symbol;Acc:HGNC:4714]	-	5037	5126	5420	3811	2916
3429	105.2308376	103.620918	143.8628057

90.34276641	76.46756704	68.02066851
ENSG00000144021 2347.058249	2918.714015	2136.659653
3397.976663	3874.223265	3492.825494	2467.477305
3588.341807	-0.539727901	0.000374695	0.005689256	CIAO1
	2	96266132	96273349	+	4031
protein_coding	cytosolic iron-sulfur assembly component 1
[Source:HGNC Symbol;Acc:HGNC:14280]	-	2587	3298	1838
	3280	3394	4049	31.4276794	38.76718743
28.36870789	45.21401658	51.75429536	46.7052818
ENSG00000271447 16.33051739	27.43484975	5.812458249	0
	0	0	16.52594179	0	6.483400473
0.000378535	0.005742636	MMP28	17	35756249
35795707	-	2858	protein_coding	matrix
metallopeptidase 28 [Source:HGNC Symbol;Acc:HGNC:14366] -	18
	31	5	0	0	0	0.308417466
0.513955964	0.10884654	0	0	0
ENSG00000182010 510.7822938	485.8623391	425.4719439
289.0352101	243.1377594	94.02765594	474.0388589
208.7335418	1.18687798	0.000384164	0.005823037	RTKN2
	10	62183035	62268707	-	9548
protein_coding	rhotekin 2 [Source:HGNC Symbol;Acc:HGNC:19364]	-
	563	549	366	279	213	109	2.887517786
	2.724497257	2.384929369	1.623692694	1.37124341
	0.530817345
ENSG00000136518 2215.506859	2027.523896	2281.971109
1695.880426	1483.939377	1264.62884	2175.000621
1481.482881	0.554267241	0.000385449	0.005837509	ACTL6A
	3	179562880	179588408	+	3675
protein_coding	actin like 6A [Source:HGNC Symbol;Acc:HGNC:24124]
	-	2442	2291	1963	1637	1300	1466
32.53995888	29.53889102	33.23301953	24.75160579
21.74369608	18.54845091
ENSG00000088387 1314.60665	1160.228646	1084.604709
1662.729434	1685.98343	1807.2288	1186.480002
1718.647221	-0.534250352	0.000386215	0.005844099	DOCK9
	13	98793429	99086625	-	14816
protein_coding	dedicator of cytokinesis 9 [Source:HGNC
Symbol;Acc:HGNC:14132]	-	1449	1311	933	1605	1477
	2095	4.789234471	4.192742234	3.917937656
6.019440293	6.12769141	6.574823157
ENSG00000110042 14.51601546	16.81490791	27.8997996
17.61146441	148.3939377	151.824472	19.74357432
105.9432913	-2.431657525	0.000386585	0.005844707	DTX4
	11	59171430	59208587	+	6072
protein_coding	deltex E3 ubiquitin ligase 4 [Source:HGNC
Symbol;Acc:HGNC:29151]	-	16	19	24	17	130
	176	0.129037786	0.14826829	0.245915739
0.1555712	1.316009273	1.347758014
ENSG00000204387 355.6423787	507.9872179	537.0711423
160.5751167	297.9293672	244.1268498	466.9002463

234.2104446	0.995382339	0.00038902	0.005876498
C6orf48 6	31834608	31839766	+	1801
protein_coding	chromosome 6 open reading frame 48 [Source:HGNC
Symbol;Acc:HGNC:19078]	-	392	574	462	155	261
283	10.65862144	15.10165764	15.96008167
4.782225259	8.907875832	7.306404983
ENSG00000187951 205.9459693	184.0789918	97.64929859
76.66166862	44.5181813	50.03306463	162.5580866
57.07097152	1.512521465	0.000389893	0.005884663
AC091057.1	15	30658717	30772993	+	5637
processed_transcript	OTU deubiquitinase 7A pseudogene
[Source:NCBI gene;Acc:100288637]	-	227	208	84
74	39	58	1.971998162	1.748403797
0.927124585	0.729450336	0.425269202	0.478421818
ENSG00000184787 3282.433995	3655.029982	2214.546593
4029.917445	4771.435842	5666.675888	3050.67019
4822.676392	-0.660366938	0.000393581	0.005935252	UBE2G2
21	44768580	44802019	-	7388
protein_coding	ubiquitin conjugating enzyme E2 G2 [Source:HGNC
Symbol;Acc:HGNC:12483]	-	3618	4130	1905	3890	4180
6569	23.98116953	26.48802671	16.04260682
29.25733458	34.77737155	41.34313383
ENSG00000167779 97.07585336	100.8894475	231.3358383
56.97826722	33.10326302	43.13195226	143.1003797
44.40449417	1.683342546	0.000396102	0.00596818	IGFBP6
12	53097436	53102345	+	1346
protein_coding	insulin like growth factor binding protein 6
[Source:HGNC Symbol;Acc:HGNC:5475]	-	107	114	199
55	29	50	3.892847594	4.013157758
9.198454472	2.270542698	1.324342445	1.727253027
ENSG00000279484 0	0	0	8.287747959	4.565967313
38.81875704	0	17.22415744	-6.587567049
0.000398626	0.006001101	KLHL30-AS1	2	238152889
238155994	-	3106	TEC	KLHL30 antisense RNA 1
[Source:HGNC Symbol;Acc:HGNC:31018]	-	0	0	0
8	4	45	0	0	0	0.143120083
0.079160021	0.673662047
ENSG00000154832 1574.987677	2175.318086	1803.024549
2403.446908	2771.542159	3177.099604	1851.110104
2784.029557	-0.588864785	0.000400681	0.006026915	CXXC1
18	50282343	50288304	-	4319
protein_coding	CXXC finger protein 1 [Source:HGNC
Symbol;Acc:HGNC:24343]	-	1736	2458	1551	2320	2428
3683	19.68317711	26.96653307	22.342689
29.84810917	34.55515446	39.65057895
ENSG00000198727 22814.64004	23627.6006	16920.06596
26397.51322	31102.22784	35080.07942	21120.76887
30859.94016	-0.547023255	0.000403893	0.006070058	MT-CYB
MT	14747	15887	+	1141	protein_coding
mitochondrially encoded cytochrome b [Source:HGNC

Symbol;Acc:HGNC:7427]	-	25147	26698	14555	25481	27247
	40666	1079.267802	1108.713984	793.658025
1240.91738	1467.845709	1657.207271
ENSG00000106462 1167.631993	1008.009479	857.9188376
759.3649068	536.5011592	546.0505157	1011.18677
613.9721939	0.720579398	0.000408693	0.006136984	EZH2
	7	148807383	148884321	-	4522
protein_coding	enhancer of zeste 2 polycomb repressive complex 2
subunit [Source:HGNC Symbol;Acc:HGNC:3527]	-	1287	1139
	738	733	470	633	13.93723659	11.9349226
	10.15389459	9.007109868	6.388731849	6.508848607
ENSG00000204256 4563.472359	5076.332198	4576.729626
5734.085619	6925.430922	7021.01919	4738.844728
6560.178577	-0.469154238	0.000409459	0.006143268	BRD2
	6	32968660	32981505	+	7337
protein_coding	bromodomain containing 2 [Source:HGNC
Symbol;Acc:HGNC:1103]	-	5030	5736	3937	5535	6067
	8139	33.57207228	37.04393081	33.38518155
41.91902278	50.82797833	51.58026244
ENSG00000148843 2714.49489	2961.193782	2261.046259
3380.365199	3689.301589	4977.427291	2645.578311
4015.698026	-0.602011814	0.00041408	0.006207344	PDCD11
	10	103396626	103446292	+	7670
protein_coding	programmed cell death 11 [Source:HGNC
Symbol;Acc:HGNC:13408]	-	2992	3346	1945	3263	3232
	5770	19.10270675	20.67078708	15.77724205
23.63925326	25.90140653	34.97932844
ENSG00000011426 9938.934332	6871.102369	8913.985971
4339.672025	2393.708364	1501.854578	8574.674224
2745.078322	1.643300427	0.000415937	0.006229901	ANLN
	7	36389806	36453791	+	6012
protein_coding	anillin actin binding protein [Source:HGNC
Symbol;Acc:HGNC:14082]	-	10955	7764	7668	4189	2097
	1741	89.23230129	61.19176719	79.35421118
38.71715455	21.4401006	13.46513827
ENSG00000163755 1207.551036	880.5701774	1097.392117
814.271237	560.4724876	500.3306463	1061.837777
625.0247903	0.764949971	0.000419725	0.006281313	HPS3
	3	149129584	149173732	+	6256
protein_coding	"HPS3, biogenesis of lysosomal organelles complex 2
subunit 1 [Source:HGNC Symbol;Acc:HGNC:15597]"	-	1331	995
	944	786	491	580	10.41861522	7.536206352
	9.388194983	6.981324896	4.824275624	4.310843649
ENSG00000213676 1242.026572	1046.949266	1182.254008
885.7530631	696.3100152	533.973569	1157.076616
705.3455491	0.714908761	0.000423354	0.006330269	ATF6B
	6	32115264	32128253	-	3629
protein_coding	activating transcription factor 6 beta [Source:HGNC
Symbol;Acc:HGNC:2349]	TF_bZIP 1369	1183	1017	855	610
	619	18.47332894	15.44629117	17.43575795

13.09155366	10.33213869	7.931123251
ENSG00000280287 88.91059467	100.8894475	54.63710754
168.8628647	189.4876435	233.7751813	81.47904989
197.3752298	-1.272087718	0.00042567	0.006355199
AC131212.3	12	132550729	132554947	+	4219
	TEC	novel transcript	-	98	114	47
	163	166	271	1.137483836	1.280329543
0.693098851	2.146792763	2.41849906	2.986694372
ENSG00000067596 2767.115446	2724.900076	2885.304275
3509.861261	3869.657298	4100.986021	2792.439933
3826.83486	-0.454872522	0.000425859	0.006355199	DHX8
	17	43483865	43610338	+	10439
protein_coding	DEAH-box helicase 8 [Source:HGNC Symbol;Acc:HGNC:2749]
	-	3050	3079	2482	3388	3390	4754
14.30769341	13.97581786	14.79277754	18.03418613
19.96126962	21.17538416
ENSG00000196204 777.5140778	868.1802452	777.7069138
1105.378384	1247.650568	1231.848557	807.8004123
1194.95917	-0.564552993	0.000426099	0.006355199
RNF216P1	7	4973988 5040675 +	5510
transcribed_unprocessed_pseudogene	ring finger protein 216
pseudogene 1 [Source:HGNC Symbol;Acc:HGNC:33610]	-	857
	981	669	1067	1093	1428	7.616543146
8.436141379	7.554076272	10.760312	12.19315019
12.05057102
ENSG00000174945 119.7571275	152.2191663	115.0866733
7.251779464	22.82983656	60.38473317	129.0209891
30.15544973	2.085659406	0.000432805	0.006449776	AMZ1
	7	2679522 2775500 +	7011	protein_coding
archaelysin family metallopeptidase 1 [Source:HGNC
Symbol;Acc:HGNC:22231]	-	132	172	99	7	20
	70	0.921982436	1.16245171	0.878541081
0.055479191	0.175346616	0.464246984
ENSG00000276071 0.907250966	0	0	7.251779464
47.94265678	11.21430759	0.302416989
22.13624795	-5.978253284	0.000433482	0.006454435
AC074138.1	19	36668102	36669404	+	1303
	lincRNA novel transcript	-	1	0	0
	7	42	13	0.037582379	0	0
0.298514663	1.981309103	0.463905963
ENSG00000162733 3262.474474	2573.565905	2878.329325
2320.569429	1844.650794	1384.535668	2904.789901
1849.91863	0.651425346	0.000435387	0.006477349	DDR2
	1	162631373	162787400	+	10730
protein_coding	discoidin domain receptor tyrosine kinase 2
[Source:HGNC Symbol;Acc:HGNC:2731]	-	3596	2908	2476
	2240	1616	1605	16.41151389	12.84165864
14.35680379	11.60006279	9.257399259	6.955147312
ENSG00000178209 15161.97814	16279.48584	16245.82081
12843.9374	11063.3388	11884.57813	15895.7616

11930.61811	0.413929724	0.000442036	0.006570729	PLEC
	8	143915147	143976734	-	16991
protein_coding	plectin [Source:HGNC Symbol;Acc:HGNC:9069]	-
	16712	18395	13975	12398	9692	13777	48.16573264
	51.29881028	51.17286393	40.54569375	35.062415
	37.7022038
ENSG00000142871 3215.297423	3907.253601	3807.160153
2945.258431	2223.626081	2352.416676	3643.237059
2507.100396	0.539110948	0.000442737	0.006572074	CYR61
	1	85580761	85583962	+	2455
protein_coding	cysteine rich angiogenic inducer 61 [Source:HGNC
Symbol;Acc:HGNC:2654]	-	3544	4415	3275	2843	1948
	2727	70.6921028	85.21296017	82.99781408
64.34834875	48.77360045	51.64932613
ENSG00000167112 1180.333507	1325.722739	1367.09018
1527.017561	2161.985523	2244.586796	1291.048809
1977.863293	-0.615790667	0.000442869	0.006572074	TRUB2
	9	128305161	128322742	-	6125
protein_coding	TruB pseudouridine synthase family member 2
[Source:HGNC Symbol;Acc:HGNC:17170]	-	1301	1498	1176
	1474	1894	2602	10.40159373	11.58863171
11.94560294	13.37221757	19.00733556	19.7529615
ENSG00000033050 538.9070738	556.6619513	549.8585504
774.9044342	788.7708533	1074.84825	548.4758585
879.507846	-0.682284785	0.000443937	0.006580849	ABCF2
	7	151212490	151227230	-	4177
protein_coding	ATP binding cassette subfamily F member 2 [Source:HGNC
Symbol;Acc:HGNC:71]	-	594	629	473	748	691
	1246	6.963869969	7.135306136	7.045365235
9.950597656	10.16859458	13.87025946
ENSG00000149016 465.4197455	539.8470434	455.6967268
585.3221996	920.0424135	1042.067967	486.9878386
849.1441933	-0.802105575	0.000444205	0.006580849	TUT1
	11	62575045	62592177	-	4082
protein_coding	"terminal uridylyl transferase 1, U6 snRNA-specific
[Source:HGNC Symbol;Acc:HGNC:26184]"	-	513	610	392
	565	806	1208	6.154220439	7.080815452
5.974752412	7.691082875	12.1369455	13.76020652
ENSG00000115053 17637.86603	17068.01652	17364.13777
14390.63836	11693.44229	12561.74978	17356.67344
12881.94348	0.430120192	0.000445896	0.006600374	NCL
	2	231453531	231483641	-	6929
protein_coding	nucleolin [Source:HGNC Symbol;Acc:HGNC:7667]	-
	19441	19286	14937	13891	10244	14562	137.396833
	131.8858012	134.1218905	111.3973844	90.87540694
	97.71955566
ENSG00000117226 518.0403016	406.2127753	567.2959251
346.0134773	228.2983656	227.736708	497.1830007
267.349517	0.894611277	0.000448539	0.006633948	GBP3
	1	89006666	89022894	-	4297

protein_coding guanylate binding protein 3 [Source:HGNC
Symbol;Acc:HGNC:4184] - 571 459 488 334 200
264 6.507279158 5.061436268 7.065799585
4.31909898 2.860961425 2.856732603
ENSG00000126883 1565.915167 1745.210442 1357.790247
2175.533839 2198.513261 2330.8507 1556.305285
2234.965934 -0.521574835 0.00045013 0.006651926 NUP214
9 131125561 131234670 + 13062
protein_coding nucleoporin 214 [Source:HGNC Symbol;Acc:HGNC:8064]
- 1726 1972 1168 2100 1926 2702
6.470827098 7.153583849 5.563396462 8.933500354
9.063458772 9.618485251
ENSG00000198682 620.5596607 469.9324263 747.4821309
308.7186115 336.7400893 395.9513218 612.6580726
347.1366742 0.817364576 0.0004521 0.006675462 PAPSS2
10 87659613 87747705 + 4083
protein_coding 3'-phosphoadenosine 5'-phosphosulfate synthase 2
[Source:HGNC Symbol;Acc:HGNC:8604] - 684 531 643
298 295 459 8.203617547 6.162282188
9.798022662 4.055542228 4.441094314 5.227142307
ENSG00000161681 154.2326642 246.9136477 163.9113226
277.6395566 347.0135158 628.863864 188.3525449
417.8389788 -1.14981832 0.000453433 0.006689561 SHANK1
19 50661827 50719450 - 7567
protein_coding SH3 and multiple ankyrin repeat domains 1 [Source:HGNC
Symbol;Acc:HGNC:15474] - 170 279 141 268 304
729 1.100154985 1.747056338 1.159317056
1.967990981 2.469432786 4.479554373
ENSG00000154813 1212.994542 1322.182759 1249.678524
1834.700204 1662.012102 1871.926728 1261.618608
1789.546345 -0.504510801 0.000455489 0.006710249 DPH3
3 16257978 16264972 - 3562
protein_coding diphthamide biosynthesis 3 [Source:HGNC
Symbol;Acc:HGNC:27717] - 1337 1494 1075 1771 1456
2170 18.38087475 19.87390105 18.77679201
27.62718843 25.12550619 28.32675569
ENSG00000123130 524.3910583 614.1866363 623.0955243
362.5889732 355.0039586 366.6215942 587.2244063
361.404842 0.699676057 0.000455594 0.006710249 ACOT9
X 23702253 23766475 - 5046
protein_coding acyl-CoA thioesterase 9 [Source:HGNC
Symbol;Acc:HGNC:17152] - 578 694 536 350 311
425 5.609307852 6.516864109 6.608827102
3.854187529 3.788440782 3.916270687
ENSG00000055483 1811.780179 2062.038707 1433.352204
2521.547317 2421.104168 3059.780694 1769.05703
2667.477393 -0.592122389 0.000458849 0.006752568 USP36
17 78787381 78841441 - 9235
protein_coding ubiquitin specific peptidase 36 [Source:HGNC
Symbol;Acc:HGNC:20062] - 1997 2330 1233 2434 2121

3547	10.58936331	11.95488663	8.306786022
14.64521385	14.11728326	17.85892473
ENSG00000130177 1673.878032	1967.344225	1834.411824
2956.654084	2665.383419	2279.954997	1825.21136
2633.9975	-0.528908409	0.000461926	0.006792194	CDC16
13	114234887	114272723	+	3959
protein_coding	cell division cycle 16 [Source:HGNC
Symbol;Acc:HGNC:1720]	-	1845	2223	1578	2854	2335
2643	22.8212565	26.60604993	24.79866739
40.05719279	36.25340004	31.04149859
ENSG00000125352 275.8042937	405.3277801	442.9093186
475.5095392	743.1111801	919.5732223	374.6804641
712.7313139	-0.929424415	0.000466229	0.006849777
RNF113A X	119870475	119871827	-	1353
protein_coding	ring finger protein 113A [Source:HGNC
Symbol;Acc:HGNC:12974]	-	304	458	381	459	651
1066	11.00283171	16.03962174	17.51999693
18.85067616	29.5753949	36.6345133
ENSG00000176678 1462.488557	1318.642778	2085.51002
947.9111728	1198.56642	778.1004188	1622.213785
974.8593371	0.734888559	0.000466693	0.006850898	FOXL1
16	86576368	86582160	+	3742
protein_coding	forkhead box L1 [Source:HGNC Symbol;Acc:HGNC:3817]
Fork	1612	1490	1794	915	1050	902
21.09550556	18.86726399	29.82809394	13.5871808
17.24776698	11.20814582
ENSG00000071539 1727.405839	1381.477434	1490.314295
1142.67325	1034.191596	862.6390453	1533.065856
1013.167964	0.598245962	0.000472214	0.006926197	TRIP13
5	892643	919357	+	3560	protein_coding
thyroid hormone receptor interactor 13 [Source:HGNC
Symbol;Acc:HGNC:12307]	-	1904	1561	1282	1103	906
1000	26.19061097	20.77683285	22.40499616
17.21621026	15.64319863	13.06113806
ENSG00000112144 1418.940511	1073.499121	1412.427355
833.9546384	956.570152	661.6441477	1301.622329
817.389646	0.67197499	0.000475694	0.006966274	ICK
6	53001279	53061802	-	6259
protein_coding	intestinal cell kinase [Source:HGNC
Symbol;Acc:HGNC:21219]	-	1564	1213	1215	805	838
767	12.23659203	9.182951491	12.07753134
7.146657553	8.22974592	5.697986691
ENSG00000177674 908.1582169	1192.973466	1048.567468
1363.334539	1627.767347	1804.640883	1049.899717
1598.580923	-0.606755441	0.000475734	0.006966274	AGTRAP
1	11736084	11754802	+	2435
protein_coding	angiotensin II receptor associated protein
[Source:HGNC Symbol;Acc:HGNC:13539]	-	1001	1348	902
1316	1426	2092	20.130928	26.23115221
23.04700089	30.03094284	35.99713362	39.94787965

ENSG00000197563 521.6693054 487.6323294 624.258016
331.5099184 351.5794831 205.3080928 544.5198836
296.1324981 0.88052207 0.000476564 0.006972663 PIGN
18 61905255 62187118 - 19003
protein_coding phosphatidylinositol glycan anchor biosynthesis class
N [Source:HGNC Symbol;Acc:HGNC:8967] - 575 551 537
320 308 238 1.481748035 1.373902366
1.758162303 0.935706947 0.996267374 0.582352316
ENSG00000174720 1062.390881 869.9502355 833.506513
636.0846559 448.6062885 613.3363612 921.9492099
566.0091018 0.703238704 0.000480598 0.007025871 LARP7
4 112636964 112657592 + 4083
protein_coding La ribonucleoprotein domain family member 7
[Source:HGNC Symbol;Acc:HGNC:24912] - 1171 983 717
614 393 711 14.04449729 11.40776533
10.92563336 8.356050094 5.9164409 8.096945926
ENSG00000117461 356.5496296 298.2433666 272.0230461
110.848629 170.0822824 176.8410043 308.9386808
152.5906385 1.017625512 0.000483956 0.007069109 PIK3R3
1 46040140 46133036 - 7410
protein_coding phosphoinositide-3-kinase regulatory subunit 3
[Source:HGNC Symbol;Acc:HGNC:8981] - 393 337 234
107 149 205 2.597185837 2.154954643
1.964737326 0.802375415 1.235991319 1.286372275
ENSG00000244313 1702.910063 1994.779075 2328.470775
2901.747754 2919.936096 2835.494542 2008.719971
2885.726131 -0.523212351 0.000484776 0.007075248
AC024293.1 11 46428653 46429150 - 498
processed_pseudogene ribosomal protein S10 (RPS10)
pseudogene - 1877 2254 2003 2801 2558 3287
184.571063 214.4623249 250.2409921 312.5329489
315.7319687 306.9031737
ENSG00000137710 3633.540119 2589.495818 3379.363226
2500.827947 2050.119323 1730.453925 3200.799721
2093.800398 0.612470657 0.000490016 0.007145826 RDX
11 109864295 110296722 - 8528
protein_coding radixin [Source:HGNC Symbol;Acc:HGNC:9944] -
4005 2926 2907 2414 1796 2006 22.99767924
16.25749282 21.2082428 15.72903641 12.94513252
10.93741662
ENSG00000152620 1381.743221 1165.538617 1236.891115
978.9902277 761.3750494 716.8530466 1261.390984
819.0727746 0.623369276 0.000490921 0.007149889 NADK2
5 36192592 36242279 - 4885
protein_coding "NAD kinase 2, mitochondrial [Source:HGNC
Symbol;Acc:HGNC:26404]" - 1523 1317 1064 945 667
831 15.26736255 12.77460992 13.55139195
10.74927773 8.392833858 7.909835905
ENSG00000260032 7044.803751 6369.310117 6139.118403
8625.473689 8522.377989 8920.550367 6517.74409

8689.467348	-0.414828036	0.000491103	0.007149889	NORAD
	20	36045622	36050960	-	5339
lincRNA non-coding RNA activated by DNA damage [Source:HGNC
Symbol;Acc:HGNC:44311]	-	7765	7197	5281	8326	7466
	10341	71.22135349	63.87310413	61.54079372
86.65398295	85.95584715	90.06035103
ENSG00000198034 23031.47302	24716.14464	28204.37241
33438.99108	34962.75321	33278.02645	25317.33002
33893.25691	-0.420917844	0.000494134	0.007188101	RPS4X
	X	72255679	72277300	-	2982
protein_coding	ribosomal protein S4 X-linked [Source:HGNC
Symbol;Acc:HGNC:10424]	-	25386	27928	24262	32278	30629
	38577	416.8840892	443.7707133	506.2042719
601.4659161	631.3534223	601.5224352
ENSG00000026751 0	0.884995153	0	15.53952742
17.12237742	25.87917136	0.294998384
19.51369207	-5.802118976	0.000497701	0.00723405	SLAMF7
	1	160739057	160754821	+	4833
protein_coding	SLAM family member 7 [Source:HGNC
Symbol;Acc:HGNC:21394]	-	0	1	0	15	15
	30	0	0.009804143	0	0.172459256
0.190775159	0.288626018
ENSG00000179933 931.7467421	1032.789344	932.3183032
1331.219516	1360.658259	1505.305134	965.6181297
1399.06097	-0.534963269	0.000501783	0.007287382
C14orf119	14	23094765	23100462	+	3676
	protein_coding	chromosome 14 open reading frame 119
[Source:HGNC Symbol;Acc:HGNC:20270]	-	1027	1167	802
	1285	1192	1745	13.68118213	15.04256145
13.57393334	19.42404469	19.93187307	22.07247058
ENSG00000119335 9177.750772	8258.774769	8724.499832
7077.736757	6352.402024	5915.115933	8720.341791
6448.418238	0.435517167	0.000504517	0.007321084	SET
	9	128683424	128696400	+	4355
protein_coding	SET nuclear proto-oncogene [Source:HGNC
Symbol;Acc:HGNC:10760]	-	10116	9332	7505	6832	5565
	6857	113.7494603	101.5343544	107.2184153
87.17094257	78.54605359	73.21111282
ENSG00000168389 235.8852512	337.1831533	352.2349699
164.7189907	171.2237742	124.2200225	308.4344581
153.3875958	1.009170803	0.000513365	0.007443377	MFSD2A
	1	39955112	39969968	+	4007
protein_coding	major facilitator superfamily domain containing 2A
[Source:HGNC Symbol;Acc:HGNC:25897]	-	260	381	303
	159	150	144	3.177479001	4.505386715
4.704680471	2.204904712	2.301014084	1.670991219
ENSG00000138385 2345.243747	2072.658649	2073.885103
1618.182789	1335.545439	1586.393204	2163.929166
1513.373811	0.515608907	0.000514993	0.007460861	SSB
	2	169791933	169812064	+	5113

protein_coding	Sjogren syndrome antigen B [Source:HGNC
Symbol;Acc:HGNC:11316]	-	2585	2342	1784	1562	1170
	1839	24.75787912	21.70388796	21.70830446
16.97529305	14.06557301	16.72387661
ENSG00000074695 3090.09679	2379.751967	3683.936039
2249.087602	2038.704405	1607.95918	3051.261598
1965.250396	0.634672529	0.000516022	0.007469657	LMAN1
	18	59327823	59359962	-	6767
protein_coding	"lectin, mannose binding 1 [Source:HGNC
Symbol;Acc:HGNC:6631]"	-	3406	2689	3169	2171	1786
	1864	24.64774264	18.82873168	29.13619998
17.82689435	16.22305491	12.80798321
ENSG00000182551 1582.245685	1522.191663	1777.449733
1293.92465	963.419103	985.1337897	1627.295694
1080.825848	0.590071166	0.000517153	0.007479206	ADI1
	2	3497361 3519736 -	5560	protein_coding
acireductone dioxygenase 1 [Source:HGNC Symbol;Acc:HGNC:30576]	-
	1744	1720	1529	1249	844	1142	15.36032386
	14.65818154	17.10958755	12.48244584	9.330716951
	9.550416909
ENSG00000145555 4445.529733	3681.579837	4175.670006
3181.459248	2445.075496	2998.533321	4100.926526
2875.022688	0.512077957	0.000517552	0.007479206	MYO10
	5	16661914	16936276	-	13963
protein_coding	myosin X [Source:HGNC Symbol;Acc:HGNC:7593]	-
	4900	4160	3592	3071	2142	3476	17.18486107
	14.1169551	16.00532276	12.22117948	9.42948749
	11.57529446
ENSG00000102893 665.922209	527.4571113	606.8206412
366.7328472	402.9466153	314.8632515	600.0666538
361.514238	0.732337442	0.000517951	0.007479206	PHKB
	16	47461123	47701523	+	11822
protein_coding	phosphorylase kinase regulatory subunit beta
[Source:HGNC Symbol;Acc:HGNC:8927]	-	734	596	522
	354	353	365	3.040421455	2.388810754
2.747175436	1.663889	1.835401619	1.435598274
ENSG00000107614 349.2916219	299.1283618	288.2979292
484.8332556	523.9447491	550.3637109	312.2393043
519.7139052	-0.734479218	0.00052003	0.007503111	TRDMT1
	10	17142254	17202054	-	10535
protein_coding	tRNA aspartic acid methyltransferase 1 [Source:HGNC
Symbol;Acc:HGNC:2977]	-	385	338	248	468	459
	638	1.789595476	1.520227559	1.464616719
2.468444441	2.678092084	2.81589954
ENSG00000179950 5098.750429	5832.118059	5471.848196
6332.875409	8061.215291	9112.918874	5467.572228
7835.669858	-0.519245503	0.000521254	0.007514634	PUF60
	8	143816344	143829859	-	5424
protein_coding	poly(U) binding splicing factor 60 [Source:HGNC
Symbol;Acc:HGNC:17042]	-	5620	6590	4707	6113	7062

10564	50.73939894	57.56946249	53.99224229
62.62485864	80.03047464	90.56069145
ENSG00000160606 174.1921855	247.7986429	156.9363727
363.6249417	299.070859	501.1932853	192.9757337
387.9630287	-1.006903914	0.00052314	0.007535693	TLCD1
17	28724348	28727935	-	1311
protein_coding	TLC domain containing 1 [Source:HGNC
Symbol;Acc:HGNC:25177]	-	192	280	135	351	262
581	7.171784323	10.12002955	6.406752152
14.87703787	12.28417401	20.6065107
ENSG00000123219 662.2932052	577.901835	514.9838009
413.3514295	297.9293672	170.802531	585.0596137
294.0277759	0.99508458	0.000524344	0.007546878	CENPK
5	65517766	65563171	-	4312
protein_coding	centromere protein K [Source:HGNC
Symbol;Acc:HGNC:29479]	-	730	653	443	399	261
198	8.29034858	7.175643764	6.39192721
5.141693532	3.720566877	2.135096242
ENSG00000204209 2319.84072	2941.723889	2637.693554
3325.458869	3705.282474	4363.228291	2633.086054
3797.989878	-0.52867282	0.000534021	0.007679915	DAXX
6	33318558	33323016	-	3084
protein_coding	death domain associated protein [Source:HGNC
Symbol;Acc:HGNC:2681]	-	2557	3324	2269	3210	3246
5058	40.60177706	51.07085005	45.77485456
57.83659363	64.69660723	76.25976695
ENSG00000108592 4397.445432	4944.46792	3819.947562
5506.17255	6154.923938	7068.464337	4387.286971
6243.186942	-0.508823319	0.000534474	0.007680194	FTSJ3
17	63819433	63830012	-	5212
protein_coding	FtsJ RNA methyltransferase homolog 3 [Source:HGNC
Symbol;Acc:HGNC:17136]	-	4847	5587	3286	5315	5392
8194	45.54044775	50.79263051	39.22563643
56.66447971	63.5905874	73.10087421
ENSG00000128791 1408.96075	1082.349072	1290.365731
819.4510795	913.1934625	774.6498627	1260.558518
835.7648016	0.593330424	0.000545301	0.00782679	TWSG1
18	9334767 9402420 +	4435	protein_coding
twisted gastrulation BMP signaling modulator 1 [Source:HGNC
Symbol;Acc:HGNC:12429]	-	1553	1223	1110	791	800
898	17.1477252	13.06650003	15.57170621
9.910484873	11.0877576	9.414857057
ENSG00000149823 2359.759763	3165.627663	2750.455244
3132.768729	4845.632811	4755.729057	2758.614223
4244.710199	-0.62172145	0.000545561	0.00782679	VPS51
11	65089324	65111860	+	4878
protein_coding	"VPS51, GARP complex subunit [Source:HGNC
Symbol;Acc:HGNC:1172]"	-	2601	3577	2366	3024	4245
5513	26.11122456	34.74590157	30.17725905
34.44704993	53.49131307	52.55054381

ENSG00000142621 175.0994364 134.5192633 105.7867401
227.9130689 297.9293672 344.1929791 138.4684799
290.011805 -1.064385252 0.000550391 0.007889681 FHAD1
1 15247272 15400283 + 12805
protein_coding forkhead associated phosphopeptide binding domain 1
[Source:HGNC Symbol;Acc:HGNC:29408] - 193 152 91
220 261 399 0.738085052 0.56245845
0.442149009 0.954674103 1.252876562 1.448853022
ENSG00000079931 176.9139384 109.739399 120.8991316
68.37392066 26.25431205 38.81875704 135.850823
44.48232992 1.609973303 0.000555748 0.007957047 MOXD1
6 132296055 132401545 - 5293
protein_coding monooxygenase DBH like 1 [Source:HGNC
Symbol;Acc:HGNC:21063] - 195 124 104 66 23
45 1.8041033 1.110059434 1.22247023
0.692873713 0.26709964 0.395313493
ENSG00000174173 1351.803939 1768.220316 1343.840347
2300.886027 1930.262681 2403.31238 1487.954868
2211.48703 -0.571531419 0.00055599 0.007957047
TRMT10C 3 101561862 101566446 + 1888
protein_coding "tRNA methyltransferase 10C, mitochondrial RNase P
subunit [Source:HGNC Symbol;Acc:HGNC:26022]" - 1490 1998
1156 2221 1691 2786 38.6467486 50.14411084
38.09453623 65.36700882 55.05401259 68.61358955
ENSG00000165704 2205.527098 1966.45923 2170.37191
1418.24087 1546.721427 1569.140423 2114.119413
1511.367573 0.483973579 0.000558804 0.007990858 HPRT1
X 134460153 134520513 + 1624
protein_coding hypoxanthine phosphoribosyltransferase 1 [Source:HGNC
Symbol;Acc:HGNC:5157] - 2431 2222 1867 1369 1355
1819 73.30399054 64.83126124 71.52619785
46.84135686 51.28621286 52.08080547
ENSG00000169764 1237.490318 1123.943844 1236.891115
972.7744167 607.2736526 640.9408106 1199.441759
740.3296266 0.696094411 0.000559625 0.007994318 UGP2
2 63840940 63891562 + 6501
protein_coding UDP-glucose pyrophosphorylase 2 [Source:HGNC
Symbol;Acc:HGNC:12527] - 1364 1270 1064 939 532
743 10.27455184 9.256567997 10.18282567
8.025968851 5.030125567 5.314221668
ENSG00000254995 16.33051739 14.15992245 13.9498998 0
0 0 14.81344655 0 6.322237469
0.000560034 0.007994318 STX16-NPEPL1 20 58651434
58715410 + 3859 protein_coding STX16-NPEPL1
readthrough (NMD candidate) [Source:HGNC Symbol;Acc:HGNC:41993] -
18 16 12 0 0 0 0.228415941
0.196458871 0.193469859 0 0 0
ENSG00000238228 26.31027801 45.13475281 32.5497662
148.1434948 107.3002318 75.91223598 34.66493234
110.4519875 -1.668727624 0.000560402 0.007994318 OR7E7P

7	97946987	97947998	-	1012
unprocessed_pseudogene	olfactory receptor family 7 subfamily E member
7 pseudogene [Source:HGNC Symbol;Acc:HGNC:8457] -	29	51
28	143	94	88	1.403285924	2.387899829
1.721410174	7.851769985	5.709455617	4.043274043
ENSG00000158710 30910.04041	38197.2758	48763.03724
25927.18352	29723.30571	24875.92215	39290.11782
26842.13713	0.549648674	0.000562391	0.008016224	TAGLN2
1	159918107	159925732	-	2487
protein_coding	transgelin 2 [Source:HGNC Symbol;Acc:HGNC:11554]
-	34070	43161	41947	25027	26039	28837
670.8493941	822.3224631	1049.37803	559.1715308
643.5701263	539.1446628
ENSG00000134717 2262.683909	2197.442965	2254.071309
2891.388069	3311.467794	2976.967345	2238.066061
3059.941069	-0.451119016	0.000565042	0.008047526	BTF3L4
1	52056125	52090716	+	6022
protein_coding	basic transcription factor 3 like 4 [Source:HGNC
Symbol;Acc:HGNC:30547]	-	2494	2483	1939	2791	2901
3451	20.28076728	19.53720391	20.03290375
25.75319575	29.61108615	26.6461965
ENSG00000115159 1997.766627	1354.927579	1875.099031
1299.104493	958.8531357	1079.161446	1742.597746
1112.373025	0.647175413	0.000566014	0.008054884	GPD2
2	156435290	156613735	+	7155
protein_coding	glycerol-3-phosphate dehydrogenase 2 [Source:HGNC
Symbol;Acc:HGNC:4456]	-	2202	1531	1613	1254	840
1251	15.07080186	10.13892694	14.02592705
9.738676554	7.216340353	8.129778061
ENSG00000101639 840.1143945	641.621486	741.6696726
567.7107352	366.4188768	315.7258906	741.1351844
416.6185009	0.831763935	0.000575833	0.008188031	CEP192
18	12991362	13125052	+	9901
protein_coding	centrosomal protein 192 [Source:HGNC
Symbol;Acc:HGNC:25515]	-	926	725	638	548	321
366	4.57994866	3.469647757	4.009114546
3.075484493	1.992844132	1.718830467
ENSG00000090520 1534.161383	1362.007541	1428.702238
1222.442824	622.1130464	603.8473317	1441.623721
816.1344007	0.82111372	0.000578075	0.008213303
DNAJB11 3	186567403	186585800	+	5731
protein_coding	DnaJ heat shock protein family (Hsp40) member B11
[Source:HGNC Symbol;Acc:HGNC:14889]	-	1691	1539	1229
1180	545	700	14.44913613	12.72432204
13.34222687	11.44099096	5.845389485	5.679350209
ENSG00000113810 6316.281225	3633.790099	4498.842685
2621.000292	1430.289261	1274.11787	4816.30467
1775.135808	1.440065872	0.000582672	0.008271976	SMC4
3	160399274	160434962	+	8457
protein_coding	structural maintenance of chromosomes 4 [Source:HGNC

Symbol;Acc:HGNC:14013]	-	6962	4106	3870	2530	1253
	1477	40.31311634	23.00536114	28.47091869
16.6232614	9.10714184	8.120732088
ENSG00000121005 215.0184789	140.7142293	161.5863393
92.20119605	62.78205055	51.75834272	172.4396825
68.9138631	1.325717864	0.000585329	0.008303039
CRISPLD1	8	74984515	75034558	+	4543
	protein_coding	cysteine rich secretory protein LCCL domain
containing 1 [Source:HGNC Symbol;Acc:HGNC:18206]	-	237
	159	139	89	55	60	2.554666969
1.658367692	1.903614087	1.0885774	0.744161698
0.614100614
ENSG00000204291 56.24955989	100.0044523	131.3615564
40.4027713	17.12237742	9.489029498	95.87185621
22.33805941	2.105585972	0.000588548	0.008340175
COL15A1 9	98943179	99070792	+	6653
protein_coding	collagen type XV alpha 1 chain [Source:HGNC
Symbol;Acc:HGNC:2192]	-	62	113	113	39	15
	11	0.456355038	0.804798875	1.056739075
0.325731026	0.138586554	0.076878726
ENSG00000276180 106.148363	120.3593408	112.76169
269.3518087	220.3079228	205.3080928	113.089798
231.6559414	-1.03389022	0.00058889	0.008340175
HIST1H4I	6	27138588	27139881	+	1294
	protein_coding	histone cluster 1 H4 family member i
[Source:HGNC Symbol;Acc:HGNC:4793]	-	117	136	97
	260	193	238	4.427721221	4.980019839
4.663847105	11.16480431	9.16791109	8.552118282
ENSG00000080845 1245.655576	1174.388568	1585.63861
999.7095976	826.4400836	755.6718037	1335.227585
860.6071616	0.633296705	0.000589631	0.008343986	DLGAP4
	20	36306336	36528637	+	8789
protein_coding	DLG associated protein 4 [Source:HGNC
Symbol;Acc:HGNC:24476]	-	1373	1327	1364	965	724
	876	7.649970428	7.154147653	9.65565528
6.100984091	5.063449255	4.634422882
ENSG00000202111 30.84653284	56.6396898	37.1997328
90.12925906	94.74382174	216.5224004	41.56198515
133.7984937	-1.687512232	0.000595333	0.008417946
VTRNA1-2	5	140718925	140719013	+	89
	misc_RNA	vault RNA 1-2 [Source:HGNC
Symbol;Acc:HGNC:12655]	-	34	64	32	87	83
	251	18.70757926	34.07347348	22.37004297
54.31768968	57.32386254	131.1338261
ENSG00000138119 3404.912875	2566.485944	3335.188544
2659.331126	1511.335181	1361.244413	3102.195788
1843.97024	0.75057001	0.000596306	0.008422294	MYOF
	10	93306429	93482317	-	8473
protein_coding	myoferlin [Source:HGNC Symbol;Acc:HGNC:3656]	-
	3753	2900	2869	2567	1324	1578	21.69052389

16.21762419	21.0668784	16.83451876	9.605017023
8.659659397
ENSG00000140465 3.629003864	7.964956378	0	26.93518087
46.80116496	44.85723035	3.864653414	39.53119206
-3.313219831	0.000596593	0.008422294	CYP1A1	15
74719542	74725610	-	3435	protein_coding
cytochrome P450 family 1 subfamily A member 1 [Source:HGNC
Symbol;Acc:HGNC:2595]	-	4	9	0	26	41
52	0.057024559	0.124148709	0	0.420589717
0.733676275	0.703894579
ENSG00000106803 686.7889812	858.4452985	891.6310955
454.7901693	438.332862	598.6714974	812.2884584
497.2648429	0.706015048	0.00060186	0.008481317	SEC61B
9	99222064	99230615	+	1190
protein_coding	Sec61 translocon beta subunit [Source:HGNC
Symbol;Acc:HGNC:16993]	-	757	970	767	439	384
694	31.15140231	38.62346331	40.10100428
20.49885496	19.83497344	27.11711777
ENSG00000151640 420.0571972	434.5326202	476.6215765
562.5308927	738.5452128	975.6447602	443.7371313
758.9069553	-0.77569211	0.000602112	0.008481317	DPYSL4
10	132186900	132205776	+	3176
protein_coding	dihydropyrimidinase like 4 [Source:HGNC
Symbol;Acc:HGNC:3016]	-	463	491	410	543	647
1131	7.13886519	7.325334135	8.031750511
9.500170059	12.52192641	16.5582002
ENSG00000166794 2074.882959	2445.241608	3195.689546
1760.110473	1860.63168	1266.354118	2571.938038
1629.03209	0.658989395	0.000602212	0.008481317	PPIB
15	64155812	64163205	-	1911
protein_coding	peptidylprolyl isomerase B [Source:HGNC
Symbol;Acc:HGNC:9255]	-	2287	2763	2749	1699	1630
1468	58.60493129	68.50884389	89.49955983
49.40202322	52.42932634	35.71876107
ENSG00000178252 4136.157154	4349.751178	3761.822979
4807.929785	6028.218345	6419.759775	4082.577104
5751.969301	-0.494491903	0.000606129	0.008529681	WDR6
3	49007062	49015953	+	5304
protein_coding	WD repeat domain 6 [Source:HGNC Symbol;Acc:HGNC:12758]
-	4559	4915	3236	4641	5281	7442
42.09153465	43.90828228	37.9587449	48.62057568
61.20121052	65.24048312
ENSG00000050344 200.5024635	232.7537253	176.6987308
104.632818	90.17785443	90.57709975	203.3183065
95.12925739	1.097192652	0.000611839	0.008603192	NFE2L3
7	26152240	26187125	+	4249
protein_coding	"nuclear factor, erythroid 2 like 3 [Source:HGNC
Symbol;Acc:HGNC:7783]"	TF_bZIP 221	263	152	101	79
105	2.547030973	2.932887862	2.225685023
1.320829276	1.142846021	1.149035869

ENSG00000089157 30715.8887 37099.88181 42573.93169
44408.86147 57907.88044 55167.49222 36796.5674
52494.74471 -0.5126331 0.000612577 0.00860673 RPLP0
12 120196686 120201235 - 4001
protein_coding ribosomal protein lateral stalk subunit P0
[Source:HGNC Symbol;Acc:HGNC:10371] - 33856 41921 36623
42867 50730 63952 414.3771301 496.4660135
569.4980118 595.3420838 779.3699758 743.2186475
ENSG00000122203 1710.168071 1910.704536 1951.82348
2264.62713 2691.637731 3101.187368 1857.565362
2685.817409 -0.53240491 0.000617185 0.008664588
KIAA1191 5 176346061 176361968 - 3973
protein_coding KIAA1191 [Source:HGNC Symbol;Acc:HGNC:29209]
- 1885 2159 1679 2186 2358 3595
23.23386561 25.74900894 26.29292955 30.57339281
36.48149237 42.07376218
ENSG00000137509 1232.046812 1315.102798 1737.925017
1102.270479 784.204886 800.529034 1428.358209
895.6681328 0.67273092 0.000619188 0.008685815 PRCP
11 82823502 82970584 - 12798
protein_coding prolylcarboxypeptidase [Source:HGNC
Symbol;Acc:HGNC:9344] - 1358 1486 1495 1064 687
928 5.196205852 5.501779039 7.267849633
4.619676514 3.299605292 3.371606547
ENSG00000087111 1233.861314 1493.871818 1531.001503
969.6665112 954.2871684 996.3480973 1419.578212
973.4339256 0.54378817 0.000620612 0.008698893 PIGS
17 28553383 28571872 - 5990
protein_coding phosphatidylinositol glycan anchor biosynthesis class
S [Source:HGNC Symbol;Acc:HGNC:14937] - 1360 1688 1317
936 836 1155 11.11836096 13.35279129
13.67936063 8.682825439 8.578805376 8.965740815
ENSG00000141458 4083.536598 3530.245666 3230.564295
6587.723659 4774.860317 4613.393614 3614.782186
5325.325863 -0.558719725 0.000622173 0.008713872 NPC1
18 23506184 23586898 - 10102
protein_coding NPC intracellular cholesterol transporter 1
[Source:HGNC Symbol;Acc:HGNC:7897] - 4501 3989 2779
6359 4183 5348 21.81877342 18.71040176
17.11543846 34.97788169 25.45234847 24.61586223
ENSG00000168495 2093.027979 2568.255934 2318.00835
3023.992037 3377.67432 3294.418514 2326.430754
3232.02829 -0.474258484 0.000623815 0.008729952 POLR3D
8 22245104 22254600 + 5611
protein_coding RNA polymerase III subunit D [Source:HGNC
Symbol;Acc:HGNC:1080] - 2307 2902 1994 2919 2959
3819 20.13427562 24.50662923 22.11015219
28.90718953 32.41545013 31.64757282
ENSG00000136938 3334.1473 3466.526015 3644.411322
2771.215724 2429.09461 2461.971835 3481.694879

2554.094056	0.4468601	0.000629407	0.008801254	ANP32B
	9	97983361	98015943	+	1850
protein_coding	acidic nuclear phosphoprotein 32 family member B
[Source:HGNC Symbol;Acc:HGNC:16677]	-	3675	3917	3135
	2675	2128	2854	97.27792507	100.3247957
105.432053	80.34597062	70.70453256	71.73205264
ENSG00000128050 7692.580941	6876.41234	7242.322979
6189.911757	4748.606005	4423.613024	7270.438753
5120.710262	0.505792302	0.000631639	0.008825482	PAICS
	4	56435741	56464579	+	7517
protein_coding	phosphoribosylaminoimidazole carboxylase and
phosphoribosylaminoimidazolesuccinocarboxamide synthase [Source:HGNC
Symbol;Acc:HGNC:8587]	-	8479	7770	6230	5975	4160
	5128	55.23683277	48.97821005	51.56444444
44.16776293	34.01701023	31.72009537
ENSG00000007376 1405.331746	1924.864458	1578.663661
1657.549592	3054.632132	3833.567917	1636.286622
2848.583214	-0.799947996	0.000636008	0.008879518	RPUSD1
	16	784974	788397	-	2769	protein_coding	RNA
pseudouridylate synthase domain containing 1 [Source:HGNC
Symbol;Acc:HGNC:14173]	-	1549	2175	1358	1600	2676
	4444	27.39410686	37.21883255	30.5129123
32.10769069	59.40329203	74.62461656
ENSG00000260442 15.42326642	71.6846074	37.1997328	0
	0	7.763751407	41.43586887	2.587917136
3.931104807	0.000639224	0.008917381	ATP2A1-AS1	16
	28878957	28879920	-	583	antisense
	ATP2A1 antisense RNA 1 [Source:HGNC Symbol;Acc:HGNC:51370]
	-	17	81	32	0	0	9
1.427937011	6.583288762	3.41498083	0	0
0.71780251
ENSG00000133316 1073.277893	1477.941906	1245.028557
1424.45668	2139.155686	2746.64272	1265.416118
2103.418362	-0.733388802	0.000642935	0.008956574	WDR74
	11	62832342	62841809	-	4247
protein_coding	WD repeat domain 74 [Source:HGNC
Symbol;Acc:HGNC:25529]	-	1183	1670	1071	1375	1874
	3184	13.64052755	18.63205043	15.68967893
17.99005457	27.12281026	34.85955318
ENSG00000108100 2033.149415	1953.184303	2078.53507
2386.871412	3205.309054	3078.758753	2021.622929
2890.313073	-0.515824604	0.000643046	0.008956574	CCNY
	10	35247025	35572669	+	5743
protein_coding	cyclin Y [Source:HGNC Symbol;Acc:HGNC:23354]	-
	2241	2207	1788	2304	2808	3569	19.10872559
	18.20916192	19.37026422	22.29234225	30.05423289
	28.89606794
ENSG00000089737 3135.459338	3160.317692	3496.774883
4069.284248	4625.324888	4586.651804	3264.183971
4427.08698	-0.439880552	0.000648468	0.009024982	DDX24

14	94048291	94081245	-	8636
protein_coding	DEAD-box helicase 24 [Source:HGNC
Symbol;Acc:HGNC:13266]	-	3456	3571	3008	3928	4052
5317	19.59700862	19.59312266	21.67065533
25.27382006	28.8405915	28.62760688
ENSG00000182919 283.9695524	272.5785072	318.5227121
517.9842475	534.2181756	422.6931322	291.6902572
491.6318517	-0.753329293	0.000649162	0.009027538
C11orf54	11	93741591	93764749	+	6269
protein_coding	chromosome 11 open reading frame 54
[Source:HGNC Symbol;Acc:HGNC:30204]	-	313	308	274
500	468	490	2.444976849	2.327978084
2.719312629	4.431836992	4.588755768	3.634367401
ENSG00000173083 137.9021468	148.6791857	129.0365731
73.55376314	39.95221399	42.26931322	138.5393019
51.92509678	1.41699005	0.000653585	0.00908191	HPSE
4	83292461	83335153	-	4721
protein_coding	heparanase [Source:HGNC Symbol;Acc:HGNC:5164]	-
152	168	111	71	35	49	1.576660804
1.686171413	1.462836624	0.835673041	0.455702493
0.482606423
ENSG00000113645 507.15329	455.7725039	623.0955243
395.7399651	220.3079228	173.3904481	528.6737727
263.146112	1.007017769	0.000654634	0.009089347	WWC1
5	168291651	168472303	+	10076
protein_coding	WW and C2 domain containing 1 [Source:HGNC
Symbol;Acc:HGNC:29435]	-	559	515	536	382	193
201	2.71676662	2.421840352	3.309660734
2.106625068	1.17737961	0.927553389
ENSG00000177700 531.6490661	1041.639295	1054.379926
336.6897608	530.7937001	527.0724567	875.8894292
464.8519725	0.913299282	0.000657728	0.009125141	POLR2L
11	837356	842545	-	1142	protein_coding	RNA
polymerase II subunit L [Source:HGNC Symbol;Acc:HGNC:9199]	-
586	1177	907	325	465	611	25.12813147
48.83563057	49.41377459	15.81354726	25.02846466
24.87746503
ENSG00000188191 255.8447724	432.7626299	301.0853373
426.8190199	591.292767	965.2930917	329.8975799
661.1349595	-1.00332504	0.000665831	0.009222056
PRKAR1B 7	549197	727650	-	4491	protein_coding
protein kinase cAMP-dependent type I regulatory subunit beta
[Source:HGNC Symbol;Acc:HGNC:9390]	-	282	489	259
412	518	1119	3.074926482	5.159317382
3.588091881	5.097605288	7.089801319	11.58558718
ENSG00000196372 662.2932052	745.1659189	675.4076486
971.7384482	991.9563987	1137.820901	694.2889242
1033.838583	-0.574616307	0.000666208	0.009222056	ASB13
10	5638867 5666595 -	3003	protein_coding
ankyrin repeat and SOCS box containing 13 [Source:HGNC

Symbol;Acc:HGNC:19765]	-	730	842	581	938	869
1319	11.90409027	13.28566202	12.03726015
17.35639598	17.78737268	20.4230444
ENSG00000100418 926.3032363	1237.223224	1012.530227
1214.155076	1756.755924	2314.460558	1058.685562
1761.790519	-0.735015656	0.000666278	0.009222056	DESI1
22	41598028	41621096	-	4972
protein_coding	desumoylating isopeptidase 1 [Source:HGNC
Symbol;Acc:HGNC:24577]	-	1021	1398	871	1172	1539
2683	10.05595464	13.32301425	10.89918142
13.09810704	19.02632277	25.09115024
ENSG00000180104 1089.60841	1229.258268	1106.692051
1513.549971	1902.866878	1566.552506	1141.85291
1660.989785	-0.54004217	0.000667841	0.009236464	EXOC3
5	443158	471937	+	8182	protein_coding
exocyst complex component 3 [Source:HGNC Symbol;Acc:HGNC:30378] -
1201	1389	952	1461	1667	1816	7.188068643
8.043947203	7.239095731	9.922081372	12.52343554
10.32018273
ENSG00000049323 1469.746565	1128.36882	1617.025885
999.7095976	941.7307582	791.9026436	1405.04709
911.1143331	0.624873456	0.00067118	0.009275389	LTBP1
2	32946972	33399509	+	7329
protein_coding	latent transforming growth factor beta binding protein
1 [Source:HGNC Symbol;Acc:HGNC:6714]	-	1620	1275	1391
965	825	918	10.82427896	8.243125347
11.80835103	7.316352732	6.9192112	5.824102071
ENSG00000144713 13571.5672	18481.35378	18205.78173
20616.80902	24564.90414	26383.8152	16752.9009
23855.17612	-0.509945493	0.000682564	0.009425346	RPL32
3	12834485	12841588	-	3720
protein_coding	ribosomal protein L32 [Source:HGNC
Symbol;Acc:HGNC:10336]	-	14959	20883	15661	19901	21520
30585	196.9193102	265.9967862	261.9288863
297.2651541	355.587665	382.2931912
ENSG00000163527 5451.671055	4670.119423	5261.437207
4426.693379	3031.802296	2835.494542	5127.742562
3431.330072	0.579636149	0.000685931	0.009464455	STT3B
3	31532638	31637622	+	5012
protein_coding	"STT3B, catalytic subunit of the
oligosaccharyltransferase complex [Source:HGNC
Symbol;Acc:HGNC:30611]" -	6009	5277	4526	4273	2656
3287	58.711047	49.88873279	56.18369967
47.3733257	32.57349571	30.49436961
ENSG00000127022 12744.15432	10105.75965	12867.62007
10158.70706	7340.933947	5874.571898	11905.84468
7791.404302	0.611769398	0.000686958	0.009471233	CANX
5	179678628	179730925	+	6235
protein_coding	calnexin [Source:HGNC Symbol;Acc:HGNC:1473]	-
14047	11419	11069	9806	6431	6810	110.3254756

86.77968232 110.4533205 87.3911541 63.40002249
50.78572682
ENSG00000142039 1098.68092 1242.533195 1025.317635
1409.953122 1892.593451 1690.772529 1122.17725
1664.4397 -0.568055383 0.000688888 0.009490456 CCDC97
19 41310189 41324883 + 3821
protein_coding coiled-coil domain containing 97 [Source:HGNC
Symbol;Acc:HGNC:28289] - 1211 1404 882 1361 1658
1960 15.52014552 17.41071117 14.36145342
19.79215718 26.67195494 23.85118999
ENSG00000197063 1375.392464 1687.685757 1429.864729
1800.513244 2377.727478 2486.988368 1497.64765
2221.74303 -0.568911067 0.000692735 0.009536027 MAFG
17 81918270 81927714 - 5220
protein_coding MAF bZIP transcription factor G [Source:HGNC
Symbol;Acc:HGNC:6781] TF_bZIP 1516 1907 1230 1738 2083
2883 14.22189218 17.31038116 14.66025266
18.50083427 24.52822533 25.68059948
ENSG00000135317 1163.095738 897.3850853 976.4929859
793.5518671 555.9065203 422.6931322 1012.324603
590.7171732 0.778137399 0.000694582 0.009554011 SNX14
6 85505496 85594156 - 7105
protein_coding sorting nexin 14 [Source:HGNC Symbol;Acc:HGNC:14977]
- 1282 1014 840 766 487 490
8.835937321 6.762391554 7.355666838 5.990688404
4.213201587 3.206734586
ENSG00000184060 1.814501932 3.539980612 11.6249165
53.87036173 61.64055872 19.84069804 5.659799681
45.11720617 -3.028478763 0.00069513 0.009554119 ADAP2
17 30906344 30959322 + 3750
protein_coding ArfGAP with dual PH domains 2 [Source:HGNC
Symbol;Acc:HGNC:16487] - 2 4 10 52 54
23 0.026117248 0.050542319 0.165911152
0.770520361 0.88513569 0.285185596
ENSG00000197712 1151.301476 987.6545909 1474.039412
912.688244 698.5929988 579.6934384 1204.331826
730.3248938 0.721496563 0.000697782 0.009578958
FAM114A1 4 38867677 38945739 + 4871
protein_coding family with sequence similarity 114 member A1
[Source:HGNC Symbol;Acc:HGNC:25087] - 1269 1116 1268
881 612 672 12.75769385 10.85606677
16.19600755 10.05008703 7.722904292 6.414785836
ENSG00000115255 287.5985562 382.3179061 259.2356379
484.8332556 535.3596674 575.3802432 309.7173668
531.8577221 -0.778549923 0.00069802 0.009578958 REEP6
19 1490747 1497927 + 2449 protein_coding
receptor accessory protein 6 [Source:HGNC Symbol;Acc:HGNC:30078]
- 317 432 223 468 469 667
6.338684862 8.35836635 5.665300158 10.61864524
11.77148945 12.66391733

ENSG00000104419 7076.557535	8862.341463	17915.15882
25812.19102	40509.262	34952.40884	11284.68594
33757.95395	-1.580903027	0.00069876	0.009579106	NDRG1
8	133237171	133302022	-	9823
protein_coding	N-myc downstream regulated 1 [Source:HGNC
Symbol;Acc:HGNC:7679]	-	7800	10014	15411	24916	35488
40518	38.88473488	48.30475501	97.6098225
140.9438796	222.0673656	191.793937
ENSG00000132275 728.5225257	905.3500416	669.5951903
1037.004463	1322.989029	1188.716604	767.8225859
1182.903366	-0.622360045	0.000699645	0.009579106	RRP8
11	6595075 6603620 -	6922	protein_coding
ribosomal RNA processing 8 [Source:HGNC Symbol;Acc:HGNC:29030]	-
803	1023	576	1001	1159	1378	5.680840998
7.002779949	5.17723329	8.035529988	10.29198634
9.256539117
ENSG00000177030 1258.35709	1415.992245	1190.391449
1675.161056	1828.669909	2050.493011	1288.246928
1851.441325	-0.523079295	0.000699655	0.009579106	DEAF1
11	644233	706715	-	4452
protein_coding	"DEAF1, transcription factor [Source:HGNC
Symbol;Acc:HGNC:14677]" SAND	1387	1600	1024	1617	1602
2377	15.25632701	17.02908322	14.31039586
20.18212575	22.11845136	24.82590243
ENSG00000055163 129.7368881	121.244336	117.4116566
269.3518087	198.6195781	270.8686602	122.7976269
246.2800157	-1.004568513	0.000700635	0.009585094	CYFIP2
5	157266079	157395595	+	10498
protein_coding	cytoplasmic FMR1 interacting protein 2 [Source:HGNC
Symbol;Acc:HGNC:13760]	-	143	137	101	260	174
314	0.667049638	0.618358649	0.598579242
1.376191348	1.018802589	1.390766105
ENSG00000150637 9.979760626	7.964956378	26.73730795	0
0	0	14.89400832	0	6.325289135
0.000702673	0.009605548	CD226	18	69831158
69961803	-	13638	protein_coding	CD226 molecule
[Source:HGNC Symbol;Acc:HGNC:16961]	-	11	9	23
0	0	0	0.039497598	0.031269308
0.104926213	0	0	0
ENSG00000187624 9.979760626	34.51481097	27.8997996
70.44585765	99.30978905	103.5166854	24.13145706
91.09077738	-1.919120386	0.000703693	0.009612065
C17orf97	17	410327	431062	+	2710	protein_coding
chromosome 17 open reading frame 97 [Source:HGNC
Symbol;Acc:HGNC:33800]	-	11	39	24	68	87
120	0.198770568	0.681901675	0.550996446
1.394285354	1.973319111	2.058936598
ENSG00000145425 17160.65202	18976.06607	24834.30912
26950.72039	30827.12831	28439.48404	20323.67574
28739.11092	-0.499931683	0.000704977	0.009622178	RPS3A

4	151099573	151104652	+	2341
protein_coding	ribosomal protein S3A [Source:HGNC
Symbol;Acc:HGNC:10421]	-	18915	21442	21363	26015	27006
32968	395.6704487	434.0005889	567.7637666
617.4964429	709.0979173	654.8204078
ENSG00000163877 800.195352	821.2755021	823.0440881
1017.321062	1263.631454	1383.673029	814.8383141
1221.541848	-0.584543125	0.000707863	0.009654122	SNIP1
1	37534449	37554344	-	5769
protein_coding	Smad nuclear interacting protein 1 [Source:HGNC
Symbol;Acc:HGNC:30587]	-	882	928	708	982	1107
1604	7.486808586	7.622086588	7.635536637
9.458515774	11.79490486	12.92810418
ENSG00000172216 2529.415693	4209.921943	4101.270541
4054.780689	6528.191765	7401.443008	3613.536059
5994.805154	-0.730498597	0.000709624	0.009664252	CEBPB
20	50190734	50192689	+	1956
protein_coding	CCAAT enhancer binding protein beta [Source:HGNC
Symbol;Acc:HGNC:1834]	C/EBP	2788	4757	3528	3914	5719
8580	69.79954675	115.2366811	112.2190461
111.1895607	179.7209089	203.9620909
ENSG00000162385 611.4871511	964.6447169	848.6189044
391.5960911	497.6904371	545.1878766	808.2502575
478.1581349	0.756616517	0.000709699	0.009664252	MAGOH
1	53226892	53238610	-	1463
protein_coding	"mago homolog, exon junction complex subunit
[Source:HGNC Symbol;Acc:HGNC:6815]"	-	674	1090	730
378	436	632	22.56026797	35.30275613
31.04455083	14.35686172	18.31848374	20.08647693
ENSG00000117360 1839.904959	2079.73861	1902.998831
2477.000671	2848.022111	2710.41188	1940.8808
2678.478221	-0.46452195	0.000710497	0.009667672	PRPF3
1	150321476	150353195	+	5373
protein_coding	pre-mRNA processing factor 3 [Source:HGNC
Symbol;Acc:HGNC:17348]	-	2028	2350	1637	2391	2495
3142	18.48331196	20.7241851	18.95565018
24.72719076	28.54309543	27.19069812
ENSG00000151725 1306.441391	1152.263689	1038.105043
836.0265754	783.0633941	738.4190228	1165.603375
785.8363308	0.569639119	0.00071851	0.009762564	CENPU
4	184694618	184734133	-	2953
protein_coding	centromere protein U [Source:HGNC
Symbol;Acc:HGNC:21348]	-	1440	1302	893	807	686
856	23.87963744	20.89170949	18.81459432
15.18525647	14.27933653	13.47849295
ENSG00000145916 401.004927	522.1471403	431.2844021
640.2285298	722.5643272	810.8807026	451.4788231
724.5578532	-0.682393278	0.000718679	0.009762564	RMND5B
5	178130996	178150565	+	7701
protein_coding	required for meiotic nuclear division 5 homolog B

[Source:HGNC Symbol;Acc:HGNC:26181]	-	442	590	371
	618	633	940	2.810630984	3.630206492
2.997323597	4.459163488	5.052472366	5.675599586
ENSG00000122970 605.1363943	301.7833472	404.5470942
261.0640607	186.063168	216.5224004	437.1556119
221.216543	0.982457655	0.000719126	0.009762564	IFT81
	12	110124335	110218797	+	7005
protein_coding	intraflagellar transport 81 [Source:HGNC
Symbol;Acc:HGNC:14313]	-	667	341	348	252	163
	251	4.662795599	2.306602085	3.090850155
1.99896157	1.430298967	1.666082873
ENSG00000103316 42.6407954	30.08983521	22.08734135
84.94941658	141.5449867	81.9507093	31.60599065
102.8150375	-1.693835079	0.000720876	0.009778819	CRYM
	16	21238874	21303083	-	3782
protein_coding	crystallin mu [Source:HGNC Symbol;Acc:HGNC:2418]
	-	47	34	19	82	124	95
0.608562261	0.425974727	0.312563976	1.204770629
2.01533627	1.167973795
ENSG00000182492 8521.808323	7928.671577	16384.15731
2868.596762	4668.701577	1695.085724	10944.87907
3077.461355	1.83051108	0.000723602	0.009804776	BGN
	X	153494939	153509554	+	2453
protein_coding	biglycan [Source:HGNC Symbol;Acc:HGNC:1044]	-
	9393	8959	14094	2769	4090	1965	187.5147597
	173.0567045	357.4732638	62.72453511	102.488024
	37.2474053
ENSG00000170035 1665.712774	1439.887114	1828.599365
1138.529376	1065.011876	1205.106746	1644.733084
1136.215999	0.532699806	0.00072417	0.009804776	UBE2E3
	2	180967248	181076585	+	2973
protein_coding	ubiquitin conjugating enzyme E2 E3 [Source:HGNC
Symbol;Acc:HGNC:12479]	-	1836	1627	1573	1099	933
	1397	30.24171743	25.93098922	32.91854719
20.54068047	19.29008293	21.84904781
ENSG00000196369 273.9897917	359.3080322	356.8849365
209.265636	166.6578069	144.9233596	330.0609201
173.6156008	0.9275593	0.000724453	0.009804776
SRGAP2B 1	144887265	145095528	-	7230
protein_coding	SLIT-ROBO Rho GTPase activating protein 2B
[Source:HGNC Symbol;Acc:HGNC:35237]	-	302	406	307
	202	146	168	2.045489852	2.660811918
2.641842514	1.552476789	1.241257594	1.080443354
ENSG00000004776 175.0994364	108.8544038	86.02438209
42.47470829	47.94265678	38.81875704	123.3260741
43.07870737	1.521628837	0.000728628	0.009853752	HSPB6
	19	35754569	35758079	-	1721
protein_coding	heat shock protein family B (small) member 6
[Source:HGNC Symbol;Acc:HGNC:26511]	-	193	123	74
	41	42	45	5.491678726	3.386496896

2.675208872	1.323777606	1.500084696	1.215801463
ENSG00000185624 10476.93416	11344.75287	14399.78407
9406.593934	8564.613187	7669.723752	12073.8237
8546.976957	0.498332573	0.00073154	0.009885569	P4HB
	17	81843159	81860694	-	5303
protein_coding	prolyl 4-hydroxylase subunit beta [Source:HGNC
Symbol;Acc:HGNC:8548]	-	11548	12819	12387	9080	7503
	8891	106.6384519	114.5404701	145.3286894
95.14287377	86.96823966	77.95787658
ENSG00000129682 34.47553671	18.58489822	9.299933199
49.72648776	113.007691	99.20349021	20.78678937
87.31255632	-2.059458962	0.000733225	0.009900138	FGF13
	X	138614731	139222777	-	20681
protein_coding	fibroblast growth factor 13 [Source:HGNC
Symbol;Acc:HGNC:3670]	-	38	21	8	48	99
	115	0.089978914	0.048114303	0.024067185
0.128967935	0.294246307	0.25855761
ENSG00000136859 118.8498765	133.6342681	224.3608884
81.8415111	15.98088559	43.13195226	158.9483444
46.98478299	1.753800709	0.000733737	0.009900138
ANGPTL2 9	127087332	127122883	-	3889
protein_coding	angiopoietin like 2 [Source:HGNC Symbol;Acc:HGNC:490]
	-	131	151	193	79	14	50
1.649536904	1.8397781	3.087636829	1.128758911
0.2212776	0.597809868
ENSG00000168243 736.6877844	549.5819901	835.8314963
486.9051926	423.4934683	299.3357487	707.3670902
403.2448032	0.811377074	0.000737236	0.009939766	GNG4
	1	235547687	235650754	-	5401
protein_coding	G protein subunit gamma 4 [Source:HGNC
Symbol;Acc:HGNC:4407]	-	812	621	719	470	371
	347	7.362249573	5.448084862	8.282502196
4.835436944	4.222280607	2.987351429
ENSG00000117724 4411.054197	2724.900076	3022.47829
1616.110852	638.0939319	496.017451	3386.144188
916.740745	1.885180481	0.000742717	0.01000603	CENPF
	1	214603195	214664588	+	11690
protein_coding	centromere protein F [Source:HGNC
Symbol;Acc:HGNC:1857]	-	4862	3079	2600	1560	559
	575	20.36709677	12.48020211	13.83775648
7.415187394	2.939305024	2.287095775
ENSG00000140379 2.721752898	17.69990306	16.2748831
78.73360561	79.90442797	39.68139608	12.23217969
66.10647656	-2.439165403	0.000744742	0.010025682	BCL2A1
	15	79960889	79971446	-	1089
protein_coding	BCL2 related protein A1 [Source:HGNC
Symbol;Acc:HGNC:991]	-	3	20	14	76	70
	46	0.13490314	0.870218991	0.799847152
3.877910273	3.951095441	1.964088126
ENSG00000138336 86.18884177	76.10958317	127.8740815

29.00711786 37.66923033 25.01653231 96.72416881
30.5642935 1.664136661 0.000748148 0.010063871 TET1
10 68560656 68694482 + 9288
protein_coding tet methylcytosine dioxygenase 1 [Source:HGNC
Symbol;Acc:HGNC:29484] - 95 86 110 28 33
29 0.500875838 0.438735408 0.736847009
0.16751275 0.218393191 0.145180006
ENSG00000102384 938.0974988 650.4714375 743.9946559
615.365286 279.6654979 181.1541995 777.5211974
358.7283278 1.117106016 0.000749971 0.010071164 CENPI
X 101098218 101163681 + 4321
protein_coding centromere protein I [Source:HGNC
Symbol;Acc:HGNC:3968] - 1034 735 640 594 245
210 11.71830928 8.059897404 9.215153086
7.638607982 3.48521182 2.259779406
ENSG00000124207 7864.051373 6432.144773 7281.847695
6281.076985 4116.219532 3925.007656 7192.68128
4774.101391 0.591357685 0.000750481 0.010071164 CSE1L
20 49046246 49096960 + 3800
protein_coding chromosome segregation 1 like [Source:HGNC
Symbol;Acc:HGNC:2431] - 8668 7268 6264 6063 3606
4550 111.702782 90.62703317 102.5592884
88.65760912 58.32966551 55.67481956
ENSG00000165271 3096.447547 3521.395714 2862.054442
3794.752597 4393.602047 5909.940099 3159.965901
4699.431581 -0.572637629 0.00075072 0.010071164 NOL6
9 33461441 33473930 - 5906
protein_coding nucleolar protein 6 [Source:HGNC
Symbol;Acc:HGNC:19910] - 3413 3979 2462 3663 3849
6851 28.29902867 31.92323812 25.93590774
34.4631936 40.05915911 53.93759066
ENSG00000189369 361.9931354 369.927974 395.247161
572.8905777 741.9696883 533.973569 375.7227568
616.277945 -0.713343448 0.000750968 0.010071164 GSPT2
X 51743431 51746232 + 2802
protein_coding G1 to S phase transition 2 [Source:HGNC
Symbol;Acc:HGNC:4622] - 399 418 340 553 650
619 6.973221304 7.068619292 7.549490322
10.96652528 14.25911547 10.27196512
ENSG00000004660 430.9442088 596.4867332 381.2972612
642.3004668 737.403721 1155.936321 469.5760677
845.2135028 -0.847735233 0.000753204 0.0100935 CAMKK1
17 3860315 3894891 - 7424 protein_coding
calcium/calmodulin dependent protein kinase kinase 1 [Source:HGNC
Symbol;Acc:HGNC:1469] - 475 674 328 620 646
1340 3.133172672 4.301781764 2.748797373
4.64051061 5.348622106 8.392625675
ENSG00000151914 6250.051905 4703.749239 6418.116399
4651.498542 3778.337951 3537.682725 5790.639181
3989.173073 0.537580744 0.000757516 0.010135757 DST

6 56457987 56954628 - 42341
protein_coding dystonin [Source:HGNC Symbol;Acc:HGNC:1090] -
6889 5315 5521 4490 3310 4101 7.967530918
5.947967664 8.11266388 5.892468557 4.8052307
4.503598611
ENSG00000115295 291.2275601 240.7186816 220.8734135
379.1644691 566.1799468 410.6161856 250.9398851
451.9868672 -0.846715901 0.000757769 0.010135757 CLIP4
2 29097705 29189643 + 7775
protein_coding CAP-Gly domain containing linker protein family member
4 [Source:HGNC Symbol;Acc:HGNC:26108] - 321 272 190
366 496 476 2.02177731 1.657658051
1.520407663 2.615728904 3.921287085 2.846672941
ENSG00000126243 681.3454755 867.2952501 750.9696058
931.3356769 1302.442176 1394.024697 766.5367771
1209.267517 -0.65779274 0.000758076 0.010135757 LRFN3
19 35935358 35945767 + 4044
protein_coding leucine rich repeat and fibronectin type III domain
containing 3 [Source:HGNC Symbol;Acc:HGNC:28370] - 751
980 646 899 1141 1616 9.094052847
11.48262996 9.938668787 12.35266286 17.3429055
18.5806639
ENSG00000166801 1933.351809 1466.436969 1653.063126
1377.838098 916.617938 915.260027 1684.283968
1069.905354 0.654837097 0.000760354 0.010158536
FAM111A 11 59142748 59155039 + 7221
protein_coding family with sequence similarity 111 member A
[Source:HGNC Symbol;Acc:HGNC:24725] - 2131 1657 1422
1330 803 1061 14.45156193 10.87305549
12.25205952 10.23449315 6.835425598 6.832018867
ENSG00000110104 1662.991021 2212.487883 2141.309619
2420.022404 3135.678052 3392.759365 2005.596174
2982.81994 -0.572959849 0.000762095 0.010166648 CCDC86
11 60842071 60851081 + 3384
protein_coding coiled-coil domain containing 86 [Source:HGNC
Symbol;Acc:HGNC:28359] - 1833 2500 1842 2336 2747
3933 26.52532991 35.00548467 33.86617265
38.35787396 49.89714135 54.04115347
ENSG00000101335 948.0772594 1277.933001 1414.752338
776.9763712 866.3922976 672.8584553 1213.587533
772.075708 0.652660878 0.000762111 0.010166648 MYL9
20 36541484 36551447 + 2821
protein_coding myosin light chain 9 [Source:HGNC
Symbol;Acc:HGNC:15754] - 1045 1444 1217 750 759
780 18.14019235 24.25440069 26.84073095
14.77305181 16.53811661 12.8564935
ENSG00000184445 1488.798835 1025.709382 1105.529559
930.2997084 672.3386868 442.5338302 1206.679259
681.7240751 0.824913174 0.000767786 0.010234641 KNTC1
12 122527246 122626396 + 11325

protein_coding	kinetochore associated 1 [Source:HGNC
Symbol;Acc:HGNC:17255]	-	1641	1159	951	898	589
	513	7.095762222	4.849217526	5.224553164
4.406057593	3.196866085	2.106251234
ENSG00000171617 1222.974302	1078.809092	1141.5668
923.047929	668.9142113	590.907746	1147.783398
727.6232954	0.658163804	0.000774044	0.010303045	ENC1
	5	74627406	74641424	-	5858
protein_coding	ectodermal-neural cortex 1 [Source:HGNC
Symbol;Acc:HGNC:3345]	-	1348	1219	982	891	586
	685	11.26858042	9.86008773	10.42963157
8.451628138	6.148874214	5.437161365
ENSG00000213030 77.11633211	89.38451047	79.04943219
35.22292883	12.55641011	5.175834272	81.85009159
17.6517244	2.221159858	0.000774083	0.010303045	CGB8
	19	49047638	49049106	-	882
protein_coding	chorionic gonadotropin subunit beta 8 [Source:HGNC
Symbol;Acc:HGNC:16453]	-	85	101	68	34	11
	6	4.71931563	5.425993004	4.796750994
2.142014348	0.766604669	0.316310554
ENSG00000170442 20.86677222	63.71965102	46.499666	0
	0	9.489029498	43.69536308	3.163009833
3.724192417	0.000776579	0.010328502	KRT86	12
52249300	52309163	+	2394	protein_coding
keratin 86 [Source:HGNC Symbol;Acc:HGNC:6463]	-	23	72
	40	0	0	11	0.470470475	1.425065385
	1.039543559	0	0	0.213648357
ENSG00000068308 1153.115978	1301.82787	1354.302772
1529.089498	1902.866878	2293.757221	1269.748873
1908.571199	-0.588634294	0.000778573	0.010347246	OTUD5
	X	48922028	48958386	-	3592
protein_coding	OTU deubiquitinase 5 [Source:HGNC
Symbol;Acc:HGNC:25402]	-	1271	1471	1165	1476	1667
	2659	17.32757974	19.4045147	20.17885149
22.8329525	28.52637796	34.42017131
ENSG00000127666 537.0925719	651.3564327	757.9445557
791.4799301	1162.038681	1243.925503	648.7978534
1065.814705	-0.717342296	0.000779306	0.010349205	TICAM1
	19	4815932 4831704 -	2676	protein_coding	toll
like receptor adaptor molecule 1 [Source:HGNC Symbol;Acc:HGNC:18348]
	-	592	736	652	764	1018	1442
10.83338759	13.0322123	15.15892252	15.86424079
23.38347378	25.05590937
ENSG00000156802 4746.737054	3406.346344	3969.908984
3505.717387	2047.83634	1614.860293	4040.997461
2389.47134	0.758248271	0.000780544	0.010357874	ATAD2
	8	123319850	123416350	-	7118
protein_coding	"ATPase family, AAA domain containing 2 [Source:HGNC
Symbol;Acc:HGNC:30123]" -	5232	3849	3415	3384	1794
	1872	35.9946898	25.62219713	29.84967253

26.41705585   15.49215435   12.22866024
ENSG00000233922 721.264518   808.88557   396.4096526
945.8392358   1106.105581   1348.304828   642.1865802
1133.416548   -0.818178797   0.000782085   0.010370538
LINC01694   21   45593654   45603056   +   6810
lincRNA long intergenic non-protein coding RNA 1694
[Source:HGNC Symbol;Acc:HGNC:52481]   -   795   914   341
913   969   1563   5.716743416   6.359537384
3.115402139   7.449647257   8.746293066   10.67192794
ENSG00000102554 224.0909886   192.0439482   239.4732799
319.0782964   472.5776169   393.3634046   218.5360722
395.0064393   -0.854684641   0.000785024   0.010401712   KLF5
13   73054976   73077542   +   3893
protein_coding Kruppel like factor 5 [Source:HGNC
Symbol;Acc:HGNC:6349]   zf-C2H2 247   217   206   308   414
456   3.106999856   2.641202933   3.292226173
4.396209257   6.536771404   5.446424116
ENSG00000267260 17.23776835   7.079961225   10.46242485
29.00711786   94.74382174   58.65945508   11.59338481
60.80346489   -2.387706136   0.000785817   0.010404428
AC020928.1   19   36773153   36777078   +   2050
lincRNA uncharacterized LOC728485 [Source:NCBI
gene;Acc:728485]   -   19   8   9   28   83
68   0.453866808   0.184910923   0.273146409
0.758955328   2.48869452   1.542361123
ENSG00000108846 113.4063707   130.0942875   152.2864061
45.58261378   47.94265678   61.24737221   131.9290215
51.59088092   1.350211652   0.000787335   0.010416736   ABCC3
17   50634777   50692252   +   9614
protein_coding ATP binding cassette subfamily C member 3 [Source:HGNC
Symbol;Acc:HGNC:54]   -   125   147   131   44   42
71   0.636699603   0.724502115   0.847762153
0.25430834   0.268529827   0.343388107
ENSG00000197622 2044.036426   1772.645292   2087.835003
1287.708839   1287.602782   1551.025003   1968.17224
1375.445542   0.516112893   0.000792438   0.010476414
CDC42SE1   1   151050971   151070325   -   4184
protein_coding CDC42 small effector 1 [Source:HGNC
Symbol;Acc:HGNC:17719] -   2253   2003   1796   1243   1128
1798   26.36927561   22.68379502   26.70677841
16.50788734   16.57161305   19.98154335
ENSG00000136631 1012.492078   911.5450077   1018.342685
758.3289383   559.3309958   598.6714974   980.793257
638.7771438   0.618345352   0.000796919   0.010527778   VPS45
1   150067279   150145329   +   6637
protein_coding vacuolar protein sorting 45 homolog [Source:HGNC
Symbol;Acc:HGNC:14579] -   1116   1030   876   732   490
694   8.234193348   7.353461922   8.21181459
6.128459312   4.538074514   4.862041606
ENSG00000197363 243.1432589   380.5479158   262.7231129

418.5312719   538.7841429   659.9188696   295.4714292
539.0780948   -0.867061201   0.000801143   0.010575681   ZNF517
        8   144798876   144811169   +   5308
protein_coding zinc finger protein 517 [Source:HGNC
Symbol;Acc:HGNC:27984] zf-C2H2 268   430   226   404   472
        765   2.472478725   3.838521542   2.64901472
4.229241592   5.465859257   6.701338244
ENSG00000224877 497.1735294   631.0015442   580.0833333
319.0782964   369.8433523   362.308399   569.4194689
350.4100159   0.700376432   0.000802848   0.010590284
NDUFAF8 17   81239239   81241281   +   1376
protein_coding NADH:ubiquinone oxidoreductase complex assembly factor
8 [Source:HGNC Symbol;Acc:HGNC:33551]   -   548   713   499
        308   324   420   19.50252342   24.55260273
22.56259035   12.43782168   14.47351237   14.19259711
ENSG00000136104 1278.316611   1311.562817   1332.215431
976.9182907   940.5892664   762.572916   1307.364953
893.3601577   0.550109959   0.00081188   0.010701447
RNASEH2B   13   50909678   51024120   +   26291
        protein_coding ribonuclease H2 subunit B [Source:HGNC
Symbol;Acc:HGNC:25671] -   1409   1482   1146   943   824
        884   2.624415364   2.670960954   2.711966741
1.993042828   1.926493139   1.563421853
ENSG00000245648 21.77402318   25.66485944   1.16249165   0
        0   0   16.20045809   0   6.455356868
0.000815255   0.01073794   AC022075.1   12   10363769
        10398506   +   2830   antisense
uncharacterized LOC101928100 [Source:NCBI gene;Acc:101928100]   -
        24   29   1   0   0   0   0.415291928
        0.485554522   0.021984693   0   0   0
ENSG00000123374 1256.542588   1169.078597   1356.627755
958.2708578   837.8550019   791.0400045   1260.749647
862.3886214   0.547781281   0.000819436   0.010777534   CDK2
        12   55966769   55972784   +   3894
protein_coding cyclin dependent kinase 2 [Source:HGNC
Symbol;Acc:HGNC:1771]   -   1385   1321   1167   925   734
        917   17.41736727   16.07434596   18.64583151
13.1995106   11.58637213   10.94975512
ENSG00000242372 2825.179508   4577.194932   4293.081663
4893.91517   6154.923938   6870.919996   3898.485368
5973.253034   -0.615789481   0.00081948   0.010777534   EIF6
        20   35278911   35284985   -   1631
protein_coding eukaryotic translation initiation factor 6
[Source:HGNC Symbol;Acc:HGNC:6159]   -   3114   5172   3693
        4724   5392   7965   93.49606452   150.2557138
140.8744369   160.9414729   203.209161   227.0716089
ENSG00000092978 635.0756762   654.8964133   687.0325651
951.0190783   885.7976587   1132.645066   659.0015515
989.8206011   -0.58803621   0.00082363   0.010824068
GPATCH2 1   217426992   217631082   -   8833

protein_coding G-patch domain containing 2 [Source:HGNC
Symbol;Acc:HGNC:25499] - 700 740 591 918 776
1313 3.880775261 3.969629096 4.162805283
5.774926942 5.400088172 6.911741925
ENSG00000184937 47.17705023 12.38993214 38.36222445
4.14387398 3.424475485 0 32.64306894 2.522783155
3.732081126 0.000825854 0.010845237 WT1 11
32387775 32435630 - 4113 protein_coding
Wilms tumor 1 [Source:HGNC Symbol;Acc:HGNC:12796] zf-
C2H2 52 14 33 4 3 0 0.619117839
0.161285664 0.499185632 0.054039749 0.04483425
0
ENSG00000014641 2063.088697 2077.083624 2380.782899
1738.355134 1442.845671 1401.788449 2173.65174
1527.663085 0.508617211 0.000830713 0.010900947 MDH1
2 63588609 63607197 + 4990
protein_coding malate dehydrogenase 1 [Source:HGNC
Symbol;Acc:HGNC:6970] - 2274 2347 2048 1678 1264
1625 22.31611539 22.28635196 25.535023
18.6854454 15.57018915 15.14202078
ENSG00000122299 1184.869762 954.9097702 966.0305611
768.6886232 676.9046541 561.5780185 1035.270031
669.0570986 0.630894472 0.000838218 0.010991288 ZC3H7A
16 11750586 11797267 - 7076
protein_coding zinc finger CCCH-type containing 7A [Source:HGNC
Symbol;Acc:HGNC:30959] - 1306 1079 831 742 593
651 9.038243474 7.225369496 7.306679303
5.826773343 5.151268999 4.277836507
ENSG00000120913 260.3810272 208.8588561 439.4218437
84.94941658 131.2715602 176.8410043 302.8872423
131.0206604 1.20454496 0.000840349 0.01101106 PDLIM2
8 22578279 22598025 + 12388
protein_coding PDZ and LIM domain 2 [Source:HGNC
Symbol;Acc:HGNC:13992] - 287 236 378 82 115
205 1.134512757 0.902687123 1.898442509
0.367810988 0.570616077 0.769455809
ENSG00000112651 890.9204486 1068.18915 1036.942552
1179.968116 1690.549398 1707.162671 998.68405
1525.893395 -0.611837871 0.000842769 0.011034601 MRPL2
6 43054029 43059806 - 2539
protein_coding mitochondrial ribosomal protein L2 [Source:HGNC
Symbol;Acc:HGNC:14056] - 982 1207 892 1139 1481
1979 18.93989079 22.52532211 21.85792846
24.92717524 35.85417368 36.24216318
ENSG00000132383 3980.109988 3442.631146 3964.096526
2983.589265 2735.01442 2656.928259 3795.612553
2791.843982 0.443064894 0.000845977 0.011059069 RPA1
17 1829702 1900082 + 5492 protein_coding
replication protein A1 [Source:HGNC Symbol;Acc:HGNC:10289] -
4387 3890 3410 2880 2396 3080 39.11702246

33.56182075	38.63053271	29.13895702	26.81659576	26.07661446
ENSG00000185033 798.3808501	710.651108	1880.91149
608.1135065	553.6235367	577.1055213	1129.981149
579.6141882	0.961804973	0.000846188	0.011059069	SEMA4B
15	90160604	90229679	+	7006
protein_coding	semaphorin 4B [Source:HGNC Symbol;Acc:HGNC:10730]
-	880	803	1618	587	485	669
6.150936205	5.430900588	14.36862568	4.655646658
4.255190089	4.440041229
ENSG00000144283 1361.7837	1054.914223	1179.929025
834.9906069	831.0060509	770.3366674	1198.875649
812.1111084	0.562286218	0.000846514	0.011059069	PKP4
2	158456964	158682879	+	9525
protein_coding	plakophilin 4 [Source:HGNC Symbol;Acc:HGNC:9026]
-	1501	1192	1015	806	728	893
7.716926991	5.929768134	6.629914145	4.70199433
4.698008034	4.359307379
ENSG00000148513 64.41481858	60.17967041	134.8490314
33.15099184	19.40536108	7.763751407	86.48117346
20.10670144	2.109842819	0.000849288	0.011087121
ANKRD30A	10	37125788	37384111	+	5015
protein_coding	ankyrin repeat domain 30A [Source:HGNC
Symbol;Acc:HGNC:17234]	Others	71	68	116	32	17
9	0.69329185	0.642487106	1.439109693
0.354561099	0.208365275	0.083445436
ENSG00000181284 219.5547338	263.7285556	289.4604208
317.0063594	555.9065203	571.929687	257.5812367
481.6141889	-0.904235757	0.000855502	0.011160011
TMEM102 17	7435443 7437679 +	2129	protein_coding
transmembrane protein 102 [Source:HGNC Symbol;Acc:HGNC:26722]	-
242	298	249	306	487	663	5.566322799
6.632343997	7.276634016	7.98652414	14.06049661
14.48001078
ENSG00000105974 9276.641127	8774.726943	11051.80812
8058.798922	6648.048407	5079.218699	9701.058729
6595.355343	0.556746179	0.000859134	0.011199134	CAV1
7	116524785	116561184	+	4082
protein_coding	caveolin 1 [Source:HGNC Symbol;Acc:HGNC:1527]	-
10225	9915	9507	7779	5824	5888	122.6645302
115.0922708	144.9029877	105.891918	87.69921907
67.06961588
ENSG00000166847 1894.340017	2001.859036	1836.736807
2429.346121	2560.366171	2930.384837	1910.97862
2640.032376	-0.466338955	0.000860629	0.011210356	DCTN5
16	23641392	23677455	+	12740
protein_coding	dynactin subunit 5 [Source:HGNC Symbol;Acc:HGNC:24594]
-	2088	2262	1580	2345	2243	3397
8.025826183	8.412975291	7.716040625	10.2278762
10.82199193	12.39815715

ENSG00000054654 976.2020394 704.4561419 919.5308951
626.7609394 507.9638635 273.4565774 866.7296921
469.3937934 0.886186804 0.000865769 0.01126901 SYNE2
14 63852983 64226433 + 31374
protein_coding spectrin repeat containing nuclear envelope protein 2
[Source:HGNC Symbol;Acc:HGNC:17084] - 1076 796 791
605 445 317 1.679465406 1.202180326
1.568604433 1.071513201 0.871841382 0.469807979
ENSG00000135046 7759.717512 7789.727338 8438.526887
6626.054493 5641.252615 5699.456172 7995.990579
5988.921093 0.416918368 0.000868151 0.011291708 ANXA1
9 73151757 73170393 + 4561
protein_coding annexin A1 [Source:HGNC Symbol;Acc:HGNC:533] -
8553 8802 7259 6396 4942 6607 91.83052841
91.44242459 99.02014792 77.92206021 66.60242299
67.35583939
ENSG00000285106 61.69306569 65.48964133 49.98714095
113.9565344 144.9694622 177.7036433 59.05661599
145.5432133 -1.299596007 0.000879926 0.011436454
AC016831.6 7 130791264 131110161 - 11088
lincRNA novel transcript - 68 74 43
110 127 206 0.300320086 0.316231365
0.241280423 0.551253692 0.704040859 0.863863294
ENSG00000129103 1677.507036 1738.130481 2075.047595
1336.399358 1272.763388 1266.354118 1830.228371
1291.838955 0.502073059 0.000882975 0.011467658 SUMF2
7 56064002 56080670 + 4101
protein_coding sulfatase modifying factor 2 [Source:HGNC
Symbol;Acc:HGNC:20415] - 1849 1964 1785 1290 1115
1468 22.0788183 22.69228111 27.08041389
17.47881496 16.71215513 16.64436781
ENSG00000050438 273.0825408 108.8544038 145.3114562
95.30910153 36.5277385 16.39014186 175.7494669
49.40899396 1.835007128 0.000884684 0.011481421 SLC4A8
12 51391317 51515763 + 17980
protein_coding solute carrier family 4 member 8 [Source:HGNC
Symbol;Acc:HGNC:11034] - 301 123 125 92 32
19 0.819795428 0.324146894 0.432540893
0.284321815 0.109397564 0.049135449
ENSG00000153140 210.4822241 266.3835411 269.6980628
164.7189907 92.46083808 76.77487503 248.8546093
111.3182346 1.161927364 0.000886732 0.011499556 CETN3
5 90392261 90409786 - 3106
protein_coding centrin 3 [Source:HGNC Symbol;Acc:HGNC:1868] -
232 301 232 159 81 89 3.65776009
4.591890097 4.647221579 2.844511648 1.602990423
1.332353826
ENSG00000134815 1069.648889 1141.643748 869.5437541
1358.154697 1439.421195 1900.393817 1026.945464
1565.989903 -0.608501128 0.000893236 0.01157542 DHX34

19	47349281	47382704	+	5696
protein_coding	DExH-box helicase 34 [Source:HGNC
Symbol;Acc:HGNC:16719]	-	1179	1290	748	1311	1261
2203	10.13613784	10.73114765	8.170308662
12.78924051	13.60794252	17.98355447
ENSG00000007968 716.7282631	659.3213891	631.2329659
452.7182323	267.1090878	449.4349426	669.094206
389.7540876	0.77807465	0.000901787	0.011677685	E2F2
1	23506430	23531220	-	5457
protein_coding	E2F transcription factor 2 [Source:HGNC
Symbol;Acc:HGNC:3114]	E2F	790	745	543	437	234
521	7.089274962	6.468875009	6.190884795
4.449790114	2.635780641	4.439302993
ENSG00000189043 2686.37011	3406.346344	3836.222445
2468.712923	2228.192049	2229.059293	3309.6463
2308.654755	0.519322408	0.000911444	0.0117941	NDUFA4
7	10931951	10940256	-	3556
protein_coding	"NDUFA4, mitochondrial complex associated [Source:HGNC
Symbol;Acc:HGNC:7687]"	-	2961	3849	3300	2383	1952
2584	40.77606742	51.28762632	57.73764078
37.2369699	33.74158047	33.78794473
ENSG00000041802 2028.61316	2321.342287	1579.826152
2564.022025	2906.238195	3286.654763	1976.593866
2918.971661	-0.561978501	0.000913163	0.011807635	LSG1
3	194640788	194672477	-	4818
protein_coding	large 60S subunit nuclear export GTPase 1 [Source:HGNC
Symbol;Acc:HGNC:25652]	-	2236	2623	1359	2475	2546
3810	22.72655912	25.7963307	17.54928826
28.54436928	32.48171593	36.76962479
ENSG00000096080 449.0892282	512.4121937	462.6716767
632.9767504	812.7421817	775.5125017	474.7243662
740.4104779	-0.640882238	0.000913825	0.011807635
MRPS18A 6	43671303	43687791	-	1284
protein_coding	mitochondrial ribosomal protein S18A [Source:HGNC
Symbol;Acc:HGNC:14515]	-	495	579	398	611	712
899	18.87855975	21.3668244	19.28523321
26.44163039	34.08492401	32.55559867
ENSG00000275966 10.88701159	17.69990306	12.78740815	0
0	0	13.79144093	0	6.219344522
0.000916987	0.011839847	AC110285.6	17	81345476
81345966	-	491	lincRNA novel transcript
-	12	20	11	0	0	0
1.196818896	1.930078373	1.39385642	0	0	0
ENSG00000085644 365.6221393	417.7177123	446.3967936
545.9553968	751.1016229	690.1112362	409.912215
662.3894187	-0.693020351	0.000918655	0.011852728	ZNF213
16	3129777 3142805 +	6443	protein_coding	zinc
finger protein 213 [Source:HGNC Symbol;Acc:HGNC:13005]	zf-C2H2 403
472	384	527	658	800	3.062990137
3.471205363	3.708087209	4.545006697	6.277476889

5.773416297
ENSG00000125735 0 1.769990306 2.3249833 10.35968495
42.23519764 23.29125422 1.364991202 25.29537894
-4.249062868 0.000921891 0.011885806 TNFSF14 19
6661253 6670588 - 4778 protein_coding TNF
superfamily member 14 [Source:HGNC Symbol;Acc:HGNC:11930] -
0 2 2 10 37 27 0
0.019833999 0.026042981 0.1162963 0.475995601
0.262753577
ENSG00000078269 2456.835616 2129.298338 2777.192552
1758.038536 1877.754057 1454.40943 2454.442169
1696.734008 0.532795018 0.000923369 0.01188978 SYNJ2
6 157981887 158099176 + 12493
protein_coding synaptojanin 2 [Source:HGNC Symbol;Acc:HGNC:11504]
- 2708 2406 2389 1697 1645 1686
10.61477037 9.125471726 11.89751487 7.547917524
8.093689184 6.2751173
ENSG00000283787 35.38278767 47.78973827 43.01219105 0
10.27342645 0 42.06157233 3.424475485
3.67366869 0.000923543 0.01188978 PRR33 11
1888577 1891895 - 3319 protein_coding proline rich 33
[Source:HGNC Symbol;Acc:HGNC:35118] - 39 54 37
0 9 0 0.575421438 0.770926453
0.693587597 0 0.166679664 0
ENSG00000064601 1550.491901 1763.79534 1782.099699
1253.521879 1256.782503 1089.513114 1698.795647
1199.939165 0.501855875 0.000924237 0.011890055 CTSA
20 45890144 45898820 + 5856
protein_coding cathepsin A [Source:HGNC Symbol;Acc:HGNC:9251] -
1709 1993 1533 1210 1101 1263 14.29123229
16.12622339 16.28725641 11.48143961 11.55669392
10.02843816
ENSG00000197747 2619.233539 3409.886325 5063.813627
2124.771383 2832.041226 1703.712114 3697.644497
2220.174908 0.736002596 0.000927589 0.011924504
S100A10 1 151982915 151994390 - 1543
protein_coding S100 calcium binding protein A10 [Source:HGNC
Symbol;Acc:HGNC:10487] - 2887 3853 4356 2051 2481
1975 91.62406195 118.3203713 175.642169
73.86042086 98.83441554 59.51578854
ENSG00000100083 1045.153113 1208.018384 863.7312959
1223.478792 1763.604875 1934.899379 1038.967598
1640.661015 -0.658616894 0.000930762 0.01195661 GGA1
22 37608475 37633564 + 9369
protein_coding "golgi associated, gamma adaptin ear containing, ARF
binding protein 1 [Source:HGNC Symbol;Acc:HGNC:17842]" - 1152
1365 743 1181 1545 2243 6.02126753
6.903444747 4.934037222 7.00436392 10.1363735
11.13184249
ENSG00000138160 2406.029562 1867.339773 2477.269706

1216.227013   647.2258666   402.8524341   2250.213014
755.4351046   1.574897861   0.000932878   0.011975094   KIF11
        10   92593286   92655395   +   4860
protein_coding kinesin family member 11 [Source:HGNC
Symbol;Acc:HGNC:6388]   -   2652   2110   2131   1174   567
        467   26.72181383   20.57181579   27.28060686
13.42282324   7.171238226   4.467984208
ENSG00000269974 17.23776835   29.20484005   1.16249165   0
        0   0   15.86836669   0   6.425561622
0.000936226   0.01200935   AC091057.4   15   30648797
        30649529   +   733   lincRNA novel transcript
        -       19   33   1   0   0   0
1.269341005   2.13322373   0.084879512   0   0   0
ENSG00000162396 290.3203091   267.2685362   276.6730127
387.4522171   442.8988293   646.1166449   278.087286
492.1558971   -0.825001427   0.000939788   0.012046317   PARS2
        1   54756898   54764514   -   2347
protein_coding "prolyl-tRNA synthetase 2, mitochondrial [Source:HGNC
Symbol;Acc:HGNC:30563]" -   320   302   238   374   388
        749   6.676757029   6.097057548   6.309147983
8.854632809   10.16169127   14.83883297
ENSG00000105193 17901.87606   22628.44107   24151.92652
26928.96506   31586.22038   30492.56497   21560.74788
29669.25014   -0.460606108   0.000953447   0.012204591   RPS16
        19   39433207   39435948   -   2603
protein_coding ribosomal protein S16 [Source:HGNC
Symbol;Acc:HGNC:10396] -   19732   25569   20776   25994   27671
        35348   371.2150901   465.4424778   496.5861642
554.8952282   653.4284604   631.4248887
ENSG00000135829 8421.103466   7438.384262   8120.004174
6774.197988   5498.566136   5207.751916   7993.163968
5826.83868   0.456103681   0.000953516   0.012204591   DHX9
        1   182839369   182887751   +   5645
protein_coding DExH-box helicase 9 [Source:HGNC Symbol;Acc:HGNC:2750]
        -       9282   8405   6985   6539   4817   6037
80.52047003   70.5505189   76.98556666   64.36643186
52.45175938   49.72654067
ENSG00000205208 929.0249892   894.7300998   878.8436873
726.2139149   493.1244698   458.061333   900.8662588
559.1332392   0.688746754   0.000954753   0.012211582
C4orf46 4   158666679   158672255   -   3730
protein_coding chromosome 4 open reading frame 46 [Source:HGNC
Symbol;Acc:HGNC:27320] -   1024   1011   756   701   432
        531   13.44373083   12.84306749   12.61013716
10.44290266   7.119053803   6.619370763
ENSG00000213551 1202.10753   1208.903379   1025.317635
750.0411903   852.6943956   746.1827742   1145.442848
782.9727867   0.549996316   0.000956313   0.012222697   DNAJC9
        10   73183362   73248862   -   3955
protein_coding DnaJ heat shock protein family (Hsp40) member C9

[Source:HGNC Symbol;Acc:HGNC:19123]	-	1325	1366	882
	724	747	865	16.40582498	16.36555177
13.87487068	10.17194778	11.60971274	10.16952429
ENSG00000240875 2.721752898	0	1.16249165	15.53952742
	22.82983656	34.50556181	1.294748183	24.29164193
	-4.231483327	0.000958667	0.01224394	LINC00886
	3	156747346	156817062	-	3822
lincRNA long intergenic non-protein coding RNA 886 [Source:HGNC
Symbol;Acc:HGNC:48572]	-	3	0	1	15	20
	40	0.038437865	0	0.016278567	0.218078384
	0.321652309	0.486631622
ENSG00000102144 24993.85686	30140.27993	34893.34936
39485.93918	47597.92625	38746.29536	30009.16205
41943.38693	-0.48306311	0.000960921	0.012263872	PGK1
	X	77910739	78129296	+	5512
protein_coding	phosphoglycerate kinase 1 [Source:HGNC
Symbol;Acc:HGNC:8896]	-	27549	34057	30016	38115	41698
	44916	244.7514727	292.7680104	338.805502
384.2366249	465.0004533	378.8984969
ENSG00000175575 547.9795835	507.1022227	544.0460921
661.9838682	928.0328563	920.4358613	533.0426328
836.8175286	-0.650988053	0.000961695	0.012264903	PAAF1
	11	73876699	73931124	+	7937
protein_coding	proteasomal ATPase associated factor 1 [Source:HGNC
Symbol;Acc:HGNC:25687]	-	604	573	468	639	813
	1067	3.726569643	3.420776362	3.668565853
4.473593529	6.296243645	6.25084971
ENSG00000161692 436.3877146	423.9126783	466.1591516
272.4597142	278.5240061	256.2037964	442.1531815
269.0625056	0.716743287	0.000966066	0.012311778	DBF4B
	17	44708608	44752264	+	7146
protein_coding	DBF4 zinc finger B [Source:HGNC Symbol;Acc:HGNC:17883]
	-	481	479	401	263	244	297
3.296178696	3.176134918	3.491308352	2.045054001
2.098815074	1.93252204
ENSG00000198933 198.6879615	251.3386235	224.3608884
300.4308635	426.9179437	514.99551	224.7958245
414.1147724	-0.882186602	0.000975734	0.012426027	TBKBP1
	17	47694081	47712050	+	4280
protein_coding	TBK1 binding protein 1 [Source:HGNC
Symbol;Acc:HGNC:30140]	-	219	284	193	290	374
	597	2.505699748	3.144133746	2.805565333
3.765011201	5.371247857	6.485770548
ENSG00000111961 1045.153113	803.575599	863.7312959
624.6890024	619.8300627	365.7589552	904.1533359
536.7593401	0.754108643	0.000978825	0.012456433	SASH1
	6	148272304	148552050	+	11143
protein_coding	SAM and SH3 domain containing 1 [Source:HGNC
Symbol;Acc:HGNC:19182]	-	1152	908	743	603	543
	424	5.062663151	3.86109208	4.148523264

3.006957053	2.995332642	1.769272569
ENSG00000089289 1155.837731	1059.339198	1413.589846
1757.002567	1624.342871	1875.377284	1209.588925
1752.240908	-0.535919286	0.000985127	0.012520838	IGBP1
X	70133449	70166324	+	1862
protein_coding	immunoglobulin binding protein 1 [Source:HGNC
Symbol;Acc:HGNC:5461]	-	1274	1197	1216	1696	1423
2174	33.50567988	30.46077261	40.63130253
50.61254955	46.9756268	54.28887989
ENSG00000107159 802.0098539	1432.807153	1443.814629
1517.693845	2349.190182	2305.834168	1226.210545
2057.572732	-0.747211787	0.000985302	0.012520838	CA9
9	35673856	35681159	+	1670
protein_coding	carbonic anhydrase 9 [Source:HGNC
Symbol;Acc:HGNC:1383]	-	884	1619	1242	1465	2058
2673	25.92175953	45.93638536	46.27132877
48.74535046	75.74888761	74.4240853
ENSG00000154277 4436.457224	5202.886505	4084.995658
5924.703822	6086.434428	6731.17247	4574.779796
6247.436907	-0.449417818	0.000986642	0.012528868	UCHL1
4	41256413	41268455	+	2282
protein_coding	ubiquitin C-terminal hydrolase L1 [Source:HGNC
Symbol;Acc:HGNC:12513]	-	4890	5879	3514	5719	5332
7803	104.9353711	122.0714943	95.80605634
139.2568285	143.6222946	158.9926269
ENSG00000105877 103.4266101	84.9595347	95.32431529
159.5391482	237.4303003	206.1707318	94.57015337
201.0467268	-1.087889301	0.000989322	0.01255389	DNAH11
7	21543215	21901839	+	15998
protein_coding	dynein axonemal heavy chain 11 [Source:HGNC
Symbol;Acc:HGNC:2942]	-	114	96	82	154	208
239	0.348953728	0.284336086	0.318900358
0.534893194	0.799180728	0.6946455
ENSG00000171792 668.6439619	511.5271985	638.2079158
461.0059802	303.6368263	215.6597613	606.1263587
326.7675226	0.892522492	0.000990695	0.012562294	RHNO1
12	2876258 2889523 +	2643	protein_coding	RAD9-
HUS1-RAD1 interacting nuclear orphan 1 [Source:HGNC
Symbol;Acc:HGNC:28206]	-	737	578	549	445	266
250	13.65522965	10.36232278	12.92355597
9.355669932	6.186312204	4.398188753
ENSG00000119013 329.3321007	429.2226493	427.7969272
264.1719662	211.1759882	188.9179509	395.450559
221.4219684	0.83715082	0.000995194	0.012597352	NDUFB3
2	201071433	201085750	+	1103
protein_coding	NADH:ubiquinone oxidoreductase subunit B3 [Source:HGNC
Symbol;Acc:HGNC:7698]	-	363	485	368	255	185
219	16.11609416	20.83495981	20.75769626
12.84626012	10.3096418	9.232081304
ENSG00000161618 1198.478526	1161.998636	1418.239813

594.6459161	874.3827404	935.1007251	1259.572325
801.3764605	0.651508918	0.000995381	0.012597352
ALDH16A1	19	49453169	49471048	+	4643
	protein_coding	aldehyde dehydrogenase 16 family member A1
[Source:HGNC Symbol;Acc:HGNC:28114]	-	1321	1313	1220
	574	766	1084	13.93262081	13.39962003
16.34812665	6.869501969	10.14092209	10.85579458
ENSG00000240583 150.6036604	83.18954439	254.5856713
32.11502334	87.89487077	27.60444945	162.7929587
49.20478119	1.731352617	0.000995968	0.012597352	AQP1
	7	30911694	30925516	+	3435
protein_coding	aquaporin 1 (Colton blood group) [Source:HGNC
Symbol;Acc:HGNC:633]	-	166	94	219	31	77
	32	2.366519188	1.296664297	3.966653089
0.501472355	1.377879834	0.433165895
ENSG00000136541 49.89880313	53.09970919	60.44956579
152.2873688	100.4512809	155.2750281	54.4826927
136.0045593	-1.323479243	0.000996308	0.012597352	ERMN
	2	157318625	157327713	-	4393
protein_coding	ermin [Source:HGNC Symbol;Acc:HGNC:29208]	-
	55	60	52	147	88	180	0.613098382
	0.647167185	0.7364597	1.859380085	1.231314033
	1.905207664
ENSG00000163040 278.5260466	232.7537253	262.7231129
114.9925029	118.7151501	161.3135015	258.0009616
131.6737182	0.96737816	0.001009539	0.012755526
CCDC74A 2	131527675	131533666	+	3077
protein_coding	coiled-coil domain containing 74A [Source:HGNC
Symbol;Acc:HGNC:25197]	-	307	263	226	111	104
	187	4.885843623	4.049996921	4.569701051
2.004506765	2.077558221	2.825824124
ENSG00000090432 1261.986094	1626.621091	1434.514696
1642.010064	2536.394842	2408.488214	1441.040627
2195.63104	-0.607474934	0.001012198	0.012779992	MUL1
	1	20499448	20508161	-	2438
protein_coding	mitochondrial E3 ubiquitin protein ligase 1
[Source:HGNC Symbol;Acc:HGNC:25762]	-	1391	1838	1234
	1585	2222	2792	27.93972404	35.72220403
31.49113437	36.12497947	56.02188446	53.24915627
ENSG00000170144 7592.783334	7799.462284	8243.228289
6843.607877	4982.61183	5116.312178	7878.491303
5647.510628	0.480278053	0.001018769	0.012851608
HNRNPA3 2	177212563	177223958	+	6033
protein_coding	heterogeneous nuclear ribonucleoprotein A3
[Source:HGNC Symbol;Acc:HGNC:24941]	-	8369	8813	7091
	6606	4365	5931	67.93114364	69.21765559
73.12754717	60.84393416	44.4731901	45.71151517
ENSG00000188342 1336.380673	1853.179851	1601.913494
1198.615549	1023.91817	1021.36463	1597.158006
1081.299449	0.562866822	0.001019323	0.012851608	GTF2F2

13	45120515	45284909	+	3619
protein_coding	general transcription factor IIF subunit 2
[Source:HGNC Symbol;Acc:HGNC:4653]	-	1473	2094	1378
1157	897	1184	19.93163141	27.41665929
23.69013203	17.76465671	15.23530736	15.21227394
ENSG00000116774 208.6677222	150.449176	282.4854709
118.1004084	89.0363626	39.68139608	213.8674564
82.27272237	1.381065356	0.001021258	0.012866829	OLFML3
1	113979391	114035572	+	2821
protein_coding	olfactomedin like 3 [Source:HGNC
Symbol;Acc:HGNC:24956]	-	230	170	243	114	78
46	3.992578221	2.855434984	5.359324256
2.245503875	1.699569296	0.758203463
ENSG00000231079 0	5.309970919	3.48747495	25.89921237
21.68834474	51.75834272	2.932481956	33.11529994
-3.504850251	0.001024944	0.012902342	AC105402.3
2	148866470	148888823	-	4156
antisense	novel transcript	-	0	6	3
25	19	60	0	0.068407253	0.044910983
0.334253923	0.28101236	0.67128467
ENSG00000168653 2485.867647	3216.957382	3367.73831
2045.001809	2180.249392	2195.41637	3023.521113
2140.222524	0.498169207	0.001026956	0.012902342	NDUFS5
1	39026318	39034636	+	548
protein_coding	NADH:ubiquinone oxidoreductase subunit S5 [Source:HGNC
Symbol;Acc:HGNC:7712]	-	2740	3635	2897	1974	1910
2545	244.8491992	314.3042818	328.9082623
200.1606181	214.2398073	215.9425603
ENSG00000181458 464.5124946	533.6520773	592.8707414
945.8392358	728.2717864	799.666395	530.3451045
824.5924724	-0.637882182	0.001027036	0.012902342
TMEM45A 3	100492619	100577444	+	6005
protein_coding	transmembrane protein 45A [Source:HGNC
Symbol;Acc:HGNC:25480]	-	512	603	510	913	638
927	4.175280265	4.75806906	5.284014625
8.448309379	6.53062922	7.177905569
ENSG00000175221 2220.950365	2297.447417	2255.233801
2631.359977	3199.601594	3888.776816	2257.877194
3239.912796	-0.521275825	0.001027465	0.012902342	MED16
19	867630	893218	-	4222	protein_coding
mediator complex subunit 16 [Source:HGNC Symbol;Acc:HGNC:17556] -
2448	2596	1940	2540	2803	4508	28.39369207
29.13485761	28.58843275	33.42931914	40.80865009
49.64742136
ENSG00000065154 1386.279476	1269.968045	1478.689379
1077.407235	933.7403154	747.0454132	1378.3123
919.3976545	0.584608781	0.001027723	0.012902342	OAT
10	124397303	124418976	-	3388
protein_coding	ornithine aminotransferase [Source:HGNC
Symbol;Acc:HGNC:8091]	-	1528	1435	1272	1040	818

866	22.08557121	20.06942548	23.35880151
17.05697376	14.84079828	11.88517302
ENSG00000160813 420.0571972	394.7078383	455.6967268
278.6755251	194.0536108	259.6543526	423.4872541
244.1278295	0.792618489	0.001032143	0.012947193
PPP1R35 7	100435282	100436565	-	1187
protein_coding	protein phosphatase 1 regulatory subunit 35
[Source:HGNC Symbol;Acc:HGNC:28320]	-	463	446	392
269	170	301	19.10112539	17.80371283
20.54670543	12.5925477	8.803301228	11.79089562
ENSG00000125351 1165.817491	1126.59883	1025.317635
839.1344809	654.0748175	750.4959694	1105.911319
747.9017559	0.564296095	0.00103276	0.012947193	UPF3B
X	119805311	119852998	-	3081
protein_coding	"UPF3B, regulator of nonsense mediated mRNA decay
[Source:HGNC Symbol;Acc:HGNC:20439]"	-	1285	1273	882
810	573	870	20.4239676	19.57776658
17.81081257	14.60849123	11.43168592	13.12981396
ENSG00000157551 114.3136217	106.1994184	105.7867401
234.1288798	154.1013968	321.7643639	108.7665934
236.6648802	-1.123733908	0.001036417	0.012983833	KCNJ15
21	38155549	38307357	+	10342
protein_coding	potassium voltage-gated channel subfamily J member 15
[Source:HGNC Symbol;Acc:HGNC:6261]	-	126	120	91
226	135	373	0.596615724	0.549797997
0.547449049	1.214271912	0.802373534	1.677008703
ENSG00000103489 958.964271	838.09041	1372.902639
765.5807177	555.9065203	609.023166	1056.65244
643.503468	0.714287571	0.001042488	0.013050648	XYLT1
16	17101769	17470881	-	11004
protein_coding	xylosyltransferase 1 [Source:HGNC
Symbol;Acc:HGNC:15516]	-	1057	947	1181	739	487
706	4.703845938	4.077799217	6.677381084
3.731692935	2.720355987	2.983219008
ENSG00000133398 868.2391744	981.4596248	904.4185036
1275.277217	1232.811174	1431.980815	918.039101
1313.356402	-0.516933285	0.001050358	0.013139874	MED10
5	6371881 6378594 -	1880	protein_coding
mediator complex subunit 10 [Source:HGNC Symbol;Acc:HGNC:28760] -
957	1109	778	1231	1080	1660	24.9277323
27.9511794	25.74711628	36.38415116	35.31126421
41.05643696
ENSG00000116521 3749.668242	4671.889413	4190.782398
5132.187924	5880.965899	6324.86948	4204.113351
5779.341101	-0.459162628	0.001052008	0.013151218	SCAMP3
1	155255979	155262430	-	2442
protein_coding	secretory carrier membrane protein 3 [Source:HGNC
Symbol;Acc:HGNC:10565]	-	4133	5279	3605	4954	5152
7332	82.8797494	102.4312431	91.84731312
112.7255561	129.6813596	139.6071993

ENSG00000130479 1395.351986 1479.711896 1483.339345
1668.945245 2114.042866 2963.165121 1452.801076
2248.717744 -0.630820022 0.001057564 0.013211338 MAP1S
19 17719242 17734516 + 5040
protein_coding microtubule associated protein 1S [Source:HGNC
Symbol;Acc:HGNC:15715] - 1538 1672 1276 1611 1852
3435 14.94357414 15.7192629 15.75168378
17.76139397 22.58696121 31.69036367
ENSG00000167325 4276.781054 3524.0507 4173.345023
3417.660065 2491.876661 1999.597307 3991.392259
2636.378011 0.598536497 0.001060032 0.013232826 RRM1
11 4094707 4138876 + 4177 protein_coding
ribonucleotide reductase catalytic subunit M1 [Source:HGNC
Symbol;Acc:HGNC:10451] - 4714 3982 3590 3299 2183
2318 55.26545965 45.17136571 53.47327948
43.8863926 32.12451803 25.8035806
ENSG00000082684 0.907250966 0 2.3249833 9.323716454
44.5181813 17.25278091 1.077411422 23.69822622
-4.536002039 0.001063115 0.013261946 SEMA5B 3
122909082 123028605 - 8517 protein_coding
semaphorin 5B [Source:HGNC Symbol;Acc:HGNC:10737] -
1 0 2 9 39 20 0.005749658
0 0.014609999 0.058717547 0.281465597
0.109187863
ENSG00000113593 722.1717689 684.1012534 581.2458249
501.4087515 296.7878753 376.9732628 662.5062824
391.7232965 0.758119916 0.00106451 0.013269993 PPWD1
5 65563236 65587549 + 3777
protein_coding peptidylprolyl isomerase domain and WD repeat
containing 1 [Source:HGNC Symbol;Acc:HGNC:28954] - 796
773 500 484 260 437 10.32035809
9.69748128 8.236256553 7.120498847 4.231299078
5.37979182
ENSG00000130176 15.42326642 8.849951531 16.2748831 0
0 0 13.51603368 0 6.188376475
0.001065918 0.013278183 CNN1 19 11538717
11550323 + 2887 protein_coding calponin 1
[Source:HGNC Symbol;Acc:HGNC:2155] - 17 10 14
0 0 0 0.288357214 0.164126859
0.301708884 0 0 0
ENSG00000171425 427.315205 531.882087 447.5592852
510.732468 841.2794774 1229.26064 468.9188591
860.424195 -0.876260738 0.001071308 0.013329893 ZNF581
19 55635459 55645622 + 1736
protein_coding zinc finger protein 581 [Source:HGNC
Symbol;Acc:HGNC:25017] zf-C2H2 471 601 385 493 737
1425 13.28617198 16.40405407 13.79805448
15.78008151 26.09547024 38.16771508
ENSG00000262943 17.23776835 19.46989337 15.11239145 0
1.141491828 0 17.27335106 0.380497276

5.582631906	0.00107374	0.013329893	ALOX12P2	17
	6853861 6954107 +	5655
transcribed_unprocessed_pseudogene	arachidonate 12-lipoxygenase
pseudogene 2 [Source:HGNC Symbol;Acc:HGNC:432]	-	19	22
	13	0	1	0	0.164531734	0.184338697
	0.143026855	0	0.01086963	0
ENSG00000164484 314.8160852	377.0079352	368.509853
701.350671	688.3195724	431.3195226	353.4446245
606.9965887	-0.779242303	0.001073883	0.013329893
TMEM200A	6	130365734	130443063	+	6137
	protein_coding	transmembrane protein 200A [Source:HGNC
Symbol;Acc:HGNC:21075]	-	347	426	317	677	603
	500	2.768866616	3.2891215	3.21373443
6.129775783	6.039605182	3.788304668
ENSG00000158290 3795.938042	3005.44354	3444.462759
2894.495975	2055.826783	2041.86662	3415.281447
2330.729792	0.551280721	0.001074024	0.013329893	CUL4B
	X	120524609	120575794	-	7031
protein_coding	cullin 4B [Source:HGNC Symbol;Acc:HGNC:2555]	-
	4184	3396	2963	2794	1801	2367	29.14092019
	22.88637578	26.21931856	22.08113269	15.7450475
	15.65352597
ENSG00000196507 420.0571972	557.5469465	510.3338343
326.3300759	312.7687609	221.6982346	495.979326
286.9323571	0.7913846	0.001074211	0.013329893	TCEAL3
	X	103607451	103629690	+	2305
protein_coding	transcription elongation factor A like 3 [Source:HGNC
Symbol;Acc:HGNC:28247]	-	463	630	439	315	274
	257	9.836458066	12.95078402	11.84951124
7.593669086	7.306796184	5.184336848
ENSG00000179454 489.9155216	399.1328141	345.26002
661.9838682	731.6962619	599.5341365	411.4361186
664.4047555	-0.689319479	0.00107459	0.013329893	KLHL28
	14	44924319	45042322	-	8076
protein_coding	kelch like family member 28 [Source:HGNC
Symbol;Acc:HGNC:19741]	-	540	451	297	639	641
	695	3.27435779	2.646102557	2.288057771
4.396596315	4.878755787	4.001469514
ENSG00000189266 1904.319778	1751.405408	1951.82348
1416.168933	1405.17644	1133.507705	1869.182889
1318.28436	0.50421743	0.001076653	0.013346122	PNRC2
	1	23956839	23963462	+	2779
protein_coding	proline rich nuclear receptor coactivator 2
[Source:HGNC Symbol;Acc:HGNC:23158]	-	2099	1979	1679
	1367	1231	1314	36.98729536	33.74299971
37.5897118	27.33329644	27.2280705	21.98557541
ENSG00000133466 275.8042937	251.3386235	320.8476954
95.30910153	36.5277385	184.6047557	282.6635375
105.4805319	1.415094504	0.001079791	0.013375644
C1QTNF6 22	37180167	37199385	-	7143

protein_coding C1q and TNF related 6 [Source:HGNC
Symbol;Acc:HGNC:14343] -   304   284   276   92   32
        214   2.084114701   1.883927262   2.404004513
0.71568056   0.27537004   1.393041778
ENSG00000171763 379.2309038   365.5029982   392.9221777
449.6103268   725.9888027   757.3970818   379.2186932
644.3320704   -0.765420588   0.001085891   0.013435422
SPATA5L1   15   45402331   45421419   +   3406
        protein_coding spermatogenesis associated 5 like 1
[Source:HGNC Symbol;Acc:HGNC:28762]   -   418   413   338
        434   636   878   6.009804184   5.745553181
6.17417455   7.080389177   11.47783117   11.98618262
ENSG00000109323 361.9931354   311.5182939   409.1970608
250.7043758   176.9312334   143.1980815   360.90283
190.2778969   0.924173907   0.001086136   0.013435422   MANBA
        4   102630770   102760994   -   11743
protein_coding mannosidase beta [Source:HGNC Symbol;Acc:HGNC:6831]
        -       399   352   352   242   155   166
1.66388198   1.420332561   1.864963984   1.14511301
0.811334601   0.657294571
ENSG00000185774 57.15681086   65.48964133   92.99933199
135.7118728   231.7228411   150.0991939   71.88192806
172.5113026   -1.265980652   0.00108808   0.013444418   KCNIP4
        4   20728616   21948799   -   4256
protein_coding potassium voltage-gated channel interacting protein 4
[Source:HGNC Symbol;Acc:HGNC:30083]   -   63   74   80
        131   203   174   0.724882498   0.823865926
1.169486504   1.710337114   2.931850214   1.900984812
ENSG00000081760 593.3421318   646.9314569   584.7332999
405.0636815   420.0689928   269.1433821   608.3356295
364.7586855   0.740337432   0.001088383   0.013444418   AACS
        12   125065379   125143333   +   16094
protein_coding acetoacetyl-CoA synthetase [Source:HGNC
Symbol;Acc:HGNC:21298] -   654   731   503   391   368
        312   1.98995124   2.152186093   1.944512927
1.349972134   1.405501074   0.90140843
ENSG00000114473 589.7131279   783.2207105   552.1835337
879.5372522   1061.5874   1048.969079   641.7057907
996.6979105   -0.634184441   0.001091589   0.013474617   IQCG
        3   197889075   197960142   -   7474
protein_coding IQ motif containing G [Source:HGNC
Symbol;Acc:HGNC:25251] -   650   885   475   849   930
        1216   4.258816683   5.610694446   3.954097398
6.311994916   7.648516629   7.565044718
ENSG00000198301 1553.213654   1512.456717   1390.340013
2028.426313   2150.570604   1940.937852   1485.336795
2039.978256   -0.457147102   0.00109556   0.013514201   SDAD1
        4   75940950   75990962   -   3491
protein_coding SDA1 domain containing 1 [Source:HGNC
Symbol;Acc:HGNC:25537] -   1712   1709   1196   1958   1884

2250 24.01500023 23.19629668 21.31513941
31.16555622 33.17251582 29.96840902
ENSG00000254087 434.5732127 377.0079352 433.6093854
295.251021 220.3079228 182.0168386 415.0635111
232.5252608 0.837005491 0.001097294 0.013526159 LYN
8 55879813 56014168 + 6185
protein_coding "LYN proto-oncogene, Src family tyrosine kinase
[Source:HGNC Symbol;Acc:HGNC:6735]" - 479 426 373
285 193 211 3.792490425 3.263595578
3.752113563 2.560455308 1.918072264 1.586257796
ENSG00000173267 9.979760626 9.734946684 20.9248497 0
0 0 13.546519 0 6.189784169
0.00110773 0.013641711 SNCG 10 86958618
86963260 + 1247 protein_coding synuclein gamma
[Source:HGNC Symbol;Acc:HGNC:11141] - 11 11 18
0 0 0 0.431971322 0.417977277
0.898075602 0 0 0
ENSG00000105705 695.8614909 829.2404585 721.9073146
888.8609686 1334.403947 1260.315645 749.003088
1161.19352 -0.632280072 0.001108968 0.013641711 SUGP1
19 19276018 19320844 - 5356
protein_coding SURP and G-patch domain containing 1 [Source:HGNC
Symbol;Acc:HGNC:18643] - 767 937 621 858 1169
1461 7.012671238 8.289445172 7.2136967
8.901409139 13.41594605 12.68354534
ENSG00000165525 688.6034832 466.3924457 574.270875
386.4162486 336.7400893 318.3138077 576.422268
347.1567152 0.73188946 0.001108982 0.013641711 NEMF
14 49782083 49853203 - 9426
protein_coding nuclear export mediator factor [Source:HGNC
Symbol;Acc:HGNC:10663] - 759 527 494 373 295
369 3.94314751 2.649168733 3.260666339
2.198839044 1.923720357 1.820245428
ENSG00000154845 2249.075145 1862.029802 2333.120741
1532.197404 1604.93751 1463.89846 2148.075229
1533.677791 0.485947577 0.001111718 0.013665862 PPP4R1
18 9546791 9615240 - 8131 protein_coding
protein phosphatase 4 regulatory subunit 1 [Source:HGNC
Symbol;Acc:HGNC:9320] - 2479 2104 2007 1479 1406
1697 14.93004956 12.26106558 15.35713698
10.1073256 10.62890976 9.704404697
ENSG00000160714 1889.803762 2350.547127 1929.736139
2656.223221 2889.115817 3028.725688 2056.695676
2858.021575 -0.47450259 0.001114953 0.013696109 UBE2Q1
1 154548577 154559028 - 4161
protein_coding ubiquitin conjugating enzyme E2 Q1 [Source:HGNC
Symbol;Acc:HGNC:15698] - 2083 2656 1660 2564 2531
3511 24.51434183 30.24522333 24.82088251
34.23988905 37.38882264 39.23413949
ENSG00000267041 241.328757 198.2389143 176.6987308

415.4233665	360.7114177	322.6270029	205.422134
366.253929	-0.832021548	0.001118089	0.013725101	ZNF850
	19	36714383	36772825	-	7850
protein_coding	zinc finger protein 850 [Source:HGNC
Symbol;Acc:HGNC:27994]	zf-C2H2	266	224	152	401	316
	374	1.659360178	1.352087514	1.20470518
2.83848602	2.474370824	2.215302122
ENSG00000152402	381.0454057	306.208323	398.7346359
250.7043758	179.214217	149.2365548	361.9961215
193.0517159	0.907685916	0.001120542	0.013739263
GUCY1A2	11	106674012	107018524	-	16361
protein_coding	guanylate cyclase 1 soluble subunit alpha 2
[Source:HGNC Symbol;Acc:HGNC:4684]	-	420	346	343
	242	157	173	1.257095088	1.00205762
1.304340928	0.821897321	0.589844003	0.491662717
ENSG00000105372	17417.40404	22613.39615	24179.82632
25129.48778	32250.56862	33830.11544	21403.54217
30403.39061	-0.506435818	0.001120796	0.013739263	RPS19
	19	41859918	41872926	+	3799
protein_coding	ribosomal protein S19 [Source:HGNC
Symbol;Acc:HGNC:10402]	-	19198	25552	20800	24257	28253
	39217	247.4659082	318.6999872	340.6441131
354.7969177	457.1330657	479.994314
ENSG00000117475	1082.350402	1029.249363	860.2438209
1575.708081	1287.602782	1463.035821	990.6145288
1442.115561	-0.541193711	0.001126107	0.013794807	BLZF1
	1	169367970	169396540	+	3820
protein_coding	basic leucine zipper nuclear factor 1 [Source:HGNC
Symbol;Acc:HGNC:1065]	Others	1193	1163	740	1521	1128
	1696	15.29346045	14.42589586	12.05244625
22.12472569	18.15068822	20.64398349
ENSG00000079819	2108.451245	1721.315573	1969.260855
1511.478034	1310.432619	1278.431065	1933.009224
1366.780573	0.500103053	0.001128471	0.013814197
EPB41L2	6	130839347	131063322	-	10320
protein_coding	erythrocyte membrane protein band 4.1 like 2
[Source:HGNC Symbol;Acc:HGNC:3379]	-	2324	1945	1694
	1459	1148	1482	11.02770424	8.930306181
10.21269955	7.855749713	6.837692262	6.677279024
ENSG00000109107	2297.159446	3038.188361	3231.726787
3651.788945	6173.187807	3670.529138	2855.691531
4498.501963	-0.655528637	0.001132184	0.013845258	ALDOC
	17	28573115	28577264	-	2928
protein_coding	"aldolase, fructose-bisphosphate C [Source:HGNC
Symbol;Acc:HGNC:418]"	-	2532	3433	2780	3525	5408
	4255	42.34686969	55.55577008	59.07184972
66.89599112	113.5306098	67.57086992
ENSG00000089234	501.7097842	430.9926396	416.1720107
680.6313012	686.0365887	697.8749876	449.6248115
688.1809592	-0.613406693	0.001132573	0.013845258	BRAP

12 111642146 111685986 - 4686
protein_coding BRCA1 associated protein [Source:HGNC
Symbol;Acc:HGNC:1099] - 553 487 358 657 601
809 5.778984513 4.924397677 4.753216423
7.790675744 7.88350864 8.027443462
ENSG00000179051 6191.080592 6843.667519 7470.171342
4831.75706 4688.106938 5620.09338 6834.973151
5046.65246 0.437281756 0.00113704 0.01389026 RCC2
1 17406760 17439724 - 4126
protein_coding regulator of chromosome condensation 2 [Source:HGNC
Symbol;Acc:HGNC:30297] - 6824 7733 6426 4664 4107
6515 80.99132017 88.80659676 96.89878782
62.81181773 61.18470063 73.42031011
ENSG00000144935 448.1819772 288.5084199 442.9093186
168.8628647 253.4111859 209.621288 393.1999052
210.6317795 0.900568861 0.001140279 0.013920215 TRPC1
3 142724074 142807888 + 4768
protein_coding transient receptor potential cation channel subfamily
C member 1 [Source:HGNC Symbol;Acc:HGNC:12333] - 494 326
381 163 222 243 5.073636929 3.239722366
4.971593088 1.899605426 2.861963482 2.369741886
ENSG00000113594 410.9846876 320.3682454 383.6222445
238.2727538 203.1855454 198.4069804 371.6583925
213.2884266 0.801198913 0.001147367 0.013997083 LIFR
5 38474963 38608354 - 11864
protein_coding LIF receptor alpha [Source:HGNC Symbol;Acc:HGNC:6597]
- 453 362 330 230 178 230
1.869802549 1.445785528 1.730571903 1.077230749
0.922223585 0.901421093
ENSG00000233672 5.443505796 9.734946684 26.73730795 0
0 0 13.97192014 0 6.232646757
0.001154416 0.014073379 RNASEH2B-AS1 13 50862172
50910764 - 4642 antisense RNASEH2B
antisense RNA 1 [Source:HGNC Symbol;Acc:HGNC:39967] - 6
11 23 0 0 0 0.063295786
0.112282995 0.308268782 0 0 0
ENSG00000130309 5925.256059 6794.992786 7292.31012
4677.397754 5141.279194 5111.136343 6670.852988
4976.604431 0.422561564 0.001160763 0.014141003
COLGALT1 19 17555594 17583162 + 4900
protein_coding collagen beta(1-O)galactosyltransferase 1
[Source:HGNC Symbol;Acc:HGNC:26182] - 6531 7678 6273
4515 4504 5925 65.2698008 74.24692447
79.65005025 51.20044296 56.50015796 56.22420105
ENSG00000125037 1525.088874 1729.280529 1656.550601
2088.512486 2424.528643 2239.410962 1636.973335
2250.817363 -0.459320313 0.001162055 0.014147004 EMC3
3 9962537 10011116 - 4270 protein_coding
ER membrane protein complex subunit 3 [Source:HGNC
Symbol;Acc:HGNC:23999] - 1681 1954 1425 2016 2124

2596	19.27829058	21.6831875	20.76317842
26.23461507	30.57553026	28.26882981
ENSG00000167685 565.2173518	677.0212921	642.8578824
832.9186699	928.0328563	1091.238392	628.3655088
950.7299728	-0.598149259	0.00116543	0.014178325	ZNF444
19	56132599	56160893	+	7239
protein_coding	zinc finger protein 444 [Source:HGNC
Symbol;Acc:HGNC:16052]	zf-C2H2 623	765	553	804	813
1265	4.214423293	5.007365576	4.752842264
6.171482698	6.903341043	8.125366646
ENSG00000186106 282.1550504	352.2280709	310.3852705
409.2075555	598.141718	592.6330241	314.9227973
533.3274325	-0.759937439	0.001175586	0.014292054
ANKRD46 8	100509752	100559784	-	5820
protein_coding	ankyrin repeat domain 46 [Source:HGNC
Symbol;Acc:HGNC:27229]	-	311	398	267	395	524
687	2.616773228	3.240309755	2.854270463
3.77125722	5.534210354	5.488640306
ENSG00000067334 3678.902667	3654.144987	2896.929192
5297.942883	4351.366849	4573.712218	3409.992282
4741.007317	-0.47507849	0.001176568	0.014294174
DNTTIP2 1	93866283	93879918	-	7519
protein_coding	deoxynucleotidyltransferase terminal interacting
protein 2 [Source:HGNC Symbol;Acc:HGNC:24013]	-	4055	4129
2492	5114	3812	5302	26.40945611	26.02023646
20.62029147	37.79311444	31.16306513	32.78767765
ENSG00000230838 13.60876449	6.194966072	20.9248497	0
0	0	13.57619342	0	6.192897451
0.001177539	0.014296154	LINC01614	2	215718043
215719424	+	648	lincRNA long intergenic non-
protein coding RNA 1614 [Source:HGNC Symbol;Acc:HGNC:51847]	-
15	7	18	0	0	0	1.133561107
0.511857976	1.728241167	0	0	0
ENSG00000168078 1208.458287	1083.234067	1306.640614
558.3870188	303.6368263	129.3958568	1199.444323
330.4732339	1.860309594	0.001179976	0.014315915	PBK
8	27809620	27838095	-	2165
protein_coding	PDZ binding kinase [Source:HGNC Symbol;Acc:HGNC:18282]
-	1332	1224	1124	539	266	150
30.1283264	26.78859633	32.30094715	13.83384509
7.552158501	3.221546293
ENSG00000160293 749.3892979	725.6960256	838.1564796
570.8186407	503.3978962	401.1271561	771.080601
491.781231	0.649511661	0.001183497	0.014348783	VAV2
9	133761894	133992604	-	5682
protein_coding	vav guanine nucleotide exchange factor 2 [Source:HGNC
Symbol;Acc:HGNC:12658]	-	826	820	721	551	441
465	7.118811633	6.838156939	7.894795446
5.388432081	4.770728703	3.805246031
ENSG00000100626 129.7368881	113.2793796	120.8991316

60.0861727	35.38624667	10.35166854	121.3051331
35.27469597	1.789370677	0.001191053	0.014430503
GALNT16 14	69259277	69357033	+	10802
protein_coding	polypeptide N-acetylgalactosaminyltransferase 16
[Source:HGNC Symbol;Acc:HGNC:23233]	-	143	128	104
58	31	12	0.648276902	0.561477345
0.599012676	0.298356748	0.176402559	0.051654492
ENSG00000115806 2105.729492	1954.069298	2184.32181
2956.654084	2561.507662	2979.555262	2081.373533
2832.572336	-0.445052341	0.001194466	0.014456369
GORASP2 2	170928464	170967129	+	5340
protein_coding	golgi reassembly stacking protein 2 [Source:HGNC
Symbol;Acc:HGNC:17500]	-	2321	2208	1879	2854	2244
3454	21.2844566	19.59224725	21.89234934
29.69783263	25.83027059	30.07544724
ENSG00000004478 2868.727554	3399.266383	3148.027388
3512.969166	4865.038172	5133.564958	3138.673775
4503.857432	-0.521088091	0.001194822	0.014456369	FKBP4
12	2794953 2805423 +	5180	protein_coding	FK506
binding protein 4 [Source:HGNC Symbol;Acc:HGNC:3720]	-	3162
3841	2708	3391	4262	5951	29.8924003
35.13508336	32.52563221	36.3755923	50.57443572
53.41844094
ENSG00000085999 537.0925719	577.901835	542.8836005
331.5099184	310.4857773	395.9513218	552.6260024
345.9823391	0.67418949	0.001198139	0.014486586	RAD54L
1	46247688	46278473	+	3212
protein_coding	RAD54 like [Source:HGNC Symbol;Acc:HGNC:9826]	-
592	653	467	320	272	459	9.025574465
9.633056011	9.045825186	5.535877679	5.20523963
6.644589676
ENSG00000173581 584.2696221	817.7355215	685.8700734
900.2566221	1083.275745	1268.079397	695.9584057
1083.870588	-0.639405711	0.001208822	0.014605769
CCDC106 19	55641062	55653161	+	2709
protein_coding	coiled-coil domain containing 106 [Source:HGNC
Symbol;Acc:HGNC:30181]	-	644	924	590	869	949
1470	11.64140895	16.16178805	13.55032941
17.82472405	21.53300135	25.23128376
ENSG00000227500 2343.429245	2776.229795	2726.042919
3244.653326	3641.358932	3817.177775	2615.233986
3567.730011	-0.448288658	0.001215619	0.014677873	SCAMP4
19	1905214 1926013 +	6953	protein_coding
secretory carrier membrane protein 4 [Source:HGNC
Symbol;Acc:HGNC:30385]	-	2583	3137	2345	3132	3190
4425	18.1920173	21.37808159	20.98347754
25.03004138	28.20108476	29.59184638
ENSG00000151023 25.40302705	26.54985459	12.78740815
56.97826722	49.08414861	152.687111	21.5800966
86.24984228	-1.994522448	0.001226093	0.014794248	ENKUR

10	24981979	25062279	-	4076
protein_coding	"enkurin, TRPC channel interacting protein
[Source:HGNC Symbol;Acc:HGNC:28388]"	-	28	30	11
55	43	177	0.33639733	0.348749441
0.167905668	0.749791578	0.648457683	2.019157094
ENSG00000134030 604.2291433	536.3070628	669.5951903
730.3577889	1053.596957	1061.046026	603.3771322
948.3335907	-0.65330623	0.001231298	0.014846919	CTIF
18	48539046	48863217	+	8100
protein_coding	cap binding complex dependent translation initiation
factor [Source:HGNC Symbol;Acc:HGNC:23925]	-	666	606
576	705	923	1230	4.026409053	3.544982096
4.424297387	4.836332395	7.004288764	7.060754487
ENSG00000184009 107860.3456	112744.8425	126486.0665
148354.8323	151132.3766	148605.9657	115697.0849
149364.3915	-0.368493903	0.001240924	0.014952795	ACTG1
17	81509971	81523847	-	2993
protein_coding	actin gamma 1 [Source:HGNC Symbol;Acc:HGNC:144] -
118887	127396	108806	143204	132399	172269	1945.1645
2016.858901	2261.793619	2658.645762	2719.101054
2676.279294
ENSG00000144857 340.2191122	301.7833472	332.4726119
132.6039673	232.8643329	142.3354425	324.8250238
169.2679143	0.943198045	0.001244508	0.014976824	BOC
3	113211003	113287459	+	10330
protein_coding	"BOC cell adhesion associated, oncogene regulated
[Source:HGNC Symbol;Acc:HGNC:17173]"	-	375	341	286
128	204	165	1.777704737	1.564157561
1.722552861	0.688528136	1.213884053	0.742702081
ENSG00000198176 5596.831209	5805.568204	5460.22328
4471.240024	4350.225357	4095.810187	5620.874231
4305.758523	0.384698314	0.001244611	0.014976824	TFDP1
13	113584721	113641470	+	3741
protein_coding	transcription factor Dp-1 [Source:HGNC
Symbol;Acc:HGNC:11749]	E2F	6169	6560	4697	4316	3811
4748	80.75245708	83.08881631	78.11594637
64.10704689	62.61791472	59.01385975
ENSG00000224543 23.58852512	16.81490791	18.5998664
1.035968495	0	0.862639045	19.66776647	0.63286918
4.909178359	0.0012486	0.015009872	SNRPGP15
19	14489388	14489609	+	222
processed_pseudogene	small nuclear ribonucleoprotein polypeptide G
pseudogene 15 [Source:HGNC Symbol;Acc:HGNC:49371]	-	26
19	16	1	0	1	5.735206467
4.055338095	4.48408519	0.250298974	0	0.209448881
ENSG00000082898 5789.168414	4618.789704	5180.062792
4366.607206	3395.938189	3368.605472	5196.00697
3710.383622	0.485881259	0.001249055	0.015009872	XPO1
2	61477849	61538626	-	11708
protein_coding	exportin 1 [Source:HGNC Symbol;Acc:HGNC:12825]	-

6381 5219 4456 4215 2975 3905 26.68914827
21.12180476 23.67932482 20.00446351 15.61894216
15.50848387
ENSG00000088826 293.949313 243.3736671 446.3967936
123.2802509 154.1013968 205.3080928 327.9065912
160.8965802 1.023001841 0.001254253 0.015053467 SMOX
20 4120980 4187747 + 2946 protein_coding
spermine oxidase [Source:HGNC Symbol;Acc:HGNC:15862] - 324
275 384 119 135 238 5.385685032
4.423096271 8.109710078 2.244534383 2.81675054
3.756429415
ENSG00000102908 1858.049978 1497.411799 1504.264195
2513.259569 1973.639371 2474.911421 1619.908657
2320.603453 -0.518631304 0.001254508 0.015053467 NFAT5
16 69565094 69704666 + 16401
protein_coding nuclear factor of activated T cells 5 [Source:HGNC
Symbol;Acc:HGNC:7774] RHD 2048 1692 1294 2426 1729
2869 6.114885188 4.888284464 4.908748644
8.21925608 6.479955521 8.133757829
ENSG00000164125 316.6305871 141.5992245 210.4109886
2.07193699 45.65967313 21.56597613 222.8802668
23.09919542 3.273483849 0.001255236 0.015053467
FAM198B 4 158124474 158173318 - 7880
protein_coding family with sequence similarity 198 member B
[Source:HGNC Symbol;Acc:HGNC:25312] - 349 160 181
2 40 25 2.168841891 0.962099981
1.429088762 0.01410314 0.312019067 0.14751793
ENSG00000065308 2953.101894 2798.354674 2696.980628
2421.058373 1425.723293 1783.074907 2816.145732
1876.618858 0.585484596 0.001262875 0.015127878 TRAM2
6 52497402 52576915 - 6908
protein_coding translocation associated membrane protein 2
[Source:HGNC Symbol;Acc:HGNC:16855] - 3255 3162 2320
2337 1249 2067 23.07423692 21.68882265
20.89500612 18.79829355 11.1136693 13.91294813
ENSG00000125170 124.2933823 153.1041615 163.9113226
319.0782964 253.4111859 256.2037964 147.1029555
276.2310929 -0.910472898 0.001263151 0.015127878 DOK4
16 57471922 57487327 - 4788
protein_coding docking protein 4 [Source:HGNC Symbol;Acc:HGNC:19868]
- 137 173 141 308 222 297
1.401183805 1.71205772 1.832195523 3.574444995
2.850008747 2.884252819
ENSG00000158545 4292.20432 4552.415068 4186.132431
5065.88594 5985.983147 6697.529548 4343.58394
5916.466212 -0.445895314 0.0012729 0.015234327 ZC3H18
16 88570381 88631966 + 8648
protein_coding zinc finger CCCH-type containing 18 [Source:HGNC
Symbol;Acc:HGNC:25091] - 4731 5144 3601 4890 5244
7764 26.78958282 28.18458989 25.9068307

31.41993063	37.27300111	41.74465382
ENSG00000268001 107.055614	125.6693117	104.6242485
212.3735415	207.7515127	240.6762936	112.4497247
220.2671159	-0.969457675	0.001286342	0.01537687	CARD8-
AS1	19	48255675	48258199	+	1969
antisense	CARD8 antisense RNA 1 [Source:HGNC
Symbol;Acc:HGNC:51408]	-	118	142	90	205	182
	279	2.934708532	3.417189546	2.843830056
5.785224125	5.681631098	6.588544829
ENSG00000163347 27.21752898	30.97483036	31.38727455
32.11502334	138.1205112	157.0003062	29.85987796
109.0786136	-1.870772595	0.001286549	0.01537687	CLDN1
	3	190305701	190322475	-	3663
protein_coding	claudin 1 [Source:HGNC Symbol;Acc:HGNC:2032]	-
	30	35	27	31	121	182	0.401063389
	0.45274907	0.458599622	0.470258678	2.030466422
	2.310284623
ENSG00000278540 2761.67194	2369.132025	2462.157314
2021.174534	1732.784595	1025.677825	2530.987093
1593.212318	0.668483374	0.001288429	0.015388942	ACACA
	17	37084988	37406818	-	13008
protein_coding	acetyl-CoA carboxylase alpha [Source:HGNC
Symbol;Acc:HGNC:84]	-	3044	2677	2118	1951	1518
	1189	11.45942439	9.751339652	10.13029924
8.334101489	7.173128378	4.250131276
ENSG00000207751 3.629003864	23.89486913	13.9498998	0
	0	0	13.82459093	0	6.222537452
0.001292007	0.015421267	AP000553.1	22	21652270
	21670237	+	1093	processed_transcript	novel
transcript	-	4	27	12	0	0	0
	0.179212589	1.170496294	0.683074276	0	0
	0
ENSG00000196498 5655.802522	4733.839074	6028.681696
4384.21867	3717.838884	3910.342792	5472.774431
4004.133449	0.450572916	0.001300391	0.01551087	NCOR2
	12	124324415	124567589	-	13975
protein_coding	nuclear receptor corepressor 2 [Source:HGNC
Symbol;Acc:HGNC:7673]	MYB	6234	5349	5186	4232	3257
	4533	21.84457828	18.13623866	23.08806532
16.82696867	14.32561589	15.08220782
ENSG00000173281 513.5040467	587.6367817	646.3453573
953.0910153	760.2335576	942.8644765	582.4953953
885.3963498	-0.605520345	0.001303825	0.015540185
PPP1R3B 8	9136255 9151574 -	5776	protein_coding
protein phosphatase 1 regulatory subunit 3B [Source:HGNC
Symbol;Acc:HGNC:14942]	-	566	664	556	920	666
	1093	4.798637352	5.447124926	5.989001938
8.850599451	7.087521752	8.79881113
ENSG00000130772 514.4112977	592.9467526	563.8084502
716.8901985	967.9850703	866.0896015	557.0555002

850.3216234	-0.609992635	0.001304606	0.015540185	MED18
	1	28329002	28335967	+	2019
protein_coding	mediator complex subunit 18 [Source:HGNC
Symbol;Acc:HGNC:25944]	-	567	670	485	692	848
	1004	13.75230272	15.72406841	14.94556254
19.04503693	25.81706651	23.12216053
ENSG00000253729 14036.98695	11821.76526	11673.74115
11036.17238	7311.255159	7787.905301	12510.83112
8711.777612	0.522174656	0.001307392	0.015562894	PRKDC
	8	47773108	47960183	-	15417
protein_coding	"protein kinase, DNA-activated, catalytic subunit
[Source:HGNC Symbol;Acc:HGNC:9413]"	-	15472	13358	10042
	10653	6405	9028	49.14453927	41.05518444
40.5253889	38.39583337	25.53680863	27.228436
ENSG00000152104 4941.796012	4249.746725	5181.225284
4107.615082	3123.121642	2753.543833	4790.922674
3328.093519	0.525661501	0.001308616	0.015566986	PTPN14
	1	214348696	214552449	-	18756
protein_coding	"protein tyrosine phosphatase, non-receptor type 14
[Source:HGNC Symbol;Acc:HGNC:9647]"	-	5447	4802	4457
	3965	2736	3192	14.22151405	12.13132876
14.78458902	11.74667657	8.966505706	7.913227958
ENSG00000125755 2635.564056	2869.154286	2678.380761
3326.494837	3543.190635	4654.800288	2727.699701
3841.495253	-0.494254481	0.001316411	0.015649191	SYMPK
	19	45815410	45863290	-	10628
protein_coding	symplekin [Source:HGNC Symbol;Acc:HGNC:22935]	-
	2905	3242	2304	3211	3104	5396	13.38515099
	14.45399518	13.48769621	16.78807124	17.95219376
	23.60757692
ENSG00000092621 4812.059124	6396.744967	6564.590347
7371.95181	8103.450488	9658.96939	5924.464812
8378.123896	-0.500160618	0.001320468	0.015686871	PHGDH
	1	119648411	119744226	+	9217
protein_coding	phosphoglycerate dehydrogenase [Source:HGNC
Symbol;Acc:HGNC:8923]	-	5304	7228	5647	7116	7099
	11197	28.18010529	37.15822817	38.11843369
42.90010899	47.34290999	56.48629748
ENSG00000091409 890.9204486	700.0311661	666.1077154
598.7897901	218.0249392	358.8578428	752.35311
391.8908574	0.940622091	0.001344297	0.015959241	ITGA6
	2	172427354	172506282	+	6533
protein_coding	integrin subunit alpha 6 [Source:HGNC
Symbol;Acc:HGNC:6142]	-	982	791	573	578	191
	416	7.360842295	5.737071549	5.456935373
4.916173754	1.797082724	2.960817852
ENSG00000233968 123.3861314	101.7744426	118.5741483
62.15810969	30.82027936	18.978059	114.5782408
37.31881602	1.622663709	0.001359428	0.01612805
AL157895.1	10	19710328	19728550	-	2151

antisense	uncharacterized LOC101928834 [Source:NCBI gene;Acc:101928834]	-	136	115	102	60	27	22	3.096186991	2.533283945	2.950302912	1.549968541	0.771561794	0.475568728
ENSG00000225163 16.33051739	15.92991276	6.974949899	0	0	0	13.07846001	0	6.144889216	0.001363444	0.016164858	LINC00618	14	96931367	96945394	+	700	sense_overlapping	long intergenic non-protein coding RNA 618 [Source:HGNC Symbol;Acc:HGNC:20110]	-	18	18	6	0	0	0	1.259224453	1.218430904	0.533285846	0	0	0
ENSG00000152894 3258.84547	2706.315178	3619.998998	2716.309394	1949.668043	1732.179203	3195.053215	2132.71888	0.583132026	0.001364511	0.016166665	PTPRK	6	127968779	128520674	-	9000	protein_coding	"protein tyrosine phosphatase, receptor type K [Source:HGNC Symbol;Acc:HGNC:9674]"	-	3592	3058	3114	2622	1708	2008	19.54440719	16.09983453	21.52697197	16.18833643	11.66521418	10.37414269
ENSG00000109472 578.8261163	602.6816993	906.7434869	416.459335	473.7191087	360.5831209	696.0837675	416.9205215	0.739151839	0.001369239	0.016211832	CPE	4	165361194	165498320	+	2958	protein_coding	carboxypeptidase E [Source:HGNC Symbol;Acc:HGNC:2303]	-	638	681	780	402	415	418	10.56212232	10.9087599	16.40602162	7.551616506	8.623772424	6.570662044
ENSG00000216636 0	0	0	8.287747959	9.131934625	21.56597613	0	12.99521957	-6.178854799	0.001371162	0.016223733	RPL7P25 6	34616538	34617258	+	721	processed_pseudogene	ribosomal protein L7 pseudogene 25 [Source:HGNC Symbol;Acc:HGNC:36455]	-	0	0	0	8	8	25	0	0	0	0.616547819	0.682027808	1.612262535
ENSG00000182179 416.4281934	370.8129692	344.0975284	273.4956827	167.7992987	179.4289214	377.112897	206.9079676	0.866700862	0.001372423	0.016227805	UBA7	3	49805207	49813946	-	4545	protein_coding	ubiquitin like modifier activating enzyme 7 [Source:HGNC Symbol;Acc:HGNC:12471]	-	459	419	296	264	147	208	4.945468973	4.368240854	4.051955528	3.227617659	1.988066043	2.127945327
ENSG00000198815 2397.864303	1887.694662	1893.698898	3042.63947	2806.928405	2734.565774	2059.752621	2861.377883	-0.473904508	0.001387133	0.016390782	FOXJ3	1	42176539	42335877	-	6893	protein_coding	forkhead box J3 [Source:HGNC Symbol;Acc:HGNC:29178]	Fork	2643	2133	1629	2937	2459	3170	18.77662653	14.66253351	14.70346366	23.67596622

21.9279287 21.3836581
ENSG00000104221 255.8447724 288.5084199 318.5227121
421.6391774 461.1626986 537.4241252 287.6253015
473.4086671 -0.720853013 0.001393859 0.016438473 BRF2
8 37843268 37849904 - 2760
protein_coding "BRF2, RNA polymerase III transcription initiation
factor subunit [Source:HGNC Symbol;Acc:HGNC:17298]" - 282
326 274 407 404 623 5.003440157
5.596737769 6.176583648 8.194026625 8.997454171
10.49566554
ENSG00000171490 4947.239517 5160.406738 5023.126419
6138.113332 6885.478708 6669.062459 5043.590892
6564.218166 -0.380140413 0.001394267 0.016438473 RSL1D1
16 11833850 11851585 - 6167
protein_coding ribosomal L1 domain containing 1 [Source:HGNC
Symbol;Acc:HGNC:24534] - 5453 5831 4321 5925 6032
7731 43.30023295 44.80180731 43.59304085
53.38588541 60.12218348 58.28982386
ENSG00000087510 93.4468495 66.37463648 79.04943219
17.61146441 28.5372957 27.60444945 79.62363939
24.58440319 1.694912078 0.001394598 0.016438473 TFAP2C
20 56629302 56639283 + 3005
protein_coding transcription factor AP-2 gamma [Source:HGNC
Symbol;Acc:HGNC:11744] AP-2 103 75 68 17 25
32 1.678500334 1.182614577 1.407898295
0.314352189 0.511379003 0.4951497
ENSG00000112977 3193.5234 3119.607915 3915.271877
2835.445771 2244.172934 2093.624963 3409.467731
2391.081223 0.51174345 0.001394887 0.016438473 DAP
5 10679230 10761272 - 3361
protein_coding death associated protein [Source:HGNC
Symbol;Acc:HGNC:2672] - 3520 3525 3368 2737 1966
2427 51.28647314 49.6954983 62.34626153
45.24997344 35.95525401 33.57625712
ENSG00000167283 599.6928885 774.370759 859.0813293
508.660531 481.7095515 427.0063274 744.3816589
472.4588033 0.655652364 0.001395971 0.016440306 ATP5MG
11 118401154 118431496 + 3382
protein_coding ATP synthase membrane subunit g [Source:HGNC
Symbol;Acc:HGNC:14247] - 661 875 739 491 422
495 9.570982888 12.25916501 13.5949521
8.06714629 7.669838298 6.805540358
ENSG00000040531 548.8868344 774.370759 544.0460921
1075.335298 945.1552337 881.6171043 622.4345618
967.3692119 -0.634909639 0.001404721 0.016532346 CTNS
17 3636468 3661542 + 3398
protein_coding "cystinosin, lysosomal cystine transporter
[Source:HGNC Symbol;Acc:HGNC:2518]" - 605 875 468
1038 828 1022 8.718879664 12.20144086
8.568983866 16.97407132 14.97801711 13.98487341

ENSG00000141644 1700.18831 1793.00018 1618.188377
2227.332264 2390.283888 2273.053884 1703.792289
2296.890012 -0.430536146 0.00140824 0.01656274 MBD1
18 50266882 50281774 - 6734
protein_coding methyl-CpG binding domain protein 1 [Source:HGNC
Symbol;Acc:HGNC:6916] MBD 1874 2026 1392 2150 2094
2635 13.62778138 14.2558386 12.86094763
17.74097123 19.11397112 18.19443298
ENSG00000100934 1976.899855 1655.825931 2393.570307
1484.542853 1441.704179 884.2050214 2008.765364
1270.150685 0.661715038 0.001420362 0.01669421 SEC23A
14 39031919 39109646 - 8945
protein_coding "Sec23 homolog A, coat complex II component
[Source:HGNC Symbol;Acc:HGNC:10701]" - 2179 1871 2059
1433 1263 1025 11.92904203 9.911054937
14.32131339 8.901801159 8.679013539 5.328126639
ENSG00000167767 858.2594138 813.3105457 816.0691382
664.0558052 485.134027 435.6327179 829.2130326
528.2741834 0.651188426 0.001422244 0.016705224 KRT80
12 52168996 52192000 - 4350
protein_coding keratin 80 [Source:HGNC Symbol;Acc:HGNC:27056] -
946 919 702 641 425 505 10.64953298
10.01042913 10.04048523 8.188056226 6.005470435
5.39800322
ENSG00000225648 508.0605409 506.2172276 606.8206412
311.826517 218.0249392 397.6765999 540.3661366
309.1760187 0.80215385 0.001429214 0.016775959 SBDSP1
7 72829425 72836701 + 1239
transcribed_unprocessed_pseudogene "SBDS, ribosome maturation
factor pseudogene 1 [Source:HGNC Symbol;Acc:HGNC:21646]" -
560 572 522 301 191 461 22.13326094
21.87515622 26.21235513 13.49917517 9.475658949
17.30057897
ENSG00000137876 2695.44262 3169.167643 3514.212258
4012.305981 4382.187128 4398.596492 3126.274174
4264.3632 -0.448243473 0.001433481 0.016803475
RSL24D1 15 55180806 55197067 - 3009
protein_coding ribosomal L24 domain containing 1 [Source:HGNC
Symbol;Acc:HGNC:18479] - 2971 3581 3023 3873 3839
5099 48.35141048 56.39084132 62.50615809
71.52162165 78.4229698 78.79412595
ENSG00000136718 1800.893167 2124.873363 1859.98664
2173.461902 2818.343324 3711.935812 1928.58439
2901.247013 -0.589364432 0.001433725 0.016803475 IMP4
2 130342225 130347810 + 3861
protein_coding "IMP4, U3 small nucleolar ribonucleoprotein
[Source:HGNC Symbol;Acc:HGNC:30856]" - 1985 2401 1600
2098 2469 4303 25.17615438 29.46583817
25.78261881 30.19379665 39.30688684 51.82061497
ENSG00000103245 525.2983093 718.6160643 652.1578156

792.5158986 1085.558729 1073.985611 632.0240631
984.0200795 -0.638925533 0.001434408 0.016803475 CIAO3
16 729753 741329 - 8101 protein_coding
cytosolic iron-sulfur assembly component 3 [Source:HGNC
Symbol;Acc:HGNC:14179] - 579 812 561 765 951
1245 3.5000046 4.749455664 4.30854939
5.247287339 7.215879048 7.145979029
ENSG00000153179 534.370819 489.4023197 482.4340347
382.2723746 267.1090878 213.9344832 502.0690578
287.7719819 0.804497412 0.001442746 0.016889959 RASSF3
12 64507001 64697567 + 4226
protein_coding Ras association domain family member 3 [Source:HGNC
Symbol;Acc:HGNC:14271] - 589 553 415 369 234
248 6.825185911 6.200433862 6.109778285
4.851867331 3.40356246 2.728683761
ENSG00000167840 309.3725794 288.5084199 261.5606212
447.5383898 519.3787818 432.1821617 286.4805402
466.3664444 -0.701241636 0.001453477 0.017003458 ZNF232
17 5105541 5123116 - 2794 protein_coding zinc
finger protein 232 [Source:HGNC Symbol;Acc:HGNC:13026] zf-C2H2 341
326 225 432 455 501 5.976633995
5.52863144 5.010291142 8.591507799 10.0099603
8.337624696
ENSG00000250657 55.34230892 68.14462679 17.43737475
118.1004084 136.9790194 161.3135015 46.97477015
138.7976431 -1.554060282 0.001454363 0.017003458
AC097451.1 4 43340875 43345600 + 681
processed_transcript novel transcript - 61
77 15 114 120 187 4.386432055
5.35759714 1.370411498 9.301859664 10.83132268
12.76807758
ENSG00000088836 8.165258694 4.424975766 11.6249165
31.07905485 46.80116496 53.48362081 8.071716986
43.78794687 -2.455654818 0.001455707 0.017003984
SLC4A11 20 3227417 3239559 - 4346 protein_coding solute
carrier family 4 member 11 [Source:HGNC Symbol;Acc:HGNC:16438] -
9 5 10 30 41 62 0.101410161
0.054513833 0.143158495 0.383569067 0.579884493
0.663335111
ENSG00000130717 1014.30658 1157.57366 1052.054943
1352.974854 1521.608607 1711.475866 1074.645061
1528.686442 -0.508628288 0.001456331 0.017003984 UCK1
9 131523801 131531268 - 2688
protein_coding uridine-cytidine kinase 1 [Source:HGNC
Symbol;Acc:HGNC:14859] - 1118 1308 905 1306 1333
1984 20.36766404 23.0571126 20.94720879
26.99764959 30.48233595 34.31969515
ENSG00000154122 2540.302705 2293.022442 3487.47495
2045.001809 1873.18809 1780.486989 2773.600032
1899.558963 0.545636115 0.001457344 0.017004586 ANKH

5	14704800	14871785	-	12702
protein_coding	ANKH inorganic pyrophosphate transport regulator
[Source:HGNC Symbol;Acc:HGNC:15492]	-	2800	2591	3000
1974	1641	2064	10.79480015	9.665442586
14.69453992	8.635491948	7.941157924	7.55559382
ENSG00000119801 524.3910583	448.6925426	661.4577488
850.5301343	877.8072159	797.0784778	544.8471166
841.805276	-0.629162049	0.001462707	0.017055913	YPEL5
2	30146941	30160533	+	3421
protein_coding	yippee like 5 [Source:HGNC Symbol;Acc:HGNC:18329]
-	578	507	569	821	769	924
8.273770074	7.022331481	10.34822919	13.33527962
13.81721851	12.55885121
ENSG00000090013 897.2712054	1273.508025	1200.853874
1675.161056	1562.702313	1668.343914	1123.877702
1635.402428	-0.541570366	0.001464892	0.017060032	BLVRB
19	40447765	40465840	-	1689
protein_coding	biliverdin reductase B [Source:HGNC
Symbol;Acc:HGNC:1063]	-	989	1439	1033	1617	1369
1934	28.67446512	40.36989178	38.05200267
53.19764586	49.82200016	53.24242629
ENSG00000262001 93.4468495	64.60464618	89.51185704
294.2150526	130.1300684	163.9014186	82.52111757
196.0821799	-1.250113178	0.001466206	0.017060032
DLGAP1-AS2	18	3603000 3608336 +	3171	antisense
DLGAP1 antisense RNA 2 [Source:HGNC Symbol;Acc:HGNC:28146]
-	103	73	77	284	114	190
1.59063182	1.090819917	1.510780358	4.976616117
2.209815266	2.786046605
ENSG00000225285 12.70151352	15.0449176	10.46242485	0
0	0	12.73628533	0	6.10506556
0.001466319	0.017060032	LINC01770	1	1430539
1434573 -	851	lincRNA long intergenic non-protein coding RNA
1770 [Source:HGNC Symbol;Acc:HGNC:52560]	-	14	17
9	0	0	0	0.805614286	0.946554887
0.657990762	0	0	0
ENSG00000180370 4123.45564	3961.238305	4103.595524
5474.057527	5423.227676	4984.328404	4062.763157
5293.871202	-0.381785689	0.001466918	0.017060032	PAK2
3	196739857	196832647	+	6270
protein_coding	p21 (RAC1) activated kinase 2 [Source:HGNC
Symbol;Acc:HGNC:8591]	-	4545	4476	3530	5284	4751
5778	35.49727624	33.82587019	35.02789274
46.82818353	46.57628545	42.84903196
ENSG00000005102 1.814501932	19.46989337	20.9248497	0
0	0	14.06974833	0	6.245438762
0.001473478	0.017125067	MEOX1	17	43640388
43661954	-	2707	protein_coding	mesenchyme homeobox 1
[Source:HGNC Symbol;Acc:HGNC:7013]	Homeobox	2	22
18	0	0	0	0.036180155	0.385088781

0.413705311 0 0 0
ENSG00000174444 32366.17821 30497.81797 36625.46192
42025.09796 44207.69552 42854.18249 33163.1527
43028.99199 -0.375764933 0.001474891 0.017130232 RPL4
15 66498015 66524532 - 5637
protein_coding ribosomal protein L4 [Source:HGNC
Symbol;Acc:HGNC:10353] - 35675 34461 31506 40566 38728
49678 309.9164513 289.6718425 347.7379427
399.8767881 422.3032221 409.7765356
ENSG00000135842 1482.448078 1535.466591 1364.765197
2139.274942 1942.819092 1911.608124 1460.893289
1997.900719 -0.451154543 0.001479739 0.017170069
FAM129A 1 184790724 184974550 - 8372
protein_coding family with sequence similarity 129 member A
[Source:HGNC Symbol;Acc:HGNC:16784] - 1634 1735 1174
2065 1702 2216 9.557658658 9.81966564
8.724603998 13.70575234 12.4961922 12.30754846
ENSG00000167671 2330.727732 2716.050125 3010.853373
3118.26517 4133.34191 4208.815902 2685.877077
3820.140994 -0.508590456 0.001480262 0.017170069 UBXN6
19 4444999 4457822 - 5275 protein_coding UBX
domain protein 6 [Source:HGNC Symbol;Acc:HGNC:14928] - 2569
3069 2590 3010 3621 4879 23.84900825
27.56772103 30.548096 31.70706736 42.19426451
43.00702212
ENSG00000126351 635.9829272 493.8272954 831.1815297
313.898454 458.8797149 374.3853456 653.6639174
382.3878382 0.772923309 0.001496765 0.017340536 THRA
17 40058290 40093867 + 6959
protein_coding thyroid hormone receptor alpha [Source:HGNC
Symbol;Acc:HGNC:11796] THR-like 701 558 715 303
402 434 4.932872212 3.79938937 6.392431044
2.419400888 3.550802989 2.899839165
ENSG00000241945 12.70151352 20.35488852 211.5734803
15.53952742 11.41491828 8.626390453 81.54329411
11.86027872 2.779619223 0.001496919 0.017340536 PWP2
21 44107290 44131181 + 6922
protein_coding "PWP2, small subunit processome component [Source:HGNC
Symbol;Acc:HGNC:9711]" - 14 23 182 15 10
10 0.099043305 0.157442755 1.635861908
0.120412537 0.088800572 0.067173724
ENSG00000132254 1472.468318 1773.530287 1451.952071
1629.578442 2794.371995 2913.994695 1565.983558
2445.981711 -0.6432439 0.001499601 0.017351734 ARFIP2
11 6474683 6481479 - 5312 protein_coding ADP
ribosylation factor interacting protein 2 [Source:HGNC
Symbol;Acc:HGNC:17160] - 1623 2004 1249 1573 2448
3378 14.96198231 17.87582489 14.62888476
16.4544246 28.32700814 29.56872492
ENSG00000028310 1206.643785 1238.993214 1135.754342

1361.262602	1977.063846	1912.470763	1193.797114
1750.265737	-0.551805161	0.001499847	0.017351734	BRD9
	5	850291	892824	-	9572	protein_coding
bromodomain containing 9 [Source:HGNC Symbol;Acc:HGNC:25818]	-
	1330	1400	977	1314	1732	2217	6.804208836
	6.930296038	6.350365474	7.627895223	11.12224757
	10.76946233
ENSG00000154065 146.0674055	123.8993214	77.88694054
223.7691949	262.5431205	228.599347	115.9512225
238.3038875	-1.034375241	0.00151025	0.017460662
ANKRD29 18	23598926	23662885	-	6391
protein_coding	ankyrin repeat domain 29 [Source:HGNC
Symbol;Acc:HGNC:27110]	-	161	140	67	216	230
	265	1.233632329	1.037972049	0.652248114
1.878006008	2.212108266	1.928004639
ENSG00000027847 399.190425	484.0923488	476.6215765
697.2067971	690.602556	672.8584553	453.3014501
686.8892695	-0.599959231	0.001524692	0.01761612
B4GALT7 5	177600100	177610347	+	4927
protein_coding	"beta-1,4-galactosyltransferase 7 [Source:HGNC
Symbol;Acc:HGNC:930]"	-	440	547	410	673	605
	780	4.373194546	5.260550631	5.177357342
7.590048405	7.547796329	7.361105778
ENSG00000168906 4714.98327	5112.617	4160.557615
5542.431448	6256.51671	7712.855704	4662.719295
6503.934621	-0.480119401	0.0015303	0.017665481	MAT2A
	2	85539165	85545280	+	4487
protein_coding	methionine adenosyltransferase 2A [Source:HGNC
Symbol;Acc:HGNC:6904]	-	5197	5777	3579	5350	5481
	8941	56.71857758	61.00602647	49.62636615
66.25364192	75.08463825	92.65333231
ENSG00000101974 1975.992604	1254.923127	1578.663661
1223.478792	1006.795792	905.7709975	1603.193131
1045.348527	0.617277291	0.001531859	0.017665481	ATP11C
	X	139726346	139945276	-	7407
protein_coding	ATPase phospholipid transporting 11C [Source:HGNC
Symbol;Acc:HGNC:13554]	-	2178	1418	1358	1181	882
	1050	14.39939397	9.071107778	11.40681169
8.859711836	7.319368298	6.591404627
ENSG00000210077 160.583421	123.8993214	119.7366399
189.5822346	229.4398575	472.7261968	134.7397941
297.2494296	-1.14274386	0.00153196	0.017665481	MT-TV
	MT	1602	1670	+	69	Mt_tRNA
mitochondrially encoded tRNA valine [Source:HGNC
Symbol;Acc:HGNC:7500]	-	177	140	103	183	201
	548	125.6182848	96.14028069	92.87417749
147.3716828	179.0582464	369.285696
ENSG00000135956 1979.621608	2419.576749	2039.010354
2634.467883	3050.066165	3224.544751	2146.06957
2969.692933	-0.468521325	0.001534893	0.017687764

TMEM127 2 96248516 96265994 - 6367
protein_coding transmembrane protein 127 [Source:HGNC
Symbol;Acc:HGNC:26038] - 2182 2734 1754 2543 2672
3738 16.78218792 20.3465182 17.13963566
22.19338535 25.7957978 27.29829139
ENSG00000133424 9.07250966 10.61994184 18.5998664 0
0 0 12.76410597 0 6.104470912
0.001537975 0.017711741 LARGE1 22 33162226
33922841 - 10687 protein_coding LARGE xylosyl- and
glucuronyltransferase 1 [Source:HGNC Symbol;Acc:HGNC:6511] -
10 12 16 0 0 0 0.045821877
0.05320493 0.093147461 0 0 0
ENSG00000103194 3249.77296 3148.812755 3359.600868
3746.062078 4768.011366 4737.613637 3252.728861
4417.229027 -0.441619662 0.001546499 0.01779832 USP10
16 84699978 84779922 + 5083
protein_coding ubiquitin specific peptidase 10 [Source:HGNC
Symbol;Acc:HGNC:12608] - 3582 3558 2890 3616 4177
5492 34.50914151 33.16746464 35.37403325
39.52941214 50.51166983 50.23905214
ENSG00000182580 131.5513901 66.37463648 156.9363727
34.18696033 57.07459141 13.80222472 118.2874664
35.02125882 1.764392909 0.001556397 0.017900587 EPHB3
3 184561784 184582409 + 4532
protein_coding EPH receptor B3 [Source:HGNC Symbol;Acc:HGNC:3394]
- 145 75 135 33 50 16
1.566775546 0.784147574 1.853321287 0.404609506
0.67815265 0.16415764
ENSG00000089220 4132.52815 4501.970344 4823.177855
3160.739878 3656.198326 3257.325035 4485.892116
3358.087746 0.41775689 0.001557624 0.017903065 PEBP1
12 118135858 118145584 + 1780
protein_coding phosphatidylethanolamine binding protein 1
[Source:HGNC Symbol;Acc:HGNC:8630] - 4555 5087 4149
3051 3203 3776 125.3132699 135.4154372
145.0207942 95.24325932 110.6074287 98.63771464
ENSG00000128524 748.4820469 1055.799218 902.0935203
1025.60881 1495.354295 1808.091439 902.1249283
1443.018181 -0.678035678 0.001561109 0.017921501
ATP6V1F 7 128862826 128865844 + 789
protein_coding ATPase H+ transporting V1 subunit F [Source:HGNC
Symbol;Acc:HGNC:16832] - 825 1193 776 990 1310
2096 51.20420515 71.64565893 61.19156557
69.72206398 102.0567309 123.5222783
ENSG00000047410 5820.014947 4245.32175 4591.842017
4078.607964 3236.129333 2735.428413 4885.726238
3350.055237 0.544634803 0.001562266 0.017921501 TPR
1 186311652 186375693 - 12350
protein_coding "translocated promoter region, nuclear basket protein
[Source:HGNC Symbol;Acc:HGNC:12017]" - 6415 4797 3950

3937 2835 3171 25.43656053 18.40471945
19.89926267 17.71374958 14.11020963 11.93878971
ENSG00000148308 1200.293028 1428.382177 1288.040748
1630.614411 1864.056155 1999.597307 1305.571984
1831.422624 -0.488382027 0.001562268 0.017921501 GTF3C5
9 133030675 133058503 + 3592
protein_coding general transcription factor IIIC subunit 5
[Source:HGNC Symbol;Acc:HGNC:4668] - 1323 1614 1108
1574 1633 2318 18.03649724 21.29088152
19.19156004 24.34896154 27.94455621 30.00600116
ENSG00000116863 1487.891584 2149.653227 1668.175518
2193.145304 2690.496239 2875.175938 1768.573443
2586.272494 -0.548178051 0.001567067 0.017964907
ADPRHL2 1 36088875 36093932 + 1668
protein_coding ADP-ribosylhydrolase like 2 [Source:HGNC
Symbol;Acc:HGNC:21304] - 1640 2429 1435 2117 2357
3333 48.14780415 69.0014011 53.52574261
70.52398678 86.85821427 92.91167413
ENSG00000166681 1981.43611 3203.682454 2808.579826
1777.721937 1843.509302 1819.305746 2664.56613
1813.512329 0.55505244 0.001569665 0.017975038 BEX3
X 103376340 103378077 + 1080
protein_coding brain expressed X-linked 3 [Source:HGNC
Symbol;Acc:HGNC:13388] - 2184 3620 2416 1716 1615
2109 99.02789833 158.8222177 139.181022
88.28879139 91.91706138 90.79958057
ENSG00000160570 596.0638846 1054.029227 956.7306279
996.6016921 1640.323757 1780.486989 868.9412466
1472.470813 -0.761375872 0.001569983 0.017975038 DEDD2
19 42198598 42220140 - 2631
protein_coding death effector domain containing 2 [Source:HGNC
Symbol;Acc:HGNC:24450] - 657 1191 823 962 1437
2064 12.22850048 21.44950895 19.46192675
20.31731283 33.5724689 36.47706298
ENSG00000131849 354.7351277 289.3934151 244.1232465
529.3799009 455.4552394 486.5284215 296.0839297
490.4545206 -0.726433245 0.001571996 0.017986442 ZNF132
19 58432814 58440222 - 3527
protein_coding zinc finger protein 132 [Source:HGNC
Symbol;Acc:HGNC:12916] zf-C2H2 391 327 210 511 399
564 5.428751736 4.393076174 3.704423936
8.050585822 6.953681523 7.43540557
ENSG00000166402 419.1499463 373.4679546 346.4225117
278.6755251 133.5545439 186.3300338 379.6801375
199.5200343 0.927835702 0.001580791 0.018075376 TUB
11 8019244 8106112 + 6722 protein_coding tubby
bipartite transcription factor [Source:HGNC Symbol;Acc:HGNC:12406]
Tub 462 422 298 269 117 216
3.3656748 2.974680891 2.758192686 2.22364685
1.069879125 1.49412269

ENSG00000145715 2435.968844 1813.355069 1982.048263
1782.90178 1172.312108 1016.188795 2077.124058
1323.800894 0.650304234 0.001583134 0.018090476 RASA1
5 87267888 87391931 + 5232
protein_coding RAS p21 protein activator 1 [Source:HGNC
Symbol;Acc:HGNC:9871] - 2685 2049 1705 1721 1027
1178 25.13073776 18.55669646 20.27512286
18.27785294 12.06563181 10.46908132
ENSG00000167700 363.8076374 407.9827656 385.9472278
158.5031797 160.9503478 293.2972754 385.9125436
204.2502676 0.914212205 0.001584359 0.018092783 MFSD3
8 144509074 144511213 + 1823
protein_coding major facilitator superfamily domain containing 3
[Source:HGNC Symbol;Acc:HGNC:25157] - 401 461 332
153 141 340 10.77175303 11.98231404
11.33073967 4.663551809 4.754225796 8.672079819
ENSG00000198464 310.2798304 230.0987398 291.7854041
158.5031797 158.6673641 133.709052 277.3879914
150.2931986 0.884975667 0.001590291 0.018132506 ZNF480
19 52297177 52325922 + 4836
protein_coding zinc finger protein 480 [Source:HGNC
Symbol;Acc:HGNC:23305] zf-C2H2 342 260 251 153 139
155 3.463127631 2.547495917 3.229195034
1.757993165 1.766753125 1.490309343
ENSG00000196550 247.6795137 190.2739579 202.2735471
137.7838098 78.76293614 85.40126548 213.4090062
100.6493371 1.084800836 0.001590362 0.018132506 FAM72A
1 206186179 206204414 - 3783
protein_coding family with sequence similarity 72 member A
[Source:HGNC Symbol;Acc:HGNC:24044] - 273 215 174
133 69 99 3.533905967 2.692951671
2.861671337 1.953562649 1.121140676 1.216829897
ENSG00000132842 1879.824002 1369.972497 1812.324482
1342.615169 1100.398122 884.2050214 1687.37366
1109.072771 0.605736262 0.001590912 0.018132506 AP3B1
5 78000525 78294755 - 7310
protein_coding adaptor related protein complex 3 subunit beta 1
[Source:HGNC Symbol;Acc:HGNC:566] - 2072 1548 1559
1296 964 1025 13.88037047 10.03413686
13.26892028 9.851438902 8.106007797 6.519848535
ENSG00000179134 4030.916042 3850.613911 4076.858216
4947.785532 5041.969405 5751.214515 3986.12939
5246.989817 -0.39673843 0.001606804 0.018301835 SAMD4B
19 39342396 39385710 + 8057
protein_coding sterile alpha motif domain containing 4B [Source:HGNC
Symbol;Acc:HGNC:25492] - 4443 4351 3507 4776 4417
6667 27.00421973 25.58834282 27.08128383
32.93843784 33.69778816 38.47583996
ENSG00000139734 1845.348465 1509.801731 1954.148463
944.8032674 465.7286659 270.8686602 1769.76622

560.4668645	1.659123295	0.001611012	0.01832721	DIAPH3
13	59665583	60163987	-	7442
protein_coding	diaphanous related formin 3 [Source:HGNC
Symbol;Acc:HGNC:15480]	-	2034	1706	1681	912	408
314	13.38412446	10.86215015	14.05351283
6.809531235	3.369906549	1.961873498
ENSG00000166451 1127.712951	975.2646587	882.3311623
805.983489	553.6235367	342.467701	995.1029239
567.3582422	0.812046068	0.001613081	0.01832721	CENPN
16	81006498	81033114	+	3568
protein_coding	centromere protein N [Source:HGNC
Symbol;Acc:HGNC:30873]	-	1243	1102	759	778	485
397	17.0598405	14.63467862	13.23499485
12.11621008	8.355342423	5.173645641
ENSG00000204899 2427.803585	2472.676458	2597.006346
1972.484014	1907.432845	1645.052659	2499.16213
1841.656506	0.440712461	0.001613543	0.01832721	MZT1
13	72708357	72727687	-	2299
protein_coding	mitotic spindle organizing protein 1 [Source:HGNC
Symbol;Acc:HGNC:33830]	-	2676	2794	2234	1904	1671
1907	57.00012675	57.5855967	60.45761966
46.01930086	44.67708597	38.5693873
ENSG00000132646 4320.3291	4287.801517	4565.104709
3658.004756	3184.762201	3071.85764	4391.078442
3304.874865	0.409984198	0.001613548	0.01832721	PCNA
20	5114953 5126626 -	1471	protein_coding
proliferating cell nuclear antigen [Source:HGNC Symbol;Acc:HGNC:8729]
-	4762	4845	3927	3531	2790	3561
158.5277888	156.0657305	166.0944325	133.381958
116.5839836	112.5616159
ENSG00000060138 3237.978698	3655.029982	4294.244155
4792.390257	5322.776395	5105.960509	3729.084278
5073.709054	-0.444543494	0.001614212	0.01832721	YBX3
12	10699089	10723312	-	8610
protein_coding	Y-box binding protein 3 [Source:HGNC
Symbol;Acc:HGNC:2428]	CSD	3569	4130	3694	4626	4663
5919	20.29888018	22.7286343	26.69319667
29.85482437	33.28968028	31.96511025
ENSG00000173540 601.5073904	718.6160643	568.4584168
404.027713	336.7400893	449.4349426	629.5272905
396.7342483	0.665318321	0.001617207	0.01834944	GMPPB
3	49716844	49723951	-	6742
protein_coding	GDP-mannose pyrophosphorylase B [Source:HGNC
Symbol;Acc:HGNC:22932]	-	663	812	489	390	295
521	4.815633908	5.706814051	4.512601231
3.214311059	2.689556227	3.593188435
ENSG00000135052 1809.058426	1899.199599	2145.959586
1652.369749	1148.340779	1196.480356	1951.40587
1332.396961	0.550249562	0.001621898	0.018390866	GOLM1
9	86026146	86100173	-	4233

protein_coding	golgi membrane protein 1 [Source:HGNC
Symbol;Acc:HGNC:15451]	-	1994	2146	1846	1595	1006
	1387	23.06776769	24.02192961	27.13252893
20.93748256	14.60821232	15.23558768
ENSG00000138172 502.6170352	453.1175184	669.5951903
262.1000292	413.2200418	230.3246251	541.7765813
301.8815654	0.845035113	0.001624391	0.018407332	CALHM2
	10	103446786	103452402	-	3108
protein_coding	calcium homeostasis modulator family member 2
[Source:HGNC Symbol;Acc:HGNC:23493]	-	554	512	576
	253	362	267	8.728858194	7.805763551
11.53050477	4.523260028	7.159371863	3.994489366
ENSG00000100151 455.4399849	597.3717284	337.1225785
766.6166862	636.9524401	948.9029498	463.3114306
784.1573587	-0.758136024	0.001629598	0.018447962	PICK1
	22	38056311	38075701	+	4371
protein_coding	protein interacting with PRKCA 1 [Source:HGNC
Symbol;Acc:HGNC:9394]	-	502	675	290	740	558
	1100	5.624081353	7.317275506	4.127851243
9.407255875	7.846947603	11.70153664
ENSG00000142552 2320.747971	3276.252057	4167.532565
1516.657877	2618.582254	1936.624657	3254.844198
2023.954929	0.685367887	0.001630062	0.018447962	RCN3
	19	49527618	49546962	+	2093
protein_coding	reticulocalbin 3 [Source:HGNC Symbol;Acc:HGNC:21145]
	-	2558	3702	3585	1464	2294	2245
59.8494268	83.80957279	106.5679909	38.867257
67.37077533	49.8744518
ENSG00000115266 564.3101008	799.1506233	632.3954575
863.9977247	1124.369451	1074.84825	665.2853939
1021.071809	-0.61757339	0.001639494	0.018542842	APC2
	19	1446302 1473244 +	12610	protein_coding	"APC2,
WNT signaling pathway regulator [Source:HGNC Symbol;Acc:HGNC:24036]"
	-	622	903	544	834	985	1246
2.41548298	3.393119106	2.684050358	3.675047932
4.80140681	4.5944547
ENSG00000131094 799.288101	1410.682274	1278.740815
862.9617563	695.1685234	523.6219005	1162.90373
693.9173934	0.745413755	0.001648482	0.018632584	C1QL1
	17	44959693	44968071	-	1295
protein_coding	complement C1q like 1 [Source:HGNC
Symbol;Acc:HGNC:24182]	-	881	1594	1100	833	609
	607	33.3146169	58.32368953	52.84814688
35.74269347	28.90645833	21.79465209
ENSG00000125971 2514.899678	2605.425731	2923.666499
3295.415782	3978.099021	3594.616902	2681.330636
3622.710568	-0.43431527	0.001650454	0.018642964
DYNLRB1 20	34516409	34540958	+	2460
protein_coding	dynein light chain roadblock-type 1 [Source:HGNC
Symbol;Acc:HGNC:15468]	-	2772	2944	2515	3181	3485

4167 55.18064879 56.70601643 63.60770538
71.85228861 87.07932131 78.76248528
ENSG00000138764 379.2309038 243.3736671 496.3839345
218.5893524 202.0440536 159.5882234 372.9961685
193.4072098 0.946982786 0.001652801 0.018657557 CCNG2
4 77157151 77433388 + 7471
protein_coding cyclin G2 [Source:HGNC Symbol;Acc:HGNC:1593] -
418 275 427 211 177 185 2.73984648
1.744136209 3.55595278 1.569335368 1.456269957
1.151394128
ENSG00000175505 234.0707492 205.3188755 267.3730795
133.6399358 127.8470848 54.34625985 235.5875681
105.2777602 1.166107264 0.001657526 0.018694278 CLCF1
11 67364168 67374177 - 1975
protein_coding cardiotrophin like cytokine factor 1 [Source:HGNC
Symbol;Acc:HGNC:17412] - 258 232 230 129 112
63 6.397072748 5.566052851 7.245487018
3.629398488 3.485766429 1.483216225
ENSG00000188120 450.9037301 163.7241033 205.761022
138.8197783 122.1396256 92.30237784 273.4629518
117.7539273 1.21801128 0.001658167 0.018694278 DAZ1
Y 23129355 23199094 - 4787
protein_coding deleted in azoospermia 1 [Source:HGNC
Symbol;Acc:HGNC:2682] - 497 185 177 134 107
107 5.08418851 1.831195624 2.30047059
1.555440542 1.373939819 1.039324987
ENSG00000181104 2406.029562 2123.103372 3012.015865
1792.225496 1888.027484 1604.508624 2513.716266
1761.587201 0.512731048 0.001660722 0.018711152 F2R
5 76716043 76735781 + 4066
protein_coding coagulation factor II thrombin receptor [Source:HGNC
Symbol;Acc:HGNC:3537] - 2652 2399 2591 1730 1654
1860 31.93999391 27.95691941 39.64668546
23.64235709 25.0043455 21.2704456
ENSG00000131943 944.4482556 1154.918675 953.2431529
1299.104493 1465.675507 1625.211961 1017.536694
1463.330654 -0.524043372 0.001662562 0.01871996
C19orf12 19 29698886 29715789 - 5378
protein_coding chromosome 19 open reading frame 12
[Source:HGNC Symbol;Acc:HGNC:25443] - 1041 1305 820
1254 1284 1884 9.478914702 11.49783719
9.486366538 12.95653231 14.67545537 16.28887606
ENSG00000102981 129.7368881 127.439302 151.1239145
193.7261085 336.7400893 267.418104 136.1000349
265.961434 -0.96730216 0.001666029 0.018747064 PARD6A
16 67660946 67662778 + 1488
protein_coding par-6 family cell polarity regulator alpha
[Source:HGNC Symbol;Acc:HGNC:15943] - 143 144 130
187 295 310 4.706106919 4.585492651
5.435597218 6.983139517 12.18614791 9.687010729

ENSG00000155660 4615.185664 4619.674699 4777.840681
4348.995742 2203.079228 2290.306665 4670.900348
2947.460545 0.664269534 0.001667954 0.01875679 PDIA4
7 149003062 149028641 - 3277
protein_coding protein disulfide isomerase family A member 4
[Source:HGNC Symbol;Acc:HGNC:30167] - 5087 5220 4110
4198 1930 2655 76.01756949 75.47802062
78.03190816 71.1832867 36.20163854 37.67203684
ENSG00000204396 15.42326642 11.50493699 10.46242485 0
0 0 12.46354275 0 6.073603644
0.00167385 0.018811141 VWA7 6 31765590
31777294 - 3638 protein_coding von Willebrand factor
A domain containing 7 [Source:HGNC Symbol;Acc:HGNC:13939] -
17 13 9 0 0 0 0.228831027
0.169319547 0.153917025 0 0 0
ENSG00000132382 5554.190414 5702.023772 4776.678189
6232.386465 7218.794321 8823.934794 5344.297458
7425.038527 -0.474382699 0.001683348 0.01890587
MYBBP1A 17 4538897 4555631 - 6203 protein_coding MYB
binding protein 1a [Source:HGNC Symbol;Acc:HGNC:7546] - 6122
6443 4109 6016 6324 10229 48.33038198
49.21673403 41.21366216 53.89122927 62.6667887
76.67652381
ENSG00000163872 1358.154696 1113.323903 1598.426019
850.5301343 1028.484137 860.9137672 1356.634872
913.3093462 0.570624932 0.001690698 0.018976363 YEATS2
3 183697818 183812625 + 10212
protein_coding YEATS domain containing 2 [Source:HGNC
Symbol;Acc:HGNC:25489] - 1497 1258 1375 821 901
998 7.17859873 5.837088471 8.377197195
4.467292556 5.42327148 4.544130062
ENSG00000105835 3894.828397 4626.754661 4847.59018
5722.689966 6053.331165 6005.693033 4456.391079
5927.238055 -0.411671604 0.001693623 0.018997148 NAMPT
7 106248285 106286326 - 9049
protein_coding nicotinamide phosphoribosyltransferase [Source:HGNC
Symbol;Acc:HGNC:30092] - 4293 5228 4170 5524 5303
6962 23.23212757 27.37546038 28.67096519
33.92072495 36.02204788 35.77374845
ENSG00000101199 2948.565639 3479.800942 2440.069973
3362.753734 4471.223491 5351.812637 2956.145518
4395.263287 -0.57206865 0.001694895 0.018999368
ARFGAP1 20 63272785 63289793 + 6613
protein_coding ADP ribosylation factor GTPase activating protein 1
[Source:HGNC Symbol;Acc:HGNC:15852] - 3250 3932 2099
3246 3917 6204 24.0665325 28.17354051
19.74789287 27.274829 36.40846834 43.62187054
ENSG00000112299 12.70151352 14.15992245 6.974949899
64.23004668 23.97132839 75.91223598 11.27879529
54.70453702 -2.270906677 0.00171309 0.019186277 VNN1

6	132681590	132714049	-	3106
protein_coding	vanin 1 [Source:HGNC Symbol;Acc:HGNC:12705]	-
14	16	6	62	21	88	0.220726902
0.244087181	0.120186765	1.109180643	0.41559011
1.317383558
ENSG00000186352 135.1803939	130.9792827	192.9736139
50.76245625	58.21608324	84.53862644	153.0444302
64.50572197	1.240329106	0.001713738	0.019186277
ANKRD37 4	185396021	185400628	+	2580
protein_coding	ankyrin repeat domain 37 [Source:HGNC
Symbol;Acc:HGNC:29593]	-	149	148	166	49	51
98	2.828103153	2.718118899	4.003088842
1.055330325	1.215060297	1.766189863
ENSG00000137337 1868.029739	1768.220316	1961.123413
1442.068145	1343.535882	1336.227881	1865.791156
1373.943969	0.44126676	0.001720057	0.019244834	MDC1
6	30699807	30717889	-	8064
protein_coding	mediator of DNA damage checkpoint 1 [Source:HGNC
Symbol;Acc:HGNC:21163]	-	2059	1998	1687	1392	1177
1549	12.50358386	11.74008944	13.01581629
9.591814249	8.971670272	8.931654535
ENSG00000132600 774.7923249	869.9502355	943.9432197
1045.292211	1275.046372	1564.827228	862.8952601
1295.055271	-0.58683505	0.001728652	0.019328772	PRMT7
16	68310974	68358563	+	9458
protein_coding	protein arginine methyltransferase 7 [Source:HGNC
Symbol;Acc:HGNC:25557]	-	854	983	812	1009	1117
1814	4.421679342	4.924709859	5.341504101
5.927941378	7.2594083	8.918031277
ENSG00000133134 17.23776835	11.50493699	12.78740815
61.1221412	43.37668947	62.11001126	13.84337116
55.53628064	-2.004005275	0.001733972	0.019366657	BEX2
X	103309346	103311046	-	1176
protein_coding	brain expressed X-linked 2 [Source:HGNC
Symbol;Acc:HGNC:30933]	-	19	13	11	59	38
72	0.791179385	0.523796354	0.58195876
2.787768673	1.986203007	2.84679499
ENSG00000173692 3814.083061	3329.351766	3997.808784
2782.611377	2846.880619	2738.01633	3713.74787
2789.169442	0.412887456	0.001734229	0.019366657	PSMD1
2	231056864	231172827	+	5187
protein_coding	"proteasome 26S subunit, non-ATPase 1 [Source:HGNC
Symbol;Acc:HGNC:9554]"	-	4204	3762	3439	2686	2494
3174	39.68945569	34.36599986	41.24988807
28.77410367	29.55476846	28.45258258
ENSG00000110400 303.9290736	272.5785072	332.4726119
190.6182031	158.6673641	164.7640576	302.9933975
171.3498749	0.821403796	0.00173625	0.019366989
NECTIN1 11	119623408	119729084	-	7701
protein_coding	nectin cell adhesion molecule 1 [Source:HGNC

Symbol;Acc:HGNC:9706]	-	335	308	286	184	139
	191	2.130229365	1.895090847	2.310605253
1.327647382	1.109468655	1.153233533
ENSG00000116675 398.2831741	407.0977704	433.6093854
575.9984832	639.2354238	663.3694258	412.9967766
626.2011109	-0.601295704	0.001736449	0.019366989	DNAJC6
	1	65248219	65415869	+	6954
protein_coding	DnaJ heat shock protein family (Hsp40) member C6
[Source:HGNC Symbol;Acc:HGNC:15469]	-	439	460	373
	556	560	769	3.091423596	3.134365123
3.33719045	4.442752796	4.949948732	5.141888698
ENSG00000122140 904.5292131	1115.093893	940.4557448
1073.263361	1618.635412	1988.382999	986.6929503
1560.093924	-0.661158125	0.001739792	0.019392049	MRPS2
	9	135499984	135504673	+	2464
protein_coding	mitochondrial ribosomal protein S2 [Source:HGNC
Symbol;Acc:HGNC:14495]	-	997	1260	809	1036	1418
	2305	19.81450094	24.23016003	20.42747392
23.36313377	35.37389542	43.49719428
ENSG00000164054 2940.400381	3344.396684	3204.989479
3866.234423	4285.160323	4433.964693	3163.262181
4195.119813	-0.407396726	0.001741429	0.019398074	SHISA5
	3	48467798	48504826	-	4664
protein_coding	shisa family member 5 [Source:HGNC
Symbol;Acc:HGNC:30376]	-	3241	3779	2757	3732	3754
	5140	34.02899891	38.39235838	36.77774277
44.46262887	49.47469058	51.24312365
ENSG00000085563 1.814501932	0	1.16249165	41.4387398
	13.69790194	9.489029498	0.992331194	21.54189041
	-4.455558339	0.001749244	0.019472863	ABCB1	7
	87503633	87713323	-	5806	protein_coding
	ATP binding cassette subfamily B member 1 [Source:HGNC
Symbol;Acc:HGNC:40]	-	2	0	1	40	12
	11	0.016868701	0	0.010715929	0.382820339
	0.127043244	0.088094069
ENSG00000183684 2701.793377	3044.383327	3223.589345
1925.865432	2117.467341	2458.521279	2989.922016
2167.284684	0.463660281	0.001758694	0.019554257	ALYREF
	17	81887844	81891586	-	1574
protein_coding	Aly/REF export factor [Source:HGNC
Symbol;Acc:HGNC:19071]	-	2978	3440	2773	1859	1855
	2850	92.65068807	103.5571657	109.6104569
65.62762765	72.44135183	84.19206275
ENSG00000078808 4017.307277	4819.683604	5411.39863
3349.286144	3643.641916	3460.045211	4749.46317
3484.324423	0.446698538	0.001758767	0.019554257	SDF4
	1	1216908 1232031 -	4709	protein_coding
stromal cell derived factor 4 [Source:HGNC Symbol;Acc:HGNC:24188]
	-	4428	5446	4655	3233	3192	4011
46.04766421	54.79934751	61.50321825	38.14951823

41.66597534	39.60545342
ENSG00000139998 975.2947884	848.7103518	674.2451569
561.4949242	593.5757507	417.5172979	832.7500991
524.1959909	0.669994556	0.001763697	0.019596759	RAB15
14	64945814	64973226	-	5015
protein_coding	"RAB15, member RAS oncogene family [Source:HGNC
Symbol;Acc:HGNC:20150]" -	1075	959	580	542	520
484	10.49702449	9.06095786	7.195548467
6.005378611	6.373526069	4.487510135
ENSG00000136932 164.2124248	155.7591469	160.4238477
283.8553676	335.5985975	245.8521279	160.1318065
288.4353643	-0.847662594	0.001772773	0.019685233	TRMO
9	97904489	97922570	-	4203
protein_coding	tRNA methyltransferase O [Source:HGNC
Symbol;Acc:HGNC:30967]	-	181	176	138	274	294
285	2.108860578	1.984173836	2.042803264
3.622456813	4.299671741	3.152945676
ENSG00000113312 1371.763461	1360.23755	1263.628423
1688.628647	1795.566646	2003.910502	1331.876478
1829.368598	-0.457889679	0.001777279	0.019722699	TTC1
5	160009113	160065543	+	1938
protein_coding	tetratricopeptide repeat domain 1 [Source:HGNC
Symbol;Acc:HGNC:12391]	-	1512	1537	1087	1630	1573
2323	38.20557163	37.57911392	34.89656003
46.73539045	49.89101163	55.73480105
ENSG00000143458 242.2360079	287.6234248	170.8862725
342.9055718	391.5316971	554.6769061	233.5819017
429.704725	-0.878015408	0.001778377	0.019722699	GABPB2
1	151070578	151125542	+	9189
protein_coding	GA binding protein transcription factor subunit beta 2
[Source:HGNC Symbol;Acc:HGNC:28441]	-	267	325	147
331	343	643	1.4228912	1.675874722
0.995304413	2.001574622	2.294421633	3.253671772
ENSG00000186815 1344.545932	1101.818966	906.7434869
736.5735999	773.9314595	734.1058275	1117.702795
748.203629	0.580042496	0.001789113	0.019829338	TPCN1
12	113221050	113298585	+	8728
protein_coding	two pore segment channel 1 [Source:HGNC
Symbol;Acc:HGNC:18182]	-	1482	1245	780	711	678
851	8.31499801	6.75897834	5.560152608
4.526545673	4.774878405	4.533627569
ENSG00000171940 1669.341777	1305.367851	2113.40982
1199.651517	1216.830289	873.8533529	1696.039816
1096.778386	0.628934867	0.001791602	0.019844218	ZNF217
20	53567065	53609907	-	6181
protein_coding	zinc finger protein 217 [Source:HGNC
Symbol;Acc:HGNC:13009]	zf-C2H2 1840	1475	1818	1158	1066
1013	14.57765819	11.3073209	18.29961622
10.41026679	10.60097527	7.620469337
ENSG00000130803 889.1059467	961.9897314	882.3311623

1276.313186	1278.470848	1241.337586	911.1422801
1265.373873	-0.473420888	0.001792699	0.019844218	ZNF317
	19	9140380 9163424 +	5065	protein_coding	zinc
finger protein 317 [Source:HGNC Symbol;Acc:HGNC:13507]	zf-C2H2 980
	1087	759	1232	1120	1439	9.474914716
10.16895991	9.323289564	13.51584809	13.59208035
13.21029033
ENSG00000073008 2284.457932	2683.305304	2293.596025
3162.811815	3135.678052	3444.517708	2420.453087
3247.669192	-0.424051279	0.001803176	0.019947706	PVR
	19	44643798	44663583	+	3519
protein_coding	poliovirus receptor [Source:HGNC Symbol;Acc:HGNC:9705]
	-	2518	3032	1973	3053	2747	3993
35.04008431	40.82595673	34.88306723	48.20805181
47.98292877	52.76076227
ENSG00000163171 1653.01126	1357.582565	1465.901971
1298.068524	777.355935	763.4355551	1492.165265
946.2866714	0.657318038	0.001806034	0.019966832
CDC42EP3	2	37641882	37738468	-	10406
	protein_coding	CDC42 effector protein 3 [Source:HGNC
Symbol;Acc:HGNC:16943]	-	1822	1534	1261	1253	681
	885	8.574192598	6.985025226	7.539423026
6.690819178	4.022635209	3.954489869
ENSG00000175832 1032.451599	903.5800513	1045.079993
657.8399943	631.244981	758.2597208	993.7038813
682.448232	0.540877153	0.001807896	0.019974936	ETV4
	17	43527844	43579620	-	3607
protein_coding	ETS variant 4 [Source:HGNC Symbol;Acc:HGNC:3493]
	ETS	1138	1021	899	635	553	879
15.44986907	13.41238591	15.5067361	9.782269573
9.423806263	11.33114379
ENSG00000175104 249.4940156	246.0286526	281.3229793
390.5601226	448.6062885	421.8304931	258.9485492
420.3323014	-0.699813114	0.001816526	0.020057757	TRAF6
	11	36487027	36510272	-	4871
protein_coding	TNF receptor associated factor 6 [Source:HGNC
Symbol;Acc:HGNC:12036]	-	275	278	242	377	393
	489	2.764669668	2.704289035	3.091036141
4.300661532	4.959315991	4.667902193
ENSG00000173068 544.3505796	427.452659	554.508517
286.9632731	377.8337951	207.0333709	508.7705852
290.6101464	0.810148664	0.001818372	0.020065619	BNC2
	9	16409503	16870843	-	15323
protein_coding	basonuclin 2 [Source:HGNC Symbol;Acc:HGNC:30988]
	zf-C2H2 600	483	477	277	331	240
1.917503354	1.493584404	1.93678505	1.004495536
1.3277966	0.728280125
ENSG00000177971 563.4028499	747.8209044	776.5444221
803.911552	1328.696488	1199.930912	695.9227255
1110.846317	-0.675138493	0.001820898	0.020080962	IMP3

15	75639085	75648706	-	2565
protein_coding	"IMP3, U3 small nucleolar ribonucleoprotein
[Source:HGNC Symbol;Acc:HGNC:14497]"	-	621	845	668
776	1164	1391	11.85585596	15.60974399
16.20301894	16.81072313	27.89413966	25.21568547
ENSG00000129250 4002.791262	3465.64102	3209.639445
2702.841803	2604.884352	2647.43923	3559.357242
2651.721795	0.424913453	0.001824326	0.020106232	KIF1C
17	4997948 5028401 +	8580	protein_coding
kinesin family member 1C [Source:HGNC Symbol;Acc:HGNC:6317]	-
4412	3916	2761	2609	2282	3069	25.18122766
21.62628072	20.02100921	16.89658101	16.34841721
16.63185227
ENSG00000011007 2732.63991	2551.441026	2555.156646
3554.407906	3293.203924	3439.341874	2613.079194
3428.984568	-0.392006309	0.001826668	0.020110367	ELOA
1	23743155	23762059	+	5391
protein_coding	elongin A [Source:HGNC Symbol;Acc:HGNC:11620]	-
3012	2883	2198	3431	2885	3987	27.35988825
25.33971648	25.36677185	35.36416676	32.89454214
34.38807949
ENSG00000151012 586.084124	827.4704682	691.6825317
912.688244	1438.279703	991.172263	701.745708
1114.046737	-0.665972914	0.001826975	0.020110367
SLC7A11 4	138164097	138242349	-	9645
protein_coding	solute carrier family 7 member 11 [Source:HGNC
Symbol;Acc:HGNC:11059]	-	646	935	595	881	1260
1149	3.279887665	4.593416433	3.83814679
5.075580499	8.030002368	5.539222558
ENSG00000105556 570.6608576	710.651108	778.8694054
951.0190783	1107.247073	998.9360144	686.7271237
1019.067389	-0.570150868	0.001836379	0.020201309	MIER2
19	301444	344815	-	7939	protein_coding	MIER
family member 2 [Source:HGNC Symbol;Acc:HGNC:29210]	-	629
803	670	918	970	1158	3.879837417
4.792655185	5.25068358	6.425233617	7.510230953
6.782249709
ENSG00000164087 730.3370276	802.6906039	814.9066466
629.8688449	359.5699259	460.6492502	782.6447594
483.3626737	0.69456985	0.001846003	0.020294562	POC1A
3	52075253	52154690	-	2247
protein_coding	POC1 centriolar protein A [Source:HGNC
Symbol;Acc:HGNC:24488]	-	805	907	701	608	315
534	17.54371209	19.12628643	19.4098327
15.03531566	8.616975171	11.05017619
ENSG00000159399 693.139738	621.2665975	1050.892452
590.5020421	392.6731889	407.1656294	788.432929
463.4469535	0.765438406	0.001854398	0.02037419	HK2
2	74833981	74893359	+	6064
protein_coding	hexokinase 2 [Source:HGNC Symbol;Acc:HGNC:4923] -

764	702	904	570	344	472	6.169682987
5.485350212	9.275046262	5.223092374	3.486957147
3.619210341
ENSG00000131381 1118.640441	1162.883631	1022.992652
1478.327042	1507.910705	1557.063477	1101.505575
1514.433741	-0.458876416	0.001859774	0.020418662	RBSN
3	15070073	15099163	-	7921
protein_coding	"rabenosyn, RAB effector [Source:HGNC
Symbol;Acc:HGNC:20759]" -	1233	1314	880	1427	1321
1805	7.622751232	7.860348341	6.912091928
10.01050538	10.25109279	10.59566481
ENSG00000138735 151.5109113	138.944239	165.0738143
64.23004668	71.91398517	74.18695789	151.8429882
70.11032992	1.11347745	0.001860754	0.020418662	PDE5A
4	119494395	119628991	-	9775
protein_coding	phosphodiesterase 5A [Source:HGNC
Symbol;Acc:HGNC:8784]	-	167	157	142	62	63
86	0.836620282	0.76104323	0.90381267
0.35244144	0.396160475	0.409084197
ENSG00000172943 1203.014781	1163.768626	956.7306279
1557.060648	1572.975739	1525.145832	1107.838012
1551.727406	-0.485170556	0.001865276	0.020455588	PHF8
X	53936676	54048958	-	9015
protein_coding	PHD finger protein 8 [Source:HGNC
Symbol;Acc:HGNC:20672]	-	1326	1315	823	1503	1378
1768	7.202884927	6.911725195	5.679903415
9.264143918	9.395736891	9.119006972
ENSG00000141076 4000.97676	4081.597646	3991.996326
4987.152334	5686.912288	5064.553835	4024.856911
5246.206152	-0.382185518	0.00187642	0.020562221	UTP4
16	69131291	69231130	+	5681
protein_coding	"UTP4, small subunit processome component [Source:HGNC
Symbol;Acc:HGNC:1983]"	-	4410	4612	3434	4814	4982
5871	38.01390489	38.46723319	37.60818272
47.08616719	53.90465789	48.0527569
ENSG00000131711 3809.546806	2616.045673	3510.724783
2580.597521	2317.228411	2028.064395	3312.105754
2308.630109	0.520788474	0.001877641	0.020562221	MAP1B
5	72107234	72209570	+	12630
protein_coding	microtubule associated protein 1B [Source:HGNC
Symbol;Acc:HGNC:6836]	-	4199	2956	3020	2491	2030
2351	16.28063004	11.08989719	14.87683133
10.95929004	9.879615608	8.655263553
ENSG00000168135 17.23776835	15.92991276	4.6499666	0
0	0	12.60588257	0	6.092613792
0.001878487	0.020562221	KCNJ4	22	38426327
38455199	-	2065	protein_coding	potassium voltage-
gated channel subfamily J member 4 [Source:HGNC Symbol;Acc:HGNC:6265]
-	19	18	4	0	0	0
0.450569955	0.413027425	0.120516575	0	0	0

ENSG00000233117 461.7907417 339.8381388 463.8341683
271.4237457 182.6386925 267.418104 421.8210163
240.4935141 0.808128114 0.001880343 0.020569818
LINC00702 10 4201141 4243912 - 6785 lincRNA long
intergenic non-protein coding RNA 702 [Source:HGNC
Symbol;Acc:HGNC:44676] - 509 384 399 262 160
310 3.673640158 2.681685311 3.658726031
2.145672736 1.449497567 2.124432125
ENSG00000121210 244.9577608 211.5138416 242.9607548
158.5031797 90.17785443 47.44514749 233.1441191
98.70872721 1.243123998 0.001886446 0.020623816
TMEM131L 4 153466346 153636711 + 6393
protein_coding transmembrane 131 like [Source:HGNC
Symbol;Acc:HGNC:29146] - 270 239 209 153 79
55 2.068177187 1.771412224 2.033988196
1.329838096 0.759573399 0.400026722
ENSG00000169554 2399.678805 2160.273169 2469.132264
1881.318787 1668.861053 1631.250435 2343.028079
1727.143425 0.439883067 0.001890143 0.020651478 ZEB2
2 144364364 144524583 - 25058
protein_coding zinc finger E-box binding homeobox 2 [Source:HGNC
Symbol;Acc:HGNC:14881] zf-C2H2 2645 2441 2124 1816 1462
1891 5.169016935 4.615808848 5.273694332
4.02699864 3.586314135 3.508941615
ENSG00000198053 296.6710659 292.0484005 624.258016
193.7261085 171.2237742 240.6762936 404.3258275
201.8753921 0.99814096 0.001893067 0.02067066 SIRPA
20 1894167 1940592 + 5068 protein_coding signal
regulatory protein alpha [Source:HGNC Symbol;Acc:HGNC:9662] -
327 330 537 187 150 279 3.159656201
3.085345292 6.592414806 2.050298264 1.819290338
2.559756268
ENSG00000273270 134.273143 145.1392051 160.4238477
217.5533839 285.372957 309.6874173 146.6120653
270.8712527 -0.887510067 0.001905908 0.020798034
AC090114.2 7 128524016 128531069 - 7054
lincRNA novel transcript - 148 164 138
210 250 359 1.02743639 1.101627664
1.217167865 1.654229963 2.178471655 2.366410106
ENSG00000131165 4382.022166 5707.333742 4966.164328
5498.920771 7626.306904 8495.269318 5018.506745
7206.832331 -0.522161833 0.001931808 0.021061089 CHMP1A
16 89644431 89657845 - 3208
protein_coding charged multivesicular body protein 1A [Source:HGNC
Symbol;Acc:HGNC:8740] - 4830 6449 4272 5308 6681
9848 73.72952818 95.25427111 82.85214013
91.94086772 128.013117 142.7396733
ENSG00000066557 1091.422912 808.0005748 1007.88026
747.9692533 606.1321608 552.088989 969.1012491
635.396801 0.609225468 0.001932396 0.021061089 LRRC40

1 70144805 70205620 - 2884
protein_coding leucine rich repeat containing 40 [Source:HGNC
Symbol;Acc:HGNC:26004] - 1203 913 867 722 531
640 20.42673971 15.00036968 18.7038361
13.91086017 11.31739894 10.31848022
ENSG00000187801 54.43505796 39.82478189 30.2247829
154.3593057 115.2906746 75.91223598 41.49487425
115.1874055 -1.466750581 0.001934746 0.02107372 ZFP69B
1 40450102 40463718 + 2386
protein_coding ZFP69 zinc finger protein B [Source:HGNC
Symbol;Acc:HGNC:28053] zf-C2H2 60 45 26 149 101
88 1.231429334 0.893652172 0.677968873
3.4699872 2.601946093 1.714917574
ENSG00000175213 356.5496296 505.3322324 454.5342351
591.5380106 712.2909008 778.1004188 438.8053657
693.9764434 -0.661884449 0.001936288 0.021077539 ZNF408
11 46700818 46705912 + 3078
protein_coding zinc finger protein 408 [Source:HGNC
Symbol;Acc:HGNC:20041] zf-C2H2 393 571 391 571 624
902 6.252484423 8.790102383 7.903418669
10.30812168 12.46129951 13.62601743
ENSG00000166881 1682.950542 1254.038132 1449.627087
1249.378005 803.610247 723.754159 1462.205254
925.5808036 0.660085601 0.001942934 0.02113688 NEMP1
12 57055643 57088063 - 5708
protein_coding nuclear envelope integral membrane protein 1
[Source:HGNC Symbol;Acc:HGNC:29001] - 1855 1417 1247
1206 704 839 15.91434003 11.76284371
13.59218684 11.74019707 7.58116685 6.834535671
ENSG00000203434 5.443505796 4.424975766 1.16249165
22.79130689 53.65011592 18.978059 3.67699107
31.80649394 -3.082461993 0.001950643 0.021207701
AL353740.1 10 108547975 108561815 - 575
lincRNA novel transcript - 6 5 1
22 47 22 0.510989633 0.412029774
0.108202925 2.126017719 5.024320943 1.779040579
ENSG00000271216 182.3574442 166.3790888 86.02438209
229.9850059 337.8815811 320.9017248 144.920305
296.256104 -1.027247208 0.001953316 0.021223714
LINC01050 13 42810366 42812562 - 760
lincRNA long intergenic non-protein coding RNA 1050
[Source:HGNC Symbol;Acc:HGNC:49044] - 201 188 74
222 296 372 12.95123396 11.72116279
6.05794009 16.23122977 23.94007348 22.75937679
ENSG00000154898 32.66103478 23.00987398 17.43737475
4.14387398 0 0 24.36942783 1.381291327
4.160611959 0.001964231 0.021329213 CCDC144CP 17
20321164 20403557 + 8145
transcribed_processed_pseudogene "coiled-coil domain containing
144C, pseudogene [Source:HGNC Symbol;Acc:HGNC:29073]" - 36

26	15	4	0	0	0.216441281
0.151254638	0.114579525	0.027288581	0	0
ENSG00000130724 1680.228789	2132.838319	1984.373246
2157.922375	2974.727704	3341.001022	1932.480118
2824.550367	-0.547815408	0.0019661	0.02133641	CHMP2A
19	58551566	58555124	-	2129
protein_coding	charged multivesicular body protein 2A [Source:HGNC
Symbol;Acc:HGNC:30216]	-	1852	2410	1707	2083	2606
3873	42.59847035	53.63741286	49.88439464
54.36578361	75.23953627	84.58685029
ENSG00000123472 1605.83421	1691.225738	1529.839011
1128.169691	1208.839846	1203.381468	1608.96632
1180.130335	0.447436781	0.001967643	0.021340056	ATPAF1
1	46632737	46673867	-	5677
protein_coding	ATP synthase mitochondrial F1 complex assembly factor
1 [Source:HGNC Symbol;Acc:HGNC:18803]	-	1770	1911	1316
1089	1059	1395	15.2680318	15.95027715
14.42260939	10.65911209	11.46632972	11.42579247
ENSG00000101474 2366.110519	2569.14093	2714.418002
1841.951984	1525.033082	2099.663436	2549.889817
1822.216168	0.483915891	0.001969243	0.021344329	APMAP
20	24962925	24992979	-	2454
protein_coding	adipocyte plasma membrane associated protein
[Source:HGNC Symbol;Acc:HGNC:13238]	-	2608	2903	2335
1778	1336	2434	52.04292677	56.05300735
59.19965464	40.25958019	33.46410852	46.11869753
ENSG00000120539 725.8007728	549.5819901	582.4083166
456.8621063	355.0039586	357.1325647	619.2636932
389.6662099	0.668801936	0.001975086	0.021394542	MASTL
10	27154824	27186924	+	3631
protein_coding	microtubule associated serine/threonine kinase like
[Source:HGNC Symbol;Acc:HGNC:19042]	-	800	621	501
441	311	414	10.78927895	8.103857433
8.584565597	6.748766219	5.264795424	5.30157745
ENSG00000116489 8376.648169	6612.683784	7291.147628
6143.293175	5310.219985	5090.433006	7426.826527
5514.648722	0.42954629	0.001980691	0.021438056	CAPZA1
1	112619173	112671619	+	4130
protein_coding	capping actin protein of muscle Z-line subunit alpha 1
[Source:HGNC Symbol;Acc:HGNC:1488]	-	9233	7472	6272
5930	4652	5901	109.4766419	85.72613669
94.48499504	79.78416154	69.23680434	66.43647494
ENSG00000135775 775.6995759	806.2305845	723.0698062
1233.838477	1061.5874	1008.425044	768.3333222
1101.283641	-0.518653358	0.00198167	0.021438056	COG2
1	230642489	230693982	+	10938
protein_coding	component of oligomeric golgi complex 2 [Source:HGNC
Symbol;Acc:HGNC:6546]	-	855	911	622	1191	930
1169	3.827867348	3.946452671	3.538012087
6.050425059	5.226276585	4.969441818

ENSG00000138398 1786.377152 1376.167463 1408.93988
1195.507643 979.3999886 990.309624 1523.828165
1055.072419 0.530680773 0.001982738 0.021438056 PPIG
2 169584340 169641406 + 7368
protein_coding peptidylprolyl isomerase G [Source:HGNC
Symbol;Acc:HGNC:14650] - 1969 1555 1212 1154 858
1148 13.08653836 10.00016618 10.2343402
8.702985006 7.157890179 7.244748089
ENSG00000108828 5491.590097 5730.343616 7062.136773
8216.266133 8908.202227 7523.937753 6094.690162
8216.135371 -0.431043996 0.001994706 0.021551771 VAT1
17 43014605 43025123 - 3501
protein_coding vesicle amine transport 1 [Source:HGNC
Symbol;Acc:HGNC:16919] - 6053 6475 6075 7931 7804
8722 84.66564996 87.63429613 107.9595382
125.8774344 137.0163866 115.8390507
ENSG00000156603 604.2291433 879.6851822 909.0684702
1017.321062 1220.254764 1488.914992 797.6609319
1242.163606 -0.640110313 0.001995692 0.021551771 MED19
11 57703714 57712323 - 1877
protein_coding mediator complex subunit 19 [Source:HGNC
Symbol;Acc:HGNC:29600] - 666 994 782 982 1069
1726 17.37555318 25.09276692 25.92085526
29.07095232 35.00747544 42.75703063
ENSG00000250786 74.39457921 106.1994184 142.9864729
40.4027713 17.12237742 48.30778654 107.8601568
35.27764509 1.602460314 0.002003239 0.021620066 SNHG18
5 9546200 9550609 + 1951 lincRNA small
nucleolar RNA host gene 18 [Source:HGNC Symbol;Acc:HGNC:49007] -
82 120 123 39 15 56 2.058189065
2.91440845 3.922425365 1.110757825 0.472586542
1.334632744
ENSG00000133703 1123.176696 860.2152888 1069.492318
768.6886232 648.3673584 649.5672011 1017.628101
688.8743942 0.56268016 0.002036474 0.021952455 KRAS
12 25204789 25250936 - 7302
protein_coding "KRAS proto-oncogene, GTPase [Source:HGNC
Symbol;Acc:HGNC:6407]" - 1238 972 920 742 568
753 8.302473529 6.307407311 7.838858867
5.646432234 4.781386679 4.794950915
ENSG00000242808 8.165258694 8.849951531 19.76235805 0
0 0 12.25918942 0 6.045360067
0.002036519 0.021952455 SOX2-OT 3 180989762
181836880 + 31629 sense_overlapping SOX2
overlapping transcript [Source:HGNC Symbol;Acc:HGNC:20209] -
9 10 17 0 0 0 0.013934318
0.014981006 0.033440311 0 0 0
ENSG00000075240 368.3438922 396.4778286 401.0596192
239.3087223 252.269694 224.2861518 388.6271133
238.6215227 0.704269309 0.002050136 0.022082428 GRAMD4

22 46576012 46679790 + 4885
protein_coding GRAM domain containing 4 [Source:HGNC
Symbol;Acc:HGNC:29113] - 406 448 345 231 221
260 4.069960076 4.345501326 4.394013366
2.627601224 2.780834007 2.474798237
ENSG00000169188 1260.171592 1683.260781 1250.841015
1903.074125 1870.905106 2227.334015 1398.091129
2000.437749 -0.516644062 0.002051401 0.022082428 APEX2
X 55000357 55009057 + 3245
protein_coding apurinic/apyrimidinic endodeoxyribonuclease 2
[Source:HGNC Symbol;Acc:HGNC:17889] - 1389 1902 1076
1837 1639 2582 20.96120417 27.77296535
20.63024648 31.45621748 31.04642602 36.997515
ENSG00000273291 15.42326642 7.079961225 13.9498998 0
0 0 12.15104248 0 6.035066757
0.002052321 0.022082428 AC092042.3 3 42809446
43055871 + 1654 protein_coding novel protein
- 17 8 12 0 0 0
0.50331758 0.22918222 0.45139068 0 0 0
ENSG00000263513 144.2529036 89.38451047 118.5741483
35.22292883 65.06503421 21.56597613 117.4038541
40.61797972 1.540292062 0.002061159 0.022164042 FAM72C
1 143955364 143971965 - 2297
protein_coding family with sequence similarity 72 member C
[Source:HGNC Symbol;Acc:HGNC:30602] - 159 101 102
34 57 25 3.389727703 2.083467928
2.762778217 0.822488748 1.525320899 0.506069346
ENSG00000113732 1030.637097 1246.073176 1212.478791
1483.506885 1663.153594 1717.514339 1163.063021
1621.391606 -0.479649573 0.002070415 0.022239191
ATP6V0E1 5 172983757 173035445 + 1623
protein_coding ATPase H+ transporting V0 subunit e1
[Source:HGNC Symbol;Acc:HGNC:863] - 1136 1408 1043
1432 1457 1991 34.27587064 41.10650713
39.98274759 49.02713801 55.18085078 57.04055708
ENSG00000162711 146.0674055 118.5893505 180.1862057
266.2439032 239.7132839 325.2149201 148.2809873
277.0573691 -0.906115395 0.002070662 0.022239191 NLRP3
1 247416156 247449108 + 4757
protein_coding NLR family pyrin domain containing 3 [Source:HGNC
Symbol;Acc:HGNC:16400] - 161 134 155 257 210
377 1.657377383 1.33474434 2.027241058
3.002009177 2.713522978 3.685014634
ENSG00000181666 341.1263632 340.723134 291.7854041
424.7470829 591.292767 555.5395452 324.5449671
523.8597984 -0.689611092 0.002072475 0.0222445 HKR1
19 37312837 37369365 + 7203
protein_coding "HKR1, GLI-Kruppel zinc finger family member
[Source:HGNC Symbol;Acc:HGNC:4928]" zf-C2H2 376 385 251
410 518 644 2.556248754 2.532641713

2.168039315	3.162878329	4.42042173	4.15722443
ENSG00000171302 2530.322944	2839.064451	2943.428858
3464.278647	3699.575015	3837.881112	2770.938751
3667.244925	-0.404610299	0.002073672	0.0222445	CANT1
	17	78991717	79009867	-	4421
protein_coding	calcium activated nucleotidase 1 [Source:HGNC
Symbol;Acc:HGNC:19721]	-	2789	3208	2532	3344	3241
	4449	30.89275804	34.38272435	35.63280679
42.02984588	45.06152407	46.79214013
ENSG00000189184 289.4130581	292.0484005	323.1726787
164.7189907	205.4685291	107.8298807	301.5447124
159.3391335	0.923753436	0.002083097	0.022324547	PCDH18
	4	137518918	137532494	-	6686
protein_coding	protocadherin 18 [Source:HGNC Symbol;Acc:HGNC:14268]
	-	319	330	278	159	180	125
2.336431186	2.338697269	2.586933532	1.321425842
1.65483041	0.869309967
ENSG00000115524 6752.66894	5313.510899	5953.119739
5151.871325	4144.756828	3240.072254	6006.433193
4178.900136	0.523593967	0.002083658	0.022324547	SF3B1
	2	197389784	197435091	-	9164
protein_coding	splicing factor 3b subunit 1 [Source:HGNC
Symbol;Acc:HGNC:10768]	-	7443	6004	5121	4973	3631
	3756	39.77329964	31.04431231	34.76774646
30.15403415	24.35502213	19.05774542
ENSG00000134996 476.3067571	433.647625	599.8456913
357.4091307	224.8738902	305.374222	503.2666912
295.8857476	0.764047948	0.002102043	0.022507891	OSTF1
	9	75088543	75147265	+	1319
protein_coding	osteoclast stimulating factor 1 [Source:HGNC
Symbol;Acc:HGNC:8510]	-	525	490	516	345	197
	354	19.49140706	17.60263668	24.33950562
14.53403973	9.180551915	12.47927872
ENSG00000133083 468.1414984	393.8228431	540.5586172
350.1573513	221.4494147	241.5389327	467.5076529
271.0485662	0.7854748	0.002115724	0.022640676	DCLK1
	13	35768652	36131306	-	14375
protein_coding	doublecortin like kinase 1 [Source:HGNC
Symbol;Acc:HGNC:2700]	-	516	445	465	338	194
	280	1.757804338	1.466825997	2.012574409
1.306534526	0.829547458	0.905693386
ENSG00000112319 303.0218226	334.5281679	215.0609552
526.2719954	447.4647966	453.7481378	284.2036486
475.82831	-0.740870359	0.002120451	0.022677542	EYA4
	6	133240598	133532120	+	8968
protein_coding	EYA transcriptional coactivator and phosphatase 4
[Source:HGNC Symbol;Acc:HGNC:3522]	-	334	378	185
	508	392	526	1.823809824	1.997204984
1.283461883	3.147604491	2.686815392	2.727226214
ENSG00000159363 1919.743044	1723.970558	2180.834335

1207.939265 1471.382967 1468.211655 1941.515979
1382.511296 0.489353346 0.002121939 0.022679747
ATP13A2 1 16985958 17011928 - 5253
protein_coding ATPase cation transporting 13A2 [Source:HGNC
Symbol;Acc:HGNC:30213] - 2116 1948 1876 1166 1289
1702 19.7259054 17.57146584 22.21939757
12.33397868 15.08317871 15.06548693
ENSG00000124659 409.1701857 522.1471403 489.4089846
584.2862311 818.4496408 825.5455663 473.5754369
742.7604794 -0.649672657 0.002127457 0.022724997 TBCC
6 42744481 42746096 - 1616
protein_coding tubulin folding cofactor C [Source:HGNC
Symbol;Acc:HGNC:11580] - 451 590 421 564 717
957 13.66670654 17.29964121 16.20867768
19.39321406 27.27251312 27.53604733
ENSG00000131149 2669.132342 2370.902015 2821.367234
1965.232235 1779.58576 2065.157874 2620.467197
1936.658623 0.435636959 0.002130031 0.022738756 GSE1
16 85169525 85676204 + 13387
protein_coding Gse1 coiled-coil protein [Source:HGNC
Symbol;Acc:HGNC:28979] - 2942 2679 2427 1897 1559
2394 10.7618786 9.482348028 11.27959119
7.874012704 7.158305218 8.315184708
ENSG00000006459 75.30183018 169.9190694 95.32431529
153.3233372 269.3920714 364.8963162 113.5150716
262.5372416 -1.208810634 0.002150794 0.022946562 KDM7A
7 140084746 140177035 - 9430
protein_coding lysine demethylase 7A [Source:HGNC
Symbol;Acc:HGNC:22224] - 83 192 82 148 236
423 0.431017678 0.964752642 0.541014626
0.872091526 1.538323485 2.085737708
ENSG00000143494 307.5580775 197.3539191 282.4854709
169.8988332 117.5736583 115.5936321 262.4658225
134.3553745 0.965981304 0.002162781 0.023060542 VASH2
1 212950520 212992037 + 9086
protein_coding vasohibin 2 [Source:HGNC Symbol;Acc:HGNC:25723] -
339 223 243 164 103 134 1.827071946
1.162943382 1.663950443 1.002958952 0.696805953
0.685745686
ENSG00000139083 797.4735991 501.7922518 628.9079826
465.1498542 327.6081547 375.2479847 642.7246112
389.3353312 0.722885758 0.00216473 0.023067416 ETV6
12 11649854 11895402 + 7075
protein_coding ETS variant 6 [Source:HGNC Symbol;Acc:HGNC:3495]
ETS 879 567 541 449 287 435
6.084026744 3.797371228 4.757487628 3.526402985
2.493462337 2.858866205
ENSG00000173531 46.26979926 46.90474312 51.1496326
65.26601518 158.6673641 161.3135015 48.10805833
128.4156269 -1.418007248 0.002166056 0.02306765 MST1

3	49683947	49689501	-	5084
protein_coding	macrophage stimulating 1 [Source:HGNC
Symbol;Acc:HGNC:7380]	-	51	53	44	63	139
187	0.491239542	0.493965672	0.538460663
0.688568342	1.680570046	1.710279471
ENSG00000135404 5060.645888	6153.3713	7627.107715
4380.074796	5007.72465	3920.694461	6280.374968
4436.164636	0.501507607	0.002181251	0.023215495	CD63
12	55725323	55729707	-	2633
protein_coding	CD63 molecule [Source:HGNC Symbol;Acc:HGNC:1692]
-	5578	6953	6561	4228	4387	4545
103.7424104	125.126072	155.0336691	89.22697367
102.4151335	80.26275202
ENSG00000126016 320.259591	251.3386235	290.6229125
137.7838098	182.6386925	160.4508624	287.4070423
160.2911216	0.842988271	0.002182633	0.023216233	AMOT
X	112774503	112840815	-	8502
protein_coding	angiomotin [Source:HGNC Symbol;Acc:HGNC:17810]	-
353	284	250	133	160	186	2.03321024
1.582791394	1.829471948	0.869245766	1.156767936
1.017238671
ENSG00000162631 204.1314673	153.9891566	173.2112558
77.69763712	102.7342645	75.04959694	177.1106266
85.16049953	1.059157122	0.002199163	0.023378003	NTNG1
1	107140007	107483458	+	9138
protein_coding	netrin G1 [Source:HGNC Symbol;Acc:HGNC:23319]	-
225	174	149	75	90	87	1.205757711
0.902245107	1.01447643	0.456060179	0.605394841
0.442689394
ENSG00000129911 1056.040124	1323.067754	1232.241149
1377.838098	1752.189956	2340.33973	1203.783009
1823.455928	-0.599665223	0.002217431	0.023545514	KLF16
19	1852399 1863568 -	2955	protein_coding
Kruppel like factor 16 [Source:HGNC Symbol;Acc:HGNC:16857]	zf-
C2H2	1164	1495	1060	1330	1535	2713	19.28964249
23.97232452	22.31799761	25.00956854	31.9299512
42.689722
ENSG00000231595 19.05227029	2.654985459	16.2748831	0
0	0	12.66071295	0	6.093566089
0.002217583	0.023545514	AC005224.1	17	14210488
14217922	+	1537	lincRNA novel transcript
-	21	3	14	0	0	0
0.669073934	0.092485538	0.566710181	0	0	0
ENSG00000174371 1776.397391	1600.956232	1492.639278
1371.622287	995.3808742	988.5843459	1623.330967
1118.529169	0.537790543	0.002230683	0.023670396	EXO1
1	241847967	241895148	+	4140
protein_coding	exonuclease 1 [Source:HGNC Symbol;Acc:HGNC:3511]
-	1958	1809	1284	1324	872	1146
23.16013198	20.70449616	19.29618833	17.77050164

12.94683174	12.87108904
ENSG00000128617 218.6474828	201.7788949	226.6858717
102.560881	128.9885766	109.5551588	215.7040831
113.7015388	0.924563609	0.002237132	0.023724592	OPN1SW
7	128772491	128775790	-	1096
protein_coding	"opsin 1, short wave sensitive [Source:HGNC
Symbol;Acc:HGNC:1012]"	-	241	228	195	99	113
127	10.7680031	9.857135661	11.0695739
5.019225226	6.337460267	5.387957792
ENSG00000196967 35.38278767	72.56960256	60.44956579
132.6039673	133.5545439	139.7475253	56.13398534
135.3020122	-1.270287306	0.002240477	0.023745833
ZNF585A 19	37106734	37172741	-	10758
protein_coding	zinc finger protein 585A [Source:HGNC
Symbol;Acc:HGNC:26305]	zf-C2H2 39	82	52	128	117
162	0.177525911	0.361167575	0.300731313
0.661135494	0.668500416	0.700187725
ENSG00000101680 1.814501932	4.424975766	2.3249833
4.14387398	14.83939377	81.9507093	2.854820332
33.64465902	-3.555578127	0.002242744	0.023755624	LAMA1
18	6941744 7117814 -	15872	protein_coding
laminin subunit alpha 1 [Source:HGNC Symbol;Acc:HGNC:6481]	-
2	5	2	4	13	95	0.006170595
0.014926734	0.007839804	0.014003622	0.050345314
0.278306256
ENSG00000174516 204.1314673	285.8534345	196.4610888
291.1071471	406.3710908	582.2813556	228.8153302
426.5865312	-0.898537847	0.00226131	0.023937947	PELI3
11	66466327	66477337	+	3926
protein_coding	pellino E3 ubiquitin protein ligase family member 3
[Source:HGNC Symbol;Acc:HGNC:30010]	-	225	323	169
281	356	675	2.806473246	3.898330609
2.678201543	3.977114261	5.573744578	7.994374621
ENSG00000177352 788.4010894	1022.169402	835.8314963
1040.112369	1437.138212	1472.52485	882.1339959
1316.59181	-0.577540664	0.002275137	0.024069913	CCDC71
3	49162535	49166321	-	1781
protein_coding	coiled-coil domain containing 71 [Source:HGNC
Symbol;Acc:HGNC:25760]	-	869	1155	719	1004	1259
1707	23.89376239	30.72872251	25.11723434
31.32433335	43.45193997	44.56568844
ENSG00000123933 581.5478692	739.855948	924.1808617
822.558985	1359.516767	1513.931524	748.5282263
1232.002426	-0.72011286	0.002288437	0.024196151	MXD4
4	2247432 2262294 -	7058	protein_coding	MAX
dimerization protein 4 [Source:HGNC Symbol;Acc:HGNC:13906]	bHLH
641	836	795	794	1191	1755	4.447388401
5.612431639	7.007971407	6.251020052	10.37235728
11.56182748
ENSG00000103021 25.40302705	13.2749273	0	56.97826722

90.17785443	66.42320649	12.89265145	71.19310938
-2.445926411	0.002297911	0.024281805	CCDC113 16
58231157	58283836	+	5927	protein_coding
coiled-coil domain containing 113 [Source:HGNC
Symbol;Acc:HGNC:25002]	-	28	15	0	55	79
77	0.231340563	0.119917557	0	0.515631934
0.819293528	0.604069372
ENSG00000123572 35.38278767	33.62981582	44.1746827
6.215810969	0	8.626390453	37.7290954	4.947400474
2.900016098	0.002304546	0.024337384	NRK	X
105822543	105958610	+	9573	protein_coding
Nik related kinase [Source:HGNC Symbol;Acc:HGNC:25391]	-
39	38	38	6	0	10	0.199501071
0.188088385	0.246968966	0.034826933	0
0.048571661
ENSG00000116132 5195.826282	5390.505478	5733.408817
7164.758111	7203.954928	6625.067868	5439.913526
6997.926969	-0.363381121	0.002308986	0.024369718	PRRX1
1	170662728	170739419	+	8246
protein_coding	paired related homeobox 1 [Source:HGNC
Symbol;Acc:HGNC:9142]	Homeobox	5727	6091	4932	6916
6311	7680	34.01046237	35.00029541	37.21230605
46.60405411	47.04377995	43.30608338
ENSG00000119986 131.5513901	145.1392051	142.9864729
191.6541716	336.7400893	271.7312993	139.892356
266.70852	-0.930518725	0.002316735	0.024436922	AVPI1
10	97677424	97687323	-	1457
protein_coding	arginine vasopressin induced 1 [Source:HGNC
Symbol;Acc:HGNC:30898]	-	145	164	123	185	295
315	4.87345695	5.333480813	5.252334857
7.055441906	12.44542765	10.05268375
ENSG00000166016 165.1196758	92.03949592	165.0738143
37.29486582	83.32890346	39.68139608	140.7443287
53.43505512	1.401896976	0.002340353	0.024671339	ABTB2
11	34150988	34358008	-	4973
protein_coding	ankyrin repeat and BTB domain containing 2
[Source:HGNC Symbol;Acc:HGNC:23842]	-	182	104	142
36	73	46	1.792179942	0.990926222
1.776547123	0.40225003	0.90230167	0.430100939
ENSG00000152518 1186.684264	1107.128937	1257.815965
874.3574097	831.0060509	848.8368206	1183.876388
851.4000937	0.475142276	0.002359176	0.0248522
ZFP36L2 2	43222402	43226609	-	3696
protein_coding	ZFP36 ring finger protein like 2 [Source:HGNC
Symbol;Acc:HGNC:1108]	Others	1308	1251	1082	844	728
984	17.33023552	16.0380583	18.21386632
12.68885772	12.10728532	12.37924488
ENSG00000146410 347.47712	274.3484975	235.9858049
173.0067386	158.6673641	141.4728034	285.9371408
157.7156354	0.860632637	0.00236032	0.0248522	MTFR2

6 136231024 136250335 - 2450
protein_coding mitochondrial fission regulator 2 [Source:HGNC
Symbol;Acc:HGNC:21115] - 383 310 203 167 139
164 7.655285166 5.995453656 5.155096509
3.787585371 3.487354332 3.112495855
ENSG00000157216 1330.937167 1240.763205 1725.137608
976.9182907 940.5892664 1059.320748 1432.279327
992.2761016 0.528261991 0.002362805 0.024863573 SSBP3
1 54225432 54413479 - 5227
protein_coding single stranded DNA binding protein 3 [Source:HGNC
Symbol;Acc:HGNC:15674] - 1467 1402 1484 943 824
1228 13.74378325 12.70930946 17.66396711
10.02469657 9.689961952 10.9238791
ENSG00000128609 1600.390704 1461.126998 1575.176186
1220.370887 1102.681106 1062.771304 1545.564629
1128.607766 0.453675341 0.002374155 0.024968149 NDUFA5
7 123536997 123558255 - 9851
protein_coding NADH:ubiquinone oxidoreductase subunit A5 [Source:HGNC
Symbol;Acc:HGNC:7688] - 1764 1651 1355 1178 966
1232 8.768936906 7.941329115 8.557872715
6.644725049 6.027596438 5.815156497
ENSG00000140830 771.1633211 757.5558511 730.0447561
1030.788652 947.4382174 1332.777325 752.9213094
1103.668065 -0.5524664 0.002376956 0.024982754 TXNL4B
16 72044289 72094431 - 3170
protein_coding thioredoxin like 4B [Source:HGNC
Symbol;Acc:HGNC:26041] - 850 856 628 995 830
1545 13.13071415 12.79501924 12.32557612
17.44117992 16.09408128 22.6621046
ENSG00000178078 356.5496296 328.3332018 358.0474282
468.2577597 561.6139795 587.4571898 347.6434199
539.109643 -0.633680487 0.002382457 0.025018499 STAP2
19 4324043 4342786 - 2766 protein_coding signal
transducing adaptor family member 2 [Source:HGNC
Symbol;Acc:HGNC:30430] - 393 371 308 452 492
681 6.957753816 6.355477341 6.927960252
9.080260389 10.93352714 11.44790335
ENSG00000269927 23.58852512 23.89486913 30.2247829
5.179842475 0 0 25.90272572 1.726614158
3.922056534 0.002383186 0.025018499 AC004817.3 14
71141125 71143253 - 2129 lincRNA novel
transcript - 26 27 26 5 0 0
0.598034681 0.600917074 0.759809174 0.13049876
0 0
ENSG00000048471 614.208904 625.6915733 630.0704742
1124.025817 1027.342645 711.6772124 623.3236505
954.3485582 -0.613699924 0.002391199 0.025087737 SNX29
16 11976737 12574289 + 11298
protein_coding sorting nexin 29 [Source:HGNC Symbol;Acc:HGNC:30542]
- 677 707 542 1085 900 825

2.934376134	2.965133723	2.984726646	5.336299685
4.896528642	3.395340988
ENSG00000240038 9.979760626	20.35488852	9.299933199
33.15099184	58.21608324	78.50015312	13.21152745
56.6224094	-2.09289343	0.002399291	0.025157711	AMY2B
1	103553815	103579534	+	7491
protein_coding	"amylase, alpha 2B (pancreatic) [Source:HGNC
Symbol;Acc:HGNC:478]"	-	11	23	8	32	51
91	0.071908722	0.145483748	0.066444194
0.23736803	0.418482922	0.564849324
ENSG00000182054 1670.249028	1732.82051	2492.382097
1338.471295	1523.891591	1046.381162	1965.150545
1302.914683	0.592957498	0.002407951	0.025233555	IDH2
15	90083045	90102504	-	2814
protein_coding	"isocitrate dehydrogenase (NADP(+)) 2, mitochondrial
[Source:HGNC Symbol;Acc:HGNC:5383]"	-	1841	1958	2144
1292	1335	1213	32.03748228	32.96970302
47.40318629	25.51234999	29.16114234	20.04323073
ENSG00000077232 2219.135863	1827.514991	1889.048931
1689.664615	1278.470848	1034.304215	1978.566595
1334.146559	0.569124099	0.002412343	0.025264616
DNAJC10 2	182716041	182794464	+	21835
protein_coding	DnaJ heat shock protein family (Hsp40) member C10
[Source:HGNC Symbol;Acc:HGNC:24637]	-	2446	2065	1625
1631	1120	1199	5.485698568	4.481189406
4.630277456	4.150618414	3.152914448	2.553271543
ENSG00000067141 1046.967615	962.8747266	1191.553941
779.0483082	787.6293614	468.4130016	1067.132094
678.3635571	0.654660439	0.002434357	0.025466601	NEO1
15	73051710	73305206	+	8117
protein_coding	neogenin 1 [Source:HGNC Symbol;Acc:HGNC:7754]	-
1154	1088	1025	752	690	543	6.962078991
6.351258516	7.856609468	5.147950215	5.225175778
3.110536499
ENSG00000090857 951.7062633	1086.774048	875.3562124
1412.025059	1348.101849	1310.34871	971.2788412
1356.825206	-0.481424086	0.002434509	0.025466601	PDPR
16	70113626	70162537	+	10385
protein_coding	pyruvate dehydrogenase phosphatase regulatory subunit
[Source:HGNC Symbol;Acc:HGNC:30264]	-	1049	1228	753
1363	1181	1519	4.946496099	5.602970124
4.511233659	7.292919144	6.990218594	6.801149025
ENSG00000110013 216.8329809	180.5390112	240.6357715
128.4600934	105.0172482	94.02765594	212.6692545
109.1683325	0.962078305	0.002435991	0.025467046	SIAE
11	124633113	124695707	-	8139
protein_coding	sialic acid acetylesterase [Source:HGNC
Symbol;Acc:HGNC:18187]	-	239	204	207	124	92
109	1.437988908	1.187642033	1.582363088
0.846569622	0.694806926	0.6227109

ENSG00000214022 2044.036426 1939.909376 1958.79843
1499.046412 1587.815133 1255.139811 1980.914744
1447.333785 0.453487297 0.002439168 0.0254852 REPIN1
7 150368189 150374044 + 4649
protein_coding replication initiator 1 [Source:HGNC
Symbol;Acc:HGNC:17922] zf-C2H2 2253 2192 1685 1447 1391
1455 23.73178084 22.34124877 22.55003424
17.29501841 18.39140652 14.55239469
ENSG00000145390 1095.959167 955.7947654 1074.142285
1628.542474 1586.673641 1234.436474 1041.965406
1483.21753 -0.509055437 0.002443762 0.025518131 USP53
4 119212587 119295517 + 11950
protein_coding ubiquitin specific peptidase 53 [Source:HGNC
Symbol;Acc:HGNC:29255] - 1208 1080 924 1572 1390
1431 4.950256612 4.282351295 4.810729219
7.30965164 7.149805954 5.568045129
ENSG00000082458 215.9257299 147.7941906 203.4360387
123.2802509 62.78205055 69.87376267 189.0519864
85.31202137 1.147577883 0.002450181 0.025570055 DLG3
X 70444861 70505490 + 7844
protein_coding discs large MAGUK scaffold protein 3 [Source:HGNC
Symbol;Acc:HGNC:2902] - 238 167 175 119 55
81 1.485826349 1.008800589 1.388057031
0.842988054 0.430995231 0.480151679
ENSG00000119333 1987.786866 2266.472587 2363.345524
1709.348017 1385.771079 1704.574753 2205.868326
1599.89795 0.462686367 0.002465439 0.025714124 WDR34
9 128633661 128656787 - 2604
protein_coding WD repeat domain 34 [Source:HGNC
Symbol;Acc:HGNC:28296] - 2191 2561 2033 1650 1214
1976 41.20311792 46.60097888 48.57393031
35.20910681 28.65662675 35.28393217
ENSG00000125650 152.4181623 180.5390112 149.9614228
190.6182031 369.8433523 374.3853456 160.9728655
311.6156337 -0.95247081 0.002474464 0.025793044 PSPN
19 6375148 6379058 - 3911 protein_coding
persephin [Source:HGNC Symbol;Acc:HGNC:9579] - 168 204
129 184 324 434 2.103536971 2.471546537
2.052148294 2.614219505 5.09218947 5.159800755
ENSG00000272325 203.2242164 315.9432697 259.2356379
102.560881 98.16829722 180.2915605 259.467708
127.0069129 1.026455029 0.002480893 0.025844824 NUDT3
6 34279679 34392674 - 9905
protein_coding nudix hydrolase 3 [Source:HGNC Symbol;Acc:HGNC:8050]
- 224 357 223 99 86 209
1.107445141 1.707812457 1.400739029 0.555383225
0.533692785 0.981121571
ENSG00000115363 685.8817303 914.1999932 1171.791583
581.1783256 576.4533732 617.6495564 923.9577688
591.7604184 0.641444558 0.002485134 0.025873766 EVA1A

2 75469302 75569722 - 2744
protein_coding "eva-1 homolog A, regulator of programmed cell death
[Source:HGNC Symbol;Acc:HGNC:25816]" - 756 1033 1008
561 505 716 13.49169057 17.83785607
22.85510768 11.3603261 11.31239683 12.13276912
ENSG00000105429 1991.41587 2193.017989 2986.441049
1323.967736 1671.144036 1858.987143 2390.291636
1618.032972 0.562147843 0.002492416 0.025934322 MEGF8
19 42325609 42378769 + 11372
protein_coding multiple EGF like domains 8 [Source:HGNC
Symbol;Acc:HGNC:3233] - 2195 2478 2569 1278 1464
2155 9.452057548 10.32501977 14.05510518
6.244620443 7.913189862 8.811329492
ENSG00000164266 0 0.884995153 0 39.36680281 0
18.978059 0.294998384 19.44828727 -5.797083149
0.002508992 0.02609145 SPINK1 5 147824568
147831786 - 1480 protein_coding "serine
peptidase inhibitor, Kazal type 1 [Source:HGNC
Symbol;Acc:HGNC:11244]" - 0 1 0 38 0
22 0 0.032015827 0 1.426704151 0
0.691181306
ENSG00000163159 1261.986094 1513.341712 1583.313627
1725.923513 2351.473166 2098.800797 1452.880478
2058.732492 -0.503065956 0.002516482 0.026153965 VPS72
1 151169987 151195321 - 4545
protein_coding vacuolar protein sorting 72 homolog [Source:HGNC
Symbol;Acc:HGNC:11644] - 1391 1710 1362 1666 2060
2433 14.98724911 17.82742687 18.64447104
20.36822356 27.85997312 24.89082202
ENSG00000198774 202.3169654 107.9694087 148.7989312
62.15810969 55.93309958 80.22543121 153.0284351
66.10554683 1.207722222 0.002524541 0.026211886 RASSF9
12 85800697 85836570 - 5682
protein_coding Ras association domain family member 9 [Source:HGNC
Symbol;Acc:HGNC:15739] - 223 122 128 60 49
93 1.921906773 1.017384325 1.401572562
0.586762114 0.530080967 0.761049206
ENSG00000185164 585.1768731 761.0958317 1053.217435
486.9051926 567.3214386 401.9897951 799.8300465
485.4054754 0.720431454 0.002525019 0.026211886 NOMO2
16 18417325 18562211 - 6801
protein_coding NODAL modulator 2 [Source:HGNC Symbol;Acc:HGNC:22652]
- 645 860 906 470 497 466
4.644250359 5.991728376 8.288239067 3.840052189
4.491909255 3.185988178
ENSG00000127554 459.9762398 565.5119028 519.6337675
300.4308635 386.9657298 273.4565774 515.0406367
320.2843902 0.687206339 0.002528036 0.02622782 GFER
16 1984207 1987749 + 2687
protein_coding "growth factor, augmenter of liver regeneration

[Source:HGNC Symbol;Acc:HGNC:4236]"	-	507	639	447
	290	339	317	9.239936284	11.26833196
10.35015142	5.997114975	7.754956963	5.485580769
ENSG00000240996 10.88701159	8.849951531	15.11239145	0
	0	0	11.61645152	0	5.969365176
0.002540748	0.026323561	AL157400.4	10	89694295
	89697928	-	667	lincRNA novel transcript
	-	12	10	13	0	0	0
0.881016609	0.710396163	1.21261899	0	0	0
ENSG00000170006 238.6070041	273.4635023	142.9864729
406.09965	406.3710908	363.1710381	218.3523264
391.880593	-0.83978429	0.002541483	0.026323561
TMEM154 4	152618632	152680165	-	11587
protein_coding	transmembrane protein 154 [Source:HGNC
Symbol;Acc:HGNC:26489]	-	263	309	123	392	356
	421	1.11151013	1.2636125	0.660451531
1.879866911	1.888540711	1.689437411
ENSG00000137252 35.38278767	46.01974796	49.98714095
8.287747959	0	11.21430759	43.79655886	6.500685183
	2.725912014	0.002541729	0.026323561	HCRTR2	6
	55106460	55282620	+	1964	protein_coding
	hypocretin receptor 2 [Source:HGNC Symbol;Acc:HGNC:4849]
	-	39	52	43	8	0	13
0.972415353	1.254550942	1.362177865	0.226339602	0
	0.307774679
ENSG00000257167 139.7166488	101.7744426	144.1489646
44.54664528	41.09370581	71.59904076	128.5466853
52.41313062	1.28744592	0.002545757	0.026349856	TMPO-
AS1	12	98512973	98516422	-	3357
antisense	TMPO antisense RNA 1 [Source:HGNC
Symbol;Acc:HGNC:44158]	-	154	115	124	43	36
	83	2.246456757	1.623203386	2.29814375
0.711752757	0.659171648	1.149629155
ENSG00000033170 1011.584827	855.7903131	1055.542418
754.1850643	433.7668947	664.2320649	974.3058527
617.3946746	0.656829681	0.002547914	0.026356756	FUT8
	14	65410592	65744121	+	6700
protein_coding	fucosyltransferase 8 [Source:HGNC
Symbol;Acc:HGNC:4019]	-	1115	967	908	728	380
	770	8.14945842	6.8387718	8.431753322
6.037659547	3.48623095	5.343759948
ENSG00000137434 66.22932052	69.9146171	52.31212425
117.0644399	117.5736583	207.0333709	62.81868729
147.223823	-1.2285942	0.002557804	0.026443597
C6orf52 6	10671418	10694797	-	884
protein_coding	chromosome 6 open reading frame 52 [Source:HGNC
Symbol;Acc:HGNC:20881]	-	73	79	45	113	103
	240	4.043889488	4.234491516	3.16713879
7.102941243	7.161967071	12.62379679
ENSG00000224032 182.3574442	261.0735702	233.6608216

370.8767212	355.0039586	413.2041027	225.6972787
379.6949275	-0.751343482	0.002565219	0.026504766
EPB41L4A-AS1	5	112160526	112164276	+	3344
	lincRNA	EPB41L4A antisense RNA 1 [Source:HGNC
Symbol;Acc:HGNC:30749]	-	201	295	201	358	311
	479	2.943462263	4.180056847	3.739698888
5.948792239	5.71664838	6.660399243
ENSG00000179954	291.2275601	192.9289434	383.6222445
155.3952742	100.4512809	172.5278091	289.2595826
142.7914547	1.01384921	0.002569583	0.02653436	SSC5D
	19	55488404	55519098	+	7314
protein_coding	scavenger receptor cysteine rich family member with 5
domains [Source:HGNC Symbol;Acc:HGNC:26641]	-	321	218
	330	150	88	200	2.149209541	1.412303315
	2.807151362	1.139589258	0.739562831	1.271469825
ENSG00000283041	3449.368173	3576.265414	4397.705912
4353.139616	6106.981281	5438.93918	3807.779833
5299.686692	-0.477184495	0.002574065	0.026549666
AC008038.1	7	133034607	133035920	-	1314
	processed_pseudogene	eukaryotic translation elongation
factor 1 gamma (	-	3802	4041	3783	4202	5350
	6305	141.6920328	145.7202562	179.1215434
177.6939848	250.2682616	223.1108772
ENSG00000140199	650.4989426	623.9215829	647.507849
859.8538508	860.6848384	1049.831718	640.6427915
923.4568024	-0.528370987	0.002574067	0.026549666
SLC12A6	15	34229996	34338060	-	10056
protein_coding	solute carrier family 12 member 6 [Source:HGNC
Symbol;Acc:HGNC:10914]	-	717	705	557	830	754
	1217	3.491584642	3.321928596	3.446170632
4.58632547	4.608859207	5.627251579
ENSG00000130726	14400.79458	16138.77161	16076.09703
16867.63903	22092.43284	24266.89898	15538.55441
21075.65695	-0.439772975	0.002586147	0.026658716	TRIM28
	19	58544091	58550722	+	4034
protein_coding	tripartite motif containing 28 [Source:HGNC
Symbol;Acc:HGNC:16384]	-	15873	18236	13829	16282	19354
	28131	192.6867297	214.2003275	213.2856954
224.2765672	294.9050456	324.2502316
ENSG00000112531	3983.738992	3217.842377	3773.447896
2914.179376	2717.892043	2587.917136	3658.343088
2739.996185	0.417025613	0.002588935	0.026671909	QKI
	6	163414000	163578596	+	17368
protein_coding	"QKI, KH domain containing RNA binding [Source:HGNC
Symbol;Acc:HGNC:21100]"	-	4391	3636	3246	2813	2381
	3000	12.38061761	9.919744925	11.62801415
8.999781495	8.426688597	8.031607237
ENSG00000157873	47.17705023	25.66485944	24.41232465
45.58261378	119.856642	144.9233596	32.41807811
103.4542051	-1.671633666	0.002592268	0.02667899

TNFRSF14 1 2555639 2565382 + 6219 protein_coding
TNF receptor superfamily member 14 [Source:HGNC
Symbol;Acc:HGNC:11912] - 52 29 21 44 105
168 0.409459989 0.220955025 0.210090098
0.393137221 1.037805821 1.256087064
ENSG00000117682 627.8176685 794.7256475 764.9195056
1000.745566 1244.226093 966.1557307 729.1542739
1070.375796 -0.553468488 0.002592639 0.02667899 DHDDS
1 26432282 26471294 + 5596
protein_coding dehydrodolichyl diphosphate synthase subunit
[Source:HGNC Symbol;Acc:HGNC:20603] - 692 898 658
966 1090 1120 6.055598492 7.603701716
7.315685625 9.59205067 11.9728117 9.30617757
ENSG00000167565 382.8599076 412.4077414 519.6337675
292.1431156 284.2314652 204.4454537 438.3004722
260.2733448 0.752592787 0.002604681 0.026787325
SERTAD3 19 40440844 40444705 - 2218
protein_coding SERTA domain containing 3 [Source:HGNC
Symbol;Acc:HGNC:17931] - 422 466 447 282 249
237 9.317075027 9.95521894 12.53870913
7.064795744 6.900573173 4.96841452
ENSG00000151247 1263.800596 1221.293311 1097.392117
975.8823222 751.1016229 765.1608332 1194.162008
830.7149261 0.524064075 0.00260843 0.026810293 EIF4E
4 98871684 98930637 - 14067
protein_coding eukaryotic translation initiation factor 4E
[Source:HGNC Symbol;Acc:HGNC:3287] - 1393 1380 944
942 658 887 4.849291739 4.648405857
4.175200669 3.721015328 2.875224539 2.931926984
ENSG00000237596 43.54804637 31.85982551 11.6249165
109.8126605 103.8757564 68.14848458 29.01092946
93.9456338 -1.683225787 0.002613154 0.026839281
AL138828.1 6 135991936 136225751 - 6585
antisense novel transcript - 48 36
10 106 91 79 0.356955552 0.259043776
0.094482433 0.894462483 0.849440519 0.557830595
ENSG00000119927 790.2155914 667.2863455 691.6825317
475.5095392 444.0403212 527.0724567 716.3948228
482.207439 0.570358853 0.002614285 0.026839281 GPAM
10 112149864 112215377 - 9808
protein_coding "glycerol-3-phosphate acyltransferase, mitochondrial
[Source:HGNC Symbol;Acc:HGNC:24865]" - 871 754 595
459 389 611 4.348769422 3.642649035
3.774360297 2.600424637 2.437903463 2.896621642
ENSG00000213614 469.0487494 578.7868301 652.1578156
225.8411319 327.6081547 431.3195226 566.664465
328.2562697 0.785389146 0.002618415 0.02685514 HEXA
15 72340919 72376476 - 12611
protein_coding hexosaminidase subunit alpha [Source:HGNC
Symbol;Acc:HGNC:4878] - 517 654 561 218 287

500	2.007565395	2.457280099	2.767707446
0.960547866	1.398877649	1.843535465
ENSG00000145868 951.7062633	793.8406523	899.768537
1259.73769	1272.763388	1162.837433	881.7718176
1231.779504	-0.482202178	0.002618866	0.02685514	FBXO38
5	148383935	148442836	+	8722
protein_coding	F-box protein 38 [Source:HGNC Symbol;Acc:HGNC:28844]
-	1049	897	774	1216	1115	1348
5.889631046	4.873071701	5.521177697	7.746928296
7.857893624	7.186291472
ENSG00000061273 1706.539067	1763.79534	1729.787575
1111.594195	1389.195555	1296.546485	1733.373994
1265.778745	0.453710778	0.002627896	0.026924997	HDAC7
12	47782722	47833132	-	9928
protein_coding	histone deacetylase 7 [Source:HGNC
Symbol;Acc:HGNC:14067]	-	1881	1993	1488	1073	1217
1503	9.278028679	9.512002835	9.324982154
6.00551142	7.534877047	7.039279835
ENSG00000261620 9.07250966	17.69990306	8.137441549	0
0	0	11.63661809	0	5.97561647
0.002628722	0.026924997	HMGN2P41	16	35802869
35803280	-	412	processed_pseudogene	high
mobility group nucleosomal binding domain 2 pseudogene 41 [Source:HGNC
Symbol;Acc:HGNC:39412]	-	10	20	7	0	0
0	1.188588345	2.300166216	1.057079549	0
0	0
ENSG00000100258 1660.269268	2008.938998	2162.234469
1423.420712	1506.769213	1245.650781	1943.814245
1391.946902	0.481911933	0.002634188	0.026954646	LMF2
22	50502949	50507691	-	3054
protein_coding	lipase maturation factor 2 [Source:HGNC
Symbol;Acc:HGNC:25096]	-	1830	2270	1860	1374	1320
1444	29.34342072	35.21950642	37.89228177
24.9994091	26.56759601	21.98513712
ENSG00000091651 885.4769428	861.100284	720.7448229
636.0846559	452.030764	542.5999595	822.4406832
543.5717931	0.597584708	0.002634665	0.026954646	ORC6
16	46689643	46698394	+	4309
protein_coding	origin recognition complex subunit 6 [Source:HGNC
Symbol;Acc:HGNC:17151]	-	976	973	620	614	396
629	11.09179941	10.69948285	8.952040576
7.917788938	5.648928165	6.787426965
ENSG00000251562 2751.69218	2607.195721	1553.088844
3502.609481	3239.553808	3524.743139	2303.992248
3422.302143	-0.570199909	0.002637606	0.02696914	MALAT1
11	65497688	65506516	+	8829
lincRNA metastasis associated lung adenocarcinoma transcript 1
[Source:HGNC Symbol;Acc:HGNC:29665]	-	3033	2946	1336
3381	2838	4086	16.82246282	15.81057507
9.41459816	21.27872969	19.758239	21.51879081

ENSG00000231638 4.53625483	35.39980612	1.16249165	0
	0	0	13.69951753	0	6.213574302
0.002640599	0.026972264	LUARIS	7	43508728
43522542	-	2916	antisense	lncRNA upregulator of
antiviral response interferon signaling [Source:HGNC
Symbol;Acc:HGNC:40992]	-	5	40	1	0	0
	0	0.083967489	0.649978382	0.021336311	0
	0	0
ENSG00000146223 7770.604524	8092.39568	7498.071142
9760.895159	9574.833455	10324.06409	7787.023782
9886.597569	-0.344391425	0.002640961	0.026972264	RPL7L1
	6	42879618	42889925	+	7809
protein_coding	ribosomal protein L7 like 1 [Source:HGNC
Symbol;Acc:HGNC:21370]	-	8565	9144	6450	9422	8388
	11968	53.71067719	55.48393259	51.38911499
67.04396964	66.02529635	71.26186364
ENSG00000244045 265.824533	346.0331049	366.1848697
485.8692241	619.8300627	473.5888359	326.0141692
526.4293742	-0.691565647	0.002654176	0.027085259
TMEM199 17	28357581	28363683	+	4620
protein_coding	transmembrane protein 199 [Source:HGNC
Symbol;Acc:HGNC:18085]	-	293	391	315	469	543
	549	3.105663002	4.010155586	4.2420465
5.640828693	7.224457062	5.525370276
ENSG00000198952 4449.158737	4694.014292	4197.757348
5199.525876	6091.000395	6180.808759	4446.976792
5823.778343	-0.389031843	0.002655087	0.027085259	SMG5
	1	156249224	156282825	-	5304
protein_coding	"SMG5, nonsense mediated mRNA decay factor
[Source:HGNC Symbol;Acc:HGNC:24644]"	-	4904	5304	3611
	5019	5336	7165	45.27679007	47.38342406
42.35754878	52.58062256	61.83860241	62.81215554
ENSG00000133247 167.8414287	242.488672	196.4610888
69.40988916	66.20652603	138.0222472	202.2637298
91.21288748	1.143426855	0.002675994	0.027270205	KMT5C
	19	55339853	55348120	+	4775
protein_coding	lysine methyltransferase 5C [Source:HGNC
Symbol;Acc:HGNC:28405]	-	185	274	169	67	58
	160	1.89726081	2.718965066	2.202014504
0.779674751	0.746624055	1.55803649
ENSG00000285644 0	0	0	6.215810969	10.27342645
	18.11541995	0	11.53488579	-6.006144273
0.0026763	0.027270205	AC108448.3	11	3057538
3064707 +	2507	antisense	"novel transcript, antisense
to CARS"	-	0	0	0	6	9	21
	0	0	0	0.13298693	0.220666057
0.389489701
ENSG00000109805 2319.84072	1778.840258	2297.0835
1251.449942	497.6904371	392.5007656	2131.921493
713.8803815	1.578527257	0.00267921	0.027284136	NCAPG

        4   17810902   17844862   +   4921
protein_coding non-SMC condensin I complex subunit G [Source:HGNC
Symbol;Acc:HGNC:24304] -   2557   2010   1976   1208   436
        455   25.44521041   19.35392854   24.98276034
13.6403531   5.446035707   4.299213866
ENSG00000121774 6868.797063   6524.184269   8057.229625
5955.782877   5285.107164   4856.657825   7150.070319
5365.849289   0.41407942   0.002698372   0.027461867
KHDRBS1 1   32013829   32060850   +   3588
protein_coding "KH RNA binding domain containing, signal transduction
associated 1 [Source:HGNC Symbol;Acc:HGNC:18116]"   -   7571
        7372   6931   5749   4630   5630   103.3307295
97.35524028   120.185012   89.03318668   79.31876012
72.96036175
ENSG00000135723 2619.233539   2622.240639   2642.34352
2134.095099   1966.79042   1899.531178   2627.939233
2000.138899   0.393945267   0.002699768   0.027461867   FHOD1
        16   67229387   67247658   -   5407
protein_coding formin homology 2 domain containing 1 [Source:HGNC
Symbol;Acc:HGNC:17905] -   2887   2963   2273   2060   1723
        2202   26.14683329   25.9658009   26.15471023
21.17009927   19.58737636   18.93616212
ENSG00000012822 276.7115446   333.6431727   292.9478958
440.2866103   460.0212068   521.8966224   301.100871
474.0681465   -0.655044599   0.002706912   0.027518712
CALCOCO1   12   53708517   53727745   -   8898
        protein_coding calcium binding and coiled-coil domain 1
[Source:HGNC Symbol;Acc:HGNC:29306]   -   305   377   252
        425   403   605   1.678557108   2.007591691
1.762036847   2.654046773   2.783940858   3.161505862
ENSG00000213073 34.47553671   35.39980612   22.08734135
66.30198367   126.7055929   77.63751407   30.65422806
90.21503023   -1.549661698   0.002717099   0.027606408
AL353625.1   6   160093082   160096212   +   3131
        transcribed_processed_pseudogene   calcineurin like EF-
hand protein 1 pseudogene [Source:NCBI gene;Acc:729603] -   38
        40   19   64   111   90   0.594332135
0.605345564   0.377552526   1.135818531   2.179150732
1.336566156
ENSG00000160285 3540.093269   3393.071417   3173.602204
2825.086086   2462.197873   1648.503216   3368.922297
2311.929058   0.543775924   0.002722648   0.027646908   LSS
        21   46188141   46228824   -   7238
protein_coding lanosterol synthase [Source:HGNC Symbol;Acc:HGNC:6708]
        -       3902   3834   2730   2727   2157   1911
26.39960141   25.09920528   23.46664021   20.93527176
18.31803677   12.27645925
ENSG00000161091 1985.065114   2349.662132   2100.622411
2610.640607   2854.871062   3229.720586   2145.116552
2898.410752   -0.434332632   0.002732973   0.027735837   MFSD12

19	3538261	3574290	-	5277	protein_coding	major facilitator superfamily domain containing 12 [Source:HGNC Symbol;Acc:HGNC:28299]	-	2188	2655	1807	2520	2501	3744	20.3043414	23.83986941	21.30482175	26.53539093	29.13224527	32.98980618
ENSG00000198373	1522.367121	1428.382177	1492.639278	2003.563069	1902.866878	1988.382999	1481.129526	1964.937649	-0.407952008	0.002745949	0.027842799	WWP2	16	69762306	69941741	+	9015	protein_coding	WW domain containing E3 ubiquitin protein ligase 2 [Source:HGNC Symbol;Acc:HGNC:16804]	-	1678	1614	1284	1934	1667	2305	9.114962977	8.483288566	8.861477504	11.9207281	11.36625065	11.88875061
ENSG00000026508	12051.92183	11204.92363	14059.17401	10334.82171	9522.324831	7856.916424	12438.67316	9238.020987	0.429186573	0.002746661	0.027842799	CD44	11	35138870	35232402	+	10004	protein_coding	CD44 molecule (Indian blood group) [Source:HGNC Symbol;Acc:HGNC:1681]	-	13284	12661	12094	9976	8342	9108	65.02552502	59.96816593	75.21476931	55.41084857	51.25589988	42.33312773
ENSG00000203923	2.721752898	1.769990306	0	8.287747959	21.68834474	37.95611799	1.497247735	22.64407023	-3.871108117	0.002770383	0.028067187	SPANXN1	X	145246828	145256112	+	1276	protein_coding	SPANX family member N1 [Source:HGNC Symbol;Acc:HGNC:33174]	-	3	2	0	8	19	44	0.115132852	0.07426869	0	0.348378509	0.915272232	1.603367293
ENSG00000113441	1144.950719	894.7300998	960.2181028	833.9546384	590.1512752	494.2921729	999.9663072	639.4660288	0.645822739	0.002783224	0.028181142	LNPEP	5	96935394	97037515	+	15266	protein_coding	leucyl and cystinyl aminopeptidase [Source:HGNC Symbol;Acc:HGNC:6656]	-	1262	1011	826	805	517	573	4.048207642	3.137995658	3.366368357	2.930101508	2.081673652	1.745260992
ENSG00000163536	156.0471661	105.3144232	155.7738811	85.98538508	42.23519764	35.36820086	139.0451568	54.52959453	1.351782965	0.002790605	0.028239707	SERPINI1	3	167735243	167825568	+	2418	protein_coding	serpin family I member 1 [Source:HGNC Symbol;Acc:HGNC:8943]	-	172	119	134	83	37	41	3.483379839	2.33193857	3.447905454	1.907365133	0.940573606	0.788421717
ENSG00000267317	393.7469192	446.0375572	352.2349699	497.2648776	624.39603	786.7268093	397.3398154	636.129239	-0.678821092	0.00279885	0.02830695	AC027307.2	19	1457670	1458580	-	911	antisense	"novel transcript, antisense to APC2"	-	434	504

303	480	547	912	23.32921019	26.21432022
20.69336405	29.27756164	36.90764287	46.54869173
ENSG00000111011 1040.616858	997.3895376	1109.017034
773.8684657	765.9410167	713.4024904	1049.00781
751.0706576	0.481984424	0.002809983	0.028403311	RSRC2
12	122503458	122527000	-	8741
protein_coding	arginine and serine rich coiled-coil 2 [Source:HGNC
Symbol;Acc:HGNC:30559]	-	1147	1127	954	747	671
827	6.425855885	6.109268838	6.790380349
4.748664917	4.718552159	4.399217228
ENSG00000115211 532.556317	659.3213891	678.8951235
775.9404027	1088.983204	942.0018374	623.5909432
935.6418147	-0.585642574	0.002813889	0.028426551	EIF2B4
2	27364352	27370486	-	5341
protein_coding	eukaryotic translation initiation factor 2B subunit
delta [Source:HGNC Symbol;Acc:HGNC:3260]	-	587	745
584	749	954	1092	5.382006362	6.609371077
6.802947443	7.79240082	10.9792622	9.506728223
ENSG00000058668 1662.08377	1342.537647	1793.724616
1257.665753	1095.832155	1026.540464	1599.448678
1126.679457	0.505250118	0.002818822	0.028460129	ATP2B4
1	203626561	203744081	+	9721
protein_coding	ATPase plasma membrane Ca2+ transporting 4
[Source:HGNC Symbol;Acc:HGNC:817]	-	1832	1517	1543
1214	960	1190	9.228756977	7.394368305
9.875562219	6.939365894	6.07026499	5.692028113
ENSG00000165661 1229.325059	1309.792827	1264.790915
977.9542592	792.1953288	952.353506	1267.9696
907.5010313	0.48196408	0.002824764	0.028503852	QSOX2
9	136206333	136245841	-	4530
protein_coding	quiescin sulfhydryl oxidase 2 [Source:HGNC
Symbol;Acc:HGNC:30249]	-	1355	1480	1088	944	694
1104	14.64771147	15.48067717	14.94299117
11.57939412	9.416914529	11.33187798
ENSG00000177602 469.0487494	400.0178092	474.2965932
314.9344225	223.7323983	286.396163	447.7877173
275.0209946	0.701645166	0.002830142	0.028541837	HASPIN
17	3723903 3726699 +	2797	protein_coding
histone H3 associated protein kinase [Source:HGNC
Symbol;Acc:HGNC:19682]	-	517	452	408	304	196
332	9.051629315	7.657242643	9.075583217
6.039391187	4.307357962	5.519206399
ENSG00000160703 360.1786335	346.0331049	426.6344355
494.1569721	696.3100152	585.7319117	377.6153913
592.0662997	-0.649633638	0.002832539	0.028549731	NLRX1
11	119166568	119184016	+	5320
protein_coding	NLR family member X1 [Source:HGNC
Symbol;Acc:HGNC:29890]	-	397	391	367	477	610
679	3.654328273	3.482503535	4.292015469
4.982172846	7.047994604	5.934568678

ENSG00000165572 777.5140778 749.5908947 711.4448897
959.3068263 1219.113272 1017.051434 746.1832874
1065.157178 -0.512613198 0.002839015 0.028598707 KBTBD6
13 41127569 41132746 - 5178
protein_coding kelch repeat and BTB domain containing 6 [Source:HGNC
Symbol;Acc:HGNC:25340] - 857 847 612 926 1068
1179 8.104896241 7.750822745 7.353535996
9.937130293 12.67816988 10.58724046
ENSG00000177613 956.2425181 1260.233098 1033.455077
1390.26972 1464.534016 1817.580468 1083.310231
1557.461401 -0.523938555 0.002849718 0.02869019 CSTF2T
10 51695487 51699591 - 4105
protein_coding cleavage stimulation factor subunit 2 tau variant
[Source:HGNC Symbol;Acc:HGNC:17086] - 1054 1424 889
1342 1283 2107 12.57349846 16.437027
13.47396597 18.16566906 19.21148142 23.86615145
ENSG00000155959 2469.537129 2223.107825 2583.056446
1995.275321 1779.58576 1570.003062 2425.2338
1781.621381 0.445048091 0.002868834 0.028865965 VBP1
X 155197007 155239817 + 2018
protein_coding VHL binding protein 1 [Source:HGNC
Symbol;Acc:HGNC:12662] - 2722 2512 2222 1926 1559
1820 66.05347077 58.98273599 68.5061781
53.03311837 47.48673536 41.93544387
ENSG00000162458 849.1869042 935.4398768 1333.377922
638.1565929 807.0347225 525.3471786 1039.334901
656.8461647 0.662260948 0.002870441 0.028865965 FBLIM1
1 15756607 15786594 + 4716
protein_coding filamin binding LIM protein 1 [Source:HGNC
Symbol;Acc:HGNC:24686] - 936 1057 1147 616 707
609 9.719204852 10.62007617 15.13200472
7.25803335 9.214949883 6.004467719
ENSG00000011275 3225.277184 3437.321175 3174.764696
3778.177101 4371.913702 5062.828557 3279.121018
4404.306453 -0.425713498 0.002873451 0.028879814 RNF216
7 5620047 5781739 - 7619 protein_coding ring
finger protein 216 [Source:HGNC Symbol;Acc:HGNC:21698] - 3555
3884 2731 3647 3830 5869 22.84916401
24.15503597 22.30132019 26.59805216 30.89926583
35.81765542
ENSG00000166848 2052.201685 2462.056516 2234.308951
2588.885269 3174.488774 3526.468417 2249.522384
3096.614153 -0.461219012 0.002923614 0.0293673
TERF2IP 16 75647786 75761872 + 3379
protein_coding TERF2 interacting protein [Source:HGNC
Symbol;Acc:HGNC:19246] - 2262 2782 1922 2499 2781
4088 32.78182234 39.01174481 35.38930536
41.09510628 50.58947323 56.25403946
ENSG00000144161 299.3928188 421.2576929 239.4732799
591.5380106 450.8892721 576.2428822 320.0412638

539.5567217	-0.751807382	0.002942247	0.029530124	ZC3H8
2	112211525	112255136	-	6600
protein_coding	zinc finger CCCH-type containing 8 [Source:HGNC
Symbol;Acc:HGNC:30941]	-	330	476	206	571	395
668	2.448491992	3.417349977	1.94191462
4.80733311	3.678752077	4.70612594
ENSG00000183496 277.6187956	198.2389143	315.0352371
125.3521879	140.4034949	158.7255843	263.6309823
141.4937557	0.895075014	0.002943163	0.029530124	MEX3B
15	82041778	82046141	-	4364
protein_coding	mex-3 RNA binding family member B [Source:HGNC
Symbol;Acc:HGNC:25297]	-	306	224	271	121	123
184	3.433723875	2.432146423	3.863593223
1.540680806	1.732478005	1.960487598
ENSG00000185480 499.8952823	386.7428819	423.1469606
338.7616978	220.3079228	137.1596082	436.5950416
232.0764096	0.913769486	0.002945162	0.029533434	PARPBP
12	102120185	102197520	+	4745
protein_coding	PARP1 binding protein [Source:HGNC
Symbol;Acc:HGNC:26074]	-	551	437	364	327	193
159	5.686487196	4.363868559	4.772786565
3.829336925	2.500163741	1.558087795
ENSG00000137154 18382.71907	24532.06564	24451.84936
27781.56713	32629.54391	29660.11829	22455.54469
30023.74311	-0.41905051	0.002949	0.029555157	RPS6
9	19375715	19380254	-	2714
protein_coding	ribosomal protein S6 [Source:HGNC
Symbol;Acc:HGNC:10429]	-	20262	27720	21034	26817	28585
34383	365.5957607	483.960396	482.1907477
549.0506276	647.4044995	589.0673366
ENSG00000147614 19.95952125	11.50493699	15.11239145
44.54664528	77.62144432	50.89570367	15.52561656
57.68793109	-1.891794455	0.002966724	0.02971596
ATP6V0D2	8	85987323	86154228	+	3010
protein_coding	ATPase H+ transporting V0 subunit d2
[Source:HGNC Symbol;Acc:HGNC:18266]	-	22	13	13
43	68	59	0.357919095	0.204646018
0.268709922	0.793805317	1.38864034	0.91141576
ENSG00000132196 408.2629347	382.3179061	373.1598196
284.8913361	149.5354295	225.1487908	387.9135535
219.8585188	0.81790191	0.002970705	0.029738989
HSD17B7 1	162790702	162812817	+	4778
protein_coding	hydroxysteroid 17-beta dehydrogenase 7 [Source:HGNC
Symbol;Acc:HGNC:5215]	-	450	432	321	275	131
261	4.612061098	4.284143824	4.179898477
3.198148254	1.685281721	2.539951244
ENSG00000121440 360.1786335	303.5533375	387.1097194
108.776692	197.4780863	245.8521279	350.2805635
184.0356354	0.926062297	0.002976197	0.029777122	PDZRN3
3	73382433	73624940	-	6136

protein_coding	PDZ domain containing ring finger 3 [Source:HGNC Symbol;Acc:HGNC:17704]	-	397	343	333	105	173	285	3.168355022	2.648714871	3.376492032	0.950858716	1.733038107	2.159685573
ENSG00000156869 221.3692357	158.4141324	229.010855	100.488944	125.5641011	56.93417699	202.9314077	94.32907403	1.109451913	0.002985083	0.029849145	FRRS1	1	99708632	99766631	-	3958
protein_coding	ferric chelate reductase 1 [Source:HGNC Symbol;Acc:HGNC:27622]	-	244	179	197	97	110	66	3.018858241	2.142908769	3.0966868	1.36178325	1.708300451	0.77535245
ENSG00000246575 3.629003864	2.654985459	4.6499666	16.57549592	18.26386925	51.75834272	3.644651974	28.86590263	-3.001881567	0.002990698	0.029888394
AC093162.2	2	85315041	85316529	+	704	unprocessed_pseudogene	"retinol saturase (all-trans-retinol 13,14-reductase) (RESAT) pseudogene"	-	4	3	4	16	16	60	0.278237726	0.201918	0.353503875	1.262872096	1.39699446	3.962868026
ENSG00000120686 2374.275778	2402.761841	2540.044255	3025.028005	3239.553808	3231.445864	2439.027291	3165.342559	-0.376255584	0.00299667	0.02993117	UFM1	13	38349849	38363619	+	4382
protein_coding	ubiquitin fold modifier 1 [Source:HGNC Symbol;Acc:HGNC:20597]	-	2617	2715	2185	2920	2838	3746	29.24556614	29.35782663	31.02315157	37.02734067	39.80956005	39.74901929
ENSG00000168710 5131.411464	4156.822234	4918.502171	3559.587748	3905.043544	3108.08848	4735.578623	3524.239924	0.426434117	0.003000034	0.0299373	AHCYL1	1	109984686	110023741	+	5113
protein_coding	adenosylhomocysteinase like 1 [Source:HGNC Symbol;Acc:HGNC:344]	-	5656	4697	4231	3436	3421	3603	54.17043108	43.52825011	51.4842131	37.34129765	41.12677372	32.76570279
ENSG00000166250 4369.320652	4517.900257	4894.089846	5968.214499	6046.482214	5652.873664	4593.770252	5889.190126	-0.358483648	0.003000669	0.0299373	CLMP	11	123069865	123195281	-	5097
protein_coding	CXADR like membrane protein [Source:HGNC Symbol;Acc:HGNC:24039]	-	4816	5105	4210	5761	5297	6553	46.2701096	47.45779474	51.38949014	62.80515406	63.87967524	59.78008834
ENSG00000185250 25.40302705	28.3198449	44.1746827	91.16522755	86.75337894	88.85182166	32.63251821	88.92347605	-1.453802845	0.003006112	0.029974699	PPIL6	6	109390215	109441171	-	4481
protein_coding	peptidylprolyl isomerase like 6 [Source:HGNC Symbol;Acc:HGNC:21557]	-	28	32	38	88	76

103	0.305993197	0.338377498	0.527613014
1.091238731	1.042523873	1.068792257
ENSG00000177370 677.7164716	885.8801483	671.9201736
927.1918029	1167.74614	1205.969385	745.1722645
1100.302443	-0.561706452	0.003009292	0.029989499	TIMM22
17	997117	1003671 +	3194	protein_coding
translocase of inner mitochondrial membrane 22 [Source:HGNC
Symbol;Acc:HGNC:17317]	-	747	1001	578	895	1023
1398	11.45287112	14.84997103	11.25899881
15.57041425	19.68738717	20.35182116
ENSG00000138944 1395.351986	1128.36882	1000.905311
787.3360561	696.3100152	910.9468318	1174.875372
798.1976344	0.557394442	0.003013795	0.030017467
SHISAL1 22	44243667	44312851	-	6751
protein_coding	shisa like 1 [Source:HGNC Symbol;Acc:HGNC:29335]
-	1538	1275	861	760	610	1056
11.15621592	8.948876562	7.934907896	6.255435176
5.554041075	7.273221743
ENSG00000119705 938.0974988	1046.064271	1028.80511
735.5376314	578.7363569	766.8861113	1004.322293
693.7200332	0.532632761	0.003016841	0.030030893	SLIRP
14	77708071	77761104	+	2631
protein_coding	SRA stem-loop interacting RNA binding protein
[Source:HGNC Symbol;Acc:HGNC:20495]	-	1034	1182	885
710	507	889	19.24546347	21.28742198
20.92807433	14.99510614	11.84498381	15.71129311
ENSG00000068784 352.0133748	275.2334926	365.0223781
204.0857935	207.7515127	169.0772529	330.7564152
193.6381864	0.772972948	0.003024032	0.030085549	SRBD1
2	45388680	45612165	-	5045
protein_coding	S1 RNA binding domain 1 [Source:HGNC
Symbol;Acc:HGNC:25521]	-	388	311	314	197	182
196	3.766164094	2.920960333	3.872356422
2.169786982	2.217469104	1.80644989
ENSG00000143740 1117.73319	1077.924097	1038.105043
1446.212019	1445.128654	1466.486377	1077.920777
1452.609017	-0.430170321	0.003028788	0.030113017	SNAP47
1	227728539	227781231	+	6580
protein_coding	synaptosome associated protein 47 [Source:HGNC
Symbol;Acc:HGNC:30669]	-	1232	1218	893	1396	1266
1700	9.168821075	8.77097424	8.443692558
11.78885343	11.82647103	12.01307106
ENSG00000109943 10.88701159	8.849951531	13.9498998	0
0	0	11.22895431	0	5.920759172
0.003030198	0.030113017	CRTAM	11	122838500
122872639	+	2675	protein_coding	cytotoxic and
regulatory T cell molecule [Source:HGNC Symbol;Acc:HGNC:24313]	-
12	10	12	0	0	0	0.219677786
0.177134296	0.279102873	0	0	0
ENSG00000276023 958.0570201	1021.284407	991.6053774

1532.197404 1284.178307 1286.194817 990.3156014
1367.523509 -0.465516218 0.003033832 0.030132208 DUSP14
17 37489831 37513501 + 2499
protein_coding dual specificity phosphatase 14 [Source:HGNC
Symbol;Acc:HGNC:17007] - 1056 1154 853 1479 1125
1491 20.6931376 21.88094092 21.23682663
32.88622028 27.67155892 27.74229627
ENSG00000165476 1750.087113 1583.256329 1708.862725
1426.528617 1142.63332 957.5293403 1680.735389
1175.563759 0.516212861 0.003037648 0.030153179 REEP3
10 63521363 63625123 + 5248
protein_coding receptor accessory protein 3 [Source:HGNC
Symbol;Acc:HGNC:23711] - 1929 1789 1470 1377 1001
1110 17.99977535 16.15261921 17.42730994
14.57981984 11.72431859 9.834678576
ENSG00000166685 684.9744793 791.1856669 806.769205
1014.213157 1280.753831 996.3480973 760.9764504
1097.105028 -0.527523171 0.003043466 0.030193982 COG1
17 73192632 73208507 + 5317
protein_coding component of oligomeric golgi complex 1 [Source:HGNC
Symbol;Acc:HGNC:6545] - 755 894 694 979 1122
1155 6.953588315 7.967045534 8.120815744
10.23123536 12.97100291 10.10058068
ENSG00000114648 1064.205383 1110.668917 1035.78006
1410.98909 1320.706045 1791.701297 1070.21812
1507.798811 -0.495068933 0.003047295 0.030215018 KLHL18
3 47282917 47346816 + 7064
protein_coding kelch like family member 18 [Source:HGNC
Symbol;Acc:HGNC:29120] - 1173 1255 891 1362 1157
2077 8.131599961 8.418204585 7.847545819
10.71367482 10.0676945 13.67152069
ENSG00000267414 4.53625483 23.89486913 6.974949899 0
0 0 11.80202462 0 5.996636775
0.003054731 0.030271784 AC120049.1 18 44676927
44679717 - 2065 lincRNA uncharacterized
LOC101927943 [Source:NCBI gene;Acc:101927943] - 5 27
6 0 0 0 0.118571041 0.619541138
0.180774863 0 0 0
ENSG00000142541 37269.86968 43445.29706 44403.69355
49983.40794 52305.43855 60407.16178 41706.28677
54232.00276 -0.378912583 0.003058393 0.030291103 RPL13A
19 49487554 49492308 + 2369
protein_coding ribosomal protein L13a [Source:HGNC
Symbol;Acc:HGNC:10304] - 41080 49091 38197 48248 45822
70026 849.1688562 981.8909541 1003.161926
1131.686925 1188.93015 1374.438389
ENSG00000108946 4497.243038 4187.797065 5390.473781
3733.630456 3674.462195 2756.994389 4691.837961
3388.362346 0.469658332 0.003064526 0.030334858
PRKAR1A 17 68511780 68551319 + 6821

protein_coding protein kinase cAMP-dependent type I regulatory subunit alpha [Source:HGNC Symbol;Acc:HGNC:9388] - 4957
4732 4637 3604 3219 3196 35.58766985
32.87177285 42.29566844 29.3595082 29.00816702
21.78661401
ENSG00000217165 108.8701159 126.5543069 88.34936539
198.905951 234.0058248 190.643229 107.9245961
207.8516683 -0.941688131 0.003070503 0.030377025
ANKRD18EP 6 39110321 39112952 + 2632
processed_pseudogene "ankyrin repeat domain 18E, pseudogene [Source:HGNC Symbol;Acc:HGNC:43609]" - 120 143 76
192 205 221 2.23266747 2.574403359
1.796530331 4.05347396 4.787572198 3.904248093
ENSG00000152475 59.87856375 133.6342681 98.81179024
165.7549592 205.4685291 257.0664355 97.44154071
209.4299746 -1.104603874 0.003083681 0.030490345 ZNF837
19 58367618 58381060 - 2076
protein_coding zinc finger protein 837 [Source:HGNC Symbol;Acc:HGNC:25164] zf-C2H2 66 151 85 160 180
298 1.556844619 3.446482193 2.5474075
4.282572039 5.329574239 6.674518375
ENSG00000165646 12.70151352 7.079961225 13.9498998 0
0 0 11.24379152 0 5.922607716
0.003097687 0.030611727 SLC18A2 10 117241073
117279430 + 4145 protein_coding solute carrier family 18 member A2 [Source:HGNC Symbol;Acc:HGNC:10935] - 14
8 12 0 0 0 0.165398735
0.091451723 0.180120672 0 0 0
ENSG00000228222 17.23776835 14.15992245 3.48747495 0
0 0 11.62838858 0 5.976461345
0.003100824 0.030615006 AC073050.1 2 167293171
167558333 + 462 lincRNA novel transcript (hCG1660379) - 19 16 3 0 0 0
2.013911162 1.640984383 0.404004429 0 0
0
ENSG00000075420 2198.269091 2023.983915 2578.406479
1894.786377 1582.107674 1289.645373 2266.886495
1588.846475 0.512813716 0.003101481 0.030615006 FNDC3B
3 172039628 172401665 + 13270
protein_coding fibronectin type III domain containing 3B [Source:HGNC Symbol;Acc:HGNC:24670] - 2423 2287 2218 1829 1386
1495 8.941516346 8.16623141 10.39914097
7.658695912 6.420068585 5.238431725
ENSG00000140941 3452.997177 3713.439662 4343.068804
5459.553968 5048.818356 4739.338915 3836.501881
5082.570413 -0.40600627 0.003111584 0.030697609
MAP1LC3B 16 87383995 87404779 + 5313
protein_coding microtubule associated protein 1 light chain 3 beta [Source:HGNC Symbol;Acc:HGNC:13352] - 3806 4196
3736 5270 4423 5494 35.07984385 37.42157864

43.74958102 55.11665377 51.17106828 48.08170475
ENSG00000033100 1305.53414 1733.705505 1554.251336
1702.096237 2321.794379 2706.961324 1531.16366
2243.617313 -0.551485478 0.003117038 0.030734277 CHPF2
7 151232489 151238827 + 4205
protein_coding chondroitin polymerizing factor 2 [Source:HGNC
Symbol;Acc:HGNC:29270] - 1439 1959 1337 1643 2034
3138 16.75804983 22.07470338 19.78209366
21.71118895 29.73256032 34.69907976
ENSG00000160447 492.6372745 531.882087 532.4211757
295.251021 377.8337951 338.1545057 518.9801791
337.079774 0.622938915 0.003126906 0.030814395 PKN3
9 128702523 128720918 + 3375
protein_coding protein kinase N3 [Source:HGNC Symbol;Acc:HGNC:17999]
- 543 601 458 285 331 392
7.87870312 8.437759365 8.44303418 4.69227143
6.028393277 5.400616115
ENSG00000134318 2196.454589 1648.74597 1643.763193
1476.255105 1255.641011 1068.809777 1829.654584
1266.901964 0.530961389 0.003129748 0.030825229 ROCK2
2 11179761 11348330 - 10145
protein_coding Rho associated coiled-coil containing protein kinase 2
[Source:HGNC Symbol;Acc:HGNC:10252] - 2421 1863 1414
1425 1100 1239 11.68614906 8.70136215
8.671699197 7.805035031 6.66481339 5.678717615
ENSG00000205531 2620.14079 2950.573841 2800.442385
2282.238594 2064.958717 1997.872029 2790.385672
2115.023113 0.39991841 0.003145184 0.030925997 NAP1L4
11 2944431 2992377 - 5200 protein_coding
nucleosome assembly protein 1 like 4 [Source:HGNC
Symbol;Acc:HGNC:7640] - 2888 3334 2409 2203 1809
2316 27.19709566 30.38006458 28.82307441
23.5409073 21.38368673 20.70933863
ENSG00000278635 9.979760626 14.15992245 9.299933199 0
0 0 11.14653876 0 5.912687492
0.003145671 0.030925997 AC141557.2 12 9415641
9416718 + 482 unprocessed_pseudogene WAS protein family
homolog 1 (WASH1) pseudogene - 11 16 8 0
0 0 1.117568959 1.572893745 1.032642025
0 0 0
ENSG00000167716 1391.722982 1616.886145 1121.804442
1448.283956 2231.616524 2728.5273 1376.804523
2136.142593 -0.633444737 0.003147392 0.030925997 WDR81
17 1716523 1738599 + 7913 protein_coding WD
repeat domain 81 [Source:HGNC Symbol;Acc:HGNC:26600] - 1534
1827 965 1398 1955 3163 9.493205396
10.94016375 7.587400244 9.816983235 15.18633431
18.58613316
ENSG00000163545 97.07585336 70.79961225 153.4488978
36.25889732 50.22564044 32.78028372 107.1081211

39.75494049	1.430559211	0.003148572	0.030925997	NUAK2
	1	205302059	205321791	-	3441
protein_coding	NUAK family kinase 2 [Source:HGNC
Symbol;Acc:HGNC:29558]	-	107	80	132	35	44
	38	1.522747126	1.101619856	2.386690504
0.565191231	0.785987002	0.513487578
ENSG00000196954 847.3724022	1185.00851	953.2431529
1404.773279	1333.262455	1514.794164	995.2080217
1417.609966	-0.510344744	0.00314913	0.030925997	CASP4
	11	104942866	104969436	-	7256
protein_coding	caspase 4 [Source:HGNC Symbol;Acc:HGNC:1505]	-
	934	1339	820	1356	1168	1756	6.303449615
	8.743991843	7.031102432	10.38423383	9.89447895
	11.25273925
ENSG00000134343 6.350756762	18.58489822	2.3249833
96.34507003	30.82027936	30.19236658	9.086879426
52.45257199	-2.509234304	0.003153055	0.030925997	ANO3
	11	26309599	26663288	+	7700
protein_coding	anoctamin 3 [Source:HGNC Symbol;Acc:HGNC:14004] -
	7	21	2	93	27	35	0.044518036
	0.12922752	0.016160177	0.671126314	0.215536288
	0.211352961
ENSG00000157557 2147.463036	1860.259812	2359.858049
1495.938507	1615.210937	1587.255843	2122.526966
1566.135096	0.4381437	0.003153911	0.030925997	ETS2
	21	38805307	38824955	+	4595
protein_coding	"ETS proto-oncogene 2, transcription factor
[Source:HGNC Symbol;Acc:HGNC:3489]"	ETS	2367	2102	2030
	1444	1415	1840	25.2255954	21.67572522
27.48636876	17.46198944	18.92859087	18.61929897
ENSG00000123131 1949.682326	2674.455353	2835.317134
1742.499008	1929.12119	1607.95918	2486.484938
1759.859793	0.498657746	0.003154153	0.030925997	PRDX4
	X	23664262	23686399	+	1904
protein_coding	peroxiredoxin 4 [Source:HGNC Symbol;Acc:HGNC:17169]
	-	2149	3022	2439	1682	1690	1864
55.27110599	75.20625394	79.6987854	49.08751998
54.55909034	45.52081008
ENSG00000112139 53.52780699	45.13475281	106.9492318
9.323716454	25.11282022	20.70333709	68.53726386
18.37995792	1.896198625	0.003158284	0.030925997	MDGA1
	6	37630679	37699306	-	14187
protein_coding	MAM domain containing glycosylphosphatidylinositol
anchor 1 [Source:HGNC Symbol;Acc:HGNC:19267]	-	59	51
	92	9	22	24	0.203652679	0.170335845
	0.403463364	0.035250395	0.095318999	0.078659592
ENSG00000110080 1491.520588	1649.630965	1782.099699
1247.306068	1186.010009	1199.068273	1641.083751
1210.794783	0.438279137	0.003158794	0.030925997
ST3GAL4 11	126355640	126440344	+	4465

protein_coding "ST3 beta-galactoside alpha-2,3-sialyltransferase 4
[Source:HGNC Symbol;Acc:HGNC:10864]" - 1644 1864 1533
1204 1039 1390 18.03055245 19.78112037
21.36129306 14.98363094 14.30347116 14.47519274
ENSG00000165072 168.7486797 110.6243941 218.5484302
96.34507003 26.25431205 66.42320649 165.9738347
63.00752952 1.392487418 0.003159211 0.030925997 MAMDC2
9 70043581 70226970 + 3854
protein_coding MAM domain containing 2 [Source:HGNC
Symbol;Acc:HGNC:23673] - 186 125 188 93 23
77 2.363360199 1.536826156 3.034960098
1.340859526 0.366828851 0.928987848
ENSG00000176485 246.7722627 177.8840258 161.5863393
131.5679989 55.93309958 75.91223598 195.4142093
87.80444481 1.154459754 0.003181058 0.031122646
PLA2G16 11 63573195 63616883 - 3116
protein_coding phospholipase A2 group XVI [Source:HGNC
Symbol;Acc:HGNC:17825] - 272 201 139 127 49
88 4.274645839 3.056504568 2.775391142
2.264739817 0.96659822 1.313155755
ENSG00000112245 16.33051739 7.964956378 9.299933199 0
0 0 11.19846899 0 5.919125837
0.003188641 0.031162776 PTP4A1 6 63521746
63583377 + 5281 protein_coding "protein tyrosine
phosphatase type IVA, member 1 [Source:HGNC Symbol;Acc:HGNC:9634]"
- 18 9 8 0 0 0
0.166911024 0.080751906 0.09424985 0 0 0
ENSG00000173402 3447.553671 3447.056121 4086.158149
3040.567533 2753.27829 2168.67456 3660.25598
2654.173461 0.463814821 0.003188684 0.031162776 DAG1
3 49468703 49535618 + 6923
protein_coding dystroglycan 1 [Source:HGNC Symbol;Acc:HGNC:2666]
- 3800 3895 3515 2935 2412 2514
26.87929963 26.65873706 31.58914304 23.55731654
21.41560422 16.88503479
ENSG00000070759 63.50756762 53.09970919 86.02438209
27.97114936 9.131934625 19.84069804 67.5438863
18.98126068 1.82394722 0.003193659 0.031194168 TESK2
1 45343883 45491166 - 3322
protein_coding testis associated actin remodelling kinase 2
[Source:HGNC Symbol;Acc:HGNC:11732] - 70 60 74
27 8 23 1.031875012 0.855811392
1.385922477 0.451623133 0.148025903 0.321928352
ENSG00000213096 286.6913052 261.0735702 322.010187
210.3016045 143.8279703 100.0661293 289.9250208
151.398568 0.938932718 0.003195835 0.03119819 ZNF254
19 24033405 24129961 + 7602
protein_coding zinc finger protein 254 [Source:HGNC
Symbol;Acc:HGNC:13047] zf-C2H2 316 295 277 203 126
116 2.035578714 1.838741133 2.267037742

1.483816569	1.018802589	0.709514282
ENSG00000127863 776.6068269	615.9566266	807.9316967
574.9625147	461.1626986	359.7204819	733.4983834
465.2818984	0.657248204	0.003214093	0.031359128
TNFRSF19	13	23570370	23676104	+	5113
protein_coding	TNF receptor superfamily member 19
[Source:HGNC Symbol;Acc:HGNC:11915]	-	856	696	695
555	404	417	8.198353784	6.45000257
8.456990806	6.03155419	4.856830337	3.792200406
ENSG00000135372 1973.270851	2031.063876	1909.973781
2339.216861	2840.031668	2676.768957	1971.436169
2618.672496	-0.409372143	0.003233492	0.031522367	NAT10
11	34105602	34147670	+	5242
protein_coding	N-acetyltransferase 10 [Source:HGNC
Symbol;Acc:HGNC:29830]	-	2175	2295	1643	2258	2488
3103	20.31846655	20.7449367	19.50057393
23.93530493	29.17431848	27.52426795
ENSG00000108798 150.6036604	171.6890597	168.5612892
89.09329056	85.61188711	32.78028372	163.6180031
69.16182047	1.248251671	0.003234388	0.031522367	ABI3
17	49210227	49223225	+	2456
protein_coding	ABI family member 3 [Source:HGNC
Symbol;Acc:HGNC:29859]	-	166	194	145	86	75
38	3.309850738	3.74282747	3.673216161
1.945728017	1.877069103	0.719426204
ENSG00000120217 278.5260466	277.8884781	245.2857381
440.2866103	404.0881072	415.7920198	267.2334209
420.0555791	-0.651470148	0.003253154	0.031687795	CD274
9	5450503 5470566 +	4108	protein_coding	CD274
molecule [Source:HGNC Symbol;Acc:HGNC:17635]	-	307	314
211	425	354	482	3.659625323	3.621809921
3.195647493	5.748711827	5.296880648	5.455664076
ENSG00000210196 288.5058072	242.488672	261.5606212
394.7039966	430.3424192	426.1436884	264.1850335
417.063368	-0.658701571	0.003256583	0.031697267	MT-TP
MT	15956	16023	-	68	Mt_tRNA
mitochondrially encoded tRNA proline [Source:HGNC
Symbol;Acc:HGNC:7494]	-	318	274	225	381	377
494	229.0060157	190.9273263	205.8640213
311.3351148	340.784472	337.7917624
ENSG00000112761 2.721752898	0	1.16249165	11.39565344
20.54685291	29.32972754	1.294748183	20.42407796
-3.981177332	0.003259201	0.031697267	WISP3	6
112054072	112070969	+	3202	protein_coding
WNT1 inducible signaling pathway protein 3 [Source:HGNC
Symbol;Acc:HGNC:12771]	-	3	0	1	11	18
34	0.045880549	0	0.019430569	0.190890098
0.345540166	0.493728967
ENSG00000117305 574.2898615	683.2162582	743.9946559
917.8680865	949.721201	1000.661293	667.1669252

956.0835267	-0.520197168	0.003259502	0.031697267	HMGCL
1	23801885	23838620	-	5658
protein_coding	3-hydroxy-3-methylglutaryl-CoA lyase [Source:HGNC
Symbol;Acc:HGNC:5005]	-	633	772	640	886	832
1160	5.4785982	6.465182639	7.037588633
8.701273554	9.038736864	9.532922542
ENSG00000141424 2634.656805	2177.088077	2923.666499
1953.836581	2020.440536	1565.689867	2578.47046
1846.655661	0.481724132	0.003264465	0.031728091
SLC39A6 18	36108532	36129385	-	3682
protein_coding	solute carrier family 39 member 6 [Source:HGNC
Symbol;Acc:HGNC:18607]	-	2904	2460	2515	1886	1770
1815	38.62260045	31.6575837	42.49727193
28.46229711	29.54859547	22.92048818
ENSG00000253738 624.1886646	685.8712437	617.2830661
449.6103268	470.2946332	365.7589552	642.4476581
428.5546384	0.585791371	0.003270561	0.03176913
OTUD6B-AS1	8	91059909	91070189	-	6009
antisense	OTUD6B antisense RNA 1 (head to head)
[Source:HGNC Symbol;Acc:HGNC:50466]	-	688	775	531
434	412	424	5.606798104	6.111192152
5.497929463	4.013281001	4.214464231	3.28091267
ENSG00000117318 3675.273663	3905.483611	4888.277388
2594.065111	3038.651247	3409.149507	4156.344887
3013.955288	0.463161986	0.003273799	0.03176913	ID3
1	23557918	23559794	-	1797
protein_coding	"inhibitor of DNA binding 3, HLH protein [Source:HGNC
Symbol;Acc:HGNC:5362]"	bHLH	4051	4413	4205	2504	2662
3952	110.393334	116.3622985	145.5877283
77.42804452	91.05574127	102.2586081
ENSG00000145757 9.07250966	6.194966072	18.5998664	0
0	0	11.28911404	0	5.926158928
0.003274076	0.03176913	SPATA9	5	95652181
95698711	-	2811	protein_coding	spermatogenesis
associated 9 [Source:HGNC Symbol;Acc:HGNC:22988]	-	10
7	16	0	0	0	0.174207897
0.117995008	0.354132662	0	0	0
ENSG00000181649 304.8363246	457.5424942	654.4827989
266.2439032	302.4953345	217.3850394	472.2872059
262.0414257	0.849550628	0.003277074	0.031780789	PHLDA2
11	2928273 2929455 -	955	protein_coding
pleckstrin homology like domain family A member 2 [Source:HGNC
Symbol;Acc:HGNC:12385]	-	336	517	563	257	265
252	17.22917925	25.65154999	36.67852562
14.95346351	17.0564978	12.26953736
ENSG00000073536 977.1092904	1360.23755	1357.790247
1566.384364	1797.849629	1905.569651	1231.712363
1756.601215	-0.512695664	0.003283342	0.031807243	NLE1
17	35128753	35142315	-	5463
protein_coding	notchless homolog 1 [Source:HGNC

Symbol;Acc:HGNC:19889] -   1077   1537   1168   1512   1575
        2209   9.654130972   13.33119582   13.30204733
15.37916068   17.72134652   18.80163137
ENSG00000114520 653.2206955   660.2063842   668.4326987
491.0490666   405.229599   451.1602207   660.6199261
449.1462954   0.556099808   0.003283398   0.031807243   SNX4
        3   125446644   125520197   -   2709
protein_coding sorting nexin 4 [Source:HGNC Symbol;Acc:HGNC:11175]
        -       720   746   575   474   355   523
13.01523982   13.04837001   13.20582951   9.722576753
8.055021579   8.976844494
ENSG00000058804 3160.862365   2606.310726   2650.480962
2461.461144   1746.482497   1755.470457   2805.884684
1987.804699   0.497472142   0.003288411   0.031838369   NDC1
        1   53765460   53838860   -   5135
protein_coding NDC1 transmembrane nucleoporin [Source:HGNC
Symbol;Acc:HGNC:25525] -   3484   2945   2280   2376   1530
        2035   33.22510652   27.17510883   27.62493378
25.71094457   18.31463817   18.42701476
ENSG00000160445 729.4297766   739.855948   776.5444221
944.8032674   974.8340213   1407.826922   748.6100489
1109.154737   -0.568367755   0.003319104   0.032117958   ZER1
        9   128729786   128772414   -   4370
protein_coding zyg-11 related cell cycle regulator [Source:HGNC
Symbol;Acc:HGNC:30960] -   804   836   668   912   854
        1632   9.009554056   9.064655037   9.510467638
11.59646029   12.01223428   17.364798
ENSG00000198331 278.5260466   222.1337834   212.7359719
100.488944   140.4034949   141.4728034   237.7986006
127.4550808   0.900049318   0.003323166   0.032139684   HYLS1
        11   125883614   125900648   +   2999
protein_coding "HYLS1, centriolar and ciliogenesis associated
[Source:HGNC Symbol;Acc:HGNC:26558]"   -   307   251   183
        97   123   164   5.012917915   3.965735058
3.796483097   1.797245116   2.521018344   2.542719188
ENSG00000164236 104.3338611   107.0844135   133.6865397
34.18696033   68.48950969   31.91764468   115.0349381
44.8647049   1.364204313   0.003328847   0.032177032
ANKRD33B   5   10564330   10657816   +   9783
        protein_coding ankyrin repeat domain 33B [Source:HGNC
Symbol;Acc:HGNC:35240] -   115   121   115   33   60
        37   0.575644647   0.586056865   0.731362407
0.187436398   0.376987159   0.175857416
ENSG00000122862 620.5596607   717.7310692   798.6317635
509.6964995   446.3233048   485.6657825   712.3074978
480.5618623   0.566656473   0.003336551   0.032233891   SRGN
        10   69088106   69104811   +   1220
protein_coding serglycin [Source:HGNC Symbol;Acc:HGNC:9361]   -
        684   811   687   492   391   563   27.45522168
        31.49832534   35.03513159   22.40873371   19.69991203

21.45752278
ENSG00000101654 1751.901615 1425.727192 1684.450401
2267.735035 2002.176667 2314.460558 1620.693069
2194.790753 -0.437962716 0.003341845 0.032267415 RNMT
18 13726660 13764558 + 6982
protein_coding RNA guanine-7 methyltransferase [Source:HGNC
Symbol;Acc:HGNC:10075] - 1931 1611 1449 2189 1754
2683 13.54350626 10.9330702 12.9120556
17.42119575 15.4417709 17.86783142
ENSG00000105499 20.86677222 69.02962194 51.1496326
113.9565344 102.7342645 166.4893357 47.01534225
127.7267116 -1.44414988 0.003353626 0.032363493
PLA2G4C 19 48047843 48110817 - 6613
protein_coding phospholipase A2 group IVC [Source:HGNC
Symbol;Acc:HGNC:9037] - 23 78 44 110 90
193 0.170316999 0.558885086 0.413962499
0.924285641 0.836548928 1.357031111
ENSG00000157538 1536.883136 1563.786436 1589.126085
2075.044895 2209.928179 1927.998266 1563.265219
2070.990447 -0.405528592 0.003357337 0.032373392 VPS26C
21 37223420 37267919 - 9301
protein_coding VPS26 endosomal protein sorting factor C [Source:HGNC
Symbol;Acc:HGNC:3044] - 1694 1767 1367 2003 1936
2235 8.918923629 9.001882626 9.144200011
11.96639539 12.79449264 11.17323418
ENSG00000227496 7.258007728 16.81490791 9.299933199 0
0 0 11.12428295 0 5.909894713
0.003358312 0.032373392 AC099066.2 1 225700604
225752243 + 1530 antisense novel
transcript - 8 19 8 0 0 0
0.25605145 0.588421606 0.325315984 0 0
0
ENSG00000133265 2340.707492 3028.453414 2902.74165
3244.653326 3989.513939 4050.952957 2757.300852
3761.706741 -0.448299985 0.003364252 0.032412992 HSPBP1
19 55262231 55280381 - 2159
protein_coding HSPA (Hsp70) binding protein 1 [Source:HGNC
Symbol;Acc:HGNC:24989] - 2580 3422 2497 3132 3495
4696 58.51884519 75.10239792 71.95694996
80.60855847 99.50431125 101.1361609
ENSG00000169689 1456.1378 1936.369395 1779.774716
1301.17643 1312.715602 1054.144913 1724.09397
1222.678982 0.496319296 0.003369622 0.032447056 CENPX
17 82018702 82024107 - 1531
protein_coding centromere protein X [Source:HGNC
Symbol;Acc:HGNC:11422] - 1605 2188 1531 1256 1150
1222 51.33676873 67.71713379 62.216682
45.58547582 46.1710775 37.11308304
ENSG00000198355 1468.839314 1846.984885 1690.262859
1680.340899 2693.920714 3242.660171 1668.695686

2538.973928	-0.605790521	0.00338265	0.032554222	PIM3
	22	49960513	49964080	+	2461
protein_coding	"Pim-3 proto-oncogene, serine/threonine kinase
[Source:HGNC Symbol;Acc:HGNC:19310]"	-	1619	2087	1454
	1622	2360	3759	32.21542897	40.18252987
36.75865731	36.62277762	58.94510552	71.02180902
ENSG00000136950 1082.350402	1399.177337	1120.64195
1473.1472	1725.935644	1877.102563	1200.72323
1692.061802	-0.494713596	0.003384432	0.032554222	ARPC5L
	9	124862130	124877733	+	3610
protein_coding	actin related protein 2/3 complex subunit 5 like
[Source:HGNC Symbol;Acc:HGNC:23366]	-	1193	1581	964
	1422	1512	2176	16.18310773	20.75157713
16.61409459	21.88791725	25.74494388	28.02739326
ENSG00000136754 1100.495422	1011.54946	1248.516032
1469.003326	1619.776904	1513.068885	1120.186971
1533.949705	-0.45413275	0.003386328	0.032554761	ABI1
	10	26746593	26861087	-	4218
protein_coding	abl interactor 1 [Source:HGNC Symbol;Acc:HGNC:11320]
	-	1213	1143	1074	1418	1419	1754
14.08260211	12.8400317	15.84180097	18.68020763
20.6786975	19.33543877
ENSG00000182183 0	0	0	21.75533839	0
19.84069804	0	13.86534548	-6.271886673	0.003391663
	0.032588331	SHISAL2A	1	52633344
52669683	+	2508	protein_coding	shisa like 2A
[Source:HGNC Symbol;Acc:HGNC:28757]	-	0	0	0
	21	0	23	0	0	0	0.465268667
	0	0.426413869
ENSG00000102977 964.4077768	1238.108219	1031.130093
1436.888302	1560.419329	1480.288602	1077.88203
1492.532078	-0.469109701	0.003393872	0.032591859	ACD
	16	67657512	67660815	-	3223
protein_coding	"ACD, shelterin complex subunit and telomerase
recruitment factor [Source:HGNC Symbol;Acc:HGNC:25070]" -	1063
	1399	887	1387	1367	1716	16.15108276
20.567611	17.12261773	23.91267708	26.07087271
24.75642878
ENSG00000154144 940.8192517	869.0652404	703.3074482
1136.457439	1122.086467	1346.57955	837.7306468
1201.707819	-0.519981428	0.003397425	0.03260827	TBRG1
	11	124622836	124635398	+	6627
protein_coding	transforming growth factor beta regulator 1
[Source:HGNC Symbol;Acc:HGNC:29551]	-	1037	982	605
	1097	983	1561	7.662852559	7.021355428
5.679959652	9.198175691	9.117670796	10.95259303
ENSG00000121680 660.4787032	874.3752113	780.0318971
882.6451577	1294.451733	1293.095929	771.6286039
1156.73094	-0.584187027	0.003415139	0.032760518	PEX16
	11	45909669	45918812	-	3013

protein_coding	peroxisomal biogenesis factor 16 [Source:HGNC Symbol;Acc:HGNC:8857]	-	728	988	671	852	1134
1499	11.83207547	15.53761134	13.85575626
15.71276107	23.13456208	23.13308317
ENSG00000010361 540.7215757	683.2162582	577.75835
732.4297259	899.4955606	1079.161446	600.5653946
903.6955774	-0.589799503	0.003422218	0.032810627	FUZ
19	49806869	49817376	-	2950
protein_coding	fuzzy planar cell polarity protein [Source:HGNC Symbol;Acc:HGNC:26219]	-	596	772	497	707	788
1251	9.893567641	12.40000114	10.48192914
13.31709327	16.4191837	19.71815662
ENSG00000245857 22.68127415	7.079961225	4.6499666	0
0	0	11.47040066	0	5.955865666
0.003443831	0.032999962	GS1-24F4.2	8	6835554
6885276 +	1433	lincRNA uncharacterized LOC100652791
[Source:NCBI gene;Acc:100652791]	-	25	8	4
0	0	0	0.854323793	0.264527141
0.173668338	0	0	0
ENSG00000134851 1310.070395	1457.587017	1529.839011
1071.191424	1119.803483	916.1226661	1432.498808
1035.705858	0.468324918	0.00344633	0.033006026
TMEM165 4	55395957	55453397	+	7045
protein_coding	transmembrane protein 165 [Source:HGNC Symbol;Acc:HGNC:30760]	-	1444	1647	1316	1034	981
1062	10.03725319	11.07743072	11.62202321
8.155518645	8.559243272	7.00929821
ENSG00000135631 1384.464974	1469.091954	1646.088176
1750.786756	2276.134705	2169.537199	1499.881702
2065.48622	-0.462055493	0.003451807	0.033040596
RAB11FIP5	2	73073382	73156721	-	7058
protein_coding	RAB11 family interacting protein 5
[Source:HGNC Symbol;Acc:HGNC:24845]	-	1526	1660	1416
1690	1994	2515	10.58769844	11.14430206
12.48212266	13.30506787	17.36564266	16.56865876
ENSG00000136213 673.1802168	829.2404585	841.6439545
887.8250001	1231.669683	1394.024697	781.3548766
1171.173127	-0.584688685	0.003453752	0.033041326	CHST12
7	2403560 2448483 +	16143	protein_coding
carbohydrate sulfotransferase 12 [Source:HGNC Symbol;Acc:HGNC:17423]
-	742	937	724	857	1079	1616
2.250859267	2.750310868	2.790365965	2.949908999
4.108511984	4.654661762
ENSG00000132507 10701.02514	13296.16718	14092.88627
8940.408111	8901.353276	10702.76263	12696.69287
9514.841341	0.41603254	0.003462246	0.033096962	EIF5A
17	7306999 7312463 +	2815	protein_coding
eukaryotic translation initiation factor 5A [Source:HGNC Symbol;Acc:HGNC:3300]	-	11795	15024	12123	8630	7798
12407	205.1862383	252.8911414	267.9406167

170.3509031	170.2755108	204.9365407
ENSG00000280206 155.1399152	218.5938028	267.3730795
100.488944	136.9790194	63.83528935	213.7022658
100.4344176	1.092626294	0.003463309	0.033096962
AC026401.3	16	15701237	15702118	+	882
	lincRNA novel transcript	-	171	247	230
	97	120	74	9.49415262	13.26950764
16.22430483	6.111040934	8.36296003	3.901163505
ENSG00000204574 5243.910583	5638.304121	5109.150801
6313.192008	6872.922297	7389.366062	5330.455168
6858.493456	-0.363620458	0.003466059	0.033104097	ABCF1
	6	30571376	30597179	+	4523
protein_coding	ATP binding cassette subfamily F member 1 [Source:HGNC
Symbol;Acc:HGNC:70]	-	5780	6371	4395	6094	6021
	8566	62.57918953	66.74326656	60.45596228
74.86656471	81.8256379	88.06077443
ENSG00000074842 2595.645014	3554.140535	3432.837842
2434.525963	2313.803936	2287.718748	3194.207797
2345.349549	0.445540887	0.003467799	0.033104097	MYDGF
	19	4641374 4670370 -	1678	protein_coding
myeloid derived growth factor [Source:HGNC Symbol;Acc:HGNC:16948]
	-	2861	4016	2953	2350	2027	2652
83.49386875	113.4039517	109.4909785	77.8194128
74.25217036	73.48734909
ENSG00000249249 13.60876449	12.38993214	6.974949899	0
	0	0	10.99121551	0	5.893482466
0.003478213	0.033185606	AC010226.1	5	115602057
	115620659	+	1218	antisense
uncharacterized LOC101927100 [Source:NCBI gene;Acc:101927100]	-
	15	14	6	0	0	0	0.603076845
	0.544637058	0.306486118	0	0	0
ENSG00000116478 3161.769616	3129.342861	3653.711256
2433.489995	2688.213255	2444.719054	3314.941244
2522.140768	0.394208973	0.003482511	0.033208702	HDAC1
	1	32292086	32333635	+	3963
protein_coding	histone deacetylase 1 [Source:HGNC
Symbol;Acc:HGNC:4852]	-	3485	3536	3143	2349	2355
	2834	43.06330856	42.27801854	49.34318232
32.93601017	36.52701638	33.25115931
ENSG00000169213 1282.852866	1005.354494	1155.5167
983.1341017	667.7727195	416.6546589	1147.90802
689.18716	0.736989165	0.003492675	0.033287673	RAB3B
	1	51907956	51990764	-	12844
protein_coding	"RAB3B, member RAS oncogene family [Source:HGNC
Symbol;Acc:HGNC:9778]"	-	1414	1136	994	949	585
	483	5.39110507	4.19087276	4.814962777
4.105612521	2.799644767	1.748549181
ENSG00000177600 7598.22684	10954.47001	10470.56229
10799.97156	14790.30962	15241.96929	9674.419712
13610.75016	-0.492563533	0.003495285	0.033294613	RPLP2

11 809647 812880 + 1955 protein_coding
ribosomal protein lateral stalk subunit P2 [Source:HGNC
Symbol;Acc:HGNC:10377] - 8375 12378 9007 10425 12957
17669 209.7812832 300.0061499 286.6422787
296.3066139 407.3850217 420.2388769
ENSG00000136999 935.3757459 845.1703712 689.3575484
1457.607672 1007.937284 1186.128687 823.3012218
1217.224548 -0.56339928 0.003505599 0.033374891 NOV
8 119416306 119424353 + 2673
protein_coding nephroblastoma overexpressed [Source:HGNC
Symbol;Acc:HGNC:7885] - 1031 955 593 1407 883
1375 18.88810508 16.92898241 13.80265336
29.24874137 20.30528572 23.91854501
ENSG00000132768 1785.469901 2248.772684 1536.813961
2099.908139 3036.368263 2963.165121 1857.018849
2699.813841 -0.539389624 0.003516325 0.033458992 DPH2
1 43970000 43973369 + 2930
protein_coding DPH2 homolog [Source:HGNC Symbol;Acc:HGNC:3004] -
1968 2541 1322 2027 2660 3435 32.89168764
41.09258721 28.07182717 38.44130937 55.80349199
54.51175184
ENSG00000138071 7704.375203 6445.4197 8001.430026
6269.681331 5412.954249 4949.822842 7383.741643
5544.152807 0.413372406 0.003521624 0.033491401 ACTR2
2 65227753 65271253 + 4347
protein_coding ARP2 actin related protein 2 homolog [Source:HGNC
Symbol;Acc:HGNC:169] - 8492 7283 6883 6052 4742
5738 95.66410855 79.38658325 98.51332465
77.36086602 67.0531631 61.37647212
ENSG00000104228 901.8074602 1121.288859 1016.017702
1236.946383 1430.289261 1620.036127 1013.038007
1429.09059 -0.496747376 0.003526759 0.033522218 TRIM35
8 27284887 27311319 - 4269
protein_coding tripartite motif containing 35 [Source:HGNC
Symbol;Acc:HGNC:16285] - 994 1267 874 1194 1253
1878 11.40220679 14.06296516 12.73773251
15.54140277 18.04148478 20.45504556
ENSG00000153071 915.4162247 843.4003809 940.4557448
1215.191045 1340.111406 1159.386877 899.7574501
1238.229776 -0.460621774 0.003533688 0.033555535 DAB2
5 39371675 39462300 - 7717
protein_coding "DAB2, clathrin adaptor protein [Source:HGNC
Symbol;Acc:HGNC:2662]" - 1009 953 809 1173 1174
1344 6.402820836 5.851548934 6.52239157
8.446203783 9.351191629 8.09807485
ENSG00000107104 713.0992593 449.5775378 603.3331663
355.3371938 351.5794831 405.4403513 588.6699878
370.785676 0.665876409 0.003535457 0.033555535 KANK1
9 470291 746106 + 11908 protein_coding KN
motif and ankyrin repeat domains 1 [Source:HGNC

Symbol;Acc:HGNC:19309]	-	786	508	519	343	308
	470	3.232305518	2.021395652	2.711660897
1.600542968	1.589861347	1.835228099
ENSG00000160124 344.7553671	350.4580806	404.5470942
242.4166278	139.262003	242.4015717	366.5868473
208.0267342	0.814181892	0.003535956	0.033555535	CCDC58
	3	122359591	122383231	-	1746
protein_coding	coiled-coil domain containing 58 [Source:HGNC
Symbol;Acc:HGNC:31136]	-	380	396	348	234	122
	281	10.65781165	10.74675597	12.40057579
7.447039574	4.294997857	7.48329901
ENSG00000230910 20.86677222	7.079961225	5.812458249	0
	0	0	11.2530639	0	5.927750142
0.00354443	0.033588211	AL391807.1	6	68628117
	68635161	-	5924	processed_transcript
uncharacterized LOC101928307 [Source:NCBI gene;Acc:101928307]	-
	23	8	5	0	0	0	0.190125982
	0.063988419	0.052512392	0	0	0
ENSG00000104969 4453.694992	4262.136657	4008.271209
4669.110007	5874.116948	6663.886625	4241.367619
5735.704526	-0.435475366	0.003544665	0.033588211	SGTA
	19	2754714 2783371 -	3801	protein_coding	small
glutamine rich tetratricopeptide repeat containing alpha [Source:HGNC
Symbol;Acc:HGNC:10819]	-	4909	4816	3448	4507	5146
	7725	63.24465765	60.03645626	56.43860025
65.88730321	83.21838293	94.4999626
ENSG00000120656 657.7569503	829.2404585	745.1571476
978.9902277	1063.870384	1116.254925	744.0515188
1053.038512	-0.501220719	0.003545095	0.033588211	TAF12
	1	28589323	28643085	-	2337
protein_coding	TATA-box binding protein associated factor 12
[Source:HGNC Symbol;Acc:HGNC:11545]	-	725	937	641
	945	932	1294	15.19175605	18.99797533
17.06499494	22.4690722	24.51345691	25.74581131
ENSG00000128581 300.3000697	301.7833472	344.0975284
477.5814762	587.8682915	424.4184103	315.3936484
496.622726	-0.654782464	0.003557516	0.033687846	IFT22
	7	101313367	101321823	-	4132
protein_coding	intraflagellar transport 22 [Source:HGNC
Symbol;Acc:HGNC:21895]	-	331	341	296	461	515
	492	3.922801787	3.910393902	4.456954955
6.199442784	7.661155482	5.536506423
ENSG00000145362 528.0200622	508.872213	444.0718103
848.4581973	768.2240004	609.885805	493.6546952
742.1893342	-0.586642732	0.003566462	0.033742169	ANK2
	4	112818109	113383740	+	18224
protein_coding	ankyrin 2 [Source:HGNC Symbol;Acc:HGNC:493]	-
	582	575	382	819	673	707	1.563896334
	1.495032311	1.304146868	2.497193746	2.269962683
	1.803876186

ENSG00000258441 472.6777533 452.2325232 418.496994
683.7392066 561.6139795 777.2377798 447.8024235
674.1969886 -0.590780799 0.003567439 0.033742169
LINC00641 14 21200079 21206900 - 5715
processed_transcript long intergenic non-protein coding RNA
641 [Source:HGNC Symbol;Acc:HGNC:27511] - 521 511 360
660 492 901 4.464267113 4.236733105
3.919161071 6.41711385 5.291712346 7.330600875
ENSG00000162946 171.4704326 169.9190694 191.8111222
77.69763712 95.88531357 99.20349021 177.7335414
90.92881363 0.965487441 0.003568975 0.033742169 DISC1
1 231626815 232041272 + 12906
protein_coding DISC1 scaffold protein [Source:HGNC
Symbol;Acc:HGNC:2888] - 189 192 165 75 84
115 0.717131546 0.704913793 0.795424805
0.322910113 0.400069078 0.41432124
ENSG00000186960 782.9575836 807.1155796 963.7055778
714.8182615 463.4456822 474.4514749 851.2595803
550.9051395 0.627397619 0.003573464 0.033766561
LINC01551 14 28772704 28813453 + 2772
lincRNA long intergenic non-protein coding RNA 1551
[Source:HGNC Symbol;Acc:HGNC:19828] - 863 912 829
690 406 550 15.24566081 15.58935164
18.60664841 13.83145628 9.002853184 9.225724504
ENSG00000166813 578.8261163 606.2216799 607.9831329
353.2652568 364.1358932 464.9624454 597.6769764
394.1211985 0.599066774 0.003587192 0.033878187 KIF7
15 89608789 89655451 - 5001
protein_coding kinesin family member 7 [Source:HGNC
Symbol;Acc:HGNC:30497] - 638 685 523 341 319
539 6.247302102 6.490231049 6.506563625
3.78886881 3.920858675 5.011444543
ENSG00000125651 2948.565639 4112.572477 3697.885938
3975.011115 5388.982921 5858.181756 3586.341351
5074.058597 -0.500747105 0.003590297 0.033889416 GTF2F1
19 6379569 6393981 - 3134 protein_coding
general transcription factor IIF subunit 1 [Source:HGNC
Symbol;Acc:HGNC:4652] - 3250 4647 3181 3837 4721
6791 50.78238017 70.25870185 63.14973371
68.03068607 92.59389825 100.7548026
ENSG00000255197 9.07250966 9.734946684 2.3249833
31.07905485 26.25431205 62.11001126 7.044146548
39.81445938 -2.481967506 0.003592733 0.033894321
AC090559.1 11 47383148 47409190 - 1113
antisense novel transcript - 10 11
2 30 23 72 0.439980591 0.468299789
0.111799968 1.497745881 1.270223174 3.007934329
ENSG00000167770 2384.255539 2624.895624 2826.017201
2968.049738 3797.743312 3810.276663 2611.722788
3525.356571 -0.433057391 0.003608253 0.034007845 OTUB1

11	63985853	64001811	+	5336
protein_coding	"OTU deubiquitinase, ubiquitin aldehyde binding 1
[Source:HGNC Symbol;Acc:HGNC:23077]"	-	2628	2966	2431
2865	3327	4417	24.11782966	26.33793773
28.34496888	29.83464325	38.32519208	38.48952899
ENSG00000120837 648.6844407	546.0420095	554.508517
390.5601226	420.0689928	351.9567305	583.0783224
387.5286153	0.59072012	0.003608611	0.034007845	NFYB
12	104117077	104138289	-	4956
protein_coding	nuclear transcription factor Y subunit beta
[Source:HGNC Symbol;Acc:HGNC:7805]	NF-YB	715	617	477
377	368	408	7.064857845	5.899025957
5.988167336	4.226901195	4.564191745	3.827893828
ENSG00000121957 1804.522171	1290.322933	1606.56346
1004.88944	420.0689928	371.7974285	1567.136188
598.9186204	1.387845466	0.003612866	0.034029808	GPSM2
1	108875350	108934545	+	9130
protein_coding	G protein signaling modulator 2 [Source:HGNC
Symbol;Acc:HGNC:29501]	-	1989	1458	1382	970	368
431	10.66823783	7.56681624	9.417683957
5.903546663	2.477561258	2.195015093
ENSG00000092847 1574.080426	1517.766688	1379.877588
1179.968116	1034.191596	1079.161446	1490.574901
1097.773719	0.441584542	0.003616547	0.034046357	AGO1
1	35869808	35930528	+	13886
protein_coding	"argonaute 1, RISC catalytic component [Source:HGNC
Symbol;Acc:HGNC:3262]"	-	1735	1715	1187	1139	906
1251	6.118585057	5.852122444	5.318392737
4.557835081	4.010498857	4.18900778
ENSG00000198542 864.6101706	782.3357154	1213.641282
763.5087807	575.3118814	428.7316055	953.5290561
589.1840892	0.694534893	0.003623734	0.034095869	ITGBL1
13	101452593	101720856	+	6174
protein_coding	integrin subunit beta like 1 [Source:HGNC
Symbol;Acc:HGNC:6164]	-	953	884	1044	737	504
497	7.558836631	6.784409923	10.52060512
6.63304443	5.017776018	3.743008227
ENSG00000203943 34.47553671	13.2749273	41.8496994
5.179842475	4.565967313	0	29.86672113	3.248603262
3.230948793	0.003626704	0.034105674	SAMD13	1
84298366	84389957	+	3455	protein_coding
sterile alpha motif domain containing 13 [Source:HGNC
Symbol;Acc:HGNC:24582]	-	38	15	36	5	4
0	0.53859737	0.205716747	0.648278018
0.080414432	0.071163828	0
ENSG00000011198 939.0047498	894.7300998	928.8308283
1133.349533	1536.448001	1226.672722	920.855226
1298.823419	-0.495715187	0.003638606	0.034199418	ABHD5
3	43690108	43734371	+	7549
protein_coding	abhydrolase domain containing 5 [Source:HGNC

Symbol;Acc:HGNC:21396]	-	1035	1011	799	1094	1346
	1422	6.713973271	6.345826165	6.585127689
8.05267071	10.95980923	8.758731015
ENSG00000229163 1.814501932	2.654985459	2.3249833
17.61146441	13.69790194	36.2308399	2.264823564
22.51340208	-3.317965174	0.003661333	0.034394757
NAP1L1P2	Y	2929001 2931120 -	1089
processed_pseudogene	nucleosome assembly protein 1 like 1
pseudogene 2 [Source:HGNC Symbol;Acc:HGNC:38005]	-	2
	3	2	17	12	42	0.089935427
0.130532849	0.114263879	0.867427298	0.677330647
1.793297854
ENSG00000196628 1917.928542	1426.612187	2000.648129
1252.48591	1413.166883	1055.870191	1781.729619
1240.507662	0.522601685	0.003672117	0.03447775	TCF4
	18	55222331	55664787	-	27828
protein_coding	transcription factor 4 [Source:HGNC
Symbol;Acc:HGNC:11634]	bHLH	2114	1612	1721	1209	1238
	1224	3.720074795	2.74479228	3.847740036
2.41410608	2.734550891	2.04517484
ENSG00000125485 585.1768731	697.3761807	562.6459585
742.7894108	862.9678221	1261.178284	615.0663374
955.6451724	-0.63614488	0.003679475	0.034528505	DDX31
	9	132592997	132670401	-	5546
protein_coding	DEAD-box helicase 31 [Source:HGNC
Symbol;Acc:HGNC:16715]	-	645	788	484	717	756
	1462	5.695194138	6.732444673	5.429656345
7.18375205	8.37894405	12.2574047
ENSG00000101596 2573.87099	1969.114216	2207.571643
1911.361873	1227.103715	1541.535974	2250.185616
1560.000521	0.528253731	0.00369671	0.034671842	SMCHD1
	18	2655738 2805017 +	12435	protein_coding
structural maintenance of chromosomes flexible hinge domain containing
1 [Source:HGNC Symbol;Acc:HGNC:29090]	-	2837	2225	1899
	1845	1075	1787	11.17229076	8.478336833
9.50136543	8.244467769	5.313859102	6.682050923
ENSG00000140563 273.9897917	230.0987398	365.0223781
171.9707702	179.214217	75.91223598	289.7036365
142.3657411	1.027644877	0.003703544	0.03471754	MCTP2
	15	94231538	94483952	+	12978
protein_coding	multiple C2 and transmembrane domain containing 2
[Source:HGNC Symbol;Acc:HGNC:25636]	-	302	260	314
	166	157	88	1.139535493	0.949274946
1.505319629	0.710742625	0.743599763	0.315286896
ENSG00000144134 69.85832438	90.26950562	79.04943219
139.8557468	157.5258723	189.78059	79.72575406
162.387403	-1.027095999	0.003707545	0.034722393	RABL2A
	2	113627229	113643396	+	6008
protein_coding	"RAB, member of RAS oncogene family like 2A
[Source:HGNC Symbol;Acc:HGNC:9799]"	-	77	102	68

135	138	220	0.627609465	0.804445615
0.704183485	1.248578603	1.411875892	1.702643697
ENSG00000048828 3204.410412	2846.144412	3092.227789
2583.705426	2262.436803	1929.723544	3047.594204
2258.621925	0.432514383	0.003707988	0.034722393
FAM120A 9	93451722	93566107	+	7204
protein_coding	family with sequence similarity 120A [Source:HGNC Symbol;Acc:HGNC:13247]	-	3532	3216	2660	2494	1982
2237	24.0090886	21.1528445	22.97284483
19.23688677	16.91131216	14.43854059
ENSG00000159335 3729.708721	4937.387959	5752.008684
3332.710648	3675.603687	3413.462702	4806.368455
3473.925679	0.468182494	0.003712481	0.034732454	PTMS
12	6765516 6770952 +	1475	protein_coding
parathymosin [Source:HGNC Symbol;Acc:HGNC:9629] -	4111	5579
4948	3217	3220	3957	136.4847536	179.2217782
208.7106051	121.1911996	134.1872373	124.7398013
ENSG00000198554 1113.196935	861.9852791	1059.029893
871.2495042	557.0480121	518.4460662	1011.404036
648.9145275	0.640472992	0.003712989	0.034732454	WDHD1
14	54938950	55027105	-	6385
protein_coding	WD repeat and HMG-box DNA binding protein 1 [Source:HGNC Symbol;Acc:HGNC:23170]	HMG	1227	974	911
841	488	601	9.410492322	7.228105721
8.876961207	7.318922321	4.697927179	4.376677925
ENSG00000132603 3160.862365	3653.259992	3242.189212
4407.009977	4290.867782	4266.612718	3352.103856
4321.496826	-0.366321204	0.003718838	0.034768778	NIP7
16	69337996	69343111	+	4749
protein_coding	"NIP7, nucleolar pre-rRNA processing protein [Source:HGNC Symbol;Acc:HGNC:24328]"	-	3484	4128	2789
4254	3759	4946	35.92565213	41.18736039
36.53870838	49.77455198	48.65388411	48.42648649
ENSG00000139613 1499.685847	1408.027289	1498.451737
1204.83136	956.570152	1050.694357	1468.721624
1070.698623	0.455697948	0.003731157	0.034865525
SMARCC2 12	56162983	56189567	-	8555
protein_coding	"SWI/SNF related, matrix associated, actin dependent regulator of chromatin subfamily c member 2 [Source:HGNC Symbol;Acc:HGNC:11105]" MYB	1653	1591	1289	1163	838
1218	9.461969052	8.812042977	9.374319474
7.553908927	6.021037956	6.62000462
ENSG00000129245 1771.861137	2037.258842	2042.497829
2010.814849	3127.687609	3338.413105	1950.539269
2825.638521	-0.534955338	0.003739249	0.034922692	FXR2
17	7591230 7614871 -	4331	protein_coding	FMR1 autosomal homolog 2 [Source:HGNC Symbol;Acc:HGNC:4024]	-	1953
2302	1757	1941	2740	3870	22.08222055
25.18509401	25.24006241	24.90286965	38.8874745
41.54835172

ENSG00000006652 1647.567754 1648.74597 1569.363727
1196.543612 1183.727026 1274.980509 1621.892484
1218.417049 0.412548898 0.003760918 0.035106529 IFRD1
7 112422968 112481017 + 7489
protein_coding interferon related developmental regulator 1
[Source:HGNC Symbol;Acc:HGNC:5456] - 1816 1863 1350
1155 1037 1478 11.8746467 11.78733062
11.21545209 8.569790345 8.511425184 9.176596197
ENSG00000177426 555.2375912 642.5064812 691.6825317
788.3720246 1014.786235 939.4139203 629.808868
914.1907267 -0.538181592 0.003793334 0.035374311 TGIF1
18 3411608 3459978 + 8222 protein_coding TGFB
induced factor homeobox 1 [Source:HGNC Symbol;Acc:HGNC:11776]
Homeobox 612 726 595 761 889 1089
3.645042809 4.18394136 4.502423473 5.143032017
6.646173106 6.158591886
ENSG00000188486 7082.00104 7587.948443 8236.253339
6008.61727 5874.116948 6107.484441 7635.400941
5996.739553 0.34836225 0.003793604 0.035374311 H2AFX
11 119093854 119095467 - 1614
protein_coding H2A histone family member X [Source:HGNC
Symbol;Acc:HGNC:4739] - 7806 8574 7085 5800 5146
7080 236.8392625 251.7134311 273.1135019
199.6808914 195.9808386 203.9673932
ENSG00000015285 63.50756762 134.5192633 183.6736807
47.65455077 63.92354238 22.42861518 127.2335039
44.66890277 1.514908411 0.003799177 0.03540468 WAS
X 48676596 48691427 + 2381
protein_coding Wiskott-Aldrich syndrome [Source:HGNC
Symbol;Acc:HGNC:12731] - 70 152 158 46 56
26 1.439684497 3.024897294 4.128616446
1.073520841 1.445692712 0.507744199
ENSG00000143153 853.723159 643.3914763 581.2458249
575.9984832 213.4589719 352.8193695 692.7868201
380.7589415 0.863314538 0.00380235 0.03540468 ATP1B1
1 169105697 169132722 + 2962
protein_coding ATPase Na+/K+ transporting subunit beta 1 [Source:HGNC
Symbol;Acc:HGNC:804] - 941 727 500 556 187
409 15.55726512 11.6298951 10.50247839
10.43041963 3.880644974 6.420506233
ENSG00000150672 8.165258694 5.309970919 19.76235805 0
0 0 11.07919589 0 5.898359852
0.003803604 0.03540468 DLG2 11 83455012
85627922 - 16709 protein_coding discs large MAGUK
scaffold protein 2 [Source:HGNC Symbol;Acc:HGNC:2901] - 9
6 17 0 0 0 0.026376717
0.017014815 0.063300233 0 0 0
ENSG00000160214 1547.770148 1874.419734 1650.738143
1820.196646 2540.96081 2937.285949 1690.976008
2432.814468 -0.524931018 0.003804867 0.03540468 RRP1

21	43789513	43805293	+	4251
protein_coding	ribosomal RNA processing 1 [Source:HGNC
Symbol;Acc:HGNC:18785]	-	1706	2118	1420	1757	2226
3405	19.65244572	23.60811389	20.78280133
22.96638813	32.18706783	37.24406101
ENSG00000213015 915.4162247	848.7103518	998.5803273
701.350671	606.1321608	643.5287278	920.9023013
650.3371865	0.501115633	0.00381941	0.035521315	ZNF580
19	55635016	55643469	+	2692
protein_coding	zinc finger protein 580 [Source:HGNC
Symbol;Acc:HGNC:29473]	zf-C2H2 1009	959	859	677	531
746	18.3545945	16.87990478	19.85294571
13.97415824	12.12458341	12.88530759
ENSG00000100991 2084.86272	2314.262325	2650.480962
2730.812953	3373.108352	3485.061743	2349.868669
3196.327683	-0.44431796	0.00382148	0.035521893
TRPC4AP 20	35002404	35092871	-	3226
protein_coding	transient receptor potential cation channel subfamily
C member 4 associated protein [Source:HGNC Symbol;Acc:HGNC:16181]
-	2298	2615	2280	2636	2955	4040
34.88304152	38.40906817	43.97211251	45.40389248
56.30415984	58.23016493
ENSG00000111863 304.8363246	339.8381388	167.3987976
438.2146733	474.8606005	495.154812	270.691087
469.4100286	-0.791041966	0.003827443	0.035558631	ADTRP
6	11712054	11807046	-	4150
protein_coding	androgen dependent TFPI regulating protein
[Source:HGNC Symbol;Acc:HGNC:21214]	-	336	384	144
423	416	574	3.964787032	4.384393937
2.158843906	5.663753118	6.16158713	6.431241436
ENSG00000178741 1245.655576	1505.376755	2148.284569
1032.860589	1244.226093	982.5458726	1633.105634
1086.544185	0.587525896	0.003858495	0.035803035	COX5A
15	74919791	74938168	-	1411
protein_coding	cytochrome c oxidase subunit 5A [Source:HGNC
Symbol;Acc:HGNC:2267]	-	1373	1701	1848	997	1090
1139	47.65102062	57.12204417	81.48577487
39.2627024	47.48395059	37.5342488
ENSG00000104427 509.8750429	442.4975766	433.6093854
362.5889732	248.8452185	238.0883765	461.9940016
283.1741894	0.707133535	0.003859401	0.035803035
ZC2HC1A 8	78666047	78719765	+	4181
protein_coding	zinc finger C2HC-type containing 1A [Source:HGNC
Symbol;Acc:HGNC:24277]	-	562	500	373	350	218
276	6.582408511	5.666518065	5.550543503
4.651573851	3.204967916	3.069445543
ENSG00000234769 53.52780699	60.17967041	41.8496994
110.848629	150.6769213	100.9287683	51.85239227
120.8181062	-1.214938984	0.003859822	0.035803035	WASH4P
16	14381	18068	-	1246	unprocessed_pseudogene

"WAS protein family homolog 4, pseudogene [Source:HGNC
Symbol;Acc:HGNC:14126]" - 59 68 36 107 132
117 2.318796589 2.585933255 1.797592738
4.771751064 6.511832922 4.366151866
ENSG00000175806 584.2696221 703.5711467 811.4191716
442.3585473 503.3978962 456.336055 699.7533135
467.3641662 0.581758869 0.003867916 0.035859311 MSRA
8 10054268 10428891 + 4162
protein_coding methionine sulfoxide reductase A [Source:HGNC
Symbol;Acc:HGNC:7377] - 644 795 698 427 441
529 7.577264981 9.050894311 10.4342249
5.700826749 6.513041925 5.909960991
ENSG00000117152 1252.913584 931.8998962 1299.665665
978.9902277 671.197195 365.7589552 1161.493048
671.9821259 0.790261236 0.003882027 0.035971277 RGS4
1 163068775 163076802 + 4836
protein_coding regulator of G protein signaling 4 [Source:HGNC
Symbol;Acc:HGNC:10000] - 1381 1053 1118 945 588
424 13.98414988 10.31735846 14.38342648
10.85819308 7.473747034 4.07671717
ENSG00000214460 0 0 0 0 21.68834474
18.978059 0 13.55546791 -6.236713858 0.00388509
0.035980816 TPT1P6 6 85427453 85427966
- 514 processed_pseudogene "tumor protein,
translationally-controlled 1 pseudogene 6 [Source:HGNC
Symbol;Acc:HGNC:49297]" - 0 0 0 0 19
22 0 0 0 0 2.272154413
1.990171854
ENSG00000137699 14.51601546 4.424975766 13.9498998 0
0 0 10.96363034 0 5.885965198
0.003895534 0.03605866 TRIM29 11 120111275
120185529 - 10218 protein_coding tripartite motif
containing 29 [Source:HGNC Symbol;Acc:HGNC:17274] - 16
5 12 0 0 0 0.076680117
0.023186252 0.073067154 0 0 0
ENSG00000019505 4.53625483 50.44472373 30.2247829 0
5.707459141 0 28.40192048 1.90248638
3.963502901 0.003913298 0.036204148 SYT13 11
45240301 45286319 - 5424 protein_coding
synaptotagmin 13 [Source:HGNC Symbol;Acc:HGNC:14962] - 5
57 26 0 5 0 0.045141814
0.497945275 0.298236308 0 0.056662755 0
ENSG00000134352 3534.649763 2806.319631 3260.789078
2656.223221 2378.86897 2022.888561 3200.586157
2352.660251 0.444280819 0.003923587 0.036280365 IL6ST
5 55935095 55994993 - 9292
protein_coding interleukin 6 signal transducer [Source:HGNC
Symbol;Acc:HGNC:6021] - 3896 3171 2805 2564 2084
2345 20.53233921 16.17012889 18.78151023
15.33277855 13.7859238 11.73450202

ENSG00000273893 43.54804637 58.40968011 36.03724115
95.30910153 120.9981338 114.730993 45.99832254
110.3460761 -1.257877501 0.003943731 0.036447577
AL133520.1 20 46681676 46682375 - 700
sense_intronic "novel transcript, sense intronic to SLC13A3"
- 48 66 31 92 106 133
3.357931874 4.467579982 2.755310203 7.303008918
9.307974513 8.834553785
ENSG00000085733 4418.312204 4770.123875 4275.644288
3739.846267 3191.611152 3470.396879 4488.026789
3467.284766 0.372297198 0.003968345 0.036653745 CTTN
11 70398404 70436584 + 6949
protein_coding cortactin [Source:HGNC Symbol;Acc:HGNC:3338] -
4870 5390 3678 3610 2796 4023 34.31905598
36.75300844 32.93034342 28.86668638 24.73216958
26.91898863
ENSG00000141540 4.53625483 19.46989337 12.78740815
55.94229872 30.82027936 74.18695789 12.26451878
53.64984533 -2.131554779 0.003970183 0.036653745 TTYH2
17 74213514 74262020 + 5375
protein_coding tweety family member 2 [Source:HGNC
Symbol;Acc:HGNC:13877] - 5 22 11 54 27
86 0.045553339 0.193941457 0.127327163
0.558248204 0.308768264 0.743962424
ENSG00000071205 551.6085873 578.7868301 613.7955911
727.2498834 1076.426794 795.3531997 581.3970029
866.3432924 -0.575047297 0.003994971 0.036859403
ARHGAP10 4 147732063 148072780 + 10759
protein_coding Rho GTPase activating protein 10 [Source:HGNC
Symbol;Acc:HGNC:26099] - 608 654 528 702 943
922 2.767326203 2.880263903 3.053295669
3.625577961 5.387498291 3.984648637
ENSG00000271204 0 0 0 10.35968495 12.55641011
8.626390453 0 10.51416184 -5.868923559
0.003997459 0.036859403 AC016831.4 7 130930209
130932206 - 1998 lincRNA novel transcript
- 0 0 0 10 11 10 0
0 0 0.278109971 0.33841107 0.232720978
ENSG00000127445 1284.667368 1721.315573 2065.747662
968.6305427 1227.103715 1250.826616 1690.576868
1148.853625 0.556596355 0.003998711 0.036859403 PIN1
19 9835257 9849682 + 4570
protein_coding "peptidylprolyl cis/trans isomerase, NIMA-interacting
1 [Source:HGNC Symbol;Acc:HGNC:8988]" - 1416 1945 1777
935 1075 1450 15.1731495 20.16646822
24.19235097 11.36861226 14.4590455 14.75308417
ENSG00000250251 329.3321007 290.2784102 289.4604208
223.7691949 148.3939377 145.7859987 303.0236439
172.6497104 0.812479108 0.004002462 0.036874762 PKD1P6
16 15125242 15154564 - 6015

transcribed_unprocessed_pseudogene	"polycystin 1, transient receptor potential channel interacting pseudogene 6 [Source:HGNC Symbol;Acc:HGNC:30070]" -	363	328	249	216	130
	169	2.955287092	2.583834263	2.57555342
1.995400897	1.328480184	1.306417806
ENSG00000160345 48.99155216	46.01974796	47.66215765
78.73360561	106.15874	163.9014186	47.55781926
116.2645881	-1.292131776	0.004012876	0.036944384
C9orf116	9	135495181	135501734	-	1864
	protein_coding	chromosome 9 open reading frame 116 [Source:HGNC Symbol;Acc:HGNC:28435]	-	54	52	41
	76	93	190	1.418654158	1.321855178
1.36849998	2.265581699	3.066792558	4.739567481
ENSG00000196961 3518.319246	3185.097556	3819.947562
2631.359977	2703.052649	2752.681193	3507.788121
2695.69794	0.379583384	0.004014196	0.036944384	AP2A1
	19	49766968	49807113	+	8746
protein_coding	adaptor related protein complex 2 subunit alpha 1 [Source:HGNC Symbol;Acc:HGNC:561]	-	3878	3599	3286
	2540	2368	3191	21.71335912	19.49839277
23.37571656	16.13750119	16.64253907	16.96478458
ENSG00000257218 389.2106644	312.4032891	369.6723447
571.8546092	486.2755188	570.2044089	357.0954327
542.778179	-0.605097452	0.004020474	0.036982922	GATC
	12	120446438	120463749	+	4422
protein_coding	glutamyl-tRNA amidotransferase subunit C [Source:HGNC Symbol;Acc:HGNC:25068]	-	429	353	318	552	426
	661	4.750805357	3.782530233	4.474198299
6.936372107	5.921588455	6.950463058
ENSG00000164258 655.0351974	774.370759	874.1937207
550.0992708	503.3978962	521.0339833	767.866559
524.8437168	0.548015953	0.004033201	0.037080715	NDUFS4
	5	53560633	53683340	+	1176
protein_coding	NADH:ubiquinone oxidoreductase subunit S4 [Source:HGNC Symbol;Acc:HGNC:7711]	-	722	875	752	531	441
	604	30.06481663	35.25552385	39.78481706
25.08991806	23.05040858	23.88144686
ENSG00000113300 906.343715	839.8604003	977.6554776
628.8328764	705.4419498	584.0066337	907.9531976
639.4271533	0.506193929	0.00403765	0.037102333	CNOT6
	5	180494412	180578405	+	6586
protein_coding	CCR4-NOT transcription complex subunit 6 [Source:HGNC Symbol;Acc:HGNC:14099]	-	999	949	841	607	618
	677	7.428009413	6.827644918	7.944766105
5.121285747	5.767852011	4.779670523
ENSG00000140406 1142.228966	1130.138811	1496.126753
785.2641191	763.658033	1029.99102	1256.164843
859.6377241	0.545480263	0.004047886	0.037177076	TLNRD1
	15	81000944	81005788	+	4845
protein_coding	talin rod domain containing 1 [Source:HGNC

Symbol;Acc:HGNC:13519] - 1259 1277 1287 758 669
1194 12.72508325 12.48888184 16.5269081
8.693356064 8.487498227 11.45886396
ENSG00000204178 1103.217175 969.9546878 1109.017034
1319.823863 1583.249166 1446.645679 1060.729632
1449.906236 -0.451056103 0.004053259 0.037207107 MACO1
1 25430858 25500209 + 3979
protein_coding macoilin 1 [Source:HGNC Symbol;Acc:HGNC:25572] -
1216 1096 954 1274 1387 1677 14.96539966
13.05157898 14.91699287 17.79129384 21.42643324
19.59702477
ENSG00000278535 263.1027801 263.7285556 209.248497
181.2944866 103.8757564 63.83528935 245.3599442
116.3351774 1.080079915 0.004073718 0.037375517 DHRS11
17 36591798 36600806 + 5462
protein_coding dehydrogenase/reductase 11 [Source:HGNC
Symbol;Acc:HGNC:28639] - 290 298 180 175 91
74 2.600009804 2.58518132 2.050348363
1.780321336 1.02408748 0.629957197
ENSG00000114544 1057.854626 1073.499121 1109.017034
1308.428209 1434.855228 1731.316564 1080.123594
1491.533334 -0.466307028 0.004082639 0.037437943
SLC41A3 3 126006355 126101561 - 6592
protein_coding solute carrier family 41 member 3 [Source:HGNC
Symbol;Acc:HGNC:31046] - 1166 1213 954 1263 1257
2007 8.661837567 8.719067564 9.004052584
10.64628764 11.72102087 14.15667272
ENSG00000114062 1880.731252 1599.186242 1868.124081
2573.345741 2212.211163 2337.751813 1782.680525
2374.436239 -0.413857734 0.004107181 0.037643484 UBE3A
15 25333728 25439056 - 12813
protein_coding ubiquitin protein ligase E3A [Source:HGNC
Symbol;Acc:HGNC:12496] - 2073 1807 1607 2484 1938
2710 7.922772026 6.682419985 7.803184889
10.77240838 9.297160037 9.834436554
ENSG00000165280 9237.629336 9221.649496 9942.791082
11657.75347 12196.84018 11690.48434 9467.356638
11848.35933 -0.323703881 0.004110121 0.037650925 VCP
9 35056064 35073249 - 5438
protein_coding valosin containing protein [Source:HGNC
Symbol;Acc:HGNC:12666] - 10182 10420 8553 11253 10685
13552 91.69012673 90.7935415 97.85569717
114.984992 120.7765677 115.876457
ENSG00000138035 1326.400912 1037.214319 1079.954743
905.4364645 710.0079171 796.2158388 1147.856658
803.8867402 0.51384285 0.004149347 0.037990582 PNPT1
2 55634265 55693910 - 5134
protein_coding polyribonucleotide nucleotidyltransferase 1
[Source:HGNC Symbol;Acc:HGNC:23166] - 1462 1172 929
874 622 923 13.94505373 10.81678477

11.25814133	9.459487593	7.447009032	8.359433645
ENSG00000104897 2161.071801	2916.944025	2723.717936
2994.984919	3674.462195	4169.997145	2600.57792
3613.148086	-0.474679952	0.004159762	0.038066236	SF3A2
	19	2236504 2248679 +	3134	protein_coding
splicing factor 3a subunit 2 [Source:HGNC Symbol;Acc:HGNC:10766]
	-	2382	3296	2343	2891	3219	4834
37.21957833	49.83272677	46.51362027	51.25793939
63.13487788	71.71973432
ENSG00000223891 109.7773669	89.38451047	101.1367735
49.72648776	43.37668947	23.29125422	100.0995503
38.79814382	1.373160193	0.004177488	0.038208679	OSER1-
DT	20	44210960	44226027	+	2186
lincRNA OSER1 divergent transcript [Source:HGNC Symbol;Acc:HGNC:48585]
	-	121	101	87	48	38	27
2.710590402	2.189261587	2.476144252	1.220121622
1.068515433	0.574307681
ENSG00000146701 4435.549973	4973.672761	5164.9504
5726.83384	6829.545608	6330.045314	4858.057711
6295.474921	-0.374011182	0.004180239	0.038214082	MDH2
	7	76048051	76067508	+	3641
protein_coding	malate dehydrogenase 2 [Source:HGNC
Symbol;Acc:HGNC:6971]	-	4889	5620	4443	5528	5983
	7338	65.75488793	73.13783114	75.92109809
84.36443437	101.0056538	93.71045501
ENSG00000153015 557.9593441	614.1866363	691.6825317
457.8980747	399.5221399	396.8139608	621.2761707
418.0780585	0.570835741	0.004204357	0.03841471	CWC27
	5	64768930	65018763	+	3740
protein_coding	CWC27 spliceosome associated protein homolog
[Source:HGNC Symbol;Acc:HGNC:10664]	-	615	694	595
	442	350	460	8.052527138	8.792539116
9.898108501	6.566934897	5.752330128	5.718962484
ENSG00000090097 990.7180548	1479.711896	1249.678524
666.1277422	979.3999886	843.6609863	1240.036158
829.7295724	0.580010846	0.004206891	0.038418012	PCBP4
	3	51957454	51974016	-	3738
protein_coding	poly(rC) binding protein 4 [Source:HGNC
Symbol;Acc:HGNC:8652]	-	1092	1672	1075	643	858
	978	14.30579591	21.19451178	17.8927055
9.558367396	14.10897133	12.16551717
ENSG00000089006 1969.641847	1840.789918	1576.338677
1520.801751	1187.151501	1149.897847	1795.590148
1285.950366	0.482227795	0.004210524	0.038431356	SNX5
	20	17941597	17968980	-	7953
protein_coding	sorting nexin 5 [Source:HGNC Symbol;Acc:HGNC:14969]
	-	2171	2080	1356	1468	1040	1333
13.36772567	12.39249617	10.60804989	10.25668734
8.038031745	7.793457745
ENSG00000145604 1171.260997	1035.444329	859.0813293

764.5447492	704.300458	674.5837334	1021.928885
714.4763135	0.517392535	0.004214168	0.038444771	SKP2
5	36151989	36184319	+	3837
protein_coding	S-phase kinase associated protein 2 [Source:HGNC
Symbol;Acc:HGNC:10901]	-	1291	1170	739	738	617
782	16.47643034	14.44842485	11.98283242
10.68751178	9.884181805	9.476456469
ENSG00000272391 2593.830512	2361.167069	2445.882431
3147.272288	3093.442854	3281.478928	2466.960004
3174.06469	-0.363688725	0.004224616	0.038514185
POM121C 7	75416787	75486271	-	8767
protein_coding	POM121 transmembrane nucleoporin C [Source:HGNC
Symbol;Acc:HGNC:34005]	-	2859	2668	2104	3038	2710
3804	15.96951889	14.41986716	14.93143595
19.25523426	19.0005269	20.17532409
ENSG00000177311 3091.911292	2492.146351	3437.487809
2394.123192	2288.691115	1355.20594	3007.181817
2012.673416	0.579653642	0.004226222	0.038514185	ZBTB38
3	141324213	141449792	+	14243
protein_coding	zinc finger and BTB domain containing 38 [Source:HGNC
Symbol;Acc:HGNC:26636]	ZBTB	3408	2816	2957	2311	2005
1571	11.71727966	9.368231562	12.9168524
9.015929661	8.652871672	5.128681493
ENSG00000139722 667.736711	1063.764174	850.9438877
1091.910794	1300.159192	1445.78304	860.8149242
1279.284342	-0.571678845	0.004228308	0.038514185	VPS37B
12	122865328	122896444	-	5953
protein_coding	"VPS37B, ESCRT-I subunit [Source:HGNC
Symbol;Acc:HGNC:25754]" -	736	1202	732	1054	1139
1676	6.054393099	9.567424108	7.650363048
9.838225483	11.76075497	13.09088929
ENSG00000185414 946.2627575	903.5800513	760.269539
1109.522258	1211.12283	1326.738852	870.0374493
1215.794646	-0.4821871	0.004238062	0.038582146	MRPL30
2	99181079	99197626	+	2898
protein_coding	mitochondrial ribosomal protein L30 [Source:HGNC
Symbol;Acc:HGNC:14036]	-	1043	1021	654	1071	1061
1538	17.62441095	16.69374602	14.04061768
20.53539842	22.50424063	24.67680746
ENSG00000105245 1024.286341	1363.777531	1097.392117
1330.183547	1870.905106	1810.679356	1161.818663
1670.589337	-0.523689439	0.004240132	0.038582146	NUMBL
19	40665905	40690972	-	5823
protein_coding	"NUMB like, endocytic adaptor protein [Source:HGNC
Symbol;Acc:HGNC:8061]"	-	1129	1541	944	1284	1639
2099	9.494581688	12.53955976	10.08630393
12.25265703	17.30133135	16.76087421
ENSG00000164930 2083.048218	1791.23019	2175.021877
1655.477655	1451.977605	1353.480662	2016.433428
1486.978641	0.439353244	0.004249092	0.038627763	FZD6

8	103298433	103332866	+	4065
protein_coding	frizzled class receptor 6 [Source:HGNC
Symbol;Acc:HGNC:4044]	-	2296	2024	1871	1598	1272
1569	27.65922565	23.5926323	28.63650972
21.84380388	19.23419088	17.94706401
ENSG00000095015 509.8750429	429.2226493	412.6845357
319.0782964	240.8547757	295.0225535	450.594076
284.9852086	0.660553673	0.004249512	0.038627763	MAP3K1
5	56815574	56896152	+	7716
protein_coding	mitogen-activated protein kinase kinase kinase 1
[Source:HGNC Symbol;Acc:HGNC:6848]	-	562	485	355
308	211	342	3.566750905	2.978351564
2.862483426	2.218045961	1.680883432	2.060937897
ENSG00000166435 315.7233362	319.4832503	408.0345691
475.5095392	511.388339	639.2155325	347.7470518
542.0378036	-0.642987191	0.004252347	0.038633673	XRRA1
11	74807739	74949200	-	9086
protein_coding	X-ray radiation resistance associated 1 [Source:HGNC
Symbol;Acc:HGNC:18868]	-	348	361	351	459	448
741	1.875578281	1.88261238	2.403483974
2.807061946	3.030767641	3.792071292
ENSG00000255327 8.165258694	10.61994184	12.78740815	0
0	0	10.52420289	0	5.827509457
0.004260611	0.0386885	AP003062.1	11	134950708
134951568	+	762	lincRNA uncharacterized
LOC105369586 [Source:NCBI gene;Acc:105369586]	-	9	12
11	0	0	0	0.578383935	0.746195654
0.898141079	0	0	0
ENSG00000180573 237.6997531	176.9990306	223.1983968
311.826517	463.4456822	315.7258906	212.6323935
363.6660299	-0.773622043	0.004262756	0.0386885
HIST1H2AC	6	26124145	26139116	+	1668
protein_coding	histone cluster 1 H2A family member c
[Source:HGNC Symbol;Acc:HGNC:4733]	-	262	200	192
301	406	366	7.691905297	5.681465714
7.161632461	10.02726501	14.96157615	10.20272209
ENSG00000105053 643.2409349	563.7419125	742.8321643
861.9257878	942.8722501	984.2711507	649.9383372
929.6897295	-0.517856595	0.004267921	0.038715512	VRK3
19	49976467	50025946	-	9796
protein_coding	vaccinia related kinase 3 [Source:HGNC
Symbol;Acc:HGNC:18996]	-	709	637	639	832	826
1141	3.544264643	3.081180188	4.058438118
4.719397884	5.182969236	5.415865696
ENSG00000120694 10881.56809	10105.75965	9410.369906
12683.36228	13148.84437	12497.91449	10132.56588
12776.70705	-0.334403464	0.004281429	0.038818141	HSPH1
13	31134974	31162388	-	8390
protein_coding	heat shock protein family H (Hsp110) member 1
[Source:HGNC Symbol;Acc:HGNC:16969]	-	11994	11419	8095

12243 11519 14488 70.0052752 64.49002614
60.02908711 81.08451667 84.39178592 80.29296483
ENSG00000163900 567.0318537 700.0311661 576.5958583
738.6455369 1005.654301 967.0183698 614.5529594
903.7727357 -0.555896219 0.00428524 0.038832796
TMEM41A 3 185476496 185499057 - 8668
protein_coding transmembrane protein 41A [Source:HGNC
Symbol;Acc:HGNC:30544] - 625 791 496 713 881
1121 3.530935613 4.323983437 3.560160853
4.570699513 6.247472685 6.013367251
ENSG00000134986 671.3657148 514.182184 929.9933199
308.7186115 525.086241 430.4568836 705.1804062
421.4205787 0.742006516 0.004295263 0.038903693 NREP
5 111662621 111997464 - 9337
protein_coding neuronal regeneration related protein [Source:HGNC
Symbol;Acc:HGNC:16834] - 740 581 800 298 460
499 3.881084018 2.948459824 5.330764229
1.773458168 3.028292584 2.48498748
ENSG00000153815 1186.684264 922.1649496 1682.125417
967.5945742 744.252672 736.6937447 1263.65821
816.1803303 0.62978724 0.004318314 0.039089008 CMIP
16 81445170 81711762 + 12715
protein_coding c-Maf inducing protein [Source:HGNC
Symbol;Acc:HGNC:24319] - 1308 1042 1447 934 652
854 5.037558042 3.883093029 7.080419887
4.081713853 3.151944715 3.123003884
ENSG00000170322 1318.235654 1234.568239 1085.767201
1621.290695 1630.050331 1639.014186 1212.857031
1630.118404 -0.425922256 0.004320143 0.039089008 NFRKB
11 129863636 129895590 - 6335
protein_coding nuclear factor related to kappaB binding protein
[Source:HGNC Symbol;Acc:HGNC:7802] Others 1453 1395 934
1565 1428 1900 11.23175648 10.4340768
9.172909391 13.72713062 13.85571521 13.94562555
ENSG00000138162 407.3556837 388.5128722 416.1720107
298.3589265 152.959905 251.0279622 404.0135222
234.1155979 0.785191246 0.004327524 0.039128942 TACC2
10 121989174 122254545 + 18844
protein_coding transforming acidic coiled-coil containing protein 2
[Source:HGNC Symbol;Acc:HGNC:11523] - 449 439 358
288 134 291 1.1668148 1.103869834
1.181998098 0.849241944 0.437098245 0.718043759
ENSG00000111237 680.4382245 619.4966072 840.4814629
466.1858227 465.7286659 513.2702319 713.4720982
481.7282402 0.564951954 0.004328981 0.039128942 VPS29
12 110491097 110502117 - 3211
protein_coding "VPS29, retromer complex component [Source:HGNC
Symbol;Acc:HGNC:14340]" - 750 700 723 450 408
595 11.43798813 10.32961596 14.00892591
7.787252411 7.810291043 8.61603944

ENSG00000197324 2636.471307 2755.874907 3674.636105
2181.74965 2414.255217 1971.130218 3022.32744
2189.045028 0.465204722 0.004337348 0.03917289 LRP10
14 22871613 22881580 + 7112
protein_coding LDL receptor related protein 10 [Source:HGNC
Symbol;Acc:HGNC:14553] - 2906 3114 3161 2106 2115
2285 20.00932994 20.74690418 27.65283068
16.45427163 18.27957036 14.93913578
ENSG00000139644 10154.86006 10478.34261 11300.58133
13553.57582 13943.32268 12679.93133 10644.59467
13392.27661 -0.331304734 0.004338272 0.03917289 TMBIM6
12 49707725 49764934 + 6186
protein_coding transmembrane BAX inhibitor motif containing 6
[Source:HGNC Symbol;Acc:HGNC:11723] - 11193 11840 9721
13083 12215 14699 88.60643667 90.691843
97.77050853 117.519374 121.3754675 110.4864176
ENSG00000142634 3402.191122 3856.808877 5167.275384
2720.453268 3250.968727 2974.379428 4142.091794
2981.933807 0.473813066 0.004341607 0.039182999 EFHD2
1 15409895 15430343 + 2531
protein_coding EF-hand domain family member D2 [Source:HGNC
Symbol;Acc:HGNC:28670] - 3750 4358 4445 2626 2848
3448 72.5550768 81.58710472 109.2663578
57.65203216 69.16640447 63.34409418
ENSG00000182923 822.8766261 684.1012534 695.1700066
498.300846 568.4629304 418.379937 734.0492954
495.0479045 0.570003238 0.004357639 0.039300446 CEP63
3 134485721 134575017 + 10385
protein_coding centrosomal protein 63 [Source:HGNC
Symbol;Acc:HGNC:25815] - 907 773 598 481 498
485 4.276903681 3.526951064 3.582626465
2.573656719 2.947611227 2.171532111
ENSG00000147955 2347.058249 2181.513052 2181.996827
1657.549592 1725.935644 1766.684765 2236.856043
1716.723334 0.381812648 0.004359064 0.039300446
SIGMAR1 9 34634722 34637809 - 2196
protein_coding sigma non-opioid intracellular receptor 1 [Source:HGNC
Symbol;Acc:HGNC:8157] - 2587 2465 1877 1600 1512
2048 57.68896887 53.18767773 53.17883066
40.48551709 42.32206166 43.36393
ENSG00000103111 1556.842658 1383.247424 1505.426687
1797.405339 2054.685291 2058.256762 1481.838923
1970.115797 -0.4110649 0.004368649 0.039366805 MON1B
16 77190835 77202405 + 6942
protein_coding "MON1 homolog B, secretory trafficking associated
[Source:HGNC Symbol;Acc:HGNC:25020]" - 1716 1563 1295
1735 1800 2386 12.10490423 10.66843731
11.60625226 13.88759086 15.93805261 15.98147457
ENSG00000138185 176.0066874 161.954113 112.76169
225.8411319 325.325171 268.2807431 150.2408302

273.1490153	-0.858684005	0.00438096	0.039457636	ENTPD1
	10	95711779	95869695	+	6975
protein_coding	ectonucleoside triphosphate diphosphohydrolase 1
[Source:HGNC Symbol;Acc:HGNC:3363]	-	194	183	97
	218	285	311	1.36202852	1.243178007
0.865235578	1.736698085	2.511585738	2.073228619
ENSG00000166086 1112.289684	1085.004058	1246.191049
761.4368438	893.7881015	837.622513	1147.828264
830.9491527	0.465762887	0.004387663	0.0394979	JAM3
	11	134068925	134152001	+	5451
protein_coding	junctional adhesion molecule 3 [Source:HGNC
Symbol;Acc:HGNC:15532]	-	1226	1226	1072	735	783
	971	11.01394673	10.65714142	12.23560505
7.492438739	8.829435538	8.282740709
ENSG00000087008 456.3472359	587.6367817	535.9086506
796.6597726	757.9505739	731.5179104	526.6308894
762.0427523	-0.532983706	0.004395072	0.039525782	ACOX3
	4	8366282 8440723 -	3846	protein_coding	"acyl-
CoA oxidase 3, pristanoyl [Source:HGNC Symbol;Acc:HGNC:121]"	-
	503	664	461	769	664	848	6.404531834
	8.180601553	7.457589809	11.11038487	10.61221792
	10.25221229
ENSG00000183741 1588.596441	2114.253421	1714.675184
2410.698688	2027.289487	3653.276357	1805.841682
2697.088177	-0.579108989	0.004395229	0.039525782	CBX6
	22	38861450	38872314	-	8030
protein_coding	chromobox 6 [Source:HGNC Symbol;Acc:HGNC:1556]	-
	1751	2389	1475	2327	1776	4235	10.67823033
	14.09701122	11.42834445	16.1024842	13.59486115
	24.52273401
ENSG00000198836 2672.761346	2210.717892	2277.321142
2037.750029	1687.124922	1457.859987	2386.93346
1727.578313	0.466865529	0.004405457	0.039597623	OPA1
	3	193593144	193697823	+	9052
protein_coding	"OPA1, mitochondrial dynamin like GTPase [Source:HGNC
Symbol;Acc:HGNC:8140]"	-	2946	2498	1959	1967	1478
	1690	15.93737827	13.07598247	13.46470173
12.07457514	10.03638353	8.681068387
ENSG00000267493 41.73354444	27.43484975	18.5998664
3.107905485	0	6.901112362	29.25608686	3.336339282
	3.093249392	0.004421479	0.039711083	CIRBP-AS1
	19	1267814 1270241 -	994	antisense	CIRBP
antisense RNA 1 [Source:HGNC Symbol;Acc:HGNC:28588]	-	46
	31	16	3	0	8	2.266209892
1.477752662	1.001475767	0.167705349	0	0.374226571
ENSG00000154217 92.53959853	45.13475281	60.44956579
98.41700702	142.6864785	233.7751813	66.04130571
158.2928889	-1.262508289	0.00442257	0.039711083
PITPNC1 17	67377281	67697261	+	7819
protein_coding	phosphatidylinositol transfer protein cytoplasmic 1

[Source:HGNC Symbol;Acc:HGNC:21045]	-	102	51	52
	95	125	271	0.638818732	0.309061853
0.413769979	0.675125382	0.982666521	1.611569709
ENSG00000106608 1109.567931	1375.282468	1390.340013
1531.161435	1836.660352	2024.613839	1291.730138
1797.478542	-0.477259575	0.004426195	0.039723472	URGCP
	7	43875894	43926411	-	7830
protein_coding	upregulator of cell proliferation [Source:HGNC
Symbol;Acc:HGNC:30890]	-	1223	1554	1196	1478	1609
	2347	7.648801292	9.404066537	9.503339933
10.48877371	12.63111363	13.93741866
ENSG00000149182 1855.328225	2116.908406	2080.860053
2273.950846	2890.257309	3025.275132	2017.698895
2729.827762	-0.436327047	0.004443022	0.039854267
ARFGAP2 11	47164299	47177125	-	5918
protein_coding	ADP ribosylation factor GTPase activating protein 2
[Source:HGNC Symbol;Acc:HGNC:13504]	-	2045	2392	1790
	2195	2532	3507	16.9218186	19.15193483
18.81849625	20.60969702	26.2988102	27.55445485
ENSG00000148339 361.0858845	461.9674699	433.6093854
235.1648483	265.967596	290.7093583	418.8875799
263.9472675	0.665347191	0.00444611	0.039861758
SLC25A25	9	128068201	128109245	+	4936
	protein_coding	solute carrier family 25 member 25
[Source:HGNC Symbol;Acc:HGNC:20663]	-	398	522	373
	227	233	337	3.94854057	5.010969886
4.701544244	2.555422709	2.901537115	3.174576288
ENSG00000132823 582.4551202	615.9566266	709.1199064
807.0194575	883.514675	1064.496582	635.8438844
918.3435715	-0.53191726	0.004448689	0.039864667	OSER1
	20	44196496	44210791	-	1712
protein_coding	oxidative stress responsive serine rich 1 [Source:HGNC
Symbol;Acc:HGNC:16105]	-	642	696	610	779	774
	1234	18.36368994	19.26335464	22.16832712
25.28399763	27.78974493	33.51524647
ENSG00000183648 202.3169654	232.7537253	215.0609552
133.6399358	90.17785443	126.8079397	216.7105486
116.8752433	0.888726771	0.004452411	0.039873069	NDUFB1
	14	92116122	92121917	-	2263
protein_coding	NADH:ubiquinone oxidoreductase subunit B1 [Source:HGNC
Symbol;Acc:HGNC:7695]	-	223	263	185	129	79
	147	4.825574142	5.506778845	5.086206881
3.167504204	2.145803244	3.020395391
ENSG00000163902 6737.245673	7010.931603	7791.019038
6254.141804	5401.539331	4013.859478	7179.732105
5223.180204	0.45913626	0.004454134	0.039873069	RPN1
	3	128619970	128681075	-	3992
protein_coding	ribophorin I [Source:HGNC Symbol;Acc:HGNC:10381]
	-	7426	7922	6702	6037	4732	4653
91.09469704	94.03093321	104.4529566	84.03161047

72.86207977	54.19678668
ENSG00000137312 2243.631639	2385.946933	3378.200735
1710.383985	2214.494147	1635.56363	2669.259769
1853.480587	0.526117767	0.004456413	0.03987329	FLOT1
	6	30727709	30742733	-	3186
protein_coding	flotillin 1 [Source:HGNC Symbol;Acc:HGNC:3757]	-
	2473	2696	2906	1651	1940	1896	38.0108016
	40.09595457	56.74880035	28.7947522	37.42857723
	27.67091878
ENSG00000225697 690.4179851	547.8119998	480.1090514
386.4162486	234.0058248	414.0667417	572.7796788
344.829605	0.730832616	0.00448905	0.040135866
SLC26A6 3	48625723	48635493	-	6373
protein_coding	solute carrier family 26 member 6 [Source:HGNC
Symbol;Acc:HGNC:14472]	-	761	619	413	373	205
	480	5.847489112	4.60228142	4.031929965
3.25219784	1.977230508	3.50209834
ENSG00000167395 738.5022863	739.855948	702.1449565
831.8827014	1148.340779	1155.936321	726.834397
1045.3866	-0.524249569	0.004490298	0.040135866	ZNF646
	16	31074422	31084196	+	8635
protein_coding	zinc finger protein 646 [Source:HGNC
Symbol;Acc:HGNC:29004]	zf-C2H2 814	836	604	803	1006
	1340	4.616265156	4.587439781	4.351925412
5.167318689	7.161153765	7.215617025
ENSG00000247626 280.3405485	476.1273924	297.5978624
537.6676489	577.5948651	589.1824679	351.3552677
568.1483273	-0.691953335	0.00449471	0.040141137	MARS2
	2	197705369	197708387	+	3019
protein_coding	"methionyl-tRNA synthetase 2, mitochondrial
[Source:HGNC Symbol;Acc:HGNC:25133]"	-	309	538	256
	519	506	683	5.012149887	8.443949037
5.27574382	9.55248333	10.30231356	10.51934282
ENSG00000134779 2249.075145	1967.344225	2251.746326
1744.570945	1612.927953	1523.420554	2156.055232
1626.973151	0.40620502	0.004495426	0.040141137	TPGS2
	18	36777647	36829216	-	10571
protein_coding	tubulin polyglutamylase complex subunit 2 [Source:HGNC
Symbol;Acc:HGNC:24561]	-	2479	2223	1937	1684	1413
	1766	11.483893	9.96436966	11.40040801
8.851931775	8.216246291	7.767936103
ENSG00000171861 366.5293903	515.9521743	339.4475618
478.6174446	668.9142113	877.303909	407.3097088
674.9451883	-0.728259508	0.004498124	0.040144958	MRM3
	17	782273	792509	+	2065	protein_coding
mitochondrial rRNA methyltransferase 3 [Source:HGNC
Symbol;Acc:HGNC:18485]	-	404	583	292	462	586
	1017	9.580540093	13.37749938	8.797709998
12.43179853	17.44315019	22.8998119
ENSG00000127526 1936.980812	2229.302791	2102.947395

2542.266686	2674.515353	3069.269723	2089.743666
2762.017254	-0.402671719	0.004503935	0.040176558
SLC35E1 19	16549831	16572382	-	7257
protein_coding	solute carrier family 35 member E1 [Source:HGNC
Symbol;Acc:HGNC:20803]	-	2135	2519	1809	2454	2343
	3558	14.40686345	16.44740874	15.5091605
18.79011677	19.84552195	22.79711231
ENSG00000188229 8213.342995	10111.95462	9996.265697
10337.92961	14469.55041	13282.91602	9440.521104
12696.79868	-0.427552802	0.00450913	0.040202624	TUBB4B
	9	137241213	137243707	+	2111
protein_coding	tubulin beta 4B class IVb [Source:HGNC
Symbol;Acc:HGNC:20771]	-	9053	11426	8599	9979	12676
	15398	210.006613	256.4675525	253.4349827
262.6702171	369.0977157	339.1619317
ENSG00000138496 327.5175987	292.0484005	319.6852037
421.6391774	526.2277328	489.1163387	313.0837343
478.9944163	-0.613568057	0.004533791	0.040402127	PARP9
	3	122527924	122564577	-	8938
protein_coding	poly(ADP-ribose) polymerase family member 9
[Source:HGNC Symbol;Acc:HGNC:24118]	-	361	330	275
	407	461	567	1.977859944	1.74944394
1.914252355	2.53026555	3.170355294	2.949672007
ENSG00000139318 958.964271	934.5548817	1047.404977
1269.061406	1235.094158	1554.47556	980.3080431
1352.877041	-0.465832586	0.004541606	0.040450845	DUSP6
	12	89347232	89353271	-	4632
protein_coding	dual specificity phosphatase 6 [Source:HGNC
Symbol;Acc:HGNC:3072]	-	1057	1056	901	1225	1082
	1802	11.17468064	10.80243864	12.10216548
14.69533807	14.35840074	18.08911226
ENSG00000108424 15646.45016	13382.89671	14597.40765
12846.00934	10811.0691	9471.776717	14542.2515
11042.95172	0.397187467	0.004545466	0.040450845	KPNB1
	17	47649476	47685505	+	7678
protein_coding	karyopherin subunit beta 1 [Source:HGNC
Symbol;Acc:HGNC:6400]	-	17246	15122	12557	12400	9471
	10980	109.9939903	93.32275834	101.7523933
89.73990822	75.82197436	66.49442739
ENSG00000111639 899.9929583	1282.357977	1365.927689
673.3795217	828.7230673	890.2434947	1182.759541
797.4486946	0.567824709	0.004546118	0.040450845	MRPL51
	12	6491886 6493841 -	1551	protein_coding
mitochondrial ribosomal protein L51 [Source:HGNC
Symbol;Acc:HGNC:14044]	-	992	1449	1175	650	726
	1032	31.32049072	44.26729946	47.13385
23.28700318	28.77214121	30.93847605
ENSG00000143061 147.8819075	165.4940936	155.7738811
93.23716454	79.90442797	43.99459131	156.3832941
72.37872794	1.116127919	0.004558415	0.040539868	IGSF3

1 116574399 116667755 - 7400
protein_coding immunoglobulin superfamily member 3 [Source:HGNC
Symbol;Acc:HGNC:5950] - 163 187 134 90 70
51 1.078659985 1.197391932 1.126626404
0.675807229 0.581451748 0.320456787
ENSG00000161526 1558.65716 1662.905893 1927.411156
1975.59192 2496.442628 2566.35116 1716.324736
2346.128569 -0.451556536 0.004565716 0.040584401
SAP30BP 17 75667116 75708062 + 8268
protein_coding SAP30 binding protein [Source:HGNC
Symbol;Acc:HGNC:30785] - 1718 1879 1658 1907 2187
2975 10.17539729 10.76843902 12.47644639
12.81628832 16.25906904 16.7308313
ENSG00000180917 897.2712054 867.2952501 934.6432865
1108.48629 1219.113272 1385.398307 899.7365806
1237.665956 -0.460804288 0.004582149 0.040696047 CMTR2
16 71281389 71289715 - 7582
protein_coding cap methyltransferase 2 [Source:HGNC
Symbol;Acc:HGNC:25635] - 989 980 804 1070 1068
1606 6.387651226 6.12447317 6.597495691
7.841732822 8.658343925 9.849014549
ENSG00000230202 602.4146414 769.9457832 791.6568136
896.1127481 1049.03099 1188.716604 721.3390794
1044.620114 -0.535253383 0.004582999 0.040696047
AL450405.1 6 117998975 117999448 + 474
processed_pseudogene ribosomal protein L29 (RPL29)
pseudogene - 664 870 681 865 919 1378
68.59910052 86.9695758 89.38725832 101.4027678
119.1748269 135.1767168
ENSG00000174574 1485.169831 1315.102798 1372.902639
1842.987952 1691.690889 2061.707318 1391.058422
1865.462053 -0.423757235 0.004585179 0.040696047
AKIRIN1 1 38991223 39006059 + 2741
protein_coding akirin 1 [Source:HGNC Symbol;Acc:HGNC:25744] -
1637 1486 1181 1779 1482 2390 29.2461247
25.68835029 26.80696879 36.06442034 33.23429942
40.54337362
ENSG00000152642 921.7669814 808.88557 911.3934535
669.2356477 635.8109483 566.7538527 880.6820016
623.9334829 0.497581611 0.004587693 0.040697945 GPD1L
3 32105689 32168713 + 4802
protein_coding glycerol-3-phosphate dehydrogenase 1 like [Source:HGNC
Symbol;Acc:HGNC:28956] - 1016 914 784 646 557
657 10.3609657 9.018835816 10.15782563
7.475192929 7.129850107 6.361715334
ENSG00000085978 756.6473056 745.1659189 687.0325651
879.5372522 1015.927727 1241.337586 729.6152632
1045.600855 -0.519392714 0.00459052 0.040702613
ATG16L1 2 233210051 233295674 + 6047
protein_coding autophagy related 16 like 1 [Source:HGNC

Symbol;Acc:HGNC:21498] -   834   842   591   849   890
        1439   6.75390217   6.597791145   6.080710942
7.801529684   9.04685018   11.06501083
ENSG00000105287 739.4095373   810.6555603   899.768537
988.3139441   1200.849403   1258.590367   816.6112115
1149.251238   -0.493896741   0.004596149   0.040732106   PRKD2
        19   46674275   46717127   -   5446
protein_coding protein kinase D2 [Source:HGNC Symbol;Acc:HGNC:17293]
        -       815   916   774   954   1052   1459
7.328391381   7.969742276   8.84240027   9.733808131
11.87368335   12.45686257
ENSG00000205517 68.95107341   54.86969949   109.2742151
34.18696033   12.55641011   26.7418104   77.69832933
24.49506028   1.657484008   0.004602146   0.04076483   RGL3
        19   11384341   11419342   -   4358
protein_coding ral guanine nucleotide dissociation stimulator like 3
[Source:HGNC Symbol;Acc:HGNC:30282]   -   76   62   94
        33   11   31   0.853994453   0.674110209
1.341984421   0.420764177   0.155150371   0.33075429
ENSG00000258102 39.9190425   67.25963164   54.63710754
127.4241249   133.5545439   106.1046026   53.9385939
122.3610904   -1.18051894   0.004607004   0.040775048
MAP1LC3B2   12   116548105   116576448   +   1767
        protein_coding microtubule associated protein 1 light chain 3
beta 2 [Source:HGNC Symbol;Acc:HGNC:34390]   -   44   76
        47   123   117   123   1.219396125   2.037996734
        1.654886279   3.86794781   4.07002121   3.236678627
ENSG00000169136 849.1869042   1051.374242   856.756346
902.3285591   1777.302776   1623.486683   919.1058307
1434.372673   -0.641808451   0.00460791   0.040775048   ATF5
        19   49928702   49933935   +   2954
protein_coding activating transcription factor 5 [Source:HGNC
Symbol;Acc:HGNC:790]   TF_bZIP 936   1188   737   871   1557
        1882   15.51650985   19.05602836   15.52257774
16.38399126   32.39854314   29.62375766
ENSG00000104967 41.73354444   38.93978674   120.8991316
20.7193699   14.83939377   0   67.19082092   11.85292122
        2.513955203   0.004611586   0.040783115   NOVA2   19
        45933734   45973546   -   8684   protein_coding
        NOVA alternative splicing regulator 2 [Source:HGNC
Symbol;Acc:HGNC:7887]   -   46   44   104   20   13
        0   0.259398046   0.240081835   0.745109964
0.127974141   0.092017599   0
ENSG00000203446 17.23776835   16.81490791   8.137441549
58.01423571   41.09370581   57.79681603   14.0633726
52.30158585   -1.885394583   0.004613433   0.040783115
AC004988.1   7   40538127   40546928   -   1771
        antisense   novel transcript   -   19   19
        7   56   36   67   0.525368129   0.508348423
        0.245915739   1.757039437   1.249485728   1.759086759

ENSG00000100644 4304.905834 3141.732794 3719.97328
3128.624855 2639.129107 2388.647516 3722.203969
2718.800493 0.453335477 0.004618736 0.040809604 HIF1A
14 61695513 61748259 + 5151
protein_coding hypoxia inducible factor 1 subunit alpha [Source:HGNC
Symbol;Acc:HGNC:4910] Others 4745 3550 3200 3020 2312
2769 45.11005436 32.6560193 38.65140408
32.57822637 27.58948794 24.99553427
ENSG00000039523 1711.982573 1517.766688 1949.498497
1220.370887 1357.233784 1232.711196 1726.415919
1270.105289 0.44252821 0.004628012 0.040871142 RIPOR1
16 67518418 67546788 + 6729
protein_coding RHO family interacting cell polarization regulator 1
[Source:HGNC Symbol;Acc:HGNC:25836] - 1887 1715 1677
1178 1189 1429 13.73251416 12.07647084
15.50562873 9.727624677 10.8612219 9.874445533
ENSG00000173486 451.8109811 777.9107396 755.6195724
406.09965 335.5985975 477.039392 661.780431
406.2458798 0.702183705 0.004633645 0.040900468 FKBP2
11 64241003 64244132 + 1608
protein_coding FK506 binding protein 2 [Source:HGNC
Symbol;Acc:HGNC:3718] - 498 879 650 392 294
553 15.16603249 25.90175979 25.14977817
13.54603103 11.23850767 15.99079682
ENSG00000112033 1352.71119 1066.41916 1535.65147
784.2281506 852.6943956 1073.985611 1318.260606
903.6360526 0.543411329 0.004649114 0.041007483 PPARD
6 35342558 35428191 + 4457
protein_coding peroxisome proliferator activated receptor delta
[Source:HGNC Symbol;Acc:HGNC:9235] THR-like 1491 1205
1321 757 747 1245 16.38187821 12.81064079
18.44025958 9.437680897 10.30208972 12.98846222
ENSG00000136490 541.6288267 754.0158705 886.9811289
300.4308635 551.340553 467.5503625 727.541942
439.7739264 0.725848862 0.004650405 0.041007483 LIMD2
17 63695902 63701172 - 4745
protein_coding LIM domain containing 2 [Source:HGNC
Symbol;Acc:HGNC:28142] - 597 852 763 290 483
542 6.161221155 8.508045795 10.00449491
3.396048038 6.25688646 5.311217516
ENSG00000131067 508.0605409 693.8362 607.9831329
360.5170362 396.0976644 438.220635 603.2932913
398.2784452 0.598447976 0.004659628 0.041055406 GGT7
20 34844720 34872860 - 2966
protein_coding gamma-glutamyltransferase 7 [Source:HGNC
Symbol;Acc:HGNC:4259] - 560 784 523 348 347
508 9.24582276 12.52481607 10.97077704
6.519587838 7.191271547 7.963859394
ENSG00000196141 2744.434172 2152.308212 2566.781563
2165.174154 1685.98343 1448.370957 2487.841316

1766.509514	0.494222052	0.004660481	0.041055406
SPATS2L 2	200305881	200482263	+	10437
protein_coding	spermatogenesis associated serine rich 2 like
[Source:HGNC Symbol;Acc:HGNC:24574]	-	3025	2432	2208
2090	1477	1679	14.19313648	11.04115046
13.16225293	11.12711679	8.698656313	7.48007635
ENSG00000104852 4087.165602	5101.112063	4479.080327
3286.092066	3419.909517	3745.578735	4555.785997
3483.860106	0.386914855	0.004678183	0.041190828
SNRNP70 19	49085419	49108605	+	4684
protein_coding	small nuclear ribonucleoprotein U1 subunit 70
[Source:HGNC Symbol;Acc:HGNC:11150]	-	4505	5764	3853
3172	2996	4342	47.09844758	58.30872251
51.1786669	37.62949031	39.31626764	43.10264791
ENSG00000125868 5184.939271	5023.232489	5778.745992
4670.145975	3939.288299	3260.775591	5328.972584
3956.736622	0.429641624	0.004684536	0.041226249	DSTN
20	17569863	17609919	+	3851
protein_coding	"destrin, actin depolymerizing factor [Source:HGNC
Symbol;Acc:HGNC:15750]" -	5715	5676	4971	4508	3451
3780	72.67271738	69.83856529	80.31138048
65.04627522	55.08315417	45.64038501
ENSG00000171241 1363.598202	994.7345521	1375.227622
740.7174739	357.2869422	219.1103175	1244.520125
439.0382445	1.503470334	0.004694639	0.041294612	SHCBP1
16	46580554	46621626	-	4459
protein_coding	SHC binding and spindle associated 1 [Source:HGNC
Symbol;Acc:HGNC:29547]	-	1503	1124	1183	715	313
254	16.5063174	11.94415085	16.50646665
8.910059683	4.314735971	2.648666401
ENSG00000196591 3170.842126	3268.2871	3766.472946
2828.193991	2617.440762	2205.768039	3401.867391
2550.467597	0.415625174	0.004700631	0.04132677	HDAC2
6	113933028	114011308	-	15461
protein_coding	histone deacetylase 2 [Source:HGNC
Symbol;Acc:HGNC:4853]	-	3495	3693	3240	2730	2293
2557	11.069762	11.31796035	13.03809907
9.811538459	9.116199794	7.689961509
ENSG00000205609 56.24955989	76.99457832	159.261356
1329.147579	98.16829722	629.7265031	97.50183142
685.6807931	-2.815252087	0.004708119	0.041372045	EIF3CL
16	28379579	28403879	-	3091
protein_coding	eukaryotic translation initiation factor 3 subunit C
like [Source:HGNC Symbol;Acc:HGNC:26347]	-	62	87
137	1283	86	730	0.982248486	1.333664799
2.757581829	23.06426902	1.710199623	10.98132824
ENSG00000089486 169.6559306	182.3090015	119.7366399
214.4454784	273.9580388	386.4622923	157.2338574
291.6219365	-0.889992696	0.004713439	0.041398224	CDIP1
16	4510675 4538828 -	4268	protein_coding	cell

death inducing p53 target 1 [Source:HGNC Symbol;Acc:HGNC:13234] zf-LITAF-like 187 206 103 207 240 448
2.145585766 2.28701625 1.501480376 2.69499509
3.456481137 4.88072818
ENSG00000163565 3701.583941 3793.974221 4267.506847
5308.302568 4966.630944 4803.174204 3921.02167
5026.035905 -0.358383334 0.004716227 0.041402153 IFI16
1 158999968 159055155 + 5294
protein_coding interferon gamma inducible protein 16 [Source:HGNC Symbol;Acc:HGNC:5395] - 4080 4287 3671 5124 4351
5568 37.7402619 38.37037003 43.14269733
53.7820346 50.51873958 48.90421676
ENSG00000140280 287.5985562 457.5424942 317.3602204
198.905951 231.7228411 194.9564242 354.1670903
208.5284055 0.766028561 0.004724831 0.041457114 LYSMD2
15 51723011 51751585 - 2074
protein_coding LysM domain containing 2 [Source:HGNC Symbol;Acc:HGNC:28571] - 317 517 273 192 203
226 7.484782655 11.81158642 8.189563253
5.144042171 6.01637151 5.066764339
ENSG00000182405 221.3692357 165.4940936 101.1367735
331.5099184 299.070859 276.9071335 162.666701
302.4959703 -0.890815462 0.004738484 0.041548913 PGBD4
15 34102073 34108684 + 6612
protein_coding piggyBac transposable element derived 4 [Source:HGNC Symbol;Acc:HGNC:19401] - 244 187 87 320 262
321 1.807114477 1.340093814 0.818640553
2.689237614 2.435655192 2.257372373
ENSG00000065485 387.3961625 429.2226493 445.2343019
298.3589265 247.7037267 265.6928259 420.6177045
270.5851597 0.635699344 0.004741683 0.041548913 PDIA5
3 123067062 123225227 + 4030
protein_coding protein disulfide isomerase family A member 5 [Source:HGNC Symbol;Acc:HGNC:24811] - 427 485 383
288 217 308 5.188615784 5.70247163
5.912900548 3.970996326 3.309802258 3.553666665
ENSG00000140395 577.0116144 633.6565296 747.4821309
459.9700117 376.6923033 471.8635578 652.7167583
436.1752909 0.579602982 0.00474234 0.041548913 WDR61
15 78277835 78299794 - 7279
protein_coding WD repeat domain 61 [Source:HGNC Symbol;Acc:HGNC:30300] - 636 716 643 444 330
547 4.278722095 4.660878091 5.495992105
3.389403662 2.786695913 3.494190874
ENSG00000137574 1329.122665 1259.348103 1210.153808
1753.894662 1566.126788 1722.690173 1266.208192
1680.903875 -0.408663706 0.004760063 0.041677556 TGS1
8 55773142 55825448 + 3869
protein_coding trimethylguanosine synthase 1 [Source:HGNC Symbol;Acc:HGNC:17843] - 1465 1423 1041 1693 1372

1997 18.54246972 17.42740047 16.74013077
24.31477595 21.79730202 23.9999509
ENSG00000147454 2641.914813 2470.906468 2657.455912
1688.628647 2268.144263 1812.404634 2590.092397
1923.059181 0.429944857 0.004761736 0.041677556
SLC25A37 8 23528805 23575463 + 8147
protein_coding solute carrier family 25 member 37
[Source:HGNC Symbol;Acc:HGNC:29786] - 2912 2792 2286
1630 1987 2101 17.50339678 16.23843377
17.45763288 11.11736672 14.9915836 11.9911091
ENSG00000129534 1071.463391 654.0114182 766.0819973
625.7249709 519.3787818 211.3465661 830.5189354
452.1501063 0.878974096 0.004770144 0.041730501
MIS18BP1 14 45203190 45253540 - 6250
protein_coding MIS18 binding protein 1 [Source:HGNC
Symbol;Acc:HGNC:20190] - 1181 739 659 604 455
245 9.253340935 5.60261606 6.560126951
5.36993421 4.474852653 1.822707939
ENSG00000085872 2345.243747 2276.207534 2301.733467
2842.69755 2793.230504 3485.924382 2307.728249
3040.617479 -0.398261439 0.004793718 0.041916002 CHERP
19 16517889 16542530 - 6113
protein_coding calcium homeostasis endoplasmic reticulum protein
[Source:HGNC Symbol;Acc:HGNC:16930] - 2585 2572 1980
2744 2447 4041 20.70784164 19.9362288
20.15197618 24.94260189 24.60520194 30.73728279
ENSG00000152413 505.3387881 466.3924457 441.746827
565.6387982 724.8473109 825.5455663 471.1593536
705.3438918 -0.582200503 0.00481546 0.042085315 HOMER1
5 79372636 79514217 - 6154
protein_coding homer scaffold protein 1 [Source:HGNC
Symbol;Acc:HGNC:17512] - 557 527 380 546 635
957 4.432271821 4.057696535 3.841784069
4.930003124 6.342545531 7.230785259
ENSG00000126947 950.7990123 969.0696927 796.3067802
1101.23451 1377.780637 1311.211349 905.3918284
1263.408832 -0.479842201 0.004821064 0.042104911 ARMCX1
X 101550531 101554700 + 2141
protein_coding armadillo repeat containing X-linked 1 [Source:HGNC
Symbol;Acc:HGNC:18073] - 1048 1095 685 1063 1207
1520 23.97029059 24.23393243 19.90585109
27.5885351 34.65277055 33.01094362
ENSG00000147251 698.5832438 458.4274893 573.1083834
462.0419487 319.6177119 185.4673947 576.7063722
322.3756851 0.840740388 0.004822463 0.042104911 DOCK11
X 118495898 118686163 + 6721
protein_coding dedicator of cytokinesis 11 [Source:HGNC
Symbol;Acc:HGNC:23483] - 770 518 493 446 280
215 5.610292616 3.651928829 4.563729241
3.687338492 2.560775441 1.487426733

ENSG00000110851 900.9002092 999.1595279 890.4686038
1443.104113 1120.944975 1294.821207 930.1761136
1286.290099 -0.467594544 0.004828391 0.042120594 PRDM4
12 107732866 107761272 - 4777
protein_coding PR/SET domain 4 [Source:HGNC Symbol;Acc:HGNC:9348]
zf-C2H2 993 1129 766 1393 982 1501
10.17941196 11.19863633 9.976549804 16.20346587
12.63582512 14.61021036
ENSG00000158863 830.1346339 1081.464077 877.6811957
1060.831739 1384.629588 1628.662517 929.7599689
1358.041281 -0.546686018 0.004829022 0.042120594
FAM160B2 8 22089159 22104898 + 8278
protein_coding family with sequence similarity 160 member B2
[Source:HGNC Symbol;Acc:HGNC:16492] - 915 1222 755
1024 1213 1888 5.412829602 6.994750446
5.674510137 6.873636764 9.00705343 10.60492462
ENSG00000274825 10.88701159 19.46989337 2.3249833 0
0 0 10.89396275 0 5.88291822
0.004833444 0.042138391 AL023803.2 20 38955910
38956547 + 638 lincRNA novel transcript
- 12 22 2 0 0 0
0.921062818 1.633911174 0.195036621 0 0 0
ENSG00000224982 0 0 0 0 25.11282022
13.80222472 0 12.97168165 -6.171811986 0.004850399
0.042265371 TMEM233 12 119593459 119643066
+ 3146 protein_coding transmembrane protein 233
[Source:HGNC Symbol;Acc:HGNC:37219] - 0 0 0
0 22 16 0 0 0 0
0.429844449 0.236478838
ENSG00000150938 2063.088697 1412.452264 1750.712425
1596.427451 906.3445116 803.1169512 1742.084462
1101.962971 0.661011869 0.004860434 0.042331962 CRIM1
2 36355926 36551135 + 7122
protein_coding cysteine rich transmembrane BMP regulator 1
[Source:HGNC Symbol;Acc:HGNC:2359] - 2274 1596 1506
1541 794 931 15.63569444 10.61835788
13.15618128 12.02299629 6.852765858 6.078252393
ENSG00000176697 164.2124248 161.954113 216.2234469
98.41700702 55.93309958 107.8298807 180.7966616
87.39332909 1.043113155 0.004880217 0.042483345 BDNF
11 27654893 27722058 - 7740
protein_coding brain derived neurotrophic factor [Source:HGNC
Symbol;Acc:HGNC:1033] - 181 183 186 95 49
125 1.145160337 1.120305763 1.495129568
0.682016196 0.389136958 0.750931064
ENSG00000138650 168.7486797 133.6342681 191.8111222
219.6253209 300.2123508 362.308399 164.7313567
294.0486902 -0.838990618 0.004885988 0.042512659 PCDH10
4 133149315 133208606 + 10161
protein_coding protocadherin 10 [Source:HGNC Symbol;Acc:HGNC:13404]

-	186	151	165	212	263	420
0.89640687	0.704152842	1.010309274	1.159341689
1.590987096	1.921957842
ENSG00000172965 1271.058603	999.1595279	1083.442218
933.4076139	740.8281965	609.023166	1117.886783
761.0863255	0.555442036	0.004896712	0.042585026
MIR4435-2HG	2	111036776	111523376	-	13094
lincRNA MIR4435-2 host gene [Source:HGNC
Symbol;Acc:HGNC:35163]	-	1401	1129	932	901	649
706	5.23955595	4.085526635	4.428436507
3.823529964	3.046630043	2.507052235
ENSG00000140319 3003.907948	3098.368031	3440.975284
2798.150905	2238.465475	2020.300644	3181.083754
2352.305675	0.435484175	0.00491513	0.042724191	SRP14
15	40035739	40039188	-	2005
protein_coding	signal recognition particle 14 [Source:HGNC
Symbol;Acc:HGNC:11299]	-	3311	3501	2960	2701	1961
2342	80.86740134	82.73783921	91.85106171
74.85524754	60.11883788	54.31296749
ENSG00000187164 369.2511432	222.1337834	258.0731463
166.7909277	182.6386925	97.47821212	283.152691
148.9692774	0.930334533	0.004928919	0.042823014	SHTN1
10	116881482	117126586	-	8247
protein_coding	shootin 1 [Source:HGNC Symbol;Acc:HGNC:29319]	-
407	251	222	161	160	113	2.416724241
1.442129191	1.674803372	1.084780638	1.192535588
0.637108599
ENSG00000100395 611.4871511	689.4112243	633.5579492
807.0194575	922.3253972	998.0733754	644.8187748
909.13941	-0.495780736	0.004933886	0.042845124
L3MBTL2 22	41205205	41231271	+	6451
protein_coding	"L3MBTL2, polycomb repressive complex 1 subunit
[Source:HGNC Symbol;Acc:HGNC:18594]"	-	674	779	545
779	808	1157	5.116365222	5.721855114
5.256253556	6.709999061	7.69895319	8.339448579
ENSG00000172809 3982.831741	5229.43636	4913.852204
5730.977714	6196.017643	6682.864684	4708.706768
6203.28668	-0.397835871	0.004939672	0.042863565	RPL38
17	74203582	74210655	+	3829
protein_coding	ribosomal protein L38 [Source:HGNC
Symbol;Acc:HGNC:10349]	-	4390	5909	4227	5532	5428
7747	56.14458001	73.12317909	68.68370719
80.28027449	87.13684533	94.07607892
ENSG00000153187 17913.67032	17450.33443	17891.90898
15693.88673	12307.56489	13640.04858	17751.97125
13880.50007	0.354886991	0.004941819	0.042863565	HNRNPU
1	244840638	244864560	-	22512
protein_coding	heterogeneous nuclear ribonucleoprotein U [Source:HGNC
Symbol;Acc:HGNC:5048]	-	19745	19718	15391	15149	10782
15812	42.95084788	41.5025922	42.53628966

37.39227845	29.43964764	32.65906474
ENSG00000145723 184.1719461	184.0789918	185.998664
119.1363769	91.31934625	80.22543121	184.7498673
96.89371813	0.932514353	0.00494328	0.042863565	GIN1
	5	103086000	103120151	-	4182
protein_coding	gypsy retrotransposon integrase 1 [Source:HGNC
Symbol;Acc:HGNC:25959]	-	203	208	160	115	80
	93	2.377063005	2.356707844	2.380360861
1.5280088	1.175853778	1.034022379
ENSG00000047634 532.556317	492.9423003	416.1720107
305.610706	302.4953345	335.5665886	480.556876
314.557543	0.611721839	0.004948126	0.042875319	SCML1
	X	17737449	17754988	+	5497
protein_coding	Scm polycomb group protein like 1 [Source:HGNC
Symbol;Acc:HGNC:10580]	-	587	557	358	295	265
	389	5.22926978	4.801267455	4.051950547
2.982004693	2.963244569	3.290446868
ENSG00000197561 12.70151352	12.38993214	5.812458249	0
	0	0	10.30130131	0	5.800377638
0.004949483	0.042875319	ELANE	19	851014	856247	+
	1028	protein_coding	"elastase, neutrophil expressed
[Source:HGNC Symbol;Acc:HGNC:3309]"	-	14	14	5
	0	0	0	0.666904434	0.645299549
0.302610321	0	0	0
ENSG00000139977 635.9829272	673.4813115	625.4205076
941.6953619	818.4496408	937.6886422	644.9615821
899.2778816	-0.479682215	0.004956098	0.042911613	NAA30
	14	57390544	57415917	+	7653
protein_coding	"N(alpha)-acetyltransferase 30, NatC catalytic subunit
[Source:HGNC Symbol;Acc:HGNC:19844]"	-	701	761	538
	909	717	1087	4.485542627	4.711719026
4.37378478	6.600004225	5.758837215	6.604331266
ENSG00000049167 222.2764867	215.0538222	235.9858049
148.1434948	114.1491828	114.730993	224.4387046
125.6745569	0.836484857	0.004961574	0.042938005	ERCC8
	5	60873831	60945073	-	5448
protein_coding	"ERCC excision repair 8, CSA ubiquitin ligase complex
subunit [Source:HGNC Symbol;Acc:HGNC:3439]"	-	245	243
	203	143	100	133	2.202204618	2.113467703
	2.318279451	1.458515276	1.128262779	1.135129892
ENSG00000134321 5.443505796	12.38993214	12.78740815
5.179842475	95.88531357	69.87376267	10.2069487
56.97963957	-2.484968078	0.004979381	0.043071035	RSAD2
	2	6865806 6898239 +	4834	protein_coding
radical S-adenosyl methionine domain containing 2 [Source:HGNC
Symbol;Acc:HGNC:30908]	-	6	14	11	5	84
	81	0.060781762	0.13722961	0.141577059
0.057474526	1.068119885	0.779129038
ENSG00000146409 43.54804637	33.62981582	26.73730795
142.9636523	155.2428886	30.19236658	34.63839004

109.4663025	-1.653893825	0.004987655	0.043087586
SLC18B1 6	132769370	132813339	-	2760
protein_coding	solute carrier family 18 member B1 [Source:HGNC
Symbol;Acc:HGNC:21573]	-	48	38	23	138	136
35	0.851649388	0.652380476	0.51847235
2.77831861	3.028845959	0.589644131
ENSG00000204054 407.3556837	438.0726008	497.5464262
502.44472	667.7727195	1017.051434	447.6582369
729.0896246	-0.705505862	0.004988425	0.043087586
LINC00963	9	129483451	129513686	+	13171
processed_transcript	long intergenic non-protein coding RNA
963 [Source:HGNC Symbol;Acc:HGNC:48716] -	449	495	428
485	585	1179	1.669384108	1.780790745
2.021770549	2.046138525	2.730137225	4.162229984
ENSG00000170604 1730.127592	2151.423217	1946.011022
1830.55633	3156.224905	3905.166958	1942.52061
2963.982731	-0.60982416	0.004988602	0.043087586
IRF2BP1 19	45883607	45886170	-	2564
protein_coding	interferon regulatory factor 2 binding protein 1
[Source:HGNC Symbol;Acc:HGNC:21728]	-	1907	2431	1674
1767	2765	4527	36.42179585	44.92554753
40.62040783	38.29398584	66.28640638	82.0962825
ENSG00000124313 153.3254133	132.749273	198.7860721
105.6687865	63.92354238	56.07153794	161.6202528
75.22128893	1.103293336	0.005007085	0.043226125	IQSEC2
X	53225828	53321350	-	13115
protein_coding	IQ motif and Sec7 domain 2 [Source:HGNC
Symbol;Acc:HGNC:29059]	-	169	150	171	102	56
65	0.631025767	0.541937751	0.811212552
0.432159357	0.262462398	0.230449664
ENSG00000257467 13.60876449	0.884995153	19.76235805	0
0	0	11.4187059	0	5.942578409
0.005017195	0.043292274	PPFIA2-AS1	12	81270675
81312422	+	2024	lincRNA PPFIA2 antisense RNA 1
[Source:HGNC Symbol;Acc:HGNC:53397]	-	15	1	17
0	0	0	0.362918773	0.023410783
0.522570946	0	0	0
ENSG00000050405 1341.824179	888.5351337	1515.889111
958.2708578	828.7230673	672.8584553	1248.749475
819.9507935	0.606733058	0.005021229	0.043305957	LIMA1
12	50175788	50283546	-	6317
protein_coding	LIM domain and actin binding 1 [Source:HGNC
Symbol;Acc:HGNC:24636]	-	1479	1004	1304	925	726
780	11.46531472	7.530941547	12.84320933
8.136598747	7.064364574	5.741359533
ENSG00000188976 6501.360422	7740.167609	6595.977621
7291.146267	10004.03438	11218.62078	6945.835218
9504.600478	-0.452490683	0.005025727	0.043323625	NOC2L
1	944204	959309	-	5539	protein_coding	NOC2
like nucleolar associated transcriptional repressor [Source:HGNC

Symbol;Acc:HGNC:24517]	-	7166	8746	5674	7038	8764
	13005	63.35401196	74.81773367	63.7330662
70.60410319	97.25643898	109.171684
ENSG00000100316 20863.14321	24393.12141	25300.46827
26990.0872	32618.12899	31194.75316	23518.91096
30267.65645	-0.363983227	0.005028606	0.043327333	RPL3
	22	39312882	39320389	-	4787
protein_coding	ribosomal protein L3 [Source:HGNC
Symbol;Acc:HGNC:10332]	-	22996	27563	21764	26053	28575
	36162	235.2434587	272.8283512	282.8669035
302.4171078	366.9189751	351.2529922
ENSG00000088888 2149.277538	2160.273169	1790.237141
2553.66234	2512.423514	3137.418208	2033.262616
2734.501354	-0.427393438	0.005047632	0.043470091	MAVS
	20	3846799 3876123 +	11771	protein_coding
mitochondrial antiviral signaling protein [Source:HGNC
Symbol;Acc:HGNC:29233]	-	2369	2441	1540	2465	2201
	3637	9.855539084	9.826092781	8.139808877
11.63631871	11.49354613	14.36683022
ENSG00000141002 3544.629524	3718.749633	4433.743153
2635.503851	3314.892269	2819.967039	3899.04077
2923.454386	0.415373438	0.005053887	0.043483223	TCF25
	16	89873570	89913627	+	7967
protein_coding	transcription factor 25 [Source:HGNC
Symbol;Acc:HGNC:29181]	Others	3907	4202	3814	2544	2904
	3269	24.01470619	24.99123232	29.78466489
17.74329746	22.40521703	19.07880291
ENSG00000172164 442.7384714	241.6036768	409.1970608
277.6395566	167.7992987	117.3189102	364.5130697
187.5859218	0.959432263	0.005054074	0.043483223	SNTB1
	8	120535745	120813273	-	6868
protein_coding	syntrophin beta 1 [Source:HGNC Symbol;Acc:HGNC:11168]
	-	488	273	352	268	147	136
3.479511042	1.883470409	3.188740837	2.168285928
1.31563194	0.920745574
ENSG00000135677 3065.601014	2611.620697	3526.999666
2586.813332	2203.079228	1888.31687	3068.073792
2226.06981	0.462779532	0.005057125	0.043488322	GNS
	12	64713445	64759447	-	6284
protein_coding	glucosamine (N-acetyl)-6-sulfatase [Source:HGNC
Symbol;Acc:HGNC:4422]	-	3379	2951	3034	2497	1930
	2189	26.33180916	22.25150929	30.03905366
22.07976311	18.87854384	16.19722456
ENSG00000260916 282.1550504	287.6234248	255.748163
152.2873688	181.4972007	158.7255843	275.1755461
164.1700513	0.746761167	0.005061818	0.043507535	CCPG1
	15	55340032	55408510	-	8790
protein_coding	cell cycle progression 1 [Source:HGNC
Symbol;Acc:HGNC:24227]	-	311	325	220	147	159
	184	1.73260753	1.751946851	1.55718658

0.929266975	1.111874089	0.973329679
ENSG00000153944 1378.114217	1051.374242	1164.816633
1074.299329	630.1034892	628.001225	1198.101697
777.4680144	0.624177285	0.00507648	0.043612358	MSI2
	17	57255851	57684685	+	21479
protein_coding	musashi RNA binding protein 2 [Source:HGNC
Symbol;Acc:HGNC:18585]	-	1519	1188	1002	1037	552
	728	3.463158746	2.620769485	2.902421685
2.682728617	1.579691859	1.575971428
ENSG00000266094 592.4348808	516.8371694	482.4340347
271.4237457	415.5030255	330.3907543	530.568695
339.1058418	0.647358413	0.005082247	0.043640711	RASSF5
	1	206507530	206589448	+	6976
protein_coding	Ras association domain family member 5 [Source:HGNC
Symbol;Acc:HGNC:17609]	-	653	584	415	262	364
	383	4.583902725	3.966731601	3.701250435
2.086925103	3.207319849	2.552838378
ENSG00000171502 70.76557535	50.44472373	54.63710754
12.43162194	10.27342645	26.7418104	58.61580221
16.48228627	1.817596514	0.005085517	0.0436476
COL24A1 1	85729233	86156943	-	9568
protein_coding	collagen type XXIV alpha 1 chain [Source:HGNC
Symbol;Acc:HGNC:20821]	-	78	57	47	12	9
	31	0.399210651	0.282280014	0.305621243
0.069690266	0.057818751	0.150650836
ENSG00000226853 0	0	0	4.14387398	13.69790194
	12.93958568	0	10.26045387	-5.835064188
0.005092897	0.043666953	AC010894.2	2	174487389
	174488386	+	791	lincRNA novel transcript
	-	0	0	0	4	12	15	0
	0	0	0.280993033	0.932507048	0.881750661
ENSG00000126785 258.5665253	197.3539191	361.5349031
158.5031797	159.8088559	127.6705787	272.4851159
148.6608715	0.873320427	0.005094543	0.043666953	RHOJ
	14	63204114	63293219	+	3793
protein_coding	ras homolog family member J [Source:HGNC
Symbol;Acc:HGNC:688]	-	285	223	311	153	140
	148	3.679516043	2.785790552	5.101341446
2.241406524	2.268780878	1.814303301
ENSG00000130741 4219.624243	3490.420884	3659.523714
3026.063974	2967.878753	2768.208696	3789.85628
2920.717141	0.376025133	0.005095177	0.043666953	EIF2S3
	X	24054716	24077971	+	3472
protein_coding	eukaryotic translation initiation factor 2 subunit
gamma [Source:HGNC Symbol;Acc:HGNC:3267]	-	4651	3944
	3148	2921	2600	3209	65.59871113	53.82494945
	56.41074739	46.74809136	46.03000178	42.97550797
ENSG00000183114 36.29003864	47.78973827	32.5497662
107.7407235	78.76293614	100.9287683	38.87651437
95.8108093	-1.298736316	0.005102363	0.043707358	FAM43B

1	20552439	20555010	+	2572
protein_coding	family with sequence similarity 43 member B
[Source:HGNC Symbol;Acc:HGNC:31791]	-	40	54	28
104	69	117	0.761583823	0.994830832
0.677320022	2.246851753	1.649018343	2.115173105
ENSG00000013288 672.2729658	542.5020289	606.8206412
405.0636815	471.436125	278.6324116	607.1985453
385.0440727	0.659318886	0.005111751	0.043766586	MAN2B2
4	6575175 6623362 +	8898	protein_coding
mannosidase alpha class 2B member 2 [Source:HGNC
Symbol;Acc:HGNC:29623]	-	741	613	522	391	413
323	4.078068253	3.26433344	3.649933469
2.441723031	2.853021277	1.687878336
ENSG00000107937 3371.34459	3380.681485	2786.492485
3936.680281	4016.909743	4524.541792	3179.506186
4159.377272	-0.387363746	0.005118906	0.043806644	GTPBP4
10	988019	1019936 +	6540	protein_coding	GTP
binding protein 4 [Source:HGNC Symbol;Acc:HGNC:21535]	-	3716
3820	2397	3800	3519	5245	27.82445334
27.67655656	22.80327015	32.28627131	33.07416424
37.29054772
ENSG00000170345 149.6964094	127.439302	105.7867401
511.7684365	125.5641011	227.736708	127.6408172
288.3564152	-1.174802192	0.005124103	0.043822566	FOS
14	75278774	75282230	+	3238
protein_coding	"Fos proto-oncogene, AP-1 transcription factor subunit
[Source:HGNC Symbol;Acc:HGNC:3796]"	TF_bZIP 165	144	91
494	110	264	2.495374791	2.107230718
1.748523182	8.477389706	2.088157253	3.791037676
ENSG00000101361 4183.334204	5240.056302	4198.919839
5401.539732	5971.143753	6604.364531	4540.770115
5992.349339	-0.400121236	0.005125722	0.043822566	NOP56
20	2652145 2658393 +	5398	protein_coding	NOP56
ribonucleoprotein [Source:HGNC Symbol;Acc:HGNC:15911]	-	4611
5921	3612	5214	5231	7656	41.83029482
51.97429675	41.63146636	53.67229801	59.56610463
65.94776211
ENSG00000176340 1205.736534	2216.027863	2293.596025
1004.88944	1309.291127	1391.43678	1905.120141
1235.205782	0.624619574	0.00513108	0.043847189	COX8A
11	63974607	63976543	+	507
protein_coding	cytochrome c oxidase subunit 8A [Source:HGNC
Symbol;Acc:HGNC:2294]	-	1329	2504	1973	970	1147
1613	128.3647281	234.0199089	242.1173838
106.3104162	139.0601901	147.9303982
ENSG00000254838 13.60876449	23.89486913	19.76235805	0
3.424475485	0	19.08866389	1.141491828
4.149810214	0.005152511	0.044009063	GVINP1	11
6713536 6746439 -	8438	transcribed_unprocessed_pseudogene
"GTPase, very large interferon inducible pseudogene 1

[Source:HGNC Symbol;Acc:HGNC:25813]"	-	15	27	17
	0	3	0	0.087052334	0.151617972
0.125347665	0	0.021853907	0
ENSG00000075568 640.519182	628.3465587	694.007515
559.4229872	364.1358932	320.0390858	654.2910852
414.5326554	0.658920057	0.005176926	0.044192636
TMEM131 2	97756333	97995891	-	12985
protein_coding	transmembrane protein 131 [Source:HGNC
Symbol;Acc:HGNC:30366]	-	706	710	597	540	319
	371	2.662511122	2.590853375	2.86048203
2.310807931	1.510066556	1.328504329
ENSG00000134954 2995.74269	2735.520018	3538.624582
2643.791599	2221.343098	1868.476172	3089.96243
2244.536956	0.461161532	0.005179	0.044192636	ETS1
	11	128458761	128587558	-	7149
protein_coding	"ETS proto-oncogene 1, transcription factor
[Source:HGNC Symbol;Acc:HGNC:3488]"	ETS	3302	3091	3044
	2552	1946	2166	22.6183258	20.48708403
26.49147853	19.83569476	16.73188608	14.08783231
ENSG00000233927 870.0536764	1297.402894	1291.528223
1729.031418	1418.874342	1840.009084	1152.994931
1662.638281	-0.528917532	0.00518506	0.044223009	RPS28
	19	8321158 8323340 +	2119	protein_coding
ribosomal protein S28 [Source:HGNC Symbol;Acc:HGNC:10418]	-
	959	1466	1111	1669	1243	2133	22.16237678
	32.78154774	32.62045007	43.7660572	36.05682916
	46.80485637
ENSG00000124217 510.7822938	587.6367817	562.6459585
622.6170654	870.9582649	1006.699766	553.6883447
833.4250321	-0.590620594	0.005191143	0.044233907	MOCS3
	20	50958826	50963931	+	5106
protein_coding	molybdenum cofactor synthesis 3 [Source:HGNC
Symbol;Acc:HGNC:15765]	-	563	664	484	601	763
	1167	5.399533847	6.161886716	5.897546825
6.540421013	9.185252251	10.62725407
ENSG00000125247 243.1432589	226.5587592	269.6980628
340.8336348	530.7937001	350.2314524	246.4666936
407.2862624	-0.724276833	0.005195292	0.044233907	TMTC4
	13	100603927	100675093	-	6272
protein_coding	transmembrane and tetratricopeptide repeat containing
4 [Source:HGNC Symbol;Acc:HGNC:25904]	-	268	256	232
	329	465	406	2.092461268	1.934017308
2.30138237	2.914753899	4.55715986	3.009892619
ENSG00000182973 1480.633576	1477.941906	1382.202572
1955.908518	1745.341005	2022.025922	1446.926018
1907.758482	-0.398954804	0.005196177	0.044233907	CNOT10
	3	32685145	32773875	+	4846
protein_coding	CCR4-NOT transcription complex subunit 10 [Source:HGNC
Symbol;Acc:HGNC:23817]	-	1632	1670	1189	1888	1529
	2344	16.49170008	16.32899673	15.26529816

21.64864026	19.39418061	22.49081616
ENSG00000137275 1579.523932	1635.471043	1547.276386
1909.289936	2354.897642	2046.179815	1587.423787
2103.455798	-0.405671587	0.005197413	0.044233907	RIPK1
	6	3063991 3115187 +	4231	protein_coding
receptor interacting serine/threonine kinase 1 [Source:HGNC
Symbol;Acc:HGNC:10019]	-	1741	1848	1331	1843	2063
	2372	20.15043516	20.69595076	19.57230058
24.20440179	29.97116074	26.06769779
ENSG00000111843 2849.675284	2981.548671	3778.097862
2771.215724	2252.163377	1596.744873	3203.107272
2206.707991	0.537717989	0.005198841	0.044233907
TMEM14C 6	10722915	10731129	+	1321
protein_coding	transmembrane protein 14C [Source:HGNC
Symbol;Acc:HGNC:20952]	-	3141	3369	3250	2675	1973
	1851	116.4377494	120.8438726	153.0690511
112.5208521	91.80611887	65.15303022
ENSG00000164754 9661.315537	7720.697716	9446.407147
7662.022988	6754.207147	5848.692727	8942.8068
6754.974288	0.404824688	0.005210559	0.044299635	RAD21
	8	116845935	116874866	-	6192
protein_coding	RAD21 cohesin complex component [Source:HGNC
Symbol;Acc:HGNC:9811]	-	10649	8724	8126	7396	5917
	6780	84.21831789	66.75920405	81.64934722
66.37094458	58.73784134	50.91312616
ENSG00000143198 997.0688116	1099.16398	1235.728624
885.7530631	811.6006898	590.045107	1110.653805
762.4662866	0.543262174	0.005211574	0.044299635	MGST3
	1	165630861	165661796	+	8401
protein_coding	microsomal glutathione S-transferase 3 [Source:HGNC
Symbol;Acc:HGNC:7064]	-	1099	1242	1063	855	711
	684	6.406124744	7.005143754	7.872435778
5.655189648	5.202187201	3.785786648
ENSG00000139278 1803.61492	1425.727192	1427.539746
1430.672491	724.8473109	835.8972349	1552.293953
997.1390124	0.638681756	0.005214448	0.044302772	GLIPR1
	12	75480680	75503853	+	6273
protein_coding	GLI pathogenesis related 1 [Source:HGNC
Symbol;Acc:HGNC:17001]	-	1988	1611	1228	1381	635
	969	15.51921594	12.16877031	12.17951307
12.23292843	6.222226239	7.182564053
ENSG00000270562 14.51601546	23.89486913	0	0	0
	0	12.8036282	0	6.115670274	0.00521854
	0.044316254	AC097634.1	3	71584943
71587409	+	2467	lincRNA novel transcript	-
	16	27	0	0	0	0	0.317599285
	0.518586319	0	0	0	0
ENSG00000047644 1077.814148	1072.614126	1345.002839
910.616307	551.340553	865.2269624	1165.143704
775.7279408	0.585366496	0.005234662	0.044427697	WWC3

X	10015562	10144478	+	7114
protein_coding	WWC family member 3 [Source:HGNC
Symbol;Acc:HGNC:29237]	-	1188	1212	1157	879	483
1003	8.177701676	8.07263283	10.11873785
6.865735334	4.173309847	6.555685192
ENSG00000167363 104.3338611	96.46447169	110.4367067
281.7834306	216.8834474	127.6705787	103.7450132
208.7791522	-1.007988357	0.005236687	0.044427697	FN3K
17	82735575	82751197	+	3872
protein_coding	fructosamine 3 kinase [Source:HGNC
Symbol;Acc:HGNC:24822]	-	115	109	95	272	190
148	1.454424478	1.333882547	1.526494006
3.903422841	3.016238038	1.777286266
ENSG00000130830 1056.040124	977.9196442	1162.49165
793.5518671	835.5720182	492.5668949	1065.483806
707.2302601	0.59240295	0.005253502	0.044548995	MPP1
X	154778684	154821007	-	5396
protein_coding	membrane palmitoylated protein 1 [Source:HGNC
Symbol;Acc:HGNC:7219]	-	1164	1105	1000	766	732
571	10.56354588	9.7032401	11.53014863
7.888035787	8.338472489	4.920340809
ENSG00000119681 1817.223685	1338.112672	1435.677188
1246.270099	1027.342645	992.8975411	1530.337848
1088.836762	0.491430537	0.005256463	0.04455274	LTBP2
14	74498170	74612378	-	9091
protein_coding	latent transforming growth factor beta binding protein
2 [Source:HGNC Symbol;Acc:HGNC:6715]	-	2003	1512	1235
1203	900	1151	10.78941692	7.880732282
8.45205173	7.353024503	6.085247013	5.887008786
ENSG00000149269 1530.53238	1488.561848	1456.602037
2072.972958	2174.541933	1727.003369	1491.898755
1991.506087	-0.416094684	0.005265251	0.044587091	PAK1
11	77321707	77474635	-	6299
protein_coding	p21 (RAC1) activated kinase 1 [Source:HGNC
Symbol;Acc:HGNC:8590]	-	1687	1682	1253	2001	1905
2002	13.11511665	12.65263046	12.37617123
17.65174008	18.5896294	14.77826612
ENSG00000197956 4597.947896	8144.610394	11487.74248
4055.816658	4168.728156	3950.024188	8076.766925
4058.189667	0.992880139	0.005267635	0.044587091	S100A6
1	153534599	153536244	-	971
protein_coding	S100 calcium binding protein A6 [Source:HGNC
Symbol;Acc:HGNC:10496]	-	5068	9203	9882	3915	3652
4579	255.5912958	449.093359	633.1876947
224.0394925	231.1845991	219.2716233
ENSG00000106211 5494.31185	7814.507202	8151.391449
7564.64195	10717.46677	12566.06297	7153.4035
10282.7239	-0.523672457	0.005270037	0.044587091	HSPB1
7	76302544	76304295	+	1634
protein_coding	heat shock protein family B (small) member 1

[Source:HGNC Symbol;Acc:HGNC:5246]	-	6056	8830	7012
	7302	9389	14567	181.4940943	256.0560798
266.9910491	248.3143512	353.1950815	414.5234329
ENSG00000053501 434.5732127	490.2873148	598.6831997
724.1419779	652.9333257	908.3589147	507.8479091
761.8114061	-0.587361612	0.005270598	0.044587091	USE1
	19	17215346	17219829	+	2431
protein_coding	unconventional SNARE in the ER 1 [Source:HGNC
Symbol;Acc:HGNC:30882]	-	479	554	515	699	572
	1053	9.64893183	10.79819701	13.18041597
15.97733203	14.46300146	20.14069397
ENSG00000198929 13.60876449	15.92991276	2.3249833	0
	0	0	10.62122018	0	5.846234921
0.005296563	0.044785326	NOS1AP	1	162069774
162370475	+	7771	protein_coding	nitric oxide synthase
1 adaptor protein [Source:HGNC Symbol;Acc:HGNC:16859]	-	15
	18	2	0	0	0	0.094524205
0.109754425	0.016012529	0	0	0
ENSG00000106554 1882.545754	1876.189725	2035.522879
1500.082381	1364.082735	1544.123891	1931.419453
1469.429669	0.39378479	0.005307219	0.044853987	CHCHD3
	7	132784868	133082088	-	3428
protein_coding	coiled-coil-helix-coiled-coil-helix domain containing
3 [Source:HGNC Symbol;Acc:HGNC:21906]	-	2075	2120	1751
	1448	1195	1790	29.64189546	29.30363448
31.77987462	23.4714431	21.42764547	24.2796955
ENSG00000134905 1685.672295	1922.209473	1754.1999
2107.159919	2519.272465	2455.070723	1787.360556
2360.501035	-0.401146155	0.005311552	0.044869171	CARS2
	13	110641412	110713603	-	10460
protein_coding	"cysteinyl-tRNA synthetase 2, mitochondrial
[Source:HGNC Symbol;Acc:HGNC:25695]"	-	1858	2172	1509
	2034	2207	2846	8.698466769	9.839081936
8.975618848	10.80516263	12.96934397	12.65127306
ENSG00000117501 1.814501932	1.769990306	2.3249833
23.82727538	9.131934625	27.60444945	1.969825179
20.18788649	-3.369797681	0.005316631	0.044890636	MROH9
	1	170935471	171064765	+	3831
protein_coding	maestro heat like repeat family member 9 [Source:HGNC
Symbol;Acc:HGNC:26287]	-	2	2	2	23	8
	32	0.025565043	0.024736844	0.032480648
0.333601295	0.128358666	0.38839072
ENSG00000176407 1268.33685	1323.952749	1476.364395
1998.383227	1700.822824	1737.355037	1356.217998
1812.187029	-0.418630352	0.005349006	0.045142447	KCMF1
	2	84971093	85059472	+	7968
protein_coding	potassium channel modulatory factor 1 [Source:HGNC
Symbol;Acc:HGNC:20589]	-	1398	1496	1270	1929	1490
	2014	8.591846898	8.89628544	9.916564526
13.4522505	11.49434698	11.75279494

ENSG00000135845 703.1194986	747.8209044	654.4827989
511.7684365	483.9925351	486.5284215	701.807734
494.0964644	0.506844645	0.005393126	0.045493085	PIGC
	1	172370189	172444086	-	5225
protein_coding	phosphatidylinositol glycan anchor biosynthesis class C [Source:HGNC Symbol;Acc:HGNC:8960]	-	775	845	563
	494	424	564	7.263469066	7.66296523
6.703921908	5.253547917	4.988005481	5.01907664
ENSG00000175606 937.1902479	1183.23852	956.7306279
1366.442445	1467.958491	1371.596082	1025.719798
1401.999006	-0.450182672	0.005396649	0.045496572	TMEM70
	8	73972437	73982783	+	2982
protein_coding	transmembrane protein 70 [Source:HGNC Symbol;Acc:HGNC:26050]	-	1033	1337	823	1319	1286
	1590	16.96373057	21.24468074	17.17113658
24.57815055	26.50822753	24.79251036
ENSG00000124541 2153.813793	2339.04219	2242.446393
2616.856418	3128.829101	3020.961937	2245.100792
2922.215819	-0.380243901	0.005398683	0.045496572	RRP36
	6	43021645	43034156	+	2212
protein_coding	ribosomal RNA processing 36 [Source:HGNC Symbol;Acc:HGNC:21374]	-	2374	2643	1929	2526	2741
	3502	52.55623859	56.61590858	54.25677197
63.45418453	76.16777568	73.61427466
ENSG00000101003 1248.377329	1139.873757	1196.203908
1061.867707	739.6867047	568.4791308	1194.818331
790.0111809	0.597596267	0.005422391	0.045674609	GINS1
	20	25407727	25452628	+	4068
protein_coding	GINS complex subunit 1 [Source:HGNC Symbol;Acc:HGNC:28980]	-	1376	1288	1029	1025	648
	659	16.56403629	15.00242138	15.73770054
14.00086812	9.791324	7.532436661
ENSG00000069188 15.42326642	7.964956378	6.974949899	0
	0	0	10.12105757	0	5.774008772
0.005428721	0.04570616	SDK2	17	73334384
73644089	-	11607	protein_coding	sidekick cell adhesion molecule 2 [Source:HGNC Symbol;Acc:HGNC:19308]	-	17	9
	6	0	0	0	0.071722864	0.03674083
	0.032161635	0	0	0
ENSG00000125885 1694.744804	1323.067754	1107.854542
1179.968116	777.355935	781.550975	1375.222367
912.9583419	0.59171935	0.005442114	0.04579712	MCM8
	20	5950652 5998977 +	7691	protein_coding
minichromosome maintenance 8 homologous recombination repair factor
[Source:HGNC Symbol;Acc:HGNC:16147]	-	1868	1495	953
	1139	681	906	11.89385786	9.210534256
7.709335321	8.229111681	5.442665711	5.477424556
ENSG00000177084 3847.651347	3519.625724	3073.627922
2684.19437	2679.081321	2673.318401	3480.301664
2678.864697	0.377907448	0.005468645	0.045998506	POLE

12	132623753	132687365	-	16609
protein_coding	"DNA polymerase epsilon, catalytic subunit
[Source:HGNC Symbol;Acc:HGNC:9177]"	-	4241	3977	2644
2591	2347	3099	12.50412973	11.34588943
9.904323392	8.668340681	8.685942793	8.675791559
ENSG00000135763 575.1971124	576.1318447	573.1083834
780.0842767	690.602556	1104.177978	574.8124468
858.2882702	-0.579615896	0.005474771	0.046015266	URB2
1	229626234	229660199	+	6001
protein_coding	URB2 ribosome biogenesis 2 homolog (S. cerevisiae)
[Source:HGNC Symbol;Acc:HGNC:28967]	-	634	651	493
753	605	1280	5.173617473	5.140244803
5.11128549	6.972417642	6.196965925	9.917846012
ENSG00000177707 1715.611577	1402.717318	1556.576319
1406.845216	950.8626929	727.2047152	1558.301738
1028.304208	0.600284316	0.00547584	0.046015266
NECTIN3 3	111070071	111275563	+	6184
protein_coding	nectin cell adhesion molecule 3 [Source:HGNC
Symbol;Acc:HGNC:17664]	-	1891	1585	1339	1358	833
843	14.97444488	12.14468421	13.47156164
12.20231783	8.279857848	6.338538198
ENSG00000170989 9.979760626	10.61994184	9.299933199	0
0	0	9.966545221	0	5.750597865
0.005512301	0.046281729	S1PR1	1	101236865
101243713	+	5223	protein_coding	sphingosine-1-
phosphate receptor 1 [Source:HGNC Symbol;Acc:HGNC:3165] -	11
12	8	0	0	0	0.103133877
0.108864846	0.095296469	0	0	0
ENSG00000069424 342.0336142	452.2325232	403.3846025
534.5597434	711.149409	569.3417699	399.2169133
605.0169741	-0.599103781	0.005515393	0.046281729	KCNAB2
1	5991466 6101193 +	9534	protein_coding
potassium voltage-gated channel subfamily A regulatory beta subunit 2
[Source:HGNC Symbol;Acc:HGNC:6229]	-	377	511	347
516	623	660	1.936399163	2.539640203
2.264441856	3.007368162	4.016615495	3.218843087
ENSG00000073969 1574.987677	1179.698539	1520.539078
1220.370887	938.3062828	684.9354019	1425.075098
947.8708572	0.588791269	0.005515399	0.046281729	NSF
17	46590669	46757464	+	5005
protein_coding	"N-ethylmaleimide sensitive factor, vesicle fusing
ATPase [Source:HGNC Symbol;Acc:HGNC:8016]"	-	1736	1333
1308	1178	822	794	16.98534305	12.61980105
16.25962439	13.07835893	10.09520392	7.376450608
ENSG00000058063 1691.115801	1422.187211	1497.289245
1199.651517	1127.793926	1121.430759	1536.864086
1149.625401	0.418976971	0.005531438	0.04639431	ATP11B
3	182793500	182921635	+	9196
protein_coding	ATPase phospholipid transporting 11B (putative)
[Source:HGNC Symbol;Acc:HGNC:13553]	-	1864	1607	1288

1158 988 1300 9.92603104 8.280248201
8.71412423 6.997157352 6.603973809 6.573178224
ENSG00000260260 40.82629347 87.61452016 83.69939879
26.93518087 21.68834474 21.56597613 70.71340414
23.39650058 1.594950946 0.005539789 0.046422769 SNHG19
16 2154797 2155358 - 342 lincRNA small
nucleolar RNA host gene 19 [Source:HGNC Symbol;Acc:HGNC:49574] -
45 99 72 26 19 25 6.443399978
13.71625433 13.09824884 4.224344086 3.414875346
3.398951133
ENSG00000129824 3663.479401 3922.298519 3768.797929
4590.376401 4962.064977 4762.630169 3784.858616
4771.690516 -0.334194503 0.005540079 0.046422769 RPS4Y1
Y 2841486 2932000 + 2275 protein_coding
ribosomal protein S4 Y-linked 1 [Source:HGNC Symbol;Acc:HGNC:10425]
- 4038 4432 3242 4431 4347 5521
86.91877505 92.30915842 88.66219036 108.2261957
117.4506973 112.8411138
ENSG00000268628 3.629003864 27.43484975 2.3249833 0
0 0 11.1296123 0 5.913752349
0.005551019 0.046484611 AL121761.1 20 19756390
19758037 - 1648 lincRNA novel transcript
- 4 31 2 0 0 0
0.118858835 0.891314409 0.075505682 0 0 0
ENSG00000226380 29.03203091 61.94966072 15.11239145
121.2083139 65.06503421 128.5332177 35.36469436
104.935522 -1.561610448 0.005552715 0.046484611 LINC-
PINT 7 130876809 130913310 - 6619
lincRNA "long intergenic non-protein coding RNA, p53 induced
transcript [Source:NCBI gene;Acc:378805]" - 32 70
13 117 57 149 0.236747979 0.501108881
0.122196233 0.982212653 0.529334054 1.046706462
ENSG00000092199 10104.05401 11042.08453 12542.12241
8816.091892 8740.402928 8948.154817 11229.42031
8834.883212 0.345868756 0.005569451 0.046602654 HNRNPC
14 21209136 21269494 - 7309
protein_coding heterogeneous nuclear ribonucleoprotein C (C1/C2)
[Source:HGNC Symbol;Acc:HGNC:5035] - 11137 12477 10789
8510 7657 10373 74.61719882 80.88698617
91.83961994 64.69692536 64.39439176 65.98989451
ENSG00000172936 1057.854626 1185.00851 1009.042752
1292.888682 1795.566646 1439.744567 1083.968629
1509.399965 -0.476827148 0.005610954 0.046927738 MYD88
3 38138478 38143022 + 3989
protein_coding "MYD88, innate immune signal transduction adaptor
[Source:HGNC Symbol;Acc:HGNC:7562]" - 1166 1339 868
1248 1573 1669 14.31407201 15.90534089
13.53825018 17.38451554 24.23885198 19.45464536
ENSG00000159202 3746.946489 4412.585833 4073.370741
4487.81552 5704.034665 6009.143589 4077.634355

5400.331258	-0.405378437	0.005614151	0.046932287	UBE2Z
	17	48908369	48929056	+	5753
protein_coding	ubiquitin conjugating enzyme E2 Z [Source:HGNC
Symbol;Acc:HGNC:25847]	-	4130	4986	3504	4332	4997
	6966	35.15477812	41.06618327	37.89453394
41.84139134	53.39029686	56.30151927
ENSG00000183137 367.4366412	310.6332987	352.2349699
200.977888	247.7037267	188.0553119	343.43497
212.2456422	0.695855324	0.005624595	0.046979718
CEP57L1 6	109095110	109163932	+	6432
protein_coding	centrosomal protein 57 like 1 [Source:HGNC
Symbol;Acc:HGNC:21561]	-	405	351	303	194	217
	218	3.0834554	2.585755884	2.930916456
1.675975778	2.073772248	1.575946522
ENSG00000075391 1873.473245	1831.054972	1393.827488
2347.504609	2143.721653	2411.076132	1699.451902
2300.767465	-0.436480754	0.005627377	0.046979718	RASAL2
	1	178094141	178484147	+	16044
protein_coding	RAS protein activator like 2 [Source:HGNC
Symbol;Acc:HGNC:9874]	-	2065	2069	1199	2266	1878
	2795	6.302837151	6.110465244	4.649576273
7.848005448	7.194991659	8.100282719
ENSG00000259330 409.1701857	437.1876056	437.0968604
318.0423279	232.8643329	274.3192164	427.8182172
275.0752924	0.636322759	0.005631214	0.046979718	INAFM2
	15	40323664	40326715	+	3052
protein_coding	InaF motif containing 2 [Source:HGNC
Symbol;Acc:HGNC:35165]	-	451	494	376	307	204
	318	7.236368861	7.669531941	7.664964755
5.589408999	4.108591831	4.84477496
ENSG00000034152 1406.238997	1795.655166	1613.53841
1878.210881	2131.165243	2670.730484	1605.144191
2226.702203	-0.4725912	0.00563273	0.046979718	MAP2K3
	17	21284672	21315240	+	3783
protein_coding	mitogen-activated protein kinase kinase 3 [Source:HGNC
Symbol;Acc:HGNC:6843]	-	1550	2029	1388	1813	1867
	3096	20.06430128	25.41394856	22.82758515
26.63014348	30.33579193	38.05358949
ENSG00000151466 413.7064405	253.1086138	288.2979292
252.7763128	130.1300684	94.89029498	318.3709945
159.2655587	1.001216119	0.005633104	0.046979718	SCLT1
	4	128864921	129093607	-	8292
protein_coding	sodium channel and clathrin linker 1 [Source:HGNC
Symbol;Acc:HGNC:26406]	-	456	286	248	244	114
	110	2.69298685	1.634305268	1.860798015
1.635093442	0.845070455	0.616828469
ENSG00000149636 772.070572	724.8110304	723.0698062
654.7320888	372.126336	384.7370142	739.9838029
470.531813	0.653538585	0.005637456	0.046993854	DSN1
	20	36751791	36773818	-	2739

protein_coding "DSN1 homolog, MIS12 kinetochore complex component
[Source:HGNC Symbol;Acc:HGNC:16165]" - 851 819 622
632 326 446 15.21479872 14.16831847
14.12879745 12.82144842 7.315987049 7.571359098
ENSG00000100077 315.7233362 230.0987398 302.247829
214.4454784 131.2715602 116.4562711 282.6899683
154.0577699 0.876384698 0.005645714 0.047027151 GRK3
22 25564849 25729294 + 9303
protein_coding G protein-coupled receptor kinase 3 [Source:HGNC
Symbol;Acc:HGNC:290] - 348 260 260 207 115
135 1.831828901 1.324270693 1.738830197
1.236401058 0.759840048 0.674748248
ENSG00000178802 448.1819772 501.7922518 682.3825985
813.2352685 687.1780806 980.8205945 544.1189425
827.0779812 -0.606629731 0.005646767 0.047027151 MPI
15 74890005 74902219 + 9090
protein_coding mannose phosphate isomerase [Source:HGNC
Symbol;Acc:HGNC:7216] - 494 567 587 785 602
1137 2.661287225 2.955599718 4.01773293
4.798636103 4.070801897 5.816042877
ENSG00000112290 743.9457921 648.7014472 490.5714763
973.8103852 849.2699202 929.0622518 627.7395719
917.3808524 -0.546024126 0.005663775 0.047146593 WASF1
6 110099819 110180004 - 3260
protein_coding WAS protein family member 1 [Source:HGNC
Symbol;Acc:HGNC:12732] - 820 733 422 940 744
1077 12.31756707 10.65400302 8.053815891
16.02220548 14.02822412 15.36134069
ENSG00000160685 539.8143248 515.0671791 846.2939211
358.4450992 420.0689928 427.0063274 633.7251417
401.8401398 0.655352922 0.005677785 0.047240987 ZBTB7B
1 155002630 155018522 + 4303
protein_coding zinc finger and BTB domain containing 7B [Source:HGNC
Symbol;Acc:HGNC:18668] ZBTB 595 582 728 346 368
495 6.771335045 6.408820079 10.52608517
4.468037365 5.256828791 5.348904832
ENSG00000263432 21.77402318 6.194966072 3.48747495 0
0 0 10.48548807 0 5.826673751
0.005688654 0.047309165 RN7SL689P 5 123022487
123022783 - 297 misc_RNA "RNA, 7SL,
cytoplasmic 689, pseudogene [Source:HGNC Symbol;Acc:HGNC:46705]"
- 24 7 3 0 0 0
3.957158774 1.116781038 0.628451333 0 0 0
ENSG00000137393 36.29003864 54.86969949 25.5748163
66.30198367 133.5545439 106.9672416 38.91151814
102.2745897 -1.387323815 0.005713911 0.047479788
RNF144B 6 18387350 18468874 + 5175
protein_coding ring finger protein 144B [Source:HGNC
Symbol;Acc:HGNC:21578] - 40 62 22 64 117
124 0.378510839 0.567685467 0.264496039

0.687197647	1.389705793	1.114146625
ENSG00000225706 8.165258694	8.849951531	12.78740815	0
	0	0	9.934206125	0	5.743802236
0.005714538	0.047479788	PTPRD-AS1	9	8858130
8862255 +	2494	lincRNA PTPRD antisense RNA 1 [Source:HGNC
Symbol;Acc:HGNC:49753]	-	9	10	11	0	0
	0	0.176715541	0.189989671	0.27441199	0
	0	0
ENSG00000110330 2954.009145	2429.311695	2726.042919
2011.850817	2199.654753	1988.382999	2703.121253
2066.629523	0.387508718	0.005733258	0.047597222	BIRC2
	11	102347211	102378670	+	4532
protein_coding	baculoviral IAP repeat containing 2 [Source:HGNC
Symbol;Acc:HGNC:590]	-	3256	2745	2345	1942	1927
	2305	35.18221502	28.6998012	32.19287716
23.8106564	26.13600314	23.64896
ENSG00000119900 1627.608233	1243.41819	1531.001503
1156.14084	1066.153368	976.5073993	1467.342642
1066.267202	0.460726444	0.005735151	0.047597222	OGFRL1
	6	71288803	71308950	+	9075
protein_coding	opioid growth factor receptor like 1 [Source:HGNC
Symbol;Acc:HGNC:21378]	-	1794	1405	1317	1116	934
	1132	9.680649329	7.335946093	9.029131703
6.833286102	6.326268243	5.800037631
ENSG00000136044 1450.694295	1439.887114	1560.063794
2100.944108	1904.008369	1833.107971	1483.548401
1946.020149	-0.391646951	0.005736953	0.047597222	APPL2
	12	105173296	105236238	-	5124
protein_coding	"adaptor protein, phosphotyrosine interacting with PH
domain and leucine zipper 2 [Source:HGNC Symbol;Acc:HGNC:18242]"
	-	1599	1627	1342	2028	1668	2125
15.2815718	15.04543929	16.29484529	21.99231125
20.0094101	19.28327663
ENSG00000182324 148.7891584	110.6243941	80.21192384
137.7838098	198.6195781	405.4403513	113.2084921
247.2812464	-1.12680606	0.005739436	0.047597222	KCNJ14
	19	48455509	48466980	+	4340
protein_coding	potassium voltage-gated channel subfamily J member 14
[Source:HGNC Symbol;Acc:HGNC:6260]	-	164	125	69
	133	174	470	1.85047321	1.364729956
0.98915923	1.702840438	2.46437548	5.035459955
ENSG00000173812 8997.20783	11444.75732	12186.39997
14330.55219	13316.64367	14919.34229	10876.12171
14188.84605	-0.383724626	0.005753681	0.047692997	EIF1
	17	41688885	41692668	+	3623
protein_coding	eukaryotic translation initiation factor 1
[Source:HGNC Symbol;Acc:HGNC:3249]	-	9917	12932	10483
	13833	11666	17295	134.041927	169.1312282
180.0213849	212.1583292	197.9251571	221.9643618
ENSG00000206503 2022.262403	2808.974616	3832.73497

1421.348775 2542.102301 1580.354731 2887.990663
1847.935269 0.644221991 0.005759383 0.047710658 HLA-A
6 29941260 29945884 + 2710
protein_coding "major histocompatibility complex, class I, A
[Source:HGNC Symbol;Acc:HGNC:4931]" - 2229 3174 3297
1372 2227 1832 40.27814501 55.49630552
75.69313674 28.13175744 50.51243288 31.43309873
ENSG00000184378 41.73354444 53.09970919 26.73730795
70.44585765 113.007691 125.0826616 40.52352052
102.8454034 -1.338243358 0.005763217 0.047710658 ACTRT3
3 169766921 169769895 - 2005
protein_coding actin related protein T3 [Source:HGNC
Symbol;Acc:HGNC:24022] - 46 60 23 68 99
145 1.123497572 1.417957827 0.713707574
1.884545292 3.035066267 3.362673051
ENSG00000087302 1567.729669 1691.225738 1806.512024
1341.579201 1122.086467 1317.249822 1688.489144
1260.305163 0.421218942 0.005767299 0.047710658 RTRAF
14 51989475 52010691 + 8323
protein_coding "RNA transcription, translation and transport factor
[Source:HGNC Symbol;Acc:HGNC:23169]" - 1728 1911 1554
1295 983 1527 10.16699306 10.87945733
11.61657141 8.645734952 7.2597386 8.530807863
ENSG00000173705 158.768919 176.1140355 259.2356379
96.34507003 130.1300684 64.6979284 198.0395308
97.05768894 1.031385775 0.005768749 0.047710658 SUSD5
3 33150042 33219215 - 5008
protein_coding sushi domain containing 5 [Source:HGNC
Symbol;Acc:HGNC:29061] - 175 199 223 93 114
75 1.711206464 1.882847721 2.770431327
1.031883509 1.39922608 0.696350612
ENSG00000172340 1809.058426 1643.435999 1741.412492
1519.765782 1187.151501 1018.776712 1731.302306
1241.897999 0.479749042 0.005769297 0.047710658 SUCLG2
3 67360460 67654614 - 3090
protein_coding succinate-CoA ligase GDP-forming beta subunit
[Source:HGNC Symbol;Acc:HGNC:11450] - 1994 1857 1498
1467 1040 1181 31.60060214 28.47605776
30.16200312 26.38053981 20.68817685 17.7714325
ENSG00000277203 260.3810272 215.9388174 296.4353707
325.2941074 366.4188768 659.9188696 257.5850718
450.5439513 -0.809616348 0.005772688 0.047716393 F8A1
X 154886349 154888061 + 1713
protein_coding coagulation factor VIII associated 1 [Source:HGNC
Symbol;Acc:HGNC:3547] - 287 244 255 314 321
765 8.204520742 6.749302668 9.261677706
10.18554633 11.51847621 20.76515084
ENSG00000119720 308.4653284 353.9980612 282.4854709
437.1787048 547.9160775 461.5118892 314.9829535
482.2022239 -0.612520677 0.005781887 0.047753336 NRDE2

14	90267856	90332137	-	17565
protein_coding	"NRDE-2, necessary for RNA interference, domain containing [Source:HGNC Symbol;Acc:HGNC:20186]" -	340	400
243	422	480	535	0.947893285	1.079041823
0.860726088	1.33498486	1.679733731	1.416239314
ENSG00000180611 107.962865	143.3692148	152.2864061
174.0427071	288.7974325	282.0829678	134.5394953
248.3077025	-0.885780831	0.005782557	0.047753336	MB21D2
3	192796815	192918161	-	3368
protein_coding	Mab-21 domain containing 2 [Source:HGNC Symbol;Acc:HGNC:30438]	-	119	162	131	168	253
327	1.730228901	2.279131442	2.419948142
2.771719279	4.617381925	4.514469133
ENSG00000161204 1168.539244	1465.551974	1103.204576
1607.823104	1602.654527	1953.014799	1245.765264
1721.164143	-0.466154444	0.005822114	0.04805757	ABCF3
3	184186023	184194012	+	5207
protein_coding	ATP binding cassette subfamily F member 3 [Source:HGNC Symbol;Acc:HGNC:72]	-	1288	1656	949	1552	1404
2264	12.11314648	15.06951224	11.33928005
16.56212976	16.57398305	20.21714672
ENSG00000250906 0	0	0	3.107905485	12.55641011
14.66486377	0	10.10972645	-5.814609078
0.005827288	0.048077844	AC131953.1	4	40812779
40826151	+	477	antisense	novel
transcript	-	0	0	0	3	11	17
0	0	0	0.349474039	1.417495426
1.657149005
ENSG00000022840 2053.108936	2113.368426	2413.332665
2566.093962	3102.574789	2981.28054	2193.270009
2883.31643	-0.395011051	0.005868288	0.048393543	RNF10
12	120533480	120577594	+	5911
protein_coding	ring finger protein 10 [Source:HGNC Symbol;Acc:HGNC:10055]	-	2263	2388	2076	2477	2718
3456	18.74788488	19.14255061	21.85109657
23.2850455	28.26414505	27.18590485
ENSG00000131873 1720.147831	1564.671431	2041.335337
1378.874067	1362.941243	1173.189102	1775.384866
1305.00147	0.443922029	0.005874986	0.048412247	CHSY1
15	101175723	101251932	-	5555
protein_coding	chondroitin sulfate synthase 1 [Source:HGNC Symbol;Acc:HGNC:17198]	-	1896	1768	1756	1331	1194
1360	16.71409835	15.08080895	19.66741559
13.31392284	13.21197136	11.38376346
ENSG00000135045 913.6017227	892.0751143	1074.142285
800.8036466	607.2736526	596.0835803	959.9397072
668.0536265	0.52261427	0.005876392	0.048412247
C9orf40 9	74946581	74952886	-	2327
protein_coding	chromosome 9 open reading frame 40 [Source:HGNC Symbol;Acc:HGNC:23433]	-	1007	1008	924	773	532

691 21.19150353 20.52535086 24.70486213
18.4584468 14.05279171 13.80742466
ENSG00000158747 437.2949656 819.5055118 1526.351536
438.2146733 562.7554713 519.3087053 927.7173379
506.7596166 0.871268415 0.005878914 0.048412247 NBL1
1 19596979 19658456 + 3596
protein_coding "NBL1, DAN family BMP antagonist [Source:HGNC
Symbol;Acc:HGNC:7650]" - 482 926 1313 423 493
602 6.563810567 12.201627 22.71704768
6.536311302 8.427031095 7.7840896
ENSG00000106615 1620.350225 1715.120607 1501.939212
1323.967736 1038.757564 1215.458415 1612.470015
1192.727905 0.434946833 0.005881504 0.048412247 RHEB
7 151466012 151520120 - 3535
protein_coding "Ras homolog, mTORC1 binding [Source:HGNC
Symbol;Acc:HGNC:10011]" - 1786 1938 1292 1278 910
1409 24.74119772 25.97710773 22.73944926
20.08877615 15.82338279 18.53329306
ENSG00000232480 3.629003864 10.61994184 16.2748831 0
0 0 10.1746096 0 5.776756054
0.00589095 0.048466354 TGFB2-AS1 1 218344196
218345678 - 557 antisense TGFB2
antisense RNA 1 (head to head) [Source:HGNC Symbol;Acc:HGNC:50628]
- 4 12 14 0 0 0
0.351668509 1.020827807 1.563794521 0 0 0
ENSG00000051108 1754.623368 1993.89408 1641.43821
3268.480601 2032.996946 2331.713339 1796.651886
2544.396962 -0.501793095 0.005898286 0.048466354
HERPUD1 16 56932048 56944863 + 5155
protein_coding homocysteine inducible ER protein with ubiquitin like
domain 1 [Source:HGNC Symbol;Acc:HGNC:13744] - 1934 2253
1412 3155 1781 2703 18.37200199 20.70899212
17.04169835 34.00812886 21.23648377 24.38082483
ENSG00000251669 75.30183018 116.8193602 72.07448229
203.049825 176.9312334 147.5112767 88.06522423
175.8307784 -0.993678912 0.005898944 0.048466354
FAM86EP 4 3941760 3955419 - 3476
transcribed_unprocessed_pseudogene "family with sequence
similarity 86 member E, pseudogene [Source:HGNC
Symbol;Acc:HGNC:28017]" - 83 132 62 196 155
171 1.16930285 1.799370534 1.109733684
3.133201655 2.740938497 2.28742762
ENSG00000113387 3227.091686 3196.602493 3408.425517
2756.712165 2513.565006 2401.587102 3277.373232
2557.288091 0.357930734 0.005899037 0.048466354 SUB1
5 32531633 32604079 + 9324
protein_coding "SUB1 homolog, transcriptional regulator [Source:HGNC
Symbol;Acc:HGNC:19985]" PC4 3557 3612 2932 2661 2202
2784 18.68143718 18.35574085 19.56449074
15.85822785 14.51651643 13.88346866

ENSG00000104763 1222.974302	1140.758752	1275.25334
1027.680747	838.9964937	531.3856519	1212.995465
799.3542975	0.602601438	0.005923708	0.048646456	ASAH1
	8	18055992	18084998	-	15378
protein_coding	N-acylsphingosine amidohydrolase 1 [Source:HGNC
Symbol;Acc:HGNC:735]	-	1348	1289	1097	992	735
	616	4.292583177	3.971728028	4.438268966
3.584460998	2.937885344	1.86256687
ENSG00000130702 137.9021468	129.2092924	642.8578824
21.75533839	81.0459198	8.626390453	303.3231072
37.14254955	3.032925473	0.005952231	0.048858012	LAMA5
	20	62307955	62367312	-	14291
protein_coding	laminin subunit alpha 5 [Source:HGNC
Symbol;Acc:HGNC:6485]	-	152	146	553	21	71
	10	0.520846383	0.484079484	2.407516979
0.081652356	0.305381757	0.032536318
ENSG00000067369 1342.73143	1092.969014	1188.066466
1004.88944	716.8568681	841.9357082	1207.922303
854.5606721	0.498970821	0.005956744	0.048862776
TP53BP1 15	43403061	43510728	-	12991
protein_coding	tumor protein p53 binding protein 1 [Source:HGNC
Symbol;Acc:HGNC:11999]	-	1480	1235	1022	970	628
	976	5.578890228	4.504543816	4.894576939
4.148978603	2.971422593	3.493319057
ENSG00000157833 156.9544171	203.5488852	106.9492318
293.1790841	228.2983656	332.1160324	155.8175114
284.5311607	-0.866290488	0.005958336	0.048862776	GAREM2
	2	26173091	26189663	+	5939
protein_coding	GRB2 associated regulator of MAPK1 subtype 2
[Source:HGNC Symbol;Acc:HGNC:27172]	-	173	230	92
	283	200	385	1.426466121	1.835020632
0.963787632	2.647799854	2.069969901	3.01424412
ENSG00000119725 16.33051739	12.38993214	2.3249833	0
	0	0	10.34847761	0	5.808550491
0.005975158	0.048978018	ZNF410	14	73886617
73932511	+	6486	protein_coding	zinc finger protein
410 [Source:HGNC Symbol;Acc:HGNC:20144] zf-C2H2 18	14	2
	0	0	0	0.135901498	0.102276894
0.019184916	0	0	0
ENSG00000279412 35.38278767	18.58489822	11.6249165
59.05020421	49.08414861	108.6925197	21.8642008
72.27562418	-1.719584003	0.005984759	0.049033994
AC020763.3	16	70713987	70716890	+	2904
	TEC	novel transcript	-	39	21	10
	57	43	126	0.657652808	0.342648728
0.214244773	1.090662264	0.910163057	2.017460086
ENSG00000100629 297.5783168	209.7438513	312.7102538
203.049825	47.94265678	119.9068273	273.3441407
123.633103	1.142254024	0.006016198	0.049252768	CEP128
	14	80476983	80959517	-	9400

protein_coding	centrosomal protein 128 [Source:HGNC
Symbol;Acc:HGNC:20359]	-	328	237	269	196	42
	139	1.708734837	1.194667181	1.780456113
1.158617974	0.274643166	0.687571655
ENSG00000145217 177.8211893	277.8884781	145.3114562
275.5676196	360.7114177	440.8085521	200.3403745
359.0291965	-0.839698436	0.006019471	0.049252768
SLC26A1 4	979073	993440	-	4293	protein_coding	solute
carrier family 26 member 1 [Source:HGNC Symbol;Acc:HGNC:10993]	-
	196	314	125	266	316	511	2.235753228
	3.465733788	1.81157355	3.442966458	4.524530856
	5.534661057
ENSG00000115165 9.07250966	7.079961225	12.78740815
58.01423571	22.82983656	43.99459131	9.646626345
41.61288786	-2.120015043	0.006019814	0.049252768	CYTIP
	2	157414619	157488961	-	3428
protein_coding	cytohesin 1 interacting protein [Source:HGNC
Symbol;Acc:HGNC:9506]	-	10	8	11	56	20
	51	0.142852508	0.110579753	0.199645129
0.907735369	0.358621682	0.691767861
ENSG00000182173 626.0031665	783.2207105	626.5829993
845.3502918	1010.220268	1031.716298	678.6022921
962.4289526	-0.503602275	0.00602601	0.049280674	TSEN54
	17	75516060	75524739	+	3440
protein_coding	tRNA splicing endonuclease subunit 54 [Source:HGNC
Symbol;Acc:HGNC:27561]	-	690	885	539	816	885
	1196	9.822438803	12.19021229	9.74848593
13.18086038	15.8136524	16.16604395
ENSG00000144655 485.3792668	565.5119028	685.8700734
723.1060094	1019.352203	832.4466787	578.9204144
858.3016302	-0.568928002	0.00604413	0.049406017	CSRNP1
	3	39141855	39154562	-	3214
protein_coding	cysteine and serine rich nuclear protein 1
[Source:HGNC Symbol;Acc:HGNC:14300]	-	535	639	590
	698	893	965	8.151482362	9.420662094
11.42123285	12.0676191	17.07862673	13.96086923
ENSG00000161791 2219.135863	1840.789918	2302.895958
1583.995829	1525.033082	1682.146138	2120.94058
1597.05835	0.408670522	0.006050412	0.049415827	FMNL3
	12	49636499	49708165	-	13424
protein_coding	formin like 3 [Source:HGNC Symbol;Acc:HGNC:23698]
	-	2446	2080	1981	1529	1336	1950
8.922841793	7.341889305	9.181409941	6.329036286
6.117470375	6.754351939
ENSG00000222032 194.1517067	140.7142293	192.9736139
33.15099184	73.055477	119.9068273	175.9465166
75.37109871	1.217823091	0.006050918	0.049415827
AC112721.2	2	237428920	237434822	-	541
	lincRNA	novel protein (LOC728009)	-	214	159
	166	32	64	139	19.3706945	13.92599709

19.0905161 3.286735509 7.271601475 11.94671637
ENSG00000246250 1.814501932 4.424975766 0 27.97114936
30.82027936 9.489029498 2.079825899 22.76015274
-3.402954606 0.006058473 0.049454701 AC087521.2
11 43829709 43880726 - 3296
antisense novel transcript - 2 5 0
27 27 11 0.029714709 0.071880194 0
0.455185695 0.503528343 0.155180269
ENSG00000100092 98.89035529 114.1643748 116.249165
14.50355893 23.97132839 72.4616798 109.767965
36.97885571 1.558486471 0.006070279 0.049528216 SH3BP1
22 37634654 37656119 + 4892
protein_coding SH3 domain binding protein 1 [Source:HGNC
Symbol;Acc:HGNC:10824] - 109 129 100 14 21
84 1.091110495 1.249481133 1.27180462
0.159020689 0.263864039 0.798406117
ENSG00000179869 88.91059467 161.954113 62.77454909
173.0067386 293.3633998 198.4069804 104.5464189
221.592373 -1.07815645 0.006096026 0.049696178 ABCA13
7 48171458 48647496 + 19074
protein_coding ATP binding cassette subfamily A member 13
[Source:HGNC Symbol;Acc:HGNC:14638] - 98 183 54
167 257 230 0.251601358 0.454606616
0.176140339 0.48650436 0.828206635 0.560682596
ENSG00000182220 1297.368881 1267.313059 1684.450401
1066.011581 1092.40768 876.44127 1416.377447
1011.620177 0.485435115 0.006096484 0.049696178
ATP6AP2 X 40579372 40606848 + 9560
protein_coding ATPase H+ transporting accessory protein 2
[Source:HGNC Symbol;Acc:HGNC:18305] - 1430 1432 1449
1029 957 1016 7.324986502 7.097600758
9.430122618 5.980941108 6.153205304 4.941591415
ENSG00000196821 2995.74269 3715.209653 3494.4499
3601.026488 4807.96358 5704.632006 3401.800747
4704.540692 -0.4679569 0.006107271 0.049761176
C6orf106 6 34587288 34696859 - 4551
protein_coding chromosome 6 open reading frame 106
[Source:HGNC Symbol;Acc:HGNC:21215] - 3302 4198 3006
3476 4212 6613 35.53030348 43.70811123
41.09500024 42.44093821 56.88907695 67.56514379
ENSG00000088756 158.768919 146.9091954 304.5728123
119.1363769 105.0172482 62.97265031 203.4169756
95.70875847 1.087910273 0.006116863 0.04981253
ARHGAP28 18 6729718 6915716 + 11910 protein_coding
Rho GTPase activating protein 28 [Source:HGNC
Symbol;Acc:HGNC:25509] - 175 166 262 115 92
73 0.719540048 0.660423879 1.368662526
0.53653508 0.474813902 0.2849982
ENSG00000076003 3049.270497 3109.872968 3444.462759
2801.25881 2377.727478 1872.789367 3201.202074

2350.591885	0.445822497	0.006119206	0.04981253	MCM6
	2	135839626	135876426	-	4118
protein_coding	minichromosome maintenance complex component 6
[Source:HGNC Symbol;Acc:HGNC:6949]	-	3361	3514	2963
	2704	2083	2171	39.96785616	40.43354836
44.76639844	36.4865154	31.09211661	24.51345347
ENSG00000169047 1828.110696	1862.914797	2025.060454
1273.20528	1437.138212	1587.255843	1905.361983
1432.533112	0.410857834	0.006152753	0.050047059	IRS1
	2	226731317	226799759	-	9953
protein_coding	insulin receptor substrate 1 [Source:HGNC
Symbol;Acc:HGNC:6125]	-	2015	2105	1742	1229	1259
	1840	9.914018614	10.02131093	10.88932584
6.861355515	7.775334581	8.59596893
ENSG00000237523 71.67282631	38.93978674	40.68720775
19.6834014	5.707459141	0	50.4332736	8.463620181
	2.587646849	0.006153675	0.050047059	LINC00857
	10	80207710	80219657	+	2171
lincRNA long intergenic non-protein coding RNA 857 [Source:HGNC
Symbol;Acc:HGNC:45114]	-	79	44	35	19	5
	0	1.781951795	0.960327342	1.003032644
0.486301737	0.141565537	0
ENSG00000205413 394.6541702	269.9235217	377.8097862
221.6972579	182.6386925	226.0114299	347.4624927
210.1157934	0.723712767	0.006176057	0.050206007	SAMD9
	7	93099513	93118023	-	6855
protein_coding	sterile alpha motif domain containing 9 [Source:HGNC
Symbol;Acc:HGNC:1348]	-	435	305	325	214	160
	262	3.107495307	2.108234039	2.949733283
1.734675952	1.434695988	1.777153128
ENSG00000106683 1334.566171	1168.193602	1608.888443
855.7099768	1132.359894	918.7105832	1370.549406
968.9268179	0.50014341	0.006181677	0.050228612	LIMK1
	7	74082933	74122525	+	6902
protein_coding	LIM domain kinase 1 [Source:HGNC Symbol;Acc:HGNC:6613]
	-	1471	1320	1384	826	992	1065
10.43677693	9.062028362	12.47578787	6.649930953
8.834542766	7.174731795
ENSG00000110063 505.3387881	562.8569174	541.7211088
395.7399651	325.325171	370.0721504	536.6389381
363.7124288	0.560508915	0.006187176	0.050243854	DCPS
	11	126303752	126345754	+	3936
protein_coding	"decapping enzyme, scavenger [Source:HGNC
Symbol;Acc:HGNC:29812]" -	557	636	466	382	285
	429	6.929929062	7.656467911	7.366101071
5.392874538	4.450790275	5.067960491
ENSG00000276805 78.93083404	64.60464618	61.61205744
113.9565344	162.0918396	145.7859987	68.38251255
140.6114576	-1.038140291	0.006189234	0.050243854
AL133216.2	10	38403189	38452153	+	7213

transcribed_unprocessed_pseudogene general transcription
factor IIi (GTF2I) pseudogene - 87 73 53 110
142 169 0.590652442 0.479549419 0.457158484
0.847400657 1.210095852 1.089436171
ENSG00000174365 162.3979229 204.4338804 141.8239813
297.322958 234.0058248 348.5061743 169.5519282
293.278319 -0.789919494 0.006210286 0.050391625 SNHG11
20 38446578 38450921 + 2813
processed_transcript small nucleolar RNA host gene 11 [Source:HGNC
Symbol;Acc:HGNC:25046] - 179 231 122 287 205
404 3.116104277 3.891066817 2.698341701
5.669231718 4.479520094 6.677942128
ENSG00000185386 171.4704326 215.9388174 203.4360387
258.9921237 331.0326302 397.6765999 196.9484296
329.2337846 -0.742571607 0.006239431 0.050604904 MAPK11
22 50263713 50270767 - 2688
protein_coding mitogen-activated protein kinase 11 [Source:HGNC
Symbol;Acc:HGNC:6873] - 189 244 175 250 290
461 3.443191863 4.30117391 4.050565235
5.168003367 6.631565961 7.974485618
ENSG00000057657 88.91059467 68.14462679 198.7860721
22.79130689 70.77249335 29.32972754 118.6137645
40.96450926 1.53674389 0.006253122 0.050692704 PRDM1
6 106086320 106109939 + 6392
protein_coding PR/SET domain 1 [Source:HGNC Symbol;Acc:HGNC:9346]
zf-C2H2 98 77 171 22 62 34
0.750789159 0.570795315 1.664432513 0.191248465
0.596214156 0.247327934
ENSG00000232479 3.629003864 10.61994184 3.48747495
24.86324388 47.94265678 26.7418104 5.912140217
33.18257035 -2.471723023 0.006260849 0.05073209
AC010900.1 2 202924609 202925904 + 1296
processed_pseudogene keratin 8 (KRT8) pseudogene -
4 12 3 24 42 31 0.151141481
0.438735408 0.144020097 1.029006893 1.992010618
1.112212343
ENSG00000106636 4194.221216 5032.967436 4139.632765
5049.310444 5948.313917 6633.694258 4455.607139
5877.106206 -0.399449465 0.006270174 0.050784392 YKT6
7 44200968 44214294 + 3707
protein_coding YKT6 v-SNARE homolog [Source:HGNC
Symbol;Acc:HGNC:16959] - 4623 5687 3561 4874 5211
7690 61.07029122 72.69207785 59.76628126
73.05921179 86.40638728 96.45722688
ENSG00000104738 8725.939791 8536.663247 8253.690714
7976.957411 5641.252615 5341.460968 8505.431251
6319.890331 0.42857575 0.006276528 0.050812584 MCM4
8 47960185 47978160 + 6973
protein_coding minichromosome maintenance complex component 4
[Source:HGNC Symbol;Acc:HGNC:6947] - 9618 9646 7100

7700 4942 6192 67.54509099 65.5471832
63.34984113 61.35968248 43.5642695 41.28975449
ENSG00000169155 322.0740929 409.7527559 305.7353039
568.7467037 571.8874059 449.4349426 345.8540509
530.0230174 -0.613746696 0.006288384 0.050885283 ZBTB43
9 126805006 126838210 + 7052
protein_coding zinc finger and BTB domain containing 43 [Source:HGNC
Symbol;Acc:HGNC:17908] ZBTB 355 463 263 549 501
521 2.465157847 3.110965022 2.320332865
4.325856259 4.36689533 3.435234888
ENSG00000087365 10729.14992 10904.02528 11035.53323
12933.03069 13342.89798 14071.36811 10889.56948
13449.09893 -0.304614754 0.006295586 0.050920264 SF3B2
11 66050729 66069308 + 9795
protein_coding splicing factor 3b subunit 2 [Source:HGNC
Symbol;Acc:HGNC:10769] - 11826 12321 9493 12484 11689
16312 59.12376987 59.60297782 60.29841371
70.82088725 73.35340505 77.43437379
ENSG00000110619 1802.707669 2131.953324 2292.433534
2465.605018 3018.104394 2800.126341 2075.698176
2761.278584 -0.412027542 0.006302071 0.050949421 CARS
11 3000922 3057613 - 10037 protein_coding
cysteinyl-tRNA synthetase [Source:HGNC Symbol;Acc:HGNC:1493] -
1987 2409 1972 2380 2644 3246 9.694437755
11.37258828 12.22390126 13.17604522 16.19216374
15.03749893
ENSG00000138629 1775.49014 1654.940936 1532.163995
1804.657118 2433.660578 2543.059905 1654.198357
2260.4592 -0.450355218 0.006306466 0.050957604 UBL7
15 74445977 74461182 - 1885
protein_coding ubiquitin like 7 [Source:HGNC Symbol;Acc:HGNC:28221]
- 1957 1870 1318 1742 2132 2948
50.84030586 47.00636763 43.50216811 51.35099224
69.52215186 72.71887354
ENSG00000182544 504.4315371 515.0671791 516.1462926
527.3079639 883.514675 967.0183698 511.8816696
792.6136696 -0.631272677 0.006308845 0.050957604 MFSD5
12 53251251 53254405 + 2581
protein_coding major facilitator superfamily domain containing 5
[Source:HGNC Symbol;Acc:HGNC:28156] - 556 582 444
509 774 1121 10.54910149 10.68467757
10.70290849 10.95826558 18.43318222 20.19522175
ENSG00000186184 1890.711013 2320.457291 2022.735471
2379.619633 3046.641689 2898.467192 2077.967925
2774.909505 -0.417112423 0.006327187 0.051082429 POLR1D
13 27620742 27744237 + 11210
protein_coding RNA polymerase I and III subunit D [Source:HGNC
Symbol;Acc:HGNC:20422] - 2084 2622 1740 2297 2669
3360 9.103759698 11.08290258 9.657183469
11.3859016 14.63491894 13.93685183

ENSG00000179922 213.203977 256.6485944 240.6357715
305.610706 409.7955663 420.9678541 236.8294476
378.7913755 -0.677968951 0.006333914 0.051113417 ZNF784
19 55620742 55624601 - 2058
protein_coding zinc finger protein 784 [Source:HGNC
Symbol;Acc:HGNC:33111] zf-C2H2 235 290 207 295 359
488 5.59179415 6.676964517 6.257946149
7.965053353 10.72250947 11.02568218
ENSG00000127585 446.3674753 459.3124845 578.9208416
312.8624855 358.428434 306.2368611 494.8669338
325.8425935 0.602473966 0.006343085 0.051138304 FBXL16
16 692498 705829 - 3989 protein_coding F-box
and leucine rich repeat protein 16 [Source:HGNC
Symbol;Acc:HGNC:14150] - 492 519 498 302 314
355 6.039900024 6.164952892 7.767337087
4.206829883 4.838524806 4.138046198
ENSG00000161654 788.4010894 837.2054149 884.6561456
709.638419 440.6158457 542.5999595 836.7542166
564.2847414 0.567670595 0.006345355 0.051138304 LSM12
17 44034635 44067619 - 2811
protein_coding LSM12 homolog [Source:HGNC Symbol;Acc:HGNC:26407]
- 869 946 761 685 386 629
15.13866624 15.94618255 16.84343472 13.54072037
8.440609712 10.4044905
ENSG00000160145 146.0674055 92.92449108 185.998664
73.55376314 71.91398517 45.7198694 141.6635202
63.7292059 1.153637163 0.006347191 0.051138304 KALRN
3 124080023 124726325 + 24490
protein_coding kalirin RhoGEF kinase [Source:HGNC
Symbol;Acc:HGNC:4814] - 161 105 160 71 63
53 0.321933206 0.203154738 0.406478935
0.161094832 0.158124485 0.100627829
ENSG00000140262 2779.81696 2073.543644 3109.665163
2103.016045 2010.167109 1551.025003 2654.341922
1888.069386 0.491475047 0.006348562 0.051138304 TCF12
15 56918623 57299281 + 9446
protein_coding transcription factor 12 [Source:HGNC
Symbol;Acc:HGNC:11623] bHLH 3064 2343 2675 2030 1761
1798 15.88435203 11.75305553 17.61905826
11.94153457 11.45931809 8.850601037
ENSG00000112837 102.5193592 51.32971888 132.5240481
53.87036173 25.11282022 12.93958568 95.45770871
30.64092254 1.641998375 0.006358548 0.05119543 TBX18
6 84687351 84764519 - 8469
protein_coding T-box 18 [Source:HGNC Symbol;Acc:HGNC:11595] T-box
113 58 114 52 22 15 0.653393777
0.324505679 0.837489875 0.341179756 0.159675362
0.082355033
ENSG00000259744 0 0 0 0 14.83939377
21.56597613 0 12.1351233 -6.078617503 0.006369547

0.051260651	AC009269.4	15	70848883
70849770	-	755	sense_intronic	"novel transcript,
sense intronic to LARP6"	-	0	0	0	0
13	25	0	0	0	0	1.058385206
1.539657334
ENSG00000206053 2376.09028	2328.422248	2980.62859
1890.642503	1996.469207	1921.097154	2561.713706
1936.069622	0.403528491	0.006382707	0.051343201	JPT2
16	1678256 1702280 +	4768	protein_coding
Jupiter microtubule associated homolog 2 [Source:HGNC
Symbol;Acc:HGNC:14137]	-	2619	2631	2564	1825	1749
2227	26.89849214	26.1463483	33.45712514
21.26858835	22.54763121	21.71775795
ENSG00000124831 2038.592921	1860.259812	2111.084836
1557.060648	1539.872476	1530.321666	2003.312523
1542.418263	0.376952135	0.006386495	0.051350326
LRRFIP1 2	237627576	237813682	+	11901
protein_coding	LRR binding FLII interacting protein 1 [Source:HGNC
Symbol;Acc:HGNC:6702]	-	2247	2102	1816	1503	1349
1774	9.245881027	8.369041036	9.493781575
7.01758318	6.967481988	6.931084259
ENSG00000095383 158.768919	200.8938998	233.6608216
279.7114936	295.6463835	443.3964693	197.7745468
339.5847821	-0.783368225	0.006390329	0.051357809	TBC1D2
9	98198999	98255721	-	3681
protein_coding	TBC1 domain family member 2 [Source:HGNC
Symbol;Acc:HGNC:18026]	-	175	227	201	270	259
514	2.328096162	2.922042179	3.397324934
4.075773022	4.324952149	6.4927446
ENSG00000131473 10900.62036	9187.134685	11144.80745
8766.365404	8410.51179	6503.435762	10410.85416
7893.437652	0.399448404	0.00641124	0.05150246	ACLY
17	41866908	41930542	-	5217
protein_coding	ATP citrate lyase [Source:HGNC Symbol;Acc:HGNC:115]
-	12015	10381	9587	8462	7368	7539
112.7798784	94.28547539	114.3322466	90.12893264
86.81127618	67.19298345
ENSG00000077238 1246.562827	1182.353525	1413.589846
928.2277714	1034.191596	859.1884891	1280.835399
940.5359523	0.44565036	0.006442886	0.051733186	IL4R
16	27313668	27364778	+	5894
protein_coding	interleukin 4 receptor [Source:HGNC
Symbol;Acc:HGNC:6015]	-	1374	1336	1216	896	906
996	11.41577196	10.74045717	12.83601719
8.447144468	9.448555673	7.857424651
ENSG00000164346 1335.473422	1435.462138	1403.127421
1156.14084	961.1361193	994.6228192	1391.354327
1037.299926	0.423614586	0.006447449	0.051746336	NSA2
5	74766991	74780113	+	4962
protein_coding	"NSA2, ribosome biogenesis homolog [Source:HGNC

Symbol;Acc:HGNC:30728]" - 1472 1622 1207 1116 842
1153 14.52712701 15.48889839 15.1341264
12.49739447 10.4304415 10.80447243
ENSG00000134363 186.893699 110.6243941 181.3486974
75.62570013 93.60232991 22.42861518 159.6222635
63.88554841 1.327327203 0.006476613 0.051945811 FST
5 53480409 53487134 + 2969
protein_coding follistatin [Source:HGNC Symbol;Acc:HGNC:3971] -
206 125 156 73 82 26 3.39770529
1.994923546 3.269047623 1.366232796 1.697661169
0.407187248
ENSG00000080189 2205.527098 2462.056516 2166.884435
2300.886027 3409.636091 3936.221964 2278.156017
3215.581361 -0.497291389 0.006478176 0.051945811
SLC35C2 20 46345980 46364458 - 7708
protein_coding solute carrier family 35 member C2 [Source:HGNC
Symbol;Acc:HGNC:17117] - 2431 2782 1864 2221 2987
4563 15.44443184 17.10180147 15.04565325
16.01101617 23.81995172 27.52578928
ENSG00000213793 136.9948959 87.61452016 81.37441549
44.54664528 47.94265678 36.2308399 101.9946105
42.90671399 1.253545728 0.006527203 0.052315221 ZNF888
19 52904417 52923481 - 4444
protein_coding zinc finger protein 888 [Source:HGNC
Symbol;Acc:HGNC:38695] - 151 99 70 43 42
42 1.6639167 1.055571328 0.980010743
0.537658417 0.580928389 0.439446751
ENSG00000173818 485.3792668 620.3816023 551.021042
661.9838682 829.8645591 929.0622518 552.2606371
806.9702264 -0.547548834 0.006534169 0.052340747 ENDOV
17 80415165 80438086 + 7253
protein_coding endonuclease V [Source:HGNC Symbol;Acc:HGNC:26640]
- 535 701 474 639 727 1077
3.612141777 4.579591929 4.066001278 4.895479365
6.161182789 6.904449285
ENSG00000268520 0 0 2.3249833 18.64743291
11.41491828 17.25278091 0.774994433
15.7717107 -4.486317983 0.006536306 0.052340747
AC008750.5 19 51361712 51365544 - 473
sense_intronic "novel transcript, sense intronic to ETFB"
- 0 0 2 18 10 20 0
0 0.263072651 2.114576532 1.29952973
1.966074059
ENSG00000130414 726.7080237 681.4462679 880.006179
467.2217912 606.1321608 498.6053682 762.7201569
523.98644 0.541487851 0.006544705 0.052384295
NDUFA10 2 239892450 240025402 - 9517
protein_coding NADH:ubiquinone oxidoreductase subunit A10
[Source:HGNC Symbol;Acc:HGNC:7684] - 801 770 757
451 531 578 4.121555291 3.833690924

4.948831384	2.633228314	3.429586903	2.823961602
ENSG00000259207 77.11633211	78.76456863	132.5240481
42.47470829	35.38624667	3.450556181	96.13498294
27.10383705	1.834666772	0.006550737	0.052408855	ITGB3
	17	47253846	47311816	+	4394
protein_coding	integrin subunit beta 3 [Source:HGNC
Symbol;Acc:HGNC:6156]	-	85	89	114	41	31
	4	0.947300042	0.959746186	1.614178823
0.518484584	0.433659637	0.042328313
ENSG00000139233 601.5073904	836.3204197	590.5457581
967.5945742	885.7976587	1080.886724	676.1245228
978.0929855	-0.532232135	0.006555984	0.052427128	LLPH
	12	66116555	66130768	-	7993
protein_coding	"LLP homolog, long-term synaptic facilitation factor
[Source:HGNC Symbol;Acc:HGNC:28229]"	-	663	945	508
	934	776	1253	4.061929665	5.602068777
3.954219249	6.493055378	5.967593998	7.289072605
ENSG00000150051 9.979760626	13.2749273	6.974949899
48.69051926	59.35757507	21.56597613	10.07654594
43.20469015	-2.087926321	0.006565459	0.05247917	MKX
	10	27672875	27746060	-	4106
protein_coding	mohawk homeobox [Source:HGNC Symbol;Acc:HGNC:23729]
	Others	11	15	6	47	52	25
0.131190511	0.173100672	0.090915755	0.63604956
0.778451857	0.283107961
ENSG00000260000 46.26979926	35.39980612	25.5748163
6.215810969	10.27342645	0	35.74814056	5.496412474
	2.732578937	0.006570587	0.052496436	AL133338.1
	6	100881471	100882987	+	1517
antisense	"novel transcript, antisense to ASCC3"	-	51
	40	22	6	9	0	1.646316303
1.249398129	0.902285434	0.219774709	0.36467357	0
ENSG00000104412 652.3134445	604.4516896	618.4455577
461.0059802	460.0212068	376.9732628	625.0702306
432.6668166	0.53197465	0.006573895	0.052499155	EMC2
	8	108443601	108486918	+	3781
protein_coding	ER membrane protein complex subunit 2 [Source:HGNC
Symbol;Acc:HGNC:28963]	-	719	683	532	445	403
	437	9.312170018	8.559343727	8.754106011
6.539813708	6.55157518	5.374100425
ENSG00000135930 1183.962511	1215.98334	1253.165999
1464.859452	1768.170842	1608.821819	1217.70395
1613.950704	-0.406417506	0.006577668	0.052505584	EIF4E2
	2	232550052	232583644	+	7177
protein_coding	eukaryotic translation initiation factor 4E family
member 2 [Source:HGNC Symbol;Acc:HGNC:3293]	-	1305	1374
	1078	1414	1549	1865	8.90422753	9.071314568
	9.345072203	10.94758956	13.26648382	12.08278111
ENSG00000108588 3278.804991	2947.918855	3368.900801
2837.517708	2044.411864	2292.031943	3198.541549

2391.320505	0.419378204	0.006588329	0.052566961	CCDC47
	17	63745250	63776351	-	4537
protein_coding	coiled-coil domain containing 47 [Source:HGNC
Symbol;Acc:HGNC:24856]	-	3614	3331	2898	2739	1791
	2657	39.00749419	34.78822692	39.74078564
33.54557934	24.26465757	27.23038573
ENSG00000275091 0	0	0	0	25.11282022
11.21430759	0	12.1090426	-6.071937865	0.006645214
	0.052990008	AC022098.4	19	14006422
14006773	+	352	antisense	"novel transcript,
antisense to RFX1"	-	0	0	0	0	22
	13	0	0	0	0	3.841734764
1.717242811
ENSG00000091831 2.721752898	13.2749273	3.48747495
68.37392066	34.24475485	13.80222472	6.494718381
38.80696674	-2.56236545	0.006647342	0.052990008	ESR1
	6	151656691	152129619	+	13854
protein_coding	estrogen receptor 1 [Source:HGNC Symbol;Acc:HGNC:3467]
	ESR-like	3	15	3	66	30	16
	0.010604123	0.051302971	0.013472647	0.264716368
	0.133104712	0.053700189
ENSG00000064703 1633.051739	1616.00115	1613.53841
2017.03066	2028.430979	2234.235127	1620.863766
2093.232255	-0.369204832	0.006650748	0.05299328	DDX20
	1	111755245	111768016	+	6019
protein_coding	DEAD-box helicase 20 [Source:HGNC
Symbol;Acc:HGNC:2743]	-	1800	1826	1388	1947	1777
	2590	14.64457745	14.37483508	14.34735913
17.97436894	18.14723423	20.00812716
ENSG00000182118 288.5058072	315.9432697	377.8097862
406.09965	562.7554713	547.7757938	327.419621
505.5436383	-0.6282578	0.006656617	0.053016164	FAM89A
	1	231018958	231040246	-	2394
protein_coding	family with sequence similarity 89 member A
[Source:HGNC Symbol;Acc:HGNC:25057]	-	318	357	325
	392	493	635	6.504765692	7.065949201
8.446291417	9.098587261	12.65814696	12.33333697
ENSG00000214194 649.5916916	498.2522712	560.3209752
416.459335	405.229599	307.0995001	569.3883127
376.2628114	0.59914581	0.006672859	0.0531216	SMIM30
	7	113116718	113118613	-	1896
protein_coding	small integral membrane protein 30 [Source:HGNC
Symbol;Acc:HGNC:48953]	-	716	563	482	402	355
	356	18.49282981	14.07007792	15.81668815
11.78147765	11.50899444	8.730571695
ENSG00000246273 117.0353746	70.79961225	41.8496994
196.834014	128.9885766	183.7421166	76.56156209
169.8549024	-1.145160018	0.006694196	0.053224044	SBF2-
AS1	11	9758292 9811335 +	4644	antisense	SBF2
antisense RNA 1 [Source:HGNC Symbol;Acc:HGNC:27438]	-	129

80	36	190	113	213	1.360273329
0.816251922	0.482299861	2.273387321	1.495662458
2.132644223
ENSG00000271601 2252.704149	2170.008115	2408.682699
1890.642503	1731.643103	1638.151547	2277.131654
1753.479051	0.376956178	0.006695026	0.053224044	LIX1L
1	145933423	145958001	-	3975
protein_coding	limb and CNS expressed 1 like [Source:HGNC
Symbol;Acc:HGNC:28715]	-	2483	2452	2072	1825	1517
1899	30.58921064	29.22871844	32.43093462
25.51160485	23.45826068	22.21359502
ENSG00000143537 1813.594681	1897.429608	2172.696894
1224.514761	1757.897415	1246.51342	1961.240394
1409.641866	0.476828384	0.006696096	0.053224044	ADAM15
1	155050566	155062775	+	6584
protein_coding	ADAM metallopeptidase domain 15 [Source:HGNC
Symbol;Acc:HGNC:193]	-	1999	2144	1869	1182	1540
1445	14.86796929	15.42983918	17.66144876
9.975615423	14.37733059	10.20490681
ENSG00000071553 2776.187956	2971.813724	3685.09853
2346.468641	2519.272465	2264.427494	3144.366737
2376.722867	0.403545948	0.006697763	0.053224044
ATP6AP1 X	154428632	154436516	+	4884
protein_coding	ATPase H+ transporting accessory protein 1
[Source:HGNC Symbol;Acc:HGNC:868]	-	3060	3358	3170
2265	2207	2625	30.68134928	32.57852948
40.38224446	25.76941708	27.77627723	24.99105962
ENSG00000109171 1738.292851	1594.761266	1536.813961
2095.764265	2088.930046	2111.740383	1623.289359
2098.811565	-0.370391354	0.006704995	0.053257589	SLAIN2
4	48341322	48426212	+	6524
protein_coding	SLAIN motif family member 2 [Source:HGNC
Symbol;Acc:HGNC:29282]	-	1916	1802	1322	2023	1830
2448	14.38170036	13.08781885	12.60736567
17.23034503	17.24187521	17.44731007
ENSG00000142669 3466.605941	4221.42688	4445.368069
4200.852247	5990.549114	6562.957856	4044.466964
5584.786406	-0.465780197	0.006738458	0.053499361
SH3BGRL3	1	26279176	26281522	+	1786
protein_coding	SH3 domain binding glutamate rich protein like
3 [Source:HGNC Symbol;Acc:HGNC:15568]	-	3821	4770	3824
4055	5248	7608	104.7669418	126.5503543
133.2119776	126.1599324	180.6174606	198.070623
ENSG00000166347 542.5360777	584.0968011	632.3954575
355.3371938	380.1167788	458.061333	586.3427788
397.8384352	0.557802321	0.006747199	0.053544728	CYB5A
18	74250847	74292016	-	9313
protein_coding	cytochrome b5 type A [Source:HGNC
Symbol;Acc:HGNC:2570]	-	598	660	544	343	333
531	3.144417934	3.358000631	3.634261249

2.046522674	2.197869948	2.651159986
ENSG00000166831 33.56828574	6.194966072	43.01219105	0
	6.848950969	0	27.59181429	2.282983656
3.648683263	0.006769675	0.053699002	RBPMS2	15
64739892	64775587	-	2101	protein_coding	"RNA
binding protein, mRNA processing factor 2 [Source:HGNC
Symbol;Acc:HGNC:19098]" -	37	7	37	0	6
	0	0.862391277	0.157869571	1.095676932	0
	0.175538571	0
ENSG00000003436 921.7669814	788.5306814	1390.340013
750.0411903	644.9428829	663.3694258	1033.545892
686.117833	0.589784297	0.006776889	0.053732131	TFPI
	2	187464230	187565760	-	11714
protein_coding	tissue factor pathway inhibitor [Source:HGNC
Symbol;Acc:HGNC:11760]	-	1016	891	1196	724	565
	769	4.247341409	3.604117367	6.352326419
3.434356622	2.964767139	3.052475158
ENSG00000108587 1774.582889	1615.116154	1511.239145
1983.879668	2188.239835	2238.548322	1633.646063
2136.889275	-0.387131469	0.00678185	0.053742314	GOSR1
	17	30477362	30527592	+	9855
protein_coding	golgi SNAP receptor complex member 1 [Source:HGNC
Symbol;Acc:HGNC:4430]	-	1956	1825	1300	1915	1917
	2595	9.719432442	8.774708158	8.207172664
10.79752438	11.95674162	12.24367383
ENSG00000102393 893.6422015	808.0005748	932.3183032
553.2071763	585.5853079	721.1662419	877.9870265
619.986242	0.50050125	0.006784249	0.053742314	GLA
	X	101397803	101408012	-	2275
protein_coding	galactosidase alpha [Source:HGNC Symbol;Acc:HGNC:4296]
	-	985	913	802	534	513	836
21.20232626	19.01585326	21.93308966	13.04283198
13.86064129	17.08660952
ENSG00000085719 2416.916573	2185.053033	2358.695558
3263.300759	3157.366397	2644.851313	2320.221721
3021.839489	-0.380949028	0.006792661	0.053784862	CPNE3
	8	86514427	86561498	+	6745
protein_coding	copine 3 [Source:HGNC Symbol;Acc:HGNC:2316]	-
	2664	2469	2029	3150	2766	3066	19.34109019
	17.34465144	18.71573726	25.95019606	25.20679225
	21.13592283
ENSG00000143774 3355.921323	4376.301032	4501.167668
4386.290607	6022.510885	6378.353101	4077.796675
5595.718198	-0.456744213	0.006798046	0.053803415	GUK1
	1	228139962	228148984	+	4725
protein_coding	guanylate kinase 1 [Source:HGNC Symbol;Acc:HGNC:4693]
	-	3699	4945	3872	4234	5276	7394
38.3363889	49.58963639	50.98476036	49.79217352
68.63574218	72.76267411
ENSG00000158270 114.3136217	115.9343651	232.49833

34.18696033	108.4417237	37.95611799	154.2487723
60.194934	1.361519059	0.006813986	0.053905458
COLEC12 18	316740	500722	-	7340	protein_coding
collectin subfamily member 12 [Source:HGNC Symbol;Acc:HGNC:16016]
	-	126	131	200	33	95	44
0.840626678	0.845671465	1.695277439	0.249821564
0.795563602	0.278732516
ENSG00000171453 686.7889812	869.0652404	787.006847
1028.716715	955.4286602	1374.183999	780.9536895
1119.443125	-0.520350976	0.006817615	0.053910056	POLR1C
	6	43509702	43562419	+	5550
protein_coding	RNA polymerase I and III subunit C [Source:HGNC
Symbol;Acc:HGNC:20194]	-	757	982	677	993	837
	1593	6.679309685	8.383877914	7.589314183
9.941875243	9.270002154	13.34608267
ENSG00000112996 1462.488557	1361.122545	1377.552605
1794.297433	1776.161285	1877.965202	1400.387903
1816.141306	-0.375121734	0.006830606	0.053978614	MRPS30
	5	44808925	44820428	+	6202
protein_coding	mitochondrial ribosomal protein S30 [Source:HGNC
Symbol;Acc:HGNC:8769]	-	1612	1538	1185	1732	1556
	2177	12.72805253	11.75035572	11.88757952
15.51772923	15.42144932	16.3214104
ENSG00000187189 813.8041165	688.5262291	734.6947227
996.6016921	1015.927727	1032.578937	745.6750228
1015.036119	-0.444918056	0.006832388	0.053978614	TSPYL4
	6	116249961	116254140	-	4180
protein_coding	TSPY like 4 [Source:HGNC Symbol;Acc:HGNC:21559] -
	897	778	632	962	890	1197	10.5085996
	8.819211463	9.406924169	12.78824164	13.08763231
	13.31523657
ENSG00000213390 435.4804637	410.637751	545.2085838
362.5889732	261.4016287	257.9290745	463.7755995
293.9732255	0.656948083	0.006843108	0.054026714
ARHGAP19	10	97222173	97292673	-	6167
	protein_coding	Rho GTPase activating protein 19 [Source:HGNC
Symbol;Acc:HGNC:23724]	-	480	464	469	350	229
	299	3.811500425	3.565089794	4.731575135
3.153596606	2.282490056	2.254385892
ENSG00000147586 361.9931354	385.8578868	362.6973948
222.7332264	232.8643329	262.2422698	370.1828057
239.279943	0.628752777	0.006844585	0.054026714	MRPS28
	8	79918717	80030289	-	1817
protein_coding	mitochondrial ribosomal protein S28 [Source:HGNC
Symbol;Acc:HGNC:14513]	-	399	436	312	215	204
	304	10.75342107	11.36993555	10.68332679
6.574997261	6.901168007	7.779463982
ENSG00000166508 7836.833844	9055.270407	8772.16199
6573.2201	6966.524627	6945.106954	8554.755414
6828.283894	0.325169872	0.006868451	0.054190918	MCM7

7	100092728	100101940	-	4386
protein_coding	minichromosome maintenance complex component 7
[Source:HGNC Symbol;Acc:HGNC:6950]	-	8638	10232	7546
6345	6103	8051	96.44356509	110.5397161
107.0421985	80.38500493	85.53071505	85.35170821
ENSG00000065328 1477.004573	1392.982371	1255.490982
1160.284714	988.5319232	831.5840396	1375.159309
993.4668924	0.470032361	0.006879152	0.054251147	MCM10
10	13161554	13211104	+	5454
protein_coding	minichromosome maintenance 10 replication initiation
factor [Source:HGNC Symbol;Acc:HGNC:18043]	-	1628	1574
1080	1120	866	964	14.61732659	13.6746442
12.32013505	11.4107695	9.760006763	8.218506792
ENSG00000064115 1309.163144	1274.39302	1250.841015
1016.285093	997.6638578	829.8587616	1278.132393
947.9359043	0.432030746	0.006885139	0.054261012	TM7SF3
12	26973195	27014434	-	3888
protein_coding	transmembrane 7 superfamily member 3 [Source:HGNC
Symbol;Acc:HGNC:23049]	-	1443	1440	1076	981	874
962	18.17476309	17.54941632	17.21840274
14.02021891	13.81759746	11.50482015
ENSG00000077458 653.2206955	541.6170337	563.8084502
381.2364061	409.7955663	420.9678541	586.2153931
403.9999422	0.537067328	0.006886588	0.054261012	FAM76B
11	95768942	95790409	-	6042
protein_coding	family with sequence similarity 76 member B
[Source:HGNC Symbol;Acc:HGNC:28492]	-	720	612	485
368	359	488	5.835532055	4.799512665
4.99422224	3.384380167	3.652254962	3.755520346
ENSG00000182310 68.95107341	46.90474312	82.53690714	0
11.41491828	28.46708849	66.13090789	13.29400226
2.299578302	0.006889606	0.054261012	SPACA6	19
51689128	51712387	+	4103	protein_coding
sperm acrosome associated 6 [Source:HGNC
Symbol;Acc:HGNC:27113]	-	76	53	71	0	10
33	0.907069907	0.612069577	1.076623062	0
0.149811738	0.373975749
ENSG00000275496 103.4266101	80.53455893	87.18687374
15.53952742	50.22564044	1.725278091	90.38268093
22.49681532	2.0213888	0.006897213	0.054296748
CU633906.1	21	6228966 6267317 -	2878	lincRNA
uncharacterized LOC102724701 [Source:NCBI gene;Acc:102724701]	-
114	91	75	15	44	2	1.939736533
1.498225014	1.621352033	0.289609306	0.93974332
0.032312475
ENSG00000162298 1583.152936	1660.250907	1394.98998
2077.116832	1901.725386	2102.251353	1546.131274
2027.03119	-0.390365676	0.006901484	0.054306209	SYVN1
11	65121780	65134533	-	5496
protein_coding	synoviolin 1 [Source:HGNC Symbol;Acc:HGNC:20738]

- 1745 1876 1200 2005 1666 2437
15.54810235 16.17381796 13.58442839 20.27121111
18.63269321 20.61768135
ENSG00000115758 5201.269788 5494.934906 5478.823146
6062.487632 7047.570547 7438.536487 5391.675947
6849.531556 -0.345370455 0.006908452 0.054336866 ODC1
2 10439968 10448504 - 2968
protein_coding ornithine decarboxylase 1 [Source:HGNC
Symbol;Acc:HGNC:8109] - 5733 6209 4713 5852 6174
8623 94.59032741 99.1252291 98.79623392
109.5601112 127.8645306 135.0907173
ENSG00000185504 1333.65892 1728.395534 1342.677856
1640.974096 2056.968274 2527.532403 1468.244103
2075.158258 -0.499155215 0.006911774 0.054338828
FAAP100 17 81539885 81553961 - 5354
protein_coding FA core complex associated protein 100 [Source:HGNC
Symbol;Acc:HGNC:26171] - 1470 1953 1155 1584 1802
2930 13.44521191 17.28424116 13.42179076
16.43950945 20.68825116 25.44604387
ENSG00000150593 587.898626 478.7823778 540.5586172
416.459335 288.7974325 351.9567305 535.7465403
352.4044993 0.603505139 0.006921002 0.054379163 PDCD4
10 110871795 110900006 + 4547
protein_coding programmed cell death 4 [Source:HGNC
Symbol;Acc:HGNC:8763] - 648 541 465 402 253
408 6.978767585 5.637658327 6.362603284
4.912619667 3.420132466 4.172210647
ENSG00000160439 206.8532202 230.0987398 204.5985304
320.1142649 320.7592037 370.9347895 213.8501635
337.2694194 -0.657415365 0.006923052 0.054379163 RDH13
19 55039108 55071291 - 8594
protein_coding retinol dehydrogenase 13 [Source:HGNC
Symbol;Acc:HGNC:19978] - 228 260 176 309 281
430 1.299176575 1.433522254 1.274160581
1.997906564 2.009825401 2.326505719
ENSG00000136048 475.3995062 433.647625 448.7217769
173.0067386 240.8547757 381.286458 452.589636
265.0493241 0.769336531 0.006927575 0.054390307 DRAM1
12 101877351 102012130 + 4180
protein_coding DNA damage regulated autophagy modulator 1
[Source:HGNC Symbol;Acc:HGNC:25645] - 524 490 386
167 211 442 6.138802888 5.554516217
5.745368243 2.21999621 3.102798221 4.916737312
ENSG00000127564 824.6911281 976.1496539 979.9804609
765.5807177 644.9428829 502.0559244 926.9404143
637.5265083 0.540740579 0.006930621 0.054390307 PKMYT1
16 2968024 2980539 - 5034
protein_coding "protein kinase, membrane associated tyrosine/
threonine 1 [Source:HGNC Symbol;Acc:HGNC:29650]" - 909
1103 843 739 565 582 8.842587288

10.38218449	10.41888417	8.157240576	6.898943636
5.375771389
ENSG00000106346 799.288101	679.6762776	741.6696726
1081.551109	1011.36176	946.3150327	740.2113504
1013.075967	-0.452540412	0.006957539	0.054577339	USP42
	7	6104884 6161564 +	7072	protein_coding
ubiquitin specific peptidase 42 [Source:HGNC Symbol;Acc:HGNC:20068]
	-	881	768	638	1044	886	1097
6.100456574	5.14571121	5.612873744	8.202954267
7.700852943	7.212658894
ENSG00000104067 1050.596619	815.9655312	1112.504509
901.2925906	514.8128145	475.3141139	993.0222196
630.473173	0.655413961	0.006970414	0.054654105	TJP1
	15	29699367	29968865	-	11265
protein_coding	tight junction protein 1 [Source:HGNC
Symbol;Acc:HGNC:11827]	-	1158	922	957	870	451
	551	5.033916957	3.878163957	5.285518391
4.291410902	2.460892859	2.274319217
ENSG00000085185 254.9375214	240.7186816	258.0731463
391.5960911	326.4666629	463.2371673	251.2431165
393.7666404	-0.650051393	0.006978636	0.054694329	BCORL1
	X	129981107	130058083	+	7996
protein_coding	BCL6 corepressor like 1 [Source:HGNC
Symbol;Acc:HGNC:25657]	-	281	272	222	378	286
	537	1.720926087	1.611842339	1.727376614
2.626824499	2.198571571	3.122716215
ENSG00000185838 356.5496296	461.0824748	406.8720775
388.4881856	687.1780806	1023.089908	408.1680606
699.5853913	-0.778214408	0.006981793	0.054694832	GNB1L
	22	19783224	19854939	-	6988
protein_coding	G protein subunit beta 1 like [Source:HGNC
Symbol;Acc:HGNC:4397]	-	393	521	350	375	602
	1186	2.754027913	3.532736682	3.116176116
2.981881737	5.2953047	7.891559055
ENSG00000185860 7.258007728	15.92991276	5.812458249	0
	0	0	9.666792911	0	5.708647509
0.006988024	0.054719416	CCDC190 1	162824458
162868815	-	4316	protein_coding	coiled-coil domain
containing 190 [Source:HGNC Symbol;Acc:HGNC:28736]	-	8
	18	5	0	0	0	0.090768934
0.197613909	0.072076786	0	0	0
ENSG00000062822 2186.474828	2119.563392	2015.760521
1729.031418	1608.361986	1555.338199	2107.266247
1630.910534	0.370027235	0.006991235	0.054720333	POLD1
	19	50384204	50418016	+	4932
protein_coding	"DNA polymerase delta 1, catalytic subunit
[Source:HGNC Symbol;Acc:HGNC:9175]"	-	2410	2395	1734
	1669	1409	1803	23.92889578	23.00959055
21.87423491	18.80378654	17.56043563	16.99822905
ENSG00000110427 1344.545932	1200.053428	1184.578991

1050.472054 854.9773793 810.0180635 1243.05945
905.1558322 0.458184418 0.007003102 0.054788978
KIAA1549L 11 33376466 33674102 + 13012
protein_coding KIAA1549 like [Source:HGNC
Symbol;Acc:HGNC:24836] - 1482 1356 1019 1014 749
939 5.577413359 4.937897558 4.872333151
4.330179943 3.538222365 3.355463784
ENSG00000117592 4109.846876 4199.302002 4881.302438
3345.14227 3691.584572 3234.033781 4396.817105
3423.586874 0.360897477 0.007012377 0.054837282 PRDX6
1 173477266 173488807 + 2367
protein_coding peroxiredoxin 6 [Source:HGNC Symbol;Acc:HGNC:16753]
- 4530 4745 4199 3229 3234 3749
93.71921185 94.98704991 110.3708693 75.80220357
83.98256173 73.6458367
ENSG00000092853 1930.630056 1506.261751 1433.352204
1537.377246 797.9027879 793.6279217 1623.41467
1042.969319 0.638733342 0.007021973 0.054888063 CLSPN
1 35720218 35769967 - 5411
protein_coding claspin [Source:HGNC Symbol;Acc:HGNC:19715] -
2128 1702 1233 1484 699 920 19.25851398
14.90419289 14.17726278 15.23941903 7.940484494
7.905717867
ENSG00000073060 1037.895105 1140.758752 1053.217435
681.6672696 868.6752812 802.2543121 1077.290431
784.1989543 0.458477532 0.007049015 0.055075097 SCARB1
12 124776856 124882668 - 7741
protein_coding scavenger receptor class B member 1 [Source:HGNC
Symbol;Acc:HGNC:1664] - 1144 1289 906 658 761
930 7.236984468 7.890096061 7.281787094
4.72324931 6.042754487 5.586205386
ENSG00000101574 309.3725794 239.8336865 388.2722111
221.6972579 179.214217 83.67598739 312.4928257
161.5291541 0.953976911 0.007053436 0.055085311 METTL4
18 2537525 2571509 - 5724 protein_coding
methyltransferase like 4 [Source:HGNC Symbol;Acc:HGNC:24726] -
341 271 334 214 157 97 2.917315755
2.243345199 3.630393394 2.07742901 1.685960469
0.787958105
ENSG00000111196 496.2662784 549.5819901 485.9215097
405.0636815 302.4953345 308.8247782 510.589926
338.7945981 0.592311466 0.007059464 0.055108059 MAGOHB
12 10604190 10613623 - 4856
protein_coding "mago homolog B, exon junction complex subunit
[Source:HGNC Symbol;Acc:HGNC:25504]" - 547 621 418
391 265 358 5.51616606 6.059535902
5.355554588 4.474145702 3.354397734 3.427957009
ENSG00000128283 1706.539067 2026.638901 2213.384101
1175.824242 1606.079002 1538.085418 1982.187356
1439.996221 0.46069447 0.007077982 0.055190517

CDC42EP1 22 37560447 37569405 + 2573
protein_coding CDC42 effector protein 1 [Source:HGNC
Symbol;Acc:HGNC:17014] - 1881 2290 1904 1135 1407
1783 35.79956033 42.17179988 46.03986107
24.5114001 33.61256627 32.22126414
ENSG00000148572 399.190425 367.2729885 437.0968604
636.0846559 603.8491771 517.5834272 401.186758
585.8390867 -0.546551633 0.007078772 0.055190517 NRBF2
10 63133247 63155031 + 1901
protein_coding nuclear receptor binding factor 2 [Source:HGNC
Symbol;Acc:HGNC:19692] - 440 415 376 614 529
600 11.33441848 10.3440931 12.30587714
17.94726593 17.1049148 14.67574482
ENSG00000182472 3.629003864 17.69990306 8.137441549 0
0 0 9.822116159 0 5.73042259
0.007079387 0.055190517 CAPN12 19 38730187
38769904 - 5966 protein_coding calpain 12
[Source:HGNC Symbol;Acc:HGNC:13249] - 4 20 7
0 0 0 0.032832611 0.158844868
0.072999795 0 0 0
ENSG00000188786 899.9929583 968.1846975 718.4198396
1442.068145 1173.453599 1055.870191 862.1991651
1223.797312 -0.503975886 0.007097966 0.055301975 MTF1
1 37809567 37859620 - 8024
protein_coding metal regulatory transcription factor 1 [Source:HGNC
Symbol;Acc:HGNC:7428] zf-C2H2 992 1094 618 1392 1028
1224 6.054097846 6.460302333 4.791863096
9.639629874 7.874981729 7.092862093
ENSG00000127483 4550.770845 3786.89426 4098.945558
3483.962048 3280.647514 2760.444945 4145.536888
3175.018169 0.385069738 0.007099937 0.055301975 HP1BP3
1 20742661 20787323 - 7442
protein_coding heterochromatin protein 1 binding protein 3
[Source:HGNC Symbol;Acc:HGNC:24973] - 5016 4279 3526
3363 2874 3200 33.00627742 27.24451378
29.47810007 25.11014643 23.73801819 19.99361526
ENSG00000182534 1983.250612 2354.087107 2612.118737
1522.873688 1991.90324 1593.294317 2316.485485
1702.690415 0.444195797 0.007108313 0.0553193 MXRA7
17 76672551 76711016 - 9257
protein_coding matrix remodeling associated 7 [Source:HGNC
Symbol;Acc:HGNC:7541] - 2186 2660 2247 1470 1745
1847 11.56401317 13.61563228 15.10218045
8.823870275 11.58704058 9.277429223
ENSG00000120334 646.8699387 558.4319416 567.2959251
444.4304843 429.2009274 300.1983878 590.8659352
391.2765998 0.596593938 0.007108416 0.0553193 CENPL
1 173799550 173824720 - 4914
protein_coding centromere protein L [Source:HGNC
Symbol;Acc:HGNC:17879] - 713 631 488 429 376

348	7.105310501	6.084440492	6.178620435
4.851032494	4.703271538	3.292873977
ENSG00000228672 97.98310433	120.3593408	116.249165
169.8988332	195.1951026	241.5389327	111.5305367
202.2109562	-0.860175613	0.007117362	0.055364561	PROB1
5	139390592	139395713	-	5122
protein_coding	proline rich basic protein 1 [Source:HGNC
Symbol;Acc:HGNC:41906]	-	108	136	100	164	171
280	1.032554217	1.258130744	1.21469508
1.779165373	2.052125403	2.541847407
ENSG00000127586 1165.817491	1385.017415	868.3812625
913.7242125	584.443816	779.8256969	1139.738723
759.3312418	0.586183086	0.007125912	0.055382209	CHTF18
16	788046	800737	+	5916	protein_coding
chromosome transmission fidelity factor 18 [Source:HGNC
Symbol;Acc:HGNC:18435]	-	1285	1565	747	882	512
904	10.63662004	12.53466171	7.855960355
8.284236018	5.319724676	7.105117809
ENSG00000226200 28.12477995	40.70977704	22.08734135
88.05732207	100.4512809	56.93417699	30.30729945
81.81425998	-1.424671431	0.007125929	0.055382209	SGMS1-
AS1	10	50624951	50641451	+	12420
antisense	SGMS1 antisense RNA 1 [Source:HGNC
Symbol;Acc:HGNC:49683]	-	31	46	19	85	88
66	0.122227459	0.175494163	0.095178499
0.380285156	0.435520334	0.247088969
ENSG00000107672 803.8243559	784.1057057	795.1442885
664.0558052	539.9256347	420.9678541	794.3581167
541.6497647	0.553531106	0.007129023	0.055382209
NSMCE4A 10	121957088	121975217	-	3546
protein_coding	"NSE4 homolog A, SMC5-SMC6 complex component
[Source:HGNC Symbol;Acc:HGNC:25935]"	-	886	886	684
641	473	488	12.23555502	11.83917476
12.00118739	10.04456982	8.199167708	6.398999981
ENSG00000227775 15.42326642	5.309970919	8.137441549	0
0	0	9.62355963	0	5.700295705
0.007136721	0.055417671	AL031282.1	1	1724512
1737251 +	2759	transcribed_processed_pseudogene
ribosomal protein S7 (RPS7) pseudogene	-	17	6	7
0	0	0	0.301735149	0.103044779
0.157853126	0	0	0
ENSG00000155876 1428.01302	1723.970558	1793.724616
1994.239353	2389.142396	2194.553731	1648.569398
2192.64516	-0.411704825	0.007151004	0.055504218	RRAGA
9	19049395	19050983	+	1589
protein_coding	Ras related GTP binding A [Source:HGNC
Symbol;Acc:HGNC:16963]	-	1574	1948	1543	1925	2093
2544	48.50756947	58.08867845	60.41556975
67.31608967	80.96413705	74.44306194
ENSG00000269190 1013.399329	1166.423612	1070.65481

1380.946004	1416.591359	1507.893051	1083.492583
1435.143471	-0.405588153	0.007155897	0.055511393	FBXO17
	19	38941401	38975910	-	4960
protein_coding	F-box protein 17 [Source:HGNC Symbol;Acc:HGNC:18754]
	-	1117	1318	921	1333	1241	1748
11.02808691	12.59099857	11.5527347	14.93346253
15.37933175	16.38667234
ENSG00000068137 430.9442088	397.3628238	631.2329659
325.2941074	315.0517446	285.533524	486.5133328
308.6264587	0.655294055	0.007160195	0.055511393
PLEKHH3 17	42667914	42676994	-	4803
protein_coding	"pleckstrin homology, MyTH4 and FERM domain containing
H3 [Source:HGNC Symbol;Acc:HGNC:26105]" -	475	449	543
	314	276	331	4.842946892	4.429555986
7.033865986	3.632696413	3.532188365	3.204397803
ENSG00000137168 1923.372048	2023.983915	2250.583834
2621.000292	2568.356613	2794.950507	2065.979932
2661.435804	-0.365950126	0.00716181	0.055511393	PPIL1
	6	36854827	36875024	-	1810
protein_coding	peptidylprolyl isomerase like 1 [Source:HGNC
Symbol;Acc:HGNC:9260]	-	2120	2287	1936	2530	2250
	3240	57.35693947	59.87065791	66.54778804
77.67012247	76.4101942	83.23336512
ENSG00000171051 47.17705023	20.35488852	53.4746159	0
	0	13.80222472	40.33551822	4.600741575
3.086992308	0.007164481	0.055511393	FPR1	19
51745172	51804110	-	2594	protein_coding	formyl
peptide receptor 1 [Source:HGNC Symbol;Acc:HGNC:3826]	-	52
	23	46	0	0	16	0.981662171
0.420130591	1.103302765	0	0	0.286801243
ENSG00000067177 401.9121779	269.0385265	251.0981964
502.44472	480.5680597	468.4130016	307.3496336
483.8085938	-0.652860458	0.007173049	0.055519231	PHKA1
	X	72578814	72714319	-	6350
protein_coding	phosphorylase kinase regulatory subunit alpha 1
[Source:HGNC Symbol;Acc:HGNC:8925]	-	443	304	216
	485	421	543	3.41632111	2.268434789
2.116346978	4.244045751	4.075263838	3.976098388
ENSG00000196505 906.343715	920.3949592	782.3568804
1264.917532	1079.851269	1198.205634	869.6985182
1180.991479	-0.44089654	0.007173104	0.055519231	GDAP2
	1	117863485	117929630	-	10496
protein_coding	ganglioside induced differentiation associated protein
2 [Source:HGNC Symbol;Acc:HGNC:18010]	-	999	1040	673
	1221	946	1389	4.660906059	4.695003908
3.989312785	6.464037772	5.540062632	6.153319163
ENSG00000144218 167.8414287	123.8993214	170.8862725
71.48182615	92.46083808	70.73640171	154.2090076
78.22635531	0.980305056	0.007174909	0.055519231	AFF3
	2	99545419	100142739	-	12737

protein_coding AF4/FMR2 family member 3 [Source:HGNC
Symbol;Acc:HGNC:6473] AF-4 185 140 147 69 81
82 0.711267988 0.52081961 0.718053879
0.301019053 0.390899604 0.299348938
ENSG00000058056 1589.503692 1417.762235 1478.689379
1316.715957 1018.210711 885.9302995 1495.318435
1073.618989 0.478482557 0.007190657 0.055616762 USP13
3 179652755 179789401 + 8440
protein_coding ubiquitin specific peptidase 13 [Source:HGNC
Symbol;Acc:HGNC:12611] - 1752 1602 1272 1271 892
1027 10.16530324 8.993867931 9.376732169
8.367874297 6.496355278 5.657948826
ENSG00000127804 1225.696055 1224.833292 924.1808617
1567.420333 1454.260589 1574.316258 1124.903403
1531.99906 -0.444758095 0.007205238 0.055684904
METTL16 17 2405562 2511891 - 8934 protein_coding
methyltransferase like 16 [Source:HGNC Symbol;Acc:HGNC:28484] -
1351 1384 795 1513 1274 1825 7.40522203
7.340346865 5.53640723 9.410333685 8.765381847
9.498344973
ENSG00000053900 678.6237226 547.8119998 573.1083834
509.6964995 325.325171 322.6270029 599.8480352
385.8828912 0.63699402 0.007207211 0.055684904 ANAPC4
4 25377213 25418498 + 7494
protein_coding anaphase promoting complex subunit 4 [Source:HGNC
Symbol;Acc:HGNC:19990] - 748 619 493 492 285
374 4.887835628 3.913843006 4.092984285
3.648072474 2.337644852 2.32053932
ENSG00000104973 1104.124426 1265.543069 1399.639946
911.6522755 918.9009217 954.0787841 1256.435814
928.2106604 0.436066554 0.007208911 0.055684904 MED25
19 49818279 49840383 + 7687
protein_coding mediator complex subunit 25 [Source:HGNC
Symbol;Acc:HGNC:28845] - 1217 1430 1204 880 805
1106 7.752867839 8.814660648 9.744879034
6.361182196 6.437042248 6.690048466
ENSG00000095587 0 0 0 5.179842475 31.96177119
0 0 12.38053789 -6.101002727 0.007240888
0.055907492 TLL2 10 96364606 96513918
- 8151 protein_coding tolloid like 2 [Source:HGNC
Symbol;Acc:HGNC:11844] - 0 0 0 5 28
0 0 0 0 0.034085617 0.211151659
0
ENSG00000115461 79.83808501 153.1041615 94.16182364
360.5170362 157.5258723 170.802531 109.03469
229.6151465 -1.072575111 0.007254367 0.055970145 IGFBP5
2 216672105 216695525 - 6442
protein_coding insulin like growth factor binding protein 5
[Source:HGNC Symbol;Acc:HGNC:5474] - 88 173 81
348 138 198 0.668945344 1.272482515

0.782296064	3.001722683	1.316757274	1.429142347
ENSG00000007516 0	1.769990306	0	5.179842475
29.67878753	12.07694663	0.589996769
15.64519221	-4.651084918	0.007255331	0.055970145	BAIAP3
	16	1333601 1349441 +	6146	protein_coding	BAI1
associated protein 3 [Source:HGNC Symbol;Acc:HGNC:948]	-	0
	2	0	5	26	14	0	0.015419272
	0	0.045205314	0.260032812	0.105917202
ENSG00000116903 526.2055603	484.0923488	488.246493
618.4731915	755.6675903	754.8091646	499.5148007
709.6499821	-0.506509488	0.007265189	0.056009051	EXOC8
	1	231332753	231337852	-	5100
protein_coding	exocyst complex component 8 [Source:HGNC
Symbol;Acc:HGNC:24659]	-	580	547	420	597	662
	875	5.56911904	5.082104502	5.123726753
6.504534158	7.978755807	7.977538248
ENSG00000205138 515.3185487	735.4309722	704.4699398
812.1993	964.5605948	1055.870191	651.7398203
944.2100287	-0.535590514	0.007266707	0.056009051	SDHAF1
	19	35995199	35996315	+	1117
protein_coding	succinate dehydrogenase complex assembly factor 1
[Source:HGNC Symbol;Acc:HGNC:33867]	-	568	831	606
	784	845	1224	24.90140468	35.25123133
33.75408173	39.00092731	46.49977977	50.95176852
ENSG00000073756 232.2562473	160.1841227	303.4103206
437.1787048	399.5221399	332.9786715	231.9502302
389.8931721	-0.751723107	0.00730551	0.056283609	PTGS2
	1	186671791	186680427	-	5269
protein_coding	prostaglandin-endoperoxide synthase 2 [Source:HGNC
Symbol;Acc:HGNC:9605]	-	256	181	261	422	350
	386	2.37925204	1.627709196	3.081904347
4.45037181	4.083073577	3.406356705
ENSG00000285103 9.07250966	15.0449176	4.6499666	0
	0	0	9.589131287	0	5.697603803
0.007323347	0.056396469	AL451123.1	9	27529085
	27543902	+	9804	antisense	novel
transcript	-	10	17	4	0	0	0
	0.049948837	0.0821622	0.025384203	0	0
	0
ENSG00000147041 116.1281236	59.29467526	91.83684034
49.72648776	28.5372957	18.11541995	89.08654642
32.12640114	1.475347725	0.007350093	0.056577803	SYTL5
	X	38006582	38128819	+	4791
protein_coding	synaptotagmin like 5 [Source:HGNC
Symbol;Acc:HGNC:15589]	-	128	67	79	48	25
	21	1.308315487	0.66263607	1.025906466
0.556707549	0.320745962	0.203809368
ENSG00000163191 2931.327871	3886.898713	4546.504843
4335.528151	5811.334897	5537.280032	3788.243809
5228.047693	-0.465031686	0.007364614	0.056664922

S100A11 1	152032506	152047907	-	680
protein_coding	S100 calcium binding protein A11 [Source:HGNC
Symbol;Acc:HGNC:10488]	-	3231	4392	3911	4185	5091
	6419	232.6787537	306.0411742	357.8374166
341.9783348	460.1946278	438.9241544
ENSG00000165655 201.4097144	203.5488852	240.6357715
321.1502334	404.0881072	307.0995001	215.1981237
344.1126136	-0.677555624	0.00737058	0.05668617	ZNF503
	10	75397830	75401906	-	3347
protein_coding	zinc finger protein 503 [Source:HGNC
Symbol;Acc:HGNC:23589]	-	222	230	207	310	354
	356	3.248074228	3.256106224	3.847879646
5.146571671	6.501220706	4.945671925
ENSG00000108298 22246.70094	26625.07918	29092.51603
30483.37296	34931.93293	34405.49568	25988.09872
33273.60052	-0.356571086	0.007409112	0.056957748	RPL19
	17	39200283	39204732	+	1761
protein_coding	ribosomal protein L19 [Source:HGNC
Symbol;Acc:HGNC:10312]	-	24521	30085	25026	29425	30602
	39884	681.8792973	809.5004615	884.1764247
928.4727439	1068.1637	1053.101836
ENSG00000213553 948.0772594	1400.947327	1564.713761
1441.032176	2159.702539	2175.575672	1304.579449
1925.436796	-0.562245839	0.007434694	0.057129581
RPLP0P6 2	38481851	38482804	+	954
processed_pseudogene	ribosomal protein lateral stalk subunit P0
pseudogene 6 [Source:HGNC Symbol;Acc:HGNC:36404]	-	1045
	1583	1346	1391	1892	2522	53.64096711
78.62469631	87.78160794	81.01973138	121.9046067
122.9214644
ENSG00000114166 420.9644482	346.9181	410.3595524
335.6537923	175.7897415	102.6540464	392.7473669
204.6991934	0.941779881	0.007438664	0.057135274	KAT2B
	3	20040023	20154404	+	5634
protein_coding	lysine acetyltransferase 2B [Source:HGNC
Symbol;Acc:HGNC:8638]	-	464	392	353	324	154
	119	4.033014853	3.296823257	3.898205316
3.195510223	1.680162311	0.98211227
ENSG00000197429 528.0200622	557.5469465	391.759686
717.926167	764.7995249	672.8584553	492.4422316
718.5280491	-0.542900134	0.007449752	0.057195601	IPP
	1	45694324	45750650	-	3705
protein_coding	intracisternal A particle-promoted polypeptide
[Source:HGNC Symbol;Acc:HGNC:6108]	-	582	630	337
	693	670	780	7.692428281	8.057100447
5.659115205	10.39338622	11.11562663	9.788979263
ENSG00000114107 361.0858845	292.0484005	333.6351035
203.049825	236.2888084	88.85182166	328.9231295
176.063485	0.90553922	0.007469287	0.0573207	CEP70
	3	138494344	138594538	-	4420

protein_coding centrosomal protein 70 [Source:HGNC
Symbol;Acc:HGNC:29972] - 398 330 287 196 207
103 4.409501415 3.537676457 4.039861479
2.464029175 2.878693561 1.083542558
ENSG00000118985 1707.446318 1647.860975 1792.562124
2038.785998 2522.69694 2173.850394 1715.956472
2245.111111 -0.387736711 0.007491444 0.057465809 ELL2
5 95885098 95962071 - 7328
protein_coding elongation factor for RNA polymerase II 2 [Source:HGNC
Symbol;Acc:HGNC:17064] - 1882 1862 1542 1968 2210
2520 12.57658823 12.03983837 13.09199286
14.92284668 18.53762845 15.98991291
ENSG00000270574 0.907250966 6.194966072 1.16249165
22.79130689 19.40536108 27.60444945 2.754902896
23.26703914 -3.052800051 0.007517664 0.057641942
AC010680.2 2 178578790 178580906 + 2117
antisense "novel transcript, antisense to titin" -
1 7 1 22 17 32 0.023131715
0.156676414 0.02938908 0.57744931 0.49360031
0.702845937
ENSG00000238121 0.907250966 0 0 0 36.5277385
9.489029498 0.302416989 15.33892267 -5.449881581
0.007567996 0.058002724 LINC00426 13
30340270 30377145 - 3972 lincRNA long
intergenic non-protein coding RNA 426 [Source:HGNC
Symbol;Acc:HGNC:42761] - 1 0 0 0 32
11 0.012328761 0 0 0 0.495208509
0.128769931
ENSG00000163964 800.195352 875.2602064 823.0440881
669.2356477 559.3309958 571.067048 832.8332155
599.8778972 0.473512003 0.007596346 0.058189232 PIGX
3 196639775 196736007 + 4446
protein_coding phosphatidylinositol glycan anchor biosynthesis class
X [Source:HGNC Symbol;Acc:HGNC:26046] - 882 989 708
646 490 662 9.714664582 10.54030733
9.907649766 8.073746388 6.774449066 6.923402
ENSG00000279425 9.979760626 15.0449176 10.46242485
1.035968495 0 0 11.82903436 0.345322832
5.036455959 0.00759891 0.058189232 AC092279.2 19
24078481 24080669 + 2189 TEC TEC
- 11 17 9 1 0 0
0.246079597 0.367984563 0.255801799 0.025384364 0
0
ENSG00000165526 867.3319235 926.5899253 970.6805277
1078.443203 1295.593225 1385.398307 921.5341255
1253.144912 -0.444079822 0.007603814 0.058201588 RPUSD4
11 126202094 126211692 - 4083
protein_coding RNA pseudouridylate synthase domain containing 4
[Source:HGNC Symbol;Acc:HGNC:25898] - 956 1047 835
1041 1135 1606 11.46587482 12.15048861

12.72371528   14.16717939   17.08692219   18.28930402
ENSG00000080493 78.02358307   42.47976735   83.69939879
16.57549592   25.11282022   27.60444945   68.06758307
23.09758853   1.555126695   0.007634107   0.058401837   SLC4A4
        4   71062667   71572087   +   9622
protein_coding solute carrier family 4 member 4 [Source:HGNC
Symbol;Acc:HGNC:11030] -   86   48   72   16   22
        32   0.43768512   0.236375427   0.465558211
0.092398873   0.140541534   0.154637793
ENSG00000180596 11.79426256   1.769990306   16.2748831   0
        0   0   9.946378654   0   5.74359995
0.007636579   0.058401837   HIST1H2BC   6   26114873
        26123926   -   834   protein_coding histone
cluster 1 H2B family member c [Source:HGNC Symbol;Acc:HGNC:4757]
        -       13   2   14   0   0   0
0.763318846   0.113629314   1.044404734   0   0   0
ENSG00000065802 1159.466735   1347.847618   1171.791583
1578.815986   1601.513035   1654.541689   1226.368645
1611.62357   -0.393887816   0.007643585   0.058430156   ASB1
        2   238426742   238452250   +   7410
protein_coding ankyrin repeat and SOCS box containing 1 [Source:HGNC
Symbol;Acc:HGNC:16011] -   1278   1523   1008   1524   1403
        1918   8.445810433   9.738860302   8.463483868
11.42822554   11.63822699   12.0354245
ENSG00000259363 9.07250966   5.309970919   13.9498998   0
        0   0   9.444126792   0   5.669608539
0.007666092   0.058559507   AC090825.1   15   99807023
        99877148   +   1291   lincRNA ubiquitin conjugating
enzyme E2 Q2 pseudogene [Source:NCBI gene;Acc:400464]   -   10
        6   12   0   0   0   0.379317117
0.220217308   0.578311529   0   0   0
ENSG00000185338 29.93928188   40.70977704   69.74949899
16.57549592   7.990442797   0   46.7995193   8.188646239
        2.527485343   0.007667127   0.058559507   SOCS1   16
        11254405   11256200   -   1796   protein_coding
        suppressor of cytokine signaling 1 [Source:HGNC
Symbol;Acc:HGNC:19383] -   33   46   60   16   7
        0   0.899779908   1.213606629   2.078508308
0.49502336   0.23957366   0
ENSG00000184967 1153.115978   1276.163011   1261.30344
1362.298571   1748.765481   1923.685071   1230.194143
1678.249708   -0.448474985   0.007676588   0.058606457   NOC4L
        12   132144448   132152473   +   2029
protein_coding nucleolar complex associated 4 homolog [Source:HGNC
Symbol;Acc:HGNC:28461] -   1271   1442   1085   1315   1532
        2230   30.67553791   33.67515894   33.27013306
36.01270549   46.41133688   51.10387523
ENSG00000224184 215.0184789   198.2389143   202.2735471
109.8126605   134.6960357   106.1046026   205.1769801
116.8710996   0.813960774   0.007682562   0.058626762

MIR3681HG 2 11848622 12578348 + 3876
lincRNA MIR3681 host gene [Source:HGNC Symbol;Acc:HGNC:52001]
- 237 224 174 106 118 123
2.994285872 2.738360936 2.793008944 1.51961699
1.8713094 1.475544668
ENSG00000163864 16.33051739 22.12487883 11.6249165
34.18696033 46.80116496 91.4397388 16.69343757
57.4759547 -1.78008411 0.00770757 0.058792242 NMNAT3
3 139560180 139678017 - 12983
protein_coding nicotinamide nucleotide adenylyltransferase 3
[Source:HGNC Symbol;Acc:HGNC:20989] - 18 25 10
33 41 106 0.067893177 0.091241285
0.047921653 0.141237794 0.194113688 0.379631138
ENSG00000178031 1362.690951 1326.607735 1275.25334
1016.285093 1028.484137 968.7436478 1321.517342
1004.504293 0.396177924 0.007717528 0.058842823
ADAMTSL1 9 17906563 18910950 + 13652
protein_coding ADAMTS like 1 [Source:HGNC
Symbol;Acc:HGNC:14632] - 1502 1499 1097 981 901
1123 5.387686744 5.202736058 4.999392042
3.99286633 4.056727831 3.824850764
ENSG00000164707 8.165258694 17.69990306 10.46242485
23.82727538 38.81072216 84.53862644 12.10919554
49.05887466 -2.018199966 0.007725217 0.058876071
SLC13A4 7 135681237 135729258 - 8015
protein_coding solute carrier family 13 member 4 [Source:HGNC
Symbol;Acc:HGNC:15827] - 9 20 9 23 34
98 0.054987967 0.118236866 0.069862775
0.159454343 0.260749059 0.568530237
ENSG00000155252 799.288101 1034.559334 905.5809953
1205.867328 1088.983204 1593.294317 913.1428101
1296.048283 -0.505917773 0.007730229 0.0588889 PI4K2A
10 97640686 97676434 + 4189
protein_coding phosphatidylinositol 4-kinase type 2 alpha
[Source:HGNC Symbol;Acc:HGNC:30031] - 881 1169 779
1164 954 1847 10.2989804 13.22301808
11.57001558 15.44026193 13.99862483 20.50159043
ENSG00000065427 6465.070384 6454.269652 6954.02505
7900.295742 8425.351184 8273.571083 6624.455028
8199.739336 -0.307868002 0.007734608 0.058896894 KARS
16 75627474 75648643 - 4065
protein_coding lysyl-tRNA synthetase [Source:HGNC
Symbol;Acc:HGNC:6215] - 7126 7293 5982 7626 7381
9591 85.84479179 85.01040877 91.55724274
104.2433344 111.6097192 109.7070051
ENSG00000089154 5041.593618 4565.689995 4143.12024
5736.157556 5500.84912 6294.677113 4583.467951
5843.894597 -0.350415261 0.00774883 0.058966808 GCN1
12 120127203 120194710 - 9654
protein_coding "GCN1, eIF2 alpha kinase activator homolog

[Source:HGNC Symbol;Acc:HGNC:4199]"	-	5557	5159	3564
	5537	4819	7297	28.18783923	25.32122278
22.96874401	31.8697952	30.6829415	35.14536596
ENSG00000182902 17.23776835	3.539980612	5.812458249
8.287747959	70.77249335	64.6979284	8.863402405
47.9193899	-2.42880404	0.007750457	0.058966808
SLC25A18	22	17563439	17590994	+	4741
	protein_coding	solute carrier family 25 member 18
[Source:HGNC Symbol;Acc:HGNC:10988]	-	19	4	5
	8	62	75	0.196251204	0.039977578
0.065615568	0.093763125	0.803839039	0.735567151
ENSG00000136731 2691.813616	2369.132025	2373.807949
2161.03028	1775.019793	1701.124197	2478.251197
1879.05809	0.399597027	0.007763741	0.059035685	UGGT1
	2	128091200	128195677	+	11349
protein_coding	UDP-glucose glycoprotein glucosyltransferase 1
[Source:HGNC Symbol;Acc:HGNC:15663]	-	2967	2677	2042
	2086	1555	1972	12.80231869	11.17679321
11.19450742	10.21336262	8.42209542	8.079422747
ENSG00000110107 6895.107341	7973.80633	7209.773213
8139.604464	9967.506644	10161.88795	7359.562295
9422.999687	-0.356560639	0.007766185	0.059035685	PRPF19
	11	60890730	60906588	-	2739
protein_coding	pre-mRNA processing factor 19 [Source:HGNC
Symbol;Acc:HGNC:17896]	-	7600	9010	6202	7857	8732
	11780	135.8783435	155.8688028	140.8791025
159.3957599	195.9607329	199.9789466
ENSG00000178149 471.7705023	684.1012534	606.8206412
850.5301343	802.4687552	868.6775186	587.5641323
840.5588027	-0.516964909	0.007776133	0.059085918	DALRD3
	3	49015488	49022293	-	3682
protein_coding	DALR anticodon binding domain containing 3
[Source:HGNC Symbol;Acc:HGNC:25536]	-	520	773	522
	821	703	1007	6.915892643	9.947687886
8.820507335	12.39000314	11.73596758	12.71676672
ENSG00000160688 756.6473056	927.4749205	814.9066466
917.8680865	1359.516767	1276.705787	833.0096242
1184.69688	-0.507906371	0.007837295	0.059525083	FLAD1
	1	154983338	154993111	+	4019
protein_coding	flavin adenine dinucleotide synthetase 1 [Source:HGNC
Symbol;Acc:HGNC:24671]	-	834	1048	701	886	1191
	1480	10.16194238	12.35576721	10.85192687
12.24976506	18.21550079	17.12279777
ENSG00000121653 633.2611743	600.0267138	684.7075818
617.437223	1288.744274	1070.535055	639.3318233
992.2388507	-0.634266402	0.007846916	0.059572575
MAPK8IP1	11	45885651	45906465	+	3498
	protein_coding	mitogen-activated protein kinase 8 interacting
protein 1 [Source:HGNC Symbol;Acc:HGNC:6882]	-	698	678
	589	596	1129	1241	9.771568954	9.184094199

10.47616515	9.46756942	19.83907855	16.4961651
ENSG00000241697	15.42326642	9.734946684	3.48747495	0
0	0	9.548562685	0	5.691816846
0.007850478	0.059574045	TMEFF1	9	100473113
100577636	+	2611	protein_coding	transmembrane protein
with EGF like and two follistatin like domains 1 [Source:HGNC
Symbol;Acc:HGNC:11866]	-	17	11	3	0	0
0	0.318838482	0.19962377	0.071486038	0
0	0
ENSG00000229152	216.8329809	140.7142293	81.37441549
262.1000292	285.372957	284.6708849	146.3072086
277.3812904	-0.918737475	0.007867898	0.059663226
ANKRD10-IT1	13	110894639	110899172	-	800
sense_intronic	ANKRD10 intronic transcript 1 [Source:HGNC
Symbol;Acc:HGNC:39891]	-	239	159	70	253	250
330	14.62973965	9.417455531	5.443959675
17.57286521	19.20867382	19.18028124
ENSG00000065978	21969.98939	27264.93068	29359.88911
19031.77722	21672.36385	20495.44108	26198.26973
20399.86072	0.360881009	0.007868975	0.059663226	YBX1
1	42682427	42702349	+	1930
protein_coding	Y-box binding protein 1 [Source:HGNC
Symbol;Acc:HGNC:8014]	CSD	24216	30808	25256	18371	18986
23759	614.4319385	756.3671131	814.1681454
528.9170071	604.6771086	572.4029544
ENSG00000137522	653.2206955	766.4058026	717.257348
1005.925409	945.1552337	953.216145	712.2946154
968.0989291	-0.4426773	0.007882754	0.059742086	RNF121
11	71928701	71997597	+	3567
protein_coding	ring finger protein 121 [Source:HGNC
Symbol;Acc:HGNC:21070]	-	720	866	617	971	828
1105	9.884576585	11.50379737	10.76189874
15.12614169	14.26837739	14.40423462
ENSG00000125871	626.0031665	615.0716314	628.9079826
466.1858227	471.436125	337.2918667	623.3275935
424.9712715	0.554255659	0.007898209	0.059833583	MGME1
20	17968913	17991122	+	2336
protein_coding	mitochondrial genome maintenance exonuclease 1
[Source:HGNC Symbol;Acc:HGNC:16205]	-	690	695	541
450	413	391	14.46455029	14.09738002
14.4089148	10.70413848	10.867373	7.782783278
ENSG00000164587	11241.74672	14755.52419	15770.36172
14672.42179	21522.82842	20695.57334	13922.54421
18963.60785	-0.445868081	0.00790583	0.05986568	RPS14
5	150442635	150449756	-	5099
protein_coding	ribosomal protein S14 [Source:HGNC
Symbol;Acc:HGNC:10387]	-	12391	16673	13566	14163	18855
23991	119.0008404	154.937013	165.5288308
154.3413472	227.294478	218.7733197
ENSG00000112081	9237.629336	10225.234	9340.620407

10290.27506	12857.76395	14131.75284	9601.161247
12426.59728	-0.372176114	0.007931854	0.060037043	SRSF3
	6	36594353	36605600	+	7118
protein_coding	serine and arginine rich splicing factor 3
[Source:HGNC Symbol;Acc:HGNC:10785]	-	10182	11554	8035
	9933	11264	16382	70.04929884	76.91318931
70.23195278	77.54155312	97.27069487	107.0138419
ENSG00000165209 539.8143248	507.1022227	531.258684
439.2506418	297.9293672	297.6104706	526.0584105
344.9301599	0.609192249	0.007936007	0.060042786	STRBP
	9	123109500	123268576	-	8097
protein_coding	spermatid perinuclear RNA binding protein [Source:HGNC
Symbol;Acc:HGNC:16462]	-	595	573	457	424	261
	345	3.598500025	3.353180435	3.511550411
2.909737164	1.981361538	1.981189301
ENSG00000184432 3642.612628	3578.920399	3926.896793
3505.717387	2391.42538	2202.317483	3716.143274
2699.820083	0.461014811	0.007955353	0.060157938	COPB2
	3	139355600	139389732	-	8177
protein_coding	coatomer protein complex subunit beta 2 [Source:HGNC
Symbol;Acc:HGNC:2232]	-	4015	4044	3378	3384	2095
	2553	24.04474831	23.433847	25.70232993
22.99579351	15.74843455	14.51736631
ENSG00000053372 3357.735825	3871.853795	3437.487809
4119.010736	4583.08969	4950.685481	3555.692476
4550.928636	-0.356040232	0.007958029	0.060157938	MRTO4
	1	19251539	19260128	+	2822
protein_coding	"MRT4 homolog, ribosome maturation factor [Source:HGNC
Symbol;Acc:HGNC:18477]" -	3701	4375	2957	3976	4015
	5739	64.22302524	73.45941894	65.1930293
78.28911973	87.45323927	94.5606031
ENSG00000183814 776.6068269	568.1668883	699.8199732
590.5020421	390.3902052	295.8851925	681.5312295
425.59248	0.680199797	0.007986131	0.060319512	LIN9
	1	226231149	226309869	-	4442
protein_coding	lin-9 DREAM MuvB core complex component [Source:HGNC
Symbol;Acc:HGNC:30830]	-	856	642	602	570	342
	343	9.436781381	6.848302171	8.431887115
7.130308905	4.732546742	3.590430992
ENSG00000149295 9.979760626	15.0449176	3.48747495	0
	0	0	9.504051059	0	5.685341021
0.007986223	0.060319512	DRD2	11	113409615
113475691	-	4098	protein_coding	dopamine receptor D2
[Source:HGNC Symbol;Acc:HGNC:3023]	-	11	17	3
	0	0	0	0.131446617	0.196563741
0.045546619	0	0	0
ENSG00000271751 17.23776835	7.964956378	3.48747495	0
	0	0	9.563399894	0	5.693939793
0.007999827	0.060396472	AP003392.5	11	119065263
	119065677	-	415	lincRNA novel transcript

- 19 9 3 0 0 0
2.241992667 1.027592329 0.449759147 0 0 0
ENSG00000135362 212.296726 153.9891566 265.0480962
44.54664528 116.4321665 133.709052 210.4446596
98.22928792 1.096142075 0.008013705 0.060464177 PRR5L
11 36296288 36465204 + 6616
protein_coding proline rich 5 like [Source:HGNC
Symbol;Acc:HGNC:25878] - 234 174 228 43 102
155 1.732004613 1.246178323 2.144105728
0.361147824 0.947658878 1.089349453
ENSG00000148926 2404.21506 2649.675488 3243.351703
3181.459248 3795.460329 4108.749773 2765.747417
3695.223116 -0.418546756 0.008015631 0.060464177 ADM
11 10304680 10307397 + 2718
protein_coding adrenomedullin [Source:HGNC Symbol;Acc:HGNC:259]
- 2650 2994 2790 3071 3325 4763
47.74469299 52.19498588 63.86480603 62.78304968
75.19510281 81.4820876
ENSG00000240024 96.16860239 122.1293311 92.99933199
68.37392066 19.40536108 30.19236658 103.7657552
39.32388278 1.399901984 0.00803018 0.06054528
LINC00888 3 183447608 183456013 + 2131
transcribed_unprocessed_pseudogene long intergenic non-
protein coding RNA 888 [Source:HGNC Symbol;Acc:HGNC:48575] -
106 138 80 66 17 35 2.435853131
3.068471384 2.335680225 1.720966948 0.490357511
0.763687378
ENSG00000110719 1082.350402 1207.133389 941.6182364
697.2067971 828.7230673 816.0565368 1077.034009
780.6621337 0.46495635 0.008033228 0.06054528 TCIRG1
11 68039016 68050895 + 4384
protein_coding "T cell immune regulator 1, ATPase H+ transporting V0
subunit a3 [Source:HGNC Symbol;Acc:HGNC:11647]" - 1193 1364
810 673 726 946 13.32596234 14.74247044
11.49532674 8.530147922 10.17919503 10.03348046
ENSG00000134780 397.3759231 538.0770531 452.2092518
206.1577305 349.2964994 308.8247782 462.554076
288.0930027 0.683621515 0.008060202 0.060722703 DAGLA
11 61680433 61747001 + 5757
protein_coding diacylglycerol lipase alpha [Source:HGNC
Symbol;Acc:HGNC:1165] - 438 608 389 199 306
358 3.7256887 5.004189999 4.203975908
1.92074137 3.267176203 2.891464172
ENSG00000117448 1263.800596 1448.737066 1440.327154
1781.865811 2048.977832 1666.618635 1384.288272
1832.487426 -0.404407145 0.008079931 0.060845425 AKR1A1
1 45550543 45570049 + 3121
protein_coding aldo-keto reductase family 1 member A1 [Source:HGNC
Symbol;Acc:HGNC:380] - 1393 1637 1239 1720 1795
1932 21.85677247 24.85314488 24.69928517

30.62292861	35.35233016	28.78355101
ENSG00000104946 1089.60841	1208.903379	1121.804442
1290.816745	1634.616298	1687.321973	1140.10541
1537.585005	-0.431549676	0.008087389	0.060873118
TBC1D17 19	49877425	49888749	+	4745
protein_coding	TBC1 domain family member 17 [Source:HGNC
Symbol;Acc:HGNC:25699]	-	1201	1366	965	1246	1432
1956	12.39468443	13.64083399	12.65312922
14.59129605	18.5504377	19.16741967
ENSG00000188021 1890.711013	1932.829414	2135.497161
2581.633489	2875.417915	2309.284724	1986.345863
2588.77871	-0.382093852	0.008092947	0.060873118	UBQLN2
X	56563639	56567868	+	4230
protein_coding	ubiquilin 2 [Source:HGNC Symbol;Acc:HGNC:12509] -
2084	2184	1837	2492	2519	2677	24.12603929
24.46463313	27.01939594	32.73555544	36.60455743
29.42652791
ENSG00000064102 1105.031677	807.1155796	899.768537
661.9838682	627.8205055	710.8145733	937.3052644
666.8729823	0.490745916	0.008093933	0.060873118	INTS13
12	26905181	26938326	-	3345
protein_coding	integrator complex subunit 13 [Source:HGNC
Symbol;Acc:HGNC:20174]	-	1218	912	774	639	550
824	17.83117038	12.91888871	14.39632642
10.61492133	10.10680596	11.45413
ENSG00000225190 740.3167882	929.2449108	875.3562124
936.5155194	1245.367585	1463.035821	848.3059705
1214.972975	-0.518862315	0.008102012	0.060907986
PLEKHM1 17	45435900	45490749	-	9233
protein_coding	pleckstrin homology and RUN domain containing M1
[Source:HGNC Symbol;Acc:HGNC:29017]	-	816	1050	753
904	1091	1696	4.327887935	5.38856225
5.074099594	5.440485267	7.263221275	8.541104402
ENSG00000128245 1686.579546	1781.495243	1929.736139
1263.881564	1572.975739	1177.502297	1799.270309
1338.119867	0.427685148	0.008116388	0.060985001	YWHAH
22	31944461	31957603	+	2453
protein_coding	tyrosine 3-monooxygenase/tryptophan 5-monooxygenase
activation protein eta [Source:HGNC Symbol;Acc:HGNC:12853]	-
1859	2013	1660	1220	1378	1365	37.11167234
38.88415517	42.10342117	27.63594541	34.53019489
25.87415177
ENSG00000138442 1637.587994	1741.670461	1469.389445
2001.491132	2201.937737	2094.487602	1616.215967
2099.30549	-0.376701307	0.008122295	0.060985001	WDR12
2	202874782	203014798	-	9098
protein_coding	WD repeat domain 12 [Source:HGNC
Symbol;Acc:HGNC:14098]	-	1805	1968	1264	1932	1929
2428	9.715383699	10.24956898	8.643865251
11.79976161	13.0326777	12.40891381

ENSG00000174799 738.5022863 470.8174215 444.0718103
418.5312719 287.6559407 325.2149201 551.130506
343.8007109 0.681452475 0.008122599 0.060985001 CEP135
4 55948871 56033363 + 8640
protein_coding centrosomal protein 135 [Source:HGNC
Symbol;Acc:HGNC:29086] - 814 532 382 404 252
377 4.613593706 2.917590463 2.750783857
2.598242404 1.792809556 2.02889058
ENSG00000186318 2238.188133 2177.973072 2195.946727
1691.736552 1684.841938 1805.503522 2204.035977
1727.360671 0.351361819 0.008135876 0.061058767 BACE1
11 117285207 117316259 - 7190
protein_coding beta-secretase 1 [Source:HGNC Symbol;Acc:HGNC:933]
- 2467 2461 1889 1633 1476 2093
16.80230805 16.2184432 16.34594052 12.6202901
12.6184156 13.53540815
ENSG00000155438 1694.744804 1793.885175 1263.628423
2163.102217 1987.337273 2289.444026 1584.086134
2146.627839 -0.437808953 0.008170289 0.061291027 NIFK
2 121726945 121736923 - 2447
protein_coding nucleolar protein interacting with the FHA domain of
MKI67 [Source:HGNC Symbol;Acc:HGNC:17838] - 1868 2027
1087 2088 1741 2654 37.38277924 39.25059279
27.63773328 47.41421543 43.73329121 50.43104499
ENSG00000163214 771.1633211 615.0716314 639.3704074
927.1918029 991.9563987 883.3423824 675.2017866
934.163528 -0.467629769 0.008182213 0.061354455 DHX57
2 38797729 38875934 - 7985
protein_coding DExH-box helicase 57 [Source:HGNC
Symbol;Acc:HGNC:20086] - 850 695 550 895 869
1024 5.212819519 4.124167779 4.285432073
6.228165701 6.68947779 5.962879792
ENSG00000115568 695.8614909 773.4857638 718.4198396
934.4435824 922.3253972 1155.936321 729.2556981
1004.2351 -0.462211925 0.008186443 0.061360165 ZNF142
2 218637916 218659655 - 6994
protein_coding zinc finger protein 142 [Source:HGNC
Symbol;Acc:HGNC:12927] zf-C2H2 767 874 618 902 808
1340 5.37029842 5.92123429 5.497556402
7.166266475 7.101222051 8.908614957
ENSG00000103152 687.6962322 824.8154827 1252.003507
587.3941366 642.6598993 602.1220536 921.505074
610.7253632 0.592276281 0.008211701 0.061523414 MPG
16 77007 85853 + 1661 protein_coding N-
methylpurine DNA glycosylase [Source:HGNC Symbol;Acc:HGNC:7211] -
758 932 1077 567 563 698 22.34746453
26.58720724 40.34158129 18.96817161 20.83464585
19.53965126
ENSG00000077463 511.6895448 831.8954439 512.6588176
718.9621355 948.5797092 1239.612308 618.7479354

969.0513842	-0.646891388	0.008226407	0.061607499	SIRT6
	19	4174109 4182604 -	2787	protein_coding
sirtuin 6 [Source:HGNC Symbol;Acc:HGNC:14934]	-	564	940
	441	694	831	1437	9.909935294	15.98149215
	9.844835581	13.83676437	18.3278455	23.9745695
ENSG00000146373 264.0100311	261.0735702	182.511189
317.0063594	420.0689928	401.9897951	235.8649301
379.6883824	-0.684361298	0.00824078	0.061689018	RNF217
	6	124962545	125092633	+	12779
protein_coding	ring finger protein 217 [Source:HGNC
Symbol;Acc:HGNC:21487]	-	291	295	157	306	368
	466	1.11512821	1.093834423	0.764380552
1.330566546	1.770102065	1.695586947
ENSG00000109654 339.3118613	202.6638901	312.7102538
222.7332264	105.0172482	125.9453006	284.8953351
151.2319251	0.912947907	0.008244964	0.061694235	TRIM2
	4	153152342	153339320	+	9425
protein_coding	tripartite motif containing 2 [Source:HGNC
Symbol;Acc:HGNC:15974]	-	374	229	269	215	92
	146	1.943206376	1.151278951	1.775733417
1.267561806	0.600003562	0.720281922
ENSG00000143933 7529.275767	7829.55212	8479.214094
6602.227218	6354.685007	6251.545161	7946.013994
6402.819129	0.311443279	0.008258285	0.061713334	CALM2
	2	47160082	47176601	-	6466
protein_coding	calmodulin 2 [Source:HGNC Symbol;Acc:HGNC:1445] -
	8299	8847	7294	6373	5567	7247	62.85194877
	64.83160418	70.18380429	54.76716518	52.92158968
	52.11390047
ENSG00000185825 4958.126529	6290.545548	6271.642451
6580.47188	8404.804331	7927.652826	5840.104843
7637.643012	-0.387207999	0.008258471	0.061713334	BCAP31
	X	153700492	153724746	-	3533
protein_coding	B cell receptor associated protein 31 [Source:HGNC
Symbol;Acc:HGNC:16695]	-	5465	7108	5395	6352	7363
	9190	75.74870498	95.33013818	95.00679293
99.90308413	128.1027707	120.9491699
ENSG00000150281 57.15681086	73.45459771	67.42451569
123.2802509	134.6960357	137.1596082	66.01197475
131.7119649	-0.99695934	0.008265894	0.061713334	CTF1
	16	30896607	30903560	+	1664
protein_coding	cardiotrophin 1 [Source:HGNC Symbol;Acc:HGNC:2499]
	-	63	83	58	119	118	159
1.854026388	2.36347608	2.16861031	3.973797051
4.358891367	4.442984729
ENSG00000169220 320.259591	376.1229401	241.7982632
511.7684365	369.8433523	640.0781716	312.7269314
507.2299868	-0.697272798	0.008269595	0.061713334	RGS14
	5	177357837	177372601	+	3797
protein_coding	regulator of G protein signaling 14 [Source:HGNC

Symbol;Acc:HGNC:9996]	-	353	425	208	494	324
	742	4.55263457	5.303648992	3.408235411
7.229335757	5.245075853	9.086451781
ENSG00000139178 104.3338611	149.5641809	146.4739479
203.049825	256.8356613	238.0883765	133.45733
232.6579543	-0.802775936	0.008270506	0.061713334	C1RL
	12	7089587 7109273 -	5014	protein_coding
complement C1r subcomponent like [Source:HGNC Symbol;Acc:HGNC:21265]
	-	115	169	126	196	225	276
1.123161464	1.597087887	1.563482635	2.172119855
2.758325718	2.559503752
ENSG00000149474 412.7991895	441.6125814	401.0596192
474.4735707	652.9333257	757.3970818	418.4904634
628.2679927	-0.58649763	0.008270741	0.061713334	KAT14
	20	18138118	18188387	+	4251
protein_coding	lysine acetyltransferase 14 [Source:HGNC
Symbol;Acc:HGNC:15904]	-	455	499	345	458	572
	878	5.241420165	5.562062716	5.049342576
5.986685126	8.270890745	9.603608096
ENSG00000006634 1017.935584	879.6851822	903.256012
793.5518671	632.3864728	536.5614862	933.6255927
654.1666087	0.514063213	0.0082741	0.061713334	DBF4
	7	87876216	87909541	+	4583
protein_coding	DBF4 zinc finger [Source:HGNC Symbol;Acc:HGNC:17364]
	-	1122	994	777	766	554	622
11.9886887	10.27691981	10.54819156	9.287331684
7.43031572	6.310612968
ENSG00000171217 0	0	1.16249165	5.179842475
26.25431205	5.175834272	0.387497217
12.2033296	-5.118227314	0.008276759	0.061713334	CLDN20
	6	155264013	155276548	+	1209
protein_coding	claudin 20 [Source:HGNC Symbol;Acc:HGNC:2042]	-
	0	0	1	5	23	6	0	0
	0.051461275	0.229803028	1.169361781	0.230757576
ENSG00000127946 623.2814136	479.667373	601.008183
363.6249417	441.7573375	341.6050619	567.9856565
382.3291137	0.571856719	0.008281518	0.061713334	HIP1
	7	75533300	75738962	-	8953
protein_coding	huntingtin interacting protein 1 [Source:HGNC
Symbol;Acc:HGNC:4913]	-	687	542	517	351	387
	396	3.757654414	2.868515117	3.59276495
2.178464944	2.656988904	2.056636881
ENSG00000175283 246.7722627	373.4679546	338.2850701
409.2075555	519.3787818	566.7538527	319.5084292
498.44673	-0.642418178	0.008282405	0.061713334	DOLK
	9	128945530	128947619	-	2090
protein_coding	dolichol kinase [Source:HGNC Symbol;Acc:HGNC:23406]
	-	272	422	291	395	455	657
6.373108342	9.56737079	8.662705485	10.50177848
13.3817364	14.61672585

ENSG00000154380 1495.149592	1133.678791	1319.428023
1080.51514	945.1552337	833.3093177	1316.085469
952.9932305	0.46612299	0.008301731	0.061827238	ENAH
1	225486835	225653142	-	15044
protein_coding	"ENAH, actin regulator [Source:HGNC
Symbol;Acc:HGNC:18271]" -	1648	1281	1135	1043	828
966	5.364417445	4.034709267	4.693959989
3.852414664	3.383096394	2.98569073
ENSG00000212719 751.2037998	934.5548817	832.3440213
1010.069283	1260.206978	1186.991326	839.3675676
1152.422529	-0.457083171	0.008304682	0.061827238
C17orf51	17	21428381	21574517	-	14794
protein_coding	chromosome 17 open reading frame 51
[Source:HGNC Symbol;Acc:HGNC:27904]	-	828	1056	716
975	1104	1376	2.740775137	3.382242517
3.011162925	3.662107131	4.587021959	4.324778185
ENSG00000162976 166.0269268	200.0089046	169.7237809
307.682643	294.5048917	263.9675479	178.5865374
288.7183608	-0.691640351	0.008316476	0.061867254	PQLC3
2	11155198	11178874	+	2275
protein_coding	PQ loop repeat containing 3 [Source:HGNC
Symbol;Acc:HGNC:28503]	-	183	226	146	297	258
306	3.939112391	4.707100587	3.992806845
7.25415936	6.970848837	6.254189608
ENSG00000007062 0.907250966	0	0	12.43162194
21.68834474	2.587917136	0.302416989
12.23596127	-5.122173847	0.008317052	0.061867254	PROM1
4	15963076	16084378	-	7434
protein_coding	prominin 1 [Source:HGNC Symbol;Acc:HGNC:9454]	-
1	0	0	12	19	3	0.00658728
0	0	0.089695516	0.157100803	0.018764185
ENSG00000170222 162.3979229	144.25421	183.6736807
199.9419195	288.7974325	364.0336771	163.4419379
284.2576764	-0.8011225	0.008370691	0.062206472	ADPRM
17	10697594	10711233	+	1620
protein_coding	"ADP-ribose/CDP-alcohol diphosphatase, manganese
dependent [Source:HGNC Symbol;Acc:HGNC:30925]"	-	179	163
158	193	253	422	5.410865018	4.767591433
6.068046763	6.619944343	9.599594027	12.11235119
ENSG00000112379 156.9544171	96.46447169	54.63710754
236.2008168	224.8738902	171.66517	102.6853321
210.9132923	-1.032761213	0.008372292	0.062206472
ARFGEF3 6	138161921	138344663	+	14877
protein_coding	ARFGEF family member 3 [Source:HGNC
Symbol;Acc:HGNC:21213]	-	173	109	47	228	197
199	0.569455017	0.347166312	0.196557374
0.851591911	0.813950929	0.621968989
ENSG00000125378 455.4399849	478.7823778	478.9465598
416.459335	230.5813493	189.78059	471.0563075
278.9404247	0.756955466	0.008375512	0.062206472	BMP4

14	53949736	53958761	-	3082
protein_coding	bone morphogenetic protein 4 [Source:HGNC
Symbol;Acc:HGNC:1071]	-	502	541	412	402	202
220	7.976268525	8.317466714	8.317090521
7.247787679	4.028710823	3.319105558
ENSG00000125945 362.9003864	296.4733763	416.1720107
182.3304551	284.2314652	179.4289214	358.5152578
215.3302806	0.736842119	0.008381074	0.062206472	ZNF436
1	23359448	23369442	-	4392
protein_coding	zinc finger protein 436 [Source:HGNC
Symbol;Acc:HGNC:20814]	zf-C2H2 400	335	358	176	249
208	4.459912553	3.614172828	5.071396211
2.22670344	3.484852299	2.20207457
ENSG00000112763 535.2780699	524.8021258	533.5836673
248.6324388	385.8242379	401.9897951	531.2212877
345.4821573	0.620104083	0.008387725	0.062206472	BTN2A1
6	26457904	26476621	+	3787
protein_coding	butyrophilin subfamily 2 member A1 [Source:HGNC
Symbol;Acc:HGNC:1136]	-	590	593	459	240	338
466	7.629312253	7.419691171	7.540918152
3.521502331	5.486163613	5.721654502
ENSG00000164542 403.7266799	274.3484975	331.3101202
510.732468	496.5489453	511.5449538	336.4617658
506.2754557	-0.589593685	0.008391341	0.062206472
KIAA0895	7	36324221	36390125	-	6728
protein_coding	KIAA0895 [Source:HGNC Symbol;Acc:HGNC:22206]
-	445	310	285	493	435	593
3.238938574	2.183243379	2.635516405	4.071673825
3.974208376	4.09826209
ENSG00000149483 1112.289684	1185.893505	833.506513
1432.744428	1442.845671	1416.453312	1043.896567
1430.681137	-0.453518112	0.008393548	0.062206472
TMEM138 11	61362001	61369509	+	6348
protein_coding	transmembrane protein 138 [Source:HGNC
Symbol;Acc:HGNC:26944]	-	1226	1340	717	1383	1264
1642	9.457628172	10.00217206	7.027309546
12.10590623	12.23932638	12.02727532
ENSG00000129636 607.8581472	511.5271985	747.4821309
423.7111144	465.7286659	360.5831209	622.2891589
416.6743004	0.578599715	0.008396583	0.062206472	ITFG1
16	47154387	47464149	-	7770
protein_coding	integrin alpha FG-GAP repeat containing 1 [Source:HGNC
Symbol;Acc:HGNC:30697]	-	670	578	643	409	408
418	4.222624541	3.524790103	5.148690673
2.924922295	3.22765052	2.50141806
ENSG00000237441 1561.378912	1712.465621	2051.797762
1298.068524	1457.685065	1195.617717	1775.214099
1317.123768	0.430543803	0.008397526	0.062206472	RGL2
6	33291654	33299324	-	4541
protein_coding	ral guanine nucleotide dissociation stimulator like 2

[Source:HGNC Symbol;Acc:HGNC:9769]	-	1721	1935	1765
	1253	1277	1386	18.55914872	20.19091071
24.18243641	15.33245196	17.28569141	14.19197203
ENSG00000197903 169.6559306	319.4832503	284.8104542
376.0565636	449.7477803	446.8470255	257.9832117
424.2171231	-0.718311583	0.008397821	0.062206472
HIST1H2BK	6	27146418	27146798	-	381
	protein_coding	histone cluster 1 H2B family member k
[Source:HGNC Symbol;Acc:HGNC:13954]	-	187	361	245
	363	394	518	24.03506574	44.89610521
40.0081026	52.94118926	63.56508124	63.21727947
ENSG00000010803 1150.394225	1181.468529	1027.642619
724.1419779	905.2030197	839.3477911	1119.835124
822.8975962	0.44516424	0.00840499	0.062213704	SCMH1
	1	41027200	41242154	-	4447
protein_coding	Scm polycomb group protein homolog 1 [Source:HGNC
Symbol;Acc:HGNC:19003]	-	1268	1335	884	699	793
	973	13.96306654	14.22461685	12.36778657
8.734179035	10.96108178	10.17364851
ENSG00000136169 422.7789501	466.3924457	474.2965932
640.2285298	729.4132782	578.8307994	454.4893297
649.4908691	-0.514591912	0.008405832	0.062213704	SETDB2
	13	49444374	49495003	+	6678
protein_coding	SET domain bifurcated 2 [Source:HGNC
Symbol;Acc:HGNC:20263]	MBD	466	527	408	618	639
	671	3.417182594	3.739302857	3.801198901
5.14226086	5.881685568	4.672046145
ENSG00000137166 1958.754836	1695.650713	2372.645457
1232.802509	1625.484363	1528.596388	2009.017002
1462.29442	0.457764988	0.008415031	0.062233938	FOXP4
	6	41546426	41602384	+	6170
protein_coding	forkhead box P4 [Source:HGNC Symbol;Acc:HGNC:20842]
	Fork	2159	1916	2041	1190	1424	1772
17.13547556	14.71420429	20.58091539	10.71701506
14.18639949	13.35394464
ENSG00000189149 19.05227029	8.849951531	9.299933199
58.01423571	50.22564044	31.05500563	12.40071834
46.43162726	-1.897324286	0.008415602	0.062233938	CRYM-
AS1	16	21300849	21339037	+	2746
transcribed_unitary_pseudogene	CRYM antisense RNA 1 [Source:HGNC
Symbol;Acc:HGNC:34405]	-	21	10	8	56	44
	36	0.374496226	0.172554348	0.181257631
1.133181662	0.984916706	0.609583195
ENSG00000105486 1973.270851	1866.454778	2000.648129
1675.161056	1420.015834	1364.69497	1946.791253
1486.623953	0.389128881	0.008422851	0.062261519	LIG1
	19	48115445	48170603	-	6576
protein_coding	DNA ligase 1 [Source:HGNC Symbol;Acc:HGNC:6598] -
	2175	2109	1721	1617	1244	1582	16.19668516
	15.19641748	16.28268092	13.66344645	11.62802444

11.18602261
ENSG00000105552 834.6708887 1072.614126 986.9554108
1235.910414 1313.857094 1351.755384 964.7468083
1300.507631 -0.431049855 0.008435904 0.062331959 BCAT2
19 48795062 48811029 - 4553
protein_coding branched chain amino acid transaminase 2 [Source:HGNC
Symbol;Acc:HGNC:977] - 920 1212 849 1193 1151
1567 9.895069766 12.61337798 11.60157325
14.55978081 15.53907037 16.00303534
ENSG00000175602 4371.135154 5333.865788 4455.830494
5200.561844 5887.81485 8304.626089 4720.277145
6464.334261 -0.453753337 0.008442526 0.062354032
CCDC85B 11 65890112 65891635 + 1524
protein_coding coiled-coil domain containing 85B [Source:HGNC
Symbol;Acc:HGNC:24926] - 4818 6027 3833 5020 5158
9627 154.8140999 187.3883837 156.4806707
183.0335882 208.0385083 293.7223694
ENSG00000169914 594.2493827 458.4274893 406.8720775
326.3300759 343.5890403 286.396163 486.5163165
318.7717597 0.611991481 0.008445942 0.062354032 OTUD3
1 19882513 19912945 + 6767
protein_coding OTU deubiquitinase 3 [Source:HGNC
Symbol;Acc:HGNC:29038] - 655 518 350 315 301
332 4.739950508 3.627104132 3.217945722
2.586583012 2.734120677 2.281250229
ENSG00000172167 445.4602243 407.9827656 387.1097194
315.9703909 271.6750551 205.3080928 413.5175698
264.3178463 0.647830345 0.008487915 0.062612736 MTBP
8 120445400 120542133 + 8326
protein_coding MDM2 binding protein [Source:HGNC
Symbol;Acc:HGNC:7417] - 491 461 333 305 238
238 2.887844266 2.623559751 2.488368377
2.035520481 1.757065335 1.329142572
ENSG00000147383 1002.512317 1274.39302 1188.066466
1301.17643 1781.868744 1685.596694 1154.990601
1589.547289 -0.460797805 0.008490735 0.062612736 NSDHL
X 152830967 152869729 + 1996
protein_coding NAD(P) dependent steroid dehydrogenase-like
[Source:HGNC Symbol;Acc:HGNC:13398] - 1105 1440 1022
1256 1561 1954 27.11005662 34.18443419
31.85643738 34.96561297 48.07172718 45.519244
ENSG00000109743 0.907250966 2.654985459 4.6499666
30.04308635 28.5372957 8.626390453 2.737401008
22.4022575 -3.056791422 0.008494253 0.062612736 BST1
4 15702950 15738313 + 2980
protein_coding bone marrow stromal cell antigen 1 [Source:HGNC
Symbol;Acc:HGNC:1118] - 1 3 4 29 25
10 0.016432832 0.047701434 0.083512325
0.540746575 0.515669096 0.156032388
ENSG00000188070 558.866595 614.1866363 688.1950567

784.2281506	821.8741163	1016.188795	620.416096
874.0970208	-0.496145388	0.008495142	0.062612736
C11orf95	11	63759892	63768775	-	5948
	protein_coding	chromosome 11 open reading frame 95
[Source:HGNC Symbol;Acc:HGNC:28449]	-	616	694	592
	757	720	1178	5.071523426	5.528597225
6.192379917	7.07191388	7.440616086	9.208848935
ENSG00000163166 895.4567034	822.1604972	759.1070474
587.3941366	628.9619973	584.0066337	825.5747493
600.1209225	0.461034901	0.008503141	0.062645587	IWS1
	2	127436207	127526886	-	4150
protein_coding	"IWS1, SUPT6H interacting protein [Source:HGNC
Symbol;Acc:HGNC:25467]" -	987	929	653	567	551
	677	11.64656191	10.60703637	9.789757434
7.591839286	8.161140645	7.585279534
ENSG00000214253 709.4702554	928.3599156	985.7929191
668.1996792	541.0671266	626.2759469	874.54103
611.8475842	0.51427074	0.008513196	0.062693557	FIS1
	7	101239458	101252316	-	1795
protein_coding	"fission, mitochondrial 1 [Source:HGNC
Symbol;Acc:HGNC:21689]" -	782	1049	848	645	474
	726	21.33393579	27.69092581	29.39261635
19.96674656	16.23159691	18.80629247
ENSG00000169299 1568.63692	1185.893505	1134.59185
1144.745187	801.3272634	707.3640171	1296.374092
884.4788225	0.552358116	0.008537057	0.062843111	PGM2
	4	37826633	37862937	+	3704
protein_coding	phosphoglucomutase 2 [Source:HGNC
Symbol;Acc:HGNC:8906]	-	1729	1340	976	1105	702
	820	22.85876163	17.14195147	16.39402852
16.57690099	11.64966654	10.29375654
ENSG00000188895 1725.591337	1716.890597	1985.535738
1254.557847	1442.845671	1435.431371	1809.339224
1377.61163	0.392811679	0.008544176	0.062869356	MSL1
	17	40122298	40136916	+	7397
protein_coding	male specific lethal 1 homolog [Source:HGNC
Symbol;Acc:HGNC:27905]	-	1902	1940	1708	1211	1264
	1664	12.59167708	12.42717895	14.36610692
9.097049714	10.5036155	10.45992863
ENSG00000110955 11327.02831	11631.4913	13536.05277
9462.536232	10240.32319	9410.529345	12164.85746
9704.462923	0.32594229	0.008547951	0.062870978
ATP5F1B 12	56638175	56646068	-	2322
protein_coding	ATP synthase F1 subunit beta [Source:HGNC
Symbol;Acc:HGNC:830]	-	12485	13143	11644	9134	8971
	10909	263.3025195	268.1999752	311.9944209
218.5802083	237.479432	218.4508528
ENSG00000260920 40.82629347	24.77986429	66.26202404
107.7407235	95.88531357	112.1430759	43.9560606
105.256371	-1.269084093	0.008552185	0.062875977

AL031985.3	1	40464319	40466767	+	2449
sense_overlapping	"novel transcript, sense overlapping
with ZNF643"	-	45	28	57	104	84	130
0.899813308	0.541745967	1.448081206	2.359698942
2.108326469	2.468229765
ENSG00000031698 3530.113509	4593.124845	5253.299766
4760.275234	6969.949103	6763.952754	4458.84604
6164.725697	-0.46758868	0.008558004	0.06289262	SARS
1	109213918	109238169	+	3836
protein_coding	seryl-tRNA synthetase [Source:HGNC
Symbol;Acc:HGNC:10537]	-	3891	5190	4519	4595	6106
7841	49.67196214	64.1084387	73.29436548
66.56086556	97.84205409	95.04381789
ENSG00000196352 146.9746565	146.0242003	72.07448229
287.9992416	181.4972007	230.3246251	121.691113
233.2736891	-0.93473114	0.008566441	0.062928477	CD55
1	207321376	207386804	+	5908
protein_coding	CD55 molecule (Cromer blood group) [Source:HGNC
Symbol;Acc:HGNC:2665]	-	162	165	62	278	159
267	1.34277489	1.323335303	0.65291711
2.614666803	1.654260873	2.101366444
ENSG00000180537 667.736711	615.9566266	834.6690046
566.6747667	457.7382231	417.5172979	706.1207807
480.6434292	0.554446793	0.008597915	0.063133463	RNF182
6	13924446	13980302	+	4090
protein_coding	ring finger protein 182 [Source:HGNC
Symbol;Acc:HGNC:28522]	-	736	696	718	547	401
484	8.812176557	8.063291722	10.92214613
7.43149281	6.026545292	5.502411571
ENSG00000255306 0.907250966	0.884995153	1.16249165
38.33083431	0	18.11541995	0.98491259	18.81541809
-4.26716544	0.008603154	0.06314134	AC004923.4
11	68024809	68030461	-	739
antisense	novel transcript	-	1	1	1
37	0	21	0.066265007	0.0641183
0.084190368	2.782078175	0	1.321313507
ENSG00000213380 1015.213831	1011.54946	914.8809285
1070.155455	1377.780637	1688.184612	980.5480731
1378.706901	-0.491856136	0.008606126	0.06314134	COG8
16	69320140	69339667	-	7450
protein_coding	component of oligomeric golgi complex 8 [Source:HGNC
Symbol;Acc:HGNC:18623]	-	1119	1143	787	1033	1207
1957	7.355335674	7.269698483	6.572419965
7.704706374	9.958601578	12.2142153
ENSG00000118898 136.9948959	76.10958317	45.33717435
43.51067679	27.39580388	18.978059	86.14721779
29.96151322	1.530012428	0.008615512	0.063183996	PPL
16	4882507 4960741 -	7686	protein_coding
periplakin [Source:HGNC Symbol;Acc:HGNC:9273]	-	151	86
39	42	24	22	0.962066851	0.53018143

0.31569745	0.303641378	0.191936788	0.133092419
ENSG00000238123 0	3.539980612	4.6499666	12.43162194
22.82983656	35.36820086	2.729982404	23.54321979
-3.135860862	0.008628971	0.063256473	MID1IP1-AS1
X	38801568	38803883	-	376
antisense	MID1IP1 antisense RNA 1 [Source:HGNC
Symbol;Acc:HGNC:40932]	-	0	4	4	12	20
41	0	0.504078979	0.661879596	1.773394858
3.269561501	5.070222637
ENSG00000140297 0	2.654985459	2.3249833	34.18696033
6.848950969	17.25278091	1.659989586	19.42956407
-3.565589067	0.008636405	0.063284744	GCNT3	15
59594875	59640239	+	6475	protein_coding
"glucosaminyl (N-acetyl) transferase 3, mucin type
[Source:HGNC Symbol;Acc:HGNC:4205]"	-	0	3	2
33	6	20	0	0.02195371	0.019217508
0.28319541	0.056958539	0.14362209
ENSG00000147853 1175.797252	869.9502355	1196.203908
970.7024797	642.6598993	444.2591083	1080.650465
685.8738291	0.656416948	0.008658429	0.063419853	AK3
9	4709559 4742043 -	4483	protein_coding
adenylate kinase 3 [Source:HGNC Symbol;Acc:HGNC:17376]	-	1296
983	1029	937	563	515	14.1567951
10.38989646	14.28083109	11.61402872	7.719461689
5.341577185
ENSG00000236829 46.26979926	31.85982551	9.299933199
79.76957411	122.1396256	62.11001126	29.14318599
88.00640366	-1.582046913	0.008665136	0.063442712
Z97634.1	16	382097	392960	+	2291
transcribed_processed_pseudogene	uncharacterized LOC100134368
[Source:NCBI gene;Acc:100134368]	-	51	36	8
77	107	72	1.090118652	0.744567117
0.217255983	1.8675734	2.870820565	1.461296773
ENSG00000144647 710.3775064	1038.099315	784.6818637
1108.48629	1389.195555	1101.590061	844.3862282
1199.757302	-0.505814813	0.008681463	0.063530347
POMGNT2 3	43079232	43106076	-	2668
protein_coding	"protein O-linked mannose N-
acetylglucosaminyltransferase 2 (beta 1,4-) [Source:HGNC
Symbol;Acc:HGNC:25902]" -	783	1173	675	1070	1217
1277	14.37158343	20.83236747	15.74072727
22.28486441	28.03832808	22.25543514
ENSG00000168394 657.7569503	838.09041	838.1564796
928.2277714	1118.661992	1213.733137	778.00128
1086.8743	-0.483105597	0.008684289	0.063530347	TAP1
6	32845209	32853978	-	4805
protein_coding	"transporter 1, ATP binding cassette subfamily B
member [Source:HGNC Symbol;Acc:HGNC:43]"	-	725	947
721	896	980	1407	7.388789569	9.338626968
9.335739381	10.36159615	12.53660793	13.61544134

ENSG00000197448 756.6473056   877.0301967   883.4936539
581.1783256   632.3864728   616.7869174   839.0570521
610.1172386   0.459423126   0.008694094   0.063575789   GSTK1
        7   143244093   143270854   +   4228
protein_coding glutathione S-transferase kappa 1 [Source:HGNC
Symbol;Acc:HGNC:16906] -   834   991   760   561   554
        715   9.659613628   11.10619045   11.18369875
7.37292687   8.054195115   7.863249958
ENSG00000280407 60.78581472   48.67473342   30.2247829
15.53952742   13.69790194   10.35166854   46.56177701
13.19636597   1.825984593   0.008703551   0.06361864
AC132872.4   17   82249067   82251844   +   2778
        TEC   TEC   -   67   55   26   15   12
        12   1.181058052   0.938116747   0.582301559
0.300034407   0.265519465   0.200853786
ENSG00000117569 599.6928885   461.9674699   484.759018
469.2937282   212.31748   206.1707318   515.4731255
295.9273134   0.801390812   0.008734269   0.063816807   PTBP2
        1   96721665   96823738   +   12937
protein_coding polypyrimidine tract binding protein 2 [Source:HGNC
Symbol;Acc:HGNC:17662] -   661   522   417   453   186
        239   2.502053345   1.911892043   2.005438386
1.945703533   0.883744505   0.859004306
ENSG00000243147 243.1432589   384.9728916   402.2221109
177.1506126   165.5163151   257.0664355   343.4460871
199.9111211   0.777277698   0.008771093   0.064059401   MRPL33
        2   27771717   27988087   +   1539
protein_coding mitochondrial ribosomal protein L33 [Source:HGNC
Symbol;Acc:HGNC:14487] -   268   435   346   171   145
        298   8.527561452   13.39297561   13.98763611
6.174041356   5.791309066   9.00344389
ENSG00000147274 7395.002624   6780.832863   7966.555277
6280.041016   5867.267997   5437.213902   7380.796921
5861.507638   0.332470147   0.008786103   0.064142542   RBMX
        X   136848004   136880764   -   5882
protein_coding RNA binding motif protein X-linked [Source:HGNC
Symbol;Acc:HGNC:9910]   -   8151   7662   6853   6062   5140
        6303   67.86010957   61.72250852   72.48740595
57.26680522   53.71374821   49.8256881
ENSG00000267278 45.3625483   18.58489822   6.974949899   0
        5.707459141   0   23.6407988   1.90248638
3.698911079   0.008791207   0.064153326   MAP3K14-AS1   17
        45247925   45268630   +   3775   antisense
        MAP3K14 antisense RNA 1 [Source:HGNC Symbol;Acc:HGNC:44359]
        -       50   21   6   0   5   0
0.64860715   0.263589909   0.098887442   0   0.081414247
        0
ENSG00000143819 626.0031665   687.641234   789.3318303
548.0273338   478.285076   447.7096645   700.9920769
491.3406914   0.512304899   0.008809794   0.064262454   EPHX1

1	225810092	225845563	+	2508
protein_coding	epoxide hydrolase 1 [Source:HGNC Symbol;Acc:HGNC:3401]
-	690	777	679	529	419	519
13.47256359	14.67979286	16.84414955	11.72033927
10.26913471	9.622121662
ENSG00000205707 193.2444558	184.0789918	166.2363059
114.9925029	90.17785443	96.61557307	181.1865845
100.5953101	0.849450507	0.008816585	0.064278602	ETFRF1
12	25195216	25209645	+	2466
protein_coding	electron transfer flavoprotein regulatory factor 1
[Source:HGNC Symbol;Acc:HGNC:27052]	-	213	208	143
111	79	112	4.229755022	3.996655395
3.607861122	2.501162739	1.969161696	2.111815478
ENSG00000161671 548.8868344	889.4201289	834.6690046
485.8692241	390.3902052	599.5341365	757.658656
491.9311886	0.621319239	0.008824556	0.064278602	EMC10
19	50476400	50490870	+	10748
protein_coding	ER membrane protein complex subunit 10 [Source:HGNC
Symbol;Acc:HGNC:27609]	-	605	1005	718	469	342
695	2.756489868	4.430623481	4.156268857
2.424695624	1.955896225	3.00668662
ENSG00000238279 13.60876449	7.964956378	5.812458249	0
0	0	9.128726372	0	5.625476754
0.008826471	0.064278602	BX470102.1	1	153533603
153535115	+	531	antisense	novel
transcript	-	15	9	5	0	0	0
1.383328809	0.803108882	0.585844463	0	0
0
ENSG00000104823 1437.992781	1866.454778	1943.686039
2071.93699	2506.716055	2465.422391	1749.377866
2348.025145	-0.425009567	0.008830962	0.064278602	ECH1
19	38815422	38832005	-	2467
protein_coding	enoyl-CoA hydratase 1 [Source:HGNC
Symbol;Acc:HGNC:3149]	-	1585	2109	1672	2000	2196
2858	31.46217922	40.5073536	42.16712295
45.04772777	54.71552195	53.86716173
ENSG00000127184 2318.933469	3039.073356	2651.643453
1920.68559	1959.941469	2211.806512	2669.883426
2030.81119	0.39449751	0.008831099	0.064278602	COX7C
5	86617904	86620962	+	1993
protein_coding	cytochrome c oxidase subunit 7C [Source:HGNC
Symbol;Acc:HGNC:2292]	-	2556	3434	2281	1854	1717
2564	62.80326674	81.64308992	71.20735156
51.69094534	52.95541266	59.81935697
ENSG00000254470 840.1143945	948.7148041	809.0941883
870.2135357	1418.874342	1509.618329	865.9744623
1266.235402	-0.548070751	0.008833811	0.064278602	AP5B1
11	65775893	65780802	-	4811
protein_coding	adaptor related protein complex 5 subunit beta 1
[Source:HGNC Symbol;Acc:HGNC:25104]	-	926	1072	696

840	1243	1750	9.425498168	10.55810239
9.000792076	9.701881657	15.8811933	16.91350865
ENSG00000103404 410.0774366	452.2325232	373.1598196
516.948279	593.5757507	695.2870705	411.8232598
601.9370334	-0.54736637	0.008840442	0.064300406	USP31
16	23061406	23149270	-	12017
protein_coding	ubiquitin specific peptidase 31 [Source:HGNC
Symbol;Acc:HGNC:20060]	-	452	511	321	499	520
806	1.841921245	2.014889714	1.661941826
2.307366209	2.65983467	3.118674137
ENSG00000139437 716.7282631	690.2962194	685.8700734
586.3581681	348.1550076	469.2756406	697.6315187
467.9296054	0.575459007	0.008844214	0.064301395	TCHP
12	109900264	109983841	+	9054
protein_coding	trichoplein keratin filament binding [Source:HGNC
Symbol;Acc:HGNC:28135]	-	790	780	590	566	305
544	4.272826758	4.082070992	4.054323214
3.473665415	2.07065006	2.79376214
ENSG00000136156 2247.260643	2376.211986	2787.654976
2145.490753	1849.216762	1378.497194	2470.375868
1791.068236	0.464133885	0.008858992	0.064382377	ITM2B
13	48232612	48270357	+	10706
protein_coding	integral membrane protein 2B [Source:HGNC
Symbol;Acc:HGNC:6174]	-	2477	2685	2398	2071	1620
1598	11.32993585	11.88347596	13.93569993
10.7489218	9.301117605	6.940336923
ENSG00000178974 457.2544869	481.4373633	639.3704074
410.243524	214.6004637	345.9182572	526.0207525
323.5874149	0.698590939	0.00886438	0.064395077	FBXO34
14	55271303	55361918	+	4133
protein_coding	F-box protein 34 [Source:HGNC Symbol;Acc:HGNC:20201]
-	504	544	550	396	188	401
5.971642699	6.23677297	8.279500388	5.324046308
2.796016978	4.511385979
ENSG00000099204 11.79426256	15.0449176	6.974949899
39.36680281	29.67878753	61.24737221	11.27137669
43.43098752	-1.939063687	0.008879546	0.064429245	ABLIM1
10	114431113	114685003	-	10039
protein_coding	actin binding LIM protein 1 [Source:HGNC
Symbol;Acc:HGNC:78]	-	13	17	6	38	26
71	0.063413479	0.080238889	0.037184988
0.21033192	0.159195304	0.328850807
ENSG00000136689 5.443505796	1.769990306	6.974949899
21.75533839	19.40536108	42.26931322	4.729482
27.81000423	-2.576744646	0.008879815	0.064429245	IL1RN
2	113107214	113134016	+	3256
protein_coding	interleukin 1 receptor antagonist [Source:HGNC
Symbol;Acc:HGNC:6000]	-	6	2	6	21	17
49	0.090239263	0.029105297	0.114649905
0.358382622	0.32093116	0.699749669

ENSG00000103121 589.7131279 738.9709529 631.2329659
494.1569721 375.5508115 477.9020311 653.3056822
449.2032715 0.539694667 0.008882439 0.064429245 CMC2
16 80966448 81020270 - 12781
protein_coding C-X9-C motif containing 2 [Source:HGNC
Symbol;Acc:HGNC:24447] - 650 835 543 477 329
554 2.490446435 3.095623119 2.643271914
2.073793877 1.582262092 2.01546819
ENSG00000110944 24.49577608 5.309970919 3.48747495
13.46759043 49.08414861 105.2419635 11.09774065
55.93123419 -2.324674712 0.008885384 0.064429245 IL23A
12 56334174 56340410 + 1187
protein_coding interleukin 23 subunit alpha [Source:HGNC
Symbol;Acc:HGNC:15488] - 27 6 3 13 43
122 1.113888522 0.239511832 0.157245195
0.608561785 2.226717369 4.779034105
ENSG00000183605 364.7148883 406.2127753 361.5349031
606.0415695 466.8701577 583.1439946 377.4875222
552.0185739 -0.54851772 0.008887295 0.064429245 SFXN4
10 119140767 119165667 - 1868
protein_coding sideroflexin 4 [Source:HGNC Symbol;Acc:HGNC:16088]
- 402 459 311 585 409 676
10.53847731 11.64292914 10.35834481 17.40167438
13.45841129 16.82677324
ENSG00000119523 518.0403016 602.6816993 553.3460253
736.5735999 757.9505739 821.2323711 558.0226754
771.9188483 -0.468335628 0.00890367 0.064521516 ALG2
9 99216426 99221956 - 2983
protein_coding "ALG2, alpha-1,3/1,6-mannosyltransferase [Source:HGNC
Symbol;Acc:HGNC:23159]" - 571 681 476 711 664
952 9.373710541 10.8173355 9.927972053
13.24428114 13.68239696 14.83934436
ENSG00000166135 1421.662264 1464.666978 1256.653474
1818.124709 1761.321891 1796.014492 1380.994239
1791.820364 -0.375192928 0.008910613 0.064545387 HIF1AN
10 100529072 100559998 + 13950
protein_coding hypoxia inducible factor 1 subunit alpha inhibitor
[Source:HGNC Symbol;Acc:HGNC:17113] - 1567 1655 1081
1755 1543 2082 5.500769822 5.621474323
4.821235358 6.990608116 6.798906656 6.939649493
ENSG00000037241 563.4028499 584.0968011 538.2336339
422.6751459 393.8146807 363.1710381 561.9110949
393.2202882 0.515944323 0.0089398 0.064730296
RPL26L1 5 172958729 172969771 + 1164
protein_coding ribosomal protein L26 like 1 [Source:HGNC
Symbol;Acc:HGNC:17050] - 621 660 463 408 345
421 26.12566197 26.86688993 24.74770083
19.47687273 18.21853599 16.81744955
ENSG00000140104 282.1550504 339.8381388 240.6357715
475.5095392 404.0881072 438.220635 287.5429869

439.2727604	-0.609660052	0.008953452	0.064802624	CLBA1
	14	104985775	105010482	+	5225
protein_coding	clathrin binding box of aftiphilin containing 1
[Source:HGNC Symbol;Acc:HGNC:20126]	-	311	384	207
	459	354	508	2.914759845	3.482341596
2.464852282	4.881332984	4.16451401	4.520728605
ENSG00000167674 1617.628472	2016.018959	2370.320474
1311.536115	1510.193689	1576.041536	2001.322635
1465.92378	0.448458621	0.008962313	0.064840226	HDGFL2
	19	4472287 4502211 +	4299	protein_coding	HDGF
like 2 [Source:HGNC Symbol;Acc:HGNC:14680]	-	1783	2278
	2039	1266	1323	1827	20.31012431	25.10803443
	29.50914506	16.36357925	18.91645533	19.76069069
ENSG00000172379 995.2543097	990.3095763	1200.853874
909.5803385	700.8759825	653.8803963	1062.139253
754.7789058	0.492618548	0.00896618	0.064841682	ARNT2
	15	80404350	80597937	+	7741
protein_coding	aryl hydrocarbon receptor nuclear translocator 2
[Source:HGNC Symbol;Acc:HGNC:16876]	bHLH	1097	1119	1033
	878	614	758	6.939660806	6.849509303
8.302523254	6.302451207	4.875494422	4.553057723
ENSG00000253986 8.165258694	13.2749273	5.812458249	0
	0	0	9.084214747	0	5.618702341
0.008985252	0.064934052	AC104561.3	8	23493009
	23494198	-	715	lincRNA novel transcript
	-	9	15	5	0	0	0
0.616403578	0.994057847	0.435081692	0	0	0
ENSG00000172728 206.8532202	167.2640839	159.261356
253.8122813	308.2027936	307.0995001	177.7928867
289.7048583	-0.703243087	0.008986294	0.064934052	FUT10
	8	33370824	33473422	-	5606
protein_coding	fucosyltransferase 10 [Source:HGNC
Symbol;Acc:HGNC:19234]	-	228	189	137	245	270
	356	1.991638152	1.597478977	1.520457623
2.428426898	2.960452048	2.952758461
ENSG00000071082 6554.888229	9264.129263	8674.512691
9125.846472	11422.90872	12077.80927	8164.510061
10875.52149	-0.413722568	0.008990759	0.064939783	RPL31
	2	101001715	101024032	+	5791
protein_coding	ribosomal protein L31 [Source:HGNC
Symbol;Acc:HGNC:10334]	-	7225	10468	7462	8809	10007
	14001	61.09602707	85.65181886	80.16938027
84.52498234	106.2178961	112.4181693
ENSG00000261423 5.443505796	14.15992245	15.11239145	0
	1.141491828	0	11.5719399	0.380497276
5.002063875	0.009008934	0.065044502	TMEM202-AS1	15
	72407778	72475168	-	2713	lincRNA
TMEM202 antisense RNA 1 [Source:HGNC Symbol;Acc:HGNC:53265]	-
	6	16	13	0	1	0	0.10830042
	0.279445184	0.298126379	0	0.022656748	0

ENSG00000100129 3935.65469	3266.51711	3719.97328
4541.685882	4674.409036	4569.399023	3640.715027
4595.164647	-0.335956964	0.009014672	0.065059378	EIF3L
22	37848868	37889407	+	4296
protein_coding	eukaryotic translation initiation factor 3 subunit L
[Source:HGNC Symbol;Acc:HGNC:18138]	-	4338	3691	3200
4384	4095	5297	49.44859525	40.71047909
46.34389721	56.70460329	58.5918207	57.33195065
ENSG00000069431 208.6677222	192.9289434	96.48680694
330.4739499	240.8547757	330.3907543	166.0278242
300.57316	-0.85304889	0.009025022	0.065107513	ABCC9
12	21797401	21942529	-	11616
protein_coding	ATP binding cassette subfamily C member 9 [Source:HGNC
Symbol;Acc:HGNC:60]	-	230	218	83	319	211
383	0.969616319	0.889255031	0.444557903
1.525970449	1.116537238	1.533109549
ENSG00000146411 10.88701159	7.964956378	8.137441549	0
0	0	8.99646984	0	5.602934441
0.009037675	0.065172211	SLC2A12 6	133988697
134052636	-	4468	protein_coding	solute carrier family
2 member 12 [Source:HGNC Symbol;Acc:HGNC:18067] -	12	9
7	0	0	0	0.131521504	0.095445572
0.097474658	0	0	0
ENSG00000284693 20.86677222	38.93978674	23.249833	0
1.141491828	8.626390453	27.68546398	3.25596076
3.043229764	0.009043943	0.065190836	AL928921.2
1	9425094 9440564 -	2623	lincRNA
uncharacterized LOC100506022 [Source:NCBI gene;Acc:100506022]	-
23	44	20	0	1	10	0.429396232
0.794842035	0.474393305	0	0.023434143
0.177268973
ENSG00000111667 2136.576025	2449.666584	2445.882431
1839.880047	1860.63168	1790.838658	2344.04168
1830.450128	0.356752584	0.009050468	0.065211294	USP5
12	6852128 6866632 +	3738	protein_coding
ubiquitin specific peptidase 5 [Source:HGNC Symbol;Acc:HGNC:12628]
-	2355	2768	2104	1776	1630	2076
30.85178513	35.08756495	35.01976965	26.40071617
26.80375673	25.82373582
ENSG00000167083 49.89880313	23.89486913	36.03724115	0
0	12.93958568	36.61030447	4.313195226
3.040216938	0.009103847	0.065569199	GNGT2	17
49202791	49210574	-	1689	protein_coding	G
protein subunit gamma transducin 2 [Source:HGNC Symbol;Acc:HGNC:4412]
-	55	27	31	0	0	15
1.594636584	0.757461486	1.141928444	0	0
0.412945395
ENSG00000173511 1416.218758	1681.490791	1442.652138
1977.663857	1878.895549	2046.179815	1513.453895
1967.57974	-0.378466906	0.009117235	0.065638899	VEGFB

11	64234538	64238793	+	1545
protein_coding	vascular endothelial growth factor B [Source:HGNC Symbol;Acc:HGNC:12681]	-	1561	1900	1241	1909	1646
2372	49.47697086	58.27087748	49.97469409
68.65773756	65.48603672	71.38668567
ENSG00000100311 6.350756762	7.964956378	12.78740815	0
0	0	9.034373763	0	5.606094678
0.00913731	0.065736184	PDGFB	22	39223359
39244751	-	3761	protein_coding	platelet derived growth factor subunit B [Source:HGNC Symbol;Acc:HGNC:8800]	-
7	9	11	0	0	0	0.091143015
0.113387614	0.181968493	0	0	0
ENSG00000253719 1516.016364	1526.616639	1528.67652
1664.801371	2219.060114	2187.652619	1523.769841
2023.838035	-0.409515587	0.009138181	0.065736184
ATXN7L3B	12	74537827	74545430	+	7604
protein_coding	ataxin 7 like 3B [Source:HGNC Symbol;Acc:HGNC:37931]	-	1671	1725	1315	1607	1944
2536	10.76125754	10.74913289	10.75946039
11.74318255	15.7145342	15.50737036
ENSG00000162804 396.4686721	256.6485944	340.6100534
208.2296675	192.912119	32.78028372	331.24244
144.6406901	1.198961817	0.009153456	0.065819301	SNED1
2	240998838	241095568	+	13592
protein_coding	"sushi, nidogen and EGF like domains 1 [Source:HGNC Symbol;Acc:HGNC:24696]" -	437	290	293	201	169
38	1.574442319	1.010976529	1.341192453
0.821721661	0.764276839	0.129996377
ENSG00000169019 855.5376609	854.9053179	749.8071142
556.3150818	630.1034892	603.8473317	820.0833643
596.7553009	0.459365302	0.009164196	0.065869753	COMMD8
4	47450796	47463719	-	1766
protein_coding	COMM domain containing 8 [Source:HGNC Symbol;Acc:HGNC:26036]	-	943	966	645	537	552
700	26.14867438	25.9186793	22.72353335
16.89645633	19.21302459	18.43055269
ENSG00000122406 19641.98341	21669.10632	24253.06329
28675.60794	27177.77894	26066.36403	21854.71768
27306.58364	-0.321348869	0.009194438	0.066049359	RPL5
1	92831983	92841924	+	3717
protein_coding	ribosomal protein L5 [Source:HGNC Symbol;Acc:HGNC:10360]	-	21650	24485	20863	27680	23809
30217	285.2292258	312.1289045	349.2135154
413.7953141	393.7276857	377.9982608
ENSG00000198055 873.6826802	946.0598187	914.8809285
1063.939644	1366.365718	1255.139811	911.5411425
1228.481724	-0.430338929	0.009196652	0.066049359	GRK6
5	177403204	177442901	+	4355
protein_coding	G protein-coupled receptor kinase 6 [Source:HGNC Symbol;Acc:HGNC:4545]	-	963	1069	787	1027	1197

1455	10.82846286	11.63097137	11.24329018
13.10371165	16.89481153	15.53480664
ENSG00000104524 498.9880313	673.4813115	547.5335671
616.4012545	880.0901995	1112.804368	573.3343033
869.7652741	-0.601602891	0.009206701	0.066094694	PYCR3
8	143603913	143609773	-	3244
protein_coding	pyrroline-5-carboxylate reductase 3 [Source:HGNC
Symbol;Acc:HGNC:25846]	-	550	761	471	595	771
1290	8.302531414	11.11553197	9.033309872
10.19173596	14.60901358	18.49012652
ENSG00000227124 103.4266101	117.7043554	82.53690714
171.9707702	218.0249392	166.4893357	101.2226242
185.495015	-0.869763294	0.00921325	0.066114879	ZNF717
3	75678660	75785583	-	7641
protein_coding	zinc finger protein 717 [Source:HGNC
Symbol;Acc:HGNC:29448]	zf-C2H2 114	133	71	166	191
193	0.730606169	0.824760555	0.578116009
1.207174164	1.536492794	1.174459722
ENSG00000132031 57.15681086	18.58489822	54.63710754
13.46759043	9.131934625	0	43.45960554	7.53317502
2.544343521	0.009218908	0.066128658	MATN3	2
19992111	20012694	-	3118	protein_coding
matrilin 3 [Source:HGNC Symbol;Acc:HGNC:6909]	-	63
21	47	13	8	0	0.989448335
0.319131464	0.937839658	0.23167506	0.157710728	0
ENSG00000128272 4149.765918	5749.81351	5282.362057
5909.164295	6240.535825	8360.697627	5060.647162
6836.799249	-0.434205942	0.009233129	0.066187844	ATF4
22	39519695	39522685	+	2514
protein_coding	activating transcription factor 4 [Source:HGNC
Symbol;Acc:HGNC:786]	TF_bZIP 4574	6497	4544	5704	5467
9692	89.09627979	122.4542984	112.4552916
126.0742192	133.6691421	179.2582491
ENSG00000155367 8.165258694	6.194966072	17.43737475
25.89921237	62.78205055	39.68139608	10.59919984
42.787553	-2.028209831	0.009234643	0.066187844	PPM1J
1	112709994	112715477	-	2874
protein_coding	"protein phosphatase, Mg2+/Mn2+ dependent 1J
[Source:HGNC Symbol;Acc:HGNC:20785]"	-	9	7	15
25	55	46	0.153350229	0.115408479
0.324721722	0.483353968	1.176314054	0.744221284
ENSG00000186716 883.6624409	833.6654342	785.8443553
910.616307	1238.518634	1356.931218	834.3907435
1168.68872	-0.486118032	0.009244777	0.066218059	BCR
22	23179704	23318037	+	12389
protein_coding	"BCR, RhoGEF and GTPase activating protein
[Source:HGNC Symbol;Acc:HGNC:1014]"	-	974	942	676
879	1085	1573	3.849917184	3.60280777
3.394824202	3.942436126	5.383204092	5.903689225
ENSG00000157426 221.3692357	213.2838319	182.511189

315.9703909	350.4379912	302.7863049	205.7214189
323.0648957	-0.64896072	0.009246346	0.066218059	AASDH
4	56338287	56387508	-	4654
protein_coding	aminoadipate-semialdehyde dehydrogenase [Source:HGNC
Symbol;Acc:HGNC:23993]	-	244	241	157	305	307
351	2.567391689	2.453675375	2.098843806
3.641543516	4.054705879	3.506806119
ENSG00000162736 1966.012843	1782.380238	2294.758517
1545.664994	1624.342871	1405.239005	2014.383866
1525.08229	0.40129484	0.009278327	0.066420202	NCSTN
1	160343272	160358952	+	4175
protein_coding	nicastrin [Source:HGNC Symbol;Acc:HGNC:17091]	-
2167	2014	1974	1492	1423	1629	25.4173995
22.85753678	29.41694138	19.85749158	20.95056697
18.14243696
ENSG00000139117 523.4838074	394.7078383	397.5721443
385.3802801	204.3270372	136.2969692	438.58793
242.0014288	0.859650655	0.009284843	0.066439962	CPNE8
12	38646822	38907430	-	5178
protein_coding	copine 8 [Source:HGNC Symbol;Acc:HGNC:23498]	-
577	446	342	372	179	158	5.456855462
4.0813069	4.109328939	3.992022105	2.124899259
1.41881594
ENSG00000196091 0	0	0	9.323716454	4.565967313
12.93958568	0	8.943089815	-5.639418407
0.009337644	0.066790773	MYBPC1	12	101568353
101686018	+	7385	protein_coding	"myosin binding
protein C, slow type [Source:HGNC Symbol;Acc:HGNC:7549]"	-
0	0	0	9	4	15	0	0
0	0.067717989	0.033293301	0.094443436
ENSG00000105732 736.6877844	998.2745327	936.9682698
991.4218496	1373.214669	1425.942342	890.643529
1263.526287	-0.504929462	0.009368252	0.066961596	ZNF574
19	42068477	42081549	+	4666
protein_coding	zinc finger protein 574 [Source:HGNC
Symbol;Acc:HGNC:26166]	zf-C2H2 812	1128	806	957	1203
1653	8.521969555	11.45488691	10.74724511
11.39670343	15.84777341	16.47248563
ENSG00000105058 2427.803585	3026.683424	2599.331329
3032.279785	3510.087372	4111.33769	2684.606113
3551.234949	-0.403721353	0.009369097	0.066961596	FAM32A
19	16185380	16192046	+	1921
protein_coding	family with sequence similarity 32 member A
[Source:HGNC Symbol;Acc:HGNC:24563]	-	2676	3420	2236
2927	3075	4766	68.216185	84.35778775
72.4187928	84.66568008	98.39320686	115.3606492
ENSG00000113916 525.2983093	580.5568204	696.3324983
703.422608	980.5414804	901.4578023	600.7292093
861.8072969	-0.521651452	0.009396129	0.067127678	BCL6
3	187721377	187745727	-	5938

protein_coding B cell CLL/lymphoma 6 [Source:HGNC
Symbol;Acc:HGNC:1001]   ZBTB   579   656   599   679   859
        1045   4.774930492   5.234679384   6.276152328
6.353918276   8.892017951   8.182897578
ENSG00000105810 3375.880844   2597.460774   2684.19322
2526.727159   2107.193915   1708.02531   2885.844946
2113.982128   0.449498015   0.009413006   0.0672211   CDK6
        7   92604921   92836594   -   12260
protein_coding cyclin dependent kinase 6 [Source:HGNC
Symbol;Acc:HGNC:1777]   -   3721   2935   2309   2439   1846
        1980   14.86270587   11.34342166   11.71764427
11.05435414   9.255259215   7.509408644
ENSG00000120327 49.89880313   100.0044523   104.6242485
210.3016045   143.8279703   159.5882234   84.84250131
171.2392661   -1.016035153   0.0094262   0.067288156
PCDHB14 5   141222932   141227759   +   4828
protein_coding protocadherin beta 14 [Source:HGNC
Symbol;Acc:HGNC:8685]   -   55   113   90   203   126
        185   0.557858573   1.109015517   1.159797303
2.336365691   1.604170937   1.781703713
ENSG00000186684 168.7486797   171.6890597   283.6479626
106.704755   67.34801786   141.4728034   208.0285673
105.1751921   0.977489498   0.009436853   0.067337029
CYP27C1 2   127184120   127220078   -   4491
protein_coding cytochrome P450 family 27 subfamily C member 1
[Source:HGNC Symbol;Acc:HGNC:33480]   -   186   194   244
        103   59   164   2.028142999   2.046845751
3.380287332   1.274401322   0.807525633   1.697977031
ENSG00000143621 6193.802345   6863.137412   7422.509185
5009.943641   5957.445851   5162.032047   6826.482981
5376.473847   0.344487748   0.009442787   0.067352202   ILF2
        1   153661788   153671048   -   2281
protein_coding interleukin enhancer binding factor 2 [Source:HGNC
Symbol;Acc:HGNC:6037]   -   6827   7755   6385   4836   5219
        5984   146.5660222   161.0953326   174.1576127
117.80753   140.6401665   121.9824404
ENSG00000253598 14.51601546   26.54985459   13.9498998
79.76957411   75.33846066   27.60444945   18.33858995
60.90416141   -1.723017429   0.009449841   0.067365314
SLC10A5 8   81693607   81696174   -   2568
protein_coding solute carrier family 10 member 5 [Source:HGNC
Symbol;Acc:HGNC:22981] -   16   30   12   77   66
        32   0.305108036   0.553544674   0.290732159
1.666125646   1.579778781   0.579409988
ENSG00000104998 118.8498765   130.9792827   137.1740147
161.6110852   285.372957   241.5389327   129.001058
229.5076583   -0.831460462   0.009452242   0.067365314   IL27RA
        19   14031748   14053216   +   2962
protein_coding interleukin 27 receptor subunit alpha [Source:HGNC
Symbol;Acc:HGNC:17290] -   131   148   118   156   250

280	2.165782923	2.367571492	2.478584901
2.926520616	5.18802804	4.39545659
ENSG00000111674 1715.611577	2399.22186	2874.84185
2683.158402	3496.38947	3640.336771	2329.891762
3273.294881	-0.491021616	0.009456098	0.067365653	ENO2
12	6913745 6923698 +	4281	protein_coding
enolase 2 [Source:HGNC Symbol;Acc:HGNC:3353]	-	1891	2711
2473	2590	3063	4220	21.63091967	30.00618141
35.9406341	33.61759028	43.97938269	45.83510613
ENSG00000188015 68.95107341	73.45459771	124.3866065
25.89921237	52.5086241	23.29125422	88.93075922
33.8996969	1.395883739	0.009464127	0.067395706	S100A3
1	153547329	153549372	-	955
protein_coding	S100 calcium binding protein A3 [Source:HGNC
Symbol;Acc:HGNC:10493]	-	76	83	107	25	46
27	3.897076259	4.11814052	6.970874319
1.454617073	2.960750561	1.314593288
ENSG00000247157 4.53625483	9.734946684	12.78740815	0
0	0	9.019536554	0	5.603838262
0.009537184	0.067888631	LINC01252	12	11548030
11590369	+	3793	lincRNA long intergenic non-
protein coding RNA 1252 [Source:HGNC Symbol;Acc:HGNC:27888]	-
5	11	11	0	0	0	0.064552913
0.137415677	0.180433299	0	0	0
ENSG00000107186 986.1818	798.2656281	919.5308951
734.5016629	644.9428829	548.6384328	901.3261077
642.6943262	0.488505509	0.009547634	0.067912158	MPDZ
9	13105704	13279590	-	11273
protein_coding	multiple PDZ domain crumbs cell polarity complex
component [Source:HGNC Symbol;Acc:HGNC:7208]	-	1087	902
791	709	565	636	4.721921041	3.791346447
4.365598817	3.494771391	3.080748892	2.623304032
ENSG00000106538 2.721752898	13.2749273	11.6249165	0
0	0	9.207198898	0	5.634641004
0.009548168	0.067912158	RARRES2 7	150338317
150341674	-	1921	protein_coding	retinoic acid receptor
responder 2 [Source:HGNC Symbol;Acc:HGNC:9868]	-	3	15
10	0	0	0	0.076475544	0.369990297
0.323876533	0	0	0
ENSG00000006576 1858.957229	1499.181789	1758.849866
1607.823104	1035.333088	791.9026436	1705.662962
1145.019612	0.575387916	0.009563562	0.067994314	PHTF2
7	77798792	77957503	+	8646
protein_coding	putative homeodomain transcription factor 2
[Source:HGNC Symbol;Acc:HGNC:13411]	-	2049	1694	1513
1552	907	918	11.60527433	9.283775197
10.88755955	9.974440164	6.448213612	4.936947036
ENSG00000196843 429.1297069	477.8973827	419.6594856
472.4016337	660.9237685	926.4743346	442.2288584
686.5999123	-0.635363713	0.009574005	0.068041211	ARID5A

2	96536743	96552638	+	3565
protein_coding	AT-rich interaction domain 5A [Source:HGNC
Symbol;Acc:HGNC:17361]	ARID	473	540	361	456	579
1074	6.497260657	7.177292844	6.300202582
7.107507917	9.983122258	14.00798814
ENSG00000106034 420.9644482	254.8786041	284.8104542
225.8411319	151.8184131	188.0553119	320.2178355
188.571619	0.763722086	0.009586943	0.068105796	CPED1
7	120988677	121297444	+	7705
protein_coding	cadherin like and PC-esterase domain containing 1
[Source:HGNC Symbol;Acc:HGNC:26159]	-	464	288	245
218	133	218	2.948994897	1.77111306
1.978337066	1.572156929	1.061026811	1.315572748
ENSG00000268516 26.31027801	15.92991276	25.5748163
42.47470829	74.19696883	77.63751407	22.60500236
64.7697304	-1.522517519	0.00960629	0.068211093
AC020915.3	19	58257270	58278808	-	2708
antisense	uncharacterized LOC105372482 [Source:NCBI
gene;Acc:105372482]	-	29	18	22	41	65
90	0.524418521	0.31495629	0.505453103
0.841292932	1.475407738	1.545342923
ENSG00000230160 0	0	0	5.179842475	13.69790194
7.763751407	0	8.880498607	-5.624717826
0.00961485	0.068211093	AC004854.1	7	44919669
44920329	+	349	processed_pseudogene
mitochondrial ribosomal protein S23 (MRPS23) pseudogene -	0
0	0	5	12	9	0	0	0
0.796079831	2.113504512	1.199079838
ENSG00000132837 1.814501932	4.424975766	5.812458249
21.75533839	11.41491828	44.85723035	4.017311982
26.00916234	-2.714378159	0.009615732	0.068211093	DMGDH
5	78997606	79236038	-	5254
protein_coding	dimethylglycine dehydrogenase [Source:HGNC
Symbol;Acc:HGNC:24475]	-	2	5	5	21	10
52	0.018640974	0.045092714	0.059208871
0.222096273	0.116992303	0.460197541
ENSG00000168066 6151.161549	6992.346705	6471.591015
6890.22646	9018.926934	9572.705485	6538.366423
8493.95296	-0.377543509	0.00961719	0.068211093	SF1
11	64764606	64778786	-	5972
protein_coding	splicing factor 1 [Source:HGNC Symbol;Acc:HGNC:12950]
-	6780	7901	5567	6651	7901	11097
55.59536404	62.68861913	57.99736583	61.88411613
81.32229436	86.40060929
ENSG00000258648 0	0	0	12.43162194	7.990442797
6.038473317	0	8.820179351	-5.615618015
0.009630247	0.068276328	UBE2CP1 14	30683045
30683598	-	554	processed_pseudogene	ubiquitin
conjugating enzyme E2 C pseudogene 1 [Source:HGNC
Symbol;Acc:HGNC:19670]	-	0	0	0	12	7

7	0	0	0	1.20360373	0.7766684
0.587515452
ENSG00000005194 1680.228789	1929.289434	1900.673848
2090.584423	2604.884352	2491.301563	1836.73069
2395.590112	-0.383340174	0.009638346	0.068289291
CIAPIN1 16	57428169	57447528	-	3509
protein_coding	cytokine induced apoptosis inhibitor 1 [Source:HGNC
Symbol;Acc:HGNC:28050]	-	1852	2180	1635	2018	2282
2888	25.8455809	29.43740793	28.98953408
31.95581052	39.97418629	38.26879952
ENSG00000171872 5.443505796	9.734946684	11.6249165	0
0	0	8.934456326	0	5.590805388
0.009639796	0.068289291	KLF17	1	44118850
44135140	+	3131	protein_coding	Kruppel like factor 17
[Source:HGNC Symbol;Acc:HGNC:18830]	zf-C2H2 6	11	10
0	0	0	0.093841916	0.16647003
0.198711856	0	0	0
ENSG00000133110 53.52780699	96.46447169	38.36222445
135.7118728	89.0363626	213.9344832	62.78483438
146.2275729	-1.217164823	0.009683333	0.068570244	POSTN
13	37562583	37598844	-	5464
protein_coding	periostin [Source:HGNC Symbol;Acc:HGNC:16953]	-
59	109	33	131	78	248	0.528773893
0.945240341	0.375759609	1.332209875	0.877467969
2.110435134
ENSG00000164828 5190.382776	5153.326777	5493.935537
4868.015958	3781.762427	3523.8805	5279.21503
4057.886295	0.379650286	0.009706482	0.068687162	SUN1
7	816615	896435	+	11811	protein_coding	Sad1
and UNC84 domain containing 1 [Source:HGNC Symbol;Acc:HGNC:18587]
-	5721	5823	4726	4699	3313	4085
23.71996052	23.36073815	24.89510111	22.10705131
17.24178108	16.0818649
ENSG00000139405 530.7418151	699.146171	634.7204408
542.8474913	1215.688797	1238.749669	621.5361423
999.0953191	-0.685004558	0.009710894	0.068687162	RITA1
12	113185526	113192368	+	2309
protein_coding	RBPJ interacting and tubulin associated 1 [Source:HGNC
Symbol;Acc:HGNC:25925]	-	585	790	546	524	1065
1436	12.40682386	16.21173885	14.71213009
12.61012518	28.35130376	28.91755199
ENSG00000261801 410.0774366	341.6081291	453.3717435
306.6466745	200.9025618	255.3411574	401.6857697
254.2967979	0.65771129	0.009711493	0.068687162	LOXL1-
AS1	15	73908071	73928248	-	4221
antisense	LOXL1 antisense RNA 1 [Source:HGNC
Symbol;Acc:HGNC:44169]	-	452	386	390	296	176
296	5.243868184	4.333096822	5.748520725
3.896623116	2.562976805	3.260673974
ENSG00000130332 915.4162247	1540.776562	1394.98998

578.0704202	788.7708533	1117.980203	1283.727589
828.2738254	0.631119719	0.009725281	0.068757192	LSM7
	19	2321517 2328620 -	1698	protein_coding	"LSM7
homolog, U6 small nuclear RNA and mRNA degradation associated
[Source:HGNC Symbol;Acc:HGNC:20470]"	-	1009	1741	1200
	558	691	1296	29.09927467	48.58335764
43.96938658	18.26032726	25.01426357	35.48937358
ENSG00000167130 511.6895448	500.9072567	539.3961256
412.315461	335.5985975	311.4126953	517.3309757
353.1089179	0.55127806	0.009735223	0.068772483	DOLPP1
	9	129081100	129090438	+	2249
protein_coding	dolichyldiphosphatase 1 [Source:HGNC
Symbol;Acc:HGNC:29565]	-	564	566	464	398	294
	361	12.28056455	11.9248635	12.83616739
9.833444258	8.035358083	7.463607022
ENSG00000248428 0	0	0	14.50355893	0
18.11541995	0	10.87299296	-5.921930052	0.009736848
	0.068772483	AC010395.1	5	110970951
111008899	-	697	lincRNA novel transcript	-
	0	0	0	14	0	21	0	0
	0	1.116110776	0	1.400933546
ENSG00000108953 16194.42974	15900.70792	17131.63944
14488.0194	12725.3509	12630.7609	16408.9257
13281.37707	0.305055827	0.009739107	0.068772483	YWHAE
	17	1344272 1400378 -	3847	protein_coding
tyrosine 3-monooxygenase/tryptophan 5-monooxygenase activation protein
epsilon [Source:HGNC Symbol;Acc:HGNC:12851]	-	17850	17967
	14737	13985	11148	14642	227.2190385	221.299189
	238.3382487	202.0004459	178.1238748	176.9739052
ENSG00000075711 2352.501755	1898.314603	2301.733467
1966.268203	1518.184131	1244.788142	2184.183275
1576.413492	0.470709741	0.009750194	0.0688233	DLG1
	3	197042560	197299300	-	11001
protein_coding	discs large MAGUK scaffold protein 1 [Source:HGNC
Symbol;Acc:HGNC:2900]	-	2593	2145	1980	1898	1330
	1443	11.54247747	9.238927788	11.19798476
9.586853417	7.431334949	6.099092002
ENSG00000153982 60.78581472	44.24975766	48.8246493
103.5968495	94.74382174	131.1211349	51.28674056
109.820602	-1.099552606	0.009781005	0.069013244	GDPD1
	17	59220467	59275967	+	2970
protein_coding	glycerophosphodiester phosphodiesterase domain
containing 1 [Source:HGNC Symbol;Acc:HGNC:20883]	-	67
	50	42	100	83	152	1.104706824
0.797700742	0.879831867	1.870921623	1.71778578
2.379677787
ENSG00000143776 2052.201685	1594.761266	1749.549933
1528.05353	1333.262455	1105.903256	1798.837628
1322.406414	0.444453336	0.00978866	0.069039713
CDC42BPA	1	226989865	227318474	-	14055

protein_coding	CDC42 binding protein kinase alpha
[Source:HGNC Symbol;Acc:HGNC:1737]	-	2262	1802	1505
1475	1168	1282	7.881165258	6.075057286
6.662120698	5.831405123	5.108099556	4.241194537
ENSG00000117419 1935.16631	2633.745576	2504.007014
2634.467883	3165.35684	3847.370142	2357.639633
3215.731621	-0.448174721	0.009797005	0.06906188	ERI3
1	44221070	44355260	-	2785
protein_coding	ERI1 exoribonuclease family member 3 [Source:HGNC
Symbol;Acc:HGNC:17276]	-	2133	2976	2154	2543	2773
4460	37.50544645	50.63305924	48.1201914
50.73798367	61.20290413	74.46302538
ENSG00000148700 700.3977457	415.0627268	442.9093186
331.5099184	318.4762201	349.3688133	519.4565971
333.1183173	0.641242405	0.009799611	0.06906188	ADD3
10	109996368	110135565	+	6541
protein_coding	adducin 3 [Source:HGNC Symbol;Acc:HGNC:245]	-
772	469	381	320	279	405	5.779653929
3.397466119	3.623995695	2.718428238	2.621847422
2.879001507
ENSG00000081870 613.301653	596.4867332	573.1083834
370.8767212	414.3615336	464.9624454	594.2989232
416.7335667	0.511488498	0.009804944	0.069071317	HSPB11
1	53916574	53945929	-	3236
protein_coding	heat shock protein family B (small) member 11
[Source:HGNC Symbol;Acc:HGNC:25019]	-	676	674	493
358	363	539	10.22979349	9.869106246
9.478623062	6.147330423	6.895177845	7.744818961
ENSG00000271856 0	0	0	10.35968495	0
22.42861518	0	10.92943338	-5.930389621	0.00980876
0.069071317	LINC01215	3	108125821
108138610	+	2592	lincRNA long intergenic non-protein
coding RNA 1215 [Source:HGNC Symbol;Acc:HGNC:49651]	-	0
0	0	10	0	26	0	0	0
0.214376436	0	0.466411628
ENSG00000136942 9943.470587	13753.70967	13901.07515
14779.12655	16717.14782	17638.38056	12532.7518
16378.21831	-0.386156793	0.00981993	0.069122459	RPL35
9	124857880	124861981	-	1535
protein_coding	ribosomal protein L35 [Source:HGNC
Symbol;Acc:HGNC:10344]	-	10960	15541	11958	14266	14645
20447	349.6478466	479.7301585	484.682139
516.4233654	586.4464429	619.3729513
ENSG00000143889 962.5932749	981.4596248	1063.67986
725.1779464	823.0156081	673.7210944	1002.577586
740.6382163	0.43735242	0.009846248	0.069238432
HNRNPLL 2	38561978	38603586	-	5923
protein_coding	heterogeneous nuclear ribonucleoprotein L like
[Source:HGNC Symbol;Acc:HGNC:25127]	-	1061	1109	915
700	721	781	8.772074972	8.871892162

9.61139018 6.567020183 7.482399499 6.131127101
ENSG00000118113 6.350756762 9.734946684 10.46242485 0
0 0 8.849376098 0 5.577672916
0.009847086 0.069238432 MMP8 11 102711795
102727050 - 3438 protein_coding matrix
metallopeptidase 8 [Source:HGNC Symbol;Acc:HGNC:7175] - 7
11 9 0 0 0 0.099705898
0.151604905 0.162870895 0 0 0
ENSG00000100106 764.8125643 904.4650465 875.3562124
557.3510503 739.6867047 413.2041027 848.2112744
570.0806192 0.575005543 0.009848148 0.069238432 TRIOBP
22 37696988 37776556 + 12254
protein_coding TRIO and F-actin binding protein [Source:HGNC
Symbol;Acc:HGNC:17009] - 843 1022 753 538 648
479 3.368824464 3.951840981 3.823172968
2.439587746 3.250457486 1.817559578
ENSG00000285922 25.40302705 34.51481097 27.8997996
3.107905485 0 9.489029498 29.27254587 4.198978328
2.762994839 0.00985734 0.069259763 AC104453.1
1 98052077 98054592 - 1784
lincRNA novel transcript - 28 39 24 3
0 11 0.76858493 1.035848396 0.836995722
0.093441209 0 0.286700766
ENSG00000249992 17263.17138 21972.65966 19819.32014
29628.69895 25515.76684 21562.52558 19685.05039
25568.99712 -0.377261049 0.009859013 0.069259763
TMEM158 3 45224466 45226278 - 1813
protein_coding transmembrane protein 158 (gene/pseudogene)
[Source:HGNC Symbol;Acc:HGNC:30293] - 19028 24828 17049
28600 22353 24996 513.9537299 648.8889423
585.0701663 876.5572228 757.8536982 641.0674555
ENSG00000259488 21.77402318 12.38993214 9.299933199
2.07193699 0 0 14.48796284 0.690645663
4.414955628 0.00986443 0.069270307 AC023355.1 15
48312353 48331856 - 4710 antisense
"novel transcript, antisense to DUT" - 24 14
8 2 0 0 0.249527846 0.140842449
0.105675893 0.023595063 0 0
ENSG00000221968 431.8514598 563.7419125 631.2329659
362.5889732 417.7860091 279.4950507 542.2754461
353.290011 0.619092734 0.009873455 0.069306169 FADS3
11 61873519 61892051 - 6283
protein_coding fatty acid desaturase 3 [Source:HGNC
Symbol;Acc:HGNC:3576] - 476 637 543 350 366
324 3.709954442 4.803953704 5.3769948
3.095373272 3.580645993 2.397777986
ENSG00000112759 2388.791793 2077.96862 2580.731463
1765.290315 1943.960583 1717.514339 2349.163959
1808.921746 0.376933443 0.009883649 0.069350205
SLC29A1 6 44219505 44234151 + 4408

protein_coding solute carrier family 29 member 1 (Augustine blood group) [Source:HGNC Symbol;Acc:HGNC:11003] - 2633 2348
2220 1704 1703 1991 29.25081404 25.23962788
31.33417288 21.48028544 23.74763812 21.00200185
ENSG00000226053 21.77402318 4.424975766 2.3249833 0
0 0 9.507994083 0 5.685780125
0.009891798 0.069371317 LINC01776 1 98210747
98272658 + 2229 lincRNA long intergenic non-protein coding RNA 1776 [Source:HGNC Symbol;Acc:HGNC:52566] -
24 5 2 0 0 0 0.527266109
0.106288524 0.055824748 0 0 0
ENSG00000104643 927.2104872 692.9512049 877.6811957
1219.334919 1086.70022 1108.491173 832.6142959
1138.175437 -0.451461998 0.009894501 0.069371317 MTMR9
8 11284416 11328146 + 8804
protein_coding myotubularin related protein 9 [Source:HGNC Symbol;Acc:HGNC:14596] - 1022 783 755 1177 952
1285 5.684595219 4.21413233 5.335483293
7.428625634 6.646672413 6.786629052
ENSG00000160255 74.39457921 102.6594378 41.8496994
23.82727538 20.54685291 33.64292277 72.96790546
26.00568369 1.486467652 0.009902727 0.069401478 ITGB2
21 44885953 44931989 - 6797
protein_coding integrin subunit beta 2 [Source:HGNC Symbol;Acc:HGNC:6155] - 82 116 36 23 18
39 0.590779295 0.808662232 0.329527814
0.188028036 0.162780581 0.266795411
ENSG00000241255 3.629003864 10.61994184 12.78740815 0
0 0 9.01211795 0 5.602721983
0.009907292 0.069405974 AL136126.1 11 33237008
33237409 + 402 processed_pseudogene
ribosomal protein S24 (RPS24) pseudogene - 4 12
11 0 0 0 0.487262088 1.414430569
1.702446523 0 0 0
ENSG00000089472 42.6407954 68.14462679 213.8984636
48.69051926 34.24475485 28.46708849 108.2279619
37.13412087 1.54023899 0.009922116 0.069482309 HEPH
X 66162549 66268867 + 7113
protein_coding hephaestin [Source:HGNC Symbol;Acc:HGNC:4866] -
47 77 184 47 30 33 0.323574086
0.51293739 1.609429142 0.367161464 0.259248234
0.215720863
ENSG00000165118 253.1230195 246.9136477 369.6723447
216.5174154 163.2333314 104.3793245 289.903004
161.3766904 0.845921133 0.009977868 0.069806311
C9orf64 9 83938311 83956986 - 2729
protein_coding chromosome 9 open reading frame 64 [Source:HGNC Symbol;Acc:HGNC:28144] - 279 279 318 209 143
121 5.006443867 4.844256252 7.249873535
4.255541147 3.220919435 2.061640099

ENSG00000115526 457.2544869 615.9566266 409.1970608
686.8471121 731.6962619 732.3805494 494.1360581
716.9746411 -0.535529089 0.009981197 0.069806311 CHST10
2 100391860 100417656 - 4487
protein_coding carbohydrate sulfotransferase 10 [Source:HGNC
Symbol;Acc:HGNC:19650] - 504 696 352 663 641
849 5.50051243 7.349869209 4.880827293
8.210498054 8.781108031 8.797973284
ENSG00000104783 1322.771908 1532.811605 1411.264863
1706.240111 1838.943335 2012.536893 1422.282792
1852.573446 -0.381504199 0.009984983 0.069806311 KCNN4
19 43766533 43781261 - 3408
protein_coding potassium calcium-activated channel subfamily N member
4 [Source:HGNC Symbol;Acc:HGNC:6293] - 1458 1732 1214
1647 1611 2333 20.95012514 24.08101246
22.16286736 26.85381896 29.05650096 31.83069864
ENSG00000157837 784.7720856 764.6358123 795.1442885
1040.112369 961.1361193 1132.645066 781.5173955
1044.631185 -0.419360911 0.009986822 0.069806311 SPPL3
12 120762510 120904371 - 6672
protein_coding signal peptide peptidase like 3 [Source:HGNC
Symbol;Acc:HGNC:30424] - 865 864 684 1004 842
1313 6.348757712 6.135982971 6.378328911
8.361606369 7.757171873 9.150392149
ENSG00000172270 14302.81148 16881.28255 21739.75634
12804.5706 14664.74552 12639.38729 17641.28346
13369.5678 0.399948663 0.009988115 0.069806311 BSG
19 571277 583493 + 3797 protein_coding
basigin (Ok blood group) [Source:HGNC Symbol;Acc:HGNC:1116] -
15765 19075 18701 12360 12847 14652 203.3209178
238.0402459 306.4298578 180.8797367 207.973733
179.4268079
ENSG00000127220 688.6034832 946.0598187 838.1564796
1014.213157 1195.141944 1201.65619 824.2732605
1137.003764 -0.464148867 0.010003588 0.069886837 ABHD8
19 17292131 17310236 - 2210
protein_coding abhydrolase domain containing 8 [Source:HGNC
Symbol;Acc:HGNC:23759] - 759 1069 721 979 1047
1393 16.81814861 22.91985535 20.2978406
24.61514859 29.12069718 29.30824821
ENSG00000160789 23889.73244 26523.30474 32536.97879
22192.5171 23220.22677 18601.94837 27650.00532
21338.23075 0.373833107 0.010009664 0.069901677 LMNA
1 156082573 156140089 + 6985
protein_coding lamin A/C [Source:HGNC Symbol;Acc:HGNC:6636] -
26332 29970 27989 21422 20342 21564 184.6061306
203.3043978 249.30318 170.4141482 179.0088901
143.5469373
ENSG00000099991 2159.257299 1792.115185 2190.134268
1729.031418 1498.77877 1438.881928 2047.168917

1555.564039	0.396074041	0.010016977	0.069913533	CABIN1
	22	24011192	24178628	+	9388
protein_coding	calcineurin binding protein 1 [Source:HGNC
Symbol;Acc:HGNC:24187]	-	2380	2025	1884	1669	1313
	1668	12.4145951	10.22064697	12.48575084
9.8785977	8.596843195	8.261406338
ENSG00000134186	2322.562473	2011.593983	1906.486306
1742.499008	1580.966182	1437.156649	2080.214254
1586.873947	0.391122142	0.010019267	0.069913533
PRPF38B	1	108692323	108701803	+	5817
protein_coding	pre-mRNA processing factor 38B [Source:HGNC
Symbol;Acc:HGNC:25512]	-	2560	2273	1640	1682	1385
	1666	21.55110709	18.51513201	17.54089023
16.06715455	14.63518005	13.31701692
ENSG00000171606	285.7840543	296.4733763	227.8483634
365.6968787	369.8433523	538.2867643	270.0352647
424.6089984	-0.65289577	0.010040008	0.069982591	ZNF274
	19	58183029	58213562	+	8333
protein_coding	zinc finger protein 274 [Source:HGNC
Symbol;Acc:HGNC:13068]	zf-C2H2	315	335	196	353	324
	624	1.851133991	1.904889843	1.463394897
2.353885682	2.38996196	3.48188342
ENSG00000014164	571.5681086	704.4561419	595.1957247
708.6024505	916.617938	1053.282274	623.7399917
892.8342209	-0.51759722	0.010040765	0.069982591	ZC3H3
	8	143437655	143541453	-	3731
protein_coding	zinc finger CCCH-type containing 3 [Source:HGNC
Symbol;Acc:HGNC:28972]	-	630	796	512	684	803
	1221	8.268828489	10.10914113	8.537909726
10.18692002	13.22932411	15.21673344
ENSG00000105514	15.42326642	27.43484975	26.73730795
85.98538508	37.66923033	78.50015312	23.19847471
67.38492284	-1.54317295	0.010047166	0.069982591	RAB3D
	19	11322046	11346270	-	4417
protein_coding	"RAB3D, member RAS oncogene family [Source:HGNC
Symbol;Acc:HGNC:9779]"	-	17	31	23	83	33
	91	0.188473461	0.332552897	0.323971856
1.044149625	0.459233859	0.957954785
ENSG00000189343	1765.51038	1882.384691	1761.17485
1947.62077	2618.582254	2577.565467	1803.023307
2381.256164	-0.401244922	0.01004824	0.069982591
RPS2P46	17	19445970	19446852	-	883
processed_pseudogene	ribosomal protein S2 pseudogene 46
[Source:HGNC Symbol;Acc:HGNC:35839]	-	1946	2127	1515
	1880	2294	2988	107.9222065	114.1387803
106.7477613	118.3066588	159.6908638	157.3442612
ENSG00000022567	348.3843709	314.1732794	241.7982632
427.8549884	372.126336	659.9188696	301.4519712
486.633398	-0.691022909	0.010049097	0.069982591
SLC45A4	8	141207166	141308305	-	8608

protein_coding solute carrier family 45 member 4 [Source:HGNC
Symbol;Acc:HGNC:29196] -   384   355   208   413   326
        765   2.184528171   1.954125876   1.503377074
2.665998109   2.327891325   4.132284317
ENSG00000141499 921.7669814   915.9699835   839.3189712
1019.392999   1225.962223   1405.239005   892.3519787
1216.864742   -0.447576259   0.010052901   0.069982591   WRAP53
        17   7686071 7703502 +   5087   protein_coding WD
repeat containing antisense to TP53 [Source:HGNC
Symbol;Acc:HGNC:25522] -   1016   1035   722   984   1074
        1629   9.780490912   9.640621957   8.830439239
10.74843921   12.97746613   14.88985144
ENSG00000280054 0.907250966   0   0   19.6834014
7.990442797   6.038473317   0.302416989
11.23743917   -5.003049462   0.010060295   0.070006516
AC004241.5   12   47728151   47730598   -   2448
        TEC   TEC   -   1   0   0   19   7
        7   0.02000402   0   0   0.431274948
0.175765643   0.132958971
ENSG00000166199 628.7249194   685.8712437   731.2072478
951.0190783   836.7135101   998.0733754   681.9344703
928.6019879   -0.446576974   0.0100678   0.070031186   ALKBH3
        11   43880811   43920266   +   2372
protein_coding "alkB homolog 3, alpha-ketoglutaratedependent
dioxygenase [Source:HGNC Symbol;Acc:HGNC:30141]"   -   693
        775   629   918   733   1157   14.30695574
15.48151503   16.49843718   21.50502938   18.99488419
22.68034687
ENSG00000236939 0   0   0   7.251779464   4.565967313
        14.66486377   0   8.827536849   -5.621428524
0.010104212   0.070256842   BAALC-AS2   8   103132963
        103141475   -   513   antisense   BAALC
antisense RNA 2 [Source:HGNC Symbol;Acc:HGNC:28595]   -   0
        0   0   7   4   17   0   0   0
        0.758215605   0.47928075   1.540857847
ENSG00000105613 87.09609273   78.76456863   131.3615564
199.9419195   182.6386925   166.4893357   99.0740726
183.0233159   -0.889751456   0.010118299   0.070327147   MAST1
        19   12833951   12874951   +   6533
protein_coding microtubule associated serine/threonine kinase 1
[Source:HGNC Symbol;Acc:HGNC:19034]   -   96   89   113
        193   160   193   0.719593544   0.645511211
1.076149559   1.641559748   1.505409612   1.373648667
ENSG00000242641 12.70151352   15.0449176   0   53.87036173
        26.25431205   60.38473317   9.248810376   46.83646898
        -2.320657641   0.010154152   0.070548623   LINC00971
        3   84638406   84881679   -   8031
lincRNA long intergenic non-protein coding RNA 971 [Source:HGNC
Symbol;Acc:HGNC:48737] -   14   17   0   52   23
        70   0.085366425   0.100301109   0   0.359787244

0.176037653	0.405283975
ENSG00000042832 12.70151352	7.964956378	5.812458249	0
0	0	8.826309384	0	5.57677445
0.010174198	0.070634774	TG	8	132866958
133134903	+	11132	protein_coding	thyroglobulin
[Source:HGNC Symbol;Acc:HGNC:11764]	-	14	9	5
0	0	0	0.061586216	0.038308553
0.02794497	0	0	0
ENSG00000108439 840.1143945	644.2764715	633.5579492
507.6245625	513.6713227	468.4130016	705.9829384
496.5696289	0.508682828	0.010177783	0.070634774	PNPO
17	47941506	47949308	+	5226
protein_coding	pyridoxamine 5'-phosphate oxidase [Source:HGNC
Symbol;Acc:HGNC:30260]	-	926	728	545	490	450
543	8.677013334	6.600675988	6.488345138
5.210011936	5.292860754	4.831271482
ENSG00000133739 744.8530431	532.7670822	498.7089178
494.1569721	326.4666629	319.1764468	592.109681
379.9333606	0.641178866	0.010178531	0.070634774	LRRCC1
8	85107147	85146076	+	5065
protein_coding	leucine rich repeat and coiled-coil centrosomal
protein 1 [Source:HGNC Symbol;Acc:HGNC:29373]	-	821	602
429	477	286	370	7.937658145	5.631751487
5.269685406	5.233002871	3.470834803	3.396669507
ENSG00000104901 0.907250966	0	0	18.64743291
3.424475485	12.07694663	0.302416989
11.38295168	-5.024796587	0.010182598	0.070635286	DKKL1
19	49361783	49375116	+	1461
protein_coding	dickkopf like acrosomal protein 1 [Source:HGNC
Symbol;Acc:HGNC:16528]	-	1	0	0	18	3
14	0.033518029	0	0	0.684595961
0.126217159	0.445562711
ENSG00000151131 553.4230892	582.3268108	526.6087174
1030.788652	636.9524401	755.6718037	554.1195391
807.8042987	-0.543645754	0.01019731	0.070709609
C12orf45	12	104986310	105074197	+	25887
protein_coding	chromosome 12 open reading frame 45
[Source:HGNC Symbol;Acc:HGNC:28628]	-	610	658	453
995	558	876	1.153922907	1.204399623
1.088737859	2.135764683	1.324951055	1.573451644
ENSG00000122884 1199.385777	1100.93397	2266.858717
947.9111728	1150.623763	873.8533529	1522.392822
990.7960962	0.619120681	0.010208011	0.070756075	P4HA1
10	73007217	73096974	-	3093
protein_coding	prolyl 4-hydroxylase subunit alpha 1 [Source:HGNC
Symbol;Acc:HGNC:8546]	-	1322	1244	1950	915	1008
1013	20.93052967	19.05754269	39.22487226
16.43816054	20.03216886	15.22861978
ENSG00000138757 4010.956521	3810.789129	3883.884602
5220.245246	4828.510433	4541.794573	3901.876751

4863.516751	-0.317728152	0.010259901	0.0710657	G3BP2
	4	75642782	75724525	-	7183
protein_coding	G3BP stress granule assembly factor 2 [Source:HGNC
Symbol;Acc:HGNC:30291]	-	4421	4306	3341	5039	4230
	5265	30.14000583	28.40498733	28.93859593
38.98078094	36.19777374	34.08187876
ENSG00000173548 481.7502629	508.872213	1068.329826
444.4304843	343.5890403	447.7096645	686.3174341
411.9097297	0.734137129	0.010260716	0.0710657	SNX33
	15	75647906	75662301	+	8008
protein_coding	sorting nexin 33 [Source:HGNC Symbol;Acc:HGNC:28468]
	-	531	575	919	429	301	519
3.247125993	3.402281323	7.140001344	2.976769939
2.310413914	3.013521619
ENSG00000086589 1117.73319	1250.498151	1470.551937
1650.297812	1834.377368	1614.860293	1279.594426
1699.845158	-0.410235671	0.010271269	0.071110953	RBM22
	5	150690794	150701107	-	3615
protein_coding	RNA binding motif protein 22 [Source:HGNC
Symbol;Acc:HGNC:25503]	-	1232	1413	1265	1593	1607
	1872	16.68902979	18.52082384	21.77153603
24.48609431	27.32467061	24.0784519
ENSG00000273416 0	7.079961225	6.974949899	39.36680281
	37.66923033	15.52750281	4.684970375	30.85451198
	-2.729636657	0.010289489	0.071209221	AL732292.2
	1	235104180	235104609	-	430
lincRNA novel transcript	-	0	8	6	38
	33	18	0	0.881552075	0.868139749
4.910516613	4.717292919	1.946413319
ENSG00000144959 1385.372225	1047.834261	1156.679192
1046.32818	778.4974268	714.2651295	1196.628559
846.3635787	0.500097587	0.010304235	0.071283387	NCEH1
	3	172630249	172711218	-	4808
protein_coding	neutral cholesterol ester hydrolase 1 [Source:HGNC
Symbol;Acc:HGNC:29260]	-	1527	1184	995	1010	682
	828	15.55260928	11.66846383	12.87554047
11.67263642	8.71901201	8.007499052
ENSG00000196653 68.04382245	52.21471403	69.74949899
126.3881564	90.17785443	182.8794776	63.33601183
133.1484961	-1.076551866	0.010319318	0.071359819	ZNF502
	3	44712643	44723831	+	3273
protein_coding	zinc finger protein 502 [Source:HGNC
Symbol;Acc:HGNC:23718]	zf-C2H2 75	59	60	122	79
	212	1.122131985	0.85414666	1.140544125
2.071218273	1.483639701	3.011763556
ENSG00000101972 4823.853386	3701.04973	4820.852872
3803.040345	3362.834926	3001.983878	4448.58533
3389.286383	0.392363542	0.010337013	0.071454249	STAG2
	X	123960212	124422664	+	8187
protein_coding	stromal antigen 2 [Source:HGNC Symbol;Acc:HGNC:11355]

-	5317	4182	4147	3671	2946	3480
31.80318046	24.20391833	31.51491148	24.91561651
22.11848172	19.7644836
ENSG00000183644 0.907250966	25.66485944	3.48747495	0
0	0	10.01986179	0	5.761446137
0.01034172	0.071458859	C11orf88	11	111514785
111537031	+	3280	protein_coding	chromosome 11
open reading frame 88 [Source:HGNC Symbol;Acc:HGNC:25061]	-
1	29	3	0	0	0	0.014929829
0.41893881	0.056905502	0	0	0
ENSG00000185721 614.208904	770.8307784	632.3954575
828.7747959	970.2680539	987.7217068	672.47838
928.9215189	-0.46565979	0.010365698	0.071596576	DRG1
22	31399523	31530634	+	2279
protein_coding	developmentally regulated GTP binding protein 1
[Source:HGNC Symbol;Acc:HGNC:3029]	-	677	871	544
800	850	1145	14.54698621	18.10924193
14.8511957	19.50552776	22.92566599	23.36104035
ENSG00000170608 29.93928188	39.82478189	40.68720775
56.97826722	136.9790194	81.08807026	36.8170905
91.68178562	-1.315813267	0.010376964	0.071646414	FOXA3
19	45863989	45873797	+	2192
protein_coding	forkhead box A3 [Source:HGNC Symbol;Acc:HGNC:5023]
Fork	33	45	35	55	120	94
0.737228428	0.972743651	0.993423298	1.39422923
3.365023151	1.993968632
ENSG00000166507 26.31027801	3.539980612	5.812458249	0
0	0.862639045	11.88757229	0.287546348
5.045189117	0.010386301	0.0716829	NDST2	10
73801911	73811798	-	4654	protein_coding	N-
deacetylase and N-sulfotransferase 2 [Source:HGNC
Symbol;Acc:HGNC:7681]	-	29	4	5	0	0
1	0.305140815	0.040724902	0.066842159	0
0	0.009990901
ENSG00000132612 1639.402496	2084.163586	1961.123413
2207.648863	2495.301136	2785.461477	1894.896498
2496.137159	-0.397899403	0.010428121	0.071917571	VPS4A
16	69311356	69326939	+	5670
protein_coding	vacuolar protein sorting 4 homolog A [Source:HGNC
Symbol;Acc:HGNC:13488]	-	1807	2355	1687	2131	2186
3229	15.60643749	19.68041687	18.51138316
20.88393989	23.69815081	26.47987949
ENSG00000175471 495.3590274	370.8129692	408.0345691
267.2798717	317.3347282	254.4785184	424.7355219
279.6977061	0.604159873	0.010430945	0.071917571	MCTP1
5	94703741	95284575	-	12089
protein_coding	multiple C2 and transmembrane domain containing 1
[Source:HGNC Symbol;Acc:HGNC:26183]	-	546	419	351
258	278	295	2.211724092	1.642290899
1.806440184	1.185881713	1.413519417	1.134651931

ENSG00000188710 29.93928188 24.77986429 10.46242485 0
5.707459141 0 21.72719034 1.90248638
3.577938035 0.0104325 0.071917571 QRFP 9
130892702 130896812 - 1658 protein_coding
pyroglutamylated RFamide peptide [Source:HGNC Symbol;Acc:HGNC:29982]
- 33 28 9 0 5 0
0.974671118 0.800202578 0.337726259 0 0.185367178
0
ENSG00000218175 119.7571275 123.8993214 131.3615564
185.4383606 228.2983656 222.5608737 125.0060018
212.0992 -0.763530065 0.010441329 0.071950398
AC016739.1 2 176200908 176201252 - 345
processed_pseudogene "ribosomal protein, large, P1 (RPLP1)
pseudogene" - 132 140 113 179 200 258
18.73628654 19.22805614 20.37821758 28.83008877
35.63348187 34.77215677
ENSG00000168255 0.907250966 1.769990306 3.48747495
27.97114936 25.11282022 5.175834272 2.054905407
19.41993462 -3.266600794 0.010476847 0.072167036
POLR2J3 7 102537918 102572653 - 3190
protein_coding RNA polymerase II subunit J3 [Source:HGNC
Symbol;Acc:HGNC:33853] - 1 2 3 27 22
6 0.015351047 0.029707476 0.058510986
0.470310987 0.42391556 0.087456398
ENSG00000065882 1431.642024 1294.747909 1450.789579
1075.335298 1160.897189 874.7159919 1392.393171
1036.982826 0.425880996 0.010483954 0.07218788 TBC1D1
4 37891087 38139175 + 9032
protein_coding TBC1 domain family member 1 [Source:HGNC
Symbol;Acc:HGNC:11578] - 1578 1463 1248 1038 1017
1014 8.55562525 7.675149401 8.596813457
6.38594933 6.921247573 5.220174781
ENSG00000176946 1217.530796 1284.127967 1354.302772
1232.802509 2125.457784 2154.009696 1285.320512
1837.42333 -0.515850884 0.01049078 0.072206775 THAP4
2 241584405 241637449 - 3147
protein_coding THAP domain containing 4 [Source:HGNC
Symbol;Acc:HGNC:23187] THAP 1342 1451 1165 1190 1862
2497 20.88259455 21.8472667 23.03223214
21.0117518 36.36891073 36.89375144
ENSG00000228474 397.3759231 577.0168398 702.1449565
236.2008168 401.8051235 393.3634046 558.8459065
343.7897817 0.699688116 0.010495986 0.072214503 OST4
2 27070472 27071773 - 719
protein_coding "oligosaccharyltransferase complex subunit 4, non-
catalytic [Source:HGNC Symbol;Acc:HGNC:32483]" - 438 652
604 228 352 456 29.83141842 42.96800067
52.26547417 17.62049077 30.09269846 29.48947021
ENSG00000155034 1025.193592 1024.824387 960.2181028
1224.514761 1213.405813 1662.30544 1003.412027

1366.742005	-0.446375298	0.010513547	0.07228231	FBXL18
	7	5431335 5513798 -	5088	protein_coding	F-box
and leucine rich repeat protein 18 [Source:HGNC
Symbol;Acc:HGNC:21874]	-	1130	1158	826	1182	1063
	1927	10.87577025	10.78419911	10.10042833
12.90869732	12.84202533	17.61025441
ENSG00000171791 462.6979926	350.4580806	363.8598864
241.3806593	184.9216762	303.6489439	392.3386532
243.3170931	0.687091022	0.010517421	0.07228231	BCL2
	18	63123346	63320128	-	11765
protein_coding	"BCL2, apoptosis regulator [Source:HGNC
Symbol;Acc:HGNC:990]"	-	510	396	313	233	162
	352	2.122789487	1.594886182	1.655233444
1.10046449	0.846389843	1.391174954
ENSG00000161904 1721.962333	2067.348678	1906.486306
2181.74965	2474.754283	2790.637311	1898.599106
2482.380415	-0.387032933	0.0105181	0.07228231	LEMD2
	6	33771202	33789136	-	6788
protein_coding	LEM domain containing 2 [Source:HGNC
Symbol;Acc:HGNC:21244]	-	1898	2336	1640	2106	2168
	3235	13.69250972	16.30637575	15.03172635
17.23965525	19.63201171	22.15967923
ENSG00000132716 781.1430817	761.9808268	602.1706746
874.3574097	1168.887632	963.5678136	715.0981944
1002.270952	-0.485541739	0.010526718	0.072313442	DCAF8
	1	160215715	160262531	-	7587
protein_coding	DDB1 and CUL4 associated factor 8 [Source:HGNC
Symbol;Acc:HGNC:24891]	-	861	861	518	844	1024
	1117	5.557273243	5.377241085	4.247824078
6.181365248	8.296162168	6.845640797
ENSG00000215784 176.0066874	137.1742487	174.3737475
127.4241249	45.65967313	23.29125422	162.5182279
65.45835074	1.314666811	0.010553361	0.072468325	FAM72D
	1	145096000	145112696	+	2397
protein_coding	family with sequence similarity 72 member D
[Source:HGNC Symbol;Acc:HGNC:33593]	-	194	155	150
	123	40	27	3.963349573	3.064009482
3.893409387	2.851340751	1.025744785	0.523753271
ENSG00000075618 8395.700439	9933.185599	12574.67218
12062.81715	14343.98631	14304.28065	10301.18607
13570.36137	-0.397808399	0.010562976	0.072506199	FSCN1
	7	5592823 5606655 +	3692	protein_coding	fascin
actin-bundling protein 1 [Source:HGNC Symbol;Acc:HGNC:11148]	-
	9254	11224	10817	11644	12566	16582	122.7429301
	144.049716	182.2854413	175.2477893	209.2101367
	208.8364185
ENSG00000095002 2077.604712	1832.824962	1675.150467
1638.902159	1280.753831	1242.200225	1861.860047
1387.285405	0.424983403	0.010600329	0.072734378	MSH2
	2	47402969	47663146	+	13777

protein_coding	mutS homolog 2 [Source:HGNC Symbol;Acc:HGNC:7325]
	-	2290	2071	1441	1582	1122	1440
8.139720782	7.122818554	6.507529852	6.38063445
5.005939064	4.8600289
ENSG00000263307 0	0.884995153	0	4.14387398
13.69790194	15.52750281	0.294998384
11.12309291	-4.99059528	0.010608811	0.072764351
AC007216.4	16	11851649	11895611	+	562
	antisense	"novel transcript, antisense to GSPT1"	-
	0	1	0	4	12	18	0
0.084312142	0	0.395490194	1.31247878	1.489248625
ENSG00000072274 9335.61244	9500.422969	8208.35354
8749.789908	5544.225809	5671.851723	9014.796316
6655.289147	0.437881358	0.010614702	0.072776535	TFRC
	3	196027183	196082189	-	7887
protein_coding	transferrin receptor [Source:HGNC
Symbol;Acc:HGNC:11763]	-	10290	10735	7061	8446	4857
	6575	63.88990134	64.49360432	55.70077236
59.50470136	37.85328921	38.76278162
ENSG00000233966 191.4299538	259.3035799	277.8355043
74.58973163	184.9216762	125.9453006	242.856346
128.4855695	0.91967388	0.010624528	0.072815682
UBE2SP1 17	15704232	15704900	-	669
processed_pseudogene	ubiquitin conjugating enzyme E2 S pseudogene 1
[Source:HGNC Symbol;Acc:HGNC:30864]	-	211	293	239
	72	162	146	15.44489717	20.75238154
22.2268864	5.980237367	14.88456877	10.14746954
ENSG00000155111 873.6826802	609.7616605	952.0806613
650.5882148	495.4074534	508.0943977	811.8416673
551.3633553	0.557409067	0.010644993	0.072927685	CDK19
	6	110609978	110815958	-	7492
protein_coding	cyclin dependent kinase 19 [Source:HGNC
Symbol;Acc:HGNC:19338]	-	963	689	819	628	434
	589	6.294441505	4.357605336	6.801316412
4.657725807	3.560732274	3.65551478
ENSG00000161013 3097.354798	3468.296005	4036.171008
2773.287661	2717.892043	2772.521892	3533.940604
2754.567198	0.359070247	0.010669266	0.073065681	MGAT4B
	5	179797597	179806952	-	5495
protein_coding	"alpha-1,3-mannosyl-glycoprotein 4-beta-N-
acetylglucosaminyltransferase B [Source:HGNC Symbol;Acc:HGNC:7048]"
	-	3414	3919	3472	2677	2381	3214
30.42457383	33.79356485	39.3114322	27.07027814
26.63416334	27.19626059
ENSG00000118156 5.443505796	15.0449176	12.78740815	0
	1.141491828	0	11.09194385	0.380497276
4.941973582	0.010689602	0.073176614	ZNF541	19
47520685	47555856	-	4655	protein_coding	zinc
finger protein 541 [Source:HGNC Symbol;Acc:HGNC:25294]	zf-C2H2 6
	17	11	0	1	0	0.06311902

0.173043654	0.14702116	0	0.013204674	0
ENSG00000248487 147.8819075	146.0242003	181.3486974
224.8051634	280.8069897	272.5939383	158.4182684
259.4020305	-0.713488597	0.010696587	0.073196102
ABHD14A 3	51971426	51981199	+	2378
protein_coding	abhydrolase domain containing 14A [Source:HGNC
Symbol;Acc:HGNC:24538]	-	163	165	156	217	246
	316	3.356637465	3.287748095	4.081498063
5.070606715	6.358733402	6.178830056
ENSG00000117748 2367.01777	2492.146351	2370.320474
2833.373834	3054.632132	3202.978775	2409.828199
3030.328247	-0.33058203	0.01070348	0.073214948	RPA2
	1	27891524	27914746	-	2019
protein_coding	replication protein A2 [Source:HGNC
Symbol;Acc:HGNC:10290]	-	2609	2816	2039	2735	2676
	3713	63.2799961	66.08802483	62.83299386
75.27193065	81.46989383	85.51053988
ENSG00000170925 0	0	1.16249165	0	21.68834474
	18.978059	0.387497217	13.55546791	-5.274941164
	0.010722291	0.07331527	TEX13B	X	107980864
	107982370	-	1248	protein_coding	testis
expressed 13B [Source:HGNC Symbol;Acc:HGNC:11736]	-	0
	0	1	0	19	22	0	0
0.049853111	0	0.935807186	0.819670139
ENSG00000181929 1265.615098	1364.662526	1304.315631
1695.880426	1518.184131	1957.327994	1311.531085
1723.797517	-0.394875342	0.010745597	0.073446237	PRKAG1
	12	49002274	49018825	-	4184
protein_coding	protein kinase AMP-activated non-catalytic subunit
gamma 1 [Source:HGNC Symbol;Acc:HGNC:9385]	-	1395	1542
	1122	1637	1330	2269	16.3271813	17.46301145
	16.68430144	21.74047593	19.53922461	25.21586311
ENSG00000231948 3.629003864	12.38993214	10.46242485	0
	0	0	8.827120286	0	5.574200442
0.010788231	0.073671844	HS1BP3-IT1	2	20590775
	20592548	-	600	sense_intronic	HS1BP3
intronic transcript 1 [Source:HGNC Symbol;Acc:HGNC:41474]	-
	4	14	9	0	0	0	0.326465599
	1.105613228	0.93325023	0	0	0
ENSG00000166526 520.7620545	579.6718253	505.6838677
654.7320888	785.3463778	794.4905607	535.3725825
744.8563424	-0.47600984	0.010788292	0.073671844	ZNF3
	7	100064033	100082548	-	5424
protein_coding	zinc finger protein 3 [Source:HGNC
Symbol;Acc:HGNC:13089]	zf-C2H2 574	655	435	632	688
	921	5.182280248	5.722002721	4.989722838
6.474547793	7.796795037	7.895342373
ENSG00000168575 1697.466557	1736.36049	1483.339345
2038.785998	1966.79042	2411.938771	1639.055464
2139.17173	-0.384155228	0.010791099	0.073671844

SLC20A2 8	42416475	42541926	-	6641
protein_coding	solute carrier family 20 member 2 [Source:HGNC
Symbol;Acc:HGNC:10947]	-	1871	1962	1276	1968	1723
	2796	13.79650208	13.99883722	11.95429698
16.46658944	15.9477404	19.5764845
ENSG00000087263 1807.243924	2096.553518	1904.161323
2182.785619	2681.364304	2672.455762	1935.986255
2512.201895	-0.375837624	0.010809114	0.073766362	OGFOD1
	16	56451490	56479100	+	5806
protein_coding	2-oxoglutarate and iron dependent oxygenase domain
containing 1 [Source:HGNC Symbol;Acc:HGNC:25585]	-	1992
	2369	1638	2107	2349	3098	16.80122648
19.3336775	17.5526912	20.16506136	24.86871501
24.81049334
ENSG00000181830 797.4735991	938.9798575	1049.72996
1147.853092	1353.809308	1261.178284	928.7278055
1254.280228	-0.434204498	0.010835953	0.073921009
SLC35C1 11	45804072	45813015	+	4505
protein_coding	solute carrier family 35 member C1 [Source:HGNC
Symbol;Acc:HGNC:20197]	-	879	1061	903	1108	1186
	1462	9.554825574	11.15955892	12.47095757
13.66649065	16.18218843	15.08980388
ENSG00000120942 1161.281236	1439.002119	1119.479459
1474.183168	1767.02935	1731.316564	1239.920938
1657.509694	-0.4182195	0.010847431	0.073963832	UBIAD1
	1	11273206	11296049	+	5082
protein_coding	UbiA prenyltransferase domain containing 1
[Source:HGNC Symbol;Acc:HGNC:30791]	-	1280	1626	963
	1423	1548	2007	12.33400137	15.16045799
11.78958378	15.55902157	18.72335431	18.36300405
ENSG00000134684 3971.037478	4963.052819	5200.987642
5518.604172	6250.809251	6396.468521	4711.692646
6055.293981	-0.362163412	0.010850593	0.073963832	YARS
	1	32775237	32818153	-	8212
protein_coding	tyrosyl-tRNA synthetase [Source:HGNC
Symbol;Acc:HGNC:12840]	-	4377	5608	4474	5327	5476
	7415	26.10094848	32.35828569	33.89642417
36.0450639	40.9884843	41.9849106
ENSG00000105778 1753.716117	1753.175398	1614.700902
2267.735035	2481.603234	1922.822432	1707.197472
2224.053567	-0.380829801	0.010868926	0.0740296	AVL9
	7	32495426	32588721	+	14757
protein_coding	AVL9 cell migration associated [Source:HGNC
Symbol;Acc:HGNC:28994]	-	1933	1981	1389	2189	2174
	2229	6.414494843	6.360816091	5.856134126
8.242514654	9.055424681	7.023328942
ENSG00000164758 407.3556837	460.1974796	442.9093186
314.9344225	324.1836792	222.5608737	436.8208273
287.2263251	0.606922831	0.010871596	0.0740296	MED30
	8	117520713	117540262	+	1440

protein_coding mediator complex subunit 30 [Source:HGNC
Symbol;Acc:HGNC:23032] -   449   520   381   304   284
        258   15.26906811   17.11068091   16.46149711
11.73067858   12.12280748   8.330829227
ENSG00000177822 191.4299538   169.9190694   166.2363059
133.6399358   73.055477   63.83528935   175.8617764
90.17690073   0.965553148   0.010873932   0.0740296
AC098864.1   4   181874438   182145249   -   5551
        antisense   uncharacterized LOC90768 [Source:NCBI
gene;Acc:90768] -   211   192   143   129   64   74
        1.861400866   1.638915046   1.602771668   1.291310037
        0.708689677   0.619857001
ENSG00000020577 299.3928188   316.8282648   415.009519
211.337573   254.5526777   177.7036433   343.7435342
214.531298   0.680759109   0.010880697   0.0740296   SAMD4A
        14   54567097   54793315   +   12170
protein_coding sterile alpha motif domain containing 4A [Source:HGNC
Symbol;Acc:HGNC:23023] -   330   358   357   204   223
        206   1.327859256   1.393859146   1.825090836
0.931433026   1.126319609   0.787059672
ENSG00000131115 304.8363246   257.5335896   291.7854041
446.5024213   410.9370581   404.5777122   284.7184394
420.6723972   -0.563418917   0.010881167   0.0740296   ZNF227
        19   44207547   44237268   +   5069
protein_coding zinc finger protein 227 [Source:HGNC
Symbol;Acc:HGNC:13020] zf-C2H2 336   291   251   431   360
        469   3.24597873   2.72017684   3.080762909
4.724621507   4.365435439   4.302110585
ENSG00000070018 992.5325568   725.6960256   760.269539
641.2644983   545.6330939   570.2044089   826.1660405
585.700667   0.496568831   0.010910351   0.074158532   LRP6
        12   12116025   12267012   -   10337
protein_coding LDL receptor related protein 6 [Source:HGNC
Symbol;Acc:HGNC:6698]   -   1094   820   654   619   478
        661   5.18264533   3.758770216   3.936317116
3.327424242   2.84237085   2.973294732
ENSG00000178700 395.5614212   465.5074505   373.1598196
634.0127189   531.9351919   602.1220536   411.4095638
589.3566548   -0.517926359   0.01091373   0.074158532   DHFR2
        3   94047836   94063389   -   4659
protein_coding dihydrofolate reductase 2 [Source:HGNC
Symbol;Acc:HGNC:27309] -   436   526   321   612   466
        698   4.582710918   5.349577389   4.286661284
7.29912423   6.148094956   6.966164573
ENSG00000111669 13875.49627   17663.61826   19868.14479
19275.22982   24473.5848   23547.45802   17135.75311
22432.09088   -0.388620201   0.010915908   0.074158532   TPI1
        12   6867119 6870948 +   2700   protein_coding
triosephosphate isomerase 1 [Source:HGNC Symbol;Acc:HGNC:12009] -
        15294   19959   17091   18606   21440   27297   277.3869372

350.2688003 393.8315971 382.9140449 488.0995161
470.0912567
ENSG00000148110 1709.26082 1637.241033 1665.850534
1328.11161 1408.600916 979.9579554 1670.784129
1238.890161 0.432424276 0.010920428 0.074158532
MFSD14B 9 94374551 94461042 + 3428
protein_coding major facilitator superfamily domain containing 14B
[Source:HGNC Symbol;Acc:HGNC:23376] - 1884 1850 1433
1282 1234 1136 26.91341256 25.57156782
26.00831544 20.78065612 22.12695775 15.40879
ENSG00000185049 731.2442786 806.2305845 916.0434201
983.1341017 1073.002318 1323.288295 817.8394277
1126.474905 -0.463316834 0.01092108 0.074158532 NELFA
4 1982714 2041903 - 5935 protein_coding
negative elongation factor complex member A [Source:HGNC
Symbol;Acc:HGNC:12768] - 806 911 788 949 940
1534 6.650327027 7.273175959 8.260614224
8.885002059 9.735415475 12.0180956
ENSG00000213204 0 2.654985459 0 21.75533839
12.55641011 12.07694663 0.884995153
15.46289838 -4.063103384 0.010947296 0.074278531
AL049697.1 6 87408012 87511634 + 3530
protein_coding "novel transcript, C6orf165-SLC35A1
readthrough" - 0 3 0 21 11 14
0 0.040269199 0 0.33056482 0.191542583
0.184409949
ENSG00000133895 1253.820835 1444.31209 1428.702238
1544.629026 1841.226319 2120.366773 1375.611721
1835.407373 -0.416547004 0.010953353 0.074278531 MEN1
11 64803510 64811294 - 4246
protein_coding menin 1 [Source:HGNC Symbol;Acc:HGNC:7010] -
1382 1632 1229 1491 1613 2458 15.93884094
18.2123759 18.00854974 19.51235538 23.35079858
26.91738751
ENSG00000104324 331.1466026 398.2478189 556.8335003
304.5747375 214.6004637 282.0829678 428.7426406
267.0860563 0.680046443 0.010954626 0.074278531 CPQ
8 96645227 97149654 + 3457
protein_coding carboxypeptidase Q [Source:HGNC Symbol;Acc:HGNC:16910]
- 365 450 479 294 188 327
5.170376494 6.167931971 8.620708904 4.725633042
3.342764874 4.398244733
ENSG00000120705 4691.394745 4842.693478 4590.679525
5490.633023 5809.051914 6148.891115 4708.255916
5816.192017 -0.304893833 0.010955548 0.074278531 ETF1
5 138506095 138543300 - 4601
protein_coding eukaryotic translation termination factor 1
[Source:HGNC Symbol;Acc:HGNC:3477] - 5171 5472 3949
5300 5089 7128 55.03652288 56.35342239
53.40006026 64.00820967 67.98726612 72.03548357

ENSG00000227057 3398.562119 4268.331623 3452.6002
4059.960532 4764.586891 6079.017352 3706.497981
4967.854925 -0.422640884 0.010983723 0.074441024 WDR46
6 33279108 33289527 - 3115
protein_coding WD repeat domain 46 [Source:HGNC
Symbol;Acc:HGNC:13923] - 3746 4823 2970 3919 4174
7047 58.88957304 73.36444758 59.32056037
69.90838288 82.36482005 105.1906742
ENSG00000146197 50.80605409 51.32971888 41.8496994 0
13.69790194 16.39014186 47.99515746 10.02934793
2.25344737 0.011047079 0.074841741 SCUBE3 6
35214419 35253079 + 7356 protein_coding
"signal peptide, CUB domain and EGF like domain containing 3
[Source:HGNC Symbol;Acc:HGNC:13655]" - 56 58 36
0 12 19 0.372799216 0.373605029
0.304486209 0 0.100273664 0.12009997
ENSG00000169020 109.7773669 275.2334926 185.998664
88.05732207 113.007691 86.26390453 190.3365078
95.77630586 0.992794223 0.011073588 0.074992611 ATP5ME
4 672436 674338 - 940 protein_coding ATP
synthase membrane subunit e [Source:HGNC Symbol;Acc:HGNC:846] -
121 311 160 85 99 100 6.303564489
15.67685626 10.59007353 5.024618763 6.473731772
4.94655867
ENSG00000169359 341.1263632 389.3978674 374.3223113
651.6241833 414.3615336 587.4571898 368.2821806
551.1476356 -0.582595443 0.011130359 0.075324961
SLC33A1 3 155821024 155854456 - 10040
protein_coding solute carrier family 33 member 1 [Source:HGNC
Symbol;Acc:HGNC:95] - 376 440 322 629 363
681 1.833930257 2.076564401 1.995395578
3.481200011 2.222389991 3.153874569
ENSG00000184602 245.8650118 192.9289434 289.4604208
114.9925029 189.4876435 53.48362081 242.7514587
119.3212557 1.029017514 0.011132922 0.075324961 SNN
16 11668414 11679159 + 3312
protein_coding stannin [Source:HGNC Symbol;Acc:HGNC:11149] -
271 218 249 111 166 62 4.006892088
3.118836487 4.677522288 1.862278779 3.080811453
0.870427051
ENSG00000131467 5660.338777 5743.618544 5951.957247
6306.976197 7992.725781 7557.580676 5785.304856
7285.760884 -0.332717076 0.011141015 0.075324961 PSME3
17 42824385 42843758 + 5152
protein_coding proteasome activator subunit 3 [Source:HGNC
Symbol;Acc:HGNC:9570] - 6239 6490 5120 6088 7002
8761 59.30179168 59.68913473 61.83024299
65.66150504 83.53983483 79.06947298
ENSG00000038002 364.7148883 362.8480128 389.4347027
242.4166278 218.0249392 276.9071335 372.3325346

245.7829002	0.597170708	0.011141805	0.075324961	AGA
	4	177430770	177442503	-	2675
protein_coding	aspartylglucosaminidase [Source:HGNC
Symbol;Acc:HGNC:318]	-	402	410	335	234	191
	321	7.359205836	7.262506117	7.791621859
4.860759288	4.388912687	5.57971818
ENSG00000187713 958.0570201	1111.553912	990.4428857
1294.960619	1443.987163	1287.920095	1020.017939
1342.289292	-0.395541176	0.011143955	0.075324961
TMEM203 9	137204082	137205638	-	1557
protein_coding	transmembrane protein 203 [Source:HGNC
Symbol;Acc:HGNC:28217]	-	1056	1256	852	1250	1265
	1493	33.2126852	38.22323739	34.045352
44.6101254	49.94008453	44.58638002
ENSG00000264268 5.443505796	8.849951531	11.6249165	0
	0	0	8.639457942	0	5.542076885
0.011162978	0.075424721	MIR4767 X	7147860 7147937 +
	78	miRNA	microRNA 4767 [Source:HGNC
Symbol;Acc:HGNC:41548]	-	6	10	10	0	0
	0	3.766910756	6.074797956	7.976497693	0
	0	0
ENSG00000284052 7.258007728	6.194966072	9.299933199
40.4027713	13.69790194	49.17042558	7.584302333
34.42369961	-2.192820171	0.011173705	0.075468371
AC006460.2	2	190607660	190649840	-	7811
	bidirectional_promoter_lncRNA	novel transcript	-
	8	7	8	39	12	57	0.050154746
	0.042463701	0.063722117	0.277440599	0.094432605
	0.339312013
ENSG00000122591 2361.574264	1995.66407	2056.447729
1618.182789	1810.406039	1517.382081	2137.895354
1648.65697	0.375461207	0.011230969	0.075826189
FAM126A 7	22889371	23014130	-	14499
protein_coding	family with sequence similarity 126 member A
[Source:HGNC Symbol;Acc:HGNC:24587]	-	2603	2255	1769
	1562	1586	1759	8.79153687	7.369447634
7.590958719	5.986252388	6.723764492	5.641035174
ENSG00000139579 1418.03326	1525.731644	1383.365063
1173.752305	1149.482271	965.2930917	1442.376656
1096.175889	0.396830987	0.011235823	0.075830014	NABP2
	12	56222015	56229854	+	2218
protein_coding	nucleic acid binding protein 2 [Source:HGNC
Symbol;Acc:HGNC:28412]	-	1563	1724	1190	1133	1007
	1119	34.508503	36.83003745	33.38045608
28.38444531	27.90713729	23.45846349
ENSG00000135324 3.629003864	18.58489822	4.6499666	0
	0	0	8.954622893	0	5.598761953
0.011275872	0.076059982	MRAP2	6	84033756
84090881	+	2153	protein_coding	melanocortin 2
receptor accessory protein 2 [Source:HGNC Symbol;Acc:HGNC:21232]

-	4	21	4	0	0	0
0.09097973	0.462169951	0.115590677	0	0	0
ENSG00000126698 3754.204497	4047.96783	4095.458083
3439.415403	3169.922807	2775.109809	3965.876803
3128.14934	0.342502604	0.011278497	0.076059982	DNAJC8
1	28199456	28233025	-	2888
protein_coding	DnaJ heat shock protein family (Hsp40) member C8
[Source:HGNC Symbol;Acc:HGNC:15470]	-	4138	4574	3523
3320	2777	3217	70.1652345	75.04563076
75.8965965	63.87823951	59.10524897	51.7946485
ENSG00000116288 2405.122311	3127.572871	3180.577154
2121.663478	2425.670135	2102.251353	2904.424112
2216.528322	0.389970358	0.011288125	0.076095898	PARK7
1	7954291 7985505 +	2397	protein_coding
Parkinsonism associated deglycase [Source:HGNC Symbol;Acc:HGNC:16369]
-	2651	3534	2736	2048	2125	2437
54.15896762	69.85941619	71.01578722	47.47598259
54.49269168	47.27358227
ENSG00000229931 9.07250966	6.194966072	10.46242485	0
0	0	8.576633527	0	5.532101043
0.011311621	0.076193867	AL137003.1	6	16761138
16762652	+	698	antisense
uncharacterized LOC101928433 [Source:NCBI gene;Acc:101928433]	-
10	7	9	0	0	0	0.701573637
0.475191932	0.802220828	0	0	0
ENSG00000106263 13355.64147	15639.63435	14593.92017
15344.76535	19567.45292	20956.95297	14529.732
18623.05708	-0.358110019	0.011313429	0.076193867	EIF3B
7	2354086 2380745 +	8998	protein_coding
eukaryotic translation initiation factor 3 subunit B [Source:HGNC
Symbol;Acc:HGNC:3280]	-	14721	17672	12554	14812	17142
24294	80.11613827	93.06066569	86.80464835
91.47022728	117.1016089	125.5405585
ENSG00000026025 41904.10762	46190.55203	53097.9686
64185.50004	61294.6867	52830.60305	47064.20941
59436.92993	-0.336741605	0.01131558	0.076193867	VIM
10	17228259	17237593	+	5158
protein_coding	vimentin [Source:HGNC Symbol;Acc:HGNC:12692]	-
46188	52193	45676	61957	53697	61243	438.5069721
479.4655005	550.9517579	667.4536104	639.9057979
552.0852406
ENSG00000142544 644.1481858	964.6447169	604.4956579
744.8613478	1447.411638	1225.810083	737.7628536
1139.361023	-0.626071526	0.011320729	0.076199535	CTU1
19	51097606	51108370	-	2087
protein_coding	cytosolic thiouridylase subunit 1 [Source:HGNC
Symbol;Acc:HGNC:29590]	-	710	1090	520	719	1268
1421	16.65960052	24.74745195	15.50200031
19.14337404	37.34600617	31.6593976
ENSG00000251520 1.814501932	8.849951531	0	15.53952742

	12.55641011	58.65945508	3.554817821	28.9184642
	-2.995666752	0.011345804	0.076339268	AL162431.3
	1	180964511	180965909	+	537
unprocessed_pseudogene	"pseudogene similar to part of major
histocompatibility complex, class I-related MR1"	-	2
	10	0	15	11	68	0.182383016
0.882372887	0	1.552133302	1.259116049	5.887970767
ENSG00000167785 43.54804637	36.28480128	109.2742151
29.00711786	7.990442797	0	63.03568758	12.33252022
	2.359489857	0.011358328	0.076379372	ZNF558	19
	8806170 8832328 -	8184	protein_coding	zinc finger
protein 558 [Source:HGNC Symbol;Acc:HGNC:26422] zf-C2H2 48	41
	94	28	7	0	0.287213137	0.237380301
	0.714609984	0.190109778	0.05257506	0
ENSG00000218809 0	0	0	10.35968495	3.424475485
	12.07694663	0	8.620369022	-5.586446251
0.011360401	0.076379372	AL391903.1	6	41269875
	41270239	-	365	unprocessed_pseudogene
triggering receptor expressed on myeloid cells 1 (TREM1) pseudogene
	-	0	0	0	10	3	14	0
	0	0	1.522366362	0.505214435	1.783471564
ENSG00000115738 110.6846178	114.1643748	165.0738143
211.337573	181.4972007	301.0610268	129.974269
231.2986001	-0.836832542	0.011365923	0.076387466	ID2
	2	8678845 8684453 +	2205	protein_coding
inhibitor of DNA binding 2 [Source:HGNC Symbol;Acc:HGNC:5361]	bHLH
	122	129	142	204	159	349	2.709442385
	2.772091475	4.006697889	5.140834435	4.432368816
	7.359492233
ENSG00000129351 14473.37466	13819.19932	13982.44956
11964.40015	10135.30594	12059.69385	14091.67451
11386.46665	0.307441127	0.011371695	0.076397231	ILF3
	19	10654261	10692417	+	11022
protein_coding	interleukin enhancer binding factor 3 [Source:HGNC
Symbol;Acc:HGNC:6038]	-	15953	15615	12028	11549	8879
	13980	70.87786743	67.12866691	67.89532309
58.22319294	49.51662198	58.97633533
ENSG00000118418 653.2206955	707.9961225	570.7834001
430.9628939	525.086241	391.6381266	644.0000727
449.2290871	0.521760612	0.011390318	0.076493294	HMGN3
	6	79201245	79234738	-	1014
protein_coding	high mobility group nucleosomal binding domain 3
[Source:HGNC Symbol;Acc:HGNC:12312]	Others	720	800	491
	416	460	454	34.7714839	37.38337204
30.12661821	22.79646039	27.88478093	20.81847513
ENSG00000188985 362.9003864	330.9881873	335.9600868
245.5245333	236.2888084	98.34085116	343.2828868
193.384731	0.831452922	0.011403935	0.076555674	DHFRP1
	18	26170726	26171284	-	559
processed_pseudogene	dihydrofolate reductase pseudogene 1

[Source:HGNC Symbol;Acc:HGNC:2862]	-	400	374	289
	237	207	114	35.04103029	31.70196887
32.1656907	23.55855136	22.76176304	9.482526424
ENSG00000137720 322.9813439	341.6081291	337.1225785
560.4589557	430.3424192	470.1382797	333.9040172
486.9798849	-0.544693346	0.011448679	0.076826887
C11orf1 11	111878935	111885975	+	3831
protein_coding	chromosome 11 open reading frame 1 [Source:HGNC
Symbol;Acc:HGNC:1163]	-	356	386	290	541	377
	545	4.550577651	4.774210829	4.709694018
7.846882632	6.048902139	6.614779449
ENSG00000205534 201.4097144	185.8489822	44.1746827
59.05020421	7.990442797	71.59904076	143.8111264
46.21322925	1.633763151	0.011497203	0.077123258	SMG1P2
	16	29527568	29594966	-	4221
unprocessed_pseudogene	SMG1 pseudogene 2 [Source:HGNC
Symbol;Acc:HGNC:49859]	-	222	210	38	57	7
	83	2.575528179	2.357384281	0.560112276
0.750363235	0.101936577	0.914310608
ENSG00000198887 1485.169831	1256.693117	1128.779392
1144.745187	745.3941638	857.463211	1290.214114
915.8675206	0.494664851	0.011508048	0.07716674	SMC5
	9	70258962	70354888	+	6637
protein_coding	structural maintenance of chromosomes 5 [Source:HGNC
Symbol;Acc:HGNC:20465]	-	1637	1420	971	1105	653
	994	12.07829257	10.13778246	9.102365259
9.251294453	6.047678893	6.963788698
ENSG00000140365 1013.399329	1319.527773	1369.415164
815.3072055	852.6943956	1023.089908	1234.114089
897.0305029	0.459148607	0.011512983	0.077170578	COMMD4
	15	75335891	75343224	+	6779
protein_coding	COMM domain containing 4 [Source:HGNC
Symbol;Acc:HGNC:26027]	-	1117	1491	1178	787	747
	1186	8.068935107	10.42169719	10.8115137
6.450912365	6.77333145	8.134859814
ENSG00000083520 3472.956698	3520.510719	2720.230461
4586.232527	4293.150766	3742.128178	3237.899293
4207.17049	-0.377246568	0.011521542	0.077198695	DIS3
	13	72752169	72782096	-	10835
protein_coding	"DIS3 homolog, exosome endoribonuclease and 3'-5'
exoribonuclease [Source:HGNC Symbol;Acc:HGNC:20604]"	-	3828
	3978	2340	4427	3761	4338	17.30101955
17.39651693	13.43673612	22.70349144	21.33643112
18.61622632
ENSG00000260643 46.26979926	39.82478189	33.71225785
66.30198367	98.16829722	111.2804368	39.935613
91.91690591	-1.200881902	0.011542621	0.077310648
AC092718.3	16	81053587	81096296	-	3177
	protein_coding	novel protein	-	51	45	29
	64	86	129	0.786106966	0.671153315

0.56792061	1.119372937	1.663905268	1.888006624
ENSG00000121895 3.629003864	23.89486913	2.3249833
85.98538508	22.82983656	38.81875704	9.949618766
49.21132623	-2.289720917	0.011563135	0.077418736
TMEM156 4	38966744	39032922	-	2562
protein_coding	transmembrane protein 156 [Source:HGNC
Symbol;Acc:HGNC:26260]	-	4	27	2	83	20
45	0.076455644	0.499356928	0.048568838
1.800159599	0.479841969	0.816703481
ENSG00000127990 921.7669814	725.6960256	886.9811289
729.3218204	569.6044223	391.6381266	844.814712
563.5214564	0.585118205	0.011570729	0.077440265	SGCE
7	94524204	94656572	-	20884
protein_coding	sarcoglycan epsilon [Source:HGNC
Symbol;Acc:HGNC:10808]	-	1016	820	763	704	499
454	2.382367232	1.860486867	2.273095593
1.873143365	1.468703809	1.010818511
ENSG00000114354 3108.241809	3252.357188	3052.703073
3810.292124	4048.871515	3807.688746	3137.767357
3888.950795	-0.30944269	0.011596469	0.077583186	TFG
3	100709331	100748966	+	4994
protein_coding	TRK-fused gene [Source:HGNC Symbol;Acc:HGNC:11758]
-	3426	3675	2626	3678	3547	4414
33.59444759	34.86865907	32.71545994	40.92373187
43.6576154	41.09744368
ENSG00000260022 0.907250966	1.769990306	0	0
11.41491828	44.85723035	0.892413757
18.75738288	-4.346166978	0.011604066	0.077604659
AL031716.1	16	883780	885090	+	530	antisense
"novel transcript, antisense to LMF1"	-	1	2
0	0	10	52	0.092395924	0.178805374
0	0	1.159768985	4.562033732
ENSG00000257093 792.9373443	581.4418156	568.4584168
458.9340432	472.5776169	414.9293808	647.6125256
448.8136803	0.530316	0.011614356	0.077644122
KIAA1147	7	141656728	141702153	-	7356
protein_coding	KIAA1147 [Source:HGNC Symbol;Acc:HGNC:29472]
-	874	657	489	443	414	481
5.818330616	4.232043176	4.13593767	3.346370702
3.459441419	3.040425553
ENSG00000136231 1189.406016	846.0553664	978.8179692
871.2495042	672.3386868	498.6053682	1004.759784
680.7311864	0.562622097	0.011632751	0.077727958
IGF2BP3 7	23310209	23470467	-	8511
protein_coding	insulin like growth factor 2 mRNA binding protein 3
[Source:HGNC Symbol;Acc:HGNC:28868]	-	1311	956	842
841	589	578	7.54311597	5.322353824
6.15514584	5.490696631	4.25384895	3.157753797
ENSG00000100416 966.2222788	865.5252598	796.3067802
578.0704202	647.2258666	695.2870705	876.0181062

640.1944524	0.452550616	0.011636435	0.077727958	TRMU
	22	46330875	46357340	+	9219
protein_coding	tRNA 5-methylaminomethyl-2-thiouridylate
methyltransferase [Source:HGNC Symbol;Acc:HGNC:25481]	-	1065
	978	685	558	567	806	5.657108083
5.026682799	4.622890462	3.363275376	3.780477034
4.065203071
ENSG00000266865 48.0843012	53.98470434	81.37441549
29.00711786	15.98088559	18.978059	61.14780701
21.32202082	1.516654869	0.011640592	0.077727958
AC138207.8	17	31008497	31093127	+	2147
	transcribed_unprocessed_pseudogene	SMAD specific E3
ubiquitin protein ligase 2 (SMURF2) pseudogene	-	53	61
	70	28	14	22	1.208850261	1.346245397
	2.028489865	0.724666242	0.400814433	0.476454743
ENSG00000130720 85.2815908	70.79961225	227.8483634	0
	12.55641011	0	127.9765221	4.185470037
4.949498544	0.011644473	0.077727958	FIBCD1	9
130902438	130939286	-	3831	protein_coding
fibrinogen C domain containing 1 [Source:HGNC Symbol;Acc:HGNC:25922]
	-	94	80	196	0	11	0
1.20155702	0.989473747	3.183103543	0	0.176493166
	0
ENSG00000124767 5736.547858	5571.044489	5884.532732
6928.557294	7411.70644	6752.738446	5730.70836
7031.000727	-0.294992706	0.011664229	0.077830461	GLO1
	6	38675925	38703141	-	2352
protein_coding	glyoxalase I [Source:HGNC Symbol;Acc:HGNC:4323] -
	6323	6295	5062	6688	6493	7828	131.6480856
	126.8191558	133.9034202	158.0050584	169.6896859
	154.7549388
ENSG00000145569 29.03203091	12.38993214	40.68720775	0
	4.565967313	6.901112362	27.3697236	3.822359892
	2.821846208	0.011709776	0.078104912	OTULINL 5
	14581775	14615007	+	4074	protein_coding
	OTU deubiquitinase with linear linkage specificity like
[Source:HGNC Symbol;Acc:HGNC:25629]	-	32	14	35
	0	4	8	0.384642826	0.162829636
0.534507577	0	0.060351258	0.091306139
ENSG00000096433 2931.327871	2187.708019	2287.783567
2276.022783	1607.220494	1439.744567	2468.939819
1774.329281	0.476979283	0.011736629	0.078254513	ITPR3
	6	33620365	33696574	+	9870
protein_coding	"inositol 1,4,5-trisphosphate receptor type 3
[Source:HGNC Symbol;Acc:HGNC:6182]"	-	3231	2472	1968
	2197	1408	1669	16.03055243	11.8674594
12.40551471	12.3687254	8.768652559	7.862672781
ENSG00000132361 4022.750783	4386.920974	3728.110721
4753.023455	4750.888989	6166.143896	4045.927493
5223.352113	-0.368581393	0.011752606	0.078331518	CLUH

17	2689386 2712663 -	6900	protein_coding
clustered mitochondria homolog [Source:HGNC Symbol;Acc:HGNC:29094]
-	4434	4957	3207	4588	4162	7148
31.4684449	34.04052653	28.91723177	36.94761097
37.07663788	48.16887144
ENSG00000166197 4811.151873	4799.328715	4147.770207
5519.640141	5704.034665	5894.412596	4586.083598
5706.029134	-0.315032071	0.011773008	0.078437942	NOLC1
10	102152176	102163871	+	3970
protein_coding	nucleolar and coiled-body phosphoprotein 1
[Source:HGNC Symbol;Acc:HGNC:15608]	-	5303	5423	3568
5328	4997	6833	65.41235784	64.72551855
55.91665526	74.5737106	77.36886091	80.02983695
ENSG00000106415 159.67617	159.2991276	144.1489646
78.73360561	103.8757564	63.83528935	154.3747541
82.14821711	0.914718818	0.011809329	0.078650311	GLCCI1
7	7968794 8094272 +	6885	protein_coding
glucocorticoid induced 1 [Source:HGNC Symbol;Acc:HGNC:18713]	-
176	180	124	76	91	74	1.251807089
1.238782328	1.120532835	0.613368814	0.81242786
0.499756893
ENSG00000175040 1801.800418	2070.888658	2007.623079
2237.691949	2493.018153	2910.544139	1960.104052
2547.084747	-0.37830513	0.011822916	0.078711162	CHST2
3	143119331	143124014	+	4582
protein_coding	carbohydrate sulfotransferase 2 [Source:HGNC
Symbol;Acc:HGNC:1970]	-	1986	2340	1727	2160	2184
3374	21.2252514	24.19843132	23.45006762
26.194536	29.29846783	34.23899523
ENSG00000197467 313.9088342	274.3484975	351.0724783
175.0786756	253.4111859	117.3189102	313.1099367
181.9362572	0.786314425	0.011832946	0.078748299
COL13A1 10	69801867	69964275	+	5888
protein_coding	collagen type XIII alpha 1 chain [Source:HGNC
Symbol;Acc:HGNC:2190]	-	346	310	302	169	222
136	2.877643441	2.494711525	3.191140959
1.594890778	2.317568254	1.073994668
ENSG00000261040 45.3625483	104.4294281	46.499666
14.50355893	30.82027936	20.70333709	65.43054745
22.00905846	1.578040653	0.011844441	0.078795157
WFDC21P 17	60083566	60091885	-	1099
transcribed_unitary_pseudogene	"WAP four-disulfide core domain 21,
pseudogene [Source:HGNC Symbol;Acc:HGNC:50357]" -	50	118
40	14	27	24	2.227927199	5.087574193
2.264483421	0.707851875	1.510126859	1.015417321
ENSG00000163659 431.8514598	415.947722	475.4590848
614.3293175	623.2545382	602.1220536	441.0860889
613.2353031	-0.476109456	0.01185013	0.078803363	TIPARP
3	156673235	156706770	+	5048
protein_coding	TCDD inducible poly(ADP-ribose) polymerase

[Source:HGNC Symbol;Acc:HGNC:23696]	-	476	470	409
	593	546	698	4.617599794	4.411689641
5.040931644	6.52750767	6.648453823	6.429350386
ENSG00000258770 2.721752898	0	2.3249833	8.287747959
	17.12237742	28.46708849	1.682245399	17.95907129
	-3.4401655	0.011857	0.078819418	LINC02330
	14	87634379	87655294	-	555
lincRNA long intergenic non-protein coding RNA 2330 [Source:HGNC
Symbol;Acc:HGNC:53250]	-	3	0	2	8	15
	33	0.264701837	0	0.224204259	0.800956716
	1.661290709	2.764725224
ENSG00000140374 1799.985917	1785.920219	2062.260187
1414.096996	1439.421195	1538.085418	1882.722107
1463.86787	0.362366521	0.011867749	0.07884064	ETFA
	15	76215355	76311472	-	5031
protein_coding	electron transfer flavoprotein subunit alpha
[Source:HGNC Symbol;Acc:HGNC:3481]	-	1984	2018	1774
	1365	1261	1783	19.31150114	19.00611205
21.93846032	15.0761475	15.40664691	16.47889339
ENSG00000237685 3.629003864	0	0	11.39565344
19.40536108	20.70333709	1.209667955
17.1681172	-3.785394058	0.011869106	0.07884064
AL139039.3	6	10511036	10514546	-	1475
	antisense	"novel transcript, antisense to GCNT2"	-
	4	0	0	11	17	24	0.132799566
	0	0	0.414393284	0.708441936	0.756571957
ENSG00000169738 968.9440317	1208.903379	1392.664997
936.5155194	888.0806423	759.9849989	1190.170802
861.5270535	0.466026923	0.011876215	0.078848133	DCXR
	17	82035136	82037732	-	1902
protein_coding	dicarbonyl and L-xylulose reductase [Source:HGNC
Symbol;Acc:HGNC:18985]	-	1068	1366	1198	904	778
	881	27.49726022	34.03036659	39.18800475
26.41009488	25.14296232	21.53755572
ENSG00000127838 457.2544869	481.4373633	671.9201736
387.4522171	384.6827461	294.1599144	536.8706746
355.4316259	0.59479533	0.011879149	0.078848133	PNKD
	2	218270392	218346793	+	5184
protein_coding	"PNKD, MBL domain containing [Source:HGNC
Symbol;Acc:HGNC:9153]"	-	504	544	578	374	337
	341	4.76095665	4.972334623	6.936968017
4.008839353	3.995878443	3.058583943
ENSG00000170860 1393.537484	1640.781014	1656.550601
1368.514382	1083.275745	1053.282274	1563.623033
1168.357467	0.420389104	0.011889674	0.078888387	LSM3
	3	14178358	14201119	+	3815
protein_coding	"LSM3 homolog, U6 small nuclear RNA and mRNA
degradation associated [Source:HGNC Symbol;Acc:HGNC:17874]"	-
	1536	1854	1425	1321	949	1221	19.71629724
	23.02722626	23.2395208	19.24067567	15.29040646

14.8816861
ENSG00000042286 486.2865178 506.2172276 540.5586172
354.3012253 320.7592037 391.6381266 511.0207875
355.5661852 0.521604089 0.011905257 0.078962166 AIFM2
10 70098223 70132934 - 5135
protein_coding "apoptosis inducing factor, mitochondria associated 2
[Source:HGNC Symbol;Acc:HGNC:21411]" - 536 572 465
342 281 454 5.111554849 5.278153566
5.634032548 3.700817779 3.363668841 4.110990025
ENSG00000186197 19.05227029 22.12487883 6.974949899
2.07193699 0 1.725278091 16.05069967 1.26573836
3.63603191 0.011914383 0.078993072 EDARADD 1
236348257 236502915 + 3985 protein_coding
EDAR associated death domain [Source:HGNC
Symbol;Acc:HGNC:14341] - 21 25 6 2 0
2 0.258059382 0.29726113 0.093676309
0.027887765 0 0.023336337
ENSG00000171084 107.055614 115.0493699 104.6242485
162.6470537 154.1013968 284.6708849 108.9097441
200.4731118 -0.882418636 0.011935282 0.079088276
FAM86JP 3 125916620 125930024 + 2519
transcribed_unprocessed_pseudogene "family with sequence
similarity 86 member J, pseudogene [Source:HGNC
Symbol;Acc:HGNC:44097]" - 118 130 90 157 135
330 2.293942477 2.445353365 2.222906463
3.463247493 3.294222743 6.091395393
ENSG00000130640 2812.477995 3121.377905 3792.047762
4311.700876 4604.778035 3749.029291 3241.967887
4221.836067 -0.381194927 0.011937684 0.079088276
TUBGCP2 10 133278630 133312337 - 7697
protein_coding tubulin gamma complex associated protein 2
[Source:HGNC Symbol;Acc:HGNC:18599] - 3100 3527 3262
4162 4034 4346 19.72281454 21.71252912
26.36752198 30.04641303 32.2152694 26.25422807
ENSG00000254419 3.629003864 14.15992245 8.137441549 0
0 0 8.642122621 0 5.545104029
0.011953479 0.079144713 AL139349.1 20 58710795
58711633 + 577 sense_intronic novel
transcript - 4 16 7 0 0 0
0.339478959 1.313925104 0.754795102 0 0
0
ENSG00000103507 909.0654679 1072.614126 1078.792251
1087.76692 1571.834247 1557.063477 1020.157282
1405.554881 -0.462695956 0.011955151 0.079144713 BCKDK
16 31106107 31112791 + 3873
protein_coding branched chain ketoacid dehydrogenase kinase
[Source:HGNC Symbol;Acc:HGNC:16902] - 1002 1212 928
1050 1377 1805 12.66919171 14.82796539
14.90758608 15.06446961 21.85414416 21.67009062
ENSG00000177105 1030.637097 1265.543069 1076.467268

1397.5215 1464.534016 1572.59098 1124.215811
1478.215498 -0.394834561 0.011964224 0.07914726 RHOG
11 3826978 3840983 - 1782 protein_coding ras
homolog family member G [Source:HGNC Symbol;Acc:HGNC:672] -
1136 1430 926 1349 1283 1823 31.21758589
38.02373535 32.33032971 42.06455449 44.25540473
47.56746279
ENSG00000175029 1206.643785 984.9996054 1267.115898
906.472433 842.4209692 825.5455663 1152.919763
858.1463229 0.425544237 0.011964485 0.07914726 CTBP2
10 124984317 125161170 - 10946
protein_coding C-terminal binding protein 2 [Source:HGNC
Symbol;Acc:HGNC:2495] - 1330 1113 1090 875 738
957 5.950108439 4.817992963 6.195521961
4.441857818 4.144272254 4.065252374
ENSG00000135549 31.75378381 49.55972857 47.66215765
89.09329056 115.2906746 83.67598739 42.99189001
96.0199842 -1.159464139 0.01198135 0.0792292 PKIB
6 122471917 122726373 + 3432
protein_coding cAMP-dependent protein kinase inhibitor beta
[Source:HGNC Symbol;Acc:HGNC:9018] - 35 56 41
86 101 97 0.499401047 0.773156103
0.743264558 1.392397439 1.808928723 1.314181875
ENSG00000103264 1686.579546 1883.269686 1898.348864
2153.778501 2300.106034 2541.334627 1822.732699
2331.739721 -0.35572663 0.011998825 0.079315105 FBXO31
16 87326987 87392142 - 6897
protein_coding F-box protein 31 [Source:HGNC Symbol;Acc:HGNC:16510]
- 1859 2128 1633 2079 2015 2946
13.19920723 14.61967905 14.73101953 16.749672
17.95817439 19.86111082
ENSG00000143575 1661.176519 1899.199599 1794.887107
1959.016424 2483.886218 2556.86213 1785.087742
2333.254924 -0.386468236 0.012019447 0.079421742 HAX1
1 154272511 154275875 + 2155
protein_coding HCLS1 associated protein X-1 [Source:HGNC
Symbol;Acc:HGNC:16915] - 1831 2146 1544 1891 2176
2964 41.60732099 47.18553505 44.57659258
48.7591693 62.06674596 63.95315037
ENSG00000084710 0.907250966 15.0449176 11.6249165 0
0 0 9.192361689 0 5.632485304
0.012076853 0.079745142 EFR3B 2 25042130
25159137 + 10591 protein_coding EFR3 homolog B
[Source:HGNC Symbol;Acc:HGNC:29155] - 1 17 10
0 0 0 0.004623722 0.07605686
0.058744861 0 0 0
ENSG00000159423 117.0353746 59.29467526 149.9614228
15.53952742 38.81072216 66.42320649 108.7638242
40.25781869 1.425939758 0.012077406 0.079745142
ALDH4A1 1 18871430 18902781 - 3689

protein_coding aldehyde dehydrogenase 4 family member A1 [Source:HGNC
Symbol;Acc:HGNC:406]   -   129   67   129   15   34
        77   1.71241782   0.860582654   2.175644342
0.225940792   0.566523099   0.970539215
ENSG00000280537 14.51601546   11.50493699   1.16249165   0
        0   0   9.061148032   0   5.617289821
0.012091   0.079805114   AC068946.1   2   219075329
        219170827   -   4650   protein_coding "novel
protein, SLC23A3-NHEJ1 readthrough"   -   16   13   1
        0   0   0   0.168498374   0.132469788
0.013379932   0   0   0
ENSG00000256973 3.629003864   6.194966072   16.2748831   0
        0   0   8.699617678   0   5.549138446
0.012106292   0.079876243   AC053513.1   12   22460519
        22463914   -   738   lincRNA novel transcript
        -       4   7   14   0   0   0
0.265419186   0.449436272   1.18026226   0   0   0
ENSG00000197885 655.9424484   603.5666944   478.9465598
661.9838682   854.9773793   997.2107363   579.4852342
838.057328   -0.531686768   0.012129173   0.07999737
NKIRAS1 3   23891660   23946591   -   3473
protein_coding NFKB inhibitor interacting Ras like 1 [Source:HGNC
Symbol;Acc:HGNC:17899] -   723   682   412   639   749
        1156   10.19441238   9.304778349   7.380729337
10.2237005   13.2563632   15.47690329
ENSG00000164463 268.5462859   248.683638   254.5856713
401.955776   433.7668947   327.8028372   257.2718651
387.841836   -0.590845457   0.012137257   0.080020847   CREBRF
        5   173056352   173139284   +   12333
protein_coding CREB3 regulatory factor [Source:HGNC
Symbol;Acc:HGNC:24050] -   296   281   219   388   380
        380   1.175307921   1.079602867   1.104796348
1.748135281   1.893922595   1.432669064
ENSG00000184281 636.8901781   902.6950562   730.0447561
851.5661028   966.8435785   1601.058068   756.5433301
1139.822583   -0.592016912   0.012147969   0.080061631   TSSC4
        11   2400488 2403878 +   2659   protein_coding tumor
suppressing subtransferable candidate 4 [Source:HGNC
Symbol;Acc:HGNC:12386] -   702   1020   628   822   847
        1856   12.92847971   18.1764169   14.69427465
17.17772018   19.57998854   32.45567551
ENSG00000154473 3589.992072   3612.550215   3878.072144
2853.057235   3035.226771   3037.352078   3693.538144
2975.212028   0.311834126   0.01215791   0.080097304   BUB3
        10   123154277   123170467   +   8300
protein_coding "BUB3, mitotic checkpoint protein [Source:HGNC
Symbol;Acc:HGNC:1151]" -   3957   4082   3336   2754   2659
        3521   23.34622364   23.30351048   25.00660857
18.43732398   19.69189925   19.72508806
ENSG00000165490 1154.93048   920.3949592   771.8944555

856.7459453   496.5489453   539.1494033   949.0732982
630.8147646   0.589958221   0.012170443   0.080150026   DDIAS
        11   82899975   82958277   +   5339
protein_coding DNA damage induced apoptosis suppressor [Source:HGNC
Symbol;Acc:HGNC:26351] -   1273   1040   664   827   435
        625   11.67608281   9.229960857   7.737755544
8.607115529   5.008142715   5.443160177
ENSG00000127418 3116.407068   2307.182364   3079.440381
2066.757147   2303.530509   2124.679969   2834.343271
2164.989208   0.388524442   0.012202151   0.080328935   FGFRL1
        4   1009936 1026897 +   4145   protein_coding
fibroblast growth factor receptor like 1 [Source:HGNC
Symbol;Acc:HGNC:3693]   -   3435   2607   2649   1995   2018
        2463   40.58176112   29.80183028   39.76163827
26.74424911   29.92567721   27.62936445
ENSG00000096092 630.5394214   524.8021258   564.9709418
468.2577597   388.1072216   320.9017248   573.4374963
392.4222354   0.548353857   0.012209046   0.080344424
TMEM14A 6   52671109   52686588   +   992
protein_coding transmembrane protein 14A [Source:HGNC
Symbol;Acc:HGNC:21076] -   695   593   486   452   340
        372   34.30850674   28.32497023   30.48115671
25.31854862   21.06757774   17.43661931
ENSG00000115541 591.5276298   966.4147072   733.5322311
474.4735707   466.8701577   609.023166   763.824856
516.7889648   0.56290142   0.012216204   0.080361637   HSPE1
        2   197499994   197503457   +   1977
protein_coding heat shock protein family E (Hsp10) member 1
[Source:HGNC Symbol;Acc:HGNC:5269]   -   652   1092   631
        458   409   706   16.14989154   26.17233134
19.85772703   12.87273569   12.71639469   16.60462416
ENSG00000010704 187.80095   244.2586623   134.8490314
130.5320304   77.62144432   91.4397388   188.9695479
99.86440449   0.921825773   0.012237629   0.080472649   HFE
        6   26087281   26098343   +   5675
protein_coding homeostatic iron regulator [Source:HGNC
Symbol;Acc:HGNC:4886]   -   207   276   116   126   68
        106   1.78621266   2.304462562   1.27174187
1.233720334   0.736529942   0.868502389
ENSG00000119943 84.37433984   74.33959286   81.37441549
13.46759043   23.97132839   50.03306463   80.0294494
29.15732782   1.447132012   0.012258   0.080576648
PYROXD2 10   98383565   98415184   -   3867
protein_coding pyridine nucleotide-disulphide oxidoreductase domain 2
[Source:HGNC Symbol;Acc:HGNC:23517]   -   93   84   70
        13   21   58   1.177707552   1.029275309
1.126239395   0.186801872   0.333804727   0.697404651
ENSG00000085063 3022.960219   3478.915947   4151.257682
3822.723746   5172.099473   5169.795798   3551.044616
4721.539673   -0.411350354   0.012295416   0.080792577   CD59

11 33703010 33736491 - 10358
protein_coding CD59 molecule (CD59 blood group) [Source:HGNC
Symbol;Acc:HGNC:1689] - 3332 3931 3571 3690 4531
5993 15.75280038 17.98264529 21.44967865
19.79531893 26.88843439 26.90291808
ENSG00000137936 1660.269268 1823.975011 1493.80177
2199.361115 2098.06198 2084.998572 1659.348683
2127.473889 -0.357953293 0.012308709 0.080849889 BCAR3
1 93561786 93847150 - 6866
protein_coding "BCAR3, NSP family adaptor protein [Source:HGNC
Symbol;Acc:HGNC:973]" - 1830 2061 1285 2123 1838
2417 13.05196721 14.22330862 11.64410667
17.18138773 16.45466588 16.36831105
ENSG00000167264 325.7030968 338.0681485 333.6351035
349.1213828 545.6330939 651.2924792 332.4687829
515.3489853 -0.633160262 0.012330325 0.080961811 DUS2
16 67987746 68079320 + 4472
protein_coding dihydrouridine synthase 2 [Source:HGNC
Symbol;Acc:HGNC:26014] - 359 382 287 337 478
755 3.931165586 4.047510731 3.992886345
4.187358549 6.570122422 7.850117818
ENSG00000131015 217.7402318 231.8687301 238.3107882
383.3083431 281.9484816 396.8139608 229.3065834
354.0235952 -0.628292378 0.012375732 0.081229802 ULBP2
6 149942000 149949235 + 1349
protein_coding UL16 binding protein 2 [Source:HGNC
Symbol;Acc:HGNC:14894] - 240 262 205 370 247
460 8.712202787 9.202710973 9.454721876
15.24059134 11.25465959 15.85538895
ENSG00000196588 569.7536066 717.7310692 678.8951235
827.7388274 913.1934625 938.5512813 655.4599331
893.1611904 -0.446785869 0.012408251 0.081410918 MRTFA
22 40410281 40636702 - 6533
protein_coding myocardin related transcription factor A [Source:HGNC
Symbol;Acc:HGNC:14334] - 628 811 584 799 800
1088 4.707341101 5.882130248 5.561693294
6.795887248 7.52704806 7.743677458
ENSG00000133028 2056.73794 2266.472587 1653.063126
2393.087223 2635.704631 2817.379122 1992.091218
2615.390325 -0.392244646 0.01241253 0.081410918 SCO1
17 10672474 10698375 - 10325
protein_coding "SCO1, cytochrome c oxidase assembly protein
[Source:HGNC Symbol;Acc:HGNC:10603]" - 2267 2561 1422
2310 2309 3266 10.75202198 11.75292484
8.568728505 12.43179853 13.74615488 14.70811911
ENSG00000021355 271.2680388 346.0331049 282.4854709
197.8699825 173.5067579 200.9948975 299.9288715
190.790546 0.652292104 0.012444558 0.081590726
SERPINB1 6 2832332 2842006 - 3094 protein_coding
serpin family B member 1 [Source:HGNC Symbol;Acc:HGNC:3311]

- 299 391 243 191 152 233
4.73237948 5.988015128 4.886442704 3.430244696
3.019747558 3.501600776
ENSG00000169777 0 0 0 0 5.707459141
25.87917136 0 10.52887683 -5.876609309 0.012471966
0.081740131 TAS2R1 5 9628997 9712378 - 1727
protein_coding taste 2 receptor member 1 [Source:HGNC
Symbol;Acc:HGNC:14909] - 0 0 0 0 5
30 0 0 0 0 0.177961078
0.807718324
ENSG00000157954 2376.09028 2623.125634 2819.042251
2805.402684 3385.664762 4171.722423 2606.086055
3454.26329 -0.406970405 0.012520274 0.082026341 WIPI2
7 5190188 5233826 + 8977 protein_coding "WD
repeat domain, phosphoinositide interacting 2 [Source:HGNC
Symbol;Acc:HGNC:32225]" - 2619 2964 2425 2708 2966
4836 14.28673393 15.64492246 16.80689026
16.76214057 20.30894118 25.04875155
ENSG00000119446 795.6590972 908.8900223 657.9702738
977.9542592 1141.491828 1112.804368 787.5064644
1077.416819 -0.451038498 0.012550081 0.082169978 RBM18
9 122237624 122264839 - 5193
protein_coding RNA binding motif protein 18 [Source:HGNC
Symbol;Acc:HGNC:28413] - 877 1027 566 944 1000
1290 8.270084639 9.370840844 6.781175046
10.10103126 11.83665631 11.55054312
ENSG00000176170 3551.887532 4440.020683 3795.535237
4296.161348 5033.978962 6169.594452 3929.147817
5166.578254 -0.395102403 0.012555904 0.082169978 SPHK1
17 76376584 76387860 + 4416
protein_coding sphingosine kinase 1 [Source:HGNC
Symbol;Acc:HGNC:11240] - 3915 5017 3265 4147 4410
7152 43.41415827 53.83211922 46.00033214
52.18155469 61.38424025 75.30597906
ENSG00000205220 143.3456526 188.5039676 258.0731463
61.1221412 159.8088559 59.52209412 196.6409222
93.48436376 1.076459578 0.012556134 0.082169978 PSMB10
16 67934502 67937087 - 1853
protein_coding proteasome subunit beta 10 [Source:HGNC
Symbol;Acc:HGNC:9538] - 158 213 222 59 140
69 4.175517913 5.446664503 7.453914412
1.769247685 4.644083039 1.731429009
ENSG00000211592 39.9190425 40.70977704 31.38727455
4.14387398 0 15.52750281 37.33869803 6.557125598
2.478673448 0.012570433 0.082233133 IGKC 2
88857161 88857683 - 523 IG_C_gene
immunoglobulin kappa constant [Source:HGNC
Symbol;Acc:HGNC:5716] - 44 46 27 4 0
18 4.119833561 4.167566934 3.211951079
0.424981814 0 1.600301581

ENSG00000111846 1020.657337 690.2962194 938.1307615
746.9332848 579.8778487 483.9405044 883.0281059
603.5838793 0.549267905 0.012593017 0.082350415 GCNT2
6 10492223 10629368 + 9072
protein_coding glucosaminyl (N-acetyl) transferase 2 (I blood group)
[Source:HGNC Symbol;Acc:HGNC:4204] - 1125 780 807
721 508 561 6.072648789 4.073971645
5.534486594 4.416154581 3.441977531 2.875350804
ENSG00000136807 966.2222788 1176.158558 953.2431529
1237.982351 1358.375276 1543.261252 1031.874663
1379.87296 -0.419138652 0.012603271 0.082387013 CDK9
9 127785679 127790787 + 3000
protein_coding cyclin dependent kinase 9 [Source:HGNC
Symbol;Acc:HGNC:1780] - 1065 1329 820 1195 1190
1789 17.38429314 20.99085686 17.00589308
22.13393826 24.38220997 27.72809951
ENSG00000119714 362.9003864 437.1876056 444.0718103
227.9130689 285.372957 310.5500563 414.7199341
274.6120274 0.59346232 0.012609815 0.082399344 GPR68
14 91232532 91253925 - 3278
protein_coding G protein-coupled receptor 68 [Source:HGNC
Symbol;Acc:HGNC:4519] - 400 494 382 220 250
360 5.97557533 7.140760062 7.250388202
3.729286725 4.687900871 5.106514503
ENSG00000160352 220.4619847 138.944239 149.9614228
116.0284714 82.18741163 68.14848458 169.7892155
88.78812254 0.937752713 0.012619432 0.082431736 ZNF714
19 21082159 21125270 + 8033
protein_coding zinc finger protein 714 [Source:HGNC
Symbol;Acc:HGNC:27124] zf-C2H2 243 157 129 112 72
79 1.481348323 0.926079619 0.999122617
0.774733435 0.55093719 0.457278037
ENSG00000182199 3112.778064 3978.053213 4173.345023
2969.085706 3095.725838 2602.582 3754.725434
2889.131181 0.378109744 0.012626295 0.082436772 SHMT2
12 57229327 57234935 + 4838
protein_coding serine hydroxymethyltransferase 2 [Source:HGNC
Symbol;Acc:HGNC:10852] - 3431 4495 3590 2866 2712
3017 34.72830105 44.02407836 46.16740149
32.91716055 34.45650163 28.99615845
ENSG00000076067 707.6557535 530.1120967 676.5701402
406.09965 413.2200418 509.8196758 638.1126635
443.0464559 0.524774795 0.012629524 0.082436772 RBMS2
12 56521929 56596196 + 8808
protein_coding RNA binding motif single stranded interacting protein
2 [Source:HGNC Symbol;Acc:HGNC:9909] - 780 599 582
392 362 591 4.336566198 3.222374093
4.111047789 2.472981143 2.526263369 3.119903728
ENSG00000108669 541.6288267 561.0869271 563.8084502
430.9628939 402.9466153 340.7424229 555.508068

391.550644 0.505557966 0.01265093 0.082546038 CYTH1
17 78674048 78782297 - 7754
protein_coding cytohesin 1 [Source:HGNC Symbol;Acc:HGNC:9501] -
597 634 485 416 353 395 3.770311372
3.874270164 3.891551557 2.98112082 2.798312864
2.368657769
ENSG00000131016 10.88701159 15.0449176 17.43737475
50.76245625 28.5372957 62.97265031 14.45643465
47.42413409 -1.721645347 0.012671691 0.082651012 AKAP12
6 151239999 151358557 + 8741
protein_coding A-kinase anchoring protein 12 [Source:HGNC
Symbol;Acc:HGNC:370] - 12 17 15 49 25
73 0.067227786 0.092154011 0.106766987
0.311492076 0.175802987 0.388322682
ENSG00000170909 153.3254133 230.983735 306.8977956
260.0280922 394.9561725 551.2263499 230.4023146
402.0702049 -0.806663453 0.012683242 0.082695863 OSCAR
19 54094668 54102692 - 2084
protein_coding "osteoclast associated, immunoglobulin-like receptor
[Source:HGNC Symbol;Acc:HGNC:29960]" - 169 261 264
251 346 639 3.971162635 5.934296391
7.881575839 6.692494925 10.20529926 14.25719736
ENSG00000068697 2396.957052 2277.092529 2719.067969
2132.023163 1907.432845 1632.113074 2464.372517
1890.523027 0.382480476 0.012723311 0.082926545
LAPTM4A 2 20032650 20052028 - 1946
protein_coding lysosomal protein transmembrane 4 alpha [Source:HGNC
Symbol;Acc:HGNC:6924] - 2642 2573 2339 2058 1671
1892 66.4842327 62.65033407 74.78151038
58.76443679 52.78140835 45.20737751
ENSG00000230291 39.01179154 37.16979643 45.33717435
94.27313304 77.62144432 95.75293403 40.5062541
89.21583713 -1.142527944 0.012787944 0.083317106
AC078817.1 12 80102899 80103333 - 435
processed_pseudogene ribosomal protein L26 (RPL26)
pseudogene - 43 42 39 91 68 111
4.840696811 4.574951288 5.578047352 11.62422959
9.608752696 11.86491797
ENSG00000051128 1219.345298 1386.787405 1354.302772
1092.946762 1012.503252 904.9083585 1320.145158
1003.452791 0.396085767 0.012826758 0.083539218 HOMER3
19 18929201 18941261 - 3422
protein_coding homer scaffold protein 3 [Source:HGNC
Symbol;Acc:HGNC:17514] - 1344 1567 1165 1055 887
1049 19.23304054 21.69778653 21.18130758
17.13107033 15.93275855 14.25366348
ENSG00000125826 1188.498765 1387.6724 1134.59185
1341.579201 1812.689023 1856.399225 1236.921005
1670.222483 -0.433003774 0.01283622 0.083570074 RBCK1
20 407498 430966 + 4024 protein_coding

RANBP2-type and C3HC4-type zinc finger containing 1 [Source:HGNC
Symbol;Acc:HGNC:15864]	-	1310	1568	976	1295	1588
	2152	15.94197072	18.4635211	15.09032844
17.88231909	24.25715628	24.86653728
ENSG00000104728 39.9190425	30.08983521	13.9498998
6.215810969	6.848950969	3.450556181	27.98625917
5.50510604	2.360886055	0.012858689	0.083685561
ARHGEF10	8	1823976 1958641 +	10792	protein_coding
	Rho guanine nucleotide exchange factor 10 [Source:HGNC
Symbol;Acc:HGNC:14103]	-	44	34	12	6	6
	4	0.199654647	0.149280617	0.069180892
0.030893091	0.034174068	0.017234118
ENSG00000177963 2366.110519	2557.635993	2192.459252
2510.151663	3329.731663	3501.451885	2372.068588
3113.778404	-0.392407323	0.012868638	0.083694032	RIC8A
	11	207511	215113	+	6685	protein_coding	RIC8
guanine nucleotide exchange factor A [Source:HGNC
Symbol;Acc:HGNC:29550]	-	2608	2890	1886	2423	2917
	4059	19.10446407	20.48438228	17.55282906
20.14021239	26.82145773	28.23245586
ENSG00000171608 1419.847762	1782.380238	2330.795758
1482.470916	1332.120963	1164.562711	1844.341253
1326.384864	0.475226773	0.012869454	0.083694032	PIK3CD
	1	9651732 9729114 +	6332
protein_coding	"phosphatidylinositol-4,5-bisphosphate 3-kinase
catalytic subunit delta [Source:HGNC Symbol;Acc:HGNC:8977]"	-
	1565	2014	2005	1431	1167	1350	12.10325321
	15.07110171	19.7006392	12.5577193	11.32862784
	9.913428542
ENSG00000140848 1067.834387	1136.333777	1327.565464
894.0408111	973.6925294	727.2047152	1177.244543
864.9793519	0.445063194	0.012899507	0.08385865	CPNE2
	16	57092537	57148367	+	7781
protein_coding	copine 2 [Source:HGNC Symbol;Acc:HGNC:2315]	-
	1177	1284	1142	863	853	843	7.407467097
	7.819087069	9.131403528	6.162932684	6.738464986
	5.037594167
ENSG00000198060 1071.463391	1047.834261	1092.742151
1418.24087	1268.197421	1480.288602	1070.679934
1388.908964	-0.376014051	0.012915543	0.083923522	5-Mar
	10	92291163	92353964	+	4632
protein_coding	membrane associated ring-CH-type finger 5 [Source:HGNC
Symbol;Acc:HGNC:26025]	-	1181	1184	940	1369	1111
	1716	12.48561763	12.11182515	12.6260106
16.42279006	14.74323773	17.2258139
ENSG00000111859 331.1466026	247.7986429	242.9607548
131.5679989	205.4685291	157.0003062	273.9686668
164.6789447	0.736643874	0.012918975	0.083923522	NEDD9
	6	11183298	11382348	-	10359
protein_coding	"neural precursor cell expressed, developmentally

down-regulated 9 [Source:HGNC Symbol;Acc:HGNC:7733]"	-	365
	280	209	127	180	182	1.725455308
1.280756708	1.255264653	0.681236535	1.068075695
0.816929489
ENSG00000134569 392.8396683	396.4778286	316.1977288
324.2581389	159.8088559	170.802531	368.5050752
218.2898419	0.756515177	0.012926906	0.083944213	LRP4
	11	46856868	46918642	-	8597
protein_coding	LDL receptor related protein 4 [Source:HGNC
Symbol;Acc:HGNC:6696]	-	433	448	272	313	140
	198	2.466434878	2.469207163	1.968470106
2.02306322	1.000987073	1.070900895
ENSG00000135823 970.7585336	877.0301967	842.8064462
1052.543991	1235.094158	1289.645373	896.8650588
1192.427841	-0.410754413	0.012963081	0.084148232	STX6
	1	180972712	181023121	-	6477
protein_coding	syntaxin 6 [Source:HGNC Symbol;Acc:HGNC:11441]	-
	1070	991	725	1016	1082	1495	8.089814516
	7.249802878	6.964195531	8.716293679	10.26835143
	10.73243616
ENSG00000049249 63.50756762	106.1994184	218.5484302
67.33795217	53.65011592	43.99459131	129.4184721
54.9942198	1.232367804	0.012987838	0.084278011
TNFRSF9 1	7915894 7943165 -	6331	protein_coding	TNF
receptor superfamily member 9 [Source:HGNC Symbol;Acc:HGNC:11924]
	-	70	120	188	65	47	51
0.541445078	0.89812208	1.847533757	0.570496635
0.456323573	0.374566455
ENSG00000126010 19.95952125	10.61994184	16.2748831
3.107905485	0	0	15.6181154	1.035968495
3.931281329	0.01301666	0.084434064	GRPR	X
16123556	16153021	+	1929	protein_coding
gastrin releasing peptide receptor [Source:HGNC Symbol;Acc:HGNC:4609]
	-	22	12	14	3	0	0
0.558494804	0.294764691	0.451546681	0.086417375	0
	0
ENSG00000119599 579.7333673	671.7113212	611.4706078
876.4293467	795.6198042	839.3477911	620.9717654
837.132314	-0.430876695	0.01302207	0.084438198	DCAF4
	14	72926332	72959703	+	5581
protein_coding	DDB1 and CUL4 associated factor 4 [Source:HGNC
Symbol;Acc:HGNC:20229]	-	639	759	526	846	697
	973	5.606831688	6.444009829	5.86381916
8.423069501	7.676585932	8.106471046
ENSG00000197024 381.9526567	422.142688	359.2099198
483.7972871	611.8396199	566.7538527	387.7684215
554.1302533	-0.514014581	0.013028642	0.084449854	ZNF398
	7	149126416	149182802	+	6041
protein_coding	zinc finger protein 398 [Source:HGNC
Symbol;Acc:HGNC:18373]	zf-C2H2 421	477	309	467	536

657	3.412730106	3.741415871	3.182412637
4.295562956	5.45385157	5.056937102
ENSG00000107338 194.1517067	348.6880903	373.1598196
210.3016045	159.8088559	166.4893357	305.3332056
178.8665987	0.770306638	0.013040208	0.084459545	SHB
9	37919134	38069211	-	2783
protein_coding	SH2 domain containing adaptor protein B [Source:HGNC
Symbol;Acc:HGNC:10838]	-	214	394	321	203	140
193	3.765557213	6.708253352	7.176268388
4.05317052	3.09216165	3.224594588
ENSG00000116044 1503.314851	1241.6482	1384.527555
1969.376109	1735.067579	1667.481275	1376.496868
1790.641654	-0.379411807	0.01304167	0.084459545	NFE2L2
2	177227595	177392697	-	5979
protein_coding	"nuclear factor, erythroid 2 like 2 [Source:HGNC
Symbol;Acc:HGNC:7782]"	TF_bZIP 1657	1403	1191	1901	1520
1933	13.57133711	11.11873958	12.39338824
17.66711382	15.62652441	15.03260752
ENSG00000171155 489.9155216	565.5119028	582.4083166
410.243524	417.7860091	269.1433821	545.945247
365.7243051	0.579654323	0.013054244	0.084459545
C1GALT1C1	X	120625793	120630150	-	1785
protein_coding	C1GALT1 specific chaperone 1 [Source:HGNC
Symbol;Acc:HGNC:24338]	-	540	639	501	396	366
312	14.81440533	16.96246945	17.4624973
12.32732963	12.6034727	8.127320598
ENSG00000279861 7.258007728	10.61994184	6.974949899	0
0	0	8.284299822	0	5.484511628
0.013054942	0.084459545	AC073548.1	19	47487638
47489519	-	1882	TEC	TEC	-	8
12	6	0	0	0	0.208160849
0.302125977	0.198352865	0	0	0
ENSG00000227082 7.258007728	10.61994184	6.974949899	0
0	0	8.284299822	0	5.484511628
0.013054942	0.084459545	AC244021.1	1	121396754
121463429	+	4658	lincRNA novel transcript
-	8	12	6	0	0	0
0.084104491	0.122069791	0.080141711	0	0	0
ENSG00000247151 12.70151352	1.769990306	11.6249165	0
0	0	8.698806776	0	5.551751403
0.01305931	0.084459545	CSTF3-DT	11	33161657
33191598	+	4012	processed_transcript	CSTF3
divergent transcript [Source:HGNC Symbol;Acc:HGNC:27882]	-
14	2	10	0	0	0	0.170881794
0.023620849	0.155076476	0	0	0
ENSG00000063244 5725.660846	6791.452805	6680.839512
4359.355427	5160.684555	5570.922954	6399.317721
5030.320979	0.347107034	0.013064122	0.084459545	U2AF2
19	55654146	55674715	+	4115
protein_coding	U2 small nuclear RNA auxiliary factor 2 [Source:HGNC

Symbol;Acc:HGNC:23156]	-	6311	7674	5747	4208	4521
	6458	75.10295484	88.36461633	86.89168201
56.82218572	67.53237567	72.972499
ENSG00000136425 56.24955989	67.25963164	81.37441549
26.93518087	25.11282022	29.32972754	68.29453567
27.12590954	1.328543174	0.013068335	0.084459545	CIB2
	15	78104606	78131544	-	2146
protein_coding	calcium and integrin binding family member 2
[Source:HGNC Symbol;Acc:HGNC:24579]	-	62	76	70
	26	22	34	1.41478568	1.678070936
2.029435107	0.67321793	0.630144752	0.73668227
ENSG00000132819 581.5478692	800.0356184	726.5572812
873.3214412	972.5510376	1061.046026	702.7135896
968.9728348	-0.463988437	0.013094432	0.084597302	RBM38
	20	57391407	57409333	+	2686
protein_coding	RNA binding motif protein 38 [Source:HGNC
Symbol;Acc:HGNC:15818]	-	641	904	625	843	852
	1230	11.68639886	15.94736238	14.47707604
17.43948316	19.49759058	21.2926699
ENSG00000128944 954.4280162	823.9304876	870.7062458
680.6313012	714.5738844	512.4075929	883.0215832
635.8709262	0.475087613	0.013102749	0.084620125	KNSTRN
	15	40382721	40394246	+	3654
protein_coding	kinetochore localized astrin (SPAG5) binding protein
[Source:HGNC Symbol;Acc:HGNC:30767]	-	1052	931	749
	657	626	594	14.09859647	12.07278812
12.75322792	9.990997958	10.53060082	7.558731525
ENSG00000225151 14.51601546	10.61994184	4.6499666
20.7193699	47.94265678	50.89570367	9.928641298
39.85257678	-1.992952875	0.013157794	0.084944605
GOLGA2P7	15	84199311	84230136	-	4915
	transcribed_unprocessed_pseudogene	GOLGA2 pseudogene 7
[Source:HGNC Symbol;Acc:HGNC:49516]	-	16	12	4
	20	42	59	0.159413517	0.115686895
0.050634126	0.226109348	0.525258548	0.558161025
ENSG00000144406 7.258007728	7.079961225	10.46242485	0
	0	0	8.266797934	0	5.478818324
0.013178226	0.085022671	UNC80	2	209771993
209999300	+	19258	protein_coding	"unc-80 homolog, NALCN
channel complex subunit [Source:HGNC Symbol;Acc:HGNC:26582]"	-
	8	8	9	0	0	0	0.020342648
	0.019683632	0.029076235	0	0	0
ENSG00000116954 740.3167882	850.4803421	735.8572144
1059.79577	1052.455466	976.5073993	775.5514483
1029.586212	-0.408020021	0.013179499	0.085022671	RRAGC
	1	38838198	38859823	-	5167
protein_coding	Ras related GTP binding C [Source:HGNC
Symbol;Acc:HGNC:19902]	-	816	961	633	1023	922
	1132	7.733576409	8.81274831	7.622055295
11.00143193	10.96831261	10.18682824

ENSG00000121966 4.53625483 7.964956378 0 15.53952742
23.97132839 43.99459131 4.167070403 27.83514904
-2.710960686 0.013191237 0.085067366 CXCR4 2
136114349 136118165 - 2224 protein_coding
C-X-C motif chemokine receptor 4 [Source:HGNC
Symbol;Acc:HGNC:2561] - 5 9 0 15 21
51 0.110094064 0.191749468 0 0.374773194
0.580405972 1.066268087
ENSG00000107223 1119.547692 2230.187786 2318.00835
1027.680747 1172.312108 1551.025003 1889.247943
1250.339286 0.594714265 0.013198308 0.085081952 EDF1
9 136862119 136866286 - 1200
protein_coding endothelial differentiation related factor 1
[Source:HGNC Symbol;Acc:HGNC:3164] - 1234 2520 1994
992 1027 1798 50.35731863 99.50519052
103.3833866 45.93486769 52.6061547 69.66898116
ENSG00000103335 9545.187413 10442.05781 11011.12091
8484.581973 7776.983825 8858.440356 10332.78871
8373.335385 0.303190653 0.013223913 0.085215958 PIEZO1
16 88715338 88785220 - 11292
protein_coding piezo type mechanosensitive ion channel component 1
[Source:HGNC Symbol;Acc:HGNC:28993] - 10521 11799 9472
8190 6813 10269 45.62625619 49.51089448
52.18884272 40.30185869 37.08641721 42.28519157
ENSG00000276710 61.69306569 78.76456863 81.37441549
140.8917153 105.0172482 186.3300338 73.9440166
144.0796658 -0.966227878 0.013230618 0.085228114
CSPG4P10 15 82459472 82477258 + 7178
transcribed_unprocessed_pseudogene chondroitin sulfate
proteoglycan 4 pseudogene 10 [Source:HGNC Symbol;Acc:HGNC:48361]
- 68 89 70 136 92 216
0.463910436 0.58750693 0.606738331 1.052803931
0.787828583 1.399204893
ENSG00000166167 427.315205 463.7374602 419.6594856
636.0846559 541.0671266 656.4683135 436.9040503
611.2066986 -0.48461352 0.013238398 0.085234038 BTRC
10 101354033 101557321 + 6613
protein_coding beta-transducin repeat containing E3 ubiquitin protein
ligase [Source:HGNC Symbol;Acc:HGNC:1144] - 471 524
361 614 474 761 3.487795941 3.75456135
3.396374142 5.159194395 4.405824353 5.350780703
ENSG00000165943 320.259591 383.2029013 290.6229125
459.9700117 549.0575694 450.2975816 331.3618016
486.4417209 -0.551731558 0.013241175 0.085234038 MOAP1
14 93182196 93184928 - 2537
protein_coding modulator of apoptosis 1 [Source:HGNC
Symbol;Acc:HGNC:16658] - 353 433 250 444 481
522 6.813698644 8.087119675 6.13093043
9.724662695 11.65391831 9.567116312
ENSG00000058866 385.5816605 305.3233278 201.1110554

520.0561844	392.6731889	502.9185634	297.3386813
471.8826456	-0.664355257	0.013265647	0.08535444	DGKG
	3	186105668	186362237	-	10978
protein_coding	diacylglycerol kinase gamma [Source:HGNC
Symbol;Acc:HGNC:2853]	-	425	345	173	502	344
	583	1.895808155	1.489094671	0.980459645
2.540929026	1.92611661	2.469314158
ENSG00000151474 1425.291268	1212.44336	1533.326486
1257.665753	894.9295933	866.9522405	1390.353705
1006.515862	0.465918923	0.01326953	0.08535444	FRMD4A
	10	13643706	14462142	-	14646
protein_coding	FERM domain containing 4A [Source:HGNC
Symbol;Acc:HGNC:25491]	-	1571	1370	1319	1214	784
	1005	5.252739204	4.432288062	5.603154688
4.605870262	3.290367396	3.190641797
ENSG00000163399 9946.19234	9554.407673	11471.4676
8789.156711	8388.823445	7742.185431	10324.02254
8306.721862	0.31359709	0.013286951	0.08543543	ATP1A1
	1	116372668	116410261	+	7261
protein_coding	ATPase Na+/K+ transporting subunit alpha 1
[Source:HGNC Symbol;Acc:HGNC:799]	-	10963	10796	9868
	8484	7349	8975	73.93697205	70.45192757
84.55505	64.92564409	62.21271732	57.47368437
ENSG00000159082 768.4415682	683.2162582	620.770541
1049.436085	891.5051178	876.44127	690.8094558
939.127491	-0.442126216	0.013291873	0.085436028	SYNJ1
	21	32628759	32728048	-	8070
protein_coding	synaptojanin 1 [Source:HGNC Symbol;Acc:HGNC:11503]
	-	847	772	534	1013	781	1016
5.139709336	4.532838088	4.116940296	6.975060104
5.948738241	5.853979421
ENSG00000121851 254.0302705	215.0538222	212.7359719
107.7407235	86.75337894	182.0168386	227.2733549
125.503647	0.852226297	0.01330631	0.085497756
POLR3GL 1	145964702	145978848	+	1799
protein_coding	RNA polymerase III subunit G like [Source:HGNC
Symbol;Acc:HGNC:28466]	-	280	243	183	104	76
	211	7.621764954	6.40031798	6.328878714
3.212286108	2.5967479	5.453587808
ENSG00000155886 0	0	1.16249165	7.251779464	0
	31.91764468	0.387497217	13.05647471	-5.22650743
	0.013354248	0.085774619	SLC24A2 9	19507452
	19786928	-	10749	protein_coding	solute carrier
family 24 member 2 [Source:HGNC Symbol;Acc:HGNC:10976]	-	0
	0	1	7	0	37	0	0
0.005788137	0.03618612	0	0.160053317
ENSG00000144810 938.0974988	972.6096733	1321.753006
970.7024797	514.8128145	680.6222067	1077.486726
722.0458337	0.576479288	0.013375127	0.085877541	COL8A1
	3	99638475	99799226	+	9408

protein_coding collagen type VIII alpha 1 chain [Source:HGNC
Symbol;Acc:HGNC:2215]   -   1034   1099   1137   937   451
        789   5.382101869   5.535117245   7.519171709
5.53419332   2.946636698   3.899516054
ENSG00000197646 196.8734596   259.3035799   199.9485638
346.0134773   344.7305321   318.3138077   218.7085344
336.3526057   -0.619342454   0.013384271   0.085889017
PDCD1LG2   9   5510570 5571254 +   2365   protein_coding
        programmed cell death 1 ligand 2 [Source:HGNC
Symbol;Acc:HGNC:18731] -   217   293   172   334   302
        369   4.493215748   5.870335412   4.5248496
7.847428463   7.849159568   7.254813278
ENSG00000076650 421.8716992   392.937848   477.7840681
555.2791133   523.9447491   837.622513   430.8645384
638.9487918   -0.570763575   0.013391176   0.085889017
GPATCH1 19   33080880   33130542   +   4488
protein_coding G-patch domain containing 1 [Source:HGNC
Symbol;Acc:HGNC:24658] -   465   444   411   536   459
        971   5.073746774   4.687664947   5.697650692
6.636269051   6.286475068   10.05998654
ENSG00000136811 1088.701159   1054.914223   1135.754342
920.975992   743.1111801   803.9795902   1093.123241
822.6889208   0.409616965   0.013391481   0.085889017   ODF2
        9   128455186   128501292   +   6393
protein_coding outer dense fiber of sperm tails 2 [Source:HGNC
Symbol;Acc:HGNC:8114]   -   1200   1192   977   889   651
        932   9.191898608   8.834825821   9.508164918
7.72696776   6.259269404   6.778634631
ENSG00000282917 0   0   0   5.179842475   26.25431205
        0   0   10.47805151   -5.860157789   0.013406769
        0.085955901   AC107068.2   4   47831385
47896709   +   550   antisense   uncharacterized
LOC101927179 [Source:NCBI gene;Acc:101927179]   -   0   0
        0   5   23   0   0   0   0
0.505148838   2.570469806   0
ENSG00000142937 19301.7643   25182.53708   26396.69789
27969.07743   29880.83159   32353.27739   23626.99976
30067.7288   -0.347835613   0.013480321   0.086396158   RPS8
        1   44775251   44778779   +   2847
protein_coding ribosomal protein S8 [Source:HGNC
Symbol;Acc:HGNC:10441] -   21275   28455   22707   26998   26177
        37505   365.9407595   473.5845913   496.2255702
526.9339363   565.1708657   612.5375551
ENSG00000261024 0   0   0   3.107905485   11.41491828
        10.35166854   0   8.291497437   -5.527530342
0.013524393   0.086639438   GS1-279B7.1   1   185321157
        185335039   -   1205
transcribed_processed_pseudogene   microtubule associated protein
1 light chain 3 beta pseudogene [Source:NCBI gene;Acc:100288079]
        -       0   0   0   3   10   12   0

0	0	0.138339516	0.510105861	0.463047152
ENSG00000186567 10.88701159	15.92991276	10.46242485
39.36680281	37.66923033	48.30778654	12.42644973
41.78127322	-1.746169338	0.013528076	0.086639438
CEACAM19	19	44662278	44684359	+	5203
protein_coding	carcinoembryonic antigen related cell adhesion
molecule 19 [Source:HGNC Symbol;Acc:HGNC:31951]	-	12	18
9	38	33	56	0.112942164	0.163924973
0.10762063	0.405827819	0.389858919	0.500455215
ENSG00000210127 16.33051739	7.079961225	2.3249833	0
0	0	8.578487304	0	5.537583249
0.013545882	0.086722082	MT-TA	MT	5587	5655	-
69	Mt_tRNA mitochondrially encoded tRNA alanine
[Source:HGNC Symbol;Acc:HGNC:7475]	-	18	8	2
0	0	0	12.77474083	5.493730325
1.803382087	0	0	0
ENSG00000140832 4.53625483	15.92991276	19.76235805	0
0	1.725278091	13.40950854	0.575092697
4.42844588	0.013565811	0.086818242	MARVELD3	16
71626161	71642114	+	6446	protein_coding
MARVEL domain containing 3 [Source:HGNC Symbol;Acc:HGNC:30525]
-	5	18	17	0	0	2
0.037984673	0.132314867	0.16408371	0	0
0.014426823
ENSG00000077348 1145.85797	1495.641809	1327.565464
1444.140082	1805.840072	2165.224004	1323.021748
1805.068053	-0.448574478	0.013586936	0.086921991	EXOSC5
19	41386374	41397479	-	1488
protein_coding	exosome component 5 [Source:HGNC
Symbol;Acc:HGNC:24662]	-	1263	1690	1142	1394	1582
2510	41.56512615	53.81585125	47.74963095
52.05613095	65.35079996	78.43353848
ENSG00000256060 153.3254133	181.4240064	131.3615564
213.4095099	335.5985975	235.5004594	155.3703254
261.5028556	-0.747819397	0.013593485	0.086932451
TRAPPC2B	19	57363511	57365353	+	1843
protein_coding	trafficking protein particle complex 2B
[Source:HGNC Symbol;Acc:HGNC:10710]	-	169	205	113
206	294	273	4.490451943	5.270538216
3.814696184	6.210891304	9.805491226	6.887606543
ENSG00000273328 0.907250966	0.884995153	4.6499666
3.107905485	20.54685291	37.09347895	2.14740424
20.24941245	-3.287983205	0.013599934	0.086942257
AC099329.2	3	42809414	42908105	+	3933
processed_transcript	novel transcript	-	1
1	4	3	18	43	0.012451014
0.012047654	0.063276564	0.042384723	0.281316962
0.508364865
ENSG00000275700 2706.329632	3373.601524	3043.403139
3217.718145	3990.655431	4958.449232	3041.111432

4055.607603	-0.415536362	0.013645264	0.08720053	AATF
	17	36948875	37056871	+	4714
protein_coding	apoptosis antagonizing transcription factor
[Source:HGNC Symbol;Acc:HGNC:19235]	-	2983	3812	2618
	3106	3496	5748	30.98791519	38.3168461
34.55309153	36.61203905	45.58576066	56.69675452
ENSG00000136854 799.288101	621.2665975	685.8700734
788.3720246	1103.822598	1043.793245	702.1415907
978.6626224	-0.478934018	0.013661791	0.087247532	STXBP1
	9	127579370	127696027	+	9373
protein_coding	syntaxin binding protein 1 [Source:HGNC
Symbol;Acc:HGNC:11444]	-	881	702	590	761	967
	1210	4.602841021	3.548827877	3.916338673
4.5114701	6.341547025	6.002577437
ENSG00000090861 5616.79073	6615.33877	7248.135437
7350.196471	9090.84092	8350.345958	6493.421646
8263.79445	-0.347940828	0.013662483	0.087247532	AARS
	16	70252295	70289543	-	4498
protein_coding	alanyl-tRNA synthetase [Source:HGNC
Symbol;Acc:HGNC:20]	-	6191	7475	6235	7095	7964
	9680	67.40157368	78.74412957	86.24299962
87.64860177	108.8326391	100.0660886
ENSG00000165684 645.9626878	959.334746	464.99666
875.3933782	1013.644743	1255.139811	690.0980312
1048.059311	-0.601774654	0.013667893	0.087250579	SNAPC4
	9	136375577	136400168	-	5045
protein_coding	small nuclear RNA activating complex polypeptide 4
[Source:HGNC Symbol;Acc:HGNC:11137]	MYB	712	1084	400
	845	888	1455	6.911105245	10.18109647
4.932938117	9.306954313	10.81929981	13.41012546
ENSG00000143612 4604.298652	5092.262111	5869.42034
6520.385707	6772.471017	6236.017658	5188.660368
6509.624794	-0.327391793	0.013677591	0.08728099
C1orf43 1	154206696	154220637	-	2670
protein_coding	chromosome 1 open reading frame 43 [Source:HGNC
Symbol;Acc:HGNC:29876]	-	5075	5754	5049	6294	5933
	7229	93.07937721	102.1139408	117.6524447
130.9867965	136.5873399	125.8919561
ENSG00000164050 637.7974291	778.7957347	802.1192384
614.3293175	485.134027	478.7646701	739.5708008
526.0760049	0.491190538	0.013687608	0.08728543	PLXNB1
	3	48403854	48430051	-	9733
protein_coding	plexin B1 [Source:HGNC Symbol;Acc:HGNC:9103]	-
	703	880	690	593	425	555	3.537018124
	4.284127522	4.410717208	3.385478138	2.684043604
	2.651412368
ENSG00000129521 212.296726	182.3090015	392.9221777
161.6110852	164.3748233	101.7914073	262.5093017
142.5924386	0.880144659	0.013692443	0.08728543	EGLN3
	14	33924231	34462774	-	7359

protein_coding egl-9 family hypoxia inducible factor 3 [Source:HGNC
Symbol;Acc:HGNC:14661] -   234   206   338   156   144
        118   1.557133105   1.326401054   2.857621758
1.177925542   1.202793436   0.745579953
ENSG00000068971 283.0623014   366.3879934   402.2221109
385.3802801   597.0002261   629.7265031   350.5574685
537.3690031   -0.617720666   0.01369309   0.08728543
PPP2R5B 11   64917553   64934473   +   3376
protein_coding protein phosphatase 2 regulatory subunit B'beta
[Source:HGNC Symbol;Acc:HGNC:9310]   -   312   414   346
        372   523   730   4.525648705   5.810645012
6.376472741   6.122834852   9.522404177   10.05429076
ENSG00000101442 367.4366412   338.0681485   283.6479626
494.1569721   505.6808799   438.220635   329.7175841
479.352829   -0.537737092   0.013715179   0.087369348   ACTR5
        20   38748442   38772520   +   2565
protein_coding ARP5 actin related protein 5 homolog [Source:HGNC
Symbol;Acc:HGNC:14671] -   405   382   244   477   443
        508   7.732079973   7.056712666   5.918467996
10.33339553   10.61606862   9.208891603
ENSG00000101152 3802.288798   4104.60752   3880.397127
3850.694896   5708.600633   6128.187778   3929.097815
5229.161102   -0.412448469   0.013717485   0.087369348   DNAJC5
        20   63895182   63936031   +   5287
protein_coding DnaJ heat shock protein family (Hsp40) member C5
[Source:HGNC Symbol;Acc:HGNC:16235]   -   4191   4638   3338
        3717   5001   7104   38.81834665   41.56692279
39.28112058   39.06567155   58.14266103   62.47764635
ENSG00000118816 5292.902136   4418.7808   5821.758183
6577.363974   6796.442345   6249.819883   5177.813706
6541.208734   -0.337389944   0.013722327   0.087369348   CCNI
        4   77047158   77076005   -   3741
protein_coding cyclin I [Source:HGNC Symbol;Acc:HGNC:1595]   -
        5834   4993   5008   6349   5954   7245   76.36729366
        63.24122863   83.2881966   94.30390193   97.82919554
        90.04958169
ENSG00000099326 435.4804637   555.7769562   296.4353707
504.516657   827.5815754   679.7595677   429.2309302
670.6192667   -0.641475583   0.013726012   0.087369348   MZF1
        19   58561931   58573575   -   6691
protein_coding myeloid zinc finger 1 [Source:HGNC
Symbol;Acc:HGNC:13108] zf-C2H2 480   628   255   487   725
        788   3.513005996   4.447285952   2.37113345
4.04436157   6.660308363   5.47603488
ENSG00000173852 3074.673524   2402.761841   2958.541249
2641.719662   1888.027484   1608.821819   2811.992204
2046.189655   0.458811163   0.013739436   0.087423338
DPY19L1 7   34928876   35038271   -   6278
protein_coding dpy-19 like C-mannosyltransferase 1 [Source:HGNC
Symbol;Acc:HGNC:22205] -   3389   2715   2545   2550   1654

1865 26.43497725 20.49155723 25.22163997
22.56996641 16.19427665 13.81301689
ENSG00000156011 2192.825585 1559.36146 2398.220274
1677.232993 1552.428886 1234.436474 2050.135773
1488.032784 0.462295601 0.013780896 0.087655616 PSD3
8 18527301 19084730 - 15213
protein_coding pleckstrin and Sec7 domain containing 3 [Source:HGNC
Symbol;Acc:HGNC:19093] - 2417 1762 2063 1619 1360
1431 7.780194759 5.488042673 8.437061393
5.913492184 5.495046898 4.373768441
ENSG00000176476 355.6423787 396.4778286 495.2214429
497.2648776 606.1321608 729.7926323 415.78055
611.0632235 -0.557781367 0.01378818 0.087670426 SGF29
16 28553915 28591790 + 2124
protein_coding SAGA complex associated factor 29 [Source:HGNC
Symbol;Acc:HGNC:25156] - 392 448 426 480 531
846 9.037748218 9.994243869 12.47848707
12.55737225 15.36693905 18.52025102
ENSG00000125046 6.350756762 8.849951531 9.299933199 0
0 0 8.166880497 0 5.462117506
0.013833442 0.087926613 SSUH2 3 8619400 8745040 -
6279 protein_coding ssu-2 homolog (C. elegans)
[Source:HGNC Symbol;Acc:HGNC:24809] - 7 10 8
0 0 0 0.05459291 0.075463329
0.079269542 0 0 0
ENSG00000178105 1675.692534 1601.841227 1692.587842
2018.066628 2059.251258 2175.575672 1656.707201
2084.297853 -0.331538354 0.013855931 0.088037915 DDX10
11 108665025 108940930 + 8653
protein_coding DEAD-box helicase 10 [Source:HGNC
Symbol;Acc:HGNC:2735] - 1847 1810 1456 1948 1804
2522 10.45270937 9.911475505 10.46891124
12.509337 12.81495807 13.55218734
ENSG00000168434 244.9577608 295.5883811 347.5850033
433.0348309 537.6426511 376.9732628 296.0437151
449.2169149 -0.602021429 0.013916606 0.088391683 COG7
16 23388493 23453180 - 4343
protein_coding component of oligomeric golgi complex 7 [Source:HGNC
Symbol;Acc:HGNC:18622] - 270 334 299 418 471
437 3.044406345 3.64403952 4.283395791
5.348087401 6.666201515 4.678672278
ENSG00000229927 0 2.654985459 4.6499666 26.93518087
22.82983656 11.21430759 2.43498402 20.32644167
-3.093578163 0.013928467 0.088427052 RHEBP1 10
46634911 46635466 - 556
processed_pseudogene RHEB pseudogene 1 [Source:HGNC
Symbol;Acc:HGNC:10010] - 0 3 4 26 20
13 0 0.255665957 0.447602029 2.598427477
2.211070368 1.087175305
ENSG00000158859 188.7082009 165.4940936 204.5985304

131.5679989	106.15874	51.75834272	186.2669416
96.4950272	0.95231849	0.013932172	0.088427052
ADAMTS4 1	161184308	161199056	-	10334
protein_coding	ADAM metallopeptidase with thrombospondin type 1 motif
4 [Source:HGNC Symbol;Acc:HGNC:220]	-	208	187	176
127	93	60	0.985651895	0.857431807
1.059622221	0.682884582	0.553174117	0.269968946
ENSG00000137078 17.23776835	3.539980612	4.6499666	0
0	0	8.475905189	0	5.518591829
0.013944351	0.088472609	SIT1	9	35649295
35650950	-	1327	protein_coding	signaling threshold
regulating transmembrane adaptor 1 [Source:HGNC
Symbol;Acc:HGNC:17710]	-	19	4	4	0	0
0	0.701150683	0.142828709	0.187540865	0
0	0
ENSG00000104312 701.3049967	769.0607881	747.4821309
862.9617563	1044.465023	1063.633943	739.2826385
990.353574	-0.422041093	0.013950904	0.088482446	RIPK2
8	89757747	89791063	+	3117
protein_coding	receptor interacting serine/threonine kinase 2
[Source:HGNC Symbol;Acc:HGNC:10020]	-	773	869	643
833	915	1233	12.14426891	13.21020068
12.83456097	14.84978763	18.04395154	18.39319997
ENSG00000131828 1061.48363	1079.694087	1119.479459
850.5301343	752.2431148	875.578631	1086.885725
826.1172933	0.394981213	0.013958798	0.088500782	PDHA1
X	19343893	19361705	+	4493
protein_coding	pyruvate dehydrogenase E1 alpha 1 subunit [Source:HGNC
Symbol;Acc:HGNC:8806]	-	1170	1220	963	821	659
1015	12.75199479	12.86618681	13.33511346
10.15357035	9.015635733	10.50414339
ENSG00000072609 354.7351277	313.2882842	326.6601536
524.2000584	476.0020923	430.4568836	331.5611885
476.8863448	-0.523736207	0.013989447	0.088663323	CHFR
12	132822187	132956304	-	17127
protein_coding	checkpoint with forkhead and ring finger domains
[Source:HGNC Symbol;Acc:HGNC:20455]	-	391	354	281
506	417	499	1.117954538	0.979373627
1.020779333	1.641652615	1.496587513	1.354722257
ENSG00000215914 0.907250966	5.309970919	20.9248497	0
0	0	9.047357194	0	5.604038299
0.014026549	0.08886663	MMP23A	1	1699942 1701782 +
1017	unprocessed_pseudogene	matrix metallopeptidase 23A
(pseudogene) [Source:HGNC Symbol;Acc:HGNC:7170] -	1	6
18	0	0	0	0.048151268	0.279548225
1.101180212	0	0	0
ENSG00000170323 22.68127415	15.0449176	32.5497662
7.251779464	0	0	23.42531932	2.417259821
3.285290241	0.01404579	0.088946674	FABP4	8
81478419	81483263	-	1396	protein_coding	fatty

acid binding protein 4 [Source:HGNC Symbol;Acc:HGNC:3559]	-
	25	17	28	7	0	0	0.876967046
	0.577018774	1.247899066	0.278627941	0	0
ENSG00000196704 1584.060187	1583.256329	1671.662993
1923.793495	1959.941469	2292.031943	1612.993169
2058.588969	-0.352499973	0.01404924	0.088946674	AMZ2
	17	68247574	68257164	+	4214
protein_coding	archaelysin family metallopeptidase 2 [Source:HGNC
Symbol;Acc:HGNC:28041]	-	1746	1789	1438	1857	1717
	2657	20.28982922	20.11602886	21.23103672
24.48665239	25.04512042	29.31757476
ENSG00000138738 125.2006333	77.87957347	108.1117234
56.97826722	52.5086241	33.64292277	103.7306434
47.70993803	1.123982928	0.014055847	0.088946959	PRDM5
	4	120684919	120922870	-	9517
protein_coding	PR/SET domain 5 [Source:HGNC Symbol;Acc:HGNC:9349]
	zf-C2H2 138	88	93	55	46	39
0.710080687	0.438136106	0.607980606	0.321125404
0.29710169	0.190544122
ENSG00000167670 2130.225268	2233.727766	1992.510688
1790.153559	1571.834247	1647.640576	2118.821241
1669.876128	0.34370741	0.014059342	0.088946959	CHAF1A
	19	4402662 4445018 +	4299	protein_coding
chromatin assembly factor 1 subunit A [Source:HGNC
Symbol;Acc:HGNC:1910]	-	2348	2524	1714	1728	1377
	1910	26.74603022	27.81943762	24.80562758
22.33512239	19.68855555	20.65841227
ENSG00000176087 2124.781762	1979.734158	1921.598697
2282.238594	2656.251484	2705.236046	2008.704872
2547.908708	-0.342950878	0.014072405	0.088997772
SLC35A4 5	140564456	140569103	+	4023
protein_coding	solute carrier family 35 member A4 [Source:HGNC
Symbol;Acc:HGNC:20753]	-	2342	2237	1653	2203	2327
	3136	28.50792068	26.34768074	25.56405055
30.42821724	35.55442921	36.24574574
ENSG00000198791 3012.073207	2553.211017	2521.444389
3593.774709	3360.551942	3243.52281	2695.576204
3399.283154	-0.334361996	0.014080736	0.089018631	CNOT7
	8	17224964	17246878	-	10943
protein_coding	CCR4-NOT transcription complex subunit 7 [Source:HGNC
Symbol;Acc:HGNC:14101]	-	3320	2885	2169	3469	2944
	3760	14.85697416	12.49211171	12.33190014
17.61489036	16.53669691	15.97653017
ENSG00000139354 919.9524795	622.1515926	695.1700066
690.9909861	380.1167788	357.9952038	745.7580263
476.3676562	0.647249612	0.014116478	0.089212715	GAS2L3
	12	100573683	100628286	+	6799
protein_coding	growth arrest specific 2 like 3 [Source:HGNC
Symbol;Acc:HGNC:27475]	-	1014	703	598	667	333
	415	7.303341313	4.899330359	5.472212949

5.451209039	3.010554908	2.838141693
ENSG00000137494 493.5445255	554.891961	417.3345023
613.293349	788.7708533	671.9958163	488.5903296
691.3533395	-0.499027475	0.014139379	0.089325524
ANKRD42 11	83193739	83260694	+	6724
protein_coding	ankyrin repeat domain 42 [Source:HGNC
Symbol;Acc:HGNC:26752]	-	544	627	359	592	691
	779	3.961866875	4.418412683	3.321800839
4.892220753	6.31680838	5.38692304
ENSG00000271743 32.66103478	15.92991276	2.3249833
3.107905485	0	0	16.97197694	1.035968495
4.054284957	0.014157812	0.089410045	AF287957.1	8
	6615604 6617198 -	1595	lincRNA novel transcript
	-	36	18	2	3	0	0
1.105275382	0.534734566	0.078014648	0.104513553	0
	0
ENSG00000069696 9.979760626	28.3198449	2.3249833
79.76957411	18.26386925	73.32431885	13.54152961
57.11925407	-2.063505103	0.014204247	0.089654984	DRD4
	11	637293	640706	+	1477	protein_coding
dopamine receptor D4 [Source:HGNC Symbol;Acc:HGNC:3025] -	11
	32	2	77	16	85	0.364704291
1.026587386	0.084247369	2.896825091	0.665866012
2.675897344
ENSG00000068097 1053.318372	1088.544038	1069.492318
1256.629784	1459.968048	1438.019288	1070.451576
1384.872374	-0.371538415	0.014208274	0.089654984	HEATR6
	17	60043194	60078931	-	4868
protein_coding	HEAT repeat containing 6 [Source:HGNC
Symbol;Acc:HGNC:24076]	-	1161	1230	920	1213	1279
	1667	11.67912573	11.97239351	11.7582883
13.84593457	16.14980694	15.92267565
ENSG00000168209 704.0267496	654.8964133	3248.00167
208.2296675	529.6522083	395.9513218	1535.641611
377.9443992	2.022488552	0.014211802	0.089654984	DDIT4
	10	72273920	72276036	+	2058
protein_coding	DNA damage inducible transcript 4 [Source:HGNC
Symbol;Acc:HGNC:24944]	-	776	740	2794	201	464
	459	18.46481813	17.03777153	84.4671572
5.427036352	13.85861948	10.37046746
ENSG00000260296 9.979760626	2.654985459	2.3249833
9.323716454	18.26386925	63.83528935	4.986576462
30.47429169	-2.603440349	0.014228563	0.089728714
AC095057.3	4	40166675	40167831	+	1157
	sense_overlapping	"novel transcript, overlapping N4BP2"
	-	11	3	2	9	16	74
0.46557324	0.122861082	0.107548283	0.432236257
0.850029472	2.973920666
ENSG00000151240 562.4955989	612.416646	789.3318303
456.8621063	424.6349601	496.8800901	654.748025

459.4590521 0.508981606 0.01423935 0.08976474 DIP2C
10 274190 689668 - 8563 protein_coding disco
interacting protein 2 homolog C [Source:HGNC Symbol;Acc:HGNC:29150]
- 620 692 679 441 372 576
3.545638292 3.829187136 4.933449385 2.861703859
2.670326441 3.12771777
ENSG00000170759 5722.939093 4305.50142 5312.58684
4643.210794 3820.573149 2986.456375 5113.675784
3816.746773 0.4221806 0.014254789 0.089830053 KIF5B
10 32009010 32056431 - 5877
protein_coding kinesin family member 5B [Source:HGNC
Symbol;Acc:HGNC:6324] - 6308 4865 4570 4482 3347
3462 52.56112807 39.22415485 48.3801662
42.37680453 35.00639443 27.39065331
ENSG00000175197 207.7604712 299.1283618 268.5355711
418.5312719 358.428434 395.9513218 258.4748014
390.9703426 -0.597623066 0.014261915 0.089842951 DDIT3
12 57516588 57521737 - 1307
protein_coding DNA damage inducible transcript 3 [Source:HGNC
Symbol;Acc:HGNC:2726] TF_bZIP 229 338 231 404 314
459 8.580025495 12.25370875 10.99621541
17.17583349 14.76731098 16.32932061
ENSG00000171877 497.1735294 444.2675669 412.6845357
373.9846267 292.221908 212.2092051 451.3752106
292.8052466 0.626527521 0.014275878 0.0898989 FRMD5
15 43870761 44195252 - 6673
protein_coding FERM domain containing 5 [Source:HGNC
Symbol;Acc:HGNC:28214] - 548 502 355 361 256
246 4.021500409 3.564585475 3.309893918
3.006063295 2.358121623 1.714134912
ENSG00000184481 219.5547338 186.7339773 277.8355043
300.4308635 358.428434 408.8909075 228.0414051
355.916735 -0.645406806 0.014304545 0.090036517 FOXO4
X 71096197 71103535 + 3447
protein_coding forkhead box O4 [Source:HGNC Symbol;Acc:HGNC:7139]
Fork 242 211 239 290 314 474
3.43797541 2.90046489 4.313834348 4.674861601
5.599325632 6.39393293
ENSG00000174099 734.8732824 632.7715345 823.0440881
625.7249709 445.181813 461.5118892 730.229635
510.8062244 0.514936473 0.014308134 0.090036517 MSRB3
12 65278643 65491430 + 7405
protein_coding methionine sulfoxide reductase B3 [Source:HGNC
Symbol;Acc:HGNC:27375] - 810 715 708 604 390
535 5.356592878 4.575171938 5.948603762
4.532355005 3.237329497 3.35938468
ENSG00000122779 788.4010894 637.1965102 646.3453573
515.9123105 405.229599 542.5999595 690.6476523
487.9139563 0.500456194 0.014313002 0.090036517 TRIM24
7 138460334 138589993 + 9223

protein_coding	tripartite motif containing 24 [Source:HGNC
Symbol;Acc:HGNC:11812]	-	869	720	556	498	355
	629	4.613985776	3.699020419	3.750674964
3.000331059	2.36593879	3.171096475
ENSG00000164330 116.1281236	73.45459771	123.2241149
38.33083431	66.20652603	36.2308399	104.2689454
46.92273342	1.155482649	0.014358548	0.090290921	EBF1
	5	158695916	159099761	-	6178
protein_coding	early B cell factor 1 [Source:HGNC
Symbol;Acc:HGNC:3126]	COE	128	83	106	37	58
	42	1.014590401	0.636585335	1.06749244
0.332786625	0.577068608	0.316105756
ENSG00000100949 563.4028499	664.63136	639.3704074
786.3000876	803.610247	946.3150327	622.4682058
845.4084558	-0.442455586	0.014383739	0.090417185
RABGGTA 14	24265538	24271739	-	3545
protein_coding	Rab geranylgeranyltransferase subunit alpha
[Source:HGNC Symbol;Acc:HGNC:9795]	-	621	751	550
	759	704	1097	8.578355581	10.03806811
9.652799747	11.89700324	12.20685483	14.38869498
ENSG00000005812 1129.527453	1079.694087	1183.4165
852.6020713	921.1839053	823.8202882	1130.879346
865.868755	0.385384472	0.014412336	0.090542462	FBXL3
	13	76992598	77027195	-	7082
protein_coding	F-box and leucine rich repeat protein 3 [Source:HGNC
Symbol;Acc:HGNC:13599]	-	1245	1220	1018	823	807
	955	8.608789973	8.162634474	8.943318594
6.457374234	7.004303766	6.27015775
ENSG00000067900 2219.135863	1404.487308	1963.448397
1665.83734	1158.614206	1130.919788	1862.357189
1318.457111	0.498257059	0.014413905	0.090542462	ROCK1
	18	20946906	21111851	-	11113
protein_coding	Rho associated coiled-coil containing protein kinase 1
[Source:HGNC Symbol;Acc:HGNC:10251]	-	2446	1587	1689
	1608	1015	1311	10.77838821	6.766624132
9.45595032	8.040198551	5.614125129	5.485325395
ENSG00000115128 1377.206966	1755.830384	1475.201904
1708.312048	2076.373635	2342.065008	1536.079751
2042.25023	-0.410978991	0.014439645	0.09067195	SF3B6
	2	24067584	24076443	-	1197
protein_coding	splicing factor 3b subunit 6 [Source:HGNC
Symbol;Acc:HGNC:30096]	-	1518	1984	1269	1649	1819
	2715	62.10210264	78.53693678	65.95903882
76.54882854	93.40839479	105.464598
ENSG00000156504 2306.231956	1880.6147	1856.499165
1865.779259	1322.989029	1251.689255	2014.448607
1480.152514	0.445037551	0.014447389	0.090688382
FAM122B X	134769566	134797232	-	4404
protein_coding	family with sequence similarity 122B [Source:HGNC
Symbol;Acc:HGNC:30490]	-	2542	2125	1597	1801	1159

1451	28.26551609	22.86325525	22.56131725
22.72366856	16.17645991	15.31973032
ENSG00000267280 22.68127415	37.16979643	13.9498998	0
7.990442797	0	24.60032346	2.663480932
3.259250336	0.014463305	0.090756085	TBX2-AS1	17
61393456	61411555	-	1206	antisense
TBX2 antisense RNA 1 [Source:HGNC Symbol;Acc:HGNC:50355]
-	25	42	12	0	7	0
1.01512935	1.650168997	0.619071463	0	0.356778021
0
ENSG00000155755 1010.677576	943.4048332	1054.379926
834.9906069	698.5929988	728.0673542	1002.820779
753.8836533	0.411256143	0.014474238	0.090764802
TMEM237 2	201620184	201643570	-	7647
protein_coding	transmembrane protein 237 [Source:HGNC
Symbol;Acc:HGNC:14432]	-	1114	1066	907	806	612
844	7.133830466	6.605300123	7.37943384
5.856740682	4.919349654	5.131949505
ENSG00000119042 866.4246725	781.4507202	711.4448897
675.4514587	553.6235367	396.8139608	786.4400941
541.9629854	0.538800012	0.014476439	0.090764802	SATB2
2	199269500	199471266	-	10958
protein_coding	SATB homeobox 2 [Source:HGNC Symbol;Acc:HGNC:21637]
CUT	955	883	612	652	485	460
4.267767571	3.818175163	3.474777276	3.306194075
2.720556832	1.951899953
ENSG00000087460 14211.17913	13143.06302	20271.52939
12951.67812	12878.31081	9753.859685	15875.25718
11861.28287	0.42048952	0.014480088	0.090764802	GNAS
20	58839718	58911192	+	13320
protein_coding	GNAS complex locus [Source:HGNC Symbol;Acc:HGNC:4392]
-	15664	14851	17438	12502	11282	11307
57.58735519	52.82967197	81.4515391	52.15396286
52.06300493	39.47064155
ENSG00000165527 3202.59591	3364.751572	3421.212926
2726.669079	2685.930272	2687.120626	3329.520136
2699.906659	0.302320051	0.014487674	0.090780183	ARF6
14	49893092	49897054	+	3865
protein_coding	ADP ribosylation factor 6 [Source:HGNC
Symbol;Acc:HGNC:659]	-	3530	3802	2943	2632	2353
3115	44.72536471	46.61106811	47.3748241
37.83976498	37.42137914	37.47482133
ENSG00000147894 323.8885949	307.0933181	287.1354375
401.955776	624.39603	380.423819	306.0391168
468.9252083	-0.613555906	0.01451045	0.090890705
C9orf72 9	27546545	27573866	-	6332
protein_coding	chromosome 9 open reading frame 72 [Source:HGNC
Symbol;Acc:HGNC:28337]	-	357	347	247	388	547
441	2.7609338	2.596659531	2.426961537
3.404888252	5.309990943	3.238386657

ENSG00000244161 10.88701159 8.849951531 4.6499666 0
0 0 8.128976574 0 5.458618873
0.014538254 0.091032627 FLNB-AS1 3 58162547
58170636 - 3709 antisense FLNB antisense
RNA 1 [Source:HGNC Symbol;Acc:HGNC:40239] - 12 10
4 0 0 0 0.158435718 0.127752559
0.067098066 0 0 0
ENSG00000054148 693.139738 1076.154106 1204.341349
504.516657 743.1111801 753.9465256 991.2117312
667.1914542 0.570239382 0.014564691 0.091165895 PHPT1
9 136848724 136851027 + 1612
protein_coding phosphohistidine phosphatase 1 [Source:HGNC
Symbol;Acc:HGNC:30033] - 764 1216 1036 487 651
874 23.20903079 35.74332733 39.98541102
16.78711121 24.82351693 25.21026514
ENSG00000255346 5.443505796 0 25.5748163 0 0
0 10.3394407 0 5.797181252 0.014584762
0.091259235 NOX5 15 68930525 69062743
+ 12511 protein_coding NADPH oxidase 5 [Source:HGNC
Symbol;Acc:HGNC:14874] - 6 0 22 0 0
0 0.023484856 0 0.109405084 0 0
0
ENSG00000119689 2068.532202 2135.493304 2222.684035
2517.403443 3131.112085 2546.510462 2142.236514
2731.67533 -0.350490412 0.014617091 0.091429178 DLST
14 74881891 74903745 + 4550
protein_coding dihydrolipoamide S-succinyltransferase [Source:HGNC
Symbol;Acc:HGNC:2911] - 2280 2413 1912 2430 2743
2952 24.53873293 25.12883566 26.14468044
29.67610647 37.05627589 30.16726752
ENSG00000153936 1707.446318 1478.826901 1771.637274
1376.80213 1273.90488 1170.601184 1652.636831
1273.769398 0.375621747 0.014625834 0.091429205 HS2ST1
1 86914648 87109998 + 7429
protein_coding heparan sulfate 2-O-sulfotransferase 1 [Source:HGNC
Symbol;Acc:HGNC:5193] - 1882 1671 1524 1329 1116
1357 12.40560487 10.65792187 12.76325527
9.940464215 9.23381558 8.493379067
ENSG00000105048 205.0387183 290.2784102 381.2972612
338.7616978 489.6999943 589.1824679 292.2047966
472.5480533 -0.696326678 0.014627432 0.091429205 TNNT1
19 55132794 55149354 - 2171
protein_coding "troponin T1, slow skeletal type [Source:HGNC
Symbol;Acc:HGNC:11948]" - 226 328 328 327 429
683 5.097735515 7.158803819 9.399848778
8.369508849 12.14632309 14.62823398
ENSG00000174748 27509.66379 30460.64818 32619.5157
35391.79169 37414.67765 37642.98002 30196.60922
36816.48312 -0.286000874 0.014645445 0.09150946 RPL15
3 23916545 23924374 + 7222

protein_coding ribosomal protein L15 [Source:HGNC
Symbol;Acc:HGNC:10306] - 30322 34419 28060 34163 32777
43637 205.6028086 225.8224969 241.7336052
262.851561 278.9710116 280.949601
ENSG00000264301 218.6474828 162.8391082 136.011523
109.8126605 100.4512809 69.87376267 172.4993713
93.37923467 0.889718147 0.014651215 0.091511751
LINC01444 18 14969001 14970468 - 1336
lincRNA long intergenic non-protein coding RNA 1444
[Source:HGNC Symbol;Acc:HGNC:50769] - 241 184 117
106 88 81 8.833631287 6.525860798
5.448616613 4.408709172 4.048774362 2.81909414
ENSG00000122912 436.3877146 492.0573051 446.3967936
701.350671 597.0002261 605.5726098 458.2806044
634.641169 -0.469337768 0.014656159 0.091511751
SLC25A16 10 68477999 68527474 - 7815
protein_coding solute carrier family 25 member 16
[Source:HGNC Symbol;Acc:HGNC:10986] - 481 556 384
677 523 702 3.014010615 3.37110477
3.057096083 4.813619191 4.113581126 4.176756411
ENSG00000213639 3523.762752 3216.072386 3533.974616
3006.380572 2683.647288 2477.499338 3424.603251
2722.509066 0.331086973 0.014665246 0.091514167 PPP1CB
2 28751640 28802940 + 5760
protein_coding protein phosphatase 1 catalytic subunit beta
[Source:HGNC Symbol;Acc:HGNC:9282] - 3884 3634 3040
2902 2351 2872 33.02063505 29.89433386
32.83658217 27.99541877 25.0886623 23.18424568
ENSG00000143393 1801.800418 2034.603857 2002.973113
1933.117211 2825.192275 3144.31932 1946.459129
2634.209602 -0.436792526 0.014666893 0.091514167 PI4KB
1 151291797 151327715 - 5456
protein_coding phosphatidylinositol 4-kinase beta [Source:HGNC
Symbol;Acc:HGNC:8984] - 1986 2299 1723 1866 2475
3645 17.82516531 19.96599925 19.64797344
19.00418815 27.88355877 31.06377194
ENSG00000008324 389.2106644 461.0824748 311.5477622
291.1071471 262.5431205 184.6047557 387.2803005
246.0850077 0.657385585 0.014737178 0.091920287 SS18L2
3 42581840 42595114 + 1298
protein_coding SS18 like 2 [Source:HGNC Symbol;Acc:HGNC:15593] -
429 521 268 281 230 214 16.18494706
19.01907853 12.84597132 12.02939183 10.89182121
7.666022666
ENSG00000106355 1327.308163 1380.592439 1400.802438
1203.795391 981.6829722 941.1391984 1369.56768
1042.205854 0.394238271 0.014801616 0.092232948 LSM5
7 32485332 32495283 - 4701
protein_coding "LSM5 homolog, U6 small nuclear RNA and mRNA
degradation associated [Source:HGNC Symbol;Acc:HGNC:17162]" -

1463 1560 1205 1162 860 1091 15.2399225
15.72391864 15.94790509 13.73497649 11.24489903
10.79109504
ENSG00000142102 221.3692357 160.1841227 101.1367735
98.41700702 84.47039528 62.11001126 160.8967107
81.66580452 0.983027205 0.014801678 0.092232948 PGGHG
11 289135 296107 + 5260 protein_coding
protein-glucosylgalactosylhydroxylysine glucosidase [Source:HGNC
Symbol;Acc:HGNC:26210] - 244 181 87 95 74
72 2.271604737 1.63049425 1.029059189
1.003575163 0.864755506 0.636469754
ENSG00000104635 4712.261517 5188.726583 6260.017535
4862.836115 3942.712775 3575.638843 5387.001878
4127.062577 0.384276802 0.014807355 0.092232948
SLC39A14 8 22367249 22434129 + 6942
protein_coding solute carrier family 39 member 14
[Source:HGNC Symbol;Acc:HGNC:20858] - 5194 5863 5385
4694 3454 4145 36.63920312 40.01858474
48.26229222 37.57253689 30.58335206 27.76329091
ENSG00000231672 158.768919 186.7339773 53.4746159
301.466832 246.5622349 230.3246251 132.9925041
259.4512307 -0.958774506 0.014808162 0.092232948 DIRC3
2 217284019 217756593 - 4665
lincRNA disrupted in renal carcinoma 3 [Source:HGNC
Symbol;Acc:HGNC:17805] - 175 211 46 291 216
267 1.837025074 2.143173092 0.613497829
3.466198137 2.846095465 2.661280375
ENSG00000198743 4741.293548 4401.080896 4691.816299
6364.990433 5455.189447 5351.812637 4611.396915
5723.997505 -0.31182222 0.014846392 0.092438518 SLC5A3
21 34073570 34106262 + 11576
protein_coding solute carrier family 5 member 3 [Source:HGNC
Symbol;Acc:HGNC:11038] - 5226 4973 4036 6144 4779
6204 22.1074968 20.35571595 21.69199452
29.49203445 25.37615817 24.91978489
ENSG00000156463 117.9426256 70.79961225 113.9241817
40.4027713 23.97132839 63.83528935 100.8888065
42.73646301 1.230414687 0.014854082 0.092453853 SH3RF2
5 145936579 146081791 + 7232
protein_coding SH3 domain containing ring finger 2 [Source:HGNC
Symbol;Acc:HGNC:26299] - 130 80 98 39 21
74 0.880265373 0.524152921 0.8430911
0.299652726 0.178487677 0.475777961
ENSG00000161203 10301.83472 10908.45026 12007.37625
8624.43772 9774.594525 8515.972655 11072.55374
8971.6683 0.303542061 0.014872353 0.092535018 AP2M1
3 184174689 184184091 + 4756
protein_coding adaptor related protein complex 2 subunit mu 1
[Source:HGNC Symbol;Acc:HGNC:564] - 11355 12326 10329
8325 8563 9872 116.9160074 122.8023728

135.121133	97.26451821	110.6703946	96.51488974
ENSG00000177200 1974.178102	1677.065815	1690.262859
1538.413215	1321.847537	1244.788142	1780.502259
1368.349631	0.380268523	0.014881221	0.092557639	CHD9
	16	53055033	53329150	+	16504
protein_coding	chromodomain helicase DNA binding protein 9
[Source:HGNC Symbol;Acc:HGNC:25701]	-	2176	1895	1454
	1485	1158	1443	6.456517903	5.440595527
5.481280637	4.999761435	4.312873346	4.065445414
ENSG00000114126 1991.41587	1855.834836	1461.252004
2546.41056	2268.144263	2141.932749	1769.500903
2318.829191	-0.389262957	0.014904833	0.09267191	TFDP2
	3	141944428	142149544	-	11897
protein_coding	transcription factor Dp-2 [Source:HGNC
Symbol;Acc:HGNC:11751]	E2F	2195	2097	1257	2458	1987
	2483	9.034949855	8.351940846	6.57362102
11.48038521	10.26615379	9.704435461
ENSG00000273175 0	0	0	0	23.97132839
6.038473317	0	10.00326724	-5.794890678	0.014931668
	0.092786448	BX323046.1	1	244864738
244865272	+	535	lincRNA novel transcript	-
	0	0	0	0	21	7	0	0
	0	0	2.412753048	0.608380487
ENSG00000272155 0.907250966	15.92991276	9.299933199	0
	0	0	8.71236564	0	5.556175989
0.014933745	0.092786448	AC055822.1	8	65714334
	65714778	-	445	antisense	"novel
transcript, antisense to MTFR1" -	1	18	8	0
	0	0	0.110044584	1.916632883	1.118502148
	0	0	0
ENSG00000167695 1124.083947	1452.277046	1207.828824
1647.189907	1499.920262	1841.734362	1261.396606
1662.948177	-0.398878299	0.014949009	0.092848672	FAM57A
	17	732412	742972	+	2740	protein_coding	family
with sequence similarity 57 member A [Source:HGNC
Symbol;Acc:HGNC:29646]	-	1239	1641	1039	1590	1314
	2135	22.14366115	28.37817477	23.59238416
32.24471964	29.4776028	36.23083429
ENSG00000284753 110.6846178	210.6288464	120.8991316
234.1288798	237.4303003	296.7478316	147.4041986
256.1023372	-0.795527252	0.014960244	0.09288584
EEF1AKMT4	3	184249650	184259585	+	1823
	protein_coding	EEF1A lysine methyltransferase 4 [Source:HGNC
Symbol;Acc:HGNC:53611]	-	122	238	104	226	208
	344	3.277191695	6.186097052	3.549388332
6.888645155	7.013325998	8.774104287
ENSG00000101773 1933.351809	1696.535709	1669.338009
1564.312427	1106.105581	1321.563017	1766.408509
1330.660342	0.408560688	0.014970126	0.092914579	RBBP8
	18	22798261	23026488	+	5091

protein_coding "RB binding protein 8, endonuclease [Source:HGNC
Symbol;Acc:HGNC:9891]" -   2131   1917   1436   1510   969
        1532   20.49788424   17.84207895   17.54923499
16.48108859   11.69951989   13.99222198
ENSG00000151689 509.8750429   484.9773439   535.9086506
416.459335   313.9102527   335.5665886   510.2536791
355.3120588   0.521631195   0.015037639   0.093300878   INPP1
        2   190343470   190371665   +   3676
protein_coding inositol polyphosphate-1-phosphatase [Source:HGNC
Symbol;Acc:HGNC:6071]   -   562   548   461   402   275
        389   7.486683892   7.063687808   7.802472906
6.07662721   4.598376757   4.920453328
ENSG00000254999 2361.574264   2894.819146   3136.402471
2128.915257   2046.694848   2332.575978   2797.598627
2169.395361   0.366322152   0.015067743   0.093428652   BRK1
        3   10115592   10127190   +   1233
protein_coding "BRICK1, SCAR/WAVE actin nucleating complex subunit
[Source:HGNC Symbol;Acc:HGNC:23057]"   -   2603   3271   2698
        2055   1793   2704   103.380773   125.702498
136.1399903   92.61062034   89.38498532   101.9705188
ENSG00000214530 604.2291433   653.126423   813.7441549
731.3937574   1350.384833   959.2546183   690.3665738
1013.677736   -0.554463841   0.015068797   0.093428652
STARD10 11   72754729   72793681   -   3710
protein_coding StAR related lipid transfer domain containing 10
[Source:HGNC Symbol;Acc:HGNC:10666]   -   666   738   700
        706   1183   1112   8.790812218   9.425597561
11.7389966   10.57408592   19.60009585   13.93676239
ENSG00000141522 6039.569681   7672.907978   8175.803774
6718.255689   11195.75185   12489.2881   7296.093811
10134.43188   -0.474190947   0.015094098   0.093548138
ARHGDIA 17   81867721   81871406   -   2903
protein_coding Rho GDP dissociation inhibitor alpha [Source:HGNC
Symbol;Acc:HGNC:678]   -   6657   8670   7033   6485   9808
        14478   112.2949445   141.5137053   150.730253
124.1294949   207.6733562   231.8956247
ENSG00000136867 7.258007728   8.849951531   3.48747495
40.4027713   35.38624667   15.52750281   6.531811403
30.43884026   -2.203440175   0.01510114   0.093548138
SLC31A2 9   113150942   113164137   +   2504
protein_coding solute carrier family 31 member 2 [Source:HGNC
Symbol;Acc:HGNC:11017] -   8   10   3   39   31
        18   0.156453162   0.189230927   0.074540753
0.865450685   0.760982605   0.334248294
ENSG00000138678 55.34230892   79.64956378   65.09953239
100.488944   130.1300684   162.1761405   66.69713503
130.9317176   -0.973870605   0.015103934   0.093548138   GPAT3
        4   83535914   83605875   +   3468
protein_coding glycerol-3-phosphate acyltransferase 3 [Source:HGNC
Symbol;Acc:HGNC:28157] -   61   90   56   97   114

	188	0.861349547	1.229673635	1.004652305
1.554192071	2.020566381	2.520633934
ENSG00000101493 246.7722627	242.488672	387.1097194
211.337573	113.007691	182.8794776	292.1235514
169.0749138	0.785176057	0.015114612	0.09356432	ZNF516
	18	76358190	76495190	-	8217
protein_coding	zinc finger protein 516 [Source:HGNC
Symbol;Acc:HGNC:28990]	zf-C2H2 272	274	333	204	99
	212	1.621004799	1.58002412	2.521377036
1.379522932	0.740575376	1.199647331
ENSG00000166477 697.6759928	613.3016411	803.2817301
591.5380106	455.4552394	445.1217474	704.7531213
497.3716658	0.50222915	0.015117125	0.09356432	LEO1
	15	51938025	51971806	-	2679
protein_coding	"LEO1 homolog, Paf1/RNA polymerase II complex
component [Source:HGNC Symbol;Acc:HGNC:30401]"	-	769	693
	691	571	399	516	14.05666548	12.25707834
	16.04767722	11.84337384	9.154772203	8.955874645
ENSG00000159063 1454.323298	1385.017415	1503.101703
1319.823863	995.3808742	921.2985004	1447.480805
1078.834412	0.424256788	0.015138017	0.093660853	ALG8
	11	78100936	78139660	-	3448
protein_coding	"ALG8, alpha-1,3-glucosyltransferase [Source:HGNC
Symbol;Acc:HGNC:23161]" -	1603	1565	1293	1274	872
	1068	22.76643076	21.50668754	23.33125575
20.53119437	15.54520981	14.40240481
ENSG00000149600 561.5883479	635.4265199	604.4956579
795.6238041	904.0615279	740.1443008	600.5035086
813.2765443	-0.436885725	0.015159761	0.093756227	COMMD7
	20	32702691	32743997	-	2584
protein_coding	COMM domain containing 7 [Source:HGNC
Symbol;Acc:HGNC:16223]	-	619	718	520	768	792
	858	11.7307782	13.16613718	12.52038492
16.51508276	18.83996243	15.43923568
ENSG00000164405 1321.864657	2015.133964	1976.235805
1308.428209	1287.602782	1329.326769	1771.078142
1308.452587	0.436386343	0.015164032	0.093756227	UQCRQ
	5	132866560	132868031	+	1472
protein_coding	ubiquinol-cytochrome c reductase complex III subunit
VII [Source:HGNC Symbol;Acc:HGNC:29594] -	1457	2277	1700
	1263	1128	1541	48.47082652	73.29623409
71.85350503	47.67685332	47.10300884	48.67722891
ENSG00000089820 106.148363	69.02962194	61.61205744
118.1004084	134.6960357	213.9344832	78.93001414
155.5769758	-0.978472107	0.015213845	0.094031343
ARHGAP4 X	153907367	153934999	-	5580
protein_coding	Rho GTPase activating protein 4 [Source:HGNC
Symbol;Acc:HGNC:674]	-	117	78	53	114	118
	248	1.026786964	0.662348938	0.59094698
1.135226959	1.299855777	2.066562289

ENSG00000177406 15.42326642   17.69990306   43.01219105
42.47470829   90.17785443   80.22543121   25.37845351
70.95933131   -1.495437194   0.015237222   0.094142936
AC021054.1   12   630891 663706 +   3530   antisense
        uncharacterized LOC100049716 [Source:NCBI gene;Acc:100049716]
        -       17   20   37   41   79   93
0.23583209   0.268461326   0.652129528   0.645388459
1.375624006   1.225008949
ENSG00000165775 1518.738117   1693.880723   1776.287241
2222.152422   1921.130747   2225.608737   1662.968694
2122.963968   -0.352912599   0.015276324   0.094351572   FUNDC2
        X   155025980   155060303   +   10252
protein_coding FUN14 domain containing 2 [Source:HGNC
Symbol;Acc:HGNC:24925] -   1674   1914   1528   2145   1683
        2580   7.99605071   8.846261573   9.273028687
11.62601135   10.0907368   11.70151589
ENSG00000178878 304.8363246   290.2784102   455.6967268
246.5605018   218.0249392   50.03306463   350.2704872
171.5395019   1.032304297   0.015287281   0.094386288   APOLD1
        12   12725917   12829975   +   7243
protein_coding apolipoprotein L domain containing 1 [Source:HGNC
Symbol;Acc:HGNC:25268] -   336   328   392   238   191
        58   2.271692142   2.145763232   3.367242765
1.825872785   1.620922468   0.372340713
ENSG00000176022 1910.670534   2187.708019   2197.109218
2061.577305   2924.502064   3648.100522   2098.495924
2878.059964   -0.456133916   0.015306978   0.094474922
B3GALT6 1   1232226 1235041 +   2816
protein_coding "beta-1,3-galactosyltransferase 6 [Source:HGNC
Symbol;Acc:HGNC:17978]" -   2106   2472   1890   1990   2562
        4229   36.62304073   41.59510805   41.75764524
39.26742922   55.92343446   69.829037
ENSG00000138443 1315.513901   1253.153137   1504.264195
1272.169312   877.8072159   741.8695789   1357.643744
963.9487022   0.494238215   0.015332337   0.094598432   ABI2
        2   203328219   203447723   +   23331
protein_coding abl interactor 2 [Source:HGNC Symbol;Acc:HGNC:24011]
        -       1450   1416   1294   1228   769   860
3.043430104   2.875784513   3.450704492   2.924671256
2.026004223   1.713941978
ENSG00000104517 3139.088342   2431.966681   2649.31847
2554.698308   1741.91653   1824.481581   2740.124498
2040.365473   0.42549249   0.015375194   0.094829782   UBR5
        8   102253012   102412841   -   13085
protein_coding ubiquitin protein ligase E3 component n-recognin 5
[Source:HGNC Symbol;Acc:HGNC:16806]   -   3460   2748   2279
        2466   1526   2115   12.94884569   9.951062232
10.8362108   10.47204233   7.168497974   7.51566931
ENSG00000044090 1777.304642   1885.039676   1992.510688
2191.073367   2651.685517   2338.614452   1884.951669

2393.791112	-0.344810927	0.015398469	0.094940228	CUL7
	6	43037617	43053945	-	5628
protein_coding	cullin 7 [Source:HGNC Symbol;Acc:HGNC:21024]	-
	1959	2130	1714	2115	2323	2711	17.04547197
	17.932959	18.9480087	20.88181898	25.37128602
	22.39785594
ENSG00000184990 1113.196935	1748.750423	1504.264195
848.4581973	1006.795792	1247.376059	1455.403851
1034.210016	0.492244296	0.015412426	0.094993174	SIVA1
	14	104753100	104768494	+	4776
protein_coding	SIVA1 apoptosis inducing factor [Source:HGNC
Symbol;Acc:HGNC:17712]	-	1227	1976	1294	819	882
	1446	12.5808194	19.60419722	16.85686485
9.528655535	11.35145749	14.07780655
ENSG00000119401 455.4399849	499.1372664	497.5464262
645.4083723	780.7804105	593.4956631	484.0412258
673.2281486	-0.47521874	0.015436723	0.095109787	TRIM32
	9	116687302	116701300	+	3780
protein_coding	tripartite motif containing 32 [Source:HGNC
Symbol;Acc:HGNC:16380]	-	502	564	428	623	684
	688	6.503402009	7.069907716	7.044640185
9.158161344	11.12273684	8.463064612
ENSG00000160075 3650.777887	3901.94363	3714.160821
4043.385036	4932.38619	5225.004697	3755.627446
4733.591974	-0.333948203	0.015446951	0.095139666	SSU72
	1	1541673 1574869 -	6925	protein_coding	"SSU72
homolog, RNA polymerase II CTD phosphatase [Source:HGNC
Symbol;Acc:HGNC:25016]" -	4024	4409	3195	3903	4321
	6057	28.45554303	30.16801685	28.70502513
31.31776905	38.35410464	40.66949821
ENSG00000167615 1490.613337	1703.61567	1160.166667
1044.256243	1147.199287	1053.282274	1451.465224
1081.579268	0.425408343	0.015547339	0.095724633	LENG8
	19	54448887	54462037	+	6469
protein_coding	leukocyte receptor cluster member 8 [Source:HGNC
Symbol;Acc:HGNC:15500]	-	1643	1925	998	1008	1005
	1221	12.43738551	14.10002957	9.598430768
8.658355724	9.549404081	8.776261011
ENSG00000140829 2580.221747	2863.844315	3031.778223
3380.365199	3369.683877	3904.304319	2825.281429
3551.451132	-0.33047384	0.015555094	0.095739059	DHX38
	16	72093562	72112912	+	6361
protein_coding	DEAH-box helicase 38 [Source:HGNC
Symbol;Acc:HGNC:17211]	-	2844	3236	2608	3263	2952
	4526	21.89439152	24.10513445	25.50874181
28.50386299	28.52583184	33.08416455
ENSG00000238178 9.07250966	20.35488852	0	0	0
	0	9.809132727	0	5.731592795	0.015574873
	0.095827452	AC078993.1	X	16152941
16170869	-	1219	antisense	"novel transcript,

antisense to MAGEB17"	-	10	23	0	0	0
	0	0.40172141	0.894026869	0	0	0
	0
ENSG00000237758 5.443505796	18.58489822	12.78740815	0
	1.141491828	0.862639045	12.27193739	0.668043624
	4.203056335	0.015589981	0.095887056	BANF1P3 2
	230725143	230725403	-	261
processed_pseudogene	barrier to autointegration factor 1 pseudogene
3 [Source:HGNC Symbol;Acc:HGNC:43883]	-	6	21	11
	0	1	1	1.125743444	3.812459407
2.622159012	0	0.235508645	0.178151921
ENSG00000107821 143.3456526	183.1939967	147.6364395
74.58973163	115.2906746	62.97265031	158.0586963
84.28435219	0.912231069	0.015614542	0.096004741
KAZALD1 10	101061841	101068131	+	3613
protein_coding	Kazal type serine peptidase inhibitor domain 1
[Source:HGNC Symbol;Acc:HGNC:25460]	-	158	207	127
	72	101	73	2.14149867	2.714743642
2.186968894	1.107328757	1.718307052	0.93947649
ENSG00000155962 80.74533597	64.60464618	89.51185704
208.2296675	92.46083808	161.3135015	78.28727973
154.0013357	-0.979485655	0.015643276	0.096147989	CLIC2
	X	155276211	155334657	-	2840
protein_coding	chloride intracellular channel 2 [Source:HGNC
Symbol;Acc:HGNC:2063]	-	89	73	77	201	81
	187	1.53461822	1.21795421	1.686860744
3.932690427	1.753129667	3.061641137
ENSG00000162062 599.6928885	795.6106427	623.0955243
494.1569721	413.2200418	521.0339833	672.7996852
476.1369991	0.498352702	0.015705404	0.096496317	TEDC2
	16	2460080 2464963 +	2854	protein_coding
tubulin epsilon and delta complex 2 [Source:HGNC
Symbol;Acc:HGNC:25849]	-	661	899	536	477	362
	604	11.34164826	14.92561255	11.68470272
9.287021563	7.796540908	9.840427998
ENSG00000134668 551.6085873	674.3663067	580.0833333
745.8973163	819.5911326	887.6555776	602.0194091
817.7146755	-0.441756296	0.015717667	0.096508642	SPOCD1
	1	31790422	31816051	-	7085
protein_coding	SPOC domain containing 1 [Source:HGNC
Symbol;Acc:HGNC:26338]	-	608	762	499	720	718
	1029	4.202351816	5.096142432	4.38195121
5.646829638	6.229195337	6.753152208
ENSG00000186501 1207.551036	1708.925641	1325.240481
1473.1472	2047.83634	2377.433209	1413.905719
1966.138916	-0.475709946	0.015718322	0.096508642
TMEM222 1	27322145	27336400	+	4476
protein_coding	transmembrane protein 222 [Source:HGNC
Symbol;Acc:HGNC:25363]	-	1331	1931	1140	1422	1794
	2756	14.56185362	20.44177655	15.84607182

17.65312361	24.63654036	28.62992125
ENSG00000164062 2715.402141	2905.439088	2663.26837
2263.591161	2350.331674	1970.267579	2761.369866
2194.730138	0.3318766	0.015741206	0.096584369	APEH
	3	49674002	49683963	+	4669
protein_coding	acylaminoacyl-peptide hydrolase [Source:HGNC
Symbol;Acc:HGNC:586]	-	2993	3283	2291	2185	2059
	2284	31.39146083	33.31758003	30.52868247
26.00396729	27.10689871	22.7459062
ENSG00000147082 17.23776835	29.20484005	16.2748831
50.76245625	45.65967313	79.36279217	20.9058305
58.59497385	-1.484418733	0.015741576	0.096584369	CCNB3
	X	50202713	50351910	+	5408
protein_coding	cyclin B3 [Source:HGNC Symbol;Acc:HGNC:18709]	-
	19	33	14	49	40	92	0.172046405
	0.289137018	0.161063896	0.5034675	0.454643167
	0.791010344
ENSG00000116337 2853.304288	3242.622241	3501.424849
3396.940695	4649.296216	4377.030516	3199.117126
4141.089142	-0.372523997	0.015756707	0.096643687	AMPD2
	1	109616104	109632051	+	6823
protein_coding	adenosine monophosphate deaminase 2 [Source:HGNC
Symbol;Acc:HGNC:469]	-	3145	3664	3012	3279	4073
	5074	22.57220376	25.44523901	27.46543254
26.70410882	36.69326852	34.57849681
ENSG00000148300 1075.999646	1428.382177	1109.017034
1148.889061	1779.58576	2320.499032	1204.466286
1749.657951	-0.538830702	0.015777734	0.096739003	REXO4
	9	133406059	133418096	-	2654
protein_coding	"REX4 homolog, 3'-5' exonuclease [Source:HGNC
Symbol;Acc:HGNC:12820]" -	1186	1614	954	1109	1559
	2690	21.88328185	28.81569195	22.36424817
23.21895508	36.10709568	47.12836569
ENSG00000106052 4502.686544	3576.265414	4069.883266
3680.796062	2881.125374	2851.022045	4049.611741
3137.647827	0.36813875	0.015783185	0.096739003
TAX1BP1 7	27739331	27844564	+	9165
protein_coding	Tax1 binding protein 1 [Source:HGNC
Symbol;Acc:HGNC:11575]	-	4963	4041	3501	3553	2524
	3305	26.5179831	20.89213493	23.76656887
21.54144249	16.92794508	16.76756554
ENSG00000167778 596.0638846	644.2764715	714.9323647
753.1490958	860.6848384	1099.002144	651.7575736
904.2786926	-0.473908299	0.015791697	0.09675765	SPRYD3
	12	53064316	53079420	-	3479
protein_coding	SPRY domain containing 3 [Source:HGNC
Symbol;Acc:HGNC:25920]	-	657	728	615	727	754
	1274	9.247825458	9.915243666	10.99834994
11.61159891	13.32184196	17.027309
ENSG00000268916 95.26135143	150.449176	77.88694054

175.0786756	265.967596	161.3135015	107.8658227
200.786591	-0.891214573	0.015798515	0.096765905	CSAG3
X	152753921	152760222	+	1083
protein_coding	CSAG family member 3 [Source:HGNC
Symbol;Acc:HGNC:26237]	-	105	170	67	169	233
187	4.747768405	7.437841264	3.849046809
8.67102207	13.22436491	8.028680361
ENSG00000138468 413.7064405	315.9432697	413.8470274
336.6897608	157.5258723	194.0937852	381.1655792
229.4364728	0.731645441	0.015805333	0.096774156	SENP7
3	101324205	101513241	-	6396
protein_coding	SUMO specific peptidase 7 [Source:HGNC
Symbol;Acc:HGNC:30402]	-	456	357	356	325	138
225	3.49128314	2.644759598	3.462967291
2.823494522	1.326227386	1.635705376
ENSG00000105875 347.47712	311.5182939	340.6100534
215.4814469	256.8356613	182.8794776	333.2018224
218.398862	0.611397911	0.015851144	0.097021068	WDR91
7	135183839	135211534	-	6032
protein_coding	WD repeat domain 91 [Source:HGNC
Symbol;Acc:HGNC:24997]	-	383	352	293	208	225
212	3.109325042	2.765080449	3.022129945
1.9160818	2.292812525	1.63420128
ENSG00000156110 759.3690585	742.5109335	968.3555444
716.8901985	555.9065203	438.220635	823.4118455
570.3391179	0.52990656	0.015864755	0.097048439	ADK
10	74151185	74709303	+	3184
protein_coding	adenosine kinase [Source:HGNC Symbol;Acc:HGNC:257]
-	837	839	833	692	487	508
12.87303893	12.48577035	16.27716586	12.07661104
9.401632311	7.418595151
ENSG00000071054 7194.50016	6536.574201	7608.507849
5946.459161	6009.954475	5355.263193	7113.19407
5770.558943	0.301793336	0.015866589	0.097048439	MAP4K4
2	101696850	101894689	+	11961
protein_coding	mitogen-activated protein kinase kinase kinase kinase
4 [Source:HGNC Symbol;Acc:HGNC:6866]	-	7930	7386	6545
5740	5265	6208	32.46641835	29.25959118
34.04466046	26.66591225	27.05691301	24.133218
ENSG00000116017 160.583421	145.1392051	195.2985972
72.51779464	39.95221399	129.3958568	167.0070744
80.62195514	1.042858003	0.015915099	0.097311507	ARID3A
19	925781	975934	+	7210	protein_coding	AT-
rich interaction domain 3A [Source:HGNC Symbol;Acc:HGNC:3031]	ARID
177	164	168	70	35	150	1.202172212
1.07779217	1.449709095	0.539479342	0.298387166
0.967357521
ENSG00000155760 520.7620545	345.1481097	399.8971276
284.8913361	236.2888084	311.4126953	421.9357639
277.5309466	0.603216087	0.015925157	0.097339357	FZD7

2	202034587	202038445	+	3859
protein_coding	frizzled class receptor 7 [Source:HGNC
Symbol;Acc:HGNC:4045]	-	574	390	344	275	207
361	7.283930568	4.788684991	5.546135944
3.959769981	3.297182052	4.349741433
ENSG00000152778 412.7991895	438.0726008	391.759686
482.7613186	772.7899677	558.1274623	414.2104921
604.5595829	-0.544118415	0.015935457	0.097348049	IFIT5
10	89414586	89421001	+	4015
protein_coding	interferon induced protein with tetratricopeptide
repeats 5 [Source:HGNC Symbol;Acc:HGNC:13328]	-	455	495
337	466	677	647	5.549508624	5.841792007
5.222172313	6.449297496	10.36455068	7.492896767
ENSG00000106133 13.60876449	7.079961225	3.48747495	0
0	0	8.058733555	0	5.446706705
0.015937585	0.097348049	NSUN5P2 7	72947581
72954790	-	2576	transcribed_unprocessed_pseudogene
NOP2/Sun RNA methyltransferase family member 5 pseudogene 2
[Source:HGNC Symbol;Acc:HGNC:16609]	-	15	8	3
0	0	0	0.285150465	0.147153491
0.072457316	0	0	0
ENSG00000119844 580.6406182	557.5469465	478.9465598
666.1277422	776.2144432	769.4740284	539.0447081
737.2720713	-0.450830439	0.015949192	0.097385315	AFTPH
2	64524305	64593005	+	5767
protein_coding	aftiphilin [Source:HGNC Symbol;Acc:HGNC:25951]	-
640	630	412	643	680	892	5.434488901
5.176271398	4.444819314	6.195452978	7.247802017
7.191937773
ENSG00000067836 117.0353746	146.0242003	97.64929859
72.51779464	62.78205055	44.85723035	120.2362912
60.05235852	1.006345175	0.015998749	0.097654198	ROGDI
16	4796968 4802950 -	3683	protein_coding	rogdi
homolog [Source:HGNC Symbol;Acc:HGNC:29478]	-	129	165
84	70	55	52	1.715207531	2.122797982
1.419006595	1.056108079	0.917927394	0.656496844
ENSG00000100985 185.986448	469.0474312	699.8199732
33.15099184	211.1759882	62.97265031	451.6179508
102.4332101	2.141616311	0.01600998	0.097689046	MMP9
20	46008908	46016561	+	2336
protein_coding	matrix metallopeptidase 9 [Source:HGNC
Symbol;Acc:HGNC:7176]	-	205	530	602	32	185
73	4.297438855	10.75052001	16.03357987
0.761183181	4.867951584	1.453051609
ENSG00000159259 1143.136217	1145.183728	1289.20324
1052.543991	819.5911326	805.7048683	1192.507728
892.6133306	0.41769597	0.016029837	0.097776476	CHAF1B
21	36385378	36419015	+	5118
protein_coding	chromatin assembly factor 1 subunit B [Source:HGNC
Symbol;Acc:HGNC:1911]	-	1260	1294	1109	1016	718

934 12.05588085 11.98009979 13.48149675
11.03076087 8.623260837 8.485503419
ENSG00000076928 3988.275246 5048.897349 4216.357214
4922.922288 6159.489905 5788.307994 4417.84327
5623.573395 -0.348009978 0.016064084 0.097951597
ARHGEF1 19 41883161 41930150 + 8636
protein_coding Rho guanine nucleotide exchange factor 1 [Source:HGNC
Symbol;Acc:HGNC:681] - 4396 5705 3627 4752 5396
6710 24.92721351 31.30181036 26.13014192
30.57566011 38.40667121 36.12774914
ENSG00000173875 528.9273132 523.0321355 513.8213093
852.6020713 538.7841429 853.1500158 521.9269193
748.1787433 -0.520459701 0.016125998 0.098295238 ZNF791
19 12610918 12633840 + 6642
protein_coding zinc finger protein 791 [Source:HGNC
Symbol;Acc:HGNC:26895] zf-C2H2 583 591 442 823 472
989 4.298316263 4.216140261 4.140285072
6.88514368 4.368079033 6.923543712
ENSG00000118939 106.148363 91.15450077 148.7989312
36.25889732 62.78205055 65.56056744 115.367265
54.86717177 1.068686145 0.016168232 0.098512281 UCHL3
13 75549480 75606020 + 1905
protein_coding ubiquitin C-terminal hydrolase L3 [Source:HGNC
Symbol;Acc:HGNC:12515] - 117 103 128 35 55
76 3.007596462 2.561938414 4.180438476
1.020904476 1.774659628 1.855024417
ENSG00000100028 2299.881199 2749.679941 2694.655644
2176.569808 1949.668043 1996.146751 2581.405595
2040.794867 0.338884191 0.016172744 0.098512281 SNRPD3
22 24555503 24609980 + 2544
protein_coding small nuclear ribonucleoprotein D3 polypeptide
[Source:HGNC Symbol;Acc:HGNC:11160] - 2535 3107 2318
2101 1708 2314 48.79659747 57.86961421
56.6895711 45.89030975 41.26844639 42.29385439
ENSG00000074964 610.5799001 399.1328141 626.5829993
365.6968787 337.8815811 401.1271561 545.4319045
368.2352053 0.565002391 0.016187636 0.09856905
ARHGEF10L 1 17539835 17697874 + 7817
protein_coding Rho guanine nucleotide exchange factor 10 like
[Source:HGNC Symbol;Acc:HGNC:25540] - 673 451 539
353 296 465 4.21602945 2.733775649
4.289982295 2.509265625 2.327549679 2.765947032
ENSG00000152926 406.4484328 291.1634054 201.1110554
480.6893816 461.1626986 461.5118892 299.5742979
467.7879898 -0.640361108 0.016198194 0.098599399 ZNF117
7 64971776 65006684 - 10691
protein_coding zinc finger protein 117 [Source:HGNC
Symbol;Acc:HGNC:12897] zf-C2H2 448 329 173 464 404
535 2.052052029 1.458156067 1.006780094
2.411635647 2.322792397 2.32683973

ENSG00000229915 0.907250966	3.539980612	2.3249833
13.46759043	33.10326302	8.626390453	2.257404959
18.3990813	-3.02104752	0.016204108	0.09860147
AC016999.1	2	238427077	238427729	-	452
antisense	novel transcript	-	1	4
2	13	29	10	0.108340354	0.419322337
0.275295053	1.598147873	3.943727722	1.028709104
ENSG00000178425 753.0183018	633.6565296	704.4699398
617.437223	463.4456822	320.9017248	697.0482571
467.2615433	0.578265601	0.01624087	0.098744084	NT5DC1
6	116100849	116249497	+	7309
protein_coding	5'-nucleotidase domain containing 1 [Source:HGNC
Symbol;Acc:HGNC:21556]	-	830	716	606	596	406
372	5.560947744	4.641747383	5.158477123
4.531065513	3.41440813	2.366551698
ENSG00000253683 66.22932052	113.2793796	94.16182364
44.54664528	20.54685291	50.03306463	91.22350792
38.37552094	1.242830747	0.016241953	0.098744084
AC027309.2	5	172656522	172656713	-	192
processed_pseudogene	coiled-coil domain containing 72
(CCDC72) pseudogene	-	73	128	81	43	18
58	18.61874119	31.58894937	26.24766272
12.44455211	5.762602146	14.04616556
ENSG00000122550 698.5832438	608.8766653	657.9702738
818.415111	986.2489395	835.0345958	655.1433943
879.8995488	-0.425088161	0.016244292	0.098744084	KLHL7
7	23105758	23177914	+	7066
protein_coding	kelch like family member 7 [Source:HGNC
Symbol;Acc:HGNC:15646]	-	770	688	566	790	864
968	5.336368054	4.61361389	4.983674217
6.212487127	7.516012082	6.369901875
ENSG00000179277 48.99155216	15.92991276	29.06229125
4.14387398	11.41491828	0	31.32791872	5.186264087
2.625287821	0.016284158	0.098924263	MEIS3P1 17
15786618	15787575	+	958
processed_pseudogene	Meis homeobox 3 pseudogene 1 [Source:HGNC
Symbol;Acc:HGNC:7002]	-	54	18	25	4	10
0	2.760304124	0.89029398	1.623608612
0.232009905	0.641625848	0
ENSG00000267152 47.17705023	32.74482067	24.41232465
61.1221412	106.15874	81.08807026	34.77806518
82.78965049	-1.245210638	0.016285118	0.098924263
AC093227.1	19	37728586	37730643	-	1360
lincRNA novel transcript	-	52	37	21
59	93	94	1.872376229	1.28910786
0.960698766	2.410599971	4.2033098	3.213808265
ENSG00000139679 22.68127415	42.47976735	8.137441549
87.02135357	67.34801786	58.65945508	24.43282768
71.00960884	-1.526770038	0.016301564	0.098932971	LPAR6
13	48389567	48444704	-	4348

protein_coding lysophosphatidic acid receptor 6 [Source:HGNC
Symbol;Acc:HGNC:15520] -   25   48   7   84   59
        68   0.281565316   0.523092078   0.100164851
1.073499371   0.834084089   0.727194182
ENSG00000165689 1530.53238   1821.320025   1312.453073
1727.995449   2132.306735   2386.059599   1554.768492
2082.120595   -0.420987608   0.016302605   0.098932971   ENTR1
        9   136401922   136410609   -   3133
protein_coding endosome associated trafficking regulator 1
[Source:HGNC Symbol;Acc:HGNC:10667]   -   1687   2058   1129
        1668   1868   2766   26.36837529   31.12514737
22.42024704   29.58337339   36.64914415   41.05091096
ENSG00000265972 1242.026572   1845.214894   2324.9833
2065.721179   5068.223717   4357.189818   1804.074922
3830.378238   -1.086351942   0.016306909   0.098932971   TXNIP
        1   145992435   145996600   -   3625
protein_coding thioredoxin interacting protein [Source:HGNC
Symbol;Acc:HGNC:16952] -   1369   2085   2000   1994   4440
        5051   18.4937133   27.25363839   34.32644524
30.56533687   75.28740348   64.78886558
ENSG00000223842 5.443505796   8.849951531   9.299933199   0
        0   0   7.864463509   0   5.40745202
0.016308923   0.098932971   AL360093.1   1   219222248
        219225497   -   1126   lincRNA novel transcript
        -       6   10   8   0   0   0
0.260940532   0.420811937   0.442036817   0   0   0
ENSG00000148218 605.1363943   600.0267138   666.1077154
479.6534131   495.4074534   267.418104   623.7569412
414.1596569   0.592637675   0.01633039   0.099021632   ALAD
        9   113386317   113401333   -   4983
protein_coding aminolevulinate dehydratase [Source:HGNC
Symbol;Acc:HGNC:395]   -   667   678   573   463   434
        310   6.554863168   6.447112484   7.15435657
5.163000267   5.353603492   2.892689537
ENSG00000176402 9.07250966   10.61994184   8.137441549
23.82727538   35.38624667   44.85723035   9.276631015
34.6902508   -1.900918433   0.016334735   0.099021632   GJC3
        7   99923269   99929620   -   1116
protein_coding gap junction protein gamma 3 [Source:HGNC
Symbol;Acc:HGNC:17495] -   10   12   7   23   31
        52   0.438797848   0.509499183   0.390248005
1.14518509   1.707437673   2.166557238
ENSG00000264895 9.979760626   4.424975766   9.299933199   0
        0   0   7.90155653   0   5.413997147
0.016363951   0.099127001   AC006141.1   17   51249579
        51251748   -   2170   sense_intronic "novel
transcript, sense intronic to MBTD1"   -   11   5   8
        0   0   0   0.248234211   0.109178396
0.229370256   0   0   0
ENSG00000135926 2181.938573   2631.09059   3086.41533

3365.86164	3607.114177	3221.956834	2633.148165
3398.310884	-0.368371009	0.016367038	0.099127001	TMBIM1
	2	218274192	218292586	-	5098
protein_coding	transmembrane BAX inhibitor motif containing 1
[Source:HGNC Symbol;Acc:HGNC:23410]	-	2405	2973	2655
	3249	3160	3735	23.10169965	27.63258527
32.40197935	35.41293513	38.10084536	34.06605107
ENSG00000083812 377.4164018	473.4724069	362.6973948
533.5237749	522.8032573	690.1112362	404.5287345
582.1460895	-0.525154857	0.016368928	0.099127001	ZNF324
	19	58467045	58475436	+	5664
protein_coding	zinc finger protein 324 [Source:HGNC
Symbol;Acc:HGNC:14096]	zf-C2H2 416	535	312	515	458
	800	3.596654903	4.475658875	3.42719011
5.052380241	4.970380005	6.567464901
ENSG00000148341 774.7923249	771.7157735	813.7441549
634.0127189	624.39603	392.5007656	786.7507511
550.3031715	0.517174424	0.016383986	0.099142038
SH3GLB2 9	129007036	129028303	-	6814
protein_coding	"SH3 domain containing GRB2 like, endophilin B2
[Source:HGNC Symbol;Acc:HGNC:10834]"	-	854	872	700
	612	547	455	6.137399944	6.063743143
6.391499472	4.990698531	4.934379608	3.104847583
ENSG00000139496 3536.464265	3600.160283	3235.214262
4344.851868	4507.751229	3971.590164	3457.279603
4274.731087	-0.305817523	0.016389918	0.099142038	NUP58
	13	25301524	25349800	+	10422
protein_coding	nucleoporin 58 [Source:HGNC Symbol;Acc:HGNC:20261]
	-	3898	4068	2783	4194	3949	4604
18.31552827	18.49508435	16.61380023	22.36090626
23.29074739	20.54070116
ENSG00000221829 710.3775064	607.9916702	613.7955911
472.4016337	509.1053554	415.7920198	644.0549226
465.7663363	0.468927723	0.016391477	0.099142038	FANCG
	9	35073835	35080016	-	3614
protein_coding	FA complementation group G [Source:HGNC
Symbol;Acc:HGNC:3588]	-	783	687	528	456	446
	482	10.6096803	9.007308336	9.089764277
7.011141595	7.585672184	6.201402331
ENSG00000137054 1493.33509	1771.760297	1476.364395
1913.43381	1939.394616	2244.586796	1580.486594
2032.471741	-0.362839653	0.01639383	0.099142038	POLR1E
	9	37485935	37503697	+	2512
protein_coding	RNA polymerase I subunit E [Source:HGNC
Symbol;Acc:HGNC:17631]	-	1646	2002	1270	1847	1699
	2602	32.08772148	37.76338175	31.45509003
40.85632542	41.57393225	48.16357054
ENSG00000114982 1435.271028	1404.487308	1435.677188
1642.010064	1797.849629	2019.438005	1425.145175
1819.7659	-0.353038048	0.016400611	0.099149145	KANSL3

2	96593170	96642787	-	9015
protein_coding	KAT8 regulatory NSL complex subunit 3 [Source:HGNC
Symbol;Acc:HGNC:25473]	-	1582	1587	1235	1585	1575
2341	8.593487145	8.341374817	8.523305854
9.769572927	10.73895907	12.07443174
ENSG00000075790 1887.98926	1514.226707	1789.074649
1609.895041	1168.887632	1010.150322	1730.430205
1262.977665	0.454574116	0.016411898	0.099169804	BCAP29
7	107579977	107629170	+	8409
protein_coding	B cell receptor associated protein 29 [Source:HGNC
Symbol;Acc:HGNC:24131]	-	2081	1711	1539	1554	1024
1171	12.11871051	9.641222328	11.38678483
10.2687766	7.485192338	6.475056476
ENSG00000099341 4616.092915	5813.533161	5961.257181
6081.135065	7533.846066	7267.733956	5463.627752
6960.905029	-0.34953649	0.01641524	0.099169804	PSMD8
19	38374536	38383824	+	3105
protein_coding	"proteasome 26S subunit, non-ATPase 8 [Source:HGNC
Symbol;Acc:HGNC:9566]"	-	5088	6569	5128	5870	6600
8425	80.24429793	100.2453181	102.7527038
105.0481819	130.6561002	126.1651252
ENSG00000161036 1045.153113	1292.977919	1033.455077
1099.162573	1813.830515	1821.031025	1123.862036
1578.008038	-0.489413933	0.016464568	0.099433848	LRWD1
7	102464929	102473168	+	5926
protein_coding	leucine rich repeats and WD repeat domain containing 1
[Source:HGNC Symbol;Acc:HGNC:21769]	-	1152	1461	889
1061	1589	2111	9.519617868	11.68194103
9.333552194	9.948687294	16.48198863	16.56370947
ENSG00000144597 777.5140778	889.4201289	783.519372
1023.536873	997.6638578	1235.299113	816.8178596
1085.499948	-0.410617936	0.016483025	0.09951134	EAF1
3	15427355	15450635	+	5152
protein_coding	ELL associated factor 1 [Source:HGNC
Symbol;Acc:HGNC:20907]	-	857	1005	674	988	874
1432	8.145798279	9.243078644	8.139371831
10.65597355	10.42756579	12.92403667
ENSG00000167962 1574.987677	1979.734158	1524.026553
1804.657118	2377.727478	2624.147976	1692.916129
2268.844191	-0.422288307	0.016491043	0.099525778	ZNF598
16	1997654 2009823 -	4701	protein_coding	zinc
finger protein 598 [Source:HGNC Symbol;Acc:HGNC:28079]	-	1736
2237	1311	1742	2083	3042	18.08373579
22.54769615	17.35079134	20.59064462	27.23619149
30.08846115
ENSG00000245694 421.8716992	529.2271016	440.5843353
270.3877772	237.4303003	401.1271561	463.8943787
302.9817445	0.612286958	0.016514667	0.099634359	CRNDE
16	54845189	54929189	-	5846
lincRNA colorectal neoplasia differentially expressed [Source:HGNC

Symbol;Acc:HGNC:37078] - 465 598 379 261 208
465 3.89513779 4.846953059 4.033548149
2.480811349 2.187015616 3.698496057
ENSG00000261122 16.33051739 0.884995153 0 39.36680281
57.07459141 16.39014186 5.73850418 37.61051203
-2.690647807 0.016526315 0.099670637 LINC02167
16 35743268 35756515 + 1989
lincRNA long intergenic non-protein coding RNA 2167 [Source:HGNC
Symbol;Acc:HGNC:53028] - 18 1 0 38 50
19 0.443165971 0.023822737 0 1.061599871
1.545192464 0.444170628
ENSG00000076356 714.0065102 710.651108 626.5829993
556.3150818 328.7496465 520.1713443 683.7468725
468.4120242 0.544728964 0.016533279 0.099678654 PLXNA2
1 208022242 208244320 - 13781
protein_coding plexin A2 [Source:HGNC Symbol;Acc:HGNC:9100] -
787 803 539 537 288 603 2.796550609
2.760967239 2.433407706 2.165237782 1.284573964
2.034546394
ENSG00000107372 2868.727554 2880.659223 3114.31513
3559.587748 3679.028162 3645.512605 2954.567303
3628.042839 -0.296447896 0.01654148 0.09969412 ZFAND5
9 72351425 72365235 - 7053
protein_coding zinc finger AN1-type containing 5 [Source:HGNC
Symbol;Acc:HGNC:13008] - 3162 3255 2679 3436 3223
4226 21.95415193 21.86772229 23.63228287
27.07019068 28.08883855 27.86035378
ENSG00000172260 74.39457921 64.60464618 77.88694054
46.61858227 15.98088559 19.84069804 72.29538864
27.4800553 1.395287934 0.01658661 0.099932068 NEGR1
1 71395940 72282734 - 13406
protein_coding neuronal growth regulator 1 [Source:HGNC
Symbol;Acc:HGNC:17302] - 82 73 67 45 14
23 0.299532065 0.258018048 0.310944181
0.186519972 0.064191301 0.079773682
ENSG00000177119 2017.726148 1570.866397 2252.908817
1671.017182 1461.10954 1180.090214 1947.167121
1437.405645 0.437907803 0.016599861 0.099977846 ANO6
12 45215987 45440404 + 8235
protein_coding anoctamin 6 [Source:HGNC Symbol;Acc:HGNC:25240] -
2224 1775 1938 1613 1280 1368 13.22512736
10.21318491 14.64188582 10.88385651 9.554186759
7.724200031
ENSG00000160401 36.29003864 53.09970919 40.68720775
80.8055426 93.60232991 103.5166854 43.35898519
92.64151932 -1.094565567 0.016614867 0.10000958
CFAP157 9 127706989 127716002 + 4703
protein_coding cilia and flagella associated protein 157 [Source:HGNC
Symbol;Acc:HGNC:27843] - 40 60 35 78 82
120 0.416498744 0.604508918 0.463020172

0.921577085	1.071732088	1.186416794
ENSG00000244723 3.629003864	15.92991276	4.6499666	0
	0	0	8.06962774	0	5.448344664
0.016616437	0.10000958	ASLP1	22	23649213
23653334	-	637	unprocessed_pseudogene
argininosuccinate lyase pseudogene 1 [Source:HGNC
Symbol;Acc:HGNC:747]	-	4	18	4	0	0
	0	0.307502919	1.33893506	0.390685601	0
	0	0
ENSG00000149357 2122.060009	2624.895624	2395.89529
2496.684073	3185.903692	3758.51832	2380.950308
3147.035362	-0.402693019	0.016625315	0.10002898
LAMTOR1 11	72085895	72103387	-	2778
protein_coding	"late endosomal/lysosomal adaptor, MAPK and MTOR
activator 1 [Source:HGNC Symbol;Acc:HGNC:26068]"	-	2339
	2966	2061	2410	2791	4357	41.23126543
50.59007766	46.15859669	48.20552808	61.75540231
72.9266622
ENSG00000260300 0	0.884995153	0	4.14387398	0
	33.64292277	0.294998384	12.59559892	-5.175111346
	0.016649503	0.100134838	AC009119.2	16
83908132	83951445	+	3055	protein_coding	novel
protein -	0	1	0	4	0	39	0
	0.015510122	0	0.072754661	0	0.59358704
ENSG00000175182 58.97131279	59.29467526	86.02438209
14.50355893	41.09370581	20.70333709	68.09679005
25.43353394	1.425661901	0.016654231	0.100134838
FAM131A 3	184335926	184348421	+	8925
protein_coding	family with sequence similarity 131 member A
[Source:HGNC Symbol;Acc:HGNC:28308]	-	65	67	74
	14	36	24	0.356643091	0.355707497
0.515858204	0.087162937	0.247937168	0.125035702
ENSG00000159792 878.2189351	1202.708413	1032.292585
1113.666132	1467.958491	1720.102256	1037.739978
1433.90896	-0.46682202	0.016664286	0.100161252	PSKH1
	16	67893272	67929678	+	3977
protein_coding	protein serine kinase H1 [Source:HGNC
Symbol;Acc:HGNC:9529]	-	968	1359	888	1075	1286
	1994	11.91923685	16.19162014	13.8919823
15.01982653	19.87617161	23.31312977
ENSG00000145041 1407.146248	1495.641809	1162.49165
1965.232235	1665.436577	1675.245026	1355.093236
1768.637946	-0.383467493	0.016694365	0.100306804	DCAF1
	3	51395867	51500002	-	6849
protein_coding	DDB1 and CUL4 associated factor 1 [Source:HGNC
Symbol;Acc:HGNC:30911]	-	1551	1690	1000	1897	1459
	1942	11.08953447	11.69192388	9.084053439
15.39048154	13.09409495	13.1841786
ENSG00000144227 71.67282631	39.82478189	63.93704074
31.07905485	11.41491828	18.11541995	58.47821632

20.20313103	1.530381545	0.016703383	0.100306804	NXPH2
	2	138670772	138780348	-	1052
protein_coding	neurexophilin 2 [Source:HGNC Symbol;Acc:HGNC:8076]
	-	79	45	55	30	10	21
3.677392915	2.026857493	3.252773299	1.584592363
0.584294261	0.928185058
ENSG00000066583 568.8463557	440.7275863	602.1706746
735.5376314	760.2335576	724.616798	537.2482055
740.129329	-0.463172322	0.016705514	0.100306804	ISOC1
	5	129094751	129114028	+	1940
protein_coding	isochorismatase domain containing 1 [Source:HGNC
Symbol;Acc:HGNC:24254]	-	627	498	518	710	666
	840	15.8268503	12.1633738	16.6124955
20.33614653	21.10181734	20.13300374
ENSG00000213693 58.97131279	66.37463648	26.73730795
141.9276838	136.9790194	68.14848458	50.69441907
115.6850626	-1.181504066	0.016716988	0.100341636
SEC14L1P1	11	43897456	43899636	+	2181
	processed_pseudogene	SEC14 like 1 pseudogene 1 [Source:HGNC
Symbol;Acc:HGNC:44189]	-	65	75	23	137	120
	79	1.459440435	1.629416233	0.656113565
3.490414026	3.38199484	1.684234052
ENSG00000228430 1.814501932	0	0	0	11.41491828
	36.2308399	0.604833977	15.88191939	-4.671057871
	0.016723029	0.100343846	AL162726.3	9
81977614	82780194	+	4429
transcribed_unprocessed_pseudogene	serine palmitoyltransferase
long chain base subunit 1 (SPTLC1) pseudogene	-	2	0
	0	0	10	42	0.022113272	0	0
	0	0.138784728	0.440935056
ENSG00000101188 176.9139384	192.0439482	189.4861389
393.6680281	289.9389244	219.9729565	186.1480085
301.193303	-0.693242129	0.016758884	0.100524889	NTSR1
	20	62708837	62762771	+	4237
protein_coding	neurotensin receptor 1 [Source:HGNC
Symbol;Acc:HGNC:8039]	-	195	217	163	380	254
	255	2.253745284	2.426764933	2.393514082
4.983531139	3.68487375	2.79841896
ENSG00000253730 2.721752898	3.539980612	0	27.97114936
	21.68834474	6.901112362	2.087244503	18.85353549
	-3.131586006	0.016786553	0.100645675	AC015909.2
	17	50158333	50161276	-	547
antisense	novel transcript	-	3	4	0
	27	19	8	0.268573162	0.346496702	0
	2.742764259	2.135077456	0.680038779
ENSG00000268592 63.50756762	91.15450077	46.499666
13.46759043	10.27342645	42.26931322	67.05391146
22.00344337	1.595677073	0.016793915	0.100645675
RAET1E-AS1	6	149863494	149919507	+	1210
	antisense	RAET1E antisense RNA 1 [Source:NCBI

gene;Acc:100652739]	-	70	103	40	13	9
	49	2.832965941	4.033465023	2.056749818
0.596994082	0.457198187	1.882962747
ENSG00000088280 176.0066874	65.48964133	127.8740815
66.30198367	63.92354238	37.09347895	123.1234701
55.77300167	1.14572686	0.016797754	0.100645675	ASAP3
	1	23428563	23484568	-	4988
protein_coding	"ArfGAP with SH3 domain, ankyrin repeat and PH domain
3 [Source:HGNC Symbol;Acc:HGNC:14987]"	-	194	74	110
	64	56	43	1.904600827	0.702961784
1.372059948	0.71296067	0.690095098	0.400841823
ENSG00000140575 8081.791605	6133.901406	8832.611556
7078.772726	5524.820448	4640.998064	7682.768189
5748.197079	0.418506376	0.01680178	0.100645675	IQGAP1
	15	90388218	90502243	+	11535
protein_coding	IQ motif containing GTPase activating protein 1
[Source:HGNC Symbol;Acc:HGNC:6110]	-	8908	6931	7598
	6833	4840	5380	37.81736742	28.47113239
40.98156479	32.91590995	25.79141223	21.68681102
ENSG00000154589 227.7199925	274.3484975	166.2363059
405.0636815	293.3633998	356.2699257	222.7682653
351.565669	-0.656037297	0.016854145	0.100897854	LY96
	8	73991352	74029087	+	607
protein_coding	lymphocyte antigen 96 [Source:HGNC
Symbol;Acc:HGNC:17156]	-	251	310	143	391	257
	413	20.24947248	24.19911278	14.65730729
35.79316562	26.02506318	31.63678759
ENSG00000257557 9.979760626	8.849951531	4.6499666	0
	0	0	7.826559586	0	5.403829271
0.016855286	0.100897854	PPP1R12A-AS1	12	79934901
	79942712	+	3234	bidirectional_promoter_lncRNA
	PPP1R12A antisense RNA 1 [Source:HGNC Symbol;Acc:HGNC:51903]
	-	11	10	4	0	0	0
0.166564081	0.146516463	0.076953224	0	0	0
ENSG00000136930 2889.594327	3756.804425	3567.686874
3927.356564	4361.640275	4717.772939	3404.695208
4335.589926	-0.348899638	0.016866983	0.100933714	PSMB7
	9	124353466	124415444	-	1787
protein_coding	proteasome subunit beta 7 [Source:HGNC
Symbol;Acc:HGNC:9544]	-	3185	4245	3069	3791	3821
	5469	87.27976489	112.5588333	106.8511455
117.8803117	131.4316153	142.3031092
ENSG00000185359 3628.096613	4267.446628	4210.544756
4276.477947	5448.340496	5740.862846	4035.362666
5155.227096	-0.353492828	0.01689329	0.101030681	HGS
	17	81683326	81703138	+	8254
protein_coding	hepatocyte growth factor-regulated tyrosine kinase
substrate [Source:HGNC Symbol;Acc:HGNC:4897]	-	3999	4822
	3622	4128	4773	6655	23.72551363	27.68147211
	27.30177153	27.78991815	35.54465719	37.48992861

ENSG00000110917 4662.362714   4158.592225   4202.407314
3847.58699   3374.249844   3239.209615   4341.120751
3487.015483   0.316235542   0.01689461   0.101030681   MLEC
        12   120686869   120701864   +   6723
protein_coding malectin [Source:HGNC Symbol;Acc:HGNC:28973]   -
        5139   4699   3615   3714   2956   3755   37.43209979
        33.11835633   33.45430692   30.69663935   27.02642978
        25.97035273
ENSG00000140943 3537.371516   3282.447023   3622.323981
2976.337486   2821.767799   2636.224922   3480.714173
2811.443402   0.308097454   0.016914068   0.101112859   MBTPS1
        16   84053761   84116906   -   10238
protein_coding "membrane bound transcription factor peptidase, site 1
[Source:HGNC Symbol;Acc:HGNC:15456]"   -   3899   3709   3116
        2873   2472   3056   18.64948286   17.16596208
18.93604035   15.59310289   14.84159927   13.87935368
ENSG00000239713 2.721752898   5.309970919   16.2748831   0
        0   0   8.102202305   0   5.445788398
0.016955959   0.101329041   APOBEC3G   22   39077005
        39087743   +   5437   protein_coding apolipoprotein
B mRNA editing enzyme catalytic subunit 3G [Source:HGNC
Symbol;Acc:HGNC:17357] -   3   6   14   0   0
        0   0.027020327   0.052289966   0.160204809   0
        0   0
ENSG00000276527 8.165258694   10.61994184   4.6499666   0
        0   0   7.811722377   0   5.401196892
0.01702792   0.101724713   AL356515.1   13   44684487
        44715393   -   415   lincRNA novel transcript
        -       9   12   4   0   0   0
1.061996526   1.370123105   0.599678863   0   0   0
ENSG00000143727 1255.635337   1511.571722   1421.727288
1126.097754   983.9659559   1115.392286   1396.311449
1075.151998   0.376645291   0.017039737   0.101759472   ACP1
        2   264140 278283 +   4160   protein_coding acid
phosphatase 1 [Source:HGNC Symbol;Acc:HGNC:122] -   1384   1708
        1223   1087   862   1293   16.29188902   19.45454045
        18.29110627   14.5193862   12.73682833   14.45227485
ENSG00000266338 736.6877844   707.9961225   585.8957915
731.3937574   1144.916304   974.7821212   676.8598995
950.3640607   -0.488515453   0.017045244   0.101759472   NBPF15
        1   144421386   144461674   -   5306
protein_coding NBPF member 15 [Source:HGNC Symbol;Acc:HGNC:28791]
        -       812   800   504   706   1003   1130
7.494065199   7.144127261   5.90976399   7.393490157
11.61932897   9.902439916
ENSG00000213401 1981.43611   2201.867941   1786.749666
2070.901021   3069.471526   2763.895501   1990.017906
2634.756016   -0.404480141   0.01709443   0.101992064
MAGEA12 X   152733779   152737669   +   1943
protein_coding MAGE family member A12 [Source:HGNC

Symbol;Acc:HGNC:6799]	-	2184	2488	1537	1999	2689
	3204	55.04381379	60.67419406	49.21618129
57.16787341	85.06783143	76.67445981
ENSG00000136738 730.3370276	624.8065781	698.6574816
930.2997084	937.1647909	854.0126548	684.6003624
907.1590514	-0.405982861	0.017095736	0.101992064	STAM
	10	17644125	17715914	+	3246
protein_coding	signal transducing adaptor molecule [Source:HGNC
Symbol;Acc:HGNC:11357]	-	805	706	601	898	821
	990	12.14439959	10.30582174	11.51947809
15.37233587	15.54683545	14.18135397
ENSG00000105662 612.394402	783.2207105	769.5694722
839.1344809	979.3999886	1146.447291	721.7281949
988.3272535	-0.454449405	0.017110873	0.102021967	CRTC1
	19	18683677	18782333	+	6965
protein_coding	CREB regulated transcription coactivator 1
[Source:HGNC Symbol;Acc:HGNC:16062]	-	675	885	662
	810	858	1329	4.745820802	6.020722224
5.913487938	6.462133738	7.572050946	8.872272626
ENSG00000256292 6.350756762	2.654985459	15.11239145	0
	0	0	8.03937789	0	5.435057481
0.017112283	0.102021967	LINC02376	12	127274467
	127284283	-	724	lincRNA long intergenic non-
protein coding RNA 2376 [Source:HGNC Symbol;Acc:HGNC:53298]	-
	7	3	13	0	0	0	0.473465302
	0.196340155	1.117150367	0	0	0
ENSG00000237149 41.73354444	68.14462679	79.04943219
138.8197783	108.4417237	126.8079397	62.97586781
124.6898139	-0.98974482	0.017128478	0.10203947
ZNF503-AS2	10	75401519	75408982	+	3348
	antisense	ZNF503 antisense RNA 2 [Source:HGNC
Symbol;Acc:HGNC:23525]	-	46	77	68	134	95
	147	0.672823367	1.089762142	1.263660208
2.223982639	1.744156763	2.041563552
ENSG00000178623 1.814501932	2.654985459	3.48747495
1.035968495	7.990442797	62.97265031	2.65232078
23.9996872	-3.193731613	0.017128719	0.10203947	GPR35
	2	240605431	240631259	+	5089
protein_coding	G protein-coupled receptor 35 [Source:HGNC
Symbol;Acc:HGNC:4492]	-	2	3	3	1	7
	73	0.019245368	0.02793285	0.036677156
0.010918918	0.084549871	0.666993232
ENSG00000180739 8.165258694	9.734946684	34.8747495
4.14387398	0	0	17.59165162	1.381291327
3.679548237	0.017137248	0.10203947	S1PR5	19
10512742	10517931	-	2806	protein_coding
sphingosine-1-phosphate receptor 5 [Source:HGNC
Symbol;Acc:HGNC:14299]	-	9	11	30	4	0
	0	0.157066486	0.185751128	0.665181917
0.079210794	0	0

ENSG00000126368 304.8363246 263.7285556 337.1225785
258.9921237 731.6962619 529.6603738 301.8958196
506.7829198 -0.747517422 0.017138293 0.10203947 NR1D1
17 40092787 40100725 - 2772
protein_coding nuclear receptor subfamily 1 group D member 1
[Source:HGNC Symbol;Acc:HGNC:7962] THR-like 336 298
290 250 641 614 5.935738161 5.093889022
6.508960239 5.011397204 14.21386426 10.29926336
ENSG00000185361 413.7064405 385.8578868 567.2959251
249.6684073 357.2869422 297.6104706 455.6200841
301.52194 0.594756824 0.017150974 0.102063481
TNFAIP8L1 19 4639518 4655568 + 6667 protein_coding
TNF alpha induced protein 8 like 1 [Source:HGNC
Symbol;Acc:HGNC:28279] - 456 436 488 241 313
345 3.349369576 3.098720997 4.554033421
2.008623924 2.885766866 2.406133158
ENSG00000136448 3181.729138 3199.257479 3810.647628
3948.075934 4192.699485 4653.07501 3397.211415
4264.61681 -0.32854372 0.017153866 0.102063481 NMT1
17 45051610 45109016 + 6669
protein_coding N-myristoyltransferase 1 [Source:HGNC
Symbol;Acc:HGNC:7857] - 3507 3615 3278 3811 3673
5394 25.75157119 25.68467206 30.58123911
31.75340298 33.85381146 37.608087
ENSG00000226808 10.88701159 1.769990306 11.6249165 0
0 0 8.093972799 0 5.44722547
0.017163932 0.102089037 LINC00840 10 43845261
43895433 + 3825 lincRNA long intergenic non-
protein coding RNA 840 [Source:HGNC Symbol;Acc:HGNC:44987] -
12 2 10 0 0 0 0.15363087
0.024775647 0.162657992 0 0 0
ENSG00000108785 114.3136217 162.8391082 180.1862057
211.337573 257.9771532 282.9456069 152.4463119
250.7534443 -0.720636407 0.017183518 0.102144611
HSD17B1P1 17 42546764 42548706 + 955
unprocessed_pseudogene hydroxysteroid 17-beta dehydrogenase 1
pseudogene 1 [Source:HGNC Symbol;Acc:HGNC:5216] - 126 184
155 204 226 328 6.460942219 9.129371755
10.09799551 11.86967532 14.54629624 15.96987402
ENSG00000218052 3.629003864 6.194966072 13.9498998 0
0 0 7.924623245 0 5.415091143
0.017184825 0.102144611 ADAMTS7P4 15 85255369
85330334 - 5516
transcribed_unprocessed_pseudogene ADAMTS7 pseudogene 4
[Source:HGNC Symbol;Acc:HGNC:49410] - 4 7 12
0 0 0 0.035511124 0.060131249
0.135351738 0 0 0
ENSG00000133422 923.5814834 935.4398768 864.8937875
982.0981332 1221.396256 1487.189714 907.9717159
1230.228034 -0.438528963 0.017195182 0.10217184 MORC2

22	30925130	30968298	-	6258
protein_coding	MORC family CW-type zinc finger 2 [Source:HGNC
Symbol;Acc:HGNC:23573]	-	1018	1057	744	948	1070
1724	7.966011018	8.003240528	7.396805914
8.417532894	10.50982729	12.80951601
ENSG00000112237 1080.5359	903.5800513	856.756346
1281.493028	1211.12283	1226.672722	946.9574326
1239.76286	-0.388094419	0.017245985	0.102415311	CCNC
6	99542380	99568973	-	5765
protein_coding	cyclin C [Source:HGNC Symbol;Acc:HGNC:1581]	-
1191	1021	737	1237	1061	1422	10.11675269
8.391756455	7.953806529	11.92291456	11.31262608
11.46915185
ENSG00000126467 36.29003864	14.15992245	5.812458249
71.48182615	70.77249335	40.54403513	18.75413978
60.93278487	-1.687051799	0.017247737	0.102415311	TSKS
19	49739753	49763330	-	2391
protein_coding	testis specific serine kinase substrate [Source:HGNC
Symbol;Acc:HGNC:30719]	-	40	16	5	69	62
47	0.819236133	0.317078538	0.130105985
1.6035465	1.593894139	0.914006533
ENSG00000163029 1507.851105	1217.753331	1147.379258
1117.810006	971.4095458	674.5837334	1290.994565
921.2677617	0.488022699	0.017279492	0.102569439	SMC6
2	17663812	17800242	-	9824
protein_coding	structural maintenance of chromosomes 6 [Source:HGNC
Symbol;Acc:HGNC:20466]	-	1662	1376	987	1079	851
782	8.284596274	6.636766236	6.250800605
6.103024797	5.324619355	3.701258497
ENSG00000246695 140.6238997	173.45905	118.5741483
221.6972579	172.3652661	368.3468723	144.2190327
254.1364654	-0.817962028	0.017295865	0.10260696
RASSF8-AS1	12	25939329	25959765	-	3981
antisense	RASSF8 antisense RNA 1 [Source:HGNC
Symbol;Acc:HGNC:48637]	-	155	196	102	214	151
427	1.906637823	2.332868906	1.594097354
2.986989111	2.331482338	4.987314039
ENSG00000007047 694.9542399	873.4902161	828.8565464
856.7459453	1333.262455	1134.370345	799.1003341
1108.126248	-0.47156425	0.017297414	0.10260696	MARK4
19	45079288	45305283	+	6668
protein_coding	microtubule affinity regulating kinase 4 [Source:HGNC
Symbol;Acc:HGNC:13538]	-	766	987	713	827	1168
1315	5.625509495	7.013713189	6.652743592
6.891630146	10.76699749	9.169827793
ENSG00000126705 1026.100843	1100.93397	1076.467268
809.0913945	762.5165412	883.3423824	1067.834027
818.3167727	0.383268132	0.017303657	0.102609583	AHDC1
1	27534035	27604431	-	8297
protein_coding	AT-hook DNA binding motif containing 1 [Source:HGNC

Symbol;Acc:HGNC:25230]	-	1131	1244	926	781	668
	1024	6.67529093	7.104372608	6.94379264
5.230485319	4.948832247	5.738651939
ENSG00000173535 7.258007728	14.15992245	17.43737475
29.00711786	37.66923033	63.83528935	12.95176831
43.50387918	-1.758401296	0.017354395	0.102875969
TNFRSF10C	8	23102590	23117437	+	1868
	protein_coding	TNF receptor superfamily member 10c
[Source:HGNC Symbol;Acc:HGNC:11906]	-	8	16	15
	28	33	74	0.209720941	0.405853739
0.499598624	0.832900654	1.085886486	1.841984053
ENSG00000135298 57.15681086	31.85982551	53.4746159
11.39565344	18.26386925	17.25278091	47.49708409
15.63743453	1.601428464	0.017366244	0.102881512	ADGRB3
	6	68635367	69389511	+	6490
protein_coding	adhesion G protein-coupled receptor B3 [Source:HGNC
Symbol;Acc:HGNC:945]	-	63	36	46	11	16
	20	0.475362082	0.262835634	0.440981105
0.094180292	0.151538382	0.143290143
ENSG00000100908 241.328757	200.0089046	209.248497
76.66166862	192.912119	62.11001126	216.8620528
110.5612663	0.977509853	0.017366962	0.102881512	EMC9
	14	24138959	24141588	-	1588
protein_coding	ER membrane protein complex subunit 9 [Source:HGNC
Symbol;Acc:HGNC:20273]	-	266	226	180	74	169
	72	8.202756546	6.743484784	7.052268741
2.589364951	6.541593703	2.108205861
ENSG00000112576 1791.820658	2156.733188	2169.209419
1663.765403	1545.579935	1598.470151	2039.254422
1602.605163	0.347328828	0.0174132	0.10312089	CCND3
	6	41934933	42050357	-	5802
protein_coding	cyclin D3 [Source:HGNC Symbol;Acc:HGNC:1585]	-
	1975	2437	1866	1606	1354	1853	16.66932673
	19.90234478	20.00970848	15.38083312	14.3445953
	14.85007725
ENSG00000065615 1059.669128	900.9250659	746.3196392
1073.263361	1341.252898	1247.376059	902.3046111
1220.630773	-0.434869088	0.017441416	0.103253417	CYB5R4
	6	83859643	83967424	+	9635
protein_coding	cytochrome b5 reductase 4 [Source:HGNC
Symbol;Acc:HGNC:20147]	-	1168	1018	642	1036	1175
	1446	5.936354221	5.006364887	4.145626346
5.974754707	7.496067832	6.978267158
ENSG00000136830 13083.46618	15753.79872	18348.7682
12569.40575	13243.58819	11474.82458	15728.6777
12429.27284	0.339620215	0.01747984	0.103446265
FAM129B 9	127505339	127578989	-	4629
protein_coding	family with sequence similarity 129 member B
[Source:HGNC Symbol;Acc:HGNC:25282]	-	14421	17801	15784
	12133	11602	13302	152.558665	182.2148048

212.1469235	145.6441551	154.0611164	133.6167121
ENSG00000049449 2501.290913	2677.995333	2548.181697
2088.512486	2062.675734	2101.388714	2575.822648
2084.192311	0.305553104	0.01749143	0.103480235	RCN1
	11	32090904	32105755	+	4675
protein_coding	reticulocalbin 1 [Source:HGNC Symbol;Acc:HGNC:9934]
	-	2757	3026	2192	2016	1807	2436
28.87911196	30.66999812	29.17197154	23.96188371
23.75876695	24.22850889
ENSG00000197355 798.3808501	784.9907008	780.0318971
521.0921529	689.4610642	518.4460662	787.8011493
576.3330945	0.452192092	0.017504148	0.103520852	UAP1L1
	9	137077501	137084539	+	5283
protein_coding	UDP-N-acetylglucosamine pyrophosphorylase 1 like 1
[Source:HGNC Symbol;Acc:HGNC:28082]	-	880	887	671
	503	604	601	8.15700531	7.955536085
7.902213444	5.290532882	7.027545856	5.289624939
ENSG00000155714 9.979760626	6.194966072	6.974949899	0
	0	0	7.716558866	0	5.38149382
0.017508446	1	PDZD9	16	21983865	22001110
	-	1331	protein_coding	PDZ domain containing 9
[Source:HGNC Symbol;Acc:HGNC:28740]	-	11	7	6
	0	0	0	0.40470942	0.249199075
0.280465884	0	0	0
ENSG00000261327 30.84653284	19.46989337	63.93704074	0
	15.98088559	0	38.08448899	5.326961865
2.866890297	0.01751832	0.103564131	AC134312.5	16
	88177298	88178941	-	1507	lincRNA novel
transcript	-	34	22	55	0	14	0
	1.104827176	0.691728818	2.270681825	0
0.571034232	0
ENSG00000227214 13.60876449	10.61994184	8.137441549
41.4387398	28.5372957	42.26931322	10.78871596
37.41511624	-1.789581241	0.017529854	0.103564131	HCG15
	6	28986203	28987484	+	1032
antisense	HLA complex group 15 [Source:HGNC
Symbol;Acc:HGNC:18361]	-	15	12	7	40	25
	49	0.711770928	0.550970047	0.422012378
2.153735357	1.489044482	2.207737329
ENSG00000253659 5.443505796	3.539980612	0	17.61146441
	30.82027936	16.39014186	2.994495469	21.60729521
	-2.814893249	0.017534293	0.103564131	AC138646.1
	8	79297717	79314471	+	633
lincRNA novel transcript	-	6	4	0	17
	27	19	0.464169098	0.299421321	0
1.492303835	2.621847422	1.395664105
ENSG00000115009 22.68127415	50.44472373	8.137441549
46.61858227	60.49906689	141.4728034	27.08781314
82.8634842	-1.605755929	0.017534885	0.103564131	CCL20
	2	227805739	227817564	+	1594

protein_coding C-C motif chemokine ligand 20 [Source:HGNC
Symbol;Acc:HGNC:10619] -   25   57   7   45   53
        164   0.768033874   1.694388439   0.273222568
1.568686794   2.043783613   4.783949088
ENSG00000151612 391.9324173   248.683638   453.3717435
255.8842182   243.1377594   182.0168386   364.6625996
227.0129387   0.683713215   0.0175489   0.103612312   ZNF827
        4   145757627   145938635   -   8433
protein_coding zinc finger protein 827 [Source:HGNC
Symbol;Acc:HGNC:27193] zf-C2H2 432   281   390   247   213
        211   2.508593716   1.578885588   2.877327876
1.627522108   1.552547382   1.163406198
ENSG00000065029 939.9120008   876.1452016   889.3061122
664.0558052   743.1111801   642.6660887   901.7877715
683.2776914   0.401053853   0.017584994   0.103750458   ZNF76
        6   35258909   35295985   +   6789
protein_coding zinc finger protein 76 [Source:HGNC
Symbol;Acc:HGNC:13149] zf-C2H2 1036   990   765   641   651
        745   7.47278746   6.90964646   7.010717592
5.246434612   5.894168405   5.10248201
ENSG00000162409 303.9290736   228.3287495   203.4360387
404.027713   397.2391562   322.6270029   245.2312873
374.6312907   -0.608794551   0.017585974   0.103750458   PRKAA2
        1   56645322   56715335   +   9354
protein_coding protein kinase AMP-activated catalytic subunit alpha 2
[Source:HGNC Symbol;Acc:HGNC:9377]   -   335   258   175
        390   348   374   1.753784086   1.306919329
1.163985391   2.316750605   2.286805555   1.859110719
ENSG00000278272 9.979760626   11.50493699   2.3249833   0
        0   0   7.936560305   0   5.425649404
0.017589893   0.103750458   HIST1H3C   6   26045411
        26045821   +   411   protein_coding histone
cluster 1 H3 family member c [Source:HGNC Symbol;Acc:HGNC:4768] -
        11   13   2   0   0   0   1.310628317
        1.498745773   0.302757577   0   0   0
ENSG00000108591 758.4618076   999.1595279   902.0935203
1107.450321   1214.547305   1193.0298   886.5716186
1171.675809   -0.402292742   0.017621112   0.103899947   DRG2
        17   18087886   18107971   +   7411
protein_coding developmentally regulated GTP binding protein 2
[Source:HGNC Symbol;Acc:HGNC:3030]   -   836   1129   776
        1069   1064   1383   5.524056956   7.218443632
6.514659997   8.015173645   8.824948403   8.677135613
ENSG00000276317 0   2.654985459   2.3249833   0
19.40536108   44.85723035   1.659989586
21.42086381   -3.705082211   0.017628885   0.103911147
AL357033.3   20   62651272   62652186   -   614
        antisense   "novel transcript, antisense to SLCO4A1"
        -       0   3   2   0   17   52   0
        0.231515101   0.202660202   0   1.701875987

3.937911853
ENSG00000187144 0 24.77986429 4.6499666 0 0
0 9.809943629 0 5.729847384 0.01765879
0.104052744 SPATA21 1 16387117 16437424
- 4506 protein_coding spermatogenesis associated 21
[Source:HGNC Symbol;Acc:HGNC:28026] - 0 28 4
0 0 0 0 0.294437611 0.055230077
0 0 0
ENSG00000180801 1013.399329 1072.614126 1236.891115
908.54437 802.4687552 813.4686197 1107.634857
841.493915 0.395822151 0.017678253 0.104132738 ARSJ
4 113900284 113979727 - 5146
protein_coding arylsulfatase family member J [Source:HGNC
Symbol;Acc:HGNC:26286] - 1117 1212 1064 877 703
943 10.62948136 11.15987368 12.86407883
9.469822857 8.397169184 8.520653977
ENSG00000007923 1046.060364 1180.583534 1252.003507
1269.061406 1596.947068 1732.179203 1159.549135
1532.729226 -0.403209039 0.017688589 0.104158936
DNAJC11 1 6634168 6701924 - 5083 protein_coding DnaJ
heat shock protein family (Hsp40) member C11 [Source:HGNC
Symbol;Acc:HGNC:25570] - 1153 1334 1077 1225 1399
2008 11.10805141 12.43546876 13.18264146
13.39146291 16.91784201 18.36853909
ENSG00000103126 1197.571275 1251.383147 1172.954075
1450.355893 1401.751965 1915.92132 1207.302832
1589.343059 -0.397208519 0.017712872 0.104267221 AXIN1
16 287440 352673 - 7487 protein_coding axin 1
[Source:HGNC Symbol;Acc:HGNC:903] - 1320 1414 1009
1400 1228 2221 8.633656816 8.94886625
8.384751188 10.3903995 10.08179573 13.79341311
ENSG00000257298 6.350756762 9.734946684 6.974949899 0
0 0 7.686884449 0 5.376150456
0.01773226 1 AC008147.2 12 50185580
50191363 - 4713 sense_intronic "novel transcript,
sense intronic to LIMA1" - 7 11 6 0
0 0 0.072732629 0.110591484 0.07920647
0 0 0
ENSG00000106006 19.05227029 30.97483036 22.08734135
5.179842475 0 6.038473317 24.03814733 3.739438597
2.659129369 0.017744351 0.104415541 HOXA6 7
27145396 27150603 - 1285 protein_coding
homeobox A6 [Source:HGNC Symbol;Acc:HGNC:5107] Homeobox
21 35 19 5 0 7 0.80028532
1.290599099 0.919935376 0.216211565 0
0.2532946
ENSG00000233328 15.42326642 28.3198449 32.5497662
50.76245625 70.77249335 74.18695789 25.43095917
65.24063583 -1.364815116 0.017749875 0.104415541 PFN1P1
1 171670517 171670939 - 423

processed_pseudogene   profilin 1 pseudogene 1 [Source:HGNC
Symbol;Acc:HGNC:42989] -   17   32   28   49   62
        86   1.968055029   3.584561631   4.118361929
6.43676652   9.009458358   9.453423236
ENSG00000110700 6490.473411   7618.038278   7698.019706
8778.797026   9204.990102   8728.18186   7268.843798
8903.989663   -0.2927758   0.017757789   0.104427369   RPS13
        11   17074389   17077787   -   2186
protein_coding ribosomal protein S13 [Source:HGNC
Symbol;Acc:HGNC:10386] -   7154   8608   6622   8474   8064
        10118   160.2608573   186.5857796   188.4715774
215.4023047   226.7502224   215.2164858
ENSG00000068394 348.3843709   393.8228431   504.5213761
549.0633023   690.602556   551.2263499   415.5761967
596.9640694   -0.523651516   0.0177808   0.104527943   GPKOW
        X   49113389   49123801   -   1742
protein_coding G-patch domain and KOW motifs [Source:HGNC
Symbol;Acc:HGNC:30677] -   384   445   434   530   605
        639   10.79472933   12.1042616   15.50059701
16.9059571   21.34787171   17.05625678
ENSG00000186017 170.5631816   242.488672   168.5612892
339.7976663   243.1377594   344.1929791   193.8710476
309.0428016   -0.672230488   0.017832376   0.104768362   ZNF566
        19   36445119   36489902   -   6481
protein_coding zinc finger protein 566 [Source:HGNC
Symbol;Acc:HGNC:25919] zf-C2H2 188   274   145   328   213
        399   1.420510706   2.003249219   1.391979462
2.812184861   2.02015616   2.862608077
ENSG00000163749 5.443505796   13.2749273   4.6499666   0
        0   0   7.789466564   0   5.397205139
0.017833543   0.104768362   CCDC158 4   76312997
76421868   -   5860   protein_coding coiled-coil domain
containing 158 [Source:HGNC Symbol;Acc:HGNC:26374]   -   6
        15   4   0   0   0   0.050139768
0.121288628   0.042468725   0   0   0
ENSG00000166106 4.53625483   7.079961225   11.6249165   0
        0   0   7.747044185   0   5.383847108
0.017857861   1   ADAMTS15   11   130448974
130476641   +   5673   protein_coding ADAM metallopeptidase
with thrombospondin type 1 motif 15 [Source:HGNC
Symbol;Acc:HGNC:16305] -   5   8   10   0   0
        0   0.043160444   0.066819565   0.109671571   0
        0   0
ENSG00000075213 247.6795137   169.0340742   142.9864729
269.3518087   386.9657298   265.6928259   186.566687
307.3367881   -0.71671683   0.017870724   0.104951936   SEMA3A
        7   83955777   84492724   -   9371
protein_coding semaphorin 3A [Source:HGNC Symbol;Acc:HGNC:10723]
        -       273   191   123   260   339   308
1.426610423   0.965770355   0.816631297   1.541698514

2.223622811	1.528254899
ENSG00000138964 13.60876449	9.734946684	1.16249165	0
	0	0	8.168734275	0	5.467684048
0.017882419	0.104985766	PARVG	22	44172956
44219533	+	8656	protein_coding	parvin gamma
[Source:HGNC Symbol;Acc:HGNC:14654]	-	15	11	1
	0	0	0	0.084859935	0.06021461
0.007187694	0	0	0
ENSG00000108852 234.9780002	184.963987	292.9478958
161.6110852	152.959905	109.5551588	237.629961
141.375383	0.749568915	0.017894393	0.105021206	MPP2
	17	43875357	43909711	-	6114
protein_coding	membrane palmitoylated protein 2 [Source:HGNC
Symbol;Acc:HGNC:7220]	-	259	209	252	156	134
	127	2.074450199	1.619747404	2.564377472
1.417787711	1.347183404	0.965849156
ENSG00000124209 2861.469547	2743.484975	2597.006346
3464.278647	3032.943787	3788.710687	2733.986956
3428.644374	-0.32677912	0.017913261	0.105097074	RAB22A
	20	58309696	58367507	+	9285
protein_coding	"RAB22A, member RAS oncogene family [Source:HGNC
Symbol;Acc:HGNC:9764]"	-	3154	3100	2234	3344	2657
	4392	16.63445071	15.8199908	14.96952801
20.01227234	17.58964225	21.99436569
ENSG00000257698 127.9223862	137.1742487	195.2985972
75.62570013	75.33846066	99.20349021	153.4650774
83.389217	0.875168625	0.017928045	0.105148932
AC084033.3	12	57931528	57936175	-	1312
	lincRNA uncharacterized LOC100506844 [Source:NCBI
gene;Acc:100506844]	-	141	155	168	73	66
	115	5.262764799	5.597889275	7.966770257
3.091726502	3.092127981	4.075632563
ENSG00000232491 0	0	0	0	0	35.36820086
	0	11.78940029	-6.040135386	0.017961818
0.105312098	SAPCD2P3	7	66556216	66557065
	+	850	processed_pseudogene	suppressor APC domain
containing 2 pseudogene 3 [Source:HGNC Symbol;Acc:HGNC:51279]	-
	0	0	0	0	0	41	0	0
	0	0	0	2.242827896
ENSG00000005961 6.350756762	0	5.812458249	20.7193699
	25.11282022	29.32972754	4.054405004	25.05397255
	-2.643954138	0.017995817	0.105476474	ITGA2B	17
	44372180	44389505	-	5551	protein_coding
	integrin subunit alpha 2b [Source:HGNC Symbol;Acc:HGNC:6138]
	-	7	0	5	20	22	34
0.061752635	0	0.056040967	0.200203106	0.243612077
	0.284799162
ENSG00000276045 762.9980624	863.7552694	886.9811289
931.3356769	1318.423062	1135.232984	837.9114869
1128.330574	-0.429361071	0.018006816	0.105505987	ORAI1

12	121626550	121642677	+	2262
protein_coding	ORAI calcium release-activated calcium modulator 1
[Source:HGNC Symbol;Acc:HGNC:25896]	-	841	976	763
899	1155	1316	18.20673532	20.44483726
20.98644048	22.084071	31.38605589	27.05168407
ENSG00000083807 119.7571275	162.8391082	173.2112558
89.09329056	98.16829722	31.05500563	151.9358305
72.7721978	1.067294476	0.018036883	0.105632933
SLC27A5 19	58479512	58512413	-	8025
protein_coding	solute carrier family 27 member 5 [Source:HGNC
Symbol;Acc:HGNC:10999]	-	132	184	149	86	86
36	0.805485216	1.086423679	1.155175778
0.595477634	0.65871988	0.208587596
ENSG00000132670 1625.793731	1443.427095	1926.248664
1329.147579	1312.715602	1193.892439	1665.156497
1278.585207	0.380729784	0.018040426	0.105632933	PTPRA
20	2864184 3039076 +	4469
protein_coding	"protein tyrosine phosphatase, receptor type A
[Source:HGNC Symbol;Acc:HGNC:9664]"	-	1792	1631	1657
1283	1150	1384	19.63614969	17.29298828
23.06848111	15.95248502	15.81739084	14.39980973
ENSG00000255139 4.53625483	0	3.48747495	3.107905485
35.38624667	29.32972754	2.674576593	22.6079599
-3.09218908	0.018083441	0.105818643	AP000442.2
11	59616429	59639861	+	760
antisense	novel transcript	-	5	0	3
3	31	34	0.322169999	0	0.245592166
0.219340943	2.507237425	2.080158093
ENSG00000135454 222.2764867	231.8687301	282.4854709
189.5822346	136.9790194	122.4947444	245.5435626
149.6853328	0.713950202	0.018084106	0.105818643
B4GALNT1	12	57623410	57633355	-	8003
protein_coding	"beta-1,4-N-acetyl-galactosaminyltransferase 1
[Source:HGNC Symbol;Acc:HGNC:4117]"	-	245	262	243
183	120	142	1.499139168	1.551225428
1.889123295	1.270604288	0.921670717	0.82502393
ENSG00000255201 0	3.539980612	1.16249165	9.323716454
19.40536108	19.84069804	1.567490754	16.18992519
-3.352753175	0.018126723	0.105994539	AC087623.1
8	38421889	38426096	+	534
antisense	novel transcript	-	0	4	1
9	17	23	0	0.354932015	0.11651064
0.936511891	1.956838681	2.002707836
ENSG00000111785 550.7013363	533.6520773	524.2837341
679.5953327	757.9505739	715.9904076	536.2123826
717.845438	-0.420467272	0.018127555	0.105994539	RIC8B
12	106774595	106889316	+	7795
protein_coding	RIC8 guanine nucleotide exchange factor B [Source:HGNC
Symbol;Acc:HGNC:25555]	-	607	603	451	656	664
830	3.813302473	3.665452817	3.599707965

4.67627199	5.235996168	4.951000737
ENSG00000077380 1967.827345	1654.940936	2416.82014
1668.945245	1602.654527	1188.716604	2013.196141
1486.772125	0.437331309	0.018133803	0.105994539
DYNC1I2 2	171687409	171748420	+	4695
protein_coding	dynein cytoplasmic 1 intermediate chain 2 [Source:HGNC
Symbol;Acc:HGNC:2964]	-	2169	1870	2079	1611	1404
1378	22.62312728	18.87263109	27.55026238
19.06654433	18.38141208	13.64723403
ENSG00000126461 4050.875563	5145.36182	5031.263861
4627.671267	6394.637221	8261.494137	4742.500415
6427.934208	-0.438913631	0.018138135	0.105994539	SCAF1
19	49642125	49658642	+	4548
protein_coding	SR-related CTD associated factor 1 [Source:HGNC
Symbol;Acc:HGNC:30403]	-	4465	5814	4328	4467	5602
9577	48.07615102	60.57326901	59.20707997
54.57673365	75.71292224	97.91293061
ENSG00000196792 413.7064405	392.0528528	406.8720775
251.7403443	309.3442854	279.4950507	404.2104569
280.1932268	0.52913169	0.018170626	0.106149343	STRN3
14	30893799	31026401	-	4965
protein_coding	striatin 3 [Source:HGNC Symbol;Acc:HGNC:15720]	-
456	443	350	243	271	324	4.497532117
4.227765731	4.385868822	2.719562627	3.355037651
3.034287832
ENSG00000198522 948.9845104	1108.013932	1172.954075
1138.529376	1577.541707	1632.975713	1076.650839
1449.682265	-0.429775744	0.018204014	0.106309277	GPN1
2	27628247	27651508	+	3746
protein_coding	GPN-loop GTPase 1 [Source:HGNC Symbol;Acc:HGNC:17030]
-	1046	1252	1009	1099	1382	1893
13.67390616	15.83663826	16.75831077	16.30204032
22.67710601	23.49707803
ENSG00000057704 31.75378381	58.40968011	27.8997996
101.5249125	73.055477	94.02765594	39.35442117
89.53601515	-1.181337778	0.018245768	0.106517949	TMCC3
12	94567124	94650562	-	6515
protein_coding	transmembrane and coiled-coil domain family 3
[Source:HGNC Symbol;Acc:HGNC:29199]	-	35	66	24
98	64	109	0.263076653	0.480016268
0.229194224	0.835841055	0.603827536	0.777934615
ENSG00000279852 9.979760626	9.734946684	3.48747495	0
0	0	7.734060753	0	5.387506653
0.018251836	1	AC083964.2	8	486117	488512	-
2396	TEC	TEC	-	11	11	3	0
0	0	0.224819799	0.217536588	0.077900687
0	0	0
ENSG00000174804 151.5109113	137.1742487	132.5240481
75.62570013	77.62144432	81.9507093	140.4030694
78.39928458	0.840605736	0.018268496	0.106615448	FZD4

11 86945679 86955391 - 7383
protein_coding frizzled class receptor 4 [Source:HGNC
Symbol;Acc:HGNC:4042] - 167 155 114 73 68
95 1.107674828 0.994775935 0.960680177
0.549416927 0.566139432 0.598303791
ENSG00000099957 92.53959853 111.5093893 79.04943219
72.51779464 10.27342645 12.07694663 94.36614001
31.62272258 1.579789664 0.018276782 0.106628627 P2RX6
22 21009808 21028830 + 4730
protein_coding purinergic receptor P2X 6 [Source:HGNC
Symbol;Acc:HGNC:8538] - 102 126 68 70 9
14 1.056009231 1.26222229 0.894447014
0.822335318 0.116957676 0.137625184
ENSG00000167987 949.8917614 1154.918675 1009.042752
1274.241249 1291.027258 1490.64027 1037.951063
1351.969592 -0.381518077 0.01833813 0.106951262 VPS37C
11 61130256 61161617 - 3960
protein_coding "VPS37C, ESCRT-I subunit [Source:HGNC
Symbol;Acc:HGNC:26097]" - 1047 1305 868 1230 1131
1728 12.94732886 15.61499202 13.63739393
17.25925197 17.55556371 20.28988429
ENSG00000101868 1061.48363 902.6950562 866.0562792
928.2277714 507.9638635 439.083274 943.4116552
625.0916363 0.594578952 0.018359327 0.107039599 POLA1
X 24693919 24996986 + 6336
protein_coding "DNA polymerase alpha 1, catalytic subunit
[Source:HGNC Symbol;Acc:HGNC:9173]" - 1170 1020 745
896 445 509 9.042726105 7.628013342
7.315566303 7.857870816 4.317100934 3.735370046
ENSG00000185053 1906.13428 1671.755844 1186.903975
2157.922375 2078.656619 2132.44372 1588.264699
2123.007571 -0.417815273 0.018377963 0.107112945 SGCZ
8 14089864 15238339 - 2234
protein_coding sarcoglycan zeta [Source:HGNC Symbol;Acc:HGNC:14075]
- 2101 1889 1021 2083 1821 2472
46.0544465 40.06593019 28.43475037 51.81054311
50.10420057 51.45129566
ENSG00000128191 1453.416047 2029.293886 1340.352872
1892.71444 2078.656619 2572.389633 1607.687602
2181.253564 -0.439916091 0.018402769 0.107126019 DGCR8
22 20080232 20111877 + 7315
protein_coding "DGCR8, microprocessor complex subunit [Source:HGNC
Symbol;Acc:HGNC:2847]" - 1602 2293 1153 1827 1821
2982 10.72449534 14.85306786 9.80667592
13.87829966 15.301816 18.95502348
ENSG00000177728 1457.952302 1240.763205 1231.078657
1048.400117 1036.47458 952.353506 1309.931388
1012.409401 0.372325541 0.018403465 0.107126019 TMEM94
17 75441159 75500090 + 8854
protein_coding transmembrane protein 94 [Source:HGNC

Symbol;Acc:HGNC:28983]	-	1607	1402	1059	1012	908
	1104	8.888020399	7.502999834	7.441548028
6.351159777	6.303673215	5.797764542
ENSG00000139631 45.3625483	76.99457832	53.4746159
149.1794633	101.5927727	103.5166854	58.61058084
118.0963071	-1.008749874	0.018403476	0.107126019	CSAD
	12	53157663	53180909	-	4390
protein_coding	cysteine sulfinic acid decarboxylase [Source:HGNC
Symbol;Acc:HGNC:18966]	-	50	87	46	144	89
	120	0.55774305	0.939033689	0.651928786
1.822678268	1.246157245	1.271006419
ENSG00000166133 316.6305871	318.5982551	349.9099866
317.0063594	597.0002261	605.5726098	328.3796096
506.5263985	-0.626056184	0.018404431	0.107126019	RPUSD2
	15	40569300	40574943	+	2482
protein_coding	RNA pseudouridylate synthase domain containing 2
[Source:HGNC Symbol;Acc:HGNC:24180]	-	349	360	301
	306	523	702	6.885767164	6.872696479
7.545214055	6.850648628	12.95231124	13.15122939
ENSG00000251350 31.75378381	15.0449176	19.76235805
7.251779464	0	0	22.18701982	2.417259821
3.20896696	0.018410984	0.107128914	LINC02475	4
	44016861	44022063	+	2823	lincRNA long
intergenic non-protein coding RNA 2475 [Source:HGNC
Symbol;Acc:HGNC:53418]	-	35	17	17	7	0
	0	0.607135811	0.2853412	0.374666523
0.137784132	0	0
ENSG00000183562 0.907250966	0	0	18.64743291
4.565967313	6.038473317	0.302416989
9.750624513	-4.799264416	0.018426235	0.107164429
AC131971.1	11	2989863 2991344 +	1482	antisense
	"novel transcript, antisense to NAP1L4" -	1	0
	0	18	4	7	0.033043077	0	0
	0.674895209	0.165904875	0.219624535
ENSG00000106511 301.2073207	304.4383327	270.8605544
224.8051634	130.1300684	193.2311461	292.1687359
182.722126	0.675957237	0.018429204	0.107164429	MEOX2
	7	15611212	15686812	-	2500
protein_coding	mesenchyme homeobox 2 [Source:HGNC
Symbol;Acc:HGNC:7014]	Homeobox	332	344	233	217
	114	224	6.50319473	6.51995915	5.798594763
	4.823161107	2.802929684	4.166189574
ENSG00000196453 501.7097842	528.3421064	576.5958583
613.293349	690.602556	977.3700383	535.5492496
760.4219811	-0.507438687	0.018438444	0.107182924	ZNF777
	7	149431363	149461123	-	3233
protein_coding	zinc finger protein 777 [Source:HGNC
Symbol;Acc:HGNC:22213]	zf-C2H2 553	597	496	592	605
	1133	8.37622067	8.749738373	9.545151338
10.17485071	11.50262682	16.29503221

ENSG00000141556 2334.356735 2272.667553 2705.118069
1875.102976 1808.123056 2094.487602 2437.380786
1925.904545 0.339043271 0.018477729 0.107376004 TBCD
17 82752064 82945922 + 13546
protein_coding tubulin folding cofactor D [Source:HGNC
Symbol;Acc:HGNC:11581] - 2573 2568 2327 1810 1584
2428 9.301594411 8.982772256 10.68789451
7.424710888 7.187725222 8.334290406
ENSG00000074416 2396.957052 2585.955837 2556.319138
3179.387311 3074.037493 3011.472907 2513.077343
3088.299237 -0.297367245 0.018556887 0.107800582 MGLL
3 127689062 128052190 - 11849
protein_coding monoglyceride lipase [Source:HGNC
Symbol;Acc:HGNC:17038] - 2642 2922 2199 3069 2693
3491 10.91892285 11.68489873 11.54650044
14.39220156 13.97018039 13.69932495
ENSG00000163728 617.8379078 647.8164521 362.6973948
805.983489 778.4974268 777.2377798 542.7839182
787.2395652 -0.534312297 0.018564802 0.107811159 TTC14
3 180602130 180617828 + 7783
protein_coding tetratricopeptide repeat domain 14 [Source:HGNC
Symbol;Acc:HGNC:24697] - 681 732 312 778 682
901 4.284782337 4.456464912 2.494103146
5.554495384 5.386227643 5.38280663
ENSG00000168395 679.5309735 778.7957347 588.2207748
391.5960911 522.8032573 536.5614862 682.1824944
483.6536115 0.496702916 0.018583876 0.107886507 ING5
2 241687085 241729478 + 10259
protein_coding inhibitor of growth family member 5 [Source:HGNC
Symbol;Acc:HGNC:19421] - 749 880 506 378 458
622 3.57524223 4.064471505 3.068685164
2.047381684 2.744149756 2.819138243
ENSG00000108639 1041.524109 1254.038132 981.1429525
1259.73769 1515.901148 1536.36014 1092.235064
1437.332992 -0.395587142 0.018602853 0.107961242 SYNGR2
17 78168558 78173527 + 4074
protein_coding synaptogyrin 2 [Source:HGNC Symbol;Acc:HGNC:11499]
- 1148 1417 844 1216 1328 1781
13.7990614 16.48068529 12.88926844 16.58534821
20.03661764 20.32702929
ENSG00000204520 1153.115978 1372.627482 1233.403641
1058.759802 905.2030197 922.1611394 1253.049034
962.0413203 0.381424468 0.018643899 0.108163965 MICA
6 31399784 31415315 + 2386
protein_coding MHC class I polypeptide-related sequence A
[Source:HGNC Symbol;Acc:HGNC:7090] - 1271 1551 1061
1022 793 1069 26.08577805 30.80121153
27.66634518 23.8008518 20.42914111 20.83235099
ENSG00000174502 9.07250966 3.539980612 10.46242485 0
0 0 7.691638374 0 5.374078258

0.018680111 1 SLC26A9 1 205913048 205943460
- 5628 protein_coding solute carrier family 26
member 9 [Source:HGNC Symbol;Acc:HGNC:14469] - 10 4
9 0 0 0 0.087011087 0.033676918
0.099493628 0 0 0
ENSG00000181191 522.5765564 559.3169368 649.8328323
693.0629231 824.1570999 832.4466787 577.2421085
783.2222339 -0.441440517 0.018688975 0.108389926 PJA1
X 69160851 69165793 - 3117
protein_coding praja ring finger ubiquitin ligase 1 [Source:HGNC
Symbol;Acc:HGNC:16648] - 576 632 559 669 722
965 9.049287053 9.607418673 11.15788426
11.92617998 14.23795957 14.39532682
ENSG00000253616 9.07250966 34.51481097 52.31212425
4.14387398 11.41491828 0 31.96648163 5.186264087
2.649626915 0.01869739 0.108403187 AC107959.3
8 23071377 23074488 - 881
antisense novel transcript - 10 39 45
4 10 0 0.555843812 2.097563607
3.177923598 0.252287729 0.697704384 0
ENSG00000108509 749.3892979 990.3095763 813.7441549
1009.033314 1088.983204 1363.832331 851.1476764
1153.949616 -0.439393184 0.01871293 0.108425027 CAMTA2
17 4967992 4987652 - 5999 protein_coding
calmodulin binding transcription activator 2 [Source:HGNC
Symbol;Acc:HGNC:18807] CG-1 826 1119 700 974 954
1581 6.74263839 8.838481667 7.259822871
9.021778051 9.775002406 12.25417353
ENSG00000108829 6361.643773 6541.884172 6834.28841
7163.722142 8193.628343 8990.42413 6579.272118
8115.924872 -0.302976377 0.018713415 0.108425027 LRRC59
17 50375059 50397553 - 4570
protein_coding leucine rich repeat containing 59 [Source:HGNC
Symbol;Acc:HGNC:28817] - 7012 7392 5879 6915 7178
10422 75.13709341 76.64294762 80.03760908
84.07909492 96.54607312 106.0390643
ENSG00000233913 3103.705555 3803.709168 4175.670006
4605.915928 4516.883164 4850.619352 3694.361576
4657.806148 -0.33468858 0.018728078 0.108474449
RPL10P9 5 168616352 168616996 + 645
processed_pseudogene ribosomal protein L10 pseudogene 9
[Source:HGNC Symbol;Acc:HGNC:35579] - 3421 4298 3592
4446 3957 5623 259.7299567 315.7425684
346.4842198 383.0202958 377.097537 405.3585959
ENSG00000132286 697.6759928 792.070662 551.021042
994.5297551 807.0347225 1006.699766 680.255899
936.0880812 -0.459763638 0.018748892 0.108559462
TIMM10B 11 6481447 6484679 + 3041 protein_coding
translocase of inner mitochondrial membrane 10B [Source:HGNC
Symbol;Acc:HGNC:4022] - 769 895 474 960 707

1167	12.38336298	13.9454668	9.697700516
17.5415052	14.2905964	17.84372223
ENSG00000260123 12.70151352	15.0449176	9.299933199
2.07193699	0	0	12.34878811	0.690645663
4.182528984	0.018758859	0.108581626	AC013565.1	15
89041223	89082819	+	597	lincRNA
uncharacterized LOC102724566 [Source:NCBI gene;Acc:102724566]	-
14	17	8	2	0	0	1.148371453
1.349276732	0.833724382	0.186152001	0	0
ENSG00000173559 533.463568	408.8677607	237.1482966
643.3364353	502.2564044	701.3255438	393.1598751
615.6394612	-0.645334499	0.01876681	0.108592119	NABP1
2	191678068	191696659	+	13806
protein_coding	nucleic acid binding protein 1 [Source:HGNC
Symbol;Acc:HGNC:26232]	-	588	462	204	621	440
813	2.085634204	1.585625229	0.919325158
2.499400053	1.958989768	2.738127674
ENSG00000167635 2511.270674	2334.617214	2206.409151
2119.591541	1659.729118	1744.25615	2350.76568
1841.192269	0.352667117	0.018801431	0.108756873	ZNF146
19	36214602	36238774	+	4146
protein_coding	zinc finger protein 146 [Source:HGNC
Symbol;Acc:HGNC:12931]	zf-C2H2 2768	2638	1898	2046	1454
2022	32.69380527	30.14893214	28.48221477
27.42132116	21.55670949	22.67685753
ENSG00000088448 1652.104009	1776.185272	1300.828156
2201.433052	2052.402307	1895.217982	1576.372479
2049.684447	-0.377855551	0.01881268	0.108786369
ANKRD10 13	110878540	110915069	-	4563
protein_coding	ankyrin repeat domain 10 [Source:HGNC
Symbol;Acc:HGNC:20265]	-	1821	2007	1119	2125	1798
2197	19.54286179	20.84122991	15.25760841
25.87739227	24.22069377	22.38775813
ENSG00000064933 520.7620545	418.6027074	452.2092518
341.8696033	365.277385	195.8190633	463.8580046
300.9886839	0.626629821	0.018888156	0.109187119	PMS1
2	189784085	189877629	+	5454
protein_coding	"PMS1 homolog 1, mismatch repair system component
[Source:HGNC Symbol;Acc:HGNC:9121]"	HMG	574	473	389
330	320	227	5.153774856	4.109343524
4.437530125	3.362101728	3.606469012	1.93527079
ENSG00000175215 2392.420797	2182.398048	3073.627922
2089.548454	2087.788554	1596.744873	2549.482256
1924.69396	0.405532068	0.018905021	0.109248908	CTDSP2
12	57819927	57846739	-	5442
protein_coding	CTD small phosphatase 2 [Source:HGNC
Symbol;Acc:HGNC:17077]	-	2637	2466	2644	2017	1829
1851	23.72904587	21.47143031	30.22802411
20.59488657	20.65867808	15.81535335
ENSG00000257103 3030.218226	2735.520018	2801.604876

2394.123192	2312.662444	2229.921932	2855.78104
2312.235856	0.304756777	0.018928836	0.109350808	LSM14A
	19	34172504	34229515	+	4748
protein_coding	"LSM14A, mRNA processing body assembly factor
[Source:HGNC Symbol;Acc:HGNC:24489]"	-	3340	3091	2410
	2311	2026	2585	34.44803392	30.847128
31.58007659	27.04589009	26.22865925	25.31517041
ENSG00000143951 82.5598379	43.3647625	62.77454909
158.5031797	118.7151501	100.0661293	62.8997165
125.7614864	-0.999037968	0.018985208	0.109640663	WDPCP
	2	63121383	63827843	-	11776
protein_coding	WD repeat containing planar cell polarity effector
[Source:HGNC Symbol;Acc:HGNC:28027]	-	91	49	54
	153	104	116	0.378418429	0.197162685
0.285300682	0.721947601	0.542853825	0.458027138
ENSG00000143294 1889.803762	2070.888658	2025.060454
2033.606155	2520.413957	3559.248701	1995.250958
2704.422938	-0.439216298	0.01901078	0.109752507	PRCC
	1	156750610	156800817	+	4108
protein_coding	proline rich mitotic checkpoint control factor
[Source:HGNC Symbol;Acc:HGNC:9343]	-	2083	2340	1742
	1963	2208	4126	24.83061742	26.99055801
26.38302338	26.55228545	33.03817082	46.70138999
ENSG00000234745 1340.916928	1985.929124	2587.706413
1196.543612	1505.627721	1493.228187	1971.517488
1398.466507	0.494814356	0.019051004	0.109936059	HLA-B
	6	31269491	31357188	-	3051
protein_coding	"major histocompatibility complex, class I, B
[Source:HGNC Symbol;Acc:HGNC:4932]"	-	1478	2244	2226
	1155	1319	1731	23.72252483	34.85034532
45.39309543	21.03545064	26.57357275	26.38067347
ENSG00000130066 315.7233362	385.8578868	533.5836673
472.4016337	799.0442797	602.9846926	411.7216301
624.810202	-0.603037625	0.019055004	0.109936059	SAT1
	X	23783173	23786226	+	2498
protein_coding	spermidine/spermine N1-acetyltransferase 1
[Source:HGNC Symbol;Acc:HGNC:10540]	-	348	436	459
	456	700	699	6.822059352	8.270285384
11.43212852	10.14342103	17.22475154	13.01115228
ENSG00000152253 644.1481858	512.4121937	542.8836005
357.4091307	149.5354295	61.24737221	566.4813267
189.3973108	1.581366783	0.019062856	0.109945505	SPC25
	2	168834132	168913371	-	1841
protein_coding	"SPC25, NDC80 kinetochore complex component
[Source:HGNC Symbol;Acc:HGNC:24031]"	-	710	579	467
	345	131	71	18.88570683	14.90222842
15.78228707	10.41303553	4.373859894	1.793228276
ENSG00000106266 2166.515307	2397.45187	2029.710421
2668.654843	2543.243793	3142.594042	2197.892532
2784.830893	-0.341518286	0.019077555	0.109994419	SNX8

7	2251770 2354318 -	5773	protein_coding
sorting nexin 8 [Source:HGNC Symbol;Acc:HGNC:14972]	-	2388
2709	1746	2576	2228	3643	20.25636195
22.23483384	18.81696289	24.79455652	23.72252916
29.34192697
ENSG00000198720 501.7097842	570.8218738	430.1219105
634.0127189	683.7536051	753.9465256	500.8845228
690.5709498	-0.462419121	0.019097731	0.110032682
ANKRD13B	17	29589769	29614761	+	3971
protein_coding	ankyrin repeat domain 13B [Source:HGNC
Symbol;Acc:HGNC:26363]	-	553	645	370	612	599
874	6.81952189	7.696375854	5.797071856
8.563742077	9.272018629	10.23393287
ENSG00000188517 0	0	2.3249833	8.287747959
5.707459141	25.01653231	0.774994433
13.00391314	-4.194794286	0.019098661	0.110032682
COL25A1 4	108808725	109302752	-	8744
protein_coding	collagen type XXV alpha 1 chain [Source:HGNC
Symbol;Acc:HGNC:18603]	-	0	0	2	8	5
29	0	0	0.014230714	0.050838401
0.035148534	0.154212248
ENSG00000166326 3012.073207	2581.530862	2817.879759
3522.292883	3499.813945	3357.391164	2803.827943
3459.832664	-0.303274301	0.019102853	0.110032682	TRIM44
11	35662805	35818007	+	13077
protein_coding	tripartite motif containing 44 [Source:HGNC
Symbol;Acc:HGNC:19016]	-	3320	2917	2424	3400	3066
3892	12.43250503	10.56950738	11.53270912
14.44717179	14.41157303	13.83871374
ENSG00000198768 811.0823636	1081.464077	1464.739479
659.9119313	906.3445116	775.5125017	1119.095307
780.5896482	0.519037616	0.01912705	0.110111666
APCDD1L 20	58459101	58515131	-	3942
protein_coding	APC down-regulated 1 like [Source:HGNC
Symbol;Acc:HGNC:26892]	-	894	1222	1260	637	794
899	11.1057932	14.68862105	19.88661069
8.979142337	12.38087226	10.60410672
ENSG00000104549 2592.01601	2322.227282	2538.881763
2083.332643	2086.647062	1745.981428	2484.375018
1971.987044	0.333572491	0.019129015	0.110111666	SQLE
8	124998497	125022283	+	4305
protein_coding	squalene epoxidase [Source:HGNC Symbol;Acc:HGNC:11279]
-	2857	2624	2184	2011	1828	2024
32.49868348	28.88132514	31.56358502	25.9567885
26.10059428	21.86091676
ENSG00000117899 1164.91024	1208.018384	1221.778724
960.3427948	778.4974268	1004.111849	1198.235783
914.3173568	0.389189589	0.019147806	0.110183974	MESD
15	80946289	80989878	-	7510
protein_coding	mesoderm development LRP chaperone [Source:HGNC

Symbol;Acc:HGNC:13520]	-	1284	1365	1051	927	682
	1164	8.372473281	8.612300111	8.707021676
6.85885846	5.582025265	7.206826411
ENSG00000130734 371.9728961	418.6027074	544.0460921
458.9340432	736.2622292	796.2158388	444.8738985
663.8040371	-0.579159155	0.019161905	0.110229248	ATG4D
	19	10543895	10553418	+	2278
protein_coding	autophagy related 4D cysteine peptidase [Source:HGNC
Symbol;Acc:HGNC:20789]	-	410	473	468	443	645
	923	8.813711295	9.838612633	12.78200491
10.80592752	17.40417154	18.83991762
ENSG00000147168 1.814501932	1.769990306	8.137441549
19.6834014	14.83939377	33.64292277	3.907311262
22.72190598	-2.579818185	0.019202672	0.110367966	IL2RG
	X	71107404	71112108	-	2760
protein_coding	interleukin 2 receptor subunit gamma [Source:HGNC
Symbol;Acc:HGNC:6010]	-	2	2	7	19	13
	39	0.035485391	0.034335815	0.157795933
0.382522127	0.28952204	0.657032032
ENSG00000100564 408.2629347	544.2720192	255.748163
607.077538	602.7076853	626.2759469	402.7610389
612.0203901	-0.60125008	0.0192051	0.110367966	PIGH
	14	67581955	67600287	-	2915
protein_coding	phosphatidylinositol glycan anchor biosynthesis class
H [Source:HGNC Symbol;Acc:HGNC:8964]	-	450	615	220
	586	528	726	7.559666526	9.996845899
4.695598642	11.1704611	11.13378226	11.58054717
ENSG00000130202 1543.233893	1725.740549	2329.633266
879.5372522	1490.788328	1552.750281	1866.202569
1307.691954	0.512406116	0.019209407	0.110367966
NECTIN2 19	44846175	44889228	+	3747
protein_coding	nectin cell adhesion molecule 2 [Source:HGNC
Symbol;Acc:HGNC:9707]	-	1701	1950	2004	849	1306
	1800	22.23050375	24.6591078	33.27521504
12.59029891	21.42431001	22.33674211
ENSG00000139197 735.7805334	905.3500416	928.8308283
983.1341017	1259.065486	1187.853965	856.6538011
1143.351184	-0.416905169	0.019216893	0.110367966	PEX5
	12	7188685 7218574 +	5488	protein_coding
peroxisomal biogenesis factor 5 [Source:HGNC Symbol;Acc:HGNC:9719]
	-	811	1023	799	949	1103	1377
7.23661445	8.832587975	9.058150314	9.608689362
12.35403337	11.6667759
ENSG00000130203 144.2529036	114.1643748	115.0866733
69.40988916	28.5372957	79.36279217	124.5013172
59.10332568	1.069361818	0.019217216	0.110367966	APOE
	19	44905754	44909393	+	2191
protein_coding	apolipoprotein E [Source:HGNC Symbol;Acc:HGNC:613]
	-	159	129	99	67	25	92
3.553721832	2.78980452	2.811251264	1.69919988

0.701366456	1.952434476
ENSG00000132434 1301.905136	1339.882662	1262.465932
1499.046412	1642.606741	1836.558527	1301.41791
1659.403893	-0.350746492	0.019230853	0.110373146	LANCL2
	7	55365448	55433742	+	5235
protein_coding	LanC like 2 [Source:HGNC Symbol;Acc:HGNC:6509]	-
	1435	1514	1086	1447	1439	2129	13.42344225
	13.70363019	12.90684177	15.35903354	16.8962944
	18.90993315
ENSG00000136826 166.0269268	197.3539191	146.4739479
313.898454	227.1568738	266.555465	169.9515979
269.2035976	-0.662178249	0.019231334	0.110373146	KLF4
	9	107484852	107490482	-	3659
protein_coding	Kruppel like factor 4 [Source:HGNC
Symbol;Acc:HGNC:6348]	zf-C2H2 183	223	126	303	199
	309	2.449161161	2.887811851	2.142471149
4.601424099	3.343012705	3.926694264
ENSG00000103257 12370.36692	16956.50713	15479.73881
14484.9115	21075.36362	24239.29453	14935.53762
19933.18988	-0.416465736	0.019236837	0.110373146	SLC7A5
	16	87830023	87869488	-	4745
protein_coding	solute carrier family 7 member 5 [Source:HGNC
Symbol;Acc:HGNC:11063]	-	13635	19160	13316	13982	18463
	28099	140.7173374	191.3311707	174.6000711
163.7363575	239.1736951	275.3503708
ENSG00000158042 1267.429599	1635.471043	1397.314963
1640.974096	1703.105808	2476.636699	1433.405202
1940.238868	-0.43726253	0.019259832	0.110469249	MRPL17
	11	6680782 6683401 -	2276	protein_coding
mitochondrial ribosomal protein L17 [Source:HGNC
Symbol;Acc:HGNC:14053]	-	1397	1848	1202	1584	1492
	2871	30.05749835	38.47300864	32.85784348
38.6718513	40.29432877	58.65323263
ENSG00000198160 1418.940511	1056.684213	1146.216767
1131.277596	662.0652603	764.2981941	1207.280497
852.547017	0.501939365	0.019308103	0.11059978	MIER1
	1	66924895	66988619	+	8273
protein_coding	MIER1 transcriptional regulator [Source:HGNC
Symbol;Acc:HGNC:29657]	Others	1564	1194	986	1092	580
	886	9.25768518	6.838608524	7.415163599
7.334519333	4.309355567	4.979683214
ENSG00000260686 52.62055603	38.93978674	55.79959919
65.26601518	122.1396256	124.2200225	49.11998065
103.8752211	-1.083356616	0.01931276	0.11059978
AC008669.1	5	122832356	122834533	+	2178
	sense_overlapping	"novel transcript, overlapping SNX2"
	-	58	44	48	63	107	144
1.304063687	0.95724089	1.371166546	1.607291758
3.019765801	3.074224893
ENSG00000145451 3.629003864	2.654985459	1.16249165

29.00711786	18.26386925	7.763751407	2.482160324
18.34491284	-2.862582	0.019316676	0.11059978	GLRA3
	4	174636914	174829314	-	9599
protein_coding	glycine receptor alpha 3 [Source:HGNC
Symbol;Acc:HGNC:4328]	-	4	3	1	28	16
	9	0.020406226	0.014808863	0.00648158
0.162085469	0.102456933	0.04359609
ENSG00000106571 2051.294434	1559.36146	2054.122745
1601.607293	1458.826556	1244.788142	1888.259546
1435.073997	0.396007536	0.019316914	0.11059978	GLI3
	7	41960950	42237870	-	10950
protein_coding	GLI family zinc finger 3 [Source:HGNC
Symbol;Acc:HGNC:4319]	zf-C2H2 2261	1762	1767	1546	1278
	1443	10.1114893	7.624620382	10.03989745
7.845261317	7.174044973	6.127498732
ENSG00000174343 48.0843012	40.70977704	31.38727455
119.1363769	85.61188711	62.97265031	40.06045093
89.24030478	-1.150369855	0.019319523	0.11059978	CHRNA9
	4	40335329	40355217	+	3168
protein_coding	cholinergic receptor nicotinic alpha 9 subunit
[Source:HGNC Symbol;Acc:HGNC:14079]	-	53	46	27
	115	75	73	0.819255527	0.68801689
0.530255813	2.017087375	1.455202562	1.071442096
ENSG00000103326 2189.196581	2753.219921	1954.148463
2567.12993	2893.681784	3734.364427	2298.854989
3065.058714	-0.414910943	0.019320104	0.11059978	CAPN15
	16	527717	554636	+	7987	protein_coding
calpain 15 [Source:HGNC Symbol;Acc:HGNC:11182]	Others	2413	3111
	1681	2478	2535	4329	14.79456911	18.45622039
	13.09455896	17.2396983	19.50929786	25.20199491
ENSG00000184108 0	2.654985459	0	9.323716454
5.707459141	27.60444945	0.884995153
14.21187501	-3.951704548	0.019343531	0.110698068	TRIML1
	4	188139419	188147743	+	2113
protein_coding	tripartite motif family like 1 [Source:HGNC
Symbol;Acc:HGNC:26698]	-	0	3	0	9	5
	32	0	0.067274147	0	0.236676455
0.145451387	0.704176454
ENSG00000101882 660.4787032	687.641234	767.2444889
929.2637399	993.0978905	865.2269624	705.1214754
929.1961976	-0.398373964	0.019395858	0.110961621	NKAP
	X	119920672	119943772	-	7894
protein_coding	NFKB activating protein [Source:HGNC
Symbol;Acc:HGNC:29873]	-	728	777	660	897	870
	1003	4.516093666	4.663911894	5.201800117
6.31404052	6.774379011	5.907923037
ENSG00000148204 1.814501932	0	0	32.11502334	0
	12.93958568	0.604833977	15.01820301	-4.588182833
	0.019402788	0.11096538	CRB2	9	123356170
	123380324	+	6349	protein_coding	"crumbs 2,

cell polarity complex component [Source:HGNC Symbol;Acc:HGNC:18688]"
- 2 0 0 31 0 15
0.015426001 0 0 0.27131163 0 0.109854272
ENSG00000199473 0 0 0 0 0 34.50556181
0 11.50185394 -6.004550384 0.019430423
0.111087507 RF00092 7 17373201 17373326 -
126 snoRNA - 0 0 0 0
0 40 0 0 0 0 0
14.76115921
ENSG00000184677 1349.989437 1231.913253 946.268203
1673.089119 1423.44031 1576.041536 1176.056965
1557.523655 -0.40450011 0.019446979 0.111120818 ZBTB40
1 22428838 22531157 + 9169
protein_coding zinc finger and BTB domain containing 40 [Source:HGNC
Symbol;Acc:HGNC:29045] ZBTB 1488 1392 814 1615 1247
1827 7.947117643 7.193557235 5.523435397
9.787293173 8.359722108 9.265046274
ENSG00000125319 347.47712 441.6125814 360.3724115
228.9490374 215.7419555 314.8632515 383.1540376
253.1847481 0.595924002 0.019448813 0.111120818
C17orf53 17 44141906 44162476 + 3005
protein_coding chromosome 17 open reading frame 53
[Source:HGNC Symbol;Acc:HGNC:28460] - 383 499 310
221 189 365 6.241413862 7.868328986
6.418359874 4.086578455 3.866025266 5.647801264
ENSG00000186312 142.4384017 130.0942875 137.1740147
199.9419195 224.8738902 228.599347 136.5689013
217.8050522 -0.673734192 0.019456628 0.111129574 CA5BP1
X 15674932 15703724 + 2409
transcribed_unprocessed_pseudogene carbonic anhydrase 5B
pseudogene 1 [Source:HGNC Symbol;Acc:HGNC:29544] - 157
147 118 193 197 265 3.191475655
2.891392003 3.047558521 4.4517683 5.026628466
5.114934681
ENSG00000203786 0 0.884995153 0 8.287747959
14.83939377 5.175834272 0.294998384
9.434325332 -4.74845321 0.019466329 0.111149092 KPRP
1 152759561 152762052 + 2492
protein_coding keratinocyte proline rich protein [Source:HGNC
Symbol;Acc:HGNC:31823] - 0 1 0 8 13
6 0 0.019014215 0 0.178383217
0.320658439 0.111952612
ENSG00000107758 953.5207652 898.2700804 1149.704242
1236.946383 1253.358027 1443.195123 1000.498362
1311.166511 -0.391485992 0.019477385 0.111176337 PPP3CB
10 73436428 73496024 - 5094
protein_coding protein phosphatase 3 catalytic subunit beta
[Source:HGNC Symbol;Acc:HGNC:9315] - 1051 1015 989
1194 1098 1673 10.10351426 9.44133793
12.07936759 13.02439113 13.24923367 15.27101903

ENSG00000182263 230.4417454 159.2991276 130.1990648
131.5679989 75.33846066 68.14848458 173.3133126
91.68498136 0.921387256 0.019532705 0.111456134 FIGN
2 163593396 163736012 - 9942
protein_coding "fidgetin, microtubule severing factor [Source:HGNC
Symbol;Acc:HGNC:13285]" - 254 180 112 127 66
79 1.251090255 0.857877321 0.700892012
0.709809824 0.408053904 0.369474398
ENSG00000285091 0 0 0 12.43162194 0
14.66486377 0 9.032161903 -5.654330204 0.019555496
0.1115502 AL109840.2 20 58916000
58981169 - 1925 bidirectional_promoter_lncRNA novel
transcript - 0 0 0 12 0 17
0 0 0 0.346387775 0 0.410628611
ENSG00000249852 9.07250966 5.309970919 8.137441549 0
0 0 7.506640709 0 5.340621736
0.019578468 1 AC145676.1 7 227554 229557 -
2004 TEC TEC - 10 6 7 0
0 0 0.244360478 0.141866539 0.21732374
0 0 0
ENSG00000081791 1041.524109 940.7498478 1068.329826
819.4510795 765.9410167 759.1223598 1016.867928
781.5048187 0.379535239 0.01958097 0.11165801 DELE1
5 141923808 141942047 + 6047
protein_coding DAP3 binding cell death enhancer 1 [Source:HGNC
Symbol;Acc:HGNC:28969] - 1148 1063 919 791 671
880 9.296738238 8.329515424 9.45545407
7.268562992 6.820715135 6.766650127
ENSG00000230513 97.07585336 115.9343651 94.16182364
140.8917153 165.5163151 231.1872641 102.3906807
179.1984315 -0.808100004 0.01958702 0.11165801 THAP7-
AS1 22 21001886 21010342 + 2975
antisense THAP7 antisense RNA 1 [Source:HGNC
Symbol;Acc:HGNC:41013] - 107 131 81 136 145
268 1.761268189 2.086463379 1.693966804
2.540177015 2.995907446 4.188696001
ENSG00000232218 4.53625483 3.539980612 15.11239145 0
0 0 7.729542297 0 5.377954192
0.019625209 1 AL021937.2 22 32386668
32386868 + 201 unprocessed_pseudogene Ral-GDS
related protein Rgr (Rgr) pseudogene - 5 4 13
0 0 0 1.21815522 0.942953713
4.023964508 0 0 0
ENSG00000261061 279.4332975 291.1634054 237.1482966
221.6972579 87.89487077 155.2750281 269.2483332
154.9557189 0.796124356 0.019664415 0.112055953
AC092718.4 16 81030770 81031485 + 716
sense_intronic novel transcript - 308 329
204 214 77 180 21.06523836 21.772551
17.72654068 16.60782633 6.610359258 11.68935373

ENSG00000144354 889.1059467 888.5351337 784.6818637
740.7174739 595.8587343 560.7153794 854.107648
632.4305292 0.43441563 0.019669497 0.112055953 CDCA7
2 173354820 173368997 + 3934
protein_coding cell division cycle associated 7 [Source:HGNC
Symbol;Acc:HGNC:14628] - 980 1004 675 715 522
650 12.19889248 12.09277015 10.67520599
10.09912459 8.156118136 7.682631793
ENSG00000198746 290.3203091 369.0429789 416.1720107
427.8549884 473.7191087 718.5783247 358.5117662
540.0508073 -0.593574425 0.019684107 0.112092401
GPATCH3 1 26890488 26900466 - 2225
protein_coding G-patch domain containing 3 [Source:HGNC
Symbol;Acc:HGNC:25720] - 320 417 358 413 415
833 7.042853369 8.880399025 10.01059423
10.31411763 11.46477251 17.40788481
ENSG00000132591 1236.583067 1648.74597 1520.539078
1614.038915 2067.241701 2125.542608 1468.622705
1935.607741 -0.398537258 0.019688569 0.112092401 ERAL1
17 28854938 28861067 + 2318
protein_coding Era like 12S mitochondrial rRNA chaperone 1
[Source:HGNC Symbol;Acc:HGNC:3424] - 1363 1863 1308
1558 1811 2464 28.79460384 38.08253624
35.10760141 37.34788951 48.0233419 49.42632153
ENSG00000056558 466.3269965 427.452659 552.1835337
507.6245625 741.9696883 836.7598739 481.9877297
695.4513749 -0.53053292 0.019708074 0.112151641 TRAF1
9 120902393 120929173 - 6449
protein_coding TNF receptor associated factor 1 [Source:HGNC
Symbol;Acc:HGNC:12031] - 514 483 475 490 650
970 3.903007858 3.548797305 4.582559149
4.221975869 6.19538557 6.993754374
ENSG00000138802 1193.942271 1046.064271 1179.929025
1675.161056 1442.845671 1317.249822 1139.978522
1478.41885 -0.375002086 0.019711654 0.112151641 SEC24B
4 109433772 109540896 + 5219
protein_coding "SEC24 homolog B, COPII coat complex component
[Source:HGNC Symbol;Acc:HGNC:10704]" - 1316 1182 1015
1617 1264 1527 12.34801863 10.73140587
12.10000618 17.21609961 14.88699825 13.60450543
ENSG00000154370 975.2947884 1116.863883 971.8430193
1083.623046 1441.704179 1569.140423 1021.333897
1364.822549 -0.418275103 0.019746083 0.11228744 TRIM11
1 228393673 228406840 - 8493
protein_coding tripartite motif containing 11 [Source:HGNC
Symbol;Acc:HGNC:16281] - 1075 1262 836 1046 1263
1819 6.198348972 7.040843184 6.124237155
6.843568271 9.140913235 9.958698702
ENSG00000248968 4.53625483 11.50493699 2.3249833
36.25889732 26.25431205 23.29125422 6.122058373

28.60148786	-2.204220866	0.019752585	0.11228744
AC012640.1	5	10248325	10249915	+	688
	antisense	novel transcript	-	5	13
	2	35	23	27	0.355885464	0.895326327
	0.180862448	2.826777656	2.054881385	1.824762486
ENSG00000104980 1310.977646	1773.530287	1551.926353
1652.369749	2221.343098	2286.856109	1545.478095
2053.522985	-0.410134349	0.019760569	0.11228744	TIMM44
	19	7926718 7943920 -	4888	protein_coding
translocase of inner mitochondrial membrane 44 [Source:HGNC
Symbol;Acc:HGNC:17316]	-	1445	2004	1335	1595	1946
	2651	14.47655862	19.42642836	16.99248578
18.13182563	24.47141031	25.21793661
ENSG00000237296 484.4720158	320.3682454	355.7224449
580.1423571	574.1703896	514.132871	386.8542354
556.1485392	-0.522720141	0.019760914	0.11228744	SMG1P1
	16	22437008	22492220	+	7046
transcribed_unprocessed_pseudogene	SMG1 pseudogene 1 [Source:HGNC
Symbol;Acc:HGNC:44186]	-	534	362	306	560	503
	596	3.711310598	2.434402428	2.702001802
4.416288452	4.388061507	3.933096834
ENSG00000233421 0	0	0	10.35968495	17.12237742
	0	0	9.160687457	-5.667045207	0.019771107
	0.112309285	LINC01783	1	16533886
16536172	-	2068	lincRNA long intergenic non-protein
coding RNA 1783 [Source:HGNC Symbol;Acc:HGNC:52573]	-	0
	0	0	10	15	0	0	0	0
	0.268696191	0.445849296	0
ENSG00000137760 502.6170352	471.7024166	484.759018
694.0988916	658.6407849	612.4737221	486.3594899
655.0711329	-0.429261581	0.019933781	0.113196999	ALKBH8
	11	107502726	107565746	-	4506
protein_coding	"alkB homolog 8, tRNA methyltransferase [Source:HGNC
Symbol;Acc:HGNC:25189]" -	554	533	417	670	577
	710	6.020703788	5.604830231	5.757735552
8.262199151	7.871037581	7.326527422
ENSG00000174977 95.26135143	131.8642778	181.3486974
149.1794633	292.221908	287.2588021	136.1581089
242.8867245	-0.838387521	0.019959087	0.113304331
AC026271.1	17	18650195	18651542	+	1348
	processed_pseudogene	poly(A) binding protein interacting
protein 1 (PAIP1) pseudogene	-	105	149	156	144
	256	333	3.814416307	5.2374853	7.200150143
	5.935873588	11.67340177	11.48643765
ENSG00000141577 688.6034832	738.9709529	604.4956579
452.7182323	465.7286659	561.5780185	677.356698
493.3416389	0.457044836	0.019968685	0.11332245	CEP131
	17	81189593	81222999	-	3966
protein_coding	centrosomal protein 131 [Source:HGNC
Symbol;Acc:HGNC:29511]	-	759	835	520	437	408

651	9.371686443	9.976086507	8.157507474
6.122668848	6.323460549	7.632367909
ENSG00000157450 811.0823636	672.5963164	854.4313627
1125.061785	996.522366	973.9194821	779.3700142
1031.834545	-0.405470132	0.019977856	0.11333813	RNF111
15	58865175	59097419	+	7181
protein_coding	ring finger protein 111 [Source:HGNC
Symbol;Acc:HGNC:17384]	-	894	760	735	1086	873
1129	6.096509791	5.014817196	6.368090972
8.403436877	7.472685027	7.31038136
ENSG00000140538 44.45529733	22.12487883	37.1997328
5.179842475	0	14.66486377	34.59330299	6.614902081
2.358074493	0.02000216	0.113439631	NTRK3	15
87859751	88256768	-	26750	protein_coding
neurotrophic receptor tyrosine kinase 3 [Source:HGNC
Symbol;Acc:HGNC:8033]	-	49	25	32	5	0
17	0.089701763	0.044283574	0.074427433
0.010386238	0	0.029549909
ENSG00000166173 740.3167882	800.9206136	981.1429525
825.6668904	1496.495787	1264.62884	840.7934514
1195.597173	-0.508434757	0.020113334	0.11403358	LARP6
15	70829130	70854159	-	5554
protein_coding	La ribonucleoprotein domain family member 6
[Source:HGNC Symbol;Acc:HGNC:24012]	-	816	905	844
797	1311	1466	7.194704592	7.720921637
9.45460562	7.973784416	14.5092237	12.27323678
ENSG00000125457 229.5344944	258.4185847	244.1232465
329.4379814	327.6081547	425.2810493	244.0254419
360.7757285	-0.565308537	0.020132587	0.114106162	MIF4GD
17	75266228	75271227	-	2766
protein_coding	MIF4G domain containing [Source:HGNC
Symbol;Acc:HGNC:24030]	-	253	292	210	318	287
493	4.47916467	5.002154673	4.723609263
6.388324787	6.37789083	8.287542368
ENSG00000006757 17.23776835	10.61994184	52.31212425
9.323716454	0	0	26.72327815	3.107905485
3.108319843	0.020179745	0.114336259	PNPLA4	X
7898247 7927739 -	3441	protein_coding	patatin like
phospholipase domain containing 4 [Source:HGNC Symbol;Acc:HGNC:24887]
-	19	12	45	9	0	0
0.27039435	0.165242978	0.81364449	0.145334888	0
0
ENSG00000101928 749.3892979	726.5810207	628.9079826
1048.400117	848.1284283	906.6336366	701.6261004
934.3873939	-0.41261075	0.020186112	0.114336259	MOSPD1
X	134887626	134915267	-	2675
protein_coding	motile sperm domain containing 1 [Source:HGNC
Symbol;Acc:HGNC:25235]	-	826	821	541	1012	743
1051	15.12115428	14.54272566	12.58288784
21.0217453	17.07310014	18.26879691

ENSG00000253846 99.79760626   76.99457832   211.5734803
63.19407819   78.76293614   28.46708849   129.4552216
56.80803428   1.190428881   0.020213063   0.114408653
PCDHGA10   5   141412987   141512979   +   6643
        protein_coding "protocadherin gamma subfamily A, 10
[Source:HGNC Symbol;Acc:HGNC:8697]"   -   110   87   182
        61   69   33   0.810880985   0.62055666
1.70456663   0.510243671   0.638457802   0.230983366
ENSG00000128578 456.3472359   414.1777317   244.1232465
479.6534131   575.3118814   629.7265031   371.5494047
561.5639325   -0.593803105   0.020217269   0.114408653   STRIP2
        7   129434433   129488399   +   5876
protein_coding striatin interacting protein 2 [Source:HGNC
Symbol;Acc:HGNC:22209] -   503   468   210   463   504
        730   4.191938297   3.773901031   2.223536967
4.378357782   5.272251384   5.776597276
ENSG00000101189 1279.223862   1550.511508   1596.101035
910.616307   1114.096024   1294.821207   1475.278802
1106.511179   0.414142454   0.020221262   0.114408653   MRGBP
        20   62796453   62801738   +   2781
protein_coding MRG domain binding protein [Source:HGNC
Symbol;Acc:HGNC:15866] -   1410   1752   1373   879   976
        1501   24.82828988   29.85104601   30.71683006
17.56304968   21.57228697   25.09635919
ENSG00000271936 65.32206955   61.06466557   45.33717435
35.22292883   5.707459141   13.80222472   57.24130315
18.24420423   1.64901586   0.020224764   0.114408653
AC012073.1   2   24825610   24826717   +   1108
        antisense   "novel transcript, antisense to ADCY3" -
        72   69   39   34   5   16   3.182155657
        2.950772798   2.189937363   1.705105284   0.277381571
        0.671446231
ENSG00000105229 762.9980624   854.0203228   881.1686706
961.3787633   1068.436351   1285.332177   832.7290186
1105.049097   -0.409179463   0.020231303   0.114409054   PIAS4
        19   4007646 4039386 +   3646   protein_coding
protein inhibitor of activated STAT 4 [Source:HGNC
Symbol;Acc:HGNC:17002] zf-MIZ 841   965   758   928   936
        1490   11.29556646   12.54114213   12.93479017
14.14305908   15.77998349   19.00205725
ENSG00000277149 38.10454057   38.93978674   40.68720775
103.5968495   62.78205055   87.98918262   39.24384502
84.78936089   -1.113286845   0.020255727   0.114510566   TYW1B
        7   72558744   72828198   -   3258
protein_coding tRNA-yW synthesizing protein 1 homolog B [Source:HGNC
Symbol;Acc:HGNC:33908] -   42   44   35   100   55
        102   0.63128707   0.639923468   0.668380562
1.705536286   1.037669304   1.455727579
ENSG00000111052 25.40302705   25.66485944   45.33717435
11.39565344   6.848950969   0   32.13502028   6.081534804

2.417395742	0.020270268	0.114556154	LIN7A	12
80792520	80937925	-	6795	protein_coding
"lin-7 homolog A, crumbs cell polarity complex component
[Source:HGNC Symbol;Acc:HGNC:17787]"	-	28	29	39
11	6	0	0.201788891	0.202225062
0.357093539	0.08995292	0.054276164	0
ENSG00000272566 3.629003864	4.424975766	0	23.82727538
15.98088559	18.978059	2.684659877	19.59540666
-2.831092825	0.020302063	0.114676991	AF250324.1
4	189703881	189704490	-	610
lincRNA novel transcript	-	4	5	0	23
14	22	0.321113704	0.388388722	0
2.095125509	1.410735389	1.67696448
ENSG00000198056 628.7249194	551.3519804	491.7339679
414.387398	453.1722558	232.9125422	557.2702892
366.8240653	0.606152136	0.020304615	0.114676991	PRIM1
12	56731596	56752373	-	2437
protein_coding	DNA primase subunit 1 [Source:HGNC
Symbol;Acc:HGNC:9369]	-	693	623	423	400	397
270	13.92535864	12.11320196	10.7992025
9.120455019	10.01341782	5.151565821
ENSG00000122870 638.70468	438.0726008	657.9702738
530.4158694	329.8911383	208.758649	578.2491849
356.3552189	0.699173179	0.020340283	0.114841767	BICC1
10	58513140	58831437	+	6569
protein_coding	BicC family RNA binding protein 1 [Source:HGNC
Symbol;Acc:HGNC:19351]	-	704	495	566	512	289
242	5.248099748	3.570527463	5.360731012
4.33094574	2.704244413	1.712959608
ENSG00000179965 201.4097144	277.0034829	189.4861389
375.0205952	328.7496465	314.0006125	222.6331121
339.2569514	-0.605556245	0.020363872	0.114938269	ZNF771
16	30407297	30431108	+	5637
protein_coding	zinc finger protein 771 [Source:HGNC
Symbol;Acc:HGNC:29653]	zf-C2H2 222	313	163	362	288
364	1.92856208	2.631011483	1.799063184
3.568392183	3.140449493	3.002509339
ENSG00000074582 476.3067571	579.6718253	490.5714763
570.8186407	741.9696883	856.600572	515.5166862
723.1296337	-0.488416084	0.020379364	0.114958389	BCS1L
2	218658764	218663443	+	3379
protein_coding	"BCS1 homolog, ubiquinol-cytochrome c reductase
complex chaperone [Source:HGNC Symbol;Acc:HGNC:1020]"	-	525
655	422	551	650	993	7.608513143
9.185008214	7.770180469	9.060985819	11.82422064
13.66444745
ENSG00000031691 723.0790199	612.416646	640.5328991
445.4664528	575.3118814	383.8743751	658.6761883
468.2175698	0.494235648	0.020380435	0.114958389	CENPQ
6	49463378	49493107	+	1733

protein_coding centromere protein Q [Source:HGNC
Symbol;Acc:HGNC:21347] -   797   692   551   430   504
        445   22.52104001   18.9205594   19.78153017
13.78738606   17.87636996   11.93967393
ENSG00000142751 1337.287924   1491.216833   1131.104375
1524.945624   1792.14217   1833.107971   1319.869711
1716.731922   -0.378633326   0.020392161   0.114987864   GPN2
        1   26876133   26890297   -   4979
protein_coding GPN-loop GTPase 2 [Source:HGNC Symbol;Acc:HGNC:25513]
        -       1474   1685   973   1472   1570   2125
14.49719701   16.03556327   12.15843173   16.42773647
19.38228103   19.84485026
ENSG00000078687 518.9475525   442.4975766   509.1713427
381.2364061   323.0421874   343.33034   490.2054906
349.2029778   0.488741675   0.020404467   0.115020593   TNRC6C
        17   77959240   78108835   +   11111
protein_coding trinucleotide repeat containing 6C [Source:HGNC
Symbol;Acc:HGNC:29318] -   572   500   438   368   283
        398   2.520992564   2.132275405   2.452606131
1.840376651   1.565599407   1.665562532
ENSG00000151092 873.6826802   908.0050271   741.6696726
1198.615549   1082.134253   1029.128381   841.1191267
1103.292728   -0.390341812   0.020428221   0.115088734   NGLY1
        3   25718944   25790039   -   8326
protein_coding N-glycanase 1 [Source:HGNC Symbol;Acc:HGNC:17646]
        -       963   1026   638   1157   948   1193
5.663938957   5.838985477   4.767504578   7.721630151
6.998730831   6.662466759
ENSG00000253954 73.48732824   36.28480128   79.04943219
30.04308635   15.98088559   25.01653231   62.94052057
23.68016809   1.404873037   0.020429568   0.115088734
HMGN1P38   15   92711808   92712110   +   303
        processed_pseudogene   high mobility group nucleosome binding
domain 1 pseudogene 38 [Source:HGNC Symbol;Acc:HGNC:39422]   -
        81   41   68   29   14   29   13.09094728
        6.411618437   13.96281972   5.318233643   2.840094347
        4.450270276
ENSG00000187079 4418.312204   2765.609853   3500.262358
3208.394429   2449.641463   2267.015411   3561.394805
2641.683768   0.431104774   0.020475092   0.115252185   TEAD1
        11   12674591   12944483   +   10279
protein_coding TEA domain transcription factor 1 [Source:HGNC
Symbol;Acc:HGNC:11714] TEA   4870   3125   3011   3097   2146
        2628   23.20100399   14.4054091   18.22496639
16.741809   12.83294142   11.88791012
ENSG00000235897 41.73354444   16.81490791   13.9498998
48.69051926   66.20652603   82.81334835   24.16611738
65.90346455   -1.441841778   0.020476315   0.115252185
TM4SF19-AS1   3   196318330   196325570   +   2136
        antisense   TM4SF19 antisense RNA 1 [Source:HGNC

Symbol;Acc:HGNC:41085]	-	46	19	12	47	58
	96	1.054593929	0.421481768	0.349531921
1.222668302	1.669068287	2.08978209
ENSG00000205758 541.6288267	521.2621452	588.2207748
724.1419779	838.9964937	665.957343	550.3705822
743.0319382	-0.432841461	0.020478129	0.115252185	CRYZL1
	21	33589341	33643926	-	6177
protein_coding	crystallin zeta like 1 [Source:HGNC
Symbol;Acc:HGNC:2420]	-	597	589	506	699	735
	772	4.732879129	4.518186299	5.096590755
6.287986752	7.314036073	5.811265494
ENSG00000223496 2111.172998	2416.921763	2016.923013
2758.784102	2450.782955	3095.148894	2181.672591
2768.23865	-0.343591909	0.020512443	0.115408588	EXOSC6
	16	70246778	70251930	-	5153
protein_coding	exosome component 6 [Source:HGNC
Symbol;Acc:HGNC:19055]	-	2327	2731	1735	2663	2147
	3588	22.11387877	25.11238717	20.94817452
28.71594201	25.61057105	32.37600885
ENSG00000131737 0	0	3.48747495	8.287747959
1.141491828	42.26931322	1.16249165
17.232851	-3.987289502	0.020554915	0.115610775	KRT34
	17	41377650	41382403	-	1751
protein_coding	keratin 34 [Source:HGNC Symbol;Acc:HGNC:6452]	-
	0	0	3	8	1	49	0	0
	0.106596257	0.253872631	0.035104372	1.301190704
ENSG00000147804 0	0	0	34.18696033	0	0
	0	11.39565344	-5.985460343	0.020566215
0.115631335	SLC39A4 8	144409742	144416895	-
	5033	protein_coding	solute carrier family 39 member 4
[Source:HGNC Symbol;Acc:HGNC:17129]	-	0	0	0
	33	0	0	0	0	0	0.364333456
	0	0
ENSG00000103145 705.8412515	1011.54946	888.1436205
1045.292211	1180.30255	1274.980509	868.511444
1166.858424	-0.426312197	0.020571644	0.115631335
HCFC1R1 16	3022620 3024286 -	1433	protein_coding	host
cell factor C1 regulator 1 [Source:HGNC Symbol;Acc:HGNC:21198]	-
	778	1143	764	1009	1034	1478	26.58655645
	37.79431521	33.17065251	39.12524044	44.35286806
	47.95780106
ENSG00000136816 1011.584827	1120.403864	967.1930527
1403.737311	1273.90488	1293.095929	1033.060581
1323.579373	-0.357029902	0.020588539	0.11568954	TOR1B
	9	129803153	129811281	+	2911
protein_coding	torsin family 1 member B [Source:HGNC
Symbol;Acc:HGNC:11995]	-	1115	1266	832	1355	1116
	1499	18.7569122	20.60715041	17.78230142
25.86480053	23.56510338	23.94365496
ENSG00000162194 71.67282631	77.87957347	38.36222445

118.1004084	120.9981338	131.1211349	62.63820808
123.406559	-0.972697371	0.020604694	0.115743545	LBHD1
	11	62662817	62672255	-	3362
protein_coding	LBH domain containing 1 [Source:HGNC
Symbol;Acc:HGNC:28351]	-	79	88	33	114	106
	152	1.150689276	1.240256192	0.61069319
1.884166101	1.938007781	2.102213869
ENSG00000101040 1871.658743	1434.577143	1893.698898
1475.219137	1292.168749	1209.419941	1733.311595
1325.602609	0.386729028	0.020617646	0.115779538	ZMYND8
	20	47209214	47356889	-	7625
protein_coding	zinc finger MYND-type containing 8 [Source:HGNC
Symbol;Acc:HGNC:9397]	-	2063	1621	1629	1424	1132
	1402	13.24915142	10.07324989	13.29193115
10.37724774	9.125442628	8.549469823
ENSG00000102753 3663.479401	3118.72292	3086.41533
3960.507556	4089.96522	4120.826719	3289.539217
4057.099832	-0.302408836	0.020626663	0.11579341	KPNA3
	13	49699307	49792921	-	4474
protein_coding	karyopherin subunit alpha 3 [Source:HGNC
Symbol;Acc:HGNC:6396]	-	4038	3524	2655	3823	3583
	4777	44.19763371	37.3221248	36.92116467
47.48105519	49.22641273	49.6466878
ENSG00000064687 51.71330506	57.52468495	61.61205744
26.93518087	14.83939377	0	56.95001582	13.92485821
	2.042538429	0.020658932	0.11593777	ABCA7	19
	1040101 1065572 +	8367	protein_coding	ATP binding
cassette subfamily A member 7 [Source:HGNC Symbol;Acc:HGNC:37]	-
	57	65	53	26	13	0	0.333605936
	0.368103569	0.39410591	0.172669497	0.095503864
	0
ENSG00000130684 47.17705023	61.06466557	29.06229125
45.58261378	131.2715602	146.6486377	45.76800235
107.8342706	-1.232346798	0.020672494	0.115977085	ZNF337
	20	25674215	25696841	-	3823
protein_coding	zinc finger protein 337 [Source:HGNC
Symbol;Acc:HGNC:15809]	zf-C2H2 52	69	25	44	115
	170	0.666082048	0.855206974	0.406857716
0.639529264	1.849016993	2.06764341
ENSG00000153048 520.7620545	660.2063842	752.1320975
405.0636815	518.23729	446.8470255	644.3668454
456.715999	0.496191453	0.02071328	0.116146938
CARHSP1 16	8852942 8869012 -	5577	protein_coding
calcium regulated heat stable protein 1 [Source:HGNC
Symbol;Acc:HGNC:17150]	CSD	574	746	647	391	454
	518	5.040109031	6.338180804	7.217893717
3.895723782	5.003830254	4.318770571
ENSG00000171931 0	0	0	3.107905485	25.11282022
	0	0	9.406908568	-5.70430182	0.020715902
	0.116146938	FBXW10	17	18744024	18779349

        +   4390   protein_coding F-box and WD repeat domain
containing 10 [Source:HGNC Symbol;Acc:HGNC:1211]   -   0
        0   0   3   22   0   0   0   0
        0.037972464   0.308038869   0
ENSG00000202474 29.93928188   22.12487883   32.5497662
55.94229872   50.22564044   101.7914073   28.2046423
69.31978217   -1.303946463   0.020767836   0.116401218
RNA5SP283   9   62802320   62802438   -   119
        rRNA_pseudogene "RNA, 5S ribosomal pseudogene 283 [Source:HGNC
Symbol;Acc:HGNC:43183]" -   33   25   28   54   44
        118   13.57987155   9.954500852   14.6392193
25.21499243   22.72757373   46.10691493
ENSG00000026950 175.0994364   153.9891566   260.3981296
119.1363769   65.06503421   136.2969692   196.4955742
106.8327934   0.873209931   0.020803377   0.116563035   BTN3A1
        6   26402237   26415216   +   5547
protein_coding butyrophilin subfamily 3 member A1 [Source:HGNC
Symbol;Acc:HGNC:1138]   -   193   174   224   115   57
        158   1.703836143   1.486337802   2.512445785
1.151997981   0.631631892   1.324432835
ENSG00000085721 1419.847762   1309.792827   1143.891783
1737.319166   1638.040773   1572.59098   1291.177457
1649.316973   -0.352404203   0.020811592   0.116563035   RRN3
        16   15060022   15094317   -   4156
protein_coding "RRN3 homolog, RNA polymerase I transcription factor
[Source:HGNC Symbol;Acc:HGNC:30346]"   -   1565   1480   984
        1677   1435   1823   18.44027895   16.87378912
14.73080248   22.42175317   21.22382824   20.3958659
ENSG00000204568 1896.154519   1989.469104   2137.822144
2414.842562   2481.603234   2553.411574   2007.815256
2483.28579   -0.307013062   0.020816475   0.116563035
MRPS18B 6   30617709   30626395   +   1888
protein_coding mitochondrial ribosomal protein S18B [Source:HGNC
Symbol;Acc:HGNC:14516] -   2090   2248   1839   2331   2174
        2960   54.2091977   56.41839898   60.6019482
68.60445636   70.77907946   72.8988604
ENSG00000132003 329.3321007   421.2576929   478.9465598
287.9992416   301.3538426   242.4015717   409.8454511
277.251552   0.563952578   0.020871949   0.116836679   ZSWIM4
        19   13795460   13832230   +   4713
protein_coding zinc finger SWIM-type containing 4 [Source:HGNC
Symbol;Acc:HGNC:25704] -   363   476   412   278   264
        281   3.771706314   4.785595131   5.438844258
3.277626028   3.443133385   2.772297915
ENSG00000147255 5.443505796   9.734946684   6.974949899   0
        0   0   7.38446746   0   5.318057279
0.020879221   1   IGSF1   X   131273506   131578899
        -       7051   protein_coding immunoglobulin superfamily
member 1 [Source:HGNC Symbol;Acc:HGNC:5948]   -   6   11
        6   0   0   0   0.041670549   0.073921098

0.052942858	0	0	0
ENSG00000133812 926.3032363	768.1757929	1192.716433
795.6238041	672.3386868	608.1605269	962.3984873
692.0410059	0.475107699	0.020976461	0.117384569	SBF2
11	9778667 10294207	-	16767	protein_coding
SET binding factor 2 [Source:HGNC Symbol;Acc:HGNC:2135] -
1021	868	1026	768	589	705	2.981941103
2.452961894	3.807139962	2.545176469	2.159271689
1.95508107
ENSG00000131351 432.7587108	589.406772	440.5843353
317.0063594	255.6941695	408.0282684	487.5832727
326.9095991	0.575264441	0.02100528	0.117497621	HAUS8
19	17049729	17075625	-	5533
protein_coding	HAUS augmin like complex subunit 8 [Source:HGNC
Symbol;Acc:HGNC:30532]	-	477	666	379	306	224
473	4.221690511	5.703481009	4.261724648
3.073072455	2.488483172	3.974948339
ENSG00000111912 397.3759231	371.6979643	356.8849365
563.5668612	518.23729	477.9020311	375.319608
519.9020608	-0.469168223	0.021009948	0.117497621	NCOA7
6	125781161	125932030	+	7820
protein_coding	nuclear receptor coactivator 7 [Source:HGNC
Symbol;Acc:HGNC:21081]	-	438	420	307	544	454
554	2.742812001	2.544889783	2.442521915
3.865486762	3.568588405	3.294079147
ENSG00000225806 3.629003864	0.884995153	1.16249165
18.64743291	20.54685291	8.626390453	1.892163556
15.94022542	-3.057506433	0.02102452	0.11754195
AL121917.1	20	58863528	58888809	-	1521
antisense	uncharacterized LOC101927932 [Source:NCBI
gene;Acc:101927932]	-	4	1	1	18	18
10	0.128783274	0.03115281	0.040905116
0.657590204	0.727429068	0.305704481
ENSG00000185567 1033.35885	1045.179276	873.0312291
1635.794253	1133.501385	1199.068273	983.8564517
1322.787971	-0.426450962	0.02106265	0.117717917	AHNAK2
14	104937244	104978357	-	18771
protein_coding	AHNAK nucleoprotein 2 [Source:HGNC
Symbol;Acc:HGNC:20125]	-	1139	1181	751	1579	993
1390	2.971426539	2.981185009	2.489197602
4.674194327	3.251690476	3.443169548
ENSG00000116996 0.907250966	0	1.16249165	0
33.10326302	9.489029498	0.689914205
14.19743084	-4.401422807	0.021082295	0.117790499	ZP4
1	237877864	237890922	-	2474
protein_coding	zona pellucida glycoprotein 4 [Source:HGNC
Symbol;Acc:HGNC:15770]	-	1	0	1	0	29
11	0.019793791	0	0.025148214	0
0.720519374	0.20673976
ENSG00000158373 100.7048572	100.8894475	117.4116566

174.0427071   173.5067579   182.0168386   106.3353204
176.5221012   -0.733169663   0.021114663   0.117934094
HIST1H2BD   6   26158146   26171349   +   871
        protein_coding histone cluster 1 H2B family member d
[Source:HGNC Symbol;Acc:HGNC:4747]   -   111   114   101
        168   152   211   6.240702895   6.201734033
7.214563585   10.71773884   10.72686446   11.26406942
ENSG00000101544 1395.351986   1282.357977   1199.691383
1506.298192   1689.407906   1732.179203   1292.467115
1642.628433   -0.34558396   0.021143696   0.118058976   ADNP2
        18   80109031   80147523   +   5893
protein_coding ADNP homeobox 2 [Source:HGNC Symbol;Acc:HGNC:23803]
        Others 1538   1449   1032   1454   1480   2008
12.78052158   11.65087077   10.8955737   13.71008064
15.43734587   15.84376111
ENSG00000127452 449.0892282   543.387024   514.9838009
425.7830514   333.3156138   285.533524   502.4866844
348.2107297   0.529979035   0.021167896   0.118156807   FBXL12
        19   9810267 9827816 -   2913   protein_coding F-box
and leucine rich repeat protein 12 [Source:HGNC
Symbol;Acc:HGNC:13611] -   495   614   443   411   292
        331   8.321342505   9.987443313   9.46171992
7.839951588   6.161546453   5.283461259
ENSG00000149927 3.629003864   3.539980612   0   24.86324388
        14.83939377   15.52750281   2.389661492   18.41004682
        -2.907512509   0.021174866   0.118158423   DOC2A   16
        30005509   30023270   -   6426   protein_coding
        double C2 domain alpha [Source:HGNC Symbol;Acc:HGNC:2985]
        -       4   4   0   24   13   18
0.030482315   0.029494817   0   0.207530802   0.124351203
        0.130245522
ENSG00000104976 861.8884177   993.849557   1105.529559
1190.327801   1230.528191   1467.349016   987.0891779
1296.068336   -0.394088506   0.021190411   0.118207877   SNAPC2
        19   7920316 7923250 +   2000   protein_coding small
nuclear RNA activating complex polypeptide 2 [Source:HGNC
Symbol;Acc:HGNC:11135] -   950   1123   951   1149   1078
        1701   23.26067392   26.60579261   29.58403229
31.92288083   33.1311206   39.5462526
ENSG00000262370 6.350756762   5.309970919   10.46242485   0
        0   0   7.374384177   0   5.313066279
0.021207893   1   AC108134.3   16   3076911 3087100 -
        1326   lincRNA uncharacterized LOC105371058 [Source:NCBI
gene;Acc:105371058]   -   7   6   9   0   0
        0   0.258513483   0.214404634   0.422285172   0
        0   0
ENSG00000260641 10.88701159   15.92991276   0   0   0
        0   8.938974783   0   5.597586726   0.021238922
        0.118441136   AC114811.2   4   98658904
98664550   +   5647   antisense   "novel transcript,

antisense TSPAN5"	-	12	18	0	0	0
0	0.104061994	0.151036237	0	0	0
0
ENSG00000284726 18.14501932	18.58489822	12.78740815	0
0	4.313195226	16.50577523	1.437731742
3.447943707	0.02126486	0.11854841	AL109936.6	1
23297797	23302866	+	604	lincRNA novel
transcript	-	20	21	11	0	0	5
1.621517875	1.647436929	1.133085268	0	0
0.384914334
ENSG00000116793 1554.120905	1397.407347	1317.103039
1276.313186	1013.644743	973.9194821	1422.877097
1087.959137	0.387733328	0.021305345	0.118714448	PHTF1
1	113696831	113759489	-	7170
protein_coding	putative homeodomain transcription factor 1
[Source:HGNC Symbol;Acc:HGNC:8939]	-	1713	1579	1133
1232	888	1129	11.69948893	10.434927
9.831450587	9.547806214	7.61274303	7.321596728
ENSG00000137221 784.7720856	926.5899253	913.7184368
942.7313304	1122.086467	1550.162364	875.0268159
1204.993387	-0.462598832	0.021308066	0.118714448	TJAP1
6	43477523	43506556	+	6741
protein_coding	tight junction associated protein 1 [Source:HGNC
Symbol;Acc:HGNC:17949]	-	865	1047	786	910	983
1797	6.283772653	7.359508231	7.254459584
7.501171741	8.963477876	12.39523509
ENSG00000139921 2336.171237	2275.322539	2011.110554
2089.548454	1486.22236	1472.52485	2207.534777
1682.765222	0.39197534	0.021326322	0.11877875	TMX1
14	51240162	51257546	+	4499
protein_coding	thioredoxin related transmembrane protein 1
[Source:HGNC Symbol;Acc:HGNC:15487]	-	2575	2571	1730
2017	1302	1707	28.02785898	27.07774689
23.92417423	24.91161874	17.78862383	17.64202959
ENSG00000143196 35.38278767	23.89486913	30.2247829	0
12.55641011	0	29.83414657	4.185470037
2.870516968	0.021393097	0.119113156	DPT	1
168695459	168729264	-	1786	protein_coding
dermatopontin [Source:HGNC Symbol;Acc:HGNC:3011]	-	39
27	26	0	11	0	1.069330209
0.71632276	0.905729973	0	0.378580805	0
ENSG00000233589 9.979760626	16.81490791	0	0	0
0	8.931556178	0	5.596416401	0.021406924
0.119152635	AL138789.1	1	68479129
68483539	+	960	antisense	novel transcript
-	11	19	0	0	0	0
0.561112748	0.937796934	0	0	0	0
ENSG00000269834 9.979760626	14.15992245	34.8747495
59.05020421	38.81072216	73.32431885	19.67147752
57.06174841	-1.553101222	0.02142674	0.11922542

ZNF528-AS1 19 52388842 52397766 - 4160
processed_transcript ZNF528 antisense RNA 1 [Source:HGNC
Symbol;Acc:HGNC:51305] - 11 16 30 57 34
85 0.129487557 0.182243939 0.448677995
0.761366158 0.5023807 0.950072206
ENSG00000170876 3258.84547 3647.065026 3450.275217
4245.398892 4379.904145 4012.996839 3452.061904
4212.766625 -0.287166089 0.021454489 0.119342281 TMEM43
3 14124940 14143679 + 3572
protein_coding transmembrane protein 43 [Source:HGNC
Symbol;Acc:HGNC:28472] - 3592 4121 2968 4098 3837
4652 49.24402706 54.66603878 51.69628001
63.7488783 66.0279341 60.55629193
ENSG00000285517 170.5631816 124.7843166 137.1740147
326.3300759 196.3365944 200.1322585 144.1738376
240.9329763 -0.740139987 0.021495947 0.119535303
LINC00941 12 30795458 30879268 + 18914
lincRNA long intergenic non-protein coding RNA 941
[Source:NCBI gene;Acc:100287314] - 188 141 118
315 172 232 0.486746848 0.353233731
0.388155254 0.925420707 0.558975049 0.570342347
ENSG00000171530 1105.938928 1300.942875 1493.80177
940.6593934 997.6638578 1041.205328 1300.227858
993.1761929 0.387768385 0.021503716 0.119540927 TBCA
5 77691166 77868780 - 3058
protein_coding tubulin folding cofactor A [Source:HGNC
Symbol;Acc:HGNC:11579] - 1219 1470 1285 908 874
1207 19.52067847 22.77751254 26.14402759
16.49910594 17.5679591 18.35273557
ENSG00000105997 21.77402318 12.38993214 26.73730795
2.07193699 0 6.038473317 20.30042109 2.703470102
2.863399667 0.021514525 0.11956344 HOXA3 7
27106184 27152581 - 4306 protein_coding
homeobox A3 [Source:HGNC Symbol;Acc:HGNC:5104] Homeobox
24 14 23 2 0 7 0.272939191
0.15405665 0.332323197 0.025808812 0
0.075588379
ENSG00000205403 4.53625483 7.079961225 10.46242485 0
0 0 7.359546968 0 5.31027471
0.021547682 1 CFI 4 109740694 109802179
- 4875 protein_coding complement factor I
[Source:HGNC Symbol;Acc:HGNC:5394] - 5 8 9
0 0 0 0.050225477 0.077757414
0.114861567 0 0 0
ENSG00000174738 1392.630233 1054.914223 1075.304776
1112.630164 760.2335576 571.067048 1174.283077
814.6435897 0.528440572 0.02156116 0.119784976 NR1D2
3 23945260 23980618 + 5820
protein_coding nuclear receptor subfamily 1 group D member 2
[Source:HGNC Symbol;Acc:HGNC:7963] THR-like 1535 1192

925 1074 666 662 12.9155849 9.704646301
9.888390181 10.25400064 7.033939114 5.288908126
ENSG00000091483 2521.250434 2395.681879 2921.341516
2083.332643 2175.683424 2010.811615 2612.757943
2089.942561 0.321869619 0.021605687 0.119994659 FH
1 241497603 241519761 - 2142
protein_coding fumarate hydratase [Source:HGNC Symbol;Acc:HGNC:3700]
- 2779 2707 2513 2011 1906 2331
63.53276606 59.8818529 72.99277399 52.16805532
54.69539839 50.60038546
ENSG00000165669 909.9727189 826.585473 982.3054442
707.566482 678.0461459 690.1112362 906.2878787
691.9079547 0.388730342 0.021616342 0.120016155
FAM204A 10 118297930 118342328 - 15018
protein_coding family with sequence similarity 204 member A
[Source:HGNC Symbol;Acc:HGNC:25794] - 1003 934 845
683 594 800 3.270525326 2.946871625
3.50067228 2.52708964 2.431205699 2.476902464
ENSG00000149262 1250.191831 1026.594378 1156.679192
1449.319924 1342.39439 1640.739464 1144.488467
1477.484593 -0.36905938 0.021652575 0.120179603 INTS4
11 77878720 77994678 - 6074
protein_coding integrator complex subunit 4 [Source:HGNC
Symbol;Acc:HGNC:25048] - 1378 1160 995 1399 1176
1902 11.10972 9.049188657 10.19189967
12.79837911 11.90090242 14.56017997
ENSG00000167107 542.5360777 582.3268108 602.1706746
473.4376022 416.6445173 357.1325647 575.6778544
415.7382281 0.470209475 0.021660399 0.120185317 ACSF2
17 50426158 50474845 + 5797
protein_coding acyl-CoA synthetase family member 2 [Source:HGNC
Symbol;Acc:HGNC:26101] - 598 658 518 457 365
414 5.05157223 5.378349669 5.559468911
4.380512696 3.87023133 3.320687894
ENSG00000152133 895.4567034 634.5415248 742.8321643
628.8328764 478.285076 530.5230128 757.6101308
545.8803218 0.472668746 0.021716548 0.120453714
GPATCH11 2 37084451 37099244 + 4040
protein_coding G-patch domain containing 11 [Source:HGNC
Symbol;Acc:HGNC:26768] - 987 717 639 607 419
615 11.96367127 8.409384913 9.840707872
8.348709883 6.374997489 7.078231602
ENSG00000071859 2628.306048 3194.832503 3359.600868
3213.574271 3886.779675 4960.17451 3060.91314
4020.176152 -0.393722515 0.021722389 0.120453714 FAM50A
X 154444126 154450654 + 2567
protein_coding family with sequence similarity 50 member A
[Source:HGNC Symbol;Acc:HGNC:18786] - 2897 3610 2890
3102 3405 5750 55.26514452 66.63582425
70.04527113 67.14720942 81.53397348 104.1532903

ENSG00000138771 241.328757 229.2137447 242.9607548
197.8699825 93.60232991 130.2584958 237.8344188
140.5769361 0.757496488 0.021763527 0.120641071
SHROOM3 4 76435100 76783253 + 13958
protein_coding shroom family member 3 [Source:HGNC
Symbol;Acc:HGNC:30422] - 266 259 209 191 82
151 0.933226637 0.879231038 0.931600984
0.760365173 0.361108756 0.503019442
ENSG00000169551 15.42326642 2.654985459 4.6499666 0
0 0 7.576072827 0 5.356303036
0.02176563 1 CT55 X 135156536 135171398
- 1732 protein_coding cancer/testis antigen 55
[Source:HGNC Symbol;Acc:HGNC:26047] - 17 3 4
0 0 0 0.480650853 0.082072905
0.143687487 0 0 0
ENSG00000158417 3697.954937 2983.318661 3003.878423
2729.776984 2424.528643 2566.35116 3228.384007
2573.552262 0.327143092 0.021769817 0.120641071 EIF5B
2 99337353 99401326 + 7883
protein_coding eukaryotic translation initiation factor 5B
[Source:HGNC Symbol;Acc:HGNC:30793] - 4076 3371 2584
2635 2124 2975 25.3204449 20.26252981
20.39425426 18.5738159 16.56190717 17.54795296
ENSG00000156475 4.53625483 0.884995153 18.5998664 0
0 0 8.007038794 0 5.427390316
0.021782397 0.120672977 PPP2R2B 5 146581146
147084784 - 13206 protein_coding protein phosphatase 2
regulatory subunit Bbeta [Source:HGNC Symbol;Acc:HGNC:9305] -
5 1 16 0 0 0 0.018540754
0.003588022 0.075379896 0 0 0
ENSG00000260398 19.05227029 8.849951531 17.43737475
34.18696033 31.96177119 75.04959694 15.11319886
47.06610949 -1.646783688 0.02180963 0.12078602
AC068700.1 8 78605952 78609705 + 3754
sense_overlapping "novel transcript, overlapping to
PKIA" - 21 10 15 33 28 87
0.273938902 0.126221162 0.248601553 0.488463048
0.458470212 1.077596079
ENSG00000111832 942.6337537 829.2404585 914.8809285
668.1996792 659.7822767 724.616798 895.5850469
684.1995846 0.387750393 0.021825618 0.120791692 RWDD1
6 116571367 116597675 + 5909
protein_coding RWD domain containing 1 [Source:HGNC
Symbol;Acc:HGNC:20993] - 1039 937 787 645 578
840 8.610537077 7.513668699 8.286432347
6.065376556 6.012584717 6.609921689
ENSG00000155265 320.259591 264.6135508 420.8219773
11.39565344 143.8279703 87.98918262 335.2317063
81.07093547 2.048403081 0.021827837 0.120791692
GOLGA7B 10 97850239 97871580 + 6458

protein_coding golgin A7 family member B [Source:HGNC
Symbol;Acc:HGNC:31668] - 353 299 362 11 126
102 2.676734819 2.193812913 3.487525377
0.094646964 1.199277994 0.734400813
ENSG00000215838 0 0 0 0 6.848950969
19.84069804 0 8.89654967 -5.632820877 0.02183114
0.120791692 AL451074.1 1 165698750
165709968 + 1854 transcribed_processed_pseudogene
carbonic anhydrase 14 pseudogene [Source:NCBI gene;Acc:440700]
- 0 0 0 0 6 23 0
0 0 0 0.198924777 0.576831707
ENSG00000183527 1570.451422 1493.871818 1550.763861
1452.42783 1063.870384 950.6282279 1538.362367
1155.642147 0.413063603 0.021881026 0.121029854 PSMG1
21 39174769 39183851 - 2515
protein_coding proteasome assembly chaperone 1 [Source:HGNC
Symbol;Acc:HGNC:3043] - 1731 1688 1334 1402 932
1102 33.70449016 31.80247308 33.00081662
30.97576693 22.77850847 20.37392125
ENSG00000183735 894.5494525 648.7014472 583.5708082
569.7826722 464.5871741 471.8635578 708.9405693
502.0778013 0.49869171 0.021892983 0.121058134 TBK1
12 64451880 64502108 + 4762
protein_coding TANK binding kinase 1 [Source:HGNC
Symbol;Acc:HGNC:11584] - 986 733 502 550 407
547 10.13949225 7.293584593 6.558751442
6.417787634 5.253544053 5.341078406
ENSG00000135365 589.7131279 569.0518835 803.2817301
407.1356185 486.2755188 502.0559244 654.0155805
465.1556872 0.489952348 0.021907941 0.121102988 PHF21A
11 45929323 46121178 - 11190
protein_coding PHD finger protein 21A [Source:HGNC
Symbol;Acc:HGNC:24156] - 650 643 691 393 426
582 2.8445394 2.722747245 3.841977414
1.951526745 2.340059352 2.418376512
ENSG00000100207 1191.220518 815.9655312 913.7184368
1330.183547 1380.06362 1189.579243 973.6348288
1299.942137 -0.41641481 0.021923547 0.121132409 TCF20
22 42160013 42343616 - 7548
protein_coding transcription factor 20 [Source:HGNC
Symbol;Acc:HGNC:11631] - 1313 922 786 1284 1209
1379 8.518468429 5.787959324 6.478843675
9.452467131 9.845590523 8.495000188
ENSG00000154174 4362.062644 3981.593194 3368.900801
5401.539732 4810.246564 4496.937343 3904.185547
4902.90788 -0.328239673 0.021926959 0.121132409 TOMM70
3 100363431 100401398 - 5387
protein_coding translocase of outer mitochondrial membrane 70
[Source:HGNC Symbol;Acc:HGNC:11985] - 4808 4499 2898
5214 4214 5213 43.70651381 39.57267957

33.47019574   53.78189431   48.08337195   44.99577822
ENSG00000104835 58.06406182   87.61452016   46.499666
19.6834014   22.82983656   33.64292277   64.05941599
25.38538691   1.332933339   0.022036489   0.121684834   SARS2
        19   38915266   38930896   -   3231
protein_coding "seryl-tRNA synthetase 2, mitochondrial [Source:HGNC
Symbol;Acc:HGNC:17697]" -   64   99   40   19   20
        39   0.969999922   1.45185979   0.77024676
0.326759849   0.380487504   0.561252989
ENSG00000020129 1796.356913   2185.053033   1648.41316
2251.159539   2575.205564   2359.317789   1876.607702
2395.227631   -0.351390344   0.022040715   0.121684834   NCDN
        1   35557473   35567274   +   4917
protein_coding neurochondrin [Source:HGNC Symbol;Acc:HGNC:17597]
        -       1980   2469   1418   2173   2256   2735
19.71939859   23.79289689   17.94249646   24.55678804
28.20241164   25.86355031
ENSG00000254604 3.629003864   7.964956378   10.46242485   0
        0   0   7.352128364   0   5.308892746
0.022058262   1   AP002336.2   11   70282367
70363368   -   539   antisense   novel transcript
        -       4   9   9   0   0   0
0.36341254   0.791188899   1.038868531   0   0   0
ENSG00000167513 2962.174404   3849.728916   4036.171008
3911.817037   4812.529548   5290.565265   3616.024776
4671.637283   -0.369831291   0.022086513   0.121899636   CDT1
        16   88803213   88809258   +   3336
protein_coding chromatin licensing and DNA replication factor 1
[Source:HGNC Symbol;Acc:HGNC:24576]   -   3265   4350   3472
        3776   4216   6133   47.92761602   61.78593964
64.7530935   62.89527021   77.68227225   85.48264288
ENSG00000010244 5186.753773   4773.663856   4891.764863
6063.523601   6442.579878   5562.296564   4950.727497
6022.800014   -0.282624154   0.022099702   0.121934382   ZNF207
        17   32350117   32381886   +   15834
protein_coding zinc finger protein 207 [Source:HGNC
Symbol;Acc:HGNC:12998] -   5717   5394   4208   5853   5644
        6448   17.68097602   16.141606   16.53453315
20.53997578   21.91006796   18.93500422
ENSG00000197980 33.56828574   29.20484005   31.38727455
14.50355893   3.424475485   5.175834272   31.38680011
7.701289562   2.027101826   0.022144274   0.122125396   LEKR1
        3   156825481   157046129   +   5602
protein_coding "leucine, glutamate and lysine rich 1 [Source:HGNC
Symbol;Acc:HGNC:33765]" -   37   33   27   14   3
        6   0.323435215   0.279124062   0.299866193
0.138866335   0.032917399   0.049801126
ENSG00000132386 184.1719461   186.7339773   209.248497
82.87747959   94.74382174   155.2750281   193.3848068
110.9654432   0.796616508   0.02214813   0.122125396

SERPINF1 17 1761959 1777574 + 3069 protein_coding
serpin family F member 1 [Source:HGNC Symbol;Acc:HGNC:8824]
- 203 211 180 80 83 180
3.239125932 3.2577069 3.649072258 1.44845545
1.662373335 2.727134985
ENSG00000101849 708.5630044 730.1210013 710.2823981
874.3574097 954.2871684 956.6667012 716.3221346
928.4370931 -0.374171717 0.02216042 0.122155085 TBL1X
X 9463295 9741037 + 9092 protein_coding
transducin beta like 1 X-linked [Source:HGNC Symbol;Acc:HGNC:11585]
- 781 825 611 844 836 1109
4.206494161 4.299529789 4.181081468 5.158163016
5.651896634 5.671567918
ENSG00000167969 405.5411818 592.0617574 769.5694722
464.1138857 397.2391562 302.7863049 589.0574705
388.0464489 0.601896823 0.022201904 0.122345632 ECI1
16 2239395 2252300 - 3234 protein_coding enoyl-
CoA delta isomerase 1 [Source:HGNC Symbol;Acc:HGNC:2703] -
447 669 662 448 348 351 6.768558567
9.801951358 12.73575865 7.697506106 6.614341115
5.046591118
ENSG00000146574 841.9288964 834.5504294 855.5938543
1034.932526 1498.77877 931.6501689 844.0243934
1155.120489 -0.451796212 0.022254237 0.122591675 CCZ1B
7 6794134 6826770 - 7316 protein_coding "CCZ1
homolog B, vacuolar protein trafficking and biogenesis associated
[Source:HGNC Symbol;Acc:HGNC:21717]" - 928 943 736
999 1313 1080 6.211592587 6.107513516
6.259086653 7.587589643 11.03159704 6.86406009
ENSG00000138395 33.56828574 18.58489822 15.11239145 0
7.990442797 0 22.42185847 2.663480932
3.122633432 0.022261191 0.122591675 CDK15 2
201790461 201895550 + 6141 protein_coding cyclin
dependent kinase 15 [Source:HGNC Symbol;Acc:HGNC:14434] - 37
21 13 0 7 0 0.295047073
0.162034181 0.131707681 0 0.070065835 0
ENSG00000277782 38.10454057 38.05479158 37.1997328
11.39565344 15.98088559 7.763751407 37.78635498
11.71343015 1.699239958 0.022267344 0.122591675
AC068870.2 15 80999593 80999981 - 389
lincRNA novel transcript - 42 43 32
11 14 9 5.287232064 5.237756387
5.11808181 1.571285589 2.212207165 1.07578114
ENSG00000131263 4024.565285 3641.755055 3467.712592
4872.159832 4273.745405 4524.541792 3711.344311
4556.815676 -0.29595633 0.022319849 0.122839035 RLIM
X 74585217 74614617 - 8379
protein_coding "ring finger protein, LIM domain interacting
[Source:HGNC Symbol;Acc:HGNC:13429]" - 4436 4115 2983
4703 3744 5245 25.92555311 23.27041294

22.14970312	31.1885247	27.46572136	29.10612031
ENSG00000116209 3359.550327	3570.955443	3880.397127
3068.538682	3074.037493	2510.279622	3603.634299
2884.285266	0.321396739	0.022332302	0.122839035	TMEM59
	1	54026681	54053504	-	9149
protein_coding	transmembrane protein 59 [Source:HGNC
Symbol;Acc:HGNC:1239]	-	3703	4035	3338	2962	2693
	2910	19.82023357	20.89759712	22.6996704
17.98968133	18.09297929	14.78939402
ENSG00000129675 1425.291268	1126.59883	1558.901303
1214.155076	1017.069219	756.5344427	1370.2638
995.9195792	0.460605451	0.02234074	0.122839035
ARHGEF6 X	136665547	136782088	-	6434
protein_coding	Rac/Cdc42 guanine nucleotide exchange factor 6
[Source:HGNC Symbol;Acc:HGNC:685]	-	1571	1273	1341
	1172	891	877	11.95704358	9.375054216
12.96744958	10.12181974	8.512242896	6.33796089
ENSG00000174744 1563.193414	2114.253421	1972.74833
1880.282818	2545.526777	3287.517402	1883.398388
2571.108999	-0.449470144	0.022351719	0.122839035	BRMS1
	11	66337333	66345125	-	2514
protein_coding	"BRMS1, transcriptional repressor and anoikis
regulator [Source:HGNC Symbol;Acc:HGNC:17262]"	-	1723	2389
	1697	1815	2230	3811	33.56206604	45.02744633
	41.99749776	40.11653363	54.52390468	70.48629668
ENSG00000258754 12.70151352	1.769990306	8.137441549	0
	0	0	7.536315126	0	5.346149008
0.02235444	1	LINC01579	15	93718542
94070820	-	6795	lincRNA long intergenic non-protein
coding RNA 1579 [Source:HGNC Symbol;Acc:HGNC:27519]	-	14
	2	7	0	0	0	0.100894446
0.013946556	0.064093712	0	0	0
ENSG00000153914 1584.967438	1425.727192	1188.066466
1088.802888	1128.935418	1017.051434	1399.587032
1078.263247	0.377308436	0.02235726	0.122839035	SREK1
	5	66139971	66183615	+	18240
protein_coding	splicing regulatory glutamic acid and lysine rich
protein 1 [Source:HGNC Symbol;Acc:HGNC:17882]	-	1747	1611
	1022	1051	989	1179	4.690258234	4.185016236
	3.486044354	3.201768486	3.332873405	3.005522539
ENSG00000166986 4271.337548	4286.031527	4373.293587
4774.778793	5596.734434	5373.378613	4310.220887
5248.29728	-0.284102778	0.02236006	0.122839035	MARS
	12	57475445	57517569	+	7028
protein_coding	methionyl-tRNA synthetase [Source:HGNC
Symbol;Acc:HGNC:6898]	-	4708	4843	3762	4609	4903
	6229	32.80449714	32.65195258	33.3038073
36.44072417	42.8822437	41.21142163
ENSG00000141562 1284.667368	1604.496213	1682.125417
1737.319166	2060.39275	2103.113992	1523.762999

1966.941969	-0.368830776	0.022360885	0.122839035	NARF
	17	82458180	82490537	+	7962
protein_coding	nuclear prelamin A recognition factor [Source:HGNC
Symbol;Acc:HGNC:29916]	-	1416	1813	1447	1677	1805
	2438	8.709029541	10.78951869	11.30715133
11.70369332	13.93485305	14.23778879
ENSG00000113522 177.8211893	153.9891566	168.5612892
122.2442824	69.63100152	93.16501689	166.7905451
95.0134336	0.810371452	0.022411414	0.12307839	RAD50
	5	132556019	132646344	+	9771
protein_coding	RAD50 double strand break repair protein [Source:HGNC
Symbol;Acc:HGNC:9816]	-	196	174	145	118	61
	108	0.982303613	0.843794472	0.923285118
0.671050243	0.383740981	0.513943953
ENSG00000073578 1261.986094	1274.39302	1771.637274
1140.601313	974.8340213	1123.156037	1436.005463
1079.530457	0.410432762	0.02242594	0.123116677	SDHA
	5	218241	256700	+	8383	protein_coding
succinate dehydrogenase complex flavoprotein subunit A [Source:HGNC
Symbol;Acc:HGNC:10680]	-	1391	1440	1524	1101	854
	1302	8.125616987	8.13934518	11.31077459
7.297933412	6.261894765	7.221751432
ENSG00000105976 1936.980812	1502.72177	1747.22495
1695.880426	1117.5205	849.6994596	1728.975844
1221.033462	0.502264459	0.02243562	0.123116677	MET
	7	116672390	116798386	+	7039
protein_coding	"MET proto-oncogene, receptor tyrosine kinase
[Source:HGNC Symbol;Acc:HGNC:7029]"	-	2135	1698	1503
	1637	979	985	14.85304845	11.43018242
13.28479515	12.92259572	8.549074206	6.50663258
ENSG00000111581 2407.844064	1870.879754	1778.612224
1816.052772	1530.740542	1104.177978	2019.112014
1483.657097	0.445376351	0.022439265	0.123116677	NUP107
	12	68686734	68745809	+	8320
protein_coding	nucleoporin 107 [Source:HGNC Symbol;Acc:HGNC:29914]
	-	2654	2114	1530	1753	1341	1280
15.62090804	12.0394902	11.44128888	11.70767434
9.90724292	7.153484846
ENSG00000187796 27.21752898	38.93978674	22.08734135
64.23004668	67.34801786	75.04959694	29.41488569
68.87588716	-1.222826755	0.022501784	0.123421416	CARD9
	9	136361903	136373681	-	6699
protein_coding	caspase recruitment domain family member 9
[Source:HGNC Symbol;Acc:HGNC:16391]	-	30	44	19
	62	59	87	0.219300671	0.311221176
0.176461704	0.514273037	0.541364027	0.603865604
ENSG00000099282 24.49577608	27.43484975	22.08734135	0
	6.848950969	6.038473317	24.67265573	4.295808095
	2.5212609	0.022522843	0.123476728	TSPAN15 10
	69451473	69507669	+	2646	protein_coding

tetraspanin 15 [Source:HGNC Symbol;Acc:HGNC:23298]	-
27	31	19	0	6	7	0.499692243
0.555134598	0.44675622	0	0.139382667
0.12300966
ENSG00000154263 33.56828574	0	0	0	0	0
11.18942858	0	5.919689445	0.02252583
0.123476728	ABCA10	17	69147214	69244846	-
8405	protein_coding	ATP binding cassette subfamily A
member 10 [Source:HGNC Symbol;Acc:HGNC:30]	-	37	0
0	0	0	0	0.215572168	0	0
0	0	0
ENSG00000164741 2112.080249	1741.670461	1985.535738
1628.542474	1317.28157	1614.860293	1946.428816
1520.228112	0.355990708	0.022575092	0.123708429	DLC1
8	13083361	13604610	-	12794
protein_coding	DLC1 Rho GTPase activating protein [Source:HGNC
Symbol;Acc:HGNC:2897]	-	2328	1968	1708	1572	1154
1872	8.910566447	7.288617988	8.305931911
6.827445451	5.544301288	6.803470659
ENSG00000081026 751.2037998	508.872213	649.8328323
435.1067679	527.3692246	379.5611799	636.6362817
447.3457241	0.510206847	0.022583691	0.123717221	MAGI3
1	113390749	113685923	+	8180
protein_coding	"membrane associated guanylate kinase, WW and PDZ
domain containing 3 [Source:HGNC Symbol;Acc:HGNC:29647]"	-
828	575	559	420	462	440	4.956849313
3.330741911	4.251726802	2.853041116	3.471650779
2.501096169
ENSG00000165632 540.7215757	454.0025136	435.9343687
684.7751751	633.5279646	623.6880297	476.8861527
647.3303898	-0.439972745	0.022601442	0.123738153	TAF3
10	7818504 8016627 +	4872	protein_coding	TATA-
box binding protein associated factor 3 [Source:HGNC
Symbol;Acc:HGNC:17303]	-	596	513	375	661	555
723	5.990563329	4.989264479	4.788845598
7.538869463	7.002176663	6.900205672
ENSG00000131378 762.0908114	1012.434455	986.9554108
1111.594195	1135.784369	1445.78304	920.4935591
1231.053868	-0.42037279	0.022607002	0.123738153	RFTN1
3	16313574	16514026	-	4776
protein_coding	"raftlin, lipid raft linker 1 [Source:HGNC
Symbol;Acc:HGNC:30278]" -	840	1144	849	1073	995
1676	8.6127859	11.34979839	11.059875
12.48381855	12.80578255	16.31701506
ENSG00000092108 792.9373443	841.6303906	896.2810621
707.566482	589.0097833	617.6495564	843.6162656
638.0752739	0.40237139	0.022608497	0.123738153	SCFD1
14	30622112	30735812	+	6571
protein_coding	sec1 family domain containing 1 [Source:HGNC
Symbol;Acc:HGNC:20726]	-	874	951	771	683	516

	716	6.513413485	6.857652759	7.300115938
5.775655488	4.826869915	5.066552804
ENSG00000231607 214.111228	148.6791857	119.7366399
69.40988916	115.2906746	77.63751407	160.8423512
87.44602596	0.883589671	0.022620017	0.123762908	DLEU2
	13	49982552	50125720	-	5103
antisense	deleted in lymphocytic leukemia 2 [Source:HGNC
Symbol;Acc:HGNC:13748]	-	236	168	103	67	101
	90	2.264723143	1.559948117	1.255794287
0.729560442	1.216586984	0.8200644
ENSG00000068078 12.70151352	0	13.9498998	0	0
	0	8.883804441	0	5.581563334	0.022641271
	0.123840889	FGFR3	4	1793293 1808872 +	4848
	protein_coding	fibroblast growth factor receptor 3
[Source:HGNC Symbol;Acc:HGNC:3690]	-	14	0	12
	0	0	0	0.141414554	0	0.154001688
	0	0	0
ENSG00000213366 153.3254133	148.6791857	113.9241817
50.76245625	89.0363626	83.67598739	138.6429269
74.49160208	0.897636519	0.02266609	0.123938319	GSTM2
	1	109668022	109709551	+	9116
protein_coding	glutathione S-transferase mu 2 [Source:HGNC
Symbol;Acc:HGNC:4634]	-	169	168	98	49	78
	97	0.907843674	0.873235546	0.668849807
0.298678394	0.525941749	0.494764392
ENSG00000163257 1495.149592	1488.561848	1159.004175
1931.045274	1662.012102	1745.981428	1380.905205
1779.679601	-0.365259374	0.022683136	0.123993197	DCAF16
	4	17800655	17810758	-	4816
protein_coding	DDB1 and CUL4 associated factor 16 [Source:HGNC
Symbol;Acc:HGNC:25987]	-	1648	1682	997	1864	1456
	2024	16.75712127	16.54877892	12.87999002
21.50658592	18.58327514	19.54137181
ENSG00000169976 1366.319955	1910.704536	2018.085504
1938.297054	2515.847989	2590.505053	1765.036665
2348.216699	-0.41235733	0.022695081	0.124020169	SF3B5
	6	144094881	144095573	-	693
protein_coding	splicing factor 3b subunit 5 [Source:HGNC
Symbol;Acc:HGNC:21083]	-	1506	2159	1736	1871	2204
	3003	106.4193056	147.6202201	155.8559307
150.0211867	195.4905263	201.4898232
ENSG00000166123 676.8092206	860.2152888	797.4692718
528.3439324	574.1703896	631.4517811	778.1645938
577.988701	0.428392739	0.022729275	0.124166542	GPT2
	16	46884378	46931297	+	6354
protein_coding	glutamic--pyruvic transaminase 2 [Source:HGNC
Symbol;Acc:HGNC:18062]	-	746	972	686	510	503
	732	5.749370556	7.248455805	6.717129974
4.460001546	4.865955521	5.356669956
ENSG00000125726 166.0269268	220.3637931	174.3737475

112.9205659	116.4321665	15.52750281	186.9214891
81.62674508	1.200997061	0.022735905	0.124166542	CD70
	19	6583183 6604103 -	1532	protein_coding	CD70
molecule [Source:HGNC Symbol;Acc:HGNC:11937]	-	183	249
	150	109	102	18	5.849530476	7.701352865
	6.091711685	3.953482095	4.09250074	0.546317054
ENSG00000047648 69.85832438	61.06466557	53.4746159
17.61146441	30.82027936	26.7418104	61.46586861
25.05785139	1.296598254	0.022804266	0.124453223
ARHGAP6 X	11137543	11665701	-	7885
protein_coding	Rho GTPase activating protein 6 [Source:HGNC
Symbol;Acc:HGNC:676]	-	77	69	46	17	27
	31	0.478208962	0.414642519	0.362963522
0.119800676	0.210479317	0.182806239
ENSG00000100284 518.0403016	608.8766653	547.5335671
597.7538216	771.6484758	978.2326773	558.150178
782.5449916	-0.48818329	0.022807701	0.124453223	TOM1
	22	35299275	35347994	+	5871
protein_coding	target of myb1 membrane trafficking protein
[Source:HGNC Symbol;Acc:HGNC:11982]	-	571	688	471
	577	676	1134	4.762694353	5.552681954
4.991322981	5.461045267	7.077534186	8.981150877
ENSG00000182287 1425.291268	1146.068723	1314.778056
1030.788652	1120.944975	651.2924792	1295.379349
934.3420356	0.472492128	0.022809506	0.124453223	AP1S2
	X	15825806	15854931	-	5371
protein_coding	adaptor related protein complex 1 subunit sigma 2
[Source:HGNC Symbol;Acc:HGNC:560]	-	1571	1295	1131
	995	982	755	14.3235186	11.42460141
13.10129722	10.29390064	11.23837956	6.536162145
ENSG00000113273 600.6001395	537.1920579	740.507181
503.4806885	474.8606005	326.0775591	626.0997928
434.8062827	0.526536627	0.02282031	0.124473781	ARSB
	5	78777209	78986087	-	9053
protein_coding	arylsulfatase B [Source:HGNC Symbol;Acc:HGNC:714]
	-	662	607	637	486	416	378
3.580916157	3.177039479	4.377778243	2.983017441
2.824542868	1.941468272
ENSG00000155926 8.165258694	5.309970919	8.137441549	0
	0	0	7.204223721	0	5.281047493
0.022825541	1	SLA	8	133036724	133103054
	-	7637	protein_coding	Src like adaptor [Source:HGNC
Symbol;Acc:HGNC:10902]	-	9	6	7	0	0
	0	0.057709645	0.037226731	0.057027206	0
	0	0
ENSG00000265491 1448.879793	1324.837744	1222.941216
1768.398221	1650.597184	1624.349322	1332.219584
1681.114909	-0.335037653	0.022935271	0.125062142	RNF115
	1	145738868	145824077	-	11041
protein_coding	ring finger protein 115 [Source:HGNC

Symbol;Acc:HGNC:18154] -   1597   1497   1052   1707   1446
        1883   7.083129627   6.424507364   5.928081647
8.590870152   8.050210623   7.92999527
ENSG00000184857 234.9780002   217.7088077   167.3987976
347.0494458   232.8643329   395.9513218   206.6952018
325.2883668   -0.654016992   0.022942317   0.125062142
TMEM186 16   8780384 8797648 -   1978   protein_coding
transmembrane protein 186 [Source:HGNC Symbol;Acc:HGNC:24530]   -
        259   246   144   335   204   459   6.412127663
        5.892983983   4.529424777   9.410887102   6.33944503
        10.78989992
ENSG00000129055 1316.421152   1166.423612   1175.279058
1813.980835   1457.685065   1439.744567   1219.374607
1570.470155   -0.364765998   0.022970097   0.125174996
ANAPC13 3   134477706   134486716   -   2359
protein_coding anaphase promoting complex subunit 13 [Source:HGNC
Symbol;Acc:HGNC:24540] -   1451   1318   1011   1751   1277
        1669   30.12091462   26.47365532   26.66429229
41.24489942   33.2744064   32.89723627
ENSG00000127362 0   0   0   19.6834014   6.848950969
        0   0   8.844117457   -5.618154084   0.022981062
        0.125196184   TAS2R3 7   141764097   141765197
        +   1101   protein_coding taste 2 receptor member 3
[Source:HGNC Symbol;Acc:HGNC:14910]   -   0   0   0
        19   6   0   0   0   0   0.958911055
        0.334974148   0
ENSG00000277157 1.814501932   17.69990306   3.48747495   0
        0   0   7.667293315   0   5.375193265
0.022987218   1   HIST1H4D   6   26188765
26189076   -   312   protein_coding histone cluster 1 H4
family member d [Source:HGNC Symbol;Acc:HGNC:4782]   -   2
        20   3   0   0   0   0.31390923
3.037398978   0.598237327   0   0   0
ENSG00000059758 1007.955823   929.2449108   1020.667669
1051.508022   1462.251032   1375.909277   985.9561342
1296.55611   -0.395270314   0.023000628   0.125264196   CDK17
        12   96278261   96400560   -   6547
protein_coding cyclin dependent kinase 17 [Source:HGNC
Symbol;Acc:HGNC:8750]   -   1111   1050   878   1015   1281
        1595   8.309988094   7.599296664   8.343706553
8.614612461   12.02691244   11.32789891
ENSG00000204335 7.258007728   0.884995153   15.11239145   0
        0   0   7.75179811   0   5.382363472
0.023006099   1   SP5   2   170715351   170718078
        +   2060   protein_coding Sp5 transcription factor
[Source:HGNC Symbol;Acc:HGNC:14529]   zf-C2H2 8   1   13
        0   0   0   0.190174135   0.023001662
0.392629547   0   0   0
ENSG00000151135 943.5410046   898.2700804   1159.004175
852.6020713   696.3100152   698.7376267   1000.271753

749.2165711 0.416197192 0.023038842 0.125433695
TMEM263 12 106955719 106978778 + 4768
protein_coding transmembrane protein 263 [Source:HGNC
Symbol;Acc:HGNC:28281] - 1040 1015 997 823 610
810 10.6813409 10.08686565 13.00965435
9.591259296 7.863953711 7.899139621
ENSG00000109814 979.8310433 891.1901192 880.006179
805.983489 666.6312277 584.8692727 917.0091138
685.8279965 0.41994006 0.023047413 0.125441752 UGDH
4 39498755 39528311 - 4045
protein_coding UDP-glucose 6-dehydrogenase [Source:HGNC
Symbol;Acc:HGNC:12525] - 1080 1007 757 778 584
678 13.07476564 11.7960712 11.6435175
10.68742585 8.874454792 7.793673107
ENSG00000157036 510.7822938 564.6269077 444.0718103
628.8328764 707.7249335 722.0288809 506.4936706
686.1955636 -0.437038097 0.023072605 0.125540235 EXOG
3 38496127 38542161 + 5223
protein_coding exo/endonuclease G [Source:HGNC Symbol;Acc:HGNC:3347]
- 563 638 382 607 620 837
5.278579327 5.787980959 4.550406381 6.45774228
7.296574546 7.451375513
ENSG00000100814 699.4904948 693.8362 802.1192384
1164.428588 848.1284283 924.7490565 731.8153111
979.1020244 -0.420742484 0.023080155 0.125542699
CCNB1IP1 14 20311368 20333312 - 3680
protein_coding cyclin B1 interacting protein 1 [Source:HGNC
Symbol;Acc:HGNC:19437] - 771 784 690 1124 743
1072 10.25971373 10.09472947 11.66562788
16.97190281 12.41047361 13.54496805
ENSG00000135318 7571.009311 7487.943991 7983.992651
8895.861466 9559.994061 9124.995821 7680.981984
9193.617116 -0.259381972 0.02308899 0.125552152 NT5E
6 85449584 85495791 + 4533
protein_coding 5'-nucleotidase ecto [Source:HGNC
Symbol;Acc:HGNC:8021] - 8345 8461 6868 8587 8375
10578 90.15074198 88.44278644 94.2652045
105.2610717 113.5655103 108.5047778
ENSG00000268205 456.3472359 558.4319416 381.2972612
566.6747667 633.5279646 773.7872236 465.3588129
657.9966517 -0.498987986 0.023103541 0.125581243
AC005261.1 19 57304305 57308562 + 4258
lincRNA novel transcript - 503 631 328
547 555 897 5.784835471 7.021827285
4.792642484 7.138282197 8.011884618 9.795301408
ENSG00000135900 576.1043634 534.5370725 587.0582832
595.6818846 838.9964937 910.9468318 565.8999064
781.87507 -0.467117615 0.023108539 0.125581243 MRPL44
2 223957404 223967714 + 1748
protein_coding mitochondrial ribosomal protein L44 [Source:HGNC

Symbol;Acc:HGNC:16650] - 635 604 505 575 735
1056 17.7893869 16.37276209 17.97449909
18.27841191 25.84599589 28.09011441
ENSG00000163161 1486.077082 1600.071237 1457.764529
2076.080864 1772.736809 1852.08603 1514.637616
1900.301234 -0.327071583 0.023123209 0.125622371 ERCC3
2 127257290 127294176 - 8422
protein_coding "ERCC excision repair 3, TFIIH core complex helicase subunit [Source:HGNC Symbol;Acc:HGNC:3435]" - 1638 1808
1254 2004 1553 2147 9.524174501 10.17207679
9.263799482 13.22191996 11.33453163 11.85353334
ENSG00000070814 4040.895802 4040.002874 3750.198063
2882.064353 3147.09297 3549.759671 3943.698913
3192.972331 0.304529631 0.023141452 0.125682884 TCOF1
5 150357629 150400308 + 11439
protein_coding treacle ribosome biogenesis factor 1 [Source:HGNC Symbol;Acc:HGNC:11654] - 4454 4565 3226 2782 2757
4115 19.06737185 18.90946156 17.546203
13.51391271 14.81480933 16.72679744
ENSG00000121988 195.9662087 165.4940936 230.1733467
146.0715578 116.4321665 84.53862644 197.2112163
115.6807836 0.770559996 0.023198359 0.125935622 ZRANB3
2 135136916 135531236 - 9115
protein_coding zinc finger RANBP2-type containing 3 [Source:HGNC Symbol;Acc:HGNC:25249] - 216 187 198 141 102
98 1.160448207 0.972100965 1.351497865
0.859556608 0.687845434 0.499919895
ENSG00000184205 242.2360079 345.1481097 338.2850701
372.9486582 436.0498784 565.8912137 308.5563959
458.2965834 -0.572543396 0.023202227 0.125935622 TSPYL2
X 53082367 53088540 + 4948
protein_coding TSPY like 2 [Source:HGNC Symbol;Acc:HGNC:24358] -
267 390 291 360 382 656 2.642471147
3.73474846 3.65906517 4.04282417 4.745489668
6.164603756
ENSG00000163428 4262.265038 3296.606945 2872.516867
5167.410853 4217.812305 4150.156447 3477.129617
4511.793201 -0.37546397 0.023225517 0.126009176 LRRC58
3 120324509 120349339 - 7903
protein_coding leucine rich repeat containing 58 [Source:HGNC Symbol;Acc:HGNC:26968] - 4698 3725 2471 4988 3695
4811 29.11050329 22.33370297 19.45304583
35.07086733 28.73887881 28.30573217
ENSG00000196935 548.8868344 513.2971888 435.9343687
878.5012837 494.2659616 793.6279217 499.3727973
722.1317223 -0.532203758 0.023230026 0.126009176 SRGAP1
12 63844293 64162221 + 26641
protein_coding SLIT-ROBO Rho GTPase activating protein 1 [Source:HGNC Symbol;Acc:HGNC:17382] - 605 580 375 848 433
920 1.112073612 1.031582371 0.875765015

1.768713022 0.999044272 1.605714477
ENSG00000135912 693.139738 510.6422034 459.1842017
865.0336932 619.8300627 872.1280748 554.3220477
785.6639436 -0.502890277 0.02327805 0.126230883 TTLL4
2 218710845 218755416 + 7685
protein_coding tubulin tyrosine ligase like 4 [Source:HGNC
Symbol;Acc:HGNC:28976] - 764 577 395 835 543
1011 4.868309386 3.557610368 3.197864592
6.037465295 4.34313489 6.116997484
ENSG00000157191 1679.321538 2248.772684 2157.584502
2171.389965 2772.683651 3005.434434 2028.559575
2649.836017 -0.385764792 0.02328517 0.126230883 NECAP2
1 16440672 16460078 + 5559
protein_coding NECAP endocytosis associated 2 [Source:HGNC
Symbol;Acc:HGNC:25528] - 1851 2541 1856 2096 2429
3484 16.30566172 21.65880204 20.77247019
20.95109123 26.85828024 29.14153945
ENSG00000170606 5873.542754 5495.819901 5182.387775
6516.241833 6748.499688 6691.491074 5517.250143
6652.077532 -0.269720775 0.023300003 0.126261747 HSPA4
5 133051962 133106449 + 5153
protein_coding heat shock protein family A (Hsp70) member 4
[Source:HGNC Symbol;Acc:HGNC:5237] - 6474 6210 4458
6290 5912 7757 61.52352864 57.10286501
53.82533832 67.82699033 70.52151655 69.99462113
ENSG00000166073 2561.169477 2533.741123 2636.531062
2952.51021 3308.043318 3187.451272 2577.147221
3149.334934 -0.289329716 0.023305139 0.126261747 GPR176
15 39799032 39920892 - 5119
protein_coding G protein-coupled receptor 176 [Source:HGNC
Symbol;Acc:HGNC:32370] - 2823 2863 2268 2850 2898
3695 27.0056374 26.50102424 27.56542973
30.93654245 34.79850704 33.56296587
ENSG00000125124 402.8194289 361.0780225 346.4225117
298.3589265 207.7515127 247.577406 370.1066543
251.2292818 0.558743265 0.023327282 0.126343017 BBS2
16 56466836 56520283 - 8337
protein_coding Bardet-Biedl syndrome 2 [Source:HGNC
Symbol;Acc:HGNC:967] - 444 408 298 288 182
287 2.607965561 2.318872138 2.223890037
1.91952923 1.341865375 1.600674821
ENSG00000221890 162.3979229 129.2092924 237.1482966
109.8126605 89.0363626 7.763751407 176.2518373
68.87092482 1.35959725 0.023389012 0.126638575 NPTXR
22 38818452 38843982 - 5784
protein_coding neuronal pentraxin receptor [Source:HGNC
Symbol;Acc:HGNC:7954] - 179 146 204 106 78
9 1.51549124 1.196054618 2.194364303
1.018332547 0.828922024 0.072351117
ENSG00000230836 29.03203091 1.769990306 8.137441549

2.07193699	0	0	12.97982092	0.690645663
4.252284295	0.023471291	0.127045187	LINC01293	2
	74940258	74942670	+	1266	lincRNA long
intergenic non-protein coding RNA 1293 [Source:HGNC
Symbol;Acc:HGNC:50362]	-	32	2	7	2	0
	0	1.237784261	0.07485533	0.34401009
0.087782579	0	0
ENSG00000151694 927.2104872	707.1111273	861.4063126
798.7317096	474.8606005	441.6711912	831.909309
571.7545004	0.541299213	0.023487668	0.12705971	ADAM17
	2	9488486 9556732 -	10750	protein_coding	ADAM
metallopeptidase domain 17 [Source:HGNC Symbol;Acc:HGNC:195]	-
	1022	799	741	771	416	512	4.655551284
	3.521800541	4.28861036	3.985271904	2.378659217
	2.21458582
ENSG00000226688 136.0876449	138.0592439	122.0616232
191.6541716	186.063168	263.9675479	132.069504
213.8949625	-0.696382744	0.02348834	0.12705971
ENTPD1-AS1	10	95753206	96090238	-	12528
	antisense	ENTPD1 antisense RNA 1 [Source:HGNC
Symbol;Acc:HGNC:45203]	-	150	156	105	185	163
	306	0.586324711	0.59002348	0.521452076
0.820544289	0.799748105	1.135718499
ENSG00000140988 30754.9005	38182.23089	38084.38894
38622.97743	45061.53141	49175.60142	35673.84011
44286.70342	-0.312036401	0.023522198	0.127203962	RPS2
	16	1962052 1964860 -	2413	protein_coding
ribosomal protein S2 [Source:HGNC Symbol;Acc:HGNC:10404]	-
	33899	43144	32761	37282	39476	57006	687.9521759
	847.206982	844.7081306	858.5269326	1005.59517
	1098.485338
ENSG00000130749 1732.849345	1483.251877	1719.32515
1227.622666	1135.784369	1465.623738	1645.142124
1276.343591	0.3652341	0.023551463	0.127315803	ZC3H4
	19	47064187	47113752	-	6502
protein_coding	zinc finger CCCH-type containing 4 [Source:HGNC
Symbol;Acc:HGNC:17808]	-	1910	1676	1479	1185	995
	1699	14.38517288	12.21387553	14.15233354
10.12706107	9.406400713	12.15003228
ENSG00000141295 425.500703	540.7320386	475.4590848
287.9992416	415.5030255	291.5719973	480.5639421
331.6914214	0.536707997	0.023557274	0.127315803	SCRN2
	17	47837692	47841333	-	3043
protein_coding	secernin 2 [Source:HGNC Symbol;Acc:HGNC:30381]	-
	469	611	409	278	364	338	7.547438344
	9.514055898	8.362347335	5.076388916	7.352699068
	5.164707922
ENSG00000273056 0	0	0	13.46759043	12.55641011
	0	0	8.674666848	-5.589051196	0.023584946
	0.127403031	AL354694.1	9	7786105 7786688 -

584	lincRNA novel transcript	-	0	0
0	13	11	0	0	0	0
1.236922669	1.157783079	0
ENSG00000221819 2.721752898	2.654985459	4.6499666
11.39565344	9.131934625	40.54403513	3.342234986
20.35720773	-2.62663905	0.023587819	0.127403031	GAS8-
AS1	16	90028908	90029901	-	994
antisense	GAS8 antisense RNA 1 [Source:HGNC
Symbol;Acc:HGNC:1197]	-	3	3	4	11	8
47	0.147796297	0.143008322	0.250368942
0.614919612	0.494710312	2.198581107
ENSG00000099260 1069.648889	1078.809092	1562.388777
1381.981972	2020.440536	1648.503216	1236.948919
1683.641908	-0.445515932	0.023620931	0.127523217	PALMD
1	99645943	99694541	+	8244
protein_coding	palmdelphin [Source:HGNC Symbol;Acc:HGNC:15846] -
1179	1219	1344	1334	1770	1911	7.003328622
7.006355401	10.14303986	8.991453241	13.19722568
10.77838574
ENSG00000160172 77.11633211	70.79961225	97.64929859
141.9276838	149.5354295	138.8848863	81.85508098
143.4493332	-0.812223055	0.023627574	0.127523217
FAM86C2P	11	67791648	67805336	-	3402
transcribed_unprocessed_pseudogene	"family with sequence
similarity 86 member C2, pseudogene [Source:HGNC
Symbol;Acc:HGNC:42392]" -	85	80	84	137	131
161	1.223526274	1.114248655	1.53621437
2.237681655	2.366924181	2.200506141
ENSG00000100439 293.042062	325.6782163	385.9472278
481.7253501	399.5221399	560.7153794	334.8891687
480.6542898	-0.523982893	0.023631698	0.127523217	ABHD4
14	22598237	22613215	+	4070
protein_coding	abhydrolase domain containing 4 [Source:HGNC
Symbol;Acc:HGNC:20154]	-	323	368	332	465	350
650	3.886304242	4.284299767	5.075169146
6.348492156	5.285924982	7.425914859
ENSG00000125952 786.5865875	892.9601095	892.7935871
903.3645276	1188.292993	1360.381774	857.4467614
1150.679765	-0.425043745	0.023702911	0.127868497	MAX
14	65006174	65102695	-	4430
protein_coding	MYC associated factor X [Source:HGNC
Symbol;Acc:HGNC:6913]	bHLH	867	1009	768	872	1041
1577	9.583939308	10.79229681	10.78609747
10.93766965	14.44422894	16.55232425
ENSG00000236530 0	0	0	20.7193699	5.707459141
0	0	8.808943013	-5.612629889	0.023713562
0.127886949	KPNA2P1 4	80079532	80081122
+	1591	processed_pseudogene	karyopherin subunit
alpha 2 pseudogene 1 [Source:HGNC Symbol;Acc:HGNC:52871]	-
0	0	0	20	5	0	0	0

0	0.698508764	0.193173338	0
ENSG00000165264 649.5916916	803.575599	925.3433533
591.5380106	576.4533732	588.3198289	792.8368813
585.4370709	0.436473112	0.023734518	0.127960956	NDUFB6
9	32552999	32573184	-	1388
protein_coding	NADH:ubiquinone oxidoreductase subunit B6 [Source:HGNC
Symbol;Acc:HGNC:7701]	-	716	908	796	571	505
682	25.26109893	30.99722553	35.68046029
22.85907675	22.36398911	22.84682876
ENSG00000273450 1.814501932	7.079961225	3.48747495
15.53952742	10.27342645	43.99459131	4.127312702
23.26918173	-2.494216254	0.023751084	0.12801125
AL139124.1	10	94314907	94315327	-	421
antisense	"novel transcript, antisense to PLCE1"	-
2	8	3	15	9	51	0.232635819
0.900397607	0.443349278	1.979799485	1.314037544
5.632732129
ENSG00000225282 8.165258694	6.194966072	6.974949899	0
0	0	7.111724888	0	5.263328953
0.02376471	1	AP000350.2	22	23926900
23929574	+	1763	processed_pseudogene	kelch-like 5
(Drosophila) (KLHL5) pseudogene -	9	7	6	0
0	0	0.249987838	0.188136114	0.211741402
0	0	0
ENSG00000169221 1604.019708	1793.885175	1908.811289
1820.196646	2644.836566	2438.680581	1768.905391
2301.237931	-0.37976266	0.023808552	0.128281898
TBC1D10B	16	30357102	30370264	-	6699
protein_coding	TBC1 domain family member 10B [Source:HGNC
Symbol;Acc:HGNC:24510]	-	1768	2027	1642	1757	2317
2827	12.92411954	14.33739372	15.25000625
14.5738343	21.26000764	19.62216163
ENSG00000008311 388.3034134	279.6584684	223.1983968
233.0929114	159.8088559	160.4508624	297.0534262
184.4508766	0.689339033	0.023826123	0.128331604	AASS
7	122075647	122144280	-	4591
protein_coding	aminoadipate-semialdehyde synthase [Source:HGNC
Symbol;Acc:HGNC:17366]	-	428	316	192	225	140
186	4.565256251	3.26141625	2.601960999
2.723248474	1.874425152	1.883808142
ENSG00000121579 6570.311496	6933.05203	5513.697895
7769.763712	7302.123225	8365.873461	6339.020474
7812.586799	-0.301424149	0.023837725	0.128331604	NAA50
3	113716460	113746300	-	6436
protein_coding	"N(alpha)-acetyltransferase 50, NatE catalytic subunit
[Source:HGNC Symbol;Acc:HGNC:29533]"	-	7242	7834	4743
7500	6397	9698	55.10248292	57.67584587
45.8504852	64.752609	61.09528225	70.06436051
ENSG00000163947 284.8768033	280.5434635	237.1482966
557.3510503	339.023073	328.6654763	267.5228545

408.3465332	-0.608748016	0.023839542	0.128331604
ARHGEF3 3	56727418	57079329	-	6594
protein_coding	Rho guanine nucleotide exchange factor 3 [Source:HGNC
Symbol;Acc:HGNC:683]	-	314	317	204	538	297
	381	2.331897135	2.277911044	1.924810908
4.533622724	2.768565908	2.686624996
ENSG00000108786 118.8498765	139.8292342	119.7366399
178.1865811	175.7897415	273.4565774	126.1385836
209.1443	-0.730916947	0.023850073	0.128349239
HSD17B1 17	42549214	42555213	+	5658
protein_coding	hydroxysteroid 17-beta dehydrogenase 1 [Source:HGNC
Symbol;Acc:HGNC:5210]	-	131	158	103	172	154
	317	1.133801523	1.323185048	1.132611921
1.689186288	1.673035429	2.605117626
ENSG00000146109 655.0351974	819.5055118	712.6073814
831.8827014	1021.635186	1055.870191	729.0493635
969.7960263	-0.41163759	0.023919384	0.128683084	ABT1
	6	26596952	26600744	+	2949
protein_coding	activator of basal transcription 1 [Source:HGNC
Symbol;Acc:HGNC:17369]	-	722	926	613	803	895
	1224	11.98922494	14.8786201	12.93279962
15.13048385	18.65501588	19.29912697
ENSG00000101440 0	19.46989337	6.974949899	0	0
	0	8.814947756	0	5.574294817	0.023980788
	0.128936501	ASIP	20	34194569	34269344
	+	778	protein_coding	agouti signaling protein
[Source:HGNC Symbol;Acc:HGNC:745]	-	0	22	6
	0	0	0	0	1.339891169	0.47982017
	0	0	0
ENSG00000137094 202.3169654	144.25421	231.3358383
122.2442824	67.34801786	133.709052	192.6356712
107.7671174	0.832878196	0.023981263	0.128936501	DNAJB5
	9	34989641	34998900	+	5891
protein_coding	DnaJ heat shock protein family (Hsp40) member B5
[Source:HGNC Symbol;Acc:HGNC:14887]	-	223	163	199
	118	59	155	1.853721657	1.311067411
2.101700852	1.113025279	0.615616638	1.223414697
ENSG00000176531 124.2933823	192.9289434	167.3987976
130.5320304	351.5794831	392.5007656	161.5403744
291.5374263	-0.852552864	0.023988356	0.128936501	PHLDB3
	19	43474954	43504935	-	4553
protein_coding	pleckstrin homology like domain family B member 3
[Source:HGNC Symbol;Acc:HGNC:30499]	-	137	218	144
	126	308	455	1.473504954	2.268742904
1.967758008	1.537747177	4.158152628	4.646701391
ENSG00000198900 3807.732304	3116.952929	3923.409318
2977.373454	2950.756376	2802.714258	3616.031517
2910.281363	0.313104354	0.024012911	0.129013326	TOP1
	20	41028818	41124487	+	3738
protein_coding	DNA topoisomerase I [Source:HGNC

Symbol;Acc:HGNC:11986]	-	4197	3522	3375	2874	2585
	3249	54.98299031	44.64537708	56.17477308
42.72278055	42.50779824	40.41489292
ENSG00000164929 47.17705023	57.52468495	77.88694054
20.7193699	25.11282022	28.46708849	60.86289191
24.7664262	1.292680611	0.024017236	0.129013326	BAALC
	8	103140710	103230305	+	3997
protein_coding	"BAALC, MAP3K1 and KLF4 binding [Source:HGNC
Symbol;Acc:HGNC:14333]"	-	52	65	67	20	22
	33	0.637085732	0.77055856	1.042911607
0.278040391	0.338326404	0.383893545
ENSG00000279583 3.629003864	5.309970919	12.78740815	0
	0	0	7.242127644	0	5.285069242
0.024026791	1	AC009086.3	16	29595130
29596137	-	1008	TEC	novel transcript	-
	4	6	11	0	0	0	0.194324761
	0.282044191	0.678951887	0	0	0
ENSG00000171853 605.1363943	673.4813115	621.9330327
679.5953327	990.8149069	908.3589147	633.5169128
859.5897181	-0.439972827	0.024049051	0.129145012
TRAPPC12	2	3379675 3485094 +	9193	protein_coding
	trafficking protein particle complex 12 [Source:HGNC
Symbol;Acc:HGNC:24284]	-	667	761	535	656	868
	1053	3.55301677	3.922417677	3.620790261
3.965140886	5.803765082	5.32601186
ENSG00000182197 1599.483453	1708.925641	1483.339345
1606.787136	2324.077362	2348.96612	1597.24948
2093.276873	-0.390002064	0.024075525	0.129247937	EXT1
	8	117794490	118111853	-	8325
protein_coding	exostosin glycosyltransferase 1 [Source:HGNC
Symbol;Acc:HGNC:3512]	-	1763	1931	1276	1551	2036
	2723	10.37042974	10.9906777	9.536154503
10.35236556	15.03283503	15.20878139
ENSG00000111276 578.8261163	608.8766653	753.2945891
343.9415403	445.181813	564.1659356	646.9991236
451.0964296	0.518225813	0.024101819	0.12934984	CDKN1B
	12	12715058	12722371	+	2811
protein_coding	cyclin dependent kinase inhibitor 1B [Source:HGNC
Symbol;Acc:HGNC:1785]	-	638	688	648	332	390
	654	11.11446383	11.59722367	14.3423728
6.562801697	8.52807717	10.81802351
ENSG00000255857 12.70151352	46.90474312	46.499666
12.43162194	7.990442797	7.763751407	35.36864088
9.395272048	1.911819237	0.024150103	0.129532825	PXN-
AS1	12	120201291	120213138	+	2380
antisense	PXN antisense RNA 1 [Source:HGNC
Symbol;Acc:HGNC:44123]	-	14	53	40	12	7
	9	0.288057881	1.05517709	1.045658521
0.280166583	0.180787518	0.175831455
ENSG00000163517 88.0033437	59.29467526	125.5490982

52.83439324	42.23519764	28.46708849	90.94903905
41.17889313	1.143527556	0.02415056	0.129532825	HDAC11
	3	13479724	13506424	+	5281
protein_coding	histone deacetylase 11 [Source:HGNC
Symbol;Acc:HGNC:19086]	-	97	67	108	51	37
	33	0.899464962	0.601153079	1.27237297
0.536619008	0.430658394	0.290555292
ENSG00000122778 571.5681086	565.5119028	508.008851
498.300846	316.1932364	326.9401982	548.3629541
380.4780935	0.527972048	0.024166586	0.129579492
KIAA1549	7	138831381	138981318	-	12427
	protein_coding	KIAA1549 [Source:HGNC Symbol;Acc:HGNC:22219]
	-	630	639	437	481	277	379
2.482578184	2.43646962	2.187872378	2.150754408
1.37012702	1.418090442
ENSG00000234405 9.979760626	4.424975766	6.974949899	0
	0	0	7.126562097	0	5.266220442
0.024210777	1	Z69733.1	X	103497523
103500317	+	632	antisense	novel transcript
	-	11	5	6	0	0	0
0.852323162	0.374868861	0.590664703	0	0	0
ENSG00000114790 323.8885949	352.2280709	285.9729459
246.5605018	180.3557089	216.5224004	320.6965372
214.479537	0.580475245	0.024229378	0.12987681
ARHGEF26	3	154121003	154257827	+	5489
	protein_coding	Rho guanine nucleotide exchange factor 26
[Source:HGNC Symbol;Acc:HGNC:24490]	-	357	398	246
	238	158	251	3.184957701	3.435708285
2.788359223	2.409327124	1.769339676	2.126236204
ENSG00000152240 713.0992593	811.5405554	852.1063794
695.1348601	448.6062885	576.2428822	792.2487313
573.3280103	0.465583266	0.024314405	0.130293098	HAUS1
	18	46104332	46128333	+	2165
protein_coding	HAUS augmin like complex subunit 1 [Source:HGNC
Symbol;Acc:HGNC:25174]	-	786	917	733	671	393
	668	17.77842684	20.06956114	21.06458564
17.22172552	11.15788831	14.34661949
ENSG00000198382 663.2004561	659.3213891	631.2329659
879.5372522	752.2431148	940.2765594	651.2516037
857.3523088	-0.397157785	0.024327946	0.130326175	UVRAG
	11	75815167	76143195	+	6352
protein_coding	UV radiation resistance associated [Source:HGNC
Symbol;Acc:HGNC:12640]	-	731	745	543	849	659
	1090	5.635540447	5.557407261	5.318586009
7.426928526	6.37708617	7.978973573
ENSG00000158555 406.4484328	424.7976735	511.496326
523.1640899	623.2545382	716.8530466	447.5808107
621.0905582	-0.474545747	0.024378385	0.130556842	GDPD5
	11	75434640	75525903	-	9867
protein_coding	glycerophosphodiester phosphodiesterase domain

containing 5 [Source:HGNC Symbol;Acc:HGNC:28804] - 448
480 440 505 546 831 2.223420315
2.305061675 2.77443398 2.843926012 3.401377815
3.916038147
ENSG00000133678 198.6879615 199.1239095 192.9736139
211.337573 334.4571057 378.6985409 196.928495
308.1644065 -0.646637799 0.024406365 0.130667126
TMEM254 10 80078646 80092557 + 2996
protein_coding transmembrane protein 254 [Source:HGNC
Symbol;Acc:HGNC:25804] - 219 225 166 204 293
439 3.579571069 3.558501473 3.447252741
3.783558054 6.011366012 6.813240657
ENSG00000218725 0 0 0 8.287747959 0
17.25278091 0 8.513509622 -5.57015048 0.024422042
0.130711497 B3GALNT2P1 6 129800908
129802214 + 1295 processed_pseudogene "beta-1,3-N-
acetylgalactosaminyltransferase 2 pseudogene 1 [Source:HGNC
Symbol;Acc:HGNC:51315]" - 0 0 0 8 0
20 0 0 0 0.343267164 0
0.718110448
ENSG00000232821 9.979760626 7.964956378 3.48747495 0
0 0 7.144063985 0 5.272770272
0.02442739 1 AC003986.2 7 19112474
19114271 + 1711 antisense novel transcript
- 11 9 3 0 0 0
0.314826556 0.249240688 0.109088279 0 0 0
ENSG00000254302 9.979760626 7.964956378 3.48747495 0
0 0 7.144063985 0 5.272770272
0.02442739 1 AC087855.2 8 34174886
34184895 + 537 lincRNA novel transcript -
11 9 3 0 0 0 1.003106589
0.794135599 0.347579229 0 0 0
ENSG00000176887 1362.690951 1443.427095 1608.888443
880.5732207 1275.046372 1190.441882 1471.66883
1115.353825 0.399664663 0.024512247 0.131140266 SOX11
2 5692667 5701385 + 8719 protein_coding SRY-
box 11 [Source:HGNC Symbol;Acc:HGNC:11191] HMG 1502 1631
1384 850 1117 1380 8.435910016 8.863672971
9.875890342 5.417068055 7.874697064 7.359417258
ENSG00000068885 329.3321007 226.5587592 203.4360387
176.1146441 124.4226093 167.3519748 253.1089662
155.9630761 0.698401516 0.024521734 0.131140266 IFT80
3 160256986 160399880 - 7175
protein_coding intraflagellar transport 80 [Source:HGNC
Symbol;Acc:HGNC:29262] - 363 256 175 170 109
194 2.477498517 1.690614154 1.517480049
1.31655516 0.933795878 1.25721873
ENSG00000120948 2700.886126 2608.080716 2613.281229
2251.159539 2101.486456 2133.306359 2640.749357
2161.984118 0.288616186 0.024524394 0.131140266 TARDBP

1	11012344	11026420	+	6071
protein_coding	TAR DNA binding protein [Source:HGNC
Symbol;Acc:HGNC:11571]	-	2977	2947	2248	2173	1841
2473	24.0130478	23.00098018	23.03790169
19.8889354	18.63978574	18.94065099
ENSG00000185238 638.70468	553.1219707	560.3209752
467.2217912	454.3137476	337.2918667	584.0492087
419.6091352	0.478766591	0.024609942	0.13155795	PRMT3
11	20387530	20509294	+	5467
protein_coding	protein arginine methyltransferase 3 [Source:HGNC
Symbol;Acc:HGNC:30163]	-	704	625	482	451	398
391	6.305975351	5.416981898	5.485355904
4.583946198	4.474879637	3.325513396
ENSG00000008735 9.07250966	7.964956378	2.3249833
33.15099184	6.848950969	52.62098176	6.454149779
30.87364152	-2.2473059	0.024631929	0.131592808
MAPK8IP2	22	50600685	50613981	+	5901
protein_coding	mitogen-activated protein kinase 8 interacting
protein 2 [Source:HGNC Symbol;Acc:HGNC:6883]	-	10	9
2	32	6	61	0.082985663	0.072267551
0.021086827	0.301325862	0.06249899	0.480656963
ENSG00000220472 33.56828574	38.93978674	34.8747495
73.55376314	68.48950969	87.98918262	35.79427399
76.67748515	-1.099840888	0.024634088	0.131592808
AL139095.2	6	7338839 7339568 -	730
processed_pseudogene	ribosomal protein S3 (RPS3) pseudogene	-
37	44	30	71	60	102	2.482032978
2.855987203	2.556849945	5.404400584	5.052144347
6.496932127
ENSG00000257242 13.60876449	14.15992245	16.2748831
31.07905485	13.69790194	107.8298807	14.68119001
50.86894581	-1.800530156	0.02463878	0.131592808
LINC01619	12	91984976	92142914	-	3642
processed_transcript	long intergenic non-protein coding RNA
1619 [Source:HGNC Symbol;Acc:HGNC:27409]	-	15	16
14	30	12	125	0.201687973	0.208164411
0.239163522	0.457713115	0.202529675	1.595883151
ENSG00000095261 931.7467421	952.2547848	1029.967602
1218.29895	1201.990895	1270.667314	971.3230429
1230.319053	-0.341666727	0.024678105	0.131763057	PSMD5
9	120815496	120842984	-	4432
protein_coding	"proteasome 26S subunit, non-ATPase 5 [Source:HGNC
Symbol;Acc:HGNC:9563]"	-	1027	1076	886	1176	1053
1473	11.34747868	11.50373743	12.43772118
14.74414569	14.60413973	15.45375466
ENSG00000108256 4428.291965	3973.628238	3756.010521
4787.210415	4849.057286	5187.048579	4052.643574
4941.105427	-0.285874507	0.02472647	0.131981448	NUFIP2
17	29255836	29294118	-	10850
protein_coding	"NUFIP2, FMR1 interacting protein 2 [Source:HGNC

Symbol;Acc:HGNC:17634]" -	4881	4490	3231	4621	4248
	6013	22.0296579	19.60844	18.52738245
23.6656411	24.06590124	25.76869848
ENSG00000224330 0	0	0	18.64743291	0
6.901112362	0	8.516181757	-5.567174369	0.024840794
	0.132551673	AC005019.2	7	13854355
13859025	-	359	lincRNA novel transcript	-
	0	0	0	18	0	8	0	0
	0	2.786057659	0	1.036159365
ENSG00000157315 17.23776835	41.5947722	27.8997996
68.37392066	150.6769213	25.01653231	28.91078005
81.35579143	-1.487611927	0.024851203	0.132567226	TMED6
	16	69343248	69351809	-	1411
protein_coding	transmembrane p24 trafficking protein 6 [Source:HGNC
Symbol;Acc:HGNC:28331]	-	19	47	24	66	132
	29	0.659409608	1.578328087	1.058256817
2.599135766	5.750349979	0.955656905
ENSG00000129003 1212.087291	739.855948	861.4063126
781.1202452	679.1876378	523.6219005	937.7831837
661.3099278	0.504928446	0.024868506	0.132619536	VPS13C
	15	61852389	62060473	-	19703
protein_coding	vacuolar protein sorting 13 homolog C [Source:HGNC
Symbol;Acc:HGNC:23594]	-	1336	836	741	754	595
	607	3.320494646	2.010482795	2.339875215
2.126429713	1.856230775	1.432475991
ENSG00000198833 1161.281236	1097.39399	1122.966934
972.7744167	912.0519707	612.4737221	1127.214053
832.4333699	0.438530804	0.024877402	0.132626994	UBE2J1
	6	89326625	89352848	-	4290
protein_coding	ubiquitin conjugating enzyme E2 J1 [Source:HGNC
Symbol;Acc:HGNC:17598]	-	1280	1240	966	939	799
	710	14.61104778	13.69590812	14.00963049
12.16242972	11.44819049	7.695415516
ENSG00000197859 46.26979926	26.54985459	11.6249165	0
	11.41491828	0	28.14819012	3.804972761
2.92661563	0.025021673	0.133355942	ADAMTSL2	9
	133532164	133575519	+	4677	protein_coding
	ADAMTS like 2 [Source:HGNC Symbol;Acc:HGNC:14631]	-
	51	30	10	0	10	0	0.53398799
	0.303934728	0.133026902	0	0.131425607	0
ENSG00000183309 1244.748325	1412.452264	1041.592518
1536.341278	1607.220494	1610.547098	1232.931036
1584.702957	-0.361258151	0.025074958	0.13359968	ZNF623
	8	143636013	143656418	+	7146
protein_coding	zinc finger protein 623 [Source:HGNC
Symbol;Acc:HGNC:29084]	zf-C2H2 1372	1596	896	1483	1408
	1867	9.401989959	10.58269588	7.801028138
11.53161629	12.11119518	12.14821094
ENSG00000159176 3923.860428	4753.308967	4973.139278
5124.936144	5812.476389	6003.105116	4550.102891

5646.839216	-0.311764169	0.025087273	0.133625042	CSRP1
	1	201483530	201509456	-	10318
protein_coding	cysteine and glycine rich protein 1 [Source:HGNC
Symbol;Acc:HGNC:2469]	-	4325	5371	4278	4947	5092
	6959	20.52670646	24.66528112	25.79598426
26.6414851	30.33473684	31.36045326
ENSG00000198586 1145.85797	861.9852791	1005.555277
840.1704494	633.5279646	774.6498627	1004.466175
749.4494256	0.421911334	0.025101194	0.133635163	TLK1
	2	170990823	171231314	-	9405
protein_coding	tousled like kinase 1 [Source:HGNC
Symbol;Acc:HGNC:11841]	-	1263	974	865	811	555
	898	6.576173068	4.907119089	5.722214772
4.791528746	3.62728386	4.439648171
ENSG00000108262 2719.938396	2877.119243	2888.79175
2910.035502	3597.982242	4338.211759	2828.616463
3615.409834	-0.354370298	0.025104282	0.133635163	GIT1
	17	29573469	29594054	-	5635
protein_coding	GIT ArfGAP 1 [Source:HGNC Symbol;Acc:HGNC:4272] -
	2998	3251	2485	2809	3152	5029	26.05351904
	27.33691422	27.43717033	27.69936815	34.38267393
	41.49719421
ENSG00000134109 2542.117207	2523.121182	2071.56012
2441.777742	1407.459424	1250.826616	2378.932836
1700.021261	0.485198717	0.02511743	0.133664925	EDEM1
	3	5187646 5219957 +	6758	protein_coding	ER
degradation enhancing alpha-mannosidase like protein 1 [Source:HGNC
Symbol;Acc:HGNC:18967]	-	2802	2851	1782	2357	1233
	1450	20.30386079	19.98966292	16.40576019
19.3799851	11.2148185	9.976560325
ENSG00000130045 6.350756762	19.46989337	0	0	0
	0	8.606883377	0	5.543093408	0.025138828
	0.133738565	NXNL2	9	88534033	88584274
	+	2498	protein_coding	nucleoredoxin like 2
[Source:HGNC Symbol;Acc:HGNC:30482]	-	7	22	0
	0	0	0	0.137225332	0.417307978	0
	0	0	0
ENSG00000198455 406.4484328	366.3879934	433.6093854
485.8692241	648.3673584	538.2867643	402.1486039
557.5077823	-0.47144317	0.025221719	0.134111426	ZXDB
	X	57591652	57597545	+	5894
protein_coding	zinc finger X-linked duplicated B [Source:HGNC
Symbol;Acc:HGNC:13199]	zf-C2H2 448	414	373	469	568
	624	3.722173099	3.328255439	3.937363826
4.421552182	5.923597817	4.922723878
ENSG00000132801 188.7082009	163.7241033	194.1361055
248.6324388	262.5431205	315.7258906	182.1894699
275.6338166	-0.59930431	0.025224078	0.134111426	ZSWIM3
	20	45857617	45879122	+	2773
protein_coding	zinc finger SWIM-type containing 3 [Source:HGNC

Symbol;Acc:HGNC:16157]	-	208	185	167	240	230
	366	3.673179475	3.16117326	3.74691161
4.809206393	5.09829929	6.13708635
ENSG00000134070 471.7705023	506.2172276	834.6690046
381.2364061	490.8414861	343.33034	604.2189115
405.1360774	0.576171856	0.025266986	0.134299193	IRAK2
	3	10164865	10243743	+	3483
protein_coding	interleukin 1 receptor associated kinase 2
[Source:HGNC Symbol;Acc:HGNC:6113]	-	520	572	718
	368	430	398	7.311029777	7.781601654
12.8256037	5.870923046	7.588611879	5.313254464
ENSG00000123684 3038.383485	2677.995333	2853.917
2399.303034	2219.060114	2369.669457	2856.765273
2329.344202	0.294311388	0.025281028	0.134333463	LPGAT1
	1	211743457	211830772	-	8799
protein_coding	lysophosphatidylglycerol acyltransferase 1
[Source:HGNC Symbol;Acc:HGNC:28985]	-	3349	3026	2455
	2316	1944	2747	18.63848092	16.29528824
17.35901288	14.62572088	13.58032937	14.5163142
ENSG00000006534 422.7789501	361.0780225	463.8341683
393.6680281	184.9216762	206.1707318	415.897047
261.586812	0.668528558	0.025294472	0.134364537
ALDH3B1 11	68008578	68029282	+	6626
protein_coding	aldehyde dehydrogenase 3 family member B1 [Source:HGNC
Symbol;Acc:HGNC:410]	-	466	408	399	380	162
	239	3.444000205	2.917663298	3.746522203
3.186722221	1.502833762	1.677171553
ENSG00000169981 414.6136915	410.637751	394.0846693
522.1281214	650.6503421	504.6438415	406.4453706
559.1407683	-0.458865484	0.025319223	0.134455638	ZNF35
	3	44648727	44660791	+	2663
protein_coding	zinc finger protein 35 [Source:HGNC
Symbol;Acc:HGNC:13099]	zf-C2H2 457	464	339	504	570
	585	8.403761473	8.256067879	7.920185956
10.51650454	13.15682352	10.21446719
ENSG00000174891 1052.411121	814.1955409	964.8680694
825.6668904	599.2832098	676.3090115	943.8249103
700.4197039	0.42989782	0.025338071	0.134515348	RSRC1
	3	158105855	158545730	+	5117
protein_coding	arginine and serine rich coiled-coil 1 [Source:HGNC
Symbol;Acc:HGNC:24152]	-	1160	920	830	797	525
	784	11.10123397	8.519200729	10.09182061
8.654758383	6.306541336	7.124127179
ENSG00000261592 1.814501932	10.61994184	9.299933199	0
	0	0	7.244792323	0	5.288706047
0.025347733	1	AC010531.3	16	87396704
87400295	-	513	antisense	"novel transcript,
antisense to MAP1LC3B"	-	2	12	8	0	0
	0	0.190915555	1.108384188	0.970240655	0
	0	0

ENSG00000136861 1434.363777 1388.557395 1624.000835
1309.464178 1189.434485 763.4355551 1482.307336
1087.444739 0.447553031 0.02535911 0.134586646
CDK5RAP2 9 120388869 120580170 - 10080
protein_coding CDK5 regulatory subunit associated protein 2
[Source:HGNC Symbol;Acc:HGNC:18672] - 1581 1569 1397
1264 1042 885 7.680686188 7.37545559
8.622688964 6.967846673 6.354107339 4.082383093
ENSG00000121940 1087.793908 1031.019353 903.256012
812.1993 829.8645591 652.1551182 1007.356425
764.7396591 0.399035688 0.025397216 0.13471322 CLCC1
1 108929508 108963457 - 4964
protein_coding chloride channel CLIC like 1 [Source:HGNC
Symbol;Acc:HGNC:29675] - 1199 1165 777 784 727
756 11.82813013 11.12040472 9.738590233
8.77599432 9.002227794 7.081431211
ENSG00000227036 148.7891584 105.3144232 189.4861389
117.0644399 54.79160775 54.34625985 147.8632402
75.40076918 0.970637024 0.02539819 0.13471322
LINC00511 17 72290091 72640472 - 27605
processed_transcript long intergenic non-protein coding RNA
511 [Source:HGNC Symbol;Acc:HGNC:43564] - 164 119 163
113 48 63 0.290927503 0.204261093
0.367372547 0.227458796 0.106881083 0.10611672
ENSG00000128335 202.3169654 307.0933181 275.510521
338.7616978 438.332862 386.4622923 261.6402682
387.852284 -0.56790926 0.025420378 0.134790489 APOL2
22 36226203 36239954 - 3484
protein_coding apolipoprotein L2 [Source:HGNC Symbol;Acc:HGNC:619]
- 223 347 237 327 384 448
3.134407085 4.719301994 4.232305865 5.215328275
6.774861765 5.97903211
ENSG00000248749 11.79426256 0 13.9498998 0 0
0 8.581387452 0 5.531350452 0.025447752
0.13485785 AC104063.1 4 84371393
84380189 - 702 lincRNA novel transcript -
13 0 12 0 0 0 0.906848886
0 1.063533026 0 0 0
ENSG00000101343 1102.309924 1037.214319 1070.65481
1344.687106 1338.969914 1342.266354 1070.059684
1341.974458 -0.326696547 0.025452115 0.13485785 CRNKL1
20 20034368 20056046 - 4567
protein_coding crooked neck pre-mRNA splicing factor 1 [Source:HGNC
Symbol;Acc:HGNC:15762] - 1215 1172 921 1298 1173
1556 13.02788601 12.15970506 12.54687193
15.79267596 15.78753625 15.84198505
ENSG00000176845 633.2611743 700.0311661 1407.777388
513.8403735 762.5165412 500.3306463 913.6899095
592.229187 0.625024549 0.025455953 0.13485785 METRNL
17 83079691 83095119 + 2626

protein_coding "meteorin like, glial cell differentiation regulator [Source:HGNC Symbol;Acc:HGNC:27584]" - 698 791 1211
496 668 580 13.01635499 14.27276787
28.69169913 10.49540008 15.63612382 10.26985448
ENSG00000164307 699.4904948 608.8766653 797.4692718
559.4229872 553.6235367 414.0667417 701.9454773
509.0377552 0.464008449 0.025490189 0.13499879 ERAP1
5 96760810 96808100 - 7945
protein_coding endoplasmic reticulum aminopeptidase 1 [Source:HGNC Symbol;Acc:HGNC:18173] - 771 688 686 540 485
480 4.752139271 4.103183858 5.372013072
3.776694901 3.752279643 2.809172149
ENSG00000236801 65.32206955 54.86969949 63.93704074
159.5391482 89.0363626 104.3793245 61.37626993
117.6516118 -0.939727904 0.025516361 0.135096949
RPL24P8 9 70217195 70217668 + 474
processed_pseudogene ribosomal protein L24 pseudogene 8
[Source:HGNC Symbol;Acc:HGNC:36409] - 72 62 55
154 78 121 7.438456683 6.197831838
7.219235254 18.05320953 10.11494723 11.86965365
ENSG00000242759 37.19728961 61.06466557 24.41232465
101.5249125 66.20652603 104.3793245 40.89142661
90.70358767 -1.144206954 0.025537027 0.135165908
LINC00882 3 106836811 107240641 - 3882
lincRNA long intergenic non-protein coding RNA 882
[Source:HGNC Symbol;Acc:HGNC:48568] - 41 69 21
98 58 121 0.517198205 0.842209237
0.336566286 1.402757464 0.918374513 1.44930856
ENSG00000166682 22.68127415 3.539980612 0 0 0
0 8.740418254 0 5.564518905 0.025570776
0.135304056 TMPRSS5 11 113687546 113706373
- 2666 protein_coding transmembrane serine protease 5 [Source:HGNC Symbol;Acc:HGNC:14908] - 25 4 0
0 0 0 0.45920705 0.071092909 0
0 0 0
ENSG00000146457 1592.225445 1592.10628 1613.53841
1885.462661 1937.111632 2107.427188 1599.290045
1976.66716 -0.305936482 0.025587902 0.135354186 WTAP
6 159725585 159756319 + 6419
protein_coding WT1 associated protein [Source:HGNC Symbol;Acc:HGNC:16846] - 1755 1799 1388 1820 1697
2443 13.38870056 13.27976007 13.45330342
15.75491469 16.25031661 17.69648896
ENSG00000242296 0 0 0 0 31.96177119 0
0 10.65392373 -5.884819423 0.025608101
0.135418577 DEFB109A 8 12393209 12400366
- 265 unprocessed_pseudogene defensin beta 109A (pseudogene) [Source:HGNC Symbol;Acc:HGNC:18090] - 0
0 0 0 28 0 0 0 0
0 6.494706318 0

ENSG00000259776 4.53625483	0	0	15.53952742
26.25431205	7.763751407	1.512084943
16.51919696	-3.411542728	0.025615386	0.135418577
AC093426.1	1	239247808	239250818	-	3011
	lincRNA novel transcript	-	5	0	0
	15	23	9	0.081318233	0	0
0.276816866	0.469531183	0.138983349
ENSG00000266970 0	0	0	0	14.83939377
10.35166854	0	8.39702077	-5.545030644	0.025654579
	0.135585253	AC061992.1	17	78360453
78373911	+	1188	lincRNA uncharacterized LOC101928674
[Source:NCBI gene;Acc:101928674]	-	0	0	0
	0	13	12	0	0	0	0
0.672626962	0.469673247
ENSG00000143157 2640.100311	2471.791463	2176.184369
2916.251313	3095.725838	2979.555262	2429.358714
2997.177471	-0.302574191	0.025698652	0.135777613	POGK
	1	166839447	166856344	+	8448
protein_coding	pogo transposable element derived with KRAB domain
[Source:HGNC Symbol;Acc:HGNC:18800]	-	2910	2793	1872
	2815	2712	3454	16.86816216	15.66547152
13.78665113	18.51554661	19.73254674	19.01075856
ENSG00000250497 5.443505796	3.539980612	0	4.14387398
	43.37668947	18.978059	2.994495469	22.16620748
	-2.855487372	0.02571696	0.135833774	AC007126.1
	4	13777377	13782157	-	4781
lincRNA novel transcript	-	6	4	0	4
	38	22	0.061455561	0.039643107	0
0.04648933	0.488553595	0.213961166
ENSG00000234616 1078.721399	993.849557	852.1063794
1125.061785	1295.593225	1378.497194	974.892445
1266.384068	-0.376868552	0.02572599	0.135840905	JRK
	8	142657460	142681968	-	11229
protein_coding	Jrk helix-turn-helix protein [Source:HGNC
Symbol;Acc:HGNC:6199]	-	1189	1123	733	1086	1135
	1598	5.185247089	4.738764379	4.061343656
5.374038669	6.213011249	6.617084967
ENSG00000013619 459.0689888	359.3080322	382.4597528
287.9992416	324.1836792	168.2146138	400.2789246
260.1325115	0.624783653	0.025753107	0.135943513	MAMLD1
	X	150361422	150514178	+	5958
protein_coding	mastermind like domain containing 1 [Source:HGNC
Symbol;Acc:HGNC:2568]	-	506	406	329	278	284
	195	4.158902141	3.228880525	3.435597244
2.592724316	2.92998368	1.521826459
ENSG00000162430 2161.071801	2031.063876	2211.059118
1793.261465	1594.664084	1783.074907	2134.398265
1723.666818	0.307900095	0.025763864	0.135951035
SELENON 1	25800176	25818224	+	4424
protein_coding	selenoprotein N [Source:HGNC Symbol;Acc:HGNC:15999]

-	2382	2295	1902	1731	1397	2067
26.36667235	24.58068676	26.74867296	21.74172475
19.41013911	21.72482949
ENSG00000173209 333.8683555	307.9783133	184.8361723
158.5031797	179.214217	181.1541995	275.560947
172.9571988	0.6740864	0.025769903	0.135951035	AHSA2P
2	61177418	61191203	+	9083
transcribed_unitary_pseudogene	"activator of HSP90 ATPase homolog 2,
pseudogene [Source:HGNC Symbol;Acc:HGNC:20437]" -	368	348
159	153	157	210	1.984025218	1.815416886
1.0891173	0.935996361	1.062472501	1.075031027
ENSG00000131196 245.8650118	144.25421	256.9106546
162.6470537	106.15874	108.6925197	215.6766255
125.8327711	0.776228399	0.025812751	0.13611101	NFATC1
18	79395856	79529325	+	9068
protein_coding	nuclear factor of activated T cells 1 [Source:HGNC
Symbol;Acc:HGNC:7775]	RHD	271	163	221	157	93
126	1.463478892	0.851731156	1.516308637
0.962055628	0.630403764	0.646085585
ENSG00000033178 1936.980812	1385.017415	1634.46326
1263.881564	1378.922128	1180.090214	1652.153829
1274.297969	0.375028128	0.02582191	0.13611101	UBA6
4	67612652	67701179	-	11432
protein_coding	ubiquitin like modifier activating enzyme 6
[Source:HGNC Symbol;Acc:HGNC:25581]	-	2135	1565	1406
1220	1208	1368	9.145434573	6.486621645
7.65191173	5.929931253	6.495193274	5.564099655
ENSG00000106400 1316.421152	1672.640839	1603.075985
1749.750788	1880.037041	2294.61986	1530.712659
1974.802563	-0.368076216	0.025827821	0.13611101	ZNHIT1
7	101217668	101224190	+	5510
protein_coding	zinc finger HIT-type containing 1 [Source:HGNC
Symbol;Acc:HGNC:21688]	-	1451	1890	1379	1689	1647
2660	12.8956874	16.25311642	15.57110789
17.03295874	18.37339283	22.4471421
ENSG00000102921 537.9998228	560.2019319	585.8957915
766.6166862	667.7727195	790.1773655	561.3658488
741.5222571	-0.402558313	0.025831006	0.13611101	N4BP1
16	48538726	48620148	-	7839
protein_coding	NEDD4 binding protein 1 [Source:HGNC
Symbol;Acc:HGNC:29850]	-	593	633	504	740	585
916	3.704441258	3.826216026	4.000154067
5.245454194	4.587145987	5.433326799
ENSG00000267565 4.53625483	19.46989337	17.43737475
3.107905485	0	0	13.81450765	1.035968495
3.751442672	0.025872101	0.136286953	AC011477.3	19
19892433	19895847	-	403	antisense
"novel transcript, antisense to ZNF253" -	5	22
15	3	0	0	0.607566251	2.586688162
2.315757395	0.413645451	0	0

ENSG00000267321 200.5024635 166.3790888 155.7738811
93.23716454 114.1491828 107.8298807 174.2184778
105.072076 0.730709372 0.025880213 0.136289098
LINC02001 17 35568109 35574843 + 1504
lincRNA long intergenic non-protein coding RNA 2001
[Source:HGNC Symbol;Acc:HGNC:52836] - 221 188 134
90 100 125 7.195701199 5.922928007
5.543241615 3.325115358 4.086951876 3.864498961
ENSG00000254635 387.3961625 331.8731824 288.2979292
361.5530047 530.7937001 584.8692727 335.855758
492.4053258 -0.551410495 0.025894034 0.136321297 WAC-
AS1 10 28522652 28532743 - 2943
antisense WAC antisense RNA 1 (head to head) [Source:HGNC
Symbol;Acc:HGNC:27347] - 427 375 248 349 465
678 7.105036224 6.037643228 5.242860053
6.589420285 9.712030799 10.71199719
ENSG00000251191 5.443505796 0.884995153 16.2748831 0
0 0 7.534461349 0 5.340287413
0.025937662 1 LINC00589 8 29673922
29748109 - 2074 lincRNA long intergenic non-protein
coding RNA 589 [Source:HGNC Symbol;Acc:HGNC:32299] - 6
1 14 0 0 0 0.141667811
0.022846395 0.419977603 0 0 0
ENSG00000198590 5.443505796 4.424975766 3.48747495
7.251779464 29.67878753 31.91764468 4.451985504
22.94940389 -2.360597648 0.025962425 0.136625691
C3orf35 3 37386269 37435497 + 3072
processed_transcript chromosome 3 open reading frame 35
[Source:HGNC Symbol;Acc:HGNC:24082] - 6 5 3
7 26 37 0.095644218 0.077121458
0.060758479 0.126616082 0.520234916 0.560030308
ENSG00000130695 1091.422912 953.1397799 918.3684034
676.4874272 886.9391505 665.0947039 987.6436985
742.8404272 0.412280666 0.0259673 0.136625691 CEP85
1 26234200 26278808 + 5145
protein_coding centrosomal protein 85 [Source:HGNC
Symbol;Acc:HGNC:25309] - 1203 1077 790 653 777
771 11.45009083 9.91874591 9.553193155
7.05244724 9.282885633 6.967869642
ENSG00000160298 423.6862011 440.7275863 398.7346359
217.5533839 175.7897415 34.50556181 421.0494744
142.6162291 1.563368984 0.026053583 0.137011539
C21orf58 21 46300181 46323875 - 5315
protein_coding chromosome 21 open reading frame 58
[Source:HGNC Symbol;Acc:HGNC:1300] - 467 498 343
210 154 40 4.302712173 4.439688651
4.01511231 2.195472843 1.781003661 0.349935289
ENSG00000137700 861.8884177 867.2952501 732.3697394
634.0127189 582.1608324 655.6056744 820.5178024
623.9264086 0.39536817 0.026057479 0.137011539

SLC37A4 11	119024114	119030906	-	6186
protein_coding	solute carrier family 37 member 4 [Source:HGNC
Symbol;Acc:HGNC:4061]	-	950	980	630	612	510
	760	7.520424804	7.506588357	6.336325519
5.497352051	5.067661764	5.712611565
ENSG00000171497 780.2358307	814.1955409	759.1070474
644.3724038	521.6617655	615.9242783	784.5128063
593.9861492	0.400950846	0.026063872	0.137011539	PPID
	4	158709134	158723396	-	2509
protein_coding	peptidylprolyl isomerase D [Source:HGNC
Symbol;Acc:HGNC:9257]	-	860	920	653	622	457
	714	16.78519819	17.37455167	16.19270361
13.77532224	11.19600024	13.23209373
ENSG00000122257 1486.077082	1122.173854	1177.604041
1144.745187	827.5815754	854.8752939	1261.951659
942.4006854	0.421525832	0.026113844	0.137233446	RBBP6
	16	24537693	24572863	+	10475
protein_coding	"RB binding protein 6, ubiquitin ligase [Source:HGNC
Symbol;Acc:HGNC:9889]"	-	1638	1268	1013	1105	725
	991	7.657527221	5.735769136	6.016754069
5.861655492	4.254331576	4.398966361
ENSG00000279530 4.53625483	13.2749273	3.48747495	0
	0	0	7.099552359	0	5.264044883
0.026131524	1	AC092881.1	12	70321542
70324274	-	2733	TEC	TEC	-	5	15
	3	0	0	0	0.089589901	0.260062701
	0.068294931	0	0	0
ENSG00000174353 79.83808501	49.55972857	31.38727455
32.11502334	11.41491828	10.35166854	53.59502938
17.96053672	1.582544193	0.026139443	0.137327179
STAG3L3 7	72969696	73005922	-	3644
transcribed_unprocessed_pseudogene	stromal antigen 3-like 3
(pseudogene) [Source:HGNC Symbol;Acc:HGNC:33845]	-	88
	56	27	31	10	12	1.182586692
0.728175562	0.460990783	0.472710631	0.168682097
0.153120696
ENSG00000198040 778.4213288	715.9610789	582.4083166
1168.572462	749.9601311	941.1391984	692.2635748
953.2239306	-0.460908775	0.026152257	0.137353704	ZNF84
	12	133037292	133063304	+	9520
protein_coding	zinc finger protein 84 [Source:HGNC
Symbol;Acc:HGNC:13159]	zf-C2H2 858	809	501	1128	657
	1091	4.413458254	4.026595595	3.274218244
6.58391469	4.242049983	5.328669936
ENSG00000159322 921.7669814	991.1945715	1006.717769
690.9909861	809.3177062	759.9849989	973.2264406
753.4312304	0.369289228	0.026177514	0.137445547	ADPGK
	15	72751369	72785846	-	5931
protein_coding	ADP dependent glucokinase [Source:HGNC
Symbol;Acc:HGNC:25250]	-	1016	1120	866	667	709

881	8.388696218	8.947805588	9.084411839
6.248991782	7.347941184	6.90683375
ENSG00000150768 2220.950365	1987.699114	2285.458584
1783.937748	1845.792286	1573.453619	2164.702687
1734.394551	0.319920855	0.026201732	0.137509093	DLAT
11	112024814	112064390	+	4831
protein_coding	dihydrolipoamide S-acetyltransferase [Source:HGNC
Symbol;Acc:HGNC:2896]	-	2448	2246	1966	1722	1617
1824	24.81435891	22.02922178	25.31939491
19.80651893	20.57407613	17.55572683
ENSG00000103047 690.4179851	620.3816023	719.5823313
754.1850643	1036.47458	915.260027	676.7939729
901.9732238	-0.414593094	0.026211414	0.137509093	TANGO6
16	68843604	69085180	+	6839
protein_coding	transport and golgi organization 6 homolog
[Source:HGNC Symbol;Acc:HGNC:25749]	-	761	701	619
728	908	1061	5.449049293	4.856818287
5.631251084	5.914946478	8.160947894	7.213628928
ENSG00000150636 208.6677222	114.1643748	117.4116566
70.44585765	83.32890346	86.26390453	146.7479179
80.01288855	0.875915282	0.026214172	0.137509093
CCDC102B	18	68715209	69055189	+	5805
protein_coding	coiled-coil domain containing 102B
[Source:HGNC Symbol;Acc:HGNC:26295]	-	230	129	101
68	73	100	1.940234825	1.052964979
1.082495242	0.650906686	0.772979536	0.800993135
ENSG00000251127 32.66103478	53.98470434	26.73730795
137.7838098	63.92354238	61.24737221	37.79434902
87.6515748	-1.208158226	0.026220712	0.137509093
AC091173.1	8	61759094	61765969	-	2750
lincRNA novel transcript	-	36	61	23
133	56	71	0.641059721	1.051050497
0.520357704	2.687391819	1.251707036	1.200484821
ENSG00000129910 11.79426256	15.0449176	30.2247829
6.215810969	0	0	19.02132102	2.07193699
3.206211005	0.026302681	0.13789808	CDH15	16
89171767	89195492	+	4628	protein_coding
cadherin 15 [Source:HGNC Symbol;Acc:HGNC:1754]	-	13	17
26	6	0	0	0.13755573	0.1740532
0.349531921	0.072039376	0	0
ENSG00000189046 415.5209424	556.6619513	519.6337675
475.5095392	800.1857715	904.0457194	497.2722204
726.5803434	-0.547762926	0.026313535	0.137914112	ALKBH2
12	109088188	109093631	-	2290
protein_coding	"alkB homolog 2, alpha-ketoglutarate dependent
dioxygenase [Source:HGNC Symbol;Acc:HGNC:32487]"	-	458
629	447	459	701	1048	9.793967966
13.01492303	12.14447898	11.13753923	18.81611228
21.27927457
ENSG00000047056 635.9829272	676.136297	483.5965264

894.0408111 760.2335576 775.5125017 598.5719168
809.9289568 -0.43475991 0.026336642 0.137994331 WDR37
10 1049538 1132384 + 6000 protein_coding WD
repeat domain 37 [Source:HGNC Symbol;Acc:HGNC:31406] - 701
764 416 863 666 899 5.72130962
6.03348933 4.313689952 7.992296535 6.82292094
6.966898116
ENSG00000204536 1201.200279 1316.872788 1136.916834
873.3214412 1101.539614 826.4082054 1218.329967
933.7564203 0.385049684 0.026401107 0.138291142 CCHCR1
6 31142439 31158238 - 5086
protein_coding coiled-coil alpha-helical rod protein 1 [Source:HGNC
Symbol;Acc:HGNC:13930] - 1324 1488 978 843 965
958 12.74794887 13.86286571 11.96380554
9.210077028 11.66267887 8.758307144
ENSG00000114416 3906.62266 3555.02553 3936.196727
3472.566395 2963.312786 2782.87356 3799.281639
3072.91758 0.306163306 0.026503995 0.138788975 FXR1
3 180868141 180982753 + 12054
protein_coding FMR1 autosomal homolog 1 [Source:HGNC
Symbol;Acc:HGNC:4023] - 4306 4017 3386 3352 2596
3226 17.49329105 15.79054376 17.47682805
15.45200594 13.2379538 12.4441201
ENSG00000139618 2037.68567 1352.272594 1343.840347
1530.125467 763.658033 984.2711507 1577.93287
1092.684884 0.530261614 0.026527586 0.138871403 BRCA2
13 32315474 32400266 + 12273
protein_coding "BRCA2, DNA repair associated [Source:HGNC
Symbol;Acc:HGNC:1101]" - 2246 1528 1156 1477 669
1141 8.961644281 5.899280694 5.860220353
6.68716139 3.350601231 4.322807819
ENSG00000124783 7206.294423 6103.811571 6609.927521
6321.479756 4793.124186 4782.470867 6640.011172
5299.024936 0.325495646 0.026642536 0.139431898 SSR1
6 7268306 7347446 - 11349 protein_coding signal
sequence receptor subunit 1 [Source:HGNC Symbol;Acc:HGNC:11323] -
7943 6897 5686 6102 4199 5544 34.27327851
28.79579485 31.17138549 29.87628894 22.7423657
22.71415807
ENSG00000268182 7.258007728 5.309970919 8.137441549 0
0 0 6.901806732 0 5.218905881
0.026678402 1 SMIM17 19 56643145 56655766
+ 1076 protein_coding small integral membrane
protein 17 [Source:HGNC Symbol;Acc:HGNC:27114] - 8 6
7 0 0 0 0.364088028 0.264219837
0.404755366 0 0 0
ENSG00000160392 890.0131976 1060.224193 868.3812625
1029.752684 1265.914437 1414.728034 939.5395512
1236.798385 -0.396478763 0.026711732 0.139734855
C19orf47 19 40319536 40348527 - 4875

protein_coding	chromosome 19 open reading frame 47
[Source:HGNC Symbol;Acc:HGNC:26723]	-	981	1198	747
994	1109	1640	9.854238538	11.64417272
9.533510043	11.32984081	13.98312649	15.64228686
ENSG00000151176 1123.176696	938.0948623	1110.179526
857.7819138	833.2890346	762.572916	1057.150361
817.8812881	0.370268888	0.026716224	0.139734855	PLBD2
12	113358566	113391625	+	5260
protein_coding	phospholipase B domain containing 2 [Source:HGNC
Symbol;Acc:HGNC:27283]	-	1238	1060	955	828	730
884	11.52560109	9.548750855	11.29599455
8.746949845	8.530696205	7.814434206
ENSG00000166887 1389.90848	1395.637356	1355.465264
1751.822725	1610.64497	1774.448516	1380.337033
1712.305404	-0.311050853	0.02673793	0.139807046	VPS39
15	42158701	42208316	-	8354
protein_coding	"VPS39, HOPS complex subunit [Source:HGNC
Symbol;Acc:HGNC:20593]" -	1532	1577	1166	1691	1411
2057	8.980344101	8.94465642	8.683822267
11.24763411	10.38197319	11.44908656
ENSG00000187446 1225.696055	806.2305845	1061.354876
888.8609686	715.7153763	659.0562306	1031.093839
754.5441918	0.450657557	0.026785921	0.139989861	CHP1
15	41230839	41281890	+	3950
protein_coding	calcineurin like EF-hand protein 1 [Source:HGNC
Symbol;Acc:HGNC:17433]	-	1351	911	913	858	627
764	16.74892497	10.92817704	14.38071663
12.06986009	9.757033709	8.993469809
ENSG00000162814 93.4468495	133.6342681	97.64929859
145.0355893	167.7992987	244.9894889	108.2434721
185.941459	-0.781041849	0.026790072	0.139989861
SPATA17 1	217631324	217871696	+	6515
protein_coding	spermatogenesis associated 17 [Source:HGNC
Symbol;Acc:HGNC:25184]	-	103	151	84	140	147
284	0.774197007	1.098219038	0.802179783
1.194058651	1.386916372	2.026912207
ENSG00000171812 36.29003864	17.69990306	47.66215765
8.287747959	0	12.93958568	33.88403312	7.075777879
2.236266121	0.026796635	0.139989861	COL8A2	1
36095236	36125220	-	4670	protein_coding
collagen type VIII alpha 2 chain [Source:HGNC
Symbol;Acc:HGNC:2216]	-	40	20	41	8	0
15	0.419441883	0.20292687	0.546227829
0.095188646	0	0.149350058
ENSG00000157077 814.7113674	653.126423	678.8951235
905.4364645	888.0806423	1023.089908	715.577638
938.8690049	-0.391975669	0.026828649	0.140096684	ZFYVE9
1	52142094	52346686	+	5809
protein_coding	zinc finger FYVE-type containing 9 [Source:HGNC
Symbol;Acc:HGNC:6775]	-	898	738	584	874	778

1186 7.570135336 6.019791178 6.254870424
8.36030458 8.232383257 9.493237163
ENSG00000149527 53.52780699 26.54985459 30.2247829
66.30198367 113.007691 69.01112362 36.76748149
82.77359943 -1.166286945 0.026832922 0.140096684 PLCH2
1 2425980 2505530 + 10781 protein_coding
phospholipase C eta 2 [Source:HGNC Symbol;Acc:HGNC:29037] -
59 30 26 64 99 80 0.267991889
0.131852585 0.150044869 0.329862519 0.564447441
0.345034052
ENSG00000136630 129.7368881 163.7241033 237.1482966
174.0427071 369.8433523 355.4072867 176.8697627
299.7644487 -0.764052587 0.026851699 0.140153352 HLX
1 220879400 220885059 + 5660
protein_coding H2.0 like homeobox [Source:HGNC Symbol;Acc:HGNC:4978]
Homeobox 143 185 204 168 324 412
1.237223869 1.548751493 2.242438715 1.649319882
3.518648943 3.384634703
ENSG00000063854 311.1870813 331.8731824 409.1970608
235.1648483 231.7228411 256.2037964 350.7524415
241.0304953 0.539128516 0.026898621 0.140356847 HAGH
16 1795620 1827194 - 5975 protein_coding
hydroxyacylglutathione hydrolase [Source:HGNC Symbol;Acc:HGNC:4805]
- 343 375 352 227 203 297
2.811155659 2.973855066 3.665317501 2.111057153
2.088360588 2.311264016
ENSG00000134138 350.1988729 276.1184878 381.2972612
276.6035881 194.0536108 194.9564242 335.8715406
221.8712077 0.597457709 0.0269165 0.140408721 MEIS2
15 36889204 37101299 - 10798
protein_coding Meis homeobox 2 [Source:HGNC Symbol;Acc:HGNC:7001]
Homeobox 386 312 328 267 170 226
1.750542524 1.369108011 1.889893656 1.373978642
0.967727223 0.973186631
ENSG00000178035 9050.735637 10119.91958 9848.629258
10279.91537 12339.52666 12752.39301 9673.094823
11790.61168 -0.285635508 0.026945387 0.140512324 IMPDH2
3 49024325 49029408 - 2773
protein_coding inosine monophosphate dehydrogenase 2 [Source:HGNC
Symbol;Acc:HGNC:6053] - 9976 11435 8472 9923 10810
14783 176.1713387 195.3946823 190.0828453
198.840646 239.6200666 247.8812774
ENSG00000203706 117.0353746 78.76456863 58.12458249
24.86324388 36.5277385 51.75834272 84.64150858
37.7164417 1.164251967 0.026952248 0.140512324
SERTAD4-AS1 1 210231456 210234047 - 1004
antisense SERTAD4 antisense RNA 1 [Source:HGNC
Symbol;Acc:HGNC:32019] - 129 89 50 24 32
60 6.291941572 4.200323447 3.098440339
1.328279814 1.959131672 2.778744114

ENSG00000004777 241.328757 219.478798 254.5856713
173.0067386 58.21608324 166.4893357 238.4644088
132.5707192 0.843132921 0.02698309 0.140631668
ARHGAP33 19 35774532 35788822 + 5260
protein_coding Rho GTPase activating protein 33 [Source:HGNC
Symbol;Acc:HGNC:23085] - 266 248 219 167 51
193 2.476421558 2.23404737 2.590390372
1.764179498 0.595980146 1.706092536
ENSG00000078098 543.4433286 464.6224554 481.2715431
359.4810677 422.3519764 227.736708 496.4457757
336.5232507 0.563578614 0.027024868 0.140807921 FAP
2 162170684 162245151 - 7003
protein_coding fibroblast activation protein alpha [Source:HGNC
Symbol;Acc:HGNC:3590] - 599 525 414 347 370
264 4.188624027 3.552234418 3.678096009
2.753324454 3.247618135 1.752874482
ENSG00000226950 891.8276996 702.6861516 739.3446893
645.4083723 369.8433523 620.2374736 777.9528468
545.1630661 0.511857134 0.027038494 0.140835092 DANCR
4 52712404 52720351 + 2285
processed_transcript differentiation antagonizing non-protein
coding RNA [Source:HGNC Symbol;Acc:HGNC:28964] - 983 794
636 623 324 719 21.06667508 16.46496223
17.31720339 15.15004371 8.715778125 14.63098968
ENSG00000148541 99.79760626 66.37463648 90.67434869
64.23004668 25.11282022 12.07694663 85.61553048
33.80660451 1.34459602 0.027047175 0.140835092 FAM13C
10 59246129 59363181 - 6753
protein_coding family with sequence similarity 13 member C
[Source:HGNC Symbol;Acc:HGNC:19371] - 110 75 78
62 22 14 0.797672498 0.526248601
0.718628935 0.510160681 0.200250353 0.09639673
ENSG00000175198 420.9644482 367.2729885 462.6716767
387.4522171 238.5717921 145.7859987 416.9697045
257.2700026 0.697916627 0.027053968 0.140835092 PCCA
13 100089015 100530437 + 7288
protein_coding propionyl-CoA carboxylase subunit alpha [Source:HGNC
Symbol;Acc:HGNC:8653] - 464 415 398 374 209
169 3.117728551 2.698150519 3.39767281
2.851512514 1.762727916 1.078224904
ENSG00000283445 8.165258694 10.61994184 2.3249833 0
0 0 7.036727944 0 5.251925837
0.027060969 1 AL136985.3 1 58715609
58771295 + 4739 lincRNA novel transcript -
9 12 2 0 0 0 0.093000329
0.119983349 0.026257304 0 0 0
ENSG00000244578 9.07250966 15.92991276 0 0 0
0 8.334140805 0 5.496600175 0.027072872
0.140892038 LINC01391 3 138935189
138944020 - 3889 lincRNA long intergenic non-protein

coding RNA 1391 [Source:HGNC Symbol;Acc:HGNC:50666]	-	10
	18	0	0	0	0	0.125918848
0.219311297	0	0	0	0
ENSG00000165475 280.3405485	334.5281679	454.5342351
483.7972871	512.5298308	532.2482909	356.4676505
509.5251363	-0.517960841	0.027096125	0.140971576	CRYL1
	13	20403666	20525873	-	4327
protein_coding	crystallin lambda 1 [Source:HGNC
Symbol;Acc:HGNC:18246]	-	309	378	391	467	449
	617	3.497037326	4.139342337	5.622075957
5.997110196	6.378327373	6.630240577
ENSG00000134056 237.6997531	395.5928334	404.5470942
268.3158402	184.9216762	213.0718442	345.9465602
222.1031202	0.638016337	0.027129047	0.141101356	MRPS36
	5	69217760	69230129	+	1530
protein_coding	mitochondrial ribosomal protein S36 [Source:HGNC
Symbol;Acc:HGNC:16631]	-	262	447	348	259	162
	247	8.385684991	13.84339252	14.15124532
9.406333595	6.508350659	7.506483608
ENSG00000104774 694.046989	623.0365878	1050.892452
563.5668612	639.2354238	453.7481378	789.3253428
552.1834743	0.515126755	0.027174405	0.141257467	MAN2B1
	19	12646511	12666742	-	5374
protein_coding	mannosidase alpha class 2B member 1 [Source:HGNC
Symbol;Acc:HGNC:6826]	-	765	704	904	544	560
	526	6.970957847	6.207281454	10.46592492
5.624880253	6.405274187	4.551128524
ENSG00000256128 42.6407954	47.78973827	26.73730795
62.15810969	115.2906746	78.50015312	39.05594721
85.31631249	-1.120288137	0.027175033	0.141257467
LINC00944	12	126729787	126772411	-	1853
	lincRNA long intergenic non-protein coding RNA 944
[Source:HGNC Symbol;Acc:HGNC:48640]	-	47	54	23
	60	101	91	1.242084443	1.380844522
0.772252394	1.799234934	3.350374192	2.283478838
ENSG00000267505 4.53625483	2.654985459	8.137441549
23.82727538	23.97132839	21.56597613	5.109560613
23.12152664	-2.196769844	0.027188006	0.141283385
AC005180.2	17	44793199	44794474	+	562
	lincRNA novel transcript	-	5	3	7
	23	21	25	0.435674732	0.252936427
0.774940879	2.274068613	2.296837866	2.068400867
ENSG00000100982 1188.498765	1309.792827	1461.252004
1521.837719	1814.972007	1685.596694	1319.847865
1674.135473	-0.343489044	0.027203111	0.141320362	PCIF1
	20	45934628	45948023	+	3219
protein_coding	PDX1 C-terminal inhibiting factor 1 [Source:HGNC
Symbol;Acc:HGNC:16200]	-	1310	1480	1257	1469	1590
	1954	19.92870152	21.78548232	24.29523743
25.35787535	30.36151985	28.22504226

ENSG00000159461 2494.032905   2492.146351   2986.441049
2260.483256   1991.90324   2156.597613   2657.540102
2136.328036   0.314414642   0.027215697   0.141344233   AMFR
        16   56361452   56425538   -   5758
protein_coding autocrine motility factor receptor [Source:HGNC
Symbol;Acc:HGNC:463]   -   2749   2816   2569   2182   1745
        2500   23.37931395   23.17327581   27.75871068
21.05693368   18.62821025   20.18828217
ENSG00000247809 43.54804637   23.89486913   49.98714095
13.46759043   14.83939377   9.489029498   39.14335215
12.59867123   1.638466189   0.027285955   0.141667523   NR2F2-
AS1   15   96110040   96327361   -   6063
antisense   NR2F2 antisense RNA 1 [Source:HGNC
Symbol;Acc:HGNC:44222] -   48   27   43   13   13
        11   0.387687995   0.211009805   0.441253064
0.119142807   0.131796278   0.084359915
ENSG00000139641 3476.585702   3080.668128   3563.036907
3114.121296   2717.892043   1913.333402   3373.430246
2581.782247   0.386169257   0.0273107   0.141754391   ESYT1
        12   56118250   56144671   +   5493
protein_coding extended synaptotagmin 1 [Source:HGNC
Symbol;Acc:HGNC:29534] -   3832   3481   3065   3006   2381
        2218   34.16210199   30.02761681   34.71584386
30.40824956   26.64386083   18.77513035
ENSG00000227076 19.05227029   20.35488852   17.43737475
1.035968495   4.565967313   4.313195226   18.94817785
3.305043678   2.517781671   0.027339179   0.141860584
AL158166.1   10   127934698   127936167   +   371
        sense_intronic novel transcript   -   21   23
        15   1   4   5   2.771877726   2.937516855
        2.515499272   0.149774588   0.662725134   0.626652985
ENSG00000176834 310.2798304   232.7537253   276.6730127
165.7549592   216.8834474   148.3739158   273.2355228
177.0041074   0.628530332   0.027394204   0.142104422   VSIG10
        12   118063593   118136026   -   8350
protein_coding V-set and immunoglobulin domain containing 10
[Source:HGNC Symbol;Acc:HGNC:26078]   -   342   263   238
        160   190   172   2.005710805   1.492435991
1.773361715   1.064744857   1.398667507   0.957795935
ENSG00000275367 0.907250966   4.424975766   5.812458249
18.64743291   0   65.56056744   3.714894994   28.06933345
        -2.935449419   0.027425056   0.142222753   AC092111.1
        12   8217758 8221115 +   3358   lincRNA novel
transcript   -   1   5   5   18   0   76
        0.014583037   0.070553044   0.092639491   0.297854288
        0   1.052358998
ENSG00000169375 1696.559306   1556.706474   2036.685371
1666.873308   1184.868518   1130.057149   1763.31705
1327.266325   0.409581649   0.027458093   0.142352346   SIN3A
        15   75369379   75455842   -   12453

protein_coding	SIN3 transcription regulator family member A
[Source:HGNC Symbol;Acc:HGNC:19353]	-	1870	1759	1752
	1609	1038	1310	7.353537339	6.692960966
8.75320219	7.179498344	5.123547013	4.891345336
ENSG00000150893 108.8701159	75.22458802	86.02438209
61.1221412	36.5277385	30.19236658	90.03969534
42.6140821	1.081771268	0.027535127	0.14270989	FREM2
	13	38687129	38887131	+	16223
protein_coding	FRAS1 related extracellular matrix protein 2
[Source:HGNC Symbol;Acc:HGNC:25396]	-	120	85	74
	59	32	35	0.362225284	0.248264257
0.283796737	0.202084446	0.121245651	0.100315466
ENSG00000169955 189.6154519	185.8489822	155.7738811
269.3518087	345.8720239	213.0718442	177.0794384
276.0985589	-0.637440758	0.027543585	0.142711912	ZNF747
	16	30530367	30535347	-	3936
protein_coding	zinc finger protein 747 [Source:HGNC
Symbol;Acc:HGNC:28350]	zf-C2H2 209	210	134	260	303
	247	2.600278589	2.528079027	2.118149235
3.670542879	4.731892819	2.917916646
ENSG00000133997 471.7705023	554.0069659	455.6967268
678.5593642	641.5184074	657.3309525	493.8247316
659.1362414	-0.415806327	0.027586072	0.142878223	MED6
	14	70581257	70600690	-	4219
protein_coding	mediator complex subunit 6 [Source:HGNC
Symbol;Acc:HGNC:19970]	-	520	626	392	655	562
	762	6.035628517	7.030581526	5.780739357
8.626682577	8.187930551	8.398011482
ENSG00000178927 1336.380673	1555.821479	1412.427355
1570.528238	1941.6776	1945.251047	1434.876502
1819.152295	-0.342303394	0.027591837	0.142878223	CYBC1
	17	82442589	82450829	-	7234
protein_coding	cytochrome b-245 chaperone 1 [Source:HGNC
Symbol;Acc:HGNC:28672]	-	1473	1758	1215	1516	1701
	2255	9.971326247	11.51507596	10.44971919
11.64481895	14.45350475	14.49436054
ENSG00000150630 2340.707492	2715.16513	2487.732131
3149.344225	2907.379686	3179.687521	2514.534918
3078.803811	-0.29215337	0.027654696	0.143139362	VEGFC
	4	176683538	176792727	-	2420
protein_coding	vascular endothelial growth factor C [Source:HGNC
Symbol;Acc:HGNC:12682]	-	2580	3068	2140	3040	2547
	3686	52.20751519	60.07121694	55.01805764
69.80238492	64.69354343	70.82245596
ENSG00000111644 0	0.884995153	0	7.251779464
6.848950969	11.21430759	0.294998384
8.438346007	-4.592987192	0.027658451	0.143139362	ACRBP
	12	6638075 6647460 -	2843	protein_coding
acrosin binding protein [Source:HGNC Symbol;Acc:HGNC:17195]	-
	0	1	0	7	6	13	0

0.016666699	0	0.136814845	0.129724424	0.212616767
ENSG00000074935 303.9290736	223.9037737	201.1110554
290.0711786	460.0212068	360.5831209	242.9813009
370.2251688	-0.605333562	0.027686367	0.143224888	TUBE1
	6	112070777	112087529	-	6973
protein_coding	tubulin epsilon 1 [Source:HGNC Symbol;Acc:HGNC:20775]
	-	335	253	173	280	403	418
2.352631055	1.71920354	1.543594721	2.231261181
3.552488994	2.787325158
ENSG00000173575 1017.028333	928.3599156	902.0935203
813.2352685	505.6808799	763.4355551	949.1605896
694.1172345	0.450633586	0.027691801	0.143224888	CHD2
	15	92900189	93028005	+	16960
protein_coding	chromodomain helicase DNA binding protein 2
[Source:HGNC Symbol;Acc:HGNC:1917]	-	1121	1049	776
	785	443	885	3.23674472	2.93073183
2.846706677	2.571910506	1.605555189	2.426322027
ENSG00000177556 288.5058072	450.4625329	464.99666
247.5964703	179.214217	339.8797838	401.3216667
255.5634904	0.647703918	0.027699267	0.143224888	ATOX1
	5	151742316	151772532	-	5239
protein_coding	antioxidant 1 copper chaperone [Source:HGNC
Symbol;Acc:HGNC:798]	-	318	509	400	239	157
	394	2.972401043	4.603581379	4.750271579
2.534904172	1.842038123	3.496864801
ENSG00000176973 312.0943323	390.2828625	346.4225117
429.9269254	546.7745857	487.3910606	349.5999022
488.0308572	-0.480701032	0.027709033	0.143233515	FAM89B
	11	65572349	65574198	+	1458
protein_coding	family with sequence similarity 89 member B
[Source:HGNC Symbol;Acc:HGNC:16708]	-	344	441	298
	415	479	565	11.55392654	14.33202333
12.71644118	15.81621705	20.19413939	18.01863724
ENSG00000110852 9.07250966	0	0	11.39565344
23.97132839	36.2308399	3.024169887
23.86594058	-2.957528305	0.027750887	0.14340796	CLEC2B
	12	9852984 9870136 -	2638	protein_coding	C-type
lectin domain family 2 member B [Source:HGNC Symbol;Acc:HGNC:2053]
	-	10	0	0	11	21	42
0.185632448	0	0	0.231702083	0.489318757
0.740296195
ENSG00000076043 1027.915344	1138.103767	991.6053774
859.8538508	808.1762143	802.2543121	1052.541496
823.4281257	0.354647905	0.027776696	0.143499408	REXO2
	11	114439386	114450279	+	3699
protein_coding	RNA exonuclease 2 [Source:HGNC Symbol;Acc:HGNC:17851]
	-	1133	1286	853	830	708	930
14.99941296	16.47339371	14.34734516	12.4682587
11.76511798	11.69040711
ENSG00000184260 31.75378381	35.39980612	31.38727455

15.53952742	0	0	32.84695483	5.179842475
2.671024223	0.027788031	0.143505158	HIST2H2AC	1
149886975	149887364	+	390	protein_coding
histone cluster 2 H2A family member c [Source:HGNC
Symbol;Acc:HGNC:4738]	-	35	40	27	15	0
0	4.394729216	4.859838365	4.307308754
2.137168162	0	0
ENSG00000274641 7.258007728	6.194966072	6.974949899	0
0	0	6.8093079	0	5.200404654
0.027789734	1	HIST1H2BO	6	27893463
27893843	+	381	protein_coding	histone cluster 1 H2B
family member o [Source:HGNC Symbol;Acc:HGNC:4758]	-	8
7	6	0	0	0	1.028238107
0.870561597	0.979790268	0	0	0
ENSG00000048162 1079.62865	1485.906862	1035.78006
1362.298571	1533.023525	1928.860905	1200.438524
1608.061	-0.421603245	0.027794034	0.143505158	NOP16
5	176383938	176388975	-	2862
protein_coding	NOP16 nucleolar protein [Source:HGNC
Symbol;Acc:HGNC:26934]	-	1190	1679	891	1315	1343
2236	20.36132404	27.7976132	19.3693444
25.53102007	28.84388421	36.32730564
ENSG00000146833 657.7569503	764.6358123	796.3067802
930.2997084	873.2412486	1111.941729	739.5665143
971.8275621	-0.395304035	0.027874995	0.143881177	TRIM4
7	99876958	99919600	-	4891
protein_coding	tripartite motif containing 4 [Source:HGNC
Symbol;Acc:HGNC:16275]	-	725	864	685	898	765
1289	7.258870145	8.370328846	8.713642849
10.20212681	9.614155287	12.25423692
ENSG00000138311 264.9172821	270.8085169	174.3737475
335.6537923	358.428434	363.1710381	236.6998488
352.4177548	-0.571458254	0.027893576	0.143935083	ZNF365
10	62374192	62670301	+	5368
protein_coding	zinc finger protein 365 [Source:HGNC
Symbol;Acc:HGNC:18194]	-	292	306	150	324	314
421	2.663784134	2.70106702	1.738543648
3.353857041	3.595543117	3.646704784
ENSG00000081985 3.629003864	8.849951531	8.137441549	0
0	0	6.872132315	0	5.212936673
0.027898384	1	IL12RB2 1	67307364	67397090
+	4423	protein_coding	interleukin 12 receptor
subunit beta 2 [Source:HGNC Symbol;Acc:HGNC:5972]	-	4
10	7	0	0	0	0.044286538
0.107129604	0.098466374	0	0	0
ENSG00000124575 3.629003864	8.849951531	8.137441549	0
0	0	6.872132315	0	5.212936673
0.027898384	1	HIST1H1D	6	26234268
26234933	-	666	protein_coding	histone cluster 1 H1
family member d [Source:HGNC Symbol;Acc:HGNC:4717]	-	4

10	7	0	0	0	0.294113152
0.711462824	0.65392909	0	0	0
ENSG00000170624 107.055614	73.45459771	125.5490982
24.86324388	83.32890346	21.56597613	102.01977
43.25270782	1.245301724	0.027912504	0.143990749	SGCD
5	155870344	156767788	+	10584
protein_coding	sarcoglycan delta [Source:HGNC Symbol;Acc:HGNC:10807]
-	118	83	108	24	73	25
0.545960043	0.371582029	0.634864102	0.126000844
0.423955613	0.109830054
ENSG00000116852 1073.277893	993.849557	1332.215431
741.7534424	993.0978905	822.0950101	1133.114293
852.3154477	0.41055124	0.027931166	0.144045013	KIF21B
1	200969390	201023700	-	12150
protein_coding	kinesin family member 21B [Source:HGNC
Symbol;Acc:HGNC:29442]	-	1183	1123	1146	716	870
953	4.768009919	4.379554339	5.868338895
3.274528601	4.40139489	3.647099743
ENSG00000107560 635.0756762	572.5918641	494.0589512
688.9190491	826.4400836	758.2597208	567.2421638
757.8729512	-0.416691833	0.02796539	0.144179477
RAB11FIP2	10	118004916	118046603	-	6169
protein_coding	RAB11 family interacting protein 2
[Source:HGNC Symbol;Acc:HGNC:29152]	-	700	647	425
665	724	879	5.556636065	4.969537261
4.286284625	5.98989099	7.213917248	6.625293511
ENSG00000155366 2842.417276	3579.805394	4903.389779
2433.489995	3196.177119	2876.038577	3775.20415
2835.23523	0.412770249	0.027990053	0.144264583	RHOC
1	112701106	112707434	-	4028
protein_coding	ras homolog family member C [Source:HGNC
Symbol;Acc:HGNC:669]	-	3133	4045	4218	2349	2800
3334	38.08900402	47.58340375	65.15143116
32.40452043	42.72833104	38.48638781
ENSG00000166359 39.01179154	28.3198449	24.41232465
59.05020421	94.74382174	56.93417699	30.58132036
70.24273431	-1.193890786	0.02799829	0.144265	WDR88
19	33132090	33175795	+	1841
protein_coding	WD repeat domain 88 [Source:HGNC
Symbol;Acc:HGNC:26999]	-	43	32	21	57	83
66	1.143782245	0.823611934	0.709695992
1.720414566	2.771224208	1.666944595
ENSG00000104450 727.6152747	542.5020289	562.6459585
536.6316804	382.3997624	381.286458	610.9210874
433.4393003	0.495795948	0.028147517	0.144991678	SPAG1
8	100157906	100259278	+	5055
protein_coding	sperm associated antigen 1 [Source:HGNC
Symbol;Acc:HGNC:11212]	-	802	613	484	518	335
442	7.769300009	5.74600177	5.957047298
5.694041701	4.073530828	4.065670022

ENSG00000167721 4021.843532	3649.720011	3323.563627
4216.391774	4458.667081	4780.745589	3665.04239
4485.268148	-0.291222681	0.028204109	0.145240898	TSR1
	17	2322503 2337507 -	5545	protein_coding	"TSR1,
ribosome maturation factor [Source:HGNC Symbol;Acc:HGNC:25542]" -
	4433	4124	2859	4070	3906	5542	39.14937781
	35.24062052	32.0788988	40.78541657	43.29901818
	46.47249497
ENSG00000124422 7092.888052	6206.471009	7130.72378
5956.818846	5471.170332	5496.735996	6810.027614
5641.575058	0.271482791	0.028234398	0.14535456	USP22
	17	20999593	21043760	-	6808
protein_coding	ubiquitin specific peptidase 22 [Source:HGNC
Symbol;Acc:HGNC:12621]	-	7818	7013	6134	5750	4793
	6372	56.23475438	48.81021635	56.05715738
46.9310576	43.27481721	43.51983481
ENSG00000198742 674.0874677	715.9610789	691.6825317
869.1775672	962.2776112	853.1500158	693.9103594
894.8683981	-0.366443021	0.028319501	0.145646744	SMURF1
	7	99027438	99144100	-	6276
protein_coding	SMAD specific E3 ubiquitin protein ligase 1
[Source:HGNC Symbol;Acc:HGNC:16807]	-	743	809	595
	839	843	989	5.797417303	6.107901539
5.898490407	7.42832796	8.256424234	7.327306777
ENSG00000123500 367.4366412	462.8524651	404.5470942
190.6182031	348.1550076	284.6708849	411.6120668
274.4813652	0.585467082	0.028326449	0.145646744
COL10A1 6	116118923	116158747	-	3816
protein_coding	collagen type X alpha 1 chain [Source:HGNC
Symbol;Acc:HGNC:2185]	-	405	523	348	184	305
	330	5.197270737	6.49411184	5.673848359
2.679300966	4.912910285	4.021023322
ENSG00000136875 2326.191477	2469.136477	2147.122077
2710.093583	2997.557541	2800.126341	2314.15001
2835.925821	-0.292909315	0.028328507	0.145646744	PRPF4
	9	113275343	113292905	+	3613
protein_coding	pre-mRNA processing factor 4 [Source:HGNC
Symbol;Acc:HGNC:17349]	-	2564	2790	1847	2616	2626
	3246	34.75191512	36.590023	31.80576022
40.23294483	44.67598335	41.77452997
ENSG00000151327 712.1920083	759.3258414	647.507849
625.7249709	509.1053554	402.8524341	706.3418996
512.5609201	0.464185732	0.028335436	0.145646744
FAM177A1	14	35044907	35113130	+	8385
	protein_coding	family with sequence similarity 177 member A1
[Source:HGNC Symbol;Acc:HGNC:19829]	-	785	858	557
	604	446	467	4.584534796	4.848536415
4.132938805	4.002634324	3.269483515	2.589672421
ENSG00000186871 978.9237923	752.2458802	762.5945223
739.6815054	550.1990612	538.2867643	831.2547316

609.3891103	0.448556096	0.028335938	0.145646744	ERCC6L
	X	72204657	72239047	-	4364
protein_coding	"ERCC excision repair 6 like, spindle assembly
checkpoint helicase [Source:HGNC Symbol;Acc:HGNC:20794]"	-
	1079	850	656	714	482	624	12.10780412
	9.22912705	9.352461823	9.091290044	6.789060151
	6.648610114
ENSG00000186812 361.9931354	332.7581776	301.0853373
465.1498542	495.4074534	421.8304931	331.9455501
460.7959336	-0.471451964	0.028340556	0.145646744	ZNF397
	18	35241030	35267133	+	8710
protein_coding	zinc finger protein 397 [Source:HGNC
Symbol;Acc:HGNC:18818]	zf-C2H2 399	376	259	449	434
	489	2.243279689	2.045484207	1.850071256
2.864443297	3.062802089	2.610488127
ENSG00000265917 0	0	0	15.53952742	9.131934625
	0	0	8.223820683	-5.512605553	0.028377867
	0.145796136	MIR3685 12	95309923	95309984
	+	62	miRNA	microRNA 3685 [Source:HGNC
Symbol;Acc:HGNC:38886]	-	0	0	0	15	8
	0	0	0	0	13.44347715	7.931323383
	0
ENSG00000175764 176.9139384	188.5039676	145.3114562
143.9996208	54.79160775	77.63751407	170.2431207
92.14291421	0.885988591	0.028400089	0.145867939	TTLL11
	9	121821928	122093606	-	3535
protein_coding	tubulin tyrosine ligase like 11 [Source:HGNC
Symbol;Acc:HGNC:18113]	-	195	213	125	139	48
	90	2.701306582	2.855069116	2.200024116
2.184929487	0.834639971	1.183815738
ENSG00000189014 9.979760626	6.194966072	9.299933199	0
	0	0.862639045	8.491553299	0.287546348
4.556538353	0.028490905	0.1462388	SHLD2P3 10
47689707	47730436	+	2712	unprocessed_pseudogene
	shieldin complex subunit 2 pseudogene 3 [Source:HGNC
Symbol;Acc:HGNC:34038]	-	11	7	8	0	0
	1	0.198623982	0.122302348	0.183530035	0
	0	0.017145152
ENSG00000152291 4158.838428	3779.814299	4132.657815
3795.788565	2924.502064	2970.928872	4023.770181
3230.4065	0.31680589	0.028495969	0.1462388	TGOLN2
	2	85318020	85328425	-	7081
protein_coding	trans-golgi network protein 2 [Source:HGNC
Symbol;Acc:HGNC:15450]	-	4584	4271	3555	3664	2562
	3444	31.7014187	28.57994692	31.23574418
28.75232139	22.23985192	22.61515489
ENSG00000274191 0	16.81490791	8.137441549	0	0
	0	8.31744982	0	5.489674973	0.028500156
	0.1462388	AC026333.4	12	122634130
122634673	+	544	lincRNA novel transcript	-

        0   19   7   0   0   0   0
1.654935767   0.800582305   0   0   0
ENSG00000251131 10.88701159   6.194966072   3.48747495   0
        0   0   6.856484204   0   5.213271798
0.028500665   1   AC025171.3   5   43018429
43024247   +   553   antisense   novel transcript
        -       12   7   3   0   0   0
1.062636669   0.599790178   0.337522687   0   0   0
ENSG00000173811 0   0   0   0   30.82027936   0
        0   10.27342645   -5.832301235   0.028505364
0.1462388   CCDC13-AS1   3   42732575   42746768
        +   2164   antisense   CCDC13 antisense RNA 1
[Source:HGNC Symbol;Acc:HGNC:41142]   -   0   0   0
        0   27   0   0   0   0   0
0.766926718   0
ENSG00000284024 577.0116144   552.2369755   495.2214429
659.9119313   646.0843747   906.6336366   541.4900109
737.5433142   -0.446303179   0.028518456   0.146263544   HSPA14
        10   14838160   14847018   +   4924
protein_coding heat shock protein family A (Hsp70) member 14
[Source:NCBI gene;Acc:51182]   -   636   624   426   637
        566   1051   6.325105226   6.004723113   5.382678013
        7.1884198   7.065546308   9.924661195
ENSG00000227706 43.54804637   16.81490791   29.06229125
2.07193699   0   13.80222472   29.80841517   5.291387238
        2.459460154   0.028631402   0.146760863   AL713998.1
        6   67887646   67889339   -   1605
lincRNA novel transcript   -   48   19   25   2
        0   16   1.464518574   0.560925269   0.969107196
        0.069241585   0   0.46352799
ENSG00000103260 2074.882959   2685.96029   2849.267034
1607.823104   2288.691115   1943.525769   2536.703428
1946.679996   0.381888772   0.028636793   0.146760863   METRN
        16   715115 719655 +   3692
protein_coding "meteorin, glial cell differentiation regulator
[Source:HGNC Symbol;Acc:HGNC:14151]"   -   2287   3035   2451
        1552   2005   2253   30.3342426   38.95143337
41.30365319   23.35834498   33.38105396   28.37465028
ENSG00000167740 289.4130581   384.9728916   292.9478958
341.8696033   434.9083865   702.1881829   322.4446152
492.9887242   -0.613281195   0.028643059   0.146760863   CYB5D2
        17   4143168 4187310 +   2182   protein_coding
cytochrome b5 domain containing 2 [Source:HGNC Symbol;Acc:HGNC:28471]
        -       319   435   252   330   381   814
7.159202065   9.446282981   7.185427986   8.403713486
10.73291252   17.34605331
ENSG00000132879 84.37433984   166.3790888   169.7237809
200.977888   237.4303003   263.1049088   140.1590698
233.837699   -0.740966838   0.02864861   0.146760863   FBXO44
        1   11654375   11663327   +   3527

protein_coding F-box protein 44 [Source:HGNC Symbol;Acc:HGNC:24847]
- 93 188 146 194 208 305
1.291237625 2.525682938 2.575456641 3.05638679
3.624976834 4.020919679
ENSG00000073282 91.63234756 68.14462679 39.5247161
39.36680281 11.41491828 0 66.43389682 16.92724036
1.980496404 0.028659906 0.146776226 TP63 3
189631416 189897279 + 7454 protein_coding
tumor protein p63 [Source:HGNC Symbol;Acc:HGNC:15979] P53
101 77 34 38 10 0 0.663530161
0.489471915 0.283789534 0.283273698 0.08246278
0
ENSG00000108389 1604.926959 1177.043554 1641.43821
1419.276838 948.5797092 834.1719568 1474.469574
1067.342835 0.466199076 0.028684063 0.146857423 MTMR4
17 58489529 58517905 - 7475
protein_coding myotubularin related protein 4 [Source:HGNC
Symbol;Acc:HGNC:7452] - 1769 1330 1412 1370 831
967 11.58898283 8.430763076 11.75250234
10.18407089 6.833405407 6.015147692
ENSG00000037897 288.5058072 438.0726008 326.6601536
445.4664528 479.4265678 586.5945508 351.0795205
503.8291905 -0.52116181 0.028716546 0.146981189 METTL1
12 57768471 57772793 - 2621
protein_coding methyltransferase like 1 [Source:HGNC
Symbol;Acc:HGNC:7030] - 318 495 281 430 420
680 5.941399873 8.948796226 6.670311959
9.116192311 9.849850291 12.06348837
ENSG00000155363 1137.692711 1085.004058 1136.916834
820.487048 966.8435785 849.6994596 1119.871201
879.0100287 0.34975371 0.028726178 0.146987955 MOV10
1 112673141 112700746 + 6509
protein_coding Mov10 RISC complex RNA helicase [Source:HGNC
Symbol;Acc:HGNC:7200] - 1254 1226 978 792 847
985 9.434349232 8.924885219 9.348273929
6.761187092 7.998646415 7.036439811
ENSG00000199266 1.814501932 0 0 8.287747959
9.131934625 14.66486377 0.604833977
10.69484878 -4.095728726 0.028747389 0.14705395
SNORA60 20 38449370 38449503 + 134 snoRNA
"small nucleolar RNA, H/ACA box 60 [Source:HGNC
Symbol;Acc:HGNC:32654]" - 2 0 0 8 8
17 0.730893132 0 0 3.317395355
3.669716789 5.898955787
ENSG00000187193 169.6559306 180.5390112 204.5985304
342.9055718 301.3538426 211.3465661 184.9311574
285.2019935 -0.624701395 0.028787218 0.147215122 MT1X
16 56682424 56684196 + 1773
protein_coding metallothionein 1X [Source:HGNC Symbol;Acc:HGNC:7405]
- 187 204 176 331 264 245

5.164895684	5.451899891	6.176049652	10.37364309
9.152559302	6.425225391
ENSG00000110768 1596.7617	1363.777531	1262.465932
1711.419954	1696.256857	1935.762018	1407.668388
1781.146276	-0.339312444	0.028817442	0.147327092	GTF2H1
	11	18322295	18367044	+	6939
protein_coding	general transcription factor IIH subunit 1
[Source:HGNC Symbol;Acc:HGNC:4655]	-	1760	1541	1086
	1652	1486	2244	12.420654	10.52282122
9.737327664	13.22894464	13.16343648	15.03685401
ENSG00000273748 222.2764867	177.8840258	265.0480962
163.6830222	134.6960357	115.5936321	221.7362029
137.9908967	0.683834433	0.02885283	0.147458706
AL592183.1	GL000219.1	54224	83311	-	372
protein_coding		-	245	201	228	158	118
	134	32.25164182	25.60233397	38.1328051
23.60077099	19.49783665	16.74915403
ENSG00000185532 13.60876449	16.81490791	48.8246493
7.251779464	0	6.901112362	26.41610723	4.717630609
	2.46191092	0.028859859	0.147458706	PRKG1	10
	50990891	52298350	+	12831	protein_coding
	protein kinase cGMP-dependent 1 [Source:HGNC
Symbol;Acc:HGNC:9414]	-	15	19	42	7	0
	8	0.057247884	0.07016484	0.20365526
0.030314442	0	0.02899082
ENSG00000233912 2.721752898	9.734946684	8.137441549	0
	0	0	6.864713711	0	5.211448159
0.028872919	1	AC026202.2	3	5156905 5187329 -
	672	antisense	"novel transcript, antisense to ARL8B"
	-	3	11	7	0	0	0
0.218615356	0.775621525	0.648090438	0	0	0
ENSG00000125538 29.03203091	52.21471403	63.93704074
52.83439324	83.32890346	195.8190633	48.39459523
110.6607867	-1.200048213	0.028920904	0.147727946	IL1B
	2	112829751	112836903	-	3096
protein_coding	interleukin 1 beta [Source:HGNC Symbol;Acc:HGNC:5992]
	-	32	59	55	51	73	227
0.506148215	0.902978688	1.105270514	0.915337527
1.449336629	3.409227032
ENSG00000008516 0.907250966	17.69990306	3.48747495	0
	0	0	7.364876326	0	5.316963827
0.028951299	1	MMP25	16	3046681 3060726 +	4126
	protein_coding	matrix metallopeptidase 25 [Source:HGNC
Symbol;Acc:HGNC:14246]	-	1	20	3	0	0
	0	0.011868599	0.229682133	0.045237529	0
	0	0
ENSG00000143669 825.598379	678.7912824	652.1578156
631.9407819	518.23729	404.5777122	718.849159
518.251928	0.473520684	0.028967927	0.147899986	LYST
	1	235661041	235883640	-	19614

protein_coding lysosomal trafficking regulator [Source:HGNC
Symbol;Acc:HGNC:1968]   -   910   767   561   610   454
        469   2.271976866   1.852915583   1.779522719
1.728127207   1.422777675   1.111828212
ENSG00000179271 2360.667013   3314.306848   3018.990815
3073.718524   3877.64774   4268.337996   2897.988226
3739.90142   -0.368138642   0.028979911   0.147899986
GADD45GIP1   19   12953119   12957236   -   1782
        protein_coding GADD45G interacting protein 1 [Source:HGNC
Symbol;Acc:HGNC:29996] -   2602   3745   2597   2967   3397
        4948   71.50366063   99.57964259   90.67156182
92.51707426   117.1750661   129.1079571
ENSG00000198792 1530.53238   1656.710927   1617.025885
1328.11161   1208.839846   1325.876213   1601.423064
1287.609223   0.314337007   0.028987863   0.147899986
TMEM184B   22   38219291   38273034   -   7080
        protein_coding transmembrane protein 184B [Source:HGNC
Symbol;Acc:HGNC:1310]   -   1687   1872   1391   1282   1059
        1537   11.6683785   12.52849856   12.22364473
10.06159451   9.194117774   10.09419355
ENSG00000112874 387.3961625   340.723134   383.6222445
579.1063887   505.6808799   447.7096645   370.5805136
510.832311   -0.462885109   0.028988029   0.147899986   NUDT12
        5   103548855   103562793   -   4304
protein_coding nudix hydrolase 12 [Source:HGNC Symbol;Acc:HGNC:18826]
        -       427   385   330   559   443   519
4.85829963   4.238526548   4.770331102   7.216914977
6.32672305   5.606942641
ENSG00000155463 1582.245685   1798.310151   1507.75167
1938.297054   2087.788554   2044.454537   1629.435835
2023.513382   -0.312039929   0.029062784   0.148238634   OXA1L
        14   22766522   22773041   +   4497
protein_coding "OXA1L, mitochondrial inner membrane protein
[Source:HGNC Symbol;Acc:HGNC:8526]"   -   1744   2032   1297
        1871   1829   2370   18.99119428   21.41052205
17.94419314   23.1186752   24.99989462   24.50509986
ENSG00000198919 761.1835605   604.4516896   385.9472278
511.7684365   362.9944014   249.3026841   583.8608259
374.6885073   0.642213586   0.029086353   0.14828883   DZIP3
        3   108589682   108694846   +   6972
protein_coding DAZ interacting zinc finger protein 3 [Source:HGNC
Symbol;Acc:HGNC:30938] -   839   683   332   494   318
        289   5.892956916   4.641835719   2.962699143
3.937146854   2.803606781   1.927398348
ENSG00000144485 137.9021468   163.7241033   183.6736807
127.4241249   67.34801786   80.22543121   161.7666436
91.66585798   0.818146569   0.029089391   0.14828883   HES6
        2   238238267   238240662   -   2037
protein_coding hes family bHLH transcription factor 6 [Source:HGNC
Symbol;Acc:HGNC:18254] bHLH   152   185   158   123   59

93	3.65410685	4.303354664	4.825839841
3.355259588	1.780362109	2.122867742
ENSG00000266412 3237.071447	2765.609853	3158.489813
3821.687778	4104.804614	3372.056028	3053.723704
3766.182807	-0.302401198	0.029113614	0.148369551	NCOA4
10	46005088	46030714	-	4079
protein_coding	nuclear receptor coactivator 4 [Source:HGNC
Symbol;Acc:HGNC:7671]	-	3568	3125	2717	3689	3596
3909	42.83510383	36.30134841	41.44219784
50.25357859	54.18927467	44.55977438
ENSG00000173376 21.77402318	16.81490791	31.38727455
9.323716454	0	0	23.32540188	3.107905485
2.914526654	0.029131269	0.148391842	NDNF	4
121035613	121073021	-	3556	protein_coding	neuron
derived neurotrophic factor [Source:HGNC Symbol;Acc:HGNC:26256] -
24	19	27	9	0	0	0.330505106
0.253173526	0.472398879	0.1406348	0	0
ENSG00000137207 1999.581129	2494.801337	2671.405811
2577.489615	3335.439122	3223.682112	2388.596092
3045.53695	-0.350849445	0.029134765	0.148391842	YIPF3
6	43511827	43516990	-	3053
protein_coding	Yip1 domain family member 3 [Source:HGNC
Symbol;Acc:HGNC:21023]	-	2204	2819	2298	2488	2922
3737	35.35195774	43.75167783	46.83063716
45.28304423	58.83025997	56.9150749
ENSG00000069764 0	0	0	0	0	30.19236658
0	10.06412219	-5.812113535	0.029155968
0.148414363	PLA2G10 16	14672545	14694669	-
1116	protein_coding	phospholipase A2 group X [Source:HGNC
Symbol;Acc:HGNC:9029]	-	0	0	0	0	0
35	0	0	0	0	0	1.45825968
ENSG00000272078 0	0	0	0	0	30.19236658
0	10.06412219	-5.812113535	0.029155968
0.148414363	AL139423.1	1	10639241	10654333
+	7923	antisense	"novel transcript, antisense
to CASZ1"	-	0	0	0	0	0	35
0	0	0	0	0	0.205404241
ENSG00000116455 1571.358673	1763.79534	1600.751002
1836.772141	2176.824916	2134.168998	1645.301672
2049.255352	-0.316595196	0.029166103	0.148423244	WDR77
1	111439890	111449376	-	2805
protein_coding	WD repeat domain 77 [Source:HGNC
Symbol;Acc:HGNC:29652]	-	1732	1993	1377	1773	1907
2474	30.23734852	33.66672518	30.5427348
35.12270157	41.78930877	41.01076286
ENSG00000179041 1867.122488	2359.397078	1661.200568
2254.267445	2483.886218	2839.807737	1962.573378
2525.987133	-0.363791603	0.029179817	0.148450327	RRS1
8	66429028	66430733	+	1706
protein_coding	ribosome biogenesis regulator homolog [Source:HGNC

Symbol;Acc:HGNC:17083]	-	2058	2666	1429	2176	2176
	3292	59.07381616	74.04701555	52.11467678
70.87481003	78.40201497	89.72465928
ENSG00000167447 825.598379	746.9359092	762.5945223
867.1056302	1259.065486	972.194204	778.3762702
1032.78844	-0.407283161	0.029248976	0.148759383	SMG8
	17	59209400	59215247	+	3752
protein_coding	"SMG8, nonsense mediated mRNA decay factor
[Source:HGNC Symbol;Acc:HGNC:25551]"	-	910	844	656
	837	1103	1127	11.87701339	10.65874464
10.87796999	12.39580318	18.07007865	13.96664532
ENSG00000158079 249.4940156	199.1239095	184.8361723
157.4672112	115.2906746	123.3573835	211.1513658
132.0384231	0.678246096	0.029307114	0.149012226	PTPDC1
	9	94030794	94109856	+	6404
protein_coding	protein tyrosine phosphatase domain containing 1
[Source:HGNC Symbol;Acc:HGNC:30184]	-	275	225	159
	152	101	143	2.102858519	1.664783013
1.544730237	1.318877042	0.969432133	1.038282974
ENSG00000231845 6.350756762	7.964956378	5.812458249	0
	0	0	6.709390463	0	5.180132901
0.029370732	1	HMGB3P14	3	134170487
134171085	+	599	processed_pseudogene	high mobility
group box 3 pseudogene 14 [Source:HGNC Symbol;Acc:HGNC:39306]	-
	7	9	5	0	0	0	0.572268579
	0.711937924	0.519337913	0	0	0
ENSG00000260645 4.53625483	0	0	32.11502334
9.131934625	7.763751407	1.512084943
16.33690312	-3.398983193	0.029428324	0.149551152
AL359715.2	6	80466958	80469080	+	2123
	lincRNA novel transcript	-	5	0	0
	31	8	9	0.1153317	0	0
0.811378963	0.231626024	0.197116752
ENSG00000279794 0.907250966	8.849951531	2.3249833
6.215810969	14.83939377	52.62098176	4.027395266
24.55872883	-2.599465666	0.029430016	0.149551152
AC024580.1	19	56149817	56150490	+	674
	TEC	TEC	-	1	10	2	6	13
	61	0.072655549	0.703018161	0.184619234
0.494656132	1.185579868	4.208244424
ENSG00000049759 1121.362194	900.9250659	1078.792251
1371.622287	1208.839846	1383.673029	1033.69317
1321.378387	-0.354900808	0.029466578	0.149693942	NEDD4L
	18	58044367	58401540	+	17455
protein_coding	"neural precursor cell expressed, developmentally
down-regulated 4-like, E3 ubiquitin protein ligase [Source:HGNC
Symbol;Acc:HGNC:7728]"	-	1236	1018	928	1324	1059
	1604	3.467586481	2.763467527	3.307767453
4.214831097	3.729266906	4.272829161
ENSG00000221914 2358.852512	2413.381783	2524.931864

2988.769108   2975.869196   2863.098991   2432.388719
2942.579098   -0.274792633   0.029498348   0.149721203
PPP2R2A 8   26291491   26372680   +   7865
protein_coding protein phosphatase 2 regulatory subunit Balpha
[Source:HGNC Symbol;Acc:HGNC:9304]   -   2600   2727   2172
        2885   2607   3319   16.1883768   16.42906515
17.18177156   20.382579   20.37462689   19.62183157
ENSG00000179151 1084.164904   1394.752361   1243.866065
1277.349154   1598.088559   2122.95469   1240.927777
1666.130801   -0.425598038   0.029505016   0.149721203   EDC3
        15   74630558   74696292   -   5103
protein_coding enhancer of mRNA decapping 3 [Source:HGNC
Symbol;Acc:HGNC:26114] -   1195   1576   1070   1233   1400
        2461   11.46755998   14.633799   13.04562997
13.42608993   16.86358195   22.42420544
ENSG00000152495 625.0959156   501.7922518   540.5586172
517.9842475   337.8815811   289.8467192   555.8155949
381.9041826   0.542326766   0.0295079   0.149721203   CAMK4
        5   111223653   111494886   +   13910
protein_coding calcium/calmodulin dependent protein kinase IV
[Source:HGNC Symbol;Acc:HGNC:1464]   -   689   567   465
        500   296   336   2.425608889   1.931445107
2.079853137   1.997353422   1.308012641   1.123163976
ENSG00000145861 106.148363   113.2793796   105.7867401
25.89921237   93.60232991   17.25278091   108.4048276
45.5847744   1.259333623   0.029509626   0.149721203
C1QTNF2 5   160347751   160370641   -   2414
protein_coding C1q and TNF related 2 [Source:HGNC
Symbol;Acc:HGNC:14325] -   117   128   91   25   82
        20   2.373434656   2.512459933   2.34536788
0.57545953   2.087968521   0.385233235
ENSG00000205726 1126.8057   810.6555603   1017.180194
868.1415987   569.6044223   725.4794371   984.8804846
721.0751527   0.449103565   0.029517382   0.149721203   ITSN1
        21   33642400   33899861   +   21748
protein_coding intersectin 1 [Source:HGNC Symbol;Acc:HGNC:6183]
        -       1242   916   875   838   499   841
2.796603875   1.995733697   2.503200145   2.141098947
1.410355451   1.798074532
ENSG00000141965 144.2529036   157.5291373   115.0866733
269.3518087   144.9694622   279.4950507   138.9562381
231.2721072   -0.734975787   0.029522729   0.149721203   FEM1A
        19   4791681 4801273 +   9593   protein_coding fem-1
homolog A [Source:HGNC Symbol;Acc:HGNC:16934]   -   159   178
        99   260   127   324   0.811654804   0.879208744
        0.642077715   1.506020721   0.813760559   1.570440851
ENSG00000171049 6.350756762   3.539980612   10.46242485   0
        0   0   6.784387408   0   5.191974766
0.029527962   1   FPR2   19   51752026   51770526
        +   3700   protein_coding formyl peptide receptor 2

[Source:HGNC Symbol;Acc:HGNC:3827]	-	7	4	9
	0	0	0	0.092645643	0.051225323
0.151337875	0	0	0
ENSG00000183134 6.350756762	2.654985459	0	8.287747959
	46.80116496	9.489029498	3.001914074	21.5259808
	-2.80883845	0.029552095	0.149827172	PTGDR2	11
	60850940	60855971	-	2895	protein_coding
	prostaglandin D2 receptor 2 [Source:HGNC Symbol;Acc:HGNC:4502]
	-	7	3	0	8	41	11
0.118407212	0.049101994	0	0.153551288	0.870527808
	0.176675014
ENSG00000134308 9011.723845	8195.940113	11723.72829
8385.128998	7913.962845	6499.122567	9643.797416
7599.404803	0.343656401	0.029566642	0.149857973	YWHAQ
	2	9583972 9631014 -	2713	protein_coding
tyrosine 3-monooxygenase/tryptophan 5-monooxygenase activation protein
theta [Source:HGNC Symbol;Acc:HGNC:12854]	-	9933	9261
	10085	8094	6933	7534	179.291345	161.7463657
	231.2772717	165.7774481	157.0792311	129.1239611
ENSG00000170396 362.9003864	234.5237156	179.0237141
483.7972871	325.325171	400.264517	258.8159387
403.1289917	-0.637429032	0.029588557	0.149926093
ZNF804A 2	184598366	184939492	+	4694
protein_coding	zinc finger protein 804A [Source:HGNC
Symbol;Acc:HGNC:21711]	-	400	265	154	467	285
	464	4.172973143	2.675033527	2.041194936
5.528226634	3.732064449	4.59627403
ENSG00000170903 882.7551899	882.3401677	734.6947227
976.9182907	1167.74614	1088.650475	833.2633601
1077.771635	-0.370195906	0.029604393	0.149963375
MSANTD4 11	105995623	106022403	-	5105
protein_coding	Myb/SANT DNA binding domain containing 4 with coiled-
coils [Source:HGNC Symbol;Acc:HGNC:29383]	-	973	997
	632	943	1023	1262	9.333526769	9.253922387
	7.70243742	10.26426817	12.31763264	11.49462021
ENSG00000147394 61.69306569	77.87957347	104.6242485
138.8197783	125.5641011	166.4893357	81.39896255
143.6244051	-0.8246533	0.029619084	0.149994842	ZNF185
	X	152914442	152973480	+	4812
protein_coding	zinc finger protein 185 with LIM domain [Source:HGNC
Symbol;Acc:HGNC:12976]	-	68	88	90	134	110
	193	0.692009374	0.866529783	1.163653653
1.547359492	1.405123272	1.864930744
ENSG00000285781 0	0	0	0	10.27342645
13.80222472	0	8.025217059	-5.48192128	0.029650908
	0.150067145	AL117340.1	10	31362799
31364049	-	1251	unprocessed_pseudogene	putative
UPF0607 protein FLJ37424 [Source:NCBI gene;Acc:100505502]	-
	0	0	0	0	9	16	0	0
	0	0	0.442214074	0.594694184

ENSG00000101236 1004.326819	796.4956378	1104.367067
734.5016629	711.149409	760.8476379	968.3965082
735.4995699	0.395783496	0.029655842	0.150067145	RNF24
20	3927309 4015582 -	7668	protein_coding	ring
finger protein 24 [Source:HGNC Symbol;Acc:HGNC:13779]	-	1107
900	950	709	623	882	7.069589553
5.561434748	7.70811788	5.137787936	4.994054789
5.348321417
ENSG00000108788 1103.217175	1368.202507	1236.891115
1293.92465	1852.641237	1689.047251	1236.103599
1611.871046	-0.382799284	0.029658813	0.150067145	MLX
17	42567068	42573239	+	4259
protein_coding	"MLX, MAX dimerization protein [Source:HGNC
Symbol;Acc:HGNC:11645]" bHLH	1216	1546	1064	1249	1623
1958	13.98152741	17.1999938	15.54321429
16.29546816	23.42384793	21.37647373
ENSG00000280388 13.60876449	10.61994184	0	0	0
0	8.076235442	0	5.451072843	0.029724761
0.150352417	AC006330.1	7	76043977
76045963	-	1987	TEC	TEC	-	15	12
0	0	0	0	0.369676697	0.286160588
0	0	0	0
ENSG00000275074 153.3254133	164.6090985	115.0866733
83.91344809	77.62144432	89.71446071	144.340395
83.74978437	0.786104072	0.029741195	0.150352417	NUDT18
8	22106872	22109419	-	1756
protein_coding	nudix hydrolase 18 [Source:HGNC Symbol;Acc:HGNC:26194]
-	169	186	99	81	68	104
4.712928777	5.018973163	3.507660318	2.563141315
2.380300355	2.753847241
ENSG00000061337 435.4804637	317.71326	291.7854041
215.4814469	196.3365944	281.2203288	348.3263759
231.01279	0.591435079	0.029752524	0.150352417	LZTS1
8	20246165	20303963	-	5706
protein_coding	leucine zipper tumor suppressor 1 [Source:HGNC
Symbol;Acc:HGNC:13861]	-	480	359	251	208	172
326	4.119439733	2.981186336	2.73683617
2.025553	1.852866118	2.656543005
ENSG00000035928 1815.409183	1564.671431	1628.650801
1530.125467	1180.30255	1212.870498	1669.577138
1307.766172	0.352498434	0.029753943	0.150352417	RFC1
4	39287456	39366375	-	6719
protein_coding	replication factor C subunit 1 [Source:HGNC
Symbol;Acc:HGNC:9969]	-	2001	1768	1401	1477	1034
1406	14.58381448	12.46820862	12.97299769
12.21484324	9.459392757	9.729974402
ENSG00000171109 1463.395808	1250.498151	1213.641282
1097.090636	1110.671549	742.732218	1309.178414
983.4981343	0.413962045	0.029757692	0.150352417	MFN1
3	179347692	179394931	+	5957

protein_coding mitofusin 1 [Source:HGNC Symbol;Acc:HGNC:18262] -
1613 1413 1044 1059 973 861 13.25975351
11.239345 10.9038469 9.878258882 10.03997428
6.720577126
ENSG00000179195 1769.139384 1657.595922 1691.425351
1487.650759 1400.610473 1185.266048 1706.053552
1357.842427 0.329913378 0.029772695 0.150354502 ZNF664
12 123971845 124015439 + 6340
protein_coding zinc finger protein 664 [Source:HGNC
Symbol;Acc:HGNC:25406] zf-C2H2 1950 1873 1455 1436 1227
1374 15.06170153 13.99828916 14.27843412
12.58569566 11.89604683 10.07693583
ENSG00000163682 6169.306569 8741.982123 9242.971108
9059.544488 11035.94299 10647.55374 8051.419933
10247.68041 -0.348094687 0.029775104 0.150354502 RPL9
4 39452521 39458949 - 5022
protein_coding ribosomal protein L9 [Source:HGNC
Symbol;Acc:HGNC:10369] - 6800 9878 7951 8745 9668
12343 66.30723036 93.20060988 98.50355209
96.75984168 118.3333865 114.2812649
ENSG00000182934 2723.5674 2901.899107 3153.839846
2410.698688 2526.121416 2226.471376 2926.435451
2387.763826 0.293506162 0.029801028 0.150442462 SRPRA
11 126262919 126269144 - 4167
protein_coding SRP receptor subunit alpha [Source:HGNC
Symbol;Acc:HGNC:11307] - 3002 3279 2713 2327 2213
2581 35.27896788 37.28587652 40.5072854
31.03022513 32.64414315 28.80020123
ENSG00000235286 13.60876449 3.539980612 3.48747495 0
0 0 6.878740017 0 5.217696909
0.029808958 1 AP000925.1 11 111670956
111672404 - 1449 processed_pseudogene keratin 8
(KRT8) pseudogene - 15 4 3 0 0
0 0.50693416 0.130803103 0.128813006 0
0 0
ENSG00000135390 2138.390527 2523.121182 2946.916332
1996.31129 1983.912797 2063.432596 2536.14268
2014.552228 0.331625468 0.029840587 0.150599183
ATP5MC2 12 53632726 53677408 - 3991
protein_coding ATP synthase membrane subunit c locus 2 [Source:HGNC
Symbol;Acc:HGNC:842] - 2357 2851 2535 1927 1738
2392 28.92054936 33.84869506 39.51873938
26.82946611 26.76796801 27.86829927
ENSG00000007255 135.1803939 324.7932212 251.0981964
275.5676196 355.0039586 552.951628 237.0239372
394.5077354 -0.736377409 0.029920963 0.150961758
TRAPPC6A 19 45162928 45178237 - 933
protein_coding trafficking protein particle complex 6A
[Source:HGNC Symbol;Acc:HGNC:23069] - 149 367 216
266 311 641 7.820478172 18.63849585

14.40386207	15.84207396	20.48925207	31.94533185
ENSG00000274600 11.79426256	23.00987398	4.6499666
30.04308635	41.09370581	56.93417699	13.15136771
42.69032305	-1.686347428	0.029938389	0.151006603
RIMBP3B 22	21383374	21389478	+	6105
protein_coding	RIMS binding protein 3B [Source:HGNC
Symbol;Acc:HGNC:33891]	-	13	26	4	29	36
66	0.104276481	0.201796728	0.040764411
0.263951645	0.362463427	0.502677314
ENSG00000268199 0	0	0	3.107905485	0
21.56597613	0	8.224627206	-5.521787322	0.030060283
0.15157595	AC010335.1	19	18441419
18443597	+	599	antisense	"novel transcript,
antisense to ELL"	-	0	0	0	3	0
25	0	0	0	0.278295687	0
1.94063654
ENSG00000267534 164.2124248	209.7438513	156.9363727
66.30198367	113.007691	127.6705787	176.9642163
102.3267511	0.789662454	0.030068405	0.15157595	S1PR2
19	10221433	10231331	-	3643
protein_coding	sphingosine-1-phosphate receptor 2 [Source:HGNC
Symbol;Acc:HGNC:3169]	-	181	237	135	64	99
148	2.433033492	3.082588938	2.305586624
0.97618661	1.670411163	1.889006978
ENSG00000148296 1221.1598	1673.525835	1235.728624
1415.132964	1800.132613	2385.19696	1376.804753
1866.820846	-0.439349901	0.030120322	0.151769274	SURF6
9	133328774	133336398	-	4626
protein_coding	surfeit 6 [Source:HGNC Symbol;Acc:HGNC:11478]	-
1346	1891	1063	1366	1577	2765	14.24846615
19.36922933	14.2966565	16.40805543	20.95431292
27.79204635
ENSG00000173409 578.8261163	448.6925426	506.8463594
448.5743583	335.5985975	294.1599144	511.4550061
359.4442901	0.509653684	0.030123914	0.151769274	ARV1
1	230978981	231000595	+	1987
protein_coding	"ARV1 homolog, fatty acid homeostasis modulator
[Source:HGNC Symbol;Acc:HGNC:29561]"	-	638	507	436
433	294	341	15.72358219	12.09028485
13.65197451	12.10882696	9.094876864	7.979717746
ENSG00000027075 38.10454057	19.46989337	34.8747495
6.215810969	0	12.93958568	30.81639448	6.385132216
2.244961486	0.030165661	0.151936324	PRKCH	14
61187559	61550976	+	8833	protein_coding
protein kinase C eta [Source:HGNC Symbol;Acc:HGNC:9403] -
42	22	30	6	0	15	0.232846516
0.118016	0.211309913	0.037744621	0
0.078961256
ENSG00000143878 410.9846876	515.9521743	903.256012
371.9126897	425.7764519	417.5172979	610.0642913

405.0688132	0.588911539	0.030195493	0.152043287	RHOB
	2	20447074	20449445	+	2372
protein_coding	ras homolog family member B [Source:HGNC
Symbol;Acc:HGNC:668]	-	453	583	777	359	373
	484	9.35216587	11.64609453	20.38042239
8.409918896	9.665882407	9.487716411
ENSG00000163513 3848.558598	3501.040826	4623.229292
3558.55178	3039.792738	2970.928872	3990.942905
3189.757797	0.323022879	0.030229465	0.152171034	TGFBR2
	3	30606502	30694142	+	4704
protein_coding	transforming growth factor beta receptor 2
[Source:HGNC Symbol;Acc:HGNC:11773]	-	4242	3956	3977
	3435	2663	3444	44.16030199	39.84881496
52.60113613	40.57620929	34.797754	34.04292342
ENSG00000119396 2276.292674	2359.397078	2452.857381
2817.834306	2814.918848	2945.91234	2362.849044
2859.555165	-0.275532484	0.030240513	0.152183336	RAB14
	9	121178137	121223014	-	4304
protein_coding	"RAB14, member RAS oncogene family [Source:HGNC
Symbol;Acc:HGNC:16524]" -	2509	2666	2110	2720	2466
	3415	28.54677698	29.3504202	30.50120795
35.1162947	35.21828226	36.89346651
ENSG00000138594 2161.979052	1749.635418	2429.607548
1997.347258	1541.013968	1168.013267	2113.740673
1568.791498	0.430265415	0.030250908	0.152192352	TMOD3
	15	51829628	51947295	+	10632
protein_coding	tropomodulin 3 [Source:HGNC Symbol;Acc:HGNC:11873]
	-	2383	1977	2090	1928	1350	1354
10.97583976	8.810856787	12.2303297	10.07636998
7.804878753	5.921540644
ENSG00000150783 1.814501932	0	6.974949899	6.215810969
	35.38624667	19.84069804	2.929817277	20.48091856
	-2.848873455	0.030264904	0.152219475	TEX12	11
	112167372	112172559	+	1155	protein_coding
	testis expressed 12 [Source:HGNC Symbol;Acc:HGNC:11734] -
	2	0	6	6	31	23	0.084796259
	0	0.323203543	0.288656479	1.649783933
0.925927259
ENSG00000171017 92.53959853	107.0844135	105.7867401
110.848629	152.959905	288.9840802	101.8035841
184.2642047	-0.85907992	0.030296027	0.152332696	LRRC8E
	19	7888505 7902021 +	4329	protein_coding
leucine rich repeat containing 8 VRAC subunit E [Source:HGNC
Symbol;Acc:HGNC:26272]	-	102	121	91	107	134
	335	1.15382852	1.32441541	1.30785818
1.373435395	1.902674829	3.59822436
ENSG00000100294 468.1414984	538.0770531	512.6588176
557.3510503	657.499293	885.0676605	506.2924564
699.9726679	-0.468453154	0.03031236	0.152354533	MCAT
	22	43132206	43143394	-	2388

protein_coding malonyl-CoA-acyl carrier protein transacylase
[Source:HGNC Symbol;Acc:HGNC:29622]   -   516   608   441
        538   576   1026   10.58142268   12.06412137
11.48976414   12.51872205   14.82639346   19.97763419
ENSG00000172315 908.1582169   1019.514416   968.3555444
1103.306447   1157.472714   1499.266661   965.3427259
1253.348607   -0.377407054   0.030317596   0.152354533   TP53RK
        20   46684365   46689779   -   3520
protein_coding TP53 regulating kinase [Source:HGNC
Symbol;Acc:HGNC:16197] -   1001   1152   833   1065   1014
        1738   13.9257982   15.50730242   14.72343639
16.81198477   17.70690477   22.95821543
ENSG00000186020 335.6828574   174.3440452   235.9858049
370.8767212   437.1913702   332.9786715   248.6709025
380.3489209   -0.612049191   0.03033042   0.152363834   ZNF529
        19   36534774   36605276   -   8173
protein_coding zinc finger protein 529 [Source:HGNC
Symbol;Acc:HGNC:29328] zf-C2H2 370   197   203   358   383
        386   2.21691432   1.142118505   1.545330533
2.433960755   2.880478482   2.196022694
ENSG00000182963 2909.553848   2565.600949   2469.132264
2386.871412   1791.000678   2133.306359   2648.095687
2103.72615   0.33194283   0.03035614   0.152363834   GJC1
        17   44798448   44830816   -   8660
protein_coding gap junction protein gamma 1 [Source:HGNC
Symbol;Acc:HGNC:4280]   -   3207   2899   2124   2304   1569
        2473   18.13467394   15.86195685   15.25961115
14.78347824   11.13659463   13.27813997
ENSG00000198909 1246.562827   1349.617609   1496.126753
1020.428967   1049.03099   1161.112155   1364.102396
1076.857371   0.340221227   0.030357629   0.152363834   MAP3K3
        17   63622415   63696303   +   6107
protein_coding mitogen-activated protein kinase kinase kinase 3
[Source:HGNC Symbol;Acc:HGNC:6855]   -   1374   1525   1287
        985   919   1346   11.01761256   11.83227799
13.1116538   8.962318097   9.24985557   10.24821335
ENSG00000229994 12.70151352   10.61994184   12.78740815
68.37392066   25.11282022   23.29125422   12.03628784
38.92599837   -1.693919349   0.030359963   0.152363834   RPL5P4
        1   35350722   35351607   +   886
processed_pseudogene   ribosomal protein L5 pseudogene 4 [Source:HGNC
Symbol;Acc:HGNC:36095] -   14   12   11   66   22
        27   0.773789794   0.641761951   0.772441876
4.139255717   1.5262874   1.416971321
ENSG00000279140 9.07250966   15.0449176   0   0   0
        0   8.039142421   0   5.444625809   0.030362514
        0.152363834   AL590326.1   14   104815449
104815753   -   305   TEC   novel transcript   -
        10   17   0   0   0   0   1.605568519
        2.641043308   0   0   0   0

ENSG00000004864 891.8276996 938.0948623 946.268203
710.6743875 811.6006898 596.9462193 925.3969216
706.4070989 0.390679732 0.030400013 0.152456473
SLC25A13 7 96120220 96322147 - 3710
protein_coding solute carrier family 25 member 13
[Source:HGNC Symbol;Acc:HGNC:10983] - 983 1060 814
686 711 692 12.97502764 13.53812116
13.65077605 10.27453675 11.77993926 8.672877315
ENSG00000156587 721.264518 763.7508171 734.6947227
743.8253793 1296.734717 995.4854582 739.9033526
1012.015185 -0.451276286 0.030408082 0.152456473 UBE2L6
11 57551656 57568284 - 2197
protein_coding ubiquitin conjugating enzyme E2 L6 [Source:HGNC
Symbol;Acc:HGNC:12490] - 795 863 632 718 1136
1154 17.72008314 18.61260581 17.89756169
18.15960639 31.78305465 24.42343643
ENSG00000100124 1028.822595 1343.422642 1328.727956
1289.780776 1593.522592 2066.883152 1233.657731
1650.062174 -0.420341383 0.03041383 0.152456473
ANKRD54 22 37830855 37849327 - 4487
protein_coding ankyrin repeat domain 54 [Source:HGNC
Symbol;Acc:HGNC:25185] - 1134 1518 1143 1245 1396
2396 12.37615297 16.03031819 15.84882272
15.41790359 19.12391078 24.82914486
ENSG00000078549 0 0 2.3249833 1.035968495
25.11282022 11.21430759 0.774994433
12.45436543 -4.113622408 0.03041545 0.152456473
ADCYAP1R1 7 31052461 31111479 + 6832
protein_coding ADCYAP receptor type I [Source:HGNC
Symbol;Acc:HGNC:242] - 0 0 2 1 22
13 0 0 0.018213314 0.008133251
0.197934812 0.08847621
ENSG00000281392 24.49577608 25.66485944 20.9248497
55.94229872 39.95221399 76.77487503 23.69516174
57.55646258 -1.28111915 0.030444468 0.15251722
LINC00506 3 87089129 87157069 + 5011
lincRNA long intergenic non-protein coding RNA 506
[Source:NCBI gene;Acc:100846978] - 27 29 18
54 35 89 0.26385665 0.274220574
0.223488381 0.598799461 0.429329768 0.825841346
ENSG00000232956 436.3877146 625.6915733 430.1219105
562.5308927 660.9237685 906.6336366 497.4003995
710.0294326 -0.513453561 0.030447708 0.15251722 SNHG15
7 44983023 44986961 - 3647
lincRNA small nucleolar RNA host gene 15 [Source:HGNC
Symbol;Acc:HGNC:27797] - 481 707 370 543 579
1051 6.458594176 9.185654184 6.312084547
8.273249275 9.758659405 13.39978934
ENSG00000177548 818.3403713 906.2350368 685.8700734
895.0767796 1028.484137 1307.760793 803.4818272

1077.107236   -0.422643245   0.030453436   0.15251722   RABEP2
        16   28904421   28936526   -   4566
protein_coding "rabaptin, RAB GTPase binding effector protein 2
[Source:HGNC Symbol;Acc:HGNC:24817]"   -   902   1024   590
        864   901   1516   9.673849218   10.62650596
8.039387294   10.51453035   12.12931414   15.43811644
ENSG00000272886 987.089051   958.4497508   968.3555444
1273.20528   1154.048238   1229.26064   971.2981154
1218.838053   -0.327675889   0.030472917   0.152571591   DCP1A
        3   53283428   53347610   -   6127
protein_coding decapping mRNA 1A [Source:HGNC Symbol;Acc:HGNC:18714]
        -       1088   1083   833   1229   1011   1425
8.695803123   8.375428146   8.458706726   11.1459232
10.14263123   10.81428976
ENSG00000186153 439.1094675   434.5326202   485.9215097
361.5530047   278.5240061   336.4292277   453.1878658
325.5020795   0.47597496   0.030484793   0.15258786   WWOX
        16   78099413   79212667   +   4886
protein_coding WW domain containing oxidoreductase [Source:HGNC
Symbol;Acc:HGNC:12799] -   484   491   418   349   244
        390   4.850880573   4.761617112   5.322671526
3.969026586   3.069613696   3.711437594
ENSG00000160013 2.721752898   23.00987398   4.6499666
27.97114936   47.94265678   40.54403513   10.12719783
38.81928042   -1.925394856   0.030523252   0.152711165   PTGIR
        19   46620468   46625118   -   3546
protein_coding prostaglandin I2 receptor [Source:HGNC
Symbol;Acc:HGNC:9602]   -   3   26   4   27   42
        47   0.041429645   0.347424993   0.070182382
0.423094205   0.72804449   0.616297129
ENSG00000095739 38.10454057   58.40968011   75.56195724
52.83439324   164.3748233   146.6486377   57.35872597
121.2859514   -1.083934684   0.030526693   0.152711165   BAMBI
        10   28677342   28682939   +   2314
protein_coding BMP and activin membrane bound inhibitor [Source:HGNC
Symbol;Acc:HGNC:30251] -   42   66   65   51   144
        170   0.888821639   1.351471905   1.747659607
1.224669396   3.825132626   3.415989955
ENSG00000198467 404.6339308   538.0770531   459.1842017
263.1359977   324.1836792   391.6381266   467.2983952
326.3192678   0.51699741   0.030640144   0.153220806   TPM2
        9   35681992   35690121   -   3248
protein_coding tropomyosin 2 [Source:HGNC Symbol;Acc:HGNC:12011]
        -       446   608   395   254   284   454
6.724306824   8.869803518   7.566376044   4.345399797
5.374643709   6.499363849
ENSG00000143507 1438.900032   1372.627482   1136.916834
1389.233752   1717.945201   2061.707318   1316.148116
1722.96209   -0.388427389   0.030645894   0.153220806   DUSP10
        1   221701424   221742176   -   2914

protein_coding dual specificity phosphatase 10 [Source:HGNC
Symbol;Acc:HGNC:3065]   -   1586   1551   978   1341   1505
        2390   26.65276801   25.22020958   20.8812337
25.57120972   31.74638748   38.13637168
ENSG00000168917 205.0387183   198.2389143   262.7231129
152.2873688   150.6769213   122.4947444   222.0002485
141.8196782   0.646392056   0.030719645   0.153546142
SLC35G2 3   136818647   136855892   +   4287
protein_coding solute carrier family 35 member G2 [Source:HGNC
Symbol;Acc:HGNC:28480] -   226   224   226   147   132
        142   2.581568417   2.475830881   3.279909058
1.90535496   1.8926391   1.540160138
ENSG00000167720 176.0066874   174.3440452   162.748831
213.4095099   311.6272691   256.2037964   171.0331878
260.4135252   -0.605132493   0.030755161   0.153670233   SRR
        17   2303383 2325260 +   3498   protein_coding serine
racemase [Source:HGNC Symbol;Acc:HGNC:14398]   -   194   197
        140   206   273   297   2.715880197   2.668534745
        2.490090189   3.27234782   4.797226257   3.947913807
ENSG00000160785 1359.061947   1258.463108   1159.004175
1449.319924   1644.889724   1642.464742   1258.843077
1578.891464   -0.326397159   0.030761846   0.153670233
SLC25A44   1   156193932   156212796   +   7293
        protein_coding solute carrier family 25 member 44
[Source:HGNC Symbol;Acc:HGNC:29036]   -   1498   1422   997
        1399   1441   1904   10.05852462   9.238890581
8.505420535   10.65917383   12.14521277   12.13924701
ENSG00000198208 45.3625483   62.83465587   22.08734135
22.79130689   3.424475485   12.07694663   43.42818184
12.764243   1.764955209   0.030785875   0.153746852
RPS6KL1 14   74903954   74923396   -   7617
protein_coding ribosomal protein S6 kinase like 1 [Source:HGNC
Symbol;Acc:HGNC:20222] -   50   71   19   22   3
        14   0.321450964   0.441672983   0.155194559
0.160491032   0.024209435   0.085462403
ENSG00000103966 3030.218226   3427.586228   3890.859552
3067.502713   2668.807894   2576.702828   3449.554669
2771.004479   0.315797345   0.030805716   0.153802515   EHD4
        15   41895939   41972578   -   7879
protein_coding EH domain containing 4 [Source:HGNC
Symbol;Acc:HGNC:3245]   -   3340   3873   3347   2961   2338
        2987   20.75888628   23.29178847   26.42965283
20.88234904   18.23982917   17.62767928
ENSG00000164038 494.4517765   524.8021258   454.5342351
510.732468   785.3463778   739.2816618   491.2627125
678.4535025   -0.465141161   0.030818319   0.1538126   SLC9B2
        4   103019868   103085829   -   5374
protein_coding solute carrier family 9 member B2 [Source:HGNC
Symbol;Acc:HGNC:25143] -   545   593   391   493   688
        857   4.966237943   5.228576566   4.526744076

5.097547729	7.869336859	7.415051607
ENSG00000158163 281.2477995	162.8391082	182.511189
139.8557468	148.3939377	63.83528935	208.8660322
117.3616579	0.836219969	0.0308263	0.1538126	DZIP1L
3	138061990	138115818	-	5045
protein_coding	DAZ interacting zinc finger protein 1 like
[Source:HGNC Symbol;Acc:HGNC:26551]	-	310	184	157
135	130	74	3.009048632	1.728156596
1.936178211	1.486909861	1.583906503	0.682026999
ENSG00000231991 1132.249206	1152.263689	1677.475451
1158.212777	964.5605948	795.3531997	1320.662782
972.7088573	0.440814171	0.030833822	0.1538126
ANXA2P2 9	33624274	33625293	+	1020
processed_pseudogene	annexin A2 pseudogene 2 [Source:HGNC
Symbol;Acc:HGNC:539]	-	1248	1302	1443	1118	845
922	59.91603932	60.48354718	88.01830601
60.90510208	50.92181765	42.03023008
ENSG00000272657 8.165258694	7.079961225	4.6499666	0
0	0	6.631728839	0	5.164220403
0.030857959	1	AP000317.2	21	34073592
34360033	+	641	processed_transcript	novel
transcript	-	9	8	4	0	0	0
0.687564054	0.591368787	0.388247626	0	0
0
ENSG00000125637 649.5916916	718.6160643	676.5701402
719.998104	945.1552337	1062.771304	681.5926321
909.3082138	-0.416280481	0.030876116	0.153980156	PSD4
2	113157325	113209396	+	13442
protein_coding	pleckstrin and Sec7 domain containing 4 [Source:HGNC
Symbol;Acc:HGNC:19096]	-	716	812	582	695	828
1232	2.608421762	2.862322596	2.69380367
2.872982345	3.786289403	4.261650547
ENSG00000127325 22.68127415	36.28480128	23.249833
56.97826722	43.37668947	96.61557307	27.40530281
65.65684325	-1.261028951	0.030892142	0.154016659	BEST3
12	69643360	69699476	-	6863
protein_coding	bestrophin 3 [Source:HGNC Symbol;Acc:HGNC:17105]
-	25	41	20	55	38	112
0.178383505	0.283071599	0.181310453	0.445308243
0.340343106	0.758813488
ENSG00000183160 1320.050155	1269.968045	1207.828824
1111.594195	1043.323531	602.1220536	1265.949008
919.0132599	0.463360546	0.030967599	0.154349355
TMEM119 12	108589846	108598320	-	3189
protein_coding	transmembrane protein 119 [Source:HGNC
Symbol;Acc:HGNC:27884]	-	1455	1435	1039	1073	914
698	22.34277735	21.32179791	20.27065933
18.69636794	17.6172873	10.17728465
ENSG00000116661 1.814501932	5.309970919	6.974949899
24.86324388	37.66923033	7.763751407	4.699807583

23.4320752	-2.326120544	0.030976792	0.154351682	FBXO2
	1	11637018	11655785	-	3416
protein_coding	F-box protein 2 [Source:HGNC Symbol;Acc:HGNC:13581]
	-	2	6	6	24	33	9
0.028670866	0.083226155	0.109279886	0.390396058
0.593804437	0.122505522
ENSG00000171466 592.4348808	587.6367817	477.7840681
872.2854727	671.197195	688.3859581	552.6185769
743.9562086	-0.42778085	0.030991101	0.154379497	ZNF562
	19	9641808 9675086 -	13007	protein_coding	zinc
finger protein 562 [Source:HGNC Symbol;Acc:HGNC:25950]	zf-C2H2 653
	664	411	842	588	798	2.458468933
2.418897023	1.965945745	3.597054309	2.778737653
2.852704382
ENSG00000163762 9.07250966	8.849951531	2.3249833	0
	0	0	6.749148164	0	5.191579071
0.031020184	1	TM4SF18 3	149318498	149334414
	-	4482	protein_coding	transmembrane 4 L six family
member 18 [Source:HGNC Symbol;Acc:HGNC:25181]	-	10	10
	2	0	0	0	0.109258902	0.105719375
	0.02776291	0	0	0
ENSG00000174307 746.667545	994.7345521	1033.455077
866.0696617	1313.857094	1740.805593	924.9523913
1306.910783	-0.499632321	0.031050614	0.154495369	PHLDA3
	1	201464383	201469237	-	3138
protein_coding	pleckstrin homology like domain family A member 3
[Source:HGNC Symbol;Acc:HGNC:8934]	-	823	1124	889
	836	1151	2018	12.84326902	16.97226534
17.62607722	14.80353319	22.54601256	29.9019314
ENSG00000187634 40.82629347	28.3198449	44.1746827	0
	10.27342645	16.39014186	37.77360702	8.887856105
	2.076454762	0.03105345	0.154495369	SAMD11	1
	923928	944581	+	4166	protein_coding	sterile alpha
motif domain containing 11 [Source:HGNC Symbol;Acc:HGNC:28706]	-
	45	32	38	0	9	19	0.528958904
	0.363962931	0.567506941	0	0.1327916
0.212063221
ENSG00000133962 0	4.424975766	0	4.14387398
7.990442797	37.95611799	1.474991922
16.69681159	-3.463192755	0.031054017	0.154495369
CATSPERB	14	91580696	91780707	-	5635
	protein_coding	cation channel sperm associated auxiliary
subunit beta [Source:HGNC Symbol;Acc:HGNC:20500]	-	0
	5	0	4	7	44	0	0.042043855
	0	0.039443742	0.076357461	0.363069506
ENSG00000267002 205.0387183	173.45905	151.1239145
236.2008168	239.7132839	339.8797838	176.5405609
271.9312949	-0.623309496	0.031056297	0.154495369
AC060780.1	17	43148368	43171037	-	8450
	lincRNA novel transcript	-	226	196	130

228	210	394	1.309725894	1.099071138
0.957179723	1.499305664	1.527601042	2.168056176
ENSG00000156709 1237.490318	1262.888084	1310.128089
1041.148337	932.5988236	1058.458109	1270.16883
1010.73509	0.328970755	0.031058032	0.154495369	AIFM1
X	130129362	130165887	-	3306
protein_coding	apoptosis inducing factor mitochondria associated 1
[Source:HGNC Symbol;Acc:HGNC:8768]	-	1364	1427	1127
1005	817	1227	20.20413234	20.45255479
21.20937708	16.89177376	15.19030757	17.25729534
ENSG00000109705 95.26135143	99.11945715	60.44956579
27.97114936	33.10326302	56.93417699	84.94345812
39.33619646	1.107146081	0.031075313	0.154526395	NKX3-2
4	13540830	13545050	-	2801
protein_coding	NK3 homeobox 2 [Source:HGNC Symbol;Acc:HGNC:951]
Homeobox	105	112	52	27	29	66
1.835713382	1.894660298	1.155040151	0.535627294
0.636403045	1.095624777
ENSG00000113719 2864.1913	2862.074325	3256.139111
2496.684073	2623.148221	2159.18553	2994.134912
2426.339275	0.303478332	0.031081828	0.154526395	ERGIC1
5	172834275	172952685	+	14139
protein_coding	endoplasmic reticulum-golgi intermediate compartment 1
[Source:HGNC Symbol;Acc:HGNC:29205]	-	3157	3234	2801
2410	2298	2503	10.93413851	10.83796544
12.32540677	9.47131742	9.990303684	8.231389893
ENSG00000187621 26.31027801	21.23988367	18.5998664	0
0	8.626390453	22.05000936	2.875463484
2.889164327	0.031090477	0.154526395	TCL6	14
95650498	95679833	+	10566	processed_transcript
T cell leukemia/lymphoma 6 [Source:HGNC Symbol;Acc:HGNC:13463]
-	29	24	16	0	0	10
0.134405201	0.107628448	0.094214169	0	0
0.044006863
ENSG00000165283 3248.865709	3651.490002	3436.325317
3821.687778	4535.147033	4218.304931	3445.560343
4191.713247	-0.282709107	0.031115686	0.154565138	STOML2
9	35099776	35103195	-	1806
protein_coding	stomatin like 2 [Source:HGNC Symbol;Acc:HGNC:14559]
-	3581	4126	2956	3689	3973	4890
97.09911209	108.2524959	101.8341705	113.5018533
135.2222566	125.8989567
ENSG00000141934 1271.058603	1212.44336	1338.027889
1089.838857	996.522366	948.0403108	1273.843284
1011.467178	0.332689488	0.031115747	0.154565138	PLPP2
19	281040	291504	-	7059	protein_coding
phospholipid phosphatase 2 [Source:HGNC Symbol;Acc:HGNC:9230]	-
1401	1370	1151	1052	873	1099	9.719045984
9.196102983	10.14469486	8.281034645	7.601834705
7.23911588

ENSG00000030582 3381.32435 4024.957956 4268.669338
3148.308256 3320.599728 3033.038883 3891.650548
3167.315622 0.297042807 0.031128412 0.154584639 GRN
17 44345086 44353102 + 3680
protein_coding granulin precursor [Source:HGNC Symbol;Acc:HGNC:4601]
- 3727 4548 3672 3039 2909 3516
49.59526985 58.55973169 62.08142835 45.88755574
48.58959316 44.42547355
ENSG00000164366 435.4804637 457.5424942 433.6093854
619.50916 490.8414861 697.8749876 442.2107811
602.7418779 -0.44782185 0.031165448 0.15472512
CCDC127 5 196871 218215 - 9701 protein_coding
coiled-coil domain containing 127 [Source:HGNC Symbol;Acc:HGNC:30520]
- 480 517 373 598 430 809
2.423000012 2.525227321 2.392209297 3.425285082
2.724578412 3.877600254
ENSG00000249628 8.165258694 9.734946684 2.3249833 0
0 0 6.741729559 0 5.190052354
0.03118639 1 LINC00942 12 1500525 1504424 +
2210 lincRNA long intergenic non-protein coding RNA 942
[Source:HGNC Symbol;Acc:HGNC:48636] - 9 11 2
0 0 0 0.199424687 0.235845097
0.05630469 0 0 0
ENSG00000253540 122.4788804 96.46447169 33.71225785
130.5320304 272.8165469 117.3189102 84.21853665
173.5558291 -1.036017686 0.031235686 0.155011729
FAM86HP 3 130099092 130111472 - 1301
transcribed_unprocessed_pseudogene "family with sequence
similarity 86 member H, pseudogene [Source:HGNC
Symbol;Acc:HGNC:42359]" - 135 109 29 126 239
136 5.081420736 3.969864121 1.386843796
5.381524133 11.29192447 4.860630749
ENSG00000132613 709.4702554 618.611612 759.1070474
499.3368145 586.7267997 478.7646701 695.7296383
521.6094281 0.415822675 0.031240704 0.155011729 MTSS1L
16 70661204 70686066 - 4999
protein_coding "MTSS1L, I-BAR domain containing [Source:HGNC
Symbol;Acc:HGNC:25094]" - 782 699 653 482 514
555 7.660415033 6.625527789 8.127124095
5.357669814 6.320149369 5.162271771
ENSG00000177842 113.4063707 123.8993214 95.32431529
137.7838098 189.4876435 225.1487908 110.8766692
184.1400814 -0.731053433 0.031267169 0.155099536 ZNF620
3 40477131 40518736 + 3783
protein_coding zinc finger protein 620 [Source:HGNC
Symbol;Acc:HGNC:28742] zf-C2H2 125 140 82 133 166
261 1.618088813 1.753549925 1.348603734
1.953562649 2.69723699 3.208006091
ENSG00000159147 1236.583067 1028.364368 932.3183032
938.5874564 753.3846066 734.9684666 1065.755246

808.9801765	0.398480659	0.031277245	0.155103355	DONSON
	21	33559542	33588708	-	3513
protein_coding	downstream neighbor of SON [Source:HGNC
Symbol;Acc:HGNC:2993]	-	1363	1162	802	906	660
	852	18.99968451	15.67308248	14.20375149
14.33052468	11.5481694	11.27697099
ENSG00000189306	852.815908	1004.469499	877.6811957
1051.508022	1146.057795	1321.563017	911.6555342
1173.042945	-0.363885649	0.031285475	0.155103355	RRP7A
	22	42509968	42519802	-	4082
protein_coding	ribosomal RNA processing 7 homolog A [Source:HGNC
Symbol;Acc:HGNC:24286]	-	940	1135	755	1015	1004
	1532	11.27673921	13.1749599	11.50749508
13.8167241	15.11847801	17.45085794
ENSG00000176641	179.6356913	161.954113	219.7109218
135.7118728	34.24475485	119.0441882	187.100242
96.33360531	0.953069547	0.03131227	0.155192698	RNF152
	18	61808063	61894247	-	10632
protein_coding	ring finger protein 152 [Source:HGNC
Symbol;Acc:HGNC:26811]	-	198	183	189	131	30
	138	0.911966543	0.81557248	1.105996322
0.68464962	0.17344175	0.603524822
ENSG00000104472	1542.326642	1551.396503	1624.000835
2095.764265	1724.794152	2040.141342	1572.57466
1953.566587	-0.313477227	0.031329038	0.155232312	CHRAC1
	8	140511298	140517137	+	3301
protein_coding	chromatin accessibility complex subunit 1 [Source:HGNC
Symbol;Acc:HGNC:13544]	-	1700	1753	1397	2023	1511
	2365	25.21924499	25.16302404	26.33041647
34.05355073	28.13625557	33.31322199
ENSG00000106868	76.20908114	51.32971888	69.74949899
41.4387398	26.25431205	9.489029498	65.76276634
25.72736045	1.360535133	0.031351824	0.155301711	SUSD1
	9	112040785	112175408	-	4183
protein_coding	sushi domain containing 1 [Source:HGNC
Symbol;Acc:HGNC:25413]	-	84	58	60	40	23
	11	0.983377133	0.657001816	0.892421927
0.531354264	0.337977144	0.122274484
ENSG00000048740	145.1601546	52.21471403	29.06229125
22.79130689	43.37668947	2.587917136	75.47905328
22.91863783	1.732579791	0.03137693	0.155382561	CELF2
	10	10798397	11336675	+	10978
protein_coding	CUGBP Elav-like family member 2 [Source:HGNC
Symbol;Acc:HGNC:2550]	-	160	59	25	22	38
	3	0.713716011	0.25465677	0.14168492
0.111355455	0.212768695	0.012706591
ENSG00000118680	3255.216466	3773.619333	5247.487308
3210.466366	3160.790872	3158.121545	4092.107702
3176.459594	0.365009216	0.031445023	0.155676187	MYL12B
	18	3261479 3278431 +	1932	protein_coding	myosin

light chain 12B [Source:HGNC Symbol;Acc:HGNC:29827]	-	3588
	4264	4514	3099	2769	3661	90.94398826
104.5770808	145.3654775	89.13053181	88.09742079
88.1096802
ENSG00000005379 236.7925021	321.2532406	258.0731463
136.7478413	206.6100209	187.1926728	272.0396297
176.8501784	0.622120136	0.031489296	0.155851751
TSPOAP1 17	58301228	58328760	-	11354
protein_coding	TSPO associated protein 1 [Source:HGNC
Symbol;Acc:HGNC:16831]	-	261	363	222	132	181
	217	1.12569387	1.514900734	1.216496689
0.646006793	0.979889367	0.888672748
ENSG00000197283 351.1061238	376.1229401	355.7224449
287.9992416	178.0727252	268.2807431	360.9838363
244.7842366	0.558756706	0.031557888	0.156147546
SYNGAP1 6	33419661	33457541	+	12129
protein_coding	synaptic Ras GTPase activating protein 1 [Source:HGNC
Symbol;Acc:HGNC:11497]	-	387	425	306	278	156
	311	1.562480667	1.660314554	1.569651636
1.273596461	0.790582074	1.192247474
ENSG00000011590 2.721752898	4.424975766	0	10.35968495
	22.82983656	18.11541995	2.382242888	17.10164715
	-2.805909761	0.031571976	0.156173569	ZBTB32	19
	35704527	35717038	+	2770	protein_coding
	zinc finger and BTB domain containing 32 [Source:HGNC
Symbol;Acc:HGNC:16763]	ZBTB	3	5	0	10	20
	21	0.053035928	0.085529646	0	0.200600622
	0.443810514	0.352509271
ENSG00000043143 938.0974988	784.1057057	1093.904643
735.5376314	793.3368206	551.2263499	938.7026157
693.366934	0.437432704	0.031615532	0.1563453	JADE2
	5	134524312	134583230	+	9877
protein_coding	jade family PHD finger 2 [Source:HGNC
Symbol;Acc:HGNC:22984]	-	1034	886	941	710	695
	639	5.126537854	4.25045193	5.927498002
3.994342843	4.32520913	3.008200801
ENSG00000166924 0.907250966	0	0	0	37.66923033
	0	0.302416989	12.55641011	-5.159743843
0.031638839	0.156416831	NYAP1	7	100483927
100494799	+	3586	protein_coding	neuronal tyrosine
phosphorylated phosphoinositide-3-kinase adaptor 1 [Source:HGNC
Symbol;Acc:HGNC:22009]	-	1	0	0	0	33
	0	0.013655839	0	0	0	0.565654198
	0
ENSG00000165678 3312.373277	3612.550215	3787.397795
3107.905485	3007.830967	2664.692011	3570.773762
2926.809488	0.286983824	0.031701108	0.156635785	GHITM
	10	84139440	84153245	+	2128
protein_coding	growth hormone inducible transmembrane protein
[Source:HGNC Symbol;Acc:HGNC:17281]	-	3651	4082	3258

3000 2635 3089 84.01733328 90.8924516
95.25467574 78.33605104 76.11256468 67.49588604
ENSG00000165487 1223.881553 1106.243941 1196.203908
1073.263361 689.4610642 899.7325242 1175.443134
887.4856497 0.404772791 0.031720469 0.156635785 MICU2
13 21492691 21604214 - 3645
protein_coding mitochondrial calcium uptake 2 [Source:HGNC
Symbol;Acc:HGNC:31830] - 1349 1250 1029 1036 604
1043 18.12354292 16.24945955 17.56405097
15.79335023 10.1856035 13.3050893
ENSG00000143882 18.14501932 12.38993214 12.78740815
4.14387398 0 0 14.44078654 1.381291327
3.399946567 0.031720688 0.156635785 ATP6V1C2 2
10721100 10785110 + 3483 protein_coding
ATPase H+ transporting V1 subunit C2 [Source:HGNC
Symbol;Acc:HGNC:18264] - 20 14 11 4 0
0 0.281193453 0.190458782 0.196492536
0.063814381 0 0
ENSG00000268089 376.5091509 266.3835411 363.8598864
197.8699825 262.5431205 219.9729565 335.5841928
226.7953532 0.565432379 0.031724676 0.156635785 GABRQ
X 152638183 152657534 + 6172
protein_coding gamma-aminobutyric acid type A receptor theta subunit
[Source:HGNC Symbol;Acc:HGNC:14454] - 415 301 313
191 230 255 3.292690138 2.310824796
3.155188183 1.71956855 2.290600118 1.92107925
ENSG00000182307 2782.538713 3480.685937 3174.764696
3640.393291 3811.441214 4141.530056 3145.996449
3864.454854 -0.296873289 0.031727402 0.156635785
C8orf33 8 145052378 145066685 + 4440
protein_coding chromosome 8 open reading frame 33 [Source:HGNC
Symbol;Acc:HGNC:26104] - 3067 3933 2731 3514 3339
4801 33.82668891 41.97274928 38.26886454
43.97752971 46.22541397 50.2782038
ENSG00000134440 5979.691117 5821.498117 5922.894956
8018.396151 6937.987332 6475.831313 5908.028063
7144.071598 -0.274000838 0.031737245 0.156640661 NARS
18 57600656 57622213 - 4089
protein_coding asparaginyl-tRNA synthetase [Source:HGNC
Symbol;Acc:HGNC:7643] - 6591 6578 5095 7740 6078
7507 78.93377704 76.22601209 77.52359863
105.1806605 91.36733243 85.36509411
ENSG00000182685 40.82629347 46.90474312 32.5497662
3.107905485 7.990442797 23.29125422 40.09360093
11.46320083 1.791131821 0.031762758 0.156722853 BRICD5
16 2209253 2211950 - 2083 protein_coding
BRICHOS domain containing 5 [Source:HGNC Symbol;Acc:HGNC:28309] -
45 53 28 3 7 27 1.057917807
1.205627208 0.836326018 0.080028381 0.206564711
0.602705996

ENSG00000125898 291.2275601 269.0385265 358.0474282
161.6110852 221.4494147 228.599347 306.1045049
203.8866156 0.584351599 0.031773976 0.156734483
FAM110A 20 833715 857463 + 2722 protein_coding family
with sequence similarity 110 member A [Source:HGNC
Symbol;Acc:HGNC:16188] - 321 304 308 156 194
265 5.774914984 5.291903348 7.039947854
3.184553293 4.380876086 4.526773566
ENSG00000111775 363.8076374 837.2054149 727.7197728
309.75458 503.3978962 439.9459131 642.9109417
417.6994631 0.622050291 0.031812778 0.156882139 COX6A1
12 120438090 120440742 + 1344
protein_coding cytochrome c oxidase subunit 6A1 [Source:HGNC
Symbol;Acc:HGNC:2277] - 401 946 626 299 441
510 14.61079299 33.35172556 28.97890099
12.36186405 20.16910751 17.64419811
ENSG00000091844 468.1414984 528.3421064 341.7725451
598.7897901 791.0538369 518.4460662 446.0853833
636.0965644 -0.509167675 0.031854075 0.157042008 RGS17
6 153004459 153131249 - 7899
protein_coding regulator of G protein signaling 17 [Source:HGNC
Symbol;Acc:HGNC:14088] - 516 597 294 578 693
601 3.198941303 3.581200679 2.315698761
4.066003688 5.392727568 3.537800804
ENSG00000108091 2080.326465 1960.264264 1672.825484
2294.670216 2358.322117 2412.80141 1904.472071
2355.264581 -0.306013271 0.031892543 0.157187849 CCDC6
10 59788763 59906656 - 7345
protein_coding coiled-coil domain containing 6 [Source:HGNC
Symbol;Acc:HGNC:18782] - 2293 2215 1439 2215 2066
2797 15.28765728 14.28921501 12.18921789
16.75691143 17.28963708 17.70645762
ENSG00000167323 830.1346339 787.6456863 770.7319639
707.566482 614.1226036 380.423819 796.1707613
567.3709682 0.490391741 0.03192314 0.157274322 STIM1
11 3854527 4093210 + 7130 protein_coding
stromal interaction molecule 1 [Source:HGNC Symbol;Acc:HGNC:11386]
- 915 890 663 683 538 441
6.284348309 5.914620955 5.785366083 5.322837618
4.638099978 2.875941699
ENSG00000155011 20.86677222 11.50493699 16.2748831
5.179842475 0 0 16.21553077 1.726614158
3.242897075 0.031940912 0.157274322 DKK2 4
106921802 107283806 - 4319 protein_coding
dickkopf WNT signaling pathway inhibitor 2 [Source:HGNC
Symbol;Acc:HGNC:2892] - 23 13 14 5 0
0 0.26077942 0.142622022 0.20167482
0.064327821 0 0
ENSG00000130513 238.6070041 161.954113 325.497662
233.0929114 74.19696883 61.24737221 242.019593

122.8457508 0.97818099 0.03194948 0.157274322 GDF15
19 18374731 18389176 + 2087
protein_coding growth differentiation factor 15 [Source:HGNC
Symbol;Acc:HGNC:30142] - 263 183 280 225 65
71 6.171091459 4.154847438 8.347230935
5.990624698 1.914424607 1.581855897
ENSG00000017797 1949.682326 1708.040646 2290.10855
1712.455922 1502.203246 1477.700685 1982.610507
1564.119951 0.341573873 0.031967806 0.157274322 RALBP1
18 9475009 9538116 + 8043 protein_coding ralA
binding protein 1 [Source:HGNC Symbol;Acc:HGNC:9841] - 2149
1930 1970 1653 1316 1713 13.08419567
11.37013657 15.23894859 11.42001905 10.05738744
9.903080569
ENSG00000124120 599.6928885 674.3663067 546.3710754
867.1056302 723.7058191 802.2543121 606.8100902
797.6885871 -0.393950661 0.031968988 0.157274322 TTPAL
20 44475886 44494603 + 7044
protein_coding alpha tocopherol transfer protein like [Source:HGNC
Symbol;Acc:HGNC:16114] - 661 762 470 837 634
930 4.59526748 5.125804817 4.151311832
6.602648145 5.53244711 6.138957396
ENSG00000279118 36.29003864 49.55972857 37.1997328 0
19.40536108 11.21430759 41.0165 10.20655622
2.01258467 0.031973365 0.157274322 AC093535.2 5
126496279 126498604 + 2326 TEC tec
- 40 56 32 0 17 13
0.842129662 1.14078751 0.855947474 0 0.449248433
0.259875094
ENSG00000169258 710.3775064 889.4201289 788.1693386
843.2783549 1236.23565 1097.276866 795.9889913
1058.93029 -0.411477272 0.031979729 0.157274322 GPRIN1
5 176595802 176610133 - 4211
protein_coding G protein regulated inducer of neurite outgrowth 1
[Source:HGNC Symbol;Acc:HGNC:24835] - 783 1005 678
814 1083 1272 9.105529468 11.30855882
10.01731427 10.74116053 15.80849679 14.04536042
ENSG00000130175 7843.184601 9252.624326 10247.36389
7176.153764 7515.582197 7737.872236 9114.39094
7476.536066 0.285618775 0.031981216 0.157274322 PRKCSH
19 11435288 11450968 + 5166
protein_coding protein kinase C substrate 80K-H [Source:HGNC
Symbol;Acc:HGNC:9411] - 8645 10455 8815 6927 6584
8970 81.9481737 95.89502487 106.163386
74.50798688 78.3398581 80.73634029
ENSG00000280385 329.3321007 269.9235217 172.0487642
477.5814762 323.0421874 380.423819 257.1014622
393.6824942 -0.612297856 0.032046017 0.157508282
AP000648.3 11 90193614 90198120 + 4507
TEC novel transcript - 363 305 148

461	283	441	3.944098482	3.206555211
2.043059449	5.683624936	3.859635014	4.549692547
ENSG00000033867 1934.259059	1785.920219	1705.37525
1726.959481	1357.233784	997.2107363	1808.518176
1360.468	0.411461242	0.0320466	0.157508282	SLC4A7
3	27372721	27484420	-	9616
protein_coding	solute carrier family 4 member 7 [Source:HGNC
Symbol;Acc:HGNC:11033]	-	2132	2018	1467	1667	1189
1156	10.85728978	9.943817569	9.491667273
9.632814316	7.600370439	5.589775908
ENSG00000229644 140.6238997	173.45905	176.6987308
271.4237457	265.967596	210.483927	163.5938935
249.2917562	-0.607498654	0.032062548	0.157542891
NAMPTP1 10	36521721	36524234	-	2514
processed_pseudogene	nicotinamide phosphoribosyltransferase
pseudogene 1 [Source:HGNC Symbol;Acc:HGNC:17633]	-	155
196	152	262	233	244	3.019222424
3.694173077	3.761708697	5.790926618	5.696892283
4.512898554
ENSG00000251192 76.20908114	71.6846074	94.16182364
155.3952742	106.15874	160.4508624	80.68517073
140.6682922	-0.806197635	0.032118018	0.157771626	ZNF674
X	46497727	46545457	-	6203
protein_coding	zinc finger protein 674 [Source:HGNC
Symbol;Acc:HGNC:17625]	zf-C2H2 84	81	81	150	93
186	0.663141471	0.618742116	0.812437731
1.343697538	0.921570422	1.394254906
ENSG00000123989 2455.928365	2907.209078	3170.114729
2310.209744	2279.559181	2313.597919	2844.417391
2301.122281	0.305436257	0.032221957	0.158215571	CHPF
2	219538947	219543787	-	3354
protein_coding	chondroitin polymerizing factor [Source:HGNC
Symbol;Acc:HGNC:24291]	-	2707	3285	2727	2230	1997
2682	39.52336208	46.4086309	50.58583537
36.94484496	36.59842253	37.18148519
ENSG00000101266 5286.551379	5186.956592	5578.797428
6042.804231	6363.816942	6764.815393	5350.768466
6390.478855	-0.256355778	0.032226282	0.158215571
CSNK2A1 20	472498	543835	-	29523	protein_coding	casein
kinase 2 alpha 1 [Source:HGNC Symbol;Acc:HGNC:2457]	-	5827
5861	4799	5833	5575	7842	9.665252742
9.406708275	10.11339826	10.97851333	11.60731433
12.35086485
ENSG00000051382 850.0941551	868.1802452	749.8071142
1232.802509	1088.983204	900.5951633	822.6938382
1074.126959	-0.383565668	0.032357176	0.158793265	PIK3CB
3	138652699	138834938	-	7850
protein_coding	"phosphatidylinositol-4,5-bisphosphate 3-kinase
catalytic subunit beta [Source:HGNC Symbol;Acc:HGNC:8976]"	-
937	981	645	1190	954	1044	5.845189799

5.921418981	5.112071324	8.423437315	7.470094195
6.183891486
ENSG00000271590 31.75378381	8.849951531	2.3249833	0
3.424475485	0	14.30957288	1.141491828
3.717851	0.032361904	0.158793265	AC108463.3	2
111210995	111212476	-	1482	lincRNA novel
transcript	-	35	10	2	0	3	0
1.156507688	0.319726208	0.083963134	0
0.124428656	0
ENSG00000149571 17.23776835	24.77986429	24.41232465	0
9.131934625	0	22.1433191	3.043978208
2.90509707	0.032414532	0.159007392	KIRREL3 11
126423359	127003460	-	6594	protein_coding	kirre
like nephrin family adhesion molecule 3 [Source:HGNC
Symbol;Acc:HGNC:23204]	-	19	28	21	0	8
0	0.141102056	0.201203499	0.198142299	0
0.074574166	0
ENSG00000122042 978.0165413	985.8846006	1134.59185
1084.659014	1533.023525	1407.826922	1032.830997
1341.836487	-0.378039351	0.03245 0.159096728	UBL3	13
29764371	29850684	-	4384	protein_coding
ubiquitin like 3 [Source:HGNC Symbol;Acc:HGNC:12504]	-
1078	1114	976	1047	1343	1632	12.04139766
12.04040474	13.85115913	13.2705273	18.83010871
17.30934472
ENSG00000089063 1819.038187	1780.610248	1894.861389
2310.209744	2140.297178	2238.548322	1831.503275
2229.685081	-0.284088973	0.032454858	0.159096728
TMEM230 20	5068232 5113103 -	3051	protein_coding
transmembrane protein 230 [Source:HGNC Symbol;Acc:HGNC:15876]	-
2005	2012	1630	2230	1875	2595	32.18109763
31.24727932	33.23932864	40.61390036	37.77516975
39.54815
ENSG00000172500 1504.222102	1773.530287	1589.126085
2055.361494	1988.478765	1956.465355	1622.292825
2000.101871	-0.301835818	0.032462232	0.159096728	FIBP
11	65883741	65888539	-	3353
protein_coding	FGF1 intracellular binding protein [Source:HGNC
Symbol;Acc:HGNC:3705]	-	1658	2004	1367	1984	1742
2268	24.21473142	28.31982756	25.3654054
32.87911794	31.93463505	31.45143859
ENSG00000138801 969.8512826	962.8747266	1105.529559
801.8396151	865.2508058	680.6222067	1012.751856
782.5708758	0.3723976	0.032476245	0.159096728	PAPSS1
4	107590276	107720452	-	3478
protein_coding	3'-phosphoadenosine 5'-phosphosulfate synthase 1
[Source:HGNC Symbol;Acc:HGNC:8603]	-	1069	1088	951
774	758	789	15.051397	14.82264674
17.01209448	12.36583441	13.3963655	10.54820214
ENSG00000067704 4920.929239	3682.464832	4130.332832

4005.054201 3179.054741 2763.032862 4244.575635
3315.713935 0.356500629 0.032480943 0.159096728 IARS2
1 220094102 220148041 + 4165
protein_coding "isoleucyl-tRNA synthetase 2, mitochondrial
[Source:HGNC Symbol;Acc:HGNC:29685]" - 5424 4161 3553
3866 2785 3203 63.77248769 47.33791777
53.07463893 51.57733372 41.10148885 35.75797785
ENSG00000099940 1133.156457 1283.242972 1255.490982
1070.155455 945.1552337 871.2654357 1223.96347
962.1920416 0.347432331 0.032486708 0.159096728 SNAP29
22 20858983 20891218 + 4953
protein_coding synaptosome associated protein 29 [Source:HGNC
Symbol;Acc:HGNC:11133] - 1249 1450 1080 1033 828
1010 12.34874419 13.87158588 13.56632678
11.58894861 10.27565155 9.481653143
ENSG00000248371 6.350756762 19.46989337 8.137441549
25.89921237 10.27342645 93.16501689 11.31936389
43.11255191 -1.92899973 0.032498212 0.159109017
LINC02056 5 72574120 72660669 - 818
lincRNA long intergenic non-protein coding RNA 2056
[Source:HGNC Symbol;Acc:HGNC:52898] - 7 22 7
25 9 108 0.419057309 1.274370818
0.532416594 1.698238759 0.676295606 6.139054232
ENSG00000178150 16.33051739 15.92991276 6.974949899 0
3.424475485 0 13.07846001 1.141491828
3.591036724 0.032588325 0.159506057 ZNF114 19
48270102 48287608 + 2993 protein_coding zinc
finger protein 114 [Source:HGNC Symbol;Acc:HGNC:12894] zf-C2H2 18
18 6 0 3 0 0.29450622
0.284965464 0.124724388 0 0.061611516 0
ENSG00000135744 12.70151352 1.769990306 5.812458249 0
0 0 6.761320693 0 5.190794711
0.032648391 1 AGT 1 230702523 230714297
- 2291 protein_coding angiotensinogen [Source:HGNC
Symbol;Acc:HGNC:333] - 14 2 5 0 0
0 0.299248257 0.04136484 0.135784989 0
0 0
ENSG00000166896 137.9021468 85.84452985 138.3365063
91.16522755 57.07459141 25.01653231 120.6943943
57.75211709 1.066870617 0.03268882 0.159953674 ATP23
12 57906039 57959148 + 3327
protein_coding ATP23 metallopeptidase and ATP synthase assembly
factor homolog [Source:HGNC Symbol;Acc:HGNC:29452] - 152
97 119 88 50 29 2.23727552
1.381482457 2.225363739 1.469744741 0.923771509
0.405299637
ENSG00000161714 899.9929583 850.4803421 1025.317635
854.6740083 638.0939319 578.8307994 925.2636452
690.5329132 0.422107009 0.032707478 0.159988994 PLCD3
17 45108967 45133354 - 6740

protein_coding	phospholipase C delta 3 [Source:HGNC
Symbol;Acc:HGNC:9061]	-	992	961	882	825	559
	671	7.207430432	6.756004528	8.141708239
6.80152182	5.097993431	4.629068866
ENSG00000101856 4020.936281	4434.710712	4048.958417
5539.323542	4972.338404	4635.822229	4168.201803
5049.161392	-0.276435945	0.032714127	0.159988994	PGRMC1
	X	119236245	119244466	+	1919
protein_coding	progesterone receptor membrane component 1
[Source:HGNC Symbol;Acc:HGNC:16090]	-	4432	5011	3483
	5347	4356	5374	113.0976186	123.7302438
112.9237642	154.8271976	139.5276426	130.2128083
ENSG00000284906 47.17705023	30.08983521	23.249833
5.179842475	12.55641011	12.07694663	33.50557281
9.937733073	1.754764845	0.032743675	0.16008924
AC091057.6	15	30624548	30685606	+	2297
	protein_coding	Rho GTPase-activating protein 11B
[Source:UniProtKB/Swiss-Prot;Acc:Q3KRB8]	-	52	34
	20	5	11	14	1.108590192	0.701365441
	0.541721219	0.120954228	0.294360173	0.283398834
ENSG00000269693 13.60876449	8.849951531	27.8997996	0
	0	5.175834272	16.78617187	1.725278091
3.214922008	0.032770209	0.160174698	AC010422.6	19
	12525720	12580975	-	5857	protein_coding
	novel transcript	zf-C2H2 15	10	24	0
	0	6	0.125413624	0.080900502	0.254942866
	0	0	0.047632902
ENSG00000071537 1837.183206	1269.08305	1699.562792
1571.564207	926.8913645	998.9360144	1601.943016
1165.797195	0.458364298	0.032822257	0.160384782	SEL1L
	14	81471549	81533861	-	9559
protein_coding	"SEL1L, ERAD E3 ligase adaptor subunit [Source:HGNC
Symbol;Acc:HGNC:10717]" -	2025	1434	1462	1517	812
	1158	10.37388071	7.10825715	9.515722261
8.818305956	5.221447646	5.632836116
ENSG00000142208 2761.67194	2415.151773	2857.404475
2371.331885	2128.88226	2013.399532	2678.076063
2171.204559	0.302623411	0.032886834	0.160655951	AKT1
	14	104769349	104795751	-	11162
protein_coding	AKT serine/threonine kinase 1 [Source:HGNC
Symbol;Acc:HGNC:391]	-	3044	2729	2458	2289	1865
	2334	13.35461319	11.58478447	13.70082462
11.39503906	10.27032479	9.722766404
ENSG00000100299 79.83808501	97.34946684	103.4617568
54.90633023	54.79160775	12.07694663	93.54976956
40.59162821	1.211910532	0.032898116	0.160666696	ARSA
	22	50622754	50628173	-	2932
protein_coding	arylsulfatase A [Source:HGNC Symbol;Acc:HGNC:713]
	-	88	110	89	53	48	14
1.469763269	1.777686441	1.888569133	1.00443988

1.006293417	0.222021528
ENSG00000160888 766.6270663	1101.818966	1036.942552
976.9182907	1276.187864	1776.173794	968.4628612
1343.093316	-0.472621626	0.032939432	0.160824073	IER2
	19	13150415	13154908	+	3072
protein_coding	immediate early response 2 [Source:HGNC
Symbol;Acc:HGNC:28871]	-	845	1245	892	943	1118
	2059	13.46989409	19.20324315	18.06552095
17.05699511	22.37010138	31.16492983
ENSG00000137776 1703.817314	1483.251877	1471.714429
1214.155076	1344.677374	1161.112155	1552.927873
1239.981535	0.325358689	0.032975126	0.16095392	SLTM
	15	58879045	58933653	-	8157
protein_coding	SAFB like transcription modulator [Source:HGNC
Symbol;Acc:HGNC:20709]	-	1878	1676	1266	1172	1178
	1346	11.27440961	9.735762991	9.656285328
7.983791617	8.876917596	7.67265403
ENSG00000272173 8.165258694	7.964956378	3.48747495	0
	0	0	6.539230007	0	5.14496974
0.033008555	1	U47924.2	12	6943508 6944604 -
	1097	antisense	"novel transcript, antisense to
C12orf57"	-	9	9	3	0	0	0
	0.40175803	0.388742768	0.170145894	0	0
	0
ENSG00000213023 0	1.769990306	0	3.107905485
19.40536108	9.489029498	0.589996769
10.66743202	-4.10911639	0.033042724	0.161239378	SYT3
	19	50621307	50639827	-	3790
protein_coding	synaptotagmin 3 [Source:HGNC Symbol;Acc:HGNC:11511]
	-	0	2	0	3	17	11	0
	0.025004445	0	0.043983936	0.275712891
0.134953606
ENSG00000205097 694.046989	588.5217768	368.509853
511.7684365	323.0421874	189.78059	550.3595396
341.5304046	0.690680502	0.033101452	0.161481409	FRG2
	4	190024351	190027257	-	2103
protein_coding	FSHD region gene 2 [Source:HGNC Symbol;Acc:HGNC:19136]
	-	765	665	317	494	283	220
17.81356513	14.98334617	9.378358628	13.05268087
8.271695202	4.864233633
ENSG00000104979 1507.851105	1893.004633	2026.222946
1977.663857	2343.482723	2614.658946	1809.026228
2311.935175	-0.354503079	0.033119478	0.1615248
C19orf53	19	13774168	13778462	+	1174
	protein_coding	chromosome 19 open reading frame 53
[Source:HGNC Symbol;Acc:HGNC:24991]	-	1662	2139	1743
	1909	2053	3031	69.32527581	86.33146853
92.37110454	90.35451834	107.4900371	120.0463217
ENSG00000166454 1864.400735	1538.121576	1586.801102
1896.858314	2039.845897	2309.284724	1663.107804

2081.996312	-0.324206515	0.033214684	0.161911021	ATMIN
	16	81035847	81047358	+	6262
protein_coding	ATM interactor [Source:HGNC Symbol;Acc:HGNC:29034]
	-	2055	1738	1365	1831	1787	2677
16.07042811	13.15113239	13.56208415	16.24752914
17.54118179	19.87770889
ENSG00000185909 146.9746565	171.6890597	138.3365063
168.8628647	208.8930046	382.1490971	152.3334075
253.3016554	-0.735073426	0.033216976	0.161911021
KLHDC8B 3	49171611	49176486	+	2158
protein_coding	kelch domain containing 8B [Source:HGNC
Symbol;Acc:HGNC:28557]	-	162	194	119	163	183
	443	3.676141822	4.259677603	3.430855032
4.197089281	5.212511301	9.545162009
ENSG00000105248 589.7131279	980.5746297	790.4943219
824.6309219	1015.927727	1532.909583	786.9273598
1124.489411	-0.51564919	0.033241613	0.161934565	YJU2
	19	4247079 4269090 +	1671	protein_coding	YJU2
splicing factor homolog [Source:HGNC Symbol;Acc:HGNC:25518]	-
	650	1108	680	796	890	1777	19.04871089
	31.4188114	25.31857796	26.4696782	32.7386613
	49.44723322
ENSG00000109079 1312.792148	1557.591469	1727.462592
1743.534977	2078.656619	1987.52036	1532.615403
1936.570652	-0.33801632	0.033244221	0.161934565
TNFAIP1 17	28335602	28347009	+	3889
protein_coding	TNF alpha induced protein 1 [Source:HGNC
Symbol;Acc:HGNC:11894]	-	1447	1760	1486	1683	1821
	2304	18.22045725	21.44377124	23.77320377
24.04685123	28.78189357	27.5470787
ENSG00000103160 1222.974302	1163.768626	1109.017034
1395.449563	1465.675507	1475.112767	1165.253321
1445.412612	-0.310556659	0.03324927	0.161934565	HSDL1
	16	84122146	84145192	-	4507
protein_coding	hydroxysteroid dehydrogenase like 1 [Source:HGNC
Symbol;Acc:HGNC:16475]	-	1348	1315	954	1347	1284
	1710	14.64640428	13.82498394	13.16945077
16.60703425	17.51155957	17.64166498
ENSG00000232916 0	0	0	0	10.27342645
12.93958568	0	7.737670711	-5.429071315	0.033250433
	1	HMGN2P27	5	116218026	116218295
	-	270	processed_pseudogene	high mobility group
nucleosomal binding domain 2 pseudogene 27 [Source:HGNC
Symbol;Acc:HGNC:39394]	-	0	0	0	0	9
	15	0	0	0	0	2.048925207
2.583202861
ENSG00000134551 0	0	0	9.323716454	0
13.80222472	0	7.70864706	-5.426303558	0.033271935
	1	PRH2	12	10929236	10932341	+
	757	protein_coding	proline rich protein HaeIII subfamily

2 [Source:HGNC Symbol;Acc:HGNC:9367]	-	0	0	0
	9	0	16	0	0	0	0.660630581
	0	0.98277731
ENSG00000203930 80.74533597	33.62981582	72.07448229
68.37392066	140.4034949	171.66517	62.14987803
126.8141952	-1.032316953	0.033293486	0.162075445
LINC00632	X	140709562	140793215	+	28876
	antisense	long intergenic non-protein coding RNA 632
[Source:HGNC Symbol;Acc:HGNC:27865]	-	89	38	62
	66	123	199	0.150932115	0.062355247
0.133586171	0.127004452	0.261827608	0.32044025
ENSG00000135083 321.166842	224.7887689	312.7102538
207.193699	191.7706271	174.2530871	286.2219549
191.0724711	0.582843084	0.033302861	0.162075445	CCNJL
	5	160249106	160345396	-	9209
protein_coding	cyclin J like [Source:HGNC Symbol;Acc:HGNC:25876]
	-	354	254	269	200	168	202
1.882432761	1.306916029	1.817383805	1.206784063
1.121357698	1.019928939
ENSG00000181827 865.5174215	556.6619513	753.2945891
546.9913653	616.4055872	349.3688133	725.1579873
504.2552553	0.525569941	0.033312552	0.162075445	RFX7
	15	56087280	56243266	-	10588
protein_coding	regulatory factor X7 [Source:HGNC
Symbol;Acc:HGNC:25777]	RFX	954	629	648	528	540
	405	4.41228062	2.814901184	3.807745555
2.770971338	3.134925232	1.778574694
ENSG00000197119 511.6895448	528.3421064	418.496994
311.826517	399.5221399	343.33034	486.1762151
351.5596656	0.469479057	0.033315472	0.162075445
SLC25A29	14	100291111	100306547	-	7451
	protein_coding	solute carrier family 25 member 29
[Source:HGNC Symbol;Acc:HGNC:20116]	-	564	597	360
	301	350	398	3.706749385	3.796524515
3.006040199	2.244729302	2.887359371	2.483702227
ENSG00000060339 2140.205029	1577.061363	1753.037408
1723.851576	1312.715602	1085.199919	1823.4346
1373.922366	0.408843604	0.033324009	0.162075445	CCAR1
	10	68721012	68792377	+	6059
protein_coding	cell division cycle and apoptosis regulator 1
[Source:HGNC Symbol;Acc:HGNC:24236]	-	2359	1782	1508
	1664	1150	1258	19.06582805	13.93584117
15.4848583	15.26034714	11.66659839	9.654075852
ENSG00000121964 421.8716992	340.723134	360.3724115
516.948279	537.6426511	473.5888359	374.3224149
509.3932553	-0.443647401	0.033345416	0.162134984	GTDC1
	2	143938068	144332568	-	14162
protein_coding	glycosyltransferase like domain containing 1
[Source:HGNC Symbol;Acc:HGNC:20887]	-	465	385	310
	499	471	549	1.607892637	1.288138558

1.361896019	1.957888697	2.044295522	1.802514523
ENSG00000213214 0	16.81490791	6.974949899	0	0
	0	7.929952603	0	5.421290098	0.033373045
	0.16222473	ARHGEF35	7	144186083
144195655	-	2431	protein_coding	Rho guanine nucleotide
exchange factor 35 [Source:HGNC Symbol;Acc:HGNC:33846]	-	0
	19	6	0	0	0	0	0.370335276
	0.153558244	0	0	0
ENSG00000280062 0	0	1.16249165	0	0
36.2308399	0.387497217	12.07694663	-5.113793669
0.033401396	0.162317227	AL031719.1	16	1530715
1533301 +	2587	TEC	TEC	-	0	0	1
	0	0	42	0	0	0.024049742	0
	0	0.754890361
ENSG00000144355 192.3372048	191.1589531	211.5734803
152.2873688	123.2811174	96.61557307	198.356546
124.0613531	0.678424318	0.033410426	0.162317227	DLX1
	2	172084740	172089677	+	3656
protein_coding	distal-less homeobox 1 [Source:HGNC
Symbol;Acc:HGNC:2914]	Homeobox	212	216	182	147
	108	112	2.83960778	2.799458314	3.097219947
	2.234205885	1.815787109	1.424435713
ENSG00000102996 82.5598379	58.40968011	136.011523
27.97114936	38.81072216	60.38473317	92.32701368
42.38886823	1.11496398	0.033477323	0.162596953	MMP15
	16	58025566	58046901	+	4419
protein_coding	matrix metallopeptidase 15 [Source:HGNC
Symbol;Acc:HGNC:7161]	-	91	66	117	27	34
	70	1.008430737	0.707695403	1.64728486
0.339509402	0.472935893	0.736554787
ENSG00000182854 0	0	0	29.00711786	0	0
	0	9.669039286	-5.748377996	0.033486387
0.162596953	OR4F15	15	101812202	101820197	+
	2119	protein_coding	olfactory receptor family 4 subfamily
F member 15 [Source:HGNC Symbol;Acc:HGNC:15078] -	0	0
	0	28	0	0	0	0	0
0.734241822	0	0
ENSG00000224549 14.51601546	8.849951531	0	0	0
	0	7.788655662	0	5.398712189	0.033551091
	0.162866422	AC017067.1	9	22767175
22768316	+	447	lincRNA novel transcript	-
	16	10	0	0	0	0	1.75283543
	1.060031858	0	0	0	0
ENSG00000172432 1288.296372	1328.377725	1336.865397
1003.853472	1227.103715	788.4520874	1317.846498
1006.469758	0.390016112	0.033572994	0.162928035	GTPBP2
	6	43605316	43629162	-	4624
protein_coding	GTP binding protein 2 [Source:HGNC
Symbol;Acc:HGNC:4670]	-	1420	1501	1150	969	1075
	914	15.03831587	15.38116771	15.47343951

11.64442359 14.29018987 9.190928519
ENSG00000126814 684.9744793 676.136297 640.5328991
886.7890316 959.9946275 754.8091646 667.2145585
867.1976079 -0.377048155 0.033596739 0.162998553 TRMT5
14 60971451 60981358 - 5692
protein_coding tRNA methyltransferase 5 [Source:HGNC
Symbol;Acc:HGNC:23141] - 755 764 551 856 841
875 6.49547243 6.35996767 6.022732218
8.356432643 9.081936574 7.147829421
ENSG00000139410 50.80605409 45.13475281 60.44956579
23.82727538 18.26386925 0 52.13012423 14.03038154
1.90468996 0.033650622 0.163215203 SDSL 12
113422237 113438276 + 2216 protein_coding
serine dehydratase like [Source:HGNC Symbol;Acc:HGNC:30404]
- 56 51 52 23 16 0
1.237504978 1.09050299 1.459958242 0.576726787
0.443810514 0
ENSG00000105270 301.2073207 231.8687301 408.0345691
430.9628939 448.6062885 479.6273092 313.70354
453.0654972 -0.53333235 0.033706474 0.163441288 CLIP3
19 36014660 36033343 - 3772
protein_coding CAP-Gly domain containing linker protein 3
[Source:HGNC Symbol;Acc:HGNC:24314] - 332 262 351
416 393 556 4.310176783 3.291213442
5.789516274 6.12821072 6.404249256 6.853842586
ENSG00000226851 7.258007728 19.46989337 5.812458249
2.07193699 0 0 10.84678645 0.690645663
3.994958043 0.033718398 0.1634543 AC004112.1 7
112328189 112409623 - 707 antisense
"novel transcript, antisense to ZNF277" - 8 22
5 2 0 0 0.554114171 1.474448839
0.440004823 0.157189172 0 0
ENSG00000070047 1821.75994 1859.374817 1718.162659
1985.951605 2118.608833 2687.983265 1799.765805
2264.181234 -0.331470585 0.033729966 0.16346558 PHRF1
11 576486 612222 + 5769 protein_coding PHD
and ring finger domains 1 [Source:HGNC Symbol;Acc:HGNC:24351] -
2008 2101 1478 1917 1856 3116 17.04479778
17.25646974 15.93972196 18.46433273 19.77537798
25.11469615
ENSG00000198315 794.7518462 753.1308753 754.4570808
996.6016921 1162.038681 841.0730691 767.4466008
999.9044808 -0.380685995 0.033740973 0.163474135
ZKSCAN8 6 28141910 28159472 + 7565
protein_coding zinc finger with KRAB and SCAN domains 8 [Source:HGNC
Symbol;Acc:HGNC:12983] zf-C2H2 876 851 649 962 1018
975 5.670532676 5.330243737 5.337558046
7.066074033 8.271536792 5.992757464
ENSG00000112972 1671.156279 985.8846006 1222.941216
1165.464557 945.1552337 618.5121955 1293.327365

909.710662	0.508446473	0.033752506	0.163485237	HMGCS1
	5	43289395	43313512	-	4702
protein_coding	3-hydroxy-3-methylglutaryl-CoA synthase 1 [Source:HGNC
Symbol;Acc:HGNC:5007]	-	1842	1114	1052	1125	828
	717	19.18384623	11.22610259	13.92002328
13.29480407	10.82418166	7.090347964
ENSG00000143353 435.4804637	395.5928334	464.99666
571.8546092	601.5661934	551.2263499	432.023319
574.8823842	-0.412643728	0.033868734	0.164003294
LYPLAL1 1	219173844	219212865	+	2966
protein_coding	lysophospholipase like 1 [Source:HGNC
Symbol;Acc:HGNC:20440]	-	480	447	400	552	527
	639	7.924990937	7.141062223	8.390651653
10.34141519	10.92161414	10.0175318
ENSG00000134108 2041.314673	2017.788949	1932.061122
2844.769487	2558.083187	2091.037046	1997.054915
2497.96324	-0.322397658	0.033897065	0.164095562	ARL8B
	3	5122220 5180912 +	3576	protein_coding	ADP
ribosylation factor like GTPase 8B [Source:HGNC
Symbol;Acc:HGNC:25564]	-	2250	2280	1662	2746	2241
	2424	30.8115603	30.21090795	28.91614248
42.66925561	38.52048146	31.51854229
ENSG00000185115 573.3826105	597.3717284	595.1957247
629.8688449	842.4209692	872.9907138	588.6500212
781.760176	-0.409672645	0.033928347	0.164202061	NSMCE3
	15	29264992	29269829	-	4838
protein_coding	"NSE3 homolog, SMC5-SMC6 complex component
[Source:HGNC Symbol;Acc:HGNC:7677]"	-	632	675	512
	608	738	1012	6.397052248	6.610957263
6.584320212	6.98312408	9.376437389	9.726255336
ENSG00000145337 1110.475182	1606.266203	1233.403641
1112.630164	934.8818073	976.5073993	1316.715009
1008.006457	0.385718694	0.033938962	0.164208508	PYURF
	4	88520985	88523813	-	1361
protein_coding	PIGY upstream reading frame [Source:HGNC
Symbol;Acc:HGNC:44317]	-	1224	1815	1061	1074	819
	1132	44.04047315	63.18950379	48.50249787
43.84884919	36.98904654	38.6740202
ENSG00000185716 430.9442088	392.937848	325.497662
444.4304843	638.0939319	522.7592614	383.1265729
535.0945592	-0.480077814	0.033971553	0.164321248	MOSMO
	16	22007638	22087534	+	6122
protein_coding	modulator of smoothened [Source:HGNC
Symbol;Acc:HGNC:27087]	-	475	444	280	429	559
	606	3.799522039	3.436497922	2.845584933
3.893821247	5.612622628	4.602675075
ENSG00000173272 679.5309735	985.8846006	1136.916834
663.0198367	664.348244	734.1058275	934.1108026
687.1579694	0.441716243	0.034117806	0.16498356	MZT2A
	2	131464900	131492743	-	4093

protein_coding mitotic spindle organizing protein 2A [Source:HGNC
Symbol;Acc:HGNC:33187] -   749   1114   978   640   582
        851   8.961253368   12.89644134   14.86633643
8.688609384   8.740345497   9.667603574
ENSG00000164096 1079.62865   1147.838714   1345.002839
1500.082381   1504.48623   1472.52485   1190.823401
1492.364487   -0.326373406   0.034128997   0.164992574   C4orf3
        4   119296419   119304445   -   3148
protein_coding chromosome 4 open reading frame 3 [Source:HGNC
Symbol;Acc:HGNC:19225] -   1190   1297   1157   1448   1318
        1707   18.51147058   19.52233196   22.86680466
25.55911911   25.73522957   25.21330721
ENSG00000100387 815.6186184   895.615095   1019.505177
794.5878356   713.4323926   521.0339833   910.2462968
676.3514038   0.429022653   0.03415147   0.165056108   RBX1
        22   40951347   40973309   +   3092
protein_coding ring-box 1 [Source:HGNC Symbol;Acc:HGNC:9928]   -
        899   1012   877   767   625   604   14.23799677
        15.50841693   17.64684027   13.78376697   12.42475667
        9.082982376
ENSG00000022267 1871.658743   1702.730675   1986.69823
1772.542095   1412.025391   905.7709975   1853.695882
1363.446161   0.443671911   0.034176499   0.165131956   FHL1
        X   136146702   136211359   +   5997
protein_coding four and a half LIM domains 1 [Source:HGNC
Symbol;Acc:HGNC:3702]   -   2063   1924   1709   1711   1237
        1050   16.84588621   15.20188559   17.73025005
15.85360394   12.67894188   8.141159592
ENSG00000072121 672.2729658   540.7320386   518.4712759
857.7819138   648.3673584   815.1938978   577.1587601
773.7810567   -0.422828594   0.0342052   0.165225499
ZFYVE26 14   67727374   67816590   -   15706
protein_coding zinc finger FYVE-type containing 26 [Source:HGNC
Symbol;Acc:HGNC:20761] -   741   611   446   828   568
        945   2.310368733   1.843325614   1.766754118
2.929387252   2.22295209   2.797674816
ENSG00000057019 11325.21381   10474.80263   11235.4818
10937.75537   7735.89012   7833.62517   11011.83275
8835.756886   0.317633904   0.034251542   0.165404186   DCBLD2
        3   98795941   98901689   -   11274
protein_coding "discoidin, CUB and LCCL domain containing 2
[Source:HGNC Symbol;Acc:HGNC:24627]"   -   12483   11836   9665
        10558   6777   9081   54.22126225   49.74545034
53.33725666   52.03740977   36.94935106   37.45300455
ENSG00000115368 1810.872928   1623.966106   1456.602037
2089.548454   2084.364078   1901.256456   1630.480357
2025.056329   -0.311949185   0.034305269   0.165618421   WDR75
        2   189441433   189475565   +   5066
protein_coding WD repeat domain 75 [Source:HGNC
Symbol;Acc:HGNC:25725] -   1996   1835   1253   2017   1826

2204 19.29407823 17.16316288 15.38837397
22.12344507 22.15557103 20.22914013
ENSG00000141030 3505.617733 3739.104522 3643.248831
4348.995742 4518.024656 4147.56853 3629.323695
4338.196309 -0.257273374 0.034364696 0.165860059 COPS3
17 17246820 17281293 - 2788
protein_coding COP9 signalosome subunit 3 [Source:HGNC
Symbol;Acc:HGNC:2239] - 3864 4225 3134 4198 3958
4808 67.86924717 71.80594212 69.93797755
83.6684471 87.26304846 80.18676772
ENSG00000267353 8.165258694 15.0449176 0 0 0
0 7.736725432 0 5.389355304 0.034389254
1 AC020928.2 19 36773712 36775908
- 2197 lincRNA novel transcript - 9
17 0 0 0 0 0.200604715
0.36664461 0 0 0 0
ENSG00000186577 876.4044331 1277.048006 1326.402973
1179.968116 1638.040773 1910.745485 1159.951804
1576.251458 -0.443197313 0.034445416 0.166203991 SMIM29
6 34246380 34249470 - 2229
protein_coding small integral membrane protein 29 [Source:HGNC
Symbol;Acc:HGNC:1340] - 966 1443 1141 1139 1435
2215 21.22246087 30.67486806 31.84801892
28.39394255 39.57210865 46.20560703
ENSG00000198042 1818.130936 1923.094468 1540.301436
2269.806972 2040.987389 2244.586796 1760.508947
2185.127052 -0.311299195 0.034461078 0.166203991 MAK16
8 33484750 33501260 + 4846
protein_coding MAK16 homolog [Source:HGNC Symbol;Acc:HGNC:13703]
- 2004 2173 1325 2191 1788 2602
20.2508376 21.24725144 17.01137096 25.12297183
22.67939499 24.96634115
ENSG00000237624 61.69306569 65.48964133 62.77454909
30.04308635 35.38624667 5.175834272 63.31908537
23.53505577 1.439163015 0.034464144 0.166203991
OXCT2P1 1 39514956 39516490 + 1535
unprocessed_pseudogene 3-oxoacid CoA-transferase 2 pseudogene 1
[Source:HGNC Symbol;Acc:HGNC:21627] - 68 74 54
29 31 6 2.169347953 2.284282332
2.188730181 1.049788139 1.241368367 0.181749778
ENSG00000100528 2119.338257 2369.132025 2529.58183
2777.431535 2756.702765 3039.939996 2339.350704
2858.024765 -0.289404967 0.034521112 0.166433346 CNIH1
14 54423560 54441431 - 5060
protein_coding cornichon family AMPA receptor auxiliary protein 1
[Source:HGNC Symbol;Acc:HGNC:19431] - 2336 2677 2176
2681 2415 3524 22.60742013 25.06826605
26.75563242 29.44139207 29.33688365 32.38294938
ENSG00000143155 2139.297778 2239.037737 2261.046259
2565.057993 2693.920714 2756.13175 2213.127258

2671.703486	-0.271838943	0.034544509	0.166500766	TIPRL
	1	168178933	168202114	+	3220
protein_coding	TOR signaling pathway regulator [Source:HGNC
Symbol;Acc:HGNC:30231]	-	2358	2530	1945	2476	2360
	3195	35.86052246	37.22983319	37.58119456
42.72743403	45.05090207	46.13664489
ENSG00000145979 567.0318537	692.0662097	745.1571476
725.1779464	958.8531357	989.4469849	668.0850704
891.1593557	-0.416597808	0.034591234	0.16668056	TBC1D7
	6	13266542	13328583	-	8300
protein_coding	TBC1 domain family member 7 [Source:HGNC
Symbol;Acc:HGNC:21066]	-	625	782	641	700	840
	1147	3.687487939	4.464317785	4.804926887
4.686320547	6.22083316	6.42563931
ENSG00000256742 0	0	0	6.215810969	17.12237742
	0	0	7.779396131	-5.430588322	0.03460819
	0.166716849	AC145422.1	12	121580792
121593504	+	544	lincRNA novel transcript	-
	0	0	0	6	15	0	0	0
	0	0.612864399	1.694882984	0
ENSG00000132604 1122.269445	1134.563786	967.1930527
1240.054288	1293.310241	1581.21737	1074.675428
1371.5273	-0.351862845	0.034619297	0.166724952	TERF2
	16	69355561	69408571	-	5621
protein_coding	telomeric repeat binding factor 2 [Source:HGNC
Symbol;Acc:HGNC:11729]	MYB	1237	1282	832	1197	1133
	1833	10.7766753	10.80689373	9.209087249
11.83293854	12.38978256	15.16281715
ENSG00000068745 812.8968655	908.0050271	919.5308951
549.0633023	652.9333257	791.0400045	880.1442626
664.3455442	0.404631391	0.034633152	0.16674628	IP6K2
	3	48688003	48740353	-	5624
protein_coding	inositol hexakisphosphate kinase 2 [Source:HGNC
Symbol;Acc:HGNC:17313]	-	896	1026	791	530	572
	917	7.801738352	8.644273307	8.750603746
5.236518006	6.251699246	7.581498298
ENSG00000172482 0	0	0	0	9.131934625
13.80222472	0	7.644719783	-5.412249316	0.034637553
	1	AGXT	2	240868479	240880502	+
	4459	protein_coding	alanine--glyoxylate and serine--
pyruvate aminotransferase [Source:HGNC Symbol;Acc:HGNC:341]	-
	0	0	0	0	8	16	0	0
	0	0	0.110280792	0.166845128
ENSG00000213859 305.7435755	323.0232309	519.6337675
273.4956827	255.6941695	244.1268498	382.8001913
257.772234	0.568489345	0.03475368	0.167281048	KCTD11
	17	7351889 7354944 +	3056	protein_coding
potassium channel tetramerization domain containing 11 [Source:HGNC
Symbol;Acc:HGNC:21302]	-	337	365	447	264	224
	283	5.400142678	5.659342206	9.100411275

4.800236342	4.505489985	4.305901628
ENSG00000171612 873.6826802	826.585473	624.258016
1020.428967	995.3808742	1017.051434	774.8420564
1010.953759	-0.382473606	0.034839297	0.167647533
SLC25A33	1	9539482 9585179 +	3854	protein_coding
solute carrier family 25 member 33 [Source:HGNC
Symbol;Acc:HGNC:29681]	-	963	934	537	985	872
1179	12.23610684	11.48316504	8.669008364
14.2015767	13.90759819	14.22437237
ENSG00000018510 2770.74445	2337.272199	2638.856045
2520.511348	1793.283662	1721.827534	2582.290898
2011.874182	0.360197612	0.03485266	0.167666224	AGPS
2	177392644	177559299	+	8965
protein_coding	alkylglycerone phosphate synthase [Source:HGNC
Symbol;Acc:HGNC:327]	-	3054	2641	2270	2433	1571
1996	16.68197332	13.95868633	15.75369416
15.0800874	10.77142722	10.35240517
ENSG00000104343 596.0638846	638.9665006	650.9953239
817.3791425	873.2412486	744.4574961	628.6752364
811.692629	-0.368308369	0.034910813	0.167900319	UBE2W
8	73780097	73878910	-	9090
protein_coding	ubiquitin conjugating enzyme E2 W [Source:HGNC
Symbol;Acc:HGNC:25616]	-	657	722	560	789	765
863	3.539404265	3.763567895	3.832930905
4.823087752	5.173028989	4.414463503
ENSG00000206337 22.68127415	5.309970919	17.43737475	0
4.565967313	0	15.14287327	1.521989104
3.372880918	0.034955592	0.168069182	HCP5	6
31400702	31477506	+	3138	sense_overlapping
HLA complex P5 [Source:HGNC Symbol;Acc:HGNC:21659]	-
25	6	15	0	4	0	0.390135754
0.090599281	0.297402878	0	0.07835278	0
ENSG00000011243 806.5461088	815.080536	934.6432865
936.5155194	1124.369451	1239.612308	852.0899771
1100.165759	-0.369684248	0.034964927	0.168069182	AKAP8L
19	15380048	15419141	-	5081
protein_coding	A-kinase anchoring protein 8 like [Source:HGNC
Symbol;Acc:HGNC:29857]	-	889	921	804	904	985
1437	8.568035349	8.588886746	9.844954208
9.886242958	11.91610704	13.15038874
ENSG00000075399 507.15329	740.7409432	402.2221109
642.3004668	849.2699202	847.9741815	550.0387813
779.8481895	-0.50213413	0.034976735	0.168080269	VPS9D1
16	89707134	89720986	-	3428
protein_coding	VPS9 domain containing 1 [Source:HGNC
Symbol;Acc:HGNC:13526]	-	559	837	346	620	744
983	7.985455212	11.56940663	6.279746783
10.04992729	13.34072655	13.33348641
ENSG00000231908 2.721752898	15.0449176	2.3249833	0
0	0	6.697217934	0	5.180689107

0.035020192	1	IDH1-AS1	2	208255247
208256181	+	655	antisense	IDH1 antisense RNA 1
[Source:HGNC Symbol;Acc:HGNC:40292]	-	3	17	2
	0	0	0	0.224289343	1.229798792
0.189974602	0	0	0
ENSG00000232729 4.53625483	8.849951531	5.812458249	0
	0	0	6.39955487	0	5.111776013
0.035059597	1	AC211433.1	7	74688939
74729001	-	2847	processed_transcript
uncharacterized LOC101926943 [Source:NCBI gene;Acc:101926943]	-
	5	10	5	0	0	0	0.086002529
	0.166432821	0.109267092	0	0	0
ENSG00000272341 21.77402318	16.81490791	26.73730795	0
	9.131934625	0	21.77541301	3.043978208
2.879716541	0.03506777	0.168471968	AL137003.2	6
	16764346	16766883	+	2538	lincRNA novel
transcript	-	24	19	23	0	8	0
	0.463071771	0.354722245	0.563823359	0
0.193751793	0
ENSG00000113161 1567.729669	1497.411799	1628.650801
1395.449563	1253.358027	1087.787836	1564.597423
1245.531809	0.329326148	0.03511714	0.16866224	HMGCR
	5	75336329	75362104	+	6215
protein_coding	3-hydroxy-3-methylglutaryl-CoA reductase [Source:HGNC
Symbol;Acc:HGNC:5006]	-	1728	1692	1401	1347	1098
	1261	13.61542771	12.89987989	14.02503162
12.04310593	10.85946844	9.434197674
ENSG00000121211 283.9695524	236.2937059	322.010187
278.6755251	113.007691	75.91223598	280.7578151
155.8651507	0.850001345	0.035126445	0.16866224	MND1
	4	153344649	153415118	+	1511
protein_coding	meiotic nuclear divisions 1 [Source:HGNC
Symbol;Acc:HGNC:24839]	-	313	267	277	269	99
	88	10.14398403	8.372848592	11.40570544
9.892358784	4.027338098	2.708003529
ENSG00000137857 19.05227029	10.61994184	19.76235805
5.179842475	0	1.725278091	16.47819006	2.301706855
	2.830460458	0.035146212	0.168680355	DUOX1	15
	45129933	45165576	+	8784	protein_coding
	dual oxidase 1 [Source:HGNC Symbol;Acc:HGNC:3062]	-
	21	12	17	5	0	2	0.117072705
	0.064731454	0.120410245	0.03162931	0
0.010586897
ENSG00000144566 1555.028156	1490.331838	1637.950735
2074.008927	1882.320025	1803.778244	1561.103576
1920.035732	-0.29870646	0.035154616	0.168680355	RAB5A
	3	19947079	19985175	+	3785
protein_coding	"RAB5A, member RAS oncogene family [Source:HGNC
Symbol;Acc:HGNC:9783]"	-	1714	1684	1409	2002	1649
	2091	22.17551003	21.08155511	23.16071465

29.39072051 26.77947953 25.68734195
ENSG00000163701 15.42326642 27.43484975 17.43737475
62.15810969 45.65967313 43.99459131 20.09849697
50.60412471 -1.328431235 0.035168339 0.168680355 IL17RE
3 9902612 9916402 + 4676 protein_coding
interleukin 17 receptor E [Source:HGNC Symbol;Acc:HGNC:18439] -
17 31 15 60 40 51 0.178034063
0.314133051 0.199583026 0.712998788 0.525814852
0.507138628
ENSG00000176624 975.2947884 918.6249689 1174.116566
832.9186699 833.2890346 699.6002657 1022.678775
788.6026567 0.374833978 0.035168362 0.168680355 MEX3C
18 51174550 51218304 - 7481
protein_coding mex-3 RNA binding family member C [Source:HGNC
Symbol;Acc:HGNC:28040] - 1075 1038 1010 804 730
811 7.03683703 6.574521343 8.399792651
5.971843771 5.998056682 5.040715862
ENSG00000227627 6.350756762 2.654985459 10.46242485 0
0 0 6.489389023 0 5.127413039
0.035191107 1 AL080276.2 6 152983331
152990541 + 2146 antisense novel transcript
- 7 3 9 0 0 0
0.159733867 0.066239642 0.260927371 0 0 0
ENSG00000100650 1514.201862 1715.120607 1419.402305
1911.361873 1964.507436 1865.888255 1549.574924
1913.919188 -0.30404894 0.035199258 0.168782781 SRSF5
14 69726900 69772005 + 5357
protein_coding serine and arginine rich splicing factor 5
[Source:HGNC Symbol;Acc:HGNC:10787] - 1669 1938 1221
1845 1721 2163 15.25679721 17.1418846
14.18080432 19.13756892 19.74724817 18.77439242
ENSG00000198393 615.1161549 707.1111273 527.7712091
862.9617563 708.8664253 881.6171043 616.6661638
817.8150953 -0.406744159 0.035219227 0.168832768 ZNF26
12 132986365 133032952 + 20863
protein_coding zinc finger protein 26 [Source:HGNC
Symbol;Acc:HGNC:13053] zf-C2H2 678 799 454 833 621
1022 1.591408302 1.814664996 1.353897984
2.21860653 1.829625491 2.277745283
ENSG00000143158 744.8530431 992.0795666 857.9188376
982.0981332 1142.63332 1250.826616 864.9504824
1125.186023 -0.379674156 0.035268851 0.169024848 MPC2
1 167916729 167937040 - 2552
protein_coding mitochondrial pyruvate carrier 2 [Source:HGNC
Symbol;Acc:HGNC:24515] - 821 1121 738 948 1001
1450 15.75401195 20.8138003 17.99212826
20.64142666 24.11019748 26.41912017
ENSG00000232044 0.907250966 0 0 3.107905485
7.990442797 12.93958568 0.302416989
8.012644654 -4.519006038 0.035302044 0.169138098

LINC01105 2 5932687 6001275 + 8200
processed_transcript long intergenic non-protein coding RNA 1105
[Source:HGNC Symbol;Acc:HGNC:26403] - 1 0 0
3 7 15 0.005971932 0 0
0.020329161 0.052472475 0.08505668
ENSG00000264044 9.979760626 8.849951531 1.16249165 0
0 0 6.664067936 0 5.174032108
0.035319978 1 AC005726.2 17 28633206
28635950 + 1515 antisense "novel transcript,
antisense to KIAA0100" - 11 10 1 0 0
0 0.355556593 0.312761875 0.041067117 0
0 0
ENSG00000128656 582.4551202 474.3574021 453.3717435
427.8549884 378.975287 207.0333709 503.3947552
337.9545487 0.577377578 0.035349717 0.169320646 CHN1
2 174799363 175005369 - 4971
protein_coding chimerin 1 [Source:HGNC Symbol;Acc:HGNC:1943] -
642 536 390 413 332 240 6.324409007
5.109136048 4.881212227 4.616558383 4.105269577
2.244907737
ENSG00000129932 1009.770325 1218.638326 989.2803941
1098.126605 1435.99672 1708.02531 1072.563015
1414.049545 -0.398832425 0.035364336 0.169344815 DOHH
19 3490822 3500940 - 2031 protein_coding
deoxyhypusine hydroxylase [Source:HGNC Symbol;Acc:HGNC:28662] -
1113 1377 851 1060 1258 1980 26.83576156
32.12554157 26.06912673 29.00066693 38.07308583
45.33005907
ENSG00000226833 6.350756762 1.769990306 11.6249165 0
0 0 6.581887856 0 5.147011012
0.035380151 1 AC092164.1 2 28708953
28736205 - 1438 antisense novel transcript
- 7 2 10 0 0 0
0.238378914 0.065901842 0.43266121 0 0 0
ENSG00000138316 69.85832438 124.7843166 145.3114562
39.36680281 43.37668947 84.53862644 113.3180324
55.76070624 1.015602744 0.035410513 0.169520045
ADAMTS14 10 70672803 70762441 + 5269
protein_coding ADAM metallopeptidase with thrombospondin type
1 motif 14 [Source:HGNC Symbol;Acc:HGNC:14899] - 77 141
125 38 38 98 0.715634403 1.267994457
1.476007829 0.400744381 0.443305131 0.864826314
ENSG00000072401 585.1768731 715.9610789 559.1584836
763.5087807 856.1188711 814.3312587 620.0988118
811.3196369 -0.386750052 0.035456176 0.16969272 UBE2D1
10 58334975 58370753 + 3486
protein_coding ubiquitin conjugating enzyme E2 D1 [Source:HGNC
Symbol;Acc:HGNC:12474] - 645 809 481 737 750
944 9.06068465 10.99632532 8.584688481
11.7476811 13.22456029 12.59144665

ENSG00000166165 233.1634983 192.0439482 159.261356
285.9273046 334.4571057 254.4785184 194.8229342
291.6209762 -0.578742255 0.035586946 0.170272517 CKB
14 103519659 103523111 - 2669
protein_coding creatine kinase B [Source:HGNC Symbol;Acc:HGNC:1991]
- 257 217 137 276 293 295
4.715342389 3.852455234 3.193587649 5.746091693
6.747865332 5.139305805
ENSG00000165650 1244.748325 1288.552943 1179.929025
1638.902159 1505.627721 1453.546791 1237.743431
1532.692224 -0.307890719 0.035606077 0.170317983 PDZD8
10 117277274 117375467 - 9881
protein_coding PDZ domain containing 8 [Source:HGNC
Symbol;Acc:HGNC:26974] - 1372 1456 1015 1582 1319
1685 6.799576991 6.982113695 6.391046679
8.896468052 8.205239394 7.929211899
ENSG00000100304 1950.589577 2375.326991 1778.612224
2255.303413 2714.467567 2714.725075 2034.842931
2561.498685 -0.331644221 0.035626043 0.170367417 TTLL12
22 43166622 43187133 - 4622
protein_coding tubulin tyrosine ligase like 12 [Source:HGNC
Symbol;Acc:HGNC:28974] - 2150 2684 1530 2177 2378
3147 22.77913363 27.51560151 20.59531014
26.17221815 31.6249079 31.65904571
ENSG00000257219 53.52780699 40.70977704 8.137441549
85.98538508 91.31934625 73.32431885 34.12500853
83.54301673 -1.281069081 0.035650699 0.170439249
LINC02407 12 76259839 76305131 + 1201
lincRNA long intergenic non-protein coding RNA 2407
[Source:HGNC Symbol;Acc:HGNC:53336] - 59 46 7
83 80 85 2.405679059 1.814852212
0.362628455 3.840140627 4.094438383 3.29084128
ENSG00000230733 863.7029196 1023.939392 752.1320975
996.6016921 1186.010009 1256.865089 879.9248031
1146.492264 -0.381079498 0.035733858 0.170771076
AC092171.2 7 5475804 5479811 + 3251 lincRNA novel
transcript - 952 1157 647 962 1039 1457
14.33998386 16.86331025 12.38209574 16.44258691
19.6447243 20.83884289
ENSG00000279700 12.70151352 22.12487883 17.43737475
6.215810969 0 0 17.4212557 2.07193699
3.082275548 0.035739416 0.170771076 AC131212.2 12
132565071 132566425 + 1355 TEC novel
transcript - 14 25 15 6 0 0
0.505961445 0.874232916 0.688745557 0.246050357
0 0
ENSG00000177839 11.79426256 19.46989337 23.249833
39.36680281 55.93309958 45.7198694 18.17132964
47.0065906 -1.375932836 0.03577458 0.170892937 PCDHB9
5 141187127 141191541 + 4415

protein_coding	protocadherin beta 9 [Source:HGNC
Symbol;Acc:HGNC:8694]	-	13	22	20	38	49
	53	0.144192054	0.236112192	0.281842274
0.478260961	0.682201598	0.558182453
ENSG00000164402 1679.321538	1796.540161	1658.875584
1450.355893	1421.157326	1303.447597	1711.579094
1391.653605	0.299012437	0.035821263	0.171025405	8-Sep
	5	132750817	132807241	-	7711
protein_coding	septin 8 [Source:HGNC Symbol;Acc:HGNC:16511]	-
	1851	2030	1427	1400	1245	1511	11.75504779
	12.47417337	11.51383805	10.08856453	9.924439955
	9.111392999
ENSG00000146535 5040.686367	4794.018744	5556.710086
4412.18982	4256.623027	4180.348813	5130.471733
4283.053887	0.260295303	0.035821648	0.171025405	GNA12
	7	2728112 2844324 -	6120	protein_coding	G
protein subunit alpha 12 [Source:HGNC Symbol;Acc:HGNC:4380]	-
	5556	5417	4780	4259	3729	4846	44.45693302
	41.9405242	48.59407516	38.66947373	37.45314754
	36.81823843
ENSG00000077684 1029.729846	861.100284	1066.004843
843.2783549	662.0652603	769.4740284	985.6116578
758.2725479	0.377378621	0.035978065	0.171725845	JADE1
	4	128809623	128875224	+	9129
protein_coding	jade family PHD finger 1 [Source:HGNC
Symbol;Acc:HGNC:30027]	-	1135	973	917	814	580
	892	6.088374215	5.050287173	6.249610844
4.954652971	3.905279725	4.543313083
ENSG00000102271 289.4130581	192.9289434	291.7854041
169.8988332	190.6291353	142.3354425	258.0424686
167.621137	0.623053191	0.035992959	0.171750592	KLHL4
	X	87517749	87670050	+	5818
protein_coding	kelch like family member 4 [Source:HGNC
Symbol;Acc:HGNC:6355]	-	319	218	251	164	167
	165	2.685008406	1.775453153	2.684150427
1.566326064	1.764371827	1.318685544
ENSG00000175820 16.33051739	7.079961225	1.16249165
20.7193699	36.5277385	40.54403513	8.190990087
32.59704784	-1.975492386	0.03600437	0.171756929
CCDC168 13	102729369	102759070	-	21466
protein_coding	coiled-coil domain containing 168 [Source:HGNC
Symbol;Acc:HGNC:26851]	-	18	8	1	20	32
	47	0.041062942	0.017658967	0.002898383
0.05177152	0.091631799	0.101807026
ENSG00000136628 5116.895448	4522.325232	5382.336339
4891.843233	3608.255669	3447.968264	5007.185673
3982.689055	0.330232986	0.036018202	0.171756929	EPRS
	1	219968601	220046658	-	5450
protein_coding	glutamyl-prolyl-tRNA synthetase [Source:HGNC
Symbol;Acc:HGNC:3418]	-	5640	5110	4630	4722	3161

3997	50.67704526	44.42739393	52.85563994
48.14392836	35.65129861	34.10112166
ENSG00000004142 3664.386652	3857.693872	3811.81012
3954.291745	4691.531414	5231.043171	3777.963548
4625.62211	-0.292213065	0.036023416	0.171756929
POLDIP2 17	28347177	28357522	-	2121
protein_coding	DNA polymerase delta interacting protein 2
[Source:HGNC Symbol;Acc:HGNC:23781]	-	4039	4359	3279
3817	4110	6064	93.2527973	97.38064378
96.18505436	99.99851141	119.1100792	132.9381229
ENSG00000119906 1578.616681	1195.628452	1312.453073
1266.989469	744.252672	1023.952547	1362.232735
1011.731563	0.428755264	0.036056376	0.171867754	SLF2
10	100912569	100965136	+	9208
protein_coding	SMC5-SMC6 complex localization factor 2 [Source:HGNC
Symbol;Acc:HGNC:17814]	-	1740	1351	1129	1223	652
1187	9.253640455	6.952107504	7.628435489
7.380285969	4.352408455	5.993995692
ENSG00000054267 1005.23407	815.9655312	1020.667669
853.6380398	504.5393881	723.754159	947.28909
693.9771956	0.447807001	0.036087896	0.171971655	ARID4B
1	235131634	235328219	-	11073
protein_coding	AT-rich interaction domain 4B [Source:HGNC
Symbol;Acc:HGNC:15550]	ARID	1108	922	878	824	442
839	4.900079701	3.945409282	4.933283374
4.134985162	2.453603201	3.523121973
ENSG00000148396 2197.36184	2092.128542	2052.960254
2502.899884	2367.454052	2905.368304	2114.150212
2591.907413	-0.29421697	0.036136379	0.172156318	SEC16A
9	136440096	136483759	-	9975
protein_coding	"SEC16 homolog A, endoplasmic reticulum export factor
[Source:HGNC Symbol;Acc:HGNC:29006]"	-	2422	2364	1766
2416	2074	3368	11.89022076	11.22951523
11.01500355	13.45848173	12.78036355	15.69965817
ENSG00000247199 0	0	0	0	7.990442797
14.66486377	0	7.551768856	-5.395225656	0.036167653
1	AC091948.1	5	148268307	148383783
-	3201	antisense	uncharacterized LOC102546294
[Source:NCBI gene;Acc:102546294]	-	0	0	0
0	7	17	0	0	0	0
0.134418711	0.246941604
ENSG00000273356 18.14501932	31.85982551	32.5497662
51.79842475	79.90442797	56.93417699	27.51820368
62.8790099	-1.193707558	0.036170653	0.172273204
LINC02019	3	50669989	50672048	+	2060
lincRNA long intergenic non-protein coding RNA 2019
[Source:HGNC Symbol;Acc:HGNC:52854]	-	20	36	28
50	70	66	0.475435338	0.828059838
0.845663639	1.348698354	2.088710163	1.489730582
ENSG00000100578 887.2914447	490.2873148	781.1943887

668.1996792   436.0498784   351.9567305   719.5910494
485.402096   0.568320495   0.036199628   0.172314959
KIAA0586   14   58427385   58551289   +   10184
        protein_coding KIAA0586 [Source:HGNC Symbol;Acc:HGNC:19960]
        -       978   554   672   645   382   408
4.702720282   2.577613602   4.105421279   3.519276323
2.30564443   1.862828143
ENSG00000184220 1545.048395   1536.351586   1671.662993
1477.291074   1066.153368   1192.167161   1584.354324
1245.203867   0.347137638   0.036202944   0.172314959   CMSS1
        3   99817834   100181732   +   7210
protein_coding cms1 ribosomal small subunit homolog (yeast)
[Source:HGNC Symbol;Acc:HGNC:28666]   -   1703   1736   1438
        1426   934   1382   11.56666258   11.40882443
12.40881952   10.98996488   7.962674661   8.912587292
ENSG00000118620 233.1634983   284.0834442   238.3107882
183.3664236   155.2428886   169.0772529   251.8525769
169.228855   0.573763388   0.036208644   0.172314959   ZNF430
        19   21020620   21060050   +   6845
protein_coding zinc finger protein 430 [Source:HGNC
Symbol;Acc:HGNC:20808] zf-C2H2 257   321   205   177   136
        196   1.838604651   2.222071457   1.863319183
1.436851407   1.22127317   1.331415587
ENSG00000188747 27.21752898   19.46989337   24.41232465
6.215810969   6.848950969   5.175834272   23.69991567
6.080198737   1.966761452   0.036225854   0.172342366   NOXA1
        9   137423350   137434406   +   1678
protein_coding NADPH oxidase activator 1 [Source:HGNC
Symbol;Acc:HGNC:10668] -   30   22   21   6   6
        6   0.875503692   0.621236787   0.778635472
0.198687862   0.219789355   0.166260971
ENSG00000231528 98.89035529   104.4294281   90.67434869
70.44585765   46.80116496   33.64292277   97.99804402
50.29664846   0.96605972   0.036248988   0.172342366
FAM225A 9   113113073   113119928   +   6100
lincRNA family with sequence similarity 225 member A [Source:HGNC
Symbol;Acc:HGNC:27855] -   109   118   78   68   41
        39   0.875034843   0.916597383   0.795557573
0.619428411   0.413143935   0.297280067
ENSG00000160305 865.5174215   687.641234   940.4557448
804.9475205   523.9447491   436.4953569   831.2048001
588.4625422   0.498387222   0.036251007   0.172342366   DIP2A
        21   46458899   46569852   +   13557
protein_coding disco interacting protein 2 homolog A [Source:HGNC
Symbol;Acc:HGNC:17217] -   954   777   809   777   459
        506   3.445985631   2.715712952   3.712716364
3.184706882   2.081116774   1.73547331
ENSG00000184486 125.2006333   37.16979643   72.07448229
38.33083431   27.39580388   36.2308399   78.14830401
33.98582603   1.199666819   0.036253375   0.172342366   POU3F2

6 98834592 98839470 + 4879
protein_coding POU class 3 homeobox 2 [Source:HGNC
Symbol;Acc:HGNC:9215] Pou 138 42 62 37 24
42 1.385086677 0.407891742 0.790619857
0.421388762 0.3023624 0.400266727
ENSG00000225849 0 0 0 17.61146441 0
5.175834272 0 7.595766228 -5.401810087 0.036303973
1 MKRN7P 20 46463719 46477232 -
1994 transcribed_processed_pseudogene "makorin ring
finger protein 7, pseudogene [Source:HGNC Symbol;Acc:HGNC:7119]"
- 0 0 0 17 0 6 0
0 0 0.47373537 0 0.139912693
ENSG00000143643 825.598379 840.7453955 773.0569472
752.1131273 586.7267997 474.4514749 813.1335739
604.4304673 0.429075842 0.036402161 0.173003175 TTC13
1 230906243 230978875 - 4212
protein_coding tetratricopeptide repeat domain 13 [Source:HGNC
Symbol;Acc:HGNC:26204] - 910 950 665 726 514
550 10.57990367 10.68714455 9.822909196
9.577679539 7.501050973 6.071630656
ENSG00000223749 45.3625483 89.38451047 34.8747495
21.75533839 13.69790194 30.19236658 56.54060275
21.88186897 1.365576831 0.036534646 0.173549494
MIR503HG X 134543337 134546632 - 1678
lincRNA MIR503 host gene [Source:HGNC Symbol;Acc:HGNC:28258]
- 50 101 30 21 12 35
1.45917282 2.852041615 1.112336389 0.695407519
0.43957871 0.969855663
ENSG00000125676 3127.29408 2405.416826 2365.670508
2622.036261 1705.388791 1643.327381 2632.793804
1990.250811 0.403943374 0.036546895 0.173549494 THOC2
X 123600561 123733056 - 10277
protein_coding THO complex 2 [Source:HGNC Symbol;Acc:HGNC:19073]
- 3447 2718 2035 2531 1494 1905
16.42493314 12.53168693 12.31983535 13.68478039
8.935762167 8.619054793
ENSG00000166189 631.4466723 899.1550756 776.5444221
764.5447492 1126.652434 1266.354118 769.0487233
1052.517101 -0.453051703 0.036552792 0.173549494 HPS6
10 102065390 102068038 + 2649
protein_coding "HPS6, biogenesis of lysosomal organelles complex 2
subunit 3 [Source:HGNC Symbol;Acc:HGNC:18817]" - 696 1016
668 738 987 1468 12.86636788 18.1734839
15.68921992 15.48055217 22.90248222 25.76766795
ENSG00000204267 1144.043468 1107.128937 902.0935203
863.9977247 760.2335576 833.3093177 1051.088642
819.1802 0.360145336 0.036556358 0.173549494 TAP2
6 32821833 32838780 - 6390
protein_coding "transporter 2, ATP binding cassette subfamily B
member [Source:HGNC Symbol;Acc:HGNC:44]" - 1261 1251

776	834	666	966	9.663688267	9.27647316
7.555578284	7.252324635	6.406498536	7.029222433
ENSG00000157429 41.73354444	30.97483036	48.8246493
59.05020421	91.31934625	97.47821212	40.51100803
82.61592086	-1.032543266	0.036598588	0.173703362	ZNF19
16	71464555	71565089	-	9225
protein_coding	zinc finger protein 19 [Source:HGNC
Symbol;Acc:HGNC:12981]	zf-C2H2 46	35	42	57	80
113	0.244185651	0.179774509	0.283262942
0.343336934	0.533053712	0.569564728
ENSG00000189398 0.907250966	4.424975766	0	11.39565344
22.82983656	10.35166854	1.777408911	14.85905285
-3.018649878	0.036613574	0.173727871	OR7E12P 11
3390780 3391752 -	973	unprocessed_pseudogene
olfactory receptor family 7 subfamily E member 12 pseudogene
[Source:HGNC Symbol;Acc:HGNC:8383]	-	1	5	0
11	20	12	0.050328715	0.243491388	0
0.628191258	1.263468781	0.573455106
ENSG00000221963 581.5478692	563.7419125	521.9587508
448.5743583	441.7573375	347.6435352	555.7495108
412.6584103	0.431249083	0.036635617	0.173785848	APOL6
22	35648395	35668409	+	10121
protein_coding	apolipoprotein L6 [Source:HGNC Symbol;Acc:HGNC:14870]
-	641	637	449	433	387	403
3.101439317	2.98223902	2.760131432	2.377259081
2.350362776	1.851452777
ENSG00000198824 1644.846001	1269.968045	1374.06513
1880.282818	1677.992987	1817.580468	1429.626392
1791.952091	-0.325893791	0.036664068	0.173874182	CHAMP1
13	114314335	114337626	+	5770
protein_coding	chromosome alignment maintaining phosphoprotein 1
[Source:HGNC Symbol;Acc:HGNC:20311]	-	1813	1435	1182
1815	1470	2107	15.38688382	11.78426577
12.74525444	17.47885018	15.6598963	16.97929839
ENSG00000163251 194.1517067	151.3341712	185.998664
128.4600934	118.7151501	71.59904076	177.161514
106.2580948	0.740412987	0.036682863	0.173910588	FZD5
2	207762586	207769563	-	6708
protein_coding	frizzled class receptor 5 [Source:HGNC
Symbol;Acc:HGNC:4043]	-	214	171	160	124	104
83	1.562245934	1.207895873	1.483999571
1.027166093	0.952988469	0.575328723
ENSG00000164916 2161.071801	2393.026894	2255.233801
2638.611757	2511.282022	3303.044904	2269.777499
2817.646228	-0.312341616	0.036691408	0.173910588	FOXK1
7	4682309 4771443 +	13093	protein_coding
forkhead box K1 [Source:HGNC Symbol;Acc:HGNC:23480]	Fork	2382
2704	1940	2547	2200	3829	8.909047467
9.785746479	9.218694195	10.80940579	10.32834825
13.59806825

ENSG00000181029 42.6407954 53.98470434 22.08734135
40.4027713 90.17785443 142.3354425 39.57094703
90.97202273 -1.197317486 0.036703957 0.173915904
TRAPPC5 19 7680843 7687703 + 5669 protein_coding
trafficking protein particle complex 5 [Source:HGNC
Symbol;Acc:HGNC:23067] - 47 61 19 39 79
165 0.405994439 0.509858682 0.208523013
0.38226998 0.856580127 1.353344946
ENSG00000259172 3.629003864 2.654985459 0 5.179842475
0 58.65945508 2.094663108
21.27976585 -3.322317563 0.036712193 0.173915904
AC023024.1 15 101295419 101305737 + 2590
antisense "novel transcript, antisense to SNRPA1 and
PCSK6" - 4 3 0 5 0 68
0.075629096 0.054884275 0 0.107270989 0
1.220787761
ENSG00000173198 6.350756762 4.424975766 8.137441549 0
0 0 6.304391359 0 5.087604919
0.036777809 1 CYSLTR1 X 78271464 78327691
- 5571 protein_coding cysteinyl leukotriene receptor
1 [Source:HGNC Symbol;Acc:HGNC:17451] - 7 5 7
0 0 0 0.061530942 0.042526857
0.078175691 0 0 0
ENSG00000257126 51.71330506 52.21471403 41.8496994
33.15099184 10.27342645 6.038473317 48.59257283
16.48763054 1.565323016 0.036782689 0.174177201 FOXG1-
AS1 14 28729083 28765277 - 874
antisense FOXG1 antisense RNA 1 [Source:HGNC
Symbol;Acc:HGNC:50663] - 57 59 36 32 9
7 3.193685206 3.198652196 2.562700861
2.034466717 0.632963165 0.37240682
ENSG00000121753 146.9746565 138.0592439 180.1862057
127.4241249 52.5086241 79.36279217 155.0733687
86.43184704 0.840900992 0.036787044 0.174177201 ADGRB2
1 31727117 31764893 - 7756
protein_coding adhesion G protein-coupled receptor B2 [Source:HGNC
Symbol;Acc:HGNC:944] - 162 156 155 123 46
92 1.022835747 0.95304463 1.24337103
0.881209874 0.364558637 0.551545118
ENSG00000063046 7238.048207 6856.057451 7808.456412
9431.457178 8062.356782 8719.55547 7300.854023
8737.78981 -0.259354752 0.036813185 0.174254327 EIF4B
12 53006158 53042209 + 6146
protein_coding eukaryotic translation initiation factor 4B
[Source:HGNC Symbol;Acc:HGNC:3285] - 7978 7747 6717
9104 7063 10108 63.56677224 59.7265516
67.99698227 82.30983608 70.63891347 76.47221955
ENSG00000108423 192.3372048 156.6441421 226.6858717
106.704755 86.75337894 153.5497501 191.8890729
115.6692947 0.724891277 0.036838499 0.174311214 TUBD1

17 59859482 59892945 - 3330
protein_coding tubulin delta 1 [Source:HGNC Symbol;Acc:HGNC:16811]
- 212 177 195 103 76 178
3.117599413 2.518578396 3.643319216 1.718719621
1.40286771 2.48546005
ENSG00000253227 9.07250966 1.769990306 0 24.86324388
23.97132839 15.52750281 3.614166655 21.45402503
-2.542001823 0.036844911 0.174311214 AC090192.2
8 124271683 124277584 + 553
lincRNA novel transcript - 10 2 0 24
21 18 0.885530558 0.171368622 0
2.411560457 2.334218591 1.513485944
ENSG00000122641 552.5158383 404.442785 1053.217435
295.251021 560.4724876 415.7920198 670.058686
423.8385095 0.659802216 0.036875539 0.174409465 INHBA
7 41667168 41705834 - 8302
protein_coding inhibin subunit beta A [Source:HGNC
Symbol;Acc:HGNC:6066] - 609 457 906 285 491
482 3.592222652 2.608314237 6.789727041
1.907542288 3.635349109 2.699574563
ENSG00000216906 14.51601546 1.769990306 3.48747495 0
0 0 6.591160237 0 5.155775045
0.036968834 1 AL355312.1 6 149904243
149906418 + 2176 processed_pseudogene multimerin 2
(MMRN2) pseudogene - 16 2 3 0 0
0 0.360072352 0.043550941 0.085776676 0
0 0
ENSG00000128185 1196.664024 1353.157589 1075.304776
1189.291832 1776.161285 1795.151853 1208.375463
1586.868323 -0.392759808 0.036970806 0.174813307 DGCR6L
22 20314276 20320080 - 1154
protein_coding DiGeorge syndrome critical region gene 6 like
[Source:HGNC Symbol;Acc:HGNC:18551] - 1319 1529 925
1148 1556 2081 55.97159336 62.78098387
49.87039069 55.27746559 82.88026749 83.84888455
ENSG00000273733 2.721752898 7.079961225 9.299933199 0
0 0 6.367215774 0 5.101186698
0.037044073 1 AC011472.4 19 11221083
11221573 + 491 antisense "novel transcript,
antisense to DOCK6" - 3 8 8 0 0
0 0.299204724 0.772031349 1.01371376 0
0 0
ENSG00000178605 1788.191654 2253.19766 2044.822812
2071.93699 2676.798337 3014.923463 2028.737375
2587.886263 -0.351414915 0.037055849 0.175088595 GTPBP6
X 304529 318819 - 2964 protein_coding GTP
binding protein 6 (putative) [Source:HGNC Symbol;Acc:HGNC:30189]
- 1971 2546 1759 2000 2345 3495
32.5639522 40.7011463 36.922788 37.49417827
48.63086651 54.82769635

ENSG00000149929 348.3843709   640.7364909   654.4827989
416.459335   289.9389244   404.5777122   547.8678869
370.3253238   0.563314248   0.037057746   0.175088595   HIRIP3
16   29992321   29996436   -   3931
protein_coding HIRA interacting protein 3 [Source:HGNC
Symbol;Acc:HGNC:4917]   -   384   724   563   402   254
469   4.783622105   8.72693946   8.9107077
5.68244254   3.971714597   5.547544786
ENSG00000177576 79.83808501   96.46447169   105.7867401
164.7189907   127.8470848   174.2530871   94.02976561
155.6063875   -0.729883197   0.03705872   0.175088595
C18orf32   18   49477250   49487252   -   5740
protein_coding chromosome 18 open reading frame 32
[Source:HGNC Symbol;Acc:HGNC:31690]   -   88   109   91
159   112   202   0.750757126   0.89978976
0.986362032   1.539207871   1.199370853   1.636328502
ENSG00000127920 598.7856375   598.2567235   578.9208416
419.5672404   1068.436351   1209.419941   591.9877342
899.1411777   -0.603259503   0.037083091   0.175156957   GNG11
7   93921699   93928610   +   3055
protein_coding G protein subunit gamma 11 [Source:HGNC
Symbol;Acc:HGNC:4403]   -   660   676   498   405   936
1402   10.57940893   10.48484277   10.14203196
7.366409408   18.83267425   21.33869309
ENSG00000260793 5.443505796   11.50493699   2.3249833   0
0   0   6.424475362   0   5.120574627
0.037108769   1   AC003102.1   17   44221401
44223710   +   2012   antisense   "novel transcript,
antisense to UBTF"   -   6   13   2   0   0
0   0.14603332   0.306155324   0.061845608   0
0   0
ENSG00000108433 1046.060364   1208.018384   993.9303607
1311.536115   1392.62003   1363.832331   1082.669703
1355.996158   -0.324129087   0.037114009   0.175256194   GOSR2
17   46923075   46975524   +   14366
protein_coding golgi SNAP receptor complex member 2 [Source:HGNC
Symbol;Acc:HGNC:4431]   -   1153   1365   855   1266   1220
1581   3.930267669   4.502183895   3.702858354
4.896772046   5.220009925   5.117136783
ENSG00000100347 1009.770325   1215.98334   1071.817301
1174.788273   1496.495787   1544.123891   1099.190322
1405.135984   -0.354257978   0.037152707   0.175392111   SAMM50
22   43955421   44010531   +   6435
protein_coding SAMM50 sorting and assembly machinery component
[Source:HGNC Symbol;Acc:HGNC:24276]   -   1113   1374   922
1134   1311   1790   8.469841761   10.11729987
8.914340452   9.792115941   12.52280162   12.93407866
ENSG00000249471 277.6187956   469.0474312   331.3101202
500.372783   461.1626986   573.6549651   359.325449
511.7301489   -0.509964404   0.037165942   0.175407776

ZNF324B 19   58451604   58457833   +   4042
protein_coding zinc finger protein 324B [Source:HGNC
Symbol;Acc:HGNC:33107] zf-C2H2 306   530   285   483   404
        665   3.707266449   6.213066489   4.386876391
6.63992028   6.143734169   7.649910502
ENSG00000152208 10.88701159   0.884995153   8.137441549   0
        0   0   6.636482765   0   5.161903034
0.037204794   1   GRID2   4   92303622   93810157
        +   10951   protein_coding glutamate ionotropic receptor
delta type subunit 2 [Source:HGNC Symbol;Acc:HGNC:4576] -   12
        1   7   0   0   0   0.053660677
0.004326858   0.039769589   0   0   0
ENSG00000164733 41398.76883   53403.26252   52293.52438
64204.14747   56251.5758   57754.54672   49031.85191
59403.42333   -0.276839007   0.037214892   0.175591949   CTSB
        8   11842524   11869448   -   7428
protein_coding cathepsin B [Source:HGNC Symbol;Acc:HGNC:2527] -
        45631   60343   44984   61975   49279   66951   300.8269738
        384.9297197   376.7844943   463.6141515   407.7907322
        419.0985818
ENSG00000090924 786.5865875   654.8964133   714.9323647
516.948279   494.2659616   621.9627516   718.8051218
544.3923307   0.399800899   0.037266567   0.175788879
PLEKHG2 19   39412585   39428415   +   9200
protein_coding pleckstrin homology and RhoGEF domain containing G2
[Source:HGNC Symbol;Acc:HGNC:29515]   -   867   740   615
        499   433   721   4.614875123   3.811275413
4.159049938   3.01387171   2.892993309   3.644000732
ENSG00000188706 1081.443151   996.5045424   1169.4666
1216.227013   1703.105808   1273.255231   1082.471431
1397.52935   -0.368396481   0.037301837   0.175883471   ZDHHC9
        X   129803288   129843909   -   5243
protein_coding zinc finger DHHC-type containing 9 [Source:HGNC
Symbol;Acc:HGNC:18475] -   1192   1126   1006   1174   1492
        1476   11.13332998   10.17618453   11.93781844
12.44228895   17.49187341   13.08993584
ENSG00000160326 236.7925021   221.2487883   383.6222445
237.2367853   116.4321665   161.3135015   280.5545116
171.6608178   0.705886975   0.037306506   0.175883471   SLC2A6
        9   133471095   133479137   -   3540
protein_coding solute carrier family 2 member 6 [Source:HGNC
Symbol;Acc:HGNC:11011] -   261   250   330   229   102
        187   3.610488191   3.34628701   5.799860187
3.594547806   1.77110484   2.456231873
ENSG00000221843 69.85832438   14.15992245   31.38727455
125.3521879   65.06503421   81.9507093   38.46850713
90.78931046   -1.237582369   0.037325516   0.175926207
C2orf16 2   27537386   27582721   +   16402
protein_coding chromosome 2 open reading frame 16 [Source:HGNC
Symbol;Acc:HGNC:25275] -   77   16   27   121   57

95 0.229891334 0.046222094 0.102417413
0.409921414 0.213611883 0.269313309
ENSG00000139112 146.0674055 141.5992245 134.8490314
145.0355893 251.1282022 282.9456069 140.8385538
226.3697994 -0.685061292 0.037344358 0.175941371
GABARAPL1 12 10212458 10223130 + 4086
protein_coding GABA type A receptor associated protein like 1
[Source:HGNC Symbol;Acc:HGNC:4068] - 161 160 116
140 220 328 1.929550713 1.855444897
1.766308153 1.903889405 3.309570819 3.732557438
ENSG00000163508 14.51601546 0 2.3249833 21.75533839
13.69790194 55.2088989 5.613666252 30.22071308
-2.421990567 0.037365673 0.175941371 EOMES 3
27715949 27722711 - 3656 protein_coding
eomesodermin [Source:HGNC Symbol;Acc:HGNC:3372] T-box 16
0 2 21 12 64 0.214310021 0
0.034035384 0.319172269 0.201754123 0.813963265
ENSG00000181163 18635.84209 17272.4504 19183.43721
17056.1853 14502.65368 14638.9846 18363.9099
15399.27453 0.253986782 0.037376383 0.175941371 NPM1
5 171387116 171411137 + 3828
protein_coding nucleophosmin 1 [Source:HGNC Symbol;Acc:HGNC:7910]
- 20541 19517 16502 16464 12705 16970
262.7715465 241.5836696 268.2078596 238.9876572
204.0093633 206.1298709
ENSG00000273199 7.258007728 7.964956378 17.43737475
29.00711786 62.78205055 17.25278091 10.88677962
36.34731644 -1.747623121 0.037389176 0.175941371
AP000692.2 21 36319792 36320670 - 879
antisense "novel transcript, antisense to MORC3" -
8 9 15 28 55 20 0.445686824
0.485154513 1.061718123 1.770032334 3.846105338
1.057967042
ENSG00000153208 41.73354444 15.0449176 36.03724115
6.215810969 13.69790194 0 30.93856773 6.637904302
2.243197366 0.03738953 0.175941371 MERTK 2
111898479 112029561 + 4700 protein_coding
"MER proto-oncogene, tyrosine kinase [Source:HGNC
Symbol;Acc:HGNC:7027]" - 46 17 31 6 12
0 0.479279283 0.171386853 0.410365349
0.070935794 0.156938952 0
ENSG00000235106 646.8699387 764.6358123 761.4320307
890.9329056 1101.539614 839.3477911 724.3125939
943.9401036 -0.381623998 0.037396503 0.175941371 BRD3OS
9 134025439 134034666 + 5994
protein_coding BRD3 opposite strand [Source:HGNC
Symbol;Acc:HGNC:24742] - 713 864 655 860 965
973 5.825074374 6.830043107 6.798786572
7.972485835 9.895960085 7.547917069
ENSG00000162836 256.7520234 298.2433666 238.3107882

442.3585473 366.4188768 321.7643639 264.4353927
376.8472627 -0.50922611 0.037398358 0.175941371 ACP6
1 147629652 147670496 - 11960
protein_coding "acid phosphatase 6, lysophosphatidic [Source:HGNC
Symbol;Acc:HGNC:29609]" - 283 337 205 427 321
373 1.158734504 1.335134942 1.066423061
1.983849576 1.649761684 1.450135787
ENSG00000139637 854.63041 1044.294281 990.4428857
992.4578181 1384.629588 1388.848863 963.1225254
1255.31209 -0.382485296 0.03743014 0.176044071
C12orf10 12 53299686 53307177 + 2509
protein_coding chromosome 12 open reading frame 10
[Source:HGNC Symbol;Acc:HGNC:17590] - 942 1180 852
958 1213 1610 18.38564732 22.28475105
21.12738664 21.21665387 29.71717349 29.8370741
ENSG00000119397 536.1853209 345.1481097 387.1097194
421.6391774 195.1951026 170.802531 422.8143833
262.5456037 0.688491885 0.037517113 0.176406224 CNTRL
9 121074863 121177610 + 9417
protein_coding centriolin [Source:HGNC Symbol;Acc:HGNC:1858] -
591 390 333 407 171 198 3.073290362
1.962359072 2.200080186 2.401562439 1.116171425
0.977650525
ENSG00000256594 51.71330506 37.16979643 27.8997996
17.61146441 10.27342645 12.93958568 38.9276337
13.60815885 1.517327584 0.037532015 0.176429397
AC010186.2 12 9648047 9658412 + 2825
transcribed_unprocessed_pseudogene C-type lectin domain family 2
member D pseudogene [Source:NCBI gene;Acc:374443] - 57
42 24 17 9 15 0.988064025
0.704461526 0.528566502 0.334381709 0.19582648
0.246890185
ENSG00000115073 912.6944718 1040.7543 1136.916834
1153.032935 1348.101849 1429.392898 1030.121868
1310.175894 -0.347782305 0.037543324 0.17643567 ACTR1B
2 97655963 97664107 - 2892
protein_coding ARP1 actin related protein 1 homolog B [Source:HGNC
Symbol;Acc:HGNC:168] - 1006 1176 978 1113 1181
1657 17.03446019 19.26794837 21.04008126
21.38498349 25.10145923 26.64128926
ENSG00000226935 0 2.654985459 2.3249833 38.33083431
0 13.80222472 1.659989586
17.37768635 -3.400534765 0.037562402 0.17647844
LINC00161 21 28539318 28540355 + 870
lincRNA long intergenic non-protein coding RNA 161
[Source:HGNC Symbol;Acc:HGNC:17138] - 0 3 2
37 0 16 0 0.163391117 0.143026855
2.363167553 0 0.855129223
ENSG00000187741 1753.716117 1816.895049 1496.126753
1574.672112 1034.191596 1300.85968 1688.91264

1303.24113	0.374030963	0.037603789	0.176581596	FANCA
	16	89737549	89816657	-	9736
protein_coding	FA complementation group A [Source:HGNC
Symbol;Acc:HGNC:3582]	-	1933	2053	1287	1520	906
	1508	9.722545234	9.991595068	8.224411436
8.675111519	5.719986353	7.201978067
ENSG00000165916 5057.016884	5694.058815	6704.089345
4341.743962	4818.237007	5058.515361	5818.388348
4739.498777	0.295592554	0.037604323	0.176581596	PSMC3
	11	47418769	47426473	-	2276
protein_coding	"proteasome 26S subunit, ATPase 3 [Source:HGNC
Symbol;Acc:HGNC:9549]"	-	5574	6434	5767	4191	4221
	5864	119.9287729	133.9476935	157.6465752
102.3192732	113.996221	119.7988701
ENSG00000115594 211.3894751	176.1140355	179.0237141
104.632818	160.9503478	64.6979284	188.8424082
110.0936981	0.783807	0.037618649	0.176587733	IL1R1
	2	102064544	102179874	+	7083
protein_coding	interleukin 1 receptor type 1 [Source:HGNC
Symbol;Acc:HGNC:5993]	-	233	199	154	101	141
	75	1.610895479	1.331258137	1.352727521
0.792348382	1.223627506	0.492351244
ENSG00000146555 30.84653284	54.86969949	84.86189044
26.93518087	19.40536108	0	56.85937426	15.44684732
	1.88849025	0.037625596	0.176587733	SDK1	7
	3301448 4269000 +	12062	protein_coding	sidekick cell
adhesion molecule 1 [Source:HGNC Symbol;Acc:HGNC:19307] -	34
	62	73	26	17	0	0.1380347
0.243555985	0.376539362	0.119774969	0.086631724	0
ENSG00000169933 0	0	0	2.07193699	5.707459141
	11.21430759	0	6.331234573	-5.141437813
0.037638412	1	FRMPD4	X	12138466	12724523
	+	8478	protein_coding	FERM and PDZ domain containing
4 [Source:HGNC Symbol;Acc:HGNC:29007]	-	0	0	0
	2	5	13	0	0	0	0.013108368
	0.036251331	0.071298593
ENSG00000152207 95.26135143	114.1643748	94.16182364
130.5320304	134.6960357	257.0664355	101.1958499
174.0981672	-0.78474054	0.037796256	0.177341639
CYSLTR2 13	48653711	48711226	+	5372
protein_coding	cysteinyl leukotriene receptor 2 [Source:HGNC
Symbol;Acc:HGNC:18274]	-	105	129	81	126	118
	298	0.957154353	1.137837249	0.938114528
1.303306571	1.350185263	2.579355947
ENSG00000154269 0.907250966	6.194966072	12.78740815	0
	0	0	6.629875062	0	5.157140379
0.037818568	1	ENPP3	6	131628442	131747418
	+	3885	protein_coding	ectonucleotide
pyrophosphatase/phosphodiesterase 3 [Source:HGNC
Symbol;Acc:HGNC:3358]	-	1	7	11	0	0

0 0.012604849 0.085375539 0.17616049 0
0 0
ENSG00000175581 909.9727189 899.1550756 1028.80511
885.7530631 623.2545382 640.9408106 945.9776349
716.6494707 0.400210263 0.037818932 0.177400977 MRPL48
11 73787316 73865133 + 4386
protein_coding mitochondrial ribosomal protein L48 [Source:HGNC
Symbol;Acc:HGNC:16653] - 1003 1016 885 855 546
743 11.19852926 10.97618761 12.55398166
10.83202194 7.651936821 7.87682514
ENSG00000177302 2485.867647 2596.575779 2180.834335
2848.913361 2596.893909 3737.814983 2421.092587
3061.207418 -0.33861086 0.037840984 0.177457362 TOP3A
17 18271428 18315007 - 6476
protein_coding DNA topoisomerase III alpha [Source:HGNC
Symbol;Acc:HGNC:11992] - 2740 2934 1876 2750 2275
4333 20.7191725 21.46741294 18.02323895
23.59597337 21.59344432 31.11092093
ENSG00000197479 11.79426256 9.734946684 17.43737475 0
0 3.450556181 12.98886133 1.150185394
3.418653336 0.037883565 0.177609961 PCDHB11 5
141199582 141203779 + 4198 protein_coding
protocadherin beta 11 [Source:HGNC Symbol;Acc:HGNC:8682] -
13 11 15 0 0 4 0.151645526
0.124158567 0.222308297 0 0 0.044304575
ENSG00000236654 0 0 0 5.179842475 0
17.25278091 0 7.477541127 -5.383799892 0.037904268
1 AC079780.1 7 23480753 23480959
+ 207 processed_pseudogene chromosome 5 open
reading frame 13 (C5orf13) pseudogene - 0 0 0
5 0 20 0 0 0 1.342182903
0 4.492526715
ENSG00000150712 758.4618076 768.1757929 835.8314963
1124.025817 1116.379008 828.9961225 787.4896989
1023.133649 -0.377226816 0.037929489 0.17777815 MTMR12
5 32226994 32313009 - 5545
protein_coding myotubularin related protein 12 [Source:HGNC
Symbol;Acc:HGNC:18191] - 836 868 719 1085 978
961 7.383009215 7.417279005 8.067411066
10.87277076 10.84138243 8.058474859
ENSG00000261105 59.87856375 38.05479158 56.96209084
12.43162194 35.38624667 8.626390453 51.63181539
18.81475302 1.468287827 0.037952213 0.177822208 LMO7-
AS1 13 75604700 75635994 - 593
antisense LMO7 antisense RNA 1 [Source:HGNC
Symbol;Acc:HGNC:50277] - 66 43 49 12 31
10 5.450268851 3.435897528 5.141007451
1.12444598 3.213322838 0.784108794
ENSG00000196998 650.4989426 925.7049302 725.3947895
1126.097754 1028.484137 882.4797433 767.1995541

1012.353878 -0.39917794 0.037966087 0.177822208 WDR45
X 49071470 49101170 - 5452
protein_coding WD repeat domain 45 [Source:HGNC
Symbol;Acc:HGNC:28912] - 717 1046 624 1087 901
1023 6.440090822 9.090803661 7.120911513
11.0786219 10.15818935 8.724706068
ENSG00000172238 2.721752898 4.424975766 4.6499666
37.29486582 14.83939377 7.763751407 3.932231754
19.96600366 -2.346891188 0.037975788 0.177822208 ATOH1
4 93828753 93830964 + 2212
protein_coding atonal bHLH transcription factor 1 [Source:HGNC
Symbol;Acc:HGNC:797] bHLH 3 5 4 36 13
9 0.066414792 0.107105389 0.112507562
0.904335171 0.361248115 0.189185743
ENSG00000008988 12012.91004 14093.54781 14221.92284
15312.65032 15799.38839 17014.69253 13442.79357
16042.24375 -0.255121388 0.0379791 0.177822208 RPS20
8 56067295 56074581 - 3002
protein_coding ribosomal protein S20 [Source:HGNC
Symbol;Acc:HGNC:10405] - 13241 15925 12234 14781 13841
19724 215.9925547 251.3594364 253.5505955
273.5931204 283.4028027 305.5028908
ENSG00000116062 2867.820303 2736.405013 2405.195224
3259.156885 3056.915116 3411.737424 2669.806847
3242.603142 -0.280235362 0.037995977 0.17785415 MSH6
2 47695530 47810101 + 10993
protein_coding mutS homolog 6 [Source:HGNC Symbol;Acc:HGNC:7329]
- 3161 3092 2069 3146 2678 3955
14.08111195 13.3275309 11.709844 15.90210197
14.97413364 16.72866476
ENSG00000248309 11.79426256 2.654985459 4.6499666 0
0 0 6.366404872 0 5.104774216
0.038084529 1 MEF2C-AS1 5 88883328
89466398 + 7865 antisense MEF2C antisense RNA 1
[Source:HGNC Symbol;Acc:HGNC:48908] - 13 3 4
0 0 0 0.080941884 0.018073779
0.031642305 0 0 0
ENSG00000138231 600.6001395 515.9521743 446.3967936
671.3075847 617.547079 808.2927854 520.9830358
699.0491497 -0.423968077 0.038118999 0.178382798 DBR1
3 138161012 138174949 - 3160
protein_coding debranching RNA lariats 1 [Source:HGNC
Symbol;Acc:HGNC:15594] - 662 583 384 648 541
937 10.25887151 8.741941843 7.560508193
11.39462316 10.52343548 13.78743654
ENSG00000267523 47.17705023 14.15992245 23.249833
7.251779464 4.565967313 10.35166854 28.19560189
7.389805107 1.922255162 0.038140713 0.178437203
AC008735.2 19 55661901 55674715 - 561
antisense "novel transcript, antisense to U2AF2A" -

52	16	20	7	4	12	4.539093888
1.351398904	2.21806353	0.693341543	0.438272772
0.994602171
ENSG00000255363 6.350756762	5.309970919	6.974949899	0
0	0	6.211892527	0	5.067326364
0.038180501	1	AP001189.5	11	76607853
76630427	-	1725	lincRNA novel transcript	-
7	6	6	0	0	0	0.198718191
0.16481191	0.21640585	0	0	0
ENSG00000078902 1006.141321	1214.21335	1304.315631
1327.075642	1514.759656	1632.113074	1174.890101
1491.316124	-0.344857234	0.038220937	0.178765244	TOLLIP
11	1274371 1309654 -	5983	protein_coding	toll
interacting protein [Source:HGNC Symbol;Acc:HGNC:16476] -	1109
1372	1122	1281	1327	1892	9.07697683
10.86579606	11.66757767	11.897129	13.63324628
14.70392055
ENSG00000091640 694.046989	760.2108365	941.6182364
583.2502626	673.4801786	555.5395452	798.625354
604.0899955	0.402471325	0.038243023	0.178821263	SPAG7
17	4959226 4967872 -	2831	protein_coding	sperm
associated antigen 7 [Source:HGNC Symbol;Acc:HGNC:11216]	-
765	859	810	563	590	644	13.23275432
14.37737946	17.80131135	11.0504654	12.81030596
10.57735343
ENSG00000130560 1115.918688	1383.247424	1055.542418
1196.543612	1628.908839	1851.223391	1184.902844
1558.891947	-0.39559569	0.038260989	0.178853794	UBAC1
9	135932969	135961380	-	2979
protein_coding	UBA domain containing 1 [Source:HGNC
Symbol;Acc:HGNC:30221]	-	1230	1563	908	1155	1427
2146	20.21916851	24.86078946	18.96366138
21.54386032	29.44427262	33.49579057
ENSG00000065883 2414.19482	2493.916341	2399.382765
2835.445771	2825.192275	3131.379734	2435.831309
2930.672593	-0.266961975	0.038270653	0.178853794	CDK13
7	39949646	40099580	+	25093
protein_coding	cyclin dependent kinase 13 [Source:HGNC
Symbol;Acc:HGNC:1733]	-	2661	2818	2064	2737	2475
3630	5.193031674	5.321264456	5.117571899
6.060860029	6.062754419	6.726436653
ENSG00000076641 846.4651513	725.6960256	839.3189712
737.6095684	597.0002261	427.8689665	803.826716
587.4929203	0.453236478	0.038280313	0.178853794	PAG1
8	80967810	81112068	-	11023
protein_coding	phosphoprotein membrane anchor with glycosphingolipid
microdomains 1 [Source:HGNC Symbol;Acc:HGNC:30043]	-	933
820	722	712	523	496	4.14486624
3.524848746	4.075155984	3.589155131	2.916414452
2.092246679

ENSG00000167173 765.7198153   800.0356184   957.8931195
934.4435824   1077.568286   1265.491479   841.2161844
1092.501116   -0.378479466   0.038296953   0.17888429
C15orf39   15   75195643   75212169   +   5812
        protein_coding chromosome 15 open reading frame 39
[Source:HGNC Symbol;Acc:HGNC:24497]   -   844   904   824
        902   944   1467   7.111243086   7.37003017
8.820809699   8.623686808   9.983751182   11.73641685
ENSG00000198087 1557.749909   1373.512478   1268.27839
1285.636902   918.9009217   1059.320748   1399.846925
1087.952857   0.363785967   0.038374604   0.179176887   CD2AP
        6   47477789   47627263   +   6751
protein_coding CD2 associated protein [Source:HGNC
Symbol;Acc:HGNC:14258] -   1717   1552   1091   1241   805
        1228   12.45463116   10.89306386   10.0545697
10.21446718   7.329513221   8.457875284
ENSG00000139687 3993.718752   3654.144987   4146.607715
3672.508314   2934.77549   3041.665274   3931.490485
3216.316359   0.28953436   0.038389392   0.179176887   RB1
        13   48303726   48599436   +   6452
protein_coding RB transcriptional corepressor 1 [Source:HGNC
Symbol;Acc:HGNC:9884]   -   4402   4129   3567   3545   2571
        3526   33.41060678   30.32333508   34.39660643
30.53050054   24.49373857   25.41083682
ENSG00000264235 0   0.884995153   0   0   0
34.50556181   0.294998384   11.50185394   -5.043482551
0.038398192   0.179176887   AP005329.1   18   3255436
3261850 -   1063   antisense   uncharacterized LOC104968399
[Source:NCBI gene;Acc:104968399]   -   0   1   0
        0   0   40   0   0.044575187   0   0
        0   1.749676444
ENSG00000171223 1170.353746   1723.970558   2164.559452
1714.527859   2592.327942   2602.582   1686.294585
2303.145933   -0.450406089   0.038400112   0.179176887   JUNB
        19   12791496   12793315   +   1820
protein_coding "JunB proto-oncogene, AP-1 transcription factor
subunit [Source:HGNC Symbol;Acc:HGNC:6205]"   TF_bZIP 1290   1948
        1862   1655   2271   3017   34.70939197   50.71588465
        63.65245159   50.52876154   76.69960131   77.07879922
ENSG00000115318 288.5058072   356.6530467   326.6601536
242.4166278   257.9771532   95.75293403   323.9396692
198.7155717   0.708480859   0.038426705   0.17925369   LOXL3
        2   74532414   74555690   -   4363
protein_coding lysyl oxidase like 3 [Source:HGNC
Symbol;Acc:HGNC:13869] -   318   403   281   234   226
        111   3.569197586   4.37669491   4.007079451
2.980181319   3.183981872   1.182956525
ENSG00000197496 264.0100311   283.198449   398.7346359
206.1577305   269.3920714   103.5166854   315.314372
193.0221625   0.710316291   0.038492589   0.179473738

SLC2A10 20	46709487	46736347	+	5000
protein_coding	solute carrier family 2 member 10 [Source:HGNC
Symbol;Acc:HGNC:13444]	-	291	320	343	199	236
120	2.850044678	3.03253914	4.268064386
2.211541614	2.901278094	1.115943636
ENSG00000117713 2944.029385	2523.121182	2862.054442
2323.677334	2080.939603	2416.251966	2776.401669
2273.622968	0.287800096	0.038494169	0.179473738	ARID1A
1	26693236	26782110	+	15928
protein_coding	AT-rich interaction domain 1A [Source:HGNC
Symbol;Acc:HGNC:11110]	ARID	3245	2851	2462	2243	1823
2801	9.976590297	8.481299723	9.616867849
7.824922956	7.035140607	8.176790674
ENSG00000136783 463.6052436	503.5622421	514.9838009
317.0063594	341.3060566	426.1436884	494.0504289
361.4853681	0.448984256	0.038521053	0.17952825
NIPSNAP3A	9	104747688	104760122	+	2585
protein_coding	nipsnap homolog 3A [Source:HGNC
Symbol;Acc:HGNC:23619]	-	511	569	443	306	299
494	9.680304895	10.42985234	10.66227858
6.577682744	7.1098101	8.88581812
ENSG00000176055 410.0774366	435.4176153	385.9472278
502.44472	554.7650285	579.6934384	410.4807599
545.6343956	-0.410232466	0.03852616	0.17952825	MBLAC2
5	90458203	90474768	-	5676
protein_coding	metallo-beta-lactamase domain containing 2
[Source:HGNC Symbol;Acc:HGNC:33711]	-	452	492	332
485	486	672	3.899641932	4.107231261
3.639171675	4.748007491	5.263095406	5.505007366
ENSG00000257800 23.58852512	23.00987398	13.9498998
62.15810969	29.67878753	62.11001126	20.1827663
51.31563616	-1.342871262	0.038570744	0.179688674
FNBP1P1 2	74120680	74123218	+	1994
processed_pseudogene	formin binding protein 1 pseudogene 1
[Source:HGNC Symbol;Acc:HGNC:44528]	-	26	26	12
60	26	72	0.638523488	0.617838027
0.374423362	1.672007188	0.801485287	1.678952311
ENSG00000177380 156.0471661	251.3386235	173.2112558
247.5964703	249.9867104	408.8909075	193.5323485
302.1580294	-0.643395145	0.038593569	0.179732721	PPFIA3
19	49119389	49151026	+	6451
protein_coding	PTPRF interacting protein alpha 3 [Source:HGNC
Symbol;Acc:HGNC:9247]	-	172	284	149	239	219
474	1.305659968	2.086016498	1.437030789
2.05865183	2.086721223	3.416507024
ENSG00000134013 4617.000166	4748.883992	11018.09586
3087.186115	4277.16988	1948.701603	6794.660005
3104.352533	1.130127447	0.038606926	0.179732721	LOXL2
8	23297189	23425328	-	5707
protein_coding	lysyl oxidase like 2 [Source:HGNC

Symbol;Acc:HGNC:6666]	-	5089	5366	9478	2980	3747
	2259	43.66699052	44.55220843	103.3274421
29.01485705	40.35740013	18.40515065
ENSG00000224631 210.4822241	165.4940936	267.3730795
127.4241249	102.7342645	167.3519748	214.4497991
132.5034547	0.689592845	0.038619098	0.179732721
RPS27AP16	16	61055399	61055964	-	566
	transcribed_processed_pseudogene	ribosomal protein S27a
pseudogene 16 [Source:HGNC Symbol;Acc:HGNC:36855]	-	232
	187	230	123	90	194	20.07244318
15.65494752	25.28239728	12.0753777	9.774024841
15.93735758
ENSG00000125266 451.8109811	455.7725039	369.6723447
626.7609394	486.2755188	614.1990002	425.7519432
575.7451528	-0.434928041	0.038626703	0.179732721	EFNB2
	13	106489731	106535662	-	5019
protein_coding	ephrin B2 [Source:HGNC Symbol;Acc:HGNC:3227]	-
	498	515	318	605	426	712	4.858932105
	4.862017013	3.94200137	6.698078339	5.217227366
	6.596200014
ENSG00000272872 39.01179154	35.39980612	26.73730795
51.79842475	95.88531357	69.01112362	33.71630187
72.23162064	-1.094129139	0.038631003	0.179732721
AP000525.1	22	15823197	15823890	+	694
	sense_intronic	"novel transcript, sense intronic to novel
transcript"	-	43	40	23	50	84	80
	3.034154341	2.731033087	2.061936147	4.003340937
	7.439901329	5.359959827
ENSG00000116761 152.4181623	195.5839288	182.511189
263.1359977	302.4953345	225.1487908	176.8377601
263.5933743	-0.574799248	0.038666534	0.17985073	CTH
	1	70411218	70439851	+	2414
protein_coding	cystathionine gamma-lyase [Source:HGNC
Symbol;Acc:HGNC:2501]	-	168	221	157	254	265
	261	3.408008737	4.337919104	4.046403925
5.846668823	6.747703148	5.027293721
ENSG00000189079 733.0587805	506.2172276	545.2085838
513.8403735	417.7860091	348.5061743	594.8281973
426.7108523	0.480444776	0.038681749	0.179850862	ARID2
	12	45729665	45908040	+	12127
protein_coding	AT-rich interaction domain 2 [Source:HGNC
Symbol;Acc:HGNC:18037]	ARID	808	572	469	496	366
	404	3.26277155	2.234956589	2.406170022
2.272690741	1.855133073	1.549027064
ENSG00000145907 6171.121071	6087.881658	5978.694555
7648.555398	6948.260758	6968.398208	6079.232428
7188.404788	-0.241736288	0.038686898	0.179850862	G3BP1
	5	151771045	151812785	+	12406
protein_coding	G3BP stress granule assembly factor 1 [Source:HGNC
Symbol;Acc:HGNC:30292]	-	6802	6879	5143	7383	6087

8078 26.84933504 26.27362358 25.79239042
33.06839642 30.15913527 30.27632023
ENSG00000273568 30.84653284 53.98470434 41.8496994
67.33795217 70.77249335 121.6321054 42.22697886
86.5808503 -1.038189606 0.038708999 0.179895153
AC131009.3 12 131934642 131934928 + 287
sense_intronic "novel transcript, sense intronic to PUS1"
- 34 61 36 65 62 141
5.801305067 10.07104135 7.804183109 12.58471844
13.27874873 22.84379394
ENSG00000281849 3.629003864 13.2749273 2.3249833 0
0 0 6.409638153 0 5.117301456
0.038714812 1 AL732314.6 X 386980 405579 +
484 antisense "novel transcript, antisense to
PPP2R3B" - 4 15 2 0 0 0
0.40470942 1.468494547 0.257093727 0 0
0
ENSG00000101294 2962.174404 3086.863094 3131.752505
3532.652568 4045.447039 3478.160631 3060.263334
3685.420079 -0.268031437 0.038716764 0.179895153 HM13
20 31514410 31577923 + 10805
protein_coding histocompatibility minor 13 [Source:HGNC
Symbol;Acc:HGNC:16435] - 3265 3488 2694 3410 3544
4032 14.79745739 15.29600954 15.512424
17.53644879 20.16119649 17.35109031
ENSG00000230679 6.350756762 8.849951531 3.48747495 0
0 0 6.229394414 0 5.0748986
0.038786341 1 ENO1-AS1 1 8878835 8879894 +
456 antisense ENO1 antisense RNA 1 [Source:HGNC
Symbol;Acc:HGNC:40214] - 7 10 3 0 0
0 0.751729997 1.039110177 0.409320276 0
0 0
ENSG00000127952 303.0218226 305.3233278 365.0223781
409.2075555 416.6445173 514.99551 324.4558428
446.9491943 -0.464424347 0.038795583 0.180214041 STYXL1
7 75996338 76048004 - 2028
protein_coding serine/threonine/tyrosine interacting like 1
[Source:HGNC Symbol;Acc:HGNC:18165] - 334 345 314
395 365 597 8.065052517 8.060789595
9.633154906 10.82283877 11.06298374 13.68791812
ENSG00000206560 3696.140435 3099.253026 3268.926519
3091.329989 2750.995306 2204.042761 3354.773327
2682.122685 0.323220667 0.038864898 0.180462296
ANKRD28 3 15667236 15859771 - 11276
protein_coding ankyrin repeat domain 28 [Source:HGNC
Symbol;Acc:HGNC:29024] - 4074 3502 2812 2984 2410
2555 17.69272149 14.71592329 15.51554716
14.70468736 13.13739735 10.53578393
ENSG00000170581 690.4179851 872.605221 592.8707414
886.7890316 877.8072159 1099.864783 718.6313158

954.8203434	-0.409445402	0.03887641	0.180462296	STAT2
	12	56341597	56360155	-	6109
protein_coding	signal transducer and activator of transcription 2
[Source:HGNC Symbol;Acc:HGNC:11363]	STAT	761	986	510
	856	769	1275	6.100187938	7.647742039
5.194059228	7.786023016	7.737551896	9.704453374
ENSG00000224939 0	7.964956378	0	29.00711786
10.27342645	21.56597613	2.654985459
20.28217348	-2.901826488	0.038893689	0.180462296
LINC00184	1	234629311	234634780	+	2270
	lincRNA long intergenic non-protein coding RNA 184
[Source:HGNC Symbol;Acc:HGNC:37192]	-	0	9	0
	28	9	25	0	0.187863796	0
0.685400186	0.24370476	0.512088673
ENSG00000112183 13.60876449	23.89486913	23.249833
8.287747959	0	0	20.25115554	2.762582653
2.881877477	0.038895676	0.180462296	RBM24	6
17281346	17293875	+	3444	protein_coding	RNA
binding motif protein 24 [Source:HGNC Symbol;Acc:HGNC:21539]	-
	15	27	20	8	0	0	0.213283275
	0.371472837	0.361304774	0.129074035	0	0
ENSG00000117616 476.3067571	413.2927365	291.7854041
284.8913361	318.4762201	185.4673947	393.7949659
262.9449836	0.586398967	0.038900036	0.180462296	RSRP1
	1	25242237	25338213	-	6804
protein_coding	arginine and serine rich protein 1 [Source:HGNC
Symbol;Acc:HGNC:25234]	-	525	467	251	275	279
	215	3.778537024	3.252213262	2.295177423
2.245848377	2.520503231	1.469282051
ENSG00000127774 39.01179154	94.69448138	38.36222445
14.50355893	22.82983656	30.19236658	57.35616579
22.50858736	1.348391038	0.038997156	0.180865417	EMC6
	17	3668815 3669668 +	769	protein_coding	ER
membrane protein complex subunit 6 [Source:HGNC
Symbol;Acc:HGNC:28430]	-	43	107	33	14	20
	35	2.738235517	6.59301219	2.669896627
1.011611458	1.598641254	2.116278027
ENSG00000176853 2887.779825	2668.260387	2449.369906
3351.358081	3045.500198	3289.24268	2668.470039
3228.700319	-0.274754664	0.039092529	0.181260222
FAM91A1 8	123768456	123815452	+	7246
protein_coding	family with sequence similarity 91 member A1
[Source:HGNC Symbol;Acc:HGNC:26306]	-	3183	3015	2107
	3235	2668	3813	21.51131661	19.71584647
18.09143651	24.80778555	22.63262125	24.46805757
ENSG00000105383 612.394402	553.1219707	534.746159
435.1067679	433.7668947	421.8304931	566.7541772
430.2347186	0.398215125	0.03913711	0.181419376	CD33
	19	51225064	51243860	+	5975
protein_coding	CD33 molecule [Source:HGNC Symbol;Acc:HGNC:1659]

- 675 625 460 420 380 489
5.532157638 4.95642511 4.789903552 3.905920724
3.909246421 3.805414491
ENSG00000125356 430.9442088 854.9053179 830.019038
311.826517 505.6808799 590.045107 705.2895216
469.1841679 0.58694765 0.039208462 0.18169376 NDUFA1
X 119871487 119876662 + 766
protein_coding NADH:ubiquinone oxidoreductase subunit A1 [Source:HGNC
Symbol;Acc:HGNC:7683] - 475 966 714 301 443
684 30.36641504 59.75507525 57.99309524
21.83482772 35.54858486 41.52009612
ENSG00000150656 9.979760626 1.769990306 3.48747495
5.179842475 43.37668947 26.7418104 5.079075294
25.09944745 -2.297358193 0.039217463 0.18169376 CNDP1
18 74534440 74587212 + 7177
protein_coding carnosine dipeptidase 1 [Source:HGNC
Symbol;Acc:HGNC:20675] - 11 2 3 5 38
31 0.075054791 0.013204242 0.026006694
0.03871142 0.325452799 0.200839793
ENSG00000172244 216.8329809 188.5039676 216.2234469
164.7189907 103.8757564 127.6705787 207.1867985
132.0884419 0.648175634 0.03923217 0.18169376
C5orf34 5 43486701 43515145 - 3011
protein_coding chromosome 5 open reading frame 34 [Source:HGNC
Symbol;Acc:HGNC:24738] - 239 213 186 159 91
148 3.887011531 3.351932688 3.84334203
2.934258778 1.857710334 2.285503959
ENSG00000117020 1907.948781 1618.656135 1968.098363
1688.628647 1378.922128 1283.606899 1831.56776
1450.385891 0.336601272 0.039246151 0.18169376 AKT3
1 243488233 243851079 - 9475
protein_coding AKT serine/threonine kinase 3 [Source:HGNC
Symbol;Acc:HGNC:393] - 2103 1829 1693 1630 1208
1488 10.8689787 9.146626132 11.11692271
9.559175376 7.836733458 7.302216932
ENSG00000134259 93.4468495 61.94966072 70.91199064
24.86324388 50.22564044 31.91764468 75.43616695
35.668843 1.084927628 0.03924766 0.18169376 NGF
1 115285918 115338236 - 1047
protein_coding nerve growth factor [Source:HGNC Symbol;Acc:HGNC:7808]
- 103 70 61 24 44 37
4.817472304 3.167946212 3.624849668 1.27372773
2.583172181 1.643183482
ENSG00000124164 2538.488203 2400.106855 2356.370574
2970.121675 2949.614884 2826.868151 2431.655211
2915.534903 -0.261600224 0.039264242 0.181722964 VAPB
20 58389122 58451101 + 10585
protein_coding VAMP associated protein B and C [Source:HGNC
Symbol;Acc:HGNC:12649] - 2798 2712 2027 2867 2584
3277 12.9445075 12.14018385 11.91433296

15.05042882 15.00544942 14.39516334
ENSG00000149548 642.3336839 560.2019319 511.496326
572.8905777 332.174122 225.1487908 571.3439806
376.7378302 0.602347715 0.039359409 0.18211577 CCDC15
11 124954121 125041489 + 4823
protein_coding coiled-coil domain containing 15 [Source:HGNC
Symbol;Acc:HGNC:25798] - 708 633 440 553 291
261 7.188605972 6.218890198 5.675998358
6.371180557 3.70871181 2.516252756
ENSG00000213190 1577.70943 1575.291373 2163.39696
1397.5215 1503.344738 1258.590367 1772.132588
1386.485535 0.353705606 0.039373016 0.182126699 MLLT11
1 151057758 151068497 + 4075
protein_coding "MLLT11, transcription factor 7 cofactor [Source:HGNC
Symbol;Acc:HGNC:16997]" - 1739 1780 1861 1349 1317
1459 20.89780404 20.69754474 28.41355711
18.39485548 19.86577545 16.64787081
ENSG00000173436 214.111228 320.3682454 370.8348363
186.4743291 198.6195781 216.5224004 301.7714366
200.5387692 0.587403585 0.039382363 0.182126699 MINOS1
1 19484403 19629821 + 5480
protein_coding mitochondrial inner membrane organizing system 1
[Source:HGNC Symbol;Acc:HGNC:32068] - 236 362 319
180 174 251 2.10892011 3.130072903
3.621737511 1.82517281 1.951713427 2.129728198
ENSG00000078967 339.3118613 291.1634054 312.7102538
545.9553968 333.3156138 458.9239721 314.3951735
446.0649942 -0.505475388 0.039406884 0.182192464 UBE2D4
7 43926438 43956136 + 4713
protein_coding ubiquitin conjugating enzyme E2 D4 (putative)
[Source:HGNC Symbol;Acc:HGNC:21647] - 374 329 269
527 292 532 3.886000445 3.307690752
3.551090061 6.213341428 3.808314198 5.248620963
ENSG00000269386 43.54804637 29.20484005 30.2247829
2.07193699 14.83939377 12.93958568 34.32588977
9.950305478 1.786998941 0.039426462 0.18223535
RAB11B-AS1 19 8374373 8390685 - 1156 antisense
RAB11B antisense RNA 1 [Source:HGNC Symbol;Acc:HGNC:44178]
- 48 33 26 2 13 15
2.033349751 1.352640998 1.399337139 0.096135592
0.691246393 0.603343229
ENSG00000189241 966.2222788 978.8046394 1043.917502
1118.845974 1297.876209 1323.288295 996.3148066
1246.67016 -0.323883116 0.039482034 0.182444541 TSPYL1
6 116276578 116279903 - 3326
protein_coding TSPY like 1 [Source:HGNC Symbol;Acc:HGNC:12382] -
1065 1106 898 1080 1137 1534 15.68036062
15.75648437 16.79813002 18.04319963 21.01287998
21.4453991
ENSG00000251396 0 3.539980612 2.3249833 0

4.565967313	56.07153794	1.954987971
20.21250175	-3.378609145	0.039627176	0.183067413
LINC01301	8	60384588	60516795	-	4428
	lincRNA long intergenic non-protein coding RNA 1301
[Source:HGNC Symbol;Acc:HGNC:50464]	-	0	4	2
	0	4	65	0	0.042803454	0.028101482
	0	0.055526428	0.682553602
ENSG00000122376 1191.220518	890.305124	978.8179692
1003.853472	618.6885709	609.885805	1020.114537
744.1426158	0.455429935	0.039650743	0.183119086	SHLD2
	10	87094161	87191468	+	4242
protein_coding	shieldin complex subunit 2 [Source:HGNC
Symbol;Acc:HGNC:28773]	-	1313	1006	842	969	542
	707	15.15733138	11.23708736	12.34946871
12.69302562	7.853730286	7.749608583
ENSG00000204650 205.0387183	235.4087107	261.5606212
280.7474621	342.4475485	384.7370142	234.0026834
335.9773416	-0.523852305	0.039659066	0.183119086
LINC02210	17	45620328	45655156	+	15578
	transcribed_unitary_pseudogene	long intergenic non-protein
coding RNA 2210 [Source:HGNC Symbol;Acc:HGNC:26327]	-	226
	266	225	271	300	446	0.710436757
0.809089151	0.89862328	0.966650845	1.183741614
1.331233314
ENSG00000152558 6967.687419	6190.541096	7059.81179
6253.105835	5254.286885	5261.235537	6739.346768
5589.542752	0.269824953	0.039676622	0.183152342
TMEM123 11	102396332	102470384	-	4647
protein_coding	transmembrane protein 123 [Source:HGNC
Symbol;Acc:HGNC:30138]	-	7680	6995	6073	6036	4603
	6099	80.93143316	71.32495185	81.30878197
72.17530076	60.88575035	61.02629148
ENSG00000224239 0	0	0	0	27.39580388	0
	0	9.131934625	-5.662217041	0.039695473
0.183161861	AC090044.1	3	857124	858091	+	497
	lincRNA novel transcript	-	0	0	0
	0	24	0	0	0	0	0
2.96826187	0
ENSG00000175334 3374.973593	3972.743242	4259.369405
3143.128414	3184.762201	3235.759059	3869.028747
3187.883224	0.279106064	0.039709079	0.183161861	BANF1
	11	66002079	66004149	+	1645
protein_coding	barrier to autointegration factor 1 [Source:HGNC
Symbol;Acc:HGNC:17397]	-	3720	4489	3664	3034	2790
	3751	110.7403065	129.3034593	138.5786765
102.4853333	104.2523039	106.0259518
ENSG00000175220 3222.555431	3157.662706	3241.02672
3383.473104	3929.014873	4415.849273	3207.081619
3909.44575	-0.28593019	0.039709748	0.183161861
ARHGAP1 11	46677080	46700615	-	6138

protein_coding	Rho GTPase activating protein 1 [Source:HGNC
Symbol;Acc:HGNC:673]	-	3552	3568	2788	3266	3442
	5119	28.33836284	27.54383464	28.26003738
29.56659687	34.46921097	38.77834442
ENSG00000251093 2.721752898	15.92991276	1.16249165	0
	0	0	6.604719101	0	5.161409556
0.039726684	1	AC093281.2	5	91226475
91227071	-	597	lincRNA novel transcript	-
	3	18	1	0	0	0	0.246079597
	1.428645951	0.104215548	0	0	0
ENSG00000167191 2.721752898	15.92991276	1.16249165	0
	0	0	6.604719101	0	5.161409556
0.039726684	1	GPRC5B	16	19856691	19886167
	-	6667	protein_coding	G protein-coupled receptor
class C group 5 member B [Source:HGNC Symbol;Acc:HGNC:13308]	-
	3	18	1	0	0	0	0.022035326
	0.127928849	0.009332036	0	0	0
ENSG00000230251 0	3.539980612	0	20.7193699
7.990442797	10.35166854	1.179993537
13.02049375	-3.415658785	0.039733041	0.183208025	PHBP4
	2	154286589	154287425	+	837
processed_pseudogene	prohibitin pseudogene 4 [Source:HGNC
Symbol;Acc:HGNC:8916]	-	0	4	0	20	7
	12	0	0.226444082	0	1.327750829
0.514067256	0.666632996
ENSG00000124243 52.62055603	90.26950562	56.96209084
84.94941658	138.1205112	145.7859987	66.61738416
122.9519755	-0.88235043	0.039740471	0.183208025	BCAS4
	20	50794894	50882676	+	7583
protein_coding	breast carcinoma amplified sequence 4 [Source:HGNC
Symbol;Acc:HGNC:14367]	-	58	102	49	82	121
	169	0.374555019	0.637361104	0.402033156
0.600875975	0.980825333	1.036278927
ENSG00000115561 1163.095738	1054.914223	1235.728624
964.4866688	946.2967256	827.2708444	1151.246195
912.6847462	0.335109118	0.039764065	0.183246089	CHMP3
	2	86503431	86563479	-	7018
protein_coding	charged multivesicular body protein 3 [Source:HGNC
Symbol;Acc:HGNC:29865]	-	1282	1192	1063	931	829
	959	8.945473734	8.048025288	9.4238149
7.371372545	7.260867755	6.35383981
ENSG00000129480 323.8885949	366.3879934	292.9478958
454.7901693	414.3615336	472.7261968	327.7414947
447.2926332	-0.447969464	0.039769446	0.183246089	DTD2
	14	31446036	31457510	-	2971
protein_coding	D-tyrosyl-tRNA deacylase 2 (putative) [Source:HGNC
Symbol;Acc:HGNC:20277]	-	357	414	252	439	363
	548	5.884292434	6.602738997	5.277214361
8.210581419	7.510197074	8.576476951
ENSG00000256407 0	0	1.16249165	18.64743291	0

8.626390453	0.387497217	9.091274454	-4.700302073
0.039781837	0.183255447	AL357673.1	1
54132686	54200073	-	3952	protein_coding	novel
transcript	-	0	0	1	18	0	10
0	0	0.015743088	0.253085703	0
0.117656001
ENSG00000152049 157.8616681	149.5641809	185.998664
70.44585765	122.1396256	104.3793245	164.4748376
98.98826925	0.732146782	0.039800336	0.183292928	KCNE4
2	223051814	223198399	+	4124
protein_coding	potassium voltage-gated channel subfamily E regulatory
subunit 4 [Source:HGNC Symbol;Acc:HGNC:6244]	-	174	169
160	68	107	121	2.066137762	1.941755253
2.413838293	0.916225342	1.594822967	1.364261841
ENSG00000148730 2668.225091	2449.666584	2271.508684
2819.906243	2732.731437	3655.864274	2463.133453
3069.500651	-0.317694284	0.039830462	0.183381537
EIF4EBP2	10	70404379	70428618	+	7257
protein_coding	eukaryotic translation initiation factor 4E
binding protein 2 [Source:HGNC Symbol;Acc:HGNC:3289]	-	2941
2768	1954	2722	2394	4238	19.84570745
18.07321452	16.75229387	20.84217516	20.27749874
27.15406464
ENSG00000180660 69.85832438	29.20484005	76.72444889
3.107905485	15.98088559	37.95611799	58.59587111
19.01496969	1.611518766	0.03984031	0.183381537
MAB21L1 13	35474182	35477209	-	3028
protein_coding	mab-21 like 1 [Source:HGNC Symbol;Acc:HGNC:6757]
-	77	33	66	3	14	44
1.245270035	0.51639795	1.356109978	0.055052548
0.284197023	0.675659401
ENSG00000226650 3.629003864	3.539980612	11.6249165	0
0	0	6.264633658	0	5.075265043
0.039846684	1	KIF4B	5	155013755	155018132
+	4378	protein_coding	kinesin family member 4B
[Source:HGNC Symbol;Acc:HGNC:6322]	-	4	4	10
0	0	0	0.044741745	0.043292302
0.142112111	0	0	0
ENSG00000187889 0	2.654985459	0	27.97114936
14.83939377	0	0.884995153	14.27018104	-3.960648817
0.039876018	0.183455912	FYB2	1	56718804
56819696	-	4633	protein_coding	FYN binding
protein 2 [Source:HGNC Symbol;Acc:HGNC:27295]	-	0	3
0	27	13	0	0	0.030682122	0
0.323827336	0.172475897	0
ENSG00000163702 622.3741627	613.3016411	605.6581496
507.6245625	418.9275009	476.176753	613.7779845
467.5762721	0.392070419	0.039877211	0.183455912	IL17RC
3	9917074 9933630 +	4540	protein_coding
interleukin 17 receptor C [Source:HGNC Symbol;Acc:HGNC:18358]	-

686	693	521	490	367	552	7.399407516
7.232756139	7.139843904	5.997251625	4.96886928
5.653458949
ENSG00000136802 2340.707492	2517.811211	2286.621075
2933.862778	2730.448453	2929.522198	2381.713259
2864.611143	-0.266272629	0.039910361	0.183543774	LRRC8A
9	128882112	128918039	+	5230
protein_coding	leucine rich repeat containing 8 VRAC subunit A
[Source:HGNC Symbol;Acc:HGNC:19027]	-	2580	2845	1967
2832	2392	3396	24.15720588	25.77549549
23.39965841	30.08871244	28.11297761	30.1923565
ENSG00000273162 11.79426256	8.849951531	15.11239145
3.107905485	0	0	11.91886851	1.035968495
3.537369678	0.039917061	0.183543774	AL133215.2	10
101060029	101061005	-	977	lincRNA novel
transcript	-	13	10	13	3	0	0
0.651594593	0.484988987	0.827857591	0.170623456
0	0
ENSG00000100647 540.7215757	505.3322324	520.7962592
442.3585473	313.9102527	396.8139608	522.2833558
384.3609203	0.441450181	0.039960609	0.183696261	SUSD6
14	69611596	69715142	+	5830
protein_coding	sushi domain containing 6 [Source:HGNC
Symbol;Acc:HGNC:19956]	-	596	571	448	427	275
460	5.006179166	4.640812202	4.780973163
4.069784036	2.899422463	3.668768386
ENSG00000183011 873.6826802	1336.342681	1315.940548
614.3293175	971.4095458	979.0953164	1175.32197
854.9447265	0.458668391	0.04000776	0.18386523	NAA38
17	7856685 7885238 -	1943
protein_coding	"N(alpha)-acetyltransferase 38, NatC auxiliary subunit
[Source:HGNC Symbol;Acc:HGNC:28212]"	-	963	1510	1132
593	851	1135	24.27069262	36.82396826
36.24770151	16.95875384	26.92180162	27.16152056
ENSG00000159128 951.7062633	1240.763205	1493.80177
727.2498834	1023.91817	998.9360144	1228.757079
916.7013559	0.421911274	0.040083823	0.1841025	IFNGR2
21	33402896	33479348	+	2960
protein_coding	interferon gamma receptor 2 [Source:HGNC
Symbol;Acc:HGNC:5440]	-	1049	1402	1285	702	897
1158	17.35451418	22.44309477	27.00960688
13.17824098	18.62722207	18.19063528
ENSG00000008083 332.9611045	281.4284587	363.8598864
382.2723746	465.7286659	505.5064805	326.0831499
451.1691737	-0.470185086	0.040084934	0.1841025	JARID2
6	15246296	15522040	+	6015
protein_coding	jumonji and AT-rich interaction domain containing 2
[Source:HGNC Symbol;Acc:HGNC:6196]	ARID	367	318	313
369	408	586	2.987852239	2.505058828
3.237543054	3.408809866	4.169383963	4.529945765

ENSG00000227946 33.56828574 41.5947722 33.71225785
17.61146441 12.55641011 6.901112362 36.29177193
12.35632896 1.562159736 0.040090612 0.1841025
AC007383.2 2 206084605 206086564 + 1034
lincRNA novel transcript - 37 47 29
17 11 8 1.752305681 2.153792003
1.744955298 0.913567048 0.6539123 0.359749721
ENSG00000100228 303.9290736 384.9728916 359.2099198
473.4376022 349.2964994 712.5398514 349.3706283
511.7579843 -0.552669639 0.040130163 0.184236298 RAB36
22 23145326 23164350 + 4107
protein_coding "RAB36, member RAS oncogene family [Source:HGNC
Symbol;Acc:HGNC:9775]" - 335 435 309 457 306
826 3.994374566 5.018697216 4.681021363
6.183061139 4.579774386 9.351609481
ENSG00000096060 780.2358307 587.6367817 657.9702738
536.6316804 581.0193405 263.9675479 675.2809621
460.5395229 0.554223909 0.040173635 0.18438802 FKBP5
6 35573585 35728583 - 10628
protein_coding FK506 binding protein 5 [Source:HGNC
Symbol;Acc:HGNC:3721] - 860 664 566 518 509
306 3.962557608 2.960349414 3.313383705
2.70825939 2.943835897 1.338754362
ENSG00000128886 15.42326642 0.884995153 3.48747495 0
0 0 6.598578841 0 5.157247737
0.040181013 1 ELL3 15 43772600 43777543
- 2880 protein_coding elongation factor for RNA
polymerase II 3 [Source:HGNC Symbol;Acc:HGNC:23113] - 17
1 3 0 0 0 0.289058082
0.016452578 0.064809044 0 0 0
ENSG00000184436 820.1548732 1238.993214 967.1930527
1062.903676 1414.308375 1549.299725 1008.78038
1342.170592 -0.411963463 0.04023596 0.184626173 THAP7
22 20999104 21002196 - 2879
protein_coding THAP domain containing 7 [Source:HGNC
Symbol;Acc:HGNC:23190] THAP 904 1400 832 1026 1239
1796 15.37642765 23.04160947 17.97995117
19.80239593 26.45312607 29.00652383
ENSG00000162889 3081.931531 3309.881873 3563.036907
3276.768349 4441.544703 4733.300441 3318.283437
4150.537831 -0.323123945 0.040316131 0.184946071
MAPKAPK2 1 206684944 206734283 + 4195
protein_coding mitogen-activated protein kinase-activated
protein kinase 2 [Source:HGNC Symbol;Acc:HGNC:6887] - 3397
3740 3065 3163 3891 5487 39.65448055
42.24410154 45.45748042 41.89664726 57.01335863
60.81826312
ENSG00000113739 448.1819772 768.1757929 1165.979125
536.6316804 342.4475485 665.0947039 794.1122983
514.7246442 0.62324488 0.040347585 0.185017458 STC2

5	173314713	173329503	-	6128
protein_coding	stanniocalcin 2 [Source:HGNC Symbol;Acc:HGNC:11374]
-	494	868	1003	518	300	771
3.947633955	6.71162077	10.18331137	4.697026893
3.009191721	5.850145122
ENSG00000100227 2521.250434	2469.136477	2543.53173
2450.06549	3296.6284	3763.694155	2511.306214
3170.129348	-0.336361617	0.040352612	0.185017458
POLDIP3 22	42583721	42614962	-	3650
protein_coding	DNA polymerase delta interacting protein 3
[Source:HGNC Symbol;Acc:HGNC:23782]	-	2779	2790	2188
2365	2888	4363	37.28416024	36.21911044
37.29591787	36.00396445	48.63530958	55.5806174
ENSG00000169193 327.5175987	323.0232309	281.3229793
335.6537923	543.3501102	442.5338302	310.6212696
440.5125776	-0.502547975	0.040376159	0.18507745
CCDC126 7	23597379	23644708	+	3550
protein_coding	coiled-coil domain containing 126 [Source:HGNC
Symbol;Acc:HGNC:22398]	-	361	365	242	324	476
513	4.97974991	4.87181684	4.241249872
5.071409745	8.241873792	6.71923809
ENSG00000103855 2533.044697	2554.096012	3547.924515
2166.210123	2349.190182	2351.554037	2878.355075
2288.984781	0.329959793	0.040388688	0.185086917	CD276
15	73683966	73714518	+	5542
protein_coding	CD276 molecule [Source:HGNC Symbol;Acc:HGNC:19137]
-	2792	2886	3052	2091	2058	2726
24.67047867	24.674948	34.26295804	20.96522632
22.82581059	22.87127355
ENSG00000197050 128.8296372	129.2092924	158.0988644
187.5102976	332.174122	160.4508624	138.712598
226.7117607	-0.707724594	0.040410418	0.185138536	ZNF420
19	37007857	37130314	+	4023
protein_coding	zinc finger protein 420 [Source:HGNC
Symbol;Acc:HGNC:20649]	zf-C2H2 142	146	136	181	291
186	1.728490494	1.719607236	2.103273366
2.500003323	4.446213537	2.149779562
ENSG00000150627 261.2882782	187.6189725	140.6614896
378.1285006	251.1282022	282.0829678	196.5229134
303.7798902	-0.625920152	0.040461189	0.18529574	WDR17
4	176065834	176182818	+	7808
protein_coding	WD repeat domain 17 [Source:HGNC
Symbol;Acc:HGNC:16661]	-	288	212	121	365	220
327	1.806264584	1.286537641	0.964167331
2.597557102	1.731929607	1.947327362
ENSG00000165006 894.5494525	979.6896345	789.3318303
925.1198659	1297.876209	1243.062864	887.8569724
1155.35298	-0.379273509	0.040473469	0.18529574	UBAP1
9	34179005	34252523	+	2772
protein_coding	ubiquitin associated protein 1 [Source:HGNC

Symbol;Acc:HGNC:12461]	-	986	1107	679	893	1137
	1441	17.41856496	18.92260117	15.23994483
17.90071081	25.21242382	24.1713982
ENSG00000100345 15869.6339	16109.56677	17723.34769
15406.92346	13816.61709	12179.60068	16567.51612
13801.04707	0.263594488	0.040476157	0.18529574	MYH9
	22	36281277	36388067	-	9012
protein_coding	myosin heavy chain 9 [Source:HGNC
Symbol;Acc:HGNC:7579]	-	17492	18203	15246	14872	12104
	14119	95.04887243	95.7079969	105.2547197
91.69807894	82.55722606	72.84735259
ENSG00000261188 78.02358307	96.46447169	81.37441549
162.6470537	108.4417237	160.4508624	85.28749009
143.8465466	-0.754907061	0.040559555	0.185629488
Z95115.1	22	26512537	26514568	+	2032
	antisense	uncharacterized LOC100507599 [Source:NCBI
gene;Acc:100507599]	-	86	109	70	157	95
	186	2.072542434	2.541728948	2.143291211
4.293267931	2.873738603	4.256182667
ENSG00000184588 226.8127415	153.1041615	253.4231797
171.9707702	113.007691	103.5166854	211.1133609
129.4983822	0.704466425	0.04059309	0.18573491	PDE4B
	1	65792514	66374579	+	8644
protein_coding	phosphodiesterase 4B [Source:HGNC
Symbol;Acc:HGNC:8781]	-	250	173	218	166	99
	120	1.416295692	0.94832628	1.569092628
1.067100623	0.703992118	0.645501872
ENSG00000123094 1089.60841	779.6807299	1091.579659
1345.723075	1191.717469	1276.705787	986.9562664
1271.38211	-0.36612683	0.040622196	0.185776466	RASSF8
	12	25959029	26079892	+	7619
protein_coding	Ras association domain family member 8 [Source:HGNC
Symbol;Acc:HGNC:13232]	-	1201	881	939	1299	1044
	1480	7.71922531	5.479038797	7.667865127
9.473778382	8.422671938	9.032225255
ENSG00000185122 2537.580952	2872.694267	2917.854041
2735.992795	3542.049143	4295.079806	2776.043087
3524.373915	-0.344691266	0.040623177	0.185776466	HSF1
	8	144291591	144314722	+	5307
protein_coding	heat shock transcription factor 1 [Source:HGNC
Symbol;Acc:HGNC:5224]	HSF	2797	3246	2510	2641	3103
	4979	25.80905257	28.98183427	29.42601693
27.6523062	35.94016347	43.62385657
ENSG00000140526 3964.686721	3479.800942	4639.504175
3692.191716	2851.446587	3157.258906	4027.997279
3233.632403	0.316545325	0.040651549	0.185858166	ABHD2
	15	89087459	89202360	+	10345
protein_coding	abhydrolase domain containing 2 [Source:HGNC
Symbol;Acc:HGNC:18717]	-	4370	3932	3991	3564	2498
	3660	20.6861479	18.00982343	24.00258849

19.14340749	14.84257661	16.45059492
ENSG00000128626 1075.092395	1673.525835	1576.338677
1530.125467	1948.526551	2240.273601	1441.652302
1906.308539	-0.403552126	0.040678494	0.185933303	MRPS12
	19	38930548	38933162	+	2144
protein_coding	mitochondrial ribosomal protein S12 [Source:HGNC
Symbol;Acc:HGNC:10380]	-	1185	1891	1356	1477	1707
	2597	27.06588629	41.79200321	39.34972985
38.27963234	48.93911374	56.32201537
ENSG00000270885 4.53625483	3.539980612	10.46242485	0
	0	0	6.17955343	0	5.056412311
0.040685221	1	RASL10B 17	35731649	35743521
	+	3502	protein_coding	RAS like family 10 member B
[Source:HGNC Symbol;Acc:HGNC:30295]	-	5	4	9
	0	0	0	0.069916961	0.054121558
0.159894386	0	0	0
ENSG00000173452 3.629003864	15.92991276	18.5998664	0
	3.424475485	0	12.71959434	1.141491828
3.542113271	0.040734911	0.186143073	TMEM196 7
19719310	19773598	-	4087	protein_coding
transmembrane protein 196 [Source:HGNC Symbol;Acc:HGNC:22431]	-
	4	18	16	0	3	0	0.047927418
	0.208686477	0.2435691	0	0.045119469	0
ENSG00000183778 180.5429422	163.7241033	126.7115898
133.6399358	78.76293614	38.81875704	156.9928785
83.74054301	0.911257037	0.040795921	0.186373719
B3GALT5 21	39556442	39673137	+	14319
protein_coding	"beta-1,3-galactosyltransferase 5 [Source:HGNC
Symbol;Acc:HGNC:920]"	-	199	185	109	129	69
	45	0.680564154	0.612188941	0.473609773
0.500597948	0.296199119	0.146127126
ENSG00000278396 3.629003864	4.424975766	10.46242485	0
	0	0	6.172134826	0	5.05473927
0.040820046	1	AL122023.1	14	93334528
93335057	+	530	sense_intronic	"novel transcript,
sense intronic UNC79"	-	4	5	9	0	0
	0	0.369583697	0.447013434	1.056509694	0
	0	0
ENSG00000130052 845.5579003	702.6861516	790.4943219
510.732468	660.9237685	616.7869174	779.5794579
596.147718	0.387051338	0.040824985	0.186458341	STARD8
	X	68647666	68725842	+	5558
protein_coding	StAR related lipid transfer domain containing 8
[Source:HGNC Symbol;Acc:HGNC:19161]	-	932	794	680
	493	579	715	8.211567241	6.769060579
7.611972609	4.928791201	6.403352078	5.981615837
ENSG00000143493 1904.319778	1772.645292	1628.650801
2231.476138	2285.26664	1984.932443	1768.538624
2167.225074	-0.292613858	0.040854674	0.186545772	INTS7
	1	211940399	212035542	-	5311

protein_coding	integrator complex subunit 7 [Source:HGNC
Symbol;Acc:HGNC:24484]	-	2099	2003	1401	2154	2002
	2301	19.35373636	17.87026895	16.41227104
22.53623907	23.1704854	20.1451885
ENSG00000124299 1296.46163	1214.21335	1740.25 1020.428967
	1204.273879	1070.535055	1416.974993	1098.412634
	0.366765385	0.040890402	0.186660726	PEPD	19
	33386950	33521794	-	4157	protein_coding
	peptidase D [Source:HGNC Symbol;Acc:HGNC:8840]	-	1429
	1372	1497	985	1055	1241	16.83375057
15.63869565	22.40518955	13.16643652	15.59982747
13.88106459
ENSG00000264049 1.814501932	0	0	10.35968495
13.69790194	5.175834272	0.604833977
9.74447372	-3.958608066	0.040958844	0.186899937
MIR4737 17	60043025	60043105	-	81	miRNA
	microRNA 4737 [Source:HGNC Symbol;Acc:HGNC:41669]	-
	2	0	0	10	12	6	1.209131848
	0	0	6.860045951	9.106334255	3.444270481
ENSG00000105808 0	0	0	10.35968495	0
11.21430759	0	7.191330846	-5.325465368	0.04096128
	1	RASA4	7	102573807	102616757	-
	8077	protein_coding	RAS p21 protein activator 4
[Source:HGNC Symbol;Acc:HGNC:23181]	-	0	0	0
	10	0	13	0	0	0	0.068795806
	0	0.074838364
ENSG00000162391 11.79426256	15.0449176	10.46242485
22.79130689	53.65011592	33.64292277	12.43386834
36.69478186	-1.555319971	0.040963936	0.186899937
FAM151A 1	54609182	54623556	-	2005
protein_coding	family with sequence similarity 151 member A
[Source:HGNC Symbol;Acc:HGNC:25032]	-	13	17	9
	22	47	39	0.317510183	0.401754718
0.279276877	0.60970583	1.440890046	0.904443097
ENSG00000226803 17.23776835	11.50493699	19.76235805
40.4027713	52.5086241	34.50556181	16.16835446
42.47231907	-1.39655033	0.040990305	0.186972021
ZNF451-AS1	6	57114894	57174236	-	4918
	antisense	ZNF451 regulatory antisense RNA 1 [Source:HGNC
Symbol;Acc:HGNC:53824]	-	19	13	17	39	46
	40	0.189188076	0.125251019	0.215063765
0.440644269	0.574932246	0.37818342
ENSG00000228286 0	0	0	0	0	26.7418104
	0	8.913936801	-5.637208252	0.041019927
0.187058905	AP000593.2	11	72249856	72251288
	-	1433	processed_pseudogene	"docking protein 1,
62kDa (downstream of tyrosine kinase 1) (DOK1) pseudogene"	-
	0	0	0	0	0	31	0	0
	0	0	0	1.005880807
ENSG00000133103 786.5865875	569.9368786	814.9066466

526.2719954	577.5948651	526.2098176	723.8100376
543.3588927	0.413230424	0.041139937	0.187557821	COG6
13	39655627	39791665	+	8051
protein_coding	component of oligomeric golgi complex 6 [Source:HGNC
Symbol;Acc:HGNC:18621]	-	867	644	701	508	506
610	5.273487906	3.790203092	5.417202097
3.506113163	3.863207632	3.522986885
ENSG00000166333 70.76557535	86.72952501	112.76169
10.35968495	74.19696883	27.60444945	90.0855968
37.38703441	1.274138158	0.041163759	0.187618071	ILK
11	6603708 6610874 +	3484	protein_coding
integrin linked kinase [Source:HGNC Symbol;Acc:HGNC:6040]	-
78	98	97	10	65	32	1.096339698
1.332828805	1.732209574	0.159490161	1.146786497
0.427073722
ENSG00000207725 2.721752898	2.654985459	0	5.179842475
15.98088559	21.56597613	1.792246119	14.24223473
-2.954069393	0.041201995	0.187743972	MIR222	X
45747015	45747124	-	110	miRNA
microRNA 222 [Source:HGNC Symbol;Acc:HGNC:31602]	-	3
3	0	5	14	25	1.335541086
1.292275202	0	2.525744191	7.823168973	10.56764807
ENSG00000157445 19.95952125	20.35488852	62.77454909
17.61146441	0	0	34.36298629	5.870488138
2.55140434	0.041223778	0.187794854	CACNA2D3	3
54122547	55074557	+	4670	protein_coding
calcium voltage-gated channel auxiliary subunit alpha2delta 3
[Source:HGNC Symbol;Acc:HGNC:15460]	-	22	23	54
17	0	0	0.230693036	0.2333659
0.719422019	0.202275873	0	0
ENSG00000108255 8.165258694	0	13.9498998	0	0
0	7.371719498	0	5.310936604	0.041257436
1	CRYBA1	17	29246863	29254494	+
794	protein_coding	crystallin beta A1 [Source:HGNC
Symbol;Acc:HGNC:2394]	-	9	0	12	0	0
0	0.555073751	0	0.940302499	0	0
0
ENSG00000185000 476.3067571	726.5810207	568.4584168
722.0700409	732.8377537	937.6886422	590.4487315
797.5321456	-0.434095141	0.04128155	0.187974977	DGAT1
8	144314584	144326910	-	4131
protein_coding	diacylglycerol O-acyltransferase 1 [Source:HGNC
Symbol;Acc:HGNC:2843]	-	525	821	489	697	642
1087	6.223472746	9.417039736	7.364792423
9.375396133	9.552723189	12.23503926
ENSG00000115419 4849.256413	3694.854764	3765.310454
3643.501197	3482.691568	2612.071029	4103.140544
3246.087931	0.338447814	0.041291405	0.187974977	GLS
2	190880827	190965552	+	9827
protein_coding	glutaminase [Source:HGNC Symbol;Acc:HGNC:4331]	-

5345	4175	3239	3517	3051	3028	26.63516779
20.13084313	20.50675008	19.88673359	19.08396502
14.32735206
ENSG00000102606 1854.420974	1624.851101	1677.475451
2300.886027	1929.12119	2096.21288	1718.915842
2108.740032	-0.294911808	0.041295198	0.187974977
ARHGEF7 13	111114559	111305737	+	11768
protein_coding	Rho guanine nucleotide exchange factor 7 [Source:HGNC
Symbol;Acc:HGNC:15607]	-	2044	1836	1443	2221	1690
2430	8.505638394	7.392587234	7.629050997
10.48716117	8.827371517	9.601401525
ENSG00000197852 10.88701159	52.21471403	26.73730795
6.215810969	12.55641011	0	29.94634452	6.257407026
2.282400654	0.041310048	0.187994194	INKA2	1
111680630	111739424	-	6290	protein_coding
inka box actin regulator 2 [Source:HGNC Symbol;Acc:HGNC:28045]
-	12	59	23	6	11	0
0.093424178	0.444455011	0.227501381	0.053004489
0.107495281	0
ENSG00000131023 1020.657337	923.9349399	952.0806613
1459.679609	1216.830289	1035.166854	965.5576459
1237.225584	-0.357144106	0.041339861	0.188081481	LATS1
6	149658153	149718256	-	8643
protein_coding	large tumor suppressor kinase 1 [Source:HGNC
Symbol;Acc:HGNC:6514]	-	1125	1044	819	1409	1066
1200	6.37406801	5.723509744	5.895575907
9.05854662	7.581236623	6.455765567
ENSG00000170310 413.7064405	557.5469465	619.6080494
693.0629231	851.5529038	619.3748345	530.2871454
721.3302205	-0.44412788	0.041432014	0.188452274	STX8
17	9250471 9576591 -	3779	protein_coding
syntaxin 8 [Source:HGNC Symbol;Acc:HGNC:11443]	-	456	630
533	669	746	718	5.909035979	7.899327112
8.775202834	9.836968246	12.13414822	8.834430743
ENSG00000097021 3035.661732	3460.331049	3768.797929
2657.259189	2972.444721	2815.653844	3421.596903
2815.119251	0.281283224	0.041461211	0.18851179	ACOT7
1	6264269 6394391 -	4124	protein_coding	acyl-
CoA thioesterase 7 [Source:HGNC Symbol;Acc:HGNC:24157]	-	3346
3910	3242	2565	2604	3264	39.73159168
44.92463338	48.91039841	34.56055885	38.81232716
36.80124503
ENSG00000188130 506.246039	586.7517865	497.5464262
302.5028005	420.0689928	437.357996	530.1814172
386.6432631	0.45546601	0.041475916	0.18851179	MAPK12
22	50245450	50261825	-	9424
protein_coding	mitogen-activated protein kinase 12 [Source:HGNC
Symbol;Acc:HGNC:6874]	-	558	663	428	292	368
507	2.89952999	3.333532486	2.825630295
1.721708477	2.400268919	2.50151839

ENSG00000139826 607.8581472 643.3914763 528.9337007
757.2929698 759.0920657 776.3751407 593.3944414
764.2533921 -0.364126897 0.04147707 0.18851179 ABHD13
13 108218379 108234255 + 5338
protein_coding abhydrolase domain containing 13 [Source:HGNC
Symbol;Acc:HGNC:20293] - 670 727 455 731 665
900 6.146457978 6.453306349 5.303220366
7.609407658 7.657560488 7.839619024
ENSG00000144233 671.3657148 850.4803421 759.1070474
875.3933782 1132.359894 946.3150327 760.3177015
984.6894348 -0.372526731 0.041499284 0.188564305
AMMECR1L 2 127861630 127885922 - 4761
protein_coding AMMECR1 like [Source:HGNC
Symbol;Acc:HGNC:28658] - 740 961 653 845 992
1097 7.611359268 9.564266019 8.533394948
9.862126551 12.80739638 10.71369958
ENSG00000172250 8.165258694 19.46989337 8.137441549
1.035968495 0 2.587917136 11.92419787 1.207961877
3.253205763 0.041527392 0.188642538 SERHL 22
42500579 42512560 + 1742
transcribed_unitary_pseudogene serine hydrolase-like (pseudogene)
[Source:HGNC Symbol;Acc:HGNC:14408] - 9 22 7
1 0 3 0.253001469 0.598412933
0.250009629 0.031898032 0 0.080076323
ENSG00000110497 878.2189351 970.839683 1118.316967
1079.479172 1127.793926 1762.371569 989.1251951
1323.214889 -0.421219296 0.041537831 0.188642538 AMBRA1
11 46396414 46594125 - 7673
protein_coding autophagy and beclin 1 regulator 1 [Source:HGNC
Symbol;Acc:HGNC:25990] - 968 1097 962 1042 988
2043 6.177871101 6.774353732 7.800397249
7.545961141 7.914784719 12.38038603
ENSG00000214026 712.1920083 626.5765684 790.4943219
880.5732207 1451.977605 681.4848458 709.7542995
1004.678557 -0.500695766 0.041570771 0.188719628 MRPL23
11 1947278 1984522 + 2975 protein_coding
mitochondrial ribosomal protein L23 [Source:HGNC
Symbol;Acc:HGNC:10322] - 785 708 680 850 1272
790 12.92145354 11.27645857 14.22095589
15.87610634 26.2813398 12.34727552
ENSG00000103496 589.7131279 978.8046394 697.4949899
837.0625439 1063.870384 1180.952853 755.3375857
1027.29526 -0.44348018 0.041576143 0.188719628 STX4
16 31032889 31042975 + 4078
protein_coding syntaxin 4 [Source:HGNC Symbol;Acc:HGNC:11439] -
650 1106 600 808 932 1369 7.805393794
12.85092374 9.153999314 11.00971769 14.04805022
15.6094372
ENSG00000126003 714.9137612 570.8218738 623.0955243
789.4079931 826.4400836 839.3477911 636.2770531

818.3986226	-0.363141403	0.041589105	0.188730035	PLAGL2
	20	32192503	32207791	-	5705
protein_coding	PLAG1 like zinc finger 2 [Source:HGNC
Symbol;Acc:HGNC:9047]	zf-C2H2	788	645	536	762	724
	973	6.763932303	5.357109293	5.845423585
7.421836217	7.800640754	7.930274305
ENSG00000151743 87.09609273	130.0942875	116.249165
168.8628647	164.3748233	199.2696195	111.1465151
177.5024358	-0.676800664	0.041640219	0.188913527	AMN1
	12	31671137	31729174	-	3195
protein_coding	antagonist of mitotic exit network 1 homolog
[Source:HGNC Symbol;Acc:HGNC:27281]	-	96	147	100
	163	144	231	1.471394248	2.180082421
1.947313991	2.834841524	2.770377745	3.361802033
ENSG00000272442 9.979760626	6.194966072	2.3249833	0
	0	0	6.166569999	0	5.061036244
0.041663272	1	AL353588.1	6	44273194
44378957	+	3513	protein_coding	"novel protein,
TMEM151B-SPATS1 readtrough"	-	11	7	2	0
	0	0	0.153335678	0.09441616	0.035420827
	0	0	0
ENSG00000063660 403.7266799	500.9072567	628.9079826
400.9198075	410.9370581	251.0279622	511.1806397
354.2949426	0.529599618	0.041704595	0.18915023	GPC1
	2	240435671	240468078	+	6516
protein_coding	glypican 1 [Source:HGNC Symbol;Acc:HGNC:4449]	-
	445	566	541	387	360	291	3.344318405
	4.115871396	5.165626913	3.300212714	3.396008631
	2.076552576
ENSG00000009307 21785.81745	18519.40857	21719.99399
19619.17136	16616.69654	14805.47393	20675.07334
17013.78061	0.281203998	0.041713779	0.18915023	CSDE1
	1	114716913	114758676	-	5778
protein_coding	cold shock domain containing E1 [Source:HGNC
Symbol;Acc:HGNC:29905]	CSD	24013	20926	18684	18938	14557
	17163	203.5155355	171.6070495	201.186654
182.1246031	154.8608735	138.1168558
ENSG00000105655 35.38278767	24.77986429	29.06229125
70.44585765	117.5736583	26.7418104	29.74164774
71.58710879	-1.262653802	0.04173027	0.189176509	ISYNA1
	19	18434388	18438301	-	3284
protein_coding	inositol-3-phosphate synthase 1 [Source:HGNC
Symbol;Acc:HGNC:29821]	-	39	28	25	68	103
	31	0.581554127	0.403999961	0.473634912
1.150582616	1.927886386	0.438924238
ENSG00000078018 44.45529733	89.38451047	110.4367067
43.51067679	44.5181813	5.175834272	81.42550485
31.06823079	1.396988432	0.041758442	0.189239434	MAP2
	2	209424058	209734118	+	11559
protein_coding	microtubule associated protein 2 [Source:HGNC

Symbol;Acc:HGNC:6839]   -   49   101   95   42   39
        6   0.207589078   0.414025939   0.511340496
0.201902209   0.207391859   0.024135817
ENSG00000143162 1555.028156   1617.77114   1550.763861
1502.154318   1200.849403   977.3700383   1574.521052
1226.791253   0.360656417   0.041765546   0.189239434   CREG1
        1   167529117   167553805   -   3286
protein_coding cellular repressor of E1A stimulated genes 1
[Source:HGNC Symbol;Acc:HGNC:2351]   -   1714   1828   1334
        1450   1052   1133   25.54300227   26.35937285
25.25777657   24.51954951   19.67866085   16.03220911
ENSG00000239672 1724.684086   2087.703566   2152.934536
1465.89542   1706.530283   1636.426269   1988.440729
1602.950657   0.310674922   0.041792212   0.189311764   NME1
        17   51153536   51162428   +   3438
protein_coding NME/NM23 nucleoside diphosphate kinase 1 [Source:HGNC
Symbol;Acc:HGNC:7849]   -   1901   2359   1852   1415   1495
        1897   27.07727328   32.51236107   33.5152109
22.86981288   26.72899812   25.65620852
ENSG00000124635 14.51601546   25.66485944   53.4746159
13.46759043   6.848950969   0   31.21849693   6.772180468
        2.215148905   0.04180947   0.189341455   HIST1H2BJ
        6   27125897   27132750   -   950
protein_coding histone cluster 1 H2B family member j [Source:HGNC
Symbol;Acc:HGNC:4761]   -   16   29   46   13   6
        0   0.824755197   1.446441366   3.012597234
0.760381935   0.388217408   0
ENSG00000196678 592.4348808   429.2226493   368.509853
352.2292883   248.8452185   364.0336771   463.3891277
321.702728   0.526160561   0.041834927   0.189341578   ERI2
        16   20780193   20900349   -   6761
protein_coding ERI1 exoribonuclease family member 2 [Source:HGNC
Symbol;Acc:HGNC:30541] -   653   485   317   340   218
        422   4.729670967   3.399047577   2.917125898
2.794345001   1.981951021   2.902234719
ENSG00000149547 3540.093269   3352.36164   3227.07682
3720.162865   4092.248204   4315.783144   3373.177243
4042.731404   -0.261196671   0.041838152   0.189341578   EI24
        11   125569216   125584687   +   3558
protein_coding "EI24, autophagy associated transmembrane protein
[Source:HGNC Symbol;Acc:HGNC:13276]"   -   3902   3788   2776
        3591   3585   5003   53.70441681   50.44643348
48.54230164   56.08174328   61.93420631   65.38160496
ENSG00000259956 2230.022874   2504.536283   2627.231129
2697.661961   2900.530735   3468.671601   2453.930095
3022.288099   -0.301066651   0.041841609   0.189341578   RBM15B
        3   51391268   51397908   +   6641
protein_coding RNA binding motif protein 15B [Source:HGNC
Symbol;Acc:HGNC:24303] -   2458   2830   2260   2604   2541
        4021   18.12496105   20.19200272   21.17297114

21.7881092	23.51898337	28.15344928
ENSG00000107140 287.5985562	407.0977704	420.8219773
397.811902	586.7267997	591.7703851	371.8394346
525.4363623	-0.499966284	0.041934252	0.189712271	TESK1
9	35605305	35610041	+	3298
protein_coding	testis associated actin remodelling kinase 1
[Source:HGNC Symbol;Acc:HGNC:11731]	-	317	460	362
384	514	686	4.706925175	6.608967576
6.829120341	6.469826236	9.579874681	9.671737092
ENSG00000101955 1450.694295	1547.856523	1935.548597
1442.068145	1351.526325	1032.578937	1644.699805
1275.391136	0.366935182	0.042025019	0.190063747	SRPX
X	38149336	38220924	-	1987
protein_coding	sushi repeat containing protein X-linked [Source:HGNC
Symbol;Acc:HGNC:11309]	-	1599	1749	1665	1392	1184
1197	39.40753593	41.70790572	52.13426046
38.927222	36.6269871	28.01091537
ENSG00000284976 363.8076374	507.1022227	378.9722779
225.8411319	232.8643329	386.4622923	416.6273793
281.7225857	0.562534724	0.042033432	0.190063747
BX255925.3	9	137217452	137219361	+	1910
protein_coding	novel RING finger protein	-	401
573	326	218	204	448	10.2811025
14.21502722	10.61918237	6.34213044	6.565142549
10.90625543
ENSG00000268758 2.721752898	12.38993214	3.48747495	0
0	0	6.199719997	0	5.068182265
0.042146948	1	ADGRE4P 19	6952500 6997872 -	3321
transcribed_unprocessed_pseudogene	"adhesion G protein-
coupled receptor E4, pseudogene [Source:HGNC Symbol;Acc:HGNC:19240]"
-	3	14	3	0	0	0
0.044236531	0.199749454	0.056202965	0	0	0
ENSG00000008130 1572.265924	1385.017415	1499.614228
1340.543232	1172.312108	1048.969079	1485.632522
1187.274806	0.323772042	0.042163594	0.190470401	NADK
1	1751232 1780457 -	5570	protein_coding	NAD
kinase [Source:HGNC Symbol;Acc:HGNC:29831]	-	1733	1565
1290	1294	1027	1216	15.23603814	13.31329599
14.40924951	12.90895613	11.33346241	10.15101333
ENSG00000103202 2288.994187	2691.270261	2700.468103
2134.095099	2074.090652	2123.817329	2560.244184
2110.667694	0.278354761	0.042164041	0.190470401	NME4
16	396725	410367	+	3670	protein_coding	NME/
NM23 nucleoside diphosphate kinase 4 [Source:HGNC
Symbol;Acc:HGNC:7852]	-	2523	3041	2323	2060	1817
2462	33.66509697	39.26239579	39.3812949
31.1898438	30.43240138	31.19270245
ENSG00000120265 1207.551036	1354.927579	1449.627087
1076.371266	1118.661992	1029.99102	1337.368568
1075.008093	0.314906676	0.042168463	0.190470401	PCMT1

	6	149749443	149811420	+	2832
protein_coding	protein-L-isoaspartate (D-aspartate) O-
methyltransferase [Source:HGNC Symbol;Acc:HGNC:8728]	-	1331
	1531	1247	1039	980	1194	23.01513306
25.61582706	27.39555172	20.38610901	21.27062186
19.60388273
ENSG00000274565 64.41481858	28.3198449	51.1496326
22.79130689	0	17.25278091	47.96143203	13.34802926
	1.835790302	0.042170113	0.190470401	AC080038.1
	17	62626437	62627590	-	1154
antisense	novel transcript	-	71	32	44
	22	0	20	3.012875761	1.313925104
2.372213179	1.059324253	0	0.805851846
ENSG00000114383 807.4533597	960.2197411	694.007515
910.616307	1028.484137	1312.073988	820.5602053
1083.724811	-0.401091764	0.042177204	0.190470401	TUSC2
	3	50320027	50328251	-	2406
protein_coding	"tumor suppressor 2, mitochondrial calcium regulator
[Source:HGNC Symbol;Acc:HGNC:17034]"	-	890	1085	597
	879	901	1521	18.11436303	21.3678367
15.43780514	20.30043274	23.01847396	29.39440064
ENSG00000177169 1302.812387	1335.457686	2056.447729
1045.292211	1277.329356	1236.161752	1564.905934
1186.261106	0.398738799	0.042210011	0.190533774	ULK1
	12	131894651	131923167	+	7608
protein_coding	unc-51 like autophagy activating kinase 1 [Source:HGNC
Symbol;Acc:HGNC:12558]	-	1436	1509	1769	1009	1119
	1433	9.242992902	9.398210686	14.46652346
7.369409773	9.040801684	8.758035568
ENSG00000131781 27.21752898	21.23988367	12.78740815
56.97826722	27.39580388	72.4616798	20.41494027
52.27858363	-1.353468373	0.04221278	0.190533774	FMO5
	1	147175351	147243050	-	5986
protein_coding	flavin containing monooxygenase 5 [Source:HGNC
Symbol;Acc:HGNC:3773]	-	30	24	11	55	24
	84	0.24542185	0.189976976	0.114330689
0.510549694	0.246446066	0.652489597
ENSG00000233846 0.907250966	8.849951531	9.299933199	0
	0	0	6.352378565	0	5.098087564
0.04221471	1	SELENOTP1	9	6278667 6279153 +
	487	processed_pseudogene	selenoprotein T pseudogene 1
[Source:HGNC Symbol;Acc:HGNC:53722]	-	1	10	8
	0	0	0	0.100554086	0.972965586
1.022039951	0	0	0
ENSG00000001084 857.3521629	898.2700804	820.7191048
1020.428967	1046.748006	1159.386877	858.7804494
1075.521284	-0.324704184	0.042330472	0.191016256	GCLC
	6	53497341	53616970	-	8646
protein_coding	glutamate-cysteine ligase catalytic subunit
[Source:HGNC Symbol;Acc:HGNC:4311]	-	945	1015	706

985 917 1344 5.352359315 5.562592576
5.08038139 6.330427553 6.519307478 7.227948602
ENSG00000107890 704.0267496 436.3026105 480.1090514
367.7688157 482.8510433 251.0279622 540.1461372
367.2159404 0.559211559 0.042374314 0.191165326
ANKRD26 10 26991914 27100498 - 7941
protein_coding ankyrin repeat domain 26 [Source:HGNC
Symbol;Acc:HGNC:29186] - 776 493 413 355 423
291 4.785366542 2.941698534 3.235800235
2.484077841 3.274255243 1.703918472
ENSG00000274211 942.6337537 824.8154827 857.9188376
957.2348893 1261.34847 1128.331871 875.1226913
1115.63841 -0.34998119 0.042385239 0.191165859 SOCS7
17 38352228 38405593 + 8621
protein_coding suppressor of cytokine signaling 7 [Source:HGNC
Symbol;Acc:HGNC:29846] - 1039 932 738 924 1105
1308 5.90182851 5.12253233 5.326053975
5.955611636 7.878653361 7.054741696
ENSG00000084453 0 0 0 5.179842475 0
16.39014186 0 7.189994778 -5.327193225 0.042435397
1 SLCO1A2 12 21264600 21419594 -
11520 protein_coding solute carrier organic anion
transporter family member 1A2 [Source:HGNC Symbol;Acc:HGNC:10956]
- 0 0 0 5 0 19 0
0 0 0.024117349 0 0.076688835
ENSG00000135378 302.1145717 274.3484975 211.5734803
310.7905485 438.332862 381.286458 262.6788498
376.8032895 -0.518167682 0.042465176 0.191477567 PRRG4
11 32829943 32858123 + 5530
protein_coding proline rich and Gla domain 4 [Source:HGNC
Symbol;Acc:HGNC:30799] - 333 310 182 300 384
442 2.948816757 2.656213645 2.047637636
3.014450572 4.268285423 3.716448818
ENSG00000172183 58.97131279 118.5893505 149.9614228
196.834014 160.9503478 200.9948975 109.1740287
186.2597531 -0.775210969 0.042498764 0.191580181 ISG20
15 88636153 88656483 + 7363
protein_coding interferon stimulated exonuclease gene 20 [Source:HGNC
Symbol;Acc:HGNC:6130] - 65 134 129 190 141
233 0.432301995 0.862335845 1.090038297
1.433873519 1.177095427 1.471404699
ENSG00000280670 74.39457921 55.75469465 56.96209084
39.36680281 21.68834474 1.725278091 62.3704549
20.92680854 1.584249242 0.042535328 0.191696156
CCDC163 1 45493866 45500079 - 2757
protein_coding coiled-coil domain containing 163 [Source:HGNC
Symbol;Acc:HGNC:27003] - 82 63 49 38 19
2 1.456484173 1.082755065 1.105773456
0.76587673 0.423608041 0.033730614
ENSG00000158887 17.23776835 33.62981582 4.6499666 0

6.848950969	0	18.50585026	2.282983656
3.068265929	0.042548915	0.191708546	MPZ	1
161304735	161309972	-	2154	protein_coding	myelin
protein zero [Source:HGNC Symbol;Acc:HGNC:7225]	-	19	38
4	0	6	0	0.43195309	0.835919273
0.115537014	0	0.171219377	0
ENSG00000251768	0	0	0	0	13.69790194
7.763751407	0	7.153884449	-5.313170154	0.042571272
1	RNA5SP217	6	127317883	127317973
-	91	rRNA_pseudogene	"RNA, 5S ribosomal pseudogene
217 [Source:HGNC Symbol;Acc:HGNC:43117]"	-	0	0
0	0	12	9	0	0	0	0
8.105638183	4.59866883
ENSG00000138356	1475.190071	1110.668917	984.6304275
2076.080864	1283.036815	1435.431371	1190.163138
1598.183017	-0.42476191	0.042581943	0.191808504	AOX1
2	200585868	200677064	+	5556
protein_coding	aldehyde oxidase 1 [Source:HGNC Symbol;Acc:HGNC:553]
-	1626	1255	847	2004	1124	1664
14.33134621	10.70305925	9.484796555	20.0422984
12.43516163	13.92586251
ENSG00000173599	951.7062633	974.3796636	888.1436205
792.5158986	793.3368206	590.907746	938.0765158
725.5868217	0.372008677	0.042605889	0.191867507	PC
11	66848233	66958376	-	5927
protein_coding	pyruvate carboxylase [Source:HGNC
Symbol;Acc:HGNC:8636]	-	1049	1101	764	765	695
685	8.667008939	8.80194869	8.019832133
7.171971442	7.207708887	5.37386389
ENSG00000203950	1705.631816	1842.559909	1911.136272
2117.519604	2292.115591	2196.279009	1819.775999
2201.971401	-0.27520427	0.042640268	0.191933387	RTL8A
X	135050932	135052196	-	1265
protein_coding	retrotransposon Gag like 8A [Source:HGNC
Symbol;Acc:HGNC:24514]	-	1880	2082	1644	2044	2008
2546	72.77731137	77.98599912	80.85709503
89.78471524	97.57095217	93.58341559
ENSG00000158636	614.208904	569.9368786	642.8578824
514.876342	444.0403212	433.0448007	609.0012217
463.9871546	0.392173536	0.042642219	0.191933387	EMSY
11	76444923	76553025	+	11783
protein_coding	"EMSY, BRCA2 interacting transcriptional repressor
[Source:HGNC Symbol;Acc:HGNC:18071]"	-	677	644	553
497	389	502	2.813594294	2.589741585
2.919954608	2.343756852	2.029275835	1.980974374
ENSG00000253508	17.23776835	22.12487883	15.11239145
7.251779464	0	0	18.15834621	2.417259821
2.919374778	0.042714971	0.192211934	AC004080.2	7
27186573	27193448	-	556	lincRNA	novel
transcript	-	19	25	13	7	0	0

1.673429778	2.130549643	1.454706594	0.699576628
0	0
ENSG00000149091 3550.980281	3515.200748	3701.373413
2450.06549	3006.689475	3318.572407	3589.184814
2925.109124	0.294871867	0.042759228	0.192310241	DGKZ
11	46332905	46380554	+	10787
protein_coding	diacylglycerol kinase zeta [Source:HGNC
Symbol;Acc:HGNC:2857]	-	3914	3972	3184	2365	2634
3847	17.76842061	17.44757211	18.36450501
12.18267083	15.0093696	16.58259621
ENSG00000115687 498.9880313	426.5676638	374.3223113
291.1071471	349.2964994	304.511583	433.2926688
314.9717432	0.461835564	0.042766296	0.192310241	PASK
2	241106099	241150264	-	10686
protein_coding	PAS domain containing serine/threonine kinase
[Source:HGNC Symbol;Acc:HGNC:17270]	-	550	482	322
281	306	353	2.520439071	2.137264682
1.874768071	1.461178232	1.760165956	1.535997659
ENSG00000236618 29.03203091	17.69990306	24.41232465
2.07193699	0	11.21430759	23.71475287	4.428748193
2.384528589	0.042784097	0.192310241	PITPNA-AS1
17	1516931 1518096 +	542	antisense	PITPNA
antisense RNA 1 [Source:HGNC Symbol;Acc:HGNC:44116]	-	32
20	21	2	0	13	2.891208256
1.748465832	2.41060945	0.205041964	0	1.115257324
ENSG00000270231 662.2932052	526.5721161	441.746827
771.7965287	675.7631623	728.0673542	543.5373827
725.2090151	-0.414831654	0.042792668	0.192310241	NBPF8
1	120436353	120467739	+	5330
unprocessed_pseudogene	NBPF member 8 [Source:HGNC
Symbol;Acc:HGNC:31990]	-	730	595	380	745	592
844	6.706938664	5.289519196	4.435710912
7.766781856	6.827187933	7.362855134
ENSG00000179071 99.79760626	92.03949592	89.51185704
26.93518087	51.36713227	64.6979284	93.78298641
47.66674718	0.973485647	0.042797626	0.192310241	CCDC89
11	85684866	85686277	-	1412
protein_coding	coiled-coil domain containing 89 [Source:HGNC
Symbol;Acc:HGNC:26762]	-	110	104	77	26	45
75	3.814930865	3.489997239	3.392836058
1.023176825	1.958958236	2.469776107
ENSG00000114841 171.4704326	217.7088077	109.2742151
174.0427071	207.7515127	483.0778654	166.1511518
288.2906951	-0.794839816	0.042802047	0.192310241	DNAH1
3	52316319	52400491	+	13872
protein_coding	dynein axonemal heavy chain 1 [Source:HGNC
Symbol;Acc:HGNC:2940]	-	189	246	94	168	182
560	0.667192887	0.840276984	0.421595164
0.672949144	0.806454126	1.877067823
ENSG00000226367 0.907250966	0	0	0	13.69790194

12.93958568	0.302416989	8.879162539	-4.664874055
0.042871427	0.192573051	ST7-AS2 7	117072072
117146480	-	2866	antisense	ST7 antisense
RNA 2 [Source:HGNC Symbol;Acc:HGNC:16044]	-	1	0
0	0	12	15	0.017086476	0	0
0	0.257366739	0.24335826
ENSG00000261366 0	0	0	9.323716454	3.424475485
5.175834272	0	5.974675403	-5.055150744
0.042925967	1	MANEA-DT	6	95575183
95577450	-	2268	antisense	MANEA divergent
transcript [Source:HGNC Symbol;Acc:HGNC:43732]	-	0	0
0	9	3	6	0	0	0
0.220501477	0.081306556	0.12300966
ENSG00000100503 1756.43787	1296.517899	1245.028557
1311.536115	1073.002318	879.8918262	1432.661442
1088.14342	0.397710352	0.043024697	0.193212454	NIN
14	50719763	50831121	-	13683
protein_coding	ninein [Source:HGNC Symbol;Acc:HGNC:14906]	-
1936	1465	1071	1266	940	1020	6.928715188
5.073208817	4.86984334	5.141199094	4.222735573
3.466170031
ENSG00000100372 709.4702554	823.9304876	732.3697394
910.616307	1090.124696	893.6940509	755.2568275
964.8116846	-0.352391944	0.04312854	0.193603137
SLC25A17	22	40769630	40819399	-	3989
protein_coding	solute carrier family 25 member 17
[Source:HGNC Symbol;Acc:HGNC:10987]	-	782	931	630
879	955	1036	9.600003697	11.05890393
9.826149326	12.24438234	14.71589551	12.07610102
ENSG00000133619 175.0994364	215.0538222	172.0487642
191.6541716	309.3442854	358.8578428	187.4006743
286.6187666	-0.613002304	0.043133584	0.193603137	KRBA1
7	149714781	149734575	+	5337
protein_coding	KRAB-A domain containing 1 [Source:HGNC
Symbol;Acc:HGNC:22228]	-	193	243	148	185	271
416	1.7708786	2.15742403	1.725326763
1.926134318	3.121184549	3.624325093
ENSG00000197442 421.8716992	238.9486913	441.746827
291.1071471	198.6195781	241.5389327	367.5224058
243.7552193	0.590559641	0.04317165	0.193724841	MAP3K5
6	136557047	136792518	-	5303
protein_coding	mitogen-activated protein kinase kinase kinase 5
[Source:HGNC Symbol;Acc:HGNC:6857]	-	465	270	380
281	174	280	4.293979921	2.412506976
4.458295146	2.944399508	2.016856418	2.455090028
ENSG00000173085 457.2544869	446.0375572	355.7224449
351.1933198	220.3079228	312.2753344	419.6714963
294.5921923	0.510260832	0.043208692	0.193841886	COQ2
4	83261536	83284914	-	2774
protein_coding	"coenzyme Q2, polyprenyltransferase [Source:HGNC

Symbol;Acc:HGNC:25223]" - 504 504 306 339 193
362 8.897187915 8.608956642 6.863123538
6.790555219 4.276595873 6.067826187
ENSG00000106992 88.0033437 89.38451047 166.2363059
33.15099184 97.02680539 7.763751407 114.5413867
45.98051621 1.323835167 0.043234231 0.19390728 AK1
9 127866480 127877743 - 2667
protein_coding adenylate kinase 1 [Source:HGNC Symbol;Acc:HGNC:361]
- 97 101 143 32 85 9
1.781055292 1.794422883 3.335952578 0.666713127
1.95903985 0.15690996
ENSG00000136014 0 0 2.3249833 7.251779464
12.55641011 10.35166854 0.774994433
10.05328604 -3.8085224 0.043256634 0.19395858 USP44
12 95516560 95551490 - 5386
protein_coding ubiquitin specific peptidase 44 [Source:HGNC
Symbol;Acc:HGNC:20064] - 0 0 2 7 11
12 0 0 0.023103113 0.072217714
0.125537564 0.103596698
ENSG00000182944 4105.310621 4707.289219 4537.20491
3570.983402 3589.9918 3989.705584 4449.934917
3716.893595 0.25947543 0.043333005 0.194251781 EWSR1
22 29268009 29300525 + 10326
protein_coding EWS RNA binding protein 1 [Source:HGNC
Symbol;Acc:HGNC:3508] - 4525 5319 3903 3447 3145
4625 21.45928 24.4075569 23.51653204
18.54903012 18.72129511 20.82622876
ENSG00000104888 17.23776835 2.654985459 13.9498998
21.75533839 53.65011592 36.2308399 11.28088454
37.21209807 -1.726494174 0.043366462 0.194352508
SLC17A7 19 49429401 49442360 - 6013
protein_coding solute carrier family 17 member 7 [Source:HGNC
Symbol;Acc:HGNC:16704] - 19 3 12 21 47
42 0.154735898 0.023640491 0.124164341
0.194061835 0.480456435 0.324779871
ENSG00000222019 23.58852512 27.43484975 9.299933199
6.215810969 4.565967313 0 20.10776935 3.593926094
2.510488334 0.043380419 0.194365815 URAHP 16
90039761 90047773 - 2340
transcribed_unprocessed_pseudogene "urate (hydroxyiso-)
hydrolase, pseudogene [Source:HGNC Symbol;Acc:HGNC:43695]" -
26 31 8 6 4 0 0.544109331
0.627729122 0.212706605 0.142477877 0.105073088
0
ENSG00000143797 492.6372745 374.3529498 544.0460921
346.0134773 400.6636317 233.7751813 470.3454388
326.8174301 0.526484848 0.043395299 0.194383245 MBOAT2
2 8852690 9003813 - 8798 protein_coding
membrane bound O-acyltransferase domain containing 2 [Source:HGNC
Symbol;Acc:HGNC:25193] - 543 423 468 334 351

271 3.022348605 2.278152805 3.30954844
2.109475826 2.452282613 1.432241823
ENSG00000128709 197.7807106 273.4635023 291.7854041
252.7763128 385.8242379 514.132871 254.3432057
384.2444739 -0.597478305 0.043409725 0.194398637 HOXD9
2 176122720 176124937 + 1870
protein_coding homeobox D9 [Source:HGNC Symbol;Acc:HGNC:5140]
Homeobox 218 309 251 244 338 596
5.708783467 7.829667397 8.351009189 7.25037156
11.11021476 14.81957235
ENSG00000171960 585.1768731 780.5657251 710.2823981
457.8980747 488.5585025 607.2978879 692.0083321
517.918155 0.41689745 0.043437392 0.194473303 PPIH
1 42658425 42676758 + 1444
protein_coding peptidylprolyl isomerase H [Source:HGNC
Symbol;Acc:HGNC:14651] - 645 882 611 442 428
704 21.87364729 28.94195292 26.32575672
17.00854329 18.21897484 22.66921514
ENSG00000108883 5887.151518 5635.649135 5944.982298
6204.415316 7690.230446 6995.140018 5822.594317
6963.261927 -0.258083946 0.043458805 0.194519941 EFTUD2
17 44849943 44899662 - 10375
protein_coding elongation factor Tu GTP binding domain containing 2
[Source:HGNC Symbol;Acc:HGNC:30858] - 6489 6368 5114
5989 6737 8109 30.62797982 29.08314645
30.66757704 32.07585572 39.91405047 36.34211624
ENSG00000258860 0 0 0 13.46759043 7.990442797
0 0 7.152677744 -5.311187532 0.043460607
1 AL133163.2 14 35362232 35363628
- 624 lincRNA novel transcript - 0
0 0 13 7 0 0 0 0
1.157632754 0.689542137 0
ENSG00000175224 1554.120905 1563.786436 1742.574983
1906.182031 2106.052423 1908.157568 1620.160774
1973.464007 -0.284787287 0.043474491 0.194540925 ATG13
11 46617527 46674818 + 7001
protein_coding autophagy related 13 [Source:HGNC
Symbol;Acc:HGNC:29091] - 1713 1767 1499 1840 1845
2212 11.98190767 11.95922158 13.321355
14.60393156 16.1988302 14.69115914
ENSG00000153790 136.9948959 115.0493699 126.7115898
79.76957411 67.34801786 77.63751407 126.2519519
74.91836868 0.751748908 0.043600791 0.195056752
C7orf31 7 25134697 25180356 - 3837
protein_coding chromosome 7 open reading frame 31 [Source:HGNC
Symbol;Acc:HGNC:21722] - 151 130 109 77 59
90 1.927142511 1.605380539 1.767427245
1.115092692 0.945164873 1.09064077
ENSG00000269944 0 0 0 0 9.131934625
12.07694663 0 7.069627086 -5.298993348 0.043693859

1	AP001267.4	11	118415977	118416521
+	545	sense_intronic	novel transcript	-
0	0	0	0	8	14	0	0
0	0	0.90227899	1.194435084
ENSG00000146463 2023.169654	1777.955263	1818.13694
1563.276459	1542.15546	1515.656803	1873.087286
1540.362907	0.282327413	0.043703467	0.195466657	ZMYM4
1	35268967	35422058	+	7647
protein_coding	zinc finger MYM-type containing 4 [Source:HGNC
Symbol;Acc:HGNC:13055]	-	2230	2009	1564	1509	1351
1757	14.28046853	12.44845023	12.72484512
10.96503932	10.85954474	10.68345412
ENSG00000268912 150.6036604	147.7941906	109.2742151
198.905951	220.3079228	204.4454537	135.8906887
207.8864425	-0.61032217	0.043730219	0.19549529
AC012313.5	19	58428632	58431148	-	2517
lincRNA novel transcript	-	166	167	94
192	193	237	3.229635841	3.143834651
2.323547123	4.238674399	4.713260608	4.378205564
ENSG00000196912 80.74533597	55.75469465	60.44956579
24.86324388	39.95221399	27.60444945	65.64986547
30.80663577	1.09560998	0.043731973	0.19549529
ANKRD36B	2	97492663	97589965	-	6771
protein_coding	ankyrin repeat domain 36B [Source:HGNC
Symbol;Acc:HGNC:29333]	-	89	63	52	24	35
32	0.643673866	0.440873684	0.477812356
0.19695657	0.317733196	0.219749645
ENSG00000166797 951.7062633	964.6447169	1074.142285
825.6668904	850.411412	665.957343	996.8310882
780.6785485	0.353140595	0.043765147	0.195594158	CIAO2A
15	64072559	64094018	-	2427
protein_coding	cytosolic iron-sulfur assembly component 2A
[Source:HGNC Symbol;Acc:HGNC:26235]	-	1049	1090	924
797	745	772	21.16578574	21.2805654
23.68694445	18.24738304	18.86834709	14.79035309
ENSG00000230091 9.979760626	2.654985459	3.48747495
7.251779464	42.23519764	23.29125422	5.374073678
24.25941044	-2.164751671	0.04379746	0.195687639
TMEM254-AS1	10	80046860	80078912	-	3138
antisense	TMEM254 antisense RNA 1 [Source:HGNC
Symbol;Acc:HGNC:27340]	-	11	3	3	7	37
27	0.171659732	0.045299641	0.059480576
0.123953029	0.724763219	0.400075395
ENSG00000178567 651.4061936	650.4714375	501.0339011
803.911552	808.1762143	734.9684666	600.9705107
782.3520777	-0.378809752	0.043808498	0.195687639
EPM2AIP1	3	36985043	36993168	-	8126
protein_coding	EPM2A interacting protein 1 [Source:HGNC
Symbol;Acc:HGNC:19735]	-	718	735	431	776	708
852	4.326894536	4.285849948	3.299949538

5.306362888	5.355546567	4.875215245
ENSG00000137822 688.6034832	592.9467526	659.1327655
612.2573805	412.07855	401.9897951	646.8943338
475.4419085	0.444491268	0.043819251	0.195687639
TUBGCP4 15	43369221	43409771	+	11171
protein_coding	tubulin gamma complex associated protein 4
[Source:HGNC Symbol;Acc:HGNC:16691]	-	759	670	567
	591	361	466	3.327196172	2.841902615
3.157896222	2.939730192	1.986380807	1.939656754
ENSG00000184675 320.259591	356.6530467	299.9228457
421.6391774	470.2946332	428.7316055	325.6118278
440.2218054	-0.433723333	0.043841375	0.195696763	AMER1
	X	64185117	64205744	-	8443
protein_coding	APC membrane recruitment protein 1 [Source:HGNC
Symbol;Acc:HGNC:26837]	-	353	403	258	407	412
	497	2.047418389	2.261698436	1.90120857
2.678611096	2.999492546	2.737099703
ENSG00000216867 3.629003864	6.194966072	8.137441549	0
	0	0	5.987137162	0	5.012891858
0.043842777	1	RPL22P12	2	110633947
110634333	+	387	processed_pseudogene	ribosomal
protein L22 pseudogene 12 [Source:HGNC Symbol;Acc:HGNC:36345]	-
	4	7	7	0	0	0	0.506148215
	0.857064518	1.125366341	0	0	0
ENSG00000089050 801.102603	818.6205166	805.6067134
1026.644778	962.2776112	1035.166854	808.4432777
1008.029748	-0.318533166	0.043855421	0.195696763	RBBP9
	20	18486540	18497243	-	3861
protein_coding	"RB binding protein 9, serine hydrolase [Source:HGNC
Symbol;Acc:HGNC:9892]"	-	883	925	693	991	843
	1200	11.19926666	11.35189517	11.16709677
14.26217945	13.42069891	14.45148454
ENSG00000154518 1395.351986	1820.43503	1971.585838
1344.687106	1265.914437	1481.151241	1729.124285
1363.917595	0.341436824	0.043857652	0.195696763
ATP5MC3 2	175176258	175184607	-	6780
protein_coding	ATP synthase membrane subunit c locus 3 [Source:HGNC
Symbol;Acc:HGNC:843]	-	1538	2057	1696	1298	1109
	1717	11.10849759	14.37576745	15.563347
10.63792789	10.05423918	11.77529021
ENSG00000100867 29.93928188	42.47976735	34.8747495
5.179842475	20.54685291	0	35.76459957	8.575565127
	2.082968838	0.043865547	0.195696763	DHRS2	14
	23630115	23645639	+	7556	protein_coding
	dehydrogenase/reductase 2 [Source:HGNC Symbol;Acc:HGNC:18349]
	-	33	48	30	5	18	0
0.213870396	0.3010064	0.247022295	0.036769701
0.146429276	0
ENSG00000280417 9.07250966	3.539980612	17.43737475
39.36680281	30.82027936	27.60444945	10.01662167

32.59717721	-1.719260212	0.043886616	0.195741389
AC096887.2	3	53046166	53048122	+	1957
	TEC	TEC	-	10	4	15	38	27
	32	0.250229125	0.096849104	0.476877992
1.078958684	0.848047735	0.760309069
ENSG00000219410 0	0	1.16249165	0	9.131934625
	17.25278091	0.387497217	8.794905177	-4.653761492
	0.043932295	0.195895733	AC125494.1	12
6663260 6672069 +	2399	bidirectional_promoter_lncRNA	novel
transcript	-	0	0	1	0	8	20
	0	0	0.025934424	0	0.204977928
0.387641947
ENSG00000203837 0	0	0	0	12.55641011
8.626390453	0	7.060933521	-5.294937245	0.043989088
	1	PNLIPRP3	10	116427867	116477957
	+	2354	protein_coding	pancreatic lipase related
protein 3 [Source:HGNC Symbol;Acc:HGNC:23492]	-	0	0
	0	0	11	10	0	0	0	0
	0.287232506	0.197526132
ENSG00000151090 58.06406182	62.83465587	62.77454909
11.39565344	49.08414861	6.038473317	61.22442226
22.17275846	1.479030819	0.043992925	0.196116646	THRB
	3	24117153	24495756	-	10968
protein_coding	thyroid hormone receptor beta [Source:HGNC
Symbol;Acc:HGNC:11799]	THR-like	64	71	54	11
	43	7	0.285746695	0.306730772	0.306318456
	0.055728491	0.240984092	0.029675744
ENSG00000250697 7.258007728	2.654985459	8.137441549	0
	0	0	6.016811579	0	5.019786411
0.044162201	1	AC010343.3	5	32925639
33297910	-	979	lincRNA novel transcript	-
	8	3	7	0	0	0	0.400162123
	0.145199461	0.444858809	0	0	0
ENSG00000092470 1204.829283	1028.364368	1021.83016
903.3645276	851.5529038	839.3477911	1085.007937
864.7550741	0.327761846	0.044198247	0.19698231	WDR76
	15	43826963	43868419	+	4078
protein_coding	WD repeat domain 76 [Source:HGNC
Symbol;Acc:HGNC:25773]	-	1328	1162	879	872	746
	973	15.94701994	13.50160342	13.410609
11.88177454	11.24446938	11.09421651
ENSG00000159733 95.26135143	145.1392051	91.83684034
171.9707702	157.5258723	207.8960099	110.745799
179.1308841	-0.692566541	0.044223768	0.197030697
ZFYVE28 4	2269582 2418663 -	9624	protein_coding	zinc
finger FYVE-type containing 28 [Source:HGNC Symbol;Acc:HGNC:29334]
	-	105	164	79	166	138	241
0.534271943	0.807448207	0.510714659	0.958439088
0.881395507	1.164373858
ENSG00000079335 221.3692357	178.7690209	187.1611556

146.0715578	140.4034949	74.18695789	195.7664708
120.2206702	0.707447905	0.044240391	0.197030697	CDC14A
	1	100345001	100520278	+	6511
protein_coding	cell division cycle 14A [Source:HGNC
Symbol;Acc:HGNC:1718]	-	244	202	161	141	123
	86	1.835146816	1.470043259	1.538455814
1.203326445	1.161193982	0.614160348
ENSG00000103415 596.9711356	774.370759	785.8443553
767.6526547	942.8722501	1152.485764	719.0620833
954.3368898	-0.409504217	0.04424252	0.197030697	HMOX2
	16	4474690 4510347 +	3793	protein_coding	heme
oxygenase 2 [Source:HGNC Symbol;Acc:HGNC:5014]	-	658	875
	676	741	826	1336	8.495163356	10.93079253
	11.08844636	10.85543944	13.38580718	16.37776494
ENSG00000126882 228.6272434	315.9432697	241.7982632
137.7838098	152.959905	223.4235127	262.1229254
171.3890758	0.610981302	0.04426034	0.197060446	FAM78A
	9	131258076	131276547	-	5499
protein_coding	family with sequence similarity 78 member A
[Source:HGNC Symbol;Acc:HGNC:25465]	-	252	357	208
	133	134	259	2.244117046	3.076174284
2.353349674	1.343940262	1.497850397	2.190014864
ENSG00000130811 4470.025509	6093.191629	5361.411489
5176.734569	7057.843974	7986.312281	5308.209543
6740.296941	-0.344675217	0.044358076	0.197445901	EIF3G
	19	10115017	10119918	-	3486
protein_coding	eukaryotic translation initiation factor 3 subunit G
[Source:HGNC Symbol;Acc:HGNC:3274]	-	4927	6885	4612
	4997	6183	9258	69.21239267	93.58430138
82.31306294	79.65150944	109.023275	123.4868783
ENSG00000202523 3.629003864	0	0	23.82727538
23.97132839	0	1.209667955	15.93286792	-3.684982471
	0.044403931	0.197600284	RF00019 9	83992932
	83993033	+	102	misc_RNA		-
	4	0	0	23	21	0	1.920385876
	0	0	12.52967216	12.65512628	0
ENSG00000080293 0	0	0	14.50355893	6.848950969
	0	0	7.117503299	-5.304355082	0.044418698
	1	SCTR	2	119439843	119525301	-
	3426	protein_coding	secretin receptor [Source:HGNC
Symbol;Acc:HGNC:10608]	-	0	0	0	14	6
	0	0	0	0	0.22706632	0.10764931
	0
ENSG00000127054 2263.59116	2559.405983	2344.745658
2406.554814	3022.670361	3537.682725	2389.2476
2988.9693	-0.323289036	0.044429576	0.197664679	INTS11
	1	1311585 1324691 -	8220	protein_coding
integrator complex subunit 11 [Source:HGNC Symbol;Acc:HGNC:26052]
	-	2495	2892	2017	2323	2648	4101
14.86371659	16.67066452	15.2665508	15.70324606

19.80129178	23.19791591
ENSG00000173261 0.907250966	1.769990306	0	18.64743291
	0	18.11541995	0.892413757
12.25428429	-3.736772088	0.044491118	0.197888708
PLAC8L1 5	146084386	146104369	-	656
protein_coding	PLAC8 like 1 [Source:HGNC Symbol;Acc:HGNC:31746]
	-	1	2	0	18	0	21
0.074649146	0.144461659	0	1.524687042	0
1.488491893
ENSG00000068724 545.2578306	494.7122906	441.746827
364.6609102	408.6540745	327.8028372	493.9056494
367.039274	0.430296542	0.044572574	0.198201171	TTC7A
	2	46916157	47076137	+	6949
protein_coding	tetratricopeptide repeat domain 7A [Source:HGNC
Symbol;Acc:HGNC:19750]	-	601	559	380	352	358
	380	4.235267483	3.811675644	3.402264953
2.81470183	3.166708408	2.54268349
ENSG00000069493 53.52780699	36.28480128	34.8747495
79.76957411	67.34801786	98.34085116	41.56245259
81.81948104	-0.976612941	0.044626972	0.19839319	CLEC2D
	12	9664969 9699555 +	6957	protein_coding	C-type
lectin domain family 2 member D [Source:HGNC Symbol;Acc:HGNC:14351]
	-	59	41	30	77	59	114
0.415296902	0.279246857	0.268290996	0.615008001
0.521287569	0.761927881
ENSG00000251000 0	3.539980612	0	11.39565344
13.69790194	12.07694663	1.179993537
12.39016734	-3.343771726	0.044682663	0.198590859
AC008592.3	5	95834424	95835046	-	418
	processed_pseudogene	gamma-glutamyl cyclotransferase (GGCT)
pseudogene	-	0	4	0	11	12	14
	0	0.453429895	0	1.462272953	1.76462458
	1.55733761
ENSG00000272138 8.165258694	6.194966072	3.48747495	0
	0	0	5.949233238	0	5.008180542
0.044722934	1	LINC01607	8	79768142
79802842	+	789	lincRNA long intergenic non-protein
coding RNA 1607 [Source:HGNC Symbol;Acc:HGNC:51660]	-	9
	7	3	0	0	0	0.558591329
0.420385258	0.236565331	0	0	0
ENSG00000070808 7.258007728	3.539980612	11.6249165
1.035968495	0	0	7.474301613	0.345322832
4.369257652	0.044763181	0.198898748	CAMK2A	5
150219491	150290291	-	6150	protein_coding
calcium/calmodulin dependent protein kinase II alpha [Source:HGNC
Symbol;Acc:HGNC:1460]	-	8	4	10	1	0
	0	0.063700605	0.030818487	0.101165337
0.009035182	0	0
ENSG00000166037 1990.508619	1331.03271	1634.46326
1490.758664	1294.451733	988.5843459	1652.00153

1257.931581	0.393697195	0.044884242	0.199386581	CEP57
	11	95789965	95832693	+	7101
protein_coding	centrosomal protein 57 [Source:HGNC
Symbol;Acc:HGNC:30794]	-	2194	1504	1406	1439	1134
	1146	15.1302392	10.03586393	12.31892056
11.26038721	9.816143579	7.504056981
ENSG00000055208 1729.220341	1329.26272	1663.525551
1390.26972	1267.055929	1062.771304	1574.002871
1240.032318	0.344295148	0.044917357	0.199483585	TAB2
	6	149218641	149411613	+	6010
protein_coding	TGF-beta activated kinase 1 (MAP3K7) binding protein 2
[Source:HGNC Symbol;Acc:HGNC:17075]	-	1906	1502	1431
	1342	1110	1232	15.53020212	11.84191397
14.81398868	12.40766581	11.35261388	9.531631722
ENSG00000136147 547.9795835	462.8524651	535.9086506
583.2502626	783.0633941	672.8584553	515.580233
679.7240374	-0.398709777	0.044963352	0.19962731	PHF11
	13	49495610	49528987	+	4841
protein_coding	PHD finger protein 11 [Source:HGNC
Symbol;Acc:HGNC:17024]	-	604	523	461	563	686
	780	6.109849878	5.119093324	5.924786285
6.462273817	8.710365785	7.491875267
ENSG00000028528 2040.407422	1948.759327	2390.082832
1635.794253	1719.086693	1844.322279	2126.416527
1733.067742	0.294393584	0.044972289	0.19962731	SNX1
	15	64094123	64146090	+	9780
protein_coding	sorting nexin 1 [Source:HGNC Symbol;Acc:HGNC:11172]
	-	2249	2202	2056	1579	1506	2138
11.2610603	10.66853781	13.07949879	8.971298743
9.465280252	10.16482402
ENSG00000177699 16.33051739	1.769990306	1.16249165	0
	0	0	6.420999781	0	5.119666008
0.044988858	1	AC011944.1	15	78978889
78985926	+	1900	antisense	uncharacterized
LOC100129540 [Source:NCBI gene;Acc:100129540]	-	18	2
	1	0	0	0	0.463924798	0.049877288
	0.032745622	0	0	0
ENSG00000253688 16.33051739	1.769990306	1.16249165	0
	0	0	6.420999781	0	5.119666008
0.044988858	1	AC026904.2	8	48551527
48556441	-	1579	lincRNA novel transcript	-
	18	2	1	0	0	0	0.558237566
	0.060017003	0.039402585	0	0	0
ENSG00000196313 2410.565817	2389.486913	2270.346192
2815.762369	2611.733303	3090.835699	2356.799641
2839.44379	-0.268939775	0.044993076	0.199631472	POM121
	7	72879365	72951440	+	9822
protein_coding	POM121 transmembrane nucleoporin [Source:HGNC
Symbol;Acc:HGNC:19702]	-	2657	2700	1953	2718	2288
	3583	13.24708455	13.02537619	12.371124

15.37664423	14.3186954	16.96203271
ENSG00000148908 166.0269268	136.2892536	297.5978624
124.3162194	125.5641011	112.1430759	199.9713476
120.6744655	0.725950545	0.044995799	0.199631472	RGS10
	10	119499828	119542708	-	1252
protein_coding	regulator of G protein signaling 10 [Source:HGNC
Symbol;Acc:HGNC:9992]	-	183	154	256	120	110
	130	7.15773218	5.828312544	12.72162188
5.325850371	5.400521713	4.828030907
ENSG00000230648 6.350756762	7.964956378	3.48747495	0
	0	0	5.93439603	0	5.00472332
0.045025119	1	AL138831.1	6	4018843 4021215 -
	209	antisense	novel transcript	-	7
	9	3	0	0	0	1.640138176
2.040434529	0.893062421	0	0	0
ENSG00000180694 870.0536764	923.0499447	812.5816633
1269.061406	1299.0177	847.9741815	868.5617615
1138.684429	-0.389382801	0.045025197	0.199711813	TMEM64
	8	90621995	90791632	-	5870
protein_coding	transmembrane protein 64 [Source:HGNC
Symbol;Acc:HGNC:25441]	-	959	1043	699	1225	1138
	983	8.000353731	8.419235313	7.408766733
11.59604871	11.91657693	7.786574348
ENSG00000134313 1972.3636	1537.236581	1592.61356
1668.945245	1175.736583	1063.633943	1700.737914
1302.771924	0.385025895	0.04504902	0.199767388
KIDINS220	2	8721081 8837630 -	15362	protein_coding
	kinase D interacting substrate 220 [Source:HGNC
Symbol;Acc:HGNC:29508]	-	2174	1737	1370	1611	1030
	1233	6.930115336	5.357701314	5.548551904
5.827198647	4.121324626	3.732040379
ENSG00000285943 26.31027801	22.12487883	8.137441549
6.215810969	3.424475485	0	18.8575328	3.213428818
	2.577878278	0.045063449	0.199781289	AC112128.1
	3	113361901	113515151	-	6837
protein_coding	novel protein	-	29	25	7	6
	3	0	0.207711768	0.173261021	0.063699982
	0.048763819	0.026971372	0
ENSG00000143947 7315.164539	9225.189476	10017.19055
7457.937195	7313.538143	7106.420455	8852.514854
7292.631931	0.279543475	0.045146964	0.200101392	RPS27A
	2	55231903	55235853	+	3130
protein_coding	ribosomal protein S27a [Source:HGNC
Symbol;Acc:HGNC:10417]	-	8063	10424	8617	7199	6407
	8238	126.1481848	157.8034544	171.2847121
127.8026561	125.8223368	122.3794419
ENSG00000115641 315.7233362	335.413163	305.7353039
462.0419487	348.1550076	510.6823148	318.9572677
440.2930904	-0.466061561	0.045186568	0.200226752	FHL2
	2	105357712	105438513	-	6626

protein_coding	four and a half LIM domains 2 [Source:HGNC
Symbol;Acc:HGNC:3703]	-	348	379	263	446	305
	592	2.571914317	2.710280368	2.469512129
3.740205554	2.829409243	4.154332884
ENSG00000268061 8.165258694	4.424975766	4.6499666
4.14387398	52.5086241	20.70333709	5.746733686
25.78527839	-2.158343582	0.045204776	0.200257271	NAPA-
AS1	19	47484282	47501597	+	1894
antisense	NAPA antisense RNA 1 [Source:HGNC
Symbol;Acc:HGNC:44118]	-	9	5	4	4	46
	24	0.232697233	0.125088237	0.131397428
0.117352423	1.492881091	0.589199385
ENSG00000285035 9.979760626	4.424975766	3.48747495	0
	0	0	5.964070447	0	5.011608839
0.045209096	1	AC091057.7	15	30644633
30723495	+	675	transcribed_unprocessed_pseudogene
	"Serine/threonine-protein kinase ULK4 (ULK4), pseudogene"
	-	11	5	3	0	0	0
0.798027019	0.350988326	0.276518587	0	0	0
ENSG00000181634 58.06406182	26.54985459	33.71225785	0
	18.26386925	12.93958568	39.44205809	10.40115164
	1.925872374	0.045238865	0.200337437	TNFSF15 9
	114784635	114806126	-	7889	protein_coding
	TNF superfamily member 15 [Source:HGNC Symbol;Acc:HGNC:11931]
	-	64	30	29	0	16	15
0.397270852	0.180187948	0.228708807	0	0.124665243
	0.088409782
ENSG00000130881 1383.557723	1503.606765	1576.338677
1113.666132	1418.874342	965.2930917	1487.834389
1165.944522	0.35246834	0.045245523	0.200337437	LRP3
	19	33177603	33208867	+	5095
protein_coding	LDL receptor related protein 3 [Source:HGNC
Symbol;Acc:HGNC:6695]	-	1525	1699	1356	1075	1243
	1119	14.65731222	15.80067468	16.55855168
11.72401376	14.99596094	10.21214368
ENSG00000106077 272.1752898	213.2838319	195.2985972
308.7186115	269.3920714	419.242576	226.9192396
332.4510863	-0.551413745	0.045281558	0.200432109	ABHD11
	7	73736094	73738867	-	2289
protein_coding	abhydrolase domain containing 11 [Source:HGNC
Symbol;Acc:HGNC:16407]	-	300	241	168	298	236
	486	6.418065509	4.988818347	4.566361982
7.234066805	6.337435766	9.872371616
ENSG00000131446 2353.409006	2943.493879	2605.143787
2991.877013	3334.29763	3268.539343	2634.015557
3198.237995	-0.27993651	0.045289566	0.200432109	MGAT1
	5	180784782	180815652	-	11576
protein_coding	"mannosyl (alpha-1,3-)-glycoprotein beta-1,2-N-
acetylglucosaminyltransferase [Source:HGNC Symbol;Acc:HGNC:7044]"
	-	2594	3326	2241	2888	2921	3789

10.97337289	13.6141386	12.04453908	13.86279224
15.51030718	15.21938507
ENSG00000118096 765.7198153	891.1901192	991.6053774
913.7242125	1196.283436	1340.541076	882.8384373
1150.182908	-0.382674316	0.045380954	0.200786318	IFT46
	11	118544528	118572970	-	3814
protein_coding	intraflagellar transport 46 [Source:HGNC
Symbol;Acc:HGNC:26146]	-	844	1007	853	882	1048
	1554	10.83653509	12.510516	13.91474299
12.84990568	16.88993406	18.94529377
ENSG00000163888 155.1399152	241.6036768	184.8361723
136.7478413	134.6960357	94.02765594	193.8599214
121.8238443	0.673182795	0.045401497	0.200826978
CAMK2N2 3	184259213	184261463	-	1362
protein_coding	calcium/calmodulin dependent protein kinase II
inhibitor 2 [Source:HGNC Symbol;Acc:HGNC:24197] -	171	273
	159	132	118	109	6.14819575	9.497558566
	7.263180939	5.385287174	5.325400319	3.72117769
ENSG00000138795 739.4095373	629.2315539	591.7082498
628.8328764	433.7668947	342.467701	653.4497803
468.355824	0.481837907	0.045418607	0.200852435	LEF1
	4	108047545	108168956	-	5361
protein_coding	lymphoid enhancer binding factor 1 [Source:HGNC
Symbol;Acc:HGNC:6551]	HMG	815	711	509	607	380
	397	7.444584865	6.284203414	5.90716119
6.291510525	4.356975819	3.44330678
ENSG00000122674 930.8394911	745.1659189	854.4313627
1101.23451	1022.776678	1062.771304	843.4789242
1062.260831	-0.332972998	0.045443049	0.2008912	CCZ1
	7	5898725 5926550 +	6431	protein_coding	"CCZ1
homolog, vacuolar protein trafficking and biogenesis associated
[Source:HGNC Symbol;Acc:HGNC:21691]"	-	1026	842	735
	1063	896	1232	7.812634997	6.203831916
7.110754358	9.184738556	8.564003977	8.907651477
ENSG00000163126 0	0.884995153	3.48747495	5.179842475
	0	43.13195226	1.457490034
16.10393158	-3.520265874	0.045461179	0.2008912
ANKRD23 2	96824526	96857934	-	4116
protein_coding	ankyrin repeat domain 23 [Source:HGNC
Symbol;Acc:HGNC:24470]	-	0	1	3	5	0
	50	0	0.011512008	0.045347436	0.067500452
	0	0.564840276
ENSG00000198420 558.866595	467.2774408	597.520708
427.8549884	349.2964994	431.3195226	541.2215813
402.8236702	0.42429873	0.045461443	0.2008912	TCAF1
	7	143851375	143902198	-	6310
protein_coding	TRPM8 channel associated factor 1 [Source:HGNC
Symbol;Acc:HGNC:22201]	-	616	528	514	413	306
	500	4.780573904	3.964888732	5.06804668
3.636911524	2.98084523	3.684441482

ENSG00000168282 29.93928188 73.45459771 72.07448229
10.35968495 36.5277385 22.42861518 58.48945396
23.10534621 1.342573045 0.045482271 0.200933039 MGAT2
14 49620795 49623481 + 2687
protein_coding "mannosyl (alpha-1,6-)-glycoprotein beta-1,2-N-
acetylglucosaminyltransferase [Source:HGNC Symbol;Acc:HGNC:7045]"
- 33 83 62 10 32 26
0.601415971 1.463648752 1.435591472 0.206797068
0.732031336 0.449921451
ENSG00000182372 800.195352 864.6402646 933.4807949
959.3068263 1215.688797 1113.667007 866.1054705
1096.220877 -0.340265304 0.045518027 0.201040795 CLN8
8 1755778 1801711 + 9459 protein_coding "CLN8,
transmembrane ER and ERGIC protein [Source:HGNC
Symbol;Acc:HGNC:2079]" - 882 977 803 926 1065
1291 4.566169651 4.894133133 5.281741796
5.439735771 6.920727389 6.346174869
ENSG00000200397 0 0 0 0 11.41491828
9.489029498 0 6.967982593 -5.27647657 0.045556747
1 RF00019 7 87218611 87218712 -
102 misc_RNA - 0 0 0
0 10 11 0 0 0 0
6.02625061 5.014452613
ENSG00000088451 402.8194289 374.3529498 341.7725451
218.5893524 329.8911383 242.4015717 372.9816412
263.6273542 0.502808858 0.045574679 0.201240761 TGDS
13 94574051 94596257 - 2589
protein_coding "TDP-glucose 4,6-dehydratase [Source:HGNC
Symbol;Acc:HGNC:20324]" - 444 423 294 211 289
281 8.398072184 7.741671833 7.065162035
4.528584216 6.861406546 5.046674419
ENSG00000137198 33.56828574 28.3198449 27.8997996
70.44585765 63.92354238 54.34625985 29.92931008
62.90521996 -1.069455683 0.045752553 0.201975772 GMPR
6 16238580 16295549 + 2232
protein_coding guanosine monophosphate reductase [Source:HGNC
Symbol;Acc:HGNC:4376] - 37 32 24 68 56
63 0.811776019 0.679332245 0.668996581
1.692882307 1.542201769 1.312433712
ENSG00000184831 248.5867647 287.6234248 290.6229125
211.337573 190.6291353 173.3904481 275.611034
191.7857188 0.523486425 0.045780836 0.202050202 APOO
X 23833353 23907934 - 2353
protein_coding apolipoprotein O [Source:HGNC Symbol;Acc:HGNC:28727]
- 274 325 250 204 167 201
5.702395289 6.544671831 6.610357204 4.817484033
4.362564934 3.97196258
ENSG00000162302 1363.598202 1533.6966 1646.088176
738.6455369 1165.463157 1460.447904 1514.460993
1121.518866 0.432532264 0.045795232 0.202063321

RPS6KA4 11 64359148 64372215 + 3807
protein_coding ribosomal protein S6 kinase A4 [Source:HGNC
Symbol;Acc:HGNC:10433] - 1503 1733 1416 713 1021
1693 19.33324646 21.56960176 23.14127179
10.40683567 16.48504836 20.67783661
ENSG00000115998 205.9459693 178.7690209 195.2985972
289.0352101 218.0249392 345.0556181 193.3378625
284.0385891 -0.55703873 0.045823042 0.202135607
C2orf42 2 70149880 70248615 - 3630
protein_coding chromosome 2 open reading frame 42 [Source:HGNC
Symbol;Acc:HGNC:26056] - 227 202 168 279 191
400 3.062301279 2.636763542 2.879449746
4.270803814 3.23425384 5.123708154
ENSG00000099331 4104.40337 4312.581381 4251.231964
4970.576839 4869.604139 5063.691196 4222.738905
4967.957391 -0.234541003 0.045841182 0.202165212 MYO9B
19 17075781 17214537 + 9948
protein_coding myosin IXB [Source:HGNC Symbol;Acc:HGNC:7609] -
4524 4873 3657 4798 4266 5870 22.26975828
23.21063786 22.87157279 26.80010593 26.35921271
27.43679275
ENSG00000198498 737.5950353 704.4561419 661.4577488
577.0344517 555.9065203 504.6438415 701.169642
545.8616045 0.362207655 0.045875833 0.202168552 TMA16
4 163494442 163520539 + 3513
protein_coding translation machinery associated 16 homolog
[Source:HGNC Symbol;Acc:HGNC:25638] - 813 796 569
557 487 585 11.33290059 10.73646614
10.07722518 8.810267383 8.52114924 7.742990643
ENSG00000100413 1124.991198 1611.576174 1081.117234
1486.61479 1522.750099 1972.855497 1272.561535
1660.740129 -0.383917098 0.045876845 0.202168552 POLR3H
22 41525804 41544606 - 6594
protein_coding RNA polymerase III subunit H [Source:HGNC
Symbol;Acc:HGNC:30349] - 1240 1821 930 1435 1334
2287 9.208765755 13.08541329 8.774873258
12.09246953 12.43524216 16.12680148
ENSG00000167106 352.9206258 538.9620483 545.2085838
561.4949242 667.7727195 734.1058275 479.0304193
654.4578237 -0.45143921 0.04589964 0.202168552
FAM102A 9 127940579 127980513 - 6100
protein_coding family with sequence similarity 102 member A
[Source:HGNC Symbol;Acc:HGNC:31419] - 389 609 469
542 585 851 3.12283077 4.730574631
4.783544895 4.937208809 5.894858588 6.486803513
ENSG00000163026 532.556317 448.6925426 464.99666
723.1060094 657.499293 536.5614862 482.0818399
639.0555962 -0.405625797 0.045911572 0.202168552 WDCP
2 24029340 24049575 - 3847
protein_coding WD repeat and coiled coil containing [Source:HGNC

Symbol;Acc:HGNC:26157] - 587 507 400 698 576
622 7.472133086 6.244709123 6.469111724
10.08196719 9.203386426 7.517946252
ENSG00000154945 1732.849345 1664.675883 1580.988644
1386.125846 1319.564553 1390.574141 1659.504624
1365.421514 0.281487403 0.045921535 0.202168552
ANKRD40 17 50693190 50707924 - 4849
protein_coding ankyrin repeat domain 40 [Source:HGNC
Symbol;Acc:HGNC:28233] - 1910 1881 1360 1338 1156
1612 19.28900682 18.38074255 17.44992525
15.3326059 14.6538928 15.45766431
ENSG00000197535 1893.432766 1311.562817 1644.925685
1521.837719 1256.782503 782.4136141 1616.640423
1187.011279 0.446326875 0.045926151 0.202168552 MYO5A
15 52307283 52529050 - 12988
protein_coding myosin VA [Source:HGNC Symbol;Acc:HGNC:7602] -
2087 1482 1415 1469 1101 907 7.868806262
5.406701143 6.778303437 6.284801414 5.210655959
3.247102703
ENSG00000274762 32.66103478 11.50493699 6.974949899
6.215810969 0 0 17.04697389 2.07193699
3.052738746 0.04593036 0.202168552 AL049777.1 14
28818741 28819258 + 518 antisense
novel transcript - 36 13 6 6
0 0 3.403309332 1.189159291 0.720656548
0.643625933 0 0
ENSG00000188785 391.0251663 298.2433666 296.4353707
391.5960911 410.9370581 584.0066337 328.5679679
462.1799276 -0.492888207 0.045936403 0.202168552 ZNF548
19 57389850 57402992 + 6161
protein_coding zinc finger protein 548 [Source:HGNC
Symbol;Acc:HGNC:26561] zf-C2H2 431 337 255 378 360
677 3.425742731 2.591821767 2.575110195
3.409201216 3.591688401 5.109383228
ENSG00000133313 1437.992781 1444.31209 1973.910822
1107.450321 1437.138212 1243.925503 1618.738564
1262.838012 0.357729027 0.045944801 0.202168552 CNDP2
18 74495816 74523454 + 11848
protein_coding carnosine dipeptidase 2 [Source:HGNC
Symbol;Acc:HGNC:24437] - 1585 1632 1698 1069 1259
1442 6.551080025 6.526818709 8.916604156
5.013542529 6.531727303 5.65915036
ENSG00000100104 544.3505796 496.4822809 562.6459585
594.6459161 722.5643272 774.6498627 534.4929397
697.286702 -0.384484971 0.045967744 0.202219205 SRRD
22 26483877 26494658 + 4319
protein_coding SRR1 domain containing [Source:HGNC
Symbol;Acc:HGNC:33910] - 600 561 484 574 633
898 6.802941398 6.154688793 6.972186638
7.384833907 9.008819099 9.667721937

ENSG00000138686 434.5732127	367.2729885	424.3094522
361.5530047	227.1568738	280.3576897	408.7185512
289.6891894	0.495659437	0.046042259	0.202496647	BBS7
	4	121824440	121870497	-	4829
protein_coding	Bardet-Biedl syndrome 7 [Source:HGNC
Symbol;Acc:HGNC:18758]	-	479	415	365	349	199
	325	4.857434931	4.072089663	4.70264836
4.01587573	2.533046902	3.129371865
ENSG00000164188 150.6036604	50.44472373	77.88694054
71.48182615	29.67878753	6.901112362	92.97844154
36.02057535	1.373270336	0.046110461	0.202717056
RANBP3L 5	36248434	36302114	-	3363
protein_coding	RAN binding protein 3 like [Source:HGNC
Symbol;Acc:HGNC:26353]	-	166	57	67	69	26
	8	2.417185076	0.803108882	1.239523549
1.140077217	0.475219049	0.110609935
ENSG00000171227 18.14501932	15.0449176	12.78740815	0
	5.707459141	0	15.32578169	1.90248638
3.063277897	0.046115294	0.202717056	TMEM37	2
119429901	119438520	+	2292	protein_coding
transmembrane protein 37 [Source:HGNC Symbol;Acc:HGNC:18216]	-
	20	17	11	0	5	0	0.427310993
	0.351447735	0.298596641	0	0.134091964	0
ENSG00000143195 731.2442786	487.6323294	480.1090514
542.8474913	317.3347282	309.6874173	566.3285531
389.9565456	0.539284232	0.046137911	0.20276609	ILDR2
	1	166895711	166975482	-	13884
protein_coding	immunoglobulin like domain containing receptor 2
[Source:HGNC Symbol;Acc:HGNC:18131]	-	806	551	413
	524	278	359	2.842818417	1.88045712
1.850726712	2.097146286	1.23077184	1.202294504
ENSG00000152465 416.4281934	417.7177123	487.0840013
339.7976663	332.174122	309.6874173	440.409969
327.2197352	0.428132584	0.046172147	0.202866148	NMT2
	10	15102584	15168693	-	4700
protein_coding	N-myristoyltransferase 2 [Source:HGNC
Symbol;Acc:HGNC:7858]	-	459	472	419	328	291
	359	4.78237372	4.758505565	5.546551013
3.877823422	3.805769587	3.551629125
ENSG00000123179 877.3116841	1061.994184	739.3446893
1126.097754	1285.319799	1048.10644	892.8835191
1153.174664	-0.367542497	0.046233346	0.203035982	EBPL
	13	49660723	49691475	-	1500
protein_coding	EBP like [Source:HGNC Symbol;Acc:HGNC:18061]	-
	967	1200	636	1087	1126	1215	31.56922341
	37.90673924	26.37987317	40.26709772	46.14179567
	37.66309771
ENSG00000156239 357.4568806	449.5775378	305.7353039
469.2937282	488.5585025	564.1659356	370.9232408
507.3393888	-0.450721723	0.04624293	0.203035982	N6AMT1

21	28872191	28885371	-	4865
protein_coding	N-6 adenine-specific DNA methyltransferase 1
[Source:HGNC Symbol;Acc:HGNC:16021]	-	394	508	263
453	428	654	3.965902753	4.947744999
3.363409531	5.174011636	5.407646385	6.250660654
ENSG00000133243 3081.02428	3317.846829	3098.040247
3438.379435	3593.416275	4601.316668	3165.637119
3877.704126	-0.292963554	0.046245236	0.203035982	BTBD2
19	1985438 2034881 -	5944	protein_coding	BTB
domain containing 2 [Source:HGNC Symbol;Acc:HGNC:15504] -	3396
3749	2665	3319	3148	5334	27.97805789
29.88567577	27.89492893	31.0270507	32.55391934
41.72585348
ENSG00000111731 847.3724022	577.0168398	640.5328991
503.4806885	535.3596674	515.8581491	688.3073804
518.232835	0.409904204	0.046291375	0.203104429	C2CD5
12	22448583	22544546	-	6759
protein_coding	C2 calcium dependent domain containing 5 [Source:HGNC
Symbol;Acc:HGNC:29062]	-	934	652	551	486	469
598	6.766952271	4.570793384	5.071962093
3.99545153	4.265183854	4.113862346
ENSG00000174238 2367.925021	2510.731249	2405.195224
2806.438653	3126.546117	2788.912033	2427.950498
2907.298934	-0.259686346	0.04629164	0.203104429	PITPNA
17	1517718 1562816 -	4784	protein_coding
phosphatidylinositol transfer protein alpha [Source:HGNC
Symbol;Acc:HGNC:9001]	-	2610	2837	2069	2709	2739
3233	26.71640509	28.09924207	26.90767455
31.46515516	35.19234621	31.42284852
ENSG00000156531 2541.209956	2145.228251	2268.021209
2202.46902	1755.614432	1622.624044	2318.153139
1860.235832	0.317763226	0.046295272	0.203104429	PHF6
X	134373253	134428791	+	8016
protein_coding	PHD finger protein 6 [Source:HGNC
Symbol;Acc:HGNC:18145]	-	2801	2424	1951	2126	1538
1881	17.11134249	14.32852045	15.14280771
14.73728884	11.79358896	10.91093843
ENSG00000204592 3520.133748	4091.332593	4937.102037
4258.866483	6003.105524	5522.615168	4182.856126
5261.529058	-0.33123313	0.046344776	0.203228487	HLA-E
6	30489467	30494205	+	2975
protein_coding	"major histocompatibility complex, class I, E
[Source:HGNC Symbol;Acc:HGNC:4962]"	-	3880	4623	4247
4111	5259	6402	63.86654741	73.6314519
88.81823478	76.78432139	108.6584639	100.0598201
ENSG00000141391 150.6036604	179.6540161	179.0237141
62.15810969	115.2906746	127.6705787	169.7604635
101.7064543	0.737272939	0.046346527	0.203228487
PRELID3A	18	12407896	12432238	+	2643
protein_coding	PRELI domain containing 3A [Source:HGNC

Symbol;Acc:HGNC:24639]	-	166	203	154	60	101
	148	3.075669093	3.639362498	3.62518692
1.261438643	2.348938092	2.603727742
ENSG00000273137 12.70151352	17.69990306	11.6249165
17.61146441	46.80116496	55.2088989	14.0087777
39.87384276	-1.506791158	0.046366068	0.203263788
AL022328.2	22	50208461	50209542	-	640
	antisense	uncharacterized LOC105373095 [Source:NCBI
gene;Acc:105373095]	-	14	20	10	17	41
	64	1.071215246	1.480732002	0.972135656
1.475981762	3.937778133	4.64976515
ENSG00000166479 2976.690419	2415.151773	2690.005678
2474.928734	2177.966408	1879.69048	2693.94929
2177.528541	0.30731637	0.046398161	0.203354081	TMX3
	18	68673688	68715298	-	7637
protein_coding	thioredoxin related transmembrane protein 3
[Source:HGNC Symbol;Acc:HGNC:24718]	-	3281	2729	2314
	2389	1908	2179	21.03837168	16.93195813
18.85156503	17.38222642	15.35687821	13.26677787
ENSG00000101138 1320.050155	1283.242972	1479.85187
1530.125467	1586.673641	1976.306053	1361.048333
1697.70172	-0.319850844	0.046445982	0.203513246	CSTF1
	20	56392371	56406369	+	4800
protein_coding	cleavage stimulation factor subunit 1 [Source:HGNC
Symbol;Acc:HGNC:2483]	-	1455	1450	1273	1477	1390
	2291	14.8439827	14.31374268	16.50038254
17.09823578	17.80003774	22.19294158
ENSG00000134504 380.1381547	305.3233278	421.9844689
199.9419195	272.8165469	299.3357487	369.1486505
257.3647384	0.518459105	0.046471339	0.203516152	KCTD1
	18	26454910	26657401	-	8882
protein_coding	potassium channel tetramerization domain containing 1
[Source:HGNC Symbol;Acc:HGNC:18249]	-	419	345	363
	193	239	347	2.310106157	1.84049553
2.542744378	1.207420607	1.653996142	1.816559904
ENSG00000241015 63.50756762	84.9595347	36.03724115
121.2083139	114.1491828	105.2419635	61.50144782
113.5331534	-0.877887261	0.046480331	0.203516152	TPM3P9
	19	53431984	53444670	+	2920
transcribed_processed_pseudogene	tropomyosin 3 pseudogene 9
[Source:HGNC Symbol;Acc:HGNC:44142]	-	70	96	31
	117	100	122	1.173934517	1.557811202
0.660519569	2.226460804	2.105060145	1.942710097
ENSG00000162636 2216.41411	1500.95178	1943.686039
1783.937748	1353.809308	1209.419941	1887.017309
1449.055666	0.381149834	0.046481161	0.203516152
FAM102B 1	108560089	108644900	+	9355
protein_coding	family with sequence similarity 102 member B
[Source:HGNC Symbol;Acc:HGNC:27637]	-	2443	1696	1672
	1722	1186	1402	12.78816876	8.590303281

11.11986022	10.22825152	7.792705385	6.968434784
ENSG00000181744 744.8530431	791.1856669	617.2830661
891.9688741	970.2680539	886.7929385	717.7739254
916.3432889	-0.350958808	0.046591609	0.203949262
C3orf58 3	143971798	144048719	+	5487
protein_coding	chromosome 3 open reading frame 58 [Source:HGNC
Symbol;Acc:HGNC:28490]	-	821	894	531	861	850
1028	7.327180336	7.720207965	6.020969226
8.719272183	9.522069034	8.711424411
ENSG00000145354 1408.96075	1368.202507	1298.503173
1156.14084	1108.388565	1059.320748	1358.555477
1107.950051	0.294647462	0.046619198	0.203990158	CISD2
4	102868974	102892807	+	6352
protein_coding	CDGSH iron sulfur domain 2 [Source:HGNC
Symbol;Acc:HGNC:24212]	-	1553	1546	1117	1116	971
1228	11.97263244	11.53255252	10.94081137
9.762605696	9.396283263	8.989155548
ENSG00000173226 706.7485025	661.0913794	517.3087842
742.7894108	897.2125769	820.3697321	628.3828887
820.1239066	-0.382670075	0.046624015	0.203990158	IQCB1
3	121769763	121835079	-	3049
protein_coding	IQ motif containing B1 [Source:HGNC
Symbol;Acc:HGNC:28949]	-	779	747	445	717	786
951	12.51148089	11.60886119	9.08049311
13.06693633	15.8457384	14.50287523
ENSG00000141452 605.1363943	631.0015442	538.2336339
878.5012837	636.9524401	804.8422292	591.4571908
773.4319843	-0.386866962	0.046668933	0.204136191	RMC1
18	23503470	23531807	+	3090
protein_coding	regulator of MON1-CCZ1 [Source:HGNC
Symbol;Acc:HGNC:24326]	-	667	713	463	848	558
933	10.57051235	10.93345675	9.322434876
15.24928273	11.10000258	14.03958215
ENSG00000234918 1.814501932	2.654985459	13.9498998	0
0	0	6.13979573	0	5.044135503
0.046708264	1	AL157387.1	10	37240887
37242049	+	335	lincRNA novel transcript	-
2	3	12	0	0	0	0.292357253
0.424329171	2.228657266	0	0	0
ENSG00000197483 430.0369579	438.0726008	490.5714763
469.2937282	633.5279646	734.9684666	452.8936783
612.5967198	-0.43713578	0.046769194	0.20452417	ZNF628
19	55476332	55484487	+	3851
protein_coding	zinc finger protein 628 [Source:HGNC
Symbol;Acc:HGNC:28054]	zf-C2H2 474	495	422	453	555
852	6.027448476	6.090572555	6.817823891
6.53637149	8.858635342	10.28719789
ENSG00000181826 104.3338611	138.944239	113.9241817
72.51779464	57.07459141	77.63751407	119.0674273
69.07663338	0.783653527	0.046806072	0.204634852	RELL1

4	37590800	37686376	-	1947
protein_coding	RELT like 1 [Source:HGNC Symbol;Acc:HGNC:27379] -
115	157	98	70	50	90	2.892414782
3.820851349	3.131604949	1.997763767	1.578524813
2.149352149
ENSG00000267583 0	10.61994184	10.46242485	0	0
0	7.027455562	0	5.244181981	0.046815284
1	AC007998.3	18	35443869	35467088
-	916	lincRNA novel transcript	-	0
12	9	0	0	0	0	0.620743547
0.611299277	0	0	0
ENSG00000272953 6.350756762	5.309970919	5.812458249	0
0	0	5.82439531	0	4.975155751
0.046817765	1	AC092171.4	7	5425770 5426401 +
632	lincRNA novel transcript	-	7	6
5	0	0	0	0.542387466	0.449842633
0.492220585	0	0	0
ENSG00000167723 109.7773669	89.38451047	39.5247161
185.4383606	168.9407906	92.30237784	79.56219781
148.893843	-0.89709127	0.046831258	0.204694373	TRPV3
17	3510502 3557995 -	9780	protein_coding
transient receptor potential cation channel subfamily V member 3
[Source:HGNC Symbol;Acc:HGNC:18084]	-	121	101	34
179	148	107	0.605864072	0.489338019
0.216295214	1.017012334	0.930186904	0.508716637
ENSG00000189299 227.7199925	192.0439482	131.3615564
408.171587	208.8930046	251.8906012	183.708499
289.6517309	-0.654623134	0.046870719	0.204783207	FOXR2
X	55623400	55625325	+	1926
protein_coding	forkhead box R2 [Source:HGNC Symbol;Acc:HGNC:30469]
Fork	251	217	113	394	183	292
6.381843093	5.338630852	3.650303773	11.36716025
5.840394283	7.049488182
ENSG00000101158 3100.076551	3146.157769	3424.700401
2338.180893	2918.794605	2724.214105	3223.644907
2660.396534	0.276953636	0.046874736	0.204783207	NELFCD
20	58981208	58995133	+	6298
protein_coding	negative elongation factor complex member C/D
[Source:HGNC Symbol;Acc:HGNC:15934]	-	3417	3555	2946
2257	2557	3158	26.56874289	26.74628017
29.10294462	19.91319499	24.95602614	23.31527206
ENSG00000175536 19.95952125	32.74482067	60.44956579
70.44585765	66.20652603	103.5166854	37.71796924
80.05635637	-1.096533809	0.046888013	0.204790632	LIPT2
11	74491712	74493733	-	1187
protein_coding	lipoyl(octanoyl) transferase 2 [Source:HGNC
Symbol;Acc:HGNC:37216]	-	22	37	52	68	58
120	0.90761287	1.476989629	2.725583373
3.183246259	3.003479242	4.700689284
ENSG00000040633 1352.71119	1614.231159	1614.700902

1493.86657	2118.608833	2241.998879	1527.214417
1951.491427	-0.354039493	0.046917314	0.204868025	PHF23
	17	7235028 7239722 -	2783	protein_coding	PHD
finger protein 23 [Source:HGNC Symbol;Acc:HGNC:28428]	-	1491
	1824	1389	1442	1856	2599	26.23572806
31.05546729	31.05245106	28.79148714	40.99322873
43.42342661
ENSG00000125817 3411.263632	4187.797065	3780.422845
3760.565636	4490.628852	6293.814474	3793.161181
4848.336321	-0.354329475	0.046940512	0.204885499	CENPB
	20	3783851 3786690 -	2840	protein_coding
centromere protein B [Source:HGNC Symbol;Acc:HGNC:1852] -	3760
	4732	3252	3630	3934	7296	64.83330907
78.95012769	71.24248235	71.02321517	85.14582851
119.4531216
ENSG00000176974 1116.825939	1062.879179	1177.604041
1121.95388	765.9410167	614.1990002	1119.103053
834.031299	0.424630821	0.046944481	0.204885499	SHMT1
	17	18327860	18363563	-	7281
protein_coding	serine hydroxymethyltransferase 1 [Source:HGNC
Symbol;Acc:HGNC:10850]	-	1231	1201	1013	1083	671
	712	8.279339766	7.815889616	8.656159713
8.26512582	5.664725233	4.546947929
ENSG00000170027 8630.678439	9156.159854	9460.357047
9458.392358	11096.44206	12015.69926	9082.398447
10856.84456	-0.257564464	0.047043002	0.205264842	YWHAG
	7	76326794	76359031	-	3750
protein_coding	tyrosine 3-monooxygenase/tryptophan 5-monooxygenase
activation protein gamma [Source:HGNC Symbol;Acc:HGNC:12852]	-
	9513	10346	8138	9130	9721	13929	124.2266897
	130.7277081	135.0184955	135.2855942	159.3408155
	172.7108767
ENSG00000100024 3.629003864	10.61994184	3.48747495	0
	0	0	5.912140217	0	4.999471804
0.047077133	1	UPB1	22	24494107	24528390
	+	6793	protein_coding	beta-ureidopropionase 1
[Source:HGNC Symbol;Acc:HGNC:16297]	-	4	12	3
	0	0	0	0.028835472	0.083703973
0.027476821	0	0	0
ENSG00000197872 80.74533597	85.84452985	115.0866733
73.55376314	30.82027936	37.95611799	93.89217972
47.44338683	0.982958982	0.047084222	0.205394033	FAM49A
	2	16549459	16666331	-	5448
protein_coding	family with sequence similarity 49 member A
[Source:HGNC Symbol;Acc:HGNC:25373]	-	89	97	99
	71	27	44	0.799984535	0.843647602
1.130589486	0.724157934	0.30463095	0.375531693
ENSG00000184900 4539.883834	4697.554273	4860.377588
4182.204814	3981.523497	3754.205125	4699.271898
3972.644479	0.24236416	0.047099678	0.2054108	SUMO3

21	44805617	44818779	-	2732
protein_coding	small ubiquitin-like modifier 3 [Source:HGNC
Symbol;Acc:HGNC:11124]	-	5004	5308	4181	4037	3488
4352	89.69439184	92.06120603	95.21520771
82.1088743	78.47713532	74.06946535
ENSG00000144290 3.629003864	1.769990306	12.78740815	0
0	0	6.062134106	0	5.026481773
0.047126718	1	SLC4A10 2	161424332	161985282
+	7550	protein_coding	solute carrier family 4 member
10 [Source:HGNC Symbol;Acc:HGNC:13811]	-	4	2	11
0	0	0	0.025944286	0.0125519
0.090646821	0	0	0
ENSG00000168280 359.2713825	236.2937059	238.3107882
244.4885648	140.4034949	154.4123891	277.9586255
179.7681496	0.629594181	0.04713919	0.205450453	KIF5C
2	148875250	149026759	+	9215
protein_coding	kinesin family member 5C [Source:HGNC
Symbol;Acc:HGNC:6325]	-	396	267	205	236	123
179	2.104401147	1.37291093	1.384093306
1.423078008	0.82045947	0.903209942
ENSG00000118503 310.2798304	418.6027074	415.009519
416.459335	517.0957982	656.4683135	381.2973523
530.0078155	-0.476677375	0.047141825	0.205450453
TNFAIP3 6	137867188	137883312	+	5051
protein_coding	TNF alpha induced protein 3 [Source:HGNC
Symbol;Acc:HGNC:11896]	-	342	473	357	402	453
761	3.315716734	4.43721235	4.397417437
4.422427564	5.512748677	7.005486595
ENSG00000214826 351.1061238	303.5533375	99.97428189
116.0284714	154.1013968	163.9014186	251.5445811
144.6770956	0.800123069	0.047148129	0.205450453	DDX12P
12	9418673 9448229 -	2947	unprocessed_pseudogene
"DEAD/H-box helicase 12, pseudogene [Source:HGNC
Symbol;Acc:HGNC:2737]"	-	387	343	86	112	135
190	6.430718702	5.514935341	1.815620853
2.111786117	2.815794737	2.997812618
ENSG00000203705 199.5952125	175.2290403	194.1361055
250.7043758	269.3920714	293.2972754	189.6534528
271.1312409	-0.516571874	0.047172932	0.205450453	TATDN3
1	212791828	212816626	+	3788
protein_coding	TatD DNase domain containing 3 [Source:HGNC
Symbol;Acc:HGNC:27010]	-	220	198	167	242	236
340	2.844077287	2.476747086	2.742921303
3.54991079	3.829564537	4.173495647
ENSG00000196943 556.1448421	661.9763745	664.9452237
682.7032381	723.7058191	1155.073682	627.6888135
853.8275796	-0.445392747	0.047174634	0.205450453	NOP9
14	24299862	24309124	+	6033
protein_coding	NOP9 nucleolar protein [Source:HGNC
Symbol;Acc:HGNC:19826]	-	613	748	572	659	634

        1339   4.975718849   5.874822011   5.898879845
6.069656768   6.459565298   10.31996608
ENSG00000172409 501.7097842   708.8811176   634.7204408
798.7317096   690.602556   960.1172574   615.1037809
816.483841   -0.40961561   0.047185786   0.205450453   CLP1
        11   57648992   57661868   +   2793
protein_coding cleavage and polyadenylation factor I subunit 1
[Source:HGNC Symbol;Acc:HGNC:16999]   -   553   801   546
        771   605   1113   9.695782824   13.58901635
12.16265964   15.33894485   13.31471268   18.52913932
ENSG00000186395 637.7974291   868.1802452   768.4069806
519.0202159   638.0939319   579.6934384   758.1282183
578.9358621   0.389344483   0.047190072   0.205450453   KRT10
        17   40818117   40822595   -   2144
protein_coding keratin 10 [Source:HGNC Symbol;Acc:HGNC:6413]   -
        703   981   661   501   559   672   16.05680849
        21.68056856   19.18154235   12.98449276   16.02634129
        14.57389077
ENSG00000251495 12.70151352   20.35488852   18.5998664
4.14387398   2.282983656   4.313195226   17.21875615
3.580017621   2.256028851   0.047223275   0.205544418
PPIAP11 5   82009602   82010094   -   493
processed_pseudogene   peptidylprolyl isomerase A pseudogene 11
[Source:HGNC Symbol;Acc:HGNC:9263]   -   14   23   16
        4   2   5   1.390624255   2.210585706
2.019202661   0.450842776   0.249362094   0.471578616
ENSG00000107816 1142.228966   1103.588956   1351.977789
949.9831098   741.9696883   1089.513114   1199.265237
927.1553041   0.369657462   0.047315654   0.205895842   LZTS2
        10   100996618   101007836   +   6547
protein_coding leucine zipper tumor suppressor 2 [Source:HGNC
Symbol;Acc:HGNC:29381] -   1259   1247   1163   917   650
        1263   9.416989209   9.025069467   11.0520851
7.782856776   6.102648777   8.969991423
ENSG00000167977 1032.451599   1128.36882   1003.230294
1040.112369   1296.734717   1850.360752   1054.683571
1395.735946   -0.404742012   0.047363507   0.20605339   KCTD5
        16   2682475 2709030 +   3420   protein_coding
potassium channel tetramerization domain containing 5 [Source:HGNC
Symbol;Acc:HGNC:21423] -   1138   1275   863   1004   1136
        2145   16.29464261   17.664873   15.6997066
16.31246716   20.41735996   29.16300072
ENSG00000138449 9.07250966   15.0449176   8.137441549   0
        0   2.587917136   10.75162294   0.862639045
3.557707015   0.047391874   0.206126102   SLC40A1 2
189560579   189583758   -   4330   protein_coding solute
carrier family 40 member 1 [Source:HGNC Symbol;Acc:HGNC:10909] -
        10   17   7   0   0   3   0.113094318
        0.186031919   0.100581241   0   0   0.032215463
ENSG00000258458 8.165258694   3.539980612   5.812458249   0

0	0	5.839232519	0	4.978685301
0.047395402	1	AL160314.2	14	22701476
22766562	-	2513	processed_transcript	"novel
transcript, antisense to OXA1L"	-	9	4	5	0
0	0	0.17537945	0.075421288	0.123789658
0	0	0
ENSG00000237276 4.53625483	7.079961225	5.812458249	0
0	0	5.809558101	0	4.971618688
0.047439019	1	ANO7L1	1	16215909	16228027
-	1990	transcribed_unprocessed_pseudogene
anoctamin 7 like 1 [Source:HGNC Symbol;Acc:HGNC:32248]	-	5
8	5	0	0	0	0.123039799
0.190486127	0.156323322	0	0	0
ENSG00000108523 1711.982573	1886.809666	1712.3502
1860.599417	2285.26664	2388.647516	1770.380813
2178.171191	-0.299052873	0.047458985	0.206258222	RNF167
17	4940008 4945222 +	2744	protein_coding	ring
finger protein 167 [Source:HGNC Symbol;Acc:HGNC:24544]	-	1887
2132	1473	1796	2002	2769	33.67568796
36.81540091	33.39838651	36.36924361	44.84637316
46.92128171
ENSG00000149257 7356.898083	7846.367028	9628.918336
6445.795975	7089.805745	7038.27197	8277.394482
6857.957897	0.27120781	0.047460282	0.206258222
SERPINH1	11	75562056	75572783	+	6281
protein_coding	serpin family H member 1 [Source:HGNC
Symbol;Acc:HGNC:1546]	-	8109	8866	8283	6222	6211
8159	63.22184862	66.88448299	82.04756839
55.04441456	60.78271515	60.40030864
ENSG00000260528 1.814501932	0	0	1.035968495	0
36.2308399	0.604833977	12.42226947	-4.326109898
0.047469484	0.206258222	FAM157C 16	90102271
90178344	+	4524	lincRNA family with sequence
similarity 157 member C [Source:HGNC Symbol;Acc:HGNC:34081]	-
2	0	0	1	0	42	0.021648912
0	0	0.012282576	0	0.43167581
ENSG00000196358 282.1550504	153.9891566	168.5612892
158.5031797	86.75337894	119.9068273	201.5684988
121.7211287	0.727505288	0.04747233	0.206258222	NTNG2
9	132161676	132244534	+	7845
protein_coding	netrin G2 [Source:HGNC Symbol;Acc:HGNC:14288]	-
311	174	145	153	76	139	1.941315511
1.050951662	1.149957794	1.083703626	0.595481131
0.823858962
ENSG00000172986 368.3438922	266.3835411	353.3974616
290.0711786	215.7419555	156.1376672	329.3749649
220.6502671	0.579130386	0.047480552	0.206258222	GXYLT2
3	72888073	72998138	+	3667
protein_coding	glucoside xylosyltransferase 2 [Source:HGNC
Symbol;Acc:HGNC:33383]	-	406	301	304	280	189

181	5.421803919	3.889394775	5.157859648
4.242864526	3.168095426	2.295084516
ENSG00000163138 441.8312204	447.8075475	412.6845357
626.7609394	697.451507	444.2591083	434.1077679
589.4905182	-0.439563041	0.047544072	0.206483451	PACRGL
4	20696282	20752907	+	4032
protein_coding	parkin coregulated like [Source:HGNC
Symbol;Acc:HGNC:28442]	-	487	506	355	605	611
515	5.91475992	5.94643169	5.47790727
8.337712099	9.314682304	5.939060149
ENSG00000248664 2.721752898	0	1.16249165	5.179842475
6.848950969	24.15389327	1.294748183	12.06089557
-3.224876303	0.047565292	0.2065249	AC010273.1
5	69113112	69136394	-	946
antisense	novel transcript	-	3	0	1
5	6	28	0.155295475	0	0.065768163
0.293691185	0.389858919	1.376251841
ENSG00000243746 0	0	0	5.179842475	0
15.52750281	0	6.90244843	-5.268269941	0.047574522
1	EEF1A1P10	7	144647186	144648575
-	1390	processed_pseudogene	eukaryotic translation
elongation factor 1 alpha 1 pseudogene 10 [Source:HGNC
Symbol;Acc:HGNC:3205]	-	0	0	0	5	0
18	0	0	0	0.199879037	0
0.602127861
ENSG00000198093 149.6964094	143.3692148	185.998664
256.9201867	238.5717921	212.2092051	159.6880961
235.9003947	-0.564487062	0.047584717	0.206558542	ZNF649
19	51889224	51905040	-	4365
protein_coding	zinc finger protein 649 [Source:HGNC
Symbol;Acc:HGNC:25741]	zf-C2H2 165	162	160	248	209
246	1.851093602	1.758560068	2.280565663
3.15703558	2.943129679	2.620486201
ENSG00000184500 917.2307266	850.4803421	704.4699398
814.271237	526.2277328	463.2371673	824.0603362
601.245379	0.455934355	0.047696271	0.206980341	PROS1
3	93873033	93980003	-	5444
protein_coding	protein S [Source:HGNC Symbol;Acc:HGNC:9456]	-
1011	961	606	786	461	537	9.09414182
8.364340653	6.925662986	8.022624642	5.205113082
4.586561142
ENSG00000267871 12.70151352	30.08983521	29.06229125	0
9.131934625	6.038473317	23.95121333	5.056802647
2.247198869	0.047707292	0.206980341	ZNF460-AS1
19	57267220	57280334	-	738
antisense	ZNF460 antisense RNA 1 [Source:HGNC
Symbol;Acc:HGNC:53774]	-	14	34	25	0	8
7	0.928967151	2.182976176	2.107611179	0
0.666317141	0.441034635
ENSG00000135047 738.5022863	848.7103518	1059.029893

708.6024505 635.8109483 690.9738753 882.0808437
678.4624247 0.377289765 0.04771699 0.206980341 CTSL
9 87725519 87731393 + 2366
protein_coding cathepsin L [Source:HGNC Symbol;Acc:HGNC:2537] -
814 959 911 684 557 801 16.84761184
19.20570738 23.95578922 16.06398926 14.47064253
15.74159715
ENSG00000267731 9.07250966 1.769990306 6.974949899 0
0 0 5.939149955 0 5.002015236
0.047772605 1 AC005332.2 17 68189884
68192802 + 2919 lincRNA novel transcript -
10 2 6 0 0 0 0.167762384
0.032465518 0.127886294 0 0 0
ENSG00000001630 116.1281236 107.0844135 217.3859385
106.704755 83.32890346 15.52750281 146.8661586
68.52038708 1.102863394 0.047823929 0.207393351
CYP51A1 7 92112151 92134803 - 3584
protein_coding cytochrome P450 family 51 subfamily A member 1
[Source:HGNC Symbol;Acc:HGNC:2649] - 128 121 187
103 73 18 1.748922851 1.599719395
3.24623871 1.59691304 1.251993919 0.233526152
ENSG00000175643 269.4535369 390.2828625 351.0724783
297.322958 189.4876435 201.8575366 336.9362926
229.556046 0.553553798 0.047836141 0.207395465 RMI2
16 11249619 11381662 + 3632
protein_coding RecQ mediated genome instability 2 [Source:HGNC
Symbol;Acc:HGNC:28349] - 297 441 302 287 166
234 4.004416969 5.753328747 5.173303405
4.39084494 2.809374321 2.995718736
ENSG00000076321 655.9424484 675.2513018 577.75835
865.0336932 1018.210711 648.704562 636.3173667
843.9829887 -0.405680858 0.047859054 0.207411153 KLHL20
1 173714941 173786702 + 4254
protein_coding kelch like family member 20 [Source:HGNC
Symbol;Acc:HGNC:25056] - 723 763 497 835 892
752 8.322800705 8.498719453 7.268850718
10.90689252 12.88886661 8.219613053
ENSG00000205268 558.866595 515.0671791 519.6337675
457.8980747 413.2200418 307.0995001 531.1891806
392.7392056 0.437282402 0.047869377 0.207411153 PDE7A
8 65717510 65842322 - 7425
protein_coding phosphodiesterase 7A [Source:HGNC
Symbol;Acc:HGNC:8791] - 616 582 447 442 362
356 4.062683008 3.714094653 3.745569947
3.307789429 2.996811818 2.229382348
ENSG00000204227 958.964271 1236.338229 1163.654142
1205.867328 1518.184131 1530.321666 1119.652214
1418.124375 -0.341187384 0.047874969 0.207411153 RING1
6 33208495 33212722 + 1818
protein_coding ring finger protein 1 [Source:HGNC

Symbol;Acc:HGNC:10018]	-	1057	1397	1001	1164	1330
	1774	28.47146353	36.41069494	34.25681996
35.5771492	44.96816049	45.3722958
ENSG00000166337 219.5547338	322.1382357	231.3358383
103.5968495	219.166431	167.3519748	257.6762693
163.3717518	0.659285365	0.047895094	0.207411153	TAF10
	11	6606296 6612667 -	6103	protein_coding	TATA-
box binding protein associated factor 10 [Source:HGNC
Symbol;Acc:HGNC:11543]	-	242	364	199	100	192
	194	1.941782933	2.826080019	2.028694039
0.910476359	1.933771783	1.478050859
ENSG00000087301 118.8498765	72.56960256	156.9363727
95.30910153	28.5372957	48.30778654	116.1186173
57.38472792	1.013600131	0.047905957	0.207411153
TXNDC16 14	52430590	52552522	-	4739
protein_coding	thioredoxin domain containing 16 [Source:HGNC
Symbol;Acc:HGNC:19965]	-	131	82	135	92	25
	56	1.353671453	0.819886215	1.772368025
1.078731007	0.324265437	0.549455261
ENSG00000140451 308.4653284	287.6234248	261.5606212
68.37392066	35.38624667	0	285.8831248	34.58672245
	3.048562085	0.047918039	0.207411153	PIF1	15
	64815632	64825668	-	4656	protein_coding
	PIF1 5'-to-3' DNA helicase [Source:HGNC Symbol;Acc:HGNC:26220]
	-	340	325	225	66	31	0
3.575976276	3.30747698	3.006605123	0.787667647
0.409256968	0
ENSG00000135116 8.165258694	0	0	5.179842475
7.990442797	50.89570367	2.721752898
21.35532965	-2.954473179	0.047921837	0.207411153	HRK
	12	116856144	116881441	-	6853
protein_coding	"harakiri, BCL2 interacting protein [Source:HGNC
Symbol;Acc:HGNC:5185]"	-	9	0	0	5	7
	59	0.06431177	0	0	0.04054164
0.062786268	0.4003154
ENSG00000244754 1499.685847	1587.681305	1272.928357
1805.693087	1834.377368	1716.6517	1453.431836
1785.574052	-0.296078052	0.04796485	0.207546535
N4BP2L2 13	32432417	32538885	-	16960
protein_coding	NEDD4 binding protein 2 like 2 [Source:HGNC
Symbol;Acc:HGNC:26916]	-	1653	1794	1095	1743	1607
	1990	4.77282696	5.012138134	4.016937901
5.710624219	5.824214873	5.455797552
ENSG00000229334 4.53625483	15.92991276	19.76235805
4.14387398	0	0	13.40950854	1.381291327
3.288721285	0.048021789	0.207742094	AC046143.1	3
	194632923	194645401	+	972	antisense
	novel transcript	-	5	18	17	4
	0	0	0.251902468	0.877470816	1.088151846
	0.228668198	0	0

ENSG00000172572 103.4266101 46.01974796 161.5863393
87.02135357 19.40536108 4.313195226 103.6775658
36.91330329 1.491364086 0.04810551 0.208053392 PDE3A
12 20369245 20684381 + 8398
protein_coding phosphodiesterase 3A [Source:HGNC
Symbol;Acc:HGNC:8778] - 114 52 139 84 17
5 0.664748957 0.293395815 1.029783139
0.555796054 0.124428656 0.027683765
ENSG00000225362 0 0 0 0 13.69790194
6.901112362 0 6.8663381 -5.253599313 0.048107003
1 CT62 15 71110244 71115500 -
2855 protein_coding cancer/testis antigen 62 [Source:HGNC
Symbol;Acc:HGNC:27286] - 0 0 0 0 12
8 0 0 0 0 0.258358345
0.130291143
ENSG00000155093 727.6152747 682.3312631 631.2329659
632.9767504 465.7286659 438.220635 680.3931679
512.3086838 0.410250243 0.048123502 0.208080329 PTPRN2
7 157539056 158587788 - 4833
protein_coding "protein tyrosine phosphatase, receptor type N2
[Source:HGNC Symbol;Acc:HGNC:9677]" - 802 771 543
611 408 508 8.126176608 7.558994402
6.990204496 7.024840351 5.189084324 4.887400571
ENSG00000046651 444.5529733 380.5479158 326.6601536
544.9194283 431.483911 592.6330241 383.9203476
523.0121212 -0.445815363 0.048147904 0.208134962 OFD1
X 13734745 13769353 + 5172
protein_coding "OFD1, centriole and centriolar satellite protein
[Source:HGNC Symbol;Acc:HGNC:2567]" - 490 430 281
526 378 687 4.639447316 3.939457143
3.380295368 5.651181705 4.492423018 6.176312177
ENSG00000170889 11984.78526 16443.20994 17790.77221
16432.53227 20058.2944 20979.38158 15406.25581
19156.73608 -0.31441649 0.048181923 0.208231134 RPS9
19 54200742 54249003 + 4371
protein_coding ribosomal protein S9 [Source:HGNC
Symbol;Acc:HGNC:10442] - 13210 18580 15304 15862 17572
24320 147.9962444 201.4147836 217.8366739
201.6458009 247.1085363 258.7103373
ENSG00000233509 3.629003864 7.964956378 5.812458249 0
0 0 5.802139497 0 4.969845596
0.048204494 1 ZNF197-AS1 3 44617128
44624797 - 623 antisense ZNF197 antisense RNA 1
[Source:HGNC Symbol;Acc:HGNC:40620] - 4 9 5
0 0 0 0.314413097 0.684511744
0.499331316 0 0 0
ENSG00000210107 19.95952125 14.15992245 0 41.4387398
29.67878753 51.75834272 11.3731479 40.95862335
-1.833591983 0.0482079 0.208292513 MT-TQ MT
4329 4400 - 72 Mt_tRNA mitochondrially

encoded tRNA glutamine [Source:HGNC Symbol;Acc:HGNC:7495]	-
	22	16	0	40	26	60	14.96300662
	10.52964979	0	30.87020678	22.19668975
38.74804292
ENSG00000156973 410.0774366	401.7877995	458.0217101
482.7613186	585.5853079	609.023166	423.2956487
559.1232641	-0.402633764	0.048294512	0.208606384	PDE6D
	2	231732425	231786272	-	2850
protein_coding	phosphodiesterase 6D [Source:HGNC
Symbol;Acc:HGNC:8788]	-	452	454	394	466	513
	706	7.766444773	7.548096323	8.601183407
9.085589279	11.06419612	11.5183656
ENSG00000171148 3048.363246	3384.221466	3339.83851
3374.149388	4302.2827	4175.172979	3257.474407
3950.535022	-0.278355979	0.048309286	0.208606384	TADA3
	3	9779860 9793011 -	4234	protein_coding
transcriptional adaptor 3 [Source:HGNC Symbol;Acc:HGNC:19422]	-
	3360	3824	2873	3257	3769	4840	38.86128055
	42.79504336	42.21741318	42.74437276	54.7170461
	53.15272396
ENSG00000090581 618.7451588	903.5800513	856.756346
620.5451284	602.7076853	590.045107	793.0271854
604.4326402	0.391522978	0.048315923	0.208606384	GNPTG
	16	1351923 1364113 +	4102	protein_coding	N-
acetylglucosamine-1-phosphate transferase subunit gamma [Source:HGNC
Symbol;Acc:HGNC:23026]	-	682	1021	737	599	528
	684	8.141743239	11.79387517	11.17837509
8.114153327	7.911988124	7.753387037
ENSG00000147799 441.8312204	592.0617574	439.4218437
530.4158694	673.4801786	795.3531997	491.1049405
666.4164159	-0.440203691	0.048329492	0.20861405
ARHGAP39	8	144529179	144605816	-	4888
	protein_coding	Rho GTPase activating protein 39 [Source:HGNC
Symbol;Acc:HGNC:29351]	-	487	669	378	512	590
	922	4.8789509	6.485169945	4.811355523
5.820372866	7.41938956	8.770629027
ENSG00000230590 447.2747262	388.5128722	361.5349031
600.861727	420.0689928	609.023166	399.1075005
543.3179619	-0.445352089	0.048356427	0.208679396	FTX
	X	73946555	74293574	-	28487
lincRNA "FTX transcript, XIST regulator [Source:HGNC
Symbol;Acc:HGNC:37190]" -	493	439	311	580	368
	706	0.847478886	0.730204064	0.679235725
1.131340467	0.794051121	1.152362199
ENSG00000196284 194.1517067	176.1140355	189.4861389
330.4739499	283.0899734	209.621288	186.5839604
274.3950704	-0.555271098	0.048399174	0.208812924	SUPT3H
	6	44809317	45377953	-	3194
protein_coding	"SPT3 homolog, SAGA and STAGA complex component
[Source:HGNC Symbol;Acc:HGNC:11466]"	-	214	199	163

319	248	243	3.281009932	2.952192044
3.175115581	5.549678376	4.772699919	3.537548314
ENSG00000272068 79.83808501	114.1643748	76.72444889
202.0138565	86.75337894	185.4673947	90.24230288
158.0782101	-0.80884971	0.048430286	0.208896205
AL365181.2	1	156637783	156641004	-	3222
lincRNA novel transcript	-	88	129	66
195	76	215	1.337475452	1.897101708
1.274457173	3.362955487	1.449891208	3.102729694
ENSG00000135966 1197.571275	1215.098345	938.1307615
1137.493407	1551.287394	1633.838352	1116.933461
1440.873051	-0.366870497	0.048449584	0.208928498
TGFBRAP1	2	105264391	105330034	-	6005
protein_coding	transforming growth factor beta receptor
associated protein 1 [Source:HGNC Symbol;Acc:HGNC:16836]	-
1320	1373	807	1098	1359	1894	10.76439443
10.83387864	8.361176083	10.1601793	13.91085441
14.66553738
ENSG00000174807 427.315205	348.6880903	520.7962592
356.3731622	291.0804162	282.9456069	432.2665182
310.1330618	0.477855103	0.048473878	0.20895783	CD248
11	66314487	66317044	-	2558
protein_coding	CD248 molecule [Source:HGNC Symbol;Acc:HGNC:18219]
-	471	394	448	344	255	328
9.016729695	7.298306911	10.89643219	7.472569209
6.12755193	5.962169543
ENSG00000227848 7.258007728	7.964956378	2.3249833	0
0	0	5.849315802	0	4.984931973
0.048481549	1	SUCLA2-AS1	13	48001389
48002552	+	552	antisense	SUCLA2 antisense RNA 1
[Source:HGNC Symbol;Acc:HGNC:39965]	-	8	9	2
0	0	0	0.709707824	0.772555827
0.225422761	0	0	0
ENSG00000198753 272.1752898	247.7986429	325.497662
260.0280922	138.1205112	154.4123891	281.8238649
184.1869975	0.612654861	0.04848854	0.20895783	PLXNB3
X	153764196	153779346	+	7007
protein_coding	plexin B3 [Source:HGNC Symbol;Acc:HGNC:9105]	-
300	280	280	251	121	179	2.096610811
1.893443519	2.486181099	1.990460885	1.061452619
1.187823551
ENSG00000088832 4366.598899	4523.210228	4403.51837
4114.866862	3605.972685	3424.67701	4431.109166
3715.172186	0.25437055	0.048491825	0.20895783	FKBP1A
20	1368978 1393172 -	2577	protein_coding	FK506
binding protein 1A [Source:HGNC Symbol;Acc:HGNC:3711]	-	4813
5111	3788	3972	3159	3970	91.45977459
93.97620502	91.45393536	85.64595669	75.34988044
71.63200483
ENSG00000213463 1073.277893	1017.744426	873.0312291

1552.916774	975.9755131	1334.502603	988.0178493
1287.798297	-0.382195423	0.048574739	0.209253625
SYNJ2BP 14	70366496	70417061	-	7031
protein_coding	synaptojanin 2 binding protein [Source:HGNC
Symbol;Acc:HGNC:18955]	-	1183	1150	751	1499	855
	1547	8.239414098	7.750097805	6.645530961
11.84667785	7.474744925	10.23067371
ENSG00000100883 1507.851105	1324.837744	1406.614896
1362.298571	1066.153368	857.463211	1413.101249
1095.30505	0.368138723	0.048584128	0.209253625	SRP54
	14	34981957	35029567	+	3903
protein_coding	signal recognition particle 54 [Source:HGNC
Symbol;Acc:HGNC:11301]	-	1662	1497	1210	1315	934
	994	20.85264509	18.17396511	19.28828727
18.72143978	14.70942462	11.84183079
ENSG00000102879 97.07585336	105.3144232	96.48680694
71.48182615	54.79160775	36.2308399	99.62569451
54.16809127	0.882953491	0.048613079	0.209327351	CORO1A
	16	30182827	30189076	+	4871
protein_coding	coronin 1A [Source:HGNC Symbol;Acc:HGNC:2252]	-
	107	119	83	69	48	42	1.075707835
	1.157591349	1.060148759	0.787123729	0.605717984
	0.400924115
ENSG00000198380 1499.685847	1085.004058	1400.802438
1795.333402	1658.587626	1551.887642	1328.497448
1668.60289	-0.329001578	0.048776781	0.209981133	GFPT1
	2	69319769	69387254	-	8866
protein_coding	glutamine--fructose-6-phosphate transaminase 1
[Source:HGNC Symbol;Acc:HGNC:4241]	-	1653	1226	1205
	1733	1453	1799	9.130063754	6.552230757
8.456023214	10.86132676	10.07361265	9.434838151
ENSG00000213923 929.9322401	1362.007541	1322.915498
1377.838098	1426.864785	1901.256456	1204.951759
1568.653113	-0.381403019	0.048805035	0.210047615	CSNK1E
	22	38290691	38318084	-	6246
protein_coding	casein kinase 1 epsilon [Source:HGNC
Symbol;Acc:HGNC:2453]	-	1025	1539	1138	1330	1250
	2204	8.036196898	11.67516645	11.33566829
11.83209655	12.30142416	16.40743258
ENSG00000237976 6.350756762	8.849951531	2.3249833	0
	0	0	5.841897198	0	4.983168835
0.048808748	1	AL391069.3	1	151346967
151348027	+	618	antisense	novel transcript
	-	7	10	2	0	0	0
0.554674561	0.766722072	0.201348485	0	0	0
ENSG00000143633 534.370819	485.8623391	540.5586172
348.0854143	472.5776169	336.4292277	520.2639251
385.6974196	0.433095583	0.048815973	0.210047615
C1orf131	1	231223763	231241187	-	5413
	protein_coding	chromosome 1 open reading frame 131

[Source:HGNC Symbol;Acc:HGNC:25332]	-	589	549	465
	336	414	390	5.328512038	4.805745392
5.344680793	3.449159627	4.701210248	3.350098667
ENSG00000108651 2938.585879	2707.200173	2590.031396
3263.300759	3336.580614	3205.566692	2745.272483
3268.482688	-0.251392583	0.048846411	0.210089317	UTP6
	17	31860899	31901765	-	6450
protein_coding	"UTP6, small subunit processome component [Source:HGNC
Symbol;Acc:HGNC:18279]" -	3239	3059	2228	3150	2923
	3716	24.59121104	22.47223166	21.49128178
27.13706549	27.85585293	26.78841441
ENSG00000128274 217.7402318	180.5390112	254.5856713
105.6687865	122.1396256	186.3300338	217.6216381
138.0461486	0.65216614	0.04885697	0.210089317	A4GALT
	22	42692112	42721298	-	3407
protein_coding	"alpha 1,4-galactosyltransferase (P blood group)
[Source:HGNC Symbol;Acc:HGNC:18149]"	-	240	204	219
	102	107	216	3.449592474	2.837164223
3.999252527	1.663566177	1.930451986	2.947899244
ENSG00000139350 989.8108039	720.3860546	811.4191716
628.8328764	730.55477	572.7923261	840.5386767
644.0599908	0.38513428	0.048903177	0.210089317	NEDD1
	12	96907223	96953777	+	4463
protein_coding	"neural precursor cell expressed, developmentally
down-regulated 1 [Source:HGNC Symbol;Acc:HGNC:7723]"	-	1091
	814	698	607	640	664	11.97089295
8.64219296	9.73050505	7.557425034	8.814556124
6.917867039
ENSG00000033011 470.8632513	572.5918641	354.5599532
641.2644983	683.7536051	571.067048	466.0050229
632.0283838	-0.43720114	0.048925193	0.210089317	ALG1
	16	5033923 5087379 +	4553	protein_coding	"ALG1,
chitobiosyldiphosphodolichol beta-mannosyltransferase [Source:HGNC
Symbol;Acc:HGNC:18294]" -	519	647	305	619	599
	662	5.582110009	6.733379171	4.16782078
7.554488116	8.086796832	6.76069521
ENSG00000163885 0	0	0	0	0	25.01653231
	0	8.338844104	-5.541089696	0.048938569
0.210089317	CFAP100 3	126394939	126436556	+
	3352	protein_coding	cilia and flagella associated protein
100 [Source:HGNC Symbol;Acc:HGNC:26842] -	0	0	0
	0	0	29	0	0	0	0	0
	0.402276818
ENSG00000232282 0	0	0	0	0	25.01653231
	0	8.338844104	-5.541089696	0.048938569
0.210089317	MTND1P32	X	102785667	102786604
	+	938	processed_pseudogene	MT-ND1 pseudogene 32
[Source:HGNC Symbol;Acc:HGNC:42081]	-	0	0	0
	0	0	29	0	0	0	0	0
	1.437560654

ENSG00000188050 0	0	0	0	0	25.01653231
	0	8.338844104	-5.541089696	0.048938569
0.210089317	RNF133	7	122697712	122699156	-
	1445	protein_coding	ring finger protein 133 [Source:HGNC
Symbol;Acc:HGNC:21154]	-	0	0	0	0	0
	29	0	0	0	0	0	0.933170861
ENSG00000269014 0	0	0	0	0	25.01653231
	0	8.338844104	-5.541089696	0.048938569
0.210089317	AC008622.1	19	46826895	46828440
	-	284	processed_pseudogene	heterogeneous nuclear
ribonucleoprotein A1-like 2 (HNRNPA1L2) pseudogene	-	0
	0	0	0	0	29	0	0	0
	0	0	4.747999625
ENSG00000270617 0	0	0	0	0	25.01653231
	0	8.338844104	-5.541089696	0.048938569
0.210089317	URGCP-MRPS24	7	43866558	43906589
	-	795	protein_coding	URGCP-MRPS24 readthrough
[Source:HGNC Symbol;Acc:HGNC:49188]	-	0	0	0
	0	0	29	0	0	0	0	0
	1.696140747
ENSG00000148356 291.2275601	315.9432697	299.9228457
396.7759335	348.1550076	504.6438415	302.3645585
416.5249275	-0.463571377	0.048944433	0.210089317	LRSAM1
	9	127451486	127503501	+	4365
protein_coding	leucine rich repeat and sterile alpha motif containing
1 [Source:HGNC Symbol;Acc:HGNC:25135]	-	321	357	258
	383	305	585	3.601218462	3.875345335
3.677412132	4.875583174	4.294997857	6.231644015
ENSG00000101844 433.6659617	419.4877026	462.6716767
535.5957119	537.6426511	651.2924792	438.608447
574.843614	-0.391689844	0.048976049	0.210130969	ATG4A
	X	108091668	108154671	+	2927
protein_coding	autophagy related 4A cysteine peptidase [Source:HGNC
Symbol;Acc:HGNC:16489]	-	478	474	398	517	471
	755	7.99712451	7.673297917	8.459938312
9.814764069	9.891121688	11.99375705
ENSG00000215251 431.8514598	415.947722	317.3602204
582.2142941	445.181813	553.8142671	388.3864674
527.0701247	-0.439509209	0.048977895	0.210130969
FASTKD5 20	3146519 3159897 -	2874	protein_coding	FAST
kinase domains 5 [Source:HGNC Symbol;Acc:HGNC:25790]	-	476
	470	273	562	390	642	8.110523229
7.748855013	5.909935347	10.86579721	8.341136021
10.38674052
ENSG00000103148 1698.373808	1494.756814	1444.977121
1764.254347	1754.47294	2245.449435	1546.035914
1921.392241	-0.313836466	0.048989803	0.210131094	NPRL3
	16	84271	138860	-	6609	protein_coding	"NPR3
like, GATOR1 complex subunit [Source:HGNC Symbol;Acc:HGNC:14124]"
	-	1872	1689	1243	1703	1537	2603

13.87071269	12.10933624	11.70151849	14.31828293
14.29504332	18.3134191
ENSG00000159228 1173.98275	1459.357007	1376.390113
1390.26972	1824.103941	1834.833249	1336.576624
1683.06897	-0.332743825	0.049003007	0.210136773	CBR1
21	36069941	36073166	+	2450
protein_coding	carbonyl reductase 1 [Source:HGNC
Symbol;Acc:HGNC:1548]	-	1294	1649	1184	1342	1598
2127	25.86407051	31.89194542	30.06716387
30.43676388	40.09203038	40.36755296
ENSG00000179943 567.0318537	731.0059965	764.9195056
717.926167	932.5988236	1083.474641	687.6524519
911.3332105	-0.407433516	0.049030011	0.210179267	FIZ1
19	55591371	55601970	-	6790
protein_coding	FLT3 interacting zinc finger 1 [Source:HGNC
Symbol;Acc:HGNC:25917]	zf-C2H2 625	826	658	693	817
1256	4.507533121	5.764169112	6.029245473
5.67120706	7.396046662	8.601038333
ENSG00000173950 710.3775064	591.1767623	730.0447561
366.7328472	599.2832098	531.3856519	677.1996749
499.133903	0.439960994	0.04903668	0.210179267	XXYLT1
3	195068279	195271167	-	5409
protein_coding	xyloside xylosyltransferase 1 [Source:HGNC
Symbol;Acc:HGNC:26639]	-	783	668	628	354	525
616	7.088812089	5.85175213	7.223530468
3.636623361	5.966088374	5.295350957
ENSG00000174004 198.6879615	200.0089046	159.261356
323.2221704	247.7037267	243.2642108	185.9860741
271.3967026	-0.543150919	0.049064912	0.21024644	NRROS
3	196639686	196662004	+	2910
protein_coding	negative regulator of reactive oxygen species
[Source:HGNC Symbol;Acc:HGNC:24613]	-	219	226	137
312	217	282	3.68535908	3.679949772
2.929101524	5.957631659	4.583678041	4.50595798
ENSG00000213024 3093.725794	3378.911495	3102.690214
3665.256535	4047.730023	3673.117055	3191.775834
3795.367871	-0.249633504	0.049076124	0.21024644	NUP62
19	49906825	49929763	-	6020
protein_coding	nucleoporin 62 [Source:HGNC Symbol;Acc:HGNC:8066]
-	3410	3818	2669	3538	3546	4258
27.73872987	30.05148057	27.58410702	32.65678154
36.20675474	32.88820599
ENSG00000069849 3496.545223	3843.53395	3738.573146
3395.904726	3076.320477	2730.252578	3692.884106
3067.492594	0.267893395	0.049170528	0.210599872	ATP1B3
3	141876124	141926514	+	2584
protein_coding	ATPase Na+/K+ transporting subunit beta 3 [Source:HGNC
Symbol;Acc:HGNC:806]	-	3854	4343	3216	3278	2695
3165	73.03783387	79.63862642	77.43376522
70.49015793	64.1082055	56.95242531

ENSG00000197238 4.53625483	3.539980612	9.299933199	0
	0	0	5.792056214	0	4.963470946
0.049256895	1	HIST1H4J	6	27824108
27824480	+	373	protein_coding	histone cluster 1 H4
family member j [Source:HGNC Symbol;Acc:HGNC:4785]	-	5
	4	8	0	0	0	0.656432169
0.508133234	1.334406048	0	0	0
ENSG00000227308 1.814501932	0	0	0	12.55641011
	20.70333709	0.604833977	11.0865824	-4.156087721
	0.049284529	0.211037046	AC009502.1	2
219685381	219737937	+	2951	lincRNA
uncharacterized LOC105373886 [Source:NCBI gene;Acc:105373886]	-
	2	0	0	0	11	24	0.033188641
	0	0	0	0.229124134	0.378157789
ENSG00000108448 454.532734	672.5963164	487.0840013
579.1063887	748.8186393	867.8148795	538.0710172
731.9133025	-0.443697094	0.049311218	0.211100226
TRIM16L 17	18697998	18736118	+	4329
protein_coding	tripartite motif containing 16 like [Source:HGNC
Symbol;Acc:HGNC:32670]	-	501	760	419	559	656
	1006	5.667334201	8.318642246	6.021896456
7.175237251	9.314587221	10.80541405
ENSG00000129473 398.2831741	369.0429789	349.9099866
278.6755251	310.4857773	211.3465661	372.4120465
266.8359562	0.483585231	0.049339055	0.211168291	BCL2L2
	14	23298790	23311759	+	4189
protein_coding	BCL2 like 2 [Source:HGNC Symbol;Acc:HGNC:995]	-
	439	417	301	269	272	245	5.131955046
	4.716850759	4.470570848	3.568239227	3.991222175
	2.719485466
ENSG00000196187 627.8176685	613.3016411	435.9343687
520.0561844	287.6559407	375.2479847	559.0178928
394.3200366	0.504083501	0.049400407	0.211378644
TMEM63A 1	225845536	225882369	-	10339
protein_coding	transmembrane protein 63A [Source:HGNC
Symbol;Acc:HGNC:29118]	-	692	693	375	502	252
	435	3.277602202	3.176004727	2.256625955
2.697970679	1.498198527	1.956328311
ENSG00000169410 1262.893345	989.4245812	1597.263527
862.9617563	1083.275745	998.0733754	1283.193818
981.4369589	0.385996839	0.049412103	0.211378644	PTPN9
	15	75463251	75579289	-	8632
protein_coding	"protein tyrosine phosphatase, non-receptor type 9
[Source:HGNC Symbol;Acc:HGNC:9661]"	-	1392	1118	1374
	833	949	1157	7.896897248	6.137009742
9.903350449	5.362232165	6.757750307	6.232365939
ENSG00000214425 378.3236528	163.7241033	229.010855
202.0138565	134.6960357	141.4728034	257.0195371
159.3942319	0.689812232	0.049427581	0.211393732
LRRC37A4P	17	45506741	45550335	-	6924

transcribed_unprocessed_pseudogene "leucine rich repeat containing 37 member A4, pseudogene [Source:HGNC Symbol;Acc:HGNC:25479]" - 417 185 197 195 118 164 2.949223456 1.266021584 1.770174228 1.564910829 1.047544083 1.101330856
ENSG00000078070 482.6575139 533.6520773 503.3588844 446.5024213 270.5335633 385.5996532 506.5561585 367.5452126 0.461692538 0.049449724 0.211437315 MCCC1 3 183015218 183116075 - 4321 protein_coding methylcrotonoyl-CoA carboxylase 1 [Source:HGNC Symbol;Acc:HGNC:6936] - 532 603 433 431 237 447 6.029149454 6.612405625 6.23462701 5.54249165 3.371408985 4.810101879
ENSG00000148344 39.01179154 77.87957347 132.5240481 7.251779464 11.41491828 69.87376267 83.13847103 29.5134868 1.480297181 0.049476826 0.211499265 PTGES 9 129738331 129753047 - 1896 protein_coding prostaglandin E synthase [Source:HGNC Symbol;Acc:HGNC:9599] - 43 88 114 7 10 81 1.110602908 2.199230652 3.74087645 0.205150108 0.324197026 1.986450301
ENSG00000081154 3443.924667 3477.145957 3852.497328 3245.689295 2865.144489 2903.643026 3591.189317 3004.825603 0.256983451 0.049488126 0.211499265 PCNP 3 101574095 101594437 + 3102 protein_coding PEST proteolytic signal containing nuclear protein [Source:HGNC Symbol;Acc:HGNC:30023] - 3796 3929 3314 3133 2510 3366 59.92569697 60.01594878 66.46875698 56.12167766 49.73696586 50.45489844
ENSG00000244462 2336.171237 2272.667553 2180.834335 2582.669458 2681.364304 2837.21982 2263.224375 2700.417861 -0.25478038 0.049545326 0.21169258 RBM12 20 35648925 35664956 - 6702 protein_coding RNA binding motif protein 12 [Source:HGNC Symbol;Acc:HGNC:9898] - 2575 2568 1876 2493 2349 3289 18.81488176 18.15586884 17.41547231 20.66949655 21.54398081 22.81867738
ENSG00000185651 3130.923084 3546.175579 3639.761356 3898.349446 3839.97851 4756.591696 3438.953339 4164.973217 -0.276741608 0.049598044 0.211866445 UBE2L3 22 21549447 21624034 + 3838 protein_coding ubiquitin conjugating enzyme E2 L3 [Source:HGNC Symbol;Acc:HGNC:12488] - 3451 4007 3131 3763 3364 5514 44.03202638 49.46987498 50.75571427 54.48052595 53.87637622 66.80251443
ENSG00000183813 0 2.654985459 0 12.43162194 0 26.7418104 0.884995153 13.05781078 -3.841063957 0.049609973 0.211866445 CCR4 3 32951574 32956349 + 3095 protein_coding C-C motif chemokine receptor 4 [Source:HGNC Symbol;Acc:HGNC:1605] - 0

3	0	12	0	31	0	0.045929006
0	0.215443123	0	0.465727689
ENSG00000272716 11.79426256	3.539980612	2.3249833	0
0	0	5.886408823	0	4.993613762
0.04961539	1	AL121658.1	2	32165046
32165757	-	712	lincRNA novel transcript	-
13	4	2	0	0	0	0.894112244
0.266199012	0.174765961	0	0	0
ENSG00000047849 7549.235288	7326.874873	8952.348196
7066.341104	6885.478708	5829.714668	7942.819452
6593.844826	0.26851153	0.049625945	0.2118835	MAP4
3	47850690	48089272	-	14943
protein_coding	microtubule associated protein 4 [Source:HGNC
Symbol;Acc:HGNC:6862]	-	8321	8279	7701	6821	6032
6758	27.26882401	26.25224973	32.06388731
25.36426586	24.81252128	21.0286508
ENSG00000246308 15.42326642	7.964956378	12.78740815	0
0	3.450556181	12.05854365	1.150185394
3.316065466	0.049659193	0.211974293	AC116535.1	11
10809297	10822931	+	2335	antisense
uncharacterized LOC101928053 [Source:NCBI gene;Acc:101928053]
-	17	9	11	0	0	4
0.3565256	0.182634183	0.29309786	0	0
0.079653364
ENSG00000111203 343.8481161	370.8129692	380.1347695
529.3799009	409.7955663	526.2098176	364.9319516
488.4617616	-0.421921877	0.049694392	0.212025433	ITFG2
12	2812622 2859791 +	6250	protein_coding
integrin alpha FG-GAP repeat containing 2 [Source:HGNC
Symbol;Acc:HGNC:30879]	-	379	419	327	511	359
610	2.969531087	3.176584749	3.255176802
4.543106591	3.530707917	4.538170786
ENSG00000163961 901.8074602	993.849557	804.4442217
804.9475205	632.3864728	667.682621	900.0337463
701.6722048	0.359780309	0.049700153	0.212025433	RNF168
3	196468783	196503768	-	5347
protein_coding	ring finger protein 168 [Source:HGNC
Symbol;Acc:HGNC:26661]	-	994	1123	692	777	554
774	9.103426369	9.951671071	8.051981288
8.074634599	6.368643528	6.730724193
ENSG00000198912 758.4618076	841.6303906	746.3196392
732.4297259	527.3692246	533.973569	782.1372791
597.9241732	0.387932203	0.049707133	0.212025433
C1orf174	1	3889125 3900293 -	4388	protein_coding
chromosome 1 open reading frame 174 [Source:HGNC
Symbol;Acc:HGNC:27915]	-	836	951	642	707	462
619	9.329714243	10.26928812	9.102805343
8.952922777	6.47176467	6.559263053
ENSG00000125388 84.37433984	147.7941906	127.8740815
154.3593057	301.3538426	149.2365548	120.014204

201.6499011	-0.74682031	0.049789335	0.212206361	GRK4
	4	2963608 3040747 +	4686	protein_coding	G
protein-coupled receptor kinase 4 [Source:HGNC Symbol;Acc:HGNC:4543]
	-	93	167	110	149	264	173
0.971872622	1.688653824	1.460485493	1.766835138
3.462972181	1.716622644
ENSG00000105953 3775.97852	3462.986034	3701.373413
3156.596004	2938.199966	3136.555569	3646.779323
3077.117179	0.244872801	0.049795054	0.212206361	OGDH
	7	44606572	44709066	+	5210
protein_coding	oxoglutarate dehydrogenase [Source:HGNC
Symbol;Acc:HGNC:8124]	-	4162	3913	3184	3047	2574
	3636	39.11947666	35.58758893	38.02263253
32.49726221	30.36813906	32.45018442
ENSG00000172123 17.23776835	39.82478189	86.02438209
14.50355893	19.40536108	18.11541995	47.69564411
17.34144665	1.454344453	0.049797346	0.212206361	SLFN12
	17	35411060	35433283	-	3560
protein_coding	schlafen family member 12 [Source:HGNC
Symbol;Acc:HGNC:25500]	-	19	45	74	14	17
	21	0.261355887	0.598947776	1.293268109
0.218519441	0.293525802	0.274283899
ENSG00000128683 33.56828574	43.3647625	32.5497662
118.1004084	65.06503421	46.58250844	36.49427148
76.58265036	-1.065849263	0.049797535	0.212206361	GAD1
	2	170813213	170861151	+	8242
protein_coding	glutamate decarboxylase 1 [Source:HGNC
Symbol;Acc:HGNC:4092]	-	37	49	28	114	57
	54	0.219835486	0.281701987	0.211364608
0.768571516	0.425098532	0.304643676
ENSG00000156273 1691.115801	1608.921188	1679.800434
2069.865053	2056.968274	1871.926728	1659.945808
1999.586685	-0.268392982	0.049894783	0.21255814	BACH1
	21	29194071	29630751	+	7717
protein_coding	BTB domain and CNC homolog 1 [Source:HGNC
Symbol;Acc:HGNC:935]	TF_bZIP 1864	1818	1445	1998	1802
	2170	11.82840242	11.16276596	11.65000719
14.38662844	14.35336228	13.07501668
ENSG00000198015 1795.449662	1539.891566	1722.812625
1479.363011	1353.809308	1302.584958	1686.051284
1378.585759	0.290493269	0.049904119	0.21255814	MRPL42
	12	93467488	93516213	+	16393
protein_coding	mitochondrial ribosomal protein L42 [Source:HGNC
Symbol;Acc:HGNC:14493]	-	1979	1740	1482	1428	1186
	1510	5.911749712	5.029412423	5.624664353
4.840406241	4.447066362	4.283014321
ENSG00000071282 388.3034134	384.9728916	378.9722779
246.5605018	297.9293672	301.9236658	384.082861
282.1378449	0.444840844	0.049959803	0.212744091	LMCD1
	3	8501707 8574673 +	9130	protein_coding	LIM

and cysteine rich domains 1 [Source:HGNC Symbol;Acc:HGNC:6633] -
428 435 326 238 261 350 2.295628855
2.257589207 2.221537605 1.448499078 1.757183392
1.782494855
ENSG00000204792 9.07250966 3.539980612 4.6499666 0
0 0 5.754152291 0 4.958589462
0.049987213 1 LINC01291 2 74918148
74938418 + 4176 lincRNA long intergenic non-protein
coding RNA 1291 [Source:HGNC Symbol;Acc:HGNC:50358] - 10
4 4 0 0 0 0.117264942
0.045386422 0.059594523 0 0 0
